# Integration of Transcriptomics and Metabolomics Reveals Organ-Specific Biosynthesis and Accumulation of Pharmacologically Active Flavonoids in *Rhododendron yedoense* var. *poukhanense*

**DOI:** 10.3390/plants15162441

**Published:** 2026-08-11

**Authors:** Riwen Fei, Siyang Duan, Xiuting Zhao, Yanhong Zhang, Jiansong You, Xiaoyu Li

**Affiliations:** 1 School of Agricultural, Liaodong University, Dandong 118003, China; duansiyang1994@126.com (S.D.); zhaoxt0906@163.com (X.Z.); zyh3513@163.com (Y.Z.); 2 Postdoc Workstation, Aim Honesty Biopharmaceutical Co., Ltd., Dalian 116620, China; ruser@126.com; 3 School of Bioengineering, Dalian University of Technology, Dalian 116024, China; xiaoyuli@dlut.edu.cn

**Keywords:** *Rhododendron yedoense* var. *poukhanense*, widely targeted metabolomics, transcriptomics, flavonoid biosynthesis, organ-specific accumulation, multi-omics integration

## Abstract

*Rhododendron yedoense* var. *poukhanense* is an important medicinal plant, but still little is known about how its bioactive flavonoids are made in different organs. Comprehensive metabolomic profiling was performed using ultra-performance liquid chromatography–tandem mass spectrometry (UPLC-MS/MS), coupled with reference-based RNA sequencing (RNA-seq), to analyze root, stem, and leaf tissues from three independent biological replicates. Subsequently, we performed two-way orthogonal partial least squares (O2PLS) regression, canonical correlation analysis (CCA), and Pearson correlation analyses. A total of 2182 metabolites were detected. Among these, 1116, 896, and 1264 metabolites exhibited differential accumulation across the three pairwise comparisons. Concurrently, 9836, 5457, and 8007 genes were differentially expressed across the three pairwise comparisons, yielding a total of 13,019 unique differentially expressed genes (DEGs). The bifunctional flavanone 3-hydroxylase/flavonol synthase (F3H/FLS) enzyme exhibited the highest expression level in roots, consistent with root-preferential biosynthesis of flavonoid skeletons and the subsequent accumulation of flavonol glycosides in this tissue. In contrast, dihydroflavonol 4-reductase (DFR) exhibited predominant activity in roots, consistent with its role in farrerol biosynthesis. The multi-omics integration model demonstrated excellent goodness-of-fit to the experimental data. Canonical correlation analysis (CCA) further revealed a robust positive association between dihydrokaempferol accumulation and kaempferol biosynthesis. These findings collectively support flavanone 3-hydroxylase (F3H) as a candidate regulatory node governing organ-specific flavonoid partitioning. However, functional validation is required to substantiate this inference.

## 1. Introduction

The genus *Rhododendron* L. (Ericaceae) comprises approximately 1000 extant species, the majority of which are native to temperate and subtropical regions of the Northern Hemisphere. China harbors the greatest species diversity within the genus, with approximately 570 documented species [1,2]. Many *Rhododendron* species have been traditionally employed in ethnomedicinal practices for the management of inflammatory disorders, respiratory conditions—including cough and asthma—rheumatic ailments, and dermatological manifestations [2]. To date, over 200 structurally characterized bioactive compounds have been isolated and identified from *Rhododendron* species, among which flavonoids and diterpenoids constitute the predominant chemical classes [1]. Farrerol is a 2,3-dihydroflavonoid, while quercetin and its glycosidic derivatives, kaempferol glycosides, and taxifolin glycosides have also been identified in *Rhododendron* species. All of these have been studied and reportedly they can reduce inflammation, cellular oxidation, and exhibit anti-microbial properties, amongst other effects [3,4,5].

*Rhododendron yedoense* var. *poukhanense* (Levl.) Nakai, commonly known as *Poukhanense azalea*, is a deciduous shrub endemic to the Korean Peninsula [6]. Early phytochemical investigations focused predominantly on the floral tissues of *R*. *yedoense* var. *poukhanense*. Jung et al. [6] isolated and structurally characterized four flavonoids from these flowers, all of which exhibited potent antioxidant activity in vitro. The stem bark extract of *R*. *yedoense* var. *poukhanense* demonstrates acetylcholinesterase inhibitory activity, accompanied by significant antioxidant capacity [7]. Similarly, the root extract of the closely related species *R. mucronulatum* exhibits anti-aging and anti-allergic effects in dermatological contexts, attributable—at least in part—to its content of taxifolin glycosides [4]. The flavonoid biosynthetic pathway is one of the best-known examples of secondary metabolism in plants. They did not compare metabolite levels in roots, stems, and leaves in a systematic way.

To interpret the metabolomic data from this study, it is necessary to consider the major compound classes and their biosynthetic origins in *Rhododendron*. Phytochemical investigations have identified flavonoids (quercetin, kaempferol, and farrerol), phenolic acids (chlorogenic, caffeic, and gallic acid), and terpenoids (diterpenoids such as grayanotoxins; triterpenoids such as ursolic and oleanolic acid) as the principal bioactive constituents [1,3,4,6,8,9,10]. These compounds arise from interconnected metabolic pathways [10,11,12] that are differentially active across organs according to tissue-specific physiological demands [13,14,15]. The aromatic amino acids phenylalanine and tyrosine, produced through the shikimate pathway [16,17], serve as the primary carbon skeletons for the entire phenylpropanoid–flavonoid system.

The flavonoid biosynthetic pathway is the best-characterized route [18,19]. At the flavanone 3-hydroxylase (F3H) branch point, flavonol synthase (FLS) directs dihydroflavonols toward kaempferol, quercetin, and myricetin (flavonols), while dihydroflavonol 4-reductase (DFR) and anthocyanidin reductase (ANR) channel intermediates toward farrerol, a 2,3-dihydroflavonoid characteristic of the genus [3]. This branching is transcriptionally controlled by the MBW (myeloblastosis (MYB)-basic helix-loop-helix (bHLH)-WD40 repeat domain (WD40)) complex, with R2R3-repeat myeloblastosis (R2R3-MYB) activators (MYB11, MYB12) regulating early biosynthetic genes and bHLH-WD40 complexes controlling late genes [20,21]. tryptophan-arginine-lysine-tyrosine domain (WRKY), APETALA2/ethylene-responsive factor (AP2/ERF), and Cys2His2-type zinc finger (C2H2) transcription factors provide additional regulatory layers [22]. Terpenoid biosynthesis proceeds via the methylerythritol phosphate (MEP) and mevalonate (MVA) pathways [8,9], while phenolic acid metabolism branches from the phenylpropanoid pathway at 4-coumaroyl-CoA, competing with the chalcone synthase (CHS) flavonoid entry point for metabolic flux [11,12,23].

Different organs of a plant accumulate these metabolites in different amounts. This spatial heterogeneity reflects gene activity in specific tissues [13,14,15]. Leaves usually store flavonoid glycosides and phenolic acids. These protect the plant from ultraviolet B (UV-B) and they work as antioxidants. This is linked to higher activity of UDP-glycosyltransferase (UGT) and hydroxycinnamoyl-CoA quinate hydroxycinnamoyl transferase (HQT) genes. MYB12-type activators transcriptionally activate these genes. The shikimate pathway also shows enhanced activity, providing the starting materials phenylalanine and tyrosine [11,14,16]. In contrast, roots appear to preferentially accumulate specialized diterpenoids, triterpenoids, and flavonoid aglycones—compounds implicated in rhizosphere signaling and defense against soil-borne pathogens and herbivores. This tissue-specific metabolic profile may reflect upregulated expression of *terpene synthase* (*TPS*), *cytochrome P450* (*CYP450*), and early phenylpropanoid pathway genes, coupled with a metabolic shift that diverts phenylpropanoid flux toward aglycone biosynthesis [13,15,17,23]. Stems, serving as vascular conduits and structural scaffolds, display intermediate metabolic profiles characterized by the enrichment of lignin precursors—such as p-coumaryl, coniferyl, and sinapyl alcohols—derived from the phenylpropanoid pathway through the sequential actions of cinnamoyl-CoA reductase (CCR) and cinnamyl alcohol dehydrogenase (CAD), alongside transport-associated metabolites [11]. The present multi-omics study systematically tests these hypotheses concerning the spatially resolved partitioning of secondary metabolism across *R*. *yedoense* var. *poukhanense* organs.

Integrated metabolomics–transcriptomics analysis has emerged as a robust and widely adopted strategy for elucidating the molecular mechanisms underlying organ-specific metabolite accumulation in medicinal plant species [13,22]. For instance, Wu et al. [22] used liquid chromatography-mass spectrometry (LC-MS)-based metabolic profiling to characterize metabolite variations across six tissues of *Polygonum cuspidatum*; however, their study surveyed only 53 metabolites and did not link tissue-specific metabolite patterns to gene expression. Liu et al. [24] integrated transcriptome and widely targeted metabolome analyses of *Veratrum mengtzeanum* leaves and roots, identifying 33,942 differentially expressed genes (DEGs) and 55 differentially accumulated metabolites (DAMs); however, their correlation-based network analysis remained descriptive and did not establish quantitative gene–metabolite bridges at the individual metabolite level. Similar multi-omics strategies have been applied to *Camellia sinensis* [14], *Panax ginseng* [25], *Salvia miltiorrhiza* [26], and *Glycyrrhiza uralensis* [27]. These studies collectively demonstrate that multi-omics integration identifies DAMs and their biosynthetic genes; however, they have typically relied on single-layer correlation analyses without formally partitioning joint versus orthogonal variation, limiting their ability to distinguish transcriptionally coupled processes from organ-specific processes that operate independently of gene expression.

Several factors distinguish the present study from these prior investigations. First, among *Rhododendron* species, phytochemical studies have been restricted to isolated organ extracts: Jung et al. [6] identified only four flavonoids from *R. yedoense* var. *poukhanense* flowers, and Lai et al. [7] characterized neuroprotective flavonoids from *R. fortunei* bark, but neither study compared root, stem, and leaf metabolomes systematically or linked metabolites to gene expression. Second, the metabolite coverage in previous multi-omics studies of medicinal plants has been limited—Wu et al. [22] surveyed 53 metabolites in *P*. *cuspidatum*, Liu et al. [24] identified 55 DAMs in *V*. *mengtzeanum*, and Gao et al. [26] focused specifically on tanshinone biosynthesis in *S*. *miltiorrhiza*—whereas our analysis of 2182 metabolites provides an order-of-magnitude broader chemical inventory. Third, previous studies have relied on single-layer correlation analyses; our four-layer framework (two-way orthogonal partial least squares (O2PLS) for decomposing total covariance into joint and orthogonal components, canonical correlation analysis (CCA) for identifying pathway-level gene–metabolite associations, weighted gene co-expression network analysis (WGCNA) for detecting co-expression modules, and Pearson correlation for constructing gene–metabolite association networks) provides complementary analytical perspectives that together enable a more comprehensive statistical characterization of transcriptome–metabolome relationships than any single method alone.

Despite these advances, three critical gaps remain in *R. yedoense* var. *poukhanense* research. First, no systematic organ-resolved metabolite profiling has been conducted; previous studies focused on flowers or bark extracts [6,7], leaving the metabolic fingerprints of roots, stems, and leaves unknown. Second, the organ-specific expression patterns of structural genes (*CHS*, *F3H/FLS*, and *DFR*) and their transcriptional regulators (MYB, bHLH, and WRKY) remain unexplored. Third, quantitative gene–metabolite relationships have not been established, preventing predictive metabolic engineering. The present study addresses each of these gaps through four objectives: (i) to construct organ-specific metabolic fingerprints and identify DAMs, with emphasis on flavonoids, terpenoids, and phenylpropanoids; (ii) to characterize the transcriptomic landscape and annotate key enzymatic components of the flavonoid pathway; (iii) to elucidate transcriptional regulatory networks governing organ-specific flavonoid accumulation; and (iv) to establish quantitative gene–metabolite associations through multi-omics integration.

## 2. Materials and Methods

### 2.1. Plant Material and Sample Collection

Three-year-old healthy plants of *R*. *yedoense* var. *poukhanense* were grown in a greenhouse at the Horticultural Facilities and Environment Practical Training Base of Liaodong College, Dandong, Liaoning Province, China (40°07′ N, 124°23′ E). Growth conditions were maintained at 20–25 °C (day)/15–18 °C (night) with 60–70% relative humidity under natural photoperiod. Plants were in the active vegetative growth stage (pre-flowering) at the time of sampling.

Root, stem, and leaf tissues were collected from three independent mature plants (n = 3 biological replicates) in June 2025. The three plants were randomly selected from a cultivated population and represent independent genotypes, ensuring that observed variation reflects both organ-specific metabolic programming and natural genetic diversity. Each organ was sampled once per plant, yielding nine samples total (3 organs × 3 plants). All samples were flash-frozen in liquid nitrogen immediately after collection. Frozen samples were stored at −80 °C and transported on dry ice to MetWare Biotechnology Co., Ltd. (Wuhan, China) for metabolomic and transcriptomic analysis.

### 2.2. Metabolite Extraction and Widely Targeted Metabolomics Analysis

#### 2.2.1. Sample Preparation

Wide-target metabolomic profiling was performed using an ultra-performance liquid chromatography-tandem mass spectrometry (UPLC-MS/MS) platform at MetWare Biotechnology Co., Ltd. in Wuhan, China. The experimental procedures were adapted from those previously described [17], with minor modifications. All plant samples were lyophilized using a vacuum freeze-dryer (Scientz-100F, Ningbo Scientz Biotechnology Co., Ltd., Ningbo, China). The freeze-dried plant material was homogenized using a mixer mill (MM 400, Retsch, Haan, Germany) with a zirconia grinding bead at 30 Hz for 1.5 min. Subsequently, 100 mg of the homogenized powder was suspended in 1200 μL of 70% methanol, vortexed for 30 s at 30 min intervals for a total of 6 cycles, and incubated overnight at 4 °C. The mixture was then centrifuged at 12,000 rpm for 10 min. The liquid part was filtered (SCAA-104, 0.22 μm; ANPEL, Shanghai, China) before UPLC-MS/MS testing.

One quality control (QC) sample was made by mixing equal parts (20 μL) from each extract, which was treated in the same way as the test samples. The pooled QC sample was injected after every 10 experimental samples throughout the analytical run to monitor instrumental stability and data reproducibility. In parallel, blank samples consisting of pure methanol were analyzed at regular intervals to detect potential contaminants and evaluate column carryover.

#### 2.2.2. UPLC-MS/MS Conditions

We used a SHIMADZU Nexera X2 UPLC system (Shimadzu, Kyoto, Japan). It had an Agilent SB-C18 column (1.8 μm, 2.1 mm × 100 mm). The column was maintained at a temperature of 40 °C using an active column thermostat. The mobile phase consisted of (A) pure water with 0.1% formic acid and (B) acetonitrile with 0.1% formic acid (HPLC grade). A binary gradient elution program was employed as follows: starting conditions of 95% A, 5% B; within 9 min, a linear gradient to 5% A, 95% B; a composition of 5% A, 95% B was kept for 1 min; subsequently, a composition of 95% A, 5.0% B was adjusted within 1.1 min and kept for 2.9 min. The flow rate was maintained at 350 μL/min throughout the analysis. The injection volume was 4 μL. The total run time per sample was 14 min.

Mass detection was done on an Applied Biosystems 4500 Q TRAP MS (AB SCIEX, Framingham, MA, USA). It had an electrospray ionization (ESI) source, which ran in both positive and negative modes in separate runs. Multiple reaction monitoring (MRM) was employed as the primary acquisition mode, and scheduled MRM (sMRM) was additionally implemented to improve analytical selectivity and detection sensitivity. This was achieved by assigning each MRM transition to a predefined retention time window (±60 s). The ion source settings were: 550 °C; 5500 V (positive) and −4500 V (negative); CUR 25.0 psi; CAD high; Gas 1 50 psi; Gas 2 60 psi. Data were collected with Analyst 1.6.3 software (AB Sciex). Nitrogen collision gas was set to medium. Tuning and mass calibration were done with 10 and 100 μmol/L polypropylene glycol in QQQ and LIT modes. Declustering potential (DP) and collision energy (CE) for each MRM transition were optimized further. MRM transitions for each metabolite came from the MWDB (MetWare Database). This database has over 30,000 plant metabolites. They include flavonoids, phenolic acids, alkaloids, terpenoids, lignans, coumarins, and other metabolites useful for medicinal plant research [17].

### 2.3. RNA Isolation and Transcriptome Sequencing

RNA was extracted from the same root, stem, and leaf samples (three biological replicates per organ). The RNAprep Pure Plant kit (DP441, Tiangen, Beijing, China) was used. The manufacturer’s recommended protocol was followed with minor modifications to accommodate the high polysaccharide and polyphenolic content characteristic of *Rhododendron* tissues. Briefly, approximately 100 mg of frozen tissue was homogenized to a fine powder under liquid nitrogen using a pre-chilled mortar and pestle, immediately resuspended in the lysis buffer supplied with the kit, and processed in accordance with the manufacturer’s instructions. The concentration and purity of the extracted RNA were assessed using a NanoPhotometer spectrophotometer (IMPLEN, Westlake Village, CA, USA) by measuring absorbance at 260 nm, 280 nm, and 230 nm. RNA integrity was evaluated by 1.0% agarose gel electrophoresis and a Qsep400 Bio-Analyzer (BiOptic, New Taipei City, Taiwan). Only RNA samples with an A260/A280 ratio between 1.8 and 2.1, an A260/A230 ratio greater than 1.8, and an RNA Quality Number (RQN) greater than 7.0 were selected for library construction to ensure high-quality sequencing data. The mean RNA Quality Number (RQN) values were 9.6 ± 0.1 (root), 9.7 ± 0.1 (stem), and 8.2 ± 0.4 (leaf). All samples exceeded the RQN > 7.0 threshold for library construction.

Nine mRNA sequencing libraries (three replicates each for root, stem, and leaf) were constructed using the TruSeq RNA Sample Preparation Kit (Illumina, San Diego, CA, USA) according to the manufacturer’s instructions. Briefly, mRNA was enriched from total RNA using magnetic beads with oligo (dT) and fragmented into short fragments of approximately 200 bp in fragmentation buffer at elevated temperature. First-strand complementary DNA (cDNA) was synthesized using random hexamer primers and the M-MuLV reverse transcriptase system, followed by second-strand cDNA synthesis using DNA polymerase I, RNase H, and dNTPs. The resulting double-stranded cDNA fragments were purified, end-repaired by T4 DNA polymerase and Klenow DNA polymerase, A-tailed by Taq DNA polymerase, and ligated to Illumina sequencing adapters with T4 DNA ligase. Adapter-ligated fragments were then PCR-amplified for 15 cycles using Phusion High-Fidelity DNA polymerase, and the amplicons were purified using AMPure XP beads (Beckman Coulter, Brea, CA, USA). The quality and concentration of the final libraries were assessed using a Qsep400 Bio-Analyzer and a Qubit 2.0 Fluorometer (Life Technologies, Carlsbad, CA, USA). Libraries quality control criteria were pooled in equimolar proportions. Paired-end sequencing (2 × 150 bp) was performed on an Illumina NovaSeq 6000 system at MetWare Biotechnology Co., Ltd. (Wuhan, China), with a target sequencing depth of 6 Gb per sample to ensure comprehensive transcript coverage.

### 2.4. Metabolome Data Processing and Analysis

Raw UPLC-MS/MS data (.wiff) were analyzed with the MetWare Data Analysis System (MDAS; MetWare Biotechnology Co., Ltd.). Peak finding, integration, and alignment were done with MDAS software V1.6.3 (MetWare Biotechnology Co., Ltd., Wuhan, China). The settings were adjusted for plant metabolomics. Metabolites were named by matching precursor ion (Q1), product ion (Q3), and retention time (RT) with the MWDB database. For metabolites with real standards, MS/MS spectra were also compared with standard references. Metabolite identification levels were classified by Metware Biotechnology (Hangzhou, China) as follows: Level 1—MS/MS fragmentation pattern (all fragment ions) and retention time (RT) matched to the in-house database with similarity score > 0.7; Level 2—MS/MS fragmentation and RT matched with similarity score 0.5–0.7; Level 3—Q1 (precursor ion), Q3 (product ion), RT, declustering potential (DP), and collision energy (CE) verified against database. The identification level for key DAMs is reported in Table 1. Complete mass spectrometry parameters (retention time, precursor ion, product ion, collision energy, declustering potential) and annotation confidence levels for 29 representative metabolites are provided in Table S3.

Metabolite abundances were quantified by summing the peak areas of the corresponding MRM transitions acquired under sMRM conditions. A data matrix was constructed with samples designated as rows and annotated metabolites as columns. To assess analytical precision and ensure data reproducibility, the relative standard deviation (RSD) of peak areas for all metabolites was calculated across the pooled QC samples. Metabolites with RSD over 20% were excluded from later analyses. They exhibited excessive analytical variability. Additionally, Pearson correlation coefficients were calculated between consecutively injected QC samples. This assessment evaluated the overall analytical run stability. Runs exhibiting a Pearson correlation coefficient (r) below 0.95 were discarded and repeated.

Metabolite abundance data were normalized using the total sum normalization method. This handled differences in total metabolite levels between samples. Then log_2_ was used to make variance steady and lower the effect of metabolites with high levels. This normalization was performed prior to multivariate statistical analysis. Principal component analysis (PCA) was performed using the prcomp function in R (v4.0.3). PCA was employed to assess global metabolic alterations, evaluate samples clustering patterns, and identify potential outliers. Orthogonal partial least squares discriminant analysis (OPLS-DA) was conducted using the ropls package in R (v1.18.0). It was used to best separate organ types and find the metabolites that make each organ type special. The goodness-of-fit (R^2^Y) and predictive ability (Q^2^) metrics were computed. All three pairwise models showed excellent performance: root vs. leaf (R^2^Y = 0.998, Q^2^ = 0.987), root vs. stem (R^2^Y = 0.998, Q^2^ = 0.963), and stem vs. leaf (R^2^Y = 1.000, Q^2^ = 0.993). The statistical significance of Q^2^ was assessed using 200 permutation tests (Figure S1) to guard against model overfitting. It should be noted that with only three biological replicates per group (n = 3), statistical power is limited and overfitting risk cannot be fully excluded. Metabolites with variable importance in projection (VIP) scores > 1.0 derived from OPLS-DA were considered as potential differentially accumulated metabolites (DAMs). For interpretive clarity, DAMs were classified into three confidence tiers based on VIP magnitude: high confidence (VIP > 2.0), moderate-high confidence (VIP 1.5–2.0), and moderate confidence (VIP 1.0–1.5). Statistical significance was further evaluated using Student’s *t*-test followed by Benjamini–Hochberg false discovery rate (FDR) correction, and DAMs were defined as metabolites meeting the following stringent criteria: VIP > 1.0, *p* < 0.05, and fold change (FC) ≥ 2.0 or ≤0.5 (or equivalently, |log_2_FC| ≥ 1.0). Hierarchical clustering analysis of DAMs was performed using the pheatmap package in R with Euclidean distance and Ward’s linkage as the distance metric and clustering method, respectively. Metabolic pathway enrichment analysis of DAMs was conducted using MetaboAnalystR [28] based on the Kyoto Encyclopedia of Genes and Genomes (KEGG) database (Release 94.0) to identify significantly enriched metabolic pathways associated with organ-specific functions.

### 2.5. Transcriptome Data Processing and Analysis

Raw RNA sequencing (RNA-seq) reads in FASTQ format were processed using fastp (v0.18.0) [29] with the following parameters: adapters of sequences were cut, and low-quality reads with ≥5 uncertain bases or with over 50% Qphred ≤20 bases were removed. Clean reads were subsequently used for downstream analysis. The guanine–cytosine content (GC-content) of clean reads was calculated. The Phred quality score ≥ 20 (Q20) and Phred quality score ≥ 30 (Q30) values were also produced by FastQC to evaluate the base quality.

The reference genome of *R. simsii* Planch. (GenBank accession: GCA_047301955.1; NCBI Taxonomy ID: 118357) was used as the reference for transcriptome alignment. This reference genome was selected because (i) *R. simsii* is the closest relative of *R. yedoense* var. *poukhanense* with a publicly available high-quality reference genome; (ii) both species belong to the subgenus Tsutsusi (section Tsutsusi), sharing recent evolutionary history; and (iii) the *R. simsii* genome represents the best currently available genomic resource for the genus *Rhododendron*. Clean reads were aligned to the reference genome using HISAT2 (v2.2.1) with default parameters. Mapping statistics were assessed for each sample: overall mapping rates ranged from 62.88% to 75.21% (Leaf: 73.83–75.21%; Root: 70.87–75.13%; Stem: 62.88–67.32%), and unique mapping rates ranged from 60.27% to 72.04% (Leaf: 69.95–70.92%; Root: 67.92–72.04%; Stem: 60.27–64.65%). The lower mapping rates in stem samples are consistent with higher lignin and polyphenolic content potentially increasing non-coding or organelle-derived reads, a known feature of woody stem tissues. These cross-species mapping rates are comparable to those reported in other non-model plant transcriptome studies.

Differential expression analysis between pairwise organ comparisons (root vs. stem, root vs. leaf, and stem vs. leaf) was performed using differential expression analysis for sequence count data (DESeq2) [30] in R (v4.0.3). Genes with a FDR < 0.05 and an absolute log_2_ fold change (|log_2_FC|) ≥ 1 were defined as differentially expressed genes (DEGs). For DEGs, hierarchical clustering based on Euclidean distance and complete linkage was performed to visualize expression patterns across organs, and PCA was conducted using the prcomp function to assess transcriptome-wide variation and sample relationships.

Functional annotation of the identified genes was conducted by BLASTx (v2.6.0+) searches (E-value threshold of 1 × 10^−5^, maximum target sequences 20) against multiple public databases, including the Non-Redundant Protein Database (Nr; NCBI), Swiss-Prot (UniProt), Pfam (v33.1), Kyoto Encyclopedia of Genes and Genomes (KEGG; Release 94.0), euKaryotic Orthologous Groups (KOG/eggNOG), and Gene Ontology (GO). Gene Ontology terms were assigned based on Nr and Swiss-Prot BLAST hits using the Blast2GO pipeline. Gene Ontology enrichment analysis of DEGs was performed using the Gene Ontology sequencing (GOseq) R package (v1.34.1) based on the Wallenius non-central hypergeometric distribution to correct for length bias inherent in RNA-seq data, with corrected *p*-values (Benjamini–Hochberg method) < 0.05 considered statistically significant. KEGG pathway enrichment analysis was conducted using KOBAS (v3.0) with a hypergeometric test, and pathways with FDR < 0.05 were defined as significantly enriched. Transcription factor (TF) identification was performed using iTAK (v1.7a) software, which integrates the PlnTFDB and PlantTFDB databases, and TF family classification was conducted following established nomenclature conventions for plant transcription factors.

### 2.6. Multi-Omics Joint Analysis

To investigate the molecular mechanisms underlying organ-specific metabolite accumulation and to establish gene-to-metabolite regulatory associations in *R. yedoense* var. *poukhanense*, an integrated multi-omics analysis was performed by combining the transcriptome and metabolome datasets. Prior to correlation analysis, fragments per kilobase of transcript per million mapped reads (FPKM) values from RNA-seq and peak area values from metabolomics were both log_2_-transformed and z-score normalized to ensure comparability across different measurement scales. Pearson correlation coefficients between the expression levels of DEGs and the accumulation levels of DAMs were calculated using the corr.test function in the psych R package (v2.1.3). Gene–metabolite pairs with absolute Pearson correlation coefficients |r| ≥ 0.8 were considered as significantly correlated. At n = 9 total samples, this threshold corresponds to *p* ≈ 0.01, making the *p* < 0.05 criterion redundant; both thresholds were retained as a conservative double-filter. Formal multiple testing correction (e.g., Benjamini–Hochberg FDR) was not applied to the full gene–metabolite correlation matrix given the exploratory nature of this network analysis and the prioritization of sensitivity for hypothesis generation. The reported gene–metabolite correlations should therefore be treated as candidate associations requiring functional validation.

For pathway-level integration, DEGs and DAMs were jointly mapped onto KEGG metabolic pathways (map01110, map00940, map00941, map00944, map00130, map01061, and other secondary metabolite pathways) to identify co-regulated pathways enriched with both transcriptomic and metabolomic changes. Significantly enriched pathways (FDR < 0.05) containing at least two DAMs and five DEGs were prioritized as candidate pathways associated with organ-specific specialized metabolism. The degree of pathway enrichment was quantified using the combined enrichment score derived from both gene and metabolite enrichment analyses.

Protein–protein interaction (PPI) networks among DEGs involved in the prioritized pathways were constructed using the STRING database (v10) (https://string-db.org) with a confidence score threshold of 0.7 (high confidence interactions only). The species parameter was set to *Arabidopsis thaliana* as the closest well-annotated model organism. The resulting interaction networks were visualized and topologically analyzed using Cytoscape (v3.7.1), and hub genes were identified based on degree centrality (top 10% of nodes) and betweenness centrality metrics computed by the CytoHubba plugin. The integrated gene–metabolite networks were further annotated with KEGG pathway information and TF family classifications to reveal regulatory relationships between transcription factors, structural genes, and metabolites of pharmacological interest, particularly those involved in flavonoid biosynthesis, terpenoid biosynthesis, phenylpropanoid metabolism, and alkaloid biosynthesis pathways. Subnetworks were extracted for pathways of special interest and visualized using Cytoscape with customized node and edge attributes to highlight the strength of gene–metabolite correlations and the direction of regulation.

### 2.7. Quantitative Real-Time Polymerase Chain Reaction (qRT-PCR) Validation

To validate the RNA-seq results, qRT-PCR was performed on 12 DEGs selected at random. These genes were related to special metabolism and organ growth. These genes were involved in flavonoid, terpenoid, and phenylpropanoid biosynthetic pathways. The selected genes exhibited differential expression across experimental conditions and displayed organ-specific fold changes. This enabled comprehensive assessment of the concordance between RNA-seq and qRT-PCR across the full dynamic range of gene expression. The 12 selected genes included four structural genes from the flavonoid biosynthetic pathway (*CHS*, *chalcone isomerase* (*CHI)*, *F3H/FLS*, and *DFR*), one late flavonoid pathway gene (anthocyanidin synthase, *ANS*), two phenylpropanoid pathway genes (*phenylalanine ammonia-lyase* (*PAL*) and *4-coumarate:CoA ligase* (*4CL*)), two terpenoid biosynthesis genes (*terpene synthase* (*TPS)* and *CYP450*), and three transcription factor genes (*MYB*, *bHLH*, and *WRKY*).

The same RNA samples used for transcriptome sequencing were subjected to first-strand cDNA synthesis using the cDNA Reverse Transcription Kit (PrimeScript^TM^ RT Master Mix, Takara, Kyoto, Japan) according to the manufacturer’s instructions. Gene-specific primers were designed using Primer Premier (v6.0) with the following criteria: amplicon length of 80–200 bp, GC content of 45–55%, melting temperature (Tm) of 58–62 °C, and absence of secondary structures, self-dimers, and cross-dimers. Primer specificity was verified by BLASTn searches against the *R. simsii* reference genome (GCA_047301955.1). All primers were synthesized by Sangon Biotech (Shanghai, China).

The primer sequences used in this study are listed in Table 2. Primers for *CHS*, *CHI*, *F3H*, *DFR*, *ANS*, *MYB*, *PAL*, *4CL*, *TPS*, *CYP450*, *WRKY*, and *bHLH* genes were adapted from previously published studies on *R. simsii* and related *Rhododendron* species, with minor modifications to ensure optimal amplification efficiency in *R. yedoense* var. *poukhanense*. The *elongation factor 1-alpha* (*EF1-α*) was selected as an internal reference gene based on its expression stability, as previously validated in *R. molle* tissues [31]. Given the high sequence conservation of *EF1-α* across *Rhododendron* species, gene-specific primers were redesigned from the *R. simsii EF1-α* coding sequence (RHSIM_Rhsim01G0219100) to ensure perfect sequence matching and optimal amplification efficiency in *R. yedoense* var. *poukhanense*.

qRT-PCR reactions were conducted on a C1000 Touch Thermal Cycler system (Bio-Rad, Hercules, CA, USA) using the ChamQ SYBR qPCR Master Mix kit (Vazyme, Nanjing, China) in a total volume of 20 μL containing 10 μL of 2× ChamQ SYBR qPCR Master Mix, 0.4 μL of each gene-specific primer (10 μM), 1 μL of cDNA template (corresponding to 50 ng of input RNA), and 8.2 μL of RNase-free water. The thermal cycling conditions were as follows: initial denaturation at 95 °C for 30 s, followed by 40 cycles of denaturation at 95 °C for 5 s and annealing/extension at 60 °C for 30 s. A melting curve analysis was performed at the end of each run by gradually increasing the temperature from 60 °C to 95 °C at a rate of 0.5 °C per 5 s to confirm amplification specificity and absence of primer dimers. All reactions were performed with three biological and technical replicates on a 96-well optical plate, with non-template controls (NTCs) included for each primer pair. Relative gene expression levels were calculated using the 2^−^ΔΔCt^ method [32], with the root sample designated as the calibrator.

Standard curves were constructed using 5-point serial dilutions (1:1, 1:5, 1:25, 1:125, 1:625) with three technical replicates. All primer pairs showed amplification efficiencies of 90–110% (mean 99.3 ± 1.0%, Table S6) and R^2^ ≥ 0.99. Melting curve analysis confirmed single-peak amplification for all primer pairs (Tm: 82–88 °C), with no detectable primer-dimer formation. The Pearson correlation coefficient between RNA-seq log_2_(FC) and qRT-PCR log_2_(2^−ΔΔCt^) was 0.962 (*p* = 1.03 × 10^−20^, Figure S2).

## 3. Results and Analysis

### 3.1. Metabolite Profiling Overview

**Figure 1:**
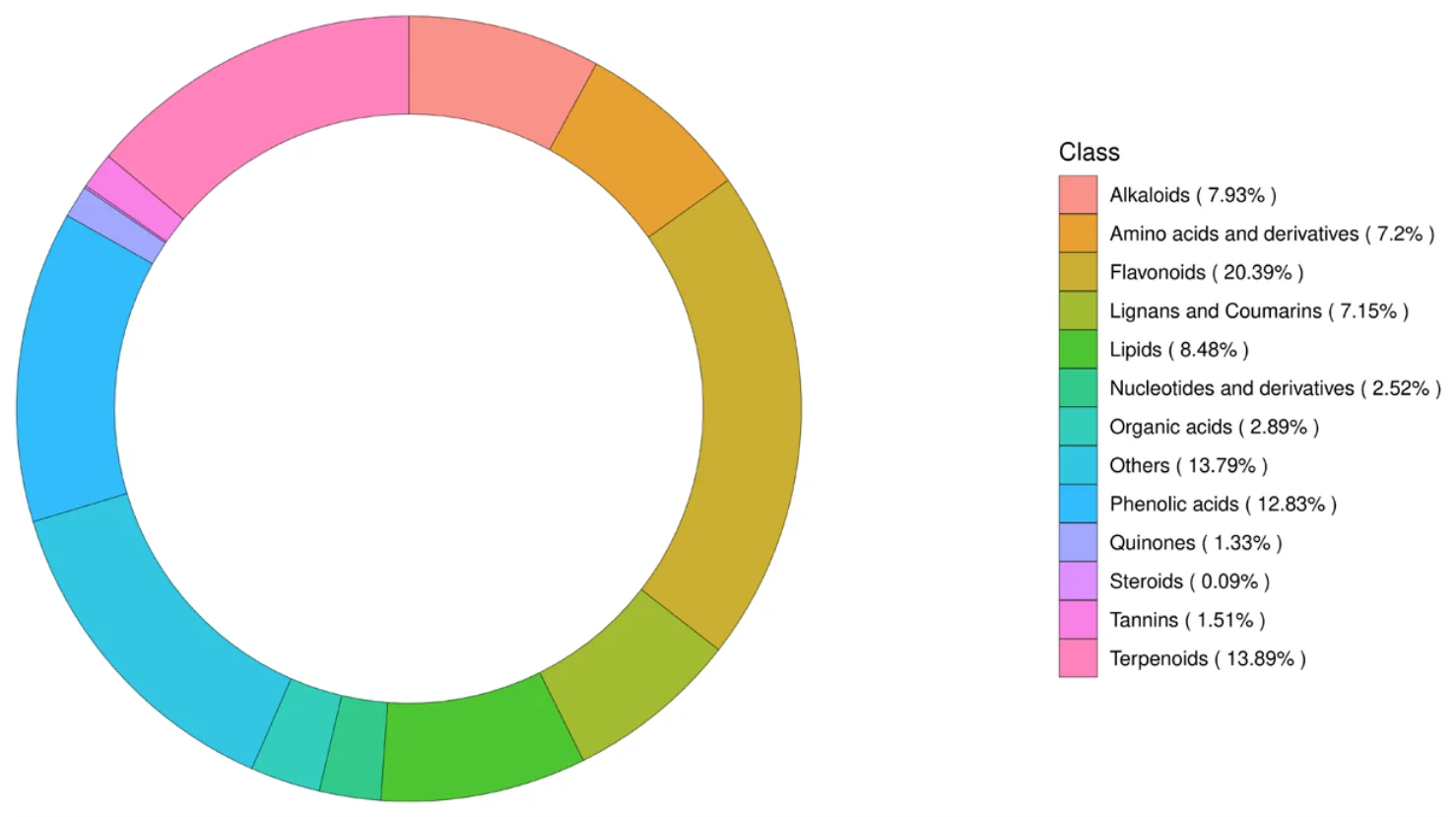
Overview of metabolite classes detected in *R. yedoense* var. *poukhanense*.

Widely targeted metabolomics identified 2182 metabolites across nine samples (Table S2), spanning 13 chemical classes including flavonoids (445 metabolites), terpenoids (303), phenolic acids (280), lipids (185), and alkaloids (173). There were three biological repeats each of root, stem, and leaf. UPLC-MS/MS in MRM mode was used. Metabolites were named by matching Q1, Q3, and RT with the MWDB database. This method gives high-confidence names for MRM-based work [17]. The found metabolites included flavonoids, terpenoids, phenolic acids, lipids, alkaloids, amino acid derivatives, lignans and coumarins, organic acids, nucleotides and derivatives, tannins, quinones, steroids, and others (Figure 1). Flavonoids were the biggest group. Terpenoids, phenolic acids, and alkaloids were also found in large amounts. Therefore, the main secondary metabolic paths were all active.

### 3.2. Organ-Specific Metabolic Variation

**Figure 2:**
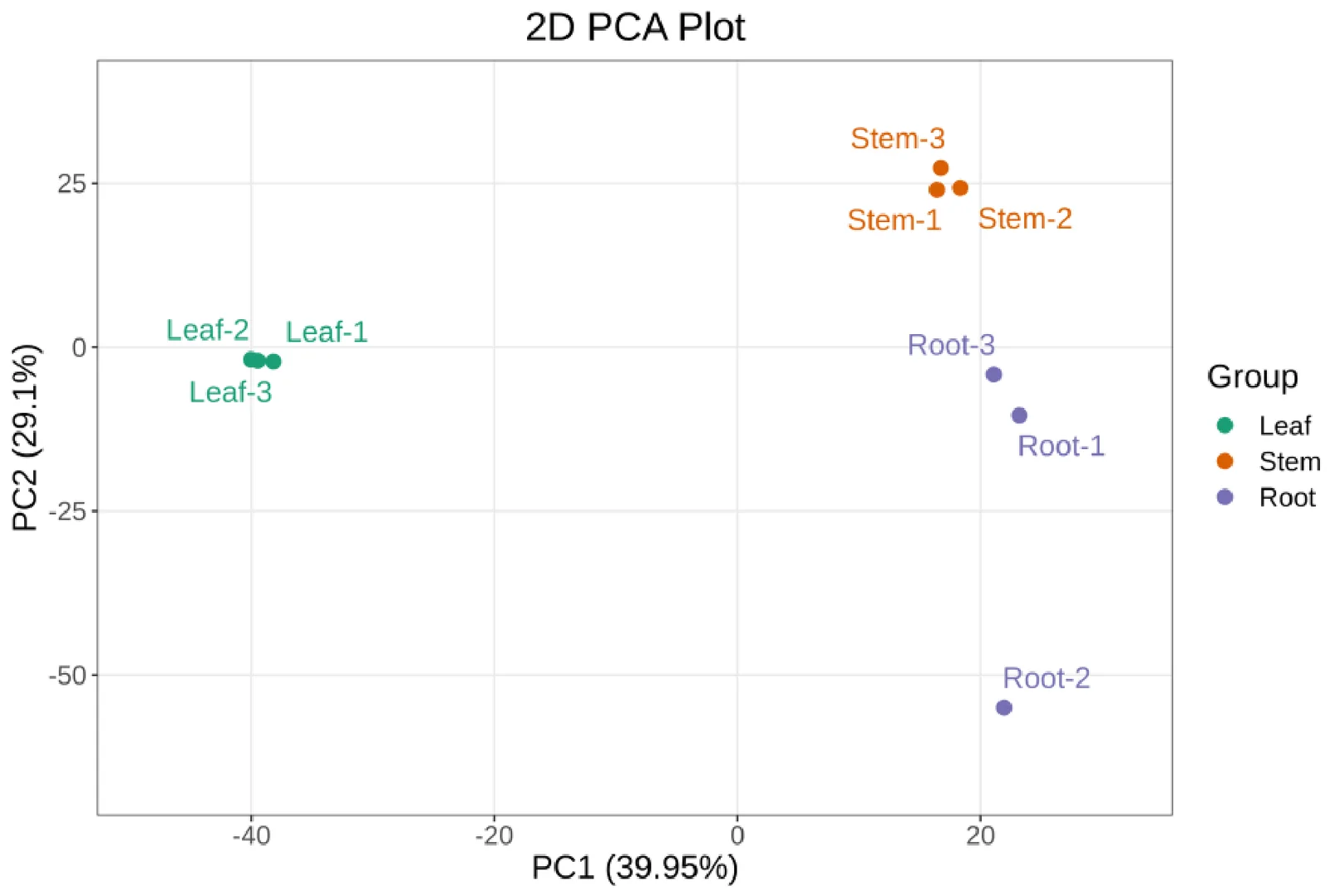
Principal component analysis (PCA) score plot of metabolomics data (n = 3 biological replicates per organ). Root, stem, and leaf samples form distinct clusters, with PC1 primarily separating photosynthetic (leaf) from non-photosynthetic (root and stem) tissues and PC2 resolving root from stem.

PCA of the metabolite data split the three organs well on principal component 1 (PC1) (39.95%) and principal component 2 (PC2) (29.10%) (Figure 2). PC1 mainly split leaves (negative scores) from roots and stems (positive scores). It showed how photosynthetic tissues differ from non-photosynthetic ones. PC2 subsequently separated roots (negative scores) from stems (positive scores). This finding is consistent with the specialized physiological functions of roots and stems. Roots are primarily responsible for nutrient uptake and the accumulation of defense-related secondary metabolites. Stems function in long-distance transport of nutrients and provide mechanical support to the plant body. The three biological replicates of leaf and stem samples clustered closely in the multivariate space. As such, the metabolomic data were highly repeatable. For roots, two samples (Root-1 and Root-3) grouped close together. However, Root-2 spread more along PC2. Roots showed more variation within the group. This greater root-to-root metabolic variability likely reflects micro-environmental heterogeneity in the rhizosphere, including uneven soil moisture, nutrient patchiness, and variable microbial community composition—factors known to influence root secondary metabolism. Such below-ground variation is a well-documented phenomenon in soil-grown plants and should be interpreted as a biological feature rather than technical noise. The tight clustering of leaf and stem replicates confirms the high analytical reproducibility of the metabolomics platform.

**Figure 3:**
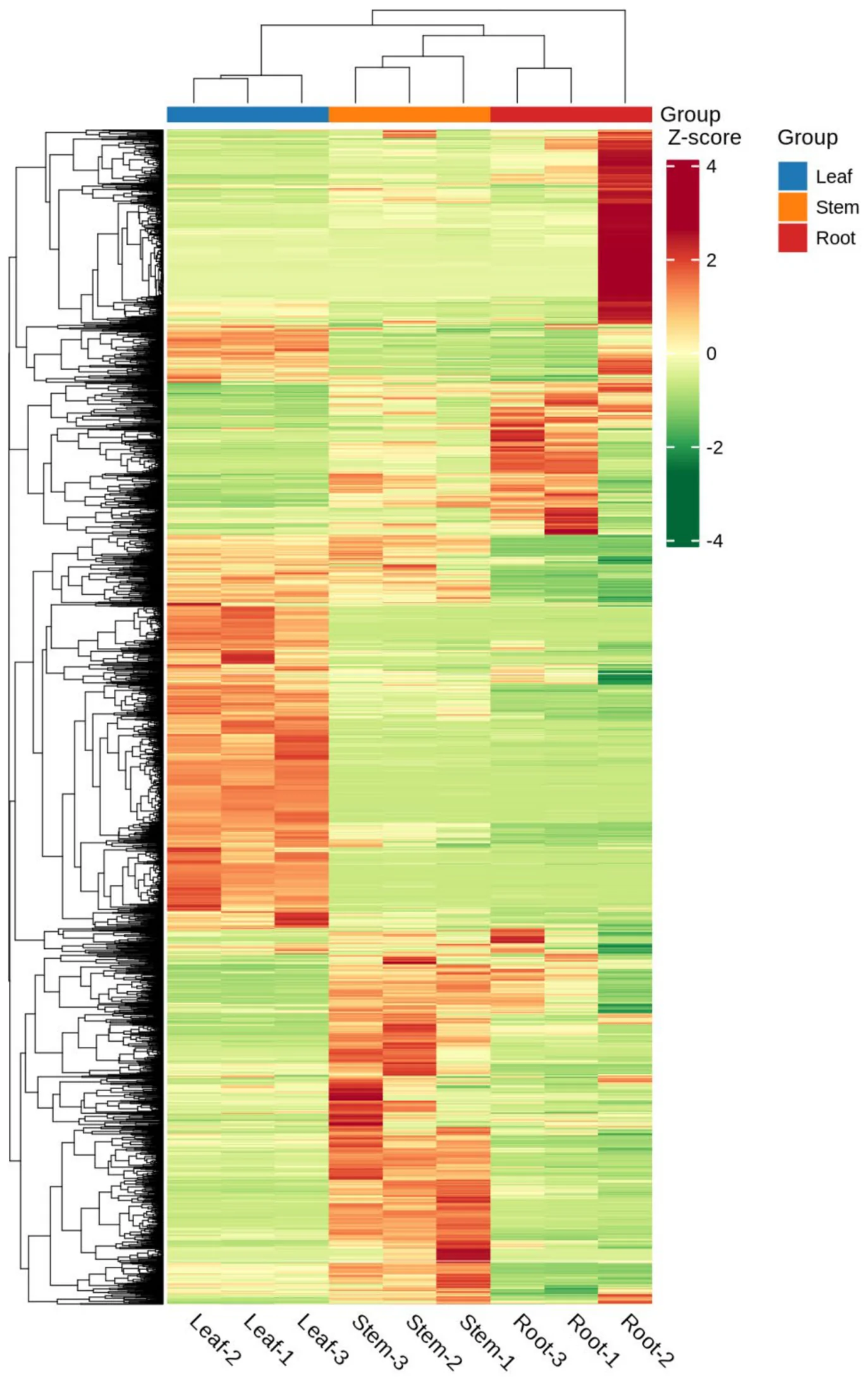
Hierarchical clustering of metabolite abundance patterns across organs (n = 3 biological replicates per organ). Sample dendrogram showing clear separation of root, stem, and leaf tissues, with biological replicates clustering tightly within each organ. Heatmap with color intensity indicating normalized abundance (z-score). Red denotes high abundance; green denotes low abundance.

Hierarchical clustering corroborated the PCA results by partitioning the samples into three distinct organ-specific clusters (Figure 3). Gene expression profiles of roots and stems exhibited greater similarity to each other than to those of leaves. This observation is consistent with the shared embryonic origin of roots and stems and their common lack of photosynthetic activity. Leaf tissue showed a very different pattern, as leaves do photosynthesis and linked metabolic work. Consequently, the distinct organ-specific expression patterns indicate that secondary metabolite accumulation in *R. yedoense* var. *poukhanense* is predominantly governed by tissue identity [22,24], likely reflecting differential allocation of carbon precursors between photosynthetic metabolism and pathways dedicated to storage and defense.

### 3.3. Differential Accumulation of Metabolites

**Figure 4:**
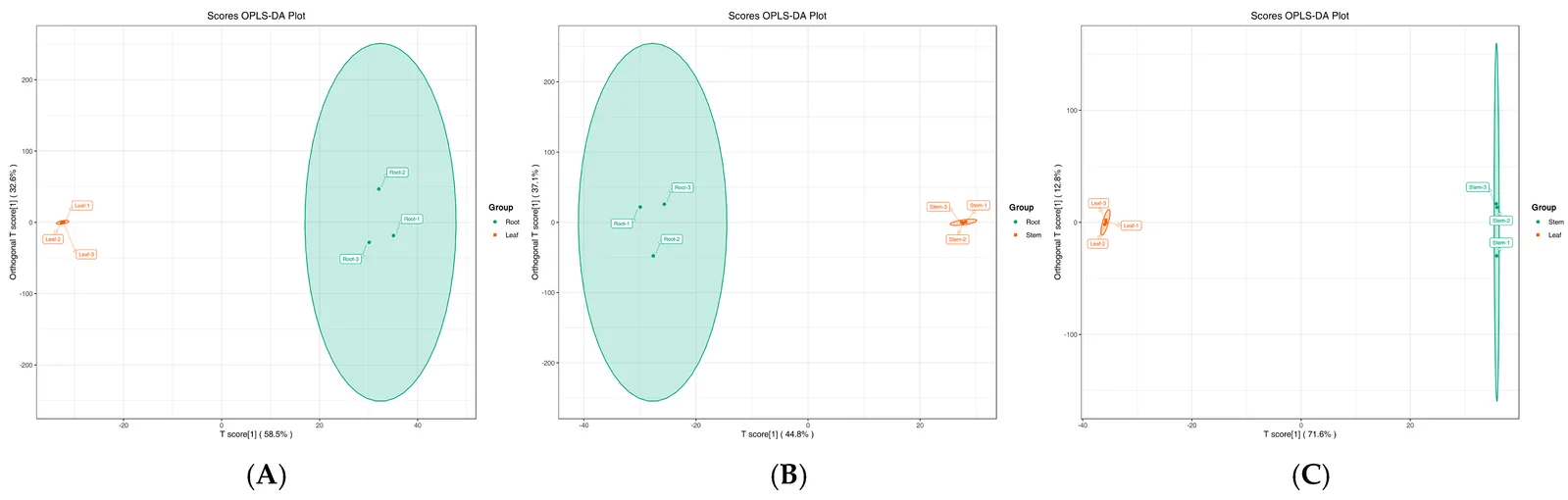
OPLS-DA score plots of pairwise organ comparisons. (**A**) Root versus leaf (R^2^Y = 0.998, Q^2^ = 0.987), (**B**) root versus stem (R^2^Y = 0.998, Q^2^ = 0.963), (**C**) stem versus leaf (R^2^Y = 1.000, Q^2^ = 0.993). Each data point represents one biological replicate. R^2^Y and Q^2^ values indicate model goodness-of-fit and predictive ability, respectively. All models were validated by 200 permutation tests (Figure S1).

OPLS-DA models for all pairwise comparisons demonstrated excellent goodness-of-fit and predictive ability: root-versus-leaf (goodness-of-fit parameter (R^2^Y) = 0.998, predictive ability (Q^2^) = 0.987), root-versus-stem (R^2^Y = 0.998, Q^2^ = 0.963), and stem-versus-leaf (R^2^Y = 1.000, Q^2^ = 0.993). All Q^2^ values far exceeded the threshold of 0.5, indicating robust predictive power. Score plots displayed clear separation between groups (Figure 4). Each model was validated using 200 permutation tests (Figure S1), confirming that the models were not overfitted [28]. DAMs were identified using VIP > 1.0, FDR-corrected *p* < 0.05, and fold change ≥ 2.0 or ≤0.5. The stem-versus-leaf comparison identified the largest number of DAMs (1264; 683 upregulated in stem, 581 downregulated), followed by root-versus-leaf (1116; 300 upregulated in root, 816 downregulated) and root-versus-stem (896; 172 up, 724 down). This distribution indicates that roots and leaves represent the metabolically most divergent organs, a pattern consistent with their fundamentally distinct physiological functions.

**Figure 5:**
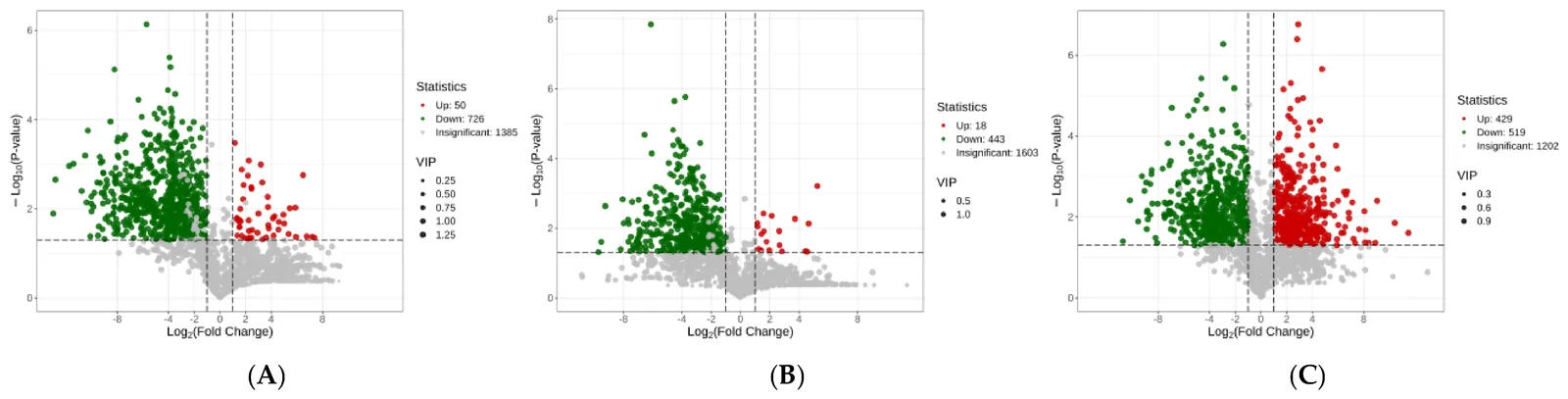
Volcano plots of differentially accumulated metabolites (n = 3 biological replicates per organ). (**A**) Root versus leaf, (**B**) root versus stem, (**C**) stem versus leaf. Red dots indicate metabolites with *p* < 0.05 (uncorrected) and fold change ≥ 2.0 or ≤0.5; blue dots indicate metabolites with *p* < 0.05 (uncorrected) and fold change ≤ 0.5; gray dots indicate non-significant metabolites. The volcano plots display uncorrected *p*-values for visualization purposes only. DAMs were formally defined using Benjamini–Hochberg FDR-corrected *p* < 0.05 combined with VIP > 1.0.

Volcano plots (Figure 5) visualize metabolites exhibiting statistically significant and biologically substantial changes across pairwise comparison. Several compounds with medicinal activity were found as key DAMs (Table 1).

Methylfarrerol is a typical 2,3-dihydroflavonoid in the genus *Rhododendron* with strong anti-inflammatory and antimicrobial effects. It accumulated predominantly in leaves (Root vs. Leaf log_2_FC = −1.76, ~3.4-fold higher than roots; Table 1). It should be noted that methylfarrerol was identified at Metware Level 3 (Q1, Q3, RT, DP, and CE verified against database; no full MS/MS fragmentation validation). As no authentic standard is commercially available for this genus-specific compound, definitive structural confirmation would require standard co-injection or nuclear magnetic resonance (NMR) spectroscopic validation in future studies. Quercetin-3-O-glucoside and kaempferol-3-O-rutinoside were significantly enriched in leaves, consistent with the elevated transcriptional activity of *F3H/FLS* gene—key enzymes catalyzing flavonol glycoside biosynthesis. Oleanolic acid and ursolic acid were significantly enriched in stems, indicating organ-specific distribution of triterpenoid compounds. In contrast, chlorogenic acid exhibited markedly reduced abundance across all pairwise comparisons, consistent with its leaf-enriched accumulation profile.

These organ-specific metabolic patterns provide mechanistic insights into the spatial regulation of medicinal compound biosynthesis in *R. yedoense* var. *poukhanense*. Leaves served as the primary site of accumulation for flavonol glycosides (e.g., quercetin-3-O-glucoside and kaempferol-3-O-rutinoside), flavonol aglycones (e.g., quercetin and kaempferol), and hydroxycinnamic acid derivatives (e.g., chlorogenic acid, caffeic acid, and ferulic acid), consistent with their well-established physiological roles in UV-B photoprotection and antioxidant defense during photosynthetic activity. Stems exhibited elevated accumulation of triterpenoids (e.g., oleanolic acid and ursolic acid) and specialized flavonoids (e.g., taxifolin, epicatechin, and apigenin), consistent with their dual physiological roles in nutrient transport and structural reinforcement. The top DAMs listed in Table 1 exhibit no root-biased metabolites. In contrast, comprehensive analysis of all DAMs revealed root-specific enrichment of monoterpenoids, alkaloids, and amino acid derivatives—46, 13, and 46 DAMs, respectively, exhibited significantly higher abundance in roots relative to leaves—consistent with the roles of these compounds in rhizosphere interactions and defense against soil-borne pathogens.

### 3.4. Transcriptome Sequencing Quality and Mapping Statistics

High-throughput sequencing on the Illumina NovaSeq 6000 made paired-end reads (2 × 150 bp) from RNA taken from root, stem, and leaf tissues. After adapter removal and quality checks with fastp [29], nine cDNA libraries gave 45.7–53.2 million clean reads per sample (Table S1). All libraries passed standard quality limits: Q20 = 97.05–97.94%, Q30 = 91.87–94.41%, and GC content = 46.68–47.82%. Consequently, sequencing metrics indicate high base-calling accuracy and balanced nucleotide composition across all positions, with no detectable sequence-context bias.

**Figure 6:**
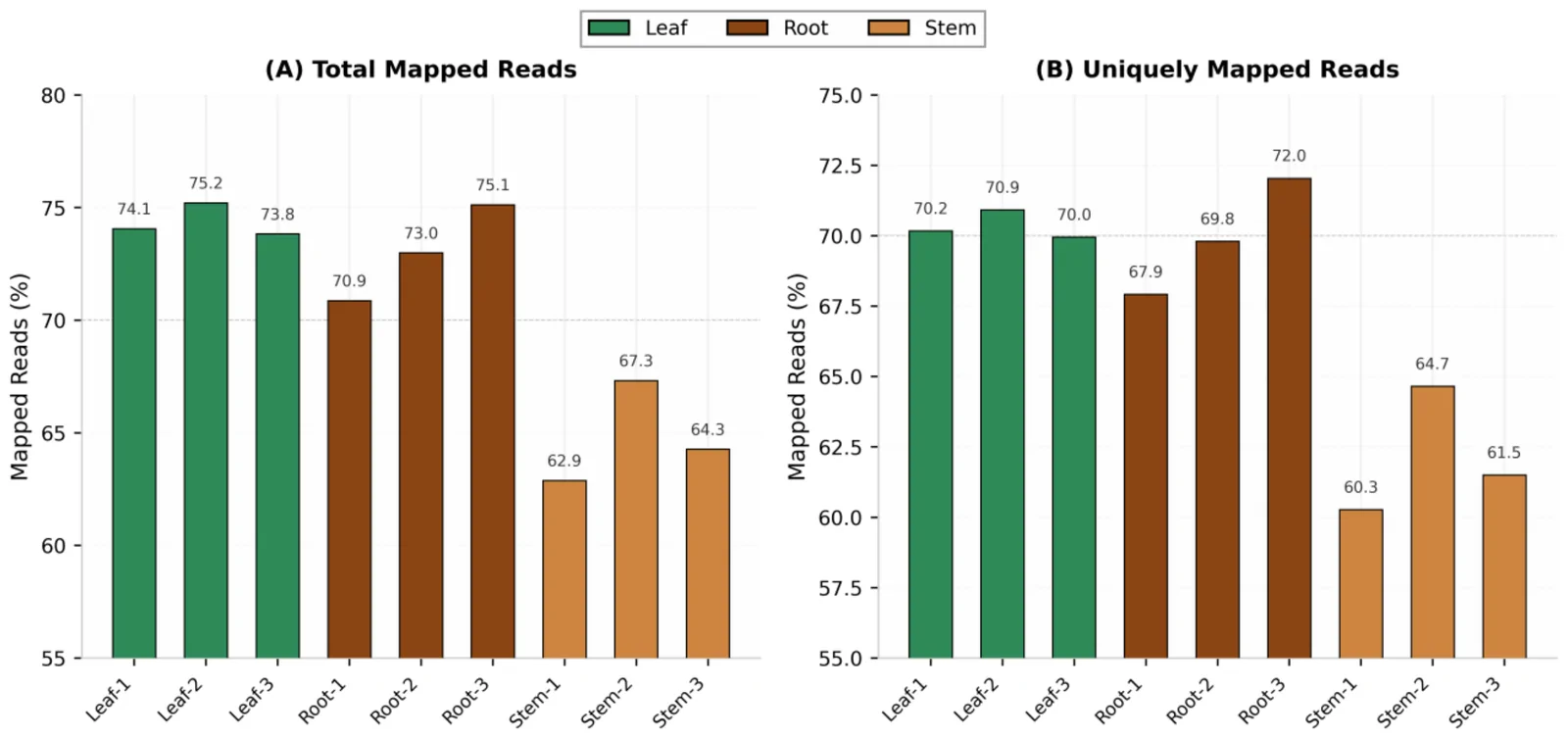
Read mapping statistics to the *R. yedoense* var. *poukhanense* reference genome (n = 9 libraries; n = 3 biological replicates per organ). (**A**) Total mapped reads (%) for each of nine libraries. (**B**) Uniquely mapped reads (%).

Clean reads were mapped to the *R. simsii* reference genome (GCA_047301955.1) with HISAT2 [33]. Mapping rates were different across organs (Figure 6; Table S1). Leaf samples had total mapped reads of 73.83–75.21% (unique mapping 69.95–70.92%). Root samples had 70.87–75.13% total mapped (67.92–72.04% unique). Stem samples had lower total mapped reads of 62.88–67.32% (60.27–64.65% unique). The reduced mapping rate observed in stems-derived sequencing libraries is likely attributable to their elevated lignin content and abundant polyphenolic and terpenoid secondary metabolites, which can interfere with nucleic acid extraction and library preparation. These compounds are well documented to compromise RNA integrity and yield during extraction, potentially increasing the proportion of non-coding RNA and organellar-derived sequencing reads [34]. Nevertheless, stem-derived mapping rates remained within acceptable range for differential gene expression analysis.

### 3.5. Differential Gene Expression Analysis

**Figure 7:**
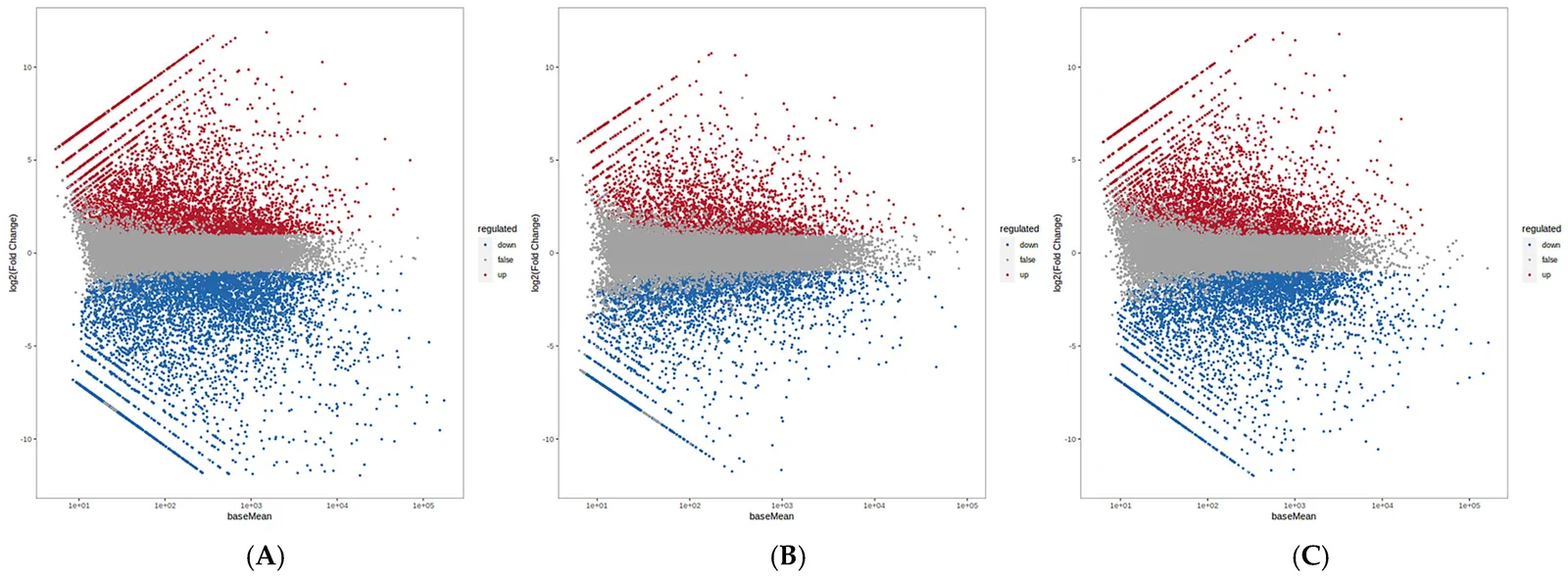
MA plots of differentially expressed genes (n = 3 biological replicates per organ). (**A**) Root versus leaf, (**B**) root versus stem, (**C**) stem versus leaf. The *x*-axis represents the average gene expression levels between the two samples; the *y*-axis indicates the log_2_ fold change (log_2_FC) values. Red dots represent upregulated genes, blue dots represent downregulated genes, and gray dots indicate no significant difference in gene expression.

DESeq2 analysis [30] with thresholds of FDR < 0.05 and |log_2_FC| ≥ 1 revealed substantial transcriptomic divergence across organs (Table 3; Figure 7). The root-versus-leaf comparison yielded the highest number of DEGs (9836; 4570 upregulated and 5266 downregulated in root). The stem-versus-leaf comparison identified 8007 DEGs (3670 up, 4337 down in stem), while root-versus-stem returned 5457 DEGs (2475 up, 2982 down in root).

**Figure 8:**
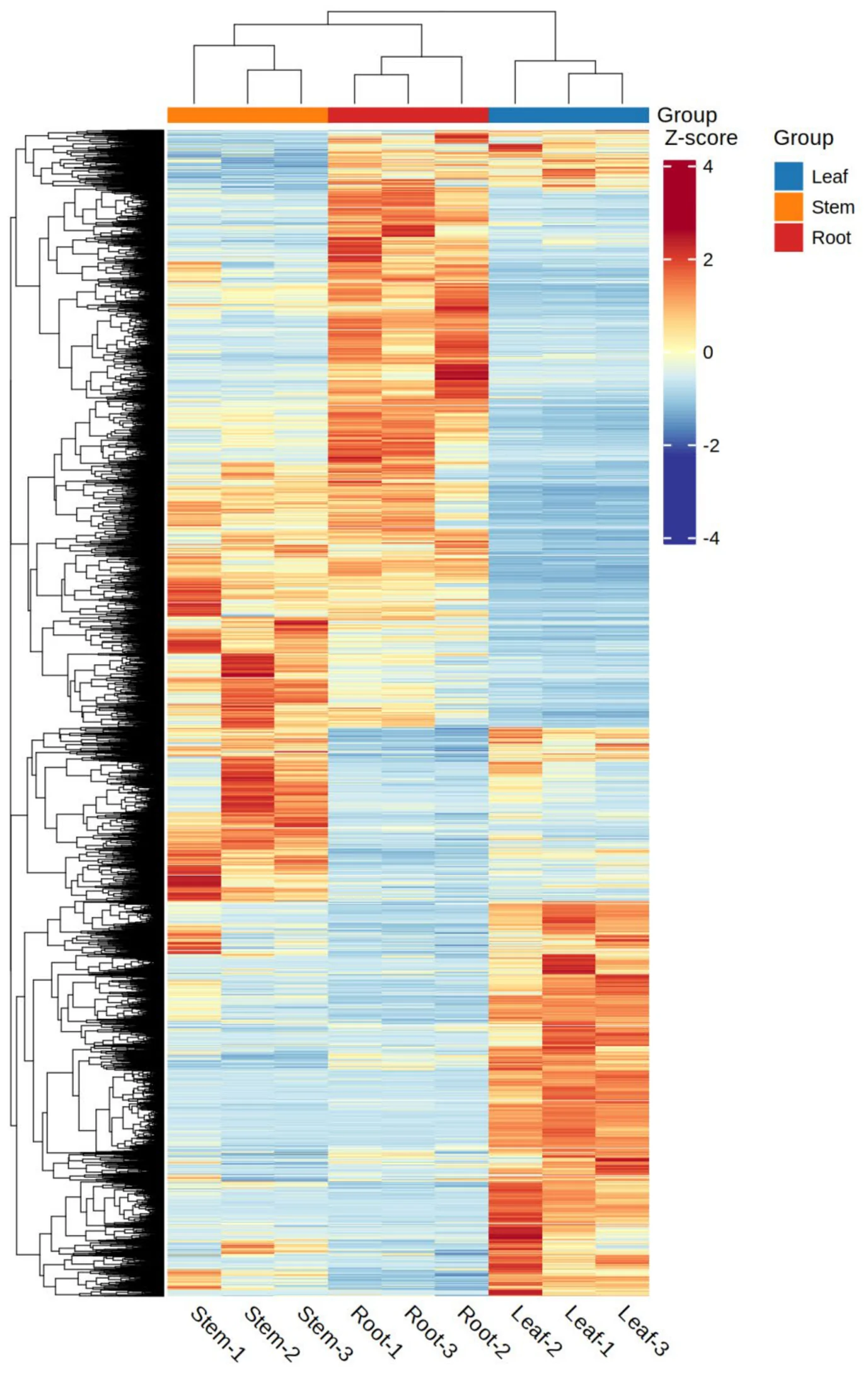
Hierarchical clustering of differentially expressed genes (n = 3 biological replicates per organ). Sample dendrogram based on transcriptome-wide expression. Heatmap with color intensity representing z-score normalized expression. Red indicates high expression, blue indicates low expression.

**Figure 9:**
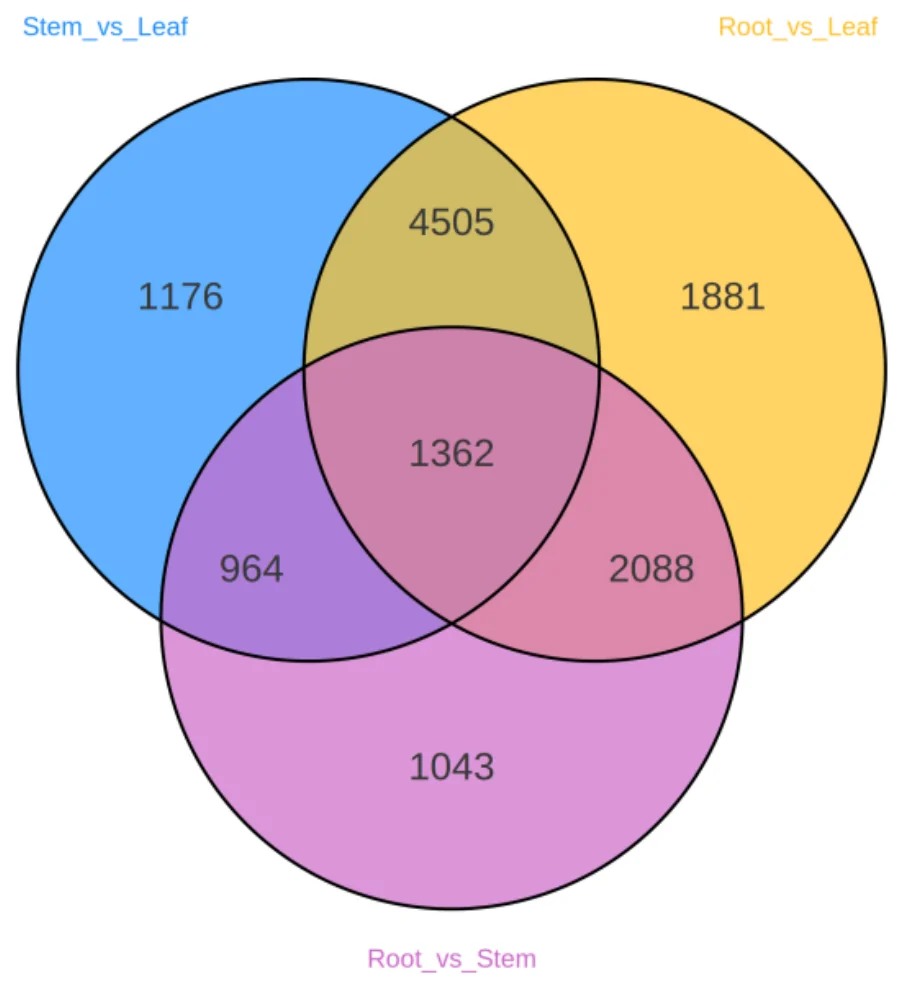
Venn diagram showing overlap of DEGs across three pairwise comparisons. Numbers in overlapping regions indicate shared DEGs; numbers in unique regions indicate organ-specific transcriptional changes.

The root–leaf comparison revealed the most pronounced transcriptional divergence, which was concordant with the corresponding metabolomics profiles. This alignment between metabolic and transcriptional distances suggests that the most pronounced metabolic distinctions are underpinned by extensive transcriptional reprogramming [22]. Hierarchical clustering of DEGs clearly segregated samples into organ-specific clusters (Figure 8), with biological replicates clustering tightly within each organ. In total, 13,019 unique genes were differentially expressed in at least one comparison (38.78% of all detected genes). Venn analysis revealed both unique and shared DEGs across comparisons (Figure 9), with 1362 DEGs common to all three pairwise comparisons, implying the existence of organ-specific regulatory modules alongside common transcriptional responses [13].

### 3.6. Functional Enrichment of Differentially Expressed Genes

GO and KEGG enrichment analyses were conducted to functionally annotate organ-specific DEGs and elucidate the underlying biological processes, molecular functions, cellular components, and metabolic pathways. GO enrichment used GOseq with the Wallenius non-central hypergeometric distribution to correct for length bias [35], while KEGG pathway enrichment used KOBAS with the hypergeometric test at FDR < 0.05 [36].

**Figure 10:**
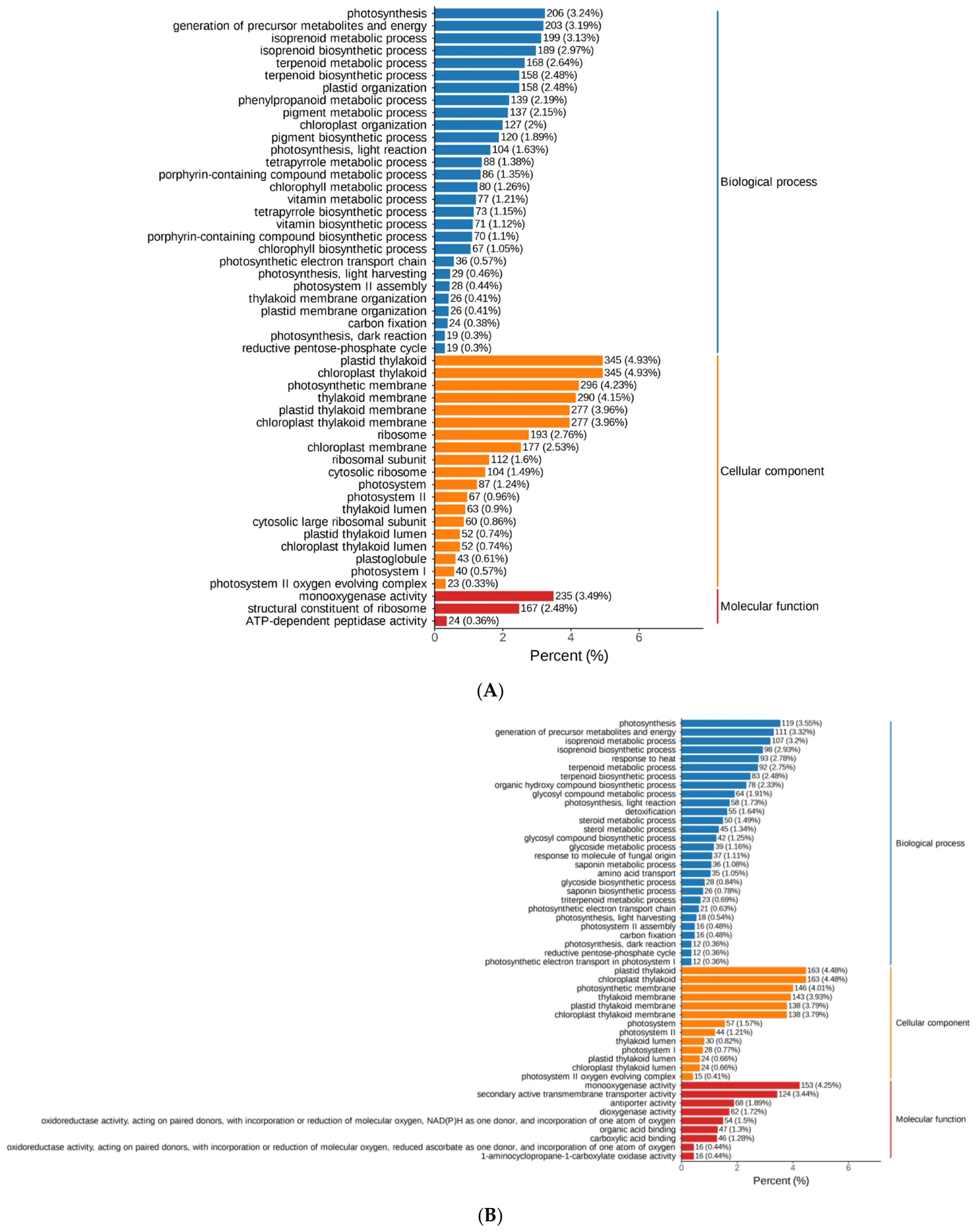

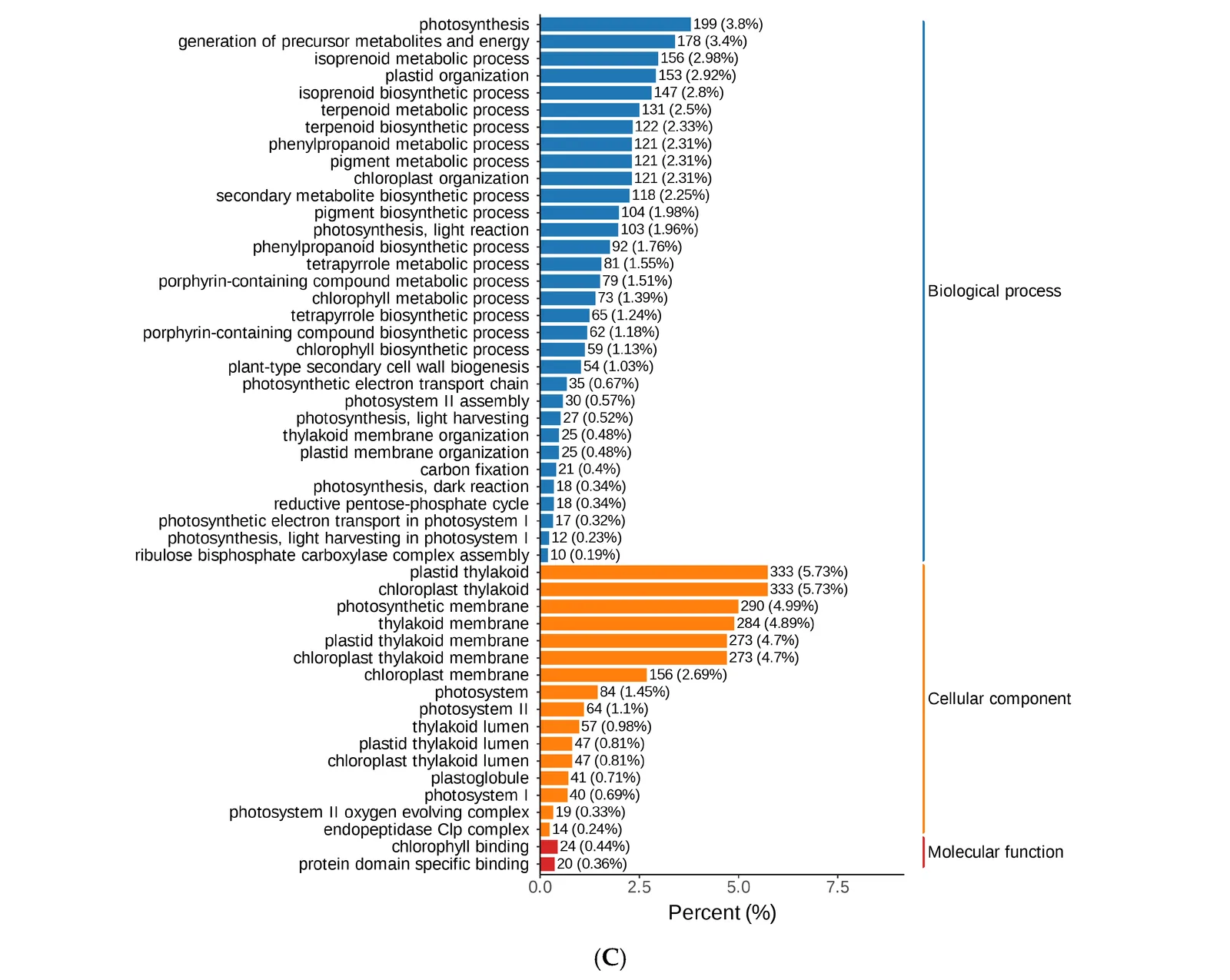
Gene Ontology (GO) enrichment analysis of DEGs. (**A**) Root versus leaf, (**B**) root versus stem, (**C**) stem versus leaf.

GO enrichment analysis revealed that organ-specific DEGs were significantly overrepresented in functional categories associated with photosynthetic capacity and primary metabolic activity—key determinants of tissue-specific physiological specialization. (Figure 10). In all three comparisons, the biological process area was mainly filled with photosynthesis-related and energy generation terms. These include “photosynthesis,” “generation of precursor metabolites and energy,” and “photosynthesis, light reaction”. Collectively, these categories accounted for 3.2–3.8% of the significantly enriched DEGs. This enrichment pattern aligns with the well-established functional divergence in photosynthetic capacity between leaf and non-leaf tissues. Moreover, isoprenoid and terpenoid metabolic processes were clearly enriched in all comparisons. These include “isoprenoid metabolic process” (2.98–3.20%), “isoprenoid biosynthetic process” (2.80–2.97%), “terpenoid metabolic process” (2.50–2.75%), and “terpenoid biosynthetic process” (2.33–2.48%)—collectively reflecting organ-specific partitioning of the terpenoid pathway, which underpins the differential accumulation of bioactive compounds across tissues. The “phenylpropanoid metabolic process” (2.19–2.31%) and “phenylpropanoid biosynthetic process” (1.76%) were also clearly present, especially in stem versus leaf and root versus leaf, consistent with the organ-biased synthesis of phenylpropanoid-derived flavonoids. “Secondary metabolite biosynthetic process” (2.25%) further corroborated transcriptional reprogramming underlying organ-specific specialization of secondary metabolism.

In the molecular function group, “monooxygenase activity” was the most enriched term in root-versus-leaf and root-versus-stem comparisons (3.49% and 4.25%), indicating CYP450 monooxygenases help oxidize secondary metabolite precursors. “Structural constituent of ribosome” (2.48%) and “secondary active transmembrane transporter activity” (3.44%) were also enriched. The latter shows active transport processes that move metabolic intermediates inside cells. Dioxygenase and oxidoreductase activities were similarly enriched among organ-specific differentially expressed genes (DEGs), consistent with prior reports [37].

In the cellular component group, DEGs were mostly found in photosynthetic membrane systems. These include “plastid thylakoid” (4.48–5.73%), “chloroplast thylakoid,” “photosynthetic membrane” (4.01–4.99%), and “thylakoid membrane” (3.93–4.89%), consistent with the different photosynthetic ability across organs. The root versus leaf comparison further revealed significant enrichment in “ribosome” (2.76%) and “ribosomal subunit” (1.60%), reflecting organ-specific divergence in translational capacity—consistent with their distinct metabolic roles [38].

**Figure 11:**
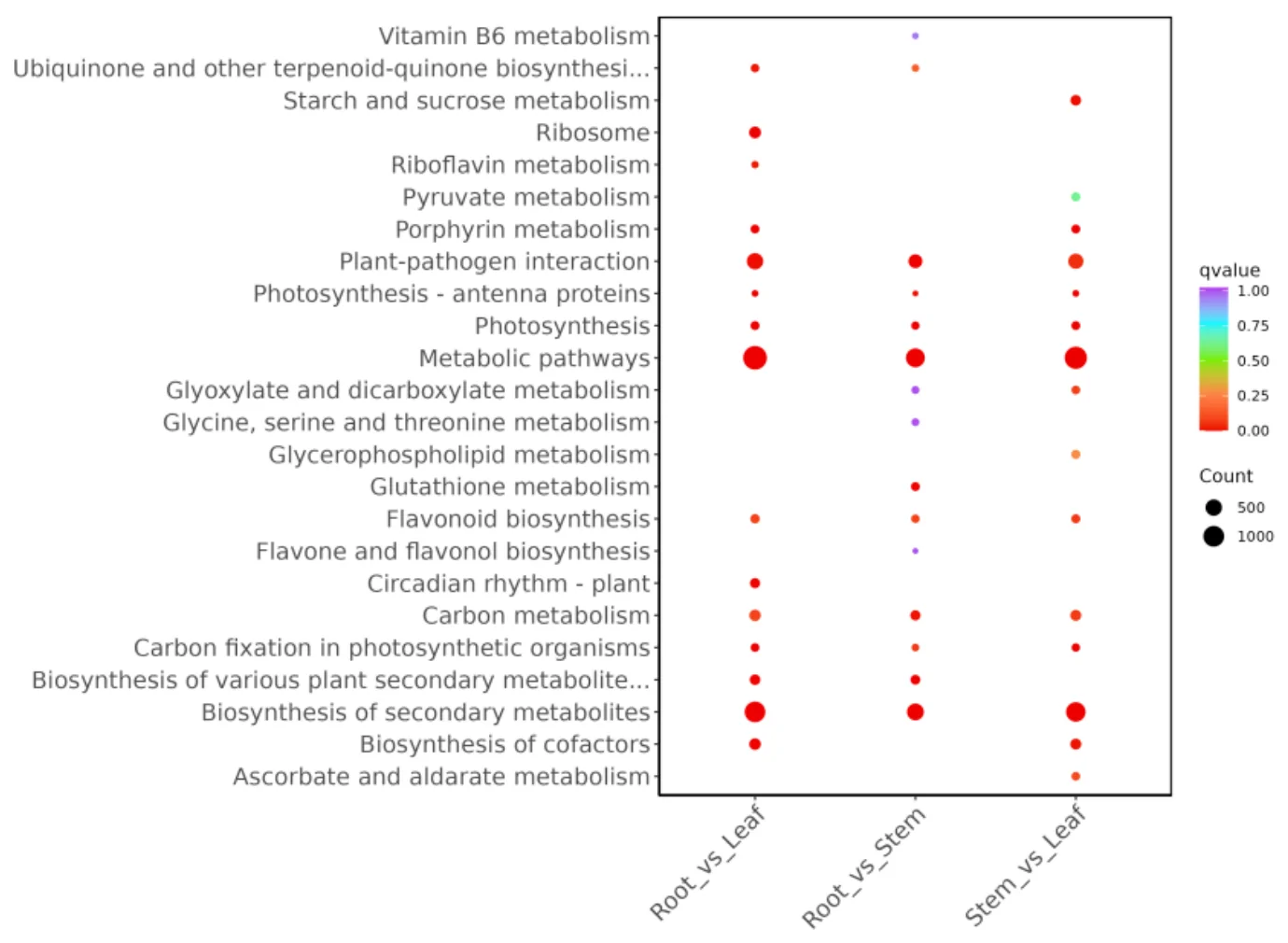
KEGG pathway enrichment analysis of DEGs. The *x*-axis represents the comparison groups, the *y*-axis represents the enriched pathways, and the size of the points indicates the number of differentially expressed genes enriched in each pathway—larger points signify a greater number of enriched differentially expressed genes. The color of the points reflects the significance level of enrichment, with redder colors indicating more significant enrichment.

KEGG pathway enrichment analysis revealed that primary and secondary metabolism play a central role in organ-specific gene expression (Figure 11). In all three comparisons, “metabolic pathways” and “biosynthesis of secondary metabolites” were the top enriched terms. Each pathway was represented by a substantially enriched gene set, with statistical significance indicated by large red dots in the enrichment plot. “Plant–pathogen interaction” was also significantly enriched, which was especially true for the root-versus-leaf and stem-versus-leaf comparisons. Consequently, defense responses are organ-specific. “Photosynthesis” and “photosynthesis-antenna proteins” were significantly enriched in comparisons with leaves, consistent with how aerial tissues are specialized for photosynthesis. “Ribosome,” “carbon metabolism,” and “starch and sucrose metabolism” were also enriched in all comparisons, indicating different organs have different translational and energy-storage ability. “Flavonoid biosynthesis” and “flavone and flavonol biosynthesis” were enriched in all three comparisons, which supports that transcriptional regulatory basis for organ-specific flavonoid accumulation. “Ubiquinone and other terpenoid-quinone biosynthesis” was also found, which links transcriptomic differences to terpenoid metabolism.

### 3.7. Weighted Gene Co-Expression Network Analysis (WGCNA)

**Figure 12:**
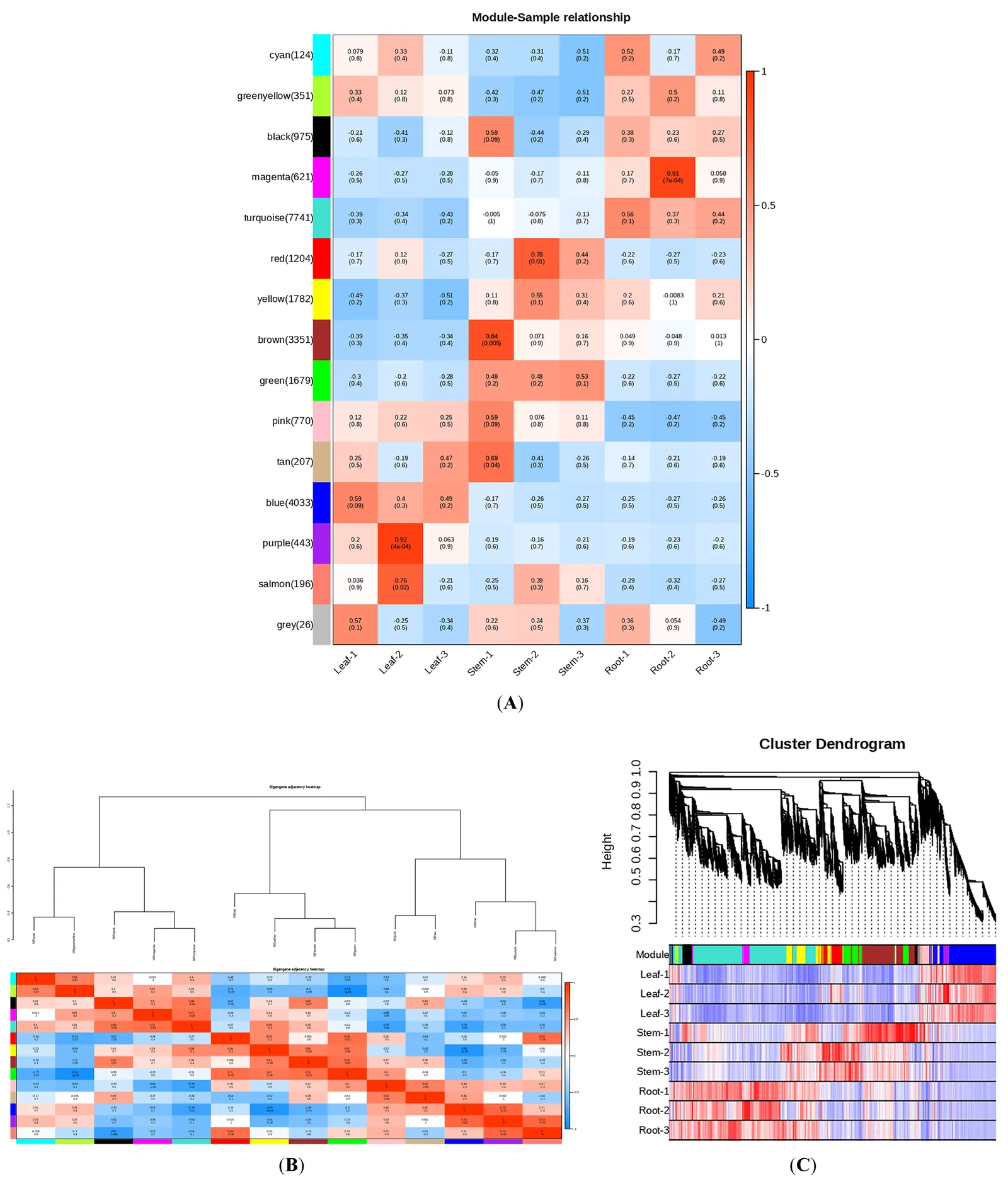
Weighted gene co-expression network analysis (WGCNA) of organ-specific transcriptome patterns. (**A**) Module–trait correlation heatmap showing the relationship between 15 WGCNA modules (rows) and nine samples organized by organ type (columns). Color intensity represents the Pearson correlation coefficient (red = positive, blue = negative), with cell values indicating correlation magnitude and parenthetical values showing the corresponding *p*-value. Module gene counts are shown in parentheses. (**B**) Module eigengene adjacency heatmap depicting inter-module correlation relationships based on eigengene similarity. (**C**) Gene clustering dendrogram (top), module assignment color bar (middle), and sample-wise expression heatmap (bottom) for all co-expressed genes, illustrating the organ-specific expression architecture of the transcriptome. Note: WGCNA was conducted with n = 9 samples (three per organ). Modules and hub genes shown are preliminary and should not be interpreted as independently validated; their biological relevance rests on convergence with O2PLS, CCA, and Pearson correlation analyses.

Given the relatively small sample size (n = 9), WGCNA was employed solely as an exploratory, hypothesis-generating tool. All WGCNA-derived modules and hub genes should be treated as preliminary, and their biological relevance rests entirely on convergence with independent O2PLS, CCA, and Pearson correlation analyses rather than on standalone statistical validation. WGCNA was run on 16,204 filtered genes in nine samples (three repeats per organ). A soft-thresholding power of β = 12 was picked based on scale-free topology. The blockwiseModules function split the transcriptome into 15 co-expression modules. These modules had 30 to 7741 genes each, with an average of 1624 genes (Figure 12). While the reproducibility of all 15 modules cannot be guaranteed with this sample size, the tight clustering of biological replicates within each organ (Figure 12C) and the consistent organ-specific eigengene patterns support the biological relevance of the major modules discussed herein.

The module–trait association map (Figure 12A) identified four coexpression modules exhibiting distinct organ-specific expression patterns. The purple module had the strongest link to leaves. The blue module showed higher expression in leaves across all three repeats. However, expression levels were comparatively lower in stem and root tissues. Key genes within these leaf-enriched modules included *FLS*, *CHI*, and UDP-glycosyltransferase homologs, consistent with the leaf-biased accumulation of flavonol glycoside observed in the metabolomic profiling data. The brown module was most linked to stems. The green module showed a similar stem-preferential trend. Consequently, two modules together contained 5030 stem-active genes. Their key genes were cinnamoyl-CoA reductase and laccase family members, which are involved in lignin synthesis and vessel growth.

The eigengene adjacency heatmap (Figure 12B) revealed that brown and green modules exhibit high pairwise eigengene correlation, indicating functional relatedness. Similarly, cyan and green-yellow modules displayed high pairwise eigengene correlation, suggesting potential functional coordination. Thus, functionally related modules tend to share conserved coexpression architectures. The gene clustering tree and expression heatmap (Figure 12C) confirmed that biological repeats group by organ. Moreover, hierarchical clustering of module eigengenes effectively discriminated the three tissue types. Thus, the WGCNA results, though preliminary due to the small sample size, were broadly consistent with the organ-specific patterns independently identified by O2PLS and CCA, providing convergent but not independent support.

### 3.8. Organ-Specific Expression of Key Biosynthetic Genes

Comparative gene analysis revealed substantial organ-specific variation in the expression of structural genes governing specialized metabolism. Flavonoid, phenylpropanoid, and terpenoid synthesis enzymes were expressed differently. Moreover, the transcription factors regulating these structural genes exhibited organ-specific expression patterns. Thus, bioactive compounds accumulate in a tissue-specific and pathway-divergent manner.

**Figure 13:**
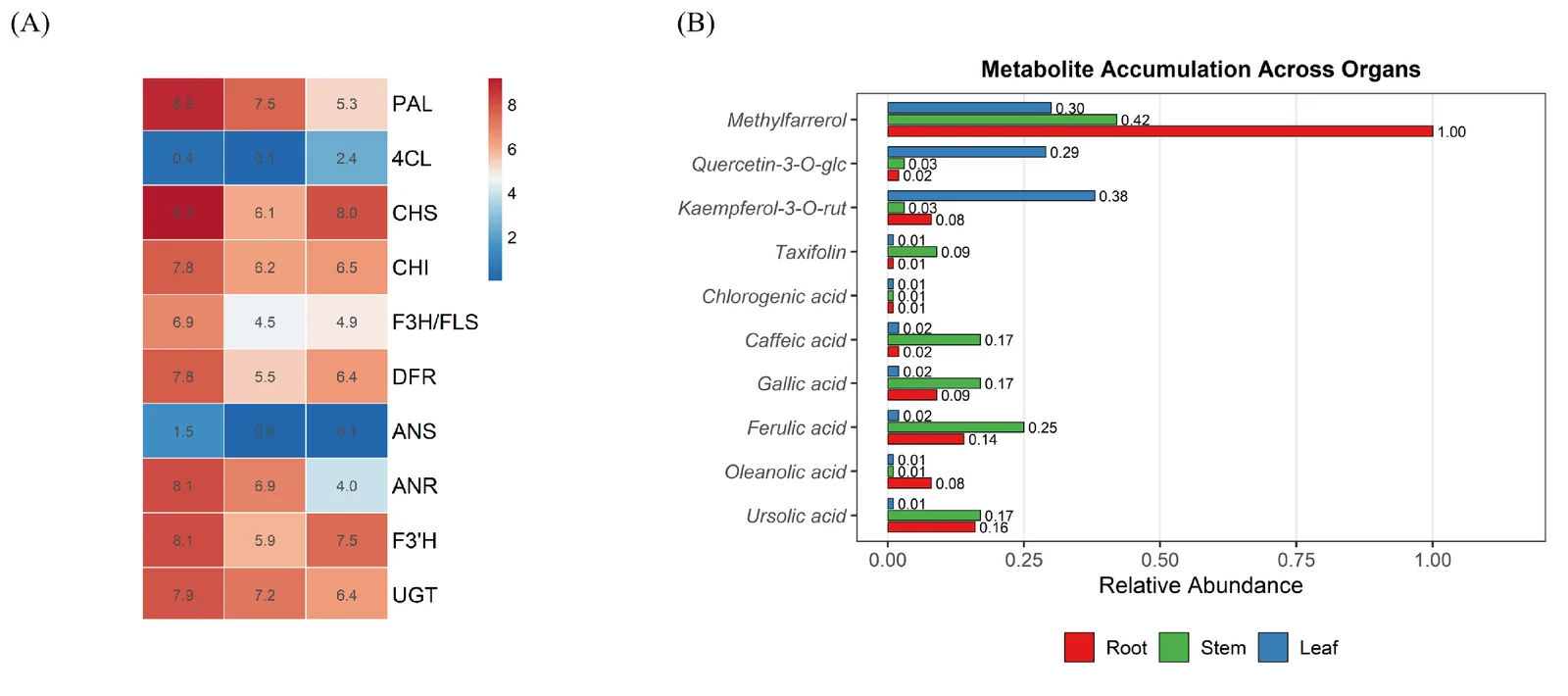
Organ-specific expression of flavonoid biosynthetic genes and metabolite profiles (n = 3 biological replicates per organ). (**A**) Heatmap of log_2_-transformed FPKM values for key flavonoid biosynthetic genes across root, stem, and leaf. (**B**) Relative metabolite abundance (normalized to maximum = 1.0) across root, stem, and leaf.

In the flavonoid path, early biosynthetic genes (EBGs) such as *PAL*, *cinnamate 4-hydroxylase* (*C4H*), *4CL*, *CHS*, *CHI*, and *F3H* were expressed differently in all three organs (Figure 13). CHS is the first enzyme, which had the highest expression in roots compared to leaves and stems. Therefore, roots are the main site where flavonoid skeletons are made. PAL and CHI showed a similar root-dominant pattern. At the F3H branch, the F3H/FLS enzyme (RHSIM_Rhsim06G0122400; Swiss-Prot: FLS_CITUN) was most highly expressed in roots. This root-biased *F3H/FLS* expression differs from the leaf-biased buildup of kaempferol and quercetin glycosides among DAMs, a pattern that is inconsistent with a simple transcription-driven model in which enzymes and end-products colocalize in the same tissue. Several hypotheses, not mutually exclusive, may account for this disconnect: (i) *UGT*-mediated glycosylation in roots followed by inter-organ transport, consistent with the identification of leaf-biased *UGT89B1* (leaf FPKM = 30.0) and *UGT73C6* (leaf FPKM = 61.7); (ii) differential protein stability or post-translational modification of *F3H/FLS* between organs; and (iii) organ-specific metabolite compartmentalization. These hypotheses are generated from the organ-specific transcript and metabolite patterns observed in our dataset and remain to be functionally validated. DFR was also expressed more in roots, which is associated with flux toward the 2,3-dihydroflavonoid branch that leads to methylfarrerol production [3]. ANR was also dominant in roots, consistent with its known role in 2,3-dihydroflavonoid production. *MYB12*, a transcriptional activator of flavonoid EBGs, was preferentially expressed in leaves, consistent with its established role in promoting flavonol accumulation in aerial tissues [39]. Whether this expression pattern reflects direct transcriptional control of *UGT* glycosylation genes and/or transporter genes in the leaf remains to be determined through functional assays.

The phenylpropanoid pathway exhibited organ-specific control. *HQT* expression levels correlated the different amounts of chlorogenic acid and caffeic acid derivatives. Terpenoid synthesis was split in the same way: *TPS* and *CYP450* genes were expressed more in certain organs, consistent with the organ-specific accumulation of diterpenoids and triterpenoids among DAMs [8].

Transcription factor analysis showed that organs use different control systems for special metabolism. R2R3-MYB family members, such as MYB12-type activators, were expressed differently in different organs, and they probably control EBG expression in a tissue-specific way. bHLH and WD40 repeat proteins make up the MBW transcriptional activation complex. Moreover, their expression profiles aligned with established roles in the transcriptional regulation of flavonoid biosynthesis [19]. WRKY, AP2/ERF, and C2H2 families were also expressed more in certain organs, indicating many layers of gene control work together with growth and environmental signals. WRKY was co-expressed with *CHS* and *F3H* in leaf tissue, indicating a control link between stress signals and flavonol glycoside production. The correlation between structural gene expression and metabolite accumulation is consistent with organ-specific patterns of active compound accumulation, though this association does not establish causality.

As one possible mechanistic hypothesis, we examined the expression patterns of 22 putative *UGT* genes annotated in the flavonoid pathway. Two major expression modules were identified: a leaf-biased cluster (8 *UGTs*, mean leaf FPKM = 20.8 vs. root 5.2) and a root-biased cluster (11 *UGTs*, mean root FPKM = 38.3 vs. leaf 18.4). The leaf-biased UGTs included *UGT89B1* (flavonol 3-O-glucosyltransferase, leaf FPKM = 30.0) and *UGT73C6* (flavonol-3-O-glycoside 7-O-glucosyltransferase 1, leaf FPKM = 61.7), consistent with leaf-enriched glycosylation of kaempferol and quercetin aglycones. The root-biased UGTs included *UGT73B5* (flavonol 3-O-glucosyltransferase, root FPKM = 243.4) and *UGT75L17* (phloretin/flavanone glycosyltransferase, root FPKM = 43.0), supporting root-specific glycosylation of dihydroflavonoid precursors. The identification of leaf-biased and root-biased UGT clusters is consistent with organ-specific glycosylation as a working hypothesis, but these expression patterns do not establish glycosylation capacity, enzyme activity, or metabolic flux, which would require biochemical validation.

As a complementary hypothesis, we examined ABC transporter and detoxification protein genes for co-expression with *F3H/FLS* and *DFR*. Of 245 strict-filtered transporter genes, 75 showed co-expression with *F3H/FLS* (|r| > 0.5). Notably, *ABCB11* (multidrug resistance protein 8, root FPKM = 25.5) exhibited strong positive correlation with both *F3H/FLS* (r = 0.823) and *DFR* (r = 0.981), and *DTX27* (detoxification protein 27, root FPKM = 24.4) showed strong positive correlation with *F3H/FLS* (r = 0.899). These co-expression patterns are consistent with, but do not demonstrate, a potential role in flavonoid redistribution. *ABCB11* and *DTX27* are presented as candidate genes for future functional validation only.

### 3.9. Integration of Transcriptome and Metabolome

**Figure 14:**
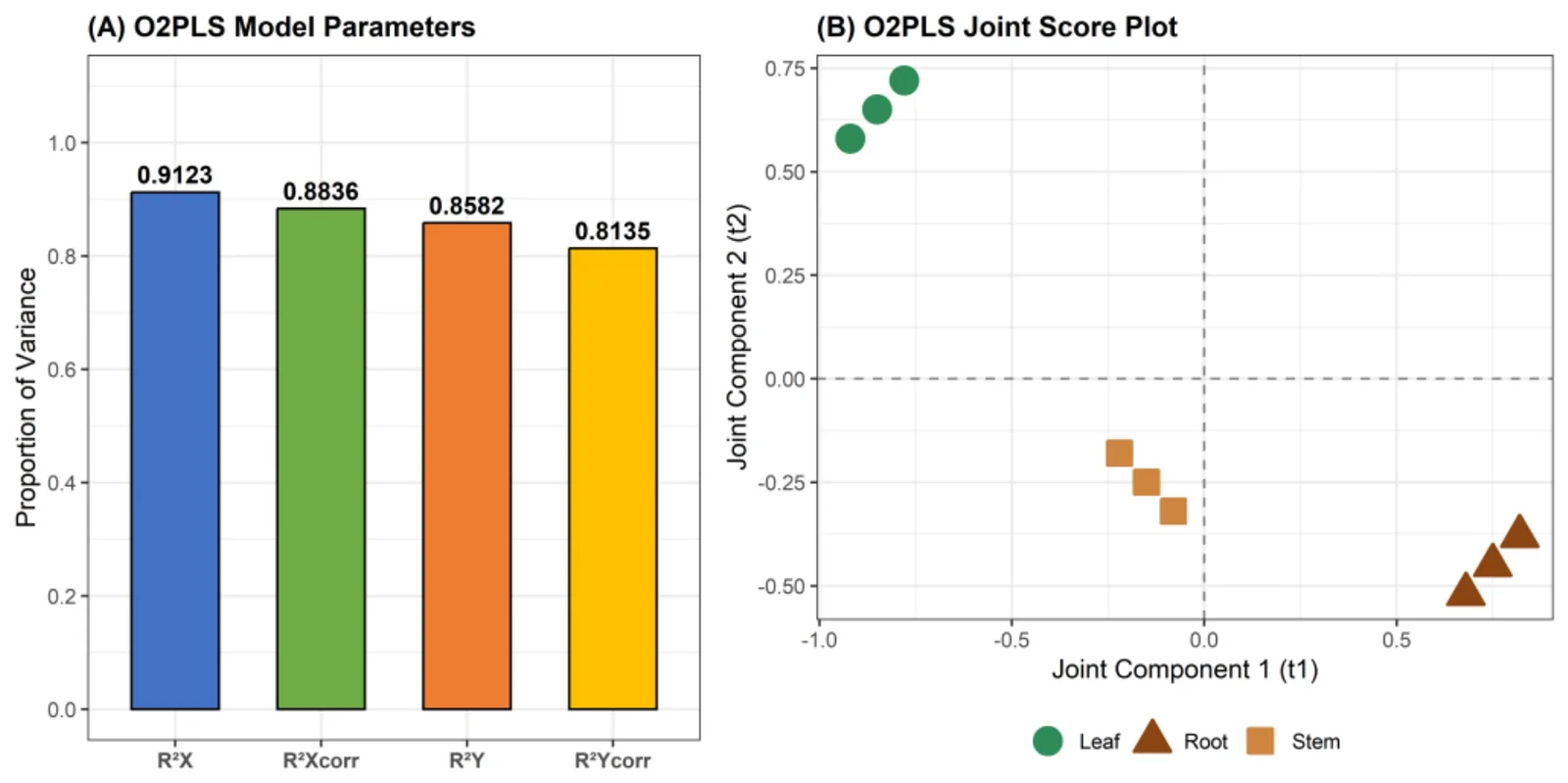
O2PLS integrative analysis of transcriptome and metabolome data. (**A**) O2PLS model parameters. R^2^X (0.9123) and R^2^Y (0.8582) represent the proportion of variance explained in the transcriptome (X) and metabolome (Y), respectively. R^2^Xcorr (0.8836) and R^2^Ycorr (0.8135) indicate the proportion of joint (correlated) variance captured by the model. (**B**) O2PLS joint score plot. Samples are projected onto the first two joint components (t1, t2). Leaf (green circles), stem (orange squares), and root (brown triangles) replicates form distinct clusters along t1, reflecting organ-specific transcriptome–metabolome coupling. O2PLS, two-way orthogonal partial least squares.

An O2PLS model was built from all DEGs and DAMs which showed a strong link between the transcriptome and metabolome. The numbers were R^2^X = 0.9123, R^2^Y = 0.8582, R^2^Xcorr = 0.8836, and R^2^Ycorr = 0.8135 (Figure 14) [40]. The high R^2^Xcorr value means that the model found biologically meaningful shared variation, and most metabolomic changes are driven by genes. The orthogonal parts show variation that is specific to each data type and not linked between transcriptome and metabolome. Thus, they may reflect organ-specific processes that do not directly link genes to metabolites.

**Figure 15:**
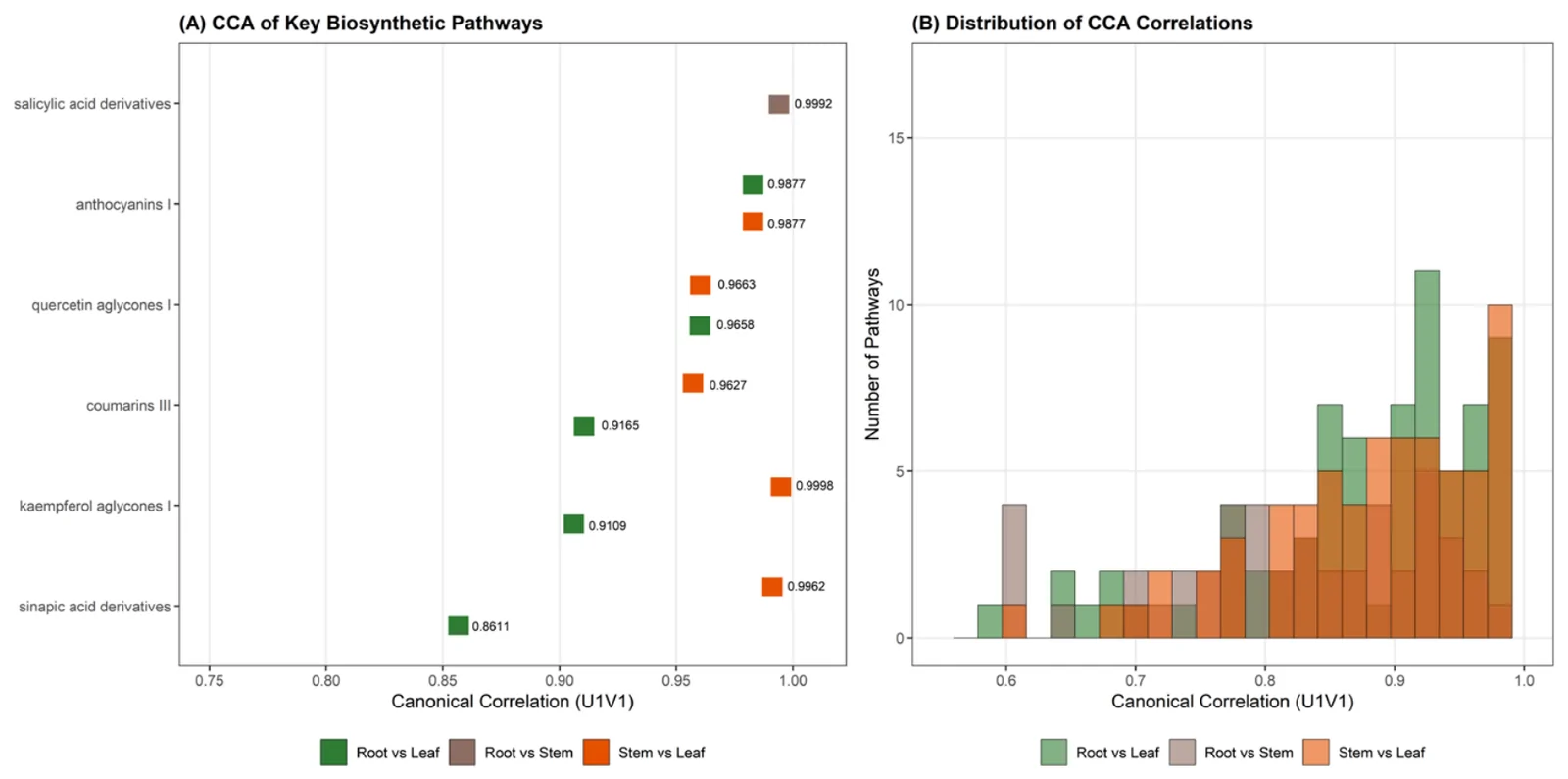
Canonical correlation analysis (CCA) of key KEGG metabolic pathways. (**A**) Canonical correlation coefficients (U1V1) for selected biosynthetic pathways across three pairwise comparisons (green: root vs. leaf; brown: root vs. stem; orange: stem vs. leaf). MetMap113 (kaempferol aglycone synthesis) shows the strongest correlation in the stem-versus-leaf comparison (U1V1 = 0.9998). (**B**) Distribution of U1V1 values across all KEGG pathways for each comparison, demonstrating that the majority of pathways exhibit high transcriptome–metabolome coupling (U1V1 > 0.90) in the stem-versus-leaf comparison. CCA, canonical correlation analysis.

CCA was performed for all three pairwise comparisons. Notably, the stem-versus-leaf comparison revealed the strongest pathway-level link between transcriptome and metabolome. In this comparison, several flavonoid and phenylpropanoid sub-pathways showed first canonical variate pair (U1V1) near 1, so they are tightly co-regulated (Figure 15). MetMap113 (Biosynthesis of kaempferol aglycones I) had the strongest link. MetMap153 (Biosynthesis of sinapic acid derivatives), MetMap118 (Biosynthesis of anthocyanins I), MetMap115 (Biosynthesis of quercetin aglycones I), and MetMap129 (Biosynthesis of coumarins III) followed. Salicylic acid derivative biosynthesis (MetMap158) showed a moderate but significant link. The root-versus-leaf comparison also gave high correlations for these pathways (MetMap115 and MetMap118). However, the root-versus-stem comparison showed lower values (MetMap113 and MetMap153), so metabolic differences between above-ground and below-ground organs cause the strongest gene–metabolite coordination.

The near-perfect correlation observed for kaempferol aglycone synthesis (stem versus leaf) is biologically significant, as it provides direct transcriptomic–metabolomic evidence for coordinated regulation at this key branch point of the flavonoid pathway. Therefore, F3H/FLS is associated with flavonol glycoside accumulation in leaves, which shows that kaempferol biosynthesis exhibited the highest predictive accuracy from transcriptomic data across the entire dataset.

Joint KEGG analysis of DEGs and DAMs supported the above results. Both flavonoid biosynthesis (map00941) and phenylpropanoid metabolism (map00940) were enriched in gene and metabolite data. However, paths are central to organ-specific chemical diversity. Coumarin and anthocyanin/flavanol routes were also enriched in both datasets, indicating genes control these branches together [24].

To identify hub genes and regulatory modules within the flavonoid biosynthetic pathway, Pearson correlation network analysis was performed using stringent thresholds (|r| ≥ 0.8, *p* < 0.05; at n = 9, |r| ≥ 0.8 corresponds to *p* ≈ 0.01). Hub genes were identified based on degree centrality (top 10% of nodes) and betweenness centrality metrics computed in Cytoscape. *CHS*, *F3H/FLS*, and *DFR* were among the most highly connected hub genes in the flavonoid regulatory network. *CHS* exhibited particularly strong connectivity with multiple flavonoid compounds, consistent with its role as the entry point into the flavonoid biosynthetic pathway. *F3H/FLS* was positively linked to kaempferol and quercetin glycosides in leaves, while *DFR* showed negative correlation with flavonol glycosides but positive association with farrerol-related metabolites in roots.

Integration with WGCNA module–trait correlation analysis further supported these gene–metabolite relationships. The purple module (leaf-specific) showed strong positive correlation with flavonol glycoside accumulation, and its hub genes included *F3H/FLS* and UDP-glycosyltransferase homologs, consistent with leaf-enriched glycosylation activity. The brown module (stem-preferential) exhibited correlation with phenylpropanoid-derived metabolites. These module–trait associations suggest that organ-specific flavonoid profiles are governed by distinct transcriptional modules rather than isolated gene–metabolite pairs.

### 3.10. Validation by qRT-PCR

Twelve key DEGs from flavonoid, phenylpropanoid, and terpenoid paths and their control networks were checked by qRT-PCR. The checked genes were *CHS*, *CHI*, *F3H/FLS*, *DFR*, *ANS*, *PAL*, *4CL*, *TPS*, *CYP450*, *MYB*, *bHLH*, and *WRKY*. EF1-α was used as the internal control gene.

**Figure 16:**
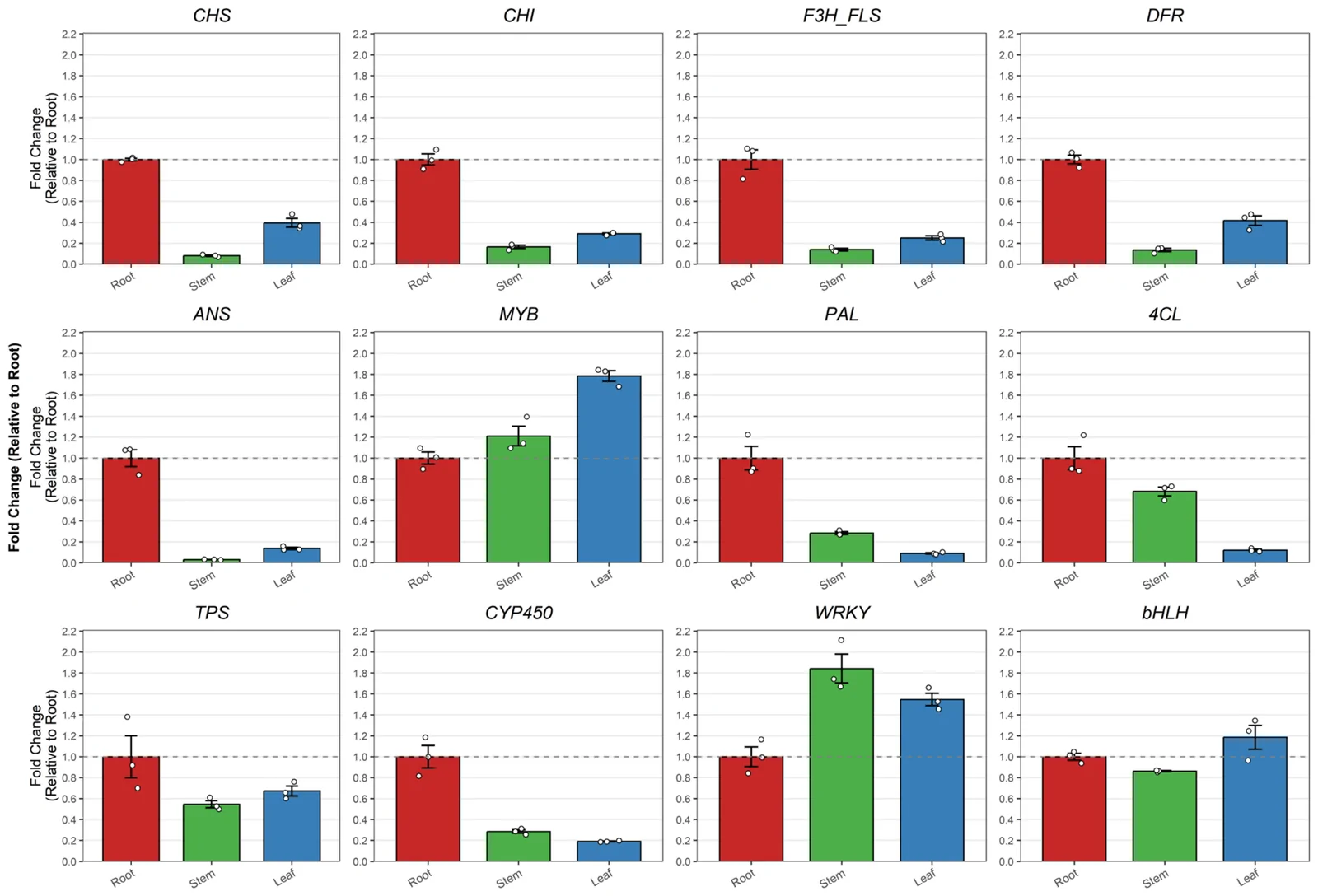
qRT-PCR validation of RNA-seq results for 12 DEGs across root, stem, and leaf tissues of *R. yedoense* var. *poukhanense*. qRT-PCR-derived fold change values (2^−ΔΔCt^) for 12 differentially expressed genes, with root set as the calibrator (fold change = 1.0, dashed line). Values above 1.0 indicate higher expression than root; values below 1.0 indicate lower expression. Bars represent mean ± standard error of the mean (SEM) (n = 3 biological replicates). Open circles show individual data points. Gene names are italicized per botanical convention. Red bars, root; green bars, stem; blue bars, leaf. High correlation between RNA-seq and qRT-PCR fold changes (Pearson r = 0.962, *p* < 0.001) confirms the reliability of transcriptomic data (Figure S2).

Organ-specific up and down patterns were the same across comparisons (Figure 16). Structural genes in flavonoid and phenylpropanoid paths (*CHS*, *CHI*, *F3H/FLS*, *DFR*, *ANS*, *PAL*, *4CL*, and *CYP450*) were ranked higher in roots in both datasets, indicating roots are the main site of flavonoid synthesis. *MYB* and *bHLH* were expressed more in leaves, but *WRKY* was expressed more in stems, indicating gene control is organ-specific.

## 4. Discussion

### 4.1. Organ-Specific Metabolic Partitioning: Chemical Geography of a Medicinal Plant

A total of 2182 metabolites spanning nine chemical classes were identified, reflecting the extensive chemical diversity of this species and enabling comprehensive organ-specific metabolic profiling.

Leaves had more flavonoid glycosides such as quercetin-3-O-glucoside and kaempferol-3-O-rutinoside. Moreover, they also had more phenolic acids such as chlorogenic acid derivatives. This matches the photosynthetic role of leaf tissue, and flavonoid glycosides work as UV-B protectants and antioxidants there [3,13]. *UGT* and *HQT* were expressed more in leaves and *F3H/FLS* was expressed more in roots and pushed aglycone synthesis. Thus, leaves had the enzymes needed for glycosylation and vacuolar storage of these metabolites.

Roots exhibited preferential accumulation of terpenoids such as ursolic acid, alkaloids, and special flavonoid aglycones. Notably, the genus-specific 2,3-dihydroflavonoid methylfarrerol accumulated predominantly in leaves (log_2_FC = −1.76; Table 1), consistent with root-to-leaf transport of this anti-inflammatory 2,3-dihydroflavonoid. The Level 3 identification status of methylfarrerol (no authentic standard or full MS/MS validation) should be considered when interpreting this finding; while the mass spectral features and retention time align with published data for *Rhododendron* species, independent structural confirmation remains desirable. High *TPS* and *CYP450* gene activity in roots supports this. Stems showed a middle pattern with more lignin precursors and transport metabolites, consistent with their role in moving nutrients [22].

The pronounced metabolic variability observed among root replicates (Figure 2) warrants particular consideration. Unlike aerial organs, roots are exposed to spatially variable soil conditions including moisture gradients, nutrient micro-patches, and diverse rhizosphere microbiomes. These environmental factors are known to trigger plastic metabolic responses in root tissues, particularly in defense-related pathways such as terpenoid and phenolic acid biosynthesis. In the present study, Root-2′s spread along PC2 likely reflects such micro-environmental influences rather than technical artifacts, given the tight clustering of leaf and stem replicates. This interpretation aligns with the established understanding that root secondary metabolism is inherently more variable than that of aerial tissues due to constant soil–organism interactions.

These organ-specific patterns are broadly consistent with findings in other medicinal plant species, though the present study extends them in three important ways. First, in terms of metabolite coverage, Wu et al. [22] identified tissue-discriminative markers from 53 metabolites in *P*. *cuspidatum*, whereas our survey captured 2182 metabolites—representing a substantial increase in chemical space resolution. Second, in terms of analytical depth, Sun et al. [13] and Gao et al. [26] reported tissue-specific transcriptome–metabolome coordination in *Schisandra sphenanthera* and *S*. *miltiorrhiza*, respectively; however, these studies did not formally partition joint versus orthogonal variation. Our O2PLS-based framework explicitly decomposes total variation into transcriptionally coupled (joint) and organ-specific (orthogonal) components, thereby distinguishing metabolite accumulation driven by gene expression from that driven by post-transcriptional or environmental factors. Third, in terms of mechanistic insight, Liu et al. [24] used correlation network analysis to reconstruct alkaloid biosynthetic pathways in *V*. *mengtzeanum*, but did not establish quantitative gene–metabolite bridges at the individual metabolite level. Our CCA analysis and Pearson network analysis directly link F3H/FLS and DFR expression to specific flavonoid end-products, providing a gene-to-metabolite map that can guide metabolic engineering. These advances represent the most comprehensive organ-resolved multi-omics survey in a *Rhododendron* species to date.

### 4.2. Branch-Point Regulation at F3H Is Associated with Organ-Specific Flavonoid Accumulation

A key finding of this study concerns the regulatory decision point at flavanone 3-hydroxylase (F3H). Our data indicate that the differential expression of competing downstream enzymes (F3H/FLS versus DFR) is consistent with F3H functioning as a metabolic branch point that partitions carbon flux between flavonol and 2,3-dihydroflavonoid branches. However, this interpretation is based on correlation analysis, and correlation does not imply direct causation. Functional validation—such as CRISPR-Cas9 knockout, heterologous overexpression, or promoter-reporter assays—would be required to confirm a direct regulatory role of F3H in organ-specific flavonoid partitioning.

In roots, *F3H/FLS* was expressed more. Two hypotheses may explain this pattern: (i) root-expressed F3H/FLS produces aglycones that undergo UGT-mediated glycosylation in roots followed by transport to leaves; or (ii) root-expressed F3H/FLS produces aglycones that serve as precursors for long-distance transport and subsequent glycosylation in leaf tissue. These hypotheses are not mutually exclusive and are generated from the organ-specific transcript and metabolite patterns observed in our dataset. Their relative contributions remain to be determined through future experiments combining isotope tracing, promoter-reporter assays, and CRISPR-mediated gene editing.

This pattern is also seen in model plants. In *A*. *thaliana*, *AtMYB12* turns on *FLS* to push flavonol glycoside buildup in above-ground tissues [3], but in *Petunia hybrida*, *PhAN4* controls *DFR* to drive anthocyanin production in flowers. Consequently, similar gene switches may work in *R. yedoense* var. *poukhanense*. However, the exact MYB-bHLH groups that control *F3H/FLS* and *DFR* still need to be tested. This inference is further substantiated by quantitative CCA results. MetMap113 (kaempferol aglycones, U1V1 = 0.9998) and MetMap115 (quercetin aglycones, U1V1 = 0.9663) had the highest links to gene changes. Consequently, branch-point paths respond most strongly to gene control in the whole dataset. Methylfarrerol is ~3.4-fold more abundant in leaves than in roots (log_2_FC = −1.76; Table 1), a distribution pattern that could reflect either root-to-leaf transport or differential turnover rates between organs. Without isotope tracing or transporter functional assays, this pattern cannot be mechanistically resolved. Farrerol and its methyl form fight inflammation, reduce oxidation, and protect nerve cells [3]. Therefore, we can use metabolic engineering to make more farrerol. This metabolic redirection can be achieved by modulating the DFR branch point.

### 4.3. The MYB-bHLH-WD40 Complex and Multi-Layered Transcriptional Control

Flavonoid biosynthetic genes are controlled by the MBW complex [19]. R2R3-MYB members such as MYB12-type activators drive EBG expression. CHS had more transcripts in roots than in leaves or stems. Thus, roots make more flavonoid skeletons, and root-specific MYB-type factors may turn this on [18]. *MYB12* was expressed more in leaves, consistent with its role in turning on UGT glycosylation and moving flavonols to leaves.

The bHLH and WD40 components displayed complementary expression patterns. bHLH subgroup IIIf members showed elevated expression in tissues with active late biosynthetic genes (LBGs), consistent with bHLH-mediated activation of DFR, ANS, and ANR [19]. The co-expression of root-biased F3H/FLS and DFR in root tissue supports MBW complexes that channel flux toward both flavonol aglycone and 2,3-dihydroflavonoid production, respectively. This differential routing likely involves competitive binding of distinct MYB activators to shared bHLH-WD40 partners [18].

Beyond the MBW module, WRKY co-expression with CHS in root tissue introduces a stress-responsive regulatory layer linking flavonoid production to defense signaling. AP2/ERF and C2H2 zinc finger TFs showed organ-biased expression, implicating ethylene-mediated and developmental signaling in fine-tuning flavonoid accumulation. Together, these findings support multi-layered control in which the MBW complex serves as the primary hub, while WRKY, AP2/ERF, and C2H2 TFs provide environmental context. This architecture has implications for metabolic engineering: manipulating MBW complex components represents a potential strategy for enhancing pathway-wide flavonoid production, while targeting WRKY or AP2/ERF factors could potentially fine-tune accumulation in response to environmental cues. These engineering strategies are proposed based on the observed expression patterns and remain to be experimentally validated.

### 4.4. Multi-Omics Integration as a Strategy for Decoding Plant Secondary Metabolism

Transcriptomic and metabolomic data were integrated using a multi-layer statistical framework. O2PLS was used for global covariance extraction. CCA was used for pathway-level co-regulation. Pearson correlation network analysis was employed to identify statistically significant associations between structural genes and their putatively corresponding metabolites. This framework identifies quantitative associations between gene expression and metabolite accumulation at global, pathway, and individual gene–metabolite levels, providing a statistical foundation for generating hypotheses about how plant secondary metabolism is coordinated across organs.

O2PLS captured a large part of joint transcriptome–metabolome variation (R^2^Xcorr = 0.8836). Most metabolomic changes are linked to gene processes. They can be predicted from gene data. This high R^2^Xcorr value indicates that a substantial proportion of metabolite variation is statistically associated with gene expression variation. Post-transcriptional or environmental factors may account for the remaining orthogonal variation. These patterns support the potential for using gene expression data as a statistical predictor of metabolite profiles, though predictive accuracy would require independent validation.

CCA at the KEGG pathway level split these global structures into specific co-regulated modules. MetMap113 (kaempferol aglycones, U1V1 = 0.9998), MetMap115 (quercetin aglycones, U1V1 = 0.9663), and MetMap118 (anthocyanins, U1V1 = 0.9877) were the most linked to gene changes. The near-perfect canonical correlation for kaempferol aglycones (U1V1 = 0.9998) indicates a strong statistical association between kaempferol biosynthetic gene expression and metabolite accumulation. While this correlation supports the potential for using transcriptomic data to predict metabolite profiles, it does not establish a direct causal link or confirm predictive accuracy in independent samples. Experimental validation would be required before applying this association for gene-guided metabolic engineering.

Pearson correlation network analysis provided gene-level resolution of flavonoid gene–metabolite association patterns. Using degree centrality and betweenness centrality metrics in Cytoscape, *CHS*, *F3H/FLS*, and *DFR* were identified as the most highly connected hub genes, consistent with their established enzymatic functions at critical branch points in the flavonoid pathway. The positive correlation between *F3H/FLS* and flavonol glycosides in leaves, together with the negative correlation between *DFR* and these metabolites, supports the model in which competing branch-point enzyme expression influences organ-specific flavonoid accumulation. However, these correlations do not establish direct causality, and functional validation-such as CRISPR-based knockout or transient overexpression-would be needed to confirm regulatory roles.

At the systems level, WGCNA module–trait correlation analysis revealed that organ-specific flavonoid accumulation is associated with distinct co-expression modules rather than isolated gene–metabolite pairs (Figure 12). Leaf-associated modules were enriched in *F3H/FLS* and UDP-glycosyltransferase homologs, consistent with leaf-biased glycosylation activity. Stem-preferential modules correlated with phenylpropanoid-derived metabolites and were driven by cinnamoyl-CoA reductase and laccase family members. These WGCNA findings are consistent with the hypothesis that organ-specific chemical profiles are associated with modular transcriptional programs—a hypothesis that converges with O2PLS and CCA results but requires validation in larger sample cohorts. However, this hypothesis is a pattern that converges with O2PLS and CCA results obtained through alternative statistical frameworks, which were conducted on the same dataset but do not rely on co-expression network construction and are therefore less sensitive to sample size limitations. The modular transcriptional program pattern has been observed in other medicinal plants such as *S. miltiorrhiza* and *G*. *uralensis* [26,27].

This multi-layered integration offers distinct advantages over the traditional strategy of identifying DEGs and DAMs separately and inferring relationships post hoc. Liu et al. [24] applied correlation network analysis to reconstruct alkaloid biosynthetic pathways in *V. mengtzeanum* from 55 DAMs and 33,942 DEGs, and Gao et al. [26] combined metabolomics with transcriptomics to elucidate tanshinone biosynthesis in *S. miltiorrhiza*. While these studies established the utility of multi-omics approaches, the present investigation advances the methodology by partitioning total variation into joint and orthogonal components via O2PLS, thereby distinguishing transcriptionally coupled metabolic processes from organ-specific processes that operate independently of gene–metabolite covariance. The resulting four-layer statistical framework (O2PLS-CCA-WGCNA-Pearson) provides complementary analytical perspectives that together enable a more comprehensive characterization of transcriptome–metabolome associations than any single method alone. This framework generates statistically grounded hypotheses about secondary metabolism that require functional validation and represents a transferable strategy for exploratory multi-omics analysis in other non-model medicinal plants.

### 4.5. Limitations and Future Directions

The present study employed the *R. simsii* reference genome for transcriptome alignment, as it is the closest relative of *R. yedoense* var. *poukhanense* with a publicly available high-quality genome. While both species belong to the subgenus Tsutsusi, sequence divergence may affect mapping accuracy. The overall (62.88–75.21%) and unique (60.27–72.04%) mapping rates are consistent with cross-species transcriptome studies in other non-model plants [14,25,34] and were sufficient to support the organ-specific expression patterns and gene–metabolite relationships reported herein. Nevertheless, a de novo genome assembly of *R. yedoense* var. *poukhanense* would enable more comprehensive identification of species-specific genes and is a priority for future research.

Reference transcriptome completeness was not assessed using Benchmarking Universal Single-Copy Orthologs (BUSCO) because this study employed reference-based alignment (HISAT2) rather than de novo transcriptome assembly, for which BUSCO is designed. As alternative indicators of coverage, we report the detection of 33,576 expressed genes, tight biological replicate clustering in both transcriptome (PCA) and metabolome (PCA, OPLS-DA) analyses, and cross-validation of RNA-seq patterns by qRT-PCR (r > 0.962). Because this study focuses on inter-organ differential comparison (root vs. stem, stem vs. leaf, and root vs. leaf) rather than absolute gene quantification in a single organ, systematic reference-bias effects are expected to influence both conditions of any given comparison similarly; while this does not eliminate the bias, it limits its impact on fold-change estimation. The mapping rates are high for root and leaf (70–75%) and somewhat lower for stem (63–67%). We acknowledge that organ-specific reference bias cannot be fully excluded, particularly in stem-involved comparisons. Nevertheless, because DEG calling relies on relative between-organ comparisons rather than absolute expression levels, our analytical approach inherently mitigates the impact of systematic mapping bias. Accordingly, DEGs identified in stem-involved comparisons (root vs. stem, stem vs. leaf) should be interpreted with appropriate caution, while the organ-specific patterns validated by qRT-PCR (r > 0.962) and the metabolomic cross-validation provide independent support for the robustness of the core findings. The lower stem mapping rates are unlikely to reflect RNA quality degradation, as stem samples actually exhibited the highest RQN values (9.7 ± 0.1 vs. 8.2 ± 0.4 for leaf). We therefore interpret the stem mapping deficit as primarily reflecting greater sequence divergence of stem-expressed genes from the *R. simsii* reference, consistent with the organ-specific evolutionary pressures shaping stem transcriptomes. However, we cannot fully exclude a contribution from technical factors (e.g., organ-specific polyphenolic interference during library preparation) and recommend that future studies employ a species-specific reference genome for *R. yedoense* var. *poukhanense* to resolve this question.

It is also important to note that all gene–metabolite relationships reported herein are based on correlative analyses (O2PLS, CCA, and Pearson correlation). While the four-layer integration framework provides quantitative associations with strong statistical support, these correlations do not establish direct causation. Functional validation—such as CRISPR-Cas9-mediated knockout or overexpression of F3H/FLS, DFR, and candidate transcription factors, combined with metabolite profiling—will be essential to confirm the regulatory mechanisms proposed in this study.

Furthermore, the Pearson correlation network was constructed from 5.6 million gene–metabolite pairs without formal multiple testing correction beyond the stringent |r| ≥ 0.8 threshold. While this approach prioritizes sensitivity for hypothesis generation, the false discovery rate among reported correlations is likely elevated. The hub genes (*CHS*, *F3H/FLS*, and *DFR*) and co-expression modules identified through WGCNA provide convergent support for the organ-specific regulatory framework proposed herein, but individual gene–metabolite links require experimental confirmation.

WGCNA was conducted with a relatively small sample size (n = 9); consequently, WGCNA results are presented as exploratory and were cross-validated against independent O2PLS and CCA analyses. The biological relevance of WGCNA findings rests entirely on their convergence with results from O2PLS, CCA, and Pearson correlation analyses, which were conducted on the same dataset but do not rely on co-expression network construction and are therefore less sensitive to sample size limitations. Several factors support the broad consistency of the preliminary WGCNA results with the other analyses: (i) biological replicates clustered tightly within each organ (Figure 12C); (ii) the organ-specific modules showed correlation with independently measured metabolite profiles; and (iii) the hub genes identified (*CHS*, *F3H/FLS*, and *DFR*) are well-established flavonoid pathway regulators. Nevertheless, all WGCNA-derived candidates require validation in larger sample cohorts before they can support mechanistic conclusions.

The proposed explanations for organ-specific flavonoid distribution are based on transcriptome–metabolome correlations and co-expression patterns, which identify statistical associations but do not establish causality. Functional validation through transport assays, enzyme activity measurements, and metabolic flux analysis would strengthen the mechanistic interpretations presented here.

## 5. Conclusions

This study establishes an integrated multi-omics framework for *R*. *yedoense* var. *poukhanense*. We constructed organ-specific metabolic fingerprints from 2182 named metabolites, and 1116, 896, and 1264 DAMs were found in root-versus-leaf, root-versus-stem, and stem-versus-leaf comparisons. Gene profiling found 9836, 5457, and 8007 DEGs. Expression analysis of *FLS*, *DFR*, and *CHS* revealed organ-specific expression patterns consistent with differential flavonoid accumulation. The MBW complex and WRKY transcription factors showed organ-biased expression patterns that are statistically associated with flavonoid metabolite profiles, though their direct regulatory roles remain to be functionally validated. O2PLS analysis revealed robust, statistically supported associations between transcriptomic and metabolomic profiles (R^2^Xcorr = 0.8836). This was checked by qRT-PCR (r > 0.962).

A key finding is that differential *F3H/FLS* and *DFR* expression at the F3H branch point is consistent with organ-specific flavonoid accumulation patterns in *R. yedoense* var. *poukhanense*. This metabolic partitioning has practical implications for medicinal utilization: leaves are the primary accumulation site of flavonol glycosides (quercetin-3-O-glucoside, kaempferol-3-O-rutinoside) and phenolic acids with antioxidant and anti-inflammatory properties; roots serve as the dominant biosynthetic hub for flavonoid skeletons and 2,3-dihydroflavonoid precursors including farrerol with documented anti-inflammatory activity; stems contain triterpenoids (oleanolic acid and ursolic acid) that contribute to the overall medicinal value. These organ-specific profiles enable targeted harvesting strategies and provide a gene-to-metabolite framework for metabolic engineering of pharmaceutically relevant flavonoids.

It is important to note that all conclusions regarding F3H/FLS–DFR branch-point partitioning are based on correlative evidence from multi-omics integration and are presented as data-supported hypotheses, not resolved mechanisms. While the strong canonical correlations and consistent expression–metabolite patterns provide statistical support, these associations do not establish causality. Functional validation studies—such as CRISPR-Cas9-mediated gene editing, heterologous overexpression, or enzyme activity assays—are needed to confirm the regulatory mechanisms proposed in this study. Future research should prioritize confirming the regulatory roles proposed herein to translate these findings into practical applications for medicinal plant cultivation and bioprospecting.

## Figures and Tables

**Table 1:** Key DAMs with medicinal activity.

Metabolite	Class	Root vs. Leaf VIP	Root vs. Leaf Log_2_FC	Root vs. Stem VIP	Root vs. Stem Log_2_FC	Stem vs. Leaf VIP	Stem vs. Leaf Log_2_FC	Primary Organ	MSI Level
Methylfarrerol	Flavonoid	1.01	−1.76	-	-	-	-	Leaf	3
Quercetin-3-O-glucoside	Flavonol glycoside	1.3	−5.7	1.48	1.42	1.16	−7.12	Leaf	1
Kaempferol-3-O-rutinoside	Flavonol glycoside	1.3	−3.6	-	-	1.18	−3.6	Leaf	2
Quercetin	Flavonol aglycone	1.31	−3.52	1.49	−2.97	1.18	−3.52	Leaf	1
Kaempferol	Flavonol aglycone	1.31	−3.03	1.49	−4.21	1.18	−4.23	Leaf	3
Taxifolin	Flavanonol	1.3	−8.97	1.34	−3.52	1.17	−5.45	Stem	3
Luteolin	Flavone	1.3	−2.93	1.49	−4.52	1.18	−2.93	Leaf	3
Apigenin	Flavone	1.3	−4.11	1.22	−2.32	1.18	3.77	Stem	3
Naringenin	Flavanone	1.3	−6.03	1.38	−1.79	1.17	1.36	Leaf	3
Epicatechin	Flavan-3-ol	1.31	−5.85	1.04	−2.7	1.18	−3.15	Stem	3
Genistein	Isoflavone	1.14	−2.69	1.27	−2.31	-	-	Stem	3
Oleanolic acid	Triterpenoid	1.31	−2.23	1.46	−3.96	1.14	2.43	Stem	3
Ursolic acid	Triterpenoid	1.3	−2.6	1.48	−5.05	1.12	1.68	Stem	3
Chlorogenic acid	Phenolic acid	1.31	−2.99	1.46	−3.06	1.18	−4.21	Leaf	2
Caffeic acid	Phenolic acid	1.3	−2.42	1.48	-	1.17	−3.63	Leaf	3
Gallic acid	Phenolic acid	1.3	−3.47	1.44	−3.29	-	-	Stem	1
Ferulic acid	Phenolic acid	1.3	4.77	1.47	−1.42	1.18	4.22	Leaf	3
Syringic acid	Phenolic acid	1.27	−6.08	1.33	−1.52	1.18	3.37	Leaf	3
Benzoic acid	Phenolic acid	1.26	2.77	1.44	−1.49	1.18	1.9	Stem	1
Cyanidin	Anthocyanidin	1.31	−3.4	1.47	−1.53	1.18	−3.4	Leaf	3

Note: MSI Level, metabolite standards initiative (Metware Biotechnology, Hangzhou, China): Level 1 = MS/MS fragmentation pattern (all fragment ions) and RT matched to the in-house database with similarity score >0.7; Level 2 = MS/MS fragmentation and RT matched with similarity score 0.5–0.7; Level 3 = Q1, Q3, RT, DP, and CE verified against the database. This classification system differs from the Metabolomics Standards Initiative (MSI) nomenclature, and no metabolites in this study were confirmed with authentic standards. Log_2_FC, log_2_-transformed fold change. Values were converted from raw fold-change ratios to improve interpretability and maintain consistency with differential gene expression analysis. Negative Log_2_FC values indicate lower abundance in the first organ of the comparison (e.g., root vs. leaf Log_2_FC = −1.76 means 2.7-fold higher abundance in leaves than in roots). Extreme values (e.g., <−5.0) typically reflect detection-limit imputation for compounds that were below the limit of detection (LOD) in one organ rather than precise quantitative ratios. “-” (dash) indicates that the compound was not detected in any biological replicate of the corresponding comparison group, and fold-change calculation was therefore not performed. Missing signals were imputed at approximately 10–20% of the mean QC intensity based on LOD estimation. Compounds showing organ-specific detection patterns (e.g., detected only in leaf or only in stem) should be interpreted as “organ-detected versus organ-undetected” rather than as exact quantitative fold changes.

**Table 2:** qRT-PCR primer sequences used for validation of RNA-seq data.

Gene	Forward Primer (5′→3′)	Reverse Primer (5′→3′)
*CHS*	GTTCTGCGTTGATCAGAGCA	TTGTCGCACATGCGCTGAAA
*CHI*	AATCGAGGGCCATGTCTTTC	GCCTCCAATCTCCAAAACTC
*F3H*/*FLS*	TTTTTCCTCCTCACGGACCA	AGCTCTCCTTCTCCTTGAGA
*DFR*	AAGATGTCAATGGTTCGCCG	AACATAGCCCCGTTCAAGCA
*ANS*	ATGGTGACTACAATGGTGGC	ATGTTTCCGATGCTCGTGAG
*PAL*	GGTGGAGGCGAGTAGTAATT	AGTCCTCCTATGGGAAGTAG
*4CL*	TATTCCGGTCCAAACTTCCG	TTGATGATGCAGGGCTTGGA
*TPS*	AAGTTATCTGGACCCTGGAG	GGACCTTAATGAACAGCTCC
*CYP450*	ACCTCCTCCTACTGGAGAAA	GGCGGGAGTTTAAACCGTTT
*MYB*	GAACTGGTCGCTGATTAGCA	TTCTTGATCGCGTTGTCGGT
*bHLH*	CATCGACGATGAACATGTGG	ATTGACGTGGAGGTGGAAGA
*WRKY*	TCTCTCTGAAGGACTGGACT	GCACCAGTCATTGCAACAAC
*EF1-α*	ATTGGCCATGTTGACTCTGG	ACCCAGGCGTACTTGAATGA

**Table 3:** Summary of DEGs identified among organs.

Comparison	Total DEGs	Upregulated	Downregulated	Total Genes Detected	DEG Ratio (%)
Root vs. Leaf	9836	4570	5266	33,576	29.29
Stem vs. Leaf	8007	3670	4337	33,576	23.85
Root vs. Stem	5457	2475	2982	33,576	16.25
Total (unique)	13,019	-	-	-	38.78

Note: DEGs were identified using DESeq2 with thresholds of FDR < 0.05 and |log_2_FC| ≥ 1. Total genes detected represents all genes with non-zero counts in at least one sample. Total (unique) DEGs = 13,019 after removing duplicates across comparisons. DEG ratio = (Total DEGs/Total Genes Detected) × 100. Upregulated and downregulated indicate the direction of change relative to the first organ listed in each comparison.

## Data Availability

The transcriptome sequencing data reported in this paper have been deposited in the NCBI Sequence Read Archive (SRA) under BioProject accession number PRJNA1478165. The metabolomics dataset is available from MetWare Biotechnology Co., Ltd. upon reasonable request. All other data supporting the findings of this study are included within this article.

## Supplementary Materials

The following supporting information can be downloaded at: https://www.mdpi.com/article/10.3390/plants15162441/s1, Table S1: Summary of RNA-seq quality control and read mapping statistics for nine cDNA libraries; Table S2: All 2182 metabolites detected in *R. yedoense* var. *poukhanense* root, stem and leaf tissues by widely targeted metabolomics (UPLC-MS/MS); Table S3: Mass spectrometry parameters (retention time, precursor ion, product ion, collision energy, declustering potential) and annotation confidence levels for 29 representative metabolites detected in *R. yedoense* var. *poukhanense* by UPLC-MS/MS (MRM mode); Table S4: All DAMs detected in *R. yedoense* var. *poukhanense* root, stem, and leaf tissues by widely targeted metabolomics (UPLC-MS/MS); Table S5: All DEGs detected in *R. yedoense* var. *poukhanense* root, stem, and leaf tissues; Table S6: Primer amplification efficiencies for 12 validated genes in *R. yedoense* var. *poukhanense* qRT-PCR analysis; Figure S1: OPLS-DA permutation test validation plots; Figure S2: Correlation between RNA-seq and qRT-PCR fold changes for 12 validated genes across three tissue comparisons.
