# Supplementary material for: Integration of Transcriptomics and Metabolomics Reveals Organ-Specific Biosynthesis and Accumulation of Pharmacologically Active Flavonoids in Rhododendron yedoense var. poukhanense
Source: Plants (Basel). 2026 Aug 11;15(16):2441. doi: 10.3390/plants15162441 (PMC13516736; doi:10.3390/plants15162441)
Supplement: Supplementary file 1 [file plants-15-02441-s001.zip › Figure S1.pdf]

(A) (B) (C)

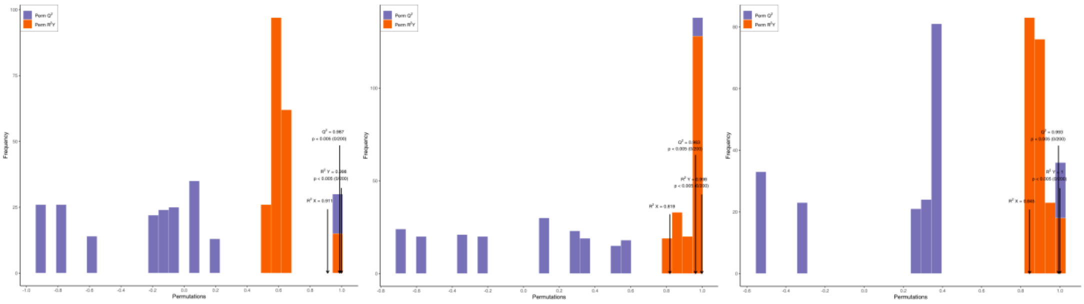

Figure S1. OPLS-DA permutation test validation plots. (A) Root versus Leaf (R^2Y = 0.998, Q^2 = 0.987), (B) Root versus Stem (R^2Y = 0.998, Q^2 = 0.963), (C) Stem versus Leaf (R^2Y = 1.000, Q^2 = 0.993). The x-axis denotes the values of R^2Y and Q^2 of the model, and the y-axis indicates the frequency of the occurrence of the corresponding model classification performance in 200 random permutation tests. In the figure, orange represents R^2Y of the randomly grouped model, purple represents Q^2 of the randomly grouped model, and the black arrows denote the R^2X, R^2Y and Q^2 values of the original model.
