# Supplementary material for: Integration of Transcriptomics and Metabolomics Reveals Organ-Specific Biosynthesis and Accumulation of Pharmacologically Active Flavonoids in Rhododendron yedoense var. poukhanense
Source: Plants (Basel). 2026 Aug 11;15(16):2441. doi: 10.3390/plants15162441 (PMC13516736; doi:10.3390/plants15162441)
Supplement: Supplementary file 1 [file plants-15-02441-s001.zip › Figure S2.pdf]

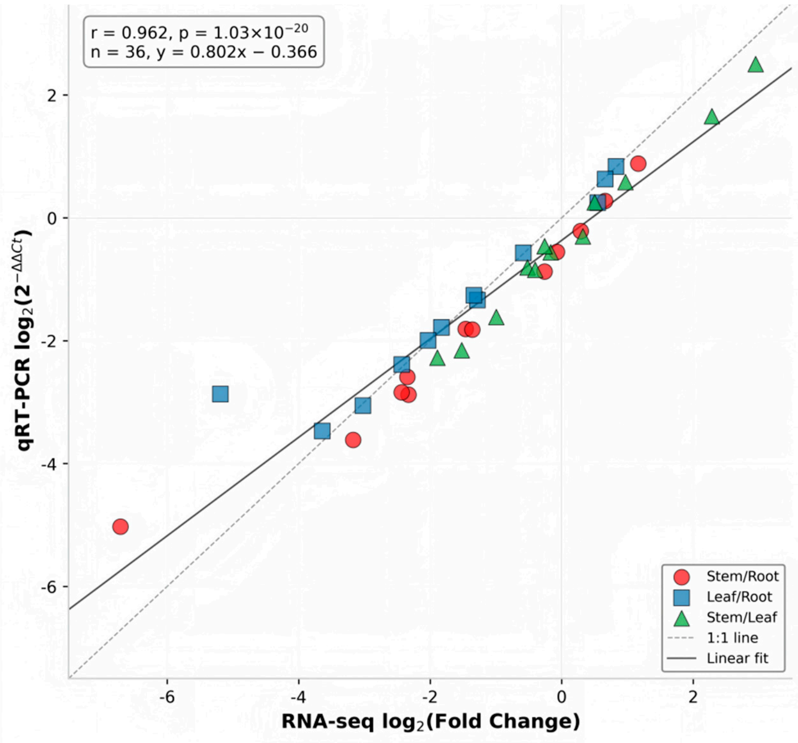

Figure S2. Correlation between RNA-seq and qRT-PCR fold changes for 12 validated genes across three tissue comparisons. Scatter plot of RNA-seq log$_2$(Fold Change) versus qRT-PCR log$_2$(2$^{\wedge-\Delta\Delta Ct}$) for 12 differentially expressed genes across three pairwise tissue comparisons: Stem/Root (red circles, n = 12), Leaf/Root (blue squares, n = 12), and Stem/Leaf (green triangles, n = 12). RNA-seq fold changes were calculated as log$_2$(FPKM_comparison / FPKM_reference), and qRT-PCR fold changes were calculated as log$_2$(2$^{\wedge-\Delta\Delta Ct}$) using the 2$^{\wedge-\Delta\Delta Ct}$ method with Root as the calibrator. The dashed line indicates the 1:1 reference. The solid line represents the linear regression fit (y = 0.802x- 0.366, R^2 = 0.925). Pearson correlation coefficient r = 0.962, p = 1.03 × 10^{-20}, n = 36. Two data points for ANS (anthocyanidin synthase) deviate from the 1:1 line due to extremely low RNA-seq read counts (FPKM < 0.05) in non-root tissues.
