# Supplementary material for: Integration of Transcriptomics and Metabolomics Reveals Organ-Specific Biosynthesis and Accumulation of Pharmacologically Active Flavonoids in Rhododendron yedoense var. poukhanense
Source: Plants (Basel). 2026 Aug 11;15(16):2441. doi: 10.3390/plants15162441 (PMC13516736; doi:10.3390/plants15162441)
Supplement: Supplementary file 1 [file plants-15-02441-s001.zip › Table S1.pdf]

**Table S1. Summary of RNA-seq quality control and read mapping statistics for nine cDNA libraries.**

| Sample | Raw Reads | Clean Reads | Clean Bases (Gb) | Error Rate (%) | Q20 (%) | Q30 (%) | GC Content (%) | Total Mapped (%) | Unique Mapped (%) | Multi Mapped (%) |
|---|---|---|---|---|---|---|---|---|---|---|
| Leaf-1 | 50,425,046 | 49,563,256 | 7.43 | 0.01 | 97.13 | 92.06 | 47.22 | 74.06 | 70.17 | 3.89 |
| Leaf-2 | 47,497,222 | 46,666,194 | 7 | 0.01 | 97.11 | 91.97 | 47.39 | 75.21 | 70.92 | 4.3 |
| Leaf-3 | 49,431,598 | 48,483,466 | 7.27 | 0.01 | 97.05 | 91.87 | 47.45 | 73.83 | 69.95 | 3.88 |
| Root-1 | 49,751,062 | 48,610,404 | 7.29 | 0.01 | 97.88 | 94.22 | 46.68 | 70.87 | 67.92 | 2.94 |
| Root-2 | 49,028,646 | 47,556,380 | 7.13 | 0.01 | 97.87 | 94.22 | 46.8 | 72.99 | 69.8 | 3.19 |
| Root-3 | 53,475,036 | 52,435,452 | 7.87 | 0.01 | 97.94 | 94.41 | 46.73 | 75.13 | 72.04 | 3.1 |
| Stem-1 | 47,839,374 | 46,892,392 | 7.03 | 0.01 | 97.77 | 93.83 | 47.01 | 62.88 | 60.27 | 2.61 |
| Stem-2 | 46,542,552 | 45,705,952 | 6.86 | 0.01 | 97.77 | 93.89 | 47.62 | 67.32 | 64.65 | 2.67 |
| Stem-3 | 54,681,168 | 53,242,576 | 7.99 | 0.01 | 97.84 | 94.07 | 47.82 | 64.27 | 61.5 | 2.77 |

**Note:** QC statistics were generated by fastp (v0.18.0). Mapping statistics were generated by HISAT2 (v2.2.1) alignment to the *R. simsii* reference genome (GCA_047301955.1). Q20 and Q30 indicate the percentage of bases with Phred quality scores ⩾ 20 and ⩾ 30, respectively.
