# Supplementary material for: Integration of Transcriptomics and Metabolomics Reveals Organ-Specific Biosynthesis and Accumulation of Pharmacologically Active Flavonoids in Rhododendron yedoense var. poukhanense
Source: Plants (Basel). 2026 Aug 11;15(16):2441. doi: 10.3390/plants15162441 (PMC13516736; doi:10.3390/plants15162441)
Supplement: Supplementary file 1 [file plants-15-02441-s001.zip › Table S2.pdf]

Table S2. All 2,182 metabolites detected in *R. yedoense* var. *poukhanense* root, stem and leaf tissues by widely targeted metabolomics (UPLC-MS/MS).

Metabolite ID	Compound Name	Class I	Class II	Q1 (m/z)	Q3 (m/z)	Molecular Weight (Da)
Lmhp013004	3-Oxolanosta-9(11),24-dien -26-oic acid (Coccinic Acid)	Terpenoids	Triterpene	455.352	315.2319	454.3447
Lasp002843	Daidzein rutinoside	Flavonoids	Isoflavones	563.1746	255.0664	562.1686
Lmhp009384	1-Linoleoylglycerol-2,3-di-O-glucoside	Lipids	Glycerol ester	679.39	263.24	678.3827
Smdp009834	Methylfarrerol	Flavonoids	Flavanones	315.12	181.05	314.1154
pmb2406	LysoPC 17:0*	Lipids	LPC	510.36	184.07	509.3481
Zmyn004449	9-Hydroxy-12-oxo-10(E),15 (Z)-octadecadienoic acid	Lipids	Free fatty acids	309.21	171.1	310.2144
Zmjp008503	5-Hydroxy-4',6,7-trimethox yflavone (Salvigenin)	Flavonoids	Flavones	329.1	296.07	328.0947

Metabolite ID	Compound Name	Class I	Class II	Q1 (m/z)	Q3 (m/z)	Molecular Weight (Da)
Lmnp002494	Nepetin-8-C-[Xylosyl-(1-2)]-glucoside	Flavonoids	Flavones	611.16	479.12	610.1534
Lmhp010757	LysoPE 20:2	Lipids	LPE	506.32	365.31	505.3168
Lafp002342	2,3-Dihydroxy-1-(4-hydroxy-3,5-dimethoxyphenyl)propan-1-one	Others	Ketone compounds	243.09	140.05	242.0785
Zbjn004445	Kaempferol-7-O-glucuronide	Flavonoids	Flavonols	461.07	285.04	462.0798
Zbnp004262	Diosmetin-8-C-(2''-O-rhamnosyl)glucoside	Flavonoids	Flavones	609.18	463.12	608.1741
Qmxp101615	Ent-3β-Acetoxyisopimar-15-8β-ol	Terpenoids	Ditepenoids	349.27	179.14	348.2664

Metabolite ID	Compound Name	Class I	Class II	Q1 (m/z)	Q3 (m/z)	Molecular Weight (Da)
Smpp012073	3-[[3-(4-Hydroxyphenyl)propenoyl]oxy]-2-hydroxyurs-12-ene-28-oic acid (Jacoumaric Acid)	Terpenoids	Triterpene	619.39	147.04	618.392
Labn003316	pyrocatechol-β-D-xylopyranosyl(1-6)-β-D-glucopyranoside	Phenolic acids	Phenolic acids	403.12	109.03	404.1319
Wcdn003654	Feruloyl Lactate	Organic acids	Organic acids	281.06	178.03	282.074
Hmln101664	Nortrachelogenin-4'-O-gentiobioside	Lignans and Coumarins	Lignans	697.24	535.18	698.2422
Lcfn121170	4,6-diethyl-3,6-dihydroxy-8-methyldodec-4-enoicacid	Lipids	Free fatty acids	299.22	281.21	300.2301
Zbqn003748	N-malonylleucine	Amino acids and	Amino acids and	216.09	130.09	217.095

Metabolite ID	Compound Name	Class I	Class II	Q1 (m/z)	Q3 (m/z)	Molecular Weight (Da)
		derivatives	derivatives			
MWS2070	Propyl 4-hydroxybenzoate	Phenolic acids	Phenolic acids	179.07	92.03	180.0786
mws1038	Pantetheine	Alkaloids	Alkaloids	277.12	146.08	278.13
Lmmp002995	Quercetin-7-O-(2''-malonyl)glucosyl-5-O-glucoside	Flavonoids	Flavonols	713.15	303.06	712.1487
Wcqp012549	2,3-Dihydroxyurs-12-en-28 -oic acid (Corosolic acid)	Terpenoids	Triterpene	473.3621	455.3553	472.3553
Zmjn004133	9S-Hydroperoxy-10E,12Z-o ctadecadienoic acid	Lipids	Free fatty acids	311.22	293.21	312.2301
Lmyp003371	3,5-Dihydroxytoluene	Others	Others	125.06	110.04	124.0524
MWS4471	N-Alpha-Acetyl-L-Aspara gine	Amino acids and derivatives	Amino acids and derivatives	173.06	58.03	174.0641

Metabolite ID	Compound Name	Class I	Class II	Q1 (m/z)	Q3 (m/z)	Molecular Weight (Da)
Zbcp002979	Petunidin-3-O-rutinoside	Flavonoids	Anthocyanidins	625.18	317.07	625.1763
mws1397	Epicatechin gallate	Flavonoids	Flavanols	441.08	169.02	442.09
mws2213	Cinnamic acid	Phenolic acids	Phenolic acids	147.05	103.06	148.0524
Lmhp010099	LysoPC 22:5	Lipids	LPC	570.36	184.07	569.3481
Zmyn004714	12-Hydroxyoctadec-9-Enoic Acid; Ricinoleic acid	Lipids	Free fatty acids	297.24	183.14	298.2508
Laxp007708	Medicarpin 3-O-(6'-malonylgluclside)	Flavonoids	Isoflavones	519.15	271.1	518.1424
pmb2789	13S-Hydroperoxy-6Z,9Z,11E-octadecatrienoic acid	Lipids	Free fatty acids	309.21	209.05	310.2144
Lmhn003799	Feruloylferuloyltartaric acid	Phenolic acids	Phenolic acids	501.1	307.05	502.1111

Metabolite ID	Compound Name	Class I	Class II	Q1 (m/z)	Q3 (m/z)	Molecular Weight (Da)
Lcyp000677	Prunetin-4'-O-glucoside(Prunitrin)*	Flavonoids	Isoflavones	447.1264	285.0769	446.1213
Smpp009685	3-O-Cafeoyltormentic acid	Terpenoids	Triterpene	651.38	163.03	650.3819
Labn003067	salicylate-6-O-β-D-glucopyranosylbenzoic acid	Phenolic acids	Phenolic acids	329.09	135.01	330.0951
Lmdn003756	Methyl caffeate	Phenolic acids	Phenolic acids	193.05	135.05	194.0579
MWSmce645	2-(Aminooxy)Acetic Acid	Alkaloids	Alkaloids	92.03	60.02	91.0269
Lmbn007891	Hydroxy ricinoleic acid	Lipids	Free fatty acids	313.24	183.14	314.2457
MWSHC20110	8-Methoxyapigenin*	Flavonoids	Flavones	301.07	286.05	300.0634
MWSmce329	Antiarol; 3,4,5-Trimethoxyphenol	Others	Others	185.08	125.06	184.0736
pmf0297	1-Eicosanol	Lipids	Free fatty acids	297.32	183.21	298.3236

Metabolite ID	Compound Name	Class I	Class II	Q1 (m/z)	Q3 (m/z)	Molecular Weight (Da)
Lmhn002926	p-Coumaroylmalic acid	Phenolic acids	Phenolic acids	279.05	163.04	280.0583
MWSslk224	Shanzhiside methyl ester	Terpenoids	Monoterpenoids	407.15	177.05	406.1475
Lmhn003074	Feruloylmalic acid	Phenolic acids	Phenolic acids	309.06	193.05	310.0689
HJN012	4,7,9,9'-Tetrahydroxy-3,3'-dimethoxy-8-O-4'-neolignan	Lignans and Coumarins	Lignans	377.16	329.14	378.1679
Lmzn001925	3,4'-Dihydroxy-3'-methoxy benzenepentanoic acid	Phenolic acids	Phenolic acids	239.09	195.1	240.0998
MWStz377	Isovitexin-2''-O-rhamnoside (2''-O-alpha-L-Rhamnopyranosyl-isovitexin)*	Flavonoids	Flavones	579.1744	433.1188	578.1636
Lmqn000213	Stachyose	Others	Saccharides	665.21	341.11	666.2219

Metabolite ID	Compound Name	Class I	Class II	Q1 (m/z)	Q3 (m/z)	Molecular Weight (Da)
Zahn005590	(7'S,8R,8'R)-3,5'-dimethoxy-3',4,9'-trihydroxy-7',9-epoxy-8,8'-lignan	Lignans and Coumarins	Lignans	359.15	329.14	360.1573
Zbpp002351	Isorhamnetin; 3'-Methoxy-3,4',5,7-Tetrahydroxyflavone	Flavonoids	Flavonols	317.06	153.02	316.0583
MWSHY0069	Hispidulin (5,7,4'-Trihydroxy-6-methoxyflavone)*	Flavonoids	Flavones	301.07	286.0486	300.0634
Zmpn003368	13S-Hydroxy-9Z,11E,15Z-octadecatrienoic acid	Lipids	Free fatty acids	293.21	195.14	294.2195
pmb0492	N',N'',N'''-p-Coumaroyl-cinnamoyl-caffeoyl	Alkaloids	Phenolamine	584.28	325	583.2682

Metabolite ID	Compound Name	Class I	Class II	Q1 (m/z)	Q3 (m/z)	Molecular Weight (Da)
	spermidine					
Zmjp004795	5,6,7-Trihydroxy-8-methoxyflavone*	Flavonoids	Flavones	301.07	286.05	300.0634
Wagp004812	Salvigenin 5-O-glucoside	Flavonoids	Flavones	491.15	329.1	490.1475
Lcsn000385	Glu-Asn	Amino acids and derivatives	Amino acids and derivatives	260.09	131.05	261.0961
zjmp120227	3-Acetoxy-11-oxoolean-12-en-28-oic acid (Lantanone)	Terpenoids	Triterpene	519.1464	315.0858	518.1424
pmf0085	9,19-Cyclolanost-24-en-3-ol (Cycloartenol)	Terpenoids	Triterpene	427.39	345.32	426.3862
Lmfn004235	Myricetin-3,7,3'-trimethyl ether	Flavonoids	Flavonols	359.08	161.02	360.0845

Metabolite ID	Compound Name	Class I	Class II	Q1 (m/z)	Q3 (m/z)	Molecular Weight (Da)
Zmhp003514	6,7,8-Tetrahydroxy-5-methoxyflavone*	Flavonoids	Flavones	301.07	286.05	300.0634
Hmgp001758	Patuletin 3-O-beta-gentiobioside	Flavonoids	Flavonols	657.17	333.06	656.1589
Smlp003276	Malvidin-3-O-(6''-O-malonyl)glucoside	Flavonoids	Anthocyanidins	579.13	331.08	579.1344
Lmyn004187	Flavogallonic Acid Dilactone	Tannins	Tannin	453.01	301	454.0172
MWS20633g	O-phosphate-L-tyrosine	Amino acids and derivatives	Amino acids and derivatives	262.05	216.04	261.0402
pmp000995	Maohuoside B	Flavonoids	Flavonols	839.2968	693.2378	838.2895
HJN084	Laricitrin-3-O-(6''-acetyl)gl	Flavonoids	Flavonols	535.11	331.05	536.1166

Metabolite ID	Compound Name	Class I	Class II	Q1 (m/z)	Q3 (m/z)	Molecular Weight (Da)
	ucoside					
Lmrn002746	2-Hydroxy-4-methylpentanoic acid	Organic acids	Organic acids	131.07	85.07	132.0786
Lskp211308	Grayanotoxin XV	Terpenoids	Ditepenoids	335.22	299.2	334.2144
Walp006571	Arteminorin B	Lignans and Coumarins	Coumarins	413.09	179.03	412.0794
Lcgn000236	Dihydrodehydrodiconiferyl alcohol-4,9-di-O-β-D-glucopyranoside	Lignans and Coumarins	Lignans	683.26	503.19	684.2629
Zbqn004177	N-(sulfonyl)phenylalanine	Amino acids and derivatives	Amino acids and derivatives	244.03	164.07	245.0358
Labn004329	6'-O-Galloylsalicin	Phenolic acids	Phenolic acids	437.11	169.01	438.1162

Metabolite ID	Compound Name	Class I	Class II	Q1 (m/z)	Q3 (m/z)	Molecular Weight (Da)
MWSmce668	ATP; Adenosine 5'-Triphosphate	Nucleotides and derivatives	Nucleotides and derivatives	505.99	158.93	506.9957
Lmmp002796	Gossypetin-3-O-rutinoside	Flavonoids	Flavonols	627.16	319.05	626.1483
mws0361	Palmitoleic Acid	Lipids	Free fatty acids	253.22	235.21	254.2246
Lmcp101905	Martynoside	Phenolic acids	Phenolic acids	653.2445	177.0532	652.2367
Hmsn003897	Idescarpin	Phenolic acids	Phenolic acids	439.13	111.04	440.1319
Lmhp008513	2-α-Linolenoyl-glycerol-1,3-di-O-glucoside	Lipids	Glycerol ester	677.37	261.22	676.367
Lmbn005662	9(10)-EpOME;(9R,10S)-(12Z)-9,10-Epoxyoctadecenoic acid	Lipids	Free fatty acids	295.23	171.1	296.2351
Latp003004	2-Methyl-5-acetonyl-7-hyd	Others	Chromone	395.13	233.08	394.1258

Metabolite ID	Compound Name	Class I	Class II	Q1 (m/z)	Q3 (m/z)	Molecular Weight (Da)
	roxychromone glucoside					
Lcsn006335	2-Hydroxymyristic acid	Lipids	Free fatty acids	243.2	197.19	244.2038
MWSHY0190	Diosmetin-7-O-rutinoside (Diosmin)	Flavonoids	Flavones	609.18	301.08	608.1741
Lmhp009773	1-α-Linolenoyl-glycerol-3-O-glucoside*	Lipids	Glycerol ester	515.32	261.22	514.3142
Lmdn001925	Olivil-4'-O-glucoside	Lignans and Coumarins	Lignans	537.2	375.15	538.205
Lssp210167	2α,5α,14β-Triacetoxy-10β-O-(Β-D glucopyranosy)taxa-4(20),11-diene	Others	Others	625.32	463.27	624.3146
zjbp110804	6,7-dimethoxy-2-[2-(4'-hyd	Others	Chromone	357.13	307.1	356.126

Metabolite ID	Compound Name	Class I	Class II	Q1 (m/z)	Q3 (m/z)	Molecular Weight (Da)
	roxy-3'-methoxyphenyl)ethyl]chromone					
mws2368	Tyrosol	Others	Alcohol compounds	137.06	119.05	138.0681
Zbsn009679	Velutin (5,4'-dihydroxy-7,3'-dimethoxyflavone)	Flavonoids	Flavones	313.07	283.03	314.079
Wcsn000472	Laminaran	Others	Saccharides	503.16	113.02	504.169
mws1068	Kaempferol (3,5,7,4'-Tetrahydroxyflavone)	Flavonoids	Flavonols	285.04	151	286.0477
pmn001691	9,12,13-Trihydroxy-10,15-octadecadienoic acid	Lipids	Free fatty acids	327.22	291.2	328.225

Metabolite ID	Compound Name	Class I	Class II	Q1 (m/z)	Q3 (m/z)	Molecular Weight (Da)
Lczn000058	6-O-Galloyl-1-O-vanilloyl-β-D-glucose	Phenolic acids	Phenolic acids	481.1	313.06	482.106
Lhyn080508	Dehydrovomifoliol	Terpenoids	Monoterpenoids	221.12	206.09	222.1256
Lasp001752	Daidzein 4',7-diglucoside	Flavonoids	Isoflavones	579.1708	255.0675	578.1636
Lmsn010896	23-Formyl-2,3,19-trihydroxyurs-12-en-28-oic acid (Pinfaensic acid)	Terpenoids	Triterpene	501.32	421.31	502.3294
Lmpp005842	6-Methoxyquercetin-3-O-rhamnoside	Flavonoids	Flavonols	479.11	333.07	478.1111
pmb2591	N-Acetyl-L-Tryptophan	Amino acids and derivatives	Amino acids and derivatives	245.09	203.08	246.1004
Lahp002664	Kaempferol triglucoside*	Flavonoids	Flavonols	773.2127	449.1094	772.2062

Metabolite ID	Compound Name	Class I	Class II	Q1 (m/z)	Q3 (m/z)	Molecular Weight (Da)
Lmfp001509	1-O-Galloyl-rhamnose	Phenolic acids	Phenolic acids	317.09	153.02	316.0794
Qmyp081607	10-Deoxygeniposidic acid	Terpenoids	Monoterpenoids	359.1332	121.1004	358.1264
Lazn006616	Buddlenol F	Lignans and Coumarins	Lignans	613.23	225.08	614.2363
Lmbn005923	Crepenynic acid	Lipids	Free fatty acids	277.22	59.01	278.2246
Zmhn002545	4-O-(6'-O-Glucosylcaffeoyl)-3,4-dihydroxybenzyl alcohol	Phenolic acids	Phenolic acids	463.12	323.08	464.1319
Zmyn004676	17-Hydroxylinolenic acid	Lipids	Free fatty acids	293.21	113.1	294.2195
Lskp211468	2,3,23-Trihydroxyolean-12,18(19)-dien-28-oic acid-glucoside	Terpenoids	Triterpene Saponin	649.3942	451.3217	648.3873
mws1417	Indole-3-carboxylic acid	Alkaloids	Plumerane	160.04	116.05	161.0477

Metabolite ID	Compound Name	Class I	Class II	Q1 (m/z)	Q3 (m/z)	Molecular Weight (Da)
Lmyp003139	Quercetin-3-O-(6''-O-galloyl)galactoside	Flavonoids	Flavonols	617.11	153.02	616.1064
Wbwp006397	(3r,4r)-3-(2h-1,3-benzodioxol-5-ylmethyl)-4-[(4-hydroxy-3-methoxyphenyl)methyl]oxolan-2-one	Lignans and Coumarins	Lignans	357.13	307.1	356.126
Lmqn005404	4-Hydroxybenzoyl-1-O-(6''-O-galloyl)glucoside	Phenolic acids	Phenolic acids	451.09	331.07	452.0955
Laxp004658	Phaseic Acid	Terpenoids	Sesquiterpenoids	279.12	139.08	280.1311
pme3382	N-Acetyl-L-threonine	Amino acids and derivatives	Amino acids and derivatives	160.06	98.02	161.0688
Lcsn003955	Maesopsin*	Flavonoids	Aurones	287.05	125.03	288.0634

Metabolite ID	Compound Name	Class I	Class II	Q1 (m/z)	Q3 (m/z)	Molecular Weight (Da)
Rfmb087	9-Hydroxy-13-oxo-10-octadecenoic Acid	Lipids	Free fatty acids	311.22	293.21	312.2301
Ymjm000116	6-O-Acetylarbutin	Others	Others	313.09	151.04	314.1002
Lskp211434	3,23-Dihydroxy-24-oxo-olean-12-en-28-oic acid-28-O-glucoside	Terpenoids	Triterpene Saponin	665.39	503.34	664.3823
Lmrn003201	Indole-3-lactic acid	Alkaloids	Plumerane	204.07	158.06	205.0739
Walp006302	4,4'-Biisofraxidin	Lignans and Coumarins	Coumarins	443.1	387.11	442.09
Lmhp008742	LysoPC 20:5	Lipids	LPC	542.32	184.07	541.3168
NK10246260	2,4-Dihydroxyquinoline	Alkaloids	Quinoline alkaloids	162.06	116.05	161.0477
pmp000781	p-Coumaroyleuscaphic acid*	Terpenoids	Triterpene	619.4	437.34	618.392

Metabolite ID	Compound Name	Class I	Class II	Q1 (m/z)	Q3 (m/z)	Molecular Weight (Da)
Lmxn006423	2(3H)-Benzothiazolone	Alkaloids	Alkaloids	150	122.01	151.0092
Wcsn010375	Methyl (4E,8E,11E)-5,9,13-trimethyltetradeca-4,8,11-trienoate	Others	Others	277.22	59.01	278.2246
Lmnn002886	1'-O-(3,4-Dihydroxyphenethyl)-O-caffeoyl-glucoside	Phenolic acids	Phenolic acids	477.14	179.03	478.1475
Lmdp002901	2,6-Dihydroxy-4-isopropylphenyl-1-O-β-D-glucoside	Others	Others	331.14	151.07	330.1315
MWSmce486	Manninotriose	Others	Saccharides	503.16	383.12	504.169
Wcsn010496	6,10,14-Trimethylpentadeca-5,9-Diene-2,13-Dione	Others	Others	277.22	233.23	278.2246
Lmtp004915	3,5-Dihydroxy-3',4'-diaceto	Others	Stilbene	491.15	329.1	490.1475

Metabolite ID	Compound Name	Class I	Class II	Q1 (m/z)	Q3 (m/z)	Molecular Weight (Da)
	xylstilbene-3-O-glucoside					
Cmsn004064	Decumbic acid	Organic acids	Organic acids	183.07	139.08	184.0736
Lacp004368	Blumenol B malonyl Glucoside*	Terpenoids	Sesquiterpenoids	475.21	209.15	474.2096
Zbyp002402	methylarbutin*	Phenolic acids	Phenolic acids	287.1122	107.049	286.1044
Hamp011640	Hexadecanamide	Alkaloids	Alkaloids	256.26	102.09	255.2562
Wchn003509	Syringoyl-D-Glucose	Phenolic acids	Phenolic acids	359.1	239.06	360.1056
Lbhp011919	Esculin 6'-Malonate	Lignans and Coumarins	Coumarins	427.0849	179.0327	426.0798
Lmdn005433	Gossypetin-8-O-β-D-glucuronide	Flavonoids	Flavonols	493.06	317.03	494.0697
mad2085	Caffeoyl-p-coumaroyltartaric acid	Phenolic acids	Phenolic acids	457.08	295.04	458.0849

Metabolite ID	Compound Name	Class I	Class II	Q1 (m/z)	Q3 (m/z)	Molecular Weight (Da)
mws0371	Cis-4,7,10,13,16,19-Docosa hexaenoic Acid	Lipids	Free fatty acids	327.23	283.24	328.2402
mws1422	Epiafzelechin	Flavonoids	Flavanols	275.09	107.05	274.0841
Waqkp05282	1-g-linolenoyl-glycerol	Lipids	Glycerol ester	353.27	261.22	352.2614
Zbdn007423	6,7-Dimethoxy-4-chroman one	Flavonoids	Other Flavonoids	207.07	192.04	208.0736
Lmsp102504	Chrysophanol-8-O-β-D-gal actoside	Quinones	Anthraquinone	417.1185	255.0607	416.1107
pme2773	L-Cystathionine	Amino acids and derivatives	Amino acids and derivatives	223.07	134.03	222.0674
Lmtn004049	Abscisic acid	Terpenoids	Sesquiterpenoids	263.13	204.12	264.1362
Smnp001524	8-C-glucosyl-noreugenin	Others	Chromone	355.1	235.06	354.0951

Metabolite ID	Compound Name	Class I	Class II	Q1 (m/z)	Q3 (m/z)	Molecular Weight (Da)
Lmbn005172	2,6-Dimethoxybenzaldehyde	Others	Aldehyde compounds	165.06	119.05	166.063
pmb2804	13S-Hydroperoxy-9Z,11E-octadecadienoic acid	Lipids	Free fatty acids	311.22	171.14	312.2301
Lssp210321	Pierisformoside B	Terpenoids	Ditepenoids	483.29	267.21	482.288
Lmhp009526	2-α-Linolenoyl-glycerol-1-O-glucoside*	Lipids	Glycerol ester	515.32	261.22	514.3142
Wayp001841	6-Hydroxypseudooxynicotine O-glucoside	Alkaloids	Pyridine alkaloids	357.1642	195.1131	356.1578
Rahp002335	Ginnalin C (2-O-Galloyl-1,5-Anhydro-D-Glucitol)	Tannins	Tannin	317.0876	153.0198	316.0794

Metabolite ID	Compound Name	Class I	Class II	Q1 (m/z)	Q3 (m/z)	Molecular Weight (Da)
mws1174	3-O-Acetylpinobanksin	Flavonoids	Flavanonols	313.07	253.05	314.079
Hmmn002781	Eriodictyol-7-O-rutinoside-5-O-(6''-O-glucosyl)glucuronide	Flavonoids	Flavanones	933.27	479.11	934.259
mws0027	Syringic acid	Phenolic acids	Phenolic acids	197.05	123.01	198.0528
Labn003405	Xylosyl tachioside	Phenolic acids	Phenolic acids	433.13	121.03	434.1424
Wahp006114	Tri-p-coumaroyl Spermidine	Alkaloids	Phenolamine	584.28	438.24	583.2677
Lmhp009890	LysoPC 20:3	Lipids	LPC	546.36	184.07	545.3481
MWS201424	Ile-Asn	Amino acids and derivatives	Amino acids and derivatives	246.15	86.1	245.1376
Cmsn002813	Methyl	Phenolic acids	Phenolic acids	317.07	165.02	318.074

Metabolite ID	Compound Name	Class I	Class II	Q1 (m/z)	Q3 (m/z)	Molecular Weight (Da)
	2-O-(4-hydroxybenzoyl)-2,4,6-trihydroxyphenylacetate					
Lmnp002413	Luteolin-6-C-glucoside-7-O-rhamnoside	Flavonoids	Flavones	595.17	449.11	594.1585
MWS0155	Adenylocuccinic Acid	Nucleotides and derivatives	Nucleotides and derivatives	462.07	96.97	463.074
Lmxn003152	8-Epiloganin	Terpenoids	Terpene	389.14	167.07	390.1526
Wmqn000820	Dehydrololiolide	Terpenoids	Monoterpenoids	193.09	163.04	194.0943
Zahp001902	Delphinidin-3-O-sambubioside	Flavonoids	Anthocyanidins	597.15	303.05	597.1456
HJN078	Pinocembrin-7-O-(2''-O-ara	Flavonoids	Flavanones	549.16	255.06	550.1686

Metabolite ID	Compound Name	Class I	Class II	Q1 (m/z)	Q3 (m/z)	Molecular Weight (Da)
	binosyl)glucoside					
pme1201	Phloretin	Flavonoids	Chalcones	273.08	167.03	274.0841
Hmpn004989	5-Hydroxylbisabolon-9-one	Terpenoids	Sesquiterpenoids	249.15	205.16	250.1569
Lmrn001951	2-Hydroxy-3-(4-Hydroxyphenyl)Propanoic Acid	Phenolic acids	Phenolic acids	181.05	135.04	182.0579
Xmyp005599	Kaempferol-3-O-galactoside-4'-O-glucoside	Flavonoids	Flavonols	611.16	287.06	610.1534
Lafp004533	(6R,9R)-3-Oxo-α-ionol-β-D-malonyl-glucoside	Terpenoids	Sesquiterpenoids	457.21	209.15	456.199
Lhqp101805	Skimmin (7-Hydroxycoumarin-7-O-	Lignans and Coumarins	Coumarins	325.09	163.04	324.0845

Metabolite ID	Compound Name	Class I	Class II	Q1 (m/z)	Q3 (m/z)	Molecular Weight (Da)
	glucoside)					
mws0744	Dihydromyricetin (Ampelopsin)	Flavonoids	Flavanonols	319.05	193.01	320.0532
Lacp004926	3,8-Dihydroxy-1-methoxy-9,10-anthraquinone glucoside	Quinones	Anthraquinone	433.1136	271.0594	432.1051
pme0085	Rosmarinic acid	Phenolic acids	Phenolic acids	359.08	161.02	360.0845
Zbqn003921	N-carboxy-N-(2-oxo-2-phenylethyl)-L-alanine	Amino acids and derivatives	Amino acids and derivatives	250.07	88.04	251.0794
Lmmn003320	5'-Methoxyisolariciresinol-9'-O-xyloside	Lignans and Coumarins	Lignans	521.2	371.15	522.2101
Lmhp002501	N-Feruloylhydroxyputresc	Alkaloids	Phenolamine	281.15	177.05	280.1423

Metabolite ID	Compound Name	Class I	Class II	Q1 (m/z)	Q3 (m/z)	Molecular Weight (Da)
	ine					
Zbjn005361	Diosmetin-7-O-(6''-malonyl)glucoside	Flavonoids	Flavones	549.1247	301.0721	548.1166
Cmsp003083	Dehydrodiconiferyl alcohol-4-O-glucoside*	Lignans and Coumarins	Lignans	521.2	131.05	520.1945
Lcsp003242	Dihydrozeatin ribonucleoside	Alkaloids	Alkaloids	354.1765	222.1344	353.1699
MWSHY0092	Eriodictyol-7-O-Rutinoside (Eriocitrin)*	Flavonoids	Flavanones	597.18	289.07	596.1741
Wayn005953	3,3'-Di-O-methylellagic acid 4'-beta-O-xylopyranoside	Tannins	Tannin	461.07	329.03	462.0798

Metabolite ID	Compound Name	Class I	Class II	Q1 (m/z)	Q3 (m/z)	Molecular Weight (Da)
Cmxp004324	Okanin-4'-O-(6''-O-malonyl)glucoside	Flavonoids	Chalcones	537.12	289.07	536.1166
MWSHC2017	Myricetin	Flavonoids	Flavonols	319.0464	153.0196	318.0376
pme2266	Biotin	Others	Vitamin	245.1	227.08	244.0882
Walp007214	2-Aminohexadecane-1,5,6-triol	Lipids	Sphingolipids	290.27	88.08	289.2617
WaYp007548	2-Aminotetradecan-1-ol	Lipids	Sphingolipids	230.25	62.06	229.2406
Zmjp012507	3,12,23,25-tetrahydroxy-20S,24S-epoxydammarane-3-O-xylosylglucoside	Terpenoids	Triterpene Saponin	787.5	625.45	786.4766
Lmhn005088	7,3'-Di-O-gallyoltricetiflavan	Flavonoids	Flavanols	593.09	289.07	594.101

Metabolite ID	Compound Name	Class I	Class II	Q1 (m/z)	Q3 (m/z)	Molecular Weight (Da)
Lahp003147	N,N-cinnamoylbutanediamine*	Alkaloids	Phenolamine	219.15	131.05	218.1419
Lhlp120627	Carvylacetate	Terpenoids	Monoterpenoids	195.14	67.05	194.1307
Lmhp008273	LysoPE 15:1(2n isomer)*	Lipids	LPE	438.26	297.24	437.2542
Zbqp000947	Lys-Pro-Ile	Amino acids and derivatives	Amino acids and derivatives	357.25	132.11	356.2424
MWSHY0040	Oroxin A	Flavonoids	Flavones	433.11	271.06	432.1056
Zahp012577	Octadecadienamide	Alkaloids	Alkaloids	280.26	81.07	279.2562
Cmsn002067	7S,8R-threo-3',9,9'-trihydroxy-3-methoxy-4',7-epoxy-neolignan-4-O-rhamnoside	Lignans and Coumarins	Lignans	491.19	315.12	492.1995
Lajp005361	5-hydroxy-6,7,8,3',4'-penta	Flavonoids	Flavones	551.1741	389.1245	550.1686

Metabolite ID	Compound Name	Class I	Class II	Q1 (m/z)	Q3 (m/z)	Molecular Weight (Da)
	methoxyflavone glucoside*					
Zbsp003407	Apigenin-6,8-di-C-glucoside (Vicenin-2)	Flavonoids	Flavones	595.17	427.1	594.1585
Smzp002615	11-O-Galloylbergenin	Lignans and Coumarins	Coumarins	481.09	153.01	480.0904
pme0183	Isoguanine	Nucleotides and derivatives	Nucleotides and derivatives	152.06	135.03	151.0494
Lmhn002895	Feruloylcaffeoyltartaric acid	Phenolic acids	Phenolic acids	487.09	193.06	488.0955
Wbtn007469	Protogenkwanone*	Flavonoids	Other Flavonoids	299.06	284.03	300.0634
pme2060	N6-Isopentenyladenine	Nucleotides and derivatives	Nucleotides and derivatives	204.12	136.06	203.1171
WaYn005157	Gentisic acid	Phenolic acids	Phenolic acids	491.12	315.07	492.1268

Metabolite ID	Compound Name	Class I	Class II	Q1 (m/z)	Q3 (m/z)	Molecular Weight (Da)
	2-O-(6''-O-Feruloyl)Glucoside*					
pme1313	N'-Formylkynurenine	Amino acids and derivatives	Amino acids and derivatives	237.09	192.07	236.0797
Lmsp003852	1-O-p-Coumaroyl-4,6-(S)-HHDP-β-D-glucose	Phenolic acids	Phenolic acids	629.12	163.04	628.1064
Safn000337	heptafluorobutanoic acid	Others	Others	273.01	169	213.9865
Zbjn003262	3,4-O-di-Caffeoyl-1-O-glucoside Quinic Acid	Phenolic acids	Phenolic acids	677.17	191.06	678.1802
Cmxp004977	Isookanin	Flavonoids	Flavanones	289.07	153.02	288.0634
Zbjp006152	1-(3,4-dihydroxyphenyl)-5-hydroxy-7-(4-hydroxy-3-m	Others	Ketone compounds	359.1477	163.0384	358.1416

Metabolite ID	Compound Name	Class I	Class II	Q1 (m/z)	Q3 (m/z)	Molecular Weight (Da)
	ethoxyphenyl)hept-1-en-3-one					
Lmsn010222	1,2,3-Trihydroxy-19-oxo-18,19-seco-urs-11,13(18)-dien-28-oic acid	Terpenoids	Triterpene	503.34	441.34	504.3451
Lmhp008440	LysoPE 15:1*	Lipids	LPE	438.26	297.24	437.2542
Hmqn003268	Dihydrotricetin	Flavonoids	Flavanones	303.05	151.03	304.0583
Lmbn001288	2-Hydroxy-2-methyl-3-oxo butanoic acid	Organic acids	Organic acids	131.03	87.04	132.0423
pmp000414	Puerarin-4'-O-glucoside	Flavonoids	Isoflavones	579.1708	297.0758	578.1636
Zmhn002032	4-O-(6'-O-Glucosylcaffeoyl glucosyl)-4-hydroxybenzyl	Phenolic acids	Phenolic acids	609.18	447.13	610.1898

Metabolite ID	Compound Name	Class I	Class II	Q1 (m/z)	Q3 (m/z)	Molecular Weight (Da)
	alcohol					
MWSprf147	Glucovanillin	Others	Aldehyde compounds	315.11	153.16	314.1002
pmb0142	Caffeic aldehyde	Others	Aldehyde compounds	165.05	95.05	164.0473
Lmbn004240	9,10-Dihydroxy-12,13-epoxyoctadecanoic acid	Lipids	Free fatty acids	329.23	199.13	330.2406
Lmjp102209	Agrimonolide-6-O-glucoside	Lignans and Coumarins	Coumarins	477.18	315.12	476.1682
MWStz282	3-Hydroxy-3-acetonyloxindole	Alkaloids	Plumerane	206.0813	118.0654	205.0739
MWStz430	Apigenin 7,4'-diglucoside	Flavonoids	Flavones	595.1624	271.0638	594.1585

Metabolite ID	Compound Name	Class I	Class II	Q1 (m/z)	Q3 (m/z)	Molecular Weight (Da)
Safn003220	Sachaloside VII	Others	Others	409.21	349.19	350.1941
Lmap001606	Helicidol*	Phenolic acids	Phenolic acids	287.11	107.05	286.1053
Wayn000884	Citric acid glucoside	Organic acids	Organic acids	353.07	111	354.0798
Hmcp001598	Isorhamnetin-3-O-(2''-O-xylosyl)glucoside	Flavonoids	Flavonols	611.16	317.06	610.1528
Zbjn002472	Caffeoylquinic acid-glucose-rhamnose-caffeoyl	Phenolic acids	Phenolic acids	823.24	191.06	824.2375
Lmzn001582	5'-Glucosyloxyjasmanic acid*	Organic acids	Organic acids	387.17	207.1	388.1733
Zmtn001624	N-Acetylisatin	Alkaloids	Plumerane	188.04	144.05	189.0426
Wdtp003420	1-O-Feruloyl-beta-D-gluco	Phenolic acids	Phenolic acids	357.12	177.05	356.1107

Metabolite ID	Compound Name	Class I	Class II	Q1 (m/z)	Q3 (m/z)	Molecular Weight (Da)
	se					
mws0242	Suberic Acid	Lipids	Free fatty acids	173.08	111.08	174.0892
Lmjp004404	Quercetagetin-3-Methyl Ether	Flavonoids	Flavonols	333.06	318.04	332.0532
Zbnn005489	Limocitrin-7-O-(6''-Acetyl) glucoside	Flavonoids	Flavonols	549.12	345.06	550.1323
Layp004192	3,4,5-Trimethoxyphenol-1-O-β-D-malonylglucoside	Phenolic acids	Phenolic acids	433.1317	185.0808	432.1268
MWStz464	25S-Inokosterone	Others	Others	481.3165	445.2978	480.3087
Lmmn003020	Lyoniresinol	Lignans and Coumarins	Lignans	419.17	404.15	420.1784
Wafn003070	Hydroxyferulic acid glucoside	Phenolic acids	Phenolic acids	371.1	209.05	372.1056

Metabolite ID	Compound Name	Class I	Class II	Q1 (m/z)	Q3 (m/z)	Molecular Weight (Da)
Lmyn002403	Mandelic acid-β-glucoside	Phenolic acids	Phenolic acids	313.09	101.02	314.1002
MWSmce690	Erythorbic Acid; Isoascorbic Acid	Others	Vitamin	175.02	59.01	176.0321
pma0791	Naringenin-7-O-(6''-malonyl)glucoside	Flavonoids	Flavanones	521.13	273.08	520.1217
Saln004708	1-O-Glucopyranosylamplexine	Terpenoids	Monoterpenoids	361.15	199.1	362.1577
Zbsp010780	Methyl [6]-Gingerdiol 5-acetate	Phenolic acids	Phenolic acids	353.2314	151.0757	352.225
Zbqp005508	N-(11-alkanoyl)nornicotine	Alkaloids	Pyridine alkaloids	347.2314	149.1076	346.2256
MWS0618	Pantothenol	Others	Vitamin	204.12	71.05	205.1314
Lssp210351	Pieroside C	Others	Others	479.26	299.2	478.2567

Metabolite ID	Compound Name	Class I	Class II	Q1 (m/z)	Q3 (m/z)	Molecular Weight (Da)
Zaxn005652	Pisiferic acid	Terpenoids	Ditepenoids	315.2	315.2	316.2038
Wayn002532	Gallic acid-1-O-xyloside	Phenolic acids	Phenolic acids	301.06	168.01	302.0638
Lsj214142	9-hydroxy-2,3-dimethoxy-1,4-anthraquinone	Quinones	Anthraquinone	285.0756	239.0698	284.0685
pmb2228	LysoPC 19:0	Lipids	LPC	538.39	184.07	537.3794
Lmhp009368	LysoPC 22:6	Lipids	LPC	568.34	184.07	567.3325
mws2101	Deoxyrhapontin	Others	Stilbene	405.15	243.1	404.1471
Lmbn004790	Methyl 3-(3-hydroxy-4-methoxyphenyl)propanoate	Phenolic acids	Phenolic acids	209.08	179.03	210.0892
mws0046	Naringenin-7-O-Neohesperidoside(Naringin)	Flavonoids	Flavanones	579.17	271.06	580.1792

Metabolite ID	Compound Name	Class I	Class II	Q1 (m/z)	Q3 (m/z)	Molecular Weight (Da)
Wccp011838	Octadec-8-enamide*	Alkaloids	Alkaloids	282.282	69.072	281.2719
Lmwp102712	5Z,8Z-tetradecadienoic acid	Lipids	Free fatty acids	225.19	67.06	224.1776
Jmgn005927	2-hydroxynaringenin	Flavonoids	Flavanones	287.06	125.03	288.0634
HJN010	Ligraminol B	Lignans and Coumarins	Lignans	597.22	387.14	598.2262
pme0422	Isoferulic Acid*	Phenolic acids	Phenolic acids	193.05	134.04	194.0579
Wbsn001677	Rhamnosylfucose	Terpenoids	Terpene	309.12	237.06	310.1264
Wchn004015	3-Hydroxy-1-(4'-hydroxy-3 '-methoxyphenyl)-propan-1-one	Others	Others	195.07	150.03	196.0736
mws1589	D-Panose*	Others	Saccharides	503.16	179.06	504.169
MWS201401	Ala-Asn	Amino acids and	Amino acids and	204.1	133.06	203.0906

Metabolite ID	Compound Name	Class I	Class II	Q1 (m/z)	Q3 (m/z)	Molecular Weight (Da)
		derivatives	derivatives			
Zmcp102703	saikolignanoside D*	Lignans and Coumarins	Lignans	521.2	131.05	520.1945
pmp000437	D-Friedoolean-14-ene-3,28-diol (Myricadoil)	Terpenoids	Triterpene	443.39	425.37	442.3811
Wbkp004181	1-(2-hydroxyethyl)-4-methoxy-9H-pyrido[3,4-b]indol-8-ol	Alkaloids	Alkaloids	259.1075	241.0971	258.1004
Zbqp002079	N-acetyl-nornicotine	Alkaloids	Pyridine alkaloids	191.1172	149.1063	190.1106
Lamp006191	Afrormosin 7-O-(6''-Malonylglucoside)*	Flavonoids	Isoflavones	547.1443	299.0943	546.1368
Lmhp008337	LysoPE 14:0(2n isomer)*	Lipids	LPE	426.26	285.24	425.2542

Metabolite ID	Compound Name	Class I	Class II	Q1 (m/z)	Q3 (m/z)	Molecular Weight (Da)
pme2563	γ-Glu-Cys	Amino acids and derivatives	Amino acids and derivatives	249.06	128.04	250.0623
Hmcp002399	Limocitrin-3-O-glucoside	Flavonoids	Flavonols	509.13	347.07	508.1217
MWS80006	9-Hydroperoxy-9Z,11E-Octadecadienoic Acid	Lipids	Free fatty acids	311.22	293.21	312.2301
pmb0883	LysoPE 18:0	Lipids	LPE	482.32	341.31	481.3168
Lmzp010525	3-O-Caffeoyl-9-hydroxylup-20(30)-en-7-one (Querspicatin A)*	Terpenoids	Triterpene	619.4	437.34	618.392
Cmsp004305	(7S,8R-Threo-3',4,9'-trihydroxy-3-methoxy-4',7-epoxy-neolignan-9-O-rhamnoside	Lignans and Coumarins	Lignans	493.21	163.08	492.1995

Metabolite ID	Compound Name	Class I	Class II	Q1 (m/z)	Q3 (m/z)	Molecular Weight (Da)
Hmqn003054	9,10,11-Trihydroxy-12-octa decenoic acid	Lipids	Free fatty acids	329.23	311.22	330.2406
Saln002505	6'-O-β-glucopyranosyl secologanol	Terpenoids	Monoterpenoids	537.18	213.08	538.1898
pme3227	Vitexin-2''-O-rhamnoside*	Flavonoids	Flavones	579.17	433.11	578.1636
Lmhp002001	L-Valyl-L-Phenylalanine	Amino acids and derivatives	Amino acids and derivatives	265.15	72.08	264.1474
Lmqp001551	cyanidin 3,3',5-tri-O-glucoside	Flavonoids	Anthocyanidins	773.21	449.11	773.2135
Wbjp005910	peganoneI	Quinones	Anthraquinone	285.0746	285.0746	284.0685
pmn001384	Isochlorogenic acid C*	Phenolic acids	Phenolic acids	515.12	353.09	516.1268
Lmbn001754	3-Isopropylmalic Acid	Organic acids	Organic acids	175.06	115.04	176.0685

Metabolite ID	Compound Name	Class I	Class II	Q1 (m/z)	Q3 (m/z)	Molecular Weight (Da)
Zbpn004791	Phloretin-2'-O-(6''-O-glucoside)glucoside	Flavonoids	Chalcones	597.18	273.08	598.1898
Zmjp004455	Trollioside	Phenolic acids	Phenolic acids	399.17	237.11	398.1577
Lhfp112906	Isosclerone;(4S)-4,8-Dihydroxy-3,4-Dihydronaphthalene-1(2H)-One	Quinones	Quinones	179.0694	115.0536	178.063
Wdbn005328	3,3'-Bis(3,4-dihydro-4-hydroxy-6,8-dimethoxy-2H-1-benzopyran)	Lignans and Coumarins	Lignans	417.16	181.05	418.1628
Smnp012194	2α-hydroxy-3β-trans-p-hydroxycinnamoyloxy oleanolic acid*	Terpenoids	Triterpene	619.39	437.34	618.392
mws2627	Tamarixetin	Flavonoids	Flavonols	317.07	285.04	316.0583

Metabolite ID	Compound Name	Class I	Class II	Q1 (m/z)	Q3 (m/z)	Molecular Weight (Da)
	(3,3',5,7-Tetrahydroxy-4'-Methoxyflavone)					
Wayn007352	9-Oxodemethylmyricanane B	Others	Others	339.12	324.1	340.1311
Hmln008119	Robinlin	Terpenoids	Monoterpenoids	197.12	153.12	198.1256
mws0103	Indole-3-carboxaldehyde	Alkaloids	Plumerane	146.06	91.05	145.0528
Labp001546	Anabasine	Alkaloids	Pyridine alkaloids	163.1244	118.0651	162.1157
Lamp007163	4'-Demethyleucomin glucoside*	Flavonoids	Other Flavonoids	447.13	285.07	446.1213
Lajp005489	5-hydroxy-6,7,3',4',5'-pentamethoxyflavone glucoside*	Flavonoids	Flavones	551.1761	389.1289	550.1686
Waptn02053	5-{[2-O-(beta-d-apiofurano	Phenolic acids	Phenolic acids	447.11	152.01	448.1217

Metabolite ID	Compound Name	Class I	Class II	Q1 (m/z)	Q3 (m/z)	Molecular Weight (Da)
	syl)-beta-d-glucopyranosyl]oxy}-2-hydroxybenzoic acid*					
Lcfn121256	Leucasperoside B	Others	Others	625.32	301.22	626.3302
pmb0808	Esculetin-6-O-quinic acid	Lignans and Coumarins	Coumarins	353.1	179	352.0794
MWStz612	Neoeriocitrin*	Flavonoids	Flavones	597.1825	289.0745	596.1741
mws0704	O-Phosphorylethanolamine	Alkaloids	Alkaloids	140.01	78.96	141.0191
Zahp003364	myricetin-3-O-β-D-xylopyranosyl-(1→2)-β-D-glucopyranoside	Flavonoids	Flavonols	613.14	319.05	612.1326
Lmrj002087	L-Isoleucyl-L-Aspartate	Amino acids and	Amino acids and	247.13	86.09	246.1216

Metabolite ID	Compound Name	Class I	Class II	Q1 (m/z)	Q3 (m/z)	Molecular Weight (Da)
		derivatives	derivatives			
Zbsp011292	Methyl [6]-Gingerdiol 3-acetate	Phenolic acids	Phenolic acids	353.2301	151.0741	352.225
Lmxn008846	Hydroxydihydrobovolide*	Others	Lactones	197.12	153.13	198.1256
Lhmp122020	3-O-caffeoyl quininyl arabinoside	Others	Others	487.1444	355.1023	486.1373
Waptn03493	3,3',4,5'-Tetrahydroxybibenzyl	Others	Stilbene	245.08	203.07	246.0892
Lmmn002828	Lyoniresinol-9'-O-xyloside (Lyoniside)	Lignans and Coumarins	Lignans	551.21	419.17	552.2207
pmb0302	2-Aminoethylphosphonate	Others	Others	126.03	96.97	125.0242
Zahp013600	Octadec-2-enamide*	Alkaloids	Alkaloids	282.28	69.07	281.2719

Metabolite ID	Compound Name	Class I	Class II	Q1 (m/z)	Q3 (m/z)	Molecular Weight (Da)
Wasyqn2417	Homomevalonic Acid Glucoside	Organic acids	Organic acids	323.14	59.02	324.142
Lmcsp00364	Succinyl arginine	Amino acids and derivatives	Amino acids and derivatives	275.14	175.12	274.1277
Sazp010510	3-O-Trans-p-Coumaroylmaslinic Acid*	Terpenoids	Triterpene	619.4	437.34	618.392
mws1360	Aromadendrin-3-O-rhamnoside; Engeletin	Flavonoids	Flavanonols	433.11	269.04	434.1213
Zbpn005529	Phloretin-2'-O-(6''-O-acetyl)glucoside	Flavonoids	Chalcones	477.14	273.08	478.1475
Wayp001214	N-(1-Deoxy-1-fructosyl)Arginine	Amino acids and derivatives	Amino acids and derivatives	337.18	175.12	336.164

Metabolite ID	Compound Name	Class I	Class II	Q1 (m/z)	Q3 (m/z)	Molecular Weight (Da)
YC512112	Anabasine-glucoside	Alkaloids	Pyridine alkaloids	325.18	163.12	324.1685
MWSslk035	Orcinol glucoside*	Phenolic acids	Phenolic acids	285.1	123.05	286.1053
Walp008158	2-AminoicoSane-1,5,19-triol	Lipids	Sphingolipids	346.33	88.08	345.3243
Lmqn000780	Uridine 5'-diphospho-N-acetylglucosamine	Nucleotides and derivatives	Nucleotides and derivatives	606.08	384.98	607.0816
Ladp004086	Glucosyl-[6''-(3-hydroxy-3-methylglutaryl)]-Isorhamnetin	Flavonoids	Flavonols	623.1598	317.0673	622.1534
Lmcp002302	N6-(2-Hydroxyethyl)adenosine*	Nucleotides and derivatives	Nucleotides and derivatives	312.13	180.09	311.123

Metabolite ID	Compound Name	Class I	Class II	Q1 (m/z)	Q3 (m/z)	Molecular Weight (Da)
Lmnp003184	Cirsimaritin-8-C-glucoside	Flavonoids	Flavones	477.14	357.09	476.1319
Lmlp011588	3,4-Secours-4(23),12-dien-3-oic acid (Roburic acid)	Terpenoids	Triterpene	441.37	217.19	440.3654
Lmmp003266	Quercetin-3-O-(2''-O-malonyl)glucoside-7-O-arabinoside	Flavonoids	Flavonols	683.15	303.05	682.1381
MWSslk133	1,14-Tetradecanedioic Acid	Lipids	Free fatty acids	257.18	195.18	258.1831
Lacp001082	cis-2,4-Dihydroxy-2-(2-hydroxyethyl)cyclohex-5-en-1-one glucoside	Others	Others	335.1316	137.0587	334.1258
Wcfp001744	N-Methylpyrrolidone	Others	Others	100.08	58.03	99.0684
pmb0532	Inosine 5'-monophosphate	Nucleotides and	Nucleotides and	349.05	137.05	348.0471

Metabolite ID	Compound Name	Class I	Class II	Q1 (m/z)	Q3 (m/z)	Molecular Weight (Da)
		derivatives	derivatives			
Wahp004852	Viscumneoside V1	Flavonoids	Flavanones	729.23	303.09	728.2158
Hmhn002787	Dihydrophaseic acid-O-β-D-glucoside	Terpenoids	Sesquiterpenoids	443.19	161.04	444.1995
mws1000	Fustin	Flavonoids	Flavanonols	287.06	149.02	288.0634
Lskp211299	Grayanotoxin III	Terpenoids	Ditepenoids	371.24	149.1	370.2355
Lmmn006306	Machilusolide D	Others	Lactones	293.21	293.21	294.2195
Lakn002165	Protocatechuic acid glucosyl xyloside*	Phenolic acids	Phenolic acids	447.12	152.01	448.1217
Lmjp003834	6-O-p-Methoxycinnamoylc atalpol	Terpenoids	Monoterpenoids	523.181	177.0552	522.1737
Zmgn004894	Methyl 4-hydroxybenzoate	Phenolic acids	Phenolic acids	151.04	136.02	152.0473

Metabolite ID	Compound Name	Class I	Class II	Q1 (m/z)	Q3 (m/z)	Molecular Weight (Da)
Lmsn003111	1-O-p-Coumaroyl-β-D-glucose*	Phenolic acids	Phenolic acids	325.09	145.03	326.1002
MWSmce682	DL-Xylose*	Others	Saccharides	149.05	59.01	150.0528
Lmtp005305	Mearnsetin-3-O-rhamnoside (Mearnsitrin)	Flavonoids	Flavonols	479.12	333.06	478.1111
Walp003030	Isofraxidin 7-O-Apiosylglucoside*	Lignans and Coumarins	Coumarins	517.15	193.05	516.1479
Lmgp001898	4,6-Dihydroxyquinoline	Alkaloids	Quinoline alkaloids	162.06	116.05	161.0477
mws0987	6-MethylCoumarin	Lignans and Coumarins	Coumarins	161.06	115.05	160.0524
Wahp005277	Tricaffeoyl Spermidine	Alkaloids	Phenolamine	632.2646	470.2321	631.253
Zbjp002681	1,7-bis(4-hydroxy-3-methoxyphenyl)hept-1-ene-3-ol	Others	Ketone compounds	359.1848	137.0597	358.178

Metabolite ID	Compound Name	Class I	Class II	Q1 (m/z)	Q3 (m/z)	Molecular Weight (Da)
mws1715	Cordycepin (3'-Deoxyadenosine)*	Nucleotides and derivatives	Nucleotides and derivatives	252.11	136.06	251.1018
Wahln02843	2,6-Dimethoxy-4-(2-Hydroxyethyl)Phenyl Beta-D-Glucopyranoside	Others	Alcohol compounds	419.16	197.08	360.142
WaYn006941	Hedyotisol B*	Lignans and Coumarins	Lignans	809.31	195.07	810.3099
WaYp004901	Reseoside*	Terpenoids	Monoterpenoids	371.21	191.14	370.1992
Wasn005803	Protocatechuic acid 4-O-(6''-O-Feruloyl)Glucoside*	Phenolic acids	Phenolic acids	491.12	315.07	492.1268
Wmhn004451	1,4,8-Trihydroxynaphthalene-1-O-[6'-O-(3'',4'',5''-trimethylbenzoyl)]glucoside	Quinones	Quinones	499.18	337.07	500.1319

Metabolite ID	Compound Name	Class I	Class II	Q1 (m/z)	Q3 (m/z)	Molecular Weight (Da)
Lmmn002260	5'-Methoxyisolariciresinol-9'-O-glucoside	Lignans and Coumarins	Lignans	551.21	389.17	552.2207
Zbsp004858	Tricin-5-O-(6'-O-malonyl)glucoside	Flavonoids	Flavones	579.1327	331.0832	578.1272
Wasn002394	Glucosyl 2,6-dimethyl-8-oxoocta-2,6-dienoic acid	Others	Others	403.16	181.09	344.1471
Lhhp102922	Fargesin	Lignans and Coumarins	Lignans	371.15	167.07	370.1416
Lhdp102526	19-Hydroxyursolic acid	Terpenoids	Triterpene	473.36	205.16	472.3553
mws1521	Salicin	Others	Others	285.1	121.03	286.1053
Xmyn008071	Gnetifolin B*	Flavonoids	Flavonols	299.06	284.03	300.0634
pmb3101	2-Isopropylmalic Acid	Organic acids	Organic acids	175.06	115.04	176.0685

Metabolite ID	Compound Name	Class I	Class II	Q1 (m/z)	Q3 (m/z)	Molecular Weight (Da)
Wbjp002336	N,N-diethyl-5-hydroxytry ptamineoxalate	Alkaloids	Alkaloids	319.1283	301.1194	318.1216
MWSmce199	L-Xylose*	Others	Saccharides	149.05	59.02	150.0528
Layp006277	Hedyotol C	Lignans and Coumarins	Lignans	585.23	359.15	584.2258
MWSmce190	4-Hydroxy-L-Isoleucine	Amino acids and derivatives	Amino acids and derivatives	148.09	74.02	147.0895
Zmzn000078	Dihydroxyacetone phosphate	Others	Ketone compounds	168.99	78.96	169.998
pmp001059	Genipin-1-O-(2''-O-apiosyl) glucoside	Terpenoids	Monoterpenoids	521.1865	209.0809	520.1792
Wayp001104	N-(1-Deoxy-1-fructosyl)Ty rosine	Amino acids and derivatives	Amino acids and derivatives	344.13	182.08	343.1262

Metabolite ID	Compound Name	Class I	Class II	Q1 (m/z)	Q3 (m/z)	Molecular Weight (Da)
Wagp005508	3-Hydroxy-Beta-Ionone 3-(6''-Malonyl)Glucoside	Others	Others	457.2	209.15	456.1995
Zbqp010721	N-(4-(tetrahydroPhoenix1-yl)butyryl)nornicotine	Alkaloids	Pyridine alkaloids	409.3187	149.1076	408.3141
MWStz411	(-)-Isolariciresinol 9'-O-glucoside	Lignans and Coumarins	Lignans	523.2168	219.1031	522.2101
WaYn003112	Erythro-Guaiacylglycerol-β-threo-syringylglycerol Ether	Lignans and Coumarins	Lignans	439.16	195.07	440.1682
MWS201478	Phe-Ser	Amino acids and derivatives	Amino acids and derivatives	253.12	120.09	252.111
Lmhn003246	Sinapoylcaffeoyltartaric acid	Phenolic acids	Phenolic acids	517.1	223.06	518.106

Metabolite ID	Compound Name	Class I	Class II	Q1 (m/z)	Q3 (m/z)	Molecular Weight (Da)
pme0109	Methyldopa	Amino acids and derivatives	Amino acids and derivatives	212.09	166.09	211.0845
Lazp004218	Malonyl Wistin*	Flavonoids	Isoflavones	547.1449	299.0919	546.1373
MWS201469	Glu-Arg	Amino acids and derivatives	Amino acids and derivatives	304.16	175.12	303.1543
Labp004779	6,7-di-O-methyl-N-deacety lisoipecosidic acid	Alkaloids	Isoquinoline alkaloids	538.23	175.07	537.221
pma0724	Naringenin-6-C-Glucoside	Flavonoids	Flavanones	435.13	285.08	434.1213
pme3967	2-(Dimethylamino)guanosi ne*	Nucleotides and derivatives	Nucleotides and derivatives	312.13	180.09	311.123
Lmlp002205	Isololiolide	Terpenoids	Monoterpenoids	197.12	161.1	196.1099
Ydyn013925	Methyl oleanolate*	Terpenoids	Triterpene	469.37	469.37	470.376

Metabolite ID	Compound Name	Class I	Class II	Q1 (m/z)	Q3 (m/z)	Molecular Weight (Da)
pmp000703	6'-O-D-Glucosylsweroside	Terpenoids	Monoterpenoids	538.213	197.0799	520.1792
Labn002716	galloyl acetyl glucoside	Phenolic acids	Phenolic acids	373.08	169.02	374.0849
Wagp003695	N-(Isoferuloyl)-Glutamic Acid	Alkaloids	Phenolamine	324.11	177.05	323.1005
Lasp006411	5'-Demethylaquillochin	Lignans and Coumarins	Lignans	387.11	191.07	386.0996
Lmyp006086	Prunetin-5-O-glucoside	Flavonoids	Isoflavones	447.13	285.07	446.1213
MWSmce425	2,3,6,23-Tetrahydroxyurs-12-en-28-oic acid (Madecassic acid)	Terpenoids	Triterpene	503.34	437.3	504.3451
Wagp002398	Tryptophan N-Rutinoside	Amino acids and derivatives	Amino acids and derivatives	513.21	205.1	512.2006
Lmbp009216	Euphatexol F	Terpenoids	Triterpene	457.36	329.25	456.3603

Metabolite ID	Compound Name	Class I	Class II	Q1 (m/z)	Q3 (m/z)	Molecular Weight (Da)
Zbrn008158	Chrysoeriol; 5,7,4'-Trihydroxy-3'-Methoxyflavone	Flavonoids	Flavones	299.06	284.03	300.0634
pmn001505	Oleanolic acid-3-O-xylosyl(1→3)glucuronide	Terpenoids	Triterpene Saponin	763.43	763.4	764.4347
Saln005033	Ibotalactone B	Terpenoids	Monoterpenoids	551.14	161.02	552.1479
Yxxp005044	Balanophonin	Lignans and Coumarins	Lignans	357.1341	137.0584	356.126
pme0500	D-Melezitose	Others	Saccharides	503.16	323.1	504.169
Lagp004883	8-Methoxy malonyl ononin*	Flavonoids	Isoflavones	547.1451	299.0929	546.1373
MWSslk208	Kaurenoic Acid*	Terpenoids	Ditepenoids	301.22	301.22	302.2246

Metabolite ID	Compound Name	Class I	Class II	Q1 (m/z)	Q3 (m/z)	Molecular Weight (Da)
Lmfn003035	6-O-p-Coumaroyl-β-D-glucose*	Phenolic acids	Phenolic acids	325.09	145.03	326.1002
Wasn002043	3,5-Di-Galloyl Sorbitol	Phenolic acids	Phenolic acids	485.09	315.07	486.101
Wahp004848	Viscumneoside I*	Flavonoids	Flavanones	597.184	303.0893	596.1736
Lmsn004092	1-O-Galloyl-3-O-Feruloyl-β-D-glucose	Phenolic acids	Phenolic acids	507.11	169.01	508.1217
pmp000724	Pseudoephedrine	Alkaloids	Alkaloids	166.1227	121.0649	165.1154
Lmsn003805	1-O-Galloyl-4-O-Feruloyl-β-D-glucose*	Phenolic acids	Phenolic acids	507.11	169.01	508.1217
Lmhn005110	Aracarpene 1	Flavonoids	Isoflavones	299.06	284.02	300.0634
Lhhp101903	3,5,4'-Tetrahydroxystilbene-2-O-β-D-glucoside*	Others	Stilbene	407.1332	245.0812	406.1264

Metabolite ID	Compound Name	Class I	Class II	Q1 (m/z)	Q3 (m/z)	Molecular Weight (Da)
Hmcp001105	5-(2-Hydroxypropyl)hygrine	Alkaloids	Pyrrole alkaloids	200.1653	140.14	199.1572
Lcsn002223	12-Hydroxyjasmonic Acid Glucoside	Organic acids	Organic acids	387.16	59.01	388.1733
Lcsp013328	linoleoyl ethanolamine	Lipids	LPE	324.29	62.06	323.2824
Lmhp206353	Tricin-4'-O-glucoside*	Flavonoids	Flavones	493.13	331.08	492.1268
Lmmn009932	3-Hydroxy-24-methylene-9,19-cyclolanostan-26-oic acid (Ambolic acid)*	Terpenoids	Triterpene	469.37	469.37	470.376
MWSslk094	Proanthocyanidins	Tannins	Proanthocyanidins	593.13	125.03	594.1373
Zmgp003455	7-(4-Hydroxy-3-methoxy-phenyl)-1-(4-hydroxy-pheny	Others	Ketone compounds	327.1597	137.0592	326.1518

Metabolite ID	Compound Name	Class I	Class II	Q1 (m/z)	Q3 (m/z)	Molecular Weight (Da)
	l)-hept-4-en-3-one					
pmf0516	Mangiferin	Flavonoids	Other Flavonoids	421.08	301.05	422.0849
Wbjp004531	peganoneI-glucose-Rhamnose	Quinones	Anthraquinone	593.1869	285.0764	592.1792
MWS20152	Syringaresinol	Lignans and Coumarins	Lignans	417.16	402.1	418.1628
Lmhn004976	Aracarpene 2*	Flavonoids	Isoflavones	299.06	284.02	300.0634
Zmdn004764	Syringopicroside	Terpenoids	Monoterpenoids	493.17	331.12	494.1788
MWSmce301	Coumarin-3-carboxylic Acid	Lignans and Coumarins	Coumarins	191.03	173.02	190.0266
Lmbn005487	12,13-DHOME; (9Z)-12,13-Dihydroxyoctadec-9-enoic acid	Lipids	Free fatty acids	313.24	251.24	314.2457

Metabolite ID	Compound Name	Class I	Class II	Q1 (m/z)	Q3 (m/z)	Molecular Weight (Da)
Lmsn003582	1-O-Galloyl-4-O-p-Coumaroyl-β-D-glucose	Phenolic acids	Phenolic acids	477.1	169.01	478.1111
Lmqp006281	Kaempferol-3-O-(4''-O-acetyl)rhamnoside	Flavonoids	Flavonols	475.12	287.06	474.1162
Wasn007507	Dehydroxy-Dihydroabscisic Acid 4'-O-glucoside	Organic acids	Organic acids	411.2	249.15	412.2097
pme3188	Uridine 5'-monophosphate	Nucleotides and derivatives	Nucleotides and derivatives	323.03	211	324.0359
MWSslk132	Hexadecanedioic acid	Lipids	Free fatty acids	285.21	223.21	286.2144
mws0884	Cyclic 3',5'-Adenylic acid	Nucleotides and derivatives	Nucleotides and derivatives	328.05	134.05	329.0525
Wbtp004927	1,3,6-trihydroxy-7-methox	Flavonoids	Other Flavonoids	453.1	291.05	452.0955

Metabolite ID	Compound Name	Class I	Class II	Q1 (m/z)	Q3 (m/z)	Molecular Weight (Da)
	y-2-{[(2s,3r,4s,5s,6r)-3,4,5-trihydroxy-6-(hydroxymethyl)oxan-2-yl]oxy}xanthen-9-one					
pme1439	p-Coumaric acid	Phenolic acids	Phenolic acids	165.05	119.05	164.0473
MA10002917	2,4-Dinitrophenol	Others	Others	183	109.02	184.012
pmn001511	3-Hydroxy-5-Methylphenol-1-O-Glucoside*	Phenolic acids	Phenolic acids	285.1	123.05	286.1053
Wayn000597	Citric acid-1-O-diglucoside	Organic acids	Organic acids	515.12	111.01	516.1326
mws0072	Apigenin-5-O-glucoside	Flavonoids	Flavones	433.11	271.06	432.1056
Lmsn003318	1-O-Feruloyl-β-D-glucose	Phenolic acids	Phenolic acids	355.1	175.04	356.1107
Zmpn000095	Sorbitol-6-phosphate	Others	Saccharides	261.04	78.96	262.0454

Metabolite ID	Compound Name	Class I	Class II	Q1 (m/z)	Q3 (m/z)	Molecular Weight (Da)
pme2651	NADP (Nicotinamide adenine dinucleotide phosphate)	Nucleotides and derivatives	Nucleotides and derivatives	742.07	620.02	743.0755
Hmbp002333	10-Dehydrogeniposide	Terpenoids	Monoterpenoids	387.13	147.04	386.1213
Lmyn007883	9,16-Dihydroxypalmitic acid	Lipids	Free fatty acids	287.22	287.22	288.2301
Lmsn006130	2,3,19-Trihydroxyurs-12-en -23,28-dioic acid-28-O-glucoside	Terpenoids	Triterpene Saponin	679.37	517.32	680.3772
pmb0864	LysoPE 14:0*	Lipids	LPE	426.26	285.24	425.2542
Wayp003359	N-Hydroxyoctanoyl nornicotine glucoside	Alkaloids	Pyridine alkaloids	453.259	291.2125	452.2517

Metabolite ID	Compound Name	Class I	Class II	Q1 (m/z)	Q3 (m/z)	Molecular Weight (Da)
Zmjp013616	12,13-Dihydroursolic acid	Terpenoids	Triterpene	459.38	423.36	458.376
Zmhn003257	5,7,2'-Trihydroxy-8-methoxyflavone*	Flavonoids	Flavones	299.06	284.04	300.0634
Waln011222	2-(2,3-dihydroxypropoxy)-3-(((2-(dimethylamino)ethoxy)(hydroxy)phosphoryl)oxy)propyl palmitate*	Lipids	LPE	554.34	255.23	555.3536
Lmdn001560	6-DeoxyCatalpol	Terpenoids	Monoterpenoids	345.12	165.06	346.1264
Wbtn004362	1,3,6,7-tetrahydroxy-2-(3,4,5-trihydroxyoxan-2-yl)xanthen-9-one	Flavonoids	Other Flavonoids	391.07	229.05	392.0743
Lhkp101525	Apiosylskimmin (Adicardin)	Lignans and Coumarins	Coumarins	457.13	163.04	456.1268

Metabolite ID	Compound Name	Class I	Class II	Q1 (m/z)	Q3 (m/z)	Molecular Weight (Da)
Lmsn003644	1-O-Galloyl-6-O-Feruloyl-β-D-glucose*	Phenolic acids	Phenolic acids	507.11	169.01	508.1217
Lssp210353	Pierisfomoside D	Others	Others	513.27	315.2	512.2621
pme0282	Phthalic acid	Phenolic acids	Phenolic acids	165.02	77.04	166.0266
Rfn057834	(2R,3R,4S,5S,6R)-2-[3-(2,4-dihydroxyphenyl)propoxy]-6-(hydroxymethyl)oxane-3,4,5-triol	Phenolic acids	Phenolic acids	329.12	59.01	330.1315
Hmgp002238	Jaceoside*	Flavonoids	Flavones	493.1354	331.08	492.1268
Wasyqn1906	5-O-Dihydrocaffeoyl Quinic Acid	Phenolic acids	Phenolic acids	355.1	181.05	356.1107
Lacp005171	3-(3'-Hydroxybutyl)-2,4,4-t	Terpenoids	Sesquiterpenoids	371.21	133.1	370.1986

Metabolite ID	Compound Name	Class I	Class II	Q1 (m/z)	Q3 (m/z)	Molecular Weight (Da)
	rimethylcyclohexa-2,5-dien one glucoside					
Lmmp003215	Icariside B2	Terpenoids	Sesquiterpenoids	387.2	207.14	386.1941
Labp005057	Limocitrin malonyl glucoside	Flavonoids	Flavonols	595.1293	347.0778	594.1221
MWSmce417	5-O-Methylvisammioside	Others	Chromone	453.17	291.13	452.1682
Lmbn005369	13(S)-HODE;13(S)-Hydroxyoctadeca-9Z,11E-dienoic acid*	Lipids	Free fatty acids	295.23	195.14	296.2351
MWSmce676	D-Arabinose*	Others	Saccharides	149.0459	59.0132	150.0528
Lakn003294	2-β-D-Glucopyranosyloxy-5-hydroxyphenylacetic	Phenolic acids	Phenolic acids	343.1	181.05	344.1107

Metabolite ID	Compound Name	Class I	Class II	Q1 (m/z)	Q3 (m/z)	Molecular Weight (Da)
	acidmethylester*					
Hmsp006409	Persicarin (Isohamnetin-3-O-sulfate)	Flavonoids	Flavones	397.0226	317.06	396.0151
Lmmp003443	Dicaffeoylspermine	Alkaloids	Phenolamine	527.29	163.04	526.2791
Lmxn002712	6''-O-Acetylharpagide	Terpenoids	Monoterpenoids	405.14	183.06	406.1475
mws1155	D-Mannitol*	Others	Saccharides	181.07	101.02	182.079
Lskp211301	Grayanotoxin IX	Terpenoids	Ditepenoids	337.24	283.21	336.2301
MWSHC20155	Scopoletin (7-Hydroxy-6-methoxycoumarin)	Lignans and Coumarins	Coumarins	191.04	176.01	192.0423
Zmgn000173	D-Ribose	Others	Saccharides	149.05	75.01	150.0528
pmb0863	LysoPC 16:2(2n isomer)	Lipids	LPC	492.31	184.07	491.3012

Metabolite ID	Compound Name	Class I	Class II	Q1 (m/z)	Q3 (m/z)	Molecular Weight (Da)
Zjmp102717	2,3-Dihydroxyolean-12-en-28-oic acid	Terpenoids	Triterpene	473.3593	189.1619	472.3553
Wamxn02513	Protocatechuic acid-1'-O-xyloside	Phenolic acids	Phenolic acids	285.06	152.01	286.0689
pmp001164	Iristectorin B	Flavonoids	Isoflavones	493.1341	316.1	492.1268
mws0014	Ferulic acid*	Phenolic acids	Phenolic acids	193.05	134.04	194.0579
pmn001399	2,4,6,4'-Tetrahydroxy-stilbene-2-O-glucoside	Others	Stilbene	405.12	243.07	406.1264
Wagn011658	1-Palmitoyl-Sn-Glycerol 3-O-Diglucoside	Lipids	Glycerol ester	653.38	397.14	654.3827
Lmqp002170	Kaempferol-3-O-sophorotrioside*	Flavonoids	Flavonols	773.21	449.11	772.2062

Metabolite ID	Compound Name	Class I	Class II	Q1 (m/z)	Q3 (m/z)	Molecular Weight (Da)
Wasn006102	Apiosylrhododendrin	Phenolic acids	Phenolic acids	459.19	165.09	460.1945
MWSHC2016	Epigallocatechin-3-O-gallate	Flavonoids	Flavanols	457.08	169.01	458.0849
pme2244	3-Indolepropionic acid	Alkaloids	Plumerane	190.09	118.07	189.079
Zmgp004297	7-(4-Hydroxyphenyl)-1-phenyl-4-hepten-3-one	Others	Ketone compounds	281.15	107.05	280.1463
pmp000596	Quercetin-3-O-(2''-O-galactosyl)glucoside	Flavonoids	Flavonols	627.16	465.1	626.1483
Lmrn003000	2-Hydroxy-3-phenylpropanoic acid	Phenolic acids	Phenolic acids	165.06	103.06	166.063
MWS1900	Undecanedioic acid	Lipids	Free fatty acids	215.13	153.13	216.1362
Layp005773	Ageconyflavone	Flavonoids	Flavones	521.17	359.11	520.1581

Metabolite ID	Compound Name	Class I	Class II	Q1 (m/z)	Q3 (m/z)	Molecular Weight (Da)
	B-4'-glucoside*					
Zahn007990	5-hydroxy-3,4-dimethyl-5-pentylfuran-2(5H)-one*	Others	Lactones	197.12	153.13	198.1256
WaYp007695	2-Aminooctadecane-1,16,18,18-tetraol	Lipids	Sphingolipids	334.3	242.25	333.2879
Wayn002636	N-(1-Deoxy-1-fructosyl)Tryptophan	Amino acids and derivatives	Amino acids and derivatives	365.13	203.08	366.1422
Lmbp011582	11-Oxo-kansenonol	Terpenoids	Triterpene	471.34	229.16	470.3396
Wmnp000001	3,12-Dihydroxyoleanan-28,13-olide (Oleanderolide)	Terpenoids	Triterpene	473.36	455.35	472.3553
Lskp211305	Grayanotoxin V	Terpenoids	Ditepenoids	369.23	145.08	368.2199
Wbtn007603	9,11-dimethoxy-2h-[1,3]dio	Flavonoids	Other Flavonoids	299.06	284.03	300.0634

Metabolite ID	Compound Name	Class I	Class II	Q1 (m/z)	Q3 (m/z)	Molecular Weight (Da)
	xolo[4,5-b]xanthen-10-one*					
mws1326	Herbacetin	Flavonoids	Flavonols	301.04	211.04	302.0427
Zbbp006101	Afrormosin-7-O-(6''-malonyl)glucoside*	Flavonoids	Isoflavones	547.1422	299.0895	546.1368
Wasn002690	Villosolside	Terpenoids	Monoterpenoids	361.15	163.08	362.1577
MWS201398	Hyp-Ser	Amino acids and derivatives	Amino acids and derivatives	219.1	86.06	218.0903
Lmmn003041	Isolariciresinol-9'-O-xyloside	Lignans and Coumarins	Lignans	491.19	359.15	492.1995
Zmsn002990	6β-diydrocormic acid	Others	Others	375.13	169.09	376.1369
Wasyqn1941	5-O-(3'-O-Glucosylcaffeoyl)Quinic Acid	Phenolic acids	Phenolic acids	515.14	179.04	516.1479

Metabolite ID	Compound Name	Class I	Class II	Q1 (m/z)	Q3 (m/z)	Molecular Weight (Da)
MWSmce489	2,3,5,4'-Tetrahydroxystilbene-2-O-glucoside*	Others	Stilbene	407.13	245.08	406.1264
Lmyn011818	3,19-Epoxy-3,22-dihydroxy dammara-20,24-dien-26-oic acid δ-lactone (Semialactone)	Terpenoids	Triterpene	467.32	467.32	468.324
Smln006854	Xanthorin	Quinones	Anthraquinone	299.05	284.03	300.0634
Wmbp000510	8-O-Acetylharpagide	Terpenoids	Monoterpenoids	407.1524	167.0712	406.1475
Waln002782	1'-Galactoyl Ferulic acid	Phenolic acids	Phenolic acids	371.1	193.05	372.1056
Lcfn121152	8alpha-acetoxy-5alpha-hydroxy-7-oxodecan-10-olide	Others	Others	257.1	197.08	258.1103
pma6455	Ribulose-5-phosphate	Others	Saccharides	229.01	96.97	230.0192

Metabolite ID	Compound Name	Class I	Class II	Q1 (m/z)	Q3 (m/z)	Molecular Weight (Da)
MWS5231	Tridecanedioic acid	Lipids	Free fatty acids	243.16	181.16	244.1675
pma6218	O-MethylNaringenin-8-C-arabinoside	Flavonoids	Flavanones	419.13	383.1	418.1264
Zmjn003630	1-p-Coumaroyl-3-feruloyl-sucrose*	Phenolic acids	Phenolic acids	663.2	145.03	664.2003
Zmsp000608	3-epi-fagomine	Alkaloids	Piperidine alkaloids	148.0966	86.0595	147.0895
Zmdp001647	γ-Glu-Val	Amino acids and derivatives	Amino acids and derivatives	247.13	184.1	246.1216
WaYn006734	Hedyotisol A*	Lignans and Coumarins	Lignans	809.31	195.07	810.3099
Wcdp004275	Lauramine oxide	Others	Others	230.25	57.07	229.2406
pmf0348	2,6-Dimethyl-7-octene-2,3,6-triol	Terpenoids	Monoterpenoids	187.13	97.06	188.1412

Metabolite ID	Compound Name	Class I	Class II	Q1 (m/z)	Q3 (m/z)	Molecular Weight (Da)
Zmyn005384	2R-Hydroxyoctadecanoic Acid*	Lipids	Free fatty acids	299.26	253.26	300.2664
Zjyp102905	7-epi-teucrenone	Terpenoids	Sesquiterpenoids	235.17	119.09	234.162
pmp000604	Rubrofusarin-6-O-glucoside	Others	Ketone compounds	435.13	273.07	434.1213
Zbjn005825	Kaempferide-3-O-(6'-O-acetyl)glucoside	Flavonoids	Flavonols	503.12	299.06	504.1268
Zbqp003165	Ile-Trp	Amino acids and derivatives	Amino acids and derivatives	318.1812	188.0704	317.1739
HJN051	Tamarixetin-3-O-rutinoside	Flavonoids	Flavonols	623.16	315.05	624.169
pmp000695	Swertiamarin	Terpenoids	Monoterpenoids	375.13	177.05	374.1213

Metabolite ID	Compound Name	Class I	Class II	Q1 (m/z)	Q3 (m/z)	Molecular Weight (Da)
Hmtp000776	4,5,6-Trihydroxy-2-cyclohexen-1-ylideneacetonitrile	Alkaloids	Alkaloids	168.07	105.03	167.0582
MWSprf050	Methylhesperidin	Flavonoids	Flavanones	625.2126	317.1048	624.2054
Zahp011321	N-Isobutyl Decanamide	Alkaloids	Alkaloids	228.23	228.24	227.2249
Zaxn007230	Isopimaradieneone	Terpenoids	Ditepenoids	285.22	285.22	286.2297
Zbyn004323	2,6-di-O-galloylarbutin	Phenolic acids	Phenolic acids	575.1	423.09	576.1096
Saln003418	4-(Beta-D-Glucopyranosyloxy)-2,4',6-Trihydroxybenzophenone	Others	Ketone compounds	407.1	287.06	408.1056
Lcsp000435	Glu-Ser	Amino acids and derivatives	Amino acids and derivatives	235.09	118.08	234.0852
Zbfn003751	3'-p-Coumaroyl-3-feruloyl-	Phenolic acids	Phenolic acids	663.19	145.03	664.2003

Metabolite ID	Compound Name	Class I	Class II	Q1 (m/z)	Q3 (m/z)	Molecular Weight (Da)
	sucrose*					
Zmyn004732	2R-hydroxy-9Z,12Z,15Z-octadecatrienoic acid	Lipids	Free fatty acids	293.21	185.12	294.2195
Wcdp007645	9-Oxo-10,12-Octadecadienoic Acid	Lipids	Free fatty acids	295.23	151.11	294.2195
Hmcp005575	Quercetin-5,4'-di-O-glucoside	Flavonoids	Flavonols	627.15	303.05	626.1483
Wmln000659	3-Methyl-4,8-dihydroxy-3,4-dihydroisocoumarin	Lignans and Coumarins	Coumarins	193.07	101.02	194.0579
Hmhp001812	2'-O-Methyladenosine	Nucleotides and derivatives	Nucleotides and derivatives	282.12	136.06	281.1124
MWS4295	DL-2-hydroxystearic acid*	Lipids	Free fatty acids	299.26	253.25	300.2664

Metabolite ID	Compound Name	Class I	Class II	Q1 (m/z)	Q3 (m/z)	Molecular Weight (Da)
Walp003622	6-beta-d-glucopyranosyloxyindole-3-carboxylic acid	Alkaloids	Plumerane	340.1	178.05	339.0954
Qmhp111702	3-Ethylidene-7-hydroxy-6-methoxyphthalide	Others	Others	207.0645	177.0533	206.0579
Zbqp004433	N-(nonanoyl)nornicotine	Alkaloids	Pyridine alkaloids	319.2001	149.1076	318.1943
Lmsn000954	Dambonitol	Others	Saccharides	207.09	59.01	208.0947
Wcsn010524	8,11-Heptadecadienoic acid	Lipids	Free fatty acids	265.22	265.22	266.2246
mws5041	L-Glycyl-L-isoleucine	Amino acids and derivatives	Amino acids and derivatives	189.1234	86.0964	188.1161
Waln010449	1-(2,3-dihydroxypropoxy)-3-(((2-(dimethylamino)etho	Lipids	LPE	552.33	253.22	553.338

Metabolite ID	Compound Name	Class I	Class II	Q1 (m/z)	Q3 (m/z)	Molecular Weight (Da)
	xy)(hydroxy)phosphoryl)oxy)propan-2-yl (E)-hexadec-9-enoate					
Lcsp002752	Asp-Pro-Phe	Amino acids and derivatives	Amino acids and derivatives	378.17	185.09	377.1587
Lhfp110342	Fukinolic acid	Tannins	Tannin	435.093	163.0382	434.0849
Waln002594	4-O-Galactoyl p-Coumaric acid*	Phenolic acids	Phenolic acids	341.09	163.04	342.0951
Zblp004317	3-(2-oxopropyl)-3-hydroxy indolin-2-one	Alkaloids	Plumerane	206.08	120.09	205.0739
Wasn002508	Glucosyl 7-methyl-3-methyleneoctane-1,2,6,7-tetraol	Terpenoids	Monoterpenoids	365.18	203.13	366.189

Metabolite ID	Compound Name	Class I	Class II	Q1 (m/z)	Q3 (m/z)	Molecular Weight (Da)
Walp009488	2-aminotetracoSane-1,6,7,23-tetraol*	Lipids	Sphingolipids	418.3863	356.3521	417.3818
MWSmce471	Fagomine	Alkaloids	Piperidine alkaloids	148.0959	86.0626	147.0895
Zbfn006560	2-(2,3,4,5-tetramethoxycinnamoyl)-6-(4-hydroxycinnamoyl)-glucoside	Phenolic acids	Phenolic acids	575.18	429.15	576.1843
Qacp004461	Sibiricose A2	Phenolic acids	Phenolic acids	563.1951	221.0826	562.1898
Walbn03176	Coniferyaldehyde Glucoside	Lignans and Coumarins	Lignans	339.11	177.05	340.1158
Zbfn006009	2-Feruloyl-6-(4-hydroxycinnamoyl)glucoside	Phenolic acids	Phenolic acids	501.14	163.04	502.1475
Lmsn010075	19-Hydroxy-2,3-secours-12	Terpenoids	Triterpene	531.33	441.3	532.34

Metabolite ID	Compound Name	Class I	Class II	Q1 (m/z)	Q3 (m/z)	Molecular Weight (Da)
	-ene-2,3,28-trioic acid 3-methyl ester (Cecropiacic acid 3-methyl ester)					
MWS5083	Flavin Single Nucleotide(FMN)	Nucleotides and derivatives	Nucleotides and derivatives	455.1	96.97	456.1046
mws0609	Guanosine 3',5'-cyclic monophosphate	Nucleotides and derivatives	Nucleotides and derivatives	344.04	150.04	345.0474
Lmqp008286	2,3,23-Trihydroxyolean-12-en-28-oic acid (Arjunolic acid)	Terpenoids	Triterpene	489.36	407.33	488.3502
Wmhp000012	Ajugoside	Terpenoids	Monoterpenoids	389.14	209.03	390.1526
Lskp211457	2,3,19-Trihydroxyolean-12-ene-24,28-dioic acid	Terpenoids	Triterpene	519.3334	455.3174	518.3244

Metabolite ID	Compound Name	Class I	Class II	Q1 (m/z)	Q3 (m/z)	Molecular Weight (Da)
	(Bartogenic acid)					
MWSslk138	2,3,4-Trihydroxybenzoic acid	Phenolic acids	Phenolic acids	169.01	151	170.0215
Lcsp000520	Alanylproline	Amino acids and derivatives	Amino acids and derivatives	187.11	70.07	186.1004
Lmbn005287	7S,8S-DiHODE; (9Z,12Z)-(7S,8S)-Dihydroxyoctadeca-9,12-dienoic acid	Lipids	Free fatty acids	311.22	249.22	312.2301
mws5035	L-Leucyl-L-phenylalanine	Amino acids and derivatives	Amino acids and derivatives	279.17	120.08	278.163
Lacp003427	Grasshopper ketone	Terpenoids	Monoterpenoids	225.15	123.12	224.1412

Metabolite ID	Compound Name	Class I	Class II	Q1 (m/z)	Q3 (m/z)	Molecular Weight (Da)
Lmsp102912	2,4,6-Trimethoxyphenyl 1-O-β-D-apiofuranosyl-(1 →6)-β-D-glucopyranoside	Others	Others	479.1764	185.0807	478.1686
Zblp004832	7,4'-Dihydroxydihydroflav onoids	Flavonoids	Flavanones	257.0814	137.02	256.0736
Waln002419	1'-O-Galactoyl p-Coumaric acid*	Phenolic acids	Phenolic acids	341.09	163.04	342.0951
Lssp210309	3-Hydroxyurs-12-en-28-oic acid (Ursolic acid)	Terpenoids	Triterpene	457.36	421.35	456.3603
pmc0066	2'-Deoxyinosine-5'-monop hosphate	Nucleotides and derivatives	Nucleotides and derivatives	333.06	137.05	332.0522
Wayn000918	Isocitric acid-1-O-diglucoside	Organic acids	Organic acids	515.12	111.01	516.1326

Metabolite ID	Compound Name	Class I	Class II	Q1 (m/z)	Q3 (m/z)	Molecular Weight (Da)
mws0028	Vanillic acid	Phenolic acids	Phenolic acids	167.03	108.02	168.0423
Lmhp001884	Kelampayoside A[3,4,5-Trimethoxyphenol-β-D-apiosyl-(1→6)-β-D-glucoside]	Phenolic acids	Phenolic acids	479.1722	185.078	478.1686
Walp009431	2-aminotetracoSane-1,5,21,23-tetraol*	Lipids	Sphingolipids	418.39	356.35	417.3818
Wmzn000227	2,2-Dimethylsuccinic acid	Organic acids	Organic acids	145.06	109.04	146.0579
MWS5209	N-Methyl-α-aminoisobutyric acid	Amino acids and derivatives	Amino acids and derivatives	235.16	118.09	117.079
MWSmce203	D-Cellobiose	Others	Saccharides	341.12	73.03	342.1162
mws1089	Sucrose-6-phosphate	Others	Saccharides	421.08	241.01	422.0825

Metabolite ID	Compound Name	Class I	Class II	Q1 (m/z)	Q3 (m/z)	Molecular Weight (Da)
pme0281	Terephthalic acid	Phenolic acids	Phenolic acids	165.02	121.03	166.0266
Labn004625	2-Phenylethyl beta-primeveroside	Others	Alcohol compounds	415.16	89.02	416.1682
mws0677	N-Acetyl-5-hydroxytryptamine	Alkaloids	Plumerane	219.11	160.08	218.1055
MWStz403	Isocarlinoside	Flavonoids	Flavones	581.1537	413.0909	580.1428
Hmcn002875	6-Hydroxyluteolin	Flavonoids	Flavones	301.04	255.02	302.0427
Lakp003115	5-O-β-D-Glucopyranosyl-3-hydrobenzo(b)fu-ran-2-one	Others	Lactones	313.09	151.04	312.0845
Lahp003408	2-(3,4-dihydroxyphenyl)-4h-chromene-3,5,7-triol-gluc	Flavonoids	Flavanols	451.12	243.06	450.1162

Metabolite ID	Compound Name	Class I	Class II	Q1 (m/z)	Q3 (m/z)	Molecular Weight (Da)
	oside					
Lmmn005168	Siegesbeckic acid	Terpenoids	Ditepenoids	335.22	335.22	336.2301
MWStz039	Coclaurine	Alkaloids	Isoquinoline alkaloids	286.1443	107.0533	285.1365
Cmqp003383	Gentianamine	Alkaloids	Pyridine alkaloids	206.08	146.06	205.0739
Zasp005752	Kaempferol-3-(2''-acetylrhamnoside)	Flavonoids	Flavonols	475.12	287.06	474.1162
MWSCX017	4-hydroxyphenyl acrylaldehyde	Others	Aldehyde compounds	165.05	119.05	164.0473
Lhmp122023	Scopoletin 7-O-(6''-Malonylglucoside)	Lignans and Coumarins	Coumarins	441.1	193.05	440.0955
Wmlp000056	3,5-Dimethoxy-4-hydroxyb	Others	Aldehyde	183.06	91.05	182.0579

Metabolite ID	Compound Name	Class I	Class II	Q1 (m/z)	Q3 (m/z)	Molecular Weight (Da)
	enzaldehyde		compounds			
Wasn000977	4-O-galactopyranosylxylose	Others	Saccharides	311.1	89.24	312.1056
pmb0034	L-α-Glutamyl-L-Glutamic Acid	Amino acids and derivatives	Amino acids and derivatives	277.1	148.06	276.0958
Lmap001823	Dhurrin	Alkaloids	Alkaloids	312.11	132.04	311.1005
Wagp004588	Dihydrovomifoliol 4-O-malonyl glucoside*	Terpenoids	Sesquiterpenoids	475.21	209.15	474.2101
Zbfn003643	3,4,5-Trimethoxyphenol-β-D-apiofuranosyl-(1→6)-O-β-D-glucopyranoside	Others	Others	477.16	293.09	478.1686
Zdcp005372	Anhydrosecoisolariciresin	Lignans and Coumarins	Lignans	345.1698	137.0609	344.1624

Metabolite ID	Compound Name	Class I	Class II	Q1 (m/z)	Q3 (m/z)	Molecular Weight (Da)
	ol (AHS)*					
Wcdp010716	Glycidyl Linoleate	Lipids	Glycerol ester	337.2735	95.0852	336.2664
pme3285	Afzelechin (3,5,7,4'-Tetrahydroxyflavan)	Flavonoids	Flavanols	275.09	139.04	274.0841
MWS20987	N5-(1-Iminoethyl)-L-ornithine	Amino acids and derivatives	Amino acids and derivatives	174.12	70.07	173.1164
Lagp003663	Quercetin 7,3',4'-trimethyl ether-3-glucoside	Flavonoids	Flavonols	507.15	345.1	506.1424
pmb0037	Hordenine	Alkaloids	Alkaloids	166.12	121.06	165.1154
Waymn03248	4-O-Feruloylquinic acid	Phenolic acids	Phenolic acids	367.1	193.05	368.1107
Lcsp001861	Leu-Pro	Amino acids and	Amino acids and	229.15	116.07	228.1474

Metabolite ID	Compound Name	Class I	Class II	Q1 (m/z)	Q3 (m/z)	Molecular Weight (Da)
		derivatives	derivatives			
Lmgp002501	Dhurrin 6'-glucoside	Alkaloids	Alkaloids	474.16	312.11	473.1533
Safp023654	PAz-PC	Lipids	LPC	666.43	184.07	665.4268
Lmbp002309	Sinapaldehyde-4-O-Glucoside	Others	Aldehyde compounds	371.1017	209.0484	370.1264
Zbtn005022	3-Hydroxyphloretin-4'-O-glucoside	Flavonoids	Chalcones	451.12	167.03	452.1319
Wbmp006300	1,2,9,10-tetraethoxy-6-methyl-5,6,6a,7-tetrahydro-4H-dibenzo[de,g]quinoline	Alkaloids	Alkaloids	412.25	412.25	411.241
Waptn02000	Fertaric acid glucoside	Phenolic acids	Phenolic acids	487.11	193.05	488.1166
pme0124	L-Glycyl-L-proline	Amino acids and	Amino acids and	173.09	116.07	172.0848

Metabolite ID	Compound Name	Class I	Class II	Q1 (m/z)	Q3 (m/z)	Molecular Weight (Da)
		derivatives	derivatives			
HJAP148	Kaempferol-3-O-sambubioside	Flavonoids	Flavonols	581.15	287.06	580.1428
Hmzp006046	Sesaminol 2-O-Beta-D-Glucoside	Lignans and Coumarins	Lignans	533.1703	371.11	532.1581
Wbkn005292	[(1S,2S,3S,7R,9R,13S,14R,15R,17S)-3,15-diacetyloxy-7-hydroxy-2,6,14-trimethyl-4,11,16-trioxo-10-oxatetracyclo[7.7.1.02,7.013,17]heptadec-5-en-17-yl]methylacetate	Terpenoids	Ditepenoids	519.18	357.13	520.1945
pma0149	Sinapoyl malate	Phenolic acids	Phenolic acids	341.09	207.07	340.0794
Walp004906	2-Aminotetradecane-1,11,1	Lipids	Sphingolipids	262.24	88.08	261.2304

Metabolite ID	Compound Name	Class I	Class II	Q1 (m/z)	Q3 (m/z)	Molecular Weight (Da)
	3-triol					
MWStz369	Candicine	Alkaloids	Phenolamine	180.1383	121.0693	180.1388
Lajp002668	Catechin-(4alpha->8)-gallocatechin-(4alpha->8)-gallocatechin	Flavonoids	Flavanols	899.2	611.14	898.1956
Wbtn004165	1,6-dihydroxy-3,7-dimethoxy-8-(3-methoxy-3-methylbutyl)-2-(3-methylbut-2-en-1-yl)xanthen-9-one	Flavonoids	Other Flavonoids	455.21	395.19	456.2148
pme2812	6-Hydroxymelatonin	Alkaloids	Plumerane	249.1234	190.0862	248.1161
Zbfn002592	3'-p-Coumaroyl-3-acetyl sucrose	Phenolic acids	Phenolic acids	529.15	145.03	530.1636

Metabolite ID	Compound Name	Class I	Class II	Q1 (m/z)	Q3 (m/z)	Molecular Weight (Da)
Wbtp007427	7,10-dihydroxy-12-methoxy-2,2-dimethyl-1,11-dioxatetracen-6-one	Others	Ketone compounds	341.1	341.0995	340.0947
Zbbp008198	6,7-Dihydroxy-4'-methoxyisoflavone; Texasin	Flavonoids	Isoflavones	285.0758	151.0749	284.0679
pmc0960	LysoPC 20:4	Lipids	LPC	544.34	184.07	543.3325
Lmyp003951	3,4-Dihydroxy-3-methoxypropiophenone	Others	Ketone compounds	227.09	181.09	226.0841
MWS201415	Pro-Asp	Amino acids and derivatives	Amino acids and derivatives	231.1	70.07	230.0903
HJN074	Procyanidin A6	Tannins	Proanthocyanidins	591.15	289.07	592.1581
Lmsn006046	2,3,19,23-Tetrahydroxyurs-	Terpenoids	Triterpene Saponin	665.39	503.34	666.3979

Metabolite ID	Compound Name	Class I	Class II	Q1 (m/z)	Q3 (m/z)	Molecular Weight (Da)
	12-en-28-oic acid-28-O-glucoside					
Smhp003736	carlinoside	Flavonoids	Flavones	581.14	413.08	580.1428
Zmcp001750	Eleutheroside B1*	Lignans and Coumarins	Coumarins	385.1133	223.0599	384.1056
Safn006393	Patuletin-7-O-Galactoside*	Flavonoids	Flavonols	493.1	331.05	494.106
MWS5147	3-(Methylthio)Propionic Acid	Organic acids	Organic acids	119.02	59.01	120.0245
Wbsp004755	Methyl Hydroxycinnamate	Phenolic acids	Phenolic acids	179.07	119.05	178.063
pmp000013	Eupatilin-7-O-glucoside	Flavonoids	Flavones	507.15	345.09	506.1424
Zbsn004080	Kakispyrone	Others	Others	391.1	229.05	392.1107
MWSslk066	3-Hydroxy-4-methoxybenzoic acid; Isovanillic Acid	Phenolic acids	Phenolic acids	169.05	65.04	168.0423

Metabolite ID	Compound Name	Class I	Class II	Q1 (m/z)	Q3 (m/z)	Molecular Weight (Da)
Zmzn000079	D-Erythrose-4-phosphate	Others	Saccharides	199	78.96	200.0086
pme1398	Delphinidin-3-O-glucoside (Mirtillin)	Flavonoids	Anthocyanidins	465.1	303.05	465.1028
WaYn006596	Buddlenol C	Lignans and Coumarins	Lignans	613.23	195.07	614.2363
mws0058	Diosmetin (5,7,3'-Trihydroxy-4'-methoxyflavone)*	Flavonoids	Flavones	299.06	284.03	300.0634
mws2220	Gallocatechin 3-O-gallate	Flavonoids	Flavanols	457.08	169.01	458.0849
pmb0736	Tricin-7-O-Glucoside*	Flavonoids	Flavones	493.13	331.08	492.1268
MWS201464	Lys-Phe	Amino acids and derivatives	Amino acids and derivatives	294.18	84.08	293.1739
Zmxn004201	Rosamultin*	Terpenoids	Triterpene Saponin	649.4	487.34	650.403

Metabolite ID	Compound Name	Class I	Class II	Q1 (m/z)	Q3 (m/z)	Molecular Weight (Da)
pma0101	Caffeoylagmatine	Alkaloids	Phenolamine	293.16	163.04	292.1535
Lcsn000560	2,5-Furandicarboxylic acid	Others	Others	155	67.02	156.0059
Walp007804	ChimSalicifoliusin C	Lignans and Coumarins	Coumarins	603.11	397.09	602.106
Wafn011571	alpha-Hydroxylinoleic acid*	Lipids	Free fatty acids	295.23	195.14	296.2351
MWS3036	3-Hydroxyglutaric acid	Organic acids	Organic acids	147.03	85.03	148.0372
pme1184	2'-Deoxyguanosine	Nucleotides and derivatives	Nucleotides and derivatives	268.1	152.06	267.0968
Rfmb091	9S-Hydroxy-10E,12Z-octadecadienoic acid*	Lipids	Free fatty acids	295.23	195.14	296.2351
MWSmce264	Hydroxytyrosol	Others	Alcohol compounds	153.06	123.05	154.063
Zbzp004346	cya-suc-glu	Flavonoids	Anthocyanidins	549.1252	287.05	549.1244

Metabolite ID	Compound Name	Class I	Class II	Q1 (m/z)	Q3 (m/z)	Molecular Weight (Da)
Lmsn008888	2,3,19-Trihydroxyolean-12-en-23,28-dioic acid	Terpenoids	Triterpene	517.32	407.3	518.3244
Wbsp011288	(3S,7aS,13aR,15bR)-4,4,7a,10,10,13a,15b-heptamethyl-2,3,4,4a,5,7,7a,8,9,9a,10,13,13a,13b,14,15,15a,15b-octadecahydro-1H-cyclohepta[1,2-a:5,4-a']dinaphthalen-3-ol	Terpenoids	Terpene	425.37	163.15	424.3705
Lssp210131	2-Deacetyldecinnamoyltaxinine E	Others	Others	479.26	299.2	478.2567
pmf0440	4-MethoxycinnaMaldehyde	Others	Aldehyde compounds	163.08	91.05	162.0681
Lmjp001367	Cyanidin	Flavonoids	Anthocyanidins	581.15	287.05	581.1501

Metabolite ID	Compound Name	Class I	Class II	Q1 (m/z)	Q3 (m/z)	Molecular Weight (Da)
	3-O-(beta-D-xylosyl-(1->2)-beta-D-galactoside)					
Sakn001946	Comoside	Phenolic acids	Phenolic acids	315.11	135.04	316.1158
pmb0962	L-Lysine-Butanoic Acid	Amino acids and derivatives	Amino acids and derivatives	235.17	118.09	234.158
Zmhp003784	Gossypetin-7-O-(3''-glucosyl)rhamnoside; Rhodioflavonoside	Flavonoids	Flavonols	627.16	465.1	626.1483
MWStz103	S-(5'-Adenosyl)-L-methionine	Amino acids and derivatives	Amino acids and derivatives	399.15	250.09	398.1372
Lmhp002031	L-Leucyl-L-Leucine	Amino acids and derivatives	Amino acids and derivatives	245.19	86.1	244.1787

Metabolite ID	Compound Name	Class I	Class II	Q1 (m/z)	Q3 (m/z)	Molecular Weight (Da)
MWSmce134	3-Oxolup-20(29)-en-28-oic acid (Betulonic acid)*	Terpenoids	Triterpene	453.35	453.35	454.3447
MWSmce366	2'-Methoxykurarinone	Flavonoids	Flavanones	453.2259	179.0419	452.2199
pmp000518	7-Hydroxyrutaecarpine	Alkaloids	Plumerane	304.11	286.14	303.1008
zjmp122403	Corehoionoside C	Terpenoids	Monoterpenoids	387.2	123.08	386.1941
Lmhp009129	LysoPC 15:0(2n isomer)*	Lipids	LPC	482.32	184.07	481.3168
Lahp003733	Clausenaglycoside A	Phenolic acids	Phenolic acids	359.17	197.12	358.1628
pme3186	DL-Glyceraldehyde-3-phosphate	Organic acids	Organic acids	168.99	78.96	169.998
MWSslk051	Scopine	Alkaloids	Tropan alkaloids	156.1022	84.0808	155.0946
Lhsp101904	polygodial	Terpenoids	Sesquiterpenoids	235.17	179.11	234.162

Metabolite ID	Compound Name	Class I	Class II	Q1 (m/z)	Q3 (m/z)	Molecular Weight (Da)
pmp001135	Buddlenoid A	Flavonoids	Flavonols	595.1446	147	594.1373
Zbzp007397	5,6,7,7a-tetrahydro-4,4,7a-trimethyl-2(4H)-benzofuranone*	Others	Others	181.12	135.12	180.115
Sasn001174	Hymenoside M	Others	Others	339.13	117.06	280.1158
pmn001606	Eicosenoic acid	Lipids	Free fatty acids	309.28	309.28	310.2872
Wahsn03220	1-O-p-Coumaroyl 3-methylglucaric acid*	Phenolic acids	Phenolic acids	369.08	223.05	370.09
mws4076	3-Hydroxy-11-oxoolean-12-en-30-oic acid (18α-Glycyrrhetinic acid)*	Terpenoids	Triterpene	469.33	425.34	470.3396
Yaap006468	Medioresinol B	Lignans and Coumarins	Lignans	389.16	327.12	388.1522

Metabolite ID	Compound Name	Class I	Class II	Q1 (m/z)	Q3 (m/z)	Molecular Weight (Da)
Walbn00429	(S)-Malic acid-1-O-beta-D-glucopyranoside	Organic acids	Organic acids	295.07	115	296.0743
Cmyp002064	Yuanhuanin	Flavonoids	Flavones	463.12	301.08	462.1162
mws1350	Syringaldehyde; 4-Hydroxy-3,5-Dimethoxybenzaldehyde	Others	Aldehyde compounds	181.05	151	182.0579
Zmcp102504	Marmesinin	Lignans and Coumarins	Coumarins	409.15	247.1	408.142
mws0791	Poncirin (Isosakuranetin-7-O-neohesperidoside)*	Flavonoids	Flavanones	593.19	285.08	594.1949
MWStz588	Dihydroactinidiolide*	Others	Lactones	181.1231	135.1173	180.115

Metabolite ID	Compound Name	Class I	Class II	Q1 (m/z)	Q3 (m/z)	Molecular Weight (Da)
Hahn000897	picrorhizin	Phenolic acids	Phenolic acids	491.14	167.03	492.1479
Wasdn02728	Strophanthobiose*	Others	Others	323.14	59.01	324.142
MWSHC20189	Epipinoresinol*	Lignans and Coumarins	Lignans	357.13	151.04	358.1416
Ladp002110	4-hydroxy-2-oxo-1,2-dihydroquinoline-3-carboxylic acid	Alkaloids	Quinoline alkaloids	206.04	160.04	205.0375
Lmmp003672	8,8'-Methylenebiscatechin	Flavonoids	Flavanols	593.17	441.11	592.1581
Wbmp007680	5,6,6a,7-tetrahydro-1,2,9,10-tetraethoxy-6-n-pentyl-4H-dibenzo[de,g]quinolin-4-ol	Alkaloids	Alkaloids	484.3	484.3	483.2985
Waptn04416	2-trans-abscisic acid	Organic acids	Organic acids	485.2	263.13	426.189

Metabolite ID	Compound Name	Class I	Class II	Q1 (m/z)	Q3 (m/z)	Molecular Weight (Da)
	D-glucosyl ester					
Zmjp001877	Cyanidin-3-O-sambubioside[Cyanidin-3-O-(2''-O-xylosyl)glucoside]	Flavonoids	Anthocyanidins	581.15	287.05	581.1501
Lahp002857	Epicatechin-(4β→8)-epigallocatechin	Flavonoids	Flavanols	595.15	409.09	594.1373
Wasn003913	Protocatechuic acid 4-O-(6''-O-Galloy)Glucoside	Phenolic acids	Phenolic acids	467.08	315.07	468.0904
HX1223	Germacra-1(10),4,7(11)-trien-9α-ol	Terpenoids	Monoterpenoids	221.19	105.07	220.1827
Lmhp012042	2-Linoleoylglycerol*	Lipids	Glycerol ester	355.28	263.24	354.277

Metabolite ID	Compound Name	Class I	Class II	Q1 (m/z)	Q3 (m/z)	Molecular Weight (Da)
Lcsn000650	Rubuside I*	Terpenoids	Triterpene Saponin	649.39	487.34	650.403
pmb0530	Nicotinic acid adenine dinucleotide	Nucleotides and derivatives	Nucleotides and derivatives	664.12	136.06	663.1091
Lmcn004052	Buddlenol D	Phenolic acids	Phenolic acids	643.24	417.16	644.2469
Lmzn206829	3-Oxoolean-12-en-28-oic Acid (Oleanonic acid)*	Terpenoids	Triterpene	453.34	453.34	454.3447
MWStz537	3-O-Feruloylquinic acid	Phenolic acids	Phenolic acids	367.1	193.05	368.1107
Zbpn005001	Phloretin-2'-O-(6''-O-xylosyl)glucoside	Flavonoids	Chalcones	567.17	273.08	568.1792
Zmdp001857	γ-Glu-Tyr	Amino acids and derivatives	Amino acids and derivatives	311.12	165.05	310.1165
Lazn003560	Methoxy-erythro-Guaiacyl	Lignans and Coumarins	Lignans	469.17	195.07	470.1788

Metabolite ID	Compound Name	Class I	Class II	Q1 (m/z)	Q3 (m/z)	Molecular Weight (Da)
	glycerol β-threo-syringyl glycerol ether					
pme0257	Uracil	Nucleotides and derivatives	Nucleotides and derivatives	111.02	67.03	112.0273
Qmyp010506	2,4,6-Trihydroxybenzophenone	Others	Ketone compounds	231.07	153.02	230.0579
Jmwn002172	3,4-dihydroxyphenylethanol-β-D-glucopyranoside	Others	Alcohol compounds	315.11	135.04	316.1158
Yxxn002325	icariside D1	Others	Ketone compounds	415.16	415.16	416.1682
Lcfn159231	3-Methoxy-2-methyl-3-oxopropylbenzoate	Phenolic acids	Phenolic acids	221.08	77.04	222.0892
Walbn06565	13-Amino-13-oxotridecano	Lipids	Free fatty acids	242.18	225.15	243.1834

Metabolite ID	Compound Name	Class I	Class II	Q1 (m/z)	Q3 (m/z)	Molecular Weight (Da)
	ic acid					
Lmqp013019	3-Hydroxyolean-12-en-29-oic acid (Epikatonic acid)	Terpenoids	Triterpene	457.3676	203.1794	456.3603
Zbsp005030	Tricin-5-O-arabinoside	Flavonoids	Flavones	463.12	331.08	462.1162
Waymn03477	(2S,3S,4S,5R,6R)-3-(3-benzoyloxy-2-hydroxypropoxy)-3,4,5-trihydroxyoxane-2-carboxylic acid	Phenolic acids	Phenolic acids	371.1	121.03	372.1056
Lmhp112042	1-Linoleoylglycerol*	Lipids	Glycerol ester	355.28	263.24	354.277
Lmbp003208	Fraxidinglucoside*	Lignans and Coumarins	Coumarins	385.12	223.06	384.1056
Wbtn005483	1,3,6-trihydroxy-2,5,7-trimethoxyxanthen-9-one	Flavonoids	Other Flavonoids	333.06	165.02	334.0689

Metabolite ID	Compound Name	Class I	Class II	Q1 (m/z)	Q3 (m/z)	Molecular Weight (Da)
mws1628	Rheic Acid	Quinones	Anthraquinone	283.02	239.04	284.0321
mws0262	L-Tartaric acid	Organic acids	Organic acids	149.01	87.01	150.0164
Hmgp001902	Pectolinarigenin-7-O-glucoside	Flavonoids	Flavones	477.14	315.09	476.1319
Zbjn007174	Kaempferol-3-O-(2''-p-Coumaroyl)glucoside	Flavonoids	Flavonols	593.13	285.04	594.1373
Lmqn004838	3-O-Methylellagic acid	Tannins	Tannin	315.01	299.99	316.0219
Labn002107	galloyl xylosyl glucoside*	Phenolic acids	Phenolic acids	463.11	168.01	464.1166
Lsjp211202	Blumenol A	Terpenoids	Monoterpenoids	225.15	123.08	224.1412
pmb2654	Anthranilate-1-O-Sophoroside	Phenolic acids	Phenolic acids	460.15	117.9	461.1533
pmb2319	LysoPC 15:0*	Lipids	LPC	482.32	184.07	481.3168

Metabolite ID	Compound Name	Class I	Class II	Q1 (m/z)	Q3 (m/z)	Molecular Weight (Da)
Lmmp003517	Solatuberenol A	Others	Others	225.15	95.08	224.1412
Lcyp000678	Prudomenin*	Flavonoids	Flavonols	493.1321	331.0823	492.1268
Zmhn002508	4-p-Cumaroyl-rhamnosyl-(1→6)-D-glucose	Phenolic acids	Phenolic acids	471.15	309.1	472.1581
Lhhp111111	Schisantherin E	Lignans and Coumarins	Lignans	539.2261	137.059	538.2203
Zbqp000676	3-(triazan-2-yl)propyl L-prolinate	Amino acids and derivatives	Amino acids and derivatives	203.15	116.07	202.143
Lcsp004886	2-Methylthio-cis-zeatin riboside	Alkaloids	Alkaloids	398.1485	266.1082	397.142
mws0463	Hesperetin	Flavonoids	Flavanones	301.07	164.01	302.079
Lskp211446	2,3,19-Trihydroxyolean-12-en-28-oic acid (Arjunic	Terpenoids	Triterpene	489.3558	443.3516	488.3502

Metabolite ID	Compound Name	Class I	Class II	Q1 (m/z)	Q3 (m/z)	Molecular Weight (Da)
	acid)					
pme3961	2'-Deoxyadenosine*	Nucleotides and derivatives	Nucleotides and derivatives	252.11	136.06	251.1018
Cmrn001591	Mussaenosidic acid*	Terpenoids	Monoterpenoids	375.13	169.09	376.1369
pmp001092	Kihadanin B	Others	Lactones	487.1963	161.1	486.189
Zmcp002924	Cyanidin 3-O-sophoroside	Flavonoids	Anthocyanidins	611.16	287.05	611.1607
Wasn002449	Protocatechuic acid 1-O-Rutinoside	Phenolic acids	Phenolic acids	461.13	152.01	462.1373
Wmmn000117	mudanoside B*	Phenolic acids	Phenolic acids	463.11	168.01	464.1166
MWSslk252	Didymin (Isosakuranetin-7-O-rutinoside)*	Flavonoids	Flavanones	593.19	285.08	594.1949

Metabolite ID	Compound Name	Class I	Class II	Q1 (m/z)	Q3 (m/z)	Molecular Weight (Da)
Lmsp102910	3-Hydroxyolean-12-ene-27, 28-dioic acid (Cincholic acid)	Terpenoids	Triterpene	487.3423	423.323	486.3345
Zmjp005293	Acacetin-7-O-neohesperid oside	Flavonoids	Flavones	593.19	285.08	592.1792
Hmbp001276	Gallacetophenone	Others	Ketone compounds	169.05	65.03	168.0423
Lmmn009550	3-Oxo-9,19-cyclolanost-24-en-26-oic acid (Mangiferonic acid)*	Terpenoids	Triterpene	453.34	453.34	454.3447
Wasdn02505	Mycarose glucoside*	Others	Others	323.13	59.01	324.142
Wafp002664	1-O-p-Coumaroyllysine	Alkaloids	Phenolamine	293.16	147.04	292.1423
MWS201471	Lys-Tyr	Amino acids and	Amino acids and	310.18	84.08	309.1689

Metabolite ID	Compound Name	Class I	Class II	Q1 (m/z)	Q3 (m/z)	Molecular Weight (Da)
		derivatives	derivatives			
Zbdp005176	Scandione	Flavonoids	Chalcones	317.07	153.05	316.0583
Rn04196	12-hydroxy jasmonoyl sulfate	Organic acids	Organic acids	305.07	96.96	306.0773
Lmbn002648	α-Hydroxycinnamic Acid*	Phenolic acids	Phenolic acids	163.04	119.05	164.0473
MWS201054	O-Acetyl-L-homoserine	Amino acids and derivatives	Amino acids and derivatives	162.08	56.05	161.0688
MWSslk248	Pedunculoside	Terpenoids	Triterpene Saponin	649.4	487.35	650.403
Safn009833	5-[(1S,2R,3R,5S)-3,5-dihydroxy-2-[(E,3S)-3-hydroxyoct-1-enyl]cyclopentyl]pentanoic acid	Lipids	Free fatty acids	327.21	171.1	328.225

Metabolite ID	Compound Name	Class I	Class II	Q1 (m/z)	Q3 (m/z)	Molecular Weight (Da)
Qmzp101901	Zanthoxyloside*	Lignans and Coumarins	Coumarins	487.1441	193.047	486.1373
mws0668	Xanthosine	Nucleotides and derivatives	Nucleotides and derivatives	283.07	151.03	284.0757
MWStz630	Quercetin-3-O-glucuronide *	Flavonoids	Flavonols	479.0849	303.0547	478.0747
Lazn002744	C-Veratroylglycol	Others	Alcohol compounds	211.06	148.02	212.0685
Zmsp004363	Quercetin-3-O-(6''-O-acetyl)glucoside	Flavonoids	Flavonols	507.12	303.05	506.106
Wmmp000180	3,23-Dihydroxylup-20(29)-en-28-oic acid (Oplopanaxogenin C)	Terpenoids	Triterpene	473.36	409.34	472.3553
pme2960	Naringenin chalcone;	Flavonoids	Chalcones	273.08	153.02	272.0685

Metabolite ID	Compound Name	Class I	Class II	Q1 (m/z)	Q3 (m/z)	Molecular Weight (Da)
	2',4,4',6'-Tetrahydroxychalcone					
Wasyqn5239	Luteolin 7-(2-sulfoglucoside)	Flavonoids	Flavones	527.05	285.04	528.0574
Lamn006527	Drimiopsin C*	Flavonoids	Other Flavonoids	287.05	125.02	288.0634
pmb1574	9,12-Octadecadien-6-Ynoic Acid	Lipids	Free fatty acids	277.22	93.07	276.2089
Ypgn004474	Resveratroloside*	Others	Stilbene	389.12	227.07	390.1315
Wayp002574	N-Hydroxyhexanoyl nornicotine glucoside	Alkaloids	Pyridine alkaloids	425.2261	263.1746	424.2204
MWSHY0142	Quercetin-3-O-(2''-O-galloyl)galactoside	Flavonoids	Flavonols	617.11	315.07	616.1064

Metabolite ID	Compound Name	Class I	Class II	Q1 (m/z)	Q3 (m/z)	Molecular Weight (Da)
Lskp211458	2,3,19,23-Tetrahydroxy-24-oxo-olean-12-en-28-oic acid	Terpenoids	Triterpene	519.3333	455.3168	518.3244
Ladp002935	3-quinolinecarboxylic acid	Alkaloids	Quinoline alkaloids	190.05	144.04	189.0426
MWStz554	Methyl 4-caffeoylquinate	Phenolic acids	Phenolic acids	369.1195	163.044	368.1107
pmb2653	DMelezitose O-rhamnoside	Others	Saccharides	649.22	145.05	650.2269
Wasn000495	6-O-alpha-L-arabinopyranosyl-D-glucopyranose	Others	Saccharides	311.1	89.02	312.1056
MWS5159	2-(2-Benzothiazolylthio)ethanol	Alkaloids	Alkaloids	212.02	168	211.0126
Wasyqn3331	5-O-p-Coumaroyl Methylglucaric Acid*	Phenolic acids	Phenolic acids	369.09	223.05	370.0895

Metabolite ID	Compound Name	Class I	Class II	Q1 (m/z)	Q3 (m/z)	Molecular Weight (Da)
Zmhn001970	Piceid (3,4',5-Trihydroxystilbene-3-O-glucoside)*	Others	Stilbene	389.12	227.08	390.1315
Lmjp003090	Isofraxidin-7-O-glucoside	Lignans and Coumarins	Coumarins	385.11	223.06	384.1056
Wagp005813	5-Hydroxy-7,8,2',3'-Tetramethoxyflavone 5-Glucoside*	Flavonoids	Flavones	521.16	359.11	520.1581
Wasn003353	Glucosyl 6-Hydroxy-2,6-Dimethyl-2E,7-Octadienoate	Terpenoids	Monoterpenoids	405.18	183.1	346.1628
pmb2826	L-Citramalic acid	Organic acids	Organic acids	147.03	87.01	148.0372
Wamxn05754	Icariside C1 Acetate	Terpenoids	Sesquiterpenoids	459.26	399.24	460.2672

Metabolite ID	Compound Name	Class I	Class II	Q1 (m/z)	Q3 (m/z)	Molecular Weight (Da)
Hmsp000364	L-Cyclopentylglycine	Amino acids and derivatives	Amino acids and derivatives	144.1	98.09	143.0946
Wmmp000110	mudanoside A	Phenolic acids	Phenolic acids	331.1015	125.0584	330.0951
mws0008	Hydrocinnamic acid	Phenolic acids	Phenolic acids	149.06	105.07	150.0681
MWS201437	Leu-Asp	Amino acids and derivatives	Amino acids and derivatives	247.13	86.1	246.1216
Lmpp003228	Petunidin-3-O-(6''-O-caffeoyl)glucoside	Flavonoids	Anthocyanidins	641.15	317.07	641.1501
mws0097	Pinoresinol*	Lignans and Coumarins	Lignans	357.13	151.04	358.1416
Lazn006735	Erythro-Guaiacylglycerol-β-O-4'-dehydrodisinapyl Ether	Lignans and Coumarins	Lignans	583.22	535.2	584.2258

Metabolite ID	Compound Name	Class I	Class II	Q1 (m/z)	Q3 (m/z)	Molecular Weight (Da)
MWS1882	N-(Carboxymethyl)glycine	Amino acids and derivatives	Amino acids and derivatives	132.03	88.04	133.0375
pme1119	Inosine	Nucleotides and derivatives	Nucleotides and derivatives	269.09	137.05	268.0808
Salp004881	Gentioluteol	Lignans and Coumarins	Lignans	407.17	181.05	406.1628
Yamp010545	quinatoside A	Terpenoids	Triterpene Saponin	589.37	571.35	588.3662
pme1194	2'-Deoxycytidine	Nucleotides and derivatives	Nucleotides and derivatives	228.1	112.05	227.0906
pma0461	14,15-Dehydrocrepenynic acid	Lipids	Free fatty acids	277.22	93.07	276.2089
pmb0464	L-Aspartic acid-O-diglucoside	Amino acids and derivatives	Amino acids and derivatives	458.15	116.03	457.1432

Metabolite ID	Compound Name	Class I	Class II	Q1 (m/z)	Q3 (m/z)	Molecular Weight (Da)
Lmyn006221	Gingerglycolipid C	Lipids	Glycerol ester	679.39	397.13	680.3983
Zbjn003801	1-O-(6'-O-feruloyl)glucoside-3-O-Caffeoyl Quinic Acid	Phenolic acids	Phenolic acids	691.18	353.09	692.1952
Cmsp005051	Isolariciresinol-9-O-glucoside	Lignans and Coumarins	Lignans	523.23	137.06	522.2101
Waptn03691	Indole-3-lactic acid glucoside	Alkaloids	Plumerane	366.12	204.07	367.1267
Hahn002315	syringaresinol-4-O-β-D-glucopyranoside*	Lignans and Coumarins	Lignans	579.21	417.16	580.2156
mws0924	2-Methylglutaric acid	Organic acids	Organic acids	145.05	101.06	146.0579
WaYp007721	2-Aminohexadecane-1,16,1	Lipids	Sphingolipids	290.27	242.25	289.2617

Metabolite ID	Compound Name	Class I	Class II	Q1 (m/z)	Q3 (m/z)	Molecular Weight (Da)
	6-triol					
pmp000608	Aurantio-obtusin-6-O-Glucoside	Quinones	Anthraquinone	493.13	331.08	492.1268
pmp000438	3,28-Dihydroxylup-20(29)-ene (Betulin)	Terpenoids	Triterpene	443.39	425.38	442.3811
Zbfp001070	N-(1-Deoxy-1-fructosyl)phenylalanine	Amino acids and derivatives	Amino acids and derivatives	328.12	166.07	327.1107
pme3081	pyridoxamine	Alkaloids	Pyridine alkaloids	169.1	134.06	168.0899
ZbWp002423	N-methylphenylalanine	Amino acids and derivatives	Amino acids and derivatives	180.1	120.08	179.0946
Wacyn02543	Methylgallic Acid 3-(6''-Sulfate)Glucoside	Phenolic acids	Phenolic acids	425.04	241	426.0468

Metabolite ID	Compound Name	Class I	Class II	Q1 (m/z)	Q3 (m/z)	Molecular Weight (Da)
Wbtp004798	8,11-dimethoxy-2h-[1,3]dioxolo[4,5-b]xanthen-10-one	Flavonoids	Other Flavonoids	301.07	286.05	300.0634
mws2523	Trehalose 6-phosphate	Others	Saccharides	421.08	241.01	422.0825
mws1086	Olean-12-ene-3,22,23-triol (Soyasapogenol B)	Terpenoids	Triterpene	457.37	375.29	458.376
Lasp004380	2',4'-Dihydroxyacetophenone*	Others	Ketone compounds	153.054	65.0388	152.0473
Walp005894	Cleomiscosin C	Lignans and Coumarins	Coumarins	417.12	367.08	416.1107
Lcwp000434	Clemiscosin D	Terpenoids	Triterpene Saponin	417.12	367.08	416.1107
Lalp013087	Bis(2-ethyl-hexyl)-phthalate	Phenolic acids	Phenolic acids	391.28	149.02	390.277
pme0075	N-Acetyl-L-glutamic acid	Amino acids and	Amino acids and	188.06	128.04	189.0637

Metabolite ID	Compound Name	Class I	Class II	Q1 (m/z)	Q3 (m/z)	Molecular Weight (Da)
		derivatives	derivatives			
Lmhn003439	Phellodenol H	Lignans and Coumarins	Coumarins	367.1	193.06	368.1107
Yajp005452	Hovetrichoside B	Lignans and Coumarins	Lignans	483.19	317.09	482.1788
Lmxp006440	2-Palmitoylglycerol*	Lipids	Glycerol ester	331.28	313.27	330.277
Lafp004228	(6R,9R)-3-Oxo-α-ionol-β-D -glucoside	Terpenoids	Sesquiterpenoids	371.2068	209.1539	370.1986
Lacp005577	3-(3'-Hydroxybutyl)-2,4,4-trimethylcyclohexa-2,5-dienone malonyl glucoside	Terpenoids	Sesquiterpenoids	457.2	133.1	456.199
Lcfn121033	(3S,5R,6R,7E,9S)-megastigm-7-ene-3,5,6,9-tetrol3-(beta-D-glucopyranoside)	Others	Others	405.18	243.12	406.1839

Metabolite ID	Compound Name	Class I	Class II	Q1 (m/z)	Q3 (m/z)	Molecular Weight (Da)
MWS4296	Gly-Phe*	Amino acids and derivatives	Amino acids and derivatives	223.11	120.08	222.1004
pmb0645	Hesperetin-6-C-glucoside-7-O-glucoside	Flavonoids	Flavanones	627.19	465.2	626.1847
Lacn005311	Phellatin	Flavonoids	Flavonols	533.17	371.11	534.1737
Lhbp111734	Isolariciresinol	Lignans and Coumarins	Lignans	361.16	137.06	360.1573
Lasp013342	Ent-16beta-Methoxy-19-Kauranoic Acid	Terpenoids	Ditepenoids	335.26	67.05	334.2503
mws0582	S-(Methyl)glutathione	Amino acids and derivatives	Amino acids and derivatives	322.11	130.05	321.0995
MWStz350	Sarmentosin	Alkaloids	Alkaloids	276.1068	114.0551	275.1005
Wdzp008195	13,17-epoxy-alisol A	Terpenoids	Triterpene	507.37	165.13	506.3607

Metabolite ID	Compound Name	Class I	Class II	Q1 (m/z)	Q3 (m/z)	Molecular Weight (Da)
Lmmn004459	Ent-15-oxo-2β,16,19-trihydroxypimar-8(14)-ene	Terpenoids	Ditepenoids	335.22	335.22	336.2301
Wcdp003754	Vanillin; 4-Hydroxy-3-Methoxybenzaldehyde*	Others	Aldehyde compounds	153.0542	65.0388	152.0473
MWSmce333	Crotonoside; 2-Hydroxyadenosine	Nucleotides and derivatives	Nucleotides and derivatives	284.1	152.06	283.0917
MWStz620	Echinocystic acid	Terpenoids	Triterpene	473.3608	427.3552	472.3553
Zmmp002167	L-Glutamyl-L-Tyrosine	Amino acids and derivatives	Amino acids and derivatives	311.12	136.07	310.1165
pme2598	3,4-Dihydroxybenzeneacetic acid*	Phenolic acids	Phenolic acids	167.03	123.05	168.0423

Metabolite ID	Compound Name	Class I	Class II	Q1 (m/z)	Q3 (m/z)	Molecular Weight (Da)
Wbtn005631	1,3,7-trihydroxy-2-[3,4,5-trihydroxy-6-(hydroxymethyl)oxan-2-yl]xanthen-9-one	Flavonoids	Other Flavonoids	405.08	243.03	406.09
Wmsn000960	Teupolioside	Amino acids and derivatives	Amino acids and derivatives	785.25	353.09	786.2582
pme3732	Cytidine	Nucleotides and derivatives	Nucleotides and derivatives	244.09	112.05	243.0855
Lmdp003110	2,6,7,4'-Tetrahydroxyisoflavanone	Flavonoids	Isoflavones	289.07	243.07	288.0634
Hmgp001888	Nepetin-5-O-diglucoside	Flavonoids	Flavones	641.17	317.06	640.1639
Hmmp003004	Vogeloside	Terpenoids	Monoterpenoids	389.14	149.06	388.1369
pmb2507	2-Deoxyribose-1-phosphat	Nucleotides and	Nucleotides and	213.02	96.97	214.0242

Metabolite ID	Compound Name	Class I	Class II	Q1 (m/z)	Q3 (m/z)	Molecular Weight (Da)
	e	derivatives	derivatives			
Zmgn002106	N-Acetyl-L-phenylalanine	Amino acids and derivatives	Amino acids and derivatives	206.08	58.03	207.0895
pmp001284	Monopalmitin*	Lipids	Glycerol ester	331.28	313.27	330.277
Rfn045100	Dihydrocoumaroyl Hexoside*	Phenolic acids	Phenolic acids	327.11	165.06	328.1158
Zbqn004590	N-(acetyl)phenylalanine	Amino acids and derivatives	Amino acids and derivatives	206.08	164.07	207.0895
Wbmp005366	Humulene epoxide II	Terpenoids	Sesquiterpenoids	221.19	135.12	220.1827
Sahp003947	Staphylionoside A	Terpenoids	Sesquiterpenoids	387.2	207.14	386.1941
Labn005411	Urolignoside	Lignans and Coumarins	Lignans	521.2	341.14	522.2101
pmn001375	1-Hydroxypinoresinol-1-O	Lignans and Coumarins	Lignans	535.18	373.13	536.1894

Metabolite ID	Compound Name	Class I	Class II	Q1 (m/z)	Q3 (m/z)	Molecular Weight (Da)
	-Glucoside					
Lhsp101912	Dehydrodiconiferyl alcohol*	Lignans and Coumarins	Lignans	359.15	327.12	358.1416
Wagp006039	2-Hexylphosphoric Acid	Others	Others	183.08	98.98	182.0708
Wasyqn3393	Trans-Ferulic Acid 4-O-[Apiosyl-(1->6)-Glucosyl] Ester	Phenolic acids	Phenolic acids	487.15	193.05	488.1524
Lmqp000873	Succinic anhydride	Organic acids	Organic acids	101.02	55.02	100.016
pmn001336	Aloeemodin-8-O-glucoside	Quinones	Anthraquinone	431.1	269	432.1056
Wcdp010708	Octadeca-2,9,12,15-tetraen-1-ol	Others	Others	263.24	81.07	262.2297
Hmcp002575	Clemaphenol A*	Lignans and Coumarins	Lignans	359.15	327.12	358.1416

Metabolite ID	Compound Name	Class I	Class II	Q1 (m/z)	Q3 (m/z)	Molecular Weight (Da)
Labn003059	galloyl malonyl glucoside	Phenolic acids	Phenolic acids	417.07	331.07	418.0747
Yacn003968	Cantleyine	Alkaloids	Alkaloids	206.08	147.04	207.0895
Lmgn002250	Dicaffeoylquinic acid-O-glucoside	Phenolic acids	Phenolic acids	677.17	191.06	678.1796
Yshs000049	6-Hydroxy-lupane-20(29)-en-3-one-28-oic acid	Terpenoids	Triterpene	471.35	407.33	470.3396
Zbtp004722	Mearnsetin	Flavonoids	Flavonols	333.06	318.03	332.0532
pmn001337	Emodin-8-O-glucoside	Quinones	Anthraquinone	431.1	269.1	432.1056
Zbdp002220	2,6-Dimethoxyhydroquinone-1-O-glucoside*	Others	Others	333.12	171.06	332.1107
MWSmce001	5-Acetylsalicylic acid	Phenolic acids	Phenolic acids	181.05	107.05	180.0423
ML10176345	3-Dehydroshikimic acid	Organic acids	Organic acids	171.03	109.03	172.0372

Metabolite ID	Compound Name	Class I	Class II	Q1 (m/z)	Q3 (m/z)	Molecular Weight (Da)
mws5042	L-Glycyl-L-phenylalanine*	Amino acids and derivatives	Amino acids and derivatives	223.11	120.09	222.1004
Zblp001862	Delphinidin-3-O-galactoside	Flavonoids	Anthocyanidins	465.1	303.05	465.1028
Wbmn010008	Cannabifolin E*	Terpenoids	Triterpene	485.33	467.31	486.3345
MWStz519	Wistin	Flavonoids	Isoflavones	461.1449	299.0932	460.1369
Zmdn011161	Chrysophanol-9-anthrone	Quinones	Anthraquinone	239.07	223.03	240.0786
Lscp211203	Sedumoside H	Others	Others	389.22	227.16	388.2097
Yshj000011	2,6-dimethoxy-4-hydroxyphenyl-1-O-beta-D-glucopyranoside*	Others	Others	333.1191	171.065	332.1107
mws2125	Phosphoenolpyruvate	Organic acids	Organic acids	166.98	78.96	167.9824

Metabolite ID	Compound Name	Class I	Class II	Q1 (m/z)	Q3 (m/z)	Molecular Weight (Da)
pmb3056	Homovanilloylquinic acid	Phenolic acids	Phenolic acids	355.1	121.03	356.1107
Lskp211307	Grayanotoxin VIII	Terpenoids	Ditepenoids	335.22	299.2	334.2144
mws0147	β-Hydroxyisovaleric acid	Organic acids	Organic acids	117.06	59.01	118.063
Wagn012030	2-Palmitoyl-Sn-Glycerol 3-O-Diglucoside	Lipids	Glycerol ester	653.38	397.14	654.3827
Wasyqn3916	1-O-p-Coumaroyl Methylglucaric Acid*	Phenolic acids	Phenolic acids	369.08	223.05	370.0895
Lsjp211201	3S-3-hydroxy-β-damascon	Terpenoids	Monoterpenoids	209.15	137.1	208.1463
Lmsp003252	Isorhamnetin-3-O-sophoro side*	Flavonoids	Flavonols	641.17	317.07	640.1639
pme1109	Guanine	Nucleotides and derivatives	Nucleotides and derivatives	152.06	135.03	151.0494

Metabolite ID	Compound Name	Class I	Class II	Q1 (m/z)	Q3 (m/z)	Molecular Weight (Da)
MWSmce295	1-Arabinosyluracil	Nucleotides and derivatives	Nucleotides and derivatives	245.07	113.03	244.0695
Hmsn002272	Demethyl coniferin	Others	Alcohol compounds	327.11	165.05	328.1158
pmp001054	Gardenoside	Terpenoids	Monoterpenoids	422.1657	225.0756	404.1319
Lakn002368	2-Hydroxy-5-methylbenzoyl-1-O-glucose	Phenolic acids	Phenolic acids	313.09	107.05	314.1002
Hmln002100	Nortrachelogenin-4-O-glucoside	Lignans and Coumarins	Lignans	535.18	373.13	536.1894
Lskp211454	2,3,19,23-Tetrahydroxyolean-12-en-28-oic acid (Sericic acid)	Terpenoids	Triterpene	505.35	405.32	504.3451
Lcfn005033	7-O-methylmyricetin	Flavonoids	Flavonols	331.05	316.03	332.0532

Metabolite ID	Compound Name	Class I	Class II	Q1 (m/z)	Q3 (m/z)	Molecular Weight (Da)
HX1340	epiloliolide	Others	Others	197.12	133.1	196.1099
Lmhp001670	L-Valyl-L-Leucine	Amino acids and derivatives	Amino acids and derivatives	231.17	72.08	230.163
MWSmce116	5'-Deoxyadenosine*	Nucleotides and derivatives	Nucleotides and derivatives	252.1	136.06	251.1018
Lmhp010908	LysoPC 19:1	Lipids	LPC	536.37	184.07	535.3638
Hmyn000254	Tababiphenyl C	Others	Chromone	297.12	59.01	298.1205
Zmfn000481	Uridine-5'-Diphosphate-D-Xylose	Nucleotides and derivatives	Nucleotides and derivatives	535.04	323.03	536.0445
MWSmce440	Guvacine	Alkaloids	Piperidine alkaloids	128.0704	99.0442	127.0633
Lmmn001643	2-Hydroxycinnamic acid*	Phenolic acids	Phenolic acids	163.04	119.05	164.0473
Cmxn004016	Dihydromarein	Flavonoids	Chalcones	451.13	289.08	452.1319

Metabolite ID	Compound Name	Class I	Class II	Q1 (m/z)	Q3 (m/z)	Molecular Weight (Da)
Lssp210325	Grayanoside C	Terpenoids	Ditepenoids	499.29	283.21	498.2829
Layp003558	3,4,5-Trimethoxyphenol-1-O-β-D-glucoside	Others	Others	347.1327	185.0817	346.1264
Zmzp006646	Capillarisin	Others	Chromone	317.07	302.04	316.0583
Zbdp006427	7,8-Dihydroxy-4'-methoxyisoflavone	Flavonoids	Other Flavonoids	285.08	253.05	284.0685
pmb3066	5-O-p-Coumaroylshikimic acid O-glucoside	Phenolic acids	Phenolic acids	481.14	319.2	482.1424
Laxp005948	7,2'-dihydroxy-4'-methoxy-isoflavanol glucoside	Flavonoids	Other Flavonoids	451.16	271.1	450.1526
Hmcn000192	Sedoheptulose	Others	Saccharides	209.07	59.01	210.074
Wchn001739	3-O-Galloyl-Glucose	Phenolic acids	Phenolic acids	331.07	211.03	332.0743

Metabolite ID	Compound Name	Class I	Class II	Q1 (m/z)	Q3 (m/z)	Molecular Weight (Da)
Lmpp003296	Delphinidin-3-O-glucuronide	Flavonoids	Anthocyanidins	479.08	303.05	479.082
Lmpn007255	Patuletin (Quercetagetin-6-methyl ether)	Flavonoids	Flavonols	331.04	316.02	332.0532
Sasn010334	3,3,14,14-tetramethylhexadecanedioic acid	Lipids	Free fatty acids	341.27	279.27	342.277
Lmsn003628	6'-O-Sinapoylsucrose*	Phenolic acids	Phenolic acids	547.17	223.06	548.1741
Zbn006783	5,6-Dihydroxylucidin	Quinones	Anthraquinone	301.04	245.05	302.0448
Rn00844	dGDP	Nucleotides and derivatives	Nucleotides and derivatives	426.02	158.93	427.0294
Lazp002140	Luteolin	Flavonoids	Flavones	757.22	449.11	756.2113

Metabolite ID	Compound Name	Class I	Class II	Q1 (m/z)	Q3 (m/z)	Molecular Weight (Da)
	7-rutinoside-4'-glucoside					
Lbhp011920	Scopoletin 7-O-β-D-Sophoroside*	Lignans and Coumarins	Coumarins	517.15	193.05	516.1479
MWSmce537	Veratraldehyde	Phenolic acids	Phenolic acids	167.07	124.05	166.063
Hmgn002038	β-Oxoacteoside	Phenolic acids	Phenolic acids	637.18	475.14	638.1847
pmn001672	Furanofructosyl-α-D-(3-mustard acyl)glucoside*	Phenolic acids	Phenolic acids	547.17	223.1	548.1741
Lcyp000689	Cyanidin-3-O-gentiobioside*	Flavonoids	Anthocyanidins	611.16	287.06	611.1612
Labn003679	(E)-caffeyl alcohol 4-O-β-D-glucopyranoside	Others	Alcohol compounds	327.11	147.05	328.1158
pmn001610	Eicosadienoic acid	Lipids	Free fatty acids	307.26	307.26	308.2715

Metabolite ID	Compound Name	Class I	Class II	Q1 (m/z)	Q3 (m/z)	Molecular Weight (Da)
MWSHY0102	Morin	Flavonoids	Flavonols	303.05	229.05	302.0427
Lssp210412	Cuscohygrine	Alkaloids	Pyrrole alkaloids	225.2	100.11	224.1889
Lmbn001467	5-Acetamidopentanoic Acid	Organic acids	Organic acids	158.08	116.07	159.0895
pmb0618	Hesperetin-8-C-glucoside-3'-O-glucoside	Flavonoids	Flavanones	627.19	465.1	626.1847
Zbqn010247	Rubiatriol	Terpenoids	Triterpene	457.37	457.37	458.376
Labp005273	Acacetin 7-apiosylglucoside	Flavonoids	Flavones	579.1719	285.0761	578.1636
MWStz497	Matairesinol	Lignans and Coumarins	Lignans	359.1498	137.0611	358.1416
Sacn003282	10-hydroxymajoroside	Terpenoids	Monoterpenoids	403.12	241.07	404.1319
Lskp211450	2,3,19,23-Tetrahydroxyolea	Terpenoids	Triterpene	505.35	423.33	504.3451

Metabolite ID	Compound Name	Class I	Class II	Q1 (m/z)	Q3 (m/z)	Molecular Weight (Da)
	n-12-en-28-oic acid (Arjungenin)*					
mws1375	Nicotianamine	Alkaloids	Alkaloids	304.15	185.09	303.143
Lmyp002078	Neolinustatin	Alkaloids	Alkaloids	424.18	262.1274	423.1741
Wtmn002164	3,4-dimethoxy-2-[(3,4,5-trihydroxy-6-{[(3,4,5-trihydroxy-6-methyloxan-2-yl)oxy]methyl}oxan-2-yl)oxy]xanthen-9-one	Flavonoids	Other Flavonoids	579.16	579.16	580.1792
MWS201414	Pro-Asn	Amino acids and derivatives	Amino acids and derivatives	230.11	70.07	229.1063
Zmyn000230	2-Dehydro-3-deoxy-L-arabinonate	Others	Saccharides	147.03	57.03	148.0372

Metabolite ID	Compound Name	Class I	Class II	Q1 (m/z)	Q3 (m/z)	Molecular Weight (Da)
Lcfn121063	N-Salicyloylasparticacid	Amino acids and derivatives	Amino acids and derivatives	252.05	136.04	253.0586
Lsjp211254	Lupa-1,20(29)-dien-3-one (Glochidone)	Terpenoids	Triterpene	423.36	203.1752	422.3549
pme2074	Jasmonoyl-L-Isoleucine	Amino acids and derivatives	Amino acids and derivatives	322.2	130.05	323.2097
MA10039492	Dehydroascorbic acid	Others	Vitamin	173.01	71.03	174.0164
Safn002073	Glu-Leu	Amino acids and derivatives	Amino acids and derivatives	259.13	130.09	260.1372
MWS2040	Methanesulfonic acid	Organic acids	Organic acids	94.98	79.96	95.9881
pmp001073	Synephrine; 4-[1-Hydroxy-2-(methylam	Alkaloids	Alkaloids	168.1	150.09	167.0946

Metabolite ID	Compound Name	Class I	Class II	Q1 (m/z)	Q3 (m/z)	Molecular Weight (Da)
	ino)ethyl]phenol					
Hmhn000927	Phloroglucinol-1-O-β-D-glucopyranoside	Others	Others	287.08	125.02	288.0845
mws1290	Kaempferol-3-O-(6''-p-Coumaroyl)glucoside (Tiliroside)	Flavonoids	Flavonols	593.13	285.04	594.1373
MWS201443	Ser-Lys	Amino acids and derivatives	Amino acids and derivatives	234.15	84.08	233.1376
Zahp007439	3,4-Methylenedioxycinnamaldehyde	Others	Aldehyde compounds	177.05	149.02	176.0473
Lmmn000774	Dihydrocaffeoylglucose*	Phenolic acids	Phenolic acids	343.1	181.05	344.1107
Wasn002933	1-O-(3,4,5-Trimethoxybenz	Phenolic acids	Phenolic acids	373.11	211.06	374.1213

Metabolite ID	Compound Name	Class I	Class II	Q1 (m/z)	Q3 (m/z)	Molecular Weight (Da)
	oyl)-B-D-Glucopyranoside					
Lmsp013238	3-Hydroxy-urs-11-en-28,13 -lactone	Terpenoids	Triterpene	455.352	301.2162	454.3447
Lmfp009701	Glycerol 9,11,13-octadecatrienoyl ester*	Lipids	Glycerol ester	353.27	261.22	352.2614
Wbjp001317	vasicinone	Alkaloids	Alkaloids	203.08	130.07	202.0742
MWSslk102	6-Chloropurine	Nucleotides and derivatives	Nucleotides and derivatives	155.01	119.04	154.0046
mws1574	Geniposidic acid	Terpenoids	Monoterpenoids	373.11	123.01	374.1213
MWS1848	Phenyl acetate	Phenolic acids	Phenolic acids	135.05	92.03	136.0524
pmp000271	1,2,3,19-Tetrahydroxyurs-1	Terpenoids	Triterpene	505.3524	423.3254	504.3451

Metabolite ID	Compound Name	Class I	Class II	Q1 (m/z)	Q3 (m/z)	Molecular Weight (Da)
	2-en-28-oic acid*					
pmp000270	Lup-12-en-15α,19β-diol-3,11-dioxo-28-oic acid	Terpenoids	Triterpene	501.3211	233.1546	500.3138
Yarn002524	5β,6β-Dihydroxyboschnaloside*	Terpenoids	Monoterpenoids	375.13	169.09	376.1369
Lman002731	Grevilloside F	Phenolic acids	Phenolic acids	341.09	135.04	342.0951
mws0011	Syringin	Others	Alcohol compounds	371.13	209.08	372.142
MWS1860	Pyrrolidin	Alkaloids	Pyrrole alkaloids	72.08	55.05	71.0735
pme1216	2-Picolinic acid	Alkaloids	Pyridine alkaloids	122.02	78.03	123.032
MWS1855	2,5-Dimethyl pyrazine	Alkaloids	Alkaloids	109.08	67.04	108.0687
pma0134	D-Threose	Others	Saccharides	121.05	93.06	120.0423

Metabolite ID	Compound Name	Class I	Class II	Q1 (m/z)	Q3 (m/z)	Molecular Weight (Da)
Lmsn004322	1,6-Di-O-caffeoyl-β-D-glucose*	Phenolic acids	Phenolic acids	503.12	179.03	504.1268
Lmhp011562	1-α-Linolenoyl-glycerol*	Lipids	Glycerol ester	353.27	261.22	352.2614
Yarn005295	Tortoside A*	Lignans and Coumarins	Lignans	579.21	417.15	580.2156
Llhp011704	3-Hydroxyolean-12-ene-28,29-dioic acid (Serratagenic Acid)	Terpenoids	Triterpene	487.34	423.33	486.3345
MWS201403	Lys-Ala	Amino acids and derivatives	Amino acids and derivatives	218.15	84.08	217.1426
Lmhp009187	LysoPE 15:0*	Lipids	LPE	440.28	299.26	439.2699
Walp001919	1-O-p-Coumaroylgalactaric acid	Phenolic acids	Phenolic acids	357.081	147.0443	356.0738

Metabolite ID	Compound Name	Class I	Class II	Q1 (m/z)	Q3 (m/z)	Molecular Weight (Da)
pme2165	Orotic acid (Vitamin B13)	Others	Vitamin	155.01	111.02	156.0171
ML10180524	Cytarabine	Nucleotides and derivatives	Nucleotides and derivatives	244.09	112.05	243.0855
Lmjp002764	Umckalin (7-hydroxy-5,6-dimethoxycoumarin)	Lignans and Coumarins	Coumarins	223.06	190.03	222.0528
Wcdp010162	1-Monolinolenoyl-Rac-Glycerol*	Lipids	Glycerol ester	353.2684	261.2229	352.2614
pmb3072	3-O-p-Coumaroylshikimic acid-O-glucoside	Phenolic acids	Phenolic acids	481.14	319.3	482.1424
Lajp003510	Furo(2,3-f)-1,3-bewnzodioxole	Phenolic acids	Phenolic acids	271.0611	149.0208	270.0528

Metabolite ID	Compound Name	Class I	Class II	Q1 (m/z)	Q3 (m/z)	Molecular Weight (Da)
Hmcp005697	Olean-13(18)-en-3-one (δ-Amyrenone)	Terpenoids	Triterpene	425.37	191.18	424.3705
Wabn003563	3'-O-Beta-D-Glucopyranosyl plumbagic acid	Phenolic acids	Phenolic acids	385.11	179.07	386.1213
Zbjn003356	1,3-O-di-Caffeoyl-4-O-glucoside Quinic Acid	Phenolic acids	Phenolic acids	677.17	353.09	678.1802
Lssp210320	3-oxo-taraxeran-14-en	Terpenoids	Triterpene	425.37	203.18	424.3705
MWSmce549	1-Monomyristin	Lipids	Glycerol ester	303.25	57.07	302.2457
pma0253	O-MethylChrysoeriol-5-O-glucoside	Flavonoids	Flavones	477.1391	315.0863	476.1319
MWStz419	3-Oxopomolic acid	Terpenoids	Triterpene	471.35	407.33	470.3396
Zbsp011022	Lup-20(29)-en-3-one	Terpenoids	Triterpene	425.38	191	424.3705

Metabolite ID	Compound Name	Class I	Class II	Q1 (m/z)	Q3 (m/z)	Molecular Weight (Da)
	(Lupenone)					
Lmsn004970	3,6-Di-O-caffeoyl glucose*	Phenolic acids	Phenolic acids	503.12	179.04	504.1268
HJN003	1-O-Sinapoyl-β-D-glucose	Phenolic acids	Phenolic acids	385.11	223.06	386.1213
Hmjp003123	Quercetin-5-O-glucuronide *	Flavonoids	Flavonols	479.08	303.06	478.0747
pmb2640	Dodecanoic acid (Lauric acid)	Lipids	Free fatty acids	199.17	199.17	200.1776
Zmhp004034	Gossypetin-7-O-L-rhamnopyranoside(Rhodiolgin)	Flavonoids	Flavonols	465.1	303.05	464.0955
Wcdn006629	3,7-dihydroxy-2-methoxy-8,8,10-trimethyl-6,7-dihydroanthracene-1,4,5-trione	Quinones	Quinones	329.1	314.08	330.1103

Metabolite ID	Compound Name	Class I	Class II	Q1 (m/z)	Q3 (m/z)	Molecular Weight (Da)
Lmhp007489	Asperic acid	Organic acids	Organic acids	285.2051	149.1326	284.1988
WaYp006462	2-Aminohexadecane-1,5,15-triol	Lipids	Sphingolipids	290.27	228.23	289.2617
Zbjp001957	Cyanidin-3,5-O-diglucoside (Cyanin)*	Flavonoids	Anthocyanidins	611.16	287.06	611.1607
Yaan006543	Benzyl 6-O-galloyl beta-D-glucopyranoside	Others	Others	421.11	313.06	422.1213
Wasyqn1721	Mevalonic Acid Glucoside	Organic acids	Organic acids	309.12	59.01	310.1258
Lmtp004044	Quercetin-3-O-apiosyl(1→2)galactoside	Flavonoids	Flavonols	597.14	303.04	596.1377
MWSmce689	Methyl linolenate	Lipids	Free fatty acids	293.25	95.09	292.2402
Lmqn005782	4,5,8-Trihydroxy-α-tetralo	Quinones	Naphthol	193.05	133.03	194.0579

Metabolite ID	Compound Name	Class I	Class II	Q1 (m/z)	Q3 (m/z)	Molecular Weight (Da)
	ne					
MWSHY0162	Quercetin-3-O-sophoroside (Baimaside)	Flavonoids	Flavonols	627.16	303.06	626.1483
pme1654	Jasmonic acid	Organic acids	Organic acids	209.12	59.02	210.1256
Lcsn000623	Tricalysioside R	Terpenoids	Ditepenoids	541.3	481.28	542.3091
Lahp003599	Homoeriodictyol-7,4'-di-O-β-D-glucopyranoside	Flavonoids	Flavanones	627.19	303.09	626.1847
pmp000275	Gambiriin A1	Tannins	Tannin	581.17	139.04	580.1581
pmp000307	Sec-O-glucosylhamaudol	Others	Chromone	439.16	259.1	438.1526
Lmgn000160	N-carbamoyl-b-alanine	Amino acids and derivatives	Amino acids and derivatives	131.05	70.03	132.0535
Lmzn001983	D-Threo-guaiacylglycerol-	Others	Alcohol compounds	375.13	135.05	376.1369

Metabolite ID	Compound Name	Class I	Class II	Q1 (m/z)	Q3 (m/z)	Molecular Weight (Da)
	7-O-β-D-glucoside					
Lmxn004090	6''-O-Caffeoylharpagide	Terpenoids	Terpene	525.16	161.02	526.1686
Lmhp011388	2-α-Linolenoyl-glycerol*	Lipids	Glycerol ester	353.27	261.22	352.2614
MWSmce501	Protocatechuic Acid Methyl Ester	Phenolic acids	Phenolic acids	167.04	108.02	168.0423
Lmmp002080	N-(4-Aminobutyl)benzamide	Alkaloids	Alkaloids	193.13	105.03	192.1263
Wcdn000881	N-Fructosyl Pyroglutamate	Amino acids and derivatives	Amino acids and derivatives	290.09	128.04	291.0954
Lssp210347	Craiobioside B	Others	Others	515.28	353.23	514.2778
Hmcp001578	Isorhamnetin-3,7-O-diglucoside	Flavonoids	Flavonols	641.17	479.11	640.1639

Metabolite ID	Compound Name	Class I	Class II	Q1 (m/z)	Q3 (m/z)	Molecular Weight (Da)
Lcsp002898	cis-Zeatin-O-glucoside riboside	Alkaloids	Alkaloids	514.2116	220.1176	513.2071
Rfn0125088	Episyringaresinol	Lignans and Coumarins	Lignans	417.15	181.06	418.1628
mws1629	Aucubin	Terpenoids	Sesquiterpenoids	345.12	183.07	346.1264
Wmzn002116	3,5-Dicaffeoylquinic acid	Phenolic acids	Phenolic acids	515.12	353.09	516.1268
Lmdn003868	Frehmaglutoside G	Terpenoids	Monoterpenoids	431.23	161.05	432.2359
Yamn006257	2-(4-(3-(hydroxymethyl)-5-(2-hydroxyvinyl)-7-methoxy-2,3-dihydrobenzofuran-2-yl)-2,6-dimethoxyphenoxy)-6-methyltetrahydro-2H-pyran-3,4,5-triol*	Lignans and Coumarins	Lignans	519.19	357.15	520.1945

Metabolite ID	Compound Name	Class I	Class II	Q1 (m/z)	Q3 (m/z)	Molecular Weight (Da)
pmb0854	LysoPC 18:3	Lipids	LPC	518.32	184.07	517.3168
Lmtn002233	Androsin	Others	Ketone compounds	327.11	165.05	328.1158
Zbcp002269	Petunidin-3-O-glucoside-5-O-arabinoside	Flavonoids	Anthocyanidins	611.16	317.07	611.1607
Zbkn004627	cyclo-Dopa 5-O-glucoside	Alkaloids	Alkaloids	356.1	150.06	357.106
MWStz607	Vicine	Alkaloids	Alkaloids	305.1098	143.0575	304.1019
Zmbp000490	Valerine	Alkaloids	Alkaloids	158.12	112.08	157.1103
Lmtp004126	Myricetin-3-O-(6''-malony) glucoside*	Flavonoids	Flavonols	567.1	319.04	566.0908
mws1060	9-(Arabinosyl)hypoxanthin e	Nucleotides and derivatives	Nucleotides and derivatives	267.07	135.03	268.0808
Lchp000218	(R)-3-ethyl-7-hydroxy-6-m	Others	Lactones	209.08	121.06	208.0736

Metabolite ID	Compound Name	Class I	Class II	Q1 (m/z)	Q3 (m/z)	Molecular Weight (Da)
	ethoxyphthalide					
Yaap006585	Resinoside B*	Flavonoids	Flavones	615.2	287.06	614.1999
Lmsp010763	Monolinolenin*	Lipids	Glycerol ester	353.27	261.22	352.2614
pmp000448	3α-Dihydrocadambine	Alkaloids	Plumerane	547.2287	367.1663	546.2213
Lcsn000648	ent-3α,16β,17,19-Tetrahydroxykauran-19-ylacetate-17-O-β-D-glucopyranoside	Terpenoids	Ditepenoids	541.3	481.28	542.3091
MWSprf117	5-Hydroxy-DL-tryptophan (5-HTP)	Amino acids and derivatives	Amino acids and derivatives	221.09	130.06	220.0848
Lmyp006119	Olean-12-en-3-one (β-Amyrone)	Terpenoids	Triterpene	425.38	217.19	424.3705
Yalp008918	Chisopanin D	Terpenoids	Triterpene	559.4	335.26	558.392

Metabolite ID	Compound Name	Class I	Class II	Q1 (m/z)	Q3 (m/z)	Molecular Weight (Da)
pmf0124	2-Hydroxymelatonin	Alkaloids	Plumerane	249.1234	190.0862	248.1161
MWS20201	1-O-Galloyl-β-D-glucose	Phenolic acids	Phenolic acids	333.08	171.03	332.0743
Wahsn03409	2-O-p-Coumaroyl 3-methylglucaric acid*	Phenolic acids	Phenolic acids	369.08	223.05	370.09
WaYp004645	3-Oxo-Alpha-Ionol 9-[Apiosyl-(1→6)-Glucoside]	Terpenoids	Monoterpenoids	503.25	209.15	502.2414
ML10171848	D-Arabinono-1,4-lactone	Others	Saccharides	147.03	85.03	148.0372
Zblp004717	3'-methoxyquercetin-3-O-L-rhamnosyl(1→2)-glucopyranoside*	Flavonoids	Flavonols	625.17	317.07	624.169
Wasyqn2983	3-[4-(Beta-D-Glucopyranos	Phenolic acids	Phenolic acids	327.11	165.06	328.1158

Metabolite ID	Compound Name	Class I	Class II	Q1 (m/z)	Q3 (m/z)	Molecular Weight (Da)
	yloxy)Phenyl]Propionic Acid*					
pmb0865	LysoPC 18:3(2n isomer)	Lipids	LPC	518.32	184.07	517.3168
mws0255	Cytosine	Nucleotides and derivatives	Nucleotides and derivatives	112.05	95.02	111.0433
mws0214	D-Sorbitol	Others	Saccharides	181.07	71.01	182.079
Lmhp008885	LysoPE 15:0(2n isomer)*	Lipids	LPE	440.28	299.26	439.2699
Ladp004043	Acacetin rhamnoside	Flavonoids	Flavones	431.13	285.08	430.1264
pmp000445	Vincoside lactam	Alkaloids	Plumerane	499.2075	337.1598	498.2002
Lcfn121061	N-Acetyl-6-hydroxy-L-tryp tophan	Amino acids and derivatives	Amino acids and derivatives	261.09	131.04	262.0954
Waptn06702	(9S)-Hydroxyoctadecadien	Lipids	Free fatty acids	295.23	171.1	296.2351

Metabolite ID	Compound Name	Class I	Class II	Q1 (m/z)	Q3 (m/z)	Molecular Weight (Da)
	oic acid					
Yalp008047	Methyl 12-phenyldodecanoate	Lipids	Free fatty acids	291.23	93.07	290.2246
pmb0496	N-Feruloylagmatine	Alkaloids	Phenolamine	307.18	177.05	306.1692
Zmzp000616	4-Aminophenol	Alkaloids	Alkaloids	110.06	93.06	109.0528
Zmmp002946	(3-(carboxyamino)-2-methylpropanoyl)phenylalanine *	Amino acids and derivatives	Amino acids and derivatives	295.1286	120.081	294.1216
Zasn010461	Methyl 14-methylpentadecanoate	Lipids	Free fatty acids	269.25	269.25	270.2559
Lmmn002230	Demethylwedelolactone	Lignans and Coumarins	Coumarins	299.02	271.03	300.027
Zmyn000247	2-Hydroxyglutaric Acid	Organic acids	Organic acids	147.03	57.03	148.0372

Metabolite ID	Compound Name	Class I	Class II	Q1 (m/z)	Q3 (m/z)	Molecular Weight (Da)
Lasp002993	Isobavachalcone glucoside	Flavonoids	Chalcones	487.19	325.14	486.189
Zmhp002730	6-Hydroxykaempferol-6,7-O-Diglucoside*	Flavonoids	Flavonols	627.1557	303.0512	626.1483
pmb0296	1-Oleoyl-Sn-Glycerol	Lipids	Glycerol ester	357.3	265.25	356.2927
Lmqp000427	N-Methyl-Trans-4-Hydroxy-L-Proline	Amino acids and derivatives	Amino acids and derivatives	146.08	82.07	145.0739
Zbfn002690	1-(4-Hydroxybenzoyl)Glucose; 25545-07-7	Phenolic acids	Phenolic acids	299.08	93.03	300.0845
HJN045	Epicatechin-(2beta->7,4beta->8)-epiafzelechin-(4beta->8)-epicatechin	Flavonoids	Flavanols	847.19	411.07	848.1952
Zbqp011279	N-(Octadecyldienyl)nornic	Alkaloids	Pyridine alkaloids	411.3392	149.1076	410.3297

Metabolite ID	Compound Name	Class I	Class II	Q1 (m/z)	Q3 (m/z)	Molecular Weight (Da)
	otine					
Lskp211491	2,3,23-Trihydroxyurs-12,19(29)-dien-28-oic acid	Terpenoids	Triterpene	487.34	405.31	486.3345
pme2122	Histamine	Alkaloids	Alkaloids	112.09	95.06	111.0796
Lmqp000329	5-Aminoimidazole ribonucleotide	Nucleotides and derivatives	Nucleotides and derivatives	296.07	104.11	295.0569
Lcfp009376	homoisocitricacid	Organic acids	Organic acids	207.05	143.03	206.0427
Cmcp006262	2,3,7,19-Tetrahydroxyurs-12-en-28-oic acid (Roxburic Acid)*	Terpenoids	Triterpene	505.35	423.33	504.3451
Hmcp002298	Limocitrin-3-O-arabinoside	Flavonoids	Flavonols	479.12	347.07	478.1111
HX1369	Lariciresinol	Lignans and Coumarins	Lignans	361.1639	189.0907	360.1573

Metabolite ID	Compound Name	Class I	Class II	Q1 (m/z)	Q3 (m/z)	Molecular Weight (Da)
Yajp005210	awabukinol	Terpenoids	Monoterpenoids	221.19	81.07	220.1827
Zjbn102543	Bletilols B	Others	Others	475.18	179.07	476.1835
pme1611	Isohemiphloin	Flavonoids	Flavanones	433.11	313.07	434.1213
Lazn006001	Dihydrobuddlenol B	Lignans and Coumarins	Lignans	585.23	537.21	586.2414
Wafn002081	Dihydroxybenzoyl xyloside	Phenolic acids	Phenolic acids	285.06	153.02	286.0689
Wbmp008389	2,3,23-Trihydroxyolean-12-en-28-oic acid	Terpenoids	Triterpene	489.36	443.35	488.3502
Lmsn002815	Kaempferol-3-O-rutinoside (Nicotiflorin)*	Flavonoids	Flavonols	593.15	285.04	594.1585
pme2954	Quercetin	Flavonoids	Flavonols	303.05	137.02	302.0427
Wahsn03908	3-O-p-Coumaroyl	Phenolic acids	Phenolic acids	369.08	223.05	370.09

Metabolite ID	Compound Name	Class I	Class II	Q1 (m/z)	Q3 (m/z)	Molecular Weight (Da)
	3-methylglucaric acid*					
Lamn006359	4'-demethyl-3,9-dihydroeucomin glucoside	Flavonoids	Other Flavonoids	447.12	285.07	448.1369
Zmxn003874	7,8-Dihydro-Buddlenol B(threo)	Lignans and Coumarins	Lignans	585.23	537.21	586.2414
Hmcp005535	α-Amyrenone	Terpenoids	Triterpene	425.38	191.17	424.3705
Rfn072621	6-Hydroxyluteolin 7-rutinoside	Flavonoids	Flavones	609.15	301.04	610.1534
pmb0889	Punicic acid (9Z,11E,13Z-octadecatrienoic acid)	Lipids	Free fatty acids	279.23	95.09	278.2246
Zbsp004438	Quercetin-3,4'-O-di-glucosi	Flavonoids	Flavonols	627.1571	303.0499	626.1483

Metabolite ID	Compound Name	Class I	Class II	Q1 (m/z)	Q3 (m/z)	Molecular Weight (Da)
	de					
Smnp004764	7-hydroxyaloin	Quinones	Anthraquinone	435.12	255.06	434.1213
Hmcn001884	6-Hydroxyluteolin 5-glucoside*	Flavonoids	Flavones	463.09	301.03	464.0955
Wbtn002952	1-(β-D-Glucopyranosyl)-3-ethyl-4-methyl-1H-pyrrole-2,5-dione	Others	Ketone compounds	300.11	138.06	301.1162
Latp009318	Virgaurenolide A	Terpenoids	Sesquiterpenoids	343.15	163.07	342.1462
Jmwn006041	Dehydrodiconiferylalcohol -9'-O-glucoside*	Lignans and Coumarins	Lignans	519.19	357.13	520.1945
MWSmce466	4-Hydroxyacetophenone	Others	Ketone compounds	135.05	92.03	136.0524
Zmxn004029	7,8-Dihydro-Buddlenol	Lignans and Coumarins	Lignans	585.23	195.07	586.2414

Metabolite ID	Compound Name	Class I	Class II	Q1 (m/z)	Q3 (m/z)	Molecular Weight (Da)
	B(erythro)					
Lmhp007598	LysoPC 19:2(2n isomer)	Lipids	LPC	534.36	184.07	533.3481
Lmjn002534	Darendoside A	Phenolic acids	Phenolic acids	431.16	149.05	432.1632
Zbfn002396	6-O-Dihydrocaffeoylglucose	Phenolic acids	Phenolic acids	343.1	163.04	344.1107
Lmjp004816	Loliolide	Terpenoids	Monoterpenoids	197.12	133.1	196.1099
Lmhp010515	LysoPC 17:0(2n isomer)*	Lipids	LPC	510.36	184.07	509.3481
Zmpn004713	Isoetin (5,7,2',4',5'-Pentahydroxyflavone)	Flavonoids	Flavones	301.04	149.03	302.0427
pme2125	Raffinose*	Others	Saccharides	503.1618	179.0551	504.169
Lmmp002143	Gossypetin-3-O-(6''-malon	Flavonoids	Flavonols	567.1	319.05	566.0908

Metabolite ID	Compound Name	Class I	Class II	Q1 (m/z)	Q3 (m/z)	Molecular Weight (Da)
	yl)glucoside*					
Wdtp003646	4-Ethylphenol glucuronide	Phenolic acids	Phenolic acids	299.1116	107.0489	298.1053
Lmhn002140	Feruloyltartaric acid (Fertaric acid)	Phenolic acids	Phenolic acids	325.06	193.05	326.0638
Wcdp009119	Octadeca-9,12,15-trienoic acid	Lipids	Free fatty acids	279.23	81.07	278.2246
Lacp003991	Blumenol B Glucoside*	Terpenoids	Sesquiterpenoids	389.21	209.15	388.2092
MWStz373	Gardoside	Terpenoids	Monoterpenoids	373.11	193.05	374.1213
mws1189	D-Galacturonic acid*	Others	Saccharides	193.04	72.99	194.0427
MWSmce583	Piperonylic acid	Phenolic acids	Phenolic acids	165.02	91.02	166.0266
Wacyp04090	Phenylethyl Primeveroside	Others	Alcohol compounds	434.2	163.06	416.1682

Metabolite ID	Compound Name	Class I	Class II	Q1 (m/z)	Q3 (m/z)	Molecular Weight (Da)
MWStz568	6-Hydroxykaempferol 3,6-diglucoside*	Flavonoids	Flavonols	627.1571	303.0535	626.1483
pmb3114	Epicatechin-(3'-O-7'')-epiafzelechin	Flavonoids	Flavanols	561.14	289.07	562.1475
Walbn07906	2,6-Di-tert-butyl-4-hydroxymethylphenol	Others	Others	235.17	217.16	236.1776
Sasn003876	(4R)-4-[(1E,3R)-4-hydroxy-3-{[(2R,3R,4S,5R,6R)-3,4,5-trihydroxy-6-(hydroxymethyl)oxan-2-yl]oxy}but-1-en-1-yl]-3,5,5-trimethylcyclohex-2-en-1-one	Others	Others	445.21	205.12	386.1941
MWSmce040	Isovanillin*	Others	Aldehyde	153.05	65.04	152.0473

Metabolite ID	Compound Name	Class I	Class II	Q1 (m/z)	Q3 (m/z)	Molecular Weight (Da)
			compounds			
pme0376	Naringenin (5,7,4'-Trihydroxyflavanone)	Flavonoids	Flavanones	271.06	151	272.0685
Lskp211249	epieudesmin	Lignans and Coumarins	Lignans	387.18	137.06	386.1729
Yaln005580	23-Hydroxytoonacilide	Terpenoids	Triterpene	585.24	537.21	586.2414
Jmlp004238	Byzantionoside A	Others	Ketone compounds	371.2	133.1	370.1992
Lazn002030	Guaiacylglycerol-β-Guaiacyl Ether glucoside	Lignans and Coumarins	Lignans	481.17	271.1	482.1788
Zbsp002083	Pelargonidin-3,5-O-diglucoside	Flavonoids	Anthocyanidins	595.17	271.06	595.1657
Zmpn000199	D-Galactaric acid	Others	Saccharides	209.03	85.03	210.0376

Metabolite ID	Compound Name	Class I	Class II	Q1 (m/z)	Q3 (m/z)	Molecular Weight (Da)
Wccp002870	γ-Glu-Phe*	Amino acids and derivatives	Amino acids and derivatives	295.13	120.08	294.1216
Smnn002694	Osmanthuside H[2-(4-Hydroxyphenyl)ethyl-β-D-apiosyl-(1→6)-β-D-glucoside]	Others	Alcohol compounds	431.15	149.05	432.1632
Lssp210307	dihydromachaeric acid lactone	Terpenoids	Triterpene	457.37	175.15	456.3603
Lmdp002381	Genipin	Terpenoids	Monoterpenoids	227.09	149.06	226.0841
Zblp002245	Petunidin-3-O-glucoside	Flavonoids	Anthocyanidins	479.12	317.07	479.1184
Cmhp002692	Madreselvin A*	Flavonoids	Flavones	641.17	317.06	640.1639
pme2237	Dulcitol*	Others	Saccharides	181.0718	101.0244	182.079

Metabolite ID	Compound Name	Class I	Class II	Q1 (m/z)	Q3 (m/z)	Molecular Weight (Da)
Wagp002530	2-Methoxy-1-benzofuran-5-carbaldehyde	Others	Aldehyde compounds	177.06	117.03	176.0473
pmp000268	2-Hydroxymethyl-A(1)-nor-19,24-dihydroxyurs-2,12-dien-28-oic acid (Rosamultic acid)	Terpenoids	Triterpene	487.34	405.31	486.3345
Lcfn121124	1-hydroxy-4-glucoside-2-naphthoicacid	Phenolic acids	Phenolic acids	365.09	202.03	366.0951
MWSmce607	DL-Threonine	Amino acids and derivatives	Amino acids and derivatives	120.07	56.05	119.0582
Hahn002084	6'-O-caffeoylcatalpol	Terpenoids	Monoterpenoids	523.15	161.03	524.153
Yaap006531	Resinoside A*	Flavonoids	Flavones	615.21	287.06	614.1999

Metabolite ID	Compound Name	Class I	Class II	Q1 (m/z)	Q3 (m/z)	Molecular Weight (Da)
Smpp010139	3-O-Feruloyleuscaphic acid	Terpenoids	Triterpene	665.4	177.05	664.3975
mws1329	Quercetin-7-O-glucoside	Flavonoids	Flavonols	463.09	301.04	464.0955
Lmmn000214	Solatriose	Others	Saccharides	487.17	341.11	488.1741
pmb3093	Esculetin-7-O-quinic acid	Lignans and Coumarins	Coumarins	351.07	105.1	352.0794
zjtp120202	Tomentodione E	Others	Others	451.32	405.32	450.3134
Qmfp122216	2,3-Dihydroxyurs-12,18-dien-28-oic acid (Goreishic Acid I)*	Terpenoids	Triterpene	471.35	201.16	470.3396
mws0126	LysoPC 18:0	Lipids	LPC	524.37	184.07	523.3638
Lmhp007840	LysoPC 19:2	Lipids	LPC	534.36	184.07	533.3481
Lskp211387	Savinin	Lignans and Coumarins	Lignans	353.1	135.04	352.0947

Metabolite ID	Compound Name	Class I	Class II	Q1 (m/z)	Q3 (m/z)	Molecular Weight (Da)
Walp004752	2-Aminododecane-1,4-diol	Lipids	Sphingolipids	218.21	88.08	217.2042
Lmgn002555	2-Propylglutaric acid	Organic acids	Organic acids	173.08	59.01	174.0892
Zmrp110404	3-Hydroxyurs-12-en-24-oic acid (β-Boswellic acid)*	Terpenoids	Triterpene	457.3676	439.3566	456.3603
pmp001251	LysoPC 18:2(2n isomer)	Lipids	LPC	520.34	184.07	519.3325
Safn005726	Creoside IV	Others	Others	455.21	395.19	396.1995
Wamxn01040	(X)-1,2-Propanediol 1-O-B-D-Glucopyranoside	Others	Alcohol compounds	297.12	59.01	238.1053
Cmsp002257	Peonidin-3-O-sambubiosid e	Flavonoids	Anthocyanidins	595.16	301.07	595.1657
pme1086	Glutathione reduced form	Amino acids and derivatives	Amino acids and derivatives	306.08	143.05	307.0838

Metabolite ID	Compound Name	Class I	Class II	Q1 (m/z)	Q3 (m/z)	Molecular Weight (Da)
Cmdn004285	6'-O-β-D-glucosylbarlerin*	Terpenoids	Monoterpenoids	609.2	447.12	610.2109
Qmjp012113	Hypericumoxide N	Others	Others	557.38	465.33	556.3764
MWS20145	Eriodictyol-7-O-glucoside*	Flavonoids	Flavanones	451.12	289.07	450.1162
Wasyqn3683	3-O-p-Coumaroyl Methylglucaric Acid*	Phenolic acids	Phenolic acids	369.08	223.05	370.0895
Wcdp005264	Tetradecasphinganine	Others	Others	246.24	88.08	245.2355
Zjmp102710	3-Hydroxy-29(or30)-al-olean-12-en-28-oic acid*	Terpenoids	Triterpene	471.35	201.16	470.3396
MWS201455	Asp-Lys*	Amino acids and derivatives	Amino acids and derivatives	262.14	84.08	261.1325
Zmrp110403	3-Hydroxyolean-12-en-24-oic acid (α-Boswellic acid)*	Terpenoids	Triterpene	457.3676	439.3566	456.3603

Metabolite ID	Compound Name	Class I	Class II	Q1 (m/z)	Q3 (m/z)	Molecular Weight (Da)
Lcgn000239	dihydrodehydrodiconiferyl alcohol-9-O-β-D-glucopyranoside	Lignans and Coumarins	Lignans	521.2	491.19	522.2101
Cmsn002480	Secoisolariciresinol-9'-O-glucoside	Lignans and Coumarins	Lignans	523.22	475.2	524.2258
Wacyp09666	6-Hydroxy-3,9-Undecadienoic acid Methyl Ester	Lipids	Free fatty acids	213.15	111.04	212.1412
Lmhp008718	LysoPC 17:2	Lipids	LPC	506.32	184.07	505.3168
Lhwp110301	19-Hydroxy-2-(hydroxymethyl)-A(1)-norursa-2,12-dien-28-oic acid (Coleonolic acid)	Terpenoids	Triterpene	471.3473	205.1587	470.3396

Metabolite ID	Compound Name	Class I	Class II	Q1 (m/z)	Q3 (m/z)	Molecular Weight (Da)
pmp000284	Fraxidin (8-Hydroxy-6,7-dimethoxy coumarin)	Lignans and Coumarins	Coumarins	223.06	208.04	222.0528
pme1292	Homogentisic acid*	Phenolic acids	Phenolic acids	167.03	123.05	168.0423
Latp002963	5-Acetonyl-6-glucosyl-7-hydroxy-2,6-methylchromone	Others	Chromone	411.13	249.08	410.1208
WaYp005128	2-Aminotetradecane-1,5,13-triol	Lipids	Sphingolipids	262.24	200.2	261.2304
pmn001681	1-(4-Methoxyphenyl)-1-propanol	Phenolic acids	Phenolic acids	165.09	149.06	166.0994
Cmrn002205	Bartsioside	Terpenoids	Monoterpenoids	329.12	209.07	330.1315
Lchp000221	(Z)-3-Ethylidene-7-hydrox	Others	Lactones	207.06	91.05	206.0579

Metabolite ID	Compound Name	Class I	Class II	Q1 (m/z)	Q3 (m/z)	Molecular Weight (Da)
	y-6-methoxyphthalide					
mws0906	Coniferin	Others	Alcohol compounds	341.12	179.07	342.1315
HJN087	Naringenin-4'-O-glucoside *	Flavonoids	Flavanones	433.12	271.06	434.1213
Hmln013535	Cryptomeridiol	Terpenoids	Sesquiterpenoids	239.2	223.02	240.2089
Walp006011	2-Aminotetradecane-1,4-diol	Lipids	Sphingolipids	246.24	88.08	245.2355
Wcfp005519	5-hydroxyindene-1,3-dione	Terpenoids	Sesquiterpenoids	163.04	133.03	162.0317
MWSmce221	5,7-Dihydroxy-4-methylcoumarin	Lignans and Coumarins	Coumarins	193.05	91.05	192.0423
Wayn006883	Quercetin 7-O-p-coumaroyl	Flavonoids	Flavonols	593.13	300.03	594.1373

Metabolite ID	Compound Name	Class I	Class II	Q1 (m/z)	Q3 (m/z)	Molecular Weight (Da)
	rhamnoside					
mws2212	Caffeic acid	Phenolic acids	Phenolic acids	179.03	135.05	180.0423
Zmhn003098	4-O-(6'-O-Glucosylcaffeoyl glucosylferuloyl)-4-hydroxybenzyl alcohol	Phenolic acids	Phenolic acids	785.23	447.13	786.2371
Zbzn008048	Embelin	Quinones	Quinones	293.18	221.15	294.1831
mws0376	Fumaric acid	Organic acids	Organic acids	115	71.01	116.011
Cmmn013378	Levopimaric acid*	Terpenoids	Ditepenoids	301.22	301.22	302.2246
pme3705	D-Glucoronic acid*	Others	Saccharides	193.04	72.99	194.0427
MWSslk178	Griffonilide	Others	Lactones	169.0494	77.0395	168.0423
pme1598	Hesperetin-5-O-glucoside	Flavonoids	Flavanones	463.12	301.07	464.1319

Metabolite ID	Compound Name	Class I	Class II	Q1 (m/z)	Q3 (m/z)	Molecular Weight (Da)
Zblp001009	3-pyridine-methanol-O-β-D-glucopyranosyl*	Alkaloids	Pyridine alkaloids	272.11	110.06	271.1056
HJAP006	Syringetin-7-O-glucoside*	Flavonoids	Flavonols	509.13	347.08	508.1217
MWSHY0077	Fisetin	Flavonoids	Flavonols	287.06	137.02	286.0477
Wasdp05671	Santalol A	Terpenoids	Sesquiterpenoids	221.19	81.07	220.1827
HJN046	Aesculitannin A	Tannins	Tannin	863.19	575.12	864.1902
Hmbp002498	Scopoletin-7-O-xylosyl(1→6)glucoside(Fabiatrin)*	Lignans and Coumarins	Coumarins	487.14	193.05	486.1373
Zbyn002745	Loganic acid	Terpenoids	Monoterpenoids	375.13	213.08	376.1366
Zahn005484	1,4,8-trihydroxynaphthalen-1-O-β-D-[6'-O-(3'',4'',5''-trihydroxybenzoyl)]glucopy	Quinones	Quinones	489.1	271.04	490.1111

Metabolite ID	Compound Name	Class I	Class II	Q1 (m/z)	Q3 (m/z)	Molecular Weight (Da)
	ranoside					
Zmyn000108	D-Saccharic acid	Others	Saccharides	209.03	85.03	210.0376
pme0368	Apigenin-7-O-rutinoside (Isorhoifolin)*	Flavonoids	Flavones	579.17	271.06	578.1636
Cmmn013275	Isopimaric acid*	Terpenoids	Ditepenoids	301.22	301.22	302.2246
Cmdn003997	6'-O-α-D-galactosylbarlerin*	Terpenoids	Monoterpenoids	609.2	447.12	610.2109
Yajp003407	Deutzioside	Terpenoids	Monoterpenoids	347.13	185.08	346.1264
pmp001048	Ixoroside	Terpenoids	Monoterpenoids	361.15	199.1	360.142
pmb3317	Quinacyl syringic acid	Phenolic acids	Phenolic acids	371.1	179.1	372.1056
Lahp003986	3,4-Dihydro-4-(4'-hydroxyphenyl)-5,7-dihydroxycou	Lignans and Coumarins	Coumarins	435.13	273.08	434.1208

Metabolite ID	Compound Name	Class I	Class II	Q1 (m/z)	Q3 (m/z)	Molecular Weight (Da)
	marin glucoside					
pmp000097	Guvacoline	Alkaloids	Piperidine alkaloids	142.09	124.07	141.079
Wbmp004211	Ovafolinin E	Lignans and Coumarins	Lignans	417.15	167.07	416.1471
Lmbn003970	9,12,13-TriHOME; 9(S),12(S),13(S)-Trihydroxy -10(E)-octadecenoic acid	Lipids	Free fatty acids	329.23	211.14	330.2406
pmp001311	6-Hydroxykaempferol-7,6-O-Diglucoside	Flavonoids	Flavonols	627.16	303.1	626.1483
pmb1207	O-Feruloyl 3-hydroxycoumarin*	Lignans and Coumarins	Coumarins	339.09	177.05	338.079
Sazp000955	Tetraphyllin A*	Alkaloids	Alkaloids	272.11	110.06	271.1056
Zmhp003716	Herbacetin-7-O-rhamnosid	Flavonoids	Flavonols	611.16	303.05	610.1534

Metabolite ID	Compound Name	Class I	Class II	Q1 (m/z)	Q3 (m/z)	Molecular Weight (Da)
	e-8-O-glucoside; Rhodionidin*					
pmb2221	4-Hydroxysphinganine; Phytosphingosine	Lipids	Sphingolipids	318.3	60.04	317.293
Safn002774	Chaenorrhinoside	Others	Others	341.09	179.04	342.0951
pmp001310	6-Hydroxykaempferol-3,6-O-Diglucoside*	Flavonoids	Flavonols	627.16	303.1	626.1483
pmn001420	1-O-Caffeoyl-β-D-glucose*	Phenolic acids	Phenolic acids	341.09	179.04	342.0951
pmd0136	LysoPC 18:0(2n isomer)	Lipids	LPC	524.37	184.07	523.3638
Lmhn002683	p-Coumaroylcaffeoyltartaric acid	Phenolic acids	Phenolic acids	457.08	163.04	458.0849
pmf0293	1-Octadecanol	Lipids	Free fatty acids	269.29	209.23	270.2923

Metabolite ID	Compound Name	Class I	Class II	Q1 (m/z)	Q3 (m/z)	Molecular Weight (Da)
Lmmn003323	2-Hydroxyhexadecanoic acid*	Lipids	Free fatty acids	271.23	225.22	272.2351
pme2596	4-Pyridoxic acid	Others	Vitamin	184.06	148.04	183.0532
MWS04412	N(6),N(6)-Dimethyl-L-lysine	Amino acids and derivatives	Amino acids and derivatives	175.12	70.07	174.1368
pme2566	5-L-Glutamyl-L-Alanine	Amino acids and derivatives	Amino acids and derivatives	217.08	199.07	218.0903
Wahsn05525	Secoisolariciresinol-sesquilignan	Lignans and Coumarins	Lignans	557.24	361.17	558.2465
Hmlp003068	Meratin*	Flavonoids	Flavonols	627.15	303.05	626.1483
Zmhn001905	HydroxyAloe-emodin-8-O-glucoside	Quinones	Anthraquinone	447.09	285.04	448.1006

Metabolite ID	Compound Name	Class I	Class II	Q1 (m/z)	Q3 (m/z)	Molecular Weight (Da)
Hmsp004125	3'-Deoxy-4-O-methylsappanol	Others	Alcohol compounds	303.0864	123.04	302.079
Jmmn002533	coleoside-B	Terpenoids	Monoterpenoids	475.18	179.07	476.1894
Hmhp004269	Nodakenin	Lignans and Coumarins	Coumarins	409.15	229.09	408.142
pme3827	3,4-Dihydroxy-L-phenylalanine (L-Dopa)	Amino acids and derivatives	Amino acids and derivatives	198.08	152.07	197.0688
Lmbp002336	Quercetin-3-O-(2''-O-rhamnosyl)galactoside*	Flavonoids	Flavonols	611.16	303.05	610.1528
pmp000100	Arecatannin B1	Tannins	Tannin	867.21	715.16	866.2058
Zmln000899	Syringaldehyde-4-O-glucoside	Others	Aldehyde compounds	343.11	181.05	344.1107
Lhkp101414	16-Hydroxy-3-oxo-5-lanost	Terpenoids	Triterpene	471.3469	425.3372	470.3396

Metabolite ID	Compound Name	Class I	Class II	Q1 (m/z)	Q3 (m/z)	Molecular Weight (Da)
	a-7,24-dien-21-oic acid (Kulonic acid)					
mws0856	Quercetin-4'-O-glucoside (Spiraeoside)*	Flavonoids	Flavonols	463.09	301.04	464.0955
Lcyp000107	Stellarin	Phenolic acids	Phenolic acids	333.08	171.03	332.0743
Wasyqn1008	Dimethylglyceric Acid Glucoside	Organic acids	Organic acids	295.1	59.01	296.1107
MWS2027	Barbituric acid;Malonylurea;2,4,6-Pyr imidinetrione	Nucleotides and derivatives	Nucleotides and derivatives	129.03	69	128.0222
Zmsn002533	6a-dihydrocornic acid	Others	Others	375.13	125.06	376.1369
Hmdn006855	3,4-Cycloopen-cycloaltin-4(	Terpenoids	Triterpene	455.35	455.35	456.3603

Metabolite ID	Compound Name	Class I	Class II	Q1 (m/z)	Q3 (m/z)	Molecular Weight (Da)
	29),25-diene-24(R)-hydroxy-3-carboxylic acid					
WaYn003084	Guaiacylglycerol 8-O-(1''-O-Glucosyl)Vanillic Acid Ether	Phenolic acids	Phenolic acids	525.16	167.03	526.1686
mws0889	D-Threonic Acid	Others	Saccharides	135.03	75.01	136.0372
MWS201412	Lys-Asp*	Amino acids and derivatives	Amino acids and derivatives	262.1383	84.0816	261.1325
Zmmp000969	5-Ribofuranosylnicotinamide	Nucleotides and derivatives	Nucleotides and derivatives	255.1	123.06	254.0903
MWSHC2054	Adenine	Nucleotides and derivatives	Nucleotides and derivatives	136.06	119.04	135.0545

Metabolite ID	Compound Name	Class I	Class II	Q1 (m/z)	Q3 (m/z)	Molecular Weight (Da)
Wayn006761	Quercetin 5-O-p-coumaroyl rhamnoside	Flavonoids	Flavonols	593.13	300.03	594.1373
pma6599	6-Hydroxymethylherniarin	Lignans and Coumarins	Coumarins	207.0652	85.0284	206.0579
Zmgn000447	3-Phospho-D-glyceric acid	Organic acids	Organic acids	184.98	78.96	185.9929
pme0490	Nicotinic acid (Vitamin B3)	Others	Vitamin	124.04	78.03	123.032
Zmhn000892	4-O-Glucosyl-3,4-dihydroxybenzyl alcohol	Others	Others	301.09	139.05	302.1002
Zmxn110201	2,6-Dimethoxy-4-hydroxyphenol-1-O-ß-D-glucopyranoside	Others	Others	331.1	108.02	332.1107
pmc0281	Ribosyladenosine	Nucleotides and	Nucleotides and	400.15	136.06	399.139

Metabolite ID	Compound Name	Class I	Class II	Q1 (m/z)	Q3 (m/z)	Molecular Weight (Da)
		derivatives	derivatives			
Zbyp002336	p-galloyl-oxyphenyl-β-D-glucoside	Phenolic acids	Phenolic acids	453.14	291.09	452.1305
Yshj000013	4,6-dimethoxy-2-methoxyphenyl-1-O-beta-D-glucopyranoside	Others	Others	319.1	125.02	318.0951
HJAP120	Rhamnetin-3-O-Rutinoside*	Flavonoids	Flavonols	625.1661	317.066	624.169
Lahn001701	4-(6-O-Sulfo-Beta-D-Glucopyranosyloxy)-3-Hydroxybenzoic Acid	Phenolic acids	Phenolic acids	395.03	241	396.0362
Lmxp001668	2-Methoxy-4-ethenylphenol	Others	Others	151.08	91.05	150.0681

Metabolite ID	Compound Name	Class I	Class II	Q1 (m/z)	Q3 (m/z)	Molecular Weight (Da)
pme3337	Succinyladenosine	Nucleotides and derivatives	Nucleotides and derivatives	384.12	252.07	383.1077
Wafp004334	N-Feruloyltyramine 4'-glucoside	Alkaloids	Phenolamine	476.2	177.05	475.1842
mws0036	Hesperetin-7-O-rutinoside (Hesperidin)	Flavonoids	Flavanones	609.18	301.09	610.1898
Walbn00444	6''-Carboxyraffinose	Others	Saccharides	517.14	175.02	518.1483
Lmxp000939	Zarzissine	Alkaloids	Alkaloids	136.0618	119.0352	135.0545
pme0137	N-Acetyl-L-Glutamine	Amino acids and derivatives	Amino acids and derivatives	187.07	125.07	188.0797
Zbsp002100	Catechin-(4alpha→8)-gallo catechin	Flavonoids	Flavanols	595.14	289.07	594.1373

Metabolite ID	Compound Name	Class I	Class II	Q1 (m/z)	Q3 (m/z)	Molecular Weight (Da)
Lmmp001673	2-Ethyl-3-methylmaleimide-N-glucoside	Alkaloids	Alkaloids	302.12	140.07	301.1162
Wmmp000181	1´-O-Benzoylsucrose	Phenolic acids	Phenolic acids	445.13	121.03	446.1424
Zmyn000155	N-α-Acetyl-L-ornithine	Amino acids and derivatives	Amino acids and derivatives	173.11	131.08	174.1004
Qmhp090808	18-Demethylparaensidimerin C	Alkaloids	Alkaloids	469.22	307.13	468.2049
lsdp210004	Elaeocarpucin B	Terpenoids	Triterpene	487.34	423.33	486.3345
NK10251888	NG,NG-Dimethyl-L-arginine*	Amino acids and derivatives	Amino acids and derivatives	203.15	70.07	202.143
pmn001694	9,10,13-Trihydroxy-11-Octadecenoic Acid	Lipids	Free fatty acids	329.23	229.15	330.2406

Metabolite ID	Compound Name	Class I	Class II	Q1 (m/z)	Q3 (m/z)	Molecular Weight (Da)
Qagp003587	N-Monocinnamoylputresci ne*	Alkaloids	Phenolamine	219.15	131.05	218.1419
Lmpp003815	Petunidin-3-O-(6''-O-p-Co umaroyl)glucoside	Flavonoids	Anthocyanidins	625.16	317.07	625.1552
Zmhn002227	4-O-Glucosyl-sinapate	Phenolic acids	Phenolic acids	385.11	223.06	386.1213
Zmhn001793	6-O-Caffeoyl-D-glucose*	Phenolic acids	Phenolic acids	341.09	179.03	342.0951
pmn001704	2,3-Dihydroxy-5(6),12(13)-diene-ursolic acid*	Terpenoids	Triterpene	469.33	423.33	470.3396
Wafn004433	1-Hexanol arabinosylglucoside	Others	Alcohol compounds	455.21	263.15	396.1995
MWSHC2041	sophorabioside*	Flavonoids	Isoflavones	579.1712	271.0615	578.1636
Sazp010264	Monogalactosyldiacylglyce	Lipids	Glycerol ester	771.5	609.45	770.4969

Metabolite ID	Compound Name	Class I	Class II	Q1 (m/z)	Q3 (m/z)	Molecular Weight (Da)
	rol					
Qmzp101810	Dimethoxysamin	Lignans and Coumarins	Lignans	267.1229	189.0848	266.1154
ZbWp000749	ethyl-L-arginine	Amino acids and derivatives	Amino acids and derivatives	203.15	70.07	202.143
Lcsp002946	Dihydrozeatin-O-glucoside riboside	Alkaloids	Alkaloids	516.23	384.19	515.2227
Hmbp000568	Asperuloside	Phenolic acids	Phenolic acids	415.1201	235.05	414.1162
MWStz355	Pratensein 7-O-glucopyranoside	Flavonoids	Isoflavones	461.11	299.06	462.1162
Zasp102439	m-Aminophenylacetylene	Alkaloids	Alkaloids	118.06	91.05	117.0578
Walp006014	Cleomiscosin A	Lignans and Coumarins	Lignans	387.11	337.07	386.1006
pme2550	Cis-Aconitic acid	Organic acids	Organic acids	173.01	129.02	174.0164

Metabolite ID	Compound Name	Class I	Class II	Q1 (m/z)	Q3 (m/z)	Molecular Weight (Da)
pme2836	5-Hydroxytryptophol	Alkaloids	Plumerane	178.09	160.08	177.079
MWStz492	Cornuside	Terpenoids	Monoterpenoids	543.1721	211.0995	542.1636
Zasp003415	Kaempferol 3-(3'',4''-diacetylglucoside)	Flavonoids	Flavonols	533.1277	287.0559	532.1217
Zbyn002532	8-Epiloganic acid	Terpenoids	Monoterpenoids	375.13	213.08	376.1371
mws1454	Persicoside*	Flavonoids	Flavanones	477.14	315.09	478.1475
Wacyn02678	Theasinensin C	Tannins	Proanthocyanidins	609.13	423.07	610.1323
pme0122	N6-Acetyl-L-lysine	Amino acids and derivatives	Amino acids and derivatives	189.12	126.09	188.1161
pma3101	Nicotinate D-ribonucleoside	Others	Vitamin	256.08	124.04	256.0816
Lmsn012627	2,3-Dihydroxyurs-12,18-die	Terpenoids	Triterpene	469.33	423.33	470.3396

Metabolite ID	Compound Name	Class I	Class II	Q1 (m/z)	Q3 (m/z)	Molecular Weight (Da)
	n-28-oic acid*					
Smhn003633	Pyrafortunoside A	Phenolic acids	Phenolic acids	475.14	167.03	476.153
MWS00327g	L-Isoserine	Amino acids and derivatives	Amino acids and derivatives	106.05	60.04	105.0426
Sasp003574	Grevilloside B	Others	Others	359.17	197.12	358.1628
pmb0981	Adenosine 5'-monophosphate	Nucleotides and derivatives	Nucleotides and derivatives	348.07	136.06	347.0631
MWStz559	Quercetin 3,7-diglucoside*	Flavonoids	Flavonols	627.1546	303.0501	626.1483
Zmyn005245	2,3'-Di-feruloyl sucrose	Phenolic acids	Phenolic acids	693.21	517.16	694.2109
MWS3020	N-Acetylcadaverine	Alkaloids	Alkaloids	145.13	86.1	144.1263
mws4052	1-Aminocyclopropane-1-carboxylic acid	Amino acids and derivatives	Amino acids and derivatives	102.06	56.05	101.0477

Metabolite ID	Compound Name	Class I	Class II	Q1 (m/z)	Q3 (m/z)	Molecular Weight (Da)
mad1424	Ditartaroyl-hydroxycoumarin	Lignans and Coumarins	Coumarins	427.05	295.05	426.0434
MWSmce165	L-Fucitol	Others	Saccharides	165.08	59.02	166.0841
pme1261	D-Panthenol	Others	Vitamin	206.14	76.08	205.1314
Wbmp002283	A-hydroxyquinoline	Alkaloids	Alkaloids	146.06	118.06	145.0528
pme0008	L-Citrulline	Amino acids and derivatives	Amino acids and derivatives	176.1	113.07	175.0957
pmb0064	DIMBOA glucoside	Alkaloids	Alkaloids	374.11	198.08	373.1009
Lmhp009769	LysoPE 17:1*	Lipids	LPE	466.29	325.27	465.2855
Zmhn001036	Choerospondin	Flavonoids	Flavanones	433.11	271.06	434.1213
MWSHC2067	Protocatechualdehyde	Others	Aldehyde compounds	139.04	65.04	138.0317

Metabolite ID	Compound Name	Class I	Class II	Q1 (m/z)	Q3 (m/z)	Molecular Weight (Da)
Zmjn004847	Ssioriside	Lignans and Coumarins	Lignans	553.23	505.21	554.2363
Zbfn002169	3'-p-Coumaroyl-sucrose	Phenolic acids	Phenolic acids	487.15	145.03	488.153
Wasn003258	Glucosyl 2-Hydroxy-4-Methylpentanoic Acid	Organic acids	Organic acids	293.12	131.07	294.1315
pmb0426	N,N-Dimethyl-5-methoxytryptamine	Alkaloids	Plumerane	219.15	174.09	218.1419
Lmlp006175	Isosalipurposide (Phlorizin Chalcone)	Flavonoids	Chalcones	435.13	273.09	434.1213
pme3184	2'-Deoxyadenosine-5'-monophosphate	Nucleotides and derivatives	Nucleotides and derivatives	332.08	136.06	331.0682
Zmsn110105	Rumexobtusifolius	Others	Others	279.07	59.01	280.0794

Metabolite ID	Compound Name	Class I	Class II	Q1 (m/z)	Q3 (m/z)	Molecular Weight (Da)
Lmbp000668	Isonicotinic acid	Others	Vitamin	124.04	78.03	123.032
pmb3041	Tricin-7-O-saccharic acid	Flavonoids	Flavones	521.09	329.2	522.101
Wcfn003882	(2S,3S,4S,5R,6R)-6-(3-benzoyloxy-2-hydroxypropoxy)-3,4,5-trihydroxyoxane-2-carboxylic acid	Others	Others	371.1	121.03	372.1056
Waqkp05977	2-g-linolenoyl-glycerol	Lipids	Glycerol ester	353.27	261.22	352.2614
Ymjm000101	6-Hydroxy-3-oxooleana-11, 13(18)-dien-28-oic acid	Terpenoids	Triterpene	469.33	407.33	468.324
Lamn005183	Astraflavonoid C	Flavonoids	Other Flavonoids	479.16	317.1	480.1637
pmn001423	Roseoside	Terpenoids	Sesquiterpenoids	385.19	223.14	386.1941
Lmgp004731	Genistein-7-O-galactoside*	Flavonoids	Isoflavones	433.11	271.06	432.1056

Metabolite ID	Compound Name	Class I	Class II	Q1 (m/z)	Q3 (m/z)	Molecular Weight (Da)
Lcsn000191	isatioxyneolignoside A	Lignans and Coumarins	Lignans	525.16	167.03	526.1686
Wbjp001169	o-Carboxy-5-hydroxytrypt amine	Alkaloids	Alkaloids	221.09	130.06	220.0848
MWSmce157	Stachydrine	Alkaloids	Pyrrole alkaloids	144.1	58.06	143.0946
Lmhp009464	LysoPE 17:1(2n isomer)*	Lipids	LPE	466.29	325.27	465.2855
Hmsn001911	2-Beta-Glucopyranosyloxy -3-Hydroxybenzyl Alcohol	Phenolic acids	Phenolic acids	301.09	139.04	302.1002
Lskp211310	Grayanotoxin XIX	Terpenoids	Ditepenoids	319.23	283.21	318.2195
Walp003918	1-O-p-Coumaroylsedohept ulose	Phenolic acids	Phenolic acids	357.12	147.04	356.1107
Lskp211268	3-Ethyl-7-hydroxyphthalid e	Others	Others	179.07	119.05	178.063

Metabolite ID	Compound Name	Class I	Class II	Q1 (m/z)	Q3 (m/z)	Molecular Weight (Da)
Wasyqn2892	Trans-Ferulic Acid 1'-O-[Apiosyl-(1->6)-Glucosyl] Ester	Phenolic acids	Phenolic acids	487.15	193.05	488.1524
MWS5164	N,N'-Dimethylarginine;SDMA*	Amino acids and derivatives	Amino acids and derivatives	203.15	70.07	202.143
Lmmn004625	Dihydrokaempferol-7-O-glucoside	Flavonoids	Flavanonols	449.11	287.06	450.1162
mws0193	L-Homocitrulline	Amino acids and derivatives	Amino acids and derivatives	190.12	84.08	189.1113
pmb0382	O-Feruloyl 4-hydroxycoumarin	Lignans and Coumarins	Coumarins	339.09	177.05	338.079
MWSHC2042	(2E)-3-(1-Hydroxy-2,6,6-trimethyl-4-oxo-2-cyclohexen	Terpenoids	Sesquiterpenoids	225.11	123.04	224.1049

Metabolite ID	Compound Name	Class I	Class II	Q1 (m/z)	Q3 (m/z)	Molecular Weight (Da)
	-1-yl)-2-propenoic acid					
Smgp105565	Cinchonain Ib*	Flavonoids	Flavanols	453.11	343.08	452.1107
Lssp210306	3-Hydroxy-18-oxoursan-28,13-olide	Terpenoids	Triterpene	471.34	207.17	470.3396
mws0179	Chlorogenic acid methyl ester	Phenolic acids	Phenolic acids	367.1	190.9	368.1107
Lssp210334	Grayanotoxin VII	Terpenoids	Ditepenoids	335.22	281.19	334.2144
Rfmb318	D-Proline betaine	Amino acids and derivatives	Amino acids and derivatives	144.1	84.08	144.1019
Zmsp000758	1-(Hydroxymethyl)hexahydro-1h-pyrrolizin-2-ol	Alkaloids	Pyrrole alkaloids	158.12	70.07	158.1174
MWSslk002	Sophoricoside*	Flavonoids	Isoflavones	433.11	271.06	432.1056

Metabolite ID	Compound Name	Class I	Class II	Q1 (m/z)	Q3 (m/z)	Molecular Weight (Da)
Wafn002827	Dihydroferulic acid glucoside	Phenolic acids	Phenolic acids	357.12	195.07	358.1264
pma0795	Tricetin-5-O-(6''-malonyl)glucoside	Flavonoids	Flavones	551.1	303.05	550.0959
Wasn001627	Glucosyl 2,3-Dihydroxy-2-Methylbutanoic Acid	Organic acids	Organic acids	295.1	89.02	296.1107
Zbnp004766	Limocitrin-7-O-glucoside*	Flavonoids	Flavonols	509.1276	347.07	508.1217
mws0885	2,4-Dihydroxybenzoic acid	Phenolic acids	Phenolic acids	153.02	109.03	154.0266
MWStz073	5-Hydroxy-2-pyrrolidinone	Alkaloids	Pyrrole alkaloids	102.05	84.05	101.0477
Lmbn001181	Fraxetin-7,8-di-O-glucosid	Lignans and Coumarins	Coumarins	531.14	531.13	532.1428

Metabolite ID	Compound Name	Class I	Class II	Q1 (m/z)	Q3 (m/z)	Molecular Weight (Da)
	e					
Haxp003920	5-[4-(3,4-dimethoxyphenyl)-hexahydrofuro[3,4-c]furan-1-yl]-4-methyl-2H-1,3-benzodioxole	Lignans and Coumarins	Lignans	385.16	206.08	384.1573
Lmtn003096	Secoisolariciresinol 4-O-glucoside*	Lignans and Coumarins	Lignans	523.22	361.14	524.2258
Wbwn013826	schisanol	Lignans and Coumarins	Lignans	453.34	453.34	454.3447
Zmzn000113	L-threo-3-Methylaspartate	Amino acids and derivatives	Amino acids and derivatives	146.05	102.06	147.0532
Walbn03514	tetrahydrodehydrodiconiferyl alcohol glucoside	Lignans and Coumarins	Lignans	523.22	361.16	524.2258

Metabolite ID	Compound Name	Class I	Class II	Q1 (m/z)	Q3 (m/z)	Molecular Weight (Da)
Sacn002893	10-acetylmonomelittoside*	Terpenoids	Monoterpenoids	403.12	223.06	404.1319
Safp008316	Quercetin 3-[6''-(3-hydroxy-3-methyl glutaryl)galactoside]	Flavonoids	Flavonols	609.15	303.05	608.1377
Wasyqp3230	4-O-Feruloyl Aminogalactitol	Phenolic acids	Phenolic acids	374.15	177.05	373.1373
zjtp120203	Tomentodione F	Others	Others	451.32	405.32	450.3134
mws1346	L-Homoglutamic acid	Amino acids and derivatives	Amino acids and derivatives	162.08	98.06	161.0688
Wdzn000513	methyl alpha-D-fructofuranoside*	Others	Saccharides	193.07	59.01	194.079
pmb3068	1-O-p-Coumaroylquinic	Phenolic acids	Phenolic acids	337.09	163.04	338.1002

Metabolite ID	Compound Name	Class I	Class II	Q1 (m/z)	Q3 (m/z)	Molecular Weight (Da)
	acid*					
pme0321	Kaempferol-7-O-rhamnoside	Flavonoids	Flavonols	431.1	285.04	432.1056
Lmmp003306	Quercetin-3-O-xylosyl(1→2)arabinoside	Flavonoids	Flavonols	567.13	303.05	566.1272
Yajn004424	2-((6-hydroxy-4-(hydroxy(methoxy)methyl)-7-methyl-1,4a,5,6,7,7a-hexahydrocyclopenta[c]pyran-1-yl)oxy)-6-(hydroxymethyl)tetrahydro-2H-pyran-3,4,5-triol	Terpenoids	Monoterpenoids	391.16	59.01	392.1682
Lmsn002288	1-O-Caffeoyl-(6-O-glucosyl)-β-D-glucose	Phenolic acids	Phenolic acids	503.14	341.09	504.1479

Metabolite ID	Compound Name	Class I	Class II	Q1 (m/z)	Q3 (m/z)	Molecular Weight (Da)
Lakn002075	4-Hydroxybenzoic acid glucosyl xyloside	Phenolic acids	Phenolic acids	431.12	137.02	432.1268
Yamn004462	6-((4-(3-hydroxy-2-(4-(3-hydroxypropyl)-2-methylphenoxy)propoxy)-2-methoxyphenoxy)methyl)tetrahydro-2H-pyran-2,3,4,5-tetraol*	Lignans and Coumarins	Lignans	523.22	361.16	524.2258
mws0232	Riboflavin (Vitamin B2)	Others	Vitamin	377.15	243.09	376.1383
MWStz413	Securoside A	Phenolic acids	Phenolic acids	693.2	175.04	694.2109
Hmhp002834	Cyanidin 3-xyloside	Flavonoids	Anthocyanidins	419.1	287.06	419.0973
Lmhp009590	LysoPC 17:1	Lipids	LPC	508.34	184.07	507.3325
Wayn001856	4-Hydroxybenzoic acid	Phenolic acids	Phenolic acids	379.03	241	380.0413

Metabolite ID	Compound Name	Class I	Class II	Q1 (m/z)	Q3 (m/z)	Molecular Weight (Da)
	4-(6-O-sulfo)glucopyranoside					
Hmjp003725	Isothankunic acid	Terpenoids	Triterpene	505.35	469.33	504.3451
mws0967	5,6,15-Trihydroxy-7,9,11,13-eicosatetraenoic acid	Lipids	Free fatty acids	351.22	271.21	352.225
Zblp002328	Cyanidin-3-O-arabinoside	Flavonoids	Anthocyanidins	419.1	287.06	419.0973
mws0224	DL-Methionine	Amino acids and derivatives	Amino acids and derivatives	150.06	61.01	149.051
Hmhp002580	Methylpicraquassioside A	Lignans and Coumarins	Coumarins	413.14	136.07	412.1369
pmn001477	4-C-Glucose-1,3,6-trihydroxy-7-methoxyxanthone	Flavonoids	Other Flavonoids	435.09	345	436.1006
Lmqn000432	1-(sn-Glycero-3-phospho)-	Others	Saccharides	333.06	333.06	334.0665

Metabolite ID	Compound Name	Class I	Class II	Q1 (m/z)	Q3 (m/z)	Molecular Weight (Da)
pmf0138	1D-myo-inositol D-Mannose*	Others	Saccharides	179.06	59.01	180.0634
Lmgp004474	Genistein 7-O-rutinoside*	Flavonoids	Isoflavones	579.17	271.06	578.1636
Lmsp004721	Tamarixetin-3-O-glucoside -7-O-rhamnoside*	Flavonoids	Flavonols	625.1763	317.0698	624.169
Zmmp003443	γ-Glu-Trp	Amino acids and derivatives	Amino acids and derivatives	334.14	188.07	333.1325
Waptn03507	Vomifoliol-D-glucopyrano side	Terpenoids	Monoterpenoids	445.21	153.09	386.1941
Smgp005565	Cinchonain Ia*	Flavonoids	Flavanols	453.11	343.08	452.1107
Zmcn009787	23-Hydroxy-3-oxoolean-12 -en-28-oic acid	Terpenoids	Triterpene	469.33	439.32	470.3396

Metabolite ID	Compound Name	Class I	Class II	Q1 (m/z)	Q3 (m/z)	Molecular Weight (Da)
	(Hederagonic acid)					
Lmmp002463	Sexangularetin-3-O-glucoside-7-O-rhamnoside*	Flavonoids	Flavonols	625.18	317.07	624.169
Zmgp004436	Okanin-4'-(6''-O-acetyl)glucoside*	Flavonoids	Chalcones	509.13	347.08	508.1217
Wdzp011630	11-deoxyalisol B	Terpenoids	Triterpene	457.37	205.16	456.3603
MWSHY0189	Apigenin-7-O-glucoside(Cosmosiin)*	Flavonoids	Flavones	433.11	271.07	432.1056
Lmsn004090	1-O-Galloyl-3-O-p-Coumaroyl-β-D-glucose	Phenolic acids	Phenolic acids	477.1	169.01	478.1111
Wdjp003498	2-(7-hydroxy-4-methoxy-9,10-dihydrophenanthren-2-	Others	Others	389.1456	155.0728	388.1522

Metabolite ID	Compound Name	Class I	Class II	Q1 (m/z)	Q3 (m/z)	Molecular Weight (Da)
	yl)-6-(hydroxymethyl)oxane-3,4,5-triol					
MWSHY0135	Isorhamnetin-3-O-rutinoside (Narcissin)*	Flavonoids	Flavonols	625.18	317.08	624.169
Wasyqn3389	1-O-P-Coumaroyl-(B-D-Glucose 6-O-Sulfate)	Phenolic acids	Phenolic acids	405.05	241	406.057
Waptp03797	2,3-dihydro-6-hydroxyltryptophan N-Glucoside	Alkaloids	Plumerane	385.16	206.08	384.1533
Hmgn001278	Verbasoside	Others	Alcohol compounds	461.17	315.1	462.1737
mws4075	Hypoglycyrrhizic acid(β)*	Terpenoids	Triterpene	469.33	425.4	470.3396
Lmgn007652	Tianshic acid	Lipids	Free fatty acids	329.23	211.13	330.2406
pme3096	Aminomalonic acid	Amino acids and	Amino acids and	118.01	74.02	119.0219

Metabolite ID	Compound Name	Class I	Class II	Q1 (m/z)	Q3 (m/z)	Molecular Weight (Da)
		derivatives	derivatives			
Wayn002279	Dihomocitric acid	Organic acids	Organic acids	219.05	111.01	220.0583
Cmsn001914	7R,8S-erythro-3',4,9,9'-tetrahydroxy-3-methoxy-8-O-4'-neolignan-7-O-glucopyr-anoside,	Lignans and Coumarins	Lignans	525.2	179.07	526.205
Zbsp004862	Syringetin-3-O-glucoside*	Flavonoids	Flavonols	509.13	347.08	508.1217
MWS00275g	DL-O-tyrosine	Amino acids and derivatives	Amino acids and derivatives	180.07	119.05	181.0739
Wasyqn1528	7-Caffeoylsedoheptulose	Phenolic acids	Phenolic acids	371.1	135.05	372.1056
Zmhp005586	Herbacetin-7-O-(3''-glucosyl)rhamnoside; Rhodiosin	Flavonoids	Flavonols	611.16	303.05	610.1534

Metabolite ID	Compound Name	Class I	Class II	Q1 (m/z)	Q3 (m/z)	Molecular Weight (Da)
Walp010787	2-aminotetracoSane-1,5,23-triol	Lipids	Sphingolipids	402.39	340.36	401.3869
mws2104	D-Pinitol*	Others	Saccharides	193.07	59.01	194.079
Lmsn004162	1-O-Galloyl-2-O-p-Coumaroyl-β-D-glucose	Phenolic acids	Phenolic acids	477.1	169.01	478.1111
mws1401	L-Theanine	Amino acids and derivatives	Amino acids and derivatives	175.11	84.04	174.1004
Lmtp004382	Sinapinaldehyde	Others	Aldehyde compounds	209.08	149.06	208.0736
mws0919	Kaempferol-3-O-rhamnoside (Afzelin)(Kaempferin)	Flavonoids	Flavonols	431.1	285.04	432.1056
Waln011524	1-(2,3-dihydroxypropoxy)-	Lipids	LPE	554.35	255.23	555.3536

Metabolite ID	Compound Name	Class I	Class II	Q1 (m/z)	Q3 (m/z)	Molecular Weight (Da)
	3-(((2-(dimethylamino)ethoxy)(hydroxy)phosphoryl)oxy)propan-2-yl palmitate*					
MWSmce285	Agarotetrol	Others	Chromone	319.11	255.1	318.1103
MWS0132	Oxiglutatione	Amino acids and derivatives	Amino acids and derivatives	611.15	306.07	612.152
mws0671	L-Homoserine*	Amino acids and derivatives	Amino acids and derivatives	120.0655	74.0601	119.0582
Cmzn002437	Scandoside	Terpenoids	Monoterpenoids	389.11	227.06	390.1162
pmp000103	Arecatannin A2	Tannins	Tannin	1155.28	867.21	1154.2692
Wasyqn0468	L-Glutamyl-L-Cysteinyl-Glycine	Amino acids and derivatives	Amino acids and derivatives	306.08	143.05	307.0838

Metabolite ID	Compound Name	Class I	Class II	Q1 (m/z)	Q3 (m/z)	Molecular Weight (Da)
Wahln03557	Dihydrosinapic Acid glucoside	Phenolic acids	Phenolic acids	387.13	207.06	388.1364
Hmcp002187	Limocitrin-3-O-galactoside *	Flavonoids	Flavonols	509.13	347.07	508.1217
pme1178	Guanosine	Nucleotides and derivatives	Nucleotides and derivatives	284.1	152.06	283.0917
Zbfn003303	3-(Hydroxycinnamoyl)-quinic acid*	Phenolic acids	Phenolic acids	337.09	163.04	338.1002
Wcfp007836	Tetradecyldiethanolamine	Alkaloids	Alkaloids	302.3	88.08	301.2981
Lmbp000728	(S)-2-Phenyloxirane	Others	Others	121.06	77.04	120.0575
Wayn001528	Homocitrate	Organic acids	Organic acids	205.03	111.01	206.0427
HJAP155	Laricitrin-3-O-xyloside*	Flavonoids	Flavonols	465.1031	333.0715	464.0955

Metabolite ID	Compound Name	Class I	Class II	Q1 (m/z)	Q3 (m/z)	Molecular Weight (Da)
Zbsp005900	7,8-Dimethoxy-6-hydroxycoumarin	Lignans and Coumarins	Coumarins	223.06	162.03	222.0528
MWSHY0019	Hesperetin-7-O-neohesperidoside(Neohesperidin)	Flavonoids	Flavanones	611.2	303.09	610.1898
Wcfp002357	N6-Threonylcarbamoyladenosine	Nucleotides and derivatives	Nucleotides and derivatives	413.14	162.04	412.1343
MWStz600	Deacetylasperulosidic acid	Terpenoids	Monoterpenoids	389.11	209.05	390.1162
mws0752	Undecylic Acid	Lipids	Free fatty acids	185.15	185.16	186.162
Wdtp002974	N-(p-hydroxybenzyl) adenosine	Nucleotides and derivatives	Nucleotides and derivatives	404.15	207.06	403.1492
Lmsp013224	Stigmasta-4,22-Dien-3-One	Steroids	Steroid	411.36	163.15	410.3549
pmp000782	Caffeoylmaslinic Acid	Terpenoids	Triterpene	635.39	435.32	634.387

Metabolite ID	Compound Name	Class I	Class II	Q1 (m/z)	Q3 (m/z)	Molecular Weight (Da)
pmb0964	Isopentenyladenine-7-N-glucoside	Nucleotides and derivatives	Nucleotides and derivatives	366.18	204.12	365.1699
Waptn06322	DGMG 18:2(2n isomer)	Lipids	Glycerol ester	737.4	397.13	678.3827
Lmtn003024	Mansorin III	Lignans and Coumarins	Coumarins	245.08	203.07	246.0892
pme0534	Gluconic acid	Others	Saccharides	195.05	75.01	196.0583
Lmhp008763	LysoPE 16:1(2n isomer)*	Lipids	LPE	452.28	311.26	451.2699
Wacyp06174	2,7-Dimethylocta-2,4-Dienal-8-ol 8-O-(beta-D-Xylopyranosyl)-beta-D-glucopyranoside	Terpenoids	Monoterpenoids	480.25	151.11	462.2101
Zbkn003828	Gambiriin B2	Flavonoids	Flavanols	561.13	289.07	562.1475
Safn001919	N5-(1-((carboxymethyl)ami	Amino acids and	Amino acids and	486.15	306.08	487.1625

Metabolite ID	Compound Name	Class I	Class II	Q1 (m/z)	Q3 (m/z)	Molecular Weight (Da)
	no)-3-((2,3-dihydroxy-1-(4-methoxyphenyl)propyl)thio)-1-oxopropan-2-yl)glutamine	derivatives	derivatives			
Yamp001702	1-O-Acetyl-N-desmethylrocaglamide	Flavonoids	Other Flavonoids	534.22	166.09	533.205
Wasn002329	Xanthurenic Acid 8-O-Glucoside	Alkaloids	Quinoline alkaloids	366.08	160.04	367.0903
Hmyp002656	Methyl dioxindole-3-acetate	Alkaloids	Plumerane	222.08	130.06	221.0688
Lmhp009034	LysoPE 16:1*	Lipids	LPE	452.28	311.26	451.2699
mws0230	L-Threonine*	Amino acids and derivatives	Amino acids and derivatives	120.07	74.06	119.0582

Metabolite ID	Compound Name	Class I	Class II	Q1 (m/z)	Q3 (m/z)	Molecular Weight (Da)
Smhn002274	Blumenol C glucoside; Byzantionoside B	Terpenoids	Terpene	373.2235	211.1699	372.2148
Lmdp003146	Rhododendrol	Others	Alcohol compounds	167.11	93.07	166.0994
Lmlp005572	Galangin 7-glucoside	Flavonoids	Flavonols	433.11	271.06	432.1056
Wahln11065	Irone	Terpenoids	Monoterpenoids	205.16	189.13	206.1671
Hasp010605	MG(18:3(9Z,12Z,15Z)/0:0/0:0)	Lipids	Glycerol ester	353.27	261.22	352.2614
Wasn004983	5-Galloyl-6-O-Benzoyl Glucose	Phenolic acids	Phenolic acids	435.09	153.02	436.1006
Waptn06182	DGMG 18:2	Lipids	Glycerol ester	737.4	397.13	678.3827
Hmgp001996	Quercetagetin; 3,3',4',5,6,7-Hexahydroxyfl	Flavonoids	Flavonols	319.05	273.04	318.0376

Metabolite ID	Compound Name	Class I	Class II	Q1 (m/z)	Q3 (m/z)	Molecular Weight (Da)
	avone					
Lajp004824	Medicarpinglucoside	Flavonoids	Isoflavones	433.15	271.1	432.142
pmn001710	Rosmarinic acid-3'-O-glucoside	Phenolic acids	Phenolic acids	521.13	359.08	522.1373
Waqkp06484	1-Linolenoylglycerol	Lipids	Glycerol ester	353.27	261.22	352.2614
pme0278	L-Diaminopimelic acid	Amino acids and derivatives	Amino acids and derivatives	191.1	128.07	190.0954
Laln002027	3,4-dihydroxybenzaldehyde-xylose-glucoside	Others	Aldehyde compounds	431.12	137.02	432.1268
Waln011704	1-(2,3-dihydroxypropoxy)-3-(((2-(dimethylamino)ethoxy)(hydroxy)phosphoryl)o	Lipids	LPE	580.36	281.25	581.3693

Metabolite ID	Compound Name	Class I	Class II	Q1 (m/z)	Q3 (m/z)	Molecular Weight (Da)
	xy)propan-2-yl (Z)-14-Octadecenoic Acid					
MWS2430	13-methylmyristic acid	Lipids	Free fatty acids	241.22	241.21	242.2246
WaYn011395	2-(2,3-dihydroxypropoxy)-3-(((2-(dimethylamino)ethoxy)(hydroxy)phosphoryl)oxy)propan-2-yl (Z)-14-Octadecenoic Acid	Lipids	LPE	580.36	281.25	581.3693
pmp000452	Annuionone D	Others	Ketone compounds	225.15	85.1	224.1412
WaXYp00973	2-[[3-Mercapto-2-(alpha-glutamylamino)-1-oxopropyl]amino]acetic acid	Amino acids and derivatives	Amino acids and derivatives	308.09	162.02	307.0834
WaYn002313	Homosyringic Acid	Phenolic acids	Phenolic acids	373.11	193.05	374.1213

Metabolite ID	Compound Name	Class I	Class II	Q1 (m/z)	Q3 (m/z)	Molecular Weight (Da)
	4'-O-Glucoside					
Lcsp000658	Rubinic acid	Terpenoids	Triterpene	471.34	425.34	470.3396
Zmtn001464	4,8-Dihydroxyquinoline-2-carboxylic acid	Alkaloids	Quinoline alkaloids	204.03	160.04	205.0375
HJN083	Lariciresinol-4'-O-glucoside*	Lignans and Coumarins	Lignans	521.2	359.15	522.2101
Lafp002938	Evofolin B glucoside	Phenolic acids	Phenolic acids	481.17	177.05	480.1626
Wafn003987	Decanoyl arabinosylglucoside	Others	Saccharides	525.26	333.19	466.2414
Ladn001032	6-(5-Carboxy-2,3-Dihydroxyphenoxy)-3,4,5-Trihydroxyoxane-2-Carboxylic Acid	Phenolic acids	Phenolic acids	345.04	169.01	346.0536

Metabolite ID	Compound Name	Class I	Class II	Q1 (m/z)	Q3 (m/z)	Molecular Weight (Da)
Zmtp102902	α-Hydrojuglone glucoside	Quinones	Quinones	339.11	177.05	338.1002
pmb1096	Indole	Alkaloids	Plumerane	118.07	91.05	117.0578
Lmhn007769	3β,6β,19α,24-Tetrahydroxy urs-12-en-28-oic acid	Terpenoids	Triterpene	503.34	485.33	504.3451
Waqkn01592	Gallocatechin-(4alpha->8)-epigallocatechin	Tannins	Proanthocyanidins	609.13	423.07	610.1323
Ymjm000148	Salidroside	Phenolic acids	Phenolic acids	299.11	137.02	300.1209
Cmmn012461	Dehydroabietic acid	Terpenoids	Ditepenoids	299.2	299.2	300.2089
Wacn003131	2-O-P-Coumaroylhydroxycitric Acid	Phenolic acids	Phenolic acids	353.05	127	354.0587
Zmdp002216	γ-Glu-Leu	Amino acids and derivatives	Amino acids and derivatives	261.14	86.1	260.1372

Metabolite ID	Compound Name	Class I	Class II	Q1 (m/z)	Q3 (m/z)	Molecular Weight (Da)
Sazp003601	Tuberonic acid glucoside*	Organic acids	Organic acids	387.17	207.11	388.1733
Safn001620	Saccharumoside C	Others	Others	461.13	167.04	462.1373
Yaap003029	3,4,5-trimethoxyphenyl acetate	Others	Others	227.09	167.07	226.0841
Lacp004935	Megastigm-5-en-3,9-diol glucoside	Terpenoids	Sesquiterpenoids	375.24	177.16	374.2299
MWStz487	3,3',4',5',7-Pentahydroxyflavone; Robinetin	Flavonoids	Flavonols	303.05	137.02	302.0427
Lmfn002465	1,2,4-Trihydroxynaphthalene-1,4-di-glucoside (Lawsoniaside)	Others	Others	499.15	337.09	500.153
mws0976	β-Pseudouridine	Nucleotides and	Nucleotides and	243.06	153.03	244.0695

Metabolite ID	Compound Name	Class I	Class II	Q1 (m/z)	Q3 (m/z)	Molecular Weight (Da)
		derivatives	derivatives			
mws0359	Pentadecanoic Acid	Lipids	Free fatty acids	241.22	241.22	242.2246
Lcsn000415	Keto-Deoxy-Nonulonic acid	Lipids	Free fatty acids	267.07	59.01	268.0794
pme1210	L-Methionine	Amino acids and derivatives	Amino acids and derivatives	150.06	61.01	149.051
MWSHY0064	Isorhamnetin-3-O-neohesperidoside*	Flavonoids	Flavonols	625.17	317.07	624.169
Zbcp002823	Cyanidin-3-O-rutinoside (Keracyanin)	Flavonoids	Anthocyanidins	595.16	287.06	595.1657
Lmmn002274	Isolariciresinol-9'-O-glucoside*	Lignans and Coumarins	Lignans	521.2	359.15	522.2101

Metabolite ID	Compound Name	Class I	Class II	Q1 (m/z)	Q3 (m/z)	Molecular Weight (Da)
Ymjm000152	p-Hydroxypheny-β-D-allo pyranoside	Others	Others	271.08	108.02	272.0896
Hmln003754	Icariside E5*	Lignans and Coumarins	Lignans	521.2	359.15	522.2101
Lssp210111	2α-Acetoxy-14β-[(S)-2-methyl-butyryloxy]-4(20),11-taxadiene	Others	Others	463.31	301.22	462.2981
MWSmce576	L-Glucose*	Others	Saccharides	179.0565	59.0141	180.0634
MWSmce506	Neoandrographolide	Terpenoids	Ditepenoids	481.28	301.21	480.2723
Lazp005033	Divanillyltetrahydrofuran*	Lignans and Coumarins	Lignans	345.17	137.06	344.1618
Lazp003665	Nagilactone C glucoside	Terpenoids	Ditepenoids	525.19	525.2	524.1888
mws1164	D-Fructose*	Others	Saccharides	179.06	59.01	180.0634
pmb0892	Trans-Zeatin-1-O-Glucosid	Alkaloids	Alkaloids	382.1721	136.3	381.1648

Metabolite ID	Compound Name	Class I	Class II	Q1 (m/z)	Q3 (m/z)	Molecular Weight (Da)
	e					
MWS2069	2-Methoxy-4-methylphenol	Others	Others	139.08	77.04	138.0681
mws4170	D-Glucose*	Others	Saccharides	179.0561	59.0139	180.0634
Zbfn007626	1-Linoleoyl-2-Lysophosphatidic Acid Monobutylamine Ester*	Lipids	Glycerol ester	504.31	279.23	505.3168
Lmyn003971	Dihydrodehydrodiconiferyl alcohol-4-O-glucoside*	Lignans and Coumarins	Lignans	521.2	359.15	522.2101
Wasn001007	Isoascorbic acid 2-O-glucoside	Others	Vitamin	337.08	277.06	338.0849
pme2617	L-Methionine Sulfoxide	Amino acids and	Amino acids and	166.05	102.06	165.046

Metabolite ID	Compound Name	Class I	Class II	Q1 (m/z)	Q3 (m/z)	Molecular Weight (Da)
		derivatives	derivatives			
Layp002880	5,7-Dihydroxychromone glucoside	Others	Chromone	341.09	179.03	340.0794
Wcjp002598	8-hydroxyquinoline	Alkaloids	Quinoline alkaloids	146.06	91.05	145.0528
Labn002293	3-methoxy-4-((3,4,5-trihydroxy-6-((sulfooxy)methyl)tetrahydro-2H-pyran-2-yl)oxy)benzoic acid	Phenolic acids	Phenolic acids	409.04	241.01	410.0519
Lakn001671	2-β-D-Glucopyranosyloxy-5-hydroxy-phenylacetic acid	Phenolic acids	Phenolic acids	329.09	123.05	330.0951
mws0749	4-Hydroxybenzoic acid	Phenolic acids	Phenolic acids	137.02	93.03	138.0317

Metabolite ID	Compound Name	Class I	Class II	Q1 (m/z)	Q3 (m/z)	Molecular Weight (Da)
MWStz618	Cichoriin*	Lignans and Coumarins	Coumarins	341.0875	179.0346	340.0794
Lasp003143	1,4-Benzodioxin-6-propano l	Lignans and Coumarins	Lignans	193.09	105.07	192.0781
Wasdn03813	Benzoylglycerol 3-glucuronide	Phenolic acids	Phenolic acids	371.1	249.06	372.1056
Sasp001236	7-Hydroxy-tryptophan*	Amino acids and derivatives	Amino acids and derivatives	221.09	130.06	220.0848
pmp000171	Andrographidine A	Flavonoids	Flavanones	463.16	301.11	462.1526
MWStz655	8-Epideoxyloganic acid	Terpenoids	Monoterpenoids	359.13	135.09	360.142
Zbfn013528	Beta-Hydroxypalmitic Acid*	Lipids	Free fatty acids	271.23	225.22	272.2351
Wacyp04942	2,7-Dimethyl-2,4-Octadien	Terpenoids	Monoterpenoids	350.22	153.13	332.1835

Metabolite ID	Compound Name	Class I	Class II	Q1 (m/z)	Q3 (m/z)	Molecular Weight (Da)
	e-1,8-Diol 8-O-B-D-Glucopyranoside					
Zasp005105	Annulatin 3'-O-β-D-xyloside*	Flavonoids	Flavones	465.1011	333.0585	464.0955
MWS0442	Maltotriose	Others	Saccharides	527.16	365.1	504.169
Hmln003605	2,3,19,23-Tetrahydroxyurs-12-ene-28-oic acid (Myrianthic acid)	Terpenoids	Triterpene	503.34	485.33	504.3451
pmb1912	10-Formyltetrahydrofolic Acid	Alkaloids	Alkaloids	474.17	327.12	473.1659
pme2679	L-Allo-isoleucine	Amino acids and derivatives	Amino acids and derivatives	132.1	86.1	131.0946

Metabolite ID	Compound Name	Class I	Class II	Q1 (m/z)	Q3 (m/z)	Molecular Weight (Da)
Safn004708	(2S,3R,4S,5S,6R)-3,4,5-trihydroxy-6-(hydroxymethyl)oxan-2-yl(2E,6E)-8-hydroxy-2,6-dimethylocta-2,6-dienoate	Terpenoids	Monoterpenoids	405.18	183.1	346.1628
mws0154	Shikimic acid	Organic acids	Organic acids	173.05	93.03	174.0528
Walbn08060	1-(((2-(dimethylamino)ethoxy)(hydroxy)phosphoryl)oxy)-3-hydroxypropan-2-yl (9Z,12Z)-octadeca-9,12-dienoate*	Lipids	LPE	504.31	279.24	505.3168
MWStz735	Ganoderal A	Terpenoids	Triterpene	437.34	391.33	436.3341
MWSHC20172	Isoscopoletin	Lignans and Coumarins	Coumarins	193.05	178.03	192.0423

Metabolite ID	Compound Name	Class I	Class II	Q1 (m/z)	Q3 (m/z)	Molecular Weight (Da)
	(6-Hydroxy-7-Methoxycoumarin)					
Lmhn002574	Caffeoyl(p-Hydroxybenzoyl)tartaric acid	Phenolic acids	Phenolic acids	431.06	137.03	432.0693
Zmhn002980	Hydroxyaloe-emodin-8-O-sulfate	Quinones	Anthraquinone	365	285.04	366.0046
Wasyqn4247	2-(3,4-Dihydroxybenzoyloxy)-4,6-Dihydroxyphenylacetic Acid	Phenolic acids	Phenolic acids	319.05	183.03	320.0532
mws1639	Isofraxidin	Lignans and Coumarins	Coumarins	223.06	190.03	222.0528
Safn000917	Mannose 6-phosphate	Others	Saccharides	259.02	78.96	260.0297
Lmyp004052	Quercetin-3-O-(6''-O-p-Co	Flavonoids	Flavonols	611.14	147.04	610.1323

Metabolite ID	Compound Name	Class I	Class II	Q1 (m/z)	Q3 (m/z)	Molecular Weight (Da)
	umaroyl)galactoside*					
Wbmn002185	6-Hydroxy-4-(4-hydroxy-3-methoxyphenyl)-7-methoxy-naphtho[2,3-c]furan-1,3-dione-1	Lignans and Coumarins	Lignans	365.06	167.03	366.074
Lmyp004516	Quercetin-3-O-(2''-O-p-Coumaroyl)galactoside	Flavonoids	Flavonols	611.14	147.04	610.1323
Lmhn003990	Phellodenol E	Lignans and Coumarins	Coumarins	367.1	191.07	368.1107
Yshs000052	6,30-Dihydroxy-3-oxo-lupane-20(29)-en-28-oic acid	Terpenoids	Triterpene	485.32	455.31	486.3345
pmp000439	3-Hydroxyurs-5(6),12,18(19)-trien-28-oic acid (Uncargenin B)	Terpenoids	Triterpene	453.34	435.33	452.329

Metabolite ID	Compound Name	Class I	Class II	Q1 (m/z)	Q3 (m/z)	Molecular Weight (Da)
Lmyn006011	Gingerglycolipid B	Lipids	Glycerol ester	677.38	397.13	678.3827
pme3011	γ-Aminobutyric acid	Organic acids	Organic acids	104.07	69.03	103.0633
mws0133	Nicotinamide	Others	Vitamin	123.06	80.05	122.048
Lhhp102923	(7'R,8'R)-7'8'-dihydro-7'-(5'-hydroxy-3'-methoxphenyl)-3-methoxy-8'-methyl-1-(E)-propenylbenzof-uran	Lignans and Coumarins	Lignans	327.16	137.06	326.1518
Safp001468	Dianthoside	Others	Others	289.09	127.04	288.0845
Lmfn003760	Quercetin-4'-O-glucuronide*	Flavonoids	Flavonols	477.07	301.04	478.0747
Sasp001756	Itoside L	Others	Others	310.15	131.07	292.1158
Yaap005268	Prodelphinidin B*	Flavonoids	Other Flavonoids	611.14	147.04	610.1323

Metabolite ID	Compound Name	Class I	Class II	Q1 (m/z)	Q3 (m/z)	Molecular Weight (Da)
pmn001687	Cis-Coutaric acid	Phenolic acids	Phenolic acids	295.05	163.04	296.0532
mws0567	4-Guanidinobutyric acid	Organic acids	Organic acids	146.09	87.04	145.0851
Lmmp004382	3-Oxo-alpha-ionol glucoside*	Terpenoids	Sesquiterpenoids	371.21	191.14	370.1992
Lskp211300	Grayanotoxin XVIII	Terpenoids	Ditepenoids	337.24	283.21	336.2301
HX1191	3,4-Secodammara-4(28),20, 24-trien-3,26-dioic acid	Terpenoids	Triterpene	471.35	407.33	470.3396
Zkep1310	4-Hydroxy-l-tryptophan*	Amino acids and derivatives	Amino acids and derivatives	221.09	130.06	220.0848
pmp001270	LysoPC 16:1*	Lipids	LPC	494.32	184.07	493.3168
Zmhn001926	1-O-Salicyloyl-β-D-glucose *	Phenolic acids	Phenolic acids	299.08	137.02	300.0845

Metabolite ID	Compound Name	Class I	Class II	Q1 (m/z)	Q3 (m/z)	Molecular Weight (Da)
Ybnn007052	Norartocarpetin	Flavonoids	Flavones	285.04	285.04	286.0477
Wcdn001780	3-methoxy-4-[3,4,5-trihydroxy-6-(sulfooxymethyl)oxan-2-yl]oxybenzoic acid	Others	Others	409.04	241	410.0519
Zjmp102713	2,3-Dihydroxylup-20(29)-en-28-oic acid	Terpenoids	Triterpene	471.3447	201.1625	470.3396
mws0913	Kaempferol-3-O-galactoside (Trifolin)*	Flavonoids	Flavonols	447.09	285.04	448.1006
Hmcn001415	Majoroside	Terpenoids	Monoterpenoids	387.13	225.07	388.1369
Waptn04321	Tricetin 7-O-glucuronide*	Flavonoids	Flavones	477.06	301.03	478.0747
Lmhp008833	LysoPC 16:1(2n isomer)*	Lipids	LPC	494.32	184.07	493.3168
Lmln010063	2,6-Di-tert-butylphenol	Others	Others	205.16	189.13	206.1671

Metabolite ID	Compound Name	Class I	Class II	Q1 (m/z)	Q3 (m/z)	Molecular Weight (Da)
Hmmn003964	7-Deoxyloganic acid	Terpenoids	Monoterpenoids	359.13	197.08	360.142
pmn001646	Procyanidin C1	Tannins	Proanthocyanidins	865.2	577.14	866.2058
Lmsp013456	3-Hydroxytaraxastan-28,20-olide	Terpenoids	Triterpene	455.35	203.18	454.3447
Zbfn008434	1-O-Linoleoyl-3-O-galactopyranosyl-L-glycerol	Lipids	Glycerol ester	515.32	279.23	516.3298
MWStz610	Diosmetin-7-O-beta-D-glucopyranoside*	Flavonoids	Flavones	463.1237	301.0718	462.1162
MWSmce331	Azetidine-2-carboxylic acid*	Alkaloids	Alkaloids	102.0543	56.0498	101.0477
Lmdp004267	Quercetin-3-O-(6''-O-p-Coumaroyl)glucoside	Flavonoids	Flavonols	611.14	147.04	610.1323

Metabolite ID	Compound Name	Class I	Class II	Q1 (m/z)	Q3 (m/z)	Molecular Weight (Da)
Lazn003953	Erythro-Guaiacylglycerol-β-dihydroconiferyl Ether glucoside	Lignans and Coumarins	Lignans	539.21	491.19	540.2207
mws0264	D-Trehalose*	Others	Saccharides	341.11	119.03	342.1162
Lmcp000611	2-Amino-4,5-dihydro-1H-imidazole-4-acetic acid	Alkaloids	Alkaloids	144.08	84.06	143.0695
mws2623	11-Octadecanoic acid(Vaccenic acid)	Lipids	Free fatty acids	281.25	281.25	282.2559
Lmtn002997	Mearnsetin-3-O-glucoside*	Flavonoids	Flavones	493.1	331.05	494.106
MWSHY0061	Kaempferol-3-O-neohesperidoside*	Flavonoids	Flavonols	595.16	287.06	594.1585
MWS2056	Delta-Hexalactone	Others	Lactones	115.08	69.07	114.0681

Metabolite ID	Compound Name	Class I	Class II	Q1 (m/z)	Q3 (m/z)	Molecular Weight (Da)
MWSslk013	Tectoridin*	Flavonoids	Isoflavones	463.12	301.09	462.1162
MWStz526	Daphnin*	Lignans and Coumarins	Coumarins	341.0881	179.0366	340.0794
Wbtn007675	Calyxanthone	Flavonoids	Other Flavonoids	285.04	285.04	286.0477
pmp000579	Diosmetin-7-O-galactoside *	Flavonoids	Flavones	463.12	301.07	462.1162
Xmzn007677	Ergotamine	Alkaloids	Alkaloids	580.36	506.33	581.2638
Hmcp001829	Quercetin-3,7-Di-O-rhamn oside	Flavonoids	Flavonols	595.16	303.04	594.1585
Lssp210311	3-Hydroxylup-12(13),20(29)-diene-28-oic acid	Terpenoids	Triterpene	455.35	207.17	454.3447
Wbmp003419	Dihydrovomifoliol-O-β-D-glucoside*	Terpenoids	Sesquiterpenoids	389.22	209.15	388.2097

Metabolite ID	Compound Name	Class I	Class II	Q1 (m/z)	Q3 (m/z)	Molecular Weight (Da)
Wacyn02305	Methyl 3-O-Beta-Glucopyranosyl-Gallate	Phenolic acids	Phenolic acids	345.08	183.03	346.09
mws0237	Azelaic acid	Lipids	Free fatty acids	187.1	125.1	188.1049
Labn004865	Hydroxy isoliquiritigenin glucoside*	Flavonoids	Chalcones	433.11	271.06	434.1213
Lmmp010562	Diisooctyl Phthalate	Phenolic acids	Phenolic acids	391.28	149.02	390.277
Wcqn007564	1beta,2alpha,3beta,23-tetrahydroxyurs-12-en-28-ursolic acid*	Terpenoids	Triterpene	503.34	485.33	504.3451
Zbln004208	2α,3α-Epoxy-5,7,3',4'-tetrahydroxyflavan-(4β-8-epicatechin)	Tannins	Proanthocyanidins	575.12	449.09	576.1268

Metabolite ID	Compound Name	Class I	Class II	Q1 (m/z)	Q3 (m/z)	Molecular Weight (Da)
Lajn004573	7,2',4'-Trihydroxy-8,3'-dimethoxyisoflavanglucoside	Flavonoids	Other Flavonoids	479.15	317.11	480.1632
pme0026	L-Lysine	Amino acids and derivatives	Amino acids and derivatives	147.11	84.08	146.1055
Labn002551	3,5-dimethoxy-4-((3,4,5-trihydroxy-6-((sulfooxy)methyl)tetrahydro-2H-pyran-2-yl)oxy)benzoic acid	Phenolic acids	Phenolic acids	439.05	241.01	440.0625
Hmln003398	2,3,19,23-Tetrahydroxyurs-12-en-28-oic acid*	Terpenoids	Triterpene	503.34	485.33	504.3451
Yshs000050	Lupane-20(29)-en-3-on-28-oic acid	Terpenoids	Triterpene	455.35	437.35	454.3447
pmf0175	2-Decanol*	Others	Alcohol compounds	157.16	157.16	158.1671

Metabolite ID	Compound Name	Class I	Class II	Q1 (m/z)	Q3 (m/z)	Molecular Weight (Da)
Rfmb320	1-Methylpiperidine-2-carboxylic acid*	Alkaloids	Piperidine alkaloids	144.1019	84.0813	143.0946
mws1179	Naringenin-7-O-glucoside (Prunin)*	Flavonoids	Flavanones	433.11	271.07	434.1213
Safn002037	2-[4-[(1R,2R)-1,2-dihydroxypropyl]phenoxy]-6-(hydroxymethyl)oxane-3,4,5-triol	Organic acids	Organic acids	329.12	167.07	330.1315
Cmxp003975	Okanin-4'-O-glucoside(Marein)*	Flavonoids	Chalcones	451.12	289.07	450.1162
MWSslk237	Hispidulin-7-O-glucoside(Homoplantaginin)*	Flavonoids	Flavones	463.13	301.07	462.1162
Wasyqp6350	Dehydrophytosphingosine	Lipids	Sphingolipids	316.29	60.04	315.2773

Metabolite ID	Compound Name	Class I	Class II	Q1 (m/z)	Q3 (m/z)	Molecular Weight (Da)
pmb2922	Uridine 5'-diphospho-D-glucose	Nucleotides and derivatives	Nucleotides and derivatives	565.05	323.03	566.055
Safp022689	Tomentosolic acid	Terpenoids	Triterpene	455.35	409.34	454.3447
Wayn004603	Tricetin 3'-glucuronide*	Flavonoids	Flavones	477.07	301.04	478.0747
Zbjn003642	1-O-rhamnose-3-O-Caffeoyl Quinic Acid	Phenolic acids	Phenolic acids	499.15	191.06	500.1554
Zasp009163	Mudanpinoic acid A	Terpenoids	Triterpene	455.35	203.18	454.3447
pmn001716	Carthamone*	Flavonoids	Chalcones	447.09	285.04	448.1006
Lmyn000160	Mucic acid Dimethyl Ester	Organic acids	Organic acids	237.06	87.01	238.0689
Zmhn001358	4-O-Glucosyl-4-hydroxybenzoic acid	Phenolic acids	Phenolic acids	299.08	137.02	300.0845
Zajp000573	N-(4-oxopentyl)-acetamide	Alkaloids	Alkaloids	144.1013	84.0805	143.0946

Metabolite ID	Compound Name	Class I	Class II	Q1 (m/z)	Q3 (m/z)	Molecular Weight (Da)
	*					
pme3388	Homoarginine	Amino acids and derivatives	Amino acids and derivatives	189.13	144.1	188.1273
Wasdn02417	3-hydroxybenzoyl-β-D-glucose*	Phenolic acids	Phenolic acids	299.08	137.02	300.0845
Waptn05947	DGMG 18:3	Lipids	Glycerol ester	735.38	397.13	676.367
Lmtp004146	Santalin	Others	Ketone compounds	275.0928	107.0489	274.0841
Lmjp003655	6-C-MethylKaempferol-3-glucoside*	Flavonoids	Flavonols	463.12	301.08	462.1162
pme1014	Menatetrenone (Vitamin K2)	Others	Vitamin	445.31	341.28	444.3028
Hmbn002692	6'-O-Feruloyl-D-sucrose	Phenolic acids	Phenolic acids	517.16	175.04	518.1636

Metabolite ID	Compound Name	Class I	Class II	Q1 (m/z)	Q3 (m/z)	Molecular Weight (Da)
Wbtn004381	1,3,6,8-tetrahydroxy-2,5-dimethoxyxanthen-9-one	Flavonoids	Other Flavonoids	319.04	183.03	320.0532
MWSmce461	L-Azetidine-2-carboxylic acid*	Alkaloids	Alkaloids	102.05	56.05	101.0477
Waptn06085	DGMG 18:3(2n isomer)	Lipids	Glycerol ester	735.38	397.13	676.367
Lajp003585	Murrayacarpin A glucoside	Lignans and Coumarins	Coumarins	369.12	177.05	368.1102
Zmhn001703	5-O-Galloyl-D-hamamelose	Phenolic acids	Phenolic acids	331.07	169.02	332.0743
MWStz656	Neoisoliquiritin	Flavonoids	Chalcones	417.12	255.07	418.1264
pme1383	Pyridoxine	Others	Vitamin	170.08	134.06	169.0739
mws0120	Choline Alfoscerate	Lipids	PC	258.11	104.11	257.1028

Metabolite ID	Compound Name	Class I	Class II	Q1 (m/z)	Q3 (m/z)	Molecular Weight (Da)
mws1526	Sweroside	Terpenoids	Monoterpenoids	359.13	197.08	358.1264
WaYn005089	Neosakuranin	Flavonoids	Chalcones	447.13	285.08	448.1369
zjbp111026	tetrahydrochromone M	Others	Chromone	343.15	163.07	342.1467
mws4532g	D-Ornithine	Amino acids and derivatives	Amino acids and derivatives	133.1	70.07	132.0899
Yshs000022	18-O-Methylvibsanin K	Terpenoids	Ditepenoids	463.26	301.07	462.2618
Wbmp003302	Frambinone	Others	Ketone compounds	165.09	91.05	164.0837
MWSmce230	Methyl gallate*	Phenolic acids	Phenolic acids	183.03	124.02	184.0372
MWS04455	Cis-L-3-hydroxyproline*	Amino acids and derivatives	Amino acids and derivatives	132.07	86.06	131.0582
Lazn006152	Isoshonanin*	Lignans and Coumarins	Lignans	343.15	105.03	342.1467

Metabolite ID	Compound Name	Class I	Class II	Q1 (m/z)	Q3 (m/z)	Molecular Weight (Da)
pmp000263	3-Hydroxyurs-12,19-dien-28-oic acid (Sanguisorbigenin)	Terpenoids	Triterpene	455.35	409.35	454.3447
HJN090	Isocoreopsin*	Flavonoids	Flavanones	433.11	271.06	434.1213
Wbsp006152	3α,20β,21β-trihydroxy-16-oxo-serrat-14-en-24-oic acid	Terpenoids	Triterpene	503.34	485.33	502.3294
pmp000593	Luteolin-7-O-rutinoside*	Flavonoids	Flavones	595.17	287.06	594.1585
Jmwn002494	3-[4-(β-D-glucopyranoside)-phenylacrylic]-acid	Phenolic acids	Phenolic acids	325.09	119.05	326.1002
pme0193	L-Glutamine	Amino acids and derivatives	Amino acids and derivatives	147.08	84.04	146.0691
Lmzn005892	Isoceanothic acid	Terpenoids	Triterpene	485.33	423.33	486.3345

Metabolite ID	Compound Name	Class I	Class II	Q1 (m/z)	Q3 (m/z)	Molecular Weight (Da)
Zblp002066	Delphinidin-3-O-arabinoside	Flavonoids	Anthocyanidins	435.09	303.05	435.0922
Qatp004621	Machilin H	Lignans and Coumarins	Lignans	375.18	177.09	374.1729
Lakp004475	Dehydrodicatechin A	Flavonoids	Flavanols	577.13	287.06	576.1268
Zmsn002575	Hastatoside*	Terpenoids	Monoterpenoids	403.12	223.07	404.1319
Sasn000277	D-inositol-4-phosphate	Others	Saccharides	259.02	78.96	260.0297
Lcsn000619	Tricalysioside N	Terpenoids	Ditepenoids	557.29	335.22	558.304
Safn006268	Pterosupin	Flavonoids	Chalcones	435.13	315.09	436.1369
Lmpp005784	6-Methoxyquercetin-3-O-Xyloside*	Flavonoids	Flavonols	465.1	333.07	464.0955
MWStz594	Sibiricose A6	Phenolic acids	Phenolic acids	547.16	205.05	548.1741

Metabolite ID	Compound Name	Class I	Class II	Q1 (m/z)	Q3 (m/z)	Molecular Weight (Da)
pme1605	Kaempferol-3-O-robinobioside(Biorobin)*	Flavonoids	Flavonols	593.15	285.04	594.1585
MWStz421	Ternatumoside II	Flavonoids	Flavonols	595.1685	287.0589	594.1585
pme2914	3-Hydroxy-3-methylpentane-1,5-dioic acid	Organic acids	Organic acids	161.05	99.05	162.0528
pme0519	D-Sucrose*	Others	Saccharides	341.11	119.03	342.1162
MWS04447	Homoproline	Amino acids and derivatives	Amino acids and derivatives	130.09	70.07	129.079
Lmln007483	6,9,10-Trihydroxyoctadec-7-enoic acid; Sanleng acid*	Lipids	Free fatty acids	329.23	171.1	330.2406
Waptn04422	Hydroxygeraniol apiosylglucoside	Terpenoids	Monoterpenoids	523.24	331.18	464.2258

Metabolite ID	Compound Name	Class I	Class II	Q1 (m/z)	Q3 (m/z)	Molecular Weight (Da)
Lmbp002962	Isofraxetin	Lignans and Coumarins	Coumarins	209.05	194.02	208.0372
HJN106	2-Carboxy-3-hydroxy-A(1)-norlupan-20(29)-en-28-oic acid (Ceanothic acid)*	Terpenoids	Triterpene	485.33	467.32	486.3345
pmn001338	Rhein-8-O-glucoside	Quinones	Anthraquinone	445.08	283	446.0849
MWSHY0201	Kaempferol-3,7-O-dirhamnoside (Kaempferitrin)	Flavonoids	Flavonols	579.17	287.06	578.1636
Zblp004882	4'-Hydroxy-3,5-dimethoxy dihydrochalcone	Flavonoids	Chalcones	287.1395	121.0283	286.1205
Lmmn003398	Kaempferol-3-O-(6''-O-acetyl)glucoside	Flavonoids	Flavonols	489.11	285.04	490.1111
mws0289	LysoPE 18:1*	Lipids	LPE	480.31	339.29	479.3012

Metabolite ID	Compound Name	Class I	Class II	Q1 (m/z)	Q3 (m/z)	Molecular Weight (Da)
mws0089	Kaempferol-7-O-glucoside *	Flavonoids	Flavonols	447.09	285.04	448.1006
MWStz593	Gossypin	Flavonoids	Flavonols	481.0997	319.0519	480.0904
Zbrn006627	Chrysoeriol-5-O-glucoside	Flavonoids	Flavones	461.11	299.06	462.1162
Zbsp004650	Tricin-5-O-Glucoside	Flavonoids	Flavones	493.13	331.08	492.1268
WaYp004929	3-O-(p-coumaroyl) 3-Hydroxy-3-methylglutari c acid	Phenolic acids	Phenolic acids	309.1	147.04	308.0896
pmb1178	O-Feruloyl 7-hydroxycoumarin*	Lignans and Coumarins	Coumarins	339.09	177.05	338.079
Lcfp001119	cis-Zeatin-O-glucoside*	Alkaloids	Alkaloids	382.17	220.12	381.1648
MWStz396	Gingerglycolipid A	Lipids	Glycerol ester	675.36	397.13	676.367

Metabolite ID	Compound Name	Class I	Class II	Q1 (m/z)	Q3 (m/z)	Molecular Weight (Da)
Lmsn011015	1-Oxo-Siaresinolic acid*	Terpenoids	Triterpene	485.33	455.32	486.3345
HJN049	2α,3α-Epoxy-5,7,3',4'-tetra hydroxyflavan-(4β→8)-catechin	Tannins	Proanthocyanidins	575.12	449.09	576.1268
Wcdn004423	Lyoniresinol-3-O-glucoside*	Lignans and Coumarins	Lignans	581.22	419.14	582.2312
Wchn005577	1-O-Galloyl-2-O-Cinnamoyl-β-D-glucose	Phenolic acids	Phenolic acids	461.11	315.07	462.1162
pmb0789	Pyridoxine-5'-O-glucoside	Others	Vitamin	332.13	152.07	331.1267
pmb0856	LysoPE 18:1(2n isomer)*	Lipids	LPE	480.31	339.29	479.3012
Lcsn000622	Tricalysioside O	Terpenoids	Ditepenoids	573.29	513.27	574.2989
Sazp001763	4-Hydroxyphenyl	Others	Others	357.12	195.07	358.1264

Metabolite ID	Compound Name	Class I	Class II	Q1 (m/z)	Q3 (m/z)	Molecular Weight (Da)
	6-O-(2-Methyl-3-Hydroxypropionyl)-Beta-D-Galactopyranoside					
HJN037	3,5-Dihydroxy-4-methoxybenzoic acid; 4-O-Methylgallic Acid*	Phenolic acids	Phenolic acids	183.03	124.02	184.0372
MWStz569	Cinnamtannin A2	Tannins	Tannin	1155.2726	579.1483	1154.2692
mws0180	2,5-Dihydroxybenzoic acid; Gentisic Acid*	Phenolic acids	Phenolic acids	153.02	109.03	154.0266
Qmzp101807	Avicennol*	Lignans and Coumarins	Coumarins	343.1546	163.0749	342.1467
pme0516	Inositol	Others	Saccharides	179.06	87.01	180.0634
HJN069	Tetrahydroxyflavan-(4α-8-	Flavonoids	Flavanols	575.12	449.09	576.1268

Metabolite ID	Compound Name	Class I	Class II	Q1 (m/z)	Q3 (m/z)	Molecular Weight (Da)
	epicatechin)					
Lmgn010741	2α,3α,23-Trihydroxyurs-12,20(30)-dien-28-oic acid*	Terpenoids	Triterpene	485.33	455.32	486.3345
Lcgp000245	dihydrodehydrodiconiferyl alcohol-9-O-β-D-xylopyranoside	Lignans and Coumarins	Lignans	493.21	331.15	492.1995
HJAP112	Quercetin-3-O-(2''-O-acetyl)rhamnoside	Flavonoids	Flavonols	491.11	303.06	490.1111
Labn002264	Grevilloside G	Phenolic acids	Phenolic acids	315.11	153.05	316.1158
Lmgn013947	2α,3α-Dihydroxyolean-12-en-28-oic acid	Terpenoids	Triterpene	471.35	471.35	472.3553

Metabolite ID	Compound Name	Class I	Class II	Q1 (m/z)	Q3 (m/z)	Molecular Weight (Da)
MWSmce675	Arbutin	Others	Others	271.08	108.02	272.0896
Ladn001231	3,5-dihydroxy-4-{[(2s,3r,4s,5s,6r)-3,4,5-trihydroxy-6-[(sulfooxy)methyl]oxan-2-yl]oxy}benzoic acid*	Phenolic acids	Phenolic acids	411.02	241	412.0312
Labn003910	Benzyl B-Primeveroside*	Others	Others	401.14	269.1	402.1526
Zblp002068	Cyanidin-3-O-glucoside*	Flavonoids	Anthocyanidins	449.11	287.06	449.1078
Lhsp101905	Actinidialactone	Terpenoids	Monoterpenoids	167.11	93.07	166.0994
MWSHC20168	Methyl cumalate*	Others	Lactones	153.02	109.03	154.0266
Lmmp003817	Kaempferol-3-O-(6''-malonyl)glucoside*	Flavonoids	Flavonols	535.11	287.05	534.101
Lmmn004951	2-Hydroxy-4-methyl-3-und	Lipids	Free fatty acids	329.23	171.1	330.2406

Metabolite ID	Compound Name	Class I	Class II	Q1 (m/z)	Q3 (m/z)	Molecular Weight (Da)
	ecanoyloxypentanoic acid methyl ester*					
Safn000900	2,3,5-trihydroxy-4-(phosphonooxy)hexanedioic acid	Others	Others	289.01	96.97	290.0039
Wasyqp2836	3-O-Feruloyl Aminogalactitol	Phenolic acids	Phenolic acids	374.14	177.06	373.1373
WaQzp02611	N-Caffeoylmethylgluconamide*	Alkaloids	Phenolamine	372.13	163.04	371.1216
MWSHC20125	5-O-Caffeoylshikimic acid	Phenolic acids	Phenolic acids	335.08	135.05	336.0845
Qatp004010	Micheliolide	Terpenoids	Sesquiterpenoids	249.15	157.1	248.1412
pme2527	L-Ornithine	Amino acids and derivatives	Amino acids and derivatives	133.1	116.07	132.0899

Metabolite ID	Compound Name	Class I	Class II	Q1 (m/z)	Q3 (m/z)	Molecular Weight (Da)
Lmjp101401	Bismurrangatin	Lignans and Coumarins	Coumarins	535.2	287.05	534.189
Wasyqn3009	5-O-p-Coumaroylgalactaric acid	Phenolic acids	Phenolic acids	355.07	209.03	356.0738
Lmdp004892	Kaempferol-3-O-(6''-malonyl)galactoside*	Flavonoids	Flavonols	535.11	287.05	534.101
Zblp102242	Cyanidin-3-O-galactoside*	Flavonoids	Anthocyanidins	449.1093	287.055	449.1078
Wahlp02159	D-Pantothenic Acid 4'-O-Beta-Glucoside	Others	Vitamin	382.17	220.12	381.1635
pmb2947	Catechin-(4alpha->8)-catechin-(4alpha->6)-catechin	Flavonoids	Flavanols	865.2	407.1	866.2058
Wbmn003453	6-O-Vanilloylajugol	Terpenoids	Monoterpenoids	497.17	437.2	498.1737
HJKP000649	N-benzylformamide*	Alkaloids	Alkaloids	136.07	91.05	135.0684

Metabolite ID	Compound Name	Class I	Class II	Q1 (m/z)	Q3 (m/z)	Molecular Weight (Da)
mws5038	Isomaltulose*	Others	Saccharides	341.1089	89.0249	342.1162
Lmsn011201	2,3-Dihydroxy-19-oxo-18,19-secours-11,13(18)-dien-28-oic acid (Swinhoeic acid)*	Terpenoids	Triterpene	485.33	455.32	486.3345
Zbln001290	β-Hydroxy-(3,4-dihydroxyphenylethanolyl)-glucoside	Others	Alcohol compounds	331.1	151.04	332.111
Lmsn000381	D-Maltose*	Others	Saccharides	341.1089	89.0244	342.1162
Labp005025	5-O-Malonylisosakuranin	Flavonoids	Flavanones	535.14	287.09	534.1373
Cmsp002787	3,4'-Dihydroxy-3',5'-dimethoxypropiophenone	Others	Ketone compounds	227.09	181.05	226.0841
Lmlp003161	N-Feruloylputrescine	Alkaloids	Phenolamine	265.15	177.06	264.1474

Metabolite ID	Compound Name	Class I	Class II	Q1 (m/z)	Q3 (m/z)	Molecular Weight (Da)
MWSslk108	O-Acetyl-L-carnitine	Alkaloids	Alkaloids	204.13	85.03	203.1158
Zbkp000668	2'-Deoxymugineic acid	Alkaloids	Alkaloids	305.13	186.08	304.1271
MWStz638	(+)-Lyoniresinol 9'-O-glucoside*	Lignans and Coumarins	Lignans	581.22	419.17	582.2312
Wdqn005743	(3b,16a,20R)-25-Acetoxy-3, 16,20,22-tetrahydroxy-5-cucurbiten-11-one 3-glucoside	Steroids	Steroidal saponins	709.42	487.34	710.4241
Zbsn004974	Procyanidin A2	Tannins	Proanthocyanidins	575.12	285.04	576.1268
Labn001995	3,5-dihydroxy-4-[(2S,3R,4S, 5S,6R)-3,4,5-trihydroxy-6-(sulfooxymethyl)oxan-2-yl]oxybenzoic acid*	Phenolic acids	Phenolic acids	411.02	241.01	412.0312

Metabolite ID	Compound Name	Class I	Class II	Q1 (m/z)	Q3 (m/z)	Molecular Weight (Da)
MWSHY0080	Luteolin-7-O-neohesperidoside (Lonicerin)*	Flavonoids	Flavones	595.17	287.06	594.1585
pmd0160	LysoPE 16:0(2n isomer)	Lipids	LPE	454.29	313.27	453.2855
MWStz539	Chrysoeriol-7-O-glucoside	Flavonoids	Flavones	461.11	299.06	462.1162
Lmqp009231	Dillapiol	Others	Others	223.1	191.07	222.0892
Lamn006238	3,9-dihydroeucomnalin glucoside*	Flavonoids	Other Flavonoids	477.13	315.09	478.1475
pmb0876	LysoPE 16:0	Lipids	LPE	454.29	313.27	453.2855
Lbhp011913	Malonyl fraxin	Lignans and Coumarins	Coumarins	457.1	209.04	456.0904
pma0702	N1,N8-Bis(sinapoyl)spermidine	Alkaloids	Phenolamine	558.28	207.07	557.2737
pmp001198	6-Deoxyfagomine	Alkaloids	Piperidine alkaloids	132.1	57.03	131.0946

Metabolite ID	Compound Name	Class I	Class II	Q1 (m/z)	Q3 (m/z)	Molecular Weight (Da)
pmb0782	Piperidine	Alkaloids	Piperidine alkaloids	86.1	69.07	85.0891
Wdsp002909	6-[(7-hydroxy-2-methyl-6-oxo-3,4-dihydroisoquinolin-1-yl)methyl]-2,3-dimethoxybenzoic acid	Alkaloids	Isoquinoline alkaloids	372.14	163.04	371.1369
Lmjn102210	Desmethylagrimonolide-6-O-β-D-glucopyranoside*	Lignans and Coumarins	Coumarins	461.14	299.09	462.1526
MWStz545	5-O-Feruloylquinic acid	Phenolic acids	Phenolic acids	369.12	177.06	368.1107
Wasyqn2830	Protocatechuic Acid 4-(6''-O-Malonyl)glucoside	Phenolic acids	Phenolic acids	401.07	152.01	402.0798
MWStz521	Quercetin-3-O-(6''-O-malonyl)glucoside	Flavonoids	Flavonols	549.09	300.03	550.0959

Metabolite ID	Compound Name	Class I	Class II	Q1 (m/z)	Q3 (m/z)	Molecular Weight (Da)
Hmhn003067	Phenylpropionic acid-O-β-D-glucopyranoside*	Phenolic acids	Phenolic acids	325.09	119.05	326.1002
MWSslk254	Kaempferol-3-O-glucorhamnoside*	Flavonoids	Flavonols	595.166	287.0598	594.1585
pmf0139	D-Galactose*	Others	Saccharides	179.06	59.01	180.0634
Wacn005438	Hydroxypentadecenoic acid glucoside	Lipids	Free fatty acids	477.27	161.04	418.2567
Hmln000873	2-O-Galloyl-D-glucose	Phenolic acids	Phenolic acids	331.07	169.01	332.0743
Lmpn006208	8-Methoxykaempferol-7-O-rhamnoside	Flavonoids	Flavonols	461.11	315.05	462.1162
pmf0174	1-Decanol*	Others	Alcohol compounds	157.16	157.16	158.1671

Metabolite ID	Compound Name	Class I	Class II	Q1 (m/z)	Q3 (m/z)	Molecular Weight (Da)
pmn001573	Desmethylagrimonolide-6-O-glucoside*	Lignans and Coumarins	Coumarins	461.15	299.09	462.1526
Zmhn002301	p-Coumaric acid-4-O-glucoside	Phenolic acids	Phenolic acids	325.09	163.04	326.1002
Li512114	2,3-Dihydroxyurs-12-en-28-oic acid methyl ester (Corosolic acid methyl ester)	Terpenoids	Triterpene	485.36	453.34	486.3709
Wcgp003276	5-(2-hydroxypropyl)-3H-2-benzofuran-1-one	Others	Others	193.09	133.06	192.0786
Sazp011041	Phyllnirurin*	Lignans and Coumarins	Lignans	343.15	163.07	342.1467
Lmxp000429	Hercynine	Alkaloids	Alkaloids	198.12	95.06	197.1164

Metabolite ID	Compound Name	Class I	Class II	Q1 (m/z)	Q3 (m/z)	Molecular Weight (Da)
mws1565	Geniposide	Terpenoids	Monoterpenoids	389.14	209.08	388.1369
Hmcp000405	Dihydroisopelletierine	Alkaloids	Piperidine alkaloids	144.14	84.08	143.131
Hmbp002730	Isoscutellarein	Flavonoids	Flavones	287.06	153.01	286.0477
Wasn002902	O-p-Coumaroylgalactaric acid	Phenolic acids	Phenolic acids	355.07	209.03	356.0738
Lmpp003789	Cyanidin 3-O-(6-O-p-coumaroyl)glucoside	Flavonoids	Anthocyanidins	595.14	287.06	595.1446
MWStz040	L-Proline*	Alkaloids	Pyrrole alkaloids	116.0703	70.0668	115.0633
MWSmce052	3-Hydroxyurs-12-en-28-oic acid (3-Epiursolic acid)*	Terpenoids	Triterpene	455.36	455.36	456.3603
pmp000587	Luteolin-7-O-(6''-malonyl)	Flavonoids	Flavones	535.11	287.05	534.101

Metabolite ID	Compound Name	Class I	Class II	Q1 (m/z)	Q3 (m/z)	Molecular Weight (Da)
	glucoside*					
WaXYp01907	N-[(R)-2-Hydroxy-3,3-dimethyl-4-(β-D-glucopyranosyloxy)butyryl]-β-alanine	Alkaloids	Alkaloids	382.17	220.12	381.1628
Wcfp003695	Oleracone B	Others	Others	207.14	95.08	206.1307
Rfn048602	Melilotoside*	Phenolic acids	Phenolic acids	325.09	119.05	326.1002
mws0344	D-Xylonic acid	Others	Saccharides	165.04	75.01	166.0477
Zkep008998	o-Phosphohomoserine	Organic acids	Organic acids	200.02	98.97	199.0246
pmn001644	Quercetin-3-O-(2''-O-acetyl)glucuronide	Flavonoids	Flavonols	519.08	300	520.0853
Cmjn004337	Benzyl-(2''-O-xylosyl)glucoside*	Others	Others	401.14	269.1	402.1526

Metabolite ID	Compound Name	Class I	Class II	Q1 (m/z)	Q3 (m/z)	Molecular Weight (Da)
Lmsp004670	Kaempferol-3-O-glucoside-7-O-rhamnoside*	Flavonoids	Flavonols	595.17	287.06	594.1585
Zaln004057	4-caffeoylshikimic acid	Phenolic acids	Phenolic acids	335.08	179.03	336.0845
Wahln03866	Ebracteatoside D	Others	Alcohol compounds	497.22	305.16	438.2096
Hahp000801	alanine betaine	Alkaloids	Alkaloids	118.08	72.08	117.079
Lmdp003808	Azaleatin (5-O-Methylquercetin)	Flavonoids	Flavonols	317.07	302.04	316.0583
Wacyp01167	(6-amino-3-oxohexan-2-yl) proline	Amino acids and derivatives	Amino acids and derivatives	229.15	142.09	228.1474
Lmtn002565	1-O-Vanilloyl-D-Glucose	Phenolic acids	Phenolic acids	329.11	167.03	330.0951
Zbqn008058	Rubianol-f	Terpenoids	Triterpene	471.35	471.35	472.3553
pmp001264	Hexadecylsphingosine	Lipids	Sphingolipids	274.27	256.27	273.2668

Metabolite ID	Compound Name	Class I	Class II	Q1 (m/z)	Q3 (m/z)	Molecular Weight (Da)
mws1333	Melibiose	Others	Saccharides	341.11	113.02	342.1162
MWSmce585	Methyl 3-aminopropanoate	Amino acids and derivatives	Amino acids and derivatives	104.07	59.01	103.0633
mws1389	3-Hydroxyolean-12-en-28-oic acid (Oleanolic acid)*	Terpenoids	Triterpene	455.35	455.35	456.3603
MWS00204g	N-Ethylglycine*	Amino acids and derivatives	Amino acids and derivatives	104.0707	58.0659	103.0633
pmn001591	3,19,23-Trihydroxyurs-12-en-28-oic acid (Rutundic acid)	Terpenoids	Triterpene	487.34	469.33	488.3502
Waptn03329	Benzyl 6-O-beta-D-apiofuranosyl-beta-D-glucoside*	Others	Alcohol compounds	461.17	269.1	402.1526

Metabolite ID	Compound Name	Class I	Class II	Q1 (m/z)	Q3 (m/z)	Molecular Weight (Da)
pmb0818	Methoxyindoleacetic acid	Alkaloids	Plumerane	206.08	145.05	205.0739
Lmxn000398	D-Lactose*	Others	Saccharides	341.11	89.02	342.1162
pme3033	N,N-Dimethylglycine*	Amino acids and derivatives	Amino acids and derivatives	104.07	58.07	103.0633
Wayp004349	N-Hydroxydecanoyl nornicotine glucoside	Alkaloids	Pyridine alkaloids	481.29	319.24	480.283
pme3017	2-Aminoisobutyric acid	Organic acids	Organic acids	104.07	58.07	103.0633
Lmhn002437	Terreic acid*	Others	Ketone compounds	153.02	109.03	154.0266
pmn001700	24,30-Dihydroxy-12(13)-en e-lupeol*	Terpenoids	Triterpene	455.35	455.35	456.3603
Wafn003429	3-Methylbutyl 6-O-(alpha-L-arabinopyran	Others	Alcohol compounds	441.2	249.13	382.1839

Metabolite ID	Compound Name	Class I	Class II	Q1 (m/z)	Q3 (m/z)	Molecular Weight (Da)
	osyl)-beta-D-glucopyranoside					
Zbln003341	2-(2-(3,4-dihydroxyphenyl)-2-methoxyethoxy)-6-(hydroxymethyl)tetrahydro-2H-pyran-3,4,5-tri	Phenolic acids	Phenolic acids	345.12	191.06	346.1286
Safn001967	2,3,4-trihydroxy-5-{[3-(4-hydroxyphenyl)prop-2-enoyl]oxy}hexanedioic acid	Phenolic acids	Phenolic acids	355.07	85.03	356.0743
pmp000101	Arecatannin C1	Tannins	Tannin	867.21	715.17	866.2058
Wasyqn5954	1-Octanol Rhamnosylglucoside	Others	Alcohol compounds	497.22	291.18	438.2465
Zmdp000972	S-Methyl-L-cysteine	Amino acids and	Amino acids and	136.04	91.05	135.0354

Metabolite ID	Compound Name	Class I	Class II	Q1 (m/z)	Q3 (m/z)	Molecular Weight (Da)
		derivatives	derivatives			
Wahlp01697	Methyl 3-oxo-2-phenylpropanoate	Phenolic acids	Phenolic acids	179.07	91.05	178.063
Lmmp003109	3'-O-Methyl-epicatechin	Flavonoids	Flavanols	305.1	139.04	304.0947
HJAP005	Laricitrin-3-O-glucoside*	Flavonoids	Flavonols	495.11	333.07	494.106
Hmln002199	Quercetin-3-O-(6''-O-acetyl)galactoside	Flavonoids	Flavonols	505.1	300.03	506.106
pmn001647	Procyanidin C2	Tannins	Proanthocyanidins	865.2	713.16	866.2058
Wafn002874	4-O-(4'-O-alpha-D-Glucopyranosyl)caffeoylquinic acid	Phenolic acids	Phenolic acids	515.14	323.08	516.1479
Wbsp011256	16-oxo-21β-hydroxyserrat-	Terpenoids	Triterpene	457.37	457.37	456.3603

Metabolite ID	Compound Name	Class I	Class II	Q1 (m/z)	Q3 (m/z)	Molecular Weight (Da)
	14-en-3α-yl acetate					
Lmzn006024	2-Aldehydo-A(1)-norlup-20(29)-en-27,28-dioic acid (Zizyberanal acid)	Terpenoids	Triterpene	483.31	483.31	484.3189
MWSmce394	2,3,19-Trihydroxyurs-12-en-28-oic acid (Tormentic acid)*	Terpenoids	Triterpene	487.34	469.33	488.3502
Wasyqn2926	3-O-Feruloyl Octose*	Phenolic acids	Phenolic acids	415.12	193.05	416.1319
pmn001426	2,3,19-trihydroxyurs-12-en-28-oic acid (Euscaphic acid)*	Terpenoids	Triterpene	487.34	469.33	488.3502
pmp000682	Matairesinol-4'-O-glucoside (Matairesinoside)	Lignans and Coumarins	Lignans	538.23	359.15	520.1945

Metabolite ID	Compound Name	Class I	Class II	Q1 (m/z)	Q3 (m/z)	Molecular Weight (Da)
Smpp009887	3-O-p-Coumaroylrotundic acid	Terpenoids	Triterpene	635.39	147.04	634.387
mws1015	Esculin (6,7-Dihydroxycoumarin-6-O-glucoside)	Lignans and Coumarins	Coumarins	339.07	177.02	340.0794
Zken113980	β-D-Fructose 2-phosphate*	Others	Saccharides	259.02	96.97	260.0297
HJN054	Secoisolariciresinol-9'-O-xyloside	Lignans and Coumarins	Lignans	493.21	361.16	494.2152
MWSHC2062	Ferulic β-glucoside	Phenolic acids	Phenolic acids	355.1	193.05	356.1107
Wbwp010459	5-[(2r,3s,4s,5s)-5-(2h-1,3-benzodioxol-5-yl)-3,4-dimethyloxolan-2-yl]-2-methoxyphenol*	Lignans and Coumarins	Lignans	343.15	163.07	342.1467

Metabolite ID	Compound Name	Class I	Class II	Q1 (m/z)	Q3 (m/z)	Molecular Weight (Da)
Zmzp004111	Selagin	Flavonoids	Flavones	317.06	302.04	316.0583
Wbmn010123	Cannabifolin F	Terpenoids	Triterpene	487.34	469.33	488.3502
Wacyp04478	4-(2,6-Dihydroxybenzoyl)-3-Formyl-5-Hydroxybenzoic acid	Phenolic acids	Phenolic acids	303.05	165.02	302.0427
Yacp000453	3-hydroxy-1-methylpyrrolidin-2-one*	Alkaloids	Pyrrole alkaloids	116.07	70.06	115.0633
WaYp005219	1-O-(p-coumaroyl) 3-Hydroxy-3-methylglutaric acid	Phenolic acids	Phenolic acids	309.1	147.04	308.0896
mws2118	Phloretin-2'-O-glucoside (Phlorizin)	Flavonoids	Chalcones	435.13	167.04	436.1369

Metabolite ID	Compound Name	Class I	Class II	Q1 (m/z)	Q3 (m/z)	Molecular Weight (Da)
Wahlp07899	Norephedrin 3-O-(2''-Phenylpropanyl)Glucoside	Alkaloids	Phenolamine	432.24	119.09	431.2308
Walbp08740	3,3'-Oxydipropyl dibenzoate	Phenolic acids	Phenolic acids	343.15	163.07	342.1467
Wahsn02657	1-O-p-Coumaroyl Octose	Phenolic acids	Phenolic acids	385.11	163.04	386.1213
Lmgp000659	2-Phenylacetamide*	Alkaloids	Alkaloids	136.08	91.05	135.0684
Cmsn002383	(7S,8S-Threo-3',4,7,9-tetrahydroxy-3-methoxy-8-O-4'-neolignan-9'-O-rhamnoside	Lignans and Coumarins	Lignans	509.2	179.07	510.2101
MWS2442	D-Fructose 6-Phosphate*	Others	Saccharides	259.02	96.97	260.0297

Metabolite ID	Compound Name	Class I	Class II	Q1 (m/z)	Q3 (m/z)	Molecular Weight (Da)
MWS00330g	1-Amino-1-cyclobutane-carboxylic-acid	Amino acids and derivatives	Amino acids and derivatives	116.07	70.07	115.0633
Wcgn003981	Methyl 3-O-glucosyl-4-hydroxybenzoate	Phenolic acids	Phenolic acids	329.09	167.03	330.0951
mws4516	Pyroglutamic acid	Amino acids and derivatives	Amino acids and derivatives	130.05	84.05	129.0426
Lajp010867	13-Hydroxy oleanolic acid-3-oxo-28-acid	Terpenoids	Triterpene	473.36	427.36	472.3547
mws1080	Galactinol	Others	Saccharides	341.11	179.06	342.1162
Safn000570	9-hydroxy-8-{[(2S,3R,4S,5S,6R)-3,4,5-trihydroxy-6-(hydroxymethyl)oxan-2-yl]ox	Others	Others	377.09	341.11	378.0951

Metabolite ID	Compound Name	Class I	Class II	Q1 (m/z)	Q3 (m/z)	Molecular Weight (Da)
	y}-1H,3H-naphtho[2,3-c]furan-1-one					
pme1474	5'-Deoxy-5'-(methylthio)adenosine	Nucleotides and derivatives	Nucleotides and derivatives	298.1	136.06	297.0896
MWStz530	Quercetin-3-O-glucose-6''-acetate	Flavonoids	Flavonols	505.1	300.03	506.106
Zmyn000453	Isocitric Acid*	Organic acids	Organic acids	191.02	111.01	192.027
Lcgn000234	davidioside C	Phenolic acids	Phenolic acids	339.07	177.02	340.0794
Ymjm000095	13,28-Epoxyolean-11-en-3-one	Terpenoids	Triterpene	439.36	203.18	438.3498
MWSmce025	Fraxetin-8-O-glucoside (Fraxin)	Lignans and Coumarins	Coumarins	369.09	207.03	370.09

Metabolite ID	Compound Name	Class I	Class II	Q1 (m/z)	Q3 (m/z)	Molecular Weight (Da)
Lmtn002796	Aromadendrin-7-O-glucoside	Flavonoids	Flavanonols	449.11	287.06	450.1162
Wasn000528	2-O-α-D-Glucopyranosyl-L-ascorbic acid	Others	Vitamin	337.08	277.06	338.0849
mws1090	D-Glucose-1-phosphate*	Others	Saccharides	259.02	96.97	260.0297
Lmjp002461	Quercetin-3-O-neohesperidoside*	Flavonoids	Flavonols	611.16	303.05	610.1534
Lcfp006038	(1-Methylpyrrolidin-2-yl)aceticacid*	Alkaloids	Pyrrole alkaloids	144.1	84.08	143.0946
Ymjm000113	Kanokoside B	Terpenoids	Terpene	477.2	181.09	478.205
Qatp010618	Lignans Machilin F*	Lignans and Coumarins	Lignans	343.15	105.03	342.1467
pmn001583	Quercetin-3-O-robinobiosi	Flavonoids	Flavonols	609.15	300.03	610.1534

Metabolite ID	Compound Name	Class I	Class II	Q1 (m/z)	Q3 (m/z)	Molecular Weight (Da)
	de					
mws1584	1,3-O-Dicaffeoylquinic Acid (Cynarin)	Phenolic acids	Phenolic acids	515.12	191.03	516.1268
Lmmn008971	3-Hydroxycycloarta-24-ene -26-oic acid (Isomangiferolic acid)*	Terpenoids	Triterpene	455.35	455.35	456.3603
Zmtn001661	Vanilloloside	Others	Alcohol compounds	315.11	153.02	316.1158
Wcdp006912	Lauryldiethanolamine	Others	Others	274.28	88.08	273.2668
Qmfp122207	24-p-Coumaroyloxy-2,3-di hydroxyurs-12-en-28-oic acid (Guavacoumaric acid)*	Terpenoids	Triterpene	635.39	453.34	634.387

Metabolite ID	Compound Name	Class I	Class II	Q1 (m/z)	Q3 (m/z)	Molecular Weight (Da)
MWSHC20176	3-Hydroxylup-20(29)-en-28-oic acid (Betulinic acid)*	Terpenoids	Triterpene	455.35	455.35	456.3603
pmp001287	N-Benzylmethylene isomethylamine	Alkaloids	Alkaloids	120.08	103.05	119.0735
pmp000589	Quercetin-7-O-(6''-malonyl)glucoside	Flavonoids	Flavonols	551.1	303.05	550.0959
Wasyqp1765	Styrylamine	Alkaloids	Alkaloids	120.08	77.04	119.0735
Walbn03092	Neocarthamin	Flavonoids	Flavanones	449.11	287.06	450.1162
Waqkn02083	Epigallocatechin-(4beta->8)-catechin	Tannins	Proanthocyanidins	593.13	407.08	594.1373
MWSmce056	DL-Tryptophan	Amino acids and derivatives	Amino acids and derivatives	205.09	146.06	204.0899

Metabolite ID	Compound Name	Class I	Class II	Q1 (m/z)	Q3 (m/z)	Molecular Weight (Da)
Safn001079	(2S,3R)-2,3-dihydroxy-5-oxohexanedioic acid*	Organic acids	Organic acids	191.02	111.01	192.027
Wacyn03531	2-[4-(3-Hydroxypropyl)-2-methoxyphenoxy]-1,3-propanediol 1-glucoside	Others	Alcohol compounds	477.2	181.09	418.1839
HJN041	Epicatechin 3-glucoside	Flavonoids	Flavanols	451.12	289.07	452.1319
Lmpp003662	Delphinidin-3-O-(6''-O-p-coumaroyl)glucoside	Flavonoids	Anthocyanidins	611.14	303.05	611.1395
mws0866	D-Glucose 6-phosphate*	Others	Saccharides	259.0225	96.9696	260.0297
Zmjp000182	N-Monomethyl-L-arginine*	Amino acids and derivatives	Amino acids and derivatives	189.13	70.07	188.1273
Lcsn002137	Homovanillic acid sulfate*	Phenolic acids	Phenolic acids	261.01	181.05	262.0147

Metabolite ID	Compound Name	Class I	Class II	Q1 (m/z)	Q3 (m/z)	Molecular Weight (Da)
Zbsn012366	Oxyallobetulin*	Terpenoids	Triterpene	455.35	455.35	456.3603
Walp007043	2-Aminohexadecane-1,4-diol	Lipids	Sphingolipids	274.27	88.08	273.2668
Lmbp000123	L-Homomethionine	Amino acids and derivatives	Amino acids and derivatives	164.07	56.05	163.0667
Lmtn000940	1-O-(3,4-Dihydroxy-5-methoxy-benzoyl)-glucoside	Phenolic acids	Phenolic acids	345.08	139.04	346.09
MWSHY0130	Taxifolin-7-O-rhamnoside	Flavonoids	Flavanonols	451.12	305.06	450.1162
Wasyqp3073	p-Coumaroyl Aminogalactitol	Phenolic acids	Phenolic acids	344.13	147.04	343.1267
Zmzp000145	Trimethyllysine	Amino acids and derivatives	Amino acids and derivatives	189.16	84.08	188.1525

Metabolite ID	Compound Name	Class I	Class II	Q1 (m/z)	Q3 (m/z)	Molecular Weight (Da)
Ylgn009115	nahagenin	Terpenoids	Triterpene	471.34	471.32	472.3553
Zkep143812	6-Deoxy-6-sulfoglucono-1, 5-lactone	Others	Lactones	243.02	225.01	242.0096
Zmmp002106	4-methyl-1,5,2,3-dioxadiazinan-2-amine	Nucleotides and derivatives	Nucleotides and derivatives	120.08	77.04	119.0695
Zbkp000480	N2-Acetyl-L-lysine	Amino acids and derivatives	Amino acids and derivatives	189.12	84.08	188.1161
Waptn02725	Feruloyloctose*	Phenolic acids	Phenolic acids	415.12	193.05	416.1319
Zmdp000292	Arginine methyl ester*	Amino acids and derivatives	Amino acids and derivatives	189.14	70.07	188.1273
Zbqn008291	3,13,15-Trihydroxyoleanane-12-one	Terpenoids	Triterpene	473.36	473.36	474.3709

Metabolite ID	Compound Name	Class I	Class II	Q1 (m/z)	Q3 (m/z)	Molecular Weight (Da)
Lmmn001294	Koaburaside	Others	Others	331.1	153.02	332.1107
pme3163	D-Sedoheptuiose 7-phosphate	Others	Saccharides	289.03	96.97	290.0403
MWSHY0066	Taxifolin(Dihydroquerceti n)	Flavonoids	Flavanonols	305.06	231.06	304.0583
Ymjm000138	9-O-Methylvibsanol	Lignans and Coumarins	Lignans	357.13	163.04	356.126
Lmmp003091	Quercetin-3-O-(4''-O-gluco syl)rhamnoside*	Flavonoids	Flavonols	611.16	303.05	610.1534
Wasn004683	Protocatechuic acid 1-O-(Glucosylvanilloyl)	Phenolic acids	Phenolic acids	465.1	303.05	466.1111
Waln011009	2-(2,3-dihydroxypropoxy)-3-(((2-(dimethylamino)etho	Lipids	LPE	578.34	279.23	579.3536

Metabolite ID	Compound Name	Class I	Class II	Q1 (m/z)	Q3 (m/z)	Molecular Weight (Da)
	xy)(hydroxy)phosphoryl)oxy)propyl (11Z,14Z)-octadeca-11,14-dienoate					
Zbsp004301	Quercetin-7-O-rutinoside*	Flavonoids	Flavonols	611.16	303.05	610.1534
WaYn000716	Citric Acid diglucoside	Organic acids	Organic acids	515.12	341.11	516.1326
pmn001667	Procyanidin B4	Tannins	Proanthocyanidins	577.14	407.08	578.1424
Wayn004278	3-O-Methylellagic acid 4'-rhamnoside	Tannins	Tannin	461.07	315.02	462.0798
Safn001820	Tichocarpol A*	Phenolic acids	Phenolic acids	261.01	181.05	262.0147
MWSHY0067	Quercetin-3-O-rutinoside (Rutin)*	Flavonoids	Flavonols	611.16	303.05	610.1534

Metabolite ID	Compound Name	Class I	Class II	Q1 (m/z)	Q3 (m/z)	Molecular Weight (Da)
pmb3081	Glucaric acid-1-Phosphate	Others	Saccharides	289	96.97	290.0039
Wasyqp2943	Caffeoylmethylgluconamide*	Alkaloids	Alkaloids	372.13	163.04	371.1211
Waln010743	1-(2,3-dihydroxypropoxy)-3-(((2-(dimethylamino)ethoxy)(hydroxy)phosphoryl)oxy)propan-2-yl (11Z,14Z)-octadeca-11,14-dienoate	Lipids	LPE	578.35	279.23	579.3536
Lmmn003663	5-Glucosyloxy-2-Hydroxybenzoic acid methyl ester	Phenolic acids	Phenolic acids	329.09	167.04	330.0951
Wbtn004512	1,8-dihydroxy-4,5-dimethoxy-3-{[(2s,3r,4s,5s,6r)-3,4,5-t	Flavonoids	Other Flavonoids	465.1	303.05	466.1111

Metabolite ID	Compound Name	Class I	Class II	Q1 (m/z)	Q3 (m/z)	Molecular Weight (Da)
	rihydroxy-6-(hydroxymethyl)oxan-2-yl]oxy}xanthen-9-one					
Lacp001431	N-(3-hydroxy-4-methoxyphenethyl)-4-hydroxybutanamide	Alkaloids	Alkaloids	268.15	136.06	267.1471
MWSHY0098	Epigallocatechin	Flavonoids	Flavanols	307.08	139.04	306.074
pmb1754	O-Phosphocholine	Alkaloids	Alkaloids	184.07	125	184.0733
Smnp010489	3-O-p-Coumaroyltormentic acid	Terpenoids	Triterpene	635.39	453.33	634.387
Lmzp009620	2-Hydroxypyracrenic acid*	Terpenoids	Triterpene	635.39	453.33	634.387
Ymjm000099	3-Oxooleana-11,13(18)-dien	Terpenoids	Triterpene	453.34	201.17	452.329

Metabolite ID	Compound Name	Class I	Class II	Q1 (m/z)	Q3 (m/z)	Molecular Weight (Da)
	-28-oic acid					
Lmcp003788	Isotamarixin*	Flavonoids	Flavonols	479.1184	317.0617	478.1111
Jmwn002117	2,6-dimethoxybenzene-1,4-diol 1-O-β-D-glucopyranoside	Phenolic acids	Phenolic acids	331.1	153.02	332.1107
Lssp210323	-Pierisformoside H	Terpenoids	Ditepenoids	499.29	283.21	498.2829
Lmzp002365	Hesperetin-7-O-glucoside*	Flavonoids	Flavanones	465.14	303.09	464.1319
Wbtp005759	1,2,5,7,8-pentahydroxy-3-methylanthracene-9,10-dione	Quinones	Quinones	303.05	303.05	302.0427
Lssp210350	Pierisfomoside C	Others	Others	479.26	317.21	478.2567
Wafn002319	4-O-(3'-O-alpha-D-Glucop	Phenolic acids	Phenolic acids	515.14	323.08	516.1479

Metabolite ID	Compound Name	Class I	Class II	Q1 (m/z)	Q3 (m/z)	Molecular Weight (Da)
	yranosyl)caffeoylquinic acid					
Zmdn002353	Epicatechin-6-C-β-D-glucopyranoside*	Flavonoids	Flavanols	451.13	289.07	452.1319
Wbtp005613	robustaquinone F	Flavonoids	Other Flavonoids	303.05	303.05	302.0427
Lamp000484	4-Methylazetidine-2-Carboxylic acid*	Alkaloids	Alkaloids	116.07	70.06	115.0633
Lmzn006582	2-Formyl-3-hydroxy-A(1)-norlup-20(29)-en-28-oic acid (Colubrinic acid)	Terpenoids	Triterpene	469.34	469.34	470.3396
Wacyp11527	Tributyl acetylcitrate	Others	Others	403.23	129.02	402.2254
MWS2044	Isophorone	Others	Ketone compounds	139.11	69.03	138.1045

Metabolite ID	Compound Name	Class I	Class II	Q1 (m/z)	Q3 (m/z)	Molecular Weight (Da)
Zmsp001773	9-Arabinosyladenine*	Nucleotides and derivatives	Nucleotides and derivatives	268.11	136.07	267.0968
Zajn002491	Catechin 4'-O-beta-D-glucopyranoside*	Flavonoids	Flavanols	451.12	289.07	452.1319
Zbpn005555	Phloretin-4'-O-glucoside (Trilobatin)*	Flavonoids	Chalcones	435.13	273.08	436.1369
mws1337	D-Pantothenic Acid	Others	Vitamin	220.12	90.05	219.1107
HJN055	Dihydrocharcone-4'-O-glucoside*	Flavonoids	Chalcones	435.13	273.08	436.1369
Lmyn012274	3,25-Epoxy-3-hydroxyolean-18-en-28-oic acid (Semimoronic acid)	Terpenoids	Triterpene	469.33	469.33	470.3396

Metabolite ID	Compound Name	Class I	Class II	Q1 (m/z)	Q3 (m/z)	Molecular Weight (Da)
Lcfp036464	Indoline	Alkaloids	Plumerane	120.08	103.05	119.0735
mws0183	3,4-Dihydroxybenzoic acid (Protocatechuic acid)*	Phenolic acids	Phenolic acids	153.02	109.03	154.0266
Lmcp003402	Tamarixetin-3-O-glucoside (Tamarixin)	Flavonoids	Flavonols	479.12	317.06	478.1111
mws1138	Betanin (Betanidin-5-O-glucoside)	Alkaloids	Plumerane	551.15	303.04	550.1435
MWSmce548	Betaine	Alkaloids	Alkaloids	118.09	58.07	117.079
Zkep161098	2-Amino-4-methylvaleric acid	Others	Others	132.1	86.1	131.0946
Lhyp111013	2(4H)-benzofuranone	Others	Others	135.04	89.04	134.0368
Lmyn003835	4-(3,4,5-Trihydroxybenzox	Phenolic acids	Phenolic acids	289.04	137.02	290.0427

Metabolite ID	Compound Name	Class I	Class II	Q1 (m/z)	Q3 (m/z)	Molecular Weight (Da)
	y)benzoic acid					
pme3207	Muconic acid	Organic acids	Organic acids	141.02	59.01	142.0266
pmb0891	Cis-Zeatin-7-N-glucoside*	Alkaloids	Alkaloids	382.1721	220.1193	381.1648
Wasyqp5995	2-Aminooctadecane-1,5,17-triol	Lipids	Sphingolipids	318.3	256.26	317.293
Sayp004048	caesalpiniaphenol A	Flavonoids	Flavones	317.1	302.04	316.0947
Zmdn002400	Epicatechin-3'-O-β-D-glucopyranoside*	Flavonoids	Flavanols	451.13	289.07	452.1319
Layp000971	Hydroxymenisdaurin D	Alkaloids	Alkaloids	332.13	152.07	331.1267
Waln010192	2-(2,3-dihydroxypropoxy)-3-(((2-(dimethylamino)ethoxy)(hydroxy)phosphoryl)o	Lipids	LPE	576.33	277.22	577.338

Metabolite ID	Compound Name	Class I	Class II	Q1 (m/z)	Q3 (m/z)	Molecular Weight (Da)
	xy)propyl (8E,11Z,14Z)-octadeca-8,11,14-trienoate					
Wcjp001256	9-Alpha-Ribofuranosyladenine*	Nucleotides and derivatives	Nucleotides and derivatives	268.1	136.06	267.0968
pmn001553	Cimidahurinine	Others	Alcohol compounds	315.11	153.06	316.1158
pme0230	Adenosine*	Nucleotides and derivatives	Nucleotides and derivatives	268.1	136.06	267.0968
Lmqn006260	2',3',4',5,7-pentahydroxyflavone	Flavonoids	Flavones	301.04	151	302.0427
MWS1777	3-Chloroaniline	Alkaloids	Alkaloids	128.03	93.06	127.0189
Wbtn004921	9-hydroxy-2-(3-hydroxy-4-	Others	Ketone compounds	451.1	341.07	452.1107

Metabolite ID	Compound Name	Class I	Class II	Q1 (m/z)	Q3 (m/z)	Molecular Weight (Da)
	methoxyphenyl)-3-(hydroxymethyl)-12-methoxy-2,3-dihydro-1,4,5-trioxatetraphen-10-one					
Waln009920	1-(2,3-dihydroxypropoxy)-3-(((2-(dimethylamino)ethoxy)(hydroxy)phosphoryl)oxy)propan-2-yl (8E,11Z,14Z)-octadeca-8,11,14-trienoate	Lipids	LPE	576.33	277.22	577.338
MWStz488	4-O-p-Coumaroylquinic acid	Phenolic acids	Phenolic acids	337.09	173.05	338.1002
Wasn006585	Geranyl	Terpenoids	Monoterpenoids	447.22	315.18	448.2308

Metabolite ID	Compound Name	Class I	Class II	Q1 (m/z)	Q3 (m/z)	Molecular Weight (Da)
	3-O-xylopyranosyl-glucopyranoside					
Ymjm000135	8R-Dihydrodehydrodiconferyl alcohol 4-O-β-D-glucopyranoside	Lignans and Coumarins	Lignans	507.18	161.02	508.1945
Lmsp004166	Quercetin-3-O-glucoside-7-O-rhamnoside*	Flavonoids	Flavonols	611.1607	303.0519	610.1534
Hmzp003994	Pedaliin*	Flavonoids	Flavones	479.12	317.08	478.1111
Zbsp002256	Pelargonidin-3-O-rutinoside	Flavonoids	Anthocyanidins	579.17	271.06	579.1708
Zmjp000966	Vidarabine	Nucleotides and derivatives	Nucleotides and derivatives	268.11	136.06	267.0968

Metabolite ID	Compound Name	Class I	Class II	Q1 (m/z)	Q3 (m/z)	Molecular Weight (Da)
pme2828	4-Nitrophenol	Others	Others	140.03	98.96	139.0269
Zmhp005139	Tamarixetin-3-O-(6''-malonyl)glucoside*	Flavonoids	Flavonols	565.12	317.07	564.1115
Zmdn002049	Epicatechin-4'-O-β-D-glucopyranoside*	Flavonoids	Flavanols	451.13	289.07	452.1319
MWSslk148	Rutin Trihydrate	Flavonoids	Flavonols	611.16	303.06	664.1851
Waptn03150	Benzyl alcohol xylopyranosyl-(1-6)-glucopyranoside*	Others	Alcohol compounds	461.16	269.1	402.1526
Lmbn000198	3-Dehydro-L-Threonic Acid	Others	Saccharides	133.01	71.01	134.0215
Wmhp000055	2,5-Dimethoxybenzoquino	Quinones	Quinones	169.05	65.04	168.0423

Metabolite ID	Compound Name	Class I	Class II	Q1 (m/z)	Q3 (m/z)	Molecular Weight (Da)
	ne*					
Zbsp004450	3'-O-Methyltricetin-7-O-glucoside*	Flavonoids	Flavones	479.12	317.07	478.1111
Lmjp003231	Patuletin-3-O-glucoside*	Flavonoids	Flavonols	495.11	333.07	494.106
Wacyp06135	3,7-Dimethyl-2,6-Octadienyl 6-O-(beta-D-Xylopyranosyl)-beta-D-glucopyranoside	Terpenoids	Monoterpenoids	466.26	81.07	448.2308
Ydhn004305	rhodomollein XIII*	Terpenoids	Ditepenoids	411.24	369.23	412.2461
Ydhn004272	grayanotoxin I*	Terpenoids	Ditepenoids	411.24	369.23	412.2461
Zmmp001941	Hydroxythreonine xyloside	Amino acids and derivatives	Amino acids and derivatives	268.1038	136.0622	267.0954

Metabolite ID	Compound Name	Class I	Class II	Q1 (m/z)	Q3 (m/z)	Molecular Weight (Da)
Xmmn006031	Borneol 7-O-[β-D-apiofuranosyl-(1 →6)]-β-D-glucopyranoside	Terpenoids	Monoterpenoids	447.22	315.18	448.2308
Zbqp000579	3-(Pyrazol-1-yl)-L-alanine	Amino acids and derivatives	Amino acids and derivatives	156.08	110.07	155.0695
Waqkn04503	Isorhamnetin 3-(2''-acetylglucoside)	Flavonoids	Flavonols	519.11	315.05	520.1217
Zbqn008164	Rubiarbonone E	Terpenoids	Triterpene	469.33	469.33	470.3396
Wbkp008219	(3S,3'S)-N,N'-((1R,2R)-5-((3,4,5-trihydroxy-6-methyltetrahydro-2H-pyran-2-yl)oxy)cyclohex-3-ene-1,2-diyl)bis(2-methyl-2,3,4,9-tetrahy	Alkaloids	Alkaloids	699.36	699.36	698.3428

Metabolite ID	Compound Name	Class I	Class II	Q1 (m/z)	Q3 (m/z)	Molecular Weight (Da)
	dro-1H-pyrido[3,4-b]indole-3-carboxamide)					
pmb2871	1-O-Gentisoyl-β-D-glucoside*	Phenolic acids	Phenolic acids	315.07	153.02	316.0794
Zbpp002027	Isorhamnetin-3-O-(6''-malonyl)glucoside*	Flavonoids	Flavonols	565.12	317.07	564.1115
Zbkp003100	Robinetinidol-(4alpha→8)-catechin-(6→4alpha)-robinetinidol	Flavonoids	Flavanols	867.21	577.13	866.2058
pmf0470	cis-4-Hydroxy-D-proline*	Amino acids and derivatives	Amino acids and derivatives	132.0655	86.0601	131.0582
Zbsp004894	3'-O-Methyltricetin-7-O-(6''-malonyl)glucoside*	Flavonoids	Flavones	565.1188	317.0656	564.1115

Metabolite ID	Compound Name	Class I	Class II	Q1 (m/z)	Q3 (m/z)	Molecular Weight (Da)
Walbn07904	Benzhydryl methyl ether*	Others	Others	197.1	182.07	198.1045
Lssp210384	2α,3α-Epoxy-5,7,3',4'-tetrahydroxyflavan-(4β→8)-epicatechin	Tannins	Proanthocyanidins	577.13	287.06	576.1268
pmb3142	Salicylic acid-2-O-glucoside	Phenolic acids	Phenolic acids	299.08	137.02	300.0845
Wbmp010544	10-Hydroxymethyllycaconitine	Alkaloids	Alkaloids	699.35	699.35	698.3415
MWS1926	DL-Leucine*	Amino acids and derivatives	Amino acids and derivatives	132.1	86.1	131.0946
mws2608	N-Acetyl-D-galactosamine	Others	Saccharides	222.1	84.05	221.0899
Lmsn009900	1β-Hydroxy-2-oxopomolic	Terpenoids	Triterpene	501.32	453.3	502.3294

Metabolite ID	Compound Name	Class I	Class II	Q1 (m/z)	Q3 (m/z)	Molecular Weight (Da)
	acid					
Ysjp000315	Pipecolic acid	Alkaloids	Piperidine alkaloids	130.1	56.05	129.079
mws0216	Trans-4-Hydroxy-L-proline*	Amino acids and derivatives	Amino acids and derivatives	132.0655	86.0601	131.0582
Zmsp001834	2,6-Dimethoxy-1,4-benzoquinone*	Quinones	Quinones	169.05	65	168.0423
Lmfn001893	Leucocyanidin; 3,4,5,7,3',4'-Hexahydroxyflavan	Flavonoids	Flavanols	305.07	125.02	306.074
Zbzp000519	Retronecine	Alkaloids	Pyrrole alkaloids	156.1	110.07	155.0946
MWSHC2022	Glucosyringic acid	Phenolic acids	Phenolic acids	359.1	197.05	360.1056
Zkep00434	Turanose	Others	Saccharides	365.11	203.05	342.1162

Metabolite ID	Compound Name	Class I	Class II	Q1 (m/z)	Q3 (m/z)	Molecular Weight (Da)
Wacyp09192	2,5-O-Di-CinnamylSorbitol	Phenolic acids	Phenolic acids	415.21	119.09	414.2042
Lmhp008589	LysoPE 18:3(2n isomer)	Lipids	LPE	476.28	335.26	475.2699
WaYn003347	Glochiflavanoside A	Flavonoids	Flavanols	479.15	317.1	480.1632
pme2827	Palmitaldehyde	Lipids	Free fatty acids	239.24	223.24	240.2453
Lmhp008801	LysoPE 18:3	Lipids	LPE	476.28	335.26	475.2699
Zbsp003857	3'-O-Methyltricetin-5-O-glucoside*	Flavonoids	Flavones	479.12	317.06	478.1111
Cmsp004086	Dihydrokaempferide	Flavonoids	Flavanonols	303.09	229.08	302.079
Wayp003781	N-Hydroxynonanoyl nornicotine glucoside	Alkaloids	Pyridine alkaloids	467.2756	305.2227	466.2674
Wbtn006715	1,2,4,5,8-pentahydroxy-6-methylanthracene-9,10-dio	Flavonoids	Other Flavonoids	301.04	151	302.0427

Metabolite ID	Compound Name	Class I	Class II	Q1 (m/z)	Q3 (m/z)	Molecular Weight (Da)
	ne					
MWS1709	D-Malic acid*	Organic acids	Organic acids	133.01	71.01	134.0215
Walbp07454	3-Hydrocinnamyl-6-CinnamylGlucose	Others	Alcohol compounds	415.21	119.09	414.2042
MWStz394	Homoeriodictyol 7-O-glucoside*	Flavonoids	Flavanones	465.1387	303.0885	464.1319
Ybnn006457	Artocarpanone	Flavonoids	Flavanones	301.07	151	302.079
mws1077	Scopoletin-7-O-glucoside (Scopolin)	Lignans and Coumarins	Coumarins	355.1024	193.0495	354.0951
pmb0855	LysoPC 16:0	Lipids	LPC	496.34	184.07	495.3325
Lmmp004257	6-C-Methylquercetin-3-O-rhamnoside	Flavonoids	Flavonols	463.12	317.06	462.1162

Metabolite ID	Compound Name	Class I	Class II	Q1 (m/z)	Q3 (m/z)	Molecular Weight (Da)
pme0274	6-Aminocaproic acid	Organic acids	Organic acids	132.1	69.07	131.0946
mws0018	Spermine	Alkaloids	Alkaloids	203.22	84.08	202.2157
Safp005121	Carbendazim	Alkaloids	Alkaloids	192.08	160.05	191.0695
pmd0132	LysoPC 16:0(2n isomer)	Lipids	LPC	496.34	184.07	495.3325
Safn011471	2-hydroxy-3-{[3,4,5-trihydroxy-6-(hydroxymethyl)oxan-2-yl]oxy}propyl octadeca-9,12,15-trienoate	Lipids	Free fatty acids	573.33	277.22	514.3142
Hmcp002316	Isorhamnetin-3-O-arabinoside	Flavonoids	Flavonols	449.11	317.06	448.1006
Cmhp005227	1,4,8-Trihydroxynaphthalene-1-O-glucoside	Quinones	Quinones	339.11	177.05	338.1002

Metabolite ID	Compound Name	Class I	Class II	Q1 (m/z)	Q3 (m/z)	Molecular Weight (Da)
Wahln09353	4-Methoxydiphenylmethane*	Others	Others	197.1	182.07	198.1045
Wbtp004961	1,2,4,5-tetrahydroxy-7-(hydroxymethyl)anthracene-9,10-dione*	Flavonoids	Other Flavonoids	303.05	229.05	302.0427
MWS04555g	D-Allo-Isoleucine*	Amino acids and derivatives	Amino acids and derivatives	132.1	86.1	131.0946
Zmyp004317	(3,4,5-trihydroxytetrahydro-2H-pyran-2-yl)methyl (E)-3-(4-hydroxy-3-methoxyphenyl)acrylate	Phenolic acids	Phenolic acids	339.1	177.05	338.1002
Hmcp002539	Isorhamnetin-3-O-rhamnoside	Flavonoids	Flavonols	463.12	317.06	462.1162

Metabolite ID	Compound Name	Class I	Class II	Q1 (m/z)	Q3 (m/z)	Molecular Weight (Da)
Hmmn002544	Ferulic acid-4-O-glucoside	Phenolic acids	Phenolic acids	355.1	193.05	356.1107
mws0256	L-Valine	Amino acids and derivatives	Amino acids and derivatives	118.09	72.08	117.079
Wbtp005060	2,3,5-trimethoxy-1-{[(2s,3r,4s,5s,6r)-3,4,5-trihydroxy-6-(hydroxymethyl)oxan-2-yl]oxy}xanthen-9-one*	Flavonoids	Other Flavonoids	465.13	303.05	464.1319
MWStz070	N-(2-Hydroxy-4-methoxyphenyl)acetamide	Alkaloids	Alkaloids	182.08	91.05	181.0739
Lmqn003292	4,5,8-Trihydroxy-α-tetralone-5-O-glucoside	Quinones	Naphthol	355.1	193.05	356.1107
mws0227	L-Leucine*	Amino acids and derivatives	Amino acids and derivatives	132.1019	86.0964	131.0946

Metabolite ID	Compound Name	Class I	Class II	Q1 (m/z)	Q3 (m/z)	Molecular Weight (Da)
MWSprf134	Plantagoside	Flavonoids	Flavanones	467.12	305.07	466.1111
mws1361	Taxifolin-3-O-rhamnoside (Astilbin)	Flavonoids	Flavanonols	449.11	303.08	450.1162
Hmyp002315	3,5,7,4'-Tetrahydroxy-Coumaronochromone	Others	Chromone	303.04	257.04	302.0427
MWS20147	Luteolin-3'-O-glucoside	Flavonoids	Flavones	449.11	287.06	448.1006
MWSHC20163	Cetostearic acid	Lipids	Free fatty acids	255.24	255.24	256.2402
Lmcp000282	L-Carnitine	Alkaloids	Alkaloids	162.11	85.03	161.1052
pma3649	5-Aminolevulinic Acid	Alkaloids	Alkaloids	132.07	86.06	131.0582
MWSslk090	Monotropein	Terpenoids	Monoterpenoids	389.11	147.04	390.1162
mws0748	1-Caffeoylquinic acid	Phenolic acids	Phenolic acids	353.09	191.06	354.0951

Metabolite ID	Compound Name	Class I	Class II	Q1 (m/z)	Q3 (m/z)	Molecular Weight (Da)
mws0470	Methylmalonic acid*	Organic acids	Organic acids	117.02	73.03	118.0266
Wacyp04721	Rhodioloside F	Terpenoids	Monoterpenoids	482.26	153.13	464.2258
Waptp02966	Procyanidin B6*	Tannins	Proanthocyanidins	579.15	127.04	578.1424
HJN043	Cinnamtannin B1	Tannins	Proanthocyanidins	863.19	711.14	864.1902
mws2108	Cryptochlorogenic acid (4-O-Caffeoylquinic acid)*	Phenolic acids	Phenolic acids	353.09	191.06	354.0951
mws0049	Gallocatechin	Flavonoids	Flavanols	307.08	139.04	306.074
Hmpp003270	Luteolin-4'-O-glucoside*	Flavonoids	Flavones	449.11	287.05	448.1006
pme1816	Neochlorogenic acid (5-O-Caffeoylquinic acid)*	Phenolic acids	Phenolic acids	353.09	191.06	354.0951
Zbkp000561	L-4-Hydroxyglutamate semialdehyde	Alkaloids	Alkaloids	148.06	84.05	147.0532

Metabolite ID	Compound Name	Class I	Class II	Q1 (m/z)	Q3 (m/z)	Molecular Weight (Da)
Ylyn009578	wilforic acid E	Terpenoids	Triterpene	485.32	485.29	486.3345
mws0367	α-Linolenic Acid*	Lipids	Free fatty acids	277.22	277.22	278.2246
Waptp03252	Procyanidin B7	Tannins	Proanthocyanidins	579.15	127.04	578.1424
Lajp003377	Fisetinidol-(4α,6)-gallocate chin	Flavonoids	Flavanols	579.15	127.04	578.1424
mws0001	L-Asparagine	Amino acids and derivatives	Amino acids and derivatives	133.06	74.02	132.0535
Safp021800	LPC(18:1/0:0)	Lipids	LPC	522.35	184.08	522.356
HX1367	coumarin	Lignans and Coumarins	Coumarins	147.04	91.06	146.0368
mws0178	Chlorogenic acid (3-O-Caffeoylquinic acid)*	Phenolic acids	Phenolic acids	353.09	191.06	354.0951
pmp001281	LysoPC 18:1	Lipids	LPC	522.36	184.07	521.3481

Metabolite ID	Compound Name	Class I	Class II	Q1 (m/z)	Q3 (m/z)	Molecular Weight (Da)
Wbtn006053	9-hydroxy-2-(2-hydroxy-3-methoxyphenyl)-3-(hydroxymethyl)-12-methoxy-2,3-dihydro-1,4,5-trioxatetraphen-10-one	Others	Ketone compounds	451.1	341.07	452.1107
Lmsn011830	2,19-Dihydroxy-3-oxours-12-en-28-oic acid	Terpenoids	Triterpene	485.33	485.33	486.3345
Lmhp010190	LysoPC 18:1(2n isomer)	Lipids	LPC	522.36	184.07	521.3481
Lmtp003948	3',5',5,7-Tetrahydroxy-4'-methoxyflavanone-3'-O-glucoside	Flavonoids	Flavanones	481.13	319.05	480.1268
Hmjn008136	2,3-Dihydroxyoleana-11,13(18)-dien-28-oic acid	Terpenoids	Triterpene	469.33	469.33	470.3396

Metabolite ID	Compound Name	Class I	Class II	Q1 (m/z)	Q3 (m/z)	Molecular Weight (Da)
	(Camaldulenic acid)					
Yhjp010205	Virgatic acid	Terpenoids	Triterpene	471.35	471.35	470.3396
pmn001648	Cinnamtannin D1	Tannins	Tannin	863.18	411.07	864.1902
pmn001417	Cinchonain Ic	Flavonoids	Flavanols	451.1	341.1	452.1107
Wcdp003593	2-(3,4-dihydroxyphenyl)-4-[2-(3,4-dihydroxyphenyl)-3,5,7-trihydroxy-3,4-dihydro-2h-1-benzopyran-8-yl]-3,4-dihydro-2h-1-benzopyran-3,5,7-triol	Others	Others	579.15	409.09	578.1424
MWSHY0104	Luteolin-7-O-glucoside (Cynaroside)	Flavonoids	Flavones	449.11	287.06	448.1006

Metabolite ID	Compound Name	Class I	Class II	Q1 (m/z)	Q3 (m/z)	Molecular Weight (Da)
Waptp02666	Procyanidin B5*	Tannins	Proanthocyanidins	579.15	127.04	578.1424
mws0192	Succinic acid*	Organic acids	Organic acids	117.02	73.03	118.0266
pmb0881	LysoPE 18:2	Lipids	LPE	478.29	337.27	477.2855
Safn000385	D-α-D-Hep-(1→6)-β-D-Glc	Others	Saccharides	371.12	191.06	372.1268
MWS201444	Gln-Gly	Amino acids and derivatives	Amino acids and derivatives	204.1	84.04	203.0906
pmb0874	LysoPE 18:2(2n isomer)	Lipids	LPE	478.29	337.27	477.2855
Wasyqn2135	Gallic Acid 4-O-Glucoside	Phenolic acids	Phenolic acids	331.07	168.01	332.0743
MWS201419	Lys-Gly	Amino acids and derivatives	Amino acids and derivatives	204.13	84.08	203.127
Zbsp003145	Leucopelargonidin; 3,4,5,7,4'-Pentahydroxyflav	Others	Others	291.09	123.04	290.079

Metabolite ID	Compound Name	Class I	Class II	Q1 (m/z)	Q3 (m/z)	Molecular Weight (Da)
	an					
Wasyqn2170	Vanillic acid glucoside	Phenolic acids	Phenolic acids	329.09	167.03	330.0951
mws0254	L-Histidine	Amino acids and derivatives	Amino acids and derivatives	156.08	110.07	155.0695
mws0282	L-Tryptophan	Amino acids and derivatives	Amino acids and derivatives	203.08	116.05	204.0899
Zmwp002963	Aesculitannin B	Tannins	Tannin	865.19	713.15	864.1902
pmn001416	Catechin-(7,8-bc)-4α-(3,4-di hydroxyphenyl)-dihydro-2 -(3H)-one*	Flavonoids	Flavanols	451.1	341.06	452.1107
pma2987	Histidinol	Alkaloids	Alkaloids	142.1	124.09	141.0902
Lhhp120812	Methylillicinone F*	Others	Others	279.1582	149.0231	278.1518

Metabolite ID	Compound Name	Class I	Class II	Q1 (m/z)	Q3 (m/z)	Molecular Weight (Da)
pmn001418	Cinchonain Id*	Flavonoids	Flavanols	451.1	341.1	452.1107
mws0836	Procyanidin B1	Tannins	Proanthocyanidins	577.14	425.09	578.1424
pmn001415	Catechin-(7,8-bc)-4β-(3,4-dihydroxyphenyl)-dihydro-2-(3H)-one	Flavonoids	Flavanols	451.1	341.06	452.1107
Zmmp000635	4,5-Dehydro-L-leucine*	Amino acids and derivatives	Amino acids and derivatives	130.091	84.0802	129.079
Laxp005049	Bracteatin*	Flavonoids	Aurones	303.05	229.05	302.0427
Ljmp121501	Mansonone N	Terpenoids	Sesquiterpenoids	279.16	149.02	278.1518
Lmsp004915	Cernuoside	Flavonoids	Aurones	449.11	287.06	448.1006
Zahp011689	2,2-dimethylchromene-6-carboxylic acid*	Others	Others	205.09	149.02	204.0786

Metabolite ID	Compound Name	Class I	Class II	Q1 (m/z)	Q3 (m/z)	Molecular Weight (Da)
Lmcn003163	Epitheaflavic acid-3'-Gallate	Flavonoids	Flavanols	579.08	289.07	580.0853
MWSHY0136	Kaempferol-3-O-glucoside (Astragalin)*	Flavonoids	Flavonols	449.1078	287.0553	448.1006
Zbkp003359	Fisetinidol-4β-ol	Flavonoids	Flavonols	291.09	123.04	290.079
Yalp000545	N-Methyl-L-proline*	Amino acids and derivatives	Amino acids and derivatives	130.09	84.08	129.079
Zmcp102202	Senkyunolide C*	Others	Lactones	205.09	149.02	204.0786
Latp011219	3,5-Dimethyl-2,3-dihydrobenzofuran	Others	Others	149.1	121.06	148.0883
Lmmp001947	Gossypetin-3-O-glucoside*	Flavonoids	Flavonols	481.1	319.05	480.0904
Lmbp003668	Kaempferol-3-O-arabinosi	Flavonoids	Flavonols	419.1	287.05	418.09

Metabolite ID	Compound Name	Class I	Class II	Q1 (m/z)	Q3 (m/z)	Molecular Weight (Da)
	de					
Lmpp003465	Myricetin-3-O-β-D-glucoside*	Flavonoids	Flavonols	481.1	319.04	480.0904
MWS0811	L-Pipecolic Acid	Alkaloids	Piperidine alkaloids	130.09	84.08	129.079
mws0366	γ-Linolenic Acid*	Lipids	Free fatty acids	277.22	277.22	278.2246
pmp001230	Senkyunolide M	Others	Lactones	279.1591	121.0284	278.1518
MWS04559g	3-Hydroxy-L-phenylalanine*	Amino acids and derivatives	Amino acids and derivatives	182.08	136.08	181.0739
ML10181668	Cycloleucine	Amino acids and derivatives	Amino acids and derivatives	130.09	84.08	129.079
mws0275	L-Malic acid*	Organic acids	Organic acids	133.01	71.01	134.0215
mws0250	L-Tyrosine*	Amino acids and	Amino acids and	182.08	136.08	181.0739

Metabolite ID	Compound Name	Class I	Class II	Q1 (m/z)	Q3 (m/z)	Molecular Weight (Da)
		derivatives	derivatives			
Zmtp102903	Kaempferol-3-O-arabinoside (Juglanin)	Flavonoids	Flavonols	419.1	287.06	418.09
Wbtp003341	(3,5-dihydroxyphenyl)-(4-hydroxy-2,6-dimethoxyphenyl)methanone	Others	Ketone compounds	291.0867	139.038	290.079
pmb0484	Choline	Alkaloids	Alkaloids	104.11	60.08	104.107
Wayn005907	Quercetin 3-(2''-acetylrhamnoside)	Flavonoids	Flavonols	489.1	300.03	490.1111
pmp000607	1-desmethylaurantio-obtusin-2-O-β-D-glucoside	Quinones	Anthraquinone	479.12	317.06	478.1111
pme0436	Procyanidin B3	Tannins	Proanthocyanidins	577.14	407.08	578.1424

Metabolite ID	Compound Name	Class I	Class II	Q1 (m/z)	Q3 (m/z)	Molecular Weight (Da)
Ladp004020	tenuiflorin C glucoside	Others	Chromone	479.122	317.062	478.1111
Wcdn001824	2,5-Dihydroxybenzoic acid-5-O-glucoside	Phenolic acids	Phenolic acids	315.07	153.02	316.0794
Lmdp002969	Myricetin-3-O-galactoside*	Flavonoids	Flavonols	481.1	319.06	480.0904
Lmln001856	5,7-Dihydroxy-4-oxo-2-(3,4,5-trihydroxyphenyl)-4H-chromen-3-yl-alloside	Others	Others	479.09	316.02	480.0904
Lcyp000676	Isoscopolin	Lignans and Coumarins	Coumarins	355.1	163.04	354.0951
Hmgp002148	Nepetin-7-O-alloside*	Flavonoids	Flavones	479.12	317.06	478.1111
Lmjp002906	Rhamnetin-3-O-Glucoside*	Flavonoids	Flavonols	479.1184	317.0651	478.1111
pme0014	L-Glutamic acid	Amino acids and derivatives	Amino acids and derivatives	148.06	84.04	147.0532

Metabolite ID	Compound Name	Class I	Class II	Q1 (m/z)	Q3 (m/z)	Molecular Weight (Da)
MWStz446	Isorhamnetin 7-O-glucoside	Flavonoids	Flavonols	479.12	317.07	478.1111
Wbmn010483	Cannabifolin D	Terpenoids	Triterpene	485.33	485.33	486.3345
Hmgp002036	Nepetin-7-O-glucoside(Nepitrin)*	Flavonoids	Flavones	479.12	317.06	478.1111
pmn001367	Protocatechuic acid-4-O-glucoside*	Phenolic acids	Phenolic acids	315.07	153.02	316.0794
Hmcp003852	Elemol	Terpenoids	Sesquiterpenoids	223.21	207.03	222.1984
mws0281	Citric Acid	Organic acids	Organic acids	191.02	111.01	192.027
Lmdp003171	Azalein (Azaleatin-3-O-rhamnoside)	Flavonoids	Flavonols	463.12	317.07	462.1162

Metabolite ID	Compound Name	Class I	Class II	Q1 (m/z)	Q3 (m/z)	Molecular Weight (Da)
MWStz602	Quercetagetin-7-O-glucoside*	Flavonoids	Flavonols	481.1	319.05	480.0904
Wbmn00859	vitelignin A	Lignans and Coumarins	Lignans	383.08	191.06	384.0845
Wbmn009702	2,3,23-Trihydroxyurs-12-en-28-oic acid (Asiatic acid)*	Terpenoids	Triterpene	487.34	487.34	488.3502
Lmjp003295	6-Methoxykaempferol-3-O-glucoside*	Flavonoids	Flavonols	479.12	317.07	478.1111
Wbsp103327	(8R)-9-hydroxy-8-(hydroxymethyl)-6-methoxy-8-methylpyrano[2,3-f]chromen-2-one	Terpenoids	Terpene	291.09	139.04	290.079
Hmjn003948	2,3,6-Trihydroxyurs-12-en-28-oic acid (Madasiatic	Terpenoids	Triterpene	487.35	487.35	488.3502

Metabolite ID	Compound Name	Class I	Class II	Q1 (m/z)	Q3 (m/z)	Molecular Weight (Da)
	acid)*					
MWSHY0065	Catechin*	Flavonoids	Flavanols	291.09	139.04	290.079
pme0434	Procyanidin B2	Tannins	Proanthocyanidins	577.14	407.08	578.1424
Zbpp001992	Isorhamnetin-3-O-Glucoside*	Flavonoids	Flavonols	479.12	317.07	478.1111
Zbzn008660	Cordianol B*	Terpenoids	Triterpene	487.34	487.34	488.3502
Wbmn009160	Cannabifolin C*	Terpenoids	Triterpene	487.34	487.34	488.3502
Hajp003819	rhusopolyphenol F	Phenolic acids	Phenolic acids	291.09	139.04	290.079
Zmjp002244	5,7,3',4',5'-Pentahydroxyflavan (Tricetiflavan)*	Flavonoids	Flavanols	291.09	139.04	290.079
mws0260	L-Arginine	Amino acids and derivatives	Amino acids and derivatives	175.12	116.07	174.1117

Metabolite ID	Compound Name	Class I	Class II	Q1 (m/z)	Q3 (m/z)	Molecular Weight (Da)
Ybmn003693	cagayanone A	Lignans and Coumarins	Lignans	337.09	191.06	338.1154
MWSHY0174	Epicatechin*	Flavonoids	Flavanols	291.0863	139.0397	290.079
Lahp002608	3,5-Dihydro-2H-Furo[3,2-C]Quinolin-4-One*	Alkaloids	Quinoline alkaloids	188.07	118.07	187.0633
Zazp002547	cyclo-(Gly-Phe)	Amino acids and derivatives	Amino acids and derivatives	205.1	146.06	204.0899
Lahp001956	Pyran[3,4-b]indole-2-keton e	Alkaloids	Plumerane	188.07	118.07	187.0633
Wamxn05520	1-Octen-3-Yl Primeveroside	Others	Alcohol compounds	481.23	289.17	422.2152
zjgp122323	4-O-acetyl-3-O-caffeoyl-2-C-methyl-D-erythronate	Others	Others	355.1	163.04	354.0951

Metabolite ID	Compound Name	Class I	Class II	Q1 (m/z)	Q3 (m/z)	Molecular Weight (Da)
Hmgp002327	3-amino-2-naphthoic acid*	Alkaloids	Alkaloids	188.07	118.06	187.0633
Wcdp001953	2-methyl-1-nitronaphthalene	Others	Others	188.0707	118.0662	187.0633
Hmmp001310	3-Indoleacrylic acid*	Alkaloids	Plumerane	188.0706	118.0651	187.0633
Smgp004575	Quercetin-5-O-β-D-glucoside*	Flavonoids	Flavonols	465.1	303.05	464.0955
pme0021	L-Phenylalanine	Amino acids and derivatives	Amino acids and derivatives	166.09	120.08	165.079
mws0056	Myricetin-3-O-rhamnoside (Myricitrin)	Flavonoids	Flavonols	463.09	316.03	464.0955
Zbhp004510	Hesperetin-3'-O-glucoside*	Flavonoids	Flavanones	465.14	303.09	464.1319
Smcp001137	naphthisoxazol A*	Alkaloids	Alkaloids	188.07	118.06	187.0633

Metabolite ID	Compound Name	Class I	Class II	Q1 (m/z)	Q3 (m/z)	Molecular Weight (Da)
mws0258	L-Isoleucine*	Amino acids and derivatives	Amino acids and derivatives	132.1019	86.0964	131.0946
Zmcp102204	Senkyunolide Q*	Others	Lactones	279.1597	149.0243	278.1518
pmb3074	5-O-p-Coumaroylquinic acid	Phenolic acids	Phenolic acids	337.09	191.06	338.1002
MWSmce341	Butyl isobutyl phthalate*	Phenolic acids	Phenolic acids	279.16	149.03	278.1518
mws1587	L-Norleucine*	Amino acids and derivatives	Amino acids and derivatives	132.1	86.1	131.0946
mws0045	Quercetin-3-O-rhamnoside (Quercitrin)	Flavonoids	Flavonols	449.11	303.05	448.1006
FQtp001338	2-Caffeoylisocitrate	Phenolic acids	Phenolic acids	355.07	163.04	354.0587
Lhzp102808	Sesamin	Lignans and Coumarins	Lignans	355.1	163.04	354.1103

Metabolite ID	Compound Name	Class I	Class II	Q1 (m/z)	Q3 (m/z)	Molecular Weight (Da)
Zbzp011453	Des-O-Methyllasiodiplodin*	Others	Lactones	279.16	149.03	278.1518
MWSHC20164	Dibutyl phthalate*	Phenolic acids	Phenolic acids	279.16	149.02	278.1518
Lmxp011770	Diisobutyl phthalate*	Phenolic acids	Phenolic acids	279.1591	149.0233	278.1518
pmn001640	Myricetin-3-O-arabinoside*	Flavonoids	Flavonols	449.07	316	450.0798
Lmzn006169	3,19-Dihydroxyurs-12-en-28-oic acid (Pomolic acid)*	Terpenoids	Triterpene	471.35	471.35	472.3553
pmn001421	3-O-p-Coumaroylquinic acid	Phenolic acids	Phenolic acids	337.09	191.06	338.1002
HX1341	7-Hydroxycoumarin;Umbelliferone	Lignans and Coumarins	Coumarins	163.04	89.04	162.0317

Metabolite ID	Compound Name	Class I	Class II	Q1 (m/z)	Q3 (m/z)	Molecular Weight (Da)
pmn001705	3,24-Dihydroxy-17,21-semi acetal-12(13)oleanolic fruit*	Terpenoids	Triterpene	471.35	471.35	472.3553
pmn001706	2,3-Dihydroxyolean-12-en-28-oic acid (2-Hydroxyoleanolic acid)*	Terpenoids	Triterpene	471.35	471.35	472.3553
Lmfn003630	Myricetin-3-O-xyloside*	Flavonoids	Flavonols	449.07	316.02	450.0798
mws4183	Quercetin-3-O-arabinoside	Flavonoids	Flavonols	433.08	300.03	434.0849
Zmjn012003	Gypensapogenin F*	Terpenoids	Triterpene	471.34	471.34	472.3553
Lmfn004036	Morin 3-alpha-L-lyxopyranoside	Flavonoids	Flavonols	433.08	300.03	434.0849
pmn001427	16,23:16,30-Diepoxydammar-24-ene-3,20-diol	Terpenoids	Triterpene	471.35	471.34	472.3553

Metabolite ID	Compound Name	Class I	Class II	Q1 (m/z)	Q3 (m/z)	Molecular Weight (Da)
	(Jujubogenin)*					
Smpn011792	2,3-Dihydroxy-12-ursen-28 -oic acid*	Terpenoids	Triterpene	471.35	471.34	472.3553
pmb1650	Octadeca-11E,13E,15Z-trie noic acid	Lipids	Free fatty acids	279.23	149.13	278.2246
Lmfp004055	Morin-3-O-xyloside*	Flavonoids	Flavonols	435.09	303.05	434.0849
mws0091	Quercetin-3-O-glucoside (Isoquercitrin)	Flavonoids	Flavonols	463.09	300.03	464.0955
Lmdp003509	Quercetin-3-O-xyloside (Reynoutrin)*	Flavonoids	Flavonols	435.09	303.06	434.0849
Lmdp003286	Quercetin-3-O-alloside; Isohyperoside*	Flavonoids	Flavonols	465.1019	303.0613	464.0955

Metabolite ID	Compound Name	Class I	Class II	Q1 (m/z)	Q3 (m/z)	Molecular Weight (Da)
mws2186	Avicularin(Quercetin-3-O-α-L-arabinofuranoside)*	Flavonoids	Flavonols	435.09	303.05	434.0849
Lmzn006284	2-Hydroxyursolic acid*	Terpenoids	Triterpene	471.35	471.35	472.3553
mws0061	Quercetin-3-O-galactoside (Hyperin)	Flavonoids	Flavonols	463.09	300.03	464.0955
mws0277	Quinic Acid	Organic acids	Organic acids	191.06	85.03	192.0634
