# Supplementary material for: Integration of Transcriptomics and Metabolomics Reveals Organ-Specific Biosynthesis and Accumulation of Pharmacologically Active Flavonoids in Rhododendron yedoense var. poukhanense
Source: Plants (Basel). 2026 Aug 11;15(16):2441. doi: 10.3390/plants15162441 (PMC13516736; doi:10.3390/plants15162441)
Supplement: Supplementary file 1 [file plants-15-02441-s001.zip › Table S3.pdf]

Table S3. Mass spectrometry parameters (retention time, precursor ion, product ion, collision energy, declustering potential) and annotation confidence levels for 29 representative metabolites detected in *R. yedoense* var. *poukhanense* by UPLC-MS/MS (MRM mode).

Metabolite_ID	Compound	RT_min	Q1_Da	Q3_Da	CE_eV	DP_V	MSI_Level
Lmhp013004	3-Oxolanosta-9(11),24-dien -26-oic acid (Coccinic Acid)	10.9	455.352	315.2319	30	50	3
Lasp002843	Daidzein rutinoside	3.4	563.1746	255.0664	30	80	3
Lmhp009384	1-Linoleoylglycerol-2,3-di- O-glucoside	8.1	679.39	263.24	30	50	3
Smdp009834	Methylfarrerol	8	315.12	181.05	30	50	3
pmb2406	LysoPC 17:0*	9.3	510.36	184.07	40	40	1
Zmyn004449	9-Hydroxy-12-oxo-10(E),15 (Z)-octadecadienoic acid	8.4	309.21	171.1	-30	-50	3
Zmjp008503	5-Hydroxy-4',6,7-trimethox	8	329.1	296.07	30	50	3

Metabolite_ID	Compound	RT_min	Q1_Da	Q3_Da	CE_eV	DP_V	MSI_Level
	yflavone (Salvigenin)						
Lmnp002494	Nepetin-8-C-[Xylosyl-(1-2)]-glucoside	3.4	611.16	479.12	30	50	3
Lmhp010757	LysoPE 20:2	9.3	506.32	365.31	30	50	3
Lafp002342	2,3-Dihydroxy-1-(4-hydroxy-3,5-dimethoxyphenyl)propan-1-one	2.7	243.09	140.05	30	80	2
Zbjn004445	Kaempferol-7-O-glucuronide	3.7	461.07	285.04	-30	-50	3
Zbnp004262	Diosmetin-8-C-(2''-O-rhamnosyl)glucoside	4.1	609.18	463.12	30	50	3
Qmxp101615	Ent-3β-Acetoxyisopimar-1	9.8	349.27	179.14	30	80	3

Metabolite_ID	Compound	RT_min	Q1_Da	Q3_Da	CE_eV	DP_V	MSI_Level
	5-8β-ol						
Smpp012073	3-[[3-(4-Hydroxyphenyl)propenoyl]oxy]-2-hydroxyurs-12-ene-28-oic acid (Jacoumaric Acid)	10.2	619.39	147.04	30	50	1
Labn003316	pyrocatechol-β-D-xylopyranosyl(1-6)-β-D-glucopyranoside	2.8	403.12	109.03	-30	-80	3
Wcdn003654	Feruloyl Lactate	3.3	281.06	178.03	-30	-50	3
Hmln101664	Nortrachelogenin-4'-O-gentiobioside	3.5	697.24	535.18	-30	-50	3
Lcfn121170	4,6-diethyl-3,6-dihydroxy-8-methyldodec-4-enoicacid	8.7	299.22	281.21	-30	-50	1

Metabolite_ID	Compound	RT_min	Q1_Da	Q3_Da	CE_eV	DP_V	MSI_Level
Zbqn003748	N-malonylleucine	3.4	216.09	130.09	-30	-50	3
MWS2070	Propyl 4-hydroxybenzoate	6.3	179.07	92.03	-30	-50	3
mws1038	Pantetheine	2.9	277.12	146.08	-30	-50	3
Lmmp002995	Quercetin-7-O-(2''-malonyl)glucosyl-5-O-glucoside	3.6	713.15	303.06	30	50	3
Wcqp012549	2,3-Dihydroxyurs-12-en-28 -oic acid (Corosolic acid)	11.1	473.3621	455.3553	30	50	3
Zmjn004133	9S-Hydroperoxy-10E,12Z-o ctadecadienoic acid	8.4	311.22	293.21	-30	-50	3
Lmyp003371	3,5-Dihydroxytoluene	2.6	125.06	110.04	30	50	3
MWS4471	N-Alpha-Acetyl-L-Aspara gine	0.9	173.06	58.03	-30	-50	3

Metabolite_ID	Compound	RT_min	Q1_Da	Q3_Da	CE_eV	DP_V	MSI_Level
Zbcp002979	Petunidin-3-O-rutinoside	2.7	625.18	317.07	30	50	3
mws1397	Epicatechin gallate	3.8	441.08	169.02	-30	-50	3
mws2213	Cinnamic acid	4.9	147.05	103.06	-30	-40	3
