# Supplementary material for: Integration of Transcriptomics and Metabolomics Reveals Organ-Specific Biosynthesis and Accumulation of Pharmacologically Active Flavonoids in Rhododendron yedoense var. poukhanense
Source: Plants (Basel). 2026 Aug 11;15(16):2441. doi: 10.3390/plants15162441 (PMC13516736; doi:10.3390/plants15162441)
Supplement: Supplementary file 1 [file plants-15-02441-s001.zip › Table S4.pdf]

Table S4. All DAMs detected in *R. yedoense* var. *poukhanense* root, stem, and leaf tissues by widely targeted metabolomics (UPLC-MS/MS).

Comparison	Compound	Class I	Class II	VIP	P-value	FDR	Fold Change	Log_2FC	MSI Level
Root vs Leaf	1-Linoleoylglycerol-2,3-di-O-glucoside	Lipids	Glycerol ester	1.236594478	0.120105494	0.238683014	9.611164141	3.264711186	3
Root vs Leaf	Methylfarrerol	Flavonoids	Flavanones	1.005827394	0.115573734	0.23276313	0.295880827	-1.756911883	3
Root vs Leaf	LysoPC 17:0*	Lipids	LPC	1.298918287	0.027690988	0.083692622	13.98557045	3.805867195	1
Root vs Leaf	9-Hydroxy-12-oxo-10(E),15(Z)-octadecadienoic acid	Lipids	Free fatty acids	1.180206393	0.222082304	0.350243387	34.01777006	5.088216667	3
Root vs Leaf	5-Hydroxy-4',6,7-trimethoxyflavone (Salvigenin)	Flavonoids	Flavones	1.216382525	0.186854329	0.325374259	14.53233653	3.861194775	3

Comparison	Compound	Class I	Class II	VIP	P-value	FDR	Fold Change	Log_2FC	MSI Level
Root vs Leaf	Nepetin-8-C-[Xylosyl-(1-2)]-glucoside	Flavonoids	Flavones	1.300613306	0.010268666	0.047156746	0.134058701	-2.899063238	3
Root vs Leaf	LysoPE 20:2	Lipids	LPE	1.259118663	0.096852029	0.20394798	25.8227378	4.690570062	3
Root vs Leaf	2,3-Dihydroxy-1-(4-hydroxy-3,5-dimethoxyphenyl)propan-1-one	Others	Ketone compounds	1.300052161	0.01633876	0.061192478	17.52502664	4.131344732	2
Root vs Leaf	Diosmetin-8-C-(2''-O-rhamnosyl)glucoside	Flavonoids	Flavones	1.259715714	0.212412126	0.342298735	63.8080906	5.995667458	3
Root vs Leaf	Ent-3β-Acetoxyisopimar-15-8β-ol	Terpenoids	Ditepenoids	1.235050675	0.146108885	0.27720922	0.108237131	-3.207732586	3

Comparison	Compound	Class I	Class II	VIP	P-value	FDR	Fold Change	Log_2FC	MSI Level
Root vs Leaf	Feruloyl Lactate	Organic acids	Organic acids	1.283973203	0.053385859	0.132301424	16.88443749	4.077622211	3
Root vs Leaf	Propyl 4-hydroxybenzoate	Phenolic acids	Phenolic acids	1.079766869	0.233552442	0.361078556	4.315627327	2.109570287	3
Root vs Leaf	2,3-Dihydroxyurs-12-en-28-oic acid (Corosolic acid)	Terpenoids	Triterpene	1.2835136	0.039471531	0.107293055	0.143044593	-2.805463128	3
Root vs Leaf	N-Alpha-Acetyl-L-Asparagine	Amino acids and derivatives	Amino acids and derivatives	1.305696856	0.000319727	0.01464066	0.036867427	-4.76150947	3
Root vs Leaf	Cinnamic acid	Phenolic acids	Phenolic acids	1.074930129	0.062060109	0.148189941	2.112858882	1.079196413	3

Comparison	Compound	Class I	Class II	VIP	P-value	FDR	Fold Change	Log_2FC	MSI Level
Root vs Leaf	LysoPC 22:5	Lipids	LPC	1.305744707	0.005398666	0.036457866	13.20816321	3.723357947	3
Root vs Leaf	Feruloylferuloyltartaric acid	Phenolic acids	Phenolic acids	1.060140971	0.060154685	0.144438082	0.208696869	-2.260519136	3
Root vs Leaf	salicylate-6-O-β-D-glucopyranosyl benzoic acid	Phenolic acids	Phenolic acids	1.154261124	0.19488757	0.330315325	6.801129278	2.765774315	3
Root vs Leaf	Antiarol; 3,4,5-Trimethoxy phenol	Others	Others	1.182740546	0.25654435	0.38322719	18.16330851	4.182955114	3
Root vs Leaf	p-Coumaroylmalic acid	Phenolic acids	Phenolic acids	1.304919822	0.003045887	0.028128894	0.070399536	-3.828290276	3
Root vs Leaf	Shanzhiside methyl ester	Terpenoids	Monoterpenoids	1.115803021	0.017426766	0.063613584	0.252742339	-1.984260732	3

Comparison	Compound	Class I	Class II	VIP	P-value	FDR	Fold Change	Log_2FC	MSI Level
Root vs Leaf	(7'S,8R,8'R)-3,5'-dimethoxy-3',4,9'-trihydroxy-7',9-epoxy-8,8'-lignan	Lignans and Coumarins	Lignans	1.306571542	0.013606261	0.055373126	30.90469141	4.949753955	3
Root vs Leaf	N',N'',N'''-p-Coumaroyl-cinnamoyl-caffeoyl spermidine	Alkaloids	Phenolamine	1.011009882	0.073141669	0.167258356	0.341384103	-1.550532214	3
Root vs Leaf	Myricetin-3,7,3'-trimethyl ether	Flavonoids	Flavonols	1.214595886	0.197599805	0.33127477	40.09726179	5.325431815	3
Root vs Leaf	Patuletin 3-O-beta-gentiobioside	Flavonoids	Flavonols	1.242713982	0.12169661	0.241271903	0.132934132	-2.911216521	3

Comparison	Compound	Class I	Class II	VIP	P-value	FDR	Fold Change	Log_2FC	MSI Level
Root vs Leaf	Flavogallonic Acid Dilactone	Tannins	Tannin	1.292187669	0.022493863	0.073762122	0.147814122	-2.758143985	3
Root vs Leaf	Maohuoside B	Flavonoids	Flavonols	1.305648388	0.000451124	0.014948097	0.041869092	-4.577970566	3
Root vs Leaf	2-Hydroxy-4-met hylpentanoic acid	Organic acids	Organic acids	1.299309457	0.037906081	0.104085185	0.014197839	-6.138184807	3
Root vs Leaf	Grayanotoxin XV	Terpenoids	Ditepenoids	1.304834144	0.005252306	0.036144451	0.020065297	-5.639153693	2
Root vs Leaf	N-(sulfonyl)phen ylalanine	Amino acids and derivatives	Amino acids and derivatives	1.258779788	0.046899199	0.120941729	0.179894642	-2.474775878	3
Root vs Leaf	Gossypetin-3-O-r utinoside	Flavonoids	Flavonols	1.29771142	0.030068663	0.087808622	0.053065627	-4.236078524	3
Root vs Leaf	2-α-Linolenoyl-gl	Lipids	Glycerol ester	1.236292576	0.027229712	0.082994932	0.200492679	-2.318378539	2

Comparison	Compound	Class I	Class II	VIP	P-value	FDR	Fold Change	Log_2FC	MSI Level
	ycerol-1,3-di-O-glucoside								
Root vs Leaf	9(10)-EpOME;(9R,10S)-(12Z)-9,10-Epoxyoctadecenoic acid	Lipids	Free fatty acids	1.173969083	0.265108084	0.39132416	18.67438175	4.222988575	1
Root vs Leaf	Diosmetin-7-O-rutinoside (Diosmin)	Flavonoids	Flavones	1.265733947	0.166390605	0.304462402	43.33683716	5.43752196	3
Root vs Leaf	2α,5α,14β-Triacetoxy-10β-O-(Β-D glucopyranosy)taxa-4(20),11-diene	Others	Others	1.30094194	0.023663146	0.075994966	0.041158453	-4.602667429	2

Comparison	Compound	Class I	Class II	VIP	P-value	FDR	Fold Change	Log_2FC	MSI Level
Root vs Leaf	6,7-dimethoxy-2-[2-(4'-hydroxy-3'-methoxyphenyl)ethyl]chromone	Others	Chromone	1.109547257	0.330856885	0.449674043	32.95005064	5.042208778	3
Root vs Leaf	Tyrosol	Others	Alcohol compounds	1.162927817	0.039805427	0.108064733	2.550860756	1.350984148	3
Root vs Leaf	Velutin (5,4'-dihydroxy-7,3'-dimethoxyflavone)	Flavonoids	Flavones	1.251664752	0.139575207	0.26645055	13.59355101	3.764850472	3
Root vs Leaf	Kaempferol (3,5,7,4'-Tetrahydroxyflavone)	Flavonoids	Flavonols	1.305616369	0.000565563	0.016207027	0.122274595	-3.031803409	2

Comparison	Compound	Class I	Class II	VIP	P-value	FDR	Fold Change	Log_2FC	MSI Level
Root vs Leaf	9,12,13-Trihydroxy-10,15-octadecadienoic acid	Lipids	Free fatty acids	1.082322586	0.349313912	0.464533762	27.21814627	4.766496908	1
Root vs Leaf	Dehydrovomifoliol	Terpenoids	Monoterpenoids	1.296229765	0.036877215	0.102563272	40.64714758	5.345082209	3
Root vs Leaf	Daidzein 4',7-diglucoside	Flavonoids	Isoflavones	1.117007362	0.017981461	0.064871349	0.189183464	-2.402142104	3
Root vs Leaf	6-Methoxyquercetin-3-O-rhamnoside	Flavonoids	Flavonols	1.299940523	0.013866077	0.055973347	0.087363963	-3.516817897	2
Root vs Leaf	Kaempferol triglucoside*	Flavonoids	Flavonols	1.305269236	0.001855291	0.023320316	0.053224967	-4.23175304	3
Root vs Leaf	Crepenynic acid	Lipids	Free fatty acids	1.194832952	0.092934054	0.198057684	6.127420052	2.615279756	3

Comparison	Compound	Class I	Class II	VIP	P-value	FDR	Fold Change	Log_2FC	MSI Level
Root vs Leaf	4-O-(6'-O-Glucosylcaffeoyl)-3,4-dihydroxybenzyl alcohol	Phenolic acids	Phenolic acids	1.287412587	0.029969397	0.087808622	0.139177355	-2.845003597	3
Root vs Leaf	17-Hydroxylinolenic acid	Lipids	Free fatty acids	1.263220146	0.091069409	0.195047348	7.625790201	2.93088684	2
Root vs Leaf	Indole-3-carboxylic acid	Alkaloids	Plumerane	1.244515369	0.180832786	0.324029561	18.97290204	4.245868461	3
Root vs Leaf	Quercetin-3-O-(6''-O-galloyl)galactoside	Flavonoids	Flavonols	1.302899679	0.00651061	0.038973485	0.126767633	-2.979741661	3
Root vs Leaf	(3r,4r)-3-(2h-1,3-benzodioxol-5-ylm	Lignans and Coumarins	Lignans	1.101210728	0.33961982	0.457148508	39.08379524	5.288498662	3

Comparison	Compound	Class I	Class II	VIP	P-value	FDR	Fold Change	Log_2FC	MSI Level
	ethyl)-4-[(4-hydroxy-3-methoxyphenyl)methyl]oxolan-2-one								
Root vs Leaf	4-Hydroxybenzoyl-1-O-(6''-O-galloyl)glucoside	Phenolic acids	Phenolic acids	1.294089539	0.02868715	0.085744027	0.107943821	-3.211647435	3
Root vs Leaf	Phaseic Acid	Terpenoids	Sesquiterpenoids	1.255935256	0.100976427	0.209615811	0.111566261	-3.164027294	3
Root vs Leaf	9-Hydroxy-13-oxo-10-octadecenoic Acid	Lipids	Free fatty acids	1.234897939	0.106077021	0.217582589	40.39933907	5.336259786	3
Root vs Leaf	Indole-3-lactic	Alkaloids	Plumerane	1.305714683	0.000368211	0.01464066	0.024110635	-5.374186528	1

Comparison	Compound	Class I	Class II	VIP	P-value	FDR	Fold Change	Log_2FC	MSI Level
	acid								
Root vs Leaf	LysoPC 20:5	Lipids	LPC	1.283298405	0.109757902	0.223406516	71.6694131	6.163285635	3
Root vs Leaf	Methyl (4E,8E,11E)-5,9,13-trimethyltetradeca-4,8,11-trienoate	Others	Others	1.149556098	0.114045704	0.230480964	5.062502497	2.339850714	1
Root vs Leaf	1'-O-(3,4-Dihydroxyphenethyl)-O-caffeoyl-glucoside	Phenolic acids	Phenolic acids	1.303455418	0.006887212	0.040009515	0.064940154	-3.944745374	3
Root vs Leaf	6,10,14-Trimethyl pentadeca-5,9-Diene-2,13-Dione	Others	Others	1.159648038	0.080699809	0.179551108	5.635462241	2.49453395	3
Root vs Leaf	Decumbic acid	Organic	Organic acids	1.29867977	0.028159365	0.084634753	0.058496031	-4.095517447	3

Comparison	Compound	Class I	Class II	VIP	P-value	FDR	Fold Change	Log_2FC	MSI Level
		acids							
Root vs Leaf	Blumenol B malonyl Glucoside*	Terpenoids	Sesquiterpenoids	1.135875567	0.17113314	0.31120559	86.88968187	6.441112962	3
Root vs Leaf	methylarbutin*	Phenolic acids	Phenolic acids	1.303943115	0.007897809	0.042229995	0.035150977	-4.830291387	3
Root vs Leaf	Gossypetin-8-O-β-D-glucuronide	Flavonoids	Flavonols	1.291600741	0.024991329	0.078611736	0.149716126	-2.739698468	3
Root vs Leaf	Chrysophanol-8-O-β-D-galactoside	Quinones	Anthraquinone	1.30563545	0.000569983	0.016207027	0.094851955	-3.398178676	3
Root vs Leaf	8-C-glucosyl-noreugenin	Others	Chromone	1.182105769	0.002804253	0.027080964	0.165319757	-2.596668951	3

Comparison	Compound	Class I	Class II	VIP	P-value	FDR	Fold Change	Log_2FC	MSI Level
Root vs Leaf	13S-Hydroperoxy-9Z,11E-octadecadienoic acid	Lipids	Free fatty acids	1.19783318	0.169151883	0.308210135	28.820033	4.849000082	3
Root vs Leaf	Pierisformoside B	Terpenoids	Ditepenoids	1.179330528	0.00194897	0.023320316	0.13757327	-2.861727907	3
Root vs Leaf	3-O-Acetylpinobanksin	Flavonoids	Flavanonols	1.208658932	0.064962063	0.152756275	0.068482209	-3.868126954	3
Root vs Leaf	Eriodictyol-7-O-rutinoside-5-O-(6''-O-glucosyl)glucuronide	Flavonoids	Flavanones	1.287772316	0.028249331	0.084787227	0.14163339	-2.819766671	3
Root vs Leaf	Tri-p-coumaroyl Spermidine	Alkaloids	Phenolamine	1.300957594	0.022123206	0.073224284	0.035177908	-4.82918649	3
Root vs Leaf	LysoPC 20:3	Lipids	LPC	1.281439921	0.132154843	0.255444201	51.80379164	5.694985791	3

Comparison	Compound	Class I	Class II	VIP	P-value	FDR	Fold Change	Log_2FC	MSI Level
Root vs Leaf	Luteolin-6-C-glucoside-7-O-rhamnoside	Flavonoids	Flavones	1.261859686	0.086363317	0.188050555	0.131380934	-2.92817217	3
Root vs Leaf	Adenylocuccinic Acid	Nucleotides and derivatives	Nucleotides and derivatives	1.289743402	0.012870293	0.053295824	8.020255553	3.003648207	3
Root vs Leaf	8-Epiloganin	Terpenoids	Terpene	1.197127232	0.00020082	0.013805514	0.086659461	-3.528498929	3
Root vs Leaf	Pinocembrin-7-O-(2''-O-arabinosyl)glucoside	Flavonoids	Flavanones	1.304325758	0.002878215	0.02727992	0.160752908	-2.637083255	3
Root vs Leaf	2-Hydroxy-3-(4-Hydroxyphenyl)Propanoic Acid	Phenolic acids	Phenolic acids	1.304201102	0.011793295	0.050167936	0.011277312	-6.470432984	3

Comparison	Compound	Class I	Class II	VIP	P-value	FDR	Fold Change	Log_2FC	MSI Level
Root vs Leaf	(6R,9R)-3-Oxo-α-ionol-β-D-malonyl-glucoside	Terpenoids	Sesquiterpenoids	1.296424901	0.025552374	0.079565821	0.081035152	-3.625308315	3
Root vs Leaf	Skimmin (7-Hydroxycoumarin-7-O-glucoside)	Lignans and Coumarins	Coumarins	1.305266825	0.001274714	0.022037253	0.070182169	-3.832751658	3
Root vs Leaf	Dihydromyricetin (Ampelopsin)	Flavonoids	Flavanonols	1.268994313	0.134335633	0.258964587	0.037402955	-4.740703918	3
Root vs Leaf	Rosmarinic acid	Phenolic acids	Phenolic acids	1.15999026	0.150139343	0.282622927	29.75392746	4.895008209	3
Root vs Leaf	N-Feruloylhydroxyputrescine	Alkaloids	Phenolamine	1.299128539	0.019929575	0.068235021	0.053729941	-4.218129939	3

Comparison	Compound	Class I	Class II	VIP	P-value	FDR	Fold Change	Log_2FC	MSI Level
Root vs Leaf	Dehydrodiconiferyl alcohol-4-O-glucoside*	Lignans and Coumarins	Lignans	1.071035863	0.369059277	0.480464625	43.93347619	5.457248751	3
Root vs Leaf	Dihydrozeatin ribonucleoside	Alkaloids	Alkaloids	1.16661635	0.000901432	0.01967671	0.088760073	-3.493945342	3
Root vs Leaf	Eriodictyol-7-O-Rutinoside (Eriocitrin)*	Flavonoids	Flavanones	1.303002985	0.008579615	0.044144164	0.071714251	-3.801596348	3
Root vs Leaf	3,3'-Di-O-methylellagic acid 4'-beta-O-xylopyranoside	Tannins	Tannin	1.303177597	0.004253237	0.032943531	0.152621595	-2.711968983	3

Comparison	Compound	Class I	Class II	VIP	P-value	FDR	Fold Change	Log_2FC	MSI Level
Root vs Leaf	Okanin-4'-O-(6''-O-malonyl)glucoside	Flavonoids	Chalcones	1.290255594	0.02786287	0.083977212	0.159641909	-2.647088655	3
Root vs Leaf	3,12,23,25-tetrahydroxy-20S,24S-epoxydammarane-3-O-xylosylglucoside	Terpenoids	Triterpene Saponin	1.274837085	0.062616649	0.148820174	0.066909757	-3.901639584	2
Root vs Leaf	N,N-cinnamoylbutanediamine*	Alkaloids	Phenolamine	1.302198586	0.009086694	0.045345906	0.095259279	-3.39199656	3
Root vs Leaf	Carvylacetate	Terpenoids	Monoterpenoids	1.122598571	0.335570242	0.453229558	38.15766295	5.253900908	3
Root vs Leaf	LysoPE 15:1(2n	Lipids	LPE	1.29967763	0.041410278	0.110889232	108.3246243	6.759217423	3

Comparison	Compound	Class I	Class II	VIP	P-value	FDR	Fold Change	Log_2FC	MSI Level
	isomer)*								
Root vs Leaf	Oroxin A	Flavonoids	Flavones	1.046849753	0.016220322	0.061021704	0.207180315	-2.271041164	3
Root vs Leaf	Octadecadienamide	Alkaloids	Alkaloids	1.076768943	0.100728746	0.20948352	2.721592815	1.444451238	3
Root vs Leaf	5-hydroxy-6,7,8,3',4'-pentamethoxyflavone glucoside*	Flavonoids	Flavones	1.126754728	0.00557163	0.036629402	0.147223678	-2.763918377	3
Root vs Leaf	Apigenin-6,8-di-C-glucoside (Vicenin-2)	Flavonoids	Flavones	1.298067656	0.027336357	0.083085609	0.057740581	-4.114270576	3
Root vs Leaf	11-O-Galloylbergenin	Lignans and Coumarins	Coumarins	1.032554205	0.105355159	0.216830952	0.197112874	-2.342906089	3

Comparison	Compound	Class I	Class II	VIP	P-value	FDR	Fold Change	Log_2FC	MSI Level
Root vs Leaf	N6-Isopentenyladenine	Nucleotides and derivatives	Nucleotides and derivatives	1.120777033	0.072961198	0.167022403	0.175642084	-2.509289538	3
Root vs Leaf	Gentisic acid 2-O-(6''-O-Feruloyl)Glucoside*	Phenolic acids	Phenolic acids	1.280004426	0.048281336	0.123620813	0.136669325	-2.871238621	1
Root vs Leaf	1-O-p-Coumaroyl-4,6-(S)-HHDP-β-D-glucose	Phenolic acids	Phenolic acids	1.28740575	0.028811833	0.085879133	0.143392168	-2.801961868	3
Root vs Leaf	heptafluorobutanoic acid	Others	Others	1.303964019	0.009809282	0.046617244	0.031398464	-4.993162207	2
Root vs Leaf	3,4-O-di-Caffeoyl-1-O-glucoside	Phenolic acids	Phenolic acids	1.30570646	0.000486844	0.015609451	0.031582891	-4.984712967	2

Comparison	Compound	Class I	Class II	VIP	P-value	FDR	Fold Change	Log_2FC	MSI Level
	Quinic Acid								
Root vs Leaf	LysoPE 15:1*	Lipids	LPE	1.283609374	0.110078653	0.2235714	98.02242809	6.615039979	3
Root vs Leaf	Dihydrotricetin	Flavonoids	Flavanones	1.217069315	0.213535668	0.343085932	47.13159985	5.558622748	2
Root vs Leaf	2-Hydroxy-2-methyl-3-oxobutanoic acid	Organic acids	Organic acids	1.146220407	0.067941356	0.158725697	0.285636335	-1.807748582	3
Root vs Leaf	4-O-(6'-O-Glucosylcaffeoylglucosyl)-4-hydroxybenzyl alcohol	Phenolic acids	Phenolic acids	1.209719126	0.007835751	0.04213351	0.113810675	-3.135292216	3
Root vs Leaf	Caffeic aldehyde	Others	Aldehyde compounds	1.304715564	0.006600527	0.039327537	0.024069128	-5.376672285	3

Comparison	Compound	Class I	Class II	VIP	P-value	FDR	Fold Change	Log_2FC	MSI Level
Root vs Leaf	3-Hydroxy-3-acetonyloxindole	Alkaloids	Plumerane	1.199070306	0.002927485	0.027505634	3.598270473	1.847303635	3
Root vs Leaf	Apigenin 7,4'-diglucoside	Flavonoids	Flavones	1.273926971	0.08528023	0.18671791	0.078267531	-3.675442259	3
Root vs Leaf	Sachaloside VII	Others	Others	1.216273285	0.041132006	0.110555056	0.088731476	-3.494410227	3
Root vs Leaf	Helicidol*	Phenolic acids	Phenolic acids	1.305593517	0.000789407	0.018954533	0.022391413	-5.480910626	3
Root vs Leaf	Citric acid glucoside	Organic acids	Organic acids	1.096973809	0.068333309	0.159257901	2.830101844	1.500853971	2
Root vs Leaf	Isorhamnetin-3-O-(2''-O-xylosyl)glucoside	Flavonoids	Flavonols	1.292846975	0.022571437	0.073904358	0.164528145	-2.603593692	3

Comparison	Compound	Class I	Class II	VIP	P-value	FDR	Fold Change	Log_2FC	MSI Level
Root vs Leaf	Caffeoylquinic acid-glucose-rhamnose-caffeoyl	Phenolic acids	Phenolic acids	1.304592509	0.002904623	0.027410003	0.115737333	-3.111073793	2
Root vs Leaf	1-O-Feruloyl-beta-D-glucose	Phenolic acids	Phenolic acids	1.30475031	0.010935765	0.048130729	0.009411969	-6.731287725	3
Root vs Leaf	Quercetagetin-3-Methyl Ether	Flavonoids	Flavonols	1.250001576	0.137211851	0.262867739	0.087658093	-3.511968898	3
Root vs Leaf	25S-Inokosterone	Others	Others	1.12882541	0.001586333	0.023084686	0.101754959	-3.296828986	3
Root vs Leaf	1-O-Glucopyranosylamplexine	Terpenoids	Monoterpenoids	1.090301882	0.004356775	0.033039104	0.108574719	-3.203239879	3
Root vs Leaf	N-(11-alkanoyl)nornicotine	Alkaloids	Pyridine alkaloids	1.30331349	0.006903529	0.040009515	0.077273164	-3.693888724	3

Comparison	Compound	Class I	Class II	VIP	P-value	FDR	Fold Change	Log_2FC	MSI Level
Root vs Leaf	Pantothenol	Others	Vitamin	1.278555146	0.178752979	0.321636292	149.0388843	7.219544969	3
Root vs Leaf	Pieroside C	Others	Others	1.293237258	0.031886778	0.091999102	0.080531697	-3.634299451	2
Root vs Leaf	Gallic acid-1-O-xyloside	Phenolic acids	Phenolic acids	1.296014019	0.022806972	0.074337656	0.09024641	-3.469986652	1
Root vs Leaf	9-hydroxy-2,3-dimethoxy-1,4-anthraquinone	Quinones	Anthraquinone	1.304348185	0.006181437	0.038607186	0.047343303	-4.40069582	3
Root vs Leaf	LysoPC 22:6	Lipids	LPC	1.287705922	0.128255605	0.250597073	124.59716	6.961127374	3
Root vs Leaf	Methyl 3-(3-hydroxy-4-methoxyphenyl)propanoate	Phenolic acids	Phenolic acids	1.193028658	0.074102516	0.168563724	2.690106339	1.427663203	3

**Comparison**	**Compound**	**Class I**	**Class II**	**VIP**	**P-value**	**FDR**	**Fold Change**	**Log$_2$FC**	**MSI Level**
Root vs Leaf	Naringenin-7-O-Neohesperidoside(Naringin)	Flavonoids	Flavanones	1.304570121	0.008318669	0.043550477	0.015338867	-6.026664287	3
Root vs Leaf	5Z,8Z-tetradecadienoic acid	Lipids	Free fatty acids	1.063831378	0.041736067	0.11148534	0.259843044	-1.944287656	3
Root vs Leaf	Isoferulic Acid*	Phenolic acids	Phenolic acids	1.215735297	0.159312894	0.294756133	27.21422883	4.76628925	3
Root vs Leaf	3-Hydroxy-1-(4'-hydroxy-3'-methoxyphenyl)-propan-1-one	Others	Others	1.131378364	0.367966806	0.48034125	112.3867943	6.812328715	3
Root vs Leaf	Ala-Asn	Amino acids and	Amino acids and derivatives	1.283942472	0.048866903	0.124090924	0.061773504	-4.016868021	3

Comparison	Compound	Class I	Class II	VIP	P-value	FDR	Fold Change	Log_2FC	MSI Level
		derivatives							
Root vs Leaf	saikolignanoside D*	Lignans and Coumarins	Lignans	1.191079947	0.288336536	0.412753537	35.58895801	5.153357788	3
Root vs Leaf	1-(2-hydroxyethyl)-4-methoxy-9H-pyrido[3,4-b]indol-8-ol	Alkaloids	Alkaloids	1.008599245	0.152175079	0.284472617	4.60871398	2.204364236	3
Root vs Leaf	Afrormosin 7-O-(6''-Malonylglucoside)*	Flavonoids	Isoflavones	1.191747993	0.000246188	0.013805514	0.093420328	-3.42011968	3
Root vs Leaf	LysoPE 14:0(2n isomer)*	Lipids	LPE	1.306594893	0.009463797	0.046176635	58.76956678	5.876997358	3
Root vs Leaf	LysoPE 18:0	Lipids	LPE	1.004627364	0.272476083	0.398659997	4.293358201	2.102106544	3

Comparison	Compound	Class I	Class II	VIP	P-value	FDR	Fold Change	Log_2FC	MSI Level
Root vs Leaf	(7S,8R-Threo-3',4,9'-trihydroxy-3-methoxy-4',7-epoxy-neolignan-9-O-rhamnoside	Lignans and Coumarins	Lignans	1.123265602	0.000453713	0.014948097	0.108921297	-3.198642024	3
Root vs Leaf	9,10,11-Trihydroxy-12-octadecenoic acid	Lipids	Free fatty acids	1.064857701	0.380775169	0.48817444	68.62447552	6.100651313	1
Root vs Leaf	6'-O-β-glucopyranosyl secologanol	Terpenoids	Monoterpenoids	1.204652135	0.002868983	0.02727992	0.104373688	-3.260170027	3
Root vs Leaf	cyanidin 3,3',5-tri-O-glucoside	Flavonoids	Anthocyanidins	1.304844388	0.002189966	0.025163593	0.094969932	-3.396385363	3

Comparison	Compound	Class I	Class II	VIP	P-value	FDR	Fold Change	Log_2FC	MSI Level
Root vs Leaf	Isochlorogenic acid C*	Phenolic acids	Phenolic acids	1.188568108	0.003760593	0.031498613	0.125934336	-2.98925641	3
Root vs Leaf	Trollioside	Phenolic acids	Phenolic acids	1.252069319	0.003652273	0.030931932	0.019560574	-5.675907474	3
Root vs Leaf	Isosclerone;(4S)-4, 8-Dihydroxy-3,4-Dihydronaphthalene-1(2H)-One	Quinones	Quinones	1.244353469	0.000875325	0.019301816	0.309522376	-1.691884387	3
Root vs Leaf	3,3'-Bis(3,4-dihydro-4-hydroxy-6,8-dimethoxy-2H-1-benzopyran)	Lignans and Coumarins	Lignans	1.304350702	0.004239041	0.032943531	0.088522331	-3.497814757	3
Root vs Leaf	9-Oxodemethylm	Others	Others	1.301086483	0.009743072	0.046617244	43.48709222	5.442515341	2

Comparison	Compound	Class I	Class II	VIP	P-value	FDR	Fold Change	Log_2FC	MSI Level
	yricanane B								
Root vs Leaf	Robinlin	Terpenoids	Monoterpenoids	1.305124391	0.004061248	0.032253374	0.020212225	-5.628628029	3
Root vs Leaf	Anabasine	Alkaloids	Pyridine alkaloids	1.295221188	0.027484181	0.083411296	0.087966617	-3.506900054	3
Root vs Leaf	Leucasperoside B	Others	Others	1.305333794	0.001136789	0.020742409	0.066201272	-3.916997247	3
Root vs Leaf	Esculetin-6-O-quinic acid	Lignans and Coumarins	Coumarins	1.272238097	0.05486446	0.134729657	0.116826988	-3.097554503	3
Root vs Leaf	Neoeriocitrin*	Flavonoids	Flavones	1.240924503	0.107417278	0.219610915	0.08600392	-3.539453769	3
Root vs Leaf	O-Phosphorylethanolamine	Alkaloids	Alkaloids	1.189923562	0.141617522	0.269634771	4.139992781	2.049628252	3
Root vs Leaf	myricetin-3-O-β-	Flavonoids	Flavonols	1.289612114	0.054944899	0.134774038	0.03584377	-4.802133808	3

Comparison	Compound	Class I	Class II	VIP	P-value	FDR	Fold Change	Log_2FC	MSI Level
	D-xylopyranosyl-(1→2)-β-D-glucopyranoside								
Root vs Leaf	Hydroxydihydrobovolide*	Others	Lactones	1.304613233	0.007486424	0.041376376	0.013924503	-6.166230388	1
Root vs Leaf	2-Aminoethylphosphonate	Others	Others	1.305567464	0.00148715	0.023084686	0.029874407	-5.064946132	3
Root vs Leaf	Homomevalonic Acid Glucoside	Organic acids	Organic acids	1.250610243	0.009356083	0.045951128	0.257819721	-1.955565476	1
Root vs Leaf	Succinyl arginine	Amino acids and derivatives	Amino acids and derivatives	1.216916099	0.001914249	0.023320316	0.043096379	-4.536289518	3
Root vs Leaf	Aromadendrin-3-	Flavonoids	Flavanonols	1.226445915	0.005998362	0.037681573	0.071501278	-3.805887155	3

Comparison	Compound	Class I	Class II	VIP	P-value	FDR	Fold Change	Log_2FC	MSI Level
	O-rhamnoside; Engeletin								
Root vs Leaf	N-(1-Deoxy-1-fructosyl)Arginine	Amino acids and derivatives	Amino acids and derivatives	1.23312317	0.001488505	0.023084686	0.084557601	-3.563921737	3
Root vs Leaf	Uridine 5'-diphospho-N-acetylglucosamine	Nucleotides and derivatives	Nucleotides and derivatives	1.237095321	0.148784683	0.281298076	22.20110985	4.472559895	2
Root vs Leaf	Quercetin-3-O-(2''-O-malonyl)glucoside-7-O-arabinoside	Flavonoids	Flavonols	1.302207826	0.005544981	0.036629402	0.177458899	-2.494443175	3
Root vs Leaf	Grayanotoxin III	Terpenoids	Ditepenoids	1.243282843	0.038397351	0.10514736	0.056618753	-4.142576215	3

Comparison	Compound	Class I	Class II	VIP	P-value	FDR	Fold Change	Log_2FC	MSI Level
Root vs Leaf	Machilusolide D	Others	Lactones	1.227960749	0.151356132	0.283924132	27.06459278	4.758334776	3
Root vs Leaf	6-O-p-Methoxycinnamoylcatalpol	Terpenoids	Monoterpenoids	1.211262021	0.011539971	0.04967503	0.061694391	-4.018716857	3
Root vs Leaf	Methyl 4-hydroxybenzoate	Phenolic acids	Phenolic acids	1.209126496	0.18246135	0.324925253	20.86155615	4.382774873	3
Root vs Leaf	Mearnsetin-3-O-rhamnoside (Mearnsitrin)	Flavonoids	Flavonols	1.300677274	0.016471094	0.061263398	0.06078191	-4.04021418	2
Root vs Leaf	Isofraxidin 7-O-Apiosylglucoside*	Lignans and Coumarins	Coumarins	1.206171831	0.19328437	0.328736334	38.2443237	5.257173731	2
Root vs Leaf	4,6-Dihydroxyqui	Alkaloids	Quinoline	1.188259261	0.040516849	0.109173205	0.373636654	-1.420292105	3

Comparison	Compound	Class I	Class II	VIP	P-value	FDR	Fold Change	Log_2FC	MSI Level
	noline		alkaloids						
Root vs Leaf	Tricaffeoyl Spermidine	Alkaloids	Phenolamine	1.069700698	0.043663089	0.115068214	0.3490907	-1.518326172	3
Root vs Leaf	Reseoside*	Terpenoids	Monoterpenoids	1.124152885	0.001771321	0.023320316	0.10390549	-3.266656206	2
Root vs Leaf	Protocatechuic acid 4-O-(6''-O-Feruloyl)Glucoside*	Phenolic acids	Phenolic acids	1.260739167	0.092083328	0.196632482	0.131238747	-2.929734367	1
Root vs Leaf	Glucosyl 2,6-dimethyl-8-oxoocta-2,6-dienoic acid	Others	Others	1.216719477	0.01848644	0.065490488	0.080122579	-3.641647338	3

Comparison	Compound	Class I	Class II	VIP	P-value	FDR	Fold Change	Log_2FC	MSI Level
Root vs Leaf	Fargesin	Lignans and Coumarins	Lignans	1.304053176	0.004757047	0.034039662	0.06326543	-3.982438815	3
Root vs Leaf	19-Hydroxyursolic acid	Terpenoids	Triterpene	1.296693189	0.016420756	0.061222642	0.164463028	-2.6041648	3
Root vs Leaf	N,N-diethyl-5-hydroxytryptamine oxalate	Alkaloids	Alkaloids	1.029960055	0.022462598	0.073762122	0.166132375	-2.58959485	3
Root vs Leaf	4-Hydroxy-L-Isoleucine	Amino acids and derivatives	Amino acids and derivatives	1.284062725	0.006121595	0.038344249	3.510394372	1.811633118	3
Root vs Leaf	Dihydroxyacetone phosphate	Others	Ketone compounds	1.067442497	0.025114833	0.078770905	0.231783562	-2.109149841	3
Root vs Leaf	Genipin-1-O-(2''-	Terpenoids	Monoterpenoid	1.30489558	0.001832543	0.023320316	0.110117347	-3.182886334	3

Comparison	Compound	Class I	Class II	VIP	P-value	FDR	Fold Change	Log_2FC	MSI Level
	O-apiosyl)glucoside		s						
Root vs Leaf	N-(1-Deoxy-1-fructosyl)Tyrosine	Amino acids and derivatives	Amino acids and derivatives	1.303948475	0.010836346	0.047921017	0.020276741	-5.624030414	3
Root vs Leaf	3-Hydroxy-Beta-Ionone 3-(6''-Malonyl)Glucoside	Others	Others	1.293946316	0.02752071	0.083411296	0.083209317	-3.587111108	3
Root vs Leaf	N-(4-(tetrahydroPhoenix1-yl)butyryl)nornicotine	Alkaloids	Pyridine alkaloids	1.151764819	0.011179491	0.048695245	0.200480416	-2.318466779	3
Root vs Leaf	Malonyl Wistin*	Flavonoids	Isoflavones	1.287961409	0.03142581	0.091155942	0.15352591	-2.703445936	3

Comparison	Compound	Class I	Class II	VIP	P-value	FDR	Fold Change	Log_2FC	MSI Level
Root vs Leaf	Naringenin-6-C-Glucoside	Flavonoids	Flavanones	1.279537526	0.000916641	0.019808613	0.222101213	-2.170710821	3
Root vs Leaf	Isololiolide	Terpenoids	Monoterpenoids	1.298776828	0.018782567	0.066106071	0.064801796	-3.947822401	3
Root vs Leaf	6'-O-D-Glucosylsweroside	Terpenoids	Monoterpenoids	1.004149022	0.321530493	0.441160252	14.14115898	3.82182846	3
Root vs Leaf	galloyl acetyl glucoside	Phenolic acids	Phenolic acids	1.269120869	0.04885738	0.124090924	9.959830151	3.31612114	3
Root vs Leaf	N-(Isoferuloyl)-Glutamic Acid	Alkaloids	Phenolamine	1.218619805	8.92386E-05	0.012840208	0.077105903	-3.697014869	3
Root vs Leaf	Euphatexol F	Terpenoids	Triterpene	1.297039797	0.008943921	0.045060703	0.165383647	-2.596111505	3
Root vs Leaf	8-Methoxy	Flavonoids	Isoflavones	1.213089501	0.01105007	0.048436512	0.109697949	-3.188391538	3

Comparison	Compound	Class I	Class II	VIP	P-value	FDR	Fold Change	Log$_2$FC	MSI Level
	malonyl ononin*								
Root vs Leaf	6-O-p-Coumaroyl -β-D-glucose*	Phenolic acids	Phenolic acids	1.30087873	0.016427539	0.061222642	0.046677189	-4.421138498	3
Root vs Leaf	3,5-Di-Galloyl Sorbitol	Phenolic acids	Phenolic acids	1.189461342	0.033068193	0.095027081	0.151169681	-2.725759276	3
Root vs Leaf	Viscumneoside I*	Flavonoids	Flavanones	1.297413329	0.037786934	0.104066249	0.029225394	-5.096633724	3
Root vs Leaf	Aracarpene 1	Flavonoids	Isoflavones	1.115110664	0.154134177	0.287141341	12.767794	3.674437375	3
Root vs Leaf	3,5,4'-Tetrahydroxystilbene-2-O-β-D-glucoside*	Others	Stilbene	1.292780824	0.029644798	0.087397555	0.076796621	-3.70281335	3
Root vs Leaf	linoleoyl ethanolamine	Lipids	LPE	1.175312813	0.331409603	0.450003556	59.09985107	5.88508259	3

Comparison	Compound	Class I	Class II	VIP	P-value	FDR	Fold Change	Log_2FC	MSI Level
Root vs Leaf	Proanthocyanidins	Tannins	Proanthocyanidins	1.084485789	0.106123114	0.217582589	0.162069143	-2.625318655	3
Root vs Leaf	7-(4-Hydroxy-3-methoxy-phenyl)-1-(4-hydroxy-phenyl)-hept-4-en-3-one	Others	Ketone compounds	1.052079475	0.380870942	0.48817444	75.19152498	6.232498156	3
Root vs Leaf	Mangiferin	Flavonoids	Other Flavonoids	1.059575863	0.125678998	0.246902104	2.24349183	1.16574593	3
Root vs Leaf	Syringaresinol	Lignans and Coumarins	Lignans	1.229813664	0.140595697	0.26792531	13.087653	3.710134497	1
Root vs Leaf	Coumarin-3-carboxylic Acid	Lignans and Coumarins	Coumarins	1.303571012	0.014647101	0.057545761	0.010947169	-6.513298326	3
Root vs Leaf	Kaempferol-3-O-(	Flavonoids	Flavonols	1.301439586	0.024747617	0.078072408	0.014517759	-6.106037437	2

Comparison	Compound	Class I	Class II	VIP	P-value	FDR	Fold Change	Log_2FC	MSI Level
	4''-O-acetyl)rhamnoside								
Root vs Leaf	1,3,6-trihydroxy-7-methoxy-2-{[(2s,3r,4s,5s,6r)-3,4,5-trihydroxy-6-(hydroxymethyl)oxan-2-yl]oxy}xanthen-9-one	Flavonoids	Other Flavonoids	1.209139057	0.010308258	0.047156746	0.116699424	-3.099130657	2
Root vs Leaf	p-Coumaric acid	Phenolic acids	Phenolic acids	1.300994882	0.023808869	0.076110898	0.040105034	-4.640072836	3
Root vs Leaf	2,4-Dinitrophenol	Others	Others	1.238871605	0.014318545	0.056984116	0.051563677	-4.277501033	2
Root vs Leaf	Citric	Organic	Organic acids	1.21771744	0.005985102	0.037681573	0.19691037	-2.344389005	1

Comparison	Compound	Class I	Class II	VIP	P-value	FDR	Fold Change	Log_2FC	MSI Level
	acid-1-O-diglucoside	acids							
Root vs Leaf	Apigenin-5-O-glucoside	Flavonoids	Flavones	1.097951453	0.071644015	0.164529985	0.113272287	-3.142133155	3
Root vs Leaf	1-O-Feruloyl-β-D-glucose	Phenolic acids	Phenolic acids	1.22830693	0.000741725	0.018214411	0.04569145	-4.451931959	3
Root vs Leaf	NADP (Nicotinamide adenine dinucleotide phosphate)	Nucleotides and derivatives	Nucleotides and derivatives	1.240668831	0.036846278	0.102563272	0.19753619	-2.339811108	3
Root vs Leaf	10-Dehydrogeniposide	Terpenoids	Monoterpenoids	1.299963764	0.018369363	0.065182585	0.060729658	-4.041454945	3

Comparison	Compound	Class I	Class II	VIP	P-value	FDR	Fold Change	Log_2FC	MSI Level
Root vs Leaf	9,16-Dihydroxypalmitic acid	Lipids	Free fatty acids	1.296747908	0.024514644	0.077563904	0.076125689	-3.715472804	3
Root vs Leaf	LysoPE 14:0*	Lipids	LPE	1.306523966	0.001763239	0.023320316	89.30978534	6.48074635	3
Root vs Leaf	12,13-Dihydroursolic acid	Terpenoids	Triterpene	1.209990518	0.007390376	0.040957167	0.115324711	-3.116226412	3
Root vs Leaf	1,3,6,7-tetrahydroxy-2-(3,4,5-trihydroxyoxan-2-yl)xanthen-9-one	Flavonoids	Other Flavonoids	1.242715048	0.018327323	0.065140371	0.045621003	-4.454158021	3
Root vs Leaf	1-O-Galloyl-6-O-Feruloyl-β-D-glucose*	Phenolic acids	Phenolic acids	1.302596065	0.007023802	0.040284231	0.123052155	-3.022658168	3
Root vs Leaf	Pierisfomoside D	Others	Others	1.302570417	0.010387141	0.047156746	0.074484185	-3.74692205	3

Comparison	Compound	Class I	Class II	VIP	P-value	FDR	Fold Change	Log_2FC	MSI Level
Root vs Leaf	Phthalic acid	Phenolic acids	Phenolic acids	1.24101234	0.089900926	0.193694817	0.113784547	-3.13562345	3
Root vs Leaf	(2R,3R,4S,5S,6R)-2-[3-(2,4-dihydroxyphenyl)propoxy]-6-(hydroxymethyl)oxane-3,4,5-triol	Phenolic acids	Phenolic acids	1.268197192	0.075677851	0.171245902	0.103556415	-3.271511171	3
Root vs Leaf	5-O-Dihydrocaffeoyl Quinic Acid	Phenolic acids	Phenolic acids	1.303511171	0.007195166	0.040362051	0.052290746	-4.257300542	1
Root vs Leaf	3-(3'-Hydroxybutyl)-2,4,4-trimethylcyclohexa-2,5-dienone glucoside	Terpenoids	Sesquiterpenoids	1.304878282	0.008182604	0.043144133	0.010944702	-6.513623497	3

Comparison	Compound	Class I	Class II	VIP	P-value	FDR	Fold Change	Log_2FC	MSI Level
Root vs Leaf	Icariside B2	Terpenoids	Sesquiterpenoids	1.251974216	0.084150164	0.184796261	0.030980453	-5.012497944	3
Root vs Leaf	5-O-Methylvisammioside	Others	Chromone	1.249329475	0.006497467	0.038973485	0.05272718	-4.245309357	3
Root vs Leaf	13(S)-HODE;13(S)-Hydroxyoctadeca-9Z,11E-dienoic acid*	Lipids	Free fatty acids	1.17199918	0.138993787	0.265781389	3.18347353	1.670601769	1
Root vs Leaf	Persicarin (Isohamnetin-3-O-sulfate)	Flavonoids	Flavones	1.300551191	0.003319164	0.029886306	5.439345154	2.443432975	3
Root vs Leaf	Grayanotoxin IX	Terpenoids	Ditepenoids	1.30470704	0.010709857	0.047719592	0.006376996	-7.292907284	3
Root vs Leaf	Scopoletin	Lignans and	Coumarins	1.304498794	0.062897236	0.149162294	184.7199238	7.529195673	3

Comparison	Compound	Class I	Class II	VIP	P-value	FDR	Fold Change	Log_2FC	MSI Level
	(7-Hydroxy-6-methoxycoumarin)	Coumarins							
Root vs Leaf	LysoPC 16:2(2n isomer)	Lipids	LPC	1.146860276	0.300527979	0.422813126	21.42778407	4.421410758	3
Root vs Leaf	Ferulic acid*	Phenolic acids	Phenolic acids	1.271143091	0.1284346	0.250623794	22.26386157	4.476631938	3
Root vs Leaf	2,4,6,4'-Tetrahydroxy-stilbene-2-O-glucoside	Others	Stilbene	1.20917112	0.00981304	0.046617244	0.093887032	-3.412930296	3
Root vs Leaf	1-Palmitoyl-Sn-Glycerol 3-O-Diglucoside	Lipids	Glycerol ester	1.244673895	0.000559956	0.016207027	0.183073198	-2.449507499	1
Root vs Leaf	Kaempferol-3-O-s	Flavonoids	Flavonols	1.29580449	0.018704253	0.065937833	0.138380875	-2.853283527	3

Comparison	Compound	Class I	Class II	VIP	P-value	FDR	Fold Change	Log_2FC	MSI Level
	ophorotrioside*								
Root vs Leaf	Quercetin-3-O-(2''-O-galactosyl)glucoside	Flavonoids	Flavonols	1.261868	0.078089772	0.175271163	0.112436599	-3.15281637	3
Root vs Leaf	2-Hydroxy-3-phenylpropanoic acid	Phenolic acids	Phenolic acids	1.245443778	0.054311631	0.133828317	0.027596958	-5.179346964	1
Root vs Leaf	Ageconyflavone B-4'-glucoside*	Flavonoids	Flavones	1.298818042	0.020984753	0.070856331	0.08487949	-3.558440209	2
Root vs Leaf	5-hydroxy-3,4-dimethyl-5-pentylfuran-2(5H)-one*	Others	Lactones	1.277024138	0.003225404	0.029409693	0.009813797	-6.670972798	1
Root vs Leaf	11-Oxo-kansenonol	Terpenoids	Triterpene	1.28368452	0.038431279	0.10514736	0.141556015	-2.82055504	3

Comparison	Compound	Class I	Class II	VIP	P-value	FDR	Fold Change	Log_2FC	MSI Level
Root vs Leaf	Grayanotoxin V	Terpenoids	Ditepenoids	1.268857677	0.034912531	0.099104526	0.023792539	-5.393346951	3
Root vs Leaf	Herbacetin	Flavonoids	Flavonols	1.293611028	0.023726696	0.075994966	0.16699698	-2.582106084	1
Root vs Leaf	Afrormosin-7-O-(6''-malonyl)glucoside*	Flavonoids	Isoflavones	1.239969801	0.005565536	0.036629402	0.173409958	-2.527741348	3
Root vs Leaf	Villosolside	Terpenoids	Monoterpenoids	1.197048491	0.039325904	0.107166807	0.053199199	-4.232451653	2
Root vs Leaf	Hyp-Ser	Amino acids and derivatives	Amino acids and derivatives	1.071212	0.155349904	0.288908041	5.076044029	2.343704583	3
Root vs Leaf	5-O-(3'-O-Glucosylcaffeoyl)Quinic Acid	Phenolic acids	Phenolic acids	1.30210466	0.010319849	0.047156746	0.062244165	-4.005917594	1

Comparison	Compound	Class I	Class II	VIP	P-value	FDR	Fold Change	Log_2FC	MSI Level
Root vs Leaf	2,3,5,4'-Tetrahydroxystilbene-2-O-glucoside*	Others	Stilbene	1.305215318	0.004700018	0.033855797	0.016164123	-5.95106096	3
Root vs Leaf	1'-Galactoyl Ferulic acid	Phenolic acids	Phenolic acids	1.044781118	0.047765672	0.122869967	0.359867077	-1.474463974	2
Root vs Leaf	8alpha-acetoxy-5alpha-hydroxy-7-oxodecan-10-olide	Others	Others	1.286466154	0.000115123	0.012840208	0.242018352	-2.046811643	1
Root vs Leaf	1-p-Coumaroyl-3-feruloyl-sucrose*	Phenolic acids	Phenolic acids	1.305786379	0.000216569	0.013805514	0.019452051	-5.683933931	1
Root vs Leaf	3-epi-fagomine	Alkaloids	Piperidine alkaloids	1.067718917	0.296023213	0.419386688	14.82229029	3.88969648	3
Root vs Leaf	Rubrofusarin-6-O	Others	Ketone	1.113275144	0.001827764	0.023320316	0.208507078	-2.261831734	3

Comparison	Compound	Class I	Class II	VIP	P-value	FDR	Fold Change	Log_2FC	MSI Level
	-glucoside		compounds						
Root vs Leaf	Kaempferide-3-O-(6'-O-acetyl)glucoside	Flavonoids	Flavonols	1.207265913	0.009882373	0.046628403	0.064272408	-3.959656658	2
Root vs Leaf	Tamarixetin-3-O-rutinoside	Flavonoids	Flavonols	1.199671167	0.013894991	0.055973347	0.085801841	-3.542847583	3
Root vs Leaf	Swertiamarin	Terpenoids	Monoterpenoids	1.302910874	0.009871052	0.046628403	0.049255881	-4.343560214	3
Root vs Leaf	Methylhesperidin	Flavonoids	Flavanones	1.295218775	0.05364506	0.132754372	0.01637271	-5.93256304	3
Root vs Leaf	4-(Beta-D-Glucopyranosyloxy)-2,4',6-Trihydroxybenzophenone	Others	Ketone compounds	1.302532829	0.015213727	0.059024889	0.031436521	-4.991414605	2

Comparison	Compound	Class I	Class II	VIP	P-value	FDR	Fold Change	Log_2FC	MSI Level
Root vs Leaf	3'-p-Coumaroyl-3-feruloyl-sucrose*	Phenolic acids	Phenolic acids	1.303572456	0.003749587	0.031498613	0.146325887	-2.772743072	1
Root vs Leaf	2R-hydroxy-9Z,12Z,15Z-octadecatrienoic acid	Lipids	Free fatty acids	1.213478821	0.192957901	0.328736334	65.68589088	6.037511612	2
Root vs Leaf	9-Oxo-10,12-Octadecadienoic Acid	Lipids	Free fatty acids	1.214996646	0.222849402	0.351004051	28.31616853	4.823554162	3
Root vs Leaf	Quercetin-5,4'-di-O-glucoside	Flavonoids	Flavonols	1.305287995	0.001413425	0.022625269	0.083342264	-3.58480789	3
Root vs Leaf	3-Methyl-4,8-dihydroxy-3,4-dihydroisocoumarin	Lignans and Coumarins	Coumarins	1.182398172	0.002387995	0.025802284	0.091210379	-3.454658184	3
Root vs Leaf	6-beta-d-glucopyr	Alkaloids	Plumerane	1.302461656	0.026053265	0.080442578	0.008498231	-6.878621725	3

Comparison	Compound	Class I	Class II	VIP	P-value	FDR	Fold Change	Log_2FC	MSI Level
	anosyloxyindole-3-carboxylic acid								
Root vs Leaf	8,11-Heptadecadienoic acid	Lipids	Free fatty acids	1.050380788	0.21942439	0.347365541	7.834602908	2.969860154	2
Root vs Leaf	1-(2,3-dihydroxypropoxy)-3-(((2-(dimethylamino)ethoxy)(hydroxy)phosphoryl)oxy)propan-2-yl (E)-hexadec-9-enoate	Lipids	LPE	1.287629392	0.056697086	0.138443392	6.411592466	2.680682728	2
Root vs Leaf	Asp-Pro-Phe	Amino acids and	Amino acids and derivatives	1.302212171	0.00791446	0.042229995	0.10614972	-3.235827524	3

Comparison	Compound	Class I	Class II	VIP	P-value	FDR	Fold Change	Log_2FC	MSI Level
		derivatives							
Root vs Leaf	2-(2,3,4,5-tetramethoxycinnamoyl)-6-(4-hydroxycinnamoyl)-glucoside	Phenolic acids	Phenolic acids	1.262558422	0.082027504	0.181806601	0.114966681	-3.120712293	2
Root vs Leaf	Sibiricose A2	Phenolic acids	Phenolic acids	1.27428689	0.076619165	0.172833001	0.101424679	-3.301519353	3
Root vs Leaf	L-Leucyl-L-phenylalanine	Amino acids and derivatives	Amino acids and derivatives	1.015814108	0.394716488	0.499935501	70.64291985	6.142473069	3
Root vs Leaf	Grasshopper ketone	Terpenoids	Monoterpenoids	1.186822655	0.006983811	0.040284231	0.133293917	-2.907317156	3
Root vs Leaf	7,4'-Dihydroxydi	Flavonoids	Flavanones	1.197671753	0.001604556	0.023084686	0.083805212	-3.576816213	3

Comparison	Compound	Class I	Class II	VIP	P-value	FDR	Fold Change	Log_2FC	MSI Level
	hydroflavonoids								
Root vs Leaf	3-Hydroxyurs-12-en-28-oic acid (Ursolic acid)	Terpenoids	Triterpene	1.267514414	0.125346617	0.246853464	0.037637832	-4.731672657	1
Root vs Leaf	Isocitric acid-1-O-diglucoside	Organic acids	Organic acids	1.073774967	0.009387584	0.046001289	0.228989191	-2.126648594	3
Root vs Leaf	Kelampayoside A[3,4,5-Trimethoxyphenol-β-D-apiosyl-(1→6)-β-D-glucoside]	Phenolic acids	Phenolic acids	1.219201029	0.134981454	0.259284375	12.30409855	3.621067058	3
Root vs Leaf	2,2-Dimethylsucci	Organic	Organic acids	1.140858567	0.314049914	0.434760963	30.78718308	4.944257967	3

Comparison	Compound	Class I	Class II	VIP	P-value	FDR	Fold Change	Log_2FC	MSI Level
	nic acid	acids							
Root vs Leaf	Terephthalic acid	Phenolic acids	Phenolic acids	1.258876989	0.010757931	0.047787654	0.162171411	-2.624408582	3
Root vs Leaf	6-Hydroxyluteolin	Flavonoids	Flavones	1.171802026	0.00407897	0.032253374	0.095924326	-3.381959462	3
Root vs Leaf	Siegesbeckic acid	Terpenoids	Ditepenoids	1.265692581	0.015080147	0.058611864	0.008140218	-6.940716816	3
Root vs Leaf	Kaempferol-3-(2''-acetylrhamnoside)	Flavonoids	Flavonols	1.300263049	0.035068624	0.0993228	0.016278491	-5.940889197	1
Root vs Leaf	4-hydroxyphenyl acrylaldehyde	Others	Aldehyde compounds	1.304172808	0.00330396	0.029873885	0.118893644	-3.072256509	3
Root vs Leaf	3,5-Dimethoxy-4-	Others	Aldehyde	1.235350617	0.10002949	0.208250219	13.61923356	3.767573611	3

Comparison	Compound	Class I	Class II	VIP	P-value	FDR	Fold Change	Log_2FC	MSI Level
	hydroxybenzaldehyde		compounds						
Root vs Leaf	4-O-galactopyranosylxylose	Others	Saccharides	1.293400184	0.007168853	0.040362051	0.267765429	-1.900958385	1
Root vs Leaf	3,4,5-Trimethoxyphenol-β-D-apiofuranosyl-(1→6)-O-β-D-glucopyranoside	Others	Others	1.294686081	0.021298736	0.071692474	32.19396701	5.008718455	3
Root vs Leaf	Glycidyl Linoleate	Lipids	Glycerol ester	1.152638122	0.28005146	0.404539576	28.97260854	4.856617678	3
Root vs Leaf	Afzelechin (3,5,7,4'-Tetrahyd	Flavonoids	Flavanols	1.26977836	0.063019516	0.149162294	0.107527073	-3.217228147	3

Comparison	Compound	Class I	Class II	VIP	P-value	FDR	Fold Change	Log_2FC	MSI Level
	roxyflavan)								
Root vs Leaf	Hordenine	Alkaloids	Alkaloids	1.296805307	0.019220923	0.06701828	0.093735047	-3.415267629	3
Root vs Leaf	4-O-Feruloylquinic acid	Phenolic acids	Phenolic acids	1.272085404	0.050471032	0.126722156	0.118002407	-3.083111811	1
Root vs Leaf	Sinapaldehyde-4-O-Glucoside	Others	Aldehyde compounds	1.031263756	0.088235951	0.190925514	0.470058758	-1.089086988	3
Root vs Leaf	3-Hydroxyphloretin-4'-O-glucoside	Flavonoids	Chalcones	1.257537124	0.037851028	0.104066249	0.037275513	-4.745627988	2
Root vs Leaf	1,2,9,10-tetraethoxy-6-methyl-5,6,6a,7-tetrahydro-4H-dibenzo[de,g]quinoline	Alkaloids	Alkaloids	1.271744524	0.02800132	0.084276954	0.020587369	-5.602096689	2

Comparison	Compound	Class I	Class II	VIP	P-value	FDR	Fold Change	Log_2FC	MSI Level
Root vs Leaf	Kaempferol-3-O-sambubioside	Flavonoids	Flavonols	1.1965181	0.028387789	0.085084622	0.074204421	-3.752351044	3
Root vs Leaf	Sesaminol 2-O-Beta-D-Glucoside	Lignans and Coumarins	Lignans	1.305644454	0.000829038	0.019191343	0.063334457	-3.980865572	3
Root vs Leaf	Candicine	Alkaloids	Phenolamine	1.024406734	0.029224742	0.086631918	0.162482266	-2.621645827	3
Root vs Leaf	Catechin-(4alpha->8)-gallocatechin-(4alpha->8)-gallocatechin	Flavonoids	Flavanols	1.299499808	0.013909157	0.055973347	0.111341555	-3.166935954	2
Root vs Leaf	3'-p-Coumaroyl-3-acetyl sucrose	Phenolic acids	Phenolic acids	1.301578259	0.00977866	0.046617244	0.09817567	-3.348490657	2
Root vs Leaf	7,10-dihydroxy-1	Others	Ketone	1.212232209	0.08337663	0.183292875	4.040284184	2.014456772	3

Comparison	Compound	Class I	Class II	VIP	P-value	FDR	Fold Change	Log_2FC	MSI Level
	2-methoxy-2,2-dimethyl-1,11-dioxatetracen-6-one		compounds						
Root vs Leaf	6,7-Dihydroxy-4'-methoxyisoflavone; Texasin	Flavonoids	Isoflavones	1.30279358	0.011988539	0.050699087	0.05320818	-4.232208124	3
Root vs Leaf	LysoPC 20:4	Lipids	LPC	1.287975504	0.080814263	0.179551108	33.52024232	5.066960673	3
Root vs Leaf	Pro-Asp	Amino acids and derivatives	Amino acids and derivatives	1.236637544	0.012970433	0.053592937	0.447099402	-1.16133248	3
Root vs Leaf	Methyl Hydroxycinnamate	Phenolic acids	Phenolic acids	1.247001436	0.019676146	0.068137925	24.31011195	4.603484632	3

Comparison	Compound	Class I	Class II	VIP	P-value	FDR	Fold Change	Log_2FC	MSI Level
Root vs Leaf	Kakispyrone	Others	Others	1.227527831	0.012746428	0.053295824	0.080321657	-3.63806715	2
Root vs Leaf	Delphinidin-3-O-glucoside (Mirtillin)	Flavonoids	Anthocyanidins	1.304937743	0.007391622	0.040957167	0.010861321	-6.524656595	3
Root vs Leaf	Gallocatechin 3-O-gallate	Flavonoids	Flavanols	1.269985075	0.001324161	0.022042878	3.271271012	1.709851286	3
Root vs Leaf	Tricin-7-O-Glucoside*	Flavonoids	Flavones	1.292411812	0.026417008	0.081244038	0.138200891	-2.855161181	3
Root vs Leaf	Lys-Phe	Amino acids and derivatives	Amino acids and derivatives	1.13655765	0.003373845	0.030030429	0.081149745	-3.623269628	3
Root vs Leaf	Rosamultin*	Terpenoids	Triterpene Saponin	1.303630932	0.005816908	0.037400858	0.078288244	-3.675060499	1

Comparison	Compound	Class I	Class II	VIP	P-value	FDR	Fold Change	Log_2FC	MSI Level
Root vs Leaf	Caffeoylagmatine	Alkaloids	Phenolamine	1.055147079	0.004041489	0.032253374	0.173320432	-2.52848636	3
Root vs Leaf	alpha-Hydroxylinoleic acid*	Lipids	Free fatty acids	1.202849669	0.106435787	0.218016812	3.380184245	1.757101886	1
Root vs Leaf	9S-Hydroxy-10E, 12Z-octadecadienoic acid*	Lipids	Free fatty acids	1.202750088	0.102200858	0.21175077	3.117602127	1.640436821	1
Root vs Leaf	Hydroxytyrosol	Others	Alcohol compounds	1.27479284	0.014403093	0.057046076	0.183950232	-2.442612597	3
Root vs Leaf	cya-suc-glu	Flavonoids	Anthocyanidins	1.300703116	0.010607654	0.047459919	0.111015202	-3.171170853	3
Root vs Leaf	(3S,7aS,13aR,15bR)-4,4,7a,10,10,13a,15b-heptamethyl-	Terpenoids	Terpene	1.299074122	0.022245928	0.073397008	0.058295234	-4.10047826	2

Comparison	Compound	Class I	Class II	VIP	P-value	FDR	Fold Change	Log_2FC	MSI Level
	2,3,4,4a,5,7,7a,8,9,9a,10,13,13a,13b,14,15,15a,15b-octadecahydro-1H-cyclohepta[1,2-a:5,4-a']dinaphthalen-3-ol								
Root vs Leaf	2-Deacetyldecinna moyltaxinine E	Others	Others	1.292432798	0.053691495	0.132754372	0.049988601	-4.322257039	2
Root vs Leaf	Comoside	Phenolic acids	Phenolic acids	1.194284947	0.089677488	0.19340624	0.092579283	-3.433166793	3
Root vs Leaf	Gossypetin-7-O-(3''-glucosyl)rhamnoside;	Flavonoids	Flavonols	1.237577408	0.068124694	0.158982142	0.108860243	-3.19945093	3

Comparison	Compound	Class I	Class II	VIP	P-value	FDR	Fold Change	Log_2FC	MSI Level
	Rhodioflavonoside								
Root vs Leaf	7-Hydroxyrutaecarpine	Alkaloids	Plumerane	1.229358369	0.105633314	0.216990106	0.069281065	-3.851395076	3
Root vs Leaf	Corehoionoside C	Terpenoids	Monoterpenoids	1.078243752	0.033658368	0.09646649	0.204094536	-2.292690534	3
Root vs Leaf	LysoPC 15:0(2n isomer)*	Lipids	LPC	1.130199603	0.277051332	0.402087259	14.85086196	3.892474764	3
Root vs Leaf	Buddlenoid A	Flavonoids	Flavonols	1.296920872	0.01916882	0.06701828	0.10585994	-3.239771354	3
Root vs Leaf	5,6,7,7a-tetrahydro-4,4,7a-trimethyl-2(4H)-benzofuranone*	Others	Others	1.284374606	0.004625788	0.033432536	0.184193741	-2.440704056	3

Comparison	Compound	Class I	Class II	VIP	P-value	FDR	Fold Change	Log_2FC	MSI Level
Root vs Leaf	Hymenoside M	Others	Others	1.280815128	0.005943419	0.037628089	0.006610859	-7.240946565	3
Root vs Leaf	3-Hydroxy-11-oxoolean-12-en-30-oic acid (18α-Glycyrrhetinic acid)*	Terpenoids	Triterpene	1.092112696	0.001114631	0.020742409	0.1386626	-2.850349377	2
Root vs Leaf	Medioresinol B	Lignans and Coumarins	Lignans	1.241684347	0.019720832	0.068137925	0.026082973	-5.260747867	3
Root vs Leaf	Yuanhuanin	Flavonoids	Flavones	1.239755515	0.021727065	0.072309564	0.254951157	-1.971707207	2
Root vs Leaf	Syringaldehyde; 4-Hydroxy-3,5-Dimethoxybenzaldehyde	Others	Aldehyde compounds	1.282322006	0.079339581	0.177671331	38.64354949	5.27215571	3

Comparison	Compound	Class I	Class II	VIP	P-value	FDR	Fold Change	Log_2FC	MSI Level
Root vs Leaf	Marmesinin	Lignans and Coumarins	Coumarins	1.300502588	0.016431806	0.061222642	0.062986555	-3.988812288	3
Root vs Leaf	Poncirin (Isosakuranetin-7-O-neohesperidoside)*	Flavonoids	Flavanones	1.301724633	0.005955024	0.037628089	0.174420286	-2.51936025	3
Root vs Leaf	Dihydroactinidiolide*	Others	Lactones	1.296369902	0.007228188	0.040362051	0.187107976	-2.418057038	3
Root vs Leaf	picrorhizin	Phenolic acids	Phenolic acids	1.084260481	0.074217296	0.168647294	5.761111594	2.526347204	3
Root vs Leaf	2-trans-abscisic acid D-glucosyl ester	Organic acids	Organic acids	1.18320475	0.003970438	0.032250367	0.134568311	-2.893589375	2

Comparison	Compound	Class I	Class II	VIP	P-value	FDR	Fold Change	Log_2FC	MSI Level
Root vs Leaf	Epicatechin-(4β→8)-epigallocatechin	Flavonoids	Flavanols	1.305484083	0.002819628	0.027080964	0.017337183	-5.849986646	2
Root vs Leaf	2-Linoleoylglycerol*	Lipids	Glycerol ester	1.227585914	0.255322339	0.382099428	45.50285451	5.507885147	3
Root vs Leaf	Rubuside I*	Terpenoids	Triterpene Saponin	1.299290724	0.021567255	0.072038699	0.06397811	-3.966277818	1
Root vs Leaf	3-O-Feruloylquinic acid	Phenolic acids	Phenolic acids	1.30355774	0.005448374	0.03656502	0.113897721	-3.134189219	1
Root vs Leaf	γ-Glu-Tyr	Amino acids and derivatives	Amino acids and derivatives	1.033878768	0.401473115	0.505290275	195.1931845	7.60875887	3
Root vs Leaf	2,4,6-Trihydroxyb	Others	Ketone	1.234880769	0.020671983	0.070019052	0.213073132	-2.23057941	3

Comparison	Compound	Class I	Class II	VIP	P-value	FDR	Fold Change	Log_2FC	MSI Level
	enzophenone		compounds						
Root vs Leaf	3,4-dihydroxyphenylethanol-β-D-glucopyranoside	Others	Alcohol compounds	1.28123713	0.058259836	0.141301353	0.11817723	-3.080976008	3
Root vs Leaf	icariside D1	Others	Ketone compounds	1.178654361	0.010274725	0.047156746	0.102814501	-3.281884339	3
Root vs Leaf	(2S,3S,4S,5R,6R)-3-(3-benzoyloxy-2-hydroxypropoxy)-3,4,5-trihydroxyoxane-2-carboxylic acid	Phenolic acids	Phenolic acids	1.034601554	0.412098664	0.507524938	438.0124844	8.774828181	3
Root vs Leaf	1-Linoleoylglycer	Lipids	Glycerol ester	1.215447639	0.273588821	0.399837811	44.26177773	5.467989492	3

Comparison	Compound	Class I	Class II	VIP	P-value	FDR	Fold Change	Log_2FC	MSI Level
	ol*								
Root vs Leaf	1,3,6-trihydroxy-2,5,7-trimethoxyxanthen-9-one	Flavonoids	Other Flavonoids	1.301414037	0.010484666	0.047173513	0.117228001	-3.092610882	3
Root vs Leaf	L-Tartaric acid	Organic acids	Organic acids	1.22814248	0.035219653	0.099359883	0.340583197	-1.553920834	3
Root vs Leaf	Pectolinarigenin-7-O-glucoside	Flavonoids	Flavones	1.126453058	0.007753467	0.04213351	0.176530551	-2.502010211	3
Root vs Leaf	Kaempferol-3-O-(2''-p-Coumaroyl)glucoside	Flavonoids	Flavonols	1.149580072	0.070223955	0.161784614	0.066691418	-3.906355068	3
Root vs Leaf	Blumenol A	Terpenoids	Monoterpenoids	1.25898355	0.01542457	0.059628796	0.140168971	-2.834761078	3

Comparison	Compound	Class I	Class II	VIP	P-value	FDR	Fold Change	Log_2FC	MSI Level
Root vs Leaf	LysoPC 15:0*	Lipids	LPC	1.173130847	0.24888418	0.376110989	18.7556562	4.229253834	3
Root vs Leaf	Prudomenin*	Flavonoids	Flavonols	1.301471046	0.010425965	0.047173513	0.124552332	-3.005176057	3
Root vs Leaf	3-(triazan-2-yl)propyl L-prolinate	Amino acids and derivatives	Amino acids and derivatives	1.125013848	0.200287911	0.333967729	10.92925971	3.450123779	3
Root vs Leaf	2-Methylthio-cis-zeatin riboside	Alkaloids	Alkaloids	1.22603025	7.02318E-05	0.012840208	0.048089682	-4.378128818	3
Root vs Leaf	Hesperetin	Flavonoids	Flavanones	1.304210854	0.002380459	0.025802284	0.185115176	-2.433504917	3
Root vs Leaf	2,3,19-Trihydroxyolean-12-en-28-oic acid (Arjunic acid)	Terpenoids	Triterpene	1.010665644	0.021565986	0.072038699	0.216467107	-2.207780278	3

Comparison	Compound	Class I	Class II	VIP	P-value	FDR	Fold Change	Log_2FC	MSI Level
Root vs Leaf	Cyanidin 3-O-sophoroside	Flavonoids	Anthocyanidins	1.301433199	0.014466103	0.057046076	0.064157661	-3.962234653	3
Root vs Leaf	Protocatechuic acid 1-O-Rutinoside	Phenolic acids	Phenolic acids	1.258337107	0.090081832	0.193891274	19.80966886	4.308132859	1
Root vs Leaf	Didymin (Isosakuranetin-7-O-rutinoside)*	Flavonoids	Flavanones	1.284195747	0.03518616	0.099359883	0.139952454	-2.836991307	3
Root vs Leaf	1-O-p-Coumaroyl lysine	Alkaloids	Phenolamine	1.291965244	0.018644988	0.065857084	0.160313153	-2.641035299	3
Root vs Leaf	Scandione	Flavonoids	Chalcones	1.294790497	0.026277368	0.081006265	0.07203234	-3.795211424	3
Root vs Leaf	12-hydroxy jasmonoyl sulfate	Organic acids	Organic acids	1.303017121	0.008053352	0.042865255	0.067036115	-3.898917655	1

Comparison	Compound	Class I	Class II	VIP	P-value	FDR	Fold Change	Log_2FC	MSI Level
Root vs Leaf	Pedunculoside	Terpenoids	Triterpene Saponin	1.305176831	0.005689482	0.036921835	0.004857885	-7.685456037	2
Root vs Leaf	5-[(1S,2R,3R,5S)-3,5-dihydroxy-2-[(E,3S)-3-hydroxyoct-1-enyl]cyclopentyl]pentanoic acid	Lipids	Free fatty acids	1.10043914	0.357097539	0.471980295	78.24898558	6.290000144	1
Root vs Leaf	Zanthoxyloside*	Lignans and Coumarins	Coumarins	1.078647905	0.352736807	0.467646773	40.85102661	5.352300429	3
Root vs Leaf	Quercetin-3-O-glucuronide*	Flavonoids	Flavonols	1.166894162	0.134641759	0.259284375	0.302099045	-1.726906472	3
Root vs Leaf	C-Veratroylglycol	Others	Alcohol compounds	1.21966884	0.160341255	0.296405005	10.43484061	3.383336659	3

Comparison	Compound	Class I	Class II	VIP	P-value	FDR	Fold Change	Log_2FC	MSI Level
Root vs Leaf	Quercetin-3-O-(6''-O-acetyl)glucoside	Flavonoids	Flavonols	1.305324487	0.001424006	0.022627044	0.066969468	-3.900352673	3
Root vs Leaf	3,23-Dihydroxylup-20(29)-en-28-oic acid (Oplopanaxogenin C)	Terpenoids	Triterpene	1.298948161	0.011320418	0.049123342	0.17847765	-2.486184669	3
Root vs Leaf	Naringenin chalcone; 2',4,4',6'-Tetrahydroxychalcone	Flavonoids	Chalcones	1.003623792	0.005853943	0.037400858	0.200746912	-2.316550302	3
Root vs Leaf	Luteolin 7-(2-sulfoglucosid	Flavonoids	Flavones	1.287452985	0.031409628	0.091155942	0.14363253	-2.799545563	1

Comparison	Compound	Class I	Class II	VIP	P-value	FDR	Fold Change	Log_2FC	MSI Level
	e)								
Root vs Leaf	9,12-Octadecadien-6-Ynoic Acid	Lipids	Free fatty acids	1.030442356	0.149777032	0.282186718	2.907131557	1.53959636	3
Root vs Leaf	Quercetin-3-O-(2''-O-galloyl)galactoside	Flavonoids	Flavonols	1.298296077	0.014148504	0.056620218	0.176099101	-2.505540547	3
Root vs Leaf	3-quinolinecarboxylic acid	Alkaloids	Quinoline alkaloids	1.295986753	0.017253212	0.063086619	0.151647102	-2.721210167	3
Root vs Leaf	Methyl 4-caffeoylquinate	Phenolic acids	Phenolic acids	1.303611003	0.007650151	0.041959333	0.07190969	-3.79767	3
Root vs Leaf	6-O-alpha-L-arabinopyranosyl-D-glucopyranose	Others	Saccharides	1.144976739	0.013187395	0.054248987	0.461877059	-1.114419205	2

Comparison	Compound	Class I	Class II	VIP	P-value	FDR	Fold Change	Log_2FC	MSI Level
Root vs Leaf	2-(2-Benzothiazolylthio)ethanol	Alkaloids	Alkaloids	1.28307674	0.072268905	0.165613047	0.07136781	-3.808582693	3
Root vs Leaf	5-O-p-Coumaroyl Methylglucaric Acid*	Phenolic acids	Phenolic acids	1.280914891	0.012583755	0.052802902	0.013424384	-6.219000313	1
Root vs Leaf	L-Citramalic acid	Organic acids	Organic acids	1.228417932	0.023965355	0.076385151	0.156291405	-2.677689654	3
Root vs Leaf	mudanoside A	Phenolic acids	Phenolic acids	1.128689587	0.019430392	0.067506555	0.388710934	-1.363230405	3
Root vs Leaf	Hydrocinnamic acid	Phenolic acids	Phenolic acids	1.194352706	0.197282793	0.33127477	25.36592089	4.664819632	3
Root vs Leaf	N-(Carboxymethyl)glycine	Amino acids and	Amino acids and derivatives	1.272603231	0.051351734	0.128290287	5.193394854	2.376677918	3

Comparison	Compound	Class I	Class II	VIP	P-value	FDR	Fold Change	Log_2FC	MSI Level
		derivatives							
Root vs Leaf	Gentioluteol	Lignans and Coumarins	Lignans	1.302826851	0.01385183	0.055973347	0.030178945	-5.05031382	3
Root vs Leaf	quinatoside A	Terpenoids	Triterpene Saponin	1.296971192	0.033499211	0.096137841	0.061814422	-4.015912713	3
Root vs Leaf	2'-Deoxycytidine	Nucleotides and derivatives	Nucleotides and derivatives	1.190224548	0.083013344	0.183052894	6.081365614	2.604395328	3
Root vs Leaf	L-Aspartic acid-O-diglucoside	Amino acids and derivatives	Amino acids and derivatives	1.059730326	0.193153397	0.328736334	3.743852087	1.904523438	3
Root vs Leaf	Gingerglycolipid C	Lipids	Glycerol ester	1.215644619	0.062967081	0.149162294	4.786400143	2.258941011	2

Comparison	Compound	Class I	Class II	VIP	P-value	FDR	Fold Change	Log_2FC	MSI Level
Root vs Leaf	1-O-(6'-O-feruloyl)glucoside-3-O-C affeoyl Quinic Acid	Phenolic acids	Phenolic acids	1.276667268	0.05048587	0.126722156	0.125183283	-2.997886179	2
Root vs Leaf	2-Methylglutaric acid	Organic acids	Organic acids	1.168007731	0.09957922	0.207896238	2.631015067	1.39561951	3
Root vs Leaf	Aurantio-obtusin-6-O-Glucoside	Quinones	Anthraquinone	1.166464553	0.014371972	0.057046076	0.254718844	-1.973022399	3
Root vs Leaf	pyridoxamine	Alkaloids	Pyridine alkaloids	1.23572857	0.016569077	0.061416425	0.129209709	-2.952213617	3
Root vs Leaf	N-methylphenyla lanine	Amino acids and derivatives	Amino acids and derivatives	1.098077254	0.342688886	0.459113876	42.31417517	5.403069139	3

Comparison	Compound	Class I	Class II	VIP	P-value	FDR	Fold Change	Log_2FC	MSI Level
Root vs Leaf	2',4'-Dihydroxyacetophenone*	Others	Ketone compounds	1.255396122	0.066939667	0.1567244	4.72327061	2.239786194	3
Root vs Leaf	Cleomiscosin C	Lignans and Coumarins	Coumarins	1.252000518	0.127596309	0.249986967	75.0829154	6.230412765	3
Root vs Leaf	Clemiscosin D	Terpenoids	Triterpene Saponin	1.253755987	0.131161289	0.253750712	91.33239443	6.513054751	3
Root vs Leaf	Phellodenol H	Lignans and Coumarins	Coumarins	1.241416973	0.001346441	0.022042878	0.070985729	-3.816327167	3
Root vs Leaf	(6R,9R)-3-Oxo-α-ionol-β-D-glucoside	Terpenoids	Sesquiterpenoids	1.299999726	0.019832639	0.068235021	0.042425402	-4.558927866	2
Root vs Leaf	(3S,5R,6R,7E,9S)-megastigm-7-ene-	Others	Others	1.189857145	0.048388855	0.123749486	0.081479852	-3.617412829	1

Comparison	Compound	Class I	Class II	VIP	P-value	FDR	Fold Change	Log_2FC	MSI Level
	3,5,6,9-tetrol3-(beta-D-glucopyranoside)								
Root vs Leaf	Hesperetin-6-C-glucoside-7-O-glucoside	Flavonoids	Flavanones	1.299441739	0.010920815	0.048130729	0.166509406	-2.586324422	3
Root vs Leaf	Phellatin	Flavonoids	Flavonols	1.195935725	0.001010164	0.02015943	0.118754459	-3.073946409	3
Root vs Leaf	Ent-16beta-Methoxy-19-Kauranoic Acid	Terpenoids	Ditepenoids	1.1128756	0.042173772	0.112405679	0.247870381	-2.012342207	3
Root vs Leaf	S-(Methyl)glutathione	Amino acids and derivatives	Amino acids and derivatives	1.273608908	0.050718266	0.127148693	14.39263436	3.847258775	3

Comparison	Compound	Class I	Class II	VIP	P-value	FDR	Fold Change	Log_2FC	MSI Level
Root vs Leaf	Ent-15-oxo-2β,16,19-trihydroxypimar-8(14)-ene	Terpenoids	Ditepenoids	1.303895378	0.01612601	0.06081729	0.011589347	-6.43105694	3
Root vs Leaf	Vanillin; 4-Hydroxy-3-Methoxybenzaldehyde*	Others	Aldehyde compounds	1.251063362	0.034662281	0.098689312	7.606821341	2.927293721	3
Root vs Leaf	Echinocystic acid	Terpenoids	Triterpene	1.291497879	0.02367634	0.075994966	0.15476321	-2.69186554	3
Root vs Leaf	L-Glutamyl-L-Tyrosine	Amino acids and derivatives	Amino acids and derivatives	1.022845447	0.40536779	0.507524938	238.0415007	7.895069308	3
Root vs Leaf	1,3,7-trihydroxy-2-[3,4,5-trihydroxy	Flavonoids	Other Flavonoids	1.296078956	0.076776899	0.173008215	0.013292441	-6.233250087	3

Comparison	Compound	Class I	Class II	VIP	P-value	FDR	Fold Change	Log_2FC	MSI Level
	-6-(hydroxymethyl)oxan-2-yl]xanthen-9-one								
Root vs Leaf	Teupolioside	Amino acids and derivatives	Amino acids and derivatives	1.13658983	0.015598397	0.059797609	0.085312858	-3.551092997	3
Root vs Leaf	Cytidine	Nucleotides and derivatives	Nucleotides and derivatives	1.279643301	0.000334809	0.01464066	2.246448693	1.167646112	3
Root vs Leaf	2-Deoxyribose-1-phosphate	Nucleotides and derivatives	Nucleotides and derivatives	1.191096974	0.008876655	0.044923775	0.197572232	-2.339547902	2
Root vs Leaf	Humulene	Terpenoids	Sesquiterpenoi	1.303525695	0.006702218	0.039572387	0.064800753	-3.947845612	1

Comparison	Compound	Class I	Class II	VIP	P-value	FDR	Fold Change	Log_2FC	MSI Level
	epoxide II		ds						
Root vs Leaf	Staphylionoside A	Terpenoids	Sesquiterpenoids	1.050858653	0.029147732	0.086522319	0.200355673	-2.319364735	2
Root vs Leaf	Urolignoside	Lignans and Coumarins	Lignans	1.089622385	0.126691056	0.24866428	4.934846161	2.303005111	2
Root vs Leaf	Dehydrodiconiferyl alcohol*	Lignans and Coumarins	Lignans	1.095706505	0.001878192	0.023320316	0.112688265	-3.14959081	3
Root vs Leaf	Trans-Ferulic Acid 4-O-[Apiosyl-(1-> 6)-Glucosyl] Ester	Phenolic acids	Phenolic acids	1.039722927	0.055322762	0.135547039	0.394026927	-1.343633872	2
Root vs Leaf	Aloeemodin-8-O-glucoside	Quinones	Anthraquinone	1.215146083	0.008845025	0.044868778	0.029189873	-5.098388248	3

Comparison	Compound	Class I	Class II	VIP	P-value	FDR	Fold Change	Log_2FC	MSI Level
Root vs Leaf	Octadeca-2,9,12,15-tetraen-1-ol	Others	Others	1.193348343	0.25443106	0.381731712	25.69176035	4.683233839	3
Root vs Leaf	Clemaphenol A*	Lignans and Coumarins	Lignans	1.144898912	0.004339638	0.033039104	0.118522097	-3.076772034	3
Root vs Leaf	galloyl malonyl glucoside	Phenolic acids	Phenolic acids	1.273828715	0.063246277	0.149535235	0.134390386	-2.895498163	1
Root vs Leaf	Dicaffeoylquinic acid-O-glucoside	Phenolic acids	Phenolic acids	1.258347242	0.004965344	0.034838011	0.112873904	-3.147216113	2
Root vs Leaf	Mearnsetin	Flavonoids	Flavonols	1.300587395	0.009915661	0.046683538	0.119002035	-3.070941849	3
Root vs Leaf	Emodin-8-O-glucoside	Quinones	Anthraquinone	1.298226077	0.012844002	0.053295824	0.14583305	-2.777610386	3
Root vs Leaf	5-Acetylsalicylic	Phenolic	Phenolic acids	1.042285659	0.046553421	0.120625829	0.219497299	-2.187724907	3

Comparison	Compound	Class I	Class II	VIP	P-value	FDR	Fold Change	Log_2FC	MSI Level
	acid	acids							
Root vs Leaf	3-Dehydroshikimic acid	Organic acids	Organic acids	1.224321796	6.64214E-05	0.012840208	0.074129685	-3.753804807	3
Root vs Leaf	Delphinidin-3-O-galactoside	Flavonoids	Anthocyanidins	1.305599118	0.000531808	0.016186447	0.072039574	-3.795066537	3
Root vs Leaf	Cannabifolin E*	Terpenoids	Triterpene	1.304273183	0.00280951	0.027080964	0.183072058	-2.449516481	2
Root vs Leaf	Wistin	Flavonoids	Isoflavones	1.212127983	0.022126542	0.073224284	0.103963841	-3.265846258	3
Root vs Leaf	Sedumoside H	Others	Others	1.254383598	0.000531533	0.016186447	0.017960173	-5.799054955	3
Root vs Leaf	Homovanilloylquinic acid	Phenolic acids	Phenolic acids	1.297382735	0.025939659	0.080308886	0.074160669	-3.753201937	3
Root vs Leaf	Grayanotoxin VIII	Terpenoids	Ditepenoids	1.282753472	0.011716995	0.049941667	0.023808622	-5.392372064	3

Comparison	Compound	Class I	Class II	VIP	P-value	FDR	Fold Change	Log_2FC	MSI Level
Root vs Leaf	2-Palmitoyl-Sn-Glycerol 3-O-Diglucoside	Lipids	Glycerol ester	1.094367101	0.03783643	0.104066249	0.426508197	-1.229354626	1
Root vs Leaf	1-O-p-Coumaroyl Methylglucaric Acid*	Phenolic acids	Phenolic acids	1.304227724	0.01320452	0.054248987	0.009150783	-6.771889026	1
Root vs Leaf	3S-3-hydroxy-β-damascon	Terpenoids	Monoterpenoids	1.221267599	0.006387725	0.038725464	0.043289789	-4.529829421	3
Root vs Leaf	Isorhamnetin-3-O-sophoroside*	Flavonoids	Flavonols	1.038995771	0.020396449	0.069326645	0.345885027	-1.531635531	3
Root vs Leaf	Gardenoside	Terpenoids	Monoterpenoids	1.261640459	0.122444453	0.241968078	28.53578418	4.834700305	3
Root vs Leaf	7-O-methylmyric	Flavonoids	Flavonols	1.28577545	0.095574099	0.202287589	0.020103202	-5.636430908	1

Comparison	Compound	Class I	Class II	VIP	P-value	FDR	Fold Change	Log_2FC	MSI Level
	etin								
Root vs Leaf	epiloliolide	Others	Others	1.208814945	0.039173516	0.106886323	0.025352066	-5.301752874	3
Root vs Leaf	L-Valyl-L-Leucine	Amino acids and derivatives	Amino acids and derivatives	1.080166285	0.406140672	0.507524938	365.3359572	8.513079944	3
Root vs Leaf	5'-Deoxyadenosine*	Nucleotides and derivatives	Nucleotides and derivatives	1.305654901	0.000331011	0.01464066	0.056116399	-4.155433765	3
Root vs Leaf	LysoPC 19:1	Lipids	LPC	1.232646	0.233589922	0.361078556	31.45834868	4.975371037	2
Root vs Leaf	Tababiphenyl C	Others	Chromone	1.159563663	0.00366431	0.030931932	0.111772316	-3.161365192	3
Root vs Leaf	Uridine-5'-Diphosphate-D-Xylose	Nucleotides and	Nucleotides and derivatives	1.233929516	0.099667054	0.207896238	6.20513547	2.633462708	2

Comparison	Compound	Class I	Class II	VIP	P-value	FDR	Fold Change	Log_2FC	MSI Level
		derivatives							
Root vs Leaf	Dihydromarein	Flavonoids	Chalcones	1.167520835	0.048695017	0.123945739	0.228690071	-2.128534363	2
Root vs Leaf	Grayanoside C	Terpenoids	Ditepenoids	1.304715715	0.016806095	0.062082001	0.002082092	-8.907750543	2
Root vs Leaf	3,4,5-Trimethoxy phenol-1-O-β-D-g lucoside	Others	Others	1.199681701	0.05953051	0.14325772	4.545160654	2.184331289	3
Root vs Leaf	Capillarisin	Others	Chromone	1.031844247	0.015531934	0.059797609	0.301521384	-1.729667771	3
Root vs Leaf	5-O-p-Coumaroyl shikimic acid O-glucoside	Phenolic acids	Phenolic acids	1.295867538	0.000156505	0.013805514	0.397335514	-1.331570346	3
Root vs Leaf	7,2'-dihydroxy-4'-methoxy-isoflava	Flavonoids	Other Flavonoids	1.305332977	0.002732956	0.026984272	0.012995438	-6.265850895	3

Comparison	Compound	Class I	Class II	VIP	P-value	FDR	Fold Change	Log_2FC	MSI Level
	nol glucoside								
Root vs Leaf	Delphinidin-3-O-glucuronide	Flavonoids	Anthocyanidins	1.204741628	0.037447193	0.103615089	0.376596416	-1.408908826	3
Root vs Leaf	Patuletin (Quercetagetin-6-methyl ether)	Flavonoids	Flavonols	1.264832019	0.146465881	0.27764278	0.034936406	-4.839124985	2
Root vs Leaf	6'-O-Sinapoylsucrose*	Phenolic acids	Phenolic acids	1.304551806	0.00267071	0.02684374	0.093570839	-3.417797193	2
Root vs Leaf	5,6-Dihydroxylucidin	Quinones	Anthraquinone	1.298867083	0.011424058	0.04934055	0.1711223	-2.546900313	1
Root vs Leaf	dGDP	Nucleotides and derivatives	Nucleotides and derivatives	1.197712331	0.001162157	0.020755546	0.218832813	-2.192099018	1

Comparison	Compound	Class I	Class II	VIP	P-value	FDR	Fold Change	Log_2FC	MSI Level
Root vs Leaf	Luteolin 7-rutinoside-4'-glucoside	Flavonoids	Flavones	1.302017553	0.009986652	0.046813787	0.079177886	-3.658758646	2
Root vs Leaf	Veratraldehyde	Phenolic acids	Phenolic acids	1.28704419	0.064792145	0.152522686	0.055920722	-4.1604732	3
Root vs Leaf	β-Oxoacteoside	Phenolic acids	Phenolic acids	1.305538801	0.002194286	0.025163593	0.003608518	-8.114377937	3
Root vs Leaf	Furanofructosyl-α-D-(3-mustard acyl)glucoside*	Phenolic acids	Phenolic acids	1.305392812	0.003477215	0.030288731	0.011043319	-6.500682416	2
Root vs Leaf	Cyanidin-3-O-gentiobioside*	Flavonoids	Anthocyanidins	1.303138614	0.01948574	0.067590182	0.007700174	-7.020893299	3
Root vs Leaf	Morin	Flavonoids	Flavonols	1.282343185	0.04679858	0.120935476	0.152010511	-2.717757013	1

Comparison	Compound	Class I	Class II	VIP	P-value	FDR	Fold Change	Log_2FC	MSI Level
Root vs Leaf	Hesperetin-8-C-glucoside-3'-O-glucoside	Flavonoids	Flavanones	1.266712882	0.098057888	0.205531617	0.083499367	-3.582090935	3
Root vs Leaf	10-hydroxymajoroside	Terpenoids	Monoterpenoids	1.282571782	0.119756632	0.238519891	95.40722119	6.57602656	3
Root vs Leaf	Nicotianamine	Alkaloids	Alkaloids	1.231683208	0.04639322	0.12036489	4.458350443	2.156510022	3
Root vs Leaf	Neolinustatin	Alkaloids	Alkaloids	1.246760311	0.007691275	0.04197183	0.170150638	-2.555115536	3
Root vs Leaf	3,4-dimethoxy-2-[(3,4,5-trihydroxy-6-{[(3,4,5-trihydroxy-6-methyloxan-2-yl)oxy]methyl}oxan-2-yl)oxy]xant	Flavonoids	Other Flavonoids	1.117613436	0.004593291	0.033309066	0.090962013	-3.458592001	3

Comparison	Compound	Class I	Class II	VIP	P-value	FDR	Fold Change	Log_2FC	MSI Level
	hen-9-one								
Root vs Leaf	Lupa-1,20(29)-dien-3-one (Glochidone)	Terpenoids	Triterpene	1.292203148	0.036793391	0.102563272	0.06043514	-4.048468546	3
Root vs Leaf	Jasmonoyl-L-Isoleucine	Amino acids and derivatives	Amino acids and derivatives	1.206267525	0.048610614	0.123945739	0.275287737	-1.860987748	3
Root vs Leaf	Dehydroascorbic acid	Others	Vitamin	1.241250198	0.016015251	0.060659572	0.066611503	-3.908084861	2
Root vs Leaf	Glu-Leu	Amino acids and derivatives	Amino acids and derivatives	1.25167222	0.208847205	0.340104604	37.63694291	5.234077544	2
Root vs Leaf	Synephrine;	Alkaloids	Alkaloids	1.254087064	0.032065261	0.092390706	0.285900053	-1.806417206	3

Comparison	Compound	Class I	Class II	VIP	P-value	FDR	Fold Change	Log$_2$FC	MSI Level
	4-[1-Hydroxy-2-(methylamino)ethyl]phenol								
Root vs Leaf	Phloroglucinol-1-O-β-D-glucopyranoside	Others	Others	1.03029938	0.04015459	0.108467586	0.393498962	-1.345568265	1
Root vs Leaf	Kaempferol-3-O-(6''-p-Coumaroyl)glucoside (Tiliroside)	Flavonoids	Flavonols	1.291540442	0.021825189	0.0724489	0.149453251	-2.742233814	2
Root vs Leaf	Dihydrocaffeoylglucose*	Phenolic acids	Phenolic acids	1.275295659	0.016936768	0.062351544	0.361710142	-1.467094046	1
Root vs Leaf	1-O-(3,4,5-Trimet	Phenolic	Phenolic acids	1.206781026	0.025713377	0.079951955	2.917005427	1.54448807	2

Comparison	Compound	Class I	Class II	VIP	P-value	FDR	Fold Change	Log_2FC	MSI Level
	hoxybenzoyl)-B-D-Glucopyranoside	acids							
Root vs Leaf	3-Hydroxy-urs-11-en-28,13-lactone	Terpenoids	Triterpene	1.127462636	0.041477827	0.110932653	0.355637798	-1.491519431	3
Root vs Leaf	Glycerol 9,11,13-octadecatrienoyl ester*	Lipids	Glycerol ester	1.235925537	0.130332207	0.252599012	8.228811209	3.040684024	3
Root vs Leaf	Phenyl acetate	Phenolic acids	Phenolic acids	1.076914221	0.362161431	0.476474548	35.72584389	5.158896185	3
Root vs Leaf	Syringin	Others	Alcohol compounds	1.040868605	0.051481295	0.128465447	0.369999229	-1.43440583	2
Root vs Leaf	2-Picolinic acid	Alkaloids	Pyridine	1.160896794	0.208819538	0.340104604	8.018056554	3.003252593	3

Comparison	Compound	Class I	Class II	VIP	P-value	FDR	Fold Change	Log_2FC	MSI Level
			alkaloids						
Root vs Leaf	D-Threose	Others	Saccharides	1.214706831	0.174155832	0.314640775	12.16958878	3.605208514	3
Root vs Leaf	1,6-Di-O-caffeoyl-β-D-glucose*	Phenolic acids	Phenolic acids	1.250095616	0.008306037	0.043550477	0.085211107	-3.552814695	2
Root vs Leaf	1-α-Linolenoyl-glycerol*	Lipids	Glycerol ester	1.19191715	0.158461855	0.29368445	7.117333927	2.831336925	3
Root vs Leaf	LysoPE 15:0*	Lipids	LPE	1.299206691	0.044364968	0.116633451	163.9605887	7.357205265	3
Root vs Leaf	1-O-p-Coumaroyl galactaric acid	Phenolic acids	Phenolic acids	1.304946311	0.001885186	0.023320316	0.086841285	-3.525475109	3
Root vs Leaf	Umckalin (7-hydroxy-5,6-dimethoxycoumari	Lignans and Coumarins	Coumarins	1.152400316	0.218124437	0.346593315	13.31426872	3.734901286	1

Comparison	Compound	Class I	Class II	VIP	P-value	FDR	Fold Change	Log_2FC	MSI Level
	n)								
Root vs Leaf	1-Monolinolenoyl -Rac-Glycerol*	Lipids	Glycerol ester	1.201136599	0.15055791	0.282917951	8.083163508	3.014920031	3
Root vs Leaf	3-O-p-Coumaroyl shikimic acid-O-glucoside	Phenolic acids	Phenolic acids	1.015698193	0.022279559	0.073397008	0.419415547	-1.253547753	3
Root vs Leaf	Olean-13(18)-en-3 -one (δ-Amyrenone)	Terpenoids	Triterpene	1.303123109	0.008818017	0.044837025	0.051745924	-4.272410967	1
Root vs Leaf	1,3-O-di-Caffeoyl-4-O-glucoside Quinic Acid	Phenolic acids	Phenolic acids	1.171275934	0.000702176	0.017644209	0.094082046	-3.40993676	2
Root vs Leaf	3-oxo-taraxeran-1	Terpenoids	Triterpene	1.212567721	0.006732601	0.039643461	0.044454663	-4.491521441	2

Comparison	Compound	Class I	Class II	VIP	P-value	FDR	Fold Change	Log_2FC	MSI Level
	4-en								
Root vs Leaf	Lup-20(29)-en-3-one (Lupenone)	Terpenoids	Triterpene	1.304115797	0.005030576	0.034920839	0.054804954	-4.189549872	2
Root vs Leaf	3,6-Di-O-caffeoyl glucose*	Phenolic acids	Phenolic acids	1.199124843	0.034945696	0.099104526	0.059914284	-4.060956207	2
Root vs Leaf	1-O-Sinapoyl-β-D-glucose	Phenolic acids	Phenolic acids	1.219061238	0.000188006	0.013805514	0.08619949	-3.536176849	2
Root vs Leaf	Quercetin-5-O-glucuronide*	Flavonoids	Flavonols	1.126080268	0.118173548	0.236019442	0.455446814	-1.134645507	3
Root vs Leaf	Gossypetin-7-O-L-rhamnopyranoside(Rhodiolgin)	Flavonoids	Flavonols	1.297497765	0.014720197	0.057627437	0.136683063	-2.871093608	3

Comparison	Compound	Class I	Class II	VIP	P-value	FDR	Fold Change	Log_2FC	MSI Level
Root vs Leaf	3,7-dihydroxy-2-methoxy-8,8,10-trimethyl-6,7-dihydroanthracene-1,4,5-trione	Quinones	Quinones	1.172730311	0.154675751	0.287902065	36.67820053	5.196850955	3
Root vs Leaf	Cyanidin-3,5-O-diglucoside (Cyanin)*	Flavonoids	Anthocyanidins	1.305704079	0.000555357	0.016207027	0.00619736	-7.334130561	3
Root vs Leaf	Benzyl 6-O-galloyl beta-D-glucopyranoside	Others	Others	1.251977695	0.120169884	0.238683014	0.073076807	-3.774442588	3
Root vs Leaf	Mevalonic Acid Glucoside	Organic acids	Organic acids	1.041920933	0.053876842	0.133060407	0.175693682	-2.508865779	1

Comparison	Compound	Class I	Class II	VIP	P-value	FDR	Fold Change	Log_2FC	MSI Level
Root vs Leaf	Quercetin-3-O-apiosyl(1→2)galactoside	Flavonoids	Flavonols	1.300633131	0.01156249	0.04967503	0.10940354	-3.192268676	3
Root vs Leaf	4,5,8-Trihydroxy-α-tetralone	Quinones	Naphthol	1.270294999	0.005631923	0.03665839	0.005846399	-7.418235955	1
Root vs Leaf	Quercetin-3-O-sophoroside (Baimaside)	Flavonoids	Flavonols	1.235567245	0.000230218	0.013805514	0.03753964	-4.735441378	3
Root vs Leaf	Tricalysioside R	Terpenoids	Ditepenoids	1.303613334	0.006385486	0.038725464	0.07137002	-3.808538011	3
Root vs Leaf	Homoeriodictyol-7,4'-di-O-β-D-glucopyranoside	Flavonoids	Flavanones	1.303419868	0.004232927	0.032943531	0.154867261	-2.690895904	3
Root vs Leaf	Sec-O-glucosylha	Others	Chromone	1.296932605	0.021440635	0.071945982	0.072113308	-3.793590675	3

Comparison	Compound	Class I	Class II	VIP	P-value	FDR	Fold Change	Log_2FC	MSI Level
	maudol								
Root vs Leaf	N-carbamoyl-b-alanine	Amino acids and derivatives	Amino acids and derivatives	1.247257026	0.177295141	0.319545287	154.0200013	7.266973904	3
Root vs Leaf	2-α-Linolenoyl-glycerol*	Lipids	Glycerol ester	1.20084865	0.149163273	0.281767336	7.282910037	2.864515024	3
Root vs Leaf	Craiobioside B	Others	Others	1.215558433	0.111789886	0.22640857	0.079410687	-3.65452302	3
Root vs Leaf	Isorhamnetin-3,7-O-diglucoside	Flavonoids	Flavonols	1.035364339	0.033760513	0.096503265	0.279926305	-1.836881032	3
Root vs Leaf	Episyringaresinol	Lignans and Coumarins	Lignans	1.262653983	0.134944946	0.259284375	36.60959075	5.194149741	1
Root vs Leaf	Aucubin	Terpenoids	Sesquiterpenoi	1.261215045	0.016720867	0.061872934	2.448992511	1.292188362	2

Comparison	Compound	Class I	Class II	VIP	P-value	FDR	Fold Change	Log_2FC	MSI Level
			ds						
Root vs Leaf	3,5-Dicaffeoylquinic acid	Phenolic acids	Phenolic acids	1.303316336	0.006849322	0.040009515	0.061636373	-4.020074226	3
Root vs Leaf	Frehmaglutoside G	Terpenoids	Monoterpenoids	1.302657404	0.008543315	0.044062301	0.098605927	-3.34218183	3
Root vs Leaf	2-(4-(3-(hydroxymethyl)-5-(2-hydroxyvinyl)-7-methoxy-2,3-dihydrobenzofuran-2-yl)-2,6-dimethoxyphenoxy)-6-methyltetrahydro-2H-pyran-3,4,5-triol*	Lignans and Coumarins	Lignans	1.28217067	0.041283322	0.110686424	0.000907113	-10.10642943	2

Comparison	Compound	Class I	Class II	VIP	P-value	FDR	Fold Change	Log_2FC	MSI Level
Root vs Leaf	LysoPC 18:3	Lipids	LPC	1.259762082	0.058183383	0.141274484	5.752598403	2.524213757	2
Root vs Leaf	Petunidin-3-O-glucoside-5-O-arabinoside	Flavonoids	Anthocyanidins	1.305685123	0.001785174	0.023320316	0.006842753	-7.191207335	3
Root vs Leaf	cyclo-Dopa 5-O-glucoside	Alkaloids	Alkaloids	1.184254563	0.001037195	0.020290333	0.023632187	-5.403103068	3
Root vs Leaf	Vicine	Alkaloids	Alkaloids	1.07612073	0.022636648	0.074005743	0.425450516	-1.232936753	3
Root vs Leaf	Valerine	Alkaloids	Alkaloids	1.04831662	0.253432082	0.381118113	17.23652347	4.107396914	3
Root vs Leaf	Myricetin-3-O-(6''-malony)glucoside*	Flavonoids	Flavonols	1.277472919	0.045317102	0.118416273	0.126665607	-2.980903244	2
Root vs Leaf	9-(Arabinosyl)hy	Nucleotides	Nucleotides	1.039293873	0.080032682	0.178483617	0.382167869	-1.387721606	3

Comparison	Compound	Class I	Class II	VIP	P-value	FDR	Fold Change	Log_2FC	MSI Level
	poxanthine	and derivatives	and derivatives						
Root vs Leaf	Resinoside B*	Flavonoids	Flavones	1.30524592	0.004016644	0.032253374	0.010850504	-6.52609415	3
Root vs Leaf	Monolinolenin*	Lipids	Glycerol ester	1.216039515	0.151100988	0.283691777	8.331524847	3.058580564	3
Root vs Leaf	3α-Dihydrocadambine	Alkaloids	Plumerane	1.302333806	0.009573474	0.046490509	0.08155607	-3.616063936	3
Root vs Leaf	ent-3α,16β,17,19-Tetrahydroxykauran-19-ylacetate-17-O-β-D-glucopyranoside	Terpenoids	Ditepenoids	1.290309155	0.049977371	0.126022285	0.027783506	-5.169627529	3
Root vs Leaf	Olean-12-en-3-one (β-Amyrone)	Terpenoids	Triterpene	1.277242404	0.007762219	0.04213351	0.018151542	-5.783764111	1

Comparison	Compound	Class I	Class II	VIP	P-value	FDR	Fold Change	Log_2FC	MSI Level
Root vs Leaf	Chisopanin D	Terpenoids	Triterpene	1.30431145	0.01767851	0.064167898	0.006418197	-7.283616115	3
Root vs Leaf	2-Hydroxymelato nin	Alkaloids	Plumerane	1.125422338	0.190345272	0.327998206	125.9317792	6.976498586	3
Root vs Leaf	2-O-p-Coumaroyl 3-methylglucaric acid*	Phenolic acids	Phenolic acids	1.270050811	0.000963456	0.02015943	0.006303782	-7.309566661	1
Root vs Leaf	3-Oxo-Alpha-Ion ol 9-[Apiosyl-(1→6)-Glucoside]	Terpenoids	Monoterpenoid s	1.03110996	0.038438878	0.10514736	0.263755924	-1.9227246	3
Root vs Leaf	3'-methoxyquerce tin-3-O-L-rhamno syl(1→2)-glucopy	Flavonoids	Flavonols	1.116223626	0.001147565	0.020742409	0.072998485	-3.775989675	1

Comparison	Compound	Class I	Class II	VIP	P-value	FDR	Fold Change	Log_2FC	MSI Level
	ranoside*								
Root vs Leaf	LysoPC 18:3(2n isomer)	Lipids	LPC	1.238703138	0.073412294	0.167692681	5.39064566	2.430458081	2
Root vs Leaf	Cytosine	Nucleotides and derivatives	Nucleotides and derivatives	1.172469787	0.068467797	0.159266856	2.403125632	1.264912072	3
Root vs Leaf	LysoPE 15:0(2n isomer)*	Lipids	LPE	1.299237267	0.042222593	0.112405679	145.9603173	7.189432382	3
Root vs Leaf	Vincoside lactam	Alkaloids	Plumerane	1.146165453	0.003632994	0.030909053	0.23411486	-2.094711588	3
Root vs Leaf	N-Acetyl-6-hydroxy-L-tryptophan	Amino acids and derivatives	Amino acids and derivatives	1.286102462	4.05771E-06	0.004087201	0.066370709	-3.913309496	1

Comparison	Compound	Class I	Class II	VIP	P-value	FDR	Fold Change	Log_2FC	MSI Level
Root vs Leaf	(9S)-Hydroxyoctadecadienoic acid	Lipids	Free fatty acids	1.197616993	0.225751109	0.354255047	43.32189157	5.437024332	1
Root vs Leaf	Methyl 12-phenyldodecanoate	Lipids	Free fatty acids	1.226094867	0.01379121	0.055915205	0.387898808	-1.366247751	3
Root vs Leaf	N-Feruloylagmatine	Alkaloids	Phenolamine	1.162150863	0.010117267	0.047156746	0.052476961	-4.252172013	3
Root vs Leaf	4-Aminophenol	Alkaloids	Alkaloids	1.093190066	0.365855463	0.478290173	69.31579988	6.115112333	3
Root vs Leaf	(3-(carboxyamino)-2-methylpropanoyl)phenylalanine*	Amino acids and derivatives	Amino acids and derivatives	1.121400032	0.290957425	0.415296563	14.20140076	3.827961332	3
Root vs Leaf	Methyl	Lipids	Free fatty acids	1.17581769	0.107608773	0.219794479	4.294517611	2.102496087	2

Comparison	Compound	Class I	Class II	VIP	P-value	FDR	Fold Change	Log_2FC	MSI Level
	14-methylpentadecanoate								
Root vs Leaf	Demethylwedelolactone	Lignans and Coumarins	Coumarins	1.301416288	0.007226755	0.040362051	0.173217769	-2.529341163	2
Root vs Leaf	6-Hydroxykaempferol-6,7-O-Diglucoside*	Flavonoids	Flavonols	1.20057892	0.128617209	0.250623794	0.056503973	-4.145503887	3
Root vs Leaf	Histamine	Alkaloids	Alkaloids	1.102284857	0.164705578	0.302660505	2.854981341	1.513481317	3
Root vs Leaf	homoisocitricacid	Organic acids	Organic acids	1.240987484	0.014439826	0.057046076	0.032732947	-4.933112701	2
Root vs Leaf	Limocitrin-3-O-arabinoside	Flavonoids	Flavonols	1.305636961	0.000447394	0.014948097	0.107797868	-3.213599446	3

Comparison	Compound	Class I	Class II	VIP	P-value	FDR	Fold Change	Log_2FC	MSI Level
Root vs Leaf	awabukinol	Terpenoids	Monoterpenoids	1.294084709	0.030035208	0.087808622	0.069312515	-3.850740314	3
Root vs Leaf	Bletilols B	Others	Others	1.218600802	0.317086782	0.437005443	105.8287307	6.725587537	3
Root vs Leaf	Isohemiphloin	Flavonoids	Flavanones	1.082754047	0.010266833	0.047156746	0.077478211	-3.690065552	3
Root vs Leaf	Dihydrobuddlenol B	Lignans and Coumarins	Lignans	1.252853372	0.050995536	0.127695658	0.217188084	-2.202983141	3
Root vs Leaf	Kaempferol-3-O-rutinoside(Nicotiflorin)*	Flavonoids	Flavonols	1.302797939	0.007883537	0.042229995	0.082572702	-3.59819127	2
Root vs Leaf	Quercetin	Flavonoids	Flavonols	1.17432317	0.003457854	0.030288731	0.147042254	-2.765697313	1
Root vs Leaf	3-O-p-Coumaroyl 3-methylglucaric	Phenolic acids	Phenolic acids	1.292309359	0.000701648	0.017644209	0.004244251	-7.880274316	1

Comparison	Compound	Class I	Class II	VIP	P-value	FDR	Fold Change	Log_2FC	MSI Level
	acid*								
Root vs Leaf	4'-demethyl-3,9-dihydroeucomin glucoside	Flavonoids	Other Flavonoids	1.094626189	0.010342276	0.047156746	0.249874443	-2.000724742	3
Root vs Leaf	7,8-Dihydro-Buddlenol B(threo)	Lignans and Coumarins	Lignans	1.243412076	0.073520622	0.167692681	0.22034461	-2.182166489	2
Root vs Leaf	α-Amyrenone	Terpenoids	Triterpene	1.235200666	0.010152036	0.047156746	0.026461187	-5.239978435	1
Root vs Leaf	6-Hydroxyluteolin 7-rutinoside	Flavonoids	Flavones	1.278121585	0.004093572	0.032253374	0.027851518	-5.16610023	1
Root vs Leaf	Punicic acid (9Z,11E,13Z-octadecatrienoic acid)	Lipids	Free fatty acids	1.197211696	0.134964454	0.259284375	8.327501244	3.057883664	3

Comparison	Compound	Class I	Class II	VIP	P-value	FDR	Fold Change	Log_2FC	MSI Level
Root vs Leaf	Quercetin-3,4'-O-di-glucoside	Flavonoids	Flavonols	1.30108031	0.008980769	0.045060703	0.128719439	-2.957698147	3
Root vs Leaf	7-hydroxyaloin	Quinones	Anthraquinone	1.280736279	0.000440671	0.014948097	0.115487812	-3.114187485	3
Root vs Leaf	6-Hydroxyluteolin 5-glucoside*	Flavonoids	Flavones	1.303286194	0.004388725	0.033039104	0.186260562	-2.424605861	3
Root vs Leaf	1-(β-D-Glucopyranosyl)-3-ethyl-4-methyl-1H-pyrrole-2,5-dione	Others	Ketone compounds	1.24193323	0.008392338	0.043806383	0.142073679	-2.815288797	1
Root vs Leaf	Dehydrodiconiferylalcohol-9'-O-glucoside*	Lignans and Coumarins	Lignans	1.295331938	0.039923208	0.108139777	0.001385174	-9.495717047	2
Root vs Leaf	4-Hydroxyacetop	Others	Ketone	1.100917812	0.352606491	0.467646773	34.81163447	5.121497648	3

Comparison	Compound	Class I	Class II	VIP	P-value	FDR	Fold Change	Log_2FC	MSI Level
	henone		compounds						
Root vs Leaf	7,8-Dihydro-Buddlenol B(erythro)	Lignans and Coumarins	Lignans	1.111763642	0.095772814	0.202325286	0.416683489	-1.26297616	2
Root vs Leaf	LysoPC 19:2(2n isomer)	Lipids	LPC	1.153756052	0.286469861	0.410737885	21.68505083	4.43862892	3
Root vs Leaf	6-O-Dihydrocaffeoylglucose	Phenolic acids	Phenolic acids	1.215477678	0.115733155	0.232867177	4.365553255	2.126164501	1
Root vs Leaf	Loliolide	Terpenoids	Monoterpenoids	1.252711405	0.016996412	0.062464705	0.098650865	-3.341524492	3
Root vs Leaf	LysoPC 17:0(2n isomer)*	Lipids	LPC	1.087714347	0.263152633	0.388969111	6.691628937	2.742357448	1
Root vs Leaf	Isoetin	Flavonoids	Flavones	1.30566262	0.000160148	0.013805514	0.196168195	-2.349836938	3

Comparison	Compound	Class I	Class II	VIP	P-value	FDR	Fold Change	Log_2FC	MSI Level
	(5,7,2',4',5'-Pentahydroxyflavone)								
Root vs Leaf	Gossypetin-3-O-(6''-malonyl)glucoside*	Flavonoids	Flavonols	1.304537915	0.002282306	0.025163593	0.184867358	-2.435437584	2
Root vs Leaf	4-Ethylphenol glucuronide	Phenolic acids	Phenolic acids	1.118158599	0.006237045	0.038725464	0.064141978	-3.962587352	3
Root vs Leaf	Feruloyltartaric acid (Fertaric acid)	Phenolic acids	Phenolic acids	1.022753449	0.011178927	0.048695245	0.283913087	-1.816478746	1
Root vs Leaf	Octadeca-9,12,15-trienoic acid	Lipids	Free fatty acids	1.185009791	0.1447258	0.274826409	7.976708582	2.995793572	3
Root vs Leaf	Blumenol B	Terpenoids	Sesquiterpenoi	1.168378909	0.007131816	0.040362051	0.239652314	-2.060985228	1

Comparison	Compound	Class I	Class II	VIP	P-value	FDR	Fold Change	Log_2FC	MSI Level
	Glucoside*		ds						
Root vs Leaf	Gardoside	Terpenoids	Monoterpenoids	1.259148802	0.019052061	0.066847799	2.755452105	1.462289051	2
Root vs Leaf	D-Galacturonic acid*	Others	Saccharides	1.219044941	0.005242212	0.036144451	0.46262093	-1.112097557	3
Root vs Leaf	Piperonylic acid	Phenolic acids	Phenolic acids	1.215705939	0.059327141	0.142927482	2.315110902	1.211081305	3
Root vs Leaf	6-Hydroxykaempferol 3,6-diglucoside*	Flavonoids	Flavonols	1.278573958	0.006784091	0.039838097	0.064774504	-3.948430123	3
Root vs Leaf	Isovanillin*	Others	Aldehyde compounds	1.17416263	0.089567683	0.193362401	6.042172394	2.595067347	3

Comparison	Compound	Class I	Class II	VIP	P-value	FDR	Fold Change	Log_2FC	MSI Level
Root vs Leaf	23-Hydroxytoonacilide	Terpenoids	Triterpene	1.242709519	0.039095586	0.106808549	0.211982096	-2.237985677	3
Root vs Leaf	Byzantionoside A	Others	Ketone compounds	1.28375591	0.069509442	0.160824309	0.058183616	-4.103243225	3
Root vs Leaf	Pelargonidin-3,5-O-diglucoside	Flavonoids	Anthocyanidins	1.305085606	0.010215465	0.047156746	0.004474171	-7.804163811	3
Root vs Leaf	γ-Glu-Phe*	Amino acids and derivatives	Amino acids and derivatives	1.070809217	0.31918151	0.438773056	12.72790355	3.669922903	3
Root vs Leaf	Genipin	Terpenoids	Monoterpenoids	1.262134792	0.036571208	0.102238525	0.12366032	-3.015545448	3
Root vs Leaf	Petunidin-3-O-glucoside	Flavonoids	Anthocyanidins	1.250151885	0.080756173	0.179551108	0.090036582	-3.473344899	3

Comparison	Compound	Class I	Class II	VIP	P-value	FDR	Fold Change	Log_2FC	MSI Level
Root vs Leaf	Madreselvin A*	Flavonoids	Flavones	1.029367262	0.020613287	0.069929848	0.230781229	-2.11540221	3
Root vs Leaf	1-hydroxy-4-glucoside-2-naphthoic acid	Phenolic acids	Phenolic acids	1.266220098	0.016245851	0.061021704	0.041795861	-4.580496094	1
Root vs Leaf	DL-Threonine	Amino acids and derivatives	Amino acids and derivatives	1.217136654	0.071909118	0.164963487	2.499483899	1.321630234	3
Root vs Leaf	Resinoside A*	Flavonoids	Flavones	1.304333754	0.008987119	0.045060703	0.01107547	-6.496488259	3
Root vs Leaf	Quercetin-7-O-glucoside	Flavonoids	Flavonols	1.232841044	0.001241001	0.021627444	0.022351051	-5.483513538	3
Root vs Leaf	Solatriose	Others	Saccharides	1.293926687	0.073678854	0.167776611	58.30024042	5.865429928	3
Root vs Leaf	Esculetin-7-O-qui	Lignans and	Coumarins	1.277812011	0.008160599	0.043144133	0.017447421	-5.840842352	3

Comparison	Compound	Class I	Class II	VIP	P-value	FDR	Fold Change	Log_2FC	MSI Level
	nic acid	Coumarins							
Root vs Leaf	LysoPC 18:0	Lipids	LPC	1.151634228	0.173642545	0.314389477	5.094905678	2.349055438	2
Root vs Leaf	LysoPC 19:2	Lipids	LPC	1.065735324	0.325329978	0.444349267	23.04168853	4.526174538	3
Root vs Leaf	2-Propylglutaric acid	Organic acids	Organic acids	1.260060438	0.036427465	0.102115395	0.046772009	-4.418210777	3
Root vs Leaf	3-Hydroxyurs-12-en-24-oic acid (β-Boswellic acid)*	Terpenoids	Triterpene	1.258608983	0.091070233	0.195047348	0.012427089	-6.330367834	2
Root vs Leaf	LysoPC 18:2(2n isomer)	Lipids	LPC	1.255078045	0.210146861	0.340482972	49.53806082	5.63046549	2
Root vs Leaf	(X)-1,2-Propanedi	Others	Alcohol	1.250123624	0.000448527	0.014948097	0.112610392	-3.15058812	1

Comparison	Compound	Class I	Class II	VIP	P-value	FDR	Fold Change	Log_2FC	MSI Level
	ol 1-O-B-D-Glucopyranoside		compounds						
Root vs Leaf	Peonidin-3-O-sambubioside	Flavonoids	Anthocyanidins	1.299840532	0.015655711	0.05987963	0.069197799	-3.853130042	3
Root vs Leaf	6'-O-β-D-glucosylbarlerin*	Terpenoids	Monoterpenoids	1.295123538	0.000216382	0.013805514	0.022778646	-5.456174207	3
Root vs Leaf	Hypericumoxide N	Others	Others	1.304057867	0.025053485	0.078692705	0.001456556	-9.423223582	3
Root vs Leaf	Eriodictyol-7-O-glucoside*	Flavonoids	Flavanones	1.251936238	0.012506204	0.052579587	0.018246492	-5.776237058	3
Root vs Leaf	3-O-p-Coumaroyl Methylglucaric	Phenolic acids	Phenolic acids	1.300206705	0.006655278	0.039511141	0.01265907	-6.303684732	1

Comparison	Compound	Class I	Class II	VIP	P-value	FDR	Fold Change	Log_2FC	MSI Level
	Acid*								
Root vs Leaf	3-Hydroxyolean-12-en-24-oic acid (α-Boswellic acid)*	Terpenoids	Triterpene	1.282670706	0.08600194	0.187727467	0.016714033	-5.902796279	2
Root vs Leaf	Secoisolariciresinol-9'-O-glucoside	Lignans and Coumarins	Lignans	1.270891804	0.018252325	0.065065644	0.010980854	-6.508865995	3
Root vs Leaf	6-Hydroxy-3,9-Undecadienoic acid Methyl Ester	Lipids	Free fatty acids	1.304681352	0.008730332	0.044706745	0.00787444	-6.988606952	1
Root vs Leaf	LysoPC 17:2	Lipids	LPC	1.228249104	0.043546614	0.114901385	12.19364301	3.608057309	3
Root vs Leaf	Fraxidin (8-Hydroxy-6,7-di	Lignans and Coumarins	Coumarins	1.282269989	0.042633217	0.113043413	61.0483299	5.931879923	1

Comparison	Compound	Class I	Class II	VIP	P-value	FDR	Fold Change	Log_2FC	MSI Level
	methoxycoumarin)								
Root vs Leaf	1-(4-Methoxyphenyl)-1-propanol	Phenolic acids	Phenolic acids	1.243218657	0.001824494	0.023320316	0.481700996	-1.053790187	3
Root vs Leaf	Bartsioside	Terpenoids	Monoterpenoids	1.183076423	0.014198088	0.05671362	0.116655292	-3.099676341	3
Root vs Leaf	5,7-Dihydroxy-4-methylcoumarin	Lignans and Coumarins	Coumarins	1.202229632	0.001563331	0.023084686	0.165352249	-2.59638543	3
Root vs Leaf	Quercetin 7-O-p-coumaroyl rhamnoside	Flavonoids	Flavonols	1.275388513	0.012307412	0.051844675	0.003212991	-8.281867323	2
Root vs Leaf	Caffeic acid	Phenolic acids	Phenolic acids	1.003794969	0.260159462	0.386926771	0.187199944	-2.417348088	3

Comparison	Compound	Class I	Class II	VIP	P-value	FDR	Fold Change	Log_2FC	MSI Level
Root vs Leaf	4-O-(6'-O-Glucosylcaffeoylglucosylferuloyl)-4-hydroxybenzyl alcohol	Phenolic acids	Phenolic acids	1.305668315	0.002446319	0.026300972	0.003385856	-8.206263526	3
Root vs Leaf	Fumaric acid	Organic acids	Organic acids	1.194266177	0.01048688	0.047173513	0.386588588	-1.371129043	3
Root vs Leaf	Hesperetin-5-O-glucoside	Flavonoids	Flavanones	1.28184638	0.000192726	0.013805514	0.017328222	-5.850732535	3
Root vs Leaf	3-pyridine-methanol-O-β-D-glucopyranosyl*	Alkaloids	Pyridine alkaloids	1.237903387	0.000417182	0.014948097	0.123728159	-3.014754214	1
Root vs Leaf	Syringetin-7-O-glucoside*	Flavonoids	Flavonols	1.286675102	0.035123693	0.099348562	0.16139079	-2.631369841	3

Comparison	Compound	Class I	Class II	VIP	P-value	FDR	Fold Change	Log_2FC	MSI Level
Root vs Leaf	Fisetin	Flavonoids	Flavonols	1.286179813	0.004582107	0.033309066	0.008976916	-6.799564452	3
Root vs Leaf	Santalol A	Terpenoids	Sesquiterpenoids	1.197533971	0.01104244	0.048436512	0.034464063	-4.858763398	3
Root vs Leaf	Loganic acid	Terpenoids	Monoterpenoids	1.216743118	0.011690078	0.04992541	0.495517948	-1.012990781	1
Root vs Leaf	1,4,8-trihydroxynaphthalen-1-O-β-D-[6'-O-(3'',4'',5''-trihydroxybenzoyl)]glucopyranoside	Quinones	Quinones	1.298835929	0.019747649	0.068137925	0.081435462	-3.618199016	2
Root vs Leaf	6'-O-α-D-galactosylbarlerin*	Terpenoids	Monoterpenoids	1.302500436	0.007075127	0.04034129	0.02728876	-5.195549375	3

Comparison	Compound	Class I	Class II	VIP	P-value	FDR	Fold Change	Log_2FC	MSI Level
Root vs Leaf	Ixoroside	Terpenoids	Monoterpenoids	1.287766864	0.00066878	0.017564328	0.123805967	-3.013847252	3
Root vs Leaf	Quinacyl syringic acid	Phenolic acids	Phenolic acids	1.271624741	0.00441677	0.033039104	0.014744077	-6.083720687	3
Root vs Leaf	3,4-Dihydro-4-(4'-hydroxyphenyl)-5,7-dihydroxycoumarin glucoside	Lignans and Coumarins	Coumarins	1.007548744	0.002333873	0.025472219	0.17568666	-2.508923443	3
Root vs Leaf	9,12,13-TriHOME; 9(S),12(S),13(S)-Trihydroxy-10(E)-octadecenoic acid	Lipids	Free fatty acids	1.168918575	0.347157439	0.46309088	88.79291025	6.472372583	2
Root vs Leaf	6-Hydroxykaemp	Flavonoids	Flavonols	1.303869526	0.007205801	0.040362051	0.05226648	-4.257970186	3

Comparison	Compound	Class I	Class II	VIP	P-value	FDR	Fold Change	Log_2FC	MSI Level
	ferol-7,6-O-Digluc oside								
Root vs Leaf	O-Feruloyl 3-hydroxycoumarin*	Lignans and Coumarins	Coumarins	1.289308468	0.049705357	0.125664132	0.066868533	-3.902528724	2
Root vs Leaf	Tetraphyllin A*	Alkaloids	Alkaloids	1.21969576	0.001851535	0.023320316	0.118061085	-3.08239459	1
Root vs Leaf	Herbacetin-7-O-rhamnoside-8-O-glucoside; Rhodionidin*	Flavonoids	Flavonols	1.303510895	0.023866756	0.076183248	0.006162526	-7.342262528	3
Root vs Leaf	4-Hydroxysphinganine; Phytosphingosine	Lipids	Sphingolipids	1.000455382	0.014106845	0.056558242	0.229259979	-2.124943565	2

Comparison	Compound	Class I	Class II	VIP	P-value	FDR	Fold Change	Log_2FC	MSI Level
Root vs Leaf	6-Hydroxykaempferol-3,6-O-Diglucoside*	Flavonoids	Flavonols	1.255439344	0.001235349	0.021627444	0.087453784	-3.515335387	3
Root vs Leaf	1-O-Caffeoyl-β-D-glucose*	Phenolic acids	Phenolic acids	1.019150301	0.173130054	0.31413438	4.647163575	2.216350427	1
Root vs Leaf	LysoPC 18:0(2n isomer)	Lipids	LPC	1.163727174	0.167386075	0.306024796	5.179341771	2.372768761	1
Root vs Leaf	p-Coumaroylcaffeoyltartaric acid	Phenolic acids	Phenolic acids	1.235312441	0.000224232	0.013805514	0.096626294	-3.371440366	3
Root vs Leaf	2-Hydroxyhexadecanoic acid*	Lipids	Free fatty acids	1.188255525	0.074503141	0.16894154	3.698957565	1.88711875	1
Root vs Leaf	4-Pyridoxic acid	Others	Vitamin	1.27732037	0.000119112	0.012840208	0.051726545	-4.27295136	3

Comparison	Compound	Class I	Class II	VIP	P-value	FDR	Fold Change	Log_2FC	MSI Level
Root vs Leaf	N(6),N(6)-Dimethyl-L-lysine	Amino acids and derivatives	Amino acids and derivatives	1.210408391	0.232437598	0.360069999	101.8872492	6.670829704	2
Root vs Leaf	Meratin*	Flavonoids	Flavonols	1.27840499	0.103374719	0.213568613	0.044720501	-4.482919849	3
Root vs Leaf	3'-Deoxy-4-O-methylsappanol	Others	Alcohol compounds	1.299763458	0.015569647	0.059797609	0.093786219	-3.414480235	3
Root vs Leaf	coleoside-B	Terpenoids	Monoterpenoids	1.208554806	0.330110808	0.448942389	120.3202701	6.9107359	3
Root vs Leaf	Nodakenin	Lignans and Coumarins	Coumarins	1.280402099	0.009664704	0.046617244	0.031362358	-4.994822158	2
Root vs Leaf	3,4-Dihydroxy-L-phenylalanine (L-Dopa)	Amino acids and derivatives	Amino acids and derivatives	1.126690765	0.02605729	0.080442578	0.26858977	-1.896523736	3

Comparison	Compound	Class I	Class II	VIP	P-value	FDR	Fold Change	Log_2FC	MSI Level
Root vs Leaf	Quercetin-3-O-(2''-O-rhamnosyl)galactoside*	Flavonoids	Flavonols	1.298446638	0.025927248	0.080308886	0.070908234	-3.81790302	3
Root vs Leaf	Syringaldehyde-4-O-glucoside	Others	Aldehyde compounds	1.091839522	0.014228714	0.056731091	0.185383005	-2.431419107	2
Root vs Leaf	Quercetin-4'-O-glucoside (Spiraeoside)*	Flavonoids	Flavonols	1.303770253	0.010376876	0.047156746	0.017178773	-5.863229223	3
Root vs Leaf	Dimethylglyceric Acid Glucoside	Organic acids	Organic acids	1.088697221	0.021053977	0.070979164	0.356299822	-1.48883633	2
Root vs Leaf	Guaiacylglycerol 8-O-(1''-O-Glucosyl)Vanillic Acid	Phenolic acids	Phenolic acids	1.210980029	0.130128452	0.252430507	15.45984198	3.950453668	1

Comparison	Compound	Class I	Class II	VIP	P-value	FDR	Fold Change	Log_2FC	MSI Level
	Ether								
Root vs Leaf	D-Threonic Acid	Others	Saccharides	1.255852107	0.031788579	0.091874745	0.118053757	-3.082484144	3
Root vs Leaf	Quercetin 5-O-p-coumaroyl rhamnoside	Flavonoids	Flavonols	1.300539477	0.017100824	0.062635391	0.065161417	-3.939838219	1
Root vs Leaf	6-Hydroxymethyl herniarin	Lignans and Coumarins	Coumarins	1.288747165	0.00038925	0.014825611	0.054442662	-4.19911857	3
Root vs Leaf	Nicotinic acid (Vitamin B3)	Others	Vitamin	1.22601808	0.104105438	0.214872829	5.521614667	2.465090211	3
Root vs Leaf	4-O-Glucosyl-3,4-dihydroxybenzyl alcohol	Others	Others	1.218678694	0.004721028	0.03389416	0.407688156	-1.294462049	1

Comparison	Compound	Class I	Class II	VIP	P-value	FDR	Fold Change	Log_2FC	MSI Level
Root vs Leaf	2,6-Dimethoxy-4-hydroxyphenol-1-O-ß-D-glucopyranoside	Others	Others	1.258434061	0.000844735	0.019215509	0.039740832	-4.653234115	1
Root vs Leaf	Ribosyladenosine	Nucleotides and derivatives	Nucleotides and derivatives	1.120305457	0.046146158	0.120002224	0.440554634	-1.182607154	3
Root vs Leaf	p-galloyl-oxyphenyl-β-D-glucoside	Phenolic acids	Phenolic acids	1.266937134	0.003848056	0.031796549	0.157273025	-2.66865685	3
Root vs Leaf	4,6-dimethoxy-2-methoxyphenyl-1-O-beta-D-glucopyranoside	Others	Others	1.100535791	0.153410835	0.286040391	10.42361588	3.38178392	3

Comparison	Compound	Class I	Class II	VIP	P-value	FDR	Fold Change	Log_2FC	MSI Level
Root vs Leaf	Rhamnetin-3-O-Rutinoside*	Flavonoids	Flavonols	1.279439623	0.029834745	0.087808622	0.082830823	-3.593688463	1
Root vs Leaf	4-(6-O-Sulfo-Beta-D-Glucopyranosyloxy)-3-Hydroxybenzoic Acid	Phenolic acids	Phenolic acids	1.132670122	0.022280628	0.073397008	0.286098191	-1.805417722	3
Root vs Leaf	Succinyladenosine	Nucleotides and derivatives	Nucleotides and derivatives	1.142348211	0.12249564	0.241968078	3.063167679	1.615024343	2
Root vs Leaf	Hesperetin-7-O-rutinoside (Hesperidin)	Flavonoids	Flavanones	1.245027108	0.019769773	0.068137925	0.028466098	-5.134611438	3
Root vs Leaf	Catechin-(4alpha	Flavonoids	Flavanols	1.2757984	0.001953252	0.023320316	0.003434136	-8.185836991	1

Comparison	Compound	Class I	Class II	VIP	P-value	FDR	Fold Change	Log_2FC	MSI Level
	→8)-gallocatechin								
Root vs Leaf	2-Ethyl-3-methyl maleimide-N-glu coside	Alkaloids	Alkaloids	1.146522248	0.020206527	0.068983105	0.47733821	-1.066916269	2
Root vs Leaf	N-α-Acetyl-L-orn ithine	Amino acids and derivatives	Amino acids and derivatives	1.247952954	0.182583599	0.324925253	117.1496675	6.87220905	3
Root vs Leaf	NG,NG-Dimethyl -L-arginine*	Amino acids and derivatives	Amino acids and derivatives	1.136076464	0.168945468	0.308093804	9.7900482	3.291315963	3
Root vs Leaf	9,10,13-Trihydrox y-11-Octadecenoi c Acid	Lipids	Free fatty acids	1.170204723	0.340375729	0.457148508	78.52798255	6.295134928	1

Comparison	Compound	Class I	Class II	VIP	P-value	FDR	Fold Change	Log_2FC	MSI Level
Root vs Leaf	Petunidin-3-O-(6''-O-p-Coumaroyl) glucoside	Flavonoids	Anthocyanidins	1.207520081	0.023394341	0.075368078	0.085213604	-3.552772428	3
Root vs Leaf	2,3-Dihydroxy-5(6),12(13)-diene-ursolic acid*	Terpenoids	Triterpene	1.267938051	0.000397268	0.014825611	0.117772514	-3.085925213	1
Root vs Leaf	Monogalactosyldiacylglycerol	Lipids	Glycerol ester	1.300860655	0.039434843	0.107293055	0.005295376	-7.561051029	1
Root vs Leaf	Dimethoxysamin	Lignans and Coumarins	Lignans	1.230567302	0.10679682	0.218549175	14.8194946	3.889424342	3
Root vs Leaf	ethyl-L-arginine	Amino acids and derivatives	Amino acids and derivatives	1.118461015	0.174282743	0.314640775	10.60996334	3.407347767	3

Comparison	Compound	Class I	Class II	VIP	P-value	FDR	Fold Change	Log_2FC	MSI Level
Root vs Leaf	Pratensein 7-O-glucopyranoside	Flavonoids	Isoflavones	1.299884913	0.010682817	0.047697452	0.176031299	-2.506096128	2
Root vs Leaf	Cleomiscosin A	Lignans and Coumarins	Lignans	1.199063273	0.151815457	0.284292203	45.2896992	5.501111052	2
Root vs Leaf	Cis-Aconitic acid	Organic acids	Organic acids	1.223776651	0.02716693	0.082920532	0.124254904	-3.008625301	3
Root vs Leaf	Theasinensin C	Tannins	Proanthocyanidins	1.305200891	0.006397497	0.038725464	0.006279765	-7.315073678	3
Root vs Leaf	N6-Acetyl-L-lysine	Amino acids and derivatives	Amino acids and derivatives	1.069621841	0.30700728	0.426993967	10.73726042	3.424554036	3
Root vs Leaf	2,3-Dihydroxyurs	Terpenoids	Triterpene	1.284014726	0.000114278	0.012840208	0.131988816	-2.921512407	1

Comparison	Compound	Class I	Class II	VIP	P-value	FDR	Fold Change	Log_2FC	MSI Level
	-12,18-dien-28-oic acid*								
Root vs Leaf	Grevilloside B	Others	Others	1.297099309	0.00226401	0.025163593	0.03412828	-4.872888497	3
Root vs Leaf	Quercetin 3,7-diglucoside*	Flavonoids	Flavonols	1.254031539	0.027822726	0.083973338	0.013945498	-6.16405675	3
Root vs Leaf	2,3'-Di-feruloyl sucrose	Phenolic acids	Phenolic acids	1.305701494	0.001209769	0.021428776	0.003551313	-8.137431695	1
Root vs Leaf	1-Aminocyclopropane-1-carboxylic acid	Amino acids and derivatives	Amino acids and derivatives	1.052808925	0.282290176	0.406853578	6.367107114	2.670638035	3
Root vs Leaf	L-Fucitol	Others	Saccharides	1.231674529	0.00223818	0.025163593	0.138810915	-2.848807083	3
Root vs Leaf	D-Panthenol	Others	Vitamin	1.138073249	0.269078276	0.395214626	14.93164067	3.900300791	3

Comparison	Compound	Class I	Class II	VIP	P-value	FDR	Fold Change	Log_2FC	MSI Level
Root vs Leaf	L-Citrulline	Amino acids and derivatives	Amino acids and derivatives	1.095430222	0.204483402	0.335781635	10.48747811	3.390595894	3
Root vs Leaf	LysoPE 17:1*	Lipids	LPE	1.291356436	0.070055271	0.161568241	426.5978934	8.736733031	3
Root vs Leaf	Ssioriside	Lignans and Coumarins	Lignans	1.303564059	0.017553646	0.063968684	0.011316963	-6.465369371	3
Root vs Leaf	3'-p-Coumaroyl-sucrose	Phenolic acids	Phenolic acids	1.160776546	0.00035742	0.01464066	0.117944324	-3.0838221	2
Root vs Leaf	Isosalipurposide (Phlorizin Chalcone)	Flavonoids	Chalcones	1.104595648	0.015831111	0.060230686	0.127504343	-2.971381701	3
Root vs Leaf	2'-Deoxyadenosine-5'-monophosph	Nucleotides and	Nucleotides and derivatives	1.216483977	0.015063634	0.058611864	0.38318516	-1.383886406	3

Comparison	Compound	Class I	Class II	VIP	P-value	FDR	Fold Change	Log_2FC	MSI Level
	ate	derivatives							
Root vs Leaf	Rumexobtusifolius	Others	Others	1.168945428	0.004517978	0.033309066	0.261493598	-1.935152469	2
Root vs Leaf	Isonicotinic acid	Others	Vitamin	1.214507484	0.12908562	0.251309933	6.014950274	2.588552811	3
Root vs Leaf	Tricin-7-O-saccharic acid	Flavonoids	Flavones	1.286924164	0.008795891	0.044830002	0.260919149	-1.938325267	3
Root vs Leaf	2-g-linolenoyl-glycerol	Lipids	Glycerol ester	1.234285017	0.001327068	0.022042878	0.232746655	-2.103167662	2
Root vs Leaf	Astraflavonoid C	Flavonoids	Other Flavonoids	1.233317126	9.49856E-05	0.012840208	0.03410819	-4.873738001	3
Root vs Leaf	Roseoside	Terpenoids	Sesquiterpenoids	1.188939702	0.003506058	0.030288731	0.206448461	-2.276146428	3

Comparison	Compound	Class I	Class II	VIP	P-value	FDR	Fold Change	Log_2FC	MSI Level
Root vs Leaf	Genistein-7-O-galactoside*	Flavonoids	Isoflavones	1.144801034	0.003261151	0.029610702	0.155165211	-2.688122959	3
Root vs Leaf	isatioxyneolignoside A	Lignans and Coumarins	Lignans	1.254458903	0.118801247	0.237054013	30.5916023	4.935063768	3
Root vs Leaf	LysoPE 17:1(2n isomer)*	Lipids	LPE	1.296009329	0.074464933	0.16894154	240.5372288	7.910116392	3
Root vs Leaf	2-Beta-Glucopyranosyloxy-3-Hydroxybenzyl Alcohol	Phenolic acids	Phenolic acids	1.268764767	0.001591053	0.023084686	0.373303726	-1.421578188	3
Root vs Leaf	Grayanotoxin XIX	Terpenoids	Ditepenoids	1.305780039	0.000111809	0.012840208	0.002708838	-8.528110069	2
Root vs Leaf	1-O-p-Coumaroyl sedoheptulose	Phenolic acids	Phenolic acids	1.30538287	0.001042215	0.020290333	0.06478362	-3.948227104	3

Comparison	Compound	Class I	Class II	VIP	P-value	FDR	Fold Change	Log_2FC	MSI Level
Root vs Leaf	3-Ethyl-7-hydroxyphthalide	Others	Others	1.258777357	0.019065026	0.066847799	3.090982646	1.628065554	1
Root vs Leaf	N,N'-Dimethylarginine;SDMA*	Amino acids and derivatives	Amino acids and derivatives	1.131477374	0.171227879	0.31120559	10.31143492	3.366173205	3
Root vs Leaf	O-Feruloyl 4-hydroxycoumarin	Lignans and Coumarins	Coumarins	1.304979891	0.011404354	0.04934055	0.000975523	-10.00153633	2
Root vs Leaf	(2E)-3-(1-Hydroxy-2,6,6-trimethyl-4-oxo-2-cyclohexen-1-yl)-2-propenoic acid	Terpenoids	Sesquiterpenoids	1.26889629	0.081592894	0.181028997	0.0273339	-5.193164892	3

Comparison	Compound	Class I	Class II	VIP	P-value	FDR	Fold Change	Log_2FC	MSI Level
Root vs Leaf	Cinchonain Ib*	Flavonoids	Flavanols	1.260025579	0.001795329	0.023320316	0.279908466	-1.836972972	3
Root vs Leaf	3-Hydroxy-18-oxoursan-28,13-olide	Terpenoids	Triterpene	1.037242561	0.042241338	0.112405679	0.473220975	-1.079414074	1
Root vs Leaf	Chlorogenic acid methyl ester	Phenolic acids	Phenolic acids	1.305808322	0.000176483	0.013805514	0.000800936	-10.28602591	2
Root vs Leaf	Grayanotoxin VII	Terpenoids	Ditepenoids	1.11650798	0.000348069	0.01464066	0.067408697	-3.890921447	2
Root vs Leaf	Tricetin-5-O-(6''-malonyl)glucoside	Flavonoids	Flavones	1.162199864	0.002203426	0.025163593	0.128691428	-2.958012138	3
Root vs Leaf	5-[4-(3,4-dimethoxyphenyl)-hexahydrofuro[3,4-c]fura	Lignans and Coumarins	Lignans	1.03551106	0.086975477	0.189088536	0.150855373	-2.728762016	3

Comparison	Compound	Class I	Class II	VIP	P-value	FDR	Fold Change	Log_2FC	MSI Level
	n-1-yl]-4-methyl-2H-1,3-benzodioxole								
Root vs Leaf	schisanol	Lignans and Coumarins	Lignans	1.234886368	0.11543323	0.23276313	13.23354861	3.726128071	3
Root vs Leaf	L-threo-3-Methyl aspartate	Amino acids and derivatives	Amino acids and derivatives	1.215488254	0.144622439	0.274826409	6.043766281	2.595447871	3
Root vs Leaf	10-acetylmonomelittoside*	Terpenoids	Monoterpenoids	1.151440042	0.136746384	0.26220846	4.831582528	2.272495804	2
Root vs Leaf	4-O-Feruloyl Aminogalactitol	Phenolic acids	Phenolic acids	1.287982269	0.01827619	0.065065644	0.014416583	-6.116126932	2
Root vs Leaf	Tomentodione F	Others	Others	1.171693784	0.200856093	0.334656913	27.57070645	4.785064329	3

Comparison	Compound	Class I	Class II	VIP	P-value	FDR	Fold Change	Log_2FC	MSI Level
Root vs Leaf	L-Homoglutamic acid	Amino acids and derivatives	Amino acids and derivatives	1.155842924	0.216258221	0.344895952	8.27742928	3.04918278	3
Root vs Leaf	methyl alpha-D-fructofuranoside*	Others	Saccharides	1.276205393	0.002179559	0.025163593	0.354286913	-1.497009921	3
Root vs Leaf	1-O-p-Coumaroyl quinic acid*	Phenolic acids	Phenolic acids	1.28626805	0.004536674	0.033309066	0.070543065	-3.825351921	2
Root vs Leaf	Kaempferol-7-O-rhamnoside	Flavonoids	Flavonols	1.305586143	0.004499205	0.033309066	0.001898273	-9.041096941	2
Root vs Leaf	Quercetin-3-O-xylosyl(1→2)arabinoside	Flavonoids	Flavonols	1.302342077	0.009311485	0.045935918	0.085948669	-3.540380887	1

Comparison	Compound	Class I	Class II	VIP	P-value	FDR	Fold Change	Log_2FC	MSI Level
Root vs Leaf	2-((6-hydroxy-4-(hydroxy(methoxy)methyl)-7-methyl-1,4a,5,6,7,7a-hexahydrocyclopenta[c]pyran-1-yl)oxy)-6-(hydroxymethyl)tetrahydro-2H-pyran-3,4,5-triol	Terpenoids	Monoterpenoids	1.279425246	0.052904502	0.131409918	0.149955769	-2.737391071	3
Root vs Leaf	1-O-Caffeoyl-(6-O-glucosyl)-β-D-glucose	Phenolic acids	Phenolic acids	1.300119161	0.013302362	0.054443946	0.067974335	-3.878866066	1
Root vs Leaf	Securoside A	Phenolic acids	Phenolic acids	1.305427177	0.004119931	0.032257864	0.002858747	-8.450401418	1

Comparison	Compound	Class I	Class II	VIP	P-value	FDR	Fold Change	Log_2FC	MSI Level
Root vs Leaf	Cyanidin 3-xyloside	Flavonoids	Anthocyanidins	1.295394567	0.098753389	0.206588648	0.004305851	-7.859486043	3
Root vs Leaf	LysoPC 17:1	Lipids	LPC	1.290437021	0.029358625	0.086672116	23.79225431	4.572420068	2
Root vs Leaf	4-Hydroxybenzoic acid 4-(6-O-sulfo)glucopyranoside	Phenolic acids	Phenolic acids	1.038723074	0.000785596	0.018954533	0.07559976	-3.72547454	1
Root vs Leaf	5,6,15-Trihydroxy-7,9,11,13-eicosatraenoic acid	Lipids	Free fatty acids	1.137708268	0.179185986	0.321912368	77.33029976	6.2729619	3
Root vs Leaf	Cyanidin-3-O-arabinoside	Flavonoids	Anthocyanidins	1.300167351	0.045203669	0.118262868	0.00943642	-6.727544694	3
Root vs Leaf	DL-Methionine	Amino acids	Amino acids	1.023400071	0.378544982	0.486347032	35.61646393	5.154472385	3

Comparison	Compound	Class I	Class II	VIP	P-value	FDR	Fold Change	Log_2FC	MSI Level
		and derivatives	and derivatives						
Root vs Leaf	Tamarixetin-3-O-glucoside-7-O-rhamnoside*	Flavonoids	Flavonols	1.235994874	0.019385983	0.067460722	0.079650487	-3.650173008	1
Root vs Leaf	Cinchonain Ia*	Flavonoids	Flavanols	1.255017718	0.00311373	0.028511738	0.253301173	-1.981074336	3
Root vs Leaf	23-Hydroxy-3-oxoolean-12-en-28-oic acid (Hederagonic acid)	Terpenoids	Triterpene	1.073197787	0.179368641	0.321939895	13.77235397	3.783703261	1
Root vs Leaf	Sexangularetin-3-O-glucoside-7-O-r	Flavonoids	Flavonols	1.200914122	0.003541349	0.030288731	0.067056159	-3.898486335	1

Comparison	Compound	Class I	Class II	VIP	P-value	FDR	Fold Change	Log_2FC	MSI Level
	hamnoside*								
Root vs Leaf	Okanin-4'-(6''-O-acetyl)glucoside*	Flavonoids	Chalcones	1.29940575	0.009771133	0.046617244	0.167842924	-2.574816379	3
Root vs Leaf	Apigenin-7-O-glucoside(Cosmosiin)*	Flavonoids	Flavones	1.067124858	0.002739775	0.026984272	0.197819018	-2.33774696	3
Root vs Leaf	2-(7-hydroxy-4-methoxy-9,10-dihydrophenanthren-2-yl)-6-(hydroxymethyl)oxane-3,4,5-triol	Others	Others	1.111387291	0.109894089	0.223406516	3.25662642	1.703378232	3
Root vs Leaf	Isorhamnetin-3-O	Flavonoids	Flavonols	1.131122989	0.024419104	0.077488521	0.070653993	-3.82308509	1

Comparison	Compound	Class I	Class II	VIP	P-value	FDR	Fold Change	Log_2FC	MSI Level
	-rutinoside (Narcissin)*								
Root vs Leaf	1-O-P-Coumaroyl -(B-D-Glucose 6-O-Sulfate)	Phenolic acids	Phenolic acids	1.184900593	0.150557097	0.282917951	28.21363745	4.818320772	2
Root vs Leaf	2,3-dihydro-6-hy droxyltryptophan N-Glucoside	Alkaloids	Plumerane	1.074827824	0.008185606	0.043144133	0.166090275	-2.58996049	3
Root vs Leaf	Verbasoside	Others	Alcohol compounds	1.303846547	0.021607052	0.072056852	0.004004332	-7.964222859	3
Root vs Leaf	Hypoglycyrrhizic acid(β)*	Terpenoids	Triterpene	1.081922887	0.001010835	0.02015943	0.162430113	-2.622108972	2
Root vs Leaf	Tianshic acid	Lipids	Free fatty acids	1.163739559	0.348922992	0.464533762	87.86391531	6.457198884	1

Comparison	Compound	Class I	Class II	VIP	P-value	FDR	Fold Change	Log_2FC	MSI Level
Root vs Leaf	Aminomalonic acid	Amino acids and derivatives	Amino acids and derivatives	1.241710394	0.002120339	0.025038538	0.289443552	-1.788646078	3
Root vs Leaf	7R,8S-erythro-3',4,9,9'-tetrahydroxy-3-methoxy-8-O-4'-neolignan-7-O-glucopyr-anoside,	Lignans and Coumarins	Lignans	1.019951572	0.123199728	0.243136633	2.278330042	1.187976753	2
Root vs Leaf	7-Caffeoylsedoheptulose	Phenolic acids	Phenolic acids	1.293782618	0.005577644	0.036629402	0.014414954	-6.116289922	1
Root vs Leaf	Herbacetin-7-O-(3''-glucosyl)rhamnoside; Rhodiosin	Flavonoids	Flavonols	1.304973385	0.001396427	0.022625269	0.142403847	-2.811939975	3

Comparison	Compound	Class I	Class II	VIP	P-value	FDR	Fold Change	Log_2FC	MSI Level
Root vs Leaf	D-Pinitol*	Others	Saccharides	1.237337307	0.002619972	0.02684374	0.372244335	-1.4256782	3
Root vs Leaf	L-Theanine	Amino acids and derivatives	Amino acids and derivatives	1.265509798	0.000255539	0.013805514	0.347341551	-1.525573089	3
Root vs Leaf	Kaempferol-3-O-rhamnoside (Afzelin)(Kaempferin)	Flavonoids	Flavonols	1.305713052	0.001876702	0.023320316	0.003613921	-8.112219412	1
Root vs Leaf	Agarotetrol	Others	Chromone	1.303929646	0.022324961	0.073431112	0.003176116	-8.298520644	3
Root vs Leaf	Oxiglutatione	Amino acids and derivatives	Amino acids and derivatives	1.075776794	0.017910189	0.064722273	0.161602836	-2.629475579	3
Root vs Leaf	L-Homoserine*	Amino acids	Amino acids	1.265812516	0.040503985	0.109173205	3.278368114	1.712977858	3

Comparison	Compound	Class I	Class II	VIP	P-value	FDR	Fold Change	Log_2FC	MSI Level
		and derivatives	and derivatives						
Root vs Leaf	Dihydrosinapic Acid glucoside	Phenolic acids	Phenolic acids	1.295138992	0.039933152	0.108139777	18.22087841	4.187520606	2
Root vs Leaf	Limocitrin-3-O-galactoside*	Flavonoids	Flavonols	1.204470488	0.034104187	0.097356867	0.104443474	-3.259205747	3
Root vs Leaf	3-(Hydroxycinnamoyl)-quinic acid*	Phenolic acids	Phenolic acids	1.296608653	0.01602805	0.060659572	0.090311255	-3.468950393	2
Root vs Leaf	Laricitrin-3-O-xyloside*	Flavonoids	Flavonols	1.299763026	0.015826878	0.060230686	0.076816066	-3.702448101	3
Root vs Leaf	7,8-Dimethoxy-6-hydroxycoumarin	Lignans and Coumarins	Coumarins	1.273227185	0.097533957	0.204830788	350.039338	8.451373253	3

Comparison	Compound	Class I	Class II	VIP	P-value	FDR	Fold Change	Log_2FC	MSI Level
Root vs Leaf	Hesperetin-7-O-neohesperidoside(Neohesperidin)	Flavonoids	Flavanones	1.267877776	0.010554577	0.047320417	0.006949139	-7.168950043	3
Root vs Leaf	Deacetylasperulosidic acid	Terpenoids	Monoterpenoids	1.124613946	0.000225341	0.013805514	0.082170402	-3.605237365	2
Root vs Leaf	Isopentenyladenine-7-N-glucoside	Nucleotides and derivatives	Nucleotides and derivatives	1.138474959	0.161452871	0.298204833	5.130616221	2.359132114	3
Root vs Leaf	DGMG 18:2(2n isomer)	Lipids	Glycerol ester	1.198396968	0.139774983	0.266596415	6.472537682	2.694331459	1
Root vs Leaf	LysoPE 16:1(2n isomer)*	Lipids	LPE	1.273172421	0.059900698	0.143988219	87.96261834	6.458818644	3
Root vs Leaf	2,7-Dimethylocta-	Terpenoids	Monoterpenoid	1.258201757	0.000254967	0.013805514	0.036704145	-4.76791319	2

Comparison	Compound	Class I	Class II	VIP	P-value	FDR	Fold Change	Log_2FC	MSI Level
	2,4-Dienal-8-ol 8-O-(beta-D-Xylo pyranosyl)-beta-D-glucopyranosid e		s						
Root vs Leaf	1-O-Acetyl-N-des methylrocaglami de	Flavonoids	Other Flavonoids	1.232059507	0.046062521	0.119929046	0.044103905	-4.502949807	3
Root vs Leaf	Methyl dioxindole-3-acet ate	Alkaloids	Plumerane	1.131603055	0.013285407	0.054443946	0.326784144	-1.613590113	3
Root vs Leaf	LysoPE 16:1*	Lipids	LPE	1.269223632	0.06234731	0.148404896	80.91144927	6.338271959	3
Root vs Leaf	L-Threonine*	Amino acids	Amino acids	1.238598317	0.073564397	0.167692681	2.926416017	1.549134876	3

Comparison	Compound	Class I	Class II	VIP	P-value	FDR	Fold Change	Log_2FC	MSI Level
		and derivatives	and derivatives						
Root vs Leaf	Blumenol C glucoside; Byzantionoside B	Terpenoids	Terpene	1.304710194	0.003984659	0.032250367	0.062195824	-4.007038468	3
Root vs Leaf	Rhododendrol	Others	Alcohol compounds	1.291583908	0.006281625	0.038725464	0.064002516	-3.965727572	3
Root vs Leaf	Galangin 7-glucoside	Flavonoids	Flavonols	1.07368877	0.017697393	0.064167898	0.220419073	-2.181679031	3
Root vs Leaf	DGMG 18:2	Lipids	Glycerol ester	1.244322447	0.062356153	0.148404896	5.661189508	2.501105219	1
Root vs Leaf	Quercetagetin; 3,3',4',5,6,7-Hexahydroxyflavone	Flavonoids	Flavonols	1.297010868	0.022791448	0.074337656	0.086485101	-3.531404568	2

Comparison	Compound	Class I	Class II	VIP	P-value	FDR	Fold Change	Log_2FC	MSI Level
Root vs Leaf	Rosmarinic acid-3'-O-glucoside	Phenolic acids	Phenolic acids	1.233680039	0.054457853	0.134035787	3.060203468	1.613627579	3
Root vs Leaf	L-Diaminopimelic acid	Amino acids and derivatives	Amino acids and derivatives	1.240971595	0.122048232	0.241525851	10.88213238	3.443889378	3
Root vs Leaf	1-(2,3-dihydroxypropoxy)-3-(((2-(dimethylamino)ethoxy)(hydroxy)phosphoryl)oxy)propan-2-yl (Z)-14-Octadecenoic Acid	Lipids	LPE	1.304356221	0.002583074	0.02684374	9.835412236	3.297985522	1

Comparison	Compound	Class I	Class II	VIP	P-value	FDR	Fold Change	Log_2FC	MSI Level
Root vs Leaf	2-(2,3-dihydroxypropoxy)-3-(((2-(dimethylamino)ethoxy)(hydroxy)phosphoryl)oxy)propan-2-yl (Z)-14-Octadecenoic Acid	Lipids	LPE	1.302077612	0.001014834	0.02015943	9.152769416	3.194208336	1
Root vs Leaf	Annuionone D	Others	Ketone compounds	1.287062692	0.051152699	0.127940953	0.035386372	-4.820662322	3
Root vs Leaf	4,8-Dihydroxyquinoline-2-carboxylic acid	Alkaloids	Quinoline alkaloids	1.214186686	0.003804557	0.031743814	0.018087903	-5.788831032	2
Root vs Leaf	Lariciresinol-4'-O-	Lignans and	Lignans	1.287772533	0.025923487	0.080308886	2.859432649	1.515728925	2

Comparison	Compound	Class I	Class II	VIP	P-value	FDR	Fold Change	Log_2FC	MSI Level
	glucoside*	Coumarins							
Root vs Leaf	Decanoyl arabinosylglucoside	Others	Saccharides	1.220296087	0.03461021	0.098671061	0.084635105	-3.562599994	2
Root vs Leaf	α-Hydrojuglone glucoside	Quinones	Quinones	1.302480624	0.010305994	0.047156746	0.059704539	-4.066015578	1
Root vs Leaf	Gallocatechin-(4alpha->8)-epigallocatechin	Tannins	Proanthocyanidins	1.305158872	0.006905853	0.040009515	0.00773761	-7.013896225	2
Root vs Leaf	Salidroside	Phenolic acids	Phenolic acids	1.231317909	0.003437748	0.030288731	0.383292884	-1.383480881	3
Root vs Leaf	2-O-P-Coumaroyl hydroxycitric	Phenolic acids	Phenolic acids	1.30561791	0.004385126	0.033039104	0.001137107	-9.780416836	3

Comparison	Compound	Class I	Class II	VIP	P-value	FDR	Fold Change	Log_2FC	MSI Level
	Acid								
Root vs Leaf	γ-Glu-Leu	Amino acids and derivatives	Amino acids and derivatives	1.199352511	0.245495376	0.373077009	19.77809863	4.305831834	3
Root vs Leaf	Tuberonic acid glucoside*	Organic acids	Organic acids	1.289517003	0.063892795	0.150733984	15.36389758	3.941472347	1
Root vs Leaf	Saccharumoside C	Others	Others	1.254866616	0.098404746	0.206058776	21.35382358	4.416422514	3
Root vs Leaf	Megastigm-5-en-3,9-diol glucoside	Terpenoids	Sesquiterpenoids	1.128376969	0.008323159	0.043550477	0.229532181	-2.123231655	3
Root vs Leaf	3,3',4',5',7-Pentahydroxyflavone; Robinetin	Flavonoids	Flavonols	1.288634225	0.030908605	0.089897033	0.120852745	-3.048677854	1

Comparison	Compound	Class I	Class II	VIP	P-value	FDR	Fold Change	Log_2FC	MSI Level
Root vs Leaf	1,2,4-Trihydroxynaphthalene-1,4-di-glucoside (Lawsoniaside)	Others	Others	1.298497945	0.003462597	0.030288731	0.006983499	-7.161834243	2
Root vs Leaf	β-Pseudouridine	Nucleotides and derivatives	Nucleotides and derivatives	1.226326148	0.0199189	0.068235021	0.085731382	-3.544032787	3
Root vs Leaf	Keto-Deoxy-Nonulonic acid	Lipids	Free fatty acids	1.148538106	0.011898624	0.050417503	0.294859768	-1.761899109	1
Root vs Leaf	Isorhamnetin-3-O-neohesperidoside*	Flavonoids	Flavonols	1.193013401	0.000456536	0.014948097	0.060042524	-4.057871553	1
Root vs Leaf	Cyanidin-3-O-ruti	Flavonoids	Anthocyanidin	1.276716879	0.047817511	0.122869967	0.001959053	-8.99562791	3

Comparison	Compound	Class I	Class II	VIP	P-value	FDR	Fold Change	Log_2FC	MSI Level
	noside (Keracyanin)		s						
Root vs Leaf	p-Hydroxypheny -β-D-allopyranoside	Others	Others	1.230789993	0.01076936	0.047787654	0.049374078	-4.340102385	1
Root vs Leaf	Icariside E5*	Lignans and Coumarins	Lignans	1.273558412	0.010072053	0.047111917	3.033826032	1.60113836	2
Root vs Leaf	Neoandrographolide	Terpenoids	Ditepenoids	1.305747006	0.000641752	0.017121318	0.000690429	-10.50021865	2
Root vs Leaf	Nagilactone C glucoside	Terpenoids	Ditepenoids	1.082944702	0.094261366	0.200293817	2.479221095	1.309886935	2
Root vs Leaf	1-Linoleoyl-2-Lysophosphatidic	Lipids	Glycerol ester	1.298676781	0.014672705	0.057545761	19.13390475	4.258059416	1

Comparison	Compound	Class I	Class II	VIP	P-value	FDR	Fold Change	Log_2FC	MSI Level
	Acid Monobutylamine Ester*								
Root vs Leaf	Dihydrodehydrodiconiferyl alcohol-4-O-glucoside*	Lignans and Coumarins	Lignans	1.001426958	0.024624875	0.077798764	0.318789292	-1.649324924	2
Root vs Leaf	Isoascorbic acid 2-O-glucoside	Others	Vitamin	1.233683684	0.028599192	0.085599519	0.239894316	-2.05952912	1
Root vs Leaf	2-β-D-Glucopyranosyloxy-5-hydroxy-phenylacetic acid	Phenolic acids	Phenolic acids	1.173408339	0.006376174	0.038725464	0.236299725	-2.081310145	1

Comparison	Compound	Class I	Class II	VIP	P-value	FDR	Fold Change	Log_2FC	MSI Level
Root vs Leaf	8-Epideoxyloganic acid	Terpenoids	Monoterpenoids	1.244463563	0.006275481	0.038725464	0.042433748	-4.558644073	2
Root vs Leaf	Beta-Hydroxypalmitic Acid*	Lipids	Free fatty acids	1.180613334	0.082294973	0.182026036	3.43010228	1.778251596	1
Root vs Leaf	2,7-Dimethyl-2,4-Octadiene-1,8-Diol 8-O-B-D-Glucopyranoside	Terpenoids	Monoterpenoids	1.267812048	0.032907809	0.094692112	0.006421313	-7.282915896	2
Root vs Leaf	Annulatin 3'-O-β-D-xyloside*	Flavonoids	Flavones	1.300315444	0.015251482	0.059065326	0.076218567	-3.713713702	3
Root vs Leaf	Maltotriose	Others	Saccharides	1.225461977	0.088262188	0.190925514	7.306363959	2.869153622	3

Comparison	Compound	Class I	Class II	VIP	P-value	FDR	Fold Change	Log_2FC	MSI Level
Root vs Leaf	(2S,3R,4S,5S,6R)-3,4,5-trihydroxy-6-(hydroxymethyl)oxan-2-yl(2E,6E)-8-hydroxy-2,6-dimethylocta-2,6-dienoate	Terpenoids	Monoterpenoids	1.301132003	0.014807496	0.057830518	0.081624264	-3.614858113	2
Root vs Leaf	Shikimic acid	Organic acids	Organic acids	1.251897826	0.006460683	0.038890072	0.01071281	-6.544519273	1
Root vs Leaf	1-(((2-(dimethylamino)ethoxy)(hydroxy)phosphoryl)oxy)-3-hydroxypropan-2-yl	Lipids	LPE	1.302278731	0.009119085	0.045345906	13.39355978	3.74346755	1

Comparison	Compound	Class I	Class II	VIP	P-value	FDR	Fold Change	Log_2FC	MSI Level
	(9Z,12Z)-octadeca -9,12-dienoate*								
Root vs Leaf	Isoscopoletin (6-Hydroxy-7-Methoxycoumarin)	Lignans and Coumarins	Coumarins	1.275004348	0.051599595	0.12861214	32.90197622	5.040102335	1
Root vs Leaf	2-(3,4-Dihydroxybenzoyloxy)-4,6-Dihydroxyphenylacetic Acid	Phenolic acids	Phenolic acids	1.305261814	0.00903148	0.045178305	0.003283521	-8.250540546	1
Root vs Leaf	Isofraxidin	Lignans and Coumarins	Coumarins	1.259837308	0.10256741	0.21230668	16.62328347	4.05513347	1
Root vs Leaf	Mannose 6-phosphate	Others	Saccharides	1.19943847	0.096244937	0.203110653	3.1779852	1.668112406	1

Comparison	Compound	Class I	Class II	VIP	P-value	FDR	Fold Change	Log_2FC	MSI Level
Root vs Leaf	Quercetin-3-O-(6'' -O-p-Coumaroyl) galactoside*	Flavonoids	Flavonols	1.258370858	8.71702E-05	0.012840208	0.014661342	-6.091839072	3
Root vs Leaf	Quercetin-3-O-(2'' -O-p-Coumaroyl) galactoside	Flavonoids	Flavonols	1.305222666	0.000816106	0.019191343	0.171884768	-2.540486391	3
Root vs Leaf	Phellodenol E	Lignans and Coumarins	Coumarins	1.217663204	0.014428963	0.057046076	0.030459193	-5.036978485	3
Root vs Leaf	Gingerglycolipid B	Lipids	Glycerol ester	1.21441797	0.116376217	0.233725842	5.828872256	2.543216784	2
Root vs Leaf	γ-Aminobutyric acid	Organic acids	Organic acids	1.217046736	0.354034854	0.468792475	174.6470957	7.448298841	2
Root vs Leaf	Nicotinamide	Others	Vitamin	1.072182389	0.270474492	0.396381437	6.971337468	2.801435467	3

Comparison	Compound	Class I	Class II	VIP	P-value	FDR	Fold Change	Log_2FC	MSI Level
Root vs Leaf	Quercetin-4'-O-glucuronide*	Flavonoids	Flavonols	1.20948275	0.024275246	0.077258919	0.185940139	-2.427089859	1
Root vs Leaf	Itoside L	Others	Others	1.300343824	0.00337686	0.030030429	0.074375352	-3.749031604	3
Root vs Leaf	Prodelphinidin B*	Flavonoids	Other Flavonoids	1.259869471	0.000865972	0.019301816	0.019787481	-5.65926824	3
Root vs Leaf	Cis-Coutaric acid	Phenolic acids	Phenolic acids	1.173091915	0.001613044	0.023084686	0.027321445	-5.193822396	1
Root vs Leaf	3-Oxo-alpha-ionol glucoside*	Terpenoids	Sesquiterpenoids	1.158618379	0.110879167	0.224985802	0.050058212	-4.32024944	2
Root vs Leaf	Grayanotoxin XVIII	Terpenoids	Ditepenoids	1.305123421	0.009148919	0.045345906	0.003326162	-8.231925839	2
Root vs Leaf	LysoPC 16:1*	Lipids	LPC	1.273776748	0.086375767	0.188050555	14.79610956	3.887145983	1

Comparison	Compound	Class I	Class II	VIP	P-value	FDR	Fold Change	Log_2FC	MSI Level
Root vs Leaf	1-O-Salicyloyl-β-D-glucose*	Phenolic acids	Phenolic acids	1.238918907	0.007027837	0.040284231	0.278897318	-1.842194035	1
Root vs Leaf	Norartocarpetin	Flavonoids	Flavones	1.27213668	0.062036334	0.148189941	0.008676095	-6.848738487	1
Root vs Leaf	Kaempferol-3-O-galactoside (Trifolin)*	Flavonoids	Flavonols	1.17604904	2.19316E-05	0.009478854	0.060410027	-4.049068167	1
Root vs Leaf	Tricetin 7-O-glucuronide*	Flavonoids	Flavones	1.246784724	0.002950583	0.027602643	0.165415634	-2.595832504	1
Root vs Leaf	LysoPC 16:1(2n isomer)*	Lipids	LPC	1.233993974	0.077578572	0.174524606	18.87770157	4.238611216	1
Root vs Leaf	7-Deoxyloganic acid	Terpenoids	Monoterpenoids	1.087994698	0.096924838	0.20394798	0.146434798	-2.771669668	1

Comparison	Compound	Class I	Class II	VIP	P-value	FDR	Fold Change	Log_2FC	MSI Level
Root vs Leaf	1-O-Linoleoyl-3-O-galactopyranosyl-L-glycerol	Lipids	Glycerol ester	1.174849465	0.203365809	0.335389786	7.599698963	2.925942272	1
Root vs Leaf	Azetidine-2-carboxylic acid*	Alkaloids	Alkaloids	1.056514019	0.278350232	0.402842285	6.13195724	2.616347637	3
Root vs Leaf	Quercetin-3-O-(6''-O-p-Coumaroyl)glucoside	Flavonoids	Flavonols	1.300408211	0.008507259	0.04398131	0.169198067	-2.563215005	3
Root vs Leaf	Mearnsetin-3-O-glucoside*	Flavonoids	Flavones	1.264995314	0.114120607	0.230480964	7.629309592	2.931552507	2
Root vs Leaf	Kaempferol-3-O-neohesperidoside*	Flavonoids	Flavonols	1.305042431	0.002776093	0.027023138	0.077586937	-3.688042414	3

Comparison	Compound	Class I	Class II	VIP	P-value	FDR	Fold Change	Log_2FC	MSI Level
Root vs Leaf	Calyxanthone	Flavonoids	Other Flavonoids	1.275241324	0.0043859	0.033039104	0.011207539	-6.479386681	2
Root vs Leaf	Ergotamine	Alkaloids	Alkaloids	1.30182486	0.003541388	0.030288731	5.503075127	2.460238024	3
Root vs Leaf	Quercetin-3,7-Di-O-rhamnoside	Flavonoids	Flavonols	1.305749623	0.000228764	0.013805514	0.005985719	-7.384259839	2
Root vs Leaf	3-Hydroxylup-12(13),20(29)-diene-28-oic acid	Terpenoids	Triterpene	1.273675668	0.041234715	0.110686424	0.035752194	-4.805824396	1
Root vs Leaf	Dihydrovomifoliol-O-β-D-glucoside*	Terpenoids	Sesquiterpenoids	1.291611698	0.00050718	0.01588428	0.063476455	-3.977634639	1
Root vs Leaf	7,2',4'-Trihydroxy-8,3'-dimethoxyis	Flavonoids	Other Flavonoids	1.255398863	0.012785727	0.053295824	0.015695462	-5.993508716	2

Comparison	Compound	Class I	Class II	VIP	P-value	FDR	Fold Change	Log_2FC	MSI Level
	oflavanglucoside								
Root vs Leaf	L-Lysine	Amino acids and derivatives	Amino acids and derivatives	1.164121741	0.327762788	0.44639891	56.55887683	5.821681566	2
Root vs Leaf	Lupane-20(29)-en -3-on-28-oic acid	Terpenoids	Triterpene	1.070345334	0.050304606	0.126699597	0.380323071	-1.394702638	1
Root vs Leaf	Naringenin-7-O-g lucoside (Prunin)*	Flavonoids	Flavanones	1.008471961	0.005744083	0.03716456	0.169283919	-2.562483162	2
Root vs Leaf	Okanin-4'-O-gluc oside(Marein)*	Flavonoids	Chalcones	1.280455032	0.01119923	0.048695245	0.027522755	-5.183231304	3
Root vs Leaf	Dehydrophytosp hingosine	Lipids	Sphingolipids	1.14067613	0.002207937	0.025163593	0.147116226	-2.764971718	1

Comparison	Compound	Class I	Class II	VIP	P-value	FDR	Fold Change	Log_2FC	MSI Level
Root vs Leaf	Uridine 5'-diphospho-D-glucose	Nucleotides and derivatives	Nucleotides and derivatives	1.283704171	0.030020208	0.087808622	5.695554303	2.509836255	1
Root vs Leaf	Tricetin 3'-glucuronide*	Flavonoids	Flavones	1.22261588	0.002729419	0.026984272	0.174428081	-2.519295775	1
Root vs Leaf	1-O-rhamnose-3-O-Caffeoyl Quinic Acid	Phenolic acids	Phenolic acids	1.28421877	0.008504358	0.04398131	0.004883182	-7.677962804	2
Root vs Leaf	Carthamone*	Flavonoids	Chalcones	1.247930246	0.006695076	0.039572387	0.026086445	-5.260555845	1
Root vs Leaf	4-O-Glucosyl-4-hydroxybenzoic acid	Phenolic acids	Phenolic acids	1.203065788	0.00194994	0.023320316	0.161446158	-2.63087499	1
Root vs Leaf	3-hydroxybenzoy	Phenolic	Phenolic acids	1.26997361	0.000274719	0.013981217	0.214154098	-2.223278808	1

Comparison	Compound	Class I	Class II	VIP	P-value	FDR	Fold Change	Log_2FC	MSI Level
	l-β-D-glucose*	acids							
Root vs Leaf	DGMG 18:3	Lipids	Glycerol ester	1.145142483	0.008754198	0.044722984	0.268927628	-1.894710119	1
Root vs Leaf	6'-O-Feruloyl-D-sucrose	Phenolic acids	Phenolic acids	1.291150429	0.00504003	0.034920839	0.012645893	-6.305187321	2
Root vs Leaf	1,3,6,8-tetrahydroxy-2,5-dimethoxyxanthen-9-one	Flavonoids	Other Flavonoids	1.296891368	0.001115454	0.020742409	0.008348763	-6.904221876	2
Root vs Leaf	L-Azetidine-2-carboxylic acid*	Alkaloids	Alkaloids	1.078954491	0.278586276	0.402842285	7.291853931	2.866285662	3
Root vs Leaf	DGMG 18:3(2n isomer)	Lipids	Glycerol ester	1.182383698	0.008169594	0.043144133	0.261542416	-1.934883159	1
Root vs Leaf	Murrayacarpin A	Lignans and	Coumarins	1.296340592	0.001545298	0.023084686	0.001822989	-9.099478818	3

Comparison	Compound	Class I	Class II	VIP	P-value	FDR	Fold Change	Log_2FC	MSI Level
	glucoside	Coumarins							
Root vs Leaf	5-O-Galloyl-D-hamamelose	Phenolic acids	Phenolic acids	1.24113755	0.02484901	0.078278004	0.06091757	-4.036997806	1
Root vs Leaf	Neoisoliquiritin	Flavonoids	Chalcones	1.305410278	0.00257326	0.02684374	0.031380482	-4.993988686	2
Root vs Leaf	Pyridoxine	Others	Vitamin	1.077555188	0.028871724	0.085939114	0.174161424	-2.521502987	3
Root vs Leaf	Sweroside	Terpenoids	Monoterpenoids	1.294961857	0.045771717	0.119315658	0.01848998	-5.757112559	2
Root vs Leaf	Neosakuranin	Flavonoids	Chalcones	1.174451665	0.005905716	0.037536039	0.283361064	-1.819286559	3
Root vs Leaf	D-Ornithine	Amino acids and derivatives	Amino acids and derivatives	1.251210239	0.184295066	0.324925253	221.8921042	7.793714522	2
Root vs Leaf	Frambinone	Others	Ketone	1.233438027	0.002166209	0.025163593	0.034746131	-4.847003845	3

Comparison	Compound	Class I	Class II	VIP	P-value	FDR	Fold Change	Log_2FC	MSI Level
			compounds						
Root vs Leaf	Isocoreopsin*	Flavonoids	Flavanones	1.121650361	0.005326049	0.036193684	0.165940063	-2.591265854	2
Root vs Leaf	Luteolin-7-O-rutinoside*	Flavonoids	Flavones	1.282505317	0.019920175	0.068235021	0.002503658	-8.64174653	3
Root vs Leaf	3-[4-(β-D-glucopyranoside)-phenyl acrylic]-acid	Phenolic acids	Phenolic acids	1.248166484	0.011633481	0.049782083	0.012847094	-6.282414086	1
Root vs Leaf	L-Glutamine	Amino acids and derivatives	Amino acids and derivatives	1.139528577	0.3401981	0.457148508	46.59427131	5.542080684	1
Root vs Leaf	Machilin H	Lignans and Coumarins	Lignans	1.000626748	0.001529582	0.023084686	0.145571816	-2.780197033	3

Comparison	Compound	Class I	Class II	VIP	P-value	FDR	Fold Change	Log_2FC	MSI Level
Root vs Leaf	Hastatoside*	Terpenoids	Monoterpenoids	1.180860491	0.139101689	0.265781389	4.459555286	2.15689985	2
Root vs Leaf	D-inositol-4-phosphate	Others	Saccharides	1.216952947	0.068944842	0.160204089	2.644047717	1.402748214	1
Root vs Leaf	Tricalysioside N	Terpenoids	Ditepenoids	1.302233372	0.012237621	0.051651364	0.064892852	-3.945796627	3
Root vs Leaf	6-Methoxyquercetin-3-O-Xyloside*	Flavonoids	Flavonols	1.301199262	0.010354598	0.047156746	0.130774736	-2.934844235	3
Root vs Leaf	Sibiricose A6	Phenolic acids	Phenolic acids	1.296931144	0.006606153	0.039327537	0.007786263	-7.004853224	3
Root vs Leaf	Kaempferol-3-O-robinobioside(Biorobin)*	Flavonoids	Flavonols	1.30521854	0.001920762	0.023320316	0.086832706	-3.525617638	2

Comparison	Compound	Class I	Class II	VIP	P-value	FDR	Fold Change	Log_2FC	MSI Level
Root vs Leaf	Ternatumoside II	Flavonoids	Flavonols	1.305119966	0.00147226	0.023084686	0.094961858	-3.396508024	3
Root vs Leaf	Homoproline	Amino acids and derivatives	Amino acids and derivatives	1.269078516	0.228406059	0.355908435	56.23605379	5.813423456	3
Root vs Leaf	6,9,10-Trihydroxy octadec-7-enoic acid; Sanleng acid*	Lipids	Free fatty acids	1.142134694	0.39308149	0.499486107	344.4074185	8.427972409	1
Root vs Leaf	Rhein-8-O-glucoside	Quinones	Anthraquinone	1.25115831	0.123965769	0.244425206	61.72351844	5.947748397	3
Root vs Leaf	4'-Hydroxy-3,5-dimethoxydihydrochalcone	Flavonoids	Chalcones	1.304897891	0.014570409	0.057352741	0.002358447	-8.727946877	3

Comparison	Compound	Class I	Class II	VIP	P-value	FDR	Fold Change	Log_2FC	MSI Level
Root vs Leaf	Kaempferol-3-O-(6''-O-acetyl)glucoside	Flavonoids	Flavonols	1.290360659	0.000682554	0.017564328	0.018626062	-5.746533525	1
Root vs Leaf	LysoPE 18:1*	Lipids	LPE	1.278023692	0.023365683	0.075368078	13.64978065	3.770805862	3
Root vs Leaf	Kaempferol-7-O-glucoside*	Flavonoids	Flavonols	1.258348388	0.001659881	0.023320316	0.032616078	-4.938272887	1
Root vs Leaf	Chrysoeriol-5-O-glucoside	Flavonoids	Flavones	1.273641292	0.001889815	0.023320316	0.013073404	-6.257221337	3
Root vs Leaf	3-O-(p-coumaroyl) 3-Hydroxy-3-methylglutaric acid	Phenolic acids	Phenolic acids	1.275692871	0.013574473	0.055347994	0.003064418	-8.350171375	3
Root vs Leaf	O-Feruloyl	Lignans and	Coumarins	1.263014716	0.009487472	0.046176635	0.008397582	-6.895810288	2

Comparison	Compound	Class I	Class II	VIP	P-value	FDR	Fold Change	Log_2FC	MSI Level
	7-hydroxycoumarin*	Coumarins							
Root vs Leaf	Gingerglycolipid A	Lipids	Glycerol ester	1.198558871	0.00752942	0.041507847	0.253412196	-1.980442136	2
Root vs Leaf	Lyoniresinol-3-O-glucoside*	Lignans and Coumarins	Lignans	1.125837489	0.233813202	0.361165353	126.5421909	6.983474669	1
Root vs Leaf	1-O-Galloyl-2-O-Cinnamoyl-β-D-glucose	Phenolic acids	Phenolic acids	1.298125434	0.002611264	0.02684374	0.020922489	-5.578801733	2
Root vs Leaf	Pyridoxine-5'-O-glucoside	Others	Vitamin	1.087447738	0.070661588	0.16261948	0.448565812	-1.156608429	3
Root vs Leaf	LysoPE 18:1(2n isomer)*	Lipids	LPE	1.292426721	0.053197702	0.131986491	18.9574811	4.24469538	3

Comparison	Compound	Class I	Class II	VIP	P-value	FDR	Fold Change	Log_2FC	MSI Level
Root vs Leaf	Tricalysioside O	Terpenoids	Ditepenoids	1.302317381	0.010379871	0.047156746	0.062615694	-3.997331881	3
Root vs Leaf	Inositol	Others	Saccharides	1.284783392	0.001146653	0.020742409	0.094965425	-3.396453839	1
Root vs Leaf	Quercetin-3-O-(2''-O-acetyl)rhamnoside	Flavonoids	Flavonols	1.297840576	0.0229527	0.074586808	0.06880592	-3.861323498	2
Root vs Leaf	Grevilloside G	Phenolic acids	Phenolic acids	1.167661725	0.000141385	0.013805514	0.071159636	-3.812797051	3
Root vs Leaf	2α,3α-Dihydroxyolean-12-en-28-oic acid	Terpenoids	Triterpene	1.143029115	0.007804904	0.04213351	0.288690511	-1.792404406	2
Root vs Leaf	Arbutin	Others	Others	1.252845311	0.025409448	0.079261115	0.0384691	-4.700156108	1
Root vs Leaf	Cyanidin-3-O-glu	Flavonoids	Anthocyanidin	1.287693896	0.036480153	0.102116076	0.003330035	-8.230247076	3

Comparison	Compound	Class I	Class II	VIP	P-value	FDR	Fold Change	Log_2FC	MSI Level
	coside*		s						
Root vs Leaf	Actinidialactone	Terpenoids	Monoterpenoids	1.275197049	0.002075495	0.024643651	0.133416807	-2.905987679	3
Root vs Leaf	Kaempferol-3-O-(6''-malonyl)glucoside*	Flavonoids	Flavonols	1.278682474	0.005027821	0.034920839	0.023686471	-5.399792915	1
Root vs Leaf	2-Hydroxy-4-methyl-3-undecanoyloxypentanoic acid methyl ester*	Lipids	Free fatty acids	1.127165385	0.393626263	0.499780467	276.5356399	8.111321617	1
Root vs Leaf	3-O-Feruloyl Aminogalactitol	Phenolic acids	Phenolic acids	1.239430253	0.000397911	0.014825611	0.236726194	-2.078708746	1
Root vs Leaf	N-Caffeoylmethyl	Alkaloids	Phenolamine	1.302978251	0.026777929	0.082080998	0.003395289	-8.202249937	1

Comparison	Compound	Class I	Class II	VIP	P-value	FDR	Fold Change	Log_2FC	MSI Level
	gluconamide*								
Root vs Leaf	5-O-Caffeoylshikimic acid	Phenolic acids	Phenolic acids	1.194984296	0.011438971	0.04934055	0.037802704	-4.725366746	1
Root vs Leaf	Micheliolide	Terpenoids	Sesquiterpenoids	1.122479197	0.182782353	0.324925253	217.4808222	7.764744377	2
Root vs Leaf	L-Ornithine	Amino acids and derivatives	Amino acids and derivatives	1.157871146	0.239356532	0.367625776	59.84297807	5.903110067	3
Root vs Leaf	Bismurrangatin	Lignans and Coumarins	Coumarins	1.261844313	0.012873864	0.053295824	0.028840095	-5.115780256	3
Root vs Leaf	5-O-p-Coumaroyl galactaric acid	Phenolic acids	Phenolic acids	1.297651718	0.003880534	0.031859153	0.005527127	-7.499254581	2

Comparison	Compound	Class I	Class II	VIP	P-value	FDR	Fold Change	Log_2FC	MSI Level
Root vs Leaf	Kaempferol-3-O-(6''-malonyl)galactoside*	Flavonoids	Flavonols	1.293077508	0.00266691	0.02684374	0.02126473	-5.555393623	1
Root vs Leaf	Cyanidin-3-O-galactoside*	Flavonoids	Anthocyanidins	1.302432765	0.036640202	0.102299065	0.004014359	-7.960614547	3
Root vs Leaf	β-Hydroxy-(3,4-dihydroxyphenylethanolyl)-glucoside	Others	Alcohol compounds	1.184475876	0.001074689	0.020735738	0.072249846	-3.79086167	1
Root vs Leaf	5-O-Malonylisosakuranin	Flavonoids	Flavanones	1.240169959	0.018650872	0.065857084	0.04578053	-4.449122019	2
Root vs Leaf	3,4'-Dihydroxy-3',5'-dimethoxyprop	Others	Ketone compounds	1.170180763	0.090464314	0.194520778	3.107385318	1.635701148	1

Comparison	Compound	Class I	Class II	VIP	P-value	FDR	Fold Change	Log_2FC	MSI Level
	iophenone								
Root vs Leaf	(+)-Lyoniresinol 9'-O-glucoside*	Lignans and Coumarins	Lignans	1.190633206	0.205965575	0.337702281	225.0320579	7.813986731	1
Root vs Leaf	(3b,16a,20R)-25-Acetoxy-3,16,20,22-tetrahydroxy-5-cucurbiten-11-one 3-glucoside	Steroids	Steroidal saponins	1.2743815	0.014825593	0.057830518	0.028060329	-5.155324261	1
Root vs Leaf	Luteolin-7-O-neohesperidoside (Lonicerin)*	Flavonoids	Flavones	1.303799353	0.005593569	0.036629402	0.07269739	-3.781952626	3
Root vs Leaf	Chrysoeriol-7-O-glucoside	Flavonoids	Flavones	1.268871593	0.003855019	0.031796549	0.017625258	-5.826211819	2

Comparison	Compound	Class I	Class II	VIP	P-value	FDR	Fold Change	Log_2FC	MSI Level
Root vs Leaf	3,9-dihydroeucomnalin glucoside*	Flavonoids	Other Flavonoids	1.242977442	0.06650783	0.156051489	4.23641392	2.082843555	2
Root vs Leaf	6-[(7-hydroxy-2-methyl-6-oxo-3,4-dihydroisoquinolin-1-yl)methyl]-2,3-dimethoxybenzoic acid	Alkaloids	Isoquinoline alkaloids	1.260089035	0.000268929	0.013981217	0.005295848	-7.560922654	1
Root vs Leaf	Desmethylagrimonolide-6-O-β-D-glucopyranoside*	Lignans and Coumarins	Coumarins	1.273406693	0.004051578	0.032253374	0.013067825	-6.257837144	3
Root vs Leaf	5-O-Feruloylquinic acid	Phenolic acids	Phenolic acids	1.277184426	0.002266083	0.025163593	0.002206294	-8.824159355	1

Comparison	Compound	Class I	Class II	VIP	P-value	FDR	Fold Change	Log_2FC	MSI Level
Root vs Leaf	Quercetin-3-O-(6''-O-malonyl)glucoside	Flavonoids	Flavonols	1.257539177	0.000319115	0.01464066	0.157074154	-2.670482284	1
Root vs Leaf	Phenylpropionic acid-O-β-D-glucopyranoside*	Phenolic acids	Phenolic acids	1.269086997	0.001822696	0.023320316	0.008161256	-6.936993173	1
Root vs Leaf	Kaempferol-3-O-glucorhamnoside*	Flavonoids	Flavonols	1.28949244	0.007621296	0.041907431	0.001803951	-9.114624165	3
Root vs Leaf	Hydroxypentadecenoic acid glucoside	Lipids	Free fatty acids	1.304546146	0.004576134	0.033309066	0.07471237	-3.742509056	1
Root vs Leaf	2-O-Galloyl-D-gl	Phenolic	Phenolic acids	1.274195487	0.002585232	0.02684374	0.086187087	-3.536384455	2

Comparison	Compound	Class I	Class II	VIP	P-value	FDR	Fold Change	Log_2FC	MSI Level
	ucose	acids							
Root vs Leaf	8-Methoxykaempferol-7-O-rhamnoside	Flavonoids	Flavonols	1.304942957	0.006349059	0.038725464	0.023273223	-5.425185156	1
Root vs Leaf	Desmethylagrimonolide-6-O-glucoside*	Lignans and Coumarins	Coumarins	1.217432022	0.001286268	0.022042878	0.013538882	-6.206747575	3
Root vs Leaf	p-Coumaric acid-4-O-glucoside	Phenolic acids	Phenolic acids	1.301786977	0.008606535	0.044177488	0.023311037	-5.422843022	1
Root vs Leaf	2,3-Dihydroxyurs-12-en-28-oic acid methyl ester	Terpenoids	Triterpene	1.289260096	6.63768E-06	0.004087201	0.069427685	-3.848345124	3

Comparison	Compound	Class I	Class II	VIP	P-value	FDR	Fold Change	Log_2FC	MSI Level
	(Corosolic acid methyl ester)								
Root vs Leaf	Geniposide	Terpenoids	Monoterpenoids	1.264907912	0.004548933	0.033309066	0.091531134	-3.449593643	3
Root vs Leaf	Isoscutellarein	Flavonoids	Flavones	1.304695322	0.010499981	0.047173513	0.008275649	-6.916911902	3
Root vs Leaf	O-p-Coumaroylgalactaric acid	Phenolic acids	Phenolic acids	1.302012535	0.000640135	0.017121318	0.004015964	-7.960037929	1
Root vs Leaf	Cyanidin 3-O-(6-O-p-coumaroyl)glucoside	Flavonoids	Anthocyanidins	1.305202892	0.008961426	0.045060703	0.001093351	-9.837028125	3
Root vs Leaf	L-Proline*	Alkaloids	Pyrrole alkaloids	1.171580225	0.128538854	0.250623794	6.943258284	2.79561284	3

Comparison	Compound	Class I	Class II	VIP	P-value	FDR	Fold Change	Log_2FC	MSI Level
Root vs Leaf	Luteolin-7-O-(6''-malonyl)glucoside*	Flavonoids	Flavones	1.268291423	0.027026705	0.082609208	0.031604662	-4.983718784	1
Root vs Leaf	Melilotoside*	Phenolic acids	Phenolic acids	1.302169665	0.00842661	0.043879287	0.022055506	-5.502717341	1
Root vs Leaf	D-Xylonic acid	Others	Saccharides	1.138398097	0.015883279	0.060322964	0.388204325	-1.365111903	3
Root vs Leaf	Quercetin-3-O-(2''-O-acetyl)glucuronide	Flavonoids	Flavonols	1.075812528	0.000255296	0.013805514	0.082657819	-3.596704896	1
Root vs Leaf	Kaempferol-3-O-glucoside-7-O-rhamnoside*	Flavonoids	Flavonols	1.275776497	0.006875513	0.040009515	0.002372881	-8.719144823	3
Root vs Leaf	4-caffeoylshikimi	Phenolic	Phenolic acids	1.203718394	0.001867528	0.023320316	0.039467605	-4.663187225	1

Comparison	Compound	Class I	Class II	VIP	P-value	FDR	Fold Change	Log_2FC	MSI Level
	c acid	acids							
Root vs Leaf	Azaleatin (5-O-Methylquercetin)	Flavonoids	Flavonols	1.258013563	0.003494968	0.030288731	0.014002441	-6.15817784	1
Root vs Leaf	1-O-Vanilloyl-D-Glucose	Phenolic acids	Phenolic acids	1.190027933	0.061768751	0.147984779	0.047066108	-4.409167635	1
Root vs Leaf	Rubianol-f	Terpenoids	Triterpene	1.028970902	0.021568273	0.072038699	0.347392152	-1.525362934	3
Root vs Leaf	3-Hydroxyolean-12-en-28-oic acid (Oleanolic acid)*	Terpenoids	Triterpene	1.124845184	0.163861631	0.301622644	0.213030411	-2.230868699	1
Root vs Leaf	24,30-Dihydroxy-12(13)-ene-lupeol*	Terpenoids	Triterpene	1.186416431	0.015484037	0.059751792	0.416433054	-1.263843509	1

Comparison	Compound	Class I	Class II	VIP	P-value	FDR	Fold Change	Log_2FC	MSI Level
Root vs Leaf	2-(2-(3,4-dihydroxyphenyl)-2-methoxyethoxy)-6-(hydroxymethyl)tetrahydro-2H-pyran-3,4,5-tri	Phenolic acids	Phenolic acids	1.304007031	0.004104432	0.032253374	0.09428976	-3.406755087	2
Root vs Leaf	2,3,4-trihydroxy-5-{[3-(4-hydroxyphenyl)prop-2-enoyl]oxy}hexanedioic acid	Phenolic acids	Phenolic acids	1.298922335	0.028808456	0.085879133	0.001754436	-9.15477721	1
Root vs Leaf	Laricitrin-3-O-glucoside*	Flavonoids	Flavonols	1.272511987	5.62049E-05	0.012840208	0.038896872	-4.684202049	1
Root vs Leaf	Quercetin-3-O-(6''	Flavonoids	Flavonols	1.146271027	0.094485038	0.200571873	0.158854985	-2.654217734	1

Comparison	Compound	Class I	Class II	VIP	P-value	FDR	Fold Change	Log_2FC	MSI Level
	-O-acetyl)galactoside								
Root vs Leaf	4-O-(4'-O-alpha-D-Glucopyranosyl) caffeoylquinic acid	Phenolic acids	Phenolic acids	1.29569095	0.000976816	0.02015943	0.000366398	-11.41429898	1
Root vs Leaf	2,3,19-Trihydroxyurs-12-en-28-oic acid (Tormentic acid)*	Terpenoids	Triterpene	1.247200688	0.00011374	0.012840208	0.074069125	-3.754983888	2
Root vs Leaf	2,3,19-trihydroxyurs-12-en-28-oic acid (Euscaphic acid)*	Terpenoids	Triterpene	1.240005299	0.000337559	0.01464066	0.083219284	-3.586938313	2

Comparison	Compound	Class I	Class II	VIP	P-value	FDR	Fold Change	Log_2FC	MSI Level
Root vs Leaf	β-D-Fructose 2-phosphate*	Others	Saccharides	1.189687099	0.151640353	0.284210583	4.907895692	2.295104587	1
Root vs Leaf	Secoisolariciresinol-9'-O-xyloside	Lignans and Coumarins	Lignans	1.049432727	0.045488947	0.118721757	3.901446589	1.96400915	2
Root vs Leaf	Ferulic β-glucoside	Phenolic acids	Phenolic acids	1.26548475	0.00383154	0.031796549	0.006316376	-7.306687216	2
Root vs Leaf	Selagin	Flavonoids	Flavones	1.202501217	0.048485811	0.123850872	0.056617127	-4.142617644	1
Root vs Leaf	Cannabifolin F	Terpenoids	Triterpene	1.247424725	0.000224292	0.013805514	0.073222844	-3.771562383	3
Root vs Leaf	4-(2,6-Dihydroxybenzoyl)-3-Formyl-5-Hydroxybenzoic acid	Phenolic acids	Phenolic acids	1.257170765	0.029335667	0.086672116	0.016150758	-5.952254323	1

Comparison	Compound	Class I	Class II	VIP	P-value	FDR	Fold Change	Log_2FC	MSI Level
Root vs Leaf	3-hydroxy-1-methylpyrrolidin-2-one*	Alkaloids	Pyrrole alkaloids	1.144638142	0.16201298	0.298728712	10.25647781	3.358463474	3
Root vs Leaf	1-O-(p-coumaroyl) 3-Hydroxy-3-methylglutaric acid	Phenolic acids	Phenolic acids	1.274233332	0.071541108	0.164468441	0.116179033	-3.105578371	3
Root vs Leaf	1-O-p-Coumaroyl Octose	Phenolic acids	Phenolic acids	1.287425626	0.011852169	0.050319328	0.008843995	-6.821086018	1
Root vs Leaf	(7S,8S-Threo-3',4,7,9-tetrahydroxy-3-methoxy-8-O-4'-neolignan-9'-O-rhamnoside	Lignans and Coumarins	Lignans	1.300269256	0.00547626	0.036629402	0.008740595	-6.838052802	1

Comparison	Compound	Class I	Class II	VIP	P-value	FDR	Fold Change	Log_2FC	MSI Level
Root vs Leaf	D-Fructose 6-Phosphate*	Others	Saccharides	1.278796345	0.018214674	0.065061009	4.735245517	2.24343923	1
Root vs Leaf	1-Amino-1-cyclob utane-carboxylic-acid	Amino acids and derivatives	Amino acids and derivatives	1.161665592	0.129850469	0.252250347	6.723208339	2.749149856	3
Root vs Leaf	Methyl 3-O-glucosyl-4-hy droxybenzoate	Phenolic acids	Phenolic acids	1.233896432	0.000634494	0.017121318	0.073025339	-3.775459036	1
Root vs Leaf	13-Hydroxy oleanolic acid-3-oxo-28-aci d	Terpenoids	Triterpene	1.306646675	0.00099536	0.02015943	0.078211531	-3.676474867	1
Root vs Leaf	Quercetin-3-O-gl	Flavonoids	Flavonols	1.22685315	0.000592699	0.016634047	0.134731436	-2.891841586	1

Comparison	Compound	Class I	Class II	VIP	P-value	FDR	Fold Change	Log_2FC	MSI Level
	ucose-6''-acetate								
Root vs Leaf	2-O-α-D-Glucopyranosyl-L-ascorbic acid	Others	Vitamin	1.107287061	0.037733605	0.104066249	0.271569656	-1.880605808	1
Root vs Leaf	Quercetin-3-O-neohesperidoside*	Flavonoids	Flavonols	1.300641979	0.000437757	0.014948097	0.023462979	-5.413470013	1
Root vs Leaf	Quercetin-3-O-robinobioside	Flavonoids	Flavonols	1.268817423	0.008506816	0.04398131	0.024787996	-5.334214563	2
Root vs Leaf	1,3-O-Dicaffeoylquinic Acid (Cynarin)	Phenolic acids	Phenolic acids	1.305526812	0.000620661	0.017121318	0.074933149	-3.738252106	2
Root vs Leaf	Epigallocatechin-(4beta->8)-catechin	Tannins	Proanthocyanidins	1.297422743	0.002586569	0.02684374	0.001343275	-9.540029548	1

Comparison	Compound	Class I	Class II	VIP	P-value	FDR	Fold Change	Log_2FC	MSI Level
Root vs Leaf	Delphinidin-3-O-(6''-O-p-coumaroyl)glucoside	Flavonoids	Anthocyanidins	1.292065275	0.020730199	0.070106353	0.027238984	-5.198183313	3
Root vs Leaf	N-Monomethyl-L-arginine*	Amino acids and derivatives	Amino acids and derivatives	1.100169754	0.36831349	0.48034125	66.18301302	6.048389068	3
Root vs Leaf	Homovanillic acid sulfate*	Phenolic acids	Phenolic acids	1.21318982	0.005613629	0.036649706	0.248568733	-2.008283258	1
Root vs Leaf	Taxifolin-7-O-rhamnoside	Flavonoids	Flavanonols	1.298515238	0.005371325	0.036386941	0.001999835	-8.965903125	3
Root vs Leaf	p-Coumaroyl Aminogalactitol	Phenolic acids	Phenolic acids	1.296152417	0.002325316	0.025472219	0.006204847	-7.332388601	1
Root vs Leaf	6-Deoxy-6-sulfogl	Others	Lactones	1.055786036	0.057792527	0.140483296	0.352985213	-1.502320348	1

Comparison	Compound	Class I	Class II	VIP	P-value	FDR	Fold Change	Log_2FC	MSI Level
	ucono-1,5-lactone								
Root vs Leaf	Arginine methyl ester*	Amino acids and derivatives	Amino acids and derivatives	1.078487025	0.372157398	0.483022305	58.83332716	5.878561721	3
Root vs Leaf	9-O-Methylvibsanol	Lignans and Coumarins	Lignans	1.290434525	0.006417595	0.038738614	0.003307532	-8.240029173	3
Root vs Leaf	Quercetin-3-O-(4''-O-glucosyl)rhamnoside*	Flavonoids	Flavonols	1.294976059	0.007023515	0.040284231	0.020334472	-5.619928692	1
Root vs Leaf	Protocatechuic acid 1-O-(Glucosylvanilloyl)	Phenolic acids	Phenolic acids	1.286619079	0.001404977	0.022625269	0.0464816	-4.427196474	1

Comparison	Compound	Class I	Class II	VIP	P-value	FDR	Fold Change	Log_2FC	MSI Level
Root vs Leaf	2-(2,3-dihydroxypropoxy)-3-(((2-(dimethylamino)ethoxy)(hydroxy)phosphoryl)oxy)propyl (11Z,14Z)-octadeca-11,14-dienoate	Lipids	LPE	1.273651202	0.044532477	0.116789662	4.873036482	2.284821023	1
Root vs Leaf	Quercetin-7-O-rutinoside*	Flavonoids	Flavonols	1.285155466	0.009128799	0.045345906	0.019806862	-5.657855852	1
Root vs Leaf	Citric Acid diglucoside	Organic acids	Organic acids	1.25068532	0.003892095	0.031859153	0.071799331	-3.799885794	1
Root vs Leaf	3-O-Methylellagic acid	Tannins	Tannin	1.13813576	0.292412802	0.416823261	15.49038946	3.953301512	1

Comparison	Compound	Class I	Class II	VIP	P-value	FDR	Fold Change	Log_2FC	MSI Level
	4'-rhamnoside								
Root vs Leaf	Quercetin-3-O-rutinoside (Rutin)*	Flavonoids	Flavonols	1.286909806	0.007074469	0.04034129	0.022978878	-5.44354782	1
Root vs Leaf	Caffeoylmethylgluconamide*	Alkaloids	Alkaloids	1.290573266	0.027596805	0.083524784	0.004059397	-7.944519021	1
Root vs Leaf	1-(2,3-dihydroxypropoxy)-3-(((2-(dimethylamino)ethoxy)(hydroxy)phosphoryl)oxy)propan-2-yl (11Z,14Z)-octadeca-11,14-dienoate	Lipids	LPE	1.276577316	0.044509799	0.116789662	5.450531023	2.446396793	1

Comparison	Compound	Class I	Class II	VIP	P-value	FDR	Fold Change	Log_2FC	MSI Level
Root vs Leaf	5-Glucosyloxy-2-Hydroxybenzoic acid methyl ester	Phenolic acids	Phenolic acids	1.248487672	0.002543663	0.02684374	0.060839181	-4.038855465	1
Root vs Leaf	1,8-dihydroxy-4,5-dimethoxy-3-{[(2s,3r,4s,5s,6r)-3,4,5-trihydroxy-6-(hydroxymethyl)oxan-2-yl]oxy}xanthen-9-one	Flavonoids	Other Flavonoids	1.239812606	0.002243559	0.025163593	0.077921985	-3.681825762	2
Root vs Leaf	Epigallocatechin	Flavonoids	Flavanols	1.028014889	0.015606595	0.059797609	0.045008012	-4.47367436	3
Root vs Leaf	-Pierisformoside H	Terpenoids	Ditepenoids	1.283719887	0.005307005	0.036193684	0.00096935	-10.0106947	2

Comparison	Compound	Class I	Class II	VIP	P-value	FDR	Fold Change	Log_2FC	MSI Level
Root vs Leaf	Hesperetin-7-O-glucoside*	Flavonoids	Flavanones	1.27682018	0.056692255	0.138443392	0.040921186	-4.611008227	3
Root vs Leaf	1,2,5,7,8-pentahydroxy-3-methylanthracene-9,10-dione	Quinones	Quinones	1.278106936	0.005311099	0.036193684	0.014640472	-6.093894098	1
Root vs Leaf	Pierisfomoside C	Others	Others	1.051652219	0.148538229	0.281078033	0.20653914	-2.275512891	1
Root vs Leaf	4-O-(3'-O-alpha-D-Glucopyranosyl) caffeoylquinic acid	Phenolic acids	Phenolic acids	1.303090407	0.001601409	0.023084686	0.002904117	-8.427684587	1
Root vs Leaf	robustaquinone F	Flavonoids	Other Flavonoids	1.281990388	0.001758025	0.023320316	0.010979118	-6.509094033	3

Comparison	Compound	Class I	Class II	VIP	P-value	FDR	Fold Change	Log_2FC	MSI Level
Root vs Leaf	4-Methylazetidine-2-Carboxylic acid*	Alkaloids	Alkaloids	1.155023995	0.21887031	0.347365541	30.2922662	4.920877607	3
Root vs Leaf	Tamarixetin-3-O-glucoside (Tamarixin)	Flavonoids	Flavonols	1.280171963	0.026962028	0.082528247	0.23599701	-2.083159512	2
Root vs Leaf	2(4H)-benzofuranone	Others	Others	1.227583756	0.029943611	0.087808622	0.003997248	-7.966777236	3
Root vs Leaf	caesalpiniaphenol A	Flavonoids	Flavones	1.231094694	0.018092329	0.065054116	0.060857218	-4.038427816	3
Root vs Leaf	Hydroxymenisdaurin D	Alkaloids	Alkaloids	1.016951959	0.069164618	0.160369893	0.4361731	-1.197027296	3
Root vs Leaf	Cimidahurinine	Others	Alcohol	1.179946886	0.018211967	0.065061009	0.100469643	-3.31516844	1

Comparison	Compound	Class I	Class II	VIP	P-value	FDR	Fold Change	Log_2FC	MSI Level
			compounds						
Root vs Leaf	2',3',4',5,7-pentahydroxyflavone	Flavonoids	Flavones	1.289507809	0.001308969	0.022042878	0.011722667	-6.414555402	1
Root vs Leaf	9-hydroxy-2-(3-hydroxy-4-methoxyphenyl)-3-(hydroxymethyl)-12-methoxy-2,3-dihydro-1,4,5-trioxatetraphen-10-one	Others	Ketone compounds	1.148381609	0.007097051	0.040359809	0.021614546	-5.53185366	1
Root vs Leaf	4-O-p-Coumaroyl quinic acid	Phenolic acids	Phenolic acids	1.273315646	0.003352958	0.030030429	0.005459132	-7.517112622	1
Root vs Leaf	Quercetin-3-O-gl	Flavonoids	Flavonols	1.300412968	0.000491181	0.015609451	0.019291475	-5.695892721	1

Comparison	Compound	Class I	Class II	VIP	P-value	FDR	Fold Change	Log_2FC	MSI Level
	ucoside-7-O-rham noside*								
Root vs Leaf	Pedaliin*	Flavonoids	Flavones	1.179106602	0.165574344	0.303237634	0.083117605	-3.588702106	3
Root vs Leaf	Rutin Trihydrate	Flavonoids	Flavonols	1.293762323	0.00982589	0.046617244	0.021836349	-5.517124516	3
Root vs Leaf	3-Dehydro-L-Thr eonic Acid	Others	Saccharides	1.169919213	0.009836864	0.046617244	0.315198274	-1.665668459	1
Root vs Leaf	3'-O-Methyltriceti n-7-O-glucoside*	Flavonoids	Flavones	1.304743614	0.006255102	0.038725464	0.016320235	-5.937194376	1
Root vs Leaf	Patuletin-3-O-glu coside*	Flavonoids	Flavonols	1.279776567	0.001134241	0.020742409	0.024871631	-5.329355075	1
Root vs Leaf	3,7-Dimethyl-2,6- Octadienyl	Terpenoids	Monoterpenoid s	1.145115848	0.004359325	0.033039104	0.095687895	-3.385519758	2

Comparison	Compound	Class I	Class II	VIP	P-value	FDR	Fold Change	Log_2FC	MSI Level
	6-O-(beta-D-Xylo pyranosyl)-beta-D-glucopyranoside								
Root vs Leaf	Rubiarbonone E	Terpenoids	Triterpene	1.268415625	0.031478914	0.091187579	4.800108426	2.263066994	3
Root vs Leaf	Benzhydryl methyl ether*	Others	Others	1.143253679	0.085882378	0.187727467	0.498159013	-1.005321768	1
Root vs Leaf	Salicylic acid-2-O-glucoside	Phenolic acids	Phenolic acids	1.179584763	0.004810662	0.034196844	0.168591775	-2.568393941	3
Root vs Leaf	Pipecolic acid	Alkaloids	Piperidine alkaloids	1.122439483	0.30657617	0.426993967	17.39365385	4.120489124	3
Root vs Leaf	Leucocyanidin;	Flavonoids	Flavanols	1.27579087	0.017025757	0.062466316	0.003646468	-8.099284485	1

Comparison	Compound	Class I	Class II	VIP	P-value	FDR	Fold Change	Log_2FC	MSI Level
	3,4,5,7,3',4'-Hexahydroxyflavan								
Root vs Leaf	Turanose	Others	Saccharides	1.251075687	0.079712857	0.178296337	7.271004038	2.862154597	1
Root vs Leaf	LysoPE 18:3(2n isomer)	Lipids	LPE	1.288876966	0.000834792	0.019191343	4.756306236	2.249841606	2
Root vs Leaf	Palmitaldehyde	Lipids	Free fatty acids	1.259971076	0.000828524	0.019191343	0.468583613	-1.093621593	3
Root vs Leaf	LysoPE 18:3	Lipids	LPE	1.285352334	0.00180861	0.023320316	4.537821685	2.181999919	2
Root vs Leaf	3'-O-Methyltricetin-5-O-glucoside*	Flavonoids	Flavones	1.226186643	0.116140877	0.233470172	0.051211057	-4.287400849	3
Root vs Leaf	Dihydrokaempferide	Flavonoids	Flavanonols	1.226358789	0.006330532	0.038725464	0.010384646	-6.589404162	3
Root vs Leaf	N-Hydroxynonan	Alkaloids	Pyridine	1.296323539	0.000372622	0.01464066	0.015240418	-6.0359537	3

Comparison	Compound	Class I	Class II	VIP	P-value	FDR	Fold Change	Log_2FC	MSI Level
	oyl nornicotine glucoside		alkaloids						
Root vs Leaf	1,2,4,5,8-pentahydroxy-6-methylanthracene-9,10-dione	Flavonoids	Other Flavonoids	1.298285518	0.005852216	0.037400858	0.017373084	-5.847002289	2
Root vs Leaf	D-Malic acid*	Organic acids	Organic acids	1.208531695	0.004519612	0.033309066	0.309650231	-1.691288573	1
Root vs Leaf	Homoeriodictyol 7-O-glucoside*	Flavonoids	Flavanones	1.261403688	0.087408895	0.189649219	0.034914419	-4.840033216	3
Root vs Leaf	Artocarpanone	Flavonoids	Flavanones	1.29271745	0.001016834	0.02015943	0.016548174	-5.917184117	1
Root vs Leaf	6-C-Methylquercetin-3-O-rhamnosi	Flavonoids	Flavonols	1.305914249	0.002714066	0.026984272	0.003559213	-8.134225923	2

Comparison	Compound	Class I	Class II	VIP	P-value	FDR	Fold Change	Log_2FC	MSI Level
	de								
Root vs Leaf	Carbendazim	Alkaloids	Alkaloids	1.115534269	0.198588361	0.332416304	22.8796918	4.515995713	1
Root vs Leaf	Isorhamnetin-3-O -arabinoside	Flavonoids	Flavonols	1.216726394	7.44543E-05	0.012840208	0.029238147	-5.096004316	3
Root vs Leaf	1,4,8-Trihydroxynaphthalene-1-O-glucoside	Quinones	Quinones	1.305707049	0.001601721	0.023084686	0.001842915	-9.083794372	3
Root vs Leaf	1,2,4,5-tetrahydroxy-7-(hydroxymethyl)anthracene-9,10-dione*	Flavonoids	Other Flavonoids	1.239053324	0.003546066	0.030288731	0.009396776	-6.733618385	2
Root vs Leaf	(3,4,5-trihydroxytetrahydro-2H-pyr	Phenolic acids	Phenolic acids	1.198727735	0.007837886	0.04213351	0.011139045	-6.488230619	3

Comparison	Compound	Class I	Class II	VIP	P-value	FDR	Fold Change	Log_2FC	MSI Level
	an-2-yl)methyl (E)-3-(4-hydroxy-3-methoxyphenyl)acrylate								
Root vs Leaf	Isorhamnetin-3-O-rhamnoside	Flavonoids	Flavonols	1.306251219	0.00933173	0.045935918	0.00473295	-7.723044497	2
Root vs Leaf	Ferulic acid-4-O-glucoside	Phenolic acids	Phenolic acids	1.303139688	0.004418464	0.033039104	0.143864818	-2.797214266	1
Root vs Leaf	L-Valine	Amino acids and derivatives	Amino acids and derivatives	1.018379856	0.349158194	0.464533762	16.03200691	4.002883131	1
Root vs Leaf	2,3,5-trimethoxy-	Flavonoids	Other	1.262548942	0.069255105	0.16040759	0.033791372	-4.887201241	3

Comparison	Compound	Class I	Class II	VIP	P-value	FDR	Fold Change	Log_2FC	MSI Level
	1-{[(2s,3r,4s,5s,6r)-3,4,5-trihydroxy-6-(hydroxymethyl)oxan-2-yl]oxy}xanthen-9-one*		Flavonoids						
Root vs Leaf	4,5,8-Trihydroxy-α-tetralone-5-O-glucoside	Quinones	Naphthol	1.270445629	0.002747126	0.026984272	0.00459611	-7.76537086	3
Root vs Leaf	Plantagoside	Flavonoids	Flavanones	1.295213409	0.001507233	0.023084686	0.011689538	-6.418638294	3
Root vs Leaf	Taxifolin-3-O-rhamnoside (Astilbin)	Flavonoids	Flavanonols	1.276209049	0.023021472	0.074586808	0.007699161	-7.021082961	3
Root vs Leaf	3,5,7,4'-Tetrahydr	Others	Chromone	1.292769541	0.003105524	0.028511738	0.007638746	-7.032448418	1

Comparison	Compound	Class I	Class II	VIP	P-value	FDR	Fold Change	Log_2FC	MSI Level
	oxy-Coumaronochromone								
Root vs Leaf	Luteolin-3'-O-glucoside	Flavonoids	Flavones	1.288489516	0.001914899	0.023320316	0.008311843	-6.91061589	3
Root vs Leaf	Monotropein	Terpenoids	Monoterpenoids	1.143777623	0.001339746	0.022042878	0.079709031	-3.649112997	2
Root vs Leaf	1-Caffeoylquinic acid	Phenolic acids	Phenolic acids	1.251757247	0.050489485	0.126722156	0.028734637	-5.121065367	1
Root vs Leaf	Methylmalonic acid*	Organic acids	Organic acids	1.238947671	0.004929636	0.034700139	0.284818781	-1.811883815	1
Root vs Leaf	Rhodioloside F	Terpenoids	Monoterpenoids	1.290342849	0.019955823	0.068235021	0.005583022	-7.48473812	3

Comparison	Compound	Class I	Class II	VIP	P-value	FDR	Fold Change	Log_2FC	MSI Level
Root vs Leaf	Cryptochlorogenic acid (4-O-Caffeoylquinic acid)*	Phenolic acids	Phenolic acids	1.271005551	0.001691514	0.023320316	0.004443011	-7.814246492	1
Root vs Leaf	Gallocatechin	Flavonoids	Flavanols	1.216471225	0.004794869	0.034196844	0.003193284	-8.290743599	1
Root vs Leaf	Luteolin-4'-O-glucoside*	Flavonoids	Flavones	1.2819698	0.020377931	0.069326645	0.017474055	-5.838641752	1
Root vs Leaf	Neochlorogenic acid (5-O-Caffeoylquinic acid)*	Phenolic acids	Phenolic acids	1.290661563	0.000927965	0.019854771	0.001598505	-9.289061051	1
Root vs Leaf	L-4-Hydroxyglutamate	Alkaloids	Alkaloids	1.266445566	0.018178585	0.065061009	2.927167869	1.549505485	3

Comparison	Compound	Class I	Class II	VIP	P-value	FDR	Fold Change	Log_2FC	MSI Level
	semialdehyde								
Root vs Leaf	α-Linolenic Acid*	Lipids	Free fatty acids	1.198971528	0.095126969	0.201538609	4.516085769	2.175072886	2
Root vs Leaf	L-Asparagine	Amino acids and derivatives	Amino acids and derivatives	1.232152658	0.219018392	0.347365541	277.4188349	8.11592193	1
Root vs Leaf	LPC(18:1/0:0)	Lipids	LPC	1.284016037	0.061326365	0.147087985	18.8386039	4.235620148	1
Root vs Leaf	coumarin	Lignans and Coumarins	Coumarins	1.238285216	0.001538044	0.023084686	0.075314093	-3.730936341	3
Root vs Leaf	LysoPC 18:1	Lipids	LPC	1.284189842	0.064035915	0.150906883	19.23544104	4.265695004	2
Root vs Leaf	9-hydroxy-2-(2-hydroxy-3-methoxyphenyl)-3-(hydr	Others	Ketone compounds	1.135690016	0.000979654	0.02015943	0.020298285	-5.622498384	1

Comparison	Compound	Class I	Class II	VIP	P-value	FDR	Fold Change	Log_2FC	MSI Level
	oxymethyl)-12-methoxy-2,3-dihydro-1,4,5-trioxatetraphen-10-one								
Root vs Leaf	LysoPC 18:1(2n isomer)	Lipids	LPC	1.281358241	0.06982239	0.161203188	18.39861472	4.201525241	2
Root vs Leaf	3',5',5,7-Tetrahydroxy-4'-methoxyflavanone-3'-O-glucoside	Flavonoids	Flavanones	1.284707383	0.00716603	0.040362051	0.00074684	-10.38691306	3
Root vs Leaf	Virgatic acid	Terpenoids	Triterpene	1.238037671	0.000734889	0.018214411	0.198693486	-2.331383517	1
Root vs Leaf	Cinchonain Ic	Flavonoids	Flavanols	1.121602363	0.002769707	0.027023138	0.017129256	-5.867393675	3
Root vs Leaf	Luteolin-7-O-gluc	Flavonoids	Flavones	1.300331728	0.002611961	0.02684374	0.007795914	-7.003066062	1

Comparison	Compound	Class I	Class II	VIP	P-value	FDR	Fold Change	Log_2FC	MSI Level
	oside (Cynaroside)								
Root vs Leaf	Succinic acid*	Organic acids	Organic acids	1.25429987	0.001924691	0.023320316	0.305587677	-1.71034173	1
Root vs Leaf	LysoPE 18:2	Lipids	LPE	1.284115099	0.082728482	0.182757953	23.49659415	4.554379747	2
Root vs Leaf	D-α-D-Hep-(1→6)-β-D-Glc	Others	Saccharides	1.011942763	0.016264924	0.061021704	0.155569868	-2.684365442	1
Root vs Leaf	LysoPE 18:2(2n isomer)	Lipids	LPE	1.27959884	0.094591798	0.200601448	22.85846737	4.514656771	1
Root vs Leaf	Gallic Acid 4-O-Glucoside	Phenolic acids	Phenolic acids	1.238202216	0.024485061	0.077563904	0.206945062	-2.272680269	1
Root vs Leaf	Catechin-(7,8-bc)-	Flavonoids	Flavanols	1.270168592	0.009714062	0.046617244	0.005607949	-7.47831094	1

Comparison	Compound	Class I	Class II	VIP	P-value	FDR	Fold Change	Log_2FC	MSI Level
	4α-(3,4-dihydroxy phenyl)-dihydro-2-(3H)-one*								
Root vs Leaf	Cinchonain Id*	Flavonoids	Flavanols	1.28571758	0.000253894	0.013805514	0.004103348	-7.92898279	1
Root vs Leaf	Catechin-(7,8-bc)-4β-(3,4-dihydroxy phenyl)-dihydro-2-(3H)-one	Flavonoids	Flavanols	1.269695871	0.005422899	0.036507429	0.00524309	-7.57536692	1
Root vs Leaf	4,5-Dehydro-L-leucine*	Amino acids and derivatives	Amino acids and derivatives	1.133238245	0.292957623	0.417049686	15.57806897	3.961444505	2
Root vs Leaf	Bracteatin*	Flavonoids	Aurones	1.258545709	0.000307331	0.01464066	0.003905565	-8.000252886	2
Root vs Leaf	Cernuoside	Flavonoids	Aurones	1.283282621	0.003962158	0.032250367	0.010479571	-6.576276473	2

Comparison	Compound	Class I	Class II	VIP	P-value	FDR	Fold Change	Log_2FC	MSI Level
Root vs Leaf	Kaempferol-3-O-glucoside (Astragalin)*	Flavonoids	Flavonols	1.293364586	0.001151823	0.020742409	0.008750301	-6.836451621	1
Root vs Leaf	N-Methyl-L-proline*	Amino acids and derivatives	Amino acids and derivatives	1.124261974	0.29522851	0.418902698	14.77259682	3.884851549	2
Root vs Leaf	Gossypetin-3-O-glucoside*	Flavonoids	Flavonols	1.273707786	0.003999799	0.032252113	0.000573817	-10.7671205	1
Root vs Leaf	Kaempferol-3-O-arabinoside	Flavonoids	Flavonols	1.29669075	0.00439522	0.033039104	0.001681534	-9.216006352	1
Root vs Leaf	Myricetin-3-O-β-D-glucoside*	Flavonoids	Flavonols	1.29181467	0.001096471	0.020742409	0.000291254	-11.74543315	1
Root vs Leaf	L-Pipecolic Acid	Alkaloids	Piperidine	1.123727436	0.294263885	0.418083008	14.51360586	3.859334091	2

Comparison	Compound	Class I	Class II	VIP	P-value	FDR	Fold Change	Log_2FC	MSI Level
			alkaloids						
Root vs Leaf	γ-Linolenic Acid*	Lipids	Free fatty acids	1.208726022	0.084231521	0.184796261	3.621100658	1.856428281	2
Root vs Leaf	Cycloleucine	Amino acids and derivatives	Amino acids and derivatives	1.110239806	0.298201708	0.4206357	12.92084658	3.691628695	2
Root vs Leaf	L-Malic acid*	Organic acids	Organic acids	1.160129251	0.024328224	0.077313666	0.356684208	-1.487280754	1
Root vs Leaf	Kaempferol-3-O-arabinoside (Juglanin)	Flavonoids	Flavonols	1.303924978	0.007679487	0.04197183	0.000957831	-10.02794123	1
Root vs Leaf	Quercetin 3-(2''-acetylrhamnoside)	Flavonoids	Flavonols	1.305308482	0.012871966	0.053295824	0.000123114	-12.98771474	1

Comparison	Compound	Class I	Class II	VIP	P-value	FDR	Fold Change	Log_2FC	MSI Level
Root vs Leaf	Myricetin-3-O-galactoside*	Flavonoids	Flavonols	1.302299815	0.002228491	0.025163593	0.000138605	-12.81673097	1
Root vs Leaf	5,7-Dihydroxy-4-oxo-2-(3,4,5-trihydroxyphenyl)-4H-chromen-3-yl-alloside	Others	Others	1.250091602	0.002649211	0.02684374	0.003637884	-8.102684704	3
Root vs Leaf	Isoscopolin	Lignans and Coumarins	Coumarins	1.303087733	0.004394202	0.033039104	0.173766398	-2.524778965	1
Root vs Leaf	L-Glutamic acid	Amino acids and derivatives	Amino acids and derivatives	1.259408681	0.016122053	0.06081729	2.513310914	1.329589154	1
Root vs Leaf	Azalein	Flavonoids	Flavonols	1.281536356	0.013723059	0.055743477	0.037992784	-4.718130755	1

Comparison	Compound	Class I	Class II	VIP	P-value	FDR	Fold Change	Log_2FC	MSI Level
	(Azaleatin-3-O-rhamnoside)								
Root vs Leaf	Quercetagetin-7-O-glucoside*	Flavonoids	Flavonols	1.273548946	0.003396936	0.030085163	0.002080625	-8.908767298	1
Root vs Leaf	vitelignin A	Lignans and Coumarins	Lignans	1.167001154	3.59437E-05	0.011096332	0.012316447	-6.343270065	3
Root vs Leaf	L-Arginine	Amino acids and derivatives	Amino acids and derivatives	1.261273793	0.19505051	0.330332408	644.0118353	9.330943391	1
Root vs Leaf	cagayanone A	Lignans and Coumarins	Lignans	1.263790032	7.56539E-06	0.004087201	0.003358216	-8.218089348	3
Root vs Leaf	3,5-Dihydro-2H-Furo[3,2-C]Quinoli	Alkaloids	Quinoline alkaloids	1.254225698	0.002666932	0.02684374	0.30816949	-1.698204059	1

Comparison	Compound	Class I	Class II	VIP	P-value	FDR	Fold Change	Log_2FC	MSI Level
	n-4-One*								
Root vs Leaf	cyclo-(Gly-Phe)	Amino acids and derivatives	Amino acids and derivatives	1.255161057	0.00585733	0.037400858	0.302518632	-1.724904094	2
Root vs Leaf	Pyran[3,4-b]indole-2-ketone	Alkaloids	Plumerane	1.207177678	0.003042467	0.028128894	0.32166709	-1.636359757	1
Root vs Leaf	4-O-acetyl-3-O-caffeoyl-2-C-methyl-D-erythronate	Others	Others	1.132228652	0.232338293	0.360069999	0.042160375	-4.567968475	3
Root vs Leaf	3-amino-2-naphthoic acid*	Alkaloids	Alkaloids	1.245966219	0.018144402	0.065061009	0.209810482	-2.252841338	1
Root vs Leaf	2-methyl-1-nitronaphthalene	Others	Others	1.28045015	0.009789215	0.046617244	0.218390238	-2.195019724	3

Comparison	Compound	Class I	Class II	VIP	P-value	FDR	Fold Change	Log_2FC	MSI Level
Root vs Leaf	Quercetin-5-O-β-D-glucoside*	Flavonoids	Flavonols	1.275481065	2.67593E-05	0.009637805	0.08976514	-3.477700895	1
Root vs Leaf	L-Phenylalanine	Amino acids and derivatives	Amino acids and derivatives	1.137980671	0.037444852	0.103615089	0.375368945	-1.413618797	1
Root vs Leaf	Myricetin-3-O-rhamnoside (Myricitrin)	Flavonoids	Flavonols	1.281067748	0.002656898	0.02684374	0.001559595	-9.324612465	2
Root vs Leaf	Hesperetin-3'-O-glucoside*	Flavonoids	Flavanones	1.238091309	0.013149463	0.054228986	0.108740167	-3.201043144	3
Root vs Leaf	naphthisoxazol A*	Alkaloids	Alkaloids	1.275420248	0.005268627	0.036144451	0.220045459	-2.184126492	1
Root vs Leaf	5-O-p-Coumaroyl	Phenolic	Phenolic acids	1.293621104	0.002856425	0.02727992	0.002273207	-8.781055127	1

Comparison	Compound	Class I	Class II	VIP	P-value	FDR	Fold Change	Log_2FC	MSI Level
	quinic acid	acids							
Root vs Leaf	Quercetin-3-O-rhamnoside(Quercitrin)	Flavonoids	Flavonols	1.252509026	0.005592161	0.036629402	0.017068691	-5.872503734	1
Root vs Leaf	2-Caffeoylisocitrate	Phenolic acids	Phenolic acids	1.184265509	7.35241E-07	0.001588856	0.019044699	-5.714466676	2
Root vs Leaf	Sesamin	Lignans and Coumarins	Lignans	1.150133049	0.030618268	0.08917261	0.028773593	-5.119110827	3
Root vs Leaf	Myricetin-3-O-arabinoside*	Flavonoids	Flavonols	1.281152935	0.000682741	0.017564328	0.001532183	-9.350196107	1
Root vs Leaf	3-O-p-Coumaroyl quinic acid	Phenolic acids	Phenolic acids	1.248003118	0.001337629	0.022042878	0.006051673	-7.368450225	1

Comparison	Compound	Class I	Class II	VIP	P-value	FDR	Fold Change	Log_2FC	MSI Level
Root vs Leaf	7-Hydroxycoumarin;Umbelliferone	Lignans and Coumarins	Coumarins	1.120504537	0.048078257	0.123246871	0.031889712	-4.970765136	1
Root vs Leaf	Myricetin-3-O-xyloside*	Flavonoids	Flavonols	1.280599571	0.000871928	0.019301816	0.001760525	-9.149778507	1
Root vs Leaf	Quercetin-3-O-arabinoside	Flavonoids	Flavonols	1.169946783	0.000124778	0.012840208	0.071293625	-3.810083107	3
Root vs Leaf	Morin 3-alpha-L-lyxopyranoside	Flavonoids	Flavonols	1.145221734	0.002459771	0.026314679	0.09513431	-3.393890446	3
Root vs Leaf	Morin-3-O-xyloside*	Flavonoids	Flavonols	1.149394117	0.017596323	0.064016253	0.081184783	-3.622646844	1
Root vs Leaf	Quercetin-3-O-glucoside	Flavonoids	Flavonols	1.292511881	0.000278201	0.013981217	0.03116562	-5.00390076	3

Comparison	Compound	Class I	Class II	VIP	P-value	FDR	Fold Change	Log_2FC	MSI Level
	(Isoquercitrin)								
Root vs Leaf	Quercetin-3-O-xyloside (Reynoutrin)*	Flavonoids	Flavonols	1.059271455	0.004537753	0.033309066	0.166114075	-2.589753777	1
Root vs Leaf	Quercetin-3-O-alloside; Isohyperoside*	Flavonoids	Flavonols	1.280592402	0.006320321	0.038725464	0.063993749	-3.965925202	1
Root vs Leaf	Avicularin(Quercetin-3-O-α-L-arabinofuranoside)*	Flavonoids	Flavonols	1.159591682	5.63964E-05	0.012840208	0.071200708	-3.811964598	1
Root vs Leaf	Quercetin-3-O-galactoside (Hyperin)	Flavonoids	Flavonols	1.293091782	0.000349219	0.01464066	0.043443656	-4.524710679	1

Comparison	Compound	Class I	Class II	VIP	P-value	FDR	Fold Change	Log_2FC	MSI Level
Root vs Stem	3-Oxolanosta-9(11),24-dien-26-oic acid (Coccinic Acid)	Terpenoids	Triterpene	1.043924624	0.191210238	0.431546011	0.165153743	-2.598118429	3
Root vs Stem	Daidzein rutinoside	Flavonoids	Isoflavones	1.18899931	0.013966729	0.11718426	0.177831339	-2.491418504	3
Root vs Stem	1-Linoleoylglycerol-2,3-di-O-glucoside	Lipids	Glycerol ester	1.102966987	0.21216056	0.455196876	0.189716983	-2.398079263	3
Root vs Stem	9-Hydroxy-12-oxo-10(E),15(Z)-octadecadienoic acid	Lipids	Free fatty acids	1.034243011	0.25917147	0.500239506	7.296939363	2.867291465	3
Root vs Stem	Nepetin-8-C-[Xyl	Flavonoids	Flavones	1.046089259	0.188146013	0.426739968	0.056884943	-4.135809357	3

Comparison	Compound	Class I	Class II	VIP	P-value	FDR	Fold Change	Log_2FC	MSI Level
	osyl-(1-2)]-glucoside								
Root vs Stem	Kaempferol-7-O-glucuronide	Flavonoids	Flavonols	1.055433062	0.217313637	0.458674946	0.054090643	-4.208477153	3
Root vs Stem	Ent-3β-Acetoxyisopimar-15-8β-ol	Terpenoids	Ditepenoids	1.488019786	0.008447415	0.088057897	0.053757631	-4.21738662	3
Root vs Stem	4,6-diethyl-3,6-dihydroxy-8-methyldodec-4-enoicacid	Lipids	Free fatty acids	1.338087035	0.011290141	0.103109957	0.210707524	-2.246686261	1
Root vs Stem	Propyl 4-hydroxybenzoate	Phenolic acids	Phenolic acids	1.109038397	0.248811114	0.489636082	3.691497882	1.884206331	3
Root vs Stem	Quercetin-7-O-(2''	Flavonoids	Flavonols	1.185752372	0.194194577	0.433518177	0.127485713	-2.971592521	3

Comparison	Compound	Class I	Class II	VIP	P-value	FDR	Fold Change	Log_2FC	MSI Level
	-malonyl)glucosyl -5-O-glucoside								
Root vs Stem	2,3-Dihydroxyurs -12-en-28-oic acid (Corosolic acid)	Terpenoids	Triterpene	1.405926448	0.115354583	0.324375831	0.042419915	-4.559114443	3
Root vs Stem	9S-Hydroperoxy- 10E,12Z-octadeca dienoic acid	Lipids	Free fatty acids	1.331662259	0.147312693	0.369894644	0.089973742	-3.47435217	3
Root vs Stem	Petunidin-3-O-rut inoside	Flavonoids	Anthocyanidin s	1.048332862	0.259571953	0.500239506	0.0338332	-4.885416563	3
Root vs Stem	LysoPC 22:5	Lipids	LPC	1.491437965	0.005398666	0.075448846	13.20816321	3.723357947	3
Root vs Stem	Feruloylferuloylta rtaric acid	Phenolic acids	Phenolic acids	1.401981948	0.066304018	0.241360658	0.026780262	-5.222686128	3

Comparison	Compound	Class I	Class II	VIP	P-value	FDR	Fold Change	Log_2FC	MSI Level
Root vs Stem	Shanzhiside methyl ester	Terpenoids	Monoterpenoids	1.374972369	0.005483201	0.075448846	0.126629013	-2.981320099	3
Root vs Stem	3,4'-Dihydroxy-3'-methoxybenzene pentanoic acid	Phenolic acids	Phenolic acids	1.476984971	0.058600891	0.224828484	0.047863174	-4.384940106	3
Root vs Stem	Isorhamnetin; 3'-Methoxy-3,4',5,7-Tetrahydroxyflavone	Flavonoids	Flavonols	1.459771796	0.070799139	0.247257907	0.042415402	-4.559267946	1
Root vs Stem	Hispidulin (5,7,4'-Trihydroxy-6-methoxyflavone)*	Flavonoids	Flavones	1.084113575	0.141040416	0.360281459	0.290338349	-1.784192953	3

Comparison	Compound	Class I	Class II	VIP	P-value	FDR	Fold Change	Log_2FC	MSI Level
Root vs Stem	5,6,7-Trihydroxy-8-methoxyflavone*	Flavonoids	Flavones	1.032574692	0.054409505	0.218644197	0.373922408	-1.419189163	3
Root vs Stem	Glu-Asn	Amino acids and derivatives	Amino acids and derivatives	1.087071422	0.200113037	0.439865079	10.42123981	3.381455019	3
Root vs Stem	6,7,8-Tetrahydroxy-5-methoxyflavone*	Flavonoids	Flavones	1.057461897	0.069145634	0.245600966	0.336003286	-1.573452754	3
Root vs Stem	Patuletin 3-O-beta-gentiobioside	Flavonoids	Flavonols	1.488211116	0.024251587	0.144292194	0.011221166	-6.477633596	3
Root vs Stem	Laricitrin-3-O-(6''-	Flavonoids	Flavonols	1.478658601	0.031085121	0.161611311	0.089404191	-3.483513729	3

Comparison	Compound	Class I	Class II	VIP	P-value	FDR	Fold Change	Log_2FC	MSI Level
	acetyl)glucoside								
Root vs Stem	Grayanotoxin XV	Terpenoids	Ditepenoids	1.439868157	0.097484331	0.298527683	0.122596	-3.028016182	2
Root vs Stem	N-(sulfonyl)phenylalanine	Amino acids and derivatives	Amino acids and derivatives	1.385547259	0.092461365	0.288714459	0.156131201	-2.679169226	3
Root vs Stem	Martynoside	Phenolic acids	Phenolic acids	1.052690941	0.191519451	0.431546011	22.93932875	4.519751271	3
Root vs Stem	Idescarpin	Phenolic acids	Phenolic acids	1.049547372	0.20191573	0.442414085	25.71458429	4.684514926	3
Root vs Stem	2-α-Linolenoyl-glycerol-1,3-di-O-glucoside	Lipids	Glycerol ester	1.194560627	0.253715958	0.494938069	0.105621417	-3.243025691	2

Comparison	Compound	Class I	Class II	VIP	P-value	FDR	Fold Change	Log_2FC	MSI Level
Root vs Stem	2-Methyl-5-acetonyl-7-hydroxychromone glucoside	Others	Chromone	1.384668655	0.024731846	0.144292194	0.27507342	-1.862111357	3
Root vs Stem	Laminaran	Others	Saccharides	1.033670058	0.060123647	0.228116192	0.291352576	-1.77916203	3
Root vs Stem	Kaempferol (3,5,7,4'-Tetrahydroxyflavone)	Flavonoids	Flavonols	1.431933241	0.10428092	0.307154948	0.085176438	-3.553401799	2
Root vs Stem	Daidzein 4',7-diglucoside	Flavonoids	Isoflavones	1.061691528	0.170270846	0.404417752	0.406053438	-1.300258491	3
Root vs Stem	1-O-Galloyl-rhamnose	Phenolic acids	Phenolic acids	1.380388334	0.037140345	0.179106708	0.065589439	-3.930392645	3
Root vs Stem	Quercetin-3-O-(6''-O-galloyl)galacto	Flavonoids	Flavonols	1.468226232	0.030814409	0.161015038	0.141676524	-2.81932737	3

Comparison	Compound	Class I	Class II	VIP	P-value	FDR	Fold Change	Log_2FC	MSI Level
	side								
Root vs Stem	9-Hydroxy-13-oxo-10-octadecenoic Acid	Lipids	Free fatty acids	1.294480699	0.129146336	0.34544855	6.900008018	2.786598038	3
Root vs Stem	LysoPC 20:5	Lipids	LPC	1.322454009	0.13711719	0.355190258	6.894483655	2.785442509	3
Root vs Stem	1'-O-(3,4-Dihydroxyphenethyl)-O-caffeoyl-glucoside	Phenolic acids	Phenolic acids	1.490569624	0.002806288	0.056750731	0.046020599	-4.441576433	3
Root vs Stem	Gossypetin-8-O-β-D-glucuronide	Flavonoids	Flavonols	1.485553819	0.018655711	0.130970703	0.079911287	-3.645456894	3
Root vs Stem	Cis-4,7,10,13,16,19-Docosahexaenoic Acid	Lipids	Free fatty acids	1.101708195	0.264262152	0.504567144	0.163786039	-2.610115707	3

Comparison	Compound	Class I	Class II	VIP	P-value	FDR	Fold Change	Log_2FC	MSI Level
Root vs Stem	Epiafzelechin	Flavonoids	Flavanols	1.364096425	0.006896863	0.083865246	0.086373772	-3.533262893	3
Root vs Stem	6,7-Dimethoxy-4-chromanone	Flavonoids	Other Flavonoids	1.106547176	0.09031146	0.285419243	0.246587964	-2.019825709	3
Root vs Stem	Chrysophanol-8-O-β-D-galactoside	Quinones	Anthraquinone	1.490643765	0.003016239	0.056750731	0.104638425	-3.256515368	3
Root vs Stem	Abscisic acid	Terpenoids	Sesquiterpenoids	1.038213716	0.07448436	0.2528548	0.359736971	-1.474985659	3
Root vs Stem	8-C-glucosyl-noreugenin	Others	Chromone	1.407024224	0.000362626	0.021950816	0.091109877	-3.45624873	3
Root vs Stem	6-Hydroxypseudooxynicotine O-glucoside	Alkaloids	Pyridine alkaloids	1.03138538	0.30717744	0.541502716	0.036728412	-4.766959663	3

Comparison	Compound	Class I	Class II	VIP	P-value	FDR	Fold Change	Log_2FC	MSI Level
Root vs Stem	Ginnalin C (2-O-Galloyl-1,5-Anhydro-D-Glucitol)	Tannins	Tannin	1.490598061	0.004693352	0.072047814	0.033021508	-4.920450164	3
Root vs Stem	3-O-Acetylpinobanksin	Flavonoids	Flavanonols	1.203414752	0.153719589	0.380885031	0.209233839	-2.256811898	3
Root vs Stem	Eriodictyol-7-O-rutinoside-5-O-(6''-O-glucosyl)glucuronide	Flavonoids	Flavanones	1.471051772	0.061577443	0.231926719	0.066489591	-3.910727694	3
Root vs Stem	Xylosyl tachioside	Phenolic acids	Phenolic acids	1.393674237	0.027976811	0.153984366	0.090051914	-3.473099248	3
Root vs Stem	Tri-p-coumaroyl	Alkaloids	Phenolamine	1.419776098	0.123592306	0.336980871	0.130903713	-2.933422079	3

Comparison	Compound	Class I	Class II	VIP	P-value	FDR	Fold Change	Log_2FC	MSI Level
	Spermidine								
Root vs Stem	LysoPC 20:3	Lipids	LPC	1.29721929	0.173231769	0.410034831	5.526983896	2.466492409	3
Root vs Stem	Ile-Asn	Amino acids and derivatives	Amino acids and derivatives	1.365332449	0.231144555	0.473540328	26.12584911	4.707406022	3
Root vs Stem	Adenylocuccinic Acid	Nucleotides and derivatives	Nucleotides and derivatives	1.459406131	0.012137152	0.107179135	6.297387369	2.654753414	3
Root vs Stem	8-Epiloganin	Terpenoids	Terpene	1.198495158	0.022016965	0.139395751	0.269792538	-1.890077651	3
Root vs Stem	Delphinidin-3-O-sambubioside	Flavonoids	Anthocyanidins	1.043866165	0.246114748	0.487204213	2.593950358	1.37515087	3
Root vs Stem	Pinocembrin-7-O-	Flavonoids	Flavanones	1.490013714	0.004301545	0.069663321	0.078849332	-3.664757655	3

Comparison	Compound	Class I	Class II	VIP	P-value	FDR	Fold Change	Log$_2$FC	MSI Level
	(2''-O-arabinosyl) glucoside								
Root vs Stem	2-Hydroxy-3-(4-Hydroxyphenyl)Propanoic Acid	Phenolic acids	Phenolic acids	1.000976121	0.281215007	0.516775123	0.147435056	-2.761848501	3
Root vs Stem	3,8-Dihydroxy-1-methoxy-9,10-anthraquinone glucoside	Quinones	Anthraquinone	1.454212494	0.066234171	0.241360658	0.060946446	-4.036314103	3
Root vs Stem	Dehydrodiconiferyl alcohol-4-O-glucoside*	Lignans and Coumarins	Lignans	1.225998479	0.369059277	0.583066805	43.93347619	5.457248751	3

Comparison	Compound	Class I	Class II	VIP	P-value	FDR	Fold Change	Log_2FC	MSI Level
Root vs Stem	3,3'-Di-O-methylellagic acid 4'-beta-O-xylopyranoside	Tannins	Tannin	1.482058942	0.024773693	0.144292194	0.09832413	-3.346310678	3
Root vs Stem	Okanin-4'-O-(6''-O-malonyl)glucoside	Flavonoids	Chalcones	1.470114036	0.059620788	0.226679801	0.051802294	-4.27084019	3
Root vs Stem	N,N-cinnamoylbutanediamine*	Alkaloids	Phenolamine	1.444663388	0.074281105	0.2528548	0.123637101	-3.015816366	3
Root vs Stem	LysoPE 15:1(2n isomer)*	Lipids	LPE	1.335446461	0.045957443	0.197617006	7.138074421	2.835534943	3
Root vs Stem	Oroxin A	Flavonoids	Flavones	1.331358243	0.10824794	0.312480766	0.049748587	-4.329200631	3
Root vs Stem	Octadecadienami	Alkaloids	Alkaloids	1.153073325	0.105508883	0.308020275	2.564493964	1.358674176	3

Comparison	Compound	Class I	Class II	VIP	P-value	FDR	Fold Change	Log_2FC	MSI Level
	de								
Root vs Stem	7S,8R-threo-3',9,9'-trihydroxy-3-methoxy-4',7-epoxy-neolignan-4-O-rhamnoside	Lignans and Coumarins	Lignans	1.253054344	0.054228698	0.218644197	0.117354489	-3.091055067	3
Root vs Stem	Gentisic acid 2-O-(6''-O-Feruloyl)Glucoside*	Phenolic acids	Phenolic acids	1.491282925	0.001237736	0.041846281	0.018074759	-5.789879802	1
Root vs Stem	1-(3,4-dihydroxyphenyl)-5-hydroxy-7-(4-hydroxy-3-methoxyphenyl)hept-1-en-3-one	Others	Ketone compounds	1.430482359	0.130898277	0.346821621	0.084512139	-3.564697613	3

Comparison	Compound	Class I	Class II	VIP	P-value	FDR	Fold Change	Log_2FC	MSI Level
Root vs Stem	1,2,3-Trihydroxy-19-oxo-18,19-seco-urs-11,13(18)-dien-28-oic acid	Terpenoids	Triterpene	1.049170609	0.204204143	0.4456275	28.75944784	4.845964072	3
Root vs Stem	LysoPE 15:1*	Lipids	LPE	1.248135087	0.140200008	0.358578459	6.336617939	2.663713032	3
Root vs Stem	Caffeic aldehyde	Others	Aldehyde compounds	1.475507448	0.024130953	0.144292194	0.172598815	-2.534505536	3
Root vs Stem	Citric acid glucoside	Organic acids	Organic acids	1.304123949	0.087999945	0.282475719	0.318639563	-1.65000269	2
Root vs Stem	Isorhamnetin-3-O-(2''-O-xylosyl)glucoside	Flavonoids	Flavonols	1.478725055	0.038272023	0.180664966	0.064450507	-3.955664484	3
Root vs Stem	5'-Glucosyloxyjas	Organic	Organic acids	1.11639567	0.162132362	0.392310896	0.237204156	-2.075798807	1

Comparison	Compound	Class I	Class II	VIP	P-value	FDR	Fold Change	Log_2FC	MSI Level
	manic acid*	acids							
Root vs Stem	1-O-Feruloyl-beta-D-glucose	Phenolic acids	Phenolic acids	1.449444379	0.053046761	0.216380463	0.115392213	-3.115382231	3
Root vs Stem	Quercetagetin-3-Methyl Ether	Flavonoids	Flavonols	1.475666495	0.020331886	0.133966689	0.150981119	-2.727559954	3
Root vs Stem	Limocitrin-7-O-(6''-Acetyl)glucoside	Flavonoids	Flavonols	1.491644958	4.16215E-05	0.00848072	0.041730067	-4.582768942	3
Root vs Stem	3,4,5-Trimethoxyphenol-1-O-β-D-malonylglucoside	Phenolic acids	Phenolic acids	1.150117434	0.013170083	0.112792745	0.305445671	-1.711012301	3
Root vs Stem	Naringenin-7-O-(6''-malonyl)glucoside	Flavonoids	Flavanones	1.116816403	0.020909193	0.135287064	0.289447878	-1.788624517	3

Comparison	Compound	Class I	Class II	VIP	P-value	FDR	Fold Change	Log_2FC	MSI Level
Root vs Stem	Pantothenol	Others	Vitamin	1.337893718	0.198964525	0.438530981	12.91807593	3.691319301	3
Root vs Stem	Gallic acid-1-O-xyloside	Phenolic acids	Phenolic acids	1.389777171	0.175826732	0.41286277	0.102289967	-3.289263454	1
Root vs Stem	LysoPC 22:6	Lipids	LPC	1.418864579	0.130436892	0.346043374	53.39878285	5.738734953	3
Root vs Stem	Methyl 3-(3-hydroxy-4-methoxyphenyl)propanoate	Phenolic acids	Phenolic acids	1.243986314	0.030665981	0.161015038	6.405364892	2.679280758	3
Root vs Stem	Naringenin-7-O-Neohesperidoside(Naringin)	Flavonoids	Flavanones	1.379905013	0.18552349	0.424526002	0.059141221	-4.079692158	3
Root vs Stem	Rhamnosylfucose	Terpenoids	Terpene	1.048558249	0.235176188	0.477759499	4.484485328	2.164942421	3

Comparison	Compound	Class I	Class II	VIP	P-value	FDR	Fold Change	Log_2FC	MSI Level
Root vs Stem	D-Panose*	Others	Saccharides	1.051978049	0.04127272	0.187223942	0.201998657	-2.307582391	2
Root vs Stem	saikolignanoside D*	Lignans and Coumarins	Lignans	1.362520439	0.288336536	0.521583358	35.58895801	5.153357788	3
Root vs Stem	D-Friedoolean-14-ene-3,28-diol (Myricadoil)	Terpenoids	Triterpene	1.365031713	0.13961204	0.357739917	0.04510438	-4.470588658	2
Root vs Stem	Afrormosin 7-O-(6''-Malonylglucoside)*	Flavonoids	Isoflavones	1.428881749	0.011806375	0.105949381	0.019466894	-5.6828335	3
Root vs Stem	LysoPE 14:0(2n isomer)*	Lipids	LPE	1.428924805	0.007329977	0.084768723	25.18695719	4.654604937	3
Root vs Stem	9-Hydroperoxy-9Z,11E-Octadecadi	Lipids	Free fatty acids	1.341556023	0.004819395	0.072520512	0.206898457	-2.273005212	2

Comparison	Compound	Class I	Class II	VIP	P-value	FDR	Fold Change	Log_2FC	MSI Level
	enoic Acid								
Root vs Stem	6'-O-β-glucopyranosyl secologanol	Terpenoids	Monoterpenoids	1.375858706	0.008291324	0.088057897	0.096326617	-3.375921686	3
Root vs Stem	peganoneI	Quinones	Anthraquinone	1.219753254	0.370689529	0.583066805	54.61358441	5.771187942	3
Root vs Stem	Phloretin-2'-O-(6''-O-glucoside)glucoside	Flavonoids	Chalcones	1.362256051	0.0027766	0.056750731	0.120125459	-3.057386154	2
Root vs Stem	Trollioside	Phenolic acids	Phenolic acids	1.231647763	0.061477861	0.231926719	0.205326728	-2.284006652	3
Root vs Stem	Tamarixetin (3,3',5,7-Tetrahydroxy-4'-Methoxyflavone)	Flavonoids	Flavonols	1.45638549	0.074473256	0.2528548	0.055352103	-4.17521806	2

Comparison	Compound	Class I	Class II	VIP	P-value	FDR	Fold Change	Log_2FC	MSI Level
Root vs Stem	9-Oxodemethylmyricanane B	Others	Others	1.371515868	0.004410819	0.069917266	4.397292913	2.136615637	2
Root vs Stem	Robinlin	Terpenoids	Monoterpenoids	1.478228809	0.019549853	0.132733212	0.176165003	-2.505000748	3
Root vs Stem	Anabasine	Alkaloids	Pyridine alkaloids	1.484032781	0.01034934	0.100388537	0.172077829	-2.538866863	3
Root vs Stem	myricetin-3-O-β-D-xylopyranosyl-(1→2)-β-D-glucopyranoside	Flavonoids	Flavonols	1.470693766	0.030783344	0.161015038	0.13840287	-2.853054237	3
Root vs Stem	Hydroxydihydrobovolide*	Others	Lactones	1.45040486	0.069226113	0.245600966	0.142435369	-2.81162066	1
Root vs Stem	Aromadendrin-3-	Flavonoids	Flavanonols	1.331364115	0.047065368	0.201124059	0.133990755	-2.89979463	3

Comparison	Compound	Class I	Class II	VIP	P-value	FDR	Fold Change	Log_2FC	MSI Level
	O-rhamnoside; Engeletin								
Root vs Stem	Phloretin-2'-O-(6''-O-acetyl)glucoside	Flavonoids	Chalcones	1.322029201	0.006886362	0.083865246	0.091724275	-3.446552587	2
Root vs Stem	Glucosyl-[6''-(3-hydroxy-3-methylglutaryl)]-Isorhamnetin	Flavonoids	Flavonols	1.378085069	0.033613017	0.168391426	0.064811831	-3.947599007	3
Root vs Stem	3,4-Secours-4(23), 12-dien-3-oic acid (Roburic acid)	Terpenoids	Triterpene	1.380564829	0.124656458	0.33832175	0.054865361	-4.187960597	2
Root vs Stem	Quercetin-3-O-(2''	Flavonoids	Flavonols	1.485526111	0.019085425	0.131897274	0.030034232	-5.057248418	3

Comparison	Compound	Class I	Class II	VIP	P-value	FDR	Fold Change	Log_2FC	MSI Level
	-O-malonyl)glucoside-7-O-arabinoside								
Root vs Stem	cis-2,4-Dihydroxy-2-(2-hydroxyethyl)cyclohex-5-en-1-one glucoside	Others	Others	1.45523666	0.047891297	0.202954576	0.130272918	-2.940390896	3
Root vs Stem	Viscumneoside V1	Flavonoids	Flavanones	1.471046669	0.030106084	0.159330661	0.140649923	-2.829819331	2
Root vs Stem	Grayanotoxin III	Terpenoids	Ditepenoids	1.313052949	0.048870023	0.205015704	0.163113026	-2.616056093	3
Root vs Stem	6-O-p-Methoxycinnamoylcatalpol	Terpenoids	Monoterpenoids	1.174399247	0.032997	0.167336137	0.296697953	-1.752933123	3
Root vs Stem	DL-Xylose*	Others	Saccharides	1.083750818	0.139121777	0.357739917	0.434629131	-1.202143221	3

Comparison	Compound	Class I	Class II	VIP	P-value	FDR	Fold Change	Log_2FC	MSI Level
Root vs Stem	Mearnsetin-3-O-rhamnoside (Mearnsitrin)	Flavonoids	Flavonols	1.011972231	0.263278328	0.503620454	0.163757293	-2.610368934	2
Root vs Stem	Cordycepin (3'-Deoxyadenosine)*	Nucleotides and derivatives	Nucleotides and derivatives	1.235424453	0.309301784	0.542021895	11.96522034	3.580775059	3
Root vs Stem	2,6-Dimethoxy-4-(2-Hydroxyethyl)Phenyl Beta-D-Glucopyranoside	Others	Alcohol compounds	1.468749677	0.002659219	0.056495906	0.304704548	-1.714517062	1
Root vs Stem	Protocatechuic acid 4-O-(6''-O-Ferulo	Phenolic acids	Phenolic acids	1.49165296	7.18011E-05	0.01075058	0.015027405	-6.056260286	1

Comparison	Compound	Class I	Class II	VIP	P-value	FDR	Fold Change	Log_2FC	MSI Level
	yl)Glucoside*								
Root vs Stem	1,4,8-Trihydroxynaphthalene-1-O-[6'-O-(3'',4'',5''-trimethylbenzoyl)]glucoside	Quinones	Quinones	1.126105403	0.057319898	0.221136951	0.233049292	-2.101292967	3
Root vs Stem	Tricin-5-O-(6'-O-malonyl)glucoside	Flavonoids	Flavones	1.036753147	0.272920144	0.508025746	0.071669318	-3.802500569	3
Root vs Stem	Glucosyl 2,6-dimethyl-8-oxoocta-2,6-dienoic acid	Others	Others	1.301517254	0.022373251	0.14035985	0.180059866	-2.473451443	3

Comparison	Compound	Class I	Class II	VIP	P-value	FDR	Fold Change	Log_2FC	MSI Level
Root vs Stem	Fargesin	Lignans and Coumarins	Lignans	1.042960186	0.218202077	0.459091831	0.146431388	-2.771703264	3
Root vs Stem	19-Hydroxyursolic acid	Terpenoids	Triterpene	1.38918337	0.134729624	0.35228335	0.030174929	-5.0505058	3
Root vs Stem	Gnetifolin B*	Flavonoids	Flavonols	1.024089303	0.092917287	0.289262866	0.351773657	-1.507280645	3
Root vs Stem	L-Xylose*	Others	Saccharides	1.154240033	0.074728238	0.253266145	0.490946863	-1.026361209	3
Root vs Stem	4-Hydroxy-L-Isoleucine	Amino acids and derivatives	Amino acids and derivatives	1.386657373	0.003806471	0.065816097	2.962257411	1.566697012	3
Root vs Stem	Genipin-1-O-(2''-O-apiosyl)glucoside	Terpenoids	Monoterpenoids	1.449622078	0.058843848	0.225331545	0.11008492	-3.183311242	3

Comparison	Compound	Class I	Class II	VIP	P-value	FDR	Fold Change	Log_2FC	MSI Level
Root vs Stem	N-(1-Deoxy-1-fructosyl)Tyrosine	Amino acids and derivatives	Amino acids and derivatives	1.043550167	0.191314203	0.431546011	0.244001753	-2.035036584	3
Root vs Stem	N-(4-(tetrahydroPhoenix1-yl)butyryl)nornicotine	Alkaloids	Pyridine alkaloids	1.372303941	0.117029362	0.327301632	0.040375934	-4.630360556	3
Root vs Stem	Malonyl Wistin*	Flavonoids	Isoflavones	1.475759615	0.057511414	0.221461862	0.031483991	-4.98923774	3
Root vs Stem	Glu-Arg	Amino acids and derivatives	Amino acids and derivatives	1.281364283	0.099556755	0.300685165	3.833504374	1.938663826	3
Root vs Stem	Isololiolide	Terpenoids	Monoterpenoids	1.486644786	0.008423073	0.088057897	0.132552324	-2.915366134	3
Root vs Stem	N-(Isoferuloyl)-Gl	Alkaloids	Phenolamine	1.27020608	0.028537182	0.154844052	0.198218248	-2.334838313	3

Comparison	Compound	Class I	Class II	VIP	P-value	FDR	Fold Change	Log_2FC	MSI Level
	utamic Acid								
Root vs Stem	Euphatexol F	Terpenoids	Triterpene	1.408161013	0.085376454	0.276986727	0.126160477	-2.986668075	3
Root vs Stem	Chrysoeriol; 5,7,4'-Trihydroxy-3'-Methoxyflavone	Flavonoids	Flavones	1.013134471	0.060262548	0.228223667	0.367913643	-1.442560918	3
Root vs Stem	8-Methoxy malonyl ononin*	Flavonoids	Isoflavones	1.447601442	0.022267406	0.14035985	0.028578424	-5.128929847	3
Root vs Stem	3,5-Di-Galloyl Sorbitol	Phenolic acids	Phenolic acids	1.382777506	0.016322604	0.122828256	0.124065745	-3.010823254	3
Root vs Stem	Viscumneoside I*	Flavonoids	Flavanones	1.060215773	0.185935807	0.424526002	0.257052068	-1.959867478	3
Root vs Stem	Aracarpene 1	Flavonoids	Isoflavones	1.006220911	0.123958432	0.337088543	0.344340566	-1.538091942	3

Comparison	Compound	Class I	Class II	VIP	P-value	FDR	Fold Change	Log_2FC	MSI Level
Root vs Stem	5-(2-Hydroxypropyl)hygrine	Alkaloids	Pyrrole alkaloids	1.070891716	0.028412346	0.154844052	0.325261998	-1.620325821	3
Root vs Stem	12-Hydroxyjasmonic Acid Glucoside	Organic acids	Organic acids	1.345363354	0.040746689	0.186064525	0.130340977	-2.939637379	1
Root vs Stem	linoleoyl ethanolamine	Lipids	LPE	1.086993133	0.360090231	0.583066805	11.17549177	3.482266412	3
Root vs Stem	7-(4-Hydroxy-3-methoxy-phenyl)-1-(4-hydroxy-phenyl)-hept-4-en-3-one	Others	Ketone compounds	1.204300094	0.380870942	0.583066805	75.19152498	6.232498156	3
Root vs Stem	Mangiferin	Flavonoids	Other Flavonoids	1.369044734	0.015967597	0.122828256	0.314652119	-1.668170438	3

Comparison	Compound	Class I	Class II	VIP	P-value	FDR	Fold Change	Log_2FC	MSI Level
Root vs Stem	Aracarpene 2*	Flavonoids	Isoflavones	1.011194199	0.09659099	0.296672325	0.364676991	-1.455308919	3
Root vs Stem	12,13-DHOME; (9Z)-12,13-Dihydroxyoctadec-9-enoic acid	Lipids	Free fatty acids	1.428499143	0.001313031	0.041846281	0.133209343	-2.90823282	2
Root vs Stem	Kaempferol-3-O-(4''-O-acetyl)rhamnoside	Flavonoids	Flavonols	1.481213792	0.014221433	0.117955669	0.169181213	-2.56335872	2
Root vs Stem	Hexadecanedioic acid	Lipids	Free fatty acids	1.397791898	0.001449263	0.041846281	0.335698578	-1.574761667	1
Root vs Stem	2,4-Dinitrophenol	Others	Others	1.327407201	0.103066543	0.30478678	0.074447961	-3.747623853	2
Root vs Stem	Citric acid-1-O-diglucos	Organic acids	Organic acids	1.111439168	0.099621469	0.300685165	0.473980801	-1.077099471	1

Comparison	Compound	Class I	Class II	VIP	P-value	FDR	Fold Change	Log_2FC	MSI Level
	ide								
Root vs Stem	1-O-Feruloyl-β-D-glucose	Phenolic acids	Phenolic acids	1.44155231	0.002463931	0.054336171	0.011814244	-6.403328861	3
Root vs Stem	NADP (Nicotinamide adenine dinucleotide phosphate)	Nucleotides and derivatives	Nucleotides and derivatives	1.378942956	0.005076299	0.073269103	0.349766646	-1.515535374	3
Root vs Stem	9,16-Dihydroxypalmitic acid	Lipids	Free fatty acids	1.046549373	0.183870191	0.42394095	0.203327509	-2.298122682	3
Root vs Stem	LysoPE 14:0*	Lipids	LPE	1.441258656	0.000616806	0.02893383	38.27562229	5.258353928	3
Root vs Stem	N-Hydroxyoctanoyl nornicotine	Alkaloids	Pyridine alkaloids	1.409637278	0.099975792	0.300685165	0.077167486	-3.695863091	3

Comparison	Compound	Class I	Class II	VIP	P-value	FDR	Fold Change	Log_2FC	MSI Level
	glucoside								
Root vs Stem	12,13-Dihydroursolic acid	Terpenoids	Triterpene	1.440312484	0.018893659	0.131556001	0.03609017	-4.792250252	3
Root vs Stem	5,7,2'-Trihydroxy-8-methoxyflavone *	Flavonoids	Flavones	1.052728851	0.059479509	0.226679801	0.344573196	-1.537117617	3
Root vs Stem	1,3,6,7-tetrahydroxy-2-(3,4,5-trihydroxyoxan-2-yl)xanthen-9-one	Flavonoids	Other Flavonoids	1.282769908	0.011508912	0.104185939	0.209563947	-2.254537554	3
Root vs Stem	Phthalic acid	Phenolic acids	Phenolic acids	1.313896152	0.013830424	0.116862585	0.386903918	-1.369952758	3
Root vs Stem	Limocitrin	Flavonoids	Flavonols	1.489051067	0.024393375	0.144292194	0.004949121	-7.658611991	3

Comparison	Compound	Class I	Class II	VIP	P-value	FDR	Fold Change	Log_2FC	MSI Level
	malonyl glucoside								
Root vs Stem	2-β-D-Glucopyranosyloxy-5-hydroxyphenylacetic acidmethylester*	Phenolic acids	Phenolic acids	1.20986963	0.017232992	0.123933437	0.248580556	-2.008214642	1
Root vs Stem	Persicarin (Isohamnetin-3-O-sulfate)	Flavonoids	Flavones	1.491257123	0.00460396	0.071447928	0.039212605	-4.672538693	3
Root vs Stem	Dicaffeoylspermine	Alkaloids	Phenolamine	1.302740035	0.000322698	0.021248282	0.105749044	-3.241283471	3
Root vs Stem	Iristectorin B	Flavonoids	Isoflavones	1.039604805	0.27228298	0.508025746	0.058518396	-4.094965962	3
Root vs Stem	2,4,6,4'-Tetrahydr	Others	Stilbene	1.140347175	0.092165603	0.288226976	0.352649354	-1.503693697	3

Comparison	Compound	Class I	Class II	VIP	P-value	FDR	Fold Change	Log_2FC	MSI Level
	oxy-stilbene-2-O-glucoside								
Root vs Stem	Quercetin-3-O-(2''-O-galactosyl)glucoside	Flavonoids	Flavonols	1.476984897	0.038014981	0.180664966	0.048022973	-4.380131463	3
Root vs Stem	5-hydroxy-3,4-dimethyl-5-pentylfuran-2(5H)-one*	Others	Lactones	1.380628398	0.021086626	0.135585033	0.087977886	-3.506715255	1
Root vs Stem	11-Oxo-kansenonol	Terpenoids	Triterpene	1.47570387	0.069372753	0.245600966	0.006231987	-7.326091952	3
Root vs Stem	Grayanotoxin V	Terpenoids	Ditepenoids	1.350382056	0.005852579	0.077933696	0.170492167	-2.552222641	3
Root vs Stem	9,11-dimethoxy-2h-[1,3]dioxolo[4,5	Flavonoids	Other Flavonoids	1.013572581	0.079845726	0.264137371	0.369862482	-1.43493913	3

Comparison	Compound	Class I	Class II	VIP	P-value	FDR	Fold Change	Log_2FC	MSI Level
	-b]xanthen-10-one *								
Root vs Stem	Herbacetin	Flavonoids	Flavonols	1.4787813	0.03006653	0.159330661	0.073393189	-3.768209999	1
Root vs Stem	Afrormosin-7-O-(6''-malonyl)glucoside*	Flavonoids	Isoflavones	1.461405018	0.037482214	0.179497192	0.038081579	-4.714762886	3
Root vs Stem	Villosolside	Terpenoids	Monoterpenoids	1.12103774	0.139177299	0.357739917	0.185530003	-2.430275582	2
Root vs Stem	Hyp-Ser	Amino acids and derivatives	Amino acids and derivatives	1.25091211	0.150090121	0.373236158	5.589532489	2.482727621	3
Root vs Stem	Isolariciresinol-9'-O-xyloside	Lignans and Coumarins	Lignans	1.038581417	0.005409827	0.075448846	0.185738983	-2.428651453	3

Comparison	Compound	Class I	Class II	VIP	P-value	FDR	Fold Change	Log_2FC	MSI Level
Root vs Stem	3,19-Epoxy-3,22-dihydroxydammara-20,24-dien-26-oic acid δ-lactone (Semialactone)	Terpenoids	Triterpene	1.041220696	0.201139882	0.441651827	4.381028118	2.131269474	3
Root vs Stem	Xanthorin	Quinones	Anthraquinone	1.067236662	0.047545912	0.201923378	0.347449572	-1.525124492	3
Root vs Stem	8-O-Acetylharpagide	Terpenoids	Monoterpenoids	1.125667066	0.012537282	0.108824056	0.17731032	-2.495651584	3
Root vs Stem	1-p-Coumaroyl-3-feruloyl-sucrose*	Phenolic acids	Phenolic acids	1.059526893	0.185830836	0.424526002	0.257402755	-1.9579006	1
Root vs Stem	Kaempferide-3-O-(6'-O-acetyl)glucoside	Flavonoids	Flavonols	1.058415918	0.195740788	0.435824149	4.232747668	2.081594486	2

Comparison	Compound	Class I	Class II	VIP	P-value	FDR	Fold Change	Log_2FC	MSI Level
Root vs Stem	Ile-Trp	Amino acids and derivatives	Amino acids and derivatives	1.322002392	0.270682686	0.508025746	19.05845332	4.252359138	3
Root vs Stem	Tamarixetin-3-O-rutinoside	Flavonoids	Flavonols	1.058736649	0.184749953	0.42416452	3.822827452	1.934640084	3
Root vs Stem	Swertiamarin	Terpenoids	Monoterpenoids	1.47513421	0.023528971	0.142440405	0.146837846	-2.767704237	3
Root vs Stem	4,5,6-Trihydroxy-2-cyclohexen-1-ylideneacetonitrile	Alkaloids	Alkaloids	1.131031313	0.406184944	0.583066805	125.6804375	6.973616298	3
Root vs Stem	Methylhesperidin	Flavonoids	Flavanones	1.302532953	0.257699128	0.499540612	0.015369331	-6.023801774	3
Root vs Stem	4-(Beta-D-Glucopyranosyloxy)-2,4',	Others	Ketone compounds	1.467495414	0.035124536	0.172612005	0.146775881	-2.768313175	2

Comparison	Compound	Class I	Class II	VIP	P-value	FDR	Fold Change	Log_2FC	MSI Level
	6-Trihydroxybenzophenone								
Root vs Stem	Glu-Ser	Amino acids and derivatives	Amino acids and derivatives	1.355303595	0.044753311	0.195335116	0.022755231	-5.457657936	3
Root vs Stem	3-Methyl-4,8-dihydroxy-3,4-dihydroisocoumarin	Lignans and Coumarins	Coumarins	1.037747126	0.09546413	0.294526106	0.400170289	-1.321314039	3
Root vs Stem	6-beta-d-glucopyranosyloxyindole-3-carboxylic acid	Alkaloids	Plumerane	1.432904101	0.09732803	0.298491907	0.14155193	-2.820596673	3
Root vs Stem	L-Glycyl-L-isoleucine	Amino acids and	Amino acids and derivatives	1.138264675	0.403974117	0.583066805	123.90646	6.953107596	3

Comparison	Compound	Class I	Class II	VIP	P-value	FDR	Fold Change	Log_2FC	MSI Level
		derivatives							
Root vs Stem	2-(2,3,4,5-tetramethoxycinnamoyl)-6-(4-hydroxycinnamoyl)-glucoside	Phenolic acids	Phenolic acids	1.490619822	0.00188871	0.045329042	0.127317707	-2.973495013	2
Root vs Stem	Coniferyaldehyde Glucoside	Lignans and Coumarins	Lignans	1.028179771	0.28829596	0.521583358	7.092234121	2.826240162	2
Root vs Stem	19-Hydroxy-2,3-secours-12-ene-2,3,28-trioic acid 3-methyl ester (Cecropiacic acid 3-methyl ester)	Terpenoids	Triterpene	1.0592076	0.193538709	0.43342621	12.54758395	3.649337693	2

Comparison	Compound	Class I	Class II	VIP	P-value	FDR	Fold Change	Log_2FC	MSI Level
Root vs Stem	Ajugoside	Terpenoids	Monoterpenoids	1.365548048	0.068050321	0.244627395	0.256684917	-1.961929569	2
Root vs Stem	7S,8S-DiHODE; (9Z,12Z)-(7S,8S)-Dihydroxyoctadeca-9,12-dienoic acid	Lipids	Free fatty acids	1.342561629	0.008145644	0.088057897	0.177311067	-2.495645507	2
Root vs Stem	L-Leucyl-L-phenylalanine	Amino acids and derivatives	Amino acids and derivatives	1.163268406	0.394716488	0.583066805	70.64291985	6.142473069	3
Root vs Stem	Grasshopper ketone	Terpenoids	Monoterpenoids	1.125934684	0.121452852	0.334238248	0.360862687	-1.470478116	3
Root vs Stem	2,4,6-Trimethoxy	Others	Others	1.227987734	0.028466846	0.154844052	0.210466064	-2.248340464	3

Comparison	Compound	Class I	Class II	VIP	P-value	FDR	Fold Change	Log_2FC	MSI Level
	phenyl 1-O-β-D-apiofuranosyl-(1→6)-β-D-glucopyranoside								
Root vs Stem	7,4'-Dihydroxydihydroflavonoids	Flavonoids	Flavanones	1.364292962	0.02635051	0.15097024	0.08160095	-3.615270234	3
Root vs Stem	3-Hydroxyurs-12-en-28-oic acid (Ursolic acid)	Terpenoids	Triterpene	1.482053099	0.014246429	0.117955669	0.173925623	-2.523457609	1
Root vs Stem	2'-Deoxyinosine-5'-monophosphate	Nucleotides and derivatives	Nucleotides and derivatives	1.067440993	0.070096119	0.24647085	0.0825908	-3.597875107	3
Root vs Stem	Kelampayoside	Phenolic	Phenolic acids	1.167603125	0.070602596	0.247257907	0.436879837	-1.19469157	3

Comparison	Compound	Class I	Class II	VIP	P-value	FDR	Fold Change	Log_2FC	MSI Level
	A[3,4,5-Trimethoxyphenol-β-D-apiosyl-(1→6)-β-D-glucoside]	acids							
Root vs Stem	N-Methyl-α-aminoisobutyric acid	Amino acids and derivatives	Amino acids and derivatives	1.329530111	0.044584866	0.195335116	0.033539217	-4.898007202	3
Root vs Stem	Sucrose-6-phosphate	Others	Saccharides	1.136347452	0.076603832	0.2560763	0.298811049	-1.7426946	3
Root vs Stem	6-Hydroxyluteolin	Flavonoids	Flavones	1.375903443	0.063766845	0.238865279	0.043677488	-4.516966301	3
Root vs Stem	5-O-β-D-Glucopyranosyl-3-hydrob	Others	Lactones	1.333031456	0.007684821	0.086203641	0.237005643	-2.077006684	3

Comparison	Compound	Class I	Class II	VIP	P-value	FDR	Fold Change	Log_2FC	MSI Level
	enzo(b)fu-ran-2-one								
Root vs Stem	Coclaurine	Alkaloids	Isoquinoline alkaloids	1.233376847	0.009640708	0.096594275	0.204123722	-2.292484239	3
Root vs Stem	Kaempferol-3-(2''-acetylrhamnoside)	Flavonoids	Flavonols	1.056672907	0.183510931	0.42394095	0.271875474	-1.878982082	1
Root vs Stem	4-O-galactopyranosylxylose	Others	Saccharides	1.430944231	0.06777318	0.244361258	0.213721215	-2.226197973	1
Root vs Stem	Dhurrin	Alkaloids	Alkaloids	1.047090841	0.023553128	0.142440405	0.182467781	-2.454286351	3
Root vs Stem	3,4,5-Trimethoxyphenol-β-D-apiofuranosyl-(1→6)-O	Others	Others	1.378097904	0.042822993	0.191665066	2.674993877	1.419535589	3

Comparison	Compound	Class I	Class II	VIP	P-value	FDR	Fold Change	Log_2FC	MSI Level
	-β-D-glucopyranoside								
Root vs Stem	Anhydrosecoisolariciresinol (AHS)*	Lignans and Coumarins	Lignans	1.211694086	0.027120066	0.152108198	0.249818473	-2.001047936	3
Root vs Stem	4-O-Feruloylquinic acid	Phenolic acids	Phenolic acids	1.467171803	0.054661049	0.218644197	0.101927867	-3.294379559	1
Root vs Stem	Sinapaldehyde-4-O-Glucoside	Others	Aldehyde compounds	1.294091669	0.016937636	0.12322237	0.427462269	-1.226131013	3
Root vs Stem	1,2,9,10-tetraethoxy-6-methyl-5,6,6a,7-tetrahydro-4H-dibenzo[de,g]quinoline	Alkaloids	Alkaloids	1.307777556	0.086752766	0.279695007	0.170233544	-2.554412755	2

Comparison	Compound	Class I	Class II	VIP	P-value	FDR	Fold Change	Log_2FC	MSI Level
Root vs Stem	Fertaric acid glucoside	Phenolic acids	Phenolic acids	1.489914646	0.015775161	0.122405758	0.015373576	-6.023403437	2
Root vs Stem	Kaempferol-3-O-sambubioside	Flavonoids	Flavonols	1.367356126	0.011156023	0.102794781	0.080268936	-3.639014411	3
Root vs Stem	Sinapoyl malate	Phenolic acids	Phenolic acids	1.284586559	0.01387177	0.116862585	0.241588322	-2.049377376	3
Root vs Stem	LysoPC 20:4	Lipids	LPC	1.380286692	0.111423666	0.318359367	4.971657524	2.313726919	3
Root vs Stem	Procyanidin A6	Tannins	Proanthocyanidins	1.129296727	0.04658688	0.199492364	0.346643108	-1.528477018	2
Root vs Stem	carlinoside	Flavonoids	Flavones	1.0775801	0.012091766	0.107179135	0.253978982	-1.977218982	3
Root vs Stem	3-(Methylthio)Propionic Acid	Organic acids	Organic acids	1.077626894	0.173466672	0.410120517	2.46849598	1.303632296	3

Comparison	Compound	Class I	Class II	VIP	P-value	FDR	Fold Change	Log_2FC	MSI Level
Root vs Stem	Kakispyrone	Others	Others	1.020923031	0.159969133	0.388900223	0.411518216	-1.280971803	2
Root vs Stem	Tricin-7-O-Glucoside*	Flavonoids	Flavones	1.046632291	0.183978525	0.42394095	0.201310357	-2.312506698	3
Root vs Stem	2'-Deoxyguanosine	Nucleotides and derivatives	Nucleotides and derivatives	1.35284193	0.330228218	0.561442375	62.86962584	5.974291272	3
Root vs Stem	Hydroxytyrosol	Others	Alcohol compounds	1.457225115	0.019892481	0.132806228	0.192216249	-2.379197792	3
Root vs Stem	cya-suc-glu	Flavonoids	Anthocyanidins	1.480616738	0.027319035	0.15242291	0.108676965	-3.201881908	3
Root vs Stem	Cyanidin 3-O-(beta-D-xylosyl-(1->2)-beta-D-g	Flavonoids	Anthocyanidins	1.050551298	0.017349813	0.124340327	0.274199941	-1.866699834	3

Comparison	Compound	Class I	Class II	VIP	P-value	FDR	Fold Change	Log_2FC	MSI Level
	alactoside)								
Root vs Stem	Comoside	Phenolic acids	Phenolic acids	1.421301572	0.02057531	0.133966689	0.053187726	-4.232762846	3
Root vs Stem	L-Lysine-Butanoic Acid	Amino acids and derivatives	Amino acids and derivatives	1.336912627	0.056160459	0.218884523	0.020857802	-5.583269022	3
Root vs Stem	Gossypetin-7-O-(3''-glucosyl)rhamnoside; Rhodioflavonoside	Flavonoids	Flavonols	1.471135657	0.019235183	0.131898396	0.048535595	-4.364813011	3
Root vs Stem	L-Leucyl-L-Leucine	Amino acids and	Amino acids and derivatives	1.131962114	0.410123474	0.583066805	210.7803452	7.719596535	3

Comparison	Compound	Class I	Class II	VIP	P-value	FDR	Fold Change	Log_2FC	MSI Level
		derivatives							
Root vs Stem	7-Hydroxyrutaecarpine	Alkaloids	Plumerane	1.451005427	0.076042324	0.255354165	0.103130973	-3.277450412	3
Root vs Stem	LysoPC 15:0(2n isomer)*	Lipids	LPC	1.104477798	0.311422741	0.542933222	6.507641648	2.702134809	3
Root vs Stem	DL-Glyceraldehyde-3-phosphate	Organic acids	Organic acids	1.223201862	0.194495074	0.433518177	0.343522882	-1.541521896	3
Root vs Stem	Buddlenoid A	Flavonoids	Flavonols	1.490521132	0.001705041	0.042917142	0.137739842	-2.859982168	3
Root vs Stem	5,6,7,7a-tetrahydro-4,4,7a-trimethyl-2(4H)-benzofuranone*	Others	Others	1.460915805	0.00109863	0.039993013	0.246749677	-2.018879901	3

Comparison	Compound	Class I	Class II	VIP	P-value	FDR	Fold Change	Log_2FC	MSI Level
Root vs Stem	Hymenoside M	Others	Others	1.273621999	0.018919052	0.131556001	0.211349416	-2.242297972	3
Root vs Stem	1-O-p-Coumaroyl 3-methylglucaric acid*	Phenolic acids	Phenolic acids	1.479339206	0.023142207	0.142440405	0.121354156	-3.042704576	1
Root vs Stem	3-Hydroxy-11-oxoolean-12-en-30-oic acid (18α-Glycyrrhetinic acid)*	Terpenoids	Triterpene	1.285228881	0.147892506	0.37089931	0.039147691	-4.674928983	2
Root vs Stem	Medioresinol B	Lignans and Coumarins	Lignans	1.367193412	0.01083805	0.101410025	0.075605503	-3.725364947	3
Root vs Stem	(S)-Malic acid-1-O-beta-D-g	Organic acids	Organic acids	1.19335281	0.163025353	0.394009751	0.496182667	-1.011056756	2

Comparison	Compound	Class I	Class II	VIP	P-value	FDR	Fold Change	Log_2FC	MSI Level
	lucopyranoside								
Root vs Stem	Syringaldehyde; 4-Hydroxy-3,5-Dimethoxybenzaldehyde	Others	Aldehyde compounds	1.228444011	0.144876972	0.365557544	3.006362726	1.588019085	3
Root vs Stem	Poncirin (Isosakuranetin-7-O-neohesperidoside)*	Flavonoids	Flavanones	1.491200604	0.001538018	0.041846281	0.031254541	-4.999790372	3
Root vs Stem	Dihydroactinidiolide*	Others	Lactones	1.475643217	0.010802392	0.101410025	0.22628704	-2.143774134	3
Root vs Stem	picrorhizin	Phenolic acids	Phenolic acids	1.159429091	0.056158167	0.218884523	0.441378989	-1.17991014	3

Comparison	Compound	Class I	Class II	VIP	P-value	FDR	Fold Change	Log_2FC	MSI Level
Root vs Stem	4-hydroxy-2-oxo-1,2-dihydroquinoline-3-carboxylic acid	Alkaloids	Quinoline alkaloids	1.056731013	0.081940296	0.269306959	0.439728136	-1.185316248	3
Root vs Stem	2-trans-abscisic acid D-glucosyl ester	Organic acids	Organic acids	1.233810912	0.145251427	0.36605379	0.158693487	-2.655685179	2
Root vs Stem	Epicatechin-(4β→8)-epigallocatechin	Flavonoids	Flavanols	1.006909137	0.273587848	0.508025746	0.154361797	-2.695612351	2
Root vs Stem	Protocatechuic acid 4-O-(6''-O-Galloy) Glucoside	Phenolic acids	Phenolic acids	1.093019101	0.032235341	0.164687485	0.281122753	-1.830727868	2

Comparison	Compound	Class I	Class II	VIP	P-value	FDR	Fold Change	Log_2FC	MSI Level
Root vs Stem	2-Linoleoylglycerol*	Lipids	Glycerol ester	1.307932634	0.273703794	0.508025746	13.54117101	3.7592806	3
Root vs Stem	3-O-Feruloylquinic acid	Phenolic acids	Phenolic acids	1.451251078	0.069915259	0.246254427	0.094163732	-3.408684688	1
Root vs Stem	3,4-dihydroxyphenylethanol-β-D-glucopyranoside	Others	Alcohol compounds	1.489881515	0.008257187	0.088057897	0.048365548	-4.369876456	3
Root vs Stem	icariside D1	Others	Ketone compounds	1.432977534	0.001747172	0.043058694	0.018212057	-5.778962329	3
Root vs Stem	3-Methoxy-2-methyl-3-oxopropylbenzoate	Phenolic acids	Phenolic acids	1.406795907	0.04478763	0.195335116	0.392187341	-1.350385126	1
Root vs Stem	1-Linoleoylglycer	Lipids	Glycerol ester	1.290103934	0.289984956	0.523647374	14.78657845	3.886216353	3

Comparison	Compound	Class I	Class II	VIP	P-value	FDR	Fold Change	Log_2FC	MSI Level
	ol*								
Root vs Stem	Rheic Acid	Quinones	Anthraquinone	1.289625978	0.174875699	0.411569763	0.011840136	-6.40017056	3
Root vs Stem	Pectolinarigenin-7-O-glucoside	Flavonoids	Flavones	1.166475158	0.194410444	0.433518177	0.080577131	-3.633485759	3
Root vs Stem	Kaempferol-3-O-(2''-p-Coumaroyl) glucoside	Flavonoids	Flavonols	1.397121818	0.005411102	0.075448846	0.027646459	-5.176761501	3
Root vs Stem	3-O-Methylellagic acid	Tannins	Tannin	1.42402446	0.007264451	0.084768723	0.034081087	-4.87488485	3
Root vs Stem	Blumenol A	Terpenoids	Monoterpenoids	1.306538812	0.064829019	0.239797661	0.320569088	-1.641292778	3
Root vs Stem	LysoPC 15:0*	Lipids	LPC	1.143376821	0.287092662	0.520702333	6.486986657	2.697548471	3

Comparison	Compound	Class I	Class II	VIP	P-value	FDR	Fold Change	Log_2FC	MSI Level
Root vs Stem	Prudomenin*	Flavonoids	Flavonols	1.032147897	0.224897779	0.465119255	0.156949425	-2.671628348	3
Root vs Stem	Schisantherin E	Lignans and Coumarins	Lignans	1.428107354	0.088914415	0.283646603	0.076835887	-3.702075893	3
Root vs Stem	3-(triazan-2-yl)propyl L-prolinate	Amino acids and derivatives	Amino acids and derivatives	1.243684713	0.192532801	0.432884206	14.06261048	3.813792525	3
Root vs Stem	Hesperetin	Flavonoids	Flavanones	1.480592601	0.019767712	0.132806228	0.084874883	-3.558518508	3
Root vs Stem	2,3,19-Trihydroxyolean-12-en-28-oic acid (Arjunic acid)	Terpenoids	Triterpene	1.216480732	0.133629744	0.351721195	0.07856792	-3.669915829	3
Root vs Stem	2'-Deoxyadenosine*	Nucleotides and	Nucleotides and derivatives	1.19866659	0.305485399	0.540854091	10.59494688	3.405304449	3

Comparison	Compound	Class I	Class II	VIP	P-value	FDR	Fold Change	Log_2FC	MSI Level
		derivatives							
Root vs Stem	Didymin (Isosakuranetin-7-O-rutinoside)*	Flavonoids	Flavanones	1.490901592	0.003153558	0.057601272	0.031103407	-5.006783552	3
Root vs Stem	Gallacetophenone	Others	Ketone compounds	1.208828778	0.110937939	0.317581007	0.465019963	-1.104635444	3
Root vs Stem	Scandione	Flavonoids	Chalcones	1.490983881	0.001533894	0.041846281	0.163616723	-2.611607887	3
Root vs Stem	12-hydroxy jasmonoyl sulfate	Organic acids	Organic acids	1.424584713	0.112422666	0.319175218	0.10613893	-3.235974185	1
Root vs Stem	O-Acetyl-L-homo serine	Amino acids and derivatives	Amino acids and derivatives	1.163687529	0.26117445	0.50178352	3.628913851	1.859537808	3

Comparison	Compound	Class I	Class II	VIP	P-value	FDR	Fold Change	Log_2FC	MSI Level
Root vs Stem	Pedunculoside	Terpenoids	Triterpene Saponin	1.484687106	0.009881899	0.097124949	0.17666659	-2.500898866	2
Root vs Stem	Quercetin-3-O-glucuronide*	Flavonoids	Flavonols	1.477030909	0.029840462	0.158958953	0.049644455	-4.332223602	3
Root vs Stem	C-Veratroylglycol	Others	Alcohol compounds	1.076163039	0.204818632	0.4456275	0.271298719	-1.882045857	3
Root vs Stem	Quercetin-3-O-(6''-O-acetyl)glucoside	Flavonoids	Flavonols	1.457819582	0.042994635	0.191665066	0.125883759	-2.98983593	3
Root vs Stem	3,23-Dihydroxylup-20(29)-en-28-oic acid (Oplopanaxogeni	Terpenoids	Triterpene	1.44246224	0.110190499	0.316654372	0.01568028	-5.994904893	3

Comparison	Compound	Class I	Class II	VIP	P-value	FDR	Fold Change	Log_2FC	MSI Level
	n C)								
Root vs Stem	Luteolin 7-(2-sulfoglucoside)	Flavonoids	Flavones	1.490104237	0.007538821	0.085520226	0.010412101	-6.585595023	1
Root vs Stem	Resveratroloside*	Others	Stilbene	1.053918921	0.019433763	0.132380485	0.30917769	-1.693491877	2
Root vs Stem	N-Hydroxyhexanoyl nornicotine glucoside	Alkaloids	Pyridine alkaloids	1.225584783	0.050090179	0.20760267	0.244658822	-2.03115679	3
Root vs Stem	Methyl 4-caffeoylquinate	Phenolic acids	Phenolic acids	1.485399381	0.010729426	0.101410025	0.130399468	-2.938990113	3
Root vs Stem	DMelezitose O-rhamnoside	Others	Saccharides	1.069459597	0.16527797	0.396590852	0.250834816	-1.995190484	3

Comparison	Compound	Class I	Class II	VIP	P-value	FDR	Fold Change	Log_2FC	MSI Level
Root vs Stem	6-O-alpha-L-arabinopyranosyl-D-glucopyranose	Others	Saccharides	1.247045439	0.132321025	0.349693464	0.342061077	-1.547674147	2
Root vs Stem	5-O-p-Coumaroyl Methylglucaric Acid*	Phenolic acids	Phenolic acids	1.388306765	0.004792487	0.072520512	0.11842452	-3.077960276	1
Root vs Stem	Isofraxidin-7-O-glucoside	Lignans and Coumarins	Coumarins	1.292448608	0.014884083	0.119072667	2.699118626	1.432488385	3
Root vs Stem	Icariside C1 Acetate	Terpenoids	Sesquiterpenoids	1.339838526	0.192971463	0.433398368	28.14578314	4.814846886	3
Root vs Stem	mudanoside A	Phenolic acids	Phenolic acids	1.416464116	0.001353204	0.041846281	0.183016218	-2.449956599	3
Root vs Stem	Hydrocinnamic	Phenolic	Phenolic acids	1.362691339	0.197282793	0.43737023	25.36592089	4.664819632	3

Comparison	Compound	Class I	Class II	VIP	P-value	FDR	Fold Change	Log_2FC	MSI Level
	acid	acids							
Root vs Stem	Leu-Asp	Amino acids and derivatives	Amino acids and derivatives	1.10068939	0.382598433	0.583066805	25.82475823	4.690682938	3
Root vs Stem	Petunidin-3-O-(6''-O-caffeoyl)glucoside	Flavonoids	Anthocyanidins	1.336576868	0.036048684	0.175897124	0.111168616	-3.169178542	3
Root vs Stem	N-(Carboxymethyl)glycine	Amino acids and derivatives	Amino acids and derivatives	1.412691197	0.053988916	0.218644197	4.057243122	2.020499756	3
Root vs Stem	Inosine	Nucleotides and derivatives	Nucleotides and derivatives	1.487763418	0.002682461	0.056495906	0.165581261	-2.594388682	3

Comparison	Compound	Class I	Class II	VIP	P-value	FDR	Fold Change	Log_2FC	MSI Level
Root vs Stem	Gentioluteol	Lignans and Coumarins	Lignans	1.456319568	0.05552624	0.218884523	0.138147904	-2.855714426	3
Root vs Stem	2'-Deoxycytidine	Nucleotides and derivatives	Nucleotides and derivatives	1.241095991	0.115040014	0.323932591	3.212555562	1.683721406	3
Root vs Stem	1-O-(6'-O-feruloyl)glucoside-3-O-C affeoyl Quinic Acid	Phenolic acids	Phenolic acids	1.42249949	0.092060709	0.288226976	0.0902065	-3.4706248	2
Root vs Stem	Isolariciresinol-9-O-glucoside	Lignans and Coumarins	Lignans	1.486025976	0.021399218	0.136742989	0.053378162	-4.227606553	3
Root vs Stem	syringaresinol-4-O-β-D-glucopyra	Lignans and Coumarins	Lignans	1.296967167	0.009855959	0.097124949	0.271637134	-1.880247378	3

Comparison	Compound	Class I	Class II	VIP	P-value	FDR	Fold Change	Log_2FC	MSI Level
	noside*								
Root vs Stem	Aurantio-obtusin-6-O-Glucoside	Quinones	Anthraquinone	1.235432676	0.04117564	0.187194978	0.340284158	-1.555188106	3
Root vs Stem	8,11-dimethoxy-2h-[1,3]dioxolo[4,5-b]xanthen-10-one	Flavonoids	Other Flavonoids	1.222985279	0.265515484	0.505217259	0.004608488	-7.761490636	2
Root vs Stem	Cleomiscosin C	Lignans and Coumarins	Coumarins	1.202948874	0.155087063	0.382436915	7.68509539	2.942063167	3
Root vs Stem	Clemiscosin D	Terpenoids	Triterpene Saponin	1.143071663	0.163230608	0.394044415	6.886881831	2.783850923	3
Root vs Stem	Phellodenol H	Lignans and Coumarins	Coumarins	1.423804699	0.067838663	0.244361258	0.044720184	-4.482930068	3

Comparison	Compound	Class I	Class II	VIP	P-value	FDR	Fold Change	Log_2FC	MSI Level
Root vs Stem	(6R,9R)-3-Oxo-α-ionol-β-D-glucoside	Terpenoids	Sesquiterpenoids	1.470410545	0.033606178	0.168391426	0.144115453	-2.794703056	2
Root vs Stem	(3S,5R,6R,7E,9S)-megastigm-7-ene-3,5,6,9-tetrol3-(beta-D-glucopyranoside)	Others	Others	1.13765942	0.098224562	0.299904581	0.257215391	-1.95895112	1
Root vs Stem	Gly-Phe*	Amino acids and derivatives	Amino acids and derivatives	1.129085094	0.402166749	0.583066805	80.07560712	6.323290926	3
Root vs Stem	Hesperetin-6-C-glucoside-7-O-glucoside	Flavonoids	Flavanones	1.485693072	0.016269437	0.122828256	0.062865762	-3.991581679	3

Comparison	Compound	Class I	Class II	VIP	P-value	FDR	Fold Change	Log_2FC	MSI Level
Root vs Stem	S-(Methyl)glutathione	Amino acids and derivatives	Amino acids and derivatives	1.058001754	0.133674536	0.351721195	2.020820816	1.014941405	3
Root vs Stem	Echinocystic acid	Terpenoids	Triterpene	1.406596187	0.133981735	0.3518299	0.025783774	-5.277392743	3
Root vs Stem	Teupolioside	Amino acids and derivatives	Amino acids and derivatives	1.161683095	0.068409229	0.244627395	0.173284322	-2.528786964	3
Root vs Stem	Nepetin-5-O-diglucoside	Flavonoids	Flavones	1.248147643	0.000339725	0.021248282	0.06069115	-4.042370044	3
Root vs Stem	Vogeloside	Terpenoids	Monoterpenoids	1.369066673	0.010505777	0.100388537	0.092010284	-3.442061071	3
Root vs Stem	Staphylionoside A	Terpenoids	Sesquiterpenoids	1.101663167	0.075015238	0.25346202	0.295098091	-1.760733506	2

Comparison	Compound	Class I	Class II	VIP	P-value	FDR	Fold Change	Log_2FC	MSI Level
Root vs Stem	1-Hydroxypinoresinol-1-O-Glucoside	Lignans and Coumarins	Lignans	1.461184103	0.103072273	0.30478678	0.012887771	-6.277853378	2
Root vs Stem	Dehydrodiconiferyl alcohol*	Lignans and Coumarins	Lignans	1.09414008	0.035535388	0.174216251	0.235890932	-2.083808132	3
Root vs Stem	Trans-Ferulic Acid 4-O-[Apiosyl-(1->6)-Glucosyl] Ester	Phenolic acids	Phenolic acids	1.186882306	0.033717436	0.168505538	0.374480391	-1.417037918	2
Root vs Stem	Octadeca-2,9,12,15-tetraen-1-ol	Others	Others	1.352869092	0.259548892	0.500239506	18.6703384	4.222676172	3
Root vs Stem	Clemaphenol A*	Lignans and Coumarins	Lignans	1.14666898	0.012151123	0.107179135	0.269011988	-1.894257631	3

Comparison	Compound	Class I	Class II	VIP	P-value	FDR	Fold Change	Log_2FC	MSI Level
Root vs Stem	Dicaffeoylquinic acid-O-glucoside	Phenolic acids	Phenolic acids	1.388101424	0.070783988	0.247257907	0.144397412	-2.791883211	2
Root vs Stem	Cannabifolin E*	Terpenoids	Triterpene	1.435997227	0.105258498	0.307877586	0.024064275	-5.376963201	2
Root vs Stem	2,6-dimethoxy-4-hydroxyphenyl-1-O-beta-D-glucopy ranoside*	Others	Others	1.133529873	0.029509359	0.157790979	0.370128458	-1.43390203	1
Root vs Stem	Grayanotoxin VIII	Terpenoids	Ditepenoids	1.436629231	0.003064185	0.056750731	0.084277887	-3.568702051	3
Root vs Stem	2-Palmitoyl-Sn-Gl ycerol 3-O-Diglucoside	Lipids	Glycerol ester	1.053278549	0.257785571	0.499540612	2.025860376	1.018534746	1
Root vs Stem	1-O-p-Coumaroyl	Phenolic	Phenolic acids	1.016759925	0.253160162	0.494344915	0.173078179	-2.530504245	1

Comparison	Compound	Class I	Class II	VIP	P-value	FDR	Fold Change	Log_2FC	MSI Level
	Methylglucaric Acid*	acids							
Root vs Stem	3S-3-hydroxy-β-damascon	Terpenoids	Monoterpenoids	1.280476389	0.026377417	0.15097024	0.144363363	-2.792223434	3
Root vs Stem	Isorhamnetin-3-O-sophoroside*	Flavonoids	Flavonols	1.448112852	0.005522233	0.07548271	0.025465684	-5.29530174	3
Root vs Stem	Gardenoside	Terpenoids	Monoterpenoids	1.185412363	0.191795483	0.431696704	3.577554628	1.838973796	3
Root vs Stem	Nortrachelogenin-4-O-glucoside	Lignans and Coumarins	Lignans	1.367771735	0.106458184	0.309043167	0.040486604	-4.62641157	2
Root vs Stem	7-O-methylmyricetin	Flavonoids	Flavonols	1.394895207	0.120978679	0.33337783	0.066486189	-3.910801511	1

Comparison	Compound	Class I	Class II	VIP	P-value	FDR	Fold Change	Log_2FC	MSI Level
Root vs Stem	epiloliolide	Others	Others	1.318524769	0.042421893	0.191176389	0.066639953	-3.907468807	3
Root vs Stem	L-Valyl-L-Leucine	Amino acids and derivatives	Amino acids and derivatives	1.03136999	0.409990221	0.583066805	78.92315121	6.302376655	3
Root vs Stem	LysoPC 19:1	Lipids	LPC	1.019073239	0.324623536	0.556529503	3.885500215	1.958100341	2
Root vs Stem	Tababiphenyl C	Others	Chromone	1.208752705	0.068307669	0.244627395	0.201301329	-2.312571395	3
Root vs Stem	Grayanoside C	Terpenoids	Ditepenoids	1.463952571	0.038342542	0.180664966	0.129807988	-2.945548924	2
Root vs Stem	3,4,5-Trimethoxyphenol-1-O-β-D-glucoside	Others	Others	1.376599188	0.038991603	0.182078434	0.312023898	-1.680271567	3
Root vs Stem	Capillarisin	Others	Chromone	1.437301556	0.058603549	0.224828484	0.01199185	-6.381801917	3
Root vs Stem	7,2'-dihydroxy-4'-	Flavonoids	Other	1.490625236	0.001584559	0.041911677	0.187052572	-2.418484293	3

Comparison	Compound	Class I	Class II	VIP	P-value	FDR	Fold Change	Log_2FC	MSI Level
	methoxy-isoflavanol glucoside		Flavonoids						
Root vs Stem	Delphinidin-3-O-glucuronide	Flavonoids	Anthocyanidins	1.48551744	0.010959727	0.101895838	0.051598527	-4.276526313	3
Root vs Stem	Patuletin (Quercetagetin-6-methyl ether)	Flavonoids	Flavonols	1.447714586	0.05531654	0.218884523	0.113485948	-3.139414419	2
Root vs Stem	3,3,14,14-tetramethylhexadecanedioic acid	Lipids	Free fatty acids	1.48766412	0.003812274	0.065816097	0.068808858	-3.861261881	1
Root vs Stem	5,6-Dihydroxylucidin	Quinones	Anthraquinone	1.474324454	0.041849572	0.18942438	0.077758464	-3.684856463	1
Root vs Stem	β-Oxoacteoside	Phenolic	Phenolic acids	1.396825959	0.177376208	0.414034699	0.091922242	-3.443442203	3

Comparison	Compound	Class I	Class II	VIP	P-value	FDR	Fold Change	Log_2FC	MSI Level
		acids							
Root vs Stem	Morin	Flavonoids	Flavonols	1.474813166	0.037630014	0.179787845	0.061103096	-4.032610715	1
Root vs Stem	Hesperetin-8-C-glucoside-3'-O-glucoside	Flavonoids	Flavanones	1.459690212	0.065462568	0.240796437	0.110428438	-3.178816344	3
Root vs Stem	Matairesinol	Lignans and Coumarins	Lignans	1.372626248	0.209212929	0.451217852	0.019966276	-5.646290934	3
Root vs Stem	10-hydroxymajoroside	Terpenoids	Monoterpenoids	1.354501758	0.146348704	0.367921711	7.766328729	2.957232774	3
Root vs Stem	3,4-dimethoxy-2-[(3,4,5-trihydroxy-6-{[(3,4,5-trihydroxy-6-methyloxan-	Flavonoids	Other Flavonoids	1.333149948	0.078974692	0.262063929	0.041383936	-4.594785311	3

Comparison	Compound	Class I	Class II	VIP	P-value	FDR	Fold Change	Log_2FC	MSI Level
	2-yl)oxy]methyl}oxan-2-yl)oxy]xanthen-9-one								
Root vs Stem	Glu-Leu	Amino acids and derivatives	Amino acids and derivatives	1.167533016	0.270020628	0.508025746	5.042893341	2.334251712	2
Root vs Stem	Phloroglucinol-1-O-β-D-glucopyranoside	Others	Others	1.093783146	0.105238913	0.307877586	0.406300337	-1.299381532	1
Root vs Stem	Kaempferol-3-O-(6''-p-Coumaroyl)glucoside (Tiliroside)	Flavonoids	Flavonols	1.491686982	0.00020088	0.016991546	0.108195798	-3.20828362	2

Comparison	Compound	Class I	Class II	VIP	P-value	FDR	Fold Change	Log_2FC	MSI Level
Root vs Stem	Dihydrocaffeoylglucose*	Phenolic acids	Phenolic acids	1.468625311	0.003009774	0.056750731	0.406153152	-1.299904255	1
Root vs Stem	3-Hydroxy-urs-11-en-28,13-lactone	Terpenoids	Triterpene	1.367456907	0.084113563	0.273402197	0.108644448	-3.202313641	3
Root vs Stem	Glycerol 9,11,13-octadecatrienoyl ester*	Lipids	Glycerol ester	1.424843961	0.123402279	0.336980871	10.85310938	3.440036525	3
Root vs Stem	Phenyl acetate	Phenolic acids	Phenolic acids	1.233434599	0.3617842	0.583066805	37.00689172	5.209722061	3
Root vs Stem	Grevilloside F	Phenolic acids	Phenolic acids	1.417603429	0.005773144	0.07737512	0.094906484	-3.397349541	3
Root vs Stem	Pyrrolidin	Alkaloids	Pyrrole alkaloids	1.071165873	0.368284104	0.583066805	16.68440226	4.060428096	3

Comparison	Compound	Class I	Class II	VIP	P-value	FDR	Fold Change	Log_2FC	MSI Level
Root vs Stem	2-Picolinic acid	Alkaloids	Pyridine alkaloids	1.319514942	0.215354841	0.45814147	6.978110309	2.802836404	3
Root vs Stem	2,5-Dimethyl pyrazine	Alkaloids	Alkaloids	1.071509961	0.099390893	0.300685165	0.474374567	-1.075901431	3
Root vs Stem	1,6-Di-O-caffeoyl-β-D-glucose*	Phenolic acids	Phenolic acids	1.320674063	0.115592199	0.324601766	4.547357238	2.185028346	2
Root vs Stem	1-α-Linolenoyl-glycerol*	Lipids	Glycerol ester	1.389031745	0.148570361	0.371920071	9.553509617	3.256030825	3
Root vs Stem	Tortoside A*	Lignans and Coumarins	Lignans	1.274842472	0.00593667	0.078546713	0.237129002	-2.076255973	3
Root vs Stem	LysoPE 15:0*	Lipids	LPE	1.456619958	0.047234118	0.201428141	23.543641	4.557265544	3
Root vs Stem	Umckalin	Lignans and	Coumarins	1.101892331	0.126015029	0.33999349	0.435027739	-1.2008207	1

Comparison	Compound	Class I	Class II	VIP	P-value	FDR	Fold Change	Log_2FC	MSI Level
	(7-hydroxy-5,6-dimethoxycoumarin)	Coumarins							
Root vs Stem	1-Monolinolenoyl-Rac-Glycerol*	Lipids	Glycerol ester	1.401925308	0.144190701	0.365557544	10.13123437	3.340738055	3
Root vs Stem	3-O-p-Coumaroyl shikimic acid-O-glucoside	Phenolic acids	Phenolic acids	1.331076752	0.028146633	0.154507048	0.216940759	-2.204626959	3
Root vs Stem	1,3-O-di-Caffeoyl-4-O-glucoside Quinic Acid	Phenolic acids	Phenolic acids	1.300504956	0.019171115	0.131897274	0.119658138	-3.063009577	2
Root vs Stem	3-oxo-taraxeran-14-en	Terpenoids	Triterpene	1.052213492	0.22152502	0.462313085	4.900544856	2.292942161	2

Comparison	Compound	Class I	Class II	VIP	P-value	FDR	Fold Change	Log_2FC	MSI Level
Root vs Stem	3,6-Di-O-caffeoyl glucose*	Phenolic acids	Phenolic acids	1.056961621	0.203120492	0.444110906	4.433109814	2.1483191	2
Root vs Stem	1-O-Sinapoyl-β-D -glucose	Phenolic acids	Phenolic acids	1.383477949	0.006160263	0.080081749	0.090811478	-3.460981526	2
Root vs Stem	Quercetin-5-O-gl ucuronide*	Flavonoids	Flavonols	1.48774969	0.005472305	0.075448846	0.045656443	-4.453037723	3
Root vs Stem	Gossypetin-7-O-L -rhamnopyranoside(Rhodiolgin)	Flavonoids	Flavonols	1.476151382	0.040720074	0.186064525	0.044536909	-4.488854747	3
Root vs Stem	3,7-dihydroxy-2-methoxy-8,8,10-trimethyl-6,7-dihydroanthracene-1,4,	Quinones	Quinones	1.189822468	0.068288205	0.244627395	0.165532752	-2.594811402	3

Comparison	Compound	Class I	Class II	VIP	P-value	FDR	Fold Change	Log_2FC	MSI Level
	5-trione								
Root vs Stem	Mevalonic Acid Glucoside	Organic acids	Organic acids	1.083654384	0.065565697	0.240796437	0.31629709	-1.660647813	1
Root vs Stem	4,5,8-Trihydroxy-α-tetralone	Quinones	Naphthol	1.321596808	0.089694762	0.284412321	0.089377232	-3.483948829	1
Root vs Stem	Quercetin-3-O-sophoroside (Baimaside)	Flavonoids	Flavonols	1.440860663	0.012340623	0.107928159	0.011681743	-6.419600685	3
Root vs Stem	Homoeriodictyol-7,4'-di-O-β-D-glucopyranoside	Flavonoids	Flavanones	1.466039035	0.067142045	0.24269909	0.066326452	-3.91427184	3
Root vs Stem	N-carbamoyl-b-alanine	Amino acids and	Amino acids and derivatives	1.332130169	0.181995297	0.4215918	41.8646295	5.387659952	3

Comparison	Compound	Class I	Class II	VIP	P-value	FDR	Fold Change	Log_2FC	MSI Level
		derivatives							
Root vs Stem	D-Threo-guaiacyl glycerol-7-O-β-D-glucoside	Others	Alcohol compounds	1.406366603	0.014573011	0.117955669	0.119589527	-3.063837039	3
Root vs Stem	6''-O-Caffeoylharpagide	Terpenoids	Terpene	1.303731873	0.026870979	0.151534702	0.312725808	-1.67702981	2
Root vs Stem	2-α-Linolenoyl-glycerol*	Lipids	Glycerol ester	1.406225305	0.138548974	0.357456352	10.4286671	3.382482872	3
Root vs Stem	Protocatechuic Acid Methyl Ester	Phenolic acids	Phenolic acids	1.264183702	0.048386168	0.203814389	0.382393042	-1.386871822	3
Root vs Stem	N-(4-Aminobutyl)benzamide	Alkaloids	Alkaloids	1.056876778	0.038584278	0.180664966	0.338974615	-1.560750859	3

Comparison	Compound	Class I	Class II	VIP	P-value	FDR	Fold Change	Log_2FC	MSI Level
Root vs Stem	Craiobioside B	Others	Others	1.380910494	0.001013075	0.038789527	0.2161964	-2.209585597	3
Root vs Stem	Isorhamnetin-3,7-O-diglucoside	Flavonoids	Flavonols	1.43531197	0.023181326	0.142440405	0.026462754	-5.239892985	3
Root vs Stem	cis-Zeatin-O-glucoside riboside	Alkaloids	Alkaloids	1.215123997	0.001492596	0.041846281	0.158581677	-2.656702009	3
Root vs Stem	2-(4-(3-(hydroxymethyl)-5-(2-hydroxyvinyl)-7-methoxy-2,3-dihydrobenzofuran-2-yl)-2,6-dimethoxyphenoxy)-6-methyltetrahydro-2H-pyran-3,4,5-triol*	Lignans and Coumarins	Lignans	1.398472948	2.944E-05	0.00848072	0.052192048	-4.260026178	2

Comparison	Compound	Class I	Class II	VIP	P-value	FDR	Fold Change	Log_2FC	MSI Level
Root vs Stem	LysoPC 18:3	Lipids	LPC	1.251009901	0.09104461	0.286457431	2.488032933	1.315005582	2
Root vs Stem	Vicine	Alkaloids	Alkaloids	1.257752412	0.113308233	0.320806848	0.299342828	-1.740129391	3
Root vs Stem	Valerine	Alkaloids	Alkaloids	1.142520249	0.261992865	0.502092176	12.20893577	3.609865544	3
Root vs Stem	Myricetin-3-O-(6''-malony)glucoside*	Flavonoids	Flavonols	1.460194959	0.076674009	0.2560763	0.039604696	-4.658184702	2
Root vs Stem	9-(Arabinosyl)hypoxanthine	Nucleotides and derivatives	Nucleotides and derivatives	1.163090265	0.075250444	0.253785812	0.396511129	-1.334566737	3
Root vs Stem	Resinoside B*	Flavonoids	Flavones	1.463292755	0.044415258	0.195335116	0.143501095	-2.800866344	3
Root vs Stem	Monolinolenin*	Lipids	Glycerol ester	1.421384331	0.139698824	0.357739917	13.22952723	3.725689601	3
Root vs Stem	3α-Dihydrocada	Alkaloids	Plumerane	1.484675696	0.010858341	0.101410025	0.165419665	-2.595797343	3

Comparison	Compound	Class I	Class II	VIP	P-value	FDR	Fold Change	Log_2FC	MSI Level
	mbine								
Root vs Stem	Chisopanin D	Terpenoids	Triterpene	1.450916541	0.067088547	0.24269909	0.14589446	-2.77700299	3
Root vs Stem	2-O-p-Coumaroyl 3-methylglucaric acid*	Phenolic acids	Phenolic acids	1.349710155	0.090694733	0.285792257	0.050815944	-4.298574967	1
Root vs Stem	LysoPC 18:3(2n isomer)	Lipids	LPC	1.193253985	0.121829748	0.334383775	2.380473058	1.251248301	2
Root vs Stem	LysoPE 15:0(2n isomer)*	Lipids	LPE	1.457205546	0.045142854	0.195335116	21.77389404	4.444527537	3
Root vs Stem	N-Acetyl-6-hydroxy-L-tryptophan	Amino acids and derivatives	Amino acids and derivatives	1.346504519	0.196594316	0.436312546	0.083401012	-3.583791308	1

Comparison	Compound	Class I	Class II	VIP	P-value	FDR	Fold Change	Log_2FC	MSI Level
Root vs Stem	4-Aminophenol	Alkaloids	Alkaloids	1.251301047	0.365855463	0.583066805	69.31579988	6.115112333	3
Root vs Stem	Demethylwedelolactone	Lignans and Coumarins	Coumarins	1.49153666	0.002310294	0.052400519	0.001659808	-9.234767948	2
Root vs Stem	6-Hydroxykaempferol-6,7-O-Diglucoside*	Flavonoids	Flavonols	1.328291525	0.272265736	0.508025746	0.030457389	-5.03706394	3
Root vs Stem	N-Methyl-Trans-4-Hydroxy-L-Proline	Amino acids and derivatives	Amino acids and derivatives	1.227764797	0.268190305	0.507839257	6.399753788	2.678016403	3
Root vs Stem	1-(4-Hydroxybenzoyl)Glucose; 25545-07-7	Phenolic acids	Phenolic acids	1.348581034	0.000187428	0.016991546	0.092531493	-3.433911727	3
Root vs Stem	Limocitrin-3-O-ar	Flavonoids	Flavonols	1.48526861	0.014735119	0.118339637	0.076214184	-3.713796679	3

Comparison	Compound	Class I	Class II	VIP	P-value	FDR	Fold Change	Log_2FC	MSI Level
	abinoside								
Root vs Stem	Bletilols B	Others	Others	1.215544055	0.336901574	0.569971188	16.29514452	4.026370242	3
Root vs Stem	Dihydrobuddlenol B	Lignans and Coumarins	Lignans	1.221474955	0.144845033	0.365557544	0.421549879	-1.246224751	3
Root vs Stem	2,3,23-Trihydroxyolean-12-en-28-oic acid	Terpenoids	Triterpene	1.199537143	0.142617497	0.36341051	0.054002843	-4.210820822	3
Root vs Stem	Quercetin	Flavonoids	Flavonols	1.40403558	0.036554175	0.17715576	0.053414844	-4.226615472	1
Root vs Stem	3-O-p-Coumaroyl 3-methylglucaric acid*	Phenolic acids	Phenolic acids	1.438362248	0.031223717	0.161812057	0.033518953	-4.898879111	1
Root vs Stem	4'-demethyl-3,9-d	Flavonoids	Other	1.355480472	0.055162172	0.218884523	0.112160871	-3.156358639	3

Comparison	Compound	Class I	Class II	VIP	P-value	FDR	Fold Change	Log_2FC	MSI Level
	ihydroeucomin glucoside		Flavonoids						
Root vs Stem	7,8-Dihydro-Buddlenol B(threo)	Lignans and Coumarins	Lignans	1.34331135	0.062752	0.235920087	0.423748192	-1.238720883	2
Root vs Stem	α-Amyrenone	Terpenoids	Triterpene	1.054090774	0.214755623	0.457908684	4.729216233	2.241601107	1
Root vs Stem	6-Hydroxyluteolin 7-rutinoside	Flavonoids	Flavones	1.410933947	0.152466571	0.378234378	9.693839429	3.277068186	1
Root vs Stem	Quercetin-3,4'-O-di-glucoside	Flavonoids	Flavonols	1.471962347	0.040278221	0.184742773	0.070701368	-3.822118053	3
Root vs Stem	7-hydroxyaloin	Quinones	Anthraquinone	1.411860327	0.023213393	0.142440405	0.212804418	-2.232399993	3
Root vs Stem	6-Hydroxyluteolin 5-glucoside*	Flavonoids	Flavones	1.489827951	0.005059534	0.073269103	0.124012555	-3.011441907	3

Comparison	Compound	Class I	Class II	VIP	P-value	FDR	Fold Change	Log_2FC	MSI Level
Root vs Stem	1-(β-D-Glucopyranosyl)-3-ethyl-4-methyl-1H-pyrrole-2,5-dione	Others	Ketone compounds	1.454953935	0.025371262	0.14668427	0.0583035	-4.100273704	1
Root vs Stem	4-Hydroxyacetophenone	Others	Ketone compounds	1.235119971	0.3545214	0.583066805	29.71079711	4.892915406	3
Root vs Stem	Loliolide	Terpenoids	Monoterpenoids	1.382365864	0.007351551	0.084768723	0.211909436	-2.238480263	3
Root vs Stem	Isoetin (5,7,2',4',5'-Pentahydroxyflavone)	Flavonoids	Flavones	1.477329293	0.037320139	0.179136667	0.066836366	-3.903222905	3
Root vs Stem	Gossypetin-3-O-(6''-malonyl)gluco	Flavonoids	Flavonols	1.472075883	0.055513029	0.218884523	0.05844132	-4.096867424	2

Comparison	Compound	Class I	Class II	VIP	P-value	FDR	Fold Change	Log_2FC	MSI Level
	side*								
Root vs Stem	Feruloyltartaric acid (Fertaric acid)	Phenolic acids	Phenolic acids	1.367129312	0.027323874	0.15242291	0.075556364	-3.726302911	1
Root vs Stem	Blumenol B Glucoside*	Terpenoids	Sesquiterpenoids	1.44516499	0.000502068	0.025274818	0.07242075	-3.787453066	1
Root vs Stem	Gardoside	Terpenoids	Monoterpenoids	1.485467458	0.009731695	0.097034876	0.113025054	-3.145285484	2
Root vs Stem	6-Hydroxykaempferol 3,6-diglucoside*	Flavonoids	Flavonols	1.476996556	0.003667855	0.064704719	0.022017968	-5.505174831	3
Root vs Stem	Epicatechin-(3'-O-7'')-epiafzelechin	Flavonoids	Flavanols	1.038714974	0.008328612	0.088057897	0.232044541	-2.107526338	1

Comparison	Compound	Class I	Class II	VIP	P-value	FDR	Fold Change	Log_2FC	MSI Level
Root vs Stem	23-Hydroxytoonacilide	Terpenoids	Triterpene	1.190036084	0.119333736	0.331500445	0.445740192	-1.165725043	3
Root vs Stem	Genipin	Terpenoids	Monoterpenoids	1.464802423	0.015363494	0.120411672	0.067851738	-3.881470429	3
Root vs Stem	Petunidin-3-O-glucoside	Flavonoids	Anthocyanidins	1.432681769	0.116729069	0.326904747	0.060476	-4.047493473	3
Root vs Stem	Madreselvin A*	Flavonoids	Flavones	1.427548591	0.00766009	0.086203641	0.017049138	-5.874157353	3
Root vs Stem	2-Methoxy-1-benzofuran-5-carbaldehyde	Others	Aldehyde compounds	1.37776589	0.042927355	0.191665066	0.208960623	-2.258696991	3
Root vs Stem	1-hydroxy-4-glucoside-2-naphthoic acid	Phenolic acids	Phenolic acids	1.430303301	0.021337497	0.136742989	0.066198994	-3.917046901	1

Comparison	Compound	Class I	Class II	VIP	P-value	FDR	Fold Change	Log_2FC	MSI Level
Root vs Stem	Resinoside A*	Flavonoids	Flavones	1.456845597	0.055827023	0.218884523	0.136931241	-2.868476458	3
Root vs Stem	Quercetin-7-O-glucoside	Flavonoids	Flavonols	1.426926937	0.003457346	0.061516921	0.012147751	-6.363166985	3
Root vs Stem	Solatriose	Others	Saccharides	1.183302285	0.065340447	0.240796437	0.396093406	-1.336087411	3
Root vs Stem	2-Propylglutaric acid	Organic acids	Organic acids	1.169002509	0.120107312	0.332753681	0.292732798	-1.772343701	3
Root vs Stem	3-Hydroxyurs-12-en-24-oic acid (β-Boswellic acid)*	Terpenoids	Triterpene	1.425514491	0.018551961	0.130686854	0.038328615	-4.705434319	2
Root vs Stem	LysoPC 18:2(2n isomer)	Lipids	LPC	1.061837766	0.307725473	0.541502716	3.696031202	1.885976936	2

Comparison	Compound	Class I	Class II	VIP	P-value	FDR	Fold Change	Log_2FC	MSI Level
Root vs Stem	(X)-1,2-Propanediol 1-O-B-D-Glucopyranoside	Others	Alcohol compounds	1.36060968	0.008604899	0.08880256	0.215083199	-2.21703326	1
Root vs Stem	6'-O-β-D-glucosyl barlerin*	Terpenoids	Monoterpenoids	1.382318825	0.086735036	0.279695007	0.13253956	-2.915505058	3
Root vs Stem	Hypericumoxide N	Others	Others	1.36337711	0.221012238	0.462177568	0.072232124	-3.791215592	3
Root vs Stem	Eriodictyol-7-O-glucoside*	Flavonoids	Flavanones	1.436451447	0.013523252	0.114864168	0.014259159	-6.13196726	3
Root vs Stem	3-O-p-Coumaroyl Methylglucaric Acid*	Phenolic acids	Phenolic acids	1.438390825	0.054593558	0.218644197	0.092584564	-3.433084507	1

Comparison	Compound	Class I	Class II	VIP	P-value	FDR	Fold Change	Log_2FC	MSI Level
Root vs Stem	3-Hydroxyolean-12-en-24-oic acid (α-Boswellic acid)*	Terpenoids	Triterpene	1.466392427	0.020574503	0.133966689	0.050702868	-4.301788841	2
Root vs Stem	Secoisolariciresinol-9'-O-glucoside	Lignans and Coumarins	Lignans	1.352177035	0.105310841	0.307877586	0.064901217	-3.945610666	3
Root vs Stem	6-Hydroxy-3,9-Undecadienoic acid Methyl Ester	Lipids	Free fatty acids	1.414441666	0.132867286	0.35068808	0.116591767	-3.100462172	1
Root vs Stem	Homogentisic acid*	Phenolic acids	Phenolic acids	1.192116877	0.043437815	0.193223383	0.2668371	-1.90596883	3
Root vs Stem	Bartsioside	Terpenoids	Monoterpenoids	1.357568636	0.000440068	0.023902667	0.11957462	-3.064016888	3

Comparison	Compound	Class I	Class II	VIP	P-value	FDR	Fold Change	Log_2FC	MSI Level
Root vs Stem	Coniferin	Others	Alcohol compounds	1.385941165	0.004919027	0.072520512	0.102039143	-3.292805413	3
Root vs Stem	5,7-Dihydroxy-4-methylcoumarin	Lignans and Coumarins	Coumarins	1.26479882	0.016700356	0.122828256	0.298218029	-1.745560615	3
Root vs Stem	Quercetin 7-O-p-coumaroyl rhamnoside	Flavonoids	Flavonols	1.445746396	0.039190614	0.182183393	0.00626502	-7.318465129	2
Root vs Stem	Hesperetin-5-O-glucoside	Flavonoids	Flavanones	1.467508571	1.42461E-08	2.94039E-05	0.014452934	-6.112493831	3
Root vs Stem	3-pyridine-methanol-O-β-D-glucopyranosyl*	Alkaloids	Pyridine alkaloids	1.232981823	0.031280528	0.161812057	0.366165764	-1.449431187	1
Root vs Stem	Syringetin-7-O-gl	Flavonoids	Flavonols	1.490787149	0.008331171	0.088057897	0.011769595	-6.408791483	3

Comparison	Compound	Class I	Class II	VIP	P-value	FDR	Fold Change	Log_2FC	MSI Level
	ucoside*								
Root vs Stem	6'-O-α-D-galactosylbarlerin*	Terpenoids	Monoterpenoids	1.41152093	0.109969759	0.316565665	0.116628433	-3.100008548	3
Root vs Stem	Deutzioside	Terpenoids	Monoterpenoids	1.213922991	0.052802446	0.216099896	0.191662623	-2.383359078	3
Root vs Stem	Ixoroside	Terpenoids	Monoterpenoids	1.448681848	0.01992959	0.132806228	0.188882971	-2.404435457	3
Root vs Stem	3,4-Dihydro-4-(4'-hydroxyphenyl)-5,7-dihydroxycoumarin glucoside	Lignans and Coumarins	Coumarins	1.22110333	0.000767239	0.032317992	0.121591899	-3.039880984	3
Root vs Stem	Ovafolinin E	Lignans and Coumarins	Lignans	1.37661152	0.100860062	0.301703141	0.126121621	-2.987112482	3

Comparison	Compound	Class I	Class II	VIP	P-value	FDR	Fold Change	Log_2FC	MSI Level
Root vs Stem	6-Hydroxykaempferol-7,6-O-Diglucoside	Flavonoids	Flavonols	1.480398991	0.015937757	0.122828256	0.157732797	-2.664445428	3
Root vs Stem	Tetraphyllin A*	Alkaloids	Alkaloids	1.095148925	0.137044493	0.355190258	0.462384918	-1.112833753	1
Root vs Stem	Chaenorrhinoside	Others	Others	1.288905215	0.00177371	0.043069855	0.137371921	-2.863840952	3
Root vs Stem	6-Hydroxykaempferol-3,6-O-Diglucoside*	Flavonoids	Flavonols	1.46310991	0.038457613	0.180664966	0.026113696	-5.259049542	3
Root vs Stem	1-O-Caffeoyl-β-D-glucose*	Phenolic acids	Phenolic acids	1.318305875	0.000533001	0.026059898	0.15871866	-2.655456346	1
Root vs Stem	LysoPC 18:0(2n isomer)	Lipids	LPC	1.005536318	0.261258758	0.50178352	2.408879566	1.268362266	1

Comparison	Compound	Class I	Class II	VIP	P-value	FDR	Fold Change	Log_2FC	MSI Level
Root vs Stem	p-Coumaroylcaffeoyltartaric acid	Phenolic acids	Phenolic acids	1.440377284	0.000481307	0.024835441	0.052178354	-4.260404765	3
Root vs Stem	4-Pyridoxic acid	Others	Vitamin	1.3471704	0.004911466	0.072520512	0.253559671	-1.979602792	3
Root vs Stem	N(6),N(6)-Dimethyl-L-lysine	Amino acids and derivatives	Amino acids and derivatives	1.201930275	0.244634939	0.486333033	21.19813964	4.405865753	2
Root vs Stem	5-L-Glutamyl-L-Alanine	Amino acids and derivatives	Amino acids and derivatives	1.116944437	0.017074418	0.12322237	0.257304056	-1.958453894	3
Root vs Stem	Secoisolariciresinol-sesquilignan	Lignans and Coumarins	Lignans	1.481919347	0.003826517	0.065816097	0.011584365	-6.431677275	3
Root vs Stem	Meratin*	Flavonoids	Flavonols	1.483512247	0.011563751	0.104225246	0.164137931	-2.607019426	3

Comparison	Compound	Class I	Class II	VIP	P-value	FDR	Fold Change	Log_2FC	MSI Level
Root vs Stem	coleoside-B	Terpenoids	Monoterpenoids	1.207101082	0.34633997	0.583066805	19.73824098	4.302921521	3
Root vs Stem	Nodakenin	Lignans and Coumarins	Coumarins	1.437829076	0.010440795	0.100388537	0.091325116	-3.452844504	2
Root vs Stem	3,4-Dihydroxy-L-phenylalanine (L-Dopa)	Amino acids and derivatives	Amino acids and derivatives	1.03256925	0.237957657	0.481986853	2.740977713	1.454690598	3
Root vs Stem	Syringaldehyde-4-O-glucoside	Others	Aldehyde compounds	1.12187923	0.091887834	0.288226976	0.323877946	-1.626477864	2
Root vs Stem	16-Hydroxy-3-oxo-5-lanosta-7,24-dien-21-oic acid (Kulonic acid)	Terpenoids	Triterpene	1.005648302	0.126692223	0.340955966	0.244764342	-2.030534699	3

Comparison	Compound	Class I	Class II	VIP	P-value	FDR	Fold Change	Log_2FC	MSI Level
Root vs Stem	Quercetin-4'-O-glucoside (Spiraeoside)*	Flavonoids	Flavonols	1.490692012	0.001014842	0.038789527	0.013869219	-6.171969662	3
Root vs Stem	Guaiacylglycerol 8-O-(1''-O-Glucosyl)Vanillic Acid Ether	Phenolic acids	Phenolic acids	1.25452908	0.009027389	0.091785871	0.295331247	-1.759594086	1
Root vs Stem	D-Threonic Acid	Others	Saccharides	1.395761753	0.010657106	0.101365289	0.215198308	-2.216261363	3
Root vs Stem	Lys-Asp*	Amino acids and derivatives	Amino acids and derivatives	1.306103341	0.108751934	0.313497197	2.362099628	1.240069815	3
Root vs Stem	Quercetin 5-O-p-coumaroyl	Flavonoids	Flavonols	1.454413184	0.05399758	0.218644197	0.128131178	-2.964306529	1

Comparison	Compound	Class I	Class II	VIP	P-value	FDR	Fold Change	Log_2FC	MSI Level
	rhamnoside								
Root vs Stem	6-Hydroxymethyl herniarin	Lignans and Coumarins	Coumarins	1.452289219	0.003014145	0.056750731	0.122993161	-3.023349992	3
Root vs Stem	Nicotinic acid (Vitamin B3)	Others	Vitamin	1.384397805	0.107235515	0.310426511	5.051325403	2.336661982	3
Root vs Stem	Ribosyladenosine	Nucleotides and derivatives	Nucleotides and derivatives	1.215041007	0.048102921	0.203035643	0.483211139	-1.049274383	3
Root vs Stem	p-galloyl-oxyphenyl-β-D-glucoside	Phenolic acids	Phenolic acids	1.40396267	0.003006648	0.056750731	0.272063756	-1.87798332	3
Root vs Stem	4,6-dimethoxy-2-methoxyphenyl-1-O-beta-D-glucop	Others	Others	1.065271775	0.0449807	0.195335116	0.361324211	-1.468634168	3

Comparison	Compound	Class I	Class II	VIP	P-value	FDR	Fold Change	Log_2FC	MSI Level
	yranoside								
Root vs Stem	Rhamnetin-3-O-Rutinoside*	Flavonoids	Flavonols	1.005500589	0.165443145	0.396590852	2.016663187	1.011970153	1
Root vs Stem	4-(6-O-Sulfo-Beta-D-Glucopyranosyloxy)-3-Hydroxybenzoic Acid	Phenolic acids	Phenolic acids	1.235174239	0.040174154	0.184742773	0.355310227	-1.49284888	3
Root vs Stem	2-Methoxy-4-ethenylphenol	Others	Others	1.344943307	0.003052145	0.056750731	0.1544884	-2.694429575	3
Root vs Stem	Succinyladenosine	Nucleotides and derivatives	Nucleotides and derivatives	1.301491015	0.114749387	0.323932591	3.230590078	1.691797702	2
Root vs Stem	Hesperetin-7-O-r	Flavonoids	Flavanones	1.459015316	0.001752389	0.043058694	0.005933626	-7.396870359	3

Comparison	Compound	Class I	Class II	VIP	P-value	FDR	Fold Change	Log_2FC	MSI Level
	utinoside (Hesperidin)								
Root vs Stem	2-Ethyl-3-methyl maleimide-N-glu coside	Alkaloids	Alkaloids	1.363831573	0.039783802	0.183699703	0.328327212	-1.606793767	2
Root vs Stem	N-α-Acetyl-L-orn ithine	Amino acids and derivatives	Amino acids and derivatives	1.110082248	0.217033269	0.458674946	8.120106693	3.021498684	3
Root vs Stem	NG,NG-Dimethyl -L-arginine*	Amino acids and derivatives	Amino acids and derivatives	1.270281144	0.169759505	0.404133355	9.325103901	3.2211198	3
Root vs Stem	6-O-Caffeoyl-D-gl ucose*	Phenolic acids	Phenolic acids	1.321520012	0.002500938	0.054336171	0.119440776	-3.065632646	1

Comparison	Compound	Class I	Class II	VIP	P-value	FDR	Fold Change	Log_2FC	MSI Level
Root vs Stem	2,3-Dihydroxy-5(6),12(13)-diene-ursolic acid*	Terpenoids	Triterpene	1.451516628	0.088226254	0.282570538	0.013552967	-6.205247476	1
Root vs Stem	Monogalactosyldiacylglycerol	Lipids	Glycerol ester	1.414201881	0.135204956	0.352797763	0.105254285	-3.248049121	1
Root vs Stem	ethyl-L-arginine	Amino acids and derivatives	Amino acids and derivatives	1.255767334	0.17646609	0.413423393	9.750318983	3.285449418	3
Root vs Stem	Pratensein 7-O-glucopyranoside	Flavonoids	Isoflavones	1.483841596	0.021852277	0.139207096	0.064010003	-3.965558806	2
Root vs Stem	Cornuside	Terpenoids	Monoterpenoids	1.009056609	0.094207547	0.292837917	0.364714424	-1.455160837	3

Comparison	Compound	Class I	Class II	VIP	P-value	FDR	Fold Change	Log_2FC	MSI Level
Root vs Stem	Persicoside*	Flavonoids	Flavanones	1.265723623	0.002247821	0.052028413	0.058841805	-4.087014687	2
Root vs Stem	2,3-Dihydroxyurs-12,18-dien-28-oic acid*	Terpenoids	Triterpene	1.455186686	0.082651627	0.270782474	0.017080037	-5.87154506	1
Root vs Stem	Grevilloside B	Others	Others	1.441824958	0.000654026	0.029471125	0.173177996	-2.529672459	3
Root vs Stem	Quercetin 3,7-diglucoside*	Flavonoids	Flavonols	1.402103276	0.003901171	0.06601695	0.039576007	-4.659230129	3
Root vs Stem	2,3'-Di-feruloyl sucrose	Phenolic acids	Phenolic acids	1.433912489	0.100082707	0.300685165	0.123131728	-3.021725539	1
Root vs Stem	1-Aminocyclopropane-1-carboxylic acid	Amino acids and derivatives	Amino acids and derivatives	1.115322047	0.303808792	0.539175707	4.832232901	2.27268999	3

Comparison	Compound	Class I	Class II	VIP	P-value	FDR	Fold Change	Log_2FC	MSI Level
Root vs Stem	D-Panthenol	Others	Vitamin	1.183325886	0.286951658	0.520702333	8.74871445	3.129071041	3
Root vs Stem	LysoPE 17:1*	Lipids	LPE	1.455411002	0.07367988	0.252807391	32.70580824	5.031474962	3
Root vs Stem	Ssioriside	Lignans and Coumarins	Lignans	1.482865805	0.013514636	0.114864168	0.162639721	-2.620248445	3
Root vs Stem	Isosalipurposide (Phlorizin Chalcone)	Flavonoids	Chalcones	1.294945221	0.000542915	0.026059898	0.108106432	-3.209475727	3
Root vs Stem	2'-Deoxyadenosine-5'-monophosphate	Nucleotides and derivatives	Nucleotides and derivatives	1.404419339	0.002938803	0.056750731	0.395664969	-1.337648754	3
Root vs Stem	Isonicotinic acid	Others	Vitamin	1.344094455	0.127509036	0.342680534	5.967255081	2.577067449	3
Root vs Stem	Tricin-7-O-saccha	Flavonoids	Flavones	1.478174987	0.001265237	0.041846281	0.293298582	-1.769557997	3

Comparison	Compound	Class I	Class II	VIP	P-value	FDR	Fold Change	Log_2FC	MSI Level
	ric acid								
Root vs Stem	2-g-linolenoyl-glycerol	Lipids	Glycerol ester	1.107410259	0.266814556	0.505698111	0.119867149	-3.060491773	2
Root vs Stem	Astraflavonoid C	Flavonoids	Other Flavonoids	1.279478455	0.308498776	0.541502716	16.32280483	4.028817079	3
Root vs Stem	Roseoside	Terpenoids	Sesquiterpenoids	1.287942595	0.064288394	0.239427592	0.210807165	-2.246004194	3
Root vs Stem	Genistein-7-O-galactoside*	Flavonoids	Isoflavones	1.267576333	0.008386119	0.088057897	0.202136467	-2.306598474	3
Root vs Stem	isatioxyneolignoside A	Lignans and Coumarins	Lignans	1.26224914	0.020515927	0.133966689	0.328269269	-1.607048398	3
Root vs Stem	Stachydrine	Alkaloids	Pyrrole	1.301765927	0.14963311	0.372548539	0.054535251	-4.196667112	3

Comparison	Compound	Class I	Class II	VIP	P-value	FDR	Fold Change	Log_2FC	MSI Level
			alkaloids						
Root vs Stem	LysoPE 17:1(2n isomer)*	Lipids	LPE	1.461816323	0.077371687	0.257988952	39.30236667	5.296544285	3
Root vs Stem	Grayanotoxin XIX	Terpenoids	Ditepenoids	1.467820592	0.033160805	0.167754661	0.135731519	-2.881172324	2
Root vs Stem	3-Ethyl-7-hydroxyphthalide	Others	Others	1.459179174	0.019845014	0.132806228	0.28442307	-1.813889606	1
Root vs Stem	Trans-Ferulic Acid 1'-O-[Apiosyl-(1->6)-Glucosyl] Ester	Phenolic acids	Phenolic acids	1.472227327	0.019134037	0.131897274	0.248351076	-2.009547098	1
Root vs Stem	N,N'-Dimethylarginine;SDMA*	Amino acids and derivatives	Amino acids and derivatives	1.244010539	0.176218945	0.413313525	8.639750278	3.110989614	3

Comparison	Compound	Class I	Class II	VIP	P-value	FDR	Fold Change	Log_2FC	MSI Level
Root vs Stem	Dihydrokaempferol-7-O-glucoside	Flavonoids	Flavanonols	1.046825299	0.004320206	0.069663321	0.198492776	-2.332841594	3
Root vs Stem	(2E)-3-(1-Hydroxy-2,6,6-trimethyl-4-oxo-2-cyclohexen-1-yl)-2-propenoic acid	Terpenoids	Sesquiterpenoids	1.446848829	0.024569855	0.144292194	0.144466466	-2.791193451	3
Root vs Stem	3-Hydroxy-18-oxoursan-28,13-olide	Terpenoids	Triterpene	1.417495246	0.118016569	0.329170539	0.01442298	-6.115486925	1
Root vs Stem	Chlorogenic acid methyl ester	Phenolic acids	Phenolic acids	1.45031483	0.056193573	0.218884523	0.119774716	-3.061604708	2
Root vs Stem	1-(Hydroxymethy	Alkaloids	Pyrrole	1.32693111	0.266093021	0.505258505	62.23703976	5.959701537	3

Comparison	Compound	Class I	Class II	VIP	P-value	FDR	Fold Change	Log_2FC	MSI Level
	l)hexahydro-1h-pyrrolizin-2-ol		alkaloids						
Root vs Stem	Sophoricoside*	Flavonoids	Isoflavones	1.085263087	0.01647462	0.122828256	0.211934672	-2.238308468	3
Root vs Stem	Tricetin-5-O-(6''-malonyl)glucoside	Flavonoids	Flavones	1.420797152	0.044874706	0.195335116	0.019888265	-5.651938792	3
Root vs Stem	Limocitrin-7-O-glucoside*	Flavonoids	Flavonols	1.368111988	0.029381316	0.157514378	0.034705752	-4.848681391	3
Root vs Stem	Fraxetin-7,8-di-O-glucoside	Lignans and Coumarins	Coumarins	1.229074998	0.021011388	0.13552345	0.458296198	-1.125647779	2
Root vs Stem	5-[4-(3,4-dimethoxyphenyl)-hexahydrofuro[3,4-c]fura	Lignans and Coumarins	Lignans	1.148113056	0.008096038	0.088057897	0.219042107	-2.190719865	3

Comparison	Compound	Class I	Class II	VIP	P-value	FDR	Fold Change	Log_2FC	MSI Level
	n-1-yl]-4-methyl-2H-1,3-benzodioxole								
Root vs Stem	Secoisolariciresinol 4-O-glucoside*	Lignans and Coumarins	Lignans	1.090207966	0.101600026	0.302452427	0.382352928	-1.387023174	2
Root vs Stem	L-threo-3-Methyl aspartate	Amino acids and derivatives	Amino acids and derivatives	1.044059118	0.248850897	0.489636082	2.322101402	1.215430974	3
Root vs Stem	tetrahydrodehydrodiconiferyl alcohol glucoside	Lignans and Coumarins	Lignans	1.014785296	0.220361874	0.461896029	0.476257517	-1.070186232	2
Root vs Stem	Quercetin 3-[6''-(3-hydroxy-	Flavonoids	Flavonols	1.33473606	0.008831274	0.090685326	0.046988711	-4.411541999	1

Comparison	Compound	Class I	Class II	VIP	P-value	FDR	Fold Change	Log_2FC	MSI Level
	3-methylglutaryl) galactoside]								
Root vs Stem	4-O-Feruloyl Aminogalactitol	Phenolic acids	Phenolic acids	1.447628098	0.016244431	0.122828256	0.060421485	-4.048794544	2
Root vs Stem	L-Homoglutamic acid	Amino acids and derivatives	Amino acids and derivatives	1.066401228	0.294293306	0.526818199	3.264698727	1.706949863	3
Root vs Stem	1-O-p-Coumaroyl quinic acid*	Phenolic acids	Phenolic acids	1.474827903	0.031479251	0.162027868	0.026783307	-5.222522098	2
Root vs Stem	Kaempferol-7-O-r hamnoside	Flavonoids	Flavonols	1.00121312	0.311264706	0.542933222	0.119896282	-3.060141172	2
Root vs Stem	Quercetin-3-O-xyl osyl(1→2)arabino	Flavonoids	Flavonols	1.475157286	0.023279713	0.142440405	0.148084358	-2.755508837	1

Comparison	Compound	Class I	Class II	VIP	P-value	FDR	Fold Change	Log_2FC	MSI Level
	side								
Root vs Stem	1-O-Caffeoyl-(6-O-glucosyl)-β-D-glucose	Phenolic acids	Phenolic acids	1.48795552	0.004873285	0.072520512	0.048669424	-4.360840478	1
Root vs Stem	6-((4-(3-hydroxy-2-(4-(3-hydroxypropyl)-2-methylphenoxy)propoxy)-2-methoxyphenoxy)methyl)tetrahydro-2H-pyran-2,3,4,5-tetraol*	Lignans and Coumarins	Lignans	1.040756192	0.184223721	0.42394095	0.476879607	-1.068303005	2
Root vs Stem	Securoside A	Phenolic acids	Phenolic acids	1.388010854	0.139542987	0.357739917	0.064273016	-3.959643028	1

Comparison	Compound	Class I	Class II	VIP	P-value	FDR	Fold Change	Log_2FC	MSI Level
Root vs Stem	Cyanidin 3-xyloside	Flavonoids	Anthocyanidins	1.414803192	0.096120417	0.296108271	0.082416714	-3.600919249	3
Root vs Stem	LysoPC 17:1	Lipids	LPC	1.353928259	0.063324481	0.237639507	2.422116097	1.276268018	2
Root vs Stem	Cyanidin-3-O-arabinoside	Flavonoids	Anthocyanidins	1.471639654	0.028687188	0.154844052	0.144017578	-2.795683182	3
Root vs Stem	1-(sn-Glycero-3-phospho)-1D-myo-inositol	Others	Saccharides	1.290501419	0.101280367	0.302452427	0.217791095	-2.198983128	3
Root vs Stem	D-Mannose*	Others	Saccharides	1.191376622	0.008410487	0.088057897	0.184015383	-2.442101721	1
Root vs Stem	Okanin-4'-(6''-O-acetyl)glucoside*	Flavonoids	Chalcones	1.491098233	0.00621601	0.080081749	0.007725191	-7.016213697	3
Root vs Stem	Apigenin-7-O-glu	Flavonoids	Flavones	1.217412571	0.002655152	0.056495906	0.200206665	-2.32043809	3

Comparison	Compound	Class I	Class II	VIP	P-value	FDR	Fold Change	Log_2FC	MSI Level
	coside(Cosmosiin)*								
Root vs Stem	2-(7-hydroxy-4-methoxy-9,10-dihydrophenanthren-2-yl)-6-(hydroxymethyl)oxane-3,4,5-triol	Others	Others	1.235783111	0.065556239	0.240796437	0.337877678	-1.565427055	3
Root vs Stem	1-O-P-Coumaroyl-(B-D-Glucose 6-O-Sulfate)	Phenolic acids	Phenolic acids	1.416831925	0.014149928	0.117955669	0.020788299	-5.588084484	2
Root vs Stem	2,3-dihydro-6-hydroxyltryptophan N-Glucoside	Alkaloids	Plumerane	1.1932855	0.033486985	0.168391426	0.184829031	-2.435736715	3

Comparison	Compound	Class I	Class II	VIP	P-value	FDR	Fold Change	Log_2FC	MSI Level
Root vs Stem	Hypoglycyrrhizic acid(β)*	Terpenoids	Triterpene	1.293811682	0.138237406	0.357199375	0.046341834	-4.431541036	2
Root vs Stem	Syringetin-3-O-glucoside*	Flavonoids	Flavonols	1.135682767	0.011232926	0.103043373	0.146048977	-2.775475846	3
Root vs Stem	Herbacetin-7-O-(3''-glucosyl)rhamnoside; Rhodiosin	Flavonoids	Flavonols	1.490021579	0.004437578	0.069917266	0.097649529	-3.356243105	3
Root vs Stem	Kaempferol-3-O-rhamnoside (Afzelin)(Kaempferin)	Flavonoids	Flavonols	1.042181779	0.22961459	0.471097926	0.195705991	-2.353240177	1
Root vs Stem	Agarotetrol	Others	Chromone	1.479435684	0.019686671	0.132806228	0.1663299	-2.587880561	3
Root vs Stem	Scandoside	Terpenoids	Monoterpenoid	1.108050965	0.020379279	0.133966689	0.237912659	-2.071496058	2

Comparison	Compound	Class I	Class II	VIP	P-value	FDR	Fold Change	Log_2FC	MSI Level
			s						
Root vs Stem	Limocitrin-3-O-galactoside*	Flavonoids	Flavonols	1.475608869	0.005770425	0.07737512	0.004870502	-7.681713859	3
Root vs Stem	3-(Hydroxycinnamoyl)-quinic acid*	Phenolic acids	Phenolic acids	1.477368133	0.059144757	0.226064406	0.032530789	-4.94205038	2
Root vs Stem	(S)-2-Phenyloxirane	Others	Others	1.148273218	0.405263418	0.583066805	151.109404	7.239449636	3
Root vs Stem	Hesperetin-7-O-neohesperidoside(Neohesperidin)	Flavonoids	Flavanones	1.456882469	0.001482903	0.041846281	0.003771177	-8.050769375	3
Root vs Stem	Deacetylasperulosidic acid	Terpenoids	Monoterpenoids	1.202715173	0.002959692	0.056750731	0.149806128	-2.738831456	2

Comparison	Compound	Class I	Class II	VIP	P-value	FDR	Fold Change	Log_2FC	MSI Level
Root vs Stem	Stigmasta-4,22-Dien-3-One	Steroids	Steroid	1.086710133	0.096355427	0.296389867	0.475459373	-1.072606025	2
Root vs Stem	LysoPE 16:1(2n isomer)*	Lipids	LPE	1.286047979	0.08548476	0.276986727	4.250120766	2.087503836	3
Root vs Stem	2,7-Dimethylocta-2,4-Dienal-8-ol 8-O-(beta-D-Xylopyranosyl)-beta-D-glucopyranoside	Terpenoids	Monoterpenoids	1.405964076	0.000409271	0.0228307	0.079838584	-3.64677006	2
Root vs Stem	Gambiriin B2	Flavonoids	Flavanols	1.024929543	0.014540433	0.117955669	0.248713637	-2.007442484	1
Root vs Stem	N5-(1-((carboxymethyl)amino)-3-((2	Amino acids and	Amino acids and derivatives	1.014200381	0.411065572	0.583066805	91.9826637	6.523290072	2

Comparison	Compound	Class I	Class II	VIP	P-value	FDR	Fold Change	Log_2FC	MSI Level
	,3-dihydroxy-1-(4-methoxyphenyl)propyl)thio)-1-oxopropan-2-yl)glutamine	derivatives							
Root vs Stem	Methyl dioxindole-3-acetate	Alkaloids	Plumerane	1.252395825	0.023431576	0.142440405	0.365995491	-1.450102222	3
Root vs Stem	LysoPE 16:1*	Lipids	LPE	1.277386199	0.088805115	0.283646603	4.246579855	2.086301379	3
Root vs Stem	Galangin 7-glucoside	Flavonoids	Flavonols	1.233814547	0.027063335	0.152108198	0.225170129	-2.15091264	3
Root vs Stem	Medicarpinglucoside	Flavonoids	Isoflavones	1.128653975	0.003429633	0.061516921	0.165068454	-2.598863658	3

Comparison	Compound	Class I	Class II	VIP	P-value	FDR	Fold Change	Log_2FC	MSI Level
Root vs Stem	13-methylmyristic acid	Lipids	Free fatty acids	1.081851093	0.108159847	0.312480766	0.427906923	-1.224631074	1
Root vs Stem	Annuionone D	Others	Ketone compounds	1.477366182	0.001358362	0.041846281	0.087955294	-3.507085781	3
Root vs Stem	Rubinic acid	Terpenoids	Triterpene	1.220543717	0.111518325	0.318359367	0.108502765	-3.204196286	3
Root vs Stem	4,8-Dihydroxyquinoline-2-carboxylic acid	Alkaloids	Quinoline alkaloids	1.111146526	0.024401235	0.144292194	0.243235351	-2.039575172	2
Root vs Stem	Decanoyl arabinosylglucoside	Others	Saccharides	1.441037327	0.019946672	0.132806228	0.034161262	-4.871494931	2
Root vs Stem	Salidroside	Phenolic acids	Phenolic acids	1.427809926	0.049071864	0.205029002	0.186140989	-2.42553232	3

Comparison	Compound	Class I	Class II	VIP	P-value	FDR	Fold Change	Log_2FC	MSI Level
Root vs Stem	2-O-P-Coumaroyl hydroxycitric Acid	Phenolic acids	Phenolic acids	1.469880093	0.03398099	0.169412474	0.15378888	-2.700976909	3
Root vs Stem	3,4,5-trimethoxyp henyl acetate	Others	Others	1.057751424	0.00318283	0.057625981	0.16761619	-2.576766589	3
Root vs Stem	Megastigm-5-en-3 ,9-diol glucoside	Terpenoids	Sesquiterpenoi ds	1.320403947	0.003079497	0.056750731	0.200799699	-2.316170986	3
Root vs Stem	1,2,4-Trihydroxyn aphthalene-1,4-di -glucoside (Lawsoniaside)	Others	Others	1.445262869	0.00046063	0.024377938	0.088849477	-3.492492909	2
Root vs Stem	β-Pseudouridine	Nucleotides and	Nucleotides and derivatives	1.160848454	0.047985384	0.202954576	0.358608165	-1.479519759	3

Comparison	Compound	Class I	Class II	VIP	P-value	FDR	Fold Change	Log_2FC	MSI Level
		derivatives							
Root vs Stem	Pentadecanoic Acid	Lipids	Free fatty acids	1.26922009	0.016714645	0.122828256	0.340662924	-1.553583154	2
Root vs Stem	Keto-Deoxy-Nonulonic acid	Lipids	Free fatty acids	1.156200868	0.064579462	0.23973383	0.462476955	-1.112546617	1
Root vs Stem	Cyanidin-3-O-rutinoside (Keracyanin)	Flavonoids	Anthocyanidins	1.326589805	0.271409034	0.508025746	0.02218352	-5.494367884	3
Root vs Stem	L-Glucose*	Others	Saccharides	1.126781121	0.014687323	0.118339637	0.242664919	-2.042962534	1
Root vs Stem	Neoandrographolide	Terpenoids	Ditepenoids	1.469857403	0.032052191	0.16415812	0.137492147	-2.862578877	2
Root vs Stem	Divanillyltetrahy	Lignans and	Lignans	1.358132362	0.006365757	0.08060689	0.056971328	-4.133620158	3

Comparison	Compound	Class I	Class II	VIP	P-value	FDR	Fold Change	Log_2FC	MSI Level
	drofuran*	Coumarins							
Root vs Stem	Nagilactone C glucoside	Terpenoids	Ditepenoids	1.25332121	0.129208469	0.34544855	2.14349405	1.099964412	2
Root vs Stem	D-Fructose*	Others	Saccharides	1.136003051	0.02233005	0.14035985	0.176355699	-2.503439893	1
Root vs Stem	D-Glucose*	Others	Saccharides	1.165177531	0.034342636	0.170802894	0.171591431	-2.542950584	1
Root vs Stem	Dihydrodehydro diconiferyl alcohol-4-O-gluco side*	Lignans and Coumarins	Lignans	1.100973483	0.023131631	0.142440405	0.355954888	-1.490233681	2
Root vs Stem	Isoascorbic acid 2-O-glucoside	Others	Vitamin	1.469805335	0.000180765	0.016991546	0.131455005	-2.927359021	1
Root vs Stem	2-β-D-Glucopyra	Phenolic	Phenolic acids	1.421526169	0.012019078	0.107179135	0.097737844	-3.35493891	1

Comparison	Compound	Class I	Class II	VIP	P-value	FDR	Fold Change	Log_2FC	MSI Level
	nosyloxy-5-hydroxy-phenylacetic acid	acids							
Root vs Stem	4-Hydroxybenzoic acid	Phenolic acids	Phenolic acids	1.441972229	0.000225199	0.017551761	0.106870905	-3.226058955	1
Root vs Stem	8-Epideoxyloganic acid	Terpenoids	Monoterpenoids	1.305351452	0.101446018	0.302452427	0.131761644	-2.923997636	2
Root vs Stem	10-Formyltetrahydrofolic Acid	Alkaloids	Alkaloids	1.249415986	0.035034947	0.172612005	0.352163021	-1.505684667	2
Root vs Stem	Shikimic acid	Organic acids	Organic acids	1.023958738	0.092655573	0.288883839	0.344768619	-1.536299629	1
Root vs Stem	Caffeoyl(p-Hydroxybenzoyl)tartari	Phenolic acids	Phenolic acids	1.278765695	0.06480964	0.239797661	0.101876587	-3.295105567	2

Comparison	Compound	Class I	Class II	VIP	P-value	FDR	Fold Change	Log_2FC	MSI Level
	c acid								
Root vs Stem	Hydroxyaloe-emodin-8-O-sulfate	Quinones	Anthraquinone	1.189909165	0.007272026	0.084768723	0.083445576	-3.583020622	2
Root vs Stem	2-(3,4-Dihydroxybenzoyloxy)-4,6-Dihydroxyphenylacetic Acid	Phenolic acids	Phenolic acids	1.420515357	0.128808032	0.34527244	0.130457144	-2.938352141	1
Root vs Stem	Quercetin-3-O-(6''-O-p-Coumaroyl)galactoside*	Flavonoids	Flavonols	1.447867632	0.001540851	0.041846281	0.008097125	-6.948374557	3
Root vs Stem	6-Hydroxy-4-(4-hydroxy-3-methoxyphenyl)-7-metho	Lignans and Coumarins	Lignans	1.194159507	0.064380966	0.239427592	0.330876756	-1.595634147	3

Comparison	Compound	Class I	Class II	VIP	P-value	FDR	Fold Change	Log_2FC	MSI Level
	xy-naphtho[2,3-c] furan-1,3-dione-1								
Root vs Stem	Quercetin-3-O-(2''-O-p-Coumaroyl) galactoside	Flavonoids	Flavonols	1.491339657	0.001604178	0.041911677	0.095490427	-3.388500077	3
Root vs Stem	Gingerglycolipid B	Lipids	Glycerol ester	1.057268713	0.253943515	0.494938069	0.210446327	-2.248475763	2
Root vs Stem	γ-Aminobutyric acid	Organic acids	Organic acids	1.199423052	0.367527086	0.583066805	24.63409886	4.622584792	2
Root vs Stem	Nicotinamide	Others	Vitamin	1.219795399	0.266601193	0.505698111	7.454989141	2.898206251	3
Root vs Stem	(7'R,8'R)-7'8'-dihydro-7'-(5'-hydroxy-3'-methoxphenyl	Lignans and Coumarins	Lignans	1.461739105	0.00716155	0.084465363	0.003954735	-7.982203303	3

Comparison	Compound	Class I	Class II	VIP	P-value	FDR	Fold Change	Log_2FC	MSI Level
	)-3-methoxy-8'-methyl-1-(E)-propenylbenzof-uran								
Root vs Stem	Quercetin-4'-O-glucuronide*	Flavonoids	Flavonols	1.465267055	0.002107979	0.049441691	0.04234977	-4.561502039	1
Root vs Stem	Itoside L	Others	Others	1.47534044	0.037251287	0.179136667	0.07282909	-3.779341367	3
Root vs Stem	Prodelphinidin B*	Flavonoids	Other Flavonoids	1.45256307	2.10211E-05	0.00848072	0.010673403	-6.549836007	3
Root vs Stem	Cis-Coutaric acid	Phenolic acids	Phenolic acids	1.269480797	0.026517844	0.15097024	0.06425519	-3.960043212	1
Root vs Stem	Grayanotoxin XVIII	Terpenoids	Ditepenoids	1.476941417	0.026462104	0.15097024	0.162803476	-2.618796588	2

Comparison	Compound	Class I	Class II	VIP	P-value	FDR	Fold Change	Log_2FC	MSI Level
Root vs Stem	1-O-Salicyloyl-β-D-glucose*	Phenolic acids	Phenolic acids	1.450906186	0.028584732	0.154844052	0.099881475	-3.323639069	1
Root vs Stem	Norartocarpetin	Flavonoids	Flavones	1.456173504	0.036330475	0.176854008	0.006940726	-7.170697701	1
Root vs Stem	Kaempferol-3-O-galactoside (Trifolin)*	Flavonoids	Flavonols	1.392498033	0.006745738	0.083794613	0.02647345	-5.239309986	1
Root vs Stem	Tricetin 7-O-glucuronide*	Flavonoids	Flavones	1.473929572	0.000135436	0.016272452	0.033601542	-4.895328734	1
Root vs Stem	Procyanidin C1	Tannins	Proanthocyanidins	1.082464272	0.00071276	0.031300782	0.113704159	-3.136643075	2
Root vs Stem	3-Hydroxytaraxastan-28,20-olide	Terpenoids	Triterpene	1.245622984	0.089253332	0.284288391	0.167660216	-2.576387705	1

Comparison	Compound	Class I	Class II	VIP	P-value	FDR	Fold Change	Log_2FC	MSI Level
Root vs Stem	Azetidine-2-carboxylic acid*	Alkaloids	Alkaloids	1.163112218	0.289015158	0.522353141	5.306549172	2.407773987	3
Root vs Stem	Quercetin-3-O-(6''-O-p-Coumaroyl) glucoside	Flavonoids	Flavonols	1.488732547	0.00894111	0.091358669	0.09525406	-3.392075605	3
Root vs Stem	Mearnsetin-3-O-glucoside*	Flavonoids	Flavones	1.468929585	0.003944329	0.066187756	0.014491633	-6.108635991	2
Root vs Stem	Calyxanthone	Flavonoids	Other Flavonoids	1.456418117	0.036649956	0.17715576	0.007116892	-7.134536979	2
Root vs Stem	Diosmetin-7-O-galactoside*	Flavonoids	Flavones	1.075408697	0.076086633	0.255354165	0.486513623	-1.039447893	2
Root vs Stem	Quercetin-3,7-Di-O-rhamnoside	Flavonoids	Flavonols	1.451670081	0.073582767	0.252807391	0.148983792	-2.746772704	2

Comparison	Compound	Class I	Class II	VIP	P-value	FDR	Fold Change	Log_2FC	MSI Level
Root vs Stem	3-Hydroxylup-12(13),20(29)-diene-28-oic acid	Terpenoids	Triterpene	1.453358478	0.083059048	0.271535315	0.009230498	-6.759375768	1
Root vs Stem	Dihydrovomifoliol-O-β-D-glucoside*	Terpenoids	Sesquiterpenoids	1.478366894	0.017068977	0.12322237	0.03002591	-5.057648205	1
Root vs Stem	Methyl 3-O-Beta-Glucopyranosyl-Gallate	Phenolic acids	Phenolic acids	1.470666543	3.6129E-05	0.00848072	0.147756381	-2.758707659	2
Root vs Stem	Hydroxy isoliquiritigenin glucoside*	Flavonoids	Chalcones	1.244189493	0.035058801	0.172612005	0.063015838	-3.988141729	2
Root vs Stem	7,2',4'-Trihydroxy	Flavonoids	Other	1.463312417	0.048721262	0.20480791	0.001199138	-9.703786012	2

Comparison	Compound	Class I	Class II	VIP	P-value	FDR	Fold Change	Log_2FC	MSI Level
	-8,3'-dimethoxyis oflavanglucoside		Flavonoids						
Root vs Stem	Lupane-20(29)-en -3-on-28-oic acid	Terpenoids	Triterpene	1.356287432	0.082476625	0.27063872	0.107078919	-3.223253618	1
Root vs Stem	Naringenin-7-O-g lucoside (Prunin)*	Flavonoids	Flavanones	1.235344852	0.000229602	0.017551761	0.102750939	-3.282776512	2
Root vs Stem	Okanin-4'-O-gluc oside(Marein)*	Flavonoids	Chalcones	1.452682791	0.020371757	0.133966689	0.04545736	-4.459342277	3
Root vs Stem	Tricetin 3'-glucuronide*	Flavonoids	Flavones	1.463580351	0.001123835	0.039993013	0.036575453	-4.772980449	1
Root vs Stem	1-O-rhamnose-3-O-Caffeoyl Quinic Acid	Phenolic acids	Phenolic acids	1.398230453	0.009879734	0.097124949	0.074238085	-3.75169669	2

Comparison	Compound	Class I	Class II	VIP	P-value	FDR	Fold Change	Log_2FC	MSI Level
Root vs Stem	Mudanpinoic acid A	Terpenoids	Triterpene	1.108416209	0.105946997	0.308426799	0.372443677	-1.424905824	1
Root vs Stem	Carthamone*	Flavonoids	Chalcones	1.451026937	0.014409921	0.117955669	0.008368232	-6.90086142	1
Root vs Stem	Mucic acid Dimethyl Ester	Organic acids	Organic acids	1.257052122	0.066658267	0.2417973	0.498565507	-1.004145019	1
Root vs Stem	4-O-Glucosyl-4-hydroxybenzoic acid	Phenolic acids	Phenolic acids	1.419794446	0.017070868	0.12322237	0.074658951	-3.743540958	1
Root vs Stem	Homoarginine	Amino acids and derivatives	Amino acids and derivatives	1.127602412	0.384169245	0.583066805	47.93058725	5.582874711	3
Root vs Stem	3-hydroxybenzoyl-β-D-glucose*	Phenolic acids	Phenolic acids	1.46561357	0.014296314	0.117955669	0.091810449	-3.445197841	1

Comparison	Compound	Class I	Class II	VIP	P-value	FDR	Fold Change	Log_2FC	MSI Level
Root vs Stem	DGMG 18:3	Lipids	Glycerol ester	1.065883136	0.272874646	0.508025746	0.142402215	-2.811956511	1
Root vs Stem	6'-O-Feruloyl-D-sucrose	Phenolic acids	Phenolic acids	1.448330057	0.004965884	0.072692091	0.073212755	-3.771761179	2
Root vs Stem	1,3,6,8-tetrahydroxy-2,5-dimethoxyxanthen-9-one	Flavonoids	Other Flavonoids	1.260468868	0.072144995	0.251108381	0.306492918	-1.706074355	2
Root vs Stem	L-Azetidine-2-carboxylic acid*	Alkaloids	Alkaloids	1.109254006	0.308027664	0.541502716	4.849183223	2.277741766	3
Root vs Stem	DGMG 18:3(2n isomer)	Lipids	Glycerol ester	1.077622433	0.280818087	0.516775123	0.132785117	-2.912834644	1
Root vs Stem	5-O-Galloyl-D-hamamelose	Phenolic acids	Phenolic acids	1.416113947	0.004162226	0.06928093	0.073628156	-3.763598618	1

Comparison	Compound	Class I	Class II	VIP	P-value	FDR	Fold Change	Log_2FC	MSI Level
Root vs Stem	Neoisoliquiritin	Flavonoids	Chalcones	1.480284666	0.016649211	0.122828256	0.163473396	-2.612872228	2
Root vs Stem	Sweroside	Terpenoids	Monoterpenoids	1.4640621	0.024817698	0.144292194	0.10122058	-3.304425448	2
Root vs Stem	Neosakuranin	Flavonoids	Chalcones	1.469046085	0.007951742	0.088057897	0.023369979	-5.419199748	3
Root vs Stem	D-Ornithine	Amino acids and derivatives	Amino acids and derivatives	1.463766413	0.183593914	0.42394095	517.7482432	9.016106943	2
Root vs Stem	Frambinone	Others	Ketone compounds	1.311470825	0.027816475	0.153984366	0.121309573	-3.043234687	3
Root vs Stem	Isocoreopsin*	Flavonoids	Flavanones	1.387767633	0.002101836	0.049441691	0.061640776	-4.019971174	2
Root vs Stem	3-[4-(β-D-glucopyranoside)-phenyl	Phenolic acids	Phenolic acids	1.306129172	0.036571522	0.17715576	0.092780139	-3.430040189	1

Comparison	Compound	Class I	Class II	VIP	P-value	FDR	Fold Change	Log_2FC	MSI Level
	acrylic]-acid								
Root vs Stem	Machilin H	Lignans and Coumarins	Lignans	1.157113163	0.01812534	0.128118842	0.131289187	-2.929179994	3
Root vs Stem	Sibiricose A6	Phenolic acids	Phenolic acids	1.330907725	0.120948934	0.33337783	0.137539612	-2.862080917	3
Root vs Stem	Rhein-8-O-glucoside	Quinones	Anthraquinone	1.10001705	0.174927719	0.411569763	4.709312786	2.235516547	3
Root vs Stem	4'-Hydroxy-3,5-dimethoxydihydrochalcone	Flavonoids	Chalcones	1.447076596	0.07401308	0.2528548	0.142819525	-2.807734872	3
Root vs Stem	Kaempferol-3-O-(6''-O-acetyl)glucoside	Flavonoids	Flavonols	1.474573224	0.034818392	0.172338517	0.009025911	-6.791711711	1

Comparison	Compound	Class I	Class II	VIP	P-value	FDR	Fold Change	Log_2FC	MSI Level
Root vs Stem	LysoPE 18:1*	Lipids	LPE	1.304572409	0.039361626	0.182370924	2.36855811	1.244009067	3
Root vs Stem	Kaempferol-7-O-glucoside*	Flavonoids	Flavonols	1.447163799	0.015401493	0.120411672	0.018056314	-5.791352745	1
Root vs Stem	Gossypin	Flavonoids	Flavonols	1.349090069	0.002768908	0.056750731	0.027995825	-5.158644503	3
Root vs Stem	Tricin-5-O-Glucoside	Flavonoids	Flavones	1.034075278	0.291575507	0.525457967	0.049337603	-4.341168568	2
Root vs Stem	3-O-(p-coumaroyl) 3-Hydroxy-3-methylglutaric acid	Phenolic acids	Phenolic acids	1.334661249	0.016225748	0.122828256	0.115172017	-3.118137867	3
Root vs Stem	O-Feruloyl 7-hydroxycoumarin*	Lignans and Coumarins	Coumarins	1.327240243	0.029056281	0.156177512	0.09417817	-3.408463506	2

Comparison	Compound	Class I	Class II	VIP	P-value	FDR	Fold Change	Log_2FC	MSI Level
Root vs Stem	cis-Zeatin-O-glucoside*	Alkaloids	Alkaloids	1.445633608	0.006948138	0.083865246	0.314833077	-1.667340973	1
Root vs Stem	Gingerglycolipid A	Lipids	Glycerol ester	1.088271854	0.287653394	0.521261287	0.126838119	-2.978939704	2
Root vs Stem	1-O-Galloyl-2-O-Cinnamoyl-β-D-glucose	Phenolic acids	Phenolic acids	1.422004136	0.031682085	0.162666227	0.208746874	-2.2601735	2
Root vs Stem	LysoPE 18:1(2n isomer)*	Lipids	LPE	1.150965529	0.124903669	0.33832175	2.194600468	1.133958318	3
Root vs Stem	4-Hydroxyphenyl 6-O-(2-Methyl-3-Hydroxypropionyl)-Beta-D-Galact	Others	Others	1.013976674	0.050964511	0.210803107	0.33280292	-1.587260003	2

Comparison	Compound	Class I	Class II	VIP	P-value	FDR	Fold Change	Log$_2$FC	MSI Level
	opyranoside								
Root vs Stem	2,5-Dihydroxybenzoic acid; Gentisic Acid*	Phenolic acids	Phenolic acids	1.265578678	0.001514743	0.041846281	0.190615535	-2.39126239	1
Root vs Stem	dihydrodehydrodiconiferyl alcohol-9-O-β-D-xylopyranoside	Lignans and Coumarins	Lignans	1.122450137	0.256233513	0.498459916	0.013230109	-6.240031213	3
Root vs Stem	Grevilloside G	Phenolic acids	Phenolic acids	1.231023193	0.086234852	0.278979209	0.141116993	-2.825036367	3
Root vs Stem	2α,3α-Dihydroxyolean-12-en-28-oic acid	Terpenoids	Triterpene	1.028413378	0.291751371	0.525457967	0.036867025	-4.761525188	2

Comparison	Compound	Class I	Class II	VIP	P-value	FDR	Fold Change	Log_2FC	MSI Level
Root vs Stem	Cyanidin-3-O-glucoside*	Flavonoids	Anthocyanidins	1.294836528	0.130029093	0.345818935	0.114101343	-3.131612327	3
Root vs Stem	Methyl cumalate*	Others	Lactones	1.198013353	0.00694461	0.083865246	0.194659784	-2.360973236	1
Root vs Stem	Kaempferol-3-O-(6''-malonyl)glucoside*	Flavonoids	Flavonols	1.465951482	0.051996695	0.21421393	0.008093648	-6.948994254	1
Root vs Stem	3-O-Feruloyl Aminogalactitol	Phenolic acids	Phenolic acids	1.471216311	0.002926375	0.056750731	0.045424677	-4.460379944	1
Root vs Stem	N-Caffeoylmethyl gluconamide*	Alkaloids	Phenolamine	1.415083428	0.099741268	0.300685165	0.079168193	-3.65893526	1
Root vs Stem	L-Ornithine	Amino acids and derivatives	Amino acids and derivatives	1.324525303	0.239356532	0.482902712	59.84297807	5.903110067	3

Comparison	Compound	Class I	Class II	VIP	P-value	FDR	Fold Change	Log_2FC	MSI Level
Root vs Stem	Bismurrangatin	Lignans and Coumarins	Coumarins	1.454684718	0.044613471	0.195335116	0.011393518	-6.455642854	3
Root vs Stem	5-O-p-Coumaroyl galactaric acid	Phenolic acids	Phenolic acids	1.415399058	0.020752794	0.134697381	0.130415829	-2.938809107	2
Root vs Stem	Kaempferol-3-O-(6''-malonyl)galactoside*	Flavonoids	Flavonols	1.475745673	0.045702518	0.196931102	0.00847547	-6.882490841	1
Root vs Stem	Cyanidin-3-O-galactoside*	Flavonoids	Anthocyanidins	1.466462353	0.038601381	0.180664966	0.15111878	-2.726245138	3
Root vs Stem	D-Pantothenic Acid 4'-O-Beta-Glucoside	Others	Vitamin	1.448466132	0.00111268	0.039993013	0.366622584	-1.447632437	1

Comparison	Compound	Class I	Class II	VIP	P-value	FDR	Fold Change	Log_2FC	MSI Level
Root vs Stem	Catechin-(4alpha->8)-catechin-(4alpha->6)-catechin	Flavonoids	Flavanols	1.076762643	0.012727591	0.109846592	0.159350658	-2.64972312	1
Root vs Stem	6-O-Vanilloylajugol	Terpenoids	Monoterpenoids	1.271806702	0.030431635	0.160641676	0.38630187	-1.372199435	3
Root vs Stem	β-Hydroxy-(3,4-dihydroxyphenylethanolyl)-glucoside	Others	Alcohol compounds	1.356491471	0.006632505	0.082966614	0.067154862	-3.896364335	1
Root vs Stem	5-O-Malonylisosakuranin	Flavonoids	Flavanones	1.445765454	0.052833238	0.216099896	0.014517632	-6.106050055	2
Root vs Stem	3,4'-Dihydroxy-3',5'-dimethoxyprop	Others	Ketone compounds	1.31234967	0.009587423	0.096528983	0.375524721	-1.413020212	1

Comparison	Compound	Class I	Class II	VIP	P-value	FDR	Fold Change	Log_2FC	MSI Level
	iophenone								
Root vs Stem	O-Acetyl-L-carnitine	Alkaloids	Alkaloids	1.000567874	0.090242757	0.285419243	0.359814788	-1.474673615	1
Root vs Stem	LysoPE 16:0(2n isomer)	Lipids	LPE	1.038067588	0.328465562	0.559873811	5.536726627	2.469033291	3
Root vs Stem	3,9-dihydroeucomnalin glucoside*	Flavonoids	Other Flavonoids	1.477336823	1.73449E-06	0.001549614	0.073796544	-3.76030293	2
Root vs Stem	LysoPE 16:0	Lipids	LPE	1.061016724	0.321504999	0.553449806	5.45295773	2.447038972	2
Root vs Stem	Piperidine	Alkaloids	Piperidine alkaloids	1.032623363	0.399116863	0.583066805	32.65646025	5.029296515	3
Root vs Stem	6-[(7-hydroxy-2-methyl-6-oxo-3,4-	Alkaloids	Isoquinoline alkaloids	1.117469232	0.138276309	0.357199375	0.165878527	-2.591800951	1

Comparison	Compound	Class I	Class II	VIP	P-value	FDR	Fold Change	Log_2FC	MSI Level
	dihydroisoquinolin-1-yl)methyl]-2,3-dimethoxybenzoic acid								
Root vs Stem	Quercetin-3-O-(6''-O-malonyl)glucoside	Flavonoids	Flavonols	1.478336776	0.032438069	0.164906836	0.014837672	-6.07459139	1
Root vs Stem	Phenylpropionic acid-O-β-D-glucopyranoside*	Phenolic acids	Phenolic acids	1.368527564	0.043722743	0.193241416	0.060851014	-4.03857488	1
Root vs Stem	D-Galactose*	Others	Saccharides	1.196420203	0.074426427	0.2528548	0.216174571	-2.209731266	1
Root vs Stem	2-O-Galloyl-D-glucose	Phenolic acids	Phenolic acids	1.429736061	0.007991923	0.088057897	0.146319367	-2.772807355	2

Comparison	Compound	Class I	Class II	VIP	P-value	FDR	Fold Change	Log_2FC	MSI Level
Root vs Stem	8-Methoxykaempferol-7-O-rhamnoside	Flavonoids	Flavonols	1.384451327	0.089680388	0.284412321	0.264049785	-1.921118127	1
Root vs Stem	p-Coumaric acid-4-O-glucoside	Phenolic acids	Phenolic acids	1.472785389	0.026551452	0.15097024	0.058615515	-4.092573616	1
Root vs Stem	2,3-Dihydroxyurs-12-en-28-oic acid methyl ester (Corosolic acid methyl ester)	Terpenoids	Triterpene	1.395232858	0.264115424	0.504567144	0.010524449	-6.570111444	3
Root vs Stem	Geniposide	Terpenoids	Monoterpenoids	1.464951909	2.25235E-06	0.001549614	0.043746818	-4.514678106	3

Comparison	Compound	Class I	Class II	VIP	P-value	FDR	Fold Change	Log_2FC	MSI Level
Root vs Stem	Isoscutellarein	Flavonoids	Flavones	1.059008623	0.189029949	0.42827422	0.24876763	-2.007129324	3
Root vs Stem	O-p-Coumaroylgalactaric acid	Phenolic acids	Phenolic acids	1.430769566	0.056205813	0.218884523	0.088773979	-3.493719331	1
Root vs Stem	Luteolin-7-O-(6''-malonyl)glucoside*	Flavonoids	Flavones	1.463137079	0.035744171	0.17482457	0.011177155	-6.48330321	1
Root vs Stem	N-[(R)-2-Hydroxy-3,3-dimethyl-4-(β-D-glucopyranosyloxy)butyryl]-β-alanine	Alkaloids	Alkaloids	1.166153555	0.099907791	0.300685165	0.486650323	-1.039042582	1
Root vs Stem	Oleracone B	Others	Others	1.035677564	0.210039456	0.452030684	0.375797033	-1.41197442	3
Root vs Stem	Melilotoside*	Phenolic	Phenolic acids	1.463965235	0.05607469	0.218884523	0.067403075	-3.89104177	1

Comparison	Compound	Class I	Class II	VIP	P-value	FDR	Fold Change	Log_2FC	MSI Level
		acids							
Root vs Stem	Quercetin-3-O-(2''-O-acetyl)glucuronide	Flavonoids	Flavonols	1.322957393	0.043626509	0.193241416	0.026858498	-5.218477585	1
Root vs Stem	alanine betaine	Alkaloids	Alkaloids	1.141762611	0.35262155	0.583066805	14.51290731	3.859264652	1
Root vs Stem	Azaleatin (5-O-Methylquercetin)	Flavonoids	Flavonols	1.420994966	0.014412296	0.117955669	0.023619301	-5.403889907	1
Root vs Stem	1-O-Vanilloyl-D-Glucose	Phenolic acids	Phenolic acids	1.336129786	0.000896608	0.036286244	0.075691235	-3.723729949	1
Root vs Stem	Rubianol-f	Terpenoids	Triterpene	1.296635015	0.132037997	0.349392853	0.076597089	-3.706566626	3
Root vs Stem	Melibiose	Others	Saccharides	1.123524968	0.078247911	0.260489819	0.399654265	-1.323175611	1

Comparison	Compound	Class I	Class II	VIP	P-value	FDR	Fold Change	Log_2FC	MSI Level
Root vs Stem	3-Hydroxyolean-12-en-28-oic acid (Oleanolic acid)*	Terpenoids	Triterpene	1.086613154	0.36775353	0.583066805	0.064279839	-3.959489873	1
Root vs Stem	3,19,23-Trihydroxyurs-12-en-28-oic acid (Rutundic acid)	Terpenoids	Triterpene	1.348516134	0.083886404	0.273093907	0.029915672	-5.06295473	2
Root vs Stem	Terreic acid*	Others	Ketone compounds	1.069344426	0.006246687	0.080081749	0.183031444	-2.449836573	1
Root vs Stem	24,30-Dihydroxy-12(13)-ene-lupeol *	Terpenoids	Triterpene	1.034002191	0.378457222	0.583066805	0.076659566	-3.705390353	1
Root vs Stem	2,3,4-trihydroxy-5	Phenolic	Phenolic acids	1.478250146	0.000736633	0.031675211	0.023143856	-5.433226927	1

Comparison	Compound	Class I	Class II	VIP	P-value	FDR	Fold Change	Log_2FC	MSI Level
	-{[3-(4-hydroxyphenyl)prop-2-enoyl]oxy}hexanedioic acid	acids							
Root vs Stem	Methyl 3-oxo-2-phenylpropanoate	Phenolic acids	Phenolic acids	1.23634419	0.000390131	0.0223675	0.109127338	-3.195915525	1
Root vs Stem	Laricitrin-3-O-glucoside*	Flavonoids	Flavonols	1.462144834	0.005286628	0.075448846	0.020835953	-5.584781091	1
Root vs Stem	Quercetin-3-O-(6''-O-acetyl)galactoside	Flavonoids	Flavonols	1.464477538	0.070673109	0.247257907	0.015210523	-6.038786394	1
Root vs Stem	4-O-(4'-O-alpha-D	Phenolic	Phenolic acids	1.250923067	0.051506831	0.212620198	0.220983504	-2.177989416	1

Comparison	Compound	Class I	Class II	VIP	P-value	FDR	Fold Change	Log_2FC	MSI Level
	-Glucopyranosyl) caffeoylquinic acid	acids							
Root vs Stem	2,3,19-Trihydroxy urs-12-en-28-oic acid (Tormentic acid)*	Terpenoids	Triterpene	1.449613999	0.098710863	0.300500326	0.006657579	-7.230786729	2
Root vs Stem	3-O-Feruloyl Octose*	Phenolic acids	Phenolic acids	1.388785455	0.00568434	0.077187348	0.053836713	-4.215265858	1
Root vs Stem	2,3,19-trihydroxy urs-12-en-28-oic acid (Euscaphic acid)*	Terpenoids	Triterpene	1.444101792	0.102908387	0.30478678	0.007241776	-7.10944071	2

Comparison	Compound	Class I	Class II	VIP	P-value	FDR	Fold Change	Log_2FC	MSI Level
Root vs Stem	Matairesinol-4'-O-glucoside (Matairesinoside)	Lignans and Coumarins	Lignans	1.403834824	0.024766316	0.144292194	0.023628004	-5.403358446	1
Root vs Stem	Secoisolariciresinol-9'-O-xyloside	Lignans and Coumarins	Lignans	1.491058989	0.02480569	0.144292194	0.001385287	-9.495599582	2
Root vs Stem	Selagin	Flavonoids	Flavones	1.363788018	0.218483914	0.4592167	0.000545611	-10.83983901	1
Root vs Stem	Cannabifolin F	Terpenoids	Triterpene	1.447528164	0.101696698	0.302452427	0.006675941	-7.226813087	3
Root vs Stem	4-(2,6-Dihydroxybenzoyl)-3-Formyl-5-Hydroxybenzoic acid	Phenolic acids	Phenolic acids	1.424977618	0.12069885	0.33337783	0.012867274	-6.280149724	1
Root vs Stem	1-O-p-Coumaroyl Octose	Phenolic acids	Phenolic acids	1.414409653	0.044708388	0.195335116	0.069469185	-3.847483019	1

Comparison	Compound	Class I	Class II	VIP	P-value	FDR	Fold Change	Log_2FC	MSI Level
Root vs Stem	Methyl 3-O-glucosyl-4-hydroxybenzoate	Phenolic acids	Phenolic acids	1.405352639	0.008604202	0.08880256	0.071993592	-3.795987682	1
Root vs Stem	13-Hydroxy oleanolic acid-3-oxo-28-acid	Terpenoids	Triterpene	1.45879214	0.110910742	0.317581007	0.007423946	-7.073598138	1
Root vs Stem	5'-Deoxy-5'-(methylthio)adenosine	Nucleotides and derivatives	Nucleotides and derivatives	1.225391318	0.190646884	0.431464001	3.66879105	1.875304741	1
Root vs Stem	Quercetin-3-O-glucose-6''-acetate	Flavonoids	Flavonols	1.468351775	0.023443779	0.142440405	0.013787199	-6.180526787	1
Root vs Stem	13,28-Epoxyolean	Terpenoids	Triterpene	1.220504535	0.08970563	0.284412321	0.39646248	-1.334743755	1

**Comparison**	**Compound**	**Class I**	**Class II**	**VIP**	**P-value**	**FDR**	**Fold Change**	**Log_2FC**	**MSI Level**
	-11-en-3-one								
Root vs Stem	2-O-α-D-Glucopyranosyl-L-ascorbic acid	Others	Vitamin	1.354006009	0.001695688	0.042917142	0.191561991	-2.38411676	1
Root vs Stem	Quercetin-3-O-neohesperidoside*	Flavonoids	Flavonols	1.223146813	0.012291443	0.107928159	3.031652498	1.600104395	1
Root vs Stem	Quercetin-3-O-robinobioside	Flavonoids	Flavonols	1.146692756	0.177033176	0.414034699	4.564166039	2.190351276	2
Root vs Stem	Quercetin-7-O-(6''-malonyl)glucoside	Flavonoids	Flavonols	1.425181788	0.015509493	0.120798467	0.031951359	-4.967978898	2
Root vs Stem	Epigallocatechin-(4beta->8)-catechin	Tannins	Proanthocyanidins	1.367282439	0.26040184	0.501370707	0.019806405	-5.657889128	1

Comparison	Compound	Class I	Class II	VIP	P-value	FDR	Fold Change	Log_2FC	MSI Level
Root vs Stem	Delphinidin-3-O-(6''-O-p-coumaroyl)glucoside	Flavonoids	Anthocyanidins	1.25440511	0.024541174	0.144292194	3.451738314	1.787323094	3
Root vs Stem	N-Monomethyl-L-arginine*	Amino acids and derivatives	Amino acids and derivatives	1.131170771	0.374019012	0.583066805	33.57214973	5.069193015	3
Root vs Stem	Homovanillic acid sulfate*	Phenolic acids	Phenolic acids	1.311995295	0.025207672	0.146147853	0.335139758	-1.577165248	1
Root vs Stem	L-Homomethionine	Amino acids and derivatives	Amino acids and derivatives	1.069462344	0.412389151	0.583066805	149.9312421	7.228157228	3
Root vs Stem	Taxifolin-7-O-rhamnoside	Flavonoids	Flavanonols	1.340715615	0.174615419	0.411569763	0.08734764	-3.517087462	3

Comparison	Compound	Class I	Class II	VIP	P-value	FDR	Fold Change	Log_2FC	MSI Level
Root vs Stem	p-Coumaroyl Aminogalactitol	Phenolic acids	Phenolic acids	1.452846417	0.049485475	0.205923427	0.038315957	-4.705910845	1
Root vs Stem	Feruloyloctose*	Phenolic acids	Phenolic acids	1.412652359	0.004413877	0.069917266	0.043526099	-4.521975464	1
Root vs Stem	Arginine methyl ester*	Amino acids and derivatives	Amino acids and derivatives	1.115101608	0.378447921	0.583066805	30.20892356	4.916902872	3
Root vs Stem	9-O-Methylvibsanol	Lignans and Coumarins	Lignans	1.259134373	0.072998483	0.252800116	0.198413712	-2.333416365	3
Root vs Stem	Quercetin-3-O-(4''-O-glucosyl)rhamnoside*	Flavonoids	Flavonols	1.216054521	0.08386248	0.273093907	2.925567496	1.548716503	1
Root vs Stem	Protocatechuic	Phenolic	Phenolic acids	1.477166725	0.000883291	0.036286244	0.026498192	-5.237962254	1

Comparison	Compound	Class I	Class II	VIP	P-value	FDR	Fold Change	Log_2FC	MSI Level
	acid 1-O-(Glucosylvanilloyl)	acids							
Root vs Stem	Quercetin-7-O-rutinoside*	Flavonoids	Flavonols	1.133525604	0.145428347	0.36605379	3.138238691	1.649955086	1
Root vs Stem	Citric Acid diglucoside	Organic acids	Organic acids	1.090785921	0.055818422	0.218884523	0.474003232	-1.077031197	1
Root vs Stem	3-O-Methylellagic acid 4'-rhamnoside	Tannins	Tannin	1.274594821	0.231419334	0.473540328	0.001951927	-9.000885362	1
Root vs Stem	Quercetin-3-O-rutinoside (Rutin)*	Flavonoids	Flavonols	1.101598478	0.11875162	0.330327956	3.015000475	1.592158229	1
Root vs Stem	Caffeoylmethylgl	Alkaloids	Alkaloids	1.343704266	0.003902165	0.06601695	0.176565013	-2.501728597	1

Comparison	Compound	Class I	Class II	VIP	P-value	FDR	Fold Change	Log_2FC	MSI Level
	uconamide*								
Root vs Stem	5-Glucosyloxy-2-Hydroxybenzoic acid methyl ester	Phenolic acids	Phenolic acids	1.423702378	0.008115734	0.088057897	0.059603437	-4.068460659	1
Root vs Stem	1,8-dihydroxy-4,5-dimethoxy-3-{[(2s,3r,4s,5s,6r)-3,4,5-trihydroxy-6-(hydroxymethyl)oxan-2-yl]oxy}xanthen-9-one	Flavonoids	Other Flavonoids	1.440081113	7.81292E-05	0.01075058	0.046682305	-4.420980388	2
Root vs Stem	O-Phosphocholine	Alkaloids	Alkaloids	1.058565708	0.17981878	0.418900634	0.446232937	-1.164131091	3

Comparison	Compound	Class I	Class II	VIP	P-value	FDR	Fold Change	Log_2FC	MSI Level
Root vs Stem	-Pierisformoside H	Terpenoids	Ditepenoids	1.383486202	7.5006E-05	0.01075058	0.068436063	-3.869099427	2
Root vs Stem	Hesperetin-7-O-glucoside*	Flavonoids	Flavanones	1.472274469	0.004299539	0.069663321	0.080986246	-3.626179272	3
Root vs Stem	1,2,5,7,8-pentahydroxy-3-methylanthracene-9,10-dione	Quinones	Quinones	1.463608173	0.027875775	0.153984366	0.0082933	-6.913837927	1
Root vs Stem	Pierisfomoside C	Others	Others	1.269167026	0.309765586	0.542286828	0.00268435	-8.541211518	1
Root vs Stem	4-O-(3'-O-alpha-D-Glucopyranosyl)caffeoylquinic acid	Phenolic acids	Phenolic acids	1.358145858	0.010082416	0.098626098	0.24768884	-2.013399227	1

Comparison	Compound	Class I	Class II	VIP	P-value	FDR	Fold Change	Log_2FC	MSI Level
Root vs Stem	robustaquinone F	Flavonoids	Other Flavonoids	1.465110193	0.027597489	0.153534276	0.007754117	-7.010821738	3
Root vs Stem	Indoline	Alkaloids	Plumerane	1.08894238	0.399219195	0.583066805	51.11754882	5.675746753	2
Root vs Stem	Tamarixetin-3-O-glucoside (Tamarixin)	Flavonoids	Flavonols	1.30925041	0.286814199	0.520702333	0.00565274	-7.46683399	2
Root vs Stem	Betanin (Betanidin-5-O-glucoside)	Alkaloids	Plumerane	1.444270928	0.016801981	0.122976201	0.029551336	-5.080632854	2
Root vs Stem	Betaine	Alkaloids	Alkaloids	1.334791322	0.000986571	0.038789527	0.185255722	-2.432409991	1
Root vs Stem	2-Amino-4-methylvaleric acid	Others	Others	1.025874891	0.404636395	0.583066805	44.51468224	5.476209352	1

Comparison	Compound	Class I	Class II	VIP	P-value	FDR	Fold Change	Log_2FC	MSI Level
Root vs Stem	2(4H)-benzofuranone	Others	Others	1.180627985	0.069357527	0.245600966	0.10661875	-3.229466924	3
Root vs Stem	Cis-Zeatin-7-N-glucoside*	Alkaloids	Alkaloids	1.482817339	0.008386247	0.088057897	0.307890536	-1.699510571	1
Root vs Stem	caesalpiniaphenol A	Flavonoids	Flavones	1.352383167	0.24619864	0.487204213	0.000561408	-10.79866195	3
Root vs Stem	Hydroxymenisdaurin D	Alkaloids	Alkaloids	1.221685919	0.027956792	0.153984366	0.449953478	-1.152152252	3
Root vs Stem	Cimidahurinine	Others	Alcohol compounds	1.131590215	0.122156607	0.33483564	0.21907307	-2.190515944	1
Root vs Stem	2',3',4',5,7-pentahydroxyflavone	Flavonoids	Flavones	1.473017301	0.031380445	0.161923098	0.006182265	-7.337648701	1

Comparison	Compound	Class I	Class II	VIP	P-value	FDR	Fold Change	Log_2FC	MSI Level
Root vs Stem	9-hydroxy-2-(3-hydroxy-4-methoxyphenyl)-3-(hydroxymethyl)-12-methoxy-2,3-dihydro-1,4,5-trioxatetraphen-10-one	Others	Ketone compounds	1.206907606	0.001067604	0.039993013	0.073753685	-3.761141065	1
Root vs Stem	4-O-p-Coumaroyl quinic acid	Phenolic acids	Phenolic acids	1.384535249	4.51976E-05	0.00848072	0.060453761	-4.048024091	1
Root vs Stem	8R-Dihydrodehydrodiconferyl alcohol 4-O-β-D-glucopyranoside	Lignans and Coumarins	Lignans	1.150797222	0.000202262	0.016991546	0.09392678	-3.412319639	3

Comparison	Compound	Class I	Class II	VIP	P-value	FDR	Fold Change	Log_2FC	MSI Level
Root vs Stem	Quercetin-3-O-glucoside-7-O-rhamnoside*	Flavonoids	Flavonols	1.141789148	0.052873279	0.216099896	2.675410199	1.419760105	1
Root vs Stem	Pedaliin*	Flavonoids	Flavones	1.382746382	0.271033434	0.508025746	0.004609852	-7.761063699	3
Root vs Stem	4-Nitrophenol	Others	Others	1.459928841	0.018055674	0.128064987	0.498637987	-1.003935301	3
Root vs Stem	Tamarixetin-3-O-(6''-malonyl)glucoside*	Flavonoids	Flavonols	1.191471963	0.001502758	0.041846281	0.076678242	-3.705038938	1
Root vs Stem	Rutin Trihydrate	Flavonoids	Flavonols	1.37787921	0.042556176	0.191363722	3.913460781	1.968444987	3
Root vs Stem	2,5-Dimethoxybenzoquinone*	Quinones	Quinones	1.379155374	0.004567315	0.071416195	0.205379046	-2.283639095	3
Root vs Stem	3'-O-Methyltriceti	Flavonoids	Flavones	1.233621053	0.310316709	0.542745606	0.001862817	-9.068298391	1

Comparison	Compound	Class I	Class II	VIP	P-value	FDR	Fold Change	Log_2FC	MSI Level
	n-7-O-glucoside*								
Root vs Stem	Patuletin-3-O-glucoside*	Flavonoids	Flavonols	1.475208458	0.017584356	0.125585157	0.00560192	-7.479862824	1
Root vs Stem	3-(Pyrazol-1-yl)-L-alanine	Amino acids and derivatives	Amino acids and derivatives	1.037380382	0.377195994	0.583066805	25.16691375	4.653456403	2
Root vs Stem	Isorhamnetin 3-(2''-acetylglucoside)	Flavonoids	Flavonols	1.280633774	0.006286581	0.080095694	0.059148448	-4.079515878	1
Root vs Stem	1-O-Gentisoyl-β-D-glucoside*	Phenolic acids	Phenolic acids	1.119592035	0.083144535	0.271535315	0.270065128	-1.888620731	1
Root vs Stem	Isorhamnetin-3-O-(6''-malonyl)gluc	Flavonoids	Flavonols	1.208270688	5.52814E-05	0.009508392	0.076440985	-3.709509827	1

Comparison	Compound	Class I	Class II	VIP	P-value	FDR	Fold Change	Log_2FC	MSI Level
	oside*								
Root vs Stem	3'-O-Methyltricetin-7-O-(6''-malonyl)glucoside*	Flavonoids	Flavones	1.28719443	0.00471243	0.072047814	0.04635375	-4.431170144	1
Root vs Stem	Salicylic acid-2-O-glucoside	Phenolic acids	Phenolic acids	1.199008608	0.049335452	0.205713883	0.339850631	-1.557027292	3
Root vs Stem	10-Hydroxymethyllycaconitine	Alkaloids	Alkaloids	1.00072979	0.244567997	0.486333033	0.211533646	-2.24104094	1
Root vs Stem	DL-Leucine*	Amino acids and derivatives	Amino acids and derivatives	1.023226163	0.401733743	0.583066805	36.15376041	5.176073807	1
Root vs Stem	2,6-Dimethoxy-1,	Quinones	Quinones	1.383805873	0.0015988	0.041911677	0.192964769	-2.373590624	3

Comparison	Compound	Class I	Class II	VIP	P-value	FDR	Fold Change	Log_2FC	MSI Level
	4-benzoquinone*								
Root vs Stem	Retronecine	Alkaloids	Pyrrole alkaloids	1.057703296	0.377411826	0.583066805	25.08984554	4.649031684	3
Root vs Stem	Glucosyringic acid	Phenolic acids	Phenolic acids	1.32749489	0.014469943	0.117955669	0.349322136	-1.517370027	1
Root vs Stem	LysoPE 18:3(2n isomer)	Lipids	LPE	1.324796355	0.009110032	0.092172087	2.259850603	1.1762274	2
Root vs Stem	Glochiflavanoside A	Flavonoids	Flavanols	1.260360497	0.038556293	0.180664966	0.174789671	-2.516308161	3
Root vs Stem	LysoPE 18:3	Lipids	LPE	1.326523338	0.007079597	0.084369056	2.234369859	1.159868017	2
Root vs Stem	3'-O-Methyltricetin-5-O-glucoside*	Flavonoids	Flavones	1.40774122	0.232402459	0.474882436	0.006740896	-7.212843907	3

Comparison	Compound	Class I	Class II	VIP	P-value	FDR	Fold Change	Log_2FC	MSI Level
Root vs Stem	Dihydrokaempferide	Flavonoids	Flavanonols	1.3449115	0.030693752	0.161015038	0.03363778	-4.893773715	3
Root vs Stem	N-Hydroxynonanoyl nornicotine glucoside	Alkaloids	Pyridine alkaloids	1.48031063	0.007112508	0.084369056	0.013550893	-6.205468242	3
Root vs Stem	1,2,4,5,8-pentahydroxy-6-methylanthracene-9,10-dione	Flavonoids	Other Flavonoids	1.482933723	0.024530199	0.144292194	0.007408118	-7.076677202	2
Root vs Stem	Homoeriodictyol 7-O-glucoside*	Flavonoids	Flavanones	1.453384666	0.095066656	0.29402987	0.017726844	-5.817920485	3
Root vs Stem	Artocarpanone	Flavonoids	Flavanones	1.477617459	0.022687133	0.141827246	0.007711059	-7.018855325	1
Root vs Stem	6-C-Methylquerce	Flavonoids	Flavonols	1.427957436	0.134837135	0.35228335	0.057036471	-4.131971471	2

Comparison	Compound	Class I	Class II	VIP	P-value	FDR	Fold Change	Log_2FC	MSI Level
	tin-3-O-rhamnoside								
Root vs Stem	6-Aminocaproic acid	Organic acids	Organic acids	1.019657326	0.398273796	0.583066805	27.97338964	4.805983176	3
Root vs Stem	Carbendazim	Alkaloids	Alkaloids	1.112753396	0.212654879	0.455783666	11.10086629	3.472600361	1
Root vs Stem	Isorhamnetin-3-O-arabinoside	Flavonoids	Flavonols	1.410054006	0.000656818	0.029471125	0.018293906	-5.772493059	3
Root vs Stem	1,4,8-Trihydroxynaphthalene-1-O-glucoside	Quinones	Quinones	1.425567449	0.090438074	0.285419243	0.094841335	-3.398340213	3
Root vs Stem	1,2,4,5-tetrahydroxy-7-(hydroxymethyl)anthracene-9,	Flavonoids	Other Flavonoids	1.357006967	0.01254851	0.108824056	0.037714328	-4.728743473	2

Comparison	Compound	Class I	Class II	VIP	P-value	FDR	Fold Change	Log_2FC	MSI Level
	10-dione*								
Root vs Stem	D-Allo-Isoleucine *	Amino acids and derivatives	Amino acids and derivatives	1.012512972	0.401283764	0.583066805	31.69760847	4.98630209	1
Root vs Stem	Isorhamnetin-3-O -rhamnoside	Flavonoids	Flavonols	1.425590723	0.175076672	0.411569763	0.054256505	-4.204060065	2
Root vs Stem	L-Valine	Amino acids and derivatives	Amino acids and derivatives	1.141562662	0.352264666	0.583066805	14.26288523	3.834193948	1
Root vs Stem	2,3,5-trimethoxy- 1-{[(2s,3r,4s,5s,6r)- 3,4,5-trihydroxy-6 -(hydroxymethyl)	Flavonoids	Other Flavonoids	1.473682971	0.006084389	0.079988394	0.022825017	-5.453240285	3

Comparison	Compound	Class I	Class II	VIP	P-value	FDR	Fold Change	Log_2FC	MSI Level
	oxan-2-yl]oxy}xanthen-9-one*								
Root vs Stem	N-(2-Hydroxy-4-methoxyphenyl)acetamide	Alkaloids	Alkaloids	1.019644058	0.387151939	0.583066805	16.49978332	4.044375174	1
Root vs Stem	4,5,8-Trihydroxy-α-tetralone-5-O-glucoside	Quinones	Naphthol	1.322635876	0.030939715	0.161261543	0.090652028	-3.463516887	3
Root vs Stem	L-Leucine*	Amino acids and derivatives	Amino acids and derivatives	1.012896777	0.399513809	0.583066805	28.47650777	4.831700327	1
Root vs Stem	Plantagoside	Flavonoids	Flavanones	1.480174755	0.002475753	0.054336171	0.01129789	-6.467802812	3
Root vs Stem	Taxifolin-3-O-rha	Flavonoids	Flavanonols	1.193521719	0.201796128	0.442414085	0.097328078	-3.36100012	3

Comparison	Compound	Class I	Class II	VIP	P-value	FDR	Fold Change	Log_2FC	MSI Level
	mnoside (Astilbin)								
Root vs Stem	3,5,7,4'-Tetrahydroxy-Coumaronochromone	Others	Chromone	1.435907562	0.075465789	0.254096882	0.031261285	-4.999479109	1
Root vs Stem	Luteolin-3'-O-glucoside	Flavonoids	Flavones	1.470460369	0.024063725	0.144292194	0.007331891	-7.091599045	3
Root vs Stem	Monotropein	Terpenoids	Monoterpenoids	1.189025306	0.010499444	0.100388537	0.187817856	-2.412593871	2
Root vs Stem	1-Caffeoylquinic acid	Phenolic acids	Phenolic acids	1.364073652	0.099533046	0.300685165	0.090011025	-3.473754472	1
Root vs Stem	Rhodioloside F	Terpenoids	Monoterpenoids	1.44081661	0.042792801	0.191665066	0.037550616	-4.735019596	3

Comparison	Compound	Class I	Class II	VIP	P-value	FDR	Fold Change	Log_2FC	MSI Level
Root vs Stem	Cryptochlorogenic acid (4-O-Caffeoylquinic acid)*	Phenolic acids	Phenolic acids	1.287253765	0.049818364	0.206891555	0.119813591	-3.061136529	1
Root vs Stem	Luteolin-4'-O-glucoside*	Flavonoids	Flavones	1.467196836	0.015132978	0.120132564	0.015851505	-5.979236344	1
Root vs Stem	Neochlorogenic acid (5-O-Caffeoylquinic acid)*	Phenolic acids	Phenolic acids	1.464851958	0.040221558	0.184742773	0.003462317	-8.174046499	1
Root vs Stem	L-Asparagine	Amino acids and derivatives	Amino acids and derivatives	1.361095765	0.222503025	0.463416996	67.1131204	6.068522931	1

Comparison	Compound	Class I	Class II	VIP	P-value	FDR	Fold Change	Log_2FC	MSI Level
Root vs Stem	LPC(18:1/0:0)	Lipids	LPC	1.138723433	0.144743408	0.365557544	2.208025727	1.142756982	1
Root vs Stem	Chlorogenic acid (3-O-Caffeoylquinic acid)*	Phenolic acids	Phenolic acids	1.213405095	0.016722258	0.122828256	0.173165241	-2.529778721	1
Root vs Stem	LysoPC 18:1	Lipids	LPC	1.12129952	0.155530207	0.382615432	2.167529586	1.116051686	2
Root vs Stem	9-hydroxy-2-(2-hydroxy-3-methoxyphenyl)-3-(hydroxymethyl)-12-methoxy-2,3-dihydro-1,4,5-trioxatetraphen-10-one	Others	Ketone compounds	1.230430876	0.001314561	0.041846281	0.048411979	-4.368492105	1
Root vs Stem	LysoPC 18:1(2n	Lipids	LPC	1.107190619	0.168040327	0.40050258	2.174136052	1.120442223	2

Comparison	Compound	Class I	Class II	VIP	P-value	FDR	Fold Change	Log_2FC	MSI Level
	isomer)								
Root vs Stem	3',5',5,7-Tetrahydroxy-4'-methoxyflavanone-3'-O-glucoside	Flavonoids	Flavanones	1.359565396	0.149205607	0.372178475	0.034502435	-4.857158022	3
Root vs Stem	2,3-Dihydroxyoleana-11,13(18)-dien-28-oic acid (Camaldulenic acid)	Terpenoids	Triterpene	1.122499933	0.111789256	0.318692024	0.21218233	-2.236623575	1
Root vs Stem	Virgatic acid	Terpenoids	Triterpene	1.450364343	0.059635238	0.226679801	0.035169089	-4.829548241	1
Root vs Stem	Cinchonain Ic	Flavonoids	Flavanols	1.215764455	1.52402E-05	0.00786396	0.041330467	-4.596650521	3
Root vs Stem	Luteolin-7-O-gluc	Flavonoids	Flavones	1.484401781	0.011049299	0.102267947	0.008447827	-6.887204075	1

Comparison	Compound	Class I	Class II	VIP	P-value	FDR	Fold Change	Log_2FC	MSI Level
	oside (Cynaroside)								
Root vs Stem	LysoPE 18:2	Lipids	LPE	1.261618177	0.164139923	0.39531482	2.690169775	1.427697223	2
Root vs Stem	LysoPE 18:2(2n isomer)	Lipids	LPE	1.206352472	0.191360322	0.431546011	2.576172857	1.365229399	1
Root vs Stem	Gallic Acid 4-O-Glucoside	Phenolic acids	Phenolic acids	1.44322096	0.000332553	0.021248282	0.171141612	-2.546737514	1
Root vs Stem	Vanillic acid glucoside	Phenolic acids	Phenolic acids	1.385822703	0.004318396	0.069663321	0.242682092	-2.042860443	1
Root vs Stem	L-Histidine	Amino acids and derivatives	Amino acids and derivatives	1.058357822	0.375817452	0.583066805	25.65525115	4.681182243	2

Comparison	Compound	Class I	Class II	VIP	P-value	FDR	Fold Change	Log_2FC	MSI Level
Root vs Stem	Catechin-(7,8-bc)-4α-(3,4-dihydroxy phenyl)-dihydro-2-(3H)-one*	Flavonoids	Flavanols	1.319964925	0.005315069	0.075448846	0.118272222	-3.079816823	1
Root vs Stem	Cinchonain Id*	Flavonoids	Flavanols	1.377132946	0.007005488	0.084065858	0.110832432	-3.173547992	1
Root vs Stem	Catechin-(7,8-bc)-4β-(3,4-dihydroxy phenyl)-dihydro-2-(3H)-one	Flavonoids	Flavanols	1.337356717	0.000372228	0.021950816	0.097996506	-3.351125878	1
Root vs Stem	Bracteatin*	Flavonoids	Aurones	1.3910705	0.078453413	0.260753372	0.013366532	-6.225230945	2
Root vs Stem	Cernuoside	Flavonoids	Aurones	1.466964284	0.01277286	0.109846592	0.00914601	-6.772641813	2
Root vs Stem	Kaempferol-3-O-glucoside	Flavonoids	Flavonols	1.475941024	0.016631938	0.122828256	0.00854616	-6.870507948	1

Comparison	Compound	Class I	Class II	VIP	P-value	FDR	Fold Change	Log_2FC	MSI Level
	(Astragalin)*								
Root vs Stem	Gossypetin-3-O-glucoside*	Flavonoids	Flavonols	1.345327245	0.094744772	0.29402987	0.032374998	-4.948976073	1
Root vs Stem	Kaempferol-3-O-arabinoside	Flavonoids	Flavonols	1.472817678	0.019357547	0.132297937	0.007722824	-7.016655756	1
Root vs Stem	Myricetin-3-O-β-D-glucoside*	Flavonoids	Flavonols	1.412981867	0.112065449	0.318787221	0.015954313	-5.969909664	1
Root vs Stem	3-Hydroxy-L-phenylalanine*	Amino acids and derivatives	Amino acids and derivatives	1.190324711	0.155946117	0.383181888	3.137506776	1.649618575	1
Root vs Stem	L-Tyrosine*	Amino acids and derivatives	Amino acids and derivatives	1.039363403	0.371805634	0.583066805	11.32899919	3.501948513	1

Comparison	Compound	Class I	Class II	VIP	P-value	FDR	Fold Change	Log_2FC	MSI Level
Root vs Stem	Kaempferol-3-O-arabinoside (Juglanin)	Flavonoids	Flavonols	1.487770864	0.007415972	0.08503648	0.005060473	-7.626511961	1
Root vs Stem	Quercetin 3-(2''-acetylrhamnoside)	Flavonoids	Flavonols	1.360479962	0.225998501	0.465529846	0.092277345	-3.437879691	1
Root vs Stem	1-desmethylaurantio-obtusin-2-O-β-D-glucoside	Quinones	Anthraquinone	1.360937754	0.001534032	0.041846281	0.036903438	-4.760100964	3
Root vs Stem	tenuiflorin C glucoside	Others	Chromone	1.312234295	0.000205808	0.016991546	0.049431649	-4.338421165	3
Root vs Stem	5,7-Dihydroxy-4-oxo-2-(3,4,5-trihy	Others	Others	1.175048096	0.011407272	0.103720745	0.184456236	-2.438649527	3

Comparison	Compound	Class I	Class II	VIP	P-value	FDR	Fold Change	Log_2FC	MSI Level
	droxyphenyl)-4H-chromen-3-yl-allo side								
Root vs Stem	Nepetin-7-O-allos ide*	Flavonoids	Flavones	1.324474651	0.001170098	0.040933583	0.038537223	-4.697603582	1
Root vs Stem	Rhamnetin-3-O-G lucoside*	Flavonoids	Flavonols	1.312920391	0.000314154	0.021248282	0.041993167	-4.573701583	1
Root vs Stem	Isorhamnetin 7-O-glucoside	Flavonoids	Flavonols	1.329758726	0.000279432	0.02036426	0.036150243	-4.789850848	2
Root vs Stem	Nepetin-7-O-gluc oside(Nepitrin)*	Flavonoids	Flavones	1.314383416	0.002880583	0.056750731	0.041156904	-4.602721732	1
Root vs Stem	Azalein (Azaleatin-3-O-rh	Flavonoids	Flavonols	1.437520744	0.136239959	0.354155259	0.003642713	-8.100771076	1

Comparison	Compound	Class I	Class II	VIP	P-value	FDR	Fold Change	Log_2FC	MSI Level
	amnoside)								
Root vs Stem	Quercetagetin-7-O-glucoside*	Flavonoids	Flavonols	1.439684207	0.045104111	0.195335116	0.005453758	-7.518533578	1
Root vs Stem	6-Methoxykaempferol-3-O-glucoside*	Flavonoids	Flavonols	1.289285849	3.51797E-05	0.00848072	0.054911224	-4.186755109	1
Root vs Stem	Isorhamnetin-3-O-Glucoside*	Flavonoids	Flavonols	1.273578105	0.000163713	0.016991546	0.057562328	-4.118731249	1
Root vs Stem	L-Arginine	Amino acids and derivatives	Amino acids and derivatives	1.179391238	0.215974471	0.458612458	14.02318694	3.809742351	1
Root vs Stem	4-O-acetyl-3-O-caffeoyl-2-C-methyl	Others	Others	1.215230237	0.068505152	0.244627395	0.139935445	-2.837166661	3

Comparison	Compound	Class I	Class II	VIP	P-value	FDR	Fold Change	Log_2FC	MSI Level
	-D-erythronate								
Root vs Stem	Quercetin-5-O-β-D-glucoside*	Flavonoids	Flavonols	1.458934021	0.001461502	0.041846281	0.081593698	-3.615398469	1
Root vs Stem	Hesperetin-3'-O-glucoside*	Flavonoids	Flavanones	1.431413257	0.000286126	0.02036426	0.092443322	-3.43528709	3
Root vs Stem	5-O-p-Coumaroyl quinic acid	Phenolic acids	Phenolic acids	1.407039474	0.002268681	0.052028413	0.108478356	-3.20452087	1
Root vs Stem	L-Norleucine*	Amino acids and derivatives	Amino acids and derivatives	1.223409544	0.20816299	0.450364436	5.069489612	2.341840506	1
Root vs Stem	Quercetin-3-O-rhamnoside(Quercitrin)	Flavonoids	Flavonols	1.248445022	0.177299742	0.414034699	0.080235441	-3.639616547	1

Comparison	Compound	Class I	Class II	VIP	P-value	FDR	Fold Change	Log_2FC	MSI Level
Root vs Stem	2-Caffeoylisocitrate	Phenolic acids	Phenolic acids	1.161448504	0.052763454	0.216099896	0.126305269	-2.985013276	2
Root vs Stem	Sesamin	Lignans and Coumarins	Lignans	1.134875074	0.002340634	0.052511621	0.153067491	-2.707760184	3
Root vs Stem	7-Hydroxycoumarin;Umbelliferone	Lignans and Coumarins	Coumarins	1.144640149	0.034552987	0.171435974	0.112089364	-3.157278701	1
Root vs Stem	Quercetin-3-O-arabinoside	Flavonoids	Flavonols	1.363746607	0.000203138	0.016991546	0.050497636	-4.307640337	3
Root vs Stem	Morin 3-alpha-L-lyxopyranoside	Flavonoids	Flavonols	1.363479232	0.000141911	0.016272452	0.05210187	-4.26252105	3
Root vs Stem	Morin-3-O-xyloside*	Flavonoids	Flavonols	1.327676816	0.007541028	0.085520226	0.074630076	-3.74409903	1

Comparison	Compound	Class I	Class II	VIP	P-value	FDR	Fold Change	Log_2FC	MSI Level
Root vs Stem	Quercetin-3-O-glucoside (Isoquercitrin)	Flavonoids	Flavonols	1.477402688	0.001627172	0.041981047	0.028187571	-5.148797012	3
Root vs Stem	Quercetin-3-O-xyloside (Reynoutrin)*	Flavonoids	Flavonols	1.331250082	4.47173E-05	0.00848072	0.065248856	-3.937903577	1
Root vs Stem	Quercetin-3-O-alloside; Isohyperoside*	Flavonoids	Flavonols	1.460264632	0.006240621	0.080081749	0.077682728	-3.686262332	1
Root vs Stem	Avicularin(Quercetin-3-O-α-L-arabinofuranoside)*	Flavonoids	Flavonols	1.346018276	8.78178E-05	0.011328498	0.056104047	-4.155751351	1
Root vs Stem	Quercetin-3-O-gal	Flavonoids	Flavonols	1.163660317	0.323979888	0.55631821	0.102082472	-3.292192924	1

Comparison	Compound	Class I	Class II	VIP	P-value	FDR	Fold Change	Log_2FC	MSI Level
	actoside (Hyperin)								
Stem vs Leaf	Daidzein rutinoside	Flavonoids	Isoflavones	1.109860966	0.020577973	0.05664871	5.219875764	2.38401547	3
Stem vs Leaf	1-Linoleoylglycerol-2,3-di-O-glucoside	Lipids	Glycerol ester	1.122959718	0.161696208	0.217551219	50.66053647	5.662790449	3
Stem vs Leaf	LysoPC 17:0*	Lipids	LPC	1.134331737	0.078037164	0.129359987	10.63176229	3.410308848	1
Stem vs Leaf	LysoPE 20:2	Lipids	LPE	1.107129718	0.114934121	0.170302109	15.52457075	3.956481474	3
Stem vs Leaf	2,3-Dihydroxy-1-(4-hydroxy-3,5-dimethoxyphenyl)propan-1-one	Others	Ketone compounds	1.171781297	0.041251072	0.086274128	28.7731823	4.846652886	2

Comparison	Compound	Class I	Class II	VIP	P-value	FDR	Fold Change	Log_2FC	MSI Level
Stem vs Leaf	Diosmetin-8-C-(2''-O-rhamnosyl)glucoside	Flavonoids	Flavones	1.085022577	0.192781458	0.249837333	18.50085512	4.209520049	3
Stem vs Leaf	Feruloyl Lactate	Organic acids	Organic acids	1.177639385	0.01639288	0.050482096	15.75065822	3.977340215	3
Stem vs Leaf	Nortrachelogenin-4'-O-gentiobioside	Lignans and Coumarins	Lignans	1.168219485	0.043070933	0.088614838	11.28446597	3.49626624	3
Stem vs Leaf	4,6-diethyl-3,6-dihydroxy-8-methyldodec-4-enoicacid	Lipids	Free fatty acids	1.094513754	0.01408131	0.046517843	4.009070389	2.003267748	1
Stem vs Leaf	N-malonylleucine	Amino acids and	Amino acids and derivatives	1.053088038	0.051060911	0.098458259	8.537133695	3.093751773	3

Comparison	Compound	Class I	Class II	VIP	P-value	FDR	Fold Change	Log_2FC	MSI Level
		derivatives							
Stem vs Leaf	Pantetheine	Alkaloids	Alkaloids	1.170373581	0.031009525	0.071688686	0.09882581	-3.338968322	3
Stem vs Leaf	9S-Hydroperoxy-10E,12Z-octadecadienoic acid	Lipids	Free fatty acids	1.149420398	0.136812965	0.191253495	26.49489631	4.727642576	3
Stem vs Leaf	3,5-Dihydroxytoluene	Others	Others	1.161172291	0.008887081	0.037911157	7.013774013	2.810190947	3
Stem vs Leaf	N-Alpha-Acetyl-L-Asparagine	Amino acids and derivatives	Amino acids and derivatives	1.181656938	0.000319727	0.010948842	0.036867427	-4.76150947	3
Stem vs Leaf	Cinnamic acid	Phenolic acids	Phenolic acids	1.05190678	0.040201403	0.08486876	2.584122346	1.369674377	3

Comparison	Compound	Class I	Class II	VIP	P-value	FDR	Fold Change	Log_2FC	MSI Level
Stem vs Leaf	Medicarpin 3-O-(6'-malonylgl uclside)	Flavonoids	Isoflavones	1.115682564	0.103006358	0.157737657	13.73392178	3.779671747	3
Stem vs Leaf	Feruloylferuloylta rtaric acid	Phenolic acids	Phenolic acids	1.092979831	0.079401967	0.131116919	7.792936127	2.962166992	3
Stem vs Leaf	Prunetin-4'-O-glu coside(Prunitrin)*	Flavonoids	Isoflavones	1.101852236	0.121763326	0.176290337	0.046018172	-4.441652524	3
Stem vs Leaf	3-O-Cafeoyltorme ntic acid	Terpenoids	Triterpene	1.170957583	0.00748955	0.035468132	0.22528585	-2.150171393	3
Stem vs Leaf	salicylate-6-O-β-D-glucopyranosyl benzoic acid	Phenolic acids	Phenolic acids	1.139049538	0.00405364	0.028204938	3.728951517	1.89877004	3
Stem vs Leaf	Hydroxy	Lipids	Free fatty acids	1.145646706	0.081171538	0.132613075	8.252310067	3.044798029	3

Comparison	Compound	Class I	Class II	VIP	P-value	FDR	Fold Change	Log_2FC	MSI Level
	ricinoleic acid								
Stem vs Leaf	8-Methoxyapigenin*	Flavonoids	Flavones	1.174449894	0.024703461	0.063137988	13.64612035	3.77041894	3
Stem vs Leaf	Antiarol; 3,4,5-Trimethoxyphenol	Others	Others	1.166924619	0.028176757	0.067762894	8.236001606	3.041944111	3
Stem vs Leaf	p-Coumaroylmalic acid	Phenolic acids	Phenolic acids	1.052908185	0.003880785	0.028194402	0.164265583	-2.605897855	3
Stem vs Leaf	Feruloylmalic acid	Phenolic acids	Phenolic acids	1.099165642	0.040759647	0.08548536	0.067872018	-3.88103929	3
Stem vs Leaf	4,7,9,9'-Tetrahydroxy-3,3'-dimethoxy-8-O-4'-neoligna	Lignans and Coumarins	Lignans	1.177383565	0.019866999	0.055784421	20.24229689	4.339301097	3

Comparison	Compound	Class I	Class II	VIP	P-value	FDR	Fold Change	Log_2FC	MSI Level
	n								
Stem vs Leaf	3,4'-Dihydroxy-3'-methoxybenzene pentanoic acid	Phenolic acids	Phenolic acids	1.068356633	0.058158169	0.10650772	8.954095372	3.162547685	3
Stem vs Leaf	Isovitexin-2''-O-rhamnoside (2''-O-alpha-L-Rhamnopyranosyl-isovitexin)*	Flavonoids	Flavones	1.177971444	0.017504198	0.052561488	23.61768416	4.561795603	3
Stem vs Leaf	Stachyose	Others	Saccharides	1.036886883	0.082810425	0.134880617	2.875746619	1.523936566	3
Stem vs Leaf	(7'S,8R,8'R)-3,5'-dimethoxy-3',4,9'-trihydroxy-7',9-epox	Lignans and Coumarins	Lignans	1.092362033	0.211363642	0.267627698	20.61076634	4.365326242	3

Comparison	Compound	Class I	Class II	VIP	P-value	FDR	Fold Change	Log_2FC	MSI Level
	y-8,8'-lignan								
Stem vs Leaf	Isorhamnetin; 3'-Methoxy-3,4',5,7-Tetrahydroxyflavone	Flavonoids	Flavonols	1.158087495	0.070799139	0.12091944	23.5763413	4.559267946	1
Stem vs Leaf	Hispidulin (5,7,4'-Trihydroxy-6-methoxyflavone)*	Flavonoids	Flavones	1.15130519	0.097238564	0.151447189	14.85499473	3.892876188	3
Stem vs Leaf	5,6,7-Trihydroxy-8-methoxyflavone*	Flavonoids	Flavones	1.172115061	0.032961901	0.074363156	13.14439615	3.716375961	3
Stem vs Leaf	3-Acetoxy-11-oxo	Terpenoids	Triterpene	1.14694281	0.058402822	0.106773866	9.03490862	3.17551001	3

Comparison	Compound	Class I	Class II	VIP	P-value	FDR	Fold Change	Log_2FC	MSI Level
	olean-12-en-28-oic acid (Lantanone)								
Stem vs Leaf	6,7,8-Tetrahydroxy-5-methoxyflavone*	Flavonoids	Flavones	1.16844315	0.048753754	0.096253968	14.0975248	3.817369976	3
Stem vs Leaf	Patuletin 3-O-beta-gentiobioside	Flavonoids	Flavonols	1.140472694	0.02507496	0.063574486	11.84673071	3.566417075	3
Stem vs Leaf	Flavogallonic Acid Dilactone	Tannins	Tannin	1.16906327	0.022493863	0.059674669	0.147814122	-2.758143985	3
Stem vs Leaf	Maohuoside B	Flavonoids	Flavonols	1.181619347	0.000451124	0.012932233	0.041869092	-4.577970566	3
Stem vs Leaf	Laricitrin-3-O-(6''-	Flavonoids	Flavonols	1.170422565	0.031085121	0.071786263	11.18515799	3.483513729	3

Comparison	Compound	Class I	Class II	VIP	P-value	FDR	Fold Change	Log_2FC	MSI Level
	acetyl)glucoside								
Stem vs Leaf	2-Hydroxy-4-methylpentanoic acid	Organic acids	Organic acids	1.011824046	0.035232268	0.077678455	0.104226751	-3.262202486	3
Stem vs Leaf	Grayanotoxin XV	Terpenoids	Ditepenoids	1.129352387	0.001153418	0.016734829	0.163670077	-2.61113751	2
Stem vs Leaf	Gossypetin-3-O-rutinoside	Flavonoids	Flavonols	1.174660695	0.030068663	0.07033949	0.053065627	-4.236078524	3
Stem vs Leaf	Palmitoleic Acid	Lipids	Free fatty acids	1.106324447	0.089403833	0.142278491	4.15046481	2.053272913	1
Stem vs Leaf	2-Hydroxymyristic acid	Lipids	Free fatty acids	1.125861521	0.00373868	0.028089239	2.050979248	1.036312895	3
Stem vs Leaf	Olivil-4'-O-glucoside	Lignans and Coumarins	Lignans	1.045401667	0.028407772	0.068014153	5.071653515	2.342456187	3
Stem vs Leaf	2α,5α,14β-Triacet	Others	Others	1.177216789	0.023663146	0.06144416	0.041158453	-4.602667429	2

Comparison	Compound	Class I	Class II	VIP	P-value	FDR	Fold Change	Log_2FC	MSI Level
	oxy-10β-O-(Β-D glucopyranosy)taxa-4(20),11-diene								
Stem vs Leaf	6,7-dimethoxy-2-[2-(4'-hydroxy-3'-methoxyphenyl)ethyl]chromone	Others	Chromone	1.179997972	0.008887048	0.037911157	29.5083423	4.883050971	3
Stem vs Leaf	Laminaran	Others	Saccharides	1.094179137	0.034751394	0.077026286	6.30223328	2.655863157	3
Stem vs Leaf	9,12,13-Trihydroxy-10,15-octadecadienoic acid	Lipids	Free fatty acids	1.135966759	0.104312754	0.159175083	12.98372343	3.698632269	1
Stem vs Leaf	Dehydrovomifoliol	Terpenoids	Monoterpenoids	1.144634726	0.134805871	0.189309356	24.66726579	4.624525906	3

Comparison	Compound	Class I	Class II	VIP	P-value	FDR	Fold Change	Log_2FC	MSI Level
Stem vs Leaf	23-Formyl-2,3,19-trihydroxyurs-12-en-28-oic acid (Pinfaensic acid)	Terpenoids	Triterpene	1.08342205	0.122768189	0.177148728	15.10975276	3.917408148	2
Stem vs Leaf	6-Methoxyquercetin-3-O-rhamnoside	Flavonoids	Flavonols	1.176592145	0.013866077	0.04641581	0.087363963	-3.516817897	2
Stem vs Leaf	Kaempferol triglucoside*	Flavonoids	Flavonols	1.181281001	0.001855291	0.020636181	0.053224967	-4.23175304	3
Stem vs Leaf	1-O-Galloyl-rhamnose	Phenolic acids	Phenolic acids	1.175257186	0.035497838	0.078035541	60.66665029	5.92283175	3
Stem vs Leaf	Buddlenol F	Lignans and Coumarins	Lignans	1.001521092	0.001856943	0.020636181	0.206924947	-2.272820504	3

Comparison	Compound	Class I	Class II	VIP	P-value	FDR	Fold Change	Log$_2$FC	MSI Level
Stem vs Leaf	4-O-(6'-O-Glucosylcaffeoyl)-3,4-dihydroxybenzyl alcohol	Phenolic acids	Phenolic acids	1.164685586	0.029969397	0.070266307	0.139177355	-2.845003597	3
Stem vs Leaf	17-Hydroxylinolenic acid	Lipids	Free fatty acids	1.133719114	0.102124385	0.15683856	9.279762073	3.214087816	2
Stem vs Leaf	2,3,23-Trihydroxyolean-12,18(19)-dien-28-oic acid-glucoside	Terpenoids	Triterpene Saponin	1.046836476	0.026825655	0.06576415	0.1552659	-2.687187078	3
Stem vs Leaf	Indole-3-carboxylic acid	Alkaloids	Plumerane	1.101223577	0.181357356	0.238920536	10.18548546	3.348442838	3
Stem vs Leaf	(3r,4r)-3-(2h-1,3-b	Lignans and	Lignans	1.177291175	0.025689669	0.064000914	31.41188036	4.973238402	3

Comparison	Compound	Class I	Class II	VIP	P-value	FDR	Fold Change	Log_2FC	MSI Level
	enzodioxol-5-ylmethyl)-4-[(4-hydroxy-3-methoxyphenyl)methyl]oxolan-2-one	Coumarins							
Stem vs Leaf	4-Hydroxybenzoyl-1-O-(6''-O-galloyl)glucoside	Phenolic acids	Phenolic acids	1.171105809	0.02868715	0.06817466	0.107943821	-3.211647435	3
Stem vs Leaf	N-Acetyl-L-threonine	Amino acids and derivatives	Amino acids and derivatives	1.040455466	0.078788625	0.130404576	2.619268049	1.389163709	3
Stem vs Leaf	Indole-3-lactic acid	Alkaloids	Plumerane	1.18164762	0.000368211	0.011641955	0.024110635	-5.374186528	1

Comparison	Compound	Class I	Class II	VIP	P-value	FDR	Fold Change	Log_2FC	MSI Level
Stem vs Leaf	4,4'-Biisofraxidin	Lignans and Coumarins	Coumarins	1.091966136	0.172011755	0.229083742	17.27733642	4.110808915	1
Stem vs Leaf	LysoPC 20:5	Lipids	LPC	1.135372072	0.080228963	0.13177408	10.3951821	3.377843126	3
Stem vs Leaf	Decumbic acid	Organic acids	Organic acids	1.175175494	0.028159365	0.067762894	0.058496031	-4.095517447	3
Stem vs Leaf	Blumenol B malonyl Glucoside*	Terpenoids	Sesquiterpenoids	1.165082781	0.047459104	0.094391372	14.91740024	3.898924224	3
Stem vs Leaf	methylarbutin*	Phenolic acids	Phenolic acids	1.180160969	0.007897809	0.036127578	0.035150977	-4.830291387	3
Stem vs Leaf	Esculin 6'-Malonate	Lignans and Coumarins	Coumarins	1.170565503	0.015749109	0.049215964	6.734108438	2.751486953	3

Comparison	Compound	Class I	Class II	VIP	P-value	FDR	Fold Change	Log_2FC	MSI Level
Stem vs Leaf	Epiafzelechin	Flavonoids	Flavanols	1.078298459	0.005459617	0.031195162	10.20882373	3.351744741	3
Stem vs Leaf	1-g-linolenoyl-glycerol	Lipids	Glycerol ester	1.130066983	0.114547461	0.17028188	7.741738116	2.952657506	2
Stem vs Leaf	L-Cystathionine	Amino acids and derivatives	Amino acids and derivatives	1.177148861	0.028696775	0.06817466	38.42917987	5.264130283	3
Stem vs Leaf	2,6-Dimethoxybenzaldehyde	Others	Aldehyde compounds	1.13203683	0.05647692	0.104496883	0.160219716	-2.641876405	1
Stem vs Leaf	13S-Hydroperoxy-9Z,11E-octadecadienoic acid	Lipids	Free fatty acids	1.152667976	0.071050351	0.121104397	8.999545868	3.169852202	3
Stem vs Leaf	Pierisformoside B	Terpenoids	Ditepenoids	1.083276343	0.000743726	0.014479152	0.114380036	-3.128092829	3

Comparison	Compound	Class I	Class II	VIP	P-value	FDR	Fold Change	Log_2FC	MSI Level
Stem vs Leaf	Ginnalin C (2-O-Galloyl-1,5-Anhydro-D-Glucitol)	Tannins	Tannin	1.180921291	0.004693352	0.029574357	30.28329252	4.920450164	3
Stem vs Leaf	Syringic acid	Phenolic acids	Phenolic acids	1.044163187	0.133069838	0.187976447	10.34018345	3.370189876	3
Stem vs Leaf	Xylosyl tachioside	Phenolic acids	Phenolic acids	1.010233411	0.021587085	0.058015292	7.110754097	2.830002566	3
Stem vs Leaf	Tri-p-coumaroyl Spermidine	Alkaloids	Phenolamine	1.062384936	0.019071502	0.054964785	0.268731173	-1.895764411	3
Stem vs Leaf	LysoPC 20:3	Lipids	LPC	1.13797584	0.08771499	0.140422359	9.3728863	3.228493382	3
Stem vs Leaf	Methyl 2-O-(4-hydroxybe	Phenolic acids	Phenolic acids	1.152851002	0.029784372	0.069908734	0.129118678	-2.953230385	3

Comparison	Compound	Class I	Class II	VIP	P-value	FDR	Fold Change	Log_2FC	MSI Level
	nzoyl)-2,4,6-trihydroxyphenylacetate								
Stem vs Leaf	Luteolin-6-C-glucoside-7-O-rhamnoside	Flavonoids	Flavones	1.142530804	0.086363317	0.138464676	0.131380934	-2.92817217	3
Stem vs Leaf	8-Epiloganin	Terpenoids	Terpene	1.154396686	0.000194816	0.008450724	0.321207775	-1.638421279	3
Stem vs Leaf	Pinocembrin-7-O-(2''-O-arabinosyl)glucoside	Flavonoids	Flavanones	1.154923179	0.007659812	0.03555507	2.038735194	1.0276744	3
Stem vs Leaf	2-Hydroxy-3-(4-Hydroxyphenyl)Propanoic Acid	Phenolic acids	Phenolic acids	1.051885352	0.006470103	0.032910525	0.07649003	-3.708584483	3

Comparison	Compound	Class I	Class II	VIP	P-value	FDR	Fold Change	Log_2FC	MSI Level
Stem vs Leaf	Kaempferol-3-O-galactoside-4'-O-glucoside	Flavonoids	Flavonols	1.180128049	0.005118505	0.030653997	0.094078812	-3.409986349	3
Stem vs Leaf	(6R,9R)-3-Oxo-α-ionol-β-D-malonyl-glucoside	Terpenoids	Sesquiterpenoids	1.173505644	0.025552374	0.063904927	0.081035152	-3.625308315	3
Stem vs Leaf	Skimmin (7-Hydroxycoumarin-7-O-glucoside)	Lignans and Coumarins	Coumarins	1.181281351	0.001274714	0.017456272	0.070182169	-3.832751658	3
Stem vs Leaf	3,8-Dihydroxy-1-methoxy-9,10-anthraquinone glucoside	Quinones	Anthraquinone	1.153982181	0.066234171	0.116254461	16.40784773	4.036314103	3

Comparison	Compound	Class I	Class II	VIP	P-value	FDR	Fold Change	Log_2FC	MSI Level
Stem vs Leaf	N-Feruloylhydroxyputrescine	Alkaloids	Phenolamine	1.175856643	0.019929575	0.055784421	0.053729941	-4.218129939	3
Stem vs Leaf	Diosmetin-7-O-(6''-malonyl)glucoside	Flavonoids	Flavones	1.080604126	0.001315614	0.017778885	6.078469786	2.603708181	3
Stem vs Leaf	Dihydrozeatin ribonucleoside	Alkaloids	Alkaloids	1.018442491	0.001164033	0.016734829	0.1477709	-2.758565906	3
Stem vs Leaf	Eriodictyol-7-O-Rutinoside (Eriocitrin)*	Flavonoids	Flavanones	1.179036337	0.008579615	0.037420256	0.071714251	-3.801596348	3
Stem vs Leaf	Okanin-4'-O-(6''-O-malonyl)glucoside	Flavonoids	Chalcones	1.048182184	0.103447774	0.158188275	3.081753639	1.623751535	3

Comparison	Compound	Class I	Class II	VIP	P-value	FDR	Fold Change	Log_2FC	MSI Level
Stem vs Leaf	3,12,23,25-tetrahydroxy-20S,24S-epoxydammarane-3-O-xylosylglucoside	Terpenoids	Triterpene Saponin	1.153189106	0.062616649	0.112375455	0.066909757	-3.901639584	2
Stem vs Leaf	Carvylacetate	Terpenoids	Monoterpenoids	1.163597324	0.054925936	0.102929846	11.71750002	3.550592892	3
Stem vs Leaf	LysoPE 15:1(2n isomer)*	Lipids	LPE	1.104206074	0.141801892	0.196186659	15.1756087	3.92368248	3
Stem vs Leaf	Lys-Pro-Ile	Amino acids and derivatives	Amino acids and derivatives	1.052186342	0.076403088	0.127635306	0.10400971	-3.265209877	3
Stem vs Leaf	7S,8R-threo-3',9,9'	Lignans and	Lignans	1.15772322	0.057353508	0.105483354	11.37581139	3.507897545	3

Comparison	Compound	Class I	Class II	VIP	P-value	FDR	Fold Change	Log_2FC	MSI Level
	-trihydroxy-3-methoxy-4',7-epoxy-neolignan-4-O-rhamnoside	Coumarins							
Stem vs Leaf	5-hydroxy-6,7,8,3',4'-pentamethoxyflavone glucoside*	Flavonoids	Flavones	1.090947273	0.006603919	0.033249269	0.097085675	-3.364597751	3
Stem vs Leaf	Apigenin-6,8-di-C-glucoside (Vicenin-2)	Flavonoids	Flavones	1.174515092	0.027336357	0.066709658	0.057740581	-4.114270576	3
Stem vs Leaf	11-O-Galloylbergenin	Lignans and Coumarins	Coumarins	1.023595356	0.099146754	0.153598711	0.129543546	-2.94849095	3
Stem vs Leaf	Protogenkwanon	Flavonoids	Other	1.150696302	0.074719621	0.125505614	16.42423668	4.037754417	3

Comparison	Compound	Class I	Class II	VIP	P-value	FDR	Fold Change	Log_2FC	MSI Level
	e*		Flavonoids						
Stem vs Leaf	Gentisic acid 2-O-(6''-O-Feruloyl)Glucoside*	Phenolic acids	Phenolic acids	1.159593911	6.34258E-05	0.005050575	7.561336089	2.918641181	1
Stem vs Leaf	1-O-p-Coumaroyl-4,6-(S)-HHDP-β-D-glucose	Phenolic acids	Phenolic acids	1.165327658	0.028811833	0.068372452	0.143392168	-2.801961868	3
Stem vs Leaf	heptafluorobutanoic acid	Others	Others	1.179972696	0.009809282	0.039494299	0.031398464	-4.993162207	2
Stem vs Leaf	3,4-O-di-Caffeoyl-1-O-glucoside Quinic Acid	Phenolic acids	Phenolic acids	1.181653616	0.000486844	0.013133103	0.031582891	-4.984712967	2
Stem vs Leaf	1-(3,4-dihydroxyp	Others	Ketone	1.094842544	0.150282504	0.206348026	6.254168124	2.644818002	3

Comparison	Compound	Class I	Class II	VIP	P-value	FDR	Fold Change	Log_2FC	MSI Level
	henyl)-5-hydroxy -7-(4-hydroxy-3-methoxyphenyl)hept-1-en-3-one		compounds						
Stem vs Leaf	LysoPE 15:1*	Lipids	LPE	1.106708134	0.12188407	0.1763464	15.46920282	3.951326947	3
Stem vs Leaf	Dihydrotricetin	Flavonoids	Flavanones	1.151508985	0.116482337	0.171885398	48.94871606	5.613199113	2
Stem vs Leaf	Puerarin-4'-O-glucoside	Flavonoids	Isoflavones	1.101670782	0.043353499	0.089025809	4.183604435	2.06474645	3
Stem vs Leaf	4-O-(6'-O-Glucosylcaffeoylglucosyl)-4-hydroxybenzyl alcohol	Phenolic acids	Phenolic acids	1.17688752	0.016613846	0.050805983	0.048776003	-4.357684637	3
Stem vs Leaf	Caffeic aldehyde	Others	Aldehyde	1.168952548	0.006258947	0.032737441	0.139451296	-2.842166748	3

Comparison	Compound	Class I	Class II	VIP	P-value	FDR	Fold Change	Log_2FC	MSI Level
			compounds						
Stem vs Leaf	Apigenin 7,4'-diglucoside	Flavonoids	Flavones	1.153359505	0.08528023	0.137342692	0.078267531	-3.675442259	3
Stem vs Leaf	Sachaloside VII	Others	Others	1.036553487	0.037075635	0.080436544	0.118824921	-3.073090649	3
Stem vs Leaf	Helicidol*	Phenolic acids	Phenolic acids	1.122352537	2.10731E-05	0.00257283	0.05224663	-4.258518204	3
Stem vs Leaf	Citric acid glucoside	Organic acids	Organic acids	1.13076208	0.058835762	0.107119274	8.881828169	3.150856661	2
Stem vs Leaf	Isorhamnetin-3-O -(2''-O-xylosyl)glu coside	Flavonoids	Flavonols	1.070996427	0.075447569	0.126505911	2.552782799	1.352070792	3
Stem vs Leaf	Caffeoylquinic	Phenolic	Phenolic acids	1.180646848	0.002904623	0.025283157	0.115737333	-3.111073793	2

Comparison	Compound	Class I	Class II	VIP	P-value	FDR	Fold Change	Log_2FC	MSI Level
	acid-glucose-rhamnose-caffeoyl	acids							
Stem vs Leaf	1-O-Feruloyl-beta-D-glucose	Phenolic acids	Phenolic acids	1.15683737	0.011036346	0.041052153	0.081565027	-3.615905494	3
Stem vs Leaf	Limocitrin-7-O-(6''-Acetyl)glucoside	Flavonoids	Flavonols	1.181714139	4.16215E-05	0.003890702	23.9635367	4.582768942	3
Stem vs Leaf	3,4,5-Trimethoxyphenol-1-O-β-D-malonylglucoside	Phenolic acids	Phenolic acids	1.177465733	0.01233143	0.042842	13.94891831	3.802081345	3
Stem vs Leaf	25S-Inokosterone	Others	Others	1.036474096	0.000399374	0.012093721	0.103702638	-3.269475501	3
Stem vs Leaf	Lyoniresinol	Lignans and Coumarins	Lignans	1.135807782	0.035096214	0.077487102	4.792500209	2.260778495	3

Comparison	Compound	Class I	Class II	VIP	P-value	FDR	Fold Change	Log_2FC	MSI Level
Stem vs Leaf	Hydroxyferulic acid glucoside	Phenolic acids	Phenolic acids	1.174982969	0.028532896	0.068161918	18.65803437	4.221725101	3
Stem vs Leaf	Erythorbic Acid; Isoascorbic Acid	Others	Vitamin	1.138733885	0.104613597	0.159517112	9.228312131	3.206066801	1
Stem vs Leaf	Naringenin-7-O-(6''-malonyl)glucoside	Flavonoids	Flavanones	1.155152709	0.000397737	0.012093721	2.560827793	1.35661024	3
Stem vs Leaf	1-O-Glucopyranosylamplexine	Terpenoids	Monoterpenoids	1.088993891	0.008839822	0.037911157	0.134836115	-2.890721125	3
Stem vs Leaf	N-(11-alkanoyl)nornicotine	Alkaloids	Pyridine alkaloids	1.179612452	0.006903529	0.033887185	0.077273164	-3.693888724	3
Stem vs Leaf	Pantothenol	Others	Vitamin	1.125044431	0.100327541	0.155182888	11.5372355	3.528225669	3

Comparison	Compound	Class I	Class II	VIP	P-value	FDR	Fold Change	Log_2FC	MSI Level
Stem vs Leaf	Pieroside C	Others	Others	1.170621972	0.031886778	0.072855019	0.080531697	-3.634299451	2
Stem vs Leaf	9-hydroxy-2,3-dimethoxy-1,4-anthraquinone	Quinones	Anthraquinone	1.180312937	0.006181437	0.032737441	0.047343303	-4.40069582	3
Stem vs Leaf	LysoPC 19:0	Lipids	LPC	1.130034273	0.117180874	0.172544123	8.437507024	3.076816798	3
Stem vs Leaf	5Z,8Z-tetradecadienoic acid	Lipids	Free fatty acids	1.070304439	0.020818854	0.05701979	0.468264269	-1.094605138	3
Stem vs Leaf	Ligraminol B	Lignans and Coumarins	Lignans	1.144531817	0.090319331	0.143099898	8.278519433	3.049372773	3
Stem vs Leaf	Isoferulic Acid*	Phenolic acids	Phenolic acids	1.066596651	0.199702803	0.256794873	22.78390655	4.509943229	3
Stem vs Leaf	Rhamnosylfucose	Terpenoids	Terpene	1.08353088	0.006100318	0.032545122	0.438705023	-1.188676871	3

Comparison	Compound	Class I	Class II	VIP	P-value	FDR	Fold Change	Log_2FC	MSI Level
Stem vs Leaf	3-Hydroxy-1-(4'-hydroxy-3'-methoxyphenyl)-propan-1-one	Others	Others	1.100367729	0.050103737	0.097483935	15.91139284	3.991988225	3
Stem vs Leaf	D-Panose*	Others	Saccharides	1.136068562	0.049073585	0.096461148	8.860002586	3.14730712	2
Stem vs Leaf	Ala-Asn	Amino acids and derivatives	Amino acids and derivatives	1.162174229	0.048866903	0.096388845	0.061773504	-4.016868021	3
Stem vs Leaf	D-Friedoolean-14-ene-3,28-diol (Myricadoil)	Terpenoids	Triterpene	1.084972319	0.13961204	0.193905611	22.17079592	4.470588658	2
Stem vs Leaf	1-(2-hydroxyethyl)-4-methoxy-9H-p	Alkaloids	Alkaloids	1.133015292	0.023565509	0.061264624	8.776880245	3.133708223	3

Comparison	Compound	Class I	Class II	VIP	P-value	FDR	Fold Change	Log_2FC	MSI Level
	yrido[3,4-b]indol-8-ol								
Stem vs Leaf	N-acetyl-nornicotine	Alkaloids	Pyridine alkaloids	1.067577716	0.068529371	0.118629748	0.329302431	-1.602514933	3
Stem vs Leaf	Afrormosin 7-O-(6''-Malonylglucoside)*	Flavonoids	Isoflavones	1.166524601	0.018200798	0.053557932	4.798933495	2.26271382	3
Stem vs Leaf	γ-Glu-Cys	Amino acids and derivatives	Amino acids and derivatives	1.150114496	0.064235121	0.113854502	0.086341965	-3.533794261	3
Stem vs Leaf	9-Hydroperoxy-9Z,11E-Octadecadienoic Acid	Lipids	Free fatty acids	1.163106656	0.011382531	0.041808314	9.262083168	3.211336712	2

Comparison	Compound	Class I	Class II	VIP	P-value	FDR	Fold Change	Log_2FC	MSI Level
Stem vs Leaf	3-O-Caffeoyl-9-hydroxylup-20(30)-en-7-one (Querspicatin A)*	Terpenoids	Triterpene	1.093272913	0.00404836	0.028204938	2.460171233	1.298758733	1
Stem vs Leaf	(7S,8R-Threo-3',4,9'-trihydroxy-3-methoxy-4',7-epoxy-neolignan-9-O-rhamnoside	Lignans and Coumarins	Lignans	1.111299286	0.001761467	0.020144441	0.0591435	-4.079636561	3
Stem vs Leaf	9,10,11-Trihydroxy-12-octadecenoic acid	Lipids	Free fatty acids	1.163926702	0.053663752	0.10176487	10.39146267	3.377326832	1
Stem vs Leaf	cyanidin 3,3',5-tri-O-glucos	Flavonoids	Anthocyanidins	1.180944422	0.002189966	0.022314818	0.094969932	-3.396385363	3

Comparison	Compound	Class I	Class II	VIP	P-value	FDR	Fold Change	Log_2FC	MSI Level
	ide								
Stem vs Leaf	peganoneI	Quinones	Anthraquinone	1.180113626	0.014019861	0.046517843	0.012273928	-6.348259107	3
Stem vs Leaf	Isochlorogenic acid C*	Phenolic acids	Phenolic acids	1.178637464	0.012276924	0.042842	0.053971858	-4.211648832	3
Stem vs Leaf	3-Isopropylmalic Acid	Organic acids	Organic acids	1.084528134	0.001892999	0.020636181	0.166283065	-2.588286848	3
Stem vs Leaf	Phloretin-2'-O-(6''-O-glucoside)glucoside	Flavonoids	Chalcones	1.094114267	0.003461735	0.027080766	2.905525454	1.538799094	2
Stem vs Leaf	Trollioside	Phenolic acids	Phenolic acids	1.15840774	0.002438871	0.023017917	0.095265601	-3.391900822	3
Stem vs Leaf	Isosclerone;(4S)-4,	Quinones	Quinones	1.102883936	0.001388197	0.017778885	0.214010812	-2.22424441	3

Comparison	Compound	Class I	Class II	VIP	P-value	FDR	Fold Change	Log_2FC	MSI Level
	8-Dihydroxy-3,4-Dihydronaphthalene-1(2H)-One								
Stem vs Leaf	3,3'-Bis(3,4-dihydro-4-hydroxy-6,8-dimethoxy-2H-1-benzopyran)	Lignans and Coumarins	Lignans	1.180307534	0.004239041	0.028481053	0.088522331	-3.497814757	3
Stem vs Leaf	Tamarixetin (3,3',5,7-Tetrahydroxy-4'-Methoxyflavone)	Flavonoids	Flavonols	1.155405623	0.074473256	0.125189602	18.06616093	4.17521806	2
Stem vs Leaf	9-Oxodemethylmyricanane B	Others	Others	1.136209749	0.084811434	0.136870736	9.889514545	3.305899704	2

Comparison	Compound	Class I	Class II	VIP	P-value	FDR	Fold Change	Log_2FC	MSI Level
Stem vs Leaf	Robinlin	Terpenoids	Monoterpenoids	1.173759513	0.003632537	0.027890607	0.114734623	-3.123627281	3
Stem vs Leaf	4'-Demethyleucomin glucoside*	Flavonoids	Other Flavonoids	1.136340267	0.077437299	0.128728271	0.096912859	-3.367168079	3
Stem vs Leaf	Leucasperoside B	Others	Others	1.181346502	0.001136789	0.016734829	0.066201272	-3.916997247	3
Stem vs Leaf	Esculetin-6-O-quinic acid	Lignans and Coumarins	Coumarins	1.15083597	0.05486446	0.102929846	0.116826988	-3.097554503	3
Stem vs Leaf	Neoeriocitrin*	Flavonoids	Flavones	1.123606417	0.107417278	0.162181985	0.08600392	-3.539453769	3
Stem vs Leaf	O-Phosphorylethanolamine	Alkaloids	Alkaloids	1.158854522	0.027598813	0.066972289	3.854003814	1.946357999	3
Stem vs Leaf	myricetin-3-O-β-D-xylopyranosyl-	Flavonoids	Flavonols	1.067955312	0.084154663	0.136039493	0.258981407	-1.949079571	3

Comparison	Compound	Class I	Class II	VIP	P-value	FDR	Fold Change	Log_2FC	MSI Level
	(1→2)-β-D-gluco pyranoside								
Stem vs Leaf	Hydroxydihydro bovolide*	Others	Lactones	1.158398756	0.005926258	0.032023409	0.097760147	-3.354609728	1
Stem vs Leaf	3-O-caffeoyl quininyl arabinoside	Others	Others	1.155301493	0.056835787	0.10505299	7.210768525	2.85015303	3
Stem vs Leaf	3,3',4,5'-Tetrahydr oxybibenzyl	Others	Stilbene	1.074474804	0.01154199	0.041808314	9.949919118	3.314684798	2
Stem vs Leaf	Lyoniresinol-9'-O -xyloside (Lyoniside)	Lignans and Coumarins	Lignans	1.122160178	0.023544602	0.061264624	22.3952423	4.48512037	3
Stem vs Leaf	2-Aminoethylpho	Others	Others	1.11166801	7.1854E-05	0.005327104	0.069706949	-3.842553711	3

Comparison	Compound	Class I	Class II	VIP	P-value	FDR	Fold Change	Log_2FC	MSI Level
	sphonate								
Stem vs Leaf	Homomevalonic Acid Glucoside	Organic acids	Organic acids	1.07060817	0.020686893	0.056875729	0.481850625	-1.05334212	1
Stem vs Leaf	Succinyl arginine	Amino acids and derivatives	Amino acids and derivatives	1.131789923	0.002709738	0.024430315	0.036940311	-4.758660187	3
Stem vs Leaf	3-O-Trans-p-Coumaroylmaslinic Acid*	Terpenoids	Triterpene	1.081471508	0.033819003	0.075426199	2.40197058	1.264218481	1
Stem vs Leaf	Phloretin-2'-O-(6''-O-acetyl)glucoside	Flavonoids	Chalcones	1.142232165	0.012064437	0.042522196	5.617603442	2.489954785	2
Stem vs Leaf	Orcinol	Phenolic	Phenolic acids	1.152022637	0.038709838	0.082729773	5.744139944	2.522090898	3

Comparison	Compound	Class I	Class II	VIP	P-value	FDR	Fold Change	Log_2FC	MSI Level
	glucoside*	acids							
Stem vs Leaf	Uridine 5'-diphospho-N-acetylglucosamine	Nucleotides and derivatives	Nucleotides and derivatives	1.151423153	0.064127768	0.113851941	21.98101571	4.458186147	2
Stem vs Leaf	Glucosyl-[6''-(3-hydroxy-3-methylglutaryl)]-Isorhamnetin	Flavonoids	Flavonols	1.175784312	0.035103459	0.077487102	36.00165761	5.169991428	3
Stem vs Leaf	N6-(2-Hydroxyethyl)adenosine*	Nucleotides and derivatives	Nucleotides and derivatives	1.10991098	0.010044008	0.039623149	0.104165044	-3.263056886	3
Stem vs Leaf	3,4-Secours-4(23), 12-dien-3-oic acid	Terpenoids	Triterpene	1.097051178	0.124656458	0.179151995	18.22643622	4.187960597	2

Comparison	Compound	Class I	Class II	VIP	P-value	FDR	Fold Change	Log_2FC	MSI Level
	(Roburic acid)								
Stem vs Leaf	Quercetin-3-O-(2''-O-malonyl)glucoside-7-O-arabinoside	Flavonoids	Flavonols	1.163134	0.025183052	0.063689739	5.908554572	2.562805243	3
Stem vs Leaf	cis-2,4-Dihydroxy-2-(2-hydroxyethyl)cyclohex-5-en-1-one glucoside	Others	Others	1.037199361	0.042591975	0.087966135	7.648356227	2.935149719	3
Stem vs Leaf	Viscumneoside V1	Flavonoids	Flavanones	1.1645625	0.030106084	0.07033949	7.109851024	2.829819331	2
Stem vs Leaf	Fustin	Flavonoids	Flavanonols	1.13943995	0.047433371	0.094391372	5.544146028	2.470965257	2
Stem vs Leaf	Grayanotoxin III	Terpenoids	Ditepenoids	1.015728176	0.065925284	0.116100452	0.347113619	-1.526520123	3

Comparison	Compound	Class I	Class II	VIP	P-value	FDR	Fold Change	Log_2FC	MSI Level
Stem vs Leaf	Machilusolide D	Others	Lactones	1.150931318	0.061194836	0.110655086	8.136322849	3.024376926	3
Stem vs Leaf	6-O-p-Methoxycinnamoylcatalpol	Terpenoids	Monoterpenoids	1.154744916	0.020497925	0.05650069	0.207936693	-2.265783734	3
Stem vs Leaf	Methyl 4-hydroxybenzoate	Phenolic acids	Phenolic acids	1.125032033	0.000555796	0.013412103	10.7802398	3.430317365	3
Stem vs Leaf	1-O-p-Coumaroyl-β-D-glucose*	Phenolic acids	Phenolic acids	1.072536922	0.092089943	0.145369587	0.086882667	-3.524787796	3
Stem vs Leaf	Isofraxidin 7-O-Apiosylglucoside*	Lignans and Coumarins	Coumarins	1.175924441	0.019912634	0.055784421	17.57747719	4.135656117	2
Stem vs Leaf	Tricaffeoyl Spermidine	Alkaloids	Phenolamine	1.020339971	0.000989171	0.016111501	0.183965095	-2.442496033	3

Comparison	Compound	Class I	Class II	VIP	P-value	FDR	Fold Change	Log_2FC	MSI Level
Stem vs Leaf	Cordycepin (3'-Deoxyadenosine)*	Nucleotides and derivatives	Nucleotides and derivatives	1.078319945	0.066350049	0.116256393	0.216783933	-2.205670263	3
Stem vs Leaf	2,6-Dimethoxy-4-(2-Hydroxyethyl)Phenyl Beta-D-Glucopyranoside	Others	Alcohol compounds	1.144896809	0.001105097	0.016559547	4.022949905	2.008253773	1
Stem vs Leaf	Reseoside*	Terpenoids	Monoterpenoids	1.130211338	0.004895508	0.030072406	0.038888333	-4.684518794	2
Stem vs Leaf	Protocatechuic acid 4-O-(6''-O-Feruloyl)Glucoside*	Phenolic acids	Phenolic acids	1.150095586	0.000725222	0.014479152	8.733294064	3.126525919	1

Comparison	Compound	Class I	Class II	VIP	P-value	FDR	Fold Change	Log_2FC	MSI Level
Stem vs Leaf	1,4,8-Trihydroxynaphthalene-1-O-[6'-O-(3'',4'',5''-trimethylbenzoyl)]glucoside	Quinones	Quinones	1.172381797	0.050621067	0.098049814	33.3033281	5.057594452	3
Stem vs Leaf	Glucosyl 2,6-dimethyl-8-oxoocta-2,6-dienoic acid	Others	Others	1.031310797	0.046424933	0.093109707	0.444977442	-1.168195895	3
Stem vs Leaf	Salicin	Others	Others	1.176146411	0.005980606	0.032145757	9.255094915	3.210247786	3
Stem vs Leaf	Gnetifolin B*	Flavonoids	Flavonols	1.16512449	0.058840866	0.107119274	14.51085495	3.859060618	3
Stem vs Leaf	2-Isopropylmalic Acid	Organic acids	Organic acids	1.146543055	0.001464888	0.018029486	0.258541027	-1.951534862	3

Comparison	Compound	Class I	Class II	VIP	P-value	FDR	Fold Change	Log_2FC	MSI Level
Stem vs Leaf	N,N-diethyl-5-hydroxytryptamine oxalate	Alkaloids	Alkaloids	1.011672868	0.029663662	0.0697015	0.077104286	-3.697045135	3
Stem vs Leaf	Dihydroxyacetone phosphate	Others	Ketone compounds	1.166612417	0.000327175	0.010948842	0.241089926	-2.052356726	3
Stem vs Leaf	N-(1-Deoxy-1-fructosyl)Tyrosine	Amino acids and derivatives	Amino acids and derivatives	1.085798078	0.007668141	0.03555507	0.0831008	-3.58899383	3
Stem vs Leaf	3-Hydroxy-Beta-Ionone 3-(6''-Malonyl)Glucoside	Others	Others	1.171224673	0.02752071	0.066858221	0.083209317	-3.587111108	3
Stem vs Leaf	(-)-Isolariciresinol	Lignans and	Lignans	1.088508653	0.084611252	0.136674825	4.012649037	2.004554978	3

Comparison	Compound	Class I	Class II	VIP	P-value	FDR	Fold Change	Log_2FC	MSI Level
	9'-O-glucoside	Coumarins							
Stem vs Leaf	Sinapoylcaffeoyltartaric acid	Phenolic acids	Phenolic acids	1.145270162	0.060939271	0.110285717	0.107657423	-3.215480304	3
Stem vs Leaf	Malonyl Wistin*	Flavonoids	Isoflavones	1.110746364	0.07965837	0.131237927	4.876316622	2.285791804	3
Stem vs Leaf	Glu-Arg	Amino acids and derivatives	Amino acids and derivatives	1.067756508	0.009279636	0.038406318	0.338447154	-1.562997512	3
Stem vs Leaf	Naringenin-6-C-Glucoside	Flavonoids	Flavanones	1.17414565	0.000106394	0.006182349	0.385854091	-1.373872695	3
Stem vs Leaf	2-(Dimethylamino)guanosine*	Nucleotides and derivatives	Nucleotides and derivatives	1.177801053	0.010803406	0.040787888	0.105481047	-3.244944295	3

Comparison	Compound	Class I	Class II	VIP	P-value	FDR	Fold Change	Log_2FC	MSI Level
Stem vs Leaf	Isololiolide	Terpenoids	Monoterpenoids	1.072822286	0.049073083	0.096461148	0.4888771	-1.032456267	3
Stem vs Leaf	6'-O-D-Glucosylsweroside	Terpenoids	Monoterpenoids	1.045502029	0.00416071	0.028378135	5.853559921	2.549314286	3
Stem vs Leaf	galloyl acetyl glucoside	Phenolic acids	Phenolic acids	1.156461226	0.033440091	0.07504822	6.916670912	2.790077816	3
Stem vs Leaf	N-(Isoferuloyl)-Glutamic Acid	Alkaloids	Phenolamine	1.114788544	0.003226312	0.026581091	0.388994981	-1.362176555	3
Stem vs Leaf	5'-Demethylaquillochin	Lignans and Coumarins	Lignans	1.137832961	0.094089662	0.148091342	8.767350951	3.132140999	3
Stem vs Leaf	Prunetin-5-O-glucoside	Flavonoids	Isoflavones	1.1806066	0.002276208	0.022493448	0.16041569	-2.640112839	3

Comparison	Compound	Class I	Class II	VIP	P-value	FDR	Fold Change	Log_2FC	MSI Level
Stem vs Leaf	2,3,6,23-Tetrahydroxyurs-12-en-28-oic acid (Madecassic acid)	Terpenoids	Triterpene	1.180289809	0.004563239	0.029286859	13.92601233	3.7997103	2
Stem vs Leaf	Chrysoeriol; 5,7,4'-Trihydroxy-3'-Methoxyflavone	Flavonoids	Flavones	1.171776446	0.037648626	0.081188111	14.56720375	3.864652066	3
Stem vs Leaf	Oleanolic acid-3-O-xylosyl(1→3)glucuronide	Terpenoids	Triterpene Saponin	1.14189991	0.017113544	0.052042603	5.405654259	2.434469241	3
Stem vs Leaf	Ibotalactone B	Terpenoids	Monoterpenoids	1.176476299	0.000941819	0.015696982	0.443383225	-1.173373906	3

Comparison	Compound	Class I	Class II	VIP	P-value	FDR	Fold Change	Log_2FC	MSI Level
Stem vs Leaf	Balanophonin	Lignans and Coumarins	Lignans	1.161903269	0.043037213	0.088614838	11.69445746	3.547753028	3
Stem vs Leaf	D-Melezitose	Others	Saccharides	1.103475842	0.124192349	0.178604381	7.434774063	2.894288901	3
Stem vs Leaf	8-Methoxy malonyl ononin*	Flavonoids	Isoflavones	1.134086888	0.033634682	0.07524929	3.838488456	1.940538309	3
Stem vs Leaf	6-O-p-Coumaroyl -β-D-glucose*	Phenolic acids	Phenolic acids	1.177425443	0.016427539	0.050482096	0.046677189	-4.421138498	3
Stem vs Leaf	Viscumneoside I*	Flavonoids	Flavanones	1.056563329	0.039469284	0.083769951	0.113694451	-3.136766246	3
Stem vs Leaf	1-O-Galloyl-3-O-Feruloyl-β-D-glucose	Phenolic acids	Phenolic acids	1.176375961	0.012798329	0.043885816	0.110393865	-3.179268094	3
Stem vs Leaf	1-O-Galloyl-4-O-F	Phenolic	Phenolic acids	1.162612778	0.034647122	0.076874419	0.130021519	-2.943177679	3

Comparison	Compound	Class I	Class II	VIP	P-value	FDR	Fold Change	Log_2FC	MSI Level
	eruloyl-β-D-glucose*	acids							
Stem vs Leaf	Aracarpene 1	Flavonoids	Isoflavones	1.16285182	0.071085465	0.121104397	37.07897139	5.212529317	3
Stem vs Leaf	3,5,4'-Tetrahydroxystilbene-2-O-β-D-glucoside*	Others	Stilbene	1.170107129	0.029644798	0.0697015	0.076796621	-3.70281335	3
Stem vs Leaf	5-(2-Hydroxypropyl)hygrine	Alkaloids	Pyrrole alkaloids	1.133927469	0.044401241	0.090400254	3.156824959	1.658474268	3
Stem vs Leaf	linoleoyl ethanolamine	Lipids	LPE	1.180978701	0.001414042	0.017778885	5.288344558	2.402816178	3
Stem vs Leaf	Tricin-4'-O-glucoside*	Flavonoids	Flavones	1.145372476	0.004784368	0.029643781	4.235036368	2.082374359	3

Comparison	Compound	Class I	Class II	VIP	P-value	FDR	Fold Change	Log_2FC	MSI Level
Stem vs Leaf	Proanthocyanidins	Tannins	Proanthocyanidins	1.124245268	0.097217385	0.151447189	0.069458204	-3.847711076	3
Stem vs Leaf	Mangiferin	Flavonoids	Other Flavonoids	1.158773279	0.018756974	0.054349722	7.130070633	2.833916369	3
Stem vs Leaf	Syringaresinol	Lignans and Coumarins	Lignans	1.179880077	0.003919129	0.028194402	7.14481015	2.836895677	1
Stem vs Leaf	Aracarpene 2*	Flavonoids	Isoflavones	1.163707439	0.058700191	0.107119274	13.73888752	3.780193284	3
Stem vs Leaf	Syringopicroside	Terpenoids	Monoterpenoids	1.149375621	0.050845628	0.098307645	4.922651807	2.299435697	2
Stem vs Leaf	Coumarin-3-carboxylic Acid	Lignans and Coumarins	Coumarins	1.138439465	0.014063853	0.046517843	0.025543395	-5.290905905	3
Stem vs Leaf	12,13-DHOME;	Lipids	Free fatty acids	1.139195499	0.001099308	0.016559547	4.969054776	2.312971445	2

Comparison	Compound	Class I	Class II	VIP	P-value	FDR	Fold Change	Log_2FC	MSI Level
	(9Z)-12,13-Dihydroxyoctadec-9-enoic acid								
Stem vs Leaf	1-O-Galloyl-4-O-p-Coumaroyl-β-D-glucose	Phenolic acids	Phenolic acids	1.078238744	0.010161048	0.039720462	8.8528563	3.146143004	3
Stem vs Leaf	Kaempferol-3-O-(4''-O-acetyl)rhamnoside	Flavonoids	Flavonols	1.166820939	0.028282447	0.067816745	0.085811885	-3.542678717	2
Stem vs Leaf	Dehydroxy-Dihydroabscisic Acid 4'-O-glucoside	Organic acids	Organic acids	1.131187412	0.019544167	0.055577281	0.188789382	-2.405150467	3
Stem vs Leaf	Hexadecanedioic	Lipids	Free fatty acids	1.123474691	0.00139589	0.017778885	2.449467859	1.292468362	1

Comparison	Compound	Class I	Class II	VIP	P-value	FDR	Fold Change	Log_2FC	MSI Level
	acid								
Stem vs Leaf	1,3,6-trihydroxy-7-methoxy-2-{[(2s,3r,4s,5s,6r)-3,4,5-trihydroxy-6-(hydroxymethyl)oxan-2-yl]oxy}xanthen-9-one	Flavonoids	Other Flavonoids	1.177628375	0.019287163	0.055216246	0.050014039	-4.321523078	2
Stem vs Leaf	p-Coumaric acid	Phenolic acids	Phenolic acids	1.177290461	0.023808869	0.061747973	0.040105034	-4.640072836	3
Stem vs Leaf	3-Hydroxy-5-Methylphenol-1-O-Glucoside*	Phenolic acids	Phenolic acids	1.162512156	0.053722385	0.10176487	11.66607313	3.544247118	3

Comparison	Compound	Class I	Class II	VIP	P-value	FDR	Fold Change	Log_2FC	MSI Level
Stem vs Leaf	Citric acid-1-O-diglucoside	Organic acids	Organic acids	1.06789188	0.013311034	0.044997992	0.415439548	-1.267289535	1
Stem vs Leaf	Apigenin-5-O-glucoside	Flavonoids	Flavones	1.014011371	0.083525818	0.135634826	0.165957131	-2.591117469	3
Stem vs Leaf	1-O-Feruloyl-β-D-glucose	Phenolic acids	Phenolic acids	1.173760474	0.003048741	0.025963636	3.867488232	1.951396902	3
Stem vs Leaf	Sorbitol-6-phosphate	Others	Saccharides	1.168568592	0.02584749	0.064103755	0.151779223	-2.719953784	3
Stem vs Leaf	10-Dehydrogeniposide	Terpenoids	Monoterpenoids	1.176226124	0.018369363	0.053806718	0.060729658	-4.041454945	3
Stem vs Leaf	N-Hydroxyoctanoyl nornicotine	Alkaloids	Pyridine alkaloids	1.119497218	0.099975792	0.154750146	12.95882576	3.695863091	3

Comparison	Compound	Class I	Class II	VIP	P-value	FDR	Fold Change	Log_2FC	MSI Level
	glucoside								
Stem vs Leaf	12,13-Dihydroursolic acid	Terpenoids	Triterpene	1.119676254	0.0321264	0.073115356	3.19546047	1.67602384	3
Stem vs Leaf	5,7,2'-Trihydroxy-8-methoxyflavone*	Flavonoids	Flavones	1.167583411	0.042561274	0.087966135	13.49204567	3.754037202	3
Stem vs Leaf	6-DeoxyCatalpol	Terpenoids	Monoterpenoids	1.133053428	0.066238007	0.116254461	7.147621324	2.837463204	2
Stem vs Leaf	1,3,6,7-tetrahydroxy-2-(3,4,5-trihydroxyoxan-2-yl)xanthen-9-one	Flavonoids	Other Flavonoids	1.144837179	0.026914882	0.06590774	0.217694903	-2.199620467	3
Stem vs Leaf	1-O-Galloyl-6-O-F	Phenolic	Phenolic acids	1.178656306	0.007023802	0.034165551	0.123052155	-3.022658168	3

Comparison	Compound	Class I	Class II	VIP	P-value	FDR	Fold Change	Log_2FC	MSI Level
	eruloyl-β-D-glucose*	acids							
Stem vs Leaf	Pierisfomoside D	Others	Others	1.178666508	0.010387141	0.04009399	0.074484185	-3.74692205	3
Stem vs Leaf	Phthalic acid	Phenolic acids	Phenolic acids	1.051797883	0.131818664	0.18682276	0.294089934	-1.765670692	3
Stem vs Leaf	(2R,3R,4S,5S,6R)-2-[3-(2,4-dihydroxyphenyl)propoxy]-6-(hydroxymethyl)oxane-3,4,5-triol	Phenolic acids	Phenolic acids	1.147755237	0.075677851	0.126719143	0.103556415	-3.271511171	3
Stem vs Leaf	5-O-Dihydrocaffeoyl Quinic Acid	Phenolic acids	Phenolic acids	1.17975517	0.007195166	0.034654934	0.052290746	-4.257300542	1

Comparison	Compound	Class I	Class II	VIP	P-value	FDR	Fold Change	Log_2FC	MSI Level
Stem vs Leaf	3-(3'-Hydroxybutyl)-2,4,4-trimethylcyclohexa-2,5-dienone glucoside	Terpenoids	Sesquiterpenoids	1.140078834	0.007221121	0.034654934	0.025537638	-5.291231076	3
Stem vs Leaf	Icariside B2	Terpenoids	Sesquiterpenoids	1.170354198	0.082002741	0.133767749	0.013277337	-6.234890365	3
Stem vs Leaf	Limocitrin malonyl glucoside	Flavonoids	Flavonols	1.150765043	0.024541155	0.062963583	86.5954651	6.43621957	3
Stem vs Leaf	5-O-Methylvisammioside	Others	Chromone	1.028684669	0.00331684	0.026708637	0.12038714	-3.054246805	3
Stem vs Leaf	2-β-D-Glucopyranosyloxy-5-hydro	Phenolic acids	Phenolic acids	1.134183542	0.011261364	0.041529903	8.562566145	3.098043228	1

Comparison	Compound	Class I	Class II	VIP	P-value	FDR	Fold Change	Log_2FC	MSI Level
	xyphenylacetic acidmethylester*								
Stem vs Leaf	Persicarin (Isohamnetin-3-O-sulfate)	Flavonoids	Flavones	1.18128086	0.004338393	0.02880574	138.7141996	7.115971668	3
Stem vs Leaf	Dicaffeoylspermine	Alkaloids	Phenolamine	1.138443328	9.74763E-05	0.005954146	7.178518738	2.84368618	3
Stem vs Leaf	D-Mannitol*	Others	Saccharides	1.154574682	0.082317794	0.134179876	46.39024407	5.535749531	1
Stem vs Leaf	Grayanotoxin IX	Terpenoids	Ditepenoids	1.148189773	0.010500125	0.040169517	0.014879658	-6.070514863	3
Stem vs Leaf	D-Ribose	Others	Saccharides	1.178191995	0.000680914	0.014479152	0.06088265	-4.037825029	3
Stem vs Leaf	LysoPC 16:2(2n isomer)	Lipids	LPC	1.163025679	0.037622487	0.081188111	6.970255021	2.801211441	3

Comparison	Compound	Class I	Class II	VIP	P-value	FDR	Fold Change	Log_2FC	MSI Level
Stem vs Leaf	2,3-Dihydroxyolean-12-en-28-oic acid	Terpenoids	Triterpene	1.105554905	0.027088337	0.066181732	3.901133129	1.963893232	3
Stem vs Leaf	Protocatechuic acid-1'-O-xyloside	Phenolic acids	Phenolic acids	1.144719987	0.010258463	0.040028485	0.289961422	-1.786067124	2
Stem vs Leaf	Ferulic acid*	Phenolic acids	Phenolic acids	1.046112968	0.302335144	0.359723608	22.84856367	4.514031571	3
Stem vs Leaf	2,4,6,4'-Tetrahydroxy-stilbene-2-O-glucoside	Others	Stilbene	1.109443863	0.019027633	0.05491196	0.266233386	-1.909236599	3
Stem vs Leaf	Kaempferol-3-O-sophorotrioside*	Flavonoids	Flavonols	1.172392257	0.018704253	0.054343439	0.138380875	-2.853283527	3
Stem vs Leaf	Apiosylrhododen	Phenolic	Phenolic acids	1.116397688	0.00052651	0.013142389	16.83604803	4.073481626	3

Comparison	Compound	Class I	Class II	VIP	P-value	FDR	Fold Change	Log_2FC	MSI Level
	drin	acids							
Stem vs Leaf	2-Hydroxy-3-phenylpropanoic acid	Phenolic acids	Phenolic acids	1.120554999	0.055378718	0.103474763	0.03341274	-4.903457897	1
Stem vs Leaf	Ageconyflavone B-4'-glucoside*	Flavonoids	Flavones	1.1752566	0.020984753	0.057255355	0.08487949	-3.558440209	2
Stem vs Leaf	5-hydroxy-3,4-dimethyl-5-pentylfuran-2(5H)-one*	Others	Lactones	1.169409221	0.00227441	0.022493448	0.111548457	-3.164257543	1
Stem vs Leaf	N-(1-Deoxy-1-fructosyl)Tryptophan	Amino acids and derivatives	Amino acids and derivatives	1.041704997	0.021536479	0.058015292	6.221071049	2.637162983	3
Stem vs Leaf	11-Oxo-kansenonol	Terpenoids	Triterpene	1.144773319	0.07424709	0.125004889	22.71442552	4.505536913	3

Comparison	Compound	Class I	Class II	VIP	P-value	FDR	Fold Change	Log_2FC	MSI Level
Stem vs Leaf	Grayanotoxin V	Terpenoids	Ditepenoids	1.15957809	0.044883679	0.091037652	0.139552095	-2.841124311	3
Stem vs Leaf	9,11-dimethoxy-2h-[1,3]dioxolo[4,5-b]xanthen-10-one*	Flavonoids	Other Flavonoids	1.167918098	0.048973986	0.096461148	13.64061097	3.76983636	3
Stem vs Leaf	Herbacetin	Flavonoids	Flavonols	1.052465396	0.063298157	0.113032423	2.275374342	1.186103915	1
Stem vs Leaf	Afrormosin-7-O-(6''-malonyl)glucoside*	Flavonoids	Isoflavones	1.138444332	0.054193173	0.102296157	4.553644097	2.187021538	3
Stem vs Leaf	Isolariciresinol-9'-O-xyloside	Lignans and Coumarins	Lignans	1.139134983	0.010870446	0.040787888	19.53561905	4.288035067	3
Stem vs Leaf	5-O-(3'-O-Glucosylcaffeoyl)Quinic	Phenolic acids	Phenolic acids	1.178426949	0.010319849	0.040049956	0.062244165	-4.005917594	1

Comparison	Compound	Class I	Class II	VIP	P-value	FDR	Fold Change	Log_2FC	MSI Level
	Acid								
Stem vs Leaf	2,3,5,4'-Tetrahydroxystilbene-2-O-glucoside*	Others	Stilbene	1.131121615	0.002518945	0.023444726	0.037716287	-4.728668539	3
Stem vs Leaf	Xanthorin	Quinones	Anthraquinone	1.17015665	0.035605984	0.078035541	12.60734078	3.6561921	3
Stem vs Leaf	1'-Galactoyl Ferulic acid	Phenolic acids	Phenolic acids	1.126583003	0.002574595	0.023734898	0.357803958	-1.482758749	2
Stem vs Leaf	Ribulose-5-phosphate	Others	Saccharides	1.106539049	0.00197811	0.020746033	2.518043199	1.332303034	3
Stem vs Leaf	1-p-Coumaroyl-3-feruloyl-sucrose*	Phenolic acids	Phenolic acids	1.098594745	9.49095E-05	0.005954146	0.075570484	-3.726033331	1
Stem vs Leaf	2,6-Dimethyl-7-oc	Terpenoids	Monoterpenoid	1.169363192	0.009722477	0.039440238	5.24644492	2.391340159	3

Comparison	Compound	Class I	Class II	VIP	P-value	FDR	Fold Change	Log_2FC	MSI Level
	tene-2,3,6-triol		s						
Stem vs Leaf	Rubrofusarin-6-O -glucoside	Others	Ketone compounds	1.042617395	0.001109105	0.016559547	0.209330079	-2.256148464	3
Stem vs Leaf	Kaempferide-3-O-(6'-O-acetyl)gluco side	Flavonoids	Flavonols	1.180337118	0.011790863	0.042066224	0.015184559	-6.041251145	2
Stem vs Leaf	Ile-Trp	Amino acids and derivatives	Amino acids and derivatives	1.17759197	0.019332999	0.055273867	0.030015899	-5.058129291	3
Stem vs Leaf	Tamarixetin-3-O-r utinoside	Flavonoids	Flavonols	1.179316004	0.016019662	0.049628637	0.022444602	-5.477487667	3
Stem vs Leaf	Swertiamarin	Terpenoids	Monoterpenoid s	1.126183652	0.009397972	0.03863411	0.335444042	-1.575855977	3

Comparison	Compound	Class I	Class II	VIP	P-value	FDR	Fold Change	Log_2FC	MSI Level
Stem vs Leaf	4,5,6-Trihydroxy-2-cyclohexen-1-ylideneacetonitrile	Alkaloids	Alkaloids	1.175551283	0.026655967	0.065596583	0.049342467	-4.34102633	3
Stem vs Leaf	4-(Beta-D-Glucopyranosyloxy)-2,4',6-Trihydroxybenzophenone	Others	Ketone compounds	1.14167819	0.016659636	0.050805983	0.21418043	-2.22310143	2
Stem vs Leaf	Glu-Ser	Amino acids and derivatives	Amino acids and derivatives	1.179150139	0.043751724	0.089501623	475.7539761	8.894071904	3
Stem vs Leaf	3'-p-Coumaroyl-3-feruloyl-sucrose*	Phenolic acids	Phenolic acids	1.179813205	0.003749587	0.028089239	0.146325887	-2.772743072	1
Stem vs Leaf	2R-hydroxy-9Z,12	Lipids	Free fatty acids	1.111279363	0.063987485	0.113790812	20.47728765	4.355952729	2

Comparison	Compound	Class I	Class II	VIP	P-value	FDR	Fold Change	Log_2FC	MSI Level
	Z,15Z-octadecatrienoic acid								
Stem vs Leaf	9-Oxo-10,12-Octadecadienoic Acid	Lipids	Free fatty acids	1.132687529	0.108662435	0.163602405	9.028287041	3.174452288	3
Stem vs Leaf	Quercetin-5,4'-di-O-glucoside	Flavonoids	Flavonols	1.18129317	0.001413425	0.017778885	0.083342264	-3.58480789	3
Stem vs Leaf	3-Methyl-4,8-dihydroxy-3,4-dihydroisocoumarin	Lignans and Coumarins	Coumarins	1.152388952	0.006815108	0.033761479	0.227928914	-2.133344145	3
Stem vs Leaf	2'-O-Methyladenosine	Nucleotides and derivatives	Nucleotides and derivatives	1.159637477	0.038956764	0.083174818	0.115192583	-3.117880265	3
Stem vs Leaf	6-beta-d-glucopyr	Alkaloids	Plumerane	1.15460155	0.02771644	0.067106245	0.060036136	-4.058025052	3

Comparison	Compound	Class I	Class II	VIP	P-value	FDR	Fold Change	Log_2FC	MSI Level
	anosyloxyindole-3-carboxylic acid								
Stem vs Leaf	8,11-Heptadecadienoic acid	Lipids	Free fatty acids	1.131461167	0.015371376	0.04873623	3.521127558	1.816037492	2
Stem vs Leaf	L-Glycyl-L-isoleucine	Amino acids and derivatives	Amino acids and derivatives	1.174700799	0.015408402	0.04873623	0.1229911	-3.023374178	3
Stem vs Leaf	1-(2,3-dihydroxypropoxy)-3-(((2-(dimethylamino)ethoxy)(hydroxy)phosphoryl)oxy)propan-2-yl (E)-hexadec-9-eno	Lipids	LPE	1.172245243	0.025561971	0.063904927	12.13963445	3.601653075	2

Comparison	Compound	Class I	Class II	VIP	P-value	FDR	Fold Change	Log_2FC	MSI Level
	ate								
Stem vs Leaf	Asp-Pro-Phe	Amino acids and derivatives	Amino acids and derivatives	1.178627462	0.00791446	0.036127578	0.10614972	-3.235827524	3
Stem vs Leaf	4-O-Galactoyl p-Coumaric acid*	Phenolic acids	Phenolic acids	1.094244313	0.036800221	0.080204277	0.080627682	-3.632580944	2
Stem vs Leaf	Glucosyl 7-methyl-3-methyleneoctane-1,2,6,7-tetraol	Terpenoids	Monoterpenoids	1.072377332	0.001929243	0.020636181	10.25540922	3.358313156	3
Stem vs Leaf	Sibiricose A2	Phenolic acids	Phenolic acids	1.153714645	0.076619165	0.127896898	0.101424679	-3.301519353	3
Stem vs Leaf	Coniferyaldehyde	Lignans and	Lignans	1.179390254	0.009972628	0.03961495	0.041503216	-4.590633066	2

Comparison	Compound	Class I	Class II	VIP	P-value	FDR	Fold Change	Log_2FC	MSI Level
	Glucoside	Coumarins							
Stem vs Leaf	2-Feruloyl-6-(4-hydroxycinnamoyl) glucoside	Phenolic acids	Phenolic acids	1.077555204	0.013767945	0.04625169	11.44644333	3.516827484	2
Stem vs Leaf	Flavin Single Nucleotide(FMN)	Nucleotides and derivatives	Nucleotides and derivatives	1.180881161	0.003652613	0.027890607	0.057560367	-4.118780412	3
Stem vs Leaf	Guanosine 3',5'-cyclic monophosphate	Nucleotides and derivatives	Nucleotides and derivatives	1.179075758	0.010865869	0.040787888	0.048193557	-4.375015898	3
Stem vs Leaf	Ajugoside	Terpenoids	Monoterpenoids	1.100772108	0.077569701	0.128783674	3.670598486	1.876015312	2
Stem vs Leaf	2,3,4-Trihydroxyb	Phenolic	Phenolic acids	1.096039067	0.003239184	0.026581091	0.284447185	-1.813767291	3

Comparison	Compound	Class I	Class II	VIP	P-value	FDR	Fold Change	Log_2FC	MSI Level
	enzoic acid	acids							
Stem vs Leaf	7S,8S-DiHODE; (9Z,12Z)-(7S,8S)-Dihydroxyoctadeca-9,12-dienoic acid	Lipids	Free fatty acids	1.173256102	0.017849557	0.053153113	11.91911783	3.575205557	2
Stem vs Leaf	Grasshopper ketone	Terpenoids	Monoterpenoids	1.059636925	0.01836431	0.053806718	0.369375725	-1.43683904	3
Stem vs Leaf	2,4,6-Trimethoxyphenyl 1-O-β-D-apiofuranosyl-(1→6)-β-D-glucopyranoside	Others	Others	1.17203936	0.032031795	0.073108662	20.62498539	4.366321192	3

Comparison	Compound	Class I	Class II	VIP	P-value	FDR	Fold Change	Log_2FC	MSI Level
Stem vs Leaf	3-Hydroxyurs-12-en-28-oic acid (Ursolic acid)	Terpenoids	Triterpene	1.027056411	0.172923476	0.229804605	0.216401882	-2.208215049	1
Stem vs Leaf	2'-Deoxyinosine-5'-monophosphate	Nucleotides and derivatives	Nucleotides and derivatives	1.161822295	0.064333565	0.113935062	819.3422348	9.678322373	3
Stem vs Leaf	Isocitric acid-1-O-diglucoside	Organic acids	Organic acids	1.13657434	0.001484288	0.018029486	0.372524218	-1.424593876	3
Stem vs Leaf	Kelampayoside A[3,4,5-Trimethoxyphenol-β-D-apiosyl-(1→6)-β-D-glucoside]	Phenolic acids	Phenolic acids	1.181477868	0.001167546	0.016734829	28.1635761	4.815758628	3

Comparison	Compound	Class I	Class II	VIP	P-value	FDR	Fold Change	Log_2FC	MSI Level
Stem vs Leaf	2,2-Dimethylsuccinic acid	Organic acids	Organic acids	1.1716634	0.030729347	0.071431365	12.3561516	3.627157572	3
Stem vs Leaf	N-Methyl-α-aminoisobutyric acid	Amino acids and derivatives	Amino acids and derivatives	1.17829849	0.04361574	0.089393557	335.6961884	8.391012346	3
Stem vs Leaf	D-Cellobiose	Others	Saccharides	1.109422573	0.045497053	0.09210797	7.595550586	2.925154547	3
Stem vs Leaf	Sucrose-6-phosphate	Others	Saccharides	1.084641436	0.08061883	0.132111649	4.152596605	2.054013731	3
Stem vs Leaf	Isocarlinoside	Flavonoids	Flavones	1.16699076	0.005470035	0.031195162	5.811314576	2.538864552	3
Stem vs Leaf	5-O-β-D-Glucopyranosyl-3-hydrobenzo(b)fu-ran-2-one	Others	Lactones	1.122717755	0.032883207	0.074341635	2.282685547	1.190732134	3

Comparison	Compound	Class I	Class II	VIP	P-value	FDR	Fold Change	Log_2FC	MSI Level
Stem vs Leaf	Coclaurine	Alkaloids	Isoquinoline alkaloids	1.162199925	0.026318005	0.064889578	4.307248438	2.106766539	3
Stem vs Leaf	Kaempferol-3-(2''-acetylrhamnoside)	Flavonoids	Flavonols	1.105826794	0.036689096	0.080204277	0.059874806	-4.061907115	1
Stem vs Leaf	4-hydroxyphenyl acrylaldehyde	Others	Aldehyde compounds	1.180363417	0.00330396	0.026707589	0.118893644	-3.072256509	3
Stem vs Leaf	Scopoletin 7-O-(6''-Malonylglucoside)	Lignans and Coumarins	Coumarins	1.035488814	0.003361247	0.026923741	13.40225037	3.744403359	3
Stem vs Leaf	3,5-Dimethoxy-4-hydroxybenzaldehyde	Others	Aldehyde compounds	1.168905332	0.056177876	0.104430525	20.26668413	4.341038161	3

Comparison	Compound	Class I	Class II	VIP	P-value	FDR	Fold Change	Log_2FC	MSI Level
Stem vs Leaf	Dihydrovomifoliol 4-O-malonyl glucoside*	Terpenoids	Sesquiterpenoids	1.150901195	0.063524108	0.113341769	7.821068861	2.967365786	3
Stem vs Leaf	3,4,5-Trimethoxyphenol-β-D-apiofuranosyl-(1→6)-O-β-D-glucopyranoside	Others	Others	1.177049047	0.009054356	0.037930918	12.0351554	3.589182865	3
Stem vs Leaf	Anhydrosecoisolariciresinol (AHS)*	Lignans and Coumarins	Lignans	1.135719572	0.007442955	0.035325285	5.206797222	2.380396222	3
Stem vs Leaf	Glycidyl Linoleate	Lipids	Glycerol ester	1.083045674	0.010764796	0.040769423	9.266359642	3.212002677	3
Stem vs Leaf	Afzelechin	Flavonoids	Flavanols	1.148607429	0.063019516	0.112815954	0.107527073	-3.217228147	3

Comparison	Compound	Class I	Class II	VIP	P-value	FDR	Fold Change	Log_2FC	MSI Level
	(3,5,7,4'-Tetrahydroxyflavan)								
Stem vs Leaf	Hordenine	Alkaloids	Alkaloids	1.173753194	0.019220923	0.055099978	0.093735047	-3.415267629	3
Stem vs Leaf	3-Hydroxyphloretin-4'-O-glucoside	Flavonoids	Chalcones	1.060069262	0.034497399	0.076779926	0.068035486	-3.877568761	2
Stem vs Leaf	1,2,9,10-tetraethoxy-6-methyl-5,6,6a,7-tetrahydro-4H-dibenzo[de,g]quinoline	Alkaloids	Alkaloids	1.134131076	0.030811465	0.071431365	0.120936033	-3.047683933	2
Stem vs Leaf	Fertaric acid glucoside	Phenolic acids	Phenolic acids	1.179802449	0.015775161	0.049225828	65.04667632	6.023403437	2
Stem vs Leaf	Sesaminol	Lignans and	Lignans	1.181552711	0.000829038	0.014978423	0.063334457	-3.980865572	3

Comparison	Compound	Class I	Class II	VIP	P-value	FDR	Fold Change	Log_2FC	MSI Level
	2-O-Beta-D-Glucoside	Coumarins							
Stem vs Leaf	Candicine	Alkaloids	Phenolamine	1.180841215	9.96973E-05	0.005954146	0.023831504	-5.390986178	3
Stem vs Leaf	Catechin-(4alpha->8)-gallocatechin-(4alpha->8)-gallocatechin	Flavonoids	Flavanols	1.175791779	0.013909157	0.046435849	0.111341555	-3.166935954	2
Stem vs Leaf	1,6-dihydroxy-3,7-dimethoxy-8-(3-methoxy-3-methylbutyl)-2-(3-methylbut-2-en-1-yl)xanthen-9-one	Flavonoids	Other Flavonoids	1.12426858	0.00846057	0.037420256	0.043042755	-4.538085753	3

Comparison	Compound	Class I	Class II	VIP	P-value	FDR	Fold Change	Log_2FC	MSI Level
Stem vs Leaf	6-Hydroxymelatonin	Alkaloids	Plumerane	1.155145151	0.081048552	0.132512842	11.67196437	3.54497548	3
Stem vs Leaf	3'-p-Coumaroyl-3-acetyl sucrose	Phenolic acids	Phenolic acids	1.178067164	0.00977866	0.039494299	0.09817567	-3.348490657	2
Stem vs Leaf	7,10-dihydroxy-12-methoxy-2,2-dimethyl-1,11-dioxatetracen-6-one	Others	Ketone compounds	1.156072802	0.000694871	0.014479152	3.145542393	1.653308806	3
Stem vs Leaf	6,7-Dihydroxy-4'-methoxyisoflavone; Texasin	Flavonoids	Isoflavones	1.178903591	0.011988539	0.042340248	0.05320818	-4.232208124	3
Stem vs Leaf	LysoPC 20:4	Lipids	LPC	1.166693581	0.029273347	0.069198428	6.742266972	2.753233754	3
Stem vs Leaf	Pro-Asp	Amino acids	Amino acids	1.127535212	0.008223562	0.036758126	0.336751173	-1.570245123	3

Comparison	Compound	Class I	Class II	VIP	P-value	FDR	Fold Change	Log_2FC	MSI Level
		and derivatives	and derivatives						
Stem vs Leaf	Procyanidin A6	Tannins	Proanthocyanidins	1.087917553	4.59741E-05	0.00395377	14.29153009	3.837088478	2
Stem vs Leaf	2,3,19,23-Tetrahydroxyurs-12-en-28-oic acid-28-O-glucoside	Terpenoids	Triterpene Saponin	1.07887509	0.00912832	0.038108522	0.256304096	-1.964071563	3
Stem vs Leaf	carlinoside	Flavonoids	Flavones	1.172131985	0.013986583	0.046517843	7.641145977	2.933789022	3
Stem vs Leaf	Patuletin-7-O-Galactoside*	Flavonoids	Flavonols	1.149815325	0.033715571	0.075278147	5.636994549	2.494926172	2
Stem vs Leaf	Methyl	Phenolic	Phenolic acids	1.111881864	0.001369765	0.017778885	14.68340736	3.876114887	3

Comparison	Compound	Class I	Class II	VIP	P-value	FDR	Fold Change	Log_2FC	MSI Level
	Hydroxycinnamate	acids							
Stem vs Leaf	Kakispyrone	Others	Others	1.10915311	0.014813359	0.047964941	0.195183723	-2.357095347	2
Stem vs Leaf	D-Erythrose-4-phosphate	Others	Saccharides	1.114783645	0.002988071	0.02580061	2.263416525	1.178502101	3
Stem vs Leaf	Delphinidin-3-O-glucoside (Mirtillin)	Flavonoids	Anthocyanidins	1.139761165	0.006395751	0.032812524	0.025343083	-5.302264173	3
Stem vs Leaf	Diosmetin (5,7,3'-Trihydroxy-4'-methoxyflavone)*	Flavonoids	Flavones	1.169467754	0.045820346	0.092614326	16.16279477	4.014604776	3
Stem vs Leaf	Lys-Phe	Amino acids	Amino acids	1.099688286	9.55101E-06	0.002053467	0.03958111	-4.659044131	3

Comparison	Compound	Class I	Class II	VIP	P-value	FDR	Fold Change	Log_2FC	MSI Level
		and derivatives	and derivatives						
Stem vs Leaf	Rosamultin*	Terpenoids	Triterpene Saponin	1.179882808	0.005816908	0.032023409	0.078288244	-3.675060499	1
Stem vs Leaf	Caffeoylagmatine	Alkaloids	Phenolamine	1.082360454	0.005362103	0.031033935	0.227541868	-2.135796065	3
Stem vs Leaf	ChimSalicifoliusin C	Lignans and Coumarins	Coumarins	1.149168902	0.067685427	0.117927409	8.026937675	3.004849696	3
Stem vs Leaf	2'-Deoxyguanosine	Nucleotides and derivatives	Nucleotides and derivatives	1.170856419	0.020911441	0.057148446	0.161908836	-2.626746373	3
Stem vs Leaf	2,3,19-Trihydroxy olean-12-en-23,28-dioic acid	Terpenoids	Triterpene	1.139675905	0.068515982	0.118629748	74.19238271	6.213199169	2

Comparison	Compound	Class I	Class II	VIP	P-value	FDR	Fold Change	Log_2FC	MSI Level
Stem vs Leaf	(3S,7aS,13aR,15bR)-4,4,7a,10,10,13a,15b-heptamethyl-2,3,4,4a,5,7,7a,8,9,9a,10,13,13a,13b,14,15,15a,15b-octadecahydro-1H-cyclohepta[1,2-a:5,4-a']dinaphthalen-3-ol	Terpenoids	Terpene	1.175851995	0.022245928	0.059198333	0.058295234	-4.10047826	2
Stem vs Leaf	2-Deacetyldecinna moyltaxinine E	Others	Others	1.16941137	0.053691495	0.10176487	0.049988601	-4.322257039	2
Stem vs Leaf	L-Lysine-Butanoic Acid	Amino acids and	Amino acids and derivatives	1.160478292	0.054636772	0.102929846	338.9936128	8.405114281	3

Comparison	Compound	Class I	Class II	VIP	P-value	FDR	Fold Change	Log_2FC	MSI Level
		derivatives							
Stem vs Leaf	S-(5'-Adenosyl)-L-methionine	Amino acids and derivatives	Amino acids and derivatives	1.102199384	0.024785331	0.063137988	0.035545791	-4.814177466	3
Stem vs Leaf	Corehoionoside C	Terpenoids	Monoterpenoids	1.170952332	0.000196528	0.008450724	0.377288305	-1.406260716	3
Stem vs Leaf	LysoPC 15:0(2n isomer)*	Lipids	LPC	1.164812651	0.006619979	0.033249269	2.28206511	1.190339954	3
Stem vs Leaf	Clausenaglycoside A	Phenolic acids	Phenolic acids	1.04741311	0.066347273	0.116256393	2.877044193	1.524587382	3
Stem vs Leaf	DL-Glyceraldehyde-3-phosphate	Organic acids	Organic acids	1.027622313	0.169709611	0.226771699	3.58413888	1.841626541	3

Comparison	Compound	Class I	Class II	VIP	P-value	FDR	Fold Change	Log_2FC	MSI Level
Stem vs Leaf	Hymenoside M	Others	Others	1.175331113	0.006179957	0.032737441	0.031279286	-4.998648593	3
Stem vs Leaf	1-O-p-Coumaroyl 3-methylglucaric acid*	Phenolic acids	Phenolic acids	1.17240776	0.023142207	0.060747898	8.240344062	3.042704576	1
Stem vs Leaf	Medioresinol B	Lignans and Coumarins	Lignans	1.118362536	0.03507742	0.077487102	0.34498776	-1.53538292	3
Stem vs Leaf	(S)-Malic acid-1-O-beta-D-glucopyranoside	Organic acids	Organic acids	1.101459887	0.092308334	0.145607423	3.614560999	1.853820437	2
Stem vs Leaf	Yuanhuanin	Flavonoids	Flavones	1.036058572	0.008541614	0.037420256	0.119856584	-3.060618936	2
Stem vs Leaf	Syringaldehyde; 4-Hydroxy-3,5-Dimethoxybenzalde	Others	Aldehyde compounds	1.168693033	0.034646593	0.076874419	12.85392117	3.684136625	3

Comparison	Compound	Class I	Class II	VIP	P-value	FDR	Fold Change	Log_2FC	MSI Level
	hyde								
Stem vs Leaf	Marmesinin	Lignans and Coumarins	Coumarins	1.176981457	0.016431806	0.050482096	0.062986555	-3.988812288	3
Stem vs Leaf	Poncirin (Isosakuranetin-7-O-neohesperidoside)*	Flavonoids	Flavanones	1.177267405	0.00100957	0.016277656	5.580638217	2.480430122	3
Stem vs Leaf	picrorhizin	Phenolic acids	Phenolic acids	1.089046868	0.02476661	0.063137988	13.05252796	3.706257344	3
Stem vs Leaf	Strophanthobiose*	Others	Others	1.175486546	0.000219783	0.008915744	4.104213631	2.037105827	3
Stem vs Leaf	Epipinoresinol*	Lignans and Coumarins	Lignans	1.146179563	0.07976618	0.131314921	7.32925716	2.873666985	3

Comparison	Compound	Class I	Class II	VIP	P-value	FDR	Fold Change	Log_2FC	MSI Level
Stem vs Leaf	8,8'-Methylenebis catechin	Flavonoids	Flavanols	1.004801292	0.010683954	0.040655755	2.389946375	1.256978248	2
Stem vs Leaf	5,6,6a,7-tetrahydro-1,2,9,10-tetraethoxy-6-n-pentyl-4H-dibenzo[de,g]quinolin-4-ol	Alkaloids	Alkaloids	1.177794274	2.2232E-06	0.001194972	26.96670541	4.753107369	2
Stem vs Leaf	Epicatechin-(4β→8)-epigallocatechin	Flavonoids	Flavanols	1.025323691	0.000506789	0.013133103	0.112315248	-3.154374295	2
Stem vs Leaf	Protocatechuic acid 4-O-(6''-O-Galloy) Glucoside	Phenolic acids	Phenolic acids	1.173497899	0.007612427	0.03555507	4.168069273	2.059379255	2

Comparison	Compound	Class I	Class II	VIP	P-value	FDR	Fold Change	Log_2FC	MSI Level
Stem vs Leaf	2-Linoleoylglycerol*	Lipids	Glycerol ester	1.108347367	0.022061303	0.058921494	3.360333792	1.748604547	3
Stem vs Leaf	Rubuside I*	Terpenoids	Triterpene Saponin	1.17606373	0.021567255	0.058015292	0.06397811	-3.966277818	1
Stem vs Leaf	Phloretin-2'-O-(6''-O-xylosyl)glucoside	Flavonoids	Chalcones	1.162811291	0.02918568	0.069183255	13.2319335	3.725951984	1
Stem vs Leaf	γ-Glu-Tyr	Amino acids and derivatives	Amino acids and derivatives	1.168814371	0.032455125	0.073606032	13.0292924	3.70368683	3
Stem vs Leaf	Methoxy-erythro-Guaiacyl glycerol β-threo-syringyl	Lignans and Coumarins	Lignans	1.128258791	0.011993122	0.042340248	3.824160977	1.935143255	3

Comparison	Compound	Class I	Class II	VIP	P-value	FDR	Fold Change	Log_2FC	MSI Level
	glycerol ether								
Stem vs Leaf	3,4-dihydroxyphenylethanol-β-D-glucopyranoside	Others	Alcohol compounds	1.062252283	0.009440122	0.038659548	2.443417591	1.288900448	3
Stem vs Leaf	icariside D1	Others	Ketone compounds	1.164730026	0.00084584	0.015154631	5.645408547	2.49707799	3
Stem vs Leaf	Tricin-5-O-arabinoside	Flavonoids	Flavones	1.058196541	0.183136651	0.240484048	10.31571937	3.366772527	3
Stem vs Leaf	(2S,3S,4S,5R,6R)-3-(3-benzoyloxy-2-hydroxypropoxy)-3,4,5-trihydroxyoxane-2-carboxylic	Phenolic acids	Phenolic acids	1.140378324	0.00788605	0.036127578	39.95026841	5.320133291	3

Comparison	Compound	Class I	Class II	VIP	P-value	FDR	Fold Change	Log_2FC	MSI Level
	acid								
Stem vs Leaf	1-Linoleoylglycerol*	Lipids	Glycerol ester	1.143642607	0.010844266	0.040787888	2.993375234	1.581773139	3
Stem vs Leaf	1,3,6-trihydroxy-2,5,7-trimethoxyxanthen-9-one	Flavonoids	Other Flavonoids	1.177748645	0.010484666	0.040169517	0.117228001	-3.092610882	3
Stem vs Leaf	Rheic Acid	Quinones	Anthraquinone	1.026043788	0.174875699	0.231801943	84.45849064	6.40017056	3
Stem vs Leaf	3-O-Methylellagic acid	Tannins	Tannin	1.180924798	0.007820533	0.03608186	114.3172683	6.836899538	3
Stem vs Leaf	Blumenol A	Terpenoids	Monoterpenoids	1.018815345	0.024185645	0.062199924	0.437250428	-1.193468299	3
Stem vs Leaf	Anthranilate-1-O-	Phenolic	Phenolic acids	1.132935768	0.068999075	0.119155029	21.24804538	4.409258228	3

Comparison	Compound	Class I	Class II	VIP	P-value	FDR	Fold Change	Log_2FC	MSI Level
	Sophoroside	acids							
Stem vs Leaf	LysoPC 15:0*	Lipids	LPC	1.160604575	0.005381148	0.031033935	2.891274052	1.531705362	3
Stem vs Leaf	Solatuberenol A	Others	Others	1.022485691	0.022905347	0.060425147	0.436061349	-1.197396973	3
Stem vs Leaf	4-p-Cumaroyl-rhamnosyl-(1→6)-D-glucose	Phenolic acids	Phenolic acids	1.16937796	0.010079846	0.0396917	4.440604623	2.150756124	2
Stem vs Leaf	Schisantherin E	Lignans and Coumarins	Lignans	1.099998019	0.09489123	0.149025671	8.724199174	3.125022707	3
Stem vs Leaf	2-Methylthio-cis-zeatin riboside	Alkaloids	Alkaloids	1.180735899	0.000184398	0.008306724	0.049855523	-4.326102849	3
Stem vs Leaf	Hesperetin	Flavonoids	Flavanones	1.103426899	0.05105396	0.098458259	2.181036011	1.125013591	3
Stem vs Leaf	Cyanidin	Flavonoids	Anthocyanidin	1.177595261	0.014466103	0.047339608	0.064157661	-3.962234653	3

Comparison	Compound	Class I	Class II	VIP	P-value	FDR	Fold Change	Log_2FC	MSI Level
	3-O-sophoroside		s						
Stem vs Leaf	Protocatechuic acid 1-O-Rutinoside	Phenolic acids	Phenolic acids	1.17801196	0.012334511	0.042842	13.41519048	3.745795634	1
Stem vs Leaf	Didymin (Isosakuranetin-7-O-rutinoside)*	Flavonoids	Flavanones	1.149626989	0.00059633	0.013639457	4.499585928	2.169792244	3
Stem vs Leaf	Mycarose glucoside*	Others	Others	1.169233517	0.009799601	0.039494299	6.561110455	2.713940009	3
Stem vs Leaf	1-O-p-Coumaroyl lysine	Alkaloids	Phenolamine	1.131919933	0.010953065	0.041026289	0.085614538	-3.546000393	3
Stem vs Leaf	Lys-Tyr	Amino acids and	Amino acids and derivatives	1.055123371	0.020741334	0.056952577	0.330600063	-1.596841093	3

Comparison	Compound	Class I	Class II	VIP	P-value	FDR	Fold Change	Log_2FC	MSI Level
		derivatives							
Stem vs Leaf	Scandione	Flavonoids	Chalcones	1.084296098	0.066401326	0.116256393	0.440250474	-1.183603537	3
Stem vs Leaf	O-Acetyl-L-homo serine	Amino acids and derivatives	Amino acids and derivatives	1.106898392	0.054848587	0.102929846	0.296063805	-1.756019971	3
Stem vs Leaf	Pedunculoside	Terpenoids	Triterpene Saponin	1.179442536	0.005919307	0.032023409	0.027497473	-5.184557171	2
Stem vs Leaf	5-[(1S,2R,3R,5S)-3,5-dihydroxy-2-[(E,3S)-3-hydroxyoct-1-enyl]cyclopentyl]pentanoic acid	Lipids	Free fatty acids	1.069960364	0.147949415	0.203643561	22.44087395	4.488056957	1
Stem vs Leaf	Zanthoxyloside*	Lignans and	Coumarins	1.148816314	0.025393849	0.063904927	17.64740184	4.141383891	3

Comparison	Compound	Class I	Class II	VIP	P-value	FDR	Fold Change	Log_2FC	MSI Level
		Coumarins							
Stem vs Leaf	Quercetin-3-O-glucuronide*	Flavonoids	Flavonols	1.114379267	0.0305895	0.071253982	6.085252514	2.60531713	3
Stem vs Leaf	C-Veratroylglycol	Others	Alcohol compounds	1.138114105	0.135668913	0.190261682	38.46255018	5.265382516	3
Stem vs Leaf	3,23-Dihydroxylup-20(29)-en-28-oic acid (Oplopanaxogenin C)	Terpenoids	Triterpene	1.074589922	0.124984056	0.179382991	11.38230015	3.508720224	3
Stem vs Leaf	Naringenin chalcone; 2',4,4',6'-Tetrahyd	Flavonoids	Chalcones	1.02949948	0.007348956	0.03511168	0.248300236	-2.009842463	3

Comparison	Compound	Class I	Class II	VIP	P-value	FDR	Fold Change	Log_2FC	MSI Level
	roxychalcone								
Stem vs Leaf	Luteolin 7-(2-sulfoglucoside)	Flavonoids	Flavones	1.169974925	0.007752969	0.035847061	13.79476964	3.78604946	1
Stem vs Leaf	Resveratroloside*	Others	Stilbene	1.089056875	0.015295875	0.048648122	2.385826108	1.254488896	2
Stem vs Leaf	Quercetin-3-O-(2''-O-galloyl)galactoside	Flavonoids	Flavonols	1.174860067	0.014148504	0.046614287	0.176099101	-2.505540547	3
Stem vs Leaf	2,3,19,23-Tetrahydroxy-24-oxo-olean-12-en-28-oic acid	Terpenoids	Triterpene	1.141254007	0.068379461	0.118629748	12.13334835	3.60090583	3
Stem vs Leaf	3-quinolinecarbox	Alkaloids	Quinoline	1.173087762	0.017253212	0.052065312	0.151647102	-2.721210167	3

Comparison	Compound	Class I	Class II	VIP	P-value	FDR	Fold Change	Log_2FC	MSI Level
	ylic acid		alkaloids						
Stem vs Leaf	DMelezitose O-rhamnoside	Others	Saccharides	1.135608306	0.11939061	0.173909086	19.04159817	4.251082664	3
Stem vs Leaf	2-(2-Benzothiazol ylthio)ethanol	Alkaloids	Alkaloids	1.161049269	0.072268905	0.122344996	0.07136781	-3.808582693	3
Stem vs Leaf	5-O-p-Coumaroyl Methylglucaric Acid*	Phenolic acids	Phenolic acids	1.174654278	0.015869691	0.049377476	0.113358145	-3.141040038	1
Stem vs Leaf	Piceid (3,4',5-Trihydroxy stilbene-3-O-gluc oside)*	Others	Stilbene	1.158072455	0.021819816	0.058494519	3.055911057	1.611602554	2
Stem vs Leaf	Glucosyl	Terpenoids	Monoterpenoid	1.138795508	0.00319397	0.026513654	29.63229794	4.889098604	3

Comparison	Compound	Class I	Class II	VIP	P-value	FDR	Fold Change	Log_2FC	MSI Level
	6-Hydroxy-2,6-Dimethyl-2E,7-Octadienoate		s						
Stem vs Leaf	L-Citramalic acid	Organic acids	Organic acids	1.16182172	0.028293777	0.067816745	0.118822819	-3.073116169	3
Stem vs Leaf	Icariside C1 Acetate	Terpenoids	Sesquiterpenoids	1.175383113	0.052373335	0.100358887	0.013010507	-6.264178968	3
Stem vs Leaf	mudanoside A	Phenolic acids	Phenolic acids	1.13814169	0.009324697	0.038406318	2.123915241	1.086726193	3
Stem vs Leaf	Leu-Asp	Amino acids and derivatives	Amino acids and derivatives	1.132830907	0.001886085	0.020636181	0.339777978	-1.557335744	3
Stem vs Leaf	Petunidin-3-O-(6''	Flavonoids	Anthocyanidin	1.176069209	0.035262405	0.077678455	37.4403753	5.226522992	3

Comparison	Compound	Class I	Class II	VIP	P-value	FDR	Fold Change	Log_2FC	MSI Level
	-O-caffeoyl)gluco side		s						
Stem vs Leaf	Pinoresinol*	Lignans and Coumarins	Lignans	1.177371066	0.018939847	0.054732084	20.4841865	4.356438694	3
Stem vs Leaf	Inosine	Nucleotides and derivatives	Nucleotides and derivatives	1.168756758	0.002244584	0.022493448	3.91258869	1.968123455	3
Stem vs Leaf	Gentioluteol	Lignans and Coumarins	Lignans	1.128763068	0.011897917	0.042212083	0.218453876	-2.194599394	3
Stem vs Leaf	quinatoside A	Terpenoids	Triterpene Saponin	1.173597694	0.033499211	0.075102505	0.061814422	-4.015912713	3
Stem vs Leaf	L-Aspartic acid-O-diglucosid	Amino acids and	Amino acids and derivatives	1.142826594	0.041295732	0.086283599	5.290051143	2.40328167	3

Comparison	Compound	Class I	Class II	VIP	P-value	FDR	Fold Change	Log_2FC	MSI Level
	e	derivatives							
Stem vs Leaf	Gingerglycolipid C	Lipids	Glycerol ester	1.046789645	0.256382939	0.312839568	21.91265405	4.453692328	2
Stem vs Leaf	Isolariciresinol-9-O-glucoside	Lignans and Coumarins	Lignans	1.176490161	0.021399218	0.057944985	18.73425306	4.227606553	3
Stem vs Leaf	syringaresinol-4-O-β-D-glucopyranoside*	Lignans and Coumarins	Lignans	1.030844601	0.007847331	0.036127578	4.945212903	2.306032634	3
Stem vs Leaf	2-Methylglutaric acid	Organic acids	Organic acids	1.13632082	0.001558485	0.018615239	2.260818601	1.176845241	3
Stem vs Leaf	3,28-Dihydroxylup-20(29)-ene (Betulin)	Terpenoids	Triterpene	1.089489521	0.129615546	0.18430782	20.80623949	4.378944331	2

Comparison	Compound	Class I	Class II	VIP	P-value	FDR	Fold Change	Log_2FC	MSI Level
Stem vs Leaf	pyridoxamine	Alkaloids	Pyridine alkaloids	1.173331141	0.020147904	0.055784421	0.080896062	-3.627786708	3
Stem vs Leaf	Trehalose 6-phosphate	Others	Saccharides	1.140695755	0.072610516	0.1228266	6.150541128	2.620713345	3
Stem vs Leaf	2',4'-Dihydroxyacetophenone*	Others	Ketone compounds	1.13518211	0.040317589	0.084899917	3.363621624	1.750015425	3
Stem vs Leaf	Cleomiscosin C	Lignans and Coumarins	Coumarins	1.14012317	0.071544334	0.121574604	9.76993929	3.288349597	3
Stem vs Leaf	Clemiscosin D	Terpenoids	Triterpene Saponin	1.115669286	0.114683123	0.17028188	13.26179201	3.729203829	3
Stem vs Leaf	N-Acetyl-L-glutamic acid	Amino acids and derivatives	Amino acids and derivatives	1.003310989	0.026696284	0.065596583	0.275677347	-1.85894737	3

Comparison	Compound	Class I	Class II	VIP	P-value	FDR	Fold Change	Log_2FC	MSI Level
Stem vs Leaf	Hovetrichoside B	Lignans and Coumarins	Lignans	1.157479068	0.016815515	0.051208722	3.404133399	1.767287574	3
Stem vs Leaf	(6R,9R)-3-Oxo-α-ionol-β-D-glucoside	Terpenoids	Sesquiterpenoids	1.111426454	0.025523133	0.063904927	0.294384821	-1.76422481	2
Stem vs Leaf	Hesperetin-6-C-glucoside-7-O-glucoside	Flavonoids	Flavanones	1.130347962	0.028678539	0.06817466	2.648650077	1.405257257	3
Stem vs Leaf	Phellatin	Flavonoids	Flavonols	1.180188205	0.006231944	0.032737441	0.050894768	-4.29633883	3
Stem vs Leaf	S-(Methyl)glutathione	Amino acids and derivatives	Amino acids and derivatives	1.122681299	0.079558202	0.131173416	7.122172458	2.83231737	3
Stem vs Leaf	Sarmentosin	Alkaloids	Alkaloids	1.101120862	0.095850954	0.149985117	3.100795807	1.632638525	3

Comparison	Compound	Class I	Class II	VIP	P-value	FDR	Fold Change	Log_2FC	MSI Level
Stem vs Leaf	13,17-epoxy-alisol A	Terpenoids	Triterpene	1.156723936	0.046543671	0.09326085	8.669738967	3.115988557	2
Stem vs Leaf	Ent-15-oxo-2β,16, 19-trihydroxypimar-8(14)-ene	Terpenoids	Ditepenoids	1.137083158	0.015534644	0.049008766	0.027041809	-5.208664519	3
Stem vs Leaf	Vanillin; 4-Hydroxy-3-Methoxybenzaldehyde*	Others	Aldehyde compounds	1.11286155	0.011798108	0.042066224	4.55240439	2.186628718	3
Stem vs Leaf	L-Glutamyl-L-Tyrosine	Amino acids and derivatives	Amino acids and derivatives	1.164770322	0.054767055	0.102929846	11.7206372	3.5509791	3
Stem vs Leaf	1,3,7-trihydroxy-2	Flavonoids	Other	1.124787986	0.078743765	0.130404576	0.031015697	-5.010857666	3

Comparison	Compound	Class I	Class II	VIP	P-value	FDR	Fold Change	Log_2FC	MSI Level
	-[3,4,5-trihydroxy-6-(hydroxymethyl)oxan-2-yl]xanthen-9-one		Flavonoids						
Stem vs Leaf	Nepetin-5-O-diglucoside	Flavonoids	Flavones	1.14783006	0.002397509	0.02293776	88.47866308	6.467257681	3
Stem vs Leaf	2-Deoxyribose-1-phosphate	Nucleotides and derivatives	Nucleotides and derivatives	1.172240581	3.73491E-06	0.00134698	0.148015572	-2.756179136	2
Stem vs Leaf	N-Acetyl-L-phenylalanine	Amino acids and derivatives	Amino acids and derivatives	1.175586075	0.004717884	0.029574357	0.464376939	-1.106631767	3
Stem vs Leaf	Humulene	Terpenoids	Sesquiterpenoi	1.179781223	0.006702218	0.033278913	0.064800753	-3.947845612	1

Comparison	Compound	Class I	Class II	VIP	P-value	FDR	Fold Change	Log_2FC	MSI Level
	epoxide II		ds						
Stem vs Leaf	Urolignoside	Lignans and Coumarins	Lignans	1.10281195	0.02237755	0.05947062	7.240518179	2.85609295	2
Stem vs Leaf	1-Hydroxypinoresinol-1-O-Glucoside	Lignans and Coumarins	Lignans	1.054105689	0.110708826	0.165870366	17.46730175	4.126584861	2
Stem vs Leaf	Dehydrodiconiferyl alcohol*	Lignans and Coumarins	Lignans	1.044133058	0.023940417	0.061939707	0.477713424	-1.065782678	3
Stem vs Leaf	Aloeemodin-8-O-glucoside	Quinones	Anthraquinone	1.093856127	0.00992978	0.03961495	0.031452679	-4.990673274	3
Stem vs Leaf	Clemaphenol A*	Lignans and Coumarins	Lignans	1.093634927	0.014230825	0.046711868	0.440582958	-1.182514402	3

Comparison	Compound	Class I	Class II	VIP	P-value	FDR	Fold Change	Log_2FC	MSI Level
Stem vs Leaf	galloyl malonyl glucoside	Phenolic acids	Phenolic acids	1.152755834	0.063246277	0.113032423	0.134390386	-2.895498163	1
Stem vs Leaf	Cantleyine	Alkaloids	Alkaloids	1.076518348	0.004642623	0.029444362	0.436556136	-1.195760916	3
Stem vs Leaf	6-Hydroxy-lupane-20(29)-en-3-one-28-oic acid	Terpenoids	Triterpene	1.06518977	0.112221155	0.167552419	6.96885715	2.800922082	2
Stem vs Leaf	Mearnsetin	Flavonoids	Flavonols	1.177061108	0.009915661	0.03961495	0.119002035	-3.070941849	3
Stem vs Leaf	Emodin-8-O-glucoside	Quinones	Anthraquinone	1.175052281	0.012844002	0.0439723	0.14583305	-2.777610386	3
Stem vs Leaf	3-Dehydroshikimic acid	Organic acids	Organic acids	1.173028159	0.000483711	0.013133103	0.125838156	-2.99035866	3
Stem vs Leaf	Delphinidin-3-O-	Flavonoids	Anthocyanidin	1.181574237	0.000531808	0.013142389	0.072039574	-3.795066537	3

Comparison	Compound	Class I	Class II	VIP	P-value	FDR	Fold Change	Log_2FC	MSI Level
	galactoside		s						
Stem vs Leaf	Cannabifolin E*	Terpenoids	Triterpene	1.045510197	0.127763496	0.182519281	7.607628093	2.92744672	2
Stem vs Leaf	Wistin	Flavonoids	Isoflavones	1.165648769	0.030766585	0.071431365	0.104328477	-3.26079509	3
Stem vs Leaf	Sedumoside H	Others	Others	1.131130187	0.000241763	0.009450719	0.02252561	-5.472289991	3
Stem vs Leaf	Homovanilloylquinic acid	Phenolic acids	Phenolic acids	1.174022826	0.025939659	0.064103755	0.074160669	-3.753201937	3
Stem vs Leaf	Grayanotoxin VIII	Terpenoids	Ditepenoids	1.154077764	0.018689979	0.054343439	0.282501413	-1.823670013	3
Stem vs Leaf	2-Palmitoyl-Sn-Glycerol 3-O-Diglucoside	Lipids	Glycerol ester	1.17068006	0.002898658	0.025283157	0.210531882	-2.247889371	1
Stem vs Leaf	1-O-p-Coumaroyl	Phenolic	Phenolic acids	1.083942586	0.011017247	0.041052135	0.052870809	-4.241384782	1

Comparison	Compound	Class I	Class II	VIP	P-value	FDR	Fold Change	Log_2FC	MSI Level
	Methylglucaric Acid*	acids							
Stem vs Leaf	3S-3-hydroxy-β-damascon	Terpenoids	Monoterpenoids	1.130167713	0.005274639	0.030931935	0.299866863	-1.737605988	3
Stem vs Leaf	Isorhamnetin-3-O-sophoroside*	Flavonoids	Flavonols	1.175248166	0.006273407	0.032737441	13.58239708	3.76366621	3
Stem vs Leaf	Demethyl coniferin	Others	Alcohol compounds	1.128456182	0.018031445	0.053398908	3.794461287	1.923895076	2
Stem vs Leaf	Gardenoside	Terpenoids	Monoterpenoids	1.17184977	0.024629874	0.063058464	7.976337793	2.995726508	3
Stem vs Leaf	Nortrachelogenin-4-O-glucoside	Lignans and Coumarins	Lignans	1.072376741	0.117808928	0.172861275	9.548967587	3.255344761	2

Comparison	Compound	Class I	Class II	VIP	P-value	FDR	Fold Change	Log_2FC	MSI Level
Stem vs Leaf	2,3,19,23-Tetrahydroxyolean-12-en-28-oic acid (Sericic acid)	Terpenoids	Triterpene	1.177033813	0.011505221	0.041808314	12.56527296	3.651370107	3
Stem vs Leaf	5'-Deoxyadenosine*	Nucleotides and derivatives	Nucleotides and derivatives	1.181610752	0.000331011	0.010948842	0.056116399	-4.155433765	3
Stem vs Leaf	LysoPC 19:1	Lipids	LPC	1.154853706	0.047279337	0.094208133	8.096344599	3.017270696	2
Stem vs Leaf	Uridine-5'-Diphosphate-D-Xylose	Nucleotides and derivatives	Nucleotides and derivatives	1.133953227	0.068391035	0.118629748	6.272969273	2.649148496	2
Stem vs Leaf	Guvacine	Alkaloids	Piperidine alkaloids	1.140691035	0.008886953	0.037911157	4.173318987	2.061195197	3

Comparison	Compound	Class I	Class II	VIP	P-value	FDR	Fold Change	Log_2FC	MSI Level
Stem vs Leaf	Grayanoside C	Terpenoids	Ditepenoids	1.1741612	0.017237371	0.052065312	0.016039782	-5.962201618	2
Stem vs Leaf	3,4,5-Trimethoxy phenol-1-O-β-D-g lucoside	Others	Others	1.156580508	0.029333983	0.069210338	14.56670688	3.864602856	3
Stem vs Leaf	Capillarisin	Others	Chromone	1.163604362	0.061772901	0.11113953	25.14385838	4.652134147	3
Stem vs Leaf	7,8-Dihydroxy-4'-methoxyisoflavon e	Flavonoids	Other Flavonoids	1.133231868	0.031272791	0.071910695	0.181139554	-2.464826481	3
Stem vs Leaf	5-O-p-Coumaroyl shikimic acid O-glucoside	Phenolic acids	Phenolic acids	1.164294357	0.000383746	0.01195731	0.443152233	-1.174125711	3
Stem vs Leaf	7,2'-dihydroxy-4'-methoxy-isoflava	Flavonoids	Other Flavonoids	1.180371002	0.003027172	0.025963636	0.069474791	-3.847366602	3

Comparison	Compound	Class I	Class II	VIP	P-value	FDR	Fold Change	Log_2FC	MSI Level
	nol glucoside								
Stem vs Leaf	Sedoheptulose	Others	Saccharides	1.08568714	0.066058652	0.116224306	2.23850537	1.162535779	3
Stem vs Leaf	Delphinidin-3-O-glucuronide	Flavonoids	Anthocyanidins	1.164430155	0.011261096	0.041529903	7.298588519	2.867617487	3
Stem vs Leaf	3,3,14,14-tetramethylhexadecanedioic acid	Lipids	Free fatty acids	1.174297437	0.003091484	0.025963636	11.17774127	3.482556782	1
Stem vs Leaf	6'-O-Sinapoylsucrose*	Phenolic acids	Phenolic acids	1.180647937	0.00267071	0.024430315	0.093570839	-3.417797193	2
Stem vs Leaf	5,6-Dihydroxylucidin	Quinones	Anthraquinone	1.036780048	0.101693743	0.156620019	2.200690328	1.13795615	1
Stem vs Leaf	dGDP	Nucleotides	Nucleotides	1.131061215	0.001176934	0.016757666	0.436262247	-1.196732464	1

Comparison	Compound	Class I	Class II	VIP	P-value	FDR	Fold Change	Log_2FC	MSI Level
		and derivatives	and derivatives						
Stem vs Leaf	Luteolin 7-rutinoside-4'-glucoside	Flavonoids	Flavones	1.178455848	0.009986652	0.03961495	0.079177886	-3.658758646	2
Stem vs Leaf	Scopoletin 7-O-β-D-Sophoroside*	Lignans and Coumarins	Coumarins	1.118398833	0.134478481	0.189096621	11.06727933	3.468228702	2
Stem vs Leaf	Veratraldehyde	Phenolic acids	Phenolic acids	1.164455243	0.064792145	0.114523833	0.055920722	-4.1604732	3
Stem vs Leaf	β-Oxoacteoside	Phenolic acids	Phenolic acids	1.146714997	0.000796932	0.014923977	0.039256198	-4.670935734	3
Stem vs Leaf	Furanofructosyl-	Phenolic	Phenolic acids	1.140246114	0.00224225	0.022493448	0.025767743	-5.278289995	2

Comparison	Compound	Class I	Class II	VIP	P-value	FDR	Fold Change	Log_2FC	MSI Level
	α-D-(3-mustard acyl)glucoside*	acids							
Stem vs Leaf	Cyanidin-3-O-gentiobioside*	Flavonoids	Anthocyanidins	1.143738943	0.01943228	0.055429619	0.017967072	-5.798500878	3
Stem vs Leaf	(E)-caffeyl alcohol 4-O-β-D-glucopyranoside	Others	Alcohol compounds	1.108434726	0.011876965	0.042207396	2.864952943	1.518511443	1
Stem vs Leaf	Morin	Flavonoids	Flavonols	1.015166393	0.069848009	0.119850934	2.487771018	1.314853701	1
Stem vs Leaf	5-Acetamidopentanoic Acid	Organic acids	Organic acids	1.068121802	0.139030404	0.19347273	5.424348265	2.439449808	3
Stem vs Leaf	Matairesinol	Lignans and Coumarins	Lignans	1.157470936	0.204386606	0.261099942	275.4090989	8.105432413	3

Comparison	Compound	Class I	Class II	VIP	P-value	FDR	Fold Change	Log_2FC	MSI Level
Stem vs Leaf	10-hydroxymajoroside	Terpenoids	Monoterpenoids	1.164166255	0.056418609	0.104485198	12.28472609	3.618793786	3
Stem vs Leaf	2,3,19,23-Tetrahydroxyolean-12-en-28-oic acid (Arjungenin)*	Terpenoids	Triterpene	1.109565299	0.061501061	0.110743117	3.198797645	1.677529731	2
Stem vs Leaf	Nicotianamine	Alkaloids	Alkaloids	1.003326815	0.061257255	0.110674872	2.318267433	1.213047004	3
Stem vs Leaf	Neolinustatin	Alkaloids	Alkaloids	1.160784057	0.019182856	0.055099978	0.269907599	-1.889462502	3
Stem vs Leaf	N-Salicyloylasparticacid	Amino acids and derivatives	Amino acids and derivatives	1.004885707	0.371768877	0.42927126	84.24279778	6.396481445	2
Stem vs Leaf	Lupa-1,20(29)-dien-3-one	Terpenoids	Triterpene	1.169079419	0.036793391	0.080204277	0.06043514	-4.048468546	3

Comparison	Compound	Class I	Class II	VIP	P-value	FDR	Fold Change	Log_2FC	MSI Level
	(Glochidone)								
Stem vs Leaf	Dehydroascorbic acid	Others	Vitamin	1.178517563	0.019837476	0.055784421	0.028547787	-5.130477282	2
Stem vs Leaf	Glu-Leu	Amino acids and derivatives	Amino acids and derivatives	1.131224929	0.111979745	0.167424514	7.463362869	2.899825832	2
Stem vs Leaf	Methanesulfonic acid	Organic acids	Organic acids	1.14367209	0.00162215	0.019058048	2.122027233	1.085443171	3
Stem vs Leaf	Synephrine; 4-[1-Hydroxy-2-(methylamino)ethyl]phenol	Alkaloids	Alkaloids	1.128704301	0.037888258	0.081541296	0.32331589	-1.62898368	3
Stem vs Leaf	Ser-Lys	Amino acids	Amino acids	1.093084163	0.004607303	0.029393776	0.394192558	-1.343027554	3

Comparison	Compound	Class I	Class II	VIP	P-value	FDR	Fold Change	Log_2FC	MSI Level
		and derivatives	and derivatives						
Stem vs Leaf	1-O-(3,4,5-Trimethoxybenzoyl)-B-D-Glucopyranoside	Phenolic acids	Phenolic acids	1.10206099	0.052851995	0.100826788	4.243463118	2.085242138	2
Stem vs Leaf	vasicinone	Alkaloids	Alkaloids	1.088536158	0.00026052	0.00965721	0.067901177	-3.880419606	3
Stem vs Leaf	1,2,3,19-Tetrahydroxyurs-12-en-28-oic acid*	Terpenoids	Triterpene	1.036895725	0.104761936	0.159517112	2.379975628	1.2509468	2
Stem vs Leaf	Lup-12-en-15α,19β-diol-3,11-dioxo-28-oic acid	Terpenoids	Triterpene	1.17431524	0.012188	0.04281732	13.98144647	3.805441719	3

Comparison	Compound	Class I	Class II	VIP	P-value	FDR	Fold Change	Log_2FC	MSI Level
Stem vs Leaf	Grevilloside F	Phenolic acids	Phenolic acids	1.178674133	0.008818287	0.037911157	20.59544096	4.364253111	3
Stem vs Leaf	Syringin	Others	Alcohol compounds	1.171960757	0.008929426	0.037930918	0.291062981	-1.780596731	2
Stem vs Leaf	1,6-Di-O-caffeoyl-β-D-glucose*	Phenolic acids	Phenolic acids	1.177117587	0.009984625	0.03961495	0.0187386	-5.737843041	2
Stem vs Leaf	Tortoside A*	Lignans and Coumarins	Lignans	1.167927941	0.018844012	0.054528435	4.173262536	2.061175682	3
Stem vs Leaf	3-Hydroxyolean-12-ene-28,29-dioic acid (Serratagenic Acid)	Terpenoids	Triterpene	1.085138089	0.118064154	0.172861275	11.9114708	3.574279659	1
Stem vs Leaf	LysoPE 15:0*	Lipids	LPE	1.146040091	0.079239656	0.130949469	6.964113525	2.799939721	3

Comparison	Compound	Class I	Class II	VIP	P-value	FDR	Fold Change	Log_2FC	MSI Level
Stem vs Leaf	1-O-p-Coumaroyl galactaric acid	Phenolic acids	Phenolic acids	1.180990542	0.001885186	0.020636181	0.086841285	-3.525475109	3
Stem vs Leaf	Umckalin (7-hydroxy-5,6-di methoxycoumari n)	Lignans and Coumarins	Coumarins	1.156050373	0.032136749	0.073115356	30.60556266	4.935721986	1
Stem vs Leaf	Olean-13(18)-en-3 -one (δ-Amyrenone)	Terpenoids	Triterpene	1.179425151	0.008818017	0.037911157	0.051745924	-4.272410967	1
Stem vs Leaf	3'-O-Beta-D-Gluc opyranosyl plumbagic acid	Phenolic acids	Phenolic acids	1.150213377	0.020111449	0.055784421	3.393158515	1.762628829	2
Stem vs Leaf	3-oxo-taraxeran-1	Terpenoids	Triterpene	1.180772341	0.008289645	0.036976633	0.009071371	-6.784463602	2

Comparison	Compound	Class I	Class II	VIP	P-value	FDR	Fold Change	Log_2FC	MSI Level
	4-en								
Stem vs Leaf	3-Oxopomolic acid	Terpenoids	Triterpene	1.138823649	0.072207747	0.122337791	9.992591845	3.320858928	2
Stem vs Leaf	Lup-20(29)-en-3-one (Lupenone)	Terpenoids	Triterpene	1.180280719	0.005030576	0.03038129	0.054804954	-4.189549872	2
Stem vs Leaf	3,6-Di-O-caffeoyl glucose*	Phenolic acids	Phenolic acids	1.176798583	0.033717607	0.075278147	0.013515181	-6.209275307	2
Stem vs Leaf	Quercetin-5-O-glucuronide*	Flavonoids	Flavonols	1.162575185	0.003686329	0.027962841	9.975521165	3.318392216	3
Stem vs Leaf	Gossypetin-7-O-L-rhamnopyranoside(Rhodiolgin)	Flavonoids	Flavonols	1.070875023	0.073077833	0.123229287	3.068984031	1.617761139	3

Comparison	Compound	Class I	Class II	VIP	P-value	FDR	Fold Change	Log_2FC	MSI Level
Stem vs Leaf	3,7-dihydroxy-2-methoxy-8,8,10-trimethyl-6,7-dihydroanthracene-1,4,5-trione	Quinones	Quinones	1.177291849	0.0568752	0.10505299	221.5766978	7.791662358	3
Stem vs Leaf	Cyanidin-3,5-O-diglucoside (Cyanin)*	Flavonoids	Anthocyanidins	1.149791099	0.000149202	0.007920636	0.014460506	-6.11173814	3
Stem vs Leaf	Benzyl 6-O-galloyl beta-D-glucopyranoside	Others	Others	1.133066713	0.120169884	0.174689148	0.073076807	-3.774442588	3
Stem vs Leaf	Quercetin-3-O-apiosyl(1→2)galacto	Flavonoids	Flavonols	1.17703041	0.01156249	0.041808314	0.10940354	-3.192268676	3

Comparison	Compound	Class I	Class II	VIP	P-value	FDR	Fold Change	Log_2FC	MSI Level
	side								
Stem vs Leaf	4,5,8-Trihydroxy-α-tetralone	Quinones	Naphthol	1.149346462	0.00423299	0.028481053	0.065412623	-3.934287127	1
Stem vs Leaf	Quercetin-3-O-sophoroside (Baimaside)	Flavonoids	Flavonols	1.156506599	0.02415391	0.062192702	3.213530816	1.684159307	3
Stem vs Leaf	Tricalysioside R	Terpenoids	Ditepenoids	1.179733099	0.006385486	0.032812524	0.07137002	-3.808538011	3
Stem vs Leaf	Homoeriodictyol-7,4'-di-O-β-D-glucopyranoside	Flavonoids	Flavanones	1.002867114	0.153046925	0.209720133	2.334924556	1.223375936	3
Stem vs Leaf	Gambiriin A1	Tannins	Tannin	1.15109226	0.03613699	0.079118665	10.75470654	3.426896253	2
Stem vs Leaf	Sec-O-glucosylha	Others	Chromone	1.17377749	0.021440635	0.057984107	0.072113308	-3.793590675	3

Comparison	Compound	Class I	Class II	VIP	P-value	FDR	Fold Change	Log_2FC	MSI Level
	maudol								
Stem vs Leaf	D-Threo-guaiacyl glycerol-7-O-β-D-glucoside	Others	Alcohol compounds	1.176762748	0.019568373	0.055577281	25.51608104	4.673334861	3
Stem vs Leaf	Protocatechuic Acid Methyl Ester	Phenolic acids	Phenolic acids	1.145286242	0.043458367	0.089156	4.496336281	2.168749939	3
Stem vs Leaf	N-(4-Aminobutyl)benzamide	Alkaloids	Alkaloids	1.169422663	0.019608206	0.055616943	7.559655212	2.918320436	3
Stem vs Leaf	N-Fructosyl Pyroglutamate	Amino acids and derivatives	Amino acids and derivatives	1.033501766	0.0329369	0.074363156	3.711424455	1.891973004	3
Stem vs Leaf	Isorhamnetin-3,7-O-diglucoside	Flavonoids	Flavonols	1.156688372	0.02591297	0.064103755	10.57812448	3.403011953	3

Comparison	Compound	Class I	Class II	VIP	P-value	FDR	Fold Change	Log_2FC	MSI Level
Stem vs Leaf	cis-Zeatin-O-glucoside riboside	Alkaloids	Alkaloids	1.155539242	0.02224756	0.059198333	2.098172635	1.069133386	3
Stem vs Leaf	Episyringaresinol	Lignans and Coumarins	Lignans	1.177564688	0.003888419	0.028194402	21.13073131	4.401270793	1
Stem vs Leaf	3,5-Dicaffeoylquinic acid	Phenolic acids	Phenolic acids	1.179546394	0.006849322	0.033824603	0.061636373	-4.020074226	3
Stem vs Leaf	Frehmaglutoside G	Terpenoids	Monoterpenoids	1.178872624	0.008543315	0.037420256	0.098605927	-3.34218183	3
Stem vs Leaf	2-(4-(3-(hydroxymethyl)-5-(2-hydroxyvinyl)-7-methoxy-2,3-dihydrobenzofuran-2-yl)-2,	Lignans and Coumarins	Lignans	1.175664556	0.042591062	0.087966135	0.017380299	-5.846403251	2

Comparison	Compound	Class I	Class II	VIP	P-value	FDR	Fold Change	Log_2FC	MSI Level
	6-dimethoxyphenoxy)-6-methyltetrahydro-2H-pyran-3,4,5-triol*								
Stem vs Leaf	LysoPC 18:3	Lipids	LPC	1.042904713	0.083905942	0.136013144	2.312107017	1.209208175	2
Stem vs Leaf	Petunidin-3-O-glucoside-5-O-arabinoside	Flavonoids	Anthocyanidins	1.148339305	0.001200399	0.016758819	0.015966425	-5.968814914	3
Stem vs Leaf	cyclo-Dopa 5-O-glucoside	Alkaloids	Alkaloids	1.128365921	0.002740632	0.024478889	0.012048122	-6.375047947	3
Stem vs Leaf	Resinoside B*	Flavonoids	Flavones	1.168637859	0.003234686	0.026581091	0.07561269	-3.725227805	3
Stem vs Leaf	3α-Dihydrocadambine	Alkaloids	Plumerane	1.10668083	0.016654402	0.050805983	0.493025239	-1.020266593	3

Comparison	Compound	Class I	Class II	VIP	P-value	FDR	Fold Change	Log_2FC	MSI Level
Stem vs Leaf	ent-3α,16β,17,19-Tetrahydroxykauran-19-ylacetate-17-O-β-D-glucopyranoside	Terpenoids	Ditepenoids	1.167852785	0.049977371	0.097483935	0.027783506	-5.169627529	3
Stem vs Leaf	Olean-12-en-3-one (β-Amyrone)	Terpenoids	Triterpene	1.180887855	0.008219163	0.036758126	0.007779232	-7.006156532	1
Stem vs Leaf	Chisopanin D	Terpenoids	Triterpene	1.167298359	0.018571817	0.054104886	0.043992057	-4.506613125	3
Stem vs Leaf	2-Hydroxymelatonin	Alkaloids	Plumerane	1.177823067	0.044242782	0.090163016	160.4388556	7.325879771	3
Stem vs Leaf	2-O-p-Coumaroyl 3-methylglucaric acid*	Phenolic acids	Phenolic acids	1.108774746	7.70124E-05	0.005487142	0.124051261	-3.010991694	1

Comparison	Compound	Class I	Class II	VIP	P-value	FDR	Fold Change	Log_2FC	MSI Level
Stem vs Leaf	3-Oxo-Alpha-Ionol 9-[Apiosyl-(1→6)-Glucoside]	Terpenoids	Monoterpenoids	1.120750813	0.028620329	0.06817466	0.290293615	-1.784415253	3
Stem vs Leaf	3'-methoxyquercetin-3-O-L-rhamnosyl(1→2)-glucopyranoside*	Flavonoids	Flavonols	1.126943017	0.004759849	0.029596181	0.023977567	-5.382170924	1
Stem vs Leaf	LysoPC 18:3(2n isomer)	Lipids	LPC	1.003117047	0.089600708	0.142486333	2.264527061	1.17920978	2
Stem vs Leaf	Cytosine	Nucleotides and derivatives	Nucleotides and derivatives	1.161557246	0.02016023	0.055784421	3.78759163	1.921280791	3

Comparison	Compound	Class I	Class II	VIP	P-value	FDR	Fold Change	Log_2FC	MSI Level
Stem vs Leaf	D-Sorbitol	Others	Saccharides	1.140723572	0.064080215	0.113851941	32.39550026	5.017721531	1
Stem vs Leaf	LysoPE 15:0(2n isomer)*	Lipids	LPE	1.14682561	0.076168671	0.127441746	6.703454928	2.744904845	3
Stem vs Leaf	Vincoside lactam	Alkaloids	Plumerane	1.132080638	0.011636951	0.041906997	0.37971326	-1.397017716	3
Stem vs Leaf	(9S)-Hydroxyocta decadienoic acid	Lipids	Free fatty acids	1.08150113	0.155413943	0.211883308	10.04955996	3.329060426	1
Stem vs Leaf	(3-(carboxyamino)-2-methylpropan oyl)phenylalanine *	Amino acids and derivatives	Amino acids and derivatives	1.146659366	0.040571392	0.085267344	6.413649519	2.681145518	3
Stem vs Leaf	Demethylwedelol actone	Lignans and Coumarins	Coumarins	1.180886326	0.002343313	0.022694245	104.3601263	6.705426785	2

Comparison	Compound	Class I	Class II	VIP	P-value	FDR	Fold Change	Log_2FC	MSI Level
Stem vs Leaf	1-Oleoyl-Sn-Glycerol	Lipids	Glycerol ester	1.080880249	0.1684276	0.225198595	18.13926133	4.181043802	3
Stem vs Leaf	N-Methyl-Trans-4-Hydroxy-L-Proline	Amino acids and derivatives	Amino acids and derivatives	1.102901984	0.053722275	0.10176487	0.415065594	-1.268588748	3
Stem vs Leaf	1-(4-Hydroxybenzoyl)Glucose; 25545-07-7	Phenolic acids	Phenolic acids	1.173991535	0.002411132	0.02293776	3.851033653	1.945245731	3
Stem vs Leaf	Epicatechin-(2beta->7,4beta->8)-epiafzelechin-(4beta->8)-epicatechin	Flavonoids	Flavanols	1.137162038	0.00058897	0.013615966	3.184401717	1.671022346	2
Stem vs Leaf	N-(Octadecyldien	Alkaloids	Pyridine	1.048459808	0.072022705	0.122216903	2.95415396	1.562745016	3

Comparison	Compound	Class I	Class II	VIP	P-value	FDR	Fold Change	Log_2FC	MSI Level
	yl)nornicotine		alkaloids						
Stem vs Leaf	Histamine	Alkaloids	Alkaloids	1.156401937	0.020013182	0.055784421	3.166932778	1.663086245	3
Stem vs Leaf	homoisocitricacid	Organic acids	Organic acids	1.169657843	0.015546036	0.049008766	0.052395069	-4.254425152	2
Stem vs Leaf	2,3,7,19-Tetrahydroxyurs-12-en-28-oic acid (Roxburic Acid)*	Terpenoids	Triterpene	1.168875587	0.014960873	0.048294837	10.17197418	3.3465278	2
Stem vs Leaf	awabukinol	Terpenoids	Monoterpenoids	1.170809514	0.030035208	0.07033949	0.069312515	-3.850740314	3
Stem vs Leaf	Bletilols B	Others	Others	1.170919979	0.019439038	0.055429619	6.494494757	2.699217295	3
Stem vs Leaf	Isohemiphloin	Flavonoids	Flavanones	1.1660603	0.020415469	0.056345645	0.200548841	-2.317974466	3

Comparison	Compound	Class I	Class II	VIP	P-value	FDR	Fold Change	Log_2FC	MSI Level
Stem vs Leaf	Dihydroxybenzoyl xyloside	Phenolic acids	Phenolic acids	1.133564872	0.0160106	0.049628637	0.133915825	-2.900601638	1
Stem vs Leaf	2,3,23-Trihydroxyolean-12-en-28-oic acid	Terpenoids	Triterpene	1.056649943	0.144677936	0.199651838	16.56202503	4.049807176	3
Stem vs Leaf	Kaempferol-3-O-rutinoside(Nicotiflorin)*	Flavonoids	Flavonols	1.179152654	0.007883537	0.036127578	0.082572702	-3.59819127	2
Stem vs Leaf	Quercetin	Flavonoids	Flavonols	1.072643004	0.076309681	0.127578394	2.752835037	1.460918159	1
Stem vs Leaf	3-O-p-Coumaroyl 3-methylglucaric acid*	Phenolic acids	Phenolic acids	1.160302426	2.23505E-05	0.00257283	0.126622421	-2.981395205	1
Stem vs Leaf	α-Amyrenone	Terpenoids	Triterpene	1.180811367	0.010443567	0.040169517	0.005595258	-7.481579542	1

Comparison	Compound	Class I	Class II	VIP	P-value	FDR	Fold Change	Log_2FC	MSI Level
Stem vs Leaf	6-Hydroxyluteolin 7-rutinoside	Flavonoids	Flavones	1.181423171	0.0046674	0.029514444	0.002873115	-8.443168416	1
Stem vs Leaf	1-(β-D-Glucopyranosyl)-3-ethyl-4-methyl-1H-pyrrole-2,5-dione	Others	Ketone compounds	1.090661813	0.054738086	0.102929846	2.436795034	1.284984907	1
Stem vs Leaf	Dehydrodiconiferylalcohol-9'-O-glucoside*	Lignans and Coumarins	Lignans	1.180078286	0.03986551	0.084527462	0.000593646	-10.71810947	2
Stem vs Leaf	LysoPC 19:2(2n isomer)	Lipids	LPC	1.127831045	0.008357225	0.037195976	4.521328643	2.176746787	3
Stem vs Leaf	Darendoside A	Phenolic acids	Phenolic acids	1.15817225	4.48151E-05	0.00395377	6.14808157	2.620136306	3

Comparison	Compound	Class I	Class II	VIP	P-value	FDR	Fold Change	Log_2FC	MSI Level
Stem vs Leaf	6-O-Dihydrocaffeoylglucose	Phenolic acids	Phenolic acids	1.095417054	0.052524138	0.100379463	3.190448515	1.673759253	1
Stem vs Leaf	Loliolide	Terpenoids	Monoterpenoids	1.070689082	0.041520542	0.086585029	0.465533138	-1.103044229	3
Stem vs Leaf	LysoPC 17:0(2n isomer)*	Lipids	LPC	1.023662715	0.094776847	0.14895484	2.253472417	1.172149791	1
Stem vs Leaf	Isoetin (5,7,2',4',5'-Pentahydroxyflavone)	Flavonoids	Flavones	1.116705286	0.070808175	0.12091944	2.935051803	1.553385967	3
Stem vs Leaf	Raffinose*	Others	Saccharides	1.123191113	0.070465872	0.120622313	6.019331744	2.58960333	2
Stem vs Leaf	Gossypetin-3-O-(6''-malonyl)glucoside*	Flavonoids	Flavonols	1.082988177	0.097686092	0.151913881	3.163298806	1.661429839	2

Comparison	Compound	Class I	Class II	VIP	P-value	FDR	Fold Change	Log_2FC	MSI Level
Stem vs Leaf	4-Ethylphenol glucuronide	Phenolic acids	Phenolic acids	1.17848163	0.010101426	0.039703961	0.02512806	-5.314556883	3
Stem vs Leaf	Feruloyltartaric acid (Fertaric acid)	Phenolic acids	Phenolic acids	1.128500283	0.044710284	0.090857383	3.757632992	1.909824166	1
Stem vs Leaf	Octadeca-9,12,15-trienoic acid	Lipids	Free fatty acids	1.006951108	0.166966089	0.223801179	3.4986624	1.80680346	3
Stem vs Leaf	Blumenol B Glucoside*	Terpenoids	Sesquiterpenoids	1.158370013	0.000470429	0.013133103	3.309166399	1.726467838	1
Stem vs Leaf	Gardoside	Terpenoids	Monoterpenoids	1.178711378	0.009000784	0.037930918	24.37912658	4.607574535	2
Stem vs Leaf	Piperonylic acid	Phenolic acids	Phenolic acids	1.14593689	0.000527822	0.013142389	2.301110446	1.20233023	3

Comparison	Compound	Class I	Class II	VIP	P-value	FDR	Fold Change	Log_2FC	MSI Level
Stem vs Leaf	6-Hydroxykaempferol 3,6-diglucoside*	Flavonoids	Flavonols	1.159464952	0.002778069	0.024478889	2.941892863	1.556744708	3
Stem vs Leaf	Epicatechin-(3'-O-7'')-epiafzelechin	Flavonoids	Flavanols	1.171381188	0.00365821	0.027890607	4.72815138	2.241276227	1
Stem vs Leaf	(4R)-4-[(1E,3R)-4-hydroxy-3-{[(2R,3R,4S,5R,6R)-3,4,5-trihydroxy-6-(hydroxymethyl)oxan-2-yl]oxy}but-1-en-1-yl]-3,5,5-trimethylcyclohex-2-en-1-one	Others	Others	1.1103768	0.004391949	0.02880574	0.346241723	-1.53014851	3

Comparison	Compound	Class I	Class II	VIP	P-value	FDR	Fold Change	Log_2FC	MSI Level
Stem vs Leaf	Isovanillin*	Others	Aldehyde compounds	1.079852006	0.010462484	0.040169517	5.33196409	2.414667064	3
Stem vs Leaf	Byzantionoside A	Others	Ketone compounds	1.161401621	0.069509442	0.119558219	0.058183616	-4.103243225	3
Stem vs Leaf	Pelargonidin-3,5-O-diglucoside	Flavonoids	Anthocyanidin s	1.152900218	0.010139772	0.039720462	0.010439733	-6.58177139	3
Stem vs Leaf	γ-Glu-Phe*	Amino acids and derivatives	Amino acids and derivatives	1.119522239	0.090122282	0.143039468	5.888658989	2.55793913	3
Stem vs Leaf	Osmanthuside H[2-(4-Hydroxyp henyl)ethyl-β-D-a piosyl-(1→6)-β-D	Others	Alcohol compounds	1.15232988	1.15348E-05	0.002168992	9.699611133	3.277926909	3

Comparison	Compound	Class I	Class II	VIP	P-value	FDR	Fold Change	Log_2FC	MSI Level
	-glucoside]								
Stem vs Leaf	Madreselvin A*	Flavonoids	Flavones	1.168916281	0.008106954	0.036632739	13.53623996	3.758755143	3
Stem vs Leaf	Dulcitol*	Others	Saccharides	1.164523626	0.054905032	0.102929846	52.06065902	5.702121669	1
Stem vs Leaf	2-Methoxy-1-benzofuran-5-carbaldehyde	Others	Aldehyde compounds	1.124854324	0.049854308	0.097403476	4.357811632	2.123603837	3
Stem vs Leaf	2-Hydroxymethyl-A(1)-nor-19,24-dihydroxyurs-2,12-dien-28-oic acid (Rosamultic acid)	Terpenoids	Triterpene	1.172112868	0.017202695	0.052065312	6.385969239	2.674905604	1
Stem vs Leaf	Resinoside A*	Flavonoids	Flavones	1.162351899	0.008662506	0.037603549	0.080883442	-3.628011801	3

Comparison	Compound	Class I	Class II	VIP	P-value	FDR	Fold Change	Log_2FC	MSI Level
Stem vs Leaf	Solatriose	Others	Saccharides	1.172427432	0.034587365	0.076874419	147.1881115	7.201517339	3
Stem vs Leaf	Esculetin-7-O-quinic acid	Lignans and Coumarins	Coumarins	1.180889591	0.008564172	0.037420256	0.007477466	-7.063234773	3
Stem vs Leaf	2,3-Dihydroxyurs-12,18-dien-28-oic acid (Goreishic Acid I)*	Terpenoids	Triterpene	1.005387027	0.12871506	0.18375656	4.541562311	2.183188674	1
Stem vs Leaf	LysoPC 19:2	Lipids	LPC	1.10211612	0.004444175	0.02880574	3.329521146	1.735314703	3
Stem vs Leaf	2-Propylglutaric acid	Organic acids	Organic acids	1.091790056	0.041496637	0.086585029	0.159777141	-2.645867076	3
Stem vs Leaf	LysoPC 18:2(2n isomer)	Lipids	LPC	1.169375682	0.02013735	0.055784421	13.40304183	3.744488554	2

Comparison	Compound	Class I	Class II	VIP	P-value	FDR	Fold Change	Log_2FC	MSI Level
Stem vs Leaf	Creoside IV	Others	Others	1.136129303	0.006395992	0.032812524	0.448346662	-1.157313439	3
Stem vs Leaf	Peonidin-3-O-sambubioside	Flavonoids	Anthocyanidins	1.176467673	0.015655711	0.049138362	0.069197799	-3.853130042	3
Stem vs Leaf	Glutathione reduced form	Amino acids and derivatives	Amino acids and derivatives	1.073147166	0.145729399	0.200973834	0.035674978	-4.808943658	1
Stem vs Leaf	6'-O-β-D-glucosyl barlerin*	Terpenoids	Monoterpenoids	1.129069133	0.000744262	0.014479152	0.171862996	-2.540669148	3
Stem vs Leaf	Hypericumoxide N	Others	Others	1.143208329	0.025554232	0.063904927	0.020164927	-5.63200799	3
Stem vs Leaf	3-O-p-Coumaroyl Methylglucaric Acid*	Phenolic acids	Phenolic acids	1.151334781	0.004145236	0.028378135	0.136729815	-2.870600226	1

Comparison	Compound	Class I	Class II	VIP	P-value	FDR	Fold Change	Log_2FC	MSI Level
Stem vs Leaf	3-Hydroxy-29(or30)-al-olean-12-en-28-oic acid*	Terpenoids	Triterpene	1.036345704	0.117816624	0.172861275	4.779614192	2.256894169	1
Stem vs Leaf	Asp-Lys*	Amino acids and derivatives	Amino acids and derivatives	1.164580184	0.017588504	0.052669879	0.368554959	-1.440048326	3
Stem vs Leaf	Secoisolariciresinol-9'-O-glucoside	Lignans and Coumarins	Lignans	1.097327096	0.015005094	0.048294837	0.169193338	-2.563255329	3
Stem vs Leaf	6-Hydroxy-3,9-Undecadienoic acid Methyl Ester	Lipids	Free fatty acids	1.147420758	0.006859315	0.033824603	0.06753856	-3.88814478	1
Stem vs Leaf	LysoPC 17:2	Lipids	LPC	1.090659276	0.015076769	0.048380676	8.044180112	3.007945384	3
Stem vs Leaf	Fraxidin	Lignans and	Coumarins	1.134526993	0.133961586	0.188684757	38.85938572	5.28019119	1

Comparison	Compound	Class I	Class II	VIP	P-value	FDR	Fold Change	Log_2FC	MSI Level
	(8-Hydroxy-6,7-dimethoxycoumarin)	Coumarins							
Stem vs Leaf	5-Acetonyl-6-glucosyl-7-hydroxy-2,6-methylchromone	Others	Chromone	1.177275227	0.00767328	0.03555507	15.02966598	3.909741042	3
Stem vs Leaf	Coniferin	Others	Alcohol compounds	1.178086977	0.009283753	0.038406318	30.97681711	4.953117009	3
Stem vs Leaf	Naringenin-4'-O-glucoside*	Flavonoids	Flavanones	1.147483942	0.000561437	0.013412103	0.335094014	-1.577362178	2
Stem vs Leaf	Caffeic acid	Phenolic acids	Phenolic acids	1.005246584	0.22117041	0.277235656	0.080933112	-3.627126118	3

Comparison	Compound	Class I	Class II	VIP	P-value	FDR	Fold Change	Log_2FC	MSI Level
Stem vs Leaf	4-O-(6'-O-Glucosylcaffeoylglucosylferuloyl)-4-hydroxybenzyl alcohol	Phenolic acids	Phenolic acids	1.156718978	0.002301731	0.022494185	0.007900331	-6.983871105	3
Stem vs Leaf	Fumaric acid	Organic acids	Organic acids	1.100611702	0.0180251	0.053398908	0.474628959	-1.075127968	3
Stem vs Leaf	3-pyridine-methanol-O-β-D-glucopyranosyl*	Alkaloids	Pyridine alkaloids	1.157246615	0.001998749	0.02075995	0.337902042	-1.565323026	1
Stem vs Leaf	Syringetin-7-O-glucoside*	Flavonoids	Flavonols	1.171216721	0.008587849	0.037420256	13.71251831	3.777421642	3
Stem vs Leaf	Fisetin	Flavonoids	Flavonols	1.18135972	0.004718142	0.029574357	0.00384725	-8.021956873	3
Stem vs Leaf	Santalol A	Terpenoids	Sesquiterpenoi	1.150046329	0.013108393	0.044664097	0.008280704	-6.916030874	3

Comparison	Compound	Class I	Class II	VIP	P-value	FDR	Fold Change	Log_2FC	MSI Level
			ds						
Stem vs Leaf	Scopoletin-7-O-xylosyl(1→6)glucoside(Fabiatrin)*	Lignans and Coumarins	Coumarins	1.173743966	0.00029561	0.010592684	9.05775625	3.179153716	3
Stem vs Leaf	1,4,8-trihydroxynaphthalen-1-O-β-D-[6'-O-(3'',4'',5''-trihydroxybenzoyl)]glucopyranoside	Quinones	Quinones	1.175659308	0.019747649	0.055718431	0.081435462	-3.618199016	2
Stem vs Leaf	Apigenin-7-O-rutinoside (Isorhoifolin)*	Flavonoids	Flavones	1.177341556	0.0106163	0.040469939	7.899406602	2.981744283	3

Comparison	Compound	Class I	Class II	VIP	P-value	FDR	Fold Change	Log_2FC	MSI Level
Stem vs Leaf	6'-O-α-D-galactosylbarlerin*	Terpenoids	Monoterpenoids	1.085447588	0.002448423	0.023017917	0.233980332	-2.095540828	3
Stem vs Leaf	Deutzioside	Terpenoids	Monoterpenoids	1.171458075	0.047908321	0.094933539	27.61559181	4.78741114	3
Stem vs Leaf	Quinacyl syringic acid	Phenolic acids	Phenolic acids	1.181296457	0.004816495	0.029756796	0.00631889	-7.306113109	3
Stem vs Leaf	Guvacoline	Alkaloids	Piperidine alkaloids	1.170319377	0.000816303	0.014923977	0.484211838	-1.046289743	3
Stem vs Leaf	Ovafolinin E	Lignans and Coumarins	Lignans	1.091043446	0.118108471	0.172861275	5.113649463	2.354353268	3
Stem vs Leaf	9,12,13-TriHOME; 9(S),12(S),13(S)-Trihydroxy-10(E)-oc	Lipids	Free fatty acids	1.165994498	0.056313823	0.104464814	22.4050182	4.485749993	2

Comparison	Compound	Class I	Class II	VIP	P-value	FDR	Fold Change	Log_2FC	MSI Level
	tadecenoic acid								
Stem vs Leaf	6-Hydroxykaempferol-7,6-O-Diglucoside	Flavonoids	Flavonols	1.146539309	0.006447196	0.032910525	0.331360891	-1.593524758	3
Stem vs Leaf	O-Feruloyl 3-hydroxycoumarin*	Lignans and Coumarins	Coumarins	1.166808997	0.049705357	0.09732834	0.066868533	-3.902528724	2
Stem vs Leaf	Tetraphyllin A*	Alkaloids	Alkaloids	1.178496756	5.40504E-05	0.004469549	0.255330743	-1.969560837	1
Stem vs Leaf	Herbacetin-7-O-rhamnoside-8-O-glucoside; Rhodionidin*	Flavonoids	Flavonols	1.147228418	0.023989796	0.061992862	0.014379227	-6.119870107	3
Stem vs Leaf	4-Hydroxysphing	Lipids	Sphingolipids	1.153865766	0.017324994	0.052096135	0.112819437	-3.147912452	2

Comparison	Compound	Class I	Class II	VIP	P-value	FDR	Fold Change	Log2FC	MSI Level
	anine; Phytosphingosine								
Stem vs Leaf	Chaenorrhinoside	Others	Others	1.178039043	0.007394133	0.035212759	26.42485827	4.723823829	3
Stem vs Leaf	6-Hydroxykaempferol-3,6-O-Diglucoside*	Flavonoids	Flavonols	1.119794979	0.069567157	0.119559861	3.348962347	1.743714155	3
Stem vs Leaf	1-O-Caffeoyl-β-D-glucose*	Phenolic acids	Phenolic acids	1.180596049	0.002142929	0.021939512	29.27925161	4.871806773	1
Stem vs Leaf	LysoPC 18:0(2n isomer)	Lipids	LPC	1.038103563	0.089818124	0.14272651	2.150104076	1.104406495	1
Stem vs Leaf	2-Hydroxyhexadecanoic acid*	Lipids	Free fatty acids	1.027017566	0.106587046	0.161381795	3.354238518	1.745985282	1

Comparison	Compound	Class I	Class II	VIP	P-value	FDR	Fold Change	Log_2FC	MSI Level
Stem vs Leaf	4-Pyridoxic acid	Others	Vitamin	1.173335573	0.000277642	0.010117467	0.204001467	-2.293348568	3
Stem vs Leaf	Secoisolariciresinol-sesquilignan	Lignans and Coumarins	Lignans	1.179985381	0.003987269	0.028204938	63.78399865	5.995122639	3
Stem vs Leaf	Meratin*	Flavonoids	Flavonols	1.038994523	0.160251785	0.216284582	0.272456833	-1.875900422	3
Stem vs Leaf	HydroxyAloe-emodin-8-O-glucoside	Quinones	Anthraquinone	1.05461446	0.077194356	0.128458099	0.317911232	-1.653304108	3
Stem vs Leaf	3'-Deoxy-4-O-methylsappanol	Others	Alcohol compounds	1.176213638	0.015569647	0.049011334	0.093786219	-3.414480235	3
Stem vs Leaf	coleoside-B	Terpenoids	Monoterpenoids	1.163658703	0.037438384	0.081141659	6.095794968	2.607814379	3
Stem vs Leaf	Nodakenin	Lignans and	Coumarins	1.135540172	0.01300205	0.044372077	0.343414377	-1.541977654	2

Comparison	Compound	Class I	Class II	VIP	P-value	FDR	Fold Change	Log_2FC	MSI Level
		Coumarins							
Stem vs Leaf	3,4-Dihydroxy-L-phenylalanine (L-Dopa)	Amino acids and derivatives	Amino acids and derivatives	1.16479431	0.030758879	0.071431365	0.097990498	-3.351214334	3
Stem vs Leaf	Quercetin-3-O-(2''-O-rhamnosyl)galactoside*	Flavonoids	Flavonols	1.174947959	0.025927248	0.064103755	0.070908234	-3.81790302	3
Stem vs Leaf	Arecatannin B1	Tannins	Tannin	1.125156963	0.055981078	0.104297502	3.290872059	1.718469939	3
Stem vs Leaf	16-Hydroxy-3-oxo-5-lanosta-7,24-dien-21-oic acid (Kulonic acid)	Terpenoids	Triterpene	1.093575951	0.10518462	0.159934182	9.249543814	3.209382214	3
Stem vs Leaf	Barbituric	Nucleotides	Nucleotides	1.167972129	0.001044798	0.016277656	6.426130625	2.683950307	3

Comparison	Compound	Class I	Class II	VIP	P-value	FDR	Fold Change	Log_2FC	MSI Level
	acid;Malonylurea; 2,4,6-Pyrimidinetrione	and derivatives	and derivatives						
Stem vs Leaf	Guaiacylglycerol 8-O-(1''-O-Glucosyl)Vanillic Acid Ether	Phenolic acids	Phenolic acids	1.171510715	0.009218058	0.038367347	52.34746448	5.710047755	1
Stem vs Leaf	Lys-Asp*	Amino acids and derivatives	Amino acids and derivatives	1.171449369	0.012584587	0.043429955	0.279963345	-1.836690143	3
Stem vs Leaf	6-Hydroxymethyl herniarin	Lignans and Coumarins	Coumarins	1.156284254	0.000337903	0.011007445	0.442647882	-1.175768577	3
Stem vs Leaf	2,6-Dimethoxy-4-	Others	Others	1.171178218	0.001942468	0.020667459	0.059161883	-4.079188226	1

Comparison	Compound	Class I	Class II	VIP	P-value	FDR	Fold Change	Log_2FC	MSI Level
	hydroxyphenol-1-O-ß-D-glucopyranoside								
Stem vs Leaf	4,6-dimethoxy-2-methoxyphenyl-1-O-beta-D-glucopyranoside	Others	Others	1.180835193	0.000506999	0.013133103	28.84837375	4.850418088	3
Stem vs Leaf	Rhamnetin-3-O-Rutinoside*	Flavonoids	Flavonols	1.150579863	0.027987463	0.067562482	0.041073206	-4.605658615	1
Stem vs Leaf	2-Methoxy-4-ethenylphenol	Others	Others	1.138687494	0.005063162	0.030492431	5.287952604	2.402709246	3
Stem vs Leaf	Hesperetin-7-O-rutinoside	Flavonoids	Flavanones	1.161799833	0.000218144	0.008915744	4.797420573	2.262258921	3

Comparison	Compound	Class I	Class II	VIP	P-value	FDR	Fold Change	Log_2FC	MSI Level
	(Hesperidin)								
Stem vs Leaf	Catechin-(4alpha →8)-gallocatechin	Flavonoids	Flavanols	1.122263293	0.001646605	0.019240218	0.011492677	-6.44314133	1
Stem vs Leaf	1´-O-Benzoylsucrose	Phenolic acids	Phenolic acids	1.174215255	0.023468087	0.061159258	24.60983392	4.621163016	3
Stem vs Leaf	N-α-Acetyl-L-ornithine	Amino acids and derivatives	Amino acids and derivatives	1.106681654	0.138158328	0.192383682	14.42710939	3.850710366	3
Stem vs Leaf	Elaeocarpucin B	Terpenoids	Triterpene	1.151685125	0.064919967	0.114596001	7.504172881	2.907693065	3
Stem vs Leaf	9,10,13-Trihydroxy-11-Octadecenoic Acid	Lipids	Free fatty acids	1.170816412	0.042686047	0.088075817	20.52823319	4.359537559	1

Comparison	Compound	Class I	Class II	VIP	P-value	FDR	Fold Change	Log_2FC	MSI Level
Stem vs Leaf	Petunidin-3-O-(6''-O-p-Coumaroyl)glucoside	Flavonoids	Anthocyanidins	1.163035522	0.024636795	0.063058464	0.034249528	-4.867772065	3
Stem vs Leaf	4-O-Glucosyl-sinapate	Phenolic acids	Phenolic acids	1.110288604	0.077191438	0.128458099	3.343495387	1.741357127	2
Stem vs Leaf	6-O-Caffeoyl-D-glucose*	Phenolic acids	Phenolic acids	1.17994661	0.007658545	0.03555507	32.97961996	5.043502869	1
Stem vs Leaf	2,3-Dihydroxy-5(6),12(13)-diene-ursolic acid*	Terpenoids	Triterpene	1.092751139	0.106299628	0.161102216	8.689795726	3.119322264	1
Stem vs Leaf	1-Hexanol arabinosylglucoside	Others	Alcohol compounds	1.142323524	0.018526424	0.054045877	0.391275693	-1.353742605	1

Comparison	Compound	Class I	Class II	VIP	P-value	FDR	Fold Change	Log_2FC	MSI Level
Stem vs Leaf	sophorabioside*	Flavonoids	Isoflavones	1.118813587	0.066528699	0.116384624	4.715356514	2.237366853	3
Stem vs Leaf	Monogalactosyldiacylglycerol	Lipids	Glycerol ester	1.139750812	0.042015673	0.087363344	0.050310317	-4.313001908	1
Stem vs Leaf	Dimethoxysamin	Lignans and Coumarins	Lignans	1.176619135	0.018497279	0.054034171	16.66638825	4.058869589	3
Stem vs Leaf	Pratensein 7-O-glucopyranoside	Flavonoids	Isoflavones	1.126816621	0.040049951	0.084751372	2.750059205	1.459462678	2
Stem vs Leaf	Cleomiscosin A	Lignans and Coumarins	Lignans	1.153443546	0.049427556	0.097049539	8.255585265	3.045370496	2
Stem vs Leaf	Cis-Aconitic acid	Organic acids	Organic acids	1.156398526	0.03232324	0.073384337	0.13730908	-2.86450106	3

Comparison	Compound	Class I	Class II	VIP	P-value	FDR	Fold Change	Log_2FC	MSI Level
Stem vs Leaf	5-Hydroxytryptophol	Alkaloids	Plumerane	1.096054123	0.025209288	0.063689739	3.190625124	1.673839112	3
Stem vs Leaf	Persicoside*	Flavonoids	Flavanones	1.178460217	0.005842499	0.032023409	22.18004685	4.471190508	2
Stem vs Leaf	Theasinensin C	Tannins	Proanthocyanidins	1.148989323	0.006060754	0.032414478	0.014652785	-6.092681256	3
Stem vs Leaf	N6-Acetyl-L-lysine	Amino acids and derivatives	Amino acids and derivatives	1.142535136	0.050162533	0.097483935	7.351382381	2.878015565	3
Stem vs Leaf	2,3-Dihydroxyurs-12,18-dien-28-oic acid*	Terpenoids	Triterpene	1.094479387	0.101970882	0.156822173	7.727665532	2.950032653	1
Stem vs Leaf	Grevilloside B	Others	Others	1.176362508	0.002701825	0.024430315	0.197070531	-2.343216038	3

Comparison	Compound	Class I	Class II	VIP	P-value	FDR	Fold Change	Log_2FC	MSI Level
Stem vs Leaf	Quercetin 3,7-diglucoside*	Flavonoids	Flavonols	1.116249534	0.058378639	0.106773866	0.352372533	-1.504826621	3
Stem vs Leaf	2,3'-Di-feruloyl sucrose	Phenolic acids	Phenolic acids	1.167123737	0.000706575	0.014479152	0.028841577	-5.115706156	1
Stem vs Leaf	L-Fucitol	Others	Saccharides	1.147880464	0.003381121	0.026923741	0.178773574	-2.483794598	3
Stem vs Leaf	L-Citrulline	Amino acids and derivatives	Amino acids and derivatives	1.068414977	0.102523462	0.157334363	3.010567268	1.590035353	3
Stem vs Leaf	LysoPE 17:1*	Lipids	LPE	1.134598154	0.042227264	0.087549294	13.04349033	3.705258069	3
Stem vs Leaf	Protocatechualdehyde	Others	Aldehyde compounds	1.049522277	0.120531291	0.174977905	0.42753053	-1.225900647	3
Stem vs Leaf	Ssioriside	Lignans and	Lignans	1.172155753	0.01952861	0.055577281	0.069583018	-3.845120926	3

Comparison	Compound	Class I	Class II	VIP	P-value	FDR	Fold Change	Log_2FC	MSI Level
Stem vs Leaf	3'-p-Coumaroyl-sucrose	Coumarins Phenolic acids	Phenolic acids	1.178690917	0.003073684	0.025963636	0.063492386	-3.9772726	2
Stem vs Leaf	N,N-Dimethyl-5-methoxytryptamine	Alkaloids	Plumerane	1.137545258	0.004030566	0.028204938	2.699960852	1.432938489	3
Stem vs Leaf	Astraflavonoid C	Flavonoids	Other Flavonoids	1.181628043	0.001408752	0.017778885	0.002089603	-8.90255508	3
Stem vs Leaf	isatioxyneolignoside A	Lignans and Coumarins	Lignans	1.172792072	0.017638104	0.052669879	93.19057618	6.542112166	3
Stem vs Leaf	o-Carboxy-5-hydroxytryptamine	Alkaloids	Alkaloids	1.119291064	0.002051968	0.021133472	0.101227562	-3.304325943	1

Comparison	Compound	Class I	Class II	VIP	P-value	FDR	Fold Change	Log_2FC	MSI Level
Stem vs Leaf	Stachydrine	Alkaloids	Pyrrole alkaloids	1.044884271	0.15284049	0.209570824	14.92498299	3.899657382	3
Stem vs Leaf	LysoPE 17:1(2n isomer)*	Lipids	LPE	1.175061077	0.011954569	0.042340248	6.120171613	2.613572107	3
Stem vs Leaf	Grayanotoxin XIX	Terpenoids	Ditepenoids	1.176954874	3.12744E-05	0.003237664	0.019957326	-5.646937745	2
Stem vs Leaf	1-O-p-Coumaroyl sedoheptulose	Phenolic acids	Phenolic acids	1.18139765	0.001042215	0.016277656	0.06478362	-3.948227104	3
Stem vs Leaf	3-Ethyl-7-hydroxyphthalide	Others	Others	1.174351798	0.015651882	0.049138362	10.8675525	3.44195516	1
Stem vs Leaf	Trans-Ferulic Acid 1'-O-[Apiosyl-(1->6)-Glucosyl] Ester	Phenolic acids	Phenolic acids	1.169050882	0.018159998	0.053557932	5.244643501	2.39084471	1

Comparison	Compound	Class I	Class II	VIP	P-value	FDR	Fold Change	Log_2FC	MSI Level
Stem vs Leaf	O-Feruloyl 4-hydroxycoumarin	Lignans and Coumarins	Coumarins	1.164609533	0.011424608	0.041808314	0.002276221	-8.779143913	2
Stem vs Leaf	(2E)-3-(1-Hydroxy-2,6,6-trimethyl-4-oxo-2-cyclohexen-1-yl)-2-propenoic acid	Terpenoids	Sesquiterpenoids	1.04741975	0.110412256	0.165541388	0.189205845	-2.401971441	3
Stem vs Leaf	3-Hydroxy-18-oxoursan-28,13-olide	Terpenoids	Triterpene	1.116823006	0.12128365	0.175832669	32.81020836	5.03607285	1
Stem vs Leaf	Chlorogenic acid methyl ester	Phenolic acids	Phenolic acids	1.176484185	0.000151587	0.007920636	0.006687018	-7.224421199	2

Comparison	Compound	Class I	Class II	VIP	P-value	FDR	Fold Change	Log_2FC	MSI Level
Stem vs Leaf	Grayanotoxin VII	Terpenoids	Ditepenoids	1.178062132	0.003438989	0.027080766	0.023774208	-5.394458887	2
Stem vs Leaf	1-(Hydroxymethyl)hexahydro-1h-pyrrolizin-2-ol	Alkaloids	Pyrrole alkaloids	1.04913057	0.010031872	0.039623149	0.15691652	-2.671930853	3
Stem vs Leaf	Tricetin-5-O-(6''-malonyl)glucoside	Flavonoids	Flavones	1.140193641	0.058812052	0.107119274	6.47072181	2.693926654	3
Stem vs Leaf	Limocitrin-7-O-glucoside*	Flavonoids	Flavonols	1.164715107	0.032548285	0.073739529	21.54276519	4.429131539	3
Stem vs Leaf	5-Hydroxy-2-pyrrolidinone	Alkaloids	Pyrrole alkaloids	1.170117741	0.008708206	0.037621317	0.445548456	-1.166345753	3
Stem vs Leaf	Fraxetin-7,8-di-O-glucoside	Lignans and Coumarins	Coumarins	1.142179492	0.007402868	0.035212759	4.490981392	2.167030744	2

Comparison	Compound	Class I	Class II	VIP	P-value	FDR	Fold Change	Log_2FC	MSI Level
Stem vs Leaf	Secoisolariciresinol 4-O-glucoside*	Lignans and Coumarins	Lignans	1.163145424	0.011478017	0.041808314	3.634607283	1.86179949	2
Stem vs Leaf	tetrahydrodehydrodiconiferyl alcohol glucoside	Lignans and Coumarins	Lignans	1.1567058	0.003304288	0.026707589	4.38398418	2.132242592	2
Stem vs Leaf	10-acetylmonomelittoside*	Terpenoids	Monoterpenoids	1.098230296	0.072081873	0.122220841	3.706644713	1.890113838	2
Stem vs Leaf	Quercetin 3-[6''-(3-hydroxy-3-methylglutaryl) galactoside]	Flavonoids	Flavonols	1.167278523	0.01408517	0.046517843	4.919357613	2.298469936	1
Stem vs Leaf	4-O-Feruloyl Aminogalactitol	Phenolic acids	Phenolic acids	1.14227677	0.025529038	0.063904927	0.238600275	-2.067332388	2

Comparison	Compound	Class I	Class II	VIP	P-value	FDR	Fold Change	Log_2FC	MSI Level
Stem vs Leaf	Tomentodione F	Others	Others	1.170792227	0.003463819	0.027080766	21.00931626	4.392957305	3
Stem vs Leaf	L-Homoglutamic acid	Amino acids and derivatives	Amino acids and derivatives	1.035333275	0.031241362	0.071910695	2.535434346	1.342232917	3
Stem vs Leaf	methyl alpha-D-fructofuranoside*	Others	Saccharides	1.127323268	0.002291193	0.022493448	0.393690692	-1.344865492	3
Stem vs Leaf	1-O-p-Coumaroyl quinic acid*	Phenolic acids	Phenolic acids	1.084107409	0.069444481	0.119558219	2.633844507	1.397170176	2
Stem vs Leaf	Kaempferol-7-O-rhamnoside	Flavonoids	Flavonols	1.111660696	0.003909692	0.028194402	0.015832624	-5.980955769	2
Stem vs Leaf	2-((6-hydroxy-4-(hydroxy(methoxy	Terpenoids	Monoterpenoids	1.157554801	0.052904502	0.100837482	0.149955769	-2.737391071	3

Comparison	Compound	Class I	Class II	VIP	P-value	FDR	Fold Change	Log_2FC	MSI Level
	)methyl)-7-methyl-1,4a,5,6,7,7a-hexahydrocyclopenta[c]pyran-1-yl)oxy)-6-(hydroxymethyl)tetrahydro-2H-pyran-3,4,5-triol								
Stem vs Leaf	4-Hydroxybenzoic acid glucosyl xyloside	Phenolic acids	Phenolic acids	1.154811988	0.001734062	0.01993708	4.877776004	2.286223509	3
Stem vs Leaf	6-((4-(3-hydroxy-2-(4-(3-hydroxypropyl)-2-methylphenoxy)propoxy)-2	Lignans and Coumarins	Lignans	1.171708122	0.011229718	0.041529903	3.73239831	1.900102954	2

Comparison	Compound	Class I	Class II	VIP	P-value	FDR	Fold Change	Log_2FC	MSI Level
	-methoxyphenoxy)methyl)tetrahydro-2H-pyran-2,3,4,5-tetraol*								
Stem vs Leaf	Securoside A	Phenolic acids	Phenolic acids	1.13289243	0.002697381	0.024430315	0.044478181	-4.49075839	1
Stem vs Leaf	Cyanidin 3-xyloside	Flavonoids	Anthocyanidins	1.116023074	0.107074457	0.16189176	0.05224487	-4.258566794	3
Stem vs Leaf	LysoPC 17:1	Lipids	LPC	1.156585919	0.015739763	0.049215964	9.822920683	3.29615205	2
Stem vs Leaf	4-Hydroxybenzoic acid 4-(6-O-sulfo)glucopyranoside	Phenolic acids	Phenolic acids	1.146391656	0.001196914	0.016758819	0.261290539	-1.936273209	1

Comparison	Compound	Class I	Class II	VIP	P-value	FDR	Fold Change	Log_2FC	MSI Level
Stem vs Leaf	Isothankunic acid	Terpenoids	Triterpene	1.151152426	0.015207645	0.04859067	3.964014847	1.986962366	1
Stem vs Leaf	Cyanidin-3-O-arabinoside	Flavonoids	Anthocyanidins	1.158875612	0.050117619	0.097483935	0.065522694	-3.931861512	3
Stem vs Leaf	DL-Methionine	Amino acids and derivatives	Amino acids and derivatives	1.024141841	0.122668771	0.177124148	2.33283287	1.222082953	3
Stem vs Leaf	4-C-Glucose-1,3,6-trihydroxy-7-methoxyxanthone	Flavonoids	Other Flavonoids	1.17534432	0.004485372	0.028959607	8.70166315	3.12129117	3
Stem vs Leaf	1-(sn-Glycero-3-phospho)-1D-myo-inositol	Others	Saccharides	1.142135033	0.088608929	0.141537294	8.534742252	3.093347585	3
Stem vs Leaf	D-Mannose*	Others	Saccharides	1.171148263	0.018261927	0.053638175	11.08971291	3.471150113	1

Comparison	Compound	Class I	Class II	VIP	P-value	FDR	Fold Change	Log_2FC	MSI Level
Stem vs Leaf	Tamarixetin-3-O-glucoside-7-O-rhamnoside*	Flavonoids	Flavonols	1.074889878	0.01843498	0.053925453	0.062905849	-3.990662025	1
Stem vs Leaf	Vomifoliol-D-glucopyranoside	Terpenoids	Monoterpenoids	1.105903893	0.026691791	0.065596583	0.425559024	-1.23256885	2
Stem vs Leaf	Sexangularetin-3-O-glucoside-7-O-rhamnoside*	Flavonoids	Flavonols	1.179638903	0.006305168	0.032812524	0.019671422	-5.66775494	1
Stem vs Leaf	Okanin-4'-(6''-O-acetyl)glucoside*	Flavonoids	Chalcones	1.178382697	0.006619286	0.033249269	21.72670239	4.441397318	3
Stem vs Leaf	11-deoxyalisol B	Terpenoids	Triterpene	1.10820082	0.030929269	0.071580116	0.118477185	-3.077318823	2
Stem vs Leaf	2-(7-hydroxy-4-methoxy-9,10-dihy	Others	Others	1.149702007	0.046936238	0.093959881	9.63847758	3.268805287	3

Comparison	Compound	Class I	Class II	VIP	P-value	FDR	Fold Change	Log_2FC	MSI Level
	drophenanthren-2-yl)-6-(hydroxy methyl)oxane-3,4,5-triol								
Stem vs Leaf	Isorhamnetin-3-O-rutinoside (Narcissin)*	Flavonoids	Flavonols	1.0708079	0.022690755	0.0600802	0.119658222	-3.063008562	1
Stem vs Leaf	1-O-P-Coumaroyl-(B-D-Glucose 6-O-Sulfate)	Phenolic acids	Phenolic acids	1.181131683	0.013967291	0.046517843	1357.188369	10.40640526	2
Stem vs Leaf	Verbasoside	Others	Alcohol compounds	1.152769057	0.021717009	0.058291597	0.00934344	-6.741830438	3
Stem vs Leaf	Tianshic acid	Lipids	Free fatty acids	1.168166109	0.053675891	0.10176487	22.11515027	4.46696314	1

Comparison	Compound	Class I	Class II	VIP	P-value	FDR	Fold Change	Log_2FC	MSI Level
Stem vs Leaf	Aminomalonic acid	Amino acids and derivatives	Amino acids and derivatives	1.16357004	8.29597E-05	0.005573854	0.274799896	-1.863546638	3
Stem vs Leaf	7R,8S-erythro-3',4,9,9'-tetrahydroxy-3-methoxy-8-O-4'-neolignan-7-O-glucopyr-anoside,	Lignans and Coumarins	Lignans	1.047223974	0.02198171	0.058781935	2.091575418	1.064590019	2
Stem vs Leaf	Syringetin-3-O-glucoside*	Flavonoids	Flavonols	1.134355098	0.003767614	0.028126281	21.55650701	4.430051519	3
Stem vs Leaf	7-Caffeoylsedoheptulose	Phenolic acids	Phenolic acids	1.180696884	0.005855206	0.032023409	0.023907314	-5.386404149	1
Stem vs Leaf	D-Pinitol*	Others	Saccharides	1.100780172	0.003693696	0.027962841	0.445897061	-1.165217403	3

Comparison	Compound	Class I	Class II	VIP	P-value	FDR	Fold Change	Log_2FC	MSI Level
Stem vs Leaf	L-Theanine	Amino acids and derivatives	Amino acids and derivatives	1.179003579	0.00055041	0.013412103	0.189247494	-2.401653896	3
Stem vs Leaf	Kaempferol-3-O-rhamnoside (Afzelin)(Kaempferin)	Flavonoids	Flavonols	1.129363453	0.001359366	0.017778885	0.018466071	-5.758979235	1
Stem vs Leaf	Agarotetrol	Others	Chromone	1.175751645	0.023009582	0.060551533	0.01909528	-5.710640084	3
Stem vs Leaf	Oxiglutatione	Amino acids and derivatives	Amino acids and derivatives	1.11856317	0.001478215	0.018029486	0.341827831	-1.54865823	3
Stem vs Leaf	Scandoside	Terpenoids	Monoterpenoids	1.078084921	0.073856088	0.124444035	2.11987913	1.083982008	2

Comparison	Compound	Class I	Class II	VIP	P-value	FDR	Fold Change	Log_2FC	MSI Level
Stem vs Leaf	Arecatannin A2	Tannins	Tannin	1.177941705	0.003068857	0.025963636	7.466738928	2.900478289	3
Stem vs Leaf	L-Glutamyl-L-Cysteinyl-Glycine	Amino acids and derivatives	Amino acids and derivatives	1.137593427	0.086014556	0.138111498	0.087233155	-3.518979615	1
Stem vs Leaf	Dihydrosinapic Acid glucoside	Phenolic acids	Phenolic acids	1.178146324	0.008597956	0.037420256	29.66897139	4.890883005	2
Stem vs Leaf	Limocitrin-3-O-galactoside*	Flavonoids	Flavonols	1.1685804	0.005791119	0.032023409	21.44408883	4.422508112	3
Stem vs Leaf	Guanosine	Nucleotides and derivatives	Nucleotides and derivatives	1.051705258	0.023168966	0.060747898	0.40714742	-1.296376833	3
Stem vs Leaf	3-(Hydroxycinnamoyl)-quinic	Phenolic acids	Phenolic acids	1.043890583	0.117801871	0.172861275	2.776177835	1.473099986	2

Comparison	Compound	Class I	Class II	VIP	P-value	FDR	Fold Change	Log_2FC	MSI Level
	acid*								
Stem vs Leaf	Laricitrin-3-O-xyloside*	Flavonoids	Flavonols	1.176450699	0.015826878	0.049315636	0.076816066	-3.702448101	3
Stem vs Leaf	7,8-Dimethoxy-6-hydroxycoumarin	Lignans and Coumarins	Coumarins	1.148113189	0.093659827	0.147548464	206.5345951	7.690239647	3
Stem vs Leaf	DGMG 18:2(2n isomer)	Lipids	Glycerol ester	1.08264737	0.229563145	0.285790829	29.10315409	4.86310361	1
Stem vs Leaf	Mansorin III	Lignans and Coumarins	Coumarins	1.150326181	0.005249478	0.030931935	0.386114835	-1.372898108	3
Stem vs Leaf	LysoPE 16:1(2n isomer)*	Lipids	LPE	1.117054729	0.084075102	0.136013144	20.69649857	4.371314808	3
Stem vs Leaf	2,7-Dimethylocta-	Terpenoids	Monoterpenoid	1.176751469	0.003414158	0.026986912	0.459729411	-1.12114313	2

Comparison	Compound	Class I	Class II	VIP	P-value	FDR	Fold Change	Log_2FC	MSI Level
	2,4-Dienal-8-ol 8-O-(beta-D-Xylopyranosyl)-beta-D-glucopyranoside		s						
Stem vs Leaf	Gambiriin B2	Flavonoids	Flavanols	1.171953333	0.003412893	0.026986912	5.967833897	2.577207382	1
Stem vs Leaf	N5-(1-((carboxymethyl)amino)-3-((2,3-dihydroxy-1-(4-methoxyphenyl)propyl)thio)-1-oxopropan-2-yl)glutamine	Amino acids and derivatives	Amino acids and derivatives	1.174056109	0.005247274	0.030931935	0.011236364	-6.475680872	2
Stem vs Leaf	1-O-Acetyl-N-des	Flavonoids	Other	1.158756377	0.047178242	0.094093896	0.038930824	-4.682943311	3

Comparison	Compound	Class I	Class II	VIP	P-value	FDR	Fold Change	Log_2FC	MSI Level
	methylrocaglamide		Flavonoids						
Stem vs Leaf	LysoPE 16:1*	Lipids	LPE	1.108346578	0.09014813	0.143039468	19.05332103	4.251970579	3
Stem vs Leaf	Blumenol C glucoside; Byzantionoside B	Terpenoids	Terpene	1.180639535	0.003984659	0.028204938	0.062195824	-4.007038468	3
Stem vs Leaf	Rhododendrol	Others	Alcohol compounds	1.077516824	0.004376698	0.02880574	0.040607971	-4.622093261	3
Stem vs Leaf	5-Galloyl-6-O-Benzoyl Glucose	Phenolic acids	Phenolic acids	1.177041881	0.000496816	0.013133103	7.86717415	2.97584552	3
Stem vs Leaf	DGMG 18:2	Lipids	Glycerol ester	1.072583321	0.235378797	0.29184799	27.89693359	4.802034645	1
Stem vs Leaf	Quercetagetin;	Flavonoids	Flavonols	1.173520157	0.022791448	0.060272587	0.086485101	-3.531404568	2

Comparison	Compound	Class I	Class II	VIP	P-value	FDR	Fold Change	Log_2FC	MSI Level
	3,3',4',5,6,7-Hexahydroxyflavone								
Stem vs Leaf	Rosmarinic acid-3'-O-glucoside	Phenolic acids	Phenolic acids	1.164524663	0.015414249	0.04873623	3.354620711	1.746149658	3
Stem vs Leaf	L-Diaminopimelic acid	Amino acids and derivatives	Amino acids and derivatives	1.158282336	0.059500712	0.108137388	23.51730334	4.555650735	3
Stem vs Leaf	3,4-dihydroxybenzaldehyde-xylose-glucoside	Others	Aldehyde compounds	1.174582271	2.12032E-05	0.00257283	4.838687327	2.274615716	3
Stem vs Leaf	1-(2,3-dihydroxypropoxy)-3-(((2-(di	Lipids	LPE	1.136374065	0.105035574	0.159820584	11.00980905	3.460717542	1

Comparison	Compound	Class I	Class II	VIP	P-value	FDR	Fold Change	Log_2FC	MSI Level
	methylamino)ethoxy)(hydroxy)phosphoryl)oxy)propan-2-yl (Z)-14-Octadecenoic Acid								
Stem vs Leaf	2-(2,3-dihydroxypropoxy)-3-(((2-(dimethylamino)ethoxy)(hydroxy)phosphoryl)oxy)propan-2-yl (Z)-14-Octadecenoic Acid	Lipids	LPE	1.130423827	0.107285664	0.162097103	10.11227682	3.338035957	1

Comparison	Compound	Class I	Class II	VIP	P-value	FDR	Fold Change	Log_2FC	MSI Level
Stem vs Leaf	Annuionone D	Others	Ketone compounds	1.052323328	0.118229773	0.172921097	0.402322258	-1.313576541	3
Stem vs Leaf	2-[[3-Mercapto-2-(alpha-glutamylamino)-1-oxopropyl]amino]acetic acid	Amino acids and derivatives	Amino acids and derivatives	1.177015745	0.043837282	0.089591404	0.003732927	-8.065477086	1
Stem vs Leaf	Rubinic acid	Terpenoids	Triterpene	1.052850351	0.121413935	0.175902938	7.075305394	2.822792421	3
Stem vs Leaf	4,8-Dihydroxyquinoline-2-carboxylic acid	Alkaloids	Quinoline alkaloids	1.177634813	0.005010316	0.03038129	0.074363792	-3.74925586	2
Stem vs Leaf	Lariciresinol-4'-O-glucoside*	Lignans and Coumarins	Lignans	1.168158053	0.012261717	0.042842	3.244006983	1.697776925	2

Comparison	Compound	Class I	Class II	VIP	P-value	FDR	Fold Change	Log_2FC	MSI Level
Stem vs Leaf	Evofolin B glucoside	Phenolic acids	Phenolic acids	1.172068766	0.026201157	0.064675646	18.47280129	4.207330754	1
Stem vs Leaf	Decanoyl arabinosylglucoside	Others	Saccharides	1.061968489	0.033406305	0.07504822	2.477516967	1.308894938	2
Stem vs Leaf	α-Hydrojuglone glucoside	Quinones	Quinones	1.178864488	0.010305994	0.040049956	0.059704539	-4.066015578	1
Stem vs Leaf	Gallocatechin-(4alpha->8)-epigallocatechin	Tannins	Proanthocyanidins	1.145804266	0.006397775	0.032812524	0.018054424	-5.791503803	2
Stem vs Leaf	Salidroside	Phenolic acids	Phenolic acids	1.000053008	0.114776505	0.17028188	2.059153584	1.042051439	3
Stem vs Leaf	2-O-P-Coumaroyl	Phenolic	Phenolic acids	1.178974719	0.004429996	0.02880574	0.007393945	-7.079439927	3

Comparison	Compound	Class I	Class II	VIP	P-value	FDR	Fold Change	Log_2FC	MSI Level
	hydroxycitric Acid	acids							
Stem vs Leaf	γ-Glu-Leu	Amino acids and derivatives	Amino acids and derivatives	1.151741691	0.056422007	0.104485198	7.36115017	2.879931203	3
Stem vs Leaf	Tuberonic acid glucoside*	Organic acids	Organic acids	1.176343044	0.020918989	0.057148446	25.58429625	4.677186644	1
Stem vs Leaf	Saccharumoside C	Others	Others	1.176868977	0.019178746	0.055099978	17.19381122	4.103817466	3
Stem vs Leaf	3,4,5-trimethoxyp henyl acetate	Others	Others	1.139727175	0.017221857	0.052065312	2.496226297	1.319748728	3
Stem vs Leaf	1,2,4-Trihydroxyn aphthalene-1,4-di	Others	Others	1.179251391	0.003920989	0.028194402	0.078599212	-3.669341334	2

Comparison	Compound	Class I	Class II	VIP	P-value	FDR	Fold Change	Log_2FC	MSI Level
	-glucoside (Lawsoniaside)								
Stem vs Leaf	β-Pseudouridine	Nucleotides and derivatives	Nucleotides and derivatives	1.124345674	0.025527896	0.063904927	0.239067011	-2.064513028	3
Stem vs Leaf	Pentadecanoic Acid	Lipids	Free fatty acids	1.083721756	0.007543162	0.035510671	3.740758464	1.903330816	2
Stem vs Leaf	Isorhamnetin-3-O-neohesperidoside*	Flavonoids	Flavonols	1.060582361	0.001535202	0.018439572	0.154333676	-2.695875199	1
Stem vs Leaf	Cyanidin-3-O-rutinoside (Keracyanin)	Flavonoids	Anthocyanidins	1.052735012	0.049083021	0.096461148	0.088311184	-3.501260026	3

Comparison	Compound	Class I	Class II	VIP	P-value	FDR	Fold Change	Log_2FC	MSI Level
Stem vs Leaf	p-Hydroxypheny-β-D-allopyranoside	Others	Others	1.17416092	0.014991181	0.048294837	0.061067417	-4.033453374	1
Stem vs Leaf	Icariside E5*	Lignans and Coumarins	Lignans	1.160091047	0.014530925	0.047479466	3.702180025	1.88837505	2
Stem vs Leaf	2α-Acetoxy-14β-[(S)-2-methyl-butyryloxy]-4(20),11-taxadiene	Others	Others	1.178479357	0.001016552	0.016277656	4.634480714	2.212407696	3
Stem vs Leaf	L-Glucose*	Others	Saccharides	1.170552839	0.019737315	0.055718431	5.34303849	2.41766041	1
Stem vs Leaf	Neoandrographolide	Terpenoids	Ditepenoids	1.179035168	0.00064033	0.014327755	0.005021591	-7.637639771	2
Stem vs Leaf	Divanillyltetrahy	Lignans and	Lignans	1.179278172	0.009068136	0.037930918	30.68153306	4.939298666	3

Comparison	Compound	Class I	Class II	VIP	P-value	FDR	Fold Change	Log_2FC	MSI Level
	drofuran*	Coumarins							
Stem vs Leaf	D-Fructose*	Others	Saccharides	1.152283913	0.037692061	0.081200331	6.523418019	2.705628079	1
Stem vs Leaf	Trans-Zeatin-1-O-Glucoside	Alkaloids	Alkaloids	1.166365618	0.013592214	0.045804483	5.493306743	2.457674853	3
Stem vs Leaf	D-Glucose*	Others	Saccharides	1.150506135	0.048663604	0.09616429	6.17992893	2.627590247	1
Stem vs Leaf	1-Linoleoyl-2-Lysophosphatidic Acid Monobutylamine Ester*	Lipids	Glycerol ester	1.170447457	0.007335499	0.03511168	13.81473746	3.78813624	1
Stem vs Leaf	L-Methionine Sulfoxide	Amino acids and derivatives	Amino acids and derivatives	1.148482653	0.00625232	0.032737441	0.451083732	-1.148532839	3

Comparison	Compound	Class I	Class II	VIP	P-value	FDR	Fold Change	Log_2FC	MSI Level
Stem vs Leaf	3-methoxy-4-((3,4,5-trihydroxy-6-((sulfooxy)methyl)tetrahydro-2H-pyran-2-yl)oxy)benzoic acid	Phenolic acids	Phenolic acids	1.077681395	0.004293606	0.028757798	0.376733469	-1.408383887	3
Stem vs Leaf	2-β-D-Glucopyranosyloxy-5-hydroxy-phenylacetic acid	Phenolic acids	Phenolic acids	1.140158167	0.038104012	0.081679615	2.417689151	1.273628765	1
Stem vs Leaf	7-Hydroxy-tryptophan*	Amino acids and derivatives	Amino acids and derivatives	1.023253472	0.008951077	0.037930918	0.081790239	-3.611927505	1
Stem vs Leaf	Andrographidine	Flavonoids	Flavanones	1.128639517	0.00092841	0.015696982	3.989538044	1.996221704	3

Comparison	Compound	Class I	Class II	VIP	P-value	FDR	Fold Change	Log_2FC	MSI Level
	A								
Stem vs Leaf	8-Epideoxyloganic acid	Terpenoids	Monoterpenoids	1.053935042	0.007967932	0.036263771	0.322049323	-1.634646437	2
Stem vs Leaf	Beta-Hydroxypalmitic Acid*	Lipids	Free fatty acids	1.047940359	0.072821736	0.122990363	2.821059694	1.496237194	1
Stem vs Leaf	2,7-Dimethyl-2,4-Octadiene-1,8-Diol 8-O-B-D-Glucopyranoside	Terpenoids	Monoterpenoids	1.160742529	0.032727612	0.074067753	0.003422332	-8.190804575	2
Stem vs Leaf	Annulatin 3'-O-β-D-xyloside*	Flavonoids	Flavones	1.176744345	0.015251482	0.04859067	0.076218567	-3.713713702	3

Comparison	Compound	Class I	Class II	VIP	P-value	FDR	Fold Change	Log_2FC	MSI Level
Stem vs Leaf	Maltotriose	Others	Saccharides	1.076323107	0.134719371	0.189309356	6.364321396	2.670006693	3
Stem vs Leaf	(2S,3R,4S,5S,6R)-3,4,5-trihydroxy-6-(hydroxymethyl)oxan-2-yl(2E,6E)-8-hydroxy-2,6-dimethylocta-2,6-dienoate	Terpenoids	Monoterpenoids	1.177358434	0.014807496	0.047964941	0.081624264	-3.614858113	2
Stem vs Leaf	Shikimic acid	Organic acids	Organic acids	1.169104684	0.00669262	0.033278913	0.031072462	-5.008219644	1
Stem vs Leaf	1-(((2-(dimethylamino)ethoxy)(hydroxy)phosphoryl)oxy)-3-hydroxypr	Lipids	LPE	1.174515813	0.009298906	0.038406318	11.29080452	3.497076383	1

Comparison	Compound	Class I	Class II	VIP	P-value	FDR	Fold Change	Log_2FC	MSI Level
	opan-2-yl (9Z,12Z)-octadeca -9,12-dienoate*								
Stem vs Leaf	Caffeoyl(p-Hydro xybenzoyl)tartari c acid	Phenolic acids	Phenolic acids	1.055267174	0.097278404	0.151447189	3.78861631	1.921671039	2
Stem vs Leaf	Hydroxyaloe-em odin-8-O-sulfate	Quinones	Anthraquinone	1.141771483	0.02585527	0.064103755	2.637717839	1.399290245	2
Stem vs Leaf	2-(3,4-Dihydroxy benzoyloxy)-4,6-Dihydroxyphenyl acetic Acid	Phenolic acids	Phenolic acids	1.164197747	0.008994586	0.037930918	0.025169347	-5.312188405	1
Stem vs Leaf	Isofraxidin	Lignans and	Coumarins	1.11434601	0.149899731	0.206064208	10.9151879	3.448265061	1

Comparison	Compound	Class I	Class II	VIP	P-value	FDR	Fold Change	Log_2FC	MSI Level
		Coumarins							
Stem vs Leaf	Mannose 6-phosphate	Others	Saccharides	1.132709342	0.050238233	0.097483935	4.808186887	2.265492972	1
Stem vs Leaf	6-Hydroxy-4-(4-hydroxy-3-methoxyphenyl)-7-methoxy-naphtho[2,3-c]furan-1,3-dione-1	Lignans and Coumarins	Lignans	1.176425737	6.98554E-06	0.001668767	3.375161443	1.754956512	3
Stem vs Leaf	Phellodenol E	Lignans and Coumarins	Coumarins	1.162610318	0.01504914	0.048364203	0.034823286	-4.843803841	3
Stem vs Leaf	6,30-Dihydroxy-3-oxo-lupane-20(29)-en-28-oic acid	Terpenoids	Triterpene	1.139455496	0.049810652	0.097403476	60.52059904	5.919354362	2

Comparison	Compound	Class I	Class II	VIP	P-value	FDR	Fold Change	Log_2FC	MSI Level
Stem vs Leaf	3-Hydroxyurs-5(6),12,18(19)-trien-28-oic acid (Uncargenin B)	Terpenoids	Triterpene	1.178402171	0.000169509	0.008153335	15.70263296	3.97293458	2
Stem vs Leaf	Gingerglycolipid B	Lipids	Glycerol ester	1.096207055	0.193754375	0.25079585	27.69766682	4.791692547	2
Stem vs Leaf	γ-Aminobutyric acid	Organic acids	Organic acids	1.107302896	0.076864449	0.128107415	7.089648243	2.825714049	2
Stem vs Leaf	(7'R,8'R)-7'8'-dihydro-7'-(5'-hydroxy-3'-methoxphenyl)-3-methoxy-8'-methyl-1-(E)-propenylbenzof-uran	Lignans and Coumarins	Lignans	1.160382386	0.007160983	0.034654934	264.9574063	8.049616645	3

Comparison	Compound	Class I	Class II	VIP	P-value	FDR	Fold Change	Log_2FC	MSI Level
Stem vs Leaf	Dianthoside	Others	Others	1.044920915	0.013881566	0.04641581	0.36144198	-1.468164016	3
Stem vs Leaf	Quercetin-4'-O-glucuronide*	Flavonoids	Flavonols	1.155000384	0.00069715	0.014479152	4.390581965	2.13441218	1
Stem vs Leaf	Cis-Coutaric acid	Phenolic acids	Phenolic acids	1.101204091	0.00372119	0.028072133	0.425202156	-1.233779184	1
Stem vs Leaf	3-Oxo-alpha-ionol glucoside*	Terpenoids	Sesquiterpenoids	1.13531889	0.114651333	0.17028188	0.063798391	-3.97033615	2
Stem vs Leaf	Grayanotoxin XVIII	Terpenoids	Ditepenoids	1.177469503	0.009419152	0.038647282	0.020430534	-5.613129251	2
Stem vs Leaf	3,4-Secodammara-4(28),20,24-trien-3,26-dioic acid	Terpenoids	Triterpene	1.123539432	0.10168691	0.156620019	11.94574586	3.57842503	2

Comparison	Compound	Class I	Class II	VIP	P-value	FDR	Fold Change	Log_2FC	MSI Level
Stem vs Leaf	4-Hydroxy-l-tryptophan*	Amino acids and derivatives	Amino acids and derivatives	1.039356317	0.005928054	0.032023409	0.075336732	-3.730502742	1
Stem vs Leaf	LysoPC 16:1*	Lipids	LPC	1.169419561	0.014634162	0.047704834	9.750913639	3.285537403	1
Stem vs Leaf	1-O-Salicyloyl-β-D-glucose*	Phenolic acids	Phenolic acids	1.111939793	0.057521985	0.105702792	2.792282744	1.481445035	1
Stem vs Leaf	3-methoxy-4-[3,4,5-trihydroxy-6-(sulfooxymethyl)oxan-2-yl]oxybenzoic acid	Others	Others	1.114463136	0.044235313	0.090163016	0.393499075	-1.345567852	3
Stem vs Leaf	Kaempferol-3-O-galactoside	Flavonoids	Flavonols	1.152774761	0.021344753	0.057944985	2.281909884	1.190241819	1

Comparison	Compound	Class I	Class II	VIP	P-value	FDR	Fold Change	Log_2FC	MSI Level
	(Trifolin)*								
Stem vs Leaf	Majoroside	Terpenoids	Monoterpenoids	1.152202039	0.001659496	0.019286033	4.209115223	2.073517003	1
Stem vs Leaf	Tricetin 7-O-glucuronide*	Flavonoids	Flavones	1.174463163	3.64978E-05	0.003566826	4.922858358	2.29949623	1
Stem vs Leaf	LysoPC 16:1(2n isomer)*	Lipids	LPC	1.115806921	0.007522658	0.035510671	12.36655164	3.628371362	1
Stem vs Leaf	Procyanidin C1	Tannins	Proanthocyanidins	1.177998217	0.005534964	0.031398874	8.252904285	3.044901908	2
Stem vs Leaf	1-O-Linoleoyl-3-O-galactopyranosyl-L-glycerol	Lipids	Glycerol ester	1.018064811	0.17038281	0.227529839	8.859898392	3.147290154	1

Comparison	Compound	Class I	Class II	VIP	P-value	FDR	Fold Change	Log_2FC	MSI Level
Stem vs Leaf	Diosmetin-7-O-beta-D-glucopyranoside*	Flavonoids	Flavones	1.171549905	0.011539726	0.041808314	4.888089565	2.28927072	2
Stem vs Leaf	Erythro-Guaiacyl glycerol-β-dihydroconiferyl Ether glucoside	Lignans and Coumarins	Lignans	1.080997353	0.005592111	0.031482129	2.747452728	1.458094659	2
Stem vs Leaf	Mearnsetin-3-O-glucoside*	Flavonoids	Flavones	1.181453133	0.004002879	0.028204938	526.463061	9.040188499	2
Stem vs Leaf	Kaempferol-3-O-neohesperidoside*	Flavonoids	Flavonols	1.180973393	0.002776093	0.024478889	0.077586937	-3.688042414	3
Stem vs Leaf	Tectoridin*	Flavonoids	Isoflavones	1.146535172	0.000574502	0.013573406	3.91519509	1.969084197	2

Comparison	Compound	Class I	Class II	VIP	P-value	FDR	Fold Change	Log_2FC	MSI Level
Stem vs Leaf	Diosmetin-7-O-galactoside*	Flavonoids	Flavones	1.172001331	0.005851128	0.032023409	5.790419751	2.533667934	2
Stem vs Leaf	Ergotamine	Alkaloids	Alkaloids	1.10919383	0.112179295	0.167552419	6.033136086	2.592908125	3
Stem vs Leaf	Quercetin-3,7-Di-O-rhamnoside	Flavonoids	Flavonols	1.170470138	3.75902E-06	0.00134698	0.040176978	-4.637487135	2
Stem vs Leaf	Dihydrovomifoliol-O-β-D-glucoside*	Terpenoids	Sesquiterpenoids	1.099325047	0.052764074	0.100748453	2.114055959	1.080013566	1
Stem vs Leaf	Methyl 3-O-Beta-Glucopyranosyl-Gallate	Phenolic acids	Phenolic acids	1.177014685	1.2825E-05	0.002168992	7.369590362	2.881584429	2
Stem vs Leaf	Azelaic acid	Lipids	Free fatty acids	1.16947898	0.01592683	0.049483647	4.668267243	2.222887153	1

Comparison	Compound	Class I	Class II	VIP	P-value	FDR	Fold Change	Log_2FC	MSI Level
Stem vs Leaf	Hydroxy isoliquiritigenin glucoside*	Flavonoids	Chalcones	1.072909101	0.064825816	0.114523833	2.954041593	1.56269014	2
Stem vs Leaf	2α,3α-Epoxy-5,7,3',4'-tetrahydroxyflavan-(4β-8-epicatechin)	Tannins	Proanthocyanidins	1.088454453	0.181902926	0.239492524	12.29656779	3.620183783	1
Stem vs Leaf	7,2',4'-Trihydroxy-8,3'-dimethoxyisoflavanglucoside	Flavonoids	Other Flavonoids	1.159306276	0.056097603	0.10442411	13.08894848	3.710277296	2
Stem vs Leaf	L-Lysine	Amino acids and derivatives	Amino acids and derivatives	1.141708017	0.051418825	0.098882356	10.99348101	3.458576373	2

Comparison	Compound	Class I	Class II	VIP	P-value	FDR	Fold Change	Log_2FC	MSI Level
Stem vs Leaf	3,5-dimethoxy-4-((3,4,5-trihydroxy-6-((sulfooxy)methyl)tetrahydro-2H-pyran-2-yl)oxy)benzoic acid	Phenolic acids	Phenolic acids	1.168735238	0.000237611	0.009450719	0.411970193	-1.279388137	3
Stem vs Leaf	Lupane-20(29)-en-3-on-28-oic acid	Terpenoids	Triterpene	1.006998252	0.123974144	0.178409912	3.551801559	1.82855098	1
Stem vs Leaf	2-[4-[(1R,2R)-1,2-dihydroxypropyl]phenoxy]-6-(hydroxymethyl)oxane-3,4,5-triol	Organic acids	Organic acids	1.157201717	0.004576923	0.029286859	3.22524931	1.689410685	1
Stem vs Leaf	Hispidulin-7-O-gl	Flavonoids	Flavones	1.174674995	0.005718163	0.032023409	4.082875761	2.029585668	2

Comparison	Compound	Class I	Class II	VIP	P-value	FDR	Fold Change	Log_2FC	MSI Level
	ucoside(Homoplantaginin)*								
Stem vs Leaf	Dehydrophytosphingosine	Lipids	Sphingolipids	1.176525001	0.006270233	0.032737441	0.072278965	-3.790280344	1
Stem vs Leaf	Uridine 5'-diphospho-D-glucose	Nucleotides and derivatives	Nucleotides and derivatives	1.137723426	0.044873352	0.091037652	4.261157277	2.091245301	1
Stem vs Leaf	Tricetin 3'-glucuronide*	Flavonoids	Flavones	1.179215761	0.00308221	0.025963636	4.768993027	2.253684673	1
Stem vs Leaf	1-O-rhamnose-3-O-Caffeoyl Quinic Acid	Phenolic acids	Phenolic acids	1.174277187	0.009307371	0.038406318	0.065777313	-3.926266115	2
Stem vs Leaf	Carthamone*	Flavonoids	Chalcones	1.141441367	0.025441329	0.063904927	3.117318524	1.640305575	1

Comparison	Compound	Class I	Class II	VIP	P-value	FDR	Fold Change	Log_2FC	MSI Level
Stem vs Leaf	4-O-Glucosyl-4-hydroxybenzoic acid	Phenolic acids	Phenolic acids	1.074893623	0.051283057	0.098709554	2.162448791	1.112665969	1
Stem vs Leaf	3-hydroxybenzoyl-β-D-glucose*	Phenolic acids	Phenolic acids	1.125992467	0.035587333	0.078035541	2.332567826	1.221919032	1
Stem vs Leaf	6-C-MethylKaempferol-3-glucoside*	Flavonoids	Flavonols	1.175586347	0.00898278	0.037930918	4.596136741	2.200421721	2
Stem vs Leaf	6'-O-Feruloyl-D-sucrose	Phenolic acids	Phenolic acids	1.172996821	0.005814456	0.032023409	0.172727998	-2.533426142	2
Stem vs Leaf	1,3,6,8-tetrahydroxy-2,5-dimethoxyxanthen-9-one	Flavonoids	Other Flavonoids	1.17063741	0.000898122	0.015544649	0.027239659	-5.198147521	2

Comparison	Compound	Class I	Class II	VIP	P-value	FDR	Fold Change	Log_2FC	MSI Level
Stem vs Leaf	Murrayacarpin A glucoside	Lignans and Coumarins	Coumarins	1.155938774	0.001527902	0.018439572	0.002577324	-8.599910139	3
Stem vs Leaf	Neoisoliquiritin	Flavonoids	Chalcones	1.169931388	0.001090905	0.016559547	0.191960788	-2.381116458	2
Stem vs Leaf	Pyridoxine	Others	Vitamin	1.108065967	0.03827309	0.081877755	0.103487025	-3.272478203	3
Stem vs Leaf	Choline Alfoscerate	Lipids	PC	1.136078666	0.012306151	0.042842	4.091974984	2.032797325	3
Stem vs Leaf	Sweroside	Terpenoids	Monoterpenoids	1.130569503	0.062023735	0.111497517	0.18267016	-2.452687111	2
Stem vs Leaf	Neosakuranin	Flavonoids	Chalcones	1.178158355	0.009226009	0.038367347	12.12500291	3.599913189	3
Stem vs Leaf	18-O-Methylvibsanin K	Terpenoids	Ditepenoids	1.155797658	0.000870209	0.015425252	4.57860942	2.194909501	3
Stem vs Leaf	Frambinone	Others	Ketone	1.140768553	0.000819083	0.014923977	0.286425301	-1.803769158	3

Comparison	Compound	Class I	Class II	VIP	P-value	FDR	Fold Change	Log_2FC	MSI Level
			compounds						
Stem vs Leaf	Isocoreopsin*	Flavonoids	Flavanones	1.142265647	0.002715742	0.024430315	2.692050213	1.42870532	2
Stem vs Leaf	3α,20β,21β-trihydroxy-16-oxo-serrat-14-en-24-oic acid	Terpenoids	Triterpene	1.169873235	0.023400767	0.061132017	26.44926689	4.72515583	1
Stem vs Leaf	Luteolin-7-O-rutinoside*	Flavonoids	Flavones	1.164242035	0.019910359	0.055784421	0.002139175	-8.868729598	3
Stem vs Leaf	3-[4-(β-D-glucopyranoside)-phenyl acrylic]-acid	Phenolic acids	Phenolic acids	1.150832556	0.011727713	0.042024305	0.138468153	-2.852373897	1
Stem vs Leaf	L-Glutamine	Amino acids and	Amino acids and derivatives	1.124335712	0.088443493	0.141378074	9.518450835	3.250726788	1

Comparison	Compound	Class I	Class II	VIP	P-value	FDR	Fold Change	Log_2FC	MSI Level
		derivatives							
Stem vs Leaf	Isoceanothic acid	Terpenoids	Triterpene	1.161756491	0.042165547	0.087505719	6.764228229	2.757925338	1
Stem vs Leaf	D-inositol-4-phos phate	Others	Saccharides	1.173208831	0.011543882	0.041808314	3.722086534	1.896111597	1
Stem vs Leaf	Tricalysioside N	Terpenoids	Ditepenoids	1.178425956	0.012237621	0.042842	0.064892852	-3.945796627	3
Stem vs Leaf	Pterosupin	Flavonoids	Chalcones	1.164317996	0.025665134	0.064000914	8.802133953	3.137853327	3
Stem vs Leaf	6-Methoxyquerce tin-3-O-Xyloside*	Flavonoids	Flavonols	1.177439837	0.010354598	0.04009399	0.130774736	-2.934844235	3
Stem vs Leaf	Sibiricose A6	Phenolic acids	Phenolic acids	1.144822437	0.005302313	0.030931935	0.056611058	-4.142772306	3
Stem vs Leaf	Kaempferol-3-O-r obinobioside(Bior	Flavonoids	Flavonols	1.181123128	0.001920762	0.020636181	0.086832706	-3.525617638	2

Comparison	Compound	Class I	Class II	VIP	P-value	FDR	Fold Change	Log_2FC	MSI Level
	obin)*								
Stem vs Leaf	Ternatumoside II	Flavonoids	Flavonols	1.181179811	0.00147226	0.018029486	0.094961858	-3.396508024	3
Stem vs Leaf	Homoproline	Amino acids and derivatives	Amino acids and derivatives	1.08736759	0.18356186	0.240645121	18.23448188	4.188597302	3
Stem vs Leaf	6,9,10-Trihydroxy octadec-7-enoic acid; Sanleng acid*	Lipids	Free fatty acids	1.165660997	0.045416717	0.092031991	17.98061772	4.16837068	1
Stem vs Leaf	Isofraxetin	Lignans and Coumarins	Coumarins	1.172408489	0.002840923	0.024930547	97.3976299	6.605814761	1
Stem vs Leaf	2-Carboxy-3-hydr oxy-A(1)-norlupa	Terpenoids	Triterpene	1.147006128	0.074310243	0.125013319	22.795557	4.510680756	2

Comparison	Compound	Class I	Class II	VIP	P-value	FDR	Fold Change	Log_2FC	MSI Level
	n-20(29)-en-28-oic acid (Ceanothic acid)*								
Stem vs Leaf	Rhein-8-O-glucoside	Quinones	Anthraquinone	1.167213258	0.032247767	0.073290379	13.10669332	3.71223185	3
Stem vs Leaf	Kaempferol-3,7-O-dirhamnoside (Kaempferitrin)	Flavonoids	Flavonols	1.169468215	0.012677172	0.043609471	11.11726271	3.474729706	3
Stem vs Leaf	4'-Hydroxy-3,5-dimethoxydihydrochalcone	Flavonoids	Chalcones	1.172865352	0.014901841	0.048178884	0.01651348	-5.920212005	3
Stem vs Leaf	Kaempferol-3-O-(6''-O-acetyl)gluco	Flavonoids	Flavonols	1.010886183	0.112333068	0.167603121	2.063621218	1.045178186	1

Comparison	Compound	Class I	Class II	VIP	P-value	FDR	Fold Change	Log_2FC	MSI Level
	side								
Stem vs Leaf	LysoPE 18:1*	Lipids	LPE	1.098418321	0.047111449	0.094093896	5.762907227	2.526796795	3
Stem vs Leaf	Gossypin	Flavonoids	Flavonols	1.170862157	0.003796901	0.028194402	6.310937994	2.657854449	3
Stem vs Leaf	Chrysoeriol-5-O-glucoside	Flavonoids	Flavones	1.111179078	0.000747528	0.014479152	0.024025461	-5.379292061	3
Stem vs Leaf	3-O-(p-coumaroyl) 3-Hydroxy-3-methylglutaric acid	Phenolic acids	Phenolic acids	1.175423916	0.014157735	0.046614287	0.02660731	-5.232033508	3
Stem vs Leaf	O-Feruloyl 7-hydroxycoumarin*	Lignans and Coumarins	Coumarins	1.166849085	0.009882582	0.03961495	0.089166971	-3.487346782	2

Comparison	Compound	Class I	Class II	VIP	P-value	FDR	Fold Change	Log_2FC	MSI Level
Stem vs Leaf	cis-Zeatin-O-glucoside*	Alkaloids	Alkaloids	1.139076285	0.018739595	0.054349722	2.195993639	1.134873876	1
Stem vs Leaf	1-Oxo-Siaresinolic acid*	Terpenoids	Triterpene	1.129047408	0.054060545	0.102225305	4.407624903	2.140001453	1
Stem vs Leaf	2α,3α-Epoxy-5,7,3',4'-tetrahydroxyflavan-(4β→8)-catechin	Tannins	Proanthocyanidins	1.058617992	0.182129873	0.239572075	10.84443298	3.438882716	2
Stem vs Leaf	Lyoniresinol-3-O-glucoside*	Lignans and Coumarins	Lignans	1.180687432	0.010839321	0.040787888	116.1491493	6.859834778	1
Stem vs Leaf	1-O-Galloyl-2-O-Cinnamoyl-β-D-glucose	Phenolic acids	Phenolic acids	1.170462622	0.001591789	0.018907996	0.100228991	-3.318628233	2

Comparison	Compound	Class I	Class II	VIP	P-value	FDR	Fold Change	Log_2FC	MSI Level
Stem vs Leaf	LysoPE 18:1(2n isomer)*	Lipids	LPE	1.146617979	0.055250522	0.103384353	8.638237976	3.110737062	3
Stem vs Leaf	Tricalysioside O	Terpenoids	Ditepenoids	1.178651669	0.010379871	0.04009399	0.062615694	-3.997331881	3
Stem vs Leaf	4-Hydroxyphenyl 6-O-(2-Methyl-3-Hydroxypropionyl)-Beta-D-Galactopyranoside	Others	Others	1.16851665	0.02014096	0.055784421	12.45433812	3.638576447	2
Stem vs Leaf	Cinnamtannin A2	Tannins	Tannin	1.146373291	0.061386308	0.110721947	13.76288956	3.782711495	3
Stem vs Leaf	2,5-Dihydroxybenzoic acid; Gentisic Acid*	Phenolic acids	Phenolic acids	1.170987247	0.005094108	0.030593106	23.29552449	4.541980908	1
Stem vs Leaf	Inositol	Others	Saccharides	1.173864812	0.002771815	0.024478889	0.15016909	-2.735340208	1

Comparison	Compound	Class I	Class II	VIP	P-value	FDR	Fold Change	Log_2FC	MSI Level
Stem vs Leaf	Tetrahydroxyflavan-(4α-8-epicatechin)	Flavonoids	Flavanols	1.159266582	0.056246766	0.104430525	12.7231933	3.669388902	2
Stem vs Leaf	2α,3α,23-Trihydroxyurs-12,20(30)-dien-28-oic acid*	Terpenoids	Triterpene	1.13563172	0.048519419	0.095967571	4.654762427	2.218707536	1
Stem vs Leaf	Quercetin-3-O-(2''-O-acetyl)rhamnoside	Flavonoids	Flavonols	1.17475502	0.0229527	0.060475863	0.06880592	-3.861323498	2
Stem vs Leaf	Arbutin	Others	Others	1.164896507	0.027440116	0.066813418	0.054746364	-4.191093029	1
Stem vs Leaf	3,5-dihydroxy-4-{[(2s,3r,4s,5s,6r)-3,4,5-trihydroxy-6-[	Phenolic acids	Phenolic acids	1.173947374	0.008220178	0.036758126	0.349877438	-1.515078459	1

Comparison	Compound	Class I	Class II	VIP	P-value	FDR	Fold Change	Log_2FC	MSI Level
	(sulfooxy)methyl] oxan-2-yl]oxy}ben zoic acid*								
Stem vs Leaf	Cyanidin-3-O-glu coside*	Flavonoids	Anthocyanidin s	1.150205535	0.038005828	0.081630899	0.029184886	-5.098634749	3
Stem vs Leaf	Actinidialactone	Terpenoids	Monoterpenoid s	1.176222012	0.005895908	0.032023409	0.129840388	-2.945188884	3
Stem vs Leaf	Methyl cumalate*	Others	Lactones	1.177651802	0.000345937	0.011100972	22.08515441	4.465005014	1
Stem vs Leaf	Kaempferol-3-O-(6''-malonyl)gluco side*	Flavonoids	Flavonols	1.043289687	0.106112831	0.161003943	2.926550834	1.549201338	1
Stem vs Leaf	2-Hydroxy-4-met hyl-3-undecanoyl	Lipids	Free fatty acids	1.167816029	0.043248187	0.088894457	14.64979501	3.872808573	1

Comparison	Compound	Class I	Class II	VIP	P-value	FDR	Fold Change	Log_2FC	MSI Level
	oxypentanoic acid methyl ester*								
Stem vs Leaf	3-O-Feruloyl Aminogalactitol	Phenolic acids	Phenolic acids	1.176836835	0.00418141	0.028378135	5.211400742	2.381671198	1
Stem vs Leaf	N-Caffeoylmethyl gluconamide*	Alkaloids	Phenolamine	1.141047806	0.028260398	0.067816745	0.042887033	-4.543314677	1
Stem vs Leaf	5-O-Caffeoylshiki mic acid	Phenolic acids	Phenolic acids	1.175505244	0.013177976	0.044759317	0.027290411	-5.195462074	1
Stem vs Leaf	Micheliolide	Terpenoids	Sesquiterpenoi ds	1.161035372	0.021065057	0.057401613	279.4159666	8.126270652	2
Stem vs Leaf	Bismurrangatin	Lignans and Coumarins	Coumarins	1.033816057	0.101899251	0.156822173	2.531272099	1.339862599	3

Comparison	Compound	Class I	Class II	VIP	P-value	FDR	Fold Change	Log_2FC	MSI Level
Stem vs Leaf	5-O-p-Coumaroyl galactaric acid	Phenolic acids	Phenolic acids	1.176457722	0.00390872	0.028194402	0.042380797	-4.560445473	2
Stem vs Leaf	Kaempferol-3-O-(6''-malonyl)galactoside*	Flavonoids	Flavonols	1.013347685	0.110106142	0.165197631	2.508973475	1.327097218	1
Stem vs Leaf	Cyanidin-3-O-galactoside*	Flavonoids	Anthocyanidins	1.169568677	0.038195902	0.081794012	0.026564265	-5.234369409	3
Stem vs Leaf	Catechin-(4alpha->8)-catechin-(4alpha->6)-catechin	Flavonoids	Flavanols	1.156021277	0.031853515	0.072855019	4.897112253	2.291931266	1
Stem vs Leaf	6-O-Vanilloylajugol	Terpenoids	Monoterpenoids	1.12152576	0.034485441	0.076779926	3.189814376	1.673472472	3
Stem vs Leaf	Isomaltulose*	Others	Saccharides	1.165037699	0.017267477	0.052065312	5.065936657	2.340829038	1

Comparison	Compound	Class I	Class II	VIP	P-value	FDR	Fold Change	Log_2FC	MSI Level
Stem vs Leaf	2,3-Dihydroxy-19-oxo-18,19-secours-11,13(18)-dien-28-oic acid (Swinhoeic acid)*	Terpenoids	Triterpene	1.174058563	0.012273469	0.042842	12.85522511	3.684282968	1
Stem vs Leaf	D-Maltose*	Others	Saccharides	1.13450681	0.057887849	0.106103048	6.158590395	2.622600178	1
Stem vs Leaf	5-O-Malonylisosakuranin	Flavonoids	Flavanones	1.060444407	0.09703474	0.151447189	3.153443395	1.656928037	2
Stem vs Leaf	3,4'-Dihydroxy-3',5'-dimethoxypropiophenone	Others	Ketone compounds	1.174331049	0.01242438	0.043015164	8.274782316	3.04872136	1
Stem vs Leaf	N-Feruloylputrescine	Alkaloids	Phenolamine	1.178616911	0.004954614	0.030262554	47.36338653	5.565700334	1

Comparison	Compound	Class I	Class II	VIP	P-value	FDR	Fold Change	Log_2FC	MSI Level
Stem vs Leaf	O-Acetyl-L-carnitine	Alkaloids	Alkaloids	1.129064996	0.010770713	0.040769423	2.134471335	1.093878788	1
Stem vs Leaf	(+)-Lyoniresinol 9'-O-glucoside*	Lignans and Coumarins	Lignans	1.158968064	0.020058413	0.055784421	198.442192	7.632574988	1
Stem vs Leaf	(3b,16a,20R)-25-Acetoxy-3,16,20,22-tetrahydroxy-5-cucurbiten-11-one 3-glucoside	Steroids	Steroidal saponins	1.178514968	0.01525521	0.04859067	0.03098981	-5.012062263	1
Stem vs Leaf	Procyanidin A2	Tannins	Proanthocyanidins	1.063714267	0.202165628	0.258877963	12.675319	3.66395015	3
Stem vs Leaf	3,5-dihydroxy-4-[(2S,3R,4S,5S,6R)-3	Phenolic acids	Phenolic acids	1.165766389	0.014675709	0.047704834	0.330041239	-1.599281794	1

Comparison	Compound	Class I	Class II	VIP	P-value	FDR	Fold Change	Log_2FC	MSI Level
	,4,5-trihydroxy-6-(sulfooxymethyl)oxan-2-yl]oxybenzoic acid*								
Stem vs Leaf	Luteolin-7-O-neohesperidoside (Lonicerin)*	Flavonoids	Flavones	1.179983643	0.005593569	0.031482129	0.07269739	-3.781952626	3
Stem vs Leaf	Chrysoeriol-7-O-glucoside	Flavonoids	Flavones	1.181295649	0.004448142	0.02880574	0.007553682	-7.04860424	2
Stem vs Leaf	3,9-dihydroeucomnalin glucoside*	Flavonoids	Other Flavonoids	1.180321821	0.000170651	0.008153335	57.40667078	5.843146486	2
Stem vs Leaf	Malonyl fraxin	Lignans and Coumarins	Coumarins	1.175751749	0.05365763	0.10176487	106.7185295	6.737666883	1

Comparison	Compound	Class I	Class II	VIP	P-value	FDR	Fold Change	Log_2FC	MSI Level
Stem vs Leaf	6-Deoxyfagomine	Alkaloids	Piperidine alkaloids	1.100494372	0.006482133	0.032910525	0.356622054	-1.487532171	1
Stem vs Leaf	Piperidine	Alkaloids	Piperidine alkaloids	1.120884078	0.024335369	0.062510206	0.164269411	-2.605864241	3
Stem vs Leaf	6-[(7-hydroxy-2-methyl-6-oxo-3,4-dihydroisoquinolin-1-yl)methyl]-2,3-dimethoxybenzoic acid	Alkaloids	Isoquinoline alkaloids	1.139349042	1.31148E-05	0.002168992	0.031926059	-4.969121703	1
Stem vs Leaf	Desmethylagrimonolide-6-O-β-D-glucopyranoside*	Lignans and Coumarins	Coumarins	1.181350415	0.004376131	0.02880574	0.005600496	-7.480229565	3

Comparison	Compound	Class I	Class II	VIP	P-value	FDR	Fold Change	Log_2FC	MSI Level
Stem vs Leaf	5-O-Feruloylquinic acid	Phenolic acids	Phenolic acids	1.148063094	0.00227744	0.022493448	0.001895631	-9.043106309	1
Stem vs Leaf	Protocatechuic Acid 4-(6''-O-Malonyl) glucoside	Phenolic acids	Phenolic acids	1.176202662	0.001914102	0.020636181	4.490386254	2.166839548	1
Stem vs Leaf	Quercetin-3-O-(6''-O-malonyl)glucoside	Flavonoids	Flavonols	1.167776558	0.03810449	0.081679615	10.58617208	3.404109106	1
Stem vs Leaf	Phenylpropionic acid-O-β-D-glucopyranoside*	Phenolic acids	Phenolic acids	1.149984229	0.000215416	0.008915744	0.134118644	-2.898418292	1
Stem vs Leaf	Kaempferol-3-O-	Flavonoids	Flavonols	1.160223993	0.00762137	0.03555507	0.002177834	-8.842890244	3

Comparison	Compound	Class I	Class II	VIP	P-value	FDR	Fold Change	Log_2FC	MSI Level
	glucorhamnoside *								
Stem vs Leaf	D-Galactose*	Others	Saccharides	1.131945069	0.083096346	0.135243864	5.927808309	2.567498795	1
Stem vs Leaf	Hydroxypentadecenoic acid glucoside	Lipids	Free fatty acids	1.180525684	0.004576134	0.029286859	0.07471237	-3.742509056	1
Stem vs Leaf	8-Methoxykaempferol-7-O-rhamnoside	Flavonoids	Flavonols	1.158303868	0.00476292	0.029596181	0.088139527	-3.504067029	1
Stem vs Leaf	Desmethylagrimonolide-6-O-glucoside*	Lignans and Coumarins	Coumarins	1.070790208	0.000892813	0.015544649	0.015759779	-5.987608878	3
Stem vs Leaf	p-Coumaric	Phenolic	Phenolic acids	1.090480468	0.008009869	0.036331682	0.39769397	-1.330269406	1

Comparison	Compound	Class I	Class II	VIP	P-value	FDR	Fold Change	Log_2FC	MSI Level
	acid-4-O-glucoside	acids							
Stem vs Leaf	5-(2-hydroxypropyl)-3H-2-benzofuran-1-one	Others	Others	1.019541653	0.070013666	0.119943731	3.84692249	1.943704762	3
Stem vs Leaf	Hercynine	Alkaloids	Alkaloids	1.099288149	0.060841403	0.110201362	2.322070577	1.215411822	3
Stem vs Leaf	Geniposide	Terpenoids	Monoterpenoids	1.149345152	0.002405255	0.02293776	2.09229237	1.065084463	3
Stem vs Leaf	Isoscutellarein	Flavonoids	Flavones	1.125808776	0.010155994	0.039720462	0.033266581	-4.909782578	3
Stem vs Leaf	O-p-Coumaroylgalactaric acid	Phenolic acids	Phenolic acids	1.169835784	0.000138689	0.007645679	0.045238077	-4.466318599	1
Stem vs Leaf	Cyanidin	Flavonoids	Anthocyanidin	1.164241088	0.00897496	0.037930918	0.002551152	-8.614635703	3

Comparison	Compound	Class I	Class II	VIP	P-value	FDR	Fold Change	Log_2FC	MSI Level
	3-O-(6-O-p-coumaroyl)glucoside		s						
Stem vs Leaf	L-Proline*	Alkaloids	Pyrrole alkaloids	1.149515882	0.020356497	0.0562551	6.037542926	2.593961541	3
Stem vs Leaf	Luteolin-7-O-(6''-malonyl)glucoside*	Flavonoids	Flavones	1.046262179	0.067739698	0.117927409	2.827612503	1.499584426	1
Stem vs Leaf	N-[(R)-2-Hydroxy-3,3-dimethyl-4-(β-D-glucopyranosyloxy)butyryl]-β-alanine	Alkaloids	Alkaloids	1.125887326	0.005737418	0.032023409	2.072856466	1.051620221	1
Stem vs Leaf	Melilotoside*	Phenolic	Phenolic acids	1.092203386	0.005226836	0.030931935	0.327218093	-1.611675572	1

Comparison	Compound	Class I	Class II	VIP	P-value	FDR	Fold Change	Log$_2$FC	MSI Level
		acids							
Stem vs Leaf	D-Xylonic acid	Others	Saccharides	1.105983356	0.001995548	0.02075995	0.373357622	-1.421369912	3
Stem vs Leaf	o-Phosphohomoserine	Organic acids	Organic acids	1.103809891	0.137563253	0.191893445	7.941454718	2.989403305	2
Stem vs Leaf	Quercetin-3-O-(2''-O-acetyl)glucuronide	Flavonoids	Flavonols	1.104767713	0.084859856	0.136870736	3.077529506	1.621772689	1
Stem vs Leaf	Kaempferol-3-O-glucoside-7-O-rhamnoside*	Flavonoids	Flavonols	1.165138265	0.006876038	0.033829477	0.002217259	-8.817006771	3
Stem vs Leaf	4-caffeoylshikimic acid	Phenolic acids	Phenolic acids	1.177231933	0.003521862	0.027237422	0.023790415	-5.393475771	1

Comparison	Compound	Class I	Class II	VIP	P-value	FDR	Fold Change	Log_2FC	MSI Level
Stem vs Leaf	Methyl 3-aminopropanoate	Amino acids and derivatives	Amino acids and derivatives	1.140351355	0.040263319	0.08486876	3.127484011	1.645002509	2
Stem vs Leaf	N-Ethylglycine*	Amino acids and derivatives	Amino acids and derivatives	1.152572587	0.024877094	0.063221929	3.09686999	1.630810818	2
Stem vs Leaf	3,19,23-Trihydroxyurs-12-en-28-oic acid (Rutundic acid)	Terpenoids	Triterpene	1.11089608	0.095784118	0.149985117	10.34293234	3.37057336	2
Stem vs Leaf	N,N-Dimethylglycine*	Amino acids and derivatives	Amino acids and derivatives	1.168939932	0.011248128	0.041529903	3.594444433	1.845768801	2

Comparison	Compound	Class I	Class II	VIP	P-value	FDR	Fold Change	Log_2FC	MSI Level
Stem vs Leaf	N-Hydroxydecanoyl nornicotine glucoside	Alkaloids	Pyridine alkaloids	1.177890359	0.01183868	0.042140998	0.088344689	-3.500712791	2
Stem vs Leaf	2-Aminoisobutyric acid	Organic acids	Organic acids	1.163475418	0.012608837	0.04344391	3.538902869	1.823302165	2
Stem vs Leaf	Terreic acid*	Others	Ketone compounds	1.175754809	0.014180782	0.04661878	31.20169618	4.963552553	1
Stem vs Leaf	3-Methylbutyl 6-O-(alpha-L-arabinopyranosyl)-beta-D-glucopyranoside	Others	Alcohol compounds	1.074830603	0.029476432	0.069413285	0.393145616	-1.346864329	3
Stem vs Leaf	2-(2-(3,4-dihydrox	Phenolic	Phenolic acids	1.18011911	0.004104432	0.028378135	0.09428976	-3.406755087	2

Comparison	Compound	Class I	Class II	VIP	P-value	FDR	Fold Change	Log_2FC	MSI Level
	yphenyl)-2-methoxyethoxy)-6-(hydroxymethyl)tetrahydro-2H-pyran-3,4,5-tri	acids							
Stem vs Leaf	2,3,4-trihydroxy-5-{[3-(4-hydroxyphenyl)prop-2-enoyl]oxy}hexanedioic acid	Phenolic acids	Phenolic acids	1.167355341	0.03335219	0.07504822	0.075805678	-3.721550283	1
Stem vs Leaf	Arecatannin C1	Tannins	Tannin	1.139708058	0.00050148	0.013133103	2.88394534	1.528043821	3
Stem vs Leaf	Methyl 3-oxo-2-phenylpropanoate	Phenolic acids	Phenolic acids	1.177055389	0.003967317	0.028204938	7.392813062	2.886123433	1

Comparison	Compound	Class I	Class II	VIP	P-value	FDR	Fold Change	Log_2FC	MSI Level
Stem vs Leaf	3'-O-Methyl-epicatechin	Flavonoids	Flavanols	1.176963424	0.005762225	0.032023409	7.000365939	2.80743034	3
Stem vs Leaf	Quercetin-3-O-(6''-O-acetyl)galactoside	Flavonoids	Flavonols	1.104204267	0.08088979	0.132454721	10.44375533	3.38456866	1
Stem vs Leaf	Procyanidin C2	Tannins	Proanthocyanidins	1.163691505	0.021502333	0.058015292	3.788850062	1.921760049	1
Stem vs Leaf	4-O-(4'-O-alpha-D-Glucopyranosyl) caffeoylquinic acid	Phenolic acids	Phenolic acids	1.179260035	0.000978402	0.016111501	0.001658035	-9.236309568	1
Stem vs Leaf	2-Aldehydo-A(1)-norlup-20(29)-en-	Terpenoids	Triterpene	1.047354777	0.101467141	0.156607576	3.243004221	1.697330901	3

Comparison	Compound	Class I	Class II	VIP	P-value	FDR	Fold Change	Log_2FC	MSI Level
	27,28-dioic acid (Zizyberanal acid)								
Stem vs Leaf	2,3,19-Trihydroxy urs-12-en-28-oic acid (Tormentic acid)*	Terpenoids	Triterpene	1.090690979	0.114461591	0.17028188	11.12553525	3.475802841	2
Stem vs Leaf	3-O-Feruloyl Octose*	Phenolic acids	Phenolic acids	1.166546037	0.008540872	0.037420256	7.115985814	2.831063634	1
Stem vs Leaf	2,3,19-trihydroxy urs-12-en-28-oic acid (Euscaphic acid)*	Terpenoids	Triterpene	1.088158106	0.118453692	0.173013205	11.49155715	3.522502397	2

Comparison	Compound	Class I	Class II	VIP	P-value	FDR	Fold Change	Log_2FC	MSI Level
Stem vs Leaf	Matairesinol-4'-O -glucoside (Matairesinoside)	Lignans and Coumarins	Lignans	1.165194784	0.024852018	0.063221929	75.94105626	6.24680816	1
Stem vs Leaf	β-D-Fructose 2-phosphate*	Others	Saccharides	1.093098258	0.109169384	0.163968466	4.302328312	2.105117621	1
Stem vs Leaf	Secoisolariciresin ol-9'-O-xyloside	Lignans and Coumarins	Lignans	1.171249896	0.024756608	0.063137988	2816.345728	11.45960873	2
Stem vs Leaf	Ferulic β-glucoside	Phenolic acids	Phenolic acids	1.181482409	0.003898899	0.028194402	0.000864867	-10.17523408	2
Stem vs Leaf	Cannabifolin F	Terpenoids	Triterpene	1.085584379	0.118098209	0.172861275	10.96816826	3.455250704	3
Stem vs Leaf	3-hydroxy-1-meth ylpyrrolidin-2-on e*	Alkaloids	Pyrrole alkaloids	1.153854205	0.025303908	0.063853759	5.350583482	2.419696226	3

Comparison	Compound	Class I	Class II	VIP	P-value	FDR	Fold Change	Log_2FC	MSI Level
Stem vs Leaf	1-O-(p-coumaroyl) 3-Hydroxy-3-methylglutaric acid	Phenolic acids	Phenolic acids	1.153665528	0.071541108	0.121574604	0.116179033	-3.105578371	3
Stem vs Leaf	1-O-p-Coumaroyl Octose	Phenolic acids	Phenolic acids	1.150058349	0.011675484	0.041906997	0.127308178	-2.973602998	1
Stem vs Leaf	(7S,8S-Threo-3',4,7,9-tetrahydroxy-3-methoxy-8-O-4'-neolignan-9'-O-rhamnoside	Lignans and Coumarins	Lignans	1.17966022	0.00555894	0.031451898	0.013591393	-6.20116281	1
Stem vs Leaf	D-Fructose 6-Phosphate*	Others	Saccharides	1.169853511	0.0085341	0.037420256	6.122455206	2.614110314	1

Comparison	Compound	Class I	Class II	VIP	P-value	FDR	Fold Change	Log_2FC	MSI Level
Stem vs Leaf	1-Amino-1-cyclobutane-carboxylic-acid	Amino acids and derivatives	Amino acids and derivatives	1.137371721	0.032093571	0.073115356	5.908918964	2.562894214	3
Stem vs Leaf	13-Hydroxy oleanolic acid-3-oxo-28-acid	Terpenoids	Triterpene	1.073793111	0.129198651	0.183941216	10.5350355	3.397123271	1
Stem vs Leaf	Galactinol	Others	Saccharides	1.151053347	0.063861278	0.113660387	7.737678327	2.951900754	3
Stem vs Leaf	9-hydroxy-8-{[(2S,3R,4S,5S,6R)-3,4,5-trihydroxy-6-(hydroxymethyl)oxan-2-yl]oxy}-1H,3H-naphtho[2,3-c]f	Others	Others	1.159868951	0.025656828	0.064000914	4.420368797	2.14416674	1

Comparison	Compound	Class I	Class II	VIP	P-value	FDR	Fold Change	Log_2FC	MSI Level
	uran-1-one								
Stem vs Leaf	5'-Deoxy-5'-(methylthio)adenosine	Nucleotides and derivatives	Nucleotides and derivatives	1.094344781	0.003146353	0.026321631	0.289860907	-1.786567324	1
Stem vs Leaf	Quercetin-3-O-glucose-6''-acetate	Flavonoids	Flavonols	1.169360131	0.028033945	0.067570608	9.772212267	3.288685201	1
Stem vs Leaf	Isocitric Acid*	Organic acids	Organic acids	1.071379147	0.05224256	0.100287057	2.489774383	1.316015015	1
Stem vs Leaf	Fraxetin-8-O-glucoside (Fraxin)	Lignans and Coumarins	Coumarins	1.177271757	0.042584898	0.087966135	274.6597283	8.101501582	3
Stem vs Leaf	D-Glucose-1-phosphate*	Others	Saccharides	1.18063657	0.001901363	0.020636181	0.189864087	-2.396961053	1

Comparison	Compound	Class I	Class II	VIP	P-value	FDR	Fold Change	Log_2FC	MSI Level
Stem vs Leaf	Quercetin-3-O-neohesperidoside*	Flavonoids	Flavonols	1.164686929	0.000418605	0.012500012	0.007739336	-7.013574408	1
Stem vs Leaf	(1-Methylpyrrolidin-2-yl)aceticacid*	Alkaloids	Pyrrole alkaloids	1.14637463	0.005197981	0.030931935	0.421551819	-1.246218111	2
Stem vs Leaf	Kanokoside B	Terpenoids	Terpene	1.176754281	0.000708811	0.014479152	16.6239453	4.055190907	3
Stem vs Leaf	Quercetin-3-O-robinobioside	Flavonoids	Flavonols	1.159422112	0.008675051	0.037603549	0.005431002	-7.524565839	2
Stem vs Leaf	1,3-O-Dicaffeoylquinic Acid (Cynarin)	Phenolic acids	Phenolic acids	1.181521389	0.000620661	0.014046537	0.074933149	-3.738252106	2
Stem vs Leaf	Vanilloloside	Others	Alcohol compounds	1.110869274	0.019741303	0.055718431	2.15351035	1.106690257	1

Comparison	Compound	Class I	Class II	VIP	P-value	FDR	Fold Change	Log_2FC	MSI Level
Stem vs Leaf	N-Benzylmethylene isomethylamine	Alkaloids	Alkaloids	1.041828825	0.057098991	0.105375821	0.189948923	-2.396316567	1
Stem vs Leaf	Quercetin-7-O-(6''-malonyl)glucoside	Flavonoids	Flavonols	1.166326462	0.016436031	0.050482096	18.5089776	4.2101533	2
Stem vs Leaf	Styrylamine	Alkaloids	Alkaloids	1.101172993	0.053872541	0.101959475	0.156228575	-2.67826974	1
Stem vs Leaf	Epigallocatechin-(4beta->8)-catechin	Tannins	Proanthocyanidins	1.082314888	0.000180728	0.008306724	0.067820234	-3.882140419	1
Stem vs Leaf	2-[4-(3-Hydroxypropyl)-2-methoxyphenoxy]-1,3-propanediol	Others	Alcohol compounds	1.178849078	6.92E-05	0.00531357	16.31986054	4.028556824	1

Comparison	Compound	Class I	Class II	VIP	P-value	FDR	Fold Change	Log_2FC	MSI Level
	1-glucoside								
Stem vs Leaf	Epicatechin 3-glucoside	Flavonoids	Flavanols	1.177208847	0.000646415	0.014327755	62.40260467	5.963534343	1
Stem vs Leaf	Delphinidin-3-O-(6''-O-p-coumaroyl)glucoside	Flavonoids	Anthocyanidins	1.161712631	0.019963927	0.055784421	0.007891381	-6.985506407	3
Stem vs Leaf	D-Glucose 6-phosphate*	Others	Saccharides	1.179296435	0.004830289	0.029756796	0.182628012	-2.453020028	1
Stem vs Leaf	L-Homomethionine	Amino acids and derivatives	Amino acids and derivatives	1.155588286	0.039457977	0.083769951	0.096391472	-3.374950684	3
Stem vs Leaf	Taxifolin-7-O-rhamnoside	Flavonoids	Flavanonols	1.14730294	0.004933894	0.030221858	0.022895126	-5.448815663	3

Comparison	Compound	Class I	Class II	VIP	P-value	FDR	Fold Change	Log_2FC	MSI Level
Stem vs Leaf	p-Coumaroyl Aminogalactitol	Phenolic acids	Phenolic acids	1.150959607	0.000254041	0.00965721	0.161938985	-2.626477756	1
Stem vs Leaf	Trimethyllysine	Amino acids and derivatives	Amino acids and derivatives	1.156844419	0.015338808	0.048712609	2.686039191	1.425480355	3
Stem vs Leaf	nahagenin	Terpenoids	Triterpene	1.052710993	0.106327463	0.161102216	2.803230303	1.487090276	3
Stem vs Leaf	6-Deoxy-6-sulfogl ucono-1,5-lactone	Others	Lactones	1.178041896	5.35167E-07	0.000383537	0.131676626	-2.924928816	1
Stem vs Leaf	4-methyl-1,5,2,3-d ioxadiazinan-2-a mine	Nucleotides and derivatives	Nucleotides and derivatives	1.107874407	0.053352382	0.101601082	0.169848252	-2.557681726	3
Stem vs Leaf	N2-Acetyl-L-lysin e	Amino acids and	Amino acids and derivatives	1.165721059	0.005308783	0.030931935	2.601362032	1.379267194	2

Comparison	Compound	Class I	Class II	VIP	P-value	FDR	Fold Change	Log_2FC	MSI Level
		derivatives							
Stem vs Leaf	Feruloylloctose*	Phenolic acids	Phenolic acids	1.168623834	0.005384027	0.031033935	7.676206564	2.940393535	1
Stem vs Leaf	D-Sedoheptuiose 7-phosphate	Others	Saccharides	1.058824829	0.060775477	0.110174769	0.46613697	-1.101174156	3
Stem vs Leaf	Taxifolin(Dihydroquercetin)	Flavonoids	Flavanonols	1.165943375	0.021254214	0.057843748	27.49508618	4.781101903	2
Stem vs Leaf	9-O-Methylvibsanol	Lignans and Coumarins	Lignans	1.169354992	0.006490262	0.032910525	0.016669876	-5.906612808	3
Stem vs Leaf	Quercetin-3-O-(4''-O-glucosyl)rhamnoside*	Flavonoids	Flavonols	1.169848964	0.006930904	0.033944065	0.006950608	-7.168645195	1

Comparison	Compound	Class I	Class II	VIP	P-value	FDR	Fold Change	Log_2FC	MSI Level
Stem vs Leaf	2-(2,3-dihydroxypropoxy)-3-(((2-(dimethylamino)ethoxy)(hydroxy)phosphoryl)oxy)propyl (11Z,14Z)-octadeca-11,14-dienoate	Lipids	LPE	1.169685719	0.011482902	0.041808314	4.969760127	2.31317622	1
Stem vs Leaf	Quercetin-7-O-rutinoside*	Flavonoids	Flavonols	1.164470583	0.009054019	0.037930918	0.006311458	-7.307810938	1
Stem vs Leaf	Citric Acid diglucoside	Organic acids	Organic acids	1.174744816	0.007172446	0.034654934	0.151474348	-2.722854597	1
Stem vs Leaf	Procyanidin B4	Tannins	Proanthocyanidins	1.178682561	0.00536734	0.031033935	8.531813552	3.092852438	1

Comparison	Compound	Class I	Class II	VIP	P-value	FDR	Fold Change	Log_2FC	MSI Level
Stem vs Leaf	3-O-Methylellagic acid 4'-rhamnoside	Tannins	Tannin	1.097158787	0.230845208	0.28705448	7935.948089	12.95418687	1
Stem vs Leaf	Quercetin-3-O-rutinoside (Rutin)*	Flavonoids	Flavonols	1.15577015	0.006999527	0.034124677	0.007621517	-7.035706049	1
Stem vs Leaf	Caffeoylmethylgluconamide*	Alkaloids	Alkaloids	1.176588946	0.028641503	0.06817466	0.022990945	-5.442790425	1
Stem vs Leaf	1-(2,3-dihydroxypropoxy)-3-(((2-(dimethylamino)ethoxy)(hydroxy)phosphoryl)oxy)propan-2-yl (11Z,14Z)-octadec	Lipids	LPE	1.168994944	0.014688651	0.047704834	4.988042313	2.318473703	1

Comparison	Compound	Class I	Class II	VIP	P-value	FDR	Fold Change	Log_2FC	MSI Level
	a-11,14-dienoate								
Stem vs Leaf	N-(3-hydroxy-4-methoxyphenethyl)-4-hydroxybutanamide	Alkaloids	Alkaloids	1.005315266	0.079990237	0.131583022	0.363881733	-1.458458464	3
Stem vs Leaf	O-Phosphocholine	Alkaloids	Alkaloids	1.153277151	0.000309815	0.010919717	3.14401927	1.65261006	3
Stem vs Leaf	3-Oxooleana-11,13(18)-dien-28-oic acid	Terpenoids	Triterpene	1.050882385	0.008220346	0.036758126	0.427703817	-1.225316011	1
Stem vs Leaf	-Pierisformoside H	Terpenoids	Ditepenoids	1.180942032	0.005448807	0.031195162	0.014164316	-6.141595277	2
Stem vs Leaf	4-O-(3'-O-alpha-D	Phenolic	Phenolic acids	1.179838992	0.00161798	0.019058048	0.011724861	-6.41428536	1

Comparison	Compound	Class I	Class II	VIP	P-value	FDR	Fold Change	Log_2FC	MSI Level
	-Glucopyranosyl) caffeoylquinic acid	acids							
Stem vs Leaf	4-Methylazetidine-2-Carboxylic acid*	Alkaloids	Alkaloids	1.165127547	0.000740131	0.014479152	4.936494366	2.303486881	3
Stem vs Leaf	Catechin 4'-O-beta-D-glucopyranoside*	Flavonoids	Flavanols	1.156340306	0.080402785	0.131958769	17.97791417	4.168153742	1
Stem vs Leaf	D-Pantothenic Acid	Others	Vitamin	1.018489178	0.012376482	0.042918447	0.455089015	-1.135779331	2
Stem vs Leaf	3,4-Dihydroxybenzoic acid	Phenolic acids	Phenolic acids	1.180609928	0.004232656	0.028481053	22.31360435	4.479851668	1

Comparison	Compound	Class I	Class II	VIP	P-value	FDR	Fold Change	Log_2FC	MSI Level
	(Protocatechuic acid)*								
Stem vs Leaf	Betanin (Betanidin-5-O-glucoside)	Alkaloids	Plumerane	1.174707452	0.018209697	0.053557932	18.11698649	4.179271098	2
Stem vs Leaf	Betaine	Alkaloids	Alkaloids	1.176631071	0.002287844	0.022493448	8.668411387	3.115767623	1
Stem vs Leaf	2-Amino-4-methylvaleric acid	Others	Others	1.061096349	0.05861372	0.107068392	0.163138351	-2.615832118	1
Stem vs Leaf	2(4H)-benzofuranone	Others	Others	1.16147939	0.031648398	0.072464384	0.037491041	-4.737310312	3
Stem vs Leaf	4-(3,4,5-Trihydroxybenzoxy)benzoic acid	Phenolic acids	Phenolic acids	1.178624144	0.001361317	0.017778885	6.017583019	2.58918414	3

Comparison	Compound	Class I	Class II	VIP	P-value	FDR	Fold Change	Log_2FC	MSI Level
Stem vs Leaf	Cis-Zeatin-7-N-glucoside*	Alkaloids	Alkaloids	1.15619287	0.006664095	0.033249269	2.374479398	1.247611239	1
Stem vs Leaf	Epicatechin-3'-O-β-D-glucopyranoside*	Flavonoids	Flavanols	1.034412974	0.11529693	0.170633795	2.223140481	1.152599116	1
Stem vs Leaf	9-hydroxy-2-(3-hydroxy-4-methoxyphenyl)-3-(hydroxymethyl)-12-methoxy-2,3-dihydro-1,4,5-trioxatetraphen-10-one	Others	Ketone compounds	1.163549592	0.011575187	0.041808314	0.293063948	-1.770712595	1
Stem vs Leaf	4-O-p-Coumaroyl quinic acid	Phenolic acids	Phenolic acids	1.179975996	0.004044035	0.028204938	0.090302608	-3.469088531	1

Comparison	Compound	Class I	Class II	VIP	P-value	FDR	Fold Change	Log_2FC	MSI Level
Stem vs Leaf	Geranyl 3-O-xylopyranosyl-glucopyranoside	Terpenoids	Monoterpenoids	1.171530421	0.000812468	0.014923977	3.46930172	1.794645315	2
Stem vs Leaf	8R-Dihydrodehydrodiconferyl alcohol 4-O-β-D-glucopyranoside	Lignans and Coumarins	Lignans	1.167820708	0.001164443	0.016734829	3.644118784	1.865569986	3
Stem vs Leaf	Quercetin-3-O-glucoside-7-O-rhamnoside*	Flavonoids	Flavonols	1.163893912	0.000517356	0.013142389	0.007210661	-7.115652826	1
Stem vs Leaf	4-Nitrophenol	Others	Others	1.159287072	0.005460883	0.031195162	3.098107494	1.631387202	3
Stem vs Leaf	Tamarixetin-3-O-(	Flavonoids	Flavonols	1.179841263	0.006362581	0.032812524	19.72346878	4.301841396	1

Comparison	Compound	Class I	Class II	VIP	P-value	FDR	Fold Change	Log_2FC	MSI Level
	6''-malonyl)glucoside*								
Stem vs Leaf	Rutin Trihydrate	Flavonoids	Flavonols	1.17598405	0.009588849	0.039045504	0.005579805	-7.485569503	3
Stem vs Leaf	3-Dehydro-L-Threonic Acid	Others	Saccharides	1.101455824	0.013279985	0.044992144	0.495166731	-1.014013709	1
Stem vs Leaf	2,5-Dimethoxybenzoquinone*	Quinones	Quinones	1.169651353	8.82722E-05	0.005751065	2.746725843	1.457712919	3
Stem vs Leaf	Patuletin-3-O-glucoside*	Flavonoids	Flavonols	1.162701666	0.02799915	0.067562482	4.439840191	2.150507749	1
Stem vs Leaf	3,7-Dimethyl-2,6-Octadienyl 6-O-(beta-D-Xylopyranosyl)-beta-	Terpenoids	Monoterpenoids	1.102999762	0.00837342	0.037195976	0.095886588	-3.382527148	2

Comparison	Compound	Class I	Class II	VIP	P-value	FDR	Fold Change	Log_2FC	MSI Level
	D-glucopyranoside								
Stem vs Leaf	rhodomollein XIII*	Terpenoids	Ditepenoids	1.171352243	0.023885603	0.061872346	0.106023908	-3.237538467	2
Stem vs Leaf	grayanotoxin I*	Terpenoids	Ditepenoids	1.151269512	0.050048808	0.097483935	0.088764923	-3.493866501	2
Stem vs Leaf	Borneol 7-O-[β-D-apiofuranosyl-(1→6)]-β-D-glucopyranoside	Terpenoids	Monoterpenoids	1.165229	0.003252721	0.026590686	3.511848387	1.812230563	2
Stem vs Leaf	Isorhamnetin 3-(2''-acetylglucoside)	Flavonoids	Flavonols	1.177882734	0.010735137	0.040769423	12.95356165	3.695276924	1

Comparison	Compound	Class I	Class II	VIP	P-value	FDR	Fold Change	Log_2FC	MSI Level
Stem vs Leaf	Rubiarbonone E	Terpenoids	Triterpene	1.096631704	0.09504841	0.149163563	6.559961305	2.713687305	3
Stem vs Leaf	Isorhamnetin-3-O-(6''-malonyl)glucoside*	Flavonoids	Flavonols	1.17862942	0.001104933	0.016559547	17.57459649	4.13541966	1
Stem vs Leaf	Robinetinidol-(4alpha→8)-catechin-(6→4alpha)-robinetinidol	Flavonoids	Flavanols	1.176788364	0.001261631	0.017412469	19.46364859	4.282710273	3
Stem vs Leaf	3'-O-Methyltricetin-7-O-(6''-malonyl)glucoside*	Flavonoids	Flavones	1.173873094	0.007205744	0.034654934	17.58468938	4.136247946	1
Stem vs Leaf	2α,3α-Epoxy-5,7,3',4'-tetrahydroxyfl	Tannins	Proanthocyanidins	1.097130267	0.199554435	0.256794873	22.60030119	4.498270094	1

Comparison	Compound	Class I	Class II	VIP	P-value	FDR	Fold Change	Log_2FC	MSI Level
	avan-(4β→8)-epicatechin								
Stem vs Leaf	Salicylic acid-2-O-glucoside	Phenolic acids	Phenolic acids	1.151129046	0.000585128	0.013615966	0.496076098	-1.011366648	3
Stem vs Leaf	DL-Leucine*	Amino acids and derivatives	Amino acids and derivatives	1.050620861	0.055395094	0.103474763	0.190698872	-2.390631784	1
Stem vs Leaf	1β-Hydroxy-2-oxopomolic acid	Terpenoids	Triterpene	1.118567438	0.0511962	0.098630672	2.771572115	1.470704546	2
Stem vs Leaf	Pipecolic acid	Alkaloids	Piperidine alkaloids	1.098421601	0.072934095	0.123083441	3.754418605	1.908589515	3
Stem vs Leaf	2,6-Dimethoxy-1,	Quinones	Quinones	1.167876252	0.012698052	0.043611522	2.831604416	1.501619731	3

Comparison	Compound	Class I	Class II	VIP	P-value	FDR	Fold Change	Log_2FC	MSI Level
	4-benzoquinone*								
Stem vs Leaf	Leucocyanidin; 3,4,5,7,3',4'-Hexahydroxyflavan	Flavonoids	Flavanols	1.046419529	0.01244794	0.043027447	0.046110821	-4.438750827	1
Stem vs Leaf	Glucosyringic acid	Phenolic acids	Phenolic acids	1.159401843	0.001829398	0.020636181	2.985361663	1.577905718	1
Stem vs Leaf	Turanose	Others	Saccharides	1.016278007	0.215030346	0.271471077	4.944907469	2.305943525	1
Stem vs Leaf	Dihydrokaempferide	Flavonoids	Flavanonols	1.118522798	0.003304173	0.026707589	0.308719722	-1.695630447	3
Stem vs Leaf	1,2,4,5,8-pentahydroxy-6-methylanthracene-9,10-dione	Flavonoids	Other Flavonoids	1.088677699	0.063174586	0.112999467	2.345141401	1.229674913	2

Comparison	Compound	Class I	Class II	VIP	P-value	FDR	Fold Change	Log_2FC	MSI Level
Stem vs Leaf	D-Malic acid*	Organic acids	Organic acids	1.096343758	0.005884341	0.032023409	0.498916643	-1.003129298	1
Stem vs Leaf	Artocarpanone	Flavonoids	Flavanones	1.090867188	0.070940949	0.121050033	2.146031435	1.101671209	1
Stem vs Leaf	Scopoletin-7-O-glucoside (Scopolin)	Lignans and Coumarins	Coumarins	1.133949487	0.040393566	0.08497668	3.901107461	1.96388374	3
Stem vs Leaf	6-C-Methylquercetin-3-O-rhamnoside	Flavonoids	Flavonols	1.139270838	0.000701848	0.014479152	0.06240241	-4.002254451	2
Stem vs Leaf	6-Aminocaproic acid	Organic acids	Organic acids	1.086612542	0.027498062	0.066858221	0.152282471	-2.715178213	3
Stem vs Leaf	1,4,8-Trihydroxynaphthalene-1-O-gl	Quinones	Quinones	1.16550203	0.001380743	0.017778885	0.019431564	-5.685454159	3

Comparison	Compound	Class I	Class II	VIP	P-value	FDR	Fold Change	Log_2FC	MSI Level
	ucoside								
Stem vs Leaf	1,2,4,5-tetrahydroxy-7-(hydroxymethyl)anthracene-9,10-dione*	Flavonoids	Other Flavonoids	1.15937719	0.001869401	0.020636181	0.249156668	-2.004874912	2
Stem vs Leaf	D-Allo-Isoleucine*	Amino acids and derivatives	Amino acids and derivatives	1.048103309	0.037509732	0.081188111	0.191973289	-2.381022503	1
Stem vs Leaf	(3,4,5-trihydroxytetrahydro-2H-pyran-2-yl)methyl (E)-3-(4-hydroxy-3-methoxyphenyl)acrylate	Phenolic acids	Phenolic acids	1.170619968	0.008431803	0.037378095	0.017775224	-5.813988436	3

Comparison	Compound	Class I	Class II	VIP	P-value	FDR	Fold Change	Log_2FC	MSI Level
Stem vs Leaf	Isorhamnetin-3-O-rhamnoside	Flavonoids	Flavonols	1.118820945	0.00497286	0.030287956	0.087232864	-3.518984432	2
Stem vs Leaf	Ferulic acid-4-O-glucoside	Phenolic acids	Phenolic acids	1.179399649	0.004418464	0.02880574	0.143864818	-2.797214266	1
Stem vs Leaf	4,5,8-Trihydroxy-α-tetralone-5-O-glucoside	Quinones	Naphthol	1.169491665	0.002401608	0.02293776	0.050700579	-4.301853973	3
Stem vs Leaf	L-Leucine*	Amino acids and derivatives	Amino acids and derivatives	1.036800329	0.04603159	0.092840448	0.215451191	-2.214567018	1
Stem vs Leaf	Taxifolin-3-O-rhamnoside	Flavonoids	Flavanonols	1.070080863	0.021380684	0.057944985	0.079105245	-3.66008284	3

Comparison	Compound	Class I	Class II	VIP	P-value	FDR	Fold Change	Log_2FC	MSI Level
	(Astilbin)								
Stem vs Leaf	3,5,7,4'-Tetrahydroxy-Coumaronochromone	Others	Chromone	1.067219841	0.001263416	0.017412469	0.24435164	-2.032969309	1
Stem vs Leaf	Monotropein	Terpenoids	Monoterpenoids	1.140200353	0.001773097	0.020170153	0.424395385	-1.236519126	2
Stem vs Leaf	Methylmalonic acid*	Organic acids	Organic acids	1.175168253	6.57147E-06	0.001668767	0.237358854	-2.074858226	1
Stem vs Leaf	Rhodioloside F	Terpenoids	Monoterpenoids	1.139445938	0.022833155	0.060308703	0.148679895	-2.749718524	3
Stem vs Leaf	Cinnamtannin B1	Tannins	Proanthocyanidins	1.162635313	0.016656399	0.050805983	3.412385165	1.770780497	2

Comparison	Compound	Class I	Class II	VIP	P-value	FDR	Fold Change	Log_2FC	MSI Level
Stem vs Leaf	Cryptochlorogenic acid (4-O-Caffeoylquinic acid)*	Phenolic acids	Phenolic acids	1.171469203	0.001316377	0.017778885	0.037082698	-4.753109964	1
Stem vs Leaf	Gallocatechin	Flavonoids	Flavanols	1.028051646	0.000675154	0.014479152	0.050874621	-4.296910035	1
Stem vs Leaf	Neochlorogenic acid (5-O-Caffeoylquinic acid)*	Phenolic acids	Phenolic acids	1.035423508	0.021552228	0.058015292	0.461686498	-1.115014552	1
Stem vs Leaf	wilforic acid E	Terpenoids	Triterpene	1.149822771	0.037586584	0.081188111	6.424246247	2.683527194	3
Stem vs Leaf	Procyanidin B7	Tannins	Proanthocyanidins	1.165067779	0.004423499	0.02880574	3.548588914	1.827245455	3
Stem vs Leaf	Fisetinidol-(4α,6)-	Flavonoids	Flavanols	1.164901559	3.16237E-05	0.003237664	4.376060778	2.129632776	3

Comparison	Compound	Class I	Class II	VIP	P-value	FDR	Fold Change	Log_2FC	MSI Level
	gallocatechin								
Stem vs Leaf	LPC(18:1/0:0)	Lipids	LPC	1.151703182	0.04763005	0.09458827	8.531876995	3.092863166	1
Stem vs Leaf	coumarin	Lignans and Coumarins	Coumarins	1.137254151	0.001337912	0.017778885	0.067350526	-3.892166974	3
Stem vs Leaf	LysoPC 18:1	Lipids	LPC	1.153616511	0.047113593	0.094093896	8.874361468	3.149643318	2
Stem vs Leaf	9-hydroxy-2-(2-hydroxy-3-methoxyphenyl)-3-(hydroxymethyl)-12-methoxy-2,3-dihydro-1,4,5-trioxatetraphen-10-one	Others	Ketone compounds	1.164581207	0.00073667	0.014479152	0.419282267	-1.254006279	1
Stem vs Leaf	2,19-Dihydroxy-3	Terpenoids	Triterpene	1.14939707	0.054555845	0.102890409	11.43002007	3.514756031	1

Comparison	Compound	Class I	Class II	VIP	P-value	FDR	Fold Change	Log_2FC	MSI Level
	-oxours-12-en-28-oic acid								
Stem vs Leaf	LysoPC 18:1(2n isomer)	Lipids	LPC	1.153650996	0.04621672	0.092982619	8.462494656	3.081083018	2
Stem vs Leaf	3',5',5,7-Tetrahydroxy-4'-methoxyflavanone-3'-O-glucoside	Flavonoids	Flavanones	1.146263356	0.006959343	0.03400588	0.02164601	-5.529755041	3
Stem vs Leaf	2,3-Dihydroxyoleana-11,13(18)-dien-28-oic acid (Camaldulenic acid)	Terpenoids	Triterpene	1.062360619	0.113998115	0.169734036	5.495868177	2.4583474	1

Comparison	Compound	Class I	Class II	VIP	P-value	FDR	Fold Change	Log_2FC	MSI Level
Stem vs Leaf	Virgatic acid	Terpenoids	Triterpene	1.108778794	0.079552839	0.131173416	5.649662649	2.498164724	1
Stem vs Leaf	Cinnamtannin D1	Tannins	Tannin	1.163069069	0.013288377	0.044992144	3.216514025	1.685497979	3
Stem vs Leaf	Cinchonain Ic	Flavonoids	Flavanols	1.169753588	0.007599044	0.03555507	0.41444623	-1.270743154	3
Stem vs Leaf	2-(3,4-dihydroxyphenyl)-4-[2-(3,4-dihydroxyphenyl)-3,5,7-trihydroxy-3,4-dihydro-2h-1-benzopyran-8-yl]-3,4-dihydro-2h-1-benzopyran-3,5,7-triol	Others	Others	1.151613285	0.025897558	0.064103755	3.924994949	1.972690797	3
Stem vs Leaf	Succinic acid*	Organic	Organic acids	1.166707802	7.91169E-05	0.005487142	0.299510924	-1.739319473	1

Comparison	Compound	Class I	Class II	VIP	P-value	FDR	Fold Change	Log_2FC	MSI Level
		acids							
Stem vs Leaf	LysoPE 18:2	Lipids	LPE	1.171202779	0.011672451	0.041906997	8.734242118	3.126682524	2
Stem vs Leaf	D-α-D-Hep-(1→6)-β-D-Glc	Others	Saccharides	1.01846041	0.037939843	0.081570662	0.357823903	-1.482678332	1
Stem vs Leaf	Gln-Gly	Amino acids and derivatives	Amino acids and derivatives	1.162207702	0.000915837	0.015627374	0.490738345	-1.026974091	2
Stem vs Leaf	LysoPE 18:2(2n isomer)	Lipids	LPE	1.16820314	0.015721759	0.049215964	8.873033232	3.149427372	1
Stem vs Leaf	Leucopelargonidin; 3,4,5,7,4'-Pentahydroxyflavan	Others	Others	1.169658776	0.001355125	0.017778885	0.237079026	-2.076560058	1

Comparison	Compound	Class I	Class II	VIP	P-value	FDR	Fold Change	Log_2FC	MSI Level
Stem vs Leaf	Vanillic acid glucoside	Phenolic acids	Phenolic acids	1.173863541	0.000502491	0.013133103	2.200876532	1.138078213	1
Stem vs Leaf	Catechin-(7,8-bc)-4α-(3,4-dihydroxy phenyl)-dihydro-2-(3H)-one*	Flavonoids	Flavanols	1.176568301	0.010463738	0.040169517	0.047415609	-4.398494117	1
Stem vs Leaf	Cinchonain Id*	Flavonoids	Flavanols	1.17936531	0.000155361	0.007920636	0.037022989	-4.755434798	1
Stem vs Leaf	Catechin-(7,8-bc)-4β-(3,4-dihydroxy phenyl)-dihydro-2-(3H)-one	Flavonoids	Flavanols	1.179220359	0.005952206	0.032073291	0.053502828	-4.224241042	1
Stem vs Leaf	4,5-Dehydro-L-le ucine*	Amino acids and	Amino acids and derivatives	1.059027376	0.089302385	0.142222317	3.174705145	1.666622606	2

Comparison	Compound	Class I	Class II	VIP	P-value	FDR	Fold Change	Log_2FC	MSI Level
		derivatives							
Stem vs Leaf	Bracteatin*	Flavonoids	Aurones	1.043610213	0.011589653	0.041808314	0.292189868	-1.775021941	2
Stem vs Leaf	Epitheaflavic acid-3'-Gallate	Flavonoids	Flavanols	1.177638103	0.008127356	0.036632739	9.02248139	3.173524262	3
Stem vs Leaf	Fisetinidol-4β-ol	Flavonoids	Flavonols	1.17342223	0.000185452	0.008306724	0.242300909	-2.045128276	3
Stem vs Leaf	N-Methyl-L-proline*	Amino acids and derivatives	Amino acids and derivatives	1.085609634	0.080179148	0.13177408	3.506113808	1.809872827	2
Stem vs Leaf	Gossypetin-3-O-glucoside*	Flavonoids	Flavonols	1.163572895	0.00390114	0.028194402	0.017724092	-5.818144429	1
Stem vs Leaf	Kaempferol-3-O-arabinoside	Flavonoids	Flavonols	1.157358022	0.002325124	0.022619987	0.217735629	-2.199350596	1

Comparison	Compound	Class I	Class II	VIP	P-value	FDR	Fold Change	Log_2FC	MSI Level
Stem vs Leaf	Myricetin-3-O-β-D-glucoside*	Flavonoids	Flavonols	1.160649437	0.000773042	0.014839646	0.018255519	-5.775523482	1
Stem vs Leaf	L-Pipecolic Acid	Alkaloids	Piperidine alkaloids	1.080369782	0.084033095	0.136013144	3.54070368	1.82403611	2
Stem vs Leaf	3-Hydroxy-L-phe nylalanine*	Amino acids and derivatives	Amino acids and derivatives	1.04948336	0.004015866	0.028204938	0.303849598	-1.71857071	1
Stem vs Leaf	Cycloleucine	Amino acids and derivatives	Amino acids and derivatives	1.058108499	0.09088662	0.143786779	3.062321474	1.614625741	2
Stem vs Leaf	L-Tyrosine*	Amino acids and derivatives	Amino acids and derivatives	1.079128801	0.00205437	0.021133472	0.27450458	-1.865097874	1

Comparison	Compound	Class I	Class II	VIP	P-value	FDR	Fold Change	Log_2FC	MSI Level
Stem vs Leaf	Kaempferol-3-O-arabinoside (Juglanin)	Flavonoids	Flavonols	1.168525763	0.009691015	0.039386921	0.189276963	-2.401429265	1
Stem vs Leaf	(3,5-dihydroxyphenyl)-(4-hydroxy-2,6-dimethoxyphenyl)methanone	Others	Ketone compounds	1.179320752	0.002464293	0.023035781	0.276274748	-1.855824393	3
Stem vs Leaf	Choline	Alkaloids	Alkaloids	1.162239568	0.017260225	0.052065312	2.73319709	1.450589496	3
Stem vs Leaf	Quercetin 3-(2''-acetylrhamnoside)	Flavonoids	Flavonols	1.169310926	0.012900117	0.0440942	0.001334176	-9.549835048	1
Stem vs Leaf	1-desmethylaurantio-obtusin-2-O-β-	Quinones	Anthraquinone	1.179396561	0.003509537	0.027237422	9.39106078	3.231288129	3

Comparison	Compound	Class I	Class II	VIP	P-value	FDR	Fold Change	Log_2FC	MSI Level
	D-glucoside								
Stem vs Leaf	Procyanidin B3	Tannins	Proanthocyanidins	1.179726499	0.00395383	0.028204938	6.39989628	2.678048524	1
Stem vs Leaf	tenuiflorin C glucoside	Others	Chromone	1.179585479	1.73909E-07	0.000373904	7.489570527	2.904882993	3
Stem vs Leaf	2,5-Dihydroxybenzoic acid-5-O-glucoside	Phenolic acids	Phenolic acids	1.124859807	0.002451676	0.023017917	2.107029906	1.075210791	3
Stem vs Leaf	5,7-Dihydroxy-4-oxo-2-(3,4,5-trihydroxyphenyl)-4H-chromen-3-yl-allo	Others	Others	1.17969901	0.002763892	0.024478889	0.019722207	-5.664035177	3

Comparison	Compound	Class I	Class II	VIP	P-value	FDR	Fold Change	Log2FC	MSI Level
	side								
Stem vs Leaf	Isoscopolin	Lignans and Coumarins	Coumarins	1.17941615	0.004394202	0.02880574	0.173766398	-2.524778965	1
Stem vs Leaf	Nepetin-7-O-allos ide*	Flavonoids	Flavones	1.1793226	0.003172513	0.026437605	8.728994013	3.125815398	1
Stem vs Leaf	Rhamnetin-3-O-G lucoside*	Flavonoids	Flavonols	1.180665906	4.02642E-07	0.000383537	7.073436262	2.822411244	1
Stem vs Leaf	Isorhamnetin 7-O-glucoside	Flavonoids	Flavonols	1.177620204	0.001043013	0.016277656	7.827757831	2.968599124	2
Stem vs Leaf	Cannabifolin D	Terpenoids	Triterpene	1.145671326	0.017933657	0.053329687	8.267743637	3.047493655	3
Stem vs Leaf	Nepetin-7-O-gluc oside(Nepitrin)*	Flavonoids	Flavones	1.176550368	0.006267459	0.032737441	6.697390689	2.74359913	1

Comparison	Compound	Class I	Class II	VIP	P-value	FDR	Fold Change	Log_2FC	MSI Level
Stem vs Leaf	Protocatechuic acid-4-O-glucoside*	Phenolic acids	Phenolic acids	1.159080221	0.000321285	0.010948842	2.351938929	1.2338506	1
Stem vs Leaf	Quercetagetin-7-O-glucoside*	Flavonoids	Flavonols	1.08066697	0.005282887	0.030931935	0.381502993	-1.39023372	1
Stem vs Leaf	vitelignin A	Lignans and Coumarins	Lignans	1.132091273	2.27366E-05	0.00257283	0.026768663	-5.223311105	3
Stem vs Leaf	6-Methoxykaempferol-3-O-glucoside*	Flavonoids	Flavonols	1.177434758	4.94605E-06	0.001519143	5.015748325	2.32646496	1
Stem vs Leaf	(8R)-9-hydroxy-8-(hydroxymethyl)-6-methoxy-8-met	Terpenoids	Terpene	1.170956044	0.006578617	0.033249269	0.290689335	-1.782449954	3

Comparison	Compound	Class I	Class II	VIP	P-value	FDR	Fold Change	Log$_2$FC	MSI Level
	hylpyrano[2,3-f]c hromen-2-one								
Stem vs Leaf	Catechin*	Flavonoids	Flavanols	1.160333198	0.000432501	0.012565906	0.367076313	-1.445848072	1
Stem vs Leaf	Procyanidin B2	Tannins	Proanthocyanidins	1.154693512	0.004113464	0.028378135	6.184310309	2.628612711	1
Stem vs Leaf	Isorhamnetin-3-O-Glucoside*	Flavonoids	Flavonols	1.177320103	0.001969013	0.020746033	5.386878846	2.429449619	1
Stem vs Leaf	5,7,3',4',5'-Pentahydroxyflavan (Tricetiflavan)*	Flavonoids	Flavanols	1.141333184	0.009849902	0.039583717	2.685507231	1.425194606	1
Stem vs Leaf	L-Arginine	Amino acids and derivatives	Amino acids and derivatives	1.123615284	0.13238767	0.187382153	45.92478429	5.52120104	1

Comparison	Compound	Class I	Class II	VIP	P-value	FDR	Fold Change	Log_2FC	MSI Level
Stem vs Leaf	cagayanone A	Lignans and Coumarins	Lignans	1.177227966	2.01092E-05	0.00257283	0.008238529	-6.923397514	3
Stem vs Leaf	Epicatechin*	Flavonoids	Flavanols	1.161337729	0.01511992	0.048446838	3.488948783	1.802792419	1
Stem vs Leaf	L-Phenylalanine	Amino acids and derivatives	Amino acids and derivatives	1.062412969	0.023324784	0.061034782	0.20294593	-2.300832691	1
Stem vs Leaf	L-Isoleucine*	Amino acids and derivatives	Amino acids and derivatives	1.025317537	0.033150038	0.074709206	0.218370754	-2.195148445	1
Stem vs Leaf	5-O-p-Coumaroyl quinic acid	Phenolic acids	Phenolic acids	1.18053586	0.002950094	0.025575409	0.020955398	-5.576534257	1
Stem vs Leaf	L-Norleucine*	Amino acids and	Amino acids and derivatives	1.03680846	0.047177621	0.094093896	0.21454206	-2.220667583	1

Comparison	Compound	Class I	Class II	VIP	P-value	FDR	Fold Change	Log_2FC	MSI Level
		derivatives							
Stem vs Leaf	Quercetin-3-O-rhamnoside(Quercitrin)	Flavonoids	Flavonols	1.003305274	0.00355735	0.027413271	0.212732567	-2.232887187	1
Stem vs Leaf	2-Caffeoylisocitrate	Phenolic acids	Phenolic acids	1.150781148	0.001128252	0.016729257	0.150783095	-2.7294534	2
Stem vs Leaf	Sesamin	Lignans and Coumarins	Lignans	1.154461396	0.043725446	0.089501623	0.187979775	-2.411350644	3
Stem vs Leaf	Myricetin-3-O-arabinoside*	Flavonoids	Flavonols	1.178685044	0.000689127	0.014479152	0.00278361	-8.48882719	1
Stem vs Leaf	3-O-p-Coumaroyl quinic acid	Phenolic acids	Phenolic acids	1.171899819	0.001430872	0.017885898	0.011834195	-6.400894567	1

Comparison	Compound	Class I	Class II	VIP	P-value	FDR	Fold Change	Log_2FC	MSI Level
Stem vs Leaf	7-Hydroxycoumarin;Umbelliferone	Lignans and Coumarins	Coumarins	1.07205139	0.076784617	0.128073644	0.284502565	-1.813486436	1
Stem vs Leaf	Myricetin-3-O-xyloside*	Flavonoids	Flavonols	1.175471475	0.000875293	0.015425252	0.002746738	-8.508065085	1
Stem vs Leaf	Quercetin-3-O-xyloside (Reynoutrin)*	Flavonoids	Flavonols	1.179215287	0.000110038	0.006225838	2.545854201	1.3481498	1
Stem vs Leaf	Quinic Acid	Organic acids	Organic acids	1.01902093	0.057341015	0.105483354	0.377728401	-1.404578834	1
