# Supplementary material for: Integration of Transcriptomics and Metabolomics Reveals Organ-Specific Biosynthesis and Accumulation of Pharmacologically Active Flavonoids in Rhododendron yedoense var. poukhanense
Source: Plants (Basel). 2026 Aug 11;15(16):2441. doi: 10.3390/plants15162441 (PMC13516736; doi:10.3390/plants15162441)
Supplement: Supplementary file 1 [file plants-15-02441-s001.zip › Table S5.pdf]

Table S5. All DEGs detected in *R. yedoense* var. *poukhanense* root, stem, and leaf tissues.

Comparison	Gene ID	log_2FC	P-value	FDR (padj)	Gene Name
Root vs Leaf	RHSIM_Rhsim01G0274600	-5.885923803	0	0	RHSIM_Rhsim01G0274600
Root vs Leaf	RHSIM_Rhsim04G0032000	-8.231505568	0	0	RHSIM_Rhsim04G0032000
Root vs Leaf	RHSIM_Rhsim07G0134900	-9.190633214	0	0	RHSIM_Rhsim07G0134900
Root vs Leaf	RHSIM_Rhsim12G0015600	-9.224351673	0	0	RHSIM_Rhsim12G0015600
Root vs Leaf	RHSIM_Rhsim11G0183800	-8.025729583	2.2184E-295	1.0267E-291	RHSIM_Rhsim11G0183800
Root vs Leaf	RHSIM_Rhsim07G0142300	-7.863917695	6.9354E-288	2.6749E-284	RHSIM_Rhsim07G0142300
Root vs Leaf	RHSIM_Rhsim04G0213100	-7.492794792	6.1556E-283	2.035E-279	RHSIM_Rhsim04G0213100
Root vs Leaf	RHSIM_Rhsim04G0197500	-11.38221671	3.2373E-276	9.3643E-273	RHSIM_Rhsim04G0197500
Root vs Leaf	RHSIM_Rhsim10G0057900	-5.247923929	1.4731E-265	3.7876E-262	RHSIM_Rhsim10G0057900
Root vs Leaf	RHSIM_Rhsim13G0077400	-9.531986287	6.4024E-260	1.4816E-256	RHSIM_Rhsim13G0077400
Root vs Leaf	RHSIM_Rhsim02G0202100	-5.221917977	3.1605E-259	6.6488E-256	RHSIM_Rhsim02G0202100
Root vs Leaf	RHSIM_Rhsim04G0009800	-7.123487065	3.5264E-259	6.8005E-256	RHSIM_Rhsim04G0009800
Root vs Leaf	RHSIM_Rhsim12G0004800	-5.71657885	9.0415E-252	1.6095E-248	RHSIM_Rhsim12G0004800
Root vs Leaf	RHSIM_Rhsim04G0081300	-8.071852055	1.0693E-251	1.7676E-248	RHSIM_Rhsim04G0081300
Root vs Leaf	RHSIM_Rhsim11G0098700	-8.335515322	2.434E-242	3.755E-239	RHSIM_Rhsim11G0098700
Root vs Leaf	RHSIM_Rhsim06G0035600	-7.187681175	1.3964E-237	2.0196E-234	RHSIM_Rhsim06G0035600
Root vs Leaf	novel.3541	6.452841554	2.5326E-237	3.4474E-234	--
Root vs Leaf	RHSIM_Rhsim12G0046400	-7.087564906	3.7127E-231	4.7731E-228	RHSIM_Rhsim12G0046400
Root vs Leaf	RHSIM_Rhsim09G0170400	6.134371739	7.6068E-224	9.2646E-221	RHSIM_Rhsim09G0170400
Root vs Leaf	RHSIM_Rhsim07G0103700	-4.887423629	8.3286E-224	9.6366E-221	RHSIM_Rhsim07G0103700
Root vs Leaf	RHSIM_Rhsim07G0251100	-13.04566473	6.4839E-218	7.1449E-215	RHSIM_Rhsim07G0251100
Root vs Leaf	RHSIM_Rhsim08G0226600	4.632613481	1.0485E-216	1.1029E-213	RHSIM_Rhsim08G0226600
Root vs Leaf	RHSIM_Rhsim09G0152700	-10.06125609	2.372E-213	2.3866E-210	RHSIM_Rhsim09G0152700

Comparison	Gene ID	log_2FC	P-value	FDR (padj)	Gene Name
Root vs Leaf	RHSIM_Rhsim09G0111900	-4.817645699	4.6815E-213	4.5139E-210	RHSIM_Rhsim09G0111900
Root vs Leaf	RHSIM_Rhsim04G0122300	-11.39924658	9.8807E-209	9.146E-206	RHSIM_Rhsim04G0122300
Root vs Leaf	RHSIM_RhsimUnG0040900	-5.237445647	3.7331E-206	3.3226E-203	RHSIM_RhsimUnG0040900
Root vs Leaf	RHSIM_Rhsim13G0154000	-7.272055984	1.3356E-203	1.1447E-200	RHSIM_Rhsim13G0154000
Root vs Leaf	RHSIM_Rhsim08G0052700	-6.773340231	2.0641E-202	1.7059E-199	RHSIM_Rhsim08G0052700
Root vs Leaf	RHSIM_Rhsim03G0210700	-4.361733999	1.3113E-199	1.0463E-196	RHSIM_Rhsim03G0210700
Root vs Leaf	RHSIM_Rhsim11G0077200	-10.28876875	1.4154E-189	1.0918E-186	RHSIM_Rhsim11G0077200
Root vs Leaf	RHSIM_Rhsim04G0018900	-5.785019593	4.6587E-188	3.4776E-185	RHSIM_Rhsim04G0018900
Root vs Leaf	RHSIM_Rhsim05G0231800	-5.588867865	1.2492E-187	9.0338E-185	RHSIM_Rhsim05G0231800
Root vs Leaf	RHSIM_Rhsim07G0179600	-5.226328482	1.8552E-187	1.301E-184	RHSIM_Rhsim07G0179600
Root vs Leaf	RHSIM_Rhsim11G0130100	-4.613843907	9.6457E-187	6.565E-184	RHSIM_Rhsim11G0130100
Root vs Leaf	RHSIM_Rhsim08G0042600	-4.058746845	1.789E-186	1.1829E-183	RHSIM_Rhsim08G0042600
Root vs Leaf	RHSIM_Rhsim13G0064600	-3.025163446	1.745E-184	1.1217E-181	RHSIM_Rhsim13G0064600
Root vs Leaf	RHSIM_Rhsim01G0226500	-4.454832329	9.2436E-181	5.7813E-178	RHSIM_Rhsim01G0226500
Root vs Leaf	RHSIM_Rhsim12G0138000	-5.385833323	2.7386E-180	1.6677E-177	RHSIM_Rhsim12G0138000
Root vs Leaf	RHSIM_Rhsim03G0040000	-2.952843782	1.2516E-178	7.4263E-176	RHSIM_Rhsim03G0040000
Root vs Leaf	RHSIM_Rhsim08G0033400	-9.676238277	3.8593E-177	2.2327E-174	RHSIM_Rhsim08G0033400
Root vs Leaf	RHSIM_Rhsim04G0002800	-4.840187018	7.1244E-176	4.0211E-173	RHSIM_Rhsim04G0002800
Root vs Leaf	RHSIM_Rhsim11G0008700	-5.421646047	8.4802E-175	4.6724E-172	RHSIM_Rhsim11G0008700
Root vs Leaf	RHSIM_Rhsim04G0243100	-3.726386293	4.5804E-173	2.465E-170	RHSIM_Rhsim04G0243100
Root vs Leaf	RHSIM_Rhsim06G0127100	-4.264297753	5.3118E-172	2.7936E-169	RHSIM_Rhsim06G0127100
Root vs Leaf	RHSIM_Rhsim06G0198700	-10.90165905	1.0212E-169	5.2515E-167	RHSIM_Rhsim06G0198700
Root vs Leaf	RHSIM_Rhsim12G0036500	5.666607058	1.6748E-169	8.4254E-167	RHSIM_Rhsim12G0036500
Root vs Leaf	RHSIM_Rhsim10G0211400	-5.564292488	2.7298E-169	1.344E-166	RHSIM_Rhsim10G0211400
Root vs Leaf	RHSIM_Rhsim06G0131200	-4.89795096	1.8928E-168	9.1251E-166	RHSIM_Rhsim06G0131200

Comparison	Gene ID	log_2FC	P-value	FDR (padj)	Gene Name
Root vs Leaf	RHSIM_Rhsim04G0052300	-8.367771699	4.1689E-167	1.9688E-164	RHSIM_Rhsim04G0052300
Root vs Leaf	RHSIM_Rhsim13G0215900	-4.366874797	6.759E-166	3.1282E-163	RHSIM_Rhsim13G0215900
Root vs Leaf	RHSIM_Rhsim06G0008500	-4.623878706	1.7026E-164	7.7253E-162	RHSIM_Rhsim06G0008500
Root vs Leaf	RHSIM_Rhsim12G0099800	-7.602715201	2.4239E-163	1.0787E-160	RHSIM_Rhsim12G0099800
Root vs Leaf	RHSIM_Rhsim06G0181200	-6.732798977	2.1961E-162	9.5887E-160	RHSIM_Rhsim06G0181200
Root vs Leaf	RHSIM_Rhsim13G0049600	-10.57928049	2.8537E-162	1.2229E-159	RHSIM_Rhsim13G0049600
Root vs Leaf	RHSIM_Rhsim10G0187700	-8.379586689	3.0035E-160	1.2637E-157	RHSIM_Rhsim10G0187700
Root vs Leaf	RHSIM_Rhsim04G0066500	-6.27247351	1.9038E-159	7.8672E-157	RHSIM_Rhsim04G0066500
Root vs Leaf	RHSIM_Rhsim02G0161200	4.210166406	3.0611E-158	1.2428E-155	RHSIM_Rhsim02G0161200
Root vs Leaf	novel.3139	-3.806781151	5.6746E-157	2.2641E-154	--
Root vs Leaf	novel.1475	-4.165719965	6.6418E-156	2.6051E-153	--
Root vs Leaf	RHSIM_Rhsim12G0139100	-7.755771007	8.3008E-155	3.2015E-152	RHSIM_Rhsim12G0139100
Root vs Leaf	RHSIM_Rhsim01G0126800	-4.679497879	4.2543E-154	1.6139E-151	RHSIM_Rhsim01G0126800
Root vs Leaf	RHSIM_Rhsim12G0078900	-4.628436458	1.0356E-153	3.8655E-151	RHSIM_Rhsim12G0078900
Root vs Leaf	RHSIM_Rhsim04G0167100	-3.074504098	2.6846E-153	9.8608E-151	RHSIM_Rhsim04G0167100
Root vs Leaf	RHSIM_Rhsim01G0184800	-6.103584145	1.041E-151	3.7639E-149	RHSIM_Rhsim01G0184800
Root vs Leaf	RHSIM_Rhsim11G0012900	-6.356691837	4.866E-151	1.7324E-148	RHSIM_Rhsim11G0012900
Root vs Leaf	RHSIM_Rhsim05G0043600	-3.998977419	1.3298E-148	4.6626E-146	RHSIM_Rhsim05G0043600
Root vs Leaf	RHSIM_Rhsim06G0084800	-6.933083468	2.5656E-147	8.8612E-145	RHSIM_Rhsim06G0084800
Root vs Leaf	RHSIM_Rhsim04G0052800	-3.226718708	7.6825E-146	2.6144E-143	RHSIM_Rhsim04G0052800
Root vs Leaf	RHSIM_Rhsim01G0151800	-4.801226562	4.9055E-145	1.6452E-142	RHSIM_Rhsim01G0151800
Root vs Leaf	RHSIM_Rhsim10G0180100	-5.319625982	7.9509E-144	2.6285E-141	RHSIM_Rhsim10G0180100
Root vs Leaf	RHSIM_Rhsim04G0038600	-7.209645911	6.3085E-143	2.0561E-140	RHSIM_Rhsim04G0038600
Root vs Leaf	RHSIM_Rhsim06G0004600	-7.003266718	7.9129E-143	2.5432E-140	RHSIM_Rhsim06G0004600
Root vs Leaf	RHSIM_Rhsim06G0006600	-5.572499138	5.4377E-141	1.7238E-138	RHSIM_Rhsim06G0006600

Comparison	Gene ID	log_2FC	P-value	FDR (padj)	Gene Name
Root vs Leaf	RHSIM_Rhsim11G0052900	-3.73263284	4.5188E-140	1.4131E-137	RHSIM_Rhsim11G0052900
Root vs Leaf	RHSIM_Rhsim10G0072700	-6.383965886	1.4954E-138	4.614E-136	RHSIM_Rhsim10G0072700
Root vs Leaf	RHSIM_Rhsim07G0205000	-5.247674845	1.7566E-138	5.3487E-136	RHSIM_Rhsim07G0205000
Root vs Leaf	RHSIM_Rhsim08G0096200	-4.328017048	7.9181E-138	2.3796E-135	RHSIM_Rhsim08G0096200
Root vs Leaf	RHSIM_Rhsim05G0230200	-12.8891204	7.2674E-137	2.1561E-134	RHSIM_Rhsim05G0230200
Root vs Leaf	RHSIM_Rhsim05G0221700	-8.127516173	9.9325E-136	2.9095E-133	RHSIM_Rhsim05G0221700
Root vs Leaf	RHSIM_Rhsim04G0234400	6.458931543	4.3815E-135	1.2674E-132	RHSIM_Rhsim04G0234400
Root vs Leaf	RHSIM_Rhsim05G0183000	-5.513510108	5.8924E-133	1.6834E-130	RHSIM_Rhsim05G0183000
Root vs Leaf	RHSIM_Rhsim08G0223300	-5.987365266	2.447E-132	6.9055E-130	RHSIM_Rhsim08G0223300
Root vs Leaf	novel.4723	-4.852694241	1.2733E-131	3.5502E-129	--
Root vs Leaf	RHSIM_Rhsim07G0251700	-5.934868221	5.2796E-131	1.4545E-128	RHSIM_Rhsim07G0251700
Root vs Leaf	RHSIM_Rhsim01G0260300	-7.424380167	8.5819E-131	2.3364E-128	RHSIM_Rhsim01G0260300
Root vs Leaf	RHSIM_Rhsim05G0032600	-4.550009754	1.6818E-130	4.5253E-128	RHSIM_Rhsim05G0032600
Root vs Leaf	RHSIM_Rhsim09G0211900	-6.573027125	2.2725E-129	6.0445E-127	RHSIM_Rhsim09G0211900
Root vs Leaf	RHSIM_Rhsim01G0245100	-7.170622436	3.6056E-129	9.4815E-127	RHSIM_Rhsim01G0245100
Root vs Leaf	novel.1220	8.658930469	8.2952E-128	2.1568E-125	--
Root vs Leaf	RHSIM_Rhsim05G0193900	-3.3180625	2.1146E-127	5.4371E-125	RHSIM_Rhsim05G0193900
Root vs Leaf	RHSIM_Rhsim05G0056500	-5.139158026	2.6301E-127	6.6882E-125	RHSIM_Rhsim05G0056500
Root vs Leaf	RHSIM_Rhsim07G0077100	-11.42514068	1.042E-126	2.6209E-124	RHSIM_Rhsim07G0077100
Root vs Leaf	RHSIM_Rhsim12G0088300	-3.172941999	1.1882E-126	2.9565E-124	RHSIM_Rhsim12G0088300
Root vs Leaf	RHSIM_Rhsim09G0005000	-9.901982071	7.532E-126	1.8542E-123	RHSIM_Rhsim09G0005000
Root vs Leaf	RHSIM_Rhsim03G0220900	-3.218672705	3.4301E-125	8.3555E-123	RHSIM_Rhsim03G0220900
Root vs Leaf	RHSIM_Rhsim06G0070400	-4.779747183	4.6263E-125	1.1152E-122	RHSIM_Rhsim06G0070400
Root vs Leaf	RHSIM_Rhsim01G0091800	-4.149833698	6.4592E-124	1.5409E-121	RHSIM_Rhsim01G0091800
Root vs Leaf	RHSIM_Rhsim11G0027600	-6.207450961	8.0396E-124	1.8984E-121	RHSIM_Rhsim11G0027600

Comparison	Gene ID	log_2FC	P-value	FDR (padj)	Gene Name
Root vs Leaf	RHSIM_Rhsim10G0021200	-12.66860577	4.0906E-123	9.5616E-121	RHSIM_Rhsim10G0021200
Root vs Leaf	RHSIM_Rhsim12G0214000	5.645319823	4.2623E-122	9.8633E-120	RHSIM_Rhsim12G0214000
Root vs Leaf	RHSIM_Rhsim11G0038900	-11.68067845	4.2842E-121	9.816E-119	RHSIM_Rhsim11G0038900
Root vs Leaf	RHSIM_Rhsim06G0108600	-5.981278982	5.6972E-120	1.2925E-117	RHSIM_Rhsim06G0108600
Root vs Leaf	RHSIM_Rhsim07G0049500	5.461781225	2.0121E-119	4.5206E-117	RHSIM_Rhsim07G0049500
Root vs Leaf	RHSIM_Rhsim06G0243400	-3.448311138	4.4732E-119	9.9533E-117	RHSIM_Rhsim06G0243400
Root vs Leaf	RHSIM_Rhsim09G0178500	-4.710673488	6.0025E-118	1.3229E-115	RHSIM_Rhsim09G0178500
Root vs Leaf	RHSIM_Rhsim04G0172100	-7.265610284	8.8049E-118	1.9222E-115	RHSIM_Rhsim04G0172100
Root vs Leaf	RHSIM_RhsimUnG0232700	-4.574414147	1.0199E-117	2.2058E-115	RHSIM_RhsimUnG0232700
Root vs Leaf	RHSIM_RhsimUnG0258600	-10.24674618	1.4222E-117	3.0472E-115	RHSIM_RhsimUnG0258600
Root vs Leaf	RHSIM_Rhsim13G0049700	-9.966181994	7.7419E-117	1.6436E-114	RHSIM_Rhsim13G0049700
Root vs Leaf	RHSIM_Rhsim01G0126900	-5.046123716	8.1721E-117	1.7192E-114	RHSIM_Rhsim01G0126900
Root vs Leaf	RHSIM_Rhsim09G0184500	-8.47918983	2.9153E-116	6.0777E-114	RHSIM_Rhsim09G0184500
Root vs Leaf	RHSIM_Rhsim05G0220000	-7.225741909	5.9812E-116	1.2358E-113	RHSIM_Rhsim05G0220000
Root vs Leaf	RHSIM_Rhsim03G0225500	-4.035216546	4.9949E-115	1.0229E-112	RHSIM_Rhsim03G0225500
Root vs Leaf	RHSIM_Rhsim04G0164800	-7.736913082	5.7964E-115	1.1766E-112	RHSIM_Rhsim04G0164800
Root vs Leaf	RHSIM_Rhsim09G0129500	-3.592776539	1.0695E-114	2.1522E-112	RHSIM_Rhsim09G0129500
Root vs Leaf	RHSIM_Rhsim07G0011000	4.360046667	2.1457E-114	4.2804E-112	RHSIM_Rhsim07G0011000
Root vs Leaf	RHSIM_Rhsim10G0012800	-4.080436744	4.2533E-114	8.4125E-112	RHSIM_Rhsim10G0012800
Root vs Leaf	RHSIM_Rhsim11G0135900	-5.809710096	4.2562E-113	8.3468E-111	RHSIM_Rhsim11G0135900
Root vs Leaf	RHSIM_Rhsim10G0100700	-5.449573546	8.8849E-113	1.7278E-110	RHSIM_Rhsim10G0100700
Root vs Leaf	RHSIM_Rhsim08G0088200	-7.064423983	9.0516E-113	1.7455E-110	RHSIM_Rhsim08G0088200
Root vs Leaf	RHSIM_Rhsim12G0000600	-7.604415494	9.4315E-113	1.8038E-110	RHSIM_Rhsim12G0000600
Root vs Leaf	RHSIM_RhsimUnG0008100	6.419379108	1.1821E-112	2.2422E-110	RHSIM_RhsimUnG0008100
Root vs Leaf	RHSIM_Rhsim07G0170000	-3.200181437	2.9452E-112	5.541E-110	RHSIM_Rhsim07G0170000

Comparison	Gene ID	log_2FC	P-value	FDR (padj)	Gene Name
Root vs Leaf	RHSIM_Rhsim13G0021200	-3.173357457	2.6984E-111	5.0357E-109	RHSIM_Rhsim13G0021200
Root vs Leaf	RHSIM_Rhsim01G0292400	-2.568097999	3.0899E-111	5.7203E-109	RHSIM_Rhsim01G0292400
Root vs Leaf	RHSIM_Rhsim06G0006900	3.099627386	3.7676E-111	6.9196E-109	RHSIM_Rhsim06G0006900
Root vs Leaf	RHSIM_Rhsim07G0054800	-4.726696265	7.4236E-111	1.3527E-108	RHSIM_Rhsim07G0054800
Root vs Leaf	RHSIM_Rhsim12G0144000	-3.404961064	1.0143E-110	1.8338E-108	RHSIM_Rhsim12G0144000
Root vs Leaf	RHSIM_Rhsim01G0202100	-4.136149905	1.17E-110	2.0988E-108	RHSIM_Rhsim01G0202100
Root vs Leaf	RHSIM_Rhsim06G0183300	-4.637617382	1.9714E-110	3.5092E-108	RHSIM_Rhsim06G0183300
Root vs Leaf	RHSIM_Rhsim08G0178200	-4.000655104	4.1475E-110	7.3264E-108	RHSIM_Rhsim08G0178200
Root vs Leaf	RHSIM_Rhsim12G0013500	-5.507294037	7.6803E-110	1.3464E-107	RHSIM_Rhsim12G0013500
Root vs Leaf	RHSIM_Rhsim02G0140300	-3.302678335	4.2488E-109	7.3926E-107	RHSIM_Rhsim02G0140300
Root vs Leaf	novel.4057	-3.645177289	7.1928E-109	1.2421E-106	--
Root vs Leaf	RHSIM_Rhsim06G0011800	-8.15520168	2.2821E-108	3.9119E-106	RHSIM_Rhsim06G0011800
Root vs Leaf	RHSIM_Rhsim12G0191200	-9.89690983	6.1379E-108	1.0444E-105	RHSIM_Rhsim12G0191200
Root vs Leaf	RHSIM_Rhsim09G0012200	-4.531386578	6.2494E-108	1.0556E-105	RHSIM_Rhsim09G0012200
Root vs Leaf	RHSIM_Rhsim08G0211400	-4.401292136	3.0237E-107	5.0703E-105	RHSIM_Rhsim08G0211400
Root vs Leaf	RHSIM_Rhsim04G0033100	-5.358108578	6.4405E-107	1.0722E-104	RHSIM_Rhsim04G0033100
Root vs Leaf	RHSIM_Rhsim13G0040100	-9.606811329	7.5212E-107	1.2432E-104	RHSIM_Rhsim13G0040100
Root vs Leaf	RHSIM_Rhsim07G0062900	-3.229411478	1.8264E-106	2.9975E-104	RHSIM_Rhsim07G0062900
Root vs Leaf	RHSIM_Rhsim11G0111300	-3.361729857	2.5356E-106	4.1321E-104	RHSIM_Rhsim11G0111300
Root vs Leaf	RHSIM_Rhsim03G0198900	-3.947916053	2.7903E-106	4.5155E-104	RHSIM_Rhsim03G0198900
Root vs Leaf	RHSIM_Rhsim01G0188400	-6.675661441	3.1353E-106	5.0384E-104	RHSIM_Rhsim01G0188400
Root vs Leaf	RHSIM_Rhsim02G0043000	-8.429272577	5.7114E-105	9.115E-103	RHSIM_Rhsim02G0043000
Root vs Leaf	novel.316	-3.139840606	5.8752E-105	9.3122E-103	--
Root vs Leaf	RHSIM_Rhsim07G0096500	-3.7385827	8.4596E-105	1.3317E-102	RHSIM_Rhsim07G0096500
Root vs Leaf	RHSIM_Rhsim07G0256400	-5.609601449	9.5672E-105	1.4959E-102	RHSIM_Rhsim07G0256400

Comparison	Gene ID	log_2FC	P-value	FDR (padj)	Gene Name
Root vs Leaf	RHSIM_Rhsim11G0196300	-3.389562673	1.0191E-104	1.5827E-102	RHSIM_Rhsim11G0196300
Root vs Leaf	RHSIM_Rhsim09G0004800	-5.617629838	1.0637E-103	1.641E-101	RHSIM_Rhsim09G0004800
Root vs Leaf	RHSIM_Rhsim03G0009200	-6.795045493	1.4255E-103	2.1846E-101	RHSIM_Rhsim03G0009200
Root vs Leaf	RHSIM_Rhsim05G0214000	-9.266195987	1.3749E-102	2.09313E-100	RHSIM_Rhsim05G0214000
Root vs Leaf	RHSIM_Rhsim04G0034600	-7.099781161	2.4623E-102	3.7242E-100	RHSIM_Rhsim04G0034600
Root vs Leaf	RHSIM_Rhsim08G0028400	5.558233675	3.0417E-102	4.5706E-100	RHSIM_Rhsim08G0028400
Root vs Leaf	RHSIM_Rhsim07G0004400	-2.886130579	3.6097E-102	5.38922E-100	RHSIM_Rhsim07G0004400
Root vs Leaf	RHSIM_Rhsim10G0046600	-5.095940367	5.0785E-102	7.53346E-100	RHSIM_Rhsim10G0046600
Root vs Leaf	RHSIM_Rhsim08G0155400	-2.632812946	5.1774E-102	7.63121E-100	RHSIM_Rhsim08G0155400
Root vs Leaf	RHSIM_Rhsim05G0126700	-3.56131271	5.615E-102	8.22387E-100	RHSIM_Rhsim05G0126700
Root vs Leaf	RHSIM_Rhsim03G0254900	-3.032937631	5.9616E-102	8.6766E-100	RHSIM_Rhsim03G0254900
Root vs Leaf	RHSIM_Rhsim10G0188100	-6.424087437	6.6061E-102	9.55449E-100	RHSIM_Rhsim10G0188100
Root vs Leaf	RHSIM_Rhsim12G0162200	-5.647435236	8.1921E-102	1.17747E-99	RHSIM_Rhsim12G0162200
Root vs Leaf	RHSIM_Rhsim07G0084600	-10.03879158	3.0776E-101	4.39625E-99	RHSIM_Rhsim07G0084600
Root vs Leaf	RHSIM_Rhsim11G0117800	7.498459715	3.3328E-101	4.7315E-99	RHSIM_Rhsim11G0117800
Root vs Leaf	RHSIM_Rhsim09G0107700	-3.407819809	4.7479E-101	6.69945E-99	RHSIM_Rhsim09G0107700
Root vs Leaf	RHSIM_Rhsim03G0264800	2.644766619	1.11513E-100	1.56396E-98	RHSIM_Rhsim03G0264800
Root vs Leaf	RHSIM_Rhsim10G0128500	-6.316397326	3.59219E-100	5.00765E-98	RHSIM_Rhsim10G0128500
Root vs Leaf	RHSIM_Rhsim08G0054100	-2.8252498	8.69299E-100	1.20458E-97	RHSIM_Rhsim08G0054100
Root vs Leaf	RHSIM_Rhsim09G0111700	-3.464336477	2.75152E-99	3.79005E-97	RHSIM_Rhsim09G0111700
Root vs Leaf	RHSIM_Rhsim05G0195600	-9.167216202	3.94858E-99	5.40675E-97	RHSIM_Rhsim05G0195600
Root vs Leaf	RHSIM_Rhsim11G0173000	-4.321007435	1.07532E-98	1.46376E-96	RHSIM_Rhsim11G0173000
Root vs Leaf	RHSIM_Rhsim02G0054000	-3.014666629	1.32821E-98	1.79743E-96	RHSIM_Rhsim02G0054000
Root vs Leaf	RHSIM_Rhsim09G0063200	-3.011794631	1.77055E-98	2.38211E-96	RHSIM_Rhsim09G0063200
Root vs Leaf	RHSIM_Rhsim04G0216800	-10.16376451	2.62371E-98	3.50956E-96	RHSIM_Rhsim04G0216800

Comparison	Gene ID	log_2FC	P-value	FDR (padj)	Gene Name
Root vs Leaf	RHSIM_Rhsim06G0137600	-3.947666477	3.2907E-98	4.37644E-96	RHSIM_Rhsim06G0137600
Root vs Leaf	RHSIM_Rhsim08G0197600	6.32423849	7.19698E-98	9.51688E-96	RHSIM_Rhsim08G0197600
Root vs Leaf	RHSIM_RhsimUnG0006000	-5.476127687	1.98514E-97	2.61012E-95	RHSIM_RhsimUnG0006000
Root vs Leaf	novel.4755	9.094649206	6.11298E-97	7.99212E-95	--
Root vs Leaf	RHSIM_Rhsim01G0067200	3.609591201	2.44435E-96	3.17779E-94	RHSIM_Rhsim01G0067200
Root vs Leaf	RHSIM_Rhsim03G0259400	-4.029774096	5.42684E-96	7.01578E-94	RHSIM_Rhsim03G0259400
Root vs Leaf	RHSIM_Rhsim03G0012100	-5.729580689	6.14547E-96	7.90068E-94	RHSIM_Rhsim03G0012100
Root vs Leaf	RHSIM_Rhsim08G0088300	-6.800260761	6.79656E-96	8.68946E-94	RHSIM_Rhsim08G0088300
Root vs Leaf	RHSIM_Rhsim10G0128400	-6.484873259	1.22447E-95	1.55689E-93	RHSIM_Rhsim10G0128400
Root vs Leaf	RHSIM_Rhsim07G0047700	-3.951568086	2.45355E-95	3.10261E-93	RHSIM_Rhsim07G0047700
Root vs Leaf	RHSIM_Rhsim12G0197600	-3.883190598	4.75663E-95	5.98224E-93	RHSIM_Rhsim12G0197600
Root vs Leaf	RHSIM_Rhsim07G0257000	-12.31523686	5.25452E-95	6.57269E-93	RHSIM_Rhsim07G0257000
Root vs Leaf	RHSIM_Rhsim13G0053000	-4.654009415	1.17529E-94	1.46222E-92	RHSIM_Rhsim13G0053000
Root vs Leaf	RHSIM_Rhsim12G0110200	-5.458046876	1.98617E-94	2.45786E-92	RHSIM_Rhsim12G0110200
Root vs Leaf	novel.1961	-4.245614101	2.91234E-94	3.58481E-92	--
Root vs Leaf	RHSIM_Rhsim13G0116300	-6.667181843	4.57407E-94	5.60046E-92	RHSIM_Rhsim13G0116300
Root vs Leaf	RHSIM_Rhsim07G0198600	-4.720731391	5.86808E-94	7.14701E-92	RHSIM_Rhsim07G0198600
Root vs Leaf	RHSIM_Rhsim09G0064600	-4.025687075	7.43952E-94	9.01351E-92	RHSIM_Rhsim09G0064600
Root vs Leaf	RHSIM_Rhsim11G0167000	-2.763713162	1.70843E-93	2.0591E-91	RHSIM_Rhsim11G0167000
Root vs Leaf	RHSIM_Rhsim09G0171900	-8.756313594	1.93968E-93	2.3257E-91	RHSIM_Rhsim09G0171900
Root vs Leaf	RHSIM_Rhsim08G0012300	-7.569970889	3.30555E-93	3.94298E-91	RHSIM_Rhsim08G0012300
Root vs Leaf	RHSIM_Rhsim06G0129400	-5.089911085	3.40492E-93	4.04068E-91	RHSIM_Rhsim06G0129400
Root vs Leaf	RHSIM_Rhsim01G0097100	-3.730637013	4.85506E-93	5.73219E-91	RHSIM_Rhsim01G0097100
Root vs Leaf	RHSIM_Rhsim03G0074900	5.873362676	6.36296E-93	7.47438E-91	RHSIM_Rhsim03G0074900
Root vs Leaf	RHSIM_RhsimPtG0004100	-3.931470264	2.38229E-92	2.78427E-90	RHSIM_RhsimPtG0004100

Comparison	Gene ID	log$_2$FC	P-value	FDR (padj)	Gene Name
Root vs Leaf	RHSIM_Rhsim05G0042000	-3.474064175	2.88294E-92	3.35247E-90	RHSIM_Rhsim05G0042000
Root vs Leaf	RHSIM_Rhsim10G0156600	-4.641966228	1.19635E-91	1.38424E-89	RHSIM_Rhsim10G0156600
Root vs Leaf	RHSIM_Rhsim08G0115600	-3.56261638	1.40417E-91	1.61661E-89	RHSIM_Rhsim08G0115600
Root vs Leaf	RHSIM_Rhsim03G0172900	-3.845939833	1.62542E-91	1.86208E-89	RHSIM_Rhsim03G0172900
Root vs Leaf	RHSIM_Rhsim08G0006300	-3.880944119	4.26856E-91	4.86594E-89	RHSIM_Rhsim08G0006300
Root vs Leaf	RHSIM_Rhsim12G0025700	-4.196092006	8.65581E-91	9.81883E-89	RHSIM_Rhsim12G0025700
Root vs Leaf	RHSIM_Rhsim06G0067100	-3.286010101	2.52391E-90	2.84907E-88	RHSIM_Rhsim06G0067100
Root vs Leaf	RHSIM_Rhsim09G0042300	6.048945997	4.03013E-89	4.52725E-87	RHSIM_Rhsim09G0042300
Root vs Leaf	RHSIM_Rhsim07G0007900	-5.57460228	4.38236E-89	4.89914E-87	RHSIM_Rhsim07G0007900
Root vs Leaf	RHSIM_Rhsim10G0083200	-3.607845232	5.30893E-89	5.90644E-87	RHSIM_Rhsim10G0083200
Root vs Leaf	RHSIM_RhsimUnG0254000	-4.028175522	6.20254E-89	6.86761E-87	RHSIM_RhsimUnG0254000
Root vs Leaf	RHSIM_Rhsim12G0063600	-3.032650932	7.92433E-89	8.73223E-87	RHSIM_Rhsim12G0063600
Root vs Leaf	RHSIM_Rhsim13G0142400	-4.587897747	1.10902E-88	1.21629E-86	RHSIM_Rhsim13G0142400
Root vs Leaf	RHSIM_Rhsim10G0133700	-6.255646324	2.42465E-88	2.64664E-86	RHSIM_Rhsim10G0133700
Root vs Leaf	RHSIM_Rhsim05G0161100	-3.629005651	2.56453E-88	2.78618E-86	RHSIM_Rhsim05G0161100
Root vs Leaf	RHSIM_Rhsim07G0124400	-4.479656071	6.84411E-88	7.40091E-86	RHSIM_Rhsim07G0124400
Root vs Leaf	RHSIM_Rhsim04G0206400	-6.226376081	1.29874E-87	1.39786E-85	RHSIM_Rhsim04G0206400
Root vs Leaf	RHSIM_Rhsim12G0182800	-3.468896518	1.8918E-87	2.02676E-85	RHSIM_Rhsim12G0182800
Root vs Leaf	RHSIM_Rhsim12G0050500	6.169094239	2.64911E-87	2.82503E-85	RHSIM_Rhsim12G0050500
Root vs Leaf	novel.4801	6.274371327	4.85737E-87	5.15616E-85	--
Root vs Leaf	RHSIM_Rhsim01G0235400	-3.829478433	4.97546E-87	5.2574E-85	RHSIM_Rhsim01G0235400
Root vs Leaf	RHSIM_Rhsim02G0147000	-3.027173364	5.31656E-87	5.59229E-85	RHSIM_Rhsim02G0147000
Root vs Leaf	RHSIM_Rhsim05G0022400	-3.482956476	1.494E-86	1.55798E-84	RHSIM_Rhsim05G0022400
Root vs Leaf	RHSIM_Rhsim02G0021200	-11.82774858	1.49463E-86	1.55798E-84	RHSIM_Rhsim02G0021200
Root vs Leaf	RHSIM_Rhsim01G0101900	-9.614821776	4.10237E-86	4.25708E-84	RHSIM_Rhsim01G0101900

Comparison	Gene ID	log_2FC	P-value	FDR (padj)	Gene Name
Root vs Leaf	RHSIM_Rhsim07G0256500	-4.890291302	5.10068E-86	5.26941E-84	RHSIM_Rhsim07G0256500
Root vs Leaf	RHSIM_Rhsim05G0053200	-3.042279492	5.89718E-86	6.06519E-84	RHSIM_Rhsim05G0053200
Root vs Leaf	RHSIM_RhsimUnG0074300	-4.931457166	7.43411E-86	7.61207E-84	RHSIM_RhsimUnG0074300
Root vs Leaf	RHSIM_Rhsim10G0102900	-3.198191801	6.77226E-85	6.90383E-83	RHSIM_Rhsim10G0102900
Root vs Leaf	RHSIM_Rhsim13G0157200	6.296715153	7.12241E-85	7.22894E-83	RHSIM_Rhsim13G0157200
Root vs Leaf	RHSIM_Rhsim06G0187500	-3.089112749	7.64309E-85	7.72353E-83	RHSIM_Rhsim06G0187500
Root vs Leaf	RHSIM_Rhsim12G0108700	-6.372988586	1.03224E-84	1.03856E-82	RHSIM_Rhsim12G0108700
Root vs Leaf	RHSIM_Rhsim01G0203000	-8.483320264	1.30429E-84	1.30661E-82	RHSIM_Rhsim01G0203000
Root vs Leaf	RHSIM_Rhsim12G0135200	-6.136186878	1.50205E-84	1.49823E-82	RHSIM_Rhsim12G0135200
Root vs Leaf	RHSIM_Rhsim06G0091500	-2.631242604	1.62353E-84	1.61245E-82	RHSIM_Rhsim06G0091500
Root vs Leaf	RHSIM_Rhsim04G0170900	-2.262682056	3.50785E-84	3.46903E-82	RHSIM_Rhsim04G0170900
Root vs Leaf	RHSIM_Rhsim01G0207700	-3.665684062	3.85961E-84	3.80065E-82	RHSIM_Rhsim01G0207700
Root vs Leaf	novel.3458	-6.000481617	5.29988E-84	5.19681E-82	--
Root vs Leaf	RHSIM_RhsimUnG0003000	-2.990412708	7.18705E-84	7.01753E-82	RHSIM_RhsimUnG0003000
Root vs Leaf	RHSIM_Rhsim02G0169800	-4.051968811	9.15992E-84	8.90629E-82	RHSIM_Rhsim02G0169800
Root vs Leaf	RHSIM_Rhsim04G0119100	-4.379164334	1.07459E-83	1.04047E-81	RHSIM_Rhsim04G0119100
Root vs Leaf	RHSIM_Rhsim10G0050900	-7.906794792	4.46188E-83	4.30218E-81	RHSIM_Rhsim10G0050900
Root vs Leaf	RHSIM_Rhsim03G0085600	-5.64564665	1.24073E-82	1.19136E-80	RHSIM_Rhsim03G0085600
Root vs Leaf	RHSIM_Rhsim03G0226500	-4.97310298	1.97069E-82	1.88445E-80	RHSIM_Rhsim03G0226500
Root vs Leaf	RHSIM_Rhsim13G0041600	-6.440933151	3.56386E-82	3.39388E-80	RHSIM_Rhsim13G0041600
Root vs Leaf	RHSIM_Rhsim10G0099300	-6.179665676	3.90424E-82	3.70279E-80	RHSIM_Rhsim10G0099300
Root vs Leaf	RHSIM_Rhsim11G0193400	-3.003817912	4.02159E-82	3.79852E-80	RHSIM_Rhsim11G0193400
Root vs Leaf	RHSIM_Rhsim08G0153200	-7.808668842	1.04843E-81	9.86253E-80	RHSIM_Rhsim08G0153200
Root vs Leaf	RHSIM_Rhsim05G0032000	5.128800442	1.11358E-81	1.04329E-79	RHSIM_Rhsim05G0032000
Root vs Leaf	RHSIM_Rhsim12G0096900	5.426306016	1.64093E-81	1.53116E-79	RHSIM_Rhsim12G0096900

Comparison	Gene ID	log_2FC	P-value	FDR (padj)	Gene Name
Root vs Leaf	RHSIM_Rhsim04G0167000	-3.169710153	2.85629E-81	2.65451E-79	RHSIM_Rhsim04G0167000
Root vs Leaf	RHSIM_Rhsim13G0189300	-6.968615286	3.20066E-81	2.96266E-79	RHSIM_Rhsim13G0189300
Root vs Leaf	RHSIM_Rhsim11G0088600	-2.989458101	4.37535E-81	4.03387E-79	RHSIM_Rhsim11G0088600
Root vs Leaf	RHSIM_Rhsim13G0229700	-2.549502373	6.20273E-81	5.69592E-79	RHSIM_Rhsim13G0229700
Root vs Leaf	RHSIM_Rhsim01G0002000	-3.926094112	1.21285E-80	1.10935E-78	RHSIM_Rhsim01G0002000
Root vs Leaf	RHSIM_Rhsim08G0205700	-9.00000664	2.26823E-80	2.0665E-78	RHSIM_Rhsim08G0205700
Root vs Leaf	RHSIM_RhsimUnG0024100	-9.98979179	2.84368E-80	2.58061E-78	RHSIM_RhsimUnG0024100
Root vs Leaf	RHSIM_Rhsim08G0038900	-5.129009864	2.95645E-80	2.67247E-78	RHSIM_Rhsim08G0038900
Root vs Leaf	novel.5960	-6.391523756	3.1099E-80	2.80024E-78	--
Root vs Leaf	RHSIM_Rhsim07G0150700	-2.79865983	5.18241E-80	4.6483E-78	RHSIM_Rhsim07G0150700
Root vs Leaf	RHSIM_Rhsim07G0132800	-2.566541981	8.82191E-80	7.88215E-78	RHSIM_Rhsim07G0132800
Root vs Leaf	RHSIM_Rhsim02G0239700	-8.455156442	1.49252E-79	1.3284E-77	RHSIM_Rhsim02G0239700
Root vs Leaf	RHSIM_Rhsim13G0056200	-4.703838139	1.884E-79	1.6704E-77	RHSIM_Rhsim13G0056200
Root vs Leaf	RHSIM_Rhsim11G0127300	-5.408288434	2.96024E-79	2.61462E-77	RHSIM_Rhsim11G0127300
Root vs Leaf	RHSIM_RhsimUnG0122900	-5.463279426	8.61211E-79	7.57767E-77	RHSIM_RhsimUnG0122900
Root vs Leaf	RHSIM_Rhsim12G0131400	3.586766752	1.83525E-78	1.60869E-76	RHSIM_Rhsim12G0131400
Root vs Leaf	RHSIM_Rhsim06G0077300	-2.816732871	4.09308E-78	3.57426E-76	RHSIM_Rhsim06G0077300
Root vs Leaf	RHSIM_Rhsim07G0101400	-3.047259057	5.02793E-78	4.37411E-76	RHSIM_Rhsim07G0101400
Root vs Leaf	RHSIM_RhsimPtG0003600	-3.529327891	2.14115E-77	1.85574E-75	RHSIM_RhsimPtG0003600
Root vs Leaf	RHSIM_Rhsim02G0088100	-10.51952653	2.17538E-77	1.87837E-75	RHSIM_Rhsim02G0088100
Root vs Leaf	RHSIM_Rhsim13G0216500	-3.760610327	2.74176E-77	2.35862E-75	RHSIM_Rhsim13G0216500
Root vs Leaf	RHSIM_Rhsim04G0195000	-12.43551604	3.15307E-77	2.70241E-75	RHSIM_Rhsim04G0195000
Root vs Leaf	RHSIM_Rhsim02G0235500	-2.559960421	6.2171E-77	5.30885E-75	RHSIM_Rhsim02G0235500
Root vs Leaf	RHSIM_Rhsim03G0150700	-3.277715339	1.03465E-76	8.80253E-75	RHSIM_Rhsim03G0150700
Root vs Leaf	RHSIM_Rhsim13G0152700	-4.54032719	1.06546E-76	9.03145E-75	RHSIM_Rhsim13G0152700

Comparison	Gene ID	log_2FC	P-value	FDR (padj)	Gene Name
Root vs Leaf	RHSIM_Rhsim08G0079500	-5.386124851	1.14631E-76	9.68132E-75	RHSIM_Rhsim08G0079500
Root vs Leaf	RHSIM_Rhsim06G0237200	-5.846252346	1.87891E-76	1.58108E-74	RHSIM_Rhsim06G0237200
Root vs Leaf	RHSIM_Rhsim12G0165600	-3.528472488	2.79266E-76	2.34148E-74	RHSIM_Rhsim12G0165600
Root vs Leaf	RHSIM_Rhsim09G0114100	-4.448750035	2.94E-76	2.45612E-74	RHSIM_Rhsim09G0114100
Root vs Leaf	RHSIM_Rhsim05G0030700	-4.815361251	8.35993E-76	6.95889E-74	RHSIM_Rhsim05G0030700
Root vs Leaf	RHSIM_Rhsim08G0246100	4.361462294	1.2846E-75	1.06548E-73	RHSIM_Rhsim08G0246100
Root vs Leaf	RHSIM_Rhsim05G0060400	-8.12963143	2.96466E-75	2.45018E-73	RHSIM_Rhsim05G0060400
Root vs Leaf	RHSIM_Rhsim07G0032300	-6.571890584	3.24434E-75	2.67179E-73	RHSIM_Rhsim07G0032300
Root vs Leaf	RHSIM_Rhsim02G0041500	-4.677212148	4.99166E-75	4.09617E-73	RHSIM_Rhsim02G0041500
Root vs Leaf	RHSIM_Rhsim09G0143800	-9.998104753	6.20739E-75	5.0758E-73	RHSIM_Rhsim09G0143800
Root vs Leaf	RHSIM_Rhsim12G0214300	-2.428481043	7.75867E-75	6.32195E-73	RHSIM_Rhsim12G0214300
Root vs Leaf	RHSIM_Rhsim10G0174200	8.029740807	8.08293E-75	6.56306E-73	RHSIM_Rhsim10G0174200
Root vs Leaf	RHSIM_Rhsim10G0134900	-4.864243951	9.46178E-75	7.65577E-73	RHSIM_Rhsim10G0134900
Root vs Leaf	RHSIM_Rhsim07G0032500	-5.053489768	1.09743E-74	8.84867E-73	RHSIM_Rhsim07G0032500
Root vs Leaf	RHSIM_Rhsim10G0021300	-11.95734448	1.36709E-74	1.09847E-72	RHSIM_Rhsim10G0021300
Root vs Leaf	RHSIM_Rhsim05G0148600	-6.373770448	1.4753E-74	1.18131E-72	RHSIM_Rhsim05G0148600
Root vs Leaf	RHSIM_Rhsim12G0037700	-6.090483019	2.61684E-74	2.08815E-72	RHSIM_Rhsim12G0037700
Root vs Leaf	RHSIM_Rhsim12G0112100	-4.162695618	4.39587E-74	3.4957E-72	RHSIM_Rhsim12G0112100
Root vs Leaf	RHSIM_Rhsim05G0164500	-3.548552325	6.62331E-74	5.24897E-72	RHSIM_Rhsim05G0164500
Root vs Leaf	RHSIM_Rhsim12G0171100	-5.944363004	1.01418E-73	8.00998E-72	RHSIM_Rhsim12G0171100
Root vs Leaf	RHSIM_Rhsim04G0167300	-3.162235581	1.19815E-73	9.43072E-72	RHSIM_Rhsim04G0167300
Root vs Leaf	RHSIM_Rhsim06G0027400	-3.004864661	1.8912E-73	1.48353E-71	RHSIM_Rhsim06G0027400
Root vs Leaf	RHSIM_Rhsim08G0081400	-4.67405708	4.6965E-73	3.67168E-71	RHSIM_Rhsim08G0081400
Root vs Leaf	RHSIM_Rhsim07G0014000	-3.525866156	8.48193E-73	6.60876E-71	RHSIM_Rhsim07G0014000
Root vs Leaf	RHSIM_Rhsim04G0169500	-4.180405281	8.57926E-73	6.66217E-71	RHSIM_Rhsim04G0169500

Comparison	Gene ID	log_2FC	P-value	FDR (padj)	Gene Name
Root vs Leaf	RHSIM_Rhsim08G0196000	-2.824657059	9.76454E-73	7.55723E-71	RHSIM_Rhsim08G0196000
Root vs Leaf	RHSIM_Rhsim11G0113500	-3.647048111	1.4036E-72	1.08269E-70	RHSIM_Rhsim11G0113500
Root vs Leaf	RHSIM_Rhsim08G0025300	-3.654338269	1.95303E-72	1.5015E-70	RHSIM_Rhsim08G0025300
Root vs Leaf	RHSIM_Rhsim07G0020300	-8.0194569	2.36638E-72	1.81326E-70	RHSIM_Rhsim07G0020300
Root vs Leaf	RHSIM_Rhsim04G0098600	7.359444497	2.85831E-72	2.18298E-70	RHSIM_Rhsim04G0098600
Root vs Leaf	RHSIM_Rhsim03G0153700	-2.117502433	5.59878E-72	4.26189E-70	RHSIM_Rhsim03G0153700
Root vs Leaf	RHSIM_Rhsim03G0248000	-4.655215542	6.11197E-72	4.63729E-70	RHSIM_Rhsim03G0248000
Root vs Leaf	RHSIM_RhsimUnG0205000	-4.781731947	1.24021E-71	9.37897E-70	RHSIM_RhsimUnG0205000
Root vs Leaf	RHSIM_Rhsim10G0020100	-3.145225238	1.41083E-71	1.06345E-69	RHSIM_Rhsim10G0020100
Root vs Leaf	RHSIM_Rhsim10G0148300	-2.12112638	1.78151E-71	1.33851E-69	RHSIM_Rhsim10G0148300
Root vs Leaf	RHSIM_RhsimUnG0077000	-2.93378453	1.99442E-71	1.49362E-69	RHSIM_RhsimUnG0077000
Root vs Leaf	RHSIM_Rhsim12G0132700	-4.215594788	2.58319E-71	1.92831E-69	RHSIM_Rhsim12G0132700
Root vs Leaf	RHSIM_Rhsim09G0175300	-7.640204833	3.03077E-71	2.25515E-69	RHSIM_Rhsim09G0175300
Root vs Leaf	RHSIM_Rhsim02G0106400	-1.559795708	5.25795E-71	3.89981E-69	RHSIM_Rhsim02G0106400
Root vs Leaf	RHSIM_Rhsim13G0056900	-3.048768936	5.31004E-71	3.92586E-69	RHSIM_Rhsim13G0056900
Root vs Leaf	RHSIM_Rhsim01G0155600	-3.310925661	5.38161E-71	3.96611E-69	RHSIM_Rhsim01G0155600
Root vs Leaf	RHSIM_Rhsim13G0127200	-5.727025416	5.4578E-71	4.00949E-69	RHSIM_Rhsim13G0127200
Root vs Leaf	RHSIM_Rhsim04G0043200	-2.816927835	8.21431E-71	6.01542E-69	RHSIM_Rhsim04G0043200
Root vs Leaf	RHSIM_Rhsim01G0015700	4.768498405	9.11619E-71	6.65482E-69	RHSIM_Rhsim01G0015700
Root vs Leaf	RHSIM_Rhsim10G0129800	-5.762408058	1.65785E-70	1.20642E-68	RHSIM_Rhsim10G0129800
Root vs Leaf	RHSIM_Rhsim05G0015000	-2.922821727	1.73889E-70	1.26143E-68	RHSIM_Rhsim05G0015000
Root vs Leaf	RHSIM_Rhsim03G0238900	-2.353256788	2.04627E-70	1.47977E-68	RHSIM_Rhsim03G0238900
Root vs Leaf	RHSIM_Rhsim12G0099000	-2.980297826	2.08608E-70	1.50386E-68	RHSIM_Rhsim12G0099000
Root vs Leaf	RHSIM_Rhsim13G0100500	-2.154344147	4.04879E-70	2.90972E-68	RHSIM_Rhsim13G0100500
Root vs Leaf	RHSIM_RhsimPtG0005500	-6.299073209	5.35053E-70	3.83333E-68	RHSIM_RhsimPtG0005500

Comparison	Gene ID	log_2FC	P-value	FDR (padj)	Gene Name
Root vs Leaf	RHSIM_Rhsim03G0088200	-3.775970007	6.14006E-70	4.3854E-68	RHSIM_Rhsim03G0088200
Root vs Leaf	RHSIM_Rhsim06G0171700	4.992212815	9.14352E-70	6.51047E-68	RHSIM_Rhsim06G0171700
Root vs Leaf	RHSIM_Rhsim07G0018700	-4.465883449	2.37062E-69	1.68277E-67	RHSIM_Rhsim07G0018700
Root vs Leaf	RHSIM_Rhsim04G0206300	-2.798908625	2.62327E-69	1.85643E-67	RHSIM_Rhsim04G0206300
Root vs Leaf	RHSIM_Rhsim09G0098100	2.179080725	3.4056E-69	2.40271E-67	RHSIM_Rhsim09G0098100
Root vs Leaf	RHSIM_Rhsim01G0182200	-6.686147817	4.94587E-69	3.4788E-67	RHSIM_Rhsim01G0182200
Root vs Leaf	RHSIM_Rhsim03G0074800	-5.133402835	5.04803E-69	3.53989E-67	RHSIM_Rhsim03G0074800
Root vs Leaf	RHSIM_Rhsim06G0028500	-1.911061164	5.55225E-69	3.88171E-67	RHSIM_Rhsim06G0028500
Root vs Leaf	RHSIM_Rhsim13G0233300	-4.99675307	5.64241E-69	3.93286E-67	RHSIM_Rhsim13G0233300
Root vs Leaf	RHSIM_Rhsim09G0079200	-7.606348809	5.68403E-69	3.94997E-67	RHSIM_Rhsim09G0079200
Root vs Leaf	RHSIM_Rhsim03G0095400	-9.250471305	7.2601E-69	5.03012E-67	RHSIM_Rhsim03G0095400
Root vs Leaf	RHSIM_Rhsim04G0207100	5.278387276	7.70021E-69	5.31912E-67	RHSIM_Rhsim04G0207100
Root vs Leaf	RHSIM_Rhsim01G0235600	-5.808173004	8.53733E-69	5.87488E-67	RHSIM_Rhsim01G0235600
Root vs Leaf	RHSIM_Rhsim06G0177200	-2.615784824	8.55552E-69	5.87488E-67	RHSIM_Rhsim06G0177200
Root vs Leaf	RHSIM_Rhsim06G0148500	-6.047913007	8.75863E-69	5.99655E-67	RHSIM_Rhsim06G0148500
Root vs Leaf	RHSIM_Rhsim06G0198600	-4.571613554	9.53492E-69	6.50878E-67	RHSIM_Rhsim06G0198600
Root vs Leaf	RHSIM_Rhsim01G0236500	-3.372933429	1.33098E-68	9.05888E-67	RHSIM_Rhsim01G0236500
Root vs Leaf	RHSIM_Rhsim07G0139800	-12.88084985	1.86749E-68	1.26732E-66	RHSIM_Rhsim07G0139800
Root vs Leaf	RHSIM_Rhsim12G0180300	-5.672728677	1.99352E-68	1.34889E-66	RHSIM_Rhsim12G0180300
Root vs Leaf	RHSIM_Rhsim08G0152500	-4.631925983	2.03862E-68	1.37539E-66	RHSIM_Rhsim08G0152500
Root vs Leaf	RHSIM_Rhsim03G0162100	-2.856458415	2.38246E-68	1.60269E-66	RHSIM_Rhsim03G0162100
Root vs Leaf	RHSIM_Rhsim06G0178100	-3.333905888	4.44146E-68	2.97913E-66	RHSIM_Rhsim06G0178100
Root vs Leaf	RHSIM_Rhsim07G0137000	-4.321622741	6.5357E-68	4.37117E-66	RHSIM_Rhsim07G0137000
Root vs Leaf	RHSIM_Rhsim08G0089400	-11.74588556	8.62288E-68	5.75049E-66	RHSIM_Rhsim08G0089400
Root vs Leaf	RHSIM_Rhsim07G0251400	-6.009938198	1.06542E-67	7.08472E-66	RHSIM_Rhsim07G0251400

Comparison	Gene ID	log_2FC	P-value	FDR (padj)	Gene Name
Root vs Leaf	RHSIM_Rhsim04G0225200	-5.386074002	1.20626E-67	7.99829E-66	RHSIM_Rhsim04G0225200
Root vs Leaf	RHSIM_Rhsim07G0170200	-3.309373884	1.30491E-67	8.6277E-66	RHSIM_Rhsim07G0170200
Root vs Leaf	RHSIM_Rhsim13G0208500	-4.119919757	1.35286E-67	8.91926E-66	RHSIM_Rhsim13G0208500
Root vs Leaf	RHSIM_Rhsim06G0197700	-3.642323833	2.26208E-67	1.48713E-65	RHSIM_Rhsim06G0197700
Root vs Leaf	RHSIM_Rhsim12G0183500	-3.548162648	2.55977E-67	1.67806E-65	RHSIM_Rhsim12G0183500
Root vs Leaf	RHSIM_Rhsim03G0219600	-4.261478043	3.36525E-67	2.19986E-65	RHSIM_Rhsim03G0219600
Root vs Leaf	RHSIM_Rhsim09G0131800	4.838123638	6.76888E-67	4.41235E-65	RHSIM_Rhsim09G0131800
Root vs Leaf	RHSIM_Rhsim03G0176200	-3.14278157	8.58237E-67	5.57878E-65	RHSIM_Rhsim03G0176200
Root vs Leaf	RHSIM_Rhsim06G0087100	-2.488999524	8.60751E-67	5.57945E-65	RHSIM_Rhsim06G0087100
Root vs Leaf	RHSIM_Rhsim08G0034000	-4.706327812	9.21666E-67	5.95762E-65	RHSIM_Rhsim08G0034000
Root vs Leaf	novel.4900	-10.11762883	1.37981E-66	8.89423E-65	--
Root vs Leaf	RHSIM_Rhsim08G0076500	-7.28049628	1.43132E-66	9.20058E-65	RHSIM_Rhsim08G0076500
Root vs Leaf	RHSIM_Rhsim09G0093900	-9.622225829	1.44768E-66	9.28E-65	RHSIM_Rhsim09G0093900
Root vs Leaf	RHSIM_RhsimUnG0141100	3.567768274	2.91635E-66	1.86429E-64	RHSIM_RhsimUnG0141100
Root vs Leaf	RHSIM_Rhsim02G0052500	3.224399591	3.22646E-66	2.05684E-64	RHSIM_Rhsim02G0052500
Root vs Leaf	RHSIM_Rhsim10G0010700	-6.914161901	5.33797E-66	3.39357E-64	RHSIM_Rhsim10G0010700
Root vs Leaf	RHSIM_Rhsim10G0186700	-2.901673428	6.54204E-66	4.14766E-64	RHSIM_Rhsim10G0186700
Root vs Leaf	RHSIM_Rhsim03G0219000	-2.680126275	6.62217E-66	4.18699E-64	RHSIM_Rhsim03G0219000
Root vs Leaf	RHSIM_Rhsim03G0254100	-5.128554423	6.84432E-66	4.31565E-64	RHSIM_Rhsim03G0254100
Root vs Leaf	RHSIM_Rhsim08G0109700	-5.609826144	9.5701E-66	6.01798E-64	RHSIM_Rhsim08G0109700
Root vs Leaf	RHSIM_Rhsim03G0159700	-3.031493188	9.82613E-66	6.16223E-64	RHSIM_Rhsim03G0159700
Root vs Leaf	RHSIM_Rhsim03G0141600	-4.188999581	1.19483E-65	7.47285E-64	RHSIM_Rhsim03G0141600
Root vs Leaf	RHSIM_Rhsim08G0152900	3.959717943	1.41597E-65	8.83205E-64	RHSIM_Rhsim08G0152900
Root vs Leaf	RHSIM_RhsimUnG0187900	-4.572458547	1.65009E-65	1.02449E-63	RHSIM_RhsimUnG0187900
Root vs Leaf	RHSIM_Rhsim04G0087000	-6.383897834	1.65133E-65	1.02449E-63	RHSIM_Rhsim04G0087000

Comparison	Gene ID	log_2FC	P-value	FDR (padj)	Gene Name
Root vs Leaf	RHSIM_Rhsim06G0214800	-3.241303255	1.90238E-65	1.17709E-63	RHSIM_Rhsim06G0214800
Root vs Leaf	novel.3658	-4.196357172	2.38175E-65	1.46976E-63	--
Root vs Leaf	RHSIM_Rhsim01G0107200	6.508162769	2.62269E-65	1.61414E-63	RHSIM_Rhsim01G0107200
Root vs Leaf	RHSIM_Rhsim11G0135800	-4.685379454	3.06546E-65	1.88164E-63	RHSIM_Rhsim11G0135800
Root vs Leaf	RHSIM_Rhsim09G0155900	-6.800354477	3.10535E-65	1.90108E-63	RHSIM_Rhsim09G0155900
Root vs Leaf	RHSIM_RhsimUnG0257700	-6.061259278	3.75474E-65	2.292E-63	RHSIM_RhsimUnG0257700
Root vs Leaf	RHSIM_Rhsim09G0129300	-6.366675589	3.7637E-65	2.292E-63	RHSIM_Rhsim09G0129300
Root vs Leaf	RHSIM_Rhsim13G0177900	-6.765700019	3.84502E-65	2.33537E-63	RHSIM_Rhsim13G0177900
Root vs Leaf	RHSIM_Rhsim08G0181200	5.363826749	3.99665E-65	2.42111E-63	RHSIM_Rhsim08G0181200
Root vs Leaf	RHSIM_Rhsim05G0050000	-3.838737485	6.41315E-65	3.87485E-63	RHSIM_Rhsim05G0050000
Root vs Leaf	RHSIM_Rhsim13G0187900	4.006686137	7.32591E-65	4.41482E-63	RHSIM_Rhsim13G0187900
Root vs Leaf	RHSIM_Rhsim01G0184600	-2.8075321	8.49743E-65	5.10751E-63	RHSIM_Rhsim01G0184600
Root vs Leaf	RHSIM_Rhsim07G0198500	3.992139401	1.14717E-64	6.87734E-63	RHSIM_Rhsim07G0198500
Root vs Leaf	RHSIM_Rhsim07G0052400	-6.639425564	1.44638E-64	8.64875E-63	RHSIM_Rhsim07G0052400
Root vs Leaf	RHSIM_Rhsim08G0006500	-4.284865318	2.25084E-64	1.34244E-62	RHSIM_Rhsim08G0006500
Root vs Leaf	RHSIM_Rhsim06G0103300	-10.01349846	2.70726E-64	1.61051E-62	RHSIM_Rhsim06G0103300
Root vs Leaf	RHSIM_Rhsim01G0192800	-9.292276283	2.8418E-64	1.68621E-62	RHSIM_Rhsim01G0192800
Root vs Leaf	RHSIM_Rhsim12G0005700	-5.142876636	2.87904E-64	1.70394E-62	RHSIM_Rhsim12G0005700
Root vs Leaf	RHSIM_Rhsim08G0090000	-8.161004022	2.96732E-64	1.7517E-62	RHSIM_Rhsim08G0090000
Root vs Leaf	RHSIM_Rhsim07G0101900	-6.373819823	3.15367E-64	1.85697E-62	RHSIM_Rhsim07G0101900
Root vs Leaf	RHSIM_Rhsim11G0025800	1.951015339	3.17391E-64	1.86415E-62	RHSIM_Rhsim11G0025800
Root vs Leaf	RHSIM_Rhsim04G0248400	-2.599882162	3.27447E-64	1.91834E-62	RHSIM_Rhsim04G0248400
Root vs Leaf	RHSIM_Rhsim12G0025500	-3.185871051	3.49115E-64	2.04012E-62	RHSIM_Rhsim12G0025500
Root vs Leaf	RHSIM_Rhsim02G0248400	-3.200863388	5.12771E-64	2.98893E-62	RHSIM_Rhsim02G0248400
Root vs Leaf	novel.1311	-6.962844421	5.43856E-64	3.16216E-62	--

Comparison	Gene ID	log_2FC	P-value	FDR (padj)	Gene Name
Root vs Leaf	RHSIM_Rhsim06G0131100	6.141539797	6.09984E-64	3.53776E-62	RHSIM_Rhsim06G0131100
Root vs Leaf	RHSIM_Rhsim02G0136400	-2.788112739	6.72594E-64	3.89113E-62	RHSIM_Rhsim02G0136400
Root vs Leaf	RHSIM_Rhsim03G0018300	-3.704474955	7.41001E-64	4.27618E-62	RHSIM_Rhsim03G0018300
Root vs Leaf	RHSIM_Rhsim08G0200300	2.956297767	7.87782E-64	4.53484E-62	RHSIM_Rhsim08G0200300
Root vs Leaf	RHSIM_Rhsim08G0198600	-5.476933427	1.08056E-63	6.20478E-62	RHSIM_Rhsim08G0198600
Root vs Leaf	RHSIM_Rhsim09G0054100	-5.132124477	1.3933E-63	7.98078E-62	RHSIM_Rhsim09G0054100
Root vs Leaf	RHSIM_Rhsim01G0210400	-3.33915714	1.76845E-63	1.01046E-61	RHSIM_Rhsim01G0210400
Root vs Leaf	RHSIM_Rhsim08G0192000	2.217536941	1.79568E-63	1.02349E-61	RHSIM_Rhsim08G0192000
Root vs Leaf	RHSIM_Rhsim13G0025100	-3.684666857	1.99828E-63	1.13617E-61	RHSIM_Rhsim13G0025100
Root vs Leaf	RHSIM_Rhsim11G0138200	-5.894273	2.33102E-63	1.32211E-61	RHSIM_Rhsim11G0138200
Root vs Leaf	RHSIM_Rhsim08G0067400	-5.515421757	3.01628E-63	1.70659E-61	RHSIM_Rhsim08G0067400
Root vs Leaf	RHSIM_Rhsim09G0143500	5.631331318	3.74018E-63	2.11101E-61	RHSIM_Rhsim09G0143500
Root vs Leaf	RHSIM_Rhsim01G0205500	-4.994286151	3.9708E-63	2.23573E-61	RHSIM_Rhsim01G0205500
Root vs Leaf	novel.2605	-6.75026782	4.57022E-63	2.56698E-61	--
Root vs Leaf	RHSIM_Rhsim13G0022200	-2.040270273	6.77399E-63	3.79557E-61	RHSIM_Rhsim13G0022200
Root vs Leaf	novel.569	7.755725709	9.34189E-63	5.22176E-61	--
Root vs Leaf	RHSIM_Rhsim05G0228300	5.209660115	1.46657E-62	8.1778E-61	RHSIM_Rhsim05G0228300
Root vs Leaf	RHSIM_Rhsim02G0212300	-2.419053785	1.781E-62	9.90724E-61	RHSIM_Rhsim02G0212300
Root vs Leaf	RHSIM_Rhsim08G0136700	-7.40434097	1.98809E-62	1.10327E-60	RHSIM_Rhsim08G0136700
Root vs Leaf	RHSIM_Rhsim03G0214900	-8.269271899	2.20487E-62	1.22064E-60	RHSIM_Rhsim03G0214900
Root vs Leaf	RHSIM_Rhsim06G0202500	-1.708440987	3.5843E-62	1.97958E-60	RHSIM_Rhsim06G0202500
Root vs Leaf	RHSIM_Rhsim05G0223200	-6.158687323	3.64226E-62	2.0068E-60	RHSIM_Rhsim05G0223200
Root vs Leaf	RHSIM_Rhsim01G0217200	-2.724405407	4.43517E-62	2.43787E-60	RHSIM_Rhsim01G0217200
Root vs Leaf	RHSIM_Rhsim07G0025600	-4.915596714	4.53453E-62	2.48658E-60	RHSIM_Rhsim07G0025600
Root vs Leaf	RHSIM_Rhsim03G0045800	-5.096536447	5.82369E-62	3.18596E-60	RHSIM_Rhsim03G0045800

Comparison	Gene ID	log_2FC	P-value	FDR (padj)	Gene Name
Root vs Leaf	RHSIM_Rhsim04G0167600	-2.703457374	8.43553E-62	4.60393E-60	RHSIM_Rhsim04G0167600
Root vs Leaf	RHSIM_Rhsim13G0175300	3.037035232	1.41288E-61	7.69304E-60	RHSIM_Rhsim13G0175300
Root vs Leaf	RHSIM_Rhsim12G0096200	-3.50011829	1.76892E-61	9.60903E-60	RHSIM_Rhsim12G0096200
Root vs Leaf	RHSIM_Rhsim02G0198800	-2.376322482	1.82824E-61	9.90801E-60	RHSIM_Rhsim02G0198800
Root vs Leaf	RHSIM_Rhsim05G0077800	7.747223751	2.01897E-61	1.09161E-59	RHSIM_Rhsim05G0077800
Root vs Leaf	RHSIM_Rhsim06G0166100	-4.341051931	3.48303E-61	1.8788E-59	RHSIM_Rhsim06G0166100
Root vs Leaf	RHSIM_Rhsim10G0053100	-5.909792594	3.58863E-61	1.93127E-59	RHSIM_Rhsim10G0053100
Root vs Leaf	RHSIM_Rhsim05G0090500	-1.713618694	4.54278E-61	2.43908E-59	RHSIM_Rhsim05G0090500
Root vs Leaf	RHSIM_Rhsim12G0182900	-4.003184996	4.73027E-61	2.53387E-59	RHSIM_Rhsim12G0182900
Root vs Leaf	RHSIM_Rhsim05G0094600	-2.236842545	7.0765E-61	3.78192E-59	RHSIM_Rhsim05G0094600
Root vs Leaf	RHSIM_Rhsim06G0136100	3.049542704	1.15231E-60	6.14414E-59	RHSIM_Rhsim06G0136100
Root vs Leaf	RHSIM_RhsimUnG0118600	-4.467990957	1.15705E-60	6.15522E-59	RHSIM_RhsimUnG0118600
Root vs Leaf	RHSIM_Rhsim05G0202600	-3.594964597	1.38166E-60	7.33328E-59	RHSIM_Rhsim05G0202600
Root vs Leaf	novel.4020	6.605781888	1.46265E-60	7.74535E-59	--
Root vs Leaf	novel.5556	-6.81375442	1.99887E-60	1.05607E-58	--
Root vs Leaf	RHSIM_Rhsim05G0226800	-5.572653055	2.8531E-60	1.50395E-58	RHSIM_Rhsim05G0226800
Root vs Leaf	RHSIM_Rhsim12G0025900	-2.101460624	2.97828E-60	1.56584E-58	RHSIM_Rhsim12G0025900
Root vs Leaf	RHSIM_Rhsim04G0083900	-9.867943418	2.98403E-60	1.56584E-58	RHSIM_Rhsim04G0083900
Root vs Leaf	RHSIM_RhsimUnG0255000	-1.971067	3.31818E-60	1.73724E-58	RHSIM_RhsimUnG0255000
Root vs Leaf	RHSIM_Rhsim06G0197400	-3.089820145	5.41187E-60	2.827E-58	RHSIM_Rhsim06G0197400
Root vs Leaf	RHSIM_Rhsim12G0124200	-2.889729921	7.71662E-60	4.02185E-58	RHSIM_Rhsim12G0124200
Root vs Leaf	RHSIM_Rhsim12G0155700	-10.12800513	8.10632E-60	4.21547E-58	RHSIM_Rhsim12G0155700
Root vs Leaf	RHSIM_Rhsim03G0035800	-2.96711551	9.60198E-60	4.98205E-58	RHSIM_Rhsim03G0035800
Root vs Leaf	RHSIM_Rhsim06G0229500	-3.411131934	1.0037E-59	5.1961E-58	RHSIM_Rhsim06G0229500
Root vs Leaf	RHSIM_Rhsim08G0250300	-3.247318299	1.20432E-59	6.22081E-58	RHSIM_Rhsim08G0250300

Comparison	Gene ID	log_2FC	P-value	FDR (padj)	Gene Name
Root vs Leaf	RHSIM_Rhsim02G0076500	-2.20659022	1.62464E-59	8.37323E-58	RHSIM_Rhsim02G0076500
Root vs Leaf	RHSIM_Rhsim03G0159800	-3.566128256	1.83987E-59	9.46144E-58	RHSIM_Rhsim03G0159800
Root vs Leaf	RHSIM_Rhsim06G0064800	-2.317284069	2.90739E-59	1.49179E-57	RHSIM_Rhsim06G0064800
Root vs Leaf	RHSIM_Rhsim12G0098100	-3.719715657	4.22855E-59	2.16489E-57	RHSIM_Rhsim12G0098100
Root vs Leaf	RHSIM_RhsimUnG0166600	-2.587267176	4.31153E-59	2.2025E-57	RHSIM_RhsimUnG0166600
Root vs Leaf	RHSIM_Rhsim12G0116400	-7.661446542	5.62788E-59	2.86861E-57	RHSIM_Rhsim12G0116400
Root vs Leaf	RHSIM_Rhsim02G0088400	-6.221360758	5.80355E-59	2.95165E-57	RHSIM_Rhsim02G0088400
Root vs Leaf	RHSIM_RhsimUnG0105400	6.80156028	6.01942E-59	3.05472E-57	RHSIM_RhsimUnG0105400
Root vs Leaf	RHSIM_Rhsim11G0056000	-6.355686487	6.12002E-59	3.09898E-57	RHSIM_Rhsim11G0056000
Root vs Leaf	RHSIM_Rhsim12G0005500	-6.159065759	6.45336E-59	3.26064E-57	RHSIM_Rhsim12G0005500
Root vs Leaf	RHSIM_Rhsim12G0153200	-4.632358009	6.63322E-59	3.34421E-57	RHSIM_Rhsim12G0153200
Root vs Leaf	RHSIM_Rhsim04G0192600	-11.24967654	6.97701E-59	3.50989E-57	RHSIM_Rhsim04G0192600
Root vs Leaf	RHSIM_RhsimPtG0004000	-4.686970954	7.32612E-59	3.67752E-57	RHSIM_RhsimPtG0004000
Root vs Leaf	RHSIM_Rhsim03G0066700	-2.891385486	8.0293E-59	4.02177E-57	RHSIM_Rhsim03G0066700
Root vs Leaf	RHSIM_Rhsim09G0098200	5.282511687	9.83289E-59	4.91453E-57	RHSIM_Rhsim09G0098200
Root vs Leaf	RHSIM_Rhsim03G0254300	-2.845679821	1.12996E-58	5.63545E-57	RHSIM_Rhsim03G0254300
Root vs Leaf	RHSIM_Rhsim08G0128200	-8.29654728	1.65812E-58	8.25174E-57	RHSIM_Rhsim08G0128200
Root vs Leaf	RHSIM_Rhsim09G0113200	-4.968376825	1.99371E-58	9.90054E-57	RHSIM_Rhsim09G0113200
Root vs Leaf	novel.4483	-5.760287196	2.00746E-58	9.94748E-57	--
Root vs Leaf	RHSIM_Rhsim11G0113700	-4.170121724	2.19134E-58	1.08354E-56	RHSIM_Rhsim11G0113700
Root vs Leaf	RHSIM_Rhsim01G0255600	-3.096685811	2.81202E-58	1.38748E-56	RHSIM_Rhsim01G0255600
Root vs Leaf	RHSIM_Rhsim11G0185100	-7.345479164	3.13859E-58	1.54532E-56	RHSIM_Rhsim11G0185100
Root vs Leaf	RHSIM_Rhsim10G0147700	4.632919519	3.48561E-58	1.71254E-56	RHSIM_Rhsim10G0147700
Root vs Leaf	RHSIM_Rhsim05G0022300	-3.302256058	4.59391E-58	2.25228E-56	RHSIM_Rhsim05G0022300
Root vs Leaf	RHSIM_Rhsim11G0195400	-3.584216769	8.86368E-58	4.33646E-56	RHSIM_Rhsim11G0195400

Comparison	Gene ID	log_2FC	P-value	FDR (padj)	Gene Name
Root vs Leaf	RHSIM_Rhsim02G0188600	2.409567117	9.1729E-58	4.47827E-56	RHSIM_Rhsim02G0188600
Root vs Leaf	RHSIM_Rhsim04G0019500	-3.129923688	1.03715E-57	5.05277E-56	RHSIM_Rhsim04G0019500
Root vs Leaf	RHSIM_Rhsim07G0070500	-4.438934545	1.32E-57	6.41727E-56	RHSIM_Rhsim07G0070500
Root vs Leaf	RHSIM_Rhsim01G0269100	-5.436405382	1.80913E-57	8.77676E-56	RHSIM_Rhsim01G0269100
Root vs Leaf	RHSIM_Rhsim08G0062800	-1.925630661	1.91618E-57	9.27663E-56	RHSIM_Rhsim08G0062800
Root vs Leaf	RHSIM_Rhsim13G0103500	-3.576663394	1.98355E-57	9.58274E-56	RHSIM_Rhsim13G0103500
Root vs Leaf	RHSIM_RhsimUnG0258900	-2.468448481	2.50895E-57	1.20958E-55	RHSIM_RhsimUnG0258900
Root vs Leaf	RHSIM_Rhsim01G0287300	-3.419394125	3.01782E-57	1.45188E-55	RHSIM_Rhsim01G0287300
Root vs Leaf	RHSIM_Rhsim05G0139000	-1.998917043	3.80914E-57	1.82878E-55	RHSIM_Rhsim05G0139000
Root vs Leaf	RHSIM_Rhsim08G0007000	-4.014864058	3.82019E-57	1.83029E-55	RHSIM_Rhsim08G0007000
Root vs Leaf	RHSIM_Rhsim10G0176300	8.765700241	5.26004E-57	2.51493E-55	RHSIM_Rhsim10G0176300
Root vs Leaf	RHSIM_Rhsim05G0212900	-10.17268158	5.31892E-57	2.53784E-55	RHSIM_Rhsim05G0212900
Root vs Leaf	RHSIM_Rhsim13G0015700	-2.488761816	9.64948E-57	4.59462E-55	RHSIM_Rhsim13G0015700
Root vs Leaf	RHSIM_Rhsim05G0046600	3.827000143	1.03291E-56	4.9081E-55	RHSIM_Rhsim05G0046600
Root vs Leaf	RHSIM_Rhsim09G0108100	-5.314399657	1.57113E-56	7.45033E-55	RHSIM_Rhsim09G0108100
Root vs Leaf	novel.1309	-5.883706352	1.58139E-56	7.48363E-55	--
Root vs Leaf	RHSIM_Rhsim04G0102500	-5.09501637	1.7737E-56	8.37658E-55	RHSIM_Rhsim04G0102500
Root vs Leaf	RHSIM_Rhsim11G0020200	-2.184762167	1.77734E-56	8.37667E-55	RHSIM_Rhsim11G0020200
Root vs Leaf	novel.5195	-3.257086398	2.24066E-56	1.05389E-54	--
Root vs Leaf	RHSIM_RhsimUnG0018800	-5.712663016	2.29844E-56	1.07887E-54	RHSIM_RhsimUnG0018800
Root vs Leaf	RHSIM_Rhsim08G0000900	-2.760016512	3.00181E-56	1.40617E-54	RHSIM_Rhsim08G0000900
Root vs Leaf	RHSIM_Rhsim05G0073200	3.376218149	6.57679E-56	3.07308E-54	RHSIM_Rhsim05G0073200
Root vs Leaf	RHSIM_Rhsim11G0150000	-3.934903065	6.58677E-56	3.07308E-54	RHSIM_Rhsim11G0150000
Root vs Leaf	RHSIM_Rhsim07G0234600	-3.956825306	6.94405E-56	3.23325E-54	RHSIM_Rhsim07G0234600
Root vs Leaf	RHSIM_Rhsim04G0242900	7.795098418	7.31421E-56	3.39876E-54	RHSIM_Rhsim04G0242900

Comparison	Gene ID	log_2FC	P-value	FDR (padj)	Gene Name
Root vs Leaf	RHSIM_Rhsim07G0139000	-2.343120396	7.775E-56	3.60564E-54	RHSIM_Rhsim07G0139000
Root vs Leaf	RHSIM_Rhsim05G0126900	5.766947593	1.09319E-55	5.05952E-54	RHSIM_Rhsim05G0126900
Root vs Leaf	RHSIM_Rhsim13G0166700	4.990170501	1.23078E-55	5.68495E-54	RHSIM_Rhsim13G0166700
Root vs Leaf	RHSIM_Rhsim04G0160100	-3.100583097	1.75082E-55	8.07085E-54	RHSIM_Rhsim04G0160100
Root vs Leaf	RHSIM_Rhsim02G0173200	-2.685072418	2.46658E-55	1.13477E-53	RHSIM_Rhsim02G0173200
Root vs Leaf	RHSIM_Rhsim11G0040600	-3.143149134	2.76282E-55	1.2673E-53	RHSIM_Rhsim11G0040600
Root vs Leaf	RHSIM_RhsimUnG0008300	-4.637664668	2.76561E-55	1.2673E-53	RHSIM_RhsimUnG0008300
Root vs Leaf	RHSIM_Rhsim08G0029700	-8.953266257	3.63726E-55	1.66344E-53	RHSIM_Rhsim08G0029700
Root vs Leaf	RHSIM_Rhsim11G0122000	-4.860252816	3.73302E-55	1.70386E-53	RHSIM_Rhsim11G0122000
Root vs Leaf	RHSIM_Rhsim02G0244700	-2.519300308	5.2926E-55	2.41095E-53	RHSIM_Rhsim02G0244700
Root vs Leaf	RHSIM_Rhsim04G0202000	-4.675330838	5.34199E-55	2.42866E-53	RHSIM_Rhsim04G0202000
Root vs Leaf	RHSIM_Rhsim07G0193500	-3.197594473	5.76412E-55	2.61544E-53	RHSIM_Rhsim07G0193500
Root vs Leaf	RHSIM_Rhsim08G0229600	-7.072746803	6.81804E-55	3.0876E-53	RHSIM_Rhsim08G0229600
Root vs Leaf	RHSIM_Rhsim13G0094600	3.003405406	9.6465E-55	4.35996E-53	RHSIM_Rhsim13G0094600
Root vs Leaf	RHSIM_Rhsim09G0188800	-2.222295412	1.06702E-54	4.81325E-53	RHSIM_Rhsim09G0188800
Root vs Leaf	RHSIM_Rhsim08G0237900	-3.832957228	1.15575E-54	5.20333E-53	RHSIM_Rhsim08G0237900
Root vs Leaf	RHSIM_Rhsim02G0240300	-6.649070029	1.24104E-54	5.57649E-53	RHSIM_Rhsim02G0240300
Root vs Leaf	RHSIM_Rhsim11G0021000	-2.886937362	1.27752E-54	5.72929E-53	RHSIM_Rhsim11G0021000
Root vs Leaf	RHSIM_Rhsim03G0210800	-4.176514504	1.37549E-54	6.15672E-53	RHSIM_Rhsim03G0210800
Root vs Leaf	RHSIM_Rhsim07G0043900	3.486838448	1.55779E-54	6.95924E-53	RHSIM_Rhsim07G0043900
Root vs Leaf	RHSIM_Rhsim08G0171900	-2.472593682	1.77936E-54	7.93375E-53	RHSIM_Rhsim08G0171900
Root vs Leaf	RHSIM_Rhsim01G0044000	-3.561350449	2.06767E-54	9.20154E-53	RHSIM_Rhsim01G0044000
Root vs Leaf	RHSIM_Rhsim06G0220300	-2.890546466	2.61531E-54	1.16163E-52	RHSIM_Rhsim06G0220300
Root vs Leaf	RHSIM_Rhsim01G0128600	-4.129372003	3.0093E-54	1.33406E-52	RHSIM_Rhsim01G0128600
Root vs Leaf	RHSIM_Rhsim13G0071700	-6.195273625	4.37273E-54	1.93479E-52	RHSIM_Rhsim13G0071700

Comparison	Gene ID	log_2FC	P-value	FDR (padj)	Gene Name
Root vs Leaf	RHSIM_Rhsim07G0173600	-1.899403844	5.39243E-54	2.38142E-52	RHSIM_Rhsim07G0173600
Root vs Leaf	RHSIM_Rhsim03G0041100	-6.06004432	7.59832E-54	3.3492E-52	RHSIM_Rhsim03G0041100
Root vs Leaf	RHSIM_Rhsim05G0060700	-2.259217394	9.21947E-54	4.05604E-52	RHSIM_Rhsim05G0060700
Root vs Leaf	RHSIM_Rhsim01G0171300	-2.65446311	9.70242E-54	4.26041E-52	RHSIM_Rhsim01G0171300
Root vs Leaf	RHSIM_Rhsim03G0231800	-2.307791227	9.82059E-54	4.30413E-52	RHSIM_Rhsim03G0231800
Root vs Leaf	RHSIM_Rhsim12G0070500	-4.430610629	1.21167E-53	5.30043E-52	RHSIM_Rhsim12G0070500
Root vs Leaf	novel.1312	-9.046681659	1.31129E-53	5.72539E-52	--
Root vs Leaf	RHSIM_Rhsim06G0184200	5.429585841	1.46038E-53	6.36434E-52	RHSIM_Rhsim06G0184200
Root vs Leaf	RHSIM_Rhsim06G0227300	-3.396061718	2.32314E-53	1.01052E-51	RHSIM_Rhsim06G0227300
Root vs Leaf	RHSIM_Rhsim04G0111900	4.782738584	2.48533E-53	1.07904E-51	RHSIM_Rhsim04G0111900
Root vs Leaf	novel.4572	-7.075083421	2.54406E-53	1.10247E-51	--
Root vs Leaf	RHSIM_Rhsim09G0054700	-5.521308939	2.79439E-53	1.20869E-51	RHSIM_Rhsim09G0054700
Root vs Leaf	novel.28	-4.573415269	2.95999E-53	1.27793E-51	--
Root vs Leaf	RHSIM_Rhsim13G0194200	-4.553493476	3.47769E-53	1.49864E-51	RHSIM_Rhsim13G0194200
Root vs Leaf	RHSIM_Rhsim02G0254700	-5.133568381	3.89329E-53	1.67462E-51	RHSIM_Rhsim02G0254700
Root vs Leaf	RHSIM_Rhsim11G0075300	2.083410948	4.00516E-53	1.71954E-51	RHSIM_Rhsim11G0075300
Root vs Leaf	novel.4886	-9.767205322	5.79387E-53	2.48289E-51	--
Root vs Leaf	RHSIM_Rhsim13G0142000	-3.283643923	6.3135E-53	2.70057E-51	RHSIM_Rhsim13G0142000
Root vs Leaf	RHSIM_Rhsim13G0064900	-3.741890128	1.03172E-52	4.40501E-51	RHSIM_Rhsim13G0064900
Root vs Leaf	RHSIM_Rhsim06G0236900	3.341529339	1.03669E-52	4.41807E-51	RHSIM_Rhsim06G0236900
Root vs Leaf	RHSIM_Rhsim04G0166900	-2.510742559	1.29258E-52	5.49844E-51	RHSIM_Rhsim04G0166900
Root vs Leaf	RHSIM_Rhsim01G0089700	-2.89210777	1.38168E-52	5.8667E-51	RHSIM_Rhsim01G0089700
Root vs Leaf	RHSIM_Rhsim09G0064200	-4.818270394	1.98442E-52	8.41054E-51	RHSIM_Rhsim09G0064200
Root vs Leaf	RHSIM_Rhsim08G0055400	-3.925689985	2.04888E-52	8.66785E-51	RHSIM_Rhsim08G0055400
Root vs Leaf	RHSIM_Rhsim08G0180100	5.076902669	2.43333E-52	1.02755E-50	RHSIM_Rhsim08G0180100

Comparison	Gene ID	log_2FC	P-value	FDR (padj)	Gene Name
Root vs Leaf	RHSIM_Rhsim04G0011700	7.102304328	2.50477E-52	1.05579E-50	RHSIM_Rhsim04G0011700
Root vs Leaf	RHSIM_Rhsim13G0185200	2.567290333	2.5468E-52	1.07155E-50	RHSIM_Rhsim13G0185200
Root vs Leaf	RHSIM_Rhsim01G0269800	-10.24509213	2.61039E-52	1.09632E-50	RHSIM_Rhsim01G0269800
Root vs Leaf	RHSIM_Rhsim12G0035600	-2.756765963	2.83573E-52	1.1888E-50	RHSIM_Rhsim12G0035600
Root vs Leaf	RHSIM_Rhsim01G0247800	-7.623479096	3.31146E-52	1.38572E-50	RHSIM_Rhsim01G0247800
Root vs Leaf	RHSIM_Rhsim04G0081700	3.349172265	3.6196E-52	1.51193E-50	RHSIM_Rhsim04G0081700
Root vs Leaf	RHSIM_Rhsim11G0088800	-1.992202548	5.40481E-52	2.25356E-50	RHSIM_Rhsim11G0088800
Root vs Leaf	RHSIM_RhsimUnG0024000	-3.986174685	5.99499E-52	2.49514E-50	RHSIM_RhsimUnG0024000
Root vs Leaf	novel.4113	3.375409548	8.12897E-52	3.37724E-50	--
Root vs Leaf	RHSIM_RhsimUnG0180200	-5.261851896	9.82587E-52	4.07492E-50	RHSIM_RhsimUnG0180200
Root vs Leaf	RHSIM_Rhsim11G0058800	-3.199646276	1.03266E-51	4.27491E-50	RHSIM_Rhsim11G0058800
Root vs Leaf	RHSIM_Rhsim06G0039700	6.726821945	1.29937E-51	5.36942E-50	RHSIM_Rhsim06G0039700
Root vs Leaf	RHSIM_Rhsim08G0168800	6.11767381	1.70456E-51	7.03125E-50	RHSIM_Rhsim08G0168800
Root vs Leaf	RHSIM_Rhsim03G0191800	-7.269548678	2.08815E-51	8.58904E-50	RHSIM_Rhsim03G0191800
Root vs Leaf	RHSIM_Rhsim05G0000100	-2.52251208	2.08964E-51	8.58904E-50	RHSIM_Rhsim05G0000100
Root vs Leaf	novel.5567	-6.180479033	2.28728E-51	9.38476E-50	--
Root vs Leaf	RHSIM_Rhsim12G0145700	-4.887130212	2.44157E-51	1.00001E-49	RHSIM_Rhsim12G0145700
Root vs Leaf	RHSIM_Rhsim11G0096200	-2.791286693	3.81423E-51	1.55945E-49	RHSIM_Rhsim11G0096200
Root vs Leaf	RHSIM_Rhsim11G0039200	-11.23034225	4.13047E-51	1.68577E-49	RHSIM_Rhsim11G0039200
Root vs Leaf	RHSIM_Rhsim06G0093200	-2.646011981	4.36506E-51	1.77838E-49	RHSIM_Rhsim06G0093200
Root vs Leaf	RHSIM_Rhsim02G0093900	-3.639882715	4.59278E-51	1.86787E-49	RHSIM_Rhsim02G0093900
Root vs Leaf	RHSIM_Rhsim12G0135300	9.36350496	5.06038E-51	2.05443E-49	RHSIM_Rhsim12G0135300
Root vs Leaf	novel.5423	-1.818867146	5.15168E-51	2.08783E-49	--
Root vs Leaf	RHSIM_Rhsim08G0055200	-3.023466868	5.4371E-51	2.19965E-49	RHSIM_Rhsim08G0055200
Root vs Leaf	RHSIM_Rhsim05G0116000	-1.981504296	5.90259E-51	2.3838E-49	RHSIM_Rhsim05G0116000

Comparison	Gene ID	log_2FC	P-value	FDR (padj)	Gene Name
Root vs Leaf	RHSIM_Rhsim11G0137800	-3.696203463	6.33109E-51	2.5524E-49	RHSIM_Rhsim11G0137800
Root vs Leaf	RHSIM_Rhsim04G0167200	-2.666996809	6.5754E-51	2.64628E-49	RHSIM_Rhsim04G0167200
Root vs Leaf	novel.5148	5.414096783	1.00205E-50	4.02577E-49	--
Root vs Leaf	RHSIM_Rhsim13G0219600	-5.651777571	1.03516E-50	4.15158E-49	RHSIM_Rhsim13G0219600
Root vs Leaf	RHSIM_Rhsim11G0043600	-5.396086068	1.07604E-50	4.30809E-49	RHSIM_Rhsim11G0043600
Root vs Leaf	RHSIM_Rhsim02G0037500	-4.601637283	1.34761E-50	5.38602E-49	RHSIM_Rhsim02G0037500
Root vs Leaf	RHSIM_Rhsim05G0167200	-2.277495585	1.46712E-50	5.85357E-49	RHSIM_Rhsim05G0167200
Root vs Leaf	RHSIM_Rhsim08G0006200	-2.882845794	1.66465E-50	6.63024E-49	RHSIM_Rhsim08G0006200
Root vs Leaf	RHSIM_Rhsim10G0173800	-4.256019123	2.10377E-50	8.36486E-49	RHSIM_Rhsim10G0173800
Root vs Leaf	RHSIM_Rhsim07G0144800	-3.190310147	3.88578E-50	1.54238E-48	RHSIM_Rhsim07G0144800
Root vs Leaf	RHSIM_Rhsim13G0148600	-3.854157646	4.01375E-50	1.59045E-48	RHSIM_Rhsim13G0148600
Root vs Leaf	RHSIM_Rhsim01G0191800	-3.080069392	4.14288E-50	1.63881E-48	RHSIM_Rhsim01G0191800
Root vs Leaf	RHSIM_Rhsim04G0190300	-2.795473951	4.45527E-50	1.75938E-48	RHSIM_Rhsim04G0190300
Root vs Leaf	RHSIM_Rhsim10G0019600	-2.599567629	4.96934E-50	1.95904E-48	RHSIM_Rhsim10G0019600
Root vs Leaf	RHSIM_Rhsim09G0008400	-2.547440722	6.36204E-50	2.50381E-48	RHSIM_Rhsim09G0008400
Root vs Leaf	RHSIM_Rhsim06G0022200	-4.974802383	7.05253E-50	2.77084E-48	RHSIM_Rhsim06G0022200
Root vs Leaf	RHSIM_Rhsim12G0065000	-7.609141185	7.1702E-50	2.8123E-48	RHSIM_Rhsim12G0065000
Root vs Leaf	RHSIM_Rhsim02G0034800	-12.59670869	7.32048E-50	2.86638E-48	RHSIM_Rhsim02G0034800
Root vs Leaf	RHSIM_Rhsim03G0158800	-2.327271755	7.37541E-50	2.88301E-48	RHSIM_Rhsim03G0158800
Root vs Leaf	RHSIM_Rhsim02G0028600	-4.846313782	8.40294E-50	3.27913E-48	RHSIM_Rhsim02G0028600
Root vs Leaf	RHSIM_Rhsim08G0181000	-8.014798251	1.02676E-49	4.00005E-48	RHSIM_Rhsim08G0181000
Root vs Leaf	RHSIM_Rhsim02G0027600	-3.541254678	1.12941E-49	4.39256E-48	RHSIM_Rhsim02G0027600
Root vs Leaf	RHSIM_Rhsim08G0214800	-3.861882692	1.3088E-49	5.08171E-48	RHSIM_Rhsim08G0214800
Root vs Leaf	RHSIM_Rhsim07G0054900	-7.818275148	1.38939E-49	5.38559E-48	RHSIM_Rhsim07G0054900
Root vs Leaf	RHSIM_Rhsim08G0232700	-2.620476853	1.57082E-49	6.07866E-48	RHSIM_Rhsim08G0232700

Comparison	Gene ID	log_2FC	P-value	FDR (padj)	Gene Name
Root vs Leaf	novel.1972	-4.352203898	2.20567E-49	8.52109E-48	--
Root vs Leaf	RHSIM_Rhsim01G0000500	-1.89361792	2.51372E-49	9.69498E-48	RHSIM_Rhsim01G0000500
Root vs Leaf	RHSIM_Rhsim09G0209600	-2.825558037	2.543E-49	9.7916E-48	RHSIM_Rhsim09G0209600
Root vs Leaf	RHSIM_Rhsim09G0122900	-3.706712013	5.76856E-49	2.21745E-47	RHSIM_Rhsim09G0122900
Root vs Leaf	RHSIM_Rhsim06G0157200	9.118259113	6.35926E-49	2.44046E-47	RHSIM_Rhsim06G0157200
Root vs Leaf	RHSIM_Rhsim12G0076700	-2.228365044	6.66649E-49	2.55413E-47	RHSIM_Rhsim12G0076700
Root vs Leaf	RHSIM_Rhsim12G0000700	-3.109381557	6.75769E-49	2.58479E-47	RHSIM_Rhsim12G0000700
Root vs Leaf	RHSIM_Rhsim05G0113400	-1.448613905	7.34442E-49	2.80457E-47	RHSIM_Rhsim05G0113400
Root vs Leaf	RHSIM_Rhsim10G0079900	-2.238430903	9.04213E-49	3.44718E-47	RHSIM_Rhsim10G0079900
Root vs Leaf	RHSIM_Rhsim12G0005400	5.437542091	1.08771E-48	4.13993E-47	RHSIM_Rhsim12G0005400
Root vs Leaf	RHSIM_Rhsim06G0049900	-5.263036218	1.14997E-48	4.36969E-47	RHSIM_Rhsim06G0049900
Root vs Leaf	RHSIM_Rhsim10G0163500	-3.036733933	1.25402E-48	4.75724E-47	RHSIM_Rhsim10G0163500
Root vs Leaf	RHSIM_Rhsim05G0161500	4.188555923	1.34659E-48	5.10008E-47	RHSIM_Rhsim05G0161500
Root vs Leaf	RHSIM_Rhsim13G0119400	-8.12864955	1.40154E-48	5.2995E-47	RHSIM_Rhsim13G0119400
Root vs Leaf	RHSIM_Rhsim01G0149300	2.542245641	1.49552E-48	5.64564E-47	RHSIM_Rhsim01G0149300
Root vs Leaf	RHSIM_Rhsim11G0065700	-2.416293658	1.5499E-48	5.84142E-47	RHSIM_Rhsim11G0065700
Root vs Leaf	RHSIM_RhsimUnG0105900	7.553068371	1.83195E-48	6.89319E-47	RHSIM_RhsimUnG0105900
Root vs Leaf	RHSIM_Rhsim12G0138700	-2.846466243	2.21672E-48	8.32745E-47	RHSIM_Rhsim12G0138700
Root vs Leaf	RHSIM_Rhsim10G0035200	-3.112714311	2.3942E-48	8.97959E-47	RHSIM_Rhsim10G0035200
Root vs Leaf	RHSIM_Rhsim10G0151800	-3.098303644	2.70239E-48	1.01191E-46	RHSIM_Rhsim10G0151800
Root vs Leaf	RHSIM_RhsimUnG0005900	-4.50652275	4.27474E-48	1.59809E-46	RHSIM_RhsimUnG0005900
Root vs Leaf	RHSIM_Rhsim08G0252300	-2.507735596	4.54204E-48	1.69528E-46	RHSIM_Rhsim08G0252300
Root vs Leaf	RHSIM_Rhsim04G0050100	-2.105744385	4.88656E-48	1.82093E-46	RHSIM_Rhsim04G0050100
Root vs Leaf	novel.6020	5.397387331	5.83826E-48	2.17208E-46	--
Root vs Leaf	RHSIM_Rhsim07G0048100	-3.63486495	7.79412E-48	2.89508E-46	RHSIM_Rhsim07G0048100

Comparison	Gene ID	log_2FC	P-value	FDR (padj)	Gene Name
Root vs Leaf	RHSIM_Rhsim03G0255200	-12.79885773	8.35586E-48	3.09876E-46	RHSIM_Rhsim03G0255200
Root vs Leaf	RHSIM_Rhsim05G0079000	-5.256444261	1.02521E-47	3.7959E-46	RHSIM_Rhsim05G0079000
Root vs Leaf	RHSIM_Rhsim01G0285200	-2.87803201	1.04546E-47	3.86471E-46	RHSIM_Rhsim01G0285200
Root vs Leaf	RHSIM_Rhsim05G0077900	-5.675893497	1.3367E-47	4.93344E-46	RHSIM_Rhsim05G0077900
Root vs Leaf	RHSIM_Rhsim13G0002400	-4.314924403	1.36468E-47	5.02865E-46	RHSIM_Rhsim13G0002400
Root vs Leaf	RHSIM_Rhsim06G0161200	-2.833740268	1.5002E-47	5.51927E-46	RHSIM_Rhsim06G0161200
Root vs Leaf	RHSIM_Rhsim02G0213600	4.630847125	1.53127E-47	5.62461E-46	RHSIM_Rhsim02G0213600
Root vs Leaf	RHSIM_RhsimUnG0089100	-3.874011821	1.59717E-47	5.85739E-46	RHSIM_RhsimUnG0089100
Root vs Leaf	RHSIM_Rhsim03G0222700	-3.925039234	1.70686E-47	6.24977E-46	RHSIM_Rhsim03G0222700
Root vs Leaf	RHSIM_Rhsim04G0007400	-5.367579784	1.71112E-47	6.25544E-46	RHSIM_Rhsim04G0007400
Root vs Leaf	RHSIM_Rhsim04G0213800	-3.375778257	1.97435E-47	7.20639E-46	RHSIM_Rhsim04G0213800
Root vs Leaf	RHSIM_Rhsim07G0175400	-1.848176308	1.98273E-47	7.22557E-46	RHSIM_Rhsim07G0175400
Root vs Leaf	RHSIM_RhsimUnG0139700	-3.279413846	2.09548E-47	7.62443E-46	RHSIM_RhsimUnG0139700
Root vs Leaf	RHSIM_Rhsim08G0134100	-1.886184262	2.20974E-47	8.02757E-46	RHSIM_Rhsim08G0134100
Root vs Leaf	RHSIM_Rhsim13G0094300	3.519216406	2.3513E-47	8.52843E-46	RHSIM_Rhsim13G0094300
Root vs Leaf	RHSIM_Rhsim13G0219900	-3.745183974	3.13358E-47	1.13481E-45	RHSIM_Rhsim13G0219900
Root vs Leaf	RHSIM_Rhsim10G0102500	-3.999425511	5.08995E-47	1.84041E-45	RHSIM_Rhsim10G0102500
Root vs Leaf	RHSIM_Rhsim07G0110000	9.470423295	5.28138E-47	1.90665E-45	RHSIM_Rhsim07G0110000
Root vs Leaf	RHSIM_Rhsim13G0157300	4.507411054	5.29463E-47	1.90846E-45	RHSIM_Rhsim13G0157300
Root vs Leaf	RHSIM_Rhsim11G0106200	-1.885716954	5.84537E-47	2.1037E-45	RHSIM_Rhsim11G0106200
Root vs Leaf	RHSIM_Rhsim08G0008700	4.51601781	6.54906E-47	2.35329E-45	RHSIM_Rhsim08G0008700
Root vs Leaf	RHSIM_Rhsim08G0012200	-1.895404349	8.75788E-47	3.14211E-45	RHSIM_Rhsim08G0012200
Root vs Leaf	RHSIM_Rhsim03G0046400	-1.653649699	9.1097E-47	3.26328E-45	RHSIM_Rhsim03G0046400
Root vs Leaf	RHSIM_Rhsim01G0287200	-7.15135126	9.18165E-47	3.28397E-45	RHSIM_Rhsim01G0287200
Root vs Leaf	RHSIM_Rhsim06G0021700	-6.357107455	9.5867E-47	3.42355E-45	RHSIM_Rhsim06G0021700

Comparison	Gene ID	log_2FC	P-value	FDR (padj)	Gene Name
Root vs Leaf	RHSIM_Rhsim02G0234600	-6.048252734	1.14166E-46	4.07074E-45	RHSIM_Rhsim02G0234600
Root vs Leaf	RHSIM_Rhsim06G0183900	-1.95979819	1.19501E-46	4.25443E-45	RHSIM_Rhsim06G0183900
Root vs Leaf	RHSIM_Rhsim04G0209400	-5.342836819	1.21497E-46	4.31882E-45	RHSIM_Rhsim04G0209400
Root vs Leaf	RHSIM_Rhsim09G0195900	-6.779522039	1.22264E-46	4.33943E-45	RHSIM_Rhsim09G0195900
Root vs Leaf	RHSIM_Rhsim05G0007400	6.040319979	1.46007E-46	5.17419E-45	RHSIM_Rhsim05G0007400
Root vs Leaf	RHSIM_Rhsim07G0125500	-2.621323901	1.54394E-46	5.46303E-45	RHSIM_Rhsim07G0125500
Root vs Leaf	RHSIM_Rhsim11G0102000	-6.403973883	1.5477E-46	5.468E-45	RHSIM_Rhsim11G0102000
Root vs Leaf	RHSIM_Rhsim06G0171800	-6.362009215	1.7239E-46	6.07288E-45	RHSIM_Rhsim06G0171800
Root vs Leaf	RHSIM_Rhsim06G0166200	-2.000487059	1.72416E-46	6.07288E-45	RHSIM_Rhsim06G0166200
Root vs Leaf	RHSIM_Rhsim05G0223000	-3.730014285	1.75787E-46	6.18219E-45	RHSIM_Rhsim05G0223000
Root vs Leaf	RHSIM_RhsimPtG0003500	-2.7393838	2.08564E-46	7.32379E-45	RHSIM_RhsimPtG0003500
Root vs Leaf	RHSIM_Rhsim05G0233000	-2.67692201	2.11598E-46	7.41908E-45	RHSIM_Rhsim05G0233000
Root vs Leaf	RHSIM_Rhsim03G0080600	5.496393505	2.26222E-46	7.91983E-45	RHSIM_Rhsim03G0080600
Root vs Leaf	RHSIM_Rhsim01G0291400	-1.820263806	2.51136E-46	8.77876E-45	RHSIM_Rhsim01G0291400
Root vs Leaf	RHSIM_Rhsim06G0115400	-5.696828475	2.59209E-46	9.04728E-45	RHSIM_Rhsim06G0115400
Root vs Leaf	RHSIM_Rhsim10G0052200	-3.839192085	2.8815E-46	1.00423E-44	RHSIM_Rhsim10G0052200
Root vs Leaf	RHSIM_Rhsim06G0029800	-12.51460739	2.8973E-46	1.00822E-44	RHSIM_Rhsim06G0029800
Root vs Leaf	RHSIM_Rhsim12G0146800	-4.412198357	3.59616E-46	1.24953E-44	RHSIM_Rhsim12G0146800
Root vs Leaf	RHSIM_Rhsim07G0065200	-5.710758191	3.60582E-46	1.25101E-44	RHSIM_Rhsim07G0065200
Root vs Leaf	RHSIM_Rhsim09G0010400	5.057087661	3.73924E-46	1.29536E-44	RHSIM_Rhsim09G0010400
Root vs Leaf	RHSIM_RhsimUnG0209700	3.902954305	5.0925E-46	1.76152E-44	RHSIM_RhsimUnG0209700
Root vs Leaf	RHSIM_Rhsim13G0011100	-2.504747778	5.45763E-46	1.885E-44	RHSIM_Rhsim13G0011100
Root vs Leaf	novel.3711	6.095829837	5.46665E-46	1.8853E-44	--
Root vs Leaf	RHSIM_Rhsim03G0154900	-6.178374148	6.63952E-46	2.28639E-44	RHSIM_Rhsim03G0154900
Root vs Leaf	RHSIM_Rhsim07G0037800	-2.238018832	7.4308E-46	2.55507E-44	RHSIM_Rhsim07G0037800

Comparison	Gene ID	log$_2$FC	P-value	FDR (padj)	Gene Name
Root vs Leaf	RHSIM_Rhsim07G0153500	-1.951386101	7.65385E-46	2.62786E-44	RHSIM_Rhsim07G0153500
Root vs Leaf	RHSIM_RhsimUnG0027200	-3.00205176	8.69238E-46	2.98001E-44	RHSIM_RhsimUnG0027200
Root vs Leaf	RHSIM_Rhsim05G0141700	-3.012570471	9.31977E-46	3.19037E-44	RHSIM_Rhsim05G0141700
Root vs Leaf	RHSIM_Rhsim09G0006700	-2.639309228	9.42616E-46	3.22202E-44	RHSIM_Rhsim09G0006700
Root vs Leaf	RHSIM_Rhsim13G0057000	9.072406737	9.95652E-46	3.39829E-44	RHSIM_Rhsim13G0057000
Root vs Leaf	RHSIM_Rhsim09G0211500	-2.359234855	1.06586E-45	3.63256E-44	RHSIM_Rhsim09G0211500
Root vs Leaf	RHSIM_Rhsim01G0141500	-2.556203124	1.12256E-45	3.82018E-44	RHSIM_Rhsim01G0141500
Root vs Leaf	RHSIM_Rhsim13G0003800	-1.952758065	1.15583E-45	3.92761E-44	RHSIM_Rhsim13G0003800
Root vs Leaf	RHSIM_Rhsim12G0024900	2.990252495	1.29644E-45	4.39896E-44	RHSIM_Rhsim12G0024900
Root vs Leaf	RHSIM_Rhsim02G0115100	-4.8251301	1.74631E-45	5.91675E-44	RHSIM_Rhsim02G0115100
Root vs Leaf	RHSIM_Rhsim07G0175800	-3.779501031	1.92664E-45	6.51817E-44	RHSIM_Rhsim07G0175800
Root vs Leaf	RHSIM_Rhsim09G0149700	-1.767917793	1.95377E-45	6.60032E-44	RHSIM_Rhsim09G0149700
Root vs Leaf	RHSIM_Rhsim03G0063700	-3.87836742	2.20593E-45	7.44132E-44	RHSIM_Rhsim03G0063700
Root vs Leaf	RHSIM_Rhsim04G0133000	3.18246468	2.24206E-45	7.55217E-44	RHSIM_Rhsim04G0133000
Root vs Leaf	RHSIM_Rhsim06G0103100	-2.741666619	2.51949E-45	8.47434E-44	RHSIM_Rhsim06G0103100
Root vs Leaf	RHSIM_Rhsim13G0230700	-2.767491528	2.5861E-45	8.68577E-44	RHSIM_Rhsim13G0230700
Root vs Leaf	novel.5013	-2.918073457	2.73821E-45	9.18331E-44	--
Root vs Leaf	RHSIM_Rhsim08G0223600	-3.323081877	3.37895E-45	1.13158E-43	RHSIM_Rhsim08G0223600
Root vs Leaf	RHSIM_Rhsim04G0139800	-2.913320157	4.02256E-45	1.34517E-43	RHSIM_Rhsim04G0139800
Root vs Leaf	RHSIM_Rhsim10G0193100	-3.631768807	4.14789E-45	1.38508E-43	RHSIM_Rhsim10G0193100
Root vs Leaf	RHSIM_Rhsim12G0166700	-8.232032428	5.9338E-45	1.97859E-43	RHSIM_Rhsim12G0166700
Root vs Leaf	RHSIM_Rhsim01G0181300	-5.39608265	5.99251E-45	1.99529E-43	RHSIM_Rhsim01G0181300
Root vs Leaf	RHSIM_Rhsim03G0205000	-3.930303695	6.20628E-45	2.0635E-43	RHSIM_Rhsim03G0205000
Root vs Leaf	RHSIM_Rhsim09G0153300	3.145822709	6.98147E-45	2.31791E-43	RHSIM_Rhsim09G0153300
Root vs Leaf	novel.5489	5.720947887	9.96821E-45	3.30479E-43	--

Comparison	Gene ID	log_2FC	P-value	FDR (padj)	Gene Name
Root vs Leaf	RHSIM_Rhsim04G0117700	-2.239254369	1.03496E-44	3.42633E-43	RHSIM_Rhsim04G0117700
Root vs Leaf	novel.1773	-4.450602646	1.09634E-44	3.62435E-43	--
Root vs Leaf	RHSIM_Rhsim04G0170500	4.493263247	1.15545E-44	3.81431E-43	RHSIM_Rhsim04G0170500
Root vs Leaf	RHSIM_Rhsim08G0029600	-9.439235269	1.2888E-44	4.24844E-43	RHSIM_Rhsim08G0029600
Root vs Leaf	novel.6125	6.765451719	1.38737E-44	4.56687E-43	--
Root vs Leaf	RHSIM_RhsimPtG0005300	-5.247857032	1.49556E-44	4.91601E-43	RHSIM_RhsimPtG0005300
Root vs Leaf	RHSIM_Rhsim02G0109300	-2.136069819	1.53275E-44	5.03112E-43	RHSIM_Rhsim02G0109300
Root vs Leaf	RHSIM_Rhsim10G0141000	-6.163236376	1.64985E-44	5.40781E-43	RHSIM_Rhsim10G0141000
Root vs Leaf	novel.4372	-3.499232739	2.0422E-44	6.68439E-43	--
Root vs Leaf	RHSIM_Rhsim04G0081600	-3.570103838	2.24774E-44	7.34676E-43	RHSIM_Rhsim04G0081600
Root vs Leaf	RHSIM_Rhsim01G0180300	-4.493066215	2.58956E-44	8.45205E-43	RHSIM_Rhsim01G0180300
Root vs Leaf	RHSIM_Rhsim03G0204000	-3.028587856	3.31381E-44	1.08007E-42	RHSIM_Rhsim03G0204000
Root vs Leaf	RHSIM_Rhsim12G0110600	-2.952682285	3.9881E-44	1.29801E-42	RHSIM_Rhsim12G0110600
Root vs Leaf	RHSIM_Rhsim01G0244300	-2.535344277	4.8816E-44	1.58659E-42	RHSIM_Rhsim01G0244300
Root vs Leaf	RHSIM_Rhsim12G0043600	-3.159630274	4.92535E-44	1.59856E-42	RHSIM_Rhsim12G0043600
Root vs Leaf	novel.1310	-8.851016728	5.21727E-44	1.69094E-42	--
Root vs Leaf	RHSIM_Rhsim04G0101200	-5.890684071	5.53674E-44	1.79197E-42	RHSIM_Rhsim04G0101200
Root vs Leaf	RHSIM_Rhsim10G0028700	-4.698983394	6.58295E-44	2.1276E-42	RHSIM_Rhsim10G0028700
Root vs Leaf	RHSIM_Rhsim05G0093200	-3.315714804	7.90762E-44	2.55217E-42	RHSIM_Rhsim05G0093200
Root vs Leaf	novel.1226	6.33554127	8.42674E-44	2.71592E-42	--
Root vs Leaf	RHSIM_Rhsim11G0105900	3.778109034	9.46849E-44	3.04743E-42	RHSIM_Rhsim11G0105900
Root vs Leaf	RHSIM_Rhsim12G0156100	-3.237615087	9.57159E-44	3.07634E-42	RHSIM_Rhsim12G0156100
Root vs Leaf	RHSIM_Rhsim12G0144700	-3.708424393	9.8503E-44	3.16152E-42	RHSIM_Rhsim12G0144700
Root vs Leaf	RHSIM_Rhsim09G0082600	5.063024057	1.0141E-43	3.25032E-42	RHSIM_Rhsim09G0082600
Root vs Leaf	RHSIM_Rhsim12G0077600	-1.874914716	1.03724E-43	3.31987E-42	RHSIM_Rhsim12G0077600

Comparison	Gene ID	log_2FC	P-value	FDR (padj)	Gene Name
Root vs Leaf	RHSIM_Rhsim01G0280000	-1.76769442	1.04249E-43	3.3321E-42	RHSIM_Rhsim01G0280000
Root vs Leaf	RHSIM_Rhsim10G0028000	-5.140573013	1.04625E-43	3.33948E-42	RHSIM_Rhsim10G0028000
Root vs Leaf	RHSIM_Rhsim08G0034600	-2.491886499	1.18762E-43	3.7855E-42	RHSIM_Rhsim08G0034600
Root vs Leaf	novel.4440	2.11004326	1.48134E-43	4.71522E-42	--
Root vs Leaf	RHSIM_RhsimUnG0086600	-4.70233657	1.64256E-43	5.22121E-42	RHSIM_RhsimUnG0086600
Root vs Leaf	RHSIM_RhsimUnG0053600	4.33163893	1.86509E-43	5.92046E-42	RHSIM_RhsimUnG0053600
Root vs Leaf	RHSIM_RhsimUnG0156300	-4.004338744	2.08481E-43	6.60884E-42	RHSIM_RhsimUnG0156300
Root vs Leaf	RHSIM_Rhsim10G0133100	-3.10074723	2.19385E-43	6.94498E-42	RHSIM_Rhsim10G0133100
Root vs Leaf	RHSIM_Rhsim07G0239800	-2.22435545	2.27403E-43	7.18896E-42	RHSIM_Rhsim07G0239800
Root vs Leaf	RHSIM_Rhsim11G0182100	-2.769598666	2.67695E-43	8.45119E-42	RHSIM_Rhsim11G0182100
Root vs Leaf	RHSIM_Rhsim06G0036100	-3.347459169	2.99019E-43	9.41708E-42	RHSIM_Rhsim06G0036100
Root vs Leaf	RHSIM_RhsimUnG0136700	-3.447690888	2.99103E-43	9.41708E-42	RHSIM_RhsimUnG0136700
Root vs Leaf	novel.5388	-5.71205648	3.01465E-43	9.47853E-42	--
Root vs Leaf	RHSIM_Rhsim01G0247500	-2.854459953	3.20332E-43	1.00581E-41	RHSIM_Rhsim01G0247500
Root vs Leaf	RHSIM_Rhsim13G0047000	-3.022723624	3.40119E-43	1.06649E-41	RHSIM_Rhsim13G0047000
Root vs Leaf	RHSIM_Rhsim07G0053300	-2.873065345	4.11448E-43	1.28841E-41	RHSIM_Rhsim07G0053300
Root vs Leaf	RHSIM_Rhsim13G0013500	4.236048623	4.24973E-43	1.32896E-41	RHSIM_Rhsim13G0013500
Root vs Leaf	RHSIM_RhsimUnG0018400	3.37269426	4.27889E-43	1.33627E-41	RHSIM_RhsimUnG0018400
Root vs Leaf	RHSIM_Rhsim06G0231000	-5.188465609	4.32792E-43	1.34976E-41	RHSIM_Rhsim06G0231000
Root vs Leaf	RHSIM_Rhsim04G0190700	-2.417620497	4.65453E-43	1.44967E-41	RHSIM_Rhsim04G0190700
Root vs Leaf	RHSIM_Rhsim03G0001300	-10.94367089	5.51294E-43	1.71472E-41	RHSIM_Rhsim03G0001300
Root vs Leaf	RHSIM_Rhsim03G0074400	-2.871477283	5.57951E-43	1.73309E-41	RHSIM_Rhsim03G0074400
Root vs Leaf	RHSIM_Rhsim06G0002000	1.686493493	5.6698E-43	1.75878E-41	RHSIM_Rhsim06G0002000
Root vs Leaf	RHSIM_Rhsim02G0044500	4.947706176	6.38255E-43	1.97722E-41	RHSIM_Rhsim02G0044500
Root vs Leaf	RHSIM_Rhsim05G0073400	-5.625230174	6.91722E-43	2.13999E-41	RHSIM_Rhsim05G0073400

Comparison	Gene ID	log_2FC	P-value	FDR (padj)	Gene Name
Root vs Leaf	RHSIM_RhsimUnG0227500	-2.324727453	7.47104E-43	2.30824E-41	RHSIM_RhsimUnG0227500
Root vs Leaf	RHSIM_Rhsim08G0000300	-2.227416395	7.75479E-43	2.39272E-41	RHSIM_Rhsim08G0000300
Root vs Leaf	RHSIM_Rhsim13G0210300	3.871523574	8.08849E-43	2.49235E-41	RHSIM_Rhsim13G0210300
Root vs Leaf	RHSIM_Rhsim04G0057100	-4.209819792	8.48446E-43	2.61089E-41	RHSIM_Rhsim04G0057100
Root vs Leaf	RHSIM_Rhsim04G0024300	-2.066419386	8.72745E-43	2.6821E-41	RHSIM_Rhsim04G0024300
Root vs Leaf	RHSIM_Rhsim12G0138500	-2.475678721	8.90813E-43	2.73399E-41	RHSIM_Rhsim12G0138500
Root vs Leaf	RHSIM_Rhsim09G0128900	-2.151962966	9.35119E-43	2.86617E-41	RHSIM_Rhsim09G0128900
Root vs Leaf	RHSIM_Rhsim02G0132700	-1.620336068	9.84668E-43	3.01405E-41	RHSIM_Rhsim02G0132700
Root vs Leaf	RHSIM_Rhsim02G0143000	7.973540861	1.04777E-42	3.20296E-41	RHSIM_Rhsim02G0143000
Root vs Leaf	RHSIM_Rhsim13G0193900	-3.128545058	1.09571E-42	3.3451E-41	RHSIM_Rhsim13G0193900
Root vs Leaf	RHSIM_Rhsim12G0085000	-7.929388157	1.27502E-42	3.8874E-41	RHSIM_Rhsim12G0085000
Root vs Leaf	RHSIM_Rhsim04G0140900	10.27303364	1.58967E-42	4.84032E-41	RHSIM_Rhsim04G0140900
Root vs Leaf	novel.3191	-3.46573422	1.64272E-42	4.99529E-41	--
Root vs Leaf	RHSIM_Rhsim01G0071400	-2.317615705	1.83955E-42	5.58647E-41	RHSIM_Rhsim01G0071400
Root vs Leaf	RHSIM_Rhsim12G0017000	-3.473782815	2.16103E-42	6.55419E-41	RHSIM_Rhsim12G0017000
Root vs Leaf	RHSIM_Rhsim08G0203900	-3.14370914	2.42787E-42	7.35385E-41	RHSIM_Rhsim08G0203900
Root vs Leaf	RHSIM_Rhsim11G0194300	-3.937613903	2.82023E-42	8.53111E-41	RHSIM_Rhsim11G0194300
Root vs Leaf	RHSIM_Rhsim11G0178300	4.550615006	3.46384E-42	1.0459E-40	RHSIM_Rhsim11G0178300
Root vs Leaf	RHSIM_Rhsim13G0052100	-7.572036365	3.46659E-42	1.0459E-40	RHSIM_Rhsim13G0052100
Root vs Leaf	RHSIM_RhsimUnG0094700	-1.49057571	3.79003E-42	1.14199E-40	RHSIM_RhsimUnG0094700
Root vs Leaf	RHSIM_Rhsim06G0083300	5.367176738	3.86523E-42	1.16314E-40	RHSIM_Rhsim06G0083300
Root vs Leaf	RHSIM_Rhsim01G0027300	-2.121918021	3.97427E-42	1.1944E-40	RHSIM_Rhsim01G0027300
Root vs Leaf	RHSIM_Rhsim11G0173100	5.991165977	4.3647E-42	1.31003E-40	RHSIM_Rhsim11G0173100
Root vs Leaf	novel.2048	6.789504778	4.49865E-42	1.34849E-40	--
Root vs Leaf	RHSIM_Rhsim04G0028000	-3.664183263	4.82786E-42	1.4453E-40	RHSIM_Rhsim04G0028000

Comparison	Gene ID	log_2FC	P-value	FDR (padj)	Gene Name
Root vs Leaf	RHSIM_Rhsim05G0169700	-4.939639126	5.30788E-42	1.58695E-40	RHSIM_Rhsim05G0169700
Root vs Leaf	RHSIM_RhsimUnG0241900	-5.510865042	5.34286E-42	1.59534E-40	RHSIM_RhsimUnG0241900
Root vs Leaf	RHSIM_Rhsim11G0038000	-2.779993787	5.75335E-42	1.7157E-40	RHSIM_Rhsim11G0038000
Root vs Leaf	RHSIM_Rhsim13G0149600	-2.425040553	7.0768E-42	2.10765E-40	RHSIM_Rhsim13G0149600
Root vs Leaf	RHSIM_Rhsim12G0159500	2.277358459	8.57698E-42	2.55116E-40	RHSIM_Rhsim12G0159500
Root vs Leaf	RHSIM_Rhsim05G0088400	-1.463580701	8.72138E-42	2.59078E-40	RHSIM_Rhsim05G0088400
Root vs Leaf	RHSIM_Rhsim06G0229700	-6.549164049	9.01967E-42	2.67595E-40	RHSIM_Rhsim06G0229700
Root vs Leaf	RHSIM_Rhsim02G0214300	-8.073981341	1.28894E-41	3.81912E-40	RHSIM_Rhsim02G0214300
Root vs Leaf	RHSIM_Rhsim02G0231600	6.154511234	1.29685E-41	3.83764E-40	RHSIM_Rhsim02G0231600
Root vs Leaf	RHSIM_Rhsim04G0155800	-2.568577238	1.34803E-41	3.98401E-40	RHSIM_Rhsim04G0155800
Root vs Leaf	RHSIM_Rhsim03G0053000	2.724615254	1.51458E-41	4.47051E-40	RHSIM_Rhsim03G0053000
Root vs Leaf	RHSIM_Rhsim03G0097900	-3.035769852	1.64151E-41	4.83899E-40	RHSIM_Rhsim03G0097900
Root vs Leaf	novel.3103	6.471066048	1.9666E-41	5.78996E-40	--
Root vs Leaf	RHSIM_Rhsim09G0021800	-1.58984644	2.00308E-41	5.88988E-40	RHSIM_Rhsim09G0021800
Root vs Leaf	RHSIM_Rhsim05G0170400	-2.691882293	2.31997E-41	6.81298E-40	RHSIM_Rhsim05G0170400
Root vs Leaf	RHSIM_Rhsim01G0196900	-2.719128146	2.34057E-41	6.86478E-40	RHSIM_Rhsim01G0196900
Root vs Leaf	RHSIM_Rhsim10G0072400	-4.191200672	3.24582E-41	9.50778E-40	RHSIM_Rhsim10G0072400
Root vs Leaf	RHSIM_Rhsim04G0121700	-2.413019177	3.74242E-41	1.09486E-39	RHSIM_Rhsim04G0121700
Root vs Leaf	RHSIM_Rhsim02G0049200	-3.050105426	3.76758E-41	1.10083E-39	RHSIM_Rhsim02G0049200
Root vs Leaf	RHSIM_Rhsim02G0063500	-7.23634981	4.82756E-41	1.40876E-39	RHSIM_Rhsim02G0063500
Root vs Leaf	RHSIM_Rhsim02G0188300	-4.441645185	5.76455E-41	1.68007E-39	RHSIM_Rhsim02G0188300
Root vs Leaf	RHSIM_Rhsim04G0130700	-1.724400372	6.28339E-41	1.82898E-39	RHSIM_Rhsim04G0130700
Root vs Leaf	RHSIM_Rhsim03G0119100	6.30737005	6.81487E-41	1.98119E-39	RHSIM_Rhsim03G0119100
Root vs Leaf	RHSIM_Rhsim10G0003000	-2.099402176	1.03528E-40	3.00595E-39	RHSIM_Rhsim10G0003000
Root vs Leaf	RHSIM_Rhsim08G0196800	-3.858583048	1.0481E-40	3.03792E-39	RHSIM_Rhsim08G0196800

Comparison	Gene ID	log_2FC	P-value	FDR (padj)	Gene Name
Root vs Leaf	novel.5711	-5.329605286	1.04892E-40	3.03792E-39	--
Root vs Leaf	RHSIM_Rhsim08G0167600	5.387788817	1.15006E-40	3.3267E-39	RHSIM_Rhsim08G0167600
Root vs Leaf	RHSIM_Rhsim12G0044800	-8.069682395	1.57539E-40	4.55132E-39	RHSIM_Rhsim12G0044800
Root vs Leaf	RHSIM_Rhsim08G0080800	-2.480768919	1.6182E-40	4.66498E-39	RHSIM_Rhsim08G0080800
Root vs Leaf	RHSIM_Rhsim11G0194000	-2.877663995	1.61876E-40	4.66498E-39	RHSIM_Rhsim11G0194000
Root vs Leaf	RHSIM_Rhsim10G0007000	2.940406364	1.79334E-40	5.16164E-39	RHSIM_Rhsim10G0007000
Root vs Leaf	RHSIM_Rhsim13G0046000	-2.584412377	1.79668E-40	5.16485E-39	RHSIM_Rhsim13G0046000
Root vs Leaf	RHSIM_Rhsim08G0043600	-2.763944268	1.88708E-40	5.41799E-39	RHSIM_Rhsim08G0043600
Root vs Leaf	RHSIM_Rhsim13G0026100	-2.968845803	2.02344E-40	5.80228E-39	RHSIM_Rhsim13G0026100
Root vs Leaf	RHSIM_Rhsim09G0196800	-7.760255981	2.10874E-40	6.0394E-39	RHSIM_Rhsim09G0196800
Root vs Leaf	RHSIM_Rhsim06G0109000	-6.88953294	2.31853E-40	6.63204E-39	RHSIM_Rhsim06G0109000
Root vs Leaf	novel.3214	-1.806287126	2.50342E-40	7.15206E-39	--
Root vs Leaf	RHSIM_Rhsim12G0136700	-1.769407281	2.54003E-40	7.2477E-39	RHSIM_Rhsim12G0136700
Root vs Leaf	novel.3884	-3.011692133	2.68245E-40	7.64466E-39	--
Root vs Leaf	RHSIM_Rhsim03G0005600	-1.855289049	2.74376E-40	7.80975E-39	RHSIM_Rhsim03G0005600
Root vs Leaf	RHSIM_Rhsim03G0094400	-7.111509644	3.01392E-40	8.56821E-39	RHSIM_Rhsim03G0094400
Root vs Leaf	RHSIM_Rhsim12G0070400	-5.850948261	4.06019E-40	1.15284E-38	RHSIM_Rhsim12G0070400
Root vs Leaf	RHSIM_Rhsim08G0118900	-2.775202106	4.47699E-40	1.26963E-38	RHSIM_Rhsim08G0118900
Root vs Leaf	RHSIM_Rhsim01G0290800	-5.043665534	4.89195E-40	1.38561E-38	RHSIM_Rhsim01G0290800
Root vs Leaf	RHSIM_Rhsim06G0040300	9.866209917	4.9044E-40	1.38744E-38	RHSIM_Rhsim06G0040300
Root vs Leaf	RHSIM_Rhsim03G0027400	-2.66160351	5.38572E-40	1.52175E-38	RHSIM_Rhsim03G0027400
Root vs Leaf	RHSIM_Rhsim09G0007900	-4.015896642	5.72816E-40	1.61653E-38	RHSIM_Rhsim09G0007900
Root vs Leaf	RHSIM_Rhsim07G0067500	5.322862507	7.62559E-40	2.14938E-38	RHSIM_Rhsim07G0067500
Root vs Leaf	RHSIM_Rhsim11G0060100	-2.700904473	7.79617E-40	2.19478E-38	RHSIM_Rhsim11G0060100
Root vs Leaf	RHSIM_Rhsim08G0217500	4.730477941	7.96503E-40	2.2396E-38	RHSIM_Rhsim08G0217500

Comparison	Gene ID	log_2FC	P-value	FDR (padj)	Gene Name
Root vs Leaf	RHSIM_Rhsim06G0001500	-3.640017693	9.6423E-40	2.70792E-38	RHSIM_Rhsim06G0001500
Root vs Leaf	RHSIM_Rhsim03G0171900	-1.94984137	9.74382E-40	2.73311E-38	RHSIM_Rhsim03G0171900
Root vs Leaf	novel.3979	-3.747070266	9.88553E-40	2.7695E-38	--
Root vs Leaf	RHSIM_Rhsim07G0202000	1.661021698	1.51195E-39	4.23073E-38	RHSIM_Rhsim07G0202000
Root vs Leaf	RHSIM_Rhsim01G0258700	-2.244529692	1.66828E-39	4.66253E-38	RHSIM_Rhsim01G0258700
Root vs Leaf	RHSIM_Rhsim04G0092500	-2.053681586	1.68584E-39	4.70592E-38	RHSIM_Rhsim04G0092500
Root vs Leaf	RHSIM_Rhsim12G0127500	-2.99983202	1.77223E-39	4.94109E-38	RHSIM_Rhsim12G0127500
Root vs Leaf	RHSIM_Rhsim02G0187100	-2.81635348	2.12853E-39	5.92735E-38	RHSIM_Rhsim02G0187100
Root vs Leaf	RHSIM_Rhsim13G0064200	8.269077335	2.14238E-39	5.95875E-38	RHSIM_Rhsim13G0064200
Root vs Leaf	RHSIM_Rhsim03G0169800	3.462462864	2.41828E-39	6.71805E-38	RHSIM_Rhsim03G0169800
Root vs Leaf	RHSIM_Rhsim10G0204000	-2.813735031	2.4579E-39	6.81993E-38	RHSIM_Rhsim10G0204000
Root vs Leaf	RHSIM_Rhsim11G0014500	-3.278565742	2.48113E-39	6.87613E-38	RHSIM_Rhsim11G0014500
Root vs Leaf	RHSIM_RhsimUnG0118200	-2.883070059	2.65496E-39	7.34909E-38	RHSIM_RhsimUnG0118200
Root vs Leaf	RHSIM_Rhsim13G0016900	5.629614629	2.90699E-39	8.03711E-38	RHSIM_Rhsim13G0016900
Root vs Leaf	RHSIM_Rhsim02G0223600	-1.936659218	3.48439E-39	9.62198E-38	RHSIM_Rhsim02G0223600
Root vs Leaf	RHSIM_Rhsim05G0106800	-1.545109409	4.06029E-39	1.11989E-37	RHSIM_Rhsim05G0106800
Root vs Leaf	novel.3171	6.722129413	4.15863E-39	1.14565E-37	--
Root vs Leaf	RHSIM_Rhsim13G0082400	-1.91735598	4.32194E-39	1.18923E-37	RHSIM_Rhsim13G0082400
Root vs Leaf	RHSIM_Rhsim01G0283700	-2.752727755	4.58589E-39	1.26036E-37	RHSIM_Rhsim01G0283700
Root vs Leaf	RHSIM_Rhsim04G0006200	-8.541894013	5.02515E-39	1.37944E-37	RHSIM_Rhsim04G0006200
Root vs Leaf	RHSIM_Rhsim11G0029300	-3.543273344	5.07744E-39	1.39214E-37	RHSIM_Rhsim11G0029300
Root vs Leaf	RHSIM_Rhsim08G0227200	4.556864384	5.27041E-39	1.44334E-37	RHSIM_Rhsim08G0227200
Root vs Leaf	RHSIM_Rhsim04G0166800	-2.52762957	5.52226E-39	1.51053E-37	RHSIM_Rhsim04G0166800
Root vs Leaf	novel.134	6.504445698	6.12873E-39	1.67444E-37	--
Root vs Leaf	RHSIM_Rhsim05G0192200	-7.932037374	7.08449E-39	1.93328E-37	RHSIM_Rhsim05G0192200

Comparison	Gene ID	log_2FC	P-value	FDR (padj)	Gene Name
Root vs Leaf	RHSIM_Rhsim11G0163600	-6.962355082	7.19987E-39	1.96245E-37	RHSIM_Rhsim11G0163600
Root vs Leaf	RHSIM_Rhsim09G0146900	-4.208845752	7.5707E-39	2.0611E-37	RHSIM_Rhsim09G0146900
Root vs Leaf	RHSIM_Rhsim10G0043300	-9.193223298	8.16855E-39	2.22125E-37	RHSIM_Rhsim10G0043300
Root vs Leaf	RHSIM_Rhsim01G0176500	-2.761711744	1.00456E-38	2.72847E-37	RHSIM_Rhsim01G0176500
Root vs Leaf	RHSIM_Rhsim12G0166000	-5.367195857	1.16874E-38	3.17066E-37	RHSIM_Rhsim12G0166000
Root vs Leaf	RHSIM_Rhsim06G0212900	-1.647074317	1.2089E-38	3.27579E-37	RHSIM_Rhsim06G0212900
Root vs Leaf	novel.1987	5.330586159	1.21736E-38	3.29485E-37	--
Root vs Leaf	RHSIM_Rhsim08G0173200	-1.654739427	1.35005E-38	3.64971E-37	RHSIM_Rhsim08G0173200
Root vs Leaf	RHSIM_Rhsim07G0229500	-5.932876928	1.4036E-38	3.79006E-37	RHSIM_Rhsim07G0229500
Root vs Leaf	RHSIM_Rhsim06G0244400	-2.632663514	1.40699E-38	3.79478E-37	RHSIM_Rhsim06G0244400
Root vs Leaf	RHSIM_Rhsim08G0118800	-2.930611282	1.50398E-38	4.05163E-37	RHSIM_Rhsim08G0118800
Root vs Leaf	RHSIM_Rhsim03G0050100	-2.085298739	1.73831E-38	4.67747E-37	RHSIM_Rhsim03G0050100
Root vs Leaf	RHSIM_Rhsim13G0189800	4.872359183	1.76123E-38	4.73363E-37	RHSIM_Rhsim13G0189800
Root vs Leaf	RHSIM_Rhsim10G0118200	2.329729282	1.78764E-38	4.79905E-37	RHSIM_Rhsim10G0118200
Root vs Leaf	RHSIM_Rhsim13G0062200	16.19443736	1.96509E-38	5.26931E-37	RHSIM_Rhsim13G0062200
Root vs Leaf	RHSIM_Rhsim06G0011300	-2.407815787	2.07822E-38	5.56621E-37	RHSIM_Rhsim06G0011300
Root vs Leaf	RHSIM_Rhsim07G0175500	-4.737608242	2.13673E-38	5.71632E-37	RHSIM_Rhsim07G0175500
Root vs Leaf	RHSIM_Rhsim03G0010500	-6.039793574	2.29143E-38	6.1231E-37	RHSIM_Rhsim03G0010500
Root vs Leaf	RHSIM_Rhsim05G0136600	-6.192810576	2.37517E-38	6.33953E-37	RHSIM_Rhsim05G0136600
Root vs Leaf	RHSIM_Rhsim09G0137300	6.700647289	3.47826E-38	9.27308E-37	RHSIM_Rhsim09G0137300
Root vs Leaf	RHSIM_Rhsim02G0119200	-5.406412533	3.99628E-38	1.06419E-36	RHSIM_Rhsim02G0119200
Root vs Leaf	RHSIM_Rhsim02G0047900	7.773710983	4.01893E-38	1.06899E-36	RHSIM_Rhsim02G0047900
Root vs Leaf	RHSIM_Rhsim05G0068100	-8.315628208	4.09919E-38	1.08909E-36	RHSIM_Rhsim05G0068100
Root vs Leaf	RHSIM_RhsimUnG0153300	-2.599653918	5.77022E-38	1.53129E-36	RHSIM_RhsimUnG0153300
Root vs Leaf	RHSIM_Rhsim07G0175700	-2.711390639	6.44593E-38	1.70865E-36	RHSIM_Rhsim07G0175700

Comparison	Gene ID	log_2FC	P-value	FDR (padj)	Gene Name
Root vs Leaf	RHSIM_Rhsim08G0040800	4.188326959	6.79961E-38	1.7988E-36	RHSIM_Rhsim08G0040800
Root vs Leaf	RHSIM_Rhsim03G0102000	-2.864945438	6.80156E-38	1.7988E-36	RHSIM_Rhsim03G0102000
Root vs Leaf	RHSIM_Rhsim13G0152000	1.631417339	7.1468E-38	1.88795E-36	RHSIM_Rhsim13G0152000
Root vs Leaf	RHSIM_RhsimUnG0155900	-3.287562453	7.323E-38	1.93229E-36	RHSIM_RhsimUnG0155900
Root vs Leaf	RHSIM_Rhsim06G0192600	-8.822379291	7.7422E-38	2.04057E-36	RHSIM_Rhsim06G0192600
Root vs Leaf	RHSIM_Rhsim07G0048700	7.023641604	7.83908E-38	2.06376E-36	RHSIM_Rhsim07G0048700
Root vs Leaf	RHSIM_RhsimUnG0139600	-3.26685583	7.95317E-38	2.09141E-36	RHSIM_RhsimUnG0139600
Root vs Leaf	RHSIM_Rhsim11G0087200	-3.09728729	8.20145E-38	2.15426E-36	RHSIM_Rhsim11G0087200
Root vs Leaf	RHSIM_Rhsim13G0171300	-4.71028681	8.40113E-38	2.2042E-36	RHSIM_Rhsim13G0171300
Root vs Leaf	RHSIM_Rhsim12G0036900	-5.271468622	8.62677E-38	2.26084E-36	RHSIM_Rhsim12G0036900
Root vs Leaf	RHSIM_Rhsim12G0142000	7.06871592	8.69141E-38	2.2752E-36	RHSIM_Rhsim12G0142000
Root vs Leaf	RHSIM_Rhsim02G0251400	-4.087852463	1.01278E-37	2.64821E-36	RHSIM_Rhsim02G0251400
Root vs Leaf	RHSIM_Rhsim09G0141500	-2.248818098	1.09138E-37	2.85052E-36	RHSIM_Rhsim09G0141500
Root vs Leaf	RHSIM_Rhsim06G0178000	-3.009714917	1.09279E-37	2.85098E-36	RHSIM_Rhsim06G0178000
Root vs Leaf	RHSIM_Rhsim13G0008600	-4.107381271	1.17005E-37	3.04912E-36	RHSIM_Rhsim13G0008600
Root vs Leaf	RHSIM_Rhsim13G0232300	-3.203736302	1.27238E-37	3.31206E-36	RHSIM_Rhsim13G0232300
Root vs Leaf	RHSIM_Rhsim06G0172700	3.649671993	1.36205E-37	3.54149E-36	RHSIM_Rhsim06G0172700
Root vs Leaf	RHSIM_Rhsim05G0161600	4.576092019	1.37847E-37	3.58014E-36	RHSIM_Rhsim05G0161600
Root vs Leaf	RHSIM_Rhsim02G0220400	-4.766131105	1.44116E-37	3.73878E-36	RHSIM_Rhsim02G0220400
Root vs Leaf	RHSIM_Rhsim07G0059100	2.689178737	1.49722E-37	3.87987E-36	RHSIM_Rhsim07G0059100
Root vs Leaf	RHSIM_Rhsim12G0158600	-2.191382131	1.59168E-37	4.12003E-36	RHSIM_Rhsim12G0158600
Root vs Leaf	RHSIM_Rhsim01G0012100	-2.198829282	1.63414E-37	4.2252E-36	RHSIM_Rhsim01G0012100
Root vs Leaf	novel.135	-4.054066228	2.01056E-37	5.19267E-36	--
Root vs Leaf	RHSIM_Rhsim04G0091400	-1.437189887	2.01641E-37	5.20198E-36	RHSIM_Rhsim04G0091400
Root vs Leaf	RHSIM_Rhsim12G0087800	1.451739615	2.20436E-37	5.68053E-36	RHSIM_Rhsim12G0087800

Comparison	Gene ID	log_2FC	P-value	FDR (padj)	Gene Name
Root vs Leaf	RHSIM_Rhsim09G0192400	-6.646866877	2.35301E-37	6.05684E-36	RHSIM_Rhsim09G0192400
Root vs Leaf	novel.5910	-6.512236218	2.46987E-37	6.35059E-36	--
Root vs Leaf	RHSIM_Rhsim05G0203100	-2.145428344	2.48588E-37	6.38465E-36	RHSIM_Rhsim05G0203100
Root vs Leaf	RHSIM_Rhsim03G0241500	-5.301750896	2.81987E-37	7.23444E-36	RHSIM_Rhsim03G0241500
Root vs Leaf	novel.1873	-5.737781885	2.91119E-37	7.46045E-36	--
Root vs Leaf	RHSIM_Rhsim01G0009700	-2.748474507	2.9255E-37	7.48883E-36	RHSIM_Rhsim01G0009700
Root vs Leaf	RHSIM_Rhsim10G0047200	-5.508349742	3.02547E-37	7.73618E-36	RHSIM_Rhsim10G0047200
Root vs Leaf	RHSIM_Rhsim11G0038100	-2.739175336	3.13709E-37	8.01274E-36	RHSIM_Rhsim11G0038100
Root vs Leaf	RHSIM_Rhsim01G0129200	-8.485067604	3.14681E-37	8.0287E-36	RHSIM_Rhsim01G0129200
Root vs Leaf	RHSIM_Rhsim03G0236600	-2.951960205	3.38651E-37	8.63076E-36	RHSIM_Rhsim03G0236600
Root vs Leaf	RHSIM_Rhsim13G0035600	-1.748951203	4.04043E-37	1.0286E-35	RHSIM_Rhsim13G0035600
Root vs Leaf	RHSIM_Rhsim02G0041900	3.289460039	4.14179E-37	1.05324E-35	RHSIM_Rhsim02G0041900
Root vs Leaf	RHSIM_Rhsim02G0213900	3.737727531	4.14973E-37	1.0541E-35	RHSIM_Rhsim02G0213900
Root vs Leaf	RHSIM_Rhsim09G0111600	4.417685452	4.62253E-37	1.17292E-35	RHSIM_Rhsim09G0111600
Root vs Leaf	RHSIM_Rhsim04G0216700	3.39557071	4.74439E-37	1.20252E-35	RHSIM_Rhsim04G0216700
Root vs Leaf	RHSIM_Rhsim07G0005600	-3.179130344	4.92758E-37	1.24758E-35	RHSIM_Rhsim07G0005600
Root vs Leaf	RHSIM_Rhsim12G0106200	2.532130149	5.12667E-37	1.29657E-35	RHSIM_Rhsim12G0106200
Root vs Leaf	RHSIM_Rhsim06G0199700	-2.875726002	5.55104E-37	1.40236E-35	RHSIM_Rhsim06G0199700
Root vs Leaf	RHSIM_Rhsim10G0072300	-3.618882125	5.98931E-37	1.51144E-35	RHSIM_Rhsim10G0072300
Root vs Leaf	RHSIM_Rhsim05G0064300	-2.62174964	6.28863E-37	1.58524E-35	RHSIM_Rhsim05G0064300
Root vs Leaf	RHSIM_Rhsim01G0286700	1.841656537	7.57809E-37	1.90821E-35	RHSIM_Rhsim01G0286700
Root vs Leaf	RHSIM_Rhsim06G0184100	-2.002697892	7.94696E-37	1.99892E-35	RHSIM_Rhsim06G0184100
Root vs Leaf	RHSIM_Rhsim06G0083500	7.778348061	8.18601E-37	2.05681E-35	RHSIM_Rhsim06G0083500
Root vs Leaf	RHSIM_Rhsim05G0104400	-2.138582443	1.00183E-36	2.51448E-35	RHSIM_Rhsim05G0104400
Root vs Leaf	RHSIM_Rhsim10G0177000	-2.427152051	1.14156E-36	2.86206E-35	RHSIM_Rhsim10G0177000

Comparison	Gene ID	log_2FC	P-value	FDR (padj)	Gene Name
Root vs Leaf	RHSIM_Rhsim12G0131600	-5.27459784	1.4171E-36	3.54903E-35	RHSIM_Rhsim12G0131600
Root vs Leaf	RHSIM_Rhsim01G0108800	-4.036289374	1.54254E-36	3.85903E-35	RHSIM_Rhsim01G0108800
Root vs Leaf	RHSIM_Rhsim08G0026800	3.087199364	1.82351E-36	4.55699E-35	RHSIM_Rhsim08G0026800
Root vs Leaf	RHSIM_Rhsim10G0165400	-11.09185263	1.83752E-36	4.58707E-35	RHSIM_Rhsim10G0165400
Root vs Leaf	RHSIM_Rhsim01G0233700	-5.173819755	1.95436E-36	4.87348E-35	RHSIM_Rhsim01G0233700
Root vs Leaf	RHSIM_Rhsim09G0101000	3.478100609	1.96355E-36	4.89112E-35	RHSIM_Rhsim09G0101000
Root vs Leaf	RHSIM_Rhsim04G0185800	5.764012973	2.3453E-36	5.83575E-35	RHSIM_Rhsim04G0185800
Root vs Leaf	RHSIM_Rhsim08G0188700	-1.582604818	2.61727E-36	6.50549E-35	RHSIM_Rhsim08G0188700
Root vs Leaf	RHSIM_Rhsim04G0126900	-2.425736605	2.99419E-36	7.43438E-35	RHSIM_Rhsim04G0126900
Root vs Leaf	RHSIM_Rhsim10G0148800	-5.189294721	3.00094E-36	7.44316E-35	RHSIM_Rhsim10G0148800
Root vs Leaf	RHSIM_Rhsim03G0002000	-2.633683394	3.03231E-36	7.51292E-35	RHSIM_Rhsim03G0002000
Root vs Leaf	RHSIM_Rhsim13G0210400	3.220917446	3.22806E-36	7.98937E-35	RHSIM_Rhsim13G0210400
Root vs Leaf	RHSIM_Rhsim11G0145600	-3.722630536	3.29562E-36	8.14786E-35	RHSIM_Rhsim11G0145600
Root vs Leaf	RHSIM_Rhsim12G0007700	6.620523623	3.33607E-36	8.23905E-35	RHSIM_Rhsim12G0007700
Root vs Leaf	RHSIM_Rhsim02G0018500	-3.469698017	4.00237E-36	9.87408E-35	RHSIM_Rhsim02G0018500
Root vs Leaf	novel.579	-3.491216977	4.43742E-36	1.09357E-34	--
Root vs Leaf	novel.2457	-5.187972271	4.45182E-36	1.09595E-34	--
Root vs Leaf	RHSIM_Rhsim03G0201200	-2.341436422	4.54956E-36	1.11882E-34	RHSIM_Rhsim03G0201200
Root vs Leaf	RHSIM_Rhsim10G0053700	-5.513703313	5.01667E-36	1.23239E-34	RHSIM_Rhsim10G0053700
Root vs Leaf	RHSIM_Rhsim02G0173600	-1.959319041	5.22986E-36	1.28339E-34	RHSIM_Rhsim02G0173600
Root vs Leaf	novel.3762	-2.490079647	5.78708E-36	1.41863E-34	--
Root vs Leaf	RHSIM_Rhsim04G0073000	3.511218194	6.25109E-36	1.53076E-34	RHSIM_Rhsim04G0073000
Root vs Leaf	RHSIM_Rhsim12G0209000	-2.234531415	6.59394E-36	1.61301E-34	RHSIM_Rhsim12G0209000
Root vs Leaf	RHSIM_Rhsim13G0216400	-2.208095356	7.19184E-36	1.75741E-34	RHSIM_Rhsim13G0216400
Root vs Leaf	RHSIM_Rhsim06G0207500	7.946692897	7.56349E-36	1.84627E-34	RHSIM_Rhsim06G0207500

Comparison	Gene ID	log$_2$FC	P-value	FDR (padj)	Gene Name
Root vs Leaf	RHSIM_Rhsim03G0097000	2.465619031	7.95027E-36	1.93864E-34	RHSIM_Rhsim03G0097000
Root vs Leaf	RHSIM_Rhsim01G0273700	2.847670664	8.00321E-36	1.9495E-34	RHSIM_Rhsim01G0273700
Root vs Leaf	RHSIM_Rhsim07G0119300	-2.297838437	8.19352E-36	1.99376E-34	RHSIM_Rhsim07G0119300
Root vs Leaf	RHSIM_Rhsim03G0028600	2.566474488	8.21393E-36	1.99662E-34	RHSIM_Rhsim03G0028600
Root vs Leaf	RHSIM_Rhsim02G0019900	-1.633782861	8.50885E-36	2.06614E-34	RHSIM_Rhsim02G0019900
Root vs Leaf	RHSIM_Rhsim03G0196300	-7.650898549	9.64022E-36	2.33841E-34	RHSIM_Rhsim03G0196300
Root vs Leaf	RHSIM_Rhsim01G0216100	3.237770031	1.01053E-35	2.44865E-34	RHSIM_Rhsim01G0216100
Root vs Leaf	novel.2946	5.962824775	1.0254E-35	2.4821E-34	--
Root vs Leaf	RHSIM_Rhsim13G0068500	-4.409169242	1.06981E-35	2.58687E-34	RHSIM_Rhsim13G0068500
Root vs Leaf	novel.3082	5.408235049	1.07798E-35	2.60393E-34	--
Root vs Leaf	RHSIM_Rhsim03G0229800	-4.367733981	1.17276E-35	2.82991E-34	RHSIM_Rhsim03G0229800
Root vs Leaf	RHSIM_Rhsim07G0184400	2.622506966	1.20348E-35	2.90102E-34	RHSIM_Rhsim07G0184400
Root vs Leaf	RHSIM_RhsimUnG0054600	-2.102955	1.39755E-35	3.36532E-34	RHSIM_RhsimUnG0054600
Root vs Leaf	RHSIM_Rhsim07G0011900	4.48143704	1.42667E-35	3.43186E-34	RHSIM_Rhsim07G0011900
Root vs Leaf	RHSIM_Rhsim13G0176900	-2.368036176	1.46916E-35	3.53041E-34	RHSIM_Rhsim13G0176900
Root vs Leaf	RHSIM_Rhsim13G0203000	-4.375009767	1.49204E-35	3.58168E-34	RHSIM_Rhsim13G0203000
Root vs Leaf	RHSIM_Rhsim08G0198800	-1.956031784	1.98123E-35	4.75104E-34	RHSIM_Rhsim08G0198800
Root vs Leaf	RHSIM_Rhsim03G0122000	-1.289792183	2.26007E-35	5.41412E-34	RHSIM_Rhsim03G0122000
Root vs Leaf	RHSIM_Rhsim04G0219900	5.890541909	2.36637E-35	5.6629E-34	RHSIM_Rhsim04G0219900
Root vs Leaf	RHSIM_Rhsim12G0087000	-4.814960381	2.514E-35	6.00996E-34	RHSIM_Rhsim12G0087000
Root vs Leaf	RHSIM_Rhsim07G0117800	1.75573771	2.64832E-35	6.32454E-34	RHSIM_Rhsim07G0117800
Root vs Leaf	RHSIM_Rhsim10G0067800	-5.585605674	2.67123E-35	6.37268E-34	RHSIM_Rhsim10G0067800
Root vs Leaf	novel.6150	-1.719136172	2.96932E-35	7.07652E-34	--
Root vs Leaf	RHSIM_Rhsim11G0055800	-6.192285951	3.2525E-35	7.74344E-34	RHSIM_Rhsim11G0055800
Root vs Leaf	RHSIM_Rhsim12G0018700	5.030930207	3.32061E-35	7.89746E-34	RHSIM_Rhsim12G0018700

Comparison	Gene ID	log_2FC	P-value	FDR (padj)	Gene Name
Root vs Leaf	RHSIM_Rhsim11G0184900	-2.912998888	3.59275E-35	8.53591E-34	RHSIM_Rhsim11G0184900
Root vs Leaf	RHSIM_Rhsim07G0123700	-1.809978042	3.69526E-35	8.77046E-34	RHSIM_Rhsim07G0123700
Root vs Leaf	RHSIM_Rhsim01G0293800	-2.775248517	4.21079E-35	9.9838E-34	RHSIM_Rhsim01G0293800
Root vs Leaf	RHSIM_Rhsim05G0069500	-3.449324615	4.55891E-35	1.07981E-33	RHSIM_Rhsim05G0069500
Root vs Leaf	RHSIM_Rhsim10G0068500	-3.529917049	4.56429E-35	1.07998E-33	RHSIM_Rhsim10G0068500
Root vs Leaf	RHSIM_RhsimUnG0194300	-1.804798439	4.67992E-35	1.10621E-33	RHSIM_RhsimUnG0194300
Root vs Leaf	RHSIM_Rhsim13G0077300	-2.140659561	5.27264E-35	1.24504E-33	RHSIM_Rhsim13G0077300
Root vs Leaf	RHSIM_RhsimUnG0060900	-2.647628333	5.27933E-35	1.24535E-33	RHSIM_RhsimUnG0060900
Root vs Leaf	RHSIM_Rhsim10G0117200	-3.515682358	5.31976E-35	1.25361E-33	RHSIM_Rhsim10G0117200
Root vs Leaf	RHSIM_Rhsim08G0070300	-3.109079987	5.43623E-35	1.27975E-33	RHSIM_Rhsim08G0070300
Root vs Leaf	RHSIM_Rhsim01G0060800	2.330366155	5.4593E-35	1.28388E-33	RHSIM_Rhsim01G0060800
Root vs Leaf	RHSIM_Rhsim08G0102300	-8.125679182	5.48259E-35	1.28805E-33	RHSIM_Rhsim08G0102300
Root vs Leaf	RHSIM_Rhsim08G0101500	-7.269952565	5.52834E-35	1.29748E-33	RHSIM_Rhsim08G0101500
Root vs Leaf	RHSIM_Rhsim11G0115200	3.301267055	5.61743E-35	1.31705E-33	RHSIM_Rhsim11G0115200
Root vs Leaf	RHSIM_Rhsim06G0121500	2.244048067	5.68006E-35	1.33039E-33	RHSIM_Rhsim06G0121500
Root vs Leaf	RHSIM_Rhsim10G0038600	2.58558918	5.7146E-35	1.33712E-33	RHSIM_Rhsim10G0038600
Root vs Leaf	RHSIM_Rhsim08G0126000	4.785313376	5.80049E-35	1.35585E-33	RHSIM_Rhsim08G0126000
Root vs Leaf	RHSIM_Rhsim12G0149400	-3.654739957	6.37788E-35	1.48931E-33	RHSIM_Rhsim12G0149400
Root vs Leaf	RHSIM_RhsimUnG0106100	-4.378015437	7.0879E-35	1.65344E-33	RHSIM_RhsimUnG0106100
Root vs Leaf	RHSIM_RhsimUnG0069500	-2.791673877	7.10534E-35	1.65584E-33	RHSIM_RhsimUnG0069500
Root vs Leaf	RHSIM_Rhsim12G0072500	-2.594994737	7.15045E-35	1.66389E-33	RHSIM_Rhsim12G0072500
Root vs Leaf	RHSIM_Rhsim03G0066800	-2.453365546	7.15427E-35	1.66389E-33	RHSIM_Rhsim03G0066800
Root vs Leaf	RHSIM_Rhsim05G0148200	-1.421857036	7.46241E-35	1.73381E-33	RHSIM_Rhsim05G0148200
Root vs Leaf	RHSIM_Rhsim01G0051700	-5.235548442	8.60092E-35	1.99633E-33	RHSIM_Rhsim01G0051700
Root vs Leaf	RHSIM_Rhsim11G0172000	-3.99165184	8.63874E-35	2.0031E-33	RHSIM_Rhsim11G0172000

Comparison	Gene ID	log_2FC	P-value	FDR (padj)	Gene Name
Root vs Leaf	RHSIM_Rhsim10G0003100	1.94799453	8.68573E-35	2.01198E-33	RHSIM_Rhsim10G0003100
Root vs Leaf	RHSIM_Rhsim13G0148700	-2.487460749	8.81614E-35	2.04014E-33	RHSIM_Rhsim13G0148700
Root vs Leaf	RHSIM_Rhsim04G0057400	3.609430713	9.0767E-35	2.09834E-33	RHSIM_Rhsim04G0057400
Root vs Leaf	RHSIM_Rhsim07G0235700	-7.617118565	9.23727E-35	2.13333E-33	RHSIM_Rhsim07G0235700
Root vs Leaf	RHSIM_Rhsim07G0202900	-2.614691075	9.63266E-35	2.22243E-33	RHSIM_Rhsim07G0202900
Root vs Leaf	RHSIM_RhsimUnG0242100	3.794963489	9.66131E-35	2.22682E-33	RHSIM_RhsimUnG0242100
Root vs Leaf	RHSIM_Rhsim04G0162900	-10.07725582	1.05379E-34	2.42644E-33	RHSIM_Rhsim04G0162900
Root vs Leaf	RHSIM_Rhsim06G0149300	-1.644078332	1.11406E-34	2.56267E-33	RHSIM_Rhsim06G0149300
Root vs Leaf	RHSIM_Rhsim03G0251700	1.683369838	1.13885E-34	2.6171E-33	RHSIM_Rhsim03G0251700
Root vs Leaf	RHSIM_Rhsim12G0123300	-2.656027481	1.15625E-34	2.65444E-33	RHSIM_Rhsim12G0123300
Root vs Leaf	RHSIM_Rhsim06G0054400	-2.187472067	1.23221E-34	2.82603E-33	RHSIM_Rhsim06G0054400
Root vs Leaf	RHSIM_Rhsim02G0217900	-2.497575808	1.24656E-34	2.85611E-33	RHSIM_Rhsim02G0217900
Root vs Leaf	RHSIM_Rhsim09G0002100	-4.897738426	1.27446E-34	2.91713E-33	RHSIM_Rhsim09G0002100
Root vs Leaf	RHSIM_Rhsim09G0044600	-2.837426407	1.36389E-34	3.11875E-33	RHSIM_Rhsim09G0044600
Root vs Leaf	RHSIM_Rhsim12G0201100	-2.406013959	1.37112E-34	3.12972E-33	RHSIM_Rhsim12G0201100
Root vs Leaf	RHSIM_Rhsim05G0199400	-2.970997229	1.37139E-34	3.12972E-33	RHSIM_Rhsim05G0199400
Root vs Leaf	RHSIM_Rhsim03G0027000	2.051200622	1.37292E-34	3.13012E-33	RHSIM_Rhsim03G0027000
Root vs Leaf	RHSIM_Rhsim06G0063800	-2.479971558	1.45577E-34	3.31574E-33	RHSIM_Rhsim06G0063800
Root vs Leaf	RHSIM_Rhsim13G0217800	-2.219469657	1.58849E-34	3.61448E-33	RHSIM_Rhsim13G0217800
Root vs Leaf	RHSIM_Rhsim03G0135900	-2.789738669	1.63059E-34	3.70664E-33	RHSIM_Rhsim03G0135900
Root vs Leaf	RHSIM_RhsimUnG0119200	-1.820110589	1.63436E-34	3.71155E-33	RHSIM_RhsimUnG0119200
Root vs Leaf	RHSIM_Rhsim03G0050300	-2.339733743	1.87626E-34	4.25673E-33	RHSIM_Rhsim03G0050300
Root vs Leaf	novel.2840	-4.74020183	2.00454E-34	4.5433E-33	--
Root vs Leaf	RHSIM_Rhsim08G0090300	-2.661330749	2.0078E-34	4.54622E-33	RHSIM_Rhsim08G0090300
Root vs Leaf	RHSIM_Rhsim04G0003900	-2.558439839	2.10684E-34	4.76581E-33	RHSIM_Rhsim04G0003900

Comparison	Gene ID	log2FC	P-value	FDR (padj)	Gene Name
Root vs Leaf	RHSIM_Rhsim06G0119000	-5.639802938	2.42444E-34	5.4789E-33	RHSIM_Rhsim06G0119000
Root vs Leaf	RHSIM_Rhsim01G0270000	-1.859272725	2.7768E-34	6.26908E-33	RHSIM_Rhsim01G0270000
Root vs Leaf	RHSIM_Rhsim08G0044500	-1.858005105	2.79703E-34	6.30859E-33	RHSIM_Rhsim08G0044500
Root vs Leaf	RHSIM_Rhsim04G0005900	-3.151703983	2.83282E-34	6.38242E-33	RHSIM_Rhsim04G0005900
Root vs Leaf	RHSIM_Rhsim03G0240800	-1.682051661	2.83528E-34	6.38242E-33	RHSIM_Rhsim03G0240800
Root vs Leaf	RHSIM_Rhsim10G0006600	-2.523523278	2.87031E-34	6.45019E-33	RHSIM_Rhsim10G0006600
Root vs Leaf	RHSIM_Rhsim04G0058600	-5.156787874	2.87096E-34	6.45019E-33	RHSIM_Rhsim04G0058600
Root vs Leaf	RHSIM_Rhsim02G0214600	-10.30838042	3.05721E-34	6.86197E-33	RHSIM_Rhsim02G0214600
Root vs Leaf	RHSIM_Rhsim09G0087300	-3.651287129	3.34701E-34	7.50516E-33	RHSIM_Rhsim09G0087300
Root vs Leaf	RHSIM_Rhsim03G0070300	-7.282858799	3.71747E-34	8.32779E-33	RHSIM_Rhsim03G0070300
Root vs Leaf	RHSIM_Rhsim01G0262500	5.013418887	3.72957E-34	8.3468E-33	RHSIM_Rhsim01G0262500
Root vs Leaf	RHSIM_Rhsim02G0173800	-2.47037478	4.18728E-34	9.3621E-33	RHSIM_Rhsim02G0173800
Root vs Leaf	RHSIM_Rhsim10G0109000	-5.209356699	4.39394E-34	9.8147E-33	RHSIM_Rhsim10G0109000
Root vs Leaf	RHSIM_Rhsim05G0075200	-2.414532528	4.42258E-34	9.86914E-33	RHSIM_Rhsim05G0075200
Root vs Leaf	RHSIM_Rhsim04G0216600	-3.166870628	4.54421E-34	1.01308E-32	RHSIM_Rhsim04G0216600
Root vs Leaf	RHSIM_Rhsim12G0037800	-1.806366094	4.56442E-34	1.0166E-32	RHSIM_Rhsim12G0037800
Root vs Leaf	RHSIM_Rhsim04G0181100	2.343033241	4.72607E-34	1.0516E-32	RHSIM_Rhsim04G0181100
Root vs Leaf	RHSIM_Rhsim10G0068700	-2.851028074	4.75631E-34	1.05731E-32	RHSIM_Rhsim10G0068700
Root vs Leaf	RHSIM_RhsimUnG0197900	-5.038839107	4.90553E-34	1.08943E-32	RHSIM_RhsimUnG0197900
Root vs Leaf	RHSIM_Rhsim13G0077700	-2.194206653	5.12969E-34	1.13812E-32	RHSIM_Rhsim13G0077700
Root vs Leaf	novel.4256	-4.869792527	5.24751E-34	1.16315E-32	--
Root vs Leaf	RHSIM_Rhsim01G0033300	3.614001518	5.7064E-34	1.26365E-32	RHSIM_Rhsim01G0033300
Root vs Leaf	RHSIM_Rhsim03G0226300	-1.400387141	6.05415E-34	1.33938E-32	RHSIM_Rhsim03G0226300
Root vs Leaf	RHSIM_Rhsim13G0184700	-5.963239165	6.29504E-34	1.39134E-32	RHSIM_Rhsim13G0184700
Root vs Leaf	RHSIM_Rhsim02G0188700	4.788719849	6.44039E-34	1.42211E-32	RHSIM_Rhsim02G0188700

Comparison	Gene ID	log_2FC	P-value	FDR (padj)	Gene Name
Root vs Leaf	RHSIM_Rhsim11G0101900	-1.903610966	6.82078E-34	1.50467E-32	RHSIM_Rhsim11G0101900
Root vs Leaf	RHSIM_Rhsim03G0241300	-3.10087791	7.8158E-34	1.72253E-32	RHSIM_Rhsim03G0241300
Root vs Leaf	novel.4079	-2.808147513	7.93805E-34	1.74781E-32	--
Root vs Leaf	RHSIM_Rhsim04G0008200	-1.89172117	8.17351E-34	1.79794E-32	RHSIM_Rhsim04G0008200
Root vs Leaf	RHSIM_Rhsim06G0068800	-5.777429552	8.37897E-34	1.84138E-32	RHSIM_Rhsim06G0068800
Root vs Leaf	RHSIM_Rhsim09G0200600	-5.899964018	8.50755E-34	1.86787E-32	RHSIM_Rhsim09G0200600
Root vs Leaf	RHSIM_Rhsim08G0042000	-5.23892281	8.66757E-34	1.9012E-32	RHSIM_Rhsim08G0042000
Root vs Leaf	RHSIM_Rhsim05G0146200	-3.5416743	8.96076E-34	1.96365E-32	RHSIM_Rhsim05G0146200
Root vs Leaf	RHSIM_Rhsim08G0233500	3.460121183	1.02653E-33	2.2474E-32	RHSIM_Rhsim08G0233500
Root vs Leaf	RHSIM_Rhsim02G0068600	6.800856305	1.11768E-33	2.44464E-32	RHSIM_Rhsim02G0068600
Root vs Leaf	RHSIM_Rhsim02G0193100	-1.7875979	1.20153E-33	2.62555E-32	RHSIM_Rhsim02G0193100
Root vs Leaf	RHSIM_Rhsim10G0188700	1.637924373	1.23732E-33	2.7012E-32	RHSIM_Rhsim10G0188700
Root vs Leaf	RHSIM_Rhsim03G0201600	-4.308527391	1.33444E-33	2.91049E-32	RHSIM_Rhsim03G0201600
Root vs Leaf	RHSIM_Rhsim08G0114900	-2.764119316	1.38372E-33	3.01513E-32	RHSIM_Rhsim08G0114900
Root vs Leaf	RHSIM_Rhsim06G0004000	-1.763914042	1.49954E-33	3.26443E-32	RHSIM_Rhsim06G0004000
Root vs Leaf	RHSIM_Rhsim06G0074400	-7.275383008	1.5405E-33	3.35045E-32	RHSIM_Rhsim06G0074400
Root vs Leaf	RHSIM_Rhsim01G0192900	-5.775723739	1.55057E-33	3.36918E-32	RHSIM_Rhsim01G0192900
Root vs Leaf	RHSIM_Rhsim02G0105300	-7.514766004	1.64421E-33	3.5693E-32	RHSIM_Rhsim02G0105300
Root vs Leaf	RHSIM_Rhsim09G0000900	2.762146901	1.82166E-33	3.9508E-32	RHSIM_Rhsim09G0000900
Root vs Leaf	RHSIM_Rhsim01G0100400	-1.93880515	1.86609E-33	4.04337E-32	RHSIM_Rhsim01G0100400
Root vs Leaf	RHSIM_Rhsim02G0236300	5.340637066	1.97649E-33	4.27857E-32	RHSIM_Rhsim02G0236300
Root vs Leaf	novel.1217	9.282485975	1.99081E-33	4.30554E-32	--
Root vs Leaf	RHSIM_Rhsim08G0026900	2.433552377	2.15361E-33	4.65328E-32	RHSIM_Rhsim08G0026900
Root vs Leaf	RHSIM_Rhsim04G0011000	1.680042715	2.21202E-33	4.77503E-32	RHSIM_Rhsim04G0011000
Root vs Leaf	RHSIM_Rhsim09G0120100	-2.900684573	2.52918E-33	5.4546E-32	RHSIM_Rhsim09G0120100

Comparison	Gene ID	log_2FC	P-value	FDR (padj)	Gene Name
Root vs Leaf	RHSIM_Rhsim12G0164800	-2.455423269	2.65482E-33	5.72022E-32	RHSIM_Rhsim12G0164800
Root vs Leaf	RHSIM_Rhsim03G0231400	1.358646851	2.78963E-33	6.00511E-32	RHSIM_Rhsim03G0231400
Root vs Leaf	RHSIM_Rhsim02G0142400	-1.589903572	2.8436E-33	6.11559E-32	RHSIM_Rhsim02G0142400
Root vs Leaf	RHSIM_Rhsim06G0211200	-3.501890181	2.95495E-33	6.34916E-32	RHSIM_Rhsim06G0211200
Root vs Leaf	novel.4021	8.138563325	3.23826E-33	6.95144E-32	--
Root vs Leaf	novel.2740	-2.042836768	3.40357E-33	7.29954E-32	--
Root vs Leaf	RHSIM_Rhsim04G0071000	-2.707281213	4.08168E-33	8.74575E-32	RHSIM_Rhsim04G0071000
Root vs Leaf	RHSIM_Rhsim02G0225700	7.366539242	4.61175E-33	9.8724E-32	RHSIM_Rhsim02G0225700
Root vs Leaf	RHSIM_Rhsim06G0023400	4.643918877	4.68796E-33	1.00262E-31	RHSIM_Rhsim06G0023400
Root vs Leaf	novel.4114	3.300209073	4.90194E-33	1.04742E-31	--
Root vs Leaf	RHSIM_Rhsim07G0178600	-2.266557501	5.18586E-33	1.10707E-31	RHSIM_Rhsim07G0178600
Root vs Leaf	RHSIM_Rhsim13G0157800	-2.387564037	5.64299E-33	1.20354E-31	RHSIM_Rhsim13G0157800
Root vs Leaf	RHSIM_Rhsim05G0044900	-3.472525534	5.97459E-33	1.27309E-31	RHSIM_Rhsim05G0044900
Root vs Leaf	RHSIM_Rhsim12G0124500	-1.541752889	5.98248E-33	1.2736E-31	RHSIM_Rhsim12G0124500
Root vs Leaf	RHSIM_Rhsim02G0175200	-7.799002039	6.56498E-33	1.39633E-31	RHSIM_Rhsim02G0175200
Root vs Leaf	RHSIM_Rhsim06G0211400	-2.964471126	6.58597E-33	1.3995E-31	RHSIM_Rhsim06G0211400
Root vs Leaf	RHSIM_Rhsim08G0234400	-2.110560886	6.7327E-33	1.42937E-31	RHSIM_Rhsim08G0234400
Root vs Leaf	RHSIM_Rhsim08G0211300	-2.035387877	6.7916E-33	1.44055E-31	RHSIM_Rhsim08G0211300
Root vs Leaf	RHSIM_Rhsim13G0029900	-2.440610096	8.00398E-33	1.69616E-31	RHSIM_Rhsim13G0029900
Root vs Leaf	RHSIM_Rhsim10G0173400	4.539266112	8.27053E-33	1.75104E-31	RHSIM_Rhsim10G0173400
Root vs Leaf	RHSIM_Rhsim06G0096300	-2.43073183	8.35585E-33	1.76748E-31	RHSIM_Rhsim06G0096300
Root vs Leaf	RHSIM_Rhsim07G0016400	-3.243257958	8.59395E-33	1.81619E-31	RHSIM_Rhsim07G0016400
Root vs Leaf	RHSIM_Rhsim07G0048600	-3.074037055	8.68647E-33	1.83407E-31	RHSIM_Rhsim07G0048600
Root vs Leaf	novel.1017	-6.610335288	8.81079E-33	1.85862E-31	--
Root vs Leaf	RHSIM_Rhsim03G0067900	4.476662035	1.00021E-32	2.108E-31	RHSIM_Rhsim03G0067900

Comparison	Gene ID	log_2FC	P-value	FDR (padj)	Gene Name
Root vs Leaf	RHSIM_Rhsim12G0064600	-2.211385784	1.1034E-32	2.32336E-31	RHSIM_Rhsim12G0064600
Root vs Leaf	RHSIM_Rhsim08G0197500	2.369899422	1.10467E-32	2.32392E-31	RHSIM_Rhsim08G0197500
Root vs Leaf	novel.3885	-13.1780294	1.11376E-32	2.34091E-31	--
Root vs Leaf	RHSIM_Rhsim12G0182100	3.643753814	1.15261E-32	2.42037E-31	RHSIM_Rhsim12G0182100
Root vs Leaf	RHSIM_Rhsim07G0250100	-10.08829493	1.17659E-32	2.4685E-31	RHSIM_Rhsim07G0250100
Root vs Leaf	RHSIM_Rhsim08G0005400	-2.653249367	1.18339E-32	2.48052E-31	RHSIM_Rhsim08G0005400
Root vs Leaf	RHSIM_Rhsim03G0192000	-1.824678188	1.20679E-32	2.52726E-31	RHSIM_Rhsim03G0192000
Root vs Leaf	RHSIM_Rhsim10G0081700	4.487898158	1.24907E-32	2.61345E-31	RHSIM_Rhsim10G0081700
Root vs Leaf	RHSIM_Rhsim03G0256500	-2.656483727	1.2695E-32	2.6538E-31	RHSIM_Rhsim03G0256500
Root vs Leaf	RHSIM_Rhsim04G0196300	-3.850506069	1.27132E-32	2.6552E-31	RHSIM_Rhsim04G0196300
Root vs Leaf	RHSIM_Rhsim12G0085200	-2.233430963	1.29434E-32	2.70084E-31	RHSIM_Rhsim12G0085200
Root vs Leaf	RHSIM_Rhsim04G0234700	3.694303222	1.4721E-32	3.069E-31	RHSIM_Rhsim04G0234700
Root vs Leaf	RHSIM_Rhsim10G0036100	4.192967611	1.54438E-32	3.21679E-31	RHSIM_Rhsim10G0036100
Root vs Leaf	RHSIM_Rhsim01G0072900	-2.518570409	1.67425E-32	3.48417E-31	RHSIM_Rhsim01G0072900
Root vs Leaf	RHSIM_Rhsim03G0052700	7.093528743	1.67629E-32	3.48527E-31	RHSIM_Rhsim03G0052700
Root vs Leaf	RHSIM_Rhsim11G0080300	-5.796884043	2.14097E-32	4.44742E-31	RHSIM_Rhsim11G0080300
Root vs Leaf	RHSIM_Rhsim07G0067100	8.561078386	2.17118E-32	4.50612E-31	RHSIM_Rhsim07G0067100
Root vs Leaf	RHSIM_Rhsim12G0137200	-2.028162544	2.33204E-32	4.83565E-31	RHSIM_Rhsim12G0137200
Root vs Leaf	RHSIM_Rhsim04G0173400	-8.390198804	2.41194E-32	4.99684E-31	RHSIM_Rhsim04G0173400
Root vs Leaf	RHSIM_Rhsim01G0276200	-1.751678776	2.56672E-32	5.31275E-31	RHSIM_Rhsim01G0276200
Root vs Leaf	RHSIM_Rhsim02G0022400	3.770684813	2.61435E-32	5.40649E-31	RHSIM_Rhsim02G0022400
Root vs Leaf	RHSIM_Rhsim12G0001700	3.020898235	2.66547E-32	5.50729E-31	RHSIM_Rhsim12G0001700
Root vs Leaf	RHSIM_Rhsim01G0271500	-2.262069992	2.74046E-32	5.65718E-31	RHSIM_Rhsim01G0271500
Root vs Leaf	RHSIM_Rhsim02G0074200	-5.961886886	2.76513E-32	5.70302E-31	RHSIM_Rhsim02G0074200
Root vs Leaf	RHSIM_Rhsim03G0165400	-3.3145353	2.86823E-32	5.91039E-31	RHSIM_Rhsim03G0165400

Comparison	Gene ID	log_2FC	P-value	FDR (padj)	Gene Name
Root vs Leaf	RHSIM_Rhsim12G0003000	-3.398139646	2.89293E-32	5.95599E-31	RHSIM_Rhsim12G0003000
Root vs Leaf	RHSIM_Rhsim07G0054000	-1.349286876	3.17901E-32	6.53916E-31	RHSIM_Rhsim07G0054000
Root vs Leaf	RHSIM_Rhsim11G0044100	-10.53339172	3.29656E-32	6.77494E-31	RHSIM_Rhsim11G0044100
Root vs Leaf	novel.4716	4.318502519	3.31483E-32	6.80644E-31	--
Root vs Leaf	RHSIM_Rhsim13G0173500	-3.612279212	3.37992E-32	6.93392E-31	RHSIM_Rhsim13G0173500
Root vs Leaf	RHSIM_Rhsim08G0171200	-2.033687035	3.4175E-32	7.00481E-31	RHSIM_Rhsim08G0171200
Root vs Leaf	RHSIM_Rhsim13G0028700	2.300979605	3.64604E-32	7.46663E-31	RHSIM_Rhsim13G0028700
Root vs Leaf	RHSIM_Rhsim03G0040300	-3.682586831	3.79825E-32	7.77147E-31	RHSIM_Rhsim03G0040300
Root vs Leaf	RHSIM_Rhsim05G0104700	-2.221234968	4.1258E-32	8.43419E-31	RHSIM_Rhsim05G0104700
Root vs Leaf	RHSIM_RhsimUnG0087600	-5.475561373	4.94206E-32	1.00939E-30	RHSIM_RhsimUnG0087600
Root vs Leaf	RHSIM_Rhsim04G0197800	-7.385902342	5.01224E-32	1.02282E-30	RHSIM_Rhsim04G0197800
Root vs Leaf	RHSIM_Rhsim11G0100000	2.084619947	5.02216E-32	1.02394E-30	RHSIM_Rhsim11G0100000
Root vs Leaf	RHSIM_RhsimUnG0246000	6.649535915	5.05511E-32	1.02971E-30	RHSIM_RhsimUnG0246000
Root vs Leaf	RHSIM_Rhsim09G0084100	3.562423501	5.05934E-32	1.02971E-30	RHSIM_Rhsim09G0084100
Root vs Leaf	novel.4077	-5.464445152	5.29989E-32	1.07772E-30	--
Root vs Leaf	RHSIM_Rhsim04G0242100	-2.102180384	5.33193E-32	1.08328E-30	RHSIM_Rhsim04G0242100
Root vs Leaf	RHSIM_Rhsim12G0005600	-1.176419469	5.45166E-32	1.10664E-30	RHSIM_Rhsim12G0005600
Root vs Leaf	RHSIM_Rhsim02G0234500	-5.524574085	5.65664E-32	1.14724E-30	RHSIM_Rhsim02G0234500
Root vs Leaf	novel.578	4.895884441	5.69215E-32	1.15343E-30	--
Root vs Leaf	RHSIM_Rhsim09G0142500	3.591967304	5.78568E-32	1.17136E-30	RHSIM_Rhsim09G0142500
Root vs Leaf	RHSIM_Rhsim08G0053400	-4.98033779	5.80969E-32	1.17519E-30	RHSIM_Rhsim08G0053400
Root vs Leaf	RHSIM_Rhsim02G0113400	-10.17119999	6.15338E-32	1.24363E-30	RHSIM_Rhsim02G0113400
Root vs Leaf	RHSIM_Rhsim10G0165500	-14.07609806	6.21709E-32	1.25541E-30	RHSIM_Rhsim10G0165500
Root vs Leaf	RHSIM_Rhsim03G0188900	-2.887513415	6.25733E-32	1.26243E-30	RHSIM_Rhsim03G0188900
Root vs Leaf	novel.145	3.385410478	7.17868E-32	1.44705E-30	--

Comparison	Gene ID	log_2FC	P-value	FDR (padj)	Gene Name
Root vs Leaf	RHSIM_Rhsim08G0166900	-1.775779674	8.65963E-32	1.74406E-30	RHSIM_Rhsim08G0166900
Root vs Leaf	RHSIM_Rhsim07G0028800	-2.376451036	9.30183E-32	1.87177E-30	RHSIM_Rhsim07G0028800
Root vs Leaf	RHSIM_Rhsim03G0221700	3.250957811	1.11794E-31	2.24763E-30	RHSIM_Rhsim03G0221700
Root vs Leaf	RHSIM_Rhsim05G0111800	-2.048289328	1.18893E-31	2.38829E-30	RHSIM_Rhsim05G0111800
Root vs Leaf	RHSIM_Rhsim10G0212700	-2.005167817	1.19931E-31	2.40704E-30	RHSIM_Rhsim10G0212700
Root vs Leaf	RHSIM_Rhsim12G0201300	-1.708169149	1.24659E-31	2.49977E-30	RHSIM_Rhsim12G0201300
Root vs Leaf	novel.136	-5.510099805	1.2589E-31	2.52226E-30	--
Root vs Leaf	RHSIM_Rhsim04G0095900	-2.805624382	1.27467E-31	2.55165E-30	RHSIM_Rhsim04G0095900
Root vs Leaf	RHSIM_Rhsim08G0239700	-1.839865942	1.33257E-31	2.66526E-30	RHSIM_Rhsim08G0239700
Root vs Leaf	novel.5618	-7.177076011	1.64934E-31	3.29598E-30	--
Root vs Leaf	RHSIM_RhsimPtG0005800	-3.782179586	1.70583E-31	3.40592E-30	RHSIM_RhsimPtG0005800
Root vs Leaf	RHSIM_Rhsim03G0136100	3.815563731	1.7898E-31	3.57049E-30	RHSIM_Rhsim03G0136100
Root vs Leaf	RHSIM_Rhsim05G0172500	-2.428589823	1.90053E-31	3.78813E-30	RHSIM_Rhsim05G0172500
Root vs Leaf	RHSIM_Rhsim02G0035300	5.904264154	1.97039E-31	3.924E-30	RHSIM_Rhsim02G0035300
Root vs Leaf	RHSIM_Rhsim12G0014900	-2.198400981	1.9849E-31	3.94949E-30	RHSIM_Rhsim12G0014900
Root vs Leaf	RHSIM_Rhsim11G0122200	-2.908399479	2.03956E-31	4.05151E-30	RHSIM_Rhsim11G0122200
Root vs Leaf	RHSIM_Rhsim07G0075600	-3.674146304	2.03967E-31	4.05151E-30	RHSIM_Rhsim07G0075600
Root vs Leaf	RHSIM_Rhsim11G0138000	-5.789706567	2.20159E-31	4.36937E-30	RHSIM_Rhsim11G0138000
Root vs Leaf	RHSIM_Rhsim12G0106000	-1.746296597	2.2645E-31	4.49039E-30	RHSIM_Rhsim12G0106000
Root vs Leaf	RHSIM_Rhsim11G0107000	-5.428102562	2.47099E-31	4.89564E-30	RHSIM_Rhsim11G0107000
Root vs Leaf	RHSIM_Rhsim11G0016500	-2.881091263	2.59456E-31	5.13607E-30	RHSIM_Rhsim11G0016500
Root vs Leaf	RHSIM_Rhsim03G0232900	-4.474502991	2.76189E-31	5.46264E-30	RHSIM_Rhsim03G0232900
Root vs Leaf	RHSIM_Rhsim09G0158400	-3.169806739	3.30539E-31	6.53203E-30	RHSIM_Rhsim09G0158400
Root vs Leaf	RHSIM_Rhsim02G0247000	-5.010707006	3.31637E-31	6.54812E-30	RHSIM_Rhsim02G0247000
Root vs Leaf	RHSIM_Rhsim03G0186100	-10.28274251	3.54495E-31	6.9935E-30	RHSIM_Rhsim03G0186100

Comparison	Gene ID	log_2FC	P-value	FDR (padj)	Gene Name
Root vs Leaf	RHSIM_Rhsim09G0214000	-1.919242003	4.15712E-31	8.1942E-30	RHSIM_Rhsim09G0214000
Root vs Leaf	RHSIM_Rhsim12G0020600	4.33103859	4.35309E-31	8.57318E-30	RHSIM_Rhsim12G0020600
Root vs Leaf	RHSIM_Rhsim13G0042600	-4.227803267	5.25818E-31	1.03469E-29	RHSIM_Rhsim13G0042600
Root vs Leaf	RHSIM_Rhsim13G0149800	1.602511093	5.33799E-31	1.0495E-29	RHSIM_Rhsim13G0149800
Root vs Leaf	RHSIM_Rhsim11G0191400	3.915518783	5.50064E-31	1.08056E-29	RHSIM_Rhsim11G0191400
Root vs Leaf	RHSIM_Rhsim12G0137100	-3.043396489	5.68318E-31	1.11548E-29	RHSIM_Rhsim12G0137100
Root vs Leaf	RHSIM_Rhsim06G0161000	-4.181533267	6.19199E-31	1.21431E-29	RHSIM_Rhsim06G0161000
Root vs Leaf	RHSIM_Rhsim12G0076500	-3.298735838	6.44522E-31	1.2629E-29	RHSIM_Rhsim12G0076500
Root vs Leaf	novel.2485	-3.668821102	7.00549E-31	1.37152E-29	--
Root vs Leaf	RHSIM_Rhsim06G0111800	-9.869679357	7.10537E-31	1.3899E-29	RHSIM_Rhsim06G0111800
Root vs Leaf	RHSIM_Rhsim12G0143500	-13.76945144	8.48852E-31	1.65906E-29	RHSIM_Rhsim12G0143500
Root vs Leaf	RHSIM_Rhsim12G0107100	-2.617199276	8.50744E-31	1.66136E-29	RHSIM_Rhsim12G0107100
Root vs Leaf	RHSIM_RhsimUnG0255500	2.158463311	9.23185E-31	1.8013E-29	RHSIM_RhsimUnG0255500
Root vs Leaf	RHSIM_Rhsim07G0170300	-2.665739122	9.7359E-31	1.89805E-29	RHSIM_Rhsim07G0170300
Root vs Leaf	RHSIM_Rhsim09G0095600	13.00591635	9.85513E-31	1.91968E-29	RHSIM_Rhsim09G0095600
Root vs Leaf	RHSIM_Rhsim03G0262800	-1.5081289	1.09056E-30	2.12251E-29	RHSIM_Rhsim03G0262800
Root vs Leaf	RHSIM_Rhsim13G0098500	-3.466262149	1.13284E-30	2.20294E-29	RHSIM_Rhsim13G0098500
Root vs Leaf	RHSIM_Rhsim13G0107300	-1.687081683	1.14633E-30	2.22731E-29	RHSIM_Rhsim13G0107300
Root vs Leaf	RHSIM_Rhsim04G0126500	3.589682576	1.15476E-30	2.24181E-29	RHSIM_Rhsim04G0126500
Root vs Leaf	RHSIM_Rhsim03G0045300	-2.660419353	1.19723E-30	2.32231E-29	RHSIM_Rhsim03G0045300
Root vs Leaf	RHSIM_Rhsim10G0190300	-3.6780201	1.24793E-30	2.41863E-29	RHSIM_Rhsim10G0190300
Root vs Leaf	RHSIM_Rhsim02G0032800	-3.062320235	1.27478E-30	2.46859E-29	RHSIM_Rhsim02G0032800
Root vs Leaf	RHSIM_Rhsim02G0139100	2.995182885	1.36517E-30	2.64143E-29	RHSIM_Rhsim02G0139100
Root vs Leaf	RHSIM_Rhsim05G0021900	-9.967653738	1.43257E-30	2.76952E-29	RHSIM_Rhsim05G0021900
Root vs Leaf	RHSIM_Rhsim13G0143200	-4.896613212	1.75347E-30	3.38706E-29	RHSIM_Rhsim13G0143200

Comparison	Gene ID	log_2FC	P-value	FDR (padj)	Gene Name
Root vs Leaf	RHSIM_Rhsim05G0110900	-2.275373178	1.79661E-30	3.46751E-29	RHSIM_Rhsim05G0110900
Root vs Leaf	RHSIM_RhsimUnG0179800	6.545495268	1.83909E-30	3.54653E-29	RHSIM_RhsimUnG0179800
Root vs Leaf	RHSIM_Rhsim01G0144700	-2.694362089	1.85595E-30	3.57607E-29	RHSIM_Rhsim01G0144700
Root vs Leaf	RHSIM_Rhsim06G0240900	3.786833402	1.87933E-30	3.6181E-29	RHSIM_Rhsim06G0240900
Root vs Leaf	RHSIM_Rhsim05G0142600	-4.584348842	1.99776E-30	3.84291E-29	RHSIM_Rhsim05G0142600
Root vs Leaf	RHSIM_Rhsim02G0135000	-3.755018307	2.02684E-30	3.89562E-29	RHSIM_Rhsim02G0135000
Root vs Leaf	RHSIM_Rhsim06G0064000	-1.96531385	2.05209E-30	3.94087E-29	RHSIM_Rhsim06G0064000
Root vs Leaf	RHSIM_Rhsim05G0004900	-13.77668283	2.09066E-30	4.01161E-29	RHSIM_Rhsim05G0004900
Root vs Leaf	RHSIM_Rhsim08G0021000	3.51939879	2.25229E-30	4.31816E-29	RHSIM_Rhsim08G0021000
Root vs Leaf	RHSIM_Rhsim11G0044300	-3.760220445	2.25676E-30	4.32315E-29	RHSIM_Rhsim11G0044300
Root vs Leaf	RHSIM_Rhsim07G0131300	-1.860993066	2.26249E-30	4.33054E-29	RHSIM_Rhsim07G0131300
Root vs Leaf	RHSIM_Rhsim09G0207900	-4.769856619	2.41622E-30	4.62097E-29	RHSIM_Rhsim09G0207900
Root vs Leaf	RHSIM_RhsimUnG0188100	-5.531042027	2.46009E-30	4.70099E-29	RHSIM_RhsimUnG0188100
Root vs Leaf	RHSIM_Rhsim06G0218100	7.515768555	2.61451E-30	4.99195E-29	RHSIM_Rhsim06G0218100
Root vs Leaf	RHSIM_Rhsim11G0140600	-9.852785977	2.61956E-30	4.99747E-29	RHSIM_Rhsim11G0140600
Root vs Leaf	RHSIM_Rhsim13G0053400	3.257905828	2.73928E-30	5.22155E-29	RHSIM_Rhsim13G0053400
Root vs Leaf	RHSIM_RhsimUnG0226000	-1.94258509	2.93534E-30	5.59068E-29	RHSIM_RhsimUnG0226000
Root vs Leaf	RHSIM_Rhsim03G0158600	-8.683330164	3.01993E-30	5.74705E-29	RHSIM_Rhsim03G0158600
Root vs Leaf	RHSIM_Rhsim02G0206200	-7.334622113	3.59277E-30	6.83158E-29	RHSIM_Rhsim02G0206200
Root vs Leaf	RHSIM_Rhsim06G0047000	-2.361208354	3.63351E-30	6.90337E-29	RHSIM_Rhsim06G0047000
Root vs Leaf	RHSIM_Rhsim01G0237700	7.096288244	3.65653E-30	6.9414E-29	RHSIM_Rhsim01G0237700
Root vs Leaf	RHSIM_RhsimUnG0205800	-2.030901762	4.23536E-30	8.03364E-29	RHSIM_RhsimUnG0205800
Root vs Leaf	RHSIM_Rhsim13G0114900	5.133659373	4.32335E-30	8.19383E-29	RHSIM_Rhsim13G0114900
Root vs Leaf	RHSIM_Rhsim06G0243200	5.63934153	4.73166E-30	8.96034E-29	RHSIM_Rhsim06G0243200
Root vs Leaf	RHSIM_Rhsim07G0155900	-2.246168256	4.77192E-30	9.02918E-29	RHSIM_Rhsim07G0155900

Comparison	Gene ID	log_2FC	P-value	FDR (padj)	Gene Name
Root vs Leaf	RHSIM_Rhsim07G0203500	7.686248333	4.85086E-30	9.17107E-29	RHSIM_Rhsim07G0203500
Root vs Leaf	RHSIM_Rhsim05G0200400	3.733042545	4.93716E-30	9.32659E-29	RHSIM_Rhsim05G0200400
Root vs Leaf	RHSIM_Rhsim13G0140700	1.84294174	5.02533E-30	9.48003E-29	RHSIM_Rhsim13G0140700
Root vs Leaf	RHSIM_Rhsim06G0021400	-3.842099871	5.02657E-30	9.48003E-29	RHSIM_Rhsim06G0021400
Root vs Leaf	RHSIM_Rhsim01G0233900	-5.085849328	5.0468E-30	9.51041E-29	RHSIM_Rhsim01G0233900
Root vs Leaf	RHSIM_RhsimUnG0217500	2.173807891	5.75865E-30	1.0843E-28	RHSIM_RhsimUnG0217500
Root vs Leaf	RHSIM_Rhsim08G0045300	2.68368787	6.00856E-30	1.12956E-28	RHSIM_Rhsim08G0045300
Root vs Leaf	RHSIM_Rhsim02G0070300	4.80802111	6.00878E-30	1.12956E-28	RHSIM_Rhsim02G0070300
Root vs Leaf	RHSIM_Rhsim09G0005700	-1.893780599	6.1153E-30	1.14865E-28	RHSIM_Rhsim09G0005700
Root vs Leaf	RHSIM_Rhsim12G0004100	-2.409389808	6.30838E-30	1.18396E-28	RHSIM_Rhsim12G0004100
Root vs Leaf	RHSIM_Rhsim04G0227700	1.850177904	6.3407E-30	1.18906E-28	RHSIM_Rhsim04G0227700
Root vs Leaf	RHSIM_Rhsim05G0052600	-2.212384597	6.35279E-30	1.19036E-28	RHSIM_Rhsim05G0052600
Root vs Leaf	RHSIM_Rhsim10G0118300	6.665771288	6.55388E-30	1.22705E-28	RHSIM_Rhsim10G0118300
Root vs Leaf	RHSIM_Rhsim05G0200800	-4.456376744	6.56065E-30	1.22732E-28	RHSIM_Rhsim05G0200800
Root vs Leaf	RHSIM_Rhsim03G0266200	-4.871322063	7.34559E-30	1.37306E-28	RHSIM_Rhsim03G0266200
Root vs Leaf	RHSIM_Rhsim06G0002800	3.469925954	7.53144E-30	1.40666E-28	RHSIM_Rhsim06G0002800
Root vs Leaf	RHSIM_Rhsim03G0082000	-1.995387914	7.56532E-30	1.41185E-28	RHSIM_Rhsim03G0082000
Root vs Leaf	RHSIM_Rhsim07G0095100	-2.284760078	8.12773E-30	1.51558E-28	RHSIM_Rhsim07G0095100
Root vs Leaf	RHSIM_Rhsim01G0257200	-1.523285807	9.18525E-30	1.7114E-28	RHSIM_Rhsim01G0257200
Root vs Leaf	RHSIM_Rhsim05G0002300	2.812270982	9.2335E-30	1.71901E-28	RHSIM_Rhsim05G0002300
Root vs Leaf	RHSIM_Rhsim06G0137200	-3.691234924	9.24398E-30	1.71957E-28	RHSIM_Rhsim06G0137200
Root vs Leaf	RHSIM_Rhsim04G0223100	-3.781239644	1.05526E-29	1.96143E-28	RHSIM_Rhsim04G0223100
Root vs Leaf	RHSIM_Rhsim03G0166500	-2.30409635	1.06933E-29	1.98599E-28	RHSIM_Rhsim03G0166500
Root vs Leaf	RHSIM_Rhsim12G0127900	1.744439844	1.07897E-29	2.00229E-28	RHSIM_Rhsim12G0127900
Root vs Leaf	RHSIM_Rhsim13G0042000	-2.127745987	1.12667E-29	2.08913E-28	RHSIM_Rhsim13G0042000

Comparison	Gene ID	log_2FC	P-value	FDR (padj)	Gene Name
Root vs Leaf	RHSIM_Rhsim01G0168200	-1.944818261	1.15945E-29	2.14818E-28	RHSIM_Rhsim01G0168200
Root vs Leaf	RHSIM_Rhsim09G0164300	1.51403179	1.16285E-29	2.15275E-28	RHSIM_Rhsim09G0164300
Root vs Leaf	novel.5854	-2.258355992	1.19685E-29	2.21393E-28	--
Root vs Leaf	RHSIM_Rhsim11G0172800	-9.903810323	1.20303E-29	2.22358E-28	RHSIM_Rhsim11G0172800
Root vs Leaf	RHSIM_Rhsim09G0183900	-2.187332366	1.20896E-29	2.23277E-28	RHSIM_Rhsim09G0183900
Root vs Leaf	RHSIM_RhsimUnG0258200	-4.89014271	1.2286E-29	2.26723E-28	RHSIM_RhsimUnG0258200
Root vs Leaf	RHSIM_Rhsim06G0163800	3.375716175	1.23466E-29	2.27659E-28	RHSIM_Rhsim06G0163800
Root vs Leaf	RHSIM_RhsimUnG0020200	2.829950328	1.25076E-29	2.30444E-28	RHSIM_RhsimUnG0020200
Root vs Leaf	RHSIM_Rhsim10G0009700	-1.445852895	1.26135E-29	2.32211E-28	RHSIM_Rhsim10G0009700
Root vs Leaf	RHSIM_Rhsim10G0005000	-4.756705482	1.26243E-29	2.32225E-28	RHSIM_Rhsim10G0005000
Root vs Leaf	RHSIM_Rhsim01G0120900	3.943355482	1.26934E-29	2.3331E-28	RHSIM_Rhsim01G0120900
Root vs Leaf	RHSIM_Rhsim07G0069700	-3.957918625	1.34554E-29	2.4712E-28	RHSIM_Rhsim07G0069700
Root vs Leaf	RHSIM_Rhsim07G0243900	-2.047372521	1.38593E-29	2.54337E-28	RHSIM_Rhsim07G0243900
Root vs Leaf	RHSIM_Rhsim03G0010600	-1.945989985	1.44632E-29	2.65209E-28	RHSIM_Rhsim03G0010600
Root vs Leaf	RHSIM_Rhsim13G0119700	-3.47973659	1.50023E-29	2.74877E-28	RHSIM_Rhsim13G0119700
Root vs Leaf	RHSIM_Rhsim06G0239000	-2.725730714	1.54716E-29	2.8325E-28	RHSIM_Rhsim06G0239000
Root vs Leaf	RHSIM_Rhsim10G0108800	3.815058581	1.57133E-29	2.87448E-28	RHSIM_Rhsim10G0108800
Root vs Leaf	RHSIM_Rhsim02G0105900	-4.240246411	1.58498E-29	2.89715E-28	RHSIM_Rhsim02G0105900
Root vs Leaf	RHSIM_Rhsim06G0124400	-3.971109254	1.62112E-29	2.96088E-28	RHSIM_Rhsim06G0124400
Root vs Leaf	RHSIM_Rhsim13G0003300	2.826855585	1.63841E-29	2.9901E-28	RHSIM_Rhsim13G0003300
Root vs Leaf	RHSIM_Rhsim01G0284500	-3.221329425	1.68309E-29	3.06921E-28	RHSIM_Rhsim01G0284500
Root vs Leaf	RHSIM_Rhsim03G0146900	-2.852111467	1.73234E-29	3.15655E-28	RHSIM_Rhsim03G0146900
Root vs Leaf	RHSIM_Rhsim03G0223500	-2.426871843	1.93333E-29	3.51999E-28	RHSIM_Rhsim03G0223500
Root vs Leaf	RHSIM_Rhsim01G0205800	4.508753651	1.96365E-29	3.5724E-28	RHSIM_Rhsim01G0205800
Root vs Leaf	RHSIM_Rhsim01G0090700	1.86503249	1.98777E-29	3.61343E-28	RHSIM_Rhsim01G0090700

Comparison	Gene ID	log_2FC	P-value	FDR (padj)	Gene Name
Root vs Leaf	RHSIM_Rhsim02G0029600	-3.806727111	2.09678E-29	3.80861E-28	RHSIM_Rhsim02G0029600
Root vs Leaf	RHSIM_Rhsim06G0149000	-1.353812934	2.1854E-29	3.96646E-28	RHSIM_Rhsim06G0149000
Root vs Leaf	RHSIM_Rhsim10G0105500	-2.453449911	2.23354E-29	4.05066E-28	RHSIM_Rhsim10G0105500
Root vs Leaf	RHSIM_Rhsim03G0216700	8.898078614	2.24414E-29	4.06669E-28	RHSIM_Rhsim03G0216700
Root vs Leaf	RHSIM_Rhsim12G0170600	-3.746622972	2.37659E-29	4.30057E-28	RHSIM_Rhsim12G0170600
Root vs Leaf	RHSIM_RhsimUnG0121000	-5.864894808	2.37692E-29	4.30057E-28	RHSIM_RhsimUnG0121000
Root vs Leaf	RHSIM_Rhsim08G0222400	-2.257114963	2.40066E-29	4.34013E-28	RHSIM_Rhsim08G0222400
Root vs Leaf	RHSIM_Rhsim02G0181400	1.677054394	2.53064E-29	4.57154E-28	RHSIM_Rhsim02G0181400
Root vs Leaf	RHSIM_Rhsim02G0113900	-7.926003527	2.57821E-29	4.65385E-28	RHSIM_Rhsim02G0113900
Root vs Leaf	RHSIM_Rhsim01G0198000	-7.475426253	2.87328E-29	5.18243E-28	RHSIM_Rhsim01G0198000
Root vs Leaf	RHSIM_Rhsim07G0118600	-1.242716721	3.00793E-29	5.42107E-28	RHSIM_Rhsim07G0118600
Root vs Leaf	RHSIM_Rhsim05G0104300	-2.252610359	3.71116E-29	6.68326E-28	RHSIM_Rhsim05G0104300
Root vs Leaf	RHSIM_Rhsim06G0152000	-4.816949637	3.72876E-29	6.70973E-28	RHSIM_Rhsim06G0152000
Root vs Leaf	novel.1794	-2.602669328	4.40249E-29	7.91592E-28	--
Root vs Leaf	RHSIM_Rhsim02G0237200	1.166744304	4.51111E-29	8.10494E-28	RHSIM_Rhsim02G0237200
Root vs Leaf	RHSIM_Rhsim05G0231400	-5.708811911	4.55419E-29	8.176E-28	RHSIM_Rhsim05G0231400
Root vs Leaf	RHSIM_Rhsim12G0036400	8.388054733	4.58512E-29	8.22513E-28	RHSIM_Rhsim12G0036400
Root vs Leaf	RHSIM_Rhsim06G0204400	-7.345533878	4.72949E-29	8.47754E-28	RHSIM_Rhsim06G0204400
Root vs Leaf	RHSIM_Rhsim04G0108200	-1.919453378	4.99936E-29	8.95435E-28	RHSIM_Rhsim04G0108200
Root vs Leaf	RHSIM_Rhsim10G0098900	3.246874274	5.22494E-29	9.35114E-28	RHSIM_Rhsim10G0098900
Root vs Leaf	RHSIM_Rhsim10G0029400	-2.706392395	5.46166E-29	9.76725E-28	RHSIM_Rhsim10G0029400
Root vs Leaf	RHSIM_RhsimUnG0185400	-2.638907899	5.52307E-29	9.86945E-28	RHSIM_RhsimUnG0185400
Root vs Leaf	RHSIM_Rhsim12G0022100	13.9082697	5.759E-29	1.02831E-27	RHSIM_Rhsim12G0022100
Root vs Leaf	RHSIM_Rhsim12G0126100	2.681822797	5.83332E-29	1.04078E-27	RHSIM_Rhsim12G0126100
Root vs Leaf	RHSIM_RhsimUnG0092800	-1.765480251	5.89881E-29	1.05089E-27	RHSIM_RhsimUnG0092800

Comparison	Gene ID	log_2FC	P-value	FDR (padj)	Gene Name
Root vs Leaf	RHSIM_Rhsim08G0144900	-3.271114138	5.89909E-29	1.05089E-27	RHSIM_Rhsim08G0144900
Root vs Leaf	RHSIM_Rhsim03G0111900	-3.355944789	6.14716E-29	1.09424E-27	RHSIM_Rhsim03G0111900
Root vs Leaf	RHSIM_Rhsim09G0094600	14.2034812	6.86064E-29	1.22031E-27	RHSIM_Rhsim09G0094600
Root vs Leaf	RHSIM_Rhsim12G0163200	1.878268078	7.19732E-29	1.27921E-27	RHSIM_Rhsim12G0163200
Root vs Leaf	RHSIM_Rhsim07G0172700	-2.706005505	7.50993E-29	1.33375E-27	RHSIM_Rhsim07G0172700
Root vs Leaf	RHSIM_Rhsim07G0025800	-2.995650887	7.95548E-29	1.41179E-27	RHSIM_Rhsim07G0025800
Root vs Leaf	RHSIM_RhsimUnG0115200	9.378317798	7.9906E-29	1.41694E-27	RHSIM_RhsimUnG0115200
Root vs Leaf	novel.4139	2.531833283	8.26967E-29	1.4653E-27	--
Root vs Leaf	RHSIM_Rhsim03G0110700	-3.024511671	8.56753E-29	1.51692E-27	RHSIM_Rhsim03G0110700
Root vs Leaf	RHSIM_Rhsim02G0005200	4.888281823	9.62213E-29	1.70234E-27	RHSIM_Rhsim02G0005200
Root vs Leaf	RHSIM_Rhsim08G0242300	-5.519749021	9.72848E-29	1.71984E-27	RHSIM_Rhsim08G0242300
Root vs Leaf	RHSIM_Rhsim01G0280700	-4.243869924	9.74769E-29	1.72192E-27	RHSIM_Rhsim01G0280700
Root vs Leaf	RHSIM_RhsimUnG0095500	-4.192535828	9.84721E-29	1.73817E-27	RHSIM_RhsimUnG0095500
Root vs Leaf	RHSIM_Rhsim06G0167500	-3.582308717	1.01116E-28	1.78348E-27	RHSIM_Rhsim06G0167500
Root vs Leaf	RHSIM_Rhsim04G0172800	1.436294776	1.05001E-28	1.85046E-27	RHSIM_Rhsim04G0172800
Root vs Leaf	RHSIM_Rhsim09G0208800	-2.49135062	1.05074E-28	1.85046E-27	RHSIM_Rhsim09G0208800
Root vs Leaf	RHSIM_RhsimUnG0007000	-9.794127919	1.05731E-28	1.86062E-27	RHSIM_RhsimUnG0007000
Root vs Leaf	RHSIM_Rhsim04G0191900	-4.570969105	1.13387E-28	1.99383E-27	RHSIM_Rhsim04G0191900
Root vs Leaf	RHSIM_Rhsim04G0001800	-4.238051752	1.25364E-28	2.20277E-27	RHSIM_Rhsim04G0001800
Root vs Leaf	novel.783	-2.047855186	1.27777E-28	2.24346E-27	--
Root vs Leaf	RHSIM_Rhsim13G0115100	11.87723881	1.28847E-28	2.26054E-27	RHSIM_Rhsim13G0115100
Root vs Leaf	RHSIM_Rhsim12G0014400	2.798474911	1.32739E-28	2.32706E-27	RHSIM_Rhsim12G0014400
Root vs Leaf	RHSIM_Rhsim02G0062100	2.767465605	1.42321E-28	2.49315E-27	RHSIM_Rhsim02G0062100
Root vs Leaf	RHSIM_Rhsim04G0207700	3.383789677	1.47023E-28	2.57358E-27	RHSIM_Rhsim04G0207700
Root vs Leaf	RHSIM_Rhsim06G0068700	2.528754989	1.47368E-28	2.57765E-27	RHSIM_Rhsim06G0068700

Comparison	Gene ID	log_2FC	P-value	FDR (padj)	Gene Name
Root vs Leaf	RHSIM_Rhsim01G0192200	-5.328049477	1.48625E-28	2.59768E-27	RHSIM_Rhsim01G0192200
Root vs Leaf	RHSIM_Rhsim09G0108500	-4.089641075	1.54076E-28	2.69092E-27	RHSIM_Rhsim09G0108500
Root vs Leaf	RHSIM_Rhsim11G0071200	-2.523934218	1.56072E-28	2.72373E-27	RHSIM_Rhsim11G0071200
Root vs Leaf	RHSIM_Rhsim04G0170700	-9.058468388	1.67178E-28	2.91535E-27	RHSIM_Rhsim04G0170700
Root vs Leaf	RHSIM_Rhsim07G0123800	-2.265457872	1.91354E-28	3.33443E-27	RHSIM_Rhsim07G0123800
Root vs Leaf	RHSIM_Rhsim08G0225500	-13.43076884	1.9599E-28	3.41239E-27	RHSIM_Rhsim08G0225500
Root vs Leaf	RHSIM_Rhsim10G0094600	2.297780944	1.96123E-28	3.41239E-27	RHSIM_Rhsim10G0094600
Root vs Leaf	RHSIM_Rhsim07G0023100	-2.759137821	2.04585E-28	3.55695E-27	RHSIM_Rhsim07G0023100
Root vs Leaf	RHSIM_Rhsim05G0076900	-2.270215801	2.17238E-28	3.77411E-27	RHSIM_Rhsim05G0076900
Root vs Leaf	RHSIM_Rhsim10G0127700	-2.020015438	2.2498E-28	3.90567E-27	RHSIM_Rhsim10G0127700
Root vs Leaf	RHSIM_Rhsim04G0036100	-1.454787454	2.28895E-28	3.97066E-27	RHSIM_Rhsim04G0036100
Root vs Leaf	RHSIM_Rhsim06G0218800	-1.553167473	2.37438E-28	4.11578E-27	RHSIM_Rhsim06G0218800
Root vs Leaf	RHSIM_Rhsim01G0198300	-4.224219674	2.39764E-28	4.15298E-27	RHSIM_Rhsim01G0198300
Root vs Leaf	RHSIM_Rhsim06G0218300	3.443178086	2.40248E-28	4.15825E-27	RHSIM_Rhsim06G0218300
Root vs Leaf	RHSIM_Rhsim01G0110100	-2.311958677	2.53001E-28	4.37571E-27	RHSIM_Rhsim01G0110100
Root vs Leaf	RHSIM_Rhsim11G0193300	-3.908843188	2.53353E-28	4.37852E-27	RHSIM_Rhsim11G0193300
Root vs Leaf	RHSIM_Rhsim04G0184300	6.263654565	2.63242E-28	4.54604E-27	RHSIM_Rhsim04G0184300
Root vs Leaf	RHSIM_Rhsim12G0099200	-10.17526875	2.71264E-28	4.68108E-27	RHSIM_Rhsim12G0099200
Root vs Leaf	RHSIM_Rhsim04G0138800	2.456086473	2.83064E-28	4.88107E-27	RHSIM_Rhsim04G0138800
Root vs Leaf	RHSIM_Rhsim06G0182000	-5.975750382	2.89049E-28	4.98054E-27	RHSIM_Rhsim06G0182000
Root vs Leaf	RHSIM_Rhsim03G0148300	-2.625543539	2.92141E-28	5.03008E-27	RHSIM_Rhsim03G0148300
Root vs Leaf	RHSIM_Rhsim08G0049700	-2.202492396	2.94698E-28	5.07034E-27	RHSIM_Rhsim08G0049700
Root vs Leaf	RHSIM_Rhsim10G0171700	-2.179092502	2.96734E-28	5.10158E-27	RHSIM_Rhsim10G0171700
Root vs Leaf	RHSIM_Rhsim04G0204600	-3.498788703	3.00745E-28	5.16669E-27	RHSIM_Rhsim04G0204600
Root vs Leaf	RHSIM_Rhsim07G0134000	1.359395479	3.05641E-28	5.24691E-27	RHSIM_Rhsim07G0134000

Comparison	Gene ID	log_2FC	P-value	FDR (padj)	Gene Name
Root vs Leaf	RHSIM_Rhsim12G0077000	-1.27519437	3.07579E-28	5.27628E-27	RHSIM_Rhsim12G0077000
Root vs Leaf	RHSIM_Rhsim12G0172700	2.305081478	3.12862E-28	5.36292E-27	RHSIM_Rhsim12G0172700
Root vs Leaf	RHSIM_RhsimUnG0060000	2.16110132	3.18176E-28	5.44996E-27	RHSIM_RhsimUnG0060000
Root vs Leaf	RHSIM_Rhsim06G0186600	3.185959025	3.52355E-28	6.03096E-27	RHSIM_Rhsim06G0186600
Root vs Leaf	RHSIM_Rhsim07G0124000	-2.072663811	4.93248E-28	8.43626E-27	RHSIM_Rhsim07G0124000
Root vs Leaf	RHSIM_Rhsim10G0126200	-1.96587136	5.08877E-28	8.69713E-27	RHSIM_Rhsim10G0126200
Root vs Leaf	RHSIM_Rhsim04G0024200	-4.268948658	5.20251E-28	8.88497E-27	RHSIM_Rhsim04G0024200
Root vs Leaf	RHSIM_Rhsim08G0225000	2.5935073	5.41282E-28	9.23732E-27	RHSIM_Rhsim08G0225000
Root vs Leaf	novel.2086	1.738116256	5.41941E-28	9.24175E-27	--
Root vs Leaf	RHSIM_Rhsim07G0239500	-3.137284925	5.43506E-28	9.26162E-27	RHSIM_Rhsim07G0239500
Root vs Leaf	RHSIM_Rhsim09G0184400	-3.91702276	5.45396E-28	9.28697E-27	RHSIM_Rhsim09G0184400
Root vs Leaf	RHSIM_Rhsim06G0199300	-4.116120256	5.81217E-28	9.88967E-27	RHSIM_Rhsim06G0199300
Root vs Leaf	RHSIM_Rhsim06G0027900	-3.313809768	5.91968E-28	1.00652E-26	RHSIM_Rhsim06G0027900
Root vs Leaf	RHSIM_Rhsim13G0179900	1.82493945	6.36797E-28	1.08195E-26	RHSIM_Rhsim13G0179900
Root vs Leaf	RHSIM_Rhsim12G0140300	5.328116666	6.4447E-28	1.09418E-26	RHSIM_Rhsim12G0140300
Root vs Leaf	RHSIM_Rhsim08G0062300	-5.660584405	6.81643E-28	1.15644E-26	RHSIM_Rhsim08G0062300
Root vs Leaf	RHSIM_Rhsim05G0061700	3.001042939	7.18629E-28	1.2183E-26	RHSIM_Rhsim05G0061700
Root vs Leaf	RHSIM_Rhsim12G0024300	-5.504702668	7.32767E-28	1.24136E-26	RHSIM_Rhsim12G0024300
Root vs Leaf	novel.1315	-9.580715827	7.51599E-28	1.27233E-26	--
Root vs Leaf	RHSIM_Rhsim10G0051100	-13.09632193	7.78802E-28	1.31742E-26	RHSIM_Rhsim10G0051100
Root vs Leaf	RHSIM_Rhsim10G0045100	-3.94546685	7.88702E-28	1.33319E-26	RHSIM_Rhsim10G0045100
Root vs Leaf	RHSIM_Rhsim02G0182300	-2.06771159	8.26889E-28	1.39672E-26	RHSIM_Rhsim02G0182300
Root vs Leaf	RHSIM_Rhsim06G0183200	-6.815347343	8.37671E-28	1.4139E-26	RHSIM_Rhsim06G0183200
Root vs Leaf	RHSIM_Rhsim01G0251800	3.89583158	8.66793E-28	1.46199E-26	RHSIM_Rhsim01G0251800
Root vs Leaf	RHSIM_Rhsim12G0040000	-3.07739406	8.73112E-28	1.47157E-26	RHSIM_Rhsim12G0040000

Comparison	Gene ID	log_2FC	P-value	FDR (padj)	Gene Name
Root vs Leaf	RHSIM_Rhsim02G0220000	-6.119053984	8.82003E-28	1.48547E-26	RHSIM_Rhsim02G0220000
Root vs Leaf	RHSIM_Rhsim07G0056200	5.848692275	8.96551E-28	1.50888E-26	RHSIM_Rhsim07G0056200
Root vs Leaf	RHSIM_Rhsim07G0045400	-13.13520142	9.03211E-28	1.51898E-26	RHSIM_Rhsim07G0045400
Root vs Leaf	RHSIM_Rhsim01G0210600	-2.498351437	9.59203E-28	1.61198E-26	RHSIM_Rhsim01G0210600
Root vs Leaf	RHSIM_Rhsim10G0166900	1.330595666	1.04805E-27	1.76002E-26	RHSIM_Rhsim10G0166900
Root vs Leaf	RHSIM_Rhsim09G0066700	-2.103608703	1.05392E-27	1.76858E-26	RHSIM_Rhsim09G0066700
Root vs Leaf	RHSIM_Rhsim10G0041900	-4.754815905	1.10147E-27	1.84703E-26	RHSIM_Rhsim10G0041900
Root vs Leaf	RHSIM_Rhsim01G0187900	-6.544234722	1.16453E-27	1.95137E-26	RHSIM_Rhsim01G0187900
Root vs Leaf	RHSIM_Rhsim08G0067200	-5.530787114	1.23863E-27	2.07403E-26	RHSIM_Rhsim08G0067200
Root vs Leaf	RHSIM_Rhsim04G0065900	-2.283098585	1.24403E-27	2.08157E-26	RHSIM_Rhsim04G0065900
Root vs Leaf	RHSIM_Rhsim04G0196000	-2.909377164	1.49238E-27	2.49532E-26	RHSIM_Rhsim04G0196000
Root vs Leaf	RHSIM_Rhsim02G0082500	-1.75526805	1.53745E-27	2.56882E-26	RHSIM_Rhsim02G0082500
Root vs Leaf	RHSIM_Rhsim11G0161100	3.373272929	1.68013E-27	2.80357E-26	RHSIM_Rhsim11G0161100
Root vs Leaf	RHSIM_Rhsim05G0039000	2.794637847	1.68037E-27	2.80357E-26	RHSIM_Rhsim05G0039000
Root vs Leaf	RHSIM_Rhsim01G0270400	1.644751943	1.7021E-27	2.83778E-26	RHSIM_Rhsim01G0270400
Root vs Leaf	novel.4891	-8.331572248	1.81726E-27	3.02759E-26	--
Root vs Leaf	RHSIM_Rhsim03G0088100	-4.373051501	1.8336E-27	3.05261E-26	RHSIM_Rhsim03G0088100
Root vs Leaf	RHSIM_Rhsim11G0076800	5.326061079	2.07938E-27	3.4593E-26	RHSIM_Rhsim11G0076800
Root vs Leaf	RHSIM_Rhsim08G0003400	-2.210087325	2.14757E-27	3.57019E-26	RHSIM_Rhsim08G0003400
Root vs Leaf	novel.2784	13.31446197	2.3692E-27	3.9358E-26	--
Root vs Leaf	RHSIM_Rhsim03G0207200	3.19473882	2.38041E-27	3.95159E-26	RHSIM_Rhsim03G0207200
Root vs Leaf	RHSIM_Rhsim08G0007800	-2.367919456	2.43469E-27	4.03879E-26	RHSIM_Rhsim08G0007800
Root vs Leaf	RHSIM_Rhsim04G0247700	1.678342015	2.50592E-27	4.15397E-26	RHSIM_Rhsim04G0247700
Root vs Leaf	RHSIM_RhsimUnG0136600	-2.34825486	2.57224E-27	4.26086E-26	RHSIM_RhsimUnG0136600
Root vs Leaf	RHSIM_Rhsim06G0207600	-2.826002079	2.61532E-27	4.32912E-26	RHSIM_Rhsim06G0207600

Comparison	Gene ID	log_2FC	P-value	FDR (padj)	Gene Name
Root vs Leaf	RHSIM_Rhsim08G0103500	6.08900273	2.92485E-27	4.83803E-26	RHSIM_Rhsim08G0103500
Root vs Leaf	novel.3058	6.808119404	3.04041E-27	5.02558E-26	--
Root vs Leaf	novel.3793	9.528064183	3.0976E-27	5.11646E-26	--
Root vs Leaf	RHSIM_RhsimUnG0226900	-1.745662565	3.18148E-27	5.25126E-26	RHSIM_RhsimUnG0226900
Root vs Leaf	RHSIM_Rhsim02G0001500	-1.813764066	3.2491E-27	5.35904E-26	RHSIM_Rhsim02G0001500
Root vs Leaf	RHSIM_Rhsim04G0187700	1.85399594	3.29457E-27	5.43018E-26	RHSIM_Rhsim04G0187700
Root vs Leaf	RHSIM_Rhsim02G0019800	-13.02533372	3.34275E-27	5.50566E-26	RHSIM_Rhsim02G0019800
Root vs Leaf	RHSIM_Rhsim08G0002700	-2.555536833	3.4948E-27	5.752E-26	RHSIM_Rhsim08G0002700
Root vs Leaf	RHSIM_Rhsim04G0178000	-9.195100789	3.50328E-27	5.76186E-26	RHSIM_Rhsim04G0178000
Root vs Leaf	RHSIM_Rhsim13G0163900	2.875059684	3.57474E-27	5.87523E-26	RHSIM_Rhsim13G0163900
Root vs Leaf	RHSIM_Rhsim03G0174700	2.259131926	3.646E-27	5.98808E-26	RHSIM_Rhsim03G0174700
Root vs Leaf	RHSIM_Rhsim03G0073400	3.220650262	3.71392E-27	6.0953E-26	RHSIM_Rhsim03G0073400
Root vs Leaf	RHSIM_Rhsim03G0051000	7.371214012	4.38826E-27	7.19693E-26	RHSIM_Rhsim03G0051000
Root vs Leaf	RHSIM_Rhsim07G0058600	6.276648255	4.57709E-27	7.5013E-26	RHSIM_Rhsim07G0058600
Root vs Leaf	novel.3076	-2.373617099	4.61412E-27	7.55664E-26	--
Root vs Leaf	novel.14	-12.89970352	4.81028E-27	7.87233E-26	--
Root vs Leaf	RHSIM_Rhsim12G0124100	4.409695808	4.9074E-27	8.02559E-26	RHSIM_Rhsim12G0124100
Root vs Leaf	RHSIM_Rhsim08G0031900	3.521508314	5.33326E-27	8.71589E-26	RHSIM_Rhsim08G0031900
Root vs Leaf	RHSIM_Rhsim03G0191300	-4.793319527	5.6042E-27	9.1522E-26	RHSIM_Rhsim03G0191300
Root vs Leaf	RHSIM_Rhsim07G0189000	-2.176790946	5.7707E-27	9.41748E-26	RHSIM_Rhsim07G0189000
Root vs Leaf	novel.3956	-3.926935325	6.00821E-27	9.79816E-26	--
Root vs Leaf	novel.452	-1.918899331	6.14642E-27	1.00121E-25	--
Root vs Leaf	novel.5170	5.806504825	6.14807E-27	1.00121E-25	--
Root vs Leaf	RHSIM_Rhsim01G0252300	1.435913773	6.17449E-27	1.00481E-25	RHSIM_Rhsim01G0252300
Root vs Leaf	novel.597	-7.588715878	6.22788E-27	1.01279E-25	--

Comparison	Gene ID	log_2FC	P-value	FDR (padj)	Gene Name
Root vs Leaf	RHSIM_Rhsim07G0016700	5.422973035	6.48964E-27	1.05461E-25	RHSIM_Rhsim07G0016700
Root vs Leaf	novel.4036	8.470141101	6.82566E-27	1.10844E-25	--
Root vs Leaf	RHSIM_Rhsim08G0100700	-2.584968772	6.91588E-27	1.1223E-25	RHSIM_Rhsim08G0100700
Root vs Leaf	RHSIM_Rhsim08G0171100	-2.271484378	7.08533E-27	1.149E-25	RHSIM_Rhsim08G0171100
Root vs Leaf	RHSIM_Rhsim10G0070700	6.312318765	7.17467E-27	1.16267E-25	RHSIM_Rhsim10G0070700
Root vs Leaf	novel.5093	4.601946274	7.2168E-27	1.16868E-25	--
Root vs Leaf	RHSIM_Rhsim04G0175900	-2.987959321	7.96301E-27	1.28862E-25	RHSIM_Rhsim04G0175900
Root vs Leaf	RHSIM_Rhsim08G0047300	4.528237762	7.97115E-27	1.28903E-25	RHSIM_Rhsim08G0047300
Root vs Leaf	RHSIM_Rhsim06G0071400	-6.03276147	8.18709E-27	1.32303E-25	RHSIM_Rhsim06G0071400
Root vs Leaf	RHSIM_Rhsim05G0082300	-2.382287068	8.21888E-27	1.32724E-25	RHSIM_Rhsim05G0082300
Root vs Leaf	RHSIM_Rhsim09G0187900	3.265462924	8.70717E-27	1.40511E-25	RHSIM_Rhsim09G0187900
Root vs Leaf	novel.1056	4.421730105	9.15799E-27	1.47683E-25	--
Root vs Leaf	RHSIM_Rhsim03G0215400	-2.853806872	9.35557E-27	1.50764E-25	RHSIM_Rhsim03G0215400
Root vs Leaf	RHSIM_Rhsim04G0010400	1.399958279	9.59228E-27	1.54471E-25	RHSIM_Rhsim04G0010400
Root vs Leaf	RHSIM_Rhsim05G0095100	-2.161634196	9.76226E-27	1.57099E-25	RHSIM_Rhsim05G0095100
Root vs Leaf	RHSIM_Rhsim11G0109900	8.635274298	1.0176E-26	1.63643E-25	RHSIM_Rhsim11G0109900
Root vs Leaf	RHSIM_Rhsim06G0175900	-2.825477457	1.03978E-26	1.67094E-25	RHSIM_Rhsim06G0175900
Root vs Leaf	RHSIM_Rhsim03G0161200	-2.56639298	1.05181E-26	1.6891E-25	RHSIM_Rhsim03G0161200
Root vs Leaf	RHSIM_Rhsim06G0232900	-1.918893573	1.1458E-26	1.83876E-25	RHSIM_Rhsim06G0232900
Root vs Leaf	RHSIM_Rhsim05G0069300	-2.165970848	1.23289E-26	1.97715E-25	RHSIM_Rhsim05G0069300
Root vs Leaf	RHSIM_Rhsim07G0037500	4.596355612	1.2803E-26	2.05175E-25	RHSIM_Rhsim07G0037500
Root vs Leaf	RHSIM_Rhsim09G0075000	2.173197524	1.43535E-26	2.29864E-25	RHSIM_Rhsim09G0075000
Root vs Leaf	RHSIM_Rhsim06G0108900	-6.061968676	1.48522E-26	2.37687E-25	RHSIM_Rhsim06G0108900
Root vs Leaf	RHSIM_Rhsim13G0063600	4.14387283	1.50951E-26	2.41406E-25	RHSIM_Rhsim13G0063600
Root vs Leaf	RHSIM_Rhsim01G0179000	-2.854452906	1.55029E-26	2.47757E-25	RHSIM_Rhsim01G0179000

Comparison	Gene ID	log_2FC	P-value	FDR (padj)	Gene Name
Root vs Leaf	RHSIM_Rhsim07G0207000	-2.996906411	1.58389E-26	2.52953E-25	RHSIM_Rhsim07G0207000
Root vs Leaf	RHSIM_Rhsim02G0243800	-5.202453243	1.59064E-26	2.53855E-25	RHSIM_Rhsim02G0243800
Root vs Leaf	RHSIM_Rhsim04G0134200	-1.951642457	1.60553E-26	2.56055E-25	RHSIM_Rhsim04G0134200
Root vs Leaf	novel.1469	-2.092494946	1.6169E-26	2.57691E-25	--
Root vs Leaf	RHSIM_Rhsim09G0068400	-1.924866301	1.63519E-26	2.60426E-25	RHSIM_Rhsim09G0068400
Root vs Leaf	RHSIM_Rhsim13G0220700	-2.811203055	1.69762E-26	2.70182E-25	RHSIM_Rhsim13G0220700
Root vs Leaf	RHSIM_Rhsim11G0179300	2.029595375	1.90324E-26	3.027E-25	RHSIM_Rhsim11G0179300
Root vs Leaf	RHSIM_Rhsim05G0180400	-3.562020215	1.98315E-26	3.15193E-25	RHSIM_Rhsim05G0180400
Root vs Leaf	RHSIM_Rhsim13G0143000	3.190722951	2.03407E-26	3.23063E-25	RHSIM_Rhsim13G0143000
Root vs Leaf	RHSIM_Rhsim02G0041100	-5.100737583	2.04596E-26	3.2473E-25	RHSIM_Rhsim02G0041100
Root vs Leaf	RHSIM_Rhsim12G0101300	3.501278201	2.07503E-26	3.29118E-25	RHSIM_Rhsim12G0101300
Root vs Leaf	RHSIM_Rhsim08G0167800	2.614449738	2.20676E-26	3.49772E-25	RHSIM_Rhsim08G0167800
Root vs Leaf	RHSIM_Rhsim05G0185900	2.480000051	2.25167E-26	3.56646E-25	RHSIM_Rhsim05G0185900
Root vs Leaf	RHSIM_Rhsim04G0049500	2.798274738	2.32049E-26	3.67295E-25	RHSIM_Rhsim04G0049500
Root vs Leaf	RHSIM_Rhsim11G0060200	6.397245382	2.36791E-26	3.74545E-25	RHSIM_Rhsim11G0060200
Root vs Leaf	RHSIM_Rhsim05G0188100	-1.910963369	2.52275E-26	3.98764E-25	RHSIM_Rhsim05G0188100
Root vs Leaf	novel.5387	-6.326849604	2.53011E-26	3.99654E-25	--
Root vs Leaf	RHSIM_Rhsim13G0115800	-3.941666943	2.58135E-26	4.07469E-25	RHSIM_Rhsim13G0115800
Root vs Leaf	RHSIM_Rhsim01G0016000	-1.70608536	2.5857E-26	4.07877E-25	RHSIM_Rhsim01G0016000
Root vs Leaf	RHSIM_Rhsim06G0141500	2.212051627	2.59786E-26	4.09516E-25	RHSIM_Rhsim06G0141500
Root vs Leaf	RHSIM_Rhsim02G0109900	-1.960805237	2.67514E-26	4.21412E-25	RHSIM_Rhsim02G0109900
Root vs Leaf	RHSIM_Rhsim03G0091900	3.659836876	2.73721E-26	4.30896E-25	RHSIM_Rhsim03G0091900
Root vs Leaf	RHSIM_RhsimUnG0120300	2.975694693	2.78631E-26	4.38327E-25	RHSIM_RhsimUnG0120300
Root vs Leaf	RHSIM_Rhsim11G0068500	-10.23093095	2.80113E-26	4.40359E-25	RHSIM_Rhsim11G0068500
Root vs Leaf	RHSIM_Rhsim09G0102500	3.702263837	2.87745E-26	4.5205E-25	RHSIM_Rhsim09G0102500

Comparison	Gene ID	log_2FC	P-value	FDR (padj)	Gene Name
Root vs Leaf	RHSIM_Rhsim13G0097500	4.221245927	2.8875E-26	4.53323E-25	RHSIM_Rhsim13G0097500
Root vs Leaf	RHSIM_Rhsim08G0123400	1.552826309	2.99952E-26	4.7059E-25	RHSIM_Rhsim08G0123400
Root vs Leaf	RHSIM_Rhsim06G0175100	7.647033101	3.16089E-26	4.95571E-25	RHSIM_Rhsim06G0175100
Root vs Leaf	RHSIM_Rhsim09G0061500	-1.339989653	3.17712E-26	4.97777E-25	RHSIM_Rhsim09G0061500
Root vs Leaf	RHSIM_Rhsim13G0024600	-1.879906805	3.37821E-26	5.28925E-25	RHSIM_Rhsim13G0024600
Root vs Leaf	RHSIM_RhsimUnG0027100	-1.98238333	3.38343E-26	5.29384E-25	RHSIM_RhsimUnG0027100
Root vs Leaf	RHSIM_Rhsim05G0006000	-2.972735894	3.56251E-26	5.57028E-25	RHSIM_Rhsim05G0006000
Root vs Leaf	novel.963	-1.416479543	3.59798E-26	5.62194E-25	--
Root vs Leaf	RHSIM_Rhsim01G0264200	6.660316899	3.69366E-26	5.76754E-25	RHSIM_Rhsim01G0264200
Root vs Leaf	RHSIM_Rhsim01G0024600	-2.297700395	3.75406E-26	5.85791E-25	RHSIM_Rhsim01G0024600
Root vs Leaf	RHSIM_Rhsim13G0076700	4.169971196	3.83882E-26	5.98613E-25	RHSIM_Rhsim13G0076700
Root vs Leaf	RHSIM_Rhsim04G0185200	3.348815428	3.84745E-26	5.99554E-25	RHSIM_Rhsim04G0185200
Root vs Leaf	RHSIM_Rhsim04G0083600	3.70856411	4.07802E-26	6.35057E-25	RHSIM_Rhsim04G0083600
Root vs Leaf	novel.870	7.785402074	4.65708E-26	7.24744E-25	--
Root vs Leaf	RHSIM_Rhsim01G0182400	-2.064550968	4.67518E-26	7.27072E-25	RHSIM_Rhsim01G0182400
Root vs Leaf	RHSIM_Rhsim03G0040900	-12.59052791	4.68367E-26	7.27904E-25	RHSIM_Rhsim03G0040900
Root vs Leaf	RHSIM_Rhsim03G0197200	-3.15967174	4.83608E-26	7.51086E-25	RHSIM_Rhsim03G0197200
Root vs Leaf	RHSIM_Rhsim01G0171700	-1.718064731	5.19065E-26	8.05612E-25	RHSIM_Rhsim01G0171700
Root vs Leaf	RHSIM_Rhsim12G0189400	-2.221220582	5.71046E-26	8.85695E-25	RHSIM_Rhsim12G0189400
Root vs Leaf	RHSIM_Rhsim13G0081700	2.734619403	5.71815E-26	8.86293E-25	RHSIM_Rhsim13G0081700
Root vs Leaf	RHSIM_Rhsim07G0201400	3.870043923	5.88876E-26	9.12126E-25	RHSIM_Rhsim07G0201400
Root vs Leaf	RHSIM_Rhsim03G0078700	2.695496754	6.07664E-26	9.40599E-25	RHSIM_Rhsim03G0078700
Root vs Leaf	RHSIM_Rhsim03G0161100	-4.385733851	6.24081E-26	9.65365E-25	RHSIM_Rhsim03G0161100
Root vs Leaf	RHSIM_Rhsim09G0117900	-5.560782968	6.52064E-26	1.00798E-24	RHSIM_Rhsim09G0117900
Root vs Leaf	RHSIM_Rhsim07G0124600	-3.021268543	6.54651E-26	1.0113E-24	RHSIM_Rhsim07G0124600

Comparison	Gene ID	log_2FC	P-value	FDR (padj)	Gene Name
Root vs Leaf	RHSIM_Rhsim13G0116000	-5.221023504	6.74973E-26	1.042E-24	RHSIM_Rhsim13G0116000
Root vs Leaf	RHSIM_Rhsim09G0040800	-1.510634114	6.92937E-26	1.06902E-24	RHSIM_Rhsim09G0040800
Root vs Leaf	RHSIM_RhsimUnG0254900	3.428958155	7.05543E-26	1.08774E-24	RHSIM_RhsimUnG0254900
Root vs Leaf	RHSIM_Rhsim07G0228900	-8.503738705	7.09644E-26	1.09333E-24	RHSIM_Rhsim07G0228900
Root vs Leaf	RHSIM_Rhsim11G0138400	-3.719737782	7.77843E-26	1.19761E-24	RHSIM_Rhsim11G0138400
Root vs Leaf	novel.1307	-7.565579063	8.71104E-26	1.34031E-24	--
Root vs Leaf	RHSIM_Rhsim02G0158200	-1.933168814	8.82643E-26	1.35716E-24	RHSIM_Rhsim02G0158200
Root vs Leaf	RHSIM_Rhsim01G0118700	2.790909753	9.11975E-26	1.40133E-24	RHSIM_Rhsim01G0118700
Root vs Leaf	RHSIM_Rhsim03G0073500	3.097660626	9.32155E-26	1.43139E-24	RHSIM_Rhsim03G0073500
Root vs Leaf	RHSIM_Rhsim09G0138900	-4.011377816	1.01413E-25	1.55623E-24	RHSIM_Rhsim09G0138900
Root vs Leaf	RHSIM_Rhsim03G0139800	2.315116563	1.03937E-25	1.59391E-24	RHSIM_Rhsim03G0139800
Root vs Leaf	RHSIM_Rhsim03G0140300	1.566143799	1.05733E-25	1.62037E-24	RHSIM_Rhsim03G0140300
Root vs Leaf	RHSIM_Rhsim06G0028900	4.821144384	1.06455E-25	1.63036E-24	RHSIM_Rhsim06G0028900
Root vs Leaf	RHSIM_Rhsim07G0174300	-7.662810168	1.08552E-25	1.66137E-24	RHSIM_Rhsim07G0174300
Root vs Leaf	RHSIM_Rhsim05G0087600	-2.906703659	1.095E-25	1.67478E-24	RHSIM_Rhsim05G0087600
Root vs Leaf	RHSIM_Rhsim06G0114600	-11.14920773	1.12546E-25	1.72022E-24	RHSIM_Rhsim06G0114600
Root vs Leaf	RHSIM_Rhsim07G0203600	4.477391138	1.12745E-25	1.72213E-24	RHSIM_Rhsim07G0203600
Root vs Leaf	RHSIM_Rhsim13G0033100	4.935288242	1.15162E-25	1.75789E-24	RHSIM_Rhsim13G0033100
Root vs Leaf	RHSIM_Rhsim07G0037000	5.613908739	1.29775E-25	1.97965E-24	RHSIM_Rhsim07G0037000
Root vs Leaf	RHSIM_Rhsim04G0104300	2.624228159	1.31267E-25	2.00108E-24	RHSIM_Rhsim04G0104300
Root vs Leaf	RHSIM_Rhsim10G0119200	-1.608309084	1.42723E-25	2.1743E-24	RHSIM_Rhsim10G0119200
Root vs Leaf	RHSIM_Rhsim07G0257200	-2.312267694	1.42858E-25	2.17493E-24	RHSIM_Rhsim07G0257200
Root vs Leaf	RHSIM_Rhsim06G0179600	-4.572946735	1.50967E-25	2.29686E-24	RHSIM_Rhsim06G0179600
Root vs Leaf	RHSIM_Rhsim13G0123100	-4.555075037	1.53358E-25	2.33171E-24	RHSIM_Rhsim13G0123100
Root vs Leaf	RHSIM_Rhsim04G0013500	-3.289761181	1.60931E-25	2.44523E-24	RHSIM_Rhsim04G0013500

Comparison	Gene ID	log_2FC	P-value	FDR (padj)	Gene Name
Root vs Leaf	RHSIM_Rhsim04G0231800	4.237833712	1.65401E-25	2.51151E-24	RHSIM_Rhsim04G0231800
Root vs Leaf	RHSIM_Rhsim01G0245900	-1.928958677	1.80146E-25	2.73361E-24	RHSIM_Rhsim01G0245900
Root vs Leaf	RHSIM_Rhsim01G0039300	-1.559442371	1.82361E-25	2.76542E-24	RHSIM_Rhsim01G0039300
Root vs Leaf	RHSIM_Rhsim08G0119500	-1.721014158	1.83462E-25	2.78029E-24	RHSIM_Rhsim08G0119500
Root vs Leaf	RHSIM_Rhsim08G0156700	-12.71389473	1.92049E-25	2.90851E-24	RHSIM_Rhsim08G0156700
Root vs Leaf	RHSIM_Rhsim04G0197900	-4.846338008	2.01675E-25	3.05229E-24	RHSIM_Rhsim04G0197900
Root vs Leaf	RHSIM_Rhsim07G0163500	-1.842998293	2.12189E-25	3.20933E-24	RHSIM_Rhsim07G0163500
Root vs Leaf	RHSIM_Rhsim02G0005500	1.519704244	2.1525E-25	3.2535E-24	RHSIM_Rhsim02G0005500
Root vs Leaf	RHSIM_Rhsim05G0081200	13.73518538	2.24944E-25	3.3978E-24	RHSIM_Rhsim05G0081200
Root vs Leaf	RHSIM_Rhsim06G0086900	-1.562352283	2.27713E-25	3.43737E-24	RHSIM_Rhsim06G0086900
Root vs Leaf	RHSIM_Rhsim09G0114800	-1.972655325	2.29187E-25	3.45738E-24	RHSIM_Rhsim09G0114800
Root vs Leaf	RHSIM_Rhsim03G0174400	-2.089654358	2.30512E-25	3.47509E-24	RHSIM_Rhsim03G0174400
Root vs Leaf	RHSIM_Rhsim12G0109900	-1.926433719	2.32883E-25	3.50855E-24	RHSIM_Rhsim12G0109900
Root vs Leaf	RHSIM_Rhsim10G0176600	-2.532933741	2.35267E-25	3.54217E-24	RHSIM_Rhsim10G0176600
Root vs Leaf	RHSIM_Rhsim06G0066300	3.33430443	2.41934E-25	3.64018E-24	RHSIM_Rhsim06G0066300
Root vs Leaf	RHSIM_Rhsim04G0172200	-3.534867646	2.55414E-25	3.84051E-24	RHSIM_Rhsim04G0172200
Root vs Leaf	novel.3652	-6.36157079	2.5897E-25	3.89145E-24	--
Root vs Leaf	RHSIM_Rhsim08G0079200	-2.862753066	2.65609E-25	3.98861E-24	RHSIM_Rhsim08G0079200
Root vs Leaf	RHSIM_Rhsim07G0058800	1.962253467	2.88984E-25	4.33682E-24	RHSIM_Rhsim07G0058800
Root vs Leaf	RHSIM_Rhsim07G0014300	-5.007560646	2.95211E-25	4.4274E-24	RHSIM_Rhsim07G0014300
Root vs Leaf	RHSIM_Rhsim09G0035700	5.320971982	2.95567E-25	4.42986E-24	RHSIM_Rhsim09G0035700
Root vs Leaf	RHSIM_Rhsim11G0106600	-5.689421699	3.16017E-25	4.7333E-24	RHSIM_Rhsim11G0106600
Root vs Leaf	RHSIM_Rhsim05G0114700	-5.866394935	3.23379E-25	4.84044E-24	RHSIM_Rhsim05G0114700
Root vs Leaf	novel.218	-7.982374288	3.3041E-25	4.94249E-24	--
Root vs Leaf	novel.3988	-1.972808825	3.90349E-25	5.83532E-24	--

Comparison	Gene ID	log_2FC	P-value	FDR (padj)	Gene Name
Root vs Leaf	RHSIM_Rhsim11G0076200	-6.553889075	3.93238E-25	5.87471E-24	RHSIM_Rhsim11G0076200
Root vs Leaf	RHSIM_Rhsim12G0160500	-2.036762143	4.12626E-25	6.16037E-24	RHSIM_Rhsim12G0160500
Root vs Leaf	RHSIM_Rhsim02G0193800	5.767457965	4.44721E-25	6.63526E-24	RHSIM_Rhsim02G0193800
Root vs Leaf	RHSIM_Rhsim05G0194900	-3.68973563	4.67242E-25	6.96679E-24	RHSIM_Rhsim05G0194900
Root vs Leaf	RHSIM_Rhsim10G0080000	-2.45084832	4.87312E-25	7.26136E-24	RHSIM_Rhsim10G0080000
Root vs Leaf	RHSIM_Rhsim01G0292700	1.239669305	5.30575E-25	7.90092E-24	RHSIM_Rhsim01G0292700
Root vs Leaf	RHSIM_Rhsim02G0087500	-12.46911172	5.44239E-25	8.09919E-24	RHSIM_Rhsim02G0087500
Root vs Leaf	RHSIM_Rhsim07G0064800	-6.335192361	5.47872E-25	8.14802E-24	RHSIM_Rhsim07G0064800
Root vs Leaf	RHSIM_Rhsim04G0215700	2.039155936	5.65945E-25	8.41138E-24	RHSIM_Rhsim04G0215700
Root vs Leaf	RHSIM_Rhsim03G0059500	-2.952929225	5.75918E-25	8.55411E-24	RHSIM_Rhsim03G0059500
Root vs Leaf	RHSIM_Rhsim03G0128600	2.119010948	5.85924E-25	8.69715E-24	RHSIM_Rhsim03G0128600
Root vs Leaf	RHSIM_Rhsim01G0015500	3.567819798	6.6149E-25	9.81252E-24	RHSIM_Rhsim01G0015500
Root vs Leaf	RHSIM_Rhsim12G0184600	-3.272384478	7.62828E-25	1.13085E-23	RHSIM_Rhsim12G0184600
Root vs Leaf	RHSIM_Rhsim03G0237900	-4.42130074	7.63499E-25	1.13112E-23	RHSIM_Rhsim03G0237900
Root vs Leaf	RHSIM_Rhsim05G0069600	-2.391138743	7.69053E-25	1.13862E-23	RHSIM_Rhsim05G0069600
Root vs Leaf	RHSIM_Rhsim04G0149400	4.203136858	8.22077E-25	1.21635E-23	RHSIM_Rhsim04G0149400
Root vs Leaf	RHSIM_Rhsim07G0095400	-11.65050386	8.55572E-25	1.2651E-23	RHSIM_Rhsim07G0095400
Root vs Leaf	RHSIM_Rhsim02G0034700	-12.31642824	9.27796E-25	1.37102E-23	RHSIM_Rhsim02G0034700
Root vs Leaf	RHSIM_Rhsim06G0122700	-6.491011468	9.59751E-25	1.41733E-23	RHSIM_Rhsim06G0122700
Root vs Leaf	RHSIM_Rhsim03G0171300	2.15604301	9.6428E-25	1.42311E-23	RHSIM_Rhsim03G0171300
Root vs Leaf	RHSIM_Rhsim03G0116500	-6.259889297	9.94417E-25	1.46665E-23	RHSIM_Rhsim03G0116500
Root vs Leaf	RHSIM_Rhsim11G0194700	-1.153497879	9.95861E-25	1.46785E-23	RHSIM_Rhsim11G0194700
Root vs Leaf	RHSIM_Rhsim03G0073300	3.638832675	1.00494E-24	1.48028E-23	RHSIM_Rhsim03G0073300
Root vs Leaf	RHSIM_Rhsim13G0001400	-2.751835904	1.03326E-24	1.52103E-23	RHSIM_Rhsim13G0001400
Root vs Leaf	RHSIM_Rhsim02G0022200	-11.24404685	1.05507E-24	1.55216E-23	RHSIM_Rhsim02G0022200

Comparison	Gene ID	log_2FC	P-value	FDR (padj)	Gene Name
Root vs Leaf	RHSIM_Rhsim08G0003900	-12.71385866	1.06672E-24	1.5683E-23	RHSIM_Rhsim08G0003900
Root vs Leaf	RHSIM_Rhsim13G0112300	-7.485506167	1.17759E-24	1.7302E-23	RHSIM_Rhsim13G0112300
Root vs Leaf	RHSIM_Rhsim08G0171700	-2.333405559	1.18304E-24	1.7371E-23	RHSIM_Rhsim08G0171700
Root vs Leaf	RHSIM_Rhsim07G0225700	3.343648546	1.19049E-24	1.74693E-23	RHSIM_Rhsim07G0225700
Root vs Leaf	RHSIM_Rhsim07G0016900	-4.271704071	1.19145E-24	1.74724E-23	RHSIM_Rhsim07G0016900
Root vs Leaf	novel.1066	3.132248754	1.19559E-24	1.75219E-23	--
Root vs Leaf	RHSIM_Rhsim09G0178100	-6.772536876	1.28613E-24	1.88369E-23	RHSIM_Rhsim09G0178100
Root vs Leaf	RHSIM_Rhsim01G0282700	3.842053217	1.30206E-24	1.90582E-23	RHSIM_Rhsim01G0282700
Root vs Leaf	RHSIM_Rhsim01G0210800	2.399539296	1.32341E-24	1.93584E-23	RHSIM_Rhsim01G0210800
Root vs Leaf	RHSIM_Rhsim03G0246400	-2.31734313	1.43009E-24	2.09056E-23	RHSIM_Rhsim03G0246400
Root vs Leaf	RHSIM_Rhsim06G0049600	-12.32360102	1.44009E-24	2.10386E-23	RHSIM_Rhsim06G0049600
Root vs Leaf	novel.124	-13.44318883	1.44225E-24	2.10568E-23	--
Root vs Leaf	RHSIM_Rhsim03G0082500	4.078524811	1.46087E-24	2.13152E-23	RHSIM_Rhsim03G0082500
Root vs Leaf	RHSIM_Rhsim11G0117500	-4.510571078	1.49456E-24	2.17931E-23	RHSIM_Rhsim11G0117500
Root vs Leaf	RHSIM_Rhsim13G0054900	-3.040604228	1.51849E-24	2.21281E-23	RHSIM_Rhsim13G0054900
Root vs Leaf	novel.4724	-5.343451811	1.60059E-24	2.33097E-23	--
Root vs Leaf	RHSIM_Rhsim01G0259800	1.374492358	1.61494E-24	2.3504E-23	RHSIM_Rhsim01G0259800
Root vs Leaf	RHSIM_Rhsim02G0169200	-2.219836663	1.71296E-24	2.49149E-23	RHSIM_Rhsim02G0169200
Root vs Leaf	RHSIM_Rhsim04G0185500	2.713729227	1.7201E-24	2.5003E-23	RHSIM_Rhsim04G0185500
Root vs Leaf	RHSIM_Rhsim02G0200700	2.45709403	1.72744E-24	2.5094E-23	RHSIM_Rhsim02G0200700
Root vs Leaf	RHSIM_Rhsim10G0101200	-1.538124821	1.87883E-24	2.72761E-23	RHSIM_Rhsim10G0101200
Root vs Leaf	RHSIM_Rhsim09G0032900	-2.820273502	1.9649E-24	2.85077E-23	RHSIM_Rhsim09G0032900
Root vs Leaf	RHSIM_Rhsim08G0240300	-1.333765164	1.98331E-24	2.87567E-23	RHSIM_Rhsim08G0240300
Root vs Leaf	RHSIM_Rhsim05G0197300	1.972434495	1.9907E-24	2.88459E-23	RHSIM_Rhsim05G0197300
Root vs Leaf	RHSIM_Rhsim07G0166500	-1.065049197	2.03802E-24	2.9513E-23	RHSIM_Rhsim07G0166500

Comparison	Gene ID	log_2FC	P-value	FDR (padj)	Gene Name
Root vs Leaf	RHSIM_Rhsim06G0142000	1.887978911	2.04707E-24	2.96256E-23	RHSIM_Rhsim06G0142000
Root vs Leaf	RHSIM_Rhsim12G0056800	4.621618849	2.11872E-24	3.06433E-23	RHSIM_Rhsim12G0056800
Root vs Leaf	RHSIM_Rhsim13G0225600	-6.130505368	2.15752E-24	3.1185E-23	RHSIM_Rhsim13G0225600
Root vs Leaf	RHSIM_Rhsim05G0175400	2.369317865	2.17444E-24	3.141E-23	RHSIM_Rhsim05G0175400
Root vs Leaf	RHSIM_Rhsim07G0040300	-11.88359765	2.23186E-24	3.22192E-23	RHSIM_Rhsim07G0040300
Root vs Leaf	RHSIM_Rhsim13G0166900	-2.414595423	2.23345E-24	3.22221E-23	RHSIM_Rhsim13G0166900
Root vs Leaf	RHSIM_Rhsim11G0080000	-5.905311277	2.30801E-24	3.32771E-23	RHSIM_Rhsim11G0080000
Root vs Leaf	RHSIM_Rhsim04G0222700	-2.060836521	2.36028E-24	3.40095E-23	RHSIM_Rhsim04G0222700
Root vs Leaf	RHSIM_Rhsim04G0168700	-2.790990636	2.47271E-24	3.56074E-23	RHSIM_Rhsim04G0168700
Root vs Leaf	RHSIM_Rhsim11G0169300	-2.193396726	2.54591E-24	3.66387E-23	RHSIM_Rhsim11G0169300
Root vs Leaf	novel.2291	-4.418772322	2.67544E-24	3.84788E-23	--
Root vs Leaf	RHSIM_Rhsim11G0057900	1.731248944	2.74996E-24	3.9526E-23	RHSIM_Rhsim11G0057900
Root vs Leaf	RHSIM_Rhsim04G0170600	-2.720379109	2.75208E-24	3.95319E-23	RHSIM_Rhsim04G0170600
Root vs Leaf	RHSIM_Rhsim05G0172300	-2.191126528	2.90964E-24	4.17692E-23	RHSIM_Rhsim05G0172300
Root vs Leaf	RHSIM_Rhsim06G0243000	2.187338404	3.11132E-24	4.46368E-23	RHSIM_Rhsim06G0243000
Root vs Leaf	novel.1755	2.362539372	3.11753E-24	4.46981E-23	--
Root vs Leaf	novel.4367	-2.514154284	3.1698E-24	4.54195E-23	--
Root vs Leaf	RHSIM_RhsimUnG0069700	5.430424031	3.31871E-24	4.75237E-23	RHSIM_RhsimUnG0069700
Root vs Leaf	novel.1576	4.775636754	3.44193E-24	4.92577E-23	--
Root vs Leaf	RHSIM_Rhsim10G0002200	2.045121033	3.49218E-24	4.99459E-23	RHSIM_Rhsim10G0002200
Root vs Leaf	RHSIM_Rhsim03G0069400	-3.098833156	3.55493E-24	5.08121E-23	RHSIM_Rhsim03G0069400
Root vs Leaf	RHSIM_Rhsim13G0054100	-2.18022597	3.77104E-24	5.38466E-23	RHSIM_Rhsim13G0054100
Root vs Leaf	RHSIM_Rhsim08G0234300	-1.926132659	3.77189E-24	5.38466E-23	RHSIM_Rhsim08G0234300
Root vs Leaf	RHSIM_Rhsim06G0187600	-3.751218805	3.8683E-24	5.51889E-23	RHSIM_Rhsim06G0187600
Root vs Leaf	RHSIM_Rhsim06G0087800	2.275214813	3.87579E-24	5.52617E-23	RHSIM_Rhsim06G0087800

Comparison	Gene ID	log_2FC	P-value	FDR (padj)	Gene Name
Root vs Leaf	novel.5146	3.676922871	3.90936E-24	5.57059E-23	--
Root vs Leaf	RHSIM_Rhsim08G0014200	-2.565878777	3.92958E-24	5.59596E-23	RHSIM_Rhsim08G0014200
Root vs Leaf	RHSIM_Rhsim04G0123100	5.367767003	3.9698E-24	5.64976E-23	RHSIM_Rhsim04G0123100
Root vs Leaf	RHSIM_RhsimUnG0146700	3.450441289	4.04979E-24	5.76006E-23	RHSIM_RhsimUnG0146700
Root vs Leaf	RHSIM_Rhsim10G0167700	1.866017043	4.1439E-24	5.89029E-23	RHSIM_Rhsim10G0167700
Root vs Leaf	RHSIM_RhsimUnG0035300	13.92036388	4.15242E-24	5.89878E-23	RHSIM_RhsimUnG0035300
Root vs Leaf	novel.4491	-2.392977494	4.2683E-24	6.05968E-23	--
Root vs Leaf	RHSIM_Rhsim11G0087300	-1.75064723	4.28296E-24	6.07676E-23	RHSIM_Rhsim11G0087300
Root vs Leaf	RHSIM_Rhsim10G0152000	-12.33881532	4.34933E-24	6.16714E-23	RHSIM_Rhsim10G0152000
Root vs Leaf	RHSIM_Rhsim06G0030400	4.114826308	4.46635E-24	6.3292E-23	RHSIM_Rhsim06G0030400
Root vs Leaf	RHSIM_Rhsim05G0143000	-2.480071141	4.65748E-24	6.596E-23	RHSIM_Rhsim05G0143000
Root vs Leaf	RHSIM_Rhsim13G0209200	-1.562907154	4.67474E-24	6.61641E-23	RHSIM_Rhsim13G0209200
Root vs Leaf	RHSIM_Rhsim09G0093700	-3.670898276	4.90052E-24	6.93172E-23	RHSIM_Rhsim09G0093700
Root vs Leaf	RHSIM_Rhsim03G0140800	8.052346025	4.95245E-24	7.0009E-23	RHSIM_Rhsim03G0140800
Root vs Leaf	RHSIM_Rhsim07G0081200	-2.480630323	5.10055E-24	7.20584E-23	RHSIM_Rhsim07G0081200
Root vs Leaf	RHSIM_Rhsim02G0254100	-2.12169018	5.29962E-24	7.48252E-23	RHSIM_Rhsim02G0254100
Root vs Leaf	RHSIM_Rhsim09G0022400	3.058179129	5.31605E-24	7.50114E-23	RHSIM_Rhsim09G0022400
Root vs Leaf	RHSIM_Rhsim07G0092600	4.66939451	5.38821E-24	7.59832E-23	RHSIM_Rhsim07G0092600
Root vs Leaf	RHSIM_Rhsim02G0007400	2.01854568	5.49556E-24	7.74499E-23	RHSIM_Rhsim02G0007400
Root vs Leaf	RHSIM_Rhsim12G0090500	-1.086242697	5.69159E-24	8.01637E-23	RHSIM_Rhsim12G0090500
Root vs Leaf	RHSIM_Rhsim03G0167300	-3.105923815	6.11472E-24	8.6071E-23	RHSIM_Rhsim03G0167300
Root vs Leaf	RHSIM_Rhsim01G0262300	-1.600706003	6.34016E-24	8.91901E-23	RHSIM_Rhsim01G0262300
Root vs Leaf	RHSIM_Rhsim05G0229600	-2.06437837	6.39221E-24	8.98676E-23	RHSIM_Rhsim05G0229600
Root vs Leaf	RHSIM_Rhsim06G0079300	2.696878574	6.48507E-24	9.11178E-23	RHSIM_Rhsim06G0079300
Root vs Leaf	RHSIM_Rhsim08G0014700	3.192660332	6.59153E-24	9.25574E-23	RHSIM_Rhsim08G0014700

Comparison	Gene ID	log_2FC	P-value	FDR (padj)	Gene Name
Root vs Leaf	RHSIM_Rhsim04G0136600	2.069885498	6.63604E-24	9.3126E-23	RHSIM_Rhsim04G0136600
Root vs Leaf	RHSIM_Rhsim04G0134300	-1.66233271	6.85795E-24	9.61816E-23	RHSIM_Rhsim04G0134300
Root vs Leaf	RHSIM_RhsimUnG0087700	-3.840293793	6.88326E-24	9.64782E-23	RHSIM_RhsimUnG0087700
Root vs Leaf	RHSIM_Rhsim01G0250800	-1.426989695	7.54146E-24	1.0564E-22	RHSIM_Rhsim01G0250800
Root vs Leaf	RHSIM_Rhsim04G0050200	-2.030596184	7.67436E-24	1.07436E-22	RHSIM_Rhsim04G0050200
Root vs Leaf	RHSIM_Rhsim08G0005200	1.580261828	7.85028E-24	1.09833E-22	RHSIM_Rhsim08G0005200
Root vs Leaf	RHSIM_Rhsim13G0100200	4.130886328	7.99979E-24	1.11857E-22	RHSIM_Rhsim13G0100200
Root vs Leaf	RHSIM_Rhsim03G0232600	-12.07015658	8.67394E-24	1.2121E-22	RHSIM_Rhsim03G0232600
Root vs Leaf	RHSIM_Rhsim05G0044400	5.105899594	8.84924E-24	1.23585E-22	RHSIM_Rhsim05G0044400
Root vs Leaf	RHSIM_Rhsim04G0138200	-2.502671479	8.96816E-24	1.2517E-22	RHSIM_Rhsim04G0138200
Root vs Leaf	novel.4179	2.970890733	9.11715E-24	1.27173E-22	--
Root vs Leaf	RHSIM_Rhsim02G0244000	-12.30404506	9.52872E-24	1.32834E-22	RHSIM_Rhsim02G0244000
Root vs Leaf	RHSIM_Rhsim10G0028200	-4.549234828	9.62819E-24	1.3414E-22	RHSIM_Rhsim10G0028200
Root vs Leaf	RHSIM_Rhsim12G0042700	4.297850893	1.03045E-23	1.43475E-22	RHSIM_Rhsim12G0042700
Root vs Leaf	RHSIM_Rhsim11G0012800	6.539490558	1.04318E-23	1.45161E-22	RHSIM_Rhsim11G0012800
Root vs Leaf	RHSIM_Rhsim01G0197200	-2.288839262	1.07463E-23	1.49358E-22	RHSIM_Rhsim01G0197200
Root vs Leaf	RHSIM_Rhsim12G0036300	9.816136722	1.09353E-23	1.51894E-22	RHSIM_Rhsim12G0036300
Root vs Leaf	RHSIM_Rhsim09G0117800	-3.446822053	1.10647E-23	1.53598E-22	RHSIM_Rhsim09G0117800
Root vs Leaf	RHSIM_Rhsim08G0055300	3.750414217	1.11447E-23	1.54616E-22	RHSIM_Rhsim08G0055300
Root vs Leaf	RHSIM_Rhsim01G0118100	1.821067351	1.17895E-23	1.63464E-22	RHSIM_Rhsim01G0118100
Root vs Leaf	RHSIM_Rhsim03G0039900	-2.507837466	1.23175E-23	1.70683E-22	RHSIM_Rhsim03G0039900
Root vs Leaf	RHSIM_Rhsim11G0164900	-1.542064749	1.25127E-23	1.73283E-22	RHSIM_Rhsim11G0164900
Root vs Leaf	RHSIM_Rhsim07G0214800	-2.897381499	1.25829E-23	1.74151E-22	RHSIM_Rhsim07G0214800
Root vs Leaf	RHSIM_Rhsim09G0103400	1.257816025	1.3763E-23	1.9037E-22	RHSIM_Rhsim09G0103400
Root vs Leaf	novel.5184	-2.190124346	1.38261E-23	1.91128E-22	--

Comparison	Gene ID	log_2FC	P-value	FDR (padj)	Gene Name
Root vs Leaf	RHSIM_Rhsim10G0166800	1.500715754	1.44024E-23	1.98977E-22	RHSIM_Rhsim10G0166800
Root vs Leaf	RHSIM_Rhsim02G0240600	3.561912821	1.47254E-23	2.03318E-22	RHSIM_Rhsim02G0240600
Root vs Leaf	novel.2705	-2.084310777	1.48934E-23	2.05514E-22	--
Root vs Leaf	RHSIM_Rhsim08G0232900	-2.385891414	1.54277E-23	2.12761E-22	RHSIM_Rhsim08G0232900
Root vs Leaf	RHSIM_Rhsim09G0131100	4.895119895	1.55251E-23	2.13977E-22	RHSIM_Rhsim09G0131100
Root vs Leaf	RHSIM_Rhsim06G0142500	-1.57450964	1.55477E-23	2.14161E-22	RHSIM_Rhsim06G0142500
Root vs Leaf	RHSIM_Rhsim02G0237100	-10.42713439	1.67688E-23	2.30843E-22	RHSIM_Rhsim02G0237100
Root vs Leaf	RHSIM_Rhsim09G0192800	-2.110776446	1.81707E-23	2.49993E-22	RHSIM_Rhsim09G0192800
Root vs Leaf	RHSIM_Rhsim12G0020700	2.994818002	1.90352E-23	2.61731E-22	RHSIM_Rhsim12G0020700
Root vs Leaf	RHSIM_Rhsim09G0067100	-3.254670111	1.94844E-23	2.67749E-22	RHSIM_Rhsim09G0067100
Root vs Leaf	RHSIM_Rhsim02G0233400	1.896984257	1.97862E-23	2.71735E-22	RHSIM_Rhsim02G0233400
Root vs Leaf	RHSIM_Rhsim12G0083500	-2.472448104	2.03664E-23	2.79537E-22	RHSIM_Rhsim12G0083500
Root vs Leaf	RHSIM_Rhsim08G0144800	-8.71946142	2.15978E-23	2.96262E-22	RHSIM_Rhsim08G0144800
Root vs Leaf	RHSIM_Rhsim09G0142300	2.001624854	2.18994E-23	3.00222E-22	RHSIM_Rhsim09G0142300
Root vs Leaf	RHSIM_Rhsim01G0239400	-1.66657845	2.20196E-23	3.01691E-22	RHSIM_Rhsim01G0239400
Root vs Leaf	RHSIM_Rhsim01G0230400	5.783293067	2.26142E-23	3.09653E-22	RHSIM_Rhsim01G0230400
Root vs Leaf	novel.3963	12.57631409	2.27781E-23	3.11714E-22	--
Root vs Leaf	RHSIM_Rhsim11G0023300	3.33570698	2.34034E-23	3.20082E-22	RHSIM_Rhsim11G0023300
Root vs Leaf	RHSIM_Rhsim02G0141100	3.325136704	2.45551E-23	3.35634E-22	RHSIM_Rhsim02G0141100
Root vs Leaf	RHSIM_Rhsim04G0072300	2.175355917	2.71397E-23	3.70743E-22	RHSIM_Rhsim04G0072300
Root vs Leaf	RHSIM_RhsimUnG0221400	-12.62799306	2.73293E-23	3.73114E-22	RHSIM_RhsimUnG0221400
Root vs Leaf	RHSIM_Rhsim03G0209000	3.12733501	2.81944E-23	3.84685E-22	RHSIM_Rhsim03G0209000
Root vs Leaf	RHSIM_Rhsim10G0065100	-2.964937493	2.82101E-23	3.84685E-22	RHSIM_Rhsim10G0065100
Root vs Leaf	novel.1316	-10.43234833	2.85277E-23	3.88786E-22	--
Root vs Leaf	RHSIM_Rhsim03G0141800	-2.515184826	3.29345E-23	4.48579E-22	RHSIM_Rhsim03G0141800

Comparison	Gene ID	log_2FC	P-value	FDR (padj)	Gene Name
Root vs Leaf	RHSIM_Rhsim12G0157000	1.485876489	3.35906E-23	4.57247E-22	RHSIM_Rhsim12G0157000
Root vs Leaf	novel.1138	-1.242832056	3.37227E-23	4.58775E-22	--
Root vs Leaf	RHSIM_Rhsim12G0106700	5.862583359	3.58986E-23	4.88091E-22	RHSIM_Rhsim12G0106700
Root vs Leaf	RHSIM_RhsimUnG0249500	-1.54109665	3.6027E-23	4.89548E-22	RHSIM_RhsimUnG0249500
Root vs Leaf	RHSIM_Rhsim05G0184000	-6.06913909	3.71989E-23	5.05177E-22	RHSIM_Rhsim05G0184000
Root vs Leaf	RHSIM_Rhsim01G0067800	-1.267364737	3.73971E-23	5.07569E-22	RHSIM_Rhsim01G0067800
Root vs Leaf	RHSIM_Rhsim09G0002200	-3.273781653	3.7803E-23	5.12778E-22	RHSIM_Rhsim09G0002200
Root vs Leaf	RHSIM_Rhsim05G0203700	3.422445065	3.87344E-23	5.25104E-22	RHSIM_Rhsim05G0203700
Root vs Leaf	RHSIM_Rhsim07G0226800	4.27165084	3.89783E-23	5.28101E-22	RHSIM_Rhsim07G0226800
Root vs Leaf	RHSIM_Rhsim08G0239300	-4.162383957	3.96197E-23	5.36477E-22	RHSIM_Rhsim08G0239300
Root vs Leaf	RHSIM_Rhsim08G0032000	3.70379613	4.05334E-23	5.48529E-22	RHSIM_Rhsim08G0032000
Root vs Leaf	RHSIM_Rhsim05G0086700	-1.99137681	4.16298E-23	5.63037E-22	RHSIM_Rhsim05G0086700
Root vs Leaf	RHSIM_Rhsim05G0049700	-5.36911581	4.41155E-23	5.96306E-22	RHSIM_Rhsim05G0049700
Root vs Leaf	RHSIM_Rhsim05G0145100	-8.610742838	4.42969E-23	5.9841E-22	RHSIM_Rhsim05G0145100
Root vs Leaf	RHSIM_Rhsim04G0022600	-2.722679318	4.58924E-23	6.19601E-22	RHSIM_Rhsim04G0022600
Root vs Leaf	RHSIM_Rhsim02G0001800	6.389094905	4.62125E-23	6.23559E-22	RHSIM_Rhsim02G0001800
Root vs Leaf	novel.2	1.42910389	4.68603E-23	6.31931E-22	--
Root vs Leaf	RHSIM_Rhsim10G0139200	-8.157106605	5.0905E-23	6.86076E-22	RHSIM_Rhsim10G0139200
Root vs Leaf	RHSIM_Rhsim12G0209400	-2.691551898	5.4286E-23	7.31217E-22	RHSIM_Rhsim12G0209400
Root vs Leaf	RHSIM_Rhsim11G0178600	2.243517176	5.53267E-23	7.44802E-22	RHSIM_Rhsim11G0178600
Root vs Leaf	RHSIM_Rhsim02G0132300	-2.55544209	5.53759E-23	7.45031E-22	RHSIM_Rhsim02G0132300
Root vs Leaf	RHSIM_Rhsim07G0075100	-1.456455038	5.61218E-23	7.54628E-22	RHSIM_Rhsim07G0075100
Root vs Leaf	RHSIM_Rhsim03G0154300	-11.79814894	6.08878E-23	8.18238E-22	RHSIM_Rhsim03G0154300
Root vs Leaf	RHSIM_Rhsim11G0046700	3.243738759	6.56839E-23	8.82177E-22	RHSIM_Rhsim11G0046700
Root vs Leaf	RHSIM_Rhsim13G0181400	-6.917564127	6.58281E-23	8.83601E-22	RHSIM_Rhsim13G0181400

Comparison	Gene ID	log_2FC	P-value	FDR (padj)	Gene Name
Root vs Leaf	RHSIM_Rhsim02G0014200	1.456109489	7.01061E-23	9.40478E-22	RHSIM_Rhsim02G0014200
Root vs Leaf	RHSIM_Rhsim07G0006200	-2.183012449	7.03746E-23	9.43533E-22	RHSIM_Rhsim07G0006200
Root vs Leaf	novel.4168	-8.606268049	7.09917E-23	9.51256E-22	--
Root vs Leaf	novel.2137	-6.396113561	7.11027E-23	9.52192E-22	--
Root vs Leaf	RHSIM_Rhsim06G0184000	3.48826314	7.31601E-23	9.79178E-22	RHSIM_Rhsim06G0184000
Root vs Leaf	RHSIM_Rhsim10G0069300	-2.199756886	7.34779E-23	9.82863E-22	RHSIM_Rhsim10G0069300
Root vs Leaf	RHSIM_Rhsim08G0113600	-2.095620529	7.48391E-23	1.00049E-21	RHSIM_Rhsim08G0113600
Root vs Leaf	RHSIM_Rhsim11G0160000	3.4062122	7.56537E-23	1.0108E-21	RHSIM_Rhsim11G0160000
Root vs Leaf	RHSIM_Rhsim08G0245900	2.78164549	7.63848E-23	1.01998E-21	RHSIM_Rhsim08G0245900
Root vs Leaf	RHSIM_Rhsim10G0174000	-4.382351579	7.67004E-23	1.0236E-21	RHSIM_Rhsim10G0174000
Root vs Leaf	RHSIM_RhsimUnG0234700	-2.782652018	8.67902E-23	1.15759E-21	RHSIM_RhsimUnG0234700
Root vs Leaf	RHSIM_Rhsim01G0206500	-1.712047582	8.89006E-23	1.18505E-21	RHSIM_Rhsim01G0206500
Root vs Leaf	RHSIM_Rhsim01G0175000	-1.033878623	9.04486E-23	1.20499E-21	RHSIM_Rhsim01G0175000
Root vs Leaf	RHSIM_Rhsim04G0180800	2.991748782	9.8964E-23	1.31768E-21	RHSIM_Rhsim04G0180800
Root vs Leaf	novel.2464	-3.376482874	1.02E-22	1.35656E-21	--
Root vs Leaf	RHSIM_Rhsim07G0108600	-1.576268235	1.02001E-22	1.35656E-21	RHSIM_Rhsim07G0108600
Root vs Leaf	RHSIM_Rhsim13G0042900	1.40038999	1.03275E-22	1.37271E-21	RHSIM_Rhsim13G0042900
Root vs Leaf	novel.1308	-6.658326854	1.07252E-22	1.42475E-21	--
Root vs Leaf	RHSIM_Rhsim07G0090300	-3.869558664	1.14674E-22	1.52247E-21	RHSIM_Rhsim07G0090300
Root vs Leaf	RHSIM_Rhsim02G0186800	2.0603285	1.21325E-22	1.60985E-21	RHSIM_Rhsim02G0186800
Root vs Leaf	RHSIM_Rhsim10G0040000	-1.876374628	1.24181E-22	1.6468E-21	RHSIM_Rhsim10G0040000
Root vs Leaf	RHSIM_Rhsim11G0055300	2.777461236	1.25794E-22	1.66724E-21	RHSIM_Rhsim11G0055300
Root vs Leaf	RHSIM_Rhsim07G0225300	-1.955956264	1.28727E-22	1.70514E-21	RHSIM_Rhsim07G0225300
Root vs Leaf	RHSIM_Rhsim05G0175300	-2.963306072	1.2935E-22	1.71187E-21	RHSIM_Rhsim05G0175300
Root vs Leaf	RHSIM_Rhsim02G0153500	-1.684693594	1.29384E-22	1.71187E-21	RHSIM_Rhsim02G0153500

Comparison	Gene ID	log_2FC	P-value	FDR (padj)	Gene Name
Root vs Leaf	novel.1133	-2.68843926	1.29651E-22	1.71443E-21	--
Root vs Leaf	RHSIM_Rhsim03G0131600	-3.089322657	1.37391E-22	1.81574E-21	RHSIM_Rhsim03G0131600
Root vs Leaf	RHSIM_Rhsim09G0143600	5.784912217	1.38604E-22	1.83073E-21	RHSIM_Rhsim09G0143600
Root vs Leaf	RHSIM_Rhsim01G0192300	2.435227873	1.3977E-22	1.84508E-21	RHSIM_Rhsim01G0192300
Root vs Leaf	RHSIM_Rhsim12G0081600	1.166596282	1.41127E-22	1.86193E-21	RHSIM_Rhsim12G0081600
Root vs Leaf	RHSIM_Rhsim12G0114700	-6.107166711	1.4215E-22	1.87435E-21	RHSIM_Rhsim12G0114700
Root vs Leaf	RHSIM_Rhsim13G0069000	-1.879250481	1.48099E-22	1.95168E-21	RHSIM_Rhsim13G0069000
Root vs Leaf	RHSIM_Rhsim05G0012400	-1.574701351	1.62869E-22	2.1451E-21	RHSIM_Rhsim05G0012400
Root vs Leaf	RHSIM_Rhsim06G0169600	-8.27662815	1.75549E-22	2.31079E-21	RHSIM_Rhsim06G0169600
Root vs Leaf	RHSIM_Rhsim06G0058200	-6.182585245	1.77775E-22	2.33877E-21	RHSIM_Rhsim06G0058200
Root vs Leaf	RHSIM_Rhsim03G0234800	-1.866865465	1.80113E-22	2.36818E-21	RHSIM_Rhsim03G0234800
Root vs Leaf	RHSIM_Rhsim13G0017200	-2.542871654	1.8341E-22	2.41016E-21	RHSIM_Rhsim13G0017200
Root vs Leaf	RHSIM_RhsimUnG0030200	-5.274012401	1.91741E-22	2.51821E-21	RHSIM_RhsimUnG0030200
Root vs Leaf	novel.5152	-4.566085128	1.92108E-22	2.52159E-21	--
Root vs Leaf	RHSIM_Rhsim07G0234200	2.362854257	1.99326E-22	2.61486E-21	RHSIM_Rhsim07G0234200
Root vs Leaf	RHSIM_Rhsim06G0159700	8.541694647	1.99659E-22	2.61773E-21	RHSIM_Rhsim06G0159700
Root vs Leaf	RHSIM_Rhsim01G0124300	-2.554136424	2.00884E-22	2.6323E-21	RHSIM_Rhsim01G0124300
Root vs Leaf	RHSIM_RhsimUnG0154700	3.206459612	2.02084E-22	2.64654E-21	RHSIM_RhsimUnG0154700
Root vs Leaf	novel.1577	2.009220707	2.16231E-22	2.8302E-21	--
Root vs Leaf	RHSIM_Rhsim11G0048100	2.001382763	2.17724E-22	2.84813E-21	RHSIM_Rhsim11G0048100
Root vs Leaf	RHSIM_Rhsim13G0013600	-3.133806293	2.22346E-22	2.90695E-21	RHSIM_Rhsim13G0013600
Root vs Leaf	RHSIM_Rhsim12G0074200	4.46438618	2.26375E-22	2.95796E-21	RHSIM_Rhsim12G0074200
Root vs Leaf	RHSIM_Rhsim07G0014500	-7.141212566	2.30525E-22	3.01049E-21	RHSIM_Rhsim07G0014500
Root vs Leaf	RHSIM_Rhsim11G0108000	9.753627575	2.37924E-22	3.10536E-21	RHSIM_Rhsim11G0108000
Root vs Leaf	RHSIM_RhsimUnG0074100	-2.650349671	2.38699E-22	3.11372E-21	RHSIM_RhsimUnG0074100

Comparison	Gene ID	log_2FC	P-value	FDR (padj)	Gene Name
Root vs Leaf	RHSIM_Rhsim03G0227400	-2.132851915	2.47885E-22	3.23172E-21	RHSIM_Rhsim03G0227400
Root vs Leaf	RHSIM_Rhsim12G0212200	-3.454115665	2.6443E-22	3.44548E-21	RHSIM_Rhsim12G0212200
Root vs Leaf	RHSIM_Rhsim01G0026600	-1.81607723	2.72211E-22	3.54488E-21	RHSIM_Rhsim01G0026600
Root vs Leaf	RHSIM_Rhsim05G0233100	-2.22276181	2.80599E-22	3.65205E-21	RHSIM_Rhsim05G0233100
Root vs Leaf	RHSIM_Rhsim01G0214800	2.659973897	2.81848E-22	3.66625E-21	RHSIM_Rhsim01G0214800
Root vs Leaf	RHSIM_Rhsim02G0145800	-1.650438801	2.92725E-22	3.80559E-21	RHSIM_Rhsim02G0145800
Root vs Leaf	RHSIM_Rhsim12G0192900	-1.38444817	3.03807E-22	3.94744E-21	RHSIM_Rhsim12G0192900
Root vs Leaf	RHSIM_Rhsim11G0164100	-3.175731147	3.16237E-22	4.10665E-21	RHSIM_Rhsim11G0164100
Root vs Leaf	RHSIM_Rhsim11G0145400	6.308491119	3.56692E-22	4.62939E-21	RHSIM_Rhsim11G0145400
Root vs Leaf	RHSIM_Rhsim11G0088900	-2.513114861	3.98817E-22	5.17322E-21	RHSIM_Rhsim11G0088900
Root vs Leaf	RHSIM_Rhsim11G0126700	-2.345335389	4.14742E-22	5.37677E-21	RHSIM_Rhsim11G0126700
Root vs Leaf	RHSIM_Rhsim02G0120800	1.691483089	4.21257E-22	5.45818E-21	RHSIM_Rhsim02G0120800
Root vs Leaf	RHSIM_Rhsim06G0010600	-10.35553982	4.21812E-22	5.46231E-21	RHSIM_Rhsim06G0010600
Root vs Leaf	RHSIM_Rhsim12G0025400	-5.290416759	4.35661E-22	5.6385E-21	RHSIM_Rhsim12G0025400
Root vs Leaf	RHSIM_Rhsim07G0241000	-9.175411889	4.476E-22	5.78978E-21	RHSIM_Rhsim07G0241000
Root vs Leaf	RHSIM_Rhsim01G0238200	3.033814708	4.61171E-22	5.96199E-21	RHSIM_Rhsim01G0238200
Root vs Leaf	RHSIM_Rhsim01G0059800	-1.477398603	4.66587E-22	6.02864E-21	RHSIM_Rhsim01G0059800
Root vs Leaf	RHSIM_Rhsim04G0155500	-1.47037378	4.67628E-22	6.03872E-21	RHSIM_Rhsim04G0155500
Root vs Leaf	RHSIM_Rhsim08G0198900	-1.626797105	4.92009E-22	6.35001E-21	RHSIM_Rhsim08G0198900
Root vs Leaf	RHSIM_Rhsim13G0070200	2.825729148	5.09805E-22	6.57603E-21	RHSIM_Rhsim13G0070200
Root vs Leaf	RHSIM_Rhsim08G0158800	6.271640348	5.84187E-22	7.5313E-21	RHSIM_Rhsim08G0158800
Root vs Leaf	RHSIM_Rhsim04G0108400	-1.444745615	5.97504E-22	7.69868E-21	RHSIM_Rhsim04G0108400
Root vs Leaf	RHSIM_Rhsim12G0182500	-1.106768503	6.41533E-22	8.26139E-21	RHSIM_Rhsim12G0182500
Root vs Leaf	RHSIM_Rhsim07G0073300	1.515040822	6.56985E-22	8.45567E-21	RHSIM_Rhsim07G0073300
Root vs Leaf	RHSIM_Rhsim03G0098400	-2.150449202	7.01081E-22	9.01819E-21	RHSIM_Rhsim03G0098400

Comparison	Gene ID	log_2FC	P-value	FDR (padj)	Gene Name
Root vs Leaf	RHSIM_Rhsim02G0006500	-3.202633404	7.06298E-22	9.08025E-21	RHSIM_Rhsim02G0006500
Root vs Leaf	RHSIM_Rhsim06G0174300	-3.062257892	7.30482E-22	9.38594E-21	RHSIM_Rhsim06G0174300
Root vs Leaf	RHSIM_Rhsim01G0015400	3.420856477	7.62895E-22	9.79698E-21	RHSIM_Rhsim01G0015400
Root vs Leaf	RHSIM_Rhsim06G0198100	-2.587098038	7.86172E-22	1.00903E-20	RHSIM_Rhsim06G0198100
Root vs Leaf	RHSIM_RhsimUnG0113300	-3.786135587	8.01415E-22	1.02802E-20	RHSIM_RhsimUnG0113300
Root vs Leaf	RHSIM_Rhsim13G0014200	4.683656493	8.237E-22	1.05602E-20	RHSIM_Rhsim13G0014200
Root vs Leaf	novel.4892	-7.611394579	8.67501E-22	1.11156E-20	--
Root vs Leaf	RHSIM_Rhsim03G0252700	-1.886857734	8.76558E-22	1.12255E-20	RHSIM_Rhsim03G0252700
Root vs Leaf	RHSIM_Rhsim10G0026900	-3.606061516	9.21968E-22	1.18005E-20	RHSIM_Rhsim10G0026900
Root vs Leaf	RHSIM_Rhsim12G0183700	1.634633225	9.57552E-22	1.22491E-20	RHSIM_Rhsim12G0183700
Root vs Leaf	RHSIM_Rhsim02G0234900	3.691547952	9.68219E-22	1.23744E-20	RHSIM_Rhsim02G0234900
Root vs Leaf	novel.4202	2.850793649	9.68416E-22	1.23744E-20	--
Root vs Leaf	RHSIM_Rhsim06G0068900	11.56472124	1.00681E-21	1.28579E-20	RHSIM_Rhsim06G0068900
Root vs Leaf	novel.2124	-3.502771233	1.049E-21	1.33893E-20	--
Root vs Leaf	RHSIM_Rhsim12G0207300	-1.718154306	1.07324E-21	1.36911E-20	RHSIM_Rhsim12G0207300
Root vs Leaf	RHSIM_Rhsim08G0106000	-5.072302017	1.08712E-21	1.38606E-20	RHSIM_Rhsim08G0106000
Root vs Leaf	RHSIM_Rhsim12G0160400	-3.535199719	1.10126E-21	1.40332E-20	RHSIM_Rhsim12G0160400
Root vs Leaf	RHSIM_RhsimPtG0002000	-3.709202609	1.1705E-21	1.49072E-20	RHSIM_RhsimPtG0002000
Root vs Leaf	RHSIM_Rhsim03G0167100	-4.725358642	1.181E-21	1.50276E-20	RHSIM_Rhsim03G0167100
Root vs Leaf	RHSIM_Rhsim10G0018400	-2.798007389	1.18125E-21	1.50276E-20	RHSIM_Rhsim10G0018400
Root vs Leaf	RHSIM_Rhsim06G0082800	-4.656848125	1.18579E-21	1.5069E-20	RHSIM_Rhsim06G0082800
Root vs Leaf	RHSIM_Rhsim07G0179500	-3.583176858	1.1858E-21	1.5069E-20	RHSIM_Rhsim07G0179500
Root vs Leaf	RHSIM_Rhsim03G0091000	-1.960727336	1.22869E-21	1.56054E-20	RHSIM_Rhsim03G0091000
Root vs Leaf	RHSIM_Rhsim13G0191500	-2.80732557	1.23462E-21	1.56721E-20	RHSIM_Rhsim13G0191500
Root vs Leaf	RHSIM_Rhsim12G0081400	-1.937145329	1.2709E-21	1.61239E-20	RHSIM_Rhsim12G0081400

Comparison	Gene ID	log_2FC	P-value	FDR (padj)	Gene Name
Root vs Leaf	RHSIM_RhsimUnG0211400	-5.759824096	1.27282E-21	1.61393E-20	RHSIM_RhsimUnG0211400
Root vs Leaf	RHSIM_Rhsim04G0168900	-3.374995761	1.31893E-21	1.67149E-20	RHSIM_Rhsim04G0168900
Root vs Leaf	RHSIM_RhsimUnG0024700	-11.76583603	1.34349E-21	1.70168E-20	RHSIM_RhsimUnG0024700
Root vs Leaf	novel.2690	3.280295062	1.36616E-21	1.72945E-20	--
Root vs Leaf	RHSIM_Rhsim03G0224100	-1.519892462	1.39245E-21	1.76177E-20	RHSIM_Rhsim03G0224100
Root vs Leaf	RHSIM_Rhsim12G0112400	-1.614616768	1.42135E-21	1.79734E-20	RHSIM_Rhsim12G0112400
Root vs Leaf	RHSIM_Rhsim08G0215400	-2.30998652	1.48634E-21	1.87851E-20	RHSIM_Rhsim08G0215400
Root vs Leaf	RHSIM_Rhsim12G0174100	1.334005701	1.51275E-21	1.91084E-20	RHSIM_Rhsim12G0174100
Root vs Leaf	novel.4040	3.123099156	1.53232E-21	1.93451E-20	--
Root vs Leaf	RHSIM_Rhsim07G0163600	-1.624420594	1.54274E-21	1.9466E-20	RHSIM_Rhsim07G0163600
Root vs Leaf	RHSIM_Rhsim10G0142100	-5.436265986	1.65365E-21	2.0854E-20	RHSIM_Rhsim10G0142100
Root vs Leaf	RHSIM_Rhsim10G0214500	5.004246305	1.69663E-21	2.13844E-20	RHSIM_Rhsim10G0214500
Root vs Leaf	RHSIM_Rhsim01G0284800	1.700677274	1.73261E-21	2.18259E-20	RHSIM_Rhsim01G0284800
Root vs Leaf	RHSIM_Rhsim07G0056600	-8.619797162	1.76807E-21	2.22606E-20	RHSIM_Rhsim07G0056600
Root vs Leaf	RHSIM_Rhsim09G0069900	-2.771479163	1.78354E-21	2.24431E-20	RHSIM_Rhsim09G0069900
Root vs Leaf	RHSIM_Rhsim11G0025700	11.68150679	2.0205E-21	2.54111E-20	RHSIM_Rhsim11G0025700
Root vs Leaf	RHSIM_Rhsim03G0054900	3.736044601	2.0734E-21	2.60622E-20	RHSIM_Rhsim03G0054900
Root vs Leaf	RHSIM_Rhsim02G0145200	2.494342897	2.18798E-21	2.74875E-20	RHSIM_Rhsim02G0145200
Root vs Leaf	RHSIM_Rhsim01G0265500	3.850978364	2.22331E-21	2.79163E-20	RHSIM_Rhsim01G0265500
Root vs Leaf	RHSIM_Rhsim03G0191500	-1.975341233	2.2405E-21	2.81169E-20	RHSIM_Rhsim03G0191500
Root vs Leaf	RHSIM_Rhsim07G0126300	1.768035844	2.24195E-21	2.81197E-20	RHSIM_Rhsim07G0126300
Root vs Leaf	RHSIM_Rhsim04G0008900	3.946699582	2.2724E-21	2.84862E-20	RHSIM_Rhsim04G0008900
Root vs Leaf	novel.6200	6.217375269	2.31675E-21	2.90265E-20	--
Root vs Leaf	RHSIM_Rhsim07G0100900	-1.608069089	2.31935E-21	2.90434E-20	RHSIM_Rhsim07G0100900
Root vs Leaf	RHSIM_Rhsim07G0165600	-1.87457169	2.32235E-21	2.90651E-20	RHSIM_Rhsim07G0165600

Comparison	Gene ID	log$_2$FC	P-value	FDR (padj)	Gene Name
Root vs Leaf	novel.4482	-5.417719862	2.3491E-21	2.9384E-20	--
Root vs Leaf	RHSIM_Rhsim09G0209500	-2.277339164	2.37426E-21	2.96827E-20	RHSIM_Rhsim09G0209500
Root vs Leaf	RHSIM_Rhsim07G0128900	-2.458671638	2.5267E-21	3.15715E-20	RHSIM_Rhsim07G0128900
Root vs Leaf	RHSIM_Rhsim03G0089100	2.979910064	2.60669E-21	3.25533E-20	RHSIM_Rhsim03G0089100
Root vs Leaf	RHSIM_Rhsim13G0010700	-3.573056516	2.62688E-21	3.27878E-20	RHSIM_Rhsim13G0010700
Root vs Leaf	RHSIM_RhsimUnG0085400	-2.694402453	2.68238E-21	3.34625E-20	RHSIM_RhsimUnG0085400
Root vs Leaf	RHSIM_Rhsim07G0006600	-2.434309976	2.76374E-21	3.44589E-20	RHSIM_Rhsim07G0006600
Root vs Leaf	RHSIM_Rhsim06G0063200	-6.485993485	2.81362E-21	3.50619E-20	RHSIM_Rhsim06G0063200
Root vs Leaf	RHSIM_Rhsim02G0016100	4.657447373	2.83082E-21	3.52572E-20	RHSIM_Rhsim02G0016100
Root vs Leaf	novel.3959	-4.425904117	2.90709E-21	3.61683E-20	--
Root vs Leaf	RHSIM_Rhsim10G0060600	4.349583097	2.98439E-21	3.711E-20	RHSIM_Rhsim10G0060600
Root vs Leaf	RHSIM_Rhsim09G0164200	2.112619867	3.0237E-21	3.75786E-20	RHSIM_Rhsim09G0164200
Root vs Leaf	RHSIM_Rhsim08G0250500	-1.951693178	3.04422E-21	3.78133E-20	RHSIM_Rhsim08G0250500
Root vs Leaf	novel.2562	-11.53757311	3.16133E-21	3.92469E-20	--
Root vs Leaf	RHSIM_Rhsim01G0003800	-3.408793186	3.19499E-21	3.96436E-20	RHSIM_Rhsim01G0003800
Root vs Leaf	RHSIM_Rhsim05G0113300	1.668349054	3.30971E-21	4.1045E-20	RHSIM_Rhsim05G0113300
Root vs Leaf	RHSIM_Rhsim08G0048800	-2.79077095	3.3261E-21	4.12084E-20	RHSIM_Rhsim08G0048800
Root vs Leaf	RHSIM_Rhsim12G0044700	-6.892075672	3.32645E-21	4.12084E-20	RHSIM_Rhsim12G0044700
Root vs Leaf	RHSIM_Rhsim09G0069000	-1.916647649	3.37656E-21	4.18068E-20	RHSIM_Rhsim09G0069000
Root vs Leaf	novel.2016	-1.314442904	3.50272E-21	4.33457E-20	--
Root vs Leaf	RHSIM_Rhsim09G0195600	-1.545180067	3.51962E-21	4.35315E-20	RHSIM_Rhsim09G0195600
Root vs Leaf	RHSIM_Rhsim08G0129700	-1.891659165	3.68734E-21	4.55815E-20	RHSIM_Rhsim08G0129700
Root vs Leaf	RHSIM_Rhsim09G0089100	-2.909126629	3.7176E-21	4.59311E-20	RHSIM_Rhsim09G0089100
Root vs Leaf	RHSIM_Rhsim01G0259600	-2.160643864	4.0388E-21	4.9873E-20	RHSIM_Rhsim01G0259600
Root vs Leaf	RHSIM_Rhsim07G0244600	-1.250952414	4.04909E-21	4.99734E-20	RHSIM_Rhsim07G0244600

Comparison	Gene ID	log_2FC	P-value	FDR (padj)	Gene Name
Root vs Leaf	RHSIM_RhsimUnG0046700	3.219146145	4.13616E-21	5.101E-20	RHSIM_RhsimUnG0046700
Root vs Leaf	RHSIM_Rhsim09G0118400	-2.18234058	4.13749E-21	5.101E-20	RHSIM_Rhsim09G0118400
Root vs Leaf	RHSIM_Rhsim11G0027000	-2.429775918	4.22854E-21	5.21047E-20	RHSIM_Rhsim11G0027000
Root vs Leaf	RHSIM_Rhsim02G0168500	12.96618949	4.3924E-21	5.40951E-20	RHSIM_Rhsim02G0168500
Root vs Leaf	RHSIM_Rhsim13G0019800	5.954848177	4.43182E-21	5.45515E-20	RHSIM_Rhsim13G0019800
Root vs Leaf	RHSIM_Rhsim07G0065500	-3.207510329	4.53305E-21	5.57678E-20	RHSIM_Rhsim07G0065500
Root vs Leaf	RHSIM_Rhsim03G0173200	1.51035412	4.55912E-21	5.60588E-20	RHSIM_Rhsim03G0173200
Root vs Leaf	RHSIM_Rhsim07G0142200	2.617169792	4.65465E-21	5.7203E-20	RHSIM_Rhsim07G0142200
Root vs Leaf	novel.234	-10.57324043	4.92864E-21	6.0538E-20	--
Root vs Leaf	RHSIM_Rhsim05G0202400	-4.676780989	4.93343E-21	6.05648E-20	RHSIM_Rhsim05G0202400
Root vs Leaf	RHSIM_Rhsim08G0230400	-1.579070681	5.17942E-21	6.35509E-20	RHSIM_Rhsim08G0230400
Root vs Leaf	RHSIM_Rhsim04G0188800	1.195590243	5.26459E-21	6.45617E-20	RHSIM_Rhsim04G0188800
Root vs Leaf	RHSIM_Rhsim05G0041100	-2.469523591	5.36931E-21	6.5811E-20	RHSIM_Rhsim05G0041100
Root vs Leaf	RHSIM_Rhsim07G0045200	-11.81678727	5.41981E-21	6.63948E-20	RHSIM_Rhsim07G0045200
Root vs Leaf	RHSIM_Rhsim09G0104500	-3.264268899	5.48889E-21	6.72055E-20	RHSIM_Rhsim09G0104500
Root vs Leaf	RHSIM_Rhsim08G0001800	-1.048290056	5.50075E-21	6.73152E-20	RHSIM_Rhsim08G0001800
Root vs Leaf	RHSIM_Rhsim05G0182100	2.835436807	5.53161E-21	6.7657E-20	RHSIM_Rhsim05G0182100
Root vs Leaf	RHSIM_Rhsim09G0038500	-2.248192833	5.68419E-21	6.94865E-20	RHSIM_Rhsim09G0038500
Root vs Leaf	RHSIM_Rhsim06G0207400	2.779959375	5.72964E-21	7.0005E-20	RHSIM_Rhsim06G0207400
Root vs Leaf	RHSIM_Rhsim13G0030200	3.422178286	5.90379E-21	7.20948E-20	RHSIM_Rhsim13G0030200
Root vs Leaf	RHSIM_Rhsim13G0228000	3.022118056	5.93752E-21	7.24685E-20	RHSIM_Rhsim13G0228000
Root vs Leaf	RHSIM_Rhsim07G0067700	2.760306097	5.995E-21	7.31314E-20	RHSIM_Rhsim07G0067700
Root vs Leaf	RHSIM_Rhsim10G0054600	-5.872307816	6.08603E-21	7.42028E-20	RHSIM_Rhsim10G0054600
Root vs Leaf	RHSIM_Rhsim07G0004200	-1.799185818	6.11E-21	7.44558E-20	RHSIM_Rhsim07G0004200
Root vs Leaf	novel.1789	-2.942382282	6.11902E-21	7.45264E-20	--

Comparison	Gene ID	log_2FC	P-value	FDR (padj)	Gene Name
Root vs Leaf	RHSIM_Rhsim10G0043400	-11.4301156	6.13496E-21	7.46813E-20	RHSIM_Rhsim10G0043400
Root vs Leaf	RHSIM_Rhsim09G0003700	-1.965292077	6.21532E-21	7.56197E-20	RHSIM_Rhsim09G0003700
Root vs Leaf	novel.3953	-2.640797406	6.40439E-21	7.78791E-20	--
Root vs Leaf	RHSIM_Rhsim08G0058800	-2.451041413	6.65358E-21	8.08669E-20	RHSIM_Rhsim08G0058800
Root vs Leaf	RHSIM_Rhsim12G0203100	-2.090967641	6.88286E-21	8.36096E-20	RHSIM_Rhsim12G0203100
Root vs Leaf	RHSIM_Rhsim08G0244600	2.688961119	6.97694E-21	8.47079E-20	RHSIM_Rhsim08G0244600
Root vs Leaf	RHSIM_Rhsim06G0018200	-11.86282389	7.19659E-21	8.73289E-20	RHSIM_Rhsim06G0018200
Root vs Leaf	RHSIM_Rhsim12G0161500	2.703072993	7.36365E-21	8.93093E-20	RHSIM_Rhsim12G0161500
Root vs Leaf	RHSIM_Rhsim01G0199400	-11.2457699	7.47525E-21	9.06154E-20	RHSIM_Rhsim01G0199400
Root vs Leaf	RHSIM_Rhsim04G0239100	-1.303030724	8.07602E-21	9.78466E-20	RHSIM_Rhsim04G0239100
Root vs Leaf	novel.5686	-2.268079302	8.46435E-21	1.02498E-19	--
Root vs Leaf	RHSIM_Rhsim08G0199800	1.219130992	8.46993E-21	1.02512E-19	RHSIM_Rhsim08G0199800
Root vs Leaf	RHSIM_Rhsim06G0053200	-1.940976749	8.53461E-21	1.03241E-19	RHSIM_Rhsim06G0053200
Root vs Leaf	RHSIM_Rhsim01G0062600	-3.285856686	8.72222E-21	1.05455E-19	RHSIM_Rhsim01G0062600
Root vs Leaf	RHSIM_Rhsim01G0160500	-2.281154996	8.74365E-21	1.05659E-19	RHSIM_Rhsim01G0160500
Root vs Leaf	RHSIM_RhsimUnG0147100	-11.36108196	8.77486E-21	1.05981E-19	RHSIM_RhsimUnG0147100
Root vs Leaf	RHSIM_RhsimMtG0000800	-2.615143949	9.7907E-21	1.18188E-19	RHSIM_RhsimMtG0000800
Root vs Leaf	RHSIM_RhsimUnG0008000	6.702076074	1.00379E-20	1.21109E-19	RHSIM_RhsimUnG0008000
Root vs Leaf	RHSIM_Rhsim03G0142500	-3.901822384	1.02584E-20	1.23705E-19	RHSIM_Rhsim03G0142500
Root vs Leaf	RHSIM_Rhsim03G0027200	3.055612554	1.03366E-20	1.24583E-19	RHSIM_Rhsim03G0027200
Root vs Leaf	RHSIM_Rhsim01G0274400	-2.457632628	1.03723E-20	1.24948E-19	RHSIM_Rhsim01G0274400
Root vs Leaf	RHSIM_Rhsim13G0059800	-5.473906286	1.04237E-20	1.25502E-19	RHSIM_Rhsim13G0059800
Root vs Leaf	RHSIM_Rhsim05G0224400	-4.246722398	1.04448E-20	1.25691E-19	RHSIM_Rhsim05G0224400
Root vs Leaf	RHSIM_Rhsim13G0076500	-10.14467045	1.04673E-20	1.25895E-19	RHSIM_Rhsim13G0076500
Root vs Leaf	RHSIM_Rhsim13G0167300	-2.442593128	1.05875E-20	1.27276E-19	RHSIM_Rhsim13G0167300

Comparison	Gene ID	log_2FC	P-value	FDR (padj)	Gene Name
Root vs Leaf	RHSIM_Rhsim10G0165900	-3.562456269	1.06498E-20	1.27958E-19	RHSIM_Rhsim10G0165900
Root vs Leaf	RHSIM_Rhsim12G0204400	-2.886933582	1.11983E-20	1.34478E-19	RHSIM_Rhsim12G0204400
Root vs Leaf	RHSIM_Rhsim09G0198300	5.379456467	1.13319E-20	1.36012E-19	RHSIM_Rhsim09G0198300
Root vs Leaf	RHSIM_Rhsim03G0038900	-1.498966217	1.14806E-20	1.37726E-19	RHSIM_Rhsim03G0038900
Root vs Leaf	RHSIM_RhsimUnG0083500	-2.501633353	1.21733E-20	1.4596E-19	RHSIM_RhsimUnG0083500
Root vs Leaf	RHSIM_Rhsim02G0069500	-9.835863022	1.23676E-20	1.48212E-19	RHSIM_Rhsim02G0069500
Root vs Leaf	novel.792	-2.833459151	1.34668E-20	1.61301E-19	--
Root vs Leaf	RHSIM_Rhsim01G0239300	3.055939065	1.35003E-20	1.61619E-19	RHSIM_Rhsim01G0239300
Root vs Leaf	novel.1228	9.860507559	1.35665E-20	1.62328E-19	--
Root vs Leaf	RHSIM_RhsimUnG0253500	-9.91098554	1.38803E-20	1.65913E-19	RHSIM_RhsimUnG0253500
Root vs Leaf	RHSIM_Rhsim13G0045200	2.456848669	1.38805E-20	1.65913E-19	RHSIM_Rhsim13G0045200
Root vs Leaf	novel.5951	-8.600175083	1.39387E-20	1.66523E-19	--
Root vs Leaf	novel.595	-11.46487551	1.46441E-20	1.7486E-19	--
Root vs Leaf	RHSIM_Rhsim09G0071200	-1.871900765	1.4654E-20	1.74888E-19	RHSIM_Rhsim09G0071200
Root vs Leaf	novel.5565	-5.063671338	1.48413E-20	1.77032E-19	--
Root vs Leaf	RHSIM_Rhsim01G0182900	-1.929913199	1.50216E-20	1.79091E-19	RHSIM_Rhsim01G0182900
Root vs Leaf	novel.5601	-1.784088147	1.53362E-20	1.82747E-19	--
Root vs Leaf	RHSIM_Rhsim06G0033600	-11.56441048	1.58869E-20	1.89212E-19	RHSIM_Rhsim06G0033600
Root vs Leaf	RHSIM_Rhsim11G0168700	-1.842702743	1.59615E-20	1.90002E-19	RHSIM_Rhsim11G0168700
Root vs Leaf	novel.5156	5.484357844	1.6084E-20	1.91362E-19	--
Root vs Leaf	RHSIM_Rhsim05G0146400	-2.411161477	1.62833E-20	1.93634E-19	RHSIM_Rhsim05G0146400
Root vs Leaf	RHSIM_RhsimPtG0004600	-2.386925503	1.73417E-20	2.06114E-19	RHSIM_RhsimPtG0004600
Root vs Leaf	RHSIM_Rhsim02G0213700	-4.778770876	1.83671E-20	2.1819E-19	RHSIM_Rhsim02G0213700
Root vs Leaf	RHSIM_Rhsim08G0005000	-2.10127411	1.85262E-20	2.19967E-19	RHSIM_Rhsim08G0005000
Root vs Leaf	RHSIM_Rhsim02G0040500	-4.815543444	1.8995E-20	2.25417E-19	RHSIM_Rhsim02G0040500

Comparison	Gene ID	log_2FC	P-value	FDR (padj)	Gene Name
Root vs Leaf	RHSIM_Rhsim07G0162600	-5.129462646	1.92123E-20	2.27879E-19	RHSIM_Rhsim07G0162600
Root vs Leaf	RHSIM_Rhsim07G0158900	-12.65813394	1.937E-20	2.29632E-19	RHSIM_Rhsim07G0158900
Root vs Leaf	RHSIM_Rhsim02G0042700	1.311963923	1.94481E-20	2.3044E-19	RHSIM_Rhsim02G0042700
Root vs Leaf	RHSIM_Rhsim04G0100500	-1.199411998	1.99607E-20	2.36392E-19	RHSIM_Rhsim04G0100500
Root vs Leaf	RHSIM_Rhsim05G0115700	-1.697701176	2.01717E-20	2.38768E-19	RHSIM_Rhsim05G0115700
Root vs Leaf	RHSIM_Rhsim09G0153100	-2.287786328	2.01915E-20	2.38881E-19	RHSIM_Rhsim09G0153100
Root vs Leaf	RHSIM_RhsimUnG0179200	-6.630175916	2.2033E-20	2.60534E-19	RHSIM_RhsimUnG0179200
Root vs Leaf	RHSIM_Rhsim11G0161700	4.46580363	2.21046E-20	2.61248E-19	RHSIM_Rhsim11G0161700
Root vs Leaf	RHSIM_RhsimUnG0044700	1.242696716	2.21382E-20	2.61511E-19	RHSIM_RhsimUnG0044700
Root vs Leaf	RHSIM_Rhsim02G0035000	-11.10402264	2.28065E-20	2.69267E-19	RHSIM_Rhsim02G0035000
Root vs Leaf	RHSIM_Rhsim10G0056000	2.387558185	2.31475E-20	2.73154E-19	RHSIM_Rhsim10G0056000
Root vs Leaf	novel.2741	-1.560438602	2.33605E-20	2.75527E-19	--
Root vs Leaf	RHSIM_Rhsim01G0040200	-7.392588842	2.33762E-20	2.75572E-19	RHSIM_Rhsim01G0040200
Root vs Leaf	RHSIM_RhsimUnG0048400	-5.048257294	2.34123E-20	2.75857E-19	RHSIM_RhsimUnG0048400
Root vs Leaf	RHSIM_Rhsim07G0014600	-3.490014088	2.3557E-20	2.77421E-19	RHSIM_Rhsim07G0014600
Root vs Leaf	RHSIM_Rhsim12G0086100	1.875573987	2.48891E-20	2.9296E-19	RHSIM_Rhsim12G0086100
Root vs Leaf	RHSIM_Rhsim09G0067000	3.847610479	2.49644E-20	2.93697E-19	RHSIM_Rhsim09G0067000
Root vs Leaf	RHSIM_Rhsim09G0092400	4.156099761	2.53645E-20	2.98253E-19	RHSIM_Rhsim09G0092400
Root vs Leaf	RHSIM_Rhsim06G0026900	6.863901557	2.57318E-20	3.02418E-19	RHSIM_Rhsim06G0026900
Root vs Leaf	RHSIM_RhsimUnG0168400	-9.025809909	2.59182E-20	3.04431E-19	RHSIM_RhsimUnG0168400
Root vs Leaf	RHSIM_Rhsim13G0005500	6.817779675	2.59295E-20	3.04431E-19	RHSIM_Rhsim13G0005500
Root vs Leaf	RHSIM_Rhsim02G0207600	3.453568798	2.61114E-20	3.06412E-19	RHSIM_Rhsim02G0207600
Root vs Leaf	novel.1638	-7.399467203	2.66523E-20	3.12601E-19	--
Root vs Leaf	RHSIM_Rhsim11G0188100	3.31048533	2.67927E-20	3.14089E-19	RHSIM_Rhsim11G0188100
Root vs Leaf	RHSIM_Rhsim01G0191600	-1.589796974	2.73352E-20	3.20286E-19	RHSIM_Rhsim01G0191600

Comparison	Gene ID	log_2FC	P-value	FDR (padj)	Gene Name
Root vs Leaf	RHSIM_Rhsim02G0085600	3.332463075	2.76979E-20	3.24371E-19	RHSIM_Rhsim02G0085600
Root vs Leaf	RHSIM_Rhsim01G0000700	-1.587605046	2.83281E-20	3.31584E-19	RHSIM_Rhsim01G0000700
Root vs Leaf	RHSIM_Rhsim10G0080600	-1.580218427	2.84386E-20	3.32709E-19	RHSIM_Rhsim10G0080600
Root vs Leaf	RHSIM_Rhsim10G0009000	-2.663651977	2.93558E-20	3.43265E-19	RHSIM_Rhsim10G0009000
Root vs Leaf	RHSIM_Rhsim01G0124600	-2.688620099	3.00645E-20	3.51375E-19	RHSIM_Rhsim01G0124600
Root vs Leaf	RHSIM_Rhsim05G0231700	-1.784245679	3.22189E-20	3.76365E-19	RHSIM_Rhsim05G0231700
Root vs Leaf	RHSIM_Rhsim01G0178400	-4.286137724	3.26871E-20	3.81641E-19	RHSIM_Rhsim01G0178400
Root vs Leaf	RHSIM_Rhsim04G0166600	-1.53261846	3.45417E-20	4.03091E-19	RHSIM_Rhsim04G0166600
Root vs Leaf	RHSIM_Rhsim05G0223300	-5.814691265	3.4691E-20	4.0463E-19	RHSIM_Rhsim05G0223300
Root vs Leaf	RHSIM_Rhsim02G0200800	-1.575358105	3.52219E-20	4.10615E-19	RHSIM_Rhsim02G0200800
Root vs Leaf	RHSIM_Rhsim13G0217500	1.690459755	3.5348E-20	4.11878E-19	RHSIM_Rhsim13G0217500
Root vs Leaf	RHSIM_Rhsim08G0244400	3.081372321	3.77735E-20	4.39917E-19	RHSIM_Rhsim08G0244400
Root vs Leaf	RHSIM_Rhsim12G0024400	-11.28252152	3.82144E-20	4.44829E-19	RHSIM_Rhsim12G0024400
Root vs Leaf	RHSIM_Rhsim03G0192700	-3.748167923	3.96497E-20	4.61304E-19	RHSIM_Rhsim03G0192700
Root vs Leaf	RHSIM_Rhsim05G0083700	-1.261461171	4.06956E-20	4.73235E-19	RHSIM_Rhsim05G0083700
Root vs Leaf	RHSIM_Rhsim07G0083300	1.471597088	4.33254E-20	5.03562E-19	RHSIM_Rhsim07G0083300
Root vs Leaf	RHSIM_Rhsim10G0199000	-4.10051347	4.66436E-20	5.41857E-19	RHSIM_Rhsim10G0199000
Root vs Leaf	RHSIM_Rhsim07G0231600	6.718410642	4.75632E-20	5.52263E-19	RHSIM_Rhsim07G0231600
Root vs Leaf	RHSIM_Rhsim01G0073700	-2.750503034	4.90114E-20	5.68793E-19	RHSIM_Rhsim01G0073700
Root vs Leaf	RHSIM_Rhsim04G0062100	-2.243514322	4.94365E-20	5.73439E-19	RHSIM_Rhsim04G0062100
Root vs Leaf	RHSIM_Rhsim01G0291700	-2.080298696	5.06604E-20	5.8734E-19	RHSIM_Rhsim01G0291700
Root vs Leaf	RHSIM_Rhsim06G0007500	-1.920463589	5.0806E-20	5.88734E-19	RHSIM_Rhsim06G0007500
Root vs Leaf	RHSIM_Rhsim08G0050800	3.123447717	5.29999E-20	6.13849E-19	RHSIM_Rhsim08G0050800
Root vs Leaf	RHSIM_Rhsim07G0082300	-1.194998373	5.32896E-20	6.16896E-19	RHSIM_Rhsim07G0082300
Root vs Leaf	novel.3784	1.51602912	5.47896E-20	6.33943E-19	--

Comparison	Gene ID	log_2FC	P-value	FDR (padj)	Gene Name
Root vs Leaf	RHSIM_Rhsim12G0209900	3.565544795	5.51393E-20	6.3767E-19	RHSIM_Rhsim12G0209900
Root vs Leaf	RHSIM_RhsimUnG0115600	-11.14947231	5.53182E-20	6.3942E-19	RHSIM_RhsimUnG0115600
Root vs Leaf	novel.561	-3.218339711	5.54589E-20	6.40726E-19	--
Root vs Leaf	RHSIM_Rhsim12G0023300	-1.703999029	5.66242E-20	6.53863E-19	RHSIM_Rhsim12G0023300
Root vs Leaf	RHSIM_Rhsim07G0094100	4.767465691	5.88421E-20	6.79134E-19	RHSIM_Rhsim07G0094100
Root vs Leaf	RHSIM_Rhsim02G0206400	-8.234631411	5.94486E-20	6.85792E-19	RHSIM_Rhsim02G0206400
Root vs Leaf	RHSIM_Rhsim13G0229100	-11.52018084	5.94904E-20	6.85933E-19	RHSIM_Rhsim13G0229100
Root vs Leaf	RHSIM_Rhsim05G0018700	-1.550631163	6.07759E-20	7.00406E-19	RHSIM_Rhsim05G0018700
Root vs Leaf	RHSIM_Rhsim03G0185800	-12.10573746	6.25368E-20	7.20341E-19	RHSIM_Rhsim03G0185800
Root vs Leaf	RHSIM_Rhsim12G0170000	-3.13784703	6.33404E-20	7.29234E-19	RHSIM_Rhsim12G0170000
Root vs Leaf	RHSIM_Rhsim09G0166900	-2.168837321	6.33998E-20	7.29555E-19	RHSIM_Rhsim09G0166900
Root vs Leaf	RHSIM_Rhsim07G0049400	5.129833444	6.36111E-20	7.31622E-19	RHSIM_Rhsim07G0049400
Root vs Leaf	RHSIM_Rhsim11G0105700	1.942742011	6.36991E-20	7.32271E-19	RHSIM_Rhsim11G0105700
Root vs Leaf	RHSIM_Rhsim08G0221900	1.384442701	6.38681E-20	7.33849E-19	RHSIM_Rhsim08G0221900
Root vs Leaf	RHSIM_Rhsim08G0019300	-3.711825791	6.41221E-20	7.36402E-19	RHSIM_Rhsim08G0019300
Root vs Leaf	RHSIM_Rhsim12G0140400	4.309559755	6.59837E-20	7.57405E-19	RHSIM_Rhsim12G0140400
Root vs Leaf	RHSIM_Rhsim04G0197300	6.38843092	6.61137E-20	7.58521E-19	RHSIM_Rhsim04G0197300
Root vs Leaf	RHSIM_Rhsim03G0270100	-2.520261707	6.6164E-20	7.58722E-19	RHSIM_Rhsim03G0270100
Root vs Leaf	RHSIM_Rhsim13G0220800	1.204117192	6.70735E-20	7.68771E-19	RHSIM_Rhsim13G0220800
Root vs Leaf	RHSIM_Rhsim01G0247700	-2.019586139	6.72158E-20	7.7002E-19	RHSIM_Rhsim01G0247700
Root vs Leaf	RHSIM_Rhsim08G0074900	-11.25600676	7.06184E-20	8.086E-19	RHSIM_Rhsim08G0074900
Root vs Leaf	RHSIM_Rhsim02G0071000	2.025747609	7.30758E-20	8.36324E-19	RHSIM_Rhsim02G0071000
Root vs Leaf	RHSIM_Rhsim10G0126800	-1.832477967	7.31755E-20	8.36682E-19	RHSIM_Rhsim10G0126800
Root vs Leaf	RHSIM_Rhsim02G0044700	3.864811501	7.31794E-20	8.36682E-19	RHSIM_Rhsim02G0044700
Root vs Leaf	novel.4878	5.344312859	7.41344E-20	8.47182E-19	--

Comparison	Gene ID	log_2FC	P-value	FDR (padj)	Gene Name
Root vs Leaf	RHSIM_Rhsim06G0080200	6.243574484	7.51828E-20	8.58739E-19	RHSIM_Rhsim06G0080200
Root vs Leaf	RHSIM_Rhsim09G0093500	2.825362138	7.56948E-20	8.64161E-19	RHSIM_Rhsim09G0093500
Root vs Leaf	RHSIM_Rhsim13G0221900	-1.78417838	7.92948E-20	9.04813E-19	RHSIM_Rhsim13G0221900
Root vs Leaf	RHSIM_Rhsim04G0193800	-4.179437212	8.00651E-20	9.13152E-19	RHSIM_Rhsim04G0193800
Root vs Leaf	RHSIM_Rhsim03G0197600	4.893951877	8.23761E-20	9.39046E-19	RHSIM_Rhsim03G0197600
Root vs Leaf	RHSIM_Rhsim07G0075300	-3.80221055	8.53762E-20	9.72767E-19	RHSIM_Rhsim07G0075300
Root vs Leaf	novel.5135	6.566561021	8.64074E-20	9.84032E-19	--
Root vs Leaf	RHSIM_Rhsim10G0148400	-1.658259802	8.96277E-20	1.0202E-18	RHSIM_Rhsim10G0148400
Root vs Leaf	RHSIM_Rhsim08G0174100	11.01031364	9.13697E-20	1.03952E-18	RHSIM_Rhsim08G0174100
Root vs Leaf	RHSIM_Rhsim04G0077100	-11.03882781	9.31163E-20	1.05887E-18	RHSIM_Rhsim04G0077100
Root vs Leaf	RHSIM_Rhsim05G0193500	-4.683978576	9.38762E-20	1.06699E-18	RHSIM_Rhsim05G0193500
Root vs Leaf	RHSIM_Rhsim10G0185700	-2.290095566	9.42448E-20	1.07065E-18	RHSIM_Rhsim10G0185700
Root vs Leaf	RHSIM_Rhsim12G0075600	2.795600819	9.44007E-20	1.0719E-18	RHSIM_Rhsim12G0075600
Root vs Leaf	RHSIM_Rhsim02G0007600	-3.570000916	9.50837E-20	1.07912E-18	RHSIM_Rhsim02G0007600
Root vs Leaf	RHSIM_Rhsim07G0224100	-3.291893808	9.64193E-20	1.09374E-18	RHSIM_Rhsim07G0224100
Root vs Leaf	novel.3057	2.906935276	9.6551E-20	1.0947E-18	--
Root vs Leaf	RHSIM_Rhsim01G0034300	4.857211026	9.93539E-20	1.12593E-18	RHSIM_Rhsim01G0034300
Root vs Leaf	RHSIM_Rhsim03G0169600	-2.486253355	1.00287E-19	1.13595E-18	RHSIM_Rhsim03G0169600
Root vs Leaf	RHSIM_Rhsim09G0049600	-6.863158986	1.00692E-19	1.13998E-18	RHSIM_Rhsim09G0049600
Root vs Leaf	RHSIM_Rhsim09G0065100	-2.201875244	1.04764E-19	1.1855E-18	RHSIM_Rhsim09G0065100
Root vs Leaf	RHSIM_Rhsim06G0111200	-11.23795029	1.05295E-19	1.19093E-18	RHSIM_Rhsim06G0111200
Root vs Leaf	RHSIM_Rhsim01G0080300	-2.883652537	1.06765E-19	1.20696E-18	RHSIM_Rhsim01G0080300
Root vs Leaf	RHSIM_Rhsim03G0049400	-3.120965766	1.10083E-19	1.24386E-18	RHSIM_Rhsim03G0049400
Root vs Leaf	RHSIM_Rhsim10G0005100	9.672277133	1.17158E-19	1.32316E-18	RHSIM_Rhsim10G0005100
Root vs Leaf	novel.5389	8.729952427	1.2383E-19	1.39783E-18	--

Comparison	Gene ID	log_2FC	P-value	FDR (padj)	Gene Name
Root vs Leaf	novel.4043	1.41059818	1.25262E-19	1.4133E-18	--
Root vs Leaf	RHSIM_Rhsim02G0043800	2.131375169	1.31288E-19	1.48057E-18	RHSIM_Rhsim02G0043800
Root vs Leaf	RHSIM_RhsimUnG0217100	4.925922651	1.32322E-19	1.49151E-18	RHSIM_RhsimUnG0217100
Root vs Leaf	RHSIM_Rhsim09G0125000	-3.909699069	1.3529E-19	1.52422E-18	RHSIM_Rhsim09G0125000
Root vs Leaf	RHSIM_Rhsim12G0056300	4.577392688	1.35502E-19	1.52586E-18	RHSIM_Rhsim12G0056300
Root vs Leaf	RHSIM_Rhsim03G0184700	-10.07125756	1.35592E-19	1.52614E-18	RHSIM_Rhsim03G0184700
Root vs Leaf	RHSIM_Rhsim03G0209100	3.459153852	1.36013E-19	1.53012E-18	RHSIM_Rhsim03G0209100
Root vs Leaf	novel.1318	1.504489533	1.36171E-19	1.53116E-18	--
Root vs Leaf	RHSIM_Rhsim08G0057900	-11.44217889	1.3647E-19	1.53378E-18	RHSIM_Rhsim08G0057900
Root vs Leaf	RHSIM_Rhsim02G0227300	2.780024111	1.37063E-19	1.53969E-18	RHSIM_Rhsim02G0227300
Root vs Leaf	RHSIM_Rhsim03G0130900	-2.656455367	1.37823E-19	1.54749E-18	RHSIM_Rhsim03G0130900
Root vs Leaf	RHSIM_Rhsim09G0153600	3.238190378	1.40288E-19	1.5744E-18	RHSIM_Rhsim09G0153600
Root vs Leaf	RHSIM_Rhsim01G0067900	-10.89404864	1.40962E-19	1.58119E-18	RHSIM_Rhsim01G0067900
Root vs Leaf	RHSIM_Rhsim10G0004900	-1.851344862	1.43613E-19	1.61015E-18	RHSIM_Rhsim10G0004900
Root vs Leaf	RHSIM_Rhsim08G0029800	-1.377644093	1.43948E-19	1.61312E-18	RHSIM_Rhsim08G0029800
Root vs Leaf	RHSIM_Rhsim04G0061200	-3.197760861	1.49097E-19	1.67002E-18	RHSIM_Rhsim04G0061200
Root vs Leaf	RHSIM_Rhsim09G0116000	-4.116637361	1.50378E-19	1.68355E-18	RHSIM_Rhsim09G0116000
Root vs Leaf	RHSIM_Rhsim03G0205700	-4.221344849	1.5518E-19	1.73647E-18	RHSIM_Rhsim03G0205700
Root vs Leaf	RHSIM_RhsimUnG0112800	-3.299736904	1.55275E-19	1.7367E-18	RHSIM_RhsimUnG0112800
Root vs Leaf	RHSIM_Rhsim03G0199300	-5.115568899	1.57597E-19	1.76181E-18	RHSIM_Rhsim03G0199300
Root vs Leaf	novel.618	7.894485116	1.61404E-19	1.8035E-18	--
Root vs Leaf	RHSIM_RhsimUnG0044400	1.364004681	1.62522E-19	1.81512E-18	RHSIM_RhsimUnG0044400
Root vs Leaf	RHSIM_Rhsim13G0164100	2.490090314	1.68437E-19	1.88017E-18	RHSIM_Rhsim13G0164100
Root vs Leaf	novel.1621	10.8670687	1.68509E-19	1.88017E-18	--
Root vs Leaf	RHSIM_Rhsim08G0092300	-1.552729219	1.74891E-19	1.95043E-18	RHSIM_Rhsim08G0092300

Comparison	Gene ID	log_2FC	P-value	FDR (padj)	Gene Name
Root vs Leaf	RHSIM_Rhsim12G0183300	-1.977405153	1.77812E-19	1.98206E-18	RHSIM_Rhsim12G0183300
Root vs Leaf	RHSIM_Rhsim02G0249000	-6.809900475	1.82332E-19	2.03146E-18	RHSIM_Rhsim02G0249000
Root vs Leaf	RHSIM_Rhsim10G0056300	11.08248856	1.85299E-19	2.06353E-18	RHSIM_Rhsim10G0056300
Root vs Leaf	RHSIM_Rhsim05G0167700	-3.502014438	1.89383E-19	2.10799E-18	RHSIM_Rhsim05G0167700
Root vs Leaf	RHSIM_Rhsim01G0269200	-1.663721811	1.90368E-19	2.11794E-18	RHSIM_Rhsim01G0269200
Root vs Leaf	RHSIM_Rhsim03G0200000	-1.546662621	1.91347E-19	2.12781E-18	RHSIM_Rhsim03G0200000
Root vs Leaf	RHSIM_Rhsim01G0033500	3.308815604	1.9807E-19	2.20151E-18	RHSIM_Rhsim01G0033500
Root vs Leaf	RHSIM_Rhsim05G0097700	-11.15900274	2.05026E-19	2.27773E-18	RHSIM_Rhsim05G0097700
Root vs Leaf	RHSIM_Rhsim13G0044400	6.938879234	2.0821E-19	2.31199E-18	RHSIM_Rhsim13G0044400
Root vs Leaf	novel.4832	-2.728529793	2.09426E-19	2.32438E-18	--
Root vs Leaf	RHSIM_Rhsim12G0090700	-1.079265715	2.11809E-19	2.34955E-18	RHSIM_Rhsim12G0090700
Root vs Leaf	RHSIM_Rhsim03G0130400	-2.439991674	2.11897E-19	2.34955E-18	RHSIM_Rhsim03G0130400
Root vs Leaf	RHSIM_Rhsim04G0046500	-1.788152182	2.12445E-19	2.3545E-18	RHSIM_Rhsim04G0046500
Root vs Leaf	RHSIM_Rhsim08G0044400	-1.741921295	2.12981E-19	2.3593E-18	RHSIM_Rhsim08G0044400
Root vs Leaf	RHSIM_Rhsim05G0228900	1.551993403	2.20499E-19	2.44142E-18	RHSIM_Rhsim05G0228900
Root vs Leaf	RHSIM_Rhsim10G0014500	-1.826965432	2.27045E-19	2.5127E-18	RHSIM_Rhsim10G0014500
Root vs Leaf	RHSIM_Rhsim05G0229400	7.251449194	2.3171E-19	2.5631E-18	RHSIM_Rhsim05G0229400
Root vs Leaf	RHSIM_Rhsim13G0086700	5.021760357	2.34195E-19	2.58935E-18	RHSIM_Rhsim13G0086700
Root vs Leaf	RHSIM_Rhsim01G0027800	-11.19208603	2.39221E-19	2.64366E-18	RHSIM_Rhsim01G0027800
Root vs Leaf	RHSIM_Rhsim13G0156800	-10.96697437	2.42524E-19	2.67888E-18	RHSIM_Rhsim13G0156800
Root vs Leaf	RHSIM_Rhsim03G0031300	-1.635771079	2.4479E-19	2.70262E-18	RHSIM_Rhsim03G0031300
Root vs Leaf	RHSIM_RhsimUnG0190300	-11.2182349	2.46579E-19	2.72107E-18	RHSIM_RhsimUnG0190300
Root vs Leaf	RHSIM_Rhsim09G0187300	-2.127571499	2.46791E-19	2.72212E-18	RHSIM_Rhsim09G0187300
Root vs Leaf	RHSIM_Rhsim12G0003400	1.672513651	2.47292E-19	2.72634E-18	RHSIM_Rhsim12G0003400
Root vs Leaf	novel.5524	-6.0628493	2.6063E-19	2.87202E-18	--

Comparison	Gene ID	log_2FC	P-value	FDR (padj)	Gene Name
Root vs Leaf	RHSIM_Rhsim03G0168800	-8.636308911	2.69586E-19	2.96885E-18	RHSIM_Rhsim03G0168800
Root vs Leaf	RHSIM_Rhsim09G0184000	-1.824815107	2.69674E-19	2.96885E-18	RHSIM_Rhsim09G0184000
Root vs Leaf	RHSIM_Rhsim10G0177700	4.247034023	2.71814E-19	2.99098E-18	RHSIM_Rhsim10G0177700
Root vs Leaf	RHSIM_Rhsim03G0177300	-11.21761222	2.72162E-19	2.9934E-18	RHSIM_Rhsim03G0177300
Root vs Leaf	RHSIM_Rhsim10G0053400	-10.73767675	2.73239E-19	3.00381E-18	RHSIM_Rhsim10G0053400
Root vs Leaf	RHSIM_Rhsim09G0188700	2.209740939	2.80519E-19	3.08238E-18	RHSIM_Rhsim09G0188700
Root vs Leaf	RHSIM_Rhsim07G0120500	1.607577549	2.81665E-19	3.09351E-18	RHSIM_Rhsim07G0120500
Root vs Leaf	RHSIM_Rhsim02G0239800	1.933309944	2.92023E-19	3.20574E-18	RHSIM_Rhsim02G0239800
Root vs Leaf	RHSIM_Rhsim07G0239900	-5.330033889	2.99893E-19	3.29057E-18	RHSIM_Rhsim07G0239900
Root vs Leaf	novel.981	1.501123466	3.02251E-19	3.31487E-18	--
Root vs Leaf	RHSIM_Rhsim08G0013000	3.499430187	3.04501E-19	3.33797E-18	RHSIM_Rhsim08G0013000
Root vs Leaf	RHSIM_Rhsim08G0242400	-5.367026936	3.11927E-19	3.41775E-18	RHSIM_Rhsim08G0242400
Root vs Leaf	RHSIM_Rhsim02G0256200	-1.863844694	3.19765E-19	3.50198E-18	RHSIM_Rhsim02G0256200
Root vs Leaf	RHSIM_Rhsim07G0037400	4.131925442	3.21919E-19	3.52391E-18	RHSIM_Rhsim07G0037400
Root vs Leaf	RHSIM_Rhsim05G0229300	2.894835989	3.25424E-19	3.56058E-18	RHSIM_Rhsim05G0229300
Root vs Leaf	RHSIM_Rhsim09G0206700	-10.77469714	3.31152E-19	3.62154E-18	RHSIM_Rhsim09G0206700
Root vs Leaf	RHSIM_Rhsim06G0157300	7.893821519	3.46232E-19	3.78468E-18	RHSIM_Rhsim06G0157300
Root vs Leaf	RHSIM_Rhsim13G0118300	-1.281573629	3.50775E-19	3.83253E-18	RHSIM_Rhsim13G0118300
Root vs Leaf	novel.2600	-11.53364631	3.73197E-19	4.07558E-18	--
Root vs Leaf	novel.5864	-4.349876317	3.82725E-19	4.17766E-18	--
Root vs Leaf	RHSIM_Rhsim08G0188100	1.409510198	4.20237E-19	4.58496E-18	RHSIM_Rhsim08G0188100
Root vs Leaf	RHSIM_Rhsim11G0161600	6.9118922	4.23472E-19	4.61808E-18	RHSIM_Rhsim11G0161600
Root vs Leaf	RHSIM_RhsimUnG0197300	-5.178099784	4.26305E-19	4.64679E-18	RHSIM_RhsimUnG0197300
Root vs Leaf	RHSIM_Rhsim12G0078000	-2.467054421	4.2731E-19	4.65555E-18	RHSIM_Rhsim12G0078000
Root vs Leaf	RHSIM_RhsimUnG0120700	-1.305680155	4.29659E-19	4.67894E-18	RHSIM_RhsimUnG0120700

Comparison	Gene ID	log_2FC	P-value	FDR (padj)	Gene Name
Root vs Leaf	RHSIM_Rhsim04G0214500	-2.81275835	4.34029E-19	4.7243E-18	RHSIM_Rhsim04G0214500
Root vs Leaf	RHSIM_Rhsim11G0118300	1.888283845	4.34675E-19	4.7291E-18	RHSIM_Rhsim11G0118300
Root vs Leaf	RHSIM_Rhsim03G0066200	4.739392679	4.36289E-19	4.74444E-18	RHSIM_Rhsim03G0066200
Root vs Leaf	RHSIM_Rhsim08G0201700	-5.644351374	4.41062E-19	4.79409E-18	RHSIM_Rhsim08G0201700
Root vs Leaf	RHSIM_Rhsim02G0018300	-3.649396769	4.48994E-19	4.87801E-18	RHSIM_Rhsim02G0018300
Root vs Leaf	RHSIM_Rhsim12G0150400	11.08408737	4.60677E-19	5.00259E-18	RHSIM_Rhsim12G0150400
Root vs Leaf	RHSIM_RhsimUnG0205200	-1.464273757	4.68427E-19	5.08437E-18	RHSIM_RhsimUnG0205200
Root vs Leaf	RHSIM_Rhsim02G0061500	-11.74856686	4.76967E-19	5.17463E-18	RHSIM_Rhsim02G0061500
Root vs Leaf	RHSIM_RhsimUnG0216000	3.522379184	4.78029E-19	5.18372E-18	RHSIM_RhsimUnG0216000
Root vs Leaf	RHSIM_Rhsim03G0008500	-4.688763103	4.9031E-19	5.31441E-18	RHSIM_Rhsim03G0008500
Root vs Leaf	RHSIM_RhsimUnG0151800	-11.06605674	4.99909E-19	5.41591E-18	RHSIM_RhsimUnG0151800
Root vs Leaf	RHSIM_Rhsim10G0173100	-2.592823596	5.01888E-19	5.43481E-18	RHSIM_Rhsim10G0173100
Root vs Leaf	RHSIM_Rhsim01G0221100	4.3136921	5.02923E-19	5.44347E-18	RHSIM_Rhsim01G0221100
Root vs Leaf	RHSIM_Rhsim03G0215200	-3.17356741	5.09185E-19	5.50867E-18	RHSIM_Rhsim03G0215200
Root vs Leaf	RHSIM_Rhsim10G0195400	2.426477268	5.48349E-19	5.9296E-18	RHSIM_Rhsim10G0195400
Root vs Leaf	RHSIM_Rhsim02G0008500	1.078366816	5.63847E-19	6.09435E-18	RHSIM_Rhsim02G0008500
Root vs Leaf	RHSIM_Rhsim13G0141900	2.805770776	5.66011E-19	6.11488E-18	RHSIM_Rhsim13G0141900
Root vs Leaf	RHSIM_Rhsim13G0009800	1.387054812	5.71083E-19	6.16679E-18	RHSIM_Rhsim13G0009800
Root vs Leaf	RHSIM_Rhsim06G0112600	-10.90950622	5.72302E-19	6.17708E-18	RHSIM_Rhsim06G0112600
Root vs Leaf	RHSIM_Rhsim02G0217800	-2.462983306	5.7416E-19	6.19424E-18	RHSIM_Rhsim02G0217800
Root vs Leaf	RHSIM_Rhsim13G0075500	4.817963634	5.79927E-19	6.25353E-18	RHSIM_Rhsim13G0075500
Root vs Leaf	RHSIM_Rhsim13G0016400	-1.270449774	6.0097E-19	6.47744E-18	RHSIM_Rhsim13G0016400
Root vs Leaf	RHSIM_Rhsim08G0081800	-4.403480751	6.16304E-19	6.63961E-18	RHSIM_Rhsim08G0081800
Root vs Leaf	RHSIM_Rhsim01G0188800	-6.287894243	6.20191E-19	6.67838E-18	RHSIM_Rhsim01G0188800
Root vs Leaf	RHSIM_Rhsim09G0214300	-1.060196278	6.20913E-19	6.68304E-18	RHSIM_Rhsim09G0214300

Comparison	Gene ID	log_2FC	P-value	FDR (padj)	Gene Name
Root vs Leaf	RHSIM_Rhsim04G0205300	1.681436611	6.31342E-19	6.79214E-18	RHSIM_Rhsim04G0205300
Root vs Leaf	RHSIM_RhsimUnG0223700	3.101197952	6.42734E-19	6.91148E-18	RHSIM_RhsimUnG0223700
Root vs Leaf	RHSIM_Rhsim09G0164800	-1.233658024	6.51352E-19	7.0009E-18	RHSIM_Rhsim09G0164800
Root vs Leaf	RHSIM_Rhsim10G0000700	-1.722123467	6.53279E-19	7.01835E-18	RHSIM_Rhsim10G0000700
Root vs Leaf	RHSIM_Rhsim10G0041600	-3.741425054	6.59479E-19	7.08167E-18	RHSIM_Rhsim10G0041600
Root vs Leaf	RHSIM_Rhsim10G0064600	1.288904121	6.64192E-19	7.12898E-18	RHSIM_Rhsim10G0064600
Root vs Leaf	RHSIM_Rhsim10G0107900	-2.616576365	6.77807E-19	7.27173E-18	RHSIM_Rhsim10G0107900
Root vs Leaf	RHSIM_Rhsim06G0007800	-1.900885575	6.80851E-19	7.30101E-18	RHSIM_Rhsim06G0007800
Root vs Leaf	RHSIM_Rhsim01G0253700	1.710798647	6.81813E-19	7.30794E-18	RHSIM_Rhsim01G0253700
Root vs Leaf	RHSIM_Rhsim09G0012000	-4.130568149	6.94709E-19	7.44271E-18	RHSIM_Rhsim09G0012000
Root vs Leaf	RHSIM_Rhsim02G0062900	-7.528291243	7.22133E-19	7.73294E-18	RHSIM_Rhsim02G0062900
Root vs Leaf	RHSIM_Rhsim09G0055600	-10.74419561	7.36126E-19	7.87913E-18	RHSIM_Rhsim09G0055600
Root vs Leaf	novel.2289	6.753508038	7.546E-19	8.07314E-18	--
Root vs Leaf	RHSIM_Rhsim02G0071700	-2.108241401	7.59038E-19	8.11687E-18	RHSIM_Rhsim02G0071700
Root vs Leaf	RHSIM_Rhsim02G0087400	-8.120512212	7.78478E-19	8.3209E-18	RHSIM_Rhsim02G0087400
Root vs Leaf	RHSIM_Rhsim12G0107300	-2.039983433	8.01467E-19	8.56267E-18	RHSIM_Rhsim12G0107300
Root vs Leaf	RHSIM_Rhsim04G0046000	3.907499604	8.45253E-19	9.0263E-18	RHSIM_Rhsim04G0046000
Root vs Leaf	RHSIM_Rhsim07G0224800	5.511612614	8.54804E-19	9.12408E-18	RHSIM_Rhsim07G0224800
Root vs Leaf	RHSIM_Rhsim04G0052400	2.841136071	9.02077E-19	9.62423E-18	RHSIM_Rhsim04G0052400
Root vs Leaf	RHSIM_Rhsim11G0039900	-11.81870471	9.08204E-19	9.68514E-18	RHSIM_Rhsim11G0039900
Root vs Leaf	RHSIM_Rhsim08G0045000	-1.485329561	9.30288E-19	9.91607E-18	RHSIM_Rhsim08G0045000
Root vs Leaf	RHSIM_Rhsim04G0247500	-1.86526943	9.4815E-19	1.01018E-17	RHSIM_Rhsim04G0247500
Root vs Leaf	RHSIM_Rhsim02G0034900	-10.8332748	9.5167E-19	1.01347E-17	RHSIM_Rhsim02G0034900
Root vs Leaf	novel.4766	3.167164463	9.54548E-19	1.01606E-17	--
Root vs Leaf	RHSIM_Rhsim03G0039800	-7.549351967	9.78588E-19	1.04117E-17	RHSIM_Rhsim03G0039800

Comparison	Gene ID	log_2FC	P-value	FDR (padj)	Gene Name
Root vs Leaf	RHSIM_Rhsim08G0180500	-7.116229346	9.82656E-19	1.04502E-17	RHSIM_Rhsim08G0180500
Root vs Leaf	RHSIM_Rhsim04G0174900	-2.908050485	1.00564E-18	1.06897E-17	RHSIM_Rhsim04G0174900
Root vs Leaf	novel.5066	-2.687786036	1.05718E-18	1.12324E-17	--
Root vs Leaf	RHSIM_Rhsim12G0000100	-1.380335131	1.11675E-18	1.18599E-17	RHSIM_Rhsim12G0000100
Root vs Leaf	RHSIM_Rhsim05G0214700	-4.636415857	1.14055E-18	1.21071E-17	RHSIM_Rhsim05G0214700
Root vs Leaf	RHSIM_Rhsim13G0087000	3.327396195	1.15562E-18	1.22615E-17	RHSIM_Rhsim13G0087000
Root vs Leaf	novel.5894	-7.57342536	1.16349E-18	1.23392E-17	--
Root vs Leaf	RHSIM_Rhsim04G0239000	1.754252242	1.2025E-18	1.27471E-17	RHSIM_Rhsim04G0239000
Root vs Leaf	RHSIM_Rhsim07G0074600	-1.97868992	1.23794E-18	1.31168E-17	RHSIM_Rhsim07G0074600
Root vs Leaf	RHSIM_Rhsim04G0250200	-2.579274777	1.25057E-18	1.32446E-17	RHSIM_Rhsim04G0250200
Root vs Leaf	novel.760	-12.13020864	1.28536E-18	1.36068E-17	--
Root vs Leaf	RHSIM_Rhsim13G0181800	-11.16597363	1.32553E-18	1.40256E-17	RHSIM_Rhsim13G0181800
Root vs Leaf	RHSIM_Rhsim04G0038700	1.403953542	1.32647E-18	1.40291E-17	RHSIM_Rhsim04G0038700
Root vs Leaf	RHSIM_Rhsim01G0060200	-1.677000009	1.34703E-18	1.42401E-17	RHSIM_Rhsim01G0060200
Root vs Leaf	RHSIM_Rhsim06G0104100	2.36978997	1.35467E-18	1.43144E-17	RHSIM_Rhsim06G0104100
Root vs Leaf	RHSIM_Rhsim11G0178000	4.257165599	1.39403E-18	1.47236E-17	RHSIM_Rhsim11G0178000
Root vs Leaf	RHSIM_Rhsim05G0074000	3.782312817	1.43309E-18	1.51291E-17	RHSIM_Rhsim05G0074000
Root vs Leaf	RHSIM_Rhsim06G0072500	6.730493497	1.44088E-18	1.52045E-17	RHSIM_Rhsim06G0072500
Root vs Leaf	RHSIM_Rhsim03G0249700	5.74547366	1.47477E-18	1.5555E-17	RHSIM_Rhsim03G0249700
Root vs Leaf	RHSIM_Rhsim13G0180200	-11.36149918	1.49223E-18	1.5732E-17	RHSIM_Rhsim13G0180200
Root vs Leaf	RHSIM_Rhsim11G0132800	2.842620389	1.56134E-18	1.64531E-17	RHSIM_Rhsim11G0132800
Root vs Leaf	RHSIM_Rhsim08G0227000	10.64227433	1.57818E-18	1.6623E-17	RHSIM_Rhsim08G0227000
Root vs Leaf	RHSIM_Rhsim01G0066800	1.089491451	1.59558E-18	1.67986E-17	RHSIM_Rhsim01G0066800
Root vs Leaf	novel.2764	2.254766894	1.61702E-18	1.70166E-17	--
Root vs Leaf	RHSIM_Rhsim01G0233000	3.759178429	1.63313E-18	1.71783E-17	RHSIM_Rhsim01G0233000

Comparison	Gene ID	log_2FC	P-value	FDR (padj)	Gene Name
Root vs Leaf	RHSIM_Rhsim01G0236100	-1.938891344	1.65593E-18	1.74102E-17	RHSIM_Rhsim01G0236100
Root vs Leaf	RHSIM_RhsimUnG0010900	9.427831884	1.68008E-18	1.76561E-17	RHSIM_RhsimUnG0010900
Root vs Leaf	RHSIM_Rhsim09G0112700	3.69326271	1.72177E-18	1.8086E-17	RHSIM_Rhsim09G0112700
Root vs Leaf	RHSIM_Rhsim13G0163700	-1.592430252	1.73167E-18	1.81818E-17	RHSIM_Rhsim13G0163700
Root vs Leaf	RHSIM_Rhsim03G0059100	-2.875062004	1.7589E-18	1.84593E-17	RHSIM_Rhsim03G0059100
Root vs Leaf	RHSIM_RhsimUnG0131800	-7.076227341	1.83677E-18	1.92678E-17	RHSIM_RhsimUnG0131800
Root vs Leaf	RHSIM_Rhsim12G0149300	-10.5976121	1.8508E-18	1.94061E-17	RHSIM_Rhsim12G0149300
Root vs Leaf	novel.4173	-10.79483919	1.89425E-18	1.98527E-17	--
Root vs Leaf	novel.3788	3.014812093	1.905E-18	1.99564E-17	--
Root vs Leaf	RHSIM_Rhsim03G0125000	1.870820683	1.91951E-18	2.00993E-17	RHSIM_Rhsim03G0125000
Root vs Leaf	RHSIM_Rhsim06G0235200	2.34132177	1.99278E-18	2.08571E-17	RHSIM_Rhsim06G0235200
Root vs Leaf	RHSIM_Rhsim11G0024800	-1.435985131	2.05809E-18	2.15309E-17	RHSIM_Rhsim11G0024800
Root vs Leaf	RHSIM_Rhsim03G0185600	-11.85918033	2.18083E-18	2.28046E-17	RHSIM_Rhsim03G0185600
Root vs Leaf	RHSIM_Rhsim11G0165400	5.802224557	2.18427E-18	2.28302E-17	RHSIM_Rhsim11G0165400
Root vs Leaf	RHSIM_Rhsim02G0031100	3.217579897	2.26829E-18	2.36977E-17	RHSIM_Rhsim02G0031100
Root vs Leaf	RHSIM_Rhsim08G0210900	1.874889906	2.29706E-18	2.39875E-17	RHSIM_Rhsim08G0210900
Root vs Leaf	RHSIM_Rhsim09G0207400	2.110967539	2.38797E-18	2.49255E-17	RHSIM_Rhsim09G0207400
Root vs Leaf	RHSIM_RhsimUnG0075700	8.233994269	2.40798E-18	2.51232E-17	RHSIM_RhsimUnG0075700
Root vs Leaf	RHSIM_Rhsim08G0039400	-6.787799345	2.43926E-18	2.5438E-17	RHSIM_Rhsim08G0039400
Root vs Leaf	RHSIM_Rhsim12G0203600	-6.307137094	2.44298E-18	2.54654E-17	RHSIM_Rhsim12G0203600
Root vs Leaf	RHSIM_Rhsim08G0111200	-2.860179776	2.44903E-18	2.55169E-17	RHSIM_Rhsim08G0111200
Root vs Leaf	RHSIM_Rhsim08G0028900	1.405001872	2.49336E-18	2.5967E-17	RHSIM_Rhsim08G0028900
Root vs Leaf	RHSIM_Rhsim05G0191000	-1.514021774	2.55806E-18	2.66289E-17	RHSIM_Rhsim05G0191000
Root vs Leaf	RHSIM_Rhsim11G0025100	-2.693986933	2.57247E-18	2.67669E-17	RHSIM_Rhsim11G0025100
Root vs Leaf	RHSIM_Rhsim09G0075500	-6.842551837	2.63395E-18	2.73819E-17	RHSIM_Rhsim09G0075500

Comparison	Gene ID	log_2FC	P-value	FDR (padj)	Gene Name
Root vs Leaf	RHSIM_Rhsim10G0056700	5.108196657	2.63833E-18	2.74152E-17	RHSIM_Rhsim10G0056700
Root vs Leaf	RHSIM_Rhsim01G0245400	-2.502826453	2.64542E-18	2.74765E-17	RHSIM_Rhsim01G0245400
Root vs Leaf	RHSIM_Rhsim10G0194900	1.31062754	2.6768E-18	2.779E-17	RHSIM_Rhsim10G0194900
Root vs Leaf	novel.2029	3.20551569	2.70406E-18	2.80604E-17	--
Root vs Leaf	RHSIM_Rhsim08G0249300	-1.039978386	2.70908E-18	2.80998E-17	RHSIM_Rhsim08G0249300
Root vs Leaf	RHSIM_Rhsim04G0246400	1.498482313	2.80981E-18	2.91317E-17	RHSIM_Rhsim04G0246400
Root vs Leaf	RHSIM_Rhsim08G0164000	-1.223121187	2.87409E-18	2.97848E-17	RHSIM_Rhsim08G0164000
Root vs Leaf	RHSIM_Rhsim06G0241100	5.546462508	2.9388E-18	3.04417E-17	RHSIM_Rhsim06G0241100
Root vs Leaf	RHSIM_Rhsim08G0120800	-10.52076805	3.01607E-18	3.12282E-17	RHSIM_Rhsim08G0120800
Root vs Leaf	RHSIM_Rhsim13G0190500	-9.32345259	3.08294E-18	3.19063E-17	RHSIM_Rhsim13G0190500
Root vs Leaf	RHSIM_Rhsim11G0066400	6.179588283	3.24466E-18	3.35649E-17	RHSIM_Rhsim11G0066400
Root vs Leaf	RHSIM_Rhsim09G0067900	-1.349583622	3.26288E-18	3.37383E-17	RHSIM_Rhsim09G0067900
Root vs Leaf	RHSIM_Rhsim05G0208400	-11.53919883	3.32693E-18	3.43852E-17	RHSIM_Rhsim05G0208400
Root vs Leaf	RHSIM_Rhsim09G0145500	1.619132725	3.40223E-18	3.51478E-17	RHSIM_Rhsim09G0145500
Root vs Leaf	RHSIM_Rhsim04G0068100	-1.689083744	3.42804E-18	3.53986E-17	RHSIM_Rhsim04G0068100
Root vs Leaf	RHSIM_Rhsim06G0241300	4.834848318	3.46818E-18	3.57971E-17	RHSIM_Rhsim06G0241300
Root vs Leaf	RHSIM_Rhsim11G0105800	3.032300428	3.48427E-18	3.59472E-17	RHSIM_Rhsim11G0105800
Root vs Leaf	RHSIM_Rhsim10G0068300	-5.142966644	3.55481E-18	3.66586E-17	RHSIM_Rhsim10G0068300
Root vs Leaf	RHSIM_Rhsim08G0119600	-1.61267976	3.61702E-18	3.72835E-17	RHSIM_Rhsim08G0119600
Root vs Leaf	RHSIM_Rhsim04G0011200	-11.54571163	3.75964E-18	3.87363E-17	RHSIM_Rhsim04G0011200
Root vs Leaf	RHSIM_Rhsim10G0187000	3.195958788	3.84722E-18	3.9621E-17	RHSIM_Rhsim10G0187000
Root vs Leaf	RHSIM_Rhsim08G0223100	-1.104615648	3.90832E-18	4.02324E-17	RHSIM_Rhsim08G0223100
Root vs Leaf	novel.5534	-7.932211229	4.12657E-18	4.24601E-17	--
Root vs Leaf	RHSIM_Rhsim13G0202500	-1.598654065	4.12869E-18	4.24631E-17	RHSIM_Rhsim13G0202500
Root vs Leaf	RHSIM_Rhsim08G0092100	6.340562797	4.14167E-18	4.25777E-17	RHSIM_Rhsim08G0092100

Comparison	Gene ID	log_2FC	P-value	FDR (padj)	Gene Name
Root vs Leaf	RHSIM_Rhsim09G0092100	-5.853169854	4.15614E-18	4.27074E-17	RHSIM_Rhsim09G0092100
Root vs Leaf	RHSIM_Rhsim10G0186800	2.216869847	4.21222E-18	4.32646E-17	RHSIM_Rhsim10G0186800
Root vs Leaf	RHSIM_Rhsim09G0095700	11.45142227	4.26392E-18	4.37762E-17	RHSIM_Rhsim09G0095700
Root vs Leaf	RHSIM_Rhsim08G0104400	-2.99520853	4.27025E-18	4.38216E-17	RHSIM_Rhsim08G0104400
Root vs Leaf	RHSIM_RhsimUnG0026400	-7.287586767	4.30763E-18	4.41857E-17	RHSIM_RhsimUnG0026400
Root vs Leaf	RHSIM_Rhsim11G0021500	-2.193654044	4.50998E-18	4.62408E-17	RHSIM_Rhsim11G0021500
Root vs Leaf	novel.3094	-2.091136868	4.61002E-18	4.72455E-17	--
Root vs Leaf	RHSIM_Rhsim12G0181800	-2.651157382	4.70482E-18	4.81958E-17	RHSIM_Rhsim12G0181800
Root vs Leaf	RHSIM_RhsimUnG0089000	-3.988638941	4.74938E-18	4.86307E-17	RHSIM_RhsimUnG0089000
Root vs Leaf	RHSIM_Rhsim07G0082900	2.160923103	4.79461E-18	4.90721E-17	RHSIM_Rhsim07G0082900
Root vs Leaf	RHSIM_RhsimUnG0002100	-1.611291332	4.91692E-18	5.03017E-17	RHSIM_RhsimUnG0002100
Root vs Leaf	RHSIM_Rhsim03G0075700	-10.44322702	4.97077E-18	5.08302E-17	RHSIM_Rhsim03G0075700
Root vs Leaf	RHSIM_Rhsim13G0071200	-3.349327682	5.08067E-18	5.1931E-17	RHSIM_Rhsim13G0071200
Root vs Leaf	RHSIM_Rhsim02G0249900	3.010623248	5.17754E-18	5.28977E-17	RHSIM_Rhsim02G0249900
Root vs Leaf	RHSIM_Rhsim04G0049700	3.519687004	5.22824E-18	5.33922E-17	RHSIM_Rhsim04G0049700
Root vs Leaf	RHSIM_Rhsim03G0205500	-2.706734415	5.37051E-18	5.48209E-17	RHSIM_Rhsim03G0205500
Root vs Leaf	RHSIM_Rhsim13G0174100	2.035952424	5.41677E-18	5.52687E-17	RHSIM_Rhsim13G0174100
Root vs Leaf	RHSIM_Rhsim11G0049500	-1.307589103	5.45458E-18	5.563E-17	RHSIM_Rhsim11G0049500
Root vs Leaf	RHSIM_Rhsim03G0221200	-1.553991481	5.46048E-18	5.56657E-17	RHSIM_Rhsim03G0221200
Root vs Leaf	RHSIM_Rhsim06G0012300	-1.957278637	5.52106E-18	5.62506E-17	RHSIM_Rhsim06G0012300
Root vs Leaf	RHSIM_Rhsim03G0212500	1.850016647	5.52273E-18	5.62506E-17	RHSIM_Rhsim03G0212500
Root vs Leaf	RHSIM_Rhsim03G0241100	-1.653243086	5.54866E-18	5.64899E-17	RHSIM_Rhsim03G0241100
Root vs Leaf	RHSIM_Rhsim06G0116100	-1.104749441	5.57038E-18	5.66861E-17	RHSIM_Rhsim06G0116100
Root vs Leaf	RHSIM_Rhsim13G0071400	5.361340455	5.60579E-18	5.70213E-17	RHSIM_Rhsim13G0071400
Root vs Leaf	RHSIM_Rhsim09G0009700	3.867422419	5.8682E-18	5.96644E-17	RHSIM_Rhsim09G0009700

Comparison	Gene ID	log_2FC	P-value	FDR (padj)	Gene Name
Root vs Leaf	novel.2800	9.256236005	5.87515E-18	5.97088E-17	--
Root vs Leaf	novel.6181	-4.162307441	6.07131E-18	6.16753E-17	--
Root vs Leaf	RHSIM_Rhsim13G0096800	-4.707764633	6.1871E-18	6.28239E-17	RHSIM_Rhsim13G0096800
Root vs Leaf	RHSIM_Rhsim09G0188300	5.540575535	6.2955E-18	6.38966E-17	RHSIM_Rhsim09G0188300
Root vs Leaf	RHSIM_Rhsim01G0289900	1.285688059	6.31655E-18	6.40821E-17	RHSIM_Rhsim01G0289900
Root vs Leaf	RHSIM_Rhsim05G0215200	-2.594205105	6.49105E-18	6.58236E-17	RHSIM_Rhsim05G0215200
Root vs Leaf	RHSIM_Rhsim05G0179500	2.946110807	6.52167E-18	6.61051E-17	RHSIM_Rhsim05G0179500
Root vs Leaf	RHSIM_Rhsim06G0215000	-10.85200521	6.55877E-18	6.6452E-17	RHSIM_Rhsim06G0215000
Root vs Leaf	RHSIM_Rhsim06G0154100	-1.688046547	6.62568E-18	6.71006E-17	RHSIM_Rhsim06G0154100
Root vs Leaf	RHSIM_Rhsim05G0088500	2.364892653	6.89778E-18	6.98257E-17	RHSIM_Rhsim05G0088500
Root vs Leaf	RHSIM_Rhsim05G0190000	-10.47473937	6.91988E-18	7.00188E-17	RHSIM_Rhsim05G0190000
Root vs Leaf	RHSIM_Rhsim04G0068500	2.540323255	6.92409E-18	7.00307E-17	RHSIM_Rhsim04G0068500
Root vs Leaf	novel.4679	-4.876824042	7.1072E-18	7.18514E-17	--
Root vs Leaf	RHSIM_Rhsim08G0099800	-6.042393952	7.15267E-18	7.22794E-17	RHSIM_Rhsim08G0099800
Root vs Leaf	RHSIM_Rhsim06G0052400	-4.078129834	7.17967E-18	7.25207E-17	RHSIM_Rhsim06G0052400
Root vs Leaf	novel.823	6.453494627	7.29796E-18	7.36833E-17	--
Root vs Leaf	RHSIM_Rhsim01G0215000	10.33746874	7.3221E-18	7.38948E-17	RHSIM_Rhsim01G0215000
Root vs Leaf	RHSIM_Rhsim05G0221600	-3.593744057	7.5222E-18	7.58811E-17	RHSIM_Rhsim05G0221600
Root vs Leaf	RHSIM_Rhsim07G0100700	1.85826926	7.59625E-18	7.65947E-17	RHSIM_Rhsim07G0100700
Root vs Leaf	RHSIM_Rhsim01G0237500	-1.933992871	7.63357E-18	7.69375E-17	RHSIM_Rhsim01G0237500
Root vs Leaf	RHSIM_Rhsim02G0002000	-1.07765991	7.67828E-18	7.73544E-17	RHSIM_Rhsim02G0002000
Root vs Leaf	RHSIM_Rhsim09G0202700	2.45462757	8.04356E-18	8.09991E-17	RHSIM_Rhsim09G0202700
Root vs Leaf	RHSIM_Rhsim08G0197800	5.597137475	8.51784E-18	8.57378E-17	RHSIM_Rhsim08G0197800
Root vs Leaf	novel.526	-10.39555359	8.5239E-18	8.57616E-17	--
Root vs Leaf	novel.1495	-2.043605929	8.59754E-18	8.64649E-17	--

Comparison	Gene ID	log_2FC	P-value	FDR (padj)	Gene Name
Root vs Leaf	RHSIM_Rhsim02G0236400	-4.635352641	8.63913E-18	8.68454E-17	RHSIM_Rhsim02G0236400
Root vs Leaf	RHSIM_Rhsim06G0071900	-1.624698229	8.75095E-18	8.79313E-17	RHSIM_Rhsim06G0071900
Root vs Leaf	RHSIM_Rhsim08G0112900	-1.470599515	9.01403E-18	9.05354E-17	RHSIM_Rhsim08G0112900
Root vs Leaf	RHSIM_Rhsim13G0231500	-1.402132124	9.51075E-18	9.5483E-17	RHSIM_Rhsim13G0231500
Root vs Leaf	RHSIM_Rhsim03G0267400	-1.491609772	9.58864E-18	9.62232E-17	RHSIM_Rhsim03G0267400
Root vs Leaf	RHSIM_Rhsim12G0139300	1.122882962	9.6918E-18	9.72163E-17	RHSIM_Rhsim12G0139300
Root vs Leaf	novel.2863	-4.511242892	9.7685E-18	9.79432E-17	--
Root vs Leaf	RHSIM_Rhsim01G0089900	-3.016393375	1.00506E-17	1.00728E-16	RHSIM_Rhsim01G0089900
Root vs Leaf	RHSIM_Rhsim09G0031700	1.237791608	1.00592E-17	1.00771E-16	RHSIM_Rhsim09G0031700
Root vs Leaf	novel.570	2.997145495	1.08332E-17	1.08477E-16	--
Root vs Leaf	RHSIM_Rhsim02G0228200	10.63402555	1.18044E-17	1.18151E-16	RHSIM_Rhsim02G0228200
Root vs Leaf	RHSIM_Rhsim04G0023000	-4.351311016	1.1947E-17	1.19527E-16	RHSIM_Rhsim04G0023000
Root vs Leaf	RHSIM_Rhsim07G0187000	2.130286617	1.20839E-17	1.20845E-16	RHSIM_Rhsim07G0187000
Root vs Leaf	RHSIM_Rhsim02G0252500	1.498489221	1.2122E-17	1.21173E-16	RHSIM_Rhsim02G0252500
Root vs Leaf	novel.5885	6.969584384	1.25703E-17	1.256E-16	--
Root vs Leaf	RHSIM_Rhsim01G0148500	-1.243175326	1.25959E-17	1.25801E-16	RHSIM_Rhsim01G0148500
Root vs Leaf	RHSIM_Rhsim11G0111500	-2.09092887	1.32268E-17	1.31998E-16	RHSIM_Rhsim11G0111500
Root vs Leaf	RHSIM_Rhsim10G0178400	-2.012627648	1.32277E-17	1.31998E-16	RHSIM_Rhsim10G0178400
Root vs Leaf	RHSIM_Rhsim06G0214700	2.348571887	1.34357E-17	1.34015E-16	RHSIM_Rhsim06G0214700
Root vs Leaf	RHSIM_Rhsim06G0092900	-1.506912218	1.34683E-17	1.34282E-16	RHSIM_Rhsim06G0092900
Root vs Leaf	RHSIM_Rhsim13G0039000	-2.40187681	1.38908E-17	1.38375E-16	RHSIM_Rhsim13G0039000
Root vs Leaf	RHSIM_RhsimUnG0033300	-1.744897585	1.38997E-17	1.38405E-16	RHSIM_RhsimUnG0033300
Root vs Leaf	RHSIM_Rhsim05G0161900	2.378082161	1.41353E-17	1.4069E-16	RHSIM_Rhsim05G0161900
Root vs Leaf	RHSIM_Rhsim11G0128300	-1.308883087	1.43709E-17	1.42974E-16	RHSIM_Rhsim11G0128300
Root vs Leaf	RHSIM_Rhsim11G0138900	-4.089064941	1.43777E-17	1.42979E-16	RHSIM_Rhsim11G0138900

Comparison	Gene ID	log_2FC	P-value	FDR (padj)	Gene Name
Root vs Leaf	RHSIM_Rhsim13G0125600	3.065838031	1.44666E-17	1.43802E-16	RHSIM_Rhsim13G0125600
Root vs Leaf	RHSIM_Rhsim05G0131900	1.138158741	1.44929E-17	1.44002E-16	RHSIM_Rhsim05G0131900
Root vs Leaf	RHSIM_RhsimUnG0109200	-2.261183238	1.48101E-17	1.4709E-16	RHSIM_RhsimUnG0109200
Root vs Leaf	RHSIM_Rhsim05G0067600	-2.159060714	1.49633E-17	1.48548E-16	RHSIM_Rhsim05G0067600
Root vs Leaf	RHSIM_Rhsim01G0275100	7.703679318	1.50042E-17	1.4889E-16	RHSIM_Rhsim01G0275100
Root vs Leaf	RHSIM_Rhsim02G0083600	-2.480531895	1.57491E-17	1.56215E-16	RHSIM_Rhsim02G0083600
Root vs Leaf	RHSIM_Rhsim02G0116900	10.91072004	1.61267E-17	1.59892E-16	RHSIM_Rhsim02G0116900
Root vs Leaf	RHSIM_Rhsim13G0028300	-6.143069872	1.63535E-17	1.62071E-16	RHSIM_Rhsim13G0028300
Root vs Leaf	RHSIM_Rhsim10G0069600	-2.666454389	1.67745E-17	1.66172E-16	RHSIM_Rhsim10G0069600
Root vs Leaf	RHSIM_Rhsim04G0131900	-1.620498055	1.69858E-17	1.68193E-16	RHSIM_Rhsim04G0131900
Root vs Leaf	RHSIM_Rhsim01G0069300	-4.119380402	1.73859E-17	1.72082E-16	RHSIM_Rhsim01G0069300
Root vs Leaf	RHSIM_Rhsim11G0063600	-1.162964779	1.74576E-17	1.72718E-16	RHSIM_Rhsim11G0063600
Root vs Leaf	RHSIM_Rhsim02G0138600	-3.555326397	1.75162E-17	1.73223E-16	RHSIM_Rhsim02G0138600
Root vs Leaf	RHSIM_Rhsim03G0078300	-1.364522964	1.76425E-17	1.74397E-16	RHSIM_Rhsim03G0078300
Root vs Leaf	RHSIM_Rhsim03G0145900	2.810926033	1.77031E-17	1.74922E-16	RHSIM_Rhsim03G0145900
Root vs Leaf	RHSIM_Rhsim09G0145900	1.684287251	1.87852E-17	1.85478E-16	RHSIM_Rhsim09G0145900
Root vs Leaf	novel.5225	7.744701983	1.87875E-17	1.85478E-16	--
Root vs Leaf	RHSIM_Rhsim03G0033200	-2.705731849	1.93101E-17	1.90556E-16	RHSIM_Rhsim03G0033200
Root vs Leaf	RHSIM_Rhsim11G0110200	5.881163476	1.98091E-17	1.95398E-16	RHSIM_Rhsim11G0110200
Root vs Leaf	RHSIM_Rhsim07G0226200	2.094932031	2.0555E-17	2.02669E-16	RHSIM_Rhsim07G0226200
Root vs Leaf	RHSIM_Rhsim01G0263900	-1.805968032	2.06491E-17	2.0351E-16	RHSIM_Rhsim01G0263900
Root vs Leaf	RHSIM_Rhsim06G0133700	-4.374198682	2.06776E-17	2.03704E-16	RHSIM_Rhsim06G0133700
Root vs Leaf	RHSIM_Rhsim07G0102000	-5.385686921	2.12244E-17	2.09001E-16	RHSIM_Rhsim07G0102000
Root vs Leaf	RHSIM_Rhsim08G0049300	-1.912395654	2.13383E-17	2.10034E-16	RHSIM_Rhsim08G0049300
Root vs Leaf	RHSIM_Rhsim08G0122300	3.723330919	2.16775E-17	2.13282E-16	RHSIM_Rhsim08G0122300

Comparison	Gene ID	log_2FC	P-value	FDR (padj)	Gene Name
Root vs Leaf	RHSIM_Rhsim02G0117300	7.49591747	2.16933E-17	2.13346E-16	RHSIM_Rhsim02G0117300
Root vs Leaf	RHSIM_Rhsim02G0069000	-5.917378302	2.17592E-17	2.13904E-16	RHSIM_Rhsim02G0069000
Root vs Leaf	RHSIM_Rhsim02G0171500	-1.849045499	2.19529E-17	2.15717E-16	RHSIM_Rhsim02G0171500
Root vs Leaf	RHSIM_Rhsim04G0158000	-2.879359067	2.2041E-17	2.1649E-16	RHSIM_Rhsim04G0158000
Root vs Leaf	RHSIM_Rhsim09G0209100	-1.371999645	2.36755E-17	2.32446E-16	RHSIM_Rhsim09G0209100
Root vs Leaf	novel.4450	-7.259233651	2.39165E-17	2.34712E-16	--
Root vs Leaf	RHSIM_Rhsim03G0009400	3.925839711	2.39684E-17	2.35122E-16	RHSIM_Rhsim03G0009400
Root vs Leaf	RHSIM_RhsimUnG0057600	-4.180091856	2.45301E-17	2.4053E-16	RHSIM_RhsimUnG0057600
Root vs Leaf	RHSIM_Rhsim07G0200400	-2.920360619	2.47871E-17	2.42947E-16	RHSIM_Rhsim07G0200400
Root vs Leaf	RHSIM_Rhsim10G0093100	-2.851889386	2.51693E-17	2.46589E-16	RHSIM_Rhsim10G0093100
Root vs Leaf	RHSIM_Rhsim08G0179600	-4.202021363	2.53648E-17	2.48399E-16	RHSIM_Rhsim08G0179600
Root vs Leaf	RHSIM_Rhsim13G0217100	-2.214847476	2.66131E-17	2.60513E-16	RHSIM_Rhsim13G0217100
Root vs Leaf	novel.4849	-1.918397503	2.66891E-17	2.61147E-16	--
Root vs Leaf	RHSIM_Rhsim13G0100100	2.254522264	2.79825E-17	2.73687E-16	RHSIM_Rhsim13G0100100
Root vs Leaf	RHSIM_Rhsim01G0251300	-5.500811713	2.82268E-17	2.7596E-16	RHSIM_Rhsim01G0251300
Root vs Leaf	RHSIM_Rhsim13G0091100	-10.24977264	2.88078E-17	2.81521E-16	RHSIM_Rhsim13G0091100
Root vs Leaf	RHSIM_Rhsim06G0022900	-1.325009917	2.92091E-17	2.85322E-16	RHSIM_Rhsim06G0022900
Root vs Leaf	RHSIM_Rhsim08G0101200	-4.860625925	2.93214E-17	2.86298E-16	RHSIM_Rhsim08G0101200
Root vs Leaf	RHSIM_Rhsim01G0222000	-4.447562249	2.98936E-17	2.91762E-16	RHSIM_Rhsim01G0222000
Root vs Leaf	RHSIM_Rhsim10G0098600	1.346781537	3.01782E-17	2.94416E-16	RHSIM_Rhsim10G0098600
Root vs Leaf	RHSIM_Rhsim10G0167200	6.00323297	3.0342E-17	2.95889E-16	RHSIM_Rhsim10G0167200
Root vs Leaf	RHSIM_Rhsim13G0083600	-5.554245539	3.13478E-17	3.05568E-16	RHSIM_Rhsim13G0083600
Root vs Leaf	RHSIM_Rhsim13G0000600	2.110995746	3.16681E-17	3.08561E-16	RHSIM_Rhsim13G0000600
Root vs Leaf	RHSIM_Rhsim09G0087600	-3.347046478	3.1881E-17	3.10504E-16	RHSIM_Rhsim09G0087600
Root vs Leaf	RHSIM_Rhsim03G0101200	-2.286975188	3.23631E-17	3.15068E-16	RHSIM_Rhsim03G0101200

Comparison	Gene ID	log_2FC	P-value	FDR (padj)	Gene Name
Root vs Leaf	RHSIM_Rhsim09G0180100	-1.352207044	3.25271E-17	3.1653E-16	RHSIM_Rhsim09G0180100
Root vs Leaf	RHSIM_Rhsim07G0031300	-2.269427505	3.31776E-17	3.22725E-16	RHSIM_Rhsim07G0031300
Root vs Leaf	RHSIM_Rhsim06G0242100	-1.887774626	3.33651E-17	3.24412E-16	RHSIM_Rhsim06G0242100
Root vs Leaf	RHSIM_Rhsim06G0088000	-1.557989045	3.39782E-17	3.30235E-16	RHSIM_Rhsim06G0088000
Root vs Leaf	novel.3337	2.321505366	3.42106E-17	3.32354E-16	--
Root vs Leaf	RHSIM_Rhsim10G0202700	-11.04927513	3.42792E-17	3.32881E-16	RHSIM_Rhsim10G0202700
Root vs Leaf	RHSIM_Rhsim12G0026500	-5.966826929	3.46875E-17	3.36704E-16	RHSIM_Rhsim12G0026500
Root vs Leaf	RHSIM_Rhsim03G0235800	-10.89819546	3.48266E-17	3.37913E-16	RHSIM_Rhsim03G0235800
Root vs Leaf	RHSIM_Rhsim03G0036600	2.215867852	3.54815E-17	3.44123E-16	RHSIM_Rhsim03G0036600
Root vs Leaf	RHSIM_Rhsim09G0044400	-3.059785079	3.56849E-17	3.45951E-16	RHSIM_Rhsim09G0044400
Root vs Leaf	RHSIM_Rhsim10G0053300	-6.288144079	3.60676E-17	3.49515E-16	RHSIM_Rhsim10G0053300
Root vs Leaf	novel.4890	-10.31039358	3.6196E-17	3.50612E-16	--
Root vs Leaf	RHSIM_Rhsim06G0155000	4.122930338	3.70917E-17	3.59138E-16	RHSIM_Rhsim06G0155000
Root vs Leaf	RHSIM_Rhsim06G0160900	3.109754803	3.71942E-17	3.59979E-16	RHSIM_Rhsim06G0160900
Root vs Leaf	RHSIM_Rhsim05G0217500	1.301480622	3.7412E-17	3.61936E-16	RHSIM_Rhsim05G0217500
Root vs Leaf	RHSIM_RhsimUnG0083600	-4.688099395	3.76618E-17	3.64201E-16	RHSIM_RhsimUnG0083600
Root vs Leaf	RHSIM_Rhsim01G0229400	-2.743977605	3.76927E-17	3.64347E-16	RHSIM_Rhsim01G0229400
Root vs Leaf	RHSIM_RhsimUnG0240300	-4.896683175	4.15918E-17	4.01838E-16	RHSIM_RhsimUnG0240300
Root vs Leaf	novel.3142	-10.52122713	4.1606E-17	4.01838E-16	--
Root vs Leaf	RHSIM_Rhsim09G0022000	-3.920669033	4.18923E-17	4.04434E-16	RHSIM_Rhsim09G0022000
Root vs Leaf	novel.4446	-2.31519769	4.2617E-17	4.11259E-16	--
Root vs Leaf	RHSIM_Rhsim12G0123000	-6.837758344	4.39219E-17	4.23675E-16	RHSIM_Rhsim12G0123000
Root vs Leaf	RHSIM_Rhsim13G0181700	-9.20966528	4.43492E-17	4.27619E-16	RHSIM_Rhsim13G0181700
Root vs Leaf	RHSIM_Rhsim05G0202300	-3.57081043	4.5418E-17	4.37742E-16	RHSIM_Rhsim05G0202300
Root vs Leaf	RHSIM_Rhsim11G0022900	3.356472899	4.5714E-17	4.40412E-16	RHSIM_Rhsim11G0022900

Comparison	Gene ID	log_2FC	P-value	FDR (padj)	Gene Name
Root vs Leaf	RHSIM_Rhsim06G0121900	-2.270188281	4.75842E-17	4.58238E-16	RHSIM_Rhsim06G0121900
Root vs Leaf	RHSIM_Rhsim05G0122600	2.200407896	4.81498E-17	4.63492E-16	RHSIM_Rhsim05G0122600
Root vs Leaf	novel.5470	3.250809536	4.8352E-17	4.65245E-16	--
Root vs Leaf	RHSIM_Rhsim13G0194600	1.374887037	5.0281E-17	4.83604E-16	RHSIM_Rhsim13G0194600
Root vs Leaf	RHSIM_Rhsim04G0243200	-7.902922231	5.05898E-17	4.86272E-16	RHSIM_Rhsim04G0243200
Root vs Leaf	RHSIM_Rhsim12G0163100	2.386666187	5.06003E-17	4.86272E-16	RHSIM_Rhsim12G0163100
Root vs Leaf	RHSIM_Rhsim08G0105200	6.184463512	5.07204E-17	4.87223E-16	RHSIM_Rhsim08G0105200
Root vs Leaf	RHSIM_Rhsim10G0197300	-2.143939576	5.11633E-17	4.91274E-16	RHSIM_Rhsim10G0197300
Root vs Leaf	RHSIM_Rhsim04G0188500	7.46786143	5.16766E-17	4.95997E-16	RHSIM_Rhsim04G0188500
Root vs Leaf	RHSIM_Rhsim12G0070300	-8.935016764	5.19336E-17	4.98257E-16	RHSIM_Rhsim12G0070300
Root vs Leaf	RHSIM_Rhsim11G0129100	1.305794553	5.42014E-17	5.19799E-16	RHSIM_Rhsim11G0129100
Root vs Leaf	RHSIM_Rhsim04G0233400	-1.556695159	5.67168E-17	5.43697E-16	RHSIM_Rhsim04G0233400
Root vs Leaf	RHSIM_Rhsim08G0198100	-1.13898364	6.00817E-17	5.75715E-16	RHSIM_Rhsim08G0198100
Root vs Leaf	RHSIM_Rhsim01G0071100	-3.521306632	6.08546E-17	5.82879E-16	RHSIM_Rhsim01G0071100
Root vs Leaf	RHSIM_Rhsim01G0233600	-6.248728113	6.08992E-17	5.83065E-16	RHSIM_Rhsim01G0233600
Root vs Leaf	RHSIM_Rhsim01G0251500	-4.655060001	6.22276E-17	5.95537E-16	RHSIM_Rhsim01G0251500
Root vs Leaf	RHSIM_Rhsim03G0143200	2.322868941	6.22813E-17	5.95805E-16	RHSIM_Rhsim03G0143200
Root vs Leaf	RHSIM_Rhsim08G0239900	3.249219201	6.30199E-17	6.02621E-16	RHSIM_Rhsim08G0239900
Root vs Leaf	RHSIM_RhsimUnG0173800	-2.592394744	6.32671E-17	6.04735E-16	RHSIM_RhsimUnG0173800
Root vs Leaf	RHSIM_Rhsim03G0252500	3.828854362	6.37276E-17	6.08885E-16	RHSIM_Rhsim03G0252500
Root vs Leaf	RHSIM_RhsimUnG0042300	1.45841732	6.43042E-17	6.14141E-16	RHSIM_RhsimUnG0042300
Root vs Leaf	RHSIM_Rhsim07G0026400	-11.49807786	6.46617E-17	6.17301E-16	RHSIM_Rhsim07G0026400
Root vs Leaf	RHSIM_Rhsim09G0132600	-1.870851026	6.69873E-17	6.39239E-16	RHSIM_Rhsim09G0132600
Root vs Leaf	RHSIM_Rhsim02G0171900	-1.628406402	6.71838E-17	6.40849E-16	RHSIM_Rhsim02G0171900
Root vs Leaf	RHSIM_Rhsim08G0226700	-10.72406388	6.7459E-17	6.43209E-16	RHSIM_Rhsim08G0226700

Comparison	Gene ID	log_2FC	P-value	FDR (padj)	Gene Name
Root vs Leaf	RHSIM_Rhsim12G0190600	-2.836480812	6.96191E-17	6.63532E-16	RHSIM_Rhsim12G0190600
Root vs Leaf	RHSIM_Rhsim10G0148700	-2.900675299	7.39602E-17	7.04616E-16	RHSIM_Rhsim10G0148700
Root vs Leaf	RHSIM_Rhsim08G0101800	-3.508402891	7.40499E-17	7.05181E-16	RHSIM_Rhsim08G0101800
Root vs Leaf	RHSIM_Rhsim01G0291100	2.34096792	7.88908E-17	7.50971E-16	RHSIM_Rhsim01G0291100
Root vs Leaf	novel.5469	-6.805240433	7.9241E-17	7.53996E-16	--
Root vs Leaf	novel.869	4.253705572	8.13784E-17	7.74015E-16	--
Root vs Leaf	RHSIM_Rhsim02G0098500	4.304834673	8.17547E-17	7.77275E-16	RHSIM_Rhsim02G0098500
Root vs Leaf	RHSIM_Rhsim10G0016800	1.66258322	8.20289E-17	7.79561E-16	RHSIM_Rhsim10G0016800
Root vs Leaf	RHSIM_Rhsim09G0028800	2.509972143	8.36214E-17	7.94369E-16	RHSIM_Rhsim09G0028800
Root vs Leaf	RHSIM_Rhsim04G0073600	2.474334312	8.5835E-17	8.15062E-16	RHSIM_Rhsim04G0073600
Root vs Leaf	novel.3288	-1.807028535	8.8426E-17	8.39322E-16	--
Root vs Leaf	RHSIM_Rhsim01G0193400	-4.685536354	8.88475E-17	8.42976E-16	RHSIM_Rhsim01G0193400
Root vs Leaf	RHSIM_Rhsim03G0188800	-1.650454064	8.91198E-17	8.45214E-16	RHSIM_Rhsim03G0188800
Root vs Leaf	RHSIM_Rhsim03G0093400	-1.5413277	9.10539E-17	8.63203E-16	RHSIM_Rhsim03G0093400
Root vs Leaf	RHSIM_Rhsim11G0106000	2.206328571	9.27973E-17	8.7937E-16	RHSIM_Rhsim11G0106000
Root vs Leaf	RHSIM_Rhsim02G0237500	-3.132989649	9.36124E-17	8.86732E-16	RHSIM_Rhsim02G0237500
Root vs Leaf	RHSIM_Rhsim01G0028000	-1.935525378	9.36629E-17	8.86846E-16	RHSIM_Rhsim01G0028000
Root vs Leaf	novel.5900	-6.765975683	9.5983E-17	9.08442E-16	--
Root vs Leaf	RHSIM_Rhsim03G0018600	-1.090640971	9.67338E-17	9.15175E-16	RHSIM_Rhsim03G0018600
Root vs Leaf	RHSIM_Rhsim08G0121700	2.002962413	9.74909E-17	9.2196E-16	RHSIM_Rhsim08G0121700
Root vs Leaf	RHSIM_Rhsim09G0194700	2.169234725	9.82497E-17	9.28757E-16	RHSIM_Rhsim09G0194700
Root vs Leaf	RHSIM_Rhsim12G0069900	-1.092417507	9.94842E-17	9.40043E-16	RHSIM_Rhsim12G0069900
Root vs Leaf	RHSIM_Rhsim13G0142600	-1.884786924	9.96901E-17	9.41604E-16	RHSIM_Rhsim13G0142600
Root vs Leaf	RHSIM_Rhsim11G0104900	-5.95903957	1.00974E-16	9.53346E-16	RHSIM_Rhsim11G0104900
Root vs Leaf	RHSIM_Rhsim09G0065800	-2.161581868	1.0312E-16	9.73208E-16	RHSIM_Rhsim09G0065800

Comparison	Gene ID	log_2FC	P-value	FDR (padj)	Gene Name
Root vs Leaf	RHSIM_Rhsim01G0179300	6.54092175	1.04428E-16	9.85148E-16	RHSIM_Rhsim01G0179300
Root vs Leaf	RHSIM_Rhsim01G0225400	1.0665632	1.04582E-16	9.86195E-16	RHSIM_Rhsim01G0225400
Root vs Leaf	RHSIM_Rhsim02G0236500	-8.586587161	1.05995E-16	9.99115E-16	RHSIM_Rhsim02G0236500
Root vs Leaf	RHSIM_Rhsim11G0114000	-2.742421993	1.06324E-16	1.00181E-15	RHSIM_Rhsim11G0114000
Root vs Leaf	RHSIM_Rhsim13G0199500	4.593630582	1.08381E-16	1.02078E-15	RHSIM_Rhsim13G0199500
Root vs Leaf	RHSIM_RhsimUnG0197600	-2.876399214	1.12968E-16	1.06354E-15	RHSIM_RhsimUnG0197600
Root vs Leaf	RHSIM_Rhsim12G0200100	-8.980695595	1.153E-16	1.08506E-15	RHSIM_Rhsim12G0200100
Root vs Leaf	RHSIM_Rhsim08G0193800	-3.159453352	1.15833E-16	1.08963E-15	RHSIM_Rhsim08G0193800
Root vs Leaf	RHSIM_Rhsim07G0215400	-3.670058232	1.16206E-16	1.0927E-15	RHSIM_Rhsim07G0215400
Root vs Leaf	RHSIM_Rhsim01G0089500	-2.414130283	1.16414E-16	1.09421E-15	RHSIM_Rhsim01G0089500
Root vs Leaf	RHSIM_Rhsim05G0122100	-1.441977935	1.16829E-16	1.09766E-15	RHSIM_Rhsim05G0122100
Root vs Leaf	RHSIM_Rhsim03G0195200	-5.921952003	1.19474E-16	1.12206E-15	RHSIM_Rhsim03G0195200
Root vs Leaf	RHSIM_Rhsim06G0185300	2.363750109	1.19841E-16	1.12505E-15	RHSIM_Rhsim06G0185300
Root vs Leaf	RHSIM_RhsimUnG0088600	11.23978692	1.22656E-16	1.15101E-15	RHSIM_RhsimUnG0088600
Root vs Leaf	novel.1829	-10.26748186	1.23896E-16	1.16217E-15	--
Root vs Leaf	RHSIM_RhsimUnG0158600	-3.164360152	1.27329E-16	1.19389E-15	RHSIM_RhsimUnG0158600
Root vs Leaf	RHSIM_Rhsim09G0028200	1.932781464	1.31251E-16	1.23017E-15	RHSIM_Rhsim09G0028200
Root vs Leaf	RHSIM_Rhsim03G0219900	2.234750033	1.35364E-16	1.2682E-15	RHSIM_Rhsim03G0219900
Root vs Leaf	RHSIM_Rhsim08G0058000	10.14254328	1.38702E-16	1.29895E-15	RHSIM_Rhsim08G0058000
Root vs Leaf	novel.3528	-1.355420159	1.42014E-16	1.32942E-15	--
Root vs Leaf	RHSIM_Rhsim02G0199800	-1.504020625	1.42562E-16	1.33402E-15	RHSIM_Rhsim02G0199800
Root vs Leaf	RHSIM_RhsimUnG0120600	-2.47713821	1.43486E-16	1.34213E-15	RHSIM_RhsimUnG0120600
Root vs Leaf	RHSIM_Rhsim08G0246600	-1.490645716	1.43972E-16	1.34612E-15	RHSIM_Rhsim08G0246600
Root vs Leaf	RHSIM_Rhsim05G0109100	2.55599447	1.49009E-16	1.39266E-15	RHSIM_Rhsim05G0109100
Root vs Leaf	RHSIM_Rhsim08G0062200	-4.713983773	1.50428E-16	1.40535E-15	RHSIM_Rhsim08G0062200

Comparison	Gene ID	log_2FC	P-value	FDR (padj)	Gene Name
Root vs Leaf	RHSIM_Rhsim08G0043800	-1.156576973	1.51016E-16	1.41027E-15	RHSIM_Rhsim08G0043800
Root vs Leaf	RHSIM_Rhsim02G0081500	-4.483854552	1.51481E-16	1.41405E-15	RHSIM_Rhsim02G0081500
Root vs Leaf	RHSIM_Rhsim09G0170000	-5.117215006	1.52325E-16	1.42135E-15	RHSIM_Rhsim09G0170000
Root vs Leaf	novel.2903	-10.28348862	1.52885E-16	1.426E-15	--
Root vs Leaf	RHSIM_Rhsim05G0187800	-1.286781295	1.53171E-16	1.42809E-15	RHSIM_Rhsim05G0187800
Root vs Leaf	RHSIM_Rhsim09G0191300	1.871958825	1.56081E-16	1.45464E-15	RHSIM_Rhsim09G0191300
Root vs Leaf	RHSIM_RhsimUnG0228600	-1.788254088	1.58386E-16	1.47553E-15	RHSIM_RhsimUnG0228600
Root vs Leaf	RHSIM_Rhsim07G0229800	-2.922310202	1.60763E-16	1.49707E-15	RHSIM_Rhsim07G0229800
Root vs Leaf	RHSIM_Rhsim01G0056200	5.803241156	1.60846E-16	1.49724E-15	RHSIM_Rhsim01G0056200
Root vs Leaf	RHSIM_Rhsim02G0171600	-1.2784302	1.61817E-16	1.50567E-15	RHSIM_Rhsim02G0171600
Root vs Leaf	RHSIM_Rhsim13G0109500	-10.51119199	1.6382E-16	1.5237E-15	RHSIM_Rhsim13G0109500
Root vs Leaf	RHSIM_Rhsim12G0023500	6.038603619	1.66001E-16	1.54336E-15	RHSIM_Rhsim12G0023500
Root vs Leaf	RHSIM_Rhsim07G0009600	3.972182682	1.71406E-16	1.59298E-15	RHSIM_Rhsim07G0009600
Root vs Leaf	RHSIM_Rhsim04G0033400	-10.3620859	1.78914E-16	1.66208E-15	RHSIM_Rhsim04G0033400
Root vs Leaf	novel.5231	-7.378317379	1.79485E-16	1.66672E-15	--
Root vs Leaf	RHSIM_Rhsim02G0241400	3.405729489	1.87997E-16	1.74506E-15	RHSIM_Rhsim02G0241400
Root vs Leaf	RHSIM_Rhsim04G0071900	7.227786557	1.90345E-16	1.76575E-15	RHSIM_Rhsim04G0071900
Root vs Leaf	RHSIM_Rhsim03G0264300	1.397387893	1.90378E-16	1.76575E-15	RHSIM_Rhsim03G0264300
Root vs Leaf	RHSIM_Rhsim11G0092000	11.39023367	1.93599E-16	1.7949E-15	RHSIM_Rhsim11G0092000
Root vs Leaf	RHSIM_Rhsim12G0110900	-2.549658802	2.03584E-16	1.88671E-15	RHSIM_Rhsim12G0110900
Root vs Leaf	RHSIM_Rhsim11G0175100	6.366348308	2.0787E-16	1.92567E-15	RHSIM_Rhsim11G0175100
Root vs Leaf	RHSIM_Rhsim09G0074900	1.345308801	2.09113E-16	1.9364E-15	RHSIM_Rhsim09G0074900
Root vs Leaf	RHSIM_Rhsim03G0138400	-3.023786398	2.12833E-16	1.97007E-15	RHSIM_Rhsim03G0138400
Root vs Leaf	RHSIM_Rhsim09G0024000	2.890279471	2.13764E-16	1.97789E-15	RHSIM_Rhsim09G0024000
Root vs Leaf	RHSIM_Rhsim01G0237100	-1.439072447	2.15894E-16	1.9968E-15	RHSIM_Rhsim01G0237100

Comparison	Gene ID	log_2FC	P-value	FDR (padj)	Gene Name
Root vs Leaf	RHSIM_Rhsim10G0210900	-2.547011348	2.22678E-16	2.05873E-15	RHSIM_Rhsim10G0210900
Root vs Leaf	RHSIM_Rhsim08G0010600	-4.375641648	2.26408E-16	2.09238E-15	RHSIM_Rhsim08G0010600
Root vs Leaf	RHSIM_Rhsim09G0109200	-3.136244668	2.34056E-16	2.16219E-15	RHSIM_Rhsim09G0109200
Root vs Leaf	RHSIM_Rhsim02G0029500	-11.41409923	2.36631E-16	2.1851E-15	RHSIM_Rhsim02G0029500
Root vs Leaf	RHSIM_Rhsim10G0129900	2.590030733	2.39069E-16	2.20674E-15	RHSIM_Rhsim10G0129900
Root vs Leaf	RHSIM_Rhsim03G0175000	1.586875714	2.41168E-16	2.22522E-15	RHSIM_Rhsim03G0175000
Root vs Leaf	RHSIM_Rhsim04G0003700	-1.8085538	2.41656E-16	2.22884E-15	RHSIM_Rhsim04G0003700
Root vs Leaf	RHSIM_Rhsim05G0104500	-3.864949091	2.5429E-16	2.34443E-15	RHSIM_Rhsim05G0104500
Root vs Leaf	RHSIM_Rhsim12G0149600	1.559264764	2.59021E-16	2.3871E-15	RHSIM_Rhsim12G0149600
Root vs Leaf	RHSIM_Rhsim09G0132300	6.459105538	2.63184E-16	2.4245E-15	RHSIM_Rhsim09G0132300
Root vs Leaf	RHSIM_Rhsim06G0002500	9.983059253	2.6549E-16	2.44477E-15	RHSIM_Rhsim06G0002500
Root vs Leaf	RHSIM_Rhsim05G0206200	1.92141463	2.66319E-16	2.45142E-15	RHSIM_Rhsim05G0206200
Root vs Leaf	RHSIM_Rhsim03G0179300	1.789308493	2.69569E-16	2.48035E-15	RHSIM_Rhsim03G0179300
Root vs Leaf	RHSIM_Rhsim11G0111000	1.330796557	2.82818E-16	2.60123E-15	RHSIM_Rhsim11G0111000
Root vs Leaf	RHSIM_Rhsim06G0073700	-1.601096195	2.86091E-16	2.63029E-15	RHSIM_Rhsim06G0073700
Root vs Leaf	RHSIM_Rhsim11G0121200	1.535733778	2.87359E-16	2.64089E-15	RHSIM_Rhsim11G0121200
Root vs Leaf	RHSIM_Rhsim04G0078100	-9.926134446	2.93963E-16	2.70051E-15	RHSIM_Rhsim04G0078100
Root vs Leaf	RHSIM_Rhsim05G0011200	-6.115004314	3.09149E-16	2.8389E-15	RHSIM_Rhsim05G0011200
Root vs Leaf	RHSIM_Rhsim12G0193300	9.957323903	3.2104E-16	2.94692E-15	RHSIM_Rhsim12G0193300
Root vs Leaf	RHSIM_Rhsim12G0148800	-10.02428649	3.25385E-16	2.98562E-15	RHSIM_Rhsim12G0148800
Root vs Leaf	RHSIM_Rhsim02G0241200	-2.037543934	3.26932E-16	2.99863E-15	RHSIM_Rhsim02G0241200
Root vs Leaf	RHSIM_Rhsim11G0166900	-6.493392539	3.37236E-16	3.09191E-15	RHSIM_Rhsim11G0166900
Root vs Leaf	novel.2385	-2.442870764	3.37968E-16	3.09739E-15	--
Root vs Leaf	RHSIM_Rhsim11G0159600	-11.87850292	3.40257E-16	3.11713E-15	RHSIM_Rhsim11G0159600
Root vs Leaf	RHSIM_Rhsim11G0066500	-4.018777436	3.54089E-16	3.24257E-15	RHSIM_Rhsim11G0066500

Comparison	Gene ID	log_2FC	P-value	FDR (padj)	Gene Name
Root vs Leaf	RHSIM_Rhsim12G0138800	-2.843634486	3.54429E-16	3.2444E-15	RHSIM_Rhsim12G0138800
Root vs Leaf	RHSIM_Rhsim11G0107700	5.946605283	3.62981E-16	3.32137E-15	RHSIM_Rhsim11G0107700
Root vs Leaf	RHSIM_Rhsim03G0208700	-1.553043297	3.69371E-16	3.3785E-15	RHSIM_Rhsim03G0208700
Root vs Leaf	RHSIM_Rhsim02G0035200	9.309029872	3.7081E-16	3.39032E-15	RHSIM_Rhsim02G0035200
Root vs Leaf	RHSIM_Rhsim04G0199100	1.449560316	3.72025E-16	3.40009E-15	RHSIM_Rhsim04G0199100
Root vs Leaf	RHSIM_RhsimUnG0212700	2.672718076	3.73661E-16	3.41369E-15	RHSIM_RhsimUnG0212700
Root vs Leaf	RHSIM_Rhsim12G0074100	-1.4168081	3.78884E-16	3.46004E-15	RHSIM_Rhsim12G0074100
Root vs Leaf	RHSIM_Rhsim01G0051900	-10.20322727	3.81814E-16	3.48542E-15	RHSIM_Rhsim01G0051900
Root vs Leaf	RHSIM_Rhsim01G0287600	1.812559513	3.82421E-16	3.48959E-15	RHSIM_Rhsim01G0287600
Root vs Leaf	RHSIM_Rhsim08G0244800	2.175316915	3.85283E-16	3.51432E-15	RHSIM_Rhsim08G0244800
Root vs Leaf	RHSIM_Rhsim03G0225300	-2.284380018	3.86664E-16	3.52552E-15	RHSIM_Rhsim03G0225300
Root vs Leaf	RHSIM_Rhsim13G0088200	-10.13267952	3.87947E-16	3.53584E-15	RHSIM_Rhsim13G0088200
Root vs Leaf	RHSIM_Rhsim07G0090400	1.923742113	3.91711E-16	3.56873E-15	RHSIM_Rhsim07G0090400
Root vs Leaf	RHSIM_RhsimUnG0103200	9.86060206	4.01278E-16	3.65445E-15	RHSIM_RhsimUnG0103200
Root vs Leaf	novel.3017	1.154484276	4.08825E-16	3.72172E-15	--
Root vs Leaf	RHSIM_Rhsim11G0021900	-1.069755684	4.12094E-16	3.75001E-15	RHSIM_Rhsim11G0021900
Root vs Leaf	RHSIM_Rhsim09G0010600	-1.011271828	4.39598E-16	3.99872E-15	RHSIM_Rhsim09G0010600
Root vs Leaf	RHSIM_Rhsim07G0020900	-4.117107434	4.43318E-16	4.03097E-15	RHSIM_Rhsim07G0020900
Root vs Leaf	RHSIM_Rhsim13G0234200	-1.718838255	4.56056E-16	4.14516E-15	RHSIM_Rhsim13G0234200
Root vs Leaf	RHSIM_Rhsim07G0139700	-4.276333854	4.68628E-16	4.25777E-15	RHSIM_Rhsim07G0139700
Root vs Leaf	RHSIM_Rhsim13G0014700	3.987208299	4.72421E-16	4.29054E-15	RHSIM_Rhsim13G0014700
Root vs Leaf	RHSIM_Rhsim02G0164500	-7.569550062	4.78748E-16	4.3463E-15	RHSIM_Rhsim02G0164500
Root vs Leaf	RHSIM_RhsimUnG0217800	-1.046066536	5.08159E-16	4.61149E-15	RHSIM_RhsimUnG0217800
Root vs Leaf	RHSIM_Rhsim08G0006000	6.564906543	5.12711E-16	4.65098E-15	RHSIM_Rhsim08G0006000
Root vs Leaf	RHSIM_Rhsim03G0046200	1.918689021	5.23268E-16	4.74489E-15	RHSIM_Rhsim03G0046200

Comparison	Gene ID	log_2FC	P-value	FDR (padj)	Gene Name
Root vs Leaf	RHSIM_Rhsim04G0026700	1.513399272	5.33531E-16	4.83605E-15	RHSIM_Rhsim04G0026700
Root vs Leaf	RHSIM_Rhsim05G0086800	4.082277181	5.36196E-16	4.8583E-15	RHSIM_Rhsim05G0086800
Root vs Leaf	RHSIM_Rhsim04G0201800	-3.583837405	5.42432E-16	4.91288E-15	RHSIM_Rhsim04G0201800
Root vs Leaf	RHSIM_Rhsim08G0123500	-1.535809217	5.52391E-16	5.00113E-15	RHSIM_Rhsim08G0123500
Root vs Leaf	RHSIM_Rhsim01G0227500	-1.920530179	5.53772E-16	5.01167E-15	RHSIM_Rhsim01G0227500
Root vs Leaf	RHSIM_Rhsim07G0214200	-2.028812195	5.54982E-16	5.02066E-15	RHSIM_Rhsim07G0214200
Root vs Leaf	novel.1533	4.262466653	5.64627E-16	5.10591E-15	--
Root vs Leaf	RHSIM_Rhsim12G0079300	10.44391324	5.67939E-16	5.13386E-15	RHSIM_Rhsim12G0079300
Root vs Leaf	novel.3705	1.407693672	5.69162E-16	5.1429E-15	--
Root vs Leaf	RHSIM_Rhsim13G0089900	-3.489180978	5.73558E-16	5.18061E-15	RHSIM_Rhsim13G0089900
Root vs Leaf	novel.5155	-3.519544268	5.74675E-16	5.18867E-15	--
Root vs Leaf	RHSIM_Rhsim12G0167500	1.946967522	5.78508E-16	5.22124E-15	RHSIM_Rhsim12G0167500
Root vs Leaf	RHSIM_Rhsim02G0074000	7.285986534	5.78831E-16	5.22211E-15	RHSIM_Rhsim02G0074000
Root vs Leaf	RHSIM_Rhsim12G0036800	-4.108821265	5.92964E-16	5.34754E-15	RHSIM_Rhsim12G0036800
Root vs Leaf	RHSIM_Rhsim10G0052500	-9.942891319	6.16162E-16	5.55458E-15	RHSIM_Rhsim10G0052500
Root vs Leaf	RHSIM_Rhsim08G0145100	1.910126243	6.17756E-16	5.56678E-15	RHSIM_Rhsim08G0145100
Root vs Leaf	RHSIM_Rhsim08G0081500	1.096157079	6.22213E-16	5.60476E-15	RHSIM_Rhsim08G0081500
Root vs Leaf	RHSIM_Rhsim10G0021100	-1.560178276	6.32882E-16	5.69865E-15	RHSIM_Rhsim10G0021100
Root vs Leaf	RHSIM_Rhsim07G0118200	-1.390980189	6.33785E-16	5.70456E-15	RHSIM_Rhsim07G0118200
Root vs Leaf	RHSIM_Rhsim13G0030400	1.325521198	6.39981E-16	5.75809E-15	RHSIM_Rhsim13G0030400
Root vs Leaf	RHSIM_Rhsim06G0222600	4.157593721	6.40886E-16	5.76399E-15	RHSIM_Rhsim06G0222600
Root vs Leaf	RHSIM_Rhsim08G0134300	-1.836678583	6.42755E-16	5.77855E-15	RHSIM_Rhsim08G0134300
Root vs Leaf	RHSIM_Rhsim07G0076600	-3.267534397	6.5763E-16	5.90998E-15	RHSIM_Rhsim07G0076600
Root vs Leaf	novel.2522	-9.933198707	6.58876E-16	5.91889E-15	--
Root vs Leaf	RHSIM_Rhsim06G0185600	6.508441536	6.6211E-16	5.94563E-15	RHSIM_Rhsim06G0185600

Comparison	Gene ID	log_2FC	P-value	FDR (padj)	Gene Name
Root vs Leaf	novel.2230	-5.461960118	6.92602E-16	6.21703E-15	--
Root vs Leaf	RHSIM_Rhsim08G0076400	1.029318663	7.00683E-16	6.28713E-15	RHSIM_Rhsim08G0076400
Root vs Leaf	novel.4274	6.332690877	7.11643E-16	6.383E-15	--
Root vs Leaf	novel.3147	10.6444714	7.38694E-16	6.62306E-15	--
Root vs Leaf	RHSIM_Rhsim02G0212700	-1.972773222	7.39491E-16	6.62764E-15	RHSIM_Rhsim02G0212700
Root vs Leaf	RHSIM_Rhsim10G0187900	-2.945077784	7.40315E-16	6.63245E-15	RHSIM_Rhsim10G0187900
Root vs Leaf	RHSIM_Rhsim10G0084900	-6.142574601	7.56845E-16	6.77792E-15	RHSIM_Rhsim10G0084900
Root vs Leaf	RHSIM_Rhsim04G0096800	-2.017510789	7.77802E-16	6.9629E-15	RHSIM_Rhsim04G0096800
Root vs Leaf	RHSIM_Rhsim13G0030500	-1.466726054	7.91869E-16	7.08609E-15	RHSIM_Rhsim13G0030500
Root vs Leaf	RHSIM_Rhsim12G0048700	-5.051404934	8.02349E-16	7.17659E-15	RHSIM_Rhsim12G0048700
Root vs Leaf	RHSIM_Rhsim03G0175300	-2.605760724	8.02603E-16	7.17659E-15	RHSIM_Rhsim03G0175300
Root vs Leaf	RHSIM_Rhsim01G0129100	-1.406291436	8.09095E-16	7.23185E-15	RHSIM_Rhsim01G0129100
Root vs Leaf	novel.3847	-2.202055055	8.11122E-16	7.24717E-15	--
Root vs Leaf	RHSIM_Rhsim13G0077100	1.352695021	8.15558E-16	7.28399E-15	RHSIM_Rhsim13G0077100
Root vs Leaf	RHSIM_Rhsim05G0216400	1.021126694	8.25194E-16	7.36721E-15	RHSIM_Rhsim05G0216400
Root vs Leaf	RHSIM_Rhsim09G0195700	-1.429517794	8.33223E-16	7.43603E-15	RHSIM_Rhsim09G0195700
Root vs Leaf	RHSIM_Rhsim09G0072100	-4.445074175	8.53133E-16	7.61078E-15	RHSIM_Rhsim09G0072100
Root vs Leaf	RHSIM_Rhsim01G0125200	1.419576242	8.54877E-16	7.62339E-15	RHSIM_Rhsim01G0125200
Root vs Leaf	RHSIM_RhsimUnG0021100	1.858858777	8.58664E-16	7.65421E-15	RHSIM_RhsimUnG0021100
Root vs Leaf	RHSIM_Rhsim05G0071600	3.950814601	8.63274E-16	7.69235E-15	RHSIM_Rhsim05G0071600
Root vs Leaf	RHSIM_Rhsim06G0128200	-2.653559624	8.71237E-16	7.76031E-15	RHSIM_Rhsim06G0128200
Root vs Leaf	RHSIM_Rhsim02G0028800	-10.18563927	8.76346E-16	7.80281E-15	RHSIM_Rhsim02G0028800
Root vs Leaf	RHSIM_Rhsim10G0078600	-1.27546311	8.81601E-16	7.84659E-15	RHSIM_Rhsim10G0078600
Root vs Leaf	novel.4447	-2.359917731	8.9964E-16	8.00406E-15	--
Root vs Leaf	novel.1637	-8.701235295	9.00637E-16	8.00986E-15	--

Comparison	Gene ID	log_2FC	P-value	FDR (padj)	Gene Name
Root vs Leaf	RHSIM_Rhsim08G0245600	1.86252474	9.02859E-16	8.02653E-15	RHSIM_Rhsim08G0245600
Root vs Leaf	RHSIM_Rhsim11G0088700	3.093354265	9.0351E-16	8.02924E-15	RHSIM_Rhsim11G0088700
Root vs Leaf	novel.841	-6.58831429	9.0812E-16	8.0671E-15	--
Root vs Leaf	RHSIM_Rhsim13G0122100	-10.0068841	9.24961E-16	8.21356E-15	RHSIM_Rhsim13G0122100
Root vs Leaf	RHSIM_Rhsim08G0031200	-1.739762521	9.33045E-16	8.28216E-15	RHSIM_Rhsim08G0031200
Root vs Leaf	RHSIM_Rhsim11G0022000	1.135441505	9.35668E-16	8.30226E-15	RHSIM_Rhsim11G0022000
Root vs Leaf	novel.1251	4.509581114	9.45512E-16	8.38639E-15	--
Root vs Leaf	RHSIM_Rhsim11G0141600	-4.284311174	9.63967E-16	8.54681E-15	RHSIM_Rhsim11G0141600
Root vs Leaf	RHSIM_Rhsim09G0109300	-2.937038278	9.80717E-16	8.69199E-15	RHSIM_Rhsim09G0109300
Root vs Leaf	RHSIM_Rhsim08G0106300	-10.31740997	9.90163E-16	8.77234E-15	RHSIM_Rhsim08G0106300
Root vs Leaf	RHSIM_Rhsim04G0014200	-4.104375867	1.03198E-15	9.13937E-15	RHSIM_Rhsim04G0014200
Root vs Leaf	RHSIM_RhsimUnG0255400	4.646614013	1.0455E-15	9.25555E-15	RHSIM_RhsimUnG0255400
Root vs Leaf	RHSIM_Rhsim10G0047000	9.903613049	1.06453E-15	9.42042E-15	RHSIM_Rhsim10G0047000
Root vs Leaf	RHSIM_Rhsim11G0110100	10.28422424	1.07021E-15	9.467E-15	RHSIM_Rhsim11G0110100
Root vs Leaf	RHSIM_Rhsim03G0067600	4.915618972	1.09615E-15	9.69282E-15	RHSIM_Rhsim03G0067600
Root vs Leaf	RHSIM_Rhsim09G0035900	3.118909713	1.10952E-15	9.80728E-15	RHSIM_Rhsim09G0035900
Root vs Leaf	RHSIM_Rhsim03G0045900	-2.358032903	1.11671E-15	9.86703E-15	RHSIM_Rhsim03G0045900
Root vs Leaf	RHSIM_Rhsim04G0197400	-1.268267285	1.11963E-15	9.88909E-15	RHSIM_Rhsim04G0197400
Root vs Leaf	novel.3794	9.900575858	1.14259E-15	1.0088E-14	--
Root vs Leaf	RHSIM_Rhsim06G0037000	11.22196191	1.14757E-15	1.01282E-14	RHSIM_Rhsim06G0037000
Root vs Leaf	RHSIM_Rhsim03G0244600	-2.496938616	1.18689E-15	1.04712E-14	RHSIM_Rhsim03G0244600
Root vs Leaf	RHSIM_Rhsim01G0273600	-1.981170045	1.19291E-15	1.05202E-14	RHSIM_Rhsim01G0273600
Root vs Leaf	RHSIM_Rhsim04G0205000	4.002961159	1.19662E-15	1.0549E-14	RHSIM_Rhsim04G0205000
Root vs Leaf	RHSIM_Rhsim03G0240900	-2.499752575	1.21219E-15	1.06821E-14	RHSIM_Rhsim03G0240900
Root vs Leaf	RHSIM_Rhsim04G0169700	-1.506993845	1.22242E-15	1.07682E-14	RHSIM_Rhsim04G0169700

Comparison	Gene ID	log_2FC	P-value	FDR (padj)	Gene Name
Root vs Leaf	RHSIM_Rhsim04G0178300	4.334570499	1.24362E-15	1.09508E-14	RHSIM_Rhsim04G0178300
Root vs Leaf	novel.736	-8.668839781	1.25896E-15	1.10816E-14	--
Root vs Leaf	RHSIM_RhsimUnG0251400	10.15365905	1.2624E-15	1.11077E-14	RHSIM_RhsimUnG0251400
Root vs Leaf	RHSIM_Rhsim01G0203700	-1.664285898	1.27437E-15	1.12087E-14	RHSIM_Rhsim01G0203700
Root vs Leaf	RHSIM_Rhsim10G0166700	-3.771548213	1.29933E-15	1.14239E-14	RHSIM_Rhsim10G0166700
Root vs Leaf	RHSIM_Rhsim13G0131000	1.377737804	1.32202E-15	1.16191E-14	RHSIM_Rhsim13G0131000
Root vs Leaf	RHSIM_Rhsim12G0094500	5.096491094	1.32659E-15	1.16548E-14	RHSIM_Rhsim12G0094500
Root vs Leaf	RHSIM_Rhsim11G0078100	2.336258402	1.32729E-15	1.16565E-14	RHSIM_Rhsim11G0078100
Root vs Leaf	RHSIM_Rhsim12G0211600	1.104438229	1.32995E-15	1.16754E-14	RHSIM_Rhsim12G0211600
Root vs Leaf	RHSIM_Rhsim05G0178600	1.134579305	1.33242E-15	1.16927E-14	RHSIM_Rhsim05G0178600
Root vs Leaf	RHSIM_Rhsim13G0061600	2.752447694	1.35498E-15	1.18861E-14	RHSIM_Rhsim13G0061600
Root vs Leaf	RHSIM_Rhsim02G0201600	-1.803130233	1.35602E-15	1.18907E-14	RHSIM_Rhsim02G0201600
Root vs Leaf	RHSIM_Rhsim09G0056000	-4.395801046	1.36236E-15	1.19418E-14	RHSIM_Rhsim09G0056000
Root vs Leaf	RHSIM_Rhsim12G0119400	-10.76075598	1.37906E-15	1.20836E-14	RHSIM_Rhsim12G0119400
Root vs Leaf	RHSIM_Rhsim11G0054300	1.136340467	1.40055E-15	1.22673E-14	RHSIM_Rhsim11G0054300
Root vs Leaf	RHSIM_Rhsim10G0171200	-2.262368335	1.41204E-15	1.23602E-14	RHSIM_Rhsim10G0171200
Root vs Leaf	RHSIM_Rhsim09G0153000	-8.646044728	1.41222E-15	1.23602E-14	RHSIM_Rhsim09G0153000
Root vs Leaf	RHSIM_Rhsim03G0170000	3.279945874	1.41541E-15	1.23833E-14	RHSIM_Rhsim03G0170000
Root vs Leaf	novel.320	-3.095939191	1.41754E-15	1.23973E-14	--
Root vs Leaf	RHSIM_Rhsim12G0052600	2.6562917	1.47891E-15	1.29292E-14	RHSIM_Rhsim12G0052600
Root vs Leaf	RHSIM_Rhsim12G0162400	-2.468523243	1.57613E-15	1.37738E-14	RHSIM_Rhsim12G0162400
Root vs Leaf	RHSIM_Rhsim09G0173500	2.440461065	1.57958E-15	1.37988E-14	RHSIM_Rhsim09G0173500
Root vs Leaf	RHSIM_Rhsim05G0215900	-8.711429729	1.59637E-15	1.39402E-14	RHSIM_Rhsim05G0215900
Root vs Leaf	RHSIM_Rhsim11G0098400	-2.040929244	1.62064E-15	1.41455E-14	RHSIM_Rhsim11G0098400
Root vs Leaf	RHSIM_Rhsim05G0086400	-1.1279495	1.6211E-15	1.41455E-14	RHSIM_Rhsim05G0086400

Comparison	Gene ID	log_2FC	P-value	FDR (padj)	Gene Name
Root vs Leaf	RHSIM_Rhsim08G0157600	1.929923892	1.66519E-15	1.45193E-14	RHSIM_Rhsim08G0157600
Root vs Leaf	RHSIM_Rhsim11G0136600	-2.122781136	1.71463E-15	1.49447E-14	RHSIM_Rhsim11G0136600
Root vs Leaf	novel.4653	3.071672446	1.72049E-15	1.49902E-14	--
Root vs Leaf	RHSIM_Rhsim07G0031600	1.490551397	1.7225E-15	1.5002E-14	RHSIM_Rhsim07G0031600
Root vs Leaf	RHSIM_Rhsim13G0140900	1.635076421	1.72833E-15	1.50471E-14	RHSIM_Rhsim13G0140900
Root vs Leaf	RHSIM_Rhsim05G0004300	-1.815478124	1.74878E-15	1.52194E-14	RHSIM_Rhsim05G0004300
Root vs Leaf	RHSIM_Rhsim11G0089700	-3.416060596	1.86042E-15	1.61849E-14	RHSIM_Rhsim11G0089700
Root vs Leaf	RHSIM_Rhsim11G0171000	-3.318618813	1.91053E-15	1.66147E-14	RHSIM_Rhsim11G0171000
Root vs Leaf	RHSIM_Rhsim05G0166700	1.096427866	1.92998E-15	1.67775E-14	RHSIM_Rhsim05G0166700
Root vs Leaf	RHSIM_Rhsim03G0155100	1.815053623	1.9461E-15	1.69112E-14	RHSIM_Rhsim03G0155100
Root vs Leaf	RHSIM_Rhsim07G0162200	-1.624123917	1.96088E-15	1.70333E-14	RHSIM_Rhsim07G0162200
Root vs Leaf	RHSIM_Rhsim08G0072400	-3.740624473	1.97097E-15	1.71145E-14	RHSIM_Rhsim08G0072400
Root vs Leaf	RHSIM_Rhsim08G0117500	-6.091796363	1.98087E-15	1.7194E-14	RHSIM_Rhsim08G0117500
Root vs Leaf	RHSIM_Rhsim06G0034900	-1.055622829	2.0899E-15	1.81337E-14	RHSIM_Rhsim06G0034900
Root vs Leaf	novel.1243	5.312142008	2.10874E-15	1.82903E-14	--
Root vs Leaf	novel.170	-9.75198736	2.1195E-15	1.83767E-14	--
Root vs Leaf	RHSIM_Rhsim10G0016100	-1.010819341	2.16407E-15	1.87561E-14	RHSIM_Rhsim10G0016100
Root vs Leaf	RHSIM_Rhsim05G0037700	-4.749049398	2.17475E-15	1.88416E-14	RHSIM_Rhsim05G0037700
Root vs Leaf	RHSIM_Rhsim02G0002700	2.532063253	2.18164E-15	1.88942E-14	RHSIM_Rhsim02G0002700
Root vs Leaf	RHSIM_Rhsim07G0125300	-1.389402799	2.19243E-15	1.89806E-14	RHSIM_Rhsim07G0125300
Root vs Leaf	RHSIM_Rhsim01G0288000	-2.342106138	2.22649E-15	1.92682E-14	RHSIM_Rhsim01G0288000
Root vs Leaf	RHSIM_Rhsim12G0013800	2.048479521	2.23794E-15	1.93601E-14	RHSIM_Rhsim12G0013800
Root vs Leaf	RHSIM_Rhsim11G0177700	2.577637613	2.32612E-15	2.01154E-14	RHSIM_Rhsim11G0177700
Root vs Leaf	RHSIM_Rhsim08G0196100	-2.568570949	2.33318E-15	2.01689E-14	RHSIM_Rhsim08G0196100
Root vs Leaf	RHSIM_Rhsim03G0063600	2.360971204	2.35696E-15	2.03668E-14	RHSIM_Rhsim03G0063600

Comparison	Gene ID	log_2FC	P-value	FDR (padj)	Gene Name
Root vs Leaf	RHSIM_Rhsim12G0006200	2.446766886	2.36036E-15	2.03886E-14	RHSIM_Rhsim12G0006200
Root vs Leaf	novel.2602	-10.85734816	2.36396E-15	2.04121E-14	--
Root vs Leaf	RHSIM_Rhsim05G0214800	-1.428064238	2.41046E-15	2.08058E-14	RHSIM_Rhsim05G0214800
Root vs Leaf	RHSIM_Rhsim08G0213200	-2.491366944	2.41149E-15	2.0807E-14	RHSIM_Rhsim08G0213200
Root vs Leaf	RHSIM_Rhsim02G0106600	2.102112743	2.41242E-15	2.08072E-14	RHSIM_Rhsim02G0106600
Root vs Leaf	RHSIM_Rhsim02G0016500	7.087298333	2.46567E-15	2.12586E-14	RHSIM_Rhsim02G0016500
Root vs Leaf	RHSIM_Rhsim06G0194400	-2.978707955	2.49974E-15	2.15443E-14	RHSIM_Rhsim06G0194400
Root vs Leaf	RHSIM_Rhsim07G0013000	-1.443179936	2.50699E-15	2.15988E-14	RHSIM_Rhsim07G0013000
Root vs Leaf	RHSIM_Rhsim04G0246800	2.966434985	2.50864E-15	2.16049E-14	RHSIM_Rhsim04G0246800
Root vs Leaf	RHSIM_Rhsim06G0153100	-2.614704503	2.51318E-15	2.1636E-14	RHSIM_Rhsim06G0153100
Root vs Leaf	RHSIM_Rhsim13G0184800	-3.42423798	2.52435E-15	2.17241E-14	RHSIM_Rhsim13G0184800
Root vs Leaf	RHSIM_Rhsim11G0180900	1.3838805	2.64898E-15	2.27797E-14	RHSIM_Rhsim11G0180900
Root vs Leaf	RHSIM_Rhsim05G0169000	-2.147164596	2.77639E-15	2.38664E-14	RHSIM_Rhsim05G0169000
Root vs Leaf	RHSIM_Rhsim11G0063800	2.337156111	2.79742E-15	2.40383E-14	RHSIM_Rhsim11G0063800
Root vs Leaf	RHSIM_Rhsim07G0049200	1.392359964	2.86425E-15	2.46035E-14	RHSIM_Rhsim07G0049200
Root vs Leaf	novel.4141	1.325326534	2.87922E-15	2.47228E-14	--
Root vs Leaf	RHSIM_Rhsim08G0177900	-7.086856629	2.88332E-15	2.47489E-14	RHSIM_Rhsim08G0177900
Root vs Leaf	RHSIM_Rhsim03G0119600	2.382820213	2.9081E-15	2.49523E-14	RHSIM_Rhsim03G0119600
Root vs Leaf	RHSIM_Rhsim13G0121500	-2.458452939	2.90931E-15	2.49535E-14	RHSIM_Rhsim13G0121500
Root vs Leaf	RHSIM_Rhsim07G0241500	-2.947282462	2.93057E-15	2.51264E-14	RHSIM_Rhsim07G0241500
Root vs Leaf	RHSIM_Rhsim12G0056700	-9.803997124	2.95372E-15	2.53155E-14	RHSIM_Rhsim12G0056700
Root vs Leaf	RHSIM_Rhsim07G0231700	13.60910914	2.96421E-15	2.53961E-14	RHSIM_Rhsim07G0231700
Root vs Leaf	RHSIM_Rhsim09G0104000	9.67743466	3.0425E-15	2.60572E-14	RHSIM_Rhsim09G0104000
Root vs Leaf	RHSIM_Rhsim06G0047300	-10.7167993	3.09424E-15	2.64905E-14	RHSIM_Rhsim06G0047300
Root vs Leaf	novel.5813	-6.191992112	3.11684E-15	2.66741E-14	--

Comparison	Gene ID	log_2FC	P-value	FDR (padj)	Gene Name
Root vs Leaf	RHSIM_Rhsim10G0104200	1.440803442	3.15584E-15	2.69976E-14	RHSIM_Rhsim10G0104200
Root vs Leaf	RHSIM_Rhsim01G0288900	-1.509167521	3.15698E-15	2.69976E-14	RHSIM_Rhsim01G0288900
Root vs Leaf	RHSIM_Rhsim10G0191400	1.927739866	3.2137E-15	2.74726E-14	RHSIM_Rhsim10G0191400
Root vs Leaf	RHSIM_Rhsim11G0148900	-2.750854453	3.22583E-15	2.75661E-14	RHSIM_Rhsim11G0148900
Root vs Leaf	RHSIM_Rhsim13G0008800	6.241811964	3.27444E-15	2.79712E-14	RHSIM_Rhsim13G0008800
Root vs Leaf	novel.3413	6.922651233	3.38165E-15	2.88736E-14	--
Root vs Leaf	RHSIM_Rhsim02G0254800	-3.820541882	3.38258E-15	2.88736E-14	RHSIM_Rhsim02G0254800
Root vs Leaf	RHSIM_Rhsim06G0022800	1.79568423	3.46788E-15	2.95908E-14	RHSIM_Rhsim06G0022800
Root vs Leaf	RHSIM_Rhsim13G0174700	1.526675398	3.4769E-15	2.96569E-14	RHSIM_Rhsim13G0174700
Root vs Leaf	RHSIM_Rhsim03G0172600	-8.459494409	3.53554E-15	3.01459E-14	RHSIM_Rhsim03G0172600
Root vs Leaf	RHSIM_Rhsim06G0144200	5.096870158	3.54176E-15	3.01878E-14	RHSIM_Rhsim06G0144200
Root vs Leaf	RHSIM_Rhsim13G0068200	-6.156964987	3.60278E-15	3.06966E-14	RHSIM_Rhsim13G0068200
Root vs Leaf	RHSIM_Rhsim13G0153900	-1.674400335	3.6047E-15	3.07016E-14	RHSIM_Rhsim13G0153900
Root vs Leaf	RHSIM_Rhsim04G0175100	-10.5449283	3.67907E-15	3.13235E-14	RHSIM_Rhsim04G0175100
Root vs Leaf	RHSIM_Rhsim12G0096800	1.035566571	3.69934E-15	3.14845E-14	RHSIM_Rhsim12G0096800
Root vs Leaf	RHSIM_Rhsim10G0190900	-4.128456997	3.7428E-15	3.18427E-14	RHSIM_Rhsim10G0190900
Root vs Leaf	RHSIM_Rhsim07G0104100	-9.849395318	3.74433E-15	3.1844E-14	RHSIM_Rhsim07G0104100
Root vs Leaf	RHSIM_Rhsim10G0041500	-3.171706904	3.75406E-15	3.1915E-14	RHSIM_Rhsim10G0041500
Root vs Leaf	novel.3168	5.75082044	3.8497E-15	3.27161E-14	--
Root vs Leaf	RHSIM_Rhsim04G0049600	-1.014732279	3.89621E-15	3.30992E-14	RHSIM_Rhsim04G0049600
Root vs Leaf	RHSIM_Rhsim07G0154100	-4.223625117	3.93597E-15	3.34247E-14	RHSIM_Rhsim07G0154100
Root vs Leaf	RHSIM_Rhsim02G0107700	-5.236271325	3.93913E-15	3.34392E-14	RHSIM_Rhsim02G0107700
Root vs Leaf	RHSIM_Rhsim11G0177400	-2.388419902	4.10245E-15	3.48129E-14	RHSIM_Rhsim11G0177400
Root vs Leaf	novel.677	-10.64858898	4.20886E-15	3.57028E-14	--
Root vs Leaf	RHSIM_Rhsim03G0263900	-10.08255838	4.26321E-15	3.61506E-14	RHSIM_Rhsim03G0263900

Comparison	Gene ID	log_2FC	P-value	FDR (padj)	Gene Name
Root vs Leaf	RHSIM_Rhsim02G0119000	3.684176685	4.41203E-15	3.73988E-14	RHSIM_Rhsim02G0119000
Root vs Leaf	RHSIM_Rhsim03G0188500	-2.569682783	4.48964E-15	3.80427E-14	RHSIM_Rhsim03G0188500
Root vs Leaf	RHSIM_Rhsim10G0096900	-5.504401516	4.50354E-15	3.81466E-14	RHSIM_Rhsim10G0096900
Root vs Leaf	novel.4869	-10.22121751	4.58227E-15	3.87992E-14	--
Root vs Leaf	RHSIM_Rhsim09G0115600	-4.544244285	4.60016E-15	3.89364E-14	RHSIM_Rhsim09G0115600
Root vs Leaf	RHSIM_RhsimMtG0002100	-5.232518597	4.64051E-15	3.92636E-14	RHSIM_RhsimMtG0002100
Root vs Leaf	RHSIM_Rhsim09G0200400	1.511406598	4.64624E-15	3.92977E-14	RHSIM_Rhsim09G0200400
Root vs Leaf	RHSIM_Rhsim03G0058600	-3.135349567	4.67568E-15	3.95323E-14	RHSIM_Rhsim03G0058600
Root vs Leaf	novel.776	10.12727863	4.74686E-15	4.01195E-14	--
Root vs Leaf	novel.2518	-5.346076638	4.79437E-15	4.05062E-14	--
Root vs Leaf	RHSIM_Rhsim09G0013900	-3.149638986	4.9207E-15	4.15583E-14	RHSIM_Rhsim09G0013900
Root vs Leaf	RHSIM_Rhsim03G0201500	-2.633511208	4.95958E-15	4.18714E-14	RHSIM_Rhsim03G0201500
Root vs Leaf	RHSIM_Rhsim02G0079100	-10.41119111	5.04174E-15	4.25496E-14	RHSIM_Rhsim02G0079100
Root vs Leaf	novel.5707	-8.890072106	5.13034E-15	4.32658E-14	--
Root vs Leaf	RHSIM_Rhsim13G0136200	3.887117031	5.14809E-15	4.33996E-14	RHSIM_Rhsim13G0136200
Root vs Leaf	RHSIM_Rhsim04G0044200	-5.177411364	5.37163E-15	4.52677E-14	RHSIM_Rhsim04G0044200
Root vs Leaf	RHSIM_Rhsim10G0030100	3.216938531	5.43486E-15	4.57838E-14	RHSIM_Rhsim10G0030100
Root vs Leaf	RHSIM_Rhsim11G0085900	1.36685492	5.50918E-15	4.63761E-14	RHSIM_Rhsim11G0085900
Root vs Leaf	RHSIM_Rhsim10G0174300	8.394775155	5.56977E-15	4.68691E-14	RHSIM_Rhsim10G0174300
Root vs Leaf	RHSIM_Rhsim01G0023900	-1.433999721	5.61921E-15	4.72679E-14	RHSIM_Rhsim01G0023900
Root vs Leaf	RHSIM_Rhsim12G0144400	-1.72093735	5.65966E-15	4.75909E-14	RHSIM_Rhsim12G0144400
Root vs Leaf	RHSIM_Rhsim08G0152100	-1.275467342	5.69913E-15	4.79054E-14	RHSIM_Rhsim08G0152100
Root vs Leaf	RHSIM_Rhsim03G0225800	-2.642450163	5.74654E-15	4.82864E-14	RHSIM_Rhsim03G0225800
Root vs Leaf	RHSIM_Rhsim13G0103200	10.49837643	5.80702E-15	4.87769E-14	RHSIM_Rhsim13G0103200
Root vs Leaf	RHSIM_Rhsim07G0010300	1.929352216	5.81905E-15	4.88602E-14	RHSIM_Rhsim07G0010300

Comparison	Gene ID	log_2FC	P-value	FDR (padj)	Gene Name
Root vs Leaf	RHSIM_Rhsim04G0134900	1.504159062	5.85222E-15	4.91208E-14	RHSIM_Rhsim04G0134900
Root vs Leaf	RHSIM_Rhsim03G0220500	1.985989542	5.89414E-15	4.94547E-14	RHSIM_Rhsim03G0220500
Root vs Leaf	RHSIM_Rhsim07G0203400	5.860296081	5.94294E-15	4.98461E-14	RHSIM_Rhsim07G0203400
Root vs Leaf	RHSIM_Rhsim01G0265400	-1.817977949	6.04492E-15	5.06831E-14	RHSIM_Rhsim01G0265400
Root vs Leaf	RHSIM_Rhsim04G0076000	-4.079288941	6.07436E-15	5.09115E-14	RHSIM_Rhsim04G0076000
Root vs Leaf	RHSIM_Rhsim07G0174800	5.217352152	6.08662E-15	5.09958E-14	RHSIM_Rhsim07G0174800
Root vs Leaf	RHSIM_Rhsim12G0213700	-1.222694595	6.18269E-15	5.1782E-14	RHSIM_Rhsim12G0213700
Root vs Leaf	novel.3823	-2.48364905	6.23712E-15	5.22189E-14	--
Root vs Leaf	RHSIM_Rhsim12G0116600	-1.16693266	6.26218E-15	5.24098E-14	RHSIM_Rhsim12G0116600
Root vs Leaf	RHSIM_Rhsim02G0127600	1.142207223	6.40546E-15	5.35896E-14	RHSIM_Rhsim02G0127600
Root vs Leaf	RHSIM_Rhsim06G0131600	-1.448364063	6.45182E-15	5.39384E-14	RHSIM_Rhsim06G0131600
Root vs Leaf	RHSIM_Rhsim02G0164900	-10.14280286	6.47023E-15	5.40646E-14	RHSIM_Rhsim02G0164900
Root vs Leaf	RHSIM_Rhsim08G0108400	-8.487081834	6.47158E-15	5.40646E-14	RHSIM_Rhsim08G0108400
Root vs Leaf	novel.3672	-4.018794622	6.55435E-15	5.47363E-14	--
Root vs Leaf	novel.5847	10.35545549	6.69415E-15	5.58836E-14	--
Root vs Leaf	RHSIM_Rhsim06G0233800	-6.179659061	7.0815E-15	5.90959E-14	RHSIM_Rhsim06G0233800
Root vs Leaf	novel.1237	1.528400546	7.13862E-15	5.95511E-14	--
Root vs Leaf	RHSIM_Rhsim03G0026900	-1.365257934	7.21427E-15	6.01605E-14	RHSIM_Rhsim03G0026900
Root vs Leaf	RHSIM_Rhsim07G0079100	1.487964825	7.22425E-15	6.0222E-14	RHSIM_Rhsim07G0079100
Root vs Leaf	RHSIM_Rhsim07G0229000	-5.704387848	7.37255E-15	6.14362E-14	RHSIM_Rhsim07G0229000
Root vs Leaf	RHSIM_Rhsim08G0092700	9.605468804	7.39693E-15	6.16172E-14	RHSIM_Rhsim08G0092700
Root vs Leaf	RHSIM_Rhsim04G0242600	-2.118398889	7.41947E-15	6.17826E-14	RHSIM_Rhsim04G0242600
Root vs Leaf	RHSIM_Rhsim02G0115500	-1.58244057	7.55613E-15	6.2898E-14	RHSIM_Rhsim02G0115500
Root vs Leaf	novel.1494	4.029080463	7.57202E-15	6.30076E-14	--
Root vs Leaf	RHSIM_Rhsim13G0131200	-1.938510847	7.70293E-15	6.40739E-14	RHSIM_Rhsim13G0131200

Comparison	Gene ID	log_2FC	P-value	FDR (padj)	Gene Name
Root vs Leaf	RHSIM_Rhsim01G0195300	-1.208501429	7.84727E-15	6.52511E-14	RHSIM_Rhsim01G0195300
Root vs Leaf	RHSIM_Rhsim10G0021400	-1.656077772	7.85875E-15	6.5323E-14	RHSIM_Rhsim10G0021400
Root vs Leaf	novel.1651	5.14396088	7.99526E-15	6.64339E-14	--
Root vs Leaf	RHSIM_Rhsim11G0031500	-2.177851268	8.11381E-15	6.73947E-14	RHSIM_Rhsim11G0031500
Root vs Leaf	RHSIM_Rhsim06G0018300	-10.04785572	8.56267E-15	7.10975E-14	RHSIM_Rhsim06G0018300
Root vs Leaf	RHSIM_Rhsim10G0072800	1.59354324	8.57013E-15	7.11339E-14	RHSIM_Rhsim10G0072800
Root vs Leaf	RHSIM_Rhsim02G0024100	-1.385408294	8.67617E-15	7.19883E-14	RHSIM_Rhsim02G0024100
Root vs Leaf	RHSIM_Rhsim09G0166500	1.197189187	8.72784E-15	7.2391E-14	RHSIM_Rhsim09G0166500
Root vs Leaf	RHSIM_Rhsim03G0120400	-1.548585897	8.85906E-15	7.3453E-14	RHSIM_Rhsim03G0120400
Root vs Leaf	RHSIM_Rhsim13G0227100	-4.29421395	9.07455E-15	7.52128E-14	RHSIM_Rhsim13G0227100
Root vs Leaf	RHSIM_Rhsim12G0130100	-1.737158634	9.25264E-15	7.66614E-14	RHSIM_Rhsim12G0130100
Root vs Leaf	RHSIM_Rhsim07G0137200	1.779074796	9.27655E-15	7.6832E-14	RHSIM_Rhsim07G0137200
Root vs Leaf	RHSIM_Rhsim08G0193100	-1.953713139	9.37189E-15	7.75939E-14	RHSIM_Rhsim08G0193100
Root vs Leaf	RHSIM_Rhsim08G0228100	-5.156623079	9.84406E-15	8.1474E-14	RHSIM_Rhsim08G0228100
Root vs Leaf	novel.3093	1.971691408	9.90621E-15	8.19591E-14	--
Root vs Leaf	novel.1767	-6.505012402	1.00051E-14	8.27478E-14	--
Root vs Leaf	RHSIM_RhsimUnG0151900	-8.7099293	1.01273E-14	8.37281E-14	RHSIM_RhsimUnG0151900
Root vs Leaf	RHSIM_Rhsim13G0103000	1.714498383	1.03866E-14	8.58417E-14	RHSIM_Rhsim13G0103000
Root vs Leaf	RHSIM_Rhsim02G0218800	3.530616829	1.03943E-14	8.58745E-14	RHSIM_Rhsim02G0218800
Root vs Leaf	RHSIM_Rhsim13G0012100	-1.429638427	1.04669E-14	8.64436E-14	RHSIM_Rhsim13G0012100
Root vs Leaf	RHSIM_Rhsim01G0285000	2.40087277	1.06172E-14	8.76537E-14	RHSIM_Rhsim01G0285000
Root vs Leaf	RHSIM_Rhsim10G0127400	-2.727195705	1.06334E-14	8.77562E-14	RHSIM_Rhsim10G0127400
Root vs Leaf	RHSIM_Rhsim01G0068600	1.060605272	1.07715E-14	8.88637E-14	RHSIM_Rhsim01G0068600
Root vs Leaf	RHSIM_Rhsim08G0189400	1.420510728	1.07955E-14	8.90303E-14	RHSIM_Rhsim08G0189400
Root vs Leaf	novel.4117	3.25092241	1.09368E-14	9.01632E-14	--

Comparison	Gene ID	log$_2$FC	P-value	FDR (padj)	Gene Name
Root vs Leaf	RHSIM_Rhsim07G0258700	-1.417819983	1.09724E-14	9.04248E-14	RHSIM_Rhsim07G0258700
Root vs Leaf	RHSIM_RhsimUnG0254700	1.576106266	1.09767E-14	9.04277E-14	RHSIM_RhsimUnG0254700
Root vs Leaf	RHSIM_Rhsim12G0185700	-5.595474547	1.12841E-14	9.29273E-14	RHSIM_Rhsim12G0185700
Root vs Leaf	RHSIM_Rhsim04G0080600	-4.149991322	1.1322E-14	9.32063E-14	RHSIM_Rhsim04G0080600
Root vs Leaf	RHSIM_Rhsim13G0028100	-3.008632783	1.15813E-14	9.53064E-14	RHSIM_Rhsim13G0028100
Root vs Leaf	RHSIM_Rhsim13G0070900	-5.139116495	1.16576E-14	9.59003E-14	RHSIM_Rhsim13G0070900
Root vs Leaf	RHSIM_Rhsim01G0075900	-1.440353656	1.1683E-14	9.60751E-14	RHSIM_Rhsim01G0075900
Root vs Leaf	RHSIM_Rhsim08G0004000	-4.124582325	1.17029E-14	9.62049E-14	RHSIM_Rhsim08G0004000
Root vs Leaf	RHSIM_Rhsim13G0039400	2.051194002	1.17739E-14	9.67546E-14	RHSIM_Rhsim13G0039400
Root vs Leaf	RHSIM_Rhsim04G0076500	-4.618049219	1.17889E-14	9.68431E-14	RHSIM_Rhsim04G0076500
Root vs Leaf	RHSIM_Rhsim03G0073800	1.98546758	1.23281E-14	1.01201E-13	RHSIM_Rhsim03G0073800
Root vs Leaf	RHSIM_Rhsim07G0065100	-4.403192807	1.24149E-14	1.01877E-13	RHSIM_Rhsim07G0065100
Root vs Leaf	novel.1500	1.073530568	1.24468E-14	1.02103E-13	--
Root vs Leaf	RHSIM_Rhsim10G0046900	-1.544633155	1.25022E-14	1.02521E-13	RHSIM_Rhsim10G0046900
Root vs Leaf	RHSIM_Rhsim09G0030900	5.889302533	1.25861E-14	1.03172E-13	RHSIM_Rhsim09G0030900
Root vs Leaf	RHSIM_Rhsim12G0084400	3.723999726	1.29644E-14	1.06235E-13	RHSIM_Rhsim12G0084400
Root vs Leaf	RHSIM_Rhsim01G0188300	-11.1417339	1.31243E-14	1.07508E-13	RHSIM_Rhsim01G0188300
Root vs Leaf	RHSIM_RhsimUnG0093600	11.08332224	1.31569E-14	1.07737E-13	RHSIM_RhsimUnG0093600
Root vs Leaf	RHSIM_RhsimPtG0000800	-3.150354872	1.31822E-14	1.07906E-13	RHSIM_RhsimPtG0000800
Root vs Leaf	RHSIM_Rhsim04G0036700	-1.658629141	1.32466E-14	1.08394E-13	RHSIM_Rhsim04G0036700
Root vs Leaf	RHSIM_Rhsim08G0228800	-1.916480398	1.36719E-14	1.11835E-13	RHSIM_Rhsim08G0228800
Root vs Leaf	RHSIM_Rhsim11G0045300	1.623446039	1.38107E-14	1.12931E-13	RHSIM_Rhsim11G0045300
Root vs Leaf	RHSIM_Rhsim07G0128400	-1.214748476	1.38859E-14	1.13505E-13	RHSIM_Rhsim07G0128400
Root vs Leaf	RHSIM_Rhsim11G0164700	-3.111154767	1.41785E-14	1.15856E-13	RHSIM_Rhsim11G0164700
Root vs Leaf	RHSIM_Rhsim06G0108700	3.633907263	1.43751E-14	1.17421E-13	RHSIM_Rhsim06G0108700

Comparison	Gene ID	log_2FC	P-value	FDR (padj)	Gene Name
Root vs Leaf	RHSIM_Rhsim01G0089400	1.238896145	1.44038E-14	1.17602E-13	RHSIM_Rhsim01G0089400
Root vs Leaf	novel.1879	-3.283096371	1.44074E-14	1.17602E-13	--
Root vs Leaf	RHSIM_Rhsim07G0174700	-1.35931814	1.4438E-14	1.1781E-13	RHSIM_Rhsim07G0174700
Root vs Leaf	RHSIM_RhsimUnG0053100	-4.306351386	1.45448E-14	1.1864E-13	RHSIM_RhsimUnG0053100
Root vs Leaf	RHSIM_Rhsim09G0107000	4.721167775	1.51964E-14	1.23911E-13	RHSIM_Rhsim09G0107000
Root vs Leaf	RHSIM_Rhsim06G0188300	-9.59740081	1.5271E-14	1.2445E-13	RHSIM_Rhsim06G0188300
Root vs Leaf	novel.4899	-10.71087868	1.52732E-14	1.2445E-13	--
Root vs Leaf	RHSIM_Rhsim02G0230300	-1.996136513	1.52833E-14	1.24488E-13	RHSIM_Rhsim02G0230300
Root vs Leaf	novel.4685	-3.403448705	1.54945E-14	1.26164E-13	--
Root vs Leaf	RHSIM_Rhsim02G0024500	-1.527536622	1.56499E-14	1.27385E-13	RHSIM_Rhsim02G0024500
Root vs Leaf	RHSIM_Rhsim12G0047500	2.022000918	1.57548E-14	1.28194E-13	RHSIM_Rhsim12G0047500
Root vs Leaf	RHSIM_Rhsim10G0066600	7.796645715	1.57628E-14	1.28214E-13	RHSIM_Rhsim10G0066600
Root vs Leaf	RHSIM_Rhsim02G0194200	-6.676469326	1.58067E-14	1.28525E-13	RHSIM_Rhsim02G0194200
Root vs Leaf	RHSIM_Rhsim12G0156200	-1.789024353	1.60133E-14	1.30159E-13	RHSIM_Rhsim12G0156200
Root vs Leaf	RHSIM_Rhsim06G0232300	4.674903147	1.61688E-14	1.31377E-13	RHSIM_Rhsim06G0232300
Root vs Leaf	RHSIM_Rhsim13G0044200	-3.909288159	1.61916E-14	1.31516E-13	RHSIM_Rhsim13G0044200
Root vs Leaf	RHSIM_Rhsim13G0019700	9.986475549	1.65625E-14	1.34413E-13	RHSIM_Rhsim13G0019700
Root vs Leaf	RHSIM_Rhsim10G0083700	-6.979357891	1.65657E-14	1.34413E-13	RHSIM_Rhsim10G0083700
Root vs Leaf	RHSIM_Rhsim02G0135200	3.298615891	1.6692E-14	1.35358E-13	RHSIM_Rhsim02G0135200
Root vs Leaf	RHSIM_Rhsim13G0036800	2.715703747	1.66946E-14	1.35358E-13	RHSIM_Rhsim13G0036800
Root vs Leaf	RHSIM_Rhsim02G0086700	-9.484284602	1.66997E-14	1.35358E-13	RHSIM_Rhsim02G0086700
Root vs Leaf	RHSIM_Rhsim10G0112400	-1.054028085	1.68982E-14	1.36919E-13	RHSIM_Rhsim10G0112400
Root vs Leaf	novel.6000	4.798943463	1.71098E-14	1.38585E-13	--
Root vs Leaf	RHSIM_RhsimUnG0065900	-1.97609231	1.74504E-14	1.41294E-13	RHSIM_RhsimUnG0065900
Root vs Leaf	novel.2795	4.705553336	1.78366E-14	1.44371E-13	--

Comparison	Gene ID	log_2FC	P-value	FDR (padj)	Gene Name
Root vs Leaf	RHSIM_Rhsim05G0012800	4.652644044	1.80017E-14	1.45656E-13	RHSIM_Rhsim05G0012800
Root vs Leaf	RHSIM_Rhsim06G0165300	-5.001639158	1.80163E-14	1.45723E-13	RHSIM_Rhsim06G0165300
Root vs Leaf	RHSIM_Rhsim08G0245400	-1.183988491	1.81953E-14	1.4712E-13	RHSIM_Rhsim08G0245400
Root vs Leaf	RHSIM_Rhsim07G0228500	2.804880617	1.83864E-14	1.48613E-13	RHSIM_Rhsim07G0228500
Root vs Leaf	RHSIM_Rhsim01G0141300	2.90410215	1.85878E-14	1.50189E-13	RHSIM_Rhsim01G0141300
Root vs Leaf	novel.2151	3.181830852	1.86194E-14	1.50391E-13	--
Root vs Leaf	RHSIM_Rhsim03G0270300	-3.216616093	1.89263E-14	1.52817E-13	RHSIM_Rhsim03G0270300
Root vs Leaf	RHSIM_Rhsim12G0017100	1.354275908	1.90308E-14	1.53607E-13	RHSIM_Rhsim12G0017100
Root vs Leaf	novel.3451	5.502575929	1.92672E-14	1.55461E-13	--
Root vs Leaf	RHSIM_Rhsim12G0124700	-1.627796709	1.93792E-14	1.5631E-13	RHSIM_Rhsim12G0124700
Root vs Leaf	RHSIM_Rhsim06G0057500	4.278689022	2.01475E-14	1.62451E-13	RHSIM_Rhsim06G0057500
Root vs Leaf	RHSIM_Rhsim07G0130100	2.775128159	2.04799E-14	1.65073E-13	RHSIM_Rhsim07G0130100
Root vs Leaf	RHSIM_Rhsim07G0221300	-3.023900577	2.1181E-14	1.70665E-13	RHSIM_Rhsim07G0221300
Root vs Leaf	RHSIM_Rhsim09G0204600	-1.156083018	2.16241E-14	1.74145E-13	RHSIM_Rhsim09G0204600
Root vs Leaf	RHSIM_Rhsim03G0238500	-3.842421425	2.16279E-14	1.74145E-13	RHSIM_Rhsim03G0238500
Root vs Leaf	RHSIM_Rhsim08G0063900	-1.049965699	2.30775E-14	1.85752E-13	RHSIM_Rhsim08G0063900
Root vs Leaf	RHSIM_Rhsim08G0022100	-4.282870847	2.34794E-14	1.88921E-13	RHSIM_Rhsim08G0022100
Root vs Leaf	RHSIM_Rhsim12G0091800	3.413831229	2.41227E-14	1.94029E-13	RHSIM_Rhsim12G0091800
Root vs Leaf	RHSIM_Rhsim08G0223400	-2.192034361	2.47132E-14	1.9871E-13	RHSIM_Rhsim08G0223400
Root vs Leaf	RHSIM_Rhsim08G0102800	1.888161606	2.48507E-14	1.99747E-13	RHSIM_Rhsim08G0102800
Root vs Leaf	RHSIM_Rhsim06G0210500	-10.73300837	2.49383E-14	2.00381E-13	RHSIM_Rhsim06G0210500
Root vs Leaf	RHSIM_Rhsim02G0070200	-1.350694497	2.54963E-14	2.04793E-13	RHSIM_Rhsim02G0070200
Root vs Leaf	novel.1542	1.332549607	2.5835E-14	2.07442E-13	--
Root vs Leaf	RHSIM_Rhsim05G0003400	-2.047267245	2.58683E-14	2.07637E-13	RHSIM_Rhsim05G0003400
Root vs Leaf	RHSIM_Rhsim06G0078400	3.764975841	2.59703E-14	2.08384E-13	RHSIM_Rhsim06G0078400

Comparison	Gene ID	log_2FC	P-value	FDR (padj)	Gene Name
Root vs Leaf	RHSIM_Rhsim09G0065700	-12.33022353	2.62151E-14	2.10275E-13	RHSIM_Rhsim09G0065700
Root vs Leaf	RHSIM_Rhsim05G0080700	1.615005631	2.63893E-14	2.11599E-13	RHSIM_Rhsim05G0080700
Root vs Leaf	RHSIM_Rhsim04G0014300	-2.847857527	2.6442E-14	2.11933E-13	RHSIM_Rhsim04G0014300
Root vs Leaf	RHSIM_Rhsim08G0113800	-7.076911926	2.64521E-14	2.11933E-13	RHSIM_Rhsim08G0113800
Root vs Leaf	novel.3006	-1.166493227	2.64585E-14	2.11933E-13	--
Root vs Leaf	RHSIM_Rhsim04G0030200	2.432545885	2.6544E-14	2.12545E-13	RHSIM_Rhsim04G0030200
Root vs Leaf	RHSIM_Rhsim06G0000500	-1.387108318	2.66116E-14	2.13013E-13	RHSIM_Rhsim06G0000500
Root vs Leaf	novel.3441	-4.228861325	2.6738E-14	2.1395E-13	--
Root vs Leaf	RHSIM_Rhsim06G0029100	-2.206400361	2.744E-14	2.19491E-13	RHSIM_Rhsim06G0029100
Root vs Leaf	RHSIM_Rhsim13G0019500	3.044710722	2.75104E-14	2.19978E-13	RHSIM_Rhsim13G0019500
Root vs Leaf	RHSIM_Rhsim04G0076100	-2.498053674	2.76783E-14	2.21245E-13	RHSIM_Rhsim04G0076100
Root vs Leaf	novel.2828	-3.592764929	2.7727E-14	2.21557E-13	--
Root vs Leaf	RHSIM_Rhsim06G0222200	8.504759936	2.8168E-14	2.25004E-13	RHSIM_Rhsim06G0222200
Root vs Leaf	RHSIM_RhsimUnG0111000	3.93510225	2.87493E-14	2.29568E-13	RHSIM_RhsimUnG0111000
Root vs Leaf	RHSIM_Rhsim01G0060000	1.435024948	2.89979E-14	2.31473E-13	RHSIM_Rhsim01G0060000
Root vs Leaf	RHSIM_Rhsim13G0112000	5.583627497	2.90814E-14	2.3206E-13	RHSIM_Rhsim13G0112000
Root vs Leaf	RHSIM_Rhsim08G0230000	-8.470275553	2.92947E-14	2.33681E-13	RHSIM_Rhsim08G0230000
Root vs Leaf	RHSIM_Rhsim01G0054500	-9.394372756	2.93403E-14	2.33964E-13	RHSIM_Rhsim01G0054500
Root vs Leaf	RHSIM_Rhsim12G0172500	-1.244277867	2.95501E-14	2.35556E-13	RHSIM_Rhsim12G0172500
Root vs Leaf	RHSIM_Rhsim06G0112100	-9.882679115	2.99889E-14	2.38972E-13	RHSIM_Rhsim06G0112100
Root vs Leaf	RHSIM_Rhsim11G0149600	-1.18933292	3.01023E-14	2.39793E-13	RHSIM_Rhsim11G0149600
Root vs Leaf	RHSIM_Rhsim05G0172800	1.596169736	3.02136E-14	2.40597E-13	RHSIM_Rhsim05G0172800
Root vs Leaf	RHSIM_Rhsim06G0171600	10.38077816	3.03137E-14	2.4131E-13	RHSIM_Rhsim06G0171600
Root vs Leaf	RHSIM_Rhsim07G0118000	5.660067874	3.03627E-14	2.41617E-13	RHSIM_Rhsim07G0118000
Root vs Leaf	RHSIM_Rhsim12G0048400	2.370575165	3.0497E-14	2.42603E-13	RHSIM_Rhsim12G0048400

Comparison	Gene ID	log_2FC	P-value	FDR (padj)	Gene Name
Root vs Leaf	RHSIM_Rhsim01G0285600	-1.053564403	3.07542E-14	2.44564E-13	RHSIM_Rhsim01G0285600
Root vs Leaf	novel.3095	-1.335446006	3.09347E-14	2.45832E-13	--
Root vs Leaf	RHSIM_Rhsim02G0090500	-6.702953662	3.09349E-14	2.45832E-13	RHSIM_Rhsim02G0090500
Root vs Leaf	RHSIM_Rhsim12G0045000	-9.985908298	3.18755E-14	2.5322E-13	RHSIM_Rhsim12G0045000
Root vs Leaf	RHSIM_Rhsim11G0071100	-6.463305948	3.20293E-14	2.54355E-13	RHSIM_Rhsim11G0071100
Root vs Leaf	RHSIM_Rhsim09G0121500	-10.90647646	3.2804E-14	2.60417E-13	RHSIM_Rhsim09G0121500
Root vs Leaf	novel.5339	2.865230457	3.42293E-14	2.71546E-13	--
Root vs Leaf	RHSIM_Rhsim07G0170900	1.859250263	3.42768E-14	2.7183E-13	RHSIM_Rhsim07G0170900
Root vs Leaf	RHSIM_Rhsim02G0203700	-2.507661221	3.44043E-14	2.72747E-13	RHSIM_Rhsim02G0203700
Root vs Leaf	RHSIM_Rhsim03G0262700	-3.961428288	3.50511E-14	2.7778E-13	RHSIM_Rhsim03G0262700
Root vs Leaf	RHSIM_Rhsim11G0139400	5.100340804	3.55221E-14	2.81417E-13	RHSIM_Rhsim11G0139400
Root vs Leaf	RHSIM_Rhsim09G0150400	-4.675277318	3.57562E-14	2.83174E-13	RHSIM_Rhsim09G0150400
Root vs Leaf	RHSIM_Rhsim13G0179100	-9.946904254	3.6239E-14	2.86899E-13	RHSIM_Rhsim13G0179100
Root vs Leaf	RHSIM_Rhsim11G0114400	-4.660936923	3.63931E-14	2.88021E-13	RHSIM_Rhsim11G0114400
Root vs Leaf	RHSIM_Rhsim04G0161900	1.901697718	3.70356E-14	2.93006E-13	RHSIM_Rhsim04G0161900
Root vs Leaf	RHSIM_Rhsim03G0163700	-2.365105812	3.72341E-14	2.94475E-13	RHSIM_Rhsim03G0163700
Root vs Leaf	RHSIM_Rhsim10G0022700	1.356439487	3.76986E-14	2.98047E-13	RHSIM_Rhsim10G0022700
Root vs Leaf	RHSIM_Rhsim04G0077800	-8.075121695	3.77841E-14	2.98621E-13	RHSIM_Rhsim04G0077800
Root vs Leaf	RHSIM_Rhsim05G0081100	13.15478223	3.92641E-14	3.10212E-13	RHSIM_Rhsim05G0081100
Root vs Leaf	novel.4898	-11.35709691	3.95082E-14	3.12034E-13	--
Root vs Leaf	RHSIM_Rhsim08G0098300	1.82702219	3.97179E-14	3.1349E-13	RHSIM_Rhsim08G0098300
Root vs Leaf	novel.2058	-7.003052907	3.97196E-14	3.1349E-13	--
Root vs Leaf	RHSIM_Rhsim01G0235100	-2.984359918	3.97558E-14	3.13668E-13	RHSIM_Rhsim01G0235100
Root vs Leaf	RHSIM_Rhsim08G0156400	-2.555402512	4.03468E-14	3.18223E-13	RHSIM_Rhsim08G0156400
Root vs Leaf	RHSIM_Rhsim04G0078700	-9.370879889	4.09654E-14	3.22991E-13	RHSIM_Rhsim04G0078700

Comparison	Gene ID	log_2FC	P-value	FDR (padj)	Gene Name
Root vs Leaf	RHSIM_Rhsim01G0153000	-1.246825364	4.16759E-14	3.2837E-13	RHSIM_Rhsim01G0153000
Root vs Leaf	RHSIM_Rhsim07G0037100	-1.628528189	4.1924E-14	3.30212E-13	RHSIM_Rhsim07G0037100
Root vs Leaf	RHSIM_Rhsim08G0160700	2.586355227	4.25501E-14	3.35014E-13	RHSIM_Rhsim08G0160700
Root vs Leaf	RHSIM_Rhsim05G0216700	3.221273153	4.25626E-14	3.35014E-13	RHSIM_Rhsim05G0216700
Root vs Leaf	RHSIM_Rhsim10G0138500	1.802518259	4.28141E-14	3.36879E-13	RHSIM_Rhsim10G0138500
Root vs Leaf	novel.1353	-5.697903347	4.28677E-14	3.37186E-13	--
Root vs Leaf	RHSIM_Rhsim07G0234400	-1.528654284	4.34252E-14	3.41455E-13	RHSIM_Rhsim07G0234400
Root vs Leaf	RHSIM_Rhsim12G0135100	7.187572509	4.39225E-14	3.45248E-13	RHSIM_Rhsim12G0135100
Root vs Leaf	RHSIM_Rhsim04G0015400	1.347937022	4.39889E-14	3.45653E-13	RHSIM_Rhsim04G0015400
Root vs Leaf	RHSIM_Rhsim06G0213800	1.003833284	4.43701E-14	3.4853E-13	RHSIM_Rhsim06G0213800
Root vs Leaf	RHSIM_Rhsim12G0201400	3.156455595	4.49944E-14	3.53314E-13	RHSIM_Rhsim12G0201400
Root vs Leaf	novel.1990	2.195629047	4.5025E-14	3.53365E-13	--
Root vs Leaf	RHSIM_Rhsim07G0045100	-9.410398226	4.50315E-14	3.53365E-13	RHSIM_Rhsim07G0045100
Root vs Leaf	RHSIM_RhsimUnG0108100	4.211595964	4.52886E-14	3.55262E-13	RHSIM_RhsimUnG0108100
Root vs Leaf	RHSIM_Rhsim05G0005800	-1.384654887	4.53529E-14	3.55646E-13	RHSIM_Rhsim05G0005800
Root vs Leaf	RHSIM_Rhsim13G0016100	-6.064801437	4.54287E-14	3.56119E-13	RHSIM_Rhsim13G0016100
Root vs Leaf	RHSIM_Rhsim09G0130900	2.527581688	4.56701E-14	3.57891E-13	RHSIM_Rhsim09G0130900
Root vs Leaf	novel.5955	10.7906746	4.57128E-14	3.58104E-13	--
Root vs Leaf	RHSIM_RhsimUnG0256700	-1.659716475	4.6348E-14	3.62957E-13	RHSIM_RhsimUnG0256700
Root vs Leaf	RHSIM_Rhsim06G0137100	-2.247521076	4.72432E-14	3.69843E-13	RHSIM_Rhsim06G0137100
Root vs Leaf	RHSIM_Rhsim04G0079800	-6.9257752	4.7502E-14	3.71743E-13	RHSIM_Rhsim04G0079800
Root vs Leaf	RHSIM_Rhsim06G0120400	-9.317419836	4.75478E-14	3.71915E-13	RHSIM_Rhsim06G0120400
Root vs Leaf	RHSIM_Rhsim09G0003600	-1.637454828	4.75561E-14	3.71915E-13	RHSIM_Rhsim09G0003600
Root vs Leaf	RHSIM_Rhsim02G0247900	-10.21254172	4.774E-14	3.73227E-13	RHSIM_Rhsim02G0247900
Root vs Leaf	RHSIM_Rhsim12G0211400	-1.731890618	4.92274E-14	3.84725E-13	RHSIM_Rhsim12G0211400

Comparison	Gene ID	log_2FC	P-value	FDR (padj)	Gene Name
Root vs Leaf	RHSIM_Rhsim11G0088500	6.397630176	4.96875E-14	3.8819E-13	RHSIM_Rhsim11G0088500
Root vs Leaf	RHSIM_Rhsim13G0229800	-6.118911646	5.01059E-14	3.91326E-13	RHSIM_Rhsim13G0229800
Root vs Leaf	RHSIM_Rhsim08G0216900	-1.490144426	5.0202E-14	3.91945E-13	RHSIM_Rhsim08G0216900
Root vs Leaf	RHSIM_Rhsim08G0060100	-3.371322596	5.2008E-14	4.05908E-13	RHSIM_Rhsim08G0060100
Root vs Leaf	RHSIM_Rhsim08G0252600	-4.061478632	5.27842E-14	4.11827E-13	RHSIM_Rhsim08G0252600
Root vs Leaf	RHSIM_Rhsim03G0056600	1.369290892	5.49254E-14	4.28388E-13	RHSIM_Rhsim03G0056600
Root vs Leaf	RHSIM_Rhsim05G0201700	3.795485694	5.53609E-14	4.3164E-13	RHSIM_Rhsim05G0201700
Root vs Leaf	RHSIM_Rhsim08G0135400	-1.130142287	5.56524E-14	4.33766E-13	RHSIM_Rhsim08G0135400
Root vs Leaf	RHSIM_Rhsim01G0211000	2.150168903	5.6139E-14	4.37412E-13	RHSIM_Rhsim01G0211000
Root vs Leaf	RHSIM_Rhsim01G0010600	5.814339478	5.66196E-14	4.41008E-13	RHSIM_Rhsim01G0010600
Root vs Leaf	RHSIM_Rhsim05G0080500	4.47566357	5.69046E-14	4.43079E-13	RHSIM_Rhsim05G0080500
Root vs Leaf	RHSIM_RhsimUnG0087200	1.779383	5.95387E-14	4.63433E-13	RHSIM_RhsimUnG0087200
Root vs Leaf	RHSIM_Rhsim01G0265100	-1.091510301	6.04071E-14	4.70034E-13	RHSIM_Rhsim01G0265100
Root vs Leaf	RHSIM_Rhsim07G0206100	4.260796785	6.16267E-14	4.79362E-13	RHSIM_Rhsim07G0206100
Root vs Leaf	RHSIM_Rhsim06G0028000	-1.963158125	6.21101E-14	4.82961E-13	RHSIM_Rhsim06G0028000
Root vs Leaf	RHSIM_Rhsim12G0195800	-2.239846299	6.39986E-14	4.97478E-13	RHSIM_Rhsim12G0195800
Root vs Leaf	RHSIM_Rhsim05G0170000	1.200925336	6.46815E-14	5.02617E-13	RHSIM_Rhsim05G0170000
Root vs Leaf	RHSIM_Rhsim04G0132500	-1.780297093	6.60059E-14	5.12737E-13	RHSIM_Rhsim04G0132500
Root vs Leaf	RHSIM_Rhsim06G0005500	-2.191879483	6.60899E-14	5.13217E-13	RHSIM_Rhsim06G0005500
Root vs Leaf	RHSIM_Rhsim05G0203800	-3.103634283	6.70953E-14	5.2085E-13	RHSIM_Rhsim05G0203800
Root vs Leaf	RHSIM_Rhsim07G0005400	-1.995346056	6.78066E-14	5.26195E-13	RHSIM_Rhsim07G0005400
Root vs Leaf	RHSIM_Rhsim02G0149800	1.130831355	6.83949E-14	5.30582E-13	RHSIM_Rhsim02G0149800
Root vs Leaf	RHSIM_Rhsim03G0003700	-9.333220808	6.87326E-14	5.33023E-13	RHSIM_Rhsim03G0003700
Root vs Leaf	RHSIM_Rhsim02G0025700	-1.09137321	6.97467E-14	5.40706E-13	RHSIM_Rhsim02G0025700
Root vs Leaf	RHSIM_Rhsim04G0145800	3.093148918	7.15915E-14	5.54822E-13	RHSIM_Rhsim04G0145800

Comparison	Gene ID	log_2FC	P-value	FDR (padj)	Gene Name
Root vs Leaf	RHSIM_Rhsim04G0181600	-1.022377229	7.2263E-14	5.59838E-13	RHSIM_Rhsim04G0181600
Root vs Leaf	RHSIM_Rhsim13G0031400	1.266706746	7.23817E-14	5.60571E-13	RHSIM_Rhsim13G0031400
Root vs Leaf	RHSIM_Rhsim07G0193400	1.228617378	7.31139E-14	5.66051E-13	RHSIM_Rhsim07G0193400
Root vs Leaf	novel.2606	-9.428636181	7.67263E-14	5.93821E-13	--
Root vs Leaf	RHSIM_Rhsim01G0269000	1.899078951	7.7618E-14	6.00521E-13	RHSIM_Rhsim01G0269000
Root vs Leaf	RHSIM_Rhsim07G0116300	-2.55757082	7.80047E-14	6.03311E-13	RHSIM_Rhsim07G0116300
Root vs Leaf	RHSIM_Rhsim04G0169000	-1.258480251	7.88628E-14	6.09744E-13	RHSIM_Rhsim04G0169000
Root vs Leaf	RHSIM_Rhsim09G0037400	1.222036566	7.9378E-14	6.13279E-13	RHSIM_Rhsim09G0037400
Root vs Leaf	RHSIM_Rhsim12G0163900	1.21148573	7.93861E-14	6.13279E-13	RHSIM_Rhsim12G0163900
Root vs Leaf	RHSIM_Rhsim06G0163700	1.776484405	7.93996E-14	6.13279E-13	RHSIM_Rhsim06G0163700
Root vs Leaf	RHSIM_Rhsim06G0201500	1.384890609	8.06137E-14	6.2245E-13	RHSIM_Rhsim06G0201500
Root vs Leaf	novel.2618	-1.334650973	8.15159E-14	6.29206E-13	--
Root vs Leaf	novel.3508	-3.999224486	8.32418E-14	6.42314E-13	--
Root vs Leaf	RHSIM_Rhsim13G0085200	-1.320101978	8.34052E-14	6.4336E-13	RHSIM_Rhsim13G0085200
Root vs Leaf	RHSIM_Rhsim07G0099100	-9.276114468	8.35639E-14	6.44369E-13	RHSIM_Rhsim07G0099100
Root vs Leaf	novel.163	-9.196158457	8.42113E-14	6.49145E-13	--
Root vs Leaf	RHSIM_Rhsim01G0090600	-1.155218857	8.62132E-14	6.64355E-13	RHSIM_Rhsim01G0090600
Root vs Leaf	RHSIM_Rhsim06G0083200	-1.483316078	8.62775E-14	6.6463E-13	RHSIM_Rhsim06G0083200
Root vs Leaf	RHSIM_Rhsim03G0052000	-6.541111429	8.73653E-14	6.72673E-13	RHSIM_Rhsim03G0052000
Root vs Leaf	RHSIM_Rhsim04G0106400	3.868539949	8.73797E-14	6.72673E-13	RHSIM_Rhsim04G0106400
Root vs Leaf	RHSIM_Rhsim13G0177200	5.539824687	8.7414E-14	6.72713E-13	RHSIM_Rhsim13G0177200
Root vs Leaf	RHSIM_Rhsim04G0164300	-1.533623333	8.76748E-14	6.74496E-13	RHSIM_Rhsim04G0164300
Root vs Leaf	RHSIM_Rhsim05G0094300	-1.999309255	8.81616E-14	6.78015E-13	RHSIM_Rhsim05G0094300
Root vs Leaf	RHSIM_Rhsim11G0161300	2.764631262	8.85562E-14	6.80824E-13	RHSIM_Rhsim11G0161300
Root vs Leaf	RHSIM_Rhsim07G0132500	-3.693646362	8.92099E-14	6.85621E-13	RHSIM_Rhsim07G0132500

Comparison	Gene ID	log_2FC	P-value	FDR (padj)	Gene Name
Root vs Leaf	RHSIM_Rhsim08G0097800	-3.083544044	8.95278E-14	6.87836E-13	RHSIM_Rhsim08G0097800
Root vs Leaf	RHSIM_RhsimUnG0018100	-9.20941744	8.96376E-14	6.88451E-13	RHSIM_RhsimUnG0018100
Root vs Leaf	RHSIM_Rhsim01G0184700	2.782733981	9.01272E-14	6.91982E-13	RHSIM_Rhsim01G0184700
Root vs Leaf	RHSIM_Rhsim12G0186500	-3.396092881	9.06967E-14	6.96123E-13	RHSIM_Rhsim12G0186500
Root vs Leaf	RHSIM_Rhsim07G0124800	-1.180228672	9.18429E-14	7.04687E-13	RHSIM_Rhsim07G0124800
Root vs Leaf	novel.1950	-5.405261483	9.21413E-14	7.06743E-13	--
Root vs Leaf	RHSIM_Rhsim08G0251000	1.273298711	9.23511E-14	7.08117E-13	RHSIM_Rhsim08G0251000
Root vs Leaf	RHSIM_Rhsim05G0142700	-1.618466208	9.25609E-14	7.0949E-13	RHSIM_Rhsim05G0142700
Root vs Leaf	RHSIM_Rhsim02G0169600	-2.553587053	9.50195E-14	7.28095E-13	RHSIM_Rhsim02G0169600
Root vs Leaf	RHSIM_Rhsim06G0239900	3.464528045	9.75485E-14	7.47226E-13	RHSIM_Rhsim06G0239900
Root vs Leaf	novel.1387	-2.250734816	9.77789E-14	7.48743E-13	--
Root vs Leaf	RHSIM_Rhsim02G0179400	-2.389509995	9.91013E-14	7.58618E-13	RHSIM_Rhsim02G0179400
Root vs Leaf	RHSIM_Rhsim08G0203800	3.913629009	9.99932E-14	7.65193E-13	RHSIM_Rhsim08G0203800
Root vs Leaf	RHSIM_Rhsim12G0047600	1.013649297	1.0307E-13	7.88478E-13	RHSIM_Rhsim12G0047600
Root vs Leaf	RHSIM_Rhsim01G0146900	-1.152472699	1.0325E-13	7.8959E-13	RHSIM_Rhsim01G0146900
Root vs Leaf	RHSIM_Rhsim08G0059200	8.673854397	1.03915E-13	7.94414E-13	RHSIM_Rhsim08G0059200
Root vs Leaf	RHSIM_Rhsim09G0042000	10.20379795	1.04827E-13	8.01127E-13	RHSIM_Rhsim09G0042000
Root vs Leaf	RHSIM_Rhsim09G0021700	1.168444673	1.0731E-13	8.19828E-13	RHSIM_Rhsim09G0021700
Root vs Leaf	RHSIM_Rhsim12G0010500	-5.08718507	1.07458E-13	8.20687E-13	RHSIM_Rhsim12G0010500
Root vs Leaf	RHSIM_Rhsim08G0252400	-2.723062831	1.08071E-13	8.251E-13	RHSIM_Rhsim08G0252400
Root vs Leaf	RHSIM_Rhsim13G0159200	-9.924678128	1.08537E-13	8.2838E-13	RHSIM_Rhsim13G0159200
Root vs Leaf	RHSIM_Rhsim05G0234200	-3.200466906	1.09148E-13	8.32773E-13	RHSIM_Rhsim05G0234200
Root vs Leaf	novel.2302	-1.902033759	1.10736E-13	8.44611E-13	--
Root vs Leaf	RHSIM_Rhsim08G0148500	-1.264689579	1.10982E-13	8.46204E-13	RHSIM_Rhsim08G0148500
Root vs Leaf	RHSIM_Rhsim07G0010600	4.164844488	1.11594E-13	8.5059E-13	RHSIM_Rhsim07G0010600

Comparison	Gene ID	log_2FC	P-value	FDR (padj)	Gene Name
Root vs Leaf	RHSIM_Rhsim13G0076000	1.811797296	1.13562E-13	8.65305E-13	RHSIM_Rhsim13G0076000
Root vs Leaf	RHSIM_Rhsim10G0159000	2.660537608	1.14351E-13	8.71035E-13	RHSIM_Rhsim10G0159000
Root vs Leaf	RHSIM_Rhsim12G0036200	11.03043888	1.1665E-13	8.88249E-13	RHSIM_Rhsim12G0036200
Root vs Leaf	RHSIM_Rhsim12G0090600	-1.540317857	1.16837E-13	8.89297E-13	RHSIM_Rhsim12G0090600
Root vs Leaf	novel.1035	-5.148473378	1.16864E-13	8.89297E-13	--
Root vs Leaf	RHSIM_RhsimUnG0094600	2.500781289	1.18643E-13	9.02344E-13	RHSIM_RhsimUnG0094600
Root vs Leaf	RHSIM_Rhsim05G0167600	1.165729585	1.18657E-13	9.02344E-13	RHSIM_Rhsim05G0167600
Root vs Leaf	RHSIM_Rhsim09G0033900	4.132767806	1.19182E-13	9.05932E-13	RHSIM_Rhsim09G0033900
Root vs Leaf	RHSIM_Rhsim03G0029500	-9.340226619	1.19207E-13	9.05932E-13	RHSIM_Rhsim03G0029500
Root vs Leaf	RHSIM_Rhsim02G0144300	1.93842829	1.21956E-13	9.26524E-13	RHSIM_Rhsim02G0144300
Root vs Leaf	RHSIM_Rhsim08G0246300	3.596183858	1.22274E-13	9.28613E-13	RHSIM_Rhsim08G0246300
Root vs Leaf	RHSIM_Rhsim07G0204200	4.164794786	1.22312E-13	9.28613E-13	RHSIM_Rhsim07G0204200
Root vs Leaf	novel.3092	-9.614201472	1.23547E-13	9.37685E-13	--
Root vs Leaf	RHSIM_Rhsim04G0181300	6.042762913	1.23762E-13	9.39006E-13	RHSIM_Rhsim04G0181300
Root vs Leaf	RHSIM_Rhsim07G0055200	2.830119446	1.23867E-13	9.395E-13	RHSIM_Rhsim07G0055200
Root vs Leaf	RHSIM_Rhsim03G0265300	1.501312542	1.24057E-13	9.40629E-13	RHSIM_Rhsim03G0265300
Root vs Leaf	RHSIM_Rhsim05G0187900	5.641080532	1.24946E-13	9.47057E-13	RHSIM_Rhsim05G0187900
Root vs Leaf	RHSIM_RhsimUnG0007900	5.510347983	1.25897E-13	9.53956E-13	RHSIM_RhsimUnG0007900
Root vs Leaf	RHSIM_Rhsim08G0074100	1.505332284	1.2835E-13	9.72222E-13	RHSIM_Rhsim08G0074100
Root vs Leaf	RHSIM_Rhsim10G0049100	-3.422238944	1.30052E-13	9.84792E-13	RHSIM_Rhsim10G0049100
Root vs Leaf	RHSIM_Rhsim02G0127800	-4.98914554	1.34387E-13	1.01729E-12	RHSIM_Rhsim02G0127800
Root vs Leaf	RHSIM_Rhsim06G0155400	1.568464436	1.34927E-13	1.02105E-12	RHSIM_Rhsim06G0155400
Root vs Leaf	RHSIM_Rhsim09G0172100	5.118291221	1.36252E-13	1.03073E-12	RHSIM_Rhsim09G0172100
Root vs Leaf	RHSIM_Rhsim01G0234600	-2.498628697	1.37335E-13	1.03858E-12	RHSIM_Rhsim01G0234600
Root vs Leaf	RHSIM_Rhsim05G0114800	-4.75882856	1.38784E-13	1.0492E-12	RHSIM_Rhsim05G0114800

Comparison	Gene ID	log_2FC	P-value	FDR (padj)	Gene Name
Root vs Leaf	RHSIM_Rhsim09G0030700	-1.284898271	1.38851E-13	1.04936E-12	RHSIM_Rhsim09G0030700
Root vs Leaf	RHSIM_RhsimUnG0062600	-5.085654826	1.39078E-13	1.05074E-12	RHSIM_RhsimUnG0062600
Root vs Leaf	RHSIM_Rhsim03G0049500	-1.122534949	1.3938E-13	1.05268E-12	RHSIM_Rhsim03G0049500
Root vs Leaf	RHSIM_Rhsim01G0166600	-4.651825377	1.39638E-13	1.05428E-12	RHSIM_Rhsim01G0166600
Root vs Leaf	RHSIM_Rhsim07G0039800	-5.46444537	1.41586E-13	1.06829E-12	RHSIM_Rhsim07G0039800
Root vs Leaf	RHSIM_Rhsim04G0081000	1.747437092	1.45072E-13	1.09407E-12	RHSIM_Rhsim04G0081000
Root vs Leaf	RHSIM_Rhsim05G0075000	-1.600383861	1.45097E-13	1.09407E-12	RHSIM_Rhsim05G0075000
Root vs Leaf	RHSIM_Rhsim03G0135800	1.066346963	1.46184E-13	1.1019E-12	RHSIM_Rhsim03G0135800
Root vs Leaf	RHSIM_Rhsim06G0124000	-1.111124839	1.47275E-13	1.10977E-12	RHSIM_Rhsim06G0124000
Root vs Leaf	RHSIM_RhsimUnG0098600	2.584251459	1.48911E-13	1.12173E-12	RHSIM_RhsimUnG0098600
Root vs Leaf	RHSIM_Rhsim10G0126900	3.647929912	1.49304E-13	1.12432E-12	RHSIM_Rhsim10G0126900
Root vs Leaf	RHSIM_Rhsim11G0114800	-4.321390819	1.50024E-13	1.12938E-12	RHSIM_Rhsim11G0114800
Root vs Leaf	RHSIM_Rhsim13G0144400	-3.23476241	1.50718E-13	1.13423E-12	RHSIM_Rhsim13G0144400
Root vs Leaf	RHSIM_Rhsim10G0110300	-6.863564875	1.55172E-13	1.16706E-12	RHSIM_Rhsim10G0110300
Root vs Leaf	RHSIM_Rhsim08G0200500	-2.004771571	1.55181E-13	1.16706E-12	RHSIM_Rhsim08G0200500
Root vs Leaf	RHSIM_Rhsim07G0175300	1.015858934	1.59124E-13	1.19632E-12	RHSIM_Rhsim07G0175300
Root vs Leaf	RHSIM_Rhsim11G0080500	-7.998165283	1.61615E-13	1.21466E-12	RHSIM_Rhsim11G0080500
Root vs Leaf	RHSIM_Rhsim09G0110000	-2.696570852	1.62478E-13	1.22075E-12	RHSIM_Rhsim09G0110000
Root vs Leaf	novel.4918	1.512779543	1.62626E-13	1.22147E-12	--
Root vs Leaf	RHSIM_Rhsim13G0023600	2.047086469	1.63109E-13	1.22469E-12	RHSIM_Rhsim13G0023600
Root vs Leaf	RHSIM_Rhsim05G0110100	-4.077650609	1.63372E-13	1.22627E-12	RHSIM_Rhsim05G0110100
Root vs Leaf	RHSIM_Rhsim08G0237000	1.796993309	1.65618E-13	1.24272E-12	RHSIM_Rhsim08G0237000
Root vs Leaf	RHSIM_Rhsim02G0227900	6.26285496	1.65699E-13	1.24293E-12	RHSIM_Rhsim02G0227900
Root vs Leaf	RHSIM_Rhsim08G0250400	-2.488599113	1.67081E-13	1.25289E-12	RHSIM_Rhsim08G0250400
Root vs Leaf	RHSIM_Rhsim07G0063200	-2.043914252	1.70947E-13	1.28105E-12	RHSIM_Rhsim07G0063200

Comparison	Gene ID	log_2FC	P-value	FDR (padj)	Gene Name
Root vs Leaf	RHSIM_Rhsim12G0055100	5.491888418	1.72148E-13	1.28963E-12	RHSIM_Rhsim12G0055100
Root vs Leaf	novel.2911	-3.243704213	1.73678E-13	1.30067E-12	--
Root vs Leaf	RHSIM_Rhsim01G0286900	-3.883142209	1.78138E-13	1.33364E-12	RHSIM_Rhsim01G0286900
Root vs Leaf	RHSIM_Rhsim05G0208200	-4.533735662	1.81735E-13	1.36013E-12	RHSIM_Rhsim05G0208200
Root vs Leaf	RHSIM_Rhsim06G0013000	3.211914389	1.85373E-13	1.38691E-12	RHSIM_Rhsim06G0013000
Root vs Leaf	RHSIM_Rhsim11G0136400	-2.216940722	1.87287E-13	1.40078E-12	RHSIM_Rhsim11G0136400
Root vs Leaf	RHSIM_Rhsim06G0239200	1.934837836	1.88282E-13	1.40776E-12	RHSIM_Rhsim06G0239200
Root vs Leaf	novel.1513	4.658993761	1.90438E-13	1.42343E-12	--
Root vs Leaf	RHSIM_RhsimUnG0120200	-1.438729673	1.9141E-13	1.43023E-12	RHSIM_RhsimUnG0120200
Root vs Leaf	RHSIM_RhsimUnG0208200	-2.509798167	1.94072E-13	1.44966E-12	RHSIM_RhsimUnG0208200
Root vs Leaf	RHSIM_Rhsim02G0085800	-8.956098321	1.96509E-13	1.46738E-12	RHSIM_Rhsim02G0085800
Root vs Leaf	RHSIM_Rhsim10G0179900	-9.308325099	1.96793E-13	1.46902E-12	RHSIM_Rhsim10G0179900
Root vs Leaf	RHSIM_RhsimUnG0024500	-9.655969953	2.03298E-13	1.5171E-12	RHSIM_RhsimUnG0024500
Root vs Leaf	RHSIM_Rhsim08G0146000	1.913634683	2.03891E-13	1.52104E-12	RHSIM_Rhsim08G0146000
Root vs Leaf	RHSIM_Rhsim04G0168200	4.028000216	2.04858E-13	1.52775E-12	RHSIM_Rhsim04G0168200
Root vs Leaf	RHSIM_Rhsim11G0139300	-2.914829671	2.07518E-13	1.54709E-12	RHSIM_Rhsim11G0139300
Root vs Leaf	RHSIM_Rhsim06G0208100	-1.911792729	2.12719E-13	1.58536E-12	RHSIM_Rhsim06G0208100
Root vs Leaf	RHSIM_Rhsim02G0059400	1.164996679	2.1541E-13	1.60489E-12	RHSIM_Rhsim02G0059400
Root vs Leaf	RHSIM_Rhsim08G0013100	1.333814922	2.15742E-13	1.60685E-12	RHSIM_Rhsim08G0013100
Root vs Leaf	RHSIM_Rhsim02G0195700	-1.933946251	2.16685E-13	1.61335E-12	RHSIM_Rhsim02G0195700
Root vs Leaf	RHSIM_Rhsim07G0001100	1.519078108	2.1883E-13	1.6288E-12	RHSIM_Rhsim07G0001100
Root vs Leaf	RHSIM_Rhsim09G0185300	-9.786196	2.18952E-13	1.62919E-12	RHSIM_Rhsim09G0185300
Root vs Leaf	RHSIM_Rhsim08G0198400	-2.922263029	2.21607E-13	1.64841E-12	RHSIM_Rhsim08G0198400
Root vs Leaf	novel.5516	3.029792237	2.27693E-13	1.69314E-12	--
Root vs Leaf	novel.1181	-2.983016913	2.3272E-13	1.72997E-12	--

Comparison	Gene ID	log_2FC	P-value	FDR (padj)	Gene Name
Root vs Leaf	RHSIM_Rhsim07G0123900	-2.309252718	2.33925E-13	1.73836E-12	RHSIM_Rhsim07G0123900
Root vs Leaf	RHSIM_Rhsim11G0120500	-5.262259815	2.35913E-13	1.75257E-12	RHSIM_Rhsim11G0120500
Root vs Leaf	RHSIM_Rhsim08G0041500	1.394420872	2.36833E-13	1.75884E-12	RHSIM_Rhsim08G0041500
Root vs Leaf	RHSIM_Rhsim03G0110300	1.209848532	2.38928E-13	1.77383E-12	RHSIM_Rhsim03G0110300
Root vs Leaf	RHSIM_Rhsim10G0182600	-1.676632934	2.41288E-13	1.79078E-12	RHSIM_Rhsim10G0182600
Root vs Leaf	RHSIM_Rhsim03G0161400	1.94115319	2.42103E-13	1.79625E-12	RHSIM_Rhsim03G0161400
Root vs Leaf	novel.4969	-6.261509789	2.42305E-13	1.79718E-12	--
Root vs Leaf	novel.4153	3.663253592	2.44916E-13	1.81595E-12	--
Root vs Leaf	RHSIM_Rhsim01G0023400	-2.482789385	2.45818E-13	1.82206E-12	RHSIM_Rhsim01G0023400
Root vs Leaf	RHSIM_Rhsim08G0075300	-2.412564282	2.46144E-13	1.82389E-12	RHSIM_Rhsim08G0075300
Root vs Leaf	RHSIM_Rhsim06G0028700	3.873215047	2.46786E-13	1.82807E-12	RHSIM_Rhsim06G0028700
Root vs Leaf	RHSIM_Rhsim03G0145700	5.05834574	2.49686E-13	1.84896E-12	RHSIM_Rhsim03G0145700
Root vs Leaf	RHSIM_Rhsim09G0207500	1.870084991	2.55975E-13	1.89492E-12	RHSIM_Rhsim09G0207500
Root vs Leaf	RHSIM_Rhsim01G0140100	3.45124901	2.56441E-13	1.89776E-12	RHSIM_Rhsim01G0140100
Root vs Leaf	RHSIM_Rhsim08G0055100	1.300047238	2.56765E-13	1.89955E-12	RHSIM_Rhsim08G0055100
Root vs Leaf	RHSIM_Rhsim02G0105700	1.964685012	2.57093E-13	1.90137E-12	RHSIM_Rhsim02G0105700
Root vs Leaf	RHSIM_Rhsim08G0015000	-1.499446199	2.65008E-13	1.95928E-12	RHSIM_Rhsim08G0015000
Root vs Leaf	RHSIM_Rhsim01G0224400	-2.72567977	2.75271E-13	2.03451E-12	RHSIM_Rhsim01G0224400
Root vs Leaf	novel.3792	-3.584609611	2.78649E-13	2.05882E-12	--
Root vs Leaf	RHSIM_Rhsim10G0186000	1.832652365	2.80979E-13	2.07537E-12	RHSIM_Rhsim10G0186000
Root vs Leaf	RHSIM_Rhsim02G0206900	2.257145412	2.8142E-13	2.07796E-12	RHSIM_Rhsim02G0206900
Root vs Leaf	RHSIM_Rhsim10G0173000	-2.578255274	2.83164E-13	2.09018E-12	RHSIM_Rhsim10G0173000
Root vs Leaf	RHSIM_Rhsim12G0146900	-1.752249785	2.88306E-13	2.12745E-12	RHSIM_Rhsim12G0146900
Root vs Leaf	novel.525	-8.972321994	2.89272E-13	2.1339E-12	--
Root vs Leaf	RHSIM_Rhsim13G0155200	1.857718765	2.93345E-13	2.16263E-12	RHSIM_Rhsim13G0155200

Comparison	Gene ID	log_2FC	P-value	FDR (padj)	Gene Name
Root vs Leaf	RHSIM_Rhsim03G0259200	-1.889270933	2.93353E-13	2.16263E-12	RHSIM_Rhsim03G0259200
Root vs Leaf	RHSIM_Rhsim02G0225800	5.067215731	2.95055E-13	2.17448E-12	RHSIM_Rhsim02G0225800
Root vs Leaf	RHSIM_Rhsim07G0200900	3.218048628	3.03279E-13	2.23438E-12	RHSIM_Rhsim07G0200900
Root vs Leaf	novel.5127	-3.999908988	3.07485E-13	2.26465E-12	--
Root vs Leaf	RHSIM_Rhsim12G0164900	3.906456393	3.10806E-13	2.28838E-12	RHSIM_Rhsim12G0164900
Root vs Leaf	RHSIM_Rhsim04G0047500	2.865109443	3.12131E-13	2.2974E-12	RHSIM_Rhsim04G0047500
Root vs Leaf	RHSIM_Rhsim06G0108800	3.068883383	3.15439E-13	2.32101E-12	RHSIM_Rhsim06G0108800
Root vs Leaf	RHSIM_Rhsim13G0077000	-1.40569334	3.19173E-13	2.34774E-12	RHSIM_Rhsim13G0077000
Root vs Leaf	RHSIM_Rhsim11G0137300	-3.623476243	3.23858E-13	2.38144E-12	RHSIM_Rhsim11G0137300
Root vs Leaf	RHSIM_Rhsim02G0244900	1.883001132	3.29322E-13	2.42085E-12	RHSIM_Rhsim02G0244900
Root vs Leaf	RHSIM_Rhsim04G0004400	-1.289584065	3.30814E-13	2.43028E-12	RHSIM_Rhsim04G0004400
Root vs Leaf	RHSIM_Rhsim12G0164600	3.767211479	3.35416E-13	2.4633E-12	RHSIM_Rhsim12G0164600
Root vs Leaf	RHSIM_Rhsim08G0003800	-9.296035111	3.39672E-13	2.49376E-12	RHSIM_Rhsim08G0003800
Root vs Leaf	RHSIM_Rhsim04G0060500	-9.321779706	3.46869E-13	2.5458E-12	RHSIM_Rhsim04G0060500
Root vs Leaf	RHSIM_RhsimUnG0056800	-9.630188266	3.60763E-13	2.64693E-12	RHSIM_RhsimUnG0056800
Root vs Leaf	RHSIM_Rhsim01G0195600	1.997373687	3.62294E-13	2.65732E-12	RHSIM_Rhsim01G0195600
Root vs Leaf	RHSIM_Rhsim12G0017800	1.067646677	3.65525E-13	2.68017E-12	RHSIM_Rhsim12G0017800
Root vs Leaf	RHSIM_Rhsim01G0167000	-4.133174859	3.69624E-13	2.70937E-12	RHSIM_Rhsim01G0167000
Root vs Leaf	RHSIM_Rhsim04G0110800	1.816730576	3.71196E-13	2.72003E-12	RHSIM_Rhsim04G0110800
Root vs Leaf	novel.5498	-9.082663735	3.88017E-13	2.84239E-12	--
Root vs Leaf	novel.4584	1.531453499	3.93759E-13	2.88354E-12	--
Root vs Leaf	RHSIM_Rhsim07G0207200	3.875868632	3.96866E-13	2.90537E-12	RHSIM_Rhsim07G0207200
Root vs Leaf	novel.4834	4.773377585	3.98118E-13	2.91362E-12	--
Root vs Leaf	RHSIM_Rhsim10G0132900	-3.484521114	3.99522E-13	2.92296E-12	RHSIM_Rhsim10G0132900
Root vs Leaf	RHSIM_Rhsim04G0163000	1.369642758	4.07044E-13	2.97706E-12	RHSIM_Rhsim04G0163000

Comparison	Gene ID	log_2FC	P-value	FDR (padj)	Gene Name
Root vs Leaf	RHSIM_Rhsim04G0198200	-4.635807535	4.14938E-13	3.03383E-12	RHSIM_Rhsim04G0198200
Root vs Leaf	RHSIM_Rhsim06G0209000	-2.571180279	4.17047E-13	3.04829E-12	RHSIM_Rhsim06G0209000
Root vs Leaf	RHSIM_Rhsim06G0138800	3.370577078	4.19486E-13	3.06515E-12	RHSIM_Rhsim06G0138800
Root vs Leaf	RHSIM_Rhsim02G0070800	3.044681396	4.2047E-13	3.07137E-12	RHSIM_Rhsim02G0070800
Root vs Leaf	RHSIM_Rhsim01G0101200	3.929417928	4.21436E-13	3.07746E-12	RHSIM_Rhsim01G0101200
Root vs Leaf	RHSIM_Rhsim01G0247100	-1.88604219	4.22021E-13	3.08075E-12	RHSIM_Rhsim01G0247100
Root vs Leaf	RHSIM_Rhsim08G0097300	2.0401949	4.25382E-13	3.10431E-12	RHSIM_Rhsim08G0097300
Root vs Leaf	RHSIM_Rhsim12G0204000	6.969384092	4.34092E-13	3.16688E-12	RHSIM_Rhsim12G0204000
Root vs Leaf	RHSIM_Rhsim04G0114400	-2.642200769	4.34788E-13	3.17095E-12	RHSIM_Rhsim04G0114400
Root vs Leaf	RHSIM_Rhsim07G0140500	5.36640168	4.35567E-13	3.17563E-12	RHSIM_Rhsim07G0140500
Root vs Leaf	RHSIM_Rhsim07G0024500	-5.722208777	4.36818E-13	3.18375E-12	RHSIM_Rhsim07G0024500
Root vs Leaf	RHSIM_Rhsim09G0091700	-2.858644453	4.44454E-13	3.23838E-12	RHSIM_Rhsim09G0091700
Root vs Leaf	RHSIM_Rhsim04G0023100	3.170636945	4.46225E-13	3.25027E-12	RHSIM_Rhsim04G0023100
Root vs Leaf	RHSIM_Rhsim12G0139600	-2.703242867	4.52174E-13	3.29256E-12	RHSIM_Rhsim12G0139600
Root vs Leaf	RHSIM_Rhsim04G0231000	-3.520355821	4.61209E-13	3.35729E-12	RHSIM_Rhsim04G0231000
Root vs Leaf	RHSIM_Rhsim01G0069500	-2.18798131	4.63243E-13	3.37104E-12	RHSIM_Rhsim01G0069500
Root vs Leaf	RHSIM_Rhsim02G0111100	-7.794787791	4.64479E-13	3.37897E-12	RHSIM_Rhsim02G0111100
Root vs Leaf	RHSIM_Rhsim01G0138300	9.286607059	4.69188E-13	3.41215E-12	RHSIM_Rhsim01G0138300
Root vs Leaf	RHSIM_Rhsim04G0109400	-1.142377188	4.73675E-13	3.44371E-12	RHSIM_Rhsim04G0109400
Root vs Leaf	RHSIM_Rhsim08G0078500	-1.491317414	4.84074E-13	3.5182E-12	RHSIM_Rhsim08G0078500
Root vs Leaf	RHSIM_Rhsim03G0257900	1.271220116	4.8623E-13	3.53276E-12	RHSIM_Rhsim03G0257900
Root vs Leaf	RHSIM_Rhsim13G0085100	1.487654701	4.92866E-13	3.57985E-12	RHSIM_Rhsim13G0085100
Root vs Leaf	RHSIM_Rhsim02G0226400	9.272354771	4.94178E-13	3.58826E-12	RHSIM_Rhsim02G0226400
Root vs Leaf	RHSIM_Rhsim11G0133200	8.923668643	4.95823E-13	3.59907E-12	RHSIM_Rhsim11G0133200
Root vs Leaf	RHSIM_Rhsim07G0186600	-9.407844349	5.0092E-13	3.63493E-12	RHSIM_Rhsim07G0186600

Comparison	Gene ID	log_2FC	P-value	FDR (padj)	Gene Name
Root vs Leaf	novel.1227	8.964930565	5.0797E-13	3.68493E-12	--
Root vs Leaf	RHSIM_Rhsim03G0157000	-9.00148514	5.10171E-13	3.69974E-12	RHSIM_Rhsim03G0157000
Root vs Leaf	RHSIM_Rhsim13G0208400	1.029594799	5.10342E-13	3.69982E-12	RHSIM_Rhsim13G0208400
Root vs Leaf	novel.26	9.41871743	5.12975E-13	3.71774E-12	--
Root vs Leaf	RHSIM_RhsimUnG0245900	-5.062800679	5.16087E-13	3.73913E-12	RHSIM_RhsimUnG0245900
Root vs Leaf	RHSIM_Rhsim12G0136600	-1.71437059	5.17826E-13	3.75055E-12	RHSIM_Rhsim12G0136600
Root vs Leaf	RHSIM_Rhsim04G0249000	-1.178430463	5.26504E-13	3.81221E-12	RHSIM_Rhsim04G0249000
Root vs Leaf	RHSIM_Rhsim11G0089800	-2.383074265	5.34277E-13	3.86728E-12	RHSIM_Rhsim11G0089800
Root vs Leaf	RHSIM_Rhsim05G0049500	3.139038476	5.41188E-13	3.91609E-12	RHSIM_Rhsim05G0049500
Root vs Leaf	RHSIM_Rhsim03G0234100	1.339783839	5.42036E-13	3.92099E-12	RHSIM_Rhsim03G0234100
Root vs Leaf	RHSIM_Rhsim13G0050700	1.361219888	5.46311E-13	3.95068E-12	RHSIM_Rhsim13G0050700
Root vs Leaf	RHSIM_Rhsim06G0145000	5.249498249	5.50067E-13	3.9766E-12	RHSIM_Rhsim06G0145000
Root vs Leaf	RHSIM_Rhsim07G0211900	-1.222344627	5.60076E-13	4.0477E-12	RHSIM_Rhsim07G0211900
Root vs Leaf	RHSIM_Rhsim01G0010000	-2.615338478	5.71149E-13	4.12643E-12	RHSIM_Rhsim01G0010000
Root vs Leaf	RHSIM_Rhsim13G0094500	-1.996725537	5.78495E-13	4.1782E-12	RHSIM_Rhsim13G0094500
Root vs Leaf	RHSIM_Rhsim11G0121800	1.125423449	5.80218E-13	4.18934E-12	RHSIM_Rhsim11G0121800
Root vs Leaf	RHSIM_Rhsim07G0210500	1.337245904	5.80424E-13	4.18952E-12	RHSIM_Rhsim07G0210500
Root vs Leaf	RHSIM_Rhsim10G0187800	-1.977553935	5.81792E-13	4.19808E-12	RHSIM_Rhsim10G0187800
Root vs Leaf	RHSIM_Rhsim08G0008000	-1.451760924	5.8779E-13	4.24004E-12	RHSIM_Rhsim08G0008000
Root vs Leaf	RHSIM_Rhsim05G0121700	1.127648147	5.95435E-13	4.29385E-12	RHSIM_Rhsim05G0121700
Root vs Leaf	RHSIM_Rhsim04G0126300	9.543391588	6.02936E-13	4.34659E-12	RHSIM_Rhsim04G0126300
Root vs Leaf	RHSIM_Rhsim09G0135500	-4.392385844	6.05441E-13	4.36193E-12	RHSIM_Rhsim09G0135500
Root vs Leaf	RHSIM_Rhsim08G0193400	-3.269394922	6.08631E-13	4.38355E-12	RHSIM_Rhsim08G0193400
Root vs Leaf	RHSIM_RhsimUnG0146600	-4.381690417	6.19584E-13	4.46105E-12	RHSIM_RhsimUnG0146600
Root vs Leaf	RHSIM_Rhsim06G0154000	-1.723956052	6.49632E-13	4.67448E-12	RHSIM_Rhsim06G0154000

Comparison	Gene ID	log_2FC	P-value	FDR (padj)	Gene Name
Root vs Leaf	RHSIM_Rhsim12G0114200	1.563966676	6.53589E-13	4.70149E-12	RHSIM_Rhsim12G0114200
Root vs Leaf	novel.3025	12.86108294	6.57089E-13	4.7252E-12	--
Root vs Leaf	RHSIM_Rhsim06G0177900	2.233852696	6.61563E-13	4.7559E-12	RHSIM_Rhsim06G0177900
Root vs Leaf	RHSIM_RhsimUnG0032900	-4.00673712	6.72734E-13	4.83371E-12	RHSIM_RhsimUnG0032900
Root vs Leaf	novel.523	-9.117688858	6.72806E-13	4.83371E-12	--
Root vs Leaf	RHSIM_RhsimUnG0148400	1.088811946	6.77689E-13	4.86729E-12	RHSIM_RhsimUnG0148400
Root vs Leaf	novel.3863	-2.017536285	6.79162E-13	4.87635E-12	--
Root vs Leaf	RHSIM_Rhsim03G0081900	1.044239306	7.01372E-13	5.03426E-12	RHSIM_Rhsim03G0081900
Root vs Leaf	RHSIM_Rhsim06G0176300	-2.868171746	7.03645E-13	5.04882E-12	RHSIM_Rhsim06G0176300
Root vs Leaf	RHSIM_Rhsim10G0111500	-3.584111349	7.03837E-13	5.04882E-12	RHSIM_Rhsim10G0111500
Root vs Leaf	novel.4016	-1.360239353	7.06554E-13	5.06674E-12	--
Root vs Leaf	novel.3037	9.932019764	7.08879E-13	5.08184E-12	--
Root vs Leaf	RHSIM_Rhsim04G0221700	-5.592318177	7.10049E-13	5.08865E-12	RHSIM_Rhsim04G0221700
Root vs Leaf	RHSIM_RhsimUnG0043100	2.374164831	7.19882E-13	5.15752E-12	RHSIM_RhsimUnG0043100
Root vs Leaf	RHSIM_Rhsim06G0185800	5.82083707	7.42599E-13	5.31863E-12	RHSIM_Rhsim06G0185800
Root vs Leaf	RHSIM_RhsimUnG0090500	-4.755335999	7.73476E-13	5.53806E-12	RHSIM_RhsimUnG0090500
Root vs Leaf	RHSIM_Rhsim03G0217200	-6.688634115	7.77575E-13	5.56569E-12	RHSIM_Rhsim03G0217200
Root vs Leaf	RHSIM_Rhsim05G0094500	4.348784228	7.80696E-13	5.5863E-12	RHSIM_Rhsim05G0094500
Root vs Leaf	RHSIM_Rhsim06G0157100	15.36411552	7.99221E-13	5.71708E-12	RHSIM_Rhsim06G0157100
Root vs Leaf	RHSIM_Rhsim11G0169800	1.142008083	7.9966E-13	5.71846E-12	RHSIM_Rhsim11G0169800
Root vs Leaf	RHSIM_Rhsim03G0178500	-7.76529515	8.022E-13	5.73485E-12	RHSIM_Rhsim03G0178500
Root vs Leaf	novel.1473	-5.416572509	8.06187E-13	5.76157E-12	--
Root vs Leaf	RHSIM_Rhsim08G0184400	1.27339592	8.06443E-13	5.76162E-12	RHSIM_Rhsim08G0184400
Root vs Leaf	RHSIM_Rhsim03G0142000	1.323371756	8.1109E-13	5.79304E-12	RHSIM_Rhsim03G0142000
Root vs Leaf	RHSIM_Rhsim03G0192200	-1.053257397	8.48916E-13	6.06133E-12	RHSIM_Rhsim03G0192200

Comparison	Gene ID	log_2FC	P-value	FDR (padj)	Gene Name
Root vs Leaf	RHSIM_Rhsim11G0058400	4.506036914	8.49261E-13	6.06192E-12	RHSIM_Rhsim11G0058400
Root vs Leaf	RHSIM_Rhsim03G0195300	-3.887138142	8.52066E-13	6.08006E-12	RHSIM_Rhsim03G0195300
Root vs Leaf	novel.3558	-9.13640498	8.61085E-13	6.14253E-12	--
Root vs Leaf	RHSIM_Rhsim13G0161700	-2.620161899	8.66549E-13	6.1796E-12	RHSIM_Rhsim13G0161700
Root vs Leaf	RHSIM_Rhsim10G0026400	-2.734489357	8.75952E-13	6.24474E-12	RHSIM_Rhsim10G0026400
Root vs Leaf	RHSIM_Rhsim01G0120800	4.187160499	8.79275E-13	6.26649E-12	RHSIM_Rhsim01G0120800
Root vs Leaf	novel.534	-6.6231449	8.83036E-13	6.29136E-12	--
Root vs Leaf	RHSIM_Rhsim06G0164100	2.602287173	8.85182E-13	6.30277E-12	RHSIM_Rhsim06G0164100
Root vs Leaf	RHSIM_Rhsim08G0050700	2.88525096	8.88644E-13	6.32547E-12	RHSIM_Rhsim08G0050700
Root vs Leaf	RHSIM_Rhsim12G0193900	-2.578737763	9.01997E-13	6.41855E-12	RHSIM_Rhsim12G0193900
Root vs Leaf	RHSIM_Rhsim01G0213500	-1.723413795	9.14847E-13	6.50798E-12	RHSIM_Rhsim01G0213500
Root vs Leaf	RHSIM_Rhsim04G0060200	-6.82275099	9.34381E-13	6.6449E-12	RHSIM_Rhsim04G0060200
Root vs Leaf	RHSIM_Rhsim06G0236400	1.500745532	9.37524E-13	6.66521E-12	RHSIM_Rhsim06G0236400
Root vs Leaf	novel.1499	1.149585861	9.39309E-13	6.67585E-12	--
Root vs Leaf	novel.4908	5.035015674	9.53633E-13	6.77557E-12	--
Root vs Leaf	RHSIM_Rhsim13G0052400	1.468679218	9.60533E-13	6.8225E-12	RHSIM_Rhsim13G0052400
Root vs Leaf	RHSIM_Rhsim13G0167400	3.608110593	9.83728E-13	6.98296E-12	RHSIM_Rhsim13G0167400
Root vs Leaf	novel.2531	6.190045327	9.88014E-13	7.01124E-12	--
Root vs Leaf	RHSIM_Rhsim12G0085300	-1.525606906	9.91962E-13	7.03709E-12	RHSIM_Rhsim12G0085300
Root vs Leaf	RHSIM_Rhsim01G0166200	2.440224405	1.00909E-12	7.15639E-12	RHSIM_Rhsim01G0166200
Root vs Leaf	RHSIM_Rhsim04G0135600	1.365844462	1.02142E-12	7.23944E-12	RHSIM_Rhsim04G0135600
Root vs Leaf	RHSIM_Rhsim10G0083100	-2.030556921	1.02928E-12	7.29291E-12	RHSIM_Rhsim10G0083100
Root vs Leaf	RHSIM_Rhsim13G0039600	-1.397864065	1.03141E-12	7.30571E-12	RHSIM_Rhsim13G0039600
Root vs Leaf	novel.1783	-9.297284667	1.03195E-12	7.30733E-12	--
Root vs Leaf	RHSIM_Rhsim01G0052400	-2.323242857	1.03245E-12	7.30863E-12	RHSIM_Rhsim01G0052400

Comparison	Gene ID	log_2FC	P-value	FDR (padj)	Gene Name
Root vs Leaf	RHSIM_Rhsim12G0002300	-1.79007036	1.04623E-12	7.40394E-12	RHSIM_Rhsim12G0002300
Root vs Leaf	RHSIM_Rhsim04G0124400	2.623997713	1.04726E-12	7.40892E-12	RHSIM_Rhsim04G0124400
Root vs Leaf	RHSIM_Rhsim01G0064700	-7.529242114	1.05029E-12	7.42808E-12	RHSIM_Rhsim01G0064700
Root vs Leaf	RHSIM_Rhsim13G0124500	4.720456724	1.05459E-12	7.45627E-12	RHSIM_Rhsim13G0124500
Root vs Leaf	RHSIM_Rhsim09G0129400	-1.436470441	1.09049E-12	7.70298E-12	RHSIM_Rhsim09G0129400
Root vs Leaf	RHSIM_Rhsim08G0236600	-2.957753786	1.09118E-12	7.70556E-12	RHSIM_Rhsim08G0236600
Root vs Leaf	novel.4100	7.581906083	1.10119E-12	7.77387E-12	--
Root vs Leaf	RHSIM_Rhsim11G0095900	-3.122612787	1.10544E-12	7.80149E-12	RHSIM_Rhsim11G0095900
Root vs Leaf	RHSIM_Rhsim07G0088000	-2.669401271	1.11914E-12	7.89571E-12	RHSIM_Rhsim07G0088000
Root vs Leaf	RHSIM_RhsimUnG0127800	-3.362287996	1.12845E-12	7.95903E-12	RHSIM_RhsimUnG0127800
Root vs Leaf	RHSIM_Rhsim13G0146500	-2.040437952	1.13667E-12	8.01453E-12	RHSIM_Rhsim13G0146500
Root vs Leaf	RHSIM_Rhsim08G0102400	-2.199745458	1.14852E-12	8.09562E-12	RHSIM_Rhsim08G0102400
Root vs Leaf	novel.5982	-4.086352112	1.15379E-12	8.13029E-12	--
Root vs Leaf	novel.3239	-3.691319056	1.15538E-12	8.139E-12	--
Root vs Leaf	novel.2815	9.681314566	1.16489E-12	8.20347E-12	--
Root vs Leaf	RHSIM_Rhsim06G0061300	6.897258063	1.17073E-12	8.24216E-12	RHSIM_Rhsim06G0061300
Root vs Leaf	RHSIM_Rhsim08G0058300	-9.80746582	1.17477E-12	8.26807E-12	RHSIM_Rhsim08G0058300
Root vs Leaf	RHSIM_Rhsim12G0072200	5.555112959	1.17792E-12	8.28773E-12	RHSIM_Rhsim12G0072200
Root vs Leaf	RHSIM_Rhsim06G0091400	-1.09421431	1.20373E-12	8.4667E-12	RHSIM_Rhsim06G0091400
Root vs Leaf	RHSIM_Rhsim08G0108000	-9.682408267	1.20876E-12	8.49952E-12	RHSIM_Rhsim08G0108000
Root vs Leaf	RHSIM_Rhsim11G0039500	-9.155487016	1.21272E-12	8.52476E-12	RHSIM_Rhsim11G0039500
Root vs Leaf	RHSIM_Rhsim07G0258200	1.834325087	1.21606E-12	8.54565E-12	RHSIM_Rhsim07G0258200
Root vs Leaf	novel.5096	1.068492926	1.23316E-12	8.66318E-12	--
Root vs Leaf	RHSIM_Rhsim02G0032200	-3.318575941	1.23578E-12	8.67896E-12	RHSIM_Rhsim02G0032200
Root vs Leaf	RHSIM_Rhsim07G0138500	1.616719146	1.23713E-12	8.68578E-12	RHSIM_Rhsim07G0138500

Comparison	Gene ID	log_2FC	P-value	FDR (padj)	Gene Name
Root vs Leaf	RHSIM_Rhsim13G0065700	-1.76183562	1.23993E-12	8.70286E-12	RHSIM_Rhsim13G0065700
Root vs Leaf	RHSIM_Rhsim05G0202500	-2.127843044	1.24931E-12	8.76597E-12	RHSIM_Rhsim05G0202500
Root vs Leaf	RHSIM_Rhsim13G0102300	1.150632154	1.25131E-12	8.77735E-12	RHSIM_Rhsim13G0102300
Root vs Leaf	RHSIM_Rhsim11G0014300	-1.412492192	1.25303E-12	8.78678E-12	RHSIM_Rhsim11G0014300
Root vs Leaf	RHSIM_Rhsim03G0074700	-1.543580149	1.26369E-12	8.85883E-12	RHSIM_Rhsim03G0074700
Root vs Leaf	novel.4629	-9.373735726	1.26501E-12	8.8654E-12	--
Root vs Leaf	RHSIM_Rhsim07G0011100	1.080771593	1.26747E-12	8.87999E-12	RHSIM_Rhsim07G0011100
Root vs Leaf	RHSIM_Rhsim08G0139800	-1.006115534	1.26964E-12	8.8925E-12	RHSIM_Rhsim08G0139800
Root vs Leaf	RHSIM_Rhsim03G0097500	-1.005970672	1.29417E-12	9.06153E-12	RHSIM_Rhsim03G0097500
Root vs Leaf	RHSIM_Rhsim03G0227800	-2.011531956	1.31048E-12	9.17298E-12	RHSIM_Rhsim03G0227800
Root vs Leaf	RHSIM_Rhsim04G0056900	-2.694382837	1.3111E-12	9.17454E-12	RHSIM_Rhsim04G0056900
Root vs Leaf	RHSIM_Rhsim01G0064800	-8.697186266	1.32044E-12	9.2371E-12	RHSIM_Rhsim01G0064800
Root vs Leaf	RHSIM_Rhsim07G0002400	2.660826609	1.35701E-12	9.49005E-12	RHSIM_Rhsim07G0002400
Root vs Leaf	RHSIM_Rhsim09G0043700	-2.029435797	1.37139E-12	9.5877E-12	RHSIM_Rhsim09G0043700
Root vs Leaf	RHSIM_Rhsim03G0249100	-1.686642184	1.37266E-12	9.59367E-12	RHSIM_Rhsim03G0249100
Root vs Leaf	RHSIM_Rhsim01G0063800	-2.47472709	1.41542E-12	9.8866E-12	RHSIM_Rhsim01G0063800
Root vs Leaf	RHSIM_Rhsim04G0048200	-1.135687998	1.41693E-12	9.89416E-12	RHSIM_Rhsim04G0048200
Root vs Leaf	RHSIM_Rhsim11G0026000	-1.622788779	1.4643E-12	1.02187E-11	RHSIM_Rhsim11G0026000
Root vs Leaf	RHSIM_Rhsim02G0217400	-1.095073021	1.47032E-12	1.02577E-11	RHSIM_Rhsim02G0217400
Root vs Leaf	RHSIM_Rhsim06G0012900	2.934259725	1.49262E-12	1.04101E-11	RHSIM_Rhsim06G0012900
Root vs Leaf	RHSIM_Rhsim07G0056900	-3.227062732	1.4948E-12	1.04222E-11	RHSIM_Rhsim07G0056900
Root vs Leaf	RHSIM_Rhsim08G0224500	3.135306019	1.54838E-12	1.07925E-11	RHSIM_Rhsim08G0224500
Root vs Leaf	RHSIM_Rhsim13G0038900	3.16682013	1.56467E-12	1.09027E-11	RHSIM_Rhsim13G0038900
Root vs Leaf	RHSIM_Rhsim07G0253800	3.43926266	1.59185E-12	1.10888E-11	RHSIM_Rhsim07G0253800
Root vs Leaf	novel.4111	1.469661489	1.61319E-12	1.12341E-11	--

Comparison	Gene ID	log_2FC	P-value	FDR (padj)	Gene Name
Root vs Leaf	novel.2982	-9.527904356	1.61508E-12	1.12439E-11	--
Root vs Leaf	RHSIM_Rhsim04G0244800	-3.691443847	1.61858E-12	1.12648E-11	RHSIM_Rhsim04G0244800
Root vs Leaf	RHSIM_Rhsim06G0021000	2.089677017	1.62806E-12	1.13274E-11	RHSIM_Rhsim06G0021000
Root vs Leaf	RHSIM_Rhsim08G0075800	-1.241320021	1.62991E-12	1.13369E-11	RHSIM_Rhsim08G0075800
Root vs Leaf	novel.4275	4.948211394	1.64802E-12	1.14594E-11	--
Root vs Leaf	novel.2548	-2.832010127	1.64963E-12	1.14671E-11	--
Root vs Leaf	RHSIM_Rhsim09G0132200	10.55238761	1.65933E-12	1.15311E-11	RHSIM_Rhsim09G0132200
Root vs Leaf	RHSIM_Rhsim04G0070000	-4.862912945	1.66439E-12	1.15628E-11	RHSIM_Rhsim04G0070000
Root vs Leaf	RHSIM_Rhsim05G0067100	1.380739132	1.6806E-12	1.16719E-11	RHSIM_Rhsim05G0067100
Root vs Leaf	RHSIM_Rhsim13G0179700	2.138906988	1.69792E-12	1.17851E-11	RHSIM_Rhsim13G0179700
Root vs Leaf	RHSIM_Rhsim13G0088100	-9.1115761	1.72423E-12	1.19641E-11	RHSIM_Rhsim13G0088100
Root vs Leaf	RHSIM_Rhsim07G0043100	7.659656519	1.7316E-12	1.20117E-11	RHSIM_Rhsim07G0043100
Root vs Leaf	RHSIM_Rhsim01G0170800	-2.872042529	1.74109E-12	1.20739E-11	RHSIM_Rhsim01G0170800
Root vs Leaf	RHSIM_Rhsim02G0198600	-9.731564293	1.7425E-12	1.20801E-11	RHSIM_Rhsim02G0198600
Root vs Leaf	RHSIM_Rhsim01G0220700	-2.447850518	1.76926E-12	1.22619E-11	RHSIM_Rhsim01G0220700
Root vs Leaf	RHSIM_Rhsim05G0043400	-1.182411724	1.7741E-12	1.22918E-11	RHSIM_Rhsim05G0043400
Root vs Leaf	RHSIM_Rhsim07G0172500	1.122066634	1.78231E-12	1.2345E-11	RHSIM_Rhsim07G0172500
Root vs Leaf	RHSIM_Rhsim06G0176100	-2.549725141	1.81518E-12	1.25688E-11	RHSIM_Rhsim06G0176100
Root vs Leaf	RHSIM_Rhsim09G0025000	1.482852897	1.81989E-12	1.25977E-11	RHSIM_Rhsim09G0025000
Root vs Leaf	RHSIM_Rhsim01G0285400	-4.841316141	1.83021E-12	1.26653E-11	RHSIM_Rhsim01G0285400
Root vs Leaf	RHSIM_Rhsim07G0010700	1.11220985	1.8432E-12	1.27514E-11	RHSIM_Rhsim07G0010700
Root vs Leaf	RHSIM_Rhsim01G0201200	8.73879444	1.86173E-12	1.28758E-11	RHSIM_Rhsim01G0201200
Root vs Leaf	RHSIM_RhsimUnG0124900	1.588707886	1.8709E-12	1.29353E-11	RHSIM_RhsimUnG0124900
Root vs Leaf	RHSIM_Rhsim13G0115200	-1.450556298	1.90223E-12	1.3148E-11	RHSIM_Rhsim13G0115200
Root vs Leaf	RHSIM_Rhsim01G0267800	-1.400966853	1.93242E-12	1.33527E-11	RHSIM_Rhsim01G0267800

Comparison	Gene ID	log_2FC	P-value	FDR (padj)	Gene Name
Root vs Leaf	RHSIM_Rhsim08G0036600	1.531217887	1.9667E-12	1.35855E-11	RHSIM_Rhsim08G0036600
Root vs Leaf	RHSIM_Rhsim03G0199400	-3.602606714	1.98191E-12	1.36865E-11	RHSIM_Rhsim03G0199400
Root vs Leaf	RHSIM_Rhsim06G0054600	-1.761367565	2.00259E-12	1.38252E-11	RHSIM_Rhsim06G0054600
Root vs Leaf	RHSIM_Rhsim13G0022800	-4.501313507	2.02761E-12	1.39937E-11	RHSIM_Rhsim13G0022800
Root vs Leaf	RHSIM_Rhsim01G0270500	9.438516812	2.04033E-12	1.40746E-11	RHSIM_Rhsim01G0270500
Root vs Leaf	RHSIM_Rhsim07G0017000	-1.492677015	2.04055E-12	1.40746E-11	RHSIM_Rhsim07G0017000
Root vs Leaf	RHSIM_Rhsim10G0011800	-1.750113359	2.06918E-12	1.42678E-11	RHSIM_Rhsim10G0011800
Root vs Leaf	RHSIM_Rhsim08G0131900	-1.053507997	2.07299E-12	1.42885E-11	RHSIM_Rhsim08G0131900
Root vs Leaf	RHSIM_Rhsim04G0185400	4.629364218	2.07341E-12	1.42885E-11	RHSIM_Rhsim04G0185400
Root vs Leaf	RHSIM_Rhsim08G0118300	2.124867141	2.09344E-12	1.44222E-11	RHSIM_Rhsim08G0118300
Root vs Leaf	RHSIM_Rhsim09G0137500	-4.062637314	2.10308E-12	1.44843E-11	RHSIM_Rhsim09G0137500
Root vs Leaf	RHSIM_Rhsim10G0026600	-5.175374791	2.13745E-12	1.47167E-11	RHSIM_Rhsim10G0026600
Root vs Leaf	RHSIM_RhsimUnG0162000	3.725647639	2.18835E-12	1.50626E-11	RHSIM_RhsimUnG0162000
Root vs Leaf	RHSIM_Rhsim01G0188500	-8.602580164	2.18912E-12	1.50635E-11	RHSIM_Rhsim01G0188500
Root vs Leaf	novel.839	-3.95406422	2.21246E-12	1.52195E-11	--
Root vs Leaf	novel.6019	5.765576725	2.21919E-12	1.52613E-11	--
Root vs Leaf	RHSIM_Rhsim05G0096400	-1.288460707	2.22596E-12	1.53033E-11	RHSIM_Rhsim05G0096400
Root vs Leaf	novel.3376	1.00505881	2.25321E-12	1.5486E-11	--
Root vs Leaf	RHSIM_Rhsim08G0168900	3.621457145	2.28794E-12	1.57154E-11	RHSIM_Rhsim08G0168900
Root vs Leaf	RHSIM_Rhsim08G0146400	1.051375721	2.32585E-12	1.59711E-11	RHSIM_Rhsim08G0146400
Root vs Leaf	RHSIM_Rhsim08G0241800	1.261229135	2.36445E-12	1.62313E-11	RHSIM_Rhsim08G0241800
Root vs Leaf	RHSIM_Rhsim03G0111700	-4.175543112	2.36679E-12	1.62425E-11	RHSIM_Rhsim03G0111700
Root vs Leaf	RHSIM_Rhsim06G0020300	4.682845095	2.37133E-12	1.62689E-11	RHSIM_Rhsim06G0020300
Root vs Leaf	RHSIM_Rhsim06G0216100	-8.886835981	2.37274E-12	1.62737E-11	RHSIM_Rhsim06G0216100
Root vs Leaf	RHSIM_Rhsim12G0200300	-5.782964899	2.37945E-12	1.63149E-11	RHSIM_Rhsim12G0200300

Comparison	Gene ID	log_2FC	P-value	FDR (padj)	Gene Name
Root vs Leaf	RHSIM_Rhsim07G0081800	-2.299124814	2.38757E-12	1.63657E-11	RHSIM_Rhsim07G0081800
Root vs Leaf	RHSIM_Rhsim05G0186100	-1.528413999	2.432E-12	1.66555E-11	RHSIM_Rhsim05G0186100
Root vs Leaf	RHSIM_Rhsim10G0003700	3.155262507	2.43629E-12	1.66799E-11	RHSIM_Rhsim10G0003700
Root vs Leaf	novel.6013	-4.287061011	2.46373E-12	1.68628E-11	--
Root vs Leaf	RHSIM_Rhsim02G0069700	-1.534225209	2.49518E-12	1.7073E-11	RHSIM_Rhsim02G0069700
Root vs Leaf	RHSIM_Rhsim07G0135800	-5.868932232	2.49947E-12	1.70973E-11	RHSIM_Rhsim07G0135800
Root vs Leaf	RHSIM_Rhsim04G0032700	-1.435177929	2.52473E-12	1.7265E-11	RHSIM_Rhsim04G0032700
Root vs Leaf	RHSIM_Rhsim04G0132400	-1.672888456	2.53447E-12	1.73265E-11	RHSIM_Rhsim04G0132400
Root vs Leaf	novel.2598	-4.671013406	2.53577E-12	1.73284E-11	--
Root vs Leaf	novel.2766	-3.781477006	2.53624E-12	1.73284E-11	--
Root vs Leaf	novel.5346	-4.646930114	2.57781E-12	1.76019E-11	--
Root vs Leaf	RHSIM_Rhsim01G0127700	-3.360104062	2.59091E-12	1.76862E-11	RHSIM_Rhsim01G0127700
Root vs Leaf	RHSIM_Rhsim10G0052000	-5.434158903	2.64092E-12	1.80223E-11	RHSIM_Rhsim10G0052000
Root vs Leaf	RHSIM_RhsimUnG0156400	-5.05605887	2.67503E-12	1.82443E-11	RHSIM_RhsimUnG0156400
Root vs Leaf	RHSIM_Rhsim11G0110900	8.126438797	2.68698E-12	1.83204E-11	RHSIM_Rhsim11G0110900
Root vs Leaf	RHSIM_Rhsim06G0005100	1.02721579	2.71606E-12	1.85132E-11	RHSIM_Rhsim06G0005100
Root vs Leaf	RHSIM_RhsimUnG0160300	1.937575639	2.73529E-12	1.86388E-11	RHSIM_RhsimUnG0160300
Root vs Leaf	RHSIM_Rhsim12G0027600	1.578685543	2.7788E-12	1.89241E-11	RHSIM_Rhsim12G0027600
Root vs Leaf	RHSIM_Rhsim08G0097500	-1.108148589	2.81763E-12	1.91829E-11	RHSIM_Rhsim08G0097500
Root vs Leaf	RHSIM_Rhsim08G0096900	5.517643799	2.84589E-12	1.93696E-11	RHSIM_Rhsim08G0096900
Root vs Leaf	RHSIM_Rhsim03G0225700	-1.583004741	2.86776E-12	1.95128E-11	RHSIM_Rhsim03G0225700
Root vs Leaf	RHSIM_Rhsim03G0052100	-6.6835419	2.89441E-12	1.96883E-11	RHSIM_Rhsim03G0052100
Root vs Leaf	RHSIM_Rhsim09G0058700	-5.081965789	2.91684E-12	1.9835E-11	RHSIM_Rhsim09G0058700
Root vs Leaf	RHSIM_Rhsim07G0030900	-2.534831791	2.92264E-12	1.98686E-11	RHSIM_Rhsim07G0030900
Root vs Leaf	RHSIM_Rhsim03G0180200	-7.672766622	2.94463E-12	2.00122E-11	RHSIM_Rhsim03G0180200

Comparison	Gene ID	log2FC	P-value	FDR (padj)	Gene Name
Root vs Leaf	RHSIM_Rhsim01G0055800	-4.83391536	2.96327E-12	2.0133E-11	RHSIM_Rhsim01G0055800
Root vs Leaf	novel.1544	2.475971298	2.9683E-12	2.01613E-11	--
Root vs Leaf	RHSIM_Rhsim03G0115100	1.873599986	3.04045E-12	2.06452E-11	RHSIM_Rhsim03G0115100
Root vs Leaf	RHSIM_Rhsim03G0236800	2.046587882	3.05029E-12	2.0706E-11	RHSIM_Rhsim03G0236800
Root vs Leaf	RHSIM_Rhsim07G0168000	-1.669229302	3.05966E-12	2.07635E-11	RHSIM_Rhsim07G0168000
Root vs Leaf	RHSIM_Rhsim12G0113400	9.08613559	3.09465E-12	2.09948E-11	RHSIM_Rhsim12G0113400
Root vs Leaf	RHSIM_Rhsim08G0110200	3.405630607	3.15867E-12	2.14166E-11	RHSIM_Rhsim08G0110200
Root vs Leaf	RHSIM_Rhsim09G0022600	4.039733481	3.16067E-12	2.14225E-11	RHSIM_Rhsim09G0022600
Root vs Leaf	RHSIM_Rhsim12G0047100	-1.650127875	3.16139E-12	2.14225E-11	RHSIM_Rhsim12G0047100
Root vs Leaf	RHSIM_Rhsim10G0019900	-1.58104831	3.16676E-12	2.14526E-11	RHSIM_Rhsim10G0019900
Root vs Leaf	RHSIM_Rhsim09G0108000	1.220726952	3.19082E-12	2.16093E-11	RHSIM_Rhsim09G0108000
Root vs Leaf	RHSIM_Rhsim06G0137000	-2.197915346	3.21106E-12	2.17399E-11	RHSIM_Rhsim06G0137000
Root vs Leaf	RHSIM_Rhsim04G0006400	-1.624545014	3.21226E-12	2.17417E-11	RHSIM_Rhsim04G0006400
Root vs Leaf	RHSIM_Rhsim13G0183800	-6.019828512	3.21944E-12	2.17839E-11	RHSIM_Rhsim13G0183800
Root vs Leaf	RHSIM_Rhsim13G0035300	-5.350141985	3.24884E-12	2.19764E-11	RHSIM_Rhsim13G0035300
Root vs Leaf	RHSIM_Rhsim13G0038800	-1.161435622	3.27533E-12	2.21491E-11	RHSIM_Rhsim13G0038800
Root vs Leaf	RHSIM_Rhsim07G0012000	-7.713436807	3.27914E-12	2.21684E-11	RHSIM_Rhsim07G0012000
Root vs Leaf	RHSIM_Rhsim01G0078300	2.532882729	3.31935E-12	2.24337E-11	RHSIM_Rhsim01G0078300
Root vs Leaf	RHSIM_Rhsim06G0111300	-8.780332842	3.32322E-12	2.24533E-11	RHSIM_Rhsim06G0111300
Root vs Leaf	RHSIM_Rhsim12G0211200	1.149874625	3.33963E-12	2.25576E-11	RHSIM_Rhsim12G0211200
Root vs Leaf	RHSIM_Rhsim03G0175600	5.826623403	3.35937E-12	2.26781E-11	RHSIM_Rhsim03G0175600
Root vs Leaf	novel.3174	-3.035225189	3.35943E-12	2.26781E-11	--
Root vs Leaf	RHSIM_Rhsim04G0246500	1.958700168	3.39384E-12	2.29037E-11	RHSIM_Rhsim04G0246500
Root vs Leaf	RHSIM_Rhsim04G0016100	-1.299883291	3.45157E-12	2.32798E-11	RHSIM_Rhsim04G0016100
Root vs Leaf	RHSIM_Rhsim08G0236200	-1.261354479	3.45419E-12	2.32906E-11	RHSIM_Rhsim08G0236200

Comparison	Gene ID	log_2FC	P-value	FDR (padj)	Gene Name
Root vs Leaf	RHSIM_Rhsim01G0169000	-1.122698619	3.50414E-12	2.36205E-11	RHSIM_Rhsim01G0169000
Root vs Leaf	RHSIM_Rhsim03G0064600	-1.567935526	3.51466E-12	2.36846E-11	RHSIM_Rhsim03G0064600
Root vs Leaf	RHSIM_Rhsim11G0173700	-2.708512091	3.51832E-12	2.37023E-11	RHSIM_Rhsim11G0173700
Root vs Leaf	RHSIM_Rhsim01G0255000	-2.510227641	3.61315E-12	2.43341E-11	RHSIM_Rhsim01G0255000
Root vs Leaf	RHSIM_Rhsim13G0114800	3.993129938	3.62416E-12	2.44011E-11	RHSIM_Rhsim13G0114800
Root vs Leaf	RHSIM_Rhsim06G0104800	-2.03659794	3.6561E-12	2.4609E-11	RHSIM_Rhsim06G0104800
Root vs Leaf	RHSIM_Rhsim05G0057500	8.022213236	3.6672E-12	2.46765E-11	RHSIM_Rhsim05G0057500
Root vs Leaf	RHSIM_Rhsim06G0152600	5.930323587	3.68585E-12	2.47948E-11	RHSIM_Rhsim06G0152600
Root vs Leaf	novel.1467	-5.365134779	3.71408E-12	2.49775E-11	--
Root vs Leaf	RHSIM_Rhsim10G0127200	1.339311043	3.72288E-12	2.50294E-11	RHSIM_Rhsim10G0127200
Root vs Leaf	RHSIM_Rhsim08G0200900	-1.858468573	3.73137E-12	2.50792E-11	RHSIM_Rhsim08G0200900
Root vs Leaf	RHSIM_Rhsim11G0187800	-1.194837019	3.78134E-12	2.54077E-11	RHSIM_Rhsim11G0187800
Root vs Leaf	RHSIM_Rhsim10G0198000	1.038640279	3.80907E-12	2.55866E-11	RHSIM_Rhsim10G0198000
Root vs Leaf	RHSIM_Rhsim06G0095100	1.837982087	3.81151E-12	2.55956E-11	RHSIM_Rhsim06G0095100
Root vs Leaf	RHSIM_Rhsim03G0102600	-9.178538506	3.82238E-12	2.56611E-11	RHSIM_Rhsim03G0102600
Root vs Leaf	RHSIM_Rhsim07G0045800	-1.658002397	3.83792E-12	2.57579E-11	RHSIM_Rhsim07G0045800
Root vs Leaf	RHSIM_Rhsim07G0088300	1.449448874	3.86448E-12	2.59287E-11	RHSIM_Rhsim07G0088300
Root vs Leaf	RHSIM_Rhsim03G0267700	1.575502322	3.87757E-12	2.6009E-11	RHSIM_Rhsim03G0267700
Root vs Leaf	RHSIM_Rhsim01G0240600	1.476202318	3.88914E-12	2.6079E-11	RHSIM_Rhsim01G0240600
Root vs Leaf	RHSIM_RhsimUnG0189800	-3.376013105	3.90511E-12	2.61785E-11	RHSIM_RhsimUnG0189800
Root vs Leaf	RHSIM_Rhsim13G0228600	-5.202286386	3.92911E-12	2.63318E-11	RHSIM_Rhsim13G0228600
Root vs Leaf	RHSIM_Rhsim03G0001400	-9.071549006	3.96321E-12	2.65449E-11	RHSIM_Rhsim03G0001400
Root vs Leaf	RHSIM_Rhsim09G0024300	7.371329759	4.00011E-12	2.67843E-11	RHSIM_Rhsim09G0024300
Root vs Leaf	RHSIM_Rhsim03G0267900	9.082342558	4.03325E-12	2.69984E-11	RHSIM_Rhsim03G0267900
Root vs Leaf	novel.1714	8.806102008	4.04483E-12	2.7068E-11	--

Comparison	Gene ID	log_2FC	P-value	FDR (padj)	Gene Name
Root vs Leaf	RHSIM_Rhsim01G0215900	1.366393123	4.08547E-12	2.73322E-11	RHSIM_Rhsim01G0215900
Root vs Leaf	novel.2888	-1.015556501	4.11644E-12	2.75314E-11	--
Root vs Leaf	RHSIM_Rhsim12G0197900	-1.686556716	4.16015E-12	2.78157E-11	RHSIM_Rhsim12G0197900
Root vs Leaf	RHSIM_Rhsim13G0041400	3.651589039	4.18763E-12	2.79913E-11	RHSIM_Rhsim13G0041400
Root vs Leaf	RHSIM_Rhsim05G0201500	8.729250963	4.22459E-12	2.82302E-11	RHSIM_Rhsim05G0201500
Root vs Leaf	RHSIM_Rhsim09G0072600	1.480967201	4.24841E-12	2.83812E-11	RHSIM_Rhsim09G0072600
Root vs Leaf	RHSIM_Rhsim06G0238600	-1.39751856	4.28325E-12	2.86057E-11	RHSIM_Rhsim06G0238600
Root vs Leaf	RHSIM_Rhsim12G0006600	5.610693854	4.28994E-12	2.86421E-11	RHSIM_Rhsim12G0006600
Root vs Leaf	RHSIM_Rhsim01G0257400	-4.931473596	4.44022E-12	2.96283E-11	RHSIM_Rhsim01G0257400
Root vs Leaf	RHSIM_Rhsim07G0032600	2.995002203	4.46328E-12	2.97736E-11	RHSIM_Rhsim07G0032600
Root vs Leaf	RHSIM_Rhsim09G0202500	3.401062194	4.66883E-12	3.11359E-11	RHSIM_Rhsim09G0202500
Root vs Leaf	RHSIM_Rhsim03G0162500	-2.013048789	4.68032E-12	3.12035E-11	RHSIM_Rhsim03G0162500
Root vs Leaf	RHSIM_Rhsim01G0224600	2.483654034	4.73437E-12	3.15547E-11	RHSIM_Rhsim01G0224600
Root vs Leaf	RHSIM_Rhsim09G0138700	-1.211462161	4.74004E-12	3.15834E-11	RHSIM_Rhsim09G0138700
Root vs Leaf	RHSIM_Rhsim03G0202800	-2.611482253	4.88289E-12	3.25259E-11	RHSIM_Rhsim03G0202800
Root vs Leaf	RHSIM_Rhsim06G0120000	-8.901230526	4.8934E-12	3.25865E-11	RHSIM_Rhsim06G0120000
Root vs Leaf	RHSIM_Rhsim09G0101100	1.092649169	4.93885E-12	3.2876E-11	RHSIM_Rhsim09G0101100
Root vs Leaf	RHSIM_Rhsim01G0064400	-9.508179974	4.93971E-12	3.2876E-11	RHSIM_Rhsim01G0064400
Root vs Leaf	RHSIM_Rhsim01G0224200	2.14733148	4.98593E-12	3.31741E-11	RHSIM_Rhsim01G0224200
Root vs Leaf	RHSIM_Rhsim01G0160800	-1.388623933	5.02071E-12	3.33958E-11	RHSIM_Rhsim01G0160800
Root vs Leaf	RHSIM_Rhsim07G0103500	8.868924911	5.02733E-12	3.34303E-11	RHSIM_Rhsim07G0103500
Root vs Leaf	RHSIM_Rhsim11G0158900	6.060920131	5.031E-12	3.34451E-11	RHSIM_Rhsim11G0158900
Root vs Leaf	RHSIM_Rhsim05G0200500	2.028430857	5.07213E-12	3.37089E-11	RHSIM_Rhsim05G0200500
Root vs Leaf	RHSIM_Rhsim03G0160000	5.563471375	5.15601E-12	3.42565E-11	RHSIM_Rhsim03G0160000
Root vs Leaf	RHSIM_Rhsim12G0014500	-4.299483755	5.18247E-12	3.44224E-11	RHSIM_Rhsim12G0014500

Comparison	Gene ID	log_2FC	P-value	FDR (padj)	Gene Name
Root vs Leaf	RHSIM_Rhsim01G0242300	1.408735613	5.24213E-12	3.48086E-11	RHSIM_Rhsim01G0242300
Root vs Leaf	novel.1016	-8.89478905	5.26429E-12	3.49457E-11	--
Root vs Leaf	RHSIM_Rhsim12G0041000	-3.46230936	5.27158E-12	3.49841E-11	RHSIM_Rhsim12G0041000
Root vs Leaf	RHSIM_Rhsim11G0148300	-4.05959369	5.28487E-12	3.50623E-11	RHSIM_Rhsim11G0148300
Root vs Leaf	RHSIM_Rhsim13G0190400	-8.746175158	5.34502E-12	3.54512E-11	RHSIM_Rhsim13G0190400
Root vs Leaf	RHSIM_Rhsim13G0094900	-3.318489206	5.35903E-12	3.55339E-11	RHSIM_Rhsim13G0094900
Root vs Leaf	RHSIM_Rhsim12G0005300	-4.179043446	5.39037E-12	3.57315E-11	RHSIM_Rhsim12G0005300
Root vs Leaf	RHSIM_Rhsim06G0111700	-10.21642296	5.43912E-12	3.60443E-11	RHSIM_Rhsim06G0111700
Root vs Leaf	RHSIM_Rhsim05G0170900	1.008822261	5.44934E-12	3.61017E-11	RHSIM_Rhsim05G0170900
Root vs Leaf	RHSIM_Rhsim12G0151800	-1.113747334	5.5625E-12	3.68198E-11	RHSIM_Rhsim12G0151800
Root vs Leaf	RHSIM_Rhsim01G0259100	1.042543862	5.62982E-12	3.72547E-11	RHSIM_Rhsim01G0259100
Root vs Leaf	RHSIM_RhsimUnG0131600	-5.354918808	5.7372E-12	3.79496E-11	RHSIM_RhsimUnG0131600
Root vs Leaf	RHSIM_Rhsim13G0197500	-2.751423325	5.73812E-12	3.79496E-11	RHSIM_Rhsim13G0197500
Root vs Leaf	RHSIM_Rhsim06G0193000	4.472481492	5.7509E-12	3.80233E-11	RHSIM_Rhsim06G0193000
Root vs Leaf	RHSIM_Rhsim05G0168900	1.052097547	5.78778E-12	3.82562E-11	RHSIM_Rhsim05G0168900
Root vs Leaf	RHSIM_Rhsim01G0272700	1.246738865	5.79746E-12	3.83093E-11	RHSIM_Rhsim01G0272700
Root vs Leaf	RHSIM_Rhsim07G0033800	5.845260242	5.82519E-12	3.84815E-11	RHSIM_Rhsim07G0033800
Root vs Leaf	RHSIM_Rhsim08G0185200	-1.440702603	5.86528E-12	3.87353E-11	RHSIM_Rhsim08G0185200
Root vs Leaf	RHSIM_Rhsim04G0204500	1.459696094	5.86924E-12	3.87504E-11	RHSIM_Rhsim04G0204500
Root vs Leaf	RHSIM_Rhsim13G0105200	5.307866187	6.02326E-12	3.9756E-11	RHSIM_Rhsim13G0105200
Root vs Leaf	RHSIM_Rhsim09G0061300	-1.384262606	6.05547E-12	3.99571E-11	RHSIM_Rhsim09G0061300
Root vs Leaf	RHSIM_Rhsim06G0218000	7.729379185	6.07534E-12	4.00768E-11	RHSIM_Rhsim06G0218000
Root vs Leaf	novel.1140	-1.744243043	6.09197E-12	4.01751E-11	--
Root vs Leaf	RHSIM_Rhsim10G0129100	-1.338009737	6.14525E-12	4.05149E-11	RHSIM_Rhsim10G0129100
Root vs Leaf	RHSIM_Rhsim02G0096600	-1.488630448	6.16969E-12	4.06644E-11	RHSIM_Rhsim02G0096600

Comparison	Gene ID	log_2FC	P-value	FDR (padj)	Gene Name
Root vs Leaf	RHSIM_Rhsim01G0192500	1.040270559	6.20489E-12	4.08848E-11	RHSIM_Rhsim01G0192500
Root vs Leaf	RHSIM_RhsimUnG0251300	1.598739517	6.25003E-12	4.11705E-11	RHSIM_RhsimUnG0251300
Root vs Leaf	RHSIM_Rhsim13G0196800	1.36843799	6.25927E-12	4.12196E-11	RHSIM_Rhsim13G0196800
Root vs Leaf	novel.4888	-1.294794138	6.31049E-12	4.15451E-11	--
Root vs Leaf	novel.4745	6.23887618	6.31765E-12	4.15804E-11	--
Root vs Leaf	RHSIM_Rhsim13G0222700	2.938924987	6.32786E-12	4.16357E-11	RHSIM_Rhsim13G0222700
Root vs Leaf	RHSIM_Rhsim04G0122900	-1.71656231	6.35715E-12	4.18166E-11	RHSIM_Rhsim04G0122900
Root vs Leaf	novel.1313	-1.716179035	6.37434E-12	4.19177E-11	--
Root vs Leaf	RHSIM_Rhsim02G0228500	1.774970945	6.39394E-12	4.20347E-11	RHSIM_Rhsim02G0228500
Root vs Leaf	RHSIM_Rhsim08G0193500	-2.475370564	6.41891E-12	4.21869E-11	RHSIM_Rhsim08G0193500
Root vs Leaf	RHSIM_Rhsim02G0052200	-1.1796815	6.45659E-12	4.24225E-11	RHSIM_Rhsim02G0052200
Root vs Leaf	RHSIM_Rhsim13G0174400	2.271448874	6.59287E-12	4.32933E-11	RHSIM_Rhsim13G0174400
Root vs Leaf	RHSIM_Rhsim08G0172100	1.537548808	6.59819E-12	4.3316E-11	RHSIM_Rhsim08G0172100
Root vs Leaf	RHSIM_Rhsim09G0195800	-1.802923955	6.98809E-12	4.58625E-11	RHSIM_Rhsim09G0195800
Root vs Leaf	RHSIM_RhsimUnG0014700	-3.684692214	7.05387E-12	4.62812E-11	RHSIM_RhsimUnG0014700
Root vs Leaf	RHSIM_Rhsim06G0166800	2.621849509	7.10363E-12	4.65944E-11	RHSIM_Rhsim06G0166800
Root vs Leaf	RHSIM_Rhsim11G0177200	1.207669927	7.15759E-12	4.69351E-11	RHSIM_Rhsim11G0177200
Root vs Leaf	RHSIM_Rhsim04G0076200	-3.463460281	7.19083E-12	4.71263E-11	RHSIM_Rhsim04G0076200
Root vs Leaf	novel.5345	-5.214455567	7.21995E-12	4.73037E-11	--
Root vs Leaf	RHSIM_Rhsim10G0121300	-2.032791776	7.23551E-12	4.73896E-11	RHSIM_Rhsim10G0121300
Root vs Leaf	novel.1081	8.405087336	7.23715E-12	4.73896E-11	--
Root vs Leaf	RHSIM_Rhsim03G0230400	1.896326083	7.23943E-12	4.73911E-11	RHSIM_Rhsim03G0230400
Root vs Leaf	RHSIM_Rhsim10G0188500	-1.889181418	7.24165E-12	4.73922E-11	RHSIM_Rhsim10G0188500
Root vs Leaf	RHSIM_Rhsim06G0235300	6.223885288	7.3722E-12	4.8233E-11	RHSIM_Rhsim06G0235300
Root vs Leaf	RHSIM_Rhsim07G0067000	3.766915423	7.40141E-12	4.84104E-11	RHSIM_Rhsim07G0067000

Comparison	Gene ID	log_2FC	P-value	FDR (padj)	Gene Name
Root vs Leaf	RHSIM_Rhsim10G0017100	4.824289878	7.41027E-12	4.84547E-11	RHSIM_Rhsim10G0017100
Root vs Leaf	RHSIM_Rhsim13G0228400	-1.00926819	7.43064E-12	4.85742E-11	RHSIM_Rhsim13G0228400
Root vs Leaf	novel.4634	-8.63599181	7.44263E-12	4.86388E-11	--
Root vs Leaf	RHSIM_Rhsim07G0098000	-8.653290563	7.46101E-12	4.87451E-11	RHSIM_Rhsim07G0098000
Root vs Leaf	RHSIM_Rhsim10G0148600	-2.363166143	7.47423E-12	4.88177E-11	RHSIM_Rhsim10G0148600
Root vs Leaf	RHSIM_Rhsim09G0200700	1.912700079	7.49791E-12	4.89585E-11	RHSIM_Rhsim09G0200700
Root vs Leaf	RHSIM_Rhsim11G0087000	4.94013193	7.51471E-12	4.90544E-11	RHSIM_Rhsim11G0087000
Root vs Leaf	novel.3075	-1.694208269	7.53835E-12	4.91948E-11	--
Root vs Leaf	RHSIM_Rhsim08G0096600	1.15914007	7.6034E-12	4.96054E-11	RHSIM_Rhsim08G0096600
Root vs Leaf	RHSIM_Rhsim01G0012900	-8.660719917	7.62328E-12	4.97211E-11	RHSIM_Rhsim01G0012900
Root vs Leaf	RHSIM_Rhsim06G0231500	1.724362799	7.66708E-12	4.99927E-11	RHSIM_Rhsim06G0231500
Root vs Leaf	novel.5493	-8.909210227	7.82339E-12	5.09975E-11	--
Root vs Leaf	RHSIM_Rhsim01G0187700	-7.454196715	7.83057E-12	5.10299E-11	RHSIM_Rhsim01G0187700
Root vs Leaf	RHSIM_Rhsim02G0079000	-7.684313117	7.84669E-12	5.11206E-11	RHSIM_Rhsim02G0079000
Root vs Leaf	novel.1566	-1.127638115	7.89808E-12	5.14409E-11	--
Root vs Leaf	RHSIM_Rhsim03G0189100	-8.999080685	7.92291E-12	5.15881E-11	RHSIM_Rhsim03G0189100
Root vs Leaf	RHSIM_RhsimUnG0169900	-3.647645335	7.98786E-12	5.19964E-11	RHSIM_RhsimUnG0169900
Root vs Leaf	RHSIM_Rhsim13G0065600	-1.014426606	8.10413E-12	5.27384E-11	RHSIM_Rhsim13G0065600
Root vs Leaf	RHSIM_Rhsim01G0113200	-1.550444872	8.1189E-12	5.28197E-11	RHSIM_Rhsim01G0113200
Root vs Leaf	RHSIM_Rhsim09G0037000	-1.447015587	8.14378E-12	5.29666E-11	RHSIM_Rhsim09G0037000
Root vs Leaf	RHSIM_Rhsim08G0064400	1.986072937	8.15429E-12	5.30201E-11	RHSIM_Rhsim08G0064400
Root vs Leaf	RHSIM_Rhsim13G0090000	-2.640574209	8.15794E-12	5.30289E-11	RHSIM_Rhsim13G0090000
Root vs Leaf	RHSIM_Rhsim06G0205700	-1.590815847	8.24534E-12	5.3582E-11	RHSIM_Rhsim06G0205700
Root vs Leaf	RHSIM_Rhsim05G0177800	-3.925109902	8.35539E-12	5.42819E-11	RHSIM_Rhsim05G0177800
Root vs Leaf	RHSIM_Rhsim09G0105000	-3.376992635	8.37521E-12	5.43954E-11	RHSIM_Rhsim09G0105000

Comparison	Gene ID	log_2FC	P-value	FDR (padj)	Gene Name
Root vs Leaf	RHSIM_Rhsim07G0019000	1.459129112	8.46301E-12	5.49348E-11	RHSIM_Rhsim07G0019000
Root vs Leaf	RHSIM_Rhsim12G0039900	-2.455340518	8.49348E-12	5.51171E-11	RHSIM_Rhsim12G0039900
Root vs Leaf	RHSIM_Rhsim01G0249100	2.617348029	8.50515E-12	5.51773E-11	RHSIM_Rhsim01G0249100
Root vs Leaf	novel.5295	2.62078042	8.58297E-12	5.56666E-11	--
Root vs Leaf	RHSIM_Rhsim08G0031600	2.687894375	8.60818E-12	5.58145E-11	RHSIM_Rhsim08G0031600
Root vs Leaf	RHSIM_Rhsim07G0091600	-1.784073631	8.61649E-12	5.58527E-11	RHSIM_Rhsim07G0091600
Root vs Leaf	RHSIM_Rhsim09G0065900	-2.385160025	8.86544E-12	5.74503E-11	RHSIM_Rhsim09G0065900
Root vs Leaf	RHSIM_Rhsim01G0241000	6.632281515	9.05134E-12	5.86386E-11	RHSIM_Rhsim01G0241000
Root vs Leaf	RHSIM_RhsimUnG0009200	-2.350608133	9.43326E-12	6.10958E-11	RHSIM_RhsimUnG0009200
Root vs Leaf	RHSIM_Rhsim08G0155700	-8.613018739	9.44479E-12	6.11533E-11	RHSIM_Rhsim08G0155700
Root vs Leaf	RHSIM_Rhsim12G0128500	5.228401429	9.46173E-12	6.12458E-11	RHSIM_Rhsim12G0128500
Root vs Leaf	RHSIM_Rhsim03G0094900	-6.021709115	9.46593E-12	6.12559E-11	RHSIM_Rhsim03G0094900
Root vs Leaf	RHSIM_Rhsim01G0271700	1.011200156	9.61303E-12	6.21904E-11	RHSIM_Rhsim01G0271700
Root vs Leaf	RHSIM_Rhsim01G0246400	1.739233058	9.75651E-12	6.30876E-11	RHSIM_Rhsim01G0246400
Root vs Leaf	RHSIM_Rhsim08G0221600	6.505577053	9.75717E-12	6.30876E-11	RHSIM_Rhsim08G0221600
Root vs Leaf	RHSIM_Rhsim08G0095200	-2.008094001	9.82456E-12	6.35056E-11	RHSIM_Rhsim08G0095200
Root vs Leaf	RHSIM_Rhsim02G0084500	3.217422778	9.83052E-12	6.35264E-11	RHSIM_Rhsim02G0084500
Root vs Leaf	RHSIM_Rhsim04G0096600	-1.17920145	9.92681E-12	6.41308E-11	RHSIM_Rhsim04G0096600
Root vs Leaf	RHSIM_Rhsim02G0012500	1.045514596	1.0039E-11	6.48373E-11	RHSIM_Rhsim02G0012500
Root vs Leaf	RHSIM_Rhsim12G0177000	-8.223675201	1.00507E-11	6.48948E-11	RHSIM_Rhsim12G0177000
Root vs Leaf	RHSIM_Rhsim07G0154000	1.796915903	1.01053E-11	6.52293E-11	RHSIM_Rhsim07G0154000
Root vs Leaf	RHSIM_Rhsim05G0002500	1.48912421	1.0159E-11	6.55576E-11	RHSIM_Rhsim05G0002500
Root vs Leaf	RHSIM_Rhsim12G0128400	2.122348626	1.01743E-11	6.56377E-11	RHSIM_Rhsim12G0128400
Root vs Leaf	RHSIM_Rhsim08G0214400	1.100740703	1.02145E-11	6.58788E-11	RHSIM_Rhsim08G0214400
Root vs Leaf	RHSIM_Rhsim13G0220900	1.11291987	1.02909E-11	6.63524E-11	RHSIM_Rhsim13G0220900

Comparison	Gene ID	log_2FC	P-value	FDR (padj)	Gene Name
Root vs Leaf	RHSIM_Rhsim10G0195800	1.349441023	1.02936E-11	6.63524E-11	RHSIM_Rhsim10G0195800
Root vs Leaf	RHSIM_RhsimUnG0079400	-9.667520125	1.04309E-11	6.72182E-11	RHSIM_RhsimUnG0079400
Root vs Leaf	RHSIM_Rhsim12G0202400	-9.759230381	1.04839E-11	6.75413E-11	RHSIM_Rhsim12G0202400
Root vs Leaf	RHSIM_Rhsim12G0072300	1.633812725	1.07357E-11	6.91441E-11	RHSIM_Rhsim12G0072300
Root vs Leaf	RHSIM_Rhsim09G0132800	3.851414464	1.07763E-11	6.93752E-11	RHSIM_Rhsim09G0132800
Root vs Leaf	RHSIM_Rhsim12G0023400	1.378585004	1.07856E-11	6.94075E-11	RHSIM_Rhsim12G0023400
Root vs Leaf	RHSIM_Rhsim08G0123000	5.149103364	1.08207E-11	6.96141E-11	RHSIM_Rhsim08G0123000
Root vs Leaf	novel.5976	-4.565218373	1.09047E-11	7.0135E-11	--
Root vs Leaf	RHSIM_Rhsim03G0057500	3.404407827	1.10648E-11	7.11448E-11	RHSIM_Rhsim03G0057500
Root vs Leaf	RHSIM_Rhsim10G0118800	-3.372732638	1.10817E-11	7.12341E-11	RHSIM_Rhsim10G0118800
Root vs Leaf	RHSIM_Rhsim04G0188600	3.815908029	1.11853E-11	7.18798E-11	RHSIM_Rhsim04G0188600
Root vs Leaf	RHSIM_Rhsim09G0114500	-3.896004012	1.13732E-11	7.30667E-11	RHSIM_Rhsim09G0114500
Root vs Leaf	RHSIM_Rhsim04G0169400	-3.369212313	1.1435E-11	7.34439E-11	RHSIM_Rhsim04G0169400
Root vs Leaf	RHSIM_Rhsim12G0169700	9.991539112	1.14477E-11	7.35051E-11	RHSIM_Rhsim12G0169700
Root vs Leaf	novel.2929	-5.45434468	1.14965E-11	7.3798E-11	--
Root vs Leaf	RHSIM_Rhsim08G0217400	1.960652858	1.15851E-11	7.4346E-11	RHSIM_Rhsim08G0217400
Root vs Leaf	RHSIM_Rhsim05G0135500	1.345545898	1.16738E-11	7.48942E-11	RHSIM_Rhsim05G0135500
Root vs Leaf	RHSIM_Rhsim10G0210600	-2.588076923	1.17696E-11	7.54878E-11	RHSIM_Rhsim10G0210600
Root vs Leaf	RHSIM_Rhsim09G0003200	-1.642442517	1.18329E-11	7.5873E-11	RHSIM_Rhsim09G0003200
Root vs Leaf	RHSIM_Rhsim11G0095800	-4.287516171	1.18763E-11	7.61298E-11	RHSIM_Rhsim11G0095800
Root vs Leaf	RHSIM_Rhsim06G0152700	8.807624196	1.18844E-11	7.61606E-11	RHSIM_Rhsim06G0152700
Root vs Leaf	RHSIM_RhsimUnG0191900	-3.371081808	1.20288E-11	7.70647E-11	RHSIM_RhsimUnG0191900
Root vs Leaf	RHSIM_RhsimUnG0218100	-5.123316905	1.21093E-11	7.75592E-11	RHSIM_RhsimUnG0218100
Root vs Leaf	RHSIM_Rhsim03G0216500	9.629448958	1.21388E-11	7.77265E-11	RHSIM_Rhsim03G0216500
Root vs Leaf	RHSIM_RhsimUnG0225800	-1.8068166	1.22156E-11	7.81967E-11	RHSIM_RhsimUnG0225800

Comparison	Gene ID	log_2FC	P-value	FDR (padj)	Gene Name
Root vs Leaf	RHSIM_Rhsim08G0185600	3.709135326	1.23031E-11	7.87351E-11	RHSIM_Rhsim08G0185600
Root vs Leaf	RHSIM_Rhsim07G0245700	1.178109087	1.23295E-11	7.88824E-11	RHSIM_Rhsim07G0245700
Root vs Leaf	RHSIM_Rhsim10G0073300	-4.60692845	1.2442E-11	7.95798E-11	RHSIM_Rhsim10G0073300
Root vs Leaf	RHSIM_Rhsim02G0228100	5.626375564	1.24463E-11	7.95856E-11	RHSIM_Rhsim02G0228100
Root vs Leaf	RHSIM_Rhsim13G0199700	1.077135752	1.24653E-11	7.96849E-11	RHSIM_Rhsim13G0199700
Root vs Leaf	RHSIM_Rhsim03G0162400	-1.718186199	1.25163E-11	7.99886E-11	RHSIM_Rhsim03G0162400
Root vs Leaf	RHSIM_Rhsim09G0021400	-5.432800859	1.27218E-11	8.12794E-11	RHSIM_Rhsim09G0021400
Root vs Leaf	RHSIM_Rhsim05G0231500	-3.390010245	1.27563E-11	8.14766E-11	RHSIM_Rhsim05G0231500
Root vs Leaf	RHSIM_Rhsim06G0012000	4.576288834	1.27775E-11	8.15679E-11	RHSIM_Rhsim06G0012000
Root vs Leaf	RHSIM_Rhsim13G0151900	2.368285716	1.29514E-11	8.26556E-11	RHSIM_Rhsim13G0151900
Root vs Leaf	RHSIM_Rhsim04G0096700	-1.284008948	1.30307E-11	8.31385E-11	RHSIM_Rhsim04G0096700
Root vs Leaf	novel.2966	4.380146385	1.33242E-11	8.4988E-11	--
Root vs Leaf	RHSIM_Rhsim13G0179300	1.170943999	1.36773E-11	8.72157E-11	RHSIM_Rhsim13G0179300
Root vs Leaf	RHSIM_Rhsim07G0204900	-1.790876775	1.39318E-11	8.88141E-11	RHSIM_Rhsim07G0204900
Root vs Leaf	novel.3845	-4.491869913	1.3999E-11	8.92184E-11	--
Root vs Leaf	RHSIM_Rhsim10G0113900	2.140335461	1.43044E-11	9.11396E-11	RHSIM_Rhsim10G0113900
Root vs Leaf	RHSIM_Rhsim08G0212900	2.249346103	1.43198E-11	9.12123E-11	RHSIM_Rhsim08G0212900
Root vs Leaf	RHSIM_Rhsim07G0165700	1.238613225	1.43755E-11	9.15422E-11	RHSIM_Rhsim07G0165700
Root vs Leaf	RHSIM_Rhsim10G0096200	8.676894858	1.4583E-11	9.28375E-11	RHSIM_Rhsim10G0096200
Root vs Leaf	RHSIM_Rhsim03G0118900	5.690245951	1.46959E-11	9.35307E-11	RHSIM_Rhsim03G0118900
Root vs Leaf	RHSIM_Rhsim13G0061200	-2.205864781	1.48446E-11	9.44511E-11	RHSIM_Rhsim13G0061200
Root vs Leaf	RHSIM_Rhsim03G0068000	1.776496063	1.49593E-11	9.51547E-11	RHSIM_Rhsim03G0068000
Root vs Leaf	RHSIM_Rhsim13G0181300	-5.095787123	1.50106E-11	9.54287E-11	RHSIM_Rhsim13G0181300
Root vs Leaf	RHSIM_Rhsim04G0074100	-3.215069507	1.51013E-11	9.59792E-11	RHSIM_Rhsim04G0074100
Root vs Leaf	RHSIM_Rhsim06G0212700	3.525337702	1.53377E-11	9.74547E-11	RHSIM_Rhsim06G0212700

Comparison	Gene ID	log_2FC	P-value	FDR (padj)	Gene Name
Root vs Leaf	RHSIM_Rhsim13G0096900	7.319165174	1.53589E-11	9.75625E-11	RHSIM_Rhsim13G0096900
Root vs Leaf	novel.672	-9.648859845	1.53659E-11	9.75805E-11	--
Root vs Leaf	RHSIM_Rhsim06G0001000	2.710898136	1.55439E-11	9.86837E-11	RHSIM_Rhsim06G0001000
Root vs Leaf	novel.703	-8.782920658	1.56092E-11	9.9071E-11	--
Root vs Leaf	RHSIM_Rhsim04G0222400	-1.541761033	1.56955E-11	9.95914E-11	RHSIM_Rhsim04G0222400
Root vs Leaf	RHSIM_Rhsim09G0084400	-1.409639048	1.5906E-11	1.00899E-10	RHSIM_Rhsim09G0084400
Root vs Leaf	RHSIM_Rhsim08G0167000	-2.739605689	1.60061E-11	1.01507E-10	RHSIM_Rhsim08G0167000
Root vs Leaf	RHSIM_Rhsim08G0007200	-3.471788308	1.60962E-11	1.0205E-10	RHSIM_Rhsim08G0007200
Root vs Leaf	novel.5731	-2.107909312	1.61474E-11	1.02347E-10	--
Root vs Leaf	RHSIM_Rhsim01G0095300	-2.233727791	1.62888E-11	1.03214E-10	RHSIM_Rhsim01G0095300
Root vs Leaf	RHSIM_Rhsim04G0116000	2.401211683	1.64437E-11	1.04168E-10	RHSIM_Rhsim04G0116000
Root vs Leaf	RHSIM_Rhsim06G0060000	1.778334553	1.66528E-11	1.05463E-10	RHSIM_Rhsim06G0060000
Root vs Leaf	RHSIM_Rhsim01G0034600	2.949935903	1.67914E-11	1.06312E-10	RHSIM_Rhsim01G0034600
Root vs Leaf	RHSIM_Rhsim02G0112300	1.836088255	1.68247E-11	1.06493E-10	RHSIM_Rhsim02G0112300
Root vs Leaf	novel.479	1.455124398	1.68379E-11	1.06548E-10	--
Root vs Leaf	RHSIM_Rhsim12G0149800	1.577490004	1.70221E-11	1.07684E-10	RHSIM_Rhsim12G0149800
Root vs Leaf	RHSIM_Rhsim07G0256800	-1.132662403	1.71627E-11	1.08544E-10	RHSIM_Rhsim07G0256800
Root vs Leaf	RHSIM_Rhsim06G0160500	-3.845470073	1.72124E-11	1.08829E-10	RHSIM_Rhsim06G0160500
Root vs Leaf	RHSIM_Rhsim03G0180300	-1.333688884	1.77307E-11	1.12075E-10	RHSIM_Rhsim03G0180300
Root vs Leaf	RHSIM_Rhsim02G0071400	-3.735959846	1.81077E-11	1.14395E-10	RHSIM_Rhsim02G0071400
Root vs Leaf	RHSIM_Rhsim02G0084300	1.603930352	1.82328E-11	1.15154E-10	RHSIM_Rhsim02G0084300
Root vs Leaf	RHSIM_Rhsim07G0219700	2.649527991	1.84166E-11	1.16283E-10	RHSIM_Rhsim07G0219700
Root vs Leaf	RHSIM_Rhsim13G0162100	3.05784686	1.84551E-11	1.16495E-10	RHSIM_Rhsim13G0162100
Root vs Leaf	RHSIM_Rhsim09G0199500	-1.690727093	1.8466E-11	1.16532E-10	RHSIM_Rhsim09G0199500
Root vs Leaf	RHSIM_Rhsim02G0021500	-1.010863423	1.86682E-11	1.17776E-10	RHSIM_Rhsim02G0021500

Comparison	Gene ID	log$_2$FC	P-value	FDR (padj)	Gene Name
Root vs Leaf	RHSIM_Rhsim07G0058500	-1.88780416	1.89977E-11	1.19822E-10	RHSIM_Rhsim07G0058500
Root vs Leaf	RHSIM_Rhsim08G0060900	-9.140027609	1.91574E-11	1.20796E-10	RHSIM_Rhsim08G0060900
Root vs Leaf	RHSIM_Rhsim09G0024200	8.705473279	1.93239E-11	1.21779E-10	RHSIM_Rhsim09G0024200
Root vs Leaf	novel.3808	-3.100052869	1.93651E-11	1.22006E-10	--
Root vs Leaf	RHSIM_Rhsim10G0197900	1.335070057	1.95569E-11	1.23181E-10	RHSIM_Rhsim10G0197900
Root vs Leaf	RHSIM_Rhsim10G0138000	-1.196011187	1.96369E-11	1.23651E-10	RHSIM_Rhsim10G0138000
Root vs Leaf	RHSIM_Rhsim07G0107500	-1.928246307	1.97003E-11	1.24016E-10	RHSIM_Rhsim07G0107500
Root vs Leaf	RHSIM_Rhsim08G0156800	-5.073898898	2.00509E-11	1.2617E-10	RHSIM_Rhsim08G0156800
Root vs Leaf	RHSIM_Rhsim02G0062000	1.514851672	2.00533E-11	1.2617E-10	RHSIM_Rhsim02G0062000
Root vs Leaf	novel.1517	5.492351865	2.00946E-11	1.26396E-10	--
Root vs Leaf	RHSIM_Rhsim13G0135000	5.593095858	2.01062E-11	1.26434E-10	RHSIM_Rhsim13G0135000
Root vs Leaf	RHSIM_Rhsim05G0044200	2.161231447	2.01502E-11	1.26676E-10	RHSIM_Rhsim05G0044200
Root vs Leaf	RHSIM_Rhsim03G0142300	-1.280040629	2.02211E-11	1.27088E-10	RHSIM_Rhsim03G0142300
Root vs Leaf	novel.4912	-8.73969678	2.02738E-11	1.27384E-10	--
Root vs Leaf	RHSIM_Rhsim05G0221800	-8.593545157	2.02961E-11	1.2749E-10	RHSIM_Rhsim05G0221800
Root vs Leaf	RHSIM_Rhsim08G0198700	-3.317473484	2.03025E-11	1.27495E-10	RHSIM_Rhsim08G0198700
Root vs Leaf	RHSIM_Rhsim04G0202300	-1.601457391	2.0538E-11	1.28939E-10	RHSIM_Rhsim04G0202300
Root vs Leaf	RHSIM_Rhsim13G0001700	-5.357640446	2.08001E-11	1.30549E-10	RHSIM_Rhsim13G0001700
Root vs Leaf	RHSIM_Rhsim01G0287000	-1.225553995	2.10319E-11	1.31969E-10	RHSIM_Rhsim01G0287000
Root vs Leaf	RHSIM_Rhsim06G0046600	-1.064163958	2.11313E-11	1.32556E-10	RHSIM_Rhsim06G0046600
Root vs Leaf	RHSIM_Rhsim03G0054400	-4.766984411	2.1251E-11	1.33271E-10	RHSIM_Rhsim03G0054400
Root vs Leaf	RHSIM_Rhsim01G0250300	5.150984965	2.1665E-11	1.35793E-10	RHSIM_Rhsim01G0250300
Root vs Leaf	RHSIM_Rhsim02G0227200	3.750386618	2.17502E-11	1.36291E-10	RHSIM_Rhsim02G0227200
Root vs Leaf	novel.2265	-9.420619662	2.17703E-11	1.36379E-10	--
Root vs Leaf	RHSIM_Rhsim09G0013600	-1.01281262	2.18645E-11	1.36933E-10	RHSIM_Rhsim09G0013600

Comparison	Gene ID	log_2FC	P-value	FDR (padj)	Gene Name
Root vs Leaf	RHSIM_Rhsim02G0168600	9.687784502	2.20096E-11	1.37804E-10	RHSIM_Rhsim02G0168600
Root vs Leaf	RHSIM_Rhsim12G0086300	1.350701884	2.21084E-11	1.38385E-10	RHSIM_Rhsim12G0086300
Root vs Leaf	novel.6094	-2.597102133	2.22149E-11	1.39014E-10	--
Root vs Leaf	novel.3702	1.261720423	2.23673E-11	1.3993E-10	--
Root vs Leaf	RHSIM_RhsimUnG0208500	-1.954874625	2.2545E-11	1.41004E-10	RHSIM_RhsimUnG0208500
Root vs Leaf	RHSIM_Rhsim11G0117600	-8.53518959	2.2679E-11	1.41774E-10	RHSIM_Rhsim11G0117600
Root vs Leaf	RHSIM_Rhsim02G0167900	-1.299518429	2.26804E-11	1.41774E-10	RHSIM_Rhsim02G0167900
Root vs Leaf	RHSIM_Rhsim12G0044100	2.112148894	2.2786E-11	1.42396E-10	RHSIM_Rhsim12G0044100
Root vs Leaf	RHSIM_Rhsim04G0074400	-3.143248013	2.29087E-11	1.43124E-10	RHSIM_Rhsim04G0074400
Root vs Leaf	RHSIM_Rhsim04G0084900	-1.029081745	2.30504E-11	1.4397E-10	RHSIM_Rhsim04G0084900
Root vs Leaf	RHSIM_Rhsim02G0231900	2.373015493	2.3078E-11	1.44104E-10	RHSIM_Rhsim02G0231900
Root vs Leaf	RHSIM_Rhsim08G0162000	1.067679867	2.33642E-11	1.45851E-10	RHSIM_Rhsim08G0162000
Root vs Leaf	RHSIM_Rhsim09G0051600	-1.460927064	2.37746E-11	1.48328E-10	RHSIM_Rhsim09G0051600
Root vs Leaf	RHSIM_Rhsim02G0066700	2.026174629	2.37802E-11	1.48328E-10	RHSIM_Rhsim02G0066700
Root vs Leaf	RHSIM_Rhsim12G0105500	1.34164631	2.39926E-11	1.49613E-10	RHSIM_Rhsim12G0105500
Root vs Leaf	RHSIM_Rhsim07G0203100	-1.165065177	2.42452E-11	1.51147E-10	RHSIM_Rhsim07G0203100
Root vs Leaf	RHSIM_Rhsim05G0198100	4.997493503	2.43828E-11	1.51964E-10	RHSIM_Rhsim05G0198100
Root vs Leaf	RHSIM_RhsimUnG0081200	-2.682589633	2.46677E-11	1.53698E-10	RHSIM_RhsimUnG0081200
Root vs Leaf	RHSIM_Rhsim06G0119400	-8.575877458	2.49893E-11	1.55576E-10	RHSIM_Rhsim06G0119400
Root vs Leaf	RHSIM_Rhsim10G0142200	-4.672921652	2.49978E-11	1.55587E-10	RHSIM_Rhsim10G0142200
Root vs Leaf	novel.1508	-9.295334143	2.53955E-11	1.5802E-10	--
Root vs Leaf	novel.2375	-1.690911315	2.58134E-11	1.60534E-10	--
Root vs Leaf	RHSIM_Rhsim03G0172100	3.183960533	2.59907E-11	1.61594E-10	RHSIM_Rhsim03G0172100
Root vs Leaf	RHSIM_Rhsim07G0178700	1.636515554	2.61616E-11	1.62612E-10	RHSIM_Rhsim07G0178700
Root vs Leaf	RHSIM_Rhsim01G0249900	-2.299626104	2.6344E-11	1.63702E-10	RHSIM_Rhsim01G0249900

Comparison	Gene ID	log_2FC	P-value	FDR (padj)	Gene Name
Root vs Leaf	RHSIM_Rhsim13G0113600	5.734010884	2.63616E-11	1.63737E-10	RHSIM_Rhsim13G0113600
Root vs Leaf	RHSIM_Rhsim04G0129700	1.255192582	2.63638E-11	1.63737E-10	RHSIM_Rhsim04G0129700
Root vs Leaf	RHSIM_Rhsim06G0196600	3.751454992	2.64846E-11	1.64443E-10	RHSIM_Rhsim06G0196600
Root vs Leaf	RHSIM_Rhsim06G0175500	4.759378636	2.65144E-11	1.64584E-10	RHSIM_Rhsim06G0175500
Root vs Leaf	RHSIM_Rhsim12G0187000	10.07037901	2.7022E-11	1.67645E-10	RHSIM_Rhsim12G0187000
Root vs Leaf	RHSIM_Rhsim03G0082900	6.818775302	2.74281E-11	1.70028E-10	RHSIM_Rhsim03G0082900
Root vs Leaf	RHSIM_Rhsim03G0089300	2.19943331	2.75974E-11	1.71031E-10	RHSIM_Rhsim03G0089300
Root vs Leaf	RHSIM_RhsimUnG0249700	2.836899994	2.76795E-11	1.71494E-10	RHSIM_RhsimUnG0249700
Root vs Leaf	RHSIM_Rhsim07G0129800	1.628448151	2.77061E-11	1.71613E-10	RHSIM_Rhsim07G0129800
Root vs Leaf	RHSIM_Rhsim12G0205600	5.122155556	2.78838E-11	1.72668E-10	RHSIM_Rhsim12G0205600
Root vs Leaf	RHSIM_Rhsim08G0101400	3.243108293	2.82905E-11	1.75092E-10	RHSIM_Rhsim08G0101400
Root vs Leaf	RHSIM_Rhsim07G0201100	2.931586121	2.84025E-11	1.75739E-10	RHSIM_Rhsim07G0201100
Root vs Leaf	RHSIM_Rhsim13G0150700	-1.591616723	2.84264E-11	1.7584E-10	RHSIM_Rhsim13G0150700
Root vs Leaf	novel.5210	3.037779675	2.84688E-11	1.76055E-10	--
Root vs Leaf	RHSIM_Rhsim03G0078000	-2.397432399	2.8704E-11	1.77462E-10	RHSIM_Rhsim03G0078000
Root vs Leaf	RHSIM_Rhsim01G0154500	1.789998797	2.88233E-11	1.78151E-10	RHSIM_Rhsim01G0154500
Root vs Leaf	RHSIM_Rhsim06G0016600	1.886699965	2.88849E-11	1.78485E-10	RHSIM_Rhsim06G0016600
Root vs Leaf	RHSIM_Rhsim03G0127700	3.182516264	2.89208E-11	1.78625E-10	RHSIM_Rhsim03G0127700
Root vs Leaf	novel.524	-8.644558256	2.8923E-11	1.78625E-10	--
Root vs Leaf	RHSIM_Rhsim13G0083100	2.063660857	2.91201E-11	1.79794E-10	RHSIM_Rhsim13G0083100
Root vs Leaf	novel.2521	-8.916377382	2.91345E-11	1.79835E-10	--
Root vs Leaf	RHSIM_Rhsim05G0058500	-8.72722302	2.9164E-11	1.79969E-10	RHSIM_Rhsim05G0058500
Root vs Leaf	novel.125	-7.358624009	2.93551E-11	1.811E-10	--
Root vs Leaf	RHSIM_Rhsim10G0155800	4.579895066	2.94864E-11	1.81862E-10	RHSIM_Rhsim10G0155800
Root vs Leaf	RHSIM_Rhsim13G0083300	1.824388901	2.95017E-11	1.81907E-10	RHSIM_Rhsim13G0083300

Comparison	Gene ID	log_2FC	P-value	FDR (padj)	Gene Name
Root vs Leaf	RHSIM_Rhsim05G0099000	6.442421734	3.00378E-11	1.85164E-10	RHSIM_Rhsim05G0099000
Root vs Leaf	RHSIM_Rhsim12G0140500	4.124682577	3.02723E-11	1.8656E-10	RHSIM_Rhsim12G0140500
Root vs Leaf	RHSIM_Rhsim11G0069300	3.257443933	3.03915E-11	1.87244E-10	RHSIM_Rhsim11G0069300
Root vs Leaf	RHSIM_Rhsim03G0054100	-7.344256334	3.05505E-11	1.88174E-10	RHSIM_Rhsim03G0054100
Root vs Leaf	RHSIM_Rhsim02G0178300	-1.917391089	3.06487E-11	1.88728E-10	RHSIM_Rhsim02G0178300
Root vs Leaf	RHSIM_Rhsim09G0128400	-8.534771001	3.07357E-11	1.89214E-10	RHSIM_Rhsim09G0128400
Root vs Leaf	RHSIM_Rhsim08G0061000	-9.761561088	3.08589E-11	1.89922E-10	RHSIM_Rhsim08G0061000
Root vs Leaf	RHSIM_Rhsim12G0114600	-2.126366245	3.10564E-11	1.91086E-10	RHSIM_Rhsim12G0114600
Root vs Leaf	RHSIM_Rhsim05G0165200	1.83823823	3.13392E-11	1.92775E-10	RHSIM_Rhsim05G0165200
Root vs Leaf	RHSIM_Rhsim12G0054200	7.382139598	3.16106E-11	1.94393E-10	RHSIM_Rhsim12G0054200
Root vs Leaf	RHSIM_Rhsim06G0234200	9.807082116	3.18404E-11	1.95754E-10	RHSIM_Rhsim06G0234200
Root vs Leaf	RHSIM_Rhsim01G0174800	2.500990555	3.18719E-11	1.95896E-10	RHSIM_Rhsim01G0174800
Root vs Leaf	RHSIM_Rhsim09G0093000	-8.988727785	3.21222E-11	1.97382E-10	RHSIM_Rhsim09G0093000
Root vs Leaf	novel.2610	2.80927312	3.23564E-11	1.98768E-10	--
Root vs Leaf	RHSIM_Rhsim07G0059900	-8.795187589	3.24029E-11	1.99001E-10	RHSIM_Rhsim07G0059900
Root vs Leaf	RHSIM_Rhsim13G0000700	1.125374623	3.2585E-11	2.00066E-10	RHSIM_Rhsim13G0000700
Root vs Leaf	RHSIM_RhsimUnG0241500	-8.659307419	3.28508E-11	2.01645E-10	RHSIM_RhsimUnG0241500
Root vs Leaf	RHSIM_Rhsim13G0034400	4.262082502	3.30073E-11	2.02552E-10	RHSIM_Rhsim13G0034400
Root vs Leaf	RHSIM_RhsimUnG0009500	-1.819438397	3.3195E-11	2.03649E-10	RHSIM_RhsimUnG0009500
Root vs Leaf	RHSIM_Rhsim09G0135100	-5.549381643	3.33646E-11	2.04629E-10	RHSIM_Rhsim09G0135100
Root vs Leaf	RHSIM_Rhsim02G0003800	-1.191606972	3.33723E-11	2.04629E-10	RHSIM_Rhsim02G0003800
Root vs Leaf	novel.4487	-8.437130419	3.37473E-11	2.06873E-10	--
Root vs Leaf	RHSIM_Rhsim05G0218900	4.516335947	3.4113E-11	2.09059E-10	RHSIM_Rhsim05G0218900
Root vs Leaf	RHSIM_Rhsim09G0061400	-1.44797014	3.44827E-11	2.11269E-10	RHSIM_Rhsim09G0061400
Root vs Leaf	RHSIM_Rhsim06G0004200	-1.518265977	3.47388E-11	2.12782E-10	RHSIM_Rhsim06G0004200

Comparison	Gene ID	log_2FC	P-value	FDR (padj)	Gene Name
Root vs Leaf	RHSIM_Rhsim06G0237700	1.127616727	3.48807E-11	2.13595E-10	RHSIM_Rhsim06G0237700
Root vs Leaf	RHSIM_RhsimUnG0258300	1.331639901	3.49918E-11	2.14218E-10	RHSIM_RhsimUnG0258300
Root vs Leaf	RHSIM_Rhsim04G0164000	3.190365994	3.55006E-11	2.17275E-10	RHSIM_Rhsim04G0164000
Root vs Leaf	RHSIM_Rhsim12G0195100	-1.604364704	3.58321E-11	2.19247E-10	RHSIM_Rhsim12G0195100
Root vs Leaf	RHSIM_Rhsim08G0069900	-1.438541203	3.60216E-11	2.20348E-10	RHSIM_Rhsim08G0069900
Root vs Leaf	RHSIM_Rhsim09G0052000	1.414064773	3.63143E-11	2.2208E-10	RHSIM_Rhsim09G0052000
Root vs Leaf	RHSIM_Rhsim12G0130500	-1.193208254	3.65572E-11	2.23506E-10	RHSIM_Rhsim12G0130500
Root vs Leaf	RHSIM_Rhsim12G0020300	2.794197741	3.65937E-11	2.2367E-10	RHSIM_Rhsim12G0020300
Root vs Leaf	RHSIM_Rhsim04G0147400	2.892870051	3.66292E-11	2.23828E-10	RHSIM_Rhsim04G0147400
Root vs Leaf	RHSIM_Rhsim06G0163000	4.5352008	3.69447E-11	2.25696E-10	RHSIM_Rhsim06G0163000
Root vs Leaf	RHSIM_Rhsim08G0159700	-3.312740342	3.72882E-11	2.27735E-10	RHSIM_Rhsim08G0159700
Root vs Leaf	RHSIM_Rhsim07G0001200	1.450723729	3.73359E-11	2.27966E-10	RHSIM_Rhsim07G0001200
Root vs Leaf	RHSIM_Rhsim11G0179200	-1.5744756	3.75354E-11	2.29124E-10	RHSIM_Rhsim11G0179200
Root vs Leaf	novel.2612	-2.756839589	3.76066E-11	2.29498E-10	--
Root vs Leaf	RHSIM_Rhsim01G0100100	-5.351462753	3.76969E-11	2.29988E-10	RHSIM_Rhsim01G0100100
Root vs Leaf	RHSIM_Rhsim11G0012400	-8.680743086	3.84647E-11	2.34549E-10	RHSIM_Rhsim11G0012400
Root vs Leaf	RHSIM_Rhsim10G0186400	1.570576972	3.90004E-11	2.37753E-10	RHSIM_Rhsim10G0186400
Root vs Leaf	RHSIM_Rhsim01G0208800	-1.365874595	3.90779E-11	2.38162E-10	RHSIM_Rhsim01G0208800
Root vs Leaf	RHSIM_Rhsim13G0096300	3.509492864	3.93227E-11	2.39528E-10	RHSIM_Rhsim13G0096300
Root vs Leaf	novel.5499	-4.581158774	3.95905E-11	2.41095E-10	--
Root vs Leaf	novel.3414	9.230399386	3.96765E-11	2.41556E-10	--
Root vs Leaf	RHSIM_Rhsim13G0027700	-1.117932691	3.97032E-11	2.41655E-10	RHSIM_Rhsim13G0027700
Root vs Leaf	RHSIM_Rhsim07G0121700	-2.634116245	3.99529E-11	2.43111E-10	RHSIM_Rhsim07G0121700
Root vs Leaf	RHSIM_Rhsim10G0186500	-1.544656275	4.0185E-11	2.44459E-10	RHSIM_Rhsim10G0186500
Root vs Leaf	RHSIM_Rhsim03G0075300	1.693505047	4.01981E-11	2.44474E-10	RHSIM_Rhsim03G0075300

Comparison	Gene ID	log_2FC	P-value	FDR (padj)	Gene Name
Root vs Leaf	RHSIM_Rhsim12G0110000	1.857187704	4.02796E-11	2.44905E-10	RHSIM_Rhsim12G0110000
Root vs Leaf	RHSIM_Rhsim05G0029700	2.859933631	4.0971E-11	2.49044E-10	RHSIM_Rhsim05G0029700
Root vs Leaf	RHSIM_Rhsim08G0248700	1.410340495	4.10685E-11	2.49571E-10	RHSIM_Rhsim08G0248700
Root vs Leaf	RHSIM_Rhsim13G0235500	1.610608963	4.13828E-11	2.51415E-10	RHSIM_Rhsim13G0235500
Root vs Leaf	RHSIM_Rhsim08G0099100	-1.776483992	4.16617E-11	2.53043E-10	RHSIM_Rhsim08G0099100
Root vs Leaf	RHSIM_RhsimUnG0005700	-1.41754667	4.18846E-11	2.5433E-10	RHSIM_RhsimUnG0005700
Root vs Leaf	RHSIM_Rhsim09G0168800	1.57699484	4.21055E-11	2.55604E-10	RHSIM_Rhsim09G0168800
Root vs Leaf	RHSIM_Rhsim10G0117500	-2.375782669	4.24881E-11	2.57859E-10	RHSIM_Rhsim10G0117500
Root vs Leaf	RHSIM_Rhsim11G0002100	1.330150745	4.28973E-11	2.60206E-10	RHSIM_Rhsim11G0002100
Root vs Leaf	RHSIM_Rhsim02G0070900	2.671710386	4.34281E-11	2.63357E-10	RHSIM_Rhsim02G0070900
Root vs Leaf	RHSIM_Rhsim05G0070600	-1.268277234	4.41627E-11	2.67741E-10	RHSIM_Rhsim05G0070600
Root vs Leaf	RHSIM_Rhsim03G0018100	-1.260561463	4.45324E-11	2.69841E-10	RHSIM_Rhsim03G0018100
Root vs Leaf	RHSIM_Rhsim06G0066800	-2.081816274	4.47984E-11	2.71382E-10	RHSIM_Rhsim06G0066800
Root vs Leaf	RHSIM_Rhsim04G0173200	-8.651587779	4.48909E-11	2.71872E-10	RHSIM_Rhsim04G0173200
Root vs Leaf	RHSIM_Rhsim13G0205000	1.2358229	4.49404E-11	2.721E-10	RHSIM_Rhsim13G0205000
Root vs Leaf	novel.3926	-1.551054823	4.51849E-11	2.73508E-10	--
Root vs Leaf	RHSIM_Rhsim01G0245300	1.676256983	4.54137E-11	2.74822E-10	RHSIM_Rhsim01G0245300
Root vs Leaf	RHSIM_Rhsim03G0073200	3.904275169	4.58053E-11	2.77119E-10	RHSIM_Rhsim03G0073200
Root vs Leaf	novel.3519	5.410129322	4.61861E-11	2.7935E-10	--
Root vs Leaf	RHSIM_Rhsim08G0069300	2.188678298	4.63827E-11	2.80466E-10	RHSIM_Rhsim08G0069300
Root vs Leaf	RHSIM_Rhsim11G0126300	7.083608439	4.68281E-11	2.83085E-10	RHSIM_Rhsim11G0126300
Root vs Leaf	RHSIM_Rhsim04G0020100	2.218978397	4.72498E-11	2.85559E-10	RHSIM_Rhsim04G0020100
Root vs Leaf	RHSIM_Rhsim01G0041100	-8.572272584	4.75284E-11	2.87168E-10	RHSIM_Rhsim01G0041100
Root vs Leaf	RHSIM_Rhsim13G0206000	-1.131204753	4.75715E-11	2.87353E-10	RHSIM_Rhsim13G0206000
Root vs Leaf	RHSIM_Rhsim04G0060400	-3.398354328	4.84986E-11	2.92877E-10	RHSIM_Rhsim04G0060400

Comparison	Gene ID	log_2FC	P-value	FDR (padj)	Gene Name
Root vs Leaf	RHSIM_Rhsim04G0096300	8.734480412	4.91984E-11	2.96908E-10	RHSIM_Rhsim04G0096300
Root vs Leaf	RHSIM_RhsimUnG0236100	8.033182347	4.92046E-11	2.96908E-10	RHSIM_RhsimUnG0236100
Root vs Leaf	RHSIM_Rhsim09G0142900	-1.23911913	4.92854E-11	2.97318E-10	RHSIM_Rhsim09G0142900
Root vs Leaf	RHSIM_RhsimUnG0015300	-8.739966294	4.98151E-11	3.00435E-10	RHSIM_RhsimUnG0015300
Root vs Leaf	RHSIM_Rhsim01G0202500	-1.276164718	5.02944E-11	3.03247E-10	RHSIM_Rhsim01G0202500
Root vs Leaf	RHSIM_Rhsim08G0159300	-3.450800015	5.07711E-11	3.06042E-10	RHSIM_Rhsim08G0159300
Root vs Leaf	RHSIM_Rhsim03G0235700	-1.948954233	5.14249E-11	3.09821E-10	RHSIM_Rhsim03G0235700
Root vs Leaf	RHSIM_Rhsim09G0174100	-8.737201262	5.16054E-11	3.10828E-10	RHSIM_Rhsim09G0174100
Root vs Leaf	novel.421	4.293732368	5.18063E-11	3.11907E-10	--
Root vs Leaf	RHSIM_Rhsim01G0201900	-3.003794396	5.18244E-11	3.11907E-10	RHSIM_Rhsim01G0201900
Root vs Leaf	RHSIM_Rhsim10G0012500	-2.166234509	5.1825E-11	3.11907E-10	RHSIM_Rhsim10G0012500
Root vs Leaf	RHSIM_Rhsim03G0174300	-6.154890676	5.18617E-11	3.12047E-10	RHSIM_Rhsim03G0174300
Root vs Leaf	RHSIM_Rhsim05G0076600	9.778071801	5.19284E-11	3.12367E-10	RHSIM_Rhsim05G0076600
Root vs Leaf	RHSIM_Rhsim03G0215500	-5.349228919	5.20587E-11	3.13069E-10	RHSIM_Rhsim03G0215500
Root vs Leaf	RHSIM_Rhsim09G0208900	2.533958421	5.26012E-11	3.1625E-10	RHSIM_Rhsim09G0208900
Root vs Leaf	RHSIM_Rhsim12G0148700	-2.170144786	5.40673E-11	3.24895E-10	RHSIM_Rhsim12G0148700
Root vs Leaf	RHSIM_RhsimUnG0223500	-8.411812723	5.40893E-11	3.24943E-10	RHSIM_RhsimUnG0223500
Root vs Leaf	RHSIM_Rhsim09G0097600	-1.877099907	5.41191E-11	3.25037E-10	RHSIM_Rhsim09G0097600
Root vs Leaf	RHSIM_Rhsim09G0055300	-1.44095575	5.49181E-11	3.29751E-10	RHSIM_Rhsim09G0055300
Root vs Leaf	RHSIM_Rhsim11G0125800	4.529485746	5.54264E-11	3.32717E-10	RHSIM_Rhsim11G0125800
Root vs Leaf	RHSIM_Rhsim01G0156100	1.229456183	5.55246E-11	3.33219E-10	RHSIM_Rhsim01G0156100
Root vs Leaf	RHSIM_Rhsim07G0240300	2.40229812	5.57914E-11	3.34734E-10	RHSIM_Rhsim07G0240300
Root vs Leaf	RHSIM_Rhsim07G0206200	9.036659903	5.59975E-11	3.35884E-10	RHSIM_Rhsim07G0206200
Root vs Leaf	RHSIM_Rhsim04G0119900	1.004996157	5.62308E-11	3.37195E-10	RHSIM_Rhsim04G0119900
Root vs Leaf	RHSIM_Rhsim11G0026900	-1.224449225	5.70824E-11	3.42213E-10	RHSIM_Rhsim11G0026900

Comparison	Gene ID	log$_2$FC	P-value	FDR (padj)	Gene Name
Root vs Leaf	RHSIM_Rhsim07G0136300	9.249460507	5.74803E-11	3.4451E-10	RHSIM_Rhsim07G0136300
Root vs Leaf	RHSIM_Rhsim02G0058600	7.271594104	5.79442E-11	3.472E-10	RHSIM_Rhsim02G0058600
Root vs Leaf	RHSIM_Rhsim12G0191600	-3.182845831	5.79966E-11	3.47424E-10	RHSIM_Rhsim12G0191600
Root vs Leaf	RHSIM_Rhsim06G0188000	8.069906039	5.87824E-11	3.52041E-10	RHSIM_Rhsim06G0188000
Root vs Leaf	RHSIM_Rhsim13G0076300	-1.427541162	5.9285E-11	3.54959E-10	RHSIM_Rhsim13G0076300
Root vs Leaf	RHSIM_Rhsim11G0019700	1.368681233	5.94333E-11	3.55754E-10	RHSIM_Rhsim11G0019700
Root vs Leaf	RHSIM_Rhsim13G0190700	-7.27196648	5.99636E-11	3.58835E-10	RHSIM_Rhsim13G0190700
Root vs Leaf	RHSIM_Rhsim02G0042600	-3.496420889	6.01028E-11	3.59576E-10	RHSIM_Rhsim02G0042600
Root vs Leaf	RHSIM_Rhsim02G0116300	-6.005036771	6.06625E-11	3.6283E-10	RHSIM_Rhsim02G0116300
Root vs Leaf	RHSIM_Rhsim03G0142200	2.35938441	6.07288E-11	3.63133E-10	RHSIM_Rhsim03G0142200
Root vs Leaf	RHSIM_Rhsim09G0063000	-3.69949139	6.09062E-11	3.641E-10	RHSIM_Rhsim09G0063000
Root vs Leaf	RHSIM_Rhsim08G0118700	-1.467277083	6.12538E-11	3.66083E-10	RHSIM_Rhsim08G0118700
Root vs Leaf	RHSIM_Rhsim09G0024500	-1.05941076	6.14679E-11	3.67268E-10	RHSIM_Rhsim09G0024500
Root vs Leaf	RHSIM_Rhsim12G0124300	-1.553979064	6.15379E-11	3.67591E-10	RHSIM_Rhsim12G0124300
Root vs Leaf	RHSIM_Rhsim13G0053800	-1.358342891	6.20367E-11	3.70475E-10	RHSIM_Rhsim13G0053800
Root vs Leaf	RHSIM_Rhsim06G0067800	-1.520092407	6.22563E-11	3.71691E-10	RHSIM_Rhsim06G0067800
Root vs Leaf	RHSIM_Rhsim13G0107600	2.342804648	6.23531E-11	3.72172E-10	RHSIM_Rhsim13G0107600
Root vs Leaf	RHSIM_Rhsim13G0178200	-1.325915401	6.27404E-11	3.74388E-10	RHSIM_Rhsim13G0178200
Root vs Leaf	RHSIM_Rhsim10G0203600	-5.770370869	6.30295E-11	3.7596E-10	RHSIM_Rhsim10G0203600
Root vs Leaf	RHSIM_RhsimUnG0183200	-4.17875657	6.30363E-11	3.7596E-10	RHSIM_RhsimUnG0183200
Root vs Leaf	RHSIM_Rhsim13G0091200	-8.598097055	6.3355E-11	3.77689E-10	RHSIM_Rhsim13G0091200
Root vs Leaf	RHSIM_Rhsim07G0108400	3.480120532	6.33589E-11	3.77689E-10	RHSIM_Rhsim07G0108400
Root vs Leaf	RHSIM_Rhsim04G0194700	-2.344193954	6.34922E-11	3.78386E-10	RHSIM_Rhsim04G0194700
Root vs Leaf	RHSIM_Rhsim04G0226600	-1.252717473	6.3847E-11	3.80403E-10	RHSIM_Rhsim04G0226600
Root vs Leaf	RHSIM_Rhsim07G0091900	4.742599784	6.41412E-11	3.82057E-10	RHSIM_Rhsim07G0091900

Comparison	Gene ID	log_2FC	P-value	FDR (padj)	Gene Name
Root vs Leaf	RHSIM_Rhsim10G0109500	3.22730223	6.42389E-11	3.8254E-10	RHSIM_Rhsim10G0109500
Root vs Leaf	RHSIM_RhsimUnG0131400	-2.779328399	6.4474E-11	3.83842E-10	RHSIM_RhsimUnG0131400
Root vs Leaf	RHSIM_Rhsim05G0095400	-1.209278235	6.47234E-11	3.85128E-10	RHSIM_Rhsim05G0095400
Root vs Leaf	RHSIM_Rhsim06G0050600	-1.916309967	6.55887E-11	3.90177E-10	RHSIM_Rhsim06G0050600
Root vs Leaf	RHSIM_Rhsim07G0057900	3.521286258	6.56608E-11	3.90505E-10	RHSIM_Rhsim07G0057900
Root vs Leaf	RHSIM_Rhsim02G0196800	-8.550689505	6.63092E-11	3.9426E-10	RHSIM_Rhsim02G0196800
Root vs Leaf	RHSIM_Rhsim12G0169800	-1.166842205	6.8005E-11	4.04239E-10	RHSIM_Rhsim12G0169800
Root vs Leaf	RHSIM_Rhsim06G0002300	-2.96830479	6.82569E-11	4.05632E-10	RHSIM_Rhsim06G0002300
Root vs Leaf	RHSIM_Rhsim13G0039800	2.099535733	6.86971E-11	4.08144E-10	RHSIM_Rhsim13G0039800
Root vs Leaf	RHSIM_Rhsim13G0199400	1.026019028	6.88059E-11	4.08685E-10	RHSIM_Rhsim13G0199400
Root vs Leaf	RHSIM_Rhsim12G0038300	1.003949158	6.9002E-11	4.09745E-10	RHSIM_Rhsim12G0038300
Root vs Leaf	RHSIM_Rhsim08G0132300	8.547334298	6.90197E-11	4.09745E-10	RHSIM_Rhsim08G0132300
Root vs Leaf	RHSIM_Rhsim05G0119100	-1.831390285	6.93831E-11	4.11797E-10	RHSIM_Rhsim05G0119100
Root vs Leaf	RHSIM_Rhsim09G0213800	-1.110735738	7.06797E-11	4.19277E-10	RHSIM_Rhsim09G0213800
Root vs Leaf	RHSIM_Rhsim03G0250900	1.527235587	7.07854E-11	4.19796E-10	RHSIM_Rhsim03G0250900
Root vs Leaf	RHSIM_Rhsim13G0015800	1.603320993	7.08759E-11	4.20225E-10	RHSIM_Rhsim13G0015800
Root vs Leaf	RHSIM_Rhsim13G0029800	2.866670821	7.24022E-11	4.29165E-10	RHSIM_Rhsim13G0029800
Root vs Leaf	RHSIM_Rhsim04G0011800	3.6948265	7.41615E-11	4.3948E-10	RHSIM_Rhsim04G0011800
Root vs Leaf	novel.5589	-4.455913291	7.44182E-11	4.40889E-10	--
Root vs Leaf	RHSIM_Rhsim12G0164000	1.181586686	7.46212E-11	4.41978E-10	RHSIM_Rhsim12G0164000
Root vs Leaf	RHSIM_Rhsim11G0093900	-4.131805022	7.47543E-11	4.42653E-10	RHSIM_Rhsim11G0093900
Root vs Leaf	RHSIM_Rhsim02G0182200	-1.584248463	7.49144E-11	4.43488E-10	RHSIM_Rhsim02G0182200
Root vs Leaf	RHSIM_Rhsim08G0192900	8.194060708	7.53039E-11	4.4568E-10	RHSIM_Rhsim08G0192900
Root vs Leaf	RHSIM_Rhsim06G0219100	1.195414698	7.57661E-11	4.483E-10	RHSIM_Rhsim06G0219100
Root vs Leaf	RHSIM_Rhsim01G0278200	5.415585901	7.66219E-11	4.53249E-10	RHSIM_Rhsim01G0278200

Comparison	Gene ID	log_2FC	P-value	FDR (padj)	Gene Name
Root vs Leaf	RHSIM_Rhsim05G0133100	-1.156262932	7.68327E-11	4.54379E-10	RHSIM_Rhsim05G0133100
Root vs Leaf	RHSIM_Rhsim12G0114300	-9.38354922	7.69736E-11	4.55096E-10	RHSIM_Rhsim12G0114300
Root vs Leaf	RHSIM_Rhsim07G0129200	-1.109687358	8.00506E-11	4.73117E-10	RHSIM_Rhsim07G0129200
Root vs Leaf	RHSIM_Rhsim06G0085200	-3.944307107	8.00625E-11	4.73117E-10	RHSIM_Rhsim06G0085200
Root vs Leaf	RHSIM_Rhsim02G0003700	4.013860973	8.02106E-11	4.73871E-10	RHSIM_Rhsim02G0003700
Root vs Leaf	RHSIM_Rhsim07G0038100	-5.623013563	8.12101E-11	4.79654E-10	RHSIM_Rhsim07G0038100
Root vs Leaf	RHSIM_Rhsim06G0169700	-8.485014238	8.15683E-11	4.81647E-10	RHSIM_Rhsim06G0169700
Root vs Leaf	RHSIM_Rhsim09G0208700	2.714723252	8.17593E-11	4.82651E-10	RHSIM_Rhsim09G0208700
Root vs Leaf	RHSIM_Rhsim01G0231200	5.621992461	8.20122E-11	4.8402E-10	RHSIM_Rhsim01G0231200
Root vs Leaf	novel.88	-2.660704085	8.20578E-11	4.84166E-10	--
Root vs Leaf	RHSIM_Rhsim09G0082500	3.901346763	8.25629E-11	4.87022E-10	RHSIM_Rhsim09G0082500
Root vs Leaf	RHSIM_Rhsim13G0055900	5.74919801	8.26043E-11	4.87142E-10	RHSIM_Rhsim13G0055900
Root vs Leaf	RHSIM_Rhsim13G0145600	9.300827225	8.3914E-11	4.9474E-10	RHSIM_Rhsim13G0145600
Root vs Leaf	RHSIM_Rhsim01G0209300	1.114436987	8.51404E-11	5.01843E-10	RHSIM_Rhsim01G0209300
Root vs Leaf	RHSIM_Rhsim09G0210100	2.538809213	8.56263E-11	5.04578E-10	RHSIM_Rhsim09G0210100
Root vs Leaf	novel.5074	-8.89225131	8.57188E-11	5.04994E-10	--
Root vs Leaf	RHSIM_Rhsim13G0052600	-1.396005083	8.58199E-11	5.05461E-10	RHSIM_Rhsim13G0052600
Root vs Leaf	RHSIM_Rhsim04G0074500	-3.608432276	8.60594E-11	5.06728E-10	RHSIM_Rhsim04G0074500
Root vs Leaf	RHSIM_Rhsim05G0204200	-3.029371957	8.60788E-11	5.06728E-10	RHSIM_Rhsim05G0204200
Root vs Leaf	RHSIM_Rhsim08G0184600	9.976861908	8.79731E-11	5.17748E-10	RHSIM_Rhsim08G0184600
Root vs Leaf	RHSIM_Rhsim10G0141400	1.665957452	8.87901E-11	5.22424E-10	RHSIM_Rhsim10G0141400
Root vs Leaf	RHSIM_Rhsim11G0109600	8.790581902	9.01099E-11	5.30054E-10	RHSIM_Rhsim11G0109600
Root vs Leaf	RHSIM_Rhsim03G0158200	-8.766850556	9.02869E-11	5.30961E-10	RHSIM_Rhsim03G0158200
Root vs Leaf	RHSIM_Rhsim04G0018300	-1.054936521	9.04153E-11	5.31581E-10	RHSIM_Rhsim04G0018300
Root vs Leaf	RHSIM_Rhsim13G0183900	-8.04685177	9.06944E-11	5.33086E-10	RHSIM_Rhsim13G0183900

Comparison	Gene ID	log_2FC	P-value	FDR (padj)	Gene Name
Root vs Leaf	RHSIM_Rhsim05G0181000	1.424969282	9.07671E-11	5.33378E-10	RHSIM_Rhsim05G0181000
Root vs Leaf	RHSIM_RhsimUnG0094100	-3.417575399	9.14183E-11	5.37068E-10	RHSIM_RhsimUnG0094100
Root vs Leaf	RHSIM_Rhsim01G0131200	2.181664099	9.22938E-11	5.42074E-10	RHSIM_Rhsim01G0131200
Root vs Leaf	RHSIM_Rhsim05G0091800	-3.061701007	9.25106E-11	5.43209E-10	RHSIM_Rhsim05G0091800
Root vs Leaf	RHSIM_Rhsim07G0257900	4.730658445	9.27885E-11	5.44703E-10	RHSIM_Rhsim07G0257900
Root vs Leaf	RHSIM_Rhsim03G0036400	3.337088539	9.28393E-11	5.44863E-10	RHSIM_Rhsim03G0036400
Root vs Leaf	RHSIM_Rhsim07G0143200	-1.037869711	9.38156E-11	5.50453E-10	RHSIM_Rhsim07G0143200
Root vs Leaf	RHSIM_Rhsim07G0162300	-1.441554978	9.51242E-11	5.5799E-10	RHSIM_Rhsim07G0162300
Root vs Leaf	RHSIM_Rhsim07G0187500	-1.516570814	9.63855E-11	5.65245E-10	RHSIM_Rhsim07G0187500
Root vs Leaf	RHSIM_Rhsim04G0021600	-1.799979467	9.67294E-11	5.66975E-10	RHSIM_Rhsim04G0021600
Root vs Leaf	RHSIM_RhsimUnG0194700	-2.219354423	9.67853E-11	5.67158E-10	RHSIM_RhsimUnG0194700
Root vs Leaf	RHSIM_Rhsim13G0138000	-1.547900681	9.79492E-11	5.73824E-10	RHSIM_Rhsim13G0138000
Root vs Leaf	RHSIM_Rhsim09G0153700	3.044025503	9.79724E-11	5.73824E-10	RHSIM_Rhsim09G0153700
Root vs Leaf	RHSIM_Rhsim04G0191800	-8.454863875	9.91534E-11	5.80448E-10	RHSIM_Rhsim04G0191800
Root vs Leaf	RHSIM_Rhsim08G0036100	2.977965161	1.00003E-10	5.85271E-10	RHSIM_Rhsim08G0036100
Root vs Leaf	RHSIM_Rhsim13G0189700	1.882831665	1.00727E-10	5.89359E-10	RHSIM_Rhsim13G0189700
Root vs Leaf	RHSIM_RhsimUnG0070700	2.410060642	1.03276E-10	6.04126E-10	RHSIM_RhsimUnG0070700
Root vs Leaf	RHSIM_Rhsim08G0036900	-1.133987419	1.03945E-10	6.07885E-10	RHSIM_Rhsim08G0036900
Root vs Leaf	RHSIM_Rhsim06G0006000	5.268469755	1.04854E-10	6.13041E-10	RHSIM_Rhsim06G0006000
Root vs Leaf	RHSIM_Rhsim02G0066200	-7.349695537	1.04929E-10	6.13327E-10	RHSIM_Rhsim02G0066200
Root vs Leaf	novel.1114	-2.890533442	1.05999E-10	6.19422E-10	--
Root vs Leaf	RHSIM_Rhsim01G0002700	-2.052359317	1.06408E-10	6.21658E-10	RHSIM_Rhsim01G0002700
Root vs Leaf	RHSIM_Rhsim04G0094100	-8.484878912	1.07897E-10	6.30196E-10	RHSIM_Rhsim04G0094100
Root vs Leaf	novel.5627	9.682642606	1.0843E-10	6.33152E-10	--
Root vs Leaf	novel.353	-2.026841228	1.08911E-10	6.35797E-10	--

Comparison	Gene ID	log_2FC	P-value	FDR (padj)	Gene Name
Root vs Leaf	RHSIM_Rhsim13G0159400	7.774223777	1.09332E-10	6.38098E-10	RHSIM_Rhsim13G0159400
Root vs Leaf	RHSIM_Rhsim09G0199000	-1.515948156	1.10383E-10	6.44067E-10	RHSIM_Rhsim09G0199000
Root vs Leaf	novel.1534	-3.160984604	1.10772E-10	6.46173E-10	--
Root vs Leaf	RHSIM_RhsimUnG0099700	6.690786701	1.12106E-10	6.53628E-10	RHSIM_RhsimUnG0099700
Root vs Leaf	RHSIM_Rhsim13G0222500	8.866388424	1.12229E-10	6.54181E-10	RHSIM_Rhsim13G0222500
Root vs Leaf	RHSIM_Rhsim08G0247800	1.170285909	1.13599E-10	6.61829E-10	RHSIM_Rhsim08G0247800
Root vs Leaf	RHSIM_Rhsim09G0205100	2.955369378	1.15365E-10	6.71783E-10	RHSIM_Rhsim09G0205100
Root vs Leaf	RHSIM_Rhsim10G0115500	1.366020411	1.1564E-10	6.73213E-10	RHSIM_Rhsim10G0115500
Root vs Leaf	RHSIM_Rhsim01G0138100	8.368264751	1.16731E-10	6.79396E-10	RHSIM_Rhsim01G0138100
Root vs Leaf	RHSIM_Rhsim02G0000600	8.230643944	1.17983E-10	6.86507E-10	RHSIM_Rhsim02G0000600
Root vs Leaf	novel.2369	3.700026608	1.19674E-10	6.96174E-10	--
Root vs Leaf	RHSIM_Rhsim05G0214600	-1.131958631	1.22407E-10	7.11715E-10	RHSIM_Rhsim05G0214600
Root vs Leaf	RHSIM_Rhsim11G0017900	-3.766808136	1.2378E-10	7.19337E-10	RHSIM_Rhsim11G0017900
Root vs Leaf	RHSIM_Rhsim02G0066600	4.097659673	1.24767E-10	7.24707E-10	RHSIM_Rhsim02G0066600
Root vs Leaf	RHSIM_Rhsim12G0085500	-1.324099178	1.24912E-10	7.25187E-10	RHSIM_Rhsim12G0085500
Root vs Leaf	RHSIM_RhsimUnG0208700	-2.093522263	1.2614E-10	7.32132E-10	RHSIM_RhsimUnG0208700
Root vs Leaf	RHSIM_Rhsim03G0098700	1.574473961	1.26709E-10	7.3525E-10	RHSIM_Rhsim03G0098700
Root vs Leaf	RHSIM_Rhsim10G0128200	-2.457308625	1.28542E-10	7.457E-10	RHSIM_Rhsim10G0128200
Root vs Leaf	RHSIM_Rhsim08G0210600	1.300962226	1.30129E-10	7.54714E-10	RHSIM_Rhsim08G0210600
Root vs Leaf	RHSIM_Rhsim04G0049800	2.424060181	1.30426E-10	7.56249E-10	RHSIM_Rhsim04G0049800
Root vs Leaf	RHSIM_Rhsim12G0177200	8.364137139	1.31729E-10	7.63612E-10	RHSIM_Rhsim12G0177200
Root vs Leaf	RHSIM_Rhsim08G0233300	1.349156635	1.32892E-10	7.70074E-10	RHSIM_Rhsim08G0233300
Root vs Leaf	RHSIM_Rhsim06G0094000	-1.462269823	1.3291E-10	7.70074E-10	RHSIM_Rhsim06G0094000
Root vs Leaf	RHSIM_Rhsim12G0063700	1.246870313	1.34777E-10	7.80693E-10	RHSIM_Rhsim12G0063700
Root vs Leaf	RHSIM_Rhsim05G0007300	8.669456932	1.35027E-10	7.81948E-10	RHSIM_Rhsim05G0007300

Comparison	Gene ID	log_2FC	P-value	FDR (padj)	Gene Name
Root vs Leaf	RHSIM_Rhsim03G0180700	-1.281271978	1.3556E-10	7.84838E-10	RHSIM_Rhsim03G0180700
Root vs Leaf	RHSIM_Rhsim11G0052800	-1.863703765	1.36097E-10	7.8775E-10	RHSIM_Rhsim11G0052800
Root vs Leaf	RHSIM_Rhsim03G0090900	6.006934112	1.38892E-10	8.03727E-10	RHSIM_Rhsim03G0090900
Root vs Leaf	RHSIM_Rhsim05G0087000	-1.264845092	1.40106E-10	8.10546E-10	RHSIM_Rhsim05G0087000
Root vs Leaf	RHSIM_Rhsim08G0041600	-1.285275854	1.40956E-10	8.15259E-10	RHSIM_Rhsim08G0041600
Root vs Leaf	RHSIM_Rhsim04G0079300	-3.41469488	1.41645E-10	8.19044E-10	RHSIM_Rhsim04G0079300
Root vs Leaf	RHSIM_Rhsim11G0145900	-3.264403053	1.44293E-10	8.34147E-10	RHSIM_Rhsim11G0145900
Root vs Leaf	RHSIM_Rhsim13G0037500	1.084003683	1.44732E-10	8.36474E-10	RHSIM_Rhsim13G0037500
Root vs Leaf	novel.54	-2.080523434	1.44994E-10	8.37777E-10	--
Root vs Leaf	RHSIM_Rhsim05G0105400	1.565278984	1.46003E-10	8.434E-10	RHSIM_Rhsim05G0105400
Root vs Leaf	RHSIM_Rhsim11G0160500	-2.219848076	1.46123E-10	8.43881E-10	RHSIM_Rhsim11G0160500
Root vs Leaf	RHSIM_Rhsim04G0147000	1.696290431	1.48176E-10	8.551E-10	RHSIM_Rhsim04G0147000
Root vs Leaf	RHSIM_Rhsim06G0098000	3.579150935	1.4838E-10	8.56062E-10	RHSIM_Rhsim06G0098000
Root vs Leaf	RHSIM_Rhsim03G0253400	1.044016197	1.49939E-10	8.64843E-10	RHSIM_Rhsim03G0253400
Root vs Leaf	RHSIM_Rhsim01G0262700	-1.160299655	1.5047E-10	8.67689E-10	RHSIM_Rhsim01G0262700
Root vs Leaf	RHSIM_Rhsim13G0058600	3.058630259	1.53387E-10	8.84286E-10	RHSIM_Rhsim13G0058600
Root vs Leaf	RHSIM_RhsimUnG0148600	-1.394887965	1.53446E-10	8.84339E-10	RHSIM_RhsimUnG0148600
Root vs Leaf	RHSIM_Rhsim08G0017700	3.15210945	1.53472E-10	8.84339E-10	RHSIM_Rhsim08G0017700
Root vs Leaf	RHSIM_Rhsim07G0213200	2.624441706	1.56274E-10	9.00032E-10	RHSIM_Rhsim07G0213200
Root vs Leaf	RHSIM_Rhsim03G0216900	7.353342528	1.56517E-10	9.01209E-10	RHSIM_Rhsim03G0216900
Root vs Leaf	novel.79	-7.42554371	1.57949E-10	9.09E-10	--
Root vs Leaf	RHSIM_Rhsim07G0075200	4.027200456	1.5819E-10	9.10161E-10	RHSIM_Rhsim07G0075200
Root vs Leaf	RHSIM_Rhsim08G0083900	4.226850386	1.61155E-10	9.26992E-10	RHSIM_Rhsim08G0083900
Root vs Leaf	RHSIM_Rhsim03G0060400	-4.815398017	1.62174E-10	9.32388E-10	RHSIM_Rhsim03G0060400
Root vs Leaf	RHSIM_Rhsim08G0161100	1.446233089	1.63172E-10	9.37893E-10	RHSIM_Rhsim08G0161100

Comparison	Gene ID	log_2FC	P-value	FDR (padj)	Gene Name
Root vs Leaf	RHSIM_RhsimUnG0209400	-3.873208906	1.6414E-10	9.43226E-10	RHSIM_RhsimUnG0209400
Root vs Leaf	RHSIM_Rhsim08G0182900	-1.648559671	1.65401E-10	9.50234E-10	RHSIM_Rhsim08G0182900
Root vs Leaf	RHSIM_RhsimUnG0043200	-2.51278003	1.65609E-10	9.51193E-10	RHSIM_RhsimUnG0043200
Root vs Leaf	RHSIM_Rhsim04G0021200	-2.950798348	1.67259E-10	9.60432E-10	RHSIM_Rhsim04G0021200
Root vs Leaf	novel.815	1.578795329	1.681E-10	9.6502E-10	--
Root vs Leaf	RHSIM_Rhsim01G0012000	1.080464852	1.68727E-10	9.6838E-10	RHSIM_Rhsim01G0012000
Root vs Leaf	RHSIM_Rhsim06G0216000	5.217334765	1.70807E-10	9.80073E-10	RHSIM_Rhsim06G0216000
Root vs Leaf	RHSIM_Rhsim02G0127000	1.114575831	1.70872E-10	9.80203E-10	RHSIM_Rhsim02G0127000
Root vs Leaf	RHSIM_Rhsim08G0107900	-5.3895058	1.72891E-10	9.9154E-10	RHSIM_Rhsim08G0107900
Root vs Leaf	RHSIM_Rhsim09G0051700	1.022250659	1.73038E-10	9.92141E-10	RHSIM_Rhsim09G0051700
Root vs Leaf	RHSIM_Rhsim03G0049100	1.365604504	1.73298E-10	9.93367E-10	RHSIM_Rhsim03G0049100
Root vs Leaf	RHSIM_Rhsim08G0176400	1.681771253	1.73358E-10	9.93367E-10	RHSIM_Rhsim08G0176400
Root vs Leaf	RHSIM_Rhsim07G0043200	-1.373903174	1.73926E-10	9.96244E-10	RHSIM_Rhsim07G0043200
Root vs Leaf	RHSIM_Rhsim11G0187700	-1.734373214	1.76884E-10	1.01293E-09	RHSIM_Rhsim11G0187700
Root vs Leaf	RHSIM_Rhsim02G0218200	4.080943332	1.7725E-10	1.01478E-09	RHSIM_Rhsim02G0218200
Root vs Leaf	RHSIM_Rhsim04G0249600	-1.196300515	1.77333E-10	1.015E-09	RHSIM_Rhsim04G0249600
Root vs Leaf	RHSIM_Rhsim06G0003300	2.407701984	1.7771E-10	1.01691E-09	RHSIM_Rhsim06G0003300
Root vs Leaf	RHSIM_Rhsim13G0093800	1.108738898	1.78297E-10	1.02002E-09	RHSIM_Rhsim13G0093800
Root vs Leaf	RHSIM_Rhsim09G0132900	4.576862604	1.79137E-10	1.02457E-09	RHSIM_Rhsim09G0132900
Root vs Leaf	RHSIM_Rhsim01G0068500	1.291077548	1.79928E-10	1.02884E-09	RHSIM_Rhsim01G0068500
Root vs Leaf	RHSIM_Rhsim04G0202400	-2.443521925	1.80874E-10	1.03399E-09	RHSIM_Rhsim04G0202400
Root vs Leaf	RHSIM_Rhsim04G0118500	-2.278115545	1.83213E-10	1.04711E-09	RHSIM_Rhsim04G0118500
Root vs Leaf	novel.2611	2.91527118	1.86028E-10	1.06293E-09	--
Root vs Leaf	RHSIM_RhsimUnG0085800	2.936423827	1.90883E-10	1.0904E-09	RHSIM_RhsimUnG0085800
Root vs Leaf	RHSIM_Rhsim03G0056200	7.929207712	1.91583E-10	1.09413E-09	RHSIM_Rhsim03G0056200

Comparison	Gene ID	log_2FC	P-value	FDR (padj)	Gene Name
Root vs Leaf	RHSIM_Rhsim10G0002900	1.625817544	1.92276E-10	1.09782E-09	RHSIM_Rhsim10G0002900
Root vs Leaf	RHSIM_Rhsim13G0166800	-1.46174549	1.93566E-10	1.10466E-09	RHSIM_Rhsim13G0166800
Root vs Leaf	novel.3141	-8.391090894	1.93569E-10	1.10466E-09	--
Root vs Leaf	RHSIM_Rhsim04G0024700	1.348141247	1.94775E-10	1.11127E-09	RHSIM_Rhsim04G0024700
Root vs Leaf	RHSIM_Rhsim02G0106500	1.808946995	1.95309E-10	1.11403E-09	RHSIM_Rhsim02G0106500
Root vs Leaf	RHSIM_Rhsim07G0073500	1.822914532	1.9574E-10	1.11622E-09	RHSIM_Rhsim07G0073500
Root vs Leaf	RHSIM_Rhsim06G0230600	1.648314894	1.96345E-10	1.11939E-09	RHSIM_Rhsim06G0230600
Root vs Leaf	RHSIM_Rhsim09G0213700	2.344883023	1.96979E-10	1.12273E-09	RHSIM_Rhsim09G0213700
Root vs Leaf	RHSIM_Rhsim07G0054200	-1.450812037	2.0137E-10	1.14748E-09	RHSIM_Rhsim07G0054200
Root vs Leaf	RHSIM_Rhsim10G0039400	6.342736796	2.08953E-10	1.18981E-09	RHSIM_Rhsim10G0039400
Root vs Leaf	RHSIM_Rhsim03G0073700	-8.684006204	2.09931E-10	1.19508E-09	RHSIM_Rhsim03G0073700
Root vs Leaf	RHSIM_RhsimUnG0024800	-8.340070707	2.12756E-10	1.21087E-09	RHSIM_RhsimUnG0024800
Root vs Leaf	RHSIM_Rhsim04G0042600	3.111755815	2.14075E-10	1.21807E-09	RHSIM_Rhsim04G0042600
Root vs Leaf	RHSIM_Rhsim08G0044700	-1.545018477	2.14462E-10	1.21998E-09	RHSIM_Rhsim08G0044700
Root vs Leaf	RHSIM_Rhsim03G0147700	4.114523689	2.14543E-10	1.22014E-09	RHSIM_Rhsim03G0147700
Root vs Leaf	RHSIM_Rhsim03G0141500	1.285756108	2.14762E-10	1.22108E-09	RHSIM_Rhsim03G0141500
Root vs Leaf	RHSIM_Rhsim12G0015000	2.710649648	2.15363E-10	1.2242E-09	RHSIM_Rhsim12G0015000
Root vs Leaf	RHSIM_Rhsim08G0198500	-2.464503703	2.17936E-10	1.23822E-09	RHSIM_Rhsim08G0198500
Root vs Leaf	RHSIM_Rhsim07G0050500	-2.539831478	2.1844E-10	1.24077E-09	RHSIM_Rhsim07G0050500
Root vs Leaf	RHSIM_Rhsim01G0143400	-1.290146405	2.18524E-10	1.24095E-09	RHSIM_Rhsim01G0143400
Root vs Leaf	RHSIM_Rhsim06G0110000	-1.072954304	2.19923E-10	1.24859E-09	RHSIM_Rhsim06G0110000
Root vs Leaf	RHSIM_Rhsim08G0212400	3.624116696	2.22984E-10	1.26534E-09	RHSIM_Rhsim08G0212400
Root vs Leaf	RHSIM_Rhsim13G0229200	-8.653807257	2.23184E-10	1.26617E-09	RHSIM_Rhsim13G0229200
Root vs Leaf	RHSIM_Rhsim02G0253600	2.311822392	2.24894E-10	1.27556E-09	RHSIM_Rhsim02G0253600
Root vs Leaf	novel.1609	-7.414914821	2.25552E-10	1.27898E-09	--

Comparison	Gene ID	log_2FC	P-value	FDR (padj)	Gene Name
Root vs Leaf	novel.3518	7.061955774	2.26278E-10	1.28278E-09	--
Root vs Leaf	RHSIM_Rhsim12G0202100	-1.632733482	2.26916E-10	1.28608E-09	RHSIM_Rhsim12G0202100
Root vs Leaf	novel.4046	-1.382910904	2.28594E-10	1.29528E-09	--
Root vs Leaf	RHSIM_RhsimUnG0042600	-2.156141293	2.30195E-10	1.30403E-09	RHSIM_RhsimUnG0042600
Root vs Leaf	RHSIM_Rhsim02G0206500	-8.418316321	2.30303E-10	1.30432E-09	RHSIM_Rhsim02G0206500
Root vs Leaf	RHSIM_Rhsim06G0110300	1.058067462	2.30921E-10	1.3075E-09	RHSIM_Rhsim06G0110300
Root vs Leaf	RHSIM_Rhsim04G0218700	-1.602491078	2.32439E-10	1.31577E-09	RHSIM_Rhsim04G0218700
Root vs Leaf	RHSIM_Rhsim13G0227600	8.543396392	2.33251E-10	1.32004E-09	RHSIM_Rhsim13G0227600
Root vs Leaf	RHSIM_Rhsim03G0044700	1.450464116	2.33756E-10	1.32258E-09	RHSIM_Rhsim03G0044700
Root vs Leaf	RHSIM_Rhsim10G0166500	-8.671335504	2.33956E-10	1.32339E-09	RHSIM_Rhsim10G0166500
Root vs Leaf	RHSIM_Rhsim04G0161600	-2.167089511	2.38358E-10	1.34796E-09	RHSIM_Rhsim04G0161600
Root vs Leaf	RHSIM_Rhsim03G0085300	1.721950666	2.38573E-10	1.34884E-09	RHSIM_Rhsim03G0085300
Root vs Leaf	RHSIM_Rhsim05G0223400	-1.663175509	2.40969E-10	1.36206E-09	RHSIM_Rhsim05G0223400
Root vs Leaf	RHSIM_Rhsim07G0229300	-8.233782717	2.41299E-10	1.36359E-09	RHSIM_Rhsim07G0229300
Root vs Leaf	RHSIM_Rhsim12G0087900	1.208549593	2.42093E-10	1.36774E-09	RHSIM_Rhsim12G0087900
Root vs Leaf	RHSIM_Rhsim08G0196200	-1.316042817	2.43687E-10	1.37641E-09	RHSIM_Rhsim08G0196200
Root vs Leaf	RHSIM_Rhsim08G0243100	1.168447003	2.44731E-10	1.38164E-09	RHSIM_Rhsim08G0243100
Root vs Leaf	RHSIM_Rhsim13G0111100	8.236236831	2.46308E-10	1.3902E-09	RHSIM_Rhsim13G0111100
Root vs Leaf	RHSIM_Rhsim05G0063600	3.581690513	2.46975E-10	1.39362E-09	RHSIM_Rhsim05G0063600
Root vs Leaf	novel.1225	8.803580849	2.50592E-10	1.41369E-09	--
Root vs Leaf	RHSIM_Rhsim07G0049900	-3.619865569	2.51612E-10	1.4191E-09	RHSIM_Rhsim07G0049900
Root vs Leaf	RHSIM_Rhsim09G0180500	-2.46367264	2.5246E-10	1.42353E-09	RHSIM_Rhsim09G0180500
Root vs Leaf	RHSIM_Rhsim08G0157000	-5.153267307	2.52721E-10	1.42466E-09	RHSIM_Rhsim08G0157000
Root vs Leaf	RHSIM_Rhsim03G0136600	-1.071981587	2.54815E-10	1.43611E-09	RHSIM_Rhsim03G0136600
Root vs Leaf	RHSIM_Rhsim05G0044100	4.720132769	2.55335E-10	1.43869E-09	RHSIM_Rhsim05G0044100

Comparison	Gene ID	log_2FC	P-value	FDR (padj)	Gene Name
Root vs Leaf	RHSIM_Rhsim06G0030200	3.404896463	2.55887E-10	1.44145E-09	RHSIM_Rhsim06G0030200
Root vs Leaf	RHSIM_Rhsim05G0163000	1.032079051	2.56632E-10	1.4453E-09	RHSIM_Rhsim05G0163000
Root vs Leaf	RHSIM_Rhsim07G0142600	2.289228049	2.60288E-10	1.46553E-09	RHSIM_Rhsim07G0142600
Root vs Leaf	RHSIM_Rhsim03G0161300	-1.190322638	2.68318E-10	1.51037E-09	RHSIM_Rhsim03G0161300
Root vs Leaf	novel.1134	1.435941942	2.70143E-10	1.52028E-09	--
Root vs Leaf	RHSIM_Rhsim13G0045400	1.196992277	2.71974E-10	1.53021E-09	RHSIM_Rhsim13G0045400
Root vs Leaf	RHSIM_Rhsim01G0067100	1.799813045	2.73764E-10	1.53953E-09	RHSIM_Rhsim01G0067100
Root vs Leaf	RHSIM_Rhsim12G0057400	-1.411670163	2.75816E-10	1.5507E-09	RHSIM_Rhsim12G0057400
Root vs Leaf	RHSIM_Rhsim10G0097900	-8.142953639	2.78419E-10	1.56495E-09	RHSIM_Rhsim10G0097900
Root vs Leaf	RHSIM_Rhsim06G0091300	-1.185597694	2.8182E-10	1.58368E-09	RHSIM_Rhsim06G0091300
Root vs Leaf	novel.2797	1.860787834	2.82668E-10	1.58806E-09	--
Root vs Leaf	RHSIM_Rhsim09G0136400	-8.661197771	2.85227E-10	1.60205E-09	RHSIM_Rhsim09G0136400
Root vs Leaf	RHSIM_Rhsim13G0014600	3.50693327	2.85969E-10	1.60582E-09	RHSIM_Rhsim13G0014600
Root vs Leaf	RHSIM_Rhsim08G0120400	-5.354578521	2.86883E-10	1.61057E-09	RHSIM_Rhsim08G0120400
Root vs Leaf	RHSIM_Rhsim05G0196900	1.216158698	2.87418E-10	1.61318E-09	RHSIM_Rhsim05G0196900
Root vs Leaf	novel.4387	-4.42082495	2.89909E-10	1.62677E-09	--
Root vs Leaf	novel.6091	-8.245298767	2.91364E-10	1.63454E-09	--
Root vs Leaf	RHSIM_Rhsim12G0091100	3.695736338	2.92493E-10	1.64047E-09	RHSIM_Rhsim12G0091100
Root vs Leaf	RHSIM_Rhsim01G0110400	3.928242494	2.92684E-10	1.64115E-09	RHSIM_Rhsim01G0110400
Root vs Leaf	RHSIM_Rhsim12G0046500	-1.693149848	2.92906E-10	1.64199E-09	RHSIM_Rhsim12G0046500
Root vs Leaf	RHSIM_Rhsim01G0233500	-4.56500562	3.00881E-10	1.68588E-09	RHSIM_Rhsim01G0233500
Root vs Leaf	RHSIM_Rhsim12G0153900	-1.314021917	3.03332E-10	1.69921E-09	RHSIM_Rhsim12G0153900
Root vs Leaf	RHSIM_Rhsim12G0198800	1.482493128	3.04218E-10	1.70375E-09	RHSIM_Rhsim12G0198800
Root vs Leaf	RHSIM_Rhsim01G0033000	-1.035622496	3.04492E-10	1.70487E-09	RHSIM_Rhsim01G0033000
Root vs Leaf	RHSIM_Rhsim01G0196200	1.163997748	3.05563E-10	1.71046E-09	RHSIM_Rhsim01G0196200

Comparison	Gene ID	log_2FC	P-value	FDR (padj)	Gene Name
Root vs Leaf	RHSIM_Rhsim09G0132500	-3.609486442	3.05882E-10	1.71183E-09	RHSIM_Rhsim09G0132500
Root vs Leaf	RHSIM_Rhsim04G0014800	1.895506144	3.06807E-10	1.71659E-09	RHSIM_Rhsim04G0014800
Root vs Leaf	RHSIM_RhsimUnG0089300	-3.925000861	3.07484E-10	1.71967E-09	RHSIM_RhsimUnG0089300
Root vs Leaf	RHSIM_Rhsim07G0257800	4.654901431	3.07506E-10	1.71967E-09	RHSIM_Rhsim07G0257800
Root vs Leaf	novel.6098	-7.873072648	3.09671E-10	1.73136E-09	--
Root vs Leaf	RHSIM_Rhsim06G0106000	1.855038607	3.10247E-10	1.73416E-09	RHSIM_Rhsim06G0106000
Root vs Leaf	novel.4096	-8.764354246	3.12549E-10	1.7466E-09	--
Root vs Leaf	RHSIM_Rhsim10G0190200	-1.207995563	3.16089E-10	1.76596E-09	RHSIM_Rhsim10G0190200
Root vs Leaf	RHSIM_Rhsim05G0000500	-6.044998945	3.16645E-10	1.76864E-09	RHSIM_Rhsim05G0000500
Root vs Leaf	RHSIM_Rhsim05G0192400	-1.016129422	3.1879E-10	1.78019E-09	RHSIM_Rhsim05G0192400
Root vs Leaf	RHSIM_Rhsim03G0206000	8.222868823	3.19985E-10	1.78644E-09	RHSIM_Rhsim03G0206000
Root vs Leaf	RHSIM_Rhsim03G0212600	1.741705805	3.22871E-10	1.80168E-09	RHSIM_Rhsim03G0212600
Root vs Leaf	RHSIM_Rhsim03G0150300	-8.981428903	3.23083E-10	1.80243E-09	RHSIM_Rhsim03G0150300
Root vs Leaf	RHSIM_Rhsim09G0105500	4.328350833	3.26197E-10	1.81936E-09	RHSIM_Rhsim09G0105500
Root vs Leaf	novel.2691	6.130236001	3.26414E-10	1.82013E-09	--
Root vs Leaf	RHSIM_Rhsim09G0135200	-1.976876725	3.29031E-10	1.83428E-09	RHSIM_Rhsim09G0135200
Root vs Leaf	RHSIM_Rhsim07G0068200	-1.798540024	3.29231E-10	1.83495E-09	RHSIM_Rhsim07G0068200
Root vs Leaf	RHSIM_Rhsim09G0119900	-2.854087567	3.34355E-10	1.86307E-09	RHSIM_Rhsim09G0119900
Root vs Leaf	RHSIM_Rhsim03G0076800	6.826529031	3.36392E-10	1.87396E-09	RHSIM_Rhsim03G0076800
Root vs Leaf	RHSIM_Rhsim07G0146400	3.926674622	3.41403E-10	1.90096E-09	RHSIM_Rhsim07G0146400
Root vs Leaf	novel.4177	1.411763652	3.4681E-10	1.93014E-09	--
Root vs Leaf	RHSIM_Rhsim02G0118400	1.264313114	3.47141E-10	1.93152E-09	RHSIM_Rhsim02G0118400
Root vs Leaf	RHSIM_Rhsim07G0080000	-1.941980946	3.481E-10	1.93639E-09	RHSIM_Rhsim07G0080000
Root vs Leaf	RHSIM_Rhsim10G0192700	-1.440320835	3.49441E-10	1.94338E-09	RHSIM_Rhsim10G0192700
Root vs Leaf	RHSIM_Rhsim12G0177800	8.180234225	3.50045E-10	1.94628E-09	RHSIM_Rhsim12G0177800

Comparison	Gene ID	log_2FC	P-value	FDR (padj)	Gene Name
Root vs Leaf	RHSIM_Rhsim03G0191100	-1.669044418	3.5253E-10	1.95962E-09	RHSIM_Rhsim03G0191100
Root vs Leaf	RHSIM_Rhsim08G0180900	-1.558823156	3.56168E-10	1.97936E-09	RHSIM_Rhsim08G0180900
Root vs Leaf	RHSIM_Rhsim02G0193200	-2.501773476	3.57944E-10	1.98812E-09	RHSIM_Rhsim02G0193200
Root vs Leaf	novel.827	-3.438056118	3.58E-10	1.98812E-09	--
Root vs Leaf	RHSIM_Rhsim10G0042800	1.316391409	3.59034E-10	1.99338E-09	RHSIM_Rhsim10G0042800
Root vs Leaf	RHSIM_Rhsim04G0000700	-2.082602637	3.60224E-10	1.9995E-09	RHSIM_Rhsim04G0000700
Root vs Leaf	RHSIM_Rhsim13G0017300	-3.505579004	3.62377E-10	2.01097E-09	RHSIM_Rhsim13G0017300
Root vs Leaf	RHSIM_Rhsim13G0233200	1.920664697	3.65105E-10	2.02563E-09	RHSIM_Rhsim13G0233200
Root vs Leaf	RHSIM_Rhsim10G0067900	-3.605858625	3.72529E-10	2.06632E-09	RHSIM_Rhsim10G0067900
Root vs Leaf	RHSIM_Rhsim10G0070900	-4.409065514	3.73005E-10	2.06847E-09	RHSIM_Rhsim10G0070900
Root vs Leaf	RHSIM_Rhsim07G0126600	8.705360431	3.73236E-10	2.06925E-09	RHSIM_Rhsim07G0126600
Root vs Leaf	RHSIM_Rhsim01G0196600	1.506549523	3.74336E-10	2.07486E-09	RHSIM_Rhsim01G0196600
Root vs Leaf	RHSIM_Rhsim07G0036600	9.049753075	3.75612E-10	2.08143E-09	RHSIM_Rhsim07G0036600
Root vs Leaf	novel.1067	7.952818277	3.75703E-10	2.08143E-09	--
Root vs Leaf	RHSIM_Rhsim13G0004700	-1.204132134	3.76074E-10	2.08299E-09	RHSIM_Rhsim13G0004700
Root vs Leaf	RHSIM_Rhsim06G0062300	2.435217232	3.79988E-10	2.10416E-09	RHSIM_Rhsim06G0062300
Root vs Leaf	RHSIM_Rhsim05G0000200	-1.937734544	3.83121E-10	2.121E-09	RHSIM_Rhsim05G0000200
Root vs Leaf	RHSIM_Rhsim06G0077600	-1.271087707	3.85004E-10	2.13092E-09	RHSIM_Rhsim06G0077600
Root vs Leaf	RHSIM_Rhsim13G0226400	1.974468237	3.87808E-10	2.14592E-09	RHSIM_Rhsim13G0226400
Root vs Leaf	RHSIM_Rhsim04G0130400	-1.853756986	3.89521E-10	2.15489E-09	RHSIM_Rhsim04G0130400
Root vs Leaf	novel.4435	-9.677683832	3.90974E-10	2.16241E-09	--
Root vs Leaf	RHSIM_Rhsim01G0147400	-1.353620034	3.91092E-10	2.16255E-09	RHSIM_Rhsim01G0147400
Root vs Leaf	RHSIM_Rhsim07G0229400	-9.831700515	3.92391E-10	2.16921E-09	RHSIM_Rhsim07G0229400
Root vs Leaf	RHSIM_Rhsim07G0117700	-2.730364409	3.93508E-10	2.17487E-09	RHSIM_Rhsim07G0117700
Root vs Leaf	RHSIM_Rhsim06G0234600	1.187855949	3.95868E-10	2.18739E-09	RHSIM_Rhsim06G0234600

Comparison	Gene ID	log_2FC	P-value	FDR (padj)	Gene Name
Root vs Leaf	RHSIM_Rhsim06G0155900	-9.259147973	3.96535E-10	2.19055E-09	RHSIM_Rhsim06G0155900
Root vs Leaf	RHSIM_Rhsim01G0081800	-1.547917813	3.97414E-10	2.19488E-09	RHSIM_Rhsim01G0081800
Root vs Leaf	RHSIM_RhsimUnG0138800	1.515657336	4.00336E-10	2.21049E-09	RHSIM_RhsimUnG0138800
Root vs Leaf	RHSIM_Rhsim02G0064400	-5.949716526	4.03171E-10	2.22561E-09	RHSIM_Rhsim02G0064400
Root vs Leaf	RHSIM_Rhsim07G0108300	-8.658401509	4.05765E-10	2.2394E-09	RHSIM_Rhsim07G0108300
Root vs Leaf	RHSIM_Rhsim09G0116300	2.126046504	4.0721E-10	2.24684E-09	RHSIM_Rhsim09G0116300
Root vs Leaf	RHSIM_Rhsim06G0214200	1.288140912	4.08139E-10	2.25143E-09	RHSIM_Rhsim06G0214200
Root vs Leaf	RHSIM_Rhsim07G0001300	2.110020549	4.09873E-10	2.25992E-09	RHSIM_Rhsim07G0001300
Root vs Leaf	RHSIM_Rhsim07G0054600	3.649336067	4.11144E-10	2.26638E-09	RHSIM_Rhsim07G0054600
Root vs Leaf	RHSIM_Rhsim04G0246700	7.377010823	4.11493E-10	2.26777E-09	RHSIM_Rhsim04G0246700
Root vs Leaf	RHSIM_Rhsim01G0101100	1.205246984	4.132E-10	2.27663E-09	RHSIM_Rhsim01G0101100
Root vs Leaf	RHSIM_Rhsim05G0057000	1.35032674	4.16305E-10	2.29319E-09	RHSIM_Rhsim05G0057000
Root vs Leaf	RHSIM_Rhsim09G0143700	7.11992825	4.21952E-10	2.32276E-09	RHSIM_Rhsim09G0143700
Root vs Leaf	RHSIM_Rhsim10G0099400	1.660548637	4.21974E-10	2.32276E-09	RHSIM_Rhsim10G0099400
Root vs Leaf	RHSIM_Rhsim01G0096400	-1.741111254	4.30088E-10	2.36687E-09	RHSIM_Rhsim01G0096400
Root vs Leaf	RHSIM_Rhsim04G0209900	-1.951501278	4.30695E-10	2.36964E-09	RHSIM_Rhsim04G0209900
Root vs Leaf	RHSIM_Rhsim06G0223800	6.776316524	4.31401E-10	2.37296E-09	RHSIM_Rhsim06G0223800
Root vs Leaf	novel.83	-5.906925302	4.34874E-10	2.3915E-09	--
Root vs Leaf	RHSIM_Rhsim04G0096900	2.155216747	4.36256E-10	2.39853E-09	RHSIM_Rhsim04G0096900
Root vs Leaf	RHSIM_Rhsim12G0005000	-2.14365034	4.37054E-10	2.40207E-09	RHSIM_Rhsim12G0005000
Root vs Leaf	RHSIM_Rhsim07G0071500	2.603955039	4.37108E-10	2.40207E-09	RHSIM_Rhsim07G0071500
Root vs Leaf	RHSIM_Rhsim04G0126400	-1.222234349	4.37494E-10	2.40362E-09	RHSIM_Rhsim04G0126400
Root vs Leaf	RHSIM_Rhsim06G0030300	2.201315218	4.3773E-10	2.40435E-09	RHSIM_Rhsim06G0030300
Root vs Leaf	RHSIM_Rhsim03G0263700	1.424791868	4.38195E-10	2.40633E-09	RHSIM_Rhsim03G0263700
Root vs Leaf	RHSIM_Rhsim02G0029300	-8.646707698	4.39575E-10	2.41333E-09	RHSIM_Rhsim02G0029300

Comparison	Gene ID	log_2FC	P-value	FDR (padj)	Gene Name
Root vs Leaf	RHSIM_Rhsim03G0004300	6.96104639	4.43158E-10	2.43185E-09	RHSIM_Rhsim03G0004300
Root vs Leaf	RHSIM_Rhsim08G0024100	-2.166536656	4.4332E-10	2.43216E-09	RHSIM_Rhsim08G0024100
Root vs Leaf	RHSIM_Rhsim12G0138600	-3.900783527	4.43547E-10	2.43283E-09	RHSIM_Rhsim12G0138600
Root vs Leaf	RHSIM_Rhsim12G0010200	1.441228801	4.44054E-10	2.43503E-09	RHSIM_Rhsim12G0010200
Root vs Leaf	RHSIM_Rhsim07G0019700	-3.750808255	4.48306E-10	2.45777E-09	RHSIM_Rhsim07G0019700
Root vs Leaf	RHSIM_Rhsim12G0183100	-1.043494772	4.48613E-10	2.45887E-09	RHSIM_Rhsim12G0183100
Root vs Leaf	novel.2458	-6.526804058	4.51035E-10	2.47156E-09	--
Root vs Leaf	RHSIM_Rhsim12G0112800	-2.417322495	4.51837E-10	2.47537E-09	RHSIM_Rhsim12G0112800
Root vs Leaf	novel.3430	1.009720075	4.52104E-10	2.47624E-09	--
Root vs Leaf	RHSIM_Rhsim06G0020700	-2.283955576	4.5231E-10	2.47679E-09	RHSIM_Rhsim06G0020700
Root vs Leaf	RHSIM_Rhsim13G0210800	3.29280891	4.53146E-10	2.48078E-09	RHSIM_Rhsim13G0210800
Root vs Leaf	RHSIM_Rhsim02G0232000	1.540665987	4.54113E-10	2.48549E-09	RHSIM_Rhsim02G0232000
Root vs Leaf	RHSIM_Rhsim06G0203600	-1.027670984	4.60929E-10	2.5216E-09	RHSIM_Rhsim06G0203600
Root vs Leaf	RHSIM_Rhsim03G0227300	1.630121232	4.65931E-10	2.54836E-09	RHSIM_Rhsim03G0227300
Root vs Leaf	RHSIM_Rhsim06G0108200	2.976694295	4.6894E-10	2.5636E-09	RHSIM_Rhsim06G0108200
Root vs Leaf	novel.4335	-3.045222807	4.69177E-10	2.56429E-09	--
Root vs Leaf	RHSIM_Rhsim03G0069100	1.146646953	4.69489E-10	2.5654E-09	RHSIM_Rhsim03G0069100
Root vs Leaf	novel.712	7.516043052	4.73429E-10	2.58632E-09	--
Root vs Leaf	RHSIM_Rhsim04G0095200	-8.008761782	4.74827E-10	2.59334E-09	RHSIM_Rhsim04G0095200
Root vs Leaf	novel.2695	-8.902439646	4.76079E-10	2.59956E-09	--
Root vs Leaf	RHSIM_Rhsim06G0139900	4.178924074	4.80677E-10	2.62343E-09	RHSIM_Rhsim06G0139900
Root vs Leaf	RHSIM_Rhsim03G0013100	4.330384523	4.82725E-10	2.63399E-09	RHSIM_Rhsim03G0013100
Root vs Leaf	RHSIM_Rhsim05G0039700	-1.322291704	4.84606E-10	2.64363E-09	RHSIM_Rhsim05G0039700
Root vs Leaf	RHSIM_Rhsim09G0145700	2.183974718	4.84917E-10	2.6447E-09	RHSIM_Rhsim09G0145700
Root vs Leaf	RHSIM_Rhsim04G0014400	-2.632914264	4.86123E-10	2.65066E-09	RHSIM_Rhsim04G0014400

Comparison	Gene ID	log_2FC	P-value	FDR (padj)	Gene Name
Root vs Leaf	RHSIM_Rhsim05G0048000	2.563391765	4.93017E-10	2.68761E-09	RHSIM_Rhsim05G0048000
Root vs Leaf	RHSIM_Rhsim12G0187600	2.299065641	4.95062E-10	2.69812E-09	RHSIM_Rhsim12G0187600
Root vs Leaf	RHSIM_Rhsim12G0127600	1.587452683	4.98912E-10	2.71846E-09	RHSIM_Rhsim12G0127600
Root vs Leaf	RHSIM_Rhsim07G0135000	-1.022171394	4.99055E-10	2.7186E-09	RHSIM_Rhsim07G0135000
Root vs Leaf	RHSIM_Rhsim04G0079700	-4.112895965	4.99747E-10	2.72173E-09	RHSIM_Rhsim04G0079700
Root vs Leaf	RHSIM_Rhsim01G0043700	-3.205144577	5.09448E-10	2.77391E-09	RHSIM_Rhsim01G0043700
Root vs Leaf	RHSIM_Rhsim07G0229100	-6.456926395	5.09813E-10	2.77525E-09	RHSIM_Rhsim07G0229100
Root vs Leaf	RHSIM_RhsimUnG0251700	-1.405107807	5.11768E-10	2.78524E-09	RHSIM_RhsimUnG0251700
Root vs Leaf	RHSIM_Rhsim01G0028900	2.187299456	5.1303E-10	2.79145E-09	RHSIM_Rhsim01G0028900
Root vs Leaf	RHSIM_Rhsim03G0165500	1.47779996	5.13452E-10	2.79309E-09	RHSIM_Rhsim03G0165500
Root vs Leaf	novel.3725	-2.527408954	5.13635E-10	2.79343E-09	--
Root vs Leaf	novel.2199	-3.206662309	5.21933E-10	2.83722E-09	--
Root vs Leaf	RHSIM_Rhsim10G0137700	3.248535912	5.23232E-10	2.84362E-09	RHSIM_Rhsim10G0137700
Root vs Leaf	RHSIM_Rhsim03G0154100	3.575756462	5.28321E-10	2.86992E-09	RHSIM_Rhsim03G0154100
Root vs Leaf	novel.4125	4.399598874	5.34289E-10	2.90166E-09	--
Root vs Leaf	RHSIM_Rhsim06G0143700	2.278849109	5.36237E-10	2.91088E-09	RHSIM_Rhsim06G0143700
Root vs Leaf	RHSIM_Rhsim10G0118700	-1.142582303	5.44136E-10	2.95306E-09	RHSIM_Rhsim10G0118700
Root vs Leaf	RHSIM_Rhsim01G0250700	-3.127534447	5.45832E-10	2.96157E-09	RHSIM_Rhsim01G0250700
Root vs Leaf	novel.572	5.516891687	5.46091E-10	2.96228E-09	--
Root vs Leaf	RHSIM_RhsimUnG0023200	1.283313723	5.47412E-10	2.96875E-09	RHSIM_RhsimUnG0023200
Root vs Leaf	RHSIM_Rhsim13G0190600	-5.446937996	5.48114E-10	2.97186E-09	RHSIM_Rhsim13G0190600
Root vs Leaf	RHSIM_Rhsim08G0063000	3.490621675	5.50458E-10	2.98387E-09	RHSIM_Rhsim08G0063000
Root vs Leaf	novel.1949	-7.06350727	5.6352E-10	3.05325E-09	--
Root vs Leaf	RHSIM_Rhsim12G0126600	2.299734885	5.6861E-10	3.07939E-09	RHSIM_Rhsim12G0126600
Root vs Leaf	RHSIM_Rhsim01G0201500	-1.75475219	5.71773E-10	3.09579E-09	RHSIM_Rhsim01G0201500

Comparison	Gene ID	log_2FC	P-value	FDR (padj)	Gene Name
Root vs Leaf	RHSIM_Rhsim02G0193600	-1.154030912	5.76054E-10	3.11751E-09	RHSIM_Rhsim02G0193600
Root vs Leaf	RHSIM_Rhsim09G0130100	1.51037459	5.76406E-10	3.11868E-09	RHSIM_Rhsim09G0130100
Root vs Leaf	RHSIM_Rhsim11G0013500	-8.387366957	5.87284E-10	3.1768E-09	RHSIM_Rhsim11G0013500
Root vs Leaf	RHSIM_Rhsim02G0253200	3.175964321	5.88639E-10	3.18338E-09	RHSIM_Rhsim02G0253200
Root vs Leaf	RHSIM_RhsimUnG0219100	3.223177147	5.91174E-10	3.19634E-09	RHSIM_RhsimUnG0219100
Root vs Leaf	novel.698	-8.105133534	5.95976E-10	3.2208E-09	--
Root vs Leaf	RHSIM_Rhsim10G0129200	-1.462099648	5.98181E-10	3.23196E-09	RHSIM_Rhsim10G0129200
Root vs Leaf	RHSIM_Rhsim06G0046300	-1.064750278	6.01404E-10	3.24808E-09	RHSIM_Rhsim06G0046300
Root vs Leaf	RHSIM_Rhsim02G0113200	-8.007380953	6.01445E-10	3.24808E-09	RHSIM_Rhsim02G0113200
Root vs Leaf	novel.2438	2.710452674	6.04202E-10	3.26221E-09	--
Root vs Leaf	RHSIM_Rhsim10G0117100	-2.098867127	6.10488E-10	3.29538E-09	RHSIM_Rhsim10G0117100
Root vs Leaf	RHSIM_Rhsim10G0097400	-8.3643421	6.11632E-10	3.30079E-09	RHSIM_Rhsim10G0097400
Root vs Leaf	RHSIM_RhsimUnG0138500	-3.564146989	6.25284E-10	3.37211E-09	RHSIM_RhsimUnG0138500
Root vs Leaf	RHSIM_Rhsim01G0205400	-1.543687121	6.25874E-10	3.3745E-09	RHSIM_Rhsim01G0205400
Root vs Leaf	RHSIM_Rhsim08G0078900	5.472905878	6.30551E-10	3.39892E-09	RHSIM_Rhsim08G0078900
Root vs Leaf	RHSIM_Rhsim03G0033400	-1.006257518	6.39981E-10	3.44895E-09	RHSIM_Rhsim03G0033400
Root vs Leaf	RHSIM_Rhsim11G0092400	1.624768006	6.44043E-10	3.47003E-09	RHSIM_Rhsim11G0092400
Root vs Leaf	RHSIM_Rhsim03G0246300	1.746729962	6.48186E-10	3.49155E-09	RHSIM_Rhsim03G0246300
Root vs Leaf	RHSIM_Rhsim08G0224800	1.343707977	6.50162E-10	3.50137E-09	RHSIM_Rhsim08G0224800
Root vs Leaf	RHSIM_Rhsim08G0224000	-2.061656097	6.52163E-10	3.51133E-09	RHSIM_Rhsim08G0224000
Root vs Leaf	RHSIM_Rhsim03G0130200	-1.131403255	6.53979E-10	3.51947E-09	RHSIM_Rhsim03G0130200
Root vs Leaf	RHSIM_Rhsim08G0075400	-2.375119204	6.58518E-10	3.54308E-09	RHSIM_Rhsim08G0075400
Root vs Leaf	RHSIM_Rhsim07G0032100	8.650780357	6.58697E-10	3.54321E-09	RHSIM_Rhsim07G0032100
Root vs Leaf	novel.631	-2.949814684	6.65995E-10	3.58164E-09	--
Root vs Leaf	RHSIM_Rhsim11G0040800	3.557266533	6.69039E-10	3.59634E-09	RHSIM_Rhsim11G0040800

Comparison	Gene ID	log_2FC	P-value	FDR (padj)	Gene Name
Root vs Leaf	RHSIM_Rhsim09G0181600	-1.362813949	6.74311E-10	3.62383E-09	RHSIM_Rhsim09G0181600
Root vs Leaf	RHSIM_Rhsim08G0210000	-2.192532127	6.773E-10	3.63821E-09	RHSIM_Rhsim08G0210000
Root vs Leaf	RHSIM_Rhsim10G0116000	2.988320179	6.78467E-10	3.64363E-09	RHSIM_Rhsim10G0116000
Root vs Leaf	RHSIM_Rhsim12G0133300	-1.498984505	6.78713E-10	3.6441E-09	RHSIM_Rhsim12G0133300
Root vs Leaf	RHSIM_Rhsim07G0244400	2.952835567	6.79559E-10	3.6478E-09	RHSIM_Rhsim07G0244400
Root vs Leaf	RHSIM_Rhsim02G0008900	-1.195827148	6.80125E-10	3.64999E-09	RHSIM_Rhsim02G0008900
Root vs Leaf	novel.5306	-8.732371931	6.82182E-10	3.66018E-09	--
Root vs Leaf	RHSIM_Rhsim08G0104000	7.98764918	6.87306E-10	3.68682E-09	RHSIM_Rhsim08G0104000
Root vs Leaf	RHSIM_Rhsim03G0263500	8.12430216	6.88238E-10	3.69097E-09	RHSIM_Rhsim03G0263500
Root vs Leaf	RHSIM_Rhsim09G0207200	2.184611189	6.90294E-10	3.70113E-09	RHSIM_Rhsim09G0207200
Root vs Leaf	RHSIM_RhsimUnG0205500	2.805874174	6.90895E-10	3.7035E-09	RHSIM_RhsimUnG0205500
Root vs Leaf	RHSIM_RhsimUnG0188500	-7.503764103	6.94447E-10	3.72167E-09	RHSIM_RhsimUnG0188500
Root vs Leaf	RHSIM_Rhsim05G0195500	4.879984722	7.0207E-10	3.76059E-09	RHSIM_Rhsim05G0195500
Root vs Leaf	RHSIM_Rhsim05G0220400	-6.082594196	7.02262E-10	3.76059E-09	RHSIM_Rhsim05G0220400
Root vs Leaf	RHSIM_Rhsim09G0135600	-3.894455661	7.02358E-10	3.76059E-09	RHSIM_Rhsim09G0135600
Root vs Leaf	RHSIM_Rhsim05G0018000	1.968285271	7.06319E-10	3.78092E-09	RHSIM_Rhsim05G0018000
Root vs Leaf	RHSIM_Rhsim10G0193600	-2.813947146	7.07229E-10	3.78492E-09	RHSIM_Rhsim10G0193600
Root vs Leaf	novel.694	-8.44178647	7.08122E-10	3.78882E-09	--
Root vs Leaf	RHSIM_Rhsim10G0130500	-1.163057183	7.12592E-10	3.81186E-09	RHSIM_Rhsim10G0130500
Root vs Leaf	RHSIM_Rhsim01G0110200	-2.642814143	7.14322E-10	3.82023E-09	RHSIM_Rhsim01G0110200
Root vs Leaf	novel.5994	-5.061854592	7.15875E-10	3.82765E-09	--
Root vs Leaf	RHSIM_Rhsim09G0142600	-1.360967528	7.16091E-10	3.82792E-09	RHSIM_Rhsim09G0142600
Root vs Leaf	RHSIM_Rhsim13G0102500	-3.637361007	7.19675E-10	3.84619E-09	RHSIM_Rhsim13G0102500
Root vs Leaf	RHSIM_Rhsim02G0077100	-5.274770155	7.20114E-10	3.84765E-09	RHSIM_Rhsim02G0077100
Root vs Leaf	RHSIM_Rhsim06G0227500	1.012491959	7.2047E-10	3.84866E-09	RHSIM_Rhsim06G0227500

Comparison	Gene ID	log_2FC	P-value	FDR (padj)	Gene Name
Root vs Leaf	novel.6114	3.116860594	7.34797E-10	3.92255E-09	--
Root vs Leaf	novel.3717	3.919448019	7.3481E-10	3.92255E-09	--
Root vs Leaf	RHSIM_Rhsim13G0098800	1.085475842	7.38309E-10	3.9385E-09	RHSIM_Rhsim13G0098800
Root vs Leaf	RHSIM_Rhsim13G0194300	1.613974803	7.43195E-10	3.96365E-09	RHSIM_Rhsim13G0194300
Root vs Leaf	RHSIM_Rhsim10G0035500	4.179189162	7.4419E-10	3.96804E-09	RHSIM_Rhsim10G0035500
Root vs Leaf	RHSIM_Rhsim13G0021000	-1.57632075	7.65306E-10	4.07875E-09	RHSIM_Rhsim13G0021000
Root vs Leaf	RHSIM_Rhsim11G0188600	-2.650971463	7.67286E-10	4.08836E-09	RHSIM_Rhsim11G0188600
Root vs Leaf	RHSIM_Rhsim03G0157900	-7.923168602	7.67643E-10	4.08932E-09	RHSIM_Rhsim03G0157900
Root vs Leaf	RHSIM_Rhsim01G0184500	-1.094830209	7.77464E-10	4.14069E-09	RHSIM_Rhsim01G0184500
Root vs Leaf	RHSIM_Rhsim09G0148000	-2.383190874	7.78805E-10	4.14687E-09	RHSIM_Rhsim09G0148000
Root vs Leaf	RHSIM_Rhsim02G0016400	6.855633564	7.80623E-10	4.1556E-09	RHSIM_Rhsim02G0016400
Root vs Leaf	RHSIM_Rhsim13G0203400	1.065565476	7.85203E-10	4.17902E-09	RHSIM_Rhsim13G0203400
Root vs Leaf	RHSIM_Rhsim07G0124100	-2.706670316	7.89868E-10	4.20288E-09	RHSIM_Rhsim07G0124100
Root vs Leaf	RHSIM_Rhsim13G0221400	-2.852414002	7.93249E-10	4.2199E-09	RHSIM_Rhsim13G0221400
Root vs Leaf	novel.4097	-2.89659651	7.93902E-10	4.22241E-09	--
Root vs Leaf	RHSIM_Rhsim11G0198700	2.274201227	7.97988E-10	4.24316E-09	RHSIM_Rhsim11G0198700
Root vs Leaf	RHSIM_Rhsim10G0054500	3.014985129	7.99926E-10	4.25249E-09	RHSIM_Rhsim10G0054500
Root vs Leaf	RHSIM_Rhsim07G0139900	2.708242866	8.01163E-10	4.25809E-09	RHSIM_Rhsim07G0139900
Root vs Leaf	RHSIM_Rhsim03G0031200	6.164437058	8.07951E-10	4.29318E-09	RHSIM_Rhsim03G0031200
Root vs Leaf	RHSIM_RhsimUnG0200100	-8.135223451	8.10982E-10	4.3083E-09	RHSIM_RhsimUnG0200100
Root vs Leaf	RHSIM_Rhsim03G0074600	1.410371254	8.12979E-10	4.31791E-09	RHSIM_Rhsim03G0074600
Root vs Leaf	RHSIM_Rhsim02G0019000	-1.491185742	8.14067E-10	4.3223E-09	RHSIM_Rhsim02G0019000
Root vs Leaf	RHSIM_Rhsim04G0221300	-1.939917882	8.14178E-10	4.3223E-09	RHSIM_Rhsim04G0221300
Root vs Leaf	RHSIM_Rhsim08G0004200	-1.028812204	8.16297E-10	4.33255E-09	RHSIM_Rhsim08G0004200
Root vs Leaf	novel.3647	2.731864935	8.20363E-10	4.35314E-09	--

Comparison	Gene ID	log_2FC	P-value	FDR (padj)	Gene Name
Root vs Leaf	novel.5924	-3.814455609	8.23895E-10	4.37087E-09	--
Root vs Leaf	RHSIM_Rhsim12G0113500	7.425524833	8.24278E-10	4.3719E-09	RHSIM_Rhsim12G0113500
Root vs Leaf	RHSIM_Rhsim12G0157300	1.50557833	8.28574E-10	4.39267E-09	RHSIM_Rhsim12G0157300
Root vs Leaf	RHSIM_Rhsim13G0199600	-1.249519654	8.29469E-10	4.39641E-09	RHSIM_Rhsim13G0199600
Root vs Leaf	RHSIM_Rhsim06G0051000	2.812528257	8.36123E-10	4.43067E-09	RHSIM_Rhsim06G0051000
Root vs Leaf	RHSIM_Rhsim11G0012500	1.354228787	8.44822E-10	4.47574E-09	RHSIM_Rhsim11G0012500
Root vs Leaf	RHSIM_Rhsim08G0091200	4.151634779	8.55558E-10	4.52951E-09	RHSIM_Rhsim08G0091200
Root vs Leaf	RHSIM_Rhsim08G0121500	-3.102662214	8.6632E-10	4.58543E-09	RHSIM_Rhsim08G0121500
Root vs Leaf	RHSIM_Rhsim05G0170800	-1.244182964	8.78116E-10	4.64641E-09	RHSIM_Rhsim05G0170800
Root vs Leaf	RHSIM_Rhsim01G0230900	7.729138398	8.78242E-10	4.64641E-09	RHSIM_Rhsim01G0230900
Root vs Leaf	novel.796	-4.223073174	8.89951E-10	4.70554E-09	--
Root vs Leaf	RHSIM_Rhsim12G0151900	1.401897196	8.90029E-10	4.70554E-09	RHSIM_Rhsim12G0151900
Root vs Leaf	RHSIM_Rhsim07G0048500	2.830008836	8.9278E-10	4.71901E-09	RHSIM_Rhsim07G0048500
Root vs Leaf	RHSIM_Rhsim02G0180200	6.946752131	8.9332E-10	4.72078E-09	RHSIM_Rhsim02G0180200
Root vs Leaf	RHSIM_Rhsim07G0185300	-1.904642143	8.9969E-10	4.75336E-09	RHSIM_Rhsim07G0185300
Root vs Leaf	RHSIM_Rhsim03G0145000	1.058880509	9.02757E-10	4.76848E-09	RHSIM_Rhsim03G0145000
Root vs Leaf	RHSIM_Rhsim08G0078600	1.41695114	9.15748E-10	4.836E-09	RHSIM_Rhsim08G0078600
Root vs Leaf	RHSIM_Rhsim09G0114700	2.650822972	9.17784E-10	4.84564E-09	RHSIM_Rhsim09G0114700
Root vs Leaf	RHSIM_Rhsim05G0191200	-2.415047665	9.3509E-10	4.93589E-09	RHSIM_Rhsim05G0191200
Root vs Leaf	RHSIM_Rhsim01G0133700	8.773043654	9.3615E-10	4.94035E-09	RHSIM_Rhsim01G0133700
Root vs Leaf	RHSIM_Rhsim07G0073200	1.789731172	9.5534E-10	5.04047E-09	RHSIM_Rhsim07G0073200
Root vs Leaf	RHSIM_Rhsim11G0096100	-2.649529552	9.75521E-10	5.14578E-09	RHSIM_Rhsim11G0096100
Root vs Leaf	RHSIM_Rhsim05G0074500	1.168748507	9.80057E-10	5.16853E-09	RHSIM_Rhsim05G0074500
Root vs Leaf	novel.1856	-1.048232254	9.91633E-10	5.22838E-09	--
Root vs Leaf	novel.1643	-2.253235748	9.96387E-10	5.25106E-09	--

Comparison	Gene ID	log_2FC	P-value	FDR (padj)	Gene Name
Root vs Leaf	RHSIM_Rhsim02G0059200	1.206034532	9.97123E-10	5.25374E-09	RHSIM_Rhsim02G0059200
Root vs Leaf	novel.4193	-8.563552341	1.00139E-09	5.27501E-09	--
Root vs Leaf	RHSIM_Rhsim04G0243400	-7.120325758	1.00269E-09	5.28065E-09	RHSIM_Rhsim04G0243400
Root vs Leaf	RHSIM_Rhsim05G0048900	1.156793268	1.01193E-09	5.32462E-09	RHSIM_Rhsim05G0048900
Root vs Leaf	RHSIM_Rhsim10G0025500	3.76947165	1.01196E-09	5.32462E-09	RHSIM_Rhsim10G0025500
Root vs Leaf	novel.5214	4.371607832	1.014E-09	5.33416E-09	--
Root vs Leaf	RHSIM_Rhsim03G0005100	1.339509221	1.01783E-09	5.35309E-09	RHSIM_Rhsim03G0005100
Root vs Leaf	RHSIM_Rhsim01G0027000	1.329542301	1.02661E-09	5.39679E-09	RHSIM_Rhsim01G0027000
Root vs Leaf	novel.6028	2.77756392	1.03686E-09	5.44947E-09	--
Root vs Leaf	RHSIM_Rhsim04G0059600	-10.75211836	1.0514E-09	5.52464E-09	RHSIM_Rhsim04G0059600
Root vs Leaf	RHSIM_Rhsim07G0090500	1.985731866	1.05912E-09	5.56265E-09	RHSIM_Rhsim07G0090500
Root vs Leaf	RHSIM_RhsimUnG0039400	-5.091022269	1.06034E-09	5.56653E-09	RHSIM_RhsimUnG0039400
Root vs Leaf	RHSIM_Rhsim13G0110400	-1.287254587	1.06784E-09	5.60465E-09	RHSIM_Rhsim13G0110400
Root vs Leaf	RHSIM_Rhsim13G0052300	1.693740335	1.06902E-09	5.60955E-09	RHSIM_Rhsim13G0052300
Root vs Leaf	RHSIM_Rhsim05G0196200	2.295490829	1.0693E-09	5.60977E-09	RHSIM_Rhsim05G0196200
Root vs Leaf	RHSIM_RhsimUnG0176100	8.22416068	1.07034E-09	5.61393E-09	RHSIM_RhsimUnG0176100
Root vs Leaf	novel.5309	-1.763430785	1.07094E-09	5.61581E-09	--
Root vs Leaf	RHSIM_Rhsim05G0189900	-5.199863862	1.08227E-09	5.67393E-09	RHSIM_Rhsim05G0189900
Root vs Leaf	RHSIM_Rhsim07G0190000	-1.568471232	1.08534E-09	5.68875E-09	RHSIM_Rhsim07G0190000
Root vs Leaf	RHSIM_Rhsim07G0227100	3.60646882	1.08936E-09	5.70853E-09	RHSIM_Rhsim07G0227100
Root vs Leaf	RHSIM_Rhsim10G0030200	2.485551756	1.11503E-09	5.84038E-09	RHSIM_Rhsim10G0030200
Root vs Leaf	RHSIM_Rhsim01G0023600	-3.170284067	1.11683E-09	5.84851E-09	RHSIM_Rhsim01G0023600
Root vs Leaf	RHSIM_Rhsim06G0113000	-8.299348424	1.13082E-09	5.92044E-09	RHSIM_Rhsim06G0113000
Root vs Leaf	RHSIM_Rhsim11G0051400	2.711278215	1.13403E-09	5.9359E-09	RHSIM_Rhsim11G0051400
Root vs Leaf	novel.2983	-5.21135638	1.15133E-09	6.02372E-09	--

Comparison	Gene ID	log_2FC	P-value	FDR (padj)	Gene Name
Root vs Leaf	RHSIM_Rhsim13G0171500	-6.003710915	1.15282E-09	6.03017E-09	RHSIM_Rhsim13G0171500
Root vs Leaf	novel.4175	-7.858717602	1.15672E-09	6.04919E-09	--
Root vs Leaf	RHSIM_Rhsim13G0127700	-1.41736544	1.15879E-09	6.05864E-09	RHSIM_Rhsim13G0127700
Root vs Leaf	RHSIM_Rhsim05G0071400	6.618180886	1.16172E-09	6.07259E-09	RHSIM_Rhsim05G0071400
Root vs Leaf	RHSIM_Rhsim13G0038500	-1.330941531	1.1621E-09	6.07318E-09	RHSIM_Rhsim13G0038500
Root vs Leaf	RHSIM_Rhsim04G0188300	-1.458822382	1.16362E-09	6.07977E-09	RHSIM_Rhsim04G0188300
Root vs Leaf	RHSIM_Rhsim07G0073600	1.909015878	1.16979E-09	6.11063E-09	RHSIM_Rhsim07G0073600
Root vs Leaf	RHSIM_Rhsim10G0153000	-1.930999719	1.17572E-09	6.14022E-09	RHSIM_Rhsim10G0153000
Root vs Leaf	RHSIM_Rhsim02G0116000	-7.470310043	1.186E-09	6.1925E-09	RHSIM_Rhsim02G0116000
Root vs Leaf	RHSIM_Rhsim08G0138100	1.532850011	1.18766E-09	6.19977E-09	RHSIM_Rhsim08G0138100
Root vs Leaf	novel.1640	-1.716834266	1.19765E-09	6.25052E-09	--
Root vs Leaf	RHSIM_Rhsim08G0156900	-5.813577352	1.19819E-09	6.25194E-09	RHSIM_Rhsim08G0156900
Root vs Leaf	novel.1256	-4.694081125	1.20011E-09	6.26053E-09	--
Root vs Leaf	RHSIM_Rhsim03G0250500	-3.592915857	1.2106E-09	6.31098E-09	RHSIM_Rhsim03G0250500
Root vs Leaf	RHSIM_Rhsim05G0127200	-5.33165643	1.21322E-09	6.32323E-09	RHSIM_Rhsim05G0127200
Root vs Leaf	RHSIM_Rhsim01G0002300	1.025256851	1.21389E-09	6.3253E-09	RHSIM_Rhsim01G0002300
Root vs Leaf	RHSIM_Rhsim08G0059300	-9.814453602	1.21931E-09	6.35065E-09	RHSIM_Rhsim08G0059300
Root vs Leaf	RHSIM_Rhsim13G0167100	6.336179623	1.26111E-09	6.56545E-09	RHSIM_Rhsim13G0167100
Root vs Leaf	RHSIM_Rhsim08G0248600	-2.777431264	1.26852E-09	6.60252E-09	RHSIM_Rhsim08G0248600
Root vs Leaf	novel.4328	8.018961863	1.28482E-09	6.68585E-09	--
Root vs Leaf	RHSIM_Rhsim01G0289500	1.245816963	1.29808E-09	6.75334E-09	RHSIM_Rhsim01G0289500
Root vs Leaf	novel.5685	4.164270558	1.30467E-09	6.78612E-09	--
Root vs Leaf	RHSIM_Rhsim03G0144000	-3.773496817	1.30669E-09	6.79506E-09	RHSIM_Rhsim03G0144000
Root vs Leaf	RHSIM_Rhsim11G0034000	-6.143985599	1.31382E-09	6.83065E-09	RHSIM_Rhsim11G0034000
Root vs Leaf	RHSIM_Rhsim08G0100100	-6.822469258	1.32413E-09	6.88269E-09	RHSIM_Rhsim08G0100100

Comparison	Gene ID	log_2FC	P-value	FDR (padj)	Gene Name
Root vs Leaf	novel.5629	2.879253423	1.32445E-09	6.88278E-09	--
Root vs Leaf	RHSIM_Rhsim10G0094900	1.196177146	1.32983E-09	6.9092E-09	RHSIM_Rhsim10G0094900
Root vs Leaf	RHSIM_Rhsim11G0091200	-2.272318038	1.33937E-09	6.95722E-09	RHSIM_Rhsim11G0091200
Root vs Leaf	novel.5952	-8.132245427	1.34597E-09	6.98991E-09	--
Root vs Leaf	novel.3243	-2.775626514	1.35012E-09	7.0099E-09	--
Root vs Leaf	RHSIM_Rhsim04G0180700	4.350397846	1.35577E-09	7.03768E-09	RHSIM_Rhsim04G0180700
Root vs Leaf	RHSIM_Rhsim02G0048800	-2.168277326	1.36815E-09	7.10034E-09	RHSIM_Rhsim02G0048800
Root vs Leaf	RHSIM_Rhsim07G0221500	-1.967306417	1.37688E-09	7.14403E-09	RHSIM_Rhsim07G0221500
Root vs Leaf	RHSIM_Rhsim12G0128900	9.287336334	1.38495E-09	7.18429E-09	RHSIM_Rhsim12G0128900
Root vs Leaf	RHSIM_Rhsim04G0075900	1.982459696	1.38629E-09	7.18961E-09	RHSIM_Rhsim04G0075900
Root vs Leaf	RHSIM_Rhsim08G0070900	-3.123161483	1.39022E-09	7.20841E-09	RHSIM_Rhsim08G0070900
Root vs Leaf	RHSIM_Rhsim04G0018200	-2.128709589	1.39518E-09	7.23251E-09	RHSIM_Rhsim04G0018200
Root vs Leaf	RHSIM_Rhsim09G0166300	1.186998778	1.3965E-09	7.23772E-09	RHSIM_Rhsim09G0166300
Root vs Leaf	RHSIM_Rhsim08G0170300	-5.97169559	1.40182E-09	7.26366E-09	RHSIM_Rhsim08G0170300
Root vs Leaf	RHSIM_Rhsim02G0090100	1.208023985	1.40334E-09	7.26989E-09	RHSIM_Rhsim02G0090100
Root vs Leaf	RHSIM_Rhsim01G0040300	-8.122076793	1.40984E-09	7.30197E-09	RHSIM_Rhsim01G0040300
Root vs Leaf	RHSIM_Rhsim08G0185900	3.201409026	1.41604E-09	7.3324E-09	RHSIM_Rhsim08G0185900
Root vs Leaf	RHSIM_Rhsim06G0075800	-8.282555277	1.41834E-09	7.34104E-09	RHSIM_Rhsim06G0075800
Root vs Leaf	RHSIM_Rhsim10G0205400	-1.351000009	1.42091E-09	7.35268E-09	RHSIM_Rhsim10G0205400
Root vs Leaf	RHSIM_Rhsim12G0137400	1.20652155	1.43148E-09	7.40573E-09	RHSIM_Rhsim12G0137400
Root vs Leaf	RHSIM_Rhsim07G0028000	-1.784739659	1.44756E-09	7.48725E-09	RHSIM_Rhsim07G0028000
Root vs Leaf	RHSIM_Rhsim07G0017300	1.841476785	1.46818E-09	7.58883E-09	RHSIM_Rhsim07G0017300
Root vs Leaf	RHSIM_Rhsim02G0079500	-8.438786011	1.48532E-09	7.67571E-09	RHSIM_Rhsim02G0079500
Root vs Leaf	novel.660	-8.208725311	1.48956E-09	7.69591E-09	--
Root vs Leaf	novel.5201	-1.166145295	1.50301E-09	7.76366E-09	--

Comparison	Gene ID	log$_2$FC	P-value	FDR (padj)	Gene Name
Root vs Leaf	RHSIM_Rhsim11G0000200	3.189580878	1.51326E-09	7.81486E-09	RHSIM_Rhsim11G0000200
Root vs Leaf	RHSIM_Rhsim07G0116400	-2.72996543	1.51474E-09	7.82075E-09	RHSIM_Rhsim07G0116400
Root vs Leaf	RHSIM_Rhsim06G0067700	1.30232189	1.54402E-09	7.96835E-09	RHSIM_Rhsim06G0067700
Root vs Leaf	RHSIM_Rhsim02G0248600	2.958539417	1.55611E-09	8.029E-09	RHSIM_Rhsim02G0248600
Root vs Leaf	RHSIM_RhsimUnG0183700	-3.109979928	1.55775E-09	8.03563E-09	RHSIM_RhsimUnG0183700
Root vs Leaf	RHSIM_Rhsim04G0155700	-1.32600515	1.55839E-09	8.03717E-09	RHSIM_Rhsim04G0155700
Root vs Leaf	RHSIM_Rhsim08G0057800	-8.264708281	1.56323E-09	8.06031E-09	RHSIM_Rhsim08G0057800
Root vs Leaf	RHSIM_Rhsim03G0211700	1.703647041	1.56364E-09	8.06064E-09	RHSIM_Rhsim03G0211700
Root vs Leaf	RHSIM_RhsimUnG0205900	-1.627619274	1.57208E-09	8.10055E-09	RHSIM_RhsimUnG0205900
Root vs Leaf	RHSIM_Rhsim11G0162200	-7.98899599	1.57474E-09	8.11109E-09	RHSIM_Rhsim11G0162200
Root vs Leaf	RHSIM_Rhsim07G0041400	-3.986233814	1.57483E-09	8.11109E-09	RHSIM_Rhsim07G0041400
Root vs Leaf	RHSIM_Rhsim07G0213800	1.572536411	1.57693E-09	8.12011E-09	RHSIM_Rhsim07G0213800
Root vs Leaf	RHSIM_Rhsim05G0111600	-1.364112662	1.5816E-09	8.14232E-09	RHSIM_Rhsim05G0111600
Root vs Leaf	RHSIM_Rhsim11G0159400	-1.687228466	1.59641E-09	8.21675E-09	RHSIM_Rhsim11G0159400
Root vs Leaf	RHSIM_Rhsim12G0006900	3.81667514	1.60366E-09	8.25226E-09	RHSIM_Rhsim12G0006900
Root vs Leaf	novel.1249	-8.6471617	1.60455E-09	8.25496E-09	--
Root vs Leaf	RHSIM_Rhsim12G0042600	-2.794050645	1.61131E-09	8.2879E-09	RHSIM_Rhsim12G0042600
Root vs Leaf	RHSIM_Rhsim06G0149400	1.371579941	1.61936E-09	8.32677E-09	RHSIM_Rhsim06G0149400
Root vs Leaf	novel.1668	-9.281982452	1.61958E-09	8.32677E-09	--
Root vs Leaf	novel.1661	-7.983110346	1.63089E-09	8.38303E-09	--
Root vs Leaf	novel.4761	-6.06752686	1.63967E-09	8.42632E-09	--
Root vs Leaf	RHSIM_Rhsim08G0005100	1.679254777	1.64095E-09	8.431E-09	RHSIM_Rhsim08G0005100
Root vs Leaf	RHSIM_Rhsim10G0049300	-4.314407844	1.64201E-09	8.4346E-09	RHSIM_Rhsim10G0049300
Root vs Leaf	RHSIM_Rhsim13G0077900	1.270884583	1.65243E-09	8.4862E-09	RHSIM_Rhsim13G0077900
Root vs Leaf	novel.4174	-7.998072647	1.65576E-09	8.50143E-09	--

Comparison	Gene ID	log_2FC	P-value	FDR (padj)	Gene Name
Root vs Leaf	RHSIM_Rhsim05G0115900	-1.279146133	1.66464E-09	8.54514E-09	RHSIM_Rhsim05G0115900
Root vs Leaf	RHSIM_Rhsim10G0105700	-2.597625851	1.67709E-09	8.60711E-09	RHSIM_Rhsim10G0105700
Root vs Leaf	RHSIM_Rhsim02G0090300	-1.359154248	1.69487E-09	8.69645E-09	RHSIM_Rhsim02G0090300
Root vs Leaf	novel.2642	7.556405591	1.69672E-09	8.704E-09	--
Root vs Leaf	RHSIM_Rhsim01G0254900	-2.043887555	1.70992E-09	8.76977E-09	RHSIM_Rhsim01G0254900
Root vs Leaf	RHSIM_RhsimUnG0223200	-7.93629888	1.73516E-09	8.89531E-09	RHSIM_RhsimUnG0223200
Root vs Leaf	novel.71	-1.476274705	1.73824E-09	8.908E-09	--
Root vs Leaf	RHSIM_Rhsim04G0167500	3.68571268	1.73841E-09	8.908E-09	RHSIM_Rhsim04G0167500
Root vs Leaf	novel.972	3.429161767	1.74856E-09	8.95802E-09	--
Root vs Leaf	RHSIM_Rhsim12G0007800	2.231418272	1.75429E-09	8.98484E-09	RHSIM_Rhsim12G0007800
Root vs Leaf	novel.2426	-1.648558606	1.75457E-09	8.98484E-09	--
Root vs Leaf	RHSIM_Rhsim05G0223800	1.262628322	1.76643E-09	9.04358E-09	RHSIM_Rhsim05G0223800
Root vs Leaf	RHSIM_Rhsim08G0034300	1.020804478	1.78629E-09	9.14322E-09	RHSIM_Rhsim08G0034300
Root vs Leaf	RHSIM_Rhsim06G0199100	1.235294903	1.80222E-09	9.22275E-09	RHSIM_Rhsim06G0199100
Root vs Leaf	RHSIM_Rhsim08G0036400	-5.801869011	1.8044E-09	9.23185E-09	RHSIM_Rhsim08G0036400
Root vs Leaf	novel.790	-1.817165096	1.80526E-09	9.23331E-09	--
Root vs Leaf	RHSIM_Rhsim01G0049100	1.364513296	1.80549E-09	9.23331E-09	RHSIM_Rhsim01G0049100
Root vs Leaf	RHSIM_Rhsim13G0110600	-2.104678786	1.82186E-09	9.31498E-09	RHSIM_Rhsim13G0110600
Root vs Leaf	RHSIM_Rhsim09G0120300	1.113669561	1.83158E-09	9.36262E-09	RHSIM_Rhsim09G0120300
Root vs Leaf	novel.5784	-8.760978517	1.83222E-09	9.36382E-09	--
Root vs Leaf	RHSIM_Rhsim10G0010300	2.311814233	1.83675E-09	9.38489E-09	RHSIM_Rhsim10G0010300
Root vs Leaf	novel.2819	4.541319819	1.83763E-09	9.38549E-09	--
Root vs Leaf	RHSIM_Rhsim09G0123700	-2.365928835	1.83768E-09	9.38549E-09	RHSIM_Rhsim09G0123700
Root vs Leaf	RHSIM_Rhsim10G0025400	4.099189469	1.84615E-09	9.42667E-09	RHSIM_Rhsim10G0025400
Root vs Leaf	novel.5497	-7.601591265	1.84876E-09	9.43796E-09	--

Comparison	Gene ID	log_2FC	P-value	FDR (padj)	Gene Name
Root vs Leaf	RHSIM_Rhsim12G0160900	-1.045790466	1.85528E-09	9.46915E-09	RHSIM_Rhsim12G0160900
Root vs Leaf	novel.1359	-2.052514493	1.86759E-09	9.52985E-09	--
Root vs Leaf	RHSIM_RhsimUnG0185000	-6.28958123	1.87535E-09	9.56737E-09	RHSIM_RhsimUnG0185000
Root vs Leaf	RHSIM_Rhsim12G0048200	3.574640165	1.87601E-09	9.56858E-09	RHSIM_Rhsim12G0048200
Root vs Leaf	novel.5075	-7.643611012	1.87806E-09	9.57694E-09	--
Root vs Leaf	RHSIM_Rhsim02G0082600	1.76842495	1.88238E-09	9.59688E-09	RHSIM_Rhsim02G0082600
Root vs Leaf	RHSIM_Rhsim08G0214200	1.522967386	1.91301E-09	9.74875E-09	RHSIM_Rhsim08G0214200
Root vs Leaf	RHSIM_Rhsim10G0056400	-2.130907963	1.91496E-09	9.75654E-09	RHSIM_Rhsim10G0056400
Root vs Leaf	RHSIM_RhsimUnG0065500	5.522308765	1.93337E-09	9.84813E-09	RHSIM_RhsimUnG0065500
Root vs Leaf	RHSIM_Rhsim06G0227000	-2.816684593	1.93758E-09	9.86742E-09	RHSIM_Rhsim06G0227000
Root vs Leaf	RHSIM_Rhsim04G0135000	-3.295330467	1.95282E-09	9.94283E-09	RHSIM_Rhsim04G0135000
Root vs Leaf	novel.592	2.609697614	1.9884E-09	1.01196E-08	--
Root vs Leaf	RHSIM_Rhsim06G0241400	1.208018523	1.98841E-09	1.01196E-08	RHSIM_Rhsim06G0241400
Root vs Leaf	RHSIM_Rhsim08G0002000	4.980927737	1.99405E-09	1.01461E-08	RHSIM_Rhsim08G0002000
Root vs Leaf	RHSIM_Rhsim05G0096900	2.366295334	2.00472E-09	1.01981E-08	RHSIM_Rhsim05G0096900
Root vs Leaf	RHSIM_Rhsim06G0073500	1.708555457	2.02277E-09	1.02877E-08	RHSIM_Rhsim06G0073500
Root vs Leaf	novel.548	1.152470188	2.03085E-09	1.03265E-08	--
Root vs Leaf	RHSIM_Rhsim13G0038200	1.309227385	2.03881E-09	1.03647E-08	RHSIM_Rhsim13G0038200
Root vs Leaf	RHSIM_Rhsim09G0181000	-2.300664162	2.04235E-09	1.03804E-08	RHSIM_Rhsim09G0181000
Root vs Leaf	RHSIM_Rhsim08G0051000	-7.89175457	2.04334E-09	1.03832E-08	RHSIM_Rhsim08G0051000
Root vs Leaf	RHSIM_Rhsim05G0160900	1.123837333	2.05198E-09	1.04248E-08	RHSIM_Rhsim05G0160900
Root vs Leaf	RHSIM_Rhsim08G0172200	1.358492126	2.05254E-09	1.04253E-08	RHSIM_Rhsim08G0172200
Root vs Leaf	RHSIM_Rhsim03G0153300	-4.081345599	2.07839E-09	1.05543E-08	RHSIM_Rhsim03G0153300
Root vs Leaf	RHSIM_Rhsim03G0229600	-1.654675758	2.09602E-09	1.06392E-08	RHSIM_Rhsim03G0229600
Root vs Leaf	RHSIM_Rhsim10G0113000	1.045190628	2.10405E-09	1.06776E-08	RHSIM_Rhsim10G0113000

Comparison	Gene ID	log_2FC	P-value	FDR (padj)	Gene Name
Root vs Leaf	RHSIM_Rhsim08G0033600	3.695367223	2.10581E-09	1.06842E-08	RHSIM_Rhsim08G0033600
Root vs Leaf	RHSIM_Rhsim04G0183500	8.155132425	2.13679E-09	1.0839E-08	RHSIM_Rhsim04G0183500
Root vs Leaf	RHSIM_Rhsim13G0188900	-8.102667886	2.14942E-09	1.09006E-08	RHSIM_Rhsim13G0188900
Root vs Leaf	RHSIM_Rhsim03G0057300	1.386427102	2.15272E-09	1.0915E-08	RHSIM_Rhsim03G0057300
Root vs Leaf	RHSIM_Rhsim06G0008700	5.164828519	2.17234E-09	1.10121E-08	RHSIM_Rhsim06G0008700
Root vs Leaf	RHSIM_Rhsim06G0043600	-5.623660876	2.1735E-09	1.10155E-08	RHSIM_Rhsim06G0043600
Root vs Leaf	RHSIM_Rhsim06G0033800	-1.706645201	2.18021E-09	1.10471E-08	RHSIM_Rhsim06G0033800
Root vs Leaf	RHSIM_Rhsim10G0109600	4.788313138	2.18405E-09	1.10642E-08	RHSIM_Rhsim10G0109600
Root vs Leaf	RHSIM_Rhsim12G0075200	-1.373601293	2.20706E-09	1.11783E-08	RHSIM_Rhsim12G0075200
Root vs Leaf	RHSIM_Rhsim06G0096400	8.732928038	2.20941E-09	1.11877E-08	RHSIM_Rhsim06G0096400
Root vs Leaf	RHSIM_Rhsim07G0022000	1.322539383	2.21362E-09	1.12066E-08	RHSIM_Rhsim07G0022000
Root vs Leaf	RHSIM_Rhsim04G0186700	-1.530419072	2.21584E-09	1.12129E-08	RHSIM_Rhsim04G0186700
Root vs Leaf	novel.2907	-7.073296675	2.2535E-09	1.13985E-08	--
Root vs Leaf	RHSIM_Rhsim04G0111400	-1.456424433	2.26274E-09	1.14428E-08	RHSIM_Rhsim04G0111400
Root vs Leaf	RHSIM_Rhsim13G0031500	-2.474084148	2.28581E-09	1.15569E-08	RHSIM_Rhsim13G0031500
Root vs Leaf	RHSIM_Rhsim08G0233000	3.030591575	2.30231E-09	1.16378E-08	RHSIM_Rhsim08G0233000
Root vs Leaf	RHSIM_Rhsim08G0217700	-6.184837621	2.33269E-09	1.17862E-08	RHSIM_Rhsim08G0217700
Root vs Leaf	RHSIM_Rhsim02G0083100	2.935764565	2.34071E-09	1.18241E-08	RHSIM_Rhsim02G0083100
Root vs Leaf	RHSIM_Rhsim10G0175300	5.2271676	2.35363E-09	1.18842E-08	RHSIM_Rhsim10G0175300
Root vs Leaf	novel.4954	2.165313486	2.36477E-09	1.19378E-08	--
Root vs Leaf	RHSIM_RhsimUnG0002400	-2.497085333	2.38297E-09	1.20271E-08	RHSIM_RhsimUnG0002400
Root vs Leaf	RHSIM_Rhsim04G0024400	8.748093597	2.40036E-09	1.21096E-08	RHSIM_Rhsim04G0024400
Root vs Leaf	RHSIM_Rhsim11G0168800	1.321409856	2.40159E-09	1.21131E-08	RHSIM_Rhsim11G0168800
Root vs Leaf	novel.1435	-6.254555339	2.42882E-09	1.22478E-08	--
Root vs Leaf	RHSIM_Rhsim11G0086800	5.793617791	2.43005E-09	1.22514E-08	RHSIM_Rhsim11G0086800

Comparison	Gene ID	log$_2$FC	P-value	FDR (padj)	Gene Name
Root vs Leaf	RHSIM_RhsimMtG0006700	-3.759693444	2.43189E-09	1.2258E-08	RHSIM_RhsimMtG0006700
Root vs Leaf	RHSIM_Rhsim08G0104800	2.444013088	2.44698E-09	1.23313E-08	RHSIM_Rhsim08G0104800
Root vs Leaf	RHSIM_Rhsim12G0127000	-1.972986908	2.44915E-09	1.23385E-08	RHSIM_Rhsim12G0127000
Root vs Leaf	RHSIM_Rhsim10G0082100	-1.168670106	2.44946E-09	1.23385E-08	RHSIM_Rhsim10G0082100
Root vs Leaf	RHSIM_Rhsim13G0203500	1.238623367	2.47332E-09	1.24559E-08	RHSIM_Rhsim13G0203500
Root vs Leaf	RHSIM_Rhsim08G0016900	-1.147481159	2.47703E-09	1.24692E-08	RHSIM_Rhsim08G0016900
Root vs Leaf	RHSIM_Rhsim09G0107100	8.411599976	2.5233E-09	1.26994E-08	RHSIM_Rhsim09G0107100
Root vs Leaf	RHSIM_RhsimUnG0079900	-7.657722911	2.55579E-09	1.28601E-08	RHSIM_RhsimUnG0079900
Root vs Leaf	RHSIM_Rhsim09G0206900	-2.897712533	2.56052E-09	1.28811E-08	RHSIM_Rhsim09G0206900
Root vs Leaf	RHSIM_Rhsim11G0109100	7.98754608	2.59895E-09	1.30687E-08	RHSIM_Rhsim11G0109100
Root vs Leaf	novel.4877	6.589202811	2.6009E-09	1.30757E-08	--
Root vs Leaf	RHSIM_Rhsim01G0010400	3.620245771	2.60245E-09	1.30806E-08	RHSIM_Rhsim01G0010400
Root vs Leaf	RHSIM_Rhsim01G0212600	1.289391865	2.60938E-09	1.31126E-08	RHSIM_Rhsim01G0212600
Root vs Leaf	RHSIM_Rhsim08G0195400	-4.450687106	2.62874E-09	1.3207E-08	RHSIM_Rhsim08G0195400
Root vs Leaf	novel.2755	8.670656962	2.63187E-09	1.32199E-08	--
Root vs Leaf	RHSIM_Rhsim03G0049600	1.282004954	2.63381E-09	1.32268E-08	RHSIM_Rhsim03G0049600
Root vs Leaf	RHSIM_Rhsim09G0182700	-1.538686108	2.63475E-09	1.32286E-08	RHSIM_Rhsim09G0182700
Root vs Leaf	RHSIM_Rhsim03G0211800	-1.129941893	2.64495E-09	1.3277E-08	RHSIM_Rhsim03G0211800
Root vs Leaf	RHSIM_Rhsim11G0056700	-8.653242252	2.65236E-09	1.33113E-08	RHSIM_Rhsim11G0056700
Root vs Leaf	RHSIM_Rhsim06G0133800	-2.945151253	2.65529E-09	1.33231E-08	RHSIM_Rhsim06G0133800
Root vs Leaf	RHSIM_Rhsim03G0234000	-1.02182939	2.66539E-09	1.33708E-08	RHSIM_Rhsim03G0234000
Root vs Leaf	RHSIM_Rhsim11G0181200	-6.27864688	2.73284E-09	1.37062E-08	RHSIM_Rhsim11G0181200
Root vs Leaf	novel.2622	-8.614984898	2.74562E-09	1.37673E-08	--
Root vs Leaf	RHSIM_Rhsim02G0007500	2.888423877	2.75758E-09	1.38244E-08	RHSIM_Rhsim02G0007500
Root vs Leaf	RHSIM_Rhsim07G0092200	2.080168958	2.79307E-09	1.39992E-08	RHSIM_Rhsim07G0092200

Comparison	Gene ID	log_2FC	P-value	FDR (padj)	Gene Name
Root vs Leaf	RHSIM_Rhsim03G0062100	4.55130734	2.82261E-09	1.41442E-08	RHSIM_Rhsim03G0062100
Root vs Leaf	RHSIM_Rhsim01G0205300	2.462088173	2.831E-09	1.41832E-08	RHSIM_Rhsim01G0205300
Root vs Leaf	novel.4978	-7.917674882	2.84451E-09	1.42478E-08	--
Root vs Leaf	RHSIM_Rhsim03G0035000	2.951819331	2.84744E-09	1.42594E-08	RHSIM_Rhsim03G0035000
Root vs Leaf	RHSIM_Rhsim12G0045800	1.242171813	2.84934E-09	1.42658E-08	RHSIM_Rhsim12G0045800
Root vs Leaf	RHSIM_Rhsim12G0040100	-1.1406443	2.85676E-09	1.42999E-08	RHSIM_Rhsim12G0040100
Root vs Leaf	RHSIM_Rhsim03G0034500	1.244006636	2.87789E-09	1.44025E-08	RHSIM_Rhsim03G0034500
Root vs Leaf	novel.1539	-2.380512885	2.92306E-09	1.46223E-08	--
Root vs Leaf	RHSIM_Rhsim01G0288700	-2.39049544	2.94781E-09	1.47429E-08	RHSIM_Rhsim01G0288700
Root vs Leaf	RHSIM_Rhsim12G0121900	1.014889285	2.94951E-09	1.47482E-08	RHSIM_Rhsim12G0121900
Root vs Leaf	RHSIM_Rhsim10G0210700	-2.552278229	2.95709E-09	1.47829E-08	RHSIM_Rhsim10G0210700
Root vs Leaf	RHSIM_Rhsim03G0055100	5.888102186	2.96504E-09	1.48195E-08	RHSIM_Rhsim03G0055100
Root vs Leaf	RHSIM_Rhsim08G0014300	5.748188098	2.98286E-09	1.49021E-08	RHSIM_Rhsim08G0014300
Root vs Leaf	RHSIM_Rhsim08G0025500	-4.015343952	3.00212E-09	1.49951E-08	RHSIM_Rhsim08G0025500
Root vs Leaf	RHSIM_Rhsim13G0090600	1.47302457	3.0115E-09	1.50387E-08	RHSIM_Rhsim13G0090600
Root vs Leaf	RHSIM_Rhsim01G0076800	2.14854589	3.01944E-09	1.5075E-08	RHSIM_Rhsim01G0076800
Root vs Leaf	RHSIM_Rhsim07G0060500	-6.369779472	3.03313E-09	1.51369E-08	RHSIM_Rhsim07G0060500
Root vs Leaf	RHSIM_RhsimUnG0134000	-3.764101139	3.04635E-09	1.51995E-08	RHSIM_RhsimUnG0134000
Root vs Leaf	novel.6118	-8.325799003	3.06182E-09	1.52735E-08	--
Root vs Leaf	RHSIM_Rhsim02G0247300	1.161278906	3.08843E-09	1.54029E-08	RHSIM_Rhsim02G0247300
Root vs Leaf	RHSIM_RhsimUnG0255800	6.191349465	3.09576E-09	1.54361E-08	RHSIM_RhsimUnG0255800
Root vs Leaf	novel.129	-8.862614244	3.11526E-09	1.553E-08	--
Root vs Leaf	RHSIM_Rhsim12G0123900	-1.775069451	3.16195E-09	1.57594E-08	RHSIM_Rhsim12G0123900
Root vs Leaf	novel.5081	-7.840154534	3.19038E-09	1.58942E-08	--
Root vs Leaf	RHSIM_Rhsim02G0070600	8.32758119	3.20364E-09	1.59568E-08	RHSIM_Rhsim02G0070600

Comparison	Gene ID	log_2FC	P-value	FDR (padj)	Gene Name
Root vs Leaf	RHSIM_Rhsim02G0216200	-1.356397431	3.26155E-09	1.62418E-08	RHSIM_Rhsim02G0216200
Root vs Leaf	RHSIM_Rhsim10G0140100	-3.972576613	3.27907E-09	1.63255E-08	RHSIM_Rhsim10G0140100
Root vs Leaf	RHSIM_RhsimUnG0142300	2.963937075	3.28E-09	1.63266E-08	RHSIM_RhsimUnG0142300
Root vs Leaf	RHSIM_Rhsim08G0195500	-2.629919165	3.28435E-09	1.63448E-08	RHSIM_Rhsim08G0195500
Root vs Leaf	RHSIM_Rhsim08G0099000	-1.816788219	3.29753E-09	1.64068E-08	RHSIM_Rhsim08G0099000
Root vs Leaf	RHSIM_Rhsim11G0171200	8.834994809	3.31394E-09	1.64849E-08	RHSIM_Rhsim11G0171200
Root vs Leaf	RHSIM_Rhsim08G0057600	-2.675879132	3.32207E-09	1.65218E-08	RHSIM_Rhsim08G0057600
Root vs Leaf	RHSIM_Rhsim09G0129100	-3.472841207	3.35233E-09	1.66687E-08	RHSIM_Rhsim09G0129100
Root vs Leaf	novel.1660	-1.18134142	3.35384E-09	1.66726E-08	--
Root vs Leaf	RHSIM_Rhsim12G0029700	-5.823914216	3.36117E-09	1.67055E-08	RHSIM_Rhsim12G0029700
Root vs Leaf	RHSIM_RhsimUnG0098900	7.849420751	3.39361E-09	1.68631E-08	RHSIM_RhsimUnG0098900
Root vs Leaf	RHSIM_Rhsim06G0168900	8.645769691	3.40644E-09	1.69232E-08	RHSIM_Rhsim06G0168900
Root vs Leaf	novel.207	-7.933550783	3.42306E-09	1.70021E-08	--
Root vs Leaf	RHSIM_Rhsim09G0039400	1.432028597	3.42418E-09	1.70041E-08	RHSIM_Rhsim09G0039400
Root vs Leaf	RHSIM_Rhsim09G0166700	3.294577192	3.43382E-09	1.70483E-08	RHSIM_Rhsim09G0166700
Root vs Leaf	RHSIM_RhsimUnG0249600	-2.272595839	3.4545E-09	1.71473E-08	RHSIM_RhsimUnG0249600
Root vs Leaf	RHSIM_RhsimMtG0003200	-4.865489174	3.48261E-09	1.72831E-08	RHSIM_RhsimMtG0003200
Root vs Leaf	novel.3031	-6.125197223	3.52385E-09	1.7484E-08	--
Root vs Leaf	RHSIM_Rhsim10G0050700	-7.79124568	3.62495E-09	1.79818E-08	RHSIM_Rhsim10G0050700
Root vs Leaf	novel.624	-7.837538458	3.64227E-09	1.80599E-08	--
Root vs Leaf	RHSIM_Rhsim09G0048000	2.125424621	3.64475E-09	1.80684E-08	RHSIM_Rhsim09G0048000
Root vs Leaf	RHSIM_Rhsim02G0073300	-1.899057349	3.736E-09	1.85128E-08	RHSIM_Rhsim02G0073300
Root vs Leaf	RHSIM_Rhsim12G0081500	2.309658488	3.80893E-09	1.88702E-08	RHSIM_Rhsim12G0081500
Root vs Leaf	novel.668	2.620182975	3.82713E-09	1.89563E-08	--
Root vs Leaf	RHSIM_Rhsim03G0202700	-7.378844856	3.82804E-09	1.89567E-08	RHSIM_Rhsim03G0202700

Comparison	Gene ID	log_2FC	P-value	FDR (padj)	Gene Name
Root vs Leaf	RHSIM_Rhsim08G0068800	1.622191043	3.83648E-09	1.89904E-08	RHSIM_Rhsim08G0068800
Root vs Leaf	RHSIM_Rhsim06G0213100	-4.16822117	3.84667E-09	1.9033E-08	RHSIM_Rhsim06G0213100
Root vs Leaf	RHSIM_Rhsim07G0203200	1.032590467	3.84673E-09	1.9033E-08	RHSIM_Rhsim07G0203200
Root vs Leaf	RHSIM_Rhsim11G0154100	-1.159128594	3.85472E-09	1.90684E-08	RHSIM_Rhsim11G0154100
Root vs Leaf	RHSIM_Rhsim11G0080800	-7.84365509	3.86568E-09	1.91185E-08	RHSIM_Rhsim11G0080800
Root vs Leaf	novel.2080	-1.360349973	3.86968E-09	1.91342E-08	--
Root vs Leaf	RHSIM_Rhsim09G0088900	1.087117611	3.87392E-09	1.91511E-08	RHSIM_Rhsim09G0088900
Root vs Leaf	RHSIM_Rhsim05G0194700	-1.980259736	3.888E-09	1.92166E-08	RHSIM_Rhsim05G0194700
Root vs Leaf	RHSIM_Rhsim12G0009500	4.005890846	3.91553E-09	1.93486E-08	RHSIM_Rhsim12G0009500
Root vs Leaf	RHSIM_Rhsim01G0210200	-1.361356698	3.91918E-09	1.93624E-08	RHSIM_Rhsim01G0210200
Root vs Leaf	RHSIM_Rhsim10G0200600	13.04454377	3.9312E-09	1.94177E-08	RHSIM_Rhsim10G0200600
Root vs Leaf	RHSIM_Rhsim04G0164400	2.92287998	3.934E-09	1.94274E-08	RHSIM_Rhsim04G0164400
Root vs Leaf	RHSIM_Rhsim07G0206000	-5.940365002	3.94327E-09	1.94681E-08	RHSIM_Rhsim07G0206000
Root vs Leaf	RHSIM_Rhsim09G0103100	7.641846318	3.94393E-09	1.94681E-08	RHSIM_Rhsim09G0103100
Root vs Leaf	RHSIM_Rhsim06G0057700	-1.505398327	3.97625E-09	1.96202E-08	RHSIM_Rhsim06G0057700
Root vs Leaf	RHSIM_Rhsim11G0127700	1.648997363	3.97644E-09	1.96202E-08	RHSIM_Rhsim11G0127700
Root vs Leaf	novel.3648	1.417402899	4.04684E-09	1.99591E-08	--
Root vs Leaf	RHSIM_Rhsim09G0110100	-1.687614392	4.06167E-09	2.00279E-08	RHSIM_Rhsim09G0110100
Root vs Leaf	RHSIM_Rhsim08G0179800	6.925863374	4.06792E-09	2.00545E-08	RHSIM_Rhsim08G0179800
Root vs Leaf	RHSIM_Rhsim09G0171500	1.619690957	4.07253E-09	2.00729E-08	RHSIM_Rhsim09G0171500
Root vs Leaf	novel.6063	-5.634442594	4.08471E-09	2.01287E-08	--
Root vs Leaf	RHSIM_Rhsim04G0221000	4.739636715	4.10686E-09	2.02335E-08	RHSIM_Rhsim04G0221000
Root vs Leaf	RHSIM_Rhsim05G0198200	4.169411632	4.10828E-09	2.02362E-08	RHSIM_Rhsim05G0198200
Root vs Leaf	RHSIM_Rhsim07G0204400	1.507489297	4.10945E-09	2.02377E-08	RHSIM_Rhsim07G0204400
Root vs Leaf	novel.1874	2.779861229	4.1681E-09	2.05221E-08	--

Comparison	Gene ID	log_2FC	P-value	FDR (padj)	Gene Name
Root vs Leaf	RHSIM_Rhsim02G0228900	1.006595486	4.21968E-09	2.07717E-08	RHSIM_Rhsim02G0228900
Root vs Leaf	RHSIM_RhsimUnG0109300	-1.292919173	4.24341E-09	2.08841E-08	RHSIM_RhsimUnG0109300
Root vs Leaf	RHSIM_Rhsim05G0232700	6.233929364	4.27407E-09	2.10305E-08	RHSIM_Rhsim05G0232700
Root vs Leaf	RHSIM_Rhsim03G0001900	1.874335639	4.30012E-09	2.11541E-08	RHSIM_Rhsim03G0001900
Root vs Leaf	RHSIM_Rhsim11G0040300	1.404496322	4.30721E-09	2.11845E-08	RHSIM_Rhsim11G0040300
Root vs Leaf	novel.2267	-2.336645193	4.40842E-09	2.16777E-08	--
Root vs Leaf	RHSIM_Rhsim06G0141300	8.703045048	4.41529E-09	2.17039E-08	RHSIM_Rhsim06G0141300
Root vs Leaf	RHSIM_Rhsim06G0197500	3.510887431	4.41563E-09	2.17039E-08	RHSIM_Rhsim06G0197500
Root vs Leaf	novel.4792	-6.237557342	4.42106E-09	2.1726E-08	--
Root vs Leaf	RHSIM_Rhsim03G0032100	7.954578631	4.43149E-09	2.17726E-08	RHSIM_Rhsim03G0032100
Root vs Leaf	RHSIM_Rhsim08G0093700	2.553792397	4.45631E-09	2.189E-08	RHSIM_Rhsim08G0093700
Root vs Leaf	RHSIM_Rhsim11G0194500	1.602703975	4.45814E-09	2.18942E-08	RHSIM_Rhsim11G0194500
Root vs Leaf	RHSIM_Rhsim13G0199200	-2.527288108	4.46051E-09	2.19012E-08	RHSIM_Rhsim13G0199200
Root vs Leaf	RHSIM_Rhsim06G0034200	2.557176316	4.49888E-09	2.2085E-08	RHSIM_Rhsim06G0034200
Root vs Leaf	RHSIM_Rhsim01G0039100	3.444720826	4.56284E-09	2.23895E-08	RHSIM_Rhsim01G0039100
Root vs Leaf	RHSIM_Rhsim06G0069000	5.792116506	4.57031E-09	2.24213E-08	RHSIM_Rhsim06G0069000
Root vs Leaf	RHSIM_Rhsim11G0043000	1.386257371	4.57851E-09	2.24568E-08	RHSIM_Rhsim11G0043000
Root vs Leaf	RHSIM_Rhsim12G0002100	-1.304772452	4.58046E-09	2.24616E-08	RHSIM_Rhsim12G0002100
Root vs Leaf	RHSIM_Rhsim12G0170700	2.673620046	4.60543E-09	2.25793E-08	RHSIM_Rhsim12G0170700
Root vs Leaf	RHSIM_Rhsim10G0196000	-1.148787692	4.64853E-09	2.27858E-08	RHSIM_Rhsim10G0196000
Root vs Leaf	RHSIM_Rhsim02G0131400	3.321104958	4.67146E-09	2.28933E-08	RHSIM_Rhsim02G0131400
Root vs Leaf	novel.50	-2.002101884	4.71749E-09	2.3114E-08	--
Root vs Leaf	RHSIM_Rhsim09G0097200	-1.52445074	4.74365E-09	2.32373E-08	RHSIM_Rhsim09G0097200
Root vs Leaf	RHSIM_Rhsim05G0057400	7.563305075	4.7471E-09	2.32492E-08	RHSIM_Rhsim05G0057400
Root vs Leaf	RHSIM_Rhsim01G0165900	-4.686322066	4.80248E-09	2.35155E-08	RHSIM_Rhsim01G0165900

Comparison	Gene ID	log_2FC	P-value	FDR (padj)	Gene Name
Root vs Leaf	RHSIM_Rhsim06G0112200	-8.299763641	4.81297E-09	2.35619E-08	RHSIM_Rhsim06G0112200
Root vs Leaf	RHSIM_Rhsim07G0006500	-2.322937889	4.85396E-09	2.37575E-08	RHSIM_Rhsim07G0006500
Root vs Leaf	RHSIM_Rhsim10G0045200	-1.13306676	4.92987E-09	2.41188E-08	RHSIM_Rhsim10G0045200
Root vs Leaf	RHSIM_Rhsim04G0226500	-4.385997005	4.96517E-09	2.42864E-08	RHSIM_Rhsim04G0226500
Root vs Leaf	novel.4092	-1.313360122	5.14117E-09	2.51367E-08	--
Root vs Leaf	RHSIM_Rhsim13G0055300	8.289146361	5.14321E-09	2.51413E-08	RHSIM_Rhsim13G0055300
Root vs Leaf	novel.2781	7.631253011	5.18212E-09	2.53262E-08	--
Root vs Leaf	RHSIM_Rhsim12G0063800	1.265074579	5.20432E-09	2.54293E-08	RHSIM_Rhsim12G0063800
Root vs Leaf	RHSIM_Rhsim08G0183000	1.058083752	5.28855E-09	2.58354E-08	RHSIM_Rhsim08G0183000
Root vs Leaf	RHSIM_Rhsim13G0008300	1.238936811	5.3655E-09	2.62058E-08	RHSIM_Rhsim13G0008300
Root vs Leaf	RHSIM_Rhsim10G0119800	-1.154530296	5.41967E-09	2.64648E-08	RHSIM_Rhsim10G0119800
Root vs Leaf	RHSIM_Rhsim07G0067800	-1.300096518	5.43191E-09	2.65189E-08	RHSIM_Rhsim07G0067800
Root vs Leaf	novel.1141	1.791640307	5.44542E-09	2.65793E-08	--
Root vs Leaf	RHSIM_Rhsim10G0103500	-4.106092879	5.52684E-09	2.69707E-08	RHSIM_Rhsim10G0103500
Root vs Leaf	RHSIM_Rhsim03G0141200	2.052729285	5.52882E-09	2.69707E-08	RHSIM_Rhsim03G0141200
Root vs Leaf	RHSIM_Rhsim08G0208200	3.00040762	5.52911E-09	2.69707E-08	RHSIM_Rhsim08G0208200
Root vs Leaf	RHSIM_Rhsim12G0080400	-3.988835173	5.55935E-09	2.71125E-08	RHSIM_Rhsim12G0080400
Root vs Leaf	RHSIM_Rhsim02G0029400	-6.817326408	5.62875E-09	2.74452E-08	RHSIM_Rhsim02G0029400
Root vs Leaf	RHSIM_Rhsim12G0186800	9.442980643	5.69401E-09	2.77576E-08	RHSIM_Rhsim12G0186800
Root vs Leaf	RHSIM_Rhsim07G0207300	8.384269749	5.71354E-09	2.7841E-08	RHSIM_Rhsim07G0207300
Root vs Leaf	RHSIM_Rhsim08G0208100	1.0045908	5.72166E-09	2.78747E-08	RHSIM_Rhsim08G0208100
Root vs Leaf	RHSIM_RhsimUnG0090600	-4.834854987	5.75015E-09	2.80076E-08	RHSIM_RhsimUnG0090600
Root vs Leaf	RHSIM_Rhsim10G0204400	-7.894303355	5.75615E-09	2.80309E-08	RHSIM_Rhsim10G0204400
Root vs Leaf	novel.3155	-3.395551657	5.78109E-09	2.81465E-08	--
Root vs Leaf	RHSIM_Rhsim12G0159800	-1.517424421	5.7915E-09	2.81912E-08	RHSIM_Rhsim12G0159800

Comparison	Gene ID	log_2FC	P-value	FDR (padj)	Gene Name
Root vs Leaf	RHSIM_Rhsim06G0020100	1.593197529	5.80983E-09	2.82745E-08	RHSIM_Rhsim06G0020100
Root vs Leaf	novel.4718	-7.846517399	5.81552E-09	2.82963E-08	--
Root vs Leaf	RHSIM_Rhsim06G0076900	2.190397289	5.82736E-09	2.83479E-08	RHSIM_Rhsim06G0076900
Root vs Leaf	RHSIM_Rhsim03G0196000	-8.295526306	5.84842E-09	2.84444E-08	RHSIM_Rhsim03G0196000
Root vs Leaf	RHSIM_Rhsim06G0120200	-7.785605993	5.86346E-09	2.85115E-08	RHSIM_Rhsim06G0120200
Root vs Leaf	RHSIM_Rhsim13G0050900	1.39523084	5.86623E-09	2.8519E-08	RHSIM_Rhsim13G0050900
Root vs Leaf	RHSIM_Rhsim10G0188600	1.056932793	5.88069E-09	2.85833E-08	RHSIM_Rhsim10G0188600
Root vs Leaf	RHSIM_Rhsim13G0015600	6.530318324	6.07769E-09	2.95346E-08	RHSIM_Rhsim13G0015600
Root vs Leaf	RHSIM_Rhsim02G0220200	-5.758240625	6.08114E-09	2.95452E-08	RHSIM_Rhsim02G0220200
Root vs Leaf	RHSIM_Rhsim10G0171100	1.706057349	6.12378E-09	2.97461E-08	RHSIM_Rhsim10G0171100
Root vs Leaf	RHSIM_Rhsim02G0070500	3.122671104	6.1339E-09	2.9789E-08	RHSIM_Rhsim02G0070500
Root vs Leaf	RHSIM_Rhsim08G0055700	-1.905713232	6.14929E-09	2.98575E-08	RHSIM_Rhsim08G0055700
Root vs Leaf	RHSIM_Rhsim01G0039600	-8.625801919	6.15683E-09	2.98878E-08	RHSIM_Rhsim01G0039600
Root vs Leaf	novel.2015	-3.251642108	6.16495E-09	2.99209E-08	--
Root vs Leaf	novel.52	-2.926562692	6.16692E-09	2.99242E-08	--
Root vs Leaf	novel.2649	1.683520821	6.17963E-09	2.99796E-08	--
Root vs Leaf	novel.5598	-3.889237049	6.20476E-09	3.00952E-08	--
Root vs Leaf	RHSIM_Rhsim01G0006100	2.943611374	6.21484E-09	3.01315E-08	RHSIM_Rhsim01G0006100
Root vs Leaf	RHSIM_Rhsim07G0094700	5.519290184	6.28557E-09	3.04617E-08	RHSIM_Rhsim07G0094700
Root vs Leaf	RHSIM_Rhsim13G0008400	-3.328277878	6.33884E-09	3.07134E-08	RHSIM_Rhsim13G0008400
Root vs Leaf	RHSIM_Rhsim07G0226100	8.002825707	6.48938E-09	3.14323E-08	RHSIM_Rhsim07G0226100
Root vs Leaf	RHSIM_RhsimUnG0053500	-3.249358436	6.48992E-09	3.14323E-08	RHSIM_RhsimUnG0053500
Root vs Leaf	RHSIM_Rhsim04G0232700	5.225925769	6.50475E-09	3.14921E-08	RHSIM_Rhsim04G0232700
Root vs Leaf	RHSIM_Rhsim06G0165600	2.340737542	6.50499E-09	3.14921E-08	RHSIM_Rhsim06G0165600
Root vs Leaf	RHSIM_Rhsim02G0212800	3.917968654	6.53109E-09	3.16118E-08	RHSIM_Rhsim02G0212800

Comparison	Gene ID	log_2FC	P-value	FDR (padj)	Gene Name
Root vs Leaf	RHSIM_Rhsim06G0040900	-1.516182528	6.57491E-09	3.18172E-08	RHSIM_Rhsim06G0040900
Root vs Leaf	novel.1175	7.500983255	6.64395E-09	3.21312E-08	--
Root vs Leaf	RHSIM_Rhsim13G0085300	1.027648991	6.67797E-09	3.22822E-08	RHSIM_Rhsim13G0085300
Root vs Leaf	RHSIM_Rhsim11G0172900	-3.716705866	6.73529E-09	3.25525E-08	RHSIM_Rhsim11G0172900
Root vs Leaf	novel.2971	-1.141717432	6.75646E-09	3.2648E-08	--
Root vs Leaf	RHSIM_Rhsim07G0006000	1.621293224	6.79665E-09	3.28353E-08	RHSIM_Rhsim07G0006000
Root vs Leaf	RHSIM_Rhsim07G0057400	5.652870495	6.90157E-09	3.33353E-08	RHSIM_Rhsim07G0057400
Root vs Leaf	RHSIM_Rhsim03G0155500	-1.810780437	6.91572E-09	3.33966E-08	RHSIM_Rhsim03G0155500
Root vs Leaf	RHSIM_Rhsim12G0005100	-2.484615608	6.94907E-09	3.35507E-08	RHSIM_Rhsim12G0005100
Root vs Leaf	novel.1103	-3.813877871	6.95481E-09	3.35714E-08	--
Root vs Leaf	RHSIM_Rhsim08G0200700	2.579471725	6.97188E-09	3.36468E-08	RHSIM_Rhsim08G0200700
Root vs Leaf	RHSIM_Rhsim01G0123600	4.803418651	7.02505E-09	3.38963E-08	RHSIM_Rhsim01G0123600
Root vs Leaf	RHSIM_Rhsim01G0040100	-8.299524345	7.0901E-09	3.4203E-08	RHSIM_Rhsim01G0040100
Root vs Leaf	RHSIM_Rhsim04G0188700	-5.987807608	7.12229E-09	3.43512E-08	RHSIM_Rhsim04G0188700
Root vs Leaf	RHSIM_Rhsim12G0163800	-3.973600917	7.20318E-09	3.47341E-08	RHSIM_Rhsim12G0163800
Root vs Leaf	RHSIM_Rhsim01G0196800	1.378059795	7.21098E-09	3.47644E-08	RHSIM_Rhsim01G0196800
Root vs Leaf	RHSIM_Rhsim10G0094000	5.926507421	7.25033E-09	3.49469E-08	RHSIM_Rhsim10G0094000
Root vs Leaf	RHSIM_Rhsim13G0105400	2.701403482	7.25772E-09	3.49752E-08	RHSIM_Rhsim13G0105400
Root vs Leaf	RHSIM_Rhsim01G0262800	-1.111231028	7.29589E-09	3.51518E-08	RHSIM_Rhsim01G0262800
Root vs Leaf	RHSIM_Rhsim05G0139500	1.352101847	7.42203E-09	3.57447E-08	RHSIM_Rhsim05G0139500
Root vs Leaf	RHSIM_Rhsim06G0169900	8.859670771	7.42622E-09	3.57574E-08	RHSIM_Rhsim06G0169900
Root vs Leaf	novel.171	-7.990160324	7.44591E-09	3.58447E-08	--
Root vs Leaf	RHSIM_Rhsim05G0109000	4.406667169	7.52041E-09	3.61884E-08	RHSIM_Rhsim05G0109000
Root vs Leaf	novel.96	-6.741368588	7.55564E-09	3.63503E-08	--
Root vs Leaf	RHSIM_Rhsim06G0001100	-2.275600306	7.57911E-09	3.64557E-08	RHSIM_Rhsim06G0001100

Comparison	Gene ID	log2FC	P-value	FDR (padj)	Gene Name
Root vs Leaf	RHSIM_Rhsim03G0035900	2.764235901	7.58555E-09	3.64791E-08	RHSIM_Rhsim03G0035900
Root vs Leaf	RHSIM_Rhsim08G0083500	2.29497387	7.61277E-09	3.66023E-08	RHSIM_Rhsim08G0083500
Root vs Leaf	RHSIM_Rhsim03G0250300	-1.471696585	7.62487E-09	3.66529E-08	RHSIM_Rhsim03G0250300
Root vs Leaf	RHSIM_Rhsim04G0184500	-8.451405243	7.62779E-09	3.66593E-08	RHSIM_Rhsim04G0184500
Root vs Leaf	RHSIM_Rhsim06G0034000	-2.025039506	7.67848E-09	3.68953E-08	RHSIM_Rhsim06G0034000
Root vs Leaf	RHSIM_Rhsim09G0134700	-7.458772434	7.68059E-09	3.68978E-08	RHSIM_Rhsim09G0134700
Root vs Leaf	RHSIM_Rhsim03G0034900	3.960670325	7.69204E-09	3.69451E-08	RHSIM_Rhsim03G0034900
Root vs Leaf	RHSIM_Rhsim05G0011300	-6.655180731	7.74988E-09	3.72152E-08	RHSIM_Rhsim05G0011300
Root vs Leaf	RHSIM_Rhsim04G0114000	1.340714488	7.85276E-09	3.77014E-08	RHSIM_Rhsim04G0114000
Root vs Leaf	RHSIM_Rhsim13G0227500	-1.119005813	7.86807E-09	3.77671E-08	RHSIM_Rhsim13G0227500
Root vs Leaf	RHSIM_Rhsim08G0210500	-1.386039884	7.89064E-09	3.78675E-08	RHSIM_Rhsim08G0210500
Root vs Leaf	RHSIM_Rhsim06G0049200	-4.276219195	7.89408E-09	3.78762E-08	RHSIM_Rhsim06G0049200
Root vs Leaf	RHSIM_Rhsim06G0128900	-3.032287135	7.91404E-09	3.79641E-08	RHSIM_Rhsim06G0128900
Root vs Leaf	RHSIM_Rhsim03G0173300	-1.026661717	7.96899E-09	3.82198E-08	RHSIM_Rhsim03G0173300
Root vs Leaf	novel.3581	-3.717505573	8.00277E-09	3.83738E-08	--
Root vs Leaf	RHSIM_Rhsim07G0127500	1.607161707	8.02599E-09	3.84692E-08	RHSIM_Rhsim07G0127500
Root vs Leaf	RHSIM_Rhsim01G0116400	-2.068923204	8.06109E-09	3.86295E-08	RHSIM_Rhsim01G0116400
Root vs Leaf	RHSIM_Rhsim12G0070200	-8.040540356	8.12667E-09	3.89276E-08	RHSIM_Rhsim12G0070200
Root vs Leaf	RHSIM_Rhsim10G0072000	2.294762399	8.12962E-09	3.89337E-08	RHSIM_Rhsim10G0072000
Root vs Leaf	RHSIM_Rhsim11G0110600	7.775229927	8.16068E-09	3.90662E-08	RHSIM_Rhsim11G0110600
Root vs Leaf	RHSIM_Rhsim02G0114000	-7.39479797	8.1802E-09	3.91516E-08	RHSIM_Rhsim02G0114000
Root vs Leaf	novel.498	-7.884062233	8.198E-09	3.92287E-08	--
Root vs Leaf	RHSIM_Rhsim03G0215000	-2.262389591	8.21407E-09	3.92974E-08	RHSIM_Rhsim03G0215000
Root vs Leaf	novel.5758	-1.542804174	8.22833E-09	3.93575E-08	--
Root vs Leaf	RHSIM_Rhsim04G0013600	-1.892805271	8.24538E-09	3.9431E-08	RHSIM_Rhsim04G0013600

Comparison	Gene ID	log_2FC	P-value	FDR (padj)	Gene Name
Root vs Leaf	RHSIM_Rhsim07G0002700	3.348178895	8.25431E-09	3.94655E-08	RHSIM_Rhsim07G0002700
Root vs Leaf	RHSIM_Rhsim08G0010100	9.155253685	8.26912E-09	3.95281E-08	RHSIM_Rhsim08G0010100
Root vs Leaf	RHSIM_Rhsim03G0252200	3.172054779	8.29331E-09	3.96356E-08	RHSIM_Rhsim03G0252200
Root vs Leaf	RHSIM_Rhsim07G0059300	-1.279992556	8.36949E-09	3.99914E-08	RHSIM_Rhsim07G0059300
Root vs Leaf	RHSIM_Rhsim09G0119600	-1.009360691	8.37351E-09	4.00023E-08	RHSIM_Rhsim09G0119600
Root vs Leaf	RHSIM_RhsimUnG0120000	7.612358382	8.40996E-09	4.01682E-08	RHSIM_RhsimUnG0120000
Root vs Leaf	RHSIM_Rhsim06G0073900	1.275573276	8.54353E-09	4.07978E-08	RHSIM_Rhsim06G0073900
Root vs Leaf	RHSIM_Rhsim01G0221900	1.441404083	8.55337E-09	4.08363E-08	RHSIM_Rhsim01G0221900
Root vs Leaf	RHSIM_Rhsim08G0036000	-1.686464758	8.56398E-09	4.08785E-08	RHSIM_Rhsim08G0036000
Root vs Leaf	RHSIM_Rhsim11G0187900	-12.26427554	8.66545E-09	4.13543E-08	RHSIM_Rhsim11G0187900
Root vs Leaf	RHSIM_Rhsim11G0043400	1.261261546	8.66793E-09	4.13577E-08	RHSIM_Rhsim11G0043400
Root vs Leaf	RHSIM_Rhsim12G0151000	2.703654889	8.68392E-09	4.14254E-08	RHSIM_Rhsim12G0151000
Root vs Leaf	novel.299	1.175362503	8.75469E-09	4.17544E-08	--
Root vs Leaf	RHSIM_Rhsim13G0010500	1.845271932	8.79293E-09	4.19237E-08	RHSIM_Rhsim13G0010500
Root vs Leaf	RHSIM_Rhsim13G0061300	7.423087713	8.79382E-09	4.19237E-08	RHSIM_Rhsim13G0061300
Root vs Leaf	RHSIM_Rhsim13G0198200	1.261680042	8.8287E-09	4.20813E-08	RHSIM_Rhsim13G0198200
Root vs Leaf	RHSIM_Rhsim10G0177600	5.666091032	8.8836E-09	4.23343E-08	RHSIM_Rhsim10G0177600
Root vs Leaf	RHSIM_Rhsim03G0052800	3.464876036	8.95387E-09	4.26604E-08	RHSIM_Rhsim03G0052800
Root vs Leaf	RHSIM_Rhsim03G0175200	-5.741350692	9.07053E-09	4.32073E-08	RHSIM_Rhsim03G0175200
Root vs Leaf	RHSIM_Rhsim12G0084700	7.305333525	9.13249E-09	4.34935E-08	RHSIM_Rhsim12G0084700
Root vs Leaf	RHSIM_Rhsim02G0040600	1.428506306	9.1469E-09	4.35532E-08	RHSIM_Rhsim02G0040600
Root vs Leaf	RHSIM_RhsimUnG0057300	-7.127313558	9.1567E-09	4.35908E-08	RHSIM_RhsimUnG0057300
Root vs Leaf	RHSIM_Rhsim09G0121700	-8.702376721	9.21808E-09	4.3874E-08	RHSIM_Rhsim09G0121700
Root vs Leaf	RHSIM_Rhsim08G0148000	-2.824359277	9.23988E-09	4.39688E-08	RHSIM_Rhsim08G0148000
Root vs Leaf	RHSIM_Rhsim03G0222100	-3.488338211	9.26023E-09	4.40565E-08	RHSIM_Rhsim03G0222100

Comparison	Gene ID	log_2FC	P-value	FDR (padj)	Gene Name
Root vs Leaf	RHSIM_Rhsim03G0205600	-2.419414756	9.26879E-09	4.40882E-08	RHSIM_Rhsim03G0205600
Root vs Leaf	RHSIM_Rhsim01G0223500	2.467021445	9.30813E-09	4.42662E-08	RHSIM_Rhsim01G0223500
Root vs Leaf	RHSIM_Rhsim01G0053000	-4.218441251	9.35776E-09	4.44931E-08	RHSIM_Rhsim01G0053000
Root vs Leaf	novel.4606	1.712308609	9.38687E-09	4.46223E-08	--
Root vs Leaf	RHSIM_Rhsim04G0220600	1.182512162	9.40486E-09	4.46987E-08	RHSIM_Rhsim04G0220600
Root vs Leaf	RHSIM_Rhsim05G0049900	3.185487963	9.41601E-09	4.47425E-08	RHSIM_Rhsim05G0049900
Root vs Leaf	RHSIM_Rhsim12G0149000	-1.20407876	9.57871E-09	4.54969E-08	RHSIM_Rhsim12G0149000
Root vs Leaf	RHSIM_Rhsim10G0167100	-1.82186468	9.61083E-09	4.56401E-08	RHSIM_Rhsim10G0167100
Root vs Leaf	RHSIM_Rhsim11G0128900	-7.200307425	9.75126E-09	4.62975E-08	RHSIM_Rhsim11G0128900
Root vs Leaf	RHSIM_Rhsim07G0202300	-6.193964581	9.79778E-09	4.65088E-08	RHSIM_Rhsim07G0202300
Root vs Leaf	RHSIM_Rhsim01G0238100	2.031697455	9.80279E-09	4.6523E-08	RHSIM_Rhsim01G0238100
Root vs Leaf	RHSIM_Rhsim01G0012600	-2.577768879	9.8517E-09	4.67456E-08	RHSIM_Rhsim01G0012600
Root vs Leaf	RHSIM_Rhsim03G0199700	-8.236367195	9.98491E-09	4.73679E-08	RHSIM_Rhsim03G0199700
Root vs Leaf	novel.5768	2.288501348	9.9871E-09	4.73686E-08	--
Root vs Leaf	RHSIM_Rhsim03G0029600	-3.659626141	1.0008E-08	4.7458E-08	RHSIM_Rhsim03G0029600
Root vs Leaf	RHSIM_Rhsim12G0105600	2.796226296	1.00474E-08	4.76352E-08	RHSIM_Rhsim12G0105600
Root vs Leaf	RHSIM_Rhsim05G0231000	-1.059083096	1.00862E-08	4.78092E-08	RHSIM_Rhsim05G0231000
Root vs Leaf	RHSIM_Rhsim13G0179500	-1.562102482	1.01513E-08	4.81081E-08	RHSIM_Rhsim13G0179500
Root vs Leaf	RHSIM_Rhsim13G0069600	-8.010495218	1.03261E-08	4.89256E-08	RHSIM_Rhsim13G0069600
Root vs Leaf	RHSIM_Rhsim06G0055500	-7.839208572	1.03281E-08	4.89256E-08	RHSIM_Rhsim06G0055500
Root vs Leaf	RHSIM_Rhsim12G0168600	-2.118433051	1.03364E-08	4.89492E-08	RHSIM_Rhsim12G0168600
Root vs Leaf	novel.1722	1.626526685	1.03513E-08	4.90054E-08	--
Root vs Leaf	RHSIM_Rhsim11G0105400	8.287675948	1.04189E-08	4.93154E-08	RHSIM_Rhsim11G0105400
Root vs Leaf	RHSIM_Rhsim05G0048500	1.641488407	1.0465E-08	4.95235E-08	RHSIM_Rhsim05G0048500
Root vs Leaf	RHSIM_Rhsim07G0144600	1.157000911	1.04798E-08	4.95836E-08	RHSIM_Rhsim07G0144600

Comparison	Gene ID	log_2FC	P-value	FDR (padj)	Gene Name
Root vs Leaf	RHSIM_Rhsim04G0181900	-1.271903747	1.07692E-08	5.09319E-08	RHSIM_Rhsim04G0181900
Root vs Leaf	novel.3928	-7.788711705	1.07846E-08	5.09946E-08	--
Root vs Leaf	RHSIM_Rhsim13G0151700	8.885945799	1.08042E-08	5.10765E-08	RHSIM_Rhsim13G0151700
Root vs Leaf	RHSIM_Rhsim12G0156500	1.610189763	1.08243E-08	5.11614E-08	RHSIM_Rhsim12G0156500
Root vs Leaf	RHSIM_Rhsim02G0115800	-5.257118904	1.08878E-08	5.145E-08	RHSIM_Rhsim02G0115800
Root vs Leaf	RHSIM_Rhsim13G0134600	-2.48776681	1.08898E-08	5.145E-08	RHSIM_Rhsim13G0134600
Root vs Leaf	RHSIM_Rhsim10G0044200	-1.273138915	1.09921E-08	5.19121E-08	RHSIM_Rhsim10G0044200
Root vs Leaf	RHSIM_Rhsim07G0202800	1.21210126	1.11844E-08	5.27983E-08	RHSIM_Rhsim07G0202800
Root vs Leaf	RHSIM_Rhsim12G0187700	1.404741881	1.11999E-08	5.28608E-08	RHSIM_Rhsim12G0187700
Root vs Leaf	RHSIM_Rhsim06G0184900	3.154151017	1.12186E-08	5.29382E-08	RHSIM_Rhsim06G0184900
Root vs Leaf	RHSIM_Rhsim10G0173300	2.15646386	1.12222E-08	5.29447E-08	RHSIM_Rhsim10G0173300
Root vs Leaf	RHSIM_Rhsim12G0141500	-1.053900365	1.12267E-08	5.29548E-08	RHSIM_Rhsim12G0141500
Root vs Leaf	RHSIM_Rhsim13G0204100	1.251624995	1.14492E-08	5.39934E-08	RHSIM_Rhsim13G0204100
Root vs Leaf	RHSIM_Rhsim13G0137100	2.374074399	1.14564E-08	5.40165E-08	RHSIM_Rhsim13G0137100
Root vs Leaf	RHSIM_Rhsim13G0099300	7.851440179	1.14857E-08	5.41434E-08	RHSIM_Rhsim13G0099300
Root vs Leaf	RHSIM_Rhsim07G0026300	7.472079003	1.16054E-08	5.46854E-08	RHSIM_Rhsim07G0026300
Root vs Leaf	RHSIM_Rhsim10G0122200	5.681366326	1.16561E-08	5.49131E-08	RHSIM_Rhsim10G0122200
Root vs Leaf	RHSIM_Rhsim06G0041400	-2.944002043	1.17061E-08	5.51264E-08	RHSIM_Rhsim06G0041400
Root vs Leaf	novel.2252	3.596555415	1.17427E-08	5.52874E-08	--
Root vs Leaf	RHSIM_Rhsim01G0248900	-1.266542761	1.17788E-08	5.54464E-08	RHSIM_Rhsim01G0248900
Root vs Leaf	RHSIM_Rhsim02G0056600	1.654646113	1.1922E-08	5.60976E-08	RHSIM_Rhsim02G0056600
Root vs Leaf	RHSIM_Rhsim13G0092400	1.21543209	1.19827E-08	5.63715E-08	RHSIM_Rhsim13G0092400
Root vs Leaf	RHSIM_Rhsim04G0039000	1.34082245	1.20545E-08	5.66863E-08	RHSIM_Rhsim04G0039000
Root vs Leaf	RHSIM_Rhsim04G0215100	8.327095062	1.22423E-08	5.75575E-08	RHSIM_Rhsim04G0215100
Root vs Leaf	novel.3412	-2.474523537	1.22674E-08	5.7664E-08	--

Comparison	Gene ID	log_2FC	P-value	FDR (padj)	Gene Name
Root vs Leaf	RHSIM_Rhsim01G0117000	7.98226576	1.23011E-08	5.78109E-08	RHSIM_Rhsim01G0117000
Root vs Leaf	RHSIM_Rhsim05G0138700	-1.474554919	1.23451E-08	5.80057E-08	RHSIM_Rhsim05G0138700
Root vs Leaf	RHSIM_Rhsim03G0246100	1.752790907	1.23993E-08	5.82484E-08	RHSIM_Rhsim03G0246100
Root vs Leaf	RHSIM_Rhsim02G0061700	1.262244725	1.25114E-08	5.87512E-08	RHSIM_Rhsim02G0061700
Root vs Leaf	RHSIM_Rhsim04G0248600	2.06059831	1.25769E-08	5.9047E-08	RHSIM_Rhsim04G0248600
Root vs Leaf	RHSIM_Rhsim08G0156100	-8.165748607	1.25983E-08	5.91353E-08	RHSIM_Rhsim08G0156100
Root vs Leaf	RHSIM_Rhsim01G0232100	-2.22311382	1.26103E-08	5.91793E-08	RHSIM_Rhsim01G0232100
Root vs Leaf	RHSIM_Rhsim04G0201600	-1.138710055	1.26128E-08	5.91793E-08	RHSIM_Rhsim04G0201600
Root vs Leaf	RHSIM_Rhsim01G0204900	-3.032398002	1.26167E-08	5.91857E-08	RHSIM_Rhsim01G0204900
Root vs Leaf	RHSIM_Rhsim13G0223600	1.355349531	1.26595E-08	5.93743E-08	RHSIM_Rhsim13G0223600
Root vs Leaf	RHSIM_Rhsim01G0286800	3.958709143	1.27886E-08	5.99557E-08	RHSIM_Rhsim01G0286800
Root vs Leaf	RHSIM_Rhsim01G0127000	1.074249633	1.27921E-08	5.99599E-08	RHSIM_Rhsim01G0127000
Root vs Leaf	RHSIM_Rhsim07G0030300	-2.04820335	1.28223E-08	6.00893E-08	RHSIM_Rhsim07G0030300
Root vs Leaf	RHSIM_Rhsim04G0018800	-2.925103036	1.28641E-08	6.02732E-08	RHSIM_Rhsim04G0018800
Root vs Leaf	RHSIM_Rhsim11G0052100	-1.076532837	1.29311E-08	6.05744E-08	RHSIM_Rhsim11G0052100
Root vs Leaf	RHSIM_Rhsim09G0198900	-1.312848752	1.29444E-08	6.06248E-08	RHSIM_Rhsim09G0198900
Root vs Leaf	RHSIM_Rhsim06G0008800	1.701075988	1.30906E-08	6.12722E-08	RHSIM_Rhsim06G0008800
Root vs Leaf	RHSIM_Rhsim02G0241800	2.244128336	1.31352E-08	6.14684E-08	RHSIM_Rhsim02G0241800
Root vs Leaf	RHSIM_Rhsim11G0084800	5.656210618	1.31679E-08	6.16091E-08	RHSIM_Rhsim11G0084800
Root vs Leaf	novel.1431	1.347788056	1.31758E-08	6.16335E-08	--
Root vs Leaf	novel.118	-8.841097744	1.32293E-08	6.18714E-08	--
Root vs Leaf	RHSIM_Rhsim10G0052700	-3.835195561	1.32626E-08	6.20147E-08	RHSIM_Rhsim10G0052700
Root vs Leaf	RHSIM_Rhsim13G0074100	-1.223768598	1.33541E-08	6.24299E-08	RHSIM_Rhsim13G0074100
Root vs Leaf	novel.441	-7.642318904	1.33938E-08	6.26026E-08	--
Root vs Leaf	RHSIM_Rhsim01G0211100	1.769274732	1.34341E-08	6.27782E-08	RHSIM_Rhsim01G0211100

Comparison	Gene ID	log2FC	P-value	FDR (padj)	Gene Name
Root vs Leaf	RHSIM_Rhsim09G0200200	-1.163684366	1.38859E-08	6.48634E-08	RHSIM_Rhsim09G0200200
Root vs Leaf	RHSIM_Rhsim07G0107200	-1.289885728	1.39085E-08	6.49558E-08	RHSIM_Rhsim07G0107200
Root vs Leaf	RHSIM_Rhsim12G0111500	3.499679207	1.3918E-08	6.4987E-08	RHSIM_Rhsim12G0111500
Root vs Leaf	RHSIM_Rhsim03G0199100	-4.016166988	1.39554E-08	6.51485E-08	RHSIM_Rhsim03G0199100
Root vs Leaf	RHSIM_Rhsim07G0060900	-2.823952706	1.40222E-08	6.54473E-08	RHSIM_Rhsim07G0060900
Root vs Leaf	novel.3730	1.576600105	1.40554E-08	6.55892E-08	--
Root vs Leaf	RHSIM_Rhsim07G0029400	1.679626781	1.41704E-08	6.61124E-08	RHSIM_Rhsim07G0029400
Root vs Leaf	RHSIM_Rhsim03G0190200	-1.325041274	1.42814E-08	6.6617E-08	RHSIM_Rhsim03G0190200
Root vs Leaf	RHSIM_Rhsim03G0096000	-2.027408975	1.45371E-08	6.77956E-08	RHSIM_Rhsim03G0096000
Root vs Leaf	RHSIM_Rhsim12G0188300	-5.754034931	1.45504E-08	6.78442E-08	RHSIM_Rhsim12G0188300
Root vs Leaf	RHSIM_Rhsim08G0229400	3.815519006	1.46381E-08	6.82393E-08	RHSIM_Rhsim08G0229400
Root vs Leaf	RHSIM_Rhsim11G0077900	1.599480176	1.46814E-08	6.84275E-08	RHSIM_Rhsim11G0077900
Root vs Leaf	novel.4874	-3.040196622	1.48375E-08	6.91412E-08	--
Root vs Leaf	RHSIM_Rhsim12G0087200	-3.94696208	1.48751E-08	6.92936E-08	RHSIM_Rhsim12G0087200
Root vs Leaf	RHSIM_Rhsim12G0023600	1.366391571	1.48762E-08	6.92936E-08	RHSIM_Rhsim12G0023600
Root vs Leaf	RHSIM_Rhsim03G0138700	1.655271433	1.48874E-08	6.93319E-08	RHSIM_Rhsim03G0138700
Root vs Leaf	novel.4893	-2.52803959	1.49075E-08	6.94114E-08	--
Root vs Leaf	RHSIM_Rhsim11G0148100	-3.403340652	1.49771E-08	6.97213E-08	RHSIM_Rhsim11G0148100
Root vs Leaf	RHSIM_Rhsim13G0200300	5.80973649	1.49909E-08	6.97714E-08	RHSIM_Rhsim13G0200300
Root vs Leaf	RHSIM_Rhsim04G0185700	8.620293771	1.50781E-08	7.01634E-08	RHSIM_Rhsim04G0185700
Root vs Leaf	RHSIM_Rhsim06G0140100	-3.918493088	1.51124E-08	7.0309E-08	RHSIM_Rhsim06G0140100
Root vs Leaf	RHSIM_Rhsim02G0252600	1.089148637	1.51231E-08	7.03446E-08	RHSIM_Rhsim02G0252600
Root vs Leaf	novel.3505	7.415493253	1.53436E-08	7.13412E-08	--
Root vs Leaf	RHSIM_Rhsim12G0194800	-1.589102569	1.53833E-08	7.15115E-08	RHSIM_Rhsim12G0194800
Root vs Leaf	RHSIM_Rhsim13G0047400	2.127819089	1.5426E-08	7.16955E-08	RHSIM_Rhsim13G0047400

Comparison	Gene ID	log_2FC	P-value	FDR (padj)	Gene Name
Root vs Leaf	RHSIM_Rhsim10G0006300	-1.308012989	1.55025E-08	7.20367E-08	RHSIM_Rhsim10G0006300
Root vs Leaf	RHSIM_Rhsim02G0049500	-2.151174815	1.55201E-08	7.21043E-08	RHSIM_Rhsim02G0049500
Root vs Leaf	novel.5704	-2.68017792	1.55427E-08	7.21948E-08	--
Root vs Leaf	RHSIM_Rhsim08G0017600	3.725183168	1.56468E-08	7.26635E-08	RHSIM_Rhsim08G0017600
Root vs Leaf	RHSIM_Rhsim06G0168600	1.008902348	1.57197E-08	7.29873E-08	RHSIM_Rhsim06G0168600
Root vs Leaf	RHSIM_Rhsim06G0061200	8.080350848	1.57643E-08	7.318E-08	RHSIM_Rhsim06G0061200
Root vs Leaf	RHSIM_RhsimUnG0231700	1.487941967	1.57749E-08	7.32143E-08	RHSIM_RhsimUnG0231700
Root vs Leaf	RHSIM_Rhsim05G0228100	1.049282587	1.58067E-08	7.33472E-08	RHSIM_Rhsim05G0228100
Root vs Leaf	RHSIM_Rhsim07G0249200	-4.279517809	1.58653E-08	7.36046E-08	RHSIM_Rhsim07G0249200
Root vs Leaf	novel.5833	1.115734613	1.58897E-08	7.37028E-08	--
Root vs Leaf	RHSIM_Rhsim04G0174800	1.366275675	1.59395E-08	7.3919E-08	RHSIM_Rhsim04G0174800
Root vs Leaf	RHSIM_Rhsim02G0074400	-7.916145908	1.5989E-08	7.41189E-08	RHSIM_Rhsim02G0074400
Root vs Leaf	RHSIM_Rhsim01G0243500	-1.544433924	1.60676E-08	7.44683E-08	RHSIM_Rhsim01G0243500
Root vs Leaf	RHSIM_Rhsim06G0172100	-6.414680166	1.61026E-08	7.46156E-08	RHSIM_Rhsim06G0172100
Root vs Leaf	RHSIM_Rhsim02G0181100	1.064108818	1.61192E-08	7.46774E-08	RHSIM_Rhsim02G0181100
Root vs Leaf	RHSIM_Rhsim02G0241900	-1.576351233	1.62626E-08	7.53266E-08	RHSIM_Rhsim02G0241900
Root vs Leaf	RHSIM_Rhsim13G0231200	2.441029603	1.62955E-08	7.54639E-08	RHSIM_Rhsim13G0231200
Root vs Leaf	RHSIM_Rhsim08G0042900	1.397042122	1.63229E-08	7.55759E-08	RHSIM_Rhsim08G0042900
Root vs Leaf	RHSIM_Rhsim01G0012500	-3.333819771	1.6355E-08	7.57093E-08	RHSIM_Rhsim01G0012500
Root vs Leaf	RHSIM_Rhsim03G0209200	-1.022919546	1.63944E-08	7.58722E-08	RHSIM_Rhsim03G0209200
Root vs Leaf	RHSIM_Rhsim01G0058400	6.319519869	1.63967E-08	7.58722E-08	RHSIM_Rhsim01G0058400
Root vs Leaf	RHSIM_Rhsim05G0227300	1.415033059	1.64114E-08	7.59248E-08	RHSIM_Rhsim05G0227300
Root vs Leaf	RHSIM_Rhsim04G0201100	3.819305574	1.65239E-08	7.64147E-08	RHSIM_Rhsim04G0201100
Root vs Leaf	RHSIM_Rhsim05G0096300	-2.413370717	1.65465E-08	7.65039E-08	RHSIM_Rhsim05G0096300
Root vs Leaf	novel.536	-3.050482467	1.66856E-08	7.71316E-08	--

Comparison	Gene ID	log_2FC	P-value	FDR (padj)	Gene Name
Root vs Leaf	RHSIM_Rhsim10G0064000	-7.812342558	1.67937E-08	7.76161E-08	RHSIM_Rhsim10G0064000
Root vs Leaf	RHSIM_Rhsim03G0233500	-2.815012101	1.68348E-08	7.77905E-08	RHSIM_Rhsim03G0233500
Root vs Leaf	RHSIM_Rhsim03G0137600	-2.51111226	1.68568E-08	7.78763E-08	RHSIM_Rhsim03G0137600
Root vs Leaf	RHSIM_Rhsim09G0171800	1.251733141	1.69616E-08	7.8345E-08	RHSIM_Rhsim09G0171800
Root vs Leaf	RHSIM_Rhsim12G0190700	-1.311763209	1.70035E-08	7.85229E-08	RHSIM_Rhsim12G0190700
Root vs Leaf	RHSIM_Rhsim10G0015700	1.385080899	1.70262E-08	7.86119E-08	RHSIM_Rhsim10G0015700
Root vs Leaf	RHSIM_Rhsim08G0147700	1.217425508	1.71867E-08	7.93371E-08	RHSIM_Rhsim08G0147700
Root vs Leaf	RHSIM_Rhsim09G0084600	-2.057478441	1.72601E-08	7.96603E-08	RHSIM_Rhsim09G0084600
Root vs Leaf	RHSIM_Rhsim11G0085000	3.325073381	1.73066E-08	7.98587E-08	RHSIM_Rhsim11G0085000
Root vs Leaf	RHSIM_Rhsim12G0089500	6.123031904	1.74065E-08	8.03036E-08	RHSIM_Rhsim12G0089500
Root vs Leaf	RHSIM_Rhsim13G0051000	7.640193536	1.76285E-08	8.13117E-08	RHSIM_Rhsim13G0051000
Root vs Leaf	RHSIM_RhsimUnG0219600	2.59990519	1.7751E-08	8.18607E-08	RHSIM_RhsimUnG0219600
Root vs Leaf	RHSIM_Rhsim10G0130800	-7.599736136	1.80907E-08	8.33608E-08	RHSIM_Rhsim10G0130800
Root vs Leaf	RHSIM_Rhsim01G0107800	2.834286501	1.81803E-08	8.37569E-08	RHSIM_Rhsim01G0107800
Root vs Leaf	RHSIM_Rhsim13G0168800	-4.741254676	1.82978E-08	8.42814E-08	RHSIM_Rhsim13G0168800
Root vs Leaf	RHSIM_Rhsim13G0126100	2.012810542	1.83672E-08	8.45841E-08	RHSIM_Rhsim13G0126100
Root vs Leaf	RHSIM_Rhsim13G0167000	2.150748	1.84188E-08	8.48047E-08	RHSIM_Rhsim13G0167000
Root vs Leaf	RHSIM_Rhsim06G0050500	-1.439848318	1.84614E-08	8.4984E-08	RHSIM_Rhsim06G0050500
Root vs Leaf	RHSIM_Rhsim09G0010800	-4.212559841	1.85444E-08	8.53491E-08	RHSIM_Rhsim09G0010800
Root vs Leaf	RHSIM_Rhsim03G0022400	1.422497521	1.87551E-08	8.62846E-08	RHSIM_Rhsim03G0022400
Root vs Leaf	novel.5263	3.726687932	1.88077E-08	8.65095E-08	--
Root vs Leaf	RHSIM_Rhsim10G0135800	2.374680725	1.8839E-08	8.66362E-08	RHSIM_Rhsim10G0135800
Root vs Leaf	RHSIM_Rhsim06G0103600	-4.323605265	1.88915E-08	8.68604E-08	RHSIM_Rhsim06G0103600
Root vs Leaf	RHSIM_Rhsim02G0089700	-8.566711539	1.89193E-08	8.6971E-08	RHSIM_Rhsim02G0089700
Root vs Leaf	RHSIM_Rhsim02G0012600	1.190128895	1.91363E-08	8.79511E-08	RHSIM_Rhsim02G0012600

Comparison	Gene ID	log_2FC	P-value	FDR (padj)	Gene Name
Root vs Leaf	RHSIM_Rhsim03G0232000	-1.739689918	1.93264E-08	8.88068E-08	RHSIM_Rhsim03G0232000
Root vs Leaf	RHSIM_Rhsim03G0270500	1.917180279	1.93547E-08	8.89196E-08	RHSIM_Rhsim03G0270500
Root vs Leaf	RHSIM_Rhsim03G0120900	-5.368955796	1.94788E-08	8.94541E-08	RHSIM_Rhsim03G0120900
Root vs Leaf	RHSIM_Rhsim11G0196000	-3.389303335	1.9532E-08	8.96805E-08	RHSIM_Rhsim11G0196000
Root vs Leaf	RHSIM_Rhsim08G0162600	-3.084052347	1.95942E-08	8.99483E-08	RHSIM_Rhsim08G0162600
Root vs Leaf	RHSIM_Rhsim13G0180100	-2.027012242	1.96417E-08	9.01483E-08	RHSIM_Rhsim13G0180100
Root vs Leaf	RHSIM_Rhsim07G0159600	3.763484303	1.97125E-08	9.04377E-08	RHSIM_Rhsim07G0159600
Root vs Leaf	RHSIM_Rhsim05G0168600	-6.37084202	1.97769E-08	9.07148E-08	RHSIM_Rhsim05G0168600
Root vs Leaf	RHSIM_Rhsim05G0041900	-1.073562949	1.98533E-08	9.10473E-08	RHSIM_Rhsim05G0041900
Root vs Leaf	novel.3566	2.273992185	1.99779E-08	9.16008E-08	--
Root vs Leaf	RHSIM_Rhsim08G0160000	-6.608248405	2.00695E-08	9.20022E-08	RHSIM_Rhsim08G0160000
Root vs Leaf	RHSIM_Rhsim03G0254600	1.251576716	2.01929E-08	9.25499E-08	RHSIM_Rhsim03G0254600
Root vs Leaf	RHSIM_Rhsim03G0263300	-2.231792545	2.02023E-08	9.25747E-08	RHSIM_Rhsim03G0263300
Root vs Leaf	RHSIM_Rhsim08G0206300	2.754800958	2.02731E-08	9.28807E-08	RHSIM_Rhsim08G0206300
Root vs Leaf	RHSIM_Rhsim08G0244000	-7.99300153	2.04395E-08	9.36243E-08	RHSIM_Rhsim08G0244000
Root vs Leaf	RHSIM_Rhsim09G0185400	-7.999700316	2.05016E-08	9.38904E-08	RHSIM_Rhsim09G0185400
Root vs Leaf	novel.3302	2.377248102	2.05122E-08	9.39203E-08	--
Root vs Leaf	novel.3736	-7.937015848	2.06829E-08	9.46829E-08	--
Root vs Leaf	RHSIM_Rhsim12G0122300	-8.412070194	2.07098E-08	9.47876E-08	RHSIM_Rhsim12G0122300
Root vs Leaf	RHSIM_Rhsim07G0087600	2.957678988	2.07445E-08	9.49275E-08	RHSIM_Rhsim07G0087600
Root vs Leaf	RHSIM_Rhsim12G0161800	-1.100081936	2.08176E-08	9.5243E-08	RHSIM_Rhsim12G0161800
Root vs Leaf	RHSIM_Rhsim09G0100700	2.30477203	2.09131E-08	9.56612E-08	RHSIM_Rhsim09G0100700
Root vs Leaf	RHSIM_Rhsim04G0234900	-1.370969247	2.09385E-08	9.57584E-08	RHSIM_Rhsim04G0234900
Root vs Leaf	RHSIM_Rhsim10G0152100	-8.664108773	2.10444E-08	9.62236E-08	RHSIM_Rhsim10G0152100
Root vs Leaf	RHSIM_Rhsim05G0187000	7.468640246	2.13047E-08	9.73756E-08	RHSIM_Rhsim05G0187000

Comparison	Gene ID	log$_2$FC	P-value	FDR (padj)	Gene Name
Root vs Leaf	novel.4116	3.335435063	2.13341E-08	9.74907E-08	--
Root vs Leaf	RHSIM_Rhsim11G0084600	7.44991685	2.14029E-08	9.77855E-08	RHSIM_Rhsim11G0084600
Root vs Leaf	RHSIM_Rhsim02G0145000	1.318381811	2.14311E-08	9.78757E-08	RHSIM_Rhsim02G0145000
Root vs Leaf	RHSIM_Rhsim12G0161600	3.026496135	2.14671E-08	9.8021E-08	RHSIM_Rhsim12G0161600
Root vs Leaf	RHSIM_Rhsim03G0170100	1.506263034	2.15087E-08	9.81916E-08	RHSIM_Rhsim03G0170100
Root vs Leaf	RHSIM_Rhsim09G0083700	-8.608996745	2.16008E-08	9.85926E-08	RHSIM_Rhsim09G0083700
Root vs Leaf	RHSIM_Rhsim03G0134500	-1.836166827	2.18253E-08	9.95976E-08	RHSIM_Rhsim03G0134500
Root vs Leaf	RHSIM_Rhsim05G0186700	-1.31392478	2.18643E-08	9.97559E-08	RHSIM_Rhsim05G0186700
Root vs Leaf	novel.1223	8.222179717	2.18745E-08	9.97827E-08	--
Root vs Leaf	RHSIM_Rhsim06G0051200	5.966750065	2.1913E-08	9.99387E-08	RHSIM_Rhsim06G0051200
Root vs Leaf	RHSIM_RhsimUnG0150900	1.782778629	2.20407E-08	1.00501E-07	RHSIM_RhsimUnG0150900
Root vs Leaf	RHSIM_Rhsim12G0139900	1.338082297	2.22036E-08	1.01224E-07	RHSIM_Rhsim12G0139900
Root vs Leaf	RHSIM_Rhsim10G0129700	-4.619236423	2.23159E-08	1.01696E-07	RHSIM_Rhsim10G0129700
Root vs Leaf	RHSIM_Rhsim04G0147200	-3.857783565	2.25015E-08	1.02522E-07	RHSIM_Rhsim04G0147200
Root vs Leaf	novel.2681	-7.622271302	2.25294E-08	1.02629E-07	--
Root vs Leaf	RHSIM_Rhsim08G0195000	-7.522923461	2.25558E-08	1.02729E-07	RHSIM_Rhsim08G0195000
Root vs Leaf	novel.767	2.682892756	2.27146E-08	1.03432E-07	--
Root vs Leaf	RHSIM_Rhsim09G0078400	-3.06034405	2.27264E-08	1.03465E-07	RHSIM_Rhsim09G0078400
Root vs Leaf	RHSIM_Rhsim11G0009700	1.156656209	2.27884E-08	1.03727E-07	RHSIM_Rhsim11G0009700
Root vs Leaf	RHSIM_Rhsim01G0240200	-8.211804673	2.27937E-08	1.03731E-07	RHSIM_Rhsim01G0240200
Root vs Leaf	RHSIM_Rhsim11G0129000	-6.404015015	2.29741E-08	1.04531E-07	RHSIM_Rhsim11G0129000
Root vs Leaf	RHSIM_Rhsim05G0133800	2.359754425	2.3026E-08	1.04746E-07	RHSIM_Rhsim05G0133800
Root vs Leaf	RHSIM_Rhsim13G0142100	2.944998334	2.3346E-08	1.0614E-07	RHSIM_Rhsim13G0142100
Root vs Leaf	RHSIM_Rhsim09G0024400	6.549078407	2.33939E-08	1.06336E-07	RHSIM_Rhsim09G0024400
Root vs Leaf	novel.1349	3.861347017	2.34513E-08	1.06576E-07	--

Comparison	Gene ID	log_2FC	P-value	FDR (padj)	Gene Name
Root vs Leaf	RHSIM_Rhsim11G0161500	3.888283007	2.37267E-08	1.07807E-07	RHSIM_Rhsim11G0161500
Root vs Leaf	RHSIM_Rhsim02G0043300	1.524122109	2.38063E-08	1.08147E-07	RHSIM_Rhsim02G0043300
Root vs Leaf	novel.3862	-7.582134415	2.38307E-08	1.08237E-07	--
Root vs Leaf	RHSIM_RhsimUnG0123600	-7.958126711	2.3849E-08	1.08277E-07	RHSIM_RhsimUnG0123600
Root vs Leaf	RHSIM_Rhsim13G0196600	5.024483486	2.40538E-08	1.09186E-07	RHSIM_Rhsim13G0196600
Root vs Leaf	RHSIM_Rhsim08G0190900	1.623947091	2.4098E-08	1.09365E-07	RHSIM_Rhsim08G0190900
Root vs Leaf	RHSIM_Rhsim11G0166300	-1.539648937	2.41541E-08	1.09598E-07	RHSIM_Rhsim11G0166300
Root vs Leaf	novel.1673	-8.148351641	2.42547E-08	1.10033E-07	--
Root vs Leaf	RHSIM_Rhsim10G0198600	-2.952723012	2.43672E-08	1.10522E-07	RHSIM_Rhsim10G0198600
Root vs Leaf	RHSIM_Rhsim08G0098600	7.368472101	2.44263E-08	1.10768E-07	RHSIM_Rhsim08G0098600
Root vs Leaf	RHSIM_Rhsim11G0068400	-8.083277309	2.50343E-08	1.13458E-07	RHSIM_Rhsim11G0068400
Root vs Leaf	RHSIM_Rhsim11G0024900	-1.865544901	2.51734E-08	1.14067E-07	RHSIM_Rhsim11G0024900
Root vs Leaf	RHSIM_Rhsim10G0049200	-1.375792044	2.527E-08	1.14482E-07	RHSIM_Rhsim10G0049200
Root vs Leaf	RHSIM_Rhsim13G0171400	-3.466828795	2.54882E-08	1.15448E-07	RHSIM_Rhsim13G0171400
Root vs Leaf	RHSIM_Rhsim05G0203000	-2.810823157	2.55885E-08	1.15879E-07	RHSIM_Rhsim05G0203000
Root vs Leaf	RHSIM_Rhsim03G0161500	2.924766795	2.59101E-08	1.1729E-07	RHSIM_Rhsim03G0161500
Root vs Leaf	RHSIM_Rhsim06G0196300	-2.487384644	2.60161E-08	1.17724E-07	RHSIM_Rhsim06G0196300
Root vs Leaf	RHSIM_Rhsim04G0029300	-2.1201471	2.6341E-08	1.1917E-07	RHSIM_Rhsim04G0029300
Root vs Leaf	RHSIM_Rhsim08G0197200	1.463884617	2.65515E-08	1.20076E-07	RHSIM_Rhsim08G0197200
Root vs Leaf	RHSIM_Rhsim04G0135700	1.836466229	2.65742E-08	1.20155E-07	RHSIM_Rhsim04G0135700
Root vs Leaf	RHSIM_Rhsim07G0139600	-4.486850907	2.65944E-08	1.20223E-07	RHSIM_Rhsim07G0139600
Root vs Leaf	novel.832	-8.738491105	2.66299E-08	1.2036E-07	--
Root vs Leaf	RHSIM_Rhsim11G0074600	2.377567244	2.68181E-08	1.21187E-07	RHSIM_Rhsim11G0074600
Root vs Leaf	novel.5844	7.518796186	2.69207E-08	1.21627E-07	--
Root vs Leaf	RHSIM_Rhsim04G0192200	-8.820698157	2.70533E-08	1.22178E-07	RHSIM_Rhsim04G0192200

Comparison	Gene ID	log_2FC	P-value	FDR (padj)	Gene Name
Root vs Leaf	RHSIM_Rhsim13G0023800	7.465194521	2.71453E-08	1.2257E-07	RHSIM_Rhsim13G0023800
Root vs Leaf	RHSIM_Rhsim06G0226100	-1.204789166	2.71521E-08	1.22576E-07	RHSIM_Rhsim06G0226100
Root vs Leaf	RHSIM_Rhsim03G0076000	-3.81992833	2.72115E-08	1.22821E-07	RHSIM_Rhsim03G0076000
Root vs Leaf	RHSIM_RhsimUnG0064700	1.209932711	2.72397E-08	1.22924E-07	RHSIM_RhsimUnG0064700
Root vs Leaf	novel.272	-2.274632846	2.74946E-08	1.2405E-07	--
Root vs Leaf	novel.4459	2.613000509	2.75836E-08	1.24427E-07	--
Root vs Leaf	RHSIM_Rhsim09G0060400	2.310448722	2.76724E-08	1.24803E-07	RHSIM_Rhsim09G0060400
Root vs Leaf	RHSIM_RhsimUnG0087900	-1.265643642	2.80067E-08	1.26287E-07	RHSIM_RhsimUnG0087900
Root vs Leaf	RHSIM_Rhsim12G0169500	-2.128564796	2.80423E-08	1.26422E-07	RHSIM_Rhsim12G0169500
Root vs Leaf	RHSIM_Rhsim10G0042700	3.781518222	2.8088E-08	1.26604E-07	RHSIM_Rhsim10G0042700
Root vs Leaf	novel.4760	-3.468562494	2.81413E-08	1.26819E-07	--
Root vs Leaf	novel.4439	3.761589308	2.81539E-08	1.26851E-07	--
Root vs Leaf	RHSIM_Rhsim09G0177700	2.001319571	2.82721E-08	1.27359E-07	RHSIM_Rhsim09G0177700
Root vs Leaf	RHSIM_RhsimUnG0180900	2.119951714	2.84458E-08	1.28117E-07	RHSIM_RhsimUnG0180900
Root vs Leaf	RHSIM_Rhsim13G0232800	2.343405773	2.85016E-08	1.28343E-07	RHSIM_Rhsim13G0232800
Root vs Leaf	RHSIM_Rhsim04G0178400	3.863864973	2.85139E-08	1.28374E-07	RHSIM_Rhsim04G0178400
Root vs Leaf	RHSIM_Rhsim02G0167800	5.850762044	2.85197E-08	1.28375E-07	RHSIM_Rhsim02G0167800
Root vs Leaf	RHSIM_RhsimUnG0208400	-2.290651698	2.87479E-08	1.29377E-07	RHSIM_RhsimUnG0208400
Root vs Leaf	RHSIM_Rhsim12G0150000	1.903770818	2.87863E-08	1.29524E-07	RHSIM_Rhsim12G0150000
Root vs Leaf	novel.4780	-3.114760862	2.89475E-08	1.30224E-07	--
Root vs Leaf	RHSIM_Rhsim08G0073600	-1.505038099	2.89624E-08	1.30266E-07	RHSIM_Rhsim08G0073600
Root vs Leaf	RHSIM_Rhsim02G0028300	2.645417084	2.9043E-08	1.30603E-07	RHSIM_Rhsim02G0028300
Root vs Leaf	RHSIM_Rhsim12G0020100	7.248253195	2.91126E-08	1.30891E-07	RHSIM_Rhsim12G0020100
Root vs Leaf	RHSIM_Rhsim13G0022100	1.035396498	2.93269E-08	1.31803E-07	RHSIM_Rhsim13G0022100
Root vs Leaf	RHSIM_Rhsim08G0026700	-2.818909398	2.9379E-08	1.32012E-07	RHSIM_Rhsim08G0026700

Comparison	Gene ID	log_2FC	P-value	FDR (padj)	Gene Name
Root vs Leaf	RHSIM_Rhsim09G0208500	1.562071117	2.96022E-08	1.32989E-07	RHSIM_Rhsim09G0208500
Root vs Leaf	novel.4319	7.490319419	2.96136E-08	1.33014E-07	--
Root vs Leaf	RHSIM_Rhsim05G0161400	1.937348977	2.96431E-08	1.33121E-07	RHSIM_Rhsim05G0161400
Root vs Leaf	RHSIM_Rhsim11G0034500	-1.065514039	2.97869E-08	1.33741E-07	RHSIM_Rhsim11G0034500
Root vs Leaf	RHSIM_Rhsim13G0005000	-1.201148879	2.97937E-08	1.33745E-07	RHSIM_Rhsim13G0005000
Root vs Leaf	RHSIM_Rhsim05G0018600	-3.699703911	2.98783E-08	1.34099E-07	RHSIM_Rhsim05G0018600
Root vs Leaf	RHSIM_RhsimUnG0098000	3.617480267	3.004E-08	1.34798E-07	RHSIM_RhsimUnG0098000
Root vs Leaf	RHSIM_RhsimUnG0195900	-1.746677941	3.00457E-08	1.34798E-07	RHSIM_RhsimUnG0195900
Root vs Leaf	novel.1915	-4.038763419	3.06602E-08	1.37501E-07	--
Root vs Leaf	novel.4085	3.240076405	3.0675E-08	1.37541E-07	--
Root vs Leaf	RHSIM_Rhsim13G0213100	3.247318356	3.07343E-08	1.3778E-07	RHSIM_Rhsim13G0213100
Root vs Leaf	RHSIM_Rhsim12G0042500	3.450858406	3.07511E-08	1.37829E-07	RHSIM_Rhsim12G0042500
Root vs Leaf	novel.3334	1.220774073	3.08889E-08	1.3842E-07	--
Root vs Leaf	RHSIM_Rhsim10G0200100	-7.11126228	3.10554E-08	1.39139E-07	RHSIM_Rhsim10G0200100
Root vs Leaf	RHSIM_Rhsim03G0171200	1.118593295	3.11072E-08	1.39344E-07	RHSIM_Rhsim03G0171200
Root vs Leaf	RHSIM_Rhsim06G0031900	-1.478396274	3.12351E-08	1.3989E-07	RHSIM_Rhsim06G0031900
Root vs Leaf	RHSIM_Rhsim08G0028200	1.211389438	3.13746E-08	1.40488E-07	RHSIM_Rhsim08G0028200
Root vs Leaf	novel.3179	-7.853264349	3.18255E-08	1.42424E-07	--
Root vs Leaf	RHSIM_Rhsim08G0212700	1.579851834	3.18872E-08	1.42672E-07	RHSIM_Rhsim08G0212700
Root vs Leaf	RHSIM_Rhsim04G0136700	3.353850108	3.21132E-08	1.43656E-07	RHSIM_Rhsim04G0136700
Root vs Leaf	RHSIM_Rhsim11G0192700	1.135468029	3.21385E-08	1.43741E-07	RHSIM_Rhsim11G0192700
Root vs Leaf	RHSIM_Rhsim01G0240500	4.546617718	3.2247E-08	1.44198E-07	RHSIM_Rhsim01G0240500
Root vs Leaf	RHSIM_Rhsim08G0104700	3.534766885	3.23206E-08	1.445E-07	RHSIM_Rhsim08G0104700
Root vs Leaf	RHSIM_Rhsim01G0215100	8.008991308	3.24116E-08	1.44879E-07	RHSIM_Rhsim01G0215100
Root vs Leaf	RHSIM_Rhsim07G0173800	1.069697731	3.24182E-08	1.4488E-07	RHSIM_Rhsim07G0173800

Comparison	Gene ID	log_2FC	P-value	FDR (padj)	Gene Name
Root vs Leaf	RHSIM_Rhsim09G0123000	2.381632333	3.25563E-08	1.45469E-07	RHSIM_Rhsim09G0123000
Root vs Leaf	RHSIM_Rhsim09G0100400	-1.135723476	3.26338E-08	1.45787E-07	RHSIM_Rhsim09G0100400
Root vs Leaf	RHSIM_Rhsim13G0176400	-8.399365035	3.26526E-08	1.45843E-07	RHSIM_Rhsim13G0176400
Root vs Leaf	RHSIM_Rhsim08G0124100	4.464838308	3.30311E-08	1.47505E-07	RHSIM_Rhsim08G0124100
Root vs Leaf	RHSIM_Rhsim07G0092900	1.865628025	3.3354E-08	1.48919E-07	RHSIM_Rhsim07G0092900
Root vs Leaf	RHSIM_Rhsim07G0125400	2.268697914	3.3371E-08	1.48966E-07	RHSIM_Rhsim07G0125400
Root vs Leaf	RHSIM_Rhsim01G0186600	2.094910427	3.36049E-08	1.49981E-07	RHSIM_Rhsim01G0186600
Root vs Leaf	RHSIM_Rhsim07G0247100	-1.363249875	3.36766E-08	1.50259E-07	RHSIM_Rhsim07G0247100
Root vs Leaf	RHSIM_Rhsim12G0165000	2.231263745	3.36802E-08	1.50259E-07	RHSIM_Rhsim12G0165000
Root vs Leaf	RHSIM_Rhsim08G0119700	-3.876605279	3.37392E-08	1.50493E-07	RHSIM_Rhsim08G0119700
Root vs Leaf	RHSIM_Rhsim10G0123000	-7.666573526	3.39066E-08	1.51211E-07	RHSIM_Rhsim10G0123000
Root vs Leaf	RHSIM_Rhsim03G0150800	3.540528894	3.3963E-08	1.51433E-07	RHSIM_Rhsim03G0150800
Root vs Leaf	RHSIM_Rhsim10G0017000	2.483456391	3.44265E-08	1.5347E-07	RHSIM_Rhsim10G0017000
Root vs Leaf	RHSIM_Rhsim02G0053200	3.048385313	3.44775E-08	1.53668E-07	RHSIM_Rhsim02G0053200
Root vs Leaf	RHSIM_Rhsim02G0131100	2.357328732	3.45851E-08	1.54118E-07	RHSIM_Rhsim02G0131100
Root vs Leaf	RHSIM_Rhsim12G0149200	-7.112330688	3.47184E-08	1.54655E-07	RHSIM_Rhsim12G0149200
Root vs Leaf	RHSIM_Rhsim06G0175800	3.666908424	3.47191E-08	1.54655E-07	RHSIM_Rhsim06G0175800
Root vs Leaf	RHSIM_Rhsim03G0166100	-1.77571469	3.49168E-08	1.55506E-07	RHSIM_Rhsim03G0166100
Root vs Leaf	RHSIM_Rhsim11G0198900	7.478862583	3.53035E-08	1.57168E-07	RHSIM_Rhsim11G0198900
Root vs Leaf	RHSIM_Rhsim07G0101200	1.377324391	3.55559E-08	1.58261E-07	RHSIM_Rhsim07G0101200
Root vs Leaf	RHSIM_RhsimUnG0110300	8.531146799	3.57318E-08	1.59013E-07	RHSIM_RhsimUnG0110300
Root vs Leaf	RHSIM_Rhsim13G0170800	-7.563123632	3.58286E-08	1.59413E-07	RHSIM_Rhsim13G0170800
Root vs Leaf	RHSIM_Rhsim01G0009500	1.044891648	3.58455E-08	1.59458E-07	RHSIM_Rhsim01G0009500
Root vs Leaf	RHSIM_Rhsim08G0084200	1.269529836	3.58539E-08	1.59465E-07	RHSIM_Rhsim08G0084200
Root vs Leaf	RHSIM_Rhsim09G0196500	1.007398673	3.59904E-08	1.60041E-07	RHSIM_Rhsim09G0196500

Comparison	Gene ID	log_2FC	P-value	FDR (padj)	Gene Name
Root vs Leaf	RHSIM_Rhsim03G0148500	2.543399664	3.60705E-08	1.60366E-07	RHSIM_Rhsim03G0148500
Root vs Leaf	RHSIM_Rhsim09G0183700	-4.841625685	3.61383E-08	1.60607E-07	RHSIM_Rhsim09G0183700
Root vs Leaf	RHSIM_Rhsim12G0169600	3.584862466	3.66295E-08	1.62758E-07	RHSIM_Rhsim12G0169600
Root vs Leaf	RHSIM_Rhsim02G0247800	-3.581198785	3.67383E-08	1.6321E-07	RHSIM_Rhsim02G0247800
Root vs Leaf	RHSIM_Rhsim03G0036200	-6.775873746	3.67933E-08	1.63423E-07	RHSIM_Rhsim03G0036200
Root vs Leaf	RHSIM_Rhsim13G0185900	7.648639744	3.68863E-08	1.63805E-07	RHSIM_Rhsim13G0185900
Root vs Leaf	RHSIM_Rhsim01G0258300	-2.981177093	3.69172E-08	1.6391E-07	RHSIM_Rhsim01G0258300
Root vs Leaf	RHSIM_RhsimUnG0171200	2.333752682	3.69489E-08	1.64004E-07	RHSIM_RhsimUnG0171200
Root vs Leaf	novel.4454	-2.137602218	3.69524E-08	1.64004E-07	--
Root vs Leaf	RHSIM_Rhsim09G0075700	1.197724983	3.70457E-08	1.64386E-07	RHSIM_Rhsim09G0075700
Root vs Leaf	RHSIM_Rhsim04G0166300	-1.797970522	3.70981E-08	1.64587E-07	RHSIM_Rhsim04G0166300
Root vs Leaf	RHSIM_Rhsim02G0005600	1.172305482	3.71397E-08	1.6474E-07	RHSIM_Rhsim02G0005600
Root vs Leaf	RHSIM_Rhsim02G0240700	1.89395519	3.72341E-08	1.65127E-07	RHSIM_Rhsim02G0240700
Root vs Leaf	RHSIM_Rhsim02G0062500	-4.929715346	3.73628E-08	1.65666E-07	RHSIM_Rhsim02G0062500
Root vs Leaf	novel.4033	-7.634354481	3.75461E-08	1.66368E-07	--
Root vs Leaf	RHSIM_Rhsim03G0196700	3.739248382	3.75498E-08	1.66368E-07	RHSIM_Rhsim03G0196700
Root vs Leaf	RHSIM_Rhsim13G0099800	2.145066352	3.75829E-08	1.66483E-07	RHSIM_Rhsim13G0099800
Root vs Leaf	RHSIM_Rhsim06G0129900	1.318062027	3.76124E-08	1.66581E-07	RHSIM_Rhsim06G0129900
Root vs Leaf	RHSIM_RhsimUnG0042500	-2.333731923	3.76871E-08	1.66881E-07	RHSIM_RhsimUnG0042500
Root vs Leaf	RHSIM_RhsimUnG0004900	-2.864626291	3.78746E-08	1.67679E-07	RHSIM_RhsimUnG0004900
Root vs Leaf	RHSIM_Rhsim10G0134600	-1.427419164	3.80167E-08	1.68275E-07	RHSIM_Rhsim10G0134600
Root vs Leaf	novel.5274	1.462933458	3.81415E-08	1.68796E-07	--
Root vs Leaf	RHSIM_Rhsim01G0076900	-3.469410007	3.83118E-08	1.69517E-07	RHSIM_Rhsim01G0076900
Root vs Leaf	RHSIM_Rhsim11G0024000	7.260769519	3.83816E-08	1.69793E-07	RHSIM_Rhsim11G0024000
Root vs Leaf	RHSIM_Rhsim08G0113400	-7.578596195	3.84402E-08	1.7002E-07	RHSIM_Rhsim08G0113400

Comparison	Gene ID	log_2FC	P-value	FDR (padj)	Gene Name
Root vs Leaf	RHSIM_Rhsim01G0027900	-7.612253241	3.85544E-08	1.70493E-07	RHSIM_Rhsim01G0027900
Root vs Leaf	RHSIM_Rhsim09G0095000	1.476257794	3.87833E-08	1.71472E-07	RHSIM_Rhsim09G0095000
Root vs Leaf	RHSIM_Rhsim07G0003100	1.260655124	3.88597E-08	1.71777E-07	RHSIM_Rhsim07G0003100
Root vs Leaf	RHSIM_Rhsim01G0240300	-7.818488728	3.91155E-08	1.72875E-07	RHSIM_Rhsim01G0240300
Root vs Leaf	RHSIM_RhsimUnG0022800	-1.323079202	3.91928E-08	1.73183E-07	RHSIM_RhsimUnG0022800
Root vs Leaf	RHSIM_Rhsim11G0127800	-1.650256796	3.97552E-08	1.75534E-07	RHSIM_Rhsim11G0127800
Root vs Leaf	novel.5956	3.636585161	3.98264E-08	1.75815E-07	--
Root vs Leaf	RHSIM_Rhsim12G0114000	2.088160618	4.00764E-08	1.76885E-07	RHSIM_Rhsim12G0114000
Root vs Leaf	RHSIM_Rhsim11G0175000	5.862874406	4.03487E-08	1.78053E-07	RHSIM_Rhsim11G0175000
Root vs Leaf	RHSIM_RhsimUnG0210400	-5.833961266	4.03566E-08	1.78054E-07	RHSIM_RhsimUnG0210400
Root vs Leaf	RHSIM_Rhsim06G0004800	3.829058474	4.04496E-08	1.7843E-07	RHSIM_Rhsim06G0004800
Root vs Leaf	RHSIM_Rhsim08G0082400	1.268298043	4.13716E-08	1.82428E-07	RHSIM_Rhsim08G0082400
Root vs Leaf	RHSIM_Rhsim03G0028300	8.432288755	4.14049E-08	1.8254E-07	RHSIM_Rhsim03G0028300
Root vs Leaf	novel.1657	-7.193100706	4.1546E-08	1.83127E-07	--
Root vs Leaf	RHSIM_Rhsim06G0156000	-8.640200344	4.16304E-08	1.83464E-07	RHSIM_Rhsim06G0156000
Root vs Leaf	RHSIM_RhsimUnG0095400	-1.026322704	4.1927E-08	1.847E-07	RHSIM_RhsimUnG0095400
Root vs Leaf	novel.6075	4.272848472	4.24875E-08	1.87099E-07	--
Root vs Leaf	RHSIM_RhsimUnG0204900	2.341664389	4.25449E-08	1.87316E-07	RHSIM_RhsimUnG0204900
Root vs Leaf	RHSIM_Rhsim13G0109100	-3.070050136	4.2966E-08	1.89134E-07	RHSIM_Rhsim13G0109100
Root vs Leaf	RHSIM_RhsimUnG0212600	-2.13805835	4.29868E-08	1.89189E-07	RHSIM_RhsimUnG0212600
Root vs Leaf	RHSIM_Rhsim07G0029600	1.835771824	4.33065E-08	1.90488E-07	RHSIM_Rhsim07G0029600
Root vs Leaf	RHSIM_RhsimUnG0144100	7.26496465	4.33148E-08	1.90488E-07	RHSIM_RhsimUnG0144100
Root vs Leaf	RHSIM_Rhsim11G0022400	1.950191486	4.36453E-08	1.91905E-07	RHSIM_Rhsim11G0022400
Root vs Leaf	RHSIM_Rhsim03G0173900	7.647074717	4.3984E-08	1.93358E-07	RHSIM_Rhsim03G0173900
Root vs Leaf	RHSIM_Rhsim01G0183500	-4.1815071	4.41987E-08	1.94264E-07	RHSIM_Rhsim01G0183500

Comparison	Gene ID	log_2FC	P-value	FDR (padj)	Gene Name
Root vs Leaf	RHSIM_Rhsim12G0192500	2.96822167	4.42209E-08	1.94325E-07	RHSIM_Rhsim12G0192500
Root vs Leaf	novel.5633	7.248922802	4.42417E-08	1.9438E-07	--
Root vs Leaf	RHSIM_Rhsim06G0112400	-7.540295084	4.432E-08	1.94687E-07	RHSIM_Rhsim06G0112400
Root vs Leaf	RHSIM_Rhsim05G0078100	1.766298345	4.45542E-08	1.95678E-07	RHSIM_Rhsim05G0078100
Root vs Leaf	RHSIM_Rhsim04G0013300	-5.176198314	4.45631E-08	1.9568E-07	RHSIM_Rhsim04G0013300
Root vs Leaf	novel.2790	-3.58304664	4.47599E-08	1.9647E-07	--
Root vs Leaf	RHSIM_Rhsim03G0243900	-1.840172563	4.51333E-08	1.98034E-07	RHSIM_Rhsim03G0243900
Root vs Leaf	RHSIM_Rhsim07G0088900	4.297589609	4.53034E-08	1.98742E-07	RHSIM_Rhsim07G0088900
Root vs Leaf	RHSIM_Rhsim13G0183000	-3.696878603	4.54596E-08	1.9939E-07	RHSIM_Rhsim13G0183000
Root vs Leaf	RHSIM_Rhsim02G0046900	-7.796875339	4.58198E-08	2.00893E-07	RHSIM_Rhsim02G0046900
Root vs Leaf	RHSIM_Rhsim08G0038200	1.514026418	4.61792E-08	2.02431E-07	RHSIM_Rhsim08G0038200
Root vs Leaf	RHSIM_Rhsim02G0218300	2.899114071	4.62052E-08	2.02507E-07	RHSIM_Rhsim02G0218300
Root vs Leaf	RHSIM_Rhsim04G0243600	-7.953581847	4.63716E-08	2.03197E-07	RHSIM_Rhsim04G0243600
Root vs Leaf	RHSIM_Rhsim12G0094100	-4.883390977	4.6384E-08	2.03213E-07	RHSIM_Rhsim12G0094100
Root vs Leaf	RHSIM_Rhsim10G0185100	1.164351156	4.65116E-08	2.03734E-07	RHSIM_Rhsim10G0185100
Root vs Leaf	RHSIM_Rhsim05G0073300	3.556784322	4.67922E-08	2.04924E-07	RHSIM_Rhsim05G0073300
Root vs Leaf	RHSIM_Rhsim03G0161800	-1.475119977	4.69351E-08	2.05472E-07	RHSIM_Rhsim03G0161800
Root vs Leaf	RHSIM_Rhsim13G0118100	1.693077033	4.70927E-08	2.06123E-07	RHSIM_Rhsim13G0118100
Root vs Leaf	RHSIM_Rhsim05G0005300	-3.55372784	4.71824E-08	2.06476E-07	RHSIM_Rhsim05G0005300
Root vs Leaf	RHSIM_Rhsim08G0159800	-3.080418915	4.72555E-08	2.06757E-07	RHSIM_Rhsim08G0159800
Root vs Leaf	RHSIM_Rhsim08G0177800	-2.552795852	4.74004E-08	2.07352E-07	RHSIM_Rhsim08G0177800
Root vs Leaf	RHSIM_Rhsim06G0064200	3.145274659	4.75363E-08	2.07907E-07	RHSIM_Rhsim06G0064200
Root vs Leaf	RHSIM_Rhsim11G0044200	2.419431392	4.764E-08	2.08322E-07	RHSIM_Rhsim11G0044200
Root vs Leaf	RHSIM_Rhsim10G0137000	4.077717715	4.76649E-08	2.08352E-07	RHSIM_Rhsim10G0137000
Root vs Leaf	RHSIM_Rhsim06G0057600	2.641423956	4.7862E-08	2.09174E-07	RHSIM_Rhsim06G0057600

Comparison	Gene ID	log2FC	P-value	FDR (padj)	Gene Name
Root vs Leaf	RHSIM_Rhsim10G0086000	-2.065188068	4.81397E-08	2.10348E-07	RHSIM_Rhsim10G0086000
Root vs Leaf	RHSIM_RhsimUnG0227100	7.605772252	4.87135E-08	2.12774E-07	RHSIM_RhsimUnG0227100
Root vs Leaf	RHSIM_Rhsim08G0135000	1.440003584	4.88353E-08	2.13266E-07	RHSIM_Rhsim08G0135000
Root vs Leaf	RHSIM_Rhsim10G0191200	-2.780112336	4.90051E-08	2.13967E-07	RHSIM_Rhsim10G0191200
Root vs Leaf	RHSIM_Rhsim06G0136000	1.009400312	4.91613E-08	2.14609E-07	RHSIM_Rhsim06G0136000
Root vs Leaf	RHSIM_Rhsim10G0183900	1.901715722	4.93446E-08	2.15369E-07	RHSIM_Rhsim10G0183900
Root vs Leaf	RHSIM_Rhsim07G0093200	4.407278167	4.97207E-08	2.16928E-07	RHSIM_Rhsim07G0093200
Root vs Leaf	novel.217	-7.691070047	5.02309E-08	2.19113E-07	--
Root vs Leaf	RHSIM_Rhsim03G0135400	4.936527221	5.04246E-08	2.19916E-07	RHSIM_Rhsim03G0135400
Root vs Leaf	RHSIM_Rhsim05G0162100	1.419438813	5.08567E-08	2.21759E-07	RHSIM_Rhsim05G0162100
Root vs Leaf	RHSIM_RhsimUnG0031800	2.782177018	5.08941E-08	2.2188E-07	RHSIM_RhsimUnG0031800
Root vs Leaf	RHSIM_Rhsim04G0171800	-1.070360939	5.12832E-08	2.23534E-07	RHSIM_Rhsim04G0171800
Root vs Leaf	RHSIM_Rhsim11G0095200	2.257323638	5.13751E-08	2.23893E-07	RHSIM_Rhsim11G0095200
Root vs Leaf	RHSIM_Rhsim03G0088700	1.428542769	5.19263E-08	2.2621E-07	RHSIM_Rhsim03G0088700
Root vs Leaf	RHSIM_Rhsim11G0113100	-4.836714375	5.21115E-08	2.26962E-07	RHSIM_Rhsim11G0113100
Root vs Leaf	RHSIM_Rhsim10G0098500	2.96543958	5.21185E-08	2.26962E-07	RHSIM_Rhsim10G0098500
Root vs Leaf	RHSIM_Rhsim07G0104500	1.262114225	5.24196E-08	2.2823E-07	RHSIM_Rhsim07G0104500
Root vs Leaf	RHSIM_Rhsim10G0169000	-1.693375287	5.25971E-08	2.2896E-07	RHSIM_Rhsim10G0169000
Root vs Leaf	novel.1851	7.513240437	5.29612E-08	2.30458E-07	--
Root vs Leaf	RHSIM_RhsimUnG0042800	-8.176680746	5.29795E-08	2.30494E-07	RHSIM_RhsimUnG0042800
Root vs Leaf	RHSIM_Rhsim12G0056500	3.827489916	5.31636E-08	2.31252E-07	RHSIM_Rhsim12G0056500
Root vs Leaf	novel.4883	-8.194475209	5.37945E-08	2.33952E-07	--
Root vs Leaf	RHSIM_Rhsim13G0072200	1.04753089	5.44477E-08	2.36748E-07	RHSIM_Rhsim13G0072200
Root vs Leaf	RHSIM_Rhsim02G0242200	3.730708594	5.46618E-08	2.37635E-07	RHSIM_Rhsim02G0242200
Root vs Leaf	RHSIM_RhsimUnG0129300	2.091407244	5.47597E-08	2.38015E-07	RHSIM_RhsimUnG0129300

Comparison	Gene ID	log_2FC	P-value	FDR (padj)	Gene Name
Root vs Leaf	RHSIM_Rhsim04G0087700	3.379082707	5.51374E-08	2.39612E-07	RHSIM_Rhsim04G0087700
Root vs Leaf	RHSIM_Rhsim10G0144800	1.213882074	5.52893E-08	2.40227E-07	RHSIM_Rhsim10G0144800
Root vs Leaf	novel.5616	-7.814336823	5.58064E-08	2.42428E-07	--
Root vs Leaf	RHSIM_Rhsim07G0208900	2.196282537	5.58569E-08	2.42602E-07	RHSIM_Rhsim07G0208900
Root vs Leaf	RHSIM_Rhsim09G0027800	-2.963761734	5.59792E-08	2.43088E-07	RHSIM_Rhsim09G0027800
Root vs Leaf	RHSIM_Rhsim08G0092000	2.861430705	5.61436E-08	2.43756E-07	RHSIM_Rhsim08G0092000
Root vs Leaf	RHSIM_Rhsim03G0191900	-1.134562484	5.63656E-08	2.44674E-07	RHSIM_Rhsim03G0191900
Root vs Leaf	RHSIM_Rhsim04G0236300	1.244664402	5.66036E-08	2.45661E-07	RHSIM_Rhsim04G0236300
Root vs Leaf	RHSIM_Rhsim09G0141100	1.350713732	5.67834E-08	2.46395E-07	RHSIM_Rhsim09G0141100
Root vs Leaf	RHSIM_Rhsim11G0096000	3.257404774	5.70166E-08	2.47268E-07	RHSIM_Rhsim11G0096000
Root vs Leaf	RHSIM_Rhsim02G0113800	-4.242630795	5.70787E-08	2.47446E-07	RHSIM_Rhsim02G0113800
Root vs Leaf	RHSIM_Rhsim05G0125200	1.103373322	5.7079E-08	2.47446E-07	RHSIM_Rhsim05G0125200
Root vs Leaf	RHSIM_Rhsim12G0053300	8.020677591	5.74143E-08	2.48806E-07	RHSIM_Rhsim12G0053300
Root vs Leaf	RHSIM_Rhsim01G0230500	2.424548933	5.74814E-08	2.4905E-07	RHSIM_Rhsim01G0230500
Root vs Leaf	RHSIM_Rhsim03G0189800	1.189069567	5.74959E-08	2.49066E-07	RHSIM_Rhsim03G0189800
Root vs Leaf	RHSIM_Rhsim04G0166400	7.280077189	5.75234E-08	2.49139E-07	RHSIM_Rhsim04G0166400
Root vs Leaf	novel.4194	-1.126247901	5.75812E-08	2.49342E-07	--
Root vs Leaf	RHSIM_Rhsim08G0106500	5.211896785	5.78465E-08	2.50444E-07	RHSIM_Rhsim08G0106500
Root vs Leaf	novel.1582	-1.021392002	5.81999E-08	2.51927E-07	--
Root vs Leaf	RHSIM_Rhsim13G0088300	-1.026478783	5.82317E-08	2.52018E-07	RHSIM_Rhsim13G0088300
Root vs Leaf	novel.2759	-7.462570988	5.87555E-08	2.54237E-07	--
Root vs Leaf	RHSIM_Rhsim01G0236600	-1.208659417	5.88318E-08	2.5452E-07	RHSIM_Rhsim01G0236600
Root vs Leaf	RHSIM_Rhsim03G0172200	-1.127079602	5.8843E-08	2.54521E-07	RHSIM_Rhsim03G0172200
Root vs Leaf	RHSIM_Rhsim08G0238900	-1.061067221	5.88748E-08	2.54611E-07	RHSIM_Rhsim08G0238900
Root vs Leaf	RHSIM_Rhsim09G0086200	7.276517131	5.89912E-08	2.55066E-07	RHSIM_Rhsim09G0086200

Comparison	Gene ID	log_2FC	P-value	FDR (padj)	Gene Name
Root vs Leaf	RHSIM_Rhsim09G0169300	1.059489516	5.93322E-08	2.56483E-07	RHSIM_Rhsim09G0169300
Root vs Leaf	RHSIM_Rhsim01G0049800	1.405484293	5.93409E-08	2.56483E-07	RHSIM_Rhsim01G0049800
Root vs Leaf	RHSIM_Rhsim06G0122500	6.846109245	5.99714E-08	2.59111E-07	RHSIM_Rhsim06G0122500
Root vs Leaf	RHSIM_Rhsim03G0036300	-8.019981139	6.0093E-08	2.59588E-07	RHSIM_Rhsim03G0036300
Root vs Leaf	novel.4078	4.208246778	6.01643E-08	2.59847E-07	--
Root vs Leaf	novel.5417	-3.514207692	6.02909E-08	2.60345E-07	--
Root vs Leaf	RHSIM_Rhsim11G0133100	-5.201483154	6.03179E-08	2.60413E-07	RHSIM_Rhsim11G0133100
Root vs Leaf	RHSIM_Rhsim06G0043300	-4.297340569	6.07464E-08	2.62214E-07	RHSIM_Rhsim06G0043300
Root vs Leaf	RHSIM_Rhsim11G0091400	4.496441416	6.08435E-08	2.62585E-07	RHSIM_Rhsim11G0091400
Root vs Leaf	RHSIM_Rhsim02G0046400	4.228008558	6.11831E-08	2.64001E-07	RHSIM_Rhsim02G0046400
Root vs Leaf	RHSIM_Rhsim02G0112600	2.311501009	6.13144E-08	2.64519E-07	RHSIM_Rhsim02G0112600
Root vs Leaf	novel.456	-3.89031431	6.18024E-08	2.66574E-07	--
Root vs Leaf	RHSIM_Rhsim13G0124400	8.340000036	6.19458E-08	2.67143E-07	RHSIM_Rhsim13G0124400
Root vs Leaf	RHSIM_Rhsim02G0223200	6.400253795	6.21292E-08	2.67834E-07	RHSIM_Rhsim02G0223200
Root vs Leaf	RHSIM_Rhsim06G0230500	-1.167319631	6.22066E-08	2.68117E-07	RHSIM_Rhsim06G0230500
Root vs Leaf	RHSIM_Rhsim10G0067500	1.119914539	6.23565E-08	2.68714E-07	RHSIM_Rhsim10G0067500
Root vs Leaf	RHSIM_Rhsim08G0117900	5.365698073	6.39435E-08	2.75501E-07	RHSIM_Rhsim08G0117900
Root vs Leaf	RHSIM_Rhsim10G0040400	3.3053582	6.40255E-08	2.75803E-07	RHSIM_Rhsim10G0040400
Root vs Leaf	RHSIM_Rhsim11G0075500	-1.816656386	6.40829E-08	2.75999E-07	RHSIM_Rhsim11G0075500
Root vs Leaf	RHSIM_Rhsim03G0092100	-1.79110704	6.42733E-08	2.76767E-07	RHSIM_Rhsim03G0092100
Root vs Leaf	RHSIM_Rhsim06G0123600	-3.508348087	6.42874E-08	2.76777E-07	RHSIM_Rhsim06G0123600
Root vs Leaf	RHSIM_Rhsim08G0174400	-1.121531791	6.46102E-08	2.78115E-07	RHSIM_Rhsim08G0174400
Root vs Leaf	RHSIM_RhsimUnG0190100	-3.245207634	6.48671E-08	2.79117E-07	RHSIM_RhsimUnG0190100
Root vs Leaf	novel.1514	1.89784839	6.51908E-08	2.80457E-07	--
Root vs Leaf	RHSIM_Rhsim02G0240500	-2.088174378	6.52162E-08	2.80515E-07	RHSIM_Rhsim02G0240500

Comparison	Gene ID	log_2FC	P-value	FDR (padj)	Gene Name
Root vs Leaf	RHSIM_Rhsim04G0094500	-1.656740344	6.52702E-08	2.80695E-07	RHSIM_Rhsim04G0094500
Root vs Leaf	RHSIM_Rhsim08G0129900	-1.584641906	6.56239E-08	2.82111E-07	RHSIM_Rhsim08G0129900
Root vs Leaf	novel.5057	1.39512359	6.58464E-08	2.83015E-07	--
Root vs Leaf	RHSIM_Rhsim01G0061700	3.005067186	6.60417E-08	2.83749E-07	RHSIM_Rhsim01G0061700
Root vs Leaf	novel.4176	-4.433186574	6.63602E-08	2.85053E-07	--
Root vs Leaf	RHSIM_Rhsim01G0109200	6.84028673	6.63698E-08	2.85053E-07	RHSIM_Rhsim01G0109200
Root vs Leaf	RHSIM_Rhsim03G0247400	1.313077761	6.64648E-08	2.85278E-07	RHSIM_Rhsim03G0247400
Root vs Leaf	RHSIM_Rhsim06G0040200	-7.621437544	6.64716E-08	2.85278E-07	RHSIM_Rhsim06G0040200
Root vs Leaf	RHSIM_Rhsim13G0226600	-4.141077245	6.65926E-08	2.85692E-07	RHSIM_Rhsim13G0226600
Root vs Leaf	RHSIM_Rhsim03G0093100	2.244082697	6.68389E-08	2.86695E-07	RHSIM_Rhsim03G0093100
Root vs Leaf	RHSIM_Rhsim02G0228000	2.276474477	6.71703E-08	2.8801E-07	RHSIM_Rhsim02G0228000
Root vs Leaf	RHSIM_Rhsim08G0147100	-3.020350475	6.76038E-08	2.89815E-07	RHSIM_Rhsim08G0147100
Root vs Leaf	RHSIM_Rhsim04G0117900	2.275446076	6.81554E-08	2.92125E-07	RHSIM_Rhsim04G0117900
Root vs Leaf	RHSIM_Rhsim07G0157100	-1.003255838	6.84719E-08	2.93373E-07	RHSIM_Rhsim07G0157100
Root vs Leaf	RHSIM_Rhsim11G0042500	-3.464189173	6.89649E-08	2.95431E-07	RHSIM_Rhsim11G0042500
Root vs Leaf	RHSIM_Rhsim03G0032700	-2.925369378	6.89954E-08	2.95507E-07	RHSIM_Rhsim03G0032700
Root vs Leaf	RHSIM_Rhsim13G0134900	-1.701383882	6.90998E-08	2.95899E-07	RHSIM_Rhsim13G0134900
Root vs Leaf	RHSIM_Rhsim09G0086800	3.138537818	6.94317E-08	2.97265E-07	RHSIM_Rhsim09G0086800
Root vs Leaf	RHSIM_Rhsim07G0178400	2.93607517	6.95286E-08	2.97625E-07	RHSIM_Rhsim07G0178400
Root vs Leaf	novel.5158	-1.376455633	6.95569E-08	2.97691E-07	--
Root vs Leaf	RHSIM_Rhsim08G0249200	-4.028238442	6.98674E-08	2.9891E-07	RHSIM_Rhsim08G0249200
Root vs Leaf	RHSIM_Rhsim06G0183000	-3.462331559	7.02253E-08	3.00274E-07	RHSIM_Rhsim06G0183000
Root vs Leaf	RHSIM_Rhsim08G0207400	1.193560021	7.04704E-08	3.01211E-07	RHSIM_Rhsim08G0207400
Root vs Leaf	RHSIM_Rhsim09G0055700	1.580368471	7.07832E-08	3.02492E-07	RHSIM_Rhsim09G0055700
Root vs Leaf	RHSIM_Rhsim09G0028000	-5.301739129	7.10737E-08	3.03677E-07	RHSIM_Rhsim09G0028000

Comparison	Gene ID	log_2FC	P-value	FDR (padj)	Gene Name
Root vs Leaf	RHSIM_Rhsim06G0073400	1.386931822	7.14437E-08	3.05145E-07	RHSIM_Rhsim06G0073400
Root vs Leaf	RHSIM_Rhsim12G0018200	6.149698911	7.17404E-08	3.06243E-07	RHSIM_Rhsim12G0018200
Root vs Leaf	RHSIM_Rhsim09G0022700	3.43660177	7.22532E-08	3.08318E-07	RHSIM_Rhsim09G0022700
Root vs Leaf	RHSIM_Rhsim12G0199300	1.572448548	7.24461E-08	3.09085E-07	RHSIM_Rhsim12G0199300
Root vs Leaf	RHSIM_Rhsim06G0165200	-3.996606364	7.24821E-08	3.09181E-07	RHSIM_Rhsim06G0165200
Root vs Leaf	RHSIM_Rhsim07G0051000	4.49857689	7.26571E-08	3.09871E-07	RHSIM_Rhsim07G0051000
Root vs Leaf	RHSIM_Rhsim09G0185800	-2.896631926	7.26756E-08	3.09892E-07	RHSIM_Rhsim09G0185800
Root vs Leaf	RHSIM_Rhsim04G0083400	2.39326716	7.27622E-08	3.10205E-07	RHSIM_Rhsim04G0083400
Root vs Leaf	RHSIM_Rhsim04G0249900	2.115893573	7.34107E-08	3.12912E-07	RHSIM_Rhsim04G0249900
Root vs Leaf	RHSIM_Rhsim11G0101000	-2.783754622	7.41209E-08	3.1588E-07	RHSIM_Rhsim11G0101000
Root vs Leaf	novel.3742	1.561509302	7.47983E-08	3.18709E-07	--
Root vs Leaf	RHSIM_Rhsim13G0009900	-1.606989731	7.50261E-08	3.19621E-07	RHSIM_Rhsim13G0009900
Root vs Leaf	RHSIM_RhsimUnG0149200	-1.565671984	7.55047E-08	3.216E-07	RHSIM_RhsimUnG0149200
Root vs Leaf	RHSIM_Rhsim04G0085600	-1.143361062	7.66928E-08	3.2642E-07	RHSIM_Rhsim04G0085600
Root vs Leaf	RHSIM_Rhsim09G0115900	-5.926155039	7.74971E-08	3.29783E-07	RHSIM_Rhsim09G0115900
Root vs Leaf	RHSIM_Rhsim01G0212900	-1.331420835	7.77397E-08	3.30713E-07	RHSIM_Rhsim01G0212900
Root vs Leaf	RHSIM_Rhsim11G0170400	6.895587188	7.77442E-08	3.30713E-07	RHSIM_Rhsim11G0170400
Root vs Leaf	RHSIM_Rhsim11G0045800	-1.431847274	7.80501E-08	3.31953E-07	RHSIM_Rhsim11G0045800
Root vs Leaf	novel.4059	1.344667206	7.82982E-08	3.32947E-07	--
Root vs Leaf	RHSIM_Rhsim01G0185200	-1.444091843	7.86045E-08	3.34188E-07	RHSIM_Rhsim01G0185200
Root vs Leaf	RHSIM_Rhsim06G0191400	-7.510091659	7.89305E-08	3.35451E-07	RHSIM_Rhsim06G0191400
Root vs Leaf	novel.511	-7.510091659	7.89305E-08	3.35451E-07	--
Root vs Leaf	novel.2311	2.742890486	7.9049E-08	3.35893E-07	--
Root vs Leaf	novel.4757	2.177130065	7.97952E-08	3.39002E-07	--
Root vs Leaf	RHSIM_Rhsim13G0020000	-2.168838028	7.98157E-08	3.39026E-07	RHSIM_Rhsim13G0020000

Comparison	Gene ID	log_2FC	P-value	FDR (padj)	Gene Name
Root vs Leaf	RHSIM_Rhsim13G0111800	2.279182528	8.00025E-08	3.39695E-07	RHSIM_Rhsim13G0111800
Root vs Leaf	RHSIM_Rhsim09G0154800	1.945689067	8.01944E-08	3.40385E-07	RHSIM_Rhsim09G0154800
Root vs Leaf	novel.93	1.272726657	8.02995E-08	3.40769E-07	--
Root vs Leaf	RHSIM_Rhsim09G0118000	4.516482852	8.04118E-08	3.41183E-07	RHSIM_Rhsim09G0118000
Root vs Leaf	RHSIM_RhsimUnG0135800	2.06156523	8.04842E-08	3.41427E-07	RHSIM_RhsimUnG0135800
Root vs Leaf	RHSIM_Rhsim01G0293000	-1.706271838	8.16808E-08	3.46313E-07	RHSIM_Rhsim01G0293000
Root vs Leaf	RHSIM_Rhsim03G0214300	-2.823426606	8.18475E-08	3.46956E-07	RHSIM_Rhsim03G0214300
Root vs Leaf	RHSIM_Rhsim08G0160100	1.042080687	8.19053E-08	3.47137E-07	RHSIM_Rhsim08G0160100
Root vs Leaf	RHSIM_Rhsim13G0111900	1.9928782	8.19966E-08	3.47461E-07	RHSIM_Rhsim13G0111900
Root vs Leaf	RHSIM_Rhsim09G0074000	-1.08278409	8.5946E-08	3.6413E-07	RHSIM_Rhsim09G0074000
Root vs Leaf	RHSIM_Rhsim02G0189800	-7.343868983	8.60668E-08	3.64575E-07	RHSIM_Rhsim02G0189800
Root vs Leaf	RHSIM_RhsimUnG0207000	-7.372566704	8.61086E-08	3.64685E-07	RHSIM_RhsimUnG0207000
Root vs Leaf	RHSIM_Rhsim08G0160300	-1.538457391	8.64007E-08	3.65855E-07	RHSIM_Rhsim08G0160300
Root vs Leaf	RHSIM_Rhsim02G0008000	-7.185946065	8.65187E-08	3.66288E-07	RHSIM_Rhsim02G0008000
Root vs Leaf	RHSIM_Rhsim10G0164300	1.858304299	8.73536E-08	3.69755E-07	RHSIM_Rhsim10G0164300
Root vs Leaf	RHSIM_Rhsim01G0208500	-1.465465001	8.77037E-08	3.71101E-07	RHSIM_Rhsim01G0208500
Root vs Leaf	RHSIM_RhsimMtG0004200	-3.249850905	8.80707E-08	3.72586E-07	RHSIM_RhsimMtG0004200
Root vs Leaf	RHSIM_Rhsim07G0021100	-1.556152449	8.8417E-08	3.73982E-07	RHSIM_Rhsim07G0021100
Root vs Leaf	novel.3909	-7.345532146	8.86793E-08	3.74955E-07	--
Root vs Leaf	RHSIM_Rhsim03G0146600	1.018409743	8.88056E-08	3.75413E-07	RHSIM_Rhsim03G0146600
Root vs Leaf	RHSIM_Rhsim09G0175500	-7.026260689	8.882E-08	3.75413E-07	RHSIM_Rhsim09G0175500
Root vs Leaf	RHSIM_Rhsim10G0193300	-3.313784452	8.88585E-08	3.75507E-07	RHSIM_Rhsim10G0193300
Root vs Leaf	RHSIM_Rhsim04G0005400	1.102721078	8.9217E-08	3.76953E-07	RHSIM_Rhsim04G0005400
Root vs Leaf	RHSIM_Rhsim04G0103100	1.969368475	8.95564E-08	3.78318E-07	RHSIM_Rhsim04G0103100
Root vs Leaf	novel.6146	3.38941499	8.98352E-08	3.79426E-07	--

Comparison	Gene ID	log_2FC	P-value	FDR (padj)	Gene Name
Root vs Leaf	RHSIM_Rhsim10G0169200	-2.761161868	8.99102E-08	3.79674E-07	RHSIM_Rhsim10G0169200
Root vs Leaf	RHSIM_RhsimUnG0019600	-1.594687845	9.00116E-08	3.80033E-07	RHSIM_RhsimUnG0019600
Root vs Leaf	RHSIM_Rhsim12G0041100	1.231948231	9.01102E-08	3.80379E-07	RHSIM_Rhsim12G0041100
Root vs Leaf	RHSIM_Rhsim03G0207000	7.172660583	9.03975E-08	3.81493E-07	RHSIM_Rhsim03G0207000
Root vs Leaf	novel.3760	6.845564075	9.04069E-08	3.81493E-07	--
Root vs Leaf	RHSIM_Rhsim04G0011600	-1.537460932	9.08063E-08	3.83108E-07	RHSIM_Rhsim04G0011600
Root vs Leaf	RHSIM_Rhsim09G0082700	4.501146819	9.1239E-08	3.84864E-07	RHSIM_Rhsim09G0082700
Root vs Leaf	RHSIM_Rhsim07G0217300	1.267952562	9.14676E-08	3.85757E-07	RHSIM_Rhsim07G0217300
Root vs Leaf	RHSIM_Rhsim04G0095000	-8.323272451	9.15656E-08	3.8603E-07	RHSIM_Rhsim04G0095000
Root vs Leaf	novel.1286	-8.242000928	9.20483E-08	3.87994E-07	--
Root vs Leaf	RHSIM_Rhsim04G0020400	2.69691877	9.2272E-08	3.88866E-07	RHSIM_Rhsim04G0020400
Root vs Leaf	RHSIM_Rhsim07G0039900	-1.204740855	9.23936E-08	3.89308E-07	RHSIM_Rhsim07G0039900
Root vs Leaf	RHSIM_Rhsim06G0029300	-3.890921286	9.26016E-08	3.90114E-07	RHSIM_Rhsim06G0029300
Root vs Leaf	RHSIM_Rhsim03G0085100	-1.589461852	9.27211E-08	3.90546E-07	RHSIM_Rhsim03G0085100
Root vs Leaf	RHSIM_Rhsim07G0178800	-7.263603431	9.28048E-08	3.90827E-07	RHSIM_Rhsim07G0178800
Root vs Leaf	RHSIM_Rhsim07G0019900	-4.000314555	9.29044E-08	3.91176E-07	RHSIM_Rhsim07G0019900
Root vs Leaf	RHSIM_Rhsim12G0208000	1.110143396	9.34127E-08	3.93244E-07	RHSIM_Rhsim12G0208000
Root vs Leaf	RHSIM_Rhsim04G0034700	-7.775772867	9.41181E-08	3.96142E-07	RHSIM_Rhsim04G0034700
Root vs Leaf	RHSIM_Rhsim05G0133000	-1.692965585	9.47029E-08	3.98531E-07	RHSIM_Rhsim05G0133000
Root vs Leaf	RHSIM_Rhsim13G0101700	-1.347606012	9.61831E-08	4.04686E-07	RHSIM_Rhsim13G0101700
Root vs Leaf	RHSIM_Rhsim07G0008700	1.07576504	9.63413E-08	4.05278E-07	RHSIM_Rhsim07G0008700
Root vs Leaf	RHSIM_Rhsim04G0240000	1.111923775	9.73454E-08	4.09428E-07	RHSIM_Rhsim04G0240000
Root vs Leaf	RHSIM_Rhsim07G0180400	5.134518337	9.7463E-08	4.09848E-07	RHSIM_Rhsim07G0180400
Root vs Leaf	novel.5220	7.508714104	9.77632E-08	4.11035E-07	--
Root vs Leaf	RHSIM_Rhsim08G0118200	-7.585785627	9.7832E-08	4.1125E-07	RHSIM_Rhsim08G0118200

Comparison	Gene ID	log_2FC	P-value	FDR (padj)	Gene Name
Root vs Leaf	novel.5597	-2.921694335	9.85399E-08	4.1415E-07	--
Root vs Leaf	RHSIM_Rhsim01G0286600	1.28972509	9.89614E-08	4.15846E-07	RHSIM_Rhsim01G0286600
Root vs Leaf	novel.1584	1.162749207	9.91897E-08	4.16657E-07	--
Root vs Leaf	RHSIM_Rhsim12G0016400	-4.115545226	9.93918E-08	4.17428E-07	RHSIM_Rhsim12G0016400
Root vs Leaf	RHSIM_Rhsim06G0109400	-1.345412206	9.96164E-08	4.18295E-07	RHSIM_Rhsim06G0109400
Root vs Leaf	RHSIM_Rhsim09G0196000	1.708820796	1.00177E-07	4.20574E-07	RHSIM_Rhsim09G0196000
Root vs Leaf	RHSIM_Rhsim13G0026200	1.495919755	1.00982E-07	4.23796E-07	RHSIM_Rhsim13G0026200
Root vs Leaf	RHSIM_Rhsim02G0210200	7.271646965	1.0154E-07	4.26062E-07	RHSIM_Rhsim02G0210200
Root vs Leaf	RHSIM_RhsimUnG0171300	-3.975698122	1.02001E-07	4.27918E-07	RHSIM_RhsimUnG0171300
Root vs Leaf	RHSIM_Rhsim06G0078800	1.110362145	1.02605E-07	4.30299E-07	RHSIM_Rhsim06G0078800
Root vs Leaf	RHSIM_Rhsim10G0159200	-2.740555093	1.0283E-07	4.31164E-07	RHSIM_Rhsim10G0159200
Root vs Leaf	RHSIM_Rhsim08G0125400	3.394226123	1.03238E-07	4.32796E-07	RHSIM_Rhsim08G0125400
Root vs Leaf	RHSIM_Rhsim02G0111300	-2.40385897	1.03374E-07	4.33286E-07	RHSIM_Rhsim02G0111300
Root vs Leaf	RHSIM_Rhsim06G0161700	4.667503382	1.04686E-07	4.38708E-07	RHSIM_Rhsim06G0161700
Root vs Leaf	RHSIM_Rhsim12G0141200	4.81275281	1.05101E-07	4.40366E-07	RHSIM_Rhsim12G0141200
Root vs Leaf	RHSIM_Rhsim10G0028400	-2.188406811	1.05178E-07	4.40609E-07	RHSIM_Rhsim10G0028400
Root vs Leaf	RHSIM_Rhsim05G0165700	-1.790581857	1.0526E-07	4.40871E-07	RHSIM_Rhsim05G0165700
Root vs Leaf	RHSIM_Rhsim10G0076800	1.409400336	1.05378E-07	4.41288E-07	RHSIM_Rhsim10G0076800
Root vs Leaf	RHSIM_RhsimUnG0015100	3.47531556	1.05636E-07	4.42286E-07	RHSIM_RhsimUnG0015100
Root vs Leaf	RHSIM_Rhsim11G0193000	1.059121255	1.06306E-07	4.45011E-07	RHSIM_Rhsim11G0193000
Root vs Leaf	RHSIM_Rhsim03G0151800	2.200695078	1.06939E-07	4.47488E-07	RHSIM_Rhsim03G0151800
Root vs Leaf	RHSIM_RhsimUnG0215600	2.538183224	1.06955E-07	4.47488E-07	RHSIM_RhsimUnG0215600
Root vs Leaf	RHSIM_Rhsim09G0003300	2.912405226	1.07702E-07	4.50528E-07	RHSIM_Rhsim09G0003300
Root vs Leaf	RHSIM_Rhsim01G0054300	-7.556000181	1.07963E-07	4.5154E-07	RHSIM_Rhsim01G0054300
Root vs Leaf	RHSIM_Rhsim03G0098800	3.568850119	1.09362E-07	4.5731E-07	RHSIM_Rhsim03G0098800

Comparison	Gene ID	log$_2$FC	P-value	FDR (padj)	Gene Name
Root vs Leaf	RHSIM_Rhsim08G0108700	5.438868437	1.09578E-07	4.58128E-07	RHSIM_Rhsim08G0108700
Root vs Leaf	novel.4167	7.604535968	1.09809E-07	4.59011E-07	--
Root vs Leaf	novel.2862	2.572707059	1.10574E-07	4.62128E-07	--
Root vs Leaf	RHSIM_Rhsim05G0193600	-1.110844526	1.10839E-07	4.63148E-07	RHSIM_Rhsim05G0193600
Root vs Leaf	novel.5863	-5.369251306	1.11086E-07	4.64098E-07	--
Root vs Leaf	RHSIM_Rhsim08G0093900	1.808573041	1.11137E-07	4.64229E-07	RHSIM_Rhsim08G0093900
Root vs Leaf	RHSIM_Rhsim12G0044900	-3.445199586	1.11237E-07	4.64561E-07	RHSIM_Rhsim12G0044900
Root vs Leaf	RHSIM_Rhsim11G0063700	-3.679815053	1.117E-07	4.66412E-07	RHSIM_Rhsim11G0063700
Root vs Leaf	RHSIM_Rhsim09G0179800	-2.008826915	1.12089E-07	4.67868E-07	RHSIM_Rhsim09G0179800
Root vs Leaf	RHSIM_Rhsim09G0199300	2.909365882	1.12603E-07	4.69927E-07	RHSIM_Rhsim09G0199300
Root vs Leaf	RHSIM_Rhsim03G0097200	-4.402475093	1.12974E-07	4.71305E-07	RHSIM_Rhsim03G0097200
Root vs Leaf	RHSIM_Rhsim07G0129600	5.48048294	1.13007E-07	4.71357E-07	RHSIM_Rhsim07G0129600
Root vs Leaf	RHSIM_RhsimUnG0151400	-7.398292456	1.1338E-07	4.7283E-07	RHSIM_RhsimUnG0151400
Root vs Leaf	RHSIM_Rhsim07G0027100	7.135817951	1.13548E-07	4.73443E-07	RHSIM_Rhsim07G0027100
Root vs Leaf	RHSIM_Rhsim04G0232900	4.023527795	1.14598E-07	4.77643E-07	RHSIM_Rhsim04G0232900
Root vs Leaf	RHSIM_Rhsim03G0200700	-2.77117142	1.14617E-07	4.77643E-07	RHSIM_Rhsim03G0200700
Root vs Leaf	RHSIM_RhsimUnG0024200	-7.530586944	1.14906E-07	4.7876E-07	RHSIM_RhsimUnG0024200
Root vs Leaf	RHSIM_Rhsim07G0226400	-1.857671976	1.15689E-07	4.81936E-07	RHSIM_Rhsim07G0226400
Root vs Leaf	novel.1062	-5.929846561	1.16271E-07	4.84275E-07	--
Root vs Leaf	RHSIM_Rhsim02G0004000	-1.444587708	1.16563E-07	4.85405E-07	RHSIM_Rhsim02G0004000
Root vs Leaf	RHSIM_Rhsim03G0068700	1.247606685	1.16636E-07	4.85618E-07	RHSIM_Rhsim03G0068700
Root vs Leaf	RHSIM_Rhsim08G0200800	1.799069993	1.1714E-07	4.87543E-07	RHSIM_Rhsim08G0200800
Root vs Leaf	RHSIM_Rhsim07G0029300	1.201260372	1.1746E-07	4.88787E-07	RHSIM_Rhsim07G0029300
Root vs Leaf	RHSIM_Rhsim06G0192300	5.116792898	1.17951E-07	4.90741E-07	RHSIM_Rhsim06G0192300
Root vs Leaf	novel.261	-8.83986933	1.18072E-07	4.91082E-07	--

Comparison	Gene ID	log_2FC	P-value	FDR (padj)	Gene Name
Root vs Leaf	RHSIM_Rhsim02G0227800	7.342762536	1.18075E-07	4.91082E-07	RHSIM_Rhsim02G0227800
Root vs Leaf	RHSIM_Rhsim10G0099800	1.23899706	1.18219E-07	4.91591E-07	RHSIM_Rhsim10G0099800
Root vs Leaf	RHSIM_RhsimMtG0002200	-3.581078197	1.18744E-07	4.93684E-07	RHSIM_RhsimMtG0002200
Root vs Leaf	RHSIM_Rhsim01G0246600	3.179121141	1.18801E-07	4.93795E-07	RHSIM_Rhsim01G0246600
Root vs Leaf	RHSIM_Rhsim01G0148700	1.472374733	1.18813E-07	4.93795E-07	RHSIM_Rhsim01G0148700
Root vs Leaf	RHSIM_Rhsim08G0185700	3.261406451	1.20075E-07	4.98861E-07	RHSIM_Rhsim08G0185700
Root vs Leaf	RHSIM_Rhsim09G0196100	1.622777255	1.20139E-07	4.99038E-07	RHSIM_Rhsim09G0196100
Root vs Leaf	RHSIM_Rhsim08G0079800	1.133988102	1.21385E-07	5.04121E-07	RHSIM_Rhsim08G0079800
Root vs Leaf	RHSIM_Rhsim11G0084500	5.285708095	1.21428E-07	5.0421E-07	RHSIM_Rhsim11G0084500
Root vs Leaf	RHSIM_Rhsim01G0180600	-2.742522369	1.22641E-07	5.09064E-07	RHSIM_Rhsim01G0180600
Root vs Leaf	novel.149	-8.695210737	1.22931E-07	5.10177E-07	--
Root vs Leaf	RHSIM_Rhsim01G0130800	-1.219487706	1.24209E-07	5.15388E-07	RHSIM_Rhsim01G0130800
Root vs Leaf	RHSIM_Rhsim13G0123200	2.958682572	1.25139E-07	5.19155E-07	RHSIM_Rhsim13G0123200
Root vs Leaf	RHSIM_Rhsim01G0107600	2.276939577	1.26825E-07	5.26053E-07	RHSIM_Rhsim01G0107600
Root vs Leaf	novel.5141	1.900677382	1.27245E-07	5.27704E-07	--
Root vs Leaf	RHSIM_Rhsim04G0072200	2.075260348	1.27348E-07	5.28035E-07	RHSIM_Rhsim04G0072200
Root vs Leaf	novel.1279	7.907966962	1.27868E-07	5.30076E-07	--
Root vs Leaf	novel.6047	3.588284907	1.28178E-07	5.31188E-07	--
Root vs Leaf	RHSIM_Rhsim07G0119700	1.402882927	1.2866E-07	5.32999E-07	RHSIM_Rhsim07G0119700
Root vs Leaf	novel.583	-4.298279775	1.29864E-07	5.37889E-07	--
Root vs Leaf	RHSIM_Rhsim06G0169500	-1.652773642	1.30986E-07	5.4244E-07	RHSIM_Rhsim06G0169500
Root vs Leaf	RHSIM_Rhsim02G0227700	5.158811191	1.31506E-07	5.44398E-07	RHSIM_Rhsim02G0227700
Root vs Leaf	RHSIM_Rhsim04G0147100	-7.85696288	1.31983E-07	5.46274E-07	RHSIM_Rhsim04G0147100
Root vs Leaf	RHSIM_Rhsim13G0078600	-3.383872033	1.32119E-07	5.4674E-07	RHSIM_Rhsim13G0078600
Root vs Leaf	RHSIM_RhsimUnG0258400	-2.643833723	1.34165E-07	5.55008E-07	RHSIM_RhsimUnG0258400

Comparison	Gene ID	log_2FC	P-value	FDR (padj)	Gene Name
Root vs Leaf	RHSIM_Rhsim01G0234200	1.412697996	1.34823E-07	5.57628E-07	RHSIM_Rhsim01G0234200
Root vs Leaf	novel.3173	-1.175980873	1.35139E-07	5.58837E-07	--
Root vs Leaf	RHSIM_Rhsim08G0028700	-3.60036906	1.35642E-07	5.60816E-07	RHSIM_Rhsim08G0028700
Root vs Leaf	RHSIM_Rhsim06G0115800	-4.177075317	1.35752E-07	5.61171E-07	RHSIM_Rhsim06G0115800
Root vs Leaf	RHSIM_Rhsim13G0104000	-1.252278339	1.36717E-07	5.65059E-07	RHSIM_Rhsim13G0104000
Root vs Leaf	RHSIM_Rhsim03G0153100	-2.258992836	1.3692E-07	5.65796E-07	RHSIM_Rhsim03G0153100
Root vs Leaf	RHSIM_Rhsim12G0143300	4.045003911	1.38348E-07	5.71598E-07	RHSIM_Rhsim12G0143300
Root vs Leaf	RHSIM_Rhsim08G0152600	1.511399598	1.38529E-07	5.72241E-07	RHSIM_Rhsim08G0152600
Root vs Leaf	RHSIM_Rhsim12G0089600	7.417856278	1.38569E-07	5.72307E-07	RHSIM_Rhsim12G0089600
Root vs Leaf	RHSIM_RhsimUnG0190800	-2.565552615	1.3864E-07	5.72494E-07	RHSIM_RhsimUnG0190800
Root vs Leaf	RHSIM_RhsimUnG0207300	-4.163314585	1.4122E-07	5.82942E-07	RHSIM_RhsimUnG0207300
Root vs Leaf	RHSIM_Rhsim04G0116300	2.154055614	1.41733E-07	5.84956E-07	RHSIM_Rhsim04G0116300
Root vs Leaf	RHSIM_Rhsim08G0152800	1.0153871	1.43093E-07	5.90258E-07	RHSIM_Rhsim08G0152800
Root vs Leaf	novel.3306	-1.78187928	1.43095E-07	5.90258E-07	--
Root vs Leaf	RHSIM_Rhsim02G0183100	-5.230354881	1.43255E-07	5.90816E-07	RHSIM_Rhsim02G0183100
Root vs Leaf	RHSIM_Rhsim07G0022200	-1.7773406	1.45766E-07	6.00957E-07	RHSIM_Rhsim07G0022200
Root vs Leaf	RHSIM_Rhsim04G0091600	-1.009758132	1.45842E-07	6.01164E-07	RHSIM_Rhsim04G0091600
Root vs Leaf	RHSIM_RhsimUnG0039300	-3.906588804	1.46591E-07	6.04142E-07	RHSIM_RhsimUnG0039300
Root vs Leaf	novel.1531	-2.678930297	1.46915E-07	6.05372E-07	--
Root vs Leaf	RHSIM_Rhsim02G0209400	7.363543545	1.48205E-07	6.10467E-07	RHSIM_Rhsim02G0209400
Root vs Leaf	RHSIM_Rhsim12G0178300	1.284391256	1.48668E-07	6.12268E-07	RHSIM_Rhsim12G0178300
Root vs Leaf	RHSIM_Rhsim08G0135100	2.616400592	1.4927E-07	6.14638E-07	RHSIM_Rhsim08G0135100
Root vs Leaf	RHSIM_Rhsim01G0123200	1.004144631	1.50407E-07	6.19207E-07	RHSIM_Rhsim01G0123200
Root vs Leaf	RHSIM_Rhsim04G0176700	-1.194638698	1.51236E-07	6.2251E-07	RHSIM_Rhsim04G0176700
Root vs Leaf	RHSIM_Rhsim09G0026700	-1.252059614	1.5222E-07	6.26338E-07	RHSIM_Rhsim09G0026700

Comparison	Gene ID	log_2FC	P-value	FDR (padj)	Gene Name
Root vs Leaf	RHSIM_Rhsim03G0173400	-2.366223617	1.53027E-07	6.29433E-07	RHSIM_Rhsim03G0173400
Root vs Leaf	novel.4791	-5.190260896	1.54191E-07	6.33995E-07	--
Root vs Leaf	RHSIM_Rhsim03G0117200	-1.262001145	1.55007E-07	6.37241E-07	RHSIM_Rhsim03G0117200
Root vs Leaf	RHSIM_Rhsim08G0242700	-1.431523491	1.55106E-07	6.37533E-07	RHSIM_Rhsim08G0242700
Root vs Leaf	RHSIM_Rhsim03G0160200	-2.8798728	1.55504E-07	6.39053E-07	RHSIM_Rhsim03G0160200
Root vs Leaf	RHSIM_Rhsim01G0227100	6.253308528	1.56866E-07	6.44311E-07	RHSIM_Rhsim01G0227100
Root vs Leaf	RHSIM_Rhsim04G0105900	-1.458995416	1.58043E-07	6.48915E-07	RHSIM_Rhsim04G0105900
Root vs Leaf	RHSIM_Rhsim09G0214200	1.440478237	1.58109E-07	6.49068E-07	RHSIM_Rhsim09G0214200
Root vs Leaf	RHSIM_Rhsim05G0033700	2.383845168	1.59618E-07	6.55146E-07	RHSIM_Rhsim05G0033700
Root vs Leaf	RHSIM_Rhsim03G0150200	4.063733032	1.60137E-07	6.57162E-07	RHSIM_Rhsim03G0150200
Root vs Leaf	RHSIM_Rhsim02G0239900	4.425722585	1.60417E-07	6.58193E-07	RHSIM_Rhsim02G0239900
Root vs Leaf	RHSIM_Rhsim03G0078400	1.062642326	1.61704E-07	6.63356E-07	RHSIM_Rhsim03G0078400
Root vs Leaf	RHSIM_Rhsim13G0088400	-1.757011361	1.61783E-07	6.63563E-07	RHSIM_Rhsim13G0088400
Root vs Leaf	RHSIM_Rhsim05G0209300	-4.572823114	1.62025E-07	6.64439E-07	RHSIM_Rhsim05G0209300
Root vs Leaf	RHSIM_Rhsim01G0244100	1.052777342	1.62489E-07	6.66221E-07	RHSIM_Rhsim01G0244100
Root vs Leaf	RHSIM_Rhsim04G0249500	1.221591246	1.63725E-07	6.71171E-07	RHSIM_Rhsim04G0249500
Root vs Leaf	novel.4172	-3.968336856	1.64185E-07	6.72938E-07	--
Root vs Leaf	novel.1319	1.313634196	1.64516E-07	6.74176E-07	--
Root vs Leaf	novel.2770	-7.936577399	1.65085E-07	6.76386E-07	--
Root vs Leaf	RHSIM_Rhsim09G0126100	4.45629608	1.66161E-07	6.80676E-07	RHSIM_Rhsim09G0126100
Root vs Leaf	novel.5881	5.71234212	1.66323E-07	6.81219E-07	--
Root vs Leaf	RHSIM_Rhsim08G0077700	-7.091709528	1.674E-07	6.85507E-07	RHSIM_Rhsim08G0077700
Root vs Leaf	RHSIM_Rhsim01G0258600	4.622977039	1.67554E-07	6.86016E-07	RHSIM_Rhsim01G0258600
Root vs Leaf	novel.3975	6.123215869	1.67614E-07	6.8614E-07	--
Root vs Leaf	RHSIM_Rhsim10G0138300	-1.391335418	1.67898E-07	6.87182E-07	RHSIM_Rhsim10G0138300

Comparison	Gene ID	log_2FC	P-value	FDR (padj)	Gene Name
Root vs Leaf	RHSIM_Rhsim08G0182500	2.371076716	1.67931E-07	6.87191E-07	RHSIM_Rhsim08G0182500
Root vs Leaf	RHSIM_Rhsim13G0151100	-1.167988813	1.6796E-07	6.87191E-07	RHSIM_Rhsim13G0151100
Root vs Leaf	novel.5831	-4.06645774	1.68549E-07	6.89481E-07	--
Root vs Leaf	novel.3911	-7.592533127	1.70348E-07	6.96716E-07	--
Root vs Leaf	RHSIM_Rhsim02G0095100	-1.847609909	1.70886E-07	6.98794E-07	RHSIM_Rhsim02G0095100
Root vs Leaf	RHSIM_Rhsim13G0053100	-2.132095199	1.71015E-07	6.99197E-07	RHSIM_Rhsim13G0053100
Root vs Leaf	RHSIM_Rhsim11G0134500	2.20885017	1.71104E-07	6.99437E-07	RHSIM_Rhsim11G0134500
Root vs Leaf	RHSIM_Rhsim06G0076100	2.855630869	1.72327E-07	7.0419E-07	RHSIM_Rhsim06G0076100
Root vs Leaf	novel.1200	8.16867352	1.73599E-07	7.0926E-07	--
Root vs Leaf	novel.1842	1.916925061	1.73832E-07	7.10086E-07	--
Root vs Leaf	RHSIM_RhsimUnG0178900	-2.983438386	1.74271E-07	7.11755E-07	RHSIM_RhsimUnG0178900
Root vs Leaf	RHSIM_Rhsim02G0017700	-1.88803207	1.75828E-07	7.17859E-07	RHSIM_Rhsim02G0017700
Root vs Leaf	RHSIM_Rhsim11G0029400	1.756105496	1.75862E-07	7.17873E-07	RHSIM_Rhsim11G0029400
Root vs Leaf	RHSIM_Rhsim01G0074000	8.341229243	1.7737E-07	7.23901E-07	RHSIM_Rhsim01G0074000
Root vs Leaf	RHSIM_Rhsim04G0110500	-1.309623711	1.78192E-07	7.27129E-07	RHSIM_Rhsim04G0110500
Root vs Leaf	novel.6132	2.293979745	1.78367E-07	7.27714E-07	--
Root vs Leaf	RHSIM_Rhsim13G0174000	2.583896628	1.79031E-07	7.30293E-07	RHSIM_Rhsim13G0174000
Root vs Leaf	RHSIM_Rhsim04G0108300	1.887568784	1.80713E-07	7.36997E-07	RHSIM_Rhsim04G0108300
Root vs Leaf	RHSIM_Rhsim11G0158800	5.29940826	1.80738E-07	7.36997E-07	RHSIM_Rhsim11G0158800
Root vs Leaf	RHSIM_Rhsim13G0033700	1.390793488	1.80915E-07	7.3759E-07	RHSIM_Rhsim13G0033700
Root vs Leaf	RHSIM_Rhsim11G0126500	1.048064485	1.81333E-07	7.39162E-07	RHSIM_Rhsim11G0126500
Root vs Leaf	RHSIM_Rhsim04G0036600	-3.29547474	1.81521E-07	7.39799E-07	RHSIM_Rhsim04G0036600
Root vs Leaf	RHSIM_Rhsim01G0071500	4.155090783	1.82134E-07	7.42034E-07	RHSIM_Rhsim01G0071500
Root vs Leaf	RHSIM_Rhsim09G0154100	-1.076509609	1.85526E-07	7.5559E-07	RHSIM_Rhsim09G0154100
Root vs Leaf	RHSIM_Rhsim02G0026200	2.61390633	1.85947E-07	7.57168E-07	RHSIM_Rhsim02G0026200

Comparison	Gene ID	log_2FC	P-value	FDR (padj)	Gene Name
Root vs Leaf	RHSIM_Rhsim05G0059500	-4.840654121	1.86961E-07	7.61167E-07	RHSIM_Rhsim05G0059500
Root vs Leaf	RHSIM_Rhsim04G0128200	6.116514573	1.87479E-07	7.63139E-07	RHSIM_Rhsim04G0128200
Root vs Leaf	novel.2561	-7.328195169	1.88595E-07	7.67546E-07	--
Root vs Leaf	RHSIM_Rhsim04G0097100	2.643772642	1.89351E-07	7.7049E-07	RHSIM_Rhsim04G0097100
Root vs Leaf	RHSIM_Rhsim13G0158500	-2.285596739	1.9115E-07	7.77671E-07	RHSIM_Rhsim13G0158500
Root vs Leaf	RHSIM_Rhsim07G0072500	4.922401548	1.92125E-07	7.81503E-07	RHSIM_Rhsim07G0072500
Root vs Leaf	RHSIM_Rhsim07G0215600	-1.367709942	1.92316E-07	7.82143E-07	RHSIM_Rhsim07G0215600
Root vs Leaf	RHSIM_Rhsim13G0044800	1.392377091	1.94117E-07	7.89325E-07	RHSIM_Rhsim13G0044800
Root vs Leaf	novel.512	4.460642529	1.94153E-07	7.89333E-07	--
Root vs Leaf	RHSIM_RhsimUnG0073500	3.418080398	1.97484E-07	8.02738E-07	RHSIM_RhsimUnG0073500
Root vs Leaf	RHSIM_Rhsim03G0166300	1.325429178	1.9812E-07	8.05182E-07	RHSIM_Rhsim03G0166300
Root vs Leaf	novel.4767	2.25524005	1.98307E-07	8.05799E-07	--
Root vs Leaf	RHSIM_Rhsim03G0223400	-1.122225815	1.99195E-07	8.09265E-07	RHSIM_Rhsim03G0223400
Root vs Leaf	RHSIM_Rhsim09G0128200	-5.088988255	1.99267E-07	8.09413E-07	RHSIM_Rhsim09G0128200
Root vs Leaf	RHSIM_RhsimUnG0202600	-3.94893733	2.00416E-07	8.13938E-07	RHSIM_RhsimUnG0202600
Root vs Leaf	RHSIM_RhsimUnG0161700	-5.281504214	2.00773E-07	8.15246E-07	RHSIM_RhsimUnG0161700
Root vs Leaf	RHSIM_Rhsim06G0077500	2.228671399	2.02411E-07	8.21608E-07	RHSIM_Rhsim06G0077500
Root vs Leaf	RHSIM_Rhsim05G0063200	-1.037166556	2.02484E-07	8.21759E-07	RHSIM_Rhsim05G0063200
Root vs Leaf	RHSIM_Rhsim13G0027900	-4.047342391	2.03384E-07	8.25271E-07	RHSIM_Rhsim13G0027900
Root vs Leaf	RHSIM_Rhsim08G0009100	-2.281914463	2.04204E-07	8.28449E-07	RHSIM_Rhsim08G0009100
Root vs Leaf	RHSIM_Rhsim10G0060000	-1.151859107	2.04642E-07	8.30081E-07	RHSIM_Rhsim10G0060000
Root vs Leaf	RHSIM_Rhsim08G0017000	7.474685635	2.05212E-07	8.32249E-07	RHSIM_Rhsim08G0017000
Root vs Leaf	RHSIM_Rhsim02G0064000	-1.324117423	2.05314E-07	8.32515E-07	RHSIM_Rhsim02G0064000
Root vs Leaf	RHSIM_Rhsim12G0089300	-2.492479018	2.05415E-07	8.3278E-07	RHSIM_Rhsim12G0089300
Root vs Leaf	RHSIM_Rhsim13G0206900	3.818094337	2.07637E-07	8.41639E-07	RHSIM_Rhsim13G0206900

Comparison	Gene ID	log_2FC	P-value	FDR (padj)	Gene Name
Root vs Leaf	RHSIM_Rhsim03G0203100	-5.871969995	2.08641E-07	8.45531E-07	RHSIM_Rhsim03G0203100
Root vs Leaf	novel.2465	-1.110926324	2.0867E-07	8.45531E-07	--
Root vs Leaf	novel.4488	-7.457491925	2.09536E-07	8.48893E-07	--
Root vs Leaf	RHSIM_Rhsim03G0178400	1.794625465	2.10271E-07	8.5172E-07	RHSIM_Rhsim03G0178400
Root vs Leaf	RHSIM_Rhsim11G0027400	1.017573425	2.1103E-07	8.54645E-07	RHSIM_Rhsim11G0027400
Root vs Leaf	RHSIM_Rhsim02G0057700	3.046348801	2.11274E-07	8.55484E-07	RHSIM_Rhsim02G0057700
Root vs Leaf	RHSIM_Rhsim10G0188900	1.372509979	2.11667E-07	8.56775E-07	RHSIM_Rhsim10G0188900
Root vs Leaf	RHSIM_Rhsim08G0011000	1.694700008	2.13115E-07	8.62485E-07	RHSIM_Rhsim08G0011000
Root vs Leaf	RHSIM_Rhsim10G0198900	1.82945272	2.13752E-07	8.64911E-07	RHSIM_Rhsim10G0198900
Root vs Leaf	RHSIM_Rhsim07G0129700	1.078840572	2.15315E-07	8.70933E-07	RHSIM_Rhsim07G0129700
Root vs Leaf	RHSIM_Rhsim13G0009100	1.12092321	2.1555E-07	8.7173E-07	RHSIM_Rhsim13G0009100
Root vs Leaf	novel.4797	2.636934557	2.16075E-07	8.73397E-07	--
Root vs Leaf	RHSIM_Rhsim05G0079500	-4.660854894	2.17709E-07	8.79388E-07	RHSIM_Rhsim05G0079500
Root vs Leaf	novel.6192	1.166497983	2.1974E-07	8.87436E-07	--
Root vs Leaf	RHSIM_Rhsim12G0138300	-2.128257907	2.20796E-07	8.91388E-07	RHSIM_Rhsim12G0138300
Root vs Leaf	RHSIM_Rhsim05G0142400	1.130562398	2.21816E-07	8.95352E-07	RHSIM_Rhsim05G0142400
Root vs Leaf	RHSIM_Rhsim03G0175900	5.064020957	2.25759E-07	9.10789E-07	RHSIM_Rhsim03G0175900
Root vs Leaf	RHSIM_Rhsim02G0244400	-1.571238139	2.25864E-07	9.11054E-07	RHSIM_Rhsim02G0244400
Root vs Leaf	RHSIM_Rhsim01G0243300	-1.430921093	2.26345E-07	9.12834E-07	RHSIM_Rhsim01G0243300
Root vs Leaf	RHSIM_Rhsim02G0179200	-3.931281428	2.26415E-07	9.1296E-07	RHSIM_Rhsim02G0179200
Root vs Leaf	RHSIM_RhsimUnG0043600	-1.11191111	2.27504E-07	9.1675E-07	RHSIM_RhsimUnG0043600
Root vs Leaf	RHSIM_Rhsim06G0135300	1.114867612	2.27505E-07	9.1675E-07	RHSIM_Rhsim06G0135300
Root vs Leaf	RHSIM_RhsimMtG0001000	-4.564596779	2.27514E-07	9.1675E-07	RHSIM_RhsimMtG0001000
Root vs Leaf	novel.499	1.478791717	2.28769E-07	9.21646E-07	--
Root vs Leaf	RHSIM_Rhsim08G0011100	1.719840013	2.29087E-07	9.22767E-07	RHSIM_Rhsim08G0011100

Comparison	Gene ID	log_2FC	P-value	FDR (padj)	Gene Name
Root vs Leaf	RHSIM_Rhsim03G0231300	2.042833784	2.29139E-07	9.22818E-07	RHSIM_Rhsim03G0231300
Root vs Leaf	RHSIM_Rhsim12G0106900	1.480338171	2.29632E-07	9.24643E-07	RHSIM_Rhsim12G0106900
Root vs Leaf	novel.4365	-7.644222666	2.30799E-07	9.29179E-07	--
Root vs Leaf	RHSIM_Rhsim08G0201800	-1.652704187	2.30938E-07	9.29576E-07	RHSIM_Rhsim08G0201800
Root vs Leaf	RHSIM_Rhsim07G0116700	-2.835369132	2.32021E-07	9.33612E-07	RHSIM_Rhsim07G0116700
Root vs Leaf	RHSIM_Rhsim03G0230500	2.385801562	2.33162E-07	9.38037E-07	RHSIM_Rhsim03G0230500
Root vs Leaf	RHSIM_Rhsim03G0105900	-5.099210772	2.33346E-07	9.38615E-07	RHSIM_Rhsim03G0105900
Root vs Leaf	RHSIM_Rhsim02G0146600	-5.345407075	2.3578E-07	9.47911E-07	RHSIM_Rhsim02G0146600
Root vs Leaf	RHSIM_Rhsim07G0211300	1.004745701	2.36252E-07	9.49645E-07	RHSIM_Rhsim07G0211300
Root vs Leaf	RHSIM_Rhsim10G0014700	1.428263369	2.36314E-07	9.49731E-07	RHSIM_Rhsim10G0014700
Root vs Leaf	RHSIM_RhsimUnG0113800	1.51646385	2.37326E-07	9.53631E-07	RHSIM_RhsimUnG0113800
Root vs Leaf	RHSIM_Rhsim02G0087700	-1.195376636	2.40177E-07	9.64921E-07	RHSIM_Rhsim02G0087700
Root vs Leaf	RHSIM_Rhsim02G0254900	2.104451249	2.43275E-07	9.77196E-07	RHSIM_Rhsim02G0254900
Root vs Leaf	RHSIM_Rhsim02G0119900	1.307256292	2.461E-07	9.88372E-07	RHSIM_Rhsim02G0119900
Root vs Leaf	RHSIM_Rhsim08G0044900	-1.151426249	2.46229E-07	9.88718E-07	RHSIM_Rhsim08G0044900
Root vs Leaf	RHSIM_Rhsim05G0186400	1.297644469	2.46362E-07	9.88911E-07	RHSIM_Rhsim05G0186400
Root vs Leaf	RHSIM_Rhsim12G0181400	-3.431644067	2.4689E-07	9.90859E-07	RHSIM_Rhsim12G0181400
Root vs Leaf	RHSIM_Rhsim09G0200900	2.873037846	2.47422E-07	9.92821E-07	RHSIM_Rhsim09G0200900
Root vs Leaf	novel.104	-7.171924832	2.48258E-07	9.95996E-07	--
Root vs Leaf	RHSIM_Rhsim10G0154600	3.421581154	2.483E-07	9.95996E-07	RHSIM_Rhsim10G0154600
Root vs Leaf	RHSIM_Rhsim07G0081700	4.443757036	2.51373E-07	1.00745E-06	RHSIM_Rhsim07G0081700
Root vs Leaf	RHSIM_Rhsim06G0047500	3.962454339	2.53034E-07	1.01393E-06	RHSIM_Rhsim06G0047500
Root vs Leaf	RHSIM_Rhsim10G0181400	2.292933476	2.5355E-07	1.01582E-06	RHSIM_Rhsim10G0181400
Root vs Leaf	RHSIM_Rhsim07G0118800	-1.116805764	2.5389E-07	1.01701E-06	RHSIM_Rhsim07G0118800
Root vs Leaf	RHSIM_Rhsim06G0006800	1.551152304	2.54122E-07	1.01776E-06	RHSIM_Rhsim06G0006800

Comparison	Gene ID	log_2FC	P-value	FDR (padj)	Gene Name
Root vs Leaf	RHSIM_Rhsim06G0049700	2.710658589	2.54863E-07	1.02056E-06	RHSIM_Rhsim06G0049700
Root vs Leaf	RHSIM_Rhsim07G0081100	-2.832697785	2.56987E-07	1.02888E-06	RHSIM_Rhsim07G0081100
Root vs Leaf	RHSIM_Rhsim03G0127300	-1.679972107	2.5722E-07	1.02963E-06	RHSIM_Rhsim03G0127300
Root vs Leaf	novel.3804	8.394298759	2.60298E-07	1.04142E-06	--
Root vs Leaf	novel.1116	-2.818853765	2.60652E-07	1.04265E-06	--
Root vs Leaf	RHSIM_Rhsim12G0154500	1.209140099	2.61029E-07	1.04398E-06	RHSIM_Rhsim12G0154500
Root vs Leaf	novel.854	-5.922055908	2.63801E-07	1.05488E-06	--
Root vs Leaf	RHSIM_Rhsim02G0065000	-4.329195706	2.63943E-07	1.05527E-06	RHSIM_Rhsim02G0065000
Root vs Leaf	novel.2187	-6.639476254	2.64922E-07	1.05882E-06	--
Root vs Leaf	novel.220	-3.889766065	2.66186E-07	1.06369E-06	--
Root vs Leaf	RHSIM_Rhsim04G0104800	4.16276005	2.67171E-07	1.06725E-06	RHSIM_Rhsim04G0104800
Root vs Leaf	RHSIM_Rhsim04G0081400	1.73900375	2.67513E-07	1.06844E-06	RHSIM_Rhsim04G0081400
Root vs Leaf	RHSIM_Rhsim07G0053700	-1.028029001	2.6804E-07	1.07017E-06	RHSIM_Rhsim07G0053700
Root vs Leaf	novel.2266	-8.249362851	2.68118E-07	1.0703E-06	--
Root vs Leaf	RHSIM_Rhsim02G0201900	2.990001799	2.68614E-07	1.07209E-06	RHSIM_Rhsim02G0201900
Root vs Leaf	RHSIM_RhsimUnG0196500	-1.365499859	2.73096E-07	1.08923E-06	RHSIM_RhsimUnG0196500
Root vs Leaf	RHSIM_Rhsim09G0079100	-1.59003117	2.73637E-07	1.0912E-06	RHSIM_Rhsim09G0079100
Root vs Leaf	RHSIM_Rhsim02G0129800	1.659723014	2.75934E-07	1.09979E-06	RHSIM_Rhsim02G0129800
Root vs Leaf	novel.3114	-1.240193388	2.76337E-07	1.10121E-06	--
Root vs Leaf	RHSIM_Rhsim06G0187800	-1.817224415	2.80916E-07	1.11888E-06	RHSIM_Rhsim06G0187800
Root vs Leaf	RHSIM_Rhsim06G0153800	-4.461990842	2.8116E-07	1.11966E-06	RHSIM_Rhsim06G0153800
Root vs Leaf	RHSIM_RhsimUnG0040800	-1.364614301	2.81354E-07	1.12024E-06	RHSIM_RhsimUnG0040800
Root vs Leaf	RHSIM_Rhsim06G0147900	-1.837605735	2.81743E-07	1.12159E-06	RHSIM_Rhsim06G0147900
Root vs Leaf	RHSIM_Rhsim02G0158700	-4.464688738	2.82679E-07	1.12512E-06	RHSIM_Rhsim02G0158700
Root vs Leaf	RHSIM_Rhsim06G0068500	2.404731017	2.83144E-07	1.12678E-06	RHSIM_Rhsim06G0068500

Comparison	Gene ID	log_2FC	P-value	FDR (padj)	Gene Name
Root vs Leaf	novel.1798	-7.508011942	2.83772E-07	1.12908E-06	--
Root vs Leaf	RHSIM_Rhsim01G0180700	-2.792772363	2.84027E-07	1.12991E-06	RHSIM_Rhsim01G0180700
Root vs Leaf	RHSIM_Rhsim10G0112200	-4.687852883	2.84831E-07	1.13291E-06	RHSIM_Rhsim10G0112200
Root vs Leaf	RHSIM_Rhsim05G0051100	8.327912226	2.86847E-07	1.14073E-06	RHSIM_Rhsim05G0051100
Root vs Leaf	RHSIM_Rhsim06G0081600	6.914195627	2.8762E-07	1.14341E-06	RHSIM_Rhsim06G0081600
Root vs Leaf	RHSIM_Rhsim04G0100800	1.156976437	2.88613E-07	1.14697E-06	RHSIM_Rhsim04G0100800
Root vs Leaf	RHSIM_Rhsim10G0005500	1.345564995	2.90161E-07	1.15292E-06	RHSIM_Rhsim10G0005500
Root vs Leaf	novel.1532	-2.418730729	2.91359E-07	1.15748E-06	--
Root vs Leaf	RHSIM_Rhsim13G0107500	-2.931613609	2.91968E-07	1.1597E-06	RHSIM_Rhsim13G0107500
Root vs Leaf	RHSIM_Rhsim12G0206900	-1.436980502	2.92136E-07	1.16017E-06	RHSIM_Rhsim12G0206900
Root vs Leaf	RHSIM_Rhsim09G0024100	2.420476313	2.95325E-07	1.17264E-06	RHSIM_Rhsim09G0024100
Root vs Leaf	RHSIM_Rhsim04G0087500	1.445603547	2.96032E-07	1.17524E-06	RHSIM_Rhsim04G0087500
Root vs Leaf	RHSIM_Rhsim09G0103900	-1.165996841	2.96515E-07	1.17695E-06	RHSIM_Rhsim09G0103900
Root vs Leaf	RHSIM_Rhsim13G0224900	-2.382511601	2.99594E-07	1.18897E-06	RHSIM_Rhsim13G0224900
Root vs Leaf	RHSIM_Rhsim07G0129900	1.659661397	2.99728E-07	1.18926E-06	RHSIM_Rhsim07G0129900
Root vs Leaf	RHSIM_Rhsim03G0052900	1.241918709	2.99768E-07	1.18926E-06	RHSIM_Rhsim03G0052900
Root vs Leaf	RHSIM_Rhsim13G0212200	-7.338407814	3.00168E-07	1.19043E-06	RHSIM_Rhsim13G0212200
Root vs Leaf	RHSIM_Rhsim08G0216200	-1.598710464	3.01413E-07	1.19517E-06	RHSIM_Rhsim08G0216200
Root vs Leaf	novel.5316	7.167163657	3.01465E-07	1.19517E-06	--
Root vs Leaf	RHSIM_Rhsim13G0004600	-1.111360352	3.03165E-07	1.2017E-06	RHSIM_Rhsim13G0004600
Root vs Leaf	RHSIM_Rhsim05G0230100	-7.703821873	3.03913E-07	1.20446E-06	RHSIM_Rhsim05G0230100
Root vs Leaf	RHSIM_Rhsim08G0096100	-2.197108754	3.04086E-07	1.20494E-06	RHSIM_Rhsim08G0096100
Root vs Leaf	RHSIM_Rhsim01G0242500	1.396214185	3.06303E-07	1.21352E-06	RHSIM_Rhsim01G0242500
Root vs Leaf	RHSIM_RhsimMtG0007400	-3.436036486	3.06697E-07	1.21487E-06	RHSIM_RhsimMtG0007400
Root vs Leaf	RHSIM_Rhsim04G0120900	-1.340570821	3.07082E-07	1.21619E-06	RHSIM_Rhsim04G0120900

Comparison	Gene ID	log_2FC	P-value	FDR (padj)	Gene Name
Root vs Leaf	RHSIM_Rhsim07G0188600	-4.191071597	3.07728E-07	1.21854E-06	RHSIM_Rhsim07G0188600
Root vs Leaf	RHSIM_Rhsim02G0241500	2.089967909	3.08493E-07	1.22136E-06	RHSIM_Rhsim02G0241500
Root vs Leaf	RHSIM_Rhsim07G0115900	-2.399903637	3.09637E-07	1.22568E-06	RHSIM_Rhsim07G0115900
Root vs Leaf	RHSIM_Rhsim06G0140800	2.876616979	3.10562E-07	1.22892E-06	RHSIM_Rhsim06G0140800
Root vs Leaf	novel.296	1.859536719	3.11222E-07	1.23132E-06	--
Root vs Leaf	RHSIM_Rhsim13G0063100	1.070456255	3.1192E-07	1.23387E-06	RHSIM_Rhsim13G0063100
Root vs Leaf	novel.2867	-1.861136765	3.16365E-07	1.25103E-06	--
Root vs Leaf	RHSIM_Rhsim08G0022000	-5.1045665	3.19353E-07	1.26263E-06	RHSIM_Rhsim08G0022000
Root vs Leaf	RHSIM_Rhsim06G0176400	-1.289969526	3.20641E-07	1.2675E-06	RHSIM_Rhsim06G0176400
Root vs Leaf	RHSIM_Rhsim09G0186200	-1.026012923	3.21566E-07	1.27072E-06	RHSIM_Rhsim09G0186200
Root vs Leaf	RHSIM_Rhsim01G0238000	-2.10200522	3.26674E-07	1.29035E-06	RHSIM_Rhsim01G0238000
Root vs Leaf	RHSIM_Rhsim08G0018500	-1.280226399	3.26701E-07	1.29035E-06	RHSIM_Rhsim08G0018500
Root vs Leaf	novel.1710	2.005664501	3.26936E-07	1.29106E-06	--
Root vs Leaf	RHSIM_Rhsim10G0013000	-2.723817124	3.30526E-07	1.30485E-06	RHSIM_Rhsim10G0013000
Root vs Leaf	RHSIM_Rhsim11G0086000	1.467018428	3.3054E-07	1.30485E-06	RHSIM_Rhsim11G0086000
Root vs Leaf	RHSIM_Rhsim10G0071300	7.080441743	3.32137E-07	1.31093E-06	RHSIM_Rhsim10G0071300
Root vs Leaf	RHSIM_Rhsim13G0160800	-4.58725261	3.32275E-07	1.31125E-06	RHSIM_Rhsim13G0160800
Root vs Leaf	RHSIM_Rhsim08G0047000	1.791590833	3.32331E-07	1.31125E-06	RHSIM_Rhsim08G0047000
Root vs Leaf	RHSIM_Rhsim04G0166500	8.58717459	3.33487E-07	1.31558E-06	RHSIM_Rhsim04G0166500
Root vs Leaf	RHSIM_Rhsim12G0136000	-2.460474325	3.34103E-07	1.31757E-06	RHSIM_Rhsim12G0136000
Root vs Leaf	RHSIM_Rhsim08G0222300	-2.4063626	3.35046E-07	1.32106E-06	RHSIM_Rhsim08G0222300
Root vs Leaf	RHSIM_Rhsim03G0129000	1.547083863	3.35169E-07	1.32132E-06	RHSIM_Rhsim03G0129000
Root vs Leaf	RHSIM_Rhsim07G0061900	4.178432995	3.37421E-07	1.32997E-06	RHSIM_Rhsim07G0061900
Root vs Leaf	RHSIM_Rhsim12G0042800	1.631337268	3.38686E-07	1.33473E-06	RHSIM_Rhsim12G0042800
Root vs Leaf	RHSIM_Rhsim10G0130700	-5.629180707	3.40756E-07	1.34266E-06	RHSIM_Rhsim10G0130700

Comparison	Gene ID	log_2FC	P-value	FDR (padj)	Gene Name
Root vs Leaf	RHSIM_Rhsim10G0049000	-1.21971361	3.43617E-07	1.3537E-06	RHSIM_Rhsim10G0049000
Root vs Leaf	RHSIM_Rhsim13G0141800	1.725490707	3.4374E-07	1.35395E-06	RHSIM_Rhsim13G0141800
Root vs Leaf	novel.3891	-4.677322157	3.45496E-07	1.36064E-06	--
Root vs Leaf	novel.4882	-7.131495848	3.49037E-07	1.37435E-06	--
Root vs Leaf	RHSIM_Rhsim13G0069800	-4.134389491	3.50642E-07	1.3802E-06	RHSIM_Rhsim13G0069800
Root vs Leaf	RHSIM_Rhsim02G0108100	-2.403630216	3.51468E-07	1.38322E-06	RHSIM_Rhsim02G0108100
Root vs Leaf	RHSIM_Rhsim11G0074100	6.791742549	3.52418E-07	1.38672E-06	RHSIM_Rhsim11G0074100
Root vs Leaf	RHSIM_Rhsim08G0070700	3.797743357	3.5693E-07	1.40424E-06	RHSIM_Rhsim08G0070700
Root vs Leaf	RHSIM_RhsimUnG0151500	4.979377882	3.57539E-07	1.40606E-06	RHSIM_RhsimUnG0151500
Root vs Leaf	novel.444	1.419344498	3.57577E-07	1.40606E-06	--
Root vs Leaf	RHSIM_Rhsim02G0018600	-7.678458199	3.5823E-07	1.40839E-06	RHSIM_Rhsim02G0018600
Root vs Leaf	novel.2936	7.425502412	3.58618E-07	1.40968E-06	--
Root vs Leaf	RHSIM_RhsimUnG0129000	6.210787832	3.58802E-07	1.41016E-06	RHSIM_RhsimUnG0129000
Root vs Leaf	novel.5872	-7.116919174	3.60965E-07	1.41842E-06	--
Root vs Leaf	RHSIM_RhsimUnG0043700	3.097931771	3.62514E-07	1.42403E-06	RHSIM_RhsimUnG0043700
Root vs Leaf	RHSIM_Rhsim03G0089900	-1.15991262	3.63188E-07	1.42619E-06	RHSIM_Rhsim03G0089900
Root vs Leaf	RHSIM_Rhsim01G0234400	-1.300502849	3.64161E-07	1.42977E-06	RHSIM_Rhsim01G0234400
Root vs Leaf	RHSIM_Rhsim08G0239200	-8.240021865	3.65613E-07	1.43523E-06	RHSIM_Rhsim08G0239200
Root vs Leaf	RHSIM_Rhsim06G0113700	-1.377238615	3.6604E-07	1.43666E-06	RHSIM_Rhsim06G0113700
Root vs Leaf	RHSIM_Rhsim02G0116700	6.985064485	3.66271E-07	1.43732E-06	RHSIM_Rhsim02G0116700
Root vs Leaf	RHSIM_Rhsim03G0208300	-4.077277883	3.66493E-07	1.43795E-06	RHSIM_Rhsim03G0208300
Root vs Leaf	RHSIM_Rhsim11G0196700	2.0964631	3.68343E-07	1.44484E-06	RHSIM_Rhsim11G0196700
Root vs Leaf	RHSIM_Rhsim08G0162500	-2.963225246	3.68375E-07	1.44484E-06	RHSIM_Rhsim08G0162500
Root vs Leaf	RHSIM_Rhsim06G0068200	-1.937299798	3.69383E-07	1.4483E-06	RHSIM_Rhsim06G0068200
Root vs Leaf	RHSIM_RhsimUnG0077500	-4.657934976	3.70181E-07	1.45119E-06	RHSIM_RhsimUnG0077500

Comparison	Gene ID	log_2FC	P-value	FDR (padj)	Gene Name
Root vs Leaf	RHSIM_Rhsim03G0259000	4.483043254	3.7124E-07	1.45509E-06	RHSIM_Rhsim03G0259000
Root vs Leaf	RHSIM_Rhsim04G0014100	-1.820743104	3.71406E-07	1.4555E-06	RHSIM_Rhsim04G0014100
Root vs Leaf	RHSIM_Rhsim05G0129000	-2.761692086	3.71887E-07	1.45713E-06	RHSIM_Rhsim05G0129000
Root vs Leaf	RHSIM_Rhsim01G0078200	7.433729249	3.76123E-07	1.47323E-06	RHSIM_Rhsim01G0078200
Root vs Leaf	RHSIM_Rhsim13G0119900	1.837266362	3.76704E-07	1.47526E-06	RHSIM_Rhsim13G0119900
Root vs Leaf	RHSIM_Rhsim07G0072400	1.365316956	3.76886E-07	1.47572E-06	RHSIM_Rhsim07G0072400
Root vs Leaf	RHSIM_Rhsim02G0222900	-1.911272026	3.81311E-07	1.4928E-06	RHSIM_Rhsim02G0222900
Root vs Leaf	RHSIM_RhsimUnG0101400	-1.253526	3.88449E-07	1.52023E-06	RHSIM_RhsimUnG0101400
Root vs Leaf	novel.756	3.961547556	3.88889E-07	1.52169E-06	--
Root vs Leaf	novel.1023	-2.754062289	3.89442E-07	1.52334E-06	--
Root vs Leaf	RHSIM_Rhsim10G0181200	1.933686847	3.89817E-07	1.52455E-06	RHSIM_Rhsim10G0181200
Root vs Leaf	RHSIM_Rhsim13G0087900	1.510912415	3.92881E-07	1.53627E-06	RHSIM_Rhsim13G0087900
Root vs Leaf	RHSIM_Rhsim13G0113700	-8.265374641	3.92962E-07	1.53633E-06	RHSIM_Rhsim13G0113700
Root vs Leaf	RHSIM_Rhsim02G0032400	-1.374632375	3.94438E-07	1.54184E-06	RHSIM_Rhsim02G0032400
Root vs Leaf	RHSIM_Rhsim06G0065800	3.745551901	3.95039E-07	1.54367E-06	RHSIM_Rhsim06G0065800
Root vs Leaf	RHSIM_Rhsim10G0163700	5.174687001	3.95191E-07	1.544E-06	RHSIM_Rhsim10G0163700
Root vs Leaf	RHSIM_Rhsim04G0010200	-3.617457503	3.99728E-07	1.5612E-06	RHSIM_Rhsim04G0010200
Root vs Leaf	novel.2580	-4.985526384	3.9998E-07	1.56192E-06	--
Root vs Leaf	RHSIM_Rhsim07G0123000	8.093221139	4.00723E-07	1.56456E-06	RHSIM_Rhsim07G0123000
Root vs Leaf	RHSIM_Rhsim08G0248500	-1.50794352	4.01454E-07	1.56715E-06	RHSIM_Rhsim08G0248500
Root vs Leaf	novel.1715	-7.383375627	4.01838E-07	1.56838E-06	--
Root vs Leaf	RHSIM_Rhsim02G0178400	7.849581334	4.03139E-07	1.57319E-06	RHSIM_Rhsim02G0178400
Root vs Leaf	novel.809	4.676544665	4.04299E-07	1.57746E-06	--
Root vs Leaf	RHSIM_Rhsim09G0021500	1.631992789	4.07893E-07	1.59086E-06	RHSIM_Rhsim09G0021500
Root vs Leaf	RHSIM_Rhsim07G0226900	1.933149833	4.07942E-07	1.59086E-06	RHSIM_Rhsim07G0226900

Comparison	Gene ID	log_2FC	P-value	FDR (padj)	Gene Name
Root vs Leaf	RHSIM_Rhsim07G0222400	4.598946496	4.08555E-07	1.59299E-06	RHSIM_Rhsim07G0222400
Root vs Leaf	RHSIM_Rhsim01G0176000	-2.495175202	4.09145E-07	1.59502E-06	RHSIM_Rhsim01G0176000
Root vs Leaf	RHSIM_Rhsim05G0124000	-1.425394373	4.09584E-07	1.59646E-06	RHSIM_Rhsim05G0124000
Root vs Leaf	RHSIM_Rhsim10G0133800	-1.693714413	4.11553E-07	1.60386E-06	RHSIM_Rhsim10G0133800
Root vs Leaf	RHSIM_Rhsim12G0129200	-3.289652811	4.12823E-07	1.60854E-06	RHSIM_Rhsim12G0129200
Root vs Leaf	RHSIM_Rhsim05G0075900	-1.204105142	4.13001E-07	1.60896E-06	RHSIM_Rhsim05G0075900
Root vs Leaf	RHSIM_Rhsim08G0044200	-2.302716746	4.15583E-07	1.61848E-06	RHSIM_Rhsim08G0044200
Root vs Leaf	RHSIM_Rhsim12G0156300	5.31910285	4.17925E-07	1.62733E-06	RHSIM_Rhsim12G0156300
Root vs Leaf	RHSIM_Rhsim09G0023900	1.476015849	4.19231E-07	1.63214E-06	RHSIM_Rhsim09G0023900
Root vs Leaf	RHSIM_Rhsim06G0097000	-1.900070848	4.2072E-07	1.63766E-06	RHSIM_Rhsim06G0097000
Root vs Leaf	RHSIM_RhsimUnG0079800	-2.811181956	4.22043E-07	1.64226E-06	RHSIM_RhsimUnG0079800
Root vs Leaf	RHSIM_Rhsim12G0087300	-1.638780246	4.26441E-07	1.65853E-06	RHSIM_Rhsim12G0087300
Root vs Leaf	RHSIM_Rhsim11G0160300	-1.507443653	4.27614E-07	1.66281E-06	RHSIM_Rhsim11G0160300
Root vs Leaf	RHSIM_Rhsim12G0080000	3.060848212	4.28205E-07	1.66483E-06	RHSIM_Rhsim12G0080000
Root vs Leaf	RHSIM_Rhsim11G0034600	1.722770309	4.28326E-07	1.66503E-06	RHSIM_Rhsim11G0034600
Root vs Leaf	RHSIM_Rhsim03G0200500	-1.775967355	4.28821E-07	1.66667E-06	RHSIM_Rhsim03G0200500
Root vs Leaf	RHSIM_Rhsim01G0169300	1.499190116	4.38106E-07	1.70218E-06	RHSIM_Rhsim01G0169300
Root vs Leaf	RHSIM_Rhsim05G0228000	6.0312331	4.38757E-07	1.70414E-06	RHSIM_Rhsim05G0228000
Root vs Leaf	novel.3974	4.625832145	4.4119E-07	1.7133E-06	--
Root vs Leaf	RHSIM_Rhsim04G0166000	2.291851295	4.44579E-07	1.72589E-06	RHSIM_Rhsim04G0166000
Root vs Leaf	novel.5407	-3.614707343	4.45924E-07	1.73081E-06	--
Root vs Leaf	RHSIM_Rhsim06G0156100	-6.044218508	4.46349E-07	1.73217E-06	RHSIM_Rhsim06G0156100
Root vs Leaf	RHSIM_Rhsim07G0049800	1.149100147	4.47144E-07	1.73497E-06	RHSIM_Rhsim07G0049800
Root vs Leaf	RHSIM_Rhsim13G0083000	1.292804963	4.47815E-07	1.73728E-06	RHSIM_Rhsim13G0083000
Root vs Leaf	RHSIM_Rhsim03G0141900	3.668839835	4.48852E-07	1.74101E-06	RHSIM_Rhsim03G0141900

Comparison	Gene ID	log_2FC	P-value	FDR (padj)	Gene Name
Root vs Leaf	RHSIM_Rhsim09G0194500	4.883663198	4.52458E-07	1.75471E-06	RHSIM_Rhsim09G0194500
Root vs Leaf	RHSIM_Rhsim12G0143200	4.67013621	4.52633E-07	1.75509E-06	RHSIM_Rhsim12G0143200
Root vs Leaf	novel.3330	-1.772334058	4.53561E-07	1.75839E-06	--
Root vs Leaf	novel.4056	-2.118512017	4.54028E-07	1.75961E-06	--
Root vs Leaf	novel.2057	-7.350202881	4.56921E-07	1.77023E-06	--
Root vs Leaf	RHSIM_RhsimUnG0185600	-1.092964388	4.58431E-07	1.77549E-06	RHSIM_RhsimUnG0185600
Root vs Leaf	RHSIM_Rhsim05G0117600	-7.687296138	4.61716E-07	1.78791E-06	RHSIM_Rhsim05G0117600
Root vs Leaf	novel.4081	2.43606092	4.64626E-07	1.79828E-06	--
Root vs Leaf	RHSIM_Rhsim05G0157600	-3.862519779	4.71552E-07	1.82417E-06	RHSIM_Rhsim05G0157600
Root vs Leaf	RHSIM_Rhsim07G0099200	-1.058409402	4.74612E-07	1.83539E-06	RHSIM_Rhsim07G0099200
Root vs Leaf	RHSIM_Rhsim06G0015100	-3.017086374	4.77109E-07	1.84474E-06	RHSIM_Rhsim06G0015100
Root vs Leaf	RHSIM_Rhsim02G0212900	-2.522617742	4.80581E-07	1.85786E-06	RHSIM_Rhsim02G0212900
Root vs Leaf	novel.5582	-2.983253877	4.81367E-07	1.86058E-06	--
Root vs Leaf	RHSIM_Rhsim06G0222100	-1.231669247	4.81717E-07	1.86135E-06	RHSIM_Rhsim06G0222100
Root vs Leaf	RHSIM_Rhsim10G0211500	2.208677012	4.81726E-07	1.86135E-06	RHSIM_Rhsim10G0211500
Root vs Leaf	RHSIM_Rhsim07G0223300	4.996825166	4.8264E-07	1.86457E-06	RHSIM_Rhsim07G0223300
Root vs Leaf	RHSIM_Rhsim06G0141000	7.22011089	4.82854E-07	1.86509E-06	RHSIM_Rhsim06G0141000
Root vs Leaf	novel.5530	-3.195411024	4.83709E-07	1.86802E-06	--
Root vs Leaf	RHSIM_Rhsim13G0024000	-1.040355932	4.838E-07	1.86802E-06	RHSIM_Rhsim13G0024000
Root vs Leaf	novel.3685	-6.704032228	4.83872E-07	1.86802E-06	--
Root vs Leaf	RHSIM_Rhsim12G0070000	3.828114858	4.84775E-07	1.87095E-06	RHSIM_Rhsim12G0070000
Root vs Leaf	RHSIM_Rhsim08G0252200	-5.488807339	4.85596E-07	1.8738E-06	RHSIM_Rhsim08G0252200
Root vs Leaf	RHSIM_Rhsim04G0223500	7.225657247	4.86577E-07	1.87727E-06	RHSIM_Rhsim04G0223500
Root vs Leaf	RHSIM_RhsimPtG0005000	-1.992913293	4.88026E-07	1.88255E-06	RHSIM_RhsimPtG0005000
Root vs Leaf	RHSIM_Rhsim04G0031000	-6.397412447	4.90011E-07	1.88989E-06	RHSIM_Rhsim04G0031000

Comparison	Gene ID	log_2FC	P-value	FDR (padj)	Gene Name
Root vs Leaf	RHSIM_Rhsim02G0229900	1.185045589	4.91921E-07	1.89663E-06	RHSIM_Rhsim02G0229900
Root vs Leaf	RHSIM_Rhsim10G0059900	1.316934359	4.93148E-07	1.90104E-06	RHSIM_Rhsim10G0059900
Root vs Leaf	novel.6111	-7.689249262	4.95501E-07	1.90947E-06	--
Root vs Leaf	RHSIM_Rhsim09G0172300	1.664067265	4.95967E-07	1.91078E-06	RHSIM_Rhsim09G0172300
Root vs Leaf	RHSIM_Rhsim11G0144100	4.617170247	4.96005E-07	1.91078E-06	RHSIM_Rhsim11G0144100
Root vs Leaf	RHSIM_Rhsim09G0061200	2.043137361	4.96473E-07	1.91226E-06	RHSIM_Rhsim09G0061200
Root vs Leaf	novel.4064	1.149477463	4.96612E-07	1.91248E-06	--
Root vs Leaf	RHSIM_Rhsim01G0077000	9.017052321	4.98346E-07	1.91884E-06	RHSIM_Rhsim01G0077000
Root vs Leaf	RHSIM_Rhsim08G0171300	4.430994526	4.99903E-07	1.92451E-06	RHSIM_Rhsim08G0171300
Root vs Leaf	RHSIM_Rhsim01G0005700	-1.258240625	5.00227E-07	1.92544E-06	RHSIM_Rhsim01G0005700
Root vs Leaf	RHSIM_Rhsim04G0086800	-1.971976911	5.00451E-07	1.92598E-06	RHSIM_Rhsim04G0086800
Root vs Leaf	novel.5545	-4.466912756	5.04882E-07	1.94239E-06	--
Root vs Leaf	RHSIM_RhsimUnG0236600	-1.388942733	5.07692E-07	1.95288E-06	RHSIM_RhsimUnG0236600
Root vs Leaf	novel.1213	6.910708112	5.11197E-07	1.9657E-06	--
Root vs Leaf	RHSIM_Rhsim12G0080200	3.712978362	5.1321E-07	1.97312E-06	RHSIM_Rhsim12G0080200
Root vs Leaf	RHSIM_Rhsim02G0009500	1.691142302	5.13996E-07	1.97581E-06	RHSIM_Rhsim02G0009500
Root vs Leaf	RHSIM_Rhsim01G0073600	2.901324509	5.14654E-07	1.97801E-06	RHSIM_Rhsim01G0073600
Root vs Leaf	RHSIM_Rhsim12G0208300	-2.259610503	5.18594E-07	1.99282E-06	RHSIM_Rhsim12G0208300
Root vs Leaf	RHSIM_Rhsim03G0122700	4.864037281	5.19528E-07	1.99608E-06	RHSIM_Rhsim03G0122700
Root vs Leaf	RHSIM_Rhsim11G0101700	-1.441389923	5.19848E-07	1.99698E-06	RHSIM_Rhsim11G0101700
Root vs Leaf	RHSIM_Rhsim07G0070600	-3.901846786	5.20431E-07	1.99889E-06	RHSIM_Rhsim07G0070600
Root vs Leaf	RHSIM_RhsimUnG0065400	-1.871095125	5.21494E-07	2.00264E-06	RHSIM_RhsimUnG0065400
Root vs Leaf	novel.424	1.620681755	5.2311E-07	2.00851E-06	--
Root vs Leaf	RHSIM_Rhsim06G0203000	-2.049011202	5.23581E-07	2.00998E-06	RHSIM_Rhsim06G0203000
Root vs Leaf	RHSIM_Rhsim08G0216500	1.208411998	5.23857E-07	2.01071E-06	RHSIM_Rhsim08G0216500

Comparison	Gene ID	log_2FC	P-value	FDR (padj)	Gene Name
Root vs Leaf	RHSIM_Rhsim02G0251300	1.027456432	5.23972E-07	2.01082E-06	RHSIM_Rhsim02G0251300
Root vs Leaf	novel.4632	-5.626815388	5.26528E-07	2.01996E-06	--
Root vs Leaf	novel.2399	5.188246523	5.30948E-07	2.03658E-06	--
Root vs Leaf	RHSIM_Rhsim07G0042800	2.120806509	5.31089E-07	2.03678E-06	RHSIM_Rhsim07G0042800
Root vs Leaf	RHSIM_Rhsim01G0284300	-1.064957789	5.337E-07	2.04645E-06	RHSIM_Rhsim01G0284300
Root vs Leaf	RHSIM_Rhsim01G0019600	-3.138415564	5.35533E-07	2.0528E-06	RHSIM_Rhsim01G0019600
Root vs Leaf	RHSIM_Rhsim01G0038500	-8.760921471	5.35803E-07	2.0535E-06	RHSIM_Rhsim01G0038500
Root vs Leaf	RHSIM_Rhsim07G0027700	1.463975103	5.36381E-07	2.05537E-06	RHSIM_Rhsim07G0027700
Root vs Leaf	novel.6060	6.832983029	5.38166E-07	2.06187E-06	--
Root vs Leaf	RHSIM_Rhsim12G0061500	2.429982344	5.38808E-07	2.06399E-06	RHSIM_Rhsim12G0061500
Root vs Leaf	RHSIM_Rhsim12G0110500	-1.065873982	5.40014E-07	2.06826E-06	RHSIM_Rhsim12G0110500
Root vs Leaf	RHSIM_Rhsim01G0046600	-1.101605661	5.41584E-07	2.07359E-06	RHSIM_Rhsim01G0046600
Root vs Leaf	RHSIM_RhsimUnG0205700	2.326587843	5.42511E-07	2.07645E-06	RHSIM_RhsimUnG0205700
Root vs Leaf	RHSIM_Rhsim09G0093800	-1.520950866	5.44563E-07	2.08397E-06	RHSIM_Rhsim09G0093800
Root vs Leaf	RHSIM_RhsimUnG0250800	3.066075866	5.46472E-07	2.09058E-06	RHSIM_RhsimUnG0250800
Root vs Leaf	novel.1187	1.125487147	5.50697E-07	2.10604E-06	--
Root vs Leaf	RHSIM_Rhsim10G0139500	-4.240526896	5.51718E-07	2.10925E-06	RHSIM_Rhsim10G0139500
Root vs Leaf	RHSIM_Rhsim04G0249100	-1.156021852	5.52657E-07	2.11249E-06	RHSIM_Rhsim04G0249100
Root vs Leaf	RHSIM_Rhsim03G0099400	1.411936998	5.53627E-07	2.11585E-06	RHSIM_Rhsim03G0099400
Root vs Leaf	RHSIM_Rhsim04G0157800	1.132004851	5.5663E-07	2.12698E-06	RHSIM_Rhsim04G0157800
Root vs Leaf	RHSIM_RhsimUnG0066000	-3.574140028	5.5889E-07	2.13526E-06	RHSIM_RhsimUnG0066000
Root vs Leaf	RHSIM_RhsimUnG0144200	-3.045259117	5.59383E-07	2.13679E-06	RHSIM_RhsimUnG0144200
Root vs Leaf	RHSIM_Rhsim11G0175600	6.004225962	5.60609E-07	2.14112E-06	RHSIM_Rhsim11G0175600
Root vs Leaf	RHSIM_Rhsim03G0008900	-2.669458526	5.6127E-07	2.14329E-06	RHSIM_Rhsim03G0008900
Root vs Leaf	RHSIM_Rhsim13G0008000	1.848410303	5.63574E-07	2.15138E-06	RHSIM_Rhsim13G0008000

Comparison	Gene ID	log_2FC	P-value	FDR (padj)	Gene Name
Root vs Leaf	RHSIM_Rhsim06G0052600	-2.611140386	5.64752E-07	2.15552E-06	RHSIM_Rhsim06G0052600
Root vs Leaf	RHSIM_Rhsim06G0174600	-1.376661267	5.75671E-07	2.19575E-06	RHSIM_Rhsim06G0174600
Root vs Leaf	RHSIM_Rhsim01G0067700	-1.096874587	5.77021E-07	2.20053E-06	RHSIM_Rhsim01G0067700
Root vs Leaf	RHSIM_Rhsim05G0103200	4.698278718	5.79141E-07	2.20825E-06	RHSIM_Rhsim05G0103200
Root vs Leaf	RHSIM_Rhsim02G0214700	-7.410766788	5.82215E-07	2.21925E-06	RHSIM_Rhsim02G0214700
Root vs Leaf	RHSIM_Rhsim10G0095600	2.395669245	5.83393E-07	2.22337E-06	RHSIM_Rhsim10G0095600
Root vs Leaf	RHSIM_Rhsim06G0150100	1.066971485	5.8379E-07	2.22449E-06	RHSIM_Rhsim06G0150100
Root vs Leaf	RHSIM_Rhsim05G0010100	-5.643881994	5.83879E-07	2.22449E-06	RHSIM_Rhsim05G0010100
Root vs Leaf	RHSIM_Rhsim09G0089600	1.158676245	5.85976E-07	2.23211E-06	RHSIM_Rhsim09G0089600
Root vs Leaf	RHSIM_Rhsim10G0022600	2.894385851	5.86601E-07	2.23412E-06	RHSIM_Rhsim10G0022600
Root vs Leaf	RHSIM_Rhsim07G0216300	7.335087094	5.87021E-07	2.23535E-06	RHSIM_Rhsim07G0216300
Root vs Leaf	RHSIM_Rhsim04G0195900	-1.512067339	5.87156E-07	2.2355E-06	RHSIM_Rhsim04G0195900
Root vs Leaf	novel.4607	-3.800868183	5.88764E-07	2.24089E-06	--
Root vs Leaf	RHSIM_Rhsim12G0057300	-1.763878109	5.9068E-07	2.24781E-06	RHSIM_Rhsim12G0057300
Root vs Leaf	RHSIM_Rhsim10G0034700	1.020128516	6.0319E-07	2.29466E-06	RHSIM_Rhsim10G0034700
Root vs Leaf	RHSIM_Rhsim13G0099700	-4.098424743	6.06734E-07	2.30776E-06	RHSIM_Rhsim13G0099700
Root vs Leaf	RHSIM_Rhsim12G0042300	4.278553202	6.07264E-07	2.3094E-06	RHSIM_Rhsim12G0042300
Root vs Leaf	RHSIM_Rhsim10G0062200	1.894333147	6.11495E-07	2.32511E-06	RHSIM_Rhsim10G0062200
Root vs Leaf	RHSIM_RhsimUnG0152500	-1.613785642	6.1183E-07	2.32562E-06	RHSIM_RhsimUnG0152500
Root vs Leaf	RHSIM_Rhsim10G0037000	3.400254119	6.14891E-07	2.33687E-06	RHSIM_Rhsim10G0037000
Root vs Leaf	RHSIM_Rhsim05G0232600	1.282644651	6.16366E-07	2.34209E-06	RHSIM_Rhsim05G0232600
Root vs Leaf	novel.3683	2.135918421	6.16513E-07	2.34227E-06	--
Root vs Leaf	RHSIM_Rhsim12G0185200	4.137406606	6.18272E-07	2.34856E-06	RHSIM_Rhsim12G0185200
Root vs Leaf	novel.4385	4.333130533	6.20607E-07	2.35704E-06	--
Root vs Leaf	novel.1518	6.468974167	6.21191E-07	2.35887E-06	--

Comparison	Gene ID	log_2FC	P-value	FDR (padj)	Gene Name
Root vs Leaf	RHSIM_Rhsim07G0140600	-2.448398761	6.2152E-07	2.35974E-06	RHSIM_Rhsim07G0140600
Root vs Leaf	RHSIM_Rhsim01G0099300	-6.706419442	6.24956E-07	2.37239E-06	RHSIM_Rhsim01G0099300
Root vs Leaf	RHSIM_Rhsim09G0148200	-1.035771996	6.2804E-07	2.38371E-06	RHSIM_Rhsim09G0148200
Root vs Leaf	RHSIM_Rhsim03G0056500	1.569134036	6.2833E-07	2.38442E-06	RHSIM_Rhsim03G0056500
Root vs Leaf	RHSIM_Rhsim01G0231400	-1.901379085	6.28894E-07	2.38617E-06	RHSIM_Rhsim01G0231400
Root vs Leaf	RHSIM_RhsimUnG0121800	-2.181506056	6.39367E-07	2.42511E-06	RHSIM_RhsimUnG0121800
Root vs Leaf	RHSIM_Rhsim07G0087100	-1.282694933	6.40089E-07	2.42745E-06	RHSIM_Rhsim07G0087100
Root vs Leaf	RHSIM_Rhsim05G0145600	4.428786499	6.4252E-07	2.43627E-06	RHSIM_Rhsim05G0145600
Root vs Leaf	novel.2694	-3.093587671	6.47258E-07	2.45343E-06	--
Root vs Leaf	RHSIM_Rhsim13G0094400	1.042153517	6.54263E-07	2.47917E-06	RHSIM_Rhsim13G0094400
Root vs Leaf	RHSIM_Rhsim06G0116500	-3.696565548	6.54384E-07	2.47922E-06	RHSIM_Rhsim06G0116500
Root vs Leaf	RHSIM_Rhsim02G0073800	-4.996371235	6.60554E-07	2.50219E-06	RHSIM_Rhsim02G0073800
Root vs Leaf	RHSIM_Rhsim08G0209700	1.353542919	6.69549E-07	2.53502E-06	RHSIM_Rhsim08G0209700
Root vs Leaf	RHSIM_Rhsim06G0149500	1.121626935	6.74192E-07	2.55218E-06	RHSIM_Rhsim06G0149500
Root vs Leaf	RHSIM_Rhsim02G0044100	-2.135737622	6.7872E-07	2.5689E-06	RHSIM_Rhsim02G0044100
Root vs Leaf	RHSIM_Rhsim06G0127300	-1.135821381	6.83289E-07	2.58577E-06	RHSIM_Rhsim06G0127300
Root vs Leaf	RHSIM_Rhsim06G0063900	1.145517063	6.87221E-07	2.60023E-06	RHSIM_Rhsim06G0063900
Root vs Leaf	RHSIM_Rhsim13G0018000	4.304918816	6.97562E-07	2.63892E-06	RHSIM_Rhsim13G0018000
Root vs Leaf	RHSIM_RhsimUnG0120100	1.076304481	7.10404E-07	2.68707E-06	RHSIM_RhsimUnG0120100
Root vs Leaf	RHSIM_Rhsim12G0132000	1.659955196	7.12932E-07	2.69575E-06	RHSIM_Rhsim12G0132000
Root vs Leaf	RHSIM_RhsimUnG0207700	-2.170311225	7.24294E-07	2.73781E-06	RHSIM_RhsimUnG0207700
Root vs Leaf	RHSIM_Rhsim03G0198800	-3.628025243	7.24873E-07	2.73955E-06	RHSIM_Rhsim03G0198800
Root vs Leaf	RHSIM_Rhsim09G0164900	1.018647158	7.28199E-07	2.75122E-06	RHSIM_Rhsim09G0164900
Root vs Leaf	RHSIM_Rhsim05G0222000	-4.720687462	7.42787E-07	2.80542E-06	RHSIM_Rhsim05G0222000
Root vs Leaf	RHSIM_Rhsim06G0242500	-1.325384379	7.45643E-07	2.81575E-06	RHSIM_Rhsim06G0242500

Comparison	Gene ID	log_2FC	P-value	FDR (padj)	Gene Name
Root vs Leaf	RHSIM_Rhsim04G0085500	-1.859510193	7.46414E-07	2.8182E-06	RHSIM_Rhsim04G0085500
Root vs Leaf	RHSIM_Rhsim13G0095500	7.303274082	7.51366E-07	2.83644E-06	RHSIM_Rhsim13G0095500
Root vs Leaf	RHSIM_Rhsim04G0246900	1.081501702	7.51949E-07	2.83817E-06	RHSIM_Rhsim04G0246900
Root vs Leaf	RHSIM_Rhsim06G0081900	-1.348749877	7.5561E-07	2.8506E-06	RHSIM_Rhsim06G0081900
Root vs Leaf	RHSIM_Rhsim09G0071700	4.225600713	7.57238E-07	2.85581E-06	RHSIM_Rhsim09G0071700
Root vs Leaf	RHSIM_RhsimUnG0085600	3.144592829	7.57416E-07	2.85602E-06	RHSIM_RhsimUnG0085600
Root vs Leaf	RHSIM_Rhsim06G0059300	-4.93954318	7.59395E-07	2.86301E-06	RHSIM_Rhsim06G0059300
Root vs Leaf	novel.699	-4.603021547	7.62306E-07	2.87352E-06	--
Root vs Leaf	RHSIM_Rhsim08G0208000	1.070174858	7.6332E-07	2.87687E-06	RHSIM_Rhsim08G0208000
Root vs Leaf	novel.5650	-5.093344107	7.63995E-07	2.87895E-06	--
Root vs Leaf	RHSIM_Rhsim08G0190700	-1.331991832	7.67567E-07	2.89147E-06	RHSIM_Rhsim08G0190700
Root vs Leaf	RHSIM_Rhsim01G0182500	4.33673958	7.76023E-07	2.92284E-06	RHSIM_Rhsim01G0182500
Root vs Leaf	RHSIM_Rhsim05G0108500	-1.017537229	7.76211E-07	2.92307E-06	RHSIM_Rhsim05G0108500
Root vs Leaf	novel.4142	-2.485404738	7.7683E-07	2.92493E-06	--
Root vs Leaf	RHSIM_Rhsim07G0028100	5.564146823	7.78305E-07	2.93001E-06	RHSIM_Rhsim07G0028100
Root vs Leaf	RHSIM_Rhsim09G0017900	2.873398082	7.80148E-07	2.93599E-06	RHSIM_Rhsim09G0017900
Root vs Leaf	RHSIM_Rhsim12G0069800	4.790449327	7.83038E-07	2.94543E-06	RHSIM_Rhsim12G0069800
Root vs Leaf	RHSIM_Rhsim04G0051600	-1.817544879	7.85588E-07	2.95452E-06	RHSIM_Rhsim04G0051600
Root vs Leaf	RHSIM_Rhsim12G0075500	2.045536671	7.85709E-07	2.95452E-06	RHSIM_Rhsim12G0075500
Root vs Leaf	RHSIM_Rhsim02G0044200	-7.304082421	7.87459E-07	2.96061E-06	RHSIM_Rhsim02G0044200
Root vs Leaf	novel.3041	-8.166457294	7.90372E-07	2.97109E-06	--
Root vs Leaf	RHSIM_Rhsim03G0190600	1.034040402	7.99503E-07	3.00443E-06	RHSIM_Rhsim03G0190600
Root vs Leaf	RHSIM_Rhsim01G0268800	3.371406585	8.00553E-07	3.00789E-06	RHSIM_Rhsim01G0268800
Root vs Leaf	RHSIM_Rhsim08G0060200	-6.510659106	8.01203E-07	3.00984E-06	RHSIM_Rhsim08G0060200
Root vs Leaf	RHSIM_Rhsim05G0184900	1.087307019	8.01926E-07	3.01207E-06	RHSIM_Rhsim05G0184900

Comparison	Gene ID	log_2FC	P-value	FDR (padj)	Gene Name
Root vs Leaf	RHSIM_Rhsim07G0212000	3.660086962	8.02712E-07	3.01453E-06	RHSIM_Rhsim07G0212000
Root vs Leaf	novel.1830	-2.647803071	8.03335E-07	3.01638E-06	--
Root vs Leaf	RHSIM_Rhsim13G0139800	2.744716828	8.07641E-07	3.03206E-06	RHSIM_Rhsim13G0139800
Root vs Leaf	RHSIM_Rhsim13G0110800	-1.070001767	8.10374E-07	3.04183E-06	RHSIM_Rhsim13G0110800
Root vs Leaf	RHSIM_Rhsim07G0094500	1.751299694	8.1079E-07	3.0429E-06	RHSIM_Rhsim07G0094500
Root vs Leaf	RHSIM_Rhsim07G0227000	6.321904052	8.1149E-07	3.04503E-06	RHSIM_Rhsim07G0227000
Root vs Leaf	novel.4884	-7.982011391	8.14649E-07	3.05639E-06	--
Root vs Leaf	RHSIM_RhsimUnG0188600	-3.658274304	8.1542E-07	3.05878E-06	RHSIM_RhsimUnG0188600
Root vs Leaf	RHSIM_Rhsim07G0034900	4.510449562	8.21029E-07	3.07883E-06	RHSIM_Rhsim07G0034900
Root vs Leaf	RHSIM_Rhsim13G0196500	-1.010277692	8.22165E-07	3.08259E-06	RHSIM_Rhsim13G0196500
Root vs Leaf	RHSIM_Rhsim07G0094200	2.840489628	8.2766E-07	3.10168E-06	RHSIM_Rhsim07G0094200
Root vs Leaf	RHSIM_Rhsim10G0160200	1.435748283	8.29238E-07	3.10709E-06	RHSIM_Rhsim10G0160200
Root vs Leaf	RHSIM_RhsimUnG0251100	1.115561321	8.33936E-07	3.12419E-06	RHSIM_RhsimUnG0251100
Root vs Leaf	RHSIM_Rhsim07G0063300	4.884886132	8.38495E-07	3.14076E-06	RHSIM_Rhsim07G0063300
Root vs Leaf	novel.1034	4.283727585	8.47556E-07	3.17367E-06	--
Root vs Leaf	RHSIM_Rhsim11G0009100	-2.722729185	8.51643E-07	3.18846E-06	RHSIM_Rhsim11G0009100
Root vs Leaf	RHSIM_Rhsim04G0118400	1.161592597	8.5276E-07	3.19213E-06	RHSIM_Rhsim04G0118400
Root vs Leaf	RHSIM_Rhsim05G0137900	1.885645439	8.58746E-07	3.21401E-06	RHSIM_Rhsim05G0137900
Root vs Leaf	RHSIM_Rhsim01G0003900	2.254712765	8.655E-07	3.23772E-06	RHSIM_Rhsim01G0003900
Root vs Leaf	RHSIM_Rhsim06G0174200	-1.634444206	8.66583E-07	3.24125E-06	RHSIM_Rhsim06G0174200
Root vs Leaf	RHSIM_Rhsim03G0147800	5.281046563	8.67714E-07	3.24495E-06	RHSIM_Rhsim03G0147800
Root vs Leaf	RHSIM_Rhsim07G0104200	3.049051072	8.70749E-07	3.25578E-06	RHSIM_Rhsim07G0104200
Root vs Leaf	RHSIM_Rhsim10G0135400	-5.484255593	8.72598E-07	3.26163E-06	RHSIM_Rhsim10G0135400
Root vs Leaf	RHSIM_Rhsim07G0005300	4.286349575	8.76906E-07	3.27721E-06	RHSIM_Rhsim07G0005300
Root vs Leaf	RHSIM_RhsimUnG0232300	-3.510442697	8.77578E-07	3.27919E-06	RHSIM_RhsimUnG0232300

Comparison	Gene ID	log_2FC	P-value	FDR (padj)	Gene Name
Root vs Leaf	novel.3973	-7.274450762	8.8335E-07	3.30023E-06	--
Root vs Leaf	RHSIM_Rhsim10G0007600	2.87667569	8.89906E-07	3.32418E-06	RHSIM_Rhsim10G0007600
Root vs Leaf	RHSIM_Rhsim11G0128100	-3.533289908	8.95759E-07	3.34497E-06	RHSIM_Rhsim11G0128100
Root vs Leaf	RHSIM_Rhsim11G0110400	-1.944870974	9.00378E-07	3.36167E-06	RHSIM_Rhsim11G0110400
Root vs Leaf	novel.1465	-5.473049688	9.02472E-07	3.36895E-06	--
Root vs Leaf	novel.4388	-7.321312135	9.02684E-07	3.3692E-06	--
Root vs Leaf	RHSIM_Rhsim13G0015400	-1.085451144	9.04026E-07	3.37366E-06	RHSIM_Rhsim13G0015400
Root vs Leaf	RHSIM_Rhsim04G0172500	3.451393625	9.0552E-07	3.37869E-06	RHSIM_Rhsim04G0172500
Root vs Leaf	RHSIM_Rhsim11G0004600	1.733025198	9.06382E-07	3.38136E-06	RHSIM_Rhsim11G0004600
Root vs Leaf	RHSIM_Rhsim04G0093600	-1.220457193	9.121E-07	3.40215E-06	RHSIM_Rhsim04G0093600
Root vs Leaf	RHSIM_Rhsim06G0139300	8.216828549	9.17618E-07	3.42163E-06	RHSIM_Rhsim06G0139300
Root vs Leaf	RHSIM_Rhsim06G0200000	-1.03227983	9.28615E-07	3.46207E-06	RHSIM_Rhsim06G0200000
Root vs Leaf	RHSIM_Rhsim13G0183400	-10.69128648	9.41705E-07	3.50861E-06	RHSIM_Rhsim13G0183400
Root vs Leaf	RHSIM_Rhsim09G0166000	1.671843391	9.44451E-07	3.51828E-06	RHSIM_Rhsim09G0166000
Root vs Leaf	RHSIM_Rhsim05G0159300	-1.418767808	9.47463E-07	3.52893E-06	RHSIM_Rhsim05G0159300
Root vs Leaf	RHSIM_Rhsim06G0055000	-1.30282718	9.49628E-07	3.53637E-06	RHSIM_Rhsim06G0055000
Root vs Leaf	RHSIM_Rhsim08G0031700	2.291377644	9.49765E-07	3.53637E-06	RHSIM_Rhsim08G0031700
Root vs Leaf	RHSIM_Rhsim12G0047800	3.1926773	9.53092E-07	3.54816E-06	RHSIM_Rhsim12G0047800
Root vs Leaf	RHSIM_Rhsim10G0200900	5.515412577	9.56683E-07	3.56041E-06	RHSIM_Rhsim10G0200900
Root vs Leaf	RHSIM_Rhsim08G0025100	-3.880700395	9.57102E-07	3.56139E-06	RHSIM_Rhsim08G0025100
Root vs Leaf	RHSIM_Rhsim11G0081400	-3.119914942	9.59962E-07	3.57146E-06	RHSIM_Rhsim11G0081400
Root vs Leaf	novel.2387	-1.667674973	9.60127E-07	3.5715E-06	--
Root vs Leaf	RHSIM_Rhsim08G0090600	4.244944752	9.6587E-07	3.59228E-06	RHSIM_Rhsim08G0090600
Root vs Leaf	RHSIM_Rhsim09G0100900	5.363867824	9.69442E-07	3.60499E-06	RHSIM_Rhsim09G0100900
Root vs Leaf	RHSIM_Rhsim05G0201900	6.772676685	9.72217E-07	3.61473E-06	RHSIM_Rhsim05G0201900

Comparison	Gene ID	log_2FC	P-value	FDR (padj)	Gene Name
Root vs Leaf	novel.5807	6.897150344	9.73111E-07	3.61747E-06	--
Root vs Leaf	RHSIM_Rhsim13G0087100	-1.490970133	9.73558E-07	3.61855E-06	RHSIM_Rhsim13G0087100
Root vs Leaf	RHSIM_Rhsim12G0136200	1.26605236	9.75766E-07	3.62618E-06	RHSIM_Rhsim12G0136200
Root vs Leaf	RHSIM_Rhsim12G0174000	1.055558998	9.76984E-07	3.63012E-06	RHSIM_Rhsim12G0174000
Root vs Leaf	RHSIM_Rhsim07G0211500	4.301525693	9.82401E-07	3.64966E-06	RHSIM_Rhsim07G0211500
Root vs Leaf	RHSIM_Rhsim03G0059400	-1.585503722	9.84834E-07	3.65811E-06	RHSIM_Rhsim03G0059400
Root vs Leaf	RHSIM_RhsimUnG0091100	-1.378151172	9.9041E-07	3.67823E-06	RHSIM_RhsimUnG0091100
Root vs Leaf	RHSIM_Rhsim03G0070600	-1.279336915	9.93983E-07	3.69091E-06	RHSIM_Rhsim03G0070600
Root vs Leaf	RHSIM_Rhsim05G0201300	-2.440554557	9.97722E-07	3.7042E-06	RHSIM_Rhsim05G0201300
Root vs Leaf	RHSIM_Rhsim13G0180800	-2.75365113	1.00049E-06	3.71344E-06	RHSIM_Rhsim13G0180800
Root vs Leaf	RHSIM_Rhsim08G0193000	6.712292073	1.00053E-06	3.71344E-06	RHSIM_Rhsim08G0193000
Root vs Leaf	novel.503	-7.155465058	1.00074E-06	3.71363E-06	--
Root vs Leaf	RHSIM_RhsimUnG0237000	-3.206179893	1.00102E-06	3.71405E-06	RHSIM_RhsimUnG0237000
Root vs Leaf	RHSIM_Rhsim13G0091900	3.088528856	1.00157E-06	3.71549E-06	RHSIM_Rhsim13G0091900
Root vs Leaf	RHSIM_Rhsim01G0054000	-6.95745857	1.00398E-06	3.72386E-06	RHSIM_Rhsim01G0054000
Root vs Leaf	RHSIM_RhsimUnG0210000	-9.102878481	1.00536E-06	3.72837E-06	RHSIM_RhsimUnG0210000
Root vs Leaf	RHSIM_Rhsim08G0189100	1.120486986	1.00747E-06	3.73558E-06	RHSIM_Rhsim08G0189100
Root vs Leaf	RHSIM_Rhsim01G0255200	1.178012168	1.00818E-06	3.73762E-06	RHSIM_Rhsim01G0255200
Root vs Leaf	novel.1540	-2.586290146	1.00977E-06	3.74293E-06	--
Root vs Leaf	RHSIM_Rhsim05G0125300	7.597711124	1.01202E-06	3.75066E-06	RHSIM_Rhsim05G0125300
Root vs Leaf	RHSIM_RhsimUnG0019900	-2.41840944	1.01716E-06	3.76909E-06	RHSIM_RhsimUnG0019900
Root vs Leaf	RHSIM_Rhsim03G0068100	1.209918187	1.02506E-06	3.79715E-06	RHSIM_Rhsim03G0068100
Root vs Leaf	RHSIM_Rhsim01G0186500	-1.356135101	1.0287E-06	3.80942E-06	RHSIM_Rhsim01G0186500
Root vs Leaf	RHSIM_Rhsim10G0114500	-2.26737325	1.03479E-06	3.83078E-06	RHSIM_Rhsim10G0114500
Root vs Leaf	RHSIM_Rhsim02G0205900	-1.350322659	1.03575E-06	3.8337E-06	RHSIM_Rhsim02G0205900

Comparison	Gene ID	log_2FC	P-value	FDR (padj)	Gene Name
Root vs Leaf	RHSIM_Rhsim09G0157200	1.993934775	1.03604E-06	3.83414E-06	RHSIM_Rhsim09G0157200
Root vs Leaf	RHSIM_Rhsim05G0060200	-1.985616839	1.0374E-06	3.83796E-06	RHSIM_Rhsim05G0060200
Root vs Leaf	RHSIM_Rhsim10G0082700	1.779932128	1.04071E-06	3.84959E-06	RHSIM_Rhsim10G0082700
Root vs Leaf	RHSIM_Rhsim06G0030500	2.863418896	1.04537E-06	3.86547E-06	RHSIM_Rhsim06G0030500
Root vs Leaf	RHSIM_Rhsim12G0198100	-1.674921323	1.04549E-06	3.86547E-06	RHSIM_Rhsim12G0198100
Root vs Leaf	RHSIM_Rhsim04G0205600	8.269364666	1.0455E-06	3.86547E-06	RHSIM_Rhsim04G0205600
Root vs Leaf	RHSIM_Rhsim08G0088000	2.52838141	1.04674E-06	3.86942E-06	RHSIM_Rhsim08G0088000
Root vs Leaf	RHSIM_Rhsim10G0195700	-1.181423805	1.04735E-06	3.87107E-06	RHSIM_Rhsim10G0195700
Root vs Leaf	RHSIM_Rhsim01G0228500	2.912210853	1.05118E-06	3.88459E-06	RHSIM_Rhsim01G0228500
Root vs Leaf	RHSIM_Rhsim10G0025300	1.199887487	1.06352E-06	3.92956E-06	RHSIM_Rhsim10G0025300
Root vs Leaf	RHSIM_Rhsim07G0103400	1.522177158	1.06613E-06	3.93836E-06	RHSIM_Rhsim07G0103400
Root vs Leaf	RHSIM_Rhsim01G0243600	1.893528179	1.06624E-06	3.93836E-06	RHSIM_Rhsim01G0243600
Root vs Leaf	RHSIM_Rhsim12G0017600	1.10181538	1.06682E-06	3.93988E-06	RHSIM_Rhsim12G0017600
Root vs Leaf	novel.3714	3.642838859	1.0683E-06	3.94471E-06	--
Root vs Leaf	RHSIM_Rhsim11G0088200	-1.423769615	1.06973E-06	3.94936E-06	RHSIM_Rhsim11G0088200
Root vs Leaf	RHSIM_Rhsim08G0201400	1.271630687	1.07061E-06	3.95199E-06	RHSIM_Rhsim08G0201400
Root vs Leaf	RHSIM_Rhsim12G0183800	6.760878058	1.07168E-06	3.95529E-06	RHSIM_Rhsim12G0183800
Root vs Leaf	RHSIM_Rhsim10G0014900	-1.086416084	1.07705E-06	3.97448E-06	RHSIM_Rhsim10G0014900
Root vs Leaf	novel.1591	2.081860816	1.07744E-06	3.97529E-06	--
Root vs Leaf	RHSIM_Rhsim12G0085900	-1.56304173	1.08303E-06	3.99465E-06	RHSIM_Rhsim12G0085900
Root vs Leaf	RHSIM_Rhsim08G0073700	-1.523383405	1.08763E-06	4.01034E-06	RHSIM_Rhsim08G0073700
Root vs Leaf	novel.4278	-3.456771384	1.08921E-06	4.01552E-06	--
Root vs Leaf	RHSIM_Rhsim03G0154400	3.418965266	1.09105E-06	4.02166E-06	RHSIM_Rhsim03G0154400
Root vs Leaf	RHSIM_Rhsim07G0011500	1.011971565	1.10037E-06	4.05473E-06	RHSIM_Rhsim07G0011500
Root vs Leaf	RHSIM_Rhsim13G0122900	-1.339101153	1.105E-06	4.07115E-06	RHSIM_Rhsim13G0122900

Comparison	Gene ID	log_2FC	P-value	FDR (padj)	Gene Name
Root vs Leaf	RHSIM_Rhsim06G0037500	1.662841707	1.10701E-06	4.0779E-06	RHSIM_Rhsim06G0037500
Root vs Leaf	RHSIM_Rhsim08G0019100	3.964648656	1.10857E-06	4.08299E-06	RHSIM_Rhsim08G0019100
Root vs Leaf	RHSIM_Rhsim11G0047500	1.117316788	1.10964E-06	4.08629E-06	RHSIM_Rhsim11G0047500
Root vs Leaf	RHSIM_Rhsim09G0027600	1.676233183	1.11694E-06	4.11185E-06	RHSIM_Rhsim09G0027600
Root vs Leaf	RHSIM_Rhsim04G0217000	-1.130978243	1.12265E-06	4.13221E-06	RHSIM_Rhsim04G0217000
Root vs Leaf	RHSIM_Rhsim13G0090900	-3.336961644	1.12559E-06	4.14236E-06	RHSIM_Rhsim13G0090900
Root vs Leaf	novel.4800	-2.785170467	1.13488E-06	4.17591E-06	--
Root vs Leaf	RHSIM_Rhsim13G0157700	-3.466624674	1.13594E-06	4.17913E-06	RHSIM_Rhsim13G0157700
Root vs Leaf	RHSIM_RhsimUnG0098700	3.910065077	1.14942E-06	4.22739E-06	RHSIM_RhsimUnG0098700
Root vs Leaf	novel.4320	6.89742028	1.15648E-06	4.25268E-06	--
Root vs Leaf	RHSIM_Rhsim06G0062500	2.172463995	1.15817E-06	4.25704E-06	RHSIM_Rhsim06G0062500
Root vs Leaf	RHSIM_Rhsim03G0259600	2.325280973	1.15822E-06	4.25704E-06	RHSIM_Rhsim03G0259600
Root vs Leaf	RHSIM_Rhsim03G0245000	4.093138827	1.16088E-06	4.26616E-06	RHSIM_Rhsim03G0245000
Root vs Leaf	novel.598	6.959376214	1.1796E-06	4.33357E-06	--
Root vs Leaf	novel.4255	-2.70687161	1.18241E-06	4.3432E-06	--
Root vs Leaf	RHSIM_Rhsim09G0170600	7.841357036	1.18602E-06	4.35575E-06	RHSIM_Rhsim09G0170600
Root vs Leaf	RHSIM_RhsimMtG0002600	-3.77458797	1.18803E-06	4.36246E-06	RHSIM_RhsimMtG0002600
Root vs Leaf	novel.3245	-5.086507162	1.18897E-06	4.36521E-06	--
Root vs Leaf	novel.4039	1.416645141	1.19463E-06	4.38528E-06	--
Root vs Leaf	RHSIM_Rhsim01G0215500	3.895024179	1.19606E-06	4.38984E-06	RHSIM_Rhsim01G0215500
Root vs Leaf	RHSIM_Rhsim08G0251600	-3.838679582	1.20274E-06	4.41366E-06	RHSIM_Rhsim08G0251600
Root vs Leaf	RHSIM_RhsimUnG0168000	-1.042044976	1.20747E-06	4.42961E-06	RHSIM_RhsimUnG0168000
Root vs Leaf	RHSIM_Rhsim09G0125500	3.992740642	1.20806E-06	4.43109E-06	RHSIM_Rhsim09G0125500
Root vs Leaf	RHSIM_Rhsim04G0167700	-2.339538366	1.20842E-06	4.43171E-06	RHSIM_Rhsim04G0167700
Root vs Leaf	novel.5965	4.439661135	1.21072E-06	4.43944E-06	--

Comparison	Gene ID	log_2FC	P-value	FDR (padj)	Gene Name
Root vs Leaf	RHSIM_Rhsim09G0200300	1.880736671	1.21199E-06	4.44339E-06	RHSIM_Rhsim09G0200300
Root vs Leaf	RHSIM_Rhsim01G0034400	-1.782501955	1.21501E-06	4.45306E-06	RHSIM_Rhsim01G0034400
Root vs Leaf	RHSIM_Rhsim05G0123400	-2.515355408	1.21621E-06	4.45673E-06	RHSIM_Rhsim05G0123400
Root vs Leaf	RHSIM_Rhsim08G0091900	-5.348898551	1.21792E-06	4.4623E-06	RHSIM_Rhsim08G0091900
Root vs Leaf	RHSIM_Rhsim02G0243900	2.017663301	1.21915E-06	4.46608E-06	RHSIM_Rhsim02G0243900
Root vs Leaf	RHSIM_RhsimMtG0002800	-2.531638349	1.22009E-06	4.46882E-06	RHSIM_RhsimMtG0002800
Root vs Leaf	RHSIM_Rhsim04G0139900	-1.15222749	1.22036E-06	4.46911E-06	RHSIM_Rhsim04G0139900
Root vs Leaf	novel.4535	1.570367878	1.22443E-06	4.48331E-06	--
Root vs Leaf	RHSIM_Rhsim10G0141300	1.245880298	1.23605E-06	4.52443E-06	RHSIM_Rhsim10G0141300
Root vs Leaf	RHSIM_Rhsim08G0099600	2.478467923	1.23969E-06	4.53632E-06	RHSIM_Rhsim08G0099600
Root vs Leaf	RHSIM_Rhsim03G0170900	-3.602984609	1.24009E-06	4.53706E-06	RHSIM_Rhsim03G0170900
Root vs Leaf	RHSIM_Rhsim08G0099300	3.501276736	1.24084E-06	4.53835E-06	RHSIM_Rhsim08G0099300
Root vs Leaf	RHSIM_Rhsim03G0091800	6.539603973	1.24512E-06	4.5526E-06	RHSIM_Rhsim03G0091800
Root vs Leaf	novel.3674	-2.739428877	1.25131E-06	4.57306E-06	--
Root vs Leaf	novel.5585	-6.828273012	1.25177E-06	4.57399E-06	--
Root vs Leaf	RHSIM_Rhsim08G0004400	4.473353997	1.25449E-06	4.58324E-06	RHSIM_Rhsim08G0004400
Root vs Leaf	novel.850	3.095598941	1.26284E-06	4.61299E-06	--
Root vs Leaf	RHSIM_Rhsim04G0130500	-1.299485185	1.26474E-06	4.6192E-06	RHSIM_Rhsim04G0130500
Root vs Leaf	RHSIM_Rhsim08G0030300	2.404685526	1.27298E-06	4.64811E-06	RHSIM_Rhsim08G0030300
Root vs Leaf	RHSIM_Rhsim03G0002700	-1.046479171	1.27305E-06	4.64811E-06	RHSIM_Rhsim03G0002700
Root vs Leaf	RHSIM_Rhsim10G0082900	1.605252793	1.2786E-06	4.66762E-06	RHSIM_Rhsim10G0082900
Root vs Leaf	RHSIM_Rhsim01G0206400	-3.857570933	1.28514E-06	4.6907E-06	RHSIM_Rhsim01G0206400
Root vs Leaf	RHSIM_Rhsim01G0025400	2.123823591	1.28931E-06	4.70447E-06	RHSIM_Rhsim01G0025400
Root vs Leaf	RHSIM_Rhsim13G0132700	3.179865522	1.28951E-06	4.70447E-06	RHSIM_Rhsim13G0132700
Root vs Leaf	RHSIM_Rhsim08G0165000	-7.826063985	1.29085E-06	4.70865E-06	RHSIM_Rhsim08G0165000

Comparison	Gene ID	log_2FC	P-value	FDR (padj)	Gene Name
Root vs Leaf	RHSIM_Rhsim03G0215300	1.414435683	1.2963E-06	4.72776E-06	RHSIM_Rhsim03G0215300
Root vs Leaf	RHSIM_Rhsim12G0184900	3.539648021	1.2995E-06	4.73794E-06	RHSIM_Rhsim12G0184900
Root vs Leaf	RHSIM_Rhsim02G0032500	1.427677761	1.3003E-06	4.74012E-06	RHSIM_Rhsim02G0032500
Root vs Leaf	RHSIM_Rhsim01G0289200	2.176022486	1.30053E-06	4.74019E-06	RHSIM_Rhsim01G0289200
Root vs Leaf	novel.4455	-2.508725147	1.31571E-06	4.79401E-06	--
Root vs Leaf	RHSIM_Rhsim11G0109700	6.971505871	1.31594E-06	4.79412E-06	RHSIM_Rhsim11G0109700
Root vs Leaf	novel.3667	3.945503806	1.31713E-06	4.79769E-06	--
Root vs Leaf	novel.4956	7.670306648	1.3219E-06	4.81432E-06	--
Root vs Leaf	RHSIM_Rhsim06G0055100	2.308798644	1.32258E-06	4.81604E-06	RHSIM_Rhsim06G0055100
Root vs Leaf	RHSIM_Rhsim06G0062700	-1.797568292	1.32728E-06	4.83237E-06	RHSIM_Rhsim06G0062700
Root vs Leaf	novel.3467	2.223474023	1.33201E-06	4.84808E-06	--
Root vs Leaf	RHSIM_Rhsim03G0270700	2.443707638	1.33225E-06	4.84817E-06	RHSIM_Rhsim03G0270700
Root vs Leaf	RHSIM_Rhsim13G0025800	-2.912664422	1.33379E-06	4.85304E-06	RHSIM_Rhsim13G0025800
Root vs Leaf	RHSIM_Rhsim09G0086000	1.400876388	1.34802E-06	4.90366E-06	RHSIM_Rhsim09G0086000
Root vs Leaf	RHSIM_Rhsim06G0141400	5.490255269	1.34813E-06	4.90366E-06	RHSIM_Rhsim06G0141400
Root vs Leaf	RHSIM_Rhsim11G0103500	-1.765190927	1.34887E-06	4.9056E-06	RHSIM_Rhsim11G0103500
Root vs Leaf	RHSIM_Rhsim02G0123400	-1.632926878	1.3505E-06	4.91074E-06	RHSIM_Rhsim02G0123400
Root vs Leaf	RHSIM_Rhsim03G0050000	-1.08214305	1.35678E-06	4.9328E-06	RHSIM_Rhsim03G0050000
Root vs Leaf	novel.1745	3.074459582	1.36174E-06	4.95006E-06	--
Root vs Leaf	RHSIM_Rhsim09G0087400	3.229925612	1.37115E-06	4.9819E-06	RHSIM_Rhsim09G0087400
Root vs Leaf	RHSIM_RhsimUnG0242900	-3.089652196	1.37236E-06	4.98475E-06	RHSIM_RhsimUnG0242900
Root vs Leaf	RHSIM_Rhsim06G0225700	-1.190174754	1.37262E-06	4.98491E-06	RHSIM_Rhsim06G0225700
Root vs Leaf	RHSIM_Rhsim09G0029700	-7.493780034	1.38289E-06	5.02141E-06	RHSIM_Rhsim09G0029700
Root vs Leaf	RHSIM_Rhsim01G0008300	-2.632285923	1.38509E-06	5.02862E-06	RHSIM_Rhsim01G0008300
Root vs Leaf	RHSIM_Rhsim12G0191500	-1.067545299	1.38749E-06	5.03655E-06	RHSIM_Rhsim12G0191500

Comparison	Gene ID	log_2FC	P-value	FDR (padj)	Gene Name
Root vs Leaf	RHSIM_Rhsim02G0231800	4.76855237	1.39161E-06	5.0499E-06	RHSIM_Rhsim02G0231800
Root vs Leaf	RHSIM_Rhsim07G0110200	2.831112298	1.39646E-06	5.06671E-06	RHSIM_Rhsim07G0110200
Root vs Leaf	RHSIM_RhsimUnG0125200	4.386049699	1.39688E-06	5.06744E-06	RHSIM_RhsimUnG0125200
Root vs Leaf	RHSIM_Rhsim07G0088500	4.37829718	1.39881E-06	5.07284E-06	RHSIM_Rhsim07G0088500
Root vs Leaf	RHSIM_Rhsim03G0086200	1.341182342	1.39961E-06	5.07496E-06	RHSIM_Rhsim03G0086200
Root vs Leaf	RHSIM_Rhsim09G0102300	6.730745654	1.40726E-06	5.1019E-06	RHSIM_Rhsim09G0102300
Root vs Leaf	novel.2906	-6.922148255	1.40816E-06	5.10436E-06	--
Root vs Leaf	RHSIM_Rhsim13G0029700	-1.38069151	1.41094E-06	5.11363E-06	RHSIM_Rhsim13G0029700
Root vs Leaf	RHSIM_Rhsim02G0034100	-3.22552371	1.41329E-06	5.12054E-06	RHSIM_Rhsim02G0034100
Root vs Leaf	RHSIM_Rhsim03G0057900	2.772401079	1.41626E-06	5.1289E-06	RHSIM_Rhsim03G0057900
Root vs Leaf	novel.2065	1.82788303	1.41907E-06	5.13829E-06	--
Root vs Leaf	RHSIM_Rhsim03G0252900	1.061518739	1.42039E-06	5.14224E-06	RHSIM_Rhsim03G0252900
Root vs Leaf	RHSIM_Rhsim13G0078000	-1.731694299	1.42289E-06	5.1505E-06	RHSIM_Rhsim13G0078000
Root vs Leaf	RHSIM_Rhsim07G0091500	6.367536114	1.42783E-06	5.16757E-06	RHSIM_Rhsim07G0091500
Root vs Leaf	RHSIM_Rhsim09G0060000	1.094990993	1.4292E-06	5.17171E-06	RHSIM_Rhsim09G0060000
Root vs Leaf	RHSIM_Rhsim11G0081500	-5.001877965	1.42988E-06	5.17338E-06	RHSIM_Rhsim11G0081500
Root vs Leaf	RHSIM_Rhsim06G0139500	1.542109596	1.44241E-06	5.21706E-06	RHSIM_Rhsim06G0139500
Root vs Leaf	RHSIM_Rhsim11G0164300	-7.309777856	1.44275E-06	5.2175E-06	RHSIM_Rhsim11G0164300
Root vs Leaf	RHSIM_Rhsim07G0022900	-1.17925726	1.4585E-06	5.27278E-06	RHSIM_Rhsim07G0022900
Root vs Leaf	RHSIM_Rhsim10G0159100	3.447418901	1.46767E-06	5.30428E-06	RHSIM_Rhsim10G0159100
Root vs Leaf	RHSIM_Rhsim08G0174500	2.538644313	1.47584E-06	5.33216E-06	RHSIM_Rhsim08G0174500
Root vs Leaf	RHSIM_Rhsim07G0089300	-7.72309046	1.47688E-06	5.33507E-06	RHSIM_Rhsim07G0089300
Root vs Leaf	RHSIM_Rhsim03G0218100	-1.255434211	1.48204E-06	5.35287E-06	RHSIM_Rhsim03G0218100
Root vs Leaf	RHSIM_Rhsim01G0279600	6.70898198	1.49114E-06	5.38342E-06	RHSIM_Rhsim01G0279600
Root vs Leaf	novel.2909	-1.624174911	1.49119E-06	5.38342E-06	--

Comparison	Gene ID	log_2FC	P-value	FDR (padj)	Gene Name
Root vs Leaf	RHSIM_RhsimUnG0005600	-1.079038529	1.49894E-06	5.41032E-06	RHSIM_RhsimUnG0005600
Root vs Leaf	RHSIM_Rhsim05G0228600	-1.39701032	1.519E-06	5.48038E-06	RHSIM_Rhsim05G0228600
Root vs Leaf	RHSIM_Rhsim10G0078400	-1.217830343	1.51996E-06	5.48298E-06	RHSIM_Rhsim10G0078400
Root vs Leaf	RHSIM_Rhsim04G0087100	1.396861044	1.52823E-06	5.51197E-06	RHSIM_Rhsim04G0087100
Root vs Leaf	RHSIM_Rhsim08G0039700	-1.956749797	1.53184E-06	5.52413E-06	RHSIM_Rhsim08G0039700
Root vs Leaf	RHSIM_Rhsim09G0186000	2.125999073	1.54004E-06	5.55197E-06	RHSIM_Rhsim09G0186000
Root vs Leaf	RHSIM_Rhsim12G0123500	-1.097214922	1.55928E-06	5.62045E-06	RHSIM_Rhsim12G0123500
Root vs Leaf	RHSIM_Rhsim10G0152600	-1.331633352	1.56537E-06	5.64152E-06	RHSIM_Rhsim10G0152600
Root vs Leaf	RHSIM_Rhsim07G0177100	-1.322221252	1.56724E-06	5.64738E-06	RHSIM_Rhsim07G0177100
Root vs Leaf	RHSIM_Rhsim05G0001200	-1.07672281	1.56812E-06	5.64968E-06	RHSIM_Rhsim05G0001200
Root vs Leaf	RHSIM_Rhsim11G0137400	-1.26945437	1.57223E-06	5.66359E-06	RHSIM_Rhsim11G0137400
Root vs Leaf	RHSIM_Rhsim03G0216000	2.049308081	1.57318E-06	5.66612E-06	RHSIM_Rhsim03G0216000
Root vs Leaf	RHSIM_Rhsim09G0080600	4.970731814	1.58473E-06	5.70684E-06	RHSIM_Rhsim09G0080600
Root vs Leaf	RHSIM_Rhsim01G0249800	2.230032495	1.59308E-06	5.73603E-06	RHSIM_Rhsim01G0249800
Root vs Leaf	RHSIM_Rhsim02G0082100	7.163810553	1.62204E-06	5.83849E-06	RHSIM_Rhsim02G0082100
Root vs Leaf	novel.169	-7.089413348	1.62375E-06	5.84373E-06	--
Root vs Leaf	RHSIM_Rhsim06G0121300	-1.6457196	1.62603E-06	5.85104E-06	RHSIM_Rhsim06G0121300
Root vs Leaf	RHSIM_Rhsim02G0226600	7.280385007	1.64667E-06	5.92436E-06	RHSIM_Rhsim02G0226600
Root vs Leaf	RHSIM_Rhsim12G0197300	1.010761767	1.64724E-06	5.92551E-06	RHSIM_Rhsim12G0197300
Root vs Leaf	RHSIM_RhsimUnG0071100	-3.355661061	1.65216E-06	5.94229E-06	RHSIM_RhsimUnG0071100
Root vs Leaf	RHSIM_Rhsim06G0155500	-3.081582851	1.65368E-06	5.94682E-06	RHSIM_Rhsim06G0155500
Root vs Leaf	RHSIM_Rhsim10G0123100	-7.061522319	1.65551E-06	5.95247E-06	RHSIM_Rhsim10G0123100
Root vs Leaf	RHSIM_Rhsim07G0153900	1.426671837	1.65695E-06	5.95673E-06	RHSIM_Rhsim07G0153900
Root vs Leaf	RHSIM_Rhsim07G0138600	1.753273234	1.66707E-06	5.99217E-06	RHSIM_Rhsim07G0138600
Root vs Leaf	RHSIM_RhsimUnG0141400	-1.297143264	1.66805E-06	5.99477E-06	RHSIM_RhsimUnG0141400

Comparison	Gene ID	log_2FC	P-value	FDR (padj)	Gene Name
Root vs Leaf	RHSIM_Rhsim12G0049900	-1.979937363	1.68634E-06	6.05956E-06	RHSIM_Rhsim12G0049900
Root vs Leaf	novel.3462	1.986337558	1.70108E-06	6.11157E-06	--
Root vs Leaf	novel.43	5.463527758	1.70744E-06	6.13348E-06	--
Root vs Leaf	RHSIM_Rhsim09G0112800	2.401631189	1.70856E-06	6.13657E-06	RHSIM_Rhsim09G0112800
Root vs Leaf	RHSIM_Rhsim05G0209100	-5.745998619	1.71279E-06	6.1508E-06	RHSIM_Rhsim05G0209100
Root vs Leaf	RHSIM_Rhsim06G0148000	-2.831764405	1.72254E-06	6.18486E-06	RHSIM_Rhsim06G0148000
Root vs Leaf	RHSIM_Rhsim06G0144100	5.839712114	1.72453E-06	6.19104E-06	RHSIM_Rhsim06G0144100
Root vs Leaf	RHSIM_Rhsim08G0016300	2.947992481	1.73152E-06	6.21515E-06	RHSIM_Rhsim08G0016300
Root vs Leaf	RHSIM_Rhsim10G0140300	-2.809967232	1.73445E-06	6.22376E-06	RHSIM_Rhsim10G0140300
Root vs Leaf	RHSIM_Rhsim01G0248300	-3.691318901	1.7506E-06	6.27973E-06	RHSIM_Rhsim01G0248300
Root vs Leaf	RHSIM_Rhsim04G0020500	-1.460500678	1.75517E-06	6.29517E-06	RHSIM_Rhsim04G0020500
Root vs Leaf	RHSIM_Rhsim06G0038600	1.353831032	1.75665E-06	6.2995E-06	RHSIM_Rhsim06G0038600
Root vs Leaf	RHSIM_Rhsim08G0248900	1.003434094	1.75864E-06	6.30565E-06	RHSIM_Rhsim08G0248900
Root vs Leaf	RHSIM_Rhsim12G0064200	-3.017849897	1.77002E-06	6.34449E-06	RHSIM_Rhsim12G0064200
Root vs Leaf	RHSIM_Rhsim02G0133500	3.098199406	1.77086E-06	6.34652E-06	RHSIM_Rhsim02G0133500
Root vs Leaf	RHSIM_Rhsim09G0096700	1.50983159	1.77492E-06	6.36009E-06	RHSIM_Rhsim09G0096700
Root vs Leaf	RHSIM_Rhsim04G0147900	1.086209569	1.77664E-06	6.36527E-06	RHSIM_Rhsim04G0147900
Root vs Leaf	RHSIM_Rhsim01G0241800	5.341519264	1.78514E-06	6.39474E-06	RHSIM_Rhsim01G0241800
Root vs Leaf	novel.936	-2.791765991	1.78691E-06	6.40006E-06	--
Root vs Leaf	RHSIM_Rhsim12G0036700	-3.101517223	1.78784E-06	6.4024E-06	RHSIM_Rhsim12G0036700
Root vs Leaf	RHSIM_Rhsim05G0053600	2.414123025	1.79055E-06	6.41114E-06	RHSIM_Rhsim05G0053600
Root vs Leaf	RHSIM_Rhsim07G0188300	-1.445602172	1.79459E-06	6.4236E-06	RHSIM_Rhsim07G0188300
Root vs Leaf	RHSIM_Rhsim11G0168500	-1.033354718	1.79517E-06	6.42467E-06	RHSIM_Rhsim11G0168500
Root vs Leaf	RHSIM_Rhsim09G0178600	-1.90953673	1.80177E-06	6.44731E-06	RHSIM_Rhsim09G0178600
Root vs Leaf	RHSIM_Rhsim08G0083700	4.945942162	1.81667E-06	6.4986E-06	RHSIM_Rhsim08G0083700

Comparison	Gene ID	log_2FC	P-value	FDR (padj)	Gene Name
Root vs Leaf	RHSIM_Rhsim05G0070400	-6.176765639	1.81695E-06	6.49862E-06	RHSIM_Rhsim05G0070400
Root vs Leaf	RHSIM_Rhsim12G0192800	1.52702019	1.82007E-06	6.50876E-06	RHSIM_Rhsim12G0192800
Root vs Leaf	RHSIM_RhsimUnG0163900	-1.710487437	1.82173E-06	6.51372E-06	RHSIM_RhsimUnG0163900
Root vs Leaf	RHSIM_Rhsim05G0039300	-7.53858853	1.82238E-06	6.51502E-06	RHSIM_Rhsim05G0039300
Root vs Leaf	RHSIM_Rhsim02G0165100	-9.301362928	1.82759E-06	6.53062E-06	RHSIM_Rhsim02G0165100
Root vs Leaf	RHSIM_Rhsim07G0034700	6.307739931	1.83047E-06	6.53989E-06	RHSIM_Rhsim07G0034700
Root vs Leaf	RHSIM_Rhsim05G0220200	6.316250581	1.83824E-06	6.56663E-06	RHSIM_Rhsim05G0220200
Root vs Leaf	novel.5544	-4.708823508	1.83968E-06	6.57076E-06	--
Root vs Leaf	novel.3676	7.024300415	1.84033E-06	6.57207E-06	--
Root vs Leaf	RHSIM_Rhsim06G0185900	7.397231161	1.85054E-06	6.60753E-06	RHSIM_Rhsim06G0185900
Root vs Leaf	RHSIM_Rhsim08G0180400	-1.168055069	1.85938E-06	6.63805E-06	RHSIM_Rhsim08G0180400
Root vs Leaf	novel.5268	1.227396033	1.86443E-06	6.65403E-06	--
Root vs Leaf	RHSIM_Rhsim10G0157100	6.212628702	1.8863E-06	6.73104E-06	RHSIM_Rhsim10G0157100
Root vs Leaf	RHSIM_Rhsim03G0178700	1.6827487	1.89704E-06	6.76835E-06	RHSIM_Rhsim03G0178700
Root vs Leaf	RHSIM_Rhsim09G0144600	3.5507394	1.89879E-06	6.77352E-06	RHSIM_Rhsim09G0144600
Root vs Leaf	RHSIM_Rhsim11G0179100	1.613141272	1.91625E-06	6.83476E-06	RHSIM_Rhsim11G0179100
Root vs Leaf	RHSIM_Rhsim13G0131500	-2.878699328	1.92692E-06	6.87175E-06	RHSIM_Rhsim13G0131500
Root vs Leaf	RHSIM_Rhsim04G0101900	1.321348418	1.93406E-06	6.89614E-06	RHSIM_Rhsim04G0101900
Root vs Leaf	RHSIM_Rhsim05G0040200	4.138028166	1.93632E-06	6.90315E-06	RHSIM_Rhsim05G0040200
Root vs Leaf	RHSIM_Rhsim13G0154600	-1.633530608	1.94004E-06	6.91536E-06	RHSIM_Rhsim13G0154600
Root vs Leaf	RHSIM_Rhsim10G0181500	6.614243044	1.94381E-06	6.92774E-06	RHSIM_Rhsim10G0181500
Root vs Leaf	RHSIM_Rhsim06G0069800	4.783021646	1.9458E-06	6.93375E-06	RHSIM_Rhsim06G0069800
Root vs Leaf	RHSIM_Rhsim06G0205000	6.977995857	1.94724E-06	6.9378E-06	RHSIM_Rhsim06G0205000
Root vs Leaf	RHSIM_Rhsim08G0020400	1.880412542	1.96866E-06	7.01088E-06	RHSIM_Rhsim08G0020400
Root vs Leaf	novel.415	-2.520350784	1.99541E-06	7.10395E-06	--

Comparison	Gene ID	log_2FC	P-value	FDR (padj)	Gene Name
Root vs Leaf	RHSIM_Rhsim11G0095100	-3.701691222	2.01553E-06	7.17052E-06	RHSIM_Rhsim11G0095100
Root vs Leaf	RHSIM_Rhsim02G0223500	2.359092163	2.0225E-06	7.19376E-06	RHSIM_Rhsim02G0223500
Root vs Leaf	RHSIM_RhsimUnG0252900	1.279076372	2.02596E-06	7.20387E-06	RHSIM_RhsimUnG0252900
Root vs Leaf	RHSIM_Rhsim08G0168600	-1.509541091	2.04263E-06	7.26202E-06	RHSIM_Rhsim08G0168600
Root vs Leaf	RHSIM_Rhsim08G0015900	1.659578081	2.05042E-06	7.2886E-06	RHSIM_Rhsim08G0015900
Root vs Leaf	novel.5337	1.170888896	2.06439E-06	7.33486E-06	--
Root vs Leaf	RHSIM_Rhsim08G0103100	-1.676734255	2.07142E-06	7.35874E-06	RHSIM_Rhsim08G0103100
Root vs Leaf	novel.255	-4.262544092	2.07467E-06	7.36914E-06	--
Root vs Leaf	RHSIM_Rhsim04G0133700	-2.633499841	2.07966E-06	7.38574E-06	RHSIM_Rhsim04G0133700
Root vs Leaf	RHSIM_Rhsim09G0163900	-1.520344538	2.0821E-06	7.39327E-06	RHSIM_Rhsim09G0163900
Root vs Leaf	RHSIM_Rhsim08G0171600	-11.70818711	2.08526E-06	7.40333E-06	RHSIM_Rhsim08G0171600
Root vs Leaf	novel.3837	-5.971862443	2.09948E-06	7.45267E-06	--
Root vs Leaf	RHSIM_RhsimUnG0131500	-7.333830824	2.10881E-06	7.48007E-06	RHSIM_RhsimUnG0131500
Root vs Leaf	RHSIM_Rhsim11G0139200	-6.460124672	2.1189E-06	7.51355E-06	RHSIM_Rhsim11G0139200
Root vs Leaf	RHSIM_Rhsim01G0062900	3.579723905	2.1344E-06	7.56502E-06	RHSIM_Rhsim01G0062900
Root vs Leaf	RHSIM_Rhsim07G0119000	1.06847687	2.13914E-06	7.58069E-06	RHSIM_Rhsim07G0119000
Root vs Leaf	RHSIM_Rhsim10G0141100	3.82701454	2.144E-06	7.59674E-06	RHSIM_Rhsim10G0141100
Root vs Leaf	RHSIM_Rhsim10G0176200	6.975061251	2.14768E-06	7.60861E-06	RHSIM_Rhsim10G0176200
Root vs Leaf	RHSIM_Rhsim01G0143700	-6.452841432	2.17201E-06	7.69245E-06	RHSIM_Rhsim01G0143700
Root vs Leaf	RHSIM_Rhsim06G0141200	6.069175209	2.17451E-06	7.70014E-06	RHSIM_Rhsim06G0141200
Root vs Leaf	RHSIM_Rhsim03G0146700	1.991159528	2.19492E-06	7.7712E-06	RHSIM_Rhsim03G0146700
Root vs Leaf	RHSIM_RhsimUnG0253800	2.300782964	2.20493E-06	7.80546E-06	RHSIM_RhsimUnG0253800
Root vs Leaf	RHSIM_Rhsim06G0182500	-1.833697256	2.20966E-06	7.82102E-06	RHSIM_Rhsim06G0182500
Root vs Leaf	novel.5041	5.638770569	2.21593E-06	7.84199E-06	--
Root vs Leaf	RHSIM_Rhsim05G0098000	-1.10859067	2.24213E-06	7.92964E-06	RHSIM_Rhsim05G0098000

Comparison	Gene ID	log_2FC	P-value	FDR (padj)	Gene Name
Root vs Leaf	RHSIM_RhsimUnG0248500	1.078432683	2.25145E-06	7.95918E-06	RHSIM_RhsimUnG0248500
Root vs Leaf	RHSIM_Rhsim01G0012300	6.87716991	2.26139E-06	7.99312E-06	RHSIM_Rhsim01G0012300
Root vs Leaf	RHSIM_Rhsim03G0180900	1.979914484	2.27658E-06	8.04309E-06	RHSIM_Rhsim03G0180900
Root vs Leaf	RHSIM_Rhsim03G0143900	1.072658878	2.27834E-06	8.0481E-06	RHSIM_Rhsim03G0143900
Root vs Leaf	RHSIM_Rhsim06G0201100	1.605072378	2.27944E-06	8.05077E-06	RHSIM_Rhsim06G0201100
Root vs Leaf	RHSIM_Rhsim08G0210300	-1.804104978	2.28958E-06	8.08534E-06	RHSIM_Rhsim08G0210300
Root vs Leaf	novel.3549	-2.943222748	2.29607E-06	8.10699E-06	--
Root vs Leaf	RHSIM_Rhsim10G0171300	-1.967353594	2.3051E-06	8.13642E-06	RHSIM_Rhsim10G0171300
Root vs Leaf	RHSIM_Rhsim03G0219100	1.141297015	2.30551E-06	8.13662E-06	RHSIM_Rhsim03G0219100
Root vs Leaf	RHSIM_Rhsim13G0045300	1.140910347	2.32091E-06	8.18847E-06	RHSIM_Rhsim13G0045300
Root vs Leaf	novel.4311	-6.475762905	2.32863E-06	8.21445E-06	--
Root vs Leaf	RHSIM_Rhsim05G0229800	5.64630626	2.33591E-06	8.23887E-06	RHSIM_Rhsim05G0229800
Root vs Leaf	RHSIM_Rhsim12G0163700	7.391009322	2.34082E-06	8.25493E-06	RHSIM_Rhsim12G0163700
Root vs Leaf	RHSIM_Rhsim10G0026500	-7.755495811	2.34336E-06	8.26263E-06	RHSIM_Rhsim10G0026500
Root vs Leaf	novel.4410	-7.896847621	2.34382E-06	8.26301E-06	--
Root vs Leaf	RHSIM_Rhsim07G0032900	-2.892606363	2.37119E-06	8.35567E-06	RHSIM_Rhsim07G0032900
Root vs Leaf	novel.4979	-4.693596166	2.37439E-06	8.36568E-06	--
Root vs Leaf	RHSIM_Rhsim13G0165700	-2.424673812	2.38184E-06	8.38939E-06	RHSIM_Rhsim13G0165700
Root vs Leaf	RHSIM_Rhsim07G0083200	-1.467171459	2.42137E-06	8.52601E-06	RHSIM_Rhsim07G0083200
Root vs Leaf	RHSIM_Rhsim05G0036800	-6.482382921	2.42499E-06	8.53746E-06	RHSIM_Rhsim05G0036800
Root vs Leaf	RHSIM_Rhsim03G0084800	3.264535845	2.45031E-06	8.62267E-06	RHSIM_Rhsim03G0084800
Root vs Leaf	RHSIM_RhsimUnG0062700	-3.247125206	2.45502E-06	8.63792E-06	RHSIM_RhsimUnG0062700
Root vs Leaf	RHSIM_Rhsim12G0023700	5.921378816	2.45918E-06	8.65059E-06	RHSIM_Rhsim12G0023700
Root vs Leaf	novel.239	-7.672835855	2.45937E-06	8.65059E-06	--
Root vs Leaf	RHSIM_Rhsim08G0014400	1.804183812	2.4604E-06	8.65289E-06	RHSIM_Rhsim08G0014400

Comparison	Gene ID	log$_2$FC	P-value	FDR (padj)	Gene Name
Root vs Leaf	RHSIM_Rhsim13G0231100	1.118329693	2.47047E-06	8.68699E-06	RHSIM_Rhsim13G0231100
Root vs Leaf	RHSIM_Rhsim02G0245000	-4.327119846	2.48271E-06	8.72872E-06	RHSIM_Rhsim02G0245000
Root vs Leaf	novel.780	-1.75281654	2.51352E-06	8.83568E-06	--
Root vs Leaf	RHSIM_RhsimUnG0166700	-2.978772675	2.52442E-06	8.8713E-06	RHSIM_RhsimUnG0166700
Root vs Leaf	RHSIM_Rhsim06G0157400	7.438236381	2.53655E-06	8.91259E-06	RHSIM_Rhsim06G0157400
Root vs Leaf	RHSIM_Rhsim05G0031300	1.228565897	2.54001E-06	8.9234E-06	RHSIM_Rhsim05G0031300
Root vs Leaf	RHSIM_RhsimUnG0081900	-4.062524587	2.54166E-06	8.92696E-06	RHSIM_RhsimUnG0081900
Root vs Leaf	RHSIM_Rhsim13G0065300	1.838080254	2.55252E-06	8.96325E-06	RHSIM_Rhsim13G0065300
Root vs Leaf	RHSIM_Rhsim02G0253900	-1.73263849	2.55369E-06	8.96601E-06	RHSIM_Rhsim02G0253900
Root vs Leaf	RHSIM_RhsimUnG0100000	-7.248990093	2.55555E-06	8.97116E-06	RHSIM_RhsimUnG0100000
Root vs Leaf	RHSIM_Rhsim06G0140000	2.795123494	2.56286E-06	8.99548E-06	RHSIM_Rhsim06G0140000
Root vs Leaf	novel.3060	-6.653379415	2.58607E-06	9.07419E-06	--
Root vs Leaf	RHSIM_Rhsim01G0029200	4.179893311	2.60604E-06	9.14148E-06	RHSIM_Rhsim01G0029200
Root vs Leaf	novel.3925	-1.886479737	2.60896E-06	9.15034E-06	--
Root vs Leaf	RHSIM_Rhsim07G0028400	-1.116717003	2.6185E-06	9.17963E-06	RHSIM_Rhsim07G0028400
Root vs Leaf	RHSIM_Rhsim11G0123000	3.250500372	2.62012E-06	9.1839E-06	RHSIM_Rhsim11G0123000
Root vs Leaf	RHSIM_Rhsim09G0090200	-1.101397332	2.62456E-06	9.19809E-06	RHSIM_Rhsim09G0090200
Root vs Leaf	novel.5347	-1.400813461	2.63428E-06	9.23077E-06	--
Root vs Leaf	RHSIM_Rhsim03G0089000	1.345421678	2.65339E-06	9.2963E-06	RHSIM_Rhsim03G0089000
Root vs Leaf	RHSIM_Rhsim09G0008900	1.908890053	2.65633E-06	9.30518E-06	RHSIM_Rhsim09G0008900
Root vs Leaf	RHSIM_Rhsim12G0019200	6.904286852	2.66009E-06	9.31695E-06	RHSIM_Rhsim12G0019200
Root vs Leaf	RHSIM_Rhsim12G0048600	-1.006741205	2.66694E-06	9.33953E-06	RHSIM_Rhsim12G0048600
Root vs Leaf	RHSIM_Rhsim13G0146400	-1.000116496	2.66861E-06	9.34396E-06	RHSIM_Rhsim13G0146400
Root vs Leaf	novel.5621	-1.445621861	2.67299E-06	9.35789E-06	--
Root vs Leaf	RHSIM_Rhsim11G0190900	1.476405715	2.69828E-06	9.44256E-06	RHSIM_Rhsim11G0190900

Comparison	Gene ID	log_2FC	P-value	FDR (padj)	Gene Name
Root vs Leaf	RHSIM_Rhsim07G0233900	-6.489296991	2.6984E-06	9.44256E-06	RHSIM_Rhsim07G0233900
Root vs Leaf	novel.4048	2.051130947	2.7038E-06	9.45859E-06	--
Root vs Leaf	RHSIM_Rhsim12G0053700	-2.917393868	2.70484E-06	9.45936E-06	RHSIM_Rhsim12G0053700
Root vs Leaf	RHSIM_Rhsim11G0178400	-6.447370713	2.7057E-06	9.46096E-06	RHSIM_Rhsim11G0178400
Root vs Leaf	RHSIM_Rhsim13G0035200	-6.503928025	2.7167E-06	9.49798E-06	RHSIM_Rhsim13G0035200
Root vs Leaf	RHSIM_Rhsim01G0181700	-1.423763752	2.7261E-06	9.52941E-06	RHSIM_Rhsim01G0181700
Root vs Leaf	novel.2791	-3.187905633	2.72677E-06	9.53032E-06	--
Root vs Leaf	RHSIM_Rhsim05G0161800	3.329822374	2.72805E-06	9.53222E-06	RHSIM_Rhsim05G0161800
Root vs Leaf	RHSIM_Rhsim05G0175500	7.99520628	2.72814E-06	9.53222E-06	RHSIM_Rhsim05G0175500
Root vs Leaf	RHSIM_Rhsim01G0261700	1.052876329	2.73101E-06	9.5408E-06	RHSIM_Rhsim01G0261700
Root vs Leaf	RHSIM_Rhsim06G0054300	-1.994598727	2.73734E-06	9.55883E-06	RHSIM_Rhsim06G0054300
Root vs Leaf	novel.3996	-2.672837476	2.73741E-06	9.55883E-06	--
Root vs Leaf	RHSIM_Rhsim03G0210900	3.093568602	2.74662E-06	9.58953E-06	RHSIM_Rhsim03G0210900
Root vs Leaf	novel.203	-7.346921466	2.74962E-06	9.59857E-06	--
Root vs Leaf	RHSIM_Rhsim07G0224900	2.203730476	2.75492E-06	9.61563E-06	RHSIM_Rhsim07G0224900
Root vs Leaf	RHSIM_RhsimUnG0027400	-7.104517755	2.75596E-06	9.61781E-06	RHSIM_RhsimUnG0027400
Root vs Leaf	RHSIM_Rhsim10G0194800	-6.383610831	2.76732E-06	9.65599E-06	RHSIM_Rhsim10G0194800
Root vs Leaf	RHSIM_Rhsim09G0190300	-1.741474891	2.79827E-06	9.76251E-06	RHSIM_Rhsim09G0190300
Root vs Leaf	RHSIM_Rhsim06G0152100	2.289663048	2.80118E-06	9.77121E-06	RHSIM_Rhsim06G0152100
Root vs Leaf	RHSIM_RhsimMtG0000400	-3.3956196	2.80525E-06	9.78391E-06	RHSIM_RhsimMtG0000400
Root vs Leaf	RHSIM_Rhsim13G0136000	-7.247932883	2.83497E-06	9.88458E-06	RHSIM_Rhsim13G0136000
Root vs Leaf	RHSIM_Rhsim13G0194100	2.438157224	2.849E-06	9.93202E-06	RHSIM_Rhsim13G0194100
Root vs Leaf	RHSIM_Rhsim05G0174700	1.038996367	2.8594E-06	9.96677E-06	RHSIM_Rhsim05G0174700
Root vs Leaf	RHSIM_Rhsim07G0007500	-2.267293979	2.89811E-06	1.00986E-05	RHSIM_Rhsim07G0007500
Root vs Leaf	RHSIM_Rhsim12G0152000	1.114307818	2.91906E-06	1.01686E-05	RHSIM_Rhsim12G0152000

Comparison	Gene ID	log_2FC	P-value	FDR (padj)	Gene Name
Root vs Leaf	RHSIM_Rhsim05G0108400	-2.57455229	2.9368E-06	1.02273E-05	RHSIM_Rhsim05G0108400
Root vs Leaf	RHSIM_Rhsim03G0035500	-11.07187175	2.94336E-06	1.02486E-05	RHSIM_Rhsim03G0035500
Root vs Leaf	RHSIM_Rhsim10G0004200	-5.073656972	2.94864E-06	1.02655E-05	RHSIM_Rhsim10G0004200
Root vs Leaf	novel.1333	-6.770704128	2.95554E-06	1.02879E-05	--
Root vs Leaf	RHSIM_Rhsim03G0010700	-1.029901409	2.96566E-06	1.03216E-05	RHSIM_Rhsim03G0010700
Root vs Leaf	RHSIM_Rhsim01G0249700	7.449307223	2.97447E-06	1.03507E-05	RHSIM_Rhsim01G0249700
Root vs Leaf	RHSIM_Rhsim03G0251500	1.028028868	3.00634E-06	1.04569E-05	RHSIM_Rhsim03G0251500
Root vs Leaf	RHSIM_Rhsim06G0032200	-5.150429294	3.00894E-06	1.04633E-05	RHSIM_Rhsim06G0032200
Root vs Leaf	RHSIM_Rhsim04G0027100	2.549995819	3.03847E-06	1.05601E-05	RHSIM_Rhsim04G0027100
Root vs Leaf	RHSIM_Rhsim08G0091800	-5.044046691	3.03874E-06	1.05601E-05	RHSIM_Rhsim08G0091800
Root vs Leaf	RHSIM_Rhsim04G0177100	1.904020954	3.05114E-06	1.06016E-05	RHSIM_Rhsim04G0177100
Root vs Leaf	novel.4080	-1.28359526	3.05869E-06	1.06262E-05	--
Root vs Leaf	RHSIM_Rhsim03G0192800	-1.186667378	3.08294E-06	1.07072E-05	RHSIM_Rhsim03G0192800
Root vs Leaf	RHSIM_Rhsim03G0055500	1.521413484	3.09732E-06	1.07539E-05	RHSIM_Rhsim03G0055500
Root vs Leaf	RHSIM_Rhsim08G0154200	-1.611450691	3.10972E-06	1.07954E-05	RHSIM_Rhsim08G0154200
Root vs Leaf	RHSIM_Rhsim08G0199300	1.440387356	3.11736E-06	1.08189E-05	RHSIM_Rhsim08G0199300
Root vs Leaf	novel.95	-5.574775976	3.1622E-06	1.09693E-05	--
Root vs Leaf	RHSIM_Rhsim11G0100300	1.974700885	3.1671E-06	1.09847E-05	RHSIM_Rhsim11G0100300
Root vs Leaf	RHSIM_Rhsim12G0053200	-1.172813557	3.17137E-06	1.09979E-05	RHSIM_Rhsim12G0053200
Root vs Leaf	RHSIM_Rhsim03G0219500	1.200414698	3.17196E-06	1.09982E-05	RHSIM_Rhsim03G0219500
Root vs Leaf	RHSIM_Rhsim07G0098300	2.1747941	3.18578E-06	1.10445E-05	RHSIM_Rhsim07G0098300
Root vs Leaf	RHSIM_Rhsim10G0095900	7.805894236	3.2021E-06	1.10994E-05	RHSIM_Rhsim10G0095900
Root vs Leaf	RHSIM_Rhsim10G0064200	1.69680463	3.20688E-06	1.11143E-05	RHSIM_Rhsim10G0064200
Root vs Leaf	novel.5586	-4.12682431	3.21052E-06	1.11253E-05	--
Root vs Leaf	RHSIM_Rhsim05G0039600	-1.819322635	3.21431E-06	1.11362E-05	RHSIM_Rhsim05G0039600

Comparison	Gene ID	log_2FC	P-value	FDR (padj)	Gene Name
Root vs Leaf	RHSIM_Rhsim04G0226400	1.366530031	3.21463E-06	1.11362E-05	RHSIM_Rhsim04G0226400
Root vs Leaf	RHSIM_Rhsim05G0090300	3.344362164	3.21798E-06	1.11461E-05	RHSIM_Rhsim05G0090300
Root vs Leaf	RHSIM_Rhsim04G0039200	3.76069989	3.21882E-06	1.11474E-05	RHSIM_Rhsim04G0039200
Root vs Leaf	RHSIM_Rhsim06G0236300	2.901446759	3.2228E-06	1.11595E-05	RHSIM_Rhsim06G0236300
Root vs Leaf	RHSIM_RhsimUnG0139500	1.265757897	3.2236E-06	1.11606E-05	RHSIM_RhsimUnG0139500
Root vs Leaf	RHSIM_RhsimUnG0149600	-2.521718597	3.23268E-06	1.11903E-05	RHSIM_RhsimUnG0149600
Root vs Leaf	RHSIM_Rhsim07G0015600	1.548008799	3.23746E-06	1.12052E-05	RHSIM_Rhsim07G0015600
Root vs Leaf	RHSIM_Rhsim07G0244300	4.739956821	3.23987E-06	1.12119E-05	RHSIM_Rhsim07G0244300
Root vs Leaf	RHSIM_Rhsim10G0014800	1.343202176	3.24825E-06	1.12392E-05	RHSIM_Rhsim10G0014800
Root vs Leaf	RHSIM_Rhsim01G0243000	-1.882923138	3.26022E-06	1.12789E-05	RHSIM_Rhsim01G0243000
Root vs Leaf	novel.1910	-7.18379844	3.26594E-06	1.1297E-05	--
Root vs Leaf	RHSIM_RhsimUnG0216300	1.518619309	3.28304E-06	1.13545E-05	RHSIM_RhsimUnG0216300
Root vs Leaf	RHSIM_Rhsim02G0148100	-3.199761089	3.31111E-06	1.14464E-05	RHSIM_Rhsim02G0148100
Root vs Leaf	RHSIM_Rhsim13G0075700	-2.029187533	3.31392E-06	1.14544E-05	RHSIM_Rhsim13G0075700
Root vs Leaf	novel.2026	-1.820347884	3.32875E-06	1.1504E-05	--
Root vs Leaf	novel.3229	-7.076563517	3.34393E-06	1.15547E-05	--
Root vs Leaf	RHSIM_Rhsim09G0205600	-1.277692738	3.3447E-06	1.15556E-05	RHSIM_Rhsim09G0205600
Root vs Leaf	RHSIM_Rhsim03G0073100	1.062286146	3.36032E-06	1.16062E-05	RHSIM_Rhsim03G0073100
Root vs Leaf	RHSIM_Rhsim08G0010200	6.531705297	3.37649E-06	1.16602E-05	RHSIM_Rhsim08G0010200
Root vs Leaf	RHSIM_Rhsim06G0186000	3.18110274	3.38324E-06	1.16818E-05	RHSIM_Rhsim06G0186000
Root vs Leaf	RHSIM_Rhsim01G0265300	4.528549009	3.38627E-06	1.16906E-05	RHSIM_Rhsim01G0265300
Root vs Leaf	RHSIM_Rhsim07G0040500	1.375992734	3.38768E-06	1.16919E-05	RHSIM_Rhsim07G0040500
Root vs Leaf	RHSIM_RhsimMtG0002300	-3.062632115	3.39999E-06	1.17327E-05	RHSIM_RhsimMtG0002300
Root vs Leaf	RHSIM_Rhsim09G0156500	-2.113458275	3.41551E-06	1.17844E-05	RHSIM_Rhsim09G0156500
Root vs Leaf	RHSIM_Rhsim04G0139400	-2.823497849	3.42002E-06	1.17983E-05	RHSIM_Rhsim04G0139400

Comparison	Gene ID	log_2FC	P-value	FDR (padj)	Gene Name
Root vs Leaf	RHSIM_Rhsim07G0096000	-1.064175104	3.42184E-06	1.18028E-05	RHSIM_Rhsim07G0096000
Root vs Leaf	RHSIM_RhsimUnG0258800	-2.422745829	3.43992E-06	1.18616E-05	RHSIM_RhsimUnG0258800
Root vs Leaf	RHSIM_Rhsim12G0149900	4.179098531	3.46427E-06	1.19438E-05	RHSIM_Rhsim12G0149900
Root vs Leaf	RHSIM_Rhsim08G0066200	-1.134653079	3.47565E-06	1.19812E-05	RHSIM_Rhsim08G0066200
Root vs Leaf	novel.3799	1.014053943	3.48021E-06	1.19952E-05	--
Root vs Leaf	novel.1291	3.508218321	3.48544E-06	1.20114E-05	--
Root vs Leaf	RHSIM_Rhsim09G0165900	2.62933834	3.48769E-06	1.20174E-05	RHSIM_Rhsim09G0165900
Root vs Leaf	RHSIM_Rhsim03G0056800	2.247907691	3.50705E-06	1.20805E-05	RHSIM_Rhsim03G0056800
Root vs Leaf	RHSIM_Rhsim08G0008300	-1.171323094	3.52142E-06	1.21282E-05	RHSIM_Rhsim08G0008300
Root vs Leaf	RHSIM_Rhsim05G0127100	1.755730017	3.53073E-06	1.21584E-05	RHSIM_Rhsim05G0127100
Root vs Leaf	RHSIM_Rhsim09G0075600	1.333210507	3.54394E-06	1.22021E-05	RHSIM_Rhsim09G0075600
Root vs Leaf	RHSIM_Rhsim05G0087200	6.343298711	3.54907E-06	1.22179E-05	RHSIM_Rhsim05G0087200
Root vs Leaf	RHSIM_RhsimUnG0036900	-3.302853287	3.54989E-06	1.2219E-05	RHSIM_RhsimUnG0036900
Root vs Leaf	novel.1843	3.697112952	3.58064E-06	1.23175E-05	--
Root vs Leaf	RHSIM_Rhsim01G0056800	4.630895677	3.59339E-06	1.23595E-05	RHSIM_Rhsim01G0056800
Root vs Leaf	RHSIM_Rhsim12G0154100	1.391249857	3.60592E-06	1.23971E-05	RHSIM_Rhsim12G0154100
Root vs Leaf	RHSIM_Rhsim04G0106300	4.008253261	3.60717E-06	1.23995E-05	RHSIM_Rhsim04G0106300
Root vs Leaf	RHSIM_Rhsim08G0155000	5.69525824	3.62722E-06	1.24666E-05	RHSIM_Rhsim08G0155000
Root vs Leaf	RHSIM_Rhsim11G0137600	-2.656429049	3.64225E-06	1.25164E-05	RHSIM_Rhsim11G0137600
Root vs Leaf	RHSIM_Rhsim13G0031000	-3.50306855	3.66155E-06	1.25808E-05	RHSIM_Rhsim13G0031000
Root vs Leaf	RHSIM_Rhsim05G0087300	-1.745227612	3.68191E-06	1.2647E-05	RHSIM_Rhsim05G0087300
Root vs Leaf	RHSIM_Rhsim05G0179100	-5.690157769	3.68665E-06	1.26614E-05	RHSIM_Rhsim05G0179100
Root vs Leaf	RHSIM_Rhsim08G0105000	2.861314781	3.69338E-06	1.26827E-05	RHSIM_Rhsim08G0105000
Root vs Leaf	RHSIM_Rhsim07G0203900	3.812550653	3.72766E-06	1.27985E-05	RHSIM_Rhsim07G0203900
Root vs Leaf	RHSIM_Rhsim13G0165900	-1.64722983	3.73002E-06	1.28047E-05	RHSIM_Rhsim13G0165900

Comparison	Gene ID	log_2FC	P-value	FDR (padj)	Gene Name
Root vs Leaf	RHSIM_Rhsim07G0028300	1.309847965	3.73066E-06	1.2805E-05	RHSIM_Rhsim07G0028300
Root vs Leaf	RHSIM_Rhsim05G0197000	-1.252635515	3.73336E-06	1.28123E-05	RHSIM_Rhsim05G0197000
Root vs Leaf	RHSIM_Rhsim11G0134400	-1.60529628	3.73993E-06	1.28311E-05	RHSIM_Rhsim11G0134400
Root vs Leaf	RHSIM_Rhsim10G0175200	4.546205963	3.74957E-06	1.28623E-05	RHSIM_Rhsim10G0175200
Root vs Leaf	RHSIM_Rhsim05G0206300	2.390149211	3.7627E-06	1.29016E-05	RHSIM_Rhsim05G0206300
Root vs Leaf	RHSIM_Rhsim12G0134500	1.449607334	3.77058E-06	1.29267E-05	RHSIM_Rhsim12G0134500
Root vs Leaf	novel.3895	5.418614987	3.77283E-06	1.29325E-05	--
Root vs Leaf	RHSIM_Rhsim02G0052400	6.212017384	3.77515E-06	1.29385E-05	RHSIM_Rhsim02G0052400
Root vs Leaf	RHSIM_Rhsim13G0160200	4.273113301	3.77836E-06	1.29476E-05	RHSIM_Rhsim13G0160200
Root vs Leaf	RHSIM_Rhsim12G0054500	1.198254348	3.81532E-06	1.30704E-05	RHSIM_Rhsim12G0054500
Root vs Leaf	RHSIM_Rhsim03G0204200	-1.508271744	3.8163E-06	1.30718E-05	RHSIM_Rhsim03G0204200
Root vs Leaf	RHSIM_Rhsim04G0022400	1.637710278	3.83056E-06	1.31187E-05	RHSIM_Rhsim04G0022400
Root vs Leaf	RHSIM_Rhsim05G0082500	-3.656881564	3.84014E-06	1.31495E-05	RHSIM_Rhsim05G0082500
Root vs Leaf	RHSIM_Rhsim07G0067300	1.148026872	3.84906E-06	1.31781E-05	RHSIM_Rhsim07G0067300
Root vs Leaf	RHSIM_Rhsim06G0179400	1.865124252	3.86317E-06	1.32208E-05	RHSIM_Rhsim06G0179400
Root vs Leaf	RHSIM_Rhsim04G0179400	1.893168242	3.86322E-06	1.32208E-05	RHSIM_Rhsim04G0179400
Root vs Leaf	RHSIM_Rhsim01G0178100	-6.657417962	3.87855E-06	1.32713E-05	RHSIM_Rhsim01G0178100
Root vs Leaf	RHSIM_Rhsim05G0123600	-1.960683884	3.88676E-06	1.32974E-05	RHSIM_Rhsim05G0123600
Root vs Leaf	RHSIM_Rhsim02G0108400	10.8116427	3.90461E-06	1.33565E-05	RHSIM_Rhsim02G0108400
Root vs Leaf	novel.5518	3.203936573	3.90931E-06	1.33686E-05	--
Root vs Leaf	novel.4853	-7.239382123	3.91547E-06	1.33857E-05	--
Root vs Leaf	RHSIM_Rhsim10G0045300	1.588660675	3.93606E-06	1.34541E-05	RHSIM_Rhsim10G0045300
Root vs Leaf	RHSIM_Rhsim13G0026900	4.648711987	3.93957E-06	1.34641E-05	RHSIM_Rhsim13G0026900
Root vs Leaf	RHSIM_Rhsim12G0099400	-1.29259766	3.9425E-06	1.34712E-05	RHSIM_Rhsim12G0099400
Root vs Leaf	RHSIM_Rhsim04G0149300	1.718378321	3.94646E-06	1.34817E-05	RHSIM_Rhsim04G0149300

Comparison	Gene ID	log_2FC	P-value	FDR (padj)	Gene Name
Root vs Leaf	RHSIM_Rhsim08G0001100	1.517402272	3.95212E-06	1.34991E-05	RHSIM_Rhsim08G0001100
Root vs Leaf	RHSIM_Rhsim06G0100200	1.066611258	3.96998E-06	1.3558E-05	RHSIM_Rhsim06G0100200
Root vs Leaf	RHSIM_Rhsim05G0008700	3.076540857	3.98599E-06	1.36107E-05	RHSIM_Rhsim05G0008700
Root vs Leaf	RHSIM_Rhsim07G0019500	-3.829461384	3.9898E-06	1.36217E-05	RHSIM_Rhsim07G0019500
Root vs Leaf	RHSIM_RhsimUnG0116900	-1.103902507	3.99774E-06	1.36468E-05	RHSIM_RhsimUnG0116900
Root vs Leaf	RHSIM_Rhsim11G0190700	-3.034378358	4.005E-06	1.36696E-05	RHSIM_Rhsim11G0190700
Root vs Leaf	novel.3251	6.576301555	4.02135E-06	1.37233E-05	--
Root vs Leaf	RHSIM_Rhsim06G0229300	-1.297568575	4.02287E-06	1.37245E-05	RHSIM_Rhsim06G0229300
Root vs Leaf	RHSIM_Rhsim12G0050700	-1.276283829	4.04628E-06	1.38023E-05	RHSIM_Rhsim12G0050700
Root vs Leaf	RHSIM_Rhsim05G0227600	-1.072131584	4.04912E-06	1.381E-05	RHSIM_Rhsim05G0227600
Root vs Leaf	RHSIM_Rhsim12G0189900	-1.359618003	4.07457E-06	1.38947E-05	RHSIM_Rhsim12G0189900
Root vs Leaf	RHSIM_Rhsim04G0018700	1.208680805	4.08261E-06	1.3918E-05	RHSIM_Rhsim04G0018700
Root vs Leaf	RHSIM_Rhsim02G0226300	7.216377235	4.10198E-06	1.3982E-05	RHSIM_Rhsim02G0226300
Root vs Leaf	RHSIM_RhsimUnG0155700	-2.492600345	4.1069E-06	1.39967E-05	RHSIM_RhsimUnG0155700
Root vs Leaf	RHSIM_Rhsim13G0177400	2.067308098	4.11395E-06	1.40187E-05	RHSIM_Rhsim13G0177400
Root vs Leaf	RHSIM_Rhsim12G0058000	-1.154711283	4.11512E-06	1.40206E-05	RHSIM_Rhsim12G0058000
Root vs Leaf	RHSIM_Rhsim02G0250800	-1.703038775	4.12159E-06	1.40385E-05	RHSIM_Rhsim02G0250800
Root vs Leaf	RHSIM_Rhsim11G0082700	-4.064085219	4.12878E-06	1.40589E-05	RHSIM_Rhsim11G0082700
Root vs Leaf	novel.4762	-1.854543917	4.13019E-06	1.40616E-05	--
Root vs Leaf	RHSIM_Rhsim06G0201700	-1.139346839	4.1546E-06	1.41405E-05	RHSIM_Rhsim06G0201700
Root vs Leaf	RHSIM_Rhsim01G0215300	-1.696028124	4.18063E-06	1.4227E-05	RHSIM_Rhsim01G0215300
Root vs Leaf	RHSIM_Rhsim08G0089500	1.28459733	4.19599E-06	1.42751E-05	RHSIM_Rhsim08G0089500
Root vs Leaf	novel.5887	6.65205686	4.20377E-06	1.42995E-05	--
Root vs Leaf	RHSIM_Rhsim11G0159000	5.338686768	4.21231E-06	1.43264E-05	RHSIM_Rhsim11G0159000
Root vs Leaf	RHSIM_Rhsim06G0006400	-1.970245773	4.21433E-06	1.43312E-05	RHSIM_Rhsim06G0006400

Comparison	Gene ID	log_2FC	P-value	FDR (padj)	Gene Name
Root vs Leaf	RHSIM_Rhsim08G0113000	1.085131956	4.23668E-06	1.4403E-05	RHSIM_Rhsim08G0113000
Root vs Leaf	RHSIM_Rhsim12G0199800	-1.566213463	4.26801E-06	1.45073E-05	RHSIM_Rhsim12G0199800
Root vs Leaf	RHSIM_Rhsim06G0213900	-1.479666235	4.27273E-06	1.45212E-05	RHSIM_Rhsim06G0213900
Root vs Leaf	RHSIM_Rhsim06G0186700	-6.652932494	4.27807E-06	1.45373E-05	RHSIM_Rhsim06G0186700
Root vs Leaf	RHSIM_Rhsim02G0165000	-7.856634641	4.29618E-06	1.45967E-05	RHSIM_Rhsim02G0165000
Root vs Leaf	RHSIM_Rhsim01G0151900	1.645444135	4.32254E-06	1.46819E-05	RHSIM_Rhsim01G0151900
Root vs Leaf	RHSIM_Rhsim11G0125500	1.276576871	4.33248E-06	1.47135E-05	RHSIM_Rhsim11G0125500
Root vs Leaf	RHSIM_Rhsim06G0003200	-3.347889644	4.33736E-06	1.47258E-05	RHSIM_Rhsim06G0003200
Root vs Leaf	RHSIM_Rhsim08G0154500	3.803315661	4.34685E-06	1.47493E-05	RHSIM_Rhsim08G0154500
Root vs Leaf	RHSIM_Rhsim04G0068300	-1.444261807	4.35219E-06	1.47653E-05	RHSIM_Rhsim04G0068300
Root vs Leaf	RHSIM_Rhsim01G0189700	-3.183707638	4.36067E-06	1.47919E-05	RHSIM_Rhsim01G0189700
Root vs Leaf	RHSIM_Rhsim01G0273400	1.694079451	4.39106E-06	1.48928E-05	RHSIM_Rhsim01G0273400
Root vs Leaf	RHSIM_Rhsim08G0049800	3.783030658	4.39282E-06	1.48966E-05	RHSIM_Rhsim08G0049800
Root vs Leaf	RHSIM_Rhsim08G0006900	-6.422374193	4.41462E-06	1.49661E-05	RHSIM_Rhsim08G0006900
Root vs Leaf	RHSIM_Rhsim04G0200100	3.967768163	4.44356E-06	1.50576E-05	RHSIM_Rhsim04G0200100
Root vs Leaf	RHSIM_Rhsim06G0057200	1.316690787	4.45603E-06	1.50976E-05	RHSIM_Rhsim06G0057200
Root vs Leaf	RHSIM_Rhsim06G0196500	1.774197429	4.4579E-06	1.51005E-05	RHSIM_Rhsim06G0196500
Root vs Leaf	RHSIM_Rhsim08G0190300	-5.578032678	4.52871E-06	1.53349E-05	RHSIM_Rhsim08G0190300
Root vs Leaf	RHSIM_Rhsim06G0196000	2.18535833	4.53796E-06	1.5364E-05	RHSIM_Rhsim06G0196000
Root vs Leaf	RHSIM_Rhsim11G0166600	2.376902821	4.56304E-06	1.54467E-05	RHSIM_Rhsim11G0166600
Root vs Leaf	novel.5715	-2.86506296	4.5908E-06	1.55383E-05	--
Root vs Leaf	novel.4753	-1.186271184	4.59848E-06	1.55598E-05	--
Root vs Leaf	RHSIM_Rhsim04G0149500	-1.20119331	4.60688E-06	1.55859E-05	RHSIM_Rhsim04G0149500
Root vs Leaf	RHSIM_Rhsim05G0219500	-1.298675661	4.61069E-06	1.55965E-05	RHSIM_Rhsim05G0219500
Root vs Leaf	RHSIM_Rhsim05G0191500	-4.049348081	4.62901E-06	1.56562E-05	RHSIM_Rhsim05G0191500

Comparison	Gene ID	log_2FC	P-value	FDR (padj)	Gene Name
Root vs Leaf	RHSIM_Rhsim01G0216300	-1.088693412	4.63799E-06	1.56843E-05	RHSIM_Rhsim01G0216300
Root vs Leaf	novel.3303	-4.353004288	4.65411E-06	1.57319E-05	--
Root vs Leaf	RHSIM_Rhsim11G0173400	-2.285371557	4.66271E-06	1.57587E-05	RHSIM_Rhsim11G0173400
Root vs Leaf	RHSIM_Rhsim09G0153800	4.106264692	4.67513E-06	1.57984E-05	RHSIM_Rhsim09G0153800
Root vs Leaf	RHSIM_Rhsim08G0024900	-3.841417541	4.68124E-06	1.58167E-05	RHSIM_Rhsim08G0024900
Root vs Leaf	RHSIM_Rhsim08G0009000	3.803862127	4.7325E-06	1.59876E-05	RHSIM_Rhsim08G0009000
Root vs Leaf	RHSIM_Rhsim12G0089100	2.398094833	4.74319E-06	1.60213E-05	RHSIM_Rhsim12G0089100
Root vs Leaf	RHSIM_Rhsim02G0255100	-3.828228183	4.78771E-06	1.61646E-05	RHSIM_Rhsim02G0255100
Root vs Leaf	RHSIM_Rhsim13G0168300	-1.114242366	4.8422E-06	1.63438E-05	RHSIM_Rhsim13G0168300
Root vs Leaf	RHSIM_Rhsim09G0157400	-3.009030361	4.84666E-06	1.63565E-05	RHSIM_Rhsim09G0157400
Root vs Leaf	novel.2308	-2.565242217	4.85427E-06	1.63798E-05	--
Root vs Leaf	RHSIM_RhsimUnG0088800	6.646007645	4.87739E-06	1.64554E-05	RHSIM_RhsimUnG0088800
Root vs Leaf	RHSIM_Rhsim04G0121500	-3.050641447	4.88048E-06	1.64634E-05	RHSIM_Rhsim04G0121500
Root vs Leaf	RHSIM_Rhsim10G0022200	-1.309162675	4.90495E-06	1.65412E-05	RHSIM_Rhsim10G0022200
Root vs Leaf	novel.5643	3.250050167	4.90753E-06	1.65475E-05	--
Root vs Leaf	novel.2829	6.133310233	4.91004E-06	1.65535E-05	--
Root vs Leaf	RHSIM_Rhsim06G0026000	-1.204844635	4.96951E-06	1.67483E-05	RHSIM_Rhsim06G0026000
Root vs Leaf	RHSIM_Rhsim02G0058100	-2.662407913	4.97424E-06	1.67577E-05	RHSIM_Rhsim02G0058100
Root vs Leaf	RHSIM_Rhsim08G0041100	1.333884084	4.97831E-06	1.6769E-05	RHSIM_Rhsim08G0041100
Root vs Leaf	novel.4523	-5.15707089	4.99009E-06	1.68062E-05	--
Root vs Leaf	RHSIM_Rhsim01G0009900	2.850150245	5.0003E-06	1.68382E-05	RHSIM_Rhsim01G0009900
Root vs Leaf	RHSIM_Rhsim06G0154200	-2.954367773	5.06517E-06	1.70492E-05	RHSIM_Rhsim06G0154200
Root vs Leaf	RHSIM_Rhsim09G0090300	1.245196151	5.08532E-06	1.7112E-05	RHSIM_Rhsim09G0090300
Root vs Leaf	RHSIM_Rhsim13G0184400	-7.090693679	5.08997E-06	1.71252E-05	RHSIM_Rhsim13G0184400
Root vs Leaf	RHSIM_Rhsim01G0049000	-1.651859445	5.0931E-06	1.71332E-05	RHSIM_Rhsim01G0049000

Comparison	Gene ID	log_2FC	P-value	FDR (padj)	Gene Name
Root vs Leaf	RHSIM_Rhsim09G0147600	-7.469610842	5.09767E-06	1.71461E-05	RHSIM_Rhsim09G0147600
Root vs Leaf	RHSIM_Rhsim13G0223900	1.325218261	5.10476E-06	1.71675E-05	RHSIM_Rhsim13G0223900
Root vs Leaf	RHSIM_Rhsim03G0228000	-2.460937692	5.10779E-06	1.71752E-05	RHSIM_Rhsim03G0228000
Root vs Leaf	RHSIM_Rhsim01G0244000	1.476180687	5.14701E-06	1.73045E-05	RHSIM_Rhsim01G0244000
Root vs Leaf	RHSIM_Rhsim02G0133200	1.956069105	5.15993E-06	1.73442E-05	RHSIM_Rhsim02G0133200
Root vs Leaf	RHSIM_Rhsim01G0290700	1.406347645	5.16031E-06	1.73442E-05	RHSIM_Rhsim01G0290700
Root vs Leaf	novel.685	-2.023491848	5.17566E-06	1.73932E-05	--
Root vs Leaf	RHSIM_Rhsim05G0219200	-1.116334887	5.18776E-06	1.74314E-05	RHSIM_Rhsim05G0219200
Root vs Leaf	novel.5590	-4.541086734	5.2336E-06	1.75777E-05	--
Root vs Leaf	RHSIM_Rhsim06G0148400	-1.695607679	5.24673E-06	1.76193E-05	RHSIM_Rhsim06G0148400
Root vs Leaf	novel.35	-1.68120345	5.27368E-06	1.77047E-05	--
Root vs Leaf	RHSIM_Rhsim05G0016600	-1.227034853	5.2894E-06	1.77523E-05	RHSIM_Rhsim05G0016600
Root vs Leaf	RHSIM_Rhsim13G0112800	4.220287387	5.2957E-06	1.77703E-05	RHSIM_Rhsim13G0112800
Root vs Leaf	novel.5271	1.792398381	5.29632E-06	1.77703E-05	--
Root vs Leaf	RHSIM_Rhsim06G0053100	1.573587657	5.30065E-06	1.77823E-05	RHSIM_Rhsim06G0053100
Root vs Leaf	RHSIM_Rhsim11G0159200	4.401726087	5.32385E-06	1.78576E-05	RHSIM_Rhsim11G0159200
Root vs Leaf	novel.2667	-1.311417032	5.33575E-06	1.78949E-05	--
Root vs Leaf	novel.3071	2.484917713	5.34834E-06	1.79319E-05	--
Root vs Leaf	RHSIM_Rhsim01G0043100	1.472422719	5.36053E-06	1.79702E-05	RHSIM_Rhsim01G0043100
Root vs Leaf	RHSIM_Rhsim12G0076900	1.949187166	5.39294E-06	1.80736E-05	RHSIM_Rhsim12G0076900
Root vs Leaf	RHSIM_Rhsim05G0177600	4.195808614	5.39393E-06	1.80743E-05	RHSIM_Rhsim05G0177600
Root vs Leaf	RHSIM_Rhsim03G0123500	-3.83595078	5.41442E-06	1.81403E-05	RHSIM_Rhsim03G0123500
Root vs Leaf	RHSIM_Rhsim08G0034900	-1.968500225	5.41651E-06	1.81447E-05	RHSIM_Rhsim08G0034900
Root vs Leaf	RHSIM_Rhsim01G0021100	1.045551578	5.42144E-06	1.81586E-05	RHSIM_Rhsim01G0021100
Root vs Leaf	RHSIM_Rhsim07G0008600	-1.09316469	5.42669E-06	1.81735E-05	RHSIM_Rhsim07G0008600

Comparison	Gene ID	log_2FC	P-value	FDR (padj)	Gene Name
Root vs Leaf	RHSIM_Rhsim11G0102700	-6.896965177	5.47064E-06	1.83128E-05	RHSIM_Rhsim11G0102700
Root vs Leaf	novel.2111	-3.385304663	5.48075E-06	1.83439E-05	--
Root vs Leaf	RHSIM_Rhsim11G0144900	1.010052574	5.48314E-06	1.83493E-05	RHSIM_Rhsim11G0144900
Root vs Leaf	RHSIM_Rhsim02G0228400	1.571304357	5.50618E-06	1.84237E-05	RHSIM_Rhsim02G0228400
Root vs Leaf	RHSIM_Rhsim08G0176100	-1.284706555	5.52648E-06	1.84863E-05	RHSIM_Rhsim08G0176100
Root vs Leaf	novel.4128	6.694676491	5.563E-06	1.86058E-05	--
Root vs Leaf	novel.4643	-2.345914294	5.59545E-06	1.87089E-05	--
Root vs Leaf	RHSIM_Rhsim09G0202800	3.268019265	5.60125E-06	1.87256E-05	RHSIM_Rhsim09G0202800
Root vs Leaf	RHSIM_Rhsim02G0058700	2.544041429	5.62269E-06	1.87897E-05	RHSIM_Rhsim02G0058700
Root vs Leaf	RHSIM_Rhsim06G0194800	7.591516221	5.62286E-06	1.87897E-05	RHSIM_Rhsim06G0194800
Root vs Leaf	RHSIM_Rhsim04G0196400	1.642297401	5.62533E-06	1.87952E-05	RHSIM_Rhsim04G0196400
Root vs Leaf	RHSIM_Rhsim08G0211200	2.674452709	5.64958E-06	1.88735E-05	RHSIM_Rhsim08G0211200
Root vs Leaf	RHSIM_Rhsim04G0103200	1.983643602	5.65223E-06	1.88796E-05	RHSIM_Rhsim04G0103200
Root vs Leaf	RHSIM_Rhsim13G0093600	1.924911856	5.66996E-06	1.89361E-05	RHSIM_Rhsim13G0093600
Root vs Leaf	RHSIM_Rhsim05G0007600	2.130319043	5.70875E-06	1.90574E-05	RHSIM_Rhsim05G0007600
Root vs Leaf	novel.2141	-2.421929093	5.70987E-06	1.90584E-05	--
Root vs Leaf	RHSIM_Rhsim07G0226000	2.826340354	5.71311E-06	1.90665E-05	RHSIM_Rhsim07G0226000
Root vs Leaf	RHSIM_Rhsim06G0204300	1.605981868	5.72775E-06	1.91126E-05	RHSIM_Rhsim06G0204300
Root vs Leaf	RHSIM_Rhsim13G0183300	-3.998248852	5.73153E-06	1.91224E-05	RHSIM_Rhsim13G0183300
Root vs Leaf	RHSIM_Rhsim13G0178300	2.090781986	5.76609E-06	1.9235E-05	RHSIM_Rhsim13G0178300
Root vs Leaf	RHSIM_Rhsim01G0235500	5.927439311	5.7897E-06	1.9311E-05	RHSIM_Rhsim01G0235500
Root vs Leaf	novel.3275	4.99427715	5.85448E-06	1.95242E-05	--
Root vs Leaf	RHSIM_Rhsim03G0062800	2.979758013	5.88383E-06	1.96193E-05	RHSIM_Rhsim03G0062800
Root vs Leaf	RHSIM_Rhsim07G0190100	2.205477386	5.88623E-06	1.96244E-05	RHSIM_Rhsim07G0190100
Root vs Leaf	RHSIM_Rhsim11G0045900	1.031009516	5.89947E-06	1.96657E-05	RHSIM_Rhsim11G0045900

Comparison	Gene ID	log_2FC	P-value	FDR (padj)	Gene Name
Root vs Leaf	RHSIM_Rhsim03G0235000	1.145085863	5.90535E-06	1.96797E-05	RHSIM_Rhsim03G0235000
Root vs Leaf	novel.1665	-3.309278143	5.91597E-06	1.97122E-05	--
Root vs Leaf	RHSIM_Rhsim03G0203300	-5.532464206	5.94004E-06	1.97896E-05	RHSIM_Rhsim03G0203300
Root vs Leaf	RHSIM_Rhsim06G0066700	3.936653187	5.97718E-06	1.99104E-05	RHSIM_Rhsim06G0066700
Root vs Leaf	RHSIM_Rhsim04G0126200	2.044691544	5.99113E-06	1.99541E-05	RHSIM_Rhsim04G0126200
Root vs Leaf	RHSIM_Rhsim10G0067000	1.85877251	6.00097E-06	1.9984E-05	RHSIM_Rhsim10G0067000
Root vs Leaf	RHSIM_Rhsim10G0085800	-1.321920395	6.00446E-06	1.99927E-05	RHSIM_Rhsim10G0085800
Root vs Leaf	RHSIM_RhsimUnG0119500	1.17933572	6.0365E-06	2.00965E-05	RHSIM_RhsimUnG0119500
Root vs Leaf	RHSIM_Rhsim03G0220400	1.431124887	6.04545E-06	2.01234E-05	RHSIM_Rhsim03G0220400
Root vs Leaf	RHSIM_Rhsim06G0127800	-1.056647595	6.10106E-06	2.03027E-05	RHSIM_Rhsim06G0127800
Root vs Leaf	RHSIM_Rhsim06G0076000	2.896114833	6.10532E-06	2.03139E-05	RHSIM_Rhsim06G0076000
Root vs Leaf	RHSIM_Rhsim08G0136200	1.191130076	6.18284E-06	2.05659E-05	RHSIM_Rhsim08G0136200
Root vs Leaf	novel.777	3.406815599	6.185E-06	2.05702E-05	--
Root vs Leaf	RHSIM_Rhsim13G0148000	7.18986377	6.19677E-06	2.06041E-05	RHSIM_Rhsim13G0148000
Root vs Leaf	RHSIM_Rhsim03G0028700	1.966782226	6.20197E-06	2.06177E-05	RHSIM_Rhsim03G0028700
Root vs Leaf	RHSIM_Rhsim11G0127500	2.305417593	6.20525E-06	2.06256E-05	RHSIM_Rhsim11G0127500
Root vs Leaf	RHSIM_Rhsim12G0023900	1.397241638	6.21571E-06	2.06545E-05	RHSIM_Rhsim12G0023900
Root vs Leaf	novel.1129	4.032376928	6.23161E-06	2.07043E-05	--
Root vs Leaf	RHSIM_Rhsim06G0163100	6.553245663	6.24724E-06	2.07533E-05	RHSIM_Rhsim06G0163100
Root vs Leaf	RHSIM_Rhsim12G0100200	2.391383055	6.26133E-06	2.07956E-05	RHSIM_Rhsim12G0100200
Root vs Leaf	RHSIM_Rhsim05G0063500	4.965358702	6.26178E-06	2.07956E-05	RHSIM_Rhsim05G0063500
Root vs Leaf	RHSIM_Rhsim03G0099700	-1.541656476	6.28269E-06	2.08621E-05	RHSIM_Rhsim03G0099700
Root vs Leaf	RHSIM_Rhsim03G0144600	-1.071579059	6.30674E-06	2.09389E-05	RHSIM_Rhsim03G0144600
Root vs Leaf	RHSIM_Rhsim11G0052000	-1.630113751	6.31237E-06	2.09546E-05	RHSIM_Rhsim11G0052000
Root vs Leaf	novel.5223	1.047584773	6.33484E-06	2.10262E-05	--

Comparison	Gene ID	log_2FC	P-value	FDR (padj)	Gene Name
Root vs Leaf	RHSIM_Rhsim01G0250100	-3.521809851	6.38285E-06	2.11794E-05	RHSIM_Rhsim01G0250100
Root vs Leaf	RHSIM_RhsimUnG0256500	-2.887880757	6.42352E-06	2.13113E-05	RHSIM_RhsimUnG0256500
Root vs Leaf	RHSIM_Rhsim11G0191000	3.768983069	6.43327E-06	2.13406E-05	RHSIM_Rhsim11G0191000
Root vs Leaf	novel.5150	2.454224992	6.44457E-06	2.13751E-05	--
Root vs Leaf	RHSIM_Rhsim13G0149700	6.298539146	6.45967E-06	2.14221E-05	RHSIM_Rhsim13G0149700
Root vs Leaf	novel.3614	2.094011443	6.46115E-06	2.14239E-05	--
Root vs Leaf	RHSIM_Rhsim02G0113100	-7.452144071	6.51868E-06	2.16054E-05	RHSIM_Rhsim02G0113100
Root vs Leaf	RHSIM_Rhsim10G0169500	1.463855014	6.52601E-06	2.16266E-05	RHSIM_Rhsim10G0169500
Root vs Leaf	RHSIM_Rhsim08G0232000	-1.6294971	6.54513E-06	2.16868E-05	RHSIM_Rhsim08G0232000
Root vs Leaf	RHSIM_Rhsim01G0259700	-1.283312735	6.57722E-06	2.17901E-05	RHSIM_Rhsim01G0259700
Root vs Leaf	RHSIM_Rhsim12G0064900	4.79653868	6.66254E-06	2.20601E-05	RHSIM_Rhsim12G0064900
Root vs Leaf	RHSIM_Rhsim13G0002500	-2.161877212	6.6717E-06	2.20872E-05	RHSIM_Rhsim13G0002500
Root vs Leaf	RHSIM_Rhsim09G0036800	1.239246031	6.69079E-06	2.21473E-05	RHSIM_Rhsim09G0036800
Root vs Leaf	RHSIM_Rhsim03G0178200	-1.211995582	6.69207E-06	2.21484E-05	RHSIM_Rhsim03G0178200
Root vs Leaf	novel.2486	-6.537593344	6.70172E-06	2.21739E-05	--
Root vs Leaf	RHSIM_Rhsim09G0143900	-2.119251553	6.71164E-06	2.22004E-05	RHSIM_Rhsim09G0143900
Root vs Leaf	RHSIM_Rhsim09G0099000	-1.161702968	6.73423E-06	2.2272E-05	RHSIM_Rhsim09G0099000
Root vs Leaf	RHSIM_Rhsim02G0014500	1.203479672	6.74717E-06	2.23115E-05	RHSIM_Rhsim02G0014500
Root vs Leaf	novel.2930	-2.35269598	6.76002E-06	2.23509E-05	--
Root vs Leaf	RHSIM_Rhsim07G0141300	1.194154596	6.83705E-06	2.26023E-05	RHSIM_Rhsim07G0141300
Root vs Leaf	novel.2737	2.357854372	6.86035E-06	2.26761E-05	--
Root vs Leaf	novel.37	2.024646538	6.86758E-06	2.26968E-05	--
Root vs Leaf	RHSIM_Rhsim12G0040700	1.225416959	6.86893E-06	2.2698E-05	RHSIM_Rhsim12G0040700
Root vs Leaf	novel.5727	6.534702391	6.89437E-06	2.27755E-05	--
Root vs Leaf	RHSIM_Rhsim09G0145300	-1.524873803	6.94679E-06	2.29454E-05	RHSIM_Rhsim09G0145300

Comparison	Gene ID	$\log_2FC$	P-value	FDR (padj)	Gene Name
Root vs Leaf	RHSIM_Rhsim05G0219900	4.755158429	6.98663E-06	2.30737E-05	RHSIM_Rhsim05G0219900
Root vs Leaf	RHSIM_Rhsim04G0010600	1.447305843	6.99084E-06	2.30843E-05	RHSIM_Rhsim04G0010600
Root vs Leaf	RHSIM_Rhsim01G0251700	1.874616137	7.0108E-06	2.31436E-05	RHSIM_Rhsim01G0251700
Root vs Leaf	RHSIM_Rhsim12G0164200	2.21923316	7.03115E-06	2.32042E-05	RHSIM_Rhsim12G0164200
Root vs Leaf	RHSIM_Rhsim03G0084600	1.126713695	7.03237E-06	2.32049E-05	RHSIM_Rhsim03G0084600
Root vs Leaf	RHSIM_Rhsim05G0110700	-4.390683398	7.04605E-06	2.32434E-05	RHSIM_Rhsim05G0110700
Root vs Leaf	RHSIM_Rhsim12G0129700	-1.376565824	7.1317E-06	2.35134E-05	RHSIM_Rhsim12G0129700
Root vs Leaf	novel.739	2.392634719	7.13195E-06	2.35134E-05	--
Root vs Leaf	novel.886	4.406195375	7.13877E-06	2.35325E-05	--
Root vs Leaf	RHSIM_Rhsim06G0232700	-2.568673417	7.15599E-06	2.35859E-05	RHSIM_Rhsim06G0232700
Root vs Leaf	novel.3478	6.805216754	7.19893E-06	2.37207E-05	--
Root vs Leaf	RHSIM_Rhsim05G0069400	-1.543825009	7.22925E-06	2.38172E-05	RHSIM_Rhsim05G0069400
Root vs Leaf	RHSIM_RhsimUnG0102000	2.087942215	7.2481E-06	2.38759E-05	RHSIM_RhsimUnG0102000
Root vs Leaf	RHSIM_Rhsim13G0168400	-1.945199453	7.29191E-06	2.40168E-05	RHSIM_Rhsim13G0168400
Root vs Leaf	RHSIM_Rhsim09G0191600	-2.756936356	7.29489E-06	2.40232E-05	RHSIM_Rhsim09G0191600
Root vs Leaf	RHSIM_Rhsim12G0079600	6.695218554	7.29808E-06	2.40303E-05	RHSIM_Rhsim12G0079600
Root vs Leaf	RHSIM_Rhsim01G0178700	-8.12291239	7.30294E-06	2.40429E-05	RHSIM_Rhsim01G0178700
Root vs Leaf	novel.5419	-2.051815896	7.31984E-06	2.40951E-05	--
Root vs Leaf	RHSIM_Rhsim12G0179700	1.07972118	7.36989E-06	2.42495E-05	RHSIM_Rhsim12G0179700
Root vs Leaf	novel.2693	-1.791812012	7.40447E-06	2.43564E-05	--
Root vs Leaf	RHSIM_Rhsim12G0060900	-1.959601573	7.41417E-06	2.43848E-05	RHSIM_Rhsim12G0060900
Root vs Leaf	RHSIM_Rhsim04G0039300	1.812855002	7.41914E-06	2.43977E-05	RHSIM_Rhsim04G0039300
Root vs Leaf	RHSIM_Rhsim05G0112500	1.038584008	7.42073E-06	2.43994E-05	RHSIM_Rhsim05G0112500
Root vs Leaf	RHSIM_Rhsim10G0080200	7.03005603	7.42532E-06	2.44076E-05	RHSIM_Rhsim10G0080200
Root vs Leaf	RHSIM_Rhsim10G0205800	1.010895741	7.42533E-06	2.44076E-05	RHSIM_Rhsim10G0205800

Comparison	Gene ID	log_2FC	P-value	FDR (padj)	Gene Name
Root vs Leaf	RHSIM_RhsimUnG0072900	-3.556569638	7.45168E-06	2.44908E-05	RHSIM_RhsimUnG0072900
Root vs Leaf	RHSIM_Rhsim08G0209800	1.760467574	7.62803E-06	2.50561E-05	RHSIM_Rhsim08G0209800
Root vs Leaf	RHSIM_Rhsim10G0195900	-1.285530434	7.62934E-06	2.50568E-05	RHSIM_Rhsim10G0195900
Root vs Leaf	novel.2303	1.431712136	7.63326E-06	2.50662E-05	--
Root vs Leaf	RHSIM_Rhsim08G0190800	1.064824554	7.64233E-06	2.50924E-05	RHSIM_Rhsim08G0190800
Root vs Leaf	RHSIM_Rhsim03G0199800	-3.466012593	7.65507E-06	2.51307E-05	RHSIM_Rhsim03G0199800
Root vs Leaf	RHSIM_Rhsim13G0111400	1.468097879	7.70158E-06	2.52762E-05	RHSIM_Rhsim13G0111400
Root vs Leaf	RHSIM_Rhsim07G0102900	3.64672845	7.70921E-06	2.52976E-05	RHSIM_Rhsim07G0102900
Root vs Leaf	RHSIM_Rhsim07G0232100	-1.69747533	7.71904E-06	2.53263E-05	RHSIM_Rhsim07G0232100
Root vs Leaf	RHSIM_Rhsim07G0073100	1.786253565	7.72193E-06	2.53322E-05	RHSIM_Rhsim07G0073100
Root vs Leaf	RHSIM_Rhsim01G0206300	3.785886299	7.74108E-06	2.53878E-05	RHSIM_Rhsim01G0206300
Root vs Leaf	novel.4436	-4.735138739	7.79272E-06	2.55535E-05	--
Root vs Leaf	RHSIM_Rhsim01G0049500	1.115615894	7.86971E-06	2.5795E-05	RHSIM_Rhsim01G0049500
Root vs Leaf	RHSIM_Rhsim10G0193400	-4.46657061	7.90893E-06	2.59163E-05	RHSIM_Rhsim10G0193400
Root vs Leaf	RHSIM_Rhsim09G0104400	-1.471729052	7.92867E-06	2.59699E-05	RHSIM_Rhsim09G0104400
Root vs Leaf	novel.1198	4.805717643	7.93112E-06	2.59742E-05	--
Root vs Leaf	RHSIM_Rhsim05G0234700	-2.774526146	7.99081E-06	2.61623E-05	RHSIM_Rhsim05G0234700
Root vs Leaf	RHSIM_Rhsim04G0130000	-1.239081908	8.00155E-06	2.61938E-05	RHSIM_Rhsim04G0130000
Root vs Leaf	RHSIM_Rhsim11G0169400	-1.732899365	8.01668E-06	2.62396E-05	RHSIM_Rhsim11G0169400
Root vs Leaf	RHSIM_Rhsim07G0250500	-1.064953798	8.04559E-06	2.63305E-05	RHSIM_Rhsim07G0250500
Root vs Leaf	RHSIM_Rhsim11G0113800	-3.988964519	8.09618E-06	2.64923E-05	RHSIM_Rhsim11G0113800
Root vs Leaf	RHSIM_Rhsim07G0220600	2.805911584	8.09918E-06	2.64984E-05	RHSIM_Rhsim07G0220600
Root vs Leaf	novel.1551	-5.162487392	8.11373E-06	2.65422E-05	--
Root vs Leaf	RHSIM_Rhsim04G0017600	-7.782585353	8.11824E-06	2.65532E-05	RHSIM_Rhsim04G0017600
Root vs Leaf	RHSIM_Rhsim01G0136500	3.856966358	8.13365E-06	2.65999E-05	RHSIM_Rhsim01G0136500

Comparison	Gene ID	log_2FC	P-value	FDR (padj)	Gene Name
Root vs Leaf	RHSIM_RhsimUnG0233500	7.275054198	8.1392E-06	2.66143E-05	RHSIM_RhsimUnG0233500
Root vs Leaf	RHSIM_Rhsim02G0207000	4.489375929	8.14551E-06	2.66283E-05	RHSIM_Rhsim02G0207000
Root vs Leaf	RHSIM_Rhsim03G0193700	-2.057723061	8.14726E-06	2.66294E-05	RHSIM_Rhsim03G0193700
Root vs Leaf	RHSIM_Rhsim06G0172200	2.666259474	8.15654E-06	2.66559E-05	RHSIM_Rhsim06G0172200
Root vs Leaf	RHSIM_Rhsim09G0106600	-1.304993736	8.16526E-06	2.66807E-05	RHSIM_Rhsim09G0106600
Root vs Leaf	RHSIM_Rhsim02G0238000	1.637457782	8.1809E-06	2.6728E-05	RHSIM_Rhsim02G0238000
Root vs Leaf	RHSIM_Rhsim04G0174300	-1.467043992	8.21195E-06	2.68256E-05	RHSIM_Rhsim04G0174300
Root vs Leaf	RHSIM_Rhsim12G0106800	-5.513411364	8.22699E-06	2.6871E-05	RHSIM_Rhsim12G0106800
Root vs Leaf	novel.5056	-1.93340807	8.2627E-06	2.6973E-05	--
Root vs Leaf	RHSIM_Rhsim13G0056000	4.863406192	8.2629E-06	2.6973E-05	RHSIM_Rhsim13G0056000
Root vs Leaf	novel.3809	-1.962145528	8.3136E-06	2.71273E-05	--
Root vs Leaf	RHSIM_Rhsim05G0233800	2.779078969	8.37345E-06	2.73146E-05	RHSIM_Rhsim05G0233800
Root vs Leaf	RHSIM_Rhsim06G0012400	2.627141203	8.43039E-06	2.74965E-05	RHSIM_Rhsim06G0012400
Root vs Leaf	RHSIM_Rhsim06G0237600	1.866745931	8.45863E-06	2.75799E-05	RHSIM_Rhsim06G0237600
Root vs Leaf	RHSIM_Rhsim08G0010000	4.378841593	8.45953E-06	2.75799E-05	RHSIM_Rhsim08G0010000
Root vs Leaf	RHSIM_RhsimUnG0119300	-2.501254623	8.46746E-06	2.76018E-05	RHSIM_RhsimUnG0119300
Root vs Leaf	RHSIM_Rhsim08G0002400	-1.059748674	8.48215E-06	2.76458E-05	RHSIM_Rhsim08G0002400
Root vs Leaf	RHSIM_Rhsim06G0193500	4.254964172	8.49124E-06	2.76716E-05	RHSIM_Rhsim06G0193500
Root vs Leaf	RHSIM_Rhsim06G0238400	1.294921573	8.51402E-06	2.77419E-05	RHSIM_Rhsim06G0238400
Root vs Leaf	novel.53	-1.792477695	8.53827E-06	2.7817E-05	--
Root vs Leaf	RHSIM_Rhsim02G0223000	-1.569567323	8.54096E-06	2.78218E-05	RHSIM_Rhsim02G0223000
Root vs Leaf	RHSIM_Rhsim09G0173400	1.101763971	8.56018E-06	2.78805E-05	RHSIM_Rhsim09G0173400
Root vs Leaf	RHSIM_Rhsim05G0003300	-1.772476525	8.56546E-06	2.78938E-05	RHSIM_Rhsim05G0003300
Root vs Leaf	RHSIM_Rhsim08G0228300	6.379167436	8.56745E-06	2.78963E-05	RHSIM_Rhsim08G0228300
Root vs Leaf	RHSIM_RhsimUnG0150100	2.311861975	8.58264E-06	2.79379E-05	RHSIM_RhsimUnG0150100

Comparison	Gene ID	log_2FC	P-value	FDR (padj)	Gene Name
Root vs Leaf	RHSIM_Rhsim09G0046600	-1.211509853	8.59441E-06	2.79723E-05	RHSIM_Rhsim09G0046600
Root vs Leaf	novel.1666	-7.322257632	8.62866E-06	2.80799E-05	--
Root vs Leaf	RHSIM_Rhsim06G0159100	2.621135963	8.70176E-06	2.83137E-05	RHSIM_Rhsim06G0159100
Root vs Leaf	RHSIM_Rhsim04G0229900	2.356946649	8.7044E-06	2.83184E-05	RHSIM_Rhsim04G0229900
Root vs Leaf	RHSIM_RhsimUnG0099800	-1.590150728	8.74671E-06	2.844E-05	RHSIM_RhsimUnG0099800
Root vs Leaf	RHSIM_Rhsim12G0167300	1.947514759	8.76043E-06	2.84806E-05	RHSIM_Rhsim12G0167300
Root vs Leaf	novel.4025	5.159301813	8.78435E-06	2.85544E-05	--
Root vs Leaf	RHSIM_Rhsim07G0135900	-2.765205901	8.81963E-06	2.8665E-05	RHSIM_Rhsim07G0135900
Root vs Leaf	novel.5251	1.110145229	8.87476E-06	2.8828E-05	--
Root vs Leaf	RHSIM_Rhsim03G0017700	-1.433444481	8.93028E-06	2.90002E-05	RHSIM_Rhsim03G0017700
Root vs Leaf	RHSIM_Rhsim01G0026200	-4.526989929	8.93184E-06	2.90012E-05	RHSIM_Rhsim01G0026200
Root vs Leaf	RHSIM_Rhsim01G0102400	4.564051412	8.93356E-06	2.90027E-05	RHSIM_Rhsim01G0102400
Root vs Leaf	RHSIM_Rhsim08G0038700	2.35154539	8.96267E-06	2.90891E-05	RHSIM_Rhsim08G0038700
Root vs Leaf	novel.6073	3.078040405	8.98705E-06	2.91641E-05	--
Root vs Leaf	RHSIM_Rhsim07G0155500	5.155463141	9.03428E-06	2.93133E-05	RHSIM_Rhsim07G0155500
Root vs Leaf	RHSIM_RhsimUnG0098400	6.296819041	9.05244E-06	2.93681E-05	RHSIM_RhsimUnG0098400
Root vs Leaf	novel.709	1.086602909	9.08294E-06	2.94629E-05	--
Root vs Leaf	RHSIM_Rhsim02G0235900	2.2491791	9.13366E-06	2.96191E-05	RHSIM_Rhsim02G0235900
Root vs Leaf	RHSIM_Rhsim05G0101100	6.05801507	9.14784E-06	2.9661E-05	RHSIM_Rhsim05G0101100
Root vs Leaf	RHSIM_Rhsim03G0019300	-1.129719571	9.15138E-06	2.96683E-05	RHSIM_Rhsim03G0019300
Root vs Leaf	RHSIM_Rhsim08G0066000	-1.902537995	9.18953E-06	2.97836E-05	RHSIM_Rhsim08G0066000
Root vs Leaf	novel.2049	-5.714923998	9.23376E-06	2.99186E-05	--
Root vs Leaf	RHSIM_Rhsim01G0120500	1.694056271	9.24062E-06	2.99366E-05	RHSIM_Rhsim01G0120500
Root vs Leaf	RHSIM_Rhsim07G0087700	-1.457511022	9.2965E-06	3.01134E-05	RHSIM_Rhsim07G0087700
Root vs Leaf	RHSIM_Rhsim08G0219400	-2.391904206	9.3045E-06	3.01351E-05	RHSIM_Rhsim08G0219400

Comparison	Gene ID	log_2FC	P-value	FDR (padj)	Gene Name
Root vs Leaf	RHSIM_Rhsim07G0035100	3.463336618	9.33061E-06	3.02155E-05	RHSIM_Rhsim07G0035100
Root vs Leaf	RHSIM_Rhsim08G0190500	-1.002135543	9.35175E-06	3.02754E-05	RHSIM_Rhsim08G0190500
Root vs Leaf	RHSIM_Rhsim09G0071800	1.1022106	9.37509E-06	3.03468E-05	RHSIM_Rhsim09G0071800
Root vs Leaf	RHSIM_Rhsim04G0176900	1.139264314	9.38115E-06	3.03621E-05	RHSIM_Rhsim04G0176900
Root vs Leaf	RHSIM_Rhsim03G0187500	1.184676225	9.40861E-06	3.04468E-05	RHSIM_Rhsim03G0187500
Root vs Leaf	RHSIM_Rhsim08G0118000	-2.070898234	9.41394E-06	3.04597E-05	RHSIM_Rhsim08G0118000
Root vs Leaf	RHSIM_Rhsim03G0188700	1.311476729	9.42324E-06	3.04856E-05	RHSIM_Rhsim03G0188700
Root vs Leaf	RHSIM_Rhsim10G0051000	-8.554344397	9.49654E-06	3.07055E-05	RHSIM_Rhsim10G0051000
Root vs Leaf	RHSIM_Rhsim01G0006200	2.48907729	9.50239E-06	3.07202E-05	RHSIM_Rhsim01G0006200
Root vs Leaf	RHSIM_Rhsim08G0155100	2.009697048	9.5054E-06	3.07256E-05	RHSIM_Rhsim08G0155100
Root vs Leaf	RHSIM_Rhsim08G0206100	4.042622872	9.51349E-06	3.07474E-05	RHSIM_Rhsim08G0206100
Root vs Leaf	novel.98	1.299994166	9.55705E-06	3.08839E-05	--
Root vs Leaf	RHSIM_Rhsim01G0182000	-2.732686723	9.5858E-06	3.09682E-05	RHSIM_Rhsim01G0182000
Root vs Leaf	RHSIM_Rhsim01G0012200	1.000450203	9.66149E-06	3.12084E-05	RHSIM_Rhsim01G0012200
Root vs Leaf	RHSIM_Rhsim04G0128100	7.123170661	9.70816E-06	3.13547E-05	RHSIM_Rhsim04G0128100
Root vs Leaf	RHSIM_Rhsim09G0055800	-2.059043884	9.78723E-06	3.16013E-05	RHSIM_Rhsim09G0055800
Root vs Leaf	RHSIM_Rhsim06G0119900	-7.683282545	9.79942E-06	3.16362E-05	RHSIM_Rhsim06G0119900
Root vs Leaf	RHSIM_Rhsim03G0180800	1.855672624	9.82396E-06	3.1711E-05	RHSIM_Rhsim03G0180800
Root vs Leaf	RHSIM_Rhsim13G0071600	4.065535828	9.98239E-06	3.22134E-05	RHSIM_Rhsim13G0071600
Root vs Leaf	RHSIM_Rhsim12G0213000	2.304752407	1.00058E-05	3.228E-05	RHSIM_Rhsim12G0213000
Root vs Leaf	RHSIM_Rhsim02G0109400	10.20919566	1.00139E-05	3.23015E-05	RHSIM_Rhsim02G0109400
Root vs Leaf	RHSIM_Rhsim07G0252700	1.376181789	1.00464E-05	3.24019E-05	RHSIM_Rhsim07G0252700
Root vs Leaf	RHSIM_Rhsim09G0110500	3.9036062	1.00614E-05	3.24411E-05	RHSIM_Rhsim09G0110500
Root vs Leaf	RHSIM_Rhsim05G0072400	2.258387882	1.00869E-05	3.2519E-05	RHSIM_Rhsim05G0072400
Root vs Leaf	RHSIM_Rhsim07G0243400	1.643040669	1.0111E-05	3.25922E-05	RHSIM_Rhsim07G0243400

Comparison	Gene ID	log_2FC	P-value	FDR (padj)	Gene Name
Root vs Leaf	novel.1970	-5.568398407	1.01281E-05	3.26426E-05	--
Root vs Leaf	RHSIM_Rhsim12G0167600	1.286787211	1.01314E-05	3.26489E-05	RHSIM_Rhsim12G0167600
Root vs Leaf	RHSIM_Rhsim11G0181300	2.945718883	1.01429E-05	3.26813E-05	RHSIM_Rhsim11G0181300
Root vs Leaf	RHSIM_Rhsim06G0029900	-5.211534019	1.01511E-05	3.2703E-05	RHSIM_Rhsim06G0029900
Root vs Leaf	RHSIM_Rhsim04G0035900	-7.201203669	1.01744E-05	3.27735E-05	RHSIM_Rhsim04G0035900
Root vs Leaf	RHSIM_Rhsim06G0051800	3.612351229	1.03191E-05	3.32211E-05	RHSIM_Rhsim06G0051800
Root vs Leaf	RHSIM_Rhsim03G0195800	-3.994757476	1.03304E-05	3.32531E-05	RHSIM_Rhsim03G0195800
Root vs Leaf	novel.2798	2.481554971	1.03325E-05	3.3255E-05	--
Root vs Leaf	RHSIM_Rhsim13G0095200	-1.061136114	1.0417E-05	3.35132E-05	RHSIM_Rhsim13G0095200
Root vs Leaf	RHSIM_Rhsim04G0058900	-2.146803113	1.04208E-05	3.35208E-05	RHSIM_Rhsim04G0058900
Root vs Leaf	RHSIM_RhsimUnG0192000	3.271521871	1.04378E-05	3.35708E-05	RHSIM_RhsimUnG0192000
Root vs Leaf	RHSIM_RhsimUnG0152600	-1.265897258	1.04466E-05	3.35943E-05	RHSIM_RhsimUnG0152600
Root vs Leaf	novel.1817	-7.003427393	1.05287E-05	3.38441E-05	--
Root vs Leaf	RHSIM_Rhsim02G0168800	8.004973974	1.05548E-05	3.39141E-05	RHSIM_Rhsim02G0168800
Root vs Leaf	RHSIM_Rhsim01G0121600	1.10848083	1.0599E-05	3.40466E-05	RHSIM_Rhsim01G0121600
Root vs Leaf	RHSIM_RhsimUnG0253900	-1.464256485	1.06206E-05	3.41114E-05	RHSIM_RhsimUnG0253900
Root vs Leaf	RHSIM_Rhsim01G0050100	2.798245399	1.06674E-05	3.42568E-05	RHSIM_Rhsim01G0050100
Root vs Leaf	RHSIM_Rhsim08G0134400	1.901748293	1.06765E-05	3.42812E-05	RHSIM_Rhsim08G0134400
Root vs Leaf	RHSIM_Rhsim09G0144500	4.715501832	1.07395E-05	3.44691E-05	RHSIM_Rhsim09G0144500
Root vs Leaf	RHSIM_Rhsim01G0131300	6.970950014	1.07663E-05	3.45361E-05	RHSIM_Rhsim01G0131300
Root vs Leaf	RHSIM_Rhsim12G0141600	-1.23443165	1.08784E-05	3.48859E-05	RHSIM_Rhsim12G0141600
Root vs Leaf	RHSIM_Rhsim13G0172800	4.070128508	1.09214E-05	3.50141E-05	RHSIM_Rhsim13G0172800
Root vs Leaf	RHSIM_Rhsim13G0186100	6.416575972	1.09234E-05	3.50156E-05	RHSIM_Rhsim13G0186100
Root vs Leaf	RHSIM_Rhsim04G0140800	2.663886484	1.09502E-05	3.50968E-05	RHSIM_Rhsim04G0140800
Root vs Leaf	RHSIM_Rhsim08G0009200	-1.876750353	1.09864E-05	3.52078E-05	RHSIM_Rhsim08G0009200

Comparison	Gene ID	log_2FC	P-value	FDR (padj)	Gene Name
Root vs Leaf	RHSIM_Rhsim09G0191400	1.404070697	1.10293E-05	3.53405E-05	RHSIM_Rhsim09G0191400
Root vs Leaf	RHSIM_Rhsim12G0012700	-5.482124419	1.10464E-05	3.53903E-05	RHSIM_Rhsim12G0012700
Root vs Leaf	novel.4948	-4.274640699	1.10683E-05	3.54506E-05	--
Root vs Leaf	RHSIM_Rhsim04G0083100	-1.399348294	1.10862E-05	3.55031E-05	RHSIM_Rhsim04G0083100
Root vs Leaf	RHSIM_Rhsim09G0060100	-1.864620324	1.1088E-05	3.5504E-05	RHSIM_Rhsim09G0060100
Root vs Leaf	RHSIM_Rhsim03G0011500	1.526731588	1.10959E-05	3.55245E-05	RHSIM_Rhsim03G0011500
Root vs Leaf	RHSIM_Rhsim07G0214300	-5.459076073	1.11076E-05	3.55569E-05	RHSIM_Rhsim07G0214300
Root vs Leaf	RHSIM_Rhsim08G0051200	2.210122143	1.1123E-05	3.56013E-05	RHSIM_Rhsim08G0051200
Root vs Leaf	RHSIM_Rhsim07G0211600	1.109741843	1.113E-05	3.56186E-05	RHSIM_Rhsim07G0211600
Root vs Leaf	novel.2416	-5.328720168	1.11591E-05	3.5707E-05	--
Root vs Leaf	RHSIM_Rhsim05G0182200	2.400105019	1.11729E-05	3.57413E-05	RHSIM_Rhsim05G0182200
Root vs Leaf	RHSIM_Rhsim07G0218300	-1.066596719	1.12035E-05	3.58341E-05	RHSIM_Rhsim07G0218300
Root vs Leaf	RHSIM_Rhsim13G0150300	-1.138885873	1.12514E-05	3.59776E-05	RHSIM_Rhsim13G0150300
Root vs Leaf	RHSIM_Rhsim10G0170200	2.598455269	1.1254E-05	3.59792E-05	RHSIM_Rhsim10G0170200
Root vs Leaf	RHSIM_Rhsim06G0180100	4.128637839	1.12551E-05	3.59792E-05	RHSIM_Rhsim06G0180100
Root vs Leaf	RHSIM_Rhsim09G0179700	-1.086884891	1.12675E-05	3.60141E-05	RHSIM_Rhsim09G0179700
Root vs Leaf	RHSIM_Rhsim08G0050500	3.294512034	1.12822E-05	3.60561E-05	RHSIM_Rhsim08G0050500
Root vs Leaf	RHSIM_Rhsim05G0086200	-2.187920917	1.13155E-05	3.61575E-05	RHSIM_Rhsim05G0086200
Root vs Leaf	RHSIM_Rhsim10G0041800	-4.424181464	1.13338E-05	3.6211E-05	RHSIM_Rhsim10G0041800
Root vs Leaf	RHSIM_Rhsim07G0085700	2.358208805	1.13738E-05	3.63338E-05	RHSIM_Rhsim07G0085700
Root vs Leaf	RHSIM_Rhsim02G0140700	-1.116817592	1.14037E-05	3.64242E-05	RHSIM_Rhsim02G0140700
Root vs Leaf	RHSIM_Rhsim06G0156900	7.874345116	1.14162E-05	3.64589E-05	RHSIM_Rhsim06G0156900
Root vs Leaf	RHSIM_Rhsim08G0189800	4.988759282	1.14825E-05	3.66557E-05	RHSIM_Rhsim08G0189800
Root vs Leaf	RHSIM_Rhsim06G0000700	1.047862161	1.14955E-05	3.66921E-05	RHSIM_Rhsim06G0000700
Root vs Leaf	RHSIM_Rhsim03G0214800	1.027308357	1.15105E-05	3.67349E-05	RHSIM_Rhsim03G0214800

Comparison	Gene ID	log_2FC	P-value	FDR (padj)	Gene Name
Root vs Leaf	RHSIM_Rhsim10G0034600	-1.229488229	1.16223E-05	3.70762E-05	RHSIM_Rhsim10G0034600
Root vs Leaf	RHSIM_Rhsim07G0012300	6.815844928	1.16466E-05	3.71488E-05	RHSIM_Rhsim07G0012300
Root vs Leaf	novel.1962	1.917010866	1.16623E-05	3.71936E-05	--
Root vs Leaf	RHSIM_Rhsim09G0163400	1.35575919	1.16781E-05	3.72391E-05	RHSIM_Rhsim09G0163400
Root vs Leaf	RHSIM_Rhsim08G0050400	-1.541632725	1.16824E-05	3.72474E-05	RHSIM_Rhsim08G0050400
Root vs Leaf	RHSIM_Rhsim03G0200400	3.727384943	1.17468E-05	3.74477E-05	RHSIM_Rhsim03G0200400
Root vs Leaf	RHSIM_RhsimUnG0070500	3.311828175	1.17737E-05	3.75282E-05	RHSIM_RhsimUnG0070500
Root vs Leaf	RHSIM_Rhsim06G0181400	-2.74047369	1.18321E-05	3.77093E-05	RHSIM_Rhsim06G0181400
Root vs Leaf	RHSIM_Rhsim12G0166600	-1.276113941	1.18345E-05	3.77118E-05	RHSIM_Rhsim12G0166600
Root vs Leaf	RHSIM_Rhsim13G0155300	-1.411776779	1.18572E-05	3.77788E-05	RHSIM_Rhsim13G0155300
Root vs Leaf	novel.2498	6.1597205	1.1863E-05	3.7792E-05	--
Root vs Leaf	RHSIM_Rhsim03G0226800	1.016273243	1.18721E-05	3.78102E-05	RHSIM_Rhsim03G0226800
Root vs Leaf	RHSIM_RhsimUnG0128600	4.367630781	1.18747E-05	3.78102E-05	RHSIM_RhsimUnG0128600
Root vs Leaf	RHSIM_Rhsim09G0032200	-1.472957729	1.18781E-05	3.78142E-05	RHSIM_Rhsim09G0032200
Root vs Leaf	novel.4618	-1.789736449	1.19114E-05	3.79098E-05	--
Root vs Leaf	RHSIM_Rhsim13G0123700	2.530989679	1.19141E-05	3.79131E-05	RHSIM_Rhsim13G0123700
Root vs Leaf	novel.5568	-6.785321425	1.19391E-05	3.79876E-05	--
Root vs Leaf	RHSIM_Rhsim10G0173500	1.289318331	1.19887E-05	3.81402E-05	RHSIM_Rhsim10G0173500
Root vs Leaf	novel.339	-1.847170002	1.1993E-05	3.81484E-05	--
Root vs Leaf	RHSIM_Rhsim02G0026400	-3.464036852	1.21114E-05	3.85145E-05	RHSIM_Rhsim02G0026400
Root vs Leaf	RHSIM_Rhsim11G0138700	-4.099185543	1.21184E-05	3.85313E-05	RHSIM_Rhsim11G0138700
Root vs Leaf	RHSIM_Rhsim08G0241000	1.818915375	1.21649E-05	3.86739E-05	RHSIM_Rhsim08G0241000
Root vs Leaf	RHSIM_Rhsim11G0058700	1.160013956	1.21703E-05	3.86807E-05	RHSIM_Rhsim11G0058700
Root vs Leaf	RHSIM_Rhsim05G0078400	1.8766633	1.21934E-05	3.87487E-05	RHSIM_Rhsim05G0078400
Root vs Leaf	RHSIM_Rhsim05G0213800	1.54342764	1.2289E-05	3.90362E-05	RHSIM_Rhsim05G0213800

Comparison	Gene ID	log_2FC	P-value	FDR (padj)	Gene Name
Root vs Leaf	RHSIM_RhsimUnG0212900	1.855323857	1.23059E-05	3.90848E-05	RHSIM_RhsimUnG0212900
Root vs Leaf	novel.4217	4.163740378	1.23222E-05	3.91257E-05	--
Root vs Leaf	RHSIM_Rhsim06G0051500	-2.41335464	1.23447E-05	3.91917E-05	RHSIM_Rhsim06G0051500
Root vs Leaf	RHSIM_Rhsim11G0195600	1.70577986	1.23678E-05	3.9249E-05	RHSIM_Rhsim11G0195600
Root vs Leaf	RHSIM_Rhsim12G0054800	-1.386078056	1.24521E-05	3.95055E-05	RHSIM_Rhsim12G0054800
Root vs Leaf	RHSIM_Rhsim02G0135700	3.893615844	1.24596E-05	3.95241E-05	RHSIM_Rhsim02G0135700
Root vs Leaf	novel.3135	-1.097488543	1.25619E-05	3.98376E-05	--
Root vs Leaf	RHSIM_RhsimUnG0241400	1.013370338	1.25703E-05	3.98588E-05	RHSIM_RhsimUnG0241400
Root vs Leaf	novel.1247	-1.508632107	1.25835E-05	3.98951E-05	--
Root vs Leaf	RHSIM_Rhsim11G0044600	1.251744051	1.26537E-05	4.01122E-05	RHSIM_Rhsim11G0044600
Root vs Leaf	RHSIM_Rhsim02G0024200	-1.753031354	1.26668E-05	4.01481E-05	RHSIM_Rhsim02G0024200
Root vs Leaf	RHSIM_Rhsim10G0011500	1.022610319	1.27326E-05	4.03456E-05	RHSIM_Rhsim10G0011500
Root vs Leaf	RHSIM_Rhsim09G0203200	1.280281828	1.27543E-05	4.04091E-05	RHSIM_Rhsim09G0203200
Root vs Leaf	RHSIM_Rhsim01G0102500	-1.104953041	1.27626E-05	4.04296E-05	RHSIM_Rhsim01G0102500
Root vs Leaf	RHSIM_Rhsim03G0065700	-1.432554979	1.28067E-05	4.05639E-05	RHSIM_Rhsim03G0065700
Root vs Leaf	RHSIM_Rhsim12G0169900	7.149912367	1.29227E-05	4.09201E-05	RHSIM_Rhsim12G0169900
Root vs Leaf	RHSIM_Rhsim03G0173100	2.140108611	1.29529E-05	4.101E-05	RHSIM_Rhsim03G0173100
Root vs Leaf	RHSIM_Rhsim13G0011200	2.143580893	1.29907E-05	4.11242E-05	RHSIM_Rhsim13G0011200
Root vs Leaf	RHSIM_RhsimUnG0042100	-1.198682002	1.30709E-05	4.13666E-05	RHSIM_RhsimUnG0042100
Root vs Leaf	RHSIM_Rhsim04G0112000	3.401080695	1.30784E-05	4.13847E-05	RHSIM_Rhsim04G0112000
Root vs Leaf	RHSIM_Rhsim13G0088000	-2.153046726	1.30913E-05	4.14199E-05	RHSIM_Rhsim13G0088000
Root vs Leaf	RHSIM_Rhsim10G0115900	1.157551149	1.31006E-05	4.14436E-05	RHSIM_Rhsim10G0115900
Root vs Leaf	RHSIM_Rhsim08G0228000	-1.91144069	1.32022E-05	4.17537E-05	RHSIM_Rhsim08G0228000
Root vs Leaf	RHSIM_Rhsim01G0020900	1.422531264	1.33231E-05	4.2119E-05	RHSIM_Rhsim01G0020900
Root vs Leaf	RHSIM_Rhsim13G0040300	-1.534545962	1.3334E-05	4.21475E-05	RHSIM_Rhsim13G0040300

Comparison	Gene ID	log_2FC	P-value	FDR (padj)	Gene Name
Root vs Leaf	RHSIM_Rhsim13G0012700	5.531143021	1.34264E-05	4.2428E-05	RHSIM_Rhsim13G0012700
Root vs Leaf	RHSIM_Rhsim13G0212700	1.444933463	1.34373E-05	4.24567E-05	RHSIM_Rhsim13G0212700
Root vs Leaf	RHSIM_Rhsim09G0153900	2.047714672	1.36108E-05	4.29988E-05	RHSIM_Rhsim09G0153900
Root vs Leaf	RHSIM_Rhsim07G0136900	2.522829861	1.3626E-05	4.30292E-05	RHSIM_Rhsim07G0136900
Root vs Leaf	RHSIM_Rhsim13G0228700	-3.566579754	1.37356E-05	4.33695E-05	RHSIM_Rhsim13G0228700
Root vs Leaf	RHSIM_Rhsim08G0042100	-1.768428227	1.38455E-05	4.37048E-05	RHSIM_Rhsim08G0042100
Root vs Leaf	RHSIM_Rhsim12G0148600	-1.683372137	1.38563E-05	4.37327E-05	RHSIM_Rhsim12G0148600
Root vs Leaf	RHSIM_Rhsim06G0235100	4.096301113	1.39033E-05	4.38691E-05	RHSIM_Rhsim06G0235100
Root vs Leaf	RHSIM_Rhsim07G0173700	1.075795822	1.39779E-05	4.40986E-05	RHSIM_Rhsim07G0173700
Root vs Leaf	RHSIM_Rhsim01G0041200	2.533211316	1.40018E-05	4.4168E-05	RHSIM_Rhsim01G0041200
Root vs Leaf	RHSIM_Rhsim03G0262900	1.033162149	1.40688E-05	4.43732E-05	RHSIM_Rhsim03G0262900
Root vs Leaf	RHSIM_Rhsim03G0072900	-2.030762017	1.40707E-05	4.43732E-05	RHSIM_Rhsim03G0072900
Root vs Leaf	RHSIM_Rhsim11G0162900	4.145819998	1.41131E-05	4.45007E-05	RHSIM_Rhsim11G0162900
Root vs Leaf	RHSIM_Rhsim10G0156400	1.844522725	1.41504E-05	4.46122E-05	RHSIM_Rhsim10G0156400
Root vs Leaf	novel.735	2.600166278	1.42304E-05	4.48524E-05	--
Root vs Leaf	RHSIM_Rhsim02G0032900	-5.740380146	1.42444E-05	4.48902E-05	RHSIM_Rhsim02G0032900
Root vs Leaf	RHSIM_Rhsim04G0150400	1.47149709	1.43042E-05	4.50728E-05	RHSIM_Rhsim04G0150400
Root vs Leaf	RHSIM_Rhsim02G0187400	2.321196946	1.44531E-05	4.55355E-05	RHSIM_Rhsim02G0187400
Root vs Leaf	RHSIM_Rhsim07G0030000	-1.571300254	1.4456E-05	4.55387E-05	RHSIM_Rhsim07G0030000
Root vs Leaf	RHSIM_Rhsim11G0138500	-2.868705044	1.44649E-05	4.55605E-05	RHSIM_Rhsim11G0138500
Root vs Leaf	RHSIM_RhsimUnG0198000	4.318230093	1.44897E-05	4.56322E-05	RHSIM_RhsimUnG0198000
Root vs Leaf	RHSIM_Rhsim06G0195000	-4.233819986	1.45223E-05	4.57226E-05	RHSIM_Rhsim06G0195000
Root vs Leaf	RHSIM_Rhsim04G0203400	-2.803818697	1.45721E-05	4.58732E-05	RHSIM_Rhsim04G0203400
Root vs Leaf	RHSIM_Rhsim03G0157300	-7.06660296	1.46099E-05	4.59857E-05	RHSIM_Rhsim03G0157300
Root vs Leaf	novel.5790	-7.300273465	1.46229E-05	4.60206E-05	--

Comparison	Gene ID	log_2FC	P-value	FDR (padj)	Gene Name
Root vs Leaf	RHSIM_Rhsim08G0080600	-1.042826199	1.46921E-05	4.62068E-05	RHSIM_Rhsim08G0080600
Root vs Leaf	RHSIM_Rhsim07G0222200	6.480485705	1.47153E-05	4.62735E-05	RHSIM_Rhsim07G0222200
Root vs Leaf	novel.4536	1.141516128	1.47269E-05	4.63038E-05	--
Root vs Leaf	RHSIM_Rhsim10G0190000	-3.301508988	1.48285E-05	4.66106E-05	RHSIM_Rhsim10G0190000
Root vs Leaf	RHSIM_Rhsim12G0177900	-1.150519721	1.50592E-05	4.73098E-05	RHSIM_Rhsim12G0177900
Root vs Leaf	RHSIM_Rhsim07G0254100	-5.275330134	1.50935E-05	4.74112E-05	RHSIM_Rhsim07G0254100
Root vs Leaf	novel.2459	-1.217472105	1.51343E-05	4.75264E-05	--
Root vs Leaf	novel.2145	-2.434552965	1.51976E-05	4.77122E-05	--
Root vs Leaf	RHSIM_Rhsim07G0099800	1.799949868	1.52066E-05	4.7734E-05	RHSIM_Rhsim07G0099800
Root vs Leaf	RHSIM_Rhsim11G0056100	1.675406573	1.52255E-05	4.77846E-05	RHSIM_Rhsim11G0056100
Root vs Leaf	RHSIM_Rhsim02G0030000	2.845596829	1.52446E-05	4.78339E-05	RHSIM_Rhsim02G0030000
Root vs Leaf	RHSIM_Rhsim02G0086400	3.099702214	1.5283E-05	4.79479E-05	RHSIM_Rhsim02G0086400
Root vs Leaf	novel.5312	7.903371594	1.53286E-05	4.80846E-05	--
Root vs Leaf	novel.5907	7.171280336	1.53371E-05	4.81046E-05	--
Root vs Leaf	RHSIM_Rhsim03G0119000	6.022827438	1.54056E-05	4.82932E-05	RHSIM_Rhsim03G0119000
Root vs Leaf	RHSIM_Rhsim02G0015900	-4.562625745	1.55144E-05	4.86276E-05	RHSIM_Rhsim02G0015900
Root vs Leaf	RHSIM_Rhsim08G0234500	-1.558537136	1.55275E-05	4.86622E-05	RHSIM_Rhsim08G0234500
Root vs Leaf	RHSIM_Rhsim02G0086500	-2.613962055	1.55517E-05	4.87315E-05	RHSIM_Rhsim02G0086500
Root vs Leaf	RHSIM_Rhsim04G0052500	3.173305959	1.55828E-05	4.88224E-05	RHSIM_Rhsim04G0052500
Root vs Leaf	RHSIM_Rhsim08G0027400	-1.013674711	1.55928E-05	4.88469E-05	RHSIM_Rhsim08G0027400
Root vs Leaf	RHSIM_Rhsim05G0092500	-2.700041192	1.56615E-05	4.90556E-05	RHSIM_Rhsim05G0092500
Root vs Leaf	novel.4904	-1.391936711	1.56765E-05	4.90893E-05	--
Root vs Leaf	RHSIM_Rhsim06G0161500	2.643728473	1.57124E-05	4.91951E-05	RHSIM_Rhsim06G0161500
Root vs Leaf	RHSIM_Rhsim10G0094100	6.002529999	1.5759E-05	4.93276E-05	RHSIM_Rhsim10G0094100
Root vs Leaf	RHSIM_RhsimUnG0003500	3.938385612	1.57903E-05	4.94188E-05	RHSIM_RhsimUnG0003500

Comparison	Gene ID	log$_2$FC	P-value	FDR (padj)	Gene Name
Root vs Leaf	RHSIM_Rhsim02G0118200	1.086700943	1.58816E-05	4.96979E-05	RHSIM_Rhsim02G0118200
Root vs Leaf	RHSIM_Rhsim01G0246000	-3.271512162	1.59063E-05	4.97683E-05	RHSIM_Rhsim01G0246000
Root vs Leaf	RHSIM_Rhsim07G0197600	-2.500319554	1.5911E-05	4.97764E-05	RHSIM_Rhsim07G0197600
Root vs Leaf	RHSIM_Rhsim13G0168100	-4.273775373	1.59407E-05	4.98626E-05	RHSIM_Rhsim13G0168100
Root vs Leaf	RHSIM_Rhsim04G0173000	1.545521961	1.59433E-05	4.98641E-05	RHSIM_Rhsim04G0173000
Root vs Leaf	RHSIM_Rhsim11G0048800	1.071730271	1.60099E-05	5.00587E-05	RHSIM_Rhsim11G0048800
Root vs Leaf	RHSIM_Rhsim02G0161500	-6.372049419	1.6014E-05	5.00647E-05	RHSIM_Rhsim02G0161500
Root vs Leaf	RHSIM_Rhsim07G0227900	2.157709316	1.61053E-05	5.03435E-05	RHSIM_Rhsim07G0227900
Root vs Leaf	RHSIM_Rhsim01G0004700	-1.338106392	1.61374E-05	5.0437E-05	RHSIM_Rhsim01G0004700
Root vs Leaf	RHSIM_RhsimUnG0155600	-5.497460155	1.62074E-05	5.06489E-05	RHSIM_RhsimUnG0155600
Root vs Leaf	RHSIM_Rhsim08G0148400	3.920865379	1.62605E-05	5.08079E-05	RHSIM_Rhsim08G0148400
Root vs Leaf	RHSIM_Rhsim13G0158800	6.210981338	1.62692E-05	5.08283E-05	RHSIM_Rhsim13G0158800
Root vs Leaf	RHSIM_Rhsim01G0256200	1.184312019	1.62786E-05	5.08507E-05	RHSIM_Rhsim01G0256200
Root vs Leaf	RHSIM_Rhsim06G0184300	1.51092848	1.63488E-05	5.10564E-05	RHSIM_Rhsim06G0184300
Root vs Leaf	RHSIM_Rhsim10G0162700	1.418407814	1.63811E-05	5.11502E-05	RHSIM_Rhsim10G0162700
Root vs Leaf	RHSIM_Rhsim09G0037500	-1.117254433	1.6422E-05	5.12711E-05	RHSIM_Rhsim09G0037500
Root vs Leaf	RHSIM_Rhsim10G0129000	-3.311986885	1.65494E-05	5.16549E-05	RHSIM_Rhsim10G0129000
Root vs Leaf	RHSIM_Rhsim01G0108200	-3.498439983	1.65783E-05	5.17381E-05	RHSIM_Rhsim01G0108200
Root vs Leaf	novel.5095	6.956653472	1.66395E-05	5.19155E-05	--
Root vs Leaf	RHSIM_Rhsim06G0111400	-8.03959088	1.66453E-05	5.19262E-05	RHSIM_Rhsim06G0111400
Root vs Leaf	RHSIM_Rhsim04G0167800	-1.362082501	1.66604E-05	5.19663E-05	RHSIM_Rhsim04G0167800
Root vs Leaf	RHSIM_Rhsim05G0006900	3.779764101	1.67831E-05	5.23421E-05	RHSIM_Rhsim05G0006900
Root vs Leaf	RHSIM_Rhsim07G0184300	1.640190345	1.68139E-05	5.24309E-05	RHSIM_Rhsim07G0184300
Root vs Leaf	novel.3562	7.313255506	1.68292E-05	5.24647E-05	--
Root vs Leaf	RHSIM_Rhsim13G0175600	3.25367068	1.69154E-05	5.27248E-05	RHSIM_Rhsim13G0175600

Comparison	Gene ID	log_2FC	P-value	FDR (padj)	Gene Name
Root vs Leaf	RHSIM_Rhsim13G0071000	1.325267333	1.69172E-05	5.27248E-05	RHSIM_Rhsim13G0071000
Root vs Leaf	novel.2999	6.393595615	1.72394E-05	5.37218E-05	--
Root vs Leaf	RHSIM_Rhsim13G0067800	1.563136163	1.73277E-05	5.39679E-05	RHSIM_Rhsim13G0067800
Root vs Leaf	RHSIM_Rhsim01G0281800	-4.153363523	1.7394E-05	5.41598E-05	RHSIM_Rhsim01G0281800
Root vs Leaf	RHSIM_Rhsim05G0040800	-1.096217852	1.74499E-05	5.43134E-05	RHSIM_Rhsim05G0040800
Root vs Leaf	RHSIM_Rhsim07G0253500	1.862817925	1.74504E-05	5.43134E-05	RHSIM_Rhsim07G0253500
Root vs Leaf	RHSIM_Rhsim01G0145000	-1.317569425	1.76101E-05	5.4803E-05	RHSIM_Rhsim01G0145000
Root vs Leaf	RHSIM_Rhsim07G0005100	-1.020195978	1.76524E-05	5.49272E-05	RHSIM_Rhsim07G0005100
Root vs Leaf	novel.5912	1.012160262	1.76697E-05	5.49738E-05	--
Root vs Leaf	RHSIM_Rhsim09G0183800	-3.150574707	1.77153E-05	5.51082E-05	RHSIM_Rhsim09G0183800
Root vs Leaf	RHSIM_RhsimUnG0239400	-1.50681958	1.7726E-05	5.51341E-05	RHSIM_RhsimUnG0239400
Root vs Leaf	RHSIM_Rhsim02G0065100	-3.838867855	1.77489E-05	5.51905E-05	RHSIM_Rhsim02G0065100
Root vs Leaf	novel.2228	-5.375709697	1.77756E-05	5.5266E-05	--
Root vs Leaf	RHSIM_Rhsim02G0097800	3.239323636	1.78131E-05	5.53752E-05	RHSIM_Rhsim02G0097800
Root vs Leaf	novel.5519	-6.291551801	1.7882E-05	5.55821E-05	--
Root vs Leaf	RHSIM_Rhsim11G0154000	5.139984929	1.79369E-05	5.57376E-05	RHSIM_Rhsim11G0154000
Root vs Leaf	RHSIM_Rhsim12G0128800	1.096889741	1.80939E-05	5.62102E-05	RHSIM_Rhsim12G0128800
Root vs Leaf	RHSIM_Rhsim03G0132900	1.015961306	1.82726E-05	5.6758E-05	RHSIM_Rhsim03G0132900
Root vs Leaf	RHSIM_Rhsim04G0006900	-1.052198538	1.83435E-05	5.69705E-05	RHSIM_Rhsim04G0006900
Root vs Leaf	novel.3074	-1.739694487	1.84142E-05	5.71762E-05	--
Root vs Leaf	RHSIM_Rhsim01G0086800	-1.342675223	1.84147E-05	5.71762E-05	RHSIM_Rhsim01G0086800
Root vs Leaf	RHSIM_Rhsim04G0070800	-3.518777369	1.84855E-05	5.73884E-05	RHSIM_Rhsim04G0070800
Root vs Leaf	RHSIM_Rhsim05G0160100	1.068756816	1.8593E-05	5.77003E-05	RHSIM_Rhsim05G0160100
Root vs Leaf	novel.5736	-1.175516213	1.85934E-05	5.77003E-05	--
Root vs Leaf	RHSIM_Rhsim09G0027500	1.20411465	1.86475E-05	5.78602E-05	RHSIM_Rhsim09G0027500

Comparison	Gene ID	log_2FC	P-value	FDR (padj)	Gene Name
Root vs Leaf	RHSIM_Rhsim01G0216800	2.767337876	1.86737E-05	5.79339E-05	RHSIM_Rhsim01G0216800
Root vs Leaf	RHSIM_Rhsim09G0102900	1.738556039	1.877E-05	5.82247E-05	RHSIM_Rhsim09G0102900
Root vs Leaf	RHSIM_Rhsim07G0123400	-1.500656268	1.8823E-05	5.83734E-05	RHSIM_Rhsim07G0123400
Root vs Leaf	RHSIM_Rhsim12G0156400	-1.958486303	1.89429E-05	5.87376E-05	RHSIM_Rhsim12G0156400
Root vs Leaf	RHSIM_Rhsim03G0254000	-1.355971509	1.89547E-05	5.87583E-05	RHSIM_Rhsim03G0254000
Root vs Leaf	RHSIM_Rhsim05G0023400	-3.496807567	1.89719E-05	5.88037E-05	RHSIM_Rhsim05G0023400
Root vs Leaf	RHSIM_RhsimUnG0151100	6.937202533	1.9019E-05	5.8934E-05	RHSIM_RhsimUnG0151100
Root vs Leaf	RHSIM_Rhsim09G0150500	-1.973946784	1.91065E-05	5.91972E-05	RHSIM_Rhsim09G0150500
Root vs Leaf	RHSIM_Rhsim05G0149800	1.550205323	1.91236E-05	5.92421E-05	RHSIM_Rhsim05G0149800
Root vs Leaf	RHSIM_Rhsim13G0197200	-1.291338767	1.93851E-05	6.00282E-05	RHSIM_Rhsim13G0197200
Root vs Leaf	RHSIM_Rhsim07G0072900	1.904178154	1.94076E-05	6.00898E-05	RHSIM_Rhsim07G0072900
Root vs Leaf	RHSIM_Rhsim05G0104100	1.022411662	1.95052E-05	6.03839E-05	RHSIM_Rhsim05G0104100
Root vs Leaf	RHSIM_Rhsim11G0097800	-2.105027179	1.95324E-05	6.04586E-05	RHSIM_Rhsim11G0097800
Root vs Leaf	RHSIM_Rhsim02G0104900	1.239961442	1.95345E-05	6.04586E-05	RHSIM_Rhsim02G0104900
Root vs Leaf	RHSIM_Rhsim05G0081800	2.26295566	1.95486E-05	6.04939E-05	RHSIM_Rhsim05G0081800
Root vs Leaf	RHSIM_Rhsim04G0062700	2.999287762	1.95878E-05	6.06072E-05	RHSIM_Rhsim04G0062700
Root vs Leaf	RHSIM_Rhsim09G0043800	-1.896878809	1.96191E-05	6.06877E-05	RHSIM_Rhsim09G0043800
Root vs Leaf	RHSIM_Rhsim08G0087100	-1.585081483	1.96908E-05	6.08851E-05	RHSIM_Rhsim08G0087100
Root vs Leaf	RHSIM_Rhsim12G0188600	2.032207487	1.97045E-05	6.09194E-05	RHSIM_Rhsim12G0188600
Root vs Leaf	RHSIM_Rhsim12G0087100	3.053723185	1.98265E-05	6.12803E-05	RHSIM_Rhsim12G0087100
Root vs Leaf	RHSIM_RhsimUnG0232000	-1.886386181	1.98607E-05	6.13612E-05	RHSIM_RhsimUnG0232000
Root vs Leaf	RHSIM_RhsimUnG0256600	-2.068603522	1.98794E-05	6.14028E-05	RHSIM_RhsimUnG0256600
Root vs Leaf	RHSIM_Rhsim05G0076700	3.92189481	1.99135E-05	6.14999E-05	RHSIM_Rhsim05G0076700
Root vs Leaf	RHSIM_Rhsim08G0218800	2.866796842	2.00237E-05	6.18318E-05	RHSIM_Rhsim08G0218800
Root vs Leaf	RHSIM_Rhsim01G0281700	-3.721361459	2.00887E-05	6.20243E-05	RHSIM_Rhsim01G0281700

Comparison	Gene ID	log2FC	P-value	FDR (padj)	Gene Name
Root vs Leaf	RHSIM_Rhsim12G0159400	-1.845528096	2.01115E-05	6.20865E-05	RHSIM_Rhsim12G0159400
Root vs Leaf	RHSIM_Rhsim05G0140100	3.690921783	2.01775E-05	6.2282E-05	RHSIM_Rhsim05G0140100
Root vs Leaf	novel.5213	3.548951732	2.02505E-05	6.24953E-05	--
Root vs Leaf	RHSIM_Rhsim11G0110000	6.25071521	2.04071E-05	6.29402E-05	RHSIM_Rhsim11G0110000
Root vs Leaf	RHSIM_Rhsim06G0181600	-1.021332124	2.04247E-05	6.2986E-05	RHSIM_Rhsim06G0181600
Root vs Leaf	RHSIM_Rhsim13G0031200	1.026976018	2.0492E-05	6.31854E-05	RHSIM_Rhsim13G0031200
Root vs Leaf	RHSIM_Rhsim13G0107000	2.407343269	2.05492E-05	6.33531E-05	RHSIM_Rhsim13G0107000
Root vs Leaf	RHSIM_RhsimUnG0041000	1.084243664	2.05971E-05	6.34924E-05	RHSIM_RhsimUnG0041000
Root vs Leaf	RHSIM_Rhsim01G0006000	6.476136288	2.06462E-05	6.36352E-05	RHSIM_Rhsim01G0006000
Root vs Leaf	RHSIM_Rhsim05G0095600	1.195092867	2.06681E-05	6.36944E-05	RHSIM_Rhsim05G0095600
Root vs Leaf	RHSIM_Rhsim08G0027000	1.664361537	2.07551E-05	6.39539E-05	RHSIM_Rhsim08G0027000
Root vs Leaf	RHSIM_Rhsim09G0134900	2.828504336	2.07629E-05	6.39693E-05	RHSIM_Rhsim09G0134900
Root vs Leaf	RHSIM_Rhsim03G0236000	4.232902897	2.08069E-05	6.40966E-05	RHSIM_Rhsim03G0236000
Root vs Leaf	RHSIM_Rhsim07G0216100	-2.667120535	2.08834E-05	6.43237E-05	RHSIM_Rhsim07G0216100
Root vs Leaf	RHSIM_Rhsim04G0009300	3.405022599	2.08869E-05	6.43258E-05	RHSIM_Rhsim04G0009300
Root vs Leaf	RHSIM_Rhsim06G0057900	-1.862089917	2.09444E-05	6.44943E-05	RHSIM_Rhsim06G0057900
Root vs Leaf	RHSIM_Rhsim13G0141400	-1.318257765	2.09664E-05	6.45533E-05	RHSIM_Rhsim13G0141400
Root vs Leaf	RHSIM_Rhsim05G0180500	4.640558431	2.1014E-05	6.46912E-05	RHSIM_Rhsim05G0180500
Root vs Leaf	RHSIM_Rhsim03G0053900	-3.048382619	2.10168E-05	6.46912E-05	RHSIM_Rhsim03G0053900
Root vs Leaf	RHSIM_RhsimUnG0084100	-2.285673532	2.10975E-05	6.49225E-05	RHSIM_RhsimUnG0084100
Root vs Leaf	RHSIM_Rhsim03G0162600	-6.807925119	2.13459E-05	6.56696E-05	RHSIM_Rhsim03G0162600
Root vs Leaf	RHSIM_Rhsim13G0093000	1.255723615	2.14941E-05	6.61165E-05	RHSIM_Rhsim13G0093000
Root vs Leaf	RHSIM_Rhsim08G0009500	2.795822972	2.15321E-05	6.62247E-05	RHSIM_Rhsim08G0009500
Root vs Leaf	RHSIM_Rhsim12G0076200	2.047848139	2.17333E-05	6.67991E-05	RHSIM_Rhsim12G0076200
Root vs Leaf	RHSIM_Rhsim07G0233500	1.041199433	2.17365E-05	6.68001E-05	RHSIM_Rhsim07G0233500

Comparison	Gene ID	log_2FC	P-value	FDR (padj)	Gene Name
Root vs Leaf	novel.999	-4.956302906	2.17488E-05	6.68289E-05	--
Root vs Leaf	RHSIM_Rhsim13G0185500	5.935926964	2.17552E-05	6.68397E-05	RHSIM_Rhsim13G0185500
Root vs Leaf	RHSIM_Rhsim10G0078900	-1.166225383	2.17988E-05	6.69647E-05	RHSIM_Rhsim10G0078900
Root vs Leaf	RHSIM_Rhsim10G0007700	-2.036601513	2.18356E-05	6.70689E-05	RHSIM_Rhsim10G0007700
Root vs Leaf	RHSIM_Rhsim07G0037900	-2.026680366	2.19362E-05	6.73627E-05	RHSIM_Rhsim07G0037900
Root vs Leaf	RHSIM_Rhsim05G0086300	-1.860649461	2.19927E-05	6.75247E-05	RHSIM_Rhsim05G0086300
Root vs Leaf	RHSIM_Rhsim06G0004900	-1.503229724	2.20494E-05	6.76808E-05	RHSIM_Rhsim06G0004900
Root vs Leaf	RHSIM_RhsimUnG0169200	-2.572821106	2.20569E-05	6.76949E-05	RHSIM_RhsimUnG0169200
Root vs Leaf	RHSIM_Rhsim07G0141500	-1.90003065	2.21644E-05	6.79923E-05	RHSIM_Rhsim07G0141500
Root vs Leaf	novel.3182	-7.717561832	2.21656E-05	6.79923E-05	--
Root vs Leaf	RHSIM_Rhsim04G0189100	3.767723596	2.21983E-05	6.80837E-05	RHSIM_Rhsim04G0189100
Root vs Leaf	RHSIM_Rhsim02G0186100	-3.085380894	2.2328E-05	6.84359E-05	RHSIM_Rhsim02G0186100
Root vs Leaf	novel.3056	5.463380232	2.23335E-05	6.84438E-05	--
Root vs Leaf	novel.589	2.506058199	2.23613E-05	6.85199E-05	--
Root vs Leaf	RHSIM_Rhsim05G0201200	3.325354311	2.24171E-05	6.86728E-05	RHSIM_Rhsim05G0201200
Root vs Leaf	RHSIM_Rhsim08G0197900	4.778836865	2.24837E-05	6.88586E-05	RHSIM_Rhsim08G0197900
Root vs Leaf	RHSIM_Rhsim09G0104100	2.143306739	2.25292E-05	6.89888E-05	RHSIM_Rhsim09G0104100
Root vs Leaf	RHSIM_Rhsim05G0004500	7.306343988	2.2617E-05	6.92432E-05	RHSIM_Rhsim05G0004500
Root vs Leaf	RHSIM_Rhsim05G0050400	6.781257798	2.26183E-05	6.92432E-05	RHSIM_Rhsim05G0050400
Root vs Leaf	RHSIM_Rhsim07G0105700	2.426976808	2.26282E-05	6.9257E-05	RHSIM_Rhsim07G0105700
Root vs Leaf	RHSIM_Rhsim09G0174700	-10.74969704	2.26558E-05	6.93305E-05	RHSIM_Rhsim09G0174700
Root vs Leaf	RHSIM_Rhsim13G0227900	-1.291326444	2.29337E-05	7.01532E-05	RHSIM_Rhsim13G0227900
Root vs Leaf	RHSIM_Rhsim04G0115900	-1.359015655	2.29396E-05	7.01621E-05	RHSIM_Rhsim04G0115900
Root vs Leaf	novel.5254	2.791278298	2.29656E-05	7.02323E-05	--
Root vs Leaf	RHSIM_Rhsim09G0123900	-3.552741835	2.30751E-05	7.0539E-05	RHSIM_Rhsim09G0123900

Comparison	Gene ID	log_2FC	P-value	FDR (padj)	Gene Name
Root vs Leaf	RHSIM_Rhsim12G0168400	2.348873737	2.31158E-05	7.06543E-05	RHSIM_Rhsim12G0168400
Root vs Leaf	RHSIM_Rhsim02G0146500	1.068210953	2.31253E-05	7.06654E-05	RHSIM_Rhsim02G0146500
Root vs Leaf	RHSIM_Rhsim01G0280800	-1.999396851	2.32015E-05	7.08881E-05	RHSIM_Rhsim01G0280800
Root vs Leaf	RHSIM_Rhsim11G0093200	-2.561988988	2.33916E-05	7.14218E-05	RHSIM_Rhsim11G0093200
Root vs Leaf	RHSIM_Rhsim05G0041000	-3.495649085	2.34303E-05	7.15304E-05	RHSIM_Rhsim05G0041000
Root vs Leaf	RHSIM_Rhsim11G0174700	2.474176153	2.34638E-05	7.16232E-05	RHSIM_Rhsim11G0174700
Root vs Leaf	RHSIM_Rhsim03G0169700	-1.316492557	2.35517E-05	7.1882E-05	RHSIM_Rhsim03G0169700
Root vs Leaf	RHSIM_Rhsim13G0184500	-5.540834502	2.36122E-05	7.20572E-05	RHSIM_Rhsim13G0184500
Root vs Leaf	RHSIM_Rhsim01G0235200	-1.15374587	2.36529E-05	7.2172E-05	RHSIM_Rhsim01G0235200
Root vs Leaf	RHSIM_RhsimUnG0192700	-1.191134374	2.36612E-05	7.21877E-05	RHSIM_RhsimUnG0192700
Root vs Leaf	novel.4839	4.804944106	2.3699E-05	7.22706E-05	--
Root vs Leaf	novel.1135	1.934258316	2.37009E-05	7.22706E-05	--
Root vs Leaf	RHSIM_Rhsim06G0169000	7.175880164	2.37274E-05	7.2342E-05	RHSIM_Rhsim06G0169000
Root vs Leaf	novel.4201	5.312323672	2.38757E-05	7.27846E-05	--
Root vs Leaf	RHSIM_Rhsim07G0072800	2.757684202	2.38806E-05	7.27898E-05	RHSIM_Rhsim07G0072800
Root vs Leaf	RHSIM_Rhsim03G0070900	2.233803992	2.39183E-05	7.28951E-05	RHSIM_Rhsim03G0070900
Root vs Leaf	RHSIM_Rhsim01G0249300	1.10011466	2.39609E-05	7.30154E-05	RHSIM_Rhsim01G0249300
Root vs Leaf	RHSIM_Rhsim02G0089600	-1.788517705	2.39651E-05	7.30186E-05	RHSIM_Rhsim02G0089600
Root vs Leaf	RHSIM_Rhsim02G0179600	1.217513358	2.40781E-05	7.33534E-05	RHSIM_Rhsim02G0179600
Root vs Leaf	novel.5313	-3.234604973	2.41321E-05	7.35082E-05	--
Root vs Leaf	RHSIM_Rhsim09G0167700	4.402371294	2.41589E-05	7.35705E-05	RHSIM_Rhsim09G0167700
Root vs Leaf	RHSIM_Rhsim02G0012400	-3.173263854	2.42783E-05	7.39047E-05	RHSIM_Rhsim02G0012400
Root vs Leaf	RHSIM_Rhsim06G0126000	-2.2080665	2.43541E-05	7.41257E-05	RHSIM_Rhsim06G0126000
Root vs Leaf	RHSIM_Rhsim07G0092800	7.328045529	2.43837E-05	7.4206E-05	RHSIM_Rhsim07G0092800
Root vs Leaf	RHSIM_RhsimUnG0145200	1.397541797	2.45653E-05	7.47489E-05	RHSIM_RhsimUnG0145200

Comparison	Gene ID	log_2FC	P-value	FDR (padj)	Gene Name
Root vs Leaf	RHSIM_Rhsim13G0216900	1.050247054	2.46236E-05	7.49165E-05	RHSIM_Rhsim13G0216900
Root vs Leaf	RHSIM_Rhsim06G0176600	3.308808087	2.46351E-05	7.49417E-05	RHSIM_Rhsim06G0176600
Root vs Leaf	RHSIM_Rhsim06G0244500	4.273219851	2.46886E-05	7.50944E-05	RHSIM_Rhsim06G0244500
Root vs Leaf	novel.833	-3.089900682	2.47231E-05	7.51897E-05	--
Root vs Leaf	RHSIM_Rhsim03G0031100	1.161910799	2.47887E-05	7.53692E-05	RHSIM_Rhsim03G0031100
Root vs Leaf	RHSIM_Rhsim11G0011700	1.076737683	2.48112E-05	7.54278E-05	RHSIM_Rhsim11G0011700
Root vs Leaf	novel.4032	-7.303629254	2.49557E-05	7.58472E-05	--
Root vs Leaf	novel.2682	5.962071304	2.49614E-05	7.58546E-05	--
Root vs Leaf	RHSIM_Rhsim02G0256000	-1.381944204	2.50891E-05	7.62327E-05	RHSIM_Rhsim02G0256000
Root vs Leaf	RHSIM_Rhsim06G0108400	1.439669816	2.51574E-05	7.64301E-05	RHSIM_Rhsim06G0108400
Root vs Leaf	RHSIM_Rhsim06G0167000	-2.587404159	2.51745E-05	7.64718E-05	RHSIM_Rhsim06G0167000
Root vs Leaf	RHSIM_Rhsim05G0022500	-2.162562881	2.53382E-05	7.69389E-05	RHSIM_Rhsim05G0022500
Root vs Leaf	RHSIM_Rhsim03G0096800	-1.105353957	2.54968E-05	7.74002E-05	RHSIM_Rhsim03G0096800
Root vs Leaf	novel.3532	-5.609168099	2.55658E-05	7.75805E-05	--
Root vs Leaf	novel.4109	3.118019441	2.55696E-05	7.75805E-05	--
Root vs Leaf	RHSIM_Rhsim08G0178400	3.400345631	2.56124E-05	7.77001E-05	RHSIM_Rhsim08G0178400
Root vs Leaf	RHSIM_Rhsim12G0111800	1.066203893	2.58018E-05	7.82541E-05	RHSIM_Rhsim12G0111800
Root vs Leaf	RHSIM_Rhsim02G0054500	3.987483866	2.58199E-05	7.82989E-05	RHSIM_Rhsim02G0054500
Root vs Leaf	RHSIM_Rhsim07G0145300	1.243016317	2.6007E-05	7.88559E-05	RHSIM_Rhsim07G0145300
Root vs Leaf	novel.3116	-1.341897878	2.60605E-05	7.89975E-05	--
Root vs Leaf	RHSIM_Rhsim12G0199200	5.586263238	2.61432E-05	7.92273E-05	RHSIM_Rhsim12G0199200
Root vs Leaf	RHSIM_Rhsim10G0024600	1.793867638	2.62082E-05	7.94138E-05	RHSIM_Rhsim10G0024600
Root vs Leaf	RHSIM_Rhsim02G0254000	1.3703937	2.62571E-05	7.9531E-05	RHSIM_Rhsim02G0254000
Root vs Leaf	RHSIM_Rhsim03G0233200	-1.228393201	2.62683E-05	7.95543E-05	RHSIM_Rhsim03G0233200
Root vs Leaf	RHSIM_Rhsim01G0264600	-3.287738407	2.63646E-05	7.98355E-05	RHSIM_Rhsim01G0264600

Comparison	Gene ID	log_2FC	P-value	FDR (padj)	Gene Name
Root vs Leaf	RHSIM_Rhsim06G0069700	4.424522825	2.65215E-05	8.02897E-05	RHSIM_Rhsim06G0069700
Root vs Leaf	RHSIM_Rhsim04G0208400	1.377535166	2.66116E-05	8.05414E-05	RHSIM_Rhsim04G0208400
Root vs Leaf	novel.5771	-1.171183081	2.66493E-05	8.06449E-05	--
Root vs Leaf	RHSIM_Rhsim05G0192300	1.052758788	2.67119E-05	8.08131E-05	RHSIM_Rhsim05G0192300
Root vs Leaf	RHSIM_RhsimUnG0200200	-7.115525237	2.67399E-05	8.08768E-05	RHSIM_RhsimUnG0200200
Root vs Leaf	RHSIM_Rhsim07G0018300	1.322355315	2.6756E-05	8.09149E-05	RHSIM_Rhsim07G0018300
Root vs Leaf	novel.4497	4.408310133	2.6853E-05	8.11871E-05	--
Root vs Leaf	RHSIM_Rhsim10G0028800	-1.510419542	2.70424E-05	8.1749E-05	RHSIM_Rhsim10G0028800
Root vs Leaf	RHSIM_Rhsim07G0121500	-2.576217632	2.7103E-05	8.19107E-05	RHSIM_Rhsim07G0121500
Root vs Leaf	novel.464	1.050772501	2.71372E-05	8.19891E-05	--
Root vs Leaf	novel.60	-2.866075615	2.72172E-05	8.22021E-05	--
Root vs Leaf	RHSIM_Rhsim11G0071600	-1.546777486	2.72416E-05	8.22651E-05	RHSIM_Rhsim11G0071600
Root vs Leaf	novel.2915	-1.55713563	2.72536E-05	8.22906E-05	--
Root vs Leaf	RHSIM_Rhsim07G0254200	-3.948874062	2.73013E-05	8.24238E-05	RHSIM_Rhsim07G0254200
Root vs Leaf	RHSIM_Rhsim03G0124400	6.790728985	2.73058E-05	8.24266E-05	RHSIM_Rhsim03G0124400
Root vs Leaf	RHSIM_Rhsim01G0258800	-1.255420741	2.75579E-05	8.31662E-05	RHSIM_Rhsim01G0258800
Root vs Leaf	RHSIM_Rhsim08G0163100	1.183573988	2.75919E-05	8.32577E-05	RHSIM_Rhsim08G0163100
Root vs Leaf	RHSIM_Rhsim10G0029800	3.055448183	2.76688E-05	8.34791E-05	RHSIM_Rhsim10G0029800
Root vs Leaf	novel.2608	-7.805359743	2.76902E-05	8.35326E-05	--
Root vs Leaf	RHSIM_Rhsim01G0207900	-6.114753291	2.78417E-05	8.39677E-05	RHSIM_Rhsim01G0207900
Root vs Leaf	RHSIM_Rhsim04G0098500	1.877951133	2.79718E-05	8.43489E-05	RHSIM_Rhsim04G0098500
Root vs Leaf	RHSIM_Rhsim02G0181500	3.160695176	2.79754E-05	8.43489E-05	RHSIM_Rhsim02G0181500
Root vs Leaf	RHSIM_Rhsim03G0188100	1.023984185	2.81321E-05	8.47884E-05	RHSIM_Rhsim03G0188100
Root vs Leaf	RHSIM_Rhsim01G0235000	-1.822185615	2.82097E-05	8.50001E-05	RHSIM_Rhsim01G0235000
Root vs Leaf	RHSIM_RhsimUnG0100900	6.099994434	2.82246E-05	8.5034E-05	RHSIM_RhsimUnG0100900

Comparison	Gene ID	log_2FC	P-value	FDR (padj)	Gene Name
Root vs Leaf	RHSIM_Rhsim05G0128200	-1.489428019	2.82289E-05	8.50358E-05	RHSIM_Rhsim05G0128200
Root vs Leaf	RHSIM_Rhsim11G0121500	1.442978586	2.83309E-05	8.53209E-05	RHSIM_Rhsim11G0121500
Root vs Leaf	RHSIM_Rhsim04G0131800	7.284290206	2.83556E-05	8.53841E-05	RHSIM_Rhsim04G0131800
Root vs Leaf	novel.4548	1.64353046	2.85463E-05	8.5947E-05	--
Root vs Leaf	RHSIM_Rhsim12G0161700	-1.302060545	2.86782E-05	8.63329E-05	RHSIM_Rhsim12G0161700
Root vs Leaf	novel.4828	5.025136265	2.87722E-05	8.66047E-05	--
Root vs Leaf	RHSIM_Rhsim06G0059400	2.010726159	2.88238E-05	8.67487E-05	RHSIM_Rhsim06G0059400
Root vs Leaf	RHSIM_Rhsim10G0104100	-1.324222618	2.88278E-05	8.67497E-05	RHSIM_Rhsim10G0104100
Root vs Leaf	RHSIM_Rhsim02G0147200	1.458498264	2.88452E-05	8.67858E-05	RHSIM_Rhsim02G0147200
Root vs Leaf	RHSIM_Rhsim04G0115700	-3.246948402	2.88474E-05	8.67858E-05	RHSIM_Rhsim04G0115700
Root vs Leaf	RHSIM_Rhsim03G0123400	-1.130187517	2.90907E-05	8.74611E-05	RHSIM_Rhsim03G0123400
Root vs Leaf	RHSIM_Rhsim07G0080900	-1.540898133	2.91116E-05	8.75126E-05	RHSIM_Rhsim07G0080900
Root vs Leaf	RHSIM_Rhsim08G0103600	4.099290023	2.91542E-05	8.76293E-05	RHSIM_Rhsim08G0103600
Root vs Leaf	RHSIM_Rhsim05G0077000	1.132046854	2.91801E-05	8.76958E-05	RHSIM_Rhsim05G0077000
Root vs Leaf	RHSIM_Rhsim03G0146500	-1.576668641	2.9417E-05	8.83847E-05	RHSIM_Rhsim03G0146500
Root vs Leaf	RHSIM_Rhsim09G0180300	3.115201637	2.94625E-05	8.85099E-05	RHSIM_Rhsim09G0180300
Root vs Leaf	RHSIM_Rhsim08G0030900	2.261145856	2.95204E-05	8.8661E-05	RHSIM_Rhsim08G0030900
Root vs Leaf	RHSIM_Rhsim08G0054600	-1.918699393	2.96296E-05	8.89657E-05	RHSIM_Rhsim08G0054600
Root vs Leaf	novel.3673	-3.696961921	2.96444E-05	8.89869E-05	--
Root vs Leaf	RHSIM_Rhsim09G0197500	-2.294314152	2.97188E-05	8.91872E-05	RHSIM_Rhsim09G0197500
Root vs Leaf	RHSIM_Rhsim06G0120600	-5.626599734	2.97447E-05	8.92534E-05	RHSIM_Rhsim06G0120600
Root vs Leaf	RHSIM_Rhsim11G0148600	-3.421363983	2.98462E-05	8.95464E-05	RHSIM_Rhsim11G0148600
Root vs Leaf	RHSIM_Rhsim08G0177000	-3.800713547	2.98948E-05	8.96807E-05	RHSIM_Rhsim08G0177000
Root vs Leaf	RHSIM_Rhsim07G0085000	3.982216243	2.99719E-05	8.99002E-05	RHSIM_Rhsim07G0085000
Root vs Leaf	RHSIM_Rhsim09G0203500	2.364499916	3.00438E-05	9.01042E-05	RHSIM_Rhsim09G0203500

Comparison	Gene ID	log2FC	P-value	FDR (padj)	Gene Name
Root vs Leaf	RHSIM_Rhsim05G0014900	-1.835739376	3.00882E-05	9.02194E-05	RHSIM_Rhsim05G0014900
Root vs Leaf	RHSIM_Rhsim12G0181300	-1.227305153	3.02694E-05	9.07338E-05	RHSIM_Rhsim12G0181300
Root vs Leaf	RHSIM_Rhsim07G0015900	1.238762578	3.03051E-05	9.08288E-05	RHSIM_Rhsim07G0015900
Root vs Leaf	RHSIM_Rhsim12G0067200	-2.96092035	3.05615E-05	9.15617E-05	RHSIM_Rhsim12G0067200
Root vs Leaf	RHSIM_Rhsim03G0042800	-2.522437267	3.06628E-05	9.18296E-05	RHSIM_Rhsim03G0042800
Root vs Leaf	novel.358	-2.185511588	3.06628E-05	9.18296E-05	--
Root vs Leaf	novel.4873	5.059887598	3.07289E-05	9.20156E-05	--
Root vs Leaf	novel.4409	2.758210249	3.08561E-05	9.23726E-05	--
Root vs Leaf	novel.2506	2.612663516	3.08886E-05	9.2446E-05	--
Root vs Leaf	RHSIM_Rhsim02G0014800	6.613531072	3.11292E-05	9.31422E-05	RHSIM_Rhsim02G0014800
Root vs Leaf	novel.5587	-5.364121253	3.11585E-05	9.32177E-05	--
Root vs Leaf	RHSIM_Rhsim10G0211300	3.262727948	3.11635E-05	9.32206E-05	RHSIM_Rhsim10G0211300
Root vs Leaf	RHSIM_Rhsim13G0066000	4.62052326	3.13177E-05	9.36455E-05	RHSIM_Rhsim13G0066000
Root vs Leaf	RHSIM_Rhsim02G0233500	1.062009143	3.13438E-05	9.36994E-05	RHSIM_Rhsim02G0233500
Root vs Leaf	novel.1694	-4.87477156	3.1464E-05	9.40466E-05	--
Root vs Leaf	RHSIM_Rhsim01G0265000	6.816068543	3.14801E-05	9.40826E-05	RHSIM_Rhsim01G0265000
Root vs Leaf	RHSIM_Rhsim04G0175700	2.600064979	3.14945E-05	9.41011E-05	RHSIM_Rhsim04G0175700
Root vs Leaf	RHSIM_Rhsim11G0090900	3.803832043	3.15816E-05	9.43494E-05	RHSIM_Rhsim11G0090900
Root vs Leaf	RHSIM_Rhsim11G0051500	-3.376950516	3.16599E-05	9.4566E-05	RHSIM_Rhsim11G0051500
Root vs Leaf	RHSIM_Rhsim07G0038200	5.452958222	3.16623E-05	9.4566E-05	RHSIM_Rhsim07G0038200
Root vs Leaf	RHSIM_Rhsim09G0009500	1.276244013	3.17666E-05	9.48652E-05	RHSIM_Rhsim09G0009500
Root vs Leaf	RHSIM_Rhsim03G0043100	-2.511212231	3.18914E-05	9.5201E-05	RHSIM_Rhsim03G0043100
Root vs Leaf	novel.2923	3.656964192	3.20857E-05	9.57688E-05	--
Root vs Leaf	RHSIM_Rhsim02G0111000	3.479237216	3.20965E-05	9.57888E-05	RHSIM_Rhsim02G0111000
Root vs Leaf	RHSIM_Rhsim09G0170700	1.164165696	3.22579E-05	9.62454E-05	RHSIM_Rhsim09G0170700

Comparison	Gene ID	log_2FC	P-value	FDR (padj)	Gene Name
Root vs Leaf	novel.2183	-5.714064725	3.22664E-05	9.62585E-05	--
Root vs Leaf	RHSIM_Rhsim04G0064600	-2.05014998	3.2384E-05	9.65969E-05	RHSIM_Rhsim04G0064600
Root vs Leaf	RHSIM_RhsimUnG0032200	-1.574755274	3.24485E-05	9.67767E-05	RHSIM_RhsimUnG0032200
Root vs Leaf	novel.3741	-5.075131604	3.24657E-05	9.68155E-05	--
Root vs Leaf	RHSIM_Rhsim10G0038400	-2.627909557	3.25071E-05	9.69267E-05	RHSIM_Rhsim10G0038400
Root vs Leaf	RHSIM_Rhsim02G0043700	2.204659984	3.25796E-05	9.71303E-05	RHSIM_Rhsim02G0043700
Root vs Leaf	RHSIM_Rhsim11G0163400	-1.715040785	3.26116E-05	9.72006E-05	RHSIM_Rhsim11G0163400
Root vs Leaf	novel.2142	1.095559415	3.26941E-05	9.74268E-05	--
Root vs Leaf	RHSIM_Rhsim01G0228900	5.659825569	3.26959E-05	9.74268E-05	RHSIM_Rhsim01G0228900
Root vs Leaf	RHSIM_Rhsim02G0117400	6.135704449	3.27234E-05	9.74961E-05	RHSIM_Rhsim02G0117400
Root vs Leaf	RHSIM_Rhsim01G0116500	2.248246672	3.27716E-05	9.7627E-05	RHSIM_Rhsim01G0116500
Root vs Leaf	RHSIM_Rhsim07G0240100	1.608350719	3.29926E-05	9.82602E-05	RHSIM_Rhsim07G0240100
Root vs Leaf	RHSIM_RhsimUnG0060100	1.191263574	3.30296E-05	9.83577E-05	RHSIM_RhsimUnG0060100
Root vs Leaf	RHSIM_Rhsim08G0180700	-1.739947266	3.30826E-05	9.85029E-05	RHSIM_Rhsim08G0180700
Root vs Leaf	RHSIM_Rhsim12G0201000	-3.457426655	3.32229E-05	9.89078E-05	RHSIM_Rhsim12G0201000
Root vs Leaf	RHSIM_Rhsim13G0003000	1.671713941	3.32419E-05	9.89518E-05	RHSIM_Rhsim13G0003000
Root vs Leaf	novel.714	5.694628192	3.32897E-05	9.90686E-05	--
Root vs Leaf	RHSIM_Rhsim01G0002800	-1.03011217	3.34022E-05	9.93778E-05	RHSIM_Rhsim01G0002800
Root vs Leaf	RHSIM_Rhsim05G0054700	6.110299143	3.36807E-05	0.000100168	RHSIM_Rhsim05G0054700
Root vs Leaf	RHSIM_Rhsim04G0156500	-2.044987703	3.37642E-05	0.000100403	RHSIM_Rhsim04G0156500
Root vs Leaf	RHSIM_Rhsim03G0073600	2.132812006	3.39394E-05	0.000100911	RHSIM_Rhsim03G0073600
Root vs Leaf	novel.1800	3.215021149	3.39786E-05	0.000101015	--
Root vs Leaf	RHSIM_Rhsim01G0251200	-4.092676686	3.40189E-05	0.000101122	RHSIM_Rhsim01G0251200
Root vs Leaf	RHSIM_Rhsim08G0037300	-1.557777985	3.4395E-05	0.000102213	RHSIM_Rhsim08G0037300
Root vs Leaf	RHSIM_Rhsim03G0147300	-1.049874592	3.44286E-05	0.0001023	RHSIM_Rhsim03G0147300

Comparison	Gene ID	log_2FC	P-value	FDR (padj)	Gene Name
Root vs Leaf	RHSIM_Rhsim06G0187700	6.130187103	3.45642E-05	0.000102676	RHSIM_Rhsim06G0187700
Root vs Leaf	RHSIM_Rhsim04G0122500	2.034182962	3.46751E-05	0.000102993	RHSIM_Rhsim04G0122500
Root vs Leaf	novel.2473	-3.025990183	3.50561E-05	0.000104095	--
Root vs Leaf	RHSIM_Rhsim06G0212100	-1.264328798	3.51319E-05	0.000104283	RHSIM_Rhsim06G0212100
Root vs Leaf	RHSIM_Rhsim08G0003500	-1.297883936	3.51627E-05	0.000104347	RHSIM_Rhsim08G0003500
Root vs Leaf	novel.1996	4.991765634	3.51898E-05	0.000104414	--
Root vs Leaf	RHSIM_RhsimUnG0046300	-1.424425544	3.53865E-05	0.000104971	RHSIM_RhsimUnG0046300
Root vs Leaf	RHSIM_Rhsim06G0034800	-1.0730819	3.54048E-05	0.000105012	RHSIM_Rhsim06G0034800
Root vs Leaf	RHSIM_Rhsim11G0093300	-2.73401206	3.54561E-05	0.00010515	RHSIM_Rhsim11G0093300
Root vs Leaf	RHSIM_Rhsim03G0062500	2.885871159	3.56794E-05	0.000105786	RHSIM_Rhsim03G0062500
Root vs Leaf	RHSIM_Rhsim05G0069900	2.656426305	3.56961E-05	0.000105822	RHSIM_Rhsim05G0069900
Root vs Leaf	novel.3813	-4.131894345	3.5715E-05	0.000105864	--
Root vs Leaf	RHSIM_Rhsim07G0172100	2.502988754	3.58496E-05	0.00010625	RHSIM_Rhsim07G0172100
Root vs Leaf	novel.3960	-2.47332601	3.59159E-05	0.000106432	--
Root vs Leaf	RHSIM_Rhsim10G0186100	1.68159613	3.61869E-05	0.000107194	RHSIM_Rhsim10G0186100
Root vs Leaf	RHSIM_Rhsim08G0186600	2.261308764	3.62907E-05	0.00010746	RHSIM_Rhsim08G0186600
Root vs Leaf	RHSIM_Rhsim04G0019100	5.790285281	3.67428E-05	0.000108757	RHSIM_Rhsim04G0019100
Root vs Leaf	RHSIM_Rhsim12G0191100	2.541958691	3.69395E-05	0.000109284	RHSIM_Rhsim12G0191100
Root vs Leaf	RHSIM_Rhsim05G0003600	1.877231776	3.69699E-05	0.00010936	RHSIM_Rhsim05G0003600
Root vs Leaf	RHSIM_Rhsim07G0236800	6.597028355	3.71013E-05	0.000109734	RHSIM_Rhsim07G0236800
Root vs Leaf	RHSIM_Rhsim07G0057500	-1.664505393	3.71306E-05	0.000109801	RHSIM_Rhsim07G0057500
Root vs Leaf	RHSIM_Rhsim11G0013100	2.277211061	3.71334E-05	0.000109801	RHSIM_Rhsim11G0013100
Root vs Leaf	novel.1823	6.010129037	3.72858E-05	0.000110238	--
Root vs Leaf	RHSIM_Rhsim11G0010500	1.438302759	3.72954E-05	0.000110252	RHSIM_Rhsim11G0010500
Root vs Leaf	RHSIM_RhsimUnG0204200	1.173928114	3.74462E-05	0.000110655	RHSIM_RhsimUnG0204200

Comparison	Gene ID	log_2FC	P-value	FDR (padj)	Gene Name
Root vs Leaf	RHSIM_Rhsim05G0092200	2.006113123	3.74477E-05	0.000110655	RHSIM_Rhsim05G0092200
Root vs Leaf	RHSIM_Rhsim02G0056300	-1.653708583	3.74769E-05	0.000110718	RHSIM_Rhsim02G0056300
Root vs Leaf	RHSIM_Rhsim02G0027300	-1.073045799	3.77109E-05	0.000111395	RHSIM_Rhsim02G0027300
Root vs Leaf	novel.128	-7.249399107	3.77243E-05	0.00011142	--
Root vs Leaf	RHSIM_Rhsim10G0170000	-1.767508668	3.78566E-05	0.000111783	RHSIM_Rhsim10G0170000
Root vs Leaf	RHSIM_Rhsim10G0107800	-1.538716256	3.80563E-05	0.000112344	RHSIM_Rhsim10G0107800
Root vs Leaf	RHSIM_Rhsim02G0074100	-6.041658865	3.81969E-05	0.000112744	RHSIM_Rhsim02G0074100
Root vs Leaf	RHSIM_Rhsim02G0104300	3.649848002	3.83119E-05	0.000113069	RHSIM_Rhsim02G0104300
Root vs Leaf	RHSIM_Rhsim03G0078200	-1.807686556	3.84489E-05	0.00011343	RHSIM_Rhsim03G0078200
Root vs Leaf	novel.3391	4.11912292	3.84975E-05	0.000113559	--
Root vs Leaf	RHSIM_Rhsim11G0071900	1.2051071	3.85129E-05	0.00011359	RHSIM_Rhsim11G0071900
Root vs Leaf	RHSIM_Rhsim07G0225600	1.391173579	3.89179E-05	0.000114755	RHSIM_Rhsim07G0225600
Root vs Leaf	RHSIM_Rhsim04G0199200	2.34162063	3.90424E-05	0.000115108	RHSIM_Rhsim04G0199200
Root vs Leaf	RHSIM_Rhsim07G0161300	1.199922086	3.91868E-05	0.000115519	RHSIM_Rhsim07G0161300
Root vs Leaf	RHSIM_RhsimUnG0212200	-1.197467615	3.92286E-05	0.000115624	RHSIM_RhsimUnG0212200
Root vs Leaf	RHSIM_Rhsim01G0290000	1.519105337	3.92325E-05	0.000115624	RHSIM_Rhsim01G0290000
Root vs Leaf	RHSIM_Rhsim05G0036500	-1.842076767	3.94027E-05	0.000116111	RHSIM_Rhsim05G0036500
Root vs Leaf	RHSIM_Rhsim06G0033900	-1.362399082	3.95476E-05	0.000116508	RHSIM_Rhsim06G0033900
Root vs Leaf	RHSIM_Rhsim04G0025500	2.452958012	3.96396E-05	0.000116764	RHSIM_Rhsim04G0025500
Root vs Leaf	RHSIM_Rhsim12G0185100	-3.169803772	3.98209E-05	0.000117283	RHSIM_Rhsim12G0185100
Root vs Leaf	RHSIM_RhsimUnG0118000	1.233051805	3.99351E-05	0.00011759	RHSIM_RhsimUnG0118000
Root vs Leaf	RHSIM_Rhsim08G0207100	3.330959387	4.00046E-05	0.000117779	RHSIM_Rhsim08G0207100
Root vs Leaf	RHSIM_Rhsim06G0176900	1.74825466	4.01983E-05	0.00011832	RHSIM_Rhsim06G0176900
Root vs Leaf	RHSIM_Rhsim04G0144000	-3.123177754	4.02728E-05	0.000118509	RHSIM_Rhsim04G0144000
Root vs Leaf	RHSIM_RhsimUnG0072700	-4.148950303	4.03087E-05	0.000118599	RHSIM_RhsimUnG0072700

Comparison	Gene ID	log_2FC	P-value	FDR (padj)	Gene Name
Root vs Leaf	RHSIM_Rhsim13G0147500	3.077529936	4.03794E-05	0.000118777	RHSIM_Rhsim13G0147500
Root vs Leaf	RHSIM_RhsimUnG0168200	-1.16840711	4.05543E-05	0.000119276	RHSIM_RhsimUnG0168200
Root vs Leaf	RHSIM_Rhsim02G0202900	6.12982387	4.06649E-05	0.000119586	RHSIM_Rhsim02G0202900
Root vs Leaf	novel.1342	2.782773339	4.07762E-05	0.000119899	--
Root vs Leaf	novel.881	2.347598469	4.08298E-05	0.000120041	--
Root vs Leaf	RHSIM_Rhsim06G0078500	-1.093276422	4.09946E-05	0.00012051	RHSIM_Rhsim06G0078500
Root vs Leaf	RHSIM_Rhsim02G0064800	4.32688243	4.11008E-05	0.000120792	RHSIM_Rhsim02G0064800
Root vs Leaf	RHSIM_RhsimUnG0121500	4.202835188	4.11492E-05	0.000120903	RHSIM_RhsimUnG0121500
Root vs Leaf	RHSIM_Rhsim08G0162400	-1.850121419	4.14138E-05	0.00012165	RHSIM_Rhsim08G0162400
Root vs Leaf	RHSIM_RhsimUnG0040600	-2.455309404	4.1529E-05	0.000121957	RHSIM_RhsimUnG0040600
Root vs Leaf	RHSIM_Rhsim10G0073700	-1.861225607	4.16064E-05	0.000122153	RHSIM_Rhsim10G0073700
Root vs Leaf	RHSIM_Rhsim13G0037300	-3.718048143	4.16151E-05	0.000122163	RHSIM_Rhsim13G0037300
Root vs Leaf	RHSIM_Rhsim09G0081700	1.871766572	4.18056E-05	0.000122692	RHSIM_Rhsim09G0081700
Root vs Leaf	RHSIM_Rhsim04G0036000	1.474272507	4.18057E-05	0.000122692	RHSIM_Rhsim04G0036000
Root vs Leaf	RHSIM_Rhsim01G0277300	-1.451092457	4.18598E-05	0.000122835	RHSIM_Rhsim01G0277300
Root vs Leaf	RHSIM_Rhsim01G0096100	1.061239548	4.20096E-05	0.000123259	RHSIM_Rhsim01G0096100
Root vs Leaf	RHSIM_Rhsim01G0100200	6.605330705	4.20291E-05	0.000123301	RHSIM_Rhsim01G0100200
Root vs Leaf	novel.3923	-3.576470368	4.2069E-05	0.000123402	--
Root vs Leaf	RHSIM_Rhsim02G0033500	6.216141839	4.21099E-05	0.000123506	RHSIM_Rhsim02G0033500
Root vs Leaf	RHSIM_Rhsim02G0091200	2.771099911	4.24187E-05	0.000124349	RHSIM_Rhsim02G0091200
Root vs Leaf	RHSIM_Rhsim10G0174900	4.480028329	4.26274E-05	0.000124929	RHSIM_Rhsim10G0174900
Root vs Leaf	RHSIM_Rhsim02G0035700	1.662275971	4.2725E-05	0.000125199	RHSIM_Rhsim02G0035700
Root vs Leaf	RHSIM_Rhsim11G0090400	1.898378864	4.29133E-05	0.000125735	RHSIM_Rhsim11G0090400
Root vs Leaf	RHSIM_Rhsim03G0017100	6.488723889	4.29437E-05	0.000125808	RHSIM_Rhsim03G0017100
Root vs Leaf	RHSIM_Rhsim09G0070900	1.139238944	4.30181E-05	0.00012601	RHSIM_Rhsim09G0070900

Comparison	Gene ID	$\log_2$FC	P-value	FDR (padj)	Gene Name
Root vs Leaf	RHSIM_RhsimUnG0141000	-2.176906949	4.30678E-05	0.000126124	RHSIM_RhsimUnG0141000
Root vs Leaf	novel.4652	-4.739906303	4.31294E-05	0.000126289	--
Root vs Leaf	RHSIM_Rhsim11G0027700	-1.769509661	4.32591E-05	0.000126636	RHSIM_Rhsim11G0027700
Root vs Leaf	RHSIM_RhsimUnG0056600	-6.46739073	4.32773E-05	0.000126673	RHSIM_RhsimUnG0056600
Root vs Leaf	RHSIM_Rhsim07G0241700	-1.365549205	4.33765E-05	0.000126948	RHSIM_Rhsim07G0241700
Root vs Leaf	RHSIM_Rhsim07G0035000	3.540226574	4.33834E-05	0.000126952	RHSIM_Rhsim07G0035000
Root vs Leaf	RHSIM_Rhsim03G0058200	-2.018716449	4.36732E-05	0.000127784	RHSIM_Rhsim03G0058200
Root vs Leaf	RHSIM_Rhsim12G0143600	-2.532387491	4.38402E-05	0.000128256	RHSIM_Rhsim12G0143600
Root vs Leaf	RHSIM_Rhsim06G0222800	2.089133378	4.3851E-05	0.000128272	RHSIM_Rhsim06G0222800
Root vs Leaf	novel.1644	-2.055853017	4.469E-05	0.00013066	--
Root vs Leaf	RHSIM_Rhsim06G0160700	-4.432620712	4.47999E-05	0.000130965	RHSIM_Rhsim06G0160700
Root vs Leaf	RHSIM_Rhsim07G0003200	1.592501829	4.52584E-05	0.000132288	RHSIM_Rhsim07G0003200
Root vs Leaf	RHSIM_Rhsim13G0025300	4.130208132	4.53755E-05	0.000132614	RHSIM_Rhsim13G0025300
Root vs Leaf	novel.951	-1.34907622	4.58176E-05	0.000133855	--
Root vs Leaf	RHSIM_Rhsim10G0031300	-1.027271975	4.58768E-05	0.000134011	RHSIM_Rhsim10G0031300
Root vs Leaf	RHSIM_Rhsim08G0048000	-1.263555857	4.58831E-05	0.000134013	RHSIM_Rhsim08G0048000
Root vs Leaf	RHSIM_Rhsim02G0031900	-1.303954097	4.60483E-05	0.000134478	RHSIM_Rhsim02G0031900
Root vs Leaf	novel.5571	-2.171666353	4.60946E-05	0.000134596	--
Root vs Leaf	RHSIM_RhsimUnG0158000	-1.639745051	4.6168E-05	0.000134794	RHSIM_RhsimUnG0158000
Root vs Leaf	RHSIM_Rhsim04G0005000	2.305566352	4.62178E-05	0.000134922	RHSIM_Rhsim04G0005000
Root vs Leaf	RHSIM_Rhsim02G0256400	-1.098879114	4.68231E-05	0.000136637	RHSIM_Rhsim02G0256400
Root vs Leaf	RHSIM_Rhsim10G0035600	-1.096362521	4.6833E-05	0.000136649	RHSIM_Rhsim10G0035600
Root vs Leaf	novel.2483	6.61122899	4.69379E-05	0.000136921	--
Root vs Leaf	RHSIM_Rhsim02G0215800	-2.968846917	4.75434E-05	0.000138634	RHSIM_Rhsim02G0215800
Root vs Leaf	novel.653	-1.719478005	4.77247E-05	0.000139145	--

Comparison	Gene ID	log_2FC	P-value	FDR (padj)	Gene Name
Root vs Leaf	RHSIM_Rhsim11G0176700	3.378244078	4.78295E-05	0.000139433	RHSIM_Rhsim11G0176700
Root vs Leaf	RHSIM_Rhsim10G0062700	-1.577154042	4.81952E-05	0.000140429	RHSIM_Rhsim10G0062700
Root vs Leaf	RHSIM_Rhsim07G0071200	1.369956465	4.83035E-05	0.000140709	RHSIM_Rhsim07G0071200
Root vs Leaf	novel.3426	-3.694321939	4.83722E-05	0.000140883	--
Root vs Leaf	RHSIM_Rhsim13G0186400	6.338761947	4.83812E-05	0.000140883	RHSIM_Rhsim13G0186400
Root vs Leaf	novel.3744	1.383289395	4.83814E-05	0.000140883	--
Root vs Leaf	RHSIM_Rhsim02G0094000	1.723520263	4.88966E-05	0.000142329	RHSIM_Rhsim02G0094000
Root vs Leaf	RHSIM_Rhsim06G0161800	-2.424886986	4.90749E-05	0.00014283	RHSIM_Rhsim06G0161800
Root vs Leaf	novel.734	2.022740139	4.93843E-05	0.000143713	--
Root vs Leaf	RHSIM_Rhsim08G0196900	-1.895864514	4.9409E-05	0.000143766	RHSIM_Rhsim08G0196900
Root vs Leaf	RHSIM_Rhsim02G0134600	1.561994029	4.95064E-05	0.000144032	RHSIM_Rhsim02G0134600
Root vs Leaf	RHSIM_Rhsim07G0165300	-2.560825798	4.95275E-05	0.000144066	RHSIM_Rhsim07G0165300
Root vs Leaf	RHSIM_Rhsim13G0027800	5.706922956	4.95306E-05	0.000144066	RHSIM_Rhsim13G0027800
Root vs Leaf	RHSIM_Rhsim06G0197100	1.268083483	4.95646E-05	0.000144147	RHSIM_Rhsim06G0197100
Root vs Leaf	RHSIM_Rhsim11G0189600	3.257756039	4.96474E-05	0.000144351	RHSIM_Rhsim11G0189600
Root vs Leaf	RHSIM_Rhsim07G0185900	-1.594606041	4.96693E-05	0.000144397	RHSIM_Rhsim07G0185900
Root vs Leaf	novel.241	4.920196906	4.98151E-05	0.000144802	--
Root vs Leaf	RHSIM_Rhsim07G0201200	2.865537617	4.98437E-05	0.000144867	RHSIM_Rhsim07G0201200
Root vs Leaf	novel.5875	5.060385128	4.99655E-05	0.000145203	--
Root vs Leaf	RHSIM_Rhsim10G0024100	-2.117165279	4.99935E-05	0.000145266	RHSIM_Rhsim10G0024100
Root vs Leaf	RHSIM_Rhsim07G0052300	-2.73540555	5.00443E-05	0.000145395	RHSIM_Rhsim07G0052300
Root vs Leaf	RHSIM_Rhsim07G0193600	-1.123072156	5.00933E-05	0.000145501	RHSIM_Rhsim07G0193600
Root vs Leaf	RHSIM_Rhsim09G0115100	1.204858485	5.01586E-05	0.000145673	RHSIM_Rhsim09G0115100
Root vs Leaf	RHSIM_RhsimUnG0211800	-1.146312241	5.01783E-05	0.000145712	RHSIM_RhsimUnG0211800
Root vs Leaf	RHSIM_Rhsim02G0025200	-1.106163731	5.02078E-05	0.000145779	RHSIM_Rhsim02G0025200

Comparison	Gene ID	log_2FC	P-value	FDR (padj)	Gene Name
Root vs Leaf	novel.5578	-1.084736576	5.02744E-05	0.000145954	--
Root vs Leaf	RHSIM_Rhsim10G0149000	-1.037551416	5.02935E-05	0.000145991	RHSIM_Rhsim10G0149000
Root vs Leaf	novel.2688	2.16529086	5.03864E-05	0.000146224	--
Root vs Leaf	novel.4958	-7.117713663	5.07311E-05	0.000147201	--
Root vs Leaf	RHSIM_Rhsim05G0125700	1.710152801	5.07358E-05	0.000147201	RHSIM_Rhsim05G0125700
Root vs Leaf	RHSIM_Rhsim11G0171300	6.64488736	5.11007E-05	0.000148223	RHSIM_Rhsim11G0171300
Root vs Leaf	RHSIM_Rhsim10G0111200	6.131535595	5.1159E-05	0.000148373	RHSIM_Rhsim10G0111200
Root vs Leaf	RHSIM_RhsimUnG0213900	1.128689052	5.1194E-05	0.000148456	RHSIM_RhsimUnG0213900
Root vs Leaf	RHSIM_Rhsim10G0187200	3.251230426	5.12443E-05	0.000148565	RHSIM_Rhsim10G0187200
Root vs Leaf	novel.3910	-6.817242444	5.1538E-05	0.000149342	--
Root vs Leaf	RHSIM_Rhsim06G0031500	5.466246475	5.15528E-05	0.000149366	RHSIM_Rhsim06G0031500
Root vs Leaf	RHSIM_Rhsim13G0087700	6.300965681	5.15922E-05	0.000149461	RHSIM_Rhsim13G0087700
Root vs Leaf	RHSIM_Rhsim04G0157700	1.531350196	5.16597E-05	0.000149638	RHSIM_Rhsim04G0157700
Root vs Leaf	RHSIM_Rhsim03G0259100	2.833214761	5.18196E-05	0.000150063	RHSIM_Rhsim03G0259100
Root vs Leaf	novel.2381	-1.805414714	5.18704E-05	0.000150192	--
Root vs Leaf	RHSIM_Rhsim03G0067800	-1.221425029	5.18872E-05	0.000150222	RHSIM_Rhsim03G0067800
Root vs Leaf	novel.3366	6.026089181	5.20191E-05	0.000150585	--
Root vs Leaf	RHSIM_Rhsim11G0133600	1.056234311	5.24222E-05	0.000151714	RHSIM_Rhsim11G0133600
Root vs Leaf	novel.3231	2.025183972	5.26552E-05	0.000152369	--
Root vs Leaf	RHSIM_Rhsim09G0143200	-1.578569554	5.27487E-05	0.00015262	RHSIM_Rhsim09G0143200
Root vs Leaf	RHSIM_Rhsim07G0058000	-1.103242359	5.29937E-05	0.00015331	RHSIM_Rhsim07G0058000
Root vs Leaf	RHSIM_Rhsim05G0013800	-2.055808337	5.30178E-05	0.000153361	RHSIM_Rhsim05G0013800
Root vs Leaf	RHSIM_Rhsim09G0154000	-5.307655208	5.30955E-05	0.000153566	RHSIM_Rhsim09G0154000
Root vs Leaf	RHSIM_RhsimUnG0103500	3.143775867	5.31224E-05	0.000153625	RHSIM_RhsimUnG0103500
Root vs Leaf	RHSIM_Rhsim13G0083400	3.095849727	5.31574E-05	0.000153707	RHSIM_Rhsim13G0083400

Comparison	Gene ID	log$_2$FC	P-value	FDR (padj)	Gene Name
Root vs Leaf	RHSIM_Rhsim10G0033800	-2.433115198	5.38886E-05	0.000155763	RHSIM_Rhsim10G0033800
Root vs Leaf	RHSIM_Rhsim04G0149700	1.131370235	5.41099E-05	0.000156363	RHSIM_Rhsim04G0149700
Root vs Leaf	RHSIM_Rhsim03G0089400	6.029171559	5.4118E-05	0.000156367	RHSIM_Rhsim03G0089400
Root vs Leaf	RHSIM_Rhsim09G0006200	2.956139476	5.43554E-05	0.000156994	RHSIM_Rhsim09G0006200
Root vs Leaf	RHSIM_Rhsim03G0220000	2.472883999	5.43923E-05	0.000157081	RHSIM_Rhsim03G0220000
Root vs Leaf	RHSIM_Rhsim11G0024400	6.178185742	5.44039E-05	0.000157095	RHSIM_Rhsim11G0024400
Root vs Leaf	RHSIM_RhsimUnG0097800	-4.717234045	5.45768E-05	0.000157575	RHSIM_RhsimUnG0097800
Root vs Leaf	novel.5203	-3.872117936	5.46239E-05	0.000157691	--
Root vs Leaf	novel.21	5.670830535	5.46496E-05	0.000157744	--
Root vs Leaf	RHSIM_RhsimUnG0031900	2.517474272	5.46557E-05	0.000157744	RHSIM_RhsimUnG0031900
Root vs Leaf	novel.4508	2.067491892	5.46986E-05	0.000157848	--
Root vs Leaf	RHSIM_Rhsim10G0002700	2.334934583	5.47087E-05	0.000157857	RHSIM_Rhsim10G0002700
Root vs Leaf	novel.4270	-1.549635485	5.48125E-05	0.000158117	--
Root vs Leaf	RHSIM_RhsimUnG0070300	5.761242811	5.4854E-05	0.000158217	RHSIM_RhsimUnG0070300
Root vs Leaf	RHSIM_RhsimMtG0001900	-3.275535267	5.51814E-05	0.000159142	RHSIM_RhsimMtG0001900
Root vs Leaf	RHSIM_Rhsim04G0006000	2.005779466	5.52599E-05	0.000159348	RHSIM_Rhsim04G0006000
Root vs Leaf	RHSIM_Rhsim13G0168900	1.508860568	5.52847E-05	0.0001594	RHSIM_Rhsim13G0168900
Root vs Leaf	RHSIM_Rhsim04G0156300	-1.108519895	5.53164E-05	0.000159471	RHSIM_Rhsim04G0156300
Root vs Leaf	RHSIM_Rhsim09G0194600	6.190750828	5.56118E-05	0.000160263	RHSIM_Rhsim09G0194600
Root vs Leaf	RHSIM_Rhsim09G0006400	-1.856030647	5.67599E-05	0.000163511	RHSIM_Rhsim09G0006400
Root vs Leaf	RHSIM_Rhsim09G0004100	-1.806737269	5.69345E-05	0.000163973	RHSIM_Rhsim09G0004100
Root vs Leaf	novel.2919	-1.557114634	5.69957E-05	0.000164129	--
Root vs Leaf	RHSIM_Rhsim03G0255100	-2.57035166	5.706E-05	0.000164293	RHSIM_Rhsim03G0255100
Root vs Leaf	RHSIM_Rhsim13G0006400	2.31663188	5.7154E-05	0.000164544	RHSIM_Rhsim13G0006400
Root vs Leaf	RHSIM_Rhsim07G0216200	-2.744768074	5.71925E-05	0.000164634	RHSIM_Rhsim07G0216200

Comparison	Gene ID	log_2FC	P-value	FDR (padj)	Gene Name
Root vs Leaf	RHSIM_Rhsim08G0175000	1.833660149	5.75577E-05	0.000165623	RHSIM_Rhsim08G0175000
Root vs Leaf	RHSIM_Rhsim05G0001300	1.116810276	5.75984E-05	0.00016572	RHSIM_Rhsim05G0001300
Root vs Leaf	RHSIM_Rhsim03G0115500	2.105904748	5.7685E-05	0.000165948	RHSIM_Rhsim03G0115500
Root vs Leaf	RHSIM_Rhsim09G0057700	1.646479753	5.77216E-05	0.000166033	RHSIM_Rhsim09G0057700
Root vs Leaf	RHSIM_Rhsim08G0154300	-1.794842008	5.78406E-05	0.000166293	RHSIM_Rhsim08G0154300
Root vs Leaf	novel.6143	-1.035295491	5.80035E-05	0.000166719	--
Root vs Leaf	RHSIM_RhsimMtG0005500	-4.136024935	5.83452E-05	0.00016764	RHSIM_RhsimMtG0005500
Root vs Leaf	RHSIM_Rhsim12G0070600	-4.009386825	5.83453E-05	0.00016764	RHSIM_Rhsim12G0070600
Root vs Leaf	novel.3634	1.218885982	5.85816E-05	0.000168298	--
Root vs Leaf	RHSIM_Rhsim05G0179800	6.121890451	5.89409E-05	0.000169288	RHSIM_Rhsim05G0179800
Root vs Leaf	novel.178	-1.895256003	5.9066E-05	0.000169616	--
Root vs Leaf	RHSIM_Rhsim06G0035900	1.260115611	5.90699E-05	0.000169616	RHSIM_Rhsim06G0035900
Root vs Leaf	RHSIM_Rhsim06G0219900	1.998659798	5.9421E-05	0.000170582	RHSIM_Rhsim06G0219900
Root vs Leaf	RHSIM_Rhsim08G0201300	-1.285290987	5.95191E-05	0.000170842	RHSIM_Rhsim08G0201300
Root vs Leaf	RHSIM_Rhsim03G0045100	-1.395878731	5.95271E-05	0.000170844	RHSIM_Rhsim03G0045100
Root vs Leaf	RHSIM_Rhsim03G0010300	6.108745607	5.96199E-05	0.000171089	RHSIM_Rhsim03G0010300
Root vs Leaf	RHSIM_Rhsim05G0070500	1.064853368	5.96697E-05	0.000171211	RHSIM_Rhsim05G0070500
Root vs Leaf	novel.5762	2.259549919	5.97073E-05	0.000171298	--
Root vs Leaf	RHSIM_Rhsim08G0103000	-1.702088371	5.97242E-05	0.000171325	RHSIM_Rhsim08G0103000
Root vs Leaf	novel.740	4.51812718	5.97657E-05	0.000171423	--
Root vs Leaf	RHSIM_Rhsim08G0023000	1.788393566	6.02356E-05	0.000172749	RHSIM_Rhsim08G0023000
Root vs Leaf	RHSIM_Rhsim12G0002000	1.532537208	6.09182E-05	0.000174663	RHSIM_Rhsim12G0002000
Root vs Leaf	RHSIM_Rhsim12G0134800	1.125459645	6.09548E-05	0.000174747	RHSIM_Rhsim12G0134800
Root vs Leaf	RHSIM_Rhsim06G0004100	2.055866098	6.11133E-05	0.000175139	RHSIM_Rhsim06G0004100
Root vs Leaf	novel.4626	2.207795447	6.11142E-05	0.000175139	--

Comparison	Gene ID	log_2FC	P-value	FDR (padj)	Gene Name
Root vs Leaf	RHSIM_Rhsim12G0111100	2.14270901	6.12896E-05	0.000175619	RHSIM_Rhsim12G0111100
Root vs Leaf	RHSIM_RhsimUnG0228100	1.098536656	6.14197E-05	0.00017597	RHSIM_RhsimUnG0228100
Root vs Leaf	RHSIM_Rhsim10G0162300	-1.11028412	6.17527E-05	0.000176837	RHSIM_Rhsim10G0162300
Root vs Leaf	RHSIM_Rhsim13G0093700	-1.171830155	6.19976E-05	0.000177472	RHSIM_Rhsim13G0093700
Root vs Leaf	RHSIM_Rhsim03G0249000	-1.231025863	6.21384E-05	0.000177853	RHSIM_Rhsim03G0249000
Root vs Leaf	RHSIM_RhsimUnG0126600	1.768181094	6.21751E-05	0.000177936	RHSIM_RhsimUnG0126600
Root vs Leaf	RHSIM_Rhsim04G0078500	-2.238433002	6.26361E-05	0.000179234	RHSIM_Rhsim04G0078500
Root vs Leaf	RHSIM_Rhsim07G0142900	1.93303528	6.26509E-05	0.000179254	RHSIM_Rhsim07G0142900
Root vs Leaf	RHSIM_Rhsim05G0017000	-2.071438093	6.3076E-05	0.000180425	RHSIM_Rhsim05G0017000
Root vs Leaf	RHSIM_Rhsim06G0215500	4.558321731	6.31447E-05	0.0001806	RHSIM_Rhsim06G0215500
Root vs Leaf	RHSIM_Rhsim05G0013100	-1.179487746	6.32778E-05	0.000180958	RHSIM_Rhsim05G0013100
Root vs Leaf	RHSIM_Rhsim01G0230700	1.780596136	6.36197E-05	0.000181891	RHSIM_Rhsim01G0230700
Root vs Leaf	novel.1754	-4.262486396	6.39803E-05	0.000182854	--
Root vs Leaf	novel.5990	-4.438652362	6.43305E-05	0.000183764	--
Root vs Leaf	RHSIM_Rhsim04G0227300	-4.695143918	6.45648E-05	0.000184411	RHSIM_Rhsim04G0227300
Root vs Leaf	RHSIM_Rhsim11G0162500	-2.991812034	6.60559E-05	0.000188553	RHSIM_Rhsim11G0162500
Root vs Leaf	RHSIM_Rhsim06G0227400	6.39805007	6.62316E-05	0.000189008	RHSIM_Rhsim06G0227400
Root vs Leaf	RHSIM_Rhsim11G0164500	1.075486895	6.64794E-05	0.000189692	RHSIM_Rhsim11G0164500
Root vs Leaf	RHSIM_Rhsim10G0166200	-5.79136085	6.65586E-05	0.000189894	RHSIM_Rhsim10G0166200
Root vs Leaf	RHSIM_Rhsim08G0216700	3.193972311	6.67693E-05	0.000190448	RHSIM_Rhsim08G0216700
Root vs Leaf	RHSIM_Rhsim02G0110500	1.161653149	6.79075E-05	0.000193647	RHSIM_Rhsim02G0110500
Root vs Leaf	RHSIM_Rhsim07G0109500	-2.286329006	6.79215E-05	0.000193663	RHSIM_Rhsim07G0109500
Root vs Leaf	RHSIM_Rhsim02G0235800	1.91416528	6.80598E-05	0.000193998	RHSIM_Rhsim02G0235800
Root vs Leaf	RHSIM_Rhsim08G0191800	5.423209626	6.8064E-05	0.000193998	RHSIM_Rhsim08G0191800
Root vs Leaf	RHSIM_Rhsim11G0055200	-3.698164702	6.81159E-05	0.000194122	RHSIM_Rhsim11G0055200

Comparison	Gene ID	log_2FC	P-value	FDR (padj)	Gene Name
Root vs Leaf	RHSIM_Rhsim06G0204100	1.838236778	6.82816E-05	0.00019457	RHSIM_Rhsim06G0204100
Root vs Leaf	RHSIM_Rhsim07G0104400	1.358807372	6.88683E-05	0.000196194	RHSIM_Rhsim07G0104400
Root vs Leaf	RHSIM_Rhsim04G0151800	-1.234650293	6.88905E-05	0.000196233	RHSIM_Rhsim04G0151800
Root vs Leaf	RHSIM_Rhsim02G0035600	1.817634865	6.91595E-05	0.000196951	RHSIM_Rhsim02G0035600
Root vs Leaf	RHSIM_Rhsim06G0002700	1.657434851	6.93912E-05	0.000197586	RHSIM_Rhsim06G0002700
Root vs Leaf	novel.508	1.384952598	6.98166E-05	0.000198748	--
Root vs Leaf	RHSIM_Rhsim05G0149300	1.181532043	6.98473E-05	0.000198787	RHSIM_Rhsim05G0149300
Root vs Leaf	RHSIM_Rhsim02G0061000	-5.264610979	6.98845E-05	0.000198868	RHSIM_Rhsim02G0061000
Root vs Leaf	novel.3084	-1.213690444	7.00826E-05	0.000199383	--
Root vs Leaf	RHSIM_Rhsim10G0173900	3.962947014	7.03763E-05	0.000200194	RHSIM_Rhsim10G0173900
Root vs Leaf	novel.2525	-4.009312297	7.05406E-05	0.000200637	--
Root vs Leaf	RHSIM_Rhsim04G0093700	-3.413539837	7.1484E-05	0.000203289	RHSIM_Rhsim04G0093700
Root vs Leaf	RHSIM_Rhsim01G0251100	1.696303368	7.14907E-05	0.000203289	RHSIM_Rhsim01G0251100
Root vs Leaf	novel.4976	-5.105000143	7.16572E-05	0.000203738	--
Root vs Leaf	novel.5188	4.765967021	7.16706E-05	0.000203751	--
Root vs Leaf	RHSIM_Rhsim02G0035900	1.183721919	7.17079E-05	0.000203831	RHSIM_Rhsim02G0035900
Root vs Leaf	RHSIM_Rhsim05G0133700	-1.495041236	7.22555E-05	0.000205363	RHSIM_Rhsim05G0133700
Root vs Leaf	RHSIM_Rhsim10G0069900	-1.954714002	7.29032E-05	0.000207178	RHSIM_Rhsim10G0069900
Root vs Leaf	RHSIM_Rhsim11G0179000	-1.014946986	7.30685E-05	0.000207597	RHSIM_Rhsim11G0179000
Root vs Leaf	novel.5723	-2.171273574	7.30814E-05	0.000207608	--
Root vs Leaf	RHSIM_Rhsim13G0009000	-1.351831149	7.31766E-05	0.000207853	RHSIM_Rhsim13G0009000
Root vs Leaf	RHSIM_Rhsim08G0013600	6.697858289	7.32324E-05	0.000207986	RHSIM_Rhsim08G0013600
Root vs Leaf	RHSIM_Rhsim10G0155200	1.805573612	7.32513E-05	0.000208014	RHSIM_Rhsim10G0155200
Root vs Leaf	RHSIM_Rhsim13G0233000	-1.100618644	7.34022E-05	0.000208417	RHSIM_Rhsim13G0233000
Root vs Leaf	RHSIM_Rhsim03G0036900	5.762425324	7.36508E-05	0.000209046	RHSIM_Rhsim03G0036900

Comparison	Gene ID	log_2FC	P-value	FDR (padj)	Gene Name
Root vs Leaf	RHSIM_Rhsim05G0032900	-2.541577198	7.41306E-05	0.000210356	RHSIM_Rhsim05G0032900
Root vs Leaf	RHSIM_Rhsim10G0016300	-2.653533218	7.4273E-05	0.000210735	RHSIM_Rhsim10G0016300
Root vs Leaf	RHSIM_Rhsim12G0175200	4.281956149	7.43021E-05	0.000210791	RHSIM_Rhsim12G0175200
Root vs Leaf	RHSIM_Rhsim11G0195300	2.049352192	7.43918E-05	0.000210994	RHSIM_Rhsim11G0195300
Root vs Leaf	RHSIM_Rhsim11G0004700	1.268127442	7.46514E-05	0.000211704	RHSIM_Rhsim11G0004700
Root vs Leaf	RHSIM_Rhsim01G0251900	4.917374183	7.50853E-05	0.000212831	RHSIM_Rhsim01G0251900
Root vs Leaf	RHSIM_RhsimUnG0093200	2.144115708	7.52328E-05	0.000213223	RHSIM_RhsimUnG0093200
Root vs Leaf	RHSIM_Rhsim08G0082500	3.366851323	7.56218E-05	0.000214299	RHSIM_Rhsim08G0082500
Root vs Leaf	RHSIM_Rhsim13G0042100	1.095753272	7.58816E-05	0.000214982	RHSIM_Rhsim13G0042100
Root vs Leaf	RHSIM_Rhsim07G0065800	-2.021970577	7.64739E-05	0.000216581	RHSIM_Rhsim07G0065800
Root vs Leaf	RHSIM_Rhsim09G0100800	-1.361898255	7.67733E-05	0.000217402	RHSIM_Rhsim09G0100800
Root vs Leaf	RHSIM_Rhsim04G0203000	3.579922978	7.67988E-05	0.000217448	RHSIM_Rhsim04G0203000
Root vs Leaf	RHSIM_RhsimUnG0023100	1.041715971	7.68911E-05	0.000217683	RHSIM_RhsimUnG0023100
Root vs Leaf	RHSIM_Rhsim08G0226000	-1.49974957	7.69384E-05	0.000217774	RHSIM_Rhsim08G0226000
Root vs Leaf	RHSIM_Rhsim02G0090900	-1.151902068	7.69423E-05	0.000217774	RHSIM_Rhsim02G0090900
Root vs Leaf	RHSIM_Rhsim05G0062200	-1.291318258	7.69905E-05	0.000217884	RHSIM_Rhsim05G0062200
Root vs Leaf	RHSIM_Rhsim13G0032000	1.930595688	7.71714E-05	0.000218369	RHSIM_Rhsim13G0032000
Root vs Leaf	RHSIM_Rhsim09G0116700	-1.690470077	7.72246E-05	0.000218493	RHSIM_Rhsim09G0116700
Root vs Leaf	RHSIM_Rhsim04G0235200	-1.906370734	7.74016E-05	0.00021894	RHSIM_Rhsim04G0235200
Root vs Leaf	novel.3833	6.670575159	7.74914E-05	0.000219167	--
Root vs Leaf	RHSIM_Rhsim08G0219100	-6.564540359	7.77655E-05	0.000219889	RHSIM_Rhsim08G0219100
Root vs Leaf	RHSIM_Rhsim05G0030000	1.842282567	7.79288E-05	0.000220324	RHSIM_Rhsim05G0030000
Root vs Leaf	RHSIM_Rhsim06G0109300	-1.354082364	7.86128E-05	0.000222176	RHSIM_Rhsim06G0109300
Root vs Leaf	RHSIM_Rhsim05G0011100	4.119486047	7.8657E-05	0.000222274	RHSIM_Rhsim05G0011100
Root vs Leaf	RHSIM_Rhsim13G0228900	1.221320811	7.88729E-05	0.000222802	RHSIM_Rhsim13G0228900

Comparison	Gene ID	log_2FC	P-value	FDR (padj)	Gene Name
Root vs Leaf	RHSIM_Rhsim07G0229700	-3.426357988	7.89983E-05	0.000223129	RHSIM_Rhsim07G0229700
Root vs Leaf	RHSIM_Rhsim07G0071600	4.33546608	7.93198E-05	0.000223983	RHSIM_Rhsim07G0071600
Root vs Leaf	RHSIM_Rhsim13G0170200	2.823453773	7.94282E-05	0.000224234	RHSIM_Rhsim13G0170200
Root vs Leaf	RHSIM_Rhsim04G0151500	5.689446762	7.96964E-05	0.000224964	RHSIM_Rhsim04G0151500
Root vs Leaf	RHSIM_Rhsim08G0060800	3.069948917	7.98328E-05	0.000225322	RHSIM_Rhsim08G0060800
Root vs Leaf	RHSIM_Rhsim02G0091100	-1.024902779	8.00703E-05	0.000225964	RHSIM_Rhsim02G0091100
Root vs Leaf	RHSIM_Rhsim11G0050900	1.111991648	8.02355E-05	0.000226403	RHSIM_Rhsim11G0050900
Root vs Leaf	RHSIM_RhsimUnG0009100	1.387660252	8.12089E-05	0.000229094	RHSIM_RhsimUnG0009100
Root vs Leaf	RHSIM_Rhsim10G0083000	-3.072378712	8.12639E-05	0.000229221	RHSIM_Rhsim10G0083000
Root vs Leaf	RHSIM_Rhsim08G0100300	-1.957477428	8.14107E-05	0.000229607	RHSIM_Rhsim08G0100300
Root vs Leaf	novel.1024	5.008927295	8.14847E-05	0.000229788	--
Root vs Leaf	RHSIM_Rhsim02G0020800	1.185664928	8.15551E-05	0.000229958	RHSIM_Rhsim02G0020800
Root vs Leaf	RHSIM_Rhsim10G0128000	-1.69058572	8.16398E-05	0.000230144	RHSIM_Rhsim10G0128000
Root vs Leaf	RHSIM_Rhsim07G0082800	2.273686894	8.16408E-05	0.000230144	RHSIM_Rhsim07G0082800
Root vs Leaf	RHSIM_Rhsim11G0074900	-1.167064661	8.16703E-05	0.000230199	RHSIM_Rhsim11G0074900
Root vs Leaf	RHSIM_Rhsim04G0184800	2.709794062	8.17899E-05	0.00023048	RHSIM_Rhsim04G0184800
Root vs Leaf	RHSIM_Rhsim08G0178700	2.647924111	8.1832E-05	0.00023057	RHSIM_Rhsim08G0178700
Root vs Leaf	novel.352	2.576619989	8.20138E-05	0.000231026	--
Root vs Leaf	RHSIM_Rhsim11G0058300	1.114000511	8.21193E-05	0.000231295	RHSIM_Rhsim11G0058300
Root vs Leaf	RHSIM_Rhsim07G0148200	1.279734287	8.22484E-05	0.000231603	RHSIM_Rhsim07G0148200
Root vs Leaf	RHSIM_Rhsim08G0192800	3.213774322	8.24205E-05	0.000232031	RHSIM_Rhsim08G0192800
Root vs Leaf	RHSIM_Rhsim01G0072100	3.951678165	8.27116E-05	0.000232822	RHSIM_Rhsim01G0072100
Root vs Leaf	RHSIM_Rhsim06G0153400	5.960701533	8.27877E-05	0.000233008	RHSIM_Rhsim06G0153400
Root vs Leaf	RHSIM_Rhsim06G0163400	1.752047053	8.28435E-05	0.000233136	RHSIM_Rhsim06G0163400
Root vs Leaf	RHSIM_RhsimUnG0115300	1.744583885	8.28842E-05	0.000233223	RHSIM_RhsimUnG0115300

Comparison	Gene ID	log_2FC	P-value	FDR (padj)	Gene Name
Root vs Leaf	RHSIM_Rhsim03G0182700	-1.503720779	8.31137E-05	0.00023384	RHSIM_Rhsim03G0182700
Root vs Leaf	novel.778	-1.394970218	8.31486E-05	0.00023391	--
Root vs Leaf	RHSIM_Rhsim05G0074400	3.303646694	8.35113E-05	0.000234844	RHSIM_Rhsim05G0074400
Root vs Leaf	RHSIM_Rhsim01G0236000	5.785373743	8.36353E-05	0.000235165	RHSIM_Rhsim01G0236000
Root vs Leaf	RHSIM_Rhsim12G0105200	-1.075397222	8.41067E-05	0.000236433	RHSIM_Rhsim12G0105200
Root vs Leaf	RHSIM_RhsimUnG0020500	-1.249108411	8.42972E-05	0.000236911	RHSIM_RhsimUnG0020500
Root vs Leaf	RHSIM_Rhsim11G0067700	1.279315801	8.43457E-05	0.000237018	RHSIM_Rhsim11G0067700
Root vs Leaf	novel.1907	-4.542657039	8.43591E-05	0.000237027	--
Root vs Leaf	RHSIM_Rhsim12G0148500	-1.265126372	8.44843E-05	0.00023735	RHSIM_Rhsim12G0148500
Root vs Leaf	RHSIM_Rhsim08G0105100	3.660346443	8.46726E-05	0.00023785	RHSIM_Rhsim08G0105100
Root vs Leaf	RHSIM_Rhsim01G0229000	3.195134276	8.48464E-05	0.00023828	RHSIM_Rhsim01G0229000
Root vs Leaf	RHSIM_Rhsim13G0073700	-1.05083083	8.52239E-05	0.000239311	RHSIM_Rhsim13G0073700
Root vs Leaf	RHSIM_Rhsim11G0043500	6.257929061	8.5391E-05	0.000239752	RHSIM_Rhsim11G0043500
Root vs Leaf	RHSIM_Rhsim12G0044600	1.834339865	8.57914E-05	0.000240847	RHSIM_Rhsim12G0044600
Root vs Leaf	RHSIM_Rhsim01G0260700	6.115701167	8.58065E-05	0.00024086	RHSIM_Rhsim01G0260700
Root vs Leaf	RHSIM_Rhsim09G0157800	2.206405515	8.58999E-05	0.000241093	RHSIM_Rhsim09G0157800
Root vs Leaf	RHSIM_Rhsim13G0079600	-1.154230468	8.59343E-05	0.000241154	RHSIM_Rhsim13G0079600
Root vs Leaf	novel.5090	2.882266948	8.59426E-05	0.000241154	--
Root vs Leaf	novel.6130	4.608157356	8.63322E-05	0.000242189	--
Root vs Leaf	RHSIM_Rhsim06G0193900	-2.294817766	8.65868E-05	0.000242814	RHSIM_Rhsim06G0193900
Root vs Leaf	RHSIM_Rhsim01G0108600	-3.891539138	8.66791E-05	0.000243044	RHSIM_Rhsim01G0108600
Root vs Leaf	RHSIM_Rhsim03G0150100	-5.279439798	8.67592E-05	0.000243239	RHSIM_Rhsim03G0150100
Root vs Leaf	RHSIM_Rhsim06G0195500	4.456837546	8.67742E-05	0.000243252	RHSIM_Rhsim06G0195500
Root vs Leaf	RHSIM_Rhsim04G0216100	-1.422100268	8.71437E-05	0.000244228	RHSIM_Rhsim04G0216100
Root vs Leaf	novel.5713	-4.378635052	8.79056E-05	0.000246214	--

Comparison	Gene ID	log_2FC	P-value	FDR (padj)	Gene Name
Root vs Leaf	RHSIM_Rhsim07G0117000	3.763700641	8.82298E-05	0.000247063	RHSIM_Rhsim07G0117000
Root vs Leaf	RHSIM_Rhsim04G0185100	4.597077235	8.8405E-05	0.000247523	RHSIM_Rhsim04G0185100
Root vs Leaf	RHSIM_Rhsim01G0108400	-1.756672427	8.84184E-05	0.000247531	RHSIM_Rhsim01G0108400
Root vs Leaf	RHSIM_Rhsim02G0102300	3.241178786	8.87274E-05	0.000248366	RHSIM_Rhsim02G0102300
Root vs Leaf	RHSIM_Rhsim03G0176100	1.118921962	8.89131E-05	0.000248825	RHSIM_Rhsim03G0176100
Root vs Leaf	RHSIM_Rhsim08G0043100	-1.096860415	8.93339E-05	0.000249943	RHSIM_Rhsim08G0043100
Root vs Leaf	novel.1941	-1.145680702	8.98387E-05	0.000251294	--
Root vs Leaf	novel.1906	-7.556395124	8.99282E-05	0.000251514	--
Root vs Leaf	RHSIM_Rhsim04G0104200	6.170534177	8.99906E-05	0.000251658	RHSIM_Rhsim04G0104200
Root vs Leaf	RHSIM_Rhsim12G0160200	-1.026838156	9.00607E-05	0.000251794	RHSIM_Rhsim12G0160200
Root vs Leaf	RHSIM_Rhsim12G0084800	1.227768124	9.04514E-05	0.000252855	RHSIM_Rhsim12G0084800
Root vs Leaf	novel.120	-2.609079632	9.09228E-05	0.000254112	--
Root vs Leaf	novel.3709	5.657090391	9.11553E-05	0.000254731	--
Root vs Leaf	RHSIM_Rhsim08G0162800	-2.01919803	9.16247E-05	0.000255981	RHSIM_Rhsim08G0162800
Root vs Leaf	RHSIM_Rhsim06G0221800	6.266959682	9.20319E-05	0.000257025	RHSIM_Rhsim06G0221800
Root vs Leaf	RHSIM_Rhsim06G0133400	3.288938938	9.21342E-05	0.00025728	RHSIM_Rhsim06G0133400
Root vs Leaf	RHSIM_Rhsim08G0054900	1.27323753	9.23678E-05	0.00025787	RHSIM_Rhsim08G0054900
Root vs Leaf	novel.2383	-2.951267074	9.2791E-05	0.000258958	--
Root vs Leaf	RHSIM_Rhsim12G0155200	2.029913147	9.3637E-05	0.000261256	RHSIM_Rhsim12G0155200
Root vs Leaf	RHSIM_Rhsim07G0093600	1.69925902	9.38655E-05	0.00026183	RHSIM_Rhsim07G0093600
Root vs Leaf	RHSIM_Rhsim13G0002900	-2.461444671	9.44695E-05	0.000263451	RHSIM_Rhsim13G0002900
Root vs Leaf	RHSIM_Rhsim10G0211200	1.61396182	9.50846E-05	0.000265103	RHSIM_Rhsim10G0211200
Root vs Leaf	RHSIM_Rhsim11G0029800	2.217842518	9.55831E-05	0.00026646	RHSIM_Rhsim11G0029800
Root vs Leaf	RHSIM_Rhsim08G0205900	1.904390435	9.58713E-05	0.0002672	RHSIM_Rhsim08G0205900
Root vs Leaf	RHSIM_Rhsim03G0050900	1.246529137	9.61497E-05	0.000267911	RHSIM_Rhsim03G0050900

Comparison	Gene ID	log_2FC	P-value	FDR (padj)	Gene Name
Root vs Leaf	RHSIM_Rhsim06G0240800	2.354554921	9.65269E-05	0.000268897	RHSIM_Rhsim06G0240800
Root vs Leaf	novel.2309	-1.447467278	9.69558E-05	0.000269929	--
Root vs Leaf	RHSIM_Rhsim08G0048400	-1.396300186	9.74161E-05	0.00027108	RHSIM_Rhsim08G0048400
Root vs Leaf	RHSIM_Rhsim09G0090700	2.16659237	9.74711E-05	0.000271194	RHSIM_Rhsim09G0090700
Root vs Leaf	RHSIM_Rhsim11G0037900	2.110467292	9.74802E-05	0.000271194	RHSIM_Rhsim11G0037900
Root vs Leaf	RHSIM_Rhsim01G0270200	1.205449669	9.79706E-05	0.000272525	RHSIM_Rhsim01G0270200
Root vs Leaf	RHSIM_Rhsim07G0020600	1.197656209	9.80757E-05	0.000272785	RHSIM_Rhsim07G0020600
Root vs Leaf	RHSIM_Rhsim07G0137300	2.330940996	9.81479E-05	0.000272953	RHSIM_Rhsim07G0137300
Root vs Leaf	RHSIM_Rhsim12G0214500	1.052868215	9.83228E-05	0.000273406	RHSIM_Rhsim12G0214500
Root vs Leaf	RHSIM_Rhsim13G0019000	-2.076380997	9.8476E-05	0.000273799	RHSIM_Rhsim13G0019000
Root vs Leaf	RHSIM_Rhsim04G0113500	1.235215653	9.8709E-05	0.000274414	RHSIM_Rhsim04G0113500
Root vs Leaf	RHSIM_Rhsim05G0087500	-1.733961724	9.87497E-05	0.000274462	RHSIM_Rhsim05G0087500
Root vs Leaf	RHSIM_Rhsim06G0124300	1.227904935	9.91038E-05	0.000275413	RHSIM_Rhsim06G0124300
Root vs Leaf	novel.3692	-1.209853714	9.94196E-05	0.000276257	--
Root vs Leaf	RHSIM_Rhsim04G0017800	1.129009449	9.94583E-05	0.000276331	RHSIM_Rhsim04G0017800
Root vs Leaf	novel.1723	-7.564916626	0.000100146	0.000278208	--
Root vs Leaf	RHSIM_Rhsim09G0180700	3.694268378	0.000100295	0.000278557	RHSIM_Rhsim09G0180700
Root vs Leaf	novel.5737	-1.328127672	0.000100345	0.000278628	--
Root vs Leaf	RHSIM_Rhsim10G0113600	-2.047574571	0.000100584	0.000279257	RHSIM_Rhsim10G0113600
Root vs Leaf	RHSIM_Rhsim01G0034000	4.047234654	0.000100658	0.00027943	RHSIM_Rhsim01G0034000
Root vs Leaf	RHSIM_Rhsim11G0052200	-2.021052052	0.00010097	0.000280261	RHSIM_Rhsim11G0052200
Root vs Leaf	RHSIM_Rhsim05G0224300	-1.102752401	0.000101016	0.000280357	RHSIM_Rhsim05G0224300
Root vs Leaf	RHSIM_RhsimUnG0183900	3.088082058	0.000101656	0.000282098	RHSIM_RhsimUnG0183900
Root vs Leaf	RHSIM_Rhsim07G0019600	-3.000593615	0.000102099	0.000283129	RHSIM_Rhsim07G0019600
Root vs Leaf	RHSIM_RhsimUnG0124000	3.664268993	0.000102101	0.000283129	RHSIM_RhsimUnG0124000

Comparison	Gene ID	log_2FC	P-value	FDR (padj)	Gene Name
Root vs Leaf	RHSIM_Rhsim03G0006300	1.988589705	0.000102373	0.000283815	RHSIM_Rhsim03G0006300
Root vs Leaf	RHSIM_Rhsim02G0157900	-3.235115316	0.0001024	0.000283856	RHSIM_Rhsim02G0157900
Root vs Leaf	RHSIM_Rhsim06G0190800	1.244851708	0.000102549	0.000284235	RHSIM_Rhsim06G0190800
Root vs Leaf	RHSIM_Rhsim02G0027900	1.151921317	0.00010294	0.000285253	RHSIM_Rhsim02G0027900
Root vs Leaf	RHSIM_Rhsim07G0087500	-4.40912954	0.000103101	0.000285662	RHSIM_Rhsim07G0087500
Root vs Leaf	RHSIM_Rhsim13G0225700	1.756611203	0.000103552	0.000286844	RHSIM_Rhsim13G0225700
Root vs Leaf	RHSIM_Rhsim01G0131400	6.793254952	0.000103793	0.000287476	RHSIM_Rhsim01G0131400
Root vs Leaf	RHSIM_Rhsim09G0023100	4.110945056	0.000103863	0.000287637	RHSIM_Rhsim09G0023100
Root vs Leaf	RHSIM_Rhsim06G0040500	-1.397592782	0.000104014	0.000288021	RHSIM_Rhsim06G0040500
Root vs Leaf	RHSIM_Rhsim05G0063300	2.642198137	0.000104286	0.000288705	RHSIM_Rhsim05G0063300
Root vs Leaf	RHSIM_Rhsim11G0034200	-5.599904946	0.000104398	0.00028898	RHSIM_Rhsim11G0034200
Root vs Leaf	RHSIM_Rhsim10G0029000	-2.904324679	0.00010458	0.000289448	RHSIM_Rhsim10G0029000
Root vs Leaf	RHSIM_Rhsim05G0174900	-1.547027114	0.000104758	0.000289872	RHSIM_Rhsim05G0174900
Root vs Leaf	RHSIM_Rhsim08G0115500	2.10073532	0.000105381	0.000291528	RHSIM_Rhsim08G0115500
Root vs Leaf	novel.2587	-1.23779157	0.000105659	0.00029226	--
Root vs Leaf	RHSIM_Rhsim08G0097200	5.278153867	0.000105727	0.000292415	RHSIM_Rhsim08G0097200
Root vs Leaf	RHSIM_Rhsim10G0102400	2.445380989	0.000106974	0.000295685	RHSIM_Rhsim10G0102400
Root vs Leaf	RHSIM_Rhsim08G0202900	-2.863405182	0.000107279	0.000296493	RHSIM_Rhsim08G0202900
Root vs Leaf	novel.2095	-1.467019532	0.000107933	0.000298194	--
Root vs Leaf	novel.3386	4.254764028	0.000108233	0.000298987	--
Root vs Leaf	RHSIM_Rhsim04G0204100	2.641972657	0.000108442	0.000299494	RHSIM_Rhsim04G0204100
Root vs Leaf	RHSIM_Rhsim11G0165500	-2.720911485	0.000108611	0.000299926	RHSIM_Rhsim11G0165500
Root vs Leaf	RHSIM_Rhsim04G0182600	-1.718557908	0.000109297	0.000301782	RHSIM_Rhsim04G0182600
Root vs Leaf	RHSIM_Rhsim13G0124200	2.364017112	0.000109472	0.000302229	RHSIM_Rhsim13G0124200
Root vs Leaf	novel.2538	3.822439723	0.000110045	0.000303741	--

Comparison	Gene ID	log_2FC	P-value	FDR (padj)	Gene Name
Root vs Leaf	RHSIM_Rhsim13G0163100	-1.11930105	0.000110105	0.000303869	RHSIM_Rhsim13G0163100
Root vs Leaf	RHSIM_Rhsim01G0198400	-2.564665675	0.000110489	0.00030482	RHSIM_Rhsim01G0198400
Root vs Leaf	novel.5919	2.36264881	0.000110515	0.000304854	--
Root vs Leaf	novel.5550	-1.766625153	0.000110546	0.000304904	--
Root vs Leaf	RHSIM_Rhsim09G0106800	3.046033659	0.000110781	0.000305479	RHSIM_Rhsim09G0106800
Root vs Leaf	RHSIM_Rhsim06G0151500	1.463320485	0.000110979	0.00030599	RHSIM_Rhsim06G0151500
Root vs Leaf	RHSIM_Rhsim02G0168200	1.167350376	0.000111169	0.000306476	RHSIM_Rhsim02G0168200
Root vs Leaf	RHSIM_Rhsim13G0140100	-2.496728416	0.000111601	0.000307632	RHSIM_Rhsim13G0140100
Root vs Leaf	novel.4437	3.615177809	0.000112112	0.000308893	--
Root vs Leaf	RHSIM_Rhsim12G0210300	-1.979184047	0.000112241	0.00030921	RHSIM_Rhsim12G0210300
Root vs Leaf	novel.625	3.374806817	0.000112588	0.000310093	--
Root vs Leaf	novel.408	-2.323079227	0.000112707	0.000310309	--
Root vs Leaf	RHSIM_Rhsim03G0223700	1.328287565	0.000113223	0.00031162	RHSIM_Rhsim03G0223700
Root vs Leaf	novel.924	5.065733083	0.000113268	0.000311707	--
Root vs Leaf	novel.5646	-3.260633806	0.000113415	0.000312072	--
Root vs Leaf	RHSIM_Rhsim01G0283600	1.06953187	0.000114569	0.000315062	RHSIM_Rhsim01G0283600
Root vs Leaf	RHSIM_Rhsim01G0074400	2.845618063	0.000114983	0.000316125	RHSIM_Rhsim01G0074400
Root vs Leaf	RHSIM_Rhsim10G0060800	-4.473319692	0.00011554	0.000317618	RHSIM_Rhsim10G0060800
Root vs Leaf	RHSIM_Rhsim12G0120700	5.113070418	0.000115923	0.000318634	RHSIM_Rhsim12G0120700
Root vs Leaf	RHSIM_RhsimUnG0216800	3.993791451	0.000116283	0.000319546	RHSIM_RhsimUnG0216800
Root vs Leaf	RHSIM_Rhsim01G0218700	4.775752346	0.000116528	0.000320159	RHSIM_Rhsim01G0218700
Root vs Leaf	RHSIM_Rhsim03G0239700	3.381685209	0.000116533	0.000320159	RHSIM_Rhsim03G0239700
Root vs Leaf	novel.4004	-1.893682044	0.000116736	0.000320678	--
Root vs Leaf	RHSIM_Rhsim07G0005900	-2.07254707	0.000117094	0.000321623	RHSIM_Rhsim07G0005900
Root vs Leaf	RHSIM_Rhsim11G0156500	1.547148519	0.000117155	0.000321753	RHSIM_Rhsim11G0156500

Comparison	Gene ID	log_2FC	P-value	FDR (padj)	Gene Name
Root vs Leaf	RHSIM_Rhsim02G0083700	-1.072829983	0.000117171	0.000321757	RHSIM_Rhsim02G0083700
Root vs Leaf	RHSIM_Rhsim10G0170600	-2.448993255	0.000117909	0.000323555	RHSIM_Rhsim10G0170600
Root vs Leaf	novel.2889	-1.164077762	0.000118789	0.000325814	--
Root vs Leaf	RHSIM_Rhsim05G0010700	-3.417885956	0.000119052	0.000326496	RHSIM_Rhsim05G0010700
Root vs Leaf	novel.3381	2.482263288	0.000119086	0.000326553	--
Root vs Leaf	RHSIM_Rhsim05G0011500	-2.425408727	0.000119386	0.000327335	RHSIM_Rhsim05G0011500
Root vs Leaf	RHSIM_Rhsim01G0279800	-2.617754077	0.000120185	0.000329488	RHSIM_Rhsim01G0279800
Root vs Leaf	RHSIM_Rhsim10G0023900	-3.114664033	0.000120425	0.000330066	RHSIM_Rhsim10G0023900
Root vs Leaf	RHSIM_Rhsim13G0141500	-2.575087069	0.00012057	0.000330425	RHSIM_Rhsim13G0141500
Root vs Leaf	RHSIM_RhsimUnG0101800	-1.736282936	0.000121069	0.000331675	RHSIM_RhsimUnG0101800
Root vs Leaf	novel.4443	2.034211008	0.000121085	0.000331679	--
Root vs Leaf	RHSIM_Rhsim03G0214500	-4.539271206	0.000121388	0.000332471	RHSIM_Rhsim03G0214500
Root vs Leaf	novel.656	-1.865542378	0.000121594	0.000332994	--
Root vs Leaf	RHSIM_Rhsim03G0150400	2.562442408	0.00012175	0.000333381	RHSIM_Rhsim03G0150400
Root vs Leaf	novel.6090	-4.619894639	0.000121824	0.000333545	--
Root vs Leaf	RHSIM_Rhsim02G0242400	1.339835568	0.000121927	0.000333789	RHSIM_Rhsim02G0242400
Root vs Leaf	RHSIM_Rhsim02G0023100	-1.455868428	0.000122155	0.000334333	RHSIM_Rhsim02G0023100
Root vs Leaf	RHSIM_Rhsim03G0092200	-2.482552214	0.000122256	0.000334571	RHSIM_Rhsim03G0092200
Root vs Leaf	RHSIM_Rhsim11G0043100	-2.488248764	0.000122469	0.000335113	RHSIM_Rhsim11G0043100
Root vs Leaf	RHSIM_Rhsim07G0082700	-2.771376479	0.000122595	0.000335419	RHSIM_Rhsim07G0082700
Root vs Leaf	RHSIM_Rhsim08G0244700	2.337810807	0.000123227	0.000337029	RHSIM_Rhsim08G0244700
Root vs Leaf	RHSIM_Rhsim07G0060800	-1.041041424	0.000123323	0.000337251	RHSIM_Rhsim07G0060800
Root vs Leaf	RHSIM_Rhsim06G0039200	-2.973470896	0.000123447	0.00033755	RHSIM_Rhsim06G0039200
Root vs Leaf	RHSIM_Rhsim11G0191600	2.104933086	0.000123573	0.000337856	RHSIM_Rhsim11G0191600
Root vs Leaf	RHSIM_Rhsim13G0198900	-2.850616487	0.00012491	0.000341469	RHSIM_Rhsim13G0198900

Comparison	Gene ID	log_2FC	P-value	FDR (padj)	Gene Name
Root vs Leaf	RHSIM_Rhsim02G0001900	-1.489407009	0.000125134	0.000342003	RHSIM_Rhsim02G0001900
Root vs Leaf	RHSIM_Rhsim02G0113600	-3.126002724	0.000125351	0.000342554	RHSIM_Rhsim02G0113600
Root vs Leaf	RHSIM_Rhsim06G0129100	1.146665164	0.000125706	0.000343483	RHSIM_Rhsim06G0129100
Root vs Leaf	RHSIM_Rhsim13G0052700	-1.034336146	0.000126223	0.000344855	RHSIM_Rhsim13G0052700
Root vs Leaf	novel.5408	-1.540727081	0.000126733	0.000346126	--
Root vs Leaf	RHSIM_Rhsim07G0087300	6.908435725	0.000127116	0.000347131	RHSIM_Rhsim07G0087300
Root vs Leaf	RHSIM_Rhsim02G0101200	2.762000296	0.000127337	0.000347693	RHSIM_Rhsim02G0101200
Root vs Leaf	RHSIM_Rhsim08G0232200	1.259880371	0.000128079	0.000349554	RHSIM_Rhsim08G0232200
Root vs Leaf	RHSIM_Rhsim06G0181000	-7.955930323	0.000128151	0.00034971	RHSIM_Rhsim06G0181000
Root vs Leaf	RHSIM_Rhsim09G0014700	6.262448325	0.000128294	0.00035002	RHSIM_Rhsim09G0014700
Root vs Leaf	novel.5273	1.248018991	0.000128308	0.00035002	--
Root vs Leaf	RHSIM_Rhsim11G0196500	2.798669811	0.00012831	0.00035002	RHSIM_Rhsim11G0196500
Root vs Leaf	RHSIM_Rhsim01G0131700	7.275362273	0.000128531	0.00035058	RHSIM_Rhsim01G0131700
Root vs Leaf	RHSIM_Rhsim09G0092200	1.999586187	0.000128696	0.000350989	RHSIM_Rhsim09G0092200
Root vs Leaf	RHSIM_Rhsim05G0206900	-1.087316647	0.000128729	0.000351038	RHSIM_Rhsim05G0206900
Root vs Leaf	RHSIM_Rhsim11G0125300	1.020753127	0.000128823	0.000351254	RHSIM_Rhsim11G0125300
Root vs Leaf	RHSIM_Rhsim03G0099000	1.529933601	0.000129997	0.000354303	RHSIM_Rhsim03G0099000
Root vs Leaf	RHSIM_Rhsim04G0207800	-1.517513894	0.00013023	0.000354882	RHSIM_Rhsim04G0207800
Root vs Leaf	RHSIM_Rhsim03G0151700	-6.984385451	0.000130264	0.000354933	RHSIM_Rhsim03G0151700
Root vs Leaf	RHSIM_Rhsim08G0103200	1.796749422	0.000130832	0.000356397	RHSIM_Rhsim08G0103200
Root vs Leaf	RHSIM_Rhsim13G0086100	-1.064930158	0.000130983	0.000356764	RHSIM_Rhsim13G0086100
Root vs Leaf	novel.4852	1.752720553	0.000131515	0.000358171	--
Root vs Leaf	RHSIM_Rhsim08G0026500	2.145328172	0.000131932	0.000359223	RHSIM_Rhsim08G0026500
Root vs Leaf	novel.2535	-1.439927456	0.000133061	0.000362212	--
Root vs Leaf	RHSIM_Rhsim07G0236600	1.78731255	0.000133135	0.000362372	RHSIM_Rhsim07G0236600

Comparison	Gene ID	log_2FC	P-value	FDR (padj)	Gene Name
Root vs Leaf	RHSIM_Rhsim13G0160900	-10.05969096	0.000133832	0.000364192	RHSIM_Rhsim13G0160900
Root vs Leaf	RHSIM_Rhsim02G0066800	2.225277124	0.000133836	0.000364192	RHSIM_Rhsim02G0066800
Root vs Leaf	RHSIM_Rhsim11G0075900	-3.601700589	0.000134713	0.000366365	RHSIM_Rhsim11G0075900
Root vs Leaf	RHSIM_Rhsim05G0176000	1.598467935	0.00013489	0.000366801	RHSIM_Rhsim05G0176000
Root vs Leaf	RHSIM_Rhsim01G0181900	-4.727088356	0.000135817	0.000369194	RHSIM_Rhsim01G0181900
Root vs Leaf	RHSIM_Rhsim05G0200600	1.611328909	0.000135953	0.000369519	RHSIM_Rhsim05G0200600
Root vs Leaf	RHSIM_Rhsim01G0175400	5.787392398	0.00013631	0.000370403	RHSIM_Rhsim01G0175400
Root vs Leaf	RHSIM_Rhsim12G0056900	3.850207176	0.000136497	0.000370868	RHSIM_Rhsim12G0056900
Root vs Leaf	novel.1624	1.136780461	0.000136521	0.000370889	--
Root vs Leaf	novel.2069	3.710107821	0.000136994	0.000372042	--
Root vs Leaf	RHSIM_Rhsim08G0015500	4.270147093	0.000137037	0.000372117	RHSIM_Rhsim08G0015500
Root vs Leaf	RHSIM_Rhsim13G0115300	3.235510361	0.000137123	0.00037222	RHSIM_Rhsim13G0115300
Root vs Leaf	RHSIM_Rhsim09G0171400	3.642165135	0.000137435	0.000372977	RHSIM_Rhsim09G0171400
Root vs Leaf	novel.5147	-1.62359103	0.000137853	0.000374069	--
Root vs Leaf	novel.6097	2.519844085	0.000138673	0.000376205	--
Root vs Leaf	RHSIM_Rhsim13G0225500	6.697348175	0.000138886	0.000376739	RHSIM_Rhsim13G0225500
Root vs Leaf	RHSIM_RhsimMtG0007100	-3.496085042	0.000139317	0.00037782	RHSIM_RhsimMtG0007100
Root vs Leaf	novel.5011	6.138331339	0.000139357	0.000377884	--
Root vs Leaf	RHSIM_Rhsim09G0116900	2.371289049	0.000140076	0.000379657	RHSIM_Rhsim09G0116900
Root vs Leaf	RHSIM_Rhsim03G0118600	1.439719303	0.000140202	0.000379953	RHSIM_Rhsim03G0118600
Root vs Leaf	RHSIM_Rhsim07G0233700	3.268044568	0.000140268	0.000380043	RHSIM_Rhsim07G0233700
Root vs Leaf	RHSIM_Rhsim01G0194200	5.059880339	0.000140692	0.000381147	RHSIM_Rhsim01G0194200
Root vs Leaf	novel.741	3.483970835	0.000141187	0.000382397	--
Root vs Leaf	novel.6159	-1.085469158	0.000141512	0.000383233	--
Root vs Leaf	novel.2077	-1.2228687	0.000141713	0.000383732	--

Comparison	Gene ID	log_2FC	P-value	FDR (padj)	Gene Name
Root vs Leaf	novel.39	-1.328431552	0.000141943	0.000384311	--
Root vs Leaf	RHSIM_Rhsim05G0206600	-3.083742202	0.000142616	0.000386088	RHSIM_Rhsim05G0206600
Root vs Leaf	RHSIM_Rhsim03G0182100	-4.011593373	0.000143465	0.000388211	RHSIM_Rhsim03G0182100
Root vs Leaf	novel.2814	-5.44089479	0.000143906	0.000389306	--
Root vs Leaf	RHSIM_Rhsim12G0074300	-1.249157032	0.000144233	0.000390136	RHSIM_Rhsim12G0074300
Root vs Leaf	RHSIM_Rhsim03G0095500	-1.02141287	0.000145119	0.000392359	RHSIM_Rhsim03G0095500
Root vs Leaf	RHSIM_Rhsim08G0063600	-2.309094707	0.000147448	0.000398377	RHSIM_Rhsim08G0063600
Root vs Leaf	RHSIM_Rhsim02G0206000	-1.365306422	0.000147635	0.000398834	RHSIM_Rhsim02G0206000
Root vs Leaf	RHSIM_Rhsim07G0121300	-2.525132424	0.00014797	0.000399693	RHSIM_Rhsim07G0121300
Root vs Leaf	novel.1943	-1.509539686	0.000148499	0.000401028	--
Root vs Leaf	RHSIM_Rhsim01G0292600	-1.024062776	0.000148632	0.000401342	RHSIM_Rhsim01G0292600
Root vs Leaf	RHSIM_Rhsim04G0182100	6.370600758	0.000148766	0.000401655	RHSIM_Rhsim04G0182100
Root vs Leaf	RHSIM_Rhsim08G0239000	1.992893533	0.000148977	0.000402179	RHSIM_Rhsim08G0239000
Root vs Leaf	RHSIM_Rhsim09G0184700	1.893847111	0.000149021	0.00040225	RHSIM_Rhsim09G0184700
Root vs Leaf	RHSIM_Rhsim09G0111000	1.571718284	0.000149047	0.000402275	RHSIM_Rhsim09G0111000
Root vs Leaf	novel.676	2.57589771	0.00014951	0.000403477	--
Root vs Leaf	RHSIM_Rhsim05G0187500	-2.92929107	0.000149788	0.000404179	RHSIM_Rhsim05G0187500
Root vs Leaf	RHSIM_Rhsim01G0076600	-1.486293223	0.00014983	0.000404247	RHSIM_Rhsim01G0076600
Root vs Leaf	novel.3395	-1.062473644	0.000150474	0.000405888	--
Root vs Leaf	RHSIM_Rhsim02G0015500	6.276480205	0.000150924	0.000407008	RHSIM_Rhsim02G0015500
Root vs Leaf	RHSIM_Rhsim08G0207200	-1.24629905	0.000151023	0.000407226	RHSIM_Rhsim08G0207200
Root vs Leaf	RHSIM_Rhsim03G0058500	1.415844705	0.000151141	0.000407497	RHSIM_Rhsim03G0058500
Root vs Leaf	novel.5683	2.860351742	0.000151501	0.000408326	--
Root vs Leaf	novel.763	-2.031284232	0.00015154	0.000408383	--
Root vs Leaf	novel.3703	1.379707664	0.000152119	0.000409827	--

Comparison	Gene ID	log_2FC	P-value	FDR (padj)	Gene Name
Root vs Leaf	RHSIM_Rhsim06G0017600	-2.775937932	0.000152705	0.000411284	RHSIM_Rhsim06G0017600
Root vs Leaf	RHSIM_Rhsim04G0208000	5.291254816	0.000152809	0.000411516	RHSIM_Rhsim04G0208000
Root vs Leaf	RHSIM_Rhsim05G0184300	-2.163711118	0.000153663	0.000413624	RHSIM_Rhsim05G0184300
Root vs Leaf	novel.582	-1.237642609	0.000154262	0.00041514	--
Root vs Leaf	RHSIM_Rhsim08G0214300	1.075578247	0.00015492	0.000416861	RHSIM_Rhsim08G0214300
Root vs Leaf	RHSIM_Rhsim10G0002800	-1.243356324	0.000155363	0.000417957	RHSIM_Rhsim10G0002800
Root vs Leaf	RHSIM_Rhsim01G0193200	-1.736204178	0.000155385	0.000417966	RHSIM_Rhsim01G0193200
Root vs Leaf	RHSIM_Rhsim10G0172200	6.334760974	0.000156751	0.000421299	RHSIM_Rhsim10G0172200
Root vs Leaf	novel.3300	-1.690312931	0.000156986	0.00042188	--
Root vs Leaf	RHSIM_Rhsim13G0031300	1.961940825	0.000157541	0.000423225	RHSIM_Rhsim13G0031300
Root vs Leaf	RHSIM_Rhsim02G0043600	2.810936108	0.000157794	0.000423806	RHSIM_Rhsim02G0043600
Root vs Leaf	novel.5542	3.885019046	0.000158482	0.000425554	--
Root vs Leaf	RHSIM_Rhsim11G0074800	-1.269302263	0.000158511	0.000425583	RHSIM_Rhsim11G0074800
Root vs Leaf	RHSIM_Rhsim13G0039500	1.216681727	0.000158709	0.000426015	RHSIM_Rhsim13G0039500
Root vs Leaf	RHSIM_Rhsim03G0104700	2.935847317	0.000158875	0.000426413	RHSIM_Rhsim03G0104700
Root vs Leaf	RHSIM_Rhsim03G0262000	-1.302788355	0.000158928	0.000426505	RHSIM_Rhsim03G0262000
Root vs Leaf	RHSIM_Rhsim08G0243200	1.256642648	0.000159077	0.000426857	RHSIM_Rhsim08G0243200
Root vs Leaf	RHSIM_Rhsim07G0210200	3.471184748	0.000159258	0.000427292	RHSIM_Rhsim07G0210200
Root vs Leaf	RHSIM_Rhsim08G0028800	2.163906171	0.00016052	0.000430579	RHSIM_Rhsim08G0028800
Root vs Leaf	RHSIM_RhsimUnG0165700	6.15274037	0.000160842	0.000431292	RHSIM_RhsimUnG0165700
Root vs Leaf	RHSIM_Rhsim11G0002000	1.382042234	0.000161152	0.000432072	RHSIM_Rhsim11G0002000
Root vs Leaf	RHSIM_Rhsim10G0071100	-3.133714227	0.000161491	0.00043293	RHSIM_Rhsim10G0071100
Root vs Leaf	RHSIM_Rhsim12G0045300	-9.729837325	0.000162355	0.000435148	RHSIM_Rhsim12G0045300
Root vs Leaf	RHSIM_Rhsim08G0012800	-4.0865566	0.000163259	0.000437419	RHSIM_Rhsim08G0012800
Root vs Leaf	RHSIM_Rhsim01G0038100	-7.903173688	0.000163724	0.000438611	RHSIM_Rhsim01G0038100

Comparison	Gene ID	log_2FC	P-value	FDR (padj)	Gene Name
Root vs Leaf	RHSIM_Rhsim02G0220900	2.222771937	0.000164657	0.000441009	RHSIM_Rhsim02G0220900
Root vs Leaf	RHSIM_Rhsim04G0061700	-5.544621376	0.000164886	0.000441571	RHSIM_Rhsim04G0061700
Root vs Leaf	novel.64	-1.94704891	0.000165032	0.000441912	--
Root vs Leaf	RHSIM_Rhsim02G0053300	1.165753965	0.000165068	0.000441957	RHSIM_Rhsim02G0053300
Root vs Leaf	RHSIM_Rhsim03G0238700	-3.29989551	0.00016538	0.000442743	RHSIM_Rhsim03G0238700
Root vs Leaf	RHSIM_Rhsim08G0090400	-2.388226583	0.000166402	0.000445427	RHSIM_Rhsim08G0090400
Root vs Leaf	RHSIM_Rhsim05G0232500	3.764900108	0.000166511	0.000445667	RHSIM_Rhsim05G0232500
Root vs Leaf	novel.1958	1.290929835	0.000166643	0.000445915	--
Root vs Leaf	RHSIM_Rhsim06G0091800	-1.742201895	0.000166944	0.000446617	RHSIM_Rhsim06G0091800
Root vs Leaf	RHSIM_Rhsim11G0196600	2.676348815	0.000167348	0.000447596	RHSIM_Rhsim11G0196600
Root vs Leaf	novel.5522	6.624237471	0.000168062	0.000449351	--
Root vs Leaf	RHSIM_Rhsim07G0073700	-2.343846753	0.000168226	0.000449736	RHSIM_Rhsim07G0073700
Root vs Leaf	RHSIM_RhsimUnG0137800	1.319726133	0.00016867	0.000450818	RHSIM_RhsimUnG0137800
Root vs Leaf	RHSIM_Rhsim08G0208300	1.949863251	0.000168823	0.000451124	RHSIM_Rhsim08G0208300
Root vs Leaf	RHSIM_Rhsim06G0031000	1.698607882	0.000168953	0.000451419	RHSIM_Rhsim06G0031000
Root vs Leaf	RHSIM_Rhsim12G0024800	-1.017125471	0.00016949	0.000452802	RHSIM_Rhsim12G0024800
Root vs Leaf	RHSIM_Rhsim04G0213900	1.258633149	0.000169887	0.000453811	RHSIM_Rhsim04G0213900
Root vs Leaf	RHSIM_Rhsim04G0156700	1.478169752	0.000170059	0.000454218	RHSIM_Rhsim04G0156700
Root vs Leaf	RHSIM_Rhsim10G0121100	1.422601067	0.000170784	0.000456031	RHSIM_Rhsim10G0121100
Root vs Leaf	novel.1653	6.507343337	0.000170797	0.000456031	--
Root vs Leaf	RHSIM_Rhsim11G0163700	-1.515951183	0.000171356	0.000457366	RHSIM_Rhsim11G0163700
Root vs Leaf	novel.1527	-1.510906801	0.000171739	0.00045828	--
Root vs Leaf	RHSIM_Rhsim03G0111100	1.875616585	0.000171979	0.000458869	RHSIM_Rhsim03G0111100
Root vs Leaf	RHSIM_Rhsim02G0005300	1.297654865	0.000172108	0.00045916	RHSIM_Rhsim02G0005300
Root vs Leaf	RHSIM_Rhsim05G0075500	-1.034301871	0.000172128	0.00045916	RHSIM_Rhsim05G0075500

Comparison	Gene ID	log_2FC	P-value	FDR (padj)	Gene Name
Root vs Leaf	RHSIM_Rhsim05G0232200	-2.713415612	0.000172264	0.00045947	RHSIM_Rhsim05G0232200
Root vs Leaf	RHSIM_Rhsim08G0200400	1.233314711	0.000173273	0.000462056	RHSIM_Rhsim08G0200400
Root vs Leaf	RHSIM_Rhsim09G0159100	-1.289215329	0.000173824	0.000463418	RHSIM_Rhsim09G0159100
Root vs Leaf	RHSIM_Rhsim05G0001400	-2.191355778	0.000173871	0.00046349	RHSIM_Rhsim05G0001400
Root vs Leaf	RHSIM_Rhsim01G0107700	1.890745598	0.000176286	0.000469764	RHSIM_Rhsim01G0107700
Root vs Leaf	RHSIM_Rhsim02G0006100	1.619630831	0.00017827	0.000474779	RHSIM_Rhsim02G0006100
Root vs Leaf	RHSIM_Rhsim09G0033300	2.138789974	0.000178749	0.000475944	RHSIM_Rhsim09G0033300
Root vs Leaf	novel.757	4.644375065	0.000179149	0.000476866	--
Root vs Leaf	RHSIM_Rhsim13G0195500	3.58297563	0.000179326	0.000477151	RHSIM_Rhsim13G0195500
Root vs Leaf	RHSIM_RhsimUnG0105000	1.330939259	0.00018002	0.000478944	RHSIM_RhsimUnG0105000
Root vs Leaf	RHSIM_Rhsim13G0035000	-1.938166985	0.000180444	0.000479851	RHSIM_Rhsim13G0035000
Root vs Leaf	RHSIM_Rhsim04G0221900	3.049429185	0.000180924	0.000481071	RHSIM_Rhsim04G0221900
Root vs Leaf	RHSIM_Rhsim10G0028500	-1.562470806	0.000181136	0.000481579	RHSIM_Rhsim10G0028500
Root vs Leaf	novel.3270	1.353968165	0.000181353	0.000482102	--
Root vs Leaf	RHSIM_Rhsim06G0049400	3.540658748	0.000181871	0.000483421	RHSIM_Rhsim06G0049400
Root vs Leaf	RHSIM_Rhsim11G0092800	1.500509584	0.000182486	0.000484953	RHSIM_Rhsim11G0092800
Root vs Leaf	RHSIM_Rhsim12G0041700	-1.944613351	0.000182489	0.000484953	RHSIM_Rhsim12G0041700
Root vs Leaf	RHSIM_Rhsim12G0010600	-1.292561241	0.000182668	0.000485373	RHSIM_Rhsim12G0010600
Root vs Leaf	RHSIM_Rhsim11G0074500	-1.728335911	0.000183111	0.000486495	RHSIM_Rhsim11G0074500
Root vs Leaf	RHSIM_Rhsim04G0112300	1.941764757	0.000183162	0.000486574	RHSIM_Rhsim04G0112300
Root vs Leaf	novel.5855	-5.756198566	0.000183228	0.000486693	--
Root vs Leaf	RHSIM_Rhsim09G0103200	2.391622127	0.000184846	0.000490878	RHSIM_Rhsim09G0103200
Root vs Leaf	RHSIM_Rhsim04G0231300	2.197412058	0.000185505	0.000492516	RHSIM_Rhsim04G0231300
Root vs Leaf	RHSIM_Rhsim11G0103000	-6.500674944	0.000186013	0.000493809	RHSIM_Rhsim11G0103000
Root vs Leaf	RHSIM_Rhsim10G0054900	-2.104296212	0.000186393	0.000494759	RHSIM_Rhsim10G0054900

Comparison	Gene ID	log_2FC	P-value	FDR (padj)	Gene Name
Root vs Leaf	RHSIM_Rhsim04G0190400	1.315790811	0.000186557	0.000495138	RHSIM_Rhsim04G0190400
Root vs Leaf	RHSIM_Rhsim11G0175500	6.487795344	0.00018697	0.000496065	RHSIM_Rhsim11G0175500
Root vs Leaf	novel.4206	-1.347913628	0.000187306	0.000496898	--
Root vs Leaf	RHSIM_Rhsim11G0041400	3.375971857	0.0001876	0.000497565	RHSIM_Rhsim11G0041400
Root vs Leaf	RHSIM_Rhsim13G0078200	-1.200354426	0.000187664	0.000497678	RHSIM_Rhsim13G0078200
Root vs Leaf	RHSIM_RhsimUnG0007700	-2.98290521	0.000187882	0.000498199	RHSIM_RhsimUnG0007700
Root vs Leaf	RHSIM_RhsimUnG0166400	-2.372167982	0.000187978	0.000498396	RHSIM_RhsimUnG0166400
Root vs Leaf	novel.4719	-7.253049281	0.000188368	0.000499372	--
Root vs Leaf	novel.5808	6.01569519	0.000188854	0.000500604	--
Root vs Leaf	novel.587	-1.887896151	0.000188961	0.000500829	--
Root vs Leaf	RHSIM_Rhsim12G0008400	2.015316767	0.000189024	0.00050094	RHSIM_Rhsim12G0008400
Root vs Leaf	RHSIM_Rhsim02G0073900	-7.302160318	0.000189311	0.000501643	RHSIM_Rhsim02G0073900
Root vs Leaf	RHSIM_Rhsim10G0190100	3.285261789	0.000190108	0.000503641	RHSIM_Rhsim10G0190100
Root vs Leaf	RHSIM_Rhsim01G0175600	1.112791279	0.000191501	0.000506982	RHSIM_Rhsim01G0175600
Root vs Leaf	RHSIM_Rhsim05G0018100	1.389525312	0.000192885	0.000510529	RHSIM_Rhsim05G0018100
Root vs Leaf	RHSIM_Rhsim10G0209400	1.33114551	0.000192936	0.000510605	RHSIM_Rhsim10G0209400
Root vs Leaf	RHSIM_Rhsim03G0032800	-2.971496951	0.000194613	0.000514925	RHSIM_Rhsim03G0032800
Root vs Leaf	novel.6201	-1.560151729	0.000194855	0.000515507	--
Root vs Leaf	novel.5892	1.722803885	0.000195079	0.000515923	--
Root vs Leaf	RHSIM_Rhsim07G0187200	-2.342927681	0.000195576	0.000517164	RHSIM_Rhsim07G0187200
Root vs Leaf	novel.1556	1.955208114	0.000195593	0.000517164	--
Root vs Leaf	RHSIM_Rhsim09G0210600	1.129406793	0.000196376	0.000519055	RHSIM_Rhsim09G0210600
Root vs Leaf	RHSIM_Rhsim04G0091900	4.952394437	0.00019708	0.000520739	RHSIM_Rhsim04G0091900
Root vs Leaf	RHSIM_Rhsim08G0221700	-1.245015731	0.00019743	0.000521485	RHSIM_Rhsim08G0221700
Root vs Leaf	RHSIM_Rhsim13G0186700	1.79346769	0.000197532	0.000521696	RHSIM_Rhsim13G0186700

Comparison	Gene ID	log_2FC	P-value	FDR (padj)	Gene Name
Root vs Leaf	RHSIM_Rhsim03G0164500	1.938587227	0.000198519	0.000524181	RHSIM_Rhsim03G0164500
Root vs Leaf	novel.1795	1.952871896	0.000199387	0.000526294	--
Root vs Leaf	RHSIM_Rhsim12G0007100	1.521600689	0.000199725	0.000527127	RHSIM_Rhsim12G0007100
Root vs Leaf	RHSIM_Rhsim08G0234000	-2.018155361	0.000200426	0.000528915	RHSIM_Rhsim08G0234000
Root vs Leaf	RHSIM_Rhsim04G0184100	2.600985685	0.000201222	0.000530894	RHSIM_Rhsim04G0184100
Root vs Leaf	RHSIM_Rhsim12G0185500	1.20986829	0.000201848	0.000532485	RHSIM_Rhsim12G0185500
Root vs Leaf	novel.6206	1.576990181	0.000201898	0.000532557	--
Root vs Leaf	RHSIM_Rhsim01G0164000	1.946859126	0.000203324	0.000536074	RHSIM_Rhsim01G0164000
Root vs Leaf	RHSIM_Rhsim03G0229900	1.095626614	0.00020347	0.000536398	RHSIM_Rhsim03G0229900
Root vs Leaf	RHSIM_Rhsim01G0145500	1.687789257	0.000203706	0.000536898	RHSIM_Rhsim01G0145500
Root vs Leaf	RHSIM_Rhsim03G0077400	3.459837141	0.000204042	0.000537721	RHSIM_Rhsim03G0077400
Root vs Leaf	RHSIM_RhsimUnG0179400	-2.674133265	0.000204257	0.000538226	RHSIM_RhsimUnG0179400
Root vs Leaf	RHSIM_Rhsim02G0010500	1.186368383	0.000205338	0.000541015	RHSIM_Rhsim02G0010500
Root vs Leaf	RHSIM_RhsimUnG0181700	-2.238308166	0.000205438	0.000541215	RHSIM_RhsimUnG0181700
Root vs Leaf	RHSIM_Rhsim13G0174300	1.125206238	0.000205739	0.000541885	RHSIM_Rhsim13G0174300
Root vs Leaf	RHSIM_Rhsim08G0050000	-1.153377987	0.000206593	0.000544011	RHSIM_Rhsim08G0050000
Root vs Leaf	RHSIM_Rhsim09G0141000	1.019598153	0.000207342	0.000545838	RHSIM_Rhsim09G0141000
Root vs Leaf	RHSIM_Rhsim04G0227500	1.418113107	0.000207401	0.000545889	RHSIM_Rhsim04G0227500
Root vs Leaf	RHSIM_Rhsim10G0080300	1.816118164	0.000208039	0.000547444	RHSIM_Rhsim10G0080300
Root vs Leaf	novel.5645	1.411735434	0.000208145	0.000547663	--
Root vs Leaf	novel.5094	-6.88526124	0.000208325	0.000548011	--
Root vs Leaf	RHSIM_Rhsim13G0093500	2.126255117	0.000208517	0.000548408	RHSIM_Rhsim13G0093500
Root vs Leaf	RHSIM_Rhsim12G0099700	-1.617101247	0.00020894	0.000549379	RHSIM_Rhsim12G0099700
Root vs Leaf	RHSIM_Rhsim06G0122400	1.502909605	0.000209212	0.00054997	RHSIM_Rhsim06G0122400
Root vs Leaf	RHSIM_Rhsim07G0042400	2.18951717	0.000210452	0.000552977	RHSIM_Rhsim07G0042400

Comparison	Gene ID	log_2FC	P-value	FDR (padj)	Gene Name
Root vs Leaf	RHSIM_Rhsim04G0117600	1.594704332	0.00021078	0.000553777	RHSIM_Rhsim04G0117600
Root vs Leaf	RHSIM_RhsimUnG0125400	1.767961876	0.000211284	0.000555038	RHSIM_RhsimUnG0125400
Root vs Leaf	RHSIM_Rhsim10G0168700	3.65223791	0.000212573	0.000558359	RHSIM_Rhsim10G0168700
Root vs Leaf	RHSIM_Rhsim03G0162000	-2.007146227	0.000212923	0.000559216	RHSIM_Rhsim03G0162000
Root vs Leaf	RHSIM_Rhsim13G0180900	-2.276947907	0.000213449	0.00056047	RHSIM_Rhsim13G0180900
Root vs Leaf	RHSIM_Rhsim06G0126100	1.022070974	0.000214473	0.000563096	RHSIM_Rhsim06G0126100
Root vs Leaf	RHSIM_Rhsim09G0155400	-1.111981811	0.000215426	0.000565469	RHSIM_Rhsim09G0155400
Root vs Leaf	novel.5628	6.898211492	0.000216648	0.000568484	--
Root vs Leaf	RHSIM_Rhsim12G0051900	-1.00548048	0.000218359	0.000572893	RHSIM_Rhsim12G0051900
Root vs Leaf	RHSIM_Rhsim01G0283900	1.149283342	0.000218378	0.000572893	RHSIM_Rhsim01G0283900
Root vs Leaf	RHSIM_Rhsim01G0287400	1.118272424	0.000218977	0.000574398	RHSIM_Rhsim01G0287400
Root vs Leaf	RHSIM_Rhsim08G0240800	1.049443549	0.000219093	0.000574637	RHSIM_Rhsim08G0240800
Root vs Leaf	RHSIM_Rhsim06G0030600	1.695668594	0.000219131	0.000574673	RHSIM_Rhsim06G0030600
Root vs Leaf	RHSIM_Rhsim01G0234800	3.981334669	0.000220676	0.000578593	RHSIM_Rhsim01G0234800
Root vs Leaf	RHSIM_Rhsim12G0150300	6.466296424	0.000221385	0.000580386	RHSIM_Rhsim12G0150300
Root vs Leaf	RHSIM_Rhsim13G0059200	-7.179680572	0.000221419	0.000580395	RHSIM_Rhsim13G0059200
Root vs Leaf	RHSIM_Rhsim10G0140400	-3.931868321	0.000221438	0.000580395	RHSIM_Rhsim10G0140400
Root vs Leaf	RHSIM_RhsimUnG0112900	-1.660648139	0.000221555	0.00058063	RHSIM_RhsimUnG0112900
Root vs Leaf	RHSIM_Rhsim13G0229500	1.710371602	0.000221678	0.000580825	RHSIM_Rhsim13G0229500
Root vs Leaf	RHSIM_Rhsim13G0006700	1.074550126	0.000221722	0.000580876	RHSIM_Rhsim13G0006700
Root vs Leaf	novel.2052	-2.811424213	0.000221896	0.000581265	--
Root vs Leaf	RHSIM_RhsimUnG0122800	-2.622041145	0.000222029	0.000581549	RHSIM_RhsimUnG0122800
Root vs Leaf	novel.437	-2.773290727	0.000222208	0.00058195	--
Root vs Leaf	RHSIM_Rhsim06G0072100	1.727798797	0.000222387	0.000582353	RHSIM_Rhsim06G0072100
Root vs Leaf	RHSIM_Rhsim10G0183500	-1.152876635	0.000222848	0.000583429	RHSIM_Rhsim10G0183500

Comparison	Gene ID	log_2FC	P-value	FDR (padj)	Gene Name
Root vs Leaf	RHSIM_Rhsim07G0116600	-1.563227258	0.000222885	0.000583459	RHSIM_Rhsim07G0116600
Root vs Leaf	RHSIM_Rhsim05G0172900	-2.86137828	0.000223112	0.000583988	RHSIM_Rhsim05G0172900
Root vs Leaf	RHSIM_Rhsim03G0261800	1.208587089	0.000223204	0.000584162	RHSIM_Rhsim03G0261800
Root vs Leaf	novel.5073	-1.918906764	0.000223518	0.000584919	--
Root vs Leaf	RHSIM_Rhsim05G0070300	-1.985955679	0.000224434	0.00058705	RHSIM_Rhsim05G0070300
Root vs Leaf	RHSIM_Rhsim06G0045500	3.203497839	0.000225823	0.00059043	RHSIM_Rhsim06G0045500
Root vs Leaf	RHSIM_Rhsim04G0197600	-3.808809493	0.000225863	0.000590453	RHSIM_Rhsim04G0197600
Root vs Leaf	novel.3593	-1.290917988	0.000225899	0.000590482	--
Root vs Leaf	novel.5882	5.267685376	0.000226761	0.0005926	--
Root vs Leaf	RHSIM_Rhsim06G0234800	1.168414281	0.000227742	0.00059503	RHSIM_Rhsim06G0234800
Root vs Leaf	RHSIM_Rhsim04G0206800	3.162540984	0.000229001	0.00059796	RHSIM_Rhsim04G0206800
Root vs Leaf	novel.5969	-3.750639843	0.000229875	0.000600129	--
Root vs Leaf	novel.5931	-3.039099249	0.00023	0.000600386	--
Root vs Leaf	RHSIM_Rhsim03G0216200	1.925282361	0.000230534	0.000601577	RHSIM_Rhsim03G0216200
Root vs Leaf	RHSIM_Rhsim01G0171800	-3.538303408	0.000230655	0.000601826	RHSIM_Rhsim01G0171800
Root vs Leaf	RHSIM_Rhsim13G0001000	-1.10887633	0.000231042	0.000602766	RHSIM_Rhsim13G0001000
Root vs Leaf	RHSIM_Rhsim02G0207700	2.142780012	0.000231305	0.000603385	RHSIM_Rhsim02G0207700
Root vs Leaf	RHSIM_Rhsim02G0155000	1.06325553	0.000232262	0.000605812	RHSIM_Rhsim02G0155000
Root vs Leaf	RHSIM_RhsimUnG0111100	2.893215881	0.000232632	0.00060671	RHSIM_RhsimUnG0111100
Root vs Leaf	novel.4051	-1.10490407	0.000233408	0.000608596	--
Root vs Leaf	RHSIM_Rhsim02G0254600	1.249065024	0.000234149	0.00061046	RHSIM_Rhsim02G0254600
Root vs Leaf	RHSIM_Rhsim02G0241700	2.580645111	0.000234718	0.000611806	RHSIM_Rhsim02G0241700
Root vs Leaf	RHSIM_Rhsim01G0120400	6.398794083	0.000234985	0.000612365	RHSIM_Rhsim01G0120400
Root vs Leaf	RHSIM_Rhsim02G0209600	6.098939099	0.000235295	0.000613103	RHSIM_Rhsim02G0209600
Root vs Leaf	RHSIM_Rhsim02G0171400	1.905459837	0.000235881	0.000614559	RHSIM_Rhsim02G0171400

Comparison	Gene ID	log_2FC	P-value	FDR (padj)	Gene Name
Root vs Leaf	RHSIM_Rhsim11G0009400	-3.662655826	0.000236933	0.000617162	RHSIM_Rhsim11G0009400
Root vs Leaf	novel.147	-2.728510393	0.000239287	0.000623153	--
Root vs Leaf	RHSIM_Rhsim05G0229500	2.976050122	0.000239471	0.000623563	RHSIM_Rhsim05G0229500
Root vs Leaf	novel.4842	-1.221498316	0.000239869	0.000624529	--
Root vs Leaf	novel.6172	4.616649784	0.00024153	0.000628784	--
Root vs Leaf	RHSIM_Rhsim08G0023100	2.202881647	0.000241807	0.000629433	RHSIM_Rhsim08G0023100
Root vs Leaf	novel.1776	2.022496377	0.000242416	0.000630804	--
Root vs Leaf	RHSIM_Rhsim11G0023400	-3.29247324	0.00024261	0.00063124	RHSIM_Rhsim11G0023400
Root vs Leaf	RHSIM_Rhsim11G0020500	-3.676438951	0.000242783	0.000631618	RHSIM_Rhsim11G0020500
Root vs Leaf	RHSIM_Rhsim04G0189900	-1.026331264	0.000243275	0.000632685	RHSIM_Rhsim04G0189900
Root vs Leaf	RHSIM_RhsimUnG0125700	1.4865544	0.000243617	0.000633503	RHSIM_RhsimUnG0125700
Root vs Leaf	RHSIM_Rhsim09G0114000	1.561567733	0.000243709	0.000633671	RHSIM_Rhsim09G0114000
Root vs Leaf	RHSIM_Rhsim09G0190500	-2.162020066	0.000244353	0.000635275	RHSIM_Rhsim09G0190500
Root vs Leaf	RHSIM_Rhsim05G0163100	-1.779892625	0.000244455	0.000635468	RHSIM_Rhsim05G0163100
Root vs Leaf	RHSIM_Rhsim08G0221300	1.180523112	0.000244512	0.000635544	RHSIM_Rhsim08G0221300
Root vs Leaf	novel.1619	1.445572103	0.000244581	0.000635653	--
Root vs Leaf	RHSIM_Rhsim08G0218300	1.536695745	0.000246129	0.000639461	RHSIM_Rhsim08G0218300
Root vs Leaf	RHSIM_Rhsim12G0095300	-4.579000529	0.000246398	0.000640088	RHSIM_Rhsim12G0095300
Root vs Leaf	RHSIM_Rhsim09G0023200	-2.032011161	0.000246958	0.000641398	RHSIM_Rhsim09G0023200
Root vs Leaf	RHSIM_Rhsim06G0094300	3.523595771	0.000246986	0.000641398	RHSIM_Rhsim06G0094300
Root vs Leaf	RHSIM_Rhsim13G0087300	3.156047663	0.00024746	0.000642558	RHSIM_Rhsim13G0087300
Root vs Leaf	RHSIM_Rhsim12G0174600	-2.794279124	0.0002487	0.000645633	RHSIM_Rhsim12G0174600
Root vs Leaf	RHSIM_Rhsim13G0214200	-1.161251197	0.000248947	0.0006462	RHSIM_Rhsim13G0214200
Root vs Leaf	novel.5386	-1.758653795	0.000249894	0.000648441	--
Root vs Leaf	novel.4386	4.803418679	0.000249932	0.000648466	--

Comparison	Gene ID	log_2FC	P-value	FDR (padj)	Gene Name
Root vs Leaf	RHSIM_Rhsim08G0043700	-1.258668735	0.000250266	0.00064926	RHSIM_Rhsim08G0043700
Root vs Leaf	RHSIM_Rhsim09G0177000	1.49105049	0.00025179	0.000653069	RHSIM_Rhsim09G0177000
Root vs Leaf	RHSIM_Rhsim03G0097800	3.631314248	0.000251869	0.0006532	RHSIM_Rhsim03G0097800
Root vs Leaf	novel.133	2.300142599	0.000252109	0.000653749	--
Root vs Leaf	RHSIM_Rhsim13G0014100	4.324939896	0.000252609	0.000654973	RHSIM_Rhsim13G0014100
Root vs Leaf	RHSIM_Rhsim02G0096800	1.275817727	0.000252837	0.000655491	RHSIM_Rhsim02G0096800
Root vs Leaf	RHSIM_Rhsim03G0061100	-3.835148275	0.000253452	0.000657011	RHSIM_Rhsim03G0061100
Root vs Leaf	RHSIM_Rhsim02G0109100	10.9303738	0.000253573	0.000657251	RHSIM_Rhsim02G0109100
Root vs Leaf	RHSIM_Rhsim12G0004700	5.886715357	0.000253776	0.000657704	RHSIM_Rhsim12G0004700
Root vs Leaf	RHSIM_Rhsim03G0110500	4.52106478	0.000253925	0.000658015	RHSIM_Rhsim03G0110500
Root vs Leaf	RHSIM_Rhsim06G0235800	1.410148468	0.00025428	0.000658788	RHSIM_Rhsim06G0235800
Root vs Leaf	RHSIM_Rhsim02G0238800	1.214675511	0.000255386	0.000661581	RHSIM_Rhsim02G0238800
Root vs Leaf	RHSIM_Rhsim04G0099100	1.666539844	0.000255576	0.000661996	RHSIM_Rhsim04G0099100
Root vs Leaf	RHSIM_Rhsim08G0070100	-1.514932676	0.000256137	0.000663301	RHSIM_Rhsim08G0070100
Root vs Leaf	RHSIM_RhsimUnG0188700	2.64988129	0.000256747	0.000664808	RHSIM_RhsimUnG0188700
Root vs Leaf	RHSIM_Rhsim06G0033300	-1.165953424	0.000256791	0.000664848	RHSIM_Rhsim06G0033300
Root vs Leaf	RHSIM_Rhsim06G0191600	-4.383744044	0.000257888	0.000667496	RHSIM_Rhsim06G0191600
Root vs Leaf	RHSIM_Rhsim05G0030100	1.172413623	0.000257901	0.000667496	RHSIM_Rhsim05G0030100
Root vs Leaf	RHSIM_Rhsim02G0108900	8.756846018	0.00025943	0.000671191	RHSIM_Rhsim02G0108900
Root vs Leaf	RHSIM_Rhsim03G0153900	1.313333955	0.000259444	0.000671191	RHSIM_Rhsim03G0153900
Root vs Leaf	RHSIM_Rhsim01G0003700	1.24893699	0.000259517	0.000671305	RHSIM_Rhsim01G0003700
Root vs Leaf	RHSIM_Rhsim03G0042500	-5.420519439	0.00025993	0.000672298	RHSIM_Rhsim03G0042500
Root vs Leaf	RHSIM_Rhsim01G0109700	1.851665502	0.0002602	0.000672919	RHSIM_Rhsim01G0109700
Root vs Leaf	RHSIM_Rhsim02G0067800	6.434382084	0.000261469	0.000676087	RHSIM_Rhsim02G0067800
Root vs Leaf	RHSIM_Rhsim06G0162100	-3.133572523	0.000261483	0.000676087	RHSIM_Rhsim06G0162100

Comparison	Gene ID	log_2FC	P-value	FDR (padj)	Gene Name
Root vs Leaf	RHSIM_Rhsim01G0232700	-1.07407474	0.000262263	0.000678027	RHSIM_Rhsim01G0232700
Root vs Leaf	novel.4017	1.706814568	0.000262332	0.000678054	--
Root vs Leaf	novel.1388	-1.676333692	0.000262624	0.000678733	--
Root vs Leaf	RHSIM_Rhsim01G0274000	-2.183720984	0.000262757	0.000679	RHSIM_Rhsim01G0274000
Root vs Leaf	RHSIM_Rhsim10G0188200	-1.324322253	0.000262909	0.000679243	RHSIM_Rhsim10G0188200
Root vs Leaf	RHSIM_Rhsim02G0249100	-1.278167468	0.000264939	0.000684346	RHSIM_Rhsim02G0249100
Root vs Leaf	RHSIM_Rhsim06G0083400	5.155008745	0.000265187	0.000684823	RHSIM_Rhsim06G0083400
Root vs Leaf	RHSIM_Rhsim10G0121800	5.300711155	0.000266442	0.000687909	RHSIM_Rhsim10G0121800
Root vs Leaf	RHSIM_Rhsim06G0153700	-4.113405272	0.000267896	0.000691585	RHSIM_Rhsim06G0153700
Root vs Leaf	RHSIM_Rhsim08G0097000	6.078455795	0.000268101	0.00069204	RHSIM_Rhsim08G0097000
Root vs Leaf	RHSIM_Rhsim08G0238400	4.721247846	0.000268601	0.000693096	RHSIM_Rhsim08G0238400
Root vs Leaf	RHSIM_Rhsim07G0017400	1.027194802	0.000268987	0.000693939	RHSIM_Rhsim07G0017400
Root vs Leaf	RHSIM_Rhsim04G0050300	-1.579586531	0.000269637	0.00069546	RHSIM_Rhsim04G0050300
Root vs Leaf	RHSIM_RhsimUnG0061000	4.104438821	0.000269907	0.00069608	RHSIM_RhsimUnG0061000
Root vs Leaf	RHSIM_Rhsim01G0255100	1.018907425	0.00027101	0.000698787	RHSIM_Rhsim01G0255100
Root vs Leaf	RHSIM_Rhsim09G0101600	2.393608044	0.000271498	0.000699872	RHSIM_Rhsim09G0101600
Root vs Leaf	RHSIM_Rhsim08G0145600	3.815439723	0.00027283	0.00070284	RHSIM_Rhsim08G0145600
Root vs Leaf	RHSIM_Rhsim08G0188300	1.041894055	0.000272832	0.00070284	RHSIM_Rhsim08G0188300
Root vs Leaf	RHSIM_Rhsim02G0042500	-1.238801606	0.000274445	0.000706758	RHSIM_Rhsim02G0042500
Root vs Leaf	novel.2298	4.584407882	0.000274882	0.000707806	--
Root vs Leaf	RHSIM_Rhsim07G0051300	6.958507981	0.000276481	0.000711526	RHSIM_Rhsim07G0051300
Root vs Leaf	RHSIM_Rhsim04G0043400	1.488907528	0.000277076	0.000712827	RHSIM_Rhsim04G0043400
Root vs Leaf	novel.3359	-1.046743196	0.000277079	0.000712827	--
Root vs Leaf	RHSIM_Rhsim12G0190900	1.928023587	0.000277337	0.000713412	RHSIM_Rhsim12G0190900
Root vs Leaf	RHSIM_Rhsim10G0094300	1.175031757	0.000277702	0.000714272	RHSIM_Rhsim10G0094300

Comparison	Gene ID	log_2FC	P-value	FDR (padj)	Gene Name
Root vs Leaf	RHSIM_Rhsim10G0153800	2.177400437	0.000277949	0.000714827	RHSIM_Rhsim10G0153800
Root vs Leaf	novel.4170	-1.647539817	0.000278121	0.00071519	--
Root vs Leaf	novel.3180	3.242180614	0.000278694	0.000716505	--
Root vs Leaf	RHSIM_Rhsim07G0095000	-2.703128049	0.000279658	0.000718903	RHSIM_Rhsim07G0095000
Root vs Leaf	RHSIM_Rhsim09G0169900	-1.546618789	0.000279887	0.000719352	RHSIM_Rhsim09G0169900
Root vs Leaf	RHSIM_Rhsim13G0103800	1.80205869	0.000279895	0.000719352	RHSIM_Rhsim13G0103800
Root vs Leaf	RHSIM_Rhsim02G0158600	-1.108886474	0.00028078	0.000721468	RHSIM_Rhsim02G0158600
Root vs Leaf	RHSIM_Rhsim05G0183600	2.144674234	0.000280954	0.000721833	RHSIM_Rhsim05G0183600
Root vs Leaf	RHSIM_Rhsim08G0159200	6.003811713	0.000283314	0.000727493	RHSIM_Rhsim08G0159200
Root vs Leaf	RHSIM_Rhsim08G0038400	2.729315032	0.000283649	0.000728272	RHSIM_Rhsim08G0038400
Root vs Leaf	RHSIM_Rhsim04G0111200	-3.571847929	0.000284153	0.000729487	RHSIM_Rhsim04G0111200
Root vs Leaf	novel.1850	6.151216785	0.000284554	0.000730434	--
Root vs Leaf	RHSIM_Rhsim07G0052900	-2.406906756	0.000284688	0.000730697	RHSIM_Rhsim07G0052900
Root vs Leaf	RHSIM_Rhsim07G0034100	1.047585842	0.00028615	0.000734286	RHSIM_Rhsim07G0034100
Root vs Leaf	novel.5588	-6.158635856	0.000286357	0.000734736	--
Root vs Leaf	RHSIM_Rhsim03G0246000	7.23409669	0.000286483	0.000734977	RHSIM_Rhsim03G0246000
Root vs Leaf	RHSIM_Rhsim06G0176800	2.999095519	0.000286758	0.000735602	RHSIM_Rhsim06G0176800
Root vs Leaf	RHSIM_Rhsim01G0238700	-1.709564378	0.000287785	0.000737909	RHSIM_Rhsim01G0238700
Root vs Leaf	RHSIM_Rhsim01G0191400	1.936069217	0.000289131	0.000741278	RHSIM_Rhsim01G0191400
Root vs Leaf	RHSIM_Rhsim07G0196800	1.261320696	0.000289632	0.000742471	RHSIM_Rhsim07G0196800
Root vs Leaf	novel.1855	5.553718609	0.000290668	0.000744972	--
Root vs Leaf	RHSIM_Rhsim04G0177800	1.675600662	0.000290704	0.000744983	RHSIM_Rhsim04G0177800
Root vs Leaf	RHSIM_Rhsim12G0206200	-3.505481848	0.00029163	0.000747272	RHSIM_Rhsim12G0206200
Root vs Leaf	RHSIM_Rhsim06G0058600	1.055664075	0.000292462	0.000749237	RHSIM_Rhsim06G0058600
Root vs Leaf	RHSIM_Rhsim05G0043800	1.028775611	0.000292846	0.000750137	RHSIM_Rhsim05G0043800

Comparison	Gene ID	log_2FC	P-value	FDR (padj)	Gene Name
Root vs Leaf	novel.2499	4.594352596	0.000293757	0.000752306	--
Root vs Leaf	RHSIM_Rhsim12G0006100	1.382630986	0.00029389	0.000752562	RHSIM_Rhsim12G0006100
Root vs Leaf	RHSIM_Rhsim08G0065500	-1.84626394	0.000294089	0.000752988	RHSIM_Rhsim08G0065500
Root vs Leaf	RHSIM_Rhsim08G0125700	2.627886901	0.000294538	0.000753973	RHSIM_Rhsim08G0125700
Root vs Leaf	RHSIM_Rhsim03G0136800	-1.639663961	0.00029515	0.000755454	RHSIM_Rhsim03G0136800
Root vs Leaf	novel.2785	1.26084512	0.000295209	0.000755523	--
Root vs Leaf	RHSIM_Rhsim07G0109300	2.375584596	0.000296089	0.00075769	RHSIM_Rhsim07G0109300
Root vs Leaf	RHSIM_Rhsim08G0195800	2.044882278	0.000297361	0.000760693	RHSIM_Rhsim08G0195800
Root vs Leaf	RHSIM_Rhsim08G0219300	-5.745190094	0.000297723	0.000761535	RHSIM_Rhsim08G0219300
Root vs Leaf	RHSIM_RhsimUnG0010000	6.814443665	0.000298393	0.000763079	RHSIM_RhsimUnG0010000
Root vs Leaf	RHSIM_Rhsim05G0196500	2.72052945	0.000299002	0.000764469	RHSIM_Rhsim05G0196500
Root vs Leaf	RHSIM_RhsimUnG0255900	1.85184968	0.000299157	0.000764782	RHSIM_RhsimUnG0255900
Root vs Leaf	RHSIM_RhsimUnG0070400	3.856507262	0.000299524	0.000765634	RHSIM_RhsimUnG0070400
Root vs Leaf	RHSIM_Rhsim01G0201100	2.166579728	0.000300716	0.000768512	RHSIM_Rhsim01G0201100
Root vs Leaf	novel.3992	-3.473809919	0.000301098	0.000769403	--
Root vs Leaf	RHSIM_Rhsim10G0027900	-1.923170828	0.00030118	0.000769526	RHSIM_Rhsim10G0027900
Root vs Leaf	RHSIM_Rhsim07G0038500	1.066900817	0.000301396	0.000769994	RHSIM_Rhsim07G0038500
Root vs Leaf	RHSIM_Rhsim07G0225400	1.623648004	0.000302025	0.000771517	RHSIM_Rhsim07G0225400
Root vs Leaf	RHSIM_RhsimUnG0027700	3.808914533	0.000304197	0.000776978	RHSIM_RhsimUnG0027700
Root vs Leaf	RHSIM_Rhsim05G0199800	-1.090184018	0.000304667	0.000778092	RHSIM_Rhsim05G0199800
Root vs Leaf	RHSIM_Rhsim08G0020200	-1.408621031	0.000305058	0.000779005	RHSIM_Rhsim08G0020200
Root vs Leaf	RHSIM_Rhsim13G0099600	3.749116205	0.000306131	0.000781574	RHSIM_Rhsim13G0099600
Root vs Leaf	novel.1118	-2.788588768	0.000307243	0.000784152	--
Root vs Leaf	novel.5164	6.221065282	0.000307756	0.000785375	--
Root vs Leaf	RHSIM_Rhsim06G0163900	1.59751805	0.000308161	0.000786323	RHSIM_Rhsim06G0163900

Comparison	Gene ID	log_2FC	P-value	FDR (padj)	Gene Name
Root vs Leaf	RHSIM_Rhsim03G0197300	-2.838241919	0.00030868	0.00078756	RHSIM_Rhsim03G0197300
Root vs Leaf	RHSIM_Rhsim07G0037200	1.106221803	0.000309145	0.00078866	RHSIM_Rhsim07G0037200
Root vs Leaf	RHSIM_Rhsim01G0154600	-1.115738483	0.000309854	0.000790294	RHSIM_Rhsim01G0154600
Root vs Leaf	RHSIM_Rhsim07G0166000	6.26754282	0.000310344	0.000791194	RHSIM_Rhsim07G0166000
Root vs Leaf	RHSIM_Rhsim07G0073900	1.496831949	0.000310782	0.000792137	RHSIM_Rhsim07G0073900
Root vs Leaf	RHSIM_Rhsim08G0062000	-1.693195906	0.000311713	0.000794335	RHSIM_Rhsim08G0062000
Root vs Leaf	RHSIM_Rhsim13G0159500	-1.144641571	0.000311974	0.000794911	RHSIM_Rhsim13G0159500
Root vs Leaf	RHSIM_Rhsim07G0079600	5.053384505	0.000312467	0.000796081	RHSIM_Rhsim07G0079600
Root vs Leaf	RHSIM_Rhsim04G0044700	5.150884383	0.0003135	0.000798624	RHSIM_Rhsim04G0044700
Root vs Leaf	RHSIM_Rhsim13G0114400	4.924596392	0.00031417	0.000800243	RHSIM_Rhsim13G0114400
Root vs Leaf	novel.1013	1.631606824	0.00031566	0.00080395	--
Root vs Leaf	RHSIM_Rhsim11G0106800	3.417687664	0.000316863	0.000806835	RHSIM_Rhsim11G0106800
Root vs Leaf	novel.2761	-1.274162556	0.000317478	0.000808223	--
Root vs Leaf	RHSIM_Rhsim06G0226200	1.584916361	0.000318553	0.000810872	RHSIM_Rhsim06G0226200
Root vs Leaf	RHSIM_RhsimUnG0101600	-1.020239507	0.000318892	0.000811646	RHSIM_RhsimUnG0101600
Root vs Leaf	RHSIM_RhsimUnG0126100	-1.666306978	0.000319555	0.000813243	RHSIM_RhsimUnG0126100
Root vs Leaf	RHSIM_Rhsim07G0253900	4.475196177	0.000321244	0.000817272	RHSIM_Rhsim07G0253900
Root vs Leaf	RHSIM_Rhsim09G0184600	2.800902979	0.000322015	0.000819144	RHSIM_Rhsim09G0184600
Root vs Leaf	RHSIM_RhsimUnG0048600	-2.411787063	0.000323045	0.000821674	RHSIM_RhsimUnG0048600
Root vs Leaf	RHSIM_Rhsim02G0202800	3.298050693	0.000324432	0.000825021	RHSIM_Rhsim02G0202800
Root vs Leaf	novel.5228	1.328098052	0.000324587	0.000825322	--
Root vs Leaf	novel.4861	1.903769052	0.000324685	0.000825481	--
Root vs Leaf	RHSIM_Rhsim12G0069300	-1.108502822	0.000326352	0.000829265	RHSIM_Rhsim12G0069300
Root vs Leaf	RHSIM_RhsimUnG0147500	6.437856783	0.00032737	0.000831578	RHSIM_RhsimUnG0147500
Root vs Leaf	RHSIM_Rhsim07G0080300	-3.232819433	0.000327703	0.000832331	RHSIM_Rhsim07G0080300

Comparison	Gene ID	log_2FC	P-value	FDR (padj)	Gene Name
Root vs Leaf	RHSIM_RhsimUnG0225900	-1.872486389	0.000327809	0.00083251	RHSIM_RhsimUnG0225900
Root vs Leaf	RHSIM_Rhsim07G0024200	-1.170443473	0.000328183	0.000833368	RHSIM_Rhsim07G0024200
Root vs Leaf	novel.3606	-5.458890543	0.000329689	0.000837009	--
Root vs Leaf	RHSIM_Rhsim10G0213700	2.451011043	0.000330313	0.000838384	RHSIM_Rhsim10G0213700
Root vs Leaf	RHSIM_Rhsim11G0169100	-1.752901662	0.000330339	0.000838384	RHSIM_Rhsim11G0169100
Root vs Leaf	RHSIM_Rhsim08G0091700	-2.879424853	0.000330527	0.000838768	RHSIM_Rhsim08G0091700
Root vs Leaf	RHSIM_Rhsim08G0039300	1.03480218	0.000330699	0.000839021	RHSIM_Rhsim08G0039300
Root vs Leaf	RHSIM_Rhsim06G0040000	-2.478297862	0.000331867	0.000841798	RHSIM_Rhsim06G0040000
Root vs Leaf	RHSIM_Rhsim08G0126100	1.798782938	0.000331996	0.000842033	RHSIM_Rhsim08G0126100
Root vs Leaf	RHSIM_Rhsim04G0186500	-1.066035086	0.000333578	0.000845861	RHSIM_Rhsim04G0186500
Root vs Leaf	RHSIM_Rhsim07G0033700	4.507876834	0.000334627	0.000848429	RHSIM_Rhsim07G0033700
Root vs Leaf	novel.3379	2.015652585	0.000335115	0.000849572	--
Root vs Leaf	RHSIM_Rhsim08G0010700	-1.120556278	0.000335205	0.000849708	RHSIM_Rhsim08G0010700
Root vs Leaf	RHSIM_Rhsim10G0158000	-3.890272192	0.00033696	0.00085397	RHSIM_Rhsim10G0158000
Root vs Leaf	RHSIM_Rhsim02G0100500	3.977278747	0.000338163	0.000856925	RHSIM_Rhsim02G0100500
Root vs Leaf	novel.4783	-4.903788041	0.000338415	0.000857468	--
Root vs Leaf	RHSIM_Rhsim03G0223900	-1.11211642	0.000339054	0.000858995	RHSIM_Rhsim03G0223900
Root vs Leaf	RHSIM_Rhsim05G0140200	2.074813058	0.000339621	0.000860336	RHSIM_Rhsim05G0140200
Root vs Leaf	RHSIM_Rhsim10G0079800	1.063188812	0.000340453	0.000862348	RHSIM_Rhsim10G0079800
Root vs Leaf	RHSIM_RhsimUnG0112300	-3.101383306	0.000341222	0.000864202	RHSIM_RhsimUnG0112300
Root vs Leaf	RHSIM_Rhsim05G0164100	3.758932653	0.000342415	0.00086694	RHSIM_Rhsim05G0164100
Root vs Leaf	novel.3511	-1.636373169	0.000344965	0.000873204	--
Root vs Leaf	RHSIM_Rhsim02G0115200	-1.741699474	0.000345616	0.000874756	RHSIM_Rhsim02G0115200
Root vs Leaf	RHSIM_Rhsim04G0000200	1.002599903	0.000348676	0.000882212	RHSIM_Rhsim04G0000200
Root vs Leaf	RHSIM_Rhsim10G0019200	-2.433982999	0.000348928	0.000882753	RHSIM_Rhsim10G0019200

Comparison	Gene ID	log$_2$FC	P-value	FDR (padj)	Gene Name
Root vs Leaf	novel.3522	3.584330948	0.000349417	0.000883892	--
Root vs Leaf	RHSIM_Rhsim07G0133700	1.899151899	0.00035101	0.000887826	RHSIM_Rhsim07G0133700
Root vs Leaf	RHSIM_Rhsim11G0143900	-6.543258267	0.000352769	0.000891982	RHSIM_Rhsim11G0143900
Root vs Leaf	RHSIM_Rhsim08G0001600	-1.499616799	0.000353413	0.000893417	RHSIM_Rhsim08G0001600
Root vs Leaf	RHSIM_Rhsim02G0129700	2.352534269	0.000353715	0.000894081	RHSIM_Rhsim02G0129700
Root vs Leaf	novel.1935	-1.669302102	0.000355857	0.0008993	--
Root vs Leaf	novel.1777	3.514190241	0.000356191	0.000900045	--
Root vs Leaf	RHSIM_Rhsim08G0099400	1.024614836	0.000357399	0.000903	RHSIM_Rhsim08G0099400
Root vs Leaf	RHSIM_Rhsim12G0203800	3.016748493	0.000358033	0.000904503	RHSIM_Rhsim12G0203800
Root vs Leaf	RHSIM_Rhsim06G0065200	1.876904485	0.000358268	0.000904997	RHSIM_Rhsim06G0065200
Root vs Leaf	RHSIM_Rhsim05G0003700	3.719810485	0.000359264	0.000907316	RHSIM_Rhsim05G0003700
Root vs Leaf	RHSIM_Rhsim08G0021100	2.243232293	0.000359914	0.000908857	RHSIM_Rhsim08G0021100
Root vs Leaf	RHSIM_Rhsim10G0172100	3.200327706	0.000360028	0.000909045	RHSIM_Rhsim10G0172100
Root vs Leaf	RHSIM_Rhsim02G0124200	-7.572221736	0.000360606	0.000910406	RHSIM_Rhsim02G0124200
Root vs Leaf	RHSIM_Rhsim12G0035400	-1.7110868	0.000360793	0.000910778	RHSIM_Rhsim12G0035400
Root vs Leaf	RHSIM_Rhsim06G0065900	2.424917317	0.000363634	0.000917652	RHSIM_Rhsim06G0065900
Root vs Leaf	RHSIM_Rhsim02G0217300	1.485045042	0.000366351	0.000924002	RHSIM_Rhsim02G0217300
Root vs Leaf	RHSIM_Rhsim06G0167600	1.231731962	0.000366574	0.000924465	RHSIM_Rhsim06G0167600
Root vs Leaf	RHSIM_Rhsim04G0147800	1.027442426	0.000366692	0.000924663	RHSIM_Rhsim04G0147800
Root vs Leaf	RHSIM_Rhsim12G0127100	-1.607860926	0.000367618	0.000926794	RHSIM_Rhsim12G0127100
Root vs Leaf	RHSIM_RhsimUnG0217000	1.883701876	0.000368014	0.000927691	RHSIM_RhsimUnG0217000
Root vs Leaf	RHSIM_Rhsim04G0025400	4.444737036	0.00036866	0.000929218	RHSIM_Rhsim04G0025400
Root vs Leaf	novel.1004	-6.280087738	0.000368724	0.000929279	--
Root vs Leaf	RHSIM_Rhsim10G0062600	-1.188962445	0.000369839	0.000931886	RHSIM_Rhsim10G0062600
Root vs Leaf	RHSIM_Rhsim01G0286400	1.243864192	0.000370964	0.000934619	RHSIM_Rhsim01G0286400

Comparison	Gene ID	log_2FC	P-value	FDR (padj)	Gene Name
Root vs Leaf	RHSIM_RhsimUnG0097700	-1.131872008	0.000371942	0.000936981	RHSIM_RhsimUnG0097700
Root vs Leaf	novel.3751	1.373578664	0.000375053	0.00094451	--
Root vs Leaf	RHSIM_Rhsim01G0005600	3.733128927	0.000378041	0.00095162	RHSIM_Rhsim01G0005600
Root vs Leaf	novel.3248	1.361263125	0.000378148	0.000951785	--
Root vs Leaf	novel.5267	1.4230831	0.000378426	0.000952383	--
Root vs Leaf	novel.2802	3.100969647	0.00037922	0.000954276	--
Root vs Leaf	RHSIM_Rhsim08G0146500	1.936700833	0.000381707	0.00096043	RHSIM_Rhsim08G0146500
Root vs Leaf	RHSIM_Rhsim12G0085700	1.043606669	0.000382296	0.000961808	RHSIM_Rhsim12G0085700
Root vs Leaf	RHSIM_Rhsim13G0092100	2.778225008	0.000384776	0.000967942	RHSIM_Rhsim13G0092100
Root vs Leaf	RHSIM_Rhsim07G0137100	1.012537625	0.00038516	0.000968803	RHSIM_Rhsim07G0137100
Root vs Leaf	novel.1841	-1.170772463	0.000387836	0.00097511	--
Root vs Leaf	RHSIM_RhsimPtG0003100	-1.055940276	0.000388212	0.000975844	RHSIM_RhsimPtG0003100
Root vs Leaf	RHSIM_Rhsim08G0144400	-1.047781994	0.000389757	0.000979514	RHSIM_Rhsim08G0144400
Root vs Leaf	RHSIM_Rhsim12G0151200	6.011687814	0.000389865	0.00097968	RHSIM_Rhsim12G0151200
Root vs Leaf	RHSIM_Rhsim10G0158500	4.717615925	0.00039172	0.000983914	RHSIM_Rhsim10G0158500
Root vs Leaf	RHSIM_Rhsim11G0020600	2.195652743	0.000392561	0.000985918	RHSIM_Rhsim11G0020600
Root vs Leaf	RHSIM_Rhsim13G0113000	-2.357089366	0.000393615	0.000988137	RHSIM_Rhsim13G0113000
Root vs Leaf	novel.277	2.899064583	0.000394301	0.000989751	--
Root vs Leaf	RHSIM_Rhsim07G0100300	6.373482367	0.000394773	0.000990613	RHSIM_Rhsim07G0100300
Root vs Leaf	RHSIM_Rhsim05G0140900	6.254426929	0.000398911	0.001000346	RHSIM_Rhsim05G0140900
Root vs Leaf	RHSIM_Rhsim04G0004800	1.074503062	0.000399191	0.001000941	RHSIM_Rhsim04G0004800
Root vs Leaf	RHSIM_Rhsim01G0249200	1.36733926	0.000399962	0.001002764	RHSIM_Rhsim01G0249200
Root vs Leaf	RHSIM_Rhsim03G0083200	3.589337803	0.000400754	0.001004643	RHSIM_Rhsim03G0083200
Root vs Leaf	novel.5575	-1.827723733	0.000401637	0.001006636	--
Root vs Leaf	RHSIM_RhsimUnG0123500	1.505047847	0.000403106	0.001009958	RHSIM_RhsimUnG0123500

Comparison	Gene ID	log_2FC	P-value	FDR (padj)	Gene Name
Root vs Leaf	RHSIM_Rhsim08G0009900	1.67721475	0.000403329	0.001010222	RHSIM_Rhsim08G0009900
Root vs Leaf	RHSIM_Rhsim05G0214300	-1.111859136	0.000405727	0.001016008	RHSIM_Rhsim05G0214300
Root vs Leaf	RHSIM_Rhsim06G0056700	-3.097776831	0.000408684	0.001023192	RHSIM_Rhsim06G0056700
Root vs Leaf	RHSIM_Rhsim10G0178300	1.23006264	0.000409963	0.001026173	RHSIM_Rhsim10G0178300
Root vs Leaf	RHSIM_Rhsim02G0097300	-3.906574223	0.00041173	0.001030482	RHSIM_Rhsim02G0097300
Root vs Leaf	RHSIM_Rhsim13G0013400	2.289267489	0.000412338	0.001031892	RHSIM_Rhsim13G0013400
Root vs Leaf	RHSIM_Rhsim08G0033700	4.412861162	0.000412384	0.001031897	RHSIM_Rhsim08G0033700
Root vs Leaf	novel.510	1.333860266	0.000412855	0.001032963	--
Root vs Leaf	RHSIM_Rhsim04G0080800	1.235156335	0.000413397	0.001033985	RHSIM_Rhsim04G0080800
Root vs Leaf	RHSIM_Rhsim10G0035900	-3.230922571	0.000419847	0.001049549	RHSIM_Rhsim10G0035900
Root vs Leaf	RHSIM_Rhsim09G0195000	5.701674583	0.000420016	0.001049858	RHSIM_Rhsim09G0195000
Root vs Leaf	RHSIM_Rhsim13G0225400	1.194528569	0.000420745	0.00105149	RHSIM_Rhsim13G0225400
Root vs Leaf	RHSIM_RhsimUnG0106700	3.259525725	0.00042076	0.00105149	RHSIM_RhsimUnG0106700
Root vs Leaf	RHSIM_Rhsim02G0212600	5.562165433	0.000423302	0.001057616	RHSIM_Rhsim02G0212600
Root vs Leaf	RHSIM_Rhsim03G0147200	1.156892274	0.000424461	0.001060282	RHSIM_Rhsim03G0147200
Root vs Leaf	RHSIM_Rhsim06G0157900	-1.072543166	0.000426062	0.001063936	RHSIM_Rhsim06G0157900
Root vs Leaf	RHSIM_Rhsim09G0068500	1.571444202	0.000427566	0.001067576	RHSIM_Rhsim09G0068500
Root vs Leaf	novel.3934	-1.238511089	0.000428298	0.001069289	--
Root vs Leaf	RHSIM_RhsimUnG0240600	-1.287653541	0.000429393	0.001071791	RHSIM_RhsimUnG0240600
Root vs Leaf	novel.3238	1.622389185	0.000433585	0.001081671	--
Root vs Leaf	RHSIM_Rhsim13G0118900	3.720787722	0.000435535	0.001086186	RHSIM_Rhsim13G0118900
Root vs Leaf	RHSIM_Rhsim05G0002800	5.834595757	0.000437319	0.001090164	RHSIM_Rhsim05G0002800
Root vs Leaf	RHSIM_Rhsim03G0081000	2.186728675	0.00043762	0.001090798	RHSIM_Rhsim03G0081000
Root vs Leaf	novel.1404	-1.158591223	0.00043792	0.001091427	--
Root vs Leaf	RHSIM_Rhsim05G0004700	-1.39140974	0.000438077	0.0010917	RHSIM_Rhsim05G0004700

Comparison	Gene ID	log_2FC	P-value	FDR (padj)	Gene Name
Root vs Leaf	RHSIM_Rhsim09G0142400	2.988005982	0.000440605	0.001097529	RHSIM_Rhsim09G0142400
Root vs Leaf	novel.4143	-5.590242193	0.000441489	0.001099613	--
Root vs Leaf	RHSIM_Rhsim10G0012300	1.776971807	0.000443231	0.001103833	RHSIM_Rhsim10G0012300
Root vs Leaf	novel.2355	1.80214888	0.000443354	0.001103902	--
Root vs Leaf	novel.2419	1.22489751	0.000443664	0.001104553	--
Root vs Leaf	novel.1968	2.069297544	0.000444105	0.001105522	--
Root vs Leaf	RHSIM_Rhsim10G0003400	-2.244200151	0.000445246	0.001108015	RHSIM_Rhsim10G0003400
Root vs Leaf	RHSIM_Rhsim04G0049200	-1.974480054	0.00044715	0.001112479	RHSIM_Rhsim04G0049200
Root vs Leaf	novel.636	-2.032710992	0.000447222	0.001112479	--
Root vs Leaf	RHSIM_Rhsim08G0250200	1.443621822	0.000449218	0.00111718	RHSIM_Rhsim08G0250200
Root vs Leaf	RHSIM_Rhsim07G0033100	1.162901243	0.000449363	0.001117421	RHSIM_Rhsim07G0033100
Root vs Leaf	RHSIM_Rhsim07G0199500	2.533659337	0.000449704	0.001118147	RHSIM_Rhsim07G0199500
Root vs Leaf	RHSIM_Rhsim03G0235100	1.078161336	0.000450218	0.001119186	RHSIM_Rhsim03G0235100
Root vs Leaf	novel.2325	-1.796950202	0.000450514	0.00111968	--
Root vs Leaf	RHSIM_Rhsim10G0008700	3.107988502	0.000451889	0.001122858	RHSIM_Rhsim10G0008700
Root vs Leaf	RHSIM_Rhsim11G0062300	-1.035221009	0.000452172	0.001123439	RHSIM_Rhsim11G0062300
Root vs Leaf	RHSIM_Rhsim06G0141700	1.058421552	0.000453089	0.001125598	RHSIM_Rhsim06G0141700
Root vs Leaf	RHSIM_Rhsim01G0099900	-5.718358435	0.000455671	0.001131889	RHSIM_Rhsim01G0099900
Root vs Leaf	RHSIM_Rhsim02G0199000	-1.770812495	0.000456652	0.001134204	RHSIM_Rhsim02G0199000
Root vs Leaf	novel.846	-3.578170508	0.000458441	0.001138527	--
Root vs Leaf	RHSIM_Rhsim02G0031000	2.842976448	0.00045893	0.001139618	RHSIM_Rhsim02G0031000
Root vs Leaf	RHSIM_Rhsim06G0092300	-2.381479996	0.000459085	0.001139879	RHSIM_Rhsim06G0092300
Root vs Leaf	RHSIM_Rhsim08G0206500	3.128566734	0.000464697	0.001153567	RHSIM_Rhsim08G0206500
Root vs Leaf	RHSIM_Rhsim07G0225000	-2.063713496	0.000465802	0.001156062	RHSIM_Rhsim07G0225000
Root vs Leaf	RHSIM_Rhsim05G0229200	1.41677354	0.000466281	0.001157128	RHSIM_Rhsim05G0229200

Comparison	Gene ID	log_2FC	P-value	FDR (padj)	Gene Name
Root vs Leaf	novel.4041	3.402919987	0.000467164	0.001159194	--
Root vs Leaf	RHSIM_Rhsim06G0102700	1.337042597	0.000467825	0.00116071	RHSIM_Rhsim06G0102700
Root vs Leaf	RHSIM_Rhsim09G0205000	-1.182780811	0.000469598	0.001164983	RHSIM_Rhsim09G0205000
Root vs Leaf	novel.3346	-1.625738366	0.000469672	0.001165041	--
Root vs Leaf	RHSIM_RhsimUnG0000800	6.112139694	0.000469798	0.001165229	RHSIM_RhsimUnG0000800
Root vs Leaf	RHSIM_Rhsim13G0096000	3.308405861	0.000470862	0.001167743	RHSIM_Rhsim13G0096000
Root vs Leaf	RHSIM_Rhsim01G0090800	1.183938403	0.000471264	0.00116849	RHSIM_Rhsim01G0090800
Root vs Leaf	RHSIM_Rhsim08G0011400	-1.293999803	0.000472367	0.001170473	RHSIM_Rhsim08G0011400
Root vs Leaf	RHSIM_Rhsim01G0287800	1.040382075	0.000473992	0.001174123	RHSIM_Rhsim01G0287800
Root vs Leaf	RHSIM_Rhsim06G0169100	5.944441698	0.000474417	0.00117505	RHSIM_Rhsim06G0169100
Root vs Leaf	novel.5055	-1.239382385	0.000475932	0.00117855	--
Root vs Leaf	RHSIM_Rhsim13G0218900	1.383151827	0.000477147	0.001181433	RHSIM_Rhsim13G0218900
Root vs Leaf	RHSIM_Rhsim07G0091300	1.08154284	0.000477877	0.001183114	RHSIM_Rhsim07G0091300
Root vs Leaf	RHSIM_Rhsim01G0203200	-1.681193492	0.000479131	0.001185963	RHSIM_Rhsim01G0203200
Root vs Leaf	RHSIM_Rhsim02G0256100	1.389689948	0.000481469	0.001191496	RHSIM_Rhsim02G0256100
Root vs Leaf	RHSIM_Rhsim03G0178100	5.286297384	0.000482589	0.001194012	RHSIM_Rhsim03G0178100
Root vs Leaf	novel.3396	-1.754401717	0.000484037	0.001197334	--
Root vs Leaf	RHSIM_Rhsim05G0089900	-2.837044924	0.000488022	0.00120655	RHSIM_Rhsim05G0089900
Root vs Leaf	RHSIM_Rhsim06G0074300	3.055232317	0.000488473	0.001207162	RHSIM_Rhsim06G0074300
Root vs Leaf	RHSIM_Rhsim13G0102200	-1.567814019	0.000488505	0.001207162	RHSIM_Rhsim13G0102200
Root vs Leaf	RHSIM_Rhsim09G0114200	1.188578916	0.000489516	0.00120947	RHSIM_Rhsim09G0114200
Root vs Leaf	RHSIM_Rhsim01G0082300	-1.020199615	0.000490102	0.001210789	RHSIM_Rhsim01G0082300
Root vs Leaf	RHSIM_Rhsim06G0162300	-1.863812098	0.000490712	0.001212166	RHSIM_Rhsim06G0162300
Root vs Leaf	RHSIM_RhsimUnG0088000	-1.266626254	0.000490914	0.001212535	RHSIM_RhsimUnG0088000
Root vs Leaf	RHSIM_Rhsim08G0123800	-3.095485601	0.000492743	0.001216862	RHSIM_Rhsim08G0123800

Comparison	Gene ID	log_2FC	P-value	FDR (padj)	Gene Name
Root vs Leaf	RHSIM_Rhsim04G0070900	1.041027229	0.000492771	0.001216862	RHSIM_Rhsim04G0070900
Root vs Leaf	novel.5327	4.875010501	0.000497844	0.001228865	--
Root vs Leaf	RHSIM_Rhsim01G0069000	1.910382094	0.000498009	0.001229142	RHSIM_Rhsim01G0069000
Root vs Leaf	RHSIM_Rhsim11G0113200	-7.932246254	0.000501805	0.001237717	RHSIM_Rhsim11G0113200
Root vs Leaf	RHSIM_Rhsim02G0080900	-1.404263094	0.000501992	0.001238046	RHSIM_Rhsim02G0080900
Root vs Leaf	novel.5599	-3.422941387	0.000502101	0.001238185	--
Root vs Leaf	RHSIM_Rhsim03G0252100	1.49694636	0.000505217	0.001245338	RHSIM_Rhsim03G0252100
Root vs Leaf	novel.2469	-1.767965927	0.000506182	0.001247582	--
Root vs Leaf	RHSIM_Rhsim02G0245800	-1.493663294	0.000506271	0.00124767	RHSIM_Rhsim02G0245800
Root vs Leaf	RHSIM_Rhsim04G0231100	-3.717660877	0.000507777	0.001251249	RHSIM_Rhsim04G0231100
Root vs Leaf	RHSIM_Rhsim07G0040100	-3.262162192	0.000508522	0.001252949	RHSIM_Rhsim07G0040100
Root vs Leaf	RHSIM_Rhsim06G0106300	-2.33488177	0.000509577	0.001255415	RHSIM_Rhsim06G0106300
Root vs Leaf	RHSIM_Rhsim11G0046300	2.517321362	0.000509838	0.001255924	RHSIM_Rhsim11G0046300
Root vs Leaf	RHSIM_RhsimUnG0248400	1.116859131	0.000510802	0.001258165	RHSIM_RhsimUnG0248400
Root vs Leaf	RHSIM_Rhsim11G0144300	-2.575892962	0.000511098	0.001258762	RHSIM_Rhsim11G0144300
Root vs Leaf	RHSIM_Rhsim04G0193200	-1.010845176	0.000511482	0.001259438	RHSIM_Rhsim04G0193200
Root vs Leaf	RHSIM_Rhsim07G0212700	-1.277247965	0.000513329	0.001263582	RHSIM_Rhsim07G0212700
Root vs Leaf	novel.3284	-3.440698576	0.000514051	0.001265091	--
Root vs Leaf	RHSIM_Rhsim12G0099300	2.927104985	0.000515986	0.001269313	RHSIM_Rhsim12G0099300
Root vs Leaf	novel.683	3.383555702	0.000516715	0.001270836	--
Root vs Leaf	RHSIM_Rhsim04G0174400	2.178727195	0.000516841	0.001271012	RHSIM_Rhsim04G0174400
Root vs Leaf	RHSIM_Rhsim03G0138600	-1.475811776	0.000516998	0.001271254	RHSIM_Rhsim03G0138600
Root vs Leaf	RHSIM_Rhsim07G0042200	3.591221029	0.000517104	0.001271254	RHSIM_Rhsim07G0042200
Root vs Leaf	RHSIM_Rhsim09G0135300	-9.444157057	0.000519898	0.001277442	RHSIM_Rhsim09G0135300
Root vs Leaf	RHSIM_Rhsim02G0236600	-1.601787432	0.000521377	0.001280942	RHSIM_Rhsim02G0236600

Comparison	Gene ID	log_2FC	P-value	FDR (padj)	Gene Name
Root vs Leaf	novel.4840	2.445957349	0.000521931	0.001282166	--
Root vs Leaf	RHSIM_Rhsim03G0192300	2.036047322	0.000525268	0.001289953	RHSIM_Rhsim03G0192300
Root vs Leaf	RHSIM_Rhsim11G0189700	3.907890432	0.000525362	0.001290047	RHSIM_Rhsim11G0189700
Root vs Leaf	RHSIM_Rhsim06G0031800	1.173606404	0.000528262	0.001296755	RHSIM_Rhsim06G0031800
Root vs Leaf	RHSIM_Rhsim13G0065800	1.972338695	0.000529345	0.001298726	RHSIM_Rhsim13G0065800
Root vs Leaf	RHSIM_Rhsim13G0144500	-1.758885126	0.000532186	0.00130528	RHSIM_Rhsim13G0144500
Root vs Leaf	RHSIM_Rhsim06G0040400	3.309593308	0.000533909	0.001309228	RHSIM_Rhsim06G0040400
Root vs Leaf	RHSIM_Rhsim08G0194500	1.838281372	0.000534163	0.001309713	RHSIM_Rhsim08G0194500
Root vs Leaf	RHSIM_Rhsim06G0065700	-1.320937374	0.000534797	0.0013109	RHSIM_Rhsim06G0065700
Root vs Leaf	novel.3695	2.654676444	0.000538509	0.00131939	--
Root vs Leaf	novel.707	2.911337644	0.00053963	0.001321996	--
Root vs Leaf	novel.4736	1.549306017	0.000541025	0.001325273	--
Root vs Leaf	RHSIM_RhsimUnG0182700	4.837994986	0.000542684	0.001329196	RHSIM_RhsimUnG0182700
Root vs Leaf	RHSIM_Rhsim13G0057800	5.073515524	0.00054435	0.001333136	RHSIM_Rhsim13G0057800
Root vs Leaf	RHSIM_Rhsim11G0032400	1.480399296	0.000544441	0.001333219	RHSIM_Rhsim11G0032400
Root vs Leaf	RHSIM_Rhsim12G0188400	3.252884585	0.000544828	0.001333916	RHSIM_Rhsim12G0188400
Root vs Leaf	novel.4068	2.122763563	0.00054587	0.001336294	--
Root vs Leaf	RHSIM_RhsimUnG0246700	1.525074274	0.00054888	0.001343376	RHSIM_RhsimUnG0246700
Root vs Leaf	novel.5640	-1.314529955	0.000552592	0.001352177	--
Root vs Leaf	RHSIM_Rhsim12G0078200	-2.019634345	0.000553024	0.00135309	RHSIM_Rhsim12G0078200
Root vs Leaf	RHSIM_Rhsim13G0142200	2.167735178	0.000558412	0.00136584	RHSIM_Rhsim13G0142200
Root vs Leaf	RHSIM_Rhsim07G0021900	1.565549968	0.000561713	0.001373334	RHSIM_Rhsim07G0021900
Root vs Leaf	RHSIM_Rhsim06G0004400	2.997463038	0.000561802	0.001373371	RHSIM_Rhsim06G0004400
Root vs Leaf	RHSIM_Rhsim05G0158800	-2.458698874	0.000563336	0.001376865	RHSIM_Rhsim05G0158800
Root vs Leaf	RHSIM_RhsimUnG0065100	-2.955882881	0.000563589	0.001377193	RHSIM_RhsimUnG0065100

Comparison	Gene ID	log_2FC	P-value	FDR (padj)	Gene Name
Root vs Leaf	RHSIM_RhsimUnG0058200	-2.060606881	0.000563662	0.001377227	RHSIM_RhsimUnG0058200
Root vs Leaf	RHSIM_Rhsim07G0103800	-1.165146978	0.000564663	0.001379381	RHSIM_Rhsim07G0103800
Root vs Leaf	RHSIM_Rhsim04G0029500	1.048126498	0.000565143	0.001380407	RHSIM_Rhsim04G0029500
Root vs Leaf	RHSIM_Rhsim02G0216800	1.484409873	0.000565422	0.001380943	RHSIM_Rhsim02G0216800
Root vs Leaf	RHSIM_Rhsim03G0119200	1.135017808	0.000566301	0.001382944	RHSIM_Rhsim03G0119200
Root vs Leaf	RHSIM_Rhsim08G0173300	1.616816722	0.000569945	0.001391696	RHSIM_Rhsim08G0173300
Root vs Leaf	RHSIM_Rhsim07G0071400	2.0712262	0.000571869	0.001396098	RHSIM_Rhsim07G0071400
Root vs Leaf	RHSIM_Rhsim01G0083500	-2.133668254	0.000572724	0.001398038	RHSIM_Rhsim01G0083500
Root vs Leaf	RHSIM_Rhsim05G0103300	-1.010624458	0.000573862	0.00140052	RHSIM_Rhsim05G0103300
Root vs Leaf	RHSIM_Rhsim05G0049600	1.487807889	0.000574698	0.001402265	RHSIM_Rhsim05G0049600
Root vs Leaf	RHSIM_RhsimUnG0118100	3.001493782	0.000581128	0.001417357	RHSIM_RhsimUnG0118100
Root vs Leaf	novel.2218	2.343033454	0.000582623	0.001420555	--
Root vs Leaf	RHSIM_Rhsim01G0006600	-2.118408721	0.000583041	0.001421422	RHSIM_Rhsim01G0006600
Root vs Leaf	novel.5307	4.252730154	0.000583634	0.00142257	--
Root vs Leaf	novel.4543	1.805218671	0.000583765	0.001422739	--
Root vs Leaf	RHSIM_Rhsim04G0050700	1.250142255	0.000585715	0.00142704	RHSIM_Rhsim04G0050700
Root vs Leaf	RHSIM_Rhsim06G0040100	-1.363643218	0.000586848	0.00142965	RHSIM_Rhsim06G0040100
Root vs Leaf	RHSIM_Rhsim10G0141800	1.515015599	0.000588235	0.001432879	RHSIM_Rhsim10G0141800
Root vs Leaf	novel.5416	2.520592039	0.000592664	0.001443515	--
Root vs Leaf	RHSIM_Rhsim10G0017200	3.867825835	0.00059382	0.001445875	RHSIM_Rhsim10G0017200
Root vs Leaf	novel.3072	1.344137983	0.000594151	0.001446527	--
Root vs Leaf	RHSIM_Rhsim04G0181000	-2.624077674	0.000595276	0.001448808	RHSIM_Rhsim04G0181000
Root vs Leaf	RHSIM_Rhsim04G0019600	1.274773611	0.000603787	0.001467981	RHSIM_Rhsim04G0019600
Root vs Leaf	RHSIM_Rhsim07G0179900	1.370833159	0.000604033	0.001468425	RHSIM_Rhsim07G0179900
Root vs Leaf	RHSIM_Rhsim01G0006300	4.387263289	0.000604567	0.001469414	RHSIM_Rhsim01G0006300

Comparison	Gene ID	log_2FC	P-value	FDR (padj)	Gene Name
Root vs Leaf	novel.4789	-2.825433564	0.00060525	0.001470918	--
Root vs Leaf	RHSIM_RhsimUnG0065700	-2.148781196	0.000606513	0.001473833	RHSIM_RhsimUnG0065700
Root vs Leaf	RHSIM_Rhsim08G0203600	-1.878994756	0.000607764	0.001476713	RHSIM_Rhsim08G0203600
Root vs Leaf	RHSIM_Rhsim09G0010500	1.15299287	0.000607865	0.001476713	RHSIM_Rhsim09G0010500
Root vs Leaf	RHSIM_Rhsim06G0041000	2.645404761	0.000607889	0.001476713	RHSIM_Rhsim06G0041000
Root vs Leaf	novel.4619	4.112729059	0.000609242	0.001479842	--
Root vs Leaf	RHSIM_Rhsim07G0236700	1.453160733	0.000609894	0.001481271	RHSIM_Rhsim07G0236700
Root vs Leaf	RHSIM_Rhsim08G0071300	2.504106319	0.000614645	0.001492342	RHSIM_Rhsim08G0071300
Root vs Leaf	RHSIM_Rhsim13G0164300	-1.024675998	0.000614831	0.001492636	RHSIM_Rhsim13G0164300
Root vs Leaf	RHSIM_Rhsim02G0039500	-3.064517806	0.000615207	0.001493393	RHSIM_Rhsim02G0039500
Root vs Leaf	novel.4411	-5.708555274	0.000616509	0.001496239	--
Root vs Leaf	RHSIM_Rhsim08G0146900	1.421213408	0.000617362	0.001498152	RHSIM_Rhsim08G0146900
Root vs Leaf	RHSIM_Rhsim01G0248600	6.433510464	0.000618921	0.001501776	RHSIM_Rhsim01G0248600
Root vs Leaf	novel.2739	-1.708762528	0.000619703	0.001503517	--
Root vs Leaf	RHSIM_Rhsim13G0165100	3.562458416	0.000621127	0.001506655	RHSIM_Rhsim13G0165100
Root vs Leaf	novel.4152	-1.084020367	0.000621472	0.001507178	--
Root vs Leaf	RHSIM_Rhsim11G0174600	2.927432011	0.000621669	0.001507497	RHSIM_Rhsim11G0174600
Root vs Leaf	RHSIM_Rhsim07G0113700	-1.369009224	0.000621823	0.001507712	RHSIM_Rhsim07G0113700
Root vs Leaf	RHSIM_RhsimUnG0256100	-2.821569702	0.000621993	0.001507967	RHSIM_RhsimUnG0256100
Root vs Leaf	RHSIM_Rhsim04G0205200	-2.429970047	0.000623643	0.001511649	RHSIM_Rhsim04G0205200
Root vs Leaf	novel.4427	-1.64253294	0.000625212	0.001515136	--
Root vs Leaf	RHSIM_Rhsim11G0096500	4.291220006	0.000627393	0.001519944	RHSIM_Rhsim11G0096500
Root vs Leaf	RHSIM_Rhsim03G0057100	1.605890759	0.00062806	0.0015214	RHSIM_Rhsim03G0057100
Root vs Leaf	RHSIM_Rhsim09G0047900	-1.218531591	0.000628776	0.001522975	RHSIM_Rhsim09G0047900
Root vs Leaf	RHSIM_Rhsim04G0217600	-1.214383265	0.000629819	0.001525343	RHSIM_Rhsim04G0217600

Comparison	Gene ID	log_2FC	P-value	FDR (padj)	Gene Name
Root vs Leaf	RHSIM_RhsimUnG0063400	-1.469280794	0.0006301	0.001525702	RHSIM_RhsimUnG0063400
Root vs Leaf	RHSIM_Rhsim03G0174500	3.532764803	0.000630314	0.001526061	RHSIM_Rhsim03G0174500
Root vs Leaf	RHSIM_Rhsim12G0214400	3.019353846	0.000630695	0.001526825	RHSIM_Rhsim12G0214400
Root vs Leaf	RHSIM_Rhsim10G0191700	-1.4608239	0.000630878	0.001527108	RHSIM_Rhsim10G0191700
Root vs Leaf	RHSIM_Rhsim01G0005000	1.319920694	0.000632012	0.001529533	RHSIM_Rhsim01G0005000
Root vs Leaf	RHSIM_Rhsim12G0067900	-1.243996901	0.000632086	0.001529547	RHSIM_Rhsim12G0067900
Root vs Leaf	novel.2444	1.552549776	0.00063215	0.001529547	--
Root vs Leaf	RHSIM_Rhsim03G0111800	-1.182023712	0.000632894	0.001531187	RHSIM_Rhsim03G0111800
Root vs Leaf	RHSIM_Rhsim13G0102800	-1.557760927	0.000636121	0.001538746	RHSIM_Rhsim13G0102800
Root vs Leaf	RHSIM_Rhsim13G0099100	-1.26732268	0.000640252	0.00154834	RHSIM_Rhsim13G0099100
Root vs Leaf	RHSIM_Rhsim04G0072800	-3.419378082	0.000640662	0.001549171	RHSIM_Rhsim04G0072800
Root vs Leaf	RHSIM_Rhsim04G0034800	2.924409475	0.000642003	0.001552251	RHSIM_Rhsim04G0034800
Root vs Leaf	RHSIM_Rhsim06G0188100	2.507478021	0.000643749	0.001556148	RHSIM_Rhsim06G0188100
Root vs Leaf	RHSIM_Rhsim09G0033100	1.459356138	0.000644152	0.00155696	RHSIM_Rhsim09G0033100
Root vs Leaf	RHSIM_Rhsim09G0208600	-1.215191537	0.000644286	0.001557121	RHSIM_Rhsim09G0208600
Root vs Leaf	RHSIM_Rhsim06G0094400	2.021565055	0.000648645	0.001567163	RHSIM_Rhsim06G0094400
Root vs Leaf	RHSIM_Rhsim13G0011700	-1.671081904	0.000650235	0.001570841	RHSIM_Rhsim13G0011700
Root vs Leaf	RHSIM_Rhsim09G0045600	-4.818523764	0.000650335	0.001570919	RHSIM_Rhsim09G0045600
Root vs Leaf	RHSIM_Rhsim13G0023400	4.094450422	0.000650937	0.001572208	RHSIM_Rhsim13G0023400
Root vs Leaf	RHSIM_Rhsim06G0135900	3.916000372	0.000651303	0.001572929	RHSIM_Rhsim06G0135900
Root vs Leaf	RHSIM_Rhsim12G0067300	-1.925674538	0.000651441	0.001573098	RHSIM_Rhsim12G0067300
Root vs Leaf	novel.2460	-1.563043246	0.000652798	0.001575718	--
Root vs Leaf	RHSIM_Rhsim13G0054500	-1.060324463	0.000653637	0.001577249	RHSIM_Rhsim13G0054500
Root vs Leaf	RHSIM_Rhsim06G0171100	-1.424242848	0.00065374	0.001577333	RHSIM_Rhsim06G0171100
Root vs Leaf	RHSIM_RhsimUnG0203600	-4.313888889	0.000655711	0.001581594	RHSIM_RhsimUnG0203600

Comparison	Gene ID	log_2FC	P-value	FDR (padj)	Gene Name
Root vs Leaf	RHSIM_Rhsim09G0139100	-2.656407762	0.000656381	0.001583044	RHSIM_Rhsim09G0139100
Root vs Leaf	RHSIM_Rhsim09G0003900	-1.115148924	0.000657392	0.001585319	RHSIM_Rhsim09G0003900
Root vs Leaf	RHSIM_Rhsim12G0082700	-1.396386846	0.000659107	0.001588956	RHSIM_Rhsim12G0082700
Root vs Leaf	RHSIM_Rhsim07G0187400	-7.146622636	0.000665228	0.001603212	RHSIM_Rhsim07G0187400
Root vs Leaf	RHSIM_Rhsim04G0087600	-1.98512424	0.000670222	0.001614743	RHSIM_Rhsim04G0087600
Root vs Leaf	RHSIM_Rhsim04G0120000	2.038162394	0.000671963	0.001618601	RHSIM_Rhsim04G0120000
Root vs Leaf	RHSIM_Rhsim13G0061400	1.592885857	0.000673267	0.001621572	RHSIM_Rhsim13G0061400
Root vs Leaf	RHSIM_Rhsim04G0179500	1.018739298	0.000674967	0.001625498	RHSIM_Rhsim04G0179500
Root vs Leaf	RHSIM_Rhsim03G0052600	3.912880794	0.000675081	0.001625604	RHSIM_Rhsim03G0052600
Root vs Leaf	RHSIM_Rhsim06G0220600	3.132612367	0.000677432	0.001630756	RHSIM_Rhsim06G0220600
Root vs Leaf	RHSIM_Rhsim05G0203200	1.550305291	0.000679507	0.00163558	RHSIM_Rhsim05G0203200
Root vs Leaf	RHSIM_Rhsim04G0014500	-1.89492478	0.000679833	0.001636099	RHSIM_Rhsim04G0014500
Root vs Leaf	novel.5287	1.836304107	0.000679907	0.001636099	--
Root vs Leaf	RHSIM_Rhsim03G0208000	1.591651564	0.000679934	0.001636099	RHSIM_Rhsim03G0208000
Root vs Leaf	RHSIM_Rhsim03G0176500	-2.161503319	0.000681585	0.00163973	RHSIM_Rhsim03G0176500
Root vs Leaf	RHSIM_Rhsim09G0143000	-1.52342958	0.000684153	0.001645737	RHSIM_Rhsim09G0143000
Root vs Leaf	RHSIM_RhsimUnG0069800	-1.548786059	0.000687722	0.001653805	RHSIM_RhsimUnG0069800
Root vs Leaf	RHSIM_Rhsim08G0066700	1.511172857	0.000687903	0.00165407	RHSIM_Rhsim08G0066700
Root vs Leaf	RHSIM_RhsimUnG0256400	2.503672411	0.000688506	0.001655346	RHSIM_RhsimUnG0256400
Root vs Leaf	RHSIM_Rhsim07G0049000	6.808382395	0.00068944	0.00165742	RHSIM_Rhsim07G0049000
Root vs Leaf	RHSIM_Rhsim04G0184200	2.450652137	0.000689536	0.001657479	RHSIM_Rhsim04G0184200
Root vs Leaf	RHSIM_Rhsim01G0096000	-7.869667363	0.000691415	0.001661824	RHSIM_Rhsim01G0096000
Root vs Leaf	RHSIM_Rhsim01G0290100	2.993030696	0.000692743	0.001664842	RHSIM_Rhsim01G0290100
Root vs Leaf	RHSIM_Rhsim11G0088000	-1.506730454	0.000694109	0.001667953	RHSIM_Rhsim11G0088000
Root vs Leaf	RHSIM_Rhsim07G0139500	-1.876562485	0.000694978	0.001669866	RHSIM_Rhsim07G0139500

Comparison	Gene ID	log_2FC	P-value	FDR (padj)	Gene Name
Root vs Leaf	RHSIM_Rhsim12G0072400	1.136682542	0.000695386	0.001670673	RHSIM_Rhsim12G0072400
Root vs Leaf	RHSIM_Rhsim03G0054200	3.841481737	0.000698859	0.001678669	RHSIM_Rhsim03G0054200
Root vs Leaf	RHSIM_Rhsim01G0097900	-5.816746358	0.000699406	0.001679808	RHSIM_Rhsim01G0097900
Root vs Leaf	RHSIM_Rhsim04G0035200	-2.625886672	0.000699531	0.001679935	RHSIM_Rhsim04G0035200
Root vs Leaf	RHSIM_Rhsim07G0095900	1.076258561	0.000700224	0.001681425	RHSIM_Rhsim07G0095900
Root vs Leaf	RHSIM_Rhsim11G0140900	2.619931048	0.000700464	0.001681827	RHSIM_Rhsim11G0140900
Root vs Leaf	RHSIM_Rhsim04G0187800	2.022055296	0.00070137	0.001683826	RHSIM_Rhsim04G0187800
Root vs Leaf	RHSIM_Rhsim10G0175400	2.198514872	0.000702105	0.00168524	RHSIM_Rhsim10G0175400
Root vs Leaf	RHSIM_Rhsim03G0245700	-2.124691534	0.000703301	0.001687761	RHSIM_Rhsim03G0245700
Root vs Leaf	RHSIM_Rhsim12G0145300	1.822786023	0.000704378	0.001689997	RHSIM_Rhsim12G0145300
Root vs Leaf	RHSIM_Rhsim09G0035500	3.617267222	0.000707359	0.001696973	RHSIM_Rhsim09G0035500
Root vs Leaf	novel.2537	4.027438702	0.00071005	0.001703253	--
Root vs Leaf	RHSIM_Rhsim09G0100300	1.522116214	0.00071414	0.001712353	RHSIM_Rhsim09G0100300
Root vs Leaf	RHSIM_Rhsim07G0119800	2.344740582	0.000715134	0.001714381	RHSIM_Rhsim07G0119800
Root vs Leaf	RHSIM_Rhsim10G0116100	3.542526379	0.000715519	0.001715125	RHSIM_Rhsim10G0116100
Root vs Leaf	RHSIM_Rhsim13G0180400	4.036963997	0.000716561	0.001717446	RHSIM_Rhsim13G0180400
Root vs Leaf	RHSIM_Rhsim05G0007800	3.541478952	0.000716959	0.001718222	RHSIM_Rhsim05G0007800
Root vs Leaf	RHSIM_Rhsim02G0111800	2.507226642	0.000723047	0.001731736	RHSIM_Rhsim02G0111800
Root vs Leaf	RHSIM_Rhsim13G0228300	1.090535858	0.000724921	0.001735865	RHSIM_Rhsim13G0228300
Root vs Leaf	novel.488	1.878491533	0.000727566	0.001741838	--
Root vs Leaf	novel.446	-2.062261417	0.000729611	0.00174636	--
Root vs Leaf	RHSIM_Rhsim08G0232600	-3.359880817	0.000733513	0.001754803	RHSIM_Rhsim08G0232600
Root vs Leaf	RHSIM_Rhsim11G0004400	-1.635745475	0.00073428	0.001756276	RHSIM_Rhsim11G0004400
Root vs Leaf	RHSIM_Rhsim03G0242800	1.125491693	0.000734739	0.001757194	RHSIM_Rhsim03G0242800
Root vs Leaf	RHSIM_Rhsim08G0170200	1.544225955	0.000735116	0.001757913	RHSIM_Rhsim08G0170200

Comparison	Gene ID	log_2FC	P-value	FDR (padj)	Gene Name
Root vs Leaf	RHSIM_Rhsim10G0204600	-1.004624711	0.000735494	0.001758636	RHSIM_Rhsim10G0204600
Root vs Leaf	RHSIM_Rhsim04G0018100	1.098519574	0.000737008	0.001761891	RHSIM_Rhsim04G0018100
Root vs Leaf	RHSIM_Rhsim01G0085500	-2.506990111	0.000742895	0.001775414	RHSIM_Rhsim01G0085500
Root vs Leaf	RHSIM_Rhsim11G0084700	3.827960742	0.000744012	0.001777899	RHSIM_Rhsim11G0084700
Root vs Leaf	RHSIM_Rhsim01G0065700	1.535839706	0.000744416	0.001778682	RHSIM_Rhsim01G0065700
Root vs Leaf	RHSIM_Rhsim09G0184800	1.152189573	0.000745413	0.00178088	RHSIM_Rhsim09G0184800
Root vs Leaf	RHSIM_Rhsim06G0036800	-1.103266697	0.000745773	0.001781557	RHSIM_Rhsim06G0036800
Root vs Leaf	RHSIM_Rhsim10G0178700	-1.084304039	0.00074776	0.001785565	RHSIM_Rhsim10G0178700
Root vs Leaf	RHSIM_Rhsim03G0162200	-1.35612281	0.000748301	0.001786383	RHSIM_Rhsim03G0162200
Root vs Leaf	RHSIM_Rhsim01G0006800	-1.035318428	0.000748334	0.001786383	RHSIM_Rhsim01G0006800
Root vs Leaf	RHSIM_RhsimUnG0194200	-1.361098995	0.000748763	0.001787222	RHSIM_RhsimUnG0194200
Root vs Leaf	RHSIM_Rhsim07G0029000	-1.029947498	0.000748884	0.001787327	RHSIM_Rhsim07G0029000
Root vs Leaf	RHSIM_Rhsim13G0200900	2.655827269	0.00074988	0.001789519	RHSIM_Rhsim13G0200900
Root vs Leaf	RHSIM_Rhsim13G0109600	1.145772327	0.000753815	0.001798538	RHSIM_Rhsim13G0109600
Root vs Leaf	RHSIM_Rhsim11G0142200	1.829371013	0.000754043	0.001798897	RHSIM_Rhsim11G0142200
Root vs Leaf	novel.4456	-3.195008915	0.000755635	0.001802511	--
Root vs Leaf	RHSIM_Rhsim03G0270200	1.90753866	0.000755954	0.001803086	RHSIM_Rhsim03G0270200
Root vs Leaf	RHSIM_Rhsim03G0085200	-1.51751828	0.000758377	0.001808679	RHSIM_Rhsim03G0085200
Root vs Leaf	RHSIM_Rhsim12G0109700	-1.640757086	0.000760514	0.001813587	RHSIM_Rhsim12G0109700
Root vs Leaf	RHSIM_Rhsim01G0221500	-1.606339479	0.000761818	0.001816324	RHSIM_Rhsim01G0221500
Root vs Leaf	novel.1362	-3.743202491	0.000764167	0.001821547	--
Root vs Leaf	RHSIM_Rhsim02G0118800	3.357003626	0.000764885	0.001822884	RHSIM_Rhsim02G0118800
Root vs Leaf	RHSIM_Rhsim13G0201600	2.568122415	0.000765938	0.001825206	RHSIM_Rhsim13G0201600
Root vs Leaf	RHSIM_Rhsim07G0106100	6.074899299	0.000766624	0.001826652	RHSIM_Rhsim07G0106100
Root vs Leaf	RHSIM_Rhsim08G0091400	-2.582878529	0.000768424	0.001830376	RHSIM_Rhsim08G0091400

Comparison	Gene ID	log_2FC	P-value	FDR (padj)	Gene Name
Root vs Leaf	novel.5539	2.320188071	0.000769048	0.001831674	--
Root vs Leaf	RHSIM_Rhsim11G0193200	-1.987083056	0.000771564	0.001837476	RHSIM_Rhsim11G0193200
Root vs Leaf	RHSIM_Rhsim12G0163600	3.979072059	0.000776695	0.001849125	RHSIM_Rhsim12G0163600
Root vs Leaf	novel.411	2.183814358	0.000778695	0.001853505	--
Root vs Leaf	RHSIM_Rhsim13G0185400	3.230744282	0.00077935	0.001854873	RHSIM_Rhsim13G0185400
Root vs Leaf	RHSIM_Rhsim02G0115000	2.001746022	0.000780551	0.001857371	RHSIM_Rhsim02G0115000
Root vs Leaf	RHSIM_Rhsim13G0184900	1.132345023	0.00078676	0.001871739	RHSIM_Rhsim13G0184900
Root vs Leaf	RHSIM_Rhsim04G0067500	3.524679374	0.000788153	0.001874604	RHSIM_Rhsim04G0067500
Root vs Leaf	novel.1113	-2.249293387	0.000788541	0.001875205	--
Root vs Leaf	RHSIM_Rhsim07G0022500	1.054063087	0.000792092	0.001882876	RHSIM_Rhsim07G0022500
Root vs Leaf	RHSIM_Rhsim07G0079900	-1.174772076	0.000795552	0.001890271	RHSIM_Rhsim07G0079900
Root vs Leaf	RHSIM_Rhsim12G0017400	1.144712931	0.000795611	0.001890271	RHSIM_Rhsim12G0017400
Root vs Leaf	RHSIM_Rhsim04G0066300	-3.066431508	0.000799503	0.001898933	RHSIM_Rhsim04G0066300
Root vs Leaf	novel.3525	5.862134335	0.000802468	0.001905192	--
Root vs Leaf	RHSIM_Rhsim07G0172000	-7.768792909	0.000803704	0.001907541	RHSIM_Rhsim07G0172000
Root vs Leaf	RHSIM_Rhsim02G0014400	-1.12903993	0.000805982	0.001912554	RHSIM_Rhsim02G0014400
Root vs Leaf	RHSIM_Rhsim06G0237100	-1.202630901	0.000806656	0.001913762	RHSIM_Rhsim06G0237100
Root vs Leaf	RHSIM_Rhsim13G0232900	2.392204794	0.000813272	0.001928469	RHSIM_Rhsim13G0232900
Root vs Leaf	RHSIM_Rhsim12G0015300	3.607217097	0.000814635	0.001931304	RHSIM_Rhsim12G0015300
Root vs Leaf	RHSIM_Rhsim08G0009700	1.495475993	0.000815386	0.00193269	RHSIM_Rhsim08G0009700
Root vs Leaf	novel.1597	-1.59108619	0.0008156	0.001933	--
Root vs Leaf	RHSIM_RhsimPtG0002400	-1.611840052	0.000816216	0.001934261	RHSIM_RhsimPtG0002400
Root vs Leaf	RHSIM_Rhsim04G0066700	-1.091997212	0.000816894	0.00193567	RHSIM_Rhsim04G0066700
Root vs Leaf	RHSIM_Rhsim03G0124000	-1.4555758	0.000818984	0.001940223	RHSIM_Rhsim03G0124000
Root vs Leaf	RHSIM_Rhsim04G0004600	1.190620476	0.000820662	0.001944	RHSIM_Rhsim04G0004600

Comparison	Gene ID	log_2FC	P-value	FDR (padj)	Gene Name
Root vs Leaf	novel.3375	2.360624626	0.000824018	0.00195135	--
Root vs Leaf	RHSIM_Rhsim06G0195700	2.200505067	0.000824368	0.00195198	RHSIM_Rhsim06G0195700
Root vs Leaf	novel.1611	-1.510330665	0.000826103	0.001955688	--
Root vs Leaf	RHSIM_Rhsim03G0185400	5.221376452	0.000827139	0.001957941	RHSIM_Rhsim03G0185400
Root vs Leaf	RHSIM_Rhsim12G0194500	-1.8905744	0.000833328	0.001972188	RHSIM_Rhsim12G0194500
Root vs Leaf	RHSIM_RhsimUnG0150600	-1.747654527	0.000834165	0.001973764	RHSIM_RhsimUnG0150600
Root vs Leaf	RHSIM_Rhsim10G0164800	-1.551546788	0.000835269	0.001976174	RHSIM_Rhsim10G0164800
Root vs Leaf	RHSIM_Rhsim06G0086600	1.227716989	0.000837933	0.001981667	RHSIM_Rhsim06G0086600
Root vs Leaf	RHSIM_Rhsim10G0177100	3.338416069	0.000842232	0.001991021	RHSIM_Rhsim10G0177100
Root vs Leaf	RHSIM_RhsimUnG0043300	-1.522519505	0.000844803	0.001996893	RHSIM_RhsimUnG0043300
Root vs Leaf	RHSIM_Rhsim06G0050300	1.088473453	0.000848102	0.002004486	RHSIM_Rhsim06G0050300
Root vs Leaf	RHSIM_Rhsim07G0044800	-2.73434181	0.000848636	0.002005543	RHSIM_Rhsim07G0044800
Root vs Leaf	RHSIM_Rhsim03G0215800	3.167609808	0.000853813	0.002017161	RHSIM_Rhsim03G0215800
Root vs Leaf	RHSIM_Rhsim12G0066500	1.23922637	0.000856734	0.002023236	RHSIM_Rhsim12G0066500
Root vs Leaf	RHSIM_Rhsim13G0105300	-1.126588831	0.000858353	0.002026439	RHSIM_Rhsim13G0105300
Root vs Leaf	RHSIM_Rhsim10G0009400	1.936396107	0.000860227	0.002030241	RHSIM_Rhsim10G0009400
Root vs Leaf	RHSIM_Rhsim05G0205400	-1.680821835	0.000860327	0.002030271	RHSIM_Rhsim05G0205400
Root vs Leaf	RHSIM_Rhsim03G0011700	1.322553696	0.000863943	0.002038595	RHSIM_Rhsim03G0011700
Root vs Leaf	RHSIM_Rhsim11G0181100	2.797548744	0.000865341	0.002041478	RHSIM_Rhsim11G0181100
Root vs Leaf	novel.4549	-7.769000773	0.000866596	0.002044231	--
Root vs Leaf	RHSIM_Rhsim12G0106600	3.326499442	0.000870018	0.002052094	RHSIM_Rhsim12G0106600
Root vs Leaf	RHSIM_Rhsim08G0204200	-1.372134677	0.000871626	0.002055466	RHSIM_Rhsim08G0204200
Root vs Leaf	novel.126	-2.513914204	0.000872265	0.002056764	--
Root vs Leaf	RHSIM_Rhsim07G0007000	-1.930855322	0.000876312	0.002066096	RHSIM_Rhsim07G0007000
Root vs Leaf	RHSIM_Rhsim03G0146400	-1.036833456	0.000876496	0.00206632	RHSIM_Rhsim03G0146400

Comparison	Gene ID	log_2FC	P-value	FDR (padj)	Gene Name
Root vs Leaf	novel.3192	-7.837197649	0.0008769	0.002066909	--
Root vs Leaf	novel.2609	-7.803527646	0.000876925	0.002066909	--
Root vs Leaf	RHSIM_Rhsim07G0009200	-1.517429949	0.000877795	0.00206875	RHSIM_Rhsim07G0009200
Root vs Leaf	RHSIM_Rhsim05G0021800	-2.306533708	0.000881235	0.002076222	RHSIM_Rhsim05G0021800
Root vs Leaf	RHSIM_Rhsim04G0201200	2.5417272	0.000882751	0.002079372	RHSIM_Rhsim04G0201200
Root vs Leaf	RHSIM_RhsimUnG0142400	2.886072214	0.000883903	0.002081278	RHSIM_RhsimUnG0142400
Root vs Leaf	RHSIM_Rhsim05G0180900	2.75468905	0.00088392	0.002081278	RHSIM_Rhsim05G0180900
Root vs Leaf	RHSIM_Rhsim07G0249300	-1.193041514	0.000884522	0.002082483	RHSIM_Rhsim07G0249300
Root vs Leaf	RHSIM_Rhsim05G0099300	1.663373689	0.000887957	0.00208972	RHSIM_Rhsim05G0099300
Root vs Leaf	RHSIM_Rhsim03G0096900	-1.238861347	0.000889256	0.002092563	RHSIM_Rhsim03G0096900
Root vs Leaf	novel.5674	2.885569123	0.000889507	0.002092941	--
Root vs Leaf	RHSIM_Rhsim04G0078600	1.830997428	0.000892094	0.002098176	RHSIM_Rhsim04G0078600
Root vs Leaf	RHSIM_Rhsim07G0049700	-1.700138401	0.000893985	0.002102409	RHSIM_Rhsim07G0049700
Root vs Leaf	RHSIM_Rhsim01G0128300	3.008792471	0.000895097	0.002104596	RHSIM_Rhsim01G0128300
Root vs Leaf	RHSIM_Rhsim06G0022700	-1.314477579	0.000895274	0.002104798	RHSIM_Rhsim06G0022700
Root vs Leaf	RHSIM_Rhsim04G0021000	-1.507821066	0.000895998	0.002106287	RHSIM_Rhsim04G0021000
Root vs Leaf	RHSIM_Rhsim03G0237200	1.159324832	0.000896495	0.00210724	RHSIM_Rhsim03G0237200
Root vs Leaf	RHSIM_Rhsim01G0267100	3.968978788	0.000896835	0.002107825	RHSIM_Rhsim01G0267100
Root vs Leaf	RHSIM_Rhsim08G0240200	-1.22029088	0.00090032	0.002115587	RHSIM_Rhsim08G0240200
Root vs Leaf	RHSIM_Rhsim05G0092800	-1.314840993	0.000901267	0.002117598	RHSIM_Rhsim05G0092800
Root vs Leaf	RHSIM_Rhsim09G0066000	-1.186328668	0.000904101	0.002123824	RHSIM_Rhsim09G0066000
Root vs Leaf	RHSIM_Rhsim06G0106500	-1.050931092	0.000904277	0.002123955	RHSIM_Rhsim06G0106500
Root vs Leaf	RHSIM_Rhsim08G0020100	1.018890457	0.000905681	0.002126673	RHSIM_Rhsim08G0020100
Root vs Leaf	RHSIM_Rhsim05G0004400	-1.522021309	0.000906086	0.002127035	RHSIM_Rhsim05G0004400
Root vs Leaf	novel.2405	3.118560279	0.000906111	0.002127035	--

Comparison	Gene ID	log_2FC	P-value	FDR (padj)	Gene Name
Root vs Leaf	novel.1880	6.000893069	0.000911576	0.002139647	--
Root vs Leaf	RHSIM_Rhsim13G0017900	1.507176266	0.000914983	0.002147208	RHSIM_Rhsim13G0017900
Root vs Leaf	RHSIM_Rhsim12G0131700	3.416236925	0.000916761	0.002150945	RHSIM_Rhsim12G0131700
Root vs Leaf	RHSIM_Rhsim10G0034000	-2.369698526	0.000918978	0.002155646	RHSIM_Rhsim10G0034000
Root vs Leaf	RHSIM_Rhsim05G0179300	-1.534000035	0.000920726	0.00215935	RHSIM_Rhsim05G0179300
Root vs Leaf	RHSIM_Rhsim03G0079200	1.014382367	0.000924315	0.002166911	RHSIM_Rhsim03G0079200
Root vs Leaf	RHSIM_Rhsim07G0049100	2.63826696	0.000924635	0.00216744	RHSIM_Rhsim07G0049100
Root vs Leaf	RHSIM_Rhsim05G0094700	6.44257976	0.000924959	0.00216758	RHSIM_Rhsim05G0094700
Root vs Leaf	novel.4	2.835476581	0.000926392	0.002170444	--
Root vs Leaf	RHSIM_Rhsim04G0054100	2.079193896	0.000926458	0.002170444	RHSIM_Rhsim04G0054100
Root vs Leaf	RHSIM_Rhsim12G0135400	1.271615969	0.000926479	0.002170444	RHSIM_Rhsim12G0135400
Root vs Leaf	RHSIM_Rhsim12G0040300	1.859880503	0.0009291	0.002176144	RHSIM_Rhsim12G0040300
Root vs Leaf	RHSIM_Rhsim02G0150200	2.29524878	0.000930348	0.002178846	RHSIM_Rhsim02G0150200
Root vs Leaf	RHSIM_Rhsim03G0021000	4.680358695	0.000931559	0.002181462	RHSIM_Rhsim03G0021000
Root vs Leaf	RHSIM_Rhsim07G0042600	3.689733032	0.000934073	0.002186907	RHSIM_Rhsim07G0042600
Root vs Leaf	RHSIM_Rhsim02G0237600	1.04641649	0.000934564	0.002187598	RHSIM_Rhsim02G0237600
Root vs Leaf	RHSIM_Rhsim09G0187600	1.106539643	0.000936138	0.002190854	RHSIM_Rhsim09G0187600
Root vs Leaf	RHSIM_Rhsim01G0268600	5.623334343	0.00093701	0.002192672	RHSIM_Rhsim01G0268600
Root vs Leaf	novel.3935	2.139169761	0.00093818	0.002194968	--
Root vs Leaf	RHSIM_RhsimUnG0036100	-1.679487596	0.000939689	0.002198275	RHSIM_RhsimUnG0036100
Root vs Leaf	RHSIM_Rhsim04G0191700	-3.758524042	0.000941752	0.002202657	RHSIM_Rhsim04G0191700
Root vs Leaf	RHSIM_Rhsim10G0127800	-1.055278479	0.000941994	0.002203001	RHSIM_Rhsim10G0127800
Root vs Leaf	RHSIM_RhsimUnG0202000	-1.695693927	0.000942134	0.002203104	RHSIM_RhsimUnG0202000
Root vs Leaf	RHSIM_Rhsim03G0253900	-3.841746465	0.000942251	0.002203155	RHSIM_Rhsim03G0253900
Root vs Leaf	RHSIM_Rhsim03G0067000	-1.622309053	0.00094332	0.002205432	RHSIM_Rhsim03G0067000

Comparison	Gene ID	log_2FC	P-value	FDR (padj)	Gene Name
Root vs Leaf	RHSIM_Rhsim06G0125300	1.231187902	0.000944479	0.002207697	RHSIM_Rhsim06G0125300
Root vs Leaf	RHSIM_Rhsim09G0147000	-1.515798241	0.000946828	0.002212739	RHSIM_Rhsim09G0147000
Root vs Leaf	novel.2575	2.206072427	0.000947494	0.002214072	--
Root vs Leaf	novel.554	1.411055899	0.000949715	0.002219038	--
Root vs Leaf	RHSIM_Rhsim13G0031700	-1.026429102	0.000950266	0.002220102	RHSIM_Rhsim13G0031700
Root vs Leaf	RHSIM_Rhsim02G0078100	-4.578666032	0.000950395	0.002220179	RHSIM_Rhsim02G0078100
Root vs Leaf	novel.2517	1.067500974	0.000950702	0.002220671	--
Root vs Leaf	RHSIM_Rhsim03G0223300	1.398528657	0.000951305	0.002221855	RHSIM_Rhsim03G0223300
Root vs Leaf	novel.728	2.357676188	0.000952851	0.002225118	--
Root vs Leaf	RHSIM_Rhsim05G0123700	-2.221082187	0.000952894	0.002225118	RHSIM_Rhsim05G0123700
Root vs Leaf	RHSIM_Rhsim01G0183200	5.258887229	0.000953689	0.00222675	RHSIM_Rhsim01G0183200
Root vs Leaf	RHSIM_Rhsim05G0102500	1.204014469	0.000954964	0.002229278	RHSIM_Rhsim05G0102500
Root vs Leaf	novel.1493	1.171225348	0.000955377	0.002229567	--
Root vs Leaf	novel.858	1.137570456	0.000963579	0.002247801	--
Root vs Leaf	RHSIM_Rhsim09G0096900	-1.134353962	0.00096452	0.002249769	RHSIM_Rhsim09G0096900
Root vs Leaf	RHSIM_Rhsim12G0121400	-1.441159953	0.000965679	0.002252017	RHSIM_Rhsim12G0121400
Root vs Leaf	RHSIM_Rhsim11G0063500	-3.132559563	0.00096647	0.002253636	RHSIM_Rhsim11G0063500
Root vs Leaf	RHSIM_Rhsim03G0017400	-2.779316025	0.00096841	0.002257932	RHSIM_Rhsim03G0017400
Root vs Leaf	RHSIM_Rhsim06G0166700	1.730485547	0.000971432	0.002264065	RHSIM_Rhsim06G0166700
Root vs Leaf	RHSIM_Rhsim01G0143500	-2.379230016	0.00097424	0.002269924	RHSIM_Rhsim01G0143500
Root vs Leaf	RHSIM_Rhsim13G0222800	6.277033979	0.000974877	0.00227118	RHSIM_Rhsim13G0222800
Root vs Leaf	RHSIM_Rhsim11G0104400	-1.366420885	0.000976948	0.002275649	RHSIM_Rhsim11G0104400
Root vs Leaf	RHSIM_Rhsim01G0209000	1.034319706	0.000977814	0.002277105	RHSIM_Rhsim01G0209000
Root vs Leaf	RHSIM_RhsimUnG0056400	-1.91776224	0.000980417	0.002282249	RHSIM_RhsimUnG0056400
Root vs Leaf	RHSIM_Rhsim13G0095800	1.134738416	0.000982197	0.002285931	RHSIM_Rhsim13G0095800

Comparison	Gene ID	log_2FC	P-value	FDR (padj)	Gene Name
Root vs Leaf	RHSIM_Rhsim09G0169700	1.404756318	0.000993938	0.002312559	RHSIM_Rhsim09G0169700
Root vs Leaf	RHSIM_RhsimUnG0066200	1.489102541	0.000995106	0.00231458	RHSIM_RhsimUnG0066200
Root vs Leaf	RHSIM_Rhsim07G0046600	-1.559084034	0.000999013	0.0023232	RHSIM_Rhsim07G0046600
Root vs Leaf	RHSIM_RhsimUnG0242600	-7.679242256	0.001000339	0.002325815	RHSIM_RhsimUnG0242600
Root vs Leaf	RHSIM_Rhsim07G0003500	-1.155813763	0.001000689	0.002326397	RHSIM_Rhsim07G0003500
Root vs Leaf	RHSIM_Rhsim01G0025900	-2.409380429	0.00100379	0.00233337	RHSIM_Rhsim01G0025900
Root vs Leaf	RHSIM_Rhsim02G0023700	3.171554016	0.001007534	0.002341839	RHSIM_Rhsim02G0023700
Root vs Leaf	RHSIM_Rhsim10G0190500	3.802306083	0.001010865	0.00234911	RHSIM_Rhsim10G0190500
Root vs Leaf	RHSIM_Rhsim06G0215800	2.231111998	0.001011798	0.002351042	RHSIM_Rhsim06G0215800
Root vs Leaf	RHSIM_Rhsim12G0187800	-1.003666824	0.001014525	0.002356904	RHSIM_Rhsim12G0187800
Root vs Leaf	RHSIM_Rhsim01G0141700	-1.624173639	0.001015781	0.002359586	RHSIM_Rhsim01G0141700
Root vs Leaf	RHSIM_Rhsim09G0057400	-1.063808353	0.001019037	0.002366675	RHSIM_Rhsim09G0057400
Root vs Leaf	novel.196	1.065238179	0.001023255	0.002376231	--
Root vs Leaf	RHSIM_Rhsim05G0173200	-7.716011544	0.001023526	0.002376623	RHSIM_Rhsim05G0173200
Root vs Leaf	RHSIM_Rhsim02G0081900	-2.673261339	0.001024753	0.002379232	RHSIM_Rhsim02G0081900
Root vs Leaf	novel.5060	1.91764876	0.001031747	0.00239451	--
Root vs Leaf	RHSIM_Rhsim12G0133600	1.034293236	0.001037629	0.002407436	RHSIM_Rhsim12G0133600
Root vs Leaf	RHSIM_RhsimUnG0181100	-1.555099539	0.001039086	0.002410574	RHSIM_RhsimUnG0181100
Root vs Leaf	RHSIM_Rhsim08G0217900	1.070297746	0.001039298	0.002410825	RHSIM_Rhsim08G0217900
Root vs Leaf	novel.5630	1.944948118	0.001044753	0.002423237	--
Root vs Leaf	RHSIM_RhsimUnG0189100	1.089776891	0.001045081	0.002423754	RHSIM_RhsimUnG0189100
Root vs Leaf	novel.2020	-1.404091271	0.001045625	0.002424772	--
Root vs Leaf	RHSIM_Rhsim12G0126900	2.749544701	0.001046846	0.002427361	RHSIM_Rhsim12G0126900
Root vs Leaf	RHSIM_Rhsim01G0136600	-1.639205037	0.00105115	0.002436608	RHSIM_Rhsim01G0136600
Root vs Leaf	novel.343	1.706488142	0.001052664	0.002439873	--

Comparison	Gene ID	log$_2$FC	P-value	FDR (padj)	Gene Name
Root vs Leaf	RHSIM_Rhsim10G0027600	-1.173309984	0.001056697	0.002448975	RHSIM_Rhsim10G0027600
Root vs Leaf	RHSIM_Rhsim03G0198400	-1.078990664	0.001058455	0.002452805	RHSIM_Rhsim03G0198400
Root vs Leaf	RHSIM_Rhsim11G0043200	1.058260477	0.001059217	0.002454325	RHSIM_Rhsim11G0043200
Root vs Leaf	RHSIM_Rhsim05G0213600	2.900094535	0.001065733	0.002468681	RHSIM_Rhsim05G0213600
Root vs Leaf	RHSIM_Rhsim13G0017400	-1.260905762	0.00106658	0.00247015	RHSIM_Rhsim13G0017400
Root vs Leaf	RHSIM_Rhsim03G0269300	-1.438491525	0.001067918	0.002472999	RHSIM_Rhsim03G0269300
Root vs Leaf	novel.3624	-1.014657651	0.001068576	0.002474275	--
Root vs Leaf	RHSIM_Rhsim12G0203500	2.26232614	0.001073379	0.002484901	RHSIM_Rhsim12G0203500
Root vs Leaf	novel.4779	-1.787168955	0.001076482	0.002491834	--
Root vs Leaf	RHSIM_Rhsim12G0079200	4.270354589	0.001076672	0.002492025	RHSIM_Rhsim12G0079200
Root vs Leaf	RHSIM_Rhsim01G0274700	5.10507115	0.001078156	0.002495186	RHSIM_Rhsim01G0274700
Root vs Leaf	RHSIM_Rhsim12G0056100	3.223085844	0.001079556	0.002497701	RHSIM_Rhsim12G0056100
Root vs Leaf	RHSIM_Rhsim03G0173500	1.124126072	0.001083406	0.002506357	RHSIM_Rhsim03G0173500
Root vs Leaf	novel.3538	1.948485832	0.001088243	0.002516792	--
Root vs Leaf	RHSIM_Rhsim11G0170500	1.83908474	0.001092306	0.002525181	RHSIM_Rhsim11G0170500
Root vs Leaf	RHSIM_RhsimUnG0231300	4.280513775	0.001094451	0.00252978	RHSIM_RhsimUnG0231300
Root vs Leaf	novel.427	-5.119709693	0.001094514	0.00252978	--
Root vs Leaf	RHSIM_Rhsim06G0084400	4.451466477	0.001096344	0.002533502	RHSIM_Rhsim06G0084400
Root vs Leaf	RHSIM_RhsimUnG0070800	2.740792326	0.001100533	0.002542929	RHSIM_RhsimUnG0070800
Root vs Leaf	novel.3797	-1.708119299	0.001101568	0.002545067	--
Root vs Leaf	RHSIM_Rhsim01G0283400	-2.523054818	0.001101824	0.002545404	RHSIM_Rhsim01G0283400
Root vs Leaf	novel.5752	1.337303733	0.001102416	0.002546516	--
Root vs Leaf	RHSIM_Rhsim01G0189900	2.440440707	0.001102606	0.002546702	RHSIM_Rhsim01G0189900
Root vs Leaf	RHSIM_Rhsim09G0155000	-1.550167124	0.001103244	0.002547922	RHSIM_Rhsim09G0155000
Root vs Leaf	RHSIM_Rhsim04G0155200	-1.175984622	0.001104552	0.002550687	RHSIM_Rhsim04G0155200

Comparison	Gene ID	log_2FC	P-value	FDR (padj)	Gene Name
Root vs Leaf	novel.2765	2.57967752	0.001106233	0.002554315	--
Root vs Leaf	RHSIM_Rhsim01G0289000	5.35314535	0.001113214	0.002569665	RHSIM_Rhsim01G0289000
Root vs Leaf	RHSIM_Rhsim05G0091700	-1.914053371	0.001120533	0.0025863	RHSIM_Rhsim05G0091700
Root vs Leaf	RHSIM_Rhsim04G0083200	2.29071334	0.001120826	0.002586719	RHSIM_Rhsim04G0083200
Root vs Leaf	RHSIM_Rhsim04G0109300	-1.602543099	0.001131608	0.00260952	RHSIM_Rhsim04G0109300
Root vs Leaf	RHSIM_Rhsim07G0240200	5.078526309	0.001134753	0.002616251	RHSIM_Rhsim07G0240200
Root vs Leaf	RHSIM_Rhsim02G0165400	3.457886675	0.00113508	0.002616745	RHSIM_Rhsim02G0165400
Root vs Leaf	novel.5399	1.385138178	0.001141969	0.002631727	--
Root vs Leaf	RHSIM_Rhsim03G0124500	1.795127236	0.001142034	0.002631727	RHSIM_Rhsim03G0124500
Root vs Leaf	novel.5182	-2.496023856	0.001145457	0.002639352	--
Root vs Leaf	RHSIM_Rhsim08G0045600	1.187178732	0.001146255	0.00264093	RHSIM_Rhsim08G0045600
Root vs Leaf	RHSIM_Rhsim05G0033600	-1.249200377	0.001147738	0.002644081	RHSIM_Rhsim05G0033600
Root vs Leaf	novel.3217	-7.679571715	0.001153124	0.002655696	--
Root vs Leaf	RHSIM_Rhsim02G0252100	-1.461709689	0.001156419	0.002662755	RHSIM_Rhsim02G0252100
Root vs Leaf	RHSIM_Rhsim09G0108800	-1.704583075	0.001158589	0.002667487	RHSIM_Rhsim09G0108800
Root vs Leaf	RHSIM_Rhsim09G0056200	3.521384759	0.001162494	0.002675678	RHSIM_Rhsim09G0056200
Root vs Leaf	RHSIM_Rhsim02G0069300	-7.34811966	0.001165237	0.002681726	RHSIM_Rhsim02G0069300
Root vs Leaf	RHSIM_Rhsim10G0178100	3.178008654	0.001165699	0.002682256	RHSIM_Rhsim10G0178100
Root vs Leaf	RHSIM_Rhsim06G0211800	1.598300622	0.001166132	0.002682985	RHSIM_Rhsim06G0211800
Root vs Leaf	RHSIM_Rhsim10G0037300	-1.433593825	0.001168175	0.002687419	RHSIM_Rhsim10G0037300
Root vs Leaf	RHSIM_Rhsim07G0109200	2.592768482	0.001173026	0.002697773	RHSIM_Rhsim07G0109200
Root vs Leaf	RHSIM_Rhsim08G0088100	-1.975602954	0.001175401	0.002702967	RHSIM_Rhsim08G0088100
Root vs Leaf	RHSIM_Rhsim08G0037700	-1.013348516	0.001179689	0.002711283	RHSIM_Rhsim08G0037700
Root vs Leaf	RHSIM_Rhsim13G0152900	2.988750589	0.001182466	0.002717054	RHSIM_Rhsim13G0152900
Root vs Leaf	RHSIM_Rhsim06G0193600	-1.59894265	0.00118278	0.002717506	RHSIM_Rhsim06G0193600

Comparison	Gene ID	log_2FC	P-value	FDR (padj)	Gene Name
Root vs Leaf	RHSIM_Rhsim06G0046000	3.05254322	0.001183782	0.002719537	RHSIM_Rhsim06G0046000
Root vs Leaf	RHSIM_Rhsim13G0079300	1.751712596	0.001193881	0.002741378	RHSIM_Rhsim13G0079300
Root vs Leaf	RHSIM_Rhsim12G0002700	-1.625010315	0.001197451	0.002748836	RHSIM_Rhsim12G0002700
Root vs Leaf	RHSIM_Rhsim01G0261500	2.190780337	0.001197486	0.002748836	RHSIM_Rhsim01G0261500
Root vs Leaf	novel.872	-6.728652941	0.001197629	0.002748892	--
Root vs Leaf	RHSIM_Rhsim04G0181500	1.384084898	0.001200451	0.002755096	RHSIM_Rhsim04G0181500
Root vs Leaf	RHSIM_Rhsim05G0037200	-5.858102224	0.001202898	0.002760437	RHSIM_Rhsim05G0037200
Root vs Leaf	novel.3153	3.406556476	0.001210355	0.002775624	--
Root vs Leaf	RHSIM_Rhsim02G0213000	-1.027334256	0.001210355	0.002775624	RHSIM_Rhsim02G0213000
Root vs Leaf	RHSIM_Rhsim03G0069700	-1.211222474	0.001214223	0.002783391	RHSIM_Rhsim03G0069700
Root vs Leaf	RHSIM_Rhsim03G0254800	1.387066552	0.001216383	0.002787791	RHSIM_Rhsim03G0254800
Root vs Leaf	RHSIM_Rhsim01G0245600	-1.218558723	0.00121721	0.00278941	RHSIM_Rhsim01G0245600
Root vs Leaf	RHSIM_Rhsim08G0117700	1.517821071	0.001217663	0.002790172	RHSIM_Rhsim08G0117700
Root vs Leaf	RHSIM_Rhsim11G0124000	3.360544119	0.001218082	0.002790855	RHSIM_Rhsim11G0124000
Root vs Leaf	RHSIM_Rhsim02G0122500	3.390350805	0.001221115	0.002796973	RHSIM_Rhsim02G0122500
Root vs Leaf	novel.1337	-2.016297081	0.00122258	0.002800051	--
Root vs Leaf	RHSIM_RhsimUnG0033400	-2.531316911	0.001223293	0.002801407	RHSIM_RhsimUnG0033400
Root vs Leaf	RHSIM_Rhsim11G0161400	2.9346915	0.001223639	0.002801922	RHSIM_Rhsim11G0161400
Root vs Leaf	novel.5918	1.028400166	0.001226441	0.002807783	--
Root vs Leaf	RHSIM_Rhsim05G0103600	4.81172422	0.001226717	0.002807876	RHSIM_Rhsim05G0103600
Root vs Leaf	RHSIM_Rhsim04G0170100	4.402670026	0.001226724	0.002807876	RHSIM_Rhsim04G0170100
Root vs Leaf	RHSIM_RhsimUnG0210600	-1.522463603	0.001235638	0.002826881	RHSIM_RhsimUnG0210600
Root vs Leaf	RHSIM_Rhsim04G0229700	-1.032524622	0.00123897	0.002833943	RHSIM_Rhsim04G0229700
Root vs Leaf	RHSIM_RhsimPtG0000900	-1.015515784	0.001243766	0.002844631	RHSIM_RhsimPtG0000900
Root vs Leaf	RHSIM_Rhsim09G0100100	-3.118259807	0.00124516	0.002847538	RHSIM_Rhsim09G0100100

Comparison	Gene ID	log_2FC	P-value	FDR (padj)	Gene Name
Root vs Leaf	RHSIM_Rhsim06G0104300	2.122018867	0.001254024	0.002867243	RHSIM_Rhsim06G0104300
Root vs Leaf	novel.3829	-3.572929203	0.001258158	0.002876126	--
Root vs Leaf	RHSIM_Rhsim09G0185000	2.210503825	0.001262693	0.002885638	RHSIM_Rhsim09G0185000
Root vs Leaf	RHSIM_Rhsim01G0161600	-1.928851439	0.001263838	0.00288797	RHSIM_Rhsim01G0161600
Root vs Leaf	RHSIM_Rhsim05G0186000	-1.246389305	0.001264198	0.002888508	RHSIM_Rhsim05G0186000
Root vs Leaf	novel.1350	1.911562542	0.001265319	0.002890498	--
Root vs Leaf	novel.5418	-1.506022935	0.001265957	0.002891448	--
Root vs Leaf	RHSIM_Rhsim03G0157100	-1.304790487	0.001266759	0.002892646	RHSIM_Rhsim03G0157100
Root vs Leaf	RHSIM_RhsimUnG0036700	-2.469440714	0.001269025	0.002897247	RHSIM_RhsimUnG0036700
Root vs Leaf	novel.4310	-3.048826194	0.001279058	0.002919001	--
Root vs Leaf	RHSIM_RhsimUnG0243400	-2.036376625	0.001282389	0.002926026	RHSIM_RhsimUnG0243400
Root vs Leaf	RHSIM_Rhsim03G0254500	1.057360239	0.001284626	0.002930843	RHSIM_Rhsim03G0254500
Root vs Leaf	RHSIM_Rhsim10G0077800	-1.291249851	0.001292333	0.002946393	RHSIM_Rhsim10G0077800
Root vs Leaf	RHSIM_Rhsim13G0023000	-3.96058846	0.001298336	0.002958912	RHSIM_Rhsim13G0023000
Root vs Leaf	RHSIM_Rhsim10G0029700	-1.589125958	0.001300611	0.002963805	RHSIM_Rhsim10G0029700
Root vs Leaf	novel.1239	-3.141666748	0.001307227	0.002978296	--
Root vs Leaf	RHSIM_Rhsim13G0175800	-2.526640386	0.001308396	0.002980665	RHSIM_Rhsim13G0175800
Root vs Leaf	RHSIM_Rhsim03G0213600	-1.073948001	0.00130988	0.002983289	RHSIM_Rhsim03G0213600
Root vs Leaf	RHSIM_RhsimUnG0235800	2.862966247	0.001309935	0.002983289	RHSIM_RhsimUnG0235800
Root vs Leaf	RHSIM_Rhsim11G0004500	-1.69089158	0.001310146	0.002983476	RHSIM_Rhsim11G0004500
Root vs Leaf	RHSIM_Rhsim03G0233400	4.505377052	0.00131333	0.002990138	RHSIM_Rhsim03G0233400
Root vs Leaf	RHSIM_Rhsim10G0167800	1.716341935	0.001314202	0.002991619	RHSIM_Rhsim10G0167800
Root vs Leaf	RHSIM_Rhsim11G0002900	-6.772147701	0.001314239	0.002991619	RHSIM_Rhsim11G0002900
Root vs Leaf	novel.3757	-2.66343403	0.001317176	0.00299801	--
Root vs Leaf	RHSIM_Rhsim11G0030200	2.755826218	0.001319388	0.00300275	RHSIM_Rhsim11G0030200

Comparison	Gene ID	log_2FC	P-value	FDR (padj)	Gene Name
Root vs Leaf	RHSIM_Rhsim05G0129500	-1.398765862	0.001325141	0.003015249	RHSIM_Rhsim05G0129500
Root vs Leaf	RHSIM_Rhsim10G0185000	1.730105181	0.00132926	0.003024325	RHSIM_Rhsim10G0185000
Root vs Leaf	RHSIM_Rhsim05G0002200	2.12913996	0.001330587	0.003026749	RHSIM_Rhsim05G0002200
Root vs Leaf	novel.6042	-1.465549396	0.001331398	0.003027997	--
Root vs Leaf	RHSIM_Rhsim03G0076700	-1.38579084	0.001333773	0.003032803	RHSIM_Rhsim03G0076700
Root vs Leaf	RHSIM_Rhsim01G0253900	1.277631211	0.001334464	0.003034077	RHSIM_Rhsim01G0253900
Root vs Leaf	novel.3546	1.368188587	0.00133741	0.003040327	--
Root vs Leaf	RHSIM_Rhsim13G0201200	-1.223036004	0.001337476	0.003040327	RHSIM_Rhsim13G0201200
Root vs Leaf	RHSIM_Rhsim06G0123700	3.945809775	0.001338276	0.003041847	RHSIM_Rhsim06G0123700
Root vs Leaf	RHSIM_Rhsim11G0154900	-1.545101205	0.001339651	0.003044672	RHSIM_Rhsim11G0154900
Root vs Leaf	novel.4169	3.966306453	0.001344338	0.003055025	--
Root vs Leaf	RHSIM_Rhsim03G0269100	-1.132402704	0.001348405	0.003063667	RHSIM_Rhsim03G0269100
Root vs Leaf	RHSIM_Rhsim03G0048000	1.002616993	0.0013487	0.003064036	RHSIM_Rhsim03G0048000
Root vs Leaf	RHSIM_Rhsim03G0033800	-1.98226221	0.001354109	0.003075613	RHSIM_Rhsim03G0033800
Root vs Leaf	RHSIM_Rhsim08G0153100	-1.842070526	0.001354195	0.003075613	RHSIM_Rhsim08G0153100
Root vs Leaf	RHSIM_Rhsim09G0007300	2.469048798	0.001356235	0.003079643	RHSIM_Rhsim09G0007300
Root vs Leaf	RHSIM_Rhsim12G0004000	2.920084637	0.001360245	0.003087838	RHSIM_Rhsim12G0004000
Root vs Leaf	RHSIM_Rhsim12G0061600	1.579291739	0.001360891	0.003089003	RHSIM_Rhsim12G0061600
Root vs Leaf	RHSIM_Rhsim03G0065200	-2.227818226	0.001369596	0.003106933	RHSIM_Rhsim03G0065200
Root vs Leaf	novel.4303	-1.6690212	0.00137611	0.003121404	--
Root vs Leaf	novel.3754	-7.523113613	0.001383284	0.003136754	--
Root vs Leaf	RHSIM_Rhsim12G0207000	-1.071019251	0.001384881	0.003139761	RHSIM_Rhsim12G0207000
Root vs Leaf	RHSIM_Rhsim13G0188800	-1.160302733	0.001391058	0.003153455	RHSIM_Rhsim13G0188800
Root vs Leaf	RHSIM_Rhsim06G0033700	1.163553018	0.001391235	0.003153547	RHSIM_Rhsim06G0033700
Root vs Leaf	novel.2440	-1.318560177	0.001393274	0.003157551	--

Comparison	Gene ID	log_2FC	P-value	FDR (padj)	Gene Name
Root vs Leaf	RHSIM_Rhsim01G0121700	-1.721326746	0.001400155	0.003172523	RHSIM_Rhsim01G0121700
Root vs Leaf	novel.3811	1.235745032	0.001409198	0.003191717	--
Root vs Leaf	RHSIM_Rhsim09G0102700	-6.52325014	0.001409315	0.003191717	RHSIM_Rhsim09G0102700
Root vs Leaf	RHSIM_Rhsim11G0163500	-1.440252158	0.001416187	0.003206967	RHSIM_Rhsim11G0163500
Root vs Leaf	RHSIM_Rhsim12G0025200	1.120186438	0.001420511	0.003215812	RHSIM_Rhsim12G0025200
Root vs Leaf	RHSIM_Rhsim05G0187400	3.401740696	0.001433279	0.003242497	RHSIM_Rhsim05G0187400
Root vs Leaf	RHSIM_Rhsim09G0157100	-1.504385041	0.001441222	0.003259512	RHSIM_Rhsim09G0157100
Root vs Leaf	RHSIM_Rhsim08G0109000	4.071824365	0.001447211	0.003271456	RHSIM_Rhsim08G0109000
Root vs Leaf	RHSIM_Rhsim04G0236200	-2.381999878	0.001450759	0.003279157	RHSIM_Rhsim04G0236200
Root vs Leaf	novel.2319	1.345152231	0.00145275	0.003283337	--
Root vs Leaf	RHSIM_Rhsim06G0203400	-1.877679148	0.00145363	0.003285004	RHSIM_Rhsim06G0203400
Root vs Leaf	RHSIM_RhsimUnG0072500	-1.276379185	0.001465262	0.003310644	RHSIM_RhsimUnG0072500
Root vs Leaf	RHSIM_Rhsim02G0124400	-6.717720247	0.001467263	0.003314843	RHSIM_Rhsim02G0124400
Root vs Leaf	novel.4781	-5.189307995	0.00146768	0.00331546	--
Root vs Leaf	RHSIM_Rhsim09G0053000	1.82383551	0.001468046	0.003315964	RHSIM_Rhsim09G0053000
Root vs Leaf	RHSIM_Rhsim13G0023900	1.248642451	0.001468779	0.003317295	RHSIM_Rhsim13G0023900
Root vs Leaf	novel.1963	1.183475204	0.001471236	0.003322197	--
Root vs Leaf	RHSIM_Rhsim08G0237300	-1.262913821	0.001474794	0.003329905	RHSIM_Rhsim08G0237300
Root vs Leaf	RHSIM_Rhsim02G0144600	1.562506469	0.001476339	0.003332745	RHSIM_Rhsim02G0144600
Root vs Leaf	RHSIM_Rhsim06G0115600	-2.532434788	0.001477497	0.003334708	RHSIM_Rhsim06G0115600
Root vs Leaf	novel.4611	-1.609251015	0.001479897	0.003339798	--
Root vs Leaf	novel.1361	-2.826497497	0.001488672	0.003357638	--
Root vs Leaf	novel.3186	2.508968045	0.001491158	0.003361933	--
Root vs Leaf	RHSIM_Rhsim01G0185600	-2.659443525	0.001492204	0.003363963	RHSIM_Rhsim01G0185600
Root vs Leaf	RHSIM_Rhsim13G0020800	-2.384114006	0.001493288	0.003366079	RHSIM_Rhsim13G0020800

Comparison	Gene ID	log_2FC	P-value	FDR (padj)	Gene Name
Root vs Leaf	RHSIM_Rhsim08G0014500	1.145310232	0.001506659	0.003395558	RHSIM_Rhsim08G0014500
Root vs Leaf	novel.2704	1.639244727	0.001509701	0.003402082	--
Root vs Leaf	novel.1878	1.679534568	0.00151555	0.003414599	--
Root vs Leaf	RHSIM_Rhsim09G0204800	2.340022147	0.00152615	0.003437811	RHSIM_Rhsim09G0204800
Root vs Leaf	RHSIM_RhsimMtG0004100	-2.672065028	0.001526301	0.003437818	RHSIM_RhsimMtG0004100
Root vs Leaf	RHSIM_Rhsim02G0187200	3.059138302	0.00152719	0.003439484	RHSIM_Rhsim02G0187200
Root vs Leaf	novel.1655	-4.003325213	0.001532759	0.003450011	--
Root vs Leaf	RHSIM_Rhsim05G0138300	-2.578840469	0.001536316	0.003456337	RHSIM_Rhsim05G0138300
Root vs Leaf	RHSIM_Rhsim03G0001200	-2.24653492	0.001542303	0.003468794	RHSIM_Rhsim03G0001200
Root vs Leaf	novel.5763	4.2533793	0.001543488	0.003470785	--
Root vs Leaf	RHSIM_Rhsim04G0046300	1.86843753	0.001545048	0.003473956	RHSIM_Rhsim04G0046300
Root vs Leaf	RHSIM_RhsimUnG0059700	-1.216534738	0.001547237	0.003478539	RHSIM_RhsimUnG0059700
Root vs Leaf	novel.331	-1.511863577	0.001549583	0.003483476	--
Root vs Leaf	RHSIM_Rhsim02G0236000	2.246772365	0.001550328	0.003484813	RHSIM_Rhsim02G0236000
Root vs Leaf	novel.842	2.609176473	0.001555807	0.003496787	--
Root vs Leaf	RHSIM_Rhsim01G0275600	1.455284494	0.001559439	0.003504611	RHSIM_Rhsim01G0275600
Root vs Leaf	novel.5494	-2.351851417	0.001560547	0.003506565	--
Root vs Leaf	RHSIM_Rhsim13G0162400	-1.132325839	0.001560612	0.003506565	RHSIM_Rhsim13G0162400
Root vs Leaf	RHSIM_Rhsim11G0028200	2.013199017	0.001566117	0.003517569	RHSIM_Rhsim11G0028200
Root vs Leaf	RHSIM_Rhsim04G0022700	-1.141890943	0.001569748	0.003525383	RHSIM_Rhsim04G0022700
Root vs Leaf	RHSIM_Rhsim04G0065200	1.104894524	0.001571389	0.003528041	RHSIM_Rhsim04G0065200
Root vs Leaf	RHSIM_Rhsim05G0224000	2.098802297	0.001577864	0.003541151	RHSIM_Rhsim05G0224000
Root vs Leaf	RHSIM_Rhsim08G0188500	1.782250928	0.001577994	0.003541151	RHSIM_Rhsim08G0188500
Root vs Leaf	RHSIM_Rhsim06G0215900	2.097305072	0.001579592	0.003544394	RHSIM_Rhsim06G0215900
Root vs Leaf	novel.4061	1.015052865	0.001582249	0.003549668	--

Comparison	Gene ID	log_2FC	P-value	FDR (padj)	Gene Name
Root vs Leaf	RHSIM_Rhsim04G0241600	-1.064886526	0.001583405	0.003551917	RHSIM_Rhsim04G0241600
Root vs Leaf	RHSIM_Rhsim13G0016300	1.188096749	0.001584655	0.003554376	RHSIM_Rhsim13G0016300
Root vs Leaf	RHSIM_Rhsim08G0169900	5.385803871	0.001586771	0.003558778	RHSIM_Rhsim08G0169900
Root vs Leaf	novel.5899	-3.449062891	0.001588942	0.003563301	--
Root vs Leaf	RHSIM_Rhsim01G0252800	1.105735156	0.001596054	0.003578209	RHSIM_Rhsim01G0252800
Root vs Leaf	RHSIM_Rhsim07G0059000	1.037886615	0.001596587	0.003579058	RHSIM_Rhsim07G0059000
Root vs Leaf	novel.5368	-1.689296768	0.001597924	0.003581361	--
Root vs Leaf	RHSIM_Rhsim01G0235800	-2.677703711	0.001599695	0.003584636	RHSIM_Rhsim01G0235800
Root vs Leaf	RHSIM_Rhsim08G0069000	1.547002764	0.001607253	0.003598784	RHSIM_Rhsim08G0069000
Root vs Leaf	novel.3515	1.17800818	0.00161153	0.003607313	--
Root vs Leaf	RHSIM_Rhsim11G0047700	1.062811642	0.001614802	0.00361429	RHSIM_Rhsim11G0047700
Root vs Leaf	RHSIM_Rhsim10G0007300	3.025069085	0.001629869	0.003646954	RHSIM_Rhsim10G0007300
Root vs Leaf	novel.4248	-1.154990227	0.001637956	0.00366434	--
Root vs Leaf	RHSIM_Rhsim03G0179400	4.164425748	0.001645051	0.003679146	RHSIM_Rhsim03G0179400
Root vs Leaf	novel.4457	1.389798374	0.001646934	0.003683002	--
Root vs Leaf	RHSIM_Rhsim13G0210500	1.314690373	0.001648033	0.003684747	RHSIM_Rhsim13G0210500
Root vs Leaf	RHSIM_Rhsim06G0168700	1.03761131	0.001653173	0.003695882	RHSIM_Rhsim06G0168700
Root vs Leaf	RHSIM_RhsimUnG0181000	-1.339940839	0.001654806	0.003698819	RHSIM_RhsimUnG0181000
Root vs Leaf	RHSIM_Rhsim10G0069200	-1.929189989	0.001655577	0.003700184	RHSIM_Rhsim10G0069200
Root vs Leaf	RHSIM_Rhsim04G0110600	1.613769531	0.001661278	0.003712568	RHSIM_Rhsim04G0110600
Root vs Leaf	novel.5265	1.52497877	0.001663525	0.003717231	--
Root vs Leaf	novel.4118	-1.086392581	0.001666677	0.003723914	--
Root vs Leaf	RHSIM_Rhsim07G0078600	1.259993216	0.001678807	0.003749929	RHSIM_Rhsim07G0078600
Root vs Leaf	RHSIM_Rhsim02G0080000	1.448186263	0.001680606	0.003753223	RHSIM_Rhsim02G0080000
Root vs Leaf	RHSIM_RhsimUnG0182600	2.194091936	0.001685982	0.003764503	RHSIM_RhsimUnG0182600

Comparison	Gene ID	log_2FC	P-value	FDR (padj)	Gene Name
Root vs Leaf	RHSIM_Rhsim06G0138300	1.304960303	0.001688616	0.003769792	RHSIM_Rhsim06G0138300
Root vs Leaf	RHSIM_Rhsim02G0042100	2.037780839	0.001688676	0.003769792	RHSIM_Rhsim02G0042100
Root vs Leaf	RHSIM_Rhsim06G0048800	-1.266409462	0.001691715	0.003775847	RHSIM_Rhsim06G0048800
Root vs Leaf	RHSIM_Rhsim05G0210200	-6.641781848	0.001696178	0.003784811	RHSIM_Rhsim05G0210200
Root vs Leaf	novel.4608	2.223578492	0.001698399	0.003788574	--
Root vs Leaf	RHSIM_Rhsim01G0013100	1.285699752	0.001705866	0.003804863	RHSIM_Rhsim01G0013100
Root vs Leaf	RHSIM_Rhsim09G0115200	-1.415510933	0.001706547	0.003806015	RHSIM_Rhsim09G0115200
Root vs Leaf	novel.3011	-4.459070288	0.001709019	0.003810505	--
Root vs Leaf	novel.482	1.12938326	0.001709054	0.003810505	--
Root vs Leaf	RHSIM_Rhsim01G0139700	1.079863931	0.001710017	0.003811855	RHSIM_Rhsim01G0139700
Root vs Leaf	RHSIM_Rhsim09G0194800	2.019900413	0.001710017	0.003811855	RHSIM_Rhsim09G0194800
Root vs Leaf	RHSIM_Rhsim08G0089000	2.268602822	0.001710154	0.003811855	RHSIM_Rhsim08G0089000
Root vs Leaf	RHSIM_RhsimUnG0228800	-2.343195318	0.001710995	0.003813361	RHSIM_RhsimUnG0228800
Root vs Leaf	novel.671	-7.41617414	0.001712955	0.003817363	--
Root vs Leaf	RHSIM_Rhsim13G0227700	3.779916965	0.001715701	0.003822746	RHSIM_Rhsim13G0227700
Root vs Leaf	novel.1652	4.192629065	0.001718116	0.00382739	--
Root vs Leaf	RHSIM_Rhsim11G0131400	1.191725382	0.001719216	0.003829103	RHSIM_Rhsim11G0131400
Root vs Leaf	RHSIM_Rhsim10G0009100	-3.723356569	0.001723269	0.003837391	RHSIM_Rhsim10G0009100
Root vs Leaf	RHSIM_Rhsim02G0249700	1.806019311	0.001724055	0.003838403	RHSIM_Rhsim02G0249700
Root vs Leaf	RHSIM_Rhsim01G0205900	1.226617294	0.001733385	0.003858431	RHSIM_Rhsim01G0205900
Root vs Leaf	RHSIM_Rhsim11G0120300	-2.144339514	0.001736961	0.00386602	RHSIM_Rhsim11G0120300
Root vs Leaf	novel.5256	1.878843335	0.001744795	0.003882709	--
Root vs Leaf	RHSIM_Rhsim04G0050500	4.067436572	0.001752688	0.00389915	RHSIM_Rhsim04G0050500
Root vs Leaf	novel.5108	1.699027626	0.001758878	0.003911416	--
Root vs Leaf	RHSIM_Rhsim02G0178800	5.703412987	0.001760619	0.003914911	RHSIM_Rhsim02G0178800

Comparison	Gene ID	log2FC	P-value	FDR (padj)	Gene Name
Root vs Leaf	novel.2544	1.59918663	0.001769079	0.003932212	--
Root vs Leaf	RHSIM_Rhsim13G0033500	5.142981679	0.001789059	0.003974332	RHSIM_Rhsim13G0033500
Root vs Leaf	RHSIM_Rhsim13G0186200	3.4779606	0.001790706	0.003977608	RHSIM_Rhsim13G0186200
Root vs Leaf	novel.4622	-1.367354013	0.001798157	0.003993392	--
Root vs Leaf	novel.3748	2.703101364	0.001799423	0.00399582	--
Root vs Leaf	RHSIM_Rhsim08G0011700	-3.756280131	0.001803259	0.004003954	RHSIM_Rhsim08G0011700
Root vs Leaf	RHSIM_Rhsim12G0086200	3.535161942	0.00180649	0.004010745	RHSIM_Rhsim12G0086200
Root vs Leaf	RHSIM_Rhsim01G0172800	-1.178586025	0.00181505	0.004027431	RHSIM_Rhsim01G0172800
Root vs Leaf	RHSIM_Rhsim05G0176300	-1.667249518	0.001817645	0.004032801	RHSIM_Rhsim05G0176300
Root vs Leaf	RHSIM_Rhsim10G0167900	1.171576138	0.001819487	0.004036114	RHSIM_Rhsim10G0167900
Root vs Leaf	RHSIM_Rhsim04G0150300	1.121301984	0.001821533	0.00403988	RHSIM_Rhsim04G0150300
Root vs Leaf	RHSIM_Rhsim06G0220500	2.581976107	0.001822266	0.004040731	RHSIM_Rhsim06G0220500
Root vs Leaf	RHSIM_RhsimUnG0220200	2.402008024	0.001824648	0.004045624	RHSIM_RhsimUnG0220200
Root vs Leaf	RHSIM_Rhsim03G0134400	-1.825281764	0.001830642	0.004058526	RHSIM_Rhsim03G0134400
Root vs Leaf	RHSIM_Rhsim11G0090700	-3.567511111	0.001834952	0.004066523	RHSIM_Rhsim11G0090700
Root vs Leaf	RHSIM_Rhsim10G0066900	2.178801192	0.001838692	0.004074249	RHSIM_Rhsim10G0066900
Root vs Leaf	RHSIM_Rhsim06G0055400	-1.378924177	0.001838791	0.004074249	RHSIM_Rhsim06G0055400
Root vs Leaf	RHSIM_RhsimUnG0030100	1.058005796	0.001847853	0.004093153	RHSIM_RhsimUnG0030100
Root vs Leaf	novel.1886	1.885480254	0.001849416	0.004096223	--
Root vs Leaf	RHSIM_Rhsim11G0189400	4.935740885	0.001850227	0.004097628	RHSIM_Rhsim11G0189400
Root vs Leaf	novel.1771	1.330052008	0.001853479	0.004104436	--
Root vs Leaf	RHSIM_Rhsim07G0147200	1.06559227	0.001858878	0.004115604	RHSIM_Rhsim07G0147200
Root vs Leaf	RHSIM_Rhsim04G0118100	1.052953979	0.001868371	0.004135434	RHSIM_Rhsim04G0118100
Root vs Leaf	RHSIM_Rhsim07G0076800	2.738140946	0.001886261	0.004173037	RHSIM_Rhsim07G0076800
Root vs Leaf	novel.48	-3.016595166	0.001886596	0.004173379	--

Comparison	Gene ID	log$_2$FC	P-value	FDR (padj)	Gene Name
Root vs Leaf	RHSIM_Rhsim06G0069600	2.262247234	0.001893147	0.004187069	RHSIM_Rhsim06G0069600
Root vs Leaf	novel.4332	3.019514835	0.001899381	0.004200456	--
Root vs Leaf	RHSIM_Rhsim12G0142800	1.375526541	0.001907648	0.004217126	RHSIM_Rhsim12G0142800
Root vs Leaf	RHSIM_Rhsim02G0025500	2.340611152	0.001914796	0.004232121	RHSIM_Rhsim02G0025500
Root vs Leaf	RHSIM_RhsimUnG0096000	-1.992720497	0.00192815	0.004258788	RHSIM_RhsimUnG0096000
Root vs Leaf	RHSIM_Rhsim05G0100200	1.239744415	0.001934918	0.004272922	RHSIM_Rhsim05G0100200
Root vs Leaf	RHSIM_Rhsim13G0126200	1.2210176	0.001962976	0.004331162	RHSIM_Rhsim13G0126200
Root vs Leaf	RHSIM_RhsimUnG0179500	-2.295818762	0.001964817	0.004334144	RHSIM_RhsimUnG0179500
Root vs Leaf	RHSIM_RhsimUnG0110900	-1.498878991	0.001964889	0.004334144	RHSIM_RhsimUnG0110900
Root vs Leaf	RHSIM_Rhsim12G0072100	3.70349633	0.001965341	0.004334727	RHSIM_Rhsim12G0072100
Root vs Leaf	RHSIM_Rhsim13G0040800	-1.304138082	0.001973409	0.004352106	RHSIM_Rhsim13G0040800
Root vs Leaf	RHSIM_Rhsim11G0127900	-2.532640006	0.001977327	0.004359916	RHSIM_Rhsim11G0127900
Root vs Leaf	RHSIM_Rhsim01G0288500	3.344423099	0.00198034	0.004365654	RHSIM_Rhsim01G0288500
Root vs Leaf	RHSIM_Rhsim02G0255300	3.184864764	0.001983161	0.004371115	RHSIM_Rhsim02G0255300
Root vs Leaf	RHSIM_Rhsim07G0254700	-1.269551201	0.001990499	0.004386453	RHSIM_Rhsim07G0254700
Root vs Leaf	novel.3447	-2.285190803	0.002000203	0.004407124	--
Root vs Leaf	RHSIM_Rhsim08G0099900	-1.20188689	0.00200026	0.004407124	RHSIM_Rhsim08G0099900
Root vs Leaf	RHSIM_Rhsim11G0108700	6.019144352	0.002003631	0.004413289	RHSIM_Rhsim11G0108700
Root vs Leaf	RHSIM_Rhsim08G0167200	1.394979338	0.002006145	0.004418407	RHSIM_Rhsim08G0167200
Root vs Leaf	RHSIM_Rhsim11G0152300	3.751265791	0.002008525	0.004422385	RHSIM_Rhsim11G0152300
Root vs Leaf	RHSIM_Rhsim03G0259900	-2.725286188	0.002016628	0.004439382	RHSIM_Rhsim03G0259900
Root vs Leaf	novel.3263	-4.839423901	0.00201789	0.004441315	--
Root vs Leaf	RHSIM_Rhsim01G0289700	-1.06438837	0.002024444	0.00445447	RHSIM_Rhsim01G0289700
Root vs Leaf	novel.1908	-2.526764176	0.002028092	0.004461648	--
Root vs Leaf	RHSIM_Rhsim08G0089600	4.0286108	0.002033913	0.004474029	RHSIM_Rhsim08G0089600

Comparison	Gene ID	log_2FC	P-value	FDR (padj)	Gene Name
Root vs Leaf	RHSIM_Rhsim01G0010700	-2.254673039	0.002036082	0.004478373	RHSIM_Rhsim01G0010700
Root vs Leaf	novel.285	-2.131822013	0.002040376	0.004486873	--
Root vs Leaf	novel.5239	-1.064766216	0.002049566	0.004506319	--
Root vs Leaf	novel.680	2.976721839	0.0020633	0.004535222	--
Root vs Leaf	RHSIM_Rhsim13G0233600	2.894063343	0.00206388	0.004536068	RHSIM_Rhsim13G0233600
Root vs Leaf	RHSIM_Rhsim03G0223000	3.236694548	0.002064619	0.004537261	RHSIM_Rhsim03G0223000
Root vs Leaf	RHSIM_Rhsim05G0162700	5.206824277	0.002068224	0.00454475	RHSIM_Rhsim05G0162700
Root vs Leaf	RHSIM_Rhsim03G0232500	-2.445676673	0.002076098	0.00455859	RHSIM_Rhsim03G0232500
Root vs Leaf	RHSIM_Rhsim02G0008700	2.411102482	0.002087664	0.004582683	RHSIM_Rhsim02G0008700
Root vs Leaf	novel.5179	1.436380299	0.002095295	0.004598906	--
Root vs Leaf	RHSIM_Rhsim02G0084000	1.559107455	0.002099501	0.004606917	RHSIM_Rhsim02G0084000
Root vs Leaf	novel.1811	-7.324605444	0.002100646	0.004608993	--
Root vs Leaf	RHSIM_Rhsim03G0045200	2.591989669	0.002114793	0.004638714	RHSIM_Rhsim03G0045200
Root vs Leaf	RHSIM_Rhsim07G0257600	3.39125004	0.002120788	0.004651422	RHSIM_Rhsim07G0257600
Root vs Leaf	RHSIM_Rhsim08G0065900	-2.080685887	0.002123834	0.004657662	RHSIM_Rhsim08G0065900
Root vs Leaf	RHSIM_Rhsim03G0257500	3.990977815	0.002127545	0.004665358	RHSIM_Rhsim03G0257500
Root vs Leaf	RHSIM_Rhsim12G0172000	-1.237865216	0.002132841	0.004676084	RHSIM_Rhsim12G0172000
Root vs Leaf	novel.2334	-7.276106241	0.002137522	0.004685903	--
Root vs Leaf	RHSIM_Rhsim07G0106000	-1.127644804	0.002148929	0.004710464	RHSIM_Rhsim07G0106000
Root vs Leaf	RHSIM_Rhsim07G0127300	-1.094452178	0.002159789	0.004733372	RHSIM_Rhsim07G0127300
Root vs Leaf	RHSIM_Rhsim01G0222200	-2.693346775	0.002169471	0.004753691	RHSIM_Rhsim01G0222200
Root vs Leaf	RHSIM_Rhsim03G0015100	2.677753989	0.002184831	0.004785534	RHSIM_Rhsim03G0015100
Root vs Leaf	RHSIM_Rhsim02G0173500	3.4003029	0.002204417	0.004826607	RHSIM_Rhsim02G0173500
Root vs Leaf	RHSIM_Rhsim04G0240600	1.170395998	0.002210646	0.004839331	RHSIM_Rhsim04G0240600
Root vs Leaf	novel.531	1.363526335	0.002211715	0.004841213	--

Comparison	Gene ID	log$_2$FC	P-value	FDR (padj)	Gene Name
Root vs Leaf	RHSIM_Rhsim05G0180800	1.735269274	0.002216358	0.00485	RHSIM_Rhsim05G0180800
Root vs Leaf	RHSIM_Rhsim11G0154500	1.454603818	0.002220548	0.004858708	RHSIM_Rhsim11G0154500
Root vs Leaf	RHSIM_Rhsim04G0186900	-1.035219051	0.002221407	0.004860129	RHSIM_Rhsim04G0186900
Root vs Leaf	RHSIM_Rhsim06G0163500	2.901979508	0.002223568	0.004863937	RHSIM_Rhsim06G0163500
Root vs Leaf	RHSIM_Rhsim09G0037700	2.938868022	0.002226803	0.004870554	RHSIM_Rhsim09G0037700
Root vs Leaf	novel.5238	-1.24545552	0.002230118	0.004876882	--
Root vs Leaf	RHSIM_Rhsim08G0100800	1.352826562	0.00223065	0.004877122	RHSIM_Rhsim08G0100800
Root vs Leaf	RHSIM_Rhsim04G0026900	2.854987628	0.002238155	0.004892607	RHSIM_Rhsim04G0026900
Root vs Leaf	RHSIM_Rhsim05G0027600	-1.01197598	0.002241449	0.004898883	RHSIM_Rhsim05G0027600
Root vs Leaf	RHSIM_Rhsim05G0173300	-1.674954048	0.002242017	0.004899661	RHSIM_Rhsim05G0173300
Root vs Leaf	novel.1727	1.944570689	0.002246388	0.00490875	--
Root vs Leaf	novel.329	4.276134336	0.002248351	0.004912577	--
Root vs Leaf	RHSIM_RhsimUnG0237300	-1.761501941	0.002261419	0.004939264	RHSIM_RhsimUnG0237300
Root vs Leaf	RHSIM_Rhsim02G0203100	1.785093963	0.00226276	0.004941726	RHSIM_Rhsim02G0203100
Root vs Leaf	RHSIM_Rhsim07G0232000	-1.759612797	0.002264392	0.004944824	RHSIM_Rhsim07G0232000
Root vs Leaf	RHSIM_Rhsim02G0245400	-1.118850372	0.002270246	0.004956083	RHSIM_Rhsim02G0245400
Root vs Leaf	RHSIM_Rhsim03G0011900	2.104293915	0.002270562	0.004956083	RHSIM_Rhsim03G0011900
Root vs Leaf	RHSIM_Rhsim03G0046900	2.313933989	0.002281095	0.004978479	RHSIM_Rhsim03G0046900
Root vs Leaf	RHSIM_Rhsim10G0175500	1.726272664	0.00228176	0.00497946	RHSIM_Rhsim10G0175500
Root vs Leaf	RHSIM_Rhsim05G0171000	-2.623380318	0.002282022	0.004979563	RHSIM_Rhsim05G0171000
Root vs Leaf	RHSIM_Rhsim02G0099600	1.73651313	0.002284138	0.00498371	RHSIM_Rhsim02G0099600
Root vs Leaf	RHSIM_Rhsim06G0227200	-1.009543247	0.00228588	0.00498704	RHSIM_Rhsim06G0227200
Root vs Leaf	RHSIM_Rhsim06G0164900	-6.454578582	0.002288379	0.004992023	RHSIM_Rhsim06G0164900
Root vs Leaf	RHSIM_Rhsim08G0236700	-1.601772867	0.002288629	0.004992097	RHSIM_Rhsim08G0236700
Root vs Leaf	RHSIM_Rhsim10G0070800	-2.144763488	0.00229135	0.004997561	RHSIM_Rhsim10G0070800

Comparison	Gene ID	log_2FC	P-value	FDR (padj)	Gene Name
Root vs Leaf	RHSIM_Rhsim03G0133900	1.894400966	0.002309802	0.005034485	RHSIM_Rhsim03G0133900
Root vs Leaf	RHSIM_Rhsim10G0211900	1.536828224	0.002312901	0.005040765	RHSIM_Rhsim10G0211900
Root vs Leaf	RHSIM_Rhsim07G0186400	-2.703747336	0.002321246	0.005057524	RHSIM_Rhsim07G0186400
Root vs Leaf	RHSIM_RhsimUnG0088300	4.236945479	0.002326131	0.005067213	RHSIM_RhsimUnG0088300
Root vs Leaf	RHSIM_Rhsim08G0149400	1.841668225	0.0023343	0.005084529	RHSIM_Rhsim08G0149400
Root vs Leaf	RHSIM_RhsimUnG0202900	-1.342539334	0.00233463	0.005084769	RHSIM_RhsimUnG0202900
Root vs Leaf	RHSIM_Rhsim12G0042000	-1.176443885	0.002346711	0.005109638	RHSIM_Rhsim12G0042000
Root vs Leaf	novel.3575	1.701782229	0.002350539	0.00511701	--
Root vs Leaf	RHSIM_Rhsim05G0232000	-7.251715704	0.002352267	0.005119809	RHSIM_Rhsim05G0232000
Root vs Leaf	RHSIM_Rhsim04G0033700	-1.935514136	0.002356592	0.005128257	RHSIM_Rhsim04G0033700
Root vs Leaf	RHSIM_Rhsim07G0176200	3.425286285	0.002357451	0.005129645	RHSIM_Rhsim07G0176200
Root vs Leaf	RHSIM_Rhsim09G0083800	1.509744782	0.002360913	0.005135247	RHSIM_Rhsim09G0083800
Root vs Leaf	RHSIM_Rhsim13G0039200	3.12876634	0.002363852	0.005140539	RHSIM_Rhsim13G0039200
Root vs Leaf	RHSIM_Rhsim08G0199200	1.051262177	0.002365291	0.005142351	RHSIM_Rhsim08G0199200
Root vs Leaf	novel.1040	-1.311832452	0.002366134	0.005143701	--
Root vs Leaf	RHSIM_Rhsim04G0129800	-1.004366443	0.002367177	0.005145485	RHSIM_Rhsim04G0129800
Root vs Leaf	novel.4708	1.601071689	0.002370141	0.005151446	--
Root vs Leaf	RHSIM_Rhsim12G0048300	-2.562194556	0.002373957	0.005158769	RHSIM_Rhsim12G0048300
Root vs Leaf	RHSIM_Rhsim08G0013200	1.159331106	0.002375151	0.005160394	RHSIM_Rhsim08G0013200
Root vs Leaf	RHSIM_RhsimUnG0067400	1.507183139	0.002391863	0.005194753	RHSIM_RhsimUnG0067400
Root vs Leaf	novel.443	-2.852651034	0.002392286	0.005195186	--
Root vs Leaf	RHSIM_Rhsim06G0184500	1.953565035	0.002407046	0.005225767	RHSIM_Rhsim06G0184500
Root vs Leaf	RHSIM_Rhsim07G0048300	2.552545245	0.002411992	0.005235031	RHSIM_Rhsim07G0048300
Root vs Leaf	RHSIM_Rhsim02G0214100	1.39201259	0.002417996	0.005246586	RHSIM_Rhsim02G0214100
Root vs Leaf	RHSIM_Rhsim13G0161500	-1.597348028	0.002419652	0.005249688	RHSIM_Rhsim13G0161500

Comparison	Gene ID	log_2FC	P-value	FDR (padj)	Gene Name
Root vs Leaf	novel.116	-3.751619304	0.002423097	0.005256669	--
Root vs Leaf	RHSIM_Rhsim06G0172000	2.436817354	0.002430446	0.005270636	RHSIM_Rhsim06G0172000
Root vs Leaf	RHSIM_RhsimUnG0142700	1.9441831	0.002435047	0.005278634	RHSIM_RhsimUnG0142700
Root vs Leaf	RHSIM_Rhsim02G0021300	-1.035899437	0.002437058	0.005282498	RHSIM_Rhsim02G0021300
Root vs Leaf	RHSIM_Rhsim01G0086600	-1.275899703	0.002442776	0.005293901	RHSIM_Rhsim01G0086600
Root vs Leaf	RHSIM_Rhsim07G0015400	1.447052781	0.00244748	0.005303103	RHSIM_Rhsim07G0015400
Root vs Leaf	RHSIM_Rhsim06G0050000	-1.067124761	0.002457736	0.005323331	RHSIM_Rhsim06G0050000
Root vs Leaf	RHSIM_Rhsim05G0004800	1.000080865	0.002468637	0.005345441	RHSIM_Rhsim05G0004800
Root vs Leaf	RHSIM_Rhsim07G0094400	1.425103839	0.00246965	0.005347135	RHSIM_Rhsim07G0094400
Root vs Leaf	RHSIM_Rhsim05G0086100	1.835596404	0.002474507	0.005357148	RHSIM_Rhsim05G0086100
Root vs Leaf	novel.974	-1.681882846	0.002478466	0.005364715	--
Root vs Leaf	novel.3487	6.157556337	0.002478884	0.005365118	--
Root vs Leaf	RHSIM_Rhsim02G0130100	1.414795083	0.002479573	0.005366079	RHSIM_Rhsim02G0130100
Root vs Leaf	RHSIM_Rhsim03G0194400	-2.043550359	0.002490438	0.005388615	RHSIM_Rhsim03G0194400
Root vs Leaf	RHSIM_Rhsim03G0196800	3.371968385	0.002496548	0.00540133	RHSIM_Rhsim03G0196800
Root vs Leaf	RHSIM_Rhsim13G0209500	-1.498695718	0.002498892	0.005405896	RHSIM_Rhsim13G0209500
Root vs Leaf	RHSIM_Rhsim08G0080700	-1.190575582	0.002505604	0.005419909	RHSIM_Rhsim08G0080700
Root vs Leaf	RHSIM_Rhsim07G0012600	-2.040041577	0.002510198	0.005429339	RHSIM_Rhsim07G0012600
Root vs Leaf	RHSIM_Rhsim03G0252400	1.447433841	0.002532164	0.005474631	RHSIM_Rhsim03G0252400
Root vs Leaf	RHSIM_Rhsim01G0068200	1.382503798	0.002532322	0.005474631	RHSIM_Rhsim01G0068200
Root vs Leaf	RHSIM_Rhsim09G0191700	1.423482065	0.002537937	0.005486259	RHSIM_Rhsim09G0191700
Root vs Leaf	RHSIM_Rhsim12G0116700	-1.718698902	0.002549494	0.005510212	RHSIM_Rhsim12G0116700
Root vs Leaf	RHSIM_Rhsim01G0179500	3.053512021	0.002557535	0.005526044	RHSIM_Rhsim01G0179500
Root vs Leaf	RHSIM_Rhsim02G0242300	1.360899803	0.002562794	0.005536889	RHSIM_Rhsim02G0242300
Root vs Leaf	RHSIM_Rhsim12G0133200	1.904760847	0.002574608	0.005560336	RHSIM_Rhsim12G0133200

Comparison	Gene ID	log_2FC	P-value	FDR (padj)	Gene Name
Root vs Leaf	novel.371	-1.595732549	0.002579571	0.005570016	--
Root vs Leaf	RHSIM_Rhsim10G0182500	-1.009836496	0.002583051	0.005577009	RHSIM_Rhsim10G0182500
Root vs Leaf	RHSIM_Rhsim10G0018800	1.442819705	0.002592318	0.005595451	RHSIM_Rhsim10G0018800
Root vs Leaf	RHSIM_Rhsim03G0229300	1.711457482	0.002594913	0.005600008	RHSIM_Rhsim03G0229300
Root vs Leaf	RHSIM_Rhsim04G0071500	1.186041827	0.002597139	0.005603766	RHSIM_Rhsim04G0071500
Root vs Leaf	RHSIM_Rhsim07G0033500	4.177994957	0.002599134	0.005607025	RHSIM_Rhsim07G0033500
Root vs Leaf	RHSIM_Rhsim02G0188000	3.608553218	0.002600546	0.005609548	RHSIM_Rhsim02G0188000
Root vs Leaf	novel.5471	4.056041978	0.002601162	0.005610354	--
Root vs Leaf	RHSIM_Rhsim03G0250000	-1.351248618	0.002603734	0.005614373	RHSIM_Rhsim03G0250000
Root vs Leaf	RHSIM_RhsimUnG0183300	-1.013561958	0.002603996	0.005614373	RHSIM_RhsimUnG0183300
Root vs Leaf	RHSIM_Rhsim13G0052200	2.958853755	0.002605743	0.005617617	RHSIM_Rhsim13G0052200
Root vs Leaf	RHSIM_Rhsim03G0210500	2.648950804	0.002611788	0.005629219	RHSIM_Rhsim03G0210500
Root vs Leaf	novel.5977	-2.549429109	0.002611854	0.005629219	--
Root vs Leaf	RHSIM_Rhsim05G0053900	1.318600417	0.002618692	0.005642379	RHSIM_Rhsim05G0053900
Root vs Leaf	novel.3261	1.244504915	0.002619422	0.005643427	--
Root vs Leaf	RHSIM_Rhsim11G0128500	5.095887131	0.002623297	0.00565125	RHSIM_Rhsim11G0128500
Root vs Leaf	novel.3415	-4.220961001	0.002625636	0.005655762	--
Root vs Leaf	RHSIM_Rhsim04G0127600	2.63948037	0.002635479	0.005675907	RHSIM_Rhsim04G0127600
Root vs Leaf	RHSIM_Rhsim01G0269900	-1.686917735	0.002643813	0.005692265	RHSIM_Rhsim01G0269900
Root vs Leaf	RHSIM_Rhsim09G0004300	2.405775956	0.002647128	0.005697814	RHSIM_Rhsim09G0004300
Root vs Leaf	RHSIM_Rhsim08G0014000	1.28187613	0.002663559	0.005731048	RHSIM_Rhsim08G0014000
Root vs Leaf	RHSIM_RhsimUnG0002000	-1.3860457	0.002666954	0.005737286	RHSIM_RhsimUnG0002000
Root vs Leaf	RHSIM_Rhsim13G0086500	1.715099348	0.002724463	0.00585284	RHSIM_Rhsim13G0086500
Root vs Leaf	RHSIM_Rhsim13G0200400	-1.004576104	0.002729	0.005860954	RHSIM_Rhsim13G0200400
Root vs Leaf	novel.5769	-6.140576295	0.002735387	0.005873582	--

Comparison	Gene ID	log_2FC	P-value	FDR (padj)	Gene Name
Root vs Leaf	novel.3036	2.700978017	0.002738334	0.005878274	--
Root vs Leaf	RHSIM_Rhsim01G0272500	1.116115869	0.002745355	0.005892253	RHSIM_Rhsim01G0272500
Root vs Leaf	novel.4788	-2.343127318	0.002746491	0.005894143	--
Root vs Leaf	novel.673	-7.175102351	0.002748413	0.005897721	--
Root vs Leaf	novel.3027	-2.541405869	0.002750274	0.00590062	--
Root vs Leaf	RHSIM_Rhsim07G0096400	-2.708680082	0.002751325	0.005902328	RHSIM_Rhsim07G0096400
Root vs Leaf	novel.1351	-1.862720943	0.002761485	0.005921929	--
Root vs Leaf	RHSIM_RhsimUnG0154800	1.126525092	0.002763301	0.005925274	RHSIM_RhsimUnG0154800
Root vs Leaf	RHSIM_RhsimUnG0004400	-1.522293728	0.002768772	0.005936455	RHSIM_RhsimUnG0004400
Root vs Leaf	RHSIM_Rhsim01G0012400	-4.128318244	0.002772022	0.005942872	RHSIM_Rhsim01G0012400
Root vs Leaf	RHSIM_Rhsim13G0122400	-1.744830497	0.002778596	0.005955736	RHSIM_Rhsim13G0122400
Root vs Leaf	RHSIM_Rhsim01G0292100	1.100464209	0.002780103	0.005957988	RHSIM_Rhsim01G0292100
Root vs Leaf	RHSIM_Rhsim12G0170100	-1.929825777	0.002795789	0.005990114	RHSIM_Rhsim12G0170100
Root vs Leaf	RHSIM_Rhsim06G0092800	-1.7328298	0.00279587	0.005990114	RHSIM_Rhsim06G0092800
Root vs Leaf	RHSIM_Rhsim03G0096500	-1.59697009	0.002816558	0.006031647	RHSIM_Rhsim03G0096500
Root vs Leaf	RHSIM_Rhsim10G0183600	1.144096406	0.002827735	0.006053901	RHSIM_Rhsim10G0183600
Root vs Leaf	RHSIM_Rhsim08G0041400	1.116490113	0.002838727	0.006075186	RHSIM_Rhsim08G0041400
Root vs Leaf	RHSIM_Rhsim07G0187100	-2.61964476	0.002841785	0.006080043	RHSIM_Rhsim07G0187100
Root vs Leaf	novel.474	1.481812	0.002845799	0.006087505	--
Root vs Leaf	novel.3397	-1.845285497	0.002852652	0.006100474	--
Root vs Leaf	RHSIM_RhsimUnG0182000	4.733434308	0.002855339	0.006105654	RHSIM_RhsimUnG0182000
Root vs Leaf	novel.4659	-1.456490246	0.002858553	0.006111398	--
Root vs Leaf	RHSIM_Rhsim07G0083500	2.736920413	0.00286683	0.006128527	RHSIM_Rhsim07G0083500
Root vs Leaf	RHSIM_Rhsim09G0090500	-1.643402201	0.002873781	0.006142253	RHSIM_Rhsim09G0090500
Root vs Leaf	novel.2096	1.071660468	0.002874565	0.006143361	--

Comparison	Gene ID	log_2FC	P-value	FDR (padj)	Gene Name
Root vs Leaf	RHSIM_Rhsim11G0043800	1.781099883	0.00289552	0.00618643	RHSIM_Rhsim11G0043800
Root vs Leaf	RHSIM_Rhsim08G0194300	3.749621898	0.002897348	0.006189764	RHSIM_Rhsim08G0194300
Root vs Leaf	RHSIM_Rhsim08G0015700	2.659975519	0.002900444	0.006195807	RHSIM_Rhsim08G0015700
Root vs Leaf	RHSIM_Rhsim07G0046900	1.599782884	0.002907778	0.006210326	RHSIM_Rhsim07G0046900
Root vs Leaf	novel.1263	-1.625636922	0.002911712	0.006218155	--
Root vs Leaf	RHSIM_Rhsim05G0118500	1.916607701	0.002912615	0.006219509	RHSIM_Rhsim05G0118500
Root vs Leaf	RHSIM_Rhsim04G0226700	-2.925284883	0.002918065	0.006229997	RHSIM_Rhsim04G0226700
Root vs Leaf	RHSIM_Rhsim07G0171300	1.642374733	0.002927546	0.006249086	RHSIM_Rhsim07G0171300
Root vs Leaf	RHSIM_Rhsim02G0253300	3.370945126	0.002932494	0.00625907	RHSIM_Rhsim02G0253300
Root vs Leaf	RHSIM_Rhsim02G0062400	-7.148685875	0.002932908	0.006259377	RHSIM_Rhsim02G0062400
Root vs Leaf	RHSIM_Rhsim05G0042100	1.079623634	0.002938354	0.006270422	RHSIM_Rhsim05G0042100
Root vs Leaf	RHSIM_Rhsim03G0170600	2.808444367	0.002943504	0.006280253	RHSIM_Rhsim03G0170600
Root vs Leaf	RHSIM_Rhsim06G0150300	6.033101203	0.002946457	0.006285974	RHSIM_Rhsim06G0150300
Root vs Leaf	RHSIM_Rhsim01G0268700	2.583798971	0.002948568	0.006289738	RHSIM_Rhsim01G0268700
Root vs Leaf	novel.3044	-1.496552129	0.002950481	0.006292238	--
Root vs Leaf	RHSIM_Rhsim01G0275900	-2.677748952	0.002952627	0.006295979	RHSIM_Rhsim01G0275900
Root vs Leaf	RHSIM_Rhsim07G0055500	4.694775076	0.002972959	0.006337619	RHSIM_Rhsim07G0055500
Root vs Leaf	RHSIM_Rhsim13G0179400	1.540749761	0.00297313	0.006337619	RHSIM_Rhsim13G0179400
Root vs Leaf	novel.761	-11.60405822	0.00297728	0.006345296	--
Root vs Leaf	RHSIM_Rhsim01G0126200	-1.957319775	0.002978134	0.006346533	RHSIM_Rhsim01G0126200
Root vs Leaf	RHSIM_Rhsim13G0050300	1.963027461	0.002980381	0.006350736	RHSIM_Rhsim13G0050300
Root vs Leaf	RHSIM_Rhsim02G0216300	1.200677204	0.00298861	0.006365927	RHSIM_Rhsim02G0216300
Root vs Leaf	novel.1167	1.987076306	0.002990593	0.006369565	--
Root vs Leaf	RHSIM_Rhsim11G0163300	2.352044509	0.002994957	0.006378272	RHSIM_Rhsim11G0163300
Root vs Leaf	RHSIM_Rhsim03G0065400	-1.967015214	0.002997242	0.006382552	RHSIM_Rhsim03G0065400

Comparison	Gene ID	log_2FC	P-value	FDR (padj)	Gene Name
Root vs Leaf	RHSIM_Rhsim10G0027700	-2.575682006	0.003000347	0.006388575	RHSIM_Rhsim10G0027700
Root vs Leaf	RHSIM_Rhsim13G0147000	-1.932505079	0.003005235	0.006398395	RHSIM_Rhsim13G0147000
Root vs Leaf	novel.5308	1.774545448	0.003008534	0.006404828	--
Root vs Leaf	RHSIM_Rhsim07G0083400	1.957819921	0.00301524	0.006417872	RHSIM_Rhsim07G0083400
Root vs Leaf	RHSIM_RhsimUnG0145300	4.240524939	0.003015493	0.006417872	RHSIM_RhsimUnG0145300
Root vs Leaf	novel.3817	-1.641494906	0.003023472	0.006433671	--
Root vs Leaf	RHSIM_Rhsim06G0046500	-1.43219082	0.003031087	0.006448106	RHSIM_Rhsim06G0046500
Root vs Leaf	RHSIM_Rhsim12G0040200	1.929877847	0.003033686	0.006453031	RHSIM_Rhsim12G0040200
Root vs Leaf	novel.5277	-1.819037274	0.00303967	0.006465166	--
Root vs Leaf	RHSIM_Rhsim04G0017700	-1.596582398	0.003042779	0.006471183	RHSIM_Rhsim04G0017700
Root vs Leaf	novel.1466	-2.304036751	0.00304655	0.006478013	--
Root vs Leaf	novel.3333	1.086173531	0.003048424	0.006481401	--
Root vs Leaf	RHSIM_Rhsim08G0082600	1.445963708	0.003049535	0.006483168	RHSIM_Rhsim08G0082600
Root vs Leaf	RHSIM_Rhsim12G0082400	1.084320503	0.003060815	0.006505954	RHSIM_Rhsim12G0082400
Root vs Leaf	RHSIM_Rhsim12G0186900	-1.996136902	0.003066939	0.006517774	RHSIM_Rhsim12G0186900
Root vs Leaf	RHSIM_Rhsim06G0067600	-1.44742221	0.003070407	0.006524545	RHSIM_Rhsim06G0067600
Root vs Leaf	novel.864	1.078742161	0.003074385	0.006531278	--
Root vs Leaf	RHSIM_Rhsim01G0158800	2.932319668	0.003074422	0.006531278	RHSIM_Rhsim01G0158800
Root vs Leaf	RHSIM_Rhsim05G0213200	1.104016311	0.00307961	0.006541698	RHSIM_Rhsim05G0213200
Root vs Leaf	novel.5978	-1.356739745	0.003088734	0.006559272	--
Root vs Leaf	RHSIM_Rhsim08G0218900	1.38833569	0.003111807	0.006606451	RHSIM_Rhsim08G0218900
Root vs Leaf	RHSIM_Rhsim01G0103700	-2.425496464	0.003124486	0.006631129	RHSIM_Rhsim01G0103700
Root vs Leaf	RHSIM_Rhsim05G0136800	1.065522399	0.003124577	0.006631129	RHSIM_Rhsim05G0136800
Root vs Leaf	RHSIM_Rhsim08G0191400	1.587234084	0.003127631	0.006637002	RHSIM_Rhsim08G0191400
Root vs Leaf	novel.3699	1.444987201	0.003129895	0.006641198	--

Comparison	Gene ID	log2FC	P-value	FDR (padj)	Gene Name
Root vs Leaf	novel.4164	1.989873241	0.003139172	0.006659467	--
Root vs Leaf	RHSIM_Rhsim04G0023200	-1.728738715	0.003143328	0.006666644	RHSIM_Rhsim04G0023200
Root vs Leaf	RHSIM_Rhsim07G0184200	-1.152850119	0.003150263	0.00668074	RHSIM_Rhsim07G0184200
Root vs Leaf	RHSIM_Rhsim13G0112400	2.539770535	0.003151859	0.006683512	RHSIM_Rhsim13G0112400
Root vs Leaf	novel.2108	-6.427788881	0.003153066	0.006685459	--
Root vs Leaf	RHSIM_Rhsim04G0075700	1.039819657	0.003160735	0.006701106	RHSIM_Rhsim04G0075700
Root vs Leaf	RHSIM_Rhsim13G0230400	-3.219566135	0.003182431	0.006745106	RHSIM_Rhsim13G0230400
Root vs Leaf	RHSIM_Rhsim11G0161000	2.365660439	0.003182856	0.006745106	RHSIM_Rhsim11G0161000
Root vs Leaf	RHSIM_Rhsim13G0218300	2.898745675	0.003182909	0.006745106	RHSIM_Rhsim13G0218300
Root vs Leaf	RHSIM_Rhsim10G0207400	1.162294867	0.003182946	0.006745106	RHSIM_Rhsim10G0207400
Root vs Leaf	RHSIM_RhsimUnG0103100	-1.60812514	0.003202293	0.006781136	RHSIM_RhsimUnG0103100
Root vs Leaf	RHSIM_Rhsim02G0201700	-1.521672008	0.003206246	0.006788886	RHSIM_Rhsim02G0201700
Root vs Leaf	novel.5338	3.164126844	0.003211373	0.00679912	--
Root vs Leaf	RHSIM_Rhsim11G0101600	2.162208439	0.003214694	0.006805529	RHSIM_Rhsim11G0101600
Root vs Leaf	RHSIM_Rhsim09G0210200	1.535206911	0.003227592	0.006830333	RHSIM_Rhsim09G0210200
Root vs Leaf	novel.4804	3.148071659	0.003229086	0.006832871	--
Root vs Leaf	RHSIM_Rhsim08G0115000	1.998775754	0.003236833	0.006848062	RHSIM_Rhsim08G0115000
Root vs Leaf	RHSIM_Rhsim03G0189300	4.62470858	0.003253366	0.006879846	RHSIM_Rhsim03G0189300
Root vs Leaf	RHSIM_Rhsim06G0105100	1.510315521	0.003268718	0.006911047	RHSIM_Rhsim06G0105100
Root vs Leaf	RHSIM_Rhsim13G0190900	1.563203581	0.003277099	0.0069275	RHSIM_Rhsim13G0190900
Root vs Leaf	RHSIM_Rhsim04G0113200	1.034002431	0.003278131	0.006929048	RHSIM_Rhsim04G0113200
Root vs Leaf	novel.5886	-1.87272524	0.003291992	0.006957711	--
Root vs Leaf	RHSIM_Rhsim01G0223700	-2.332854447	0.003296682	0.006966987	RHSIM_Rhsim01G0223700
Root vs Leaf	RHSIM_Rhsim11G0170600	2.197130949	0.003297869	0.006968859	RHSIM_Rhsim11G0170600
Root vs Leaf	RHSIM_Rhsim13G0130400	-2.265061932	0.003302954	0.006978331	RHSIM_Rhsim13G0130400

Comparison	Gene ID	log_2FC	P-value	FDR (padj)	Gene Name
Root vs Leaf	RHSIM_Rhsim08G0040700	1.646591732	0.003308972	0.006989441	RHSIM_Rhsim08G0040700
Root vs Leaf	novel.4330	2.229149008	0.003309297	0.006989441	--
Root vs Leaf	RHSIM_Rhsim10G0025600	1.290312507	0.003309571	0.006989441	RHSIM_Rhsim10G0025600
Root vs Leaf	RHSIM_Rhsim06G0138100	1.380030857	0.003313324	0.006994492	RHSIM_Rhsim06G0138100
Root vs Leaf	RHSIM_Rhsim02G0207900	1.594567273	0.003317021	0.007001019	RHSIM_Rhsim02G0207900
Root vs Leaf	RHSIM_Rhsim06G0064700	-2.164463648	0.003326606	0.00701997	RHSIM_Rhsim06G0064700
Root vs Leaf	novel.1252	-7.198946558	0.003334117	0.007034536	--
Root vs Leaf	RHSIM_Rhsim05G0081300	5.51516978	0.003337949	0.007041979	RHSIM_Rhsim05G0081300
Root vs Leaf	RHSIM_Rhsim06G0227800	1.675895118	0.003340176	0.007046037	RHSIM_Rhsim06G0227800
Root vs Leaf	RHSIM_Rhsim05G0047900	1.632631255	0.003342838	0.007051008	RHSIM_Rhsim05G0047900
Root vs Leaf	novel.1751	1.743270299	0.003343263	0.007051263	--
Root vs Leaf	RHSIM_Rhsim04G0033800	-1.602149843	0.003361023	0.00708549	RHSIM_Rhsim04G0033800
Root vs Leaf	RHSIM_Rhsim06G0138200	1.289919721	0.003367788	0.007097812	RHSIM_Rhsim06G0138200
Root vs Leaf	RHSIM_Rhsim07G0160800	-9.017164658	0.003373369	0.00710828	RHSIM_Rhsim07G0160800
Root vs Leaf	RHSIM_Rhsim06G0062800	-1.706413637	0.003380954	0.007122965	RHSIM_Rhsim06G0062800
Root vs Leaf	RHSIM_Rhsim13G0117700	1.996319842	0.003389245	0.007138484	RHSIM_Rhsim13G0117700
Root vs Leaf	RHSIM_Rhsim04G0186800	1.555969698	0.00339864	0.007156968	RHSIM_Rhsim04G0186800
Root vs Leaf	RHSIM_Rhsim11G0094900	3.822237958	0.00340314	0.007165141	RHSIM_Rhsim11G0094900
Root vs Leaf	RHSIM_Rhsim03G0248400	2.20061309	0.003404768	0.007167917	RHSIM_Rhsim03G0248400
Root vs Leaf	novel.3316	-1.106894993	0.003406281	0.007170449	--
Root vs Leaf	RHSIM_Rhsim02G0118600	-1.487391326	0.003430704	0.007219234	RHSIM_Rhsim02G0118600
Root vs Leaf	RHSIM_Rhsim01G0178600	3.95677413	0.003436942	0.007231704	RHSIM_Rhsim01G0178600
Root vs Leaf	RHSIM_Rhsim05G0033900	1.951962487	0.003448259	0.007253538	RHSIM_Rhsim05G0033900
Root vs Leaf	RHSIM_Rhsim05G0112000	-1.449080783	0.003453225	0.007263324	RHSIM_Rhsim05G0112000
Root vs Leaf	RHSIM_Rhsim12G0093800	2.055807201	0.003456916	0.007270425	RHSIM_Rhsim12G0093800

Comparison	Gene ID	log_2FC	P-value	FDR (padj)	Gene Name
Root vs Leaf	RHSIM_Rhsim02G0000500	1.984269126	0.003458653	0.007273419	RHSIM_Rhsim02G0000500
Root vs Leaf	novel.5860	-1.967761615	0.003463094	0.007280771	--
Root vs Leaf	novel.2487	2.436836445	0.003470234	0.007294457	--
Root vs Leaf	RHSIM_Rhsim01G0146100	2.300967561	0.003472462	0.007298477	RHSIM_Rhsim01G0146100
Root vs Leaf	RHSIM_Rhsim01G0072500	-2.203901364	0.003474199	0.007301467	RHSIM_Rhsim01G0072500
Root vs Leaf	RHSIM_Rhsim13G0102100	2.768337363	0.003485727	0.007325029	RHSIM_Rhsim13G0102100
Root vs Leaf	RHSIM_Rhsim08G0221000	1.325682617	0.003488894	0.007331018	RHSIM_Rhsim08G0221000
Root vs Leaf	novel.5981	-1.280065299	0.003490915	0.00733395	--
Root vs Leaf	RHSIM_Rhsim13G0115600	1.047197355	0.003494131	0.007339392	RHSIM_Rhsim13G0115600
Root vs Leaf	RHSIM_Rhsim13G0002700	-2.171466537	0.003494527	0.007339521	RHSIM_Rhsim13G0002700
Root vs Leaf	RHSIM_Rhsim10G0024500	4.143488493	0.00349912	0.007347835	RHSIM_Rhsim10G0024500
Root vs Leaf	RHSIM_Rhsim01G0073500	-1.289791933	0.003502043	0.007353304	RHSIM_Rhsim01G0073500
Root vs Leaf	novel.2193	2.541615938	0.003506825	0.007362009	--
Root vs Leaf	novel.6080	1.175628931	0.003523411	0.007394818	--
Root vs Leaf	novel.4559	-2.429296872	0.003533238	0.007411898	--
Root vs Leaf	RHSIM_Rhsim08G0118400	1.502057019	0.003538148	0.007421035	RHSIM_Rhsim08G0118400
Root vs Leaf	RHSIM_RhsimUnG0184000	2.087102596	0.003545501	0.007435783	RHSIM_RhsimUnG0184000
Root vs Leaf	RHSIM_RhsimUnG0212000	1.00918863	0.003549066	0.007442273	RHSIM_RhsimUnG0212000
Root vs Leaf	RHSIM_Rhsim13G0045700	-2.35443322	0.003549239	0.007442273	RHSIM_Rhsim13G0045700
Root vs Leaf	RHSIM_Rhsim13G0124300	5.380737565	0.003565248	0.007473133	RHSIM_Rhsim13G0124300
Root vs Leaf	RHSIM_Rhsim01G0021200	-1.661469471	0.003578283	0.007499099	RHSIM_Rhsim01G0021200
Root vs Leaf	novel.2148	5.596714079	0.003581279	0.007504698	--
Root vs Leaf	novel.3159	-2.610531556	0.003596061	0.007534992	--
Root vs Leaf	RHSIM_Rhsim04G0234800	-1.110252308	0.003599953	0.007541781	RHSIM_Rhsim04G0234800
Root vs Leaf	RHSIM_Rhsim03G0269000	-1.02625975	0.003602021	0.007544746	RHSIM_Rhsim03G0269000

Comparison	Gene ID	log_2FC	P-value	FDR (padj)	Gene Name
Root vs Leaf	RHSIM_Rhsim01G0244200	-1.016995691	0.003609626	0.007559307	RHSIM_Rhsim01G0244200
Root vs Leaf	RHSIM_Rhsim06G0171300	1.054731917	0.003622951	0.007585153	RHSIM_Rhsim06G0171300
Root vs Leaf	RHSIM_Rhsim09G0196700	-1.654634824	0.003625099	0.007588965	RHSIM_Rhsim09G0196700
Root vs Leaf	RHSIM_Rhsim02G0236700	3.296021351	0.003628036	0.007594427	RHSIM_Rhsim02G0236700
Root vs Leaf	novel.5424	-2.094114301	0.003631327	0.007600295	--
Root vs Leaf	RHSIM_Rhsim07G0038900	1.280622537	0.003631497	0.007600295	RHSIM_Rhsim07G0038900
Root vs Leaf	novel.2780	1.897839096	0.003633538	0.007603879	--
Root vs Leaf	RHSIM_Rhsim09G0019700	1.141645741	0.003635171	0.007606609	RHSIM_Rhsim09G0019700
Root vs Leaf	RHSIM_Rhsim01G0275300	-1.751768839	0.00364196	0.007619437	RHSIM_Rhsim01G0275300
Root vs Leaf	RHSIM_Rhsim12G0154000	1.383972415	0.003645341	0.007625821	RHSIM_Rhsim12G0154000
Root vs Leaf	RHSIM_Rhsim11G0104000	-1.007193538	0.003655901	0.007645838	RHSIM_Rhsim11G0104000
Root vs Leaf	RHSIM_Rhsim07G0072300	-1.71844194	0.003662271	0.007658468	RHSIM_Rhsim07G0072300
Root vs Leaf	RHSIM_Rhsim12G0141700	2.51957616	0.003668112	0.007669989	RHSIM_Rhsim12G0141700
Root vs Leaf	RHSIM_Rhsim06G0031700	-1.005702809	0.003668955	0.00767106	RHSIM_Rhsim06G0031700
Root vs Leaf	RHSIM_Rhsim08G0097700	1.842953321	0.003671409	0.007675496	RHSIM_Rhsim08G0097700
Root vs Leaf	novel.2112	1.788397283	0.003671797	0.007675615	--
Root vs Leaf	RHSIM_Rhsim03G0115200	2.05287432	0.003680517	0.007691065	RHSIM_Rhsim03G0115200
Root vs Leaf	RHSIM_Rhsim09G0097700	1.924214445	0.003697561	0.007722496	RHSIM_Rhsim09G0097700
Root vs Leaf	novel.2481	2.166353599	0.003709399	0.007745822	--
Root vs Leaf	novel.4458	4.313240451	0.003766668	0.00785761	--
Root vs Leaf	novel.2201	-7.008382248	0.00377271	0.007868087	--
Root vs Leaf	novel.2710	1.099784914	0.003778155	0.007878732	--
Root vs Leaf	RHSIM_Rhsim10G0061500	-2.243077892	0.003784243	0.007889711	RHSIM_Rhsim10G0061500
Root vs Leaf	RHSIM_RhsimUnG0142100	-1.425137334	0.003803718	0.007925611	RHSIM_RhsimUnG0142100
Root vs Leaf	RHSIM_Rhsim12G0107800	1.011511768	0.003804885	0.00792733	RHSIM_Rhsim12G0107800

Comparison	Gene ID	log_2FC	P-value	FDR (padj)	Gene Name
Root vs Leaf	novel.4702	-1.487008403	0.00380899	0.007935168	--
Root vs Leaf	RHSIM_Rhsim04G0223400	1.146942472	0.003812895	0.007942587	RHSIM_Rhsim04G0223400
Root vs Leaf	RHSIM_Rhsim05G0206700	-1.3556853	0.003844552	0.008002768	RHSIM_Rhsim05G0206700
Root vs Leaf	novel.619	-1.048495343	0.003869601	0.008053461	--
Root vs Leaf	RHSIM_Rhsim03G0160400	-1.786312366	0.003880168	0.008074727	RHSIM_Rhsim03G0160400
Root vs Leaf	RHSIM_Rhsim05G0092700	-1.254353812	0.003889609	0.008092919	RHSIM_Rhsim05G0092700
Root vs Leaf	RHSIM_Rhsim03G0145200	1.224558878	0.003898963	0.008111651	RHSIM_Rhsim03G0145200
Root vs Leaf	RHSIM_Rhsim08G0114000	4.284101208	0.003904747	0.008122955	RHSIM_Rhsim08G0114000
Root vs Leaf	novel.1324	-2.707677237	0.003907699	0.008128365	--
Root vs Leaf	novel.311	1.617848975	0.003912495	0.008136879	--
Root vs Leaf	RHSIM_Rhsim01G0113800	1.381810167	0.003918861	0.008148653	RHSIM_Rhsim01G0113800
Root vs Leaf	novel.2700	-6.978516417	0.003934528	0.008180496	--
Root vs Leaf	RHSIM_Rhsim06G0065300	1.804890025	0.003942963	0.008197297	RHSIM_Rhsim06G0065300
Root vs Leaf	RHSIM_Rhsim09G0040100	5.876523395	0.003945313	0.008200708	RHSIM_Rhsim09G0040100
Root vs Leaf	RHSIM_RhsimUnG0188000	-1.502075174	0.003950445	0.008209901	RHSIM_RhsimUnG0188000
Root vs Leaf	RHSIM_RhsimUnG0238300	-3.545786083	0.003973577	0.008255008	RHSIM_RhsimUnG0238300
Root vs Leaf	RHSIM_Rhsim06G0059200	5.874053242	0.003976705	0.008260765	RHSIM_Rhsim06G0059200
Root vs Leaf	RHSIM_Rhsim12G0056000	2.610175952	0.003978192	0.008263113	RHSIM_Rhsim12G0056000
Root vs Leaf	novel.1339	-6.978606345	0.003981324	0.008268135	--
Root vs Leaf	novel.4579	-4.986529652	0.00400572	0.008315067	--
Root vs Leaf	RHSIM_RhsimUnG0096800	-1.08038576	0.004006869	0.008316706	RHSIM_RhsimUnG0096800
Root vs Leaf	RHSIM_Rhsim07G0120700	-2.051235795	0.004018539	0.008338684	RHSIM_Rhsim07G0120700
Root vs Leaf	RHSIM_Rhsim10G0059700	5.871576451	0.004021496	0.008344072	RHSIM_Rhsim10G0059700
Root vs Leaf	novel.3746	-1.246089185	0.004033667	0.00836775	--
Root vs Leaf	RHSIM_Rhsim11G0192100	1.967229656	0.004033992	0.00836775	RHSIM_Rhsim11G0192100

Comparison	Gene ID	log_2FC	P-value	FDR (padj)	Gene Name
Root vs Leaf	novel.3579	1.618583089	0.004056824	0.008412499	--
Root vs Leaf	RHSIM_Rhsim06G0006200	1.784864099	0.00405702	0.008412499	RHSIM_Rhsim06G0006200
Root vs Leaf	RHSIM_Rhsim03G0034400	2.090778643	0.004063065	0.008423526	RHSIM_Rhsim03G0034400
Root vs Leaf	RHSIM_Rhsim04G0024000	-1.897530427	0.004064687	0.008426134	RHSIM_Rhsim04G0024000
Root vs Leaf	RHSIM_Rhsim09G0111400	1.345983406	0.004065229	0.008426502	RHSIM_Rhsim09G0111400
Root vs Leaf	RHSIM_Rhsim03G0097300	1.132297401	0.004079577	0.008454728	RHSIM_Rhsim03G0097300
Root vs Leaf	RHSIM_Rhsim07G0240800	1.299433967	0.004087965	0.008471354	RHSIM_Rhsim07G0240800
Root vs Leaf	RHSIM_Rhsim02G0081400	2.06014138	0.004088416	0.008471528	RHSIM_Rhsim02G0081400
Root vs Leaf	RHSIM_Rhsim06G0129000	1.458209677	0.0041031	0.008498911	RHSIM_Rhsim06G0129000
Root vs Leaf	novel.2312	-1.889827859	0.004112185	0.008516206	--
Root vs Leaf	RHSIM_Rhsim02G0008400	1.329908774	0.004112907	0.008516938	RHSIM_Rhsim02G0008400
Root vs Leaf	RHSIM_Rhsim05G0127300	-6.948018625	0.004126819	0.008544217	RHSIM_Rhsim05G0127300
Root vs Leaf	novel.433	-1.10074975	0.004128069	0.008545276	--
Root vs Leaf	novel.6096	1.435503614	0.004135761	0.008560433	--
Root vs Leaf	RHSIM_Rhsim02G0073100	2.288680159	0.00413624	0.008560658	RHSIM_Rhsim02G0073100
Root vs Leaf	RHSIM_Rhsim12G0196700	-2.305730552	0.00414515	0.008578333	RHSIM_Rhsim12G0196700
Root vs Leaf	novel.5883	4.859347492	0.004163168	0.008614079	--
Root vs Leaf	RHSIM_Rhsim10G0176400	3.20296456	0.004169706	0.008626836	RHSIM_Rhsim10G0176400
Root vs Leaf	RHSIM_Rhsim02G0131800	1.794548004	0.004170411	0.008627523	RHSIM_Rhsim02G0131800
Root vs Leaf	RHSIM_Rhsim03G0220700	3.009646671	0.004183349	0.008652741	RHSIM_Rhsim03G0220700
Root vs Leaf	RHSIM_Rhsim13G0221300	-1.087367287	0.004184402	0.008654148	RHSIM_Rhsim13G0221300
Root vs Leaf	RHSIM_RhsimUnG0013100	-1.24061605	0.004186515	0.008657743	RHSIM_RhsimUnG0013100
Root vs Leaf	RHSIM_Rhsim03G0262400	2.002535605	0.004189825	0.008663813	RHSIM_Rhsim03G0262400
Root vs Leaf	RHSIM_Rhsim03G0147400	2.322693949	0.004199803	0.00868367	RHSIM_Rhsim03G0147400
Root vs Leaf	novel.1161	-6.948202833	0.004206884	0.008697534	--

Comparison	Gene ID	log_2FC	P-value	FDR (padj)	Gene Name
Root vs Leaf	RHSIM_Rhsim10G0116200	-1.239204702	0.00421291	0.008709214	RHSIM_Rhsim10G0116200
Root vs Leaf	RHSIM_Rhsim13G0162700	1.519670247	0.004214773	0.008710731	RHSIM_Rhsim13G0162700
Root vs Leaf	RHSIM_Rhsim06G0212800	1.109711213	0.004225204	0.008730729	RHSIM_Rhsim06G0212800
Root vs Leaf	novel.2826	-2.499488202	0.00423253	0.008745088	--
Root vs Leaf	RHSIM_Rhsim12G0103500	-1.941501748	0.004244082	0.00876739	RHSIM_Rhsim12G0103500
Root vs Leaf	novel.2382	3.110320446	0.004250874	0.008780637	--
Root vs Leaf	RHSIM_Rhsim12G0201200	-1.025832143	0.004267357	0.008813112	RHSIM_Rhsim12G0201200
Root vs Leaf	RHSIM_RhsimUnG0195800	-1.062221044	0.00426921	0.008816152	RHSIM_RhsimUnG0195800
Root vs Leaf	novel.1131	-1.352179757	0.004272785	0.008822745	--
Root vs Leaf	RHSIM_RhsimUnG0078000	-1.85087995	0.004280945	0.008838019	RHSIM_RhsimUnG0078000
Root vs Leaf	RHSIM_Rhsim12G0029300	1.329721365	0.004282652	0.008840755	RHSIM_Rhsim12G0029300
Root vs Leaf	RHSIM_Rhsim02G0203300	1.346604751	0.004288203	0.00885067	RHSIM_Rhsim02G0203300
Root vs Leaf	novel.1581	-1.425687146	0.004294673	0.008863198	--
Root vs Leaf	RHSIM_Rhsim09G0197000	-1.326026617	0.004297949	0.008869167	RHSIM_Rhsim09G0197000
Root vs Leaf	RHSIM_Rhsim08G0092900	-1.215198049	0.004313017	0.008896956	RHSIM_Rhsim08G0092900
Root vs Leaf	RHSIM_Rhsim01G0207600	2.047138805	0.004313338	0.008896956	RHSIM_Rhsim01G0207600
Root vs Leaf	RHSIM_Rhsim02G0206600	1.090303912	0.004316791	0.008903284	RHSIM_Rhsim02G0206600
Root vs Leaf	RHSIM_Rhsim04G0022900	3.026957005	0.00433281	0.008933761	RHSIM_Rhsim04G0022900
Root vs Leaf	RHSIM_RhsimUnG0100100	2.696302239	0.004333111	0.008933761	RHSIM_RhsimUnG0100100
Root vs Leaf	RHSIM_Rhsim03G0150900	1.12660762	0.004346022	0.008958784	RHSIM_Rhsim03G0150900
Root vs Leaf	RHSIM_Rhsim03G0075400	-1.584657285	0.004364794	0.008996678	RHSIM_Rhsim03G0075400
Root vs Leaf	novel.6008	-8.731567359	0.004372619	0.009011201	--
Root vs Leaf	RHSIM_Rhsim12G0194400	-1.586471119	0.004389664	0.009042301	RHSIM_Rhsim12G0194400
Root vs Leaf	RHSIM_Rhsim11G0053400	1.838591239	0.004391042	0.009044335	RHSIM_Rhsim11G0053400
Root vs Leaf	RHSIM_Rhsim13G0218700	-2.128231168	0.004411026	0.009081744	RHSIM_Rhsim13G0218700

Comparison	Gene ID	log_2FC	P-value	FDR (padj)	Gene Name
Root vs Leaf	RHSIM_Rhsim11G0170900	6.200028811	0.004411167	0.009081744	RHSIM_Rhsim11G0170900
Root vs Leaf	RHSIM_Rhsim11G0025900	-2.077559291	0.004419711	0.009097716	RHSIM_Rhsim11G0025900
Root vs Leaf	novel.3524	3.042145239	0.004429031	0.00911528	--
Root vs Leaf	RHSIM_Rhsim04G0194000	-1.352090429	0.004438134	0.00913239	RHSIM_Rhsim04G0194000
Root vs Leaf	RHSIM_Rhsim09G0150900	4.22756966	0.004442946	0.009141479	RHSIM_Rhsim09G0150900
Root vs Leaf	RHSIM_Rhsim01G0232400	1.180664184	0.004453974	0.00916254	RHSIM_Rhsim01G0232400
Root vs Leaf	RHSIM_Rhsim05G0018300	1.151905214	0.004488743	0.009229962	RHSIM_Rhsim05G0018300
Root vs Leaf	novel.775	-2.056159397	0.004489501	0.009230701	--
Root vs Leaf	RHSIM_Rhsim04G0154300	2.162524905	0.004510341	0.009272726	RHSIM_Rhsim04G0154300
Root vs Leaf	RHSIM_Rhsim03G0115400	1.201637159	0.00455083	0.009351812	RHSIM_Rhsim03G0115400
Root vs Leaf	RHSIM_Rhsim07G0256300	1.601438213	0.004560111	0.009370053	RHSIM_Rhsim07G0256300
Root vs Leaf	RHSIM_Rhsim13G0089300	-3.705791476	0.004573893	0.009395868	RHSIM_Rhsim13G0089300
Root vs Leaf	novel.4178	2.688002648	0.004583212	0.009414175	--
Root vs Leaf	RHSIM_Rhsim03G0112200	2.038430733	0.004595872	0.009438506	RHSIM_Rhsim03G0112200
Root vs Leaf	RHSIM_Rhsim09G0104900	2.26705889	0.004621078	0.009486062	RHSIM_Rhsim09G0104900
Root vs Leaf	RHSIM_Rhsim07G0095500	-1.235826407	0.0046338	0.009511058	RHSIM_Rhsim07G0095500
Root vs Leaf	RHSIM_Rhsim09G0185700	1.358450283	0.004634077	0.009511058	RHSIM_Rhsim09G0185700
Root vs Leaf	RHSIM_Rhsim10G0169600	1.354939597	0.00463803	0.009518328	RHSIM_Rhsim10G0169600
Root vs Leaf	novel.3045	-2.485859376	0.004646523	0.009534065	--
Root vs Leaf	RHSIM_Rhsim06G0191100	-2.244431821	0.004650053	0.009538772	RHSIM_Rhsim06G0191100
Root vs Leaf	RHSIM_Rhsim09G0114600	-1.380160185	0.004651265	0.009540412	RHSIM_Rhsim09G0114600
Root vs Leaf	RHSIM_Rhsim11G0134600	1.34622482	0.004661928	0.009560764	RHSIM_Rhsim11G0134600
Root vs Leaf	RHSIM_Rhsim05G0110600	-1.216776509	0.004666998	0.009570138	RHSIM_Rhsim05G0110600
Root vs Leaf	RHSIM_Rhsim04G0236700	1.395672871	0.004681052	0.009597256	RHSIM_Rhsim04G0236700
Root vs Leaf	RHSIM_Rhsim07G0083100	-1.714133474	0.004687668	0.009609117	RHSIM_Rhsim07G0083100

Comparison	Gene ID	log_2FC	P-value	FDR (padj)	Gene Name
Root vs Leaf	RHSIM_Rhsim07G0144300	1.011037856	0.00472262	0.009677836	RHSIM_Rhsim07G0144300
Root vs Leaf	RHSIM_Rhsim06G0074000	2.483761002	0.004725731	0.009682853	RHSIM_Rhsim06G0074000
Root vs Leaf	RHSIM_Rhsim12G0099100	-1.205372356	0.004726871	0.009684332	RHSIM_Rhsim12G0099100
Root vs Leaf	novel.1280	1.153563866	0.00472779	0.009684827	--
Root vs Leaf	RHSIM_Rhsim06G0027500	-1.987981535	0.00472795	0.009684827	RHSIM_Rhsim06G0027500
Root vs Leaf	RHSIM_Rhsim11G0064400	-1.116476321	0.00473874	0.009705385	RHSIM_Rhsim11G0064400
Root vs Leaf	RHSIM_Rhsim03G0061600	2.885512203	0.004740574	0.009708108	RHSIM_Rhsim03G0061600
Root vs Leaf	RHSIM_Rhsim01G0273100	2.176842995	0.004753317	0.009733342	RHSIM_Rhsim01G0273100
Root vs Leaf	RHSIM_Rhsim12G0054900	-2.036534838	0.004760271	0.009745858	RHSIM_Rhsim12G0054900
Root vs Leaf	RHSIM_Rhsim04G0055900	1.290056674	0.00478453	0.009792059	RHSIM_Rhsim04G0055900
Root vs Leaf	RHSIM_Rhsim07G0089600	-2.126070631	0.00479115	0.00980474	RHSIM_Rhsim07G0089600
Root vs Leaf	RHSIM_Rhsim11G0091800	-1.177800429	0.004803991	0.00982928	RHSIM_Rhsim11G0091800
Root vs Leaf	RHSIM_Rhsim11G0077500	3.461009238	0.004826705	0.009871391	RHSIM_Rhsim11G0077500
Root vs Leaf	RHSIM_Rhsim04G0086400	-1.671899524	0.004836691	0.009890065	RHSIM_Rhsim04G0086400
Root vs Leaf	RHSIM_Rhsim03G0067100	-1.136379391	0.004866744	0.009949759	RHSIM_Rhsim03G0067100
Root vs Leaf	novel.1612	-1.358795898	0.004875395	0.009965685	--
Root vs Leaf	RHSIM_Rhsim09G0133000	-1.813907329	0.004885636	0.009983972	RHSIM_Rhsim09G0133000
Root vs Leaf	RHSIM_RhsimUnG0008700	2.831483808	0.004915497	0.010039675	RHSIM_RhsimUnG0008700
Root vs Leaf	RHSIM_Rhsim08G0107200	-1.416241533	0.004928159	0.01006376	RHSIM_Rhsim08G0107200
Root vs Leaf	RHSIM_Rhsim09G0213500	1.629519632	0.004954039	0.010111255	RHSIM_Rhsim09G0213500
Root vs Leaf	novel.3409	2.781972817	0.004955709	0.01011288	--
Root vs Leaf	novel.2523	-1.075860992	0.004980099	0.010159965	--
Root vs Leaf	RHSIM_Rhsim08G0121600	3.406455445	0.004981617	0.010162165	RHSIM_Rhsim08G0121600
Root vs Leaf	RHSIM_Rhsim02G0026500	-1.727418758	0.004994182	0.010186001	RHSIM_Rhsim02G0026500
Root vs Leaf	RHSIM_Rhsim03G0224000	-1.59928869	0.005042582	0.010279281	RHSIM_Rhsim03G0224000

Comparison	Gene ID	log$_2$FC	P-value	FDR (padj)	Gene Name
Root vs Leaf	novel.1317	-1.293110039	0.005047829	0.010288164	--
Root vs Leaf	novel.762	-1.770764144	0.00505817	0.010308332	--
Root vs Leaf	RHSIM_Rhsim10G0153900	2.334688013	0.005118049	0.010419352	RHSIM_Rhsim10G0153900
Root vs Leaf	RHSIM_Rhsim01G0051200	-3.396081913	0.005121789	0.010426048	RHSIM_Rhsim01G0051200
Root vs Leaf	RHSIM_RhsimUnG0006400	1.119909192	0.0051319	0.010444811	RHSIM_RhsimUnG0006400
Root vs Leaf	RHSIM_Rhsim07G0240000	1.444491937	0.005135551	0.010451305	RHSIM_Rhsim07G0240000
Root vs Leaf	RHSIM_Rhsim12G0169300	-2.410422175	0.005169584	0.010515017	RHSIM_Rhsim12G0169300
Root vs Leaf	RHSIM_Rhsim06G0065500	1.295688797	0.005171222	0.010515429	RHSIM_Rhsim06G0065500
Root vs Leaf	RHSIM_Rhsim07G0070400	1.015623591	0.005171605	0.010515429	RHSIM_Rhsim07G0070400
Root vs Leaf	novel.3587	1.452110429	0.005180217	0.010531091	--
Root vs Leaf	RHSIM_Rhsim06G0178300	-1.67275101	0.005212203	0.010592394	RHSIM_Rhsim06G0178300
Root vs Leaf	RHSIM_Rhsim04G0033500	-1.383154062	0.005238603	0.010644176	RHSIM_Rhsim04G0033500
Root vs Leaf	RHSIM_Rhsim12G0123200	-1.386002568	0.005288766	0.010739499	RHSIM_Rhsim12G0123200
Root vs Leaf	RHSIM_Rhsim08G0028100	5.214686839	0.005300005	0.010761378	RHSIM_Rhsim08G0028100
Root vs Leaf	RHSIM_Rhsim06G0169200	5.723426041	0.005301185	0.010761947	RHSIM_Rhsim06G0169200
Root vs Leaf	RHSIM_Rhsim06G0172300	-1.984302587	0.005301216	0.010761947	RHSIM_Rhsim06G0172300
Root vs Leaf	RHSIM_Rhsim01G0287500	1.407878829	0.005304426	0.01076752	RHSIM_Rhsim01G0287500
Root vs Leaf	RHSIM_Rhsim11G0113600	-3.766756499	0.005314169	0.010786351	RHSIM_Rhsim11G0113600
Root vs Leaf	RHSIM_Rhsim02G0240000	3.547499441	0.005316878	0.010790903	RHSIM_Rhsim02G0240000
Root vs Leaf	RHSIM_Rhsim12G0064800	-1.106552422	0.005323648	0.010802747	RHSIM_Rhsim12G0064800
Root vs Leaf	novel.2777	-2.083694992	0.00532473	0.010803997	--
Root vs Leaf	RHSIM_Rhsim05G0053000	2.049043459	0.005332281	0.010816472	RHSIM_Rhsim05G0053000
Root vs Leaf	RHSIM_Rhsim04G0125100	-1.544930228	0.00533382	0.010817698	RHSIM_Rhsim04G0125100
Root vs Leaf	RHSIM_Rhsim08G0085300	1.16678977	0.005336083	0.010821338	RHSIM_Rhsim08G0085300
Root vs Leaf	novel.4835	1.260377099	0.005361422	0.010867011	--

Comparison	Gene ID	log_2FC	P-value	FDR (padj)	Gene Name
Root vs Leaf	RHSIM_Rhsim07G0109400	-2.111289735	0.005371885	0.010887265	RHSIM_Rhsim07G0109400
Root vs Leaf	RHSIM_Rhsim10G0131100	2.48190526	0.005374292	0.010891189	RHSIM_Rhsim10G0131100
Root vs Leaf	RHSIM_Rhsim06G0180600	-4.324269073	0.005380972	0.010903773	RHSIM_Rhsim06G0180600
Root vs Leaf	RHSIM_Rhsim01G0278100	-1.144893132	0.005385724	0.010912446	RHSIM_Rhsim01G0278100
Root vs Leaf	novel.2304	1.344548342	0.005412179	0.01096029	--
Root vs Leaf	RHSIM_Rhsim06G0079900	-2.279992159	0.00543066	0.010992905	RHSIM_Rhsim06G0079900
Root vs Leaf	RHSIM_Rhsim09G0177600	-1.108310462	0.005444738	0.011019475	RHSIM_Rhsim09G0177600
Root vs Leaf	novel.2743	2.552736408	0.005468771	0.011064244	--
Root vs Leaf	RHSIM_Rhsim04G0141500	4.234988428	0.005476214	0.011077365	RHSIM_Rhsim04G0141500
Root vs Leaf	RHSIM_Rhsim11G0104700	2.366211046	0.005506585	0.011133383	RHSIM_Rhsim11G0104700
Root vs Leaf	RHSIM_Rhsim13G0192900	2.331696995	0.005519804	0.011156763	RHSIM_Rhsim13G0192900
Root vs Leaf	RHSIM_Rhsim13G0143600	-1.314556422	0.00552635	0.011168257	RHSIM_Rhsim13G0143600
Root vs Leaf	novel.5510	-1.914216855	0.00553383	0.011180231	--
Root vs Leaf	RHSIM_Rhsim13G0201900	-1.491428572	0.005536437	0.01118452	RHSIM_Rhsim13G0201900
Root vs Leaf	RHSIM_Rhsim06G0204200	-1.284890944	0.005565073	0.011238447	RHSIM_Rhsim06G0204200
Root vs Leaf	novel.1559	-1.106235101	0.005569888	0.011247188	--
Root vs Leaf	RHSIM_Rhsim04G0159300	3.623704411	0.0055712	0.011248856	RHSIM_Rhsim04G0159300
Root vs Leaf	novel.2247	-2.037771317	0.00557632	0.011258212	--
Root vs Leaf	novel.344	-1.556305358	0.005585002	0.011274755	--
Root vs Leaf	RHSIM_Rhsim10G0024700	-2.515237828	0.005597394	0.011296816	RHSIM_Rhsim10G0024700
Root vs Leaf	RHSIM_Rhsim08G0203700	-1.486806888	0.005598864	0.011298798	RHSIM_Rhsim08G0203700
Root vs Leaf	RHSIM_Rhsim13G0139700	-2.352304036	0.005606179	0.011312573	RHSIM_Rhsim13G0139700
Root vs Leaf	RHSIM_Rhsim06G0142200	2.65671859	0.005619476	0.011338416	RHSIM_Rhsim06G0142200
Root vs Leaf	RHSIM_Rhsim05G0171700	1.057338459	0.005624918	0.011347417	RHSIM_Rhsim05G0171700
Root vs Leaf	RHSIM_Rhsim13G0049800	1.427264063	0.005625834	0.011348277	RHSIM_Rhsim13G0049800

Comparison	Gene ID	log_2FC	P-value	FDR (padj)	Gene Name
Root vs Leaf	RHSIM_Rhsim04G0204800	3.51224546	0.005630831	0.011356376	RHSIM_Rhsim04G0204800
Root vs Leaf	novel.575	1.626738931	0.00563659	0.011367001	--
Root vs Leaf	novel.4933	-1.206244303	0.005638246	0.011369348	--
Root vs Leaf	RHSIM_Rhsim07G0155600	2.046229737	0.005645564	0.011383115	RHSIM_Rhsim07G0155600
Root vs Leaf	RHSIM_RhsimUnG0023300	2.240546964	0.005671081	0.011429584	RHSIM_RhsimUnG0023300
Root vs Leaf	RHSIM_Rhsim12G0121700	-1.025065252	0.005684816	0.011454273	RHSIM_Rhsim12G0121700
Root vs Leaf	RHSIM_Rhsim05G0204100	2.141577934	0.005688461	0.01146062	RHSIM_Rhsim05G0204100
Root vs Leaf	RHSIM_Rhsim07G0144000	-1.130820757	0.005691042	0.01146482	RHSIM_Rhsim07G0144000
Root vs Leaf	RHSIM_Rhsim06G0146100	-1.621016793	0.005692124	0.011466003	RHSIM_Rhsim06G0146100
Root vs Leaf	RHSIM_Rhsim09G0186300	1.101886619	0.005699657	0.011480091	RHSIM_Rhsim09G0186300
Root vs Leaf	RHSIM_Rhsim13G0192800	-1.850357113	0.005708396	0.011494779	RHSIM_Rhsim13G0192800
Root vs Leaf	RHSIM_Rhsim10G0007800	-1.516251311	0.005738862	0.011552106	RHSIM_Rhsim10G0007800
Root vs Leaf	RHSIM_Rhsim01G0269500	-1.437090998	0.005739981	0.011553354	RHSIM_Rhsim01G0269500
Root vs Leaf	RHSIM_Rhsim02G0006300	2.103592131	0.005763299	0.011594544	RHSIM_Rhsim02G0006300
Root vs Leaf	RHSIM_Rhsim13G0003700	-2.116249744	0.005767312	0.01160113	RHSIM_Rhsim13G0003700
Root vs Leaf	novel.4380	1.708101518	0.005767728	0.01160113	--
Root vs Leaf	RHSIM_Rhsim05G0209800	-2.99322495	0.005769735	0.011604157	RHSIM_Rhsim05G0209800
Root vs Leaf	novel.5153	-1.426362646	0.00579763	0.011655195	--
Root vs Leaf	RHSIM_Rhsim11G0155400	1.351222606	0.005820291	0.011699735	RHSIM_Rhsim11G0155400
Root vs Leaf	RHSIM_Rhsim08G0137600	-3.781781047	0.005828414	0.011714029	RHSIM_Rhsim08G0137600
Root vs Leaf	RHSIM_Rhsim07G0096600	2.259855557	0.005841685	0.011735605	RHSIM_Rhsim07G0096600
Root vs Leaf	RHSIM_Rhsim09G0156300	-1.179971998	0.005857162	0.011763635	RHSIM_Rhsim09G0156300
Root vs Leaf	RHSIM_Rhsim05G0049000	1.877041251	0.005869783	0.011787958	RHSIM_Rhsim05G0049000
Root vs Leaf	RHSIM_Rhsim03G0137400	-1.640031693	0.005903135	0.011848769	RHSIM_Rhsim03G0137400
Root vs Leaf	RHSIM_Rhsim04G0102200	1.47340236	0.005921711	0.011882961	RHSIM_Rhsim04G0102200

Comparison	Gene ID	log_2FC	P-value	FDR (padj)	Gene Name
Root vs Leaf	RHSIM_Rhsim09G0166100	1.104458516	0.005930053	0.011897905	RHSIM_Rhsim09G0166100
Root vs Leaf	RHSIM_Rhsim02G0098100	1.025524317	0.005935728	0.01190774	RHSIM_Rhsim02G0098100
Root vs Leaf	RHSIM_Rhsim02G0088600	1.401571236	0.005940845	0.011914126	RHSIM_Rhsim02G0088600
Root vs Leaf	RHSIM_RhsimUnG0043000	1.038683778	0.005943659	0.011918737	RHSIM_RhsimUnG0043000
Root vs Leaf	RHSIM_Rhsim09G0091500	2.249782862	0.005958778	0.011945948	RHSIM_Rhsim09G0091500
Root vs Leaf	RHSIM_Rhsim11G0072000	-1.029011831	0.005962124	0.01195162	RHSIM_Rhsim11G0072000
Root vs Leaf	RHSIM_Rhsim07G0073400	1.685251839	0.005962726	0.011951793	RHSIM_Rhsim07G0073400
Root vs Leaf	RHSIM_Rhsim13G0233400	3.095992722	0.005965231	0.011955778	RHSIM_Rhsim13G0233400
Root vs Leaf	RHSIM_Rhsim12G0213500	1.936732177	0.00599248	0.012007272	RHSIM_Rhsim12G0213500
Root vs Leaf	RHSIM_Rhsim09G0120000	-1.651279197	0.006042096	0.012101449	RHSIM_Rhsim09G0120000
Root vs Leaf	RHSIM_Rhsim06G0208700	1.708718441	0.006059129	0.012132414	RHSIM_Rhsim06G0208700
Root vs Leaf	RHSIM_Rhsim04G0009600	-1.129108119	0.006070356	0.012152791	RHSIM_Rhsim04G0009600
Root vs Leaf	RHSIM_Rhsim06G0039800	2.544470081	0.006080659	0.012168154	RHSIM_Rhsim06G0039800
Root vs Leaf	RHSIM_RhsimUnG0104500	-1.103986833	0.006084705	0.012175198	RHSIM_RhsimUnG0104500
Root vs Leaf	RHSIM_Rhsim03G0233900	3.141342985	0.006112966	0.012227517	RHSIM_Rhsim03G0233900
Root vs Leaf	RHSIM_Rhsim08G0233900	1.073741274	0.00613343	0.01226739	RHSIM_Rhsim08G0233900
Root vs Leaf	novel.5594	-3.118996121	0.006145304	0.012289016	--
Root vs Leaf	RHSIM_Rhsim09G0172900	-1.379087885	0.00615354	0.012304421	RHSIM_Rhsim09G0172900
Root vs Leaf	novel.2003	-10.69998159	0.006171358	0.012338983	--
Root vs Leaf	novel.3380	3.653013712	0.006173629	0.012342459	--
Root vs Leaf	RHSIM_RhsimUnG0215000	-2.343677098	0.006175518	0.012345168	RHSIM_RhsimUnG0215000
Root vs Leaf	RHSIM_Rhsim11G0111900	2.083282669	0.006178168	0.012349399	RHSIM_Rhsim11G0111900
Root vs Leaf	RHSIM_Rhsim06G0144700	5.7210308	0.00619795	0.012384661	RHSIM_Rhsim06G0144700
Root vs Leaf	novel.6023	-2.205906502	0.006201812	0.012391308	--
Root vs Leaf	RHSIM_Rhsim05G0159600	-1.018214566	0.006229449	0.012443305	RHSIM_Rhsim05G0159600

Comparison	Gene ID	log_2FC	P-value	FDR (padj)	Gene Name
Root vs Leaf	RHSIM_Rhsim07G0199700	2.969178507	0.006252698	0.012487588	RHSIM_Rhsim07G0199700
Root vs Leaf	RHSIM_Rhsim13G0083700	2.42735439	0.006255962	0.012493029	RHSIM_Rhsim13G0083700
Root vs Leaf	novel.2310	1.816034529	0.006268517	0.01251702	--
Root vs Leaf	RHSIM_RhsimUnG0098800	1.854712768	0.006349103	0.012669536	RHSIM_RhsimUnG0098800
Root vs Leaf	RHSIM_Rhsim02G0078300	1.367355771	0.006349277	0.012669536	RHSIM_Rhsim02G0078300
Root vs Leaf	RHSIM_Rhsim04G0091700	1.248387322	0.006364761	0.01269715	RHSIM_Rhsim04G0091700
Root vs Leaf	RHSIM_RhsimUnG0076300	-1.615995102	0.006380406	0.012725068	RHSIM_RhsimUnG0076300
Root vs Leaf	RHSIM_Rhsim07G0248800	-1.886849563	0.006402289	0.012764311	RHSIM_Rhsim07G0248800
Root vs Leaf	RHSIM_Rhsim08G0075000	-1.165403368	0.00644097	0.012838113	RHSIM_Rhsim08G0075000
Root vs Leaf	RHSIM_Rhsim04G0051800	-1.526958335	0.006476684	0.012905963	RHSIM_Rhsim04G0051800
Root vs Leaf	novel.4700	1.548161877	0.006501475	0.012952017	--
Root vs Leaf	RHSIM_Rhsim09G0142100	3.087358733	0.006509175	0.01296624	RHSIM_Rhsim09G0142100
Root vs Leaf	RHSIM_Rhsim08G0001000	-2.663665454	0.006554972	0.013049605	RHSIM_Rhsim08G0001000
Root vs Leaf	RHSIM_Rhsim11G0191800	1.429369064	0.00656496	0.013066116	RHSIM_Rhsim11G0191800
Root vs Leaf	RHSIM_Rhsim11G0101800	-1.672089448	0.006572764	0.013078275	RHSIM_Rhsim11G0101800
Root vs Leaf	novel.5617	3.445887747	0.006581372	0.013092025	--
Root vs Leaf	RHSIM_Rhsim05G0091300	1.36199507	0.00659426	0.013113155	RHSIM_Rhsim05G0091300
Root vs Leaf	RHSIM_RhsimUnG0247200	2.724497401	0.006607042	0.013137442	RHSIM_RhsimUnG0247200
Root vs Leaf	RHSIM_Rhsim02G0040700	-1.172767416	0.006628763	0.013177237	RHSIM_Rhsim02G0040700
Root vs Leaf	RHSIM_Rhsim03G0065800	1.738991721	0.00663967	0.013196651	RHSIM_Rhsim03G0065800
Root vs Leaf	RHSIM_Rhsim01G0034800	2.961629508	0.006643702	0.013201264	RHSIM_Rhsim01G0034800
Root vs Leaf	RHSIM_RhsimUnG0253100	-1.121218202	0.006655994	0.013224553	RHSIM_RhsimUnG0253100
Root vs Leaf	novel.49	-9.577656654	0.006671803	0.013252549	--
Root vs Leaf	RHSIM_Rhsim01G0008900	-2.116242805	0.006673875	0.013255526	RHSIM_Rhsim01G0008900
Root vs Leaf	novel.805	-5.649341537	0.006690752	0.013284989	--

Comparison	Gene ID	log_2FC	P-value	FDR (padj)	Gene Name
Root vs Leaf	RHSIM_Rhsim05G0029200	2.024210809	0.006691005	0.013284989	RHSIM_Rhsim05G0029200
Root vs Leaf	RHSIM_RhsimUnG0239500	-1.602196282	0.006713937	0.013329376	RHSIM_RhsimUnG0239500
Root vs Leaf	RHSIM_Rhsim08G0073300	1.567574829	0.006737309	0.013373482	RHSIM_Rhsim08G0073300
Root vs Leaf	RHSIM_Rhsim12G0160700	1.119468057	0.006739487	0.01337666	RHSIM_Rhsim12G0160700
Root vs Leaf	RHSIM_Rhsim08G0160800	2.221955203	0.006740994	0.013378502	RHSIM_Rhsim08G0160800
Root vs Leaf	novel.1613	-1.425032386	0.006747222	0.013388566	--
Root vs Leaf	novel.5536	-1.679671009	0.006769363	0.013430199	--
Root vs Leaf	novel.1872	-1.684305129	0.006780877	0.013451888	--
Root vs Leaf	novel.2877	-2.682900266	0.0068006	0.013487546	--
Root vs Leaf	RHSIM_Rhsim07G0061100	2.202736348	0.00680816	0.013499069	RHSIM_Rhsim07G0061100
Root vs Leaf	RHSIM_Rhsim02G0079800	-5.652836939	0.006819621	0.013519477	RHSIM_Rhsim02G0079800
Root vs Leaf	RHSIM_Rhsim05G0058100	1.497189627	0.006835127	0.013547895	RHSIM_Rhsim05G0058100
Root vs Leaf	RHSIM_Rhsim06G0218200	1.440744212	0.006842853	0.013560886	RHSIM_Rhsim06G0218200
Root vs Leaf	novel.3370	1.865125784	0.006845983	0.013565927	--
Root vs Leaf	RHSIM_Rhsim01G0009200	1.741103027	0.00685672	0.01358604	RHSIM_Rhsim01G0009200
Root vs Leaf	RHSIM_Rhsim07G0216900	1.798953217	0.006866225	0.013601665	RHSIM_Rhsim07G0216900
Root vs Leaf	RHSIM_Rhsim03G0220800	1.699484352	0.006866511	0.013601665	RHSIM_Rhsim03G0220800
Root vs Leaf	RHSIM_Rhsim01G0092200	-1.034578901	0.006882679	0.013629306	RHSIM_Rhsim01G0092200
Root vs Leaf	RHSIM_Rhsim11G0171100	1.547652893	0.006915515	0.013688473	RHSIM_Rhsim11G0171100
Root vs Leaf	novel.2893	-1.51694856	0.006925844	0.013706573	--
Root vs Leaf	RHSIM_Rhsim07G0137400	1.170290885	0.006935826	0.013723981	RHSIM_Rhsim07G0137400
Root vs Leaf	novel.94	-1.302961539	0.006942862	0.013736728	--
Root vs Leaf	novel.1368	-1.153035322	0.006949596	0.013748507	--
Root vs Leaf	RHSIM_Rhsim03G0259300	1.658615732	0.006950288	0.013748507	RHSIM_Rhsim03G0259300
Root vs Leaf	RHSIM_Rhsim03G0136000	-1.250156918	0.006952969	0.013750847	RHSIM_Rhsim03G0136000

Comparison	Gene ID	log_2FC	P-value	FDR (padj)	Gene Name
Root vs Leaf	RHSIM_Rhsim06G0097900	1.5135255	0.006960851	0.013764083	RHSIM_Rhsim06G0097900
Root vs Leaf	RHSIM_Rhsim01G0057600	-1.248705651	0.006964411	0.013767592	RHSIM_Rhsim01G0057600
Root vs Leaf	RHSIM_Rhsim07G0197300	-1.177799708	0.006982186	0.013801552	RHSIM_Rhsim07G0197300
Root vs Leaf	RHSIM_Rhsim07G0082600	1.949978222	0.006986634	0.013808653	RHSIM_Rhsim07G0082600
Root vs Leaf	RHSIM_Rhsim07G0247500	1.308150141	0.006986972	0.013808653	RHSIM_Rhsim07G0247500
Root vs Leaf	novel.1893	-1.485801051	0.006989238	0.013811953	--
Root vs Leaf	RHSIM_RhsimUnG0069200	6.591058236	0.007036328	0.013895518	RHSIM_RhsimUnG0069200
Root vs Leaf	novel.5330	1.276377498	0.00703901	0.013898442	--
Root vs Leaf	RHSIM_Rhsim07G0252800	1.599400649	0.007040797	0.013900783	RHSIM_Rhsim07G0252800
Root vs Leaf	RHSIM_Rhsim08G0045500	3.536378592	0.007049096	0.013915981	RHSIM_Rhsim08G0045500
Root vs Leaf	RHSIM_Rhsim11G0170000	-1.092545907	0.0070553	0.013927041	RHSIM_Rhsim11G0170000
Root vs Leaf	RHSIM_Rhsim01G0094400	-1.293476058	0.007058917	0.013931804	RHSIM_Rhsim01G0094400
Root vs Leaf	RHSIM_Rhsim03G0202400	-2.030797148	0.007099207	0.014007738	RHSIM_Rhsim03G0202400
Root vs Leaf	novel.1218	5.041935144	0.007191029	0.014176827	--
Root vs Leaf	RHSIM_Rhsim03G0121000	2.216469458	0.007229669	0.01425179	RHSIM_Rhsim03G0121000
Root vs Leaf	RHSIM_Rhsim13G0007800	2.067493098	0.007257304	0.014300176	RHSIM_Rhsim13G0007800
Root vs Leaf	RHSIM_Rhsim02G0067100	-1.105370199	0.007258854	0.014302012	RHSIM_Rhsim02G0067100
Root vs Leaf	RHSIM_Rhsim05G0192000	-2.759160911	0.007262626	0.014308227	RHSIM_Rhsim05G0192000
Root vs Leaf	RHSIM_Rhsim12G0031200	1.511783917	0.007268034	0.014316444	RHSIM_Rhsim12G0031200
Root vs Leaf	RHSIM_Rhsim01G0289300	5.78976521	0.007294826	0.014363104	RHSIM_Rhsim01G0289300
Root vs Leaf	RHSIM_Rhsim11G0033400	1.600582993	0.007323162	0.014413991	RHSIM_Rhsim11G0033400
Root vs Leaf	novel.1679	2.530814299	0.007341794	0.014446978	--
Root vs Leaf	RHSIM_Rhsim05G0130100	-1.403189297	0.007343908	0.014449908	RHSIM_Rhsim05G0130100
Root vs Leaf	RHSIM_Rhsim06G0175700	2.961954279	0.007353286	0.014466637	RHSIM_Rhsim06G0175700
Root vs Leaf	novel.3160	-2.302896782	0.007372514	0.014501934	--

Comparison	Gene ID	log_2FC	P-value	FDR (padj)	Gene Name
Root vs Leaf	novel.273	1.953394484	0.007372856	0.014501934	--
Root vs Leaf	novel.5165	2.567163212	0.007396496	0.014546417	--
Root vs Leaf	RHSIM_Rhsim01G0270100	1.230177848	0.007396728	0.014546417	RHSIM_Rhsim01G0270100
Root vs Leaf	novel.4156	-1.228971052	0.007414034	0.014576735	--
Root vs Leaf	RHSIM_Rhsim01G0281200	-3.24295165	0.007414907	0.014577212	RHSIM_Rhsim01G0281200
Root vs Leaf	RHSIM_Rhsim11G0185700	-1.799488636	0.007422176	0.014590262	RHSIM_Rhsim11G0185700
Root vs Leaf	RHSIM_Rhsim10G0105600	-2.118620293	0.007507532	0.014749283	RHSIM_Rhsim10G0105600
Root vs Leaf	RHSIM_Rhsim11G0067200	-1.95997115	0.007531686	0.014792967	RHSIM_Rhsim11G0067200
Root vs Leaf	RHSIM_Rhsim04G0237600	-1.545380904	0.007535319	0.014797591	RHSIM_Rhsim04G0237600
Root vs Leaf	RHSIM_Rhsim12G0142100	1.256523045	0.007540039	0.014805604	RHSIM_Rhsim12G0142100
Root vs Leaf	RHSIM_Rhsim03G0171500	-1.697766667	0.00766286	0.015031469	RHSIM_Rhsim03G0171500
Root vs Leaf	RHSIM_Rhsim07G0130000	1.362307479	0.007682323	0.015067093	RHSIM_Rhsim07G0130000
Root vs Leaf	novel.4399	-1.111655621	0.007684629	0.015069858	--
Root vs Leaf	novel.1834	-2.516230431	0.007685035	0.015069858	--
Root vs Leaf	RHSIM_Rhsim02G0157700	-2.466335979	0.007694679	0.015087491	RHSIM_Rhsim02G0157700
Root vs Leaf	novel.1742	-1.332834088	0.00769886	0.015093131	--
Root vs Leaf	RHSIM_Rhsim09G0188900	2.558007967	0.007723996	0.01513728	RHSIM_Rhsim09G0188900
Root vs Leaf	novel.151	-1.342235868	0.007763621	0.015209783	--
Root vs Leaf	novel.2528	3.238152308	0.007771728	0.015224376	--
Root vs Leaf	RHSIM_Rhsim04G0146900	2.225644969	0.007777453	0.015233012	RHSIM_Rhsim04G0146900
Root vs Leaf	RHSIM_Rhsim03G0019200	-1.117264573	0.007779365	0.015235468	RHSIM_Rhsim03G0019200
Root vs Leaf	novel.3273	-1.58269951	0.007788925	0.015252899	--
Root vs Leaf	RHSIM_Rhsim08G0172800	1.186015783	0.007825454	0.01532184	RHSIM_Rhsim08G0172800
Root vs Leaf	RHSIM_Rhsim05G0079800	-1.393772724	0.007844204	0.015354654	RHSIM_Rhsim05G0079800
Root vs Leaf	RHSIM_Rhsim05G0018200	-1.671705737	0.00790362	0.015467034	RHSIM_Rhsim05G0018200

Comparison	Gene ID	log_2FC	P-value	FDR (padj)	Gene Name
Root vs Leaf	RHSIM_Rhsim01G0253400	3.617082689	0.007907761	0.015472694	RHSIM_Rhsim01G0253400
Root vs Leaf	novel.5970	-1.18876157	0.007916482	0.015488275	--
Root vs Leaf	RHSIM_Rhsim10G0041300	-1.767213266	0.007921003	0.015495809	RHSIM_Rhsim10G0041300
Root vs Leaf	RHSIM_Rhsim02G0015700	1.045534657	0.007945642	0.01554007	RHSIM_Rhsim02G0015700
Root vs Leaf	novel.5197	-1.488126795	0.007951555	0.015549005	--
Root vs Leaf	RHSIM_Rhsim07G0046800	1.005626033	0.007962719	0.015569521	RHSIM_Rhsim07G0046800
Root vs Leaf	RHSIM_Rhsim04G0104700	2.213951287	0.007981585	0.015602455	RHSIM_Rhsim04G0104700
Root vs Leaf	RHSIM_RhsimUnG0086000	1.182714242	0.007998188	0.015630949	RHSIM_RhsimUnG0086000
Root vs Leaf	RHSIM_Rhsim11G0042100	1.18789919	0.008001197	0.01563419	RHSIM_Rhsim11G0042100
Root vs Leaf	RHSIM_Rhsim06G0001300	1.64006665	0.008003735	0.015637827	RHSIM_Rhsim06G0001300
Root vs Leaf	RHSIM_Rhsim01G0285700	1.892969438	0.008004953	0.015638888	RHSIM_Rhsim01G0285700
Root vs Leaf	RHSIM_Rhsim01G0179400	-1.190797049	0.008018878	0.015664769	RHSIM_Rhsim01G0179400
Root vs Leaf	RHSIM_Rhsim12G0046200	1.430048621	0.008024865	0.015675142	RHSIM_Rhsim12G0046200
Root vs Leaf	novel.952	-1.131631234	0.008085666	0.015779253	--
Root vs Leaf	RHSIM_Rhsim08G0191900	2.690654499	0.008109277	0.015822663	RHSIM_Rhsim08G0191900
Root vs Leaf	novel.4368	-1.996336089	0.008118685	0.015839685	--
Root vs Leaf	RHSIM_Rhsim13G0086600	1.27580558	0.008120377	0.01584165	RHSIM_Rhsim13G0086600
Root vs Leaf	RHSIM_Rhsim08G0053600	1.004062239	0.008128194	0.015854659	RHSIM_Rhsim08G0053600
Root vs Leaf	RHSIM_Rhsim01G0210300	2.052142959	0.008137241	0.015869198	RHSIM_Rhsim01G0210300
Root vs Leaf	novel.5463	2.915866121	0.008151085	0.015894856	--
Root vs Leaf	novel.1297	1.687225342	0.00818399	0.015953644	--
Root vs Leaf	novel.5286	1.130769597	0.008205813	0.01599349	--
Root vs Leaf	RHSIM_Rhsim02G0144000	1.036544493	0.008210861	0.016001983	RHSIM_Rhsim02G0144000
Root vs Leaf	RHSIM_Rhsim04G0080700	1.146807358	0.008237739	0.016050308	RHSIM_Rhsim04G0080700
Root vs Leaf	RHSIM_Rhsim09G0047100	2.635753369	0.008253775	0.016078846	RHSIM_Rhsim09G0047100

Comparison	Gene ID	log_2FC	P-value	FDR (padj)	Gene Name
Root vs Leaf	RHSIM_Rhsim04G0034500	-1.79036961	0.008269563	0.016105534	RHSIM_Rhsim04G0034500
Root vs Leaf	novel.4971	-1.08797406	0.00827599	0.016116695	--
Root vs Leaf	novel.6005	-2.375233631	0.008300937	0.016162954	--
Root vs Leaf	RHSIM_Rhsim03G0235400	-1.443161736	0.008355776	0.016261123	RHSIM_Rhsim03G0235400
Root vs Leaf	RHSIM_Rhsim03G0036000	-1.435214806	0.008396428	0.01633474	RHSIM_Rhsim03G0036000
Root vs Leaf	RHSIM_Rhsim07G0022800	2.721842288	0.008401524	0.016340779	RHSIM_Rhsim07G0022800
Root vs Leaf	RHSIM_Rhsim01G0180100	1.390222799	0.00840165	0.016340779	RHSIM_Rhsim01G0180100
Root vs Leaf	RHSIM_RhsimMtG0002000	-3.317273524	0.008421134	0.016377297	RHSIM_RhsimMtG0002000
Root vs Leaf	RHSIM_Rhsim01G0038700	-2.903749182	0.008480306	0.016489604	RHSIM_Rhsim01G0038700
Root vs Leaf	RHSIM_Rhsim06G0165700	1.51994699	0.008571723	0.016661758	RHSIM_Rhsim06G0165700
Root vs Leaf	RHSIM_Rhsim08G0079000	2.66371828	0.008576811	0.016670249	RHSIM_Rhsim08G0079000
Root vs Leaf	RHSIM_Rhsim12G0209800	1.333765998	0.00857945	0.016673979	RHSIM_Rhsim12G0209800
Root vs Leaf	RHSIM_Rhsim06G0041600	-2.078248627	0.008625075	0.016761241	RHSIM_Rhsim06G0041600
Root vs Leaf	novel.3716	3.693479631	0.008634249	0.01677766	--
Root vs Leaf	novel.5040	4.635828865	0.008658335	0.016821638	--
Root vs Leaf	RHSIM_Rhsim10G0028900	2.173807672	0.00869953	0.016898835	RHSIM_Rhsim10G0028900
Root vs Leaf	novel.140	4.124351297	0.008729238	0.016952274	--
Root vs Leaf	RHSIM_Rhsim03G0186300	1.076604217	0.008751136	0.016993373	RHSIM_Rhsim03G0186300
Root vs Leaf	RHSIM_Rhsim06G0229900	-1.166671147	0.008802726	0.017089252	RHSIM_Rhsim06G0229900
Root vs Leaf	RHSIM_Rhsim12G0054300	1.442272773	0.008821774	0.017124793	RHSIM_Rhsim12G0054300
Root vs Leaf	RHSIM_Rhsim07G0027300	1.009591642	0.0088267	0.01713292	RHSIM_Rhsim07G0027300
Root vs Leaf	RHSIM_RhsimUnG0222300	1.466814236	0.008835451	0.017148467	RHSIM_RhsimUnG0222300
Root vs Leaf	RHSIM_Rhsim06G0222500	6.45181619	0.008840477	0.017156783	RHSIM_Rhsim06G0222500
Root vs Leaf	RHSIM_Rhsim03G0177800	2.929968929	0.008875681	0.017219327	RHSIM_Rhsim03G0177800
Root vs Leaf	novel.4042	1.779588489	0.008893461	0.017250928	--

Comparison	Gene ID	log_2FC	P-value	FDR (padj)	Gene Name
Root vs Leaf	RHSIM_Rhsim09G0025300	1.146295739	0.008989697	0.017426267	RHSIM_Rhsim09G0025300
Root vs Leaf	RHSIM_Rhsim01G0102200	-10.20775289	0.008989878	0.017426267	RHSIM_Rhsim01G0102200
Root vs Leaf	RHSIM_Rhsim02G0162700	1.26102233	0.009033441	0.017506311	RHSIM_Rhsim02G0162700
Root vs Leaf	RHSIM_Rhsim08G0101300	4.885064723	0.009050991	0.017537811	RHSIM_Rhsim08G0101300
Root vs Leaf	RHSIM_Rhsim10G0035800	2.942154172	0.00906048	0.017554301	RHSIM_Rhsim10G0035800
Root vs Leaf	novel.4624	1.36481382	0.009070824	0.0175714	--
Root vs Leaf	RHSIM_Rhsim03G0155800	1.903297057	0.009092049	0.01761104	RHSIM_Rhsim03G0155800
Root vs Leaf	novel.6140	-2.22039398	0.009114257	0.017651103	--
Root vs Leaf	RHSIM_Rhsim03G0242200	2.135246392	0.009115736	0.01765249	RHSIM_Rhsim03G0242200
Root vs Leaf	RHSIM_Rhsim06G0223000	2.526844181	0.009144231	0.017706187	RHSIM_Rhsim06G0223000
Root vs Leaf	novel.4098	1.346189491	0.009147441	0.01771092	--
Root vs Leaf	novel.5139	1.746099671	0.009156191	0.017724896	--
Root vs Leaf	RHSIM_Rhsim13G0008700	2.012602528	0.009190855	0.017789024	RHSIM_Rhsim13G0008700
Root vs Leaf	RHSIM_Rhsim05G0181900	-1.420103847	0.009201906	0.017807435	RHSIM_Rhsim05G0181900
Root vs Leaf	novel.2581	-1.483277975	0.009205518	0.017811446	--
Root vs Leaf	RHSIM_Rhsim08G0066800	2.012494005	0.009221476	0.017839339	RHSIM_Rhsim08G0066800
Root vs Leaf	RHSIM_RhsimUnG0102400	1.472787141	0.009259364	0.017909641	RHSIM_RhsimUnG0102400
Root vs Leaf	RHSIM_Rhsim07G0186200	1.425723448	0.009264735	0.017918532	RHSIM_Rhsim07G0186200
Root vs Leaf	RHSIM_Rhsim07G0142700	-2.808187242	0.009314978	0.018011189	RHSIM_Rhsim07G0142700
Root vs Leaf	RHSIM_Rhsim06G0217900	2.944875074	0.009334381	0.018047197	RHSIM_Rhsim06G0217900
Root vs Leaf	RHSIM_Rhsim05G0012700	3.093129621	0.009347989	0.018070489	RHSIM_Rhsim05G0012700
Root vs Leaf	RHSIM_Rhsim09G0046500	3.457616348	0.009389887	0.018145418	RHSIM_Rhsim09G0046500
Root vs Leaf	RHSIM_Rhsim05G0010600	-1.177575767	0.009391423	0.018145907	RHSIM_Rhsim05G0010600
Root vs Leaf	novel.211	-2.877011084	0.009391709	0.018145907	--
Root vs Leaf	RHSIM_Rhsim09G0202200	-1.419533451	0.009409677	0.018179107	RHSIM_Rhsim09G0202200

Comparison	Gene ID	log_2FC	P-value	FDR (padj)	Gene Name
Root vs Leaf	RHSIM_Rhsim05G0161700	1.483257626	0.009460662	0.01826998	RHSIM_Rhsim05G0161700
Root vs Leaf	RHSIM_Rhsim04G0182500	3.210895786	0.009481459	0.018308616	RHSIM_Rhsim04G0182500
Root vs Leaf	novel.1954	1.805550842	0.009509978	0.018356026	--
Root vs Leaf	RHSIM_Rhsim01G0280100	1.460930231	0.009566434	0.018460377	RHSIM_Rhsim01G0280100
Root vs Leaf	RHSIM_Rhsim06G0106700	2.260166773	0.00959013	0.018501475	RHSIM_Rhsim06G0106700
Root vs Leaf	novel.3050	2.273922965	0.009597727	0.018514588	--
Root vs Leaf	RHSIM_Rhsim07G0214000	2.134315415	0.009602531	0.018522311	RHSIM_Rhsim07G0214000
Root vs Leaf	RHSIM_Rhsim03G0066500	-2.574831801	0.00965303	0.018616616	RHSIM_Rhsim03G0066500
Root vs Leaf	RHSIM_Rhsim12G0037600	1.050200088	0.009691089	0.0186869	RHSIM_Rhsim12G0037600
Root vs Leaf	RHSIM_Rhsim07G0251300	2.624703135	0.009697334	0.018695826	RHSIM_Rhsim07G0251300
Root vs Leaf	RHSIM_Rhsim04G0185900	1.26687844	0.0098331	0.018949681	RHSIM_Rhsim04G0185900
Root vs Leaf	RHSIM_RhsimUnG0068800	-3.845378959	0.009835489	0.018952706	RHSIM_RhsimUnG0068800
Root vs Leaf	novel.5647	1.750219987	0.009863628	0.019002182	--
Root vs Leaf	RHSIM_Rhsim13G0046200	1.10898536	0.009890308	0.019050409	RHSIM_Rhsim13G0046200
Root vs Leaf	RHSIM_Rhsim12G0023800	1.7542178	0.009995693	0.019243787	RHSIM_Rhsim12G0023800
Root vs Leaf	RHSIM_Rhsim03G0054000	-1.957999719	0.010008045	0.019264363	RHSIM_Rhsim03G0054000
Root vs Leaf	RHSIM_Rhsim01G0239000	2.369726188	0.010116809	0.01946239	RHSIM_Rhsim01G0239000
Root vs Leaf	RHSIM_Rhsim07G0120300	-1.893149886	0.010121234	0.019469138	RHSIM_Rhsim07G0120300
Root vs Leaf	RHSIM_Rhsim05G0138800	-1.528776073	0.010122841	0.019469138	RHSIM_Rhsim05G0138800
Root vs Leaf	RHSIM_Rhsim02G0063700	-2.381200457	0.010144741	0.019508015	RHSIM_Rhsim02G0063700
Root vs Leaf	RHSIM_Rhsim11G0008200	-1.149964676	0.010170846	0.019548467	RHSIM_Rhsim11G0008200
Root vs Leaf	novel.2144	-2.110395448	0.01018953	0.019582751	--
Root vs Leaf	RHSIM_Rhsim02G0027000	1.036904232	0.010194125	0.019588328	RHSIM_Rhsim02G0027000
Root vs Leaf	RHSIM_Rhsim02G0003000	-1.277240176	0.010203904	0.019603863	RHSIM_Rhsim02G0003000
Root vs Leaf	RHSIM_Rhsim08G0056000	-2.539843402	0.01022228	0.019637538	RHSIM_Rhsim08G0056000

Comparison	Gene ID	log_2FC	P-value	FDR (padj)	Gene Name
Root vs Leaf	RHSIM_Rhsim13G0156400	-1.306095667	0.010246845	0.019679828	RHSIM_Rhsim13G0156400
Root vs Leaf	RHSIM_Rhsim11G0084100	1.861202334	0.01026846	0.019718068	RHSIM_Rhsim11G0084100
Root vs Leaf	novel.2554	1.752095427	0.010270474	0.019720298	--
Root vs Leaf	RHSIM_Rhsim08G0239500	1.440510419	0.010285484	0.019745842	RHSIM_Rhsim08G0239500
Root vs Leaf	RHSIM_Rhsim05G0056900	-1.338955342	0.010297471	0.019767216	RHSIM_Rhsim05G0056900
Root vs Leaf	RHSIM_Rhsim11G0032800	-1.259401634	0.010302592	0.019775404	RHSIM_Rhsim11G0032800
Root vs Leaf	RHSIM_RhsimUnG0097900	-1.335108877	0.010319987	0.019805508	RHSIM_RhsimUnG0097900
Root vs Leaf	novel.314	-2.178785219	0.010334465	0.019831649	--
Root vs Leaf	RHSIM_Rhsim12G0044400	3.041345677	0.010352209	0.019860688	RHSIM_Rhsim12G0044400
Root vs Leaf	RHSIM_Rhsim06G0057800	2.319025599	0.010361679	0.019872336	RHSIM_Rhsim06G0057800
Root vs Leaf	RHSIM_Rhsim07G0093300	1.780275594	0.010387393	0.019920001	RHSIM_Rhsim07G0093300
Root vs Leaf	RHSIM_Rhsim03G0175800	2.475602028	0.010389108	0.019921641	RHSIM_Rhsim03G0175800
Root vs Leaf	RHSIM_Rhsim05G0089300	1.216270131	0.010406602	0.019951879	RHSIM_Rhsim05G0089300
Root vs Leaf	RHSIM_Rhsim12G0142700	1.233761722	0.01047972	0.020087071	RHSIM_Rhsim12G0142700
Root vs Leaf	RHSIM_Rhsim11G0177000	1.245308931	0.010494408	0.020112093	RHSIM_Rhsim11G0177000
Root vs Leaf	RHSIM_Rhsim10G0037900	-1.935305399	0.010494513	0.020112093	RHSIM_Rhsim10G0037900
Root vs Leaf	RHSIM_Rhsim12G0174800	2.17416633	0.010544374	0.020204302	RHSIM_Rhsim12G0174800
Root vs Leaf	RHSIM_Rhsim02G0205600	3.442211489	0.010553012	0.020217506	RHSIM_Rhsim02G0205600
Root vs Leaf	RHSIM_Rhsim07G0079500	1.434452541	0.010575024	0.020254646	RHSIM_Rhsim07G0079500
Root vs Leaf	RHSIM_Rhsim11G0153300	-9.986198217	0.010598303	0.020294194	RHSIM_Rhsim11G0153300
Root vs Leaf	RHSIM_Rhsim02G0132400	1.083918596	0.010644123	0.020368941	RHSIM_Rhsim02G0132400
Root vs Leaf	RHSIM_Rhsim07G0210600	1.330301795	0.010662489	0.020398533	RHSIM_Rhsim07G0210600
Root vs Leaf	RHSIM_Rhsim08G0238600	-1.286020261	0.010671753	0.020412881	RHSIM_Rhsim08G0238600
Root vs Leaf	RHSIM_Rhsim04G0017200	-1.51597062	0.010685926	0.020437159	RHSIM_Rhsim04G0017200
Root vs Leaf	RHSIM_Rhsim06G0232000	1.169448516	0.010762485	0.020572829	RHSIM_Rhsim06G0232000

Comparison	Gene ID	log2FC	P-value	FDR (padj)	Gene Name
Root vs Leaf	RHSIM_Rhsim09G0202600	1.018321431	0.010796851	0.020635112	RHSIM_Rhsim09G0202600
Root vs Leaf	novel.5974	-2.986184156	0.010820032	0.020672586	--
Root vs Leaf	novel.497	1.820008864	0.010829688	0.020689327	--
Root vs Leaf	novel.3181	-1.404807159	0.010836749	0.020701106	--
Root vs Leaf	RHSIM_Rhsim04G0179300	-1.23737859	0.010840677	0.020706901	RHSIM_Rhsim04G0179300
Root vs Leaf	RHSIM_Rhsim09G0035800	2.136554928	0.010852526	0.020724402	RHSIM_Rhsim09G0035800
Root vs Leaf	novel.2138	3.288786233	0.010906254	0.020816695	--
Root vs Leaf	RHSIM_Rhsim11G0031600	1.365417893	0.010910118	0.020822353	RHSIM_Rhsim11G0031600
Root vs Leaf	RHSIM_Rhsim13G0092900	1.750398158	0.010971346	0.020927128	RHSIM_Rhsim13G0092900
Root vs Leaf	RHSIM_Rhsim05G0063700	-1.412801885	0.011024724	0.021020279	RHSIM_Rhsim05G0063700
Root vs Leaf	RHSIM_Rhsim06G0133200	1.799116625	0.011047585	0.021060397	RHSIM_Rhsim06G0133200
Root vs Leaf	RHSIM_Rhsim12G0019900	-1.416518461	0.011050525	0.021062531	RHSIM_Rhsim12G0019900
Root vs Leaf	RHSIM_Rhsim11G0191200	1.298776537	0.011087025	0.021128621	RHSIM_Rhsim11G0191200
Root vs Leaf	RHSIM_Rhsim04G0103800	4.026708615	0.011107784	0.021164695	RHSIM_Rhsim04G0103800
Root vs Leaf	RHSIM_Rhsim05G0129300	-1.384694021	0.011115162	0.02117701	RHSIM_Rhsim05G0129300
Root vs Leaf	RHSIM_Rhsim12G0051200	1.311261046	0.011120886	0.021186171	RHSIM_Rhsim12G0051200
Root vs Leaf	RHSIM_Rhsim13G0162900	3.071435563	0.011128199	0.021198357	RHSIM_Rhsim13G0162900
Root vs Leaf	RHSIM_Rhsim03G0244800	1.315709649	0.011137783	0.021214868	RHSIM_Rhsim03G0244800
Root vs Leaf	RHSIM_Rhsim10G0062800	-1.574571851	0.011138873	0.021215199	RHSIM_Rhsim10G0062800
Root vs Leaf	RHSIM_Rhsim04G0086500	1.781160579	0.011176883	0.021278835	RHSIM_Rhsim04G0086500
Root vs Leaf	RHSIM_Rhsim13G0083900	-1.086184064	0.011179959	0.021282941	RHSIM_Rhsim13G0083900
Root vs Leaf	RHSIM_Rhsim01G0138600	1.135521051	0.011228141	0.021367632	RHSIM_Rhsim01G0138600
Root vs Leaf	RHSIM_Rhsim01G0176200	-1.30179939	0.011251099	0.021404282	RHSIM_Rhsim01G0176200
Root vs Leaf	novel.2285	4.389456624	0.011255764	0.021411395	--
Root vs Leaf	RHSIM_Rhsim05G0211500	3.335079007	0.011259585	0.021416903	RHSIM_Rhsim05G0211500

Comparison	Gene ID	log_2FC	P-value	FDR (padj)	Gene Name
Root vs Leaf	RHSIM_Rhsim05G0200900	1.495501203	0.011262732	0.021421129	RHSIM_Rhsim05G0200900
Root vs Leaf	RHSIM_Rhsim05G0029500	1.004930867	0.011281355	0.021453023	RHSIM_Rhsim05G0029500
Root vs Leaf	RHSIM_Rhsim06G0225100	-1.831765173	0.011297782	0.021478966	RHSIM_Rhsim06G0225100
Root vs Leaf	RHSIM_Rhsim12G0008700	1.713454812	0.011306506	0.02149202	RHSIM_Rhsim12G0008700
Root vs Leaf	RHSIM_Rhsim03G0148600	2.954617905	0.011312904	0.02150065	RHSIM_Rhsim03G0148600
Root vs Leaf	RHSIM_Rhsim03G0187700	1.216719673	0.011341533	0.021553291	RHSIM_Rhsim03G0187700
Root vs Leaf	RHSIM_Rhsim03G0244100	1.718532469	0.011360641	0.02158783	RHSIM_Rhsim03G0244100
Root vs Leaf	RHSIM_Rhsim08G0054500	-1.894930649	0.011399841	0.021656983	RHSIM_Rhsim08G0054500
Root vs Leaf	RHSIM_Rhsim13G0041300	3.585093292	0.011417861	0.021689186	RHSIM_Rhsim13G0041300
Root vs Leaf	RHSIM_Rhsim05G0076100	1.563322339	0.011418666	0.021689186	RHSIM_Rhsim05G0076100
Root vs Leaf	RHSIM_Rhsim02G0123200	-2.010762243	0.011427248	0.021700144	RHSIM_Rhsim02G0123200
Root vs Leaf	novel.5252	2.468762489	0.011487497	0.021805625	--
Root vs Leaf	RHSIM_Rhsim13G0118500	1.170456442	0.011505125	0.021837279	RHSIM_Rhsim13G0118500
Root vs Leaf	novel.3838	1.538812101	0.011507344	0.021839698	--
Root vs Leaf	RHSIM_Rhsim11G0059600	1.971890016	0.011523933	0.02186939	RHSIM_Rhsim11G0059600
Root vs Leaf	novel.5185	1.879200128	0.011526501	0.021872469	--
Root vs Leaf	RHSIM_Rhsim07G0060700	-1.238166528	0.011530801	0.021878834	RHSIM_Rhsim07G0060700
Root vs Leaf	RHSIM_Rhsim07G0091800	1.210147276	0.011562576	0.021928337	RHSIM_Rhsim07G0091800
Root vs Leaf	RHSIM_Rhsim03G0119800	2.557079077	0.011564655	0.021930482	RHSIM_Rhsim03G0119800
Root vs Leaf	RHSIM_RhsimUnG0052600	1.211023557	0.011566426	0.021932044	RHSIM_RhsimUnG0052600
Root vs Leaf	RHSIM_Rhsim11G0016600	1.272145969	0.0115785	0.02195314	RHSIM_Rhsim11G0016600
Root vs Leaf	RHSIM_Rhsim03G0111400	-2.157257348	0.011626711	0.022039132	RHSIM_Rhsim03G0111400
Root vs Leaf	RHSIM_Rhsim08G0130900	2.842766289	0.011644494	0.022069225	RHSIM_Rhsim08G0130900
Root vs Leaf	RHSIM_Rhsim07G0063700	1.310211805	0.011757948	0.022269657	RHSIM_Rhsim07G0063700
Root vs Leaf	RHSIM_Rhsim06G0136300	2.874574139	0.011769593	0.022288065	RHSIM_Rhsim06G0136300

Comparison	Gene ID	log_2FC	P-value	FDR (padj)	Gene Name
Root vs Leaf	RHSIM_Rhsim02G0144700	1.474601118	0.011788373	0.022321801	RHSIM_Rhsim02G0144700
Root vs Leaf	novel.1340	1.068647319	0.01181898	0.022374266	--
Root vs Leaf	novel.2742	1.442311876	0.011824876	0.022383596	--
Root vs Leaf	novel.5572	2.223862751	0.011828606	0.022388824	--
Root vs Leaf	RHSIM_Rhsim11G0053200	1.630321728	0.011832239	0.022393869	RHSIM_Rhsim11G0053200
Root vs Leaf	RHSIM_Rhsim03G0066000	2.621822672	0.011834551	0.022396414	RHSIM_Rhsim03G0066000
Root vs Leaf	RHSIM_Rhsim03G0263400	2.123500911	0.01188385	0.022484194	RHSIM_Rhsim03G0263400
Root vs Leaf	RHSIM_Rhsim11G0167700	-1.842057711	0.011941373	0.022581949	RHSIM_Rhsim11G0167700
Root vs Leaf	RHSIM_Rhsim12G0192700	1.417840419	0.011975383	0.022644415	RHSIM_Rhsim12G0192700
Root vs Leaf	RHSIM_Rhsim03G0064200	1.034123385	0.012061214	0.022797399	RHSIM_Rhsim03G0064200
Root vs Leaf	novel.2722	1.721661657	0.012069826	0.022811814	--
Root vs Leaf	RHSIM_Rhsim02G0077200	-1.395484568	0.012117245	0.022892087	RHSIM_Rhsim02G0077200
Root vs Leaf	RHSIM_Rhsim06G0150200	-1.150367207	0.012149359	0.022941518	RHSIM_Rhsim06G0150200
Root vs Leaf	RHSIM_RhsimUnG0135900	-2.276937925	0.012179246	0.022991473	RHSIM_RhsimUnG0135900
Root vs Leaf	RHSIM_Rhsim08G0084600	1.220361402	0.012179787	0.022991473	RHSIM_Rhsim08G0084600
Root vs Leaf	novel.1813	1.091756014	0.012205522	0.023034414	--
Root vs Leaf	RHSIM_Rhsim02G0247700	-1.416242034	0.012238393	0.0230908	RHSIM_Rhsim02G0247700
Root vs Leaf	RHSIM_Rhsim01G0213400	1.294814151	0.012240302	0.023092518	RHSIM_Rhsim01G0213400
Root vs Leaf	RHSIM_Rhsim04G0161100	-1.511776687	0.012267277	0.023135865	RHSIM_Rhsim04G0161100
Root vs Leaf	RHSIM_Rhsim05G0202800	1.133217303	0.012292226	0.023181028	RHSIM_Rhsim05G0202800
Root vs Leaf	RHSIM_Rhsim02G0024800	-1.275923774	0.012332983	0.0232541	RHSIM_Rhsim02G0024800
Root vs Leaf	RHSIM_RhsimUnG0011400	1.137650604	0.012376931	0.023325561	RHSIM_RhsimUnG0011400
Root vs Leaf	RHSIM_Rhsim01G0074600	-1.150920106	0.012381876	0.023332979	RHSIM_Rhsim01G0074600
Root vs Leaf	RHSIM_Rhsim08G0207600	1.254972894	0.012386624	0.023340026	RHSIM_Rhsim08G0207600
Root vs Leaf	RHSIM_Rhsim05G0154300	-1.823314508	0.012448267	0.023450452	RHSIM_Rhsim05G0154300

Comparison	Gene ID	log_2FC	P-value	FDR (padj)	Gene Name
Root vs Leaf	RHSIM_RhsimUnG0126000	1.748792731	0.012503711	0.0235434	RHSIM_RhsimUnG0126000
Root vs Leaf	RHSIM_Rhsim01G0272100	-1.270094041	0.012514441	0.02355977	RHSIM_Rhsim01G0272100
Root vs Leaf	RHSIM_Rhsim06G0211100	2.516798405	0.012529057	0.023583448	RHSIM_Rhsim06G0211100
Root vs Leaf	RHSIM_RhsimUnG0189000	1.050429509	0.012546341	0.023610219	RHSIM_RhsimUnG0189000
Root vs Leaf	RHSIM_RhsimUnG0235600	1.971909991	0.012570327	0.023651512	RHSIM_RhsimUnG0235600
Root vs Leaf	RHSIM_Rhsim01G0117700	1.274733574	0.012587432	0.023679844	RHSIM_Rhsim01G0117700
Root vs Leaf	RHSIM_RhsimUnG0021900	-2.016007821	0.012604089	0.023705398	RHSIM_RhsimUnG0021900
Root vs Leaf	novel.1815	-1.140106395	0.012608894	0.023712508	--
Root vs Leaf	novel.1345	-1.104609089	0.012664352	0.023805196	--
Root vs Leaf	RHSIM_Rhsim03G0125200	1.80066334	0.012697652	0.023863912	RHSIM_Rhsim03G0125200
Root vs Leaf	RHSIM_Rhsim04G0244500	2.016600408	0.012713754	0.023892234	RHSIM_Rhsim04G0244500
Root vs Leaf	novel.5359	1.032464812	0.01272966	0.023916299	--
Root vs Leaf	RHSIM_Rhsim04G0230900	1.07084343	0.012755241	0.02396047	RHSIM_Rhsim04G0230900
Root vs Leaf	RHSIM_Rhsim05G0214400	1.515097737	0.012769238	0.023982869	RHSIM_Rhsim05G0214400
Root vs Leaf	RHSIM_Rhsim11G0160400	-1.8165031	0.012782593	0.024006004	RHSIM_Rhsim11G0160400
Root vs Leaf	RHSIM_Rhsim03G0050600	-2.197405958	0.012827524	0.024084129	RHSIM_Rhsim03G0050600
Root vs Leaf	RHSIM_Rhsim02G0125600	-1.813359881	0.012871136	0.024155064	RHSIM_Rhsim02G0125600
Root vs Leaf	novel.1900	1.548944539	0.012894772	0.024188477	--
Root vs Leaf	RHSIM_Rhsim03G0194900	-1.418696112	0.012940218	0.024268545	RHSIM_Rhsim03G0194900
Root vs Leaf	RHSIM_Rhsim07G0159000	-9.711140056	0.012948404	0.024281929	RHSIM_Rhsim07G0159000
Root vs Leaf	novel.5202	-3.156423182	0.012993413	0.024358439	--
Root vs Leaf	RHSIM_Rhsim07G0086300	1.261727541	0.012997258	0.024363673	RHSIM_Rhsim07G0086300
Root vs Leaf	RHSIM_RhsimUnG0206500	1.058556781	0.013042791	0.024439127	RHSIM_RhsimUnG0206500
Root vs Leaf	RHSIM_Rhsim04G0238000	-2.252862739	0.013050556	0.024450901	RHSIM_Rhsim04G0238000
Root vs Leaf	RHSIM_Rhsim08G0194200	1.000107632	0.01305716	0.024460111	RHSIM_Rhsim08G0194200

Comparison	Gene ID	log_2FC	P-value	FDR (padj)	Gene Name
Root vs Leaf	novel.3822	-1.03488859	0.013058701	0.024461016	--
Root vs Leaf	novel.1840	-1.062528715	0.013069153	0.024472671	--
Root vs Leaf	RHSIM_Rhsim05G0016700	1.421749259	0.013086624	0.024503404	RHSIM_Rhsim05G0016700
Root vs Leaf	novel.3999	-1.687378187	0.01310423	0.024530414	--
Root vs Leaf	novel.4154	-2.076730644	0.013125449	0.024562187	--
Root vs Leaf	novel.2274	-2.766152666	0.013134666	0.024577448	--
Root vs Leaf	novel.2976	-1.402343259	0.01314713	0.024597306	--
Root vs Leaf	RHSIM_Rhsim05G0139700	1.761627019	0.013147404	0.024597306	RHSIM_Rhsim05G0139700
Root vs Leaf	novel.1032	1.392873716	0.01315211	0.024604121	--
Root vs Leaf	novel.2519	1.454557349	0.013156839	0.024610977	--
Root vs Leaf	RHSIM_Rhsim11G0028600	1.331120694	0.013209978	0.024702392	RHSIM_Rhsim11G0028600
Root vs Leaf	novel.2878	-1.914399793	0.013213684	0.024707326	--
Root vs Leaf	RHSIM_Rhsim01G0211600	2.368949176	0.013286916	0.02483623	RHSIM_Rhsim01G0211600
Root vs Leaf	RHSIM_Rhsim08G0015300	2.331229788	0.013300035	0.024856737	RHSIM_Rhsim08G0015300
Root vs Leaf	RHSIM_Rhsim03G0139300	2.053199434	0.013342913	0.024932844	RHSIM_Rhsim03G0139300
Root vs Leaf	RHSIM_RhsimUnG0249000	1.739819193	0.013408269	0.025042837	RHSIM_RhsimUnG0249000
Root vs Leaf	RHSIM_Rhsim13G0003600	1.325927464	0.013420369	0.025063413	RHSIM_Rhsim13G0003600
Root vs Leaf	RHSIM_Rhsim11G0176900	1.136707172	0.013452331	0.025117024	RHSIM_Rhsim11G0176900
Root vs Leaf	RHSIM_Rhsim05G0146700	2.957966281	0.013460996	0.025129147	RHSIM_Rhsim05G0146700
Root vs Leaf	RHSIM_Rhsim04G0101700	-2.813663912	0.013468542	0.025137151	RHSIM_Rhsim04G0101700
Root vs Leaf	novel.535	-1.20640918	0.013476087	0.025149204	--
Root vs Leaf	RHSIM_Rhsim02G0202300	1.835628143	0.013478831	0.025152296	RHSIM_Rhsim02G0202300
Root vs Leaf	RHSIM_RhsimUnG0010800	2.422922718	0.013487639	0.025164675	RHSIM_RhsimUnG0010800
Root vs Leaf	novel.2207	-1.353597408	0.013491185	0.02516926	--
Root vs Leaf	RHSIM_Rhsim03G0068500	1.181413468	0.01351797	0.025213132	RHSIM_Rhsim03G0068500

Comparison	Gene ID	log$_2$FC	P-value	FDR (padj)	Gene Name
Root vs Leaf	RHSIM_Rhsim01G0290500	1.244734134	0.013527832	0.025229493	RHSIM_Rhsim01G0290500
Root vs Leaf	novel.2194	1.790138601	0.013572142	0.025306015	--
Root vs Leaf	novel.1897	1.190950348	0.01372703	0.025576263	--
Root vs Leaf	RHSIM_Rhsim06G0002200	1.414825339	0.013864258	0.025825711	RHSIM_Rhsim06G0002200
Root vs Leaf	RHSIM_Rhsim13G0189900	2.564803974	0.01387313	0.025840156	RHSIM_Rhsim13G0189900
Root vs Leaf	RHSIM_Rhsim03G0254700	2.912520301	0.013904257	0.025893965	RHSIM_Rhsim03G0254700
Root vs Leaf	RHSIM_Rhsim04G0151900	1.515053611	0.013923219	0.025920935	RHSIM_Rhsim04G0151900
Root vs Leaf	RHSIM_Rhsim10G0068100	1.510241873	0.013928683	0.02592902	RHSIM_Rhsim10G0068100
Root vs Leaf	RHSIM_Rhsim03G0184300	1.374362172	0.01403234	0.026105183	RHSIM_Rhsim03G0184300
Root vs Leaf	novel.1825	2.482691269	0.014078485	0.026182611	--
Root vs Leaf	RHSIM_Rhsim08G0060000	-1.645531018	0.014120016	0.02625431	RHSIM_Rhsim08G0060000
Root vs Leaf	RHSIM_Rhsim06G0100900	-1.142742487	0.014121351	0.02625431	RHSIM_Rhsim06G0100900
Root vs Leaf	RHSIM_Rhsim07G0202100	1.253043463	0.014168294	0.026339049	RHSIM_Rhsim07G0202100
Root vs Leaf	RHSIM_Rhsim11G0152900	-1.227454951	0.014186188	0.026370196	RHSIM_Rhsim11G0152900
Root vs Leaf	novel.3859	2.238176817	0.014201859	0.026397206	--
Root vs Leaf	novel.378	1.728111208	0.014235931	0.026443544	--
Root vs Leaf	RHSIM_Rhsim01G0057500	1.039883109	0.014239108	0.026447323	RHSIM_Rhsim01G0057500
Root vs Leaf	RHSIM_RhsimUnG0159200	1.996358706	0.014269187	0.026494685	RHSIM_RhsimUnG0159200
Root vs Leaf	RHSIM_Rhsim12G0076300	2.442389455	0.014321895	0.026581887	RHSIM_Rhsim12G0076300
Root vs Leaf	novel.3642	-1.53236959	0.014334267	0.02659845	--
Root vs Leaf	RHSIM_Rhsim02G0253700	-1.150890724	0.014337731	0.026602744	RHSIM_Rhsim02G0253700
Root vs Leaf	RHSIM_Rhsim01G0038600	-1.286795948	0.014346969	0.026617751	RHSIM_Rhsim01G0038600
Root vs Leaf	RHSIM_Rhsim07G0045900	-1.299392658	0.014390112	0.026693514	RHSIM_Rhsim07G0045900
Root vs Leaf	RHSIM_Rhsim01G0038800	1.462109096	0.014413497	0.026729294	RHSIM_Rhsim01G0038800
Root vs Leaf	RHSIM_Rhsim13G0084700	1.69002835	0.014428877	0.026750656	RHSIM_Rhsim13G0084700

Comparison	Gene ID	log_2FC	P-value	FDR (padj)	Gene Name
Root vs Leaf	novel.4932	-1.016202332	0.014447299	0.026780274	--
Root vs Leaf	RHSIM_Rhsim10G0095400	1.180705466	0.014450802	0.026784622	RHSIM_Rhsim10G0095400
Root vs Leaf	novel.2094	1.194810175	0.014537419	0.026934381	--
Root vs Leaf	RHSIM_Rhsim08G0056500	1.662692018	0.014590575	0.027028537	RHSIM_Rhsim08G0056500
Root vs Leaf	novel.2104	1.249517864	0.014670794	0.027164095	--
Root vs Leaf	RHSIM_Rhsim10G0203800	1.117842816	0.014729154	0.027263426	RHSIM_Rhsim10G0203800
Root vs Leaf	RHSIM_Rhsim09G0056400	1.574725328	0.014773122	0.027333879	RHSIM_Rhsim09G0056400
Root vs Leaf	RHSIM_RhsimUnG0219400	1.061780244	0.014789044	0.027356776	RHSIM_RhsimUnG0219400
Root vs Leaf	RHSIM_Rhsim04G0079400	1.6488125	0.014792691	0.027361336	RHSIM_Rhsim04G0079400
Root vs Leaf	RHSIM_Rhsim06G0008100	1.369069713	0.014844714	0.027450977	RHSIM_Rhsim06G0008100
Root vs Leaf	RHSIM_Rhsim01G0046300	1.337027545	0.014886314	0.027519108	RHSIM_Rhsim01G0046300
Root vs Leaf	novel.3465	1.510088253	0.0149472	0.02762725	--
Root vs Leaf	RHSIM_Rhsim06G0190900	1.052727193	0.014972661	0.02766547	RHSIM_Rhsim06G0190900
Root vs Leaf	RHSIM_Rhsim01G0250600	-2.274794573	0.014990966	0.027692152	RHSIM_Rhsim01G0250600
Root vs Leaf	RHSIM_Rhsim04G0240200	-1.181752495	0.01501776	0.027731088	RHSIM_Rhsim04G0240200
Root vs Leaf	novel.4340	-1.579104488	0.015019957	0.027732931	--
Root vs Leaf	novel.1463	1.070570826	0.015111701	0.027888976	--
Root vs Leaf	RHSIM_Rhsim04G0215600	1.377668928	0.015120282	0.027902587	RHSIM_Rhsim04G0215600
Root vs Leaf	RHSIM_Rhsim01G0059200	-1.551508517	0.015178935	0.028006357	RHSIM_Rhsim01G0059200
Root vs Leaf	RHSIM_Rhsim03G0158000	-1.386007003	0.015201456	0.028038969	RHSIM_Rhsim03G0158000
Root vs Leaf	RHSIM_Rhsim09G0173600	2.096369348	0.015224194	0.02807867	RHSIM_Rhsim09G0173600
Root vs Leaf	RHSIM_Rhsim04G0000600	1.061357181	0.015263041	0.028143588	RHSIM_Rhsim04G0000600
Root vs Leaf	RHSIM_Rhsim12G0062900	2.280320531	0.015273246	0.028160162	RHSIM_Rhsim12G0062900
Root vs Leaf	RHSIM_Rhsim02G0114600	-1.877528824	0.015331816	0.028256894	RHSIM_Rhsim02G0114600
Root vs Leaf	RHSIM_Rhsim07G0077500	2.393575156	0.01540326	0.028384045	RHSIM_Rhsim07G0077500

Comparison	Gene ID	log_2FC	P-value	FDR (padj)	Gene Name
Root vs Leaf	RHSIM_Rhsim13G0229000	1.008126345	0.015473813	0.028502707	RHSIM_Rhsim13G0229000
Root vs Leaf	RHSIM_Rhsim06G0161600	-1.796278521	0.015484173	0.02851725	RHSIM_Rhsim06G0161600
Root vs Leaf	novel.4733	-1.809044961	0.01551347	0.028560466	--
Root vs Leaf	RHSIM_RhsimUnG0038200	1.914699392	0.015515043	0.028560466	RHSIM_RhsimUnG0038200
Root vs Leaf	RHSIM_Rhsim05G0079700	1.669983877	0.015535763	0.028596332	RHSIM_Rhsim05G0079700
Root vs Leaf	RHSIM_Rhsim08G0079100	-1.327172451	0.015628423	0.028750881	RHSIM_Rhsim08G0079100
Root vs Leaf	novel.912	-1.148791434	0.015689308	0.028856007	--
Root vs Leaf	RHSIM_Rhsim09G0117400	-1.311290339	0.015748247	0.028957504	RHSIM_Rhsim09G0117400
Root vs Leaf	novel.3208	1.681714956	0.015757368	0.028969671	--
Root vs Leaf	RHSIM_Rhsim02G0022100	-1.832544584	0.015760218	0.028972609	RHSIM_Rhsim02G0022100
Root vs Leaf	RHSIM_Rhsim02G0176200	-1.047439139	0.015790737	0.029024102	RHSIM_Rhsim02G0176200
Root vs Leaf	RHSIM_Rhsim02G0046300	-7.42525948	0.015802402	0.029043236	RHSIM_Rhsim02G0046300
Root vs Leaf	novel.1583	-1.12332634	0.015814693	0.029063518	--
Root vs Leaf	RHSIM_Rhsim13G0135700	2.406689209	0.01592133	0.029247877	RHSIM_Rhsim13G0135700
Root vs Leaf	RHSIM_Rhsim04G0066200	1.735277607	0.016004762	0.029389478	RHSIM_Rhsim04G0066200
Root vs Leaf	RHSIM_Rhsim13G0130200	-2.010178667	0.016068133	0.029501164	RHSIM_Rhsim13G0130200
Root vs Leaf	RHSIM_Rhsim03G0187800	1.489651279	0.016095541	0.02954914	RHSIM_Rhsim03G0187800
Root vs Leaf	novel.5690	1.553051794	0.016150722	0.029643391	--
Root vs Leaf	RHSIM_Rhsim01G0034500	1.353428131	0.016174654	0.029684961	RHSIM_Rhsim01G0034500
Root vs Leaf	RHSIM_Rhsim07G0086000	2.0453004	0.016184845	0.029701308	RHSIM_Rhsim07G0086000
Root vs Leaf	novel.2235	-2.96073943	0.016186842	0.029702618	--
Root vs Leaf	novel.3438	-1.931207609	0.016235419	0.029789394	--
Root vs Leaf	RHSIM_Rhsim05G0123500	-1.808767985	0.016252488	0.029815984	RHSIM_Rhsim05G0123500
Root vs Leaf	novel.5603	-1.521063593	0.016276954	0.029858501	--
Root vs Leaf	novel.6065	-2.067672425	0.016330965	0.029950457	--

Comparison	Gene ID	log_2FC	P-value	FDR (padj)	Gene Name
Root vs Leaf	RHSIM_Rhsim03G0209400	-1.103917491	0.016332404	0.029950722	RHSIM_Rhsim03G0209400
Root vs Leaf	novel.1185	2.649352398	0.016368141	0.030013878	--
Root vs Leaf	RHSIM_Rhsim05G0224100	1.611121084	0.016373084	0.030020564	RHSIM_Rhsim05G0224100
Root vs Leaf	RHSIM_Rhsim08G0073400	2.220646009	0.016561706	0.030339964	RHSIM_Rhsim08G0073400
Root vs Leaf	RHSIM_Rhsim08G0019600	-1.036396765	0.016590342	0.030390019	RHSIM_Rhsim08G0019600
Root vs Leaf	RHSIM_Rhsim02G0109600	1.341527181	0.016591977	0.030390608	RHSIM_Rhsim02G0109600
Root vs Leaf	novel.650	-1.16394443	0.016608532	0.030414412	--
Root vs Leaf	novel.4309	3.321795861	0.016616258	0.030425448	--
Root vs Leaf	RHSIM_RhsimUnG0233800	2.50330486	0.01665021	0.030480383	RHSIM_RhsimUnG0233800
Root vs Leaf	RHSIM_Rhsim09G0152300	-1.080426329	0.016678798	0.030527885	RHSIM_Rhsim09G0152300
Root vs Leaf	RHSIM_Rhsim05G0048100	1.498838358	0.016685097	0.030537	RHSIM_Rhsim05G0048100
Root vs Leaf	RHSIM_Rhsim11G0058500	2.687275155	0.016723552	0.030600119	RHSIM_Rhsim11G0058500
Root vs Leaf	RHSIM_Rhsim11G0142600	-1.383129604	0.016788685	0.03071628	RHSIM_Rhsim11G0142600
Root vs Leaf	RHSIM_Rhsim01G0233400	2.662531532	0.016791974	0.030718029	RHSIM_Rhsim01G0233400
Root vs Leaf	RHSIM_Rhsim12G0098300	-9.342339387	0.016818386	0.03076009	RHSIM_Rhsim12G0098300
Root vs Leaf	RHSIM_Rhsim05G0086600	-2.00150708	0.016818954	0.03076009	RHSIM_Rhsim05G0086600
Root vs Leaf	RHSIM_Rhsim04G0198700	2.96664409	0.016845018	0.030805323	RHSIM_Rhsim04G0198700
Root vs Leaf	RHSIM_Rhsim10G0044600	1.077622465	0.016847139	0.030806768	RHSIM_Rhsim10G0044600
Root vs Leaf	RHSIM_Rhsim02G0077300	1.825909627	0.016965112	0.031017592	RHSIM_Rhsim02G0077300
Root vs Leaf	RHSIM_Rhsim06G0029200	-1.201354895	0.016976147	0.031032862	RHSIM_Rhsim06G0029200
Root vs Leaf	novel.754	1.502237512	0.016983644	0.031044116	--
Root vs Leaf	RHSIM_Rhsim08G0065300	3.060334744	0.017003064	0.031074705	RHSIM_Rhsim08G0065300
Root vs Leaf	novel.3851	-1.214491257	0.017011194	0.031084653	--
Root vs Leaf	novel.5532	2.373592139	0.017041008	0.031131758	--
Root vs Leaf	RHSIM_RhsimUnG0244000	-1.08440971	0.017091985	0.031219956	RHSIM_RhsimUnG0244000

Comparison	Gene ID	log_2FC	P-value	FDR (padj)	Gene Name
Root vs Leaf	RHSIM_Rhsim02G0189300	-1.225832296	0.017119068	0.031262024	RHSIM_Rhsim02G0189300
Root vs Leaf	RHSIM_Rhsim02G0080300	1.607422136	0.017164413	0.031337074	RHSIM_Rhsim02G0080300
Root vs Leaf	RHSIM_Rhsim11G0031900	-4.979023147	0.017165583	0.031337074	RHSIM_Rhsim11G0031900
Root vs Leaf	RHSIM_Rhsim04G0006100	1.819798264	0.017189744	0.031371406	RHSIM_Rhsim04G0006100
Root vs Leaf	RHSIM_RhsimUnG0244400	-1.505694875	0.017189812	0.031371406	RHSIM_RhsimUnG0244400
Root vs Leaf	RHSIM_Rhsim11G0023700	-1.28284383	0.017202827	0.031392683	RHSIM_Rhsim11G0023700
Root vs Leaf	RHSIM_RhsimUnG0022200	1.266808845	0.017244544	0.031458889	RHSIM_RhsimUnG0022200
Root vs Leaf	novel.354	1.419542755	0.017252427	0.031470788	--
Root vs Leaf	RHSIM_RhsimUnG0184200	1.257908962	0.017261567	0.031482497	RHSIM_RhsimUnG0184200
Root vs Leaf	RHSIM_Rhsim12G0115700	-1.051672857	0.017268781	0.031493172	RHSIM_Rhsim12G0115700
Root vs Leaf	RHSIM_Rhsim04G0062800	2.471589005	0.017274108	0.031500404	RHSIM_Rhsim04G0062800
Root vs Leaf	RHSIM_Rhsim05G0110800	1.940522852	0.017280499	0.031509575	RHSIM_Rhsim05G0110800
Root vs Leaf	RHSIM_Rhsim10G0188400	4.655438843	0.01733615	0.031598601	RHSIM_Rhsim10G0188400
Root vs Leaf	RHSIM_Rhsim03G0027500	-1.120042212	0.017348011	0.031615241	RHSIM_Rhsim03G0027500
Root vs Leaf	novel.4038	1.819602211	0.017422293	0.03173312	--
Root vs Leaf	RHSIM_Rhsim02G0191600	3.972724659	0.017473317	0.031818542	RHSIM_Rhsim02G0191600
Root vs Leaf	RHSIM_Rhsim13G0070700	-2.025140388	0.017533011	0.031913135	RHSIM_Rhsim13G0070700
Root vs Leaf	RHSIM_Rhsim08G0135900	-1.508810065	0.01758359	0.031999203	RHSIM_Rhsim08G0135900
Root vs Leaf	RHSIM_Rhsim01G0268300	1.018047093	0.017594857	0.032014671	RHSIM_Rhsim01G0268300
Root vs Leaf	RHSIM_Rhsim10G0005900	2.989724243	0.017614929	0.032042335	RHSIM_Rhsim10G0005900
Root vs Leaf	RHSIM_Rhsim13G0144300	1.911571141	0.017615599	0.032042335	RHSIM_Rhsim13G0144300
Root vs Leaf	RHSIM_Rhsim12G0170200	1.421085053	0.017642213	0.032080658	RHSIM_Rhsim12G0170200
Root vs Leaf	novel.5395	1.052681736	0.0176775	0.032132162	--
Root vs Leaf	RHSIM_Rhsim05G0056100	-1.186635419	0.017703943	0.032172684	RHSIM_Rhsim05G0056100
Root vs Leaf	RHSIM_Rhsim04G0227000	-3.599979639	0.017855369	0.032427491	RHSIM_Rhsim04G0227000

Comparison	Gene ID	log_2FC	P-value	FDR (padj)	Gene Name
Root vs Leaf	RHSIM_Rhsim02G0101500	1.194557333	0.017931188	0.032549861	RHSIM_Rhsim02G0101500
Root vs Leaf	RHSIM_Rhsim09G0135000	-1.051193487	0.017963014	0.032599962	RHSIM_Rhsim09G0135000
Root vs Leaf	novel.2760	-1.055443602	0.017997459	0.032659911	--
Root vs Leaf	RHSIM_Rhsim05G0139600	1.88158657	0.01803146	0.032711353	RHSIM_Rhsim05G0139600
Root vs Leaf	RHSIM_Rhsim06G0196900	-1.149377741	0.018056315	0.032751307	RHSIM_Rhsim06G0196900
Root vs Leaf	RHSIM_Rhsim05G0199300	1.318519741	0.018081843	0.032792471	RHSIM_Rhsim05G0199300
Root vs Leaf	RHSIM_Rhsim10G0013400	1.283228752	0.018151723	0.032907191	RHSIM_Rhsim10G0013400
Root vs Leaf	RHSIM_Rhsim04G0045400	3.429378903	0.01815221	0.032907191	RHSIM_Rhsim04G0045400
Root vs Leaf	novel.4649	5.192929095	0.01816347	0.032925025	--
Root vs Leaf	novel.1486	-1.776390522	0.018179995	0.032949817	--
Root vs Leaf	RHSIM_Rhsim08G0125800	-1.538612508	0.018186314	0.032958689	RHSIM_Rhsim08G0125800
Root vs Leaf	novel.2993	-1.738681755	0.018236454	0.03304438	--
Root vs Leaf	RHSIM_Rhsim06G0142900	1.208242814	0.018283958	0.033120083	RHSIM_Rhsim06G0142900
Root vs Leaf	RHSIM_Rhsim01G0007800	-1.526860205	0.018374396	0.03325007	RHSIM_Rhsim01G0007800
Root vs Leaf	novel.993	1.391091358	0.018408767	0.033307058	--
Root vs Leaf	RHSIM_Rhsim03G0070800	2.486144178	0.01843453	0.033345849	RHSIM_Rhsim03G0070800
Root vs Leaf	RHSIM_Rhsim07G0012800	1.157628434	0.018450115	0.033371432	RHSIM_Rhsim07G0012800
Root vs Leaf	RHSIM_Rhsim03G0206400	-9.194198874	0.018638937	0.033694527	RHSIM_Rhsim03G0206400
Root vs Leaf	RHSIM_Rhsim06G0017800	1.423676022	0.018676742	0.033752322	RHSIM_Rhsim06G0017800
Root vs Leaf	RHSIM_Rhsim07G0126500	1.209244633	0.01874538	0.033860498	RHSIM_Rhsim07G0126500
Root vs Leaf	RHSIM_RhsimUnG0131300	-1.362348367	0.018763924	0.03389135	RHSIM_RhsimUnG0131300
Root vs Leaf	RHSIM_Rhsim09G0209700	2.121754552	0.018780727	0.033919052	RHSIM_Rhsim09G0209700
Root vs Leaf	RHSIM_Rhsim13G0017500	1.611665401	0.018889756	0.034102657	RHSIM_Rhsim13G0017500
Root vs Leaf	novel.5293	-2.565995532	0.018893159	0.034103926	--
Root vs Leaf	RHSIM_RhsimUnG0146800	1.269449744	0.018893407	0.034103926	RHSIM_RhsimUnG0146800

Comparison	Gene ID	log_2FC	P-value	FDR (padj)	Gene Name
Root vs Leaf	RHSIM_Rhsim02G0019100	1.356068167	0.01890955	0.034127741	RHSIM_Rhsim02G0019100
Root vs Leaf	RHSIM_Rhsim09G0018800	-1.299616733	0.018951509	0.034195467	RHSIM_Rhsim09G0018800
Root vs Leaf	RHSIM_Rhsim04G0054300	-1.971856167	0.018964853	0.034216877	RHSIM_Rhsim04G0054300
Root vs Leaf	RHSIM_Rhsim03G0138100	1.86423276	0.019002453	0.034282043	RHSIM_Rhsim03G0138100
Root vs Leaf	RHSIM_Rhsim06G0195100	1.109571636	0.019009892	0.034292791	RHSIM_Rhsim06G0195100
Root vs Leaf	RHSIM_Rhsim03G0141100	-2.118848972	0.019036418	0.034337965	RHSIM_Rhsim03G0141100
Root vs Leaf	RHSIM_Rhsim06G0241800	-1.320355446	0.019079041	0.034409484	RHSIM_Rhsim06G0241800
Root vs Leaf	RHSIM_Rhsim05G0142200	1.258350862	0.019119688	0.034477417	RHSIM_Rhsim05G0142200
Root vs Leaf	RHSIM_Rhsim10G0071000	1.498080049	0.019142948	0.034513981	RHSIM_Rhsim10G0071000
Root vs Leaf	RHSIM_Rhsim09G0162900	1.994126659	0.019299439	0.034782579	RHSIM_Rhsim09G0162900
Root vs Leaf	RHSIM_Rhsim04G0054600	1.685226585	0.019323092	0.034822496	RHSIM_Rhsim04G0054600
Root vs Leaf	RHSIM_Rhsim08G0167100	-1.119504075	0.019332571	0.034834152	RHSIM_Rhsim08G0167100
Root vs Leaf	RHSIM_Rhsim06G0062100	1.414401523	0.019353121	0.034865751	RHSIM_Rhsim06G0062100
Root vs Leaf	novel.2789	3.50181848	0.019384602	0.034917029	--
Root vs Leaf	RHSIM_Rhsim04G0102400	1.860373995	0.019400317	0.034939896	RHSIM_Rhsim04G0102400
Root vs Leaf	RHSIM_RhsimUnG0063700	-1.471032011	0.019417514	0.034965426	RHSIM_RhsimUnG0063700
Root vs Leaf	RHSIM_Rhsim13G0189500	-1.518606655	0.019437258	0.034998257	RHSIM_Rhsim13G0189500
Root vs Leaf	RHSIM_Rhsim03G0189200	3.906036732	0.019490635	0.035091635	RHSIM_Rhsim03G0189200
Root vs Leaf	RHSIM_Rhsim06G0183800	2.330311481	0.019572295	0.035232449	RHSIM_Rhsim06G0183800
Root vs Leaf	RHSIM_Rhsim13G0161800	1.082104225	0.019573414	0.035232449	RHSIM_Rhsim13G0161800
Root vs Leaf	RHSIM_Rhsim11G0102600	-4.036535895	0.019596468	0.035271203	RHSIM_Rhsim11G0102600
Root vs Leaf	novel.3151	-1.600187129	0.01962684	0.035323123	--
Root vs Leaf	RHSIM_Rhsim04G0026800	1.837572755	0.019663697	0.035383951	RHSIM_Rhsim04G0026800
Root vs Leaf	RHSIM_Rhsim07G0237700	2.372546977	0.019668142	0.035389198	RHSIM_Rhsim07G0237700
Root vs Leaf	RHSIM_RhsimUnG0254200	1.284983829	0.019733467	0.035493784	RHSIM_RhsimUnG0254200

Comparison	Gene ID	log_2FC	P-value	FDR (padj)	Gene Name
Root vs Leaf	RHSIM_Rhsim03G0236700	1.115466888	0.019792969	0.035588897	RHSIM_Rhsim03G0236700
Root vs Leaf	novel.4768	-1.667746452	0.019807099	0.035606004	--
Root vs Leaf	RHSIM_Rhsim08G0163300	-1.349949869	0.019842115	0.035666178	RHSIM_Rhsim08G0163300
Root vs Leaf	RHSIM_Rhsim06G0045400	2.097678603	0.019885016	0.035732191	RHSIM_Rhsim06G0045400
Root vs Leaf	novel.4060	1.244168092	0.01992104	0.035788587	--
Root vs Leaf	RHSIM_Rhsim04G0125500	1.970533222	0.02000083	0.035917988	RHSIM_Rhsim04G0125500
Root vs Leaf	RHSIM_Rhsim10G0150400	-1.303801756	0.02003181	0.035968041	RHSIM_Rhsim10G0150400
Root vs Leaf	novel.3420	1.371726542	0.020053546	0.036004275	--
Root vs Leaf	RHSIM_Rhsim12G0080500	2.082094165	0.020136702	0.036139555	RHSIM_Rhsim12G0080500
Root vs Leaf	RHSIM_Rhsim13G0210900	2.870317045	0.020143746	0.03614659	RHSIM_Rhsim13G0210900
Root vs Leaf	novel.5504	1.873814548	0.020148245	0.036151861	--
Root vs Leaf	novel.2224	1.632972046	0.020171818	0.036188545	--
Root vs Leaf	RHSIM_RhsimUnG0048200	5.952799503	0.020228618	0.036282007	RHSIM_RhsimUnG0048200
Root vs Leaf	RHSIM_Rhsim10G0026000	-1.158242659	0.020257978	0.036329036	RHSIM_Rhsim10G0026000
Root vs Leaf	RHSIM_Rhsim05G0128400	-1.517984997	0.020294352	0.036377728	RHSIM_Rhsim05G0128400
Root vs Leaf	RHSIM_Rhsim07G0058100	-1.408101292	0.020294562	0.036377728	RHSIM_Rhsim07G0058100
Root vs Leaf	RHSIM_Rhsim12G0042200	1.139625725	0.020306378	0.036396088	RHSIM_Rhsim12G0042200
Root vs Leaf	RHSIM_RhsimUnG0056300	-1.776860765	0.020389388	0.036527896	RHSIM_RhsimUnG0056300
Root vs Leaf	RHSIM_Rhsim13G0190000	-2.074049713	0.020436125	0.036597458	RHSIM_Rhsim13G0190000
Root vs Leaf	RHSIM_Rhsim01G0038400	1.679741575	0.020442288	0.03660283	RHSIM_Rhsim01G0038400
Root vs Leaf	novel.2117	-1.962792652	0.020531994	0.036752079	--
Root vs Leaf	novel.51	1.446598605	0.020540696	0.036764812	--
Root vs Leaf	RHSIM_Rhsim07G0176300	-1.547766195	0.02060236	0.036858076	RHSIM_Rhsim07G0176300
Root vs Leaf	novel.1037	-1.144635607	0.020696167	0.037008731	--
Root vs Leaf	RHSIM_Rhsim04G0119000	-1.843425343	0.020740871	0.037080075	RHSIM_Rhsim04G0119000

Comparison	Gene ID	log_2FC	P-value	FDR (padj)	Gene Name
Root vs Leaf	RHSIM_Rhsim03G0089200	1.659568702	0.020758602	0.037108907	RHSIM_Rhsim03G0089200
Root vs Leaf	novel.5394	-2.261679473	0.020806376	0.037191437	--
Root vs Leaf	novel.5975	2.243321869	0.02081434	0.0372028	--
Root vs Leaf	RHSIM_Rhsim04G0035500	-1.23670219	0.020818051	0.037206558	RHSIM_Rhsim04G0035500
Root vs Leaf	RHSIM_Rhsim05G0045200	-1.152466367	0.020837715	0.037238827	RHSIM_Rhsim05G0045200
Root vs Leaf	RHSIM_RhsimUnG0243600	-1.475038768	0.020860258	0.037273356	RHSIM_RhsimUnG0243600
Root vs Leaf	RHSIM_Rhsim05G0225000	2.943936234	0.020953094	0.037416123	RHSIM_Rhsim05G0225000
Root vs Leaf	novel.3289	-9.023050546	0.02095525	0.037416946	--
Root vs Leaf	RHSIM_Rhsim10G0069700	-1.946507642	0.020997333	0.037471989	RHSIM_Rhsim10G0069700
Root vs Leaf	RHSIM_RhsimUnG0028600	1.471916213	0.020999574	0.037473099	RHSIM_RhsimUnG0028600
Root vs Leaf	RHSIM_Rhsim04G0136100	1.722113772	0.02102902	0.037519858	RHSIM_Rhsim04G0136100
Root vs Leaf	novel.1524	-1.00260964	0.021043437	0.037542685	--
Root vs Leaf	RHSIM_Rhsim05G0034400	1.558361687	0.021081419	0.037598853	RHSIM_Rhsim05G0034400
Root vs Leaf	RHSIM_Rhsim10G0109900	-1.576963923	0.021083146	0.037598887	RHSIM_Rhsim10G0109900
Root vs Leaf	RHSIM_Rhsim09G0047300	1.420778811	0.021084688	0.037598887	RHSIM_Rhsim09G0047300
Root vs Leaf	RHSIM_Rhsim11G0044900	1.452120812	0.021154713	0.037717945	RHSIM_Rhsim11G0044900
Root vs Leaf	RHSIM_Rhsim01G0220900	1.075147945	0.021222973	0.037830906	RHSIM_Rhsim01G0220900
Root vs Leaf	RHSIM_Rhsim06G0157800	-2.469292323	0.021235522	0.03785036	RHSIM_Rhsim06G0157800
Root vs Leaf	RHSIM_Rhsim10G0077300	1.742002325	0.021273168	0.03791162	RHSIM_Rhsim10G0077300
Root vs Leaf	RHSIM_Rhsim02G0020300	2.789743881	0.021281071	0.037916944	RHSIM_Rhsim02G0020300
Root vs Leaf	RHSIM_Rhsim07G0107300	1.102258067	0.021360949	0.038050476	RHSIM_Rhsim07G0107300
Root vs Leaf	novel.5450	2.332068833	0.021429521	0.038155001	--
Root vs Leaf	RHSIM_Rhsim08G0080300	1.977441252	0.021452713	0.038187479	RHSIM_Rhsim08G0080300
Root vs Leaf	RHSIM_Rhsim10G0139000	1.748375925	0.021457969	0.03819382	RHSIM_Rhsim10G0139000
Root vs Leaf	RHSIM_Rhsim09G0081800	-1.535930779	0.021459576	0.03819382	RHSIM_Rhsim09G0081800

Comparison	Gene ID	log_2FC	P-value	FDR (padj)	Gene Name
Root vs Leaf	RHSIM_Rhsim04G0036300	-1.504149868	0.021468479	0.03820379	RHSIM_Rhsim04G0036300
Root vs Leaf	RHSIM_Rhsim10G0097100	1.454749438	0.021474455	0.038211486	RHSIM_Rhsim10G0097100
Root vs Leaf	novel.2281	1.532835833	0.021481586	0.038221235	--
Root vs Leaf	RHSIM_Rhsim05G0000300	-1.007545959	0.021630174	0.038479694	RHSIM_Rhsim05G0000300
Root vs Leaf	novel.5760	-1.537221032	0.02165396	0.038519047	--
Root vs Leaf	novel.2935	2.764958489	0.02167542	0.038554258	--
Root vs Leaf	RHSIM_Rhsim01G0243900	-1.396714021	0.021692983	0.038582531	RHSIM_Rhsim01G0243900
Root vs Leaf	RHSIM_Rhsim04G0135100	2.589148326	0.021696838	0.038586422	RHSIM_Rhsim04G0135100
Root vs Leaf	RHSIM_Rhsim05G0194800	-1.338849833	0.021803063	0.038769378	RHSIM_Rhsim05G0194800
Root vs Leaf	RHSIM_Rhsim01G0050900	-1.527722932	0.021822132	0.038791363	RHSIM_Rhsim01G0050900
Root vs Leaf	novel.2910	-1.24981483	0.02184402	0.038827289	--
Root vs Leaf	RHSIM_Rhsim13G0064300	2.879544261	0.021863948	0.038856741	RHSIM_Rhsim13G0064300
Root vs Leaf	novel.338	-1.571546384	0.021944306	0.03899057	--
Root vs Leaf	novel.1057	-1.229785536	0.022000492	0.039078399	--
Root vs Leaf	RHSIM_Rhsim05G0175200	1.165588321	0.022036362	0.039133103	RHSIM_Rhsim05G0175200
Root vs Leaf	RHSIM_RhsimUnG0236500	1.076610357	0.022067215	0.039178873	RHSIM_RhsimUnG0236500
Root vs Leaf	novel.662	-8.933067677	0.022271004	0.039513402	--
Root vs Leaf	RHSIM_Rhsim12G0172800	2.326177325	0.022295211	0.039553318	RHSIM_Rhsim12G0172800
Root vs Leaf	RHSIM_Rhsim13G0140300	1.74222928	0.02252834	0.039951591	RHSIM_Rhsim13G0140300
Root vs Leaf	novel.1986	1.128095455	0.022688279	0.040208823	--
Root vs Leaf	RHSIM_Rhsim12G0113300	-1.096923292	0.022817958	0.040409456	RHSIM_Rhsim12G0113300
Root vs Leaf	RHSIM_Rhsim04G0024900	2.125572163	0.022864551	0.040482676	RHSIM_Rhsim04G0024900
Root vs Leaf	novel.494	2.427156805	0.022947485	0.040623298	--
Root vs Leaf	novel.3070	1.023985831	0.022995311	0.040701736	--
Root vs Leaf	novel.513	3.2332215	0.023018923	0.040726004	--

Comparison	Gene ID	log_2FC	P-value	FDR (padj)	Gene Name
Root vs Leaf	novel.1162	-8.877813536	0.02311413	0.040883901	--
Root vs Leaf	RHSIM_Rhsim02G0143600	1.262106972	0.023138452	0.040923794	RHSIM_Rhsim02G0143600
Root vs Leaf	RHSIM_Rhsim02G0130400	-1.000086311	0.023169515	0.040975602	RHSIM_Rhsim02G0130400
Root vs Leaf	RHSIM_Rhsim03G0081200	1.178299409	0.023175352	0.040982792	RHSIM_Rhsim03G0081200
Root vs Leaf	RHSIM_Rhsim11G0157300	2.123657534	0.023194692	0.041013859	RHSIM_Rhsim11G0157300
Root vs Leaf	RHSIM_Rhsim05G0028800	-8.869249364	0.023247266	0.041100542	RHSIM_Rhsim05G0028800
Root vs Leaf	RHSIM_Rhsim04G0018600	-1.464885951	0.023255708	0.041112325	RHSIM_Rhsim04G0018600
Root vs Leaf	novel.5920	2.291836919	0.023308271	0.04118637	--
Root vs Leaf	RHSIM_RhsimUnG0168700	-1.112709479	0.023394331	0.041328972	RHSIM_RhsimUnG0168700
Root vs Leaf	RHSIM_Rhsim04G0149600	2.772066581	0.023443517	0.041412705	RHSIM_Rhsim04G0149600
Root vs Leaf	RHSIM_Rhsim04G0195600	1.630921867	0.023536475	0.041564221	RHSIM_Rhsim04G0195600
Root vs Leaf	RHSIM_Rhsim05G0061100	-1.096246479	0.023682703	0.04180969	RHSIM_Rhsim05G0061100
Root vs Leaf	RHSIM_RhsimUnG0225700	2.359980171	0.023687789	0.041812331	RHSIM_RhsimUnG0225700
Root vs Leaf	RHSIM_Rhsim04G0021700	-1.364579217	0.023687812	0.041812331	RHSIM_Rhsim04G0021700
Root vs Leaf	RHSIM_Rhsim13G0010200	1.119665819	0.023805703	0.042007609	RHSIM_Rhsim13G0010200
Root vs Leaf	novel.2834	1.119208218	0.023871221	0.042116798	--
Root vs Leaf	RHSIM_Rhsim13G0107100	1.570766302	0.023915014	0.042178686	RHSIM_Rhsim13G0107100
Root vs Leaf	RHSIM_Rhsim07G0243600	-1.002530684	0.02395001	0.042226832	RHSIM_Rhsim07G0243600
Root vs Leaf	RHSIM_Rhsim12G0153800	2.773894355	0.023992854	0.042298811	RHSIM_Rhsim12G0153800
Root vs Leaf	RHSIM_Rhsim10G0036400	1.330878684	0.02399449	0.042298811	RHSIM_Rhsim10G0036400
Root vs Leaf	RHSIM_Rhsim11G0133300	-2.268951972	0.024006455	0.042313457	RHSIM_Rhsim11G0133300
Root vs Leaf	RHSIM_Rhsim05G0074100	1.916027401	0.02407561	0.042425653	RHSIM_Rhsim05G0074100
Root vs Leaf	RHSIM_Rhsim08G0107800	-1.095809043	0.024141414	0.042528659	RHSIM_Rhsim08G0107800
Root vs Leaf	RHSIM_Rhsim08G0152300	1.464780333	0.024223293	0.042663157	RHSIM_Rhsim08G0152300
Root vs Leaf	novel.4957	-1.413615724	0.024253141	0.042712475	--

Comparison	Gene ID	log_2FC	P-value	FDR (padj)	Gene Name
Root vs Leaf	RHSIM_RhsimUnG0132700	4.991580375	0.024437466	0.04301745	RHSIM_RhsimUnG0132700
Root vs Leaf	RHSIM_Rhsim13G0229400	1.156864479	0.024440255	0.043019087	RHSIM_Rhsim13G0229400
Root vs Leaf	RHSIM_Rhsim05G0077100	1.399476387	0.024443441	0.043021423	RHSIM_Rhsim05G0077100
Root vs Leaf	RHSIM_Rhsim02G0249600	1.209464532	0.024571009	0.043232795	RHSIM_Rhsim02G0249600
Root vs Leaf	RHSIM_Rhsim12G0013200	-1.504753728	0.024649292	0.043347463	RHSIM_Rhsim12G0013200
Root vs Leaf	RHSIM_Rhsim07G0133600	1.587878037	0.024668378	0.043377731	RHSIM_Rhsim07G0133600
Root vs Leaf	RHSIM_Rhsim07G0215200	-1.357506417	0.024728177	0.043470807	RHSIM_Rhsim07G0215200
Root vs Leaf	RHSIM_RhsimUnG0174000	-1.07341424	0.024732969	0.043470807	RHSIM_RhsimUnG0174000
Root vs Leaf	RHSIM_Rhsim09G0002400	2.412851467	0.024733638	0.043470807	RHSIM_Rhsim09G0002400
Root vs Leaf	RHSIM_Rhsim09G0088800	2.244871255	0.024777147	0.043535911	RHSIM_Rhsim09G0088800
Root vs Leaf	RHSIM_Rhsim03G0083000	2.971900931	0.024901361	0.043747524	RHSIM_Rhsim03G0083000
Root vs Leaf	RHSIM_Rhsim13G0106700	2.512935648	0.024942644	0.0438001	RHSIM_Rhsim13G0106700
Root vs Leaf	novel.5666	1.554901772	0.024960299	0.043821128	--
Root vs Leaf	novel.2990	-2.48962671	0.025043597	0.043964033	--
Root vs Leaf	RHSIM_Rhsim09G0128000	-1.233890351	0.025084208	0.044028646	RHSIM_Rhsim09G0128000
Root vs Leaf	RHSIM_Rhsim11G0194400	-1.26401634	0.025246647	0.044293605	RHSIM_Rhsim11G0194400
Root vs Leaf	RHSIM_Rhsim07G0217700	1.681731947	0.025272229	0.044331765	RHSIM_Rhsim07G0217700
Root vs Leaf	RHSIM_Rhsim01G0111600	1.66503701	0.025308073	0.044387913	RHSIM_Rhsim01G0111600
Root vs Leaf	RHSIM_Rhsim11G0120700	-1.388674667	0.025376951	0.044501972	RHSIM_Rhsim11G0120700
Root vs Leaf	RHSIM_Rhsim02G0104400	1.24035365	0.025393779	0.044524734	RHSIM_Rhsim02G0104400
Root vs Leaf	RHSIM_RhsimUnG0105200	-2.83124272	0.025449699	0.044616021	RHSIM_RhsimUnG0105200
Root vs Leaf	RHSIM_Rhsim04G0247800	1.793744549	0.025493395	0.044685854	RHSIM_Rhsim04G0247800
Root vs Leaf	RHSIM_Rhsim07G0044900	-1.775658222	0.025560974	0.044787348	RHSIM_Rhsim07G0044900
Root vs Leaf	RHSIM_Rhsim04G0012700	2.82661762	0.025574282	0.044807272	RHSIM_Rhsim04G0012700
Root vs Leaf	RHSIM_Rhsim03G0243300	-1.262466793	0.025613622	0.044869404	RHSIM_Rhsim03G0243300

Comparison	Gene ID	log_2FC	P-value	FDR (padj)	Gene Name
Root vs Leaf	RHSIM_Rhsim11G0078200	1.269768047	0.025624242	0.044881212	RHSIM_Rhsim11G0078200
Root vs Leaf	novel.1669	-1.393800089	0.025665857	0.044943896	--
Root vs Leaf	RHSIM_Rhsim03G0028800	-1.409062093	0.025673351	0.044952288	RHSIM_Rhsim03G0028800
Root vs Leaf	RHSIM_Rhsim04G0196200	3.388207544	0.025844418	0.045218939	RHSIM_Rhsim04G0196200
Root vs Leaf	RHSIM_Rhsim05G0214200	1.099371062	0.025848898	0.045220278	RHSIM_Rhsim05G0214200
Root vs Leaf	RHSIM_Rhsim06G0098500	1.051544045	0.025895347	0.045290925	RHSIM_Rhsim06G0098500
Root vs Leaf	RHSIM_Rhsim05G0157000	-1.160007157	0.025920359	0.045331245	RHSIM_Rhsim05G0157000
Root vs Leaf	RHSIM_Rhsim10G0132400	2.588726211	0.025933448	0.045350708	RHSIM_Rhsim10G0132400
Root vs Leaf	RHSIM_Rhsim12G0004900	1.031209565	0.02597459	0.045419222	RHSIM_Rhsim12G0004900
Root vs Leaf	novel.635	-1.356045261	0.026001445	0.045462745	--
Root vs Leaf	RHSIM_Rhsim08G0236500	1.424016643	0.02612957	0.045655721	RHSIM_Rhsim08G0236500
Root vs Leaf	novel.5174	1.531316731	0.026200048	0.045761591	--
Root vs Leaf	RHSIM_RhsimUnG0254300	-1.134552981	0.026244424	0.045835639	RHSIM_RhsimUnG0254300
Root vs Leaf	RHSIM_Rhsim08G0218200	1.294552625	0.026247384	0.04583735	RHSIM_Rhsim08G0218200
Root vs Leaf	RHSIM_Rhsim12G0118200	1.458898016	0.026250118	0.045838664	RHSIM_Rhsim12G0118200
Root vs Leaf	RHSIM_Rhsim12G0201900	1.313922439	0.026331268	0.045976901	RHSIM_Rhsim12G0201900
Root vs Leaf	RHSIM_Rhsim12G0060500	1.147076968	0.026341263	0.045990884	RHSIM_Rhsim12G0060500
Root vs Leaf	RHSIM_Rhsim07G0215700	1.854181318	0.026377341	0.046046926	RHSIM_Rhsim07G0215700
Root vs Leaf	RHSIM_Rhsim10G0200500	-4.646261316	0.026398998	0.046081256	RHSIM_Rhsim10G0200500
Root vs Leaf	RHSIM_Rhsim02G0182100	1.178666448	0.026503153	0.046242136	RHSIM_Rhsim02G0182100
Root vs Leaf	RHSIM_Rhsim08G0018900	2.086597315	0.026553846	0.046320108	RHSIM_Rhsim08G0018900
Root vs Leaf	RHSIM_Rhsim07G0004800	1.224542804	0.026780045	0.046686531	RHSIM_Rhsim07G0004800
Root vs Leaf	novel.1748	1.389616081	0.026824726	0.04675738	--
Root vs Leaf	RHSIM_Rhsim07G0085800	1.686143441	0.026959813	0.046957477	RHSIM_Rhsim07G0085800
Root vs Leaf	novel.5949	1.094835222	0.026974639	0.046979763	--

Comparison	Gene ID	log_2FC	P-value	FDR (padj)	Gene Name
Root vs Leaf	novel.5778	-1.145118212	0.027004696	0.047028572	--
Root vs Leaf	novel.2613	1.33189912	0.027006795	0.047028689	--
Root vs Leaf	novel.2796	-1.032902133	0.027036227	0.047072855	--
Root vs Leaf	RHSIM_Rhsim07G0010900	3.553437023	0.027113947	0.047201073	RHSIM_Rhsim07G0010900
Root vs Leaf	RHSIM_Rhsim13G0169600	3.209215823	0.027148791	0.04725462	RHSIM_Rhsim13G0169600
Root vs Leaf	novel.3495	-8.632336676	0.027204202	0.047342369	--
Root vs Leaf	RHSIM_Rhsim12G0070800	1.60835219	0.027205342	0.047342369	RHSIM_Rhsim12G0070800
Root vs Leaf	RHSIM_Rhsim02G0051800	1.112176941	0.027280398	0.047456479	RHSIM_Rhsim02G0051800
Root vs Leaf	novel.4045	4.009474974	0.027306691	0.047490164	--
Root vs Leaf	novel.2039	-2.37487041	0.027337396	0.047532849	--
Root vs Leaf	RHSIM_Rhsim09G0124000	-1.564192731	0.027358979	0.047566802	RHSIM_Rhsim09G0124000
Root vs Leaf	novel.914	-1.340875303	0.027364876	0.047573481	--
Root vs Leaf	novel.1994	1.394865358	0.027381032	0.047597992	--
Root vs Leaf	RHSIM_Rhsim10G0096000	5.751751422	0.027556985	0.047882278	RHSIM_Rhsim10G0096000
Root vs Leaf	RHSIM_Rhsim02G0069900	1.409623961	0.027654787	0.048030578	RHSIM_Rhsim02G0069900
Root vs Leaf	RHSIM_Rhsim10G0170500	1.500595559	0.027701604	0.048104668	RHSIM_Rhsim10G0170500
Root vs Leaf	novel.2717	1.389079688	0.027713086	0.048120996	--
Root vs Leaf	RHSIM_Rhsim09G0167800	-1.097565428	0.027769712	0.048212086	RHSIM_Rhsim09G0167800
Root vs Leaf	novel.4557	-1.383853887	0.027941599	0.048495953	--
Root vs Leaf	RHSIM_Rhsim10G0079200	1.484673769	0.02796647	0.048535479	RHSIM_Rhsim10G0079200
Root vs Leaf	RHSIM_Rhsim10G0172000	-1.315723921	0.02801933	0.048617342	RHSIM_Rhsim10G0172000
Root vs Leaf	novel.1053	-1.458484469	0.02803472	0.048639335	--
Root vs Leaf	RHSIM_Rhsim03G0170500	1.463276682	0.028078631	0.048708215	RHSIM_Rhsim03G0170500
Root vs Leaf	RHSIM_Rhsim01G0209900	1.645113434	0.028232149	0.048952507	RHSIM_Rhsim01G0209900
Root vs Leaf	RHSIM_Rhsim04G0085400	1.05854906	0.028274161	0.049021679	RHSIM_Rhsim04G0085400

Comparison	Gene ID	log_2FC	P-value	FDR (padj)	Gene Name
Root vs Leaf	novel.4402	1.295967239	0.028452129	0.049300771	--
Root vs Leaf	novel.654	-8.563813051	0.028452176	0.049300771	--
Root vs Leaf	novel.5160	4.89066865	0.028460498	0.0493115	--
Root vs Leaf	RHSIM_RhsimUnG0112600	-1.212860888	0.028579212	0.04950134	RHSIM_RhsimUnG0112600
Root vs Leaf	RHSIM_Rhsim12G0011200	1.095914615	0.028653957	0.049624399	RHSIM_Rhsim12G0011200
Root vs Leaf	novel.5027	-3.394007315	0.02867302	0.049642552	--
Root vs Leaf	RHSIM_Rhsim12G0134300	-1.06393212	0.028694871	0.049675121	RHSIM_Rhsim12G0134300
Root vs Leaf	RHSIM_Rhsim11G0144000	4.608307627	0.028799114	0.049842219	RHSIM_Rhsim11G0144000
Root vs Leaf	RHSIM_Rhsim12G0210200	-1.070431992	0.028802863	0.049844978	RHSIM_Rhsim12G0210200
Root vs Leaf	RHSIM_RhsimUnG0246600	1.689350404	0.028813714	0.049860029	RHSIM_RhsimUnG0246600
Root vs Leaf	RHSIM_Rhsim12G0003900	2.633661178	0.028842218	0.049903035	RHSIM_Rhsim12G0003900
Root vs Leaf	novel.2986	1.19984128	0.02884288	0.049903035	--
Root vs Leaf	novel.399	-1.122559601	0.028872325	0.04994651	--
Root vs Stem	RHSIM_Rhsim09G0152700	-6.765549818	1.1735E-166	2.7053E-162	RHSIM_Rhsim09G0152700
Root vs Stem	RHSIM_Rhsim01G0233700	-4.955624052	3.0137E-143	3.4737E-139	RHSIM_Rhsim01G0233700
Root vs Stem	RHSIM_Rhsim12G0015600	-5.318169801	2.9355E-113	2.2557E-109	RHSIM_Rhsim12G0015600
Root vs Stem	RHSIM_Rhsim05G0073400	-6.128657082	1.77481E-93	1.02287E-89	RHSIM_Rhsim05G0073400
Root vs Stem	RHSIM_Rhsim06G0108600	-4.254020224	9.11406E-91	4.20213E-87	RHSIM_Rhsim06G0108600
Root vs Stem	RHSIM_Rhsim08G0223300	-2.663389637	3.0739E-90	1.18104E-86	RHSIM_Rhsim08G0223300
Root vs Stem	RHSIM_Rhsim08G0197600	4.173829524	3.77198E-89	1.24222E-85	RHSIM_Rhsim08G0197600
Root vs Stem	RHSIM_RhsimUnG0040900	-3.783817175	6.29725E-82	1.81463E-78	RHSIM_RhsimUnG0040900
Root vs Stem	RHSIM_Rhsim11G0055800	-6.83385053	1.34995E-78	3.45781E-75	RHSIM_Rhsim11G0055800
Root vs Stem	RHSIM_Rhsim13G0150200	-7.1281668	7.7537E-78	1.78746E-74	RHSIM_Rhsim13G0150200
Root vs Stem	RHSIM_Rhsim13G0068500	-4.202586575	1.48049E-75	3.10271E-72	RHSIM_Rhsim13G0068500
Root vs Stem	RHSIM_Rhsim13G0210400	3.324834007	2.32735E-74	4.47103E-71	RHSIM_Rhsim13G0210400

Comparison	Gene ID	log_2FC	P-value	FDR (padj)	Gene Name
Root vs Stem	RHSIM_Rhsim06G0207400	3.586906277	2.09949E-73	3.72303E-70	RHSIM_Rhsim06G0207400
Root vs Stem	RHSIM_Rhsim07G0100400	-6.301439831	3.1075E-70	5.11695E-67	RHSIM_Rhsim07G0100400
Root vs Stem	RHSIM_Rhsim12G0099800	-3.975505193	1.07886E-62	1.65807E-59	RHSIM_Rhsim12G0099800
Root vs Stem	novel.1069	-9.043502944	4.59098E-61	6.61474E-58	--
Root vs Stem	RHSIM_Rhsim07G0251100	-8.857987569	5.60273E-61	7.59763E-58	RHSIM_Rhsim07G0251100
Root vs Stem	RHSIM_Rhsim12G0087000	-4.555769364	7.03442E-59	9.00914E-56	RHSIM_Rhsim12G0087000
Root vs Stem	RHSIM_Rhsim01G0107200	6.922239404	2.00032E-57	2.42702E-54	RHSIM_Rhsim01G0107200
Root vs Stem	RHSIM_Rhsim11G0127300	-2.509443528	6.95825E-57	8.02042E-54	RHSIM_Rhsim11G0127300
Root vs Stem	RHSIM_Rhsim04G0032000	-4.47791056	2.12005E-56	2.32731E-53	RHSIM_Rhsim04G0032000
Root vs Stem	RHSIM_Rhsim09G0143500	6.743458521	1.22069E-54	1.27912E-51	RHSIM_Rhsim09G0143500
Root vs Stem	RHSIM_Rhsim04G0083400	3.704029268	4.31893E-52	4.32888E-49	RHSIM_Rhsim04G0083400
Root vs Stem	RHSIM_Rhsim09G0146100	-2.078644499	1.37161E-51	1.31749E-48	RHSIM_Rhsim09G0146100
Root vs Stem	RHSIM_Rhsim12G0036900	-3.445504501	1.90582E-51	1.7574E-48	RHSIM_Rhsim12G0036900
Root vs Stem	RHSIM_Rhsim09G0131800	4.078957853	1.18726E-50	1.05269E-47	RHSIM_Rhsim09G0131800
Root vs Stem	RHSIM_Rhsim11G0135900	-2.713257167	3.98641E-50	3.40365E-47	RHSIM_Rhsim11G0135900
Root vs Stem	RHSIM_Rhsim06G0171100	-3.175750746	2.05679E-49	1.69218E-46	RHSIM_Rhsim06G0171100
Root vs Stem	RHSIM_Rhsim03G0131600	-2.928376401	2.12871E-49	1.69218E-46	RHSIM_Rhsim03G0131600
Root vs Stem	RHSIM_Rhsim13G0083600	-5.673696294	2.81146E-47	2.16042E-44	RHSIM_Rhsim13G0083600
Root vs Stem	RHSIM_Rhsim11G0098700	-4.118071685	2.94163E-47	2.18753E-44	RHSIM_Rhsim11G0098700
Root vs Stem	RHSIM_Rhsim13G0041600	-3.223032155	1.10953E-46	7.99314E-44	RHSIM_Rhsim13G0041600
Root vs Stem	RHSIM_RhsimUnG0258600	-5.917579422	8.67234E-45	6.05828E-42	RHSIM_RhsimUnG0258600
Root vs Stem	RHSIM_Rhsim07G0020900	-4.058649587	9.8956E-45	6.70951E-42	RHSIM_Rhsim07G0020900
Root vs Stem	RHSIM_Rhsim08G0193800	-5.777628993	1.17662E-44	7.74986E-42	RHSIM_Rhsim08G0193800
Root vs Stem	RHSIM_RhsimUnG0122900	-4.036241159	2.92961E-44	1.87601E-41	RHSIM_RhsimUnG0122900
Root vs Stem	RHSIM_Rhsim09G0094600	7.246856054	3.09123E-44	1.926E-41	RHSIM_Rhsim09G0094600

Comparison	Gene ID	log_2FC	P-value	FDR (padj)	Gene Name
Root vs Stem	RHSIM_Rhsim06G0039700	4.918767614	6.19084E-44	3.75572E-41	RHSIM_Rhsim06G0039700
Root vs Stem	RHSIM_Rhsim13G0219600	-4.406875154	3.57174E-43	2.11127E-40	RHSIM_Rhsim13G0219600
Root vs Stem	RHSIM_RhsimUnG0255500	2.594274107	6.29618E-43	3.62865E-40	RHSIM_RhsimUnG0255500
Root vs Stem	RHSIM_Rhsim07G0124100	-3.039485005	9.5828E-43	5.38811E-40	RHSIM_Rhsim07G0124100
Root vs Stem	RHSIM_Rhsim11G0077200	-5.857999878	1.3097E-42	7.18869E-40	RHSIM_Rhsim11G0077200
Root vs Stem	RHSIM_Rhsim10G0187700	-5.331612507	1.63149E-41	8.7467E-39	RHSIM_Rhsim10G0187700
Root vs Stem	RHSIM_Rhsim03G0114800	4.136012786	1.68331E-41	8.8194E-39	RHSIM_Rhsim03G0114800
Root vs Stem	RHSIM_Rhsim02G0132700	-2.622999892	1.74888E-41	8.9593E-39	RHSIM_Rhsim02G0132700
Root vs Stem	RHSIM_Rhsim09G0084200	3.381268422	2.0499E-41	1.02731E-38	RHSIM_Rhsim09G0084200
Root vs Stem	RHSIM_Rhsim03G0097000	3.135234941	2.18126E-41	1.06989E-38	RHSIM_Rhsim03G0097000
Root vs Stem	RHSIM_Rhsim12G0149600	2.998694991	4.28104E-41	2.05606E-38	RHSIM_Rhsim12G0149600
Root vs Stem	RHSIM_Rhsim11G0137800	-3.215700163	4.71537E-41	2.21844E-38	RHSIM_Rhsim11G0137800
Root vs Stem	RHSIM_Rhsim13G0179900	2.063236097	1.657E-40	7.63976E-38	RHSIM_Rhsim13G0179900
Root vs Stem	RHSIM_Rhsim06G0083500	5.423867473	2.14256E-40	9.6848E-38	RHSIM_Rhsim06G0083500
Root vs Stem	RHSIM_Rhsim01G0203000	-5.151197336	2.36182E-40	1.04706E-37	RHSIM_Rhsim01G0203000
Root vs Stem	RHSIM_Rhsim04G0206300	-2.107263933	8.66313E-40	3.76813E-37	RHSIM_Rhsim04G0206300
Root vs Stem	RHSIM_Rhsim07G0251400	-2.175733232	1.36048E-39	5.80799E-37	RHSIM_Rhsim07G0251400
Root vs Stem	RHSIM_Rhsim10G0045100	-2.738631805	4.25132E-39	1.78192E-36	RHSIM_Rhsim10G0045100
Root vs Stem	RHSIM_RhsimUnG0057600	-4.294354307	6.52352E-39	2.68548E-36	RHSIM_RhsimUnG0057600
Root vs Stem	RHSIM_Rhsim13G0057000	6.18381395	1.40591E-38	5.68603E-36	RHSIM_Rhsim13G0057000
Root vs Stem	RHSIM_Rhsim13G0023600	2.030096383	1.9462E-38	7.73547E-36	RHSIM_Rhsim13G0023600
Root vs Stem	RHSIM_Rhsim03G0009200	-4.168853325	2.35741E-38	9.21107E-36	RHSIM_Rhsim03G0009200
Root vs Stem	RHSIM_Rhsim06G0198700	-6.568636838	5.67901E-38	2.18197E-35	RHSIM_Rhsim06G0198700
Root vs Stem	RHSIM_Rhsim08G0147100	-2.753453097	1.12744E-37	4.26081E-35	RHSIM_Rhsim08G0147100
Root vs Stem	RHSIM_Rhsim04G0230600	6.90289328	1.95954E-37	7.28603E-35	RHSIM_Rhsim04G0230600

Comparison	Gene ID	log_2FC	P-value	FDR (padj)	Gene Name
Root vs Stem	RHSIM_Rhsim08G0096200	-2.330110769	8.85465E-37	3.2401E-34	RHSIM_Rhsim08G0096200
Root vs Stem	RHSIM_Rhsim12G0153200	-3.685734613	1.68106E-35	6.05523E-33	RHSIM_Rhsim12G0153200
Root vs Stem	RHSIM_Rhsim12G0155900	-2.297327089	1.94275E-35	6.89018E-33	RHSIM_Rhsim12G0155900
Root vs Stem	RHSIM_Rhsim05G0077800	6.718020034	3.57161E-35	1.23889E-32	RHSIM_Rhsim05G0077800
Root vs Stem	novel.1961	-2.422172689	3.60063E-35	1.23889E-32	--
Root vs Stem	RHSIM_Rhsim09G0087100	4.394894215	4.08873E-35	1.38614E-32	RHSIM_Rhsim09G0087100
Root vs Stem	RHSIM_Rhsim04G0230200	6.193054905	6.82421E-35	2.27998E-32	RHSIM_Rhsim04G0230200
Root vs Stem	RHSIM_Rhsim08G0120000	-1.863142452	1.37813E-34	4.53857E-32	RHSIM_Rhsim08G0120000
Root vs Stem	RHSIM_Rhsim10G0141000	-5.990554035	2.74645E-34	8.91744E-32	RHSIM_Rhsim10G0141000
Root vs Stem	novel.1987	5.767953732	4.04778E-34	1.29602E-31	--
Root vs Stem	RHSIM_Rhsim05G0214000	-5.296125044	6.4893E-34	2.04928E-31	RHSIM_Rhsim05G0214000
Root vs Stem	RHSIM_Rhsim09G0117700	1.175359182	9.9156E-34	3.07604E-31	RHSIM_Rhsim09G0117700
Root vs Stem	RHSIM_Rhsim02G0002000	-1.47614044	1.00075E-33	3.07604E-31	RHSIM_Rhsim02G0002000
Root vs Stem	RHSIM_RhsimUnG0204600	-4.477673313	2.35795E-33	7.15234E-31	RHSIM_RhsimUnG0204600
Root vs Stem	RHSIM_Rhsim01G0233900	-3.603306575	3.04545E-33	9.11777E-31	RHSIM_Rhsim01G0233900
Root vs Stem	RHSIM_Rhsim01G0201900	-4.002392322	3.09491E-33	9.14704E-31	RHSIM_Rhsim01G0201900
Root vs Stem	RHSIM_Rhsim10G0010300	2.865313633	9.62429E-33	2.80847E-30	RHSIM_Rhsim10G0010300
Root vs Stem	RHSIM_Rhsim10G0102500	-3.915638198	1.1715E-32	3.36727E-30	RHSIM_Rhsim10G0102500
Root vs Stem	RHSIM_Rhsim09G0067100	-3.296157728	1.18314E-32	3.36727E-30	RHSIM_Rhsim09G0067100
Root vs Stem	RHSIM_Rhsim09G0093800	-2.34359666	1.29334E-32	3.63602E-30	RHSIM_Rhsim09G0093800
Root vs Stem	RHSIM_Rhsim04G0169500	-4.068174646	2.47736E-32	6.88079E-30	RHSIM_Rhsim04G0169500
Root vs Stem	RHSIM_Rhsim10G0012300	4.106647184	2.73454E-32	7.50468E-30	RHSIM_Rhsim10G0012300
Root vs Stem	RHSIM_Rhsim12G0000600	-3.65846519	3.94671E-32	1.07039E-29	RHSIM_Rhsim12G0000600
Root vs Stem	RHSIM_Rhsim09G0095600	8.359851446	4.63814E-32	1.24329E-29	RHSIM_Rhsim09G0095600
Root vs Stem	RHSIM_Rhsim04G0240600	3.014560312	7.47268E-32	1.98009E-29	RHSIM_Rhsim04G0240600

Comparison	Gene ID	log_2FC	P-value	FDR (padj)	Gene Name
Root vs Stem	RHSIM_Rhsim02G0044700	4.323534498	1.29834E-31	3.40121E-29	RHSIM_Rhsim02G0044700
Root vs Stem	RHSIM_Rhsim05G0007400	3.368673932	1.66647E-31	4.31652E-29	RHSIM_Rhsim05G0007400
Root vs Stem	RHSIM_Rhsim07G0203500	3.968746687	1.96954E-31	5.02395E-29	RHSIM_Rhsim07G0203500
Root vs Stem	RHSIM_Rhsim12G0007700	3.106808217	1.98317E-31	5.02395E-29	RHSIM_Rhsim12G0007700
Root vs Stem	RHSIM_Rhsim07G0033800	6.061250398	2.28323E-31	5.72124E-29	RHSIM_Rhsim07G0033800
Root vs Stem	novel.28	-3.88326083	4.15983E-31	1.03115E-28	--
Root vs Stem	RHSIM_Rhsim08G0055700	-2.813679874	4.38883E-31	1.07634E-28	RHSIM_Rhsim08G0055700
Root vs Stem	RHSIM_Rhsim12G0214000	2.868686397	5.04177E-31	1.22345E-28	RHSIM_Rhsim12G0214000
Root vs Stem	RHSIM_Rhsim01G0138100	-4.932398489	1.18288E-30	2.84051E-28	RHSIM_Rhsim01G0138100
Root vs Stem	RHSIM_Rhsim06G0002500	4.731135646	1.37996E-30	3.27961E-28	RHSIM_Rhsim06G0002500
Root vs Stem	RHSIM_Rhsim02G0158200	-1.736303633	1.49655E-30	3.5204E-28	RHSIM_Rhsim02G0158200
Root vs Stem	RHSIM_Rhsim02G0001800	3.718941578	2.0859E-30	4.8572E-28	RHSIM_Rhsim02G0001800
Root vs Stem	RHSIM_Rhsim02G0006500	-3.986479526	3.15966E-30	7.28396E-28	RHSIM_Rhsim02G0006500
Root vs Stem	novel.268	3.171001531	8.28672E-30	1.89142E-27	--
Root vs Stem	RHSIM_Rhsim04G0038600	-3.08012494	8.60594E-30	1.94503E-27	RHSIM_Rhsim04G0038600
Root vs Stem	novel.3076	-2.419914026	1.39445E-29	3.12099E-27	--
Root vs Stem	RHSIM_Rhsim06G0172700	2.03407907	1.54542E-29	3.3976E-27	RHSIM_Rhsim06G0172700
Root vs Stem	RHSIM_Rhsim04G0170500	6.662059349	1.54751E-29	3.3976E-27	RHSIM_Rhsim04G0170500
Root vs Stem	RHSIM_Rhsim02G0218200	2.58039241	2.25484E-29	4.90386E-27	RHSIM_Rhsim02G0218200
Root vs Stem	RHSIM_RhsimUnG0187900	-3.527443913	3.58077E-29	7.71471E-27	RHSIM_RhsimUnG0187900
Root vs Stem	RHSIM_Rhsim07G0135000	-1.980958726	3.63702E-29	7.76336E-27	RHSIM_Rhsim07G0135000
Root vs Stem	RHSIM_Rhsim06G0148000	-5.926370011	3.91405E-29	8.27804E-27	RHSIM_Rhsim06G0148000
Root vs Stem	RHSIM_Rhsim11G0138400	-3.538717599	4.08094E-29	8.55254E-27	RHSIM_Rhsim11G0138400
Root vs Stem	RHSIM_Rhsim03G0191300	-3.955879953	4.49134E-29	9.25299E-27	RHSIM_Rhsim03G0191300
Root vs Stem	novel.4118	3.824706208	4.49544E-29	9.25299E-27	--

Comparison	Gene ID	log_2FC	P-value	FDR (padj)	Gene Name
Root vs Stem	RHSIM_Rhsim07G0025800	-2.912920034	5.76528E-29	1.17617E-26	RHSIM_Rhsim07G0025800
Root vs Stem	RHSIM_Rhsim06G0237200	-3.296345451	7.66952E-29	1.55092E-26	RHSIM_Rhsim06G0237200
Root vs Stem	RHSIM_Rhsim11G0165400	6.130172557	9.90484E-29	1.98553E-26	RHSIM_Rhsim11G0165400
Root vs Stem	RHSIM_Rhsim06G0059900	5.595987768	1.22946E-28	2.44333E-26	RHSIM_Rhsim06G0059900
Root vs Stem	novel.1066	4.416479831	1.29945E-28	2.56037E-26	--
Root vs Stem	RHSIM_Rhsim11G0102000	-6.547765891	1.33889E-28	2.6157E-26	RHSIM_Rhsim11G0102000
Root vs Stem	novel.2485	-3.066008627	2.49643E-28	4.83615E-26	--
Root vs Stem	RHSIM_Rhsim09G0191800	-4.579882619	2.62408E-28	5.04108E-26	RHSIM_Rhsim09G0191800
Root vs Stem	RHSIM_Rhsim07G0226700	2.664227178	3.24146E-28	6.17565E-26	RHSIM_Rhsim07G0226700
Root vs Stem	RHSIM_Rhsim09G0170000	-5.096183567	3.45206E-28	6.52299E-26	RHSIM_Rhsim09G0170000
Root vs Stem	RHSIM_Rhsim11G0135800	-3.449429862	3.98529E-28	7.46934E-26	RHSIM_Rhsim11G0135800
Root vs Stem	RHSIM_Rhsim03G0053000	1.904835758	4.60708E-28	8.56509E-26	RHSIM_Rhsim03G0053000
Root vs Stem	RHSIM_Rhsim08G0158800	5.681300767	1.59515E-27	2.94183E-25	RHSIM_Rhsim08G0158800
Root vs Stem	RHSIM_Rhsim13G0028100	-2.455730328	2.31959E-27	4.24392E-25	RHSIM_Rhsim13G0028100
Root vs Stem	RHSIM_Rhsim10G0186800	3.041234225	3.15111E-27	5.71988E-25	RHSIM_Rhsim10G0186800
Root vs Stem	RHSIM_Rhsim06G0169500	3.878465769	3.89796E-27	7.02028E-25	RHSIM_Rhsim06G0169500
Root vs Stem	RHSIM_Rhsim06G0122400	2.368365589	5.96676E-27	1.06629E-24	RHSIM_Rhsim06G0122400
Root vs Stem	RHSIM_Rhsim11G0154100	-2.42147789	6.75325E-27	1.19756E-24	RHSIM_Rhsim11G0154100
Root vs Stem	RHSIM_Rhsim11G0145400	7.483617541	7.4309E-27	1.30767E-24	RHSIM_Rhsim11G0145400
Root vs Stem	RHSIM_Rhsim11G0183800	-3.141007373	8.07329E-27	1.40995E-24	RHSIM_Rhsim11G0183800
Root vs Stem	RHSIM_Rhsim07G0184400	2.393656604	8.2176E-27	1.42436E-24	RHSIM_Rhsim07G0184400
Root vs Stem	RHSIM_Rhsim08G0029700	-6.730978818	9.28915E-27	1.59808E-24	RHSIM_Rhsim08G0029700
Root vs Stem	RHSIM_Rhsim07G0007900	-2.943456283	1.26506E-26	2.16025E-24	RHSIM_Rhsim07G0007900
Root vs Stem	RHSIM_Rhsim13G0127200	-2.548030613	1.71092E-26	2.90014E-24	RHSIM_Rhsim13G0127200
Root vs Stem	RHSIM_Rhsim13G0171500	-6.27484344	1.89228E-26	3.18415E-24	RHSIM_Rhsim13G0171500

Comparison	Gene ID	log_2FC	P-value	FDR (padj)	Gene Name
Root vs Stem	RHSIM_Rhsim08G0064400	2.4094649	1.95563E-26	3.26689E-24	RHSIM_Rhsim08G0064400
Root vs Stem	RHSIM_Rhsim11G0107000	-5.097938833	1.97903E-26	3.2822E-24	RHSIM_Rhsim11G0107000
Root vs Stem	RHSIM_Rhsim02G0143300	2.13899798	2.32282E-26	3.82485E-24	RHSIM_Rhsim02G0143300
Root vs Stem	RHSIM_Rhsim12G0139100	-3.3620053	2.98269E-26	4.86823E-24	RHSIM_Rhsim12G0139100
Root vs Stem	RHSIM_Rhsim05G0121700	2.259042602	2.99869E-26	4.86823E-24	RHSIM_Rhsim05G0121700
Root vs Stem	RHSIM_Rhsim06G0212700	4.915302751	3.20885E-26	5.17298E-24	RHSIM_Rhsim06G0212700
Root vs Stem	RHSIM_Rhsim05G0187600	-4.276669124	3.30144E-26	5.28529E-24	RHSIM_Rhsim05G0187600
Root vs Stem	RHSIM_Rhsim06G0021000	2.568388655	3.39546E-26	5.39832E-24	RHSIM_Rhsim06G0021000
Root vs Stem	RHSIM_Rhsim12G0149800	2.928796944	3.94389E-26	6.2273E-24	RHSIM_Rhsim12G0149800
Root vs Stem	RHSIM_Rhsim03G0226500	-2.690437805	4.83787E-26	7.58691E-24	RHSIM_Rhsim03G0226500
Root vs Stem	RHSIM_RhsimUnG0136700	-2.711667486	5.29871E-26	8.25345E-24	RHSIM_RhsimUnG0136700
Root vs Stem	RHSIM_Rhsim11G0088900	-1.991068686	5.71412E-26	8.81063E-24	RHSIM_Rhsim11G0088900
Root vs Stem	RHSIM_Rhsim06G0178000	-2.336101747	5.73285E-26	8.81063E-24	RHSIM_Rhsim06G0178000
Root vs Stem	RHSIM_Rhsim02G0254500	2.529411957	6.62414E-26	1.0113E-23	RHSIM_Rhsim02G0254500
Root vs Stem	RHSIM_Rhsim04G0032800	3.660403381	6.97638E-26	1.05807E-23	RHSIM_Rhsim04G0032800
Root vs Stem	RHSIM_Rhsim08G0242400	-4.375131889	8.51025E-26	1.28227E-23	RHSIM_Rhsim08G0242400
Root vs Stem	RHSIM_Rhsim13G0021200	-1.637445945	1.04069E-25	1.55786E-23	RHSIM_Rhsim13G0021200
Root vs Stem	RHSIM_Rhsim11G0117500	-5.879659619	1.07931E-25	1.60525E-23	RHSIM_Rhsim11G0117500
Root vs Stem	RHSIM_Rhsim03G0074900	2.397506944	1.46632E-25	2.16686E-23	RHSIM_Rhsim03G0074900
Root vs Stem	RHSIM_Rhsim08G0058000	4.236003123	2.43458E-25	3.5748E-23	RHSIM_Rhsim08G0058000
Root vs Stem	RHSIM_Rhsim07G0145700	4.121776731	2.53506E-25	3.69878E-23	RHSIM_Rhsim07G0145700
Root vs Stem	RHSIM_Rhsim05G0200400	3.719856142	3.06156E-25	4.43888E-23	RHSIM_Rhsim05G0200400
Root vs Stem	RHSIM_Rhsim09G0142500	3.08644654	3.82756E-25	5.5148E-23	RHSIM_Rhsim09G0142500
Root vs Stem	RHSIM_Rhsim11G0059000	3.140173607	3.85314E-25	5.51717E-23	RHSIM_Rhsim11G0059000
Root vs Stem	RHSIM_Rhsim08G0008700	2.347686735	4.21701E-25	6.00091E-23	RHSIM_Rhsim08G0008700

Comparison	Gene ID	log_2FC	P-value	FDR (padj)	Gene Name
Root vs Stem	RHSIM_Rhsim02G0140700	-2.127218239	4.27235E-25	6.04236E-23	RHSIM_Rhsim02G0140700
Root vs Stem	RHSIM_Rhsim13G0210300	3.667795447	4.39059E-25	6.17172E-23	RHSIM_Rhsim13G0210300
Root vs Stem	novel.71	-2.29882881	4.56694E-25	6.3712E-23	--
Root vs Stem	RHSIM_Rhsim13G0119400	-4.908121679	4.58777E-25	6.3712E-23	RHSIM_Rhsim13G0119400
Root vs Stem	RHSIM_Rhsim13G0227100	-4.961217089	6.28147E-25	8.67106E-23	RHSIM_Rhsim13G0227100
Root vs Stem	RHSIM_Rhsim01G0126800	-2.150327681	6.80397E-25	9.33643E-23	RHSIM_Rhsim01G0126800
Root vs Stem	RHSIM_Rhsim10G0211400	-1.783144623	6.98183E-25	9.52379E-23	RHSIM_Rhsim10G0211400
Root vs Stem	RHSIM_Rhsim10G0072000	4.525248604	7.22584E-25	9.79866E-23	RHSIM_Rhsim10G0072000
Root vs Stem	RHSIM_Rhsim12G0185700	-4.470779793	7.76157E-25	1.04636E-22	RHSIM_Rhsim12G0185700
Root vs Stem	RHSIM_Rhsim04G0081700	2.482811584	1.14577E-24	1.53566E-22	RHSIM_Rhsim04G0081700
Root vs Stem	RHSIM_Rhsim13G0121500	-3.439885156	1.15716E-24	1.54196E-22	RHSIM_Rhsim13G0121500
Root vs Stem	RHSIM_Rhsim06G0148500	-3.204298242	1.21209E-24	1.60588E-22	RHSIM_Rhsim06G0148500
Root vs Stem	RHSIM_Rhsim03G0197600	3.035977986	1.23603E-24	1.62823E-22	RHSIM_Rhsim03G0197600
Root vs Stem	RHSIM_RhsimUnG0024000	-2.897657314	1.42029E-24	1.847E-22	RHSIM_RhsimUnG0024000
Root vs Stem	RHSIM_Rhsim07G0231600	4.176247804	1.42374E-24	1.847E-22	RHSIM_Rhsim07G0231600
Root vs Stem	RHSIM_Rhsim04G0104300	2.990894772	1.42613E-24	1.847E-22	RHSIM_Rhsim04G0104300
Root vs Stem	RHSIM_Rhsim05G0175500	12.45872474	1.88347E-24	2.42568E-22	RHSIM_Rhsim05G0175500
Root vs Stem	RHSIM_Rhsim09G0037500	-2.993637232	2.18775E-24	2.8019E-22	RHSIM_Rhsim09G0037500
Root vs Stem	RHSIM_RhsimUnG0094100	-4.210970404	2.30186E-24	2.93176E-22	RHSIM_RhsimUnG0094100
Root vs Stem	RHSIM_Rhsim01G0189000	-2.299094202	2.55285E-24	3.23356E-22	RHSIM_Rhsim01G0189000
Root vs Stem	RHSIM_Rhsim09G0109900	2.418679824	2.85276E-24	3.5937E-22	RHSIM_Rhsim09G0109900
Root vs Stem	RHSIM_Rhsim05G0011500	-5.014838347	2.91008E-24	3.64599E-22	RHSIM_Rhsim05G0011500
Root vs Stem	RHSIM_Rhsim06G0031900	-2.117585396	3.11021E-24	3.87565E-22	RHSIM_Rhsim06G0031900
Root vs Stem	novel.5470	2.89801882	3.20979E-24	3.97824E-22	--
Root vs Stem	RHSIM_Rhsim04G0162800	1.689552991	3.24796E-24	4.00403E-22	RHSIM_Rhsim04G0162800

Comparison	Gene ID	log_2FC	P-value	FDR (padj)	Gene Name
Root vs Stem	RHSIM_Rhsim11G0173100	3.789818103	3.83077E-24	4.69738E-22	RHSIM_Rhsim11G0173100
Root vs Stem	RHSIM_Rhsim05G0179500	2.418953994	4.08722E-24	4.98533E-22	RHSIM_Rhsim05G0179500
Root vs Stem	RHSIM_Rhsim10G0029800	3.21570065	4.1881E-24	5.08148E-22	RHSIM_Rhsim10G0029800
Root vs Stem	RHSIM_Rhsim01G0101900	-6.92417504	4.26803E-24	5.15136E-22	RHSIM_Rhsim01G0101900
Root vs Stem	RHSIM_Rhsim08G0038400	5.353665047	4.33922E-24	5.21E-22	RHSIM_Rhsim08G0038400
Root vs Stem	RHSIM_Rhsim12G0197900	-1.392482543	6.34102E-24	7.57407E-22	RHSIM_Rhsim12G0197900
Root vs Stem	RHSIM_Rhsim12G0114000	3.884670546	6.49898E-24	7.72273E-22	RHSIM_Rhsim12G0114000
Root vs Stem	RHSIM_Rhsim04G0106400	3.325059568	6.77774E-24	8.01267E-22	RHSIM_Rhsim04G0106400
Root vs Stem	RHSIM_Rhsim13G0062200	6.852874663	8.41271E-24	9.89481E-22	RHSIM_Rhsim13G0062200
Root vs Stem	RHSIM_Rhsim02G0090600	7.437188227	9.99588E-24	1.16972E-21	RHSIM_Rhsim02G0090600
Root vs Stem	RHSIM_Rhsim13G0116300	-3.866229612	1.04768E-23	1.2198E-21	RHSIM_Rhsim13G0116300
Root vs Stem	RHSIM_Rhsim08G0245900	2.563769885	1.25917E-23	1.45867E-21	RHSIM_Rhsim08G0245900
Root vs Stem	RHSIM_Rhsim07G0229500	-4.710371266	1.28133E-23	1.47693E-21	RHSIM_Rhsim07G0229500
Root vs Stem	RHSIM_Rhsim05G0226900	1.861506832	1.30274E-23	1.49413E-21	RHSIM_Rhsim05G0226900
Root vs Stem	RHSIM_Rhsim13G0094000	-1.664647508	1.39815E-23	1.59562E-21	RHSIM_Rhsim13G0094000
Root vs Stem	RHSIM_Rhsim06G0244400	-2.752012578	1.42145E-23	1.61422E-21	RHSIM_Rhsim06G0244400
Root vs Stem	RHSIM_Rhsim05G0071600	4.621876782	1.45204E-23	1.64088E-21	RHSIM_Rhsim05G0071600
Root vs Stem	RHSIM_Rhsim13G0126100	3.995390798	1.5531E-23	1.74652E-21	RHSIM_Rhsim13G0126100
Root vs Stem	RHSIM_Rhsim02G0211200	-12.15375125	1.6428E-23	1.8374E-21	RHSIM_Rhsim02G0211200
Root vs Stem	novel.4165	-4.320402866	1.64986E-23	1.8374E-21	--
Root vs Stem	RHSIM_Rhsim08G0014700	2.741500449	2.08952E-23	2.31585E-21	RHSIM_Rhsim08G0014700
Root vs Stem	RHSIM_RhsimUnG0115200	6.818706951	2.22807E-23	2.45759E-21	RHSIM_RhsimUnG0115200
Root vs Stem	novel.402	-2.509990829	2.39335E-23	2.62732E-21	--
Root vs Stem	novel.6020	8.638111199	3.06382E-23	3.34741E-21	--
Root vs Stem	RHSIM_Rhsim13G0154000	-2.657563833	3.75262E-23	4.07501E-21	RHSIM_Rhsim13G0154000

Comparison	Gene ID	log_2FC	P-value	FDR (padj)	Gene Name
Root vs Stem	RHSIM_Rhsim08G0123400	1.651138645	3.76513E-23	4.07501E-21	RHSIM_Rhsim08G0123400
Root vs Stem	RHSIM_Rhsim11G0066400	5.101091244	3.95658E-23	4.2622E-21	RHSIM_Rhsim11G0066400
Root vs Stem	RHSIM_Rhsim04G0225200	-3.824460166	4.67394E-23	5.01155E-21	RHSIM_Rhsim04G0225200
Root vs Stem	RHSIM_Rhsim12G0081400	-2.379810371	4.93315E-23	5.265E-21	RHSIM_Rhsim12G0081400
Root vs Stem	RHSIM_Rhsim02G0044500	3.913290022	5.58585E-23	5.93413E-21	RHSIM_Rhsim02G0044500
Root vs Stem	RHSIM_Rhsim07G0037000	4.591367574	6.63209E-23	7.01328E-21	RHSIM_Rhsim07G0037000
Root vs Stem	RHSIM_Rhsim10G0013000	-3.852620141	6.75422E-23	7.10982E-21	RHSIM_Rhsim10G0013000
Root vs Stem	novel.5389	4.647309533	7.62355E-23	7.98844E-21	--
Root vs Stem	RHSIM_Rhsim12G0164900	3.177330564	1.14412E-22	1.19345E-20	RHSIM_Rhsim12G0164900
Root vs Stem	RHSIM_Rhsim06G0087800	2.290893516	1.22002E-22	1.26256E-20	RHSIM_Rhsim06G0087800
Root vs Stem	RHSIM_Rhsim02G0116900	7.780863846	1.22132E-22	1.26256E-20	RHSIM_Rhsim02G0116900
Root vs Stem	novel.1501	-4.088827803	1.30816E-22	1.34629E-20	--
Root vs Stem	RHSIM_Rhsim05G0114700	-5.690925534	1.39634E-22	1.43066E-20	RHSIM_Rhsim05G0114700
Root vs Stem	RHSIM_Rhsim10G0188100	-3.416669762	1.69407E-22	1.72803E-20	RHSIM_Rhsim10G0188100
Root vs Stem	RHSIM_Rhsim05G0094200	2.942880421	1.7566E-22	1.78392E-20	RHSIM_Rhsim05G0094200
Root vs Stem	novel.1228	2.709860737	1.77745E-22	1.79717E-20	--
Root vs Stem	RHSIM_Rhsim09G0143600	6.745449934	1.83459E-22	1.84685E-20	RHSIM_Rhsim09G0143600
Root vs Stem	RHSIM_Rhsim12G0128500	5.282072608	1.85812E-22	1.8624E-20	RHSIM_Rhsim12G0128500
Root vs Stem	RHSIM_Rhsim05G0208200	-5.854770998	1.92243E-22	1.91851E-20	RHSIM_Rhsim05G0208200
Root vs Stem	novel.2598	-6.209037472	1.95448E-22	1.94209E-20	--
Root vs Stem	RHSIM_Rhsim01G0201200	5.68366211	2.17608E-22	2.15301E-20	RHSIM_Rhsim01G0201200
Root vs Stem	RHSIM_Rhsim02G0237600	3.607031047	2.2433E-22	2.21004E-20	RHSIM_Rhsim02G0237600
Root vs Stem	RHSIM_Rhsim13G0083000	1.905778137	3.19933E-22	3.13848E-20	RHSIM_Rhsim13G0083000
Root vs Stem	RHSIM_Rhsim06G0054800	1.737339214	3.47279E-22	3.39229E-20	RHSIM_Rhsim06G0054800
Root vs Stem	RHSIM_Rhsim04G0226500	-5.985452503	3.59127E-22	3.49323E-20	RHSIM_Rhsim04G0226500

Comparison	Gene ID	log_2FC	P-value	FDR (padj)	Gene Name
Root vs Stem	RHSIM_Rhsim12G0203100	-2.849594274	3.90398E-22	3.78145E-20	RHSIM_Rhsim12G0203100
Root vs Stem	RHSIM_Rhsim07G0087600	5.399609695	5.19929E-22	5.01503E-20	RHSIM_Rhsim07G0087600
Root vs Stem	RHSIM_Rhsim05G0054700	-5.966264573	5.53272E-22	5.31441E-20	RHSIM_Rhsim05G0054700
Root vs Stem	RHSIM_Rhsim03G0078200	-3.647947894	6.90747E-22	6.60739E-20	RHSIM_Rhsim03G0078200
Root vs Stem	RHSIM_Rhsim07G0134900	-3.299934761	7.36176E-22	7.01284E-20	RHSIM_Rhsim07G0134900
Root vs Stem	RHSIM_Rhsim04G0133000	1.964355432	7.49572E-22	7.11106E-20	RHSIM_Rhsim04G0133000
Root vs Stem	novel.5960	-2.938648929	7.91713E-22	7.48006E-20	--
Root vs Stem	RHSIM_Rhsim03G0159800	-1.78100334	1.38583E-21	1.30398E-19	RHSIM_Rhsim03G0159800
Root vs Stem	RHSIM_Rhsim13G0200900	3.276830799	1.63371E-21	1.53097E-19	RHSIM_Rhsim13G0200900
Root vs Stem	RHSIM_Rhsim04G0019500	-1.760544576	1.66455E-21	1.55356E-19	RHSIM_Rhsim04G0019500
Root vs Stem	novel.4036	6.099805942	1.72402E-21	1.60258E-19	--
Root vs Stem	RHSIM_Rhsim11G0175100	6.300198646	1.85774E-21	1.71641E-19	RHSIM_Rhsim11G0175100
Root vs Stem	RHSIM_Rhsim06G0094000	-1.681431833	1.86138E-21	1.71641E-19	RHSIM_Rhsim06G0094000
Root vs Stem	RHSIM_Rhsim05G0081100	7.715225703	1.94644E-21	1.7877E-19	RHSIM_Rhsim05G0081100
Root vs Stem	RHSIM_Rhsim04G0015400	1.917851864	2.35398E-21	2.15342E-19	RHSIM_Rhsim04G0015400
Root vs Stem	RHSIM_Rhsim02G0188700	5.295444631	2.44119E-21	2.22438E-19	RHSIM_Rhsim02G0188700
Root vs Stem	RHSIM_Rhsim07G0058600	4.940752603	2.51293E-21	2.28073E-19	RHSIM_Rhsim07G0058600
Root vs Stem	novel.2618	-2.391224066	3.00418E-21	2.7159E-19	--
Root vs Stem	RHSIM_Rhsim12G0048600	-1.813081354	3.31812E-21	2.988E-19	RHSIM_Rhsim12G0048600
Root vs Stem	novel.2946	5.282503418	3.66706E-21	3.28936E-19	--
Root vs Stem	RHSIM_Rhsim04G0164300	-2.748333255	3.83581E-21	3.4274E-19	RHSIM_Rhsim04G0164300
Root vs Stem	RHSIM_Rhsim10G0197900	1.915284719	4.32721E-21	3.85155E-19	RHSIM_Rhsim10G0197900
Root vs Stem	RHSIM_RhsimUnG0004900	-3.815636738	4.45968E-21	3.95419E-19	RHSIM_RhsimUnG0004900
Root vs Stem	RHSIM_Rhsim12G0078000	-3.508501805	4.49946E-21	3.97418E-19	RHSIM_Rhsim12G0078000
Root vs Stem	RHSIM_Rhsim13G0107100	4.393081543	4.59956E-21	4.04709E-19	RHSIM_Rhsim13G0107100

Comparison	Gene ID	log_2FC	P-value	FDR (padj)	Gene Name
Root vs Stem	RHSIM_Rhsim01G0107800	4.91436685	4.69674E-21	4.11688E-19	RHSIM_Rhsim01G0107800
Root vs Stem	RHSIM_Rhsim10G0162700	2.355584526	5.67102E-21	4.95205E-19	RHSIM_Rhsim10G0162700
Root vs Stem	RHSIM_Rhsim12G0128400	3.364944213	5.86327E-21	5.1006E-19	RHSIM_Rhsim12G0128400
Root vs Stem	RHSIM_Rhsim07G0226800	3.580386488	5.96325E-21	5.16808E-19	RHSIM_Rhsim07G0226800
Root vs Stem	RHSIM_Rhsim09G0171900	-4.616266701	6.55186E-21	5.65693E-19	RHSIM_Rhsim09G0171900
Root vs Stem	RHSIM_Rhsim12G0046400	-3.156159659	8.28617E-21	7.12765E-19	RHSIM_Rhsim12G0046400
Root vs Stem	RHSIM_Rhsim05G0004100	-2.26671722	1.03116E-20	8.83695E-19	RHSIM_Rhsim05G0004100
Root vs Stem	RHSIM_Rhsim01G0109600	2.740135088	1.1297E-20	9.64556E-19	RHSIM_Rhsim01G0109600
Root vs Stem	RHSIM_Rhsim08G0187400	1.612358378	1.29258E-20	1.09955E-18	RHSIM_Rhsim08G0187400
Root vs Stem	RHSIM_Rhsim10G0100700	-3.21992346	1.3253E-20	1.12324E-18	RHSIM_Rhsim10G0100700
Root vs Stem	RHSIM_Rhsim12G0155700	-6.103945234	1.47093E-20	1.2421E-18	RHSIM_Rhsim12G0155700
Root vs Stem	RHSIM_Rhsim09G0153100	-2.733061249	1.75011E-20	1.47245E-18	RHSIM_Rhsim09G0153100
Root vs Stem	RHSIM_Rhsim07G0059100	3.045946287	1.82865E-20	1.53294E-18	RHSIM_Rhsim07G0059100
Root vs Stem	RHSIM_Rhsim06G0030400	4.000119938	2.21985E-20	1.85413E-18	RHSIM_Rhsim06G0030400
Root vs Stem	RHSIM_Rhsim06G0157200	4.219496904	2.27255E-20	1.89131E-18	RHSIM_Rhsim06G0157200
Root vs Stem	RHSIM_Rhsim06G0080200	6.41270041	2.2905E-20	1.89938E-18	RHSIM_Rhsim06G0080200
Root vs Stem	RHSIM_Rhsim02G0211000	-11.20885357	2.80703E-20	2.31937E-18	RHSIM_Rhsim02G0211000
Root vs Stem	RHSIM_Rhsim09G0129500	-1.596621344	2.97653E-20	2.45064E-18	RHSIM_Rhsim09G0129500
Root vs Stem	RHSIM_Rhsim06G0068900	4.422687699	3.26552E-20	2.679E-18	RHSIM_Rhsim06G0068900
Root vs Stem	RHSIM_Rhsim06G0199300	-3.561483464	3.34009E-20	2.73046E-18	RHSIM_Rhsim06G0199300
Root vs Stem	RHSIM_Rhsim10G0195500	2.085058179	4.14205E-20	3.37409E-18	RHSIM_Rhsim10G0195500
Root vs Stem	RHSIM_Rhsim09G0180500	-3.07275304	5.12381E-20	4.15912E-18	RHSIM_Rhsim09G0180500
Root vs Stem	RHSIM_Rhsim04G0230700	6.124529168	5.31156E-20	4.2964E-18	RHSIM_Rhsim04G0230700
Root vs Stem	RHSIM_Rhsim03G0210700	-2.114978602	5.40557E-20	4.35715E-18	RHSIM_Rhsim03G0210700
Root vs Stem	RHSIM_Rhsim08G0234500	4.708069011	5.60154E-20	4.49939E-18	RHSIM_Rhsim08G0234500

Comparison	Gene ID	log_2FC	P-value	FDR (padj)	Gene Name
Root vs Stem	RHSIM_Rhsim10G0126900	6.103708122	5.89461E-20	4.71835E-18	RHSIM_Rhsim10G0126900
Root vs Stem	RHSIM_Rhsim10G0142100	-5.133423703	6.70823E-20	5.35103E-18	RHSIM_Rhsim10G0142100
Root vs Stem	RHSIM_Rhsim01G0107700	3.890340355	7.12805E-20	5.66631E-18	RHSIM_Rhsim01G0107700
Root vs Stem	RHSIM_Rhsim06G0154100	-1.352567986	7.43382E-20	5.88907E-18	RHSIM_Rhsim06G0154100
Root vs Stem	RHSIM_RhsimUnG0101400	-1.806411792	7.98969E-20	6.30775E-18	RHSIM_RhsimUnG0101400
Root vs Stem	RHSIM_Rhsim11G0157200	5.179410943	8.2697E-20	6.50654E-18	RHSIM_Rhsim11G0157200
Root vs Stem	RHSIM_Rhsim08G0137600	-7.861442633	8.99064E-20	7.0497E-18	RHSIM_Rhsim08G0137600
Root vs Stem	RHSIM_Rhsim08G0224500	3.756851493	9.98345E-20	7.80164E-18	RHSIM_Rhsim08G0224500
Root vs Stem	RHSIM_Rhsim13G0122100	-11.07322932	1.00619E-19	7.8364E-18	RHSIM_Rhsim13G0122100
Root vs Stem	RHSIM_Rhsim08G0148000	-4.002558927	1.15339E-19	8.95256E-18	RHSIM_Rhsim08G0148000
Root vs Stem	RHSIM_Rhsim11G0117800	4.279877308	1.31616E-19	1.01817E-17	RHSIM_Rhsim11G0117800
Root vs Stem	novel.1068	-11.04680901	1.32505E-19	1.02162E-17	--
Root vs Stem	RHSIM_Rhsim11G0137300	-4.14688802	1.37429E-19	1.05605E-17	RHSIM_Rhsim11G0137300
Root vs Stem	RHSIM_Rhsim08G0148800	3.430129903	1.5323E-19	1.17356E-17	RHSIM_Rhsim08G0148800
Root vs Stem	RHSIM_Rhsim03G0246900	3.213739385	1.54053E-19	1.17596E-17	RHSIM_Rhsim03G0246900
Root vs Stem	RHSIM_Rhsim08G0011100	1.804190512	1.56871E-19	1.19351E-17	RHSIM_Rhsim08G0011100
Root vs Stem	RHSIM_Rhsim12G0193300	7.392097848	1.74966E-19	1.3268E-17	RHSIM_Rhsim12G0193300
Root vs Stem	RHSIM_Rhsim05G0052600	-1.44661225	1.78207E-19	1.34695E-17	RHSIM_Rhsim05G0052600
Root vs Stem	RHSIM_Rhsim12G0176200	-7.757480238	2.24835E-19	1.69383E-17	RHSIM_Rhsim12G0176200
Root vs Stem	RHSIM_RhsimUnG0188100	-4.813342063	2.27774E-19	1.71038E-17	RHSIM_RhsimUnG0188100
Root vs Stem	RHSIM_Rhsim10G0056400	-2.018623991	2.29378E-19	1.71684E-17	RHSIM_Rhsim10G0056400
Root vs Stem	RHSIM_Rhsim03G0088700	1.570386126	3.45264E-19	2.57585E-17	RHSIM_Rhsim03G0088700
Root vs Stem	novel.2484	-2.385342032	4.14384E-19	3.08155E-17	--
Root vs Stem	RHSIM_Rhsim03G0198900	-1.786300537	4.63683E-19	3.43707E-17	RHSIM_Rhsim03G0198900
Root vs Stem	RHSIM_Rhsim06G0103300	-5.389234873	4.81787E-19	3.55982E-17	RHSIM_Rhsim06G0103300

Comparison	Gene ID	log_2FC	P-value	FDR (padj)	Gene Name
Root vs Stem	RHSIM_Rhsim08G0094000	1.274158087	4.86676E-19	3.58445E-17	RHSIM_Rhsim08G0094000
Root vs Stem	RHSIM_Rhsim02G0029600	-2.661653596	5.11734E-19	3.75701E-17	RHSIM_Rhsim02G0029600
Root vs Stem	RHSIM_Rhsim06G0083300	2.500073757	5.35765E-19	3.92095E-17	RHSIM_Rhsim06G0083300
Root vs Stem	novel.3956	-2.963963323	5.82646E-19	4.25055E-17	--
Root vs Stem	RHSIM_Rhsim03G0150200	4.441196111	6.39321E-19	4.6493E-17	RHSIM_Rhsim03G0150200
Root vs Stem	RHSIM_Rhsim13G0126200	4.189207423	6.83361E-19	4.95394E-17	RHSIM_Rhsim13G0126200
Root vs Stem	RHSIM_Rhsim02G0035200	4.978877567	6.90564E-19	4.99046E-17	RHSIM_Rhsim02G0035200
Root vs Stem	RHSIM_Rhsim08G0167600	4.279839081	7.01822E-19	5.05597E-17	RHSIM_Rhsim08G0167600
Root vs Stem	RHSIM_Rhsim12G0180300	-2.799848131	7.0973E-19	5.09701E-17	RHSIM_Rhsim12G0180300
Root vs Stem	RHSIM_Rhsim06G0021700	-3.906772273	8.15024E-19	5.83501E-17	RHSIM_Rhsim06G0021700
Root vs Stem	RHSIM_Rhsim07G0226000	-4.056980956	8.63271E-19	6.1613E-17	RHSIM_Rhsim07G0226000
Root vs Stem	RHSIM_Rhsim11G0130100	-1.892570545	8.87829E-19	6.31701E-17	RHSIM_Rhsim11G0130100
Root vs Stem	RHSIM_Rhsim08G0200900	-2.279896929	9.28822E-19	6.58835E-17	RHSIM_Rhsim08G0200900
Root vs Stem	RHSIM_Rhsim06G0067100	-2.354928893	9.39088E-19	6.64074E-17	RHSIM_Rhsim06G0067100
Root vs Stem	RHSIM_Rhsim10G0163100	1.462307579	9.51941E-19	6.71104E-17	RHSIM_Rhsim10G0163100
Root vs Stem	RHSIM_Rhsim01G0215000	10.63885936	9.56578E-19	6.72317E-17	RHSIM_Rhsim01G0215000
Root vs Stem	RHSIM_Rhsim11G0036000	-1.720689342	9.82731E-19	6.88599E-17	RHSIM_Rhsim11G0036000
Root vs Stem	RHSIM_Rhsim02G0115500	-1.753509672	1.0511E-18	7.34275E-17	RHSIM_Rhsim02G0115500
Root vs Stem	RHSIM_Rhsim13G0105100	-4.728252286	1.05643E-18	7.3577E-17	RHSIM_Rhsim13G0105100
Root vs Stem	RHSIM_Rhsim07G0233500	1.775734742	1.31386E-18	9.12303E-17	RHSIM_Rhsim07G0233500
Root vs Stem	novel.6075	5.126063838	1.38436E-18	9.58366E-17	--
Root vs Stem	RHSIM_Rhsim12G0190900	5.583129516	1.41223E-18	9.74738E-17	RHSIM_Rhsim12G0190900
Root vs Stem	RHSIM_Rhsim09G0133000	-4.296834671	1.53949E-18	1.0594E-16	RHSIM_Rhsim09G0133000
Root vs Stem	RHSIM_Rhsim04G0052300	-4.895023017	1.6295E-18	1.118E-16	RHSIM_Rhsim04G0052300
Root vs Stem	RHSIM_Rhsim06G0222200	4.916225266	1.85414E-18	1.26836E-16	RHSIM_Rhsim06G0222200

Comparison	Gene ID	log_2FC	P-value	FDR (padj)	Gene Name
Root vs Stem	RHSIM_Rhsim06G0213900	-2.311430822	2.60249E-18	1.77501E-16	RHSIM_Rhsim06G0213900
Root vs Stem	RHSIM_Rhsim04G0182700	1.549317924	2.81159E-18	1.91196E-16	RHSIM_Rhsim04G0182700
Root vs Stem	RHSIM_Rhsim07G0102000	-4.726026399	2.87548E-18	1.94966E-16	RHSIM_Rhsim07G0102000
Root vs Stem	RHSIM_Rhsim06G0106000	5.207469829	3.01281E-18	2.03678E-16	RHSIM_Rhsim06G0106000
Root vs Stem	RHSIM_Rhsim12G0182400	-1.989006806	3.07107E-18	2.0701E-16	RHSIM_Rhsim12G0182400
Root vs Stem	RHSIM_Rhsim08G0028400	3.149358705	3.15665E-18	2.12158E-16	RHSIM_Rhsim08G0028400
Root vs Stem	novel.3077	-2.535349243	3.21465E-18	2.15428E-16	--
Root vs Stem	RHSIM_Rhsim12G0024400	-10.61291004	3.4073E-18	2.27677E-16	RHSIM_Rhsim12G0024400
Root vs Stem	novel.1136	1.898633031	3.63006E-18	2.41861E-16	--
Root vs Stem	RHSIM_Rhsim08G0004000	-4.315014733	3.6991E-18	2.4575E-16	RHSIM_Rhsim08G0004000
Root vs Stem	RHSIM_Rhsim12G0024300	-4.474137921	3.86768E-18	2.56043E-16	RHSIM_Rhsim12G0024300
Root vs Stem	RHSIM_Rhsim02G0139100	2.656327279	3.87624E-18	2.56043E-16	RHSIM_Rhsim02G0139100
Root vs Stem	RHSIM_Rhsim05G0068700	1.352874554	3.93316E-18	2.5906E-16	RHSIM_Rhsim05G0068700
Root vs Stem	RHSIM_Rhsim07G0163600	-1.394948613	4.74289E-18	3.11504E-16	RHSIM_Rhsim07G0163600
Root vs Stem	RHSIM_Rhsim02G0043000	-4.761293506	4.97404E-18	3.25757E-16	RHSIM_Rhsim02G0043000
Root vs Stem	RHSIM_Rhsim07G0221300	-3.652917588	5.05724E-18	3.30268E-16	RHSIM_Rhsim07G0221300
Root vs Stem	RHSIM_Rhsim03G0073400	3.730253156	5.97419E-18	3.89048E-16	RHSIM_Rhsim03G0073400
Root vs Stem	RHSIM_Rhsim02G0035300	2.001554326	6.93948E-18	4.50636E-16	RHSIM_Rhsim02G0035300
Root vs Stem	RHSIM_Rhsim03G0064200	3.758604403	7.48831E-18	4.8491E-16	RHSIM_Rhsim03G0064200
Root vs Stem	RHSIM_Rhsim05G0081200	7.139998898	7.68903E-18	4.96513E-16	RHSIM_Rhsim05G0081200
Root vs Stem	RHSIM_Rhsim05G0077900	-4.924377171	9.01337E-18	5.80406E-16	RHSIM_Rhsim05G0077900
Root vs Stem	RHSIM_Rhsim06G0157100	13.4467401	9.24123E-18	5.93421E-16	RHSIM_Rhsim06G0157100
Root vs Stem	RHSIM_Rhsim04G0119100	-1.770204889	9.49214E-18	6.0784E-16	RHSIM_Rhsim04G0119100
Root vs Stem	RHSIM_Rhsim02G0037400	1.285722592	1.02901E-17	6.57114E-16	RHSIM_Rhsim02G0037400
Root vs Stem	RHSIM_Rhsim03G0073300	4.139572534	1.10602E-17	7.04338E-16	RHSIM_Rhsim03G0073300

Comparison	Gene ID	log_2FC	P-value	FDR (padj)	Gene Name
Root vs Stem	RHSIM_Rhsim11G0133200	4.673154776	1.18005E-17	7.4941E-16	RHSIM_Rhsim11G0133200
Root vs Stem	RHSIM_Rhsim11G0012900	-3.077724422	1.37853E-17	8.73053E-16	RHSIM_Rhsim11G0012900
Root vs Stem	novel.2269	-2.319039191	1.43232E-17	9.04634E-16	--
Root vs Stem	RHSIM_Rhsim04G0124400	5.162474292	1.46938E-17	9.2551E-16	RHSIM_Rhsim04G0124400
Root vs Stem	RHSIM_Rhsim02G0214900	-12.18086179	1.4752E-17	9.26644E-16	RHSIM_Rhsim02G0214900
Root vs Stem	RHSIM_Rhsim10G0064000	-10.44442653	1.49032E-17	9.33594E-16	RHSIM_Rhsim10G0064000
Root vs Stem	novel.2800	9.563594086	1.65405E-17	1.03336E-15	--
Root vs Stem	RHSIM_Rhsim05G0044200	2.692725591	1.74978E-17	1.09021E-15	RHSIM_Rhsim05G0044200
Root vs Stem	RHSIM_Rhsim07G0088000	-3.051448954	2.32466E-17	1.44449E-15	RHSIM_Rhsim07G0088000
Root vs Stem	RHSIM_Rhsim08G0019300	-2.916999469	2.36657E-17	1.46294E-15	RHSIM_Rhsim08G0019300
Root vs Stem	RHSIM_Rhsim04G0081300	-4.095859782	2.36705E-17	1.46294E-15	RHSIM_Rhsim04G0081300
Root vs Stem	RHSIM_Rhsim01G0254900	-2.138597151	2.53507E-17	1.56259E-15	RHSIM_Rhsim01G0254900
Root vs Stem	RHSIM_Rhsim02G0140300	-1.282223775	2.54412E-17	1.56399E-15	RHSIM_Rhsim02G0140300
Root vs Stem	RHSIM_Rhsim07G0023100	-2.314538156	2.60955E-17	1.59995E-15	RHSIM_Rhsim07G0023100
Root vs Stem	RHSIM_Rhsim02G0041900	2.420392005	2.94979E-17	1.80375E-15	RHSIM_Rhsim02G0041900
Root vs Stem	RHSIM_Rhsim09G0000900	2.030815649	3.33621E-17	2.02928E-15	RHSIM_Rhsim09G0000900
Root vs Stem	RHSIM_Rhsim07G0023400	-7.233520077	3.43256E-17	2.08239E-15	RHSIM_Rhsim07G0023400
Root vs Stem	RHSIM_Rhsim07G0051300	5.335362589	3.64398E-17	2.20485E-15	RHSIM_Rhsim07G0051300
Root vs Stem	RHSIM_Rhsim07G0247500	3.095632506	3.65574E-17	2.20617E-15	RHSIM_Rhsim07G0247500
Root vs Stem	RHSIM_Rhsim12G0023500	6.698571969	4.55422E-17	2.74121E-15	RHSIM_Rhsim12G0023500
Root vs Stem	RHSIM_Rhsim07G0112000	-1.514043793	4.87673E-17	2.92769E-15	RHSIM_Rhsim07G0112000
Root vs Stem	RHSIM_Rhsim01G0056200	3.553346987	5.10895E-17	3.05913E-15	RHSIM_Rhsim01G0056200
Root vs Stem	RHSIM_Rhsim04G0185500	1.965666923	5.1557E-17	3.0781E-15	RHSIM_Rhsim04G0185500
Root vs Stem	RHSIM_Rhsim13G0162700	3.46980089	5.16733E-17	3.0781E-15	RHSIM_Rhsim13G0162700
Root vs Stem	RHSIM_Rhsim11G0043100	-2.848533073	5.84901E-17	3.47519E-15	RHSIM_Rhsim11G0043100

Comparison	Gene ID	log_2FC	P-value	FDR (padj)	Gene Name
Root vs Stem	RHSIM_Rhsim01G0205800	1.993563092	5.93239E-17	3.51567E-15	RHSIM_Rhsim01G0205800
Root vs Stem	RHSIM_Rhsim02G0005400	1.003616658	6.16362E-17	3.64333E-15	RHSIM_Rhsim02G0005400
Root vs Stem	RHSIM_Rhsim03G0154900	-5.167457749	6.45495E-17	3.80578E-15	RHSIM_Rhsim03G0154900
Root vs Stem	novel.4275	4.085637977	6.66291E-17	3.91837E-15	--
Root vs Stem	RHSIM_Rhsim13G0194200	-3.193106021	7.12152E-17	4.17742E-15	RHSIM_Rhsim13G0194200
Root vs Stem	RHSIM_Rhsim06G0197700	-2.163821089	7.18785E-17	4.20562E-15	RHSIM_Rhsim06G0197700
Root vs Stem	RHSIM_Rhsim06G0004600	-2.452204282	7.25729E-17	4.2355E-15	RHSIM_Rhsim06G0004600
Root vs Stem	RHSIM_Rhsim04G0072300	1.9921336	8.42589E-17	4.9051E-15	RHSIM_Rhsim04G0072300
Root vs Stem	RHSIM_Rhsim13G0196300	4.245972886	8.97869E-17	5.21375E-15	RHSIM_Rhsim13G0196300
Root vs Stem	RHSIM_Rhsim01G0288900	-1.47697339	1.0364E-16	6.00304E-15	RHSIM_Rhsim01G0288900
Root vs Stem	RHSIM_Rhsim09G0153300	2.473945395	1.10667E-16	6.394E-15	RHSIM_Rhsim09G0153300
Root vs Stem	RHSIM_Rhsim11G0161600	4.524194018	1.22352E-16	7.05143E-15	RHSIM_Rhsim11G0161600
Root vs Stem	RHSIM_Rhsim06G0161000	-2.708474528	1.22814E-16	7.06043E-15	RHSIM_Rhsim06G0161000
Root vs Stem	RHSIM_Rhsim12G0004800	-1.837912452	1.35894E-16	7.79297E-15	RHSIM_Rhsim12G0004800
Root vs Stem	RHSIM_Rhsim03G0082500	3.264586026	1.43874E-16	8.23011E-15	RHSIM_Rhsim03G0082500
Root vs Stem	RHSIM_Rhsim12G0079300	10.7453719	1.44709E-16	8.25736E-15	RHSIM_Rhsim12G0079300
Root vs Stem	RHSIM_Rhsim02G0243900	4.570296723	1.49949E-16	8.53523E-15	RHSIM_Rhsim02G0243900
Root vs Stem	RHSIM_Rhsim02G0107100	-1.914797937	1.64729E-16	9.35345E-15	RHSIM_Rhsim02G0107100
Root vs Stem	novel.1056	-1.63202714	1.6549E-16	9.37355E-15	--
Root vs Stem	RHSIM_Rhsim13G0064200	4.745573465	1.71561E-16	9.6936E-15	RHSIM_Rhsim13G0064200
Root vs Stem	RHSIM_Rhsim07G0028800	1.283062937	1.72908E-16	9.74583E-15	RHSIM_Rhsim07G0028800
Root vs Stem	RHSIM_Rhsim08G0033400	-3.581021377	1.77433E-16	9.97647E-15	RHSIM_Rhsim08G0033400
Root vs Stem	RHSIM_Rhsim06G0046400	3.011209202	1.80128E-16	1.01034E-14	RHSIM_Rhsim06G0046400
Root vs Stem	RHSIM_Rhsim07G0124400	-1.136793604	1.83749E-16	1.02814E-14	RHSIM_Rhsim07G0124400
Root vs Stem	RHSIM_Rhsim01G0245300	3.28335144	1.97128E-16	1.10034E-14	RHSIM_Rhsim01G0245300

Comparison	Gene ID	log_2FC	P-value	FDR (padj)	Gene Name
Root vs Stem	RHSIM_Rhsim08G0109700	-2.464419688	2.01001E-16	1.11924E-14	RHSIM_Rhsim08G0109700
Root vs Stem	RHSIM_Rhsim13G0123400	-3.931985833	2.06967E-16	1.14969E-14	RHSIM_Rhsim13G0123400
Root vs Stem	RHSIM_Rhsim06G0220000	3.394663974	2.09456E-16	1.16072E-14	RHSIM_Rhsim06G0220000
Root vs Stem	RHSIM_Rhsim13G0136200	2.829305377	2.24402E-16	1.24056E-14	RHSIM_Rhsim13G0136200
Root vs Stem	RHSIM_Rhsim10G0036100	2.752596637	2.26926E-16	1.25151E-14	RHSIM_Rhsim10G0036100
Root vs Stem	RHSIM_Rhsim01G0208800	-1.60475063	2.50846E-16	1.38013E-14	RHSIM_Rhsim01G0208800
Root vs Stem	RHSIM_Rhsim09G0114500	-4.819367363	2.5857E-16	1.41924E-14	RHSIM_Rhsim09G0114500
Root vs Stem	RHSIM_Rhsim05G0201500	3.707026162	2.60947E-16	1.42888E-14	RHSIM_Rhsim05G0201500
Root vs Stem	RHSIM_Rhsim03G0142300	-1.583069626	2.86603E-16	1.56565E-14	RHSIM_Rhsim03G0142300
Root vs Stem	RHSIM_Rhsim04G0187700	1.574012712	2.94641E-16	1.60576E-14	RHSIM_Rhsim04G0187700
Root vs Stem	RHSIM_Rhsim10G0174200	3.721148269	3.30706E-16	1.79806E-14	RHSIM_Rhsim10G0174200
Root vs Stem	RHSIM_Rhsim03G0062500	3.167360087	3.32455E-16	1.80331E-14	RHSIM_Rhsim03G0062500
Root vs Stem	RHSIM_Rhsim13G0083100	2.759853654	3.49033E-16	1.88879E-14	RHSIM_Rhsim13G0083100
Root vs Stem	RHSIM_Rhsim11G0198700	3.862095575	3.51385E-16	1.89707E-14	RHSIM_Rhsim11G0198700
Root vs Stem	RHSIM_Rhsim11G0138700	-6.043101145	3.68265E-16	1.98117E-14	RHSIM_Rhsim11G0138700
Root vs Stem	RHSIM_Rhsim04G0010400	1.231083914	3.68682E-16	1.98117E-14	RHSIM_Rhsim04G0010400
Root vs Stem	RHSIM_Rhsim04G0208400	3.339862887	3.91613E-16	2.0995E-14	RHSIM_Rhsim04G0208400
Root vs Stem	novel.776	6.588459386	3.94277E-16	2.10888E-14	--
Root vs Stem	RHSIM_Rhsim01G0247800	-4.051208952	4.05418E-16	2.16345E-14	RHSIM_Rhsim01G0247800
Root vs Stem	RHSIM_Rhsim06G0196300	-3.627079799	4.22122E-16	2.24738E-14	RHSIM_Rhsim06G0196300
Root vs Stem	RHSIM_Rhsim01G0270500	5.603199695	4.28052E-16	2.27371E-14	RHSIM_Rhsim01G0270500
Root vs Stem	RHSIM_Rhsim03G0171300	1.602545751	4.29417E-16	2.27571E-14	RHSIM_Rhsim03G0171300
Root vs Stem	RHSIM_Rhsim02G0234800	-4.810734917	4.3223E-16	2.28537E-14	RHSIM_Rhsim02G0234800
Root vs Stem	RHSIM_Rhsim12G0125800	1.718334906	4.47597E-16	2.3612E-14	RHSIM_Rhsim12G0125800
Root vs Stem	RHSIM_Rhsim04G0144300	-1.753555915	4.5885E-16	2.41504E-14	RHSIM_Rhsim04G0144300

Comparison	Gene ID	log_2FC	P-value	FDR (padj)	Gene Name
Root vs Stem	RHSIM_Rhsim11G0047500	2.318190092	4.60479E-16	2.41809E-14	RHSIM_Rhsim11G0047500
Root vs Stem	RHSIM_Rhsim07G0049600	-1.726070543	5.33494E-16	2.79515E-14	RHSIM_Rhsim07G0049600
Root vs Stem	RHSIM_Rhsim07G0096600	4.208382155	5.4246E-16	2.83568E-14	RHSIM_Rhsim07G0096600
Root vs Stem	RHSIM_Rhsim12G0081500	3.56522881	5.66622E-16	2.95528E-14	RHSIM_Rhsim12G0081500
Root vs Stem	RHSIM_Rhsim04G0202900	1.186984386	6.23028E-16	3.24214E-14	RHSIM_Rhsim04G0202900
Root vs Stem	RHSIM_Rhsim08G0171700	-1.883140982	6.32619E-16	3.28463E-14	RHSIM_Rhsim08G0171700
Root vs Stem	RHSIM_Rhsim03G0141200	2.012039611	6.37024E-16	3.30007E-14	RHSIM_Rhsim03G0141200
Root vs Stem	RHSIM_Rhsim04G0096700	2.232599275	6.44811E-16	3.33292E-14	RHSIM_Rhsim04G0096700
Root vs Stem	RHSIM_Rhsim05G0200800	-3.802533165	6.88235E-16	3.54941E-14	RHSIM_Rhsim05G0200800
Root vs Stem	RHSIM_Rhsim06G0001100	-2.440286124	7.00894E-16	3.60453E-14	RHSIM_Rhsim06G0001100
Root vs Stem	RHSIM_Rhsim13G0181800	-10.01434858	7.02049E-16	3.60453E-14	RHSIM_Rhsim13G0181800
Root vs Stem	novel.4274	5.446629136	7.3588E-16	3.76983E-14	--
Root vs Stem	RHSIM_Rhsim01G0269800	-7.643787607	7.65941E-16	3.91513E-14	RHSIM_Rhsim01G0269800
Root vs Stem	RHSIM_Rhsim08G0228000	-3.050913719	8.07659E-16	4.11924E-14	RHSIM_Rhsim08G0228000
Root vs Stem	RHSIM_Rhsim04G0158900	-3.494874378	8.1815E-16	4.16353E-14	RHSIM_Rhsim04G0158900
Root vs Stem	RHSIM_Rhsim10G0099300	-2.67888748	8.3698E-16	4.24998E-14	RHSIM_Rhsim10G0099300
Root vs Stem	RHSIM_Rhsim01G0039200	3.443182779	8.39104E-16	4.2514E-14	RHSIM_Rhsim01G0039200
Root vs Stem	RHSIM_Rhsim13G0187900	2.058959984	8.81485E-16	4.45633E-14	RHSIM_Rhsim13G0187900
Root vs Stem	RHSIM_Rhsim03G0056200	5.331506587	9.38555E-16	4.73447E-14	RHSIM_Rhsim03G0056200
Root vs Stem	RHSIM_Rhsim10G0006300	-1.714892422	9.86699E-16	4.96646E-14	RHSIM_Rhsim10G0006300
Root vs Stem	RHSIM_Rhsim12G0168400	4.809147318	9.89721E-16	4.97081E-14	RHSIM_Rhsim12G0168400
Root vs Stem	RHSIM_Rhsim03G0254500	1.705791828	1.01888E-15	5.10612E-14	RHSIM_Rhsim03G0254500
Root vs Stem	RHSIM_Rhsim04G0098400	-2.237264915	1.02596E-15	5.13046E-14	RHSIM_Rhsim04G0098400
Root vs Stem	RHSIM_Rhsim12G0183300	-1.689108969	1.11943E-15	5.58578E-14	RHSIM_Rhsim12G0183300
Root vs Stem	RHSIM_Rhsim04G0181500	3.748941191	1.15714E-15	5.75742E-14	RHSIM_Rhsim04G0181500

Comparison	Gene ID	log_2FC	P-value	FDR (padj)	Gene Name
Root vs Stem	RHSIM_Rhsim08G0009100	-2.592938648	1.15883E-15	5.75742E-14	RHSIM_Rhsim08G0009100
Root vs Stem	RHSIM_Rhsim08G0203700	-3.770511147	1.18613E-15	5.88038E-14	RHSIM_Rhsim08G0203700
Root vs Stem	RHSIM_RhsimUnG0205200	-1.70700133	1.29613E-15	6.41197E-14	RHSIM_RhsimUnG0205200
Root vs Stem	RHSIM_Rhsim09G0212900	3.09146758	1.32722E-15	6.55171E-14	RHSIM_Rhsim09G0212900
Root vs Stem	RHSIM_Rhsim11G0172000	-3.604597741	1.43242E-15	7.05591E-14	RHSIM_Rhsim11G0172000
Root vs Stem	RHSIM_Rhsim10G0060300	-1.915394985	1.44439E-15	7.0997E-14	RHSIM_Rhsim10G0060300
Root vs Stem	novel.739	4.72236247	1.55054E-15	7.58909E-14	--
Root vs Stem	RHSIM_Rhsim01G0210800	1.710584477	1.56212E-15	7.62957E-14	RHSIM_Rhsim01G0210800
Root vs Stem	RHSIM_Rhsim08G0228400	-1.504665374	1.57967E-15	7.69899E-14	RHSIM_Rhsim08G0228400
Root vs Stem	novel.870	5.462095546	1.68789E-15	8.20904E-14	--
Root vs Stem	RHSIM_Rhsim11G0139400	5.67879578	1.72565E-15	8.37504E-14	RHSIM_Rhsim11G0139400
Root vs Stem	RHSIM_Rhsim04G0009800	-2.464500343	1.83838E-15	8.90342E-14	RHSIM_Rhsim04G0009800
Root vs Stem	RHSIM_Rhsim11G0019900	-1.684022367	1.93944E-15	9.37313E-14	RHSIM_Rhsim11G0019900
Root vs Stem	RHSIM_Rhsim08G0159300	-3.727239115	1.96866E-15	9.48026E-14	RHSIM_Rhsim08G0159300
Root vs Stem	novel.4113	4.368511108	1.96983E-15	9.48026E-14	--
Root vs Stem	RHSIM_Rhsim08G0213800	-3.425150271	2.03271E-15	9.76253E-14	RHSIM_Rhsim08G0213800
Root vs Stem	novel.5847	10.65640442	2.07318E-15	9.93617E-14	--
Root vs Stem	RHSIM_Rhsim02G0187400	3.900137409	2.11788E-15	1.01293E-13	RHSIM_Rhsim02G0187400
Root vs Stem	RHSIM_Rhsim13G0039000	2.376951478	2.33915E-15	1.11645E-13	RHSIM_Rhsim13G0039000
Root vs Stem	novel.3528	-1.745097116	2.35944E-15	1.12381E-13	--
Root vs Stem	RHSIM_Rhsim12G0096200	-1.636308505	2.57535E-15	1.22136E-13	RHSIM_Rhsim12G0096200
Root vs Stem	RHSIM_Rhsim03G0142900	-1.273309315	2.58015E-15	1.22136E-13	RHSIM_Rhsim03G0142900
Root vs Stem	RHSIM_Rhsim04G0052400	3.405494645	2.69441E-15	1.27283E-13	RHSIM_Rhsim04G0052400
Root vs Stem	RHSIM_Rhsim08G0233500	2.337649769	2.89865E-15	1.36652E-13	RHSIM_Rhsim08G0233500
Root vs Stem	RHSIM_Rhsim03G0008500	-4.204052712	3.16996E-15	1.49137E-13	RHSIM_Rhsim03G0008500

Comparison	Gene ID	log_2FC	P-value	FDR (padj)	Gene Name
Root vs Stem	RHSIM_Rhsim10G0045000	1.776365422	3.17755E-15	1.4919E-13	RHSIM_Rhsim10G0045000
Root vs Stem	RHSIM_Rhsim05G0044100	3.562249335	3.20326E-15	1.50091E-13	RHSIM_Rhsim05G0044100
Root vs Stem	RHSIM_Rhsim02G0237100	-8.82693655	3.21547E-15	1.50357E-13	RHSIM_Rhsim02G0237100
Root vs Stem	RHSIM_RhsimUnG0023200	1.181272429	3.22872E-15	1.50671E-13	RHSIM_RhsimUnG0023200
Root vs Stem	novel.981	3.20287751	3.47301E-15	1.61744E-13	--
Root vs Stem	RHSIM_Rhsim04G0206400	-1.864939775	3.53078E-15	1.64103E-13	RHSIM_Rhsim04G0206400
Root vs Stem	RHSIM_RhsimUnG0066200	3.162809114	3.64354E-15	1.69003E-13	RHSIM_RhsimUnG0066200
Root vs Stem	RHSIM_Rhsim01G0231100	-4.314922281	3.65837E-15	1.6935E-13	RHSIM_Rhsim01G0231100
Root vs Stem	RHSIM_Rhsim10G0114500	-2.328644006	3.94377E-15	1.82196E-13	RHSIM_Rhsim10G0114500
Root vs Stem	RHSIM_Rhsim05G0161600	2.330753285	4.07467E-15	1.87867E-13	RHSIM_Rhsim05G0161600
Root vs Stem	RHSIM_Rhsim09G0160400	-1.091055353	4.30377E-15	1.98033E-13	RHSIM_Rhsim09G0160400
Root vs Stem	novel.2605	-7.23088322	4.43132E-15	2.03496E-13	--
Root vs Stem	RHSIM_Rhsim03G0139800	1.079172544	4.68965E-15	2.14931E-13	RHSIM_Rhsim03G0139800
Root vs Stem	RHSIM_Rhsim02G0035700	3.800960392	4.74769E-15	2.1716E-13	RHSIM_Rhsim02G0035700
Root vs Stem	RHSIM_Rhsim08G0156900	-5.972473083	5.00965E-15	2.28688E-13	RHSIM_Rhsim08G0156900
Root vs Stem	RHSIM_Rhsim07G0101900	-4.768991001	5.08512E-15	2.31674E-13	RHSIM_Rhsim07G0101900
Root vs Stem	RHSIM_Rhsim05G0094500	4.066194177	5.35203E-15	2.43354E-13	RHSIM_Rhsim05G0094500
Root vs Stem	RHSIM_Rhsim08G0100400	-1.089414815	5.38281E-15	2.44272E-13	RHSIM_Rhsim08G0100400
Root vs Stem	RHSIM_Rhsim07G0131600	-2.146186681	5.43838E-15	2.46308E-13	RHSIM_Rhsim07G0131600
Root vs Stem	novel.575	-2.586903209	5.73237E-15	2.59114E-13	--
Root vs Stem	RHSIM_Rhsim12G0142000	4.255088079	5.76669E-15	2.60156E-13	RHSIM_Rhsim12G0142000
Root vs Stem	novel.5071	-2.321010256	6.20717E-15	2.7948E-13	--
Root vs Stem	novel.2106	-2.894215632	6.26561E-15	2.81561E-13	--
Root vs Stem	RHSIM_Rhsim06G0116800	1.997688491	6.57177E-15	2.94745E-13	RHSIM_Rhsim06G0116800
Root vs Stem	RHSIM_Rhsim01G0120900	3.052733328	7.50173E-15	3.35801E-13	RHSIM_Rhsim01G0120900

Comparison	Gene ID	log_2FC	P-value	FDR (padj)	Gene Name
Root vs Stem	novel.3074	-2.294992474	7.73293E-15	3.45479E-13	--
Root vs Stem	RHSIM_Rhsim09G0108100	-3.057236352	8.59938E-15	3.83446E-13	RHSIM_Rhsim09G0108100
Root vs Stem	RHSIM_Rhsim02G0070000	1.286383935	9.19179E-15	4.0907E-13	RHSIM_Rhsim02G0070000
Root vs Stem	RHSIM_Rhsim13G0065600	-1.862716257	9.2648E-15	4.11525E-13	RHSIM_Rhsim13G0065600
Root vs Stem	RHSIM_Rhsim11G0138900	-4.214855723	9.47762E-15	4.20168E-13	RHSIM_Rhsim11G0138900
Root vs Stem	RHSIM_Rhsim12G0156100	-1.425923357	9.60059E-15	4.24803E-13	RHSIM_Rhsim12G0156100
Root vs Stem	RHSIM_Rhsim10G0133700	-2.394270049	9.93979E-15	4.38969E-13	RHSIM_Rhsim10G0133700
Root vs Stem	RHSIM_Rhsim10G0181300	3.050640791	1.10879E-14	4.87805E-13	RHSIM_Rhsim10G0181300
Root vs Stem	RHSIM_Rhsim10G0118400	2.540870065	1.13253E-14	4.97298E-13	RHSIM_Rhsim10G0118400
Root vs Stem	novel.1514	3.428128774	1.13591E-14	4.97837E-13	--
Root vs Stem	RHSIM_Rhsim01G0171000	-1.597587964	1.23834E-14	5.41697E-13	RHSIM_Rhsim01G0171000
Root vs Stem	RHSIM_Rhsim08G0058800	3.738844622	1.37136E-14	5.98747E-13	RHSIM_Rhsim08G0058800
Root vs Stem	RHSIM_Rhsim11G0075500	2.058707469	1.37714E-14	6.00137E-13	RHSIM_Rhsim11G0075500
Root vs Stem	RHSIM_Rhsim13G0008600	-3.836994989	1.41324E-14	6.14705E-13	RHSIM_Rhsim13G0008600
Root vs Stem	novel.2707	-1.776965971	1.4231E-14	6.17829E-13	--
Root vs Stem	RHSIM_Rhsim13G0177900	-3.851380363	1.57303E-14	6.81635E-13	RHSIM_Rhsim13G0177900
Root vs Stem	RHSIM_Rhsim03G0188700	4.543572618	1.58147E-14	6.84008E-13	RHSIM_Rhsim03G0188700
Root vs Stem	RHSIM_Rhsim12G0212200	-2.541021427	1.61332E-14	6.96479E-13	RHSIM_Rhsim12G0212200
Root vs Stem	RHSIM_Rhsim01G0097100	-1.533712355	1.73213E-14	7.46371E-13	RHSIM_Rhsim01G0097100
Root vs Stem	RHSIM_Rhsim05G0228600	-1.736262499	1.74112E-14	7.48842E-13	RHSIM_Rhsim05G0228600
Root vs Stem	RHSIM_Rhsim08G0015900	2.985848829	1.93983E-14	8.32754E-13	RHSIM_Rhsim08G0015900
Root vs Stem	RHSIM_Rhsim03G0058200	-3.45828308	1.95642E-14	8.38317E-13	RHSIM_Rhsim03G0058200
Root vs Stem	RHSIM_Rhsim11G0118300	1.714433839	1.99453E-14	8.51699E-13	RHSIM_Rhsim11G0118300
Root vs Stem	RHSIM_Rhsim02G0046400	3.98549735	1.99504E-14	8.51699E-13	RHSIM_Rhsim02G0046400
Root vs Stem	RHSIM_Rhsim11G0096000	-4.449351786	2.21712E-14	9.44757E-13	RHSIM_Rhsim11G0096000

Comparison	Gene ID	log_2FC	P-value	FDR (padj)	Gene Name
Root vs Stem	novel.5346	-5.161470327	2.26988E-14	9.65455E-13	--
Root vs Stem	RHSIM_Rhsim08G0160700	4.184898	2.31648E-14	9.83459E-13	RHSIM_Rhsim08G0160700
Root vs Stem	RHSIM_Rhsim02G0027900	1.603833936	2.3378E-14	9.90687E-13	RHSIM_Rhsim02G0027900
Root vs Stem	RHSIM_Rhsim08G0019800	-2.985503399	2.3672E-14	1.0013E-12	RHSIM_Rhsim08G0019800
Root vs Stem	novel.3658	-1.907520506	2.42884E-14	1.0255E-12	--
Root vs Stem	RHSIM_Rhsim13G0008800	9.398703199	2.43556E-14	1.02645E-12	RHSIM_Rhsim13G0008800
Root vs Stem	novel.5210	6.764624357	2.48156E-14	1.04393E-12	--
Root vs Stem	RHSIM_Rhsim05G0214600	-1.508876595	2.81065E-14	1.18022E-12	RHSIM_Rhsim05G0214600
Root vs Stem	RHSIM_Rhsim06G0057600	4.527663464	2.84708E-14	1.19334E-12	RHSIM_Rhsim06G0057600
Root vs Stem	RHSIM_Rhsim13G0215900	-1.090233938	2.86423E-14	1.19835E-12	RHSIM_Rhsim13G0215900
Root vs Stem	RHSIM_Rhsim04G0197500	-6.26112907	2.96697E-14	1.23909E-12	RHSIM_Rhsim04G0197500
Root vs Stem	RHSIM_RhsimUnG0039400	-5.33550879	3.24515E-14	1.35281E-12	RHSIM_RhsimUnG0039400
Root vs Stem	RHSIM_Rhsim06G0054200	2.842565134	3.35788E-14	1.39728E-12	RHSIM_Rhsim06G0054200
Root vs Stem	RHSIM_Rhsim04G0230900	3.348510215	3.3649E-14	1.39767E-12	RHSIM_Rhsim04G0230900
Root vs Stem	RHSIM_Rhsim05G0217500	1.182000132	3.47964E-14	1.44274E-12	RHSIM_Rhsim05G0217500
Root vs Stem	RHSIM_Rhsim01G0207000	1.913480865	3.57874E-14	1.48116E-12	RHSIM_Rhsim01G0207000
Root vs Stem	novel.3174	-3.46794028	3.71814E-14	1.5361E-12	--
Root vs Stem	RHSIM_Rhsim08G0013000	2.513622509	3.9943E-14	1.64724E-12	RHSIM_Rhsim08G0013000
Root vs Stem	RHSIM_Rhsim08G0083400	1.250045032	4.03365E-14	1.6605E-12	RHSIM_Rhsim08G0083400
Root vs Stem	RHSIM_Rhsim01G0109700	4.200249388	4.11357E-14	1.69038E-12	RHSIM_Rhsim01G0109700
Root vs Stem	RHSIM_Rhsim13G0005500	3.777108769	4.1254E-14	1.69222E-12	RHSIM_Rhsim13G0005500
Root vs Stem	RHSIM_Rhsim12G0013800	2.647172312	4.13417E-14	1.69281E-12	RHSIM_Rhsim12G0013800
Root vs Stem	RHSIM_Rhsim13G0159400	6.268868258	4.34685E-14	1.77674E-12	RHSIM_Rhsim13G0159400
Root vs Stem	RHSIM_Rhsim03G0167300	-2.513019207	4.48871E-14	1.83147E-12	RHSIM_Rhsim03G0167300
Root vs Stem	RHSIM_Rhsim10G0022700	1.267770415	5.11917E-14	2.08502E-12	RHSIM_Rhsim10G0022700

Comparison	Gene ID	log₂FC	P-value	FDR (padj)	Gene Name
Root vs Stem	RHSIM_Rhsim09G0125000	-3.698860712	5.13738E-14	2.08875E-12	RHSIM_Rhsim09G0125000
Root vs Stem	RHSIM_Rhsim07G0058800	1.310829407	5.21907E-14	2.117E-12	RHSIM_Rhsim07G0058800
Root vs Stem	RHSIM_Rhsim05G0212900	-5.650342971	5.22523E-14	2.117E-12	RHSIM_Rhsim05G0212900
Root vs Stem	RHSIM_Rhsim09G0184700	3.509200222	5.39944E-14	2.18374E-12	RHSIM_Rhsim09G0184700
Root vs Stem	RHSIM_Rhsim09G0107700	-1.161106637	5.56586E-14	2.24711E-12	RHSIM_Rhsim09G0107700
Root vs Stem	RHSIM_Rhsim09G0123100	4.46265772	5.61055E-14	2.26119E-12	RHSIM_Rhsim09G0123100
Root vs Stem	RHSIM_Rhsim05G0060900	-3.407593864	5.72538E-14	2.30344E-12	RHSIM_Rhsim05G0060900
Root vs Stem	RHSIM_Rhsim06G0149800	-1.430734457	5.91105E-14	2.374E-12	RHSIM_Rhsim06G0149800
Root vs Stem	RHSIM_RhsimUnG0150100	4.439302765	6.17052E-14	2.47389E-12	RHSIM_RhsimUnG0150100
Root vs Stem	RHSIM_Rhsim08G0093000	-1.089924853	6.61589E-14	2.64785E-12	RHSIM_Rhsim08G0093000
Root vs Stem	RHSIM_Rhsim03G0057100	2.614612402	6.64748E-14	2.65154E-12	RHSIM_Rhsim03G0057100
Root vs Stem	novel.133	9.335068214	6.64811E-14	2.65154E-12	--
Root vs Stem	RHSIM_Rhsim01G0189500	2.370751684	6.67472E-14	2.65755E-12	RHSIM_Rhsim01G0189500
Root vs Stem	novel.3672	-3.762905387	6.90917E-14	2.74616E-12	--
Root vs Stem	RHSIM_Rhsim03G0199400	-4.242484351	6.9471E-14	2.75648E-12	RHSIM_Rhsim03G0199400
Root vs Stem	RHSIM_Rhsim02G0049500	-1.429845546	6.97751E-14	2.76256E-12	RHSIM_Rhsim02G0049500
Root vs Stem	RHSIM_Rhsim03G0214300	-3.295756647	6.98638E-14	2.76256E-12	RHSIM_Rhsim03G0214300
Root vs Stem	RHSIM_Rhsim03G0085200	-2.924623488	7.35706E-14	2.90415E-12	RHSIM_Rhsim03G0085200
Root vs Stem	novel.1433	1.648161327	7.58724E-14	2.98989E-12	--
Root vs Stem	RHSIM_Rhsim06G0085500	1.942936262	8.03437E-14	3.16069E-12	RHSIM_Rhsim06G0085500
Root vs Stem	RHSIM_Rhsim04G0219900	3.875030297	8.15167E-14	3.20137E-12	RHSIM_Rhsim04G0219900
Root vs Stem	RHSIM_Rhsim09G0028000	-6.27416294	8.34532E-14	3.27185E-12	RHSIM_Rhsim09G0028000
Root vs Stem	RHSIM_Rhsim03G0249100	-2.207320033	8.44373E-14	3.30481E-12	RHSIM_Rhsim03G0249100
Root vs Stem	RHSIM_Rhsim08G0081400	-2.760169447	8.77147E-14	3.42341E-12	RHSIM_Rhsim08G0081400
Root vs Stem	RHSIM_Rhsim13G0044300	1.754801852	8.77645E-14	3.42341E-12	RHSIM_Rhsim13G0044300

Comparison	Gene ID	log_2FC	P-value	FDR (padj)	Gene Name
Root vs Stem	RHSIM_Rhsim03G0073200	4.982148172	8.85054E-14	3.44648E-12	RHSIM_Rhsim03G0073200
Root vs Stem	RHSIM_Rhsim10G0047000	9.242707594	9.16859E-14	3.56431E-12	RHSIM_Rhsim10G0047000
Root vs Stem	novel.4446	-2.098725782	9.88682E-14	3.83705E-12	--
Root vs Stem	RHSIM_Rhsim12G0204400	-3.066322568	1.02804E-13	3.98308E-12	RHSIM_Rhsim12G0204400
Root vs Stem	RHSIM_Rhsim11G0087600	1.004237748	1.06055E-13	4.10217E-12	RHSIM_Rhsim11G0087600
Root vs Stem	novel.5170	6.304677144	1.09129E-13	4.214E-12	--
Root vs Stem	RHSIM_Rhsim04G0071900	8.528607636	1.14578E-13	4.417E-12	RHSIM_Rhsim04G0071900
Root vs Stem	RHSIM_Rhsim03G0027200	2.841234344	1.15412E-13	4.44172E-12	RHSIM_Rhsim03G0027200
Root vs Stem	novel.3434	-1.12592946	1.18645E-13	4.55855E-12	--
Root vs Stem	RHSIM_RhsimUnG0035300	5.213566197	1.20933E-13	4.63872E-12	RHSIM_RhsimUnG0035300
Root vs Stem	RHSIM_Rhsim12G0009800	3.437918433	1.25967E-13	4.82379E-12	RHSIM_Rhsim12G0009800
Root vs Stem	RHSIM_Rhsim13G0090900	-4.02021425	1.29658E-13	4.95163E-12	RHSIM_Rhsim13G0090900
Root vs Stem	RHSIM_Rhsim07G0096500	-1.669867556	1.36934E-13	5.21776E-12	RHSIM_Rhsim07G0096500
Root vs Stem	RHSIM_Rhsim12G0040000	-3.68193465	1.42611E-13	5.42509E-12	RHSIM_Rhsim12G0040000
Root vs Stem	RHSIM_Rhsim13G0024600	-1.391866076	1.44864E-13	5.50172E-12	RHSIM_Rhsim13G0024600
Root vs Stem	RHSIM_Rhsim07G0203400	5.496328608	1.4522E-13	5.50619E-12	RHSIM_Rhsim07G0203400
Root vs Stem	RHSIM_Rhsim01G0171300	-1.169288231	1.47225E-13	5.57304E-12	RHSIM_Rhsim01G0171300
Root vs Stem	RHSIM_Rhsim12G0159500	1.516133033	1.54301E-13	5.8313E-12	RHSIM_Rhsim12G0159500
Root vs Stem	RHSIM_Rhsim08G0004800	-10.26519276	1.58675E-13	5.9868E-12	RHSIM_Rhsim08G0004800
Root vs Stem	RHSIM_Rhsim08G0037200	-2.36280912	1.68735E-13	6.35598E-12	RHSIM_Rhsim08G0037200
Root vs Stem	RHSIM_Rhsim13G0077400	-3.033804648	1.72294E-13	6.47945E-12	RHSIM_Rhsim13G0077400
Root vs Stem	RHSIM_Rhsim09G0211900	-2.163888349	1.72986E-13	6.49487E-12	RHSIM_Rhsim09G0211900
Root vs Stem	RHSIM_Rhsim03G0152000	1.480767182	1.75101E-13	6.5636E-12	RHSIM_Rhsim03G0152000
Root vs Stem	RHSIM_Rhsim01G0069500	-2.361114179	1.79787E-13	6.72829E-12	RHSIM_Rhsim01G0069500
Root vs Stem	RHSIM_RhsimUnG0078000	-2.609730841	1.81465E-13	6.7801E-12	RHSIM_RhsimUnG0078000

Comparison	Gene ID	log_2FC	P-value	FDR (padj)	Gene Name
Root vs Stem	RHSIM_Rhsim08G0118200	-9.566789334	1.83822E-13	6.85703E-12	RHSIM_Rhsim08G0118200
Root vs Stem	RHSIM_RhsimUnG0131200	3.332390279	1.90451E-13	7.09283E-12	RHSIM_RhsimUnG0131200
Root vs Stem	RHSIM_Rhsim12G0013400	1.381992187	1.9217E-13	7.14532E-12	RHSIM_Rhsim12G0013400
Root vs Stem	RHSIM_Rhsim09G0103200	4.158341586	2.03948E-13	7.57103E-12	RHSIM_Rhsim09G0103200
Root vs Stem	RHSIM_Rhsim07G0203200	1.487774168	2.0536E-13	7.61118E-12	RHSIM_Rhsim07G0203200
Root vs Stem	RHSIM_Rhsim12G0182800	-1.365873771	2.1659E-13	8.01454E-12	RHSIM_Rhsim12G0182800
Root vs Stem	RHSIM_Rhsim03G0249700	5.573399571	2.20529E-13	8.1472E-12	RHSIM_Rhsim03G0249700
Root vs Stem	RHSIM_Rhsim07G0130100	3.202905295	2.46574E-13	9.09482E-12	RHSIM_Rhsim07G0130100
Root vs Stem	RHSIM_Rhsim07G0239800	-1.923940714	2.57161E-13	9.47017E-12	RHSIM_Rhsim07G0239800
Root vs Stem	RHSIM_Rhsim13G0189300	-3.114293837	2.70588E-13	9.94873E-12	RHSIM_Rhsim13G0189300
Root vs Stem	RHSIM_Rhsim07G0180700	1.408591705	2.71286E-13	9.95851E-12	RHSIM_Rhsim07G0180700
Root vs Stem	RHSIM_Rhsim12G0143800	-3.874179189	2.78605E-13	1.02109E-11	RHSIM_Rhsim12G0143800
Root vs Stem	RHSIM_Rhsim11G0102600	-5.936727242	2.82341E-13	1.03314E-11	RHSIM_Rhsim11G0102600
Root vs Stem	RHSIM_Rhsim04G0167300	-1.369433462	2.83105E-13	1.0343E-11	RHSIM_Rhsim04G0167300
Root vs Stem	RHSIM_Rhsim12G0189400	-1.620840582	2.92064E-13	1.06534E-11	RHSIM_Rhsim12G0189400
Root vs Stem	novel.6029	-2.810163043	2.95122E-13	1.07311E-11	--
Root vs Stem	RHSIM_Rhsim07G0200900	1.539455645	2.95126E-13	1.07311E-11	RHSIM_Rhsim07G0200900
Root vs Stem	RHSIM_Rhsim05G0002300	1.680689182	3.06248E-13	1.1118E-11	RHSIM_Rhsim05G0002300
Root vs Stem	RHSIM_Rhsim12G0013500	-1.568162995	3.09109E-13	1.12042E-11	RHSIM_Rhsim12G0013500
Root vs Stem	RHSIM_Rhsim04G0182600	3.031495782	3.11804E-13	1.12842E-11	RHSIM_Rhsim04G0182600
Root vs Stem	RHSIM_Rhsim01G0280700	-2.821167503	3.42953E-13	1.2392E-11	RHSIM_Rhsim01G0280700
Root vs Stem	RHSIM_Rhsim05G0068000	1.083563503	3.4832E-13	1.25662E-11	RHSIM_Rhsim05G0068000
Root vs Stem	RHSIM_Rhsim12G0036300	4.835184629	3.49676E-13	1.25955E-11	RHSIM_Rhsim12G0036300
Root vs Stem	RHSIM_Rhsim04G0216700	1.785827894	3.5491E-13	1.2764E-11	RHSIM_Rhsim04G0216700
Root vs Stem	RHSIM_Rhsim01G0015500	-2.109837782	3.57914E-13	1.2852E-11	RHSIM_Rhsim01G0015500

Comparison	Gene ID	log_2FC	P-value	FDR (padj)	Gene Name
Root vs Stem	RHSIM_Rhsim12G0136200	1.659359556	3.65103E-13	1.30898E-11	RHSIM_Rhsim12G0136200
Root vs Stem	RHSIM_Rhsim09G0129100	-4.484569857	3.66422E-13	1.31167E-11	RHSIM_Rhsim09G0129100
Root vs Stem	RHSIM_Rhsim04G0180800	3.027826091	3.90206E-13	1.39464E-11	RHSIM_Rhsim04G0180800
Root vs Stem	RHSIM_Rhsim03G0246100	1.395283101	4.15865E-13	1.48405E-11	RHSIM_Rhsim03G0246100
Root vs Stem	novel.3103	2.572170992	4.20167E-13	1.49708E-11	--
Root vs Stem	RHSIM_RhsimUnG0100000	-9.882333532	4.25336E-13	1.51316E-11	RHSIM_RhsimUnG0100000
Root vs Stem	RHSIM_Rhsim02G0002700	1.865748367	4.27905E-13	1.51995E-11	RHSIM_Rhsim02G0002700
Root vs Stem	RHSIM_Rhsim12G0085300	-1.805129507	4.9395E-13	1.75185E-11	RHSIM_Rhsim12G0085300
Root vs Stem	novel.4399	-2.465459696	5.3264E-13	1.88617E-11	--
Root vs Stem	novel.2289	4.909809855	5.41246E-13	1.9137E-11	--
Root vs Stem	RHSIM_Rhsim12G0094100	-6.072143591	5.61611E-13	1.98267E-11	RHSIM_Rhsim12G0094100
Root vs Stem	RHSIM_Rhsim01G0120800	3.574918146	5.75833E-13	2.02977E-11	RHSIM_Rhsim01G0120800
Root vs Stem	RHSIM_Rhsim04G0174300	-1.664368	5.88542E-13	2.0714E-11	RHSIM_Rhsim04G0174300
Root vs Stem	RHSIM_Rhsim03G0216700	2.56981088	5.94274E-13	2.08526E-11	RHSIM_Rhsim03G0216700
Root vs Stem	RHSIM_Rhsim04G0244400	1.06914514	5.9429E-13	2.08526E-11	RHSIM_Rhsim04G0244400
Root vs Stem	RHSIM_Rhsim02G0131400	5.614189074	6.08833E-13	2.13304E-11	RHSIM_Rhsim02G0131400
Root vs Stem	RHSIM_Rhsim05G0056500	-1.694648642	6.20477E-13	2.17054E-11	RHSIM_Rhsim05G0056500
Root vs Stem	RHSIM_Rhsim08G0199300	2.432769672	6.24821E-13	2.18242E-11	RHSIM_Rhsim08G0199300
Root vs Stem	RHSIM_Rhsim01G0236600	-1.728790838	6.46475E-13	2.25464E-11	RHSIM_Rhsim01G0236600
Root vs Stem	RHSIM_Rhsim10G0050900	-4.706033165	6.52587E-13	2.27252E-11	RHSIM_Rhsim10G0050900
Root vs Stem	RHSIM_Rhsim07G0179600	-1.371170731	6.79369E-13	2.36222E-11	RHSIM_Rhsim07G0179600
Root vs Stem	RHSIM_Rhsim08G0132300	7.370575939	6.83037E-13	2.37139E-11	RHSIM_Rhsim08G0132300
Root vs Stem	novel.3863	-2.067800811	7.08614E-13	2.45649E-11	--
Root vs Stem	novel.295	1.053787708	7.1171E-13	2.46352E-11	--
Root vs Stem	RHSIM_Rhsim04G0071500	2.194866337	7.41733E-13	2.56359E-11	RHSIM_Rhsim04G0071500

Comparison	Gene ID	log_2FC	P-value	FDR (padj)	Gene Name
Root vs Stem	RHSIM_Rhsim08G0088200	-2.441825765	7.65471E-13	2.64168E-11	RHSIM_Rhsim08G0088200
Root vs Stem	novel.5731	-2.147994947	7.83042E-13	2.69828E-11	--
Root vs Stem	RHSIM_RhsimUnG0083500	-1.79185428	7.93649E-13	2.73074E-11	RHSIM_RhsimUnG0083500
Root vs Stem	RHSIM_RhsimUnG0236100	2.68953964	8.1874E-13	2.81288E-11	RHSIM_RhsimUnG0236100
Root vs Stem	RHSIM_Rhsim08G0097800	-3.291647648	8.50857E-13	2.91453E-11	RHSIM_Rhsim08G0097800
Root vs Stem	RHSIM_Rhsim01G0211000	1.174335858	8.52358E-13	2.91534E-11	RHSIM_Rhsim01G0211000
Root vs Stem	RHSIM_Rhsim08G0156800	-5.553075795	8.89049E-13	3.03184E-11	RHSIM_Rhsim08G0156800
Root vs Stem	RHSIM_Rhsim11G0088000	1.606874388	9.05451E-13	3.08322E-11	RHSIM_Rhsim11G0088000
Root vs Stem	novel.3025	5.560416747	9.354E-13	3.1783E-11	--
Root vs Stem	RHSIM_Rhsim07G0241000	-6.369778979	9.37275E-13	3.1783E-11	RHSIM_Rhsim07G0241000
Root vs Stem	RHSIM_Rhsim01G0071400	-1.277642107	9.37512E-13	3.1783E-11	RHSIM_Rhsim01G0071400
Root vs Stem	RHSIM_Rhsim09G0052200	1.380875429	9.62961E-13	3.25978E-11	RHSIM_Rhsim09G0052200
Root vs Stem	RHSIM_Rhsim10G0110300	-7.490511059	9.80122E-13	3.31301E-11	RHSIM_Rhsim10G0110300
Root vs Stem	RHSIM_Rhsim09G0056000	-4.278598921	1.11778E-12	3.77279E-11	RHSIM_Rhsim09G0056000
Root vs Stem	RHSIM_Rhsim06G0022200	-3.110136226	1.26384E-12	4.25956E-11	RHSIM_Rhsim06G0022200
Root vs Stem	RHSIM_Rhsim02G0200900	2.0342629	1.34322E-12	4.52048E-11	RHSIM_Rhsim02G0200900
Root vs Stem	RHSIM_Rhsim12G0074200	2.137655627	1.38788E-12	4.66395E-11	RHSIM_Rhsim12G0074200
Root vs Stem	RHSIM_Rhsim05G0089900	-5.308511462	1.47541E-12	4.9509E-11	RHSIM_Rhsim05G0089900
Root vs Stem	RHSIM_Rhsim09G0051700	1.205723915	1.49162E-12	4.998E-11	RHSIM_Rhsim09G0051700
Root vs Stem	RHSIM_Rhsim04G0199400	1.270823919	1.49922E-12	5.01619E-11	RHSIM_Rhsim04G0199400
Root vs Stem	RHSIM_RhsimUnG0166800	-1.523880922	1.53909E-12	5.14211E-11	RHSIM_RhsimUnG0166800
Root vs Stem	RHSIM_Rhsim07G0233900	-9.435287581	1.54678E-12	5.16032E-11	RHSIM_Rhsim07G0233900
Root vs Stem	RHSIM_Rhsim02G0058600	6.58483543	1.56024E-12	5.19771E-11	RHSIM_Rhsim02G0058600
Root vs Stem	RHSIM_Rhsim03G0222700	-2.270004845	1.57738E-12	5.24237E-11	RHSIM_Rhsim03G0222700
Root vs Stem	RHSIM_Rhsim06G0234200	7.91054564	1.57819E-12	5.24237E-11	RHSIM_Rhsim06G0234200

Comparison	Gene ID	log_2FC	P-value	FDR (padj)	Gene Name
Root vs Stem	novel.4026	-5.331547142	1.69521E-12	5.61873E-11	--
Root vs Stem	RHSIM_Rhsim06G0121200	-1.877705222	1.69665E-12	5.61873E-11	RHSIM_Rhsim06G0121200
Root vs Stem	RHSIM_Rhsim01G0110400	2.916011807	1.6988E-12	5.61873E-11	RHSIM_Rhsim01G0110400
Root vs Stem	RHSIM_Rhsim12G0026500	-5.386086968	1.71367E-12	5.65979E-11	RHSIM_Rhsim12G0026500
Root vs Stem	novel.3075	-1.391546973	1.91926E-12	6.32972E-11	--
Root vs Stem	RHSIM_Rhsim06G0164500	-1.117034623	1.92476E-12	6.33412E-11	RHSIM_Rhsim06G0164500
Root vs Stem	RHSIM_Rhsim07G0013600	3.144260059	1.92609E-12	6.33412E-11	RHSIM_Rhsim07G0013600
Root vs Stem	RHSIM_Rhsim01G0218600	3.967619973	1.99589E-12	6.5543E-11	RHSIM_Rhsim01G0218600
Root vs Stem	RHSIM_Rhsim06G0171600	6.988927434	2.02847E-12	6.65182E-11	RHSIM_Rhsim06G0171600
Root vs Stem	RHSIM_Rhsim06G0065700	-2.532131301	2.0678E-12	6.77117E-11	RHSIM_Rhsim06G0065700
Root vs Stem	RHSIM_Rhsim11G0138500	-4.31123316	2.07695E-12	6.79149E-11	RHSIM_Rhsim11G0138500
Root vs Stem	RHSIM_Rhsim01G0260300	-3.136775983	2.13235E-12	6.95289E-11	RHSIM_Rhsim01G0260300
Root vs Stem	RHSIM_Rhsim03G0057500	3.043279863	2.14788E-12	6.99366E-11	RHSIM_Rhsim03G0057500
Root vs Stem	novel.4011	4.606706685	2.19099E-12	7.12395E-11	--
Root vs Stem	novel.2375	-1.911321452	2.23555E-12	7.25863E-11	--
Root vs Stem	RHSIM_Rhsim12G0158100	1.174824506	2.27578E-12	7.37883E-11	RHSIM_Rhsim12G0158100
Root vs Stem	RHSIM_Rhsim06G0046200	-1.613192039	2.28358E-12	7.39372E-11	RHSIM_Rhsim06G0046200
Root vs Stem	RHSIM_Rhsim06G0043300	-5.031500528	2.31591E-12	7.48788E-11	RHSIM_Rhsim06G0043300
Root vs Stem	RHSIM_RhsimUnG0039300	-4.603069944	2.34768E-12	7.57609E-11	RHSIM_RhsimUnG0039300
Root vs Stem	RHSIM_Rhsim13G0107000	4.041112126	2.34976E-12	7.57609E-11	RHSIM_Rhsim13G0107000
Root vs Stem	RHSIM_Rhsim13G0097500	1.900310396	2.35989E-12	7.59812E-11	RHSIM_Rhsim13G0097500
Root vs Stem	RHSIM_Rhsim12G0024900	1.325138259	2.5876E-12	8.31966E-11	RHSIM_Rhsim12G0024900
Root vs Stem	RHSIM_Rhsim08G0180700	-4.744562166	2.7763E-12	8.90796E-11	RHSIM_Rhsim08G0180700
Root vs Stem	RHSIM_Rhsim12G0146100	-3.344151497	2.78014E-12	8.90796E-11	RHSIM_Rhsim12G0146100
Root vs Stem	RHSIM_Rhsim01G0107600	3.757506364	2.78217E-12	8.90796E-11	RHSIM_Rhsim01G0107600

Comparison	Gene ID	log_2FC	P-value	FDR (padj)	Gene Name
Root vs Stem	RHSIM_RhsimUnG0119500	1.678383385	2.88098E-12	9.21153E-11	RHSIM_RhsimUnG0119500
Root vs Stem	RHSIM_Rhsim07G0184300	2.214281095	2.92316E-12	9.33345E-11	RHSIM_Rhsim07G0184300
Root vs Stem	RHSIM_Rhsim03G0049100	1.335011059	2.97272E-12	9.47858E-11	RHSIM_Rhsim03G0049100
Root vs Stem	RHSIM_Rhsim08G0026900	1.267572215	3.0975E-12	9.8628E-11	RHSIM_Rhsim08G0026900
Root vs Stem	RHSIM_Rhsim10G0127000	2.866589654	3.11432E-12	9.90267E-11	RHSIM_Rhsim10G0127000
Root vs Stem	RHSIM_Rhsim07G0142300	-2.692297963	3.13071E-12	9.94108E-11	RHSIM_Rhsim07G0142300
Root vs Stem	RHSIM_Rhsim01G0033300	2.129718454	3.22998E-12	1.02422E-10	RHSIM_Rhsim01G0033300
Root vs Stem	novel.5924	-3.808391325	3.42301E-12	1.08394E-10	--
Root vs Stem	RHSIM_Rhsim02G0029400	-9.818314334	3.54435E-12	1.12082E-10	RHSIM_Rhsim02G0029400
Root vs Stem	RHSIM_Rhsim12G0142800	3.316417492	3.55461E-12	1.12253E-10	RHSIM_Rhsim12G0142800
Root vs Stem	RHSIM_Rhsim01G0154500	1.348327696	3.55969E-12	1.12259E-10	RHSIM_Rhsim01G0154500
Root vs Stem	RHSIM_Rhsim09G0092400	2.714960764	3.61958E-12	1.13992E-10	RHSIM_Rhsim09G0092400
Root vs Stem	RHSIM_Rhsim04G0039300	2.743472778	3.70735E-12	1.16597E-10	RHSIM_Rhsim04G0039300
Root vs Stem	RHSIM_Rhsim03G0215400	-1.555572534	3.77741E-12	1.18638E-10	RHSIM_Rhsim03G0215400
Root vs Stem	RHSIM_Rhsim06G0164400	2.565759002	3.79005E-12	1.18873E-10	RHSIM_Rhsim06G0164400
Root vs Stem	RHSIM_Rhsim02G0023300	-1.334251249	3.86397E-12	1.20951E-10	RHSIM_Rhsim02G0023300
Root vs Stem	RHSIM_Rhsim09G0164900	1.318439963	3.86679E-12	1.20951E-10	RHSIM_Rhsim09G0164900
Root vs Stem	RHSIM_Rhsim03G0170900	-5.273799777	3.88261E-12	1.21281E-10	RHSIM_Rhsim03G0170900
Root vs Stem	novel.6028	3.849663887	4.0759E-12	1.27147E-10	--
Root vs Stem	RHSIM_Rhsim10G0030700	3.14317275	4.08176E-12	1.27158E-10	RHSIM_Rhsim10G0030700
Root vs Stem	RHSIM_Rhsim04G0111400	-1.763566093	4.1028E-12	1.27641E-10	RHSIM_Rhsim04G0111400
Root vs Stem	RHSIM_Rhsim12G0199600	-1.233931696	4.35933E-12	1.35439E-10	RHSIM_Rhsim12G0199600
Root vs Stem	RHSIM_Rhsim09G0204800	6.653916644	4.38686E-12	1.36111E-10	RHSIM_Rhsim09G0204800
Root vs Stem	RHSIM_Rhsim07G0231700	5.009481669	4.4547E-12	1.3803E-10	RHSIM_Rhsim07G0231700
Root vs Stem	RHSIM_Rhsim08G0051200	3.386820307	4.47958E-12	1.38614E-10	RHSIM_Rhsim08G0051200

Comparison	Gene ID	log_2FC	P-value	FDR (padj)	Gene Name
Root vs Stem	novel.4874	-3.049530027	4.59683E-12	1.42052E-10	--
Root vs Stem	RHSIM_Rhsim05G0216700	2.51342589	4.74234E-12	1.46352E-10	RHSIM_Rhsim05G0216700
Root vs Stem	RHSIM_Rhsim10G0055000	1.433116893	4.92298E-12	1.51724E-10	RHSIM_Rhsim10G0055000
Root vs Stem	RHSIM_Rhsim04G0008900	2.381953426	4.94283E-12	1.52132E-10	RHSIM_Rhsim04G0008900
Root vs Stem	RHSIM_Rhsim08G0033600	3.299676791	4.96922E-12	1.52741E-10	RHSIM_Rhsim08G0033600
Root vs Stem	RHSIM_Rhsim12G0020700	1.69570446	5.01429E-12	1.53921E-10	RHSIM_Rhsim12G0020700
Root vs Stem	RHSIM_Rhsim12G0052000	1.080825929	5.19796E-12	1.59346E-10	RHSIM_Rhsim12G0052000
Root vs Stem	RHSIM_Rhsim02G0200400	1.168172578	5.39943E-12	1.65303E-10	RHSIM_Rhsim02G0200400
Root vs Stem	RHSIM_Rhsim03G0067600	4.752977249	5.62731E-12	1.72051E-10	RHSIM_Rhsim03G0067600
Root vs Stem	RHSIM_RhsimUnG0217100	3.825957217	5.6363E-12	1.72097E-10	RHSIM_RhsimUnG0217100
Root vs Stem	RHSIM_Rhsim12G0169700	10.29603907	6.01381E-12	1.83382E-10	RHSIM_Rhsim12G0169700
Root vs Stem	RHSIM_Rhsim08G0088300	-2.377497106	6.09021E-12	1.85466E-10	RHSIM_Rhsim08G0088300
Root vs Stem	RHSIM_Rhsim07G0235900	-1.698935634	6.14497E-12	1.86887E-10	RHSIM_Rhsim07G0235900
Root vs Stem	RHSIM_Rhsim08G0098300	1.554037234	6.22255E-12	1.88997E-10	RHSIM_Rhsim08G0098300
Root vs Stem	RHSIM_Rhsim03G0174500	-2.925854087	6.265E-12	1.90036E-10	RHSIM_Rhsim03G0174500
Root vs Stem	RHSIM_Rhsim12G0015000	2.497546027	6.4896E-12	1.9659E-10	RHSIM_Rhsim12G0015000
Root vs Stem	RHSIM_Rhsim08G0075200	1.37512383	6.90554E-12	2.08641E-10	RHSIM_Rhsim08G0075200
Root vs Stem	RHSIM_Rhsim08G0055300	1.955483164	6.93697E-12	2.09317E-10	RHSIM_Rhsim08G0055300
Root vs Stem	RHSIM_Rhsim03G0057700	-2.264772153	7.06716E-12	2.12966E-10	RHSIM_Rhsim03G0057700
Root vs Stem	novel.1659	1.681941819	7.30496E-12	2.19845E-10	--
Root vs Stem	RHSIM_Rhsim03G0220500	1.598086411	7.38933E-12	2.22094E-10	RHSIM_Rhsim03G0220500
Root vs Stem	RHSIM_Rhsim04G0049500	1.921343758	7.4573E-12	2.238E-10	RHSIM_Rhsim04G0049500
Root vs Stem	RHSIM_Rhsim01G0076900	-4.363258942	7.4655E-12	2.238E-10	RHSIM_Rhsim01G0076900
Root vs Stem	RHSIM_Rhsim01G0249900	-1.62708254	7.60424E-12	2.27368E-10	RHSIM_Rhsim01G0249900
Root vs Stem	RHSIM_Rhsim08G0222300	-1.496183662	7.64177E-12	2.28194E-10	RHSIM_Rhsim08G0222300

Comparison	Gene ID	log_2FC	P-value	FDR (padj)	Gene Name
Root vs Stem	RHSIM_Rhsim03G0266200	-3.490893436	7.69652E-12	2.29531E-10	RHSIM_Rhsim03G0266200
Root vs Stem	RHSIM_RhsimUnG0254400	2.986234676	7.73555E-12	2.30397E-10	RHSIM_RhsimUnG0254400
Root vs Stem	RHSIM_Rhsim02G0005500	1.121093158	7.7765E-12	2.31318E-10	RHSIM_Rhsim02G0005500
Root vs Stem	RHSIM_Rhsim02G0200700	1.898929979	7.79334E-12	2.31356E-10	RHSIM_Rhsim02G0200700
Root vs Stem	RHSIM_Rhsim03G0087300	4.399195844	7.79783E-12	2.31356E-10	RHSIM_Rhsim03G0087300
Root vs Stem	RHSIM_Rhsim01G0106800	-4.176563493	7.86497E-12	2.32837E-10	RHSIM_Rhsim01G0106800
Root vs Stem	RHSIM_Rhsim01G0052300	-1.390513227	7.86795E-12	2.32837E-10	RHSIM_Rhsim01G0052300
Root vs Stem	RHSIM_RhsimUnG0255900	3.678100633	8.24847E-12	2.43785E-10	RHSIM_RhsimUnG0255900
Root vs Stem	RHSIM_Rhsim09G0123700	-3.099074012	8.28422E-12	2.44528E-10	RHSIM_Rhsim09G0123700
Root vs Stem	RHSIM_Rhsim07G0228900	-7.595928792	8.54853E-12	2.52007E-10	RHSIM_Rhsim07G0228900
Root vs Stem	RHSIM_Rhsim13G0116000	-3.101821553	8.7904E-12	2.58806E-10	RHSIM_Rhsim13G0116000
Root vs Stem	RHSIM_Rhsim04G0011800	2.737623543	8.90299E-12	2.61787E-10	RHSIM_Rhsim04G0011800
Root vs Stem	RHSIM_Rhsim12G0036500	2.668701185	8.96219E-12	2.63192E-10	RHSIM_Rhsim12G0036500
Root vs Stem	RHSIM_Rhsim01G0223500	3.728126002	9.0915E-12	2.66649E-10	RHSIM_Rhsim01G0223500
Root vs Stem	RHSIM_Rhsim06G0030600	3.701750237	9.25629E-12	2.71137E-10	RHSIM_Rhsim06G0030600
Root vs Stem	RHSIM_Rhsim08G0196800	-1.837151135	9.68793E-12	2.83421E-10	RHSIM_Rhsim08G0196800
Root vs Stem	RHSIM_Rhsim01G0230900	6.550580671	9.89214E-12	2.88684E-10	RHSIM_Rhsim01G0230900
Root vs Stem	RHSIM_Rhsim10G0079700	-1.881906558	1.009E-11	2.93694E-10	RHSIM_Rhsim10G0079700
Root vs Stem	RHSIM_Rhsim07G0199700	4.960609843	1.03241E-11	3.00128E-10	RHSIM_Rhsim07G0199700
Root vs Stem	RHSIM_RhsimUnG0138500	-4.59174675	1.04269E-11	3.02735E-10	RHSIM_RhsimUnG0138500
Root vs Stem	RHSIM_Rhsim13G0119600	1.135128384	1.1102E-11	3.21929E-10	RHSIM_Rhsim13G0119600
Root vs Stem	RHSIM_Rhsim13G0221400	-3.602512169	1.12473E-11	3.25325E-10	RHSIM_Rhsim13G0221400
Root vs Stem	RHSIM_Rhsim03G0066800	-1.419839666	1.14043E-11	3.29452E-10	RHSIM_Rhsim03G0066800
Root vs Stem	RHSIM_Rhsim07G0139700	4.134946223	1.14415E-11	3.30114E-10	RHSIM_Rhsim07G0139700
Root vs Stem	RHSIM_Rhsim05G0114800	-4.717534297	1.14577E-11	3.30168E-10	RHSIM_Rhsim05G0114800

Comparison	Gene ID	log_2FC	P-value	FDR (padj)	Gene Name
Root vs Stem	RHSIM_Rhsim02G0106100	-1.239160854	1.16177E-11	3.34304E-10	RHSIM_Rhsim02G0106100
Root vs Stem	RHSIM_Rhsim01G0136500	6.413500598	1.16302E-11	3.34304E-10	RHSIM_Rhsim01G0136500
Root vs Stem	RHSIM_Rhsim03G0221200	-1.129082666	1.18245E-11	3.39464E-10	RHSIM_Rhsim03G0221200
Root vs Stem	RHSIM_Rhsim06G0147300	-3.295791811	1.20663E-11	3.45977E-10	RHSIM_Rhsim06G0147300
Root vs Stem	RHSIM_Rhsim07G0134000	1.14792803	1.23835E-11	3.5463E-10	RHSIM_Rhsim07G0134000
Root vs Stem	RHSIM_Rhsim04G0100900	-1.459837035	1.25486E-11	3.5891E-10	RHSIM_Rhsim04G0100900
Root vs Stem	RHSIM_Rhsim12G0146800	-1.230792536	1.28438E-11	3.66898E-10	RHSIM_Rhsim12G0146800
Root vs Stem	RHSIM_Rhsim02G0228500	1.806640074	1.29329E-11	3.68987E-10	RHSIM_Rhsim02G0228500
Root vs Stem	RHSIM_Rhsim05G0161500	2.074159108	1.42812E-11	4.06951E-10	RHSIM_Rhsim05G0161500
Root vs Stem	RHSIM_Rhsim13G0157400	1.066192519	1.50303E-11	4.2777E-10	RHSIM_Rhsim13G0157400
Root vs Stem	RHSIM_Rhsim13G0029800	3.436766379	1.51207E-11	4.29811E-10	RHSIM_Rhsim13G0029800
Root vs Stem	RHSIM_Rhsim04G0214200	-1.088037943	1.52745E-11	4.33515E-10	RHSIM_Rhsim04G0214200
Root vs Stem	RHSIM_Rhsim08G0157000	-5.246896913	1.52886E-11	4.33515E-10	RHSIM_Rhsim08G0157000
Root vs Stem	RHSIM_Rhsim08G0000300	-1.494372094	1.53856E-11	4.3573E-10	RHSIM_Rhsim08G0000300
Root vs Stem	RHSIM_Rhsim04G0149300	3.264890957	1.57921E-11	4.46314E-10	RHSIM_Rhsim04G0149300
Root vs Stem	RHSIM_Rhsim05G0053600	2.830897907	1.5798E-11	4.46314E-10	RHSIM_Rhsim05G0053600
Root vs Stem	novel.1958	1.875862502	1.59772E-11	4.50271E-10	--
Root vs Stem	RHSIM_Rhsim12G0017000	-1.760376872	1.63702E-11	4.60336E-10	RHSIM_Rhsim12G0017000
Root vs Stem	RHSIM_Rhsim07G0203600	5.000768163	1.63743E-11	4.60336E-10	RHSIM_Rhsim07G0203600
Root vs Stem	RHSIM_Rhsim01G0212000	-1.114272361	1.65714E-11	4.6531E-10	RHSIM_Rhsim01G0212000
Root vs Stem	RHSIM_Rhsim09G0205600	-1.332243497	1.66239E-11	4.66218E-10	RHSIM_Rhsim09G0205600
Root vs Stem	RHSIM_Rhsim07G0018300	3.012507996	1.71102E-11	4.79272E-10	RHSIM_Rhsim07G0018300
Root vs Stem	RHSIM_Rhsim07G0216100	5.025909965	1.73948E-11	4.86653E-10	RHSIM_Rhsim07G0216100
Root vs Stem	RHSIM_Rhsim02G0132100	1.559308429	1.76905E-11	4.94325E-10	RHSIM_Rhsim02G0132100
Root vs Stem	RHSIM_Rhsim03G0055100	4.450138091	1.78997E-11	4.99567E-10	RHSIM_Rhsim03G0055100

Comparison	Gene ID	log2FC	P-value	FDR (padj)	Gene Name
Root vs Stem	RHSIM_Rhsim07G0163500	-1.529660644	1.91103E-11	5.32709E-10	RHSIM_Rhsim07G0163500
Root vs Stem	RHSIM_Rhsim08G0192900	3.944909212	1.9383E-11	5.39657E-10	RHSIM_Rhsim08G0192900
Root vs Stem	RHSIM_Rhsim01G0058400	4.813041876	2.03632E-11	5.65583E-10	RHSIM_Rhsim01G0058400
Root vs Stem	RHSIM_Rhsim06G0114200	-1.242795673	2.15986E-11	5.99173E-10	RHSIM_Rhsim06G0114200
Root vs Stem	RHSIM_Rhsim07G0023300	-9.081201711	2.24287E-11	6.21454E-10	RHSIM_Rhsim07G0023300
Root vs Stem	RHSIM_Rhsim02G0118800	3.389628559	2.28027E-11	6.31058E-10	RHSIM_Rhsim02G0118800
Root vs Stem	RHSIM_Rhsim05G0075500	1.774335826	2.31998E-11	6.41276E-10	RHSIM_Rhsim05G0075500
Root vs Stem	RHSIM_Rhsim09G0066400	1.368723054	2.34254E-11	6.46736E-10	RHSIM_Rhsim09G0066400
Root vs Stem	novel.4092	-1.389590915	2.42316E-11	6.68194E-10	--
Root vs Stem	RHSIM_Rhsim10G0108800	3.36920918	2.44761E-11	6.74132E-10	RHSIM_Rhsim10G0108800
Root vs Stem	RHSIM_Rhsim12G0116600	-2.236997385	2.4653E-11	6.78193E-10	RHSIM_Rhsim12G0116600
Root vs Stem	RHSIM_Rhsim07G0190700	-1.746653787	2.47906E-11	6.81166E-10	RHSIM_Rhsim07G0190700
Root vs Stem	RHSIM_Rhsim09G0095700	5.144626946	2.48336E-11	6.81534E-10	RHSIM_Rhsim09G0095700
Root vs Stem	RHSIM_Rhsim11G0025700	-2.118108383	2.77339E-11	7.60224E-10	RHSIM_Rhsim11G0025700
Root vs Stem	RHSIM_Rhsim08G0059200	3.575737134	2.78572E-11	7.62698E-10	RHSIM_Rhsim08G0059200
Root vs Stem	RHSIM_Rhsim12G0035400	-2.716573791	2.81348E-11	7.69385E-10	RHSIM_Rhsim12G0035400
Root vs Stem	RHSIM_Rhsim01G0188400	-2.81030821	2.81826E-11	7.69778E-10	RHSIM_Rhsim01G0188400
Root vs Stem	RHSIM_Rhsim07G0101400	-1.401817455	2.86429E-11	7.80503E-10	RHSIM_Rhsim07G0101400
Root vs Stem	RHSIM_Rhsim10G0206700	1.470410329	2.90305E-11	7.90131E-10	RHSIM_Rhsim10G0206700
Root vs Stem	novel.1227	8.304243389	2.98711E-11	8.1205E-10	--
Root vs Stem	RHSIM_Rhsim09G0021700	1.230832742	3.10685E-11	8.42615E-10	RHSIM_Rhsim09G0021700
Root vs Stem	RHSIM_Rhsim06G0054600	-1.365805706	3.15121E-11	8.5364E-10	RHSIM_Rhsim06G0054600
Root vs Stem	novel.1621	4.222591367	3.21053E-11	8.67751E-10	--
Root vs Stem	RHSIM_Rhsim06G0062700	-2.369008749	3.21083E-11	8.67751E-10	RHSIM_Rhsim06G0062700
Root vs Stem	RHSIM_Rhsim12G0084800	2.647443171	3.45302E-11	9.32113E-10	RHSIM_Rhsim12G0084800

Comparison	Gene ID	log_2FC	P-value	FDR (padj)	Gene Name
Root vs Stem	RHSIM_Rhsim07G0077100	-5.002886388	3.56784E-11	9.60859E-10	RHSIM_Rhsim07G0077100
Root vs Stem	RHSIM_Rhsim05G0221600	4.432112697	3.61307E-11	9.71904E-10	RHSIM_Rhsim05G0221600
Root vs Stem	RHSIM_Rhsim05G0197000	3.358462705	3.71594E-11	9.98409E-10	RHSIM_Rhsim05G0197000
Root vs Stem	RHSIM_Rhsim08G0105000	4.849273012	3.72944E-11	1.00087E-09	RHSIM_Rhsim08G0105000
Root vs Stem	novel.1217	2.425904879	3.85928E-11	1.03451E-09	--
Root vs Stem	RHSIM_Rhsim01G0192200	-1.721032975	3.90779E-11	1.0463E-09	RHSIM_Rhsim01G0192200
Root vs Stem	novel.3095	-1.319801321	3.93349E-11	1.05196E-09	--
Root vs Stem	RHSIM_Rhsim08G0079800	1.305547762	4.22895E-11	1.12966E-09	RHSIM_Rhsim08G0079800
Root vs Stem	RHSIM_Rhsim04G0231300	8.965171069	4.26792E-11	1.13875E-09	RHSIM_Rhsim04G0231300
Root vs Stem	RHSIM_Rhsim04G0096300	4.848061158	4.27364E-11	1.13896E-09	RHSIM_Rhsim04G0096300
Root vs Stem	RHSIM_Rhsim13G0012900	-6.321960362	4.34436E-11	1.15647E-09	RHSIM_Rhsim13G0012900
Root vs Stem	RHSIM_Rhsim04G0169400	-3.161378674	4.35622E-11	1.15829E-09	RHSIM_Rhsim04G0169400
Root vs Stem	RHSIM_RhsimUnG0093600	6.349498364	4.46204E-11	1.18506E-09	RHSIM_RhsimUnG0093600
Root vs Stem	RHSIM_Rhsim09G0190300	-1.456460085	4.5588E-11	1.20937E-09	RHSIM_Rhsim09G0190300
Root vs Stem	RHSIM_Rhsim11G0060200	3.873244122	4.62657E-11	1.22593E-09	RHSIM_Rhsim11G0060200
Root vs Stem	novel.1023	-3.135011266	4.63218E-11	1.22601E-09	--
Root vs Stem	RHSIM_Rhsim11G0158800	6.849786602	4.65163E-11	1.22975E-09	RHSIM_Rhsim11G0158800
Root vs Stem	RHSIM_Rhsim01G0053000	-6.62300484	4.67673E-11	1.23497E-09	RHSIM_Rhsim01G0053000
Root vs Stem	RHSIM_Rhsim06G0009200	1.610544551	4.69936E-11	1.23952E-09	RHSIM_Rhsim06G0009200
Root vs Stem	novel.5345	-5.838283165	4.86058E-11	1.28058E-09	--
Root vs Stem	RHSIM_Rhsim11G0195400	-2.281973236	5.02476E-11	1.32233E-09	RHSIM_Rhsim11G0195400
Root vs Stem	RHSIM_Rhsim04G0172100	-3.829245711	5.11237E-11	1.34385E-09	RHSIM_Rhsim04G0172100
Root vs Stem	RHSIM_Rhsim03G0270500	2.274183252	5.12503E-11	1.34564E-09	RHSIM_Rhsim03G0270500
Root vs Stem	RHSIM_Rhsim13G0157700	-5.079974889	5.14852E-11	1.35027E-09	RHSIM_Rhsim13G0157700
Root vs Stem	RHSIM_Rhsim10G0156600	-2.0639277	5.26822E-11	1.38009E-09	RHSIM_Rhsim10G0156600

Comparison	Gene ID	log_2FC	P-value	FDR (padj)	Gene Name
Root vs Stem	novel.5339	2.552026485	5.29207E-11	1.38477E-09	--
Root vs Stem	RHSIM_Rhsim10G0131300	-2.166704011	5.55324E-11	1.45146E-09	RHSIM_Rhsim10G0131300
Root vs Stem	novel.4641	-2.265906586	5.72861E-11	1.4956E-09	--
Root vs Stem	RHSIM_Rhsim05G0189900	-5.849803935	5.75338E-11	1.50037E-09	RHSIM_Rhsim05G0189900
Root vs Stem	RHSIM_Rhsim13G0090600	1.626573452	5.81747E-11	1.51537E-09	RHSIM_Rhsim13G0090600
Root vs Stem	RHSIM_Rhsim07G0178700	1.750851798	5.84771E-11	1.52153E-09	RHSIM_Rhsim07G0178700
Root vs Stem	RHSIM_Rhsim12G0002100	-1.101868827	6.04926E-11	1.57219E-09	RHSIM_Rhsim12G0002100
Root vs Stem	RHSIM_RhsimUnG0133400	-3.402140106	6.129E-11	1.59112E-09	RHSIM_RhsimUnG0133400
Root vs Stem	RHSIM_Rhsim05G0116000	-1.258384019	6.24785E-11	1.62015E-09	RHSIM_Rhsim05G0116000
Root vs Stem	novel.815	1.925704753	6.25689E-11	1.62067E-09	--
Root vs Stem	RHSIM_Rhsim01G0274600	-1.497116091	6.26658E-11	1.62136E-09	RHSIM_Rhsim01G0274600
Root vs Stem	RHSIM_Rhsim02G0057700	4.929058408	6.41136E-11	1.65696E-09	RHSIM_Rhsim02G0057700
Root vs Stem	RHSIM_Rhsim06G0002400	-1.965869507	6.60349E-11	1.70471E-09	RHSIM_Rhsim06G0002400
Root vs Stem	RHSIM_Rhsim04G0095900	-1.516724758	6.6665E-11	1.71905E-09	RHSIM_Rhsim04G0095900
Root vs Stem	RHSIM_Rhsim03G0056800	2.286245416	6.73233E-11	1.73408E-09	RHSIM_Rhsim03G0056800
Root vs Stem	RHSIM_Rhsim06G0138800	3.325297778	6.78842E-11	1.74658E-09	RHSIM_Rhsim06G0138800
Root vs Stem	RHSIM_Rhsim09G0111900	-1.474420457	7.23157E-11	1.85852E-09	RHSIM_Rhsim09G0111900
Root vs Stem	RHSIM_Rhsim10G0164700	2.132492533	7.36021E-11	1.88948E-09	RHSIM_Rhsim10G0164700
Root vs Stem	RHSIM_Rhsim08G0047300	2.992523299	7.57676E-11	1.94074E-09	RHSIM_Rhsim08G0047300
Root vs Stem	RHSIM_Rhsim03G0263700	-2.117001169	7.70833E-11	1.97225E-09	RHSIM_Rhsim03G0263700
Root vs Stem	novel.3155	-3.962295017	7.76889E-11	1.98555E-09	--
Root vs Stem	RHSIM_Rhsim02G0082500	1.019615278	7.81115E-11	1.99414E-09	RHSIM_Rhsim02G0082500
Root vs Stem	RHSIM_Rhsim02G0053700	3.112078978	8.07458E-11	2.05911E-09	RHSIM_Rhsim02G0053700
Root vs Stem	novel.5630	3.233833966	8.3687E-11	2.1294E-09	--
Root vs Stem	novel.2535	-2.489029076	8.51457E-11	2.16413E-09	--

Comparison	Gene ID	log_2FC	P-value	FDR (padj)	Gene Name
Root vs Stem	RHSIM_Rhsim13G0173300	2.229967509	8.54377E-11	2.16916E-09	RHSIM_Rhsim13G0173300
Root vs Stem	novel.4114	3.511459039	8.71001E-11	2.20893E-09	--
Root vs Stem	RHSIM_Rhsim01G0278000	3.792469106	8.90923E-11	2.25697E-09	RHSIM_Rhsim01G0278000
Root vs Stem	RHSIM_Rhsim13G0227700	8.418285035	9.29297E-11	2.3516E-09	RHSIM_Rhsim13G0227700
Root vs Stem	RHSIM_Rhsim04G0009100	1.163925553	9.3277E-11	2.3578E-09	RHSIM_Rhsim04G0009100
Root vs Stem	RHSIM_Rhsim08G0038900	-2.167471203	9.37656E-11	2.36755E-09	RHSIM_Rhsim08G0038900
Root vs Stem	RHSIM_Rhsim01G0269100	-1.738905011	9.59268E-11	2.41797E-09	RHSIM_Rhsim01G0269100
Root vs Stem	RHSIM_Rhsim09G0199900	2.216032511	9.59722E-11	2.41797E-09	RHSIM_Rhsim09G0199900
Root vs Stem	RHSIM_Rhsim06G0159100	3.736363688	9.9237E-11	2.4975E-09	RHSIM_Rhsim06G0159100
Root vs Stem	RHSIM_Rhsim13G0009300	-1.317014504	9.93923E-11	2.49868E-09	RHSIM_Rhsim13G0009300
Root vs Stem	novel.3467	2.836592154	1.00073E-10	2.51306E-09	--
Root vs Stem	RHSIM_Rhsim06G0011800	-2.828146014	1.00386E-10	2.51818E-09	RHSIM_Rhsim06G0011800
Root vs Stem	RHSIM_Rhsim06G0167000	-3.466026313	1.01013E-10	2.53115E-09	RHSIM_Rhsim06G0167000
Root vs Stem	RHSIM_Rhsim01G0291100	1.051336296	1.06418E-10	2.6613E-09	RHSIM_Rhsim01G0291100
Root vs Stem	RHSIM_Rhsim03G0082900	9.007345963	1.06438E-10	2.6613E-09	RHSIM_Rhsim03G0082900
Root vs Stem	RHSIM_RhsimUnG0044400	1.332170843	1.07171E-10	2.67672E-09	RHSIM_RhsimUnG0044400
Root vs Stem	RHSIM_Rhsim03G0219900	1.466107822	1.07951E-10	2.69038E-09	RHSIM_Rhsim03G0219900
Root vs Stem	RHSIM_Rhsim11G0161500	3.627058421	1.1129E-10	2.7706E-09	RHSIM_Rhsim11G0161500
Root vs Stem	RHSIM_Rhsim04G0164800	-2.668826139	1.12177E-10	2.78966E-09	RHSIM_Rhsim04G0164800
Root vs Stem	RHSIM_Rhsim06G0200000	-1.160358108	1.15549E-10	2.87042E-09	RHSIM_Rhsim06G0200000
Root vs Stem	RHSIM_Rhsim06G0237100	1.667123992	1.15748E-10	2.87226E-09	RHSIM_Rhsim06G0237100
Root vs Stem	RHSIM_Rhsim13G0095800	1.670797912	1.17413E-10	2.91046E-09	RHSIM_Rhsim13G0095800
Root vs Stem	RHSIM_Rhsim03G0206000	8.526205109	1.18174E-10	2.92616E-09	RHSIM_Rhsim03G0206000
Root vs Stem	RHSIM_Rhsim07G0045400	-10.13978731	1.18646E-10	2.93472E-09	RHSIM_Rhsim07G0045400
Root vs Stem	RHSIM_Rhsim10G0024100	-3.42528198	1.19009E-10	2.94054E-09	RHSIM_Rhsim10G0024100

Comparison	Gene ID	log_2FC	P-value	FDR (padj)	Gene Name
Root vs Stem	novel.1533	3.550764882	1.2202E-10	3.00847E-09	--
Root vs Stem	RHSIM_Rhsim06G0029300	-3.661810283	1.23304E-10	3.03689E-09	RHSIM_Rhsim06G0029300
Root vs Stem	novel.5474	-9.391172371	1.24189E-10	3.05543E-09	--
Root vs Stem	RHSIM_Rhsim10G0009900	1.552918577	1.25131E-10	3.07531E-09	RHSIM_Rhsim10G0009900
Root vs Stem	RHSIM_Rhsim03G0062800	4.151320219	1.27628E-10	3.13335E-09	RHSIM_Rhsim03G0062800
Root vs Stem	novel.6019	7.074096078	1.29069E-10	3.16534E-09	--
Root vs Stem	RHSIM_Rhsim02G0043800	1.534072296	1.30915E-10	3.20722E-09	RHSIM_Rhsim02G0043800
Root vs Stem	RHSIM_Rhsim03G0159700	-1.40622748	1.31552E-10	3.2194E-09	RHSIM_Rhsim03G0159700
Root vs Stem	RHSIM_Rhsim10G0141400	1.233730045	1.32992E-10	3.25118E-09	RHSIM_Rhsim10G0141400
Root vs Stem	RHSIM_Rhsim01G0198300	-2.930245512	1.36421E-10	3.33148E-09	RHSIM_Rhsim01G0198300
Root vs Stem	novel.5965	6.608873148	1.41122E-10	3.44262E-09	--
Root vs Stem	RHSIM_Rhsim11G0028300	1.380802385	1.44699E-10	3.52617E-09	RHSIM_Rhsim11G0028300
Root vs Stem	RHSIM_Rhsim03G0263300	-3.439782423	1.459E-10	3.55167E-09	RHSIM_Rhsim03G0263300
Root vs Stem	RHSIM_Rhsim12G0039900	-2.806480098	1.46383E-10	3.55968E-09	RHSIM_Rhsim12G0039900
Root vs Stem	novel.3774	-7.318277133	1.46669E-10	3.56286E-09	--
Root vs Stem	RHSIM_Rhsim13G0014600	3.700034833	1.48122E-10	3.59438E-09	RHSIM_Rhsim13G0014600
Root vs Stem	RHSIM_Rhsim03G0012100	-2.085100568	1.50224E-10	3.64155E-09	RHSIM_Rhsim03G0012100
Root vs Stem	RHSIM_Rhsim09G0143700	7.43219774	1.50551E-10	3.64564E-09	RHSIM_Rhsim09G0143700
Root vs Stem	RHSIM_Rhsim08G0112900	-1.020156143	1.51212E-10	3.6578E-09	RHSIM_Rhsim08G0112900
Root vs Stem	RHSIM_Rhsim08G0052400	1.40203947	1.52007E-10	3.66935E-09	RHSIM_Rhsim08G0052400
Root vs Stem	RHSIM_Rhsim08G0045800	4.013182838	1.54532E-10	3.72638E-09	RHSIM_Rhsim08G0045800
Root vs Stem	RHSIM_Rhsim04G0100800	1.319749329	1.56049E-10	3.75903E-09	RHSIM_Rhsim04G0100800
Root vs Stem	RHSIM_Rhsim01G0113200	-1.268887137	1.62692E-10	3.91088E-09	RHSIM_Rhsim01G0113200
Root vs Stem	RHSIM_Rhsim01G0290800	-2.603457271	1.64736E-10	3.9559E-09	RHSIM_Rhsim01G0290800
Root vs Stem	RHSIM_Rhsim07G0017000	-1.229147556	1.64961E-10	3.95716E-09	RHSIM_Rhsim07G0017000

Comparison	Gene ID	log_2FC	P-value	FDR (padj)	Gene Name
Root vs Stem	RHSIM_Rhsim04G0106300	6.176221371	1.72975E-10	4.14511E-09	RHSIM_Rhsim04G0106300
Root vs Stem	novel.6087	-6.699347222	1.73389E-10	4.15072E-09	--
Root vs Stem	RHSIM_Rhsim01G0263900	-1.973483348	1.7387E-10	4.1579E-09	RHSIM_Rhsim01G0263900
Root vs Stem	RHSIM_RhsimUnG0194000	1.000232267	1.79993E-10	4.29542E-09	RHSIM_RhsimUnG0194000
Root vs Stem	novel.5485	-1.436813083	1.80187E-10	4.29561E-09	--
Root vs Stem	RHSIM_Rhsim11G0063900	-2.323396534	1.91608E-10	4.56316E-09	RHSIM_Rhsim11G0063900
Root vs Stem	RHSIM_RhsimUnG0018400	1.314622091	1.92221E-10	4.57304E-09	RHSIM_RhsimUnG0018400
Root vs Stem	RHSIM_Rhsim05G0133800	4.065717994	1.93496E-10	4.59863E-09	RHSIM_Rhsim05G0133800
Root vs Stem	RHSIM_Rhsim05G0206700	-3.498444379	1.93809E-10	4.60131E-09	RHSIM_Rhsim05G0206700
Root vs Stem	novel.4116	4.136458472	2.02099E-10	4.79321E-09	--
Root vs Stem	novel.350	1.232170399	2.06129E-10	4.88376E-09	--
Root vs Stem	RHSIM_Rhsim12G0196000	-1.216793281	2.10078E-10	4.9663E-09	RHSIM_Rhsim12G0196000
Root vs Stem	RHSIM_Rhsim03G0154300	-8.788622251	2.10259E-10	4.9663E-09	RHSIM_Rhsim03G0154300
Root vs Stem	RHSIM_Rhsim07G0216200	4.642202677	2.23556E-10	5.27497E-09	RHSIM_Rhsim07G0216200
Root vs Stem	RHSIM_Rhsim13G0052600	-2.083831039	2.34445E-10	5.52624E-09	RHSIM_Rhsim13G0052600
Root vs Stem	RHSIM_Rhsim12G0177700	-1.029935244	2.38844E-10	5.62418E-09	RHSIM_Rhsim12G0177700
Root vs Stem	RHSIM_Rhsim12G0086400	-1.80784097	2.41763E-10	5.6813E-09	RHSIM_Rhsim12G0086400
Root vs Stem	RHSIM_Rhsim05G0212700	-1.193545059	2.42888E-10	5.70194E-09	RHSIM_Rhsim05G0212700
Root vs Stem	RHSIM_Rhsim08G0044000	-4.083871681	2.45563E-10	5.75887E-09	RHSIM_Rhsim08G0044000
Root vs Stem	RHSIM_Rhsim08G0190500	1.18593515	2.49059E-10	5.83199E-09	RHSIM_Rhsim08G0190500
Root vs Stem	RHSIM_Rhsim09G0209600	-3.223057853	2.49187E-10	5.83199E-09	RHSIM_Rhsim09G0209600
Root vs Stem	RHSIM_Rhsim10G0153800	-4.347217957	2.49942E-10	5.84374E-09	RHSIM_Rhsim10G0153800
Root vs Stem	RHSIM_Rhsim02G0184500	-1.175954198	2.63252E-10	6.14247E-09	RHSIM_Rhsim02G0184500
Root vs Stem	RHSIM_Rhsim06G0231500	1.522055806	2.78262E-10	6.48611E-09	RHSIM_Rhsim06G0231500
Root vs Stem	RHSIM_Rhsim07G0110000	2.910599178	2.79998E-10	6.51999E-09	RHSIM_Rhsim07G0110000

Comparison	Gene ID	log_2FC	P-value	FDR (padj)	Gene Name
Root vs Stem	RHSIM_Rhsim10G0046600	-1.817819006	2.85058E-10	6.62912E-09	RHSIM_Rhsim10G0046600
Root vs Stem	RHSIM_Rhsim02G0227700	3.682876849	2.85259E-10	6.62912E-09	RHSIM_Rhsim02G0227700
Root vs Stem	RHSIM_Rhsim07G0095900	1.890333565	3.02748E-10	7.02846E-09	RHSIM_Rhsim07G0095900
Root vs Stem	RHSIM_Rhsim10G0062700	-2.528807946	3.04871E-10	7.07061E-09	RHSIM_Rhsim10G0062700
Root vs Stem	RHSIM_Rhsim11G0148900	-2.230696668	3.08105E-10	7.13843E-09	RHSIM_Rhsim11G0148900
Root vs Stem	RHSIM_RhsimUnG0024100	-4.980456131	3.15012E-10	7.28381E-09	RHSIM_RhsimUnG0024100
Root vs Stem	RHSIM_Rhsim12G0014400	1.541131363	3.15632E-10	7.29084E-09	RHSIM_Rhsim12G0014400
Root vs Stem	RHSIM_Rhsim02G0125900	-1.057654506	3.18183E-10	7.33554E-09	RHSIM_Rhsim02G0125900
Root vs Stem	RHSIM_Rhsim06G0165600	2.6902622	3.18203E-10	7.33554E-09	RHSIM_Rhsim06G0165600
Root vs Stem	novel.1976	-2.252385775	3.26605E-10	7.52171E-09	--
Root vs Stem	RHSIM_Rhsim13G0024900	1.260656478	3.2862E-10	7.56055E-09	RHSIM_Rhsim13G0024900
Root vs Stem	RHSIM_Rhsim04G0034600	-6.130801864	3.30722E-10	7.60133E-09	RHSIM_Rhsim04G0034600
Root vs Stem	RHSIM_RhsimUnG0024200	-9.850953481	3.37354E-10	7.74604E-09	RHSIM_RhsimUnG0024200
Root vs Stem	RHSIM_Rhsim03G0111900	-1.517019707	3.39426E-10	7.78586E-09	RHSIM_Rhsim03G0111900
Root vs Stem	RHSIM_Rhsim03G0070900	4.260978103	3.41465E-10	7.82485E-09	RHSIM_Rhsim03G0070900
Root vs Stem	RHSIM_RhsimUnG0256500	-3.829086177	3.45541E-10	7.91038E-09	RHSIM_RhsimUnG0256500
Root vs Stem	RHSIM_Rhsim01G0184800	-1.853081585	3.48818E-10	7.96957E-09	RHSIM_Rhsim01G0184800
Root vs Stem	RHSIM_Rhsim02G0228100	-3.912277372	3.5578E-10	8.10462E-09	RHSIM_Rhsim02G0228100
Root vs Stem	RHSIM_Rhsim13G0228000	2.419427234	3.55784E-10	8.10462E-09	RHSIM_Rhsim13G0228000
Root vs Stem	RHSIM_Rhsim02G0228200	3.995220976	3.5641E-10	8.11088E-09	RHSIM_Rhsim02G0228200
Root vs Stem	RHSIM_Rhsim13G0053200	1.358285555	3.56951E-10	8.11519E-09	RHSIM_Rhsim13G0053200
Root vs Stem	RHSIM_Rhsim12G0043200	1.973740584	3.57593E-10	8.12177E-09	RHSIM_Rhsim12G0043200
Root vs Stem	novel.3996	-3.609032666	3.5914E-10	8.14887E-09	--
Root vs Stem	RHSIM_RhsimUnG0197900	-2.855085605	3.60204E-10	8.16498E-09	RHSIM_RhsimUnG0197900
Root vs Stem	RHSIM_Rhsim08G0179800	4.534929751	3.71911E-10	8.4138E-09	RHSIM_Rhsim08G0179800

Comparison	Gene ID	log_2FC	P-value	FDR (padj)	Gene Name
Root vs Stem	RHSIM_Rhsim03G0099600	1.13044128	3.90079E-10	8.81617E-09	RHSIM_Rhsim03G0099600
Root vs Stem	novel.579	-1.901292564	3.93517E-10	8.88517E-09	--
Root vs Stem	RHSIM_Rhsim03G0255100	-3.874467599	3.96093E-10	8.93456E-09	RHSIM_Rhsim03G0255100
Root vs Stem	RHSIM_Rhsim04G0205300	2.342300388	3.98207E-10	8.97349E-09	RHSIM_Rhsim04G0205300
Root vs Stem	RHSIM_Rhsim08G0146000	1.19228774	4.04472E-10	9.10576E-09	RHSIM_Rhsim08G0146000
Root vs Stem	RHSIM_Rhsim01G0272700	1.002878921	4.09543E-10	9.21091E-09	RHSIM_Rhsim01G0272700
Root vs Stem	RHSIM_Rhsim06G0095300	1.618314995	4.14824E-10	9.3206E-09	RHSIM_Rhsim06G0095300
Root vs Stem	RHSIM_Rhsim08G0110900	2.68414149	4.22487E-10	9.48354E-09	RHSIM_Rhsim08G0110900
Root vs Stem	RHSIM_Rhsim11G0012800	4.879626452	4.29147E-10	9.62366E-09	RHSIM_Rhsim11G0012800
Root vs Stem	RHSIM_Rhsim06G0174300	-2.264256044	4.30118E-10	9.63606E-09	RHSIM_Rhsim06G0174300
Root vs Stem	RHSIM_Rhsim04G0093600	-1.461769348	4.3911E-10	9.82796E-09	RHSIM_Rhsim04G0093600
Root vs Stem	RHSIM_Rhsim05G0208000	-1.721061917	4.42707E-10	9.89886E-09	RHSIM_Rhsim05G0208000
Root vs Stem	RHSIM_RhsimUnG0156800	2.414667378	4.44541E-10	9.93024E-09	RHSIM_RhsimUnG0156800
Root vs Stem	RHSIM_Rhsim07G0104500	1.181265168	4.49526E-10	1.00319E-08	RHSIM_Rhsim07G0104500
Root vs Stem	RHSIM_Rhsim02G0009500	2.509735594	4.50464E-10	1.00431E-08	RHSIM_Rhsim02G0009500
Root vs Stem	RHSIM_Rhsim01G0109200	8.104065834	4.52342E-10	1.00752E-08	RHSIM_Rhsim01G0109200
Root vs Stem	RHSIM_Rhsim08G0163100	2.09025578	4.53949E-10	1.01012E-08	RHSIM_Rhsim08G0163100
Root vs Stem	novel.5742	-2.437543664	4.55618E-10	1.01286E-08	--
Root vs Stem	RHSIM_Rhsim02G0042600	-4.365136132	4.5853E-10	1.01835E-08	RHSIM_Rhsim02G0042600
Root vs Stem	RHSIM_Rhsim08G0197500	1.099492209	4.63875E-10	1.02923E-08	RHSIM_Rhsim08G0197500
Root vs Stem	RHSIM_Rhsim05G0169700	-2.170165772	4.71453E-10	1.04504E-08	RHSIM_Rhsim05G0169700
Root vs Stem	novel.4878	-2.030900418	4.75741E-10	1.05291E-08	--
Root vs Stem	RHSIM_Rhsim13G0022200	-1.071249798	4.75916E-10	1.05291E-08	RHSIM_Rhsim13G0022200
Root vs Stem	RHSIM_RhsimUnG0118200	-2.337776695	4.78485E-10	1.05758E-08	RHSIM_RhsimUnG0118200
Root vs Stem	RHSIM_Rhsim08G0252300	-1.06421256	4.87895E-10	1.07631E-08	RHSIM_Rhsim08G0252300

Comparison	Gene ID	log_2FC	P-value	FDR (padj)	Gene Name
Root vs Stem	RHSIM_Rhsim13G0004700	-1.226890098	4.89136E-10	1.07802E-08	RHSIM_Rhsim13G0004700
Root vs Stem	RHSIM_Rhsim12G0048000	-4.693435387	4.91657E-10	1.08254E-08	RHSIM_Rhsim12G0048000
Root vs Stem	RHSIM_Rhsim03G0263500	4.397995569	5.06635E-10	1.11445E-08	RHSIM_Rhsim03G0263500
Root vs Stem	RHSIM_Rhsim04G0240200	5.32266316	5.08411E-10	1.11729E-08	RHSIM_Rhsim04G0240200
Root vs Stem	RHSIM_Rhsim02G0160100	2.929675562	5.13803E-10	1.12807E-08	RHSIM_Rhsim02G0160100
Root vs Stem	RHSIM_Rhsim08G0249400	-1.365520268	5.30088E-10	1.1594E-08	RHSIM_Rhsim08G0249400
Root vs Stem	RHSIM_Rhsim07G0073900	3.559102219	5.35123E-10	1.16931E-08	RHSIM_Rhsim07G0073900
Root vs Stem	RHSIM_Rhsim12G0037700	-2.047646907	5.42892E-10	1.18516E-08	RHSIM_Rhsim12G0037700
Root vs Stem	RHSIM_Rhsim13G0093000	2.13206886	5.46169E-10	1.19119E-08	RHSIM_Rhsim13G0093000
Root vs Stem	novel.3784	1.154099056	5.51459E-10	1.20159E-08	--
Root vs Stem	RHSIM_Rhsim13G0103500	-1.60330206	5.55337E-10	1.20857E-08	RHSIM_Rhsim13G0103500
Root vs Stem	RHSIM_RhsimUnG0054600	-1.212874065	5.55713E-10	1.20857E-08	RHSIM_RhsimUnG0054600
Root vs Stem	RHSIM_Rhsim07G0229300	-8.276195866	5.74454E-10	1.24698E-08	RHSIM_Rhsim07G0229300
Root vs Stem	RHSIM_Rhsim07G0193500	-1.695065927	5.7651E-10	1.25026E-08	RHSIM_Rhsim07G0193500
Root vs Stem	RHSIM_RhsimUnG0176100	8.526950915	5.85522E-10	1.26861E-08	RHSIM_RhsimUnG0176100
Root vs Stem	RHSIM_RhsimUnG0085800	-2.108948023	5.8731E-10	1.27065E-08	RHSIM_RhsimUnG0085800
Root vs Stem	RHSIM_Rhsim09G0101000	2.37311797	5.87566E-10	1.27065E-08	RHSIM_Rhsim09G0101000
Root vs Stem	RHSIM_Rhsim08G0174800	-1.344764731	5.94213E-10	1.28382E-08	RHSIM_Rhsim08G0174800
Root vs Stem	RHSIM_Rhsim01G0010200	8.157409364	6.16707E-10	1.33118E-08	RHSIM_Rhsim01G0010200
Root vs Stem	RHSIM_Rhsim13G0038200	1.32656808	6.20777E-10	1.33871E-08	RHSIM_Rhsim13G0038200
Root vs Stem	novel.2911	-3.252222256	6.41491E-10	1.38208E-08	--
Root vs Stem	RHSIM_Rhsim08G0096300	-1.160048231	6.5129E-10	1.40189E-08	RHSIM_Rhsim08G0096300
Root vs Stem	RHSIM_Rhsim06G0065600	-1.479345339	6.52872E-10	1.40398E-08	RHSIM_Rhsim06G0065600
Root vs Stem	RHSIM_Rhsim10G0146500	-1.561754434	6.56345E-10	1.41013E-08	RHSIM_Rhsim10G0146500
Root vs Stem	RHSIM_Rhsim06G0232300	2.722312818	6.77032E-10	1.45322E-08	RHSIM_Rhsim06G0232300

Comparison	Gene ID	log2FC	P-value	FDR (padj)	Gene Name
Root vs Stem	RHSIM_Rhsim04G0047500	2.244210373	6.83362E-10	1.46545E-08	RHSIM_Rhsim04G0047500
Root vs Stem	RHSIM_Rhsim03G0001300	-6.522199892	7.07605E-10	1.51602E-08	RHSIM_Rhsim03G0001300
Root vs Stem	RHSIM_Rhsim07G0026800	-1.342076991	7.11226E-10	1.52237E-08	RHSIM_Rhsim07G0026800
Root vs Stem	RHSIM_Rhsim12G0036200	6.804491466	7.14533E-10	1.52803E-08	RHSIM_Rhsim12G0036200
Root vs Stem	RHSIM_Rhsim13G0115200	-1.358317609	7.16946E-10	1.53177E-08	RHSIM_Rhsim13G0115200
Root vs Stem	RHSIM_Rhsim02G0048900	1.227841877	7.19964E-10	1.53679E-08	RHSIM_Rhsim02G0048900
Root vs Stem	RHSIM_Rhsim10G0139000	5.074640712	7.30767E-10	1.55841E-08	RHSIM_Rhsim10G0139000
Root vs Stem	RHSIM_Rhsim06G0241400	1.15477409	7.41993E-10	1.58088E-08	RHSIM_Rhsim06G0241400
Root vs Stem	RHSIM_Rhsim01G0187900	-4.625227312	7.59907E-10	1.61756E-08	RHSIM_Rhsim01G0187900
Root vs Stem	novel.5975	8.033413058	7.6959E-10	1.63666E-08	--
Root vs Stem	RHSIM_Rhsim08G0073600	-1.894735354	7.96002E-10	1.68971E-08	RHSIM_Rhsim08G0073600
Root vs Stem	RHSIM_Rhsim09G0123000	4.095585272	8.10414E-10	1.71872E-08	RHSIM_Rhsim09G0123000
Root vs Stem	RHSIM_Rhsim11G0137700	8.861230939	8.38921E-10	1.77754E-08	RHSIM_Rhsim11G0137700
Root vs Stem	novel.4328	5.240212627	8.49489E-10	1.79828E-08	--
Root vs Stem	RHSIM_Rhsim13G0146300	1.262354474	8.55537E-10	1.80942E-08	RHSIM_Rhsim13G0146300
Root vs Stem	RHSIM_Rhsim07G0002700	3.24781737	9.03128E-10	1.90832E-08	RHSIM_Rhsim07G0002700
Root vs Stem	RHSIM_Rhsim13G0086900	1.971424939	9.05457E-10	1.91149E-08	RHSIM_Rhsim13G0086900
Root vs Stem	RHSIM_Rhsim09G0003900	-1.445662492	9.12648E-10	1.92491E-08	RHSIM_Rhsim09G0003900
Root vs Stem	RHSIM_Rhsim07G0229000	-4.571839676	9.34077E-10	1.96831E-08	RHSIM_Rhsim07G0229000
Root vs Stem	RHSIM_Rhsim03G0178800	-5.487725722	9.37177E-10	1.97304E-08	RHSIM_Rhsim03G0178800
Root vs Stem	RHSIM_Rhsim08G0168800	2.531111952	9.49737E-10	1.99765E-08	RHSIM_Rhsim08G0168800
Root vs Stem	RHSIM_Rhsim02G0115100	-1.963994087	9.50823E-10	1.99811E-08	RHSIM_Rhsim02G0115100
Root vs Stem	novel.5881	5.389614004	9.60402E-10	2.01641E-08	--
Root vs Stem	RHSIM_Rhsim11G0000200	3.547049486	9.68765E-10	2.03027E-08	RHSIM_Rhsim11G0000200
Root vs Stem	RHSIM_Rhsim12G0127500	-1.016958214	9.78057E-10	2.04788E-08	RHSIM_Rhsim12G0127500

Comparison	Gene ID	log_2FC	P-value	FDR (padj)	Gene Name
Root vs Stem	RHSIM_Rhsim11G0136400	-2.042536886	9.79872E-10	2.04982E-08	RHSIM_Rhsim11G0136400
Root vs Stem	RHSIM_Rhsim01G0013100	2.261354466	9.85193E-10	2.05908E-08	RHSIM_Rhsim01G0013100
Root vs Stem	RHSIM_Rhsim04G0201100	4.832993225	9.9324E-10	2.07402E-08	RHSIM_Rhsim04G0201100
Root vs Stem	RHSIM_Rhsim03G0208000	2.019575805	1.06101E-09	2.21153E-08	RHSIM_Rhsim03G0208000
Root vs Stem	RHSIM_Rhsim04G0231000	3.111853974	1.09083E-09	2.27162E-08	RHSIM_Rhsim04G0231000
Root vs Stem	novel.4637	-1.472794843	1.10997E-09	2.30939E-08	--
Root vs Stem	RHSIM_Rhsim08G0198600	-2.417988511	1.14648E-09	2.38322E-08	RHSIM_Rhsim08G0198600
Root vs Stem	RHSIM_Rhsim13G0177200	3.760202696	1.14916E-09	2.38662E-08	RHSIM_Rhsim13G0177200
Root vs Stem	RHSIM_RhsimUnG0181100	-2.616874396	1.15887E-09	2.40462E-08	RHSIM_RhsimUnG0181100
Root vs Stem	RHSIM_Rhsim04G0172200	-1.761163497	1.16496E-09	2.4151E-08	RHSIM_Rhsim04G0172200
Root vs Stem	RHSIM_Rhsim11G0179200	-2.228677391	1.16922E-09	2.42175E-08	RHSIM_Rhsim11G0179200
Root vs Stem	RHSIM_Rhsim13G0015800	1.532595687	1.18924E-09	2.46101E-08	RHSIM_Rhsim13G0015800
Root vs Stem	novel.3742	1.494075643	1.2078E-09	2.49718E-08	--
Root vs Stem	novel.5664	-5.141046552	1.21238E-09	2.50251E-08	--
Root vs Stem	RHSIM_Rhsim12G0116700	-3.617994598	1.21255E-09	2.50251E-08	RHSIM_Rhsim12G0116700
Root vs Stem	RHSIM_Rhsim12G0098100	-1.608189284	1.2234E-09	2.52153E-08	RHSIM_Rhsim12G0098100
Root vs Stem	RHSIM_Rhsim11G0138000	-3.86939223	1.22425E-09	2.52153E-08	RHSIM_Rhsim11G0138000
Root vs Stem	RHSIM_Rhsim06G0001300	-2.034874201	1.22505E-09	2.52153E-08	RHSIM_Rhsim06G0001300
Root vs Stem	novel.6073	4.378366404	1.23413E-09	2.53795E-08	--
Root vs Stem	RHSIM_Rhsim10G0109600	6.090708902	1.2363E-09	2.54014E-08	RHSIM_Rhsim10G0109600
Root vs Stem	RHSIM_Rhsim09G0029500	1.213748612	1.24568E-09	2.55714E-08	RHSIM_Rhsim09G0029500
Root vs Stem	RHSIM_Rhsim08G0060800	6.028288992	1.28492E-09	2.63535E-08	RHSIM_Rhsim08G0060800
Root vs Stem	RHSIM_Rhsim02G0230000	1.112550908	1.29007E-09	2.64355E-08	RHSIM_Rhsim02G0230000
Root vs Stem	novel.4059	1.283686956	1.29233E-09	2.64583E-08	--
Root vs Stem	RHSIM_Rhsim11G0161700	5.187054586	1.29824E-09	2.65557E-08	RHSIM_Rhsim11G0161700

Comparison	Gene ID	log_2FC	P-value	FDR (padj)	Gene Name
Root vs Stem	RHSIM_Rhsim06G0124400	-2.660360115	1.3254E-09	2.70872E-08	RHSIM_Rhsim06G0124400
Root vs Stem	RHSIM_Rhsim06G0114600	-7.21708249	1.32662E-09	2.70882E-08	RHSIM_Rhsim06G0114600
Root vs Stem	RHSIM_Rhsim12G0144700	-1.381479165	1.33572E-09	2.72499E-08	RHSIM_Rhsim12G0144700
Root vs Stem	RHSIM_Rhsim08G0190600	1.780638907	1.34351E-09	2.73604E-08	RHSIM_Rhsim08G0190600
Root vs Stem	RHSIM_Rhsim13G0125600	1.982628396	1.36139E-09	2.77001E-08	RHSIM_Rhsim13G0125600
Root vs Stem	RHSIM_Rhsim03G0135700	-1.992338016	1.38378E-09	2.81308E-08	RHSIM_Rhsim03G0135700
Root vs Stem	RHSIM_Rhsim03G0081800	-1.069555826	1.40611E-09	2.85344E-08	RHSIM_Rhsim03G0081800
Root vs Stem	RHSIM_Rhsim03G0169800	1.522963649	1.4252E-09	2.88963E-08	RHSIM_Rhsim03G0169800
Root vs Stem	RHSIM_Rhsim13G0053100	-1.77590905	1.43931E-09	2.91568E-08	RHSIM_Rhsim13G0053100
Root vs Stem	RHSIM_Rhsim03G0223100	-1.830695227	1.44532E-09	2.92528E-08	RHSIM_Rhsim03G0223100
Root vs Stem	RHSIM_Rhsim08G0037500	-1.636836603	1.45902E-09	2.95043E-08	RHSIM_Rhsim08G0037500
Root vs Stem	RHSIM_Rhsim05G0231800	-1.720116682	1.47387E-09	2.97783E-08	RHSIM_Rhsim05G0231800
Root vs Stem	RHSIM_RhsimUnG0138800	1.185214405	1.48227E-09	2.99219E-08	RHSIM_RhsimUnG0138800
Root vs Stem	RHSIM_Rhsim09G0115100	1.445637655	1.50274E-09	3.03086E-08	RHSIM_Rhsim09G0115100
Root vs Stem	RHSIM_Rhsim07G0202900	-1.641883266	1.50876E-09	3.04033E-08	RHSIM_Rhsim07G0202900
Root vs Stem	novel.2401	-3.825619396	1.51344E-09	3.0471E-08	--
Root vs Stem	RHSIM_Rhsim08G0046700	1.63921771	1.55529E-09	3.1253E-08	RHSIM_Rhsim08G0046700
Root vs Stem	RHSIM_Rhsim12G0018700	1.734823093	1.55635E-09	3.1253E-08	RHSIM_Rhsim12G0018700
Root vs Stem	RHSIM_Rhsim06G0171700	2.388465155	1.56682E-09	3.1436E-08	RHSIM_Rhsim06G0171700
Root vs Stem	RHSIM_Rhsim13G0227500	-1.276556531	1.57045E-09	3.14814E-08	RHSIM_Rhsim13G0227500
Root vs Stem	novel.3874	-2.695800078	1.57221E-09	3.14893E-08	--
Root vs Stem	RHSIM_Rhsim07G0226900	2.854605146	1.58878E-09	3.17935E-08	RHSIM_Rhsim07G0226900
Root vs Stem	RHSIM_Rhsim07G0120500	2.455777121	1.61296E-09	3.22495E-08	RHSIM_Rhsim07G0120500
Root vs Stem	RHSIM_Rhsim01G0129200	-4.875705376	1.61726E-09	3.23073E-08	RHSIM_Rhsim01G0129200
Root vs Stem	RHSIM_Rhsim13G0203000	-2.711609222	1.62182E-09	3.23705E-08	RHSIM_Rhsim13G0203000

Comparison	Gene ID	log_2FC	P-value	FDR (padj)	Gene Name
Root vs Stem	RHSIM_Rhsim04G0228400	2.721103849	1.62481E-09	3.2402E-08	RHSIM_Rhsim04G0228400
Root vs Stem	RHSIM_Rhsim05G0004300	-1.058467524	1.62956E-09	3.24686E-08	RHSIM_Rhsim05G0004300
Root vs Stem	RHSIM_Rhsim07G0055000	-3.97679014	1.63624E-09	3.25737E-08	RHSIM_Rhsim07G0055000
Root vs Stem	RHSIM_Rhsim04G0181400	2.634788609	1.66235E-09	3.30649E-08	RHSIM_Rhsim04G0181400
Root vs Stem	RHSIM_Rhsim09G0137900	1.001459671	1.67337E-09	3.32268E-08	RHSIM_Rhsim09G0137900
Root vs Stem	RHSIM_RhsimUnG0206100	1.146930164	1.68174E-09	3.33641E-08	RHSIM_RhsimUnG0206100
Root vs Stem	RHSIM_Rhsim12G0050700	-1.423993928	1.6837E-09	3.33744E-08	RHSIM_Rhsim12G0050700
Root vs Stem	RHSIM_Rhsim12G0185900	-1.605581316	1.68912E-09	3.34529E-08	RHSIM_Rhsim12G0185900
Root vs Stem	RHSIM_Rhsim12G0042300	5.422521222	1.70569E-09	3.37522E-08	RHSIM_Rhsim12G0042300
Root vs Stem	RHSIM_Rhsim06G0067000	-1.648304461	1.7299E-09	3.42019E-08	RHSIM_Rhsim06G0067000
Root vs Stem	RHSIM_Rhsim01G0089500	-2.659614998	1.73646E-09	3.43021E-08	RHSIM_Rhsim01G0089500
Root vs Stem	RHSIM_Rhsim12G0017900	-2.320190542	1.74372E-09	3.44162E-08	RHSIM_Rhsim12G0017900
Root vs Stem	novel.5231	-5.590743827	1.76215E-09	3.47501E-08	--
Root vs Stem	RHSIM_Rhsim12G0202100	-1.468104674	1.76416E-09	3.47599E-08	RHSIM_Rhsim12G0202100
Root vs Stem	RHSIM_Rhsim01G0240500	3.967100282	1.77535E-09	3.49294E-08	RHSIM_Rhsim01G0240500
Root vs Stem	RHSIM_Rhsim07G0185300	-2.052681873	1.77579E-09	3.49294E-08	RHSIM_Rhsim07G0185300
Root vs Stem	RHSIM_Rhsim07G0190000	-1.644254644	1.79421E-09	3.52617E-08	RHSIM_Rhsim07G0190000
Root vs Stem	RHSIM_Rhsim09G0025300	2.820726857	1.84064E-09	3.61433E-08	RHSIM_Rhsim09G0025300
Root vs Stem	RHSIM_Rhsim08G0086000	-2.016762287	1.8927E-09	3.71339E-08	RHSIM_Rhsim08G0086000
Root vs Stem	novel.489	-1.799307465	1.90013E-09	3.722E-08	--
Root vs Stem	RHSIM_Rhsim03G0235400	-2.385453196	1.90032E-09	3.722E-08	RHSIM_Rhsim03G0235400
Root vs Stem	RHSIM_Rhsim04G0011700	2.644193153	1.90714E-09	3.7322E-08	RHSIM_Rhsim04G0011700
Root vs Stem	RHSIM_Rhsim10G0003400	-3.817017088	1.91099E-09	3.73404E-08	RHSIM_Rhsim10G0003400
Root vs Stem	RHSIM_Rhsim10G0205400	-1.612196837	1.91132E-09	3.73404E-08	RHSIM_Rhsim10G0205400
Root vs Stem	RHSIM_Rhsim04G0126000	3.690941312	1.91322E-09	3.73458E-08	RHSIM_Rhsim04G0126000

Comparison	Gene ID	log_2FC	P-value	FDR (padj)	Gene Name
Root vs Stem	RHSIM_Rhsim12G0039000	2.517068535	1.93109E-09	3.76628E-08	RHSIM_Rhsim12G0039000
Root vs Stem	RHSIM_Rhsim01G0138300	-4.123052538	2.01652E-09	3.92626E-08	RHSIM_Rhsim01G0138300
Root vs Stem	RHSIM_Rhsim07G0010300	1.458784401	2.07953E-09	4.0421E-08	RHSIM_Rhsim07G0010300
Root vs Stem	RHSIM_Rhsim02G0239900	4.186124451	2.1162E-09	4.10991E-08	RHSIM_Rhsim02G0239900
Root vs Stem	RHSIM_Rhsim13G0075700	-2.341688698	2.13008E-09	4.13339E-08	RHSIM_Rhsim13G0075700
Root vs Stem	novel.4842	-1.920720799	2.13191E-09	4.13346E-08	--
Root vs Stem	RHSIM_Rhsim02G0236300	1.988542542	2.14178E-09	4.14562E-08	RHSIM_Rhsim02G0236300
Root vs Stem	RHSIM_Rhsim03G0073500	2.244913197	2.29609E-09	4.42943E-08	RHSIM_Rhsim03G0073500
Root vs Stem	RHSIM_Rhsim05G0131900	1.22851521	2.4203E-09	4.66125E-08	RHSIM_Rhsim05G0131900
Root vs Stem	novel.4877	4.423654287	2.43403E-09	4.68353E-08	--
Root vs Stem	RHSIM_Rhsim09G0178500	-1.718707562	2.43593E-09	4.68353E-08	RHSIM_Rhsim09G0178500
Root vs Stem	RHSIM_Rhsim04G0209700	-3.173839234	2.45746E-09	4.72099E-08	RHSIM_Rhsim04G0209700
Root vs Stem	novel.5097	-2.979719013	2.53108E-09	4.85836E-08	--
Root vs Stem	RHSIM_Rhsim02G0029500	-6.481194669	2.54369E-09	4.87851E-08	RHSIM_Rhsim02G0029500
Root vs Stem	RHSIM_Rhsim05G0145700	1.185068243	2.54662E-09	4.88006E-08	RHSIM_Rhsim05G0145700
Root vs Stem	RHSIM_Rhsim05G0060800	1.525648511	2.59662E-09	4.97175E-08	RHSIM_Rhsim05G0060800
Root vs Stem	RHSIM_Rhsim07G0125400	-2.44580624	2.63306E-09	5.03735E-08	RHSIM_Rhsim07G0125400
Root vs Stem	RHSIM_Rhsim04G0185200	2.840807187	2.69554E-09	5.14832E-08	RHSIM_Rhsim04G0185200
Root vs Stem	RHSIM_Rhsim01G0212500	-1.423328291	2.73256E-09	5.21471E-08	RHSIM_Rhsim01G0212500
Root vs Stem	RHSIM_Rhsim13G0222500	8.204752148	2.75804E-09	5.25899E-08	RHSIM_Rhsim13G0222500
Root vs Stem	novel.145	1.762493053	2.809E-09	5.3473E-08	--
Root vs Stem	RHSIM_Rhsim03G0264100	1.225107462	2.81579E-09	5.35581E-08	RHSIM_Rhsim03G0264100
Root vs Stem	RHSIM_Rhsim08G0100700	-1.070142259	2.86205E-09	5.43931E-08	RHSIM_Rhsim08G0100700
Root vs Stem	RHSIM_Rhsim08G0031600	2.180294172	2.91972E-09	5.54369E-08	RHSIM_Rhsim08G0031600
Root vs Stem	RHSIM_Rhsim13G0100200	2.899970978	2.92178E-09	5.54369E-08	RHSIM_Rhsim13G0100200

Comparison	Gene ID	log$_2$FC	P-value	FDR (padj)	Gene Name
Root vs Stem	RHSIM_Rhsim06G0128900	-2.727906314	2.93015E-09	5.555E-08	RHSIM_Rhsim06G0128900
Root vs Stem	RHSIM_Rhsim08G0100800	-2.397366369	2.9499E-09	5.58785E-08	RHSIM_Rhsim08G0100800
Root vs Stem	RHSIM_Rhsim02G0231600	2.258477986	2.97289E-09	5.62676E-08	RHSIM_Rhsim02G0231600
Root vs Stem	RHSIM_Rhsim07G0031500	1.515733522	2.98566E-09	5.6463E-08	RHSIM_Rhsim07G0031500
Root vs Stem	RHSIM_Rhsim06G0048800	-1.957268924	3.01167E-09	5.69083E-08	RHSIM_Rhsim06G0048800
Root vs Stem	RHSIM_Rhsim02G0236600	2.736000668	3.02973E-09	5.71713E-08	RHSIM_Rhsim02G0236600
Root vs Stem	RHSIM_Rhsim04G0029500	1.758712103	3.03055E-09	5.71713E-08	RHSIM_Rhsim04G0029500
Root vs Stem	RHSIM_Rhsim04G0194700	-1.593148239	3.07993E-09	5.80552E-08	RHSIM_Rhsim04G0194700
Root vs Stem	RHSIM_Rhsim09G0150500	-2.449339125	3.10458E-09	5.8472E-08	RHSIM_Rhsim09G0150500
Root vs Stem	novel.5135	4.236446792	3.14552E-09	5.91949E-08	--
Root vs Stem	RHSIM_Rhsim06G0089700	-1.169624651	3.17451E-09	5.96917E-08	RHSIM_Rhsim06G0089700
Root vs Stem	novel.569	5.708177891	3.20775E-09	6.02676E-08	--
Root vs Stem	RHSIM_Rhsim05G0095600	1.254493786	3.22942E-09	6.05923E-08	RHSIM_Rhsim05G0095600
Root vs Stem	RHSIM_Rhsim07G0028000	-1.645818739	3.23029E-09	6.05923E-08	RHSIM_Rhsim07G0028000
Root vs Stem	novel.2065	2.787480194	3.26008E-09	6.10518E-08	--
Root vs Stem	RHSIM_Rhsim04G0006800	-1.320092436	3.26558E-09	6.1105E-08	RHSIM_Rhsim04G0006800
Root vs Stem	RHSIM_Rhsim12G0170700	-1.650806834	3.27905E-09	6.12576E-08	RHSIM_Rhsim12G0170700
Root vs Stem	RHSIM_Rhsim01G0243000	-2.392616671	3.28873E-09	6.13888E-08	RHSIM_Rhsim01G0243000
Root vs Stem	RHSIM_Rhsim12G0124200	-1.079781223	3.29844E-09	6.15202E-08	RHSIM_Rhsim12G0124200
Root vs Stem	RHSIM_Rhsim05G0226500	3.166372262	3.30683E-09	6.16269E-08	RHSIM_Rhsim05G0226500
Root vs Stem	RHSIM_Rhsim09G0184200	2.286344781	3.35504E-09	6.24747E-08	RHSIM_Rhsim09G0184200
Root vs Stem	RHSIM_Rhsim08G0096900	5.199741017	3.37394E-09	6.27759E-08	RHSIM_Rhsim08G0096900
Root vs Stem	RHSIM_Rhsim07G0067000	3.7906909	3.41757E-09	6.35365E-08	RHSIM_Rhsim07G0067000
Root vs Stem	RHSIM_Rhsim13G0120600	2.523278953	3.46277E-09	6.42732E-08	RHSIM_Rhsim13G0120600
Root vs Stem	RHSIM_Rhsim07G0014000	-1.325536709	3.47199E-09	6.43925E-08	RHSIM_Rhsim07G0014000

Comparison	Gene ID	log_2FC	P-value	FDR (padj)	Gene Name
Root vs Stem	RHSIM_Rhsim03G0083600	1.122153635	3.48521E-09	6.45856E-08	RHSIM_Rhsim03G0083600
Root vs Stem	RHSIM_RhsimUnG0160300	1.241305887	3.51197E-09	6.50292E-08	RHSIM_RhsimUnG0160300
Root vs Stem	RHSIM_Rhsim02G0254800	-3.349621387	3.5331E-09	6.5368E-08	RHSIM_Rhsim02G0254800
Root vs Stem	RHSIM_Rhsim02G0239800	2.441303	3.56517E-09	6.59085E-08	RHSIM_Rhsim02G0239800
Root vs Stem	RHSIM_Rhsim08G0097400	-1.84118636	3.58723E-09	6.62631E-08	RHSIM_Rhsim08G0097400
Root vs Stem	RHSIM_Rhsim06G0233500	-2.440711244	3.60042E-09	6.64535E-08	RHSIM_Rhsim06G0233500
Root vs Stem	RHSIM_Rhsim06G0040900	-1.088558201	3.63572E-09	6.70515E-08	RHSIM_Rhsim06G0040900
Root vs Stem	RHSIM_Rhsim04G0074400	-2.717868274	3.71586E-09	6.84746E-08	RHSIM_Rhsim04G0074400
Root vs Stem	RHSIM_Rhsim11G0069700	1.00780334	3.7841E-09	6.96207E-08	RHSIM_Rhsim11G0069700
Root vs Stem	RHSIM_Rhsim13G0015600	6.159085956	3.89648E-09	7.16083E-08	RHSIM_Rhsim13G0015600
Root vs Stem	RHSIM_Rhsim07G0123900	-2.167083537	3.89834E-09	7.16083E-08	RHSIM_Rhsim07G0123900
Root vs Stem	RHSIM_Rhsim12G0066600	1.370793095	3.93811E-09	7.22812E-08	RHSIM_Rhsim12G0066600
Root vs Stem	RHSIM_Rhsim05G0226600	2.678743106	3.97815E-09	7.29581E-08	RHSIM_Rhsim05G0226600
Root vs Stem	RHSIM_Rhsim01G0255600	-2.412754925	3.99712E-09	7.32477E-08	RHSIM_Rhsim01G0255600
Root vs Stem	RHSIM_Rhsim06G0183900	-1.034121363	4.06746E-09	7.44776E-08	RHSIM_Rhsim06G0183900
Root vs Stem	RHSIM_Rhsim08G0216900	-1.163065822	4.20491E-09	7.68722E-08	RHSIM_Rhsim08G0216900
Root vs Stem	RHSIM_Rhsim11G0159200	6.954093311	4.29373E-09	7.84337E-08	RHSIM_Rhsim11G0159200
Root vs Stem	RHSIM_Rhsim08G0008800	1.044177042	4.311E-09	7.86868E-08	RHSIM_Rhsim08G0008800
Root vs Stem	RHSIM_Rhsim03G0174700	1.050537195	4.3425E-09	7.91442E-08	RHSIM_Rhsim03G0174700
Root vs Stem	RHSIM_Rhsim03G0259000	5.271807573	4.34292E-09	7.91442E-08	RHSIM_Rhsim03G0259000
Root vs Stem	RHSIM_Rhsim02G0244700	-1.630714137	4.41167E-09	8.03335E-08	RHSIM_Rhsim02G0244700
Root vs Stem	RHSIM_Rhsim12G0124100	1.833174244	4.43489E-09	8.06926E-08	RHSIM_Rhsim12G0124100
Root vs Stem	RHSIM_Rhsim04G0144400	1.558432398	4.62833E-09	8.41458E-08	RHSIM_Rhsim04G0144400
Root vs Stem	RHSIM_Rhsim08G0118600	1.014849625	4.7509E-09	8.63061E-08	RHSIM_Rhsim08G0118600
Root vs Stem	novel.5854	-1.027548166	5.06394E-09	9.19205E-08	--

Comparison	Gene ID	log_2FC	P-value	FDR (padj)	Gene Name
Root vs Stem	RHSIM_Rhsim05G0181100	-2.296306967	5.11573E-09	9.27876E-08	RHSIM_Rhsim05G0181100
Root vs Stem	RHSIM_Rhsim03G0233500	-2.730193468	5.17969E-09	9.38737E-08	RHSIM_Rhsim03G0233500
Root vs Stem	RHSIM_Rhsim03G0241500	-3.698341065	5.19414E-09	9.40617E-08	RHSIM_Rhsim03G0241500
Root vs Stem	RHSIM_Rhsim01G0124500	1.150007543	5.21267E-09	9.43232E-08	RHSIM_Rhsim01G0124500
Root vs Stem	novel.4851	-3.099308258	5.27728E-09	9.54174E-08	--
Root vs Stem	novel.4653	3.88303942	5.32514E-09	9.62073E-08	--
Root vs Stem	RHSIM_Rhsim12G0036400	2.639945869	5.38441E-09	9.71923E-08	RHSIM_Rhsim12G0036400
Root vs Stem	RHSIM_Rhsim03G0221700	2.020097937	5.3881E-09	9.71923E-08	RHSIM_Rhsim03G0221700
Root vs Stem	RHSIM_Rhsim02G0165300	6.143465938	5.3933E-09	9.72101E-08	RHSIM_Rhsim02G0165300
Root vs Stem	RHSIM_Rhsim06G0054400	-1.034123	5.46425E-09	9.8412E-08	RHSIM_Rhsim06G0054400
Root vs Stem	novel.5148	1.574035296	5.52129E-09	9.93616E-08	--
Root vs Stem	novel.433	-1.66896115	5.58066E-09	1.00352E-07	--
Root vs Stem	RHSIM_Rhsim11G0141600	-3.520644864	5.593E-09	1.00495E-07	RHSIM_Rhsim11G0141600
Root vs Stem	RHSIM_Rhsim06G0239700	3.252202164	5.68609E-09	1.02009E-07	RHSIM_Rhsim06G0239700
Root vs Stem	RHSIM_Rhsim10G0038600	1.953690885	5.70168E-09	1.02209E-07	RHSIM_Rhsim10G0038600
Root vs Stem	RHSIM_Rhsim03G0246400	-1.692742506	5.74833E-09	1.02945E-07	RHSIM_Rhsim03G0246400
Root vs Stem	RHSIM_Rhsim01G0235600	-2.457733172	5.75165E-09	1.02945E-07	RHSIM_Rhsim01G0235600
Root vs Stem	RHSIM_Rhsim08G0026800	1.296174511	5.81808E-09	1.03892E-07	RHSIM_Rhsim08G0026800
Root vs Stem	novel.3458	-3.736594769	5.82844E-09	1.03923E-07	--
Root vs Stem	RHSIM_Rhsim01G0287300	-2.537441127	5.88442E-09	1.04833E-07	RHSIM_Rhsim01G0287300
Root vs Stem	RHSIM_Rhsim09G0130100	1.755975867	5.97826E-09	1.06422E-07	RHSIM_Rhsim09G0130100
Root vs Stem	RHSIM_Rhsim04G0241800	2.878288348	6.01588E-09	1.07009E-07	RHSIM_Rhsim04G0241800
Root vs Stem	RHSIM_Rhsim07G0229100	-5.848973537	6.1491E-09	1.0921E-07	RHSIM_Rhsim07G0229100
Root vs Stem	RHSIM_Rhsim06G0180800	2.491393927	6.1904E-09	1.09859E-07	RHSIM_Rhsim06G0180800
Root vs Stem	RHSIM_Rhsim08G0067400	-2.158635824	6.21545E-09	1.10219E-07	RHSIM_Rhsim08G0067400

Comparison	Gene ID	log_2FC	P-value	FDR (padj)	Gene Name
Root vs Stem	RHSIM_Rhsim12G0053800	3.493750183	6.40255E-09	1.1345E-07	RHSIM_Rhsim12G0053800
Root vs Stem	RHSIM_Rhsim02G0206000	-1.710417044	6.61722E-09	1.17163E-07	RHSIM_Rhsim02G0206000
Root vs Stem	RHSIM_Rhsim11G0062300	-1.451231859	6.64252E-09	1.17521E-07	RHSIM_Rhsim11G0062300
Root vs Stem	RHSIM_Rhsim08G0076500	-3.36587711	6.74012E-09	1.19156E-07	RHSIM_Rhsim08G0076500
Root vs Stem	RHSIM_RhsimUnG0205000	-1.41683568	6.76772E-09	1.19553E-07	RHSIM_RhsimUnG0205000
Root vs Stem	RHSIM_Rhsim09G0089100	-3.609999663	6.79381E-09	1.19922E-07	RHSIM_Rhsim09G0089100
Root vs Stem	RHSIM_Rhsim01G0062000	1.290143022	7.09168E-09	1.25084E-07	RHSIM_Rhsim01G0062000
Root vs Stem	RHSIM_Rhsim07G0026300	7.774559862	7.13549E-09	1.2576E-07	RHSIM_Rhsim07G0026300
Root vs Stem	novel.3703	2.232031599	7.23108E-09	1.27348E-07	--
Root vs Stem	RHSIM_Rhsim02G0122500	-5.2005891	7.28173E-09	1.28142E-07	RHSIM_Rhsim02G0122500
Root vs Stem	RHSIM_Rhsim07G0100700	1.232392437	7.33379E-09	1.28926E-07	RHSIM_Rhsim07G0100700
Root vs Stem	RHSIM_Rhsim13G0087300	3.09286509	7.34309E-09	1.28926E-07	RHSIM_Rhsim13G0087300
Root vs Stem	RHSIM_Rhsim11G0011700	-1.422592026	7.46082E-09	1.30894E-07	RHSIM_Rhsim11G0011700
Root vs Stem	RHSIM_Rhsim08G0197700	1.09683358	7.74675E-09	1.35704E-07	RHSIM_Rhsim08G0197700
Root vs Stem	novel.3306	-2.305960748	7.81451E-09	1.36787E-07	--
Root vs Stem	RHSIM_Rhsim07G0162200	-1.211265461	7.84765E-09	1.37262E-07	RHSIM_Rhsim07G0162200
Root vs Stem	novel.5360	2.754844785	7.90527E-09	1.38165E-07	--
Root vs Stem	RHSIM_Rhsim06G0055300	1.440086255	8.00036E-09	1.39721E-07	RHSIM_Rhsim06G0055300
Root vs Stem	RHSIM_Rhsim10G0063800	-7.466476391	8.10401E-09	1.41211E-07	RHSIM_Rhsim10G0063800
Root vs Stem	RHSIM_Rhsim08G0213900	-1.265999094	8.28192E-09	1.44202E-07	RHSIM_Rhsim08G0213900
Root vs Stem	RHSIM_Rhsim08G0025300	-1.383139586	8.30925E-09	1.44568E-07	RHSIM_Rhsim08G0025300
Root vs Stem	novel.1841	-1.203532404	8.32202E-09	1.44681E-07	--
Root vs Stem	RHSIM_Rhsim03G0096000	-2.202118597	8.36052E-09	1.45241E-07	RHSIM_Rhsim03G0096000
Root vs Stem	RHSIM_Rhsim10G0049200	-1.359370253	8.46345E-09	1.46919E-07	RHSIM_Rhsim10G0049200
Root vs Stem	RHSIM_Rhsim13G0231200	1.646894527	8.57223E-09	1.48669E-07	RHSIM_Rhsim13G0231200

Comparison	Gene ID	log₂FC	P-value	FDR (padj)	Gene Name
Root vs Stem	RHSIM_Rhsim05G0161400	2.03817243	8.57716E-09	1.48669E-07	RHSIM_Rhsim05G0161400
Root vs Stem	RHSIM_Rhsim07G0254100	-5.975290865	8.62105E-09	1.49317E-07	RHSIM_Rhsim07G0254100
Root vs Stem	RHSIM_Rhsim01G0213500	-1.11835206	8.6944E-09	1.50362E-07	RHSIM_Rhsim01G0213500
Root vs Stem	RHSIM_Rhsim07G0251700	-2.868695628	8.79146E-09	1.51926E-07	RHSIM_Rhsim07G0251700
Root vs Stem	RHSIM_Rhsim11G0036700	-1.128328403	8.82491E-09	1.5239E-07	RHSIM_Rhsim11G0036700
Root vs Stem	RHSIM_Rhsim01G0250200	1.663599459	8.96141E-09	1.54631E-07	RHSIM_Rhsim01G0250200
Root vs Stem	RHSIM_Rhsim09G0132500	3.61175729	9.08853E-09	1.56707E-07	RHSIM_Rhsim09G0132500
Root vs Stem	RHSIM_Rhsim13G0171300	-2.923166812	9.09824E-09	1.56758E-07	RHSIM_Rhsim13G0171300
Root vs Stem	novel.2921	3.879583184	9.15679E-09	1.57649E-07	--
Root vs Stem	RHSIM_Rhsim04G0027600	-1.579847697	9.39338E-09	1.61481E-07	RHSIM_Rhsim04G0027600
Root vs Stem	RHSIM_Rhsim09G0044400	-2.459770818	9.47047E-09	1.62685E-07	RHSIM_Rhsim09G0044400
Root vs Stem	RHSIM_Rhsim07G0042800	3.119109886	9.54069E-09	1.63769E-07	RHSIM_Rhsim07G0042800
Root vs Stem	RHSIM_Rhsim13G0063600	3.052700631	9.64412E-09	1.65421E-07	RHSIM_Rhsim13G0063600
Root vs Stem	RHSIM_Rhsim07G0200400	1.743029821	9.71256E-09	1.66471E-07	RHSIM_Rhsim07G0200400
Root vs Stem	RHSIM_Rhsim12G0126200	-1.061685798	9.81479E-09	1.68098E-07	RHSIM_Rhsim12G0126200
Root vs Stem	RHSIM_Rhsim02G0086400	5.335941058	1.0101E-08	1.72872E-07	RHSIM_Rhsim02G0086400
Root vs Stem	RHSIM_Rhsim07G0032300	-2.983175667	1.01795E-08	1.74086E-07	RHSIM_Rhsim07G0032300
Root vs Stem	RHSIM_Rhsim09G0118400	-1.730632339	1.02856E-08	1.75769E-07	RHSIM_Rhsim09G0118400
Root vs Stem	RHSIM_Rhsim11G0088700	1.497847728	1.04523E-08	1.78355E-07	RHSIM_Rhsim11G0088700
Root vs Stem	RHSIM_Rhsim11G0057900	1.037654242	1.08199E-08	1.84384E-07	RHSIM_Rhsim11G0057900
Root vs Stem	novel.371	-1.342787537	1.08615E-08	1.84926E-07	--
Root vs Stem	RHSIM_Rhsim06G0216000	4.019099459	1.089E-08	1.85275E-07	RHSIM_Rhsim06G0216000
Root vs Stem	RHSIM_Rhsim03G0093100	2.561756049	1.10174E-08	1.87304E-07	RHSIM_Rhsim03G0093100
Root vs Stem	RHSIM_Rhsim04G0190300	-1.146325885	1.11114E-08	1.88763E-07	RHSIM_Rhsim04G0190300
Root vs Stem	RHSIM_Rhsim05G0043600	-1.137903272	1.11703E-08	1.89624E-07	RHSIM_Rhsim05G0043600

Comparison	Gene ID	log_2FC	P-value	FDR (padj)	Gene Name
Root vs Stem	RHSIM_Rhsim12G0084400	2.354809859	1.11877E-08	1.89779E-07	RHSIM_Rhsim12G0084400
Root vs Stem	RHSIM_Rhsim01G0055800	-4.533892687	1.12519E-08	1.90729E-07	RHSIM_Rhsim01G0055800
Root vs Stem	RHSIM_Rhsim09G0171800	1.203532286	1.14667E-08	1.94083E-07	RHSIM_Rhsim09G0171800
Root vs Stem	novel.1714	3.557317491	1.1738E-08	1.9853E-07	--
Root vs Stem	RHSIM_Rhsim04G0207900	1.134846812	1.17625E-08	1.98798E-07	RHSIM_Rhsim04G0207900
Root vs Stem	RHSIM_Rhsim06G0113700	-1.820188897	1.17896E-08	1.9911E-07	RHSIM_Rhsim06G0113700
Root vs Stem	RHSIM_Rhsim04G0149400	3.497801314	1.18025E-08	1.99182E-07	RHSIM_Rhsim04G0149400
Root vs Stem	RHSIM_RhsimUnG0250300	-1.015748637	1.1885E-08	2.00429E-07	RHSIM_RhsimUnG0250300
Root vs Stem	RHSIM_Rhsim08G0012200	1.092003469	1.21198E-08	2.04089E-07	RHSIM_Rhsim08G0012200
Root vs Stem	RHSIM_Rhsim06G0224500	-1.880006062	1.22923E-08	2.06843E-07	RHSIM_Rhsim06G0224500
Root vs Stem	RHSIM_Rhsim06G0212100	-1.441341895	1.23146E-08	2.07067E-07	RHSIM_Rhsim06G0212100
Root vs Stem	RHSIM_Rhsim04G0231200	8.24778452	1.25894E-08	2.11532E-07	RHSIM_Rhsim04G0231200
Root vs Stem	RHSIM_Rhsim06G0222600	1.709518799	1.2726E-08	2.13673E-07	RHSIM_Rhsim06G0222600
Root vs Stem	novel.2531	4.800061881	1.27384E-08	2.13725E-07	--
Root vs Stem	novel.5402	-2.513473461	1.29103E-08	2.16452E-07	--
Root vs Stem	RHSIM_Rhsim04G0010600	1.81351961	1.29917E-08	2.17658E-07	RHSIM_Rhsim04G0010600
Root vs Stem	RHSIM_Rhsim07G0011900	2.039254028	1.31941E-08	2.2082E-07	RHSIM_Rhsim07G0011900
Root vs Stem	RHSIM_Rhsim03G0186600	1.051145494	1.31996E-08	2.2082E-07	RHSIM_Rhsim03G0186600
Root vs Stem	RHSIM_Rhsim02G0021200	-4.605186401	1.32579E-08	2.21475E-07	RHSIM_Rhsim02G0021200
Root vs Stem	novel.5976	-4.224615268	1.33598E-08	2.22965E-07	--
Root vs Stem	RHSIM_Rhsim08G0195500	3.394359365	1.35507E-08	2.25712E-07	RHSIM_Rhsim08G0195500
Root vs Stem	RHSIM_Rhsim05G0189100	-9.228137812	1.36822E-08	2.27737E-07	RHSIM_Rhsim05G0189100
Root vs Stem	RHSIM_Rhsim09G0120100	-1.300320957	1.38761E-08	2.30798E-07	RHSIM_Rhsim09G0120100
Root vs Stem	RHSIM_Rhsim09G0107100	5.264778441	1.42252E-08	2.36264E-07	RHSIM_Rhsim09G0107100
Root vs Stem	RHSIM_Rhsim10G0012800	-1.367303789	1.42519E-08	2.36537E-07	RHSIM_Rhsim10G0012800

Comparison	Gene ID	log_2FC	P-value	FDR (padj)	Gene Name
Root vs Stem	RHSIM_Rhsim08G0250300	-1.317381812	1.47199E-08	2.43953E-07	RHSIM_Rhsim08G0250300
Root vs Stem	RHSIM_Rhsim08G0194200	1.618645703	1.47602E-08	2.44444E-07	RHSIM_Rhsim08G0194200
Root vs Stem	RHSIM_Rhsim02G0212300	-1.138637911	1.5035E-08	2.48817E-07	RHSIM_Rhsim02G0212300
Root vs Stem	RHSIM_RhsimUnG0077000	-1.421892827	1.50615E-08	2.49077E-07	RHSIM_RhsimUnG0077000
Root vs Stem	RHSIM_Rhsim03G0214500	-3.448958761	1.50786E-08	2.49181E-07	RHSIM_Rhsim03G0214500
Root vs Stem	RHSIM_Rhsim07G0030900	-2.132052239	1.50936E-08	2.4925E-07	RHSIM_Rhsim07G0030900
Root vs Stem	RHSIM_Rhsim01G0241500	2.471580894	1.51362E-08	2.49774E-07	RHSIM_Rhsim01G0241500
Root vs Stem	RHSIM_Rhsim10G0071000	4.295338211	1.54169E-08	2.54225E-07	RHSIM_Rhsim10G0071000
Root vs Stem	RHSIM_Rhsim12G0054200	4.837618605	1.55833E-08	2.56785E-07	RHSIM_Rhsim12G0054200
Root vs Stem	RHSIM_RhsimUnG0087200	1.737822388	1.5708E-08	2.58655E-07	RHSIM_RhsimUnG0087200
Root vs Stem	RHSIM_Rhsim10G0200900	9.489894812	1.57905E-08	2.59828E-07	RHSIM_Rhsim10G0200900
Root vs Stem	RHSIM_Rhsim08G0182300	-1.470188308	1.58724E-08	2.60989E-07	RHSIM_Rhsim08G0182300
Root vs Stem	RHSIM_Rhsim11G0121200	1.136526481	1.60183E-08	2.63201E-07	RHSIM_Rhsim11G0121200
Root vs Stem	RHSIM_RhsimUnG0065100	-4.548829716	1.61449E-08	2.65092E-07	RHSIM_RhsimUnG0065100
Root vs Stem	novel.1493	-1.435147672	1.62534E-08	2.66683E-07	--
Root vs Stem	RHSIM_Rhsim06G0157300	2.968952826	1.62981E-08	2.67225E-07	RHSIM_Rhsim06G0157300
Root vs Stem	RHSIM_Rhsim09G0135600	-3.563824511	1.63481E-08	2.67855E-07	RHSIM_Rhsim09G0135600
Root vs Stem	RHSIM_Rhsim03G0076000	-3.426348148	1.64418E-08	2.69199E-07	RHSIM_Rhsim03G0076000
Root vs Stem	RHSIM_Rhsim09G0163800	2.685859922	1.67069E-08	2.73346E-07	RHSIM_Rhsim09G0163800
Root vs Stem	RHSIM_RhsimUnG0135600	-1.966361474	1.69078E-08	2.76437E-07	RHSIM_RhsimUnG0135600
Root vs Stem	RHSIM_RhsimUnG0125800	1.239136842	1.74149E-08	2.84123E-07	RHSIM_RhsimUnG0125800
Root vs Stem	RHSIM_Rhsim11G0016300	-1.406664105	1.75418E-08	2.85991E-07	RHSIM_Rhsim11G0016300
Root vs Stem	RHSIM_Rhsim10G0142200	-3.697990638	1.79651E-08	2.92686E-07	RHSIM_Rhsim10G0142200
Root vs Stem	novel.3072	1.964407397	1.81509E-08	2.95503E-07	--
Root vs Stem	RHSIM_Rhsim03G0089300	1.213824454	1.83884E-08	2.99159E-07	RHSIM_Rhsim03G0089300

Comparison	Gene ID	log_2FC	P-value	FDR (padj)	Gene Name
Root vs Stem	RHSIM_Rhsim03G0081300	-2.562273994	1.85421E-08	3.01447E-07	RHSIM_Rhsim03G0081300
Root vs Stem	RHSIM_Rhsim02G0035400	1.197767033	1.85771E-08	3.01802E-07	RHSIM_Rhsim02G0035400
Root vs Stem	RHSIM_Rhsim03G0255300	-7.212654778	1.8665E-08	3.03017E-07	RHSIM_Rhsim03G0255300
Root vs Stem	RHSIM_Rhsim07G0016700	4.287568703	1.91178E-08	3.10149E-07	RHSIM_Rhsim07G0016700
Root vs Stem	RHSIM_Rhsim08G0104800	-1.864615444	1.93318E-08	3.13401E-07	RHSIM_Rhsim08G0104800
Root vs Stem	RHSIM_Rhsim11G0175000	8.049560326	1.97975E-08	3.20725E-07	RHSIM_Rhsim11G0175000
Root vs Stem	RHSIM_Rhsim04G0207100	1.676431758	1.99301E-08	3.22646E-07	RHSIM_Rhsim04G0207100
Root vs Stem	RHSIM_Rhsim11G0043000	-1.296659993	1.99645E-08	3.22977E-07	RHSIM_Rhsim11G0043000
Root vs Stem	RHSIM_Rhsim07G0088300	1.117682836	2.0143E-08	3.25636E-07	RHSIM_Rhsim07G0088300
Root vs Stem	RHSIM_Rhsim04G0073000	2.968988765	2.04539E-08	3.30431E-07	RHSIM_Rhsim04G0073000
Root vs Stem	RHSIM_Rhsim08G0115500	-2.796933417	2.05422E-08	3.31624E-07	RHSIM_Rhsim08G0115500
Root vs Stem	RHSIM_Rhsim13G0169600	8.047610469	2.06105E-08	3.32495E-07	RHSIM_Rhsim13G0169600
Root vs Stem	RHSIM_Rhsim04G0180700	4.409146488	2.06634E-08	3.33114E-07	RHSIM_Rhsim04G0180700
Root vs Stem	RHSIM_Rhsim04G0033800	-2.408865585	2.07252E-08	3.33877E-07	RHSIM_Rhsim04G0033800
Root vs Stem	RHSIM_Rhsim06G0040300	2.964906429	2.09651E-08	3.37271E-07	RHSIM_Rhsim06G0040300
Root vs Stem	RHSIM_Rhsim03G0239700	4.557876052	2.11371E-08	3.39782E-07	RHSIM_Rhsim03G0239700
Root vs Stem	RHSIM_Rhsim11G0195600	3.403619168	2.11507E-08	3.39782E-07	RHSIM_Rhsim11G0195600
Root vs Stem	RHSIM_Rhsim02G0168500	5.051545343	2.1387E-08	3.43338E-07	RHSIM_Rhsim02G0168500
Root vs Stem	RHSIM_Rhsim05G0017000	1.666085854	2.14194E-08	3.4362E-07	RHSIM_Rhsim05G0017000
Root vs Stem	RHSIM_Rhsim10G0167200	2.716805543	2.157E-08	3.45795E-07	RHSIM_Rhsim10G0167200
Root vs Stem	RHSIM_Rhsim08G0180100	1.511208705	2.20784E-08	3.537E-07	RHSIM_Rhsim08G0180100
Root vs Stem	RHSIM_Rhsim03G0086400	1.087661622	2.2246E-08	3.56136E-07	RHSIM_Rhsim03G0086400
Root vs Stem	RHSIM_Rhsim12G0104400	1.597823395	2.22869E-08	3.56544E-07	RHSIM_Rhsim12G0104400
Root vs Stem	novel.4755	2.612298631	2.26073E-08	3.61419E-07	--
Root vs Stem	RHSIM_RhsimUnG0043100	2.951892503	2.27779E-08	3.63882E-07	RHSIM_RhsimUnG0043100

Comparison	Gene ID	log_2FC	P-value	FDR (padj)	Gene Name
Root vs Stem	novel.2643	3.200230012	2.27963E-08	3.63882E-07	--
Root vs Stem	RHSIM_Rhsim13G0163900	1.906253191	2.28087E-08	3.63882E-07	RHSIM_Rhsim13G0163900
Root vs Stem	RHSIM_Rhsim02G0249000	-4.550793338	2.29004E-08	3.65091E-07	RHSIM_Rhsim02G0249000
Root vs Stem	RHSIM_Rhsim10G0022600	3.401897957	2.30057E-08	3.66518E-07	RHSIM_Rhsim10G0022600
Root vs Stem	novel.5225	5.069163549	2.33105E-08	3.71116E-07	--
Root vs Stem	RHSIM_Rhsim08G0088000	2.943696631	2.35943E-08	3.75371E-07	RHSIM_Rhsim08G0088000
Root vs Stem	novel.414	1.054817645	2.40846E-08	3.82648E-07	--
Root vs Stem	RHSIM_Rhsim08G0119200	2.246153178	2.4457E-08	3.88296E-07	RHSIM_Rhsim08G0119200
Root vs Stem	RHSIM_Rhsim10G0018400	-1.671225773	2.47356E-08	3.9245E-07	RHSIM_Rhsim10G0018400
Root vs Stem	RHSIM_Rhsim02G0211400	-7.648907481	2.48858E-08	3.94562E-07	RHSIM_Rhsim02G0211400
Root vs Stem	RHSIM_Rhsim05G0179300	-2.042320182	2.50931E-08	3.97574E-07	RHSIM_Rhsim05G0179300
Root vs Stem	RHSIM_Rhsim07G0040900	-2.225975246	2.5475E-08	4.03349E-07	RHSIM_Rhsim07G0040900
Root vs Stem	RHSIM_Rhsim07G0043100	2.598551186	2.58113E-08	4.08112E-07	RHSIM_Rhsim07G0043100
Root vs Stem	RHSIM_Rhsim03G0229800	-1.331082872	2.58358E-08	4.0822E-07	RHSIM_Rhsim03G0229800
Root vs Stem	RHSIM_Rhsim06G0108700	3.350769157	2.61362E-08	4.12683E-07	RHSIM_Rhsim06G0108700
Root vs Stem	RHSIM_Rhsim07G0206200	5.695464805	2.65001E-08	4.18143E-07	RHSIM_Rhsim07G0206200
Root vs Stem	RHSIM_Rhsim11G0046700	-1.873576527	2.70704E-08	4.26557E-07	RHSIM_Rhsim11G0046700
Root vs Stem	RHSIM_RhsimUnG0073500	3.530420897	2.71889E-08	4.28132E-07	RHSIM_RhsimUnG0073500
Root vs Stem	RHSIM_Rhsim10G0188000	1.806628819	2.73663E-08	4.30526E-07	RHSIM_Rhsim10G0188000
Root vs Stem	RHSIM_Rhsim11G0107700	3.603889257	2.8193E-08	4.43036E-07	RHSIM_Rhsim11G0107700
Root vs Stem	RHSIM_Rhsim04G0087000	-2.986002175	2.86049E-08	4.48996E-07	RHSIM_Rhsim04G0087000
Root vs Stem	RHSIM_Rhsim05G0042100	2.504142838	2.86113E-08	4.48996E-07	RHSIM_Rhsim05G0042100
Root vs Stem	RHSIM_Rhsim04G0230100	8.519098892	2.98153E-08	4.66937E-07	RHSIM_Rhsim04G0230100
Root vs Stem	RHSIM_Rhsim03G0098700	1.200094917	3.01406E-08	4.71712E-07	RHSIM_Rhsim03G0098700
Root vs Stem	RHSIM_Rhsim03G0027500	-2.731688963	3.02788E-08	4.73232E-07	RHSIM_Rhsim03G0027500

Comparison	Gene ID	log_2FC	P-value	FDR (padj)	Gene Name
Root vs Stem	RHSIM_Rhsim07G0234200	1.36387273	3.03039E-08	4.73304E-07	RHSIM_Rhsim07G0234200
Root vs Stem	RHSIM_RhsimUnG0044100	-1.206956988	3.04746E-08	4.75648E-07	RHSIM_RhsimUnG0044100
Root vs Stem	RHSIM_Rhsim08G0004400	3.845968952	3.06529E-08	4.78106E-07	RHSIM_Rhsim08G0004400
Root vs Stem	RHSIM_Rhsim02G0119200	-3.2212707	3.20152E-08	4.98006E-07	RHSIM_Rhsim02G0119200
Root vs Stem	RHSIM_Rhsim13G0099100	-2.009547454	3.21745E-08	5.00147E-07	RHSIM_Rhsim13G0099100
Root vs Stem	RHSIM_Rhsim05G0128400	-2.741793638	3.23174E-08	5.02031E-07	RHSIM_Rhsim05G0128400
Root vs Stem	novel.35	-2.296831263	3.25167E-08	5.04787E-07	--
Root vs Stem	RHSIM_Rhsim04G0237600	-2.880545375	3.27327E-08	5.07798E-07	RHSIM_Rhsim04G0237600
Root vs Stem	RHSIM_Rhsim13G0200200	-3.02028814	3.27941E-08	5.08407E-07	RHSIM_Rhsim13G0200200
Root vs Stem	novel.3773	-7.798774179	3.29961E-08	5.11195E-07	--
Root vs Stem	RHSIM_Rhsim03G0218200	5.233694614	3.31107E-08	5.12626E-07	RHSIM_Rhsim03G0218200
Root vs Stem	RHSIM_Rhsim12G0089100	2.952126574	3.35035E-08	5.18313E-07	RHSIM_Rhsim12G0089100
Root vs Stem	RHSIM_Rhsim01G0231600	1.020264578	3.35229E-08	5.18313E-07	RHSIM_Rhsim01G0231600
Root vs Stem	novel.37	2.559370854	3.35891E-08	5.18988E-07	--
Root vs Stem	RHSIM_Rhsim06G0205500	-1.685059031	3.36981E-08	5.20323E-07	RHSIM_Rhsim06G0205500
Root vs Stem	RHSIM_Rhsim12G0203800	8.077011002	3.37258E-08	5.20402E-07	RHSIM_Rhsim12G0203800
Root vs Stem	RHSIM_Rhsim09G0190700	-1.374417923	3.37581E-08	5.20551E-07	RHSIM_Rhsim09G0190700
Root vs Stem	RHSIM_Rhsim03G0215600	-1.580718614	3.40348E-08	5.24468E-07	RHSIM_Rhsim03G0215600
Root vs Stem	RHSIM_RhsimUnG0254000	-1.501918203	3.42344E-08	5.27192E-07	RHSIM_RhsimUnG0254000
Root vs Stem	RHSIM_Rhsim03G0057300	1.360802327	3.43418E-08	5.28492E-07	RHSIM_Rhsim03G0057300
Root vs Stem	RHSIM_Rhsim05G0092200	2.507446531	3.48502E-08	5.35958E-07	RHSIM_Rhsim05G0092200
Root vs Stem	novel.3508	-3.872518111	3.49924E-08	5.37787E-07	--
Root vs Stem	RHSIM_RhsimUnG0195900	-1.558202946	3.50376E-08	5.38122E-07	RHSIM_RhsimUnG0195900
Root vs Stem	RHSIM_Rhsim09G0196000	2.385788424	3.50968E-08	5.38672E-07	RHSIM_Rhsim09G0196000
Root vs Stem	RHSIM_Rhsim08G0034600	-1.855388896	3.52963E-08	5.41374E-07	RHSIM_Rhsim08G0034600

Comparison	Gene ID	log_2FC	P-value	FDR (padj)	Gene Name
Root vs Stem	RHSIM_Rhsim10G0113200	1.070348184	3.55525E-08	5.44467E-07	RHSIM_Rhsim10G0113200
Root vs Stem	RHSIM_Rhsim02G0234500	-3.380045432	3.55643E-08	5.44467E-07	RHSIM_Rhsim02G0234500
Root vs Stem	RHSIM_Rhsim12G0050300	-7.339456552	3.55688E-08	5.44467E-07	RHSIM_Rhsim12G0050300
Root vs Stem	RHSIM_Rhsim09G0188300	4.033761081	3.56181E-08	5.4486E-07	RHSIM_Rhsim09G0188300
Root vs Stem	novel.4611	-2.259992687	3.60877E-08	5.51312E-07	--
Root vs Stem	novel.1851	7.81448345	3.61228E-08	5.51483E-07	--
Root vs Stem	RHSIM_Rhsim12G0106100	1.457536629	3.63243E-08	5.54192E-07	RHSIM_Rhsim12G0106100
Root vs Stem	novel.5629	3.217064473	3.68287E-08	5.61145E-07	--
Root vs Stem	RHSIM_Rhsim03G0031300	-1.26128128	3.72412E-08	5.66681E-07	RHSIM_Rhsim03G0031300
Root vs Stem	RHSIM_Rhsim08G0199200	2.243175553	3.73639E-08	5.68173E-07	RHSIM_Rhsim08G0199200
Root vs Stem	RHSIM_Rhsim02G0235400	4.268778882	3.80144E-08	5.77683E-07	RHSIM_Rhsim02G0235400
Root vs Stem	RHSIM_Rhsim06G0020100	-1.111193832	3.84053E-08	5.8324E-07	RHSIM_Rhsim06G0020100
Root vs Stem	RHSIM_Rhsim04G0058600	-2.990539815	3.88294E-08	5.89292E-07	RHSIM_Rhsim04G0058600
Root vs Stem	RHSIM_Rhsim06G0108200	2.536456432	3.89828E-08	5.91231E-07	RHSIM_Rhsim06G0108200
Root vs Stem	RHSIM_Rhsim06G0058200	-4.738021628	3.91925E-08	5.94021E-07	RHSIM_Rhsim06G0058200
Root vs Stem	RHSIM_Rhsim03G0151800	2.82771201	3.9311E-08	5.95425E-07	RHSIM_Rhsim03G0151800
Root vs Stem	RHSIM_Rhsim04G0166400	7.581036914	3.93426E-08	5.95512E-07	RHSIM_Rhsim04G0166400
Root vs Stem	RHSIM_Rhsim07G0133100	-6.197564749	3.94829E-08	5.97243E-07	RHSIM_Rhsim07G0133100
Root vs Stem	RHSIM_Rhsim04G0126300	3.298084555	4.0182E-08	6.0742E-07	RHSIM_Rhsim04G0126300
Root vs Stem	novel.2321	-7.989023603	4.06914E-08	6.14718E-07	--
Root vs Stem	RHSIM_Rhsim10G0181600	-1.40561913	4.15205E-08	6.25919E-07	RHSIM_Rhsim10G0181600
Root vs Stem	RHSIM_Rhsim09G0035700	2.035996418	4.15683E-08	6.25919E-07	RHSIM_Rhsim09G0035700
Root vs Stem	novel.3947	-2.573257722	4.15686E-08	6.25919E-07	--
Root vs Stem	RHSIM_Rhsim04G0121900	-1.229593504	4.17565E-08	6.28338E-07	RHSIM_Rhsim04G0121900
Root vs Stem	RHSIM_Rhsim07G0082600	4.984135057	4.22663E-08	6.35594E-07	RHSIM_Rhsim07G0082600

Comparison	Gene ID	log_2FC	P-value	FDR (padj)	Gene Name
Root vs Stem	RHSIM_RhsimUnG0180200	-2.706714149	4.24232E-08	6.37537E-07	RHSIM_RhsimUnG0180200
Root vs Stem	RHSIM_Rhsim09G0144600	5.97790438	4.26838E-08	6.40804E-07	RHSIM_Rhsim09G0144600
Root vs Stem	RHSIM_Rhsim05G0161700	3.559152623	4.26962E-08	6.40804E-07	RHSIM_Rhsim05G0161700
Root vs Stem	RHSIM_Rhsim02G0110500	1.466361672	4.27505E-08	6.41202E-07	RHSIM_Rhsim02G0110500
Root vs Stem	RHSIM_Rhsim12G0136600	-1.463668249	4.2874E-08	6.42636E-07	RHSIM_Rhsim12G0136600
Root vs Stem	RHSIM_Rhsim07G0227000	6.033099258	4.30175E-08	6.44368E-07	RHSIM_Rhsim07G0227000
Root vs Stem	RHSIM_Rhsim13G0181500	1.448359971	4.41968E-08	6.59461E-07	RHSIM_Rhsim13G0181500
Root vs Stem	RHSIM_Rhsim02G0022300	1.596454553	4.53536E-08	6.75847E-07	RHSIM_Rhsim02G0022300
Root vs Stem	RHSIM_Rhsim11G0077500	-4.161660475	4.62164E-08	6.8826E-07	RHSIM_Rhsim11G0077500
Root vs Stem	RHSIM_Rhsim01G0171100	-1.225940839	4.64927E-08	6.91928E-07	RHSIM_Rhsim01G0171100
Root vs Stem	RHSIM_Rhsim01G0138500	-14.32591007	4.73735E-08	7.04581E-07	RHSIM_Rhsim01G0138500
Root vs Stem	RHSIM_Rhsim01G0180600	-2.924571565	4.76674E-08	7.08495E-07	RHSIM_Rhsim01G0180600
Root vs Stem	RHSIM_Rhsim06G0222300	1.347851696	4.8326E-08	7.1736E-07	RHSIM_Rhsim06G0222300
Root vs Stem	RHSIM_Rhsim10G0180100	-1.449422455	4.85973E-08	7.20923E-07	RHSIM_Rhsim10G0180100
Root vs Stem	novel.2705	-1.309266839	4.8824E-08	7.23815E-07	--
Root vs Stem	RHSIM_Rhsim10G0037900	-3.797711883	4.88551E-08	7.23815E-07	RHSIM_Rhsim10G0037900
Root vs Stem	RHSIM_Rhsim05G0079000	-1.75208654	5.00675E-08	7.40825E-07	RHSIM_Rhsim05G0079000
Root vs Stem	RHSIM_Rhsim02G0062100	1.633800552	5.12632E-08	7.58031E-07	RHSIM_Rhsim02G0062100
Root vs Stem	RHSIM_Rhsim04G0188500	3.338146576	5.17502E-08	7.64742E-07	RHSIM_Rhsim04G0188500
Root vs Stem	RHSIM_Rhsim08G0085100	1.083356035	5.18296E-08	7.65424E-07	RHSIM_Rhsim08G0085100
Root vs Stem	RHSIM_Rhsim11G0181300	3.549765836	5.20442E-08	7.6761E-07	RHSIM_Rhsim11G0181300
Root vs Stem	novel.1380	-1.207196187	5.23832E-08	7.71624E-07	--
Root vs Stem	RHSIM_Rhsim09G0196100	2.319308512	5.28607E-08	7.78159E-07	RHSIM_Rhsim09G0196100
Root vs Stem	novel.3652	-3.634673437	5.29941E-08	7.79625E-07	--
Root vs Stem	RHSIM_Rhsim06G0070400	-1.246868822	5.38666E-08	7.91452E-07	RHSIM_Rhsim06G0070400

Comparison	Gene ID	log_2FC	P-value	FDR (padj)	Gene Name
Root vs Stem	RHSIM_Rhsim02G0162200	-6.866178237	5.41384E-08	7.94938E-07	RHSIM_Rhsim02G0162200
Root vs Stem	RHSIM_Rhsim11G0127900	-4.916350503	5.6056E-08	8.22035E-07	RHSIM_Rhsim11G0127900
Root vs Stem	RHSIM_Rhsim06G0163800	1.388759607	5.60908E-08	8.22035E-07	RHSIM_Rhsim06G0163800
Root vs Stem	RHSIM_Rhsim11G0093500	1.388280656	5.70806E-08	8.3601E-07	RHSIM_Rhsim11G0093500
Root vs Stem	RHSIM_RhsimUnG0043200	2.881239358	5.75487E-08	8.41795E-07	RHSIM_RhsimUnG0043200
Root vs Stem	RHSIM_Rhsim05G0229600	-1.027960743	5.84112E-08	8.53871E-07	RHSIM_Rhsim05G0229600
Root vs Stem	RHSIM_Rhsim13G0114900	2.117632742	5.84787E-08	8.54315E-07	RHSIM_Rhsim13G0114900
Root vs Stem	RHSIM_Rhsim09G0019700	2.775190693	5.89466E-08	8.60605E-07	RHSIM_Rhsim09G0019700
Root vs Stem	novel.4021	2.548249378	5.94889E-08	8.67973E-07	--
Root vs Stem	RHSIM_Rhsim06G0146400	2.109736365	5.97924E-08	8.71849E-07	RHSIM_Rhsim06G0146400
Root vs Stem	RHSIM_Rhsim01G0274100	-1.127081378	6.01574E-08	8.76617E-07	RHSIM_Rhsim01G0274100
Root vs Stem	RHSIM_Rhsim01G0062600	-2.881430428	6.02381E-08	8.77239E-07	RHSIM_Rhsim01G0062600
Root vs Stem	novel.5347	-2.080726692	6.04918E-08	8.80377E-07	--
Root vs Stem	RHSIM_Rhsim09G0011600	3.655504807	6.10298E-08	8.87647E-07	RHSIM_Rhsim09G0011600
Root vs Stem	RHSIM_Rhsim07G0210000	-2.407223999	6.1471E-08	8.935E-07	RHSIM_Rhsim07G0210000
Root vs Stem	RHSIM_Rhsim07G0144800	-1.693073165	6.21682E-08	9.02737E-07	RHSIM_Rhsim07G0144800
Root vs Stem	RHSIM_Rhsim04G0156700	2.201215907	6.21848E-08	9.02737E-07	RHSIM_Rhsim04G0156700
Root vs Stem	RHSIM_Rhsim02G0143000	1.164907222	6.29684E-08	9.13537E-07	RHSIM_Rhsim02G0143000
Root vs Stem	novel.1713	-9.864631736	6.3876E-08	9.26122E-07	--
Root vs Stem	RHSIM_Rhsim13G0084100	1.546559959	6.45671E-08	9.35553E-07	RHSIM_Rhsim13G0084100
Root vs Stem	novel.4125	2.995169351	6.48676E-08	9.39318E-07	--
Root vs Stem	RHSIM_Rhsim03G0207000	7.475321872	6.53014E-08	9.45006E-07	RHSIM_Rhsim03G0207000
Root vs Stem	RHSIM_Rhsim11G0033400	3.252693958	6.61903E-08	9.56069E-07	RHSIM_Rhsim11G0033400
Root vs Stem	novel.2572	6.131524517	6.71734E-08	9.69182E-07	--
Root vs Stem	RHSIM_Rhsim08G0173600	5.439405117	6.7648E-08	9.7529E-07	RHSIM_Rhsim08G0173600

Comparison	Gene ID	log_2FC	P-value	FDR (padj)	Gene Name
Root vs Stem	RHSIM_Rhsim05G0052200	5.374711751	6.78166E-08	9.77109E-07	RHSIM_Rhsim05G0052200
Root vs Stem	RHSIM_Rhsim01G0230200	-1.091711249	6.79375E-08	9.78241E-07	RHSIM_Rhsim01G0230200
Root vs Stem	RHSIM_Rhsim03G0011600	1.364598955	6.80581E-08	9.79365E-07	RHSIM_Rhsim03G0011600
Root vs Stem	RHSIM_Rhsim04G0236200	-3.891502053	6.83788E-08	9.83366E-07	RHSIM_Rhsim04G0236200
Root vs Stem	RHSIM_RhsimUnG0056400	-3.026586592	6.85831E-08	9.8569E-07	RHSIM_RhsimUnG0056400
Root vs Stem	novel.5220	7.808592878	6.91599E-08	9.92742E-07	--
Root vs Stem	RHSIM_Rhsim07G0204400	1.752746051	6.92765E-08	9.93797E-07	RHSIM_Rhsim07G0204400
Root vs Stem	RHSIM_Rhsim12G0164800	-1.154748802	7.00508E-08	1.00428E-06	RHSIM_Rhsim12G0164800
Root vs Stem	RHSIM_Rhsim13G0071400	2.657410235	7.11026E-08	1.01872E-06	RHSIM_Rhsim13G0071400
Root vs Stem	RHSIM_Rhsim05G0215900	-6.350867556	7.28272E-08	1.04279E-06	RHSIM_Rhsim05G0215900
Root vs Stem	RHSIM_Rhsim01G0233000	2.453225124	7.49574E-08	1.07196E-06	RHSIM_Rhsim01G0233000
Root vs Stem	RHSIM_Rhsim05G0219700	-4.77128431	7.58678E-08	1.0843E-06	RHSIM_Rhsim05G0219700
Root vs Stem	RHSIM_Rhsim10G0178400	1.351663296	7.62823E-08	1.08888E-06	RHSIM_Rhsim10G0178400
Root vs Stem	RHSIM_Rhsim05G0190800	1.194466935	7.65629E-08	1.09221E-06	RHSIM_Rhsim05G0190800
Root vs Stem	RHSIM_Rhsim05G0087600	-1.594950277	7.68545E-08	1.09569E-06	RHSIM_Rhsim05G0087600
Root vs Stem	RHSIM_Rhsim05G0069600	-1.467319545	7.88947E-08	1.12338E-06	RHSIM_Rhsim05G0069600
Root vs Stem	RHSIM_Rhsim12G0112400	-1.012352833	7.9129E-08	1.12602E-06	RHSIM_Rhsim12G0112400
Root vs Stem	RHSIM_Rhsim02G0120700	1.267705535	8.19167E-08	1.16426E-06	RHSIM_Rhsim02G0120700
Root vs Stem	RHSIM_Rhsim12G0163100	-1.412466288	8.29541E-08	1.17827E-06	RHSIM_Rhsim12G0163100
Root vs Stem	RHSIM_Rhsim13G0049600	-2.655094742	8.35602E-08	1.18615E-06	RHSIM_Rhsim13G0049600
Root vs Stem	RHSIM_Rhsim01G0149300	1.42794382	8.40612E-08	1.19253E-06	RHSIM_Rhsim01G0149300
Root vs Stem	RHSIM_Rhsim02G0223600	-1.038228868	8.57134E-08	1.21448E-06	RHSIM_Rhsim02G0223600
Root vs Stem	RHSIM_Rhsim12G0159400	-2.608149696	8.60146E-08	1.21799E-06	RHSIM_Rhsim12G0159400
Root vs Stem	RHSIM_Rhsim05G0218900	1.69202276	8.65347E-08	1.22461E-06	RHSIM_Rhsim05G0218900
Root vs Stem	RHSIM_RhsimUnG0109400	1.232096372	8.70095E-08	1.23003E-06	RHSIM_RhsimUnG0109400

Comparison	Gene ID	log_2FC	P-value	FDR (padj)	Gene Name
Root vs Stem	RHSIM_Rhsim03G0009800	-1.021926553	8.70244E-08	1.23003E-06	RHSIM_Rhsim03G0009800
Root vs Stem	RHSIM_RhsimUnG0183700	-3.318950256	8.80097E-08	1.24319E-06	RHSIM_RhsimUnG0183700
Root vs Stem	RHSIM_Rhsim10G0166400	-3.328329727	8.85936E-08	1.25067E-06	RHSIM_Rhsim10G0166400
Root vs Stem	RHSIM_Rhsim05G0177700	-3.721675609	9.08756E-08	1.28132E-06	RHSIM_Rhsim05G0177700
Root vs Stem	RHSIM_RhsimUnG0154700	2.069235742	9.11608E-08	1.28455E-06	RHSIM_RhsimUnG0154700
Root vs Stem	RHSIM_Rhsim08G0038700	2.978524084	9.29317E-08	1.30871E-06	RHSIM_Rhsim08G0038700
Root vs Stem	RHSIM_Rhsim10G0016900	-1.292792514	9.70169E-08	1.36457E-06	RHSIM_Rhsim10G0016900
Root vs Stem	RHSIM_Rhsim11G0146000	1.94020395	9.79626E-08	1.37703E-06	RHSIM_Rhsim11G0146000
Root vs Stem	RHSIM_Rhsim11G0144900	-1.272692362	9.81927E-08	1.37943E-06	RHSIM_Rhsim11G0144900
Root vs Stem	RHSIM_Rhsim06G0135300	1.150521125	1.00326E-07	1.40682E-06	RHSIM_Rhsim06G0135300
Root vs Stem	novel.3794	1.700137068	1.02019E-07	1.42969E-06	--
Root vs Stem	RHSIM_Rhsim11G0054300	1.033433497	1.02327E-07	1.43314E-06	RHSIM_Rhsim11G0054300
Root vs Stem	novel.4100	2.527012919	1.02779E-07	1.4386E-06	--
Root vs Stem	RHSIM_Rhsim11G0049700	2.366260097	1.03044E-07	1.44143E-06	RHSIM_Rhsim11G0049700
Root vs Stem	RHSIM_Rhsim10G0033800	-3.976165686	1.03131E-07	1.44177E-06	RHSIM_Rhsim10G0033800
Root vs Stem	RHSIM_Rhsim08G0069400	-8.174845548	1.0326E-07	1.4427E-06	RHSIM_Rhsim08G0069400
Root vs Stem	RHSIM_Rhsim09G0068700	-1.010897367	1.03656E-07	1.44647E-06	RHSIM_Rhsim09G0068700
Root vs Stem	novel.3359	-1.96543	1.04043E-07	1.451E-06	--
Root vs Stem	RHSIM_Rhsim01G0015400	-1.522984081	1.04571E-07	1.45668E-06	RHSIM_Rhsim01G0015400
Root vs Stem	RHSIM_Rhsim11G0140900	4.202032176	1.04951E-07	1.46014E-06	RHSIM_Rhsim11G0140900
Root vs Stem	RHSIM_Rhsim04G0234400	3.054771877	1.05371E-07	1.46509E-06	RHSIM_Rhsim04G0234400
Root vs Stem	novel.4622	-2.150179974	1.06225E-07	1.47607E-06	--
Root vs Stem	RHSIM_Rhsim05G0230200	-5.386667568	1.08352E-07	1.50472E-06	RHSIM_Rhsim05G0230200
Root vs Stem	RHSIM_Rhsim06G0192600	-4.99571893	1.09863E-07	1.52479E-06	RHSIM_Rhsim06G0192600
Root vs Stem	RHSIM_Rhsim12G0177200	2.958901004	1.11575E-07	1.54762E-06	RHSIM_Rhsim12G0177200

Comparison	Gene ID	log_2FC	P-value	FDR (padj)	Gene Name
Root vs Stem	RHSIM_Rhsim11G0087000	-3.817853105	1.13603E-07	1.5748E-06	RHSIM_Rhsim11G0087000
Root vs Stem	RHSIM_Rhsim08G0043900	-1.506284359	1.13895E-07	1.5779E-06	RHSIM_Rhsim08G0043900
Root vs Stem	RHSIM_Rhsim06G0029800	-6.21440222	1.15542E-07	1.59975E-06	RHSIM_Rhsim06G0029800
Root vs Stem	RHSIM_Rhsim03G0044700	1.117479159	1.15711E-07	1.60114E-06	RHSIM_Rhsim03G0044700
Root vs Stem	RHSIM_Rhsim02G0045600	-1.522757451	1.17203E-07	1.6208E-06	RHSIM_Rhsim02G0045600
Root vs Stem	RHSIM_Rhsim06G0191000	1.01375085	1.17343E-07	1.62177E-06	RHSIM_Rhsim06G0191000
Root vs Stem	RHSIM_Rhsim01G0197200	-1.34265973	1.17537E-07	1.62347E-06	RHSIM_Rhsim01G0197200
Root vs Stem	RHSIM_Rhsim12G0078900	-1.847271201	1.20382E-07	1.66178E-06	RHSIM_Rhsim12G0078900
Root vs Stem	RHSIM_Rhsim13G0020000	-1.910158271	1.20664E-07	1.66468E-06	RHSIM_Rhsim13G0020000
Root vs Stem	RHSIM_Rhsim01G0012100	-1.188190142	1.20966E-07	1.66785E-06	RHSIM_Rhsim01G0012100
Root vs Stem	RHSIM_Rhsim05G0054400	-1.151434594	1.22463E-07	1.68747E-06	RHSIM_Rhsim05G0054400
Root vs Stem	RHSIM_Rhsim09G0212800	1.145104777	1.23232E-07	1.69705E-06	RHSIM_Rhsim09G0212800
Root vs Stem	RHSIM_Rhsim02G0146400	1.250605348	1.24602E-07	1.71388E-06	RHSIM_Rhsim02G0146400
Root vs Stem	RHSIM_Rhsim01G0193100	3.611916526	1.25827E-07	1.72969E-06	RHSIM_Rhsim01G0193100
Root vs Stem	RHSIM_Rhsim12G0178300	1.781894813	1.27457E-07	1.75001E-06	RHSIM_Rhsim12G0178300
Root vs Stem	RHSIM_Rhsim01G0143700	-7.146626601	1.28476E-07	1.76295E-06	RHSIM_Rhsim01G0143700
Root vs Stem	novel.6047	4.10505824	1.28702E-07	1.765E-06	--
Root vs Stem	RHSIM_Rhsim09G0084100	3.478841754	1.33043E-07	1.82237E-06	RHSIM_Rhsim09G0084100
Root vs Stem	novel.1200	8.471272837	1.33707E-07	1.83037E-06	--
Root vs Stem	RHSIM_Rhsim11G0008500	-1.551677512	1.34278E-07	1.83709E-06	RHSIM_Rhsim11G0008500
Root vs Stem	RHSIM_Rhsim05G0137800	-1.592206793	1.35477E-07	1.85241E-06	RHSIM_Rhsim05G0137800
Root vs Stem	RHSIM_Rhsim10G0203600	-5.086174979	1.35661E-07	1.85382E-06	RHSIM_Rhsim10G0203600
Root vs Stem	RHSIM_RhsimUnG0038200	-2.480480313	1.36155E-07	1.85837E-06	RHSIM_RhsimUnG0038200
Root vs Stem	RHSIM_Rhsim10G0118300	1.355825336	1.39304E-07	1.89685E-06	RHSIM_Rhsim10G0118300
Root vs Stem	RHSIM_Rhsim02G0254400	2.011014413	1.39591E-07	1.89854E-06	RHSIM_Rhsim02G0254400

Comparison	Gene ID	log_2FC	P-value	FDR (padj)	Gene Name
Root vs Stem	novel.4153	2.394661495	1.39592E-07	1.89854E-06	--
Root vs Stem	RHSIM_Rhsim13G0040100	-2.594105622	1.41514E-07	1.92354E-06	RHSIM_Rhsim13G0040100
Root vs Stem	RHSIM_Rhsim12G0149400	-1.632428688	1.42607E-07	1.93725E-06	RHSIM_Rhsim12G0149400
Root vs Stem	RHSIM_Rhsim10G0170200	4.744850795	1.43944E-07	1.95427E-06	RHSIM_Rhsim10G0170200
Root vs Stem	RHSIM_Rhsim08G0147000	2.176681236	1.44995E-07	1.96506E-06	RHSIM_Rhsim08G0147000
Root vs Stem	RHSIM_Rhsim03G0063600	-1.900638392	1.45945E-07	1.97677E-06	RHSIM_Rhsim03G0063600
Root vs Stem	novel.4798	-1.059288548	1.46072E-07	1.97734E-06	--
Root vs Stem	RHSIM_Rhsim13G0122300	-6.10332981	1.46217E-07	1.97814E-06	RHSIM_Rhsim13G0122300
Root vs Stem	RHSIM_Rhsim04G0183500	6.584311258	1.46574E-07	1.9818E-06	RHSIM_Rhsim04G0183500
Root vs Stem	RHSIM_Rhsim09G0125400	-1.539307412	1.47347E-07	1.99108E-06	RHSIM_Rhsim09G0125400
Root vs Stem	RHSIM_Rhsim05G0032000	2.027677307	1.5099E-07	2.03792E-06	RHSIM_Rhsim05G0032000
Root vs Stem	novel.3529	-4.089718789	1.51755E-07	2.04586E-06	--
Root vs Stem	novel.2459	-1.680825945	1.53141E-07	2.06173E-06	--
Root vs Stem	RHSIM_Rhsim04G0234700	1.512833578	1.53201E-07	2.06173E-06	RHSIM_Rhsim04G0234700
Root vs Stem	RHSIM_Rhsim08G0020400	3.01263405	1.54351E-07	2.07479E-06	RHSIM_Rhsim08G0020400
Root vs Stem	RHSIM_Rhsim07G0048500	1.249436224	1.57024E-07	2.10825E-06	RHSIM_Rhsim07G0048500
Root vs Stem	RHSIM_Rhsim07G0014300	-2.894345963	1.57871E-07	2.11839E-06	RHSIM_Rhsim07G0014300
Root vs Stem	RHSIM_RhsimUnG0180800	3.407992702	1.58407E-07	2.12435E-06	RHSIM_RhsimUnG0180800
Root vs Stem	RHSIM_Rhsim12G0207700	-2.94966161	1.60628E-07	2.15065E-06	RHSIM_Rhsim12G0207700
Root vs Stem	RHSIM_Rhsim09G0098200	1.434993233	1.60637E-07	2.15065E-06	RHSIM_Rhsim09G0098200
Root vs Stem	RHSIM_Rhsim05G0020400	-1.76897238	1.60648E-07	2.15065E-06	RHSIM_Rhsim05G0020400
Root vs Stem	RHSIM_Rhsim12G0113000	-1.080822761	1.62028E-07	2.16786E-06	RHSIM_Rhsim12G0113000
Root vs Stem	RHSIM_Rhsim07G0146400	3.323681707	1.63994E-07	2.19289E-06	RHSIM_Rhsim07G0146400
Root vs Stem	RHSIM_Rhsim12G0106200	1.831450602	1.64302E-07	2.1957E-06	RHSIM_Rhsim12G0106200
Root vs Stem	RHSIM_Rhsim06G0137100	-1.459044858	1.64626E-07	2.19752E-06	RHSIM_Rhsim06G0137100

Comparison	Gene ID	log_2FC	P-value	FDR (padj)	Gene Name
Root vs Stem	RHSIM_Rhsim09G0198700	1.376876471	1.66247E-07	2.21787E-06	RHSIM_Rhsim09G0198700
Root vs Stem	novel.1175	6.840559917	1.66912E-07	2.22417E-06	--
Root vs Stem	RHSIM_Rhsim09G0203200	1.237498679	1.71604E-07	2.28537E-06	RHSIM_Rhsim09G0203200
Root vs Stem	RHSIM_Rhsim05G0061700	1.859330723	1.71759E-07	2.28612E-06	RHSIM_Rhsim05G0061700
Root vs Stem	RHSIM_Rhsim12G0143600	-3.291030707	1.72357E-07	2.29275E-06	RHSIM_Rhsim12G0143600
Root vs Stem	RHSIM_Rhsim10G0188300	1.257973602	1.72539E-07	2.29385E-06	RHSIM_Rhsim10G0188300
Root vs Stem	RHSIM_Rhsim09G0133100	-2.430340094	1.74549E-07	2.31768E-06	RHSIM_Rhsim09G0133100
Root vs Stem	RHSIM_Rhsim05G0106900	-1.126104398	1.74633E-07	2.31768E-06	RHSIM_Rhsim05G0106900
Root vs Stem	RHSIM_Rhsim11G0109100	6.439368188	1.74899E-07	2.31988E-06	RHSIM_Rhsim11G0109100
Root vs Stem	RHSIM_Rhsim10G0063500	1.053277158	1.76296E-07	2.33706E-06	RHSIM_Rhsim10G0063500
Root vs Stem	RHSIM_Rhsim09G0199700	2.111506608	1.76424E-07	2.33741E-06	RHSIM_Rhsim09G0199700
Root vs Stem	RHSIM_Rhsim09G0178100	-5.852748709	1.7704E-07	2.34422E-06	RHSIM_Rhsim09G0178100
Root vs Stem	RHSIM_Rhsim08G0153200	-3.626260968	1.77197E-07	2.34496E-06	RHSIM_Rhsim08G0153200
Root vs Stem	RHSIM_Rhsim12G0165000	1.265778013	1.78039E-07	2.35475E-06	RHSIM_Rhsim12G0165000
Root vs Stem	RHSIM_Rhsim06G0096300	-1.428323175	1.78509E-07	2.35962E-06	RHSIM_Rhsim06G0096300
Root vs Stem	RHSIM_Rhsim05G0112700	-3.83436982	1.79017E-07	2.36498E-06	RHSIM_Rhsim05G0112700
Root vs Stem	RHSIM_Rhsim04G0096100	-3.968055464	1.79606E-07	2.37139E-06	RHSIM_Rhsim04G0096100
Root vs Stem	RHSIM_Rhsim07G0045800	-1.047629285	1.81027E-07	2.38879E-06	RHSIM_Rhsim07G0045800
Root vs Stem	RHSIM_Rhsim05G0187900	4.912291135	1.81354E-07	2.39086E-06	RHSIM_Rhsim05G0187900
Root vs Stem	novel.1390	-1.011505327	1.81391E-07	2.39086E-06	--
Root vs Stem	novel.3694	-2.876914308	1.82541E-07	2.40327E-06	--
Root vs Stem	novel.1382	-1.151179021	1.84211E-07	2.42387E-06	--
Root vs Stem	RHSIM_Rhsim10G0021400	-1.176321281	1.86246E-07	2.44925E-06	RHSIM_Rhsim10G0021400
Root vs Stem	RHSIM_Rhsim13G0206900	7.015756283	1.88858E-07	2.48217E-06	RHSIM_Rhsim13G0206900
Root vs Stem	RHSIM_Rhsim05G0231400	-2.41172701	1.89349E-07	2.48722E-06	RHSIM_Rhsim05G0231400

Comparison	Gene ID	log_2FC	P-value	FDR (padj)	Gene Name
Root vs Stem	RHSIM_Rhsim13G0114700	3.123155741	1.92278E-07	2.52425E-06	RHSIM_Rhsim13G0114700
Root vs Stem	RHSIM_Rhsim06G0174600	-1.33304539	1.93343E-07	2.53468E-06	RHSIM_Rhsim06G0174600
Root vs Stem	RHSIM_Rhsim03G0214900	-3.440729377	1.93403E-07	2.53468E-06	RHSIM_Rhsim03G0214900
Root vs Stem	RHSIM_Rhsim13G0014700	3.52668899	1.95125E-07	2.55581E-06	RHSIM_Rhsim13G0014700
Root vs Stem	RHSIM_Rhsim12G0198100	-1.139542217	1.95385E-07	2.55776E-06	RHSIM_Rhsim12G0198100
Root vs Stem	RHSIM_Rhsim02G0010500	2.299397803	1.95916E-07	2.56325E-06	RHSIM_Rhsim02G0010500
Root vs Stem	RHSIM_Rhsim03G0049600	1.231919458	1.98786E-07	2.59786E-06	RHSIM_Rhsim03G0049600
Root vs Stem	novel.4908	2.702799442	2.00393E-07	2.61737E-06	--
Root vs Stem	novel.4303	-2.310297316	2.00592E-07	2.61849E-06	--
Root vs Stem	RHSIM_Rhsim13G0049800	2.219065578	2.01334E-07	2.6252E-06	RHSIM_Rhsim13G0049800
Root vs Stem	RHSIM_RhsimUnG0216000	1.579739508	2.05821E-07	2.68067E-06	RHSIM_RhsimUnG0216000
Root vs Stem	RHSIM_Rhsim09G0144500	7.467263591	2.07628E-07	2.70268E-06	RHSIM_Rhsim09G0144500
Root vs Stem	RHSIM_Rhsim11G0115200	1.604513404	2.08995E-07	2.71894E-06	RHSIM_Rhsim11G0115200
Root vs Stem	RHSIM_Rhsim01G0210400	-1.204585657	2.09692E-07	2.72647E-06	RHSIM_Rhsim01G0210400
Root vs Stem	RHSIM_Rhsim12G0085000	-3.09654883	2.10028E-07	2.7293E-06	RHSIM_Rhsim12G0085000
Root vs Stem	RHSIM_Rhsim13G0198200	1.126192431	2.11166E-07	2.74253E-06	RHSIM_Rhsim13G0198200
Root vs Stem	RHSIM_Rhsim03G0008900	-2.233166196	2.11853E-07	2.74992E-06	RHSIM_Rhsim03G0008900
Root vs Stem	RHSIM_Rhsim03G0073600	2.535353806	2.17554E-07	2.82074E-06	RHSIM_Rhsim03G0073600
Root vs Stem	novel.3960	-2.796840696	2.21254E-07	2.86548E-06	--
Root vs Stem	RHSIM_Rhsim04G0160100	-1.676800677	2.21455E-07	2.86648E-06	RHSIM_Rhsim04G0160100
Root vs Stem	RHSIM_Rhsim04G0161900	1.378485417	2.26213E-07	2.91987E-06	RHSIM_Rhsim04G0161900
Root vs Stem	RHSIM_Rhsim13G0028700	1.027172765	2.3102E-07	2.98025E-06	RHSIM_Rhsim13G0028700
Root vs Stem	RHSIM_RhsimUnG0171600	1.999672193	2.31481E-07	2.98453E-06	RHSIM_RhsimUnG0171600
Root vs Stem	RHSIM_Rhsim07G0164000	-1.54774422	2.31854E-07	2.98766E-06	RHSIM_Rhsim07G0164000
Root vs Stem	RHSIM_Rhsim13G0079300	6.348630765	2.33296E-07	3.00457E-06	RHSIM_Rhsim13G0079300

Comparison	Gene ID	log_2FC	P-value	FDR (padj)	Gene Name
Root vs Stem	novel.4894	1.381185951	2.35978E-07	3.03741E-06	--
Root vs Stem	novel.4117	4.288493829	2.37687E-07	3.0577E-06	--
Root vs Stem	RHSIM_Rhsim08G0039400	-5.900125844	2.41102E-07	3.09818E-06	RHSIM_Rhsim08G0039400
Root vs Stem	novel.421	4.757378555	2.4152E-07	3.10182E-06	--
Root vs Stem	RHSIM_Rhsim03G0016500	-1.001929091	2.4215E-07	3.10817E-06	RHSIM_Rhsim03G0016500
Root vs Stem	RHSIM_Rhsim12G0126600	-1.065889712	2.42623E-07	3.11251E-06	RHSIM_Rhsim12G0126600
Root vs Stem	novel.572	5.90513258	2.43085E-07	3.11671E-06	--
Root vs Stem	RHSIM_Rhsim12G0005700	-2.025564524	2.43623E-07	3.12187E-06	RHSIM_Rhsim12G0005700
Root vs Stem	novel.4085	4.035691083	2.43902E-07	3.12371E-06	--
Root vs Stem	RHSIM_Rhsim08G0026500	4.367592798	2.46143E-07	3.14891E-06	RHSIM_Rhsim08G0026500
Root vs Stem	RHSIM_Rhsim07G0207300	4.249829173	2.46746E-07	3.15487E-06	RHSIM_Rhsim07G0207300
Root vs Stem	RHSIM_Rhsim07G0016400	-1.702771787	2.47543E-07	3.16331E-06	RHSIM_Rhsim07G0016400
Root vs Stem	RHSIM_Rhsim04G0181000	-4.406996376	2.48148E-07	3.16802E-06	RHSIM_Rhsim04G0181000
Root vs Stem	novel.4040	1.885363728	2.48187E-07	3.16802E-06	--
Root vs Stem	RHSIM_Rhsim04G0074500	-2.848584382	2.49313E-07	3.18064E-06	RHSIM_Rhsim04G0074500
Root vs Stem	RHSIM_Rhsim03G0208100	-1.073754693	2.50851E-07	3.19849E-06	RHSIM_Rhsim03G0208100
Root vs Stem	novel.3671	-3.176718848	2.52887E-07	3.22267E-06	--
Root vs Stem	RHSIM_Rhsim09G0057400	-1.574525199	2.56113E-07	3.26197E-06	RHSIM_Rhsim09G0057400
Root vs Stem	RHSIM_Rhsim01G0265800	-4.311349273	2.57902E-07	3.28047E-06	RHSIM_Rhsim01G0265800
Root vs Stem	RHSIM_Rhsim09G0213600	-1.042059107	2.57992E-07	3.28047E-06	RHSIM_Rhsim09G0213600
Root vs Stem	RHSIM_Rhsim12G0042200	2.8891248	2.69014E-07	3.41497E-06	RHSIM_Rhsim12G0042200
Root vs Stem	RHSIM_Rhsim04G0085100	-4.80374538	2.71562E-07	3.44541E-06	RHSIM_Rhsim04G0085100
Root vs Stem	RHSIM_Rhsim06G0049700	1.893312369	2.74597E-07	3.48201E-06	RHSIM_Rhsim06G0049700
Root vs Stem	RHSIM_Rhsim02G0253600	-1.913271496	2.78385E-07	3.5281E-06	RHSIM_Rhsim02G0253600
Root vs Stem	novel.3451	2.089642268	2.8027E-07	3.54808E-06	--

Comparison	Gene ID	log_2FC	P-value	FDR (padj)	Gene Name
Root vs Stem	novel.5499	-4.035057164	2.86449E-07	3.62432E-06	--
Root vs Stem	RHSIM_Rhsim11G0147300	-1.044866085	2.86796E-07	3.62672E-06	RHSIM_Rhsim11G0147300
Root vs Stem	RHSIM_Rhsim07G0137000	-1.674071497	2.87542E-07	3.63217E-06	RHSIM_Rhsim07G0137000
Root vs Stem	RHSIM_Rhsim02G0116700	7.286628585	2.88643E-07	3.64407E-06	RHSIM_Rhsim02G0116700
Root vs Stem	RHSIM_Rhsim01G0078200	7.733532537	2.92088E-07	3.68354E-06	RHSIM_Rhsim01G0078200
Root vs Stem	RHSIM_Rhsim06G0041500	1.559488859	2.92506E-07	3.68679E-06	RHSIM_Rhsim06G0041500
Root vs Stem	RHSIM_Rhsim02G0095700	-1.513316983	2.93659E-07	3.69931E-06	RHSIM_Rhsim02G0095700
Root vs Stem	RHSIM_Rhsim07G0067100	2.726818678	2.95831E-07	3.72463E-06	RHSIM_Rhsim07G0067100
Root vs Stem	RHSIM_Rhsim04G0130000	-1.37553093	3.0255E-07	3.80507E-06	RHSIM_Rhsim04G0130000
Root vs Stem	RHSIM_Rhsim07G0179500	-1.874668918	3.06822E-07	3.85668E-06	RHSIM_Rhsim07G0179500
Root vs Stem	RHSIM_Rhsim02G0033100	-1.559362494	3.07101E-07	3.85809E-06	RHSIM_Rhsim02G0033100
Root vs Stem	RHSIM_Rhsim07G0207200	3.482096084	3.14255E-07	3.94152E-06	RHSIM_Rhsim07G0207200
Root vs Stem	RHSIM_Rhsim12G0085900	-1.89497119	3.15592E-07	3.95615E-06	RHSIM_Rhsim12G0085900
Root vs Stem	RHSIM_Rhsim12G0070000	3.221793195	3.17695E-07	3.98033E-06	RHSIM_Rhsim12G0070000
Root vs Stem	RHSIM_Rhsim06G0137200	6.158496106	3.23495E-07	4.04861E-06	RHSIM_Rhsim06G0137200
Root vs Stem	RHSIM_Rhsim09G0199500	-2.207670397	3.2567E-07	4.07362E-06	RHSIM_Rhsim09G0199500
Root vs Stem	RHSIM_Rhsim03G0269300	-2.324869232	3.25915E-07	4.07447E-06	RHSIM_Rhsim03G0269300
Root vs Stem	novel.3797	-2.791387437	3.28298E-07	4.10203E-06	--
Root vs Stem	RHSIM_Rhsim10G0183900	1.915884637	3.29902E-07	4.11984E-06	RHSIM_Rhsim10G0183900
Root vs Stem	RHSIM_Rhsim07G0209700	-6.506055588	3.30092E-07	4.11998E-06	RHSIM_Rhsim07G0209700
Root vs Stem	RHSIM_Rhsim04G0190400	2.23889312	3.31553E-07	4.13598E-06	RHSIM_Rhsim04G0190400
Root vs Stem	novel.1962	2.226516258	3.36106E-07	4.19051E-06	--
Root vs Stem	novel.4716	3.566498494	3.36707E-07	4.19574E-06	--
Root vs Stem	RHSIM_Rhsim05G0142900	-5.093576835	3.39206E-07	4.22459E-06	RHSIM_Rhsim05G0142900
Root vs Stem	RHSIM_Rhsim09G0028200	1.183717269	3.40054E-07	4.23286E-06	RHSIM_Rhsim09G0028200

Comparison	Gene ID	log_2FC	P-value	FDR (padj)	Gene Name
Root vs Stem	RHSIM_Rhsim01G0215500	3.048215084	3.49804E-07	4.3425E-06	RHSIM_Rhsim01G0215500
Root vs Stem	RHSIM_Rhsim06G0233200	-1.218454914	3.54483E-07	4.39822E-06	RHSIM_Rhsim06G0233200
Root vs Stem	RHSIM_Rhsim08G0034000	-1.979627543	3.59325E-07	4.44872E-06	RHSIM_Rhsim08G0034000
Root vs Stem	RHSIM_Rhsim04G0007400	-1.706857117	3.60872E-07	4.46547E-06	RHSIM_Rhsim04G0007400
Root vs Stem	RHSIM_Rhsim10G0128000	-2.49780665	3.61334E-07	4.46879E-06	RHSIM_Rhsim10G0128000
Root vs Stem	RHSIM_Rhsim03G0215500	-5.026249157	3.62332E-07	4.47874E-06	RHSIM_Rhsim03G0215500
Root vs Stem	RHSIM_Rhsim10G0128400	-2.75552158	3.63093E-07	4.48574E-06	RHSIM_Rhsim10G0128400
Root vs Stem	novel.3231	1.985518874	3.66321E-07	4.52319E-06	--
Root vs Stem	RHSIM_Rhsim06G0193500	5.871380023	3.70701E-07	4.57483E-06	RHSIM_Rhsim06G0193500
Root vs Stem	RHSIM_Rhsim13G0178800	1.389022874	3.71415E-07	4.58118E-06	RHSIM_Rhsim13G0178800
Root vs Stem	RHSIM_Rhsim13G0095700	3.053630026	3.73868E-07	4.60897E-06	RHSIM_Rhsim13G0095700
Root vs Stem	RHSIM_Rhsim06G0063600	1.58405068	3.7803E-07	4.6553E-06	RHSIM_Rhsim06G0063600
Root vs Stem	RHSIM_Rhsim05G0219500	-2.646583555	3.78847E-07	4.66287E-06	RHSIM_Rhsim05G0219500
Root vs Stem	RHSIM_Rhsim10G0143300	-2.483625576	3.8127E-07	4.69019E-06	RHSIM_Rhsim10G0143300
Root vs Stem	RHSIM_Rhsim05G0104500	-3.884417963	3.82083E-07	4.69768E-06	RHSIM_Rhsim05G0104500
Root vs Stem	RHSIM_Rhsim04G0246800	1.423146732	3.85134E-07	4.73267E-06	RHSIM_Rhsim04G0246800
Root vs Stem	RHSIM_Rhsim01G0088800	1.147808201	3.86411E-07	4.74583E-06	RHSIM_Rhsim01G0088800
Root vs Stem	RHSIM_Rhsim13G0081700	2.057131491	3.88784E-07	4.77244E-06	RHSIM_Rhsim13G0081700
Root vs Stem	RHSIM_Rhsim07G0041400	-2.5286724	3.9234E-07	4.81353E-06	RHSIM_Rhsim07G0041400
Root vs Stem	RHSIM_Rhsim01G0270200	1.243198716	3.93286E-07	4.82025E-06	RHSIM_Rhsim01G0270200
Root vs Stem	RHSIM_Rhsim07G0141300	1.095549408	3.93306E-07	4.82025E-06	RHSIM_Rhsim07G0141300
Root vs Stem	RHSIM_Rhsim03G0187500	1.538642064	3.93842E-07	4.82425E-06	RHSIM_Rhsim03G0187500
Root vs Stem	RHSIM_Rhsim01G0287900	1.015800051	3.94263E-07	4.82684E-06	RHSIM_Rhsim01G0287900
Root vs Stem	novel.3889	3.89141899	3.98028E-07	4.87036E-06	--
Root vs Stem	RHSIM_Rhsim03G0192800	2.237914838	4.00596E-07	4.89918E-06	RHSIM_Rhsim03G0192800

Comparison	Gene ID	log_2FC	P-value	FDR (padj)	Gene Name
Root vs Stem	RHSIM_Rhsim13G0195000	3.407161594	4.02719E-07	4.92253E-06	RHSIM_Rhsim13G0195000
Root vs Stem	RHSIM_Rhsim10G0035800	-2.157304123	4.07799E-07	4.97933E-06	RHSIM_Rhsim10G0035800
Root vs Stem	RHSIM_Rhsim03G0060700	-4.720829942	4.08356E-07	4.98299E-06	RHSIM_Rhsim03G0060700
Root vs Stem	novel.2923	5.129530439	4.08531E-07	4.98299E-06	--
Root vs Stem	RHSIM_Rhsim06G0093200	-1.105632583	4.12667E-07	5.03078E-06	RHSIM_Rhsim06G0093200
Root vs Stem	RHSIM_Rhsim01G0188300	-8.551389039	4.13107E-07	5.03348E-06	RHSIM_Rhsim01G0188300
Root vs Stem	RHSIM_Rhsim07G0121700	-2.716859931	4.13515E-07	5.03415E-06	RHSIM_Rhsim07G0121700
Root vs Stem	RHSIM_Rhsim01G0038100	-8.633458504	4.13787E-07	5.03415E-06	RHSIM_Rhsim01G0038100
Root vs Stem	RHSIM_Rhsim10G0049300	-4.748182593	4.13816E-07	5.03415E-06	RHSIM_Rhsim10G0049300
Root vs Stem	RHSIM_Rhsim01G0273700	-1.827934484	4.14118E-07	5.03516E-06	RHSIM_Rhsim01G0273700
Root vs Stem	RHSIM_Rhsim13G0065500	-1.227185264	4.16106E-07	5.05666E-06	RHSIM_Rhsim13G0065500
Root vs Stem	RHSIM_Rhsim06G0050000	-2.470886355	4.19353E-07	5.09344E-06	RHSIM_Rhsim06G0050000
Root vs Stem	novel.4020	1.991323348	4.23302E-07	5.13869E-06	--
Root vs Stem	RHSIM_RhsimUnG0077500	-3.911985775	4.27952E-07	5.19241E-06	RHSIM_RhsimUnG0077500
Root vs Stem	RHSIM_Rhsim09G0070800	1.172742037	4.28685E-07	5.19848E-06	RHSIM_Rhsim09G0070800
Root vs Stem	RHSIM_Rhsim08G0017700	1.80858374	4.28904E-07	5.19848E-06	RHSIM_Rhsim08G0017700
Root vs Stem	RHSIM_Rhsim02G0046300	-11.66673318	4.36691E-07	5.28453E-06	RHSIM_Rhsim02G0046300
Root vs Stem	RHSIM_Rhsim04G0033500	-2.047320517	4.36968E-07	5.28511E-06	RHSIM_Rhsim04G0033500
Root vs Stem	RHSIM_RhsimUnG0075600	-3.612664021	4.39144E-07	5.30616E-06	RHSIM_RhsimUnG0075600
Root vs Stem	RHSIM_Rhsim13G0164500	-1.355201578	4.39169E-07	5.30616E-06	RHSIM_Rhsim13G0164500
Root vs Stem	RHSIM_Rhsim07G0052500	2.046756586	4.45688E-07	5.38211E-06	RHSIM_Rhsim07G0052500
Root vs Stem	RHSIM_Rhsim13G0090200	1.076394857	4.49032E-07	5.41966E-06	RHSIM_Rhsim13G0090200
Root vs Stem	RHSIM_Rhsim03G0212400	1.448101783	4.50932E-07	5.43973E-06	RHSIM_Rhsim03G0212400
Root vs Stem	RHSIM_Rhsim08G0228300	7.645271321	4.52401E-07	5.4546E-06	RHSIM_Rhsim08G0228300
Root vs Stem	RHSIM_RhsimUnG0152500	-2.009487188	4.55016E-07	5.48326E-06	RHSIM_RhsimUnG0152500

Comparison	Gene ID	log_2FC	P-value	FDR (padj)	Gene Name
Root vs Stem	RHSIM_Rhsim12G0048200	2.539508701	4.56896E-07	5.50305E-06	RHSIM_Rhsim12G0048200
Root vs Stem	RHSIM_Rhsim10G0127400	-2.199305608	4.57805E-07	5.51111E-06	RHSIM_Rhsim10G0127400
Root vs Stem	RHSIM_Rhsim01G0012200	1.461645689	4.58753E-07	5.51964E-06	RHSIM_Rhsim01G0012200
Root vs Stem	RHSIM_Rhsim12G0114600	-1.563158971	4.59108E-07	5.52103E-06	RHSIM_Rhsim12G0114600
Root vs Stem	RHSIM_Rhsim06G0030500	3.895760123	4.60452E-07	5.53431E-06	RHSIM_Rhsim06G0030500
Root vs Stem	RHSIM_Rhsim02G0234900	2.06251651	4.61402E-07	5.54283E-06	RHSIM_Rhsim02G0234900
Root vs Stem	novel.1745	2.856064135	4.64038E-07	5.5687E-06	--
Root vs Stem	RHSIM_Rhsim01G0137100	2.070394774	4.66581E-07	5.5963E-06	RHSIM_Rhsim01G0137100
Root vs Stem	novel.2522	-7.457141253	4.67691E-07	5.60379E-06	--
Root vs Stem	novel.4792	-5.804722982	4.68385E-07	5.60919E-06	--
Root vs Stem	novel.52	-2.823712332	4.69204E-07	5.61607E-06	--
Root vs Stem	RHSIM_Rhsim09G0006200	3.496201907	4.699E-07	5.62149E-06	RHSIM_Rhsim09G0006200
Root vs Stem	RHSIM_Rhsim11G0182100	-1.642568686	4.71443E-07	5.63702E-06	RHSIM_Rhsim11G0182100
Root vs Stem	novel.2107	-8.897144042	4.72434E-07	5.64594E-06	--
Root vs Stem	novel.3681	-2.304225565	4.82585E-07	5.7583E-06	--
Root vs Stem	RHSIM_RhsimUnG0166700	-3.099004645	4.9014E-07	5.84239E-06	RHSIM_RhsimUnG0166700
Root vs Stem	RHSIM_Rhsim09G0116000	-2.160312302	4.92524E-07	5.86779E-06	RHSIM_Rhsim09G0116000
Root vs Stem	novel.3993	3.791052928	5.00801E-07	5.96023E-06	--
Root vs Stem	novel.3337	1.257462144	5.14694E-07	6.12241E-06	--
Root vs Stem	RHSIM_Rhsim11G0138200	-3.166374421	5.21817E-07	6.20394E-06	RHSIM_Rhsim11G0138200
Root vs Stem	RHSIM_Rhsim10G0028000	-2.159250269	5.23425E-07	6.21862E-06	RHSIM_Rhsim10G0028000
Root vs Stem	RHSIM_Rhsim09G0011700	3.842338067	5.23591E-07	6.21862E-06	RHSIM_Rhsim09G0011700
Root vs Stem	novel.5386	-2.662149852	5.25741E-07	6.24095E-06	--
Root vs Stem	RHSIM_Rhsim11G0052200	2.806206462	5.28511E-07	6.26737E-06	RHSIM_Rhsim11G0052200
Root vs Stem	RHSIM_Rhsim01G0231400	-2.056183197	5.3061E-07	6.28902E-06	RHSIM_Rhsim01G0231400

Comparison	Gene ID	log_2FC	P-value	FDR (padj)	Gene Name
Root vs Stem	novel.5066	-1.573631302	5.31283E-07	6.29377E-06	--
Root vs Stem	RHSIM_Rhsim11G0163500	6.167939874	5.39236E-07	6.3847E-06	RHSIM_Rhsim11G0163500
Root vs Stem	RHSIM_Rhsim13G0018900	1.361882889	5.4008E-07	6.39141E-06	RHSIM_Rhsim13G0018900
Root vs Stem	RHSIM_Rhsim13G0033000	6.251351538	5.41977E-07	6.41057E-06	RHSIM_Rhsim13G0033000
Root vs Stem	novel.483	1.115106463	5.45464E-07	6.4485E-06	--
Root vs Stem	RHSIM_Rhsim04G0024600	1.549830259	5.63408E-07	6.65381E-06	RHSIM_Rhsim04G0024600
Root vs Stem	RHSIM_Rhsim09G0158400	-1.551833508	5.65514E-07	6.67527E-06	RHSIM_Rhsim09G0158400
Root vs Stem	novel.3895	4.141658447	5.78174E-07	6.8114E-06	--
Root vs Stem	RHSIM_Rhsim07G0142200	1.823785871	5.8093E-07	6.83972E-06	RHSIM_Rhsim07G0142200
Root vs Stem	novel.2387	-1.764841866	5.82733E-07	6.85745E-06	--
Root vs Stem	RHSIM_Rhsim12G0170200	2.18297349	5.85584E-07	6.88749E-06	RHSIM_Rhsim12G0170200
Root vs Stem	RHSIM_Rhsim10G0083100	-1.561343703	5.86743E-07	6.89759E-06	RHSIM_Rhsim10G0083100
Root vs Stem	RHSIM_Rhsim11G0175600	4.710722291	5.87053E-07	6.89773E-06	RHSIM_Rhsim11G0175600
Root vs Stem	RHSIM_Rhsim11G0071200	-1.306489281	5.88285E-07	6.90868E-06	RHSIM_Rhsim11G0071200
Root vs Stem	RHSIM_Rhsim13G0151900	2.2695635	5.8933E-07	6.91391E-06	RHSIM_Rhsim13G0151900
Root vs Stem	RHSIM_Rhsim02G0086600	4.571505795	5.95372E-07	6.98123E-06	RHSIM_Rhsim02G0086600
Root vs Stem	RHSIM_Rhsim03G0169500	-1.066775377	5.98323E-07	7.01227E-06	RHSIM_Rhsim03G0169500
Root vs Stem	RHSIM_RhsimUnG0248100	3.181106193	6.02721E-07	7.06022E-06	RHSIM_RhsimUnG0248100
Root vs Stem	RHSIM_Rhsim04G0070900	1.488985891	6.085E-07	7.1243E-06	RHSIM_Rhsim04G0070900
Root vs Stem	RHSIM_Rhsim05G0231500	-2.04164999	6.14552E-07	7.1915E-06	RHSIM_Rhsim05G0231500
Root vs Stem	RHSIM_Rhsim01G0033500	2.464851538	6.1781E-07	7.2223E-06	RHSIM_Rhsim01G0033500
Root vs Stem	RHSIM_Rhsim09G0145500	1.035641106	6.19452E-07	7.23783E-06	RHSIM_Rhsim09G0145500
Root vs Stem	novel.1571	-1.233599649	6.20852E-07	7.2505E-06	--
Root vs Stem	RHSIM_Rhsim02G0069100	-9.886014361	6.21989E-07	7.2601E-06	RHSIM_Rhsim02G0069100
Root vs Stem	RHSIM_Rhsim08G0010100	6.040380238	6.22598E-07	7.26354E-06	RHSIM_Rhsim08G0010100

Comparison	Gene ID	log$_2$FC	P-value	FDR (padj)	Gene Name
Root vs Stem	novel.3094	-2.030847817	6.23234E-07	7.26728E-06	--
Root vs Stem	RHSIM_Rhsim06G0069100	1.97002748	6.24595E-07	7.27947E-06	RHSIM_Rhsim06G0069100
Root vs Stem	RHSIM_Rhsim07G0057400	4.380000316	6.25601E-07	7.28751E-06	RHSIM_Rhsim07G0057400
Root vs Stem	RHSIM_RhsimUnG0055300	1.29898606	6.33776E-07	7.37371E-06	RHSIM_RhsimUnG0055300
Root vs Stem	RHSIM_Rhsim07G0253800	1.24716607	6.36174E-07	7.39573E-06	RHSIM_Rhsim07G0253800
Root vs Stem	RHSIM_Rhsim06G0241100	2.873974214	6.3936E-07	7.42902E-06	RHSIM_Rhsim06G0241100
Root vs Stem	RHSIM_Rhsim08G0014200	-2.581189846	6.44912E-07	7.48221E-06	RHSIM_Rhsim08G0014200
Root vs Stem	RHSIM_Rhsim13G0194400	-5.089596312	6.5208E-07	7.56157E-06	RHSIM_Rhsim13G0194400
Root vs Stem	RHSIM_Rhsim07G0170200	-1.261688974	6.54067E-07	7.5808E-06	RHSIM_Rhsim07G0170200
Root vs Stem	RHSIM_Rhsim05G0057500	5.187253895	6.63295E-07	7.68389E-06	RHSIM_Rhsim05G0057500
Root vs Stem	RHSIM_Rhsim10G0038900	1.850104605	6.6489E-07	7.6985E-06	RHSIM_Rhsim10G0038900
Root vs Stem	RHSIM_Rhsim07G0054900	-3.521993447	6.71995E-07	7.77296E-06	RHSIM_Rhsim07G0054900
Root vs Stem	RHSIM_Rhsim10G0169900	2.264166301	6.77124E-07	7.8166E-06	RHSIM_Rhsim10G0169900
Root vs Stem	RHSIM_Rhsim13G0150800	-1.317002371	6.77891E-07	7.82153E-06	RHSIM_Rhsim13G0150800
Root vs Stem	RHSIM_Rhsim03G0172900	-1.699669224	6.85565E-07	7.90612E-06	RHSIM_Rhsim03G0172900
Root vs Stem	novel.5643	3.645233709	7.09177E-07	8.16617E-06	--
Root vs Stem	RHSIM_Rhsim12G0126100	1.506810233	7.10219E-07	8.17408E-06	RHSIM_Rhsim12G0126100
Root vs Stem	RHSIM_Rhsim13G0215300	-1.129257613	7.22954E-07	8.31235E-06	RHSIM_Rhsim13G0215300
Root vs Stem	RHSIM_Rhsim03G0172600	-6.137210603	7.32839E-07	8.41462E-06	RHSIM_Rhsim03G0172600
Root vs Stem	RHSIM_Rhsim08G0108700	2.795675213	7.32944E-07	8.41462E-06	RHSIM_Rhsim08G0108700
Root vs Stem	RHSIM_Rhsim11G0088600	-1.223039818	7.34363E-07	8.42671E-06	RHSIM_Rhsim11G0088600
Root vs Stem	RHSIM_Rhsim08G0103500	2.934089351	7.3725E-07	8.45564E-06	RHSIM_Rhsim08G0103500
Root vs Stem	RHSIM_Rhsim02G0028600	-2.447520893	7.41371E-07	8.49867E-06	RHSIM_Rhsim02G0028600
Root vs Stem	RHSIM_Rhsim04G0086100	-1.740517951	7.51021E-07	8.60502E-06	RHSIM_Rhsim04G0086100
Root vs Stem	RHSIM_Rhsim02G0090100	1.177224565	7.60889E-07	8.71374E-06	RHSIM_Rhsim02G0090100

Comparison	Gene ID	log_2FC	P-value	FDR (padj)	Gene Name
Root vs Stem	RHSIM_Rhsim11G0198900	4.124309657	7.63314E-07	8.73718E-06	RHSIM_Rhsim11G0198900
Root vs Stem	RHSIM_Rhsim08G0239500	3.008693	7.71631E-07	8.82799E-06	RHSIM_Rhsim08G0239500
Root vs Stem	novel.499	1.476685529	7.72875E-07	8.83784E-06	--
Root vs Stem	RHSIM_Rhsim11G0170100	-1.50852573	7.73902E-07	8.8452E-06	RHSIM_Rhsim11G0170100
Root vs Stem	RHSIM_Rhsim04G0087700	2.491838342	7.76795E-07	8.87387E-06	RHSIM_Rhsim04G0087700
Root vs Stem	RHSIM_Rhsim07G0065200	-3.961411517	7.7883E-07	8.8927E-06	RHSIM_Rhsim07G0065200
Root vs Stem	RHSIM_Rhsim06G0182500	-1.774947541	7.84562E-07	8.95372E-06	RHSIM_Rhsim06G0182500
Root vs Stem	RHSIM_Rhsim10G0186400	1.795749434	7.87241E-07	8.97985E-06	RHSIM_Rhsim10G0186400
Root vs Stem	RHSIM_Rhsim02G0043700	2.027970166	7.91022E-07	9.01851E-06	RHSIM_Rhsim02G0043700
Root vs Stem	RHSIM_Rhsim04G0071100	3.126291301	8.01218E-07	9.13024E-06	RHSIM_Rhsim04G0071100
Root vs Stem	RHSIM_Rhsim03G0137600	-1.915973651	8.04981E-07	9.16859E-06	RHSIM_Rhsim03G0137600
Root vs Stem	RHSIM_Rhsim03G0264400	1.342453904	8.0697E-07	9.18671E-06	RHSIM_Rhsim03G0264400
Root vs Stem	RHSIM_Rhsim05G0027600	-1.987624482	8.10105E-07	9.21784E-06	RHSIM_Rhsim05G0027600
Root vs Stem	RHSIM_Rhsim01G0274300	-5.558673472	8.27249E-07	9.40364E-06	RHSIM_Rhsim01G0274300
Root vs Stem	novel.913	-1.567950414	8.2923E-07	9.42151E-06	--
Root vs Stem	RHSIM_Rhsim06G0105600	7.559337603	8.30335E-07	9.42942E-06	RHSIM_Rhsim06G0105600
Root vs Stem	RHSIM_Rhsim09G0147000	-2.124530803	8.37823E-07	9.50977E-06	RHSIM_Rhsim09G0147000
Root vs Stem	RHSIM_RhsimUnG0121500	7.977842106	8.50299E-07	9.63623E-06	RHSIM_RhsimUnG0121500
Root vs Stem	RHSIM_RhsimUnG0105000	1.588970913	8.50636E-07	9.63623E-06	RHSIM_RhsimUnG0105000
Root vs Stem	RHSIM_Rhsim08G0177800	-2.440736851	8.5231E-07	9.64571E-06	RHSIM_Rhsim08G0177800
Root vs Stem	RHSIM_Rhsim04G0174800	2.257809727	8.55243E-07	9.67414E-06	RHSIM_Rhsim04G0174800
Root vs Stem	RHSIM_Rhsim13G0191600	1.599458965	8.56884E-07	9.68796E-06	RHSIM_Rhsim13G0191600
Root vs Stem	RHSIM_Rhsim01G0193400	-3.561053381	8.60196E-07	9.71594E-06	RHSIM_Rhsim01G0193400
Root vs Stem	novel.3505	3.548063936	8.60202E-07	9.71594E-06	--
Root vs Stem	RHSIM_RhsimUnG0008300	-2.756651503	8.70858E-07	9.82667E-06	RHSIM_RhsimUnG0008300

Comparison	Gene ID	log_2FC	P-value	FDR (padj)	Gene Name
Root vs Stem	RHSIM_Rhsim08G0111800	1.247351566	8.85055E-07	9.97223E-06	RHSIM_Rhsim08G0111800
Root vs Stem	RHSIM_Rhsim08G0198700	-3.045190229	8.87351E-07	9.99322E-06	RHSIM_Rhsim08G0198700
Root vs Stem	RHSIM_RhsimUnG0168600	-7.900053734	8.922E-07	1.00429E-05	RHSIM_RhsimUnG0168600
Root vs Stem	RHSIM_Rhsim01G0208500	-1.760005075	8.95273E-07	1.00726E-05	RHSIM_Rhsim01G0208500
Root vs Stem	RHSIM_Rhsim07G0163900	-1.315539578	9.00483E-07	1.01213E-05	RHSIM_Rhsim07G0163900
Root vs Stem	RHSIM_Rhsim03G0252900	1.106397447	9.0271E-07	1.01414E-05	RHSIM_Rhsim03G0252900
Root vs Stem	RHSIM_Rhsim07G0187200	-3.861660925	9.14175E-07	1.02652E-05	RHSIM_Rhsim07G0187200
Root vs Stem	RHSIM_RhsimUnG0117200	-1.999031865	9.22824E-07	1.03573E-05	RHSIM_RhsimUnG0117200
Root vs Stem	RHSIM_Rhsim02G0059200	1.175038753	9.2697E-07	1.03988E-05	RHSIM_Rhsim02G0059200
Root vs Stem	RHSIM_Rhsim03G0073100	1.066616552	9.29042E-07	1.04169E-05	RHSIM_Rhsim03G0073100
Root vs Stem	RHSIM_Rhsim02G0070300	3.499406604	9.32008E-07	1.04451E-05	RHSIM_Rhsim02G0070300
Root vs Stem	RHSIM_Rhsim12G0144800	-7.775360266	9.32575E-07	1.04464E-05	RHSIM_Rhsim12G0144800
Root vs Stem	novel.4167	5.041499013	9.33786E-07	1.04549E-05	--
Root vs Stem	RHSIM_Rhsim01G0237200	1.103515149	9.38069E-07	1.04977E-05	RHSIM_Rhsim01G0237200
Root vs Stem	novel.4568	-3.859239695	9.41681E-07	1.0533E-05	--
Root vs Stem	RHSIM_Rhsim13G0021900	-1.773815886	9.42322E-07	1.05351E-05	RHSIM_Rhsim13G0021900
Root vs Stem	RHSIM_Rhsim12G0112800	-2.026660474	9.43889E-07	1.05475E-05	RHSIM_Rhsim12G0112800
Root vs Stem	RHSIM_Rhsim12G0155600	-1.363219739	9.56229E-07	1.06774E-05	RHSIM_Rhsim12G0155600
Root vs Stem	novel.1655	-4.319527438	9.56437E-07	1.06774E-05	--
Root vs Stem	RHSIM_Rhsim03G0212700	1.284402943	9.5849E-07	1.06951E-05	RHSIM_Rhsim03G0212700
Root vs Stem	RHSIM_Rhsim12G0191200	-4.893732192	9.61577E-07	1.07244E-05	RHSIM_Rhsim12G0191200
Root vs Stem	RHSIM_RhsimUnG0075700	3.790859594	9.68191E-07	1.07877E-05	RHSIM_RhsimUnG0075700
Root vs Stem	RHSIM_Rhsim08G0173700	-1.938899616	9.69775E-07	1.08001E-05	RHSIM_Rhsim08G0173700
Root vs Stem	RHSIM_Rhsim02G0022400	1.40667018	9.74128E-07	1.08403E-05	RHSIM_Rhsim02G0022400
Root vs Stem	RHSIM_Rhsim02G0071300	1.134821614	9.74322E-07	1.08403E-05	RHSIM_Rhsim02G0071300

Comparison	Gene ID	log_2FC	P-value	FDR (padj)	Gene Name
Root vs Stem	RHSIM_Rhsim06G0096400	3.936255247	9.76E-07	1.08497E-05	RHSIM_Rhsim06G0096400
Root vs Stem	novel.2532	1.438421591	9.76115E-07	1.08497E-05	--
Root vs Stem	RHSIM_Rhsim03G0234500	1.102212401	9.82834E-07	1.09192E-05	RHSIM_Rhsim03G0234500
Root vs Stem	RHSIM_Rhsim03G0161100	-2.03842686	9.86132E-07	1.09505E-05	RHSIM_Rhsim03G0161100
Root vs Stem	novel.647	1.81687875	9.87874E-07	1.09646E-05	--
Root vs Stem	RHSIM_Rhsim12G0197600	-1.349254825	9.91827E-07	1.10032E-05	RHSIM_Rhsim12G0197600
Root vs Stem	RHSIM_Rhsim08G0023100	5.302331381	9.9423E-07	1.10245E-05	RHSIM_Rhsim08G0023100
Root vs Stem	RHSIM_Rhsim03G0066000	5.933286925	9.9795E-07	1.10605E-05	RHSIM_Rhsim03G0066000
Root vs Stem	RHSIM_Rhsim05G0194100	-9.30855386	1.00649E-06	1.11444E-05	RHSIM_Rhsim05G0194100
Root vs Stem	RHSIM_Rhsim13G0139800	3.365558844	1.01478E-06	1.12308E-05	RHSIM_Rhsim13G0139800
Root vs Stem	RHSIM_Rhsim06G0027900	-1.378693766	1.02103E-06	1.12945E-05	RHSIM_Rhsim06G0027900
Root vs Stem	RHSIM_Rhsim02G0255300	-2.784517656	1.02612E-06	1.13454E-05	RHSIM_Rhsim02G0255300
Root vs Stem	RHSIM_Rhsim10G0157100	6.51395538	1.02983E-06	1.13717E-05	RHSIM_Rhsim10G0157100
Root vs Stem	RHSIM_Rhsim11G0105400	6.161967368	1.02984E-06	1.13717E-05	RHSIM_Rhsim11G0105400
Root vs Stem	RHSIM_Rhsim06G0084800	2.544093867	1.03017E-06	1.13717E-05	RHSIM_Rhsim06G0084800
Root vs Stem	RHSIM_Rhsim07G0012300	8.077008031	1.03047E-06	1.13717E-05	RHSIM_Rhsim07G0012300
Root vs Stem	RHSIM_Rhsim06G0075100	2.014558659	1.0408E-06	1.14733E-05	RHSIM_Rhsim06G0075100
Root vs Stem	RHSIM_Rhsim09G0176400	1.556010313	1.04155E-06	1.14733E-05	RHSIM_Rhsim09G0176400
Root vs Stem	RHSIM_Rhsim09G0108500	-1.87892225	1.04995E-06	1.15535E-05	RHSIM_Rhsim09G0108500
Root vs Stem	RHSIM_Rhsim12G0075600	1.315798395	1.05766E-06	1.16327E-05	RHSIM_Rhsim12G0075600
Root vs Stem	RHSIM_Rhsim07G0154100	-2.669703974	1.05891E-06	1.1641E-05	RHSIM_Rhsim07G0154100
Root vs Stem	RHSIM_Rhsim08G0246100	1.454121154	1.06198E-06	1.16692E-05	RHSIM_Rhsim08G0246100
Root vs Stem	RHSIM_Rhsim06G0172300	-2.815884378	1.0654E-06	1.16978E-05	RHSIM_Rhsim06G0172300
Root vs Stem	RHSIM_Rhsim01G0063800	-2.141166635	1.06561E-06	1.16978E-05	RHSIM_Rhsim01G0063800
Root vs Stem	RHSIM_Rhsim11G0196300	-1.405254905	1.08733E-06	1.19307E-05	RHSIM_Rhsim11G0196300

Comparison	Gene ID	log_2FC	P-value	FDR (padj)	Gene Name
Root vs Stem	RHSIM_Rhsim12G0087200	-3.712487094	1.08798E-06	1.1932E-05	RHSIM_Rhsim12G0087200
Root vs Stem	RHSIM_Rhsim04G0167700	-2.525373195	1.0918E-06	1.19683E-05	RHSIM_Rhsim04G0167700
Root vs Stem	RHSIM_Rhsim10G0191500	-1.232708798	1.09892E-06	1.20348E-05	RHSIM_Rhsim10G0191500
Root vs Stem	RHSIM_Rhsim07G0020200	-1.39488693	1.1063E-06	1.21099E-05	RHSIM_Rhsim07G0020200
Root vs Stem	novel.4177	1.348261789	1.11186E-06	1.21635E-05	--
Root vs Stem	RHSIM_Rhsim13G0111800	2.038563327	1.11225E-06	1.21635E-05	RHSIM_Rhsim13G0111800
Root vs Stem	RHSIM_Rhsim04G0164100	1.015989901	1.11431E-06	1.21802E-05	RHSIM_Rhsim04G0164100
Root vs Stem	RHSIM_Rhsim11G0109700	7.27437379	1.11682E-06	1.22019E-05	RHSIM_Rhsim11G0109700
Root vs Stem	RHSIM_Rhsim03G0135900	-1.172197628	1.118E-06	1.22088E-05	RHSIM_Rhsim03G0135900
Root vs Stem	RHSIM_Rhsim06G0023400	2.374228296	1.11851E-06	1.22088E-05	RHSIM_Rhsim06G0023400
Root vs Stem	RHSIM_Rhsim12G0192500	2.656133759	1.12034E-06	1.2223E-05	RHSIM_Rhsim12G0192500
Root vs Stem	RHSIM_Rhsim06G0183300	-1.174750024	1.12174E-06	1.22325E-05	RHSIM_Rhsim06G0183300
Root vs Stem	novel.1313	-1.405924886	1.12982E-06	1.23147E-05	--
Root vs Stem	RHSIM_Rhsim07G0092200	1.562613071	1.1316E-06	1.23283E-05	RHSIM_Rhsim07G0092200
Root vs Stem	novel.5646	-4.665615149	1.14815E-06	1.25028E-05	--
Root vs Stem	RHSIM_Rhsim06G0147900	-1.662789833	1.15234E-06	1.25424E-05	RHSIM_Rhsim06G0147900
Root vs Stem	novel.2977	-7.231024111	1.15396E-06	1.25542E-05	--
Root vs Stem	RHSIM_Rhsim10G0207400	-1.478354631	1.16247E-06	1.26351E-05	RHSIM_Rhsim10G0207400
Root vs Stem	RHSIM_Rhsim05G0087500	-2.194936363	1.16279E-06	1.26351E-05	RHSIM_Rhsim05G0087500
Root vs Stem	RHSIM_Rhsim08G0057600	6.036548329	1.16305E-06	1.26351E-05	RHSIM_Rhsim08G0057600
Root vs Stem	RHSIM_Rhsim04G0094500	-1.744826788	1.17559E-06	1.27594E-05	RHSIM_Rhsim04G0094500
Root vs Stem	RHSIM_Rhsim04G0220600	1.054621867	1.17871E-06	1.27873E-05	RHSIM_Rhsim04G0220600
Root vs Stem	RHSIM_Rhsim06G0120600	-5.466766651	1.18404E-06	1.28329E-05	RHSIM_Rhsim06G0120600
Root vs Stem	RHSIM_Rhsim11G0051000	1.623047521	1.20468E-06	1.3044E-05	RHSIM_Rhsim11G0051000
Root vs Stem	RHSIM_Rhsim09G0012100	1.594258402	1.20522E-06	1.3044E-05	RHSIM_Rhsim09G0012100

Comparison	Gene ID	log_2FC	P-value	FDR (padj)	Gene Name
Root vs Stem	novel.3760	2.934175733	1.21322E-06	1.31184E-05	--
Root vs Stem	novel.5256	3.650046211	1.22069E-06	1.31929E-05	--
Root vs Stem	RHSIM_Rhsim08G0072400	-2.734487375	1.24254E-06	1.34166E-05	RHSIM_Rhsim08G0072400
Root vs Stem	RHSIM_Rhsim13G0193900	-1.277797199	1.24674E-06	1.34555E-05	RHSIM_Rhsim13G0193900
Root vs Stem	RHSIM_RhsimUnG0041500	-1.424119014	1.24918E-06	1.34756E-05	RHSIM_RhsimUnG0041500
Root vs Stem	RHSIM_Rhsim11G0113100	-5.63966906	1.26483E-06	1.36253E-05	RHSIM_Rhsim11G0113100
Root vs Stem	RHSIM_Rhsim11G0044300	-2.347397426	1.26828E-06	1.36561E-05	RHSIM_Rhsim11G0044300
Root vs Stem	novel.2644	-2.091964771	1.269E-06	1.36574E-05	--
Root vs Stem	RHSIM_Rhsim06G0181200	-3.125598366	1.27411E-06	1.3706E-05	RHSIM_Rhsim06G0181200
Root vs Stem	RHSIM_Rhsim05G0060200	-1.582005659	1.27661E-06	1.37202E-05	RHSIM_Rhsim05G0060200
Root vs Stem	RHSIM_Rhsim11G0044100	-5.693369416	1.2831E-06	1.37771E-05	RHSIM_Rhsim11G0044100
Root vs Stem	RHSIM_Rhsim08G0208700	-6.76724787	1.29514E-06	1.38999E-05	RHSIM_Rhsim08G0208700
Root vs Stem	RHSIM_Rhsim05G0188100	-1.043893686	1.29865E-06	1.3931E-05	RHSIM_Rhsim05G0188100
Root vs Stem	RHSIM_RhsimUnG0070700	-1.592167579	1.30646E-06	1.40018E-05	RHSIM_RhsimUnG0070700
Root vs Stem	RHSIM_Rhsim01G0214900	-1.229565768	1.30998E-06	1.4033E-05	RHSIM_Rhsim01G0214900
Root vs Stem	RHSIM_Rhsim12G0005400	1.675737956	1.32242E-06	1.41517E-05	RHSIM_Rhsim12G0005400
Root vs Stem	novel.740	7.267945271	1.3229E-06	1.41517E-05	--
Root vs Stem	RHSIM_Rhsim05G0102100	1.296951525	1.32748E-06	1.4194E-05	RHSIM_Rhsim05G0102100
Root vs Stem	RHSIM_Rhsim03G0153300	-4.093771651	1.34789E-06	1.44056E-05	RHSIM_Rhsim03G0153300
Root vs Stem	RHSIM_Rhsim06G0172100	-6.645768315	1.35346E-06	1.44584E-05	RHSIM_Rhsim06G0172100
Root vs Stem	RHSIM_Rhsim08G0097300	-1.49040227	1.36073E-06	1.45227E-05	RHSIM_Rhsim08G0097300
Root vs Stem	RHSIM_Rhsim13G0114800	1.89614732	1.36363E-06	1.45401E-05	RHSIM_Rhsim13G0114800
Root vs Stem	RHSIM_RhsimUnG0139300	1.449273716	1.36571E-06	1.45556E-05	RHSIM_RhsimUnG0139300
Root vs Stem	RHSIM_Rhsim03G0248200	1.471253095	1.36913E-06	1.45853E-05	RHSIM_Rhsim03G0248200
Root vs Stem	RHSIM_Rhsim03G0074400	-1.764742586	1.38187E-06	1.47074E-05	RHSIM_Rhsim03G0074400

Comparison	Gene ID	log_2FC	P-value	FDR (padj)	Gene Name
Root vs Stem	novel.4757	1.731702187	1.38448E-06	1.47284E-05	--
Root vs Stem	RHSIM_Rhsim11G0042500	-2.215543623	1.39251E-06	1.4807E-05	RHSIM_Rhsim11G0042500
Root vs Stem	RHSIM_Rhsim06G0144200	2.977771782	1.39629E-06	1.48335E-05	RHSIM_Rhsim06G0144200
Root vs Stem	RHSIM_Rhsim12G0006900	2.013329401	1.40421E-06	1.49108E-05	RHSIM_Rhsim12G0006900
Root vs Stem	RHSIM_Rhsim07G0030500	2.039724107	1.41348E-06	1.49954E-05	RHSIM_Rhsim07G0030500
Root vs Stem	RHSIM_Rhsim09G0004800	-1.761604667	1.41778E-06	1.50337E-05	RHSIM_Rhsim09G0004800
Root vs Stem	RHSIM_Rhsim07G0238800	-9.393434132	1.4184E-06	1.50337E-05	RHSIM_Rhsim07G0238800
Root vs Stem	RHSIM_Rhsim04G0185700	6.499164024	1.42314E-06	1.5077E-05	RHSIM_Rhsim04G0185700
Root vs Stem	RHSIM_Rhsim06G0137000	-1.286256085	1.42789E-06	1.5118E-05	RHSIM_Rhsim06G0137000
Root vs Stem	RHSIM_Rhsim13G0053000	-1.566571559	1.43997E-06	1.52274E-05	RHSIM_Rhsim13G0053000
Root vs Stem	RHSIM_Rhsim01G0287000	-2.189579398	1.45196E-06	1.53471E-05	RHSIM_Rhsim01G0287000
Root vs Stem	novel.3998	-1.461036938	1.4578E-06	1.54018E-05	--
Root vs Stem	RHSIM_Rhsim04G0246400	1.194130436	1.46051E-06	1.54191E-05	RHSIM_Rhsim04G0246400
Root vs Stem	RHSIM_Rhsim13G0015400	-1.363953382	1.46078E-06	1.54191E-05	RHSIM_Rhsim13G0015400
Root vs Stem	novel.4447	-1.687111103	1.46157E-06	1.54204E-05	--
Root vs Stem	RHSIM_Rhsim12G0064800	-1.73027236	1.47794E-06	1.5586E-05	RHSIM_Rhsim12G0064800
Root vs Stem	RHSIM_Rhsim02G0214600	-6.730890081	1.48859E-06	1.56912E-05	RHSIM_Rhsim02G0214600
Root vs Stem	RHSIM_Rhsim11G0146200	1.636229675	1.50554E-06	1.58625E-05	RHSIM_Rhsim11G0146200
Root vs Stem	RHSIM_RhsimUnG0211400	-3.285222836	1.52203E-06	1.6029E-05	RHSIM_RhsimUnG0211400
Root vs Stem	RHSIM_Rhsim04G0227000	-5.879861442	1.55062E-06	1.63082E-05	RHSIM_Rhsim04G0227000
Root vs Stem	novel.2015	-2.402797958	1.55066E-06	1.63082E-05	--
Root vs Stem	RHSIM_Rhsim09G0096600	1.415624553	1.5563E-06	1.636E-05	RHSIM_Rhsim09G0096600
Root vs Stem	novel.2785	1.787287564	1.56139E-06	1.63997E-05	--
Root vs Stem	RHSIM_Rhsim11G0059600	5.190492922	1.56151E-06	1.63997E-05	RHSIM_Rhsim11G0059600
Root vs Stem	novel.1001	-1.588448049	1.57083E-06	1.64901E-05	--

Comparison	Gene ID	log_2FC	P-value	FDR (padj)	Gene Name
Root vs Stem	RHSIM_Rhsim01G0131200	1.386286524	1.57448E-06	1.65209E-05	RHSIM_Rhsim01G0131200
Root vs Stem	novel.482	1.718809658	1.59428E-06	1.6721E-05	--
Root vs Stem	RHSIM_Rhsim04G0083900	-4.488730945	1.60218E-06	1.67963E-05	RHSIM_Rhsim04G0083900
Root vs Stem	novel.66	-1.354944985	1.61303E-06	1.69006E-05	--
Root vs Stem	RHSIM_Rhsim03G0010500	-3.532228012	1.61415E-06	1.69006E-05	RHSIM_Rhsim03G0010500
Root vs Stem	RHSIM_Rhsim08G0188700	-1.518293388	1.61433E-06	1.69006E-05	RHSIM_Rhsim08G0188700
Root vs Stem	RHSIM_Rhsim13G0084000	-1.687275202	1.61798E-06	1.69312E-05	RHSIM_Rhsim13G0084000
Root vs Stem	RHSIM_Rhsim09G0060000	1.098814865	1.62485E-06	1.69954E-05	RHSIM_Rhsim09G0060000
Root vs Stem	novel.734	4.311087063	1.62637E-06	1.70035E-05	--
Root vs Stem	RHSIM_Rhsim09G0104000	2.470030969	1.63959E-06	1.7134E-05	RHSIM_Rhsim09G0104000
Root vs Stem	RHSIM_Rhsim08G0220600	-4.107703071	1.64344E-06	1.71664E-05	RHSIM_Rhsim08G0220600
Root vs Stem	RHSIM_Rhsim01G0056800	6.416796452	1.6475E-06	1.7201E-05	RHSIM_Rhsim01G0056800
Root vs Stem	RHSIM_Rhsim07G0181700	-1.136128944	1.70291E-06	1.77554E-05	RHSIM_Rhsim07G0181700
Root vs Stem	RHSIM_Rhsim03G0162400	-1.23674271	1.71509E-06	1.78743E-05	RHSIM_Rhsim03G0162400
Root vs Stem	novel.4064	1.31173998	1.71883E-06	1.79052E-05	--
Root vs Stem	novel.2218	2.339127746	1.7245E-06	1.7948E-05	--
Root vs Stem	RHSIM_Rhsim01G0181300	-1.566858635	1.72923E-06	1.79891E-05	RHSIM_Rhsim01G0181300
Root vs Stem	novel.2486	-6.58471993	1.73197E-06	1.80014E-05	--
Root vs Stem	RHSIM_Rhsim12G0082400	1.949017507	1.74775E-06	1.81572E-05	RHSIM_Rhsim12G0082400
Root vs Stem	RHSIM_RhsimUnG0249100	1.706392917	1.75042E-06	1.81768E-05	RHSIM_RhsimUnG0249100
Root vs Stem	RHSIM_Rhsim09G0001200	1.078389719	1.75136E-06	1.81783E-05	RHSIM_Rhsim09G0001200
Root vs Stem	RHSIM_Rhsim05G0194800	-2.224705857	1.75586E-06	1.82168E-05	RHSIM_Rhsim05G0194800
Root vs Stem	RHSIM_Rhsim02G0032400	1.264378959	1.75685E-06	1.82189E-05	RHSIM_Rhsim02G0032400
Root vs Stem	RHSIM_Rhsim10G0054500	3.76714205	1.76252E-06	1.82695E-05	RHSIM_Rhsim10G0054500
Root vs Stem	RHSIM_Rhsim08G0057200	-1.317500958	1.77403E-06	1.83806E-05	RHSIM_Rhsim08G0057200

Comparison	Gene ID	log_2FC	P-value	FDR (padj)	Gene Name
Root vs Stem	RHSIM_RhsimUnG0090600	-3.67697295	1.79607E-06	1.85922E-05	RHSIM_RhsimUnG0090600
Root vs Stem	RHSIM_Rhsim08G0051800	1.935173096	1.79882E-06	1.86123E-05	RHSIM_Rhsim08G0051800
Root vs Stem	RHSIM_Rhsim06G0004800	2.255399887	1.80335E-06	1.86508E-05	RHSIM_Rhsim06G0004800
Root vs Stem	RHSIM_Rhsim12G0001700	1.602170769	1.80428E-06	1.8652E-05	RHSIM_Rhsim12G0001700
Root vs Stem	RHSIM_Rhsim02G0029200	-7.007486761	1.81309E-06	1.87348E-05	RHSIM_Rhsim02G0029200
Root vs Stem	RHSIM_Rhsim12G0053300	4.66978892	1.8235E-06	1.88339E-05	RHSIM_Rhsim12G0053300
Root vs Stem	RHSIM_Rhsim08G0232200	1.642098309	1.82452E-06	1.8836E-05	RHSIM_Rhsim08G0232200
Root vs Stem	novel.5674	4.517325454	1.82778E-06	1.88611E-05	--
Root vs Stem	RHSIM_Rhsim06G0172400	-1.016376086	1.8339E-06	1.89159E-05	RHSIM_Rhsim06G0172400
Root vs Stem	novel.4282	-7.234824887	1.85073E-06	1.90639E-05	--
Root vs Stem	RHSIM_Rhsim04G0115700	7.655118716	1.87977E-06	1.93371E-05	RHSIM_Rhsim04G0115700
Root vs Stem	RHSIM_Rhsim07G0126600	3.568373346	1.88154E-06	1.93466E-05	RHSIM_Rhsim07G0126600
Root vs Stem	RHSIM_Rhsim04G0185800	2.181804775	1.89798E-06	1.9507E-05	RHSIM_Rhsim04G0185800
Root vs Stem	RHSIM_Rhsim05G0051100	7.665102348	1.91601E-06	1.96835E-05	RHSIM_Rhsim05G0051100
Root vs Stem	RHSIM_Rhsim04G0161700	1.66410886	1.92218E-06	1.97381E-05	RHSIM_Rhsim04G0161700
Root vs Stem	RHSIM_Rhsim13G0002900	-3.654357044	1.92951E-06	1.98045E-05	RHSIM_Rhsim13G0002900
Root vs Stem	RHSIM_Rhsim08G0196200	-1.303193309	1.94628E-06	1.99657E-05	RHSIM_Rhsim08G0196200
Root vs Stem	RHSIM_Rhsim09G0199600	-3.239317328	1.94694E-06	1.99657E-05	RHSIM_Rhsim09G0199600
Root vs Stem	RHSIM_Rhsim09G0176900	2.50513878	1.95764E-06	2.00664E-05	RHSIM_Rhsim09G0176900
Root vs Stem	RHSIM_Rhsim07G0206100	2.993256253	1.96435E-06	2.01263E-05	RHSIM_Rhsim07G0206100
Root vs Stem	RHSIM_Rhsim13G0111900	1.505573382	1.97832E-06	2.02604E-05	RHSIM_Rhsim13G0111900
Root vs Stem	RHSIM_Rhsim06G0050200	-2.695980569	2.00679E-06	2.05338E-05	RHSIM_Rhsim06G0050200
Root vs Stem	RHSIM_Rhsim01G0176200	-2.481842821	2.01737E-06	2.06328E-05	RHSIM_Rhsim01G0176200
Root vs Stem	RHSIM_Rhsim13G0049700	-2.713639976	2.02579E-06	2.07098E-05	RHSIM_Rhsim13G0049700
Root vs Stem	RHSIM_Rhsim09G0207900	-2.515469436	2.02851E-06	2.07284E-05	RHSIM_Rhsim09G0207900

Comparison	Gene ID	log_2FC	P-value	FDR (padj)	Gene Name
Root vs Stem	novel.4278	-4.053379491	2.03732E-06	2.08091E-05	--
Root vs Stem	RHSIM_Rhsim11G0127500	1.607423504	2.05227E-06	2.09526E-05	RHSIM_Rhsim11G0127500
Root vs Stem	RHSIM_Rhsim01G0272300	-2.095138409	2.069E-06	2.10954E-05	RHSIM_Rhsim01G0272300
Root vs Stem	novel.5146	1.483434142	2.07556E-06	2.11529E-05	--
Root vs Stem	RHSIM_Rhsim07G0029100	-1.136321784	2.07913E-06	2.11799E-05	RHSIM_Rhsim07G0029100
Root vs Stem	RHSIM_Rhsim09G0194500	2.68840666	2.08483E-06	2.12125E-05	RHSIM_Rhsim09G0194500
Root vs Stem	RHSIM_Rhsim05G0087200	6.646489142	2.08508E-06	2.12125E-05	RHSIM_Rhsim05G0087200
Root vs Stem	RHSIM_Rhsim04G0029400	2.362527798	2.09444E-06	2.12982E-05	RHSIM_Rhsim04G0029400
Root vs Stem	RHSIM_Rhsim03G0170000	1.458795516	2.10552E-06	2.13921E-05	RHSIM_Rhsim03G0170000
Root vs Stem	RHSIM_Rhsim13G0081400	1.006042614	2.12116E-06	2.15319E-05	RHSIM_Rhsim13G0081400
Root vs Stem	RHSIM_Rhsim07G0033700	7.241517423	2.1343E-06	2.16558E-05	RHSIM_Rhsim07G0033700
Root vs Stem	novel.4061	1.501057256	2.15004E-06	2.18059E-05	--
Root vs Stem	RHSIM_Rhsim05G0108400	-2.658258936	2.17333E-06	2.20227E-05	RHSIM_Rhsim05G0108400
Root vs Stem	novel.4398	-6.734429101	2.18359E-06	2.2117E-05	--
Root vs Stem	RHSIM_Rhsim06G0133800	4.306499755	2.18504E-06	2.2122E-05	RHSIM_Rhsim06G0133800
Root vs Stem	RHSIM_Rhsim06G0179600	-2.888667307	2.1971E-06	2.22246E-05	RHSIM_Rhsim06G0179600
Root vs Stem	RHSIM_Rhsim12G0161600	-2.394955781	2.21436E-06	2.23795E-05	RHSIM_Rhsim12G0161600
Root vs Stem	RHSIM_Rhsim05G0220500	-1.733340132	2.21918E-06	2.24183E-05	RHSIM_Rhsim05G0220500
Root vs Stem	RHSIM_Rhsim12G0090900	-1.088731914	2.22113E-06	2.24283E-05	RHSIM_Rhsim12G0090900
Root vs Stem	RHSIM_Rhsim13G0022500	-2.801609615	2.24556E-06	2.26551E-05	RHSIM_Rhsim13G0022500
Root vs Stem	novel.4852	1.732449594	2.26204E-06	2.28014E-05	--
Root vs Stem	RHSIM_Rhsim06G0144100	4.148494075	2.26504E-06	2.28217E-05	RHSIM_Rhsim06G0144100
Root vs Stem	novel.3007	-2.152899032	2.28016E-06	2.2964E-05	--
Root vs Stem	RHSIM_Rhsim13G0046400	-1.656685538	2.29277E-06	2.30809E-05	RHSIM_Rhsim13G0046400
Root vs Stem	RHSIM_Rhsim11G0045900	1.304110703	2.29442E-06	2.30875E-05	RHSIM_Rhsim11G0045900

Comparison	Gene ID	log_2FC	P-value	FDR (padj)	Gene Name
Root vs Stem	RHSIM_Rhsim05G0146400	-1.710710094	2.3225E-06	2.33495E-05	RHSIM_Rhsim05G0146400
Root vs Stem	RHSIM_Rhsim05G0086700	-1.070135989	2.33182E-06	2.34331E-05	RHSIM_Rhsim05G0086700
Root vs Stem	RHSIM_Rhsim08G0182500	3.533240533	2.33919E-06	2.34968E-05	RHSIM_Rhsim08G0182500
Root vs Stem	RHSIM_Rhsim05G0194000	1.452725666	2.36424E-06	2.37278E-05	RHSIM_Rhsim05G0194000
Root vs Stem	novel.5934	-1.497903813	2.37336E-06	2.3809E-05	--
Root vs Stem	RHSIM_Rhsim13G0111400	1.485287088	2.38759E-06	2.39414E-05	RHSIM_Rhsim13G0111400
Root vs Stem	novel.5057	-1.318510045	2.40236E-06	2.4079E-05	--
Root vs Stem	novel.2381	-2.023390494	2.40884E-06	2.41334E-05	--
Root vs Stem	RHSIM_Rhsim08G0045300	1.030165034	2.42283E-06	2.42537E-05	RHSIM_Rhsim08G0045300
Root vs Stem	RHSIM_Rhsim01G0057600	-2.580605611	2.42295E-06	2.42537E-05	RHSIM_Rhsim01G0057600
Root vs Stem	RHSIM_Rhsim08G0102900	3.898460875	2.4557E-06	2.45602E-05	RHSIM_Rhsim08G0102900
Root vs Stem	novel.5955	3.888842713	2.47029E-06	2.46954E-05	--
Root vs Stem	RHSIM_Rhsim04G0036300	-2.667740272	2.47214E-06	2.47031E-05	RHSIM_Rhsim04G0036300
Root vs Stem	RHSIM_Rhsim10G0194500	1.214865362	2.48062E-06	2.47772E-05	RHSIM_Rhsim10G0194500
Root vs Stem	RHSIM_Rhsim07G0036300	-1.235125385	2.48573E-06	2.48175E-05	RHSIM_Rhsim07G0036300
Root vs Stem	RHSIM_Rhsim07G0047600	1.071903181	2.49362E-06	2.48747E-05	RHSIM_Rhsim07G0047600
Root vs Stem	RHSIM_RhsimUnG0181000	-1.742043207	2.49601E-06	2.48878E-05	RHSIM_RhsimUnG0181000
Root vs Stem	RHSIM_Rhsim05G0186100	-1.466143755	2.49907E-06	2.49059E-05	RHSIM_Rhsim05G0186100
Root vs Stem	RHSIM_Rhsim07G0049500	1.348618515	2.49999E-06	2.49059E-05	RHSIM_Rhsim07G0049500
Root vs Stem	RHSIM_Rhsim12G0061600	2.724529376	2.52293E-06	2.51112E-05	RHSIM_Rhsim12G0061600
Root vs Stem	RHSIM_Rhsim05G0050400	8.041554254	2.52387E-06	2.51112E-05	RHSIM_Rhsim05G0050400
Root vs Stem	RHSIM_Rhsim12G0162400	-1.750532688	2.52516E-06	2.51132E-05	RHSIM_Rhsim12G0162400
Root vs Stem	RHSIM_Rhsim04G0246500	1.34756478	2.52666E-06	2.51173E-05	RHSIM_Rhsim04G0246500
Root vs Stem	RHSIM_Rhsim13G0030500	-1.343164936	2.52794E-06	2.51192E-05	RHSIM_Rhsim13G0030500
Root vs Stem	RHSIM_Rhsim04G0033700	-2.584735629	2.53398E-06	2.51684E-05	RHSIM_Rhsim04G0033700

Comparison	Gene ID	log_2FC	P-value	FDR (padj)	Gene Name
Root vs Stem	RHSIM_Rhsim01G0245800	1.010831331	2.54321E-06	2.52492E-05	RHSIM_Rhsim01G0245800
Root vs Stem	RHSIM_Rhsim08G0252100	1.005521192	2.5472E-06	2.5278E-05	RHSIM_Rhsim08G0252100
Root vs Stem	novel.5760	-2.449267948	2.55624E-06	2.53567E-05	--
Root vs Stem	RHSIM_Rhsim06G0068800	-2.838186421	2.58381E-06	2.56191E-05	RHSIM_Rhsim06G0068800
Root vs Stem	RHSIM_Rhsim04G0026800	5.414821039	2.59543E-06	2.57122E-05	RHSIM_Rhsim04G0026800
Root vs Stem	RHSIM_Rhsim05G0127200	-4.710165705	2.61292E-06	2.58744E-05	RHSIM_Rhsim05G0127200
Root vs Stem	RHSIM_Rhsim11G0008400	-1.381083135	2.62767E-06	2.60093E-05	RHSIM_Rhsim11G0008400
Root vs Stem	RHSIM_Rhsim05G0088500	1.355053355	2.63178E-06	2.60195E-05	RHSIM_Rhsim05G0088500
Root vs Stem	RHSIM_Rhsim13G0050900	1.286298003	2.63209E-06	2.60195E-05	RHSIM_Rhsim13G0050900
Root vs Stem	RHSIM_Rhsim07G0054800	-1.45288123	2.69675E-06	2.66359E-05	RHSIM_Rhsim07G0054800
Root vs Stem	RHSIM_Rhsim01G0205300	1.280209795	2.70388E-06	2.66949E-05	RHSIM_Rhsim01G0205300
Root vs Stem	RHSIM_Rhsim07G0192200	-3.686079069	2.72326E-06	2.68747E-05	RHSIM_Rhsim07G0192200
Root vs Stem	RHSIM_Rhsim09G0186700	1.036161705	2.75714E-06	2.71858E-05	RHSIM_Rhsim09G0186700
Root vs Stem	novel.2740	-1.249778055	2.76661E-06	2.72558E-05	--
Root vs Stem	RHSIM_Rhsim02G0231700	1.094232806	2.78523E-06	2.74276E-05	RHSIM_Rhsim02G0231700
Root vs Stem	RHSIM_Rhsim05G0214400	2.985205594	2.7985E-06	2.75347E-05	RHSIM_Rhsim05G0214400
Root vs Stem	RHSIM_Rhsim09G0004900	1.922023063	2.85039E-06	2.80333E-05	RHSIM_Rhsim09G0004900
Root vs Stem	RHSIM_Rhsim12G0110200	-1.82211508	2.85881E-06	2.81041E-05	RHSIM_Rhsim12G0110200
Root vs Stem	RHSIM_Rhsim12G0151000	1.477783423	2.86134E-06	2.8117E-05	RHSIM_Rhsim12G0151000
Root vs Stem	RHSIM_RhsimUnG0198000	4.01722355	2.89324E-06	2.84184E-05	RHSIM_RhsimUnG0198000
Root vs Stem	RHSIM_Rhsim06G0187700	7.396401849	2.9024E-06	2.84961E-05	RHSIM_Rhsim06G0187700
Root vs Stem	RHSIM_Rhsim01G0282700	2.054171274	2.9064E-06	2.85233E-05	RHSIM_Rhsim01G0282700
Root vs Stem	RHSIM_Rhsim08G0179400	-6.23233885	2.91599E-06	2.85975E-05	RHSIM_Rhsim08G0179400
Root vs Stem	RHSIM_Rhsim03G0182700	-2.075759364	2.91645E-06	2.85975E-05	RHSIM_Rhsim03G0182700
Root vs Stem	RHSIM_Rhsim06G0163000	2.055663733	2.92079E-06	2.8628E-05	RHSIM_Rhsim06G0163000

Comparison	Gene ID	log2FC	P-value	FDR (padj)	Gene Name
Root vs Stem	RHSIM_Rhsim07G0011500	1.110342252	2.92851E-06	2.86914E-05	RHSIM_Rhsim07G0011500
Root vs Stem	RHSIM_Rhsim08G0042900	1.371389734	2.94732E-06	2.88536E-05	RHSIM_Rhsim08G0042900
Root vs Stem	novel.1651	3.891569639	2.96043E-06	2.89672E-05	--
Root vs Stem	RHSIM_Rhsim12G0008100	-2.139117436	3.00924E-06	2.94199E-05	RHSIM_Rhsim12G0008100
Root vs Stem	novel.3058	1.771815111	3.03333E-06	2.96256E-05	--
Root vs Stem	RHSIM_RhsimUnG0250800	2.978452358	3.03344E-06	2.96256E-05	RHSIM_RhsimUnG0250800
Root vs Stem	RHSIM_Rhsim07G0067400	1.470760697	3.03414E-06	2.96256E-05	RHSIM_Rhsim07G0067400
Root vs Stem	RHSIM_Rhsim05G0044400	1.315932138	3.03693E-06	2.96403E-05	RHSIM_Rhsim05G0044400
Root vs Stem	RHSIM_Rhsim04G0229700	1.230898427	3.06125E-06	2.9865E-05	RHSIM_Rhsim04G0229700
Root vs Stem	RHSIM_Rhsim12G0040100	-1.308086868	3.06689E-06	2.99074E-05	RHSIM_Rhsim12G0040100
Root vs Stem	novel.1513	2.487230289	3.08739E-06	3.00945E-05	--
Root vs Stem	RHSIM_Rhsim07G0073700	-2.985144137	3.09551E-06	3.01609E-05	RHSIM_Rhsim07G0073700
Root vs Stem	RHSIM_Rhsim13G0226200	-1.277053956	3.1163E-06	3.03507E-05	RHSIM_Rhsim13G0226200
Root vs Stem	RHSIM_Rhsim01G0254800	-2.049590763	3.13862E-06	3.05423E-05	RHSIM_Rhsim01G0254800
Root vs Stem	RHSIM_Rhsim05G0172800	1.501515803	3.14993E-06	3.06264E-05	RHSIM_Rhsim05G0172800
Root vs Stem	RHSIM_Rhsim11G0038000	-1.010597724	3.16815E-06	3.07776E-05	RHSIM_Rhsim11G0038000
Root vs Stem	RHSIM_Rhsim08G0036100	3.185628792	3.19423E-06	3.10049E-05	RHSIM_Rhsim08G0036100
Root vs Stem	novel.5931	-4.137631772	3.20518E-06	3.10981E-05	--
Root vs Stem	RHSIM_Rhsim06G0198600	-1.772651954	3.21278E-06	3.11587E-05	RHSIM_Rhsim06G0198600
Root vs Stem	RHSIM_RhsimUnG0258200	-3.002418729	3.222E-06	3.12349E-05	RHSIM_RhsimUnG0258200
Root vs Stem	RHSIM_Rhsim01G0239300	1.670152957	3.28583E-06	3.17835E-05	RHSIM_Rhsim01G0239300
Root vs Stem	RHSIM_Rhsim05G0139400	1.402163976	3.28771E-06	3.17835E-05	RHSIM_Rhsim05G0139400
Root vs Stem	novel.497	6.329486331	3.29548E-06	3.18305E-05	--
Root vs Stem	RHSIM_Rhsim13G0047600	-2.932535109	3.29586E-06	3.18305E-05	RHSIM_Rhsim13G0047600
Root vs Stem	RHSIM_Rhsim04G0083600	2.712234594	3.30729E-06	3.19276E-05	RHSIM_Rhsim04G0083600

Comparison	Gene ID	log_2FC	P-value	FDR (padj)	Gene Name
Root vs Stem	RHSIM_Rhsim04G0190500	2.350028366	3.31471E-06	3.19857E-05	RHSIM_Rhsim04G0190500
Root vs Stem	RHSIM_RhsimUnG0257700	-1.733205719	3.31795E-06	3.20037E-05	RHSIM_RhsimUnG0257700
Root vs Stem	RHSIM_Rhsim07G0254700	-2.285879627	3.33201E-06	3.21259E-05	RHSIM_Rhsim07G0254700
Root vs Stem	RHSIM_Rhsim09G0056100	2.102918823	3.34905E-06	3.22766E-05	RHSIM_Rhsim09G0056100
Root vs Stem	RHSIM_Rhsim06G0171300	2.180449848	3.35604E-06	3.23305E-05	RHSIM_Rhsim06G0171300
Root vs Stem	RHSIM_Rhsim11G0055900	7.07138959	3.35813E-06	3.23371E-05	RHSIM_Rhsim11G0055900
Root vs Stem	RHSIM_Rhsim02G0056600	1.451441251	3.37971E-06	3.25177E-05	RHSIM_Rhsim02G0056600
Root vs Stem	RHSIM_Rhsim06G0035600	-2.363645546	3.38186E-06	3.25248E-05	RHSIM_Rhsim06G0035600
Root vs Stem	novel.536	-2.831921235	3.39634E-06	3.26369E-05	--
Root vs Stem	RHSIM_Rhsim01G0235200	1.489263745	3.49462E-06	3.35533E-05	RHSIM_Rhsim01G0235200
Root vs Stem	RHSIM_Rhsim03G0123400	-1.184819409	3.5129E-06	3.37148E-05	RHSIM_Rhsim03G0123400
Root vs Stem	RHSIM_Rhsim10G0007000	1.490804089	3.53903E-06	3.39373E-05	RHSIM_Rhsim10G0007000
Root vs Stem	novel.3356	1.294124864	3.56643E-06	3.41858E-05	--
Root vs Stem	RHSIM_Rhsim13G0031300	1.983952874	3.57395E-06	3.42421E-05	RHSIM_Rhsim13G0031300
Root vs Stem	RHSIM_Rhsim06G0039400	1.380337653	3.57527E-06	3.42421E-05	RHSIM_Rhsim06G0039400
Root vs Stem	novel.5844	3.377496712	3.57949E-06	3.42682E-05	--
Root vs Stem	RHSIM_Rhsim06G0177900	1.167771925	3.58657E-06	3.43218E-05	RHSIM_Rhsim06G0177900
Root vs Stem	RHSIM_Rhsim03G0248000	-1.900331419	3.60015E-06	3.44375E-05	RHSIM_Rhsim03G0248000
Root vs Stem	RHSIM_Rhsim10G0035200	-1.178153661	3.61916E-06	3.46049E-05	RHSIM_Rhsim10G0035200
Root vs Stem	RHSIM_Rhsim07G0215200	-2.805406379	3.65725E-06	3.49546E-05	RHSIM_Rhsim07G0215200
Root vs Stem	RHSIM_Rhsim08G0088700	-4.056654548	3.66638E-06	3.50273E-05	RHSIM_Rhsim08G0088700
Root vs Stem	RHSIM_Rhsim02G0235600	1.023440882	3.70774E-06	3.53789E-05	RHSIM_Rhsim02G0235600
Root vs Stem	RHSIM_Rhsim11G0170000	-1.964484326	3.70932E-06	3.53789E-05	RHSIM_Rhsim11G0170000
Root vs Stem	RHSIM_Rhsim12G0140300	2.25105149	3.72146E-06	3.548E-05	RHSIM_Rhsim12G0140300
Root vs Stem	RHSIM_Rhsim12G0036700	-3.432294795	3.73215E-06	3.55673E-05	RHSIM_Rhsim12G0036700

Comparison	Gene ID	log_2FC	P-value	FDR (padj)	Gene Name
Root vs Stem	RHSIM_Rhsim03G0015900	-2.815772929	3.75488E-06	3.57543E-05	RHSIM_Rhsim03G0015900
Root vs Stem	RHSIM_Rhsim13G0067000	-4.362501412	3.76843E-06	3.58676E-05	RHSIM_Rhsim13G0067000
Root vs Stem	RHSIM_Rhsim06G0076100	2.782299621	3.76989E-06	3.58676E-05	RHSIM_Rhsim06G0076100
Root vs Stem	RHSIM_Rhsim07G0226400	1.915802098	3.79999E-06	3.61391E-05	RHSIM_Rhsim07G0226400
Root vs Stem	novel.3238	2.290822287	3.83319E-06	3.64248E-05	--
Root vs Stem	RHSIM_Rhsim08G0047800	-3.409439052	3.85902E-06	3.66291E-05	RHSIM_Rhsim08G0047800
Root vs Stem	novel.3829	-4.10947231	3.85946E-06	3.66291E-05	--
Root vs Stem	RHSIM_Rhsim01G0243500	-1.661499907	3.86998E-06	3.67138E-05	RHSIM_Rhsim01G0243500
Root vs Stem	RHSIM_Rhsim10G0141300	1.15099216	3.90804E-06	3.70292E-05	RHSIM_Rhsim10G0141300
Root vs Stem	RHSIM_Rhsim07G0184000	-2.074943698	3.92647E-06	3.71885E-05	RHSIM_Rhsim07G0184000
Root vs Stem	RHSIM_Rhsim13G0019200	1.746145851	3.9369E-06	3.72567E-05	RHSIM_Rhsim13G0019200
Root vs Stem	RHSIM_Rhsim12G0185600	-3.070687936	3.94266E-06	3.72959E-05	RHSIM_Rhsim12G0185600
Root vs Stem	RHSIM_Rhsim13G0233500	2.224390099	3.98895E-06	3.77183E-05	RHSIM_Rhsim13G0233500
Root vs Stem	RHSIM_Rhsim06G0067600	-2.559481059	4.01856E-06	3.79828E-05	RHSIM_Rhsim06G0067600
Root vs Stem	RHSIM_Rhsim11G0122000	-1.786703563	4.039E-06	3.81447E-05	RHSIM_Rhsim11G0122000
Root vs Stem	RHSIM_Rhsim09G0128100	-1.265644829	4.04189E-06	3.81563E-05	RHSIM_Rhsim09G0128100
Root vs Stem	RHSIM_Rhsim08G0082400	1.305618316	4.08668E-06	3.85633E-05	RHSIM_Rhsim08G0082400
Root vs Stem	RHSIM_Rhsim09G0114600	-2.313525632	4.08932E-06	3.85725E-05	RHSIM_Rhsim09G0114600
Root vs Stem	RHSIM_RhsimUnG0068300	6.89381155	4.09195E-06	3.85815E-05	RHSIM_RhsimUnG0068300
Root vs Stem	RHSIM_Rhsim05G0195400	2.828436638	4.09691E-06	3.86035E-05	RHSIM_Rhsim05G0195400
Root vs Stem	RHSIM_Rhsim12G0010200	1.509358799	4.12969E-06	3.88737E-05	RHSIM_Rhsim12G0010200
Root vs Stem	RHSIM_Rhsim11G0008300	-1.349029964	4.13626E-06	3.89197E-05	RHSIM_Rhsim11G0008300
Root vs Stem	RHSIM_Rhsim10G0147100	-2.157683839	4.15902E-06	3.91019E-05	RHSIM_Rhsim10G0147100
Root vs Stem	RHSIM_Rhsim11G0060300	-5.043306918	4.17113E-06	3.91998E-05	RHSIM_Rhsim11G0060300
Root vs Stem	RHSIM_Rhsim02G0189300	3.187166372	4.23505E-06	3.97196E-05	RHSIM_Rhsim02G0189300

Comparison	Gene ID	log_2FC	P-value	FDR (padj)	Gene Name
Root vs Stem	RHSIM_Rhsim04G0147400	1.846036804	4.2573E-06	3.9912E-05	RHSIM_Rhsim04G0147400
Root vs Stem	RHSIM_Rhsim12G0047500	2.011007278	4.28864E-06	4.01894E-05	RHSIM_Rhsim12G0047500
Root vs Stem	RHSIM_Rhsim06G0079300	1.135851274	4.29569E-06	4.02392E-05	RHSIM_Rhsim06G0079300
Root vs Stem	RHSIM_Rhsim09G0178300	5.426421593	4.33145E-06	4.05412E-05	RHSIM_Rhsim09G0178300
Root vs Stem	RHSIM_Rhsim08G0238400	6.889012625	4.3413E-06	4.06169E-05	RHSIM_Rhsim08G0238400
Root vs Stem	RHSIM_Rhsim10G0038400	-3.908059879	4.35491E-06	4.07276E-05	RHSIM_Rhsim10G0038400
Root vs Stem	RHSIM_Rhsim02G0041500	-2.101493881	4.40176E-06	4.11463E-05	RHSIM_Rhsim02G0041500
Root vs Stem	RHSIM_Rhsim04G0085500	-1.413151981	4.41471E-06	4.12368E-05	RHSIM_Rhsim04G0085500
Root vs Stem	RHSIM_Rhsim12G0110000	1.283194223	4.42114E-06	4.12801E-05	RHSIM_Rhsim12G0110000
Root vs Stem	RHSIM_Rhsim02G0027800	-2.106139006	4.4306E-06	4.13516E-05	RHSIM_Rhsim02G0027800
Root vs Stem	RHSIM_RhsimUnG0069700	3.446225305	4.43541E-06	4.13798E-05	RHSIM_RhsimUnG0069700
Root vs Stem	RHSIM_RhsimUnG0088800	6.946282121	4.47367E-06	4.16861E-05	RHSIM_RhsimUnG0088800
Root vs Stem	RHSIM_Rhsim13G0055300	4.587705068	4.49154E-06	4.18188E-05	RHSIM_Rhsim13G0055300
Root vs Stem	RHSIM_Rhsim02G0135000	-1.626056718	4.54887E-06	4.23185E-05	RHSIM_Rhsim02G0135000
Root vs Stem	RHSIM_Rhsim07G0187100	-3.877861441	4.56486E-06	4.24501E-05	RHSIM_Rhsim07G0187100
Root vs Stem	novel.613	-3.82798763	4.58416E-06	4.26124E-05	--
Root vs Stem	RHSIM_Rhsim07G0175500	-2.257179052	4.59547E-06	4.27003E-05	RHSIM_Rhsim07G0175500
Root vs Stem	RHSIM_Rhsim07G0065500	-1.798562866	4.6099E-06	4.28171E-05	RHSIM_Rhsim07G0065500
Root vs Stem	RHSIM_Rhsim06G0164600	6.705016443	4.62165E-06	4.29089E-05	RHSIM_Rhsim06G0164600
Root vs Stem	RHSIM_RhsimUnG0187200	-4.844960504	4.63808E-06	4.30441E-05	RHSIM_RhsimUnG0187200
Root vs Stem	RHSIM_Rhsim01G0151800	-1.225317782	4.64857E-06	4.31241E-05	RHSIM_Rhsim01G0151800
Root vs Stem	novel.1013	1.969400355	4.69854E-06	4.35527E-05	--
Root vs Stem	RHSIM_Rhsim04G0204500	1.206338359	4.70378E-06	4.35837E-05	RHSIM_Rhsim04G0204500
Root vs Stem	RHSIM_Rhsim13G0083300	1.036292585	4.78503E-06	4.43187E-05	RHSIM_Rhsim13G0083300
Root vs Stem	RHSIM_Rhsim08G0217100	-2.242437578	4.81006E-06	4.45327E-05	RHSIM_Rhsim08G0217100

Comparison	Gene ID	log_2FC	P-value	FDR (padj)	Gene Name
Root vs Stem	novel.3788	2.15174489	4.81621E-06	4.45717E-05	--
Root vs Stem	RHSIM_Rhsim07G0071400	-2.392982355	4.83266E-06	4.4706E-05	RHSIM_Rhsim07G0071400
Root vs Stem	RHSIM_Rhsim01G0256200	1.365862021	4.86244E-06	4.49634E-05	RHSIM_Rhsim01G0256200
Root vs Stem	RHSIM_Rhsim06G0138100	1.986392948	4.89369E-06	4.52342E-05	RHSIM_Rhsim06G0138100
Root vs Stem	novel.3366	7.29254278	4.91192E-06	4.53845E-05	--
Root vs Stem	RHSIM_Rhsim11G0121500	1.222242909	4.9225E-06	4.54277E-05	RHSIM_Rhsim11G0121500
Root vs Stem	RHSIM_Rhsim08G0213200	-1.215444507	5.03825E-06	4.64401E-05	RHSIM_Rhsim08G0213200
Root vs Stem	RHSIM_Rhsim02G0206900	1.356776106	5.04887E-06	4.65194E-05	RHSIM_Rhsim02G0206900
Root vs Stem	RHSIM_Rhsim07G0090400	1.02673893	5.05255E-06	4.65348E-05	RHSIM_Rhsim07G0090400
Root vs Stem	RHSIM_Rhsim12G0139600	-1.634486665	5.07737E-06	4.67447E-05	RHSIM_Rhsim12G0139600
Root vs Stem	RHSIM_Rhsim09G0194700	1.377848257	5.08786E-06	4.68225E-05	RHSIM_Rhsim09G0194700
Root vs Stem	RHSIM_Rhsim08G0109600	1.320724441	5.10646E-06	4.69562E-05	RHSIM_Rhsim08G0109600
Root vs Stem	RHSIM_Rhsim03G0101200	-1.167052661	5.14454E-06	4.72687E-05	RHSIM_Rhsim03G0101200
Root vs Stem	RHSIM_Rhsim13G0223000	1.330124083	5.16943E-06	4.74596E-05	RHSIM_Rhsim13G0223000
Root vs Stem	RHSIM_Rhsim10G0192900	-1.073520293	5.17837E-06	4.75227E-05	RHSIM_Rhsim10G0192900
Root vs Stem	RHSIM_Rhsim05G0145600	3.719607535	5.2284E-06	4.79436E-05	RHSIM_Rhsim05G0145600
Root vs Stem	RHSIM_Rhsim03G0217900	1.799531667	5.26483E-06	4.82585E-05	RHSIM_Rhsim03G0217900
Root vs Stem	RHSIM_Rhsim05G0057400	4.978014066	5.29288E-06	4.84861E-05	RHSIM_Rhsim05G0057400
Root vs Stem	RHSIM_Rhsim06G0190900	-2.519236566	5.29535E-06	4.84861E-05	RHSIM_Rhsim06G0190900
Root vs Stem	RHSIM_Rhsim10G0158100	-1.112143897	5.29597E-06	4.84861E-05	RHSIM_Rhsim10G0158100
Root vs Stem	RHSIM_RhsimUnG0061600	1.52518913	5.33665E-06	4.88198E-05	RHSIM_RhsimUnG0061600
Root vs Stem	RHSIM_Rhsim11G0171200	5.137600146	5.35646E-06	4.89816E-05	RHSIM_Rhsim11G0171200
Root vs Stem	RHSIM_Rhsim08G0055400	-1.180326022	5.37499E-06	4.91315E-05	RHSIM_Rhsim08G0055400
Root vs Stem	RHSIM_Rhsim03G0054100	-6.491812026	5.38957E-06	4.92062E-05	RHSIM_Rhsim03G0054100
Root vs Stem	RHSIM_Rhsim01G0091800	-1.176215918	5.43139E-06	4.95685E-05	RHSIM_Rhsim01G0091800

Comparison	Gene ID	log_2FC	P-value	FDR (padj)	Gene Name
Root vs Stem	novel.1252	-8.754534645	5.44429E-06	4.96664E-05	--
Root vs Stem	RHSIM_Rhsim10G0002200	1.208935781	5.47148E-06	4.98948E-05	RHSIM_Rhsim10G0002200
Root vs Stem	novel.3252	-1.294285966	5.51812E-06	5.02803E-05	--
Root vs Stem	RHSIM_Rhsim02G0233400	1.104378098	5.52317E-06	5.03065E-05	RHSIM_Rhsim02G0233400
Root vs Stem	novel.5904	-9.062489252	5.54555E-06	5.04903E-05	--
Root vs Stem	RHSIM_Rhsim02G0114900	-1.870843149	5.60657E-06	5.10258E-05	RHSIM_Rhsim02G0114900
Root vs Stem	novel.4800	-2.500114341	5.6251E-06	5.11742E-05	--
Root vs Stem	RHSIM_Rhsim12G0079200	5.793310199	5.63292E-06	5.12251E-05	RHSIM_Rhsim12G0079200
Root vs Stem	RHSIM_Rhsim01G0144200	1.081415567	5.64038E-06	5.12727E-05	RHSIM_Rhsim01G0144200
Root vs Stem	novel.2711	-1.532393008	5.705E-06	5.18397E-05	--
Root vs Stem	RHSIM_Rhsim11G0093900	-3.420250896	5.7629E-06	5.23452E-05	RHSIM_Rhsim11G0093900
Root vs Stem	RHSIM_Rhsim05G0201900	5.564639731	5.79491E-06	5.26152E-05	RHSIM_Rhsim05G0201900
Root vs Stem	novel.2611	1.719636111	5.80538E-06	5.26896E-05	--
Root vs Stem	novel.4367	-1.273877606	5.82067E-06	5.28049E-05	--
Root vs Stem	RHSIM_Rhsim07G0160800	-8.586701609	5.84199E-06	5.29593E-05	RHSIM_Rhsim07G0160800
Root vs Stem	RHSIM_Rhsim05G0045500	-6.709683225	5.86975E-06	5.319E-05	RHSIM_Rhsim05G0045500
Root vs Stem	RHSIM_Rhsim12G0184900	-3.44439133	5.87757E-06	5.324E-05	RHSIM_Rhsim12G0184900
Root vs Stem	RHSIM_Rhsim06G0146900	1.890064794	5.90135E-06	5.34343E-05	RHSIM_Rhsim06G0146900
Root vs Stem	novel.1384	-1.026643239	5.90524E-06	5.34485E-05	--
Root vs Stem	RHSIM_Rhsim06G0073500	1.245447439	5.92049E-06	5.35267E-05	RHSIM_Rhsim06G0073500
Root vs Stem	RHSIM_Rhsim01G0180700	-3.094227008	5.92084E-06	5.35267E-05	RHSIM_Rhsim01G0180700
Root vs Stem	RHSIM_Rhsim03G0127300	-1.550664481	5.933E-06	5.36156E-05	RHSIM_Rhsim03G0127300
Root vs Stem	RHSIM_Rhsim05G0195200	3.646398161	5.93623E-06	5.36238E-05	RHSIM_Rhsim05G0195200
Root vs Stem	RHSIM_Rhsim02G0052400	4.629216042	5.95665E-06	5.37872E-05	RHSIM_Rhsim02G0052400
Root vs Stem	RHSIM_Rhsim07G0195400	-2.024165042	5.99117E-06	5.40143E-05	RHSIM_Rhsim07G0195400

Comparison	Gene ID	log_2FC	P-value	FDR (padj)	Gene Name
Root vs Stem	RHSIM_Rhsim05G0167900	1.006017298	6.00156E-06	5.40656E-05	RHSIM_Rhsim05G0167900
Root vs Stem	RHSIM_Rhsim09G0055700	1.424199885	6.0347E-06	5.42962E-05	RHSIM_Rhsim09G0055700
Root vs Stem	RHSIM_Rhsim04G0046400	1.383818725	6.03861E-06	5.42962E-05	RHSIM_Rhsim04G0046400
Root vs Stem	RHSIM_Rhsim06G0167800	-2.042049275	6.04645E-06	5.43426E-05	RHSIM_Rhsim06G0167800
Root vs Stem	RHSIM_Rhsim02G0056300	-1.895478894	6.08988E-06	5.47116E-05	RHSIM_Rhsim02G0056300
Root vs Stem	RHSIM_Rhsim04G0121700	-1.181139129	6.17953E-06	5.54522E-05	RHSIM_Rhsim04G0121700
Root vs Stem	RHSIM_Rhsim06G0197500	3.658163717	6.22325E-06	5.58228E-05	RHSIM_Rhsim06G0197500
Root vs Stem	RHSIM_Rhsim08G0210100	1.452696326	6.23355E-06	5.58596E-05	RHSIM_Rhsim08G0210100
Root vs Stem	RHSIM_Rhsim03G0247400	-1.490417878	6.23463E-06	5.58596E-05	RHSIM_Rhsim03G0247400
Root vs Stem	RHSIM_Rhsim02G0228400	2.23573564	6.27299E-06	5.61815E-05	RHSIM_Rhsim02G0228400
Root vs Stem	RHSIM_Rhsim11G0070400	-1.688935589	6.29797E-06	5.63833E-05	RHSIM_Rhsim11G0070400
Root vs Stem	RHSIM_Rhsim11G0123700	1.926130448	6.31225E-06	5.64892E-05	RHSIM_Rhsim11G0123700
Root vs Stem	RHSIM_Rhsim08G0219100	-7.202927849	6.33548E-06	5.66752E-05	RHSIM_Rhsim08G0219100
Root vs Stem	RHSIM_Rhsim07G0009600	2.766689543	6.35189E-06	5.67778E-05	RHSIM_Rhsim07G0009600
Root vs Stem	RHSIM_Rhsim03G0099400	1.341588289	6.35821E-06	5.68124E-05	RHSIM_Rhsim03G0099400
Root vs Stem	RHSIM_Rhsim12G0064900	5.107287134	6.36912E-06	5.68877E-05	RHSIM_Rhsim12G0064900
Root vs Stem	RHSIM_Rhsim02G0141400	-5.283928139	6.38026E-06	5.69652E-05	RHSIM_Rhsim02G0141400
Root vs Stem	RHSIM_Rhsim10G0004300	1.007424125	6.42731E-06	5.73631E-05	RHSIM_Rhsim10G0004300
Root vs Stem	RHSIM_RhsimUnG0249600	-1.229203634	6.44813E-06	5.75266E-05	RHSIM_RhsimUnG0249600
Root vs Stem	novel.5028	-5.276926105	6.47393E-06	5.77344E-05	--
Root vs Stem	novel.5727	6.83487537	6.49359E-06	5.78873E-05	--
Root vs Stem	novel.4780	-2.182815634	6.51402E-06	5.80471E-05	--
Root vs Stem	RHSIM_Rhsim12G0192600	-1.121278289	6.52017E-06	5.80794E-05	RHSIM_Rhsim12G0192600
Root vs Stem	RHSIM_Rhsim01G0178100	-6.3038351	6.52644E-06	5.81128E-05	RHSIM_Rhsim01G0178100
Root vs Stem	RHSIM_Rhsim12G0204900	-6.711922149	6.5516E-06	5.82934E-05	RHSIM_Rhsim12G0204900

Comparison	Gene ID	log_2FC	P-value	FDR (padj)	Gene Name
Root vs Stem	RHSIM_Rhsim03G0265400	-1.608009338	6.55178E-06	5.82934E-05	RHSIM_Rhsim03G0265400
Root vs Stem	RHSIM_Rhsim10G0066900	-2.250986005	6.56021E-06	5.83459E-05	RHSIM_Rhsim10G0066900
Root vs Stem	RHSIM_Rhsim04G0230000	5.595267685	6.64508E-06	5.90514E-05	RHSIM_Rhsim04G0230000
Root vs Stem	RHSIM_Rhsim09G0171500	1.244121033	6.64723E-06	5.90514E-05	RHSIM_Rhsim09G0171500
Root vs Stem	RHSIM_Rhsim05G0173200	-9.021176492	6.67147E-06	5.92439E-05	RHSIM_Rhsim05G0173200
Root vs Stem	RHSIM_Rhsim05G0190000	-6.591311702	6.69822E-06	5.94587E-05	RHSIM_Rhsim05G0190000
Root vs Stem	RHSIM_Rhsim11G0171800	1.950533425	6.72178E-06	5.96448E-05	RHSIM_Rhsim11G0171800
Root vs Stem	RHSIM_Rhsim11G0023300	1.760135408	6.73447E-06	5.97344E-05	RHSIM_Rhsim11G0023300
Root vs Stem	RHSIM_Rhsim05G0039200	-1.024634452	6.74493E-06	5.98042E-05	RHSIM_Rhsim05G0039200
Root vs Stem	RHSIM_Rhsim10G0007600	-2.592222453	6.77016E-06	5.99948E-05	RHSIM_Rhsim10G0007600
Root vs Stem	RHSIM_Rhsim05G0030700	-1.751859172	6.77163E-06	5.99948E-05	RHSIM_Rhsim05G0030700
Root vs Stem	RHSIM_Rhsim10G0201100	1.219071982	6.78788E-06	6.01156E-05	RHSIM_Rhsim10G0201100
Root vs Stem	RHSIM_Rhsim11G0159000	5.368063167	6.79173E-06	6.01267E-05	RHSIM_Rhsim11G0159000
Root vs Stem	RHSIM_Rhsim09G0017900	3.073186117	6.83175E-06	6.04345E-05	RHSIM_Rhsim09G0017900
Root vs Stem	RHSIM_Rhsim07G0144700	-1.498171907	6.84697E-06	6.05459E-05	RHSIM_Rhsim07G0144700
Root vs Stem	RHSIM_Rhsim03G0036900	7.027616938	6.85422E-06	6.05868E-05	RHSIM_Rhsim03G0036900
Root vs Stem	RHSIM_Rhsim09G0183900	-1.236738164	6.90215E-06	6.09403E-05	RHSIM_Rhsim09G0183900
Root vs Stem	RHSIM_Rhsim02G0049200	-1.236715	6.90688E-06	6.09588E-05	RHSIM_Rhsim02G0049200
Root vs Stem	RHSIM_Rhsim06G0130000	-1.214189612	6.92647E-06	6.11083E-05	RHSIM_Rhsim06G0130000
Root vs Stem	RHSIM_Rhsim06G0175500	2.762469572	6.98812E-06	6.15815E-05	RHSIM_Rhsim06G0175500
Root vs Stem	RHSIM_Rhsim11G0139200	-5.800119245	7.03499E-06	6.19708E-05	RHSIM_Rhsim11G0139200
Root vs Stem	RHSIM_Rhsim09G0115600	-2.351060643	7.12196E-06	6.2689E-05	RHSIM_Rhsim09G0115600
Root vs Stem	novel.1359	-1.614475852	7.15148E-06	6.29248E-05	--
Root vs Stem	RHSIM_Rhsim04G0083100	2.736341279	7.16297E-06	6.30019E-05	RHSIM_Rhsim04G0083100
Root vs Stem	RHSIM_Rhsim09G0196800	-3.190504011	7.19812E-06	6.32869E-05	RHSIM_Rhsim09G0196800

Comparison	Gene ID	log_2FC	P-value	FDR (padj)	Gene Name
Root vs Stem	RHSIM_Rhsim09G0135200	-1.528987605	7.21964E-06	6.34519E-05	RHSIM_Rhsim09G0135200
Root vs Stem	RHSIM_Rhsim13G0008400	-2.866235968	7.22882E-06	6.35084E-05	RHSIM_Rhsim13G0008400
Root vs Stem	RHSIM_Rhsim09G0195900	-3.081875	7.34145E-06	6.44487E-05	RHSIM_Rhsim09G0195900
Root vs Stem	RHSIM_Rhsim10G0174300	1.402551084	7.36432E-06	6.46249E-05	RHSIM_Rhsim10G0174300
Root vs Stem	RHSIM_Rhsim11G0054900	-1.191080858	7.42814E-06	6.51354E-05	RHSIM_Rhsim11G0054900
Root vs Stem	RHSIM_Rhsim12G0136300	1.548523085	7.44635E-06	6.52702E-05	RHSIM_Rhsim12G0136300
Root vs Stem	RHSIM_Rhsim10G0182700	-2.404871272	7.49059E-06	6.56206E-05	RHSIM_Rhsim10G0182700
Root vs Stem	RHSIM_Rhsim08G0205900	3.032302462	7.49201E-06	6.56206E-05	RHSIM_Rhsim08G0205900
Root vs Stem	RHSIM_RhsimUnG0113300	-1.920540011	7.54882E-06	6.6093E-05	RHSIM_RhsimUnG0113300
Root vs Stem	novel.2069	5.368181806	7.60924E-06	6.65967E-05	--
Root vs Stem	RHSIM_Rhsim05G0199400	-1.233476887	7.61385E-06	6.66118E-05	RHSIM_Rhsim05G0199400
Root vs Stem	RHSIM_Rhsim06G0051000	2.256737941	7.64816E-06	6.68711E-05	RHSIM_Rhsim06G0051000
Root vs Stem	novel.6082	1.763282259	7.6493E-06	6.68711E-05	--
Root vs Stem	novel.4579	-6.064377269	7.71282E-06	6.73754E-05	--
Root vs Stem	RHSIM_Rhsim09G0108400	-1.017984914	7.74923E-06	6.76586E-05	RHSIM_Rhsim09G0108400
Root vs Stem	RHSIM_RhsimUnG0203600	-6.224001927	7.7754E-06	6.7845E-05	RHSIM_RhsimUnG0203600
Root vs Stem	RHSIM_Rhsim06G0206000	2.232252849	7.79542E-06	6.79939E-05	RHSIM_Rhsim06G0206000
Root vs Stem	RHSIM_Rhsim11G0010500	1.800922009	7.81646E-06	6.81259E-05	RHSIM_Rhsim11G0010500
Root vs Stem	RHSIM_Rhsim03G0269000	-1.604741322	7.86176E-06	6.84689E-05	RHSIM_Rhsim03G0269000
Root vs Stem	RHSIM_Rhsim04G0102500	-1.605472311	7.90516E-06	6.87895E-05	RHSIM_Rhsim04G0102500
Root vs Stem	novel.3674	-2.478166498	7.90748E-06	6.87895E-05	--
Root vs Stem	RHSIM_Rhsim06G0102400	-1.393134225	7.95529E-06	6.91528E-05	RHSIM_Rhsim06G0102400
Root vs Stem	RHSIM_Rhsim06G0066300	-3.405176459	7.96728E-06	6.9231E-05	RHSIM_Rhsim06G0066300
Root vs Stem	RHSIM_Rhsim07G0174200	2.22947187	7.97128E-06	6.92396E-05	RHSIM_Rhsim07G0174200
Root vs Stem	RHSIM_Rhsim05G0063800	-6.199878649	8.00433E-06	6.95005E-05	RHSIM_Rhsim05G0063800

Comparison	Gene ID	log_2FC	P-value	FDR (padj)	Gene Name
Root vs Stem	novel.531	2.410257736	8.00752E-06	6.9502E-05	--
Root vs Stem	RHSIM_Rhsim13G0200300	4.544568004	8.05158E-06	6.98056E-05	RHSIM_Rhsim13G0200300
Root vs Stem	RHSIM_Rhsim08G0174000	1.252013913	8.06777E-06	6.99196E-05	RHSIM_Rhsim08G0174000
Root vs Stem	novel.2986	2.046396509	8.10748E-06	7.02374E-05	--
Root vs Stem	RHSIM_Rhsim13G0197500	-1.599733296	8.13225E-06	7.03991E-05	RHSIM_Rhsim13G0197500
Root vs Stem	RHSIM_Rhsim03G0001900	1.477156678	8.15873E-06	7.06018E-05	RHSIM_Rhsim03G0001900
Root vs Stem	RHSIM_Rhsim01G0283900	-1.595736368	8.1674E-06	7.06296E-05	RHSIM_Rhsim01G0283900
Root vs Stem	RHSIM_Rhsim02G0168600	4.772985408	8.16807E-06	7.06296E-05	RHSIM_Rhsim02G0168600
Root vs Stem	RHSIM_Rhsim10G0071300	5.532216318	8.19105E-06	7.08018E-05	RHSIM_Rhsim10G0071300
Root vs Stem	RHSIM_Rhsim01G0102500	-1.06310783	8.2913E-06	7.16146E-05	RHSIM_Rhsim01G0102500
Root vs Stem	RHSIM_Rhsim09G0189800	1.806749109	8.33728E-06	7.19848E-05	RHSIM_Rhsim09G0189800
Root vs Stem	RHSIM_Rhsim09G0096900	-1.55590247	8.44912E-06	7.28958E-05	RHSIM_Rhsim09G0096900
Root vs Stem	RHSIM_Rhsim11G0084800	3.199380748	8.47622E-06	7.30749E-05	RHSIM_Rhsim11G0084800
Root vs Stem	RHSIM_Rhsim09G0068900	-1.073106703	8.49546E-06	7.32106E-05	RHSIM_Rhsim09G0068900
Root vs Stem	novel.5650	-5.890523392	8.49831E-06	7.32106E-05	--
Root vs Stem	RHSIM_Rhsim01G0246400	1.19177316	8.53923E-06	7.35356E-05	RHSIM_Rhsim01G0246400
Root vs Stem	novel.5567	-5.378155731	8.55853E-06	7.36743E-05	--
Root vs Stem	RHSIM_Rhsim01G0010600	3.346256441	8.59028E-06	7.392E-05	RHSIM_Rhsim01G0010600
Root vs Stem	RHSIM_Rhsim04G0017600	-10.11550082	8.65937E-06	7.44868E-05	RHSIM_Rhsim04G0017600
Root vs Stem	RHSIM_Rhsim04G0203500	2.070359398	8.66465E-06	7.44906E-05	RHSIM_Rhsim04G0203500
Root vs Stem	novel.270	-8.883458222	8.6678E-06	7.44906E-05	--
Root vs Stem	novel.5193	-1.847713016	8.67551E-06	7.45143E-05	--
Root vs Stem	RHSIM_Rhsim04G0000400	1.012036767	8.7191E-06	7.4833E-05	RHSIM_Rhsim04G0000400
Root vs Stem	RHSIM_Rhsim03G0259100	3.287251926	8.74949E-06	7.50658E-05	RHSIM_Rhsim03G0259100
Root vs Stem	RHSIM_Rhsim11G0002900	-8.393307621	8.84022E-06	7.57597E-05	RHSIM_Rhsim11G0002900

Comparison	Gene ID	log_2FC	P-value	FDR (padj)	Gene Name
Root vs Stem	RHSIM_Rhsim11G0022400	2.589782141	8.86685E-06	7.59597E-05	RHSIM_Rhsim11G0022400
Root vs Stem	RHSIM_Rhsim13G0153900	1.539865318	8.9241E-06	7.64217E-05	RHSIM_Rhsim13G0153900
Root vs Stem	RHSIM_Rhsim07G0060700	-2.024895092	8.95603E-06	7.66667E-05	RHSIM_Rhsim07G0060700
Root vs Stem	RHSIM_Rhsim03G0161200	-1.787184487	9.01814E-06	7.71366E-05	RHSIM_Rhsim03G0161200
Root vs Stem	RHSIM_RhsimUnG0136800	1.277566326	9.02096E-06	7.71366E-05	RHSIM_RhsimUnG0136800
Root vs Stem	RHSIM_Rhsim12G0132700	-1.527369934	9.02473E-06	7.71402E-05	RHSIM_Rhsim12G0132700
Root vs Stem	novel.3220	-7.588265412	9.04194E-06	7.72586E-05	--
Root vs Stem	RHSIM_Rhsim03G0028600	1.278971516	9.04957E-06	7.72952E-05	RHSIM_Rhsim03G0028600
Root vs Stem	novel.5359	2.552968514	9.11006E-06	7.77831E-05	--
Root vs Stem	RHSIM_Rhsim06G0057800	4.712735812	9.13095E-06	7.79326E-05	RHSIM_Rhsim06G0057800
Root vs Stem	novel.3999	-2.705653391	9.16861E-06	7.8225E-05	--
Root vs Stem	RHSIM_Rhsim08G0047900	-2.720544323	9.1728E-06	7.82318E-05	RHSIM_Rhsim08G0047900
Root vs Stem	RHSIM_Rhsim06G0030800	1.076998246	9.17623E-06	7.82321E-05	RHSIM_Rhsim06G0030800
Root vs Stem	RHSIM_Rhsim08G0092400	6.18430093	9.22605E-06	7.86278E-05	RHSIM_Rhsim08G0092400
Root vs Stem	RHSIM_Rhsim05G0204200	5.630678033	9.23514E-06	7.86761E-05	RHSIM_Rhsim05G0204200
Root vs Stem	RHSIM_Rhsim04G0186800	3.107376391	9.2742E-06	7.89755E-05	RHSIM_Rhsim04G0186800
Root vs Stem	novel.401	-1.791634487	9.27713E-06	7.89755E-05	--
Root vs Stem	RHSIM_Rhsim04G0081400	1.61070518	9.2834E-06	7.89997E-05	RHSIM_Rhsim04G0081400
Root vs Stem	RHSIM_Rhsim09G0113200	-1.89192951	9.30146E-06	7.91242E-05	RHSIM_Rhsim09G0113200
Root vs Stem	RHSIM_Rhsim02G0168800	8.3103933	9.34459E-06	7.94617E-05	RHSIM_Rhsim02G0168800
Root vs Stem	RHSIM_Rhsim03G0046900	2.938708714	9.36641E-06	7.9618E-05	RHSIM_Rhsim03G0046900
Root vs Stem	RHSIM_Rhsim12G0214600	1.070554726	9.42036E-06	8.0047E-05	RHSIM_Rhsim12G0214600
Root vs Stem	RHSIM_Rhsim06G0211700	-1.449046184	9.43672E-06	8.01342E-05	RHSIM_Rhsim06G0211700
Root vs Stem	RHSIM_Rhsim04G0176000	-1.505186118	9.43757E-06	8.01342E-05	RHSIM_Rhsim04G0176000
Root vs Stem	RHSIM_Rhsim03G0035200	3.192175125	9.45396E-06	8.02438E-05	RHSIM_Rhsim03G0035200

Comparison	Gene ID	log_2FC	P-value	FDR (padj)	Gene Name
Root vs Stem	RHSIM_Rhsim10G0200600	8.03841325	9.54099E-06	8.08634E-05	RHSIM_Rhsim10G0200600
Root vs Stem	RHSIM_Rhsim06G0207500	1.078266508	9.58844E-06	8.12059E-05	RHSIM_Rhsim06G0207500
Root vs Stem	RHSIM_Rhsim13G0162400	-1.817490107	9.65794E-06	8.17644E-05	RHSIM_Rhsim13G0162400
Root vs Stem	RHSIM_Rhsim02G0143400	1.368645728	9.66251E-06	8.17731E-05	RHSIM_Rhsim02G0143400
Root vs Stem	RHSIM_Rhsim05G0011100	6.31291845	9.74189E-06	8.24146E-05	RHSIM_Rhsim05G0011100
Root vs Stem	RHSIM_Rhsim07G0183600	-2.01886509	9.81989E-06	8.29831E-05	RHSIM_Rhsim07G0183600
Root vs Stem	novel.314	-3.114821923	9.83625E-06	8.30909E-05	--
Root vs Stem	novel.3363	1.241752453	9.85178E-06	8.31611E-05	--
Root vs Stem	novel.4947	-2.165413664	9.97227E-06	8.40551E-05	--
Root vs Stem	RHSIM_Rhsim08G0227200	1.946339318	1.00404E-05	8.45981E-05	RHSIM_Rhsim08G0227200
Root vs Stem	novel.4830	-4.412637187	1.01042E-05	8.51047E-05	--
Root vs Stem	novel.2199	-2.342319549	1.01335E-05	8.53204E-05	--
Root vs Stem	RHSIM_Rhsim05G0007900	-3.01761534	1.01385E-05	8.53315E-05	RHSIM_Rhsim05G0007900
Root vs Stem	RHSIM_Rhsim04G0168900	-1.688691469	1.01595E-05	8.54766E-05	RHSIM_Rhsim04G0168900
Root vs Stem	RHSIM_Rhsim10G0056000	1.026744324	1.02436E-05	8.61495E-05	RHSIM_Rhsim10G0056000
Root vs Stem	RHSIM_Rhsim09G0151100	1.021817516	1.02469E-05	8.61495E-05	RHSIM_Rhsim09G0151100
Root vs Stem	novel.602	1.042936624	1.02758E-05	8.63606E-05	--
Root vs Stem	RHSIM_Rhsim05G0028200	1.029265103	1.02816E-05	8.63786E-05	RHSIM_Rhsim05G0028200
Root vs Stem	novel.5807	6.235934464	1.03829E-05	8.71661E-05	--
Root vs Stem	RHSIM_Rhsim13G0163200	1.369612023	1.0391E-05	8.72015E-05	RHSIM_Rhsim13G0163200
Root vs Stem	novel.39	-1.653378766	1.04206E-05	8.74187E-05	--
Root vs Stem	novel.4695	-1.124806508	1.04695E-05	8.77971E-05	--
Root vs Stem	novel.6200	2.421074285	1.0547E-05	8.84142E-05	--
Root vs Stem	RHSIM_Rhsim10G0084800	3.04361565	1.05595E-05	8.84873E-05	RHSIM_Rhsim10G0084800
Root vs Stem	RHSIM_Rhsim12G0025400	-3.058057152	1.0601E-05	8.88023E-05	RHSIM_Rhsim12G0025400

Comparison	Gene ID	log_2FC	P-value	FDR (padj)	Gene Name
Root vs Stem	RHSIM_Rhsim02G0053300	1.884956686	1.06369E-05	8.90714E-05	RHSIM_Rhsim02G0053300
Root vs Stem	RHSIM_Rhsim08G0186700	1.050354057	1.06504E-05	8.91518E-05	RHSIM_Rhsim08G0186700
Root vs Stem	RHSIM_Rhsim12G0129500	-1.448617429	1.08425E-05	9.06611E-05	RHSIM_Rhsim12G0129500
Root vs Stem	novel.4762	3.102477323	1.0917E-05	9.11844E-05	--
Root vs Stem	RHSIM_Rhsim04G0006200	-3.499664564	1.09321E-05	9.12778E-05	RHSIM_Rhsim04G0006200
Root vs Stem	RHSIM_Rhsim11G0132500	-1.647687396	1.09371E-05	9.12865E-05	RHSIM_Rhsim11G0132500
Root vs Stem	novel.5322	-1.002980201	1.09501E-05	9.1315E-05	--
Root vs Stem	RHSIM_Rhsim04G0051600	-1.74218532	1.09524E-05	9.1315E-05	RHSIM_Rhsim04G0051600
Root vs Stem	RHSIM_Rhsim10G0037300	1.763975404	1.1005E-05	9.17207E-05	RHSIM_Rhsim10G0037300
Root vs Stem	RHSIM_Rhsim02G0223100	5.610896474	1.10731E-05	9.22549E-05	RHSIM_Rhsim02G0223100
Root vs Stem	novel.5758	-1.230561345	1.11169E-05	9.25527E-05	--
Root vs Stem	RHSIM_Rhsim06G0057200	1.313403806	1.11793E-05	9.30045E-05	RHSIM_Rhsim06G0057200
Root vs Stem	RHSIM_Rhsim05G0209100	-6.260158331	1.12365E-05	9.34468E-05	RHSIM_Rhsim05G0209100
Root vs Stem	RHSIM_Rhsim08G0017000	4.098692638	1.12487E-05	9.35149E-05	RHSIM_Rhsim08G0017000
Root vs Stem	novel.1840	-1.523223952	1.12876E-05	9.38041E-05	--
Root vs Stem	RHSIM_Rhsim11G0182600	-2.037124597	1.141E-05	9.47659E-05	RHSIM_Rhsim11G0182600
Root vs Stem	RHSIM_RhsimUnG0251400	2.995965909	1.14115E-05	9.47659E-05	RHSIM_RhsimUnG0251400
Root vs Stem	RHSIM_Rhsim01G0198800	-3.827696274	1.14734E-05	9.5211E-05	RHSIM_Rhsim01G0198800
Root vs Stem	RHSIM_Rhsim02G0062000	1.378427953	1.15219E-05	9.55791E-05	RHSIM_Rhsim02G0062000
Root vs Stem	RHSIM_Rhsim03G0232000	-1.575805876	1.15395E-05	9.5691E-05	RHSIM_Rhsim03G0232000
Root vs Stem	RHSIM_Rhsim05G0133700	1.866026797	1.155E-05	9.57435E-05	RHSIM_Rhsim05G0133700
Root vs Stem	RHSIM_Rhsim04G0060800	1.484929519	1.15672E-05	9.5817E-05	RHSIM_Rhsim04G0060800
Root vs Stem	RHSIM_Rhsim01G0039400	2.131895525	1.16009E-05	9.60619E-05	RHSIM_Rhsim01G0039400
Root vs Stem	RHSIM_Rhsim04G0185100	4.903132059	1.16797E-05	9.66447E-05	RHSIM_Rhsim04G0185100
Root vs Stem	RHSIM_Rhsim11G0042100	2.260105147	1.17451E-05	9.7151E-05	RHSIM_Rhsim11G0042100

Comparison	Gene ID	log_2FC	P-value	FDR (padj)	Gene Name
Root vs Stem	RHSIM_Rhsim05G0226800	-2.502390915	1.18609E-05	9.79729E-05	RHSIM_Rhsim05G0226800
Root vs Stem	RHSIM_Rhsim13G0018700	-1.41484271	1.18721E-05	9.80252E-05	RHSIM_Rhsim13G0018700
Root vs Stem	RHSIM_Rhsim13G0125100	-1.02878846	1.18796E-05	9.80524E-05	RHSIM_Rhsim13G0125100
Root vs Stem	RHSIM_Rhsim05G0030000	2.368495622	1.19551E-05	9.864E-05	RHSIM_Rhsim05G0030000
Root vs Stem	RHSIM_Rhsim03G0076700	-1.959350596	1.20001E-05	9.89763E-05	RHSIM_Rhsim03G0076700
Root vs Stem	RHSIM_Rhsim02G0132500	-2.302444947	1.2033E-05	9.9212E-05	RHSIM_Rhsim02G0132500
Root vs Stem	novel.2578	-6.06101117	1.21905E-05	0.000100398	--
Root vs Stem	RHSIM_Rhsim05G0196500	-1.925719277	1.21942E-05	0.000100398	RHSIM_Rhsim05G0196500
Root vs Stem	novel.2829	4.20516497	1.22636E-05	0.000100933	--
Root vs Stem	RHSIM_Rhsim01G0181900	-6.218534824	1.22741E-05	0.000100971	RHSIM_Rhsim01G0181900
Root vs Stem	RHSIM_Rhsim13G0094500	-1.368004215	1.22792E-05	0.000100971	RHSIM_Rhsim13G0094500
Root vs Stem	RHSIM_Rhsim04G0136600	1.069051319	1.23507E-05	0.000101497	RHSIM_Rhsim04G0136600
Root vs Stem	RHSIM_Rhsim11G0190500	-1.305501583	1.23541E-05	0.000101497	RHSIM_Rhsim11G0190500
Root vs Stem	RHSIM_Rhsim01G0211100	1.254627217	1.25664E-05	0.000103096	RHSIM_Rhsim01G0211100
Root vs Stem	RHSIM_Rhsim03G0141100	-2.884432032	1.25667E-05	0.000103096	RHSIM_Rhsim03G0141100
Root vs Stem	novel.5150	1.838358766	1.25853E-05	0.000103212	--
Root vs Stem	RHSIM_Rhsim06G0069600	-2.753007802	1.26448E-05	0.000103658	RHSIM_Rhsim06G0069600
Root vs Stem	RHSIM_RhsimUnG0161400	3.016576778	1.26601E-05	0.000103711	RHSIM_RhsimUnG0161400
Root vs Stem	RHSIM_Rhsim05G0112000	-2.240117344	1.26642E-05	0.000103711	RHSIM_Rhsim05G0112000
Root vs Stem	RHSIM_Rhsim05G0126700	-1.0993047	1.26971E-05	0.000103944	RHSIM_Rhsim05G0126700
Root vs Stem	RHSIM_Rhsim05G0133000	-3.12680104	1.27181E-05	0.000104005	RHSIM_Rhsim05G0133000
Root vs Stem	RHSIM_Rhsim08G0088900	-2.048134816	1.27395E-05	0.000104143	RHSIM_Rhsim08G0088900
Root vs Stem	RHSIM_Rhsim08G0193000	4.158978141	1.27704E-05	0.000104281	RHSIM_Rhsim08G0193000
Root vs Stem	RHSIM_Rhsim01G0210100	-1.978317623	1.28662E-05	0.000104955	RHSIM_Rhsim01G0210100
Root vs Stem	RHSIM_Rhsim02G0182300	-1.051521327	1.29231E-05	0.000105326	RHSIM_Rhsim02G0182300

Comparison	Gene ID	log_2FC	P-value	FDR (padj)	Gene Name
Root vs Stem	RHSIM_Rhsim13G0069000	1.141224416	1.29253E-05	0.000105326	RHSIM_Rhsim13G0069000
Root vs Stem	novel.4643	-2.219980177	1.29878E-05	0.000105798	--
Root vs Stem	RHSIM_Rhsim03G0176200	-1.219435512	1.30044E-05	0.000105895	RHSIM_Rhsim03G0176200
Root vs Stem	RHSIM_Rhsim05G0177600	-2.55904672	1.31276E-05	0.000106823	RHSIM_Rhsim05G0177600
Root vs Stem	RHSIM_Rhsim12G0024600	-6.145636015	1.31682E-05	0.000107078	RHSIM_Rhsim12G0024600
Root vs Stem	RHSIM_Rhsim08G0050400	-2.089473332	1.31806E-05	0.000107141	RHSIM_Rhsim08G0050400
Root vs Stem	RHSIM_Rhsim11G0113500	-2.481695933	1.32153E-05	0.000107385	RHSIM_Rhsim11G0113500
Root vs Stem	novel.5214	2.357164409	1.32458E-05	0.000107596	--
Root vs Stem	RHSIM_Rhsim08G0083500	1.387118439	1.34421E-05	0.000109151	RHSIM_Rhsim08G0083500
Root vs Stem	RHSIM_Rhsim04G0229200	1.31520991	1.34524E-05	0.000109197	RHSIM_Rhsim04G0229200
Root vs Stem	novel.2411	-1.344646803	1.34944E-05	0.000109499	--
Root vs Stem	RHSIM_Rhsim01G0263800	-4.585942287	1.35721E-05	0.00011009	RHSIM_Rhsim01G0263800
Root vs Stem	RHSIM_Rhsim07G0139600	7.212242198	1.36297E-05	0.000110519	RHSIM_Rhsim07G0139600
Root vs Stem	RHSIM_Rhsim09G0114100	-1.749383309	1.37804E-05	0.000111653	RHSIM_Rhsim09G0114100
Root vs Stem	RHSIM_Rhsim03G0209000	1.602413918	1.37841E-05	0.000111653	RHSIM_Rhsim03G0209000
Root vs Stem	novel.1349	2.226637129	1.38319E-05	0.000111922	--
Root vs Stem	RHSIM_Rhsim05G0206900	1.073455567	1.38411E-05	0.000111957	RHSIM_Rhsim05G0206900
Root vs Stem	RHSIM_Rhsim07G0001300	-1.3772593	1.38617E-05	0.000112085	RHSIM_Rhsim07G0001300
Root vs Stem	RHSIM_Rhsim09G0155000	-2.131359093	1.40029E-05	0.000113186	RHSIM_Rhsim09G0155000
Root vs Stem	RHSIM_Rhsim07G0193700	-1.419675606	1.40807E-05	0.00011372	RHSIM_Rhsim07G0193700
Root vs Stem	RHSIM_Rhsim07G0222200	6.783631248	1.42589E-05	0.000115094	RHSIM_Rhsim07G0222200
Root vs Stem	RHSIM_Rhsim08G0069300	1.442903371	1.42845E-05	0.000115261	RHSIM_Rhsim08G0069300
Root vs Stem	RHSIM_Rhsim08G0074900	-6.932893345	1.44081E-05	0.000116177	RHSIM_Rhsim08G0074900
Root vs Stem	RHSIM_Rhsim12G0053200	-1.16789845	1.44361E-05	0.000116362	RHSIM_Rhsim12G0053200
Root vs Stem	novel.5994	-4.222035308	1.45131E-05	0.000116942	--

Comparison	Gene ID	log_2FC	P-value	FDR (padj)	Gene Name
Root vs Stem	novel.2764	1.070087248	1.45193E-05	0.000116951	--
Root vs Stem	novel.3091	-1.605996835	1.4606E-05	0.000117567	--
Root vs Stem	RHSIM_Rhsim02G0127800	-3.29875758	1.46421E-05	0.000117817	RHSIM_Rhsim02G0127800
Root vs Stem	RHSIM_Rhsim05G0059800	1.96775705	1.46502E-05	0.00011784	RHSIM_Rhsim05G0059800
Root vs Stem	RHSIM_Rhsim01G0285300	1.712888694	1.47007E-05	0.000118206	RHSIM_Rhsim01G0285300
Root vs Stem	RHSIM_Rhsim04G0130400	-1.535492552	1.47307E-05	0.000118406	RHSIM_Rhsim04G0130400
Root vs Stem	RHSIM_Rhsim03G0084700	1.952815104	1.48454E-05	0.000119244	RHSIM_Rhsim03G0084700
Root vs Stem	RHSIM_Rhsim06G0009900	-1.484859512	1.48906E-05	0.000119524	RHSIM_Rhsim06G0009900
Root vs Stem	RHSIM_Rhsim01G0012300	4.685491076	1.48968E-05	0.000119532	RHSIM_Rhsim01G0012300
Root vs Stem	RHSIM_Rhsim05G0206200	1.004382767	1.49313E-05	0.000119768	RHSIM_Rhsim05G0206200
Root vs Stem	novel.5763	6.985120454	1.49624E-05	0.000119975	--
Root vs Stem	RHSIM_Rhsim01G0264200	3.357620236	1.51316E-05	0.00012129	RHSIM_Rhsim01G0264200
Root vs Stem	RHSIM_Rhsim04G0226600	-1.129453468	1.51395E-05	0.000121311	RHSIM_Rhsim04G0226600
Root vs Stem	novel.1247	-1.555623154	1.52336E-05	0.00012198	--
Root vs Stem	RHSIM_Rhsim06G0068200	-2.274506028	1.52484E-05	0.000122056	RHSIM_Rhsim06G0068200
Root vs Stem	novel.1611	-1.946813473	1.52629E-05	0.000122087	--
Root vs Stem	RHSIM_Rhsim10G0076800	1.132563597	1.53067E-05	0.000122395	RHSIM_Rhsim10G0076800
Root vs Stem	RHSIM_Rhsim12G0072300	1.461027455	1.55032E-05	0.00012388	RHSIM_Rhsim12G0072300
Root vs Stem	RHSIM_Rhsim07G0104400	1.867857136	1.55237E-05	0.000124001	RHSIM_Rhsim07G0104400
Root vs Stem	RHSIM_Rhsim01G0126900	-1.117472579	1.55806E-05	0.000124413	RHSIM_Rhsim01G0126900
Root vs Stem	RHSIM_Rhsim02G0183500	1.05931371	1.56217E-05	0.000124698	RHSIM_Rhsim02G0183500
Root vs Stem	novel.1551	-5.1353446	1.57292E-05	0.000125512	--
Root vs Stem	novel.1213	3.358263253	1.58714E-05	0.00012656	--
Root vs Stem	novel.456	-3.688886612	1.59241E-05	0.000126936	--
Root vs Stem	RHSIM_Rhsim13G0101700	-1.14807162	1.61569E-05	0.000128747	RHSIM_Rhsim13G0101700

Comparison	Gene ID	log_2FC	P-value	FDR (padj)	Gene Name
Root vs Stem	RHSIM_Rhsim06G0085000	1.489458509	1.61685E-05	0.000128795	RHSIM_Rhsim06G0085000
Root vs Stem	RHSIM_Rhsim06G0230700	1.296225241	1.61828E-05	0.000128864	RHSIM_Rhsim06G0230700
Root vs Stem	RHSIM_Rhsim08G0045500	-3.287655207	1.62016E-05	0.00012897	RHSIM_Rhsim08G0045500
Root vs Stem	RHSIM_Rhsim10G0158500	7.458117093	1.62113E-05	0.000129002	RHSIM_Rhsim10G0158500
Root vs Stem	novel.3759	1.110368958	1.62626E-05	0.000129366	--
Root vs Stem	RHSIM_Rhsim08G0063600	-2.38776956	1.62852E-05	0.000129481	RHSIM_Rhsim08G0063600
Root vs Stem	novel.5316	5.620564486	1.62888E-05	0.000129481	--
Root vs Stem	novel.1903	-1.821821822	1.62939E-05	0.000129481	--
Root vs Stem	RHSIM_Rhsim06G0033300	-1.099822846	1.63104E-05	0.000129567	RHSIM_Rhsim06G0033300
Root vs Stem	RHSIM_Rhsim09G0004300	3.202141778	1.63229E-05	0.000129622	RHSIM_Rhsim09G0004300
Root vs Stem	RHSIM_Rhsim07G0225700	1.836950853	1.64366E-05	0.00013048	RHSIM_Rhsim07G0225700
Root vs Stem	RHSIM_Rhsim07G0117000	2.202072972	1.6476E-05	0.000130748	RHSIM_Rhsim07G0117000
Root vs Stem	RHSIM_Rhsim04G0227100	-8.362481823	1.64958E-05	0.000130859	RHSIM_Rhsim04G0227100
Root vs Stem	RHSIM_Rhsim06G0205000	6.318355898	1.65198E-05	0.000131005	RHSIM_Rhsim06G0205000
Root vs Stem	RHSIM_Rhsim01G0189200	1.024866056	1.65924E-05	0.000131488	RHSIM_Rhsim01G0189200
Root vs Stem	novel.3808	-2.109270743	1.67535E-05	0.000132676	--
Root vs Stem	RHSIM_Rhsim06G0028700	1.092164722	1.67794E-05	0.000132835	RHSIM_Rhsim06G0028700
Root vs Stem	RHSIM_Rhsim06G0145000	2.537491874	1.68242E-05	0.000133144	RHSIM_Rhsim06G0145000
Root vs Stem	RHSIM_RhsimUnG0024500	-7.538474745	1.69232E-05	0.000133789	RHSIM_RhsimUnG0024500
Root vs Stem	RHSIM_Rhsim04G0188300	-1.320647166	1.70136E-05	0.000134459	RHSIM_Rhsim04G0188300
Root vs Stem	RHSIM_Rhsim05G0070500	1.205713622	1.70961E-05	0.000135064	RHSIM_Rhsim05G0070500
Root vs Stem	RHSIM_Rhsim05G0056800	-1.365273045	1.7141E-05	0.000135337	RHSIM_Rhsim05G0056800
Root vs Stem	RHSIM_Rhsim13G0047200	1.585158712	1.71673E-05	0.000135487	RHSIM_Rhsim13G0047200
Root vs Stem	novel.3847	-2.07133002	1.72396E-05	0.000135924	--
Root vs Stem	RHSIM_Rhsim07G0132400	1.622691018	1.72403E-05	0.000135924	RHSIM_Rhsim07G0132400

Comparison	Gene ID	log$_2$FC	P-value	FDR (padj)	Gene Name
Root vs Stem	novel.3263	-5.589669707	1.72667E-05	0.000136085	--
Root vs Stem	RHSIM_Rhsim04G0022600	-1.426467361	1.73903E-05	0.000137013	RHSIM_Rhsim04G0022600
Root vs Stem	RHSIM_Rhsim03G0197000	1.124929962	1.75536E-05	0.000138166	RHSIM_Rhsim03G0197000
Root vs Stem	RHSIM_Rhsim13G0122400	-2.51035458	1.76144E-05	0.000138589	RHSIM_Rhsim13G0122400
Root vs Stem	RHSIM_Rhsim08G0181200	1.846742393	1.77958E-05	0.000139968	RHSIM_Rhsim08G0181200
Root vs Stem	RHSIM_RhsimUnG0133900	1.975083055	1.79576E-05	0.000141125	RHSIM_RhsimUnG0133900
Root vs Stem	RHSIM_Rhsim05G0206000	1.423298282	1.80635E-05	0.000141866	RHSIM_Rhsim05G0206000
Root vs Stem	RHSIM_Rhsim12G0186300	-1.81431884	1.80679E-05	0.000141866	RHSIM_Rhsim12G0186300
Root vs Stem	RHSIM_Rhsim05G0174500	-1.953289285	1.81233E-05	0.000142204	RHSIM_Rhsim05G0174500
Root vs Stem	RHSIM_Rhsim06G0095600	-1.463454398	1.82464E-05	0.000143024	RHSIM_Rhsim06G0095600
Root vs Stem	novel.3167	-4.789752336	1.83692E-05	0.000143938	--
Root vs Stem	RHSIM_Rhsim10G0134300	1.123239239	1.85126E-05	0.000144963	RHSIM_Rhsim10G0134300
Root vs Stem	RHSIM_Rhsim13G0112000	2.871719836	1.86412E-05	0.00014592	RHSIM_Rhsim13G0112000
Root vs Stem	RHSIM_Rhsim03G0004900	-1.044551403	1.8926E-05	0.000147987	RHSIM_Rhsim03G0004900
Root vs Stem	RHSIM_Rhsim06G0116500	-3.842429034	1.89309E-05	0.000147987	RHSIM_Rhsim06G0116500
Root vs Stem	RHSIM_Rhsim01G0207900	-7.798857515	1.89681E-05	0.000148228	RHSIM_Rhsim01G0207900
Root vs Stem	RHSIM_Rhsim10G0151000	-1.035720681	1.90684E-05	0.000148961	RHSIM_Rhsim10G0151000
Root vs Stem	RHSIM_Rhsim10G0133800	1.874029818	1.91492E-05	0.000149491	RHSIM_Rhsim10G0133800
Root vs Stem	RHSIM_Rhsim13G0022100	1.109908441	1.9162E-05	0.00014954	RHSIM_Rhsim13G0022100
Root vs Stem	RHSIM_Rhsim10G0134400	1.31398369	1.92416E-05	0.000150111	RHSIM_Rhsim10G0134400
Root vs Stem	RHSIM_Rhsim05G0192200	-4.044344249	1.93962E-05	0.000151214	RHSIM_Rhsim05G0192200
Root vs Stem	RHSIM_Rhsim03G0079600	-1.748659894	1.95223E-05	0.000152094	RHSIM_Rhsim03G0079600
Root vs Stem	novel.4119	3.504005008	1.96822E-05	0.000153289	--
Root vs Stem	RHSIM_Rhsim02G0216200	-1.077141055	1.97922E-05	0.000154093	RHSIM_Rhsim02G0216200
Root vs Stem	RHSIM_Rhsim13G0086500	2.615015276	1.98119E-05	0.000154194	RHSIM_Rhsim13G0086500

Comparison	Gene ID	log_2FC	P-value	FDR (padj)	Gene Name
Root vs Stem	RHSIM_Rhsim03G0017700	-1.380919421	1.9843E-05	0.000154384	RHSIM_Rhsim03G0017700
Root vs Stem	RHSIM_RhsimUnG0111000	2.397387145	1.99047E-05	0.000154812	RHSIM_RhsimUnG0111000
Root vs Stem	novel.1615	-1.196626587	2.00052E-05	0.000155349	--
Root vs Stem	RHSIM_Rhsim09G0065700	-8.135534412	2.00075E-05	0.000155349	RHSIM_Rhsim09G0065700
Root vs Stem	RHSIM_Rhsim07G0178400	2.978985194	2.00273E-05	0.000155399	RHSIM_Rhsim07G0178400
Root vs Stem	RHSIM_Rhsim08G0244600	1.819325052	2.00632E-05	0.000155572	RHSIM_Rhsim08G0244600
Root vs Stem	RHSIM_Rhsim02G0007300	2.744436696	2.01019E-05	0.000155777	RHSIM_Rhsim02G0007300
Root vs Stem	RHSIM_Rhsim04G0047400	-7.743260947	2.0103E-05	0.000155777	RHSIM_Rhsim04G0047400
Root vs Stem	RHSIM_Rhsim03G0142500	-1.692362981	2.0123E-05	0.000155879	RHSIM_Rhsim03G0142500
Root vs Stem	RHSIM_Rhsim06G0147400	-2.476759143	2.04058E-05	0.000157945	RHSIM_Rhsim06G0147400
Root vs Stem	RHSIM_RhsimUnG0209700	1.327980324	2.04102E-05	0.000157945	RHSIM_RhsimUnG0209700
Root vs Stem	RHSIM_Rhsim03G0127700	2.413403939	2.04405E-05	0.000158122	RHSIM_Rhsim03G0127700
Root vs Stem	RHSIM_Rhsim11G0055000	-6.69950787	2.04469E-05	0.000158122	RHSIM_Rhsim11G0055000
Root vs Stem	novel.2469	-2.343097632	2.04799E-05	0.0001583	--
Root vs Stem	RHSIM_Rhsim06G0108800	2.878338188	2.04836E-05	0.0001583	RHSIM_Rhsim06G0108800
Root vs Stem	RHSIM_RhsimUnG0216700	1.006419628	2.06635E-05	0.000159502	RHSIM_RhsimUnG0216700
Root vs Stem	RHSIM_Rhsim12G0140800	4.393176396	2.06707E-05	0.000159502	RHSIM_Rhsim12G0140800
Root vs Stem	RHSIM_Rhsim03G0199300	-2.743113076	2.06737E-05	0.000159502	RHSIM_Rhsim03G0199300
Root vs Stem	RHSIM_Rhsim03G0180900	-1.160829297	2.07589E-05	0.000160105	RHSIM_Rhsim03G0180900
Root vs Stem	RHSIM_Rhsim06G0027100	1.150558037	2.08397E-05	0.000160675	RHSIM_Rhsim06G0027100
Root vs Stem	RHSIM_Rhsim04G0165200	-1.164039227	2.09549E-05	0.000161509	RHSIM_Rhsim04G0165200
Root vs Stem	RHSIM_Rhsim02G0143800	-4.323155764	2.09709E-05	0.000161578	RHSIM_Rhsim02G0143800
Root vs Stem	novel.4044	-1.171502546	2.1015E-05	0.000161864	--
Root vs Stem	RHSIM_RhsimUnG0060900	-1.513676871	2.10649E-05	0.000162194	RHSIM_RhsimUnG0060900
Root vs Stem	RHSIM_Rhsim10G0010400	1.140167182	2.12449E-05	0.000163525	RHSIM_Rhsim10G0010400

Comparison	Gene ID	log_2FC	P-value	FDR (padj)	Gene Name
Root vs Stem	RHSIM_Rhsim07G0087400	-5.464343399	2.13643E-05	0.000164389	RHSIM_Rhsim07G0087400
Root vs Stem	RHSIM_Rhsim01G0289200	2.164049113	2.13983E-05	0.000164597	RHSIM_Rhsim01G0289200
Root vs Stem	RHSIM_Rhsim08G0106400	4.094858276	2.14731E-05	0.000165117	RHSIM_Rhsim08G0106400
Root vs Stem	RHSIM_Rhsim06G0131600	-1.747118648	2.15373E-05	0.000165555	RHSIM_Rhsim06G0131600
Root vs Stem	novel.3984	-1.051255837	2.15939E-05	0.000165935	--
Root vs Stem	novel.4766	1.367710711	2.16076E-05	0.000165985	--
Root vs Stem	RHSIM_Rhsim02G0111800	3.378325368	2.16491E-05	0.000166248	RHSIM_Rhsim02G0111800
Root vs Stem	RHSIM_Rhsim05G0188900	-6.281651704	2.1691E-05	0.000166515	RHSIM_Rhsim05G0188900
Root vs Stem	RHSIM_Rhsim12G0172700	-1.502095135	2.17616E-05	0.000167001	RHSIM_Rhsim12G0172700
Root vs Stem	RHSIM_Rhsim07G0120300	-2.723973624	2.17705E-05	0.000167013	RHSIM_Rhsim07G0120300
Root vs Stem	RHSIM_Rhsim09G0192400	-3.64219998	2.18468E-05	0.000167543	RHSIM_Rhsim09G0192400
Root vs Stem	novel.1453	-3.354189951	2.19351E-05	0.000168165	--
Root vs Stem	RHSIM_Rhsim01G0260900	1.304890012	2.20102E-05	0.000168684	RHSIM_Rhsim01G0260900
Root vs Stem	RHSIM_Rhsim11G0111300	-1.18373953	2.20578E-05	0.000168993	RHSIM_Rhsim11G0111300
Root vs Stem	RHSIM_Rhsim13G0216200	1.116438491	2.20821E-05	0.000169066	RHSIM_Rhsim13G0216200
Root vs Stem	RHSIM_Rhsim09G0167700	2.556604787	2.23929E-05	0.000171389	RHSIM_Rhsim09G0167700
Root vs Stem	RHSIM_Rhsim06G0140800	2.939501745	2.24642E-05	0.000171821	RHSIM_Rhsim06G0140800
Root vs Stem	RHSIM_Rhsim02G0214100	1.414187276	2.24999E-05	0.000172037	RHSIM_Rhsim02G0214100
Root vs Stem	novel.631	-2.463124138	2.25183E-05	0.00017212	--
Root vs Stem	RHSIM_Rhsim11G0093300	2.128566008	2.2817E-05	0.00017423	RHSIM_Rhsim11G0093300
Root vs Stem	RHSIM_Rhsim08G0174400	-1.332879424	2.30249E-05	0.000175536	RHSIM_Rhsim08G0174400
Root vs Stem	RHSIM_Rhsim12G0041100	1.143698982	2.30262E-05	0.000175536	RHSIM_Rhsim12G0041100
Root vs Stem	RHSIM_Rhsim06G0006600	1.86473936	2.35333E-05	0.000179047	RHSIM_Rhsim06G0006600
Root vs Stem	RHSIM_Rhsim07G0080300	-3.220022732	2.39572E-05	0.000182213	RHSIM_Rhsim07G0080300
Root vs Stem	RHSIM_Rhsim06G0230600	1.050259124	2.4286E-05	0.000184531	RHSIM_Rhsim06G0230600

Comparison	Gene ID	log_2FC	P-value	FDR (padj)	Gene Name
Root vs Stem	RHSIM_RhsimUnG0061000	6.315278257	2.42994E-05	0.000184571	RHSIM_RhsimUnG0061000
Root vs Stem	RHSIM_Rhsim04G0085700	-1.039678912	2.4369E-05	0.000184978	RHSIM_Rhsim04G0085700
Root vs Stem	RHSIM_Rhsim12G0171100	-2.218613035	2.448E-05	0.000185698	RHSIM_Rhsim12G0171100
Root vs Stem	RHSIM_Rhsim12G0212600	4.896548605	2.47218E-05	0.000187348	RHSIM_Rhsim12G0212600
Root vs Stem	RHSIM_Rhsim11G0101300	-2.472195016	2.47314E-05	0.000187358	RHSIM_Rhsim11G0101300
Root vs Stem	RHSIM_Rhsim02G0081500	-2.837792636	2.50654E-05	0.000189765	RHSIM_Rhsim02G0081500
Root vs Stem	RHSIM_Rhsim04G0018700	1.04876368	2.50814E-05	0.000189823	RHSIM_Rhsim04G0018700
Root vs Stem	RHSIM_Rhsim13G0054900	-1.480237157	2.51071E-05	0.000189937	RHSIM_Rhsim13G0054900
Root vs Stem	RHSIM_Rhsim01G0273600	-1.05720674	2.51129E-05	0.000189937	RHSIM_Rhsim01G0273600
Root vs Stem	RHSIM_Rhsim01G0108900	1.645517981	2.51515E-05	0.000190166	RHSIM_Rhsim01G0108900
Root vs Stem	RHSIM_Rhsim07G0226500	3.077264494	2.51656E-05	0.00019021	RHSIM_Rhsim07G0226500
Root vs Stem	RHSIM_Rhsim04G0187500	1.21310862	2.52188E-05	0.000190488	RHSIM_Rhsim04G0187500
Root vs Stem	RHSIM_Rhsim12G0177500	-1.043767276	2.5276E-05	0.000190816	RHSIM_Rhsim12G0177500
Root vs Stem	RHSIM_Rhsim03G0259600	1.945415469	2.52844E-05	0.000190816	RHSIM_Rhsim03G0259600
Root vs Stem	RHSIM_Rhsim10G0018800	2.018278641	2.5287E-05	0.000190816	RHSIM_Rhsim10G0018800
Root vs Stem	RHSIM_Rhsim01G0229600	-1.423352035	2.54655E-05	0.0001921	RHSIM_Rhsim01G0229600
Root vs Stem	RHSIM_Rhsim12G0085200	-1.159351955	2.55933E-05	0.000193001	RHSIM_Rhsim12G0085200
Root vs Stem	RHSIM_Rhsim04G0104400	1.248006974	2.56154E-05	0.000193104	RHSIM_Rhsim04G0104400
Root vs Stem	RHSIM_Rhsim05G0175900	3.24272824	2.56323E-05	0.000193168	RHSIM_Rhsim05G0175900
Root vs Stem	RHSIM_Rhsim09G0168700	1.161299257	2.56944E-05	0.000193573	RHSIM_Rhsim09G0168700
Root vs Stem	RHSIM_Rhsim06G0032200	-4.577032995	2.58022E-05	0.000194321	RHSIM_Rhsim06G0032200
Root vs Stem	novel.561	-1.356612735	2.59065E-05	0.000195043	--
Root vs Stem	novel.1181	-2.433946824	2.59567E-05	0.000195357	--
Root vs Stem	RHSIM_Rhsim12G0205600	2.736817798	2.60158E-05	0.000195738	RHSIM_Rhsim12G0205600
Root vs Stem	RHSIM_Rhsim07G0148200	1.197228515	2.6033E-05	0.000195804	RHSIM_Rhsim07G0148200

Comparison	Gene ID	log_2FC	P-value	FDR (padj)	Gene Name
Root vs Stem	RHSIM_Rhsim05G0148600	-2.413562715	2.60609E-05	0.000195852	RHSIM_Rhsim05G0148600
Root vs Stem	RHSIM_Rhsim08G0011700	-6.74105006	2.60648E-05	0.000195852	RHSIM_Rhsim08G0011700
Root vs Stem	novel.5882	6.138808781	2.6264E-05	0.000197191	--
Root vs Stem	novel.2116	-1.05704054	2.62688E-05	0.000197191	--
Root vs Stem	novel.5683	3.411545346	2.64096E-05	0.000198184	--
Root vs Stem	RHSIM_Rhsim01G0267100	5.86054635	2.64799E-05	0.000198646	RHSIM_Rhsim01G0267100
Root vs Stem	RHSIM_Rhsim03G0194300	2.432456797	2.65171E-05	0.000198861	RHSIM_Rhsim03G0194300
Root vs Stem	novel.1243	2.058874044	2.69162E-05	0.000201723	--
Root vs Stem	RHSIM_Rhsim01G0258300	-2.578585434	2.70001E-05	0.000202286	RHSIM_Rhsim01G0258300
Root vs Stem	RHSIM_Rhsim12G0038900	1.882786095	2.70199E-05	0.000202368	RHSIM_Rhsim12G0038900
Root vs Stem	RHSIM_Rhsim06G0109400	1.445828069	2.70825E-05	0.000202706	RHSIM_Rhsim06G0109400
Root vs Stem	RHSIM_Rhsim06G0005500	-1.603632958	2.7106E-05	0.000202786	RHSIM_Rhsim06G0005500
Root vs Stem	RHSIM_Rhsim10G0181500	4.061151237	2.71154E-05	0.000202786	RHSIM_Rhsim10G0181500
Root vs Stem	RHSIM_RhsimUnG0243800	-1.107553265	2.71543E-05	0.000202979	RHSIM_RhsimUnG0243800
Root vs Stem	novel.872	-7.719034145	2.74679E-05	0.000205058	--
Root vs Stem	novel.583	-3.418562514	2.74852E-05	0.00020512	--
Root vs Stem	novel.6194	-1.388794993	2.75398E-05	0.000205461	--
Root vs Stem	RHSIM_Rhsim10G0159000	1.402507667	2.75602E-05	0.000205547	RHSIM_Rhsim10G0159000
Root vs Stem	RHSIM_Rhsim06G0041600	-2.983824801	2.75901E-05	0.000205704	RHSIM_Rhsim06G0041600
Root vs Stem	RHSIM_RhsimUnG0070400	3.91308431	2.76002E-05	0.000205712	RHSIM_RhsimUnG0070400
Root vs Stem	novel.6005	-4.124732047	2.76537E-05	0.000206044	--
Root vs Stem	novel.2637	-7.546498368	2.78906E-05	0.000207541	--
Root vs Stem	RHSIM_Rhsim11G0103000	-6.382095761	2.7974E-05	0.000208095	RHSIM_Rhsim11G0103000
Root vs Stem	novel.4801	1.382713538	2.80391E-05	0.000208511	--
Root vs Stem	RHSIM_Rhsim10G0069900	6.664336133	2.81472E-05	0.000209247	RHSIM_Rhsim10G0069900

Comparison	Gene ID	log_2FC	P-value	FDR (padj)	Gene Name
Root vs Stem	RHSIM_Rhsim03G0053700	-3.694709839	2.84637E-05	0.000211465	RHSIM_Rhsim03G0053700
Root vs Stem	novel.3692	-1.323305186	2.84842E-05	0.000211549	--
Root vs Stem	RHSIM_Rhsim05G0047900	1.909624812	2.84939E-05	0.000211552	RHSIM_Rhsim05G0047900
Root vs Stem	novel.5349	1.346740454	2.85185E-05	0.000211667	--
Root vs Stem	RHSIM_Rhsim02G0167600	-1.333053132	2.86972E-05	0.00021291	RHSIM_Rhsim02G0167600
Root vs Stem	RHSIM_Rhsim01G0237700	3.958263254	2.87084E-05	0.00021291	RHSIM_Rhsim01G0237700
Root vs Stem	RHSIM_Rhsim11G0154300	2.631006296	2.88565E-05	0.000213831	RHSIM_Rhsim11G0154300
Root vs Stem	RHSIM_Rhsim01G0091100	1.454878674	2.88759E-05	0.000213907	RHSIM_Rhsim01G0091100
Root vs Stem	RHSIM_Rhsim08G0111000	6.437750972	2.89109E-05	0.000214097	RHSIM_Rhsim08G0111000
Root vs Stem	RHSIM_Rhsim09G0034200	3.718091521	2.89843E-05	0.000214503	RHSIM_Rhsim09G0034200
Root vs Stem	RHSIM_Rhsim08G0152400	1.805489311	2.90687E-05	0.000215058	RHSIM_Rhsim08G0152400
Root vs Stem	RHSIM_Rhsim08G0184600	3.755942031	2.90813E-05	0.000215082	RHSIM_Rhsim08G0184600
Root vs Stem	RHSIM_Rhsim13G0068900	-5.534900756	2.90962E-05	0.000215123	RHSIM_Rhsim13G0068900
Root vs Stem	RHSIM_Rhsim03G0218000	1.409140516	2.92195E-05	0.000215966	RHSIM_Rhsim03G0218000
Root vs Stem	RHSIM_Rhsim13G0166700	1.680872658	2.9336E-05	0.000216688	RHSIM_Rhsim13G0166700
Root vs Stem	RHSIM_Rhsim04G0181300	4.447700382	2.94864E-05	0.000217729	RHSIM_Rhsim04G0181300
Root vs Stem	RHSIM_Rhsim03G0225500	-1.229106887	2.9808E-05	0.000219822	RHSIM_Rhsim03G0225500
Root vs Stem	RHSIM_Rhsim04G0070000	-4.915963243	2.98533E-05	0.000220086	RHSIM_Rhsim04G0070000
Root vs Stem	RHSIM_RhsimUnG0249900	-6.520782021	2.99252E-05	0.000220546	RHSIM_RhsimUnG0249900
Root vs Stem	novel.3593	-1.52485914	2.99799E-05	0.000220878	--
Root vs Stem	RHSIM_Rhsim08G0051600	-5.111542674	3.01626E-05	0.000222153	RHSIM_Rhsim08G0051600
Root vs Stem	RHSIM_RhsimUnG0258800	-2.06987036	3.01879E-05	0.000222268	RHSIM_RhsimUnG0258800
Root vs Stem	RHSIM_RhsimUnG0070500	3.306080821	3.02608E-05	0.000222733	RHSIM_RhsimUnG0070500
Root vs Stem	RHSIM_Rhsim07G0049800	1.00838643	3.03448E-05	0.000223281	RHSIM_Rhsim07G0049800
Root vs Stem	RHSIM_Rhsim11G0134500	-2.028835962	3.06326E-05	0.000225183	RHSIM_Rhsim11G0134500

Comparison	Gene ID	log_2FC	P-value	FDR (padj)	Gene Name
Root vs Stem	novel.3243	6.568533385	3.08092E-05	0.000226386	--
Root vs Stem	RHSIM_Rhsim01G0073600	2.101146507	3.08159E-05	0.000226386	RHSIM_Rhsim01G0073600
Root vs Stem	RHSIM_Rhsim05G0060400	-4.207117314	3.08745E-05	0.000226744	RHSIM_Rhsim05G0060400
Root vs Stem	RHSIM_Rhsim04G0014200	-2.341362934	3.11823E-05	0.000228932	RHSIM_Rhsim04G0014200
Root vs Stem	novel.2080	-1.045232855	3.12784E-05	0.000229564	--
Root vs Stem	RHSIM_Rhsim11G0134600	-1.455895172	3.16102E-05	0.000231778	RHSIM_Rhsim11G0134600
Root vs Stem	RHSIM_Rhsim08G0008900	-3.174002008	3.1647E-05	0.000231953	RHSIM_Rhsim08G0008900
Root vs Stem	RHSIM_Rhsim13G0123700	2.492576207	3.20354E-05	0.000234523	RHSIM_Rhsim13G0123700
Root vs Stem	RHSIM_Rhsim06G0069800	2.681778127	3.21059E-05	0.000234964	RHSIM_Rhsim06G0069800
Root vs Stem	RHSIM_Rhsim01G0072500	-2.610307742	3.22989E-05	0.000236212	RHSIM_Rhsim01G0072500
Root vs Stem	RHSIM_Rhsim04G0161100	3.76045893	3.23071E-05	0.000236212	RHSIM_Rhsim04G0161100
Root vs Stem	RHSIM_Rhsim05G0078400	-2.135505501	3.23299E-05	0.000236303	RHSIM_Rhsim05G0078400
Root vs Stem	novel.3925	-1.681018766	3.26395E-05	0.000238491	--
Root vs Stem	novel.4984	-7.742973315	3.26641E-05	0.000238595	--
Root vs Stem	RHSIM_RhsimUnG0188700	4.416732537	3.27184E-05	0.00023884	RHSIM_RhsimUnG0188700
Root vs Stem	RHSIM_Rhsim12G0036800	-2.507948383	3.27438E-05	0.00023895	RHSIM_Rhsim12G0036800
Root vs Stem	novel.2348	-1.39528574	3.29E-05	0.000240013	--
Root vs Stem	RHSIM_Rhsim12G0209800	1.445206454	3.29316E-05	0.000240168	RHSIM_Rhsim12G0209800
Root vs Stem	RHSIM_Rhsim05G0223000	5.297471131	3.3045E-05	0.000240691	RHSIM_Rhsim05G0223000
Root vs Stem	novel.1754	-4.64206324	3.32358E-05	0.000241969	--
Root vs Stem	RHSIM_Rhsim02G0115400	-1.044205229	3.33336E-05	0.000242486	RHSIM_Rhsim02G0115400
Root vs Stem	novel.630	-3.358810718	3.3357E-05	0.00024258	--
Root vs Stem	RHSIM_Rhsim05G0189700	-2.184668742	3.37724E-05	0.000245369	RHSIM_Rhsim05G0189700
Root vs Stem	RHSIM_Rhsim07G0249000	6.69509757	3.39364E-05	0.000246482	RHSIM_Rhsim07G0249000
Root vs Stem	RHSIM_Rhsim10G0148000	1.05979082	3.41852E-05	0.000248055	RHSIM_Rhsim10G0148000

Comparison	Gene ID	log_2FC	P-value	FDR (padj)	Gene Name
Root vs Stem	RHSIM_Rhsim13G0211700	-1.856099899	3.42532E-05	0.000248471	RHSIM_Rhsim13G0211700
Root vs Stem	RHSIM_Rhsim10G0045300	1.588204753	3.43221E-05	0.000248892	RHSIM_Rhsim10G0045300
Root vs Stem	novel.2819	4.237247006	3.4337E-05	0.000248922	--
Root vs Stem	novel.733	4.622057531	3.46411E-05	0.000250889	--
Root vs Stem	RHSIM_Rhsim07G0223300	2.41591741	3.46794E-05	0.000251088	RHSIM_Rhsim07G0223300
Root vs Stem	RHSIM_Rhsim13G0102500	-2.146605235	3.47607E-05	0.000251597	RHSIM_Rhsim13G0102500
Root vs Stem	RHSIM_Rhsim03G0173400	-1.7819066	3.47728E-05	0.000251606	RHSIM_Rhsim03G0173400
Root vs Stem	RHSIM_Rhsim11G0050200	4.217605664	3.51049E-05	0.000253877	RHSIM_Rhsim11G0050200
Root vs Stem	RHSIM_Rhsim12G0209900	1.693864406	3.51087E-05	0.000253877	RHSIM_Rhsim12G0209900
Root vs Stem	RHSIM_Rhsim13G0229500	-1.285896767	3.51554E-05	0.000254135	RHSIM_Rhsim13G0229500
Root vs Stem	RHSIM_Rhsim12G0203900	3.998490279	3.53641E-05	0.000255564	RHSIM_Rhsim12G0203900
Root vs Stem	RHSIM_Rhsim02G0093900	-1.291130588	3.54081E-05	0.000255801	RHSIM_Rhsim02G0093900
Root vs Stem	RHSIM_RhsimUnG0201700	-3.61496256	3.55676E-05	0.000256873	RHSIM_RhsimUnG0201700
Root vs Stem	RHSIM_Rhsim04G0115900	-1.752372931	3.56596E-05	0.000257457	RHSIM_Rhsim04G0115900
Root vs Stem	RHSIM_Rhsim04G0135400	5.724418914	3.58868E-05	0.000259016	RHSIM_Rhsim04G0135400
Root vs Stem	RHSIM_Rhsim13G0068600	-3.369520228	3.59627E-05	0.000259483	RHSIM_Rhsim13G0068600
Root vs Stem	novel.3642	-2.500353155	3.62333E-05	0.000261027	--
Root vs Stem	RHSIM_Rhsim08G0105100	3.951479051	3.64391E-05	0.000262427	RHSIM_Rhsim08G0105100
Root vs Stem	RHSIM_Rhsim13G0148600	-1.196020485	3.64518E-05	0.000262437	RHSIM_Rhsim13G0148600
Root vs Stem	RHSIM_Rhsim08G0234300	1.211765459	3.65239E-05	0.000262874	RHSIM_Rhsim08G0234300
Root vs Stem	RHSIM_Rhsim04G0220900	-2.663072073	3.65373E-05	0.000262881	RHSIM_Rhsim04G0220900
Root vs Stem	RHSIM_Rhsim10G0003000	-1.060341326	3.6618E-05	0.000263305	RHSIM_Rhsim10G0003000
Root vs Stem	RHSIM_Rhsim10G0033200	-4.149863748	3.67549E-05	0.000264207	RHSIM_Rhsim10G0033200
Root vs Stem	novel.5627	4.484619458	3.68226E-05	0.000264611	--
Root vs Stem	RHSIM_Rhsim10G0198900	1.506807968	3.70452E-05	0.000266128	RHSIM_Rhsim10G0198900

Comparison	Gene ID	log_2FC	P-value	FDR (padj)	Gene Name
Root vs Stem	RHSIM_Rhsim09G0068400	-1.239186779	3.71835E-05	0.000267038	RHSIM_Rhsim09G0068400
Root vs Stem	novel.809	2.450176398	3.75519E-05	0.000269599	--
Root vs Stem	RHSIM_Rhsim04G0086500	2.38321754	3.75674E-05	0.000269621	RHSIM_Rhsim04G0086500
Root vs Stem	RHSIM_Rhsim13G0170200	3.25435355	3.75802E-05	0.000269621	RHSIM_Rhsim13G0170200
Root vs Stem	RHSIM_Rhsim06G0017800	2.688867309	3.76017E-05	0.000269621	RHSIM_Rhsim06G0017800
Root vs Stem	RHSIM_Rhsim13G0196600	4.560323423	3.76208E-05	0.000269674	RHSIM_Rhsim13G0196600
Root vs Stem	RHSIM_RhsimUnG0209900	3.876052774	3.81135E-05	0.000273036	RHSIM_RhsimUnG0209900
Root vs Stem	RHSIM_Rhsim04G0221000	2.897481045	3.8448E-05	0.000275091	RHSIM_Rhsim04G0221000
Root vs Stem	novel.5394	-2.358387129	3.85354E-05	0.000275604	--
Root vs Stem	RHSIM_Rhsim11G0144500	-4.504728063	3.85436E-05	0.000275604	RHSIM_Rhsim11G0144500
Root vs Stem	novel.5055	-1.501510963	3.85746E-05	0.00027574	--
Root vs Stem	RHSIM_RhsimUnG0010800	7.038601217	3.86733E-05	0.000276274	RHSIM_RhsimUnG0010800
Root vs Stem	RHSIM_Rhsim01G0102200	-11.74593114	3.88257E-05	0.000277276	RHSIM_Rhsim01G0102200
Root vs Stem	RHSIM_Rhsim03G0039300	-2.2043329	3.89392E-05	0.000277915	RHSIM_Rhsim03G0039300
Root vs Stem	RHSIM_Rhsim01G0007400	-6.555625107	3.93237E-05	0.000280485	RHSIM_Rhsim01G0007400
Root vs Stem	RHSIM_Rhsim05G0098000	1.557110517	3.94136E-05	0.00028104	RHSIM_Rhsim05G0098000
Root vs Stem	RHSIM_Rhsim09G0043300	1.186581627	3.95098E-05	0.000281639	RHSIM_Rhsim09G0043300
Root vs Stem	RHSIM_Rhsim02G0069500	-5.874701783	3.97401E-05	0.000283193	RHSIM_Rhsim02G0069500
Root vs Stem	RHSIM_Rhsim05G0013800	-1.300836746	3.97814E-05	0.0002834	RHSIM_Rhsim05G0013800
Root vs Stem	RHSIM_Rhsim02G0209400	4.614887662	3.99693E-05	0.000284562	RHSIM_Rhsim02G0209400
Root vs Stem	RHSIM_Rhsim03G0013100	1.67241411	4.05054E-05	0.00028829	RHSIM_Rhsim03G0013100
Root vs Stem	RHSIM_RhsimUnG0213800	-2.292933463	4.06243E-05	0.000289047	RHSIM_RhsimUnG0213800
Root vs Stem	novel.3724	1.152922187	4.06467E-05	0.000289062	--
Root vs Stem	novel.4159	-6.301280973	4.06515E-05	0.000289062	--
Root vs Stem	novel.3606	-5.954021716	4.124E-05	0.000292885	--

Comparison	Gene ID	log_2FC	P-value	FDR (padj)	Gene Name
Root vs Stem	RHSIM_Rhsim10G0005100	1.847733034	4.1308E-05	0.000293278	RHSIM_Rhsim10G0005100
Root vs Stem	RHSIM_Rhsim08G0024500	-2.854775265	4.14491E-05	0.000294189	RHSIM_Rhsim08G0024500
Root vs Stem	RHSIM_Rhsim13G0147500	-1.752404895	4.15081E-05	0.000294517	RHSIM_Rhsim13G0147500
Root vs Stem	RHSIM_Rhsim04G0087600	-2.635744357	4.1776E-05	0.000296236	RHSIM_Rhsim04G0087600
Root vs Stem	RHSIM_Rhsim01G0092200	-1.54259352	4.22199E-05	0.000299199	RHSIM_Rhsim01G0092200
Root vs Stem	RHSIM_Rhsim09G0054100	-1.946423042	4.2261E-05	0.000299398	RHSIM_Rhsim09G0054100
Root vs Stem	RHSIM_Rhsim04G0058500	-1.022197565	4.23251E-05	0.000299755	RHSIM_Rhsim04G0058500
Root vs Stem	RHSIM_Rhsim07G0050500	-2.019410901	4.23373E-05	0.000299755	RHSIM_Rhsim07G0050500
Root vs Stem	novel.2368	-1.224832222	4.24044E-05	0.000300088	--
Root vs Stem	RHSIM_RhsimUnG0185700	-1.120762709	4.24104E-05	0.000300088	RHSIM_RhsimUnG0185700
Root vs Stem	RHSIM_Rhsim08G0102800	1.833147807	4.26162E-05	0.000301267	RHSIM_Rhsim08G0102800
Root vs Stem	RHSIM_Rhsim13G0036900	-1.032463647	4.28307E-05	0.000302583	RHSIM_Rhsim13G0036900
Root vs Stem	RHSIM_Rhsim12G0162500	1.25027231	4.28679E-05	0.000302583	RHSIM_Rhsim12G0162500
Root vs Stem	RHSIM_Rhsim07G0092600	3.505923799	4.31023E-05	0.000304051	RHSIM_Rhsim07G0092600
Root vs Stem	RHSIM_Rhsim05G0209800	-4.794502353	4.34964E-05	0.000306549	RHSIM_Rhsim05G0209800
Root vs Stem	RHSIM_Rhsim07G0081200	2.213541374	4.36114E-05	0.000307266	RHSIM_Rhsim07G0081200
Root vs Stem	RHSIM_Rhsim08G0227700	-1.288605123	4.36733E-05	0.000307514	RHSIM_Rhsim08G0227700
Root vs Stem	novel.1577	-1.172302102	4.37856E-05	0.00030821	--
Root vs Stem	RHSIM_Rhsim09G0196700	-2.173187716	4.3882E-05	0.000308795	RHSIM_Rhsim09G0196700
Root vs Stem	novel.26	1.996220032	4.4086E-05	0.000310135	--
Root vs Stem	novel.5678	-3.035112545	4.42198E-05	0.000310982	--
Root vs Stem	novel.2205	-5.118928313	4.43571E-05	0.000311852	--
Root vs Stem	RHSIM_Rhsim05G0058400	5.213775554	4.4449E-05	0.000312403	RHSIM_Rhsim05G0058400
Root vs Stem	RHSIM_Rhsim08G0005000	-1.068451414	4.47592E-05	0.000314296	RHSIM_Rhsim08G0005000
Root vs Stem	RHSIM_Rhsim03G0159300	-3.122666396	4.51424E-05	0.000316601	RHSIM_Rhsim03G0159300

Comparison	Gene ID	log_2FC	P-value	FDR (padj)	Gene Name
Root vs Stem	RHSIM_Rhsim11G0128100	-2.936062873	4.54524E-05	0.000318444	RHSIM_Rhsim11G0128100
Root vs Stem	RHSIM_Rhsim05G0103600	6.96485055	4.55963E-05	0.000319202	RHSIM_Rhsim05G0103600
Root vs Stem	novel.2325	-1.923376737	4.57778E-05	0.000320369	--
Root vs Stem	RHSIM_Rhsim05G0224400	-1.591191135	4.59553E-05	0.000321422	RHSIM_Rhsim05G0224400
Root vs Stem	RHSIM_Rhsim13G0231400	4.411006649	4.6015E-05	0.000321742	RHSIM_Rhsim13G0231400
Root vs Stem	RHSIM_Rhsim05G0160000	-4.680358489	4.60499E-05	0.000321889	RHSIM_Rhsim05G0160000
Root vs Stem	RHSIM_Rhsim03G0201100	-1.166600272	4.61276E-05	0.000322237	RHSIM_Rhsim03G0201100
Root vs Stem	novel.4535	1.08987959	4.63074E-05	0.000323003	--
Root vs Stem	RHSIM_Rhsim08G0246300	1.615300181	4.63646E-05	0.000323304	RHSIM_Rhsim08G0246300
Root vs Stem	RHSIM_Rhsim12G0035100	-2.025074033	4.64579E-05	0.000323856	RHSIM_Rhsim12G0035100
Root vs Stem	RHSIM_Rhsim03G0143700	1.135803042	4.66022E-05	0.000324764	RHSIM_Rhsim03G0143700
Root vs Stem	novel.1822	-2.408590738	4.70308E-05	0.000327652	--
Root vs Stem	RHSIM_Rhsim11G0110100	2.410051205	4.73234E-05	0.000329591	RHSIM_Rhsim11G0110100
Root vs Stem	RHSIM_Rhsim07G0027000	-1.112936627	4.74774E-05	0.000330564	RHSIM_Rhsim07G0027000
Root vs Stem	RHSIM_Rhsim03G0099000	1.535338963	4.75484E-05	0.000330958	RHSIM_Rhsim03G0099000
Root vs Stem	RHSIM_Rhsim05G0008600	-3.058062422	4.75707E-05	0.000331014	RHSIM_Rhsim05G0008600
Root vs Stem	novel.731	-1.62852436	4.79206E-05	0.000333247	--
Root vs Stem	RHSIM_Rhsim13G0011200	2.159467207	4.79736E-05	0.000333415	RHSIM_Rhsim13G0011200
Root vs Stem	RHSIM_RhsimUnG0110300	4.25305956	4.79736E-05	0.000333415	RHSIM_RhsimUnG0110300
Root vs Stem	RHSIM_Rhsim06G0148400	-1.562515203	4.8183E-05	0.000334668	RHSIM_Rhsim06G0148400
Root vs Stem	RHSIM_Rhsim10G0180600	-1.015264855	4.83451E-05	0.000335693	RHSIM_Rhsim10G0180600
Root vs Stem	RHSIM_Rhsim04G0213100	-2.329855438	4.83872E-05	0.000335884	RHSIM_Rhsim04G0213100
Root vs Stem	RHSIM_Rhsim08G0050100	-1.157694849	4.85152E-05	0.00033657	RHSIM_Rhsim08G0050100
Root vs Stem	RHSIM_Rhsim03G0031500	-1.4918303	4.86875E-05	0.000337664	RHSIM_Rhsim03G0031500
Root vs Stem	RHSIM_RhsimUnG0238300	-4.432777553	4.87151E-05	0.000337753	RHSIM_RhsimUnG0238300

Comparison	Gene ID	log_2FC	P-value	FDR (padj)	Gene Name
Root vs Stem	novel.2828	-2.062037449	4.90056E-05	0.000339563	--
Root vs Stem	RHSIM_Rhsim08G0073700	-1.306461076	4.95635E-05	0.000343119	RHSIM_Rhsim08G0073700
Root vs Stem	novel.1483	-1.275904507	4.97809E-05	0.000344417	--
Root vs Stem	novel.2981	-5.873320554	4.9988E-05	0.000345746	--
Root vs Stem	RHSIM_Rhsim09G0117500	1.111197242	5.00295E-05	0.00034593	RHSIM_Rhsim09G0117500
Root vs Stem	RHSIM_Rhsim07G0036000	-1.157768639	5.03677E-05	0.000348164	RHSIM_Rhsim07G0036000
Root vs Stem	RHSIM_Rhsim01G0265500	2.231561257	5.04868E-05	0.000348883	RHSIM_Rhsim01G0265500
Root vs Stem	novel.5769	-6.865957303	5.06848E-05	0.000350041	--
Root vs Stem	RHSIM_Rhsim13G0015700	-1.117721643	5.10315E-05	0.000352224	RHSIM_Rhsim13G0015700
Root vs Stem	RHSIM_Rhsim06G0095100	1.013755433	5.11474E-05	0.000352918	RHSIM_Rhsim06G0095100
Root vs Stem	RHSIM_Rhsim03G0270700	2.493304704	5.14651E-05	0.000355005	RHSIM_Rhsim03G0270700
Root vs Stem	novel.49	-10.34159434	5.16651E-05	0.000356065	--
Root vs Stem	novel.3533	-8.184601618	5.17131E-05	0.000356289	--
Root vs Stem	novel.886	3.857379804	5.17816E-05	0.000356654	--
Root vs Stem	RHSIM_Rhsim01G0217600	1.090424498	5.19512E-05	0.000357609	RHSIM_Rhsim01G0217600
Root vs Stem	RHSIM_Rhsim08G0159100	1.359801303	5.21149E-05	0.000358628	RHSIM_Rhsim08G0159100
Root vs Stem	RHSIM_Rhsim06G0030000	-3.52399735	5.22637E-05	0.000359438	RHSIM_Rhsim06G0030000
Root vs Stem	RHSIM_Rhsim10G0161300	-1.119547286	5.27823E-05	0.000362896	RHSIM_Rhsim10G0161300
Root vs Stem	RHSIM_Rhsim02G0229000	1.25677318	5.29264E-05	0.000363561	RHSIM_Rhsim02G0229000
Root vs Stem	novel.3793	2.146846361	5.3265E-05	0.00036556	--
Root vs Stem	RHSIM_Rhsim10G0111200	6.434526836	5.34551E-05	0.000366647	RHSIM_Rhsim10G0111200
Root vs Stem	novel.692	-7.467037246	5.36917E-05	0.00036816	--
Root vs Stem	RHSIM_Rhsim12G0203600	-5.139075878	5.38821E-05	0.000369356	RHSIM_Rhsim12G0203600
Root vs Stem	RHSIM_Rhsim07G0057900	2.299865777	5.5157E-05	0.000377534	RHSIM_Rhsim07G0057900
Root vs Stem	RHSIM_Rhsim06G0202500	-1.049477275	5.52211E-05	0.000377749	RHSIM_Rhsim06G0202500

Comparison	Gene ID	log_2FC	P-value	FDR (padj)	Gene Name
Root vs Stem	RHSIM_Rhsim07G0199000	-1.343417418	5.55496E-05	0.000379883	RHSIM_Rhsim07G0199000
Root vs Stem	RHSIM_Rhsim02G0025500	-2.43863186	5.59083E-05	0.000382081	RHSIM_Rhsim02G0025500
Root vs Stem	RHSIM_Rhsim04G0186700	1.418655803	5.59208E-05	0.000382081	RHSIM_Rhsim04G0186700
Root vs Stem	RHSIM_Rhsim06G0178400	-1.045183298	5.65509E-05	0.000386158	RHSIM_Rhsim06G0178400
Root vs Stem	novel.6130	4.897712739	5.65772E-05	0.000386223	--
Root vs Stem	RHSIM_Rhsim01G0034400	1.9526344	5.69521E-05	0.000388572	RHSIM_Rhsim01G0034400
Root vs Stem	novel.1340	2.283098947	5.69551E-05	0.000388572	--
Root vs Stem	RHSIM_Rhsim08G0175100	-3.333289617	5.73287E-05	0.000390774	RHSIM_Rhsim08G0175100
Root vs Stem	RHSIM_Rhsim10G0068500	-2.032659724	5.75836E-05	0.00039228	RHSIM_Rhsim10G0068500
Root vs Stem	RHSIM_Rhsim09G0110500	2.943608298	5.78375E-05	0.00039366	RHSIM_Rhsim09G0110500
Root vs Stem	RHSIM_Rhsim06G0101800	1.40432661	5.78768E-05	0.000393811	RHSIM_Rhsim06G0101800
Root vs Stem	RHSIM_Rhsim04G0026900	6.335221395	5.82109E-05	0.000395852	RHSIM_Rhsim04G0026900
Root vs Stem	RHSIM_Rhsim01G0284300	-2.341657104	5.8356E-05	0.000396721	RHSIM_Rhsim01G0284300
Root vs Stem	novel.2889	-1.43611714	5.9024E-05	0.000401108	--
Root vs Stem	RHSIM_Rhsim05G0203800	1.767375706	5.90361E-05	0.000401108	RHSIM_Rhsim05G0203800
Root vs Stem	RHSIM_Rhsim11G0174600	2.047749839	5.91783E-05	0.000401955	RHSIM_Rhsim11G0174600
Root vs Stem	RHSIM_Rhsim03G0122900	-12.38604147	5.93338E-05	0.000402893	RHSIM_Rhsim03G0122900
Root vs Stem	novel.5473	-6.527770599	5.96937E-05	0.000404979	--
Root vs Stem	RHSIM_Rhsim07G0187400	-8.492592054	6.0046E-05	0.00040689	RHSIM_Rhsim07G0187400
Root vs Stem	RHSIM_Rhsim10G0155200	-1.739516816	6.00716E-05	0.000406918	RHSIM_Rhsim10G0155200
Root vs Stem	RHSIM_Rhsim12G0144500	-1.548531017	6.00854E-05	0.000406918	RHSIM_Rhsim12G0144500
Root vs Stem	RHSIM_Rhsim01G0251100	2.242799938	6.01204E-05	0.000407036	RHSIM_Rhsim01G0251100
Root vs Stem	novel.3445	1.119117771	6.03195E-05	0.000408263	--
Root vs Stem	RHSIM_Rhsim09G0114800	-1.136232547	6.04465E-05	0.000409003	RHSIM_Rhsim09G0114800
Root vs Stem	RHSIM_Rhsim13G0093600	2.556454984	6.05481E-05	0.000409571	RHSIM_Rhsim13G0093600

Comparison	Gene ID	log_2FC	P-value	FDR (padj)	Gene Name
Root vs Stem	RHSIM_Rhsim03G0029800	1.144496559	6.05888E-05	0.000409725	RHSIM_Rhsim03G0029800
Root vs Stem	RHSIM_Rhsim03G0255900	-1.571431426	6.08619E-05	0.000411452	RHSIM_Rhsim03G0255900
Root vs Stem	novel.3583	-2.512129207	6.12003E-05	0.000413618	--
Root vs Stem	RHSIM_Rhsim11G0051900	-1.538812832	6.15617E-05	0.000415939	RHSIM_Rhsim11G0051900
Root vs Stem	novel.4255	-2.536222906	6.20772E-05	0.000419195	--
Root vs Stem	RHSIM_Rhsim01G0249700	4.576739407	6.22611E-05	0.000420048	RHSIM_Rhsim01G0249700
Root vs Stem	RHSIM_Rhsim07G0009500	-7.854618644	6.2418E-05	0.00042086	RHSIM_Rhsim07G0009500
Root vs Stem	RHSIM_Rhsim03G0010300	6.41194021	6.25065E-05	0.000421211	RHSIM_Rhsim03G0010300
Root vs Stem	novel.4386	4.118581445	6.27001E-05	0.000422268	--
Root vs Stem	novel.4965	-2.52035768	6.28207E-05	0.000422834	--
Root vs Stem	RHSIM_Rhsim13G0166000	2.131228426	6.29319E-05	0.000423459	RHSIM_Rhsim13G0166000
Root vs Stem	RHSIM_Rhsim05G0101300	-2.436005476	6.29616E-05	0.000423535	RHSIM_Rhsim05G0101300
Root vs Stem	RHSIM_Rhsim01G0034300	2.210938561	6.31933E-05	0.000424969	RHSIM_Rhsim01G0034300
Root vs Stem	RHSIM_Rhsim09G0202500	1.870479928	6.38626E-05	0.000429095	RHSIM_Rhsim09G0202500
Root vs Stem	RHSIM_Rhsim07G0034100	1.00203822	6.39402E-05	0.000429491	RHSIM_Rhsim07G0034100
Root vs Stem	RHSIM_Rhsim09G0014700	-2.683549492	6.39797E-05	0.000429632	RHSIM_Rhsim09G0014700
Root vs Stem	RHSIM_Rhsim03G0105800	5.724342948	6.44828E-05	0.000432632	RHSIM_Rhsim03G0105800
Root vs Stem	novel.5213	2.677555843	6.46085E-05	0.000433349	--
Root vs Stem	RHSIM_Rhsim06G0066800	-1.430538974	6.47236E-05	0.000433995	RHSIM_Rhsim06G0066800
Root vs Stem	RHSIM_Rhsim09G0079600	-2.801530739	6.50449E-05	0.000436022	RHSIM_Rhsim09G0079600
Root vs Stem	RHSIM_Rhsim10G0144200	-8.095711309	6.52226E-05	0.000437086	RHSIM_Rhsim10G0144200
Root vs Stem	RHSIM_Rhsim09G0083700	-7.09037001	6.53162E-05	0.000437586	RHSIM_Rhsim09G0083700
Root vs Stem	RHSIM_RhsimUnG0188500	-5.336499821	6.63403E-05	0.000443802	RHSIM_RhsimUnG0188500
Root vs Stem	RHSIM_Rhsim01G0006000	5.897130456	6.67053E-05	0.000445985	RHSIM_Rhsim01G0006000
Root vs Stem	RHSIM_Rhsim03G0008300	-1.390353046	6.69472E-05	0.000447473	RHSIM_Rhsim03G0008300

Comparison	Gene ID	log_2FC	P-value	FDR (padj)	Gene Name
Root vs Stem	RHSIM_Rhsim09G0114200	-2.417523554	6.71778E-05	0.000448884	RHSIM_Rhsim09G0114200
Root vs Stem	RHSIM_Rhsim10G0176600	-1.446466134	6.7322E-05	0.000449717	RHSIM_Rhsim10G0176600
Root vs Stem	RHSIM_Rhsim01G0021100	1.298317743	6.7376E-05	0.000449948	RHSIM_Rhsim01G0021100
Root vs Stem	RHSIM_RhsimUnG0089300	-2.941374678	6.76402E-05	0.000451581	RHSIM_RhsimUnG0089300
Root vs Stem	RHSIM_Rhsim08G0180600	1.125889853	6.79445E-05	0.00045335	RHSIM_Rhsim08G0180600
Root vs Stem	RHSIM_Rhsim04G0099400	1.495884582	6.80796E-05	0.000453989	RHSIM_Rhsim04G0099400
Root vs Stem	RHSIM_Rhsim10G0135400	-5.257469702	6.81844E-05	0.000454556	RHSIM_Rhsim10G0135400
Root vs Stem	RHSIM_Rhsim06G0213000	4.049214594	6.83132E-05	0.000455152	RHSIM_Rhsim06G0213000
Root vs Stem	novel.1537	-3.876404043	6.84782E-05	0.000455987	--
Root vs Stem	RHSIM_Rhsim08G0082500	3.435299995	6.88982E-05	0.000458519	RHSIM_Rhsim08G0082500
Root vs Stem	RHSIM_RhsimUnG0042600	-1.836127476	6.94108E-05	0.000461531	RHSIM_RhsimUnG0042600
Root vs Stem	RHSIM_Rhsim10G0116000	2.015877413	7.00493E-05	0.000465642	RHSIM_Rhsim10G0116000
Root vs Stem	RHSIM_Rhsim06G0152500	-1.904543192	7.03757E-05	0.000467542	RHSIM_Rhsim06G0152500
Root vs Stem	RHSIM_Rhsim04G0023900	-1.111352397	7.04251E-05	0.000467694	RHSIM_Rhsim04G0023900
Root vs Stem	RHSIM_RhsimUnG0007700	5.891200989	7.04928E-05	0.000467916	RHSIM_RhsimUnG0007700
Root vs Stem	RHSIM_Rhsim13G0168900	1.058463478	7.08594E-05	0.000469943	RHSIM_Rhsim13G0168900
Root vs Stem	RHSIM_Rhsim11G0109900	2.146380317	7.0985E-05	0.00047064	RHSIM_Rhsim11G0109900
Root vs Stem	RHSIM_Rhsim10G0167100	1.893820798	7.14168E-05	0.000473231	RHSIM_Rhsim10G0167100
Root vs Stem	novel.5041	3.363071015	7.18893E-05	0.000476088	--
Root vs Stem	RHSIM_Rhsim06G0182000	-3.384703945	7.20153E-05	0.000476786	RHSIM_Rhsim06G0182000
Root vs Stem	RHSIM_Rhsim13G0145600	3.863771475	7.21015E-05	0.000477219	RHSIM_Rhsim13G0145600
Root vs Stem	RHSIM_RhsimUnG0255600	2.839480828	7.2326E-05	0.000478568	RHSIM_RhsimUnG0255600
Root vs Stem	RHSIM_Rhsim02G0096600	-1.082037215	7.31638E-05	0.000483973	RHSIM_Rhsim02G0096600
Root vs Stem	RHSIM_Rhsim04G0232900	3.292335748	7.31883E-05	0.000483996	RHSIM_Rhsim04G0232900
Root vs Stem	RHSIM_Rhsim11G0143900	-7.267362857	7.32319E-05	0.000484145	RHSIM_Rhsim11G0143900

Comparison	Gene ID	log_2FC	P-value	FDR (padj)	Gene Name
Root vs Stem	RHSIM_Rhsim11G0157300	4.158670098	7.35603E-05	0.000485759	RHSIM_Rhsim11G0157300
Root vs Stem	RHSIM_Rhsim09G0212300	1.22462358	7.36417E-05	0.000486157	RHSIM_Rhsim09G0212300
Root vs Stem	RHSIM_Rhsim01G0077800	-3.95656172	7.42919E-05	0.000490029	RHSIM_Rhsim01G0077800
Root vs Stem	RHSIM_Rhsim13G0091900	3.193428732	7.43329E-05	0.000490159	RHSIM_Rhsim13G0091900
Root vs Stem	novel.1636	1.034713511	7.44197E-05	0.000490591	--
Root vs Stem	RHSIM_Rhsim08G0091700	-3.102550158	7.46435E-05	0.000491786	RHSIM_Rhsim08G0091700
Root vs Stem	RHSIM_Rhsim08G0030900	2.051623201	7.48091E-05	0.000492634	RHSIM_Rhsim08G0030900
Root vs Stem	RHSIM_Rhsim03G0255800	-3.274314746	7.48365E-05	0.000492634	RHSIM_Rhsim03G0255800
Root vs Stem	RHSIM_Rhsim05G0000100	-1.092025006	7.48549E-05	0.000492634	RHSIM_Rhsim05G0000100
Root vs Stem	novel.3833	6.969964004	7.48579E-05	0.000492634	--
Root vs Stem	RHSIM_Rhsim06G0031200	-2.175936373	7.49039E-05	0.000492796	RHSIM_Rhsim06G0031200
Root vs Stem	novel.4578	-7.735369786	7.49638E-05	0.00049305	--
Root vs Stem	RHSIM_Rhsim01G0245400	-1.116234888	7.51478E-05	0.000494119	RHSIM_Rhsim01G0245400
Root vs Stem	novel.5013	2.029892239	7.54015E-05	0.000495646	--
Root vs Stem	RHSIM_Rhsim13G0151700	3.886228835	7.56833E-05	0.000497356	RHSIM_Rhsim13G0151700
Root vs Stem	RHSIM_Rhsim07G0093000	3.471606638	7.58783E-05	0.000498354	RHSIM_Rhsim07G0093000
Root vs Stem	RHSIM_Rhsim06G0211100	3.909260722	7.6002E-05	0.000499024	RHSIM_Rhsim06G0211100
Root vs Stem	novel.2701	-1.671795936	7.63083E-05	0.000500673	--
Root vs Stem	RHSIM_Rhsim02G0039500	-4.008017559	7.63182E-05	0.000500673	RHSIM_Rhsim02G0039500
Root vs Stem	novel.6129	-7.474236439	7.68209E-05	0.000503448	--
Root vs Stem	RHSIM_Rhsim03G0236400	-1.330205188	7.68287E-05	0.000503448	RHSIM_Rhsim03G0236400
Root vs Stem	novel.1225	2.67499399	7.69192E-05	0.000503898	--
Root vs Stem	RHSIM_Rhsim13G0210800	1.966645107	7.72713E-05	0.000506061	RHSIM_Rhsim13G0210800
Root vs Stem	RHSIM_Rhsim10G0095900	6.262151207	7.73368E-05	0.000506346	RHSIM_Rhsim10G0095900
Root vs Stem	RHSIM_Rhsim02G0119400	-1.203624136	7.74389E-05	0.000506871	RHSIM_Rhsim02G0119400

Comparison	Gene ID	log_2FC	P-value	FDR (padj)	Gene Name
Root vs Stem	RHSIM_Rhsim08G0164200	1.982015353	7.80639E-05	0.000510817	RHSIM_Rhsim08G0164200
Root vs Stem	RHSIM_Rhsim13G0205900	1.248549454	7.85628E-05	0.000513634	RHSIM_Rhsim13G0205900
Root vs Stem	RHSIM_RhsimUnG0066500	-7.604277524	7.85829E-05	0.000513634	RHSIM_RhsimUnG0066500
Root vs Stem	novel.741	4.118490852	7.87404E-05	0.000514467	--
Root vs Stem	RHSIM_Rhsim13G0105400	2.126294185	7.99917E-05	0.000522393	RHSIM_Rhsim13G0105400
Root vs Stem	novel.1914	-6.799405973	8.01332E-05	0.000523169	--
Root vs Stem	RHSIM_RhsimUnG0227100	3.155980243	8.11087E-05	0.000529089	RHSIM_RhsimUnG0227100
Root vs Stem	RHSIM_Rhsim03G0147700	1.804508144	8.13071E-05	0.000530233	RHSIM_Rhsim03G0147700
Root vs Stem	RHSIM_Rhsim08G0052900	6.400182653	8.21889E-05	0.000535377	RHSIM_Rhsim08G0052900
Root vs Stem	RHSIM_Rhsim02G0073300	-1.892782182	8.22679E-05	0.000535741	RHSIM_Rhsim02G0073300
Root vs Stem	RHSIM_Rhsim04G0013600	1.318813875	8.32781E-05	0.00054186	RHSIM_Rhsim04G0013600
Root vs Stem	RHSIM_Rhsim06G0085600	2.306101787	8.39445E-05	0.00054572	RHSIM_Rhsim06G0085600
Root vs Stem	RHSIM_Rhsim13G0144400	-2.004048963	8.45054E-05	0.000549071	RHSIM_Rhsim13G0144400
Root vs Stem	RHSIM_RhsimUnG0103200	2.881902031	8.47515E-05	0.000550514	RHSIM_RhsimUnG0103200
Root vs Stem	RHSIM_RhsimUnG0155700	-2.232537673	8.48154E-05	0.000550775	RHSIM_RhsimUnG0155700
Root vs Stem	RHSIM_Rhsim07G0015900	1.269363916	8.54098E-05	0.000553854	RHSIM_Rhsim07G0015900
Root vs Stem	RHSIM_Rhsim13G0115800	-1.740335139	8.55409E-05	0.000554548	RHSIM_Rhsim13G0115800
Root vs Stem	novel.6099	2.447052913	8.58804E-05	0.000556593	--
Root vs Stem	RHSIM_Rhsim01G0179300	2.093270213	8.64254E-05	0.00055981	RHSIM_Rhsim01G0179300
Root vs Stem	RHSIM_Rhsim07G0050100	-3.904711335	8.65011E-05	0.000560144	RHSIM_Rhsim07G0050100
Root vs Stem	RHSIM_Rhsim08G0241000	1.777969311	8.66512E-05	0.000560958	RHSIM_Rhsim08G0241000
Root vs Stem	RHSIM_Rhsim04G0192400	-7.30391987	8.66761E-05	0.000560961	RHSIM_Rhsim04G0192400
Root vs Stem	RHSIM_Rhsim04G0042600	1.451981993	8.6894E-05	0.000562213	RHSIM_Rhsim04G0042600
Root vs Stem	RHSIM_Rhsim03G0053200	-2.979219949	8.70339E-05	0.000562961	RHSIM_Rhsim03G0053200
Root vs Stem	RHSIM_Rhsim05G0086100	2.442054308	8.70611E-05	0.000562979	RHSIM_Rhsim05G0086100

Comparison	Gene ID	log_2FC	P-value	FDR (padj)	Gene Name
Root vs Stem	RHSIM_Rhsim03G0243900	-1.606111078	8.72477E-05	0.000564027	RHSIM_Rhsim03G0243900
Root vs Stem	novel.2298	4.902336302	8.7335E-05	0.000564434	--
Root vs Stem	RHSIM_Rhsim04G0192600	-3.600241029	8.76372E-05	0.000566228	RHSIM_Rhsim04G0192600
Root vs Stem	RHSIM_Rhsim02G0134600	1.203177374	8.83895E-05	0.000570769	RHSIM_Rhsim02G0134600
Root vs Stem	novel.1608	1.131160834	8.85012E-05	0.000571109	--
Root vs Stem	RHSIM_Rhsim09G0104400	-1.137379634	8.88856E-05	0.000573169	RHSIM_Rhsim09G0104400
Root vs Stem	RHSIM_RhsimUnG0198800	-4.608691392	8.89479E-05	0.000573411	RHSIM_RhsimUnG0198800
Root vs Stem	RHSIM_Rhsim13G0110400	1.029434017	8.91841E-05	0.000574772	RHSIM_Rhsim13G0110400
Root vs Stem	RHSIM_RhsimUnG0123500	-1.424569135	8.93198E-05	0.000575486	RHSIM_RhsimUnG0123500
Root vs Stem	RHSIM_Rhsim03G0140800	-3.713982262	8.94624E-05	0.000576244	RHSIM_Rhsim03G0140800
Root vs Stem	RHSIM_Rhsim05G0063700	-2.52791774	8.99114E-05	0.000578651	RHSIM_Rhsim05G0063700
Root vs Stem	RHSIM_Rhsim09G0171400	3.102761913	9.01924E-05	0.000579973	RHSIM_Rhsim09G0171400
Root vs Stem	RHSIM_Rhsim04G0150300	-1.421628633	9.02771E-05	0.000580357	RHSIM_Rhsim04G0150300
Root vs Stem	RHSIM_Rhsim04G0246700	2.564434052	9.03423E-05	0.000580614	RHSIM_Rhsim04G0246700
Root vs Stem	RHSIM_Rhsim02G0145200	1.134502612	9.04215E-05	0.000580961	RHSIM_Rhsim02G0145200
Root vs Stem	RHSIM_Rhsim06G0038200	-1.705175629	9.07406E-05	0.000582849	RHSIM_Rhsim06G0038200
Root vs Stem	RHSIM_Rhsim13G0017900	1.526380615	9.10281E-05	0.000584532	RHSIM_Rhsim13G0017900
Root vs Stem	RHSIM_Rhsim13G0177500	1.000473197	9.10937E-05	0.00058479	RHSIM_Rhsim13G0177500
Root vs Stem	RHSIM_Rhsim04G0080100	-3.093064219	9.11948E-05	0.000585114	RHSIM_Rhsim04G0080100
Root vs Stem	novel.596	1.598222547	9.12285E-05	0.000585167	--
Root vs Stem	RHSIM_Rhsim01G0205600	-6.195678926	9.14782E-05	0.000586443	RHSIM_Rhsim01G0205600
Root vs Stem	RHSIM_Rhsim07G0139800	-4.579438222	9.1785E-05	0.000588245	RHSIM_Rhsim07G0139800
Root vs Stem	RHSIM_Rhsim01G0277100	-1.878894221	9.25987E-05	0.000593131	RHSIM_Rhsim01G0277100
Root vs Stem	RHSIM_Rhsim10G0047700	-7.545216289	9.30364E-05	0.000595769	RHSIM_Rhsim10G0047700
Root vs Stem	novel.5027	-5.324950382	9.34338E-05	0.000598147	--

Comparison	Gene ID	log_2FC	P-value	FDR (padj)	Gene Name
Root vs Stem	novel.1800	2.300397096	9.35912E-05	0.00059895	--
Root vs Stem	RHSIM_Rhsim06G0152000	-2.613012444	9.36849E-05	0.00059909	RHSIM_Rhsim06G0152000
Root vs Stem	RHSIM_Rhsim12G0192700	1.537953366	9.37429E-05	0.000599294	RHSIM_Rhsim12G0192700
Root vs Stem	RHSIM_Rhsim04G0215100	3.35846552	9.38828E-05	0.000600022	RHSIM_Rhsim04G0215100
Root vs Stem	RHSIM_Rhsim03G0054700	1.301526087	9.40899E-05	0.000601013	RHSIM_Rhsim03G0054700
Root vs Stem	novel.6172	4.338716962	9.44869E-05	0.000603047	--
Root vs Stem	RHSIM_Rhsim07G0181400	1.028299962	9.4565E-05	0.000603379	RHSIM_Rhsim07G0181400
Root vs Stem	RHSIM_Rhsim02G0102300	3.566388757	9.48588E-05	0.000604631	RHSIM_Rhsim02G0102300
Root vs Stem	RHSIM_Rhsim10G0150400	-1.738538414	9.5071E-05	0.000605769	RHSIM_Rhsim10G0150400
Root vs Stem	RHSIM_Rhsim12G0124700	-1.049896799	9.5203E-05	0.000606442	RHSIM_Rhsim12G0124700
Root vs Stem	RHSIM_Rhsim11G0185100	-3.231029643	9.55021E-05	0.000608012	RHSIM_Rhsim11G0185100
Root vs Stem	novel.4791	-4.077607872	9.5661E-05	0.000608855	--
Root vs Stem	RHSIM_Rhsim06G0028200	-1.581198318	9.61612E-05	0.000611195	RHSIM_Rhsim06G0028200
Root vs Stem	RHSIM_Rhsim08G0053700	-1.144699134	9.62712E-05	0.000611725	RHSIM_Rhsim08G0053700
Root vs Stem	RHSIM_Rhsim11G0044800	1.995834647	9.71908E-05	0.000617228	RHSIM_Rhsim11G0044800
Root vs Stem	RHSIM_Rhsim12G0141600	-1.628872938	9.72488E-05	0.000617427	RHSIM_Rhsim12G0141600
Root vs Stem	RHSIM_Rhsim08G0203900	-1.28183313	9.731E-05	0.000617475	RHSIM_Rhsim08G0203900
Root vs Stem	RHSIM_Rhsim04G0195000	-4.867349884	9.7556E-05	0.000618866	RHSIM_Rhsim04G0195000
Root vs Stem	RHSIM_Rhsim08G0071400	-1.093944446	9.76918E-05	0.000619557	RHSIM_Rhsim08G0071400
Root vs Stem	RHSIM_Rhsim01G0188000	-7.455594217	9.82732E-05	0.000623072	RHSIM_Rhsim01G0188000
Root vs Stem	RHSIM_Rhsim07G0213900	-2.889312664	9.83696E-05	0.000623512	RHSIM_Rhsim07G0213900
Root vs Stem	novel.3185	-2.481924453	9.85863E-05	0.000624682	--
Root vs Stem	RHSIM_Rhsim08G0081800	-1.906096543	9.86083E-05	0.000624682	RHSIM_Rhsim08G0081800
Root vs Stem	RHSIM_RhsimUnG0211800	-1.085643557	9.8754E-05	0.000625433	RHSIM_RhsimUnG0211800
Root vs Stem	RHSIM_Rhsim01G0240400	2.117112153	9.8963E-05	0.000626584	RHSIM_Rhsim01G0240400

Comparison	Gene ID	log_2FC	P-value	FDR (padj)	Gene Name
Root vs Stem	RHSIM_Rhsim07G0103700	-1.019607976	9.91902E-05	0.000627785	RHSIM_Rhsim07G0103700
Root vs Stem	RHSIM_Rhsim01G0243600	1.366118964	9.92272E-05	0.000627785	RHSIM_Rhsim01G0243600
Root vs Stem	RHSIM_Rhsim02G0035600	2.064720899	9.92342E-05	0.000627785	RHSIM_Rhsim02G0035600
Root vs Stem	RHSIM_Rhsim01G0068200	2.160412681	9.93047E-05	0.000627885	RHSIM_Rhsim01G0068200
Root vs Stem	RHSIM_Rhsim12G0153400	1.173287648	9.98242E-05	0.000630824	RHSIM_Rhsim12G0153400
Root vs Stem	RHSIM_RhsimUnG0173300	-7.435187564	0.000100077	0.000632247	RHSIM_RhsimUnG0173300
Root vs Stem	RHSIM_Rhsim03G0053300	-3.012176479	0.000100285	0.000633198	RHSIM_Rhsim03G0053300
Root vs Stem	RHSIM_Rhsim13G0033500	7.291250517	0.00010032	0.000633198	RHSIM_Rhsim13G0033500
Root vs Stem	RHSIM_Rhsim03G0216900	2.739646477	0.000100337	0.000633198	RHSIM_Rhsim03G0216900
Root vs Stem	RHSIM_Rhsim12G0003000	-1.065338129	0.000100447	0.00063372	RHSIM_Rhsim12G0003000
Root vs Stem	RHSIM_Rhsim11G0055200	-4.931758929	0.00010084	0.000636021	RHSIM_Rhsim11G0055200
Root vs Stem	novel.239	-7.381303015	0.000101744	0.000641026	--
Root vs Stem	RHSIM_Rhsim06G0140600	1.316818834	0.000102276	0.000644196	RHSIM_Rhsim06G0140600
Root vs Stem	novel.2752	-5.801499585	0.00010254	0.000645335	--
Root vs Stem	RHSIM_Rhsim09G0034100	3.744306253	0.000102976	0.000647722	RHSIM_Rhsim09G0034100
Root vs Stem	RHSIM_Rhsim08G0030700	1.545872754	0.000103039	0.000647944	RHSIM_Rhsim08G0030700
Root vs Stem	RHSIM_Rhsim06G0029100	-1.213886378	0.000103068	0.000647951	RHSIM_Rhsim06G0029100
Root vs Stem	RHSIM_Rhsim07G0070300	-1.355538185	0.000103211	0.000648667	RHSIM_Rhsim07G0070300
Root vs Stem	RHSIM_Rhsim06G0075900	-4.974046996	0.000103638	0.000650997	RHSIM_Rhsim06G0075900
Root vs Stem	RHSIM_Rhsim07G0088800	-1.312510026	0.000103687	0.000651127	RHSIM_Rhsim07G0088800
Root vs Stem	RHSIM_Rhsim08G0018500	-1.607313023	0.000103725	0.000651192	RHSIM_Rhsim08G0018500
Root vs Stem	RHSIM_Rhsim11G0121300	1.204853839	0.000104478	0.000655563	RHSIM_Rhsim11G0121300
Root vs Stem	RHSIM_Rhsim01G0038500	-7.895654266	0.000104675	0.000656439	RHSIM_Rhsim01G0038500
Root vs Stem	RHSIM_Rhsim03G0077700	-1.153260392	0.000104828	0.000656943	RHSIM_Rhsim03G0077700
Root vs Stem	RHSIM_Rhsim07G0155500	2.987239908	0.000104841	0.000656943	RHSIM_Rhsim07G0155500

Comparison	Gene ID	log_2FC	P-value	FDR (padj)	Gene Name
Root vs Stem	RHSIM_Rhsim03G0009400	1.593381084	0.000104884	0.000657037	RHSIM_Rhsim03G0009400
Root vs Stem	RHSIM_Rhsim03G0030600	-1.189968826	0.00010502	0.000657707	RHSIM_Rhsim03G0030600
Root vs Stem	RHSIM_Rhsim10G0061300	1.000038511	0.000105593	0.000660935	RHSIM_Rhsim10G0061300
Root vs Stem	RHSIM_Rhsim02G0105300	-4.619849332	0.000105671	0.000661066	RHSIM_Rhsim02G0105300
Root vs Stem	RHSIM_Rhsim08G0214200	1.52106671	0.000106023	0.000663092	RHSIM_Rhsim08G0214200
Root vs Stem	RHSIM_Rhsim01G0089900	-1.451853146	0.000106181	0.000663895	RHSIM_Rhsim01G0089900
Root vs Stem	RHSIM_Rhsim09G0204400	-1.441562666	0.000106342	0.000664724	RHSIM_Rhsim09G0204400
Root vs Stem	RHSIM_Rhsim12G0014500	-2.596825297	0.000106904	0.000668055	RHSIM_Rhsim12G0014500
Root vs Stem	RHSIM_Rhsim10G0003700	1.158696237	0.000107458	0.000671338	RHSIM_Rhsim10G0003700
Root vs Stem	novel.2119	-4.391297873	0.000107768	0.000673088	--
Root vs Stem	RHSIM_Rhsim07G0247000	1.023196637	0.000107863	0.000673499	RHSIM_Rhsim07G0247000
Root vs Stem	RHSIM_Rhsim13G0047100	1.099947898	0.000107946	0.000673684	RHSIM_Rhsim13G0047100
Root vs Stem	RHSIM_Rhsim10G0105600	5.060718691	0.000108155	0.000674676	RHSIM_Rhsim10G0105600
Root vs Stem	RHSIM_Rhsim08G0009200	2.689263336	0.000108168	0.000674676	RHSIM_Rhsim08G0009200
Root vs Stem	RHSIM_RhsimUnG0118100	-3.778522407	0.000108763	0.000678199	RHSIM_RhsimUnG0118100
Root vs Stem	RHSIM_Rhsim05G0079700	3.402620958	0.000109286	0.000681277	RHSIM_Rhsim05G0079700
Root vs Stem	RHSIM_Rhsim06G0106400	-1.502591466	0.000109347	0.000681476	RHSIM_Rhsim06G0106400
Root vs Stem	RHSIM_Rhsim06G0082800	-2.13613439	0.000109436	0.00068166	RHSIM_Rhsim06G0082800
Root vs Stem	RHSIM_Rhsim01G0167900	-1.92472522	0.000109544	0.000682074	RHSIM_Rhsim01G0167900
Root vs Stem	RHSIM_Rhsim07G0056300	4.516945751	0.000109862	0.00068352	RHSIM_Rhsim07G0056300
Root vs Stem	RHSIM_Rhsim02G0109100	5.23482679	0.000109883	0.00068352	RHSIM_Rhsim02G0109100
Root vs Stem	RHSIM_Rhsim10G0173800	-1.577295675	0.000110068	0.000684475	RHSIM_Rhsim10G0173800
Root vs Stem	RHSIM_Rhsim04G0070200	-2.027478166	0.000110096	0.000684475	RHSIM_Rhsim04G0070200
Root vs Stem	novel.3184	-4.634267872	0.000110765	0.000688267	--
Root vs Stem	RHSIM_Rhsim02G0229900	1.02859981	0.000111713	0.000693595	RHSIM_Rhsim02G0229900

Comparison	Gene ID	log_2FC	P-value	FDR (padj)	Gene Name
Root vs Stem	novel.5305	4.367831526	0.000111858	0.000694311	--
Root vs Stem	RHSIM_Rhsim04G0085000	1.12533158	0.000112284	0.000696578	RHSIM_Rhsim04G0085000
Root vs Stem	RHSIM_RhsimUnG0053800	1.340891162	0.00011242	0.000697235	RHSIM_RhsimUnG0053800
Root vs Stem	RHSIM_Rhsim13G0093700	1.058372928	0.000112646	0.000698262	RHSIM_Rhsim13G0093700
Root vs Stem	RHSIM_Rhsim04G0183700	1.294772308	0.00011366	0.000703978	RHSIM_Rhsim04G0183700
Root vs Stem	novel.2003	-11.4731588	0.000113921	0.000705217	--
Root vs Stem	RHSIM_RhsimUnG0048400	-2.762100018	0.000114009	0.000705522	RHSIM_RhsimUnG0048400
Root vs Stem	RHSIM_Rhsim10G0044200	-2.452877448	0.000114272	0.000706626	RHSIM_Rhsim10G0044200
Root vs Stem	novel.3435	-1.144744109	0.000114409	0.000707288	--
Root vs Stem	RHSIM_Rhsim04G0084300	2.677113881	0.000114628	0.000708258	RHSIM_Rhsim04G0084300
Root vs Stem	novel.1585	-1.841352863	0.000115798	0.000715103	--
Root vs Stem	novel.1612	-1.811460368	0.000116224	0.000717504	--
Root vs Stem	RHSIM_Rhsim02G0146500	1.425495983	0.000116249	0.000717504	RHSIM_Rhsim02G0146500
Root vs Stem	novel.4459	2.289133244	0.000116475	0.000718518	--
Root vs Stem	novel.3300	-1.604605184	0.000116668	0.000719324	--
Root vs Stem	novel.6159	-1.257128522	0.000116722	0.000719462	--
Root vs Stem	novel.5188	3.760226914	0.000117335	0.000723048	--
Root vs Stem	RHSIM_Rhsim09G0146900	-1.192495218	0.000117403	0.000723277	RHSIM_Rhsim09G0146900
Root vs Stem	RHSIM_Rhsim04G0227200	-1.792031975	0.000117786	0.000725053	RHSIM_Rhsim04G0227200
Root vs Stem	RHSIM_Rhsim03G0020300	1.930961599	0.000118611	0.000729934	RHSIM_Rhsim03G0020300
Root vs Stem	RHSIM_Rhsim04G0117400	1.665109762	0.000118711	0.000730354	RHSIM_Rhsim04G0117400
Root vs Stem	RHSIM_Rhsim13G0231600	3.045468303	0.000118801	0.000730715	RHSIM_Rhsim13G0231600
Root vs Stem	RHSIM_Rhsim02G0185200	3.907132104	0.000118928	0.000731301	RHSIM_Rhsim02G0185200
Root vs Stem	novel.408	-2.107796845	0.000118994	0.000731509	--
Root vs Stem	RHSIM_Rhsim01G0015700	-1.229208545	0.000120291	0.000738891	RHSIM_Rhsim01G0015700

Comparison	Gene ID	log_2FC	P-value	FDR (padj)	Gene Name
Root vs Stem	RHSIM_Rhsim08G0010200	5.329127273	0.000121576	0.00074639	RHSIM_Rhsim08G0010200
Root vs Stem	RHSIM_Rhsim06G0107700	-1.263941949	0.000122394	0.000751211	RHSIM_Rhsim06G0107700
Root vs Stem	RHSIM_Rhsim07G0088500	2.757977959	0.000122649	0.000752377	RHSIM_Rhsim07G0088500
Root vs Stem	RHSIM_Rhsim10G0094300	1.589277972	0.000122714	0.000752436	RHSIM_Rhsim10G0094300
Root vs Stem	RHSIM_Rhsim08G0240000	1.137600615	0.0001228	0.000752701	RHSIM_Rhsim08G0240000
Root vs Stem	RHSIM_Rhsim07G0180400	2.497161175	0.000123203	0.000754969	RHSIM_Rhsim07G0180400
Root vs Stem	RHSIM_RhsimUnG0258700	-1.005626967	0.000123541	0.000756437	RHSIM_RhsimUnG0258700
Root vs Stem	RHSIM_Rhsim10G0010500	1.087092269	0.000123811	0.000757489	RHSIM_Rhsim10G0010500
Root vs Stem	RHSIM_Rhsim13G0046200	1.531227009	0.000124271	0.000759897	RHSIM_Rhsim13G0046200
Root vs Stem	novel.1220	1.422903734	0.000124646	0.000761989	--
Root vs Stem	novel.3524	2.634427331	0.000125009	0.000763602	--
Root vs Stem	RHSIM_Rhsim04G0113500	1.42907163	0.000125558	0.000766546	RHSIM_Rhsim04G0113500
Root vs Stem	novel.1610	-2.151218971	0.000125642	0.000766655	--
Root vs Stem	novel.1040	-1.653824172	0.000127052	0.000774438	--
Root vs Stem	RHSIM_Rhsim04G0014300	-1.506988285	0.00012866	0.000783828	RHSIM_Rhsim04G0014300
Root vs Stem	RHSIM_Rhsim04G0017700	-2.103862406	0.000128778	0.00078434	RHSIM_Rhsim04G0017700
Root vs Stem	RHSIM_Rhsim11G0091400	-3.290773242	0.000128878	0.000784742	RHSIM_Rhsim11G0091400
Root vs Stem	RHSIM_Rhsim07G0073600	1.316131101	0.000130117	0.000791864	RHSIM_Rhsim07G0073600
Root vs Stem	RHSIM_Rhsim08G0101800	-2.712104481	0.000130413	0.000793455	RHSIM_Rhsim08G0101800
Root vs Stem	RHSIM_Rhsim10G0039000	1.259677559	0.000130557	0.000794121	RHSIM_Rhsim10G0039000
Root vs Stem	RHSIM_Rhsim01G0108800	-1.835881318	0.000130627	0.000794191	RHSIM_Rhsim01G0108800
Root vs Stem	novel.309	1.085290123	0.000130637	0.000794191	--
Root vs Stem	RHSIM_Rhsim05G0184300	2.76973252	0.000131301	0.000797806	RHSIM_Rhsim05G0184300
Root vs Stem	novel.4307	-2.426876114	0.000131684	0.000799921	--
Root vs Stem	RHSIM_RhsimUnG0106100	-2.52696214	0.000133606	0.000810959	RHSIM_RhsimUnG0106100

Comparison	Gene ID	log_2FC	P-value	FDR (padj)	Gene Name
Root vs Stem	RHSIM_Rhsim08G0079500	-1.177382276	0.000133903	0.000812548	RHSIM_Rhsim08G0079500
Root vs Stem	RHSIM_Rhsim06G0106500	-1.193702127	0.000134762	0.000817546	RHSIM_Rhsim06G0106500
Root vs Stem	RHSIM_Rhsim03G0136000	-1.723056541	0.000135119	0.000819494	RHSIM_Rhsim03G0136000
Root vs Stem	RHSIM_Rhsim12G0141700	-2.89242824	0.000135279	0.00082006	RHSIM_Rhsim12G0141700
Root vs Stem	RHSIM_Rhsim04G0034500	-2.552895491	0.000136686	0.000827909	RHSIM_Rhsim04G0034500
Root vs Stem	RHSIM_Rhsim13G0214200	-1.162546482	0.000137259	0.000831159	RHSIM_Rhsim13G0214200
Root vs Stem	RHSIM_Rhsim01G0049800	1.123865927	0.000137675	0.000833023	RHSIM_Rhsim01G0049800
Root vs Stem	novel.3819	-1.558520473	0.000138264	0.00083615	--
Root vs Stem	RHSIM_Rhsim13G0047000	-1.117166775	0.000139038	0.000840318	RHSIM_Rhsim13G0047000
Root vs Stem	RHSIM_Rhsim11G0194200	-2.511689522	0.000139063	0.000840318	RHSIM_Rhsim11G0194200
Root vs Stem	RHSIM_Rhsim07G0073500	1.335029727	0.000139158	0.000840672	RHSIM_Rhsim07G0073500
Root vs Stem	novel.972	2.440613447	0.000139538	0.000842536	--
Root vs Stem	RHSIM_Rhsim01G0016100	1.653535559	0.000140677	0.000848737	RHSIM_Rhsim01G0016100
Root vs Stem	RHSIM_Rhsim01G0022500	-3.975259589	0.000140936	0.000850075	RHSIM_Rhsim01G0022500
Root vs Stem	RHSIM_Rhsim03G0127900	1.910232458	0.000141052	0.000850553	RHSIM_Rhsim03G0127900
Root vs Stem	RHSIM_Rhsim02G0119000	1.311036569	0.000141097	0.000850606	RHSIM_Rhsim02G0119000
Root vs Stem	RHSIM_Rhsim01G0166200	2.60351857	0.000141168	0.000850809	RHSIM_Rhsim01G0166200
Root vs Stem	novel.4154	-2.703865481	0.000141395	0.000851957	--
Root vs Stem	novel.3610	-6.419783815	0.000142047	0.000855439	--
Root vs Stem	RHSIM_Rhsim12G0150000	1.341910925	0.000142666	0.000858939	RHSIM_Rhsim12G0150000
Root vs Stem	RHSIM_Rhsim04G0143700	-3.818900952	0.000142964	0.000860281	RHSIM_Rhsim04G0143700
Root vs Stem	RHSIM_Rhsim08G0062400	-2.725395481	0.000143559	0.000863412	RHSIM_Rhsim08G0062400
Root vs Stem	RHSIM_Rhsim13G0042000	-1.512195688	0.00014385	0.000864878	RHSIM_Rhsim13G0042000
Root vs Stem	RHSIM_Rhsim03G0162600	-7.297240631	0.000144187	0.000866515	RHSIM_Rhsim03G0162600
Root vs Stem	RHSIM_Rhsim07G0067500	1.903469961	0.000145115	0.000871631	RHSIM_Rhsim07G0067500

Comparison	Gene ID	log_2FC	P-value	FDR (padj)	Gene Name
Root vs Stem	novel.2648	1.381195329	0.000145152	0.000871631	--
Root vs Stem	RHSIM_Rhsim11G0106800	-2.978766368	0.000145592	0.000874046	RHSIM_Rhsim11G0106800
Root vs Stem	RHSIM_Rhsim06G0203000	-1.671928872	0.000145793	0.000875023	RHSIM_Rhsim06G0203000
Root vs Stem	RHSIM_Rhsim04G0164000	-1.613093347	0.000146787	0.00088076	RHSIM_Rhsim04G0164000
Root vs Stem	RHSIM_Rhsim13G0174300	-1.026165421	0.00014688	0.000881087	RHSIM_Rhsim13G0174300
Root vs Stem	RHSIM_Rhsim12G0006100	1.364010746	0.000147062	0.000881721	RHSIM_Rhsim12G0006100
Root vs Stem	RHSIM_RhsimUnG0157000	-1.275533285	0.000147225	0.000882152	RHSIM_RhsimUnG0157000
Root vs Stem	RHSIM_Rhsim12G0200300	-4.483402044	0.000147955	0.000886152	RHSIM_Rhsim12G0200300
Root vs Stem	RHSIM_Rhsim07G0130300	-1.9359132	0.000148005	0.000886221	RHSIM_Rhsim07G0130300
Root vs Stem	RHSIM_Rhsim04G0086800	-1.556944461	0.000148117	0.000886664	RHSIM_Rhsim04G0086800
Root vs Stem	novel.335	-7.757324784	0.000148582	0.000889215	--
Root vs Stem	RHSIM_Rhsim07G0132500	2.952834777	0.000148741	0.000889935	RHSIM_Rhsim07G0132500
Root vs Stem	novel.4470	3.664140794	0.000149447	0.00089393	--
Root vs Stem	novel.2004	-11.07999082	0.000149565	0.000894171	--
Root vs Stem	novel.1152	-3.578855791	0.000150138	0.000897342	--
Root vs Stem	novel.1347	-7.758407159	0.000150173	0.000897342	--
Root vs Stem	RHSIM_Rhsim09G0107000	2.182871342	0.000150509	0.000899112	RHSIM_Rhsim09G0107000
Root vs Stem	RHSIM_Rhsim13G0082900	1.052395192	0.000150909	0.000900802	RHSIM_Rhsim13G0082900
Root vs Stem	novel.3078	-5.121544515	0.00015101	0.000901171	--
Root vs Stem	RHSIM_Rhsim11G0194400	-2.524417409	0.000151381	0.000902919	RHSIM_Rhsim11G0194400
Root vs Stem	RHSIM_Rhsim04G0085400	-1.702931869	0.000151658	0.000904107	RHSIM_Rhsim04G0085400
Root vs Stem	RHSIM_Rhsim12G0007800	1.246099666	0.000152028	0.000905843	RHSIM_Rhsim12G0007800
Root vs Stem	novel.2126	-4.477807632	0.000152393	0.000907316	--
Root vs Stem	novel.347	-1.85769063	0.000152463	0.000907494	--
Root vs Stem	RHSIM_Rhsim09G0028900	-1.849556683	0.000153572	0.000913676	RHSIM_Rhsim09G0028900

Comparison	Gene ID	log_2FC	P-value	FDR (padj)	Gene Name
Root vs Stem	RHSIM_RhsimUnG0170200	4.517371974	0.000153581	0.000913676	RHSIM_RhsimUnG0170200
Root vs Stem	RHSIM_Rhsim03G0192700	-1.540913952	0.000153684	0.000914058	RHSIM_Rhsim03G0192700
Root vs Stem	RHSIM_Rhsim05G0195700	-2.450149103	0.000154354	0.000917806	RHSIM_Rhsim05G0195700
Root vs Stem	novel.757	4.242967265	0.000154684	0.000919526	--
Root vs Stem	RHSIM_Rhsim02G0256100	1.192188029	0.000155241	0.000922365	RHSIM_Rhsim02G0256100
Root vs Stem	RHSIM_Rhsim12G0087300	-1.53856236	0.000155802	0.000925459	RHSIM_Rhsim12G0087300
Root vs Stem	RHSIM_Rhsim01G0194900	-6.719096514	0.000155958	0.000926145	RHSIM_Rhsim01G0194900
Root vs Stem	RHSIM_RhsimUnG0088600	2.342865455	0.000156002	0.00092617	RHSIM_RhsimUnG0088600
Root vs Stem	novel.625	3.470776911	0.000157276	0.000933011	--
Root vs Stem	novel.2814	-6.962548704	0.000158278	0.00093775	--
Root vs Stem	RHSIM_Rhsim06G0226600	-1.706365076	0.000158621	0.000939355	RHSIM_Rhsim06G0226600
Root vs Stem	RHSIM_Rhsim05G0082100	-2.535923651	0.00015863	0.000939355	RHSIM_Rhsim05G0082100
Root vs Stem	RHSIM_Rhsim10G0121300	-1.340729258	0.000159193	0.000942442	RHSIM_Rhsim10G0121300
Root vs Stem	RHSIM_Rhsim12G0161700	1.935286488	0.000159548	0.000944059	RHSIM_Rhsim12G0161700
Root vs Stem	RHSIM_Rhsim01G0047300	4.760921802	0.000160036	0.000946462	RHSIM_Rhsim01G0047300
Root vs Stem	RHSIM_Rhsim04G0135700	1.33326238	0.00016089	0.000951023	RHSIM_Rhsim04G0135700
Root vs Stem	RHSIM_Rhsim10G0020200	-1.170109136	0.000161127	0.000952182	RHSIM_Rhsim10G0020200
Root vs Stem	novel.3709	5.924992396	0.000161383	0.00095345	--
Root vs Stem	RHSIM_Rhsim11G0174700	1.912835974	0.000161509	0.000953707	RHSIM_Rhsim11G0174700
Root vs Stem	novel.5522	6.928816818	0.000161597	0.000953979	--
Root vs Stem	RHSIM_Rhsim02G0131100	1.378995827	0.000161847	0.00095521	RHSIM_Rhsim02G0131100
Root vs Stem	RHSIM_Rhsim10G0137700	2.082986821	0.000162059	0.000956218	RHSIM_Rhsim10G0137700
Root vs Stem	RHSIM_Rhsim08G0200500	1.609509576	0.000163344	0.000962816	RHSIM_Rhsim08G0200500
Root vs Stem	RHSIM_Rhsim01G0055600	1.120115889	0.000164129	0.000967194	RHSIM_Rhsim01G0055600
Root vs Stem	RHSIM_Rhsim04G0230400	2.72627645	0.000165322	0.000973727	RHSIM_Rhsim04G0230400

Comparison	Gene ID	log_2FC	P-value	FDR (padj)	Gene Name
Root vs Stem	RHSIM_Rhsim06G0143700	1.619247145	0.000165571	0.000974695	RHSIM_Rhsim06G0143700
Root vs Stem	novel.2310	2.590262914	0.000165941	0.000976373	--
Root vs Stem	RHSIM_Rhsim06G0223800	2.533790408	0.000166539	0.000979644	RHSIM_Rhsim06G0223800
Root vs Stem	novel.4797	1.531341999	0.000167327	0.000984027	--
Root vs Stem	RHSIM_Rhsim09G0108800	-3.104476997	0.000168124	0.000988211	RHSIM_Rhsim09G0108800
Root vs Stem	RHSIM_Rhsim02G0124200	-8.029297143	0.000168348	0.000989275	RHSIM_Rhsim02G0124200
Root vs Stem	RHSIM_Rhsim11G0171300	6.070563756	0.00016897	0.000992427	RHSIM_Rhsim11G0171300
Root vs Stem	RHSIM_Rhsim10G0161600	-2.016805616	0.000170323	0.000999354	RHSIM_Rhsim10G0161600
Root vs Stem	RHSIM_RhsimUnG0254900	-1.007044341	0.000170387	0.000999473	RHSIM_RhsimUnG0254900
Root vs Stem	RHSIM_Rhsim03G0017600	-2.701215139	0.000170696	0.001001033	RHSIM_Rhsim03G0017600
Root vs Stem	RHSIM_Rhsim05G0138300	-3.198878106	0.000171612	0.001006149	RHSIM_Rhsim05G0138300
Root vs Stem	RHSIM_Rhsim02G0083100	2.171356966	0.000171706	0.001006443	RHSIM_Rhsim02G0083100
Root vs Stem	RHSIM_Rhsim03G0237700	-3.609499354	0.000171833	0.001006716	RHSIM_Rhsim03G0237700
Root vs Stem	RHSIM_Rhsim03G0178500	-5.817348047	0.000173367	0.001015149	RHSIM_Rhsim03G0178500
Root vs Stem	RHSIM_Rhsim12G0203300	2.273461103	0.000175561	0.001026949	RHSIM_Rhsim12G0203300
Root vs Stem	RHSIM_Rhsim02G0089700	-9.14693465	0.000175648	0.001027196	RHSIM_Rhsim02G0089700
Root vs Stem	RHSIM_Rhsim06G0032000	-1.663701205	0.000177496	0.001037476	RHSIM_Rhsim06G0032000
Root vs Stem	RHSIM_Rhsim09G0033300	1.808405355	0.000178926	0.001045305	RHSIM_Rhsim09G0033300
Root vs Stem	RHSIM_Rhsim03G0235700	-1.31504387	0.000179219	0.001046751	RHSIM_Rhsim03G0235700
Root vs Stem	RHSIM_Rhsim13G0220400	-3.506781292	0.000179294	0.001046928	RHSIM_Rhsim13G0220400
Root vs Stem	novel.3462	2.120698027	0.000179546	0.001047628	--
Root vs Stem	RHSIM_Rhsim03G0040400	1.059839579	0.000179551	0.001047628	RHSIM_Rhsim03G0040400
Root vs Stem	novel.775	-3.271899004	0.000180094	0.001050534	--
Root vs Stem	RHSIM_Rhsim08G0082600	2.346523492	0.000180559	0.001052977	RHSIM_Rhsim08G0082600
Root vs Stem	RHSIM_Rhsim04G0051700	1.148884411	0.000181169	0.001055735	RHSIM_Rhsim04G0051700

Comparison	Gene ID	log_2FC	P-value	FDR (padj)	Gene Name
Root vs Stem	RHSIM_Rhsim08G0082300	-1.608280357	0.000181599	0.001057612	RHSIM_Rhsim08G0082300
Root vs Stem	RHSIM_Rhsim12G0071800	1.199264122	0.000181675	0.001057612	RHSIM_Rhsim12G0071800
Root vs Stem	RHSIM_RhsimUnG0146900	1.5907692	0.000182035	0.00105944	RHSIM_RhsimUnG0146900
Root vs Stem	RHSIM_Rhsim06G0076000	3.145628516	0.000183295	0.00106651	RHSIM_Rhsim06G0076000
Root vs Stem	RHSIM_RhsimUnG0111100	4.617937331	0.000184431	0.001072305	RHSIM_RhsimUnG0111100
Root vs Stem	novel.5519	-6.10471968	0.000184731	0.001073779	--
Root vs Stem	RHSIM_Rhsim13G0192800	-2.23851051	0.000184992	0.001075024	RHSIM_Rhsim13G0192800
Root vs Stem	RHSIM_Rhsim13G0052000	-1.229568721	0.000185493	0.001077393	RHSIM_Rhsim13G0052000
Root vs Stem	RHSIM_Rhsim01G0067200	1.866884231	0.000185801	0.001078908	RHSIM_Rhsim01G0067200
Root vs Stem	RHSIM_Rhsim08G0205700	-3.111339356	0.000186471	0.001082529	RHSIM_Rhsim08G0205700
Root vs Stem	RHSIM_Rhsim07G0238100	-1.868293497	0.000186795	0.001083862	RHSIM_Rhsim07G0238100
Root vs Stem	RHSIM_Rhsim03G0269100	-1.44312525	0.000186908	0.001084245	RHSIM_Rhsim03G0269100
Root vs Stem	RHSIM_Rhsim13G0210900	5.860689782	0.000187959	0.001089519	RHSIM_Rhsim13G0210900
Root vs Stem	RHSIM_Rhsim06G0012500	-1.367781954	0.000188146	0.001090331	RHSIM_Rhsim06G0012500
Root vs Stem	RHSIM_Rhsim11G0084600	3.881909967	0.000188306	0.001090981	RHSIM_Rhsim11G0084600
Root vs Stem	RHSIM_Rhsim01G0100200	5.943526792	0.000188867	0.001093684	RHSIM_Rhsim01G0100200
Root vs Stem	RHSIM_RhsimUnG0185000	-5.966337063	0.000189448	0.001096774	RHSIM_RhsimUnG0185000
Root vs Stem	RHSIM_Rhsim13G0087900	1.034900214	0.000189741	0.001098	RHSIM_Rhsim13G0087900
Root vs Stem	RHSIM_Rhsim06G0028900	1.189802826	0.000189845	0.001098243	RHSIM_Rhsim06G0028900
Root vs Stem	RHSIM_Rhsim03G0252500	1.736270009	0.000190398	0.001101165	RHSIM_Rhsim03G0252500
Root vs Stem	RHSIM_Rhsim03G0008400	-2.446828551	0.000191013	0.001104443	RHSIM_Rhsim03G0008400
Root vs Stem	RHSIM_Rhsim12G0057500	1.01602064	0.00019126	0.001105319	RHSIM_Rhsim12G0057500
Root vs Stem	RHSIM_Rhsim08G0105200	2.549329789	0.000193978	0.001120465	RHSIM_Rhsim08G0105200
Root vs Stem	RHSIM_Rhsim05G0211300	-1.648307882	0.00019416	0.001121238	RHSIM_Rhsim05G0211300
Root vs Stem	RHSIM_Rhsim02G0123400	-1.264477613	0.000194505	0.001122949	RHSIM_Rhsim02G0123400

Comparison	Gene ID	log_2FC	P-value	FDR (padj)	Gene Name
Root vs Stem	novel.3133	1.427745113	0.000194819	0.001124479	--
Root vs Stem	novel.4860	-1.757066901	0.00019516	0.001126162	--
Root vs Stem	RHSIM_Rhsim04G0039200	2.913173422	0.000195325	0.001126836	RHSIM_Rhsim04G0039200
Root vs Stem	RHSIM_Rhsim01G0246600	2.61847484	0.000195681	0.001128605	RHSIM_Rhsim01G0246600
Root vs Stem	novel.4834	2.345708766	0.000196288	0.001131471	--
Root vs Stem	RHSIM_Rhsim07G0248800	2.830365612	0.000196959	0.001134842	RHSIM_Rhsim07G0248800
Root vs Stem	novel.3997	-1.237770625	0.000197188	0.001135875	--
Root vs Stem	novel.5808	6.315519216	0.000197608	0.001138008	--
Root vs Stem	RHSIM_Rhsim12G0112600	-4.247818188	0.000197712	0.001138328	RHSIM_Rhsim12G0112600
Root vs Stem	RHSIM_Rhsim07G0086300	-1.872403992	0.000198213	0.001140925	RHSIM_Rhsim07G0086300
Root vs Stem	RHSIM_Rhsim07G0007100	5.639585495	0.000198934	0.00114479	RHSIM_Rhsim07G0007100
Root vs Stem	RHSIM_RhsimUnG0002400	-1.837171428	0.000199331	0.001146788	RHSIM_RhsimUnG0002400
Root vs Stem	novel.1251	3.702597838	0.000199577	0.001147914	--
Root vs Stem	novel.2409	-1.298462573	0.000199819	0.001149024	--
Root vs Stem	RHSIM_Rhsim12G0213500	2.675782684	0.000200473	0.001151633	RHSIM_Rhsim12G0213500
Root vs Stem	RHSIM_Rhsim10G0083000	-3.654794077	0.000200587	0.001152	RHSIM_Rhsim10G0083000
Root vs Stem	novel.1306	-7.942307502	0.000203191	0.001166083	--
Root vs Stem	RHSIM_Rhsim03G0006400	-3.065419113	0.000203274	0.001166271	RHSIM_Rhsim03G0006400
Root vs Stem	RHSIM_Rhsim07G0217600	-1.384001408	0.000203426	0.001166854	RHSIM_Rhsim07G0217600
Root vs Stem	RHSIM_Rhsim12G0204000	4.292605557	0.00020359	0.001167502	RHSIM_Rhsim12G0204000
Root vs Stem	novel.714	4.292140865	0.000204758	0.001173911	--
Root vs Stem	RHSIM_Rhsim09G0208900	1.116039989	0.000205004	0.001175024	RHSIM_Rhsim09G0208900
Root vs Stem	novel.3399	-3.444163796	0.000205248	0.001176135	--
Root vs Stem	novel.2483	5.952324314	0.000205829	0.001178873	--
Root vs Stem	RHSIM_Rhsim09G0131700	-1.13785601	0.000206201	0.001180715	RHSIM_Rhsim09G0131700

Comparison	Gene ID	log_2FC	P-value	FDR (padj)	Gene Name
Root vs Stem	RHSIM_Rhsim02G0067000	3.540834396	0.000206599	0.00118221	RHSIM_Rhsim02G0067000
Root vs Stem	RHSIM_Rhsim01G0104500	-4.097440386	0.000207536	0.001186884	RHSIM_Rhsim01G0104500
Root vs Stem	RHSIM_Rhsim11G0064400	-1.32764593	0.000207955	0.001188985	RHSIM_Rhsim11G0064400
Root vs Stem	RHSIM_Rhsim07G0078600	1.008865285	0.000208972	0.001194504	RHSIM_Rhsim07G0078600
Root vs Stem	RHSIM_Rhsim10G0135600	-3.929270649	0.000209463	0.001196714	RHSIM_Rhsim10G0135600
Root vs Stem	RHSIM_Rhsim05G0137300	-1.477002857	0.00021148	0.001207639	RHSIM_Rhsim05G0137300
Root vs Stem	RHSIM_Rhsim07G0141700	-1.116376081	0.000211533	0.001207647	RHSIM_Rhsim07G0141700
Root vs Stem	RHSIM_Rhsim12G0156500	1.163967123	0.000212199	0.001211082	RHSIM_Rhsim12G0156500
Root vs Stem	RHSIM_Rhsim01G0021600	-4.014760965	0.00021224	0.001211082	RHSIM_Rhsim01G0021600
Root vs Stem	RHSIM_Rhsim03G0179400	3.694225703	0.000212352	0.001211419	RHSIM_Rhsim03G0179400
Root vs Stem	RHSIM_Rhsim04G0074100	-1.856917757	0.000213921	0.001220067	RHSIM_Rhsim04G0074100
Root vs Stem	novel.147	-2.52299309	0.000215258	0.001227086	--
Root vs Stem	RHSIM_Rhsim06G0162700	1.045307426	0.000215486	0.00122804	RHSIM_Rhsim06G0162700
Root vs Stem	RHSIM_Rhsim02G0041300	-7.276955442	0.000215532	0.00122804	RHSIM_Rhsim02G0041300
Root vs Stem	RHSIM_Rhsim07G0081800	-1.403972166	0.000216148	0.001230946	RHSIM_Rhsim07G0081800
Root vs Stem	novel.3839	-6.735152673	0.000216316	0.001231598	--
Root vs Stem	RHSIM_Rhsim03G0092500	-1.646661672	0.00021651	0.001232398	RHSIM_Rhsim03G0092500
Root vs Stem	RHSIM_Rhsim03G0238400	-3.286533994	0.000218377	0.001242718	RHSIM_Rhsim03G0238400
Root vs Stem	RHSIM_Rhsim10G0176300	1.557316363	0.000219382	0.00124782	RHSIM_Rhsim10G0176300
Root vs Stem	RHSIM_Rhsim09G0117800	-1.553837715	0.000219527	0.001248338	RHSIM_Rhsim09G0117800
Root vs Stem	RHSIM_Rhsim09G0175300	-3.632058639	0.000219635	0.001248644	RHSIM_Rhsim09G0175300
Root vs Stem	RHSIM_Rhsim11G0164700	-2.177873297	0.000219949	0.001250118	RHSIM_Rhsim11G0164700
Root vs Stem	novel.756	2.564470625	0.000220285	0.001251723	--
Root vs Stem	RHSIM_Rhsim09G0022000	-2.244613088	0.000220379	0.001251945	RHSIM_Rhsim09G0022000
Root vs Stem	RHSIM_Rhsim13G0228300	1.09913521	0.000220463	0.001252112	RHSIM_Rhsim13G0228300

Comparison	Gene ID	log_2FC	P-value	FDR (padj)	Gene Name
Root vs Stem	RHSIM_Rhsim08G0019900	3.092558895	0.000220762	0.001253197	RHSIM_Rhsim08G0019900
Root vs Stem	RHSIM_Rhsim11G0158900	3.729544743	0.000222797	0.001264125	RHSIM_Rhsim11G0158900
Root vs Stem	RHSIM_Rhsim03G0087500	-3.808583004	0.000223521	0.001267782	RHSIM_Rhsim03G0087500
Root vs Stem	RHSIM_Rhsim13G0018400	1.017012249	0.000223552	0.001267782	RHSIM_Rhsim13G0018400
Root vs Stem	RHSIM_Rhsim03G0063900	1.212185728	0.000224722	0.001273477	RHSIM_Rhsim03G0063900
Root vs Stem	RHSIM_Rhsim07G0063900	2.180749199	0.000227764	0.001289453	RHSIM_Rhsim07G0063900
Root vs Stem	RHSIM_Rhsim12G0169600	2.65976252	0.000228299	0.001292164	RHSIM_Rhsim12G0169600
Root vs Stem	RHSIM_Rhsim02G0164800	4.917404374	0.000228947	0.001295307	RHSIM_Rhsim02G0164800
Root vs Stem	RHSIM_Rhsim10G0128500	-2.295250093	0.000228967	0.001295307	RHSIM_Rhsim10G0128500
Root vs Stem	RHSIM_Rhsim02G0065000	-3.84578077	0.000229777	0.001299569	RHSIM_Rhsim02G0065000
Root vs Stem	RHSIM_Rhsim11G0053200	-2.40628979	0.000229934	0.001300141	RHSIM_Rhsim11G0053200
Root vs Stem	RHSIM_Rhsim01G0241700	-1.630439822	0.000230316	0.001301981	RHSIM_Rhsim01G0241700
Root vs Stem	RHSIM_Rhsim07G0024500	-3.711450672	0.000231107	0.001306133	RHSIM_Rhsim07G0024500
Root vs Stem	RHSIM_Rhsim03G0061600	3.040910289	0.00023163	0.001308764	RHSIM_Rhsim03G0061600
Root vs Stem	RHSIM_Rhsim10G0154300	1.986743283	0.000231711	0.001308905	RHSIM_Rhsim10G0154300
Root vs Stem	RHSIM_Rhsim06G0053300	1.38195203	0.000232076	0.001310645	RHSIM_Rhsim06G0053300
Root vs Stem	RHSIM_Rhsim03G0145900	1.215787197	0.00023231	0.001311644	RHSIM_Rhsim03G0145900
Root vs Stem	RHSIM_Rhsim03G0228000	-1.905852095	0.000233113	0.001315268	RHSIM_Rhsim03G0228000
Root vs Stem	RHSIM_RhsimUnG0098000	1.998073611	0.000233123	0.001315268	RHSIM_RhsimUnG0098000
Root vs Stem	RHSIM_Rhsim01G0289000	-4.121209382	0.000233465	0.001316877	RHSIM_Rhsim01G0289000
Root vs Stem	RHSIM_Rhsim13G0058400	-1.336990072	0.000234882	0.001324546	RHSIM_Rhsim13G0058400
Root vs Stem	RHSIM_Rhsim05G0162500	1.3325431	0.000235084	0.001325356	RHSIM_Rhsim05G0162500
Root vs Stem	RHSIM_Rhsim10G0138500	1.838677141	0.00023541	0.001326869	RHSIM_Rhsim10G0138500
Root vs Stem	RHSIM_Rhsim02G0158800	-1.422829935	0.000235601	0.001327626	RHSIM_Rhsim02G0158800
Root vs Stem	RHSIM_Rhsim11G0087700	-2.569859289	0.000236114	0.001329866	RHSIM_Rhsim11G0087700

Comparison	Gene ID	log_2FC	P-value	FDR (padj)	Gene Name
Root vs Stem	RHSIM_Rhsim01G0250300	1.845593381	0.0002364	0.001330824	RHSIM_Rhsim01G0250300
Root vs Stem	novel.2440	-1.404461728	0.000236492	0.00133102	--
Root vs Stem	RHSIM_Rhsim12G0199300	1.141499575	0.000236602	0.00133131	RHSIM_Rhsim12G0199300
Root vs Stem	RHSIM_Rhsim04G0111900	1.909297571	0.000237579	0.00133616	RHSIM_Rhsim04G0111900
Root vs Stem	RHSIM_Rhsim05G0077100	2.190739238	0.000237705	0.00133654	RHSIM_Rhsim05G0077100
Root vs Stem	RHSIM_Rhsim09G0003300	1.736319656	0.0002383	0.00133938	RHSIM_Rhsim09G0003300
Root vs Stem	RHSIM_Rhsim07G0005600	-1.20211228	0.00023865	0.001340871	RHSIM_Rhsim07G0005600
Root vs Stem	novel.4211	-4.449002189	0.000239218	0.001343734	--
Root vs Stem	RHSIM_Rhsim02G0113400	-4.587463245	0.000239715	0.001346201	RHSIM_Rhsim02G0113400
Root vs Stem	RHSIM_Rhsim06G0141400	-2.829085203	0.000240179	0.001348475	RHSIM_Rhsim06G0141400
Root vs Stem	novel.2457	-2.686268698	0.000240619	0.001350618	--
Root vs Stem	RHSIM_RhsimUnG0234700	1.839883435	0.000240937	0.001352075	RHSIM_RhsimUnG0234700
Root vs Stem	novel.5913	1.762283058	0.000245104	0.001373454	--
Root vs Stem	RHSIM_Rhsim10G0049100	-2.024566689	0.000246304	0.001379842	RHSIM_Rhsim10G0049100
Root vs Stem	RHSIM_Rhsim03G0162000	2.286359144	0.000248393	0.001390865	RHSIM_Rhsim03G0162000
Root vs Stem	RHSIM_RhsimUnG0118600	-1.492873783	0.000249087	0.001394417	RHSIM_RhsimUnG0118600
Root vs Stem	RHSIM_Rhsim09G0006400	-1.790392541	0.000250439	0.001401306	RHSIM_Rhsim09G0006400
Root vs Stem	novel.1089	-1.803923047	0.000250524	0.001401438	--
Root vs Stem	novel.64	-1.946469747	0.000250647	0.001401786	--
Root vs Stem	RHSIM_Rhsim13G0169200	-4.472597804	0.000251301	0.001405105	RHSIM_Rhsim13G0169200
Root vs Stem	RHSIM_Rhsim12G0167500	1.103358215	0.000251959	0.001408442	RHSIM_Rhsim12G0167500
Root vs Stem	RHSIM_Rhsim04G0086400	-2.175268832	0.000253378	0.001415684	RHSIM_Rhsim04G0086400
Root vs Stem	RHSIM_Rhsim04G0099100	1.600310498	0.000254536	0.001421505	RHSIM_Rhsim04G0099100
Root vs Stem	RHSIM_Rhsim11G0185700	-3.038597436	0.000254835	0.001422794	RHSIM_Rhsim11G0185700
Root vs Stem	RHSIM_Rhsim06G0147500	-4.546791329	0.000255019	0.001423476	RHSIM_Rhsim06G0147500

Comparison	Gene ID	log2FC	P-value	FDR (padj)	Gene Name
Root vs Stem	RHSIM_Rhsim04G0238000	-2.79913454	0.000256325	0.00143008	RHSIM_Rhsim04G0238000
Root vs Stem	novel.2721	-2.38361236	0.000257215	0.001432956	--
Root vs Stem	RHSIM_Rhsim02G0176200	1.942560742	0.000257838	0.001435732	RHSIM_Rhsim02G0176200
Root vs Stem	RHSIM_Rhsim03G0098500	-5.820920787	0.000258853	0.00144104	RHSIM_Rhsim03G0098500
Root vs Stem	RHSIM_Rhsim08G0176400	1.06384904	0.000259812	0.001445681	RHSIM_Rhsim08G0176400
Root vs Stem	RHSIM_Rhsim09G0109200	-1.79371167	0.000260141	0.001447159	RHSIM_Rhsim09G0109200
Root vs Stem	RHSIM_Rhsim12G0134800	1.080500447	0.000260812	0.001450481	RHSIM_Rhsim12G0134800
Root vs Stem	RHSIM_Rhsim07G0116400	-2.643314401	0.000260936	0.001450481	RHSIM_Rhsim07G0116400
Root vs Stem	RHSIM_Rhsim07G0183900	-1.520595074	0.00026099	0.001450481	RHSIM_Rhsim07G0183900
Root vs Stem	RHSIM_Rhsim08G0100600	1.521779427	0.000262468	0.001457996	RHSIM_Rhsim08G0100600
Root vs Stem	RHSIM_Rhsim08G0073000	5.416956378	0.000263651	0.001463506	RHSIM_Rhsim08G0073000
Root vs Stem	novel.974	-2.261793809	0.000264064	0.001465449	--
Root vs Stem	novel.5762	2.176862622	0.000264549	0.001467785	--
Root vs Stem	RHSIM_Rhsim11G0083400	-1.711189131	0.000264707	0.001468308	RHSIM_Rhsim11G0083400
Root vs Stem	RHSIM_Rhsim13G0142100	-2.029543104	0.000264896	0.001469005	RHSIM_Rhsim13G0142100
Root vs Stem	RHSIM_Rhsim12G0140500	1.535094094	0.000266021	0.001474532	RHSIM_Rhsim12G0140500
Root vs Stem	RHSIM_Rhsim07G0159000	-10.33782535	0.000266187	0.001474986	RHSIM_Rhsim07G0159000
Root vs Stem	novel.2473	-3.079721998	0.000266231	0.001474986	--
Root vs Stem	RHSIM_Rhsim07G0237700	3.388458525	0.000267531	0.00148148	RHSIM_Rhsim07G0237700
Root vs Stem	RHSIM_Rhsim02G0005300	1.136819848	0.000269584	0.001492131	RHSIM_Rhsim02G0005300
Root vs Stem	RHSIM_Rhsim07G0202000	1.035206065	0.000271585	0.001501401	RHSIM_Rhsim07G0202000
Root vs Stem	RHSIM_Rhsim05G0032900	-2.563427692	0.000273116	0.001509502	RHSIM_Rhsim05G0032900
Root vs Stem	RHSIM_Rhsim09G0038500	-1.34672349	0.000274455	0.001516542	RHSIM_Rhsim09G0038500
Root vs Stem	RHSIM_Rhsim07G0017100	-1.03711738	0.000275096	0.001519355	RHSIM_Rhsim07G0017100
Root vs Stem	RHSIM_Rhsim11G0145100	-1.265303359	0.000275368	0.001520493	RHSIM_Rhsim11G0145100

Comparison	Gene ID	log_2FC	P-value	FDR (padj)	Gene Name
Root vs Stem	novel.3718	1.053652297	0.000275824	0.001522644	--
Root vs Stem	RHSIM_Rhsim01G0193200	2.762857415	0.000277997	0.001533542	RHSIM_Rhsim01G0193200
Root vs Stem	RHSIM_Rhsim11G0165500	-2.881202242	0.000278111	0.001533801	RHSIM_Rhsim11G0165500
Root vs Stem	RHSIM_Rhsim12G0142700	1.112761429	0.000279362	0.001539965	RHSIM_Rhsim12G0142700
Root vs Stem	RHSIM_Rhsim07G0028100	2.649060804	0.000280426	0.00154546	RHSIM_Rhsim07G0028100
Root vs Stem	RHSIM_RhsimUnG0194200	1.925669576	0.000280773	0.001547001	RHSIM_RhsimUnG0194200
Root vs Stem	RHSIM_Rhsim01G0236000	5.205630432	0.00028086	0.001547113	RHSIM_Rhsim01G0236000
Root vs Stem	RHSIM_Rhsim01G0286800	1.833873977	0.000281266	0.001548978	RHSIM_Rhsim01G0286800
Root vs Stem	RHSIM_Rhsim03G0150800	2.378455717	0.000282021	0.001552736	RHSIM_Rhsim03G0150800
Root vs Stem	RHSIM_Rhsim13G0155300	-1.253203928	0.000282442	0.001554343	RHSIM_Rhsim13G0155300
Root vs Stem	RHSIM_Rhsim09G0050900	-1.950643797	0.000283819	0.001561176	RHSIM_Rhsim09G0050900
Root vs Stem	novel.2179	3.705069217	0.00028419	0.001562471	--
Root vs Stem	RHSIM_Rhsim12G0182900	-1.275993428	0.000284497	0.001563783	RHSIM_Rhsim12G0182900
Root vs Stem	RHSIM_Rhsim03G0183000	5.717385642	0.000284694	0.001564491	RHSIM_Rhsim03G0183000
Root vs Stem	RHSIM_Rhsim09G0194600	5.531641752	0.000284828	0.001564855	RHSIM_Rhsim09G0194600
Root vs Stem	RHSIM_Rhsim11G0067500	-1.228599478	0.000285076	0.001565844	RHSIM_Rhsim11G0067500
Root vs Stem	RHSIM_Rhsim12G0159600	1.067967333	0.00028599	0.001570119	RHSIM_Rhsim12G0159600
Root vs Stem	RHSIM_Rhsim05G0219900	3.178540039	0.000286414	0.001572007	RHSIM_Rhsim05G0219900
Root vs Stem	RHSIM_RhsimUnG0195700	2.210934253	0.000286914	0.001573694	RHSIM_RhsimUnG0195700
Root vs Stem	RHSIM_Rhsim04G0187000	2.081688523	0.000286914	0.001573694	RHSIM_Rhsim04G0187000
Root vs Stem	novel.3273	-2.252912916	0.000287166	0.001574326	--
Root vs Stem	RHSIM_Rhsim06G0065400	4.737552406	0.000288354	0.001580462	RHSIM_Rhsim06G0065400
Root vs Stem	RHSIM_Rhsim03G0144300	-2.123273625	0.000291084	0.001593155	RHSIM_Rhsim03G0144300
Root vs Stem	RHSIM_Rhsim08G0179500	-1.711688539	0.000292463	0.00159946	RHSIM_Rhsim08G0179500
Root vs Stem	RHSIM_Rhsim11G0090700	-5.088984948	0.000292493	0.00159946	RHSIM_Rhsim11G0090700

Comparison	Gene ID	log_2FC	P-value	FDR (padj)	Gene Name
Root vs Stem	novel.4549	-7.463990797	0.000292956	0.001600793	--
Root vs Stem	RHSIM_Rhsim12G0089300	-1.715551517	0.000292966	0.001600793	RHSIM_Rhsim12G0089300
Root vs Stem	novel.2538	3.535890828	0.000293374	0.001602264	--
Root vs Stem	RHSIM_Rhsim07G0019900	-2.736753242	0.000294164	0.001605817	RHSIM_Rhsim07G0019900
Root vs Stem	RHSIM_Rhsim04G0202400	-1.386469735	0.000295206	0.001610651	RHSIM_Rhsim04G0202400
Root vs Stem	RHSIM_Rhsim02G0254900	-2.221187732	0.000295804	0.001613243	RHSIM_Rhsim02G0254900
Root vs Stem	RHSIM_Rhsim11G0145300	1.089829943	0.000296612	0.001616884	RHSIM_Rhsim11G0145300
Root vs Stem	novel.1794	-1.016280071	0.000298242	0.001625385	--
Root vs Stem	RHSIM_Rhsim01G0228500	1.571130674	0.000298611	0.001627012	RHSIM_Rhsim01G0228500
Root vs Stem	RHSIM_Rhsim07G0089900	-4.490844376	0.000299697	0.001632395	RHSIM_Rhsim07G0089900
Root vs Stem	RHSIM_Rhsim06G0036800	-1.191320378	0.000299741	0.001632395	RHSIM_Rhsim06G0036800
Root vs Stem	RHSIM_Rhsim04G0023000	-2.139432673	0.000300774	0.001637248	RHSIM_Rhsim04G0023000
Root vs Stem	RHSIM_Rhsim08G0200400	1.089283176	0.000302412	0.001644276	RHSIM_Rhsim08G0200400
Root vs Stem	RHSIM_Rhsim12G0109500	-10.03306283	0.000303114	0.001647648	RHSIM_Rhsim12G0109500
Root vs Stem	RHSIM_Rhsim10G0034900	-3.18617311	0.000303365	0.001648628	RHSIM_Rhsim10G0034900
Root vs Stem	RHSIM_Rhsim07G0017300	1.189668702	0.000303798	0.001650203	RHSIM_Rhsim07G0017300
Root vs Stem	RHSIM_Rhsim10G0027900	-1.647977945	0.000303944	0.001650216	RHSIM_Rhsim10G0027900
Root vs Stem	RHSIM_Rhsim04G0001800	-2.395069816	0.000304132	0.001650611	RHSIM_Rhsim04G0001800
Root vs Stem	RHSIM_Rhsim05G0006000	-1.490653094	0.000304874	0.001653317	RHSIM_Rhsim05G0006000
Root vs Stem	RHSIM_Rhsim08G0114900	1.759875385	0.000305381	0.00165568	RHSIM_Rhsim08G0114900
Root vs Stem	RHSIM_Rhsim04G0036900	1.872962562	0.00030555	0.001656207	RHSIM_Rhsim04G0036900
Root vs Stem	RHSIM_Rhsim12G0094500	2.560833524	0.000308243	0.001669938	RHSIM_Rhsim12G0094500
Root vs Stem	RHSIM_Rhsim03G0034900	3.277195912	0.000308301	0.001669938	RHSIM_Rhsim03G0034900
Root vs Stem	RHSIM_Rhsim10G0060600	1.958214929	0.000308756	0.001671618	RHSIM_Rhsim10G0060600
Root vs Stem	RHSIM_Rhsim13G0131500	-2.628216173	0.000309257	0.001673937	RHSIM_Rhsim13G0131500

Comparison	Gene ID	log_2FC	P-value	FDR (padj)	Gene Name
Root vs Stem	RHSIM_Rhsim01G0251500	-2.151046284	0.000309408	0.001674361	RHSIM_Rhsim01G0251500
Root vs Stem	RHSIM_Rhsim04G0199600	1.471986407	0.000310246	0.001678108	RHSIM_Rhsim04G0199600
Root vs Stem	RHSIM_Rhsim07G0166000	6.566902167	0.000311071	0.00168182	RHSIM_Rhsim07G0166000
Root vs Stem	RHSIM_Rhsim02G0205200	-1.020926933	0.000311078	0.00168182	RHSIM_Rhsim02G0205200
Root vs Stem	novel.1037	-1.488607198	0.000311252	0.001681971	--
Root vs Stem	RHSIM_Rhsim02G0255200	-2.272366871	0.00031135	0.001682105	RHSIM_Rhsim02G0255200
Root vs Stem	novel.2859	-1.908652789	0.000311582	0.001682967	--
Root vs Stem	RHSIM_Rhsim09G0091500	3.683482159	0.000311824	0.001683881	RHSIM_Rhsim09G0091500
Root vs Stem	RHSIM_Rhsim11G0108000	2.328950282	0.000312707	0.00168825	RHSIM_Rhsim11G0108000
Root vs Stem	RHSIM_Rhsim08G0104000	1.918817585	0.000313296	0.001690641	RHSIM_Rhsim08G0104000
Root vs Stem	RHSIM_Rhsim04G0106000	1.858263814	0.000314023	0.001694165	RHSIM_Rhsim04G0106000
Root vs Stem	RHSIM_Rhsim06G0243800	-2.46557016	0.000314435	0.00169599	RHSIM_Rhsim06G0243800
Root vs Stem	novel.5737	-1.121289067	0.000316098	0.001704561	--
Root vs Stem	RHSIM_Rhsim03G0263400	-2.158032971	0.000317751	0.001713074	RHSIM_Rhsim03G0263400
Root vs Stem	RHSIM_Rhsim04G0013500	1.464627396	0.000318344	0.001715874	RHSIM_Rhsim04G0013500
Root vs Stem	novel.3116	3.730422093	0.000318906	0.001718498	--
Root vs Stem	RHSIM_RhsimUnG0147500	6.737854428	0.000320407	0.001725779	RHSIM_RhsimUnG0147500
Root vs Stem	RHSIM_Rhsim06G0008100	3.131298378	0.000321087	0.001729037	RHSIM_Rhsim06G0008100
Root vs Stem	RHSIM_Rhsim01G0290900	-2.051154749	0.000322204	0.001734361	RHSIM_Rhsim01G0290900
Root vs Stem	novel.3927	-1.452377861	0.000322226	0.001734361	--
Root vs Stem	RHSIM_Rhsim06G0191500	4.128445335	0.00032266	0.001736293	RHSIM_Rhsim06G0191500
Root vs Stem	novel.3673	-3.526232516	0.000323966	0.001742914	--
Root vs Stem	RHSIM_Rhsim10G0072300	2.036631159	0.000324072	0.00174308	RHSIM_Rhsim10G0072300
Root vs Stem	novel.2893	-1.69679851	0.000325042	0.001747889	--
Root vs Stem	novel.4194	-1.106373337	0.000327451	0.001760433	--

Comparison	Gene ID	log_2FC	P-value	FDR (padj)	Gene Name
Root vs Stem	RHSIM_Rhsim12G0141200	1.732618053	0.00032854	0.001765871	RHSIM_Rhsim12G0141200
Root vs Stem	RHSIM_Rhsim04G0144800	1.168965809	0.000329576	0.001770615	RHSIM_Rhsim04G0144800
Root vs Stem	novel.3275	-1.762670407	0.00033036	0.001774001	--
Root vs Stem	RHSIM_Rhsim13G0033600	-3.592434229	0.000330476	0.001774213	RHSIM_Rhsim13G0033600
Root vs Stem	RHSIM_Rhsim06G0074400	-3.898033784	0.000331544	0.001779114	RHSIM_Rhsim06G0074400
Root vs Stem	RHSIM_Rhsim02G0003700	1.759519547	0.000333653	0.001789599	RHSIM_Rhsim02G0003700
Root vs Stem	RHSIM_Rhsim01G0223600	2.545856712	0.000335464	0.001798479	RHSIM_Rhsim01G0223600
Root vs Stem	RHSIM_Rhsim01G0181200	-3.376092349	0.000338207	0.001811651	RHSIM_Rhsim01G0181200
Root vs Stem	RHSIM_Rhsim06G0178300	-1.78175851	0.000339746	0.001819321	RHSIM_Rhsim06G0178300
Root vs Stem	RHSIM_Rhsim07G0125500	-1.041983224	0.00034008	0.001819973	RHSIM_Rhsim07G0125500
Root vs Stem	RHSIM_RhsimUnG0202000	-1.613802814	0.000340748	0.00182299	RHSIM_RhsimUnG0202000
Root vs Stem	RHSIM_Rhsim09G0166700	2.036080673	0.000340994	0.001823462	RHSIM_Rhsim09G0166700
Root vs Stem	novel.1789	-1.368674334	0.00034122	0.001824245	--
Root vs Stem	RHSIM_Rhsim03G0141900	2.231258046	0.0003416	0.001825855	RHSIM_Rhsim03G0141900
Root vs Stem	RHSIM_Rhsim03G0026600	-1.835042151	0.000342444	0.001829278	RHSIM_Rhsim03G0026600
Root vs Stem	RHSIM_Rhsim06G0047900	1.395614496	0.000342714	0.00183011	RHSIM_Rhsim06G0047900
Root vs Stem	RHSIM_Rhsim10G0113900	1.614857254	0.000343129	0.001831904	RHSIM_Rhsim10G0113900
Root vs Stem	RHSIM_Rhsim03G0058500	1.44299743	0.000346048	0.001845347	RHSIM_Rhsim03G0058500
Root vs Stem	novel.5056	-2.178070326	0.000348616	0.001856895	--
Root vs Stem	RHSIM_Rhsim07G0121000	-2.128832655	0.000348701	0.001856919	RHSIM_Rhsim07G0121000
Root vs Stem	RHSIM_Rhsim07G0091400	-1.057107356	0.000349448	0.001860039	RHSIM_Rhsim07G0091400
Root vs Stem	RHSIM_Rhsim08G0252200	-4.771873542	0.000350751	0.001866542	RHSIM_Rhsim08G0252200
Root vs Stem	novel.6191	-5.121844041	0.000351169	0.001867907	--
Root vs Stem	RHSIM_Rhsim11G0071600	-1.366628188	0.000351307	0.001868208	RHSIM_Rhsim11G0071600
Root vs Stem	RHSIM_Rhsim12G0138700	-2.113136659	0.000352134	0.001872174	RHSIM_Rhsim12G0138700

Comparison	Gene ID	log_2FC	P-value	FDR (padj)	Gene Name
Root vs Stem	RHSIM_Rhsim08G0202000	2.29182888	0.000352717	0.001873977	RHSIM_Rhsim08G0202000
Root vs Stem	RHSIM_Rhsim09G0068800	-1.824269214	0.0003536	0.001878237	RHSIM_Rhsim09G0068800
Root vs Stem	RHSIM_Rhsim13G0070400	-3.247183601	0.000356204	0.001891197	RHSIM_Rhsim13G0070400
Root vs Stem	RHSIM_Rhsim09G0214800	-1.127963435	0.000356553	0.001892612	RHSIM_Rhsim09G0214800
Root vs Stem	RHSIM_Rhsim10G0095800	-1.872920599	0.000357516	0.00189685	RHSIM_Rhsim10G0095800
Root vs Stem	RHSIM_Rhsim02G0032500	1.06503876	0.000358083	0.001898984	RHSIM_Rhsim02G0032500
Root vs Stem	RHSIM_Rhsim02G0107700	-3.976669486	0.00035835	0.001899434	RHSIM_Rhsim02G0107700
Root vs Stem	RHSIM_Rhsim01G0012600	-1.928829635	0.000358415	0.001899434	RHSIM_Rhsim01G0012600
Root vs Stem	RHSIM_RhsimUnG0149600	-2.587413459	0.000359261	0.001902605	RHSIM_RhsimUnG0149600
Root vs Stem	RHSIM_Rhsim07G0159600	2.533119484	0.000360247	0.001906365	RHSIM_Rhsim07G0159600
Root vs Stem	RHSIM_Rhsim13G0101000	1.425288455	0.000360292	0.001906365	RHSIM_Rhsim13G0101000
Root vs Stem	RHSIM_Rhsim07G0067200	2.294687991	0.000360302	0.001906365	RHSIM_Rhsim07G0067200
Root vs Stem	novel.1067	3.909292413	0.000360471	0.001906825	--
Root vs Stem	RHSIM_Rhsim01G0213200	1.76171291	0.000360713	0.001907664	RHSIM_Rhsim01G0213200
Root vs Stem	novel.4319	3.500012132	0.000363056	0.001918822	--
Root vs Stem	novel.134	2.210898992	0.000363433	0.001920291	--
Root vs Stem	novel.5970	-1.337004249	0.000363528	0.001920351	--
Root vs Stem	novel.2548	-1.550074856	0.000365292	0.001928787	--
Root vs Stem	RHSIM_Rhsim09G0095000	1.121325498	0.000365687	0.001930429	RHSIM_Rhsim09G0095000
Root vs Stem	RHSIM_Rhsim08G0186800	3.536915225	0.00036609	0.001932113	RHSIM_Rhsim08G0186800
Root vs Stem	RHSIM_Rhsim08G0092700	2.386483076	0.000366494	0.001933804	RHSIM_Rhsim08G0092700
Root vs Stem	RHSIM_Rhsim05G0096500	1.406666235	0.000367038	0.00193623	RHSIM_Rhsim05G0096500
Root vs Stem	RHSIM_Rhsim13G0023200	-1.075807621	0.000367721	0.001939387	RHSIM_Rhsim13G0023200
Root vs Stem	novel.936	-2.207451291	0.000368588	0.001943074	--
Root vs Stem	RHSIM_Rhsim08G0068800	1.120906587	0.000368802	0.001943756	RHSIM_Rhsim08G0068800

Comparison	Gene ID	log_2FC	P-value	FDR (padj)	Gene Name
Root vs Stem	RHSIM_Rhsim08G0004900	1.008647872	0.00036977	0.001948415	RHSIM_Rhsim08G0004900
Root vs Stem	RHSIM_Rhsim10G0164300	1.58073742	0.000370013	0.001949249	RHSIM_Rhsim10G0164300
Root vs Stem	RHSIM_Rhsim12G0140400	1.472877113	0.000370234	0.001949968	RHSIM_Rhsim12G0140400
Root vs Stem	RHSIM_Rhsim01G0181700	-1.105949342	0.000373902	0.001968835	RHSIM_Rhsim01G0181700
Root vs Stem	RHSIM_Rhsim04G0138200	-1.607620481	0.000375049	0.001972621	RHSIM_Rhsim04G0138200
Root vs Stem	RHSIM_Rhsim10G0185000	1.508445733	0.000375142	0.001972659	RHSIM_Rhsim10G0185000
Root vs Stem	RHSIM_Rhsim06G0184200	1.261476677	0.00037589	0.001976143	RHSIM_Rhsim06G0184200
Root vs Stem	RHSIM_Rhsim07G0225400	-1.392063859	0.000376707	0.001979537	RHSIM_Rhsim07G0225400
Root vs Stem	RHSIM_Rhsim12G0044900	-2.185012375	0.000377458	0.001982581	RHSIM_Rhsim12G0044900
Root vs Stem	RHSIM_RhsimUnG0030600	2.186505208	0.00037825	0.001985404	RHSIM_RhsimUnG0030600
Root vs Stem	RHSIM_Rhsim07G0227900	2.084224315	0.000378254	0.001985404	RHSIM_Rhsim07G0227900
Root vs Stem	RHSIM_Rhsim13G0002500	-1.730314214	0.000378365	0.001985535	RHSIM_Rhsim13G0002500
Root vs Stem	RHSIM_Rhsim01G0053800	-8.470231019	0.000378875	0.001987758	RHSIM_Rhsim01G0053800
Root vs Stem	RHSIM_Rhsim07G0059000	2.884390656	0.000380151	0.00199264	RHSIM_Rhsim07G0059000
Root vs Stem	RHSIM_Rhsim03G0059400	-1.415364949	0.000380867	0.00199454	RHSIM_Rhsim03G0059400
Root vs Stem	RHSIM_Rhsim07G0220600	2.278855047	0.000382027	0.001999741	RHSIM_Rhsim07G0220600
Root vs Stem	novel.928	-7.658405902	0.000383035	0.002004108	--
Root vs Stem	RHSIM_Rhsim07G0228100	-1.184183879	0.000383543	0.002006312	RHSIM_Rhsim07G0228100
Root vs Stem	RHSIM_Rhsim05G0127300	-8.464960999	0.000384358	0.002010121	RHSIM_Rhsim05G0127300
Root vs Stem	novel.2691	-1.905434656	0.000385187	0.002013995	--
Root vs Stem	novel.2556	-1.495185236	0.000386299	0.002018895	--
Root vs Stem	RHSIM_Rhsim08G0147600	-1.938012356	0.000386582	0.002019916	RHSIM_Rhsim08G0147600
Root vs Stem	RHSIM_Rhsim03G0052700	1.954033693	0.000387396	0.002023255	RHSIM_Rhsim03G0052700
Root vs Stem	RHSIM_Rhsim10G0017000	1.405311893	0.000390141	0.002036205	RHSIM_Rhsim10G0017000
Root vs Stem	RHSIM_Rhsim12G0074300	-1.206164227	0.000390349	0.00203683	RHSIM_Rhsim12G0074300

Comparison	Gene ID	log_2FC	P-value	FDR (padj)	Gene Name
Root vs Stem	RHSIM_Rhsim06G0100700	-1.35599632	0.00039087	0.002039086	RHSIM_Rhsim06G0100700
Root vs Stem	RHSIM_Rhsim02G0083600	-1.406395045	0.000391949	0.002044255	RHSIM_Rhsim02G0083600
Root vs Stem	RHSIM_Rhsim07G0100300	6.672818797	0.000392274	0.002045485	RHSIM_Rhsim07G0100300
Root vs Stem	RHSIM_Rhsim01G0051700	-2.185492289	0.000393309	0.002050419	RHSIM_Rhsim01G0051700
Root vs Stem	RHSIM_Rhsim05G0140900	6.558981148	0.000394801	0.002057149	RHSIM_Rhsim05G0140900
Root vs Stem	RHSIM_Rhsim04G0236500	-2.912974836	0.000394868	0.002057149	RHSIM_Rhsim04G0236500
Root vs Stem	RHSIM_Rhsim07G0073100	1.15055649	0.000395187	0.002058346	RHSIM_Rhsim07G0073100
Root vs Stem	RHSIM_Rhsim13G0223500	-2.164040457	0.000396317	0.002062837	RHSIM_Rhsim13G0223500
Root vs Stem	RHSIM_Rhsim06G0062300	1.232280843	0.000396918	0.002065497	RHSIM_Rhsim06G0062300
Root vs Stem	novel.3145	-1.180654385	0.000397112	0.002066042	--
Root vs Stem	novel.5639	-1.538396228	0.000397539	0.002067326	--
Root vs Stem	RHSIM_Rhsim11G0110900	2.424644027	0.000397904	0.002068758	RHSIM_Rhsim11G0110900
Root vs Stem	RHSIM_Rhsim07G0003500	-1.0304385	0.000398949	0.002073259	RHSIM_Rhsim07G0003500
Root vs Stem	RHSIM_Rhsim12G0069000	-1.397280209	0.000399394	0.002075101	RHSIM_Rhsim12G0069000
Root vs Stem	RHSIM_Rhsim12G0201200	1.420444043	0.000400621	0.002080461	RHSIM_Rhsim12G0201200
Root vs Stem	RHSIM_Rhsim08G0227100	-2.741614823	0.000400696	0.002080461	RHSIM_Rhsim08G0227100
Root vs Stem	RHSIM_Rhsim11G0050000	3.624318576	0.000401859	0.00208556	RHSIM_Rhsim11G0050000
Root vs Stem	RHSIM_Rhsim04G0104200	5.511504831	0.000403857	0.002093841	RHSIM_Rhsim04G0104200
Root vs Stem	RHSIM_Rhsim03G0078000	-1.707756587	0.000403909	0.002093841	RHSIM_Rhsim03G0078000
Root vs Stem	RHSIM_Rhsim13G0190000	6.473447709	0.000405128	0.002099216	RHSIM_Rhsim13G0190000
Root vs Stem	RHSIM_Rhsim08G0206300	1.589041818	0.000406332	0.002104039	RHSIM_Rhsim08G0206300
Root vs Stem	RHSIM_Rhsim09G0093500	1.301984165	0.00040878	0.002114813	RHSIM_Rhsim09G0093500
Root vs Stem	novel.1583	-1.14484702	0.000409696	0.002118602	--
Root vs Stem	novel.5542	-2.352526577	0.000409795	0.00211864	--
Root vs Stem	RHSIM_Rhsim13G0172800	-2.036144593	0.000412563	0.002131516	RHSIM_Rhsim13G0172800

Comparison	Gene ID	log_2FC	P-value	FDR (padj)	Gene Name
Root vs Stem	RHSIM_Rhsim09G0180300	2.990405286	0.000413605	0.002136421	RHSIM_Rhsim09G0180300
Root vs Stem	RHSIM_Rhsim01G0034600	1.386076588	0.000415209	0.002144225	RHSIM_Rhsim01G0034600
Root vs Stem	RHSIM_Rhsim02G0114600	-2.256474354	0.000420429	0.002168267	RHSIM_Rhsim02G0114600
Root vs Stem	RHSIM_Rhsim04G0097300	-8.42388351	0.000420685	0.002168871	RHSIM_Rhsim04G0097300
Root vs Stem	RHSIM_Rhsim01G0053500	-8.448359294	0.000420734	0.002168871	RHSIM_Rhsim01G0053500
Root vs Stem	RHSIM_Rhsim12G0166600	-1.178060198	0.000421314	0.002171373	RHSIM_Rhsim12G0166600
Root vs Stem	RHSIM_Rhsim05G0141700	-1.309060636	0.000421564	0.002172109	RHSIM_Rhsim05G0141700
Root vs Stem	RHSIM_Rhsim05G0195600	-2.153283427	0.000421645	0.002172109	RHSIM_Rhsim05G0195600
Root vs Stem	RHSIM_Rhsim01G0130600	3.049555481	0.000422148	0.002174215	RHSIM_Rhsim01G0130600
Root vs Stem	novel.5094	-7.377095937	0.000424324	0.002184445	--
Root vs Stem	RHSIM_Rhsim07G0052800	2.735277732	0.000426626	0.002195313	RHSIM_Rhsim07G0052800
Root vs Stem	RHSIM_Rhsim01G0131300	4.365384865	0.00043079	0.002214763	RHSIM_Rhsim01G0131300
Root vs Stem	RHSIM_Rhsim11G0111100	1.163513664	0.00043195	0.002220232	RHSIM_Rhsim11G0111100
Root vs Stem	RHSIM_Rhsim11G0043200	1.317596931	0.000433832	0.002228413	RHSIM_Rhsim11G0043200
Root vs Stem	RHSIM_Rhsim07G0124700	-1.002664359	0.000436074	0.002238251	RHSIM_Rhsim07G0124700
Root vs Stem	RHSIM_Rhsim08G0152600	1.129924329	0.000436135	0.002238251	RHSIM_Rhsim08G0152600
Root vs Stem	RHSIM_Rhsim10G0188900	1.044269154	0.000436602	0.002240147	RHSIM_Rhsim10G0188900
Root vs Stem	RHSIM_Rhsim06G0081900	-1.037379472	0.00043681	0.002240714	RHSIM_Rhsim06G0081900
Root vs Stem	novel.4621	-8.417818152	0.000440927	0.002259295	--
Root vs Stem	RHSIM_Rhsim01G0292500	-2.649532036	0.000440976	0.002259295	RHSIM_Rhsim01G0292500
Root vs Stem	RHSIM_Rhsim11G0109600	3.945007754	0.00044102	0.002259295	RHSIM_Rhsim11G0109600
Root vs Stem	RHSIM_Rhsim02G0179400	1.221795916	0.000441517	0.002260836	RHSIM_Rhsim02G0179400
Root vs Stem	novel.2233	-8.44300956	0.000441799	0.002261277	--
Root vs Stem	RHSIM_Rhsim02G0117400	3.085154831	0.000442272	0.002262779	RHSIM_Rhsim02G0117400
Root vs Stem	RHSIM_Rhsim02G0247500	-5.044512918	0.000443663	0.002269307	RHSIM_Rhsim02G0247500

Comparison	Gene ID	log2FC	P-value	FDR (padj)	Gene Name
Root vs Stem	RHSIM_Rhsim09G0075500	-3.844737107	0.000446383	0.002282713	RHSIM_Rhsim09G0075500
Root vs Stem	RHSIM_Rhsim05G0040200	1.886074833	0.000447011	0.002284724	RHSIM_Rhsim05G0040200
Root vs Stem	RHSIM_Rhsim05G0109900	-2.38392925	0.000448721	0.002292633	RHSIM_Rhsim05G0109900
Root vs Stem	RHSIM_Rhsim11G0086800	3.339583034	0.000449399	0.002295549	RHSIM_Rhsim11G0086800
Root vs Stem	RHSIM_RhsimUnG0032200	-1.39498112	0.000449491	0.002295549	RHSIM_RhsimUnG0032200
Root vs Stem	RHSIM_RhsimUnG0097800	-3.952157617	0.000451792	0.002305258	RHSIM_RhsimUnG0097800
Root vs Stem	RHSIM_Rhsim06G0162200	-2.561292823	0.000452871	0.002309741	RHSIM_Rhsim06G0162200
Root vs Stem	RHSIM_Rhsim07G0067900	1.180565421	0.00045417	0.002315857	RHSIM_Rhsim07G0067900
Root vs Stem	novel.6000	2.162407334	0.00045463	0.00231769	--
Root vs Stem	novel.3511	-1.647592291	0.00045649	0.002326139	--
Root vs Stem	RHSIM_Rhsim01G0065700	1.450219562	0.000457586	0.002331211	RHSIM_Rhsim01G0065700
Root vs Stem	RHSIM_Rhsim06G0165700	2.293677982	0.000458988	0.002337326	RHSIM_Rhsim06G0165700
Root vs Stem	RHSIM_Rhsim09G0052000	1.030814528	0.00046075	0.002345773	RHSIM_Rhsim09G0052000
Root vs Stem	RHSIM_Rhsim13G0068200	-3.873620855	0.000461496	0.002348536	RHSIM_Rhsim13G0068200
Root vs Stem	RHSIM_Rhsim01G0226400	-4.4788693	0.000462514	0.00235268	RHSIM_Rhsim01G0226400
Root vs Stem	RHSIM_Rhsim03G0162700	2.291101406	0.000463602	0.002357694	RHSIM_Rhsim03G0162700
Root vs Stem	novel.5956	2.226151152	0.000464289	0.002360144	--
Root vs Stem	RHSIM_Rhsim12G0139500	-2.574278641	0.000466152	0.002369094	RHSIM_Rhsim12G0139500
Root vs Stem	RHSIM_Rhsim07G0051000	2.883685313	0.0004697	0.002384497	RHSIM_Rhsim07G0051000
Root vs Stem	RHSIM_Rhsim12G0196700	-2.917205155	0.000473654	0.002401923	RHSIM_Rhsim12G0196700
Root vs Stem	novel.2738	1.449674765	0.000474145	0.002403884	--
Root vs Stem	RHSIM_RhsimUnG0000800	6.411736945	0.000476069	0.002413109	RHSIM_RhsimUnG0000800
Root vs Stem	RHSIM_Rhsim04G0202000	-1.675087533	0.00047673	0.002415928	RHSIM_Rhsim04G0202000
Root vs Stem	RHSIM_Rhsim04G0140800	2.130918586	0.000477489	0.002418603	RHSIM_Rhsim04G0140800
Root vs Stem	novel.1796	-4.804495408	0.000479139	0.002423875	--

Comparison	Gene ID	log_2FC	P-value	FDR (padj)	Gene Name
Root vs Stem	RHSIM_Rhsim02G0123200	-2.420994859	0.000480366	0.002429014	RHSIM_Rhsim02G0123200
Root vs Stem	RHSIM_Rhsim05G0105800	-2.984744645	0.000481749	0.002435428	RHSIM_Rhsim05G0105800
Root vs Stem	RHSIM_Rhsim01G0194400	1.222404957	0.000481951	0.002435428	RHSIM_Rhsim01G0194400
Root vs Stem	RHSIM_Rhsim09G0024100	-1.858616807	0.000483234	0.002441377	RHSIM_Rhsim09G0024100
Root vs Stem	RHSIM_Rhsim06G0060000	-1.023771581	0.000485901	0.002452162	RHSIM_Rhsim06G0060000
Root vs Stem	RHSIM_Rhsim02G0247000	-1.958863426	0.000486647	0.002455387	RHSIM_Rhsim02G0247000
Root vs Stem	RHSIM_Rhsim13G0027800	4.545686619	0.000486854	0.002455481	RHSIM_Rhsim13G0027800
Root vs Stem	RHSIM_Rhsim06G0147700	-5.080400627	0.000486878	0.002455481	RHSIM_Rhsim06G0147700
Root vs Stem	RHSIM_Rhsim03G0253900	-3.822584295	0.000487939	0.002459463	RHSIM_Rhsim03G0253900
Root vs Stem	RHSIM_Rhsim12G0102900	4.59660236	0.000487988	0.002459463	RHSIM_Rhsim12G0102900
Root vs Stem	RHSIM_Rhsim04G0102700	-4.304190486	0.000488278	0.00246039	RHSIM_Rhsim04G0102700
Root vs Stem	novel.3563	-8.383862306	0.000489144	0.00246421	--
Root vs Stem	RHSIM_Rhsim07G0075900	1.001842245	0.000490613	0.002471076	RHSIM_Rhsim07G0075900
Root vs Stem	RHSIM_RhsimUnG0169300	-8.371865058	0.000492441	0.002479198	RHSIM_RhsimUnG0169300
Root vs Stem	RHSIM_RhsimUnG0183200	-2.64080388	0.00049495	0.002491285	RHSIM_RhsimUnG0183200
Root vs Stem	RHSIM_RhsimUnG0127800	4.704279747	0.000495327	0.002492639	RHSIM_RhsimUnG0127800
Root vs Stem	RHSIM_Rhsim01G0088600	-8.373290088	0.000496383	0.00249737	RHSIM_Rhsim01G0088600
Root vs Stem	novel.4266	-8.359048388	0.000496484	0.00249737	--
Root vs Stem	RHSIM_Rhsim11G0049200	-2.982191937	0.000496913	0.002498983	RHSIM_Rhsim11G0049200
Root vs Stem	RHSIM_Rhsim12G0013100	1.36185343	0.000498788	0.002506772	RHSIM_Rhsim12G0013100
Root vs Stem	RHSIM_Rhsim10G0183600	-1.338534213	0.000498994	0.00250726	RHSIM_Rhsim10G0183600
Root vs Stem	RHSIM_Rhsim11G0023400	-3.070730021	0.000499573	0.002509623	RHSIM_Rhsim11G0023400
Root vs Stem	RHSIM_Rhsim07G0093300	2.55178287	0.000501447	0.002516295	RHSIM_Rhsim07G0093300
Root vs Stem	RHSIM_Rhsim06G0153000	-2.972561075	0.000502225	0.002519102	RHSIM_Rhsim06G0153000
Root vs Stem	RHSIM_Rhsim04G0032600	-2.115835999	0.000502635	0.002520612	RHSIM_Rhsim04G0032600

Comparison	Gene ID	log_2FC	P-value	FDR (padj)	Gene Name
Root vs Stem	RHSIM_RhsimUnG0042100	-1.091949253	0.000502809	0.002520933	RHSIM_RhsimUnG0042100
Root vs Stem	RHSIM_Rhsim05G0221100	-2.728988205	0.00050303	0.002521493	RHSIM_Rhsim05G0221100
Root vs Stem	novel.5156	2.293692181	0.000503359	0.002522595	--
Root vs Stem	novel.1291	3.176981711	0.000503659	0.002523549	--
Root vs Stem	RHSIM_RhsimUnG0096600	1.124902133	0.000505231	0.002529817	RHSIM_RhsimUnG0096600
Root vs Stem	RHSIM_Rhsim07G0093800	-2.069301437	0.000508945	0.002546715	RHSIM_Rhsim07G0093800
Root vs Stem	RHSIM_Rhsim02G0124400	-7.52362703	0.000511072	0.002556247	RHSIM_Rhsim02G0124400
Root vs Stem	RHSIM_Rhsim10G0121100	1.175778737	0.000512054	0.002560601	RHSIM_Rhsim10G0121100
Root vs Stem	RHSIM_Rhsim03G0017500	-3.192964011	0.000512418	0.002561867	RHSIM_Rhsim03G0017500
Root vs Stem	RHSIM_Rhsim12G0149900	1.893690827	0.000513842	0.002567318	RHSIM_Rhsim12G0149900
Root vs Stem	RHSIM_Rhsim06G0081600	2.266019769	0.000514981	0.00257245	RHSIM_Rhsim06G0081600
Root vs Stem	RHSIM_Rhsim04G0234900	-1.152745555	0.000515922	0.002576594	RHSIM_Rhsim04G0234900
Root vs Stem	RHSIM_Rhsim09G0024000	1.720325945	0.000517117	0.002581999	RHSIM_Rhsim09G0024000
Root vs Stem	RHSIM_Rhsim13G0148400	-6.61705321	0.000523491	0.002610435	RHSIM_Rhsim13G0148400
Root vs Stem	RHSIM_Rhsim13G0117700	3.315833351	0.000523753	0.002611175	RHSIM_Rhsim13G0117700
Root vs Stem	RHSIM_Rhsim12G0084700	2.612723551	0.000525628	0.002619959	RHSIM_Rhsim12G0084700
Root vs Stem	RHSIM_Rhsim08G0165000	-8.328006489	0.000527143	0.002625806	RHSIM_Rhsim08G0165000
Root vs Stem	novel.5330	1.825667621	0.000530877	0.002642691	--
Root vs Stem	novel.2777	-2.135936121	0.000531693	0.002646182	--
Root vs Stem	RHSIM_Rhsim04G0182100	5.710943664	0.000532222	0.002647976	RHSIM_Rhsim04G0182100
Root vs Stem	RHSIM_Rhsim08G0062200	4.101556078	0.000532283	0.002647976	RHSIM_Rhsim08G0062200
Root vs Stem	novel.670	-8.331674966	0.00053326	0.002652262	--
Root vs Stem	novel.1185	4.512554058	0.000537831	0.002673846	--
Root vs Stem	RHSIM_Rhsim06G0168900	2.603825426	0.000539672	0.002682418	RHSIM_Rhsim06G0168900
Root vs Stem	RHSIM_Rhsim05G0183800	4.251234887	0.000541813	0.00269248	RHSIM_Rhsim05G0183800

Comparison	Gene ID	log_2FC	P-value	FDR (padj)	Gene Name
Root vs Stem	novel.912	-1.716257109	0.000546322	0.002710796	--
Root vs Stem	novel.4304	-6.842963179	0.000547213	0.002714178	--
Root vs Stem	RHSIM_Rhsim11G0104900	-2.862819835	0.000548118	0.002717953	RHSIM_Rhsim11G0104900
Root vs Stem	novel.4719	-8.305194759	0.000548843	0.002720965	--
Root vs Stem	novel.3036	4.82931821	0.000549805	0.00272498	--
Root vs Stem	RHSIM_Rhsim02G0080900	-1.601816252	0.00054989	0.00272498	RHSIM_Rhsim02G0080900
Root vs Stem	RHSIM_Rhsim07G0019700	-2.172382487	0.000550229	0.002726021	RHSIM_Rhsim07G0019700
Root vs Stem	RHSIM_Rhsim03G0096800	-1.094235221	0.000550336	0.002726021	RHSIM_Rhsim03G0096800
Root vs Stem	novel.5267	1.374725465	0.000552565	0.002735297	--
Root vs Stem	RHSIM_Rhsim04G0116900	-2.470130235	0.000556913	0.002753866	RHSIM_Rhsim04G0116900
Root vs Stem	RHSIM_Rhsim07G0020800	-1.603483616	0.000565463	0.002792547	RHSIM_Rhsim07G0020800
Root vs Stem	RHSIM_Rhsim06G0125300	1.35711187	0.00056563	0.002792778	RHSIM_Rhsim06G0125300
Root vs Stem	RHSIM_Rhsim08G0075500	1.011515664	0.000565775	0.002792892	RHSIM_Rhsim08G0075500
Root vs Stem	RHSIM_Rhsim06G0225500	-1.842623086	0.000570061	0.002810438	RHSIM_Rhsim06G0225500
Root vs Stem	RHSIM_Rhsim08G0005100	1.044223667	0.00057161	0.002817472	RHSIM_Rhsim08G0005100
Root vs Stem	RHSIM_Rhsim03G0227600	-1.013970134	0.000572751	0.002822389	RHSIM_Rhsim03G0227600
Root vs Stem	novel.3954	-8.303024589	0.000572852	0.002822389	--
Root vs Stem	novel.1344	-2.178784964	0.000573378	0.002823512	--
Root vs Stem	RHSIM_Rhsim06G0054300	-1.544409247	0.000573447	0.002823512	RHSIM_Rhsim06G0054300
Root vs Stem	novel.5049	-1.040096228	0.000573869	0.002824384	--
Root vs Stem	RHSIM_Rhsim13G0165300	1.128377398	0.000574274	0.002825771	RHSIM_Rhsim13G0165300
Root vs Stem	RHSIM_Rhsim09G0187600	1.360307161	0.000576013	0.002833117	RHSIM_Rhsim09G0187600
Root vs Stem	novel.3714	2.771384512	0.000579798	0.002849913	--
Root vs Stem	RHSIM_Rhsim02G0173800	-1.03340007	0.000580011	0.00285035	RHSIM_Rhsim02G0173800
Root vs Stem	RHSIM_Rhsim04G0065400	-2.873279012	0.000581891	0.002858372	RHSIM_Rhsim04G0065400

Comparison	Gene ID	log_2FC	P-value	FDR (padj)	Gene Name
Root vs Stem	RHSIM_Rhsim06G0192100	2.276533405	0.000583861	0.002867438	RHSIM_Rhsim06G0192100
Root vs Stem	novel.607	-1.708266001	0.000584566	0.002869679	--
Root vs Stem	RHSIM_Rhsim10G0035000	-1.976446633	0.000589537	0.002892845	RHSIM_Rhsim10G0035000
Root vs Stem	RHSIM_Rhsim10G0207200	-1.308518674	0.000589939	0.002894203	RHSIM_Rhsim10G0207200
Root vs Stem	novel.4170	-1.5877117	0.000593067	0.002907076	--
Root vs Stem	RHSIM_Rhsim09G0045100	-1.400095727	0.000593926	0.002910214	RHSIM_Rhsim09G0045100
Root vs Stem	RHSIM_RhsimUnG0022200	2.653837854	0.00059396	0.002910214	RHSIM_RhsimUnG0022200
Root vs Stem	RHSIM_Rhsim01G0148700	1.010018938	0.000595722	0.002916369	RHSIM_Rhsim01G0148700
Root vs Stem	novel.1351	-2.201949266	0.000596293	0.002917923	--
Root vs Stem	RHSIM_Rhsim12G0063600	-1.362958023	0.00059666	0.002918769	RHSIM_Rhsim12G0063600
Root vs Stem	RHSIM_Rhsim08G0138300	-8.282087261	0.000597035	0.002919694	RHSIM_Rhsim08G0138300
Root vs Stem	RHSIM_Rhsim12G0185800	-1.521488236	0.000603421	0.002946551	RHSIM_Rhsim12G0185800
Root vs Stem	RHSIM_Rhsim03G0229000	-1.466596425	0.000604899	0.002952516	RHSIM_Rhsim03G0229000
Root vs Stem	RHSIM_Rhsim13G0034600	2.764249358	0.000606382	0.002958501	RHSIM_Rhsim13G0034600
Root vs Stem	RHSIM_Rhsim08G0190900	1.115933698	0.000606564	0.002958763	RHSIM_Rhsim08G0190900
Root vs Stem	RHSIM_Rhsim02G0146200	3.137588694	0.000606697	0.002958785	RHSIM_Rhsim02G0146200
Root vs Stem	novel.4950	-1.920306443	0.000607321	0.002960818	--
Root vs Stem	RHSIM_Rhsim05G0050700	-5.347873811	0.00060737	0.002960818	RHSIM_Rhsim05G0050700
Root vs Stem	RHSIM_RhsimUnG0071100	-3.490392403	0.000609299	0.002968963	RHSIM_RhsimUnG0071100
Root vs Stem	novel.885	-8.28587433	0.000610379	0.002973598	--
Root vs Stem	RHSIM_Rhsim07G0056600	-4.727692971	0.000610674	0.002974408	RHSIM_Rhsim07G0056600
Root vs Stem	novel.4409	1.598230859	0.000612094	0.002980256	--
Root vs Stem	novel.6175	-1.378799578	0.000613257	0.002984467	--
Root vs Stem	RHSIM_RhsimUnG0042500	-2.229760952	0.000615464	0.002993942	RHSIM_RhsimUnG0042500
Root vs Stem	RHSIM_Rhsim03G0227800	-1.109670626	0.000617612	0.003001856	RHSIM_Rhsim03G0227800

Comparison	Gene ID	log_2FC	P-value	FDR (padj)	Gene Name
Root vs Stem	RHSIM_Rhsim12G0085700	-1.032399352	0.000618099	0.00300359	RHSIM_Rhsim12G0085700
Root vs Stem	novel.3891	-7.005011497	0.000619246	0.003007895	--
Root vs Stem	RHSIM_Rhsim12G0192800	1.11017049	0.000619547	0.003008093	RHSIM_Rhsim12G0192800
Root vs Stem	RHSIM_Rhsim13G0041100	1.959822327	0.000625096	0.003033757	RHSIM_Rhsim13G0041100
Root vs Stem	RHSIM_Rhsim09G0066000	-1.23437892	0.000625984	0.003037423	RHSIM_Rhsim09G0066000
Root vs Stem	RHSIM_Rhsim08G0067200	-2.493870619	0.000630161	0.00305512	RHSIM_Rhsim08G0067200
Root vs Stem	RHSIM_Rhsim06G0141000	-2.537425682	0.00063415	0.003072523	RHSIM_Rhsim06G0141000
Root vs Stem	RHSIM_Rhsim05G0188700	-3.729149315	0.000634366	0.003072922	RHSIM_Rhsim05G0188700
Root vs Stem	RHSIM_Rhsim06G0051500	-2.143492975	0.00063754	0.003085055	RHSIM_Rhsim06G0051500
Root vs Stem	RHSIM_Rhsim08G0110300	-1.384183437	0.000639089	0.003091257	RHSIM_Rhsim08G0110300
Root vs Stem	novel.2306	-1.331089568	0.000639544	0.003092808	--
Root vs Stem	RHSIM_Rhsim13G0165900	1.695988697	0.000640271	0.003095672	RHSIM_Rhsim13G0165900
Root vs Stem	RHSIM_Rhsim07G0061900	2.53361102	0.000640893	0.003096636	RHSIM_Rhsim07G0061900
Root vs Stem	novel.329	4.006456471	0.000641008	0.003096636	--
Root vs Stem	novel.5851	-8.233801959	0.000642352	0.003101623	--
Root vs Stem	RHSIM_Rhsim13G0152900	2.664471253	0.000642444	0.003101623	RHSIM_Rhsim13G0152900
Root vs Stem	RHSIM_Rhsim13G0167100	2.525002147	0.000643075	0.00310337	RHSIM_Rhsim13G0167100
Root vs Stem	RHSIM_Rhsim12G0042600	-2.613169021	0.000643726	0.003105865	RHSIM_Rhsim12G0042600
Root vs Stem	RHSIM_Rhsim05G0229500	3.135546082	0.000644222	0.003107608	RHSIM_Rhsim05G0229500
Root vs Stem	RHSIM_Rhsim12G0156400	3.722534305	0.000645654	0.003113209	RHSIM_Rhsim12G0156400
Root vs Stem	RHSIM_Rhsim11G0148300	2.872490511	0.00064976	0.0031317	RHSIM_Rhsim11G0148300
Root vs Stem	novel.1473	-3.94681879	0.000650712	0.003133668	--
Root vs Stem	RHSIM_RhsimUnG0086500	-1.980685164	0.000651397	0.003136311	RHSIM_RhsimUnG0086500
Root vs Stem	RHSIM_Rhsim06G0211800	1.981512905	0.00065195	0.00313832	RHSIM_Rhsim06G0211800
Root vs Stem	novel.1679	3.064462607	0.000653487	0.00314506	--

Comparison	Gene ID	log_2FC	P-value	FDR (padj)	Gene Name
Root vs Stem	novel.3624	-1.146127296	0.000655209	0.00315269	--
Root vs Stem	novel.213	-8.222067172	0.000657136	0.0031613	--
Root vs Stem	RHSIM_Rhsim07G0152700	1.211881483	0.000657451	0.003162158	RHSIM_Rhsim07G0152700
Root vs Stem	RHSIM_Rhsim05G0112300	-2.446108393	0.000657931	0.003163681	RHSIM_Rhsim05G0112300
Root vs Stem	RHSIM_Rhsim08G0050000	-1.49195493	0.000659963	0.003172254	RHSIM_Rhsim08G0050000
Root vs Stem	RHSIM_Rhsim08G0117400	1.198815966	0.000660731	0.003175284	RHSIM_Rhsim08G0117400
Root vs Stem	RHSIM_Rhsim01G0268700	-2.173724028	0.000661633	0.003178291	RHSIM_Rhsim01G0268700
Root vs Stem	RHSIM_Rhsim01G0063300	-1.208107211	0.00066412	0.003188246	RHSIM_Rhsim01G0063300
Root vs Stem	RHSIM_Rhsim04G0232200	-2.251448353	0.000664781	0.003190756	RHSIM_Rhsim04G0232200
Root vs Stem	RHSIM_Rhsim07G0219700	2.408983937	0.000665546	0.003193764	RHSIM_Rhsim07G0219700
Root vs Stem	RHSIM_Rhsim02G0251700	1.933379766	0.000666451	0.003197441	RHSIM_Rhsim02G0251700
Root vs Stem	RHSIM_Rhsim05G0216100	-2.166899374	0.000668599	0.003207076	RHSIM_Rhsim05G0216100
Root vs Stem	novel.4628	-1.12690267	0.000669714	0.003211756	--
Root vs Stem	RHSIM_Rhsim10G0005900	5.777042598	0.000669979	0.003212359	RHSIM_Rhsim10G0005900
Root vs Stem	novel.3695	1.881444543	0.000670153	0.003212526	--
Root vs Stem	RHSIM_Rhsim13G0086700	-1.099806034	0.000672027	0.003220817	RHSIM_Rhsim13G0086700
Root vs Stem	RHSIM_Rhsim01G0180300	-1.318523819	0.000672436	0.003221459	RHSIM_Rhsim01G0180300
Root vs Stem	RHSIM_Rhsim06G0198300	-1.014707524	0.000672575	0.003221459	RHSIM_Rhsim06G0198300
Root vs Stem	RHSIM_Rhsim10G0002700	-1.470701972	0.000675903	0.003236052	RHSIM_Rhsim10G0002700
Root vs Stem	RHSIM_Rhsim09G0161000	-3.262240594	0.000677062	0.003240928	RHSIM_Rhsim09G0161000
Root vs Stem	novel.1519	-4.297901759	0.000679424	0.00325156	--
Root vs Stem	RHSIM_Rhsim11G0177400	-1.288496447	0.000682199	0.003263483	RHSIM_Rhsim11G0177400
Root vs Stem	novel.4838	-8.233873748	0.000683726	0.003270112	--
Root vs Stem	RHSIM_Rhsim12G0204100	2.965401658	0.000687083	0.003284803	RHSIM_Rhsim12G0204100
Root vs Stem	novel.380	-8.234657676	0.000687636	0.003286257	--

Comparison	Gene ID	log_2FC	P-value	FDR (padj)	Gene Name
Root vs Stem	RHSIM_RhsimUnG0126600	1.685880632	0.000689329	0.003293493	RHSIM_RhsimUnG0126600
Root vs Stem	RHSIM_Rhsim10G0102400	-1.581009298	0.000689883	0.003294775	RHSIM_Rhsim10G0102400
Root vs Stem	RHSIM_Rhsim03G0056600	1.370066531	0.000690158	0.003295403	RHSIM_Rhsim03G0056600
Root vs Stem	RHSIM_Rhsim09G0150400	-3.530640419	0.000691044	0.003298951	RHSIM_Rhsim09G0150400
Root vs Stem	novel.5778	1.714954277	0.000691372	0.003299834	--
Root vs Stem	novel.2144	-2.501445598	0.000693689	0.003309523	--
Root vs Stem	RHSIM_Rhsim03G0044800	-1.807151445	0.000694689	0.003313267	RHSIM_Rhsim03G0044800
Root vs Stem	novel.5157	-1.810447062	0.000694761	0.003313267	--
Root vs Stem	RHSIM_Rhsim05G0139700	3.560919781	0.000695821	0.003317522	RHSIM_Rhsim05G0139700
Root vs Stem	RHSIM_Rhsim06G0016600	1.011539387	0.000695941	0.003317522	RHSIM_Rhsim06G0016600
Root vs Stem	RHSIM_Rhsim11G0084100	2.695380912	0.000697529	0.003324402	RHSIM_Rhsim11G0084100
Root vs Stem	novel.2096	1.326608214	0.000699977	0.003334692	--
Root vs Stem	RHSIM_Rhsim04G0226100	-8.223718743	0.000702093	0.003344082	RHSIM_Rhsim04G0226100
Root vs Stem	RHSIM_Rhsim08G0221600	2.829079773	0.000707693	0.003366582	RHSIM_Rhsim08G0221600
Root vs Stem	RHSIM_Rhsim12G0160700	1.331451507	0.000708972	0.003371272	RHSIM_Rhsim12G0160700
Root vs Stem	RHSIM_Rhsim12G0065000	-2.775040214	0.000713157	0.003389775	RHSIM_Rhsim12G0065000
Root vs Stem	RHSIM_Rhsim12G0170600	-1.371224612	0.00071391	0.003391955	RHSIM_Rhsim12G0170600
Root vs Stem	RHSIM_RhsimUnG0213700	3.974716704	0.000719254	0.003416642	RHSIM_RhsimUnG0213700
Root vs Stem	RHSIM_RhsimUnG0256800	-1.0333157	0.000722416	0.003430247	RHSIM_RhsimUnG0256800
Root vs Stem	RHSIM_Rhsim10G0017100	2.322503422	0.00072419	0.003437966	RHSIM_Rhsim10G0017100
Root vs Stem	novel.3217	-8.18391842	0.00072599	0.003445799	--
Root vs Stem	RHSIM_Rhsim08G0028800	-1.665303461	0.000726886	0.00344934	RHSIM_Rhsim08G0028800
Root vs Stem	novel.3953	-1.047432323	0.000731359	0.003469852	--
Root vs Stem	RHSIM_Rhsim12G0103500	-2.2945142	0.000732621	0.003475124	RHSIM_Rhsim12G0103500
Root vs Stem	novel.4217	2.636654176	0.000732852	0.003475505	--

Comparison	Gene ID	log_2FC	P-value	FDR (padj)	Gene Name
Root vs Stem	RHSIM_Rhsim01G0120400	5.736952385	0.000733886	0.003479696	RHSIM_Rhsim01G0120400
Root vs Stem	RHSIM_Rhsim02G0003000	-1.710344873	0.000736486	0.003491303	RHSIM_Rhsim02G0003000
Root vs Stem	novel.4109	2.151059341	0.000737328	0.00349386	--
Root vs Stem	RHSIM_Rhsim13G0023900	1.102991025	0.000742851	0.003517859	RHSIM_Rhsim13G0023900
Root vs Stem	novel.2036	-5.183823907	0.000746582	0.003534802	--
Root vs Stem	novel.1675	-1.618020255	0.000747775	0.003538997	--
Root vs Stem	RHSIM_Rhsim05G0232200	-2.567135756	0.000748792	0.003543082	RHSIM_Rhsim05G0232200
Root vs Stem	RHSIM_Rhsim12G0008700	2.521185327	0.000751175	0.003552901	RHSIM_Rhsim12G0008700
Root vs Stem	RHSIM_Rhsim10G0000500	-1.134125802	0.000752782	0.003558313	RHSIM_Rhsim10G0000500
Root vs Stem	novel.352	2.373669718	0.000754132	0.003563963	--
Root vs Stem	RHSIM_Rhsim06G0222800	3.47843601	0.000754381	0.003564408	RHSIM_Rhsim06G0222800
Root vs Stem	novel.1000	-4.249075472	0.000758284	0.00358138	--
Root vs Stem	RHSIM_RhsimUnG0212900	-1.492927601	0.000760168	0.003588354	RHSIM_RhsimUnG0212900
Root vs Stem	novel.878	-2.23382515	0.000761292	0.003592642	--
Root vs Stem	RHSIM_Rhsim05G0044900	1.182212248	0.000762617	0.003598161	RHSIM_Rhsim05G0044900
Root vs Stem	RHSIM_Rhsim10G0047200	-2.347164354	0.000763081	0.003598875	RHSIM_Rhsim10G0047200
Root vs Stem	RHSIM_Rhsim06G0162400	-2.494276785	0.000763379	0.003599546	RHSIM_Rhsim06G0162400
Root vs Stem	RHSIM_RhsimUnG0014700	-2.618411317	0.000764969	0.003605566	RHSIM_RhsimUnG0014700
Root vs Stem	novel.182	-8.16363613	0.00076556	0.003607613	--
Root vs Stem	RHSIM_Rhsim13G0018000	2.216732712	0.000768234	0.003618738	RHSIM_Rhsim13G0018000
Root vs Stem	RHSIM_Rhsim13G0029500	1.242401536	0.000773144	0.003641122	RHSIM_Rhsim13G0029500
Root vs Stem	RHSIM_RhsimUnG0010000	6.151724187	0.000775015	0.003649188	RHSIM_RhsimUnG0010000
Root vs Stem	RHSIM_RhsimUnG0123200	2.061063573	0.000778725	0.003664412	RHSIM_RhsimUnG0123200
Root vs Stem	novel.5554	-2.271547311	0.000783275	0.003685071	--
Root vs Stem	RHSIM_Rhsim13G0065400	1.078396972	0.00078507	0.003691993	RHSIM_Rhsim13G0065400

Comparison	Gene ID	log_2FC	P-value	FDR (padj)	Gene Name
Root vs Stem	RHSIM_Rhsim13G0107600	1.461331544	0.000785227	0.003691993	RHSIM_Rhsim13G0107600
Root vs Stem	RHSIM_Rhsim03G0231300	1.335867243	0.000785451	0.003692292	RHSIM_Rhsim03G0231300
Root vs Stem	RHSIM_Rhsim09G0111600	1.50629765	0.000787478	0.003701065	RHSIM_Rhsim09G0111600
Root vs Stem	RHSIM_RhsimUnG0245800	-8.135534431	0.000794097	0.003730652	RHSIM_RhsimUnG0245800
Root vs Stem	RHSIM_Rhsim02G0021000	1.338456064	0.000794334	0.003731009	RHSIM_Rhsim02G0021000
Root vs Stem	novel.2649	1.339687656	0.000802493	0.003767796	--
Root vs Stem	RHSIM_Rhsim06G0199000	-1.409229726	0.000804281	0.003774653	RHSIM_Rhsim06G0199000
Root vs Stem	RHSIM_Rhsim06G0243000	1.053370093	0.00080585	0.003781244	RHSIM_Rhsim06G0243000
Root vs Stem	RHSIM_Rhsim02G0101200	3.934130117	0.00080618	0.003782026	RHSIM_Rhsim02G0101200
Root vs Stem	RHSIM_Rhsim12G0201100	1.131284117	0.00080668	0.003783599	RHSIM_Rhsim12G0201100
Root vs Stem	RHSIM_RhsimUnG0098600	1.087982492	0.00081371	0.003814246	RHSIM_RhsimUnG0098600
Root vs Stem	RHSIM_Rhsim06G0240700	1.189204216	0.000819317	0.003838118	RHSIM_Rhsim06G0240700
Root vs Stem	RHSIM_Rhsim05G0039400	1.381598132	0.000819635	0.003838118	RHSIM_Rhsim05G0039400
Root vs Stem	RHSIM_Rhsim01G0043100	1.245640825	0.000821634	0.003844354	RHSIM_Rhsim01G0043100
Root vs Stem	RHSIM_Rhsim02G0062400	-8.129722417	0.000822337	0.003846864	RHSIM_Rhsim02G0062400
Root vs Stem	RHSIM_RhsimUnG0236600	-1.00509485	0.000824886	0.003856439	RHSIM_RhsimUnG0236600
Root vs Stem	RHSIM_Rhsim08G0180900	-1.116293132	0.0008263	0.003862265	RHSIM_Rhsim08G0180900
Root vs Stem	RHSIM_Rhsim02G0086500	4.131351508	0.000827605	0.003866796	RHSIM_Rhsim02G0086500
Root vs Stem	RHSIM_Rhsim05G0035200	-1.108190041	0.000827798	0.003866916	RHSIM_Rhsim05G0035200
Root vs Stem	RHSIM_Rhsim02G0209600	5.439991382	0.000829131	0.003872357	RHSIM_Rhsim02G0209600
Root vs Stem	RHSIM_Rhsim10G0142800	-2.745073134	0.000830828	0.003879495	RHSIM_Rhsim10G0142800
Root vs Stem	novel.3525	6.161502352	0.000831716	0.00388216	--
Root vs Stem	RHSIM_Rhsim06G0031100	1.225224014	0.000831735	0.00388216	RHSIM_Rhsim06G0031100
Root vs Stem	novel.5781	-8.17511369	0.000832831	0.003885702	--
Root vs Stem	novel.3804	4.158502693	0.00083402	0.003889675	--

Comparison	Gene ID	log_2FC	P-value	FDR (padj)	Gene Name
Root vs Stem	RHSIM_Rhsim04G0054300	-2.358268679	0.000837201	0.003902142	RHSIM_Rhsim04G0054300
Root vs Stem	RHSIM_Rhsim12G0098200	-8.116256186	0.000837656	0.003903473	RHSIM_Rhsim12G0098200
Root vs Stem	RHSIM_Rhsim10G0169800	3.58487669	0.000842544	0.003923078	RHSIM_Rhsim10G0169800
Root vs Stem	RHSIM_Rhsim06G0040000	-3.006933871	0.000843823	0.003928242	RHSIM_Rhsim06G0040000
Root vs Stem	novel.25	-8.118804233	0.000844179	0.003929105	--
Root vs Stem	RHSIM_Rhsim07G0093200	2.994122246	0.000844912	0.003931724	RHSIM_Rhsim07G0093200
Root vs Stem	RHSIM_Rhsim01G0116400	1.022571623	0.000846173	0.00393521	RHSIM_Rhsim01G0116400
Root vs Stem	RHSIM_Rhsim06G0031700	1.17860894	0.000848401	0.003943486	RHSIM_Rhsim06G0031700
Root vs Stem	novel.5302	1.283813292	0.000848466	0.003943486	--
Root vs Stem	RHSIM_RhsimUnG0212200	-1.056239444	0.000851694	0.003957286	RHSIM_RhsimUnG0212200
Root vs Stem	RHSIM_Rhsim08G0230800	1.726438353	0.000851779	0.003957286	RHSIM_Rhsim08G0230800
Root vs Stem	RHSIM_Rhsim12G0069600	-8.121348099	0.000853998	0.003965996	RHSIM_Rhsim12G0069600
Root vs Stem	RHSIM_Rhsim03G0174300	-3.599441364	0.000855959	0.003974306	RHSIM_Rhsim03G0174300
Root vs Stem	novel.654	-8.104378463	0.000857822	0.003981682	--
Root vs Stem	novel.98	1.147871162	0.000857893	0.003981682	--
Root vs Stem	RHSIM_Rhsim07G0078500	1.733172979	0.000858101	0.003981845	RHSIM_Rhsim07G0078500
Root vs Stem	RHSIM_Rhsim12G0142900	-1.001654852	0.000860161	0.003986811	RHSIM_Rhsim12G0142900
Root vs Stem	novel.589	2.110454312	0.000860209	0.003986811	--
Root vs Stem	RHSIM_Rhsim04G0185900	2.458044765	0.000866507	0.004011854	RHSIM_Rhsim04G0185900
Root vs Stem	RHSIM_Rhsim04G0102400	3.206090949	0.000866657	0.004011854	RHSIM_Rhsim04G0102400
Root vs Stem	RHSIM_RhsimUnG0233800	-2.591080701	0.000867048	0.004012861	RHSIM_RhsimUnG0233800
Root vs Stem	novel.3813	-3.786491634	0.000868054	0.004016709	--
Root vs Stem	RHSIM_Rhsim06G0008800	1.140444113	0.000871898	0.004032068	RHSIM_Rhsim06G0008800
Root vs Stem	RHSIM_Rhsim04G0169000	-1.199387754	0.000876552	0.004051152	RHSIM_Rhsim04G0169000
Root vs Stem	RHSIM_Rhsim11G0121700	2.336371898	0.000880797	0.004069142	RHSIM_Rhsim11G0121700

Comparison	Gene ID	log_2FC	P-value	FDR (padj)	Gene Name
Root vs Stem	RHSIM_Rhsim05G0056900	-1.740595687	0.000881199	0.004070181	RHSIM_Rhsim05G0056900
Root vs Stem	RHSIM_Rhsim11G0010800	-2.817300111	0.000881456	0.004070552	RHSIM_Rhsim11G0010800
Root vs Stem	RHSIM_Rhsim09G0112700	1.154263806	0.000881719	0.004070954	RHSIM_Rhsim09G0112700
Root vs Stem	RHSIM_Rhsim04G0046000	1.667896925	0.000884208	0.004080698	RHSIM_Rhsim04G0046000
Root vs Stem	RHSIM_Rhsim13G0169300	1.078043696	0.000884922	0.004081333	RHSIM_Rhsim13G0169300
Root vs Stem	novel.5525	-2.012436487	0.000885029	0.004081333	--
Root vs Stem	novel.4017	1.455682605	0.000886803	0.004088695	--
Root vs Stem	RHSIM_Rhsim08G0145600	2.890829184	0.000895481	0.004126229	RHSIM_Rhsim08G0145600
Root vs Stem	RHSIM_Rhsim02G0245100	-2.116125714	0.000897826	0.004134374	RHSIM_Rhsim02G0245100
Root vs Stem	RHSIM_Rhsim05G0047000	1.0523831	0.000897972	0.004134374	RHSIM_Rhsim05G0047000
Root vs Stem	RHSIM_Rhsim11G0029800	1.639132093	0.000898294	0.004134374	RHSIM_Rhsim11G0029800
Root vs Stem	RHSIM_Rhsim10G0166600	-2.691027396	0.000898457	0.004134374	RHSIM_Rhsim10G0166600
Root vs Stem	RHSIM_Rhsim09G0182700	-1.266219531	0.000898504	0.004134374	RHSIM_Rhsim09G0182700
Root vs Stem	RHSIM_Rhsim11G0106400	-2.393437777	0.000901738	0.004147599	RHSIM_Rhsim11G0106400
Root vs Stem	novel.3810	-6.064708228	0.000902928	0.004152245	--
Root vs Stem	RHSIM_Rhsim02G0178400	-3.53775511	0.000907546	0.004172648	RHSIM_Rhsim02G0178400
Root vs Stem	novel.4120	-2.178854812	0.000912325	0.004192112	--
Root vs Stem	RHSIM_Rhsim11G0014400	-1.091525321	0.000912825	0.004193576	RHSIM_Rhsim11G0014400
Root vs Stem	RHSIM_Rhsim02G0063500	-3.332831295	0.00091509	0.00420147	RHSIM_Rhsim02G0063500
Root vs Stem	RHSIM_Rhsim11G0172800	-4.597143019	0.000916126	0.004205387	RHSIM_Rhsim11G0172800
Root vs Stem	RHSIM_RhsimUnG0102200	-2.362593419	0.000917038	0.004207898	RHSIM_RhsimUnG0102200
Root vs Stem	RHSIM_RhsimUnG0189000	1.284961781	0.000920934	0.004223253	RHSIM_RhsimUnG0189000
Root vs Stem	RHSIM_Rhsim02G0241700	1.833592265	0.00092168	0.004225161	RHSIM_Rhsim02G0241700
Root vs Stem	RHSIM_Rhsim12G0070100	1.920905244	0.000921717	0.004225161	RHSIM_Rhsim12G0070100
Root vs Stem	RHSIM_Rhsim10G0045700	-8.087280632	0.00092323	0.004230418	RHSIM_Rhsim10G0045700

Comparison	Gene ID	log_2FC	P-value	FDR (padj)	Gene Name
Root vs Stem	RHSIM_Rhsim04G0187800	1.62183168	0.000924068	0.004233415	RHSIM_Rhsim04G0187800
Root vs Stem	RHSIM_Rhsim06G0180100	2.912042384	0.000925828	0.004240071	RHSIM_Rhsim06G0180100
Root vs Stem	RHSIM_Rhsim08G0044300	1.129556751	0.000925889	0.004240071	RHSIM_Rhsim08G0044300
Root vs Stem	novel.1339	-8.070781031	0.000927818	0.004247968	--
Root vs Stem	RHSIM_Rhsim03G0150600	1.904558771	0.000927999	0.004247968	RHSIM_Rhsim03G0150600
Root vs Stem	RHSIM_Rhsim11G0037200	1.21981527	0.000930049	0.004253208	RHSIM_Rhsim11G0037200
Root vs Stem	RHSIM_Rhsim05G0128200	-2.153250607	0.000933381	0.004264217	RHSIM_Rhsim05G0128200
Root vs Stem	RHSIM_Rhsim10G0121800	4.720638501	0.000934878	0.00427021	RHSIM_Rhsim10G0121800
Root vs Stem	RHSIM_Rhsim11G0144300	-2.529496597	0.000939041	0.004286677	RHSIM_Rhsim11G0144300
Root vs Stem	RHSIM_Rhsim04G0202800	-1.815143386	0.000940507	0.004290821	RHSIM_Rhsim04G0202800
Root vs Stem	RHSIM_Rhsim07G0042200	4.419389828	0.000942995	0.004299617	RHSIM_Rhsim07G0042200
Root vs Stem	RHSIM_Rhsim02G0070600	3.890296227	0.00094456	0.004303885	RHSIM_Rhsim02G0070600
Root vs Stem	RHSIM_Rhsim11G0159100	2.4655457	0.000944678	0.004303885	RHSIM_Rhsim11G0159100
Root vs Stem	RHSIM_Rhsim02G0203200	-2.484617142	0.000947859	0.004314983	RHSIM_Rhsim02G0203200
Root vs Stem	RHSIM_Rhsim11G0110000	3.352958068	0.000947862	0.004314983	RHSIM_Rhsim11G0110000
Root vs Stem	RHSIM_Rhsim02G0108900	5.232801307	0.000949345	0.004320024	RHSIM_Rhsim02G0108900
Root vs Stem	novel.5773	-8.119729676	0.000952063	0.004329671	--
Root vs Stem	novel.2460	-1.699812625	0.000952216	0.004329671	--
Root vs Stem	RHSIM_Rhsim03G0150900	1.574337576	0.000956842	0.004349849	RHSIM_Rhsim03G0150900
Root vs Stem	novel.1345	-1.66149195	0.000957766	0.004353192	--
Root vs Stem	novel.1424	-6.577465615	0.000958559	0.004355934	--
Root vs Stem	RHSIM_Rhsim12G0125700	1.11863442	0.000959565	0.004359649	RHSIM_Rhsim12G0125700
Root vs Stem	novel.1613	-1.741083028	0.000961043	0.004364642	--
Root vs Stem	RHSIM_Rhsim03G0038800	1.496104008	0.000966482	0.004387614	RHSIM_Rhsim03G0038800
Root vs Stem	RHSIM_Rhsim01G0279700	1.552061735	0.000966843	0.004388389	RHSIM_Rhsim01G0279700

Comparison	Gene ID	log_2FC	P-value	FDR (padj)	Gene Name
Root vs Stem	RHSIM_Rhsim07G0163800	-3.071468508	0.000967989	0.004392727	RHSIM_Rhsim07G0163800
Root vs Stem	RHSIM_Rhsim06G0230900	-1.613443219	0.000970326	0.004402465	RHSIM_Rhsim06G0230900
Root vs Stem	RHSIM_Rhsim11G0008200	-1.338866918	0.000972325	0.004410667	RHSIM_Rhsim11G0008200
Root vs Stem	RHSIM_RhsimUnG0090900	-5.172475351	0.000973182	0.004413688	RHSIM_RhsimUnG0090900
Root vs Stem	RHSIM_Rhsim09G0060400	1.807923505	0.000975054	0.004421307	RHSIM_Rhsim09G0060400
Root vs Stem	RHSIM_Rhsim10G0141100	-1.188997233	0.000975645	0.004423114	RHSIM_Rhsim10G0141100
Root vs Stem	RHSIM_Rhsim01G0023200	-3.437207156	0.000976313	0.004423779	RHSIM_Rhsim01G0023200
Root vs Stem	RHSIM_RhsimUnG0066000	-2.449030555	0.000976367	0.004423779	RHSIM_RhsimUnG0066000
Root vs Stem	RHSIM_Rhsim13G0141900	-1.217434116	0.000978536	0.004432736	RHSIM_Rhsim13G0141900
Root vs Stem	RHSIM_Rhsim04G0097100	-1.691305771	0.000979245	0.004434626	RHSIM_Rhsim04G0097100
Root vs Stem	RHSIM_Rhsim03G0191800	-2.490028867	0.00097953	0.004434626	RHSIM_Rhsim03G0191800
Root vs Stem	RHSIM_Rhsim03G0260000	4.017918042	0.000979793	0.004434942	RHSIM_Rhsim03G0260000
Root vs Stem	RHSIM_Rhsim08G0076800	-8.108735233	0.000982688	0.004447177	RHSIM_Rhsim08G0076800
Root vs Stem	RHSIM_Rhsim08G0145400	1.459957005	0.000984969	0.004453124	RHSIM_Rhsim08G0145400
Root vs Stem	RHSIM_Rhsim10G0166700	1.081867352	0.000986176	0.004457707	RHSIM_Rhsim10G0166700
Root vs Stem	RHSIM_Rhsim01G0126200	-1.962860278	0.000986411	0.004457896	RHSIM_Rhsim01G0126200
Root vs Stem	RHSIM_RhsimUnG0125400	1.283448423	0.00099114	0.004477512	RHSIM_RhsimUnG0125400
Root vs Stem	novel.1873	-2.576035878	0.000993905	0.004488247	--
Root vs Stem	RHSIM_Rhsim03G0163800	1.775898027	0.000998017	0.004505004	RHSIM_Rhsim03G0163800
Root vs Stem	RHSIM_Rhsim08G0155100	2.272250502	0.001001656	0.004518821	RHSIM_Rhsim08G0155100
Root vs Stem	RHSIM_Rhsim09G0177200	1.72352339	0.00101056	0.004556317	RHSIM_Rhsim09G0177200
Root vs Stem	RHSIM_Rhsim07G0212000	2.174753398	0.001012854	0.004564874	RHSIM_Rhsim07G0212000
Root vs Stem	RHSIM_Rhsim04G0123000	3.530360569	0.001014617	0.004570043	RHSIM_Rhsim04G0123000
Root vs Stem	RHSIM_Rhsim10G0159200	-1.168071099	0.001014894	0.004570043	RHSIM_Rhsim10G0159200
Root vs Stem	RHSIM_Rhsim02G0020300	3.217647178	0.001014992	0.004570043	RHSIM_Rhsim02G0020300

Comparison	Gene ID	log_2FC	P-value	FDR (padj)	Gene Name
Root vs Stem	RHSIM_Rhsim11G0092800	1.267750984	0.001019207	0.004584541	RHSIM_Rhsim11G0092800
Root vs Stem	novel.4062	-1.289289373	0.001020994	0.004590789	--
Root vs Stem	RHSIM_Rhsim11G0158700	4.655041591	0.001023381	0.004600625	RHSIM_Rhsim11G0158700
Root vs Stem	RHSIM_Rhsim01G0051200	-4.15240197	0.001034299	0.004645179	RHSIM_Rhsim01G0051200
Root vs Stem	RHSIM_Rhsim13G0229300	2.791875127	0.00103492	0.004647062	RHSIM_Rhsim13G0229300
Root vs Stem	RHSIM_Rhsim08G0234900	1.162749847	0.001036725	0.004654261	RHSIM_Rhsim08G0234900
Root vs Stem	RHSIM_Rhsim04G0024400	3.252360511	0.001037234	0.004655638	RHSIM_Rhsim04G0024400
Root vs Stem	RHSIM_Rhsim13G0087500	1.086504488	0.001038703	0.004660046	RHSIM_Rhsim13G0087500
Root vs Stem	RHSIM_Rhsim13G0035000	-1.937493098	0.001041836	0.004672653	RHSIM_Rhsim13G0035000
Root vs Stem	RHSIM_Rhsim04G0005000	1.526692433	0.001042079	0.004672835	RHSIM_Rhsim04G0005000
Root vs Stem	RHSIM_Rhsim05G0149900	2.575070963	0.001043317	0.004676566	RHSIM_Rhsim05G0149900
Root vs Stem	RHSIM_Rhsim03G0241400	-1.216022658	0.001050743	0.004706191	RHSIM_Rhsim03G0241400
Root vs Stem	RHSIM_Rhsim13G0179500	-1.042072293	0.001052266	0.004710271	RHSIM_Rhsim13G0179500
Root vs Stem	RHSIM_Rhsim03G0024100	-2.693591224	0.001053679	0.004715678	RHSIM_Rhsim03G0024100
Root vs Stem	RHSIM_Rhsim06G0157900	-1.051935101	0.001054389	0.004717939	RHSIM_Rhsim06G0157900
Root vs Stem	RHSIM_Rhsim04G0209400	1.722746239	0.001057071	0.004728104	RHSIM_Rhsim04G0209400
Root vs Stem	RHSIM_Rhsim02G0091100	1.086571857	0.001058754	0.004734715	RHSIM_Rhsim02G0091100
Root vs Stem	RHSIM_Rhsim12G0214500	1.113399306	0.001062065	0.004745838	RHSIM_Rhsim12G0214500
Root vs Stem	RHSIM_Rhsim04G0154200	1.371927219	0.001062701	0.004746952	RHSIM_Rhsim04G0154200
Root vs Stem	novel.4005	5.450825579	0.001062932	0.004746952	--
Root vs Stem	RHSIM_Rhsim12G0054800	-1.226604272	0.001064763	0.00475329	RHSIM_Rhsim12G0054800
Root vs Stem	RHSIM_Rhsim07G0006000	1.015207009	0.001065988	0.004757834	RHSIM_Rhsim07G0006000
Root vs Stem	RHSIM_Rhsim13G0142400	-1.496549345	0.001069447	0.004772351	RHSIM_Rhsim13G0142400
Root vs Stem	RHSIM_Rhsim01G0150000	1.336238927	0.001070423	0.00477578	RHSIM_Rhsim01G0150000
Root vs Stem	novel.3440	-7.993761997	0.00107516	0.004795062	--

Comparison	Gene ID	log_2FC	P-value	FDR (padj)	Gene Name
Root vs Stem	RHSIM_Rhsim10G0005000	-2.012773837	0.00107667	0.004800865	RHSIM_Rhsim10G0005000
Root vs Stem	RHSIM_RhsimUnG0114000	-2.273203896	0.001079576	0.004809487	RHSIM_RhsimUnG0114000
Root vs Stem	RHSIM_Rhsim06G0231200	1.230250077	0.001079647	0.004809487	RHSIM_Rhsim06G0231200
Root vs Stem	RHSIM_Rhsim05G0074000	1.422492068	0.001080705	0.004813271	RHSIM_Rhsim05G0074000
Root vs Stem	RHSIM_Rhsim05G0041400	-1.908563751	0.001081154	0.004814339	RHSIM_Rhsim05G0041400
Root vs Stem	novel.4903	-4.011630959	0.001082264	0.004816026	--
Root vs Stem	RHSIM_Rhsim02G0008700	2.719115968	0.001082368	0.004816026	RHSIM_Rhsim02G0008700
Root vs Stem	RHSIM_Rhsim09G0172900	-1.70458059	0.001083831	0.004821605	RHSIM_Rhsim09G0172900
Root vs Stem	RHSIM_Rhsim13G0013200	1.050017878	0.00108489	0.004824453	RHSIM_Rhsim13G0013200
Root vs Stem	novel.1317	-1.357111336	0.00108656	0.004830949	--
Root vs Stem	RHSIM_RhsimUnG0228400	-6.31947692	0.001090663	0.004848257	RHSIM_RhsimUnG0228400
Root vs Stem	RHSIM_Rhsim10G0030600	2.906345473	0.001092913	0.004856386	RHSIM_Rhsim10G0030600
Root vs Stem	RHSIM_Rhsim07G0032600	1.567908039	0.001093859	0.00485965	RHSIM_Rhsim07G0032600
Root vs Stem	RHSIM_Rhsim12G0042500	2.347033008	0.001094186	0.004859914	RHSIM_Rhsim12G0042500
Root vs Stem	novel.3216	1.059145833	0.00109434	0.004859914	--
Root vs Stem	RHSIM_Rhsim11G0102700	-6.358043272	0.001094615	0.0048602	RHSIM_Rhsim11G0102700
Root vs Stem	novel.5060	1.897546247	0.001095392	0.004862715	--
Root vs Stem	novel.2011	6.184898109	0.001099487	0.004878748	--
Root vs Stem	RHSIM_Rhsim07G0136300	2.274416268	0.001103834	0.004895475	RHSIM_Rhsim07G0136300
Root vs Stem	RHSIM_Rhsim03G0039700	-2.609917299	0.001106683	0.004906223	RHSIM_Rhsim03G0039700
Root vs Stem	RHSIM_RhsimUnG0245900	2.59722379	0.001109713	0.004918369	RHSIM_RhsimUnG0245900
Root vs Stem	RHSIM_RhsimUnG0210600	-1.631188617	0.001109849	0.004918369	RHSIM_RhsimUnG0210600
Root vs Stem	novel.3147	2.246494628	0.001110371	0.004919735	--
Root vs Stem	RHSIM_RhsimUnG0074400	-2.385743172	0.001112547	0.004928431	RHSIM_RhsimUnG0074400
Root vs Stem	RHSIM_Rhsim12G0107500	-1.319739054	0.001113021	0.004928634	RHSIM_Rhsim12G0107500

Comparison	Gene ID	log_2FC	P-value	FDR (padj)	Gene Name
Root vs Stem	RHSIM_Rhsim13G0033300	3.280802227	0.001117075	0.004944688	RHSIM_Rhsim13G0033300
Root vs Stem	RHSIM_Rhsim08G0159500	-3.48637904	0.001120218	0.004956695	RHSIM_Rhsim08G0159500
Root vs Stem	RHSIM_Rhsim10G0193500	-1.602242553	0.001121588	0.004961806	RHSIM_Rhsim10G0193500
Root vs Stem	RHSIM_Rhsim08G0053900	1.437392949	0.00112215	0.004963338	RHSIM_Rhsim08G0053900
Root vs Stem	RHSIM_Rhsim07G0245000	-3.117550913	0.001123075	0.004966477	RHSIM_Rhsim07G0245000
Root vs Stem	RHSIM_Rhsim01G0094300	-1.22353864	0.001130793	0.004994858	RHSIM_Rhsim01G0094300
Root vs Stem	RHSIM_Rhsim08G0160300	-1.116233325	0.001131012	0.004994869	RHSIM_Rhsim08G0160300
Root vs Stem	RHSIM_Rhsim04G0035500	-2.015255427	0.001132295	0.004999578	RHSIM_Rhsim04G0035500
Root vs Stem	RHSIM_Rhsim03G0097900	-1.382662313	0.001132602	0.004999975	RHSIM_Rhsim03G0097900
Root vs Stem	RHSIM_Rhsim04G0157100	-2.435697074	0.001135872	0.005012492	RHSIM_Rhsim04G0157100
Root vs Stem	RHSIM_Rhsim03G0088100	-3.07921918	0.001138806	0.005024479	RHSIM_Rhsim03G0088100
Root vs Stem	RHSIM_RhsimUnG0139500	-1.014568586	0.0011521	0.005076952	RHSIM_RhsimUnG0139500
Root vs Stem	RHSIM_Rhsim11G0078400	-2.483327432	0.00116	0.005107236	RHSIM_Rhsim11G0078400
Root vs Stem	RHSIM_Rhsim06G0111600	-8.009405053	0.001161368	0.005111868	RHSIM_Rhsim06G0111600
Root vs Stem	novel.4403	-3.298415885	0.001162935	0.00511625	--
Root vs Stem	RHSIM_Rhsim10G0101000	1.810106711	0.00116404	0.00512013	RHSIM_Rhsim10G0101000
Root vs Stem	RHSIM_Rhsim03G0069500	1.853537966	0.001164848	0.005122063	RHSIM_Rhsim03G0069500
Root vs Stem	RHSIM_Rhsim01G0143400	-1.035011641	0.001164923	0.005122063	RHSIM_Rhsim01G0143400
Root vs Stem	RHSIM_Rhsim01G0120500	1.111692948	0.001166715	0.005128226	RHSIM_Rhsim01G0120500
Root vs Stem	novel.1004	-6.058962204	0.001166992	0.005128226	--
Root vs Stem	RHSIM_Rhsim10G0168700	-2.234019809	0.001171141	0.005144494	RHSIM_Rhsim10G0168700
Root vs Stem	RHSIM_Rhsim01G0260700	3.562976661	0.00117225	0.005148385	RHSIM_Rhsim01G0260700
Root vs Stem	novel.2108	-6.9759578	0.001175453	0.005158523	--
Root vs Stem	RHSIM_Rhsim01G0099900	-5.652718159	0.0011784	0.005169487	RHSIM_Rhsim01G0099900
Root vs Stem	RHSIM_Rhsim03G0058600	-1.943234571	0.001180964	0.005178763	RHSIM_Rhsim03G0058600

Comparison	Gene ID	log_2FC	P-value	FDR (padj)	Gene Name
Root vs Stem	RHSIM_Rhsim10G0175200	2.397195317	0.001186117	0.005199381	RHSIM_Rhsim10G0175200
Root vs Stem	RHSIM_Rhsim02G0214700	-6.20134548	0.001189153	0.005210711	RHSIM_Rhsim02G0214700
Root vs Stem	novel.109	-1.905570072	0.001189541	0.005210897	--
Root vs Stem	RHSIM_RhsimUnG0098900	2.673996975	0.001189648	0.005210897	RHSIM_RhsimUnG0098900
Root vs Stem	novel.5242	-2.337465854	0.001189959	0.005211269	--
Root vs Stem	RHSIM_Rhsim09G0081700	1.216806952	0.001196021	0.005232845	RHSIM_Rhsim09G0081700
Root vs Stem	RHSIM_Rhsim13G0086800	1.079635833	0.001198666	0.005243423	RHSIM_Rhsim13G0086800
Root vs Stem	novel.2779	-1.162928626	0.0012011	0.005253076	--
Root vs Stem	novel.5890	-6.034016608	0.00120162	0.005253358	--
Root vs Stem	RHSIM_Rhsim04G0083200	3.062034496	0.001205624	0.005269861	RHSIM_Rhsim04G0083200
Root vs Stem	RHSIM_Rhsim07G0086400	1.062963738	0.001208155	0.005276923	RHSIM_Rhsim07G0086400
Root vs Stem	RHSIM_RhsimUnG0102400	1.911599561	0.001210133	0.005283561	RHSIM_RhsimUnG0102400
Root vs Stem	RHSIM_RhsimUnG0181700	-1.953307098	0.00121144	0.005287315	RHSIM_RhsimUnG0181700
Root vs Stem	RHSIM_Rhsim01G0145500	1.482975585	0.00121701	0.005309565	RHSIM_Rhsim01G0145500
Root vs Stem	RHSIM_Rhsim04G0123100	1.279503354	0.001219187	0.005316352	RHSIM_Rhsim04G0123100
Root vs Stem	novel.4892	-3.750450806	0.001222836	0.005329939	--
Root vs Stem	novel.4496	-7.931763909	0.001230709	0.005360384	--
Root vs Stem	novel.332	-5.297069544	0.001230751	0.005360384	--
Root vs Stem	RHSIM_Rhsim10G0161200	1.338029123	0.001233433	0.005371049	RHSIM_Rhsim10G0161200
Root vs Stem	RHSIM_Rhsim11G0027300	-3.11090914	0.001242942	0.005406327	RHSIM_Rhsim11G0027300
Root vs Stem	RHSIM_Rhsim09G0057700	1.340183277	0.001248214	0.005427057	RHSIM_Rhsim09G0057700
Root vs Stem	RHSIM_Rhsim11G0038900	-2.71343851	0.001248414	0.005427057	RHSIM_Rhsim11G0038900
Root vs Stem	RHSIM_Rhsim04G0184800	2.154818656	0.001248958	0.005428399	RHSIM_Rhsim04G0184800
Root vs Stem	novel.2909	-1.750026103	0.001253517	0.005447188	--
Root vs Stem	novel.3662	-3.414075808	0.00126181	0.005479095	--

Comparison	Gene ID	log_2FC	P-value	FDR (padj)	Gene Name
Root vs Stem	RHSIM_Rhsim02G0145700	-7.939466582	0.001263219	0.005482114	RHSIM_Rhsim02G0145700
Root vs Stem	RHSIM_Rhsim08G0232900	-1.069819829	0.001266795	0.00549453	RHSIM_Rhsim08G0232900
Root vs Stem	novel.538	2.218383573	0.001271436	0.005512586	--
Root vs Stem	RHSIM_Rhsim07G0216400	-1.574605297	0.001275156	0.005526635	RHSIM_Rhsim07G0216400
Root vs Stem	RHSIM_Rhsim12G0146400	-7.923122265	0.00127857	0.00554039	RHSIM_Rhsim12G0146400
Root vs Stem	RHSIM_Rhsim10G0041500	-1.810224743	0.001279947	0.005545315	RHSIM_Rhsim10G0041500
Root vs Stem	novel.3840	-4.352165397	0.001286016	0.005568469	--
Root vs Stem	RHSIM_Rhsim08G0175700	1.937034487	0.00128708	0.005571014	RHSIM_Rhsim08G0175700
Root vs Stem	RHSIM_Rhsim11G0054100	-7.943303894	0.001296308	0.005607187	RHSIM_Rhsim11G0054100
Root vs Stem	novel.1654	-3.346764714	0.001296417	0.005607187	--
Root vs Stem	RHSIM_Rhsim11G0022900	1.531412077	0.001304689	0.005638732	RHSIM_Rhsim11G0022900
Root vs Stem	RHSIM_Rhsim07G0138400	-2.492889958	0.001306798	0.005646683	RHSIM_Rhsim07G0138400
Root vs Stem	novel.1337	-2.13262789	0.001307019	0.005646683	--
Root vs Stem	RHSIM_Rhsim06G0122700	-3.905484888	0.001311364	0.005663335	RHSIM_Rhsim06G0122700
Root vs Stem	novel.4933	-1.561871663	0.001319553	0.005695497	--
Root vs Stem	RHSIM_Rhsim01G0131400	4.636285959	0.001322373	0.0057066	RHSIM_Rhsim01G0131400
Root vs Stem	RHSIM_Rhsim04G0052500	3.253709812	0.00132631	0.00571905	RHSIM_Rhsim04G0052500
Root vs Stem	RHSIM_Rhsim09G0092900	2.964700903	0.001331119	0.005736299	RHSIM_Rhsim09G0092900
Root vs Stem	RHSIM_Rhsim10G0169200	-2.231500563	0.001331245	0.005736299	RHSIM_Rhsim10G0169200
Root vs Stem	RHSIM_Rhsim01G0151700	-1.055049607	0.001331565	0.005736606	RHSIM_Rhsim01G0151700
Root vs Stem	RHSIM_Rhsim07G0236800	4.057863496	0.0013326	0.005738917	RHSIM_Rhsim07G0236800
Root vs Stem	RHSIM_Rhsim10G0165900	3.084575603	0.001338006	0.005758067	RHSIM_Rhsim10G0165900
Root vs Stem	RHSIM_Rhsim07G0094200	-1.278420254	0.001338451	0.005758735	RHSIM_Rhsim07G0094200
Root vs Stem	RHSIM_Rhsim04G0185400	3.187258348	0.001342829	0.005775365	RHSIM_Rhsim04G0185400
Root vs Stem	RHSIM_Rhsim13G0091700	-1.016819627	0.001343067	0.005775365	RHSIM_Rhsim13G0091700

Comparison	Gene ID	log_2FC	P-value	FDR (padj)	Gene Name
Root vs Stem	novel.4689	-2.448984046	0.001343937	0.005778027	--
Root vs Stem	RHSIM_Rhsim07G0117100	2.204904935	0.001344272	0.005778388	RHSIM_Rhsim07G0117100
Root vs Stem	RHSIM_Rhsim13G0103200	2.040260377	0.001345106	0.005780898	RHSIM_Rhsim13G0103200
Root vs Stem	novel.4605	-1.386411831	0.001348231	0.005792167	--
Root vs Stem	novel.6114	1.229060891	0.00134899	0.005793842	--
Root vs Stem	RHSIM_Rhsim02G0060900	1.213192107	0.001349124	0.005793842	RHSIM_Rhsim02G0060900
Root vs Stem	RHSIM_RhsimUnG0144100	3.219944152	0.001350254	0.005797617	RHSIM_RhsimUnG0144100
Root vs Stem	novel.2936	2.765176242	0.001359585	0.005835507	--
Root vs Stem	RHSIM_Rhsim04G0050200	2.065829433	0.001360552	0.005838571	RHSIM_Rhsim04G0050200
Root vs Stem	novel.3387	-5.958732089	0.001364198	0.005850949	--
Root vs Stem	novel.2422	-1.131787048	0.001369571	0.005871809	--
Root vs Stem	RHSIM_Rhsim07G0176800	-1.172483771	0.001379528	0.005912298	RHSIM_Rhsim07G0176800
Root vs Stem	novel.4176	-3.577769705	0.001382609	0.005921823	--
Root vs Stem	RHSIM_Rhsim07G0116600	-1.775821345	0.001390768	0.005951622	RHSIM_Rhsim07G0116600
Root vs Stem	RHSIM_Rhsim03G0210800	-1.09028723	0.001393732	0.005962089	RHSIM_Rhsim03G0210800
Root vs Stem	RHSIM_Rhsim05G0037200	-8.140858548	0.001396272	0.005971849	RHSIM_Rhsim05G0037200
Root vs Stem	RHSIM_Rhsim01G0131700	4.394584644	0.001397878	0.005975974	RHSIM_Rhsim01G0131700
Root vs Stem	RHSIM_RhsimUnG0169100	1.290510312	0.001401572	0.00598893	RHSIM_RhsimUnG0169100
Root vs Stem	RHSIM_Rhsim08G0042000	-2.036460268	0.001401825	0.00598893	RHSIM_Rhsim08G0042000
Root vs Stem	RHSIM_Rhsim08G0015800	-1.531596368	0.001408741	0.006017362	RHSIM_Rhsim08G0015800
Root vs Stem	novel.5642	-7.003082106	0.001410243	0.006022153	--
Root vs Stem	RHSIM_Rhsim08G0107800	2.936063606	0.001410385	0.006022153	RHSIM_Rhsim08G0107800
Root vs Stem	novel.4607	-2.715859769	0.001413828	0.006034618	--
Root vs Stem	novel.1674	-1.587097835	0.00141668	0.006045674	--
Root vs Stem	RHSIM_Rhsim08G0217200	-2.614645083	0.001421163	0.006062559	RHSIM_Rhsim08G0217200

Comparison	Gene ID	log$_2$FC	P-value	FDR (padj)	Gene Name
Root vs Stem	RHSIM_Rhsim08G0177900	-4.422082252	0.001422977	0.006069177	RHSIM_Rhsim08G0177900
Root vs Stem	RHSIM_RhsimUnG0085600	2.443770668	0.00142755	0.006087553	RHSIM_RhsimUnG0085600
Root vs Stem	RHSIM_Rhsim06G0221800	4.12470574	0.001430923	0.00609968	RHSIM_Rhsim06G0221800
Root vs Stem	novel.5982	-2.83940706	0.001433267	0.006107413	--
Root vs Stem	RHSIM_Rhsim06G0124500	-1.909327736	0.001437876	0.006120266	RHSIM_Rhsim06G0124500
Root vs Stem	RHSIM_Rhsim08G0111400	-1.8019318	0.001439201	0.006122625	RHSIM_Rhsim08G0111400
Root vs Stem	RHSIM_Rhsim12G0130000	-1.187984022	0.001440543	0.006125925	RHSIM_Rhsim12G0130000
Root vs Stem	novel.4004	-1.590220996	0.001443666	0.006134716	--
Root vs Stem	RHSIM_Rhsim13G0233600	3.39956593	0.001447474	0.006149762	RHSIM_Rhsim13G0233600
Root vs Stem	RHSIM_Rhsim05G0140200	2.810255252	0.001449517	0.006157308	RHSIM_Rhsim05G0140200
Root vs Stem	RHSIM_Rhsim03G0222200	1.360827157	0.001450395	0.006159905	RHSIM_Rhsim03G0222200
Root vs Stem	RHSIM_Rhsim03G0048000	1.101020425	0.001454741	0.006176085	RHSIM_Rhsim03G0048000
Root vs Stem	RHSIM_Rhsim07G0082800	1.658809506	0.001459126	0.006191282	RHSIM_Rhsim07G0082800
Root vs Stem	RHSIM_Rhsim07G0235800	-3.031342234	0.001465739	0.006215908	RHSIM_Rhsim07G0235800
Root vs Stem	RHSIM_Rhsim02G0152700	-2.119318588	0.001466277	0.006217049	RHSIM_Rhsim02G0152700
Root vs Stem	RHSIM_Rhsim08G0001000	-2.651475682	0.001466674	0.006217587	RHSIM_Rhsim08G0001000
Root vs Stem	RHSIM_Rhsim07G0209000	-1.029593481	0.001468388	0.006221888	RHSIM_Rhsim07G0209000
Root vs Stem	RHSIM_Rhsim13G0091100	-5.562378171	0.001471189	0.006232142	RHSIM_Rhsim13G0091100
Root vs Stem	RHSIM_Rhsim11G0120300	-3.407522119	0.001472826	0.006236787	RHSIM_Rhsim11G0120300
Root vs Stem	novel.4803	1.298087296	0.001473387	0.006238015	--
Root vs Stem	novel.3070	1.002793889	0.001474441	0.006240183	--
Root vs Stem	novel.2624	-4.038020039	0.001474886	0.006240925	--
Root vs Stem	novel.1816	1.333551153	0.001476516	0.006246674	--
Root vs Stem	RHSIM_Rhsim11G0177700	1.082915379	0.001479256	0.006257119	RHSIM_Rhsim11G0177700
Root vs Stem	RHSIM_RhsimUnG0125500	2.701816506	0.001480101	0.006259545	RHSIM_RhsimUnG0125500

Comparison	Gene ID	log_2FC	P-value	FDR (padj)	Gene Name
Root vs Stem	RHSIM_Rhsim10G0175400	1.861162127	0.001482588	0.006267762	RHSIM_Rhsim10G0175400
Root vs Stem	novel.636	-1.457467668	0.001483326	0.006268583	--
Root vs Stem	novel.4372	-1.054676608	0.001485863	0.006275853	--
Root vs Stem	RHSIM_Rhsim01G0069300	-1.508611992	0.001486216	0.006276194	RHSIM_Rhsim01G0069300
Root vs Stem	RHSIM_Rhsim04G0109300	1.510588982	0.001491415	0.006295841	RHSIM_Rhsim04G0109300
Root vs Stem	RHSIM_Rhsim10G0172000	-2.226281899	0.001495506	0.006309645	RHSIM_Rhsim10G0172000
Root vs Stem	RHSIM_Rhsim07G0172000	-7.83858419	0.001498123	0.00631953	RHSIM_Rhsim07G0172000
Root vs Stem	RHSIM_Rhsim07G0160000	-1.375362592	0.001503084	0.006338137	RHSIM_Rhsim07G0160000
Root vs Stem	RHSIM_Rhsim06G0184900	1.515217838	0.001503447	0.006338507	RHSIM_Rhsim06G0184900
Root vs Stem	novel.2665	-7.840641307	0.001504794	0.006341867	--
Root vs Stem	RHSIM_RhsimUnG0011500	-2.016626987	0.001509474	0.006359268	RHSIM_RhsimUnG0011500
Root vs Stem	RHSIM_Rhsim02G0180400	1.049912528	0.001521553	0.006406641	RHSIM_Rhsim02G0180400
Root vs Stem	RHSIM_Rhsim09G0148600	-6.995646385	0.001523276	0.006412724	RHSIM_Rhsim09G0148600
Root vs Stem	RHSIM_Rhsim01G0046300	1.64706685	0.001527949	0.006431222	RHSIM_Rhsim01G0046300
Root vs Stem	RHSIM_Rhsim05G0012800	1.834280463	0.001534246	0.006454191	RHSIM_Rhsim05G0012800
Root vs Stem	novel.2492	-7.822093278	0.001538221	0.006469733	--
Root vs Stem	novel.4261	-7.864023768	0.001540744	0.00647798	--
Root vs Stem	RHSIM_Rhsim12G0083100	1.076013866	0.001542894	0.006484655	RHSIM_Rhsim12G0083100
Root vs Stem	RHSIM_RhsimUnG0169200	-2.182987409	0.00154491	0.006491943	RHSIM_RhsimUnG0169200
Root vs Stem	RHSIM_Rhsim13G0053500	-2.637877086	0.001553967	0.006527623	RHSIM_Rhsim13G0053500
Root vs Stem	RHSIM_Rhsim06G0040400	2.198727927	0.001563088	0.006559962	RHSIM_Rhsim06G0040400
Root vs Stem	RHSIM_Rhsim03G0138400	-2.196007157	0.001571578	0.00659319	RHSIM_Rhsim03G0138400
Root vs Stem	novel.2444	2.995730243	0.001572032	0.006593897	--
Root vs Stem	novel.951	-1.126466893	0.001573905	0.006598823	--
Root vs Stem	RHSIM_RhsimUnG0253700	-1.894144322	0.001574205	0.006598823	RHSIM_RhsimUnG0253700

Comparison	Gene ID	log_2FC	P-value	FDR (padj)	Gene Name
Root vs Stem	RHSIM_Rhsim03G0143200	1.502426044	0.001581898	0.006628043	RHSIM_Rhsim03G0143200
Root vs Stem	RHSIM_Rhsim10G0066600	2.079504815	0.001592718	0.00666504	RHSIM_Rhsim10G0066600
Root vs Stem	RHSIM_Rhsim07G0109600	1.302855896	0.001592752	0.00666504	RHSIM_Rhsim07G0109600
Root vs Stem	RHSIM_Rhsim02G0179300	-1.28611321	0.001593451	0.006665353	RHSIM_Rhsim02G0179300
Root vs Stem	RHSIM_Rhsim09G0200600	-2.571373147	0.00159347	0.006665353	RHSIM_Rhsim09G0200600
Root vs Stem	novel.3754	-7.833501219	0.00159786	0.006680354	--
Root vs Stem	RHSIM_Rhsim08G0006300	-1.040650396	0.001599695	0.006686812	RHSIM_Rhsim08G0006300
Root vs Stem	RHSIM_Rhsim03G0253200	-1.191767513	0.001603801	0.006700333	RHSIM_Rhsim03G0253200
Root vs Stem	RHSIM_Rhsim05G0188200	-1.036524441	0.001606249	0.006709109	RHSIM_Rhsim05G0188200
Root vs Stem	RHSIM_Rhsim01G0067100	1.861386477	0.001606484	0.006709109	RHSIM_Rhsim01G0067100
Root vs Stem	RHSIM_Rhsim06G0047700	1.414679359	0.00161075	0.006724488	RHSIM_Rhsim06G0047700
Root vs Stem	novel.4439	3.775032704	0.001623188	0.00677396	--
Root vs Stem	RHSIM_Rhsim01G0223700	-2.398892023	0.00162491	0.006778694	RHSIM_Rhsim01G0223700
Root vs Stem	novel.5281	2.322154873	0.001625325	0.006779194	--
Root vs Stem	RHSIM_Rhsim08G0243900	-1.924656523	0.001640273	0.006834022	RHSIM_Rhsim08G0243900
Root vs Stem	RHSIM_Rhsim05G0175300	-1.165447363	0.001640545	0.006834022	RHSIM_Rhsim05G0175300
Root vs Stem	RHSIM_Rhsim01G0029200	-1.64696092	0.001650976	0.00687251	RHSIM_Rhsim01G0029200
Root vs Stem	RHSIM_Rhsim06G0195100	-1.437095459	0.001654571	0.006883743	RHSIM_Rhsim06G0195100
Root vs Stem	RHSIM_RhsimUnG0105500	-2.771709595	0.001655806	0.006885154	RHSIM_RhsimUnG0105500
Root vs Stem	RHSIM_Rhsim10G0185600	-2.621072098	0.001660802	0.006901451	RHSIM_Rhsim10G0185600
Root vs Stem	RHSIM_Rhsim02G0109400	5.460041001	0.001663828	0.006911637	RHSIM_Rhsim02G0109400
Root vs Stem	RHSIM_Rhsim09G0169700	1.478259701	0.001664992	0.006914622	RHSIM_Rhsim09G0169700
Root vs Stem	RHSIM_RhsimUnG0111200	2.055193984	0.001666656	0.006919041	RHSIM_RhsimUnG0111200
Root vs Stem	RHSIM_Rhsim06G0100600	-1.220934987	0.001670204	0.006930023	RHSIM_Rhsim06G0100600
Root vs Stem	RHSIM_Rhsim04G0224000	-1.930416658	0.001678214	0.006959499	RHSIM_Rhsim04G0224000

Comparison	Gene ID	log_2FC	P-value	FDR (padj)	Gene Name
Root vs Stem	RHSIM_Rhsim11G0052500	-3.388595846	0.001679031	0.006961637	RHSIM_Rhsim11G0052500
Root vs Stem	RHSIM_Rhsim03G0141600	-1.057706238	0.001681688	0.006970147	RHSIM_Rhsim03G0141600
Root vs Stem	RHSIM_Rhsim10G0113600	-1.808905978	0.001686016	0.006985574	RHSIM_Rhsim10G0113600
Root vs Stem	novel.5901	-1.094776263	0.00168927	0.006997796	--
Root vs Stem	RHSIM_RhsimUnG0117600	-1.116271842	0.001690998	0.007002438	RHSIM_RhsimUnG0117600
Root vs Stem	RHSIM_Rhsim13G0225500	4.535928985	0.001692187	0.007006104	RHSIM_Rhsim13G0225500
Root vs Stem	RHSIM_Rhsim10G0067200	-1.369280187	0.001697234	0.007023357	RHSIM_Rhsim10G0067200
Root vs Stem	RHSIM_Rhsim07G0213100	-1.026697808	0.001697268	0.007023357	RHSIM_Rhsim07G0213100
Root vs Stem	RHSIM_Rhsim08G0111200	-1.781613216	0.001703534	0.007046756	RHSIM_Rhsim08G0111200
Root vs Stem	RHSIM_Rhsim07G0049700	-1.510201925	0.001713549	0.007085639	RHSIM_Rhsim07G0049700
Root vs Stem	RHSIM_Rhsim13G0168100	-3.953186407	0.001715213	0.007091248	RHSIM_Rhsim13G0168100
Root vs Stem	RHSIM_Rhsim05G0128300	-1.631094582	0.001728627	0.007136445	RHSIM_Rhsim05G0128300
Root vs Stem	RHSIM_Rhsim02G0068600	1.673149349	0.001730663	0.007142314	RHSIM_Rhsim02G0068600
Root vs Stem	RHSIM_Rhsim01G0021500	-2.811772317	0.001734696	0.007157677	RHSIM_Rhsim01G0021500
Root vs Stem	RHSIM_Rhsim09G0056300	-1.243561508	0.001735617	0.007158915	RHSIM_Rhsim09G0056300
Root vs Stem	RHSIM_Rhsim13G0159800	1.916848467	0.001738601	0.00716994	RHSIM_Rhsim13G0159800
Root vs Stem	RHSIM_Rhsim07G0059900	-7.769372638	0.001740861	0.007177693	RHSIM_Rhsim07G0059900
Root vs Stem	RHSIM_Rhsim12G0091800	1.269663614	0.00174126	0.007177693	RHSIM_Rhsim12G0091800
Root vs Stem	RHSIM_Rhsim01G0006100	1.259309396	0.001741415	0.007177693	RHSIM_Rhsim01G0006100
Root vs Stem	novel.23	-4.568560228	0.001746597	0.007197151	--
Root vs Stem	novel.553	1.573723165	0.001753941	0.007221577	--
Root vs Stem	RHSIM_Rhsim06G0181800	-2.577550654	0.001762728	0.007253866	RHSIM_Rhsim06G0181800
Root vs Stem	RHSIM_Rhsim02G0066600	2.195618594	0.001774943	0.00730283	RHSIM_Rhsim02G0066600
Root vs Stem	RHSIM_Rhsim09G0117900	-2.528233672	0.001794564	0.007375659	RHSIM_Rhsim09G0117900
Root vs Stem	RHSIM_Rhsim01G0145900	-1.935561907	0.001796099	0.007380652	RHSIM_Rhsim01G0145900

Comparison	Gene ID	log_2FC	P-value	FDR (padj)	Gene Name
Root vs Stem	RHSIM_Rhsim08G0007000	-1.23779207	0.001802584	0.007404664	RHSIM_Rhsim08G0007000
Root vs Stem	RHSIM_RhsimUnG0246600	2.570696404	0.001804092	0.007409536	RHSIM_RhsimUnG0246600
Root vs Stem	RHSIM_Rhsim05G0202300	1.671782117	0.001806111	0.007415189	RHSIM_Rhsim05G0202300
Root vs Stem	RHSIM_Rhsim11G0014900	-2.204758367	0.001810101	0.007427246	RHSIM_Rhsim11G0014900
Root vs Stem	RHSIM_Rhsim02G0130600	-1.669194619	0.001810337	0.007427246	RHSIM_Rhsim02G0130600
Root vs Stem	novel.1996	3.988923708	0.001818411	0.007456389	--
Root vs Stem	RHSIM_Rhsim08G0129900	-1.008515268	0.001818862	0.007456913	RHSIM_Rhsim08G0129900
Root vs Stem	RHSIM_Rhsim07G0073400	1.5508555	0.001820618	0.00746146	RHSIM_Rhsim07G0073400
Root vs Stem	RHSIM_Rhsim09G0045600	-5.109151562	0.001830796	0.007501838	RHSIM_Rhsim09G0045600
Root vs Stem	RHSIM_Rhsim05G0100200	-1.33533546	0.001831909	0.007505064	RHSIM_Rhsim05G0100200
Root vs Stem	RHSIM_Rhsim01G0251300	-3.332905379	0.001834359	0.007513768	RHSIM_Rhsim01G0251300
Root vs Stem	novel.2450	-2.453633965	0.001836528	0.007521316	--
Root vs Stem	RHSIM_RhsimUnG0222500	-7.761867842	0.001851538	0.007576099	RHSIM_RhsimUnG0222500
Root vs Stem	RHSIM_Rhsim11G0092500	-1.568835543	0.001851548	0.007576099	RHSIM_Rhsim11G0092500
Root vs Stem	RHSIM_Rhsim02G0007500	2.126789472	0.001853973	0.007584675	RHSIM_Rhsim02G0007500
Root vs Stem	RHSIM_Rhsim06G0166100	-1.485130783	0.001860661	0.007607984	RHSIM_Rhsim06G0166100
Root vs Stem	RHSIM_Rhsim05G0187000	1.59312738	0.001862353	0.007612201	RHSIM_Rhsim05G0187000
Root vs Stem	RHSIM_Rhsim06G0099600	-3.577086269	0.001863151	0.007614114	RHSIM_Rhsim06G0099600
Root vs Stem	RHSIM_Rhsim05G0201700	1.888669827	0.001865243	0.007621313	RHSIM_Rhsim05G0201700
Root vs Stem	novel.4348	-1.151650902	0.001880959	0.007680082	--
Root vs Stem	RHSIM_Rhsim04G0121500	-2.532223903	0.00188614	0.007699805	RHSIM_Rhsim04G0121500
Root vs Stem	RHSIM_Rhsim10G0025400	2.054778655	0.001893663	0.007723748	RHSIM_Rhsim10G0025400
Root vs Stem	novel.2935	-2.026904235	0.001901303	0.007752164	--
Root vs Stem	novel.34	-1.508071042	0.001906147	0.007770542	--
Root vs Stem	RHSIM_RhsimUnG0179100	3.66378159	0.001917965	0.007817336	RHSIM_RhsimUnG0179100

Comparison	Gene ID	log_2FC	P-value	FDR (padj)	Gene Name
Root vs Stem	RHSIM_Rhsim13G0051800	1.041336085	0.001918939	0.007819922	RHSIM_Rhsim13G0051800
Root vs Stem	RHSIM_Rhsim04G0065800	1.058987771	0.001926669	0.00784726	RHSIM_Rhsim04G0065800
Root vs Stem	RHSIM_RhsimUnG0075500	-3.34413601	0.001930093	0.007858429	RHSIM_RhsimUnG0075500
Root vs Stem	RHSIM_Rhsim03G0017400	-3.241875932	0.001931692	0.007863148	RHSIM_Rhsim03G0017400
Root vs Stem	novel.578	1.457633267	0.001931934	0.007863148	--
Root vs Stem	RHSIM_Rhsim13G0156400	-1.512627542	0.001943607	0.007903682	RHSIM_Rhsim13G0156400
Root vs Stem	RHSIM_Rhsim10G0002900	1.02317968	0.001944241	0.007904866	RHSIM_Rhsim10G0002900
Root vs Stem	RHSIM_Rhsim07G0208800	-2.264219444	0.001948443	0.007920552	RHSIM_Rhsim07G0208800
Root vs Stem	RHSIM_Rhsim01G0112500	-2.926232738	0.001953474	0.007939604	RHSIM_Rhsim01G0112500
Root vs Stem	RHSIM_Rhsim07G0075200	2.667761522	0.001959039	0.00796082	RHSIM_Rhsim07G0075200
Root vs Stem	RHSIM_Rhsim10G0187600	-2.770041149	0.001961019	0.00796746	RHSIM_Rhsim10G0187600
Root vs Stem	RHSIM_Rhsim13G0167000	1.385395956	0.001964772	0.007978489	RHSIM_Rhsim13G0167000
Root vs Stem	RHSIM_Rhsim10G0080200	3.89781465	0.001974159	0.008013786	RHSIM_Rhsim10G0080200
Root vs Stem	RHSIM_Rhsim08G0093900	1.141248711	0.001975303	0.008015608	RHSIM_Rhsim08G0093900
Root vs Stem	RHSIM_Rhsim01G0251800	-1.252425614	0.001980121	0.008031775	RHSIM_Rhsim01G0251800
Root vs Stem	RHSIM_Rhsim01G0231200	1.89339706	0.001980681	0.008031775	RHSIM_Rhsim01G0231200
Root vs Stem	RHSIM_RhsimUnG0067800	-1.092811722	0.001981826	0.008035004	RHSIM_RhsimUnG0067800
Root vs Stem	RHSIM_Rhsim01G0286300	2.15925098	0.00198628	0.008051645	RHSIM_Rhsim01G0286300
Root vs Stem	RHSIM_RhsimUnG0158600	-1.037595799	0.001988626	0.008059739	RHSIM_RhsimUnG0158600
Root vs Stem	RHSIM_Rhsim10G0175300	2.573898572	0.001989242	0.008060818	RHSIM_Rhsim10G0175300
Root vs Stem	RHSIM_Rhsim08G0216400	-4.047802723	0.001990423	0.008063848	RHSIM_Rhsim08G0216400
Root vs Stem	novel.85	-3.497561084	0.001995122	0.008076129	--
Root vs Stem	RHSIM_Rhsim11G0040800	-1.78246556	0.00199712	0.008082795	RHSIM_Rhsim11G0040800
Root vs Stem	RHSIM_Rhsim08G0094100	1.183380958	0.002000894	0.00809665	RHSIM_Rhsim08G0094100
Root vs Stem	RHSIM_Rhsim02G0215800	-2.353688432	0.002007659	0.008121174	RHSIM_Rhsim02G0215800

Comparison	Gene ID	log_2FC	P-value	FDR (padj)	Gene Name
Root vs Stem	novel.3168	1.675027524	0.002010255	0.008130248	--
Root vs Stem	novel.3683	1.468320794	0.002011101	0.008131903	--
Root vs Stem	RHSIM_Rhsim06G0176300	-1.270831827	0.002017448	0.008154409	RHSIM_Rhsim06G0176300
Root vs Stem	RHSIM_Rhsim13G0112700	-1.040578781	0.002020716	0.008165392	RHSIM_Rhsim13G0112700
Root vs Stem	RHSIM_Rhsim05G0221700	2.435397491	0.002022905	0.008169942	RHSIM_Rhsim05G0221700
Root vs Stem	RHSIM_Rhsim03G0212500	1.487516818	0.002026374	0.008181087	RHSIM_Rhsim03G0212500
Root vs Stem	novel.4368	-1.89738155	0.002029642	0.008192844	--
Root vs Stem	novel.2303	1.121079609	0.002030724	0.008194343	--
Root vs Stem	novel.4068	-1.097720644	0.00203744	0.008218567	--
Root vs Stem	novel.1471	-1.138876908	0.002043074	0.00823697	--
Root vs Stem	RHSIM_Rhsim07G0120000	-7.719130136	0.002057533	0.008292361	RHSIM_Rhsim07G0120000
Root vs Stem	RHSIM_Rhsim05G0208800	-5.020133916	0.002058655	0.008295431	RHSIM_Rhsim05G0208800
Root vs Stem	RHSIM_Rhsim09G0004400	-2.553417244	0.002059537	0.008296558	RHSIM_Rhsim09G0004400
Root vs Stem	RHSIM_Rhsim10G0195400	1.04161455	0.002059738	0.008296558	RHSIM_Rhsim10G0195400
Root vs Stem	RHSIM_Rhsim07G0048300	2.673880525	0.002060014	0.008296558	RHSIM_Rhsim07G0048300
Root vs Stem	RHSIM_Rhsim09G0132900	1.564934571	0.002061031	0.008299203	RHSIM_Rhsim09G0132900
Root vs Stem	RHSIM_RhsimUnG0028600	1.716257798	0.00206238	0.008303186	RHSIM_RhsimUnG0028600
Root vs Stem	RHSIM_Rhsim01G0018200	-1.392369663	0.002063041	0.008304398	RHSIM_Rhsim01G0018200
Root vs Stem	RHSIM_Rhsim06G0143800	1.138543541	0.002063449	0.008304589	RHSIM_Rhsim06G0143800
Root vs Stem	novel.4875	1.000772797	0.002068036	0.0083216	--
Root vs Stem	RHSIM_Rhsim07G0120100	-7.678466559	0.002088025	0.008395521	RHSIM_Rhsim07G0120100
Root vs Stem	RHSIM_Rhsim11G0175500	4.362712413	0.002088228	0.008395521	RHSIM_Rhsim11G0175500
Root vs Stem	RHSIM_Rhsim07G0081700	1.839766172	0.002089365	0.008398629	RHSIM_Rhsim07G0081700
Root vs Stem	novel.805	-6.580665761	0.002093454	0.008410665	--
Root vs Stem	RHSIM_Rhsim08G0209900	-2.050683914	0.002104837	0.008446094	RHSIM_Rhsim08G0209900

Comparison	Gene ID	log_2FC	P-value	FDR (padj)	Gene Name
Root vs Stem	RHSIM_RhsimUnG0060600	2.391371348	0.002117369	0.008491946	RHSIM_RhsimUnG0060600
Root vs Stem	RHSIM_Rhsim01G0205100	1.017229481	0.002125183	0.008518841	RHSIM_Rhsim01G0205100
Root vs Stem	novel.178	-1.359338915	0.002139835	0.008573102	--
Root vs Stem	RHSIM_Rhsim12G0041700	1.604385161	0.002142107	0.008580711	RHSIM_Rhsim12G0041700
Root vs Stem	novel.1002	-1.653172086	0.002149466	0.008604208	--
Root vs Stem	RHSIM_Rhsim08G0012300	-2.079015353	0.002150937	0.008608604	RHSIM_Rhsim08G0012300
Root vs Stem	RHSIM_Rhsim04G0026400	1.633456241	0.002152886	0.008614742	RHSIM_Rhsim04G0026400
Root vs Stem	novel.5907	3.830305231	0.002153218	0.008614742	--
Root vs Stem	RHSIM_Rhsim10G0068700	-1.019680292	0.002155843	0.008622592	RHSIM_Rhsim10G0068700
Root vs Stem	RHSIM_Rhsim07G0133500	-2.232040285	0.002158168	0.00863005	RHSIM_Rhsim07G0133500
Root vs Stem	RHSIM_Rhsim04G0178300	2.348930117	0.002158648	0.008630472	RHSIM_Rhsim04G0178300
Root vs Stem	RHSIM_Rhsim02G0058100	3.043882343	0.002171554	0.00867756	RHSIM_Rhsim02G0058100
Root vs Stem	RHSIM_Rhsim09G0150200	-7.687634282	0.002175663	0.008690931	RHSIM_Rhsim09G0150200
Root vs Stem	RHSIM_Rhsim12G0153700	1.111205321	0.002176032	0.008690931	RHSIM_Rhsim12G0153700
Root vs Stem	RHSIM_Rhsim07G0078700	-1.413892931	0.002186526	0.008731331	RHSIM_Rhsim07G0078700
Root vs Stem	RHSIM_Rhsim12G0019200	3.195899982	0.002192705	0.008750075	RHSIM_Rhsim12G0019200
Root vs Stem	RHSIM_RhsimUnG0231300	2.572985725	0.002192738	0.008750075	RHSIM_RhsimUnG0231300
Root vs Stem	RHSIM_Rhsim04G0196300	1.829767845	0.002195477	0.00875646	RHSIM_Rhsim04G0196300
Root vs Stem	novel.2498	2.330524141	0.002196019	0.008757107	--
Root vs Stem	RHSIM_Rhsim03G0168400	1.016859539	0.002197494	0.008761472	RHSIM_Rhsim03G0168400
Root vs Stem	RHSIM_Rhsim12G0037400	-2.904599998	0.002198855	0.008762737	RHSIM_Rhsim12G0037400
Root vs Stem	RHSIM_RhsimUnG0118300	-3.671551184	0.002198952	0.008762737	RHSIM_RhsimUnG0118300
Root vs Stem	RHSIM_Rhsim10G0028700	-1.220057005	0.002203816	0.00877757	RHSIM_Rhsim10G0028700
Root vs Stem	RHSIM_RhsimUnG0187000	-1.056493863	0.002212169	0.008807796	RHSIM_RhsimUnG0187000
Root vs Stem	RHSIM_Rhsim04G0202600	-1.511866654	0.002219773	0.008835018	RHSIM_Rhsim04G0202600

Comparison	Gene ID	log_2FC	P-value	FDR (padj)	Gene Name
Root vs Stem	RHSIM_Rhsim12G0176700	-2.213183286	0.00222352	0.008846877	RHSIM_Rhsim12G0176700
Root vs Stem	RHSIM_Rhsim01G0055700	1.079839669	0.002232893	0.008879572	RHSIM_Rhsim01G0055700
Root vs Stem	RHSIM_Rhsim10G0211200	1.350038557	0.002236034	0.008889603	RHSIM_Rhsim10G0211200
Root vs Stem	RHSIM_Rhsim02G0200800	-1.071412616	0.002236187	0.008889603	RHSIM_Rhsim02G0200800
Root vs Stem	RHSIM_RhsimUnG0252400	-2.316225741	0.002236715	0.00889017	RHSIM_RhsimUnG0252400
Root vs Stem	novel.65	-1.36619323	0.002241654	0.008905256	--
Root vs Stem	RHSIM_Rhsim07G0071500	-1.206171074	0.002246518	0.008921443	RHSIM_Rhsim07G0071500
Root vs Stem	RHSIM_Rhsim08G0017600	1.627084747	0.002247869	0.008925269	RHSIM_Rhsim08G0017600
Root vs Stem	RHSIM_Rhsim03G0065500	2.218380113	0.002249306	0.008929438	RHSIM_Rhsim03G0065500
Root vs Stem	RHSIM_Rhsim03G0145700	1.350365344	0.002254348	0.008946374	RHSIM_Rhsim03G0145700
Root vs Stem	RHSIM_RhsimUnG0006000	-1.666790573	0.002263397	0.008979191	RHSIM_RhsimUnG0006000
Root vs Stem	RHSIM_Rhsim01G0234700	1.037068538	0.002277663	0.009031125	RHSIM_Rhsim01G0234700
Root vs Stem	RHSIM_Rhsim07G0225800	1.745389304	0.002279051	0.009035074	RHSIM_Rhsim07G0225800
Root vs Stem	RHSIM_Rhsim02G0205600	4.761564764	0.002281013	0.0090413	RHSIM_Rhsim02G0205600
Root vs Stem	RHSIM_RhsimUnG0101600	-1.143630012	0.002292497	0.009082135	RHSIM_RhsimUnG0101600
Root vs Stem	RHSIM_Rhsim01G0211600	3.159472498	0.002293869	0.009086008	RHSIM_Rhsim01G0211600
Root vs Stem	novel.1466	-2.172523075	0.002301278	0.009113789	--
Root vs Stem	RHSIM_Rhsim01G0049200	-1.681395219	0.002303328	0.009118078	RHSIM_Rhsim01G0049200
Root vs Stem	RHSIM_Rhsim12G0133600	1.281641532	0.002303548	0.009118078	RHSIM_Rhsim12G0133600
Root vs Stem	RHSIM_Rhsim06G0000900	-1.070354261	0.002308136	0.009134671	RHSIM_Rhsim06G0000900
Root vs Stem	RHSIM_RhsimUnG0126000	1.434880744	0.002311596	0.009145226	RHSIM_RhsimUnG0126000
Root vs Stem	RHSIM_Rhsim12G0159300	-2.030754767	0.00233172	0.009220093	RHSIM_Rhsim12G0159300
Root vs Stem	RHSIM_Rhsim09G0161600	-3.381545555	0.002333543	0.009224138	RHSIM_Rhsim09G0161600
Root vs Stem	RHSIM_Rhsim11G0196600	1.967378711	0.002339978	0.009244178	RHSIM_Rhsim11G0196600
Root vs Stem	RHSIM_Rhsim08G0066700	-1.165873897	0.002340217	0.009244178	RHSIM_Rhsim08G0066700

Comparison	Gene ID	log_2FC	P-value	FDR (padj)	Gene Name
Root vs Stem	RHSIM_Rhsim13G0227900	-1.086364036	0.002341907	0.009249268	RHSIM_Rhsim13G0227900
Root vs Stem	RHSIM_Rhsim09G0106800	3.324299648	0.002353262	0.009289341	RHSIM_Rhsim09G0106800
Root vs Stem	RHSIM_Rhsim03G0070300	-3.417429938	0.002361092	0.009312277	RHSIM_Rhsim03G0070300
Root vs Stem	RHSIM_Rhsim07G0063700	1.239350303	0.002363923	0.009320252	RHSIM_Rhsim07G0063700
Root vs Stem	RHSIM_RhsimUnG0046700	1.01396884	0.002367548	0.009331353	RHSIM_RhsimUnG0046700
Root vs Stem	RHSIM_Rhsim01G0109500	2.295868768	0.002371974	0.009345602	RHSIM_Rhsim01G0109500
Root vs Stem	RHSIM_Rhsim07G0109400	-2.427202739	0.002372874	0.00934755	RHSIM_Rhsim07G0109400
Root vs Stem	RHSIM_Rhsim13G0052900	-1.815763899	0.002373851	0.009349801	RHSIM_Rhsim13G0052900
Root vs Stem	RHSIM_Rhsim04G0056900	-1.602695873	0.002376886	0.009356959	RHSIM_Rhsim04G0056900
Root vs Stem	RHSIM_RhsimUnG0191900	-1.30817172	0.002379063	0.009363931	RHSIM_RhsimUnG0191900
Root vs Stem	RHSIM_Rhsim11G0180400	1.121429906	0.002383506	0.009375017	RHSIM_Rhsim11G0180400
Root vs Stem	novel.1159	-7.604475503	0.002386077	0.009383527	--
Root vs Stem	RHSIM_Rhsim04G0242900	2.090716804	0.002393247	0.009410117	RHSIM_Rhsim04G0242900
Root vs Stem	RHSIM_Rhsim05G0095500	4.584769638	0.002396826	0.009417767	RHSIM_Rhsim05G0095500
Root vs Stem	RHSIM_Rhsim13G0161200	-7.618913684	0.002399746	0.009427632	RHSIM_Rhsim13G0161200
Root vs Stem	RHSIM_Rhsim03G0146700	-1.074104683	0.00241098	0.009459767	RHSIM_Rhsim03G0146700
Root vs Stem	RHSIM_Rhsim06G0102900	-1.331122114	0.002412435	0.009461359	RHSIM_Rhsim06G0102900
Root vs Stem	RHSIM_Rhsim01G0155600	1.134779308	0.00241312	0.009462435	RHSIM_Rhsim01G0155600
Root vs Stem	RHSIM_Rhsim03G0213800	2.062935368	0.002418777	0.009481394	RHSIM_Rhsim03G0213800
Root vs Stem	RHSIM_Rhsim02G0111200	-3.083054194	0.002420262	0.009485599	RHSIM_Rhsim02G0111200
Root vs Stem	RHSIM_Rhsim12G0162200	-1.748328391	0.002421536	0.009488981	RHSIM_Rhsim12G0162200
Root vs Stem	RHSIM_Rhsim03G0123200	-1.310104315	0.002426783	0.009501465	RHSIM_Rhsim03G0123200
Root vs Stem	RHSIM_RhsimUnG0156000	-7.599631884	0.002435888	0.009532257	RHSIM_RhsimUnG0156000
Root vs Stem	RHSIM_Rhsim07G0194600	-1.007037495	0.002440062	0.009546971	RHSIM_Rhsim07G0194600
Root vs Stem	RHSIM_Rhsim07G0203900	2.41170032	0.002446238	0.009569512	RHSIM_Rhsim07G0203900

Comparison	Gene ID	log_2FC	P-value	FDR (padj)	Gene Name
Root vs Stem	novel.1104	-1.994165012	0.002448388	0.009576295	--
Root vs Stem	RHSIM_Rhsim10G0070900	2.538348944	0.002457873	0.009610134	RHSIM_Rhsim10G0070900
Root vs Stem	novel.2864	-1.241487195	0.002460785	0.009618256	--
Root vs Stem	RHSIM_Rhsim13G0165700	2.093452183	0.002469717	0.009651533	RHSIM_Rhsim13G0165700
Root vs Stem	novel.5025	-4.961115743	0.002474172	0.009667301	--
Root vs Stem	RHSIM_Rhsim02G0216300	1.109265524	0.002476107	0.009673225	RHSIM_Rhsim02G0216300
Root vs Stem	RHSIM_Rhsim11G0191800	1.62487943	0.002481702	0.009691797	RHSIM_Rhsim11G0191800
Root vs Stem	RHSIM_Rhsim13G0066900	-1.774138519	0.002484946	0.009699844	RHSIM_Rhsim13G0066900
Root vs Stem	novel.144	-7.644960126	0.002485025	0.009699844	--
Root vs Stem	RHSIM_Rhsim04G0142900	-1.226578083	0.002495498	0.009738936	RHSIM_Rhsim04G0142900
Root vs Stem	RHSIM_Rhsim02G0181500	2.008920393	0.002496398	0.009739291	RHSIM_Rhsim02G0181500
Root vs Stem	RHSIM_Rhsim05G0112200	-2.341785824	0.002498274	0.009744961	RHSIM_Rhsim05G0112200
Root vs Stem	RHSIM_Rhsim07G0174800	-1.446136996	0.002509364	0.009781599	RHSIM_Rhsim07G0174800
Root vs Stem	RHSIM_Rhsim12G0105600	-1.335099339	0.002514221	0.009797218	RHSIM_Rhsim12G0105600
Root vs Stem	novel.5560	-1.277005564	0.002523701	0.009830836	--
Root vs Stem	RHSIM_Rhsim04G0086900	-1.106654295	0.002530207	0.009854512	RHSIM_Rhsim04G0086900
Root vs Stem	RHSIM_Rhsim02G0042100	1.094767404	0.002534046	0.009866131	RHSIM_Rhsim02G0042100
Root vs Stem	RHSIM_Rhsim12G0072400	1.571848448	0.002540636	0.009890117	RHSIM_Rhsim12G0072400
Root vs Stem	RHSIM_Rhsim09G0192200	1.491620629	0.002547674	0.009910821	RHSIM_Rhsim09G0192200
Root vs Stem	RHSIM_RhsimUnG0011400	1.686332471	0.002550005	0.009918214	RHSIM_RhsimUnG0011400
Root vs Stem	RHSIM_Rhsim02G0202900	3.26877401	0.002551257	0.00992141	RHSIM_Rhsim02G0202900
Root vs Stem	RHSIM_Rhsim10G0064200	1.149256246	0.002555431	0.009935966	RHSIM_Rhsim10G0064200
Root vs Stem	novel.2750	-2.754440267	0.002558819	0.009946521	--
Root vs Stem	RHSIM_Rhsim05G0220900	-1.516243481	0.002570549	0.009988011	RHSIM_Rhsim05G0220900
Root vs Stem	RHSIM_Rhsim10G0169600	1.702703801	0.002579564	0.010017974	RHSIM_Rhsim10G0169600

Comparison	Gene ID	log_2FC	P-value	FDR (padj)	Gene Name
Root vs Stem	novel.4079	-1.268176222	0.002582417	0.010025888	--
Root vs Stem	RHSIM_Rhsim02G0242200	1.649616025	0.002584971	0.010032212	RHSIM_Rhsim02G0242200
Root vs Stem	RHSIM_Rhsim13G0039400	1.121258287	0.002591185	0.010052943	RHSIM_Rhsim13G0039400
Root vs Stem	novel.93	-1.787792456	0.002619676	0.01015835	--
Root vs Stem	RHSIM_Rhsim10G0173400	1.295488375	0.002623665	0.010172108	RHSIM_Rhsim10G0173400
Root vs Stem	novel.3346	-1.873310692	0.002627736	0.010181043	--
Root vs Stem	RHSIM_Rhsim03G0203100	-3.596938517	0.002631014	0.010188603	RHSIM_Rhsim03G0203100
Root vs Stem	RHSIM_Rhsim07G0168100	-2.262699357	0.002633582	0.010195126	RHSIM_Rhsim07G0168100
Root vs Stem	RHSIM_Rhsim03G0063700	-1.035497192	0.002634569	0.010197232	RHSIM_Rhsim03G0063700
Root vs Stem	novel.3739	2.519370549	0.002638439	0.010207072	--
Root vs Stem	RHSIM_Rhsim09G0199300	1.278136912	0.002675121	0.01032991	RHSIM_Rhsim09G0199300
Root vs Stem	RHSIM_Rhsim12G0195800	-1.010162398	0.002676735	0.01033441	RHSIM_Rhsim12G0195800
Root vs Stem	RHSIM_Rhsim07G0025400	-2.304582449	0.002691646	0.010386761	RHSIM_Rhsim07G0025400
Root vs Stem	RHSIM_Rhsim08G0167700	-1.204402012	0.002696488	0.010403706	RHSIM_Rhsim08G0167700
Root vs Stem	RHSIM_Rhsim11G0087800	-1.067855898	0.002698295	0.010408936	RHSIM_Rhsim11G0087800
Root vs Stem	RHSIM_Rhsim06G0196900	-1.496665315	0.002699124	0.010410391	RHSIM_Rhsim06G0196900
Root vs Stem	RHSIM_Rhsim13G0009900	1.314442301	0.002702004	0.010418014	RHSIM_Rhsim13G0009900
Root vs Stem	RHSIM_Rhsim06G0049900	-1.802023772	0.002705957	0.010431508	RHSIM_Rhsim06G0049900
Root vs Stem	RHSIM_Rhsim04G0122500	1.595887369	0.002709237	0.010442407	RHSIM_Rhsim04G0122500
Root vs Stem	RHSIM_Rhsim10G0208900	-7.550531318	0.002719256	0.010477521	RHSIM_Rhsim10G0208900
Root vs Stem	RHSIM_RhsimUnG0131400	-1.364102738	0.002720389	0.010480133	RHSIM_RhsimUnG0131400
Root vs Stem	RHSIM_Rhsim11G0151000	1.638673679	0.002729719	0.010507298	RHSIM_Rhsim11G0151000
Root vs Stem	RHSIM_Rhsim07G0083000	1.083527868	0.00273337	0.010518299	RHSIM_Rhsim07G0083000
Root vs Stem	RHSIM_Rhsim12G0117200	-5.606986707	0.002741064	0.010543925	RHSIM_Rhsim12G0117200
Root vs Stem	RHSIM_Rhsim10G0087200	-1.654041766	0.002744364	0.010554859	RHSIM_Rhsim10G0087200

Comparison	Gene ID	log_2FC	P-value	FDR (padj)	Gene Name
Root vs Stem	RHSIM_Rhsim05G0226400	1.024355178	0.002746244	0.010557269	RHSIM_Rhsim05G0226400
Root vs Stem	RHSIM_Rhsim09G0200900	1.881572071	0.002746365	0.010557269	RHSIM_Rhsim09G0200900
Root vs Stem	RHSIM_Rhsim06G0086400	1.227794186	0.002749573	0.010567839	RHSIM_Rhsim06G0086400
Root vs Stem	RHSIM_Rhsim08G0093400	-2.507112038	0.002750978	0.010571478	RHSIM_Rhsim08G0093400
Root vs Stem	RHSIM_Rhsim06G0051200	-1.507103666	0.002755624	0.0105858	RHSIM_Rhsim06G0051200
Root vs Stem	RHSIM_Rhsim10G0024200	-1.865587975	0.00276798	0.010629724	RHSIM_Rhsim10G0024200
Root vs Stem	RHSIM_RhsimUnG0215000	-2.604816274	0.00277412	0.010647983	RHSIM_RhsimUnG0215000
Root vs Stem	RHSIM_Rhsim04G0098600	2.818693868	0.002778565	0.010659722	RHSIM_Rhsim04G0098600
Root vs Stem	RHSIM_Rhsim03G0017100	4.363007852	0.002779906	0.010661318	RHSIM_Rhsim03G0017100
Root vs Stem	RHSIM_Rhsim01G0038700	-3.478826244	0.002780441	0.010661596	RHSIM_Rhsim01G0038700
Root vs Stem	novel.5369	-1.587930086	0.002782947	0.010667654	--
Root vs Stem	RHSIM_Rhsim10G0190100	2.797632508	0.00278817	0.010682347	RHSIM_Rhsim10G0190100
Root vs Stem	RHSIM_Rhsim03G0240300	-1.741537086	0.002791742	0.010694255	RHSIM_Rhsim03G0240300
Root vs Stem	RHSIM_Rhsim12G0091600	1.149891547	0.002796582	0.010709237	RHSIM_Rhsim12G0091600
Root vs Stem	RHSIM_Rhsim01G0071500	1.357726312	0.002802653	0.010730704	RHSIM_Rhsim01G0071500
Root vs Stem	RHSIM_Rhsim11G0025900	-2.182032868	0.002813583	0.010758256	RHSIM_Rhsim11G0025900
Root vs Stem	novel.3856	-7.534187634	0.002818784	0.010776356	--
Root vs Stem	novel.4458	4.642664874	0.002830624	0.010816238	--
Root vs Stem	novel.3491	-1.164518968	0.002834386	0.010827026	--
Root vs Stem	novel.4597	-7.636782805	0.002835607	0.010829895	--
Root vs Stem	RHSIM_Rhsim05G0136900	1.412265107	0.002839491	0.010842549	RHSIM_Rhsim05G0136900
Root vs Stem	RHSIM_Rhsim03G0162200	1.275416959	0.002839861	0.010842549	RHSIM_Rhsim03G0162200
Root vs Stem	novel.4542	-3.201524633	0.002842218	0.010849752	--
Root vs Stem	novel.4781	-5.090996019	0.002844648	0.010855434	--
Root vs Stem	RHSIM_Rhsim01G0076600	-1.572637279	0.002850929	0.010874002	RHSIM_Rhsim01G0076600

Comparison	Gene ID	log_2FC	P-value	FDR (padj)	Gene Name
Root vs Stem	novel.1565	2.519712495	0.002859966	0.010904861	--
Root vs Stem	RHSIM_Rhsim02G0028400	-2.760920962	0.002862311	0.010910193	RHSIM_Rhsim02G0028400
Root vs Stem	RHSIM_Rhsim08G0153100	-1.630733511	0.002870111	0.010936309	RHSIM_Rhsim08G0153100
Root vs Stem	RHSIM_Rhsim06G0159300	-1.470677742	0.002872246	0.010942637	RHSIM_Rhsim06G0159300
Root vs Stem	novel.4911	-3.195009055	0.002879043	0.010963093	--
Root vs Stem	RHSIM_Rhsim04G0179400	1.232362175	0.002881707	0.010969681	RHSIM_Rhsim04G0179400
Root vs Stem	RHSIM_Rhsim03G0236700	1.539684095	0.002882679	0.010971506	RHSIM_Rhsim03G0236700
Root vs Stem	RHSIM_Rhsim09G0179800	-1.341043471	0.002889651	0.010996224	RHSIM_Rhsim09G0179800
Root vs Stem	RHSIM_Rhsim06G0153800	-3.226593188	0.002905515	0.011051122	RHSIM_Rhsim06G0153800
Root vs Stem	RHSIM_Rhsim06G0175100	1.717881541	0.002908223	0.011059595	RHSIM_Rhsim06G0175100
Root vs Stem	RHSIM_Rhsim04G0120000	1.919163315	0.002911155	0.011068918	RHSIM_Rhsim04G0120000
Root vs Stem	RHSIM_Rhsim06G0152600	-2.496587719	0.002911816	0.011069605	RHSIM_Rhsim06G0152600
Root vs Stem	RHSIM_Rhsim10G0079300	-2.196159038	0.002924244	0.011104034	RHSIM_Rhsim10G0079300
Root vs Stem	RHSIM_Rhsim12G0045000	-6.380361088	0.002932926	0.0111298	RHSIM_Rhsim12G0045000
Root vs Stem	RHSIM_Rhsim05G0003600	1.519449546	0.00293296	0.0111298	RHSIM_Rhsim05G0003600
Root vs Stem	novel.1206	-7.518939348	0.002938195	0.011147831	--
Root vs Stem	RHSIM_Rhsim04G0188700	-4.579464089	0.002943214	0.011161359	RHSIM_Rhsim04G0188700
Root vs Stem	RHSIM_Rhsim06G0198900	-1.332884265	0.002962703	0.011227974	RHSIM_Rhsim06G0198900
Root vs Stem	RHSIM_Rhsim07G0221100	-1.457464944	0.002962728	0.011227974	RHSIM_Rhsim07G0221100
Root vs Stem	RHSIM_Rhsim11G0056000	-2.047921799	0.002971192	0.011258202	RHSIM_Rhsim11G0056000
Root vs Stem	RHSIM_Rhsim12G0211900	1.285669399	0.002977194	0.011273529	RHSIM_Rhsim12G0211900
Root vs Stem	novel.3650	-2.913565393	0.002978255	0.011275696	--
Root vs Stem	novel.4183	-2.307088929	0.002984769	0.011294793	--
Root vs Stem	RHSIM_Rhsim12G0034800	-1.726036985	0.002988584	0.011307373	RHSIM_Rhsim12G0034800
Root vs Stem	novel.4259	2.208139461	0.002991299	0.011315787	--

Comparison	Gene ID	log_2FC	P-value	FDR (padj)	Gene Name
Root vs Stem	novel.2519	1.472370361	0.003000668	0.011347505	--
Root vs Stem	novel.1694	-5.490990273	0.003004879	0.011360868	--
Root vs Stem	RHSIM_Rhsim03G0033800	-2.204038151	0.003008694	0.011372262	RHSIM_Rhsim03G0033800
Root vs Stem	RHSIM_Rhsim11G0089800	-1.014494312	0.003012445	0.011384572	RHSIM_Rhsim11G0089800
Root vs Stem	RHSIM_Rhsim11G0135200	1.044392753	0.003015829	0.011395492	RHSIM_Rhsim11G0135200
Root vs Stem	novel.351	-1.002183349	0.003020303	0.01140866	--
Root vs Stem	RHSIM_Rhsim01G0129500	-1.192666938	0.003021079	0.01140972	RHSIM_Rhsim01G0129500
Root vs Stem	RHSIM_Rhsim06G0214700	1.366671768	0.003033022	0.01145295	RHSIM_Rhsim06G0214700
Root vs Stem	RHSIM_Rhsim03G0059100	-1.214813094	0.003041077	0.011475182	RHSIM_Rhsim03G0059100
Root vs Stem	RHSIM_RhsimUnG0203500	-3.712906807	0.003041399	0.011475182	RHSIM_RhsimUnG0203500
Root vs Stem	RHSIM_Rhsim04G0129800	-1.032366896	0.003043841	0.011480641	RHSIM_Rhsim04G0129800
Root vs Stem	RHSIM_Rhsim05G0142600	-1.917909549	0.003046882	0.011488349	RHSIM_Rhsim05G0142600
Root vs Stem	RHSIM_Rhsim04G0075900	1.087347346	0.003047485	0.011488743	RHSIM_Rhsim04G0075900
Root vs Stem	novel.4122	-1.637344318	0.003050522	0.011498312	--
Root vs Stem	novel.1647	-7.494367728	0.003062944	0.011539476	--
Root vs Stem	RHSIM_Rhsim06G0184500	-1.123782982	0.003068008	0.011554778	RHSIM_Rhsim06G0184500
Root vs Stem	RHSIM_Rhsim04G0192800	-7.474787066	0.003093577	0.011637766	RHSIM_Rhsim04G0192800
Root vs Stem	RHSIM_Rhsim13G0173100	-7.477428732	0.003097263	0.011647831	RHSIM_Rhsim13G0173100
Root vs Stem	RHSIM_Rhsim06G0160500	-2.16620117	0.003113009	0.011701321	RHSIM_Rhsim06G0160500
Root vs Stem	RHSIM_Rhsim09G0093700	-1.258278369	0.003114397	0.011704629	RHSIM_Rhsim09G0093700
Root vs Stem	RHSIM_Rhsim06G0181000	-9.023527641	0.003119113	0.011716621	RHSIM_Rhsim06G0181000
Root vs Stem	RHSIM_Rhsim01G0162600	1.535679659	0.003137338	0.011778695	RHSIM_Rhsim01G0162600
Root vs Stem	novel.1509	-7.466834528	0.003142679	0.011795535	--
Root vs Stem	novel.3632	-7.507414754	0.003146047	0.011802989	--
Root vs Stem	RHSIM_Rhsim09G0173600	1.459034421	0.003148525	0.011809171	RHSIM_Rhsim09G0173600

Comparison	Gene ID	log_2FC	P-value	FDR (padj)	Gene Name
Root vs Stem	RHSIM_Rhsim05G0234200	2.075926962	0.003148873	0.011809171	RHSIM_Rhsim05G0234200
Root vs Stem	RHSIM_Rhsim04G0186900	1.584802361	0.003149938	0.011811244	RHSIM_Rhsim04G0186900
Root vs Stem	RHSIM_Rhsim05G0218700	-7.484013688	0.003151433	0.011814927	RHSIM_Rhsim05G0218700
Root vs Stem	RHSIM_Rhsim01G0124300	-1.035456692	0.003163712	0.011857106	RHSIM_Rhsim01G0124300
Root vs Stem	novel.1833	-1.736738675	0.003166034	0.011863878	--
Root vs Stem	novel.3420	1.512462837	0.003174219	0.011890683	--
Root vs Stem	RHSIM_Rhsim06G0175800	1.425785471	0.003176666	0.011897918	RHSIM_Rhsim06G0175800
Root vs Stem	RHSIM_Rhsim13G0226000	-6.597377196	0.003185988	0.011927814	RHSIM_Rhsim13G0226000
Root vs Stem	RHSIM_Rhsim03G0138600	-1.078620418	0.003187158	0.011929461	RHSIM_Rhsim03G0138600
Root vs Stem	RHSIM_Rhsim09G0129300	-1.644010558	0.003189243	0.011935328	RHSIM_Rhsim09G0129300
Root vs Stem	RHSIM_Rhsim06G0151500	1.04734488	0.003219516	0.012031043	RHSIM_Rhsim06G0151500
Root vs Stem	RHSIM_Rhsim12G0208300	-1.521464464	0.003224691	0.012048428	RHSIM_Rhsim12G0208300
Root vs Stem	RHSIM_Rhsim06G0057400	-3.455820487	0.003226242	0.012052268	RHSIM_Rhsim06G0057400
Root vs Stem	RHSIM_Rhsim11G0183200	2.880224919	0.003236269	0.012083855	RHSIM_Rhsim11G0183200
Root vs Stem	RHSIM_Rhsim05G0091800	-1.634464682	0.003251625	0.012139224	RHSIM_Rhsim05G0091800
Root vs Stem	RHSIM_Rhsim08G0010700	1.154715429	0.003256912	0.012156995	RHSIM_Rhsim08G0010700
Root vs Stem	novel.2610	1.415088056	0.003261754	0.012173097	--
Root vs Stem	RHSIM_Rhsim01G0227000	-2.168743006	0.003269545	0.012198221	RHSIM_Rhsim01G0227000
Root vs Stem	RHSIM_Rhsim08G0079400	2.003061523	0.003271408	0.0122032	RHSIM_Rhsim08G0079400
Root vs Stem	novel.2796	1.637322062	0.003277448	0.012223136	--
Root vs Stem	RHSIM_Rhsim05G0003400	-1.112396794	0.003279541	0.012227602	RHSIM_Rhsim05G0003400
Root vs Stem	RHSIM_Rhsim11G0050300	2.351486614	0.00328339	0.012238412	RHSIM_Rhsim11G0050300
Root vs Stem	RHSIM_RhsimUnG0187500	-1.296631334	0.003297593	0.012282986	RHSIM_RhsimUnG0187500
Root vs Stem	RHSIM_Rhsim08G0042800	-1.332167927	0.003317916	0.012351377	RHSIM_Rhsim08G0042800
Root vs Stem	RHSIM_Rhsim04G0173400	-3.525838612	0.003321971	0.012363802	RHSIM_Rhsim04G0173400

Comparison	Gene ID	log_2FC	P-value	FDR (padj)	Gene Name
Root vs Stem	RHSIM_Rhsim10G0171300	-1.348136008	0.003323215	0.012366438	RHSIM_Rhsim10G0171300
Root vs Stem	RHSIM_RhsimUnG0109100	-6.331982512	0.003324832	0.012370458	RHSIM_RhsimUnG0109100
Root vs Stem	novel.685	-1.500553422	0.003326491	0.012374634	--
Root vs Stem	RHSIM_Rhsim13G0148000	2.958539362	0.003329563	0.012384061	RHSIM_Rhsim13G0148000
Root vs Stem	RHSIM_Rhsim09G0185500	-1.956661534	0.003344865	0.012433166	RHSIM_Rhsim09G0185500
Root vs Stem	RHSIM_Rhsim06G0100900	-1.567015927	0.003345077	0.012433166	RHSIM_Rhsim06G0100900
Root vs Stem	RHSIM_Rhsim03G0082400	-2.518749899	0.003357362	0.012475383	RHSIM_Rhsim03G0082400
Root vs Stem	novel.2369	1.530710483	0.00336314	0.012493768	--
Root vs Stem	RHSIM_Rhsim01G0092300	-1.512406483	0.003363394	0.012493768	RHSIM_Rhsim01G0092300
Root vs Stem	RHSIM_Rhsim11G0028600	1.697479874	0.003369261	0.01251153	RHSIM_Rhsim11G0028600
Root vs Stem	RHSIM_Rhsim09G0160300	-2.285721545	0.003370744	0.012515019	RHSIM_Rhsim09G0160300
Root vs Stem	RHSIM_Rhsim09G0181700	-2.515844964	0.003371463	0.012515675	RHSIM_Rhsim09G0181700
Root vs Stem	novel.6023	-2.376342974	0.003380316	0.012542478	--
Root vs Stem	novel.2232	-7.472204364	0.003381516	0.012543954	--
Root vs Stem	RHSIM_Rhsim06G0201100	1.027017202	0.003382283	0.012543954	RHSIM_Rhsim06G0201100
Root vs Stem	RHSIM_RhsimUnG0063400	-1.313064632	0.003385822	0.012552808	RHSIM_RhsimUnG0063400
Root vs Stem	RHSIM_Rhsim05G0141100	-1.378707033	0.003388192	0.012557867	RHSIM_Rhsim05G0141100
Root vs Stem	RHSIM_Rhsim12G0190200	1.170781833	0.003388276	0.012557867	RHSIM_Rhsim12G0190200
Root vs Stem	RHSIM_Rhsim09G0191400	1.025266224	0.003390462	0.012563948	RHSIM_Rhsim09G0191400
Root vs Stem	RHSIM_Rhsim06G0055200	-2.238138229	0.003401323	0.012596097	RHSIM_Rhsim06G0055200
Root vs Stem	RHSIM_Rhsim05G0187400	3.04268001	0.003404746	0.0126027	RHSIM_Rhsim05G0187400
Root vs Stem	RHSIM_Rhsim01G0044000	-1.100759161	0.003413638	0.012633584	RHSIM_Rhsim01G0044000
Root vs Stem	RHSIM_Rhsim12G0063400	-2.390123814	0.003425764	0.012676427	RHSIM_Rhsim12G0063400
Root vs Stem	RHSIM_RhsimUnG0219800	1.573017585	0.003431303	0.012692848	RHSIM_RhsimUnG0219800
Root vs Stem	RHSIM_Rhsim04G0084000	1.149165809	0.003443422	0.01273415	RHSIM_Rhsim04G0084000

Comparison	Gene ID	log_2FC	P-value	FDR (padj)	Gene Name
Root vs Stem	RHSIM_Rhsim04G0019100	3.018155419	0.003443573	0.01273415	RHSIM_Rhsim04G0019100
Root vs Stem	novel.5856	-7.416974158	0.003451764	0.012762392	--
Root vs Stem	RHSIM_Rhsim05G0200500	1.012625284	0.00345659	0.012778187	RHSIM_Rhsim05G0200500
Root vs Stem	RHSIM_Rhsim05G0185500	1.993350391	0.003460125	0.012785105	RHSIM_Rhsim05G0185500
Root vs Stem	RHSIM_Rhsim06G0235100	3.082812949	0.003476778	0.012844578	RHSIM_Rhsim06G0235100
Root vs Stem	RHSIM_Rhsim02G0245200	-4.047690429	0.003484724	0.012869809	RHSIM_Rhsim02G0245200
Root vs Stem	RHSIM_RhsimUnG0086300	-2.065216559	0.003493156	0.012896817	RHSIM_RhsimUnG0086300
Root vs Stem	RHSIM_Rhsim13G0119700	-2.079043059	0.003496511	0.012907136	RHSIM_Rhsim13G0119700
Root vs Stem	RHSIM_Rhsim08G0159200	3.877649812	0.003497402	0.012908317	RHSIM_Rhsim08G0159200
Root vs Stem	RHSIM_Rhsim02G0194200	-4.076063049	0.003500322	0.012915001	RHSIM_Rhsim02G0194200
Root vs Stem	RHSIM_Rhsim06G0062500	1.263599244	0.003506416	0.012935416	RHSIM_Rhsim06G0062500
Root vs Stem	RHSIM_Rhsim06G0223100	1.234013491	0.003512126	0.012954406	RHSIM_Rhsim06G0223100
Root vs Stem	novel.3121	-1.394991731	0.003520261	0.012980258	--
Root vs Stem	RHSIM_Rhsim09G0135000	1.208009014	0.003527445	0.013004669	RHSIM_Rhsim09G0135000
Root vs Stem	RHSIM_Rhsim08G0101200	-2.658527639	0.003530112	0.013010338	RHSIM_Rhsim08G0101200
Root vs Stem	RHSIM_Rhsim04G0192200	-4.78235104	0.003535579	0.013028404	RHSIM_Rhsim04G0192200
Root vs Stem	RHSIM_Rhsim01G0292100	-1.054971178	0.003541208	0.013044976	RHSIM_Rhsim01G0292100
Root vs Stem	RHSIM_Rhsim11G0051400	-1.524193227	0.003543757	0.01305228	RHSIM_Rhsim11G0051400
Root vs Stem	RHSIM_Rhsim11G0168300	-1.423846419	0.003551777	0.013079732	RHSIM_Rhsim11G0168300
Root vs Stem	RHSIM_Rhsim10G0176200	2.628271177	0.003554878	0.013089061	RHSIM_Rhsim10G0176200
Root vs Stem	RHSIM_Rhsim10G0028800	-1.099554077	0.003558711	0.01310108	RHSIM_Rhsim10G0028800
Root vs Stem	novel.3851	-1.639459567	0.003563642	0.013117139	--
Root vs Stem	RHSIM_Rhsim11G0093200	-1.896190619	0.003565982	0.013120302	RHSIM_Rhsim11G0093200
Root vs Stem	RHSIM_Rhsim08G0168500	1.140650767	0.003568654	0.013125107	RHSIM_Rhsim08G0168500
Root vs Stem	novel.2629	-2.631943818	0.003576808	0.013147421	--

Comparison	Gene ID	log_2FC	P-value	FDR (padj)	Gene Name
Root vs Stem	RHSIM_RhsimUnG0058700	-4.524546592	0.003577002	0.013147421	RHSIM_RhsimUnG0058700
Root vs Stem	novel.2267	-1.972288199	0.003585178	0.013175374	--
Root vs Stem	RHSIM_Rhsim05G0109300	-1.848768151	0.003587344	0.013180279	RHSIM_Rhsim05G0109300
Root vs Stem	RHSIM_Rhsim06G0056800	-1.001641207	0.003587657	0.013180279	RHSIM_Rhsim06G0056800
Root vs Stem	RHSIM_Rhsim12G0105000	-1.706333458	0.003599598	0.013219937	RHSIM_Rhsim12G0105000
Root vs Stem	novel.5610	-2.051941859	0.003606023	0.013241326	--
Root vs Stem	novel.3151	-2.0440883	0.003606784	0.013241326	--
Root vs Stem	RHSIM_Rhsim09G0102500	1.474753115	0.003623213	0.013291842	RHSIM_Rhsim09G0102500
Root vs Stem	RHSIM_Rhsim09G0142100	3.282774355	0.003624551	0.013294635	RHSIM_Rhsim09G0142100
Root vs Stem	RHSIM_RhsimUnG0216300	1.719952933	0.003625819	0.013297168	RHSIM_RhsimUnG0216300
Root vs Stem	RHSIM_Rhsim01G0191100	1.659206622	0.003629914	0.013310069	RHSIM_Rhsim01G0191100
Root vs Stem	RHSIM_RhsimUnG0131300	2.220410416	0.003648582	0.013367891	RHSIM_RhsimUnG0131300
Root vs Stem	RHSIM_Rhsim10G0009400	2.098624196	0.003653946	0.01338329	RHSIM_Rhsim10G0009400
Root vs Stem	RHSIM_Rhsim13G0086600	1.475679563	0.003657702	0.013394919	RHSIM_Rhsim13G0086600
Root vs Stem	RHSIM_Rhsim13G0093200	-2.063266495	0.003661558	0.013404778	RHSIM_Rhsim13G0093200
Root vs Stem	novel.2372	-1.52284245	0.003682364	0.013474068	--
Root vs Stem	RHSIM_Rhsim06G0157400	3.093173747	0.003682822	0.013474068	RHSIM_Rhsim06G0157400
Root vs Stem	RHSIM_Rhsim13G0123800	-1.670591492	0.003692362	0.013506792	RHSIM_Rhsim13G0123800
Root vs Stem	novel.5234	-1.926320653	0.003692939	0.013506792	--
Root vs Stem	RHSIM_Rhsim13G0124400	2.638697265	0.003698182	0.013523786	RHSIM_Rhsim13G0124400
Root vs Stem	RHSIM_Rhsim02G0207700	2.092054784	0.003705108	0.013544856	RHSIM_Rhsim02G0207700
Root vs Stem	novel.1576	2.387550269	0.003727981	0.013617675	--
Root vs Stem	novel.3146	-1.347488312	0.003731848	0.01362964	--
Root vs Stem	RHSIM_Rhsim13G0004100	1.492318353	0.00373802	0.013650018	RHSIM_Rhsim13G0004100
Root vs Stem	RHSIM_Rhsim06G0026700	-1.09385009	0.003740759	0.013655696	RHSIM_Rhsim06G0026700

Comparison	Gene ID	log_2FC	P-value	FDR (padj)	Gene Name
Root vs Stem	RHSIM_Rhsim12G0052800	-1.131619939	0.003758509	0.013713977	RHSIM_Rhsim12G0052800
Root vs Stem	RHSIM_Rhsim08G0244800	1.804397503	0.003762721	0.013725002	RHSIM_Rhsim08G0244800
Root vs Stem	RHSIM_Rhsim12G0192000	-1.021489273	0.003781268	0.013781751	RHSIM_Rhsim12G0192000
Root vs Stem	RHSIM_Rhsim03G0067300	-2.765777866	0.003782035	0.013782365	RHSIM_Rhsim03G0067300
Root vs Stem	novel.1278	-2.203361179	0.003822981	0.013910347	--
Root vs Stem	RHSIM_Rhsim06G0217700	-2.108935631	0.003823444	0.013910347	RHSIM_Rhsim06G0217700
Root vs Stem	RHSIM_Rhsim08G0063800	-1.762747485	0.003827452	0.01391927	RHSIM_Rhsim08G0063800
Root vs Stem	RHSIM_Rhsim13G0186400	-2.119288324	0.003846473	0.013984033	RHSIM_Rhsim13G0186400
Root vs Stem	RHSIM_Rhsim11G0178300	1.157180476	0.003853965	0.014004645	RHSIM_Rhsim11G0178300
Root vs Stem	novel.2137	-4.548168923	0.003855724	0.014007342	--
Root vs Stem	RHSIM_Rhsim03G0262000	1.800256493	0.003855923	0.014007342	RHSIM_Rhsim03G0262000
Root vs Stem	RHSIM_Rhsim06G0105800	-7.359614387	0.003857116	0.014009468	RHSIM_Rhsim06G0105800
Root vs Stem	RHSIM_Rhsim01G0279600	-1.590842048	0.003857997	0.014010462	RHSIM_Rhsim01G0279600
Root vs Stem	RHSIM_Rhsim02G0084600	-3.580961979	0.00386232	0.01402395	RHSIM_Rhsim02G0084600
Root vs Stem	RHSIM_Rhsim01G0104100	-1.602509233	0.00386412	0.014028275	RHSIM_Rhsim01G0104100
Root vs Stem	RHSIM_Rhsim11G0144800	-1.27636464	0.003867957	0.014037784	RHSIM_Rhsim11G0144800
Root vs Stem	novel.1503	-1.829357593	0.003869743	0.014042056	--
Root vs Stem	RHSIM_Rhsim08G0233900	1.193327996	0.003871594	0.014046563	RHSIM_Rhsim08G0233900
Root vs Stem	RHSIM_Rhsim13G0201200	1.285483968	0.003873199	0.014048875	RHSIM_Rhsim13G0201200
Root vs Stem	RHSIM_Rhsim06G0162000	-2.069716646	0.003881156	0.014072396	RHSIM_Rhsim06G0162000
Root vs Stem	RHSIM_Rhsim13G0192900	1.98431854	0.00388375	0.014079585	RHSIM_Rhsim13G0192900
Root vs Stem	novel.4370	-7.397701108	0.003884485	0.014080036	--
Root vs Stem	RHSIM_Rhsim02G0226300	2.747803289	0.003892971	0.014104142	RHSIM_Rhsim02G0226300
Root vs Stem	novel.4172	-1.907597587	0.003918257	0.014180889	--
Root vs Stem	novel.3945	-4.890617013	0.00391846	0.014180889	--

Comparison	Gene ID	log_2FC	P-value	FDR (padj)	Gene Name
Root vs Stem	RHSIM_RhsimUnG0090500	-2.490209929	0.003919268	0.014181585	RHSIM_RhsimUnG0090500
Root vs Stem	RHSIM_Rhsim04G0173500	1.341613965	0.003926151	0.014204264	RHSIM_Rhsim04G0173500
Root vs Stem	novel.4208	-3.231416588	0.0039272	0.01420583	--
Root vs Stem	RHSIM_Rhsim13G0153700	-2.088879279	0.003929417	0.014211618	RHSIM_Rhsim13G0153700
Root vs Stem	RHSIM_Rhsim07G0118000	-1.530221914	0.003939476	0.014245764	RHSIM_Rhsim07G0118000
Root vs Stem	RHSIM_Rhsim08G0052600	1.340373731	0.003942224	0.014253464	RHSIM_Rhsim08G0052600
Root vs Stem	RHSIM_Rhsim03G0122200	-7.375273553	0.003948787	0.014272716	RHSIM_Rhsim03G0122200
Root vs Stem	RHSIM_Rhsim12G0047100	-1.147658271	0.003955177	0.014290764	RHSIM_Rhsim12G0047100
Root vs Stem	RHSIM_Rhsim12G0115600	-3.785949612	0.00395564	0.014290764	RHSIM_Rhsim12G0115600
Root vs Stem	RHSIM_Rhsim09G0083800	1.656623717	0.003978175	0.014365424	RHSIM_Rhsim09G0083800
Root vs Stem	novel.379	-7.376689655	0.003979917	0.014369463	--
Root vs Stem	RHSIM_Rhsim06G0154900	-3.84881947	0.003995028	0.014413834	RHSIM_Rhsim06G0154900
Root vs Stem	RHSIM_Rhsim09G0040100	6.17549581	0.003995356	0.014413834	RHSIM_Rhsim09G0040100
Root vs Stem	novel.3117	-1.183308388	0.003995958	0.014413834	--
Root vs Stem	RHSIM_Rhsim04G0219700	2.068144741	0.004001198	0.014423709	RHSIM_Rhsim04G0219700
Root vs Stem	RHSIM_Rhsim09G0109300	-1.236483354	0.004011107	0.014457167	RHSIM_Rhsim09G0109300
Root vs Stem	RHSIM_Rhsim12G0161500	1.24089567	0.004012712	0.014458434	RHSIM_Rhsim12G0161500
Root vs Stem	novel.3972	-7.378104594	0.004015467	0.014466099	--
Root vs Stem	RHSIM_Rhsim02G0128500	-2.147606691	0.004027453	0.014497949	RHSIM_Rhsim02G0128500
Root vs Stem	RHSIM_Rhsim04G0169800	-1.989447038	0.00403827	0.014532351	RHSIM_Rhsim04G0169800
Root vs Stem	RHSIM_Rhsim06G0112600	-7.348123822	0.004065894	0.014620349	RHSIM_Rhsim06G0112600
Root vs Stem	RHSIM_Rhsim02G0028000	-1.050433643	0.0040797	0.014665419	RHSIM_Rhsim02G0028000
Root vs Stem	novel.2569	-7.35536852	0.0040866	0.014687367	--
Root vs Stem	RHSIM_Rhsim05G0103700	-3.417571381	0.004089005	0.014691994	RHSIM_Rhsim05G0103700
Root vs Stem	RHSIM_Rhsim09G0026100	1.213076843	0.004107142	0.014745669	RHSIM_Rhsim09G0026100

Comparison	Gene ID	log_2FC	P-value	FDR (padj)	Gene Name
Root vs Stem	RHSIM_RhsimUnG0105400	1.554936223	0.004107978	0.014746375	RHSIM_RhsimUnG0105400
Root vs Stem	RHSIM_Rhsim12G0003900	-1.830117026	0.00411079	0.014751876	RHSIM_Rhsim12G0003900
Root vs Stem	novel.5879	-3.4258637	0.004115116	0.014760504	--
Root vs Stem	RHSIM_Rhsim02G0031700	-2.197085211	0.004123159	0.014787054	RHSIM_Rhsim02G0031700
Root vs Stem	RHSIM_Rhsim01G0275600	1.240171412	0.004125823	0.014794053	RHSIM_Rhsim01G0275600
Root vs Stem	novel.4305	-3.82515434	0.004126394	0.014794053	--
Root vs Stem	RHSIM_Rhsim08G0197800	1.26299699	0.004174325	0.014951915	RHSIM_Rhsim08G0197800
Root vs Stem	RHSIM_Rhsim02G0053200	1.100290465	0.004174965	0.014951915	RHSIM_Rhsim02G0053200
Root vs Stem	RHSIM_Rhsim09G0186000	1.413376539	0.004180925	0.014968607	RHSIM_Rhsim09G0186000
Root vs Stem	novel.4455	-1.316710883	0.004191426	0.015001542	--
Root vs Stem	novel.384	-7.333728391	0.004205513	0.015049625	--
Root vs Stem	RHSIM_Rhsim12G0200000	-1.548377349	0.004209768	0.015062513	RHSIM_Rhsim12G0200000
Root vs Stem	RHSIM_RhsimUnG0240600	-1.397948916	0.004215911	0.015082152	RHSIM_RhsimUnG0240600
Root vs Stem	RHSIM_Rhsim01G0251200	-3.333954532	0.004217535	0.015085622	RHSIM_Rhsim01G0251200
Root vs Stem	RHSIM_Rhsim11G0192000	-2.518293727	0.004220697	0.015092334	RHSIM_Rhsim11G0192000
Root vs Stem	novel.4936	-1.153436939	0.004229452	0.015118864	--
Root vs Stem	RHSIM_Rhsim07G0063800	-1.018754037	0.004231823	0.015124995	RHSIM_Rhsim07G0063800
Root vs Stem	novel.1261	-2.674554849	0.004252516	0.015194241	--
Root vs Stem	novel.5633	2.658511535	0.004254336	0.01519839	--
Root vs Stem	RHSIM_Rhsim12G0073600	-4.767528563	0.00426143	0.015221375	RHSIM_Rhsim12G0073600
Root vs Stem	RHSIM_Rhsim01G0023400	-1.03008452	0.004264493	0.015229956	RHSIM_Rhsim01G0023400
Root vs Stem	RHSIM_Rhsim07G0072900	1.198241378	0.00426941	0.015242792	RHSIM_Rhsim07G0072900
Root vs Stem	novel.4039	1.204392548	0.00427205	0.015249855	--
Root vs Stem	RHSIM_Rhsim09G0091400	-1.078470693	0.004276464	0.015263251	RHSIM_Rhsim09G0091400
Root vs Stem	novel.3232	-1.836156365	0.004320147	0.015406945	--

Comparison	Gene ID	log_2FC	P-value	FDR (padj)	Gene Name
Root vs Stem	RHSIM_Rhsim12G0062900	-1.808378443	0.004321403	0.015406945	RHSIM_Rhsim12G0062900
Root vs Stem	novel.5230	-1.29444277	0.004323453	0.015411868	--
Root vs Stem	RHSIM_Rhsim12G0117300	-1.958083226	0.004336335	0.015447514	RHSIM_Rhsim12G0117300
Root vs Stem	novel.2291	-2.421109367	0.004336803	0.015447514	--
Root vs Stem	RHSIM_Rhsim01G0076800	1.055315925	0.004342539	0.015465556	RHSIM_Rhsim01G0076800
Root vs Stem	RHSIM_Rhsim01G0119800	-1.425641895	0.004347212	0.015477417	RHSIM_Rhsim01G0119800
Root vs Stem	RHSIM_Rhsim09G0170800	-1.45091966	0.004355089	0.015495889	RHSIM_Rhsim09G0170800
Root vs Stem	RHSIM_Rhsim08G0227900	-1.380717661	0.004358102	0.015504217	RHSIM_Rhsim08G0227900
Root vs Stem	RHSIM_Rhsim10G0077800	1.662264327	0.004358893	0.015504637	RHSIM_Rhsim10G0077800
Root vs Stem	novel.2222	-7.340999886	0.004382578	0.015579271	--
Root vs Stem	RHSIM_Rhsim09G0079200	-2.994710579	0.004391482	0.015607849	RHSIM_Rhsim09G0079200
Root vs Stem	RHSIM_Rhsim07G0041200	-1.996388978	0.004400088	0.015629463	RHSIM_Rhsim07G0041200
Root vs Stem	RHSIM_Rhsim09G0054700	-1.800280536	0.004401901	0.015631087	RHSIM_Rhsim09G0054700
Root vs Stem	RHSIM_Rhsim13G0178200	-1.040982079	0.004404445	0.015637713	RHSIM_Rhsim13G0178200
Root vs Stem	RHSIM_Rhsim13G0147600	-1.748384807	0.004407078	0.015642244	RHSIM_Rhsim13G0147600
Root vs Stem	novel.2672	-7.365391903	0.004409177	0.015647285	--
Root vs Stem	novel.5935	-7.316191707	0.004413349	0.015659681	--
Root vs Stem	novel.3615	-1.141517715	0.004423121	0.015685333	--
Root vs Stem	RHSIM_Rhsim05G0004500	3.304886496	0.004423301	0.015685333	RHSIM_Rhsim05G0004500
Root vs Stem	novel.2826	-2.517042429	0.004429379	0.015704472	--
Root vs Stem	novel.4309	4.081701557	0.004443005	0.015747938	--
Root vs Stem	novel.5864	-2.481180324	0.004457828	0.015790767	--
Root vs Stem	novel.4718	-7.281923686	0.004477233	0.015852197	--
Root vs Stem	RHSIM_Rhsim03G0185700	-7.343899524	0.004491041	0.015888884	RHSIM_Rhsim03G0185700
Root vs Stem	RHSIM_Rhsim10G0051000	-7.38938286	0.004500261	0.015916619	RHSIM_Rhsim10G0051000

Comparison	Gene ID	log_2FC	P-value	FDR (padj)	Gene Name
Root vs Stem	RHSIM_Rhsim11G0004400	-1.816283818	0.00450585	0.015931497	RHSIM_Rhsim11G0004400
Root vs Stem	RHSIM_Rhsim07G0232100	-1.308850801	0.004512623	0.015950552	RHSIM_Rhsim07G0232100
Root vs Stem	novel.5740	-3.139244068	0.004522095	0.015976133	--
Root vs Stem	RHSIM_Rhsim09G0125200	2.512451063	0.004543922	0.016048879	RHSIM_Rhsim09G0125200
Root vs Stem	RHSIM_Rhsim10G0061500	-2.170811793	0.00454623	0.016054571	RHSIM_Rhsim10G0061500
Root vs Stem	RHSIM_Rhsim05G0025500	2.595465711	0.004550423	0.016066571	RHSIM_Rhsim05G0025500
Root vs Stem	RHSIM_RhsimUnG0062500	1.155830538	0.004551022	0.016066571	RHSIM_RhsimUnG0062500
Root vs Stem	RHSIM_Rhsim07G0019500	-2.563724217	0.004554082	0.01607245	RHSIM_Rhsim07G0019500
Root vs Stem	RHSIM_Rhsim05G0072700	-1.886680014	0.004590319	0.016185473	RHSIM_Rhsim05G0072700
Root vs Stem	RHSIM_Rhsim09G0163000	-1.988640538	0.004608548	0.01624478	RHSIM_Rhsim09G0163000
Root vs Stem	RHSIM_Rhsim08G0114300	-2.42714846	0.004619969	0.016282548	RHSIM_Rhsim08G0114300
Root vs Stem	RHSIM_Rhsim02G0243500	-2.023544594	0.004624727	0.016291844	RHSIM_Rhsim02G0243500
Root vs Stem	RHSIM_Rhsim09G0149900	1.36905169	0.004625503	0.01629209	RHSIM_Rhsim09G0149900
Root vs Stem	RHSIM_Rhsim01G0229400	-1.20695507	0.004628554	0.016300345	RHSIM_Rhsim01G0229400
Root vs Stem	RHSIM_Rhsim09G0049700	1.037766797	0.004629455	0.016301029	RHSIM_Rhsim09G0049700
Root vs Stem	novel.3915	-1.09777544	0.004642009	0.016337746	--
Root vs Stem	RHSIM_Rhsim08G0248600	-1.282189853	0.00464796	0.016351203	RHSIM_Rhsim08G0248600
Root vs Stem	RHSIM_Rhsim06G0157000	-2.29484621	0.004653741	0.016366542	RHSIM_Rhsim06G0157000
Root vs Stem	RHSIM_Rhsim13G0222900	1.317734278	0.004661891	0.016390204	RHSIM_Rhsim13G0222900
Root vs Stem	RHSIM_Rhsim05G0028100	-1.889663383	0.00467915	0.016447437	RHSIM_Rhsim05G0028100
Root vs Stem	RHSIM_Rhsim12G0041000	-1.564810718	0.004679597	0.016447437	RHSIM_Rhsim12G0041000
Root vs Stem	RHSIM_Rhsim02G0227800	2.090468389	0.004684628	0.016462612	RHSIM_Rhsim02G0227800
Root vs Stem	RHSIM_Rhsim02G0005200	1.185705726	0.004700301	0.016506669	RHSIM_Rhsim02G0005200
Root vs Stem	RHSIM_Rhsim12G0002500	1.213257849	0.004700745	0.016506669	RHSIM_Rhsim12G0002500
Root vs Stem	RHSIM_Rhsim09G0151700	1.436843656	0.004721672	0.016575102	RHSIM_Rhsim09G0151700

Comparison	Gene ID	log_2FC	P-value	FDR (padj)	Gene Name
Root vs Stem	RHSIM_Rhsim04G0156500	-1.404824486	0.004724608	0.016582478	RHSIM_Rhsim04G0156500
Root vs Stem	RHSIM_Rhsim12G0191600	-1.700967516	0.004725211	0.016582478	RHSIM_Rhsim12G0191600
Root vs Stem	RHSIM_Rhsim13G0048000	-1.412584821	0.004729326	0.016591864	RHSIM_Rhsim13G0048000
Root vs Stem	RHSIM_Rhsim07G0250600	-1.664412114	0.004749032	0.016650863	RHSIM_Rhsim07G0250600
Root vs Stem	RHSIM_Rhsim05G0157200	-2.038457899	0.004753992	0.016663186	RHSIM_Rhsim05G0157200
Root vs Stem	RHSIM_Rhsim10G0038300	-2.225760152	0.004757684	0.016672428	RHSIM_Rhsim10G0038300
Root vs Stem	novel.53	-1.439496031	0.00478328	0.016742933	--
Root vs Stem	RHSIM_Rhsim01G0227500	-1.101453372	0.004810181	0.016821771	RHSIM_Rhsim01G0227500
Root vs Stem	novel.1586	-2.304731398	0.004829154	0.016872761	--
Root vs Stem	RHSIM_Rhsim01G0224600	-1.036348355	0.004838299	0.016897491	RHSIM_Rhsim01G0224600
Root vs Stem	novel.4756	-7.246872757	0.004838707	0.016897491	--
Root vs Stem	novel.3752	-1.147751374	0.004847351	0.016922531	--
Root vs Stem	RHSIM_Rhsim12G0115400	-2.448942427	0.004848134	0.016922531	RHSIM_Rhsim12G0115400
Root vs Stem	RHSIM_RhsimUnG0179000	2.257409033	0.004851438	0.016930093	RHSIM_RhsimUnG0179000
Root vs Stem	RHSIM_Rhsim07G0246500	1.111072238	0.004860657	0.016959699	RHSIM_Rhsim07G0246500
Root vs Stem	novel.3578	-2.805102608	0.004875473	0.017006245	--
Root vs Stem	RHSIM_Rhsim01G0103900	-1.096597637	0.00488427	0.01703024	RHSIM_Rhsim01G0103900
Root vs Stem	RHSIM_Rhsim03G0170500	-1.712099589	0.00490495	0.017088382	RHSIM_Rhsim03G0170500
Root vs Stem	RHSIM_Rhsim06G0139600	-1.106304143	0.004909361	0.017098581	RHSIM_Rhsim06G0139600
Root vs Stem	RHSIM_Rhsim06G0163900	1.112569198	0.004937048	0.017179437	RHSIM_Rhsim06G0163900
Root vs Stem	RHSIM_Rhsim06G0152100	1.334835043	0.004941162	0.017188562	RHSIM_Rhsim06G0152100
Root vs Stem	RHSIM_Rhsim04G0128100	2.899769691	0.004958804	0.017242129	RHSIM_Rhsim04G0128100
Root vs Stem	RHSIM_Rhsim02G0141800	-3.325920312	0.004966012	0.017261982	RHSIM_Rhsim02G0141800
Root vs Stem	novel.339	-1.230623925	0.004968143	0.017264185	--
Root vs Stem	RHSIM_RhsimUnG0255400	-1.130928734	0.00498132	0.017304756	RHSIM_RhsimUnG0255400

Comparison	Gene ID	log_2FC	P-value	FDR (padj)	Gene Name
Root vs Stem	RHSIM_Rhsim08G0125400	2.04438859	0.005000077	0.0173673	RHSIM_Rhsim08G0125400
Root vs Stem	RHSIM_Rhsim10G0017200	2.791466245	0.005009805	0.017395846	RHSIM_Rhsim10G0017200
Root vs Stem	RHSIM_Rhsim03G0195000	-1.204589282	0.005029232	0.017460677	RHSIM_Rhsim03G0195000
Root vs Stem	novel.5550	-2.003756082	0.005034362	0.017470592	--
Root vs Stem	novel.1602	-7.21565538	0.005081854	0.017622137	--
Root vs Stem	RHSIM_Rhsim08G0213600	-1.407467766	0.005105307	0.017698143	RHSIM_Rhsim08G0213600
Root vs Stem	RHSIM_Rhsim07G0067300	1.065367208	0.005147064	0.017824135	RHSIM_Rhsim07G0067300
Root vs Stem	novel.5860	-2.099736215	0.005149795	0.017827902	--
Root vs Stem	RHSIM_Rhsim02G0115200	-1.349845842	0.005150472	0.017827902	RHSIM_Rhsim02G0115200
Root vs Stem	novel.3014	-10.9251469	0.005169767	0.01788267	--
Root vs Stem	RHSIM_Rhsim05G0061100	-1.255417031	0.005175949	0.017895803	RHSIM_Rhsim05G0061100
Root vs Stem	RHSIM_Rhsim10G0134900	-1.128738979	0.005176299	0.017895803	RHSIM_Rhsim10G0134900
Root vs Stem	RHSIM_Rhsim08G0042100	-1.609524994	0.005178445	0.017900539	RHSIM_Rhsim08G0042100
Root vs Stem	RHSIM_Rhsim05G0060000	-1.638069046	0.005186675	0.017926298	RHSIM_Rhsim05G0060000
Root vs Stem	novel.5628	3.767781822	0.005202081	0.017974157	--
Root vs Stem	RHSIM_Rhsim12G0184600	-1.496979409	0.0052041	0.017978437	RHSIM_Rhsim12G0184600
Root vs Stem	novel.324	-2.427149217	0.005205045	0.01797901	--
Root vs Stem	novel.4495	-7.262266302	0.005211374	0.017998174	--
Root vs Stem	RHSIM_Rhsim06G0229700	-1.935077552	0.005228861	0.018047753	RHSIM_Rhsim06G0229700
Root vs Stem	novel.20	-2.987836684	0.00523636	0.01807093	--
Root vs Stem	novel.3407	-3.99642778	0.005242529	0.018081393	--
Root vs Stem	RHSIM_Rhsim03G0047200	-7.206183925	0.005254944	0.018117598	RHSIM_Rhsim03G0047200
Root vs Stem	RHSIM_Rhsim02G0015500	2.915706849	0.005255385	0.018117598	RHSIM_Rhsim02G0015500
Root vs Stem	RHSIM_Rhsim05G0038400	-7.196611466	0.005258512	0.01812567	RHSIM_Rhsim05G0038400
Root vs Stem	RHSIM_Rhsim06G0215900	1.823579305	0.005263923	0.018140997	RHSIM_Rhsim06G0215900

Comparison	Gene ID	log_2FC	P-value	FDR (padj)	Gene Name
Root vs Stem	novel.4247	-7.207773719	0.005274168	0.018163341	--
Root vs Stem	RHSIM_RhsimUnG0207700	-1.180269539	0.0052789	0.018176919	RHSIM_RhsimUnG0207700
Root vs Stem	novel.413	-3.27164612	0.005303564	0.018256391	--
Root vs Stem	RHSIM_Rhsim04G0194000	-1.925525884	0.005305265	0.018259521	RHSIM_Rhsim04G0194000
Root vs Stem	RHSIM_Rhsim06G0232700	-1.77549996	0.00531744	0.018290501	RHSIM_Rhsim06G0232700
Root vs Stem	novel.3367	-1.346829406	0.00532214	0.018303937	--
Root vs Stem	RHSIM_Rhsim09G0038400	-2.463596997	0.005326147	0.018314987	RHSIM_Rhsim09G0038400
Root vs Stem	novel.5065	-1.485208558	0.005330192	0.018320511	--
Root vs Stem	RHSIM_Rhsim12G0111600	1.218076226	0.00533043	0.018320511	RHSIM_Rhsim12G0111600
Root vs Stem	RHSIM_Rhsim06G0156600	1.434846094	0.005330933	0.018320511	RHSIM_Rhsim06G0156600
Root vs Stem	RHSIM_Rhsim07G0240100	1.183938943	0.005335916	0.018334904	RHSIM_Rhsim07G0240100
Root vs Stem	novel.1024	2.968972822	0.00534327	0.018351824	--
Root vs Stem	RHSIM_Rhsim10G0164800	1.028132773	0.005343439	0.018351824	RHSIM_Rhsim10G0164800
Root vs Stem	RHSIM_Rhsim05G0031600	-2.123521828	0.005344025	0.018351824	RHSIM_Rhsim05G0031600
Root vs Stem	RHSIM_Rhsim12G0049900	-1.651959025	0.005351606	0.018374277	RHSIM_Rhsim12G0049900
Root vs Stem	RHSIM_Rhsim12G0005500	-2.199318422	0.005352859	0.018374277	RHSIM_Rhsim12G0005500
Root vs Stem	RHSIM_RhsimUnG0220200	2.075894536	0.005366645	0.01841579	RHSIM_RhsimUnG0220200
Root vs Stem	RHSIM_Rhsim11G0032600	1.374390686	0.005383285	0.018467391	RHSIM_Rhsim11G0032600
Root vs Stem	novel.6064	-2.236251374	0.005390643	0.018487129	--
Root vs Stem	RHSIM_Rhsim09G0084000	-2.276885173	0.005398082	0.01850989	RHSIM_Rhsim09G0084000
Root vs Stem	RHSIM_Rhsim08G0090900	-3.455539942	0.005423834	0.018589897	RHSIM_Rhsim08G0090900
Root vs Stem	RHSIM_RhsimUnG0155900	-1.983956207	0.005446404	0.018661704	RHSIM_RhsimUnG0155900
Root vs Stem	RHSIM_Rhsim08G0218000	-3.324057952	0.005466472	0.018717551	RHSIM_Rhsim08G0218000
Root vs Stem	RHSIM_Rhsim01G0235000	-1.415272159	0.005472702	0.018733292	RHSIM_Rhsim01G0235000
Root vs Stem	RHSIM_RhsimUnG0076600	-5.492207628	0.005472985	0.018733292	RHSIM_RhsimUnG0076600

Comparison	Gene ID	log2FC	P-value	FDR (padj)	Gene Name
Root vs Stem	novel.2670	-7.237524327	0.00547539	0.018738741	--
Root vs Stem	novel.4340	-1.798673436	0.005502482	0.018820283	--
Root vs Stem	RHSIM_Rhsim12G0088200	-3.44119214	0.00551754	0.018863391	RHSIM_Rhsim12G0088200
Root vs Stem	RHSIM_RhsimUnG0121800	2.047697606	0.005522348	0.018877029	RHSIM_RhsimUnG0121800
Root vs Stem	RHSIM_Rhsim10G0073700	-1.081062372	0.005546791	0.018949345	RHSIM_Rhsim10G0073700
Root vs Stem	RHSIM_RhsimUnG0007900	1.292383776	0.005548694	0.018953036	RHSIM_RhsimUnG0007900
Root vs Stem	novel.5017	-7.243834675	0.005555904	0.018972043	--
Root vs Stem	RHSIM_Rhsim03G0248400	2.064943381	0.005564747	0.018999425	RHSIM_Rhsim03G0248400
Root vs Stem	RHSIM_RhsimUnG0216800	3.455479921	0.005580647	0.01903737	RHSIM_RhsimUnG0216800
Root vs Stem	RHSIM_Rhsim03G0196300	-2.591499743	0.005580816	0.01903737	RHSIM_Rhsim03G0196300
Root vs Stem	novel.2066	-1.180344727	0.005594467	0.019081115	--
Root vs Stem	RHSIM_Rhsim11G0176700	-2.163290614	0.005608321	0.019119879	RHSIM_Rhsim11G0176700
Root vs Stem	RHSIM_Rhsim03G0255600	-6.324533249	0.005611819	0.019128975	RHSIM_Rhsim03G0255600
Root vs Stem	novel.3251	2.833097671	0.005612906	0.019129852	--
Root vs Stem	novel.4767	1.064654438	0.00562201	0.01915188	--
Root vs Stem	novel.3701	-7.167549608	0.005622692	0.01915188	--
Root vs Stem	RHSIM_Rhsim02G0094000	1.308332169	0.005630928	0.019171434	RHSIM_Rhsim02G0094000
Root vs Stem	novel.2639	-1.144212211	0.005632185	0.019172883	--
Root vs Stem	RHSIM_Rhsim07G0070600	-2.07251579	0.005635041	0.019179772	RHSIM_Rhsim07G0070600
Root vs Stem	RHSIM_Rhsim01G0197700	-2.19689108	0.00564966	0.019221018	RHSIM_Rhsim01G0197700
Root vs Stem	RHSIM_Rhsim06G0002200	1.556835033	0.005664792	0.019261129	RHSIM_Rhsim06G0002200
Root vs Stem	RHSIM_Rhsim03G0237600	-1.413979035	0.005672189	0.019280593	RHSIM_Rhsim03G0237600
Root vs Stem	novel.3523	-1.120876909	0.005673674	0.019282797	--
Root vs Stem	novel.1606	-1.697881112	0.005681707	0.019307253	--
Root vs Stem	novel.2678	-7.154459831	0.005690303	0.019333612	--

Comparison	Gene ID	log_2FC	P-value	FDR (padj)	Gene Name
Root vs Stem	RHSIM_Rhsim11G0024400	3.321012535	0.00570721	0.01937998	RHSIM_Rhsim11G0024400
Root vs Stem	novel.3192	-10.79972185	0.005707313	0.01937998	--
Root vs Stem	RHSIM_Rhsim03G0244200	-2.506151753	0.005728359	0.019442853	RHSIM_Rhsim03G0244200
Root vs Stem	RHSIM_Rhsim04G0147800	-1.274571995	0.005729729	0.019444642	RHSIM_Rhsim04G0147800
Root vs Stem	RHSIM_Rhsim10G0135800	1.368013413	0.00574396	0.019487196	RHSIM_Rhsim10G0135800
Root vs Stem	RHSIM_Rhsim13G0041400	1.243148218	0.005762374	0.019538414	RHSIM_Rhsim13G0041400
Root vs Stem	RHSIM_Rhsim10G0211900	1.330827964	0.005762447	0.019538414	RHSIM_Rhsim10G0211900
Root vs Stem	RHSIM_Rhsim02G0209700	-1.011719424	0.005763663	0.019539667	RHSIM_Rhsim02G0209700
Root vs Stem	RHSIM_Rhsim03G0039100	-1.269547395	0.005769511	0.019556615	RHSIM_Rhsim03G0039100
Root vs Stem	RHSIM_RhsimUnG0162000	1.419501647	0.005778015	0.019579684	RHSIM_RhsimUnG0162000
Root vs Stem	RHSIM_Rhsim03G0102000	-1.188404107	0.005779649	0.019581223	RHSIM_Rhsim03G0102000
Root vs Stem	novel.4267	-3.125152889	0.005780169	0.019581223	--
Root vs Stem	novel.907	-1.539989977	0.005791833	0.019612093	--
Root vs Stem	RHSIM_Rhsim01G0243900	-1.423665473	0.005820181	0.01969362	RHSIM_Rhsim01G0243900
Root vs Stem	RHSIM_Rhsim11G0149100	1.817588593	0.005839445	0.019747041	RHSIM_Rhsim11G0149100
Root vs Stem	RHSIM_Rhsim12G0107300	-1.751827722	0.005840252	0.019747041	RHSIM_Rhsim12G0107300
Root vs Stem	RHSIM_Rhsim09G0141900	-1.00882319	0.005855138	0.019791567	RHSIM_Rhsim09G0141900
Root vs Stem	RHSIM_Rhsim05G0033700	1.184211434	0.005875402	0.019845517	RHSIM_Rhsim05G0033700
Root vs Stem	RHSIM_Rhsim13G0030000	-2.370784266	0.0058867	0.019880764	RHSIM_Rhsim13G0030000
Root vs Stem	novel.4284	-3.008737686	0.005921767	0.019987481	--
Root vs Stem	RHSIM_Rhsim08G0120700	-7.13792098	0.005923951	0.019988999	RHSIM_Rhsim08G0120700
Root vs Stem	novel.1428	-7.13792098	0.005923951	0.019988999	--
Root vs Stem	RHSIM_Rhsim05G0206600	-2.096744843	0.005925571	0.019991541	RHSIM_Rhsim05G0206600
Root vs Stem	RHSIM_Rhsim08G0241700	-2.001262423	0.005942318	0.020045107	RHSIM_Rhsim08G0241700
Root vs Stem	RHSIM_Rhsim09G0143900	1.715825858	0.005951597	0.0200728	RHSIM_Rhsim09G0143900

Comparison	Gene ID	log_2FC	P-value	FDR (padj)	Gene Name
Root vs Stem	RHSIM_Rhsim08G0234000	1.498203786	0.00595314	0.0200728	RHSIM_Rhsim08G0234000
Root vs Stem	RHSIM_Rhsim05G0152100	1.157071349	0.00595887	0.020087313	RHSIM_Rhsim05G0152100
Root vs Stem	RHSIM_Rhsim11G0084400	1.793358725	0.005978757	0.020144443	RHSIM_Rhsim11G0084400
Root vs Stem	RHSIM_Rhsim02G0222900	-1.221106549	0.006024426	0.020277573	RHSIM_Rhsim02G0222900
Root vs Stem	RHSIM_Rhsim04G0049200	-1.930685347	0.00604195	0.020330621	RHSIM_Rhsim04G0049200
Root vs Stem	RHSIM_Rhsim11G0028200	-1.550337388	0.006060368	0.020389618	RHSIM_Rhsim11G0028200
Root vs Stem	RHSIM_Rhsim08G0103300	1.361087288	0.006075448	0.020431409	RHSIM_Rhsim08G0103300
Root vs Stem	novel.5745	-1.999093188	0.006108976	0.02053817	--
Root vs Stem	RHSIM_Rhsim03G0088300	1.543379317	0.006129187	0.020597105	RHSIM_Rhsim03G0088300
Root vs Stem	RHSIM_Rhsim10G0190300	-1.363352887	0.006142331	0.020634627	RHSIM_Rhsim10G0190300
Root vs Stem	RHSIM_RhsimUnG0094600	1.199191247	0.006145205	0.020638902	RHSIM_RhsimUnG0094600
Root vs Stem	RHSIM_Rhsim13G0095500	3.970842705	0.006165463	0.020690981	RHSIM_Rhsim13G0095500
Root vs Stem	novel.1634	3.27987555	0.006169694	0.020700037	--
Root vs Stem	RHSIM_Rhsim11G0050700	1.939910216	0.006171994	0.02070474	RHSIM_Rhsim11G0050700
Root vs Stem	novel.3973	-7.111038776	0.006175016	0.020711864	--
Root vs Stem	RHSIM_Rhsim02G0076000	-1.020573332	0.006182022	0.020730638	RHSIM_Rhsim02G0076000
Root vs Stem	novel.5454	-1.594589413	0.006186462	0.020741203	--
Root vs Stem	novel.2846	-1.513705764	0.006189377	0.020747959	--
Root vs Stem	RHSIM_Rhsim06G0144700	6.024918494	0.00619363	0.020756178	RHSIM_Rhsim06G0144700
Root vs Stem	RHSIM_Rhsim08G0001100	1.190186797	0.006197352	0.020765634	RHSIM_Rhsim08G0001100
Root vs Stem	novel.3836	-7.117814085	0.006202014	0.020778235	--
Root vs Stem	RHSIM_Rhsim08G0009000	-1.800494379	0.006203037	0.020778643	RHSIM_Rhsim08G0009000
Root vs Stem	RHSIM_Rhsim02G0237700	-1.051493055	0.006208872	0.020789124	RHSIM_Rhsim02G0237700
Root vs Stem	novel.1678	-3.507820404	0.006250485	0.020898105	--
Root vs Stem	RHSIM_Rhsim08G0044100	-1.052961944	0.006253917	0.020903514	RHSIM_Rhsim08G0044100

Comparison	Gene ID	log_2FC	P-value	FDR (padj)	Gene Name
Root vs Stem	RHSIM_Rhsim04G0117300	-1.809875992	0.006262236	0.020922222	RHSIM_Rhsim04G0117300
Root vs Stem	novel.4529	-2.745556749	0.006278798	0.020968439	--
Root vs Stem	RHSIM_Rhsim07G0004900	1.691062354	0.006291673	0.021008393	RHSIM_Rhsim07G0004900
Root vs Stem	RHSIM_Rhsim04G0179100	-3.009708792	0.006317328	0.021084894	RHSIM_Rhsim04G0179100
Root vs Stem	RHSIM_Rhsim06G0047500	-1.533727632	0.006326474	0.021100146	RHSIM_Rhsim06G0047500
Root vs Stem	RHSIM_Rhsim06G0140000	1.650525862	0.006341585	0.021147485	RHSIM_Rhsim06G0140000
Root vs Stem	novel.5127	-1.952288719	0.006347247	0.02115799	--
Root vs Stem	RHSIM_Rhsim11G0120700	-2.166191587	0.00635168	0.02116278	RHSIM_Rhsim11G0120700
Root vs Stem	RHSIM_Rhsim03G0068500	-1.095540335	0.006358163	0.021175198	RHSIM_Rhsim03G0068500
Root vs Stem	RHSIM_Rhsim01G0202700	-1.828279518	0.006361875	0.0211845	RHSIM_Rhsim01G0202700
Root vs Stem	novel.4897	-1.029343753	0.006364801	0.021188123	--
Root vs Stem	RHSIM_Rhsim12G0098300	-10.65970403	0.006366562	0.021190926	RHSIM_Rhsim12G0098300
Root vs Stem	novel.1708	-4.704658285	0.006371827	0.021203497	--
Root vs Stem	RHSIM_Rhsim13G0081800	-1.696109561	0.006372178	0.021203497	RHSIM_Rhsim13G0081800
Root vs Stem	novel.1648	-6.162688894	0.006383265	0.021234257	--
Root vs Stem	RHSIM_Rhsim05G0071400	1.045462115	0.006385229	0.021237727	RHSIM_Rhsim05G0071400
Root vs Stem	RHSIM_Rhsim07G0140500	2.443649331	0.006392189	0.02125781	RHSIM_Rhsim07G0140500
Root vs Stem	RHSIM_Rhsim07G0072500	-2.10447746	0.00639967	0.02127348	RHSIM_Rhsim07G0072500
Root vs Stem	RHSIM_Rhsim05G0072400	1.74718263	0.006411805	0.021305263	RHSIM_Rhsim05G0072400
Root vs Stem	novel.4969	-3.318838893	0.006412003	0.021305263	--
Root vs Stem	RHSIM_Rhsim12G0202000	-2.241569531	0.00641315	0.021306001	RHSIM_Rhsim12G0202000
Root vs Stem	RHSIM_Rhsim04G0143200	-1.700392527	0.006414431	0.021307188	RHSIM_Rhsim04G0143200
Root vs Stem	RHSIM_Rhsim06G0166700	1.590678231	0.006421662	0.021325061	RHSIM_Rhsim06G0166700
Root vs Stem	RHSIM_Rhsim04G0218000	-1.866146996	0.006429877	0.021346191	RHSIM_Rhsim04G0218000
Root vs Stem	RHSIM_Rhsim02G0168100	-1.583109498	0.006439083	0.0213706	RHSIM_Rhsim02G0168100

Comparison	Gene ID	log_2FC	P-value	FDR (padj)	Gene Name
Root vs Stem	RHSIM_RhsimUnG0168700	-1.568599407	0.006447479	0.021391644	RHSIM_RhsimUnG0168700
Root vs Stem	RHSIM_Rhsim11G0155400	1.290332388	0.006449136	0.021391644	RHSIM_Rhsim11G0155400
Root vs Stem	RHSIM_Rhsim04G0108300	1.384565192	0.006510445	0.02157638	RHSIM_Rhsim04G0108300
Root vs Stem	RHSIM_RhsimUnG0108500	2.066343948	0.006530721	0.021640465	RHSIM_RhsimUnG0108500
Root vs Stem	RHSIM_Rhsim07G0082100	1.014105539	0.00653758	0.021656966	RHSIM_Rhsim07G0082100
Root vs Stem	novel.3548	-7.095682823	0.00655053	0.021693632	--
Root vs Stem	novel.2851	-7.129604373	0.006554056	0.021702192	--
Root vs Stem	RHSIM_Rhsim09G0053400	1.208450154	0.006555826	0.021704936	RHSIM_Rhsim09G0053400
Root vs Stem	RHSIM_Rhsim10G0172200	2.935214219	0.006568028	0.02174221	RHSIM_Rhsim10G0172200
Root vs Stem	RHSIM_Rhsim06G0114300	-2.821555462	0.006569851	0.021745123	RHSIM_Rhsim06G0114300
Root vs Stem	novel.5459	-1.596812683	0.006590787	0.021805024	--
Root vs Stem	RHSIM_Rhsim06G0041400	-1.442430403	0.006613018	0.021872295	RHSIM_Rhsim06G0041400
Root vs Stem	RHSIM_Rhsim03G0173900	2.799661967	0.006613994	0.021872388	RHSIM_Rhsim03G0173900
Root vs Stem	RHSIM_Rhsim10G0082800	-1.793376421	0.006616009	0.02187591	RHSIM_Rhsim10G0082800
Root vs Stem	RHSIM_Rhsim04G0073100	-4.021217688	0.006621929	0.021892346	RHSIM_Rhsim04G0073100
Root vs Stem	novel.3817	-1.400010533	0.006625388	0.021900641	--
Root vs Stem	novel.1287	-1.85962166	0.006640998	0.021945948	--
Root vs Stem	RHSIM_Rhsim07G0010600	1.422892638	0.006649723	0.02197163	RHSIM_Rhsim07G0010600
Root vs Stem	RHSIM_Rhsim08G0160200	1.605224632	0.006671741	0.022038063	RHSIM_Rhsim08G0160200
Root vs Stem	RHSIM_Rhsim12G0066800	-2.313691394	0.006688985	0.022088693	RHSIM_Rhsim12G0066800
Root vs Stem	RHSIM_Rhsim08G0150900	-5.143718191	0.006703948	0.022134935	RHSIM_Rhsim08G0150900
Root vs Stem	RHSIM_Rhsim03G0186900	1.882634375	0.006725632	0.022196993	RHSIM_Rhsim03G0186900
Root vs Stem	novel.931	-1.089488232	0.006731561	0.02221338	--
Root vs Stem	RHSIM_Rhsim06G0151300	-7.069766871	0.006735183	0.022222152	RHSIM_Rhsim06G0151300
Root vs Stem	RHSIM_Rhsim04G0023200	2.080047223	0.006763977	0.022304387	RHSIM_Rhsim04G0023200

Comparison	Gene ID	log_2FC	P-value	FDR (padj)	Gene Name
Root vs Stem	RHSIM_Rhsim05G0194200	-5.402050203	0.006782519	0.022359133	RHSIM_Rhsim05G0194200
Root vs Stem	RHSIM_Rhsim09G0039000	1.510607323	0.006784211	0.022361512	RHSIM_Rhsim09G0039000
Root vs Stem	RHSIM_Rhsim10G0010100	2.837241653	0.006796553	0.022392588	RHSIM_Rhsim10G0010100
Root vs Stem	novel.2180	-3.206222786	0.006799202	0.022394913	--
Root vs Stem	novel.1912	-7.105973779	0.006806552	0.02241272	--
Root vs Stem	RHSIM_Rhsim12G0126900	2.307894739	0.006813639	0.022429646	RHSIM_Rhsim12G0126900
Root vs Stem	RHSIM_Rhsim01G0086800	-1.514357789	0.006816017	0.022431068	RHSIM_Rhsim01G0086800
Root vs Stem	novel.3767	-1.49122795	0.006832824	0.022476755	--
Root vs Stem	novel.3926	-1.037668871	0.00683431	0.022478435	--
Root vs Stem	novel.1843	-2.231499047	0.006842761	0.022503019	--
Root vs Stem	novel.850	1.297543173	0.00685934	0.022551106	--
Root vs Stem	novel.2970	2.688709679	0.006905006	0.022681832	--
Root vs Stem	RHSIM_Rhsim04G0227300	-2.924367078	0.006910787	0.02269759	RHSIM_Rhsim04G0227300
Root vs Stem	RHSIM_Rhsim03G0215000	-1.222801765	0.006917394	0.022712816	RHSIM_Rhsim03G0215000
Root vs Stem	novel.2803	-6.167625125	0.006921593	0.022720131	--
Root vs Stem	RHSIM_Rhsim08G0206400	-1.38308857	0.006922759	0.022720724	RHSIM_Rhsim08G0206400
Root vs Stem	novel.5605	-1.521400051	0.006941455	0.022778841	--
Root vs Stem	RHSIM_Rhsim03G0036000	-1.772970881	0.006945574	0.022789114	RHSIM_Rhsim03G0036000
Root vs Stem	RHSIM_Rhsim12G0169400	3.397255589	0.006966269	0.02284311	RHSIM_Rhsim12G0169400
Root vs Stem	novel.3792	-1.930716856	0.006966985	0.02284311	--
Root vs Stem	RHSIM_Rhsim04G0037100	-2.285042186	0.006968182	0.022843787	RHSIM_Rhsim04G0037100
Root vs Stem	novel.3488	3.364251964	0.006994006	0.022921925	--
Root vs Stem	RHSIM_Rhsim13G0109100	-2.03365267	0.006995712	0.022924256	RHSIM_Rhsim13G0109100
Root vs Stem	novel.1326	-7.039791432	0.007028549	0.023018771	--
Root vs Stem	RHSIM_Rhsim04G0143900	-2.499751323	0.007037417	0.023030039	RHSIM_Rhsim04G0143900

Comparison	Gene ID	log_2FC	P-value	FDR (padj)	Gene Name
Root vs Stem	RHSIM_Rhsim02G0236400	2.564626558	0.007070839	0.023130985	RHSIM_Rhsim02G0236400
Root vs Stem	RHSIM_Rhsim12G0020300	1.109864905	0.007080229	0.023151847	RHSIM_Rhsim12G0020300
Root vs Stem	RHSIM_Rhsim11G0084700	2.886642945	0.007083674	0.023159826	RHSIM_Rhsim11G0084700
Root vs Stem	RHSIM_Rhsim08G0238700	-1.365111994	0.007089186	0.023167929	RHSIM_Rhsim08G0238700
Root vs Stem	RHSIM_Rhsim05G0229300	1.099517801	0.007090172	0.023167929	RHSIM_Rhsim05G0229300
Root vs Stem	RHSIM_Rhsim04G0151500	2.285011268	0.007098819	0.023186325	RHSIM_Rhsim04G0151500
Root vs Stem	RHSIM_Rhsim11G0023700	1.261072955	0.007136437	0.023302588	RHSIM_Rhsim11G0023700
Root vs Stem	novel.4620	-7.052220754	0.007152772	0.023346008	--
Root vs Stem	RHSIM_Rhsim13G0185200	1.130291155	0.007163296	0.023377047	RHSIM_Rhsim13G0185200
Root vs Stem	RHSIM_Rhsim03G0138700	1.121583213	0.007166025	0.023382644	RHSIM_Rhsim03G0138700
Root vs Stem	RHSIM_Rhsim02G0020500	3.323315774	0.007170276	0.023389894	RHSIM_Rhsim02G0020500
Root vs Stem	novel.6060	2.60291223	0.007179725	0.023414089	--
Root vs Stem	RHSIM_Rhsim13G0130400	-1.916040861	0.007183354	0.023419298	RHSIM_Rhsim13G0130400
Root vs Stem	novel.5095	4.105644896	0.007186584	0.023426516	--
Root vs Stem	RHSIM_Rhsim13G0014200	1.349483803	0.007194748	0.023446497	RHSIM_Rhsim13G0014200
Root vs Stem	RHSIM_Rhsim02G0125800	-1.056357576	0.007216194	0.023509741	RHSIM_Rhsim02G0125800
Root vs Stem	RHSIM_Rhsim09G0195000	3.270397978	0.007222061	0.023525529	RHSIM_Rhsim09G0195000
Root vs Stem	RHSIM_RhsimUnG0046400	-1.214167277	0.007254143	0.023623359	RHSIM_RhsimUnG0046400
Root vs Stem	novel.4706	-1.340365331	0.007260956	0.023635528	--
Root vs Stem	RHSIM_Rhsim01G0223100	-3.172732218	0.007268658	0.02365392	RHSIM_Rhsim01G0223100
Root vs Stem	RHSIM_Rhsim02G0177000	-1.890376384	0.007280185	0.023684746	RHSIM_Rhsim02G0177000
Root vs Stem	RHSIM_Rhsim04G0009600	-1.626965621	0.007292865	0.023715956	RHSIM_Rhsim04G0009600
Root vs Stem	RHSIM_Rhsim09G0004500	1.112573333	0.007295872	0.023722389	RHSIM_Rhsim09G0004500
Root vs Stem	novel.3995	1.529997058	0.007301746	0.02373814	--
Root vs Stem	RHSIM_Rhsim01G0074900	-4.773374905	0.00731531	0.023775531	RHSIM_Rhsim01G0074900

Comparison	Gene ID	log_2FC	P-value	FDR (padj)	Gene Name
Root vs Stem	novel.2309	-1.082395644	0.007317936	0.02377896	--
Root vs Stem	RHSIM_RhsimUnG0061400	3.216201034	0.00731946	0.02377896	RHSIM_RhsimUnG0061400
Root vs Stem	RHSIM_Rhsim04G0072900	1.66984243	0.007326095	0.023797164	RHSIM_Rhsim04G0072900
Root vs Stem	RHSIM_RhsimUnG0163900	-1.035382604	0.007328173	0.023800559	RHSIM_RhsimUnG0163900
Root vs Stem	RHSIM_Rhsim03G0171800	-2.624006278	0.007389518	0.023966033	RHSIM_Rhsim03G0171800
Root vs Stem	RHSIM_Rhsim08G0031000	-3.52918246	0.007403751	0.024000131	RHSIM_Rhsim08G0031000
Root vs Stem	RHSIM_Rhsim06G0218900	1.788692757	0.007404196	0.024000131	RHSIM_Rhsim06G0218900
Root vs Stem	RHSIM_Rhsim03G0163600	-2.586454527	0.007421667	0.024053378	RHSIM_Rhsim03G0163600
Root vs Stem	novel.1956	-1.509477712	0.007477337	0.02421338	--
Root vs Stem	novel.1818	-1.970174453	0.007481763	0.024220906	--
Root vs Stem	RHSIM_Rhsim11G0031900	-5.763026447	0.007485269	0.024228857	RHSIM_Rhsim11G0031900
Root vs Stem	RHSIM_Rhsim08G0080300	-1.818011478	0.007493537	0.024248807	RHSIM_Rhsim08G0080300
Root vs Stem	RHSIM_Rhsim10G0026900	-1.198872433	0.007497541	0.024257783	RHSIM_Rhsim10G0026900
Root vs Stem	novel.1015	-10.44722456	0.007498415	0.024257783	--
Root vs Stem	novel.4308	-2.081187181	0.007528688	0.02433864	--
Root vs Stem	RHSIM_Rhsim11G0027800	-1.78179204	0.007555793	0.024412571	RHSIM_Rhsim11G0027800
Root vs Stem	RHSIM_Rhsim05G0058600	1.839172871	0.00756538	0.024440121	RHSIM_Rhsim05G0058600
Root vs Stem	RHSIM_RhsimUnG0060000	1.213659728	0.007567901	0.024443728	RHSIM_RhsimUnG0060000
Root vs Stem	RHSIM_Rhsim08G0001600	1.290408825	0.007611829	0.024555764	RHSIM_Rhsim08G0001600
Root vs Stem	RHSIM_Rhsim11G0153600	1.362825869	0.007615241	0.024563333	RHSIM_Rhsim11G0153600
Root vs Stem	novel.1895	-7.03266839	0.007652489	0.024674589	--
Root vs Stem	RHSIM_Rhsim07G0209500	-3.459694086	0.007660626	0.024695904	RHSIM_Rhsim07G0209500
Root vs Stem	novel.1931	-6.992717823	0.007669933	0.024715538	--
Root vs Stem	RHSIM_RhsimUnG0119300	-1.72160101	0.007699002	0.024795346	RHSIM_RhsimUnG0119300
Root vs Stem	RHSIM_Rhsim07G0088400	1.212578377	0.007704282	0.024808887	RHSIM_Rhsim07G0088400

Comparison	Gene ID	log_2FC	P-value	FDR (padj)	Gene Name
Root vs Stem	RHSIM_Rhsim03G0070800	1.456904161	0.007707958	0.024817256	RHSIM_Rhsim03G0070800
Root vs Stem	RHSIM_Rhsim13G0115300	2.309912735	0.007710833	0.024823045	RHSIM_Rhsim13G0115300
Root vs Stem	RHSIM_Rhsim09G0157100	1.783690215	0.007748313	0.02492282	RHSIM_Rhsim09G0157100
Root vs Stem	RHSIM_Rhsim03G0039200	-1.527400928	0.00776269	0.0249621	RHSIM_Rhsim03G0039200
Root vs Stem	RHSIM_Rhsim09G0157200	1.090193444	0.007783604	0.025015396	RHSIM_Rhsim09G0157200
Root vs Stem	RHSIM_Rhsim02G0006000	1.262077498	0.007785592	0.025018294	RHSIM_Rhsim02G0006000
Root vs Stem	RHSIM_Rhsim04G0198500	-1.233128845	0.007799996	0.025057298	RHSIM_Rhsim04G0198500
Root vs Stem	RHSIM_Rhsim06G0003600	1.0172567	0.00780099	0.025057298	RHSIM_Rhsim06G0003600
Root vs Stem	RHSIM_Rhsim03G0174600	-1.026022802	0.007807612	0.025075074	RHSIM_Rhsim03G0174600
Root vs Stem	RHSIM_Rhsim13G0071700	-1.109720991	0.00781213	0.025083584	RHSIM_Rhsim13G0071700
Root vs Stem	RHSIM_Rhsim07G0087900	-1.593123174	0.007818729	0.0250933	RHSIM_Rhsim07G0087900
Root vs Stem	RHSIM_Rhsim07G0072800	1.092778276	0.007821205	0.025094997	RHSIM_Rhsim07G0072800
Root vs Stem	RHSIM_RhsimUnG0112800	-1.150254323	0.007821435	0.025094997	RHSIM_RhsimUnG0112800
Root vs Stem	RHSIM_Rhsim06G0027500	-2.617370113	0.007857068	0.025198803	RHSIM_Rhsim06G0027500
Root vs Stem	RHSIM_Rhsim10G0040300	-1.121023085	0.007862439	0.025212519	RHSIM_Rhsim10G0040300
Root vs Stem	novel.3934	-1.165365469	0.007871672	0.025231599	--
Root vs Stem	RHSIM_Rhsim10G0030100	1.164945412	0.007877323	0.025242692	RHSIM_Rhsim10G0030100
Root vs Stem	RHSIM_Rhsim01G0146300	-1.22003156	0.007895507	0.025290417	RHSIM_Rhsim01G0146300
Root vs Stem	RHSIM_Rhsim01G0226700	1.26105177	0.00792107	0.025361727	RHSIM_Rhsim01G0226700
Root vs Stem	RHSIM_Rhsim01G0041200	2.400982086	0.007923467	0.025365878	RHSIM_Rhsim01G0041200
Root vs Stem	RHSIM_Rhsim03G0072100	-6.032640349	0.007925695	0.025369486	RHSIM_Rhsim03G0072100
Root vs Stem	RHSIM_Rhsim04G0032500	-4.314350403	0.007929658	0.025371602	RHSIM_Rhsim04G0032500
Root vs Stem	RHSIM_Rhsim05G0016700	1.83221092	0.007932984	0.025378723	RHSIM_Rhsim05G0016700
Root vs Stem	RHSIM_Rhsim12G0026200	-3.301214509	0.007950596	0.025428009	RHSIM_Rhsim12G0026200
Root vs Stem	RHSIM_Rhsim03G0080100	-1.059188212	0.00795619	0.025435312	RHSIM_Rhsim03G0080100

Comparison	Gene ID	log_2FC	P-value	FDR (padj)	Gene Name
Root vs Stem	RHSIM_Rhsim06G0166600	1.290417141	0.007958485	0.025436113	RHSIM_Rhsim06G0166600
Root vs Stem	novel.3835	-1.651447237	0.007958647	0.025436113	--
Root vs Stem	RHSIM_Rhsim08G0144900	1.230800215	0.007960256	0.02543773	RHSIM_Rhsim08G0144900
Root vs Stem	RHSIM_Rhsim12G0128300	-2.072228322	0.007966507	0.025454176	RHSIM_Rhsim12G0128300
Root vs Stem	RHSIM_Rhsim07G0032100	3.236186613	0.007996522	0.025532386	RHSIM_Rhsim07G0032100
Root vs Stem	RHSIM_Rhsim08G0189800	2.216572641	0.008008665	0.025567615	RHSIM_Rhsim08G0189800
Root vs Stem	RHSIM_Rhsim08G0077800	-1.684846792	0.008037283	0.025644772	RHSIM_Rhsim08G0077800
Root vs Stem	RHSIM_Rhsim04G0146900	2.189762775	0.00805172	0.025683729	RHSIM_Rhsim04G0146900
Root vs Stem	RHSIM_Rhsim13G0075500	1.41363667	0.008054927	0.025688948	RHSIM_Rhsim13G0075500
Root vs Stem	RHSIM_Rhsim06G0128200	-1.498573974	0.008055702	0.025688948	RHSIM_Rhsim06G0128200
Root vs Stem	RHSIM_Rhsim04G0029300	-1.480096838	0.008076005	0.025746944	RHSIM_Rhsim04G0029300
Root vs Stem	RHSIM_Rhsim13G0216300	-3.030064387	0.008080159	0.025756624	RHSIM_Rhsim13G0216300
Root vs Stem	RHSIM_RhsimUnG0157600	-4.818499908	0.008084092	0.025765599	RHSIM_RhsimUnG0157600
Root vs Stem	RHSIM_Rhsim11G0011500	-1.286323384	0.008110678	0.025843188	RHSIM_Rhsim11G0011500
Root vs Stem	RHSIM_Rhsim07G0024000	-1.642602011	0.00812603	0.025884947	RHSIM_Rhsim07G0024000
Root vs Stem	RHSIM_Rhsim09G0144000	2.838726142	0.008170963	0.026002929	RHSIM_Rhsim09G0144000
Root vs Stem	RHSIM_Rhsim09G0006700	1.343245294	0.008178589	0.026022856	RHSIM_Rhsim09G0006700
Root vs Stem	RHSIM_RhsimUnG0152400	-2.336939008	0.008180612	0.026022856	RHSIM_RhsimUnG0152400
Root vs Stem	RHSIM_RhsimUnG0122400	2.23216965	0.008196819	0.026070816	RHSIM_RhsimUnG0122400
Root vs Stem	RHSIM_Rhsim04G0145800	1.259008212	0.008207401	0.026094786	RHSIM_Rhsim04G0145800
Root vs Stem	novel.1328	-1.70812004	0.008207751	0.026094786	--
Root vs Stem	RHSIM_Rhsim07G0227100	1.040698703	0.008215173	0.02611118	RHSIM_Rhsim07G0227100
Root vs Stem	RHSIM_Rhsim04G0125800	-2.242621932	0.008217457	0.02611484	RHSIM_Rhsim04G0125800
Root vs Stem	novel.5321	-6.979786252	0.008223486	0.026126794	--
Root vs Stem	RHSIM_Rhsim07G0245900	1.006650766	0.008224648	0.026126886	RHSIM_Rhsim07G0245900

Comparison	Gene ID	log_2FC	P-value	FDR (padj)	Gene Name
Root vs Stem	RHSIM_RhsimUnG0097900	-1.262383968	0.008298201	0.026346017	RHSIM_RhsimUnG0097900
Root vs Stem	RHSIM_Rhsim03G0191200	-6.981644791	0.008299372	0.026346107	RHSIM_Rhsim03G0191200
Root vs Stem	RHSIM_Rhsim01G0143500	-1.911677858	0.008301483	0.026346743	RHSIM_Rhsim01G0143500
Root vs Stem	RHSIM_Rhsim11G0008700	-1.538944153	0.008333907	0.026440097	RHSIM_Rhsim11G0008700
Root vs Stem	RHSIM_Rhsim03G0136800	-1.257729824	0.008343194	0.026463353	RHSIM_Rhsim03G0136800
Root vs Stem	RHSIM_Rhsim11G0134400	-1.046958268	0.008349022	0.026474553	RHSIM_Rhsim11G0134400
Root vs Stem	RHSIM_Rhsim13G0184700	-2.047293055	0.008372264	0.026540953	RHSIM_Rhsim13G0184700
Root vs Stem	RHSIM_Rhsim08G0207600	-1.129189454	0.008378365	0.026556642	RHSIM_Rhsim08G0207600
Root vs Stem	novel.5530	-2.197401018	0.008383715	0.026567083	--
Root vs Stem	RHSIM_Rhsim07G0164700	-1.617596131	0.008417606	0.026651707	RHSIM_Rhsim07G0164700
Root vs Stem	RHSIM_Rhsim02G0030000	1.64064576	0.00843129	0.026691365	RHSIM_Rhsim02G0030000
Root vs Stem	RHSIM_Rhsim01G0083500	-1.890622426	0.00843286	0.026692672	RHSIM_Rhsim01G0083500
Root vs Stem	RHSIM_Rhsim11G0093400	-1.13599082	0.008477363	0.026815129	RHSIM_Rhsim11G0093400
Root vs Stem	RHSIM_Rhsim01G0215100	-2.140975714	0.008481832	0.026825584	RHSIM_Rhsim01G0215100
Root vs Stem	RHSIM_Rhsim09G0088700	-2.361416994	0.008488138	0.026839556	RHSIM_Rhsim09G0088700
Root vs Stem	novel.4962	-1.3391833	0.008490907	0.026839556	--
Root vs Stem	RHSIM_Rhsim08G0160800	-2.023927231	0.008501446	0.026869184	RHSIM_Rhsim08G0160800
Root vs Stem	RHSIM_Rhsim06G0218000	2.203203155	0.008508631	0.026887451	RHSIM_Rhsim06G0218000
Root vs Stem	RHSIM_Rhsim02G0098100	1.177713428	0.008533404	0.026955408	RHSIM_Rhsim02G0098100
Root vs Stem	RHSIM_Rhsim10G0016300	-2.087024736	0.008535906	0.026956769	RHSIM_Rhsim10G0016300
Root vs Stem	RHSIM_Rhsim07G0140900	-1.983243034	0.008536174	0.026956769	RHSIM_Rhsim07G0140900
Root vs Stem	RHSIM_Rhsim04G0223400	1.069583505	0.008560875	0.027023669	RHSIM_Rhsim04G0223400
Root vs Stem	RHSIM_Rhsim04G0001700	-1.400528677	0.008587965	0.027086978	RHSIM_Rhsim04G0001700
Root vs Stem	RHSIM_Rhsim01G0080300	-1.14175724	0.008595643	0.027100023	RHSIM_Rhsim01G0080300
Root vs Stem	novel.263	-3.496520171	0.00862426	0.027179093	--

Comparison	Gene ID	log_2FC	P-value	FDR (padj)	Gene Name
Root vs Stem	RHSIM_Rhsim10G0095300	-5.982131994	0.008640093	0.027221548	RHSIM_Rhsim10G0095300
Root vs Stem	RHSIM_Rhsim10G0010200	2.147463246	0.008649568	0.027243954	RHSIM_Rhsim10G0010200
Root vs Stem	RHSIM_Rhsim09G0138100	-2.550752765	0.008653814	0.027253604	RHSIM_Rhsim09G0138100
Root vs Stem	RHSIM_Rhsim04G0141500	-1.80989331	0.008656991	0.027259883	RHSIM_Rhsim04G0141500
Root vs Stem	RHSIM_Rhsim08G0237900	-1.05922925	0.008679445	0.027322875	RHSIM_Rhsim08G0237900
Root vs Stem	novel.4745	2.767065698	0.008694986	0.02736084	--
Root vs Stem	RHSIM_RhsimUnG0125100	-1.620010146	0.008703035	0.027378694	RHSIM_RhsimUnG0125100
Root vs Stem	RHSIM_Rhsim13G0181200	1.167215082	0.008748387	0.027491352	RHSIM_Rhsim13G0181200
Root vs Stem	novel.3207	-3.782460264	0.008751745	0.027498158	--
Root vs Stem	RHSIM_Rhsim09G0033700	-1.952329216	0.008775821	0.02756629	RHSIM_Rhsim09G0033700
Root vs Stem	RHSIM_Rhsim04G0049700	1.28547202	0.00878209	0.027578465	RHSIM_Rhsim04G0049700
Root vs Stem	RHSIM_Rhsim05G0186300	1.22544847	0.008799003	0.027627814	RHSIM_Rhsim05G0186300
Root vs Stem	novel.5021	-6.921030119	0.008821371	0.027679199	--
Root vs Stem	novel.5767	-3.242766176	0.008851686	0.027759205	--
Root vs Stem	RHSIM_Rhsim07G0176200	2.63507006	0.008902374	0.027887275	RHSIM_Rhsim07G0176200
Root vs Stem	RHSIM_Rhsim13G0205700	1.09820574	0.0089029	0.027887275	RHSIM_Rhsim13G0205700
Root vs Stem	RHSIM_Rhsim03G0007300	1.270510658	0.008903412	0.027887275	RHSIM_Rhsim03G0007300
Root vs Stem	RHSIM_Rhsim11G0020000	1.511729168	0.00892165	0.027940606	RHSIM_Rhsim11G0020000
Root vs Stem	RHSIM_RhsimUnG0113500	-1.72373271	0.008943014	0.02800126	RHSIM_RhsimUnG0113500
Root vs Stem	RHSIM_Rhsim07G0036600	2.032738441	0.008953079	0.02802761	RHSIM_Rhsim07G0036600
Root vs Stem	RHSIM_Rhsim04G0014400	-1.173433225	0.008970482	0.028073057	RHSIM_Rhsim04G0014400
Root vs Stem	RHSIM_Rhsim03G0049400	-1.061857843	0.008996171	0.028139585	RHSIM_Rhsim03G0049400
Root vs Stem	RHSIM_RhsimUnG0100900	2.721420133	0.009033416	0.028244586	RHSIM_RhsimUnG0100900
Root vs Stem	RHSIM_Rhsim08G0226800	-1.715774428	0.009038905	0.028250255	RHSIM_Rhsim08G0226800
Root vs Stem	RHSIM_Rhsim10G0150900	1.580201297	0.009087393	0.0283941	RHSIM_Rhsim10G0150900

Comparison	Gene ID	log_2FC	P-value	FDR (padj)	Gene Name
Root vs Stem	RHSIM_Rhsim12G0207200	-1.857800253	0.009089824	0.028397846	RHSIM_Rhsim12G0207200
Root vs Stem	novel.4723	-1.082104766	0.00910183	0.028431503	--
Root vs Stem	RHSIM_Rhsim07G0205300	-2.188246915	0.009127084	0.028498803	RHSIM_Rhsim07G0205300
Root vs Stem	RHSIM_Rhsim13G0117800	-2.665293011	0.009134034	0.028516643	RHSIM_Rhsim13G0117800
Root vs Stem	RHSIM_Rhsim07G0215700	2.276312202	0.009147734	0.028543952	RHSIM_Rhsim07G0215700
Root vs Stem	RHSIM_Rhsim01G0245100	-1.42579555	0.009154257	0.028560119	RHSIM_Rhsim01G0245100
Root vs Stem	RHSIM_Rhsim08G0239000	1.372102484	0.009157406	0.028562532	RHSIM_Rhsim08G0239000
Root vs Stem	RHSIM_Rhsim07G0183800	-1.164712034	0.009172447	0.028605576	RHSIM_Rhsim07G0183800
Root vs Stem	RHSIM_Rhsim01G0263300	-5.948342532	0.009177056	0.028616078	RHSIM_Rhsim01G0263300
Root vs Stem	novel.2329	-1.650948353	0.009200833	0.028682462	--
Root vs Stem	RHSIM_RhsimMtG0002100	-2.521455928	0.009228052	0.028759107	RHSIM_RhsimMtG0002100
Root vs Stem	RHSIM_Rhsim06G0226900	1.994149902	0.009229162	0.028759107	RHSIM_Rhsim06G0226900
Root vs Stem	RHSIM_Rhsim11G0018900	1.381882647	0.009233936	0.028770094	RHSIM_Rhsim11G0018900
Root vs Stem	RHSIM_Rhsim05G0028800	-6.895663342	0.009261405	0.028836807	RHSIM_Rhsim05G0028800
Root vs Stem	RHSIM_Rhsim04G0217600	-1.119058961	0.009261602	0.028836807	RHSIM_Rhsim04G0217600
Root vs Stem	RHSIM_Rhsim13G0141000	-1.969263116	0.009262974	0.028837184	RHSIM_Rhsim13G0141000
Root vs Stem	RHSIM_Rhsim04G0135300	1.536640218	0.009264581	0.028838293	RHSIM_Rhsim04G0135300
Root vs Stem	novel.2186	-1.098742678	0.009265973	0.028838732	--
Root vs Stem	novel.687	-1.068779783	0.009269393	0.028845481	--
Root vs Stem	novel.1524	-1.173227563	0.009273109	0.028853148	--
Root vs Stem	RHSIM_Rhsim08G0013600	3.325414013	0.009315209	0.028964596	RHSIM_Rhsim08G0013600
Root vs Stem	RHSIM_RhsimUnG0106600	-2.072603567	0.009327454	0.028990938	RHSIM_RhsimUnG0106600
Root vs Stem	novel.4162	-10.15606284	0.009342037	0.02903235	--
Root vs Stem	novel.3943	-6.881820244	0.009351504	0.029057853	--
Root vs Stem	RHSIM_Rhsim01G0104200	-1.825427716	0.009367384	0.02909514	RHSIM_Rhsim01G0104200

Comparison	Gene ID	log_2FC	P-value	FDR (padj)	Gene Name
Root vs Stem	RHSIM_RhsimUnG0238900	2.120268426	0.009396621	0.029162668	RHSIM_RhsimUnG0238900
Root vs Stem	RHSIM_Rhsim08G0222800	1.002758712	0.00940355	0.029172392	RHSIM_Rhsim08G0222800
Root vs Stem	RHSIM_Rhsim01G0024800	-1.119440642	0.009406505	0.029176108	RHSIM_Rhsim01G0024800
Root vs Stem	RHSIM_Rhsim08G0040700	2.382484165	0.009408018	0.029176108	RHSIM_Rhsim08G0040700
Root vs Stem	novel.5448	-6.901559996	0.009429517	0.029229349	--
Root vs Stem	RHSIM_Rhsim01G0174700	2.46111553	0.009434569	0.029238759	RHSIM_Rhsim01G0174700
Root vs Stem	RHSIM_Rhsim01G0268500	1.252058034	0.009471545	0.029343842	RHSIM_Rhsim01G0268500
Root vs Stem	RHSIM_Rhsim03G0083000	3.666838171	0.009490017	0.029381329	RHSIM_Rhsim03G0083000
Root vs Stem	RHSIM_Rhsim09G0045900	-6.877841788	0.009501057	0.029407608	RHSIM_Rhsim09G0045900
Root vs Stem	RHSIM_Rhsim06G0093000	-1.088934259	0.009505493	0.029410072	RHSIM_Rhsim06G0093000
Root vs Stem	novel.3913	-6.90352108	0.009516511	0.029431637	--
Root vs Stem	RHSIM_Rhsim02G0145000	1.199915927	0.009524347	0.029444116	RHSIM_Rhsim02G0145000
Root vs Stem	RHSIM_Rhsim05G0081300	4.938232479	0.009531137	0.029461154	RHSIM_Rhsim05G0081300
Root vs Stem	RHSIM_RhsimUnG0141700	1.69577469	0.00954578	0.029498508	RHSIM_RhsimUnG0141700
Root vs Stem	RHSIM_Rhsim10G0024600	1.260445473	0.009619007	0.029712846	RHSIM_Rhsim10G0024600
Root vs Stem	novel.1012	1.381250828	0.009620436	0.029713279	--
Root vs Stem	RHSIM_Rhsim05G0087900	-2.278224033	0.009658392	0.029810464	RHSIM_Rhsim05G0087900
Root vs Stem	novel.6061	-6.865828594	0.009674818	0.029837269	--
Root vs Stem	RHSIM_Rhsim06G0106700	-2.082471088	0.009682018	0.029851485	RHSIM_Rhsim06G0106700
Root vs Stem	novel.4768	-1.218116393	0.009733436	0.029996816	--
Root vs Stem	RHSIM_Rhsim05G0232000	-6.869842323	0.009734236	0.029996816	RHSIM_Rhsim05G0232000
Root vs Stem	novel.4702	-1.592165166	0.009742212	0.030017001	--
Root vs Stem	RHSIM_Rhsim03G0136200	-1.813960098	0.009808745	0.030189719	RHSIM_Rhsim03G0136200
Root vs Stem	RHSIM_Rhsim02G0019100	-1.09931426	0.009812201	0.030192621	RHSIM_Rhsim02G0019100
Root vs Stem	RHSIM_Rhsim12G0020800	-2.252407538	0.009815273	0.030197716	RHSIM_Rhsim12G0020800

Comparison	Gene ID	log_2FC	P-value	FDR (padj)	Gene Name
Root vs Stem	novel.5100	-4.794456444	0.009852493	0.030292013	--
Root vs Stem	novel.3822	1.991977344	0.009855805	0.030298157	--
Root vs Stem	novel.4508	2.130965441	0.009920284	0.03044765	--
Root vs Stem	RHSIM_Rhsim06G0100800	-1.20308695	0.009960244	0.03056216	RHSIM_Rhsim06G0100800
Root vs Stem	RHSIM_Rhsim09G0024200	-1.303037114	0.009962805	0.03056595	RHSIM_Rhsim09G0024200
Root vs Stem	RHSIM_Rhsim11G0024000	2.644969757	0.009971513	0.030588595	RHSIM_Rhsim11G0024000
Root vs Stem	novel.4313	-3.326701921	0.009984687	0.030620859	--
Root vs Stem	novel.1420	-6.869851388	0.009994165	0.030639993	--
Root vs Stem	RHSIM_Rhsim02G0206200	-3.734652565	0.010001246	0.030647179	RHSIM_Rhsim02G0206200
Root vs Stem	RHSIM_Rhsim08G0094300	-1.871388712	0.010009058	0.030662964	RHSIM_Rhsim08G0094300
Root vs Stem	RHSIM_Rhsim10G0007400	-1.424015836	0.010049496	0.030758236	RHSIM_Rhsim10G0007400
Root vs Stem	RHSIM_Rhsim11G0190700	-1.871157252	0.010061341	0.03078223	RHSIM_Rhsim11G0190700
Root vs Stem	RHSIM_Rhsim10G0177100	2.33874667	0.010062728	0.030782389	RHSIM_Rhsim10G0177100
Root vs Stem	RHSIM_Rhsim03G0021100	-5.685214352	0.010084508	0.030828558	RHSIM_Rhsim03G0021100
Root vs Stem	RHSIM_Rhsim02G0114400	-1.102901919	0.01011053	0.030891724	RHSIM_Rhsim02G0114400
Root vs Stem	RHSIM_Rhsim05G0220000	-2.304906183	0.010118841	0.030907392	RHSIM_Rhsim05G0220000
Root vs Stem	novel.30	1.303231848	0.010158129	0.031011031	--
Root vs Stem	RHSIM_Rhsim06G0222500	6.750630018	0.01017117	0.031035869	RHSIM_Rhsim06G0222500
Root vs Stem	novel.3386	1.897822834	0.01018114	0.03106218	--
Root vs Stem	RHSIM_Rhsim04G0191900	-1.61850523	0.010190798	0.031083418	RHSIM_Rhsim04G0191900
Root vs Stem	RHSIM_Rhsim13G0213100	1.320484714	0.010203522	0.031102815	RHSIM_Rhsim13G0213100
Root vs Stem	novel.966	-2.113989742	0.010203903	0.031102815	--
Root vs Stem	RHSIM_Rhsim10G0175500	2.166388946	0.010212038	0.031123493	RHSIM_Rhsim10G0175500
Root vs Stem	RHSIM_Rhsim08G0052300	1.196741743	0.010236221	0.031184829	RHSIM_Rhsim08G0052300
Root vs Stem	novel.2068	-1.199987638	0.010298903	0.031355072	--

Comparison	Gene ID	log2FC	P-value	FDR (padj)	Gene Name
Root vs Stem	RHSIM_RhsimUnG0171200	1.659943375	0.010358875	0.031516845	RHSIM_RhsimUnG0171200
Root vs Stem	RHSIM_Rhsim10G0202900	2.399586701	0.010370571	0.031543383	RHSIM_Rhsim10G0202900
Root vs Stem	RHSIM_Rhsim04G0240300	-1.096070388	0.010370759	0.031543383	RHSIM_Rhsim04G0240300
Root vs Stem	RHSIM_Rhsim02G0079400	-1.135627742	0.010371702	0.031543383	RHSIM_Rhsim02G0079400
Root vs Stem	RHSIM_Rhsim12G0086800	-1.058807255	0.010381165	0.031559671	RHSIM_Rhsim12G0086800
Root vs Stem	RHSIM_Rhsim03G0151700	-7.96162838	0.010385713	0.031565175	RHSIM_Rhsim03G0151700
Root vs Stem	RHSIM_Rhsim07G0147000	-1.985808726	0.010400307	0.031605362	RHSIM_Rhsim07G0147000
Root vs Stem	RHSIM_Rhsim04G0182500	3.380416772	0.010442209	0.031711796	RHSIM_Rhsim04G0182500
Root vs Stem	RHSIM_Rhsim04G0233000	-1.788706641	0.010444237	0.031712029	RHSIM_Rhsim04G0233000
Root vs Stem	RHSIM_Rhsim05G0199300	-1.821365695	0.010446084	0.031712029	RHSIM_Rhsim05G0199300
Root vs Stem	RHSIM_Rhsim03G0059500	-1.04881765	0.010487231	0.031810807	RHSIM_Rhsim03G0059500
Root vs Stem	novel.4366	-2.045103614	0.010538085	0.031931451	--
Root vs Stem	RHSIM_Rhsim03G0032100	2.270860814	0.010546078	0.031947271	RHSIM_Rhsim03G0032100
Root vs Stem	novel.5510	-1.734915141	0.010549062	0.0319515	--
Root vs Stem	RHSIM_Rhsim13G0144100	-1.065973465	0.010550246	0.0319515	RHSIM_Rhsim13G0144100
Root vs Stem	novel.1162	-9.990436725	0.010562553	0.031984569	--
Root vs Stem	RHSIM_Rhsim10G0032700	-2.291739842	0.010570058	0.031996988	RHSIM_Rhsim10G0032700
Root vs Stem	novel.909	-1.188780734	0.01058752	0.032028307	--
Root vs Stem	RHSIM_Rhsim05G0191200	-1.292825031	0.010588111	0.032028307	RHSIM_Rhsim05G0191200
Root vs Stem	RHSIM_Rhsim02G0014800	2.588561276	0.010637843	0.032165067	RHSIM_Rhsim02G0014800
Root vs Stem	RHSIM_Rhsim09G0104300	2.050143792	0.010638903	0.032165067	RHSIM_Rhsim09G0104300
Root vs Stem	RHSIM_Rhsim12G0076900	1.114743155	0.010641503	0.03216871	RHSIM_Rhsim12G0076900
Root vs Stem	novel.2995	-6.810520846	0.010660981	0.032210694	--
Root vs Stem	RHSIM_Rhsim03G0045000	-2.612803696	0.010667861	0.032227258	RHSIM_Rhsim03G0045000
Root vs Stem	RHSIM_Rhsim08G0100300	-1.425375925	0.01068062	0.032261575	RHSIM_Rhsim08G0100300

Comparison	Gene ID	log_2FC	P-value	FDR (padj)	Gene Name
Root vs Stem	RHSIM_Rhsim04G0052200	1.188019771	0.010757409	0.032459048	RHSIM_Rhsim04G0052200
Root vs Stem	novel.4200	-6.800050455	0.010780154	0.032511106	--
Root vs Stem	novel.4765	-6.816770413	0.010792331	0.032539316	--
Root vs Stem	RHSIM_Rhsim06G0163100	2.006739589	0.010805178	0.032573789	RHSIM_Rhsim06G0163100
Root vs Stem	RHSIM_Rhsim13G0137900	1.410989971	0.010833923	0.032647114	RHSIM_Rhsim13G0137900
Root vs Stem	novel.2281	1.472222117	0.010878662	0.032752488	--
Root vs Stem	RHSIM_Rhsim05G0001100	-2.348716037	0.010882613	0.032760103	RHSIM_Rhsim05G0001100
Root vs Stem	novel.2477	-1.107199679	0.010884208	0.032760628	--
Root vs Stem	RHSIM_Rhsim02G0207600	1.720825468	0.010917921	0.032850232	RHSIM_Rhsim02G0207600
Root vs Stem	RHSIM_Rhsim01G0121400	-1.957258331	0.01092009	0.032850232	RHSIM_Rhsim01G0121400
Root vs Stem	novel.2476	-1.377287357	0.010921103	0.032850232	--
Root vs Stem	novel.395	-6.795841965	0.010947931	0.032918046	--
Root vs Stem	RHSIM_Rhsim08G0005700	-2.016719355	0.010949894	0.032919053	RHSIM_Rhsim08G0005700
Root vs Stem	RHSIM_Rhsim11G0089700	-1.174225365	0.010951122	0.032919053	RHSIM_Rhsim11G0089700
Root vs Stem	RHSIM_Rhsim08G0097000	2.726733173	0.010968484	0.032962648	RHSIM_Rhsim08G0097000
Root vs Stem	novel.748	-6.820924124	0.010972537	0.032970529	--
Root vs Stem	novel.3021	1.401943651	0.010994395	0.033023294	--
Root vs Stem	RHSIM_RhsimUnG0171500	1.519447204	0.010999027	0.033032902	RHSIM_RhsimUnG0171500
Root vs Stem	RHSIM_Rhsim09G0012400	2.341225152	0.011000735	0.03303373	RHSIM_Rhsim09G0012400
Root vs Stem	novel.123	-2.264784778	0.011024234	0.033099984	--
Root vs Stem	RHSIM_Rhsim11G0161400	1.913542647	0.011025797	0.033100261	RHSIM_Rhsim11G0161400
Root vs Stem	RHSIM_Rhsim05G0096300	-1.188373024	0.011033722	0.03310691	RHSIM_Rhsim05G0096300
Root vs Stem	novel.4957	-1.811499044	0.011066452	0.033187838	--
Root vs Stem	RHSIM_Rhsim07G0224100	1.734800499	0.011074526	0.033205959	RHSIM_Rhsim07G0224100
Root vs Stem	novel.5571	-1.654836681	0.011106017	0.033280517	--

Comparison	Gene ID	log_2FC	P-value	FDR (padj)	Gene Name
Root vs Stem	RHSIM_Rhsim09G0163900	1.325264467	0.011112963	0.033288348	RHSIM_Rhsim09G0163900
Root vs Stem	novel.1920	-1.990397111	0.011119562	0.033299464	--
Root vs Stem	novel.5090	2.005991341	0.011130223	0.03332273	--
Root vs Stem	RHSIM_Rhsim08G0171300	1.241019359	0.011166225	0.033417498	RHSIM_Rhsim08G0171300
Root vs Stem	RHSIM_Rhsim02G0105000	-1.229753776	0.011223307	0.033557833	RHSIM_Rhsim02G0105000
Root vs Stem	RHSIM_Rhsim03G0019100	1.055733844	0.011225825	0.033561008	RHSIM_Rhsim03G0019100
Root vs Stem	novel.3476	-2.729870266	0.011232452	0.033572115	--
Root vs Stem	RHSIM_Rhsim07G0139900	-1.118031263	0.011248414	0.033611107	RHSIM_Rhsim07G0139900
Root vs Stem	RHSIM_Rhsim13G0083400	-1.170942729	0.011294663	0.033727444	RHSIM_Rhsim13G0083400
Root vs Stem	RHSIM_Rhsim02G0127200	-6.787363319	0.011309197	0.033762951	RHSIM_Rhsim02G0127200
Root vs Stem	RHSIM_Rhsim08G0146500	-1.411278149	0.011351848	0.03387188	RHSIM_Rhsim08G0146500
Root vs Stem	novel.1709	-5.823268659	0.011371004	0.033924649	--
Root vs Stem	novel.1960	-1.466051557	0.011391822	0.033973566	--
Root vs Stem	novel.3544	-1.107890933	0.011406684	0.03400909	--
Root vs Stem	RHSIM_Rhsim02G0195500	2.49025012	0.011421814	0.034049798	RHSIM_Rhsim02G0195500
Root vs Stem	novel.3937	-1.06592694	0.011423477	0.034050351	--
Root vs Stem	RHSIM_Rhsim04G0067300	-1.636151562	0.011429806	0.034064812	RHSIM_Rhsim04G0067300
Root vs Stem	RHSIM_RhsimUnG0217000	1.722518222	0.011435729	0.034078058	RHSIM_RhsimUnG0217000
Root vs Stem	RHSIM_Rhsim06G0217500	-1.049545955	0.011437313	0.034078374	RHSIM_Rhsim06G0217500
Root vs Stem	novel.2580	-3.897207369	0.011471335	0.034175069	--
Root vs Stem	RHSIM_Rhsim06G0089800	-4.518957366	0.011472731	0.034175069	RHSIM_Rhsim06G0089800
Root vs Stem	RHSIM_Rhsim08G0073300	1.652124963	0.011483616	0.034194238	RHSIM_Rhsim08G0073300
Root vs Stem	RHSIM_Rhsim09G0072100	-1.990754685	0.011506338	0.034244204	RHSIM_Rhsim09G0072100
Root vs Stem	RHSIM_Rhsim05G0181900	1.801773228	0.011571984	0.034412919	RHSIM_Rhsim05G0181900
Root vs Stem	RHSIM_Rhsim05G0228000	1.451739557	0.011607899	0.034506369	RHSIM_Rhsim05G0228000

Comparison	Gene ID	log_2FC	P-value	FDR (padj)	Gene Name
Root vs Stem	novel.4789	-2.231366955	0.011613417	0.03451777	--
Root vs Stem	RHSIM_Rhsim07G0030100	1.292904075	0.011648524	0.034613745	RHSIM_Rhsim07G0030100
Root vs Stem	RHSIM_Rhsim09G0156300	1.784611001	0.011743457	0.034859891	RHSIM_Rhsim09G0156300
Root vs Stem	RHSIM_Rhsim08G0063000	1.232453279	0.011757268	0.034888094	RHSIM_Rhsim08G0063000
Root vs Stem	RHSIM_RhsimUnG0237300	-1.342103852	0.011757498	0.034888094	RHSIM_RhsimUnG0237300
Root vs Stem	novel.476	1.026893831	0.011759203	0.034888663	--
Root vs Stem	RHSIM_Rhsim06G0167500	1.795133416	0.011796609	0.034985508	RHSIM_Rhsim06G0167500
Root vs Stem	RHSIM_Rhsim06G0155000	-1.460074911	0.011797915	0.034985508	RHSIM_Rhsim06G0155000
Root vs Stem	RHSIM_Rhsim13G0052800	-2.423405687	0.011802345	0.034989642	RHSIM_Rhsim13G0052800
Root vs Stem	RHSIM_Rhsim08G0200700	1.041490224	0.011829637	0.03505703	RHSIM_Rhsim08G0200700
Root vs Stem	RHSIM_Rhsim03G0266300	-3.177706258	0.011844734	0.035088237	RHSIM_Rhsim03G0266300
Root vs Stem	novel.3069	1.118864426	0.011857591	0.035117299	--
Root vs Stem	RHSIM_Rhsim12G0067600	-2.588351313	0.011865678	0.035136735	RHSIM_Rhsim12G0067600
Root vs Stem	RHSIM_Rhsim03G0262700	-1.631632582	0.011882293	0.035176897	RHSIM_Rhsim03G0262700
Root vs Stem	RHSIM_Rhsim01G0290500	1.420545777	0.011920826	0.035254754	RHSIM_Rhsim01G0290500
Root vs Stem	RHSIM_Rhsim05G0007300	2.08437026	0.012000364	0.035453595	RHSIM_Rhsim05G0007300
Root vs Stem	RHSIM_RhsimUnG0244100	-1.085586774	0.012070852	0.035636811	RHSIM_RhsimUnG0244100
Root vs Stem	RHSIM_RhsimUnG0163700	-2.509494485	0.012109321	0.035734278	RHSIM_RhsimUnG0163700
Root vs Stem	RHSIM_Rhsim07G0094800	1.738895574	0.012115649	0.035748376	RHSIM_Rhsim07G0094800
Root vs Stem	novel.5089	-1.673815703	0.012120303	0.035757531	--
Root vs Stem	RHSIM_Rhsim03G0199100	-2.130119664	0.012136401	0.035783048	RHSIM_Rhsim03G0199100
Root vs Stem	RHSIM_Rhsim13G0186600	-1.325048012	0.012139853	0.035783151	RHSIM_Rhsim13G0186600
Root vs Stem	RHSIM_Rhsim05G0031000	-5.573709138	0.012161886	0.035838931	RHSIM_Rhsim05G0031000
Root vs Stem	RHSIM_Rhsim06G0215800	1.327803244	0.012163826	0.035840065	RHSIM_Rhsim06G0215800
Root vs Stem	RHSIM_Rhsim12G0051900	1.68243844	0.012175544	0.035865426	RHSIM_Rhsim12G0051900

Comparison	Gene ID	log_2FC	P-value	FDR (padj)	Gene Name
Root vs Stem	RHSIM_Rhsim04G0152800	-1.329761962	0.012240148	0.036037308	RHSIM_Rhsim04G0152800
Root vs Stem	RHSIM_Rhsim08G0060100	2.662791288	0.012265382	0.036097774	RHSIM_Rhsim08G0060100
Root vs Stem	RHSIM_Rhsim03G0181200	-3.946182488	0.012284153	0.0361484	RHSIM_Rhsim03G0181200
Root vs Stem	RHSIM_Rhsim04G0068300	-1.933223603	0.012294816	0.036170545	RHSIM_Rhsim04G0068300
Root vs Stem	RHSIM_Rhsim04G0174700	-1.492589025	0.012318918	0.036232205	RHSIM_Rhsim04G0174700
Root vs Stem	RHSIM_Rhsim08G0062000	-1.405807245	0.01232081	0.036233146	RHSIM_Rhsim08G0062000
Root vs Stem	novel.4421	-4.860993483	0.012322511	0.036233525	--
Root vs Stem	novel.1872	-1.397765977	0.012334996	0.036260986	--
Root vs Stem	novel.3415	-4.424078684	0.012360097	0.036325511	--
Root vs Stem	novel.383	-6.720041163	0.012384139	0.036372372	--
Root vs Stem	RHSIM_Rhsim03G0206300	-6.722260072	0.012386327	0.036372372	RHSIM_Rhsim03G0206300
Root vs Stem	RHSIM_Rhsim07G0061100	2.244377515	0.012394189	0.036388594	RHSIM_Rhsim07G0061100
Root vs Stem	RHSIM_Rhsim13G0042700	-1.149491696	0.012407248	0.036414841	RHSIM_Rhsim13G0042700
Root vs Stem	novel.996	-6.724475941	0.012409628	0.036414841	--
Root vs Stem	RHSIM_Rhsim10G0128100	1.137721152	0.012426721	0.036448558	RHSIM_Rhsim10G0128100
Root vs Stem	RHSIM_Rhsim13G0179800	1.01119832	0.012427262	0.036448558	RHSIM_Rhsim13G0179800
Root vs Stem	RHSIM_Rhsim04G0101500	-6.72668884	0.012454505	0.03650988	RHSIM_Rhsim04G0101500
Root vs Stem	novel.5665	-6.72668884	0.012454505	0.03650988	--
Root vs Stem	novel.3938	-6.728898837	0.012521695	0.036669543	--
Root vs Stem	RHSIM_Rhsim10G0179300	-2.040986006	0.012535155	0.036699636	RHSIM_Rhsim10G0179300
Root vs Stem	RHSIM_Rhsim08G0138200	-9.754632897	0.012544901	0.036718979	RHSIM_Rhsim08G0138200
Root vs Stem	RHSIM_Rhsim06G0155600	-9.754404955	0.012546967	0.036720227	RHSIM_Rhsim06G0155600
Root vs Stem	RHSIM_Rhsim09G0007700	-1.965334929	0.012569483	0.036781452	RHSIM_Rhsim09G0007700
Root vs Stem	novel.315	-1.757758052	0.012583526	0.036810013	--
Root vs Stem	novel.2480	-6.731106007	0.012612247	0.036887862	--

Comparison	Gene ID	log_2FC	P-value	FDR (padj)	Gene Name
Root vs Stem	RHSIM_Rhsim07G0001000	-2.38635327	0.012633964	0.036942005	RHSIM_Rhsim07G0001000
Root vs Stem	RHSIM_Rhsim03G0109400	1.900202569	0.012699167	0.037099722	RHSIM_Rhsim03G0109400
Root vs Stem	novel.3180	1.880565917	0.012758976	0.037260283	--
Root vs Stem	RHSIM_Rhsim08G0150300	-2.968152913	0.012776071	0.037296031	RHSIM_Rhsim08G0150300
Root vs Stem	RHSIM_Rhsim12G0174800	1.462098669	0.01278446	0.037311071	RHSIM_Rhsim12G0174800
Root vs Stem	RHSIM_Rhsim05G0214100	1.515310592	0.012802033	0.0373529	RHSIM_Rhsim05G0214100
Root vs Stem	RHSIM_Rhsim02G0227900	1.747547482	0.012806849	0.037362225	RHSIM_Rhsim02G0227900
Root vs Stem	RHSIM_Rhsim07G0060500	-5.433897223	0.012815316	0.037382195	RHSIM_Rhsim07G0060500
Root vs Stem	novel.5501	1.98096933	0.012889992	0.037561999	--
Root vs Stem	RHSIM_Rhsim05G0234100	1.001203746	0.01289318	0.037566541	RHSIM_Rhsim05G0234100
Root vs Stem	novel.240	-3.705064017	0.012903367	0.037591473	--
Root vs Stem	RHSIM_RhsimUnG0013100	-1.182540005	0.012920873	0.037632961	RHSIM_RhsimUnG0013100
Root vs Stem	novel.4577	-1.898107832	0.012923007	0.037634423	--
Root vs Stem	novel.2554	1.708100847	0.012938925	0.03767126	--
Root vs Stem	RHSIM_Rhsim09G0122900	-1.273974708	0.013027047	0.037879984	RHSIM_Rhsim09G0122900
Root vs Stem	novel.3992	-3.003483664	0.013078982	0.03800362	--
Root vs Stem	RHSIM_Rhsim02G0135700	-1.742016595	0.013079457	0.03800362	RHSIM_Rhsim02G0135700
Root vs Stem	novel.1036	-2.987710569	0.013117249	0.038089425	--
Root vs Stem	novel.1190	-6.697625645	0.013150994	0.038148969	--
Root vs Stem	novel.4914	-6.697625645	0.013150994	0.038148969	--
Root vs Stem	novel.831	-6.70217234	0.013154497	0.038154331	--
Root vs Stem	RHSIM_Rhsim08G0033700	2.928109181	0.013181935	0.038217037	RHSIM_Rhsim08G0033700
Root vs Stem	RHSIM_Rhsim07G0244100	-3.508577585	0.01320662	0.038265288	RHSIM_Rhsim07G0244100
Root vs Stem	RHSIM_Rhsim01G0265300	1.533896324	0.013219354	0.038289454	RHSIM_Rhsim01G0265300
Root vs Stem	RHSIM_RhsimMtG0002300	1.264071631	0.013230238	0.038316166	RHSIM_RhsimMtG0002300

Comparison	Gene ID	log_2FC	P-value	FDR (padj)	Gene Name
Root vs Stem	RHSIM_Rhsim12G0080000	1.374590459	0.013268813	0.038423055	RHSIM_Rhsim12G0080000
Root vs Stem	RHSIM_Rhsim03G0077600	-3.101956551	0.013298178	0.038498417	RHSIM_Rhsim03G0077600
Root vs Stem	RHSIM_Rhsim10G0053100	-1.247146774	0.013310722	0.038529893	RHSIM_Rhsim10G0053100
Root vs Stem	RHSIM_Rhsim08G0022600	-1.560379048	0.013327163	0.038572641	RHSIM_Rhsim08G0022600
Root vs Stem	novel.1008	-9.668606634	0.013346319	0.038618387	--
Root vs Stem	RHSIM_Rhsim08G0024100	1.048692762	0.013452406	0.038900943	RHSIM_Rhsim08G0024100
Root vs Stem	RHSIM_Rhsim04G0112300	2.658347146	0.01347045	0.038948235	RHSIM_Rhsim04G0112300
Root vs Stem	RHSIM_Rhsim02G0026500	1.646579426	0.013482933	0.03897944	RHSIM_Rhsim02G0026500
Root vs Stem	RHSIM_Rhsim01G0268900	1.043537259	0.013490924	0.038992762	RHSIM_Rhsim01G0268900
Root vs Stem	RHSIM_Rhsim03G0222100	-2.388026714	0.013524827	0.039076053	RHSIM_Rhsim03G0222100
Root vs Stem	novel.1116	-1.742169689	0.013529307	0.039084101	--
Root vs Stem	novel.4699	2.714051398	0.013574745	0.039205536	--
Root vs Stem	novel.2345	-2.292764781	0.01359249	0.039235674	--
Root vs Stem	RHSIM_Rhsim03G0262400	-1.695375301	0.013636993	0.039345881	RHSIM_Rhsim03G0262400
Root vs Stem	novel.1521	-1.28804339	0.013650761	0.039380677	--
Root vs Stem	RHSIM_RhsimUnG0116300	-1.634374839	0.013677052	0.039451588	RHSIM_RhsimUnG0116300
Root vs Stem	RHSIM_RhsimUnG0130800	-1.400584434	0.013682629	0.039457801	RHSIM_RhsimUnG0130800
Root vs Stem	RHSIM_Rhsim11G0131800	1.122070874	0.013763128	0.039665133	RHSIM_Rhsim11G0131800
Root vs Stem	RHSIM_Rhsim03G0200400	1.746134336	0.013799141	0.039754011	RHSIM_Rhsim03G0200400
Root vs Stem	RHSIM_Rhsim12G0124800	1.639503578	0.01382708	0.039824546	RHSIM_Rhsim12G0124800
Root vs Stem	RHSIM_Rhsim10G0141800	1.033935604	0.013830161	0.039828444	RHSIM_Rhsim10G0141800
Root vs Stem	novel.1723	-9.614283445	0.013875424	0.039939987	--
Root vs Stem	RHSIM_Rhsim07G0001600	-1.989583314	0.013875823	0.039939987	RHSIM_Rhsim07G0001600
Root vs Stem	RHSIM_Rhsim09G0068500	-1.198946371	0.013906215	0.04002247	RHSIM_Rhsim09G0068500
Root vs Stem	RHSIM_Rhsim01G0059200	-1.631952588	0.013931713	0.040085843	RHSIM_Rhsim01G0059200

Comparison	Gene ID	log_2FC	P-value	FDR (padj)	Gene Name
Root vs Stem	RHSIM_Rhsim11G0106200	-1.272334248	0.013949455	0.040126877	RHSIM_Rhsim11G0106200
Root vs Stem	novel.5059	-6.649435901	0.013976652	0.040200096	--
Root vs Stem	RHSIM_Rhsim02G0000600	-3.190168788	0.013987884	0.040207318	RHSIM_Rhsim02G0000600
Root vs Stem	RHSIM_Rhsim04G0189100	2.507838195	0.013998671	0.040233308	RHSIM_Rhsim04G0189100
Root vs Stem	RHSIM_Rhsim05G0021700	2.093013868	0.014010598	0.040262567	RHSIM_Rhsim05G0021700
Root vs Stem	RHSIM_Rhsim03G0122700	1.561698653	0.014025816	0.040293171	RHSIM_Rhsim03G0122700
Root vs Stem	RHSIM_Rhsim06G0185900	2.544135188	0.014026491	0.040293171	RHSIM_Rhsim06G0185900
Root vs Stem	novel.5640	-1.555869032	0.014037454	0.040319639	--
Root vs Stem	RHSIM_Rhsim07G0007500	-1.164824417	0.014054647	0.040360099	RHSIM_Rhsim07G0007500
Root vs Stem	RHSIM_Rhsim03G0200700	-1.681899994	0.014055042	0.040360099	RHSIM_Rhsim03G0200700
Root vs Stem	novel.111	-1.720208353	0.014060538	0.040363802	--
Root vs Stem	RHSIM_Rhsim10G0052600	-1.129219251	0.014095096	0.040449924	RHSIM_Rhsim10G0052600
Root vs Stem	RHSIM_Rhsim13G0084700	1.727700852	0.01411278	0.040495633	RHSIM_Rhsim13G0084700
Root vs Stem	RHSIM_Rhsim13G0102200	1.231511688	0.01415492	0.040601389	RHSIM_Rhsim13G0102200
Root vs Stem	RHSIM_Rhsim13G0099300	-1.25097532	0.014157977	0.040605106	RHSIM_Rhsim13G0099300
Root vs Stem	novel.3289	-9.58558906	0.014162319	0.040612508	--
Root vs Stem	RHSIM_Rhsim03G0253800	-1.691324342	0.014182691	0.040665869	RHSIM_Rhsim03G0253800
Root vs Stem	novel.6	-1.838035645	0.01420202	0.040711161	--
Root vs Stem	RHSIM_Rhsim05G0022500	-1.194203604	0.014206516	0.040718986	RHSIM_Rhsim05G0022500
Root vs Stem	RHSIM_Rhsim12G0165700	-2.196359007	0.014240296	0.040802995	RHSIM_Rhsim12G0165700
Root vs Stem	RHSIM_Rhsim03G0217100	-1.940777907	0.014241136	0.040802995	RHSIM_Rhsim03G0217100
Root vs Stem	RHSIM_Rhsim12G0025800	-1.701752456	0.014249377	0.040821536	RHSIM_Rhsim12G0025800
Root vs Stem	RHSIM_Rhsim04G0043400	1.38923185	0.014256318	0.040836343	RHSIM_Rhsim04G0043400
Root vs Stem	novel.1465	-4.589819305	0.014267325	0.04085772	--
Root vs Stem	RHSIM_RhsimUnG0121300	-2.379983261	0.014321972	0.040983666	RHSIM_RhsimUnG0121300

Comparison	Gene ID	log_2FC	P-value	FDR (padj)	Gene Name
Root vs Stem	RHSIM_Rhsim05G0059500	-2.681232012	0.014353427	0.041058388	RHSIM_Rhsim05G0059500
Root vs Stem	RHSIM_Rhsim07G0215400	-1.335236206	0.014370911	0.041103302	RHSIM_Rhsim07G0215400
Root vs Stem	novel.179	1.364974892	0.014386466	0.041137585	--
Root vs Stem	RHSIM_Rhsim10G0029700	-2.161755958	0.014393094	0.041151431	RHSIM_Rhsim10G0029700
Root vs Stem	RHSIM_Rhsim09G0085800	-5.668635244	0.014405411	0.041176433	RHSIM_Rhsim09G0085800
Root vs Stem	RHSIM_Rhsim04G0009300	-2.4211429	0.014412846	0.041187472	RHSIM_Rhsim04G0009300
Root vs Stem	RHSIM_Rhsim10G0202200	3.25898622	0.014425812	0.041204095	RHSIM_Rhsim10G0202200
Root vs Stem	RHSIM_Rhsim12G0102800	-1.694190767	0.014435978	0.041228023	RHSIM_Rhsim12G0102800
Root vs Stem	RHSIM_Rhsim03G0167100	-1.532325988	0.014462733	0.041273754	RHSIM_Rhsim03G0167100
Root vs Stem	novel.6065	-1.868914701	0.014489884	0.04133077	--
Root vs Stem	RHSIM_Rhsim02G0206800	-1.248217264	0.014499581	0.041348199	RHSIM_Rhsim02G0206800
Root vs Stem	novel.1052	-2.554256311	0.014509948	0.041372644	--
Root vs Stem	RHSIM_Rhsim04G0103800	-2.824350187	0.014512257	0.04137411	RHSIM_Rhsim04G0103800
Root vs Stem	RHSIM_RhsimUnG0013000	-1.053005116	0.01456719	0.04150506	RHSIM_RhsimUnG0013000
Root vs Stem	RHSIM_Rhsim13G0173200	-9.544761737	0.014579553	0.041530018	RHSIM_Rhsim13G0173200
Root vs Stem	RHSIM_Rhsim11G0137900	-1.284816275	0.014586925	0.041545884	RHSIM_Rhsim11G0137900
Root vs Stem	RHSIM_Rhsim02G0212800	1.493739105	0.014601943	0.041579645	RHSIM_Rhsim02G0212800
Root vs Stem	RHSIM_Rhsim03G0096500	-1.46005387	0.014614286	0.041597538	RHSIM_Rhsim03G0096500
Root vs Stem	RHSIM_Rhsim08G0073400	-1.557980121	0.014615888	0.041597538	RHSIM_Rhsim08G0073400
Root vs Stem	RHSIM_Rhsim09G0178600	-1.042175839	0.014620273	0.041602401	RHSIM_Rhsim09G0178600
Root vs Stem	RHSIM_RhsimUnG0098400	2.060940062	0.014621206	0.041602401	RHSIM_RhsimUnG0098400
Root vs Stem	novel.2160	-9.537010662	0.014659979	0.041684717	--
Root vs Stem	RHSIM_Rhsim06G0051800	2.297943366	0.014678787	0.04172504	RHSIM_Rhsim06G0051800
Root vs Stem	RHSIM_Rhsim06G0148300	1.299039302	0.014695417	0.041762013	RHSIM_Rhsim06G0148300
Root vs Stem	RHSIM_Rhsim13G0221600	-3.006800716	0.014703137	0.041778802	RHSIM_Rhsim13G0221600

Comparison	Gene ID	log_2FC	P-value	FDR (padj)	Gene Name
Root vs Stem	RHSIM_Rhsim07G0091100	-2.569144313	0.014729806	0.041849422	RHSIM_Rhsim07G0091100
Root vs Stem	novel.516	-3.278965403	0.014747519	0.041889422	--
Root vs Stem	RHSIM_Rhsim02G0012400	-1.912491479	0.014767725	0.041926153	RHSIM_Rhsim02G0012400
Root vs Stem	RHSIM_Rhsim03G0023800	-3.051761475	0.014789172	0.041976702	RHSIM_Rhsim03G0023800
Root vs Stem	RHSIM_Rhsim11G0141300	-1.197525125	0.01481288	0.042038817	RHSIM_Rhsim11G0141300
Root vs Stem	RHSIM_Rhsim12G0033000	-3.684323508	0.014815882	0.042042163	RHSIM_Rhsim12G0033000
Root vs Stem	RHSIM_Rhsim08G0201300	1.06515005	0.014836718	0.042085746	RHSIM_Rhsim08G0201300
Root vs Stem	RHSIM_Rhsim01G0255000	-1.077680945	0.014852199	0.042119294	RHSIM_Rhsim01G0255000
Root vs Stem	novel.3113	-9.51366211	0.014904616	0.042236769	--
Root vs Stem	RHSIM_Rhsim02G0237500	-1.274808563	0.014908073	0.042238336	RHSIM_Rhsim02G0237500
Root vs Stem	novel.3495	-9.505577033	0.014990164	0.042447887	--
Root vs Stem	RHSIM_Rhsim09G0137500	-1.651673924	0.015023045	0.042525327	RHSIM_Rhsim09G0137500
Root vs Stem	RHSIM_Rhsim07G0197600	3.017804562	0.015029014	0.042536998	RHSIM_Rhsim07G0197600
Root vs Stem	RHSIM_Rhsim05G0222700	1.578321754	0.015032113	0.042540548	RHSIM_Rhsim05G0222700
Root vs Stem	RHSIM_Rhsim03G0134400	-1.729768675	0.015051768	0.042575263	RHSIM_Rhsim03G0134400
Root vs Stem	RHSIM_Rhsim11G0169900	1.368504652	0.01509288	0.042654917	RHSIM_Rhsim11G0169900
Root vs Stem	novel.2299	2.686872947	0.015114613	0.042695401	--
Root vs Stem	RHSIM_Rhsim06G0219800	-1.842594934	0.015181334	0.042868116	RHSIM_Rhsim06G0219800
Root vs Stem	RHSIM_Rhsim07G0205000	-1.097861651	0.015191228	0.042885546	RHSIM_Rhsim07G0205000
Root vs Stem	RHSIM_Rhsim06G0129600	-1.997351275	0.015197322	0.04289537	RHSIM_Rhsim06G0129600
Root vs Stem	RHSIM_Rhsim05G0224700	2.878188236	0.015198429	0.04289537	RHSIM_Rhsim05G0224700
Root vs Stem	RHSIM_Rhsim10G0203800	1.143445811	0.015252379	0.04303183	RHSIM_Rhsim10G0203800
Root vs Stem	RHSIM_Rhsim03G0135300	-1.585466607	0.015255912	0.043036533	RHSIM_Rhsim03G0135300
Root vs Stem	RHSIM_Rhsim03G0070100	1.98685188	0.015269142	0.043068582	RHSIM_Rhsim03G0070100
Root vs Stem	novel.5635	-5.639928386	0.015287963	0.043116395	--

Comparison	Gene ID	log2FC	P-value	FDR (padj)	Gene Name
Root vs Stem	novel.418	-6.594691482	0.01529634	0.043134744	--
Root vs Stem	RHSIM_Rhsim13G0169500	1.478500395	0.015308404	0.043163484	RHSIM_Rhsim13G0169500
Root vs Stem	RHSIM_RhsimUnG0090700	-1.196203639	0.015311036	0.043165624	RHSIM_RhsimUnG0090700
Root vs Stem	novel.1258	-6.599524098	0.015334607	0.043216222	--
Root vs Stem	RHSIM_Rhsim01G0095700	-5.62310383	0.015344745	0.043234223	RHSIM_Rhsim01G0095700
Root vs Stem	RHSIM_Rhsim10G0036400	2.185469495	0.015349467	0.043242241	RHSIM_Rhsim10G0036400
Root vs Stem	novel.3809	-1.265138088	0.015351852	0.043243676	--
Root vs Stem	RHSIM_Rhsim04G0076600	-6.58984421	0.015374413	0.043296647	RHSIM_Rhsim04G0076600
Root vs Stem	novel.587	-1.256169737	0.015378204	0.043302033	--
Root vs Stem	RHSIM_Rhsim13G0030800	-1.465850023	0.015391042	0.043332888	RHSIM_Rhsim13G0030800
Root vs Stem	novel.828	-5.623104256	0.015401517	0.043341208	--
Root vs Stem	novel.2679	1.415063756	0.015410187	0.043355021	--
Root vs Stem	RHSIM_Rhsim01G0052600	-1.375454197	0.01541375	0.043359753	RHSIM_Rhsim01G0052600
Root vs Stem	RHSIM_Rhsim10G0028200	-1.726936407	0.015425083	0.043381046	RHSIM_Rhsim10G0028200
Root vs Stem	RHSIM_Rhsim08G0164900	-6.587414851	0.015457409	0.043466658	RHSIM_Rhsim08G0164900
Root vs Stem	RHSIM_Rhsim09G0022600	1.244341009	0.015468292	0.043476047	RHSIM_Rhsim09G0022600
Root vs Stem	RHSIM_Rhsim05G0013500	-1.141750424	0.015472725	0.043483204	RHSIM_Rhsim05G0013500
Root vs Stem	RHSIM_Rhsim11G0189700	1.917763369	0.015507892	0.043566101	RHSIM_Rhsim11G0189700
Root vs Stem	RHSIM_Rhsim12G0109400	-1.050832223	0.015512984	0.043575098	RHSIM_Rhsim12G0109400
Root vs Stem	RHSIM_Rhsim05G0083600	1.374117771	0.015521414	0.043591013	RHSIM_Rhsim05G0083600
Root vs Stem	novel.933	-1.056591965	0.015522432	0.043591013	--
Root vs Stem	novel.5224	-2.757164435	0.015545559	0.043646017	--
Root vs Stem	RHSIM_Rhsim02G0132400	-1.659307034	0.015547414	0.043646017	RHSIM_Rhsim02G0132400
Root vs Stem	RHSIM_Rhsim08G0143100	-1.007823979	0.015607419	0.043776351	RHSIM_Rhsim08G0143100
Root vs Stem	RHSIM_Rhsim01G0023000	-3.505589528	0.015694573	0.044001875	RHSIM_Rhsim01G0023000

Comparison	Gene ID	log_2FC	P-value	FDR (padj)	Gene Name
Root vs Stem	RHSIM_Rhsim07G0244300	-1.439406648	0.01569875	0.04400575	RHSIM_Rhsim07G0244300
Root vs Stem	RHSIM_Rhsim02G0138600	2.268452354	0.015724541	0.044056616	RHSIM_Rhsim02G0138600
Root vs Stem	novel.5875	-2.362858103	0.015782299	0.044191825	--
Root vs Stem	novel.2053	-2.268120023	0.015801423	0.04422902	--
Root vs Stem	RHSIM_Rhsim07G0070400	1.30962275	0.015810228	0.044242921	RHSIM_Rhsim07G0070400
Root vs Stem	RHSIM_Rhsim05G0162700	4.045606992	0.015856097	0.044344365	RHSIM_Rhsim05G0162700
Root vs Stem	novel.1935	-1.231860862	0.015868847	0.044374641	--
Root vs Stem	novel.869	2.846379282	0.015873349	0.044381846	--
Root vs Stem	RHSIM_Rhsim11G0096200	1.073554844	0.015894017	0.044418083	RHSIM_Rhsim11G0096200
Root vs Stem	RHSIM_Rhsim11G0185400	-2.48184798	0.015921741	0.044473302	RHSIM_Rhsim11G0185400
Root vs Stem	RHSIM_Rhsim09G0090000	-5.600844405	0.015923421	0.044473302	RHSIM_Rhsim09G0090000
Root vs Stem	RHSIM_Rhsim01G0123600	1.861366117	0.015945322	0.044526505	RHSIM_Rhsim01G0123600
Root vs Stem	novel.2391	-1.75367426	0.015968326	0.044571718	--
Root vs Stem	novel.4582	-1.454758495	0.015994713	0.04462916	--
Root vs Stem	RHSIM_Rhsim05G0130800	1.010755814	0.016019908	0.044688642	RHSIM_Rhsim05G0130800
Root vs Stem	RHSIM_Rhsim04G0178400	2.773987566	0.016055328	0.044776505	RHSIM_Rhsim04G0178400
Root vs Stem	RHSIM_Rhsim13G0033700	-1.026781789	0.016057232	0.044776505	RHSIM_Rhsim13G0033700
Root vs Stem	RHSIM_Rhsim11G0004500	-1.681060349	0.016077923	0.044823361	RHSIM_Rhsim11G0004500
Root vs Stem	RHSIM_RhsimUnG0141000	-1.411239497	0.016171207	0.045061625	RHSIM_RhsimUnG0141000
Root vs Stem	novel.905	-2.162524495	0.016190097	0.045103361	--
Root vs Stem	novel.3264	-6.557857646	0.016209593	0.045146761	--
Root vs Stem	novel.1017	-2.591270935	0.016220104	0.045165124	--
Root vs Stem	novel.1233	-6.555374269	0.016283436	0.045308635	--
Root vs Stem	novel.2641	-6.555374269	0.016283436	0.045308635	--
Root vs Stem	novel.1544	1.143075473	0.016296208	0.045338701	--

Comparison	Gene ID	log_2FC	P-value	FDR (padj)	Gene Name
Root vs Stem	RHSIM_Rhsim08G0193400	1.465243173	0.016306377	0.045361518	RHSIM_Rhsim08G0193400
Root vs Stem	novel.1890	-1.10664608	0.016315728	0.045382055	--
Root vs Stem	RHSIM_Rhsim02G0232400	-1.281314693	0.01637739	0.045537086	RHSIM_Rhsim02G0232400
Root vs Stem	RHSIM_Rhsim04G0171300	-1.303237814	0.016391156	0.045569864	RHSIM_Rhsim04G0171300
Root vs Stem	RHSIM_Rhsim01G0102900	-3.042042776	0.016409207	0.045609048	RHSIM_Rhsim01G0102900
Root vs Stem	novel.1444	-1.748034623	0.016458461	0.045734921	--
Root vs Stem	RHSIM_Rhsim06G0135900	3.467546393	0.01648278	0.04578508	RHSIM_Rhsim06G0135900
Root vs Stem	RHSIM_Rhsim02G0210900	-1.413930013	0.016487541	0.04578508	RHSIM_Rhsim02G0210900
Root vs Stem	RHSIM_Rhsim12G0156300	1.373333649	0.016557087	0.045936998	RHSIM_Rhsim12G0156300
Root vs Stem	novel.1117	-1.583655923	0.016598698	0.046030287	--
Root vs Stem	RHSIM_Rhsim09G0104500	-1.393538275	0.016612816	0.046063898	RHSIM_Rhsim09G0104500
Root vs Stem	novel.628	-3.313902762	0.016617231	0.046067492	--
Root vs Stem	RHSIM_Rhsim03G0053800	-2.576459016	0.016667206	0.046179564	RHSIM_Rhsim03G0053800
Root vs Stem	RHSIM_Rhsim13G0130300	-1.642374176	0.016668526	0.046179564	RHSIM_Rhsim13G0130300
Root vs Stem	RHSIM_Rhsim05G0123500	-1.769006729	0.016745068	0.046380398	RHSIM_Rhsim05G0123500
Root vs Stem	RHSIM_Rhsim07G0248400	-5.563202713	0.016819835	0.046542749	RHSIM_Rhsim07G0248400
Root vs Stem	novel.633	1.87256634	0.01687171	0.046641506	--
Root vs Stem	RHSIM_Rhsim02G0080000	1.453520037	0.016879607	0.046652151	RHSIM_Rhsim02G0080000
Root vs Stem	RHSIM_Rhsim05G0229800	3.961231622	0.016934989	0.046777175	RHSIM_Rhsim05G0229800
Root vs Stem	RHSIM_Rhsim04G0161400	-2.21424043	0.017071781	0.047098583	RHSIM_Rhsim04G0161400
Root vs Stem	novel.2475	-6.527681088	0.017083568	0.047114187	--
Root vs Stem	novel.5942	-6.527681088	0.017083568	0.047114187	--
Root vs Stem	RHSIM_Rhsim05G0000200	-1.157832727	0.017116751	0.047183125	RHSIM_Rhsim05G0000200
Root vs Stem	RHSIM_Rhsim12G0008400	1.494078607	0.017130514	0.047205738	RHSIM_Rhsim12G0008400
Root vs Stem	RHSIM_Rhsim01G0142000	-6.532740742	0.017130778	0.047205738	RHSIM_Rhsim01G0142000

Comparison	Gene ID	log_2FC	P-value	FDR (padj)	Gene Name
Root vs Stem	novel.5174	-1.374002957	0.017131098	0.047205738	--
Root vs Stem	novel.4583	-1.02468992	0.017147875	0.047237798	--
Root vs Stem	novel.3545	-1.480252754	0.01714888	0.047237798	--
Root vs Stem	novel.597	-3.427844015	0.017188532	0.047324404	--
Root vs Stem	RHSIM_Rhsim01G0283400	-1.975838231	0.017285839	0.047549489	RHSIM_Rhsim01G0283400
Root vs Stem	RHSIM_RhsimUnG0222300	1.385773947	0.017286785	0.047549489	RHSIM_RhsimUnG0222300
Root vs Stem	RHSIM_Rhsim10G0022800	1.177302398	0.017347352	0.047693321	RHSIM_Rhsim10G0022800
Root vs Stem	novel.3218	-6.517512913	0.017405818	0.047831245	--
Root vs Stem	RHSIM_Rhsim10G0162000	-2.031578918	0.017419107	0.047859859	RHSIM_Rhsim10G0162000
Root vs Stem	novel.2167	3.402075053	0.017430011	0.047880606	--
Root vs Stem	RHSIM_Rhsim12G0009000	-1.068797136	0.017439334	0.047900508	RHSIM_Rhsim12G0009000
Root vs Stem	RHSIM_Rhsim12G0115700	-1.025495311	0.017483667	0.048010838	RHSIM_Rhsim12G0115700
Root vs Stem	RHSIM_Rhsim02G0124900	1.23064245	0.01750009	0.048037391	RHSIM_Rhsim02G0124900
Root vs Stem	RHSIM_Rhsim12G0143300	-1.953905822	0.017501672	0.048037391	RHSIM_Rhsim12G0143300
Root vs Stem	RHSIM_Rhsim05G0158900	1.256087752	0.01760893	0.04826857	RHSIM_Rhsim05G0158900
Root vs Stem	RHSIM_RhsimUnG0231400	-1.375655553	0.017669259	0.048382164	RHSIM_RhsimUnG0231400
Root vs Stem	novel.4995	-5.532289273	0.017697725	0.048448599	--
Root vs Stem	novel.683	2.660824057	0.017708929	0.048473514	--
Root vs Stem	RHSIM_Rhsim07G0193900	-1.175566391	0.017741754	0.048557599	RHSIM_Rhsim07G0193900
Root vs Stem	RHSIM_Rhsim01G0276700	1.107213607	0.01779693	0.048673939	RHSIM_Rhsim01G0276700
Root vs Stem	RHSIM_Rhsim02G0243200	1.286902011	0.017833148	0.048767208	RHSIM_Rhsim02G0243200
Root vs Stem	RHSIM_Rhsim03G0066900	1.223225601	0.01784151	0.048784288	RHSIM_Rhsim03G0066900
Root vs Stem	RHSIM_RhsimUnG0188000	-1.371577167	0.017858461	0.048819056	RHSIM_RhsimUnG0188000
Root vs Stem	novel.1653	3.122686802	0.017876025	0.048855484	--
Root vs Stem	RHSIM_Rhsim11G0120000	2.653567457	0.017914432	0.048931446	RHSIM_Rhsim11G0120000

Comparison	Gene ID	log_2FC	P-value	FDR (padj)	Gene Name
Root vs Stem	RHSIM_Rhsim05G0128100	1.0290744	0.017962473	0.049042555	RHSIM_Rhsim05G0128100
Root vs Stem	RHSIM_Rhsim12G0030600	-1.24011916	0.01796362	0.049042555	RHSIM_Rhsim12G0030600
Root vs Stem	RHSIM_RhsimUnG0003500	1.608312156	0.017972169	0.049054276	RHSIM_RhsimUnG0003500
Root vs Stem	novel.5320	-9.243521993	0.018007822	0.049145771	--
Root vs Stem	RHSIM_Rhsim02G0006100	1.205019378	0.018034062	0.049205732	RHSIM_Rhsim02G0006100
Root vs Stem	RHSIM_Rhsim12G0037300	-2.023129582	0.018070881	0.049294522	RHSIM_Rhsim12G0037300
Root vs Stem	RHSIM_Rhsim01G0038600	1.499157929	0.018074332	0.049298105	RHSIM_Rhsim01G0038600
Root vs Stem	RHSIM_Rhsim07G0065100	-1.991968935	0.018081314	0.049305482	RHSIM_Rhsim07G0065100
Root vs Stem	novel.1746	-6.491673948	0.018088397	0.049316025	--
Root vs Stem	RHSIM_Rhsim03G0263200	1.296894671	0.018176828	0.049536638	RHSIM_Rhsim03G0263200
Root vs Stem	RHSIM_Rhsim10G0198600	-2.023498487	0.018185369	0.049548198	RHSIM_Rhsim10G0198600
Root vs Stem	RHSIM_Rhsim07G0232000	-1.520520927	0.018207948	0.049584784	RHSIM_Rhsim07G0232000
Root vs Stem	RHSIM_Rhsim13G0111100	1.327139042	0.018212981	0.049584784	RHSIM_Rhsim13G0111100
Root vs Stem	novel.4169	2.822884241	0.018213519	0.049584784	--
Root vs Stem	novel.5200	-2.454121518	0.018213853	0.049584784	--
Root vs Stem	novel.712	2.473174997	0.018300873	0.04977768	--
Root vs Stem	RHSIM_Rhsim08G0191800	-1.757801565	0.018302458	0.04977768	RHSIM_Rhsim08G0191800
Root vs Stem	RHSIM_Rhsim06G0041900	1.006881423	0.018304142	0.04977768	RHSIM_Rhsim06G0041900
Root vs Stem	novel.1470	-1.234500999	0.018388744	0.049984165	--
Root vs Stem	RHSIM_Rhsim05G0003300	-1.081327171	0.018395865	0.049997626	RHSIM_Rhsim05G0003300
Stem vs Leaf	RHSIM_Rhsim02G0202100	-4.436437084	0	0	RHSIM_Rhsim02G0202100
Stem vs Leaf	RHSIM_Rhsim08G0090000	-7.937310763	0	0	RHSIM_Rhsim08G0090000
Stem vs Leaf	RHSIM_Rhsim11G0173000	-3.942436174	3.7081E-260	2.8483E-256	RHSIM_Rhsim11G0173000
Stem vs Leaf	RHSIM_Rhsim11G0038900	-8.975833301	1.3264E-201	7.6415E-198	RHSIM_Rhsim11G0038900
Stem vs Leaf	RHSIM_Rhsim06G0103300	-4.609853164	3.3912E-197	1.5629E-193	RHSIM_Rhsim06G0103300

Comparison	Gene ID	log_2FC	P-value	FDR (padj)	Gene Name
Stem vs Leaf	RHSIM_Rhsim04G0164800	-5.050894066	2.067E-196	7.9385E-193	RHSIM_Rhsim04G0164800
Stem vs Leaf	RHSIM_Rhsim08G0033400	-6.081506517	1.1266E-180	3.7088E-177	RHSIM_Rhsim08G0033400
Stem vs Leaf	novel.1226	7.21026621	1.0546E-174	3.0376E-171	--
Stem vs Leaf	RHSIM_Rhsim10G0057900	-4.884264721	1.1293E-173	2.8914E-170	RHSIM_Rhsim10G0057900
Stem vs Leaf	RHSIM_Rhsim06G0004600	-4.533480424	5.5587E-160	1.281E-156	RHSIM_Rhsim06G0004600
Stem vs Leaf	RHSIM_Rhsim06G0084800	-9.461470554	4.7985E-159	1.0052E-155	RHSIM_Rhsim06G0084800
Stem vs Leaf	RHSIM_Rhsim05G0032600	-4.188709829	1.6929E-156	3.251E-153	RHSIM_Rhsim05G0032600
Stem vs Leaf	RHSIM_Rhsim01G0191800	-2.643979721	4.579E-149	8.1168E-146	RHSIM_Rhsim01G0191800
Stem vs Leaf	RHSIM_Rhsim04G0018900	-5.34278197	1.708E-145	2.8114E-142	RHSIM_Rhsim04G0018900
Stem vs Leaf	RHSIM_Rhsim06G0070400	-3.51516317	8.9601E-143	1.3765E-139	RHSIM_Rhsim06G0070400
Stem vs Leaf	novel.1475	-3.895823984	1.8038E-140	2.5979E-137	--
Stem vs Leaf	RHSIM_Rhsim06G0169700	-9.581131191	6.1237E-140	8.3008E-137	RHSIM_Rhsim06G0169700
Stem vs Leaf	RHSIM_Rhsim02G0161200	4.283149068	3.2089E-139	4.1081E-136	RHSIM_Rhsim02G0161200
Stem vs Leaf	RHSIM_Rhsim07G0018700	-3.752732783	2.9084E-134	3.5275E-131	RHSIM_Rhsim07G0018700
Stem vs Leaf	RHSIM_Rhsim05G0044900	-4.639214775	1.1561E-132	1.3321E-129	RHSIM_Rhsim05G0044900
Stem vs Leaf	RHSIM_Rhsim08G0226600	4.086224626	2.1293E-132	2.3365E-129	RHSIM_Rhsim08G0226600
Stem vs Leaf	RHSIM_Rhsim04G0002800	-3.880596735	3.3404E-132	3.4989E-129	RHSIM_Rhsim04G0002800
Stem vs Leaf	RHSIM_Rhsim01G0236500	-3.009498439	9.1662E-131	9.1837E-128	RHSIM_Rhsim01G0236500
Stem vs Leaf	RHSIM_RhsimUnG0257700	-4.309692085	3.76E-129	3.6102E-126	RHSIM_RhsimUnG0257700
Stem vs Leaf	RHSIM_Rhsim13G0049600	-7.908057606	1.1771E-127	1.085E-124	RHSIM_Rhsim13G0049600
Stem vs Leaf	RHSIM_Rhsim13G0152700	-4.459070254	2.2721E-127	2.0137E-124	RHSIM_Rhsim13G0152700
Stem vs Leaf	RHSIM_Rhsim01G0126900	-3.909634713	1.5817E-126	1.3499E-123	RHSIM_Rhsim01G0126900
Stem vs Leaf	RHSIM_Rhsim12G0138000	-4.398732264	5.4724E-124	4.5038E-121	RHSIM_Rhsim12G0138000
Stem vs Leaf	RHSIM_Rhsim02G0021200	-7.185092127	8.5019E-124	6.7558E-121	RHSIM_Rhsim02G0021200
Stem vs Leaf	RHSIM_Rhsim02G0179400	-3.594509111	1.4498E-123	1.1136E-120	RHSIM_Rhsim02G0179400

Comparison	Gene ID	log_2FC	P-value	FDR (padj)	Gene Name
Stem vs Leaf	RHSIM_Rhsim12G0004800	-3.860701324	6.0208E-122	4.4756E-119	RHSIM_Rhsim12G0004800
Stem vs Leaf	RHSIM_Rhsim01G0015700	6.012703832	2.4292E-121	1.7493E-118	RHSIM_Rhsim01G0015700
Stem vs Leaf	RHSIM_Rhsim06G0131200	-4.137860948	3.6793E-121	2.5692E-118	RHSIM_Rhsim06G0131200
Stem vs Leaf	RHSIM_Rhsim12G0112100	-4.039747391	1.2447E-120	8.4361E-118	RHSIM_Rhsim12G0112100
Stem vs Leaf	RHSIM_Rhsim12G0025700	-4.017088786	1.6482E-120	1.0852E-117	RHSIM_Rhsim12G0025700
Stem vs Leaf	RHSIM_Rhsim08G0052700	-6.164673776	4.7909E-119	3.0667E-116	RHSIM_Rhsim08G0052700
Stem vs Leaf	RHSIM_Rhsim01G0184800	-4.235134273	6.9038E-118	4.2997E-115	RHSIM_Rhsim01G0184800
Stem vs Leaf	RHSIM_RhsimUnG0232700	-4.500331107	3.3504E-117	2.0318E-114	RHSIM_RhsimUnG0232700
Stem vs Leaf	RHSIM_Rhsim06G0177200	-3.480556108	8.6286E-117	5.0984E-114	RHSIM_Rhsim06G0177200
Stem vs Leaf	RHSIM_Rhsim05G0164500	-3.619020506	4.453E-116	2.5654E-113	RHSIM_Rhsim05G0164500
Stem vs Leaf	RHSIM_Rhsim12G0088300	-2.747010918	1.849E-115	1.0392E-112	RHSIM_Rhsim12G0088300
Stem vs Leaf	RHSIM_Rhsim04G0243100	-2.92946986	9.0797E-115	4.9817E-112	RHSIM_Rhsim04G0243100
Stem vs Leaf	RHSIM_Rhsim08G0178200	-3.400555663	1.6165E-113	8.663E-111	RHSIM_Rhsim08G0178200
Stem vs Leaf	RHSIM_Rhsim01G0226500	-3.707320469	9.8494E-113	5.1584E-110	RHSIM_Rhsim01G0226500
Stem vs Leaf	RHSIM_Rhsim13G0215900	-3.260450827	2.5554E-112	1.3086E-109	RHSIM_Rhsim13G0215900
Stem vs Leaf	RHSIM_Rhsim13G0013500	4.520444916	1.0348E-110	5.1838E-108	RHSIM_Rhsim13G0013500
Stem vs Leaf	RHSIM_Rhsim05G0183000	-5.324519648	6.047E-110	2.9648E-107	RHSIM_Rhsim05G0183000
Stem vs Leaf	RHSIM_Rhsim07G0179600	-3.839325648	1.3177E-109	6.3259E-107	RHSIM_Rhsim07G0179600
Stem vs Leaf	novel.1307	-8.659428423	6.0304E-107	2.836E-104	--
Stem vs Leaf	RHSIM_Rhsim08G0088200	-4.60818397	5.4361E-106	2.5054E-103	RHSIM_Rhsim08G0088200
Stem vs Leaf	RHSIM_Rhsim01G0274600	-4.370026561	5.4792E-104	2.4757E-101	RHSIM_Rhsim01G0274600
Stem vs Leaf	RHSIM_Rhsim07G0134900	-5.871007364	6.3467E-104	2.8125E-101	RHSIM_Rhsim07G0134900
Stem vs Leaf	RHSIM_Rhsim13G0049700	-7.240793265	1.358E-102	5.90455E-100	RHSIM_Rhsim13G0049700
Stem vs Leaf	RHSIM_Rhsim01G0217200	-2.241248671	5.7836E-102	2.46811E-99	RHSIM_Rhsim01G0217200
Stem vs Leaf	RHSIM_Rhsim06G0006600	-7.415943589	2.58058E-100	1.08121E-97	RHSIM_Rhsim06G0006600

Comparison	Gene ID	log_2FC	P-value	FDR (padj)	Gene Name
Stem vs Leaf	RHSIM_Rhsim08G0012300	-5.498041752	2.62778E-100	1.08133E-97	RHSIM_Rhsim08G0012300
Stem vs Leaf	RHSIM_Rhsim09G0184500	-7.087123324	9.64849E-100	3.9007E-97	RHSIM_Rhsim09G0184500
Stem vs Leaf	RHSIM_Rhsim02G0088100	-9.465103767	1.26982E-99	5.04514E-97	RHSIM_Rhsim02G0088100
Stem vs Leaf	RHSIM_Rhsim10G0021300	-12.9847581	1.83456E-98	7.16535E-96	RHSIM_Rhsim10G0021300
Stem vs Leaf	RHSIM_Rhsim01G0002000	-3.259321386	6.91842E-98	2.65714E-95	RHSIM_Rhsim01G0002000
Stem vs Leaf	RHSIM_Rhsim07G0048700	7.637607142	1.18319E-97	4.46974E-95	RHSIM_Rhsim07G0048700
Stem vs Leaf	RHSIM_Rhsim10G0180100	-3.850461702	5.5032E-97	2.04542E-94	RHSIM_Rhsim10G0180100
Stem vs Leaf	RHSIM_Rhsim06G0008500	-3.642614587	1.10673E-96	4.04816E-94	RHSIM_Rhsim06G0008500
Stem vs Leaf	RHSIM_Rhsim09G0132500	-7.208006402	1.16606E-95	4.19853E-93	RHSIM_Rhsim09G0132500
Stem vs Leaf	RHSIM_Rhsim06G0165300	-6.220284707	2.12908E-95	7.54807E-93	RHSIM_Rhsim06G0165300
Stem vs Leaf	RHSIM_Rhsim08G0205700	-5.839773765	9.36466E-95	3.26969E-92	RHSIM_Rhsim08G0205700
Stem vs Leaf	RHSIM_Rhsim06G0091500	-3.046107993	2.9473E-94	1.0137E-91	RHSIM_Rhsim06G0091500
Stem vs Leaf	RHSIM_Rhsim11G0183800	-4.86539038	9.52724E-94	3.22861E-91	RHSIM_Rhsim11G0183800
Stem vs Leaf	RHSIM_Rhsim04G0231000	-6.616930966	1.59547E-93	5.3284E-91	RHSIM_Rhsim04G0231000
Stem vs Leaf	RHSIM_Rhsim10G0211400	-3.764851877	5.86864E-93	1.93196E-90	RHSIM_Rhsim10G0211400
Stem vs Leaf	RHSIM_Rhsim13G0189300	-3.841089994	6.11558E-93	1.98489E-90	RHSIM_Rhsim13G0189300
Stem vs Leaf	RHSIM_Rhsim01G0207700	-3.081047814	2.31698E-92	7.41561E-90	RHSIM_Rhsim01G0207700
Stem vs Leaf	RHSIM_Rhsim04G0140900	9.541701415	2.86058E-91	9.03002E-89	RHSIM_Rhsim04G0140900
Stem vs Leaf	RHSIM_Rhsim07G0139800	-8.24274384	1.32586E-90	4.1288E-88	RHSIM_Rhsim07G0139800
Stem vs Leaf	RHSIM_Rhsim04G0167000	-2.451977214	4.05122E-90	1.24475E-87	RHSIM_Rhsim04G0167000
Stem vs Leaf	RHSIM_Rhsim07G0077100	-6.395830369	8.58313E-90	2.6025E-87	RHSIM_Rhsim07G0077100
Stem vs Leaf	RHSIM_Rhsim10G0071100	-7.123417432	2.9595E-89	8.85698E-87	RHSIM_Rhsim10G0071100
Stem vs Leaf	RHSIM_Rhsim07G0011000	3.726958349	5.15758E-88	1.52373E-85	RHSIM_Rhsim07G0011000
Stem vs Leaf	RHSIM_RhsimPtG0005500	-6.032152035	1.1148E-87	3.25183E-85	RHSIM_RhsimPtG0005500
Stem vs Leaf	RHSIM_Rhsim12G0085000	-4.815415635	2.34606E-85	6.75782E-83	RHSIM_Rhsim12G0085000

Comparison	Gene ID	log_2FC	P-value	FDR (padj)	Gene Name
Stem vs Leaf	RHSIM_Rhsim04G0209400	-7.054095173	3.39205E-85	9.65017E-83	RHSIM_Rhsim04G0209400
Stem vs Leaf	RHSIM_Rhsim06G0183300	-3.444703769	1.04885E-84	2.94753E-82	RHSIM_Rhsim06G0183300
Stem vs Leaf	RHSIM_Rhsim12G0013500	-3.923334749	1.70426E-84	4.73168E-82	RHSIM_Rhsim12G0013500
Stem vs Leaf	novel.4886	-6.907637419	1.41894E-83	3.89263E-81	--
Stem vs Leaf	RHSIM_Rhsim08G0042600	-3.31958567	3.21928E-83	8.72766E-81	RHSIM_Rhsim08G0042600
Stem vs Leaf	RHSIM_Rhsim05G0228300	5.067654624	3.77464E-83	1.01143E-80	RHSIM_Rhsim05G0228300
Stem vs Leaf	RHSIM_Rhsim02G0228100	9.560210479	9.17051E-83	2.42903E-80	RHSIM_Rhsim02G0228100
Stem vs Leaf	RHSIM_Rhsim06G0006900	3.155080753	2.56559E-82	6.71836E-80	RHSIM_Rhsim06G0006900
Stem vs Leaf	RHSIM_Rhsim12G0139100	-4.375194348	5.97225E-82	1.54634E-79	RHSIM_Rhsim12G0139100
Stem vs Leaf	RHSIM_Rhsim13G0056900	-2.864017165	8.89357E-82	2.27715E-79	RHSIM_Rhsim13G0056900
Stem vs Leaf	RHSIM_Rhsim04G0167100	-2.409190141	9.08009E-82	2.29936E-79	RHSIM_Rhsim04G0167100
Stem vs Leaf	RHSIM_Rhsim13G0039000	-4.758410023	1.46808E-80	3.67721E-78	RHSIM_Rhsim13G0039000
Stem vs Leaf	RHSIM_Rhsim13G0077400	-6.476940713	2.92325E-80	7.24338E-78	RHSIM_Rhsim13G0077400
Stem vs Leaf	RHSIM_Rhsim13G0216500	-3.915469735	3.44535E-80	8.44623E-78	RHSIM_Rhsim13G0216500
Stem vs Leaf	RHSIM_Rhsim12G0015600	-3.889839233	4.00109E-80	9.70539E-78	RHSIM_Rhsim12G0015600
Stem vs Leaf	RHSIM_Rhsim05G0214000	-3.951993302	1.04112E-79	2.4945E-77	RHSIM_Rhsim05G0214000
Stem vs Leaf	RHSIM_Rhsim04G0192600	-7.635681158	1.05002E-79	2.4945E-77	RHSIM_Rhsim04G0192600
Stem vs Leaf	RHSIM_Rhsim07G0234600	-3.363517703	2.23399E-79	5.25306E-77	RHSIM_Rhsim07G0234600
Stem vs Leaf	RHSIM_Rhsim04G0052800	-2.836619551	2.42131E-79	5.63603E-77	RHSIM_Rhsim04G0052800
Stem vs Leaf	RHSIM_Rhsim04G0216800	-12.80094733	1.88558E-78	4.34514E-76	RHSIM_Rhsim04G0216800
Stem vs Leaf	RHSIM_Rhsim11G0167000	-3.308851979	2.5024E-78	5.70944E-76	RHSIM_Rhsim11G0167000
Stem vs Leaf	RHSIM_Rhsim07G0256400	-4.723392056	2.54292E-78	5.745E-76	RHSIM_Rhsim07G0256400
Stem vs Leaf	RHSIM_Rhsim01G0224600	3.539763311	2.65657E-78	5.9435E-76	RHSIM_Rhsim01G0224600
Stem vs Leaf	RHSIM_Rhsim12G0129200	-4.44184514	2.73758E-78	6.06584E-76	RHSIM_Rhsim12G0129200
Stem vs Leaf	RHSIM_Rhsim13G0149600	-2.304576467	1.07108E-77	2.35066E-75	RHSIM_Rhsim13G0149600

Comparison	Gene ID	log_2FC	P-value	FDR (padj)	Gene Name
Stem vs Leaf	RHSIM_Rhsim06G0220000	-4.361494336	3.65577E-77	7.9475E-75	RHSIM_Rhsim06G0220000
Stem vs Leaf	RHSIM_Rhsim05G0221700	-10.55663623	7.8823E-77	1.69757E-74	RHSIM_Rhsim05G0221700
Stem vs Leaf	RHSIM_Rhsim07G0198600	-4.288631743	1.31843E-76	2.81314E-74	RHSIM_Rhsim07G0198600
Stem vs Leaf	RHSIM_Rhsim09G0092100	-4.849987194	1.3998E-76	2.95936E-74	RHSIM_Rhsim09G0092100
Stem vs Leaf	novel.14	-7.184519565	8.20148E-76	1.71813E-73	--
Stem vs Leaf	RHSIM_Rhsim11G0150000	-3.845784187	9.54708E-76	1.98201E-73	RHSIM_Rhsim11G0150000
Stem vs Leaf	RHSIM_RhsimUnG0024100	-5.027492815	1.04333E-75	2.14664E-73	RHSIM_RhsimUnG0024100
Stem vs Leaf	RHSIM_Rhsim12G0108700	-5.305926754	1.18923E-75	2.4252E-73	RHSIM_Rhsim12G0108700
Stem vs Leaf	novel.1056	6.073552797	1.493E-75	3.01795E-73	--
Stem vs Leaf	RHSIM_Rhsim03G0074800	-4.233871919	1.51589E-75	3.03758E-73	RHSIM_Rhsim03G0074800
Stem vs Leaf	RHSIM_Rhsim01G0293800	-2.637536423	3.17761E-74	6.31249E-72	RHSIM_Rhsim01G0293800
Stem vs Leaf	RHSIM_Rhsim02G0088400	-5.103639535	4.5086E-73	8.88001E-71	RHSIM_Rhsim02G0088400
Stem vs Leaf	RHSIM_Rhsim06G0127100	-3.530248856	6.29513E-73	1.22936E-70	RHSIM_Rhsim06G0127100
Stem vs Leaf	RHSIM_Rhsim07G0028800	-3.644183095	2.80158E-72	5.42518E-70	RHSIM_Rhsim07G0028800
Stem vs Leaf	RHSIM_Rhsim02G0076500	-2.047696874	7.10122E-72	1.36367E-69	RHSIM_Rhsim02G0076500
Stem vs Leaf	RHSIM_Rhsim04G0182600	-4.733260201	7.29914E-72	1.39009E-69	RHSIM_Rhsim04G0182600
Stem vs Leaf	RHSIM_Rhsim08G0089400	-10.13746703	1.00491E-71	1.89812E-69	RHSIM_Rhsim08G0089400
Stem vs Leaf	RHSIM_Rhsim13G0233300	-4.423982225	2.85509E-71	5.34901E-69	RHSIM_Rhsim13G0233300
Stem vs Leaf	RHSIM_Rhsim03G0254900	-2.817474008	4.83338E-71	8.98229E-69	RHSIM_Rhsim03G0254900
Stem vs Leaf	RHSIM_Rhsim05G0202300	-5.228783395	5.56053E-71	1.0251E-68	RHSIM_Rhsim05G0202300
Stem vs Leaf	RHSIM_Rhsim08G0212400	2.732030506	1.01553E-70	1.85728E-68	RHSIM_Rhsim08G0212400
Stem vs Leaf	RHSIM_Rhsim01G0097100	-2.180566646	2.25409E-70	4.09002E-68	RHSIM_Rhsim01G0097100
Stem vs Leaf	RHSIM_Rhsim01G0155600	-4.428809287	4.76294E-70	8.57478E-68	RHSIM_Rhsim01G0155600
Stem vs Leaf	RHSIM_Rhsim13G0154000	-4.597631943	5.75067E-70	1.02727E-67	RHSIM_Rhsim13G0154000
Stem vs Leaf	RHSIM_Rhsim08G0055200	-2.257922254	2.26738E-69	4.0192E-67	RHSIM_Rhsim08G0055200

Comparison	Gene ID	log_2FC	P-value	FDR (padj)	Gene Name
Stem vs Leaf	RHSIM_Rhsim07G0014000	-2.183573432	3.73268E-69	6.56609E-67	RHSIM_Rhsim07G0014000
Stem vs Leaf	RHSIM_Rhsim03G0027000	2.749421968	5.88318E-69	1.02706E-66	RHSIM_Rhsim03G0027000
Stem vs Leaf	RHSIM_Rhsim10G0133700	-3.842843103	8.63645E-69	1.49638E-66	RHSIM_Rhsim10G0133700
Stem vs Leaf	RHSIM_Rhsim07G0256500	-4.571471824	1.33818E-68	2.30127E-66	RHSIM_Rhsim07G0256500
Stem vs Leaf	RHSIM_Rhsim05G0193900	-3.391790534	1.9187E-68	3.27516E-66	RHSIM_Rhsim05G0193900
Stem vs Leaf	RHSIM_Rhsim12G0214300	-2.462155534	2.04207E-68	3.46011E-66	RHSIM_Rhsim12G0214300
Stem vs Leaf	RHSIM_Rhsim03G0188500	-2.770988752	4.60722E-68	7.74954E-66	RHSIM_Rhsim03G0188500
Stem vs Leaf	RHSIM_Rhsim01G0126800	-2.511903514	5.82138E-68	9.72086E-66	RHSIM_Rhsim01G0126800
Stem vs Leaf	RHSIM_RhsimUnG0205000	-3.348907509	5.98915E-68	9.92906E-66	RHSIM_RhsimUnG0205000
Stem vs Leaf	RHSIM_Rhsim05G0203800	-4.854980848	1.2923E-67	2.12712E-65	RHSIM_Rhsim05G0203800
Stem vs Leaf	RHSIM_Rhsim12G0144000	-2.729471557	2.07563E-67	3.39225E-65	RHSIM_Rhsim12G0144000
Stem vs Leaf	RHSIM_Rhsim13G0064600	-2.376747243	9.16855E-67	1.48789E-64	RHSIM_Rhsim13G0064600
Stem vs Leaf	RHSIM_Rhsim12G0000600	-3.929410235	2.91288E-66	4.69402E-64	RHSIM_Rhsim12G0000600
Stem vs Leaf	RHSIM_Rhsim09G0063200	-2.681260692	3.31811E-66	5.3099E-64	RHSIM_Rhsim09G0063200
Stem vs Leaf	RHSIM_Rhsim07G0052400	-6.054301962	3.76323E-66	5.98068E-64	RHSIM_Rhsim07G0052400
Stem vs Leaf	RHSIM_Rhsim03G0040000	-3.156547049	4.65337E-66	7.34468E-64	RHSIM_Rhsim03G0040000
Stem vs Leaf	RHSIM_Rhsim03G0264800	2.340112891	5.67594E-66	8.89771E-64	RHSIM_Rhsim03G0264800
Stem vs Leaf	RHSIM_Rhsim06G0231000	-4.906144374	6.0186E-66	9.37112E-64	RHSIM_Rhsim06G0231000
Stem vs Leaf	RHSIM_Rhsim07G0032300	-3.570305613	6.71563E-66	1.03862E-63	RHSIM_Rhsim07G0032300
Stem vs Leaf	RHSIM_Rhsim05G0231800	-3.852485729	1.31487E-65	2.01999E-63	RHSIM_Rhsim05G0231800
Stem vs Leaf	RHSIM_Rhsim11G0098700	-4.200278583	3.23305E-65	4.93394E-63	RHSIM_Rhsim11G0098700
Stem vs Leaf	RHSIM_Rhsim13G0040100	-6.992961465	3.60846E-65	5.47062E-63	RHSIM_Rhsim13G0040100
Stem vs Leaf	RHSIM_Rhsim01G0182200	-7.937215729	4.93499E-65	7.4328E-63	RHSIM_Rhsim01G0182200
Stem vs Leaf	RHSIM_Rhsim07G0132500	-6.634657742	6.17951E-65	9.2468E-63	RHSIM_Rhsim07G0132500
Stem vs Leaf	RHSIM_Rhsim13G0142100	4.989648348	6.98396E-65	1.03831E-62	RHSIM_Rhsim13G0142100

Comparison	Gene ID	log_2FC	P-value	FDR (padj)	Gene Name
Stem vs Leaf	RHSIM_Rhsim06G0002800	3.448847851	1.12006E-64	1.65453E-62	RHSIM_Rhsim06G0002800
Stem vs Leaf	RHSIM_Rhsim02G0018300	-4.588180669	1.94255E-64	2.85121E-62	RHSIM_Rhsim02G0018300
Stem vs Leaf	RHSIM_Rhsim08G0012200	-2.969026307	2.35465E-64	3.43422E-62	RHSIM_Rhsim08G0012200
Stem vs Leaf	RHSIM_Rhsim01G0292400	-2.117864978	1.11728E-63	1.61929E-61	RHSIM_Rhsim01G0292400
Stem vs Leaf	novel.4755	6.5003248	1.39085E-63	2.00317E-61	--
Stem vs Leaf	RHSIM_Rhsim08G0118900	-2.961275139	1.43755E-63	2.05757E-61	RHSIM_Rhsim08G0118900
Stem vs Leaf	RHSIM_RhsimUnG0245900	-7.644884398	4.46459E-63	6.35074E-61	RHSIM_RhsimUnG0245900
Stem vs Leaf	RHSIM_Rhsim06G0197400	-2.453395646	4.68625E-63	6.62515E-61	RHSIM_Rhsim06G0197400
Stem vs Leaf	RHSIM_Rhsim12G0135300	9.659431608	4.95087E-63	6.95657E-61	RHSIM_Rhsim12G0135300
Stem vs Leaf	RHSIM_Rhsim12G0096900	5.52488892	2.30123E-62	3.21391E-60	RHSIM_Rhsim12G0096900
Stem vs Leaf	RHSIM_Rhsim12G0116400	-6.699102639	2.99353E-62	4.1556E-60	RHSIM_Rhsim12G0116400
Stem vs Leaf	novel.6096	2.029574335	6.37935E-62	8.80274E-60	--
Stem vs Leaf	RHSIM_Rhsim04G0009800	-4.642430357	4.1846E-61	5.73988E-59	RHSIM_Rhsim04G0009800
Stem vs Leaf	RHSIM_Rhsim08G0136700	-6.834061052	5.33546E-61	7.27517E-59	RHSIM_Rhsim08G0136700
Stem vs Leaf	RHSIM_Rhsim01G0128600	-3.242182081	6.38151E-61	8.65033E-59	RHSIM_Rhsim01G0128600
Stem vs Leaf	RHSIM_Rhsim08G0076500	-3.887529508	6.7027E-61	9.03257E-59	RHSIM_Rhsim08G0076500
Stem vs Leaf	RHSIM_Rhsim08G0155400	-2.254374189	9.04922E-61	1.21239E-58	RHSIM_Rhsim08G0155400
Stem vs Leaf	RHSIM_Rhsim01G0245100	-5.728973773	1.51257E-60	2.01478E-58	RHSIM_Rhsim01G0245100
Stem vs Leaf	RHSIM_Rhsim08G0062800	-1.731011687	1.97937E-60	2.62141E-58	RHSIM_Rhsim08G0062800
Stem vs Leaf	RHSIM_Rhsim03G0175600	5.804970046	2.29473E-60	3.0217E-58	RHSIM_Rhsim03G0175600
Stem vs Leaf	RHSIM_Rhsim10G0177000	-2.565864225	2.63237E-60	3.44662E-58	RHSIM_Rhsim10G0177000
Stem vs Leaf	RHSIM_Rhsim11G0130100	-2.705952489	3.85504E-60	5.01896E-58	RHSIM_Rhsim11G0130100
Stem vs Leaf	RHSIM_Rhsim09G0111900	-3.324361715	4.06599E-60	5.26386E-58	RHSIM_Rhsim09G0111900
Stem vs Leaf	RHSIM_Rhsim04G0207700	3.241081895	4.65706E-60	5.99537E-58	RHSIM_Rhsim04G0207700
Stem vs Leaf	RHSIM_Rhsim13G0157200	5.432247711	9.85595E-60	1.26178E-57	RHSIM_Rhsim13G0157200

Comparison	Gene ID	log_2FC	P-value	FDR (padj)	Gene Name
Stem vs Leaf	novel.5322	1.627168504	1.05231E-59	1.33975E-57	--
Stem vs Leaf	RHSIM_Rhsim09G0064600	-3.812917317	1.06397E-59	1.34715E-57	RHSIM_Rhsim09G0064600
Stem vs Leaf	RHSIM_Rhsim13G0100500	-1.682463131	1.56167E-59	1.96651E-57	RHSIM_Rhsim13G0100500
Stem vs Leaf	RHSIM_Rhsim11G0012900	-3.253947941	1.66054E-59	2.07965E-57	RHSIM_Rhsim11G0012900
Stem vs Leaf	RHSIM_Rhsim08G0234500	-6.243288651	2.04532E-59	2.54769E-57	RHSIM_Rhsim08G0234500
Stem vs Leaf	RHSIM_Rhsim01G0247800	-3.554975872	2.30236E-59	2.85245E-57	RHSIM_Rhsim01G0247800
Stem vs Leaf	RHSIM_Rhsim01G0060800	2.800195242	3.97684E-59	4.90066E-57	RHSIM_Rhsim01G0060800
Stem vs Leaf	novel.4918	1.826231019	4.5745E-59	5.60717E-57	--
Stem vs Leaf	RHSIM_Rhsim09G0010800	-4.491925986	9.00954E-59	1.0985E-56	RHSIM_Rhsim09G0010800
Stem vs Leaf	RHSIM_Rhsim07G0020300	-7.988981488	1.42402E-58	1.72712E-56	RHSIM_Rhsim07G0020300
Stem vs Leaf	novel.3139	-3.27818702	3.57204E-58	4.30964E-56	--
Stem vs Leaf	RHSIM_Rhsim13G0025100	-3.552217057	3.70675E-58	4.44887E-56	RHSIM_Rhsim13G0025100
Stem vs Leaf	RHSIM_Rhsim05G0046600	3.278388767	4.65741E-58	5.56089E-56	RHSIM_Rhsim05G0046600
Stem vs Leaf	RHSIM_Rhsim05G0161100	-3.151513235	1.30874E-57	1.55456E-55	RHSIM_Rhsim05G0161100
Stem vs Leaf	RHSIM_Rhsim13G0143000	3.699996428	1.71996E-57	2.03256E-55	RHSIM_Rhsim13G0143000
Stem vs Leaf	RHSIM_Rhsim09G0211900	-4.392745093	3.36171E-57	3.95241E-55	RHSIM_Rhsim09G0211900
Stem vs Leaf	RHSIM_Rhsim02G0054000	-2.33957587	4.07247E-57	4.76375E-55	RHSIM_Rhsim02G0054000
Stem vs Leaf	RHSIM_Rhsim12G0143500	-10.05692757	6.59048E-57	7.67025E-55	RHSIM_Rhsim12G0143500
Stem vs Leaf	RHSIM_Rhsim02G0098500	4.375956853	8.37583E-57	9.69913E-55	RHSIM_Rhsim02G0098500
Stem vs Leaf	RHSIM_Rhsim02G0248400	-2.438404319	2.16462E-56	2.49407E-54	RHSIM_Rhsim02G0248400
Stem vs Leaf	RHSIM_Rhsim11G0088800	-2.527864543	2.59695E-56	2.97732E-54	RHSIM_Rhsim11G0088800
Stem vs Leaf	RHSIM_Rhsim05G0221600	-8.014229106	3.64182E-56	4.15456E-54	RHSIM_Rhsim05G0221600
Stem vs Leaf	RHSIM_Rhsim02G0018500	-4.351083578	4.72726E-56	5.36625E-54	RHSIM_Rhsim02G0018500
Stem vs Leaf	RHSIM_Rhsim05G0094600	-2.317904715	8.2786E-56	9.35157E-54	RHSIM_Rhsim05G0094600
Stem vs Leaf	RHSIM_Rhsim05G0073200	3.797687268	1.69271E-55	1.90277E-53	RHSIM_Rhsim05G0073200

Comparison	Gene ID	log_2FC	P-value	FDR (padj)	Gene Name
Stem vs Leaf	RHSIM_Rhsim10G0152000	-7.179839838	1.72479E-55	1.92942E-53	RHSIM_Rhsim10G0152000
Stem vs Leaf	RHSIM_Rhsim08G0225000	3.230342245	2.25519E-55	2.51057E-53	RHSIM_Rhsim08G0225000
Stem vs Leaf	novel.3541	6.645191679	3.64299E-55	4.03601E-53	--
Stem vs Leaf	RHSIM_Rhsim13G0219900	-3.438698202	6.98011E-55	7.69616E-53	RHSIM_Rhsim13G0219900
Stem vs Leaf	RHSIM_Rhsim01G0015500	5.692934662	8.71414E-55	9.56231E-53	RHSIM_Rhsim01G0015500
Stem vs Leaf	RHSIM_Rhsim04G0166900	-2.501709827	9.19393E-55	1.0041E-52	RHSIM_Rhsim04G0166900
Stem vs Leaf	RHSIM_Rhsim08G0128200	-7.420943052	1.09702E-54	1.19244E-52	RHSIM_Rhsim08G0128200
Stem vs Leaf	RHSIM_Rhsim07G0103700	-3.84872146	1.21686E-54	1.31649E-52	RHSIM_Rhsim07G0103700
Stem vs Leaf	RHSIM_Rhsim01G0188400	-3.857801642	1.41135E-54	1.51978E-52	RHSIM_Rhsim01G0188400
Stem vs Leaf	RHSIM_Rhsim05G0056500	-3.4273829	1.81362E-54	1.94386E-52	RHSIM_Rhsim05G0056500
Stem vs Leaf	RHSIM_Rhsim04G0096700	-3.499652404	2.97593E-54	3.17487E-52	RHSIM_Rhsim04G0096700
Stem vs Leaf	RHSIM_Rhsim04G0033100	-4.672485614	3.27175E-54	3.47439E-52	RHSIM_Rhsim04G0033100
Stem vs Leaf	RHSIM_Rhsim07G0142300	-5.149533453	4.48314E-54	4.73896E-52	RHSIM_Rhsim07G0142300
Stem vs Leaf	RHSIM_Rhsim09G0005000	-9.597288137	5.22874E-54	5.50188E-52	RHSIM_Rhsim09G0005000
Stem vs Leaf	RHSIM_Rhsim06G0187500	-2.99770368	5.80846E-54	6.0841E-52	RHSIM_Rhsim06G0187500
Stem vs Leaf	RHSIM_Rhsim07G0048100	-2.630607147	6.45708E-54	6.7329E-52	RHSIM_Rhsim07G0048100
Stem vs Leaf	RHSIM_Rhsim02G0213600	4.790552025	6.49605E-54	6.74301E-52	RHSIM_Rhsim02G0213600
Stem vs Leaf	RHSIM_Rhsim02G0217900	-2.980488603	6.90262E-54	7.13291E-52	RHSIM_Rhsim02G0217900
Stem vs Leaf	RHSIM_Rhsim12G0099800	-3.610785283	1.40945E-53	1.44997E-51	RHSIM_Rhsim12G0099800
Stem vs Leaf	RHSIM_Rhsim12G0105600	4.152482497	1.46634E-53	1.5018E-51	RHSIM_Rhsim12G0105600
Stem vs Leaf	RHSIM_Rhsim06G0001500	-3.033428623	1.55663E-53	1.58721E-51	RHSIM_Rhsim06G0001500
Stem vs Leaf	RHSIM_Rhsim11G0017900	-5.62084236	2.08888E-53	2.12053E-51	RHSIM_Rhsim11G0017900
Stem vs Leaf	RHSIM_Rhsim12G0005300	-4.003625929	2.24946E-53	2.27354E-51	RHSIM_Rhsim12G0005300
Stem vs Leaf	RHSIM_Rhsim08G0053400	-5.08667458	3.05057E-53	3.06976E-51	RHSIM_Rhsim08G0053400
Stem vs Leaf	RHSIM_Rhsim08G0225500	-9.492725185	4.66024E-53	4.66916E-51	RHSIM_Rhsim08G0225500

Comparison	Gene ID	log_2FC	P-value	FDR (padj)	Gene Name
Stem vs Leaf	RHSIM_Rhsim08G0088300	-4.409305153	6.6319E-53	6.61582E-51	RHSIM_Rhsim08G0088300
Stem vs Leaf	RHSIM_Rhsim02G0082500	-2.75780839	8.39069E-53	8.33427E-51	RHSIM_Rhsim02G0082500
Stem vs Leaf	RHSIM_Rhsim11G0145600	-4.418015985	8.49512E-53	8.40178E-51	RHSIM_Rhsim11G0145600
Stem vs Leaf	RHSIM_Rhsim06G0111800	-6.778507968	1.02151E-52	1.00597E-50	RHSIM_Rhsim06G0111800
Stem vs Leaf	RHSIM_Rhsim03G0114800	-4.915601985	1.55723E-52	1.52702E-50	RHSIM_Rhsim03G0114800
Stem vs Leaf	novel.4483	-4.513424905	2.51984E-52	2.46047E-50	--
Stem vs Leaf	RHSIM_Rhsim11G0087200	-3.1396115	4.60306E-52	4.47565E-50	RHSIM_Rhsim11G0087200
Stem vs Leaf	RHSIM_Rhsim11G0027600	-5.92698419	1.28348E-51	1.24271E-49	RHSIM_Rhsim11G0027600
Stem vs Leaf	novel.4057	-2.819429937	1.53647E-51	1.48144E-49	--
Stem vs Leaf	RHSIM_Rhsim12G0165600	-2.618264083	1.62955E-51	1.56464E-49	RHSIM_Rhsim12G0165600
Stem vs Leaf	RHSIM_Rhsim09G0042300	5.441904539	1.94204E-51	1.85695E-49	RHSIM_Rhsim09G0042300
Stem vs Leaf	RHSIM_Rhsim09G0171900	-4.119196056	1.9557E-51	1.86227E-49	RHSIM_Rhsim09G0171900
Stem vs Leaf	RHSIM_Rhsim12G0176200	7.328856269	2.99985E-51	2.8448E-49	RHSIM_Rhsim12G0176200
Stem vs Leaf	RHSIM_Rhsim07G0251100	-4.161087951	3.56928E-51	3.37092E-49	RHSIM_Rhsim07G0251100
Stem vs Leaf	RHSIM_Rhsim08G0006000	6.830499957	4.37829E-51	4.11809E-49	RHSIM_Rhsim08G0006000
Stem vs Leaf	RHSIM_Rhsim08G0156700	-9.54944294	4.63866E-51	4.34526E-49	RHSIM_Rhsim08G0156700
Stem vs Leaf	RHSIM_Rhsim09G0008400	-3.231351845	6.07442E-51	5.66716E-49	RHSIM_Rhsim09G0008400
Stem vs Leaf	RHSIM_Rhsim03G0088200	-2.987527369	6.70275E-51	6.22815E-49	RHSIM_Rhsim03G0088200
Stem vs Leaf	RHSIM_Rhsim12G0050500	6.236500374	7.71064E-51	7.1359E-49	RHSIM_Rhsim12G0050500
Stem vs Leaf	RHSIM_Rhsim03G0215200	-2.76964052	8.60367E-51	7.93052E-49	RHSIM_Rhsim03G0215200
Stem vs Leaf	RHSIM_RhsimUnG0003000	-2.297751194	9.29126E-51	8.53019E-49	RHSIM_RhsimUnG0003000
Stem vs Leaf	RHSIM_Rhsim01G0287200	-5.492733074	1.1883E-50	1.08663E-48	RHSIM_Rhsim01G0287200
Stem vs Leaf	RHSIM_Rhsim09G0084200	-3.927677327	1.24467E-50	1.13368E-48	RHSIM_Rhsim09G0084200
Stem vs Leaf	RHSIM_Rhsim07G0032500	-5.617134966	1.30682E-50	1.1856E-48	RHSIM_Rhsim07G0032500
Stem vs Leaf	RHSIM_Rhsim01G0260300	-4.262423361	1.62786E-50	1.47108E-48	RHSIM_Rhsim01G0260300

Comparison	Gene ID	log_2FC	P-value	FDR (padj)	Gene Name
Stem vs Leaf	RHSIM_Rhsim03G0240800	-2.240463528	1.7075E-50	1.53702E-48	RHSIM_Rhsim03G0240800
Stem vs Leaf	RHSIM_Rhsim03G0039800	-8.145416853	2.26614E-50	2.03194E-48	RHSIM_Rhsim03G0039800
Stem vs Leaf	RHSIM_Rhsim01G0091800	-2.953081705	2.55097E-50	2.27847E-48	RHSIM_Rhsim01G0091800
Stem vs Leaf	novel.1220	7.247884545	2.61034E-50	2.32249E-48	--
Stem vs Leaf	RHSIM_Rhsim08G0242300	-6.614086943	2.66862E-50	2.36522E-48	RHSIM_Rhsim08G0242300
Stem vs Leaf	RHSIM_Rhsim03G0198900	-2.144283857	2.71546E-50	2.39752E-48	RHSIM_Rhsim03G0198900
Stem vs Leaf	RHSIM_Rhsim12G0183500	-3.286799205	3.98116E-50	3.5016E-48	RHSIM_Rhsim12G0183500
Stem vs Leaf	novel.4572	-7.414893791	5.84313E-50	5.11974E-48	--
Stem vs Leaf	RHSIM_Rhsim01G0151800	-3.558880492	8.53977E-50	7.45419E-48	RHSIM_Rhsim01G0151800
Stem vs Leaf	RHSIM_Rhsim04G0083900	-5.396007173	1.08429E-49	9.42882E-48	RHSIM_Rhsim04G0083900
Stem vs Leaf	novel.3191	-2.780271344	1.12018E-49	9.70434E-48	--
Stem vs Leaf	RHSIM_Rhsim01G0178400	-3.299085326	1.84659E-49	1.59374E-47	RHSIM_Rhsim01G0178400
Stem vs Leaf	RHSIM_Rhsim07G0100400	6.108283965	3.67476E-49	3.15974E-47	RHSIM_Rhsim07G0100400
Stem vs Leaf	RHSIM_RhsimUnG0254900	4.450099261	3.83341E-49	3.28391E-47	RHSIM_RhsimUnG0254900
Stem vs Leaf	RHSIM_Rhsim08G0109700	-3.129290667	4.73366E-49	4.04009E-47	RHSIM_Rhsim08G0109700
Stem vs Leaf	RHSIM_Rhsim10G0101200	-1.594525697	5.0123E-49	4.26212E-47	RHSIM_Rhsim10G0101200
Stem vs Leaf	RHSIM_Rhsim03G0158800	-2.456012544	7.1179E-49	6.03032E-47	RHSIM_Rhsim03G0158800
Stem vs Leaf	RHSIM_Rhsim11G0087000	8.769908739	8.88152E-49	7.49692E-47	RHSIM_Rhsim11G0087000
Stem vs Leaf	RHSIM_Rhsim09G0107700	-2.23029464	1.35676E-48	1.14106E-46	RHSIM_Rhsim09G0107700
Stem vs Leaf	RHSIM_Rhsim13G0069000	-3.00239241	1.37764E-48	1.15441E-46	RHSIM_Rhsim13G0069000
Stem vs Leaf	RHSIM_Rhsim04G0170600	-2.810319418	1.39142E-48	1.16174E-46	RHSIM_Rhsim04G0170600
Stem vs Leaf	RHSIM_Rhsim09G0093900	-10.01724902	1.40516E-48	1.16897E-46	RHSIM_Rhsim09G0093900
Stem vs Leaf	RHSIM_Rhsim08G0211400	-3.836066079	1.52415E-48	1.2634E-46	RHSIM_Rhsim08G0211400
Stem vs Leaf	RHSIM_Rhsim07G0173600	-1.86478177	1.91694E-48	1.58329E-46	RHSIM_Rhsim07G0173600
Stem vs Leaf	RHSIM_Rhsim06G0011800	-5.306893156	2.60639E-48	2.14506E-46	RHSIM_Rhsim06G0011800

Comparison	Gene ID	log$_2$FC	P-value	FDR (padj)	Gene Name
Stem vs Leaf	novel.316	-2.342586404	3.19666E-48	2.62149E-46	--
Stem vs Leaf	RHSIM_Rhsim08G0172200	2.317705992	5.46881E-48	4.46891E-46	RHSIM_Rhsim08G0172200
Stem vs Leaf	RHSIM_Rhsim06G0178100	-2.922420596	7.12354E-48	5.80053E-46	RHSIM_Rhsim06G0178100
Stem vs Leaf	RHSIM_Rhsim02G0140300	-2.004082374	1.06512E-47	8.64244E-46	RHSIM_Rhsim02G0140300
Stem vs Leaf	RHSIM_Rhsim07G0075600	-3.730879969	1.55431E-47	1.25676E-45	RHSIM_Rhsim07G0075600
Stem vs Leaf	RHSIM_Rhsim04G0167600	-2.441068799	2.23872E-47	1.80382E-45	RHSIM_Rhsim04G0167600
Stem vs Leaf	RHSIM_Rhsim08G0171900	-2.67939426	5.22618E-47	4.19624E-45	RHSIM_Rhsim08G0171900
Stem vs Leaf	RHSIM_Rhsim10G0188100	-2.990510423	6.2089E-47	4.96798E-45	RHSIM_Rhsim10G0188100
Stem vs Leaf	RHSIM_Rhsim12G0025500	-2.51036948	1.02482E-46	8.17163E-45	RHSIM_Rhsim12G0025500
Stem vs Leaf	RHSIM_Rhsim07G0198500	3.218489175	1.36486E-46	1.08454E-44	RHSIM_Rhsim07G0198500
Stem vs Leaf	RHSIM_Rhsim12G0099200	-6.563987661	1.37739E-46	1.09074E-44	RHSIM_Rhsim12G0099200
Stem vs Leaf	RHSIM_Rhsim04G0197300	6.694106975	2.365E-46	1.86641E-44	RHSIM_Rhsim04G0197300
Stem vs Leaf	RHSIM_RhsimUnG0141100	3.083160427	2.45637E-46	1.9319E-44	RHSIM_RhsimUnG0141100
Stem vs Leaf	RHSIM_Rhsim04G0013500	-4.732585518	2.52249E-46	1.97458E-44	RHSIM_Rhsim04G0013500
Stem vs Leaf	RHSIM_Rhsim08G0118800	-2.797967401	2.52778E-46	1.97458E-44	RHSIM_Rhsim08G0118800
Stem vs Leaf	novel.4801	4.912330788	3.7999E-46	2.95827E-44	--
Stem vs Leaf	novel.1773	-4.694921923	4.2387E-46	3.28878E-44	--
Stem vs Leaf	RHSIM_RhsimPtG0004100	-3.710747135	6.75001E-46	5.21971E-44	RHSIM_RhsimPtG0004100
Stem vs Leaf	RHSIM_Rhsim10G0020100	-2.316234793	7.08066E-46	5.45708E-44	RHSIM_Rhsim10G0020100
Stem vs Leaf	RHSIM_Rhsim08G0090300	-3.260897605	7.1804E-46	5.5155E-44	RHSIM_Rhsim08G0090300
Stem vs Leaf	RHSIM_Rhsim04G0206400	-4.345422372	9.23214E-46	7.06796E-44	RHSIM_Rhsim04G0206400
Stem vs Leaf	RHSIM_Rhsim09G0111700	-2.968025054	1.34704E-45	1.02785E-43	RHSIM_Rhsim09G0111700
Stem vs Leaf	RHSIM_Rhsim06G0243400	-3.271656768	1.46628E-45	1.11514E-43	RHSIM_Rhsim06G0243400
Stem vs Leaf	RHSIM_Rhsim05G0068700	-1.895132486	1.52977E-45	1.15961E-43	RHSIM_Rhsim05G0068700
Stem vs Leaf	RHSIM_Rhsim08G0229600	-7.919748055	2.25145E-45	1.70106E-43	RHSIM_Rhsim08G0229600

Comparison	Gene ID	log_2FC	P-value	FDR (padj)	Gene Name
Stem vs Leaf	RHSIM_Rhsim08G0058800	-6.178485445	2.29454E-45	1.72795E-43	RHSIM_Rhsim08G0058800
Stem vs Leaf	novel.5195	-3.007249364	2.36275E-45	1.77353E-43	--
Stem vs Leaf	RHSIM_Rhsim08G0079200	-2.325250528	2.71219E-45	2.02921E-43	RHSIM_Rhsim08G0079200
Stem vs Leaf	RHSIM_Rhsim07G0124400	-3.327014045	3.29184E-45	2.45493E-43	RHSIM_Rhsim07G0124400
Stem vs Leaf	RHSIM_Rhsim11G0172800	-5.297110748	3.66941E-45	2.72767E-43	RHSIM_Rhsim11G0172800
Stem vs Leaf	RHSIM_Rhsim06G0052400	-4.616306441	4.74888E-45	3.51875E-43	RHSIM_Rhsim06G0052400
Stem vs Leaf	RHSIM_Rhsim09G0188700	2.523064258	5.12608E-45	3.78607E-43	RHSIM_Rhsim09G0188700
Stem vs Leaf	RHSIM_Rhsim12G0000700	-3.56985488	5.1519E-45	3.79298E-43	RHSIM_Rhsim12G0000700
Stem vs Leaf	RHSIM_Rhsim10G0129800	-5.134723391	6.50909E-45	4.77693E-43	RHSIM_Rhsim10G0129800
Stem vs Leaf	RHSIM_Rhsim06G0161200	-1.832135977	1.0058E-44	7.35798E-43	RHSIM_Rhsim06G0161200
Stem vs Leaf	RHSIM_RhsimPtG0005300	-4.928937373	1.14116E-44	8.32179E-43	RHSIM_RhsimPtG0005300
Stem vs Leaf	RHSIM_RhsimUnG0258600	-4.313172168	1.8226E-44	1.32492E-42	RHSIM_RhsimUnG0258600
Stem vs Leaf	RHSIM_Rhsim02G0113400	-5.538838662	2.29696E-44	1.6645E-42	RHSIM_Rhsim02G0113400
Stem vs Leaf	RHSIM_Rhsim03G0012100	-3.624173162	2.63918E-44	1.90649E-42	RHSIM_Rhsim03G0012100
Stem vs Leaf	RHSIM_Rhsim04G0190300	-1.631381332	4.21472E-44	3.03513E-42	RHSIM_Rhsim04G0190300
Stem vs Leaf	RHSIM_Rhsim06G0153100	-2.525559433	4.39671E-44	3.15632E-42	RHSIM_Rhsim06G0153100
Stem vs Leaf	RHSIM_Rhsim12G0110200	-3.61678589	4.54446E-44	3.25225E-42	RHSIM_Rhsim12G0110200
Stem vs Leaf	RHSIM_RhsimUnG0074300	-6.190245829	6.08527E-44	4.34146E-42	RHSIM_RhsimUnG0074300
Stem vs Leaf	RHSIM_Rhsim12G0037700	-4.02492589	6.23823E-44	4.43684E-42	RHSIM_Rhsim12G0037700
Stem vs Leaf	RHSIM_Rhsim05G0068100	-4.873456615	7.12179E-44	5.04968E-42	RHSIM_Rhsim05G0068100
Stem vs Leaf	RHSIM_Rhsim08G0192000	2.039625128	7.43929E-44	5.25862E-42	RHSIM_Rhsim08G0192000
Stem vs Leaf	RHSIM_Rhsim08G0038900	-2.940866248	7.96595E-44	5.61368E-42	RHSIM_Rhsim08G0038900
Stem vs Leaf	RHSIM_Rhsim10G0178400	-3.349908698	9.58689E-44	6.73538E-42	RHSIM_Rhsim10G0178400
Stem vs Leaf	RHSIM_Rhsim09G0132800	4.45744287	1.0235E-43	7.16888E-42	RHSIM_Rhsim09G0132800
Stem vs Leaf	RHSIM_Rhsim04G0024300	-2.291859966	1.39131E-43	9.71553E-42	RHSIM_Rhsim04G0024300

Comparison	Gene ID	log_2FC	P-value	FDR (padj)	Gene Name
Stem vs Leaf	RHSIM_Rhsim05G0126900	5.318806758	1.39777E-43	9.73121E-42	RHSIM_Rhsim05G0126900
Stem vs Leaf	novel.1309	-7.708151557	1.58489E-43	1.10007E-41	--
Stem vs Leaf	RHSIM_RhsimPtG0002000	-4.543341193	1.84738E-43	1.27841E-41	RHSIM_RhsimPtG0002000
Stem vs Leaf	RHSIM_Rhsim03G0169600	-2.461222382	2.55237E-43	1.76099E-41	RHSIM_Rhsim03G0169600
Stem vs Leaf	RHSIM_Rhsim06G0211400	-3.191069822	2.59086E-43	1.7822E-41	RHSIM_Rhsim06G0211400
Stem vs Leaf	RHSIM_Rhsim11G0046700	5.129543457	3.01435E-43	2.06734E-41	RHSIM_Rhsim11G0046700
Stem vs Leaf	RHSIM_Rhsim02G0240300	-6.289422761	3.55559E-43	2.43131E-41	RHSIM_Rhsim02G0240300
Stem vs Leaf	RHSIM_Rhsim10G0171700	-1.768288432	3.87096E-43	2.63912E-41	RHSIM_Rhsim10G0171700
Stem vs Leaf	RHSIM_Rhsim13G0225600	-4.973030689	4.92953E-43	3.35092E-41	RHSIM_Rhsim13G0225600
Stem vs Leaf	RHSIM_Rhsim08G0196000	-2.29230396	5.4714E-43	3.70833E-41	RHSIM_Rhsim08G0196000
Stem vs Leaf	RHSIM_Rhsim10G0070900	-6.932200933	6.559E-43	4.43242E-41	RHSIM_Rhsim10G0070900
Stem vs Leaf	RHSIM_Rhsim13G0142000	-2.345810164	6.70854E-43	4.52022E-41	RHSIM_Rhsim13G0142000
Stem vs Leaf	RHSIM_Rhsim05G0197300	2.663740782	6.94753E-43	4.66761E-41	RHSIM_Rhsim05G0197300
Stem vs Leaf	RHSIM_Rhsim11G0058800	-2.311790639	6.97725E-43	4.67394E-41	RHSIM_Rhsim11G0058800
Stem vs Leaf	RHSIM_Rhsim11G0077200	-4.413152201	7.1414E-43	4.77004E-41	RHSIM_Rhsim11G0077200
Stem vs Leaf	RHSIM_Rhsim11G0048100	2.168209143	8.55964E-43	5.70082E-41	RHSIM_Rhsim11G0048100
Stem vs Leaf	RHSIM_Rhsim10G0042700	4.627971322	1.18067E-42	7.84072E-41	RHSIM_Rhsim10G0042700
Stem vs Leaf	RHSIM_Rhsim02G0037500	-4.568996151	1.35747E-42	8.98892E-41	RHSIM_Rhsim02G0037500
Stem vs Leaf	RHSIM_Rhsim05G0043600	-2.842847928	1.40664E-42	9.28786E-41	RHSIM_Rhsim05G0043600
Stem vs Leaf	RHSIM_Rhsim10G0052200	-3.345237453	1.42336E-42	9.37139E-41	RHSIM_Rhsim10G0052200
Stem vs Leaf	RHSIM_Rhsim10G0012800	-2.694028958	1.432E-42	9.40142E-41	RHSIM_Rhsim10G0012800
Stem vs Leaf	RHSIM_Rhsim05G0223300	-9.254277037	1.46374E-42	9.58252E-41	RHSIM_Rhsim05G0223300
Stem vs Leaf	RHSIM_Rhsim03G0205000	-3.344488621	1.51322E-42	9.87835E-41	RHSIM_Rhsim03G0205000
Stem vs Leaf	RHSIM_Rhsim04G0071000	-2.697401219	3.32167E-42	2.16228E-40	RHSIM_Rhsim04G0071000
Stem vs Leaf	novel.4891	-4.735962021	3.45181E-42	2.2361E-40	--

Comparison	Gene ID	log_2FC	P-value	FDR (padj)	Gene Name
Stem vs Leaf	RHSIM_RhsimUnG0094700	-2.17895138	3.45448E-42	2.2361E-40	RHSIM_RhsimUnG0094700
Stem vs Leaf	RHSIM_Rhsim06G0129400	-4.518028072	4.24143E-42	2.7378E-40	RHSIM_Rhsim06G0129400
Stem vs Leaf	RHSIM_Rhsim04G0126900	-2.040220036	8.8088E-42	5.67011E-40	RHSIM_Rhsim04G0126900
Stem vs Leaf	RHSIM_Rhsim11G0038000	-1.751590154	9.05253E-42	5.81076E-40	RHSIM_Rhsim11G0038000
Stem vs Leaf	RHSIM_Rhsim10G0072300	-5.640468763	9.92445E-42	6.35275E-40	RHSIM_Rhsim10G0072300
Stem vs Leaf	novel.1017	-4.040756367	1.03403E-41	6.60059E-40	--
Stem vs Leaf	RHSIM_Rhsim01G0258700	-2.366922155	1.08191E-41	6.88719E-40	RHSIM_Rhsim01G0258700
Stem vs Leaf	RHSIM_Rhsim07G0091900	5.52000919	1.15146E-41	7.30974E-40	RHSIM_Rhsim07G0091900
Stem vs Leaf	RHSIM_Rhsim08G0223300	-3.308857764	1.38326E-41	8.7571E-40	RHSIM_Rhsim08G0223300
Stem vs Leaf	RHSIM_Rhsim06G0010600	-7.763632438	1.53866E-41	9.7142E-40	RHSIM_Rhsim06G0010600
Stem vs Leaf	RHSIM_Rhsim12G0201100	-3.51948514	1.70618E-41	1.07424E-39	RHSIM_Rhsim12G0201100
Stem vs Leaf	novel.5388	-6.068636407	1.77057E-41	1.11175E-39	--
Stem vs Leaf	RHSIM_Rhsim08G0152500	-3.86623104	2.12616E-41	1.33139E-39	RHSIM_Rhsim08G0152500
Stem vs Leaf	RHSIM_Rhsim10G0021200	-16.6114795	3.11226E-41	1.9436E-39	RHSIM_Rhsim10G0021200
Stem vs Leaf	RHSIM_Rhsim02G0042700	1.920737294	3.71348E-41	2.31279E-39	RHSIM_Rhsim02G0042700
Stem vs Leaf	RHSIM_Rhsim01G0273700	4.692374147	4.45968E-41	2.77005E-39	RHSIM_Rhsim01G0273700
Stem vs Leaf	RHSIM_Rhsim02G0105700	2.064961179	4.86451E-41	3.01338E-39	RHSIM_Rhsim02G0105700
Stem vs Leaf	RHSIM_Rhsim11G0169300	-1.850364029	5.74006E-41	3.54622E-39	RHSIM_Rhsim11G0169300
Stem vs Leaf	RHSIM_Rhsim01G0202100	-3.4517582	6.43776E-41	3.96663E-39	RHSIM_Rhsim01G0202100
Stem vs Leaf	RHSIM_Rhsim12G0005700	-3.104291477	7.21074E-41	4.43105E-39	RHSIM_Rhsim12G0005700
Stem vs Leaf	RHSIM_RhsimPtG0004000	-4.415793035	7.56041E-41	4.63357E-39	RHSIM_RhsimPtG0004000
Stem vs Leaf	RHSIM_Rhsim09G0152700	-3.280843846	8.31632E-41	5.08332E-39	RHSIM_Rhsim09G0152700
Stem vs Leaf	RHSIM_Rhsim03G0080600	5.681203089	9.61941E-41	5.86428E-39	RHSIM_Rhsim03G0080600
Stem vs Leaf	RHSIM_Rhsim03G0219000	-2.044976353	1.15159E-40	7.00194E-39	RHSIM_Rhsim03G0219000
Stem vs Leaf	RHSIM_Rhsim05G0015000	-2.691215938	1.64141E-40	9.95387E-39	RHSIM_Rhsim05G0015000

Comparison	Gene ID	log_2FC	P-value	FDR (padj)	Gene Name
Stem vs Leaf	RHSIM_Rhsim07G0200400	-4.649851098	1.69897E-40	1.02759E-38	RHSIM_Rhsim07G0200400
Stem vs Leaf	novel.1312	-10.19906538	1.75287E-40	1.05741E-38	--
Stem vs Leaf	RHSIM_Rhsim08G0029700	-2.20757073	1.91656E-40	1.15314E-38	RHSIM_Rhsim08G0029700
Stem vs Leaf	RHSIM_Rhsim12G0155900	3.212539495	2.15974E-40	1.29607E-38	RHSIM_Rhsim12G0155900
Stem vs Leaf	RHSIM_Rhsim06G0220300	-2.618641886	2.34268E-40	1.4022E-38	RHSIM_Rhsim06G0220300
Stem vs Leaf	RHSIM_Rhsim01G0181300	-3.81417061	3.27916E-40	1.95764E-38	RHSIM_Rhsim01G0181300
Stem vs Leaf	RHSIM_RhsimUnG0002100	-1.557767152	4.07787E-40	2.42818E-38	RHSIM_RhsimUnG0002100
Stem vs Leaf	RHSIM_Rhsim01G0269100	-3.682681966	5.78127E-40	3.4336E-38	RHSIM_Rhsim01G0269100
Stem vs Leaf	RHSIM_Rhsim05G0195600	-7.00492707	7.96086E-40	4.71594E-38	RHSIM_Rhsim05G0195600
Stem vs Leaf	RHSIM_Rhsim13G0094600	2.867764448	8.75822E-40	5.17498E-38	RHSIM_Rhsim13G0094600
Stem vs Leaf	RHSIM_Rhsim01G0192900	-7.844876315	1.45639E-39	8.58341E-38	RHSIM_Rhsim01G0192900
Stem vs Leaf	RHSIM_Rhsim13G0003800	-1.773606533	1.55037E-39	9.11398E-38	RHSIM_Rhsim13G0003800
Stem vs Leaf	RHSIM_Rhsim10G0081700	4.762579862	1.5622E-39	9.16013E-38	RHSIM_Rhsim10G0081700
Stem vs Leaf	RHSIM_Rhsim09G0129300	-4.714549567	1.72922E-39	1.01138E-37	RHSIM_Rhsim09G0129300
Stem vs Leaf	RHSIM_Rhsim08G0079500	-4.193214684	1.88735E-39	1.10106E-37	RHSIM_Rhsim08G0079500
Stem vs Leaf	RHSIM_Rhsim10G0009700	-1.300717134	2.17111E-39	1.26341E-37	RHSIM_Rhsim10G0009700
Stem vs Leaf	RHSIM_Rhsim11G0021000	-2.545122407	3.14279E-39	1.82424E-37	RHSIM_Rhsim11G0021000
Stem vs Leaf	RHSIM_Rhsim02G0198800	-1.806186567	3.75404E-39	2.17357E-37	RHSIM_Rhsim02G0198800
Stem vs Leaf	RHSIM_Rhsim07G0045200	-8.187648634	3.99051E-39	2.3047E-37	RHSIM_Rhsim07G0045200
Stem vs Leaf	RHSIM_Rhsim09G0054700	-3.698981549	4.32994E-39	2.49448E-37	RHSIM_Rhsim09G0054700
Stem vs Leaf	RHSIM_Rhsim02G0115100	-2.845423662	6.92021E-39	3.97679E-37	RHSIM_Rhsim02G0115100
Stem vs Leaf	RHSIM_Rhsim10G0105500	-2.415926375	7.20378E-39	4.12945E-37	RHSIM_Rhsim10G0105500
Stem vs Leaf	RHSIM_Rhsim01G0016000	-1.712593888	1.0327E-38	5.90512E-37	RHSIM_Rhsim01G0016000
Stem vs Leaf	RHSIM_Rhsim11G0059000	-3.112512109	1.19653E-38	6.81247E-37	RHSIM_Rhsim11G0059000
Stem vs Leaf	RHSIM_Rhsim13G0071700	-5.069488049	1.1973E-38	6.81247E-37	RHSIM_Rhsim13G0071700

Comparison	Gene ID	log_2FC	P-value	FDR (padj)	Gene Name
Stem vs Leaf	RHSIM_Rhsim09G0004800	-3.835165689	1.35441E-38	7.68744E-37	RHSIM_Rhsim09G0004800
Stem vs Leaf	RHSIM_Rhsim13G0166900	-2.744869351	1.48663E-38	8.41716E-37	RHSIM_Rhsim13G0166900
Stem vs Leaf	RHSIM_Rhsim08G0198400	-2.573007242	1.62147E-38	9.15811E-37	RHSIM_Rhsim08G0198400
Stem vs Leaf	RHSIM_Rhsim07G0174800	6.675860087	1.67034E-38	9.41107E-37	RHSIM_Rhsim07G0174800
Stem vs Leaf	RHSIM_Rhsim01G0216100	3.398797456	1.74366E-38	9.80023E-37	RHSIM_Rhsim01G0216100
Stem vs Leaf	RHSIM_Rhsim12G0076500	-4.061814104	1.99683E-38	1.11959E-36	RHSIM_Rhsim12G0076500
Stem vs Leaf	RHSIM_Rhsim11G0184900	-2.326446924	2.02731E-38	1.13392E-36	RHSIM_Rhsim11G0184900
Stem vs Leaf	RHSIM_RhsimPtG0003600	-3.347529533	3.07959E-38	1.71831E-36	RHSIM_RhsimPtG0003600
Stem vs Leaf	RHSIM_Rhsim07G0047700	-4.001228389	3.08715E-38	1.71837E-36	RHSIM_Rhsim07G0047700
Stem vs Leaf	RHSIM_Rhsim05G0167200	-2.056729703	4.09017E-38	2.27118E-36	RHSIM_Rhsim05G0167200
Stem vs Leaf	RHSIM_Rhsim01G0285600	-1.179031177	4.14957E-38	2.29862E-36	RHSIM_Rhsim01G0285600
Stem vs Leaf	RHSIM_Rhsim09G0010400	4.264221822	4.30702E-38	2.38012E-36	RHSIM_Rhsim09G0010400
Stem vs Leaf	RHSIM_Rhsim02G0019800	-9.89402962	4.41995E-38	2.43668E-36	RHSIM_Rhsim02G0019800
Stem vs Leaf	RHSIM_Rhsim10G0156600	-2.557791527	5.22379E-38	2.87296E-36	RHSIM_Rhsim10G0156600
Stem vs Leaf	RHSIM_Rhsim03G0119100	5.613461376	5.75924E-38	3.1599E-36	RHSIM_Rhsim03G0119100
Stem vs Leaf	RHSIM_Rhsim04G0176700	-1.384770991	6.68095E-38	3.65691E-36	RHSIM_Rhsim04G0176700
Stem vs Leaf	RHSIM_Rhsim11G0020200	-1.803921005	6.77744E-38	3.70093E-36	RHSIM_Rhsim11G0020200
Stem vs Leaf	RHSIM_Rhsim04G0092500	-1.652304209	7.95504E-38	4.33371E-36	RHSIM_Rhsim04G0092500
Stem vs Leaf	RHSIM_Rhsim12G0107100	-2.11756577	8.38606E-38	4.55774E-36	RHSIM_Rhsim12G0107100
Stem vs Leaf	RHSIM_Rhsim09G0079200	-4.645880818	8.67075E-38	4.70139E-36	RHSIM_Rhsim09G0079200
Stem vs Leaf	RHSIM_Rhsim10G0019600	-2.599263086	9.87514E-38	5.34185E-36	RHSIM_Rhsim10G0019600
Stem vs Leaf	RHSIM_Rhsim08G0181000	-7.09342937	1.05405E-37	5.6884E-36	RHSIM_Rhsim08G0181000
Stem vs Leaf	RHSIM_Rhsim09G0030900	6.334193823	1.11123E-37	5.98299E-36	RHSIM_Rhsim09G0030900
Stem vs Leaf	RHSIM_Rhsim03G0259400	-3.077108295	1.12754E-37	6.05668E-36	RHSIM_Rhsim03G0259400
Stem vs Leaf	RHSIM_Rhsim07G0070500	-3.510706847	1.32962E-37	7.12555E-36	RHSIM_Rhsim07G0070500

Comparison	Gene ID	log_2FC	P-value	FDR (padj)	Gene Name
Stem vs Leaf	RHSIM_RhsimUnG0018800	-4.291990595	1.36054E-37	7.27432E-36	RHSIM_RhsimUnG0018800
Stem vs Leaf	RHSIM_Rhsim11G0065700	-2.120562423	1.45235E-37	7.74723E-36	RHSIM_Rhsim11G0065700
Stem vs Leaf	RHSIM_Rhsim12G0170000	-3.864945429	1.6633E-37	8.85199E-36	RHSIM_Rhsim12G0170000
Stem vs Leaf	RHSIM_Rhsim12G0064600	-2.530543915	1.68146E-37	8.928E-36	RHSIM_Rhsim12G0064600
Stem vs Leaf	RHSIM_Rhsim10G0147700	4.775171254	1.91842E-37	1.01628E-35	RHSIM_Rhsim10G0147700
Stem vs Leaf	RHSIM_Rhsim06G0018200	-8.694536677	2.05808E-37	1.08776E-35	RHSIM_Rhsim06G0018200
Stem vs Leaf	RHSIM_Rhsim03G0141600	-3.110633204	2.07598E-37	1.09471E-35	RHSIM_Rhsim03G0141600
Stem vs Leaf	RHSIM_Rhsim04G0013600	-3.195151195	2.42476E-37	1.27571E-35	RHSIM_Rhsim04G0013600
Stem vs Leaf	RHSIM_Rhsim01G0235400	-3.010549599	2.47939E-37	1.30148E-35	RHSIM_Rhsim01G0235400
Stem vs Leaf	RHSIM_Rhsim12G0043600	-2.95913929	2.51092E-37	1.31504E-35	RHSIM_Rhsim12G0043600
Stem vs Leaf	RHSIM_Rhsim10G0199000	-5.337596518	3.09669E-37	1.61815E-35	RHSIM_Rhsim10G0199000
Stem vs Leaf	RHSIM_Rhsim05G0139000	-1.709904033	3.10492E-37	1.61878E-35	RHSIM_Rhsim05G0139000
Stem vs Leaf	RHSIM_Rhsim07G0013600	-3.803722497	3.51218E-37	1.82697E-35	RHSIM_Rhsim07G0013600
Stem vs Leaf	RHSIM_Rhsim06G0035600	-4.805640497	3.61726E-37	1.87739E-35	RHSIM_Rhsim06G0035600
Stem vs Leaf	RHSIM_Rhsim13G0052100	-5.352806328	3.77528E-37	1.955E-35	RHSIM_Rhsim13G0052100
Stem vs Leaf	RHSIM_Rhsim04G0043200	-2.465212696	3.81365E-37	1.97044E-35	RHSIM_Rhsim04G0043200
Stem vs Leaf	RHSIM_Rhsim01G0276200	-2.000868702	3.83725E-37	1.9782E-35	RHSIM_Rhsim01G0276200
Stem vs Leaf	RHSIM_Rhsim11G0127700	2.115844061	4.04431E-37	2.08029E-35	RHSIM_Rhsim11G0127700
Stem vs Leaf	RHSIM_Rhsim10G0163100	-1.90734646	4.28163E-37	2.19746E-35	RHSIM_Rhsim10G0163100
Stem vs Leaf	RHSIM_Rhsim09G0155900	-5.188432494	4.35801E-37	2.23169E-35	RHSIM_Rhsim09G0155900
Stem vs Leaf	RHSIM_Rhsim07G0150700	-2.44367668	4.54999E-37	2.32483E-35	RHSIM_Rhsim07G0150700
Stem vs Leaf	RHSIM_Rhsim12G0135200	-5.646479209	5.52415E-37	2.81634E-35	RHSIM_Rhsim12G0135200
Stem vs Leaf	RHSIM_Rhsim02G0213900	3.036457308	5.90633E-37	3.00454E-35	RHSIM_Rhsim02G0213900
Stem vs Leaf	RHSIM_Rhsim03G0262800	-2.071966681	7.21231E-37	3.66081E-35	RHSIM_Rhsim03G0262800
Stem vs Leaf	RHSIM_Rhsim11G0044100	-4.824498468	7.57575E-37	3.83683E-35	RHSIM_Rhsim11G0044100

Comparison	Gene ID	log_2FC	P-value	FDR (padj)	Gene Name
Stem vs Leaf	RHSIM_Rhsim10G0083200	-2.665435672	7.84277E-37	3.96335E-35	RHSIM_Rhsim10G0083200
Stem vs Leaf	RHSIM_Rhsim01G0239400	-1.405672993	9.81693E-37	4.95014E-35	RHSIM_Rhsim01G0239400
Stem vs Leaf	RHSIM_Rhsim04G0081600	-3.476457204	1.19186E-36	5.99678E-35	RHSIM_Rhsim04G0081600
Stem vs Leaf	RHSIM_Rhsim04G0032000	-3.735674387	1.34894E-36	6.77232E-35	RHSIM_Rhsim04G0032000
Stem vs Leaf	RHSIM_Rhsim07G0095400	-9.043420374	1.40393E-36	7.0331E-35	RHSIM_Rhsim07G0095400
Stem vs Leaf	RHSIM_Rhsim10G0043300	-7.68148153	1.47759E-36	7.38604E-35	RHSIM_Rhsim10G0043300
Stem vs Leaf	RHSIM_Rhsim06G0169500	-5.515833525	1.5571E-36	7.76663E-35	RHSIM_Rhsim06G0169500
Stem vs Leaf	RHSIM_Rhsim01G0251800	5.163146113	1.60274E-36	7.97699E-35	RHSIM_Rhsim01G0251800
Stem vs Leaf	RHSIM_Rhsim06G0007800	-1.745856534	1.80272E-36	8.95298E-35	RHSIM_Rhsim06G0007800
Stem vs Leaf	RHSIM_Rhsim07G0132800	-1.977236335	1.95576E-36	9.69214E-35	RHSIM_Rhsim07G0132800
Stem vs Leaf	RHSIM_Rhsim12G0085700	2.09396521	2.13903E-36	1.05776E-34	RHSIM_Rhsim12G0085700
Stem vs Leaf	RHSIM_Rhsim01G0184600	-1.908705932	2.50106E-36	1.23414E-34	RHSIM_Rhsim01G0184600
Stem vs Leaf	RHSIM_Rhsim09G0066700	-1.981734548	2.97524E-36	1.46499E-34	RHSIM_Rhsim09G0066700
Stem vs Leaf	RHSIM_Rhsim09G0012200	-3.53445361	3.81804E-36	1.87597E-34	RHSIM_Rhsim09G0012200
Stem vs Leaf	RHSIM_RhsimUnG0139700	-2.495481557	4.59789E-36	2.25433E-34	RHSIM_RhsimUnG0139700
Stem vs Leaf	RHSIM_Rhsim09G0184400	-4.924986523	5.12629E-36	2.50807E-34	RHSIM_Rhsim09G0184400
Stem vs Leaf	novel.4878	7.394199099	5.49256E-36	2.68158E-34	--
Stem vs Leaf	RHSIM_Rhsim05G0053200	-2.601476964	5.80028E-36	2.82583E-34	RHSIM_Rhsim05G0053200
Stem vs Leaf	RHSIM_Rhsim10G0053700	-5.464384032	6.73801E-36	3.27575E-34	RHSIM_Rhsim10G0053700
Stem vs Leaf	RHSIM_Rhsim13G0116300	-2.786372359	6.88199E-36	3.3387E-34	RHSIM_Rhsim13G0116300
Stem vs Leaf	novel.5686	-1.511373437	6.93279E-36	3.35629E-34	--
Stem vs Leaf	RHSIM_Rhsim02G0034800	-12.90571988	7.89942E-36	3.81623E-34	RHSIM_Rhsim02G0034800
Stem vs Leaf	RHSIM_Rhsim02G0225700	7.245947597	8.58713E-36	4.13979E-34	RHSIM_Rhsim02G0225700
Stem vs Leaf	RHSIM_Rhsim08G0115600	-3.648343685	9.13518E-36	4.39481E-34	RHSIM_Rhsim08G0115600
Stem vs Leaf	RHSIM_Rhsim12G0056800	4.989641973	1.13513E-35	5.44956E-34	RHSIM_Rhsim12G0056800

Comparison	Gene ID	log_2FC	P-value	FDR (padj)	Gene Name
Stem vs Leaf	RHSIM_Rhsim04G0024200	-5.21072389	1.42614E-35	6.8324E-34	RHSIM_Rhsim04G0024200
Stem vs Leaf	RHSIM_Rhsim08G0217500	4.772371955	1.84753E-35	8.83288E-34	RHSIM_Rhsim08G0217500
Stem vs Leaf	RHSIM_Rhsim08G0101500	-8.401699061	1.92918E-35	9.20412E-34	RHSIM_Rhsim08G0101500
Stem vs Leaf	RHSIM_Rhsim06G0087100	-1.853727924	2.00548E-35	9.5484E-34	RHSIM_Rhsim06G0087100
Stem vs Leaf	RHSIM_Rhsim04G0166800	-2.489921086	2.2155E-35	1.05266E-33	RHSIM_Rhsim04G0166800
Stem vs Leaf	RHSIM_Rhsim12G0145700	-3.964599684	2.49281E-35	1.18198E-33	RHSIM_Rhsim12G0145700
Stem vs Leaf	RHSIM_Rhsim09G0178500	-2.972958743	2.7695E-35	1.31048E-33	RHSIM_Rhsim09G0178500
Stem vs Leaf	RHSIM_Rhsim05G0043800	1.854830278	2.89358E-35	1.36639E-33	RHSIM_Rhsim05G0043800
Stem vs Leaf	RHSIM_Rhsim08G0157600	2.920093503	3.33616E-35	1.57216E-33	RHSIM_Rhsim08G0157600
Stem vs Leaf	RHSIM_Rhsim04G0167300	-1.775709453	3.5019E-35	1.64689E-33	RHSIM_Rhsim04G0167300
Stem vs Leaf	RHSIM_RhsimUnG0258900	-2.322588257	4.18696E-35	1.96506E-33	RHSIM_RhsimUnG0258900
Stem vs Leaf	RHSIM_Rhsim09G0137300	6.194122484	4.36698E-35	2.04538E-33	RHSIM_Rhsim09G0137300
Stem vs Leaf	RHSIM_Rhsim01G0235600	-3.331194129	4.78536E-35	2.23679E-33	RHSIM_Rhsim01G0235600
Stem vs Leaf	RHSIM_Rhsim05G0136600	-6.068331307	4.91736E-35	2.29384E-33	RHSIM_Rhsim05G0136600
Stem vs Leaf	RHSIM_Rhsim03G0255200	-14.96071686	4.96298E-35	2.31044E-33	RHSIM_Rhsim03G0255200
Stem vs Leaf	RHSIM_Rhsim01G0034400	-3.719842467	5.08517E-35	2.36255E-33	RHSIM_Rhsim01G0034400
Stem vs Leaf	RHSIM_Rhsim03G0191800	-4.747752845	5.11871E-35	2.37335E-33	RHSIM_Rhsim03G0191800
Stem vs Leaf	RHSIM_Rhsim10G0165500	-14.76361789	5.22237E-35	2.41655E-33	RHSIM_Rhsim10G0165500
Stem vs Leaf	RHSIM_Rhsim04G0134300	-1.752960529	5.36181E-35	2.4761E-33	RHSIM_Rhsim04G0134300
Stem vs Leaf	RHSIM_Rhsim07G0257000	-14.94089187	5.4093E-35	2.49304E-33	RHSIM_Rhsim07G0257000
Stem vs Leaf	RHSIM_Rhsim10G0079900	-2.281625891	6.12141E-35	2.81561E-33	RHSIM_Rhsim10G0079900
Stem vs Leaf	RHSIM_Rhsim10G0046600	-3.263399204	6.46949E-35	2.96978E-33	RHSIM_Rhsim10G0046600
Stem vs Leaf	RHSIM_Rhsim12G0035600	-2.207222042	7.41221E-35	3.39206E-33	RHSIM_Rhsim12G0035600
Stem vs Leaf	RHSIM_Rhsim05G0022400	-2.470093358	7.41885E-35	3.39206E-33	RHSIM_Rhsim05G0022400
Stem vs Leaf	RHSIM_Rhsim04G0167200	-2.342241939	7.60642E-35	3.47094E-33	RHSIM_Rhsim04G0167200

Comparison	Gene ID	log_2FC	P-value	FDR (padj)	Gene Name
Stem vs Leaf	RHSIM_Rhsim07G0139700	-8.403640909	8.41672E-35	3.8331E-33	RHSIM_Rhsim07G0139700
Stem vs Leaf	RHSIM_Rhsim01G0028000	-2.59918963	9.21689E-35	4.18923E-33	RHSIM_Rhsim01G0028000
Stem vs Leaf	RHSIM_Rhsim09G0211500	-2.020953619	9.41842E-35	4.2724E-33	RHSIM_Rhsim09G0211500
Stem vs Leaf	RHSIM_Rhsim04G0232700	5.430517039	9.68875E-35	4.3864E-33	RHSIM_Rhsim04G0232700
Stem vs Leaf	RHSIM_Rhsim03G0075700	-7.273558748	1.31161E-34	5.9264E-33	RHSIM_Rhsim03G0075700
Stem vs Leaf	RHSIM_Rhsim12G0180300	-2.856238112	1.32212E-34	5.96221E-33	RHSIM_Rhsim12G0180300
Stem vs Leaf	RHSIM_RhsimUnG0227500	-1.714630195	1.33625E-34	6.01418E-33	RHSIM_RhsimUnG0227500
Stem vs Leaf	novel.3711	6.479971638	1.52586E-34	6.85417E-33	--
Stem vs Leaf	RHSIM_Rhsim09G0065100	-1.502005154	1.59253E-34	7.13972E-33	RHSIM_Rhsim09G0065100
Stem vs Leaf	RHSIM_Rhsim04G0057100	-3.621750947	1.85522E-34	8.30131E-33	RHSIM_Rhsim04G0057100
Stem vs Leaf	novel.5013	-4.929875615	1.85933E-34	8.30356E-33	--
Stem vs Leaf	RHSIM_Rhsim06G0148000	3.110728027	2.05783E-34	9.17226E-33	RHSIM_Rhsim06G0148000
Stem vs Leaf	RHSIM_Rhsim01G0192800	-10.60855655	2.35497E-34	1.04764E-32	RHSIM_Rhsim01G0192800
Stem vs Leaf	RHSIM_Rhsim06G0049200	-5.275223941	2.4612E-34	1.09279E-32	RHSIM_Rhsim06G0049200
Stem vs Leaf	RHSIM_Rhsim01G0234600	-2.744635116	2.66797E-34	1.18232E-32	RHSIM_Rhsim01G0234600
Stem vs Leaf	RHSIM_Rhsim12G0099000	-2.232395095	2.81162E-34	1.24359E-32	RHSIM_Rhsim12G0099000
Stem vs Leaf	RHSIM_Rhsim04G0038600	-4.112101435	3.0162E-34	1.33152E-32	RHSIM_Rhsim04G0038600
Stem vs Leaf	novel.5711	-5.82678652	3.50553E-34	1.54458E-32	--
Stem vs Leaf	RHSIM_Rhsim02G0214300	-7.28822009	3.69484E-34	1.62488E-32	RHSIM_Rhsim02G0214300
Stem vs Leaf	RHSIM_Rhsim03G0027400	-2.013764508	3.94971E-34	1.73366E-32	RHSIM_Rhsim03G0027400
Stem vs Leaf	RHSIM_Rhsim10G0099300	-3.480699427	4.1753E-34	1.8292E-32	RHSIM_Rhsim10G0099300
Stem vs Leaf	RHSIM_Rhsim01G0203000	-3.317591739	4.25151E-34	1.85905E-32	RHSIM_Rhsim01G0203000
Stem vs Leaf	RHSIM_Rhsim07G0049500	4.13289625	5.8478E-34	2.55221E-32	RHSIM_Rhsim07G0049500
Stem vs Leaf	RHSIM_Rhsim13G0053000	-3.067755464	5.97647E-34	2.60344E-32	RHSIM_Rhsim13G0053000
Stem vs Leaf	novel.5387	-6.552716943	6.86824E-34	2.98626E-32	--

Comparison	Gene ID	log_2FC	P-value	FDR (padj)	Gene Name
Stem vs Leaf	RHSIM_Rhsim06G0049600	-10.57074849	8.16965E-34	3.54541E-32	RHSIM_Rhsim06G0049600
Stem vs Leaf	RHSIM_Rhsim06G0131100	5.294044485	8.57821E-34	3.71572E-32	RHSIM_Rhsim06G0131100
Stem vs Leaf	RHSIM_Rhsim11G0039200	-12.35615243	1.01265E-33	4.37816E-32	RHSIM_Rhsim11G0039200
Stem vs Leaf	RHSIM_Rhsim13G0013600	-2.344471079	1.12632E-33	4.86047E-32	RHSIM_Rhsim13G0013600
Stem vs Leaf	RHSIM_Rhsim10G0053100	-4.655026595	1.12963E-33	4.86565E-32	RHSIM_Rhsim10G0053100
Stem vs Leaf	RHSIM_RhsimUnG0070700	4.019709635	1.19169E-33	5.12337E-32	RHSIM_RhsimUnG0070700
Stem vs Leaf	RHSIM_RhsimUnG0246000	6.552230271	1.21934E-33	5.23249E-32	RHSIM_RhsimUnG0246000
Stem vs Leaf	RHSIM_Rhsim13G0025300	5.105466592	1.42667E-33	6.11083E-32	RHSIM_Rhsim13G0025300
Stem vs Leaf	RHSIM_Rhsim03G0069400	-3.675581369	1.53026E-33	6.54234E-32	RHSIM_Rhsim03G0069400
Stem vs Leaf	RHSIM_Rhsim06G0119000	-5.2271495	1.54665E-33	6.60017E-32	RHSIM_Rhsim06G0119000
Stem vs Leaf	RHSIM_Rhsim02G0120800	1.797672773	1.58858E-33	6.76658E-32	RHSIM_Rhsim02G0120800
Stem vs Leaf	RHSIM_Rhsim02G0217800	-2.638205064	1.70852E-33	7.26405E-32	RHSIM_Rhsim02G0217800
Stem vs Leaf	RHSIM_Rhsim03G0041100	-5.770856755	2.58107E-33	1.09536E-31	RHSIM_Rhsim03G0041100
Stem vs Leaf	RHSIM_Rhsim12G0065000	-4.794760493	2.59747E-33	1.1003E-31	RHSIM_Rhsim12G0065000
Stem vs Leaf	RHSIM_Rhsim07G0162600	-5.619511088	2.67968E-33	1.13304E-31	RHSIM_Rhsim07G0162600
Stem vs Leaf	RHSIM_Rhsim13G0076500	-8.053854658	2.99415E-33	1.26369E-31	RHSIM_Rhsim13G0076500
Stem vs Leaf	RHSIM_RhsimUnG0254000	-2.507269382	3.01089E-33	1.26843E-31	RHSIM_RhsimUnG0254000
Stem vs Leaf	RHSIM_Rhsim09G0067000	3.629429838	3.0283E-33	1.27343E-31	RHSIM_Rhsim09G0067000
Stem vs Leaf	RHSIM_Rhsim07G0090300	-2.85249264	3.21401E-33	1.34907E-31	RHSIM_Rhsim07G0090300
Stem vs Leaf	RHSIM_Rhsim01G0015400	4.958234474	3.67944E-33	1.54162E-31	RHSIM_Rhsim01G0015400
Stem vs Leaf	RHSIM_Rhsim10G0119200	-1.803826803	3.93375E-33	1.64518E-31	RHSIM_Rhsim10G0119200
Stem vs Leaf	RHSIM_Rhsim02G0239700	-6.130719492	4.05E-33	1.69073E-31	RHSIM_Rhsim02G0239700
Stem vs Leaf	RHSIM_Rhsim13G0162100	3.934933962	5.03057E-33	2.09628E-31	RHSIM_Rhsim13G0162100
Stem vs Leaf	RHSIM_Rhsim03G0186100	-9.416112958	5.14718E-33	2.141E-31	RHSIM_Rhsim03G0186100
Stem vs Leaf	RHSIM_Rhsim10G0148300	-2.078317585	5.71193E-33	2.37163E-31	RHSIM_Rhsim10G0148300

Comparison	Gene ID	log_2FC	P-value	FDR (padj)	Gene Name
Stem vs Leaf	RHSIM_Rhsim02G0087500	-9.341863035	5.91741E-33	2.45253E-31	RHSIM_Rhsim02G0087500
Stem vs Leaf	RHSIM_Rhsim02G0244000	-8.854654774	6.52361E-33	2.69892E-31	RHSIM_Rhsim02G0244000
Stem vs Leaf	RHSIM_Rhsim06G0237200	-2.53379035	6.77872E-33	2.79944E-31	RHSIM_Rhsim06G0237200
Stem vs Leaf	RHSIM_Rhsim08G0214800	-3.748404432	7.92906E-33	3.26865E-31	RHSIM_Rhsim08G0214800
Stem vs Leaf	RHSIM_Rhsim08G0029600	-5.662850172	8.34278E-33	3.43305E-31	RHSIM_Rhsim08G0029600
Stem vs Leaf	RHSIM_Rhsim11G0068500	-8.717378664	9.21025E-33	3.78326E-31	RHSIM_Rhsim11G0068500
Stem vs Leaf	RHSIM_Rhsim08G0114900	-4.501016023	9.54579E-33	3.91411E-31	RHSIM_Rhsim08G0114900
Stem vs Leaf	RHSIM_RhsimUnG0119200	-1.888534727	9.77755E-33	4.00202E-31	RHSIM_RhsimUnG0119200
Stem vs Leaf	RHSIM_Rhsim12G0046400	-3.91164435	1.1073E-32	4.52422E-31	RHSIM_Rhsim12G0046400
Stem vs Leaf	RHSIM_Rhsim06G0082800	-2.50561996	1.18588E-32	4.83672E-31	RHSIM_Rhsim06G0082800
Stem vs Leaf	RHSIM_Rhsim10G0187900	-3.747398928	1.20143E-32	4.89149E-31	RHSIM_Rhsim10G0187900
Stem vs Leaf	RHSIM_Rhsim09G0054100	-3.169638953	1.31263E-32	5.33479E-31	RHSIM_Rhsim09G0054100
Stem vs Leaf	RHSIM_Rhsim02G0175200	-6.683295493	1.47139E-32	5.96951E-31	RHSIM_Rhsim02G0175200
Stem vs Leaf	RHSIM_Rhsim07G0175400	-2.010300209	1.54209E-32	6.24531E-31	RHSIM_Rhsim07G0175400
Stem vs Leaf	RHSIM_Rhsim04G0230700	-4.842507795	1.57548E-32	6.36934E-31	RHSIM_Rhsim04G0230700
Stem vs Leaf	RHSIM_Rhsim09G0087300	-3.105190731	1.66573E-32	6.72243E-31	RHSIM_Rhsim09G0087300
Stem vs Leaf	RHSIM_Rhsim07G0053300	-2.45759388	1.69913E-32	6.84523E-31	RHSIM_Rhsim07G0053300
Stem vs Leaf	RHSIM_Rhsim01G0007000	-1.4747795	1.86997E-32	7.52034E-31	RHSIM_Rhsim01G0007000
Stem vs Leaf	RHSIM_Rhsim01G0100400	-1.954037675	2.02611E-32	8.1252E-31	RHSIM_Rhsim01G0100400
Stem vs Leaf	RHSIM_Rhsim08G0144800	-6.707815559	2.02742E-32	8.1252E-31	RHSIM_Rhsim08G0144800
Stem vs Leaf	RHSIM_RhsimUnG0205500	3.527135379	2.08875E-32	8.35645E-31	RHSIM_RhsimUnG0205500
Stem vs Leaf	RHSIM_Rhsim12G0077600	-1.83460497	2.09245E-32	8.35676E-31	RHSIM_Rhsim12G0077600
Stem vs Leaf	RHSIM_Rhsim10G0177600	6.472287408	2.19319E-32	8.74391E-31	RHSIM_Rhsim10G0177600
Stem vs Leaf	RHSIM_Rhsim06G0152600	8.441573581	2.32646E-32	9.25924E-31	RHSIM_Rhsim06G0152600
Stem vs Leaf	RHSIM_Rhsim11G0148300	-6.91932361	2.50661E-32	9.95902E-31	RHSIM_Rhsim11G0148300

Comparison	Gene ID	log_2FC	P-value	FDR (padj)	Gene Name
Stem vs Leaf	RHSIM_Rhsim12G0106700	5.772263091	2.64711E-32	1.04992E-30	RHSIM_Rhsim12G0106700
Stem vs Leaf	RHSIM_Rhsim03G0227400	-1.919829572	3.10704E-32	1.23022E-30	RHSIM_Rhsim03G0227400
Stem vs Leaf	RHSIM_Rhsim02G0143000	6.824580446	3.4704E-32	1.37173E-30	RHSIM_Rhsim02G0143000
Stem vs Leaf	RHSIM_Rhsim08G0045800	-4.133976356	3.51276E-32	1.3861E-30	RHSIM_Rhsim08G0045800
Stem vs Leaf	RHSIM_Rhsim06G0047500	5.514293775	3.93977E-32	1.55193E-30	RHSIM_Rhsim06G0047500
Stem vs Leaf	RHSIM_Rhsim02G0109300	-1.40434476	4.27711E-32	1.68194E-30	RHSIM_Rhsim02G0109300
Stem vs Leaf	RHSIM_Rhsim08G0220600	4.651624322	4.44593E-32	1.74535E-30	RHSIM_Rhsim08G0220600
Stem vs Leaf	RHSIM_Rhsim05G0090500	-1.623492857	4.49206E-32	1.76046E-30	RHSIM_Rhsim05G0090500
Stem vs Leaf	RHSIM_Rhsim03G0005600	-1.413750159	4.71301E-32	1.84392E-30	RHSIM_Rhsim03G0005600
Stem vs Leaf	RHSIM_Rhsim13G0046000	-2.159602186	5.18605E-32	2.02555E-30	RHSIM_Rhsim13G0046000
Stem vs Leaf	RHSIM_RhsimUnG0219100	3.853387052	6.40364E-32	2.49688E-30	RHSIM_RhsimUnG0219100
Stem vs Leaf	RHSIM_Rhsim12G0069800	5.984813031	6.92871E-32	2.69705E-30	RHSIM_Rhsim12G0069800
Stem vs Leaf	RHSIM_RhsimUnG0074100	-3.478768637	8.26226E-32	3.21072E-30	RHSIM_RhsimUnG0074100
Stem vs Leaf	RHSIM_Rhsim03G0148300	-2.818292771	9.13486E-32	3.54383E-30	RHSIM_Rhsim03G0148300
Stem vs Leaf	RHSIM_RhsimUnG0217500	2.00901531	1.26057E-31	4.88213E-30	RHSIM_RhsimUnG0217500
Stem vs Leaf	novel.3885	-12.90828204	1.42166E-31	5.49676E-30	--
Stem vs Leaf	RHSIM_Rhsim04G0087000	-3.387421905	1.60013E-31	6.17643E-30	RHSIM_Rhsim04G0087000
Stem vs Leaf	RHSIM_Rhsim07G0054800	-3.255557137	1.60429E-31	6.18214E-30	RHSIM_Rhsim07G0054800
Stem vs Leaf	RHSIM_Rhsim04G0134200	-1.89497063	1.66237E-31	6.39527E-30	RHSIM_Rhsim04G0134200
Stem vs Leaf	RHSIM_Rhsim06G0171800	-4.519290443	1.73189E-31	6.65161E-30	RHSIM_Rhsim06G0171800
Stem vs Leaf	RHSIM_Rhsim06G0115400	-4.977668496	2.10954E-31	8.08857E-30	RHSIM_Rhsim06G0115400
Stem vs Leaf	RHSIM_Rhsim05G0202600	-2.70680989	2.53678E-31	9.71055E-30	RHSIM_Rhsim05G0202600
Stem vs Leaf	RHSIM_Rhsim07G0118000	7.207057731	3.27146E-31	1.25021E-29	RHSIM_Rhsim07G0118000
Stem vs Leaf	RHSIM_RhsimPtG0004600	-2.703949431	3.35358E-31	1.27947E-29	RHSIM_RhsimPtG0004600
Stem vs Leaf	RHSIM_Rhsim08G0104800	4.326415552	3.36448E-31	1.28151E-29	RHSIM_Rhsim08G0104800

Comparison	Gene ID	log_2FC	P-value	FDR (padj)	Gene Name
Stem vs Leaf	RHSIM_Rhsim10G0070700	6.01721173	3.80929E-31	1.44854E-29	RHSIM_Rhsim10G0070700
Stem vs Leaf	RHSIM_Rhsim07G0040300	-10.62696223	4.172E-31	1.58385E-29	RHSIM_Rhsim07G0040300
Stem vs Leaf	RHSIM_Rhsim02G0136400	-2.502056521	4.24977E-31	1.61072E-29	RHSIM_Rhsim02G0136400
Stem vs Leaf	RHSIM_Rhsim08G0054100	-2.72947924	4.6482E-31	1.75884E-29	RHSIM_Rhsim08G0054100
Stem vs Leaf	RHSIM_Rhsim02G0105900	-3.689834215	5.48344E-31	2.07148E-29	RHSIM_Rhsim02G0105900
Stem vs Leaf	RHSIM_Rhsim12G0190600	-2.263008708	6.2049E-31	2.34019E-29	RHSIM_Rhsim12G0190600
Stem vs Leaf	RHSIM_RhsimUnG0253500	-8.309749563	6.56706E-31	2.47274E-29	RHSIM_RhsimUnG0253500
Stem vs Leaf	RHSIM_RhsimUnG0007000	-9.098704215	6.67531E-31	2.50939E-29	RHSIM_RhsimUnG0007000
Stem vs Leaf	RHSIM_Rhsim08G0234300	-3.120633337	6.94735E-31	2.60741E-29	RHSIM_Rhsim08G0234300
Stem vs Leaf	RHSIM_Rhsim05G0078400	4.024864538	7.39186E-31	2.76972E-29	RHSIM_Rhsim05G0078400
Stem vs Leaf	RHSIM_Rhsim03G0135400	5.749536356	7.43174E-31	2.78015E-29	RHSIM_Rhsim03G0135400
Stem vs Leaf	RHSIM_Rhsim04G0213800	-2.451564668	7.96834E-31	2.97605E-29	RHSIM_Rhsim04G0213800
Stem vs Leaf	RHSIM_Rhsim08G0006200	-2.682489637	8.39236E-31	3.12934E-29	RHSIM_Rhsim08G0006200
Stem vs Leaf	RHSIM_Rhsim13G0011100	-2.491723045	8.83188E-31	3.28791E-29	RHSIM_Rhsim13G0011100
Stem vs Leaf	RHSIM_Rhsim07G0005300	5.162806583	9.1083E-31	3.38535E-29	RHSIM_Rhsim07G0005300
Stem vs Leaf	novel.4077	-6.80320112	9.48527E-31	3.51978E-29	--
Stem vs Leaf	RHSIM_Rhsim05G0042000	-2.627276444	1.011E-30	3.74559E-29	RHSIM_Rhsim05G0042000
Stem vs Leaf	RHSIM_Rhsim04G0139800	-2.688356657	1.08171E-30	4.00111E-29	RHSIM_Rhsim04G0139800
Stem vs Leaf	RHSIM_Rhsim05G0194900	-3.576815176	1.11754E-30	4.127E-29	RHSIM_Rhsim05G0194900
Stem vs Leaf	RHSIM_Rhsim05G0161900	2.584173331	1.14787E-30	4.23225E-29	RHSIM_Rhsim05G0161900
Stem vs Leaf	RHSIM_Rhsim06G0198100	-3.301605483	1.17929E-30	4.34115E-29	RHSIM_Rhsim06G0198100
Stem vs Leaf	RHSIM_Rhsim02G0052500	2.900780246	1.20029E-30	4.41141E-29	RHSIM_Rhsim02G0052500
Stem vs Leaf	RHSIM_Rhsim03G0236600	-3.028289582	1.23622E-30	4.53622E-29	RHSIM_Rhsim03G0236600
Stem vs Leaf	RHSIM_Rhsim03G0045800	-7.558711848	1.57145E-30	5.75714E-29	RHSIM_Rhsim03G0045800
Stem vs Leaf	RHSIM_Rhsim06G0077300	-2.326307442	1.6817E-30	6.15127E-29	RHSIM_Rhsim06G0077300

Comparison	Gene ID	log_2FC	P-value	FDR (padj)	Gene Name
Stem vs Leaf	RHSIM_Rhsim06G0235200	2.396308338	2.05298E-30	7.49743E-29	RHSIM_Rhsim06G0235200
Stem vs Leaf	RHSIM_Rhsim08G0049700	-2.654537047	2.07346E-30	7.56025E-29	RHSIM_Rhsim08G0049700
Stem vs Leaf	novel.3979	-3.26323432	2.08131E-30	7.5732E-29	--
Stem vs Leaf	RHSIM_Rhsim09G0007900	-3.152692297	2.08358E-30	7.5732E-29	RHSIM_Rhsim09G0007900
Stem vs Leaf	RHSIM_Rhsim02G0235500	-1.663859309	2.39096E-30	8.67674E-29	RHSIM_Rhsim02G0235500
Stem vs Leaf	RHSIM_Rhsim06G0192600	-3.828464364	2.65024E-30	9.60254E-29	RHSIM_Rhsim06G0192600
Stem vs Leaf	novel.5960	-3.4315791	2.69289E-30	9.74174E-29	--
Stem vs Leaf	RHSIM_Rhsim04G0193800	-4.885195762	2.78221E-30	1.00491E-28	RHSIM_Rhsim04G0193800
Stem vs Leaf	RHSIM_Rhsim10G0065100	-2.600182422	2.97309E-30	1.07217E-28	RHSIM_Rhsim10G0065100
Stem vs Leaf	RHSIM_Rhsim05G0148600	-3.930194282	3.06222E-30	1.10259E-28	RHSIM_Rhsim05G0148600
Stem vs Leaf	novel.3171	5.697517544	3.08116E-30	1.10768E-28	--
Stem vs Leaf	RHSIM_Rhsim09G0188800	-1.729557069	3.11622E-30	1.11854E-28	RHSIM_Rhsim09G0188800
Stem vs Leaf	RHSIM_Rhsim11G0025700	13.81506847	4.11362E-30	1.47425E-28	RHSIM_Rhsim11G0025700
Stem vs Leaf	RHSIM_Rhsim06G0137600	-3.066185619	4.31959E-30	1.54566E-28	RHSIM_Rhsim06G0137600
Stem vs Leaf	RHSIM_Rhsim02G0186800	2.796984349	4.48566E-30	1.6026E-28	RHSIM_Rhsim02G0186800
Stem vs Leaf	novel.5378	-1.402869	5.0587E-30	1.80453E-28	--
Stem vs Leaf	RHSIM_Rhsim13G0177900	-2.901630734	5.09809E-30	1.81577E-28	RHSIM_Rhsim13G0177900
Stem vs Leaf	RHSIM_Rhsim12G0155700	-4.007819177	5.24079E-30	1.86372E-28	RHSIM_Rhsim12G0155700
Stem vs Leaf	RHSIM_Rhsim07G0251400	-3.821517064	5.3461E-30	1.89824E-28	RHSIM_Rhsim07G0251400
Stem vs Leaf	RHSIM_Rhsim08G0067400	-3.340782577	5.51714E-30	1.95595E-28	RHSIM_Rhsim08G0067400
Stem vs Leaf	RHSIM_Rhsim12G0070300	-6.486998708	5.53315E-30	1.95862E-28	RHSIM_Rhsim12G0070300
Stem vs Leaf	RHSIM_Rhsim04G0213100	-5.149274635	5.82679E-30	2.05939E-28	RHSIM_Rhsim04G0213100
Stem vs Leaf	RHSIM_Rhsim04G0231800	4.041529532	6.11155E-30	2.15673E-28	RHSIM_Rhsim04G0231800
Stem vs Leaf	novel.4165	5.646738889	7.67298E-30	2.70361E-28	--
Stem vs Leaf	RHSIM_Rhsim07G0052300	-2.909571554	8.19225E-30	2.88217E-28	RHSIM_Rhsim07G0052300

Comparison	Gene ID	log_2FC	P-value	FDR (padj)	Gene Name
Stem vs Leaf	RHSIM_Rhsim03G0201600	-4.014841994	8.28609E-30	2.91074E-28	RHSIM_Rhsim03G0201600
Stem vs Leaf	novel.2048	6.629422783	8.52826E-30	2.99125E-28	--
Stem vs Leaf	RHSIM_Rhsim11G0076800	5.719783831	8.87916E-30	3.10959E-28	RHSIM_Rhsim11G0076800
Stem vs Leaf	novel.1034	4.349468445	9.82959E-30	3.43723E-28	--
Stem vs Leaf	RHSIM_Rhsim08G0000900	-1.879626126	1.0415E-29	3.63642E-28	RHSIM_Rhsim08G0000900
Stem vs Leaf	RHSIM_Rhsim03G0210700	-2.229763703	1.41122E-29	4.91983E-28	RHSIM_Rhsim03G0210700
Stem vs Leaf	RHSIM_Rhsim05G0199400	-1.718670567	1.50113E-29	5.22539E-28	RHSIM_Rhsim05G0199400
Stem vs Leaf	novel.5148	3.858132788	1.61593E-29	5.61652E-28	--
Stem vs Leaf	RHSIM_Rhsim01G0262500	4.847584034	1.65111E-29	5.73015E-28	RHSIM_Rhsim01G0262500
Stem vs Leaf	RHSIM_Rhsim03G0045300	-2.438339118	1.89885E-29	6.58E-28	RHSIM_Rhsim03G0045300
Stem vs Leaf	RHSIM_Rhsim03G0051000	8.854178893	2.17297E-29	7.5186E-28	RHSIM_Rhsim03G0051000
Stem vs Leaf	RHSIM_Rhsim10G0053400	-8.571385824	2.17989E-29	7.53E-28	RHSIM_Rhsim10G0053400
Stem vs Leaf	RHSIM_Rhsim04G0230600	-6.290098225	2.1828E-29	7.53E-28	RHSIM_Rhsim04G0230600
Stem vs Leaf	RHSIM_Rhsim02G0113900	-6.511246347	2.36357E-29	8.14141E-28	RHSIM_Rhsim02G0113900
Stem vs Leaf	RHSIM_Rhsim03G0040300	-2.942758983	2.39581E-29	8.24016E-28	RHSIM_Rhsim03G0040300
Stem vs Leaf	RHSIM_Rhsim03G0066700	-2.413740066	2.41222E-29	8.28422E-28	RHSIM_Rhsim03G0066700
Stem vs Leaf	RHSIM_Rhsim03G0225500	-2.784769333	2.47225E-29	8.47775E-28	RHSIM_Rhsim03G0225500
Stem vs Leaf	RHSIM_Rhsim04G0195000	-7.490554759	2.51229E-29	8.60226E-28	RHSIM_Rhsim04G0195000
Stem vs Leaf	RHSIM_Rhsim08G0200300	3.58055701	2.76814E-29	9.46425E-28	RHSIM_Rhsim08G0200300
Stem vs Leaf	RHSIM_Rhsim03G0135900	-1.600762335	2.78767E-29	9.51691E-28	RHSIM_Rhsim03G0135900
Stem vs Leaf	RHSIM_Rhsim13G0157300	4.049691048	2.86428E-29	9.76397E-28	RHSIM_Rhsim13G0157300
Stem vs Leaf	RHSIM_Rhsim10G0167100	-3.697239871	3.05761E-29	1.04076E-27	RHSIM_Rhsim10G0167100
Stem vs Leaf	RHSIM_Rhsim09G0196800	-4.567054913	3.31168E-29	1.12558E-27	RHSIM_Rhsim09G0196800
Stem vs Leaf	RHSIM_Rhsim02G0008500	1.480032056	3.69177E-29	1.25292E-27	RHSIM_Rhsim02G0008500
Stem vs Leaf	RHSIM_Rhsim09G0067900	-1.255621915	4.41517E-29	1.49622E-27	RHSIM_Rhsim09G0067900

Comparison	Gene ID	log_2FC	P-value	FDR (padj)	Gene Name
Stem vs Leaf	RHSIM_Rhsim11G0096000	7.717010396	4.44378E-29	1.50371E-27	RHSIM_Rhsim11G0096000
Stem vs Leaf	RHSIM_Rhsim05G0094200	-2.442677509	4.7172E-29	1.59389E-27	RHSIM_Rhsim05G0094200
Stem vs Leaf	novel.2784	13.34010134	4.79434E-29	1.61758E-27	--
Stem vs Leaf	RHSIM_Rhsim11G0188100	3.997653368	4.83584E-29	1.6292E-27	RHSIM_Rhsim11G0188100
Stem vs Leaf	RHSIM_Rhsim05G0113400	-1.434761214	4.86158E-29	1.63548E-27	RHSIM_Rhsim05G0113400
Stem vs Leaf	novel.4372	-2.424848816	4.9342E-29	1.65749E-27	--
Stem vs Leaf	RHSIM_Rhsim08G0096200	-1.980472581	5.49443E-29	1.84299E-27	RHSIM_Rhsim08G0096200
Stem vs Leaf	RHSIM_Rhsim11G0056000	-4.291419256	5.56198E-29	1.86294E-27	RHSIM_Rhsim11G0056000
Stem vs Leaf	RHSIM_Rhsim04G0230200	-5.378478986	6.00113E-29	2.00711E-27	RHSIM_Rhsim04G0230200
Stem vs Leaf	RHSIM_Rhsim09G0143800	-7.089016626	7.47045E-29	2.49491E-27	RHSIM_Rhsim09G0143800
Stem vs Leaf	RHSIM_Rhsim13G0072000	-1.461141558	7.53138E-29	2.51049E-27	RHSIM_Rhsim13G0072000
Stem vs Leaf	RHSIM_Rhsim11G0052900	-2.994085434	7.53888E-29	2.51049E-27	RHSIM_Rhsim11G0052900
Stem vs Leaf	RHSIM_Rhsim10G0137000	4.028154775	8.00113E-29	2.66058E-27	RHSIM_Rhsim10G0137000
Stem vs Leaf	RHSIM_Rhsim12G0123000	-9.707856579	8.86232E-29	2.9427E-27	RHSIM_Rhsim12G0123000
Stem vs Leaf	RHSIM_Rhsim13G0123200	4.150775466	8.89169E-29	2.9482E-27	RHSIM_Rhsim13G0123200
Stem vs Leaf	RHSIM_Rhsim05G0230200	-7.459221347	9.59997E-29	3.17847E-27	RHSIM_Rhsim05G0230200
Stem vs Leaf	RHSIM_Rhsim06G0029800	-6.273686231	9.77143E-29	3.2306E-27	RHSIM_Rhsim06G0029800
Stem vs Leaf	RHSIM_Rhsim04G0173400	-4.895344266	1.02076E-28	3.36997E-27	RHSIM_Rhsim04G0173400
Stem vs Leaf	RHSIM_Rhsim11G0038100	-1.808393652	1.05094E-28	3.46465E-27	RHSIM_Rhsim11G0038100
Stem vs Leaf	RHSIM_Rhsim03G0256500	-2.679491453	1.2234E-28	4.02255E-27	RHSIM_Rhsim03G0256500
Stem vs Leaf	RHSIM_Rhsim10G0195800	2.213073226	1.22366E-28	4.02255E-27	RHSIM_Rhsim10G0195800
Stem vs Leaf	RHSIM_Rhsim05G0004900	-11.64802014	1.23962E-28	4.06922E-27	RHSIM_Rhsim05G0004900
Stem vs Leaf	RHSIM_Rhsim06G0214800	-3.138560319	1.32441E-28	4.34134E-27	RHSIM_Rhsim06G0214800
Stem vs Leaf	RHSIM_Rhsim02G0135000	-2.109519223	1.3467E-28	4.40815E-27	RHSIM_Rhsim02G0135000
Stem vs Leaf	RHSIM_Rhsim03G0171900	-1.575900868	1.43155E-28	4.67925E-27	RHSIM_Rhsim03G0171900

Comparison	Gene ID	log_2FC	P-value	FDR (padj)	Gene Name
Stem vs Leaf	novel.268	-3.05391307	1.57022E-28	5.12524E-27	--
Stem vs Leaf	RHSIM_Rhsim13G0009300	2.331617462	1.70158E-28	5.54614E-27	RHSIM_Rhsim13G0009300
Stem vs Leaf	RHSIM_Rhsim07G0118600	-1.454826279	1.7913E-28	5.83032E-27	RHSIM_Rhsim07G0118600
Stem vs Leaf	RHSIM_Rhsim08G0070300	-2.583484829	1.88229E-28	6.11783E-27	RHSIM_Rhsim08G0070300
Stem vs Leaf	RHSIM_Rhsim11G0140600	-9.574183642	1.90367E-28	6.17861E-27	RHSIM_Rhsim11G0140600
Stem vs Leaf	RHSIM_Rhsim13G0035600	-1.530268103	1.94036E-28	6.28885E-27	RHSIM_Rhsim13G0035600
Stem vs Leaf	RHSIM_Rhsim02G0022200	-7.266350284	1.98631E-28	6.42872E-27	RHSIM_Rhsim02G0022200
Stem vs Leaf	RHSIM_Rhsim04G0216600	-2.563091712	2.0304E-28	6.56221E-27	RHSIM_Rhsim04G0216600
Stem vs Leaf	RHSIM_Rhsim01G0190200	-1.070968347	2.23525E-28	7.21417E-27	RHSIM_Rhsim01G0190200
Stem vs Leaf	RHSIM_Rhsim09G0212900	-3.689501363	2.37563E-28	7.65651E-27	RHSIM_Rhsim09G0212900
Stem vs Leaf	novel.4118	-4.900189176	2.43909E-28	7.85005E-27	--
Stem vs Leaf	RHSIM_Rhsim09G0214000	-2.038564492	2.53167E-28	8.13666E-27	RHSIM_Rhsim09G0214000
Stem vs Leaf	RHSIM_Rhsim05G0185900	3.075475594	3.29414E-28	1.05724E-26	RHSIM_Rhsim05G0185900
Stem vs Leaf	RHSIM_RhsimUnG0234700	-4.608804101	3.68929E-28	1.18242E-26	RHSIM_RhsimUnG0234700
Stem vs Leaf	RHSIM_Rhsim11G0011700	2.516029913	3.87025E-28	1.23869E-26	RHSIM_Rhsim11G0011700
Stem vs Leaf	RHSIM_Rhsim13G0115100	12.54570354	4.10846E-28	1.31311E-26	RHSIM_Rhsim13G0115100
Stem vs Leaf	novel.5184	-1.576022516	4.30182E-28	1.37301E-26	--
Stem vs Leaf	RHSIM_Rhsim13G0208500	-3.212802851	4.37656E-28	1.39493E-26	RHSIM_Rhsim13G0208500
Stem vs Leaf	RHSIM_RhsimUnG0006000	-3.775765334	4.5E-28	1.43229E-26	RHSIM_RhsimUnG0006000
Stem vs Leaf	RHSIM_Rhsim04G0081300	-3.959816257	4.57551E-28	1.45432E-26	RHSIM_Rhsim04G0081300
Stem vs Leaf	RHSIM_Rhsim10G0176300	7.237139949	4.64684E-28	1.47496E-26	RHSIM_Rhsim10G0176300
Stem vs Leaf	RHSIM_Rhsim08G0092300	-2.325295575	4.98508E-28	1.58014E-26	RHSIM_Rhsim08G0092300
Stem vs Leaf	RHSIM_Rhsim04G0248400	-1.866388373	5.02758E-28	1.59142E-26	RHSIM_Rhsim04G0248400
Stem vs Leaf	RHSIM_Rhsim08G0119500	-1.568054408	5.76344E-28	1.82185E-26	RHSIM_Rhsim08G0119500
Stem vs Leaf	RHSIM_Rhsim03G0095400	-13.27694078	5.83804E-28	1.8429E-26	RHSIM_Rhsim03G0095400

Comparison	Gene ID	log_2FC	P-value	FDR (padj)	Gene Name
Stem vs Leaf	RHSIM_Rhsim03G0210800	-3.067170509	6.03848E-28	1.90357E-26	RHSIM_Rhsim03G0210800
Stem vs Leaf	RHSIM_Rhsim11G0135900	-3.079870532	6.29717E-28	1.9824E-26	RHSIM_Rhsim11G0135900
Stem vs Leaf	RHSIM_Rhsim08G0055400	-2.728533711	6.32651E-28	1.98892E-26	RHSIM_Rhsim08G0055400
Stem vs Leaf	RHSIM_Rhsim13G0016900	5.397401518	6.36622E-28	1.99868E-26	RHSIM_Rhsim13G0016900
Stem vs Leaf	RHSIM_Rhsim13G0100100	2.625771444	6.5001E-28	2.03794E-26	RHSIM_Rhsim13G0100100
Stem vs Leaf	RHSIM_Rhsim07G0054900	-4.283775914	6.56659E-28	2.05598E-26	RHSIM_Rhsim07G0054900
Stem vs Leaf	RHSIM_Rhsim07G0004200	-1.736776903	6.73125E-28	2.10468E-26	RHSIM_Rhsim07G0004200
Stem vs Leaf	RHSIM_Rhsim04G0167500	4.443388182	6.7842E-28	2.11836E-26	RHSIM_Rhsim04G0167500
Stem vs Leaf	RHSIM_Rhsim06G0085200	-4.31729016	7.1754E-28	2.23748E-26	RHSIM_Rhsim06G0085200
Stem vs Leaf	RHSIM_Rhsim01G0171300	-1.468380668	7.2868E-28	2.26915E-26	RHSIM_Rhsim01G0171300
Stem vs Leaf	RHSIM_Rhsim05G0075500	-2.787295958	7.92804E-28	2.4655E-26	RHSIM_Rhsim05G0075500
Stem vs Leaf	RHSIM_Rhsim03G0158600	-8.416006374	8.27747E-28	2.5707E-26	RHSIM_Rhsim03G0158600
Stem vs Leaf	novel.234	-6.492621486	8.53677E-28	2.64766E-26	--
Stem vs Leaf	RHSIM_Rhsim12G0166700	-5.919803282	8.71902E-28	2.70055E-26	RHSIM_Rhsim12G0166700
Stem vs Leaf	novel.3214	-1.696969239	9.08028E-28	2.80867E-26	--
Stem vs Leaf	RHSIM_Rhsim13G0105100	5.105076471	9.9344E-28	3.06875E-26	RHSIM_Rhsim13G0105100
Stem vs Leaf	RHSIM_Rhsim11G0105900	3.534885564	1.03742E-27	3.20031E-26	RHSIM_Rhsim11G0105900
Stem vs Leaf	novel.2562	-9.785754465	1.16701E-27	3.59526E-26	--
Stem vs Leaf	RHSIM_Rhsim06G0033600	-7.625217199	1.23243E-27	3.79173E-26	RHSIM_Rhsim06G0033600
Stem vs Leaf	RHSIM_Rhsim05G0126700	-2.446174306	1.45365E-27	4.4664E-26	RHSIM_Rhsim05G0126700
Stem vs Leaf	RHSIM_Rhsim06G0241300	4.521638253	1.51169E-27	4.63852E-26	RHSIM_Rhsim06G0241300
Stem vs Leaf	RHSIM_Rhsim01G0204900	-2.738673168	1.59537E-27	4.88878E-26	RHSIM_Rhsim01G0204900
Stem vs Leaf	RHSIM_Rhsim08G0097300	3.547889871	1.81494E-27	5.55423E-26	RHSIM_Rhsim08G0097300
Stem vs Leaf	RHSIM_Rhsim06G0027400	-2.123583415	1.87782E-27	5.73906E-26	RHSIM_Rhsim06G0027400
Stem vs Leaf	RHSIM_Rhsim02G0187100	-2.640933145	1.97897E-27	6.04019E-26	RHSIM_Rhsim02G0187100

Comparison	Gene ID	log_2FC	P-value	FDR (padj)	Gene Name
Stem vs Leaf	RHSIM_Rhsim04G0102500	-3.4720042	2.02196E-27	6.16322E-26	RHSIM_Rhsim04G0102500
Stem vs Leaf	RHSIM_Rhsim04G0016400	1.349053022	2.1134E-27	6.43343E-26	RHSIM_Rhsim04G0016400
Stem vs Leaf	RHSIM_RhsimUnG0156300	-3.406995332	2.12316E-27	6.45464E-26	RHSIM_RhsimUnG0156300
Stem vs Leaf	RHSIM_Rhsim07G0174300	-4.660193858	2.23398E-27	6.78258E-26	RHSIM_Rhsim07G0174300
Stem vs Leaf	novel.135	-3.066311561	2.62354E-27	7.95485E-26	--
Stem vs Leaf	novel.1577	3.197133248	2.79357E-27	8.45926E-26	--
Stem vs Leaf	RHSIM_Rhsim02G0027600	-2.879190814	2.92018E-27	8.83106E-26	RHSIM_Rhsim02G0027600
Stem vs Leaf	RHSIM_RhsimUnG0043200	-5.377467622	2.96798E-27	8.96384E-26	RHSIM_RhsimUnG0043200
Stem vs Leaf	RHSIM_Rhsim02G0240600	4.059500457	2.98326E-27	8.99818E-26	RHSIM_Rhsim02G0240600
Stem vs Leaf	RHSIM_Rhsim12G0201500	1.134546433	3.26624E-27	9.83886E-26	RHSIM_Rhsim12G0201500
Stem vs Leaf	RHSIM_Rhsim10G0102900	-2.824410022	3.37775E-27	1.01615E-25	RHSIM_Rhsim10G0102900
Stem vs Leaf	RHSIM_Rhsim08G0180500	-7.055480101	3.39831E-27	1.021E-25	RHSIM_Rhsim08G0180500
Stem vs Leaf	RHSIM_Rhsim10G0080000	-2.725478923	3.4649E-27	1.03965E-25	RHSIM_Rhsim10G0080000
Stem vs Leaf	novel.1069	13.0253997	3.54592E-27	1.06258E-25	--
Stem vs Leaf	RHSIM_Rhsim03G0254300	-2.23694991	3.56201E-27	1.06601E-25	RHSIM_Rhsim03G0254300
Stem vs Leaf	RHSIM_Rhsim08G0003900	-13.40591937	3.90077E-27	1.16588E-25	RHSIM_Rhsim08G0003900
Stem vs Leaf	RHSIM_Rhsim05G0138700	-1.822277716	3.97529E-27	1.18662E-25	RHSIM_Rhsim05G0138700
Stem vs Leaf	RHSIM_Rhsim12G0163100	3.81314519	4.18498E-27	1.24759E-25	RHSIM_Rhsim12G0163100
Stem vs Leaf	novel.3884	-2.183579755	4.38827E-27	1.3065E-25	--
Stem vs Leaf	RHSIM_Rhsim01G0072900	-1.819358388	4.66168E-27	1.38611E-25	RHSIM_Rhsim01G0072900
Stem vs Leaf	RHSIM_Rhsim08G0025300	-2.255037995	5.32607E-27	1.58162E-25	RHSIM_Rhsim08G0025300
Stem vs Leaf	RHSIM_Rhsim06G0184200	4.186515712	5.51489E-27	1.63459E-25	RHSIM_Rhsim06G0184200
Stem vs Leaf	RHSIM_Rhsim03G0223500	-1.500647163	5.51863E-27	1.63459E-25	RHSIM_Rhsim03G0223500
Stem vs Leaf	novel.5875	7.434078275	5.80528E-27	1.7166E-25	--
Stem vs Leaf	RHSIM_Rhsim06G0114600	-3.926449005	5.81041E-27	1.7166E-25	RHSIM_Rhsim06G0114600

Comparison	Gene ID	log_2FC	P-value	FDR (padj)	Gene Name
Stem vs Leaf	RHSIM_Rhsim13G0127200	-3.163928937	5.88773E-27	1.73722E-25	RHSIM_Rhsim13G0127200
Stem vs Leaf	RHSIM_Rhsim06G0121900	-2.992955327	6.11238E-27	1.8012E-25	RHSIM_Rhsim06G0121900
Stem vs Leaf	RHSIM_Rhsim13G0181300	-4.566754348	6.16458E-27	1.81426E-25	RHSIM_Rhsim13G0181300
Stem vs Leaf	RHSIM_Rhsim06G0011300	-1.571275103	6.23499E-27	1.83264E-25	RHSIM_Rhsim06G0011300
Stem vs Leaf	RHSIM_Rhsim12G0046500	-2.384461709	6.27804E-27	1.84295E-25	RHSIM_Rhsim12G0046500
Stem vs Leaf	RHSIM_Rhsim07G0205000	-4.126143098	6.30899E-27	1.84968E-25	RHSIM_Rhsim07G0205000
Stem vs Leaf	RHSIM_RhsimUnG0217800	-1.530787118	6.37626E-27	1.86702E-25	RHSIM_RhsimUnG0217800
Stem vs Leaf	RHSIM_Rhsim07G0226000	6.90442084	6.56445E-27	1.91969E-25	RHSIM_Rhsim07G0226000
Stem vs Leaf	RHSIM_Rhsim07G0007900	-2.613525476	6.92845E-27	2.02356E-25	RHSIM_Rhsim07G0007900
Stem vs Leaf	RHSIM_Rhsim06G0098000	4.455451903	7.35044E-27	2.1441E-25	RHSIM_Rhsim06G0098000
Stem vs Leaf	RHSIM_Rhsim08G0223600	-2.695796283	7.37609E-27	2.14886E-25	RHSIM_Rhsim08G0223600
Stem vs Leaf	RHSIM_Rhsim01G0180300	-3.154540342	7.97901E-27	2.32157E-25	RHSIM_Rhsim01G0180300
Stem vs Leaf	RHSIM_Rhsim12G0070400	-5.881435051	8.07977E-27	2.34792E-25	RHSIM_Rhsim12G0070400
Stem vs Leaf	novel.3006	-1.385878918	8.11139E-27	2.35414E-25	--
Stem vs Leaf	RHSIM_Rhsim08G0144900	-4.48810557	8.18937E-27	2.37378E-25	RHSIM_Rhsim08G0144900
Stem vs Leaf	RHSIM_Rhsim13G0098500	-3.770118862	8.3766E-27	2.425E-25	RHSIM_Rhsim13G0098500
Stem vs Leaf	RHSIM_Rhsim10G0117200	-2.787734681	8.59038E-27	2.48377E-25	RHSIM_Rhsim10G0117200
Stem vs Leaf	RHSIM_Rhsim04G0222700	-2.721373686	8.61128E-27	2.4867E-25	RHSIM_Rhsim04G0222700
Stem vs Leaf	RHSIM_Rhsim12G0146800	-3.159892393	9.30053E-27	2.68237E-25	RHSIM_Rhsim12G0146800
Stem vs Leaf	RHSIM_Rhsim12G0123300	-2.318439818	1.02755E-26	2.95985E-25	RHSIM_Rhsim12G0123300
Stem vs Leaf	RHSIM_Rhsim05G0233000	-2.354694202	1.05948E-26	3.04802E-25	RHSIM_Rhsim05G0233000
Stem vs Leaf	RHSIM_Rhsim10G0051100	-12.82357467	1.09886E-26	3.15738E-25	RHSIM_Rhsim10G0051100
Stem vs Leaf	RHSIM_Rhsim02G0234600	-6.019710528	1.18471E-26	3.39981E-25	RHSIM_Rhsim02G0234600
Stem vs Leaf	RHSIM_Rhsim11G0025100	-3.13148625	1.22031E-26	3.49762E-25	RHSIM_Rhsim11G0025100
Stem vs Leaf	RHSIM_Rhsim04G0164000	4.819379421	1.24443E-26	3.56232E-25	RHSIM_Rhsim04G0164000

Comparison	Gene ID	log_2FC	P-value	FDR (padj)	Gene Name
Stem vs Leaf	RHSIM_Rhsim11G0096200	-3.840125208	1.25088E-26	3.57635E-25	RHSIM_Rhsim11G0096200
Stem vs Leaf	RHSIM_Rhsim12G0182900	-2.709759325	1.27025E-26	3.62721E-25	RHSIM_Rhsim12G0182900
Stem vs Leaf	RHSIM_Rhsim11G0122000	-3.054594152	1.57527E-26	4.49263E-25	RHSIM_Rhsim11G0122000
Stem vs Leaf	novel.1972	-3.932908511	1.71398E-26	4.88221E-25	--
Stem vs Leaf	RHSIM_Rhsim06G0049400	4.928669705	1.77412E-26	5.04727E-25	RHSIM_Rhsim06G0049400
Stem vs Leaf	RHSIM_Rhsim08G0005400	-2.244216177	1.87113E-26	5.31669E-25	RHSIM_Rhsim08G0005400
Stem vs Leaf	RHSIM_Rhsim01G0138100	13.31579865	1.95138E-26	5.53788E-25	RHSIM_Rhsim01G0138100
Stem vs Leaf	novel.2903	-7.299882095	1.97566E-26	5.5999E-25	--
Stem vs Leaf	RHSIM_Rhsim05G0167700	-4.25111998	2.18501E-26	6.18568E-25	RHSIM_Rhsim05G0167700
Stem vs Leaf	novel.1469	-2.091490054	2.19812E-26	6.21514E-25	--
Stem vs Leaf	RHSIM_Rhsim09G0138900	-4.383140066	2.24938E-26	6.35229E-25	RHSIM_Rhsim09G0138900
Stem vs Leaf	RHSIM_Rhsim12G0161600	5.433700188	2.2966E-26	6.4777E-25	RHSIM_Rhsim12G0161600
Stem vs Leaf	RHSIM_Rhsim08G0190500	-2.172431701	2.49412E-26	7.02623E-25	RHSIM_Rhsim08G0190500
Stem vs Leaf	RHSIM_Rhsim07G0125400	4.725125417	2.58254E-26	7.26642E-25	RHSIM_Rhsim07G0125400
Stem vs Leaf	RHSIM_Rhsim13G0107300	-1.944831134	2.74947E-26	7.72668E-25	RHSIM_Rhsim13G0107300
Stem vs Leaf	RHSIM_Rhsim05G0172300	-1.712363793	2.7626E-26	7.75411E-25	RHSIM_Rhsim05G0172300
Stem vs Leaf	RHSIM_Rhsim13G0026900	5.282920199	3.19741E-26	8.96363E-25	RHSIM_Rhsim13G0026900
Stem vs Leaf	RHSIM_Rhsim06G0059900	-5.762574356	3.32776E-26	9.31772E-25	RHSIM_Rhsim06G0059900
Stem vs Leaf	novel.823	6.740562662	4.49234E-26	1.25633E-24	--
Stem vs Leaf	RHSIM_Rhsim03G0263700	3.557555194	4.63285E-26	1.29405E-24	RHSIM_Rhsim03G0263700
Stem vs Leaf	RHSIM_Rhsim03G0063600	4.278231955	5.35009E-26	1.49258E-24	RHSIM_Rhsim03G0063600
Stem vs Leaf	novel.5379	-1.289851187	5.47252E-26	1.5249E-24	--
Stem vs Leaf	RHSIM_Rhsim08G0050800	2.945493397	5.66203E-26	1.57579E-24	RHSIM_Rhsim08G0050800
Stem vs Leaf	RHSIM_Rhsim12G0132700	-2.664530991	7.21797E-26	2.0064E-24	RHSIM_Rhsim12G0132700
Stem vs Leaf	RHSIM_Rhsim06G0152700	10.64751659	7.49035E-26	2.07961E-24	RHSIM_Rhsim06G0152700

Comparison	Gene ID	log_2FC	P-value	FDR (padj)	Gene Name
Stem vs Leaf	novel.3652	-2.710086624	8.37595E-26	2.32269E-24	--
Stem vs Leaf	RHSIM_Rhsim01G0233600	-4.463620598	8.68981E-26	2.40683E-24	RHSIM_Rhsim01G0233600
Stem vs Leaf	RHSIM_RhsimUnG0241900	-3.966019424	8.863E-26	2.45185E-24	RHSIM_RhsimUnG0241900
Stem vs Leaf	RHSIM_Rhsim08G0250300	-1.911027671	8.97449E-26	2.47971E-24	RHSIM_Rhsim08G0250300
Stem vs Leaf	RHSIM_Rhsim04G0007400	-3.643157705	9.73509E-26	2.68665E-24	RHSIM_Rhsim04G0007400
Stem vs Leaf	RHSIM_RhsimUnG0194700	-2.309872214	9.91868E-26	2.73404E-24	RHSIM_RhsimUnG0194700
Stem vs Leaf	RHSIM_Rhsim08G0032100	-1.330425796	1.03394E-25	2.84661E-24	RHSIM_Rhsim08G0032100
Stem vs Leaf	RHSIM_Rhsim03G0078700	2.355930738	1.10241E-25	3.03149E-24	RHSIM_Rhsim03G0078700
Stem vs Leaf	RHSIM_Rhsim08G0043600	-2.074480855	1.15196E-25	3.16396E-24	RHSIM_Rhsim08G0043600
Stem vs Leaf	RHSIM_RhsimUnG0204600	4.043493098	1.2355E-25	3.38938E-24	RHSIM_RhsimUnG0204600
Stem vs Leaf	RHSIM_Rhsim13G0140700	1.702629702	1.25068E-25	3.425E-24	RHSIM_Rhsim13G0140700
Stem vs Leaf	RHSIM_Rhsim03G0116500	-6.896351564	1.25145E-25	3.425E-24	RHSIM_Rhsim03G0116500
Stem vs Leaf	novel.4723	-3.750372571	1.29958E-25	3.55249E-24	--
Stem vs Leaf	RHSIM_Rhsim13G0094000	2.103344688	1.30791E-25	3.57104E-24	RHSIM_Rhsim13G0094000
Stem vs Leaf	RHSIM_Rhsim01G0274400	-2.12274418	1.33421E-25	3.63852E-24	RHSIM_Rhsim01G0274400
Stem vs Leaf	RHSIM_Rhsim11G0160000	2.744484931	1.41866E-25	3.86426E-24	RHSIM_Rhsim11G0160000
Stem vs Leaf	RHSIM_Rhsim05G0104400	-1.254907525	1.59715E-25	4.34531E-24	RHSIM_Rhsim05G0104400
Stem vs Leaf	RHSIM_Rhsim03G0130400	-2.879695941	1.6632E-25	4.51967E-24	RHSIM_Rhsim03G0130400
Stem vs Leaf	RHSIM_Rhsim06G0030000	3.963837786	1.86313E-25	5.057E-24	RHSIM_Rhsim06G0030000
Stem vs Leaf	RHSIM_Rhsim07G0201400	2.983372334	1.88311E-25	5.10523E-24	RHSIM_Rhsim07G0201400
Stem vs Leaf	RHSIM_Rhsim10G0072400	-4.585255465	1.9606E-25	5.30906E-24	RHSIM_Rhsim10G0072400
Stem vs Leaf	RHSIM_Rhsim02G0032400	-2.624458112	2.07445E-25	5.61076E-24	RHSIM_Rhsim02G0032400
Stem vs Leaf	RHSIM_Rhsim09G0130900	3.399426884	2.51892E-25	6.80493E-24	RHSIM_Rhsim09G0130900
Stem vs Leaf	RHSIM_RhsimUnG0027200	-2.176324689	2.54523E-25	6.86794E-24	RHSIM_RhsimUnG0027200
Stem vs Leaf	RHSIM_Rhsim10G0134900	-3.714830605	2.6085E-25	7.03045E-24	RHSIM_Rhsim10G0134900

Comparison	Gene ID	log_2FC	P-value	FDR (padj)	Gene Name
Stem vs Leaf	RHSIM_Rhsim01G0214800	2.891053567	2.64994E-25	7.1338E-24	RHSIM_Rhsim01G0214800
Stem vs Leaf	RHSIM_Rhsim11G0127300	-2.883986966	3.00782E-25	8.08777E-24	RHSIM_Rhsim11G0127300
Stem vs Leaf	RHSIM_Rhsim08G0222400	-1.951768946	3.12211E-25	8.3853E-24	RHSIM_Rhsim08G0222400
Stem vs Leaf	RHSIM_Rhsim07G0170000	-2.706261216	3.16014E-25	8.47755E-24	RHSIM_Rhsim07G0170000
Stem vs Leaf	RHSIM_Rhsim01G0196900	-2.61763612	3.21049E-25	8.60261E-24	RHSIM_Rhsim01G0196900
Stem vs Leaf	novel.5423	-1.944891107	3.36538E-25	9.00719E-24	--
Stem vs Leaf	RHSIM_Rhsim07G0069700	-4.319348584	3.37993E-25	9.03563E-24	RHSIM_Rhsim07G0069700
Stem vs Leaf	RHSIM_Rhsim12G0056300	5.061385079	3.43248E-25	9.16548E-24	RHSIM_Rhsim12G0056300
Stem vs Leaf	RHSIM_Rhsim07G0072500	7.03894313	3.60802E-25	9.62306E-24	RHSIM_Rhsim07G0072500
Stem vs Leaf	RHSIM_Rhsim10G0054600	-6.722043934	3.65158E-25	9.72797E-24	RHSIM_Rhsim10G0054600
Stem vs Leaf	RHSIM_Rhsim07G0076600	-4.13634836	3.81864E-25	1.01613E-23	RHSIM_Rhsim07G0076600
Stem vs Leaf	RHSIM_Rhsim03G0214900	-4.807599351	4.10943E-25	1.09225E-23	RHSIM_Rhsim03G0214900
Stem vs Leaf	RHSIM_Rhsim13G0061200	-2.160909971	4.54459E-25	1.20651E-23	RHSIM_Rhsim13G0061200
Stem vs Leaf	RHSIM_Rhsim10G0040000	-2.230612725	4.57986E-25	1.21448E-23	RHSIM_Rhsim10G0040000
Stem vs Leaf	RHSIM_Rhsim12G0172700	3.821930673	5.19016E-25	1.37474E-23	RHSIM_Rhsim12G0172700
Stem vs Leaf	RHSIM_Rhsim13G0086700	6.137136468	5.35791E-25	1.41754E-23	RHSIM_Rhsim13G0086700
Stem vs Leaf	RHSIM_Rhsim03G0112100	1.222915009	5.38981E-25	1.42434E-23	RHSIM_Rhsim03G0112100
Stem vs Leaf	RHSIM_Rhsim06G0227300	-2.454411935	5.46063E-25	1.44141E-23	RHSIM_Rhsim06G0227300
Stem vs Leaf	novel.1637	-4.969980436	5.66138E-25	1.49269E-23	--
Stem vs Leaf	RHSIM_Rhsim02G0123500	1.55300916	5.76698E-25	1.51879E-23	RHSIM_Rhsim02G0123500
Stem vs Leaf	RHSIM_Rhsim12G0083500	-1.787352642	5.87048E-25	1.54429E-23	RHSIM_Rhsim12G0083500
Stem vs Leaf	RHSIM_Rhsim04G0144400	-1.800602697	5.87987E-25	1.54499E-23	RHSIM_Rhsim04G0144400
Stem vs Leaf	RHSIM_Rhsim05G0075000	-1.930711431	6.27068E-25	1.6458E-23	RHSIM_Rhsim05G0075000
Stem vs Leaf	RHSIM_Rhsim13G0071600	4.684471035	7.08041E-25	1.85621E-23	RHSIM_Rhsim13G0071600
Stem vs Leaf	RHSIM_Rhsim03G0009200	-2.609875519	7.23518E-25	1.89463E-23	RHSIM_Rhsim03G0009200

Comparison	Gene ID	log_2FC	P-value	FDR (padj)	Gene Name
Stem vs Leaf	RHSIM_Rhsim12G0137100	-2.900998962	7.46567E-25	1.95277E-23	RHSIM_Rhsim12G0137100
Stem vs Leaf	RHSIM_Rhsim07G0175700	-2.222184388	7.51886E-25	1.96445E-23	RHSIM_Rhsim07G0175700
Stem vs Leaf	RHSIM_Rhsim13G0153900	-3.192077465	7.83134E-25	2.04378E-23	RHSIM_Rhsim13G0153900
Stem vs Leaf	novel.5565	-5.501629719	8.36435E-25	2.18041E-23	--
Stem vs Leaf	RHSIM_Rhsim08G0120800	-5.48798463	8.62769E-25	2.24651E-23	RHSIM_Rhsim08G0120800
Stem vs Leaf	RHSIM_Rhsim08G0118700	-1.809459052	8.74282E-25	2.27392E-23	RHSIM_Rhsim08G0118700
Stem vs Leaf	RHSIM_Rhsim05G0030700	-3.044961798	8.8538E-25	2.30019E-23	RHSIM_Rhsim05G0030700
Stem vs Leaf	RHSIM_Rhsim12G0182800	-2.088091743	9.16163E-25	2.37748E-23	RHSIM_Rhsim12G0182800
Stem vs Leaf	RHSIM_Rhsim07G0178600	-1.817933726	9.51798E-25	2.46718E-23	RHSIM_Rhsim07G0178600
Stem vs Leaf	RHSIM_Rhsim07G0224100	-4.998590626	9.61739E-25	2.49015E-23	RHSIM_Rhsim07G0224100
Stem vs Leaf	RHSIM_Rhsim03G0002000	-1.999967177	9.75681E-25	2.52341E-23	RHSIM_Rhsim03G0002000
Stem vs Leaf	RHSIM_Rhsim11G0060100	-2.185195098	1.00691E-24	2.60125E-23	RHSIM_Rhsim11G0060100
Stem vs Leaf	RHSIM_Rhsim12G0003000	-2.315695105	1.05017E-24	2.70998E-23	RHSIM_Rhsim12G0003000
Stem vs Leaf	RHSIM_Rhsim06G0198700	-4.310113318	1.05357E-24	2.71572E-23	RHSIM_Rhsim06G0198700
Stem vs Leaf	RHSIM_Rhsim09G0012000	-5.864281368	1.08476E-24	2.79298E-23	RHSIM_Rhsim09G0012000
Stem vs Leaf	RHSIM_RhsimUnG0085800	5.064482143	1.12759E-24	2.90002E-23	RHSIM_RhsimUnG0085800
Stem vs Leaf	RHSIM_Rhsim06G0155000	5.597404975	1.13251E-24	2.90942E-23	RHSIM_Rhsim06G0155000
Stem vs Leaf	RHSIM_Rhsim02G0243800	-6.792953493	1.16193E-24	2.98169E-23	RHSIM_Rhsim02G0243800
Stem vs Leaf	RHSIM_Rhsim02G0254700	-7.906316016	1.19201E-24	3.05548E-23	RHSIM_Rhsim02G0254700
Stem vs Leaf	RHSIM_Rhsim05G0223200	-8.996098443	1.24882E-24	3.19754E-23	RHSIM_Rhsim05G0223200
Stem vs Leaf	RHSIM_Rhsim05G0011200	-6.620046453	1.25531E-24	3.21057E-23	RHSIM_Rhsim05G0011200
Stem vs Leaf	RHSIM_Rhsim09G0003700	-1.852443988	1.26175E-24	3.22347E-23	RHSIM_Rhsim09G0003700
Stem vs Leaf	RHSIM_Rhsim12G0131400	2.916173964	1.39878E-24	3.5696E-23	RHSIM_Rhsim12G0131400
Stem vs Leaf	RHSIM_Rhsim06G0218300	3.160365759	1.41434E-24	3.60532E-23	RHSIM_Rhsim06G0218300
Stem vs Leaf	RHSIM_Rhsim03G0150700	-2.879084785	1.56433E-24	3.98326E-23	RHSIM_Rhsim03G0150700

Comparison	Gene ID	log_2FC	P-value	FDR (padj)	Gene Name
Stem vs Leaf	RHSIM_Rhsim10G0073300	-4.732298335	1.69631E-24	4.31454E-23	RHSIM_Rhsim10G0073300
Stem vs Leaf	RHSIM_Rhsim01G0221600	-1.203754998	1.75415E-24	4.45673E-23	RHSIM_Rhsim01G0221600
Stem vs Leaf	RHSIM_Rhsim11G0194700	-1.413007083	1.80152E-24	4.57185E-23	RHSIM_Rhsim11G0194700
Stem vs Leaf	RHSIM_Rhsim12G0096200	-1.845494451	1.80342E-24	4.57185E-23	RHSIM_Rhsim12G0096200
Stem vs Leaf	RHSIM_Rhsim06G0103100	-1.765479059	1.83797E-24	4.65431E-23	RHSIM_Rhsim06G0103100
Stem vs Leaf	RHSIM_Rhsim02G0212900	-2.515584714	2.09148E-24	5.29047E-23	RHSIM_Rhsim02G0212900
Stem vs Leaf	RHSIM_Rhsim09G0141500	-1.652185172	2.23956E-24	5.65881E-23	RHSIM_Rhsim09G0141500
Stem vs Leaf	novel.4892	-3.867477467	2.45294E-24	6.1912E-23	--
Stem vs Leaf	RHSIM_Rhsim02G0016100	4.628556862	2.47188E-24	6.23216E-23	RHSIM_Rhsim02G0016100
Stem vs Leaf	RHSIM_Rhsim12G0022100	13.87655462	2.75258E-24	6.93229E-23	RHSIM_Rhsim12G0022100
Stem vs Leaf	RHSIM_Rhsim08G0121500	-3.742660106	2.80758E-24	7.0631E-23	RHSIM_Rhsim08G0121500
Stem vs Leaf	RHSIM_Rhsim12G0162200	-3.890772138	2.94534E-24	7.40156E-23	RHSIM_Rhsim12G0162200
Stem vs Leaf	RHSIM_Rhsim11G0075500	-3.859486384	3.0242E-24	7.59146E-23	RHSIM_Rhsim11G0075500
Stem vs Leaf	RHSIM_Rhsim11G0014500	-2.646747813	3.09627E-24	7.76393E-23	RHSIM_Rhsim11G0014500
Stem vs Leaf	RHSIM_Rhsim10G0165400	-12.29843039	3.21031E-24	8.04113E-23	RHSIM_Rhsim10G0165400
Stem vs Leaf	RHSIM_Rhsim05G0079000	-3.488133469	3.34253E-24	8.36322E-23	RHSIM_Rhsim05G0079000
Stem vs Leaf	RHSIM_Rhsim06G0207500	6.885889451	3.49459E-24	8.73421E-23	RHSIM_Rhsim06G0207500
Stem vs Leaf	RHSIM_Rhsim13G0002400	-4.033114671	3.64312E-24	9.09556E-23	RHSIM_Rhsim13G0002400
Stem vs Leaf	RHSIM_Rhsim12G0090700	-1.335534011	3.69231E-24	9.2084E-23	RHSIM_Rhsim12G0090700
Stem vs Leaf	novel.1961	-1.808153856	3.71971E-24	9.26671E-23	--
Stem vs Leaf	RHSIM_Rhsim09G0129500	-1.979882704	3.95972E-24	9.85397E-23	RHSIM_Rhsim09G0129500
Stem vs Leaf	RHSIM_Rhsim13G0041600	-3.201223873	4.12435E-24	1.02526E-22	RHSIM_Rhsim13G0041600
Stem vs Leaf	RHSIM_Rhsim06G0201500	1.717735463	4.18364E-24	1.03888E-22	RHSIM_Rhsim06G0201500
Stem vs Leaf	RHSIM_Rhsim08G0170400	-1.396699018	4.64522E-24	1.15225E-22	RHSIM_Rhsim08G0170400
Stem vs Leaf	RHSIM_RhsimUnG0092800	-1.630660889	4.73609E-24	1.17353E-22	RHSIM_RhsimUnG0092800

Comparison	Gene ID	log$_2$FC	P-value	FDR (padj)	Gene Name
Stem vs Leaf	novel.3518	9.088310054	5.0668E-24	1.25413E-22	--
Stem vs Leaf	RHSIM_Rhsim06G0183200	-6.775923452	5.16693E-24	1.27754E-22	RHSIM_Rhsim06G0183200
Stem vs Leaf	RHSIM_Rhsim06G0233800	-4.359636195	5.18268E-24	1.28006E-22	RHSIM_Rhsim06G0233800
Stem vs Leaf	RHSIM_Rhsim13G0150200	12.12896903	5.29833E-24	1.30722E-22	RHSIM_Rhsim13G0150200
Stem vs Leaf	RHSIM_Rhsim11G0111000	1.512783053	5.49478E-24	1.35424E-22	RHSIM_Rhsim11G0111000
Stem vs Leaf	RHSIM_Rhsim04G0076200	-4.205538463	5.88252E-24	1.44826E-22	RHSIM_Rhsim04G0076200
Stem vs Leaf	RHSIM_Rhsim13G0047000	-1.887665809	6.1986E-24	1.52445E-22	RHSIM_Rhsim13G0047000
Stem vs Leaf	RHSIM_Rhsim07G0062900	-2.5013356	6.73282E-24	1.65406E-22	RHSIM_Rhsim07G0062900
Stem vs Leaf	novel.1640	-2.643056953	6.86523E-24	1.6848E-22	--
Stem vs Leaf	RHSIM_Rhsim04G0097100	4.346923105	6.98657E-24	1.71275E-22	RHSIM_Rhsim04G0097100
Stem vs Leaf	RHSIM_Rhsim03G0040900	-10.46042197	7.48342E-24	1.8326E-22	RHSIM_Rhsim03G0040900
Stem vs Leaf	RHSIM_Rhsim13G0227300	1.229300571	7.56292E-24	1.85011E-22	RHSIM_Rhsim13G0227300
Stem vs Leaf	RHSIM_Rhsim03G0136100	2.85320438	7.66097E-24	1.8721E-22	RHSIM_Rhsim03G0136100
Stem vs Leaf	RHSIM_Rhsim12G0170700	4.345894133	7.90924E-24	1.93073E-22	RHSIM_Rhsim12G0170700
Stem vs Leaf	RHSIM_Rhsim10G0154600	3.956041288	8.11718E-24	1.97939E-22	RHSIM_Rhsim10G0154600
Stem vs Leaf	novel.3988	-2.045685447	8.15522E-24	1.98656E-22	--
Stem vs Leaf	RHSIM_Rhsim05G0021900	-12.13089001	8.54253E-24	2.07871E-22	RHSIM_Rhsim05G0021900
Stem vs Leaf	RHSIM_Rhsim04G0119100	-2.592134617	8.56875E-24	2.08289E-22	RHSIM_Rhsim04G0119100
Stem vs Leaf	RHSIM_Rhsim13G0232300	-3.417413702	8.63451E-24	2.09667E-22	RHSIM_Rhsim13G0232300
Stem vs Leaf	RHSIM_Rhsim02G0247300	1.62834448	9.4529E-24	2.29298E-22	RHSIM_Rhsim02G0247300
Stem vs Leaf	RHSIM_Rhsim01G0067900	-7.764583122	1.0374E-23	2.51376E-22	RHSIM_Rhsim01G0067900
Stem vs Leaf	RHSIM_Rhsim03G0063700	-2.823805862	1.09259E-23	2.64472E-22	RHSIM_Rhsim03G0063700
Stem vs Leaf	RHSIM_Rhsim13G0156800	-6.373957816	1.10511E-23	2.6722E-22	RHSIM_Rhsim13G0156800
Stem vs Leaf	RHSIM_Rhsim02G0034700	-12.04442223	1.13184E-23	2.73398E-22	RHSIM_Rhsim02G0034700
Stem vs Leaf	novel.963	-1.320970896	1.14947E-23	2.77364E-22	--

Comparison	Gene ID	log_2FC	P-value	FDR (padj)	Gene Name
Stem vs Leaf	RHSIM_RhsimUnG0194300	-1.584966111	1.30537E-23	3.14655E-22	RHSIM_RhsimUnG0194300
Stem vs Leaf	RHSIM_Rhsim02G0199800	-1.75951443	1.31409E-23	3.16424E-22	RHSIM_Rhsim02G0199800
Stem vs Leaf	novel.124	-13.17553026	1.32415E-23	3.18515E-22	--
Stem vs Leaf	RHSIM_RhsimUnG0139600	-2.556913225	1.36432E-23	3.27834E-22	RHSIM_RhsimUnG0139600
Stem vs Leaf	RHSIM_Rhsim08G0062200	-8.799287597	1.42112E-23	3.41128E-22	RHSIM_Rhsim08G0062200
Stem vs Leaf	RHSIM_Rhsim13G0229700	-1.620992684	1.43636E-23	3.44427E-22	RHSIM_Rhsim13G0229700
Stem vs Leaf	RHSIM_Rhsim07G0108400	3.403068526	1.44818E-23	3.469E-22	RHSIM_Rhsim07G0108400
Stem vs Leaf	RHSIM_Rhsim11G0193300	-2.911510555	1.49783E-23	3.58423E-22	RHSIM_Rhsim11G0193300
Stem vs Leaf	RHSIM_Rhsim02G0016500	6.611170128	1.50643E-23	3.60106E-22	RHSIM_Rhsim02G0016500
Stem vs Leaf	RHSIM_RhsimPtG0000800	-4.294518574	1.52124E-23	3.6327E-22	RHSIM_RhsimPtG0000800
Stem vs Leaf	RHSIM_RhsimUnG0069500	-2.122795533	1.56329E-23	3.72925E-22	RHSIM_RhsimUnG0069500
Stem vs Leaf	RHSIM_Rhsim06G0030200	3.63557103	1.57526E-23	3.75391E-22	RHSIM_Rhsim06G0030200
Stem vs Leaf	RHSIM_Rhsim10G0188700	1.861823522	1.63332E-23	3.88699E-22	RHSIM_Rhsim10G0188700
Stem vs Leaf	RHSIM_Rhsim12G0110600	-1.967339392	1.63448E-23	3.88699E-22	RHSIM_Rhsim12G0110600
Stem vs Leaf	RHSIM_Rhsim10G0127700	-1.836044357	1.6992E-23	4.03593E-22	RHSIM_Rhsim10G0127700
Stem vs Leaf	RHSIM_Rhsim05G0229400	7.318715943	1.70061E-23	4.03593E-22	RHSIM_Rhsim05G0229400
Stem vs Leaf	RHSIM_Rhsim03G0018300	-3.247932231	1.7615E-23	4.17613E-22	RHSIM_Rhsim03G0018300
Stem vs Leaf	RHSIM_Rhsim03G0155100	2.082402561	1.77129E-23	4.19503E-22	RHSIM_Rhsim03G0155100
Stem vs Leaf	RHSIM_Rhsim04G0006200	-5.035530955	1.86449E-23	4.41122E-22	RHSIM_Rhsim04G0006200
Stem vs Leaf	RHSIM_Rhsim01G0118700	2.655253353	1.9822E-23	4.6849E-22	RHSIM_Rhsim01G0118700
Stem vs Leaf	RHSIM_Rhsim01G0027300	-1.691687882	2.04415E-23	4.82638E-22	RHSIM_Rhsim01G0027300
Stem vs Leaf	RHSIM_Rhsim07G0110000	6.577745036	2.12763E-23	5.01833E-22	RHSIM_Rhsim07G0110000
Stem vs Leaf	RHSIM_Rhsim13G0184700	-3.885871998	2.17242E-23	5.11873E-22	RHSIM_Rhsim13G0184700
Stem vs Leaf	novel.1310	-12.0124605	2.18094E-23	5.13355E-22	--
Stem vs Leaf	RHSIM_Rhsim07G0058500	-2.51414865	2.21724E-23	5.21368E-22	RHSIM_Rhsim07G0058500

Comparison	Gene ID	log_2FC	P-value	FDR (padj)	Gene Name
Stem vs Leaf	RHSIM_Rhsim11G0164100	-3.942239674	2.33315E-23	5.48063E-22	RHSIM_Rhsim11G0164100
Stem vs Leaf	RHSIM_Rhsim05G0172500	-2.416661631	2.42326E-23	5.68652E-22	RHSIM_Rhsim05G0172500
Stem vs Leaf	RHSIM_Rhsim08G0210000	-2.241838757	2.54425E-23	5.96437E-22	RHSIM_Rhsim08G0210000
Stem vs Leaf	RHSIM_Rhsim03G0225800	-3.268076208	2.66024E-23	6.22994E-22	RHSIM_Rhsim03G0225800
Stem vs Leaf	RHSIM_RhsimUnG0040900	-1.436967269	2.90784E-23	6.80288E-22	RHSIM_RhsimUnG0040900
Stem vs Leaf	RHSIM_Rhsim10G0128500	-3.998408761	2.99496E-23	6.99564E-22	RHSIM_Rhsim10G0128500
Stem vs Leaf	RHSIM_Rhsim12G0014900	-2.439799292	2.99631E-23	6.99564E-22	RHSIM_Rhsim12G0014900
Stem vs Leaf	RHSIM_Rhsim07G0216200	-7.378195452	3.16889E-23	7.39108E-22	RHSIM_Rhsim07G0216200
Stem vs Leaf	RHSIM_Rhsim08G0196800	-2.005804202	3.2165E-23	7.49455E-22	RHSIM_Rhsim08G0196800
Stem vs Leaf	RHSIM_Rhsim05G0082300	-2.602472077	3.3257E-23	7.74116E-22	RHSIM_Rhsim05G0082300
Stem vs Leaf	novel.1638	-4.804039112	3.68863E-23	8.57729E-22	--
Stem vs Leaf	RHSIM_Rhsim06G0111200	-8.112003165	3.72789E-23	8.65983E-22	RHSIM_Rhsim06G0111200
Stem vs Leaf	RHSIM_Rhsim07G0250100	-12.87391115	3.78634E-23	8.78674E-22	RHSIM_Rhsim07G0250100
Stem vs Leaf	novel.4202	3.416350933	3.79827E-23	8.80558E-22	--
Stem vs Leaf	RHSIM_Rhsim07G0137000	-2.634575603	3.98249E-23	9.22338E-22	RHSIM_Rhsim07G0137000
Stem vs Leaf	RHSIM_Rhsim08G0081800	-2.479479414	4.08269E-23	9.44595E-22	RHSIM_Rhsim08G0081800
Stem vs Leaf	RHSIM_RhsimUnG0255000	-1.413710859	4.23911E-23	9.79801E-22	RHSIM_RhsimUnG0255000
Stem vs Leaf	RHSIM_Rhsim11G0092000	11.84644184	4.54167E-23	1.04868E-21	RHSIM_Rhsim11G0092000
Stem vs Leaf	RHSIM_Rhsim03G0159700	-1.609540935	4.78974E-23	1.10485E-21	RHSIM_Rhsim03G0159700
Stem vs Leaf	RHSIM_Rhsim04G0175900	-2.731496815	5.08365E-23	1.17148E-21	RHSIM_Rhsim04G0175900
Stem vs Leaf	RHSIM_Rhsim11G0088500	6.655077621	5.09847E-23	1.17372E-21	RHSIM_Rhsim11G0088500
Stem vs Leaf	RHSIM_Rhsim05G0146200	-3.314273691	5.3928E-23	1.24024E-21	RHSIM_Rhsim05G0146200
Stem vs Leaf	RHSIM_Rhsim02G0230300	-2.167550505	5.66932E-23	1.30253E-21	RHSIM_Rhsim02G0230300
Stem vs Leaf	novel.1311	-11.92355319	5.7011E-23	1.30853E-21	--
Stem vs Leaf	RHSIM_Rhsim06G0142500	-1.873800479	5.72655E-23	1.31306E-21	RHSIM_Rhsim06G0142500

Comparison	Gene ID	log_2FC	P-value	FDR (padj)	Gene Name
Stem vs Leaf	RHSIM_Rhsim11G0111200	1.394728061	5.87747E-23	1.34633E-21	RHSIM_Rhsim11G0111200
Stem vs Leaf	RHSIM_Rhsim08G0046700	-2.19922375	5.91067E-23	1.35259E-21	RHSIM_Rhsim08G0046700
Stem vs Leaf	RHSIM_Rhsim13G0157800	-2.413299869	6.06309E-23	1.38609E-21	RHSIM_Rhsim13G0157800
Stem vs Leaf	novel.3762	-2.546067021	6.42228E-23	1.46675E-21	--
Stem vs Leaf	RHSIM_Rhsim03G0259200	-2.15250457	6.99183E-23	1.59524E-21	RHSIM_Rhsim03G0259200
Stem vs Leaf	RHSIM_Rhsim10G0122200	6.174193622	7.11035E-23	1.62068E-21	RHSIM_Rhsim10G0122200
Stem vs Leaf	RHSIM_Rhsim11G0029300	-2.566893555	7.18069E-23	1.6351E-21	RHSIM_Rhsim11G0029300
Stem vs Leaf	RHSIM_Rhsim07G0100900	-2.042405001	7.78526E-23	1.77101E-21	RHSIM_Rhsim07G0100900
Stem vs Leaf	RHSIM_Rhsim04G0162900	-10.81683926	7.85247E-23	1.78454E-21	RHSIM_Rhsim04G0162900
Stem vs Leaf	RHSIM_Rhsim08G0002700	-2.380028796	8.09592E-23	1.83805E-21	RHSIM_Rhsim08G0002700
Stem vs Leaf	RHSIM_Rhsim01G0137100	-2.890011869	8.14297E-23	1.84692E-21	RHSIM_Rhsim01G0137100
Stem vs Leaf	novel.1035	-5.584671105	8.19305E-23	1.85645E-21	--
Stem vs Leaf	RHSIM_Rhsim06G0012000	4.553114052	8.46176E-23	1.91545E-21	RHSIM_Rhsim06G0012000
Stem vs Leaf	RHSIM_Rhsim07G0190700	1.319994423	9.34081E-23	2.11236E-21	RHSIM_Rhsim07G0190700
Stem vs Leaf	RHSIM_Rhsim13G0190500	-8.050821448	9.46827E-23	2.13909E-21	RHSIM_Rhsim13G0190500
Stem vs Leaf	RHSIM_Rhsim09G0093700	-2.392171268	9.66352E-23	2.18106E-21	RHSIM_Rhsim09G0093700
Stem vs Leaf	RHSIM_Rhsim05G0106800	-1.628209942	9.74251E-23	2.19673E-21	RHSIM_Rhsim05G0106800
Stem vs Leaf	novel.595	-12.15660579	1.03196E-22	2.32458E-21	--
Stem vs Leaf	RHSIM_Rhsim08G0042000	-3.182283493	1.06822E-22	2.40392E-21	RHSIM_Rhsim08G0042000
Stem vs Leaf	RHSIM_Rhsim10G0196000	-1.491347499	1.09961E-22	2.47215E-21	RHSIM_Rhsim10G0196000
Stem vs Leaf	RHSIM_Rhsim08G0249300	-1.093241863	1.12394E-22	2.52437E-21	RHSIM_Rhsim08G0249300
Stem vs Leaf	RHSIM_Rhsim05G0216100	3.047641492	1.13328E-22	2.54288E-21	RHSIM_Rhsim05G0216100
Stem vs Leaf	RHSIM_Rhsim11G0144100	5.63051344	1.15157E-22	2.5814E-21	RHSIM_Rhsim11G0144100
Stem vs Leaf	RHSIM_Rhsim08G0104400	-2.850534765	1.16139E-22	2.60088E-21	RHSIM_Rhsim08G0104400
Stem vs Leaf	RHSIM_Rhsim03G0232600	-11.79788723	1.17279E-22	2.62386E-21	RHSIM_Rhsim03G0232600

Comparison	Gene ID	log_2FC	P-value	FDR (padj)	Gene Name
Stem vs Leaf	novel.452	-1.876133471	1.25682E-22	2.80913E-21	--
Stem vs Leaf	RHSIM_Rhsim08G0006300	-2.820783879	1.38344E-22	3.08914E-21	RHSIM_Rhsim08G0006300
Stem vs Leaf	RHSIM_Rhsim01G0265400	-2.6925781	1.41349E-22	3.1532E-21	RHSIM_Rhsim01G0265400
Stem vs Leaf	RHSIM_Rhsim13G0010700	-5.052259591	1.44419E-22	3.21856E-21	RHSIM_Rhsim13G0010700
Stem vs Leaf	RHSIM_Rhsim10G0014500	-2.156683487	1.68938E-22	3.76135E-21	RHSIM_Rhsim10G0014500
Stem vs Leaf	RHSIM_Rhsim10G0128400	-3.707299102	1.73744E-22	3.86463E-21	RHSIM_Rhsim10G0128400
Stem vs Leaf	RHSIM_Rhsim07G0189000	-2.21346404	1.80473E-22	4.01044E-21	RHSIM_Rhsim07G0189000
Stem vs Leaf	RHSIM_Rhsim07G0214800	-2.963959514	1.83465E-22	4.073E-21	RHSIM_Rhsim07G0214800
Stem vs Leaf	RHSIM_Rhsim04G0014300	-1.322244474	1.8982E-22	4.21003E-21	RHSIM_Rhsim04G0014300
Stem vs Leaf	RHSIM_Rhsim09G0091700	-3.572436933	1.93283E-22	4.28271E-21	RHSIM_Rhsim09G0091700
Stem vs Leaf	RHSIM_Rhsim01G0171700	-1.837654977	1.94969E-22	4.31591E-21	RHSIM_Rhsim01G0171700
Stem vs Leaf	RHSIM_Rhsim03G0177300	-5.553295769	1.96014E-22	4.33487E-21	RHSIM_Rhsim03G0177300
Stem vs Leaf	RHSIM_Rhsim01G0090700	1.706673033	1.96805E-22	4.34819E-21	RHSIM_Rhsim01G0090700
Stem vs Leaf	RHSIM_Rhsim07G0101400	-1.625432552	2.00626E-22	4.42838E-21	RHSIM_Rhsim07G0101400
Stem vs Leaf	RHSIM_Rhsim12G0124200	-1.790275751	2.07542E-22	4.57665E-21	RHSIM_Rhsim12G0124200
Stem vs Leaf	RHSIM_Rhsim05G0232500	4.634245706	2.25152E-22	4.96023E-21	RHSIM_Rhsim05G0232500
Stem vs Leaf	RHSIM_Rhsim07G0081200	-4.682008387	2.28033E-22	5.0189E-21	RHSIM_Rhsim07G0081200
Stem vs Leaf	RHSIM_Rhsim02G0141100	3.482115437	2.2935E-22	5.04307E-21	RHSIM_Rhsim02G0141100
Stem vs Leaf	RHSIM_Rhsim11G0134500	4.253777282	2.29932E-22	5.05105E-21	RHSIM_Rhsim11G0134500
Stem vs Leaf	RHSIM_Rhsim01G0176500	-2.550807178	2.31834E-22	5.08798E-21	RHSIM_Rhsim01G0176500
Stem vs Leaf	RHSIM_Rhsim09G0098200	3.865702608	2.47864E-22	5.43462E-21	RHSIM_Rhsim09G0098200
Stem vs Leaf	RHSIM_Rhsim08G0094000	-1.290069642	2.54693E-22	5.57904E-21	RHSIM_Rhsim08G0094000
Stem vs Leaf	RHSIM_Rhsim08G0048800	-2.723734317	2.73374E-22	5.98255E-21	RHSIM_Rhsim08G0048800
Stem vs Leaf	RHSIM_Rhsim05G0041100	-2.894123291	2.74694E-22	6.00574E-21	RHSIM_Rhsim05G0041100
Stem vs Leaf	RHSIM_Rhsim02G0236400	-7.183842065	2.75342E-22	6.01419E-21	RHSIM_Rhsim02G0236400

Comparison	Gene ID	log$_2$FC	P-value	FDR (padj)	Gene Name
Stem vs Leaf	RHSIM_Rhsim10G0007600	5.48212711	2.87959E-22	6.28384E-21	RHSIM_Rhsim10G0007600
Stem vs Leaf	RHSIM_Rhsim01G0291400	-1.271864227	3.04217E-22	6.63233E-21	RHSIM_Rhsim01G0291400
Stem vs Leaf	RHSIM_RhsimUnG0221400	-12.35918293	3.12516E-22	6.80682E-21	RHSIM_RhsimUnG0221400
Stem vs Leaf	RHSIM_Rhsim08G0153200	-4.167807981	3.17609E-22	6.91121E-21	RHSIM_Rhsim08G0153200
Stem vs Leaf	RHSIM_Rhsim07G0075300	-3.014960388	3.44651E-22	7.49258E-21	RHSIM_Rhsim07G0075300
Stem vs Leaf	RHSIM_RhsimUnG0053600	3.740530879	3.46E-22	7.51482E-21	RHSIM_RhsimUnG0053600
Stem vs Leaf	novel.2840	-3.850641908	3.60641E-22	7.82543E-21	--
Stem vs Leaf	novel.136	-7.149990693	3.65058E-22	7.91382E-21	--
Stem vs Leaf	RHSIM_Rhsim13G0021200	-1.518812039	3.92736E-22	8.50583E-21	RHSIM_Rhsim13G0021200
Stem vs Leaf	RHSIM_Rhsim03G0153700	-1.608089642	4.25573E-22	9.20836E-21	RHSIM_Rhsim03G0153700
Stem vs Leaf	RHSIM_Rhsim10G0173000	-2.8184294	4.59351E-22	9.92992E-21	RHSIM_Rhsim10G0173000
Stem vs Leaf	RHSIM_Rhsim05G0212900	-4.493263627	4.63367E-22	1.00073E-20	RHSIM_Rhsim05G0212900
Stem vs Leaf	RHSIM_Rhsim12G0126600	3.38116572	4.67293E-22	1.00827E-20	RHSIM_Rhsim12G0126600
Stem vs Leaf	RHSIM_Rhsim13G0230700	-2.266669107	4.88662E-22	1.05339E-20	RHSIM_Rhsim13G0230700
Stem vs Leaf	RHSIM_Rhsim07G0131300	-1.575315473	4.94305E-22	1.06456E-20	RHSIM_Rhsim07G0131300
Stem vs Leaf	RHSIM_Rhsim07G0119300	-1.745107491	4.97451E-22	1.07033E-20	RHSIM_Rhsim07G0119300
Stem vs Leaf	RHSIM_Rhsim08G0195500	-6.01103223	5.03104E-22	1.08149E-20	RHSIM_Rhsim08G0195500
Stem vs Leaf	novel.1316	-10.1575947	5.26247E-22	1.13018E-20	--
Stem vs Leaf	RHSIM_Rhsim13G0229100	-12.21261755	5.43255E-22	1.16562E-20	RHSIM_Rhsim13G0229100
Stem vs Leaf	RHSIM_Rhsim08G0171100	-2.136952894	5.55297E-22	1.19035E-20	RHSIM_Rhsim08G0171100
Stem vs Leaf	RHSIM_Rhsim07G0001300	3.504853464	5.56641E-22	1.19212E-20	RHSIM_Rhsim07G0001300
Stem vs Leaf	RHSIM_Rhsim06G0229700	-4.60148616	5.69391E-22	1.2183E-20	RHSIM_Rhsim06G0229700
Stem vs Leaf	novel.1133	-1.961934567	5.70117E-22	1.21872E-20	--
Stem vs Leaf	RHSIM_Rhsim06G0109000	-10.18060525	6.1006E-22	1.30289E-20	RHSIM_Rhsim06G0109000
Stem vs Leaf	RHSIM_Rhsim04G0018300	-1.288944968	6.22394E-22	1.328E-20	RHSIM_Rhsim04G0018300

Comparison	Gene ID	log_2FC	P-value	FDR (padj)	Gene Name
Stem vs Leaf	novel.1727	2.973189257	6.56449E-22	1.39937E-20	--
Stem vs Leaf	RHSIM_Rhsim04G0116000	3.25172816	6.60189E-22	1.40482E-20	RHSIM_Rhsim04G0116000
Stem vs Leaf	RHSIM_Rhsim12G0005600	-1.269023366	6.60223E-22	1.40482E-20	RHSIM_Rhsim12G0005600
Stem vs Leaf	RHSIM_Rhsim01G0124600	-2.410654509	7.06176E-22	1.50121E-20	RHSIM_Rhsim01G0124600
Stem vs Leaf	novel.4491	-2.26822355	7.46083E-22	1.58459E-20	--
Stem vs Leaf	RHSIM_Rhsim04G0186700	-2.930048006	7.72565E-22	1.63932E-20	RHSIM_Rhsim04G0186700
Stem vs Leaf	RHSIM_Rhsim13G0141000	3.230373827	7.85907E-22	1.66609E-20	RHSIM_Rhsim13G0141000
Stem vs Leaf	RHSIM_Rhsim10G0118800	-2.839875073	7.99183E-22	1.69268E-20	RHSIM_Rhsim10G0118800
Stem vs Leaf	RHSIM_Rhsim02G0188300	-3.707683413	8.60909E-22	1.82174E-20	RHSIM_Rhsim02G0188300
Stem vs Leaf	RHSIM_Rhsim10G0166700	-4.840917715	8.61873E-22	1.82211E-20	RHSIM_Rhsim10G0166700
Stem vs Leaf	RHSIM_Rhsim08G0183600	1.202408329	8.95851E-22	1.89221E-20	RHSIM_Rhsim08G0183600
Stem vs Leaf	RHSIM_Rhsim12G0006600	6.729721588	9.29553E-22	1.9616E-20	RHSIM_Rhsim12G0006600
Stem vs Leaf	RHSIM_Rhsim02G0211000	11.62940937	9.75703E-22	2.0571E-20	RHSIM_Rhsim02G0211000
Stem vs Leaf	RHSIM_Rhsim07G0175800	-3.297559955	1.00742E-21	2.12202E-20	RHSIM_Rhsim07G0175800
Stem vs Leaf	RHSIM_Rhsim10G0165900	-6.640995554	1.02088E-21	2.14842E-20	RHSIM_Rhsim10G0165900
Stem vs Leaf	RHSIM_Rhsim12G0078900	-2.762637125	1.10178E-21	2.31655E-20	RHSIM_Rhsim12G0078900
Stem vs Leaf	RHSIM_Rhsim02G0211200	11.61233118	1.15063E-21	2.41707E-20	RHSIM_Rhsim02G0211200
Stem vs Leaf	RHSIM_Rhsim08G0173200	-1.411205962	1.1683E-21	2.45194E-20	RHSIM_Rhsim08G0173200
Stem vs Leaf	RHSIM_Rhsim04G0011200	-7.089951269	1.3022E-21	2.73048E-20	RHSIM_Rhsim04G0011200
Stem vs Leaf	RHSIM_Rhsim03G0231800	-1.819786683	1.35042E-21	2.829E-20	RHSIM_Rhsim03G0231800
Stem vs Leaf	novel.3953	-1.57370213	1.40552E-21	2.94176E-20	--
Stem vs Leaf	RHSIM_Rhsim03G0220900	-2.559765598	1.4077E-21	2.94366E-20	RHSIM_Rhsim03G0220900
Stem vs Leaf	RHSIM_Rhsim08G0200500	-3.59731576	1.41815E-21	2.96282E-20	RHSIM_Rhsim08G0200500
Stem vs Leaf	RHSIM_RhsimUnG0179800	5.939211024	1.44142E-21	3.00871E-20	RHSIM_RhsimUnG0179800
Stem vs Leaf	RHSIM_Rhsim07G0006200	-1.749195145	1.46986E-21	3.06528E-20	RHSIM_Rhsim07G0006200

Comparison	Gene ID	log_2FC	P-value	FDR (padj)	Gene Name
Stem vs Leaf	RHSIM_Rhsim13G0176900	-1.43980246	1.58015E-21	3.29232E-20	RHSIM_Rhsim13G0176900
Stem vs Leaf	RHSIM_Rhsim03G0033200	-3.535520626	1.62341E-21	3.37939E-20	RHSIM_Rhsim03G0033200
Stem vs Leaf	RHSIM_Rhsim06G0020100	2.720450536	1.77959E-21	3.70115E-20	RHSIM_Rhsim06G0020100
Stem vs Leaf	RHSIM_Rhsim05G0029200	3.003618131	1.80665E-21	3.75406E-20	RHSIM_Rhsim05G0029200
Stem vs Leaf	RHSIM_Rhsim09G0191800	4.28455375	1.85621E-21	3.85356E-20	RHSIM_Rhsim09G0191800
Stem vs Leaf	RHSIM_Rhsim05G0197000	-4.595155374	1.97009E-21	4.0863E-20	RHSIM_Rhsim05G0197000
Stem vs Leaf	RHSIM_Rhsim03G0082000	-2.270270982	2.00926E-21	4.1638E-20	RHSIM_Rhsim03G0082000
Stem vs Leaf	RHSIM_Rhsim05G0104700	-1.496709454	2.11757E-21	4.3843E-20	RHSIM_Rhsim05G0104700
Stem vs Leaf	RHSIM_Rhsim13G0029900	-2.103685047	2.18455E-21	4.51893E-20	RHSIM_Rhsim13G0029900
Stem vs Leaf	RHSIM_Rhsim02G0220000	-6.576583805	2.26275E-21	4.67648E-20	RHSIM_Rhsim02G0220000
Stem vs Leaf	RHSIM_Rhsim11G0106000	2.077478998	2.37821E-21	4.9107E-20	RHSIM_Rhsim11G0106000
Stem vs Leaf	RHSIM_Rhsim09G0071700	4.146139115	2.40171E-21	4.95479E-20	RHSIM_Rhsim09G0071700
Stem vs Leaf	RHSIM_Rhsim02G0029500	-4.913462534	2.43627E-21	5.0216E-20	RHSIM_Rhsim02G0029500
Stem vs Leaf	RHSIM_Rhsim12G0197600	-2.514910175	2.51077E-21	5.17053E-20	RHSIM_Rhsim12G0197600
Stem vs Leaf	RHSIM_Rhsim04G0196300	-5.658693468	2.53458E-21	5.21489E-20	RHSIM_Rhsim04G0196300
Stem vs Leaf	RHSIM_Rhsim01G0060200	-1.482646118	2.93279E-21	6.02883E-20	RHSIM_Rhsim01G0060200
Stem vs Leaf	RHSIM_Rhsim08G0092100	6.747254378	2.95863E-21	6.07652E-20	RHSIM_Rhsim08G0092100
Stem vs Leaf	RHSIM_Rhsim12G0086400	1.98164292	3.10226E-21	6.36586E-20	RHSIM_Rhsim12G0086400
Stem vs Leaf	RHSIM_Rhsim10G0067800	-4.826138881	3.12248E-21	6.40164E-20	RHSIM_Rhsim10G0067800
Stem vs Leaf	RHSIM_RhsimUnG0171600	-2.725762072	3.17003E-21	6.49334E-20	RHSIM_RhsimUnG0171600
Stem vs Leaf	RHSIM_Rhsim02G0251400	-4.10533153	3.24638E-21	6.64383E-20	RHSIM_Rhsim02G0251400
Stem vs Leaf	RHSIM_Rhsim03G0185600	-6.435408959	3.26095E-21	6.66773E-20	RHSIM_Rhsim03G0185600
Stem vs Leaf	RHSIM_Rhsim08G0036600	1.696128424	3.29757E-21	6.73663E-20	RHSIM_Rhsim08G0036600
Stem vs Leaf	RHSIM_Rhsim03G0161100	-2.331948774	3.7478E-21	7.64963E-20	RHSIM_Rhsim03G0161100
Stem vs Leaf	RHSIM_Rhsim10G0153800	6.534891889	3.86173E-21	7.87519E-20	RHSIM_Rhsim10G0153800

Comparison	Gene ID	log_2FC	P-value	FDR (padj)	Gene Name
Stem vs Leaf	RHSIM_Rhsim02G0104800	-1.36623684	3.87417E-21	7.89357E-20	RHSIM_Rhsim02G0104800
Stem vs Leaf	RHSIM_Rhsim01G0210400	-2.114746444	3.92775E-21	7.99569E-20	RHSIM_Rhsim01G0210400
Stem vs Leaf	RHSIM_Rhsim05G0182100	2.668763681	4.08992E-21	8.31845E-20	RHSIM_Rhsim05G0182100
Stem vs Leaf	RHSIM_Rhsim12G0149400	-2.004964622	4.52154E-21	9.18821E-20	RHSIM_Rhsim12G0149400
Stem vs Leaf	RHSIM_Rhsim07G0226400	-3.750592078	4.68176E-21	9.50541E-20	RHSIM_Rhsim07G0226400
Stem vs Leaf	RHSIM_Rhsim11G0178000	4.023426042	4.72724E-21	9.58931E-20	RHSIM_Rhsim11G0178000
Stem vs Leaf	RHSIM_Rhsim10G0138300	-2.008901362	4.77473E-21	9.67382E-20	RHSIM_Rhsim10G0138300
Stem vs Leaf	RHSIM_Rhsim02G0043000	-3.651064623	4.7773E-21	9.67382E-20	RHSIM_Rhsim02G0043000
Stem vs Leaf	novel.1068	11.46708369	4.96698E-21	1.00491E-19	--
Stem vs Leaf	RHSIM_Rhsim12G0173700	1.042802779	5.13822E-21	1.03864E-19	RHSIM_Rhsim12G0173700
Stem vs Leaf	RHSIM_Rhsim03G0162200	-2.618016481	5.14937E-21	1.03998E-19	RHSIM_Rhsim03G0162200
Stem vs Leaf	RHSIM_Rhsim02G0193800	5.843368925	5.24259E-21	1.05788E-19	RHSIM_Rhsim02G0193800
Stem vs Leaf	RHSIM_Rhsim11G0126700	-1.895212172	5.35015E-21	1.07864E-19	RHSIM_Rhsim11G0126700
Stem vs Leaf	RHSIM_Rhsim03G0238900	-1.699471196	5.6548E-21	1.13907E-19	RHSIM_Rhsim03G0238900
Stem vs Leaf	RHSIM_Rhsim02G0188600	1.720564229	5.7112E-21	1.14942E-19	RHSIM_Rhsim02G0188600
Stem vs Leaf	RHSIM_Rhsim02G0147000	-2.095859791	5.8716E-21	1.18067E-19	RHSIM_Rhsim02G0147000
Stem vs Leaf	RHSIM_Rhsim04G0008200	-1.478566084	5.89833E-21	1.18501E-19	RHSIM_Rhsim04G0008200
Stem vs Leaf	RHSIM_Rhsim06G0187800	-1.651901996	5.91819E-21	1.18797E-19	RHSIM_Rhsim06G0187800
Stem vs Leaf	RHSIM_Rhsim06G0137200	-9.830045085	6.82327E-21	1.36845E-19	RHSIM_Rhsim06G0137200
Stem vs Leaf	RHSIM_Rhsim13G0148600	-2.640222619	6.83369E-21	1.36935E-19	RHSIM_Rhsim13G0148600
Stem vs Leaf	RHSIM_Rhsim04G0109400	-1.664842397	6.84548E-21	1.37052E-19	RHSIM_Rhsim04G0109400
Stem vs Leaf	RHSIM_Rhsim12G0124300	-1.872565178	7.168E-21	1.43385E-19	RHSIM_Rhsim12G0124300
Stem vs Leaf	RHSIM_Rhsim10G0163500	-2.154861084	7.2389E-21	1.44678E-19	RHSIM_Rhsim10G0163500
Stem vs Leaf	RHSIM_Rhsim05G0143000	-2.240309283	7.40101E-21	1.47789E-19	RHSIM_Rhsim05G0143000
Stem vs Leaf	RHSIM_Rhsim03G0262400	3.716631701	8.05038E-21	1.60617E-19	RHSIM_Rhsim03G0262400

Comparison	Gene ID	log_2FC	P-value	FDR (padj)	Gene Name
Stem vs Leaf	novel.4173	-7.662972531	8.1114E-21	1.61695E-19	--
Stem vs Leaf	RHSIM_Rhsim05G0060700	-1.724792266	8.29228E-21	1.65158E-19	RHSIM_Rhsim05G0060700
Stem vs Leaf	RHSIM_Rhsim03G0231400	1.105048963	8.34635E-21	1.66091E-19	RHSIM_Rhsim03G0231400
Stem vs Leaf	RHSIM_Rhsim01G0189700	-4.109449979	8.38893E-21	1.66794E-19	RHSIM_Rhsim01G0189700
Stem vs Leaf	novel.5057	2.730844149	8.52385E-21	1.69331E-19	--
Stem vs Leaf	RHSIM_Rhsim06G0169600	-7.538643081	8.78025E-21	1.74274E-19	RHSIM_Rhsim06G0169600
Stem vs Leaf	RHSIM_Rhsim12G0138500	-1.860884968	8.86944E-21	1.75893E-19	RHSIM_Rhsim12G0138500
Stem vs Leaf	RHSIM_Rhsim03G0122000	-1.598680047	9.25514E-21	1.83384E-19	RHSIM_Rhsim03G0122000
Stem vs Leaf	RHSIM_Rhsim03G0226500	-2.263441534	9.37006E-21	1.85501E-19	RHSIM_Rhsim03G0226500
Stem vs Leaf	RHSIM_Rhsim09G0009700	3.654214213	9.60172E-21	1.89925E-19	RHSIM_Rhsim09G0009700
Stem vs Leaf	RHSIM_Rhsim04G0228400	-2.808553118	9.97002E-21	1.9704E-19	RHSIM_Rhsim04G0228400
Stem vs Leaf	RHSIM_Rhsim05G0004100	1.973958456	9.99099E-21	1.97286E-19	RHSIM_Rhsim05G0004100
Stem vs Leaf	RHSIM_Rhsim10G0111500	-3.981337122	1.06009E-20	2.09149E-19	RHSIM_Rhsim10G0111500
Stem vs Leaf	RHSIM_Rhsim11G0043000	2.704505263	1.07025E-20	2.10975E-19	RHSIM_Rhsim11G0043000
Stem vs Leaf	RHSIM_Rhsim04G0135000	-4.534367843	1.07898E-20	2.12512E-19	RHSIM_Rhsim04G0135000
Stem vs Leaf	RHSIM_Rhsim08G0118600	-1.96406691	1.11758E-20	2.19927E-19	RHSIM_Rhsim08G0118600
Stem vs Leaf	RHSIM_Rhsim02G0040500	-6.758684234	1.1995E-20	2.35847E-19	RHSIM_Rhsim02G0040500
Stem vs Leaf	RHSIM_Rhsim06G0073700	-1.460171938	1.22029E-20	2.3973E-19	RHSIM_Rhsim06G0073700
Stem vs Leaf	RHSIM_Rhsim13G0148700	-2.093113153	1.2219E-20	2.39841E-19	RHSIM_Rhsim13G0148700
Stem vs Leaf	RHSIM_Rhsim04G0178000	-5.743841527	1.25077E-20	2.45299E-19	RHSIM_Rhsim04G0178000
Stem vs Leaf	RHSIM_Rhsim07G0043900	2.523411168	1.27439E-20	2.4972E-19	RHSIM_Rhsim07G0043900
Stem vs Leaf	RHSIM_Rhsim01G0040200	-5.953375765	1.29369E-20	2.53286E-19	RHSIM_Rhsim01G0040200
Stem vs Leaf	RHSIM_Rhsim01G0189500	-3.177337505	1.3956E-20	2.73007E-19	RHSIM_Rhsim01G0189500
Stem vs Leaf	RHSIM_Rhsim06G0243200	5.789137821	1.40395E-20	2.74407E-19	RHSIM_Rhsim06G0243200
Stem vs Leaf	RHSIM_Rhsim03G0247400	2.82201702	1.45964E-20	2.8505E-19	RHSIM_Rhsim03G0247400

Comparison	Gene ID	log_2FC	P-value	FDR (padj)	Gene Name
Stem vs Leaf	RHSIM_Rhsim07G0201100	2.794665788	1.58844E-20	3.0994E-19	RHSIM_Rhsim07G0201100
Stem vs Leaf	novel.163	-5.037660809	1.64412E-20	3.20534E-19	--
Stem vs Leaf	RHSIM_Rhsim10G0174000	-3.415541086	1.68255E-20	3.27749E-19	RHSIM_Rhsim10G0174000
Stem vs Leaf	RHSIM_Rhsim03G0074900	3.497185072	1.79019E-20	3.48422E-19	RHSIM_Rhsim03G0074900
Stem vs Leaf	novel.2464	-4.978835191	1.84072E-20	3.57953E-19	--
Stem vs Leaf	RHSIM_Rhsim13G0098800	1.708139771	1.86004E-20	3.61407E-19	RHSIM_Rhsim13G0098800
Stem vs Leaf	RHSIM_Rhsim08G0174100	11.13382956	1.87237E-20	3.63496E-19	RHSIM_Rhsim08G0174100
Stem vs Leaf	RHSIM_Rhsim11G0091400	7.799568849	1.99098E-20	3.86196E-19	RHSIM_Rhsim11G0091400
Stem vs Leaf	RHSIM_Rhsim13G0189800	3.981235522	2.1092E-20	4.08783E-19	RHSIM_Rhsim13G0189800
Stem vs Leaf	RHSIM_Rhsim01G0071100	-2.498082345	2.19836E-20	4.25706E-19	RHSIM_Rhsim01G0071100
Stem vs Leaf	RHSIM_Rhsim03G0188900	-2.506377928	2.41204E-20	4.66693E-19	RHSIM_Rhsim03G0188900
Stem vs Leaf	RHSIM_Rhsim02G0253600	4.249442589	2.43725E-20	4.71175E-19	RHSIM_Rhsim02G0253600
Stem vs Leaf	RHSIM_Rhsim10G0109000	-3.758510231	2.47053E-20	4.77207E-19	RHSIM_Rhsim10G0109000
Stem vs Leaf	RHSIM_Rhsim01G0027800	-6.176971335	2.47773E-20	4.78198E-19	RHSIM_Rhsim01G0027800
Stem vs Leaf	RHSIM_Rhsim06G0063200	-6.206059487	2.70138E-20	5.20926E-19	RHSIM_Rhsim06G0063200
Stem vs Leaf	RHSIM_Rhsim09G0173500	3.025830908	2.74138E-20	5.28196E-19	RHSIM_Rhsim09G0173500
Stem vs Leaf	RHSIM_Rhsim03G0154100	4.258822149	2.75681E-20	5.30727E-19	RHSIM_Rhsim03G0154100
Stem vs Leaf	RHSIM_Rhsim06G0060000	2.82180727	2.8489E-20	5.47996E-19	RHSIM_Rhsim06G0060000
Stem vs Leaf	RHSIM_Rhsim02G0254100	-2.714745121	2.86728E-20	5.50717E-19	RHSIM_Rhsim02G0254100
Stem vs Leaf	RHSIM_Rhsim03G0091900	3.0473657	2.86782E-20	5.50717E-19	RHSIM_Rhsim03G0091900
Stem vs Leaf	RHSIM_Rhsim03G0229800	-3.022984781	2.92274E-20	5.60796E-19	RHSIM_Rhsim03G0229800
Stem vs Leaf	RHSIM_Rhsim08G0154500	4.163975493	2.93534E-20	5.62745E-19	RHSIM_Rhsim08G0154500
Stem vs Leaf	RHSIM_Rhsim02G0061500	-8.616356835	3.01799E-20	5.7811E-19	RHSIM_Rhsim02G0061500
Stem vs Leaf	RHSIM_Rhsim13G0105200	5.760290741	3.04588E-20	5.82967E-19	RHSIM_Rhsim13G0105200
Stem vs Leaf	RHSIM_Rhsim13G0056200	-4.181983171	3.05178E-20	5.83612E-19	RHSIM_Rhsim13G0056200

Comparison	Gene ID	log_2FC	P-value	FDR (padj)	Gene Name
Stem vs Leaf	RHSIM_Rhsim12G0143200	5.285300373	3.33693E-20	6.37613E-19	RHSIM_Rhsim12G0143200
Stem vs Leaf	RHSIM_Rhsim04G0038800	-1.044853767	3.40568E-20	6.50211E-19	RHSIM_Rhsim04G0038800
Stem vs Leaf	RHSIM_Rhsim11G0052200	-4.814521692	3.73499E-20	7.12492E-19	RHSIM_Rhsim11G0052200
Stem vs Leaf	RHSIM_Rhsim11G0194500	2.286078889	3.81403E-20	7.26969E-19	RHSIM_Rhsim11G0194500
Stem vs Leaf	RHSIM_Rhsim09G0078400	-3.177150851	3.97883E-20	7.57754E-19	RHSIM_Rhsim09G0078400
Stem vs Leaf	RHSIM_Rhsim07G0025600	-4.005193103	4.00819E-20	7.62714E-19	RHSIM_Rhsim07G0025600
Stem vs Leaf	RHSIM_Rhsim02G0025700	-1.246050287	4.16576E-20	7.92044E-19	RHSIM_Rhsim02G0025700
Stem vs Leaf	RHSIM_Rhsim04G0003700	-2.439631418	4.27259E-20	8.11685E-19	RHSIM_Rhsim04G0003700
Stem vs Leaf	RHSIM_Rhsim06G0133800	-7.237437493	4.28168E-20	8.12744E-19	RHSIM_Rhsim06G0133800
Stem vs Leaf	RHSIM_Rhsim06G0102100	-1.136263093	4.30595E-20	8.16677E-19	RHSIM_Rhsim06G0102100
Stem vs Leaf	RHSIM_Rhsim07G0124800	-1.683826276	4.33047E-20	8.20653E-19	RHSIM_Rhsim07G0124800
Stem vs Leaf	novel.5377	-1.427679737	4.42506E-20	8.37889E-19	--
Stem vs Leaf	RHSIM_Rhsim12G0156100	-1.79656971	4.75433E-20	8.99497E-19	RHSIM_Rhsim12G0156100
Stem vs Leaf	RHSIM_Rhsim08G0193400	-4.721315199	4.94506E-20	9.34815E-19	RHSIM_Rhsim08G0193400
Stem vs Leaf	RHSIM_Rhsim06G0004200	-1.591155951	5.01019E-20	9.4635E-19	RHSIM_Rhsim06G0004200
Stem vs Leaf	RHSIM_Rhsim02G0001500	-2.266991358	5.06259E-20	9.55466E-19	RHSIM_Rhsim02G0001500
Stem vs Leaf	RHSIM_Rhsim03G0192800	-3.412890875	5.10012E-20	9.61761E-19	RHSIM_Rhsim03G0192800
Stem vs Leaf	RHSIM_Rhsim02G0087400	-7.087652825	5.15686E-20	9.71666E-19	RHSIM_Rhsim02G0087400
Stem vs Leaf	RHSIM_Rhsim07G0239500	-2.652304108	5.65042E-20	1.06379E-18	RHSIM_Rhsim07G0239500
Stem vs Leaf	RHSIM_Rhsim04G0168900	-1.666397731	6.04838E-20	1.13779E-18	RHSIM_Rhsim04G0168900
Stem vs Leaf	RHSIM_Rhsim12G0048400	2.743024805	6.23358E-20	1.17167E-18	RHSIM_Rhsim12G0048400
Stem vs Leaf	RHSIM_Rhsim11G0106600	-5.156778413	6.29918E-20	1.18303E-18	RHSIM_Rhsim11G0106600
Stem vs Leaf	RHSIM_Rhsim03G0175300	-2.792148816	6.41568E-20	1.20393E-18	RHSIM_Rhsim03G0175300
Stem vs Leaf	RHSIM_Rhsim10G0043400	-11.15518192	6.71907E-20	1.25984E-18	RHSIM_Rhsim10G0043400
Stem vs Leaf	RHSIM_Rhsim13G0082400	-1.776892867	6.77345E-20	1.269E-18	RHSIM_Rhsim13G0082400

Comparison	Gene ID	log_2FC	P-value	FDR (padj)	Gene Name
Stem vs Leaf	RHSIM_Rhsim05G0231000	-1.797844821	6.89723E-20	1.29114E-18	RHSIM_Rhsim05G0231000
Stem vs Leaf	RHSIM_Rhsim13G0180200	-7.895846095	7.60741E-20	1.42293E-18	RHSIM_Rhsim13G0180200
Stem vs Leaf	RHSIM_Rhsim01G0081800	-1.585022593	7.67381E-20	1.43419E-18	RHSIM_Rhsim01G0081800
Stem vs Leaf	RHSIM_Rhsim03G0196300	-5.034177375	7.71867E-20	1.4414E-18	RHSIM_Rhsim03G0196300
Stem vs Leaf	RHSIM_Rhsim08G0186700	-2.024582972	8.17659E-20	1.52568E-18	RHSIM_Rhsim08G0186700
Stem vs Leaf	RHSIM_Rhsim13G0017200	-2.326231557	8.23989E-20	1.53625E-18	RHSIM_Rhsim13G0017200
Stem vs Leaf	RHSIM_Rhsim05G0067600	-2.022753395	8.31306E-20	1.54864E-18	RHSIM_Rhsim05G0067600
Stem vs Leaf	RHSIM_RhsimUnG0255400	5.800928491	8.82925E-20	1.64347E-18	RHSIM_RhsimUnG0255400
Stem vs Leaf	RHSIM_Rhsim09G0149700	-2.112541452	8.93974E-20	1.66269E-18	RHSIM_Rhsim09G0149700
Stem vs Leaf	RHSIM_Rhsim01G0269700	-1.535832823	9.16094E-20	1.70246E-18	RHSIM_Rhsim01G0269700
Stem vs Leaf	RHSIM_Rhsim08G0198500	-2.793713049	9.21411E-20	1.71096E-18	RHSIM_Rhsim08G0198500
Stem vs Leaf	RHSIM_Rhsim10G0010700	-5.944187982	9.28834E-20	1.72335E-18	RHSIM_Rhsim10G0010700
Stem vs Leaf	RHSIM_Rhsim04G0117700	-2.221763992	9.31087E-20	1.72614E-18	RHSIM_Rhsim04G0117700
Stem vs Leaf	RHSIM_Rhsim01G0199400	-10.97326481	9.7969E-20	1.81479E-18	RHSIM_Rhsim01G0199400
Stem vs Leaf	RHSIM_Rhsim07G0175500	-2.466094667	1.02286E-19	1.89324E-18	RHSIM_Rhsim07G0175500
Stem vs Leaf	RHSIM_Rhsim07G0139600	-11.68987833	1.10708E-19	2.04748E-18	RHSIM_Rhsim07G0139600
Stem vs Leaf	RHSIM_Rhsim12G0171100	-3.702148655	1.109E-19	2.04939E-18	RHSIM_Rhsim12G0171100
Stem vs Leaf	RHSIM_Rhsim11G0194300	-2.943304984	1.11311E-19	2.05532E-18	RHSIM_Rhsim11G0194300
Stem vs Leaf	RHSIM_Rhsim06G0229500	-3.231173243	1.11409E-19	2.0555E-18	RHSIM_Rhsim06G0229500
Stem vs Leaf	RHSIM_Rhsim12G0193900	-3.317284207	1.15569E-19	2.13054E-18	RHSIM_Rhsim12G0193900
Stem vs Leaf	RHSIM_Rhsim13G0181400	-5.722565056	1.16172E-19	2.13994E-18	RHSIM_Rhsim13G0181400
Stem vs Leaf	RHSIM_RhsimUnG0147100	-11.08982354	1.17377E-19	2.16042E-18	RHSIM_RhsimUnG0147100
Stem vs Leaf	RHSIM_Rhsim09G0082700	5.692733878	1.25099E-19	2.3007E-18	RHSIM_Rhsim09G0082700
Stem vs Leaf	RHSIM_Rhsim10G0187700	-3.033685339	1.2624E-19	2.31983E-18	RHSIM_Rhsim10G0187700
Stem vs Leaf	RHSIM_Rhsim01G0214900	1.599990221	1.3995E-19	2.56973E-18	RHSIM_Rhsim01G0214900

Comparison	Gene ID	log_2FC	P-value	FDR (padj)	Gene Name
Stem vs Leaf	RHSIM_Rhsim03G0039900	-2.513843326	1.41075E-19	2.58832E-18	RHSIM_Rhsim03G0039900
Stem vs Leaf	RHSIM_Rhsim11G0034500	-1.351096402	1.44803E-19	2.6546E-18	RHSIM_Rhsim11G0034500
Stem vs Leaf	RHSIM_Rhsim07G0158900	-12.39113845	1.46226E-19	2.67856E-18	RHSIM_Rhsim07G0158900
Stem vs Leaf	RHSIM_Rhsim07G0243900	-1.65329926	1.4719E-19	2.69408E-18	RHSIM_Rhsim07G0243900
Stem vs Leaf	RHSIM_Rhsim12G0042700	4.909351096	1.49517E-19	2.7345E-18	RHSIM_Rhsim12G0042700
Stem vs Leaf	RHSIM_Rhsim07G0094200	4.139568754	1.61609E-19	2.9533E-18	RHSIM_Rhsim07G0094200
Stem vs Leaf	RHSIM_Rhsim05G0069300	-2.391712171	1.72518E-19	3.15016E-18	RHSIM_Rhsim05G0069300
Stem vs Leaf	novel.1494	4.39737414	1.73718E-19	3.16957E-18	--
Stem vs Leaf	RHSIM_Rhsim10G0204000	-2.526955731	1.85925E-19	3.38961E-18	RHSIM_Rhsim10G0204000
Stem vs Leaf	RHSIM_RhsimUnG0089000	-6.542599447	1.90565E-19	3.47145E-18	RHSIM_RhsimUnG0089000
Stem vs Leaf	RHSIM_Rhsim09G0006700	-3.962263856	2.16174E-19	3.93485E-18	RHSIM_Rhsim09G0006700
Stem vs Leaf	RHSIM_Rhsim04G0033400	-8.275781605	2.20504E-19	4.01049E-18	RHSIM_Rhsim04G0033400
Stem vs Leaf	RHSIM_Rhsim05G0007900	3.70024127	2.3183E-19	4.21316E-18	RHSIM_Rhsim05G0007900
Stem vs Leaf	RHSIM_Rhsim13G0102200	-2.784720629	2.36817E-19	4.30041E-18	RHSIM_Rhsim13G0102200
Stem vs Leaf	RHSIM_Rhsim07G0207000	-3.083071517	2.41665E-19	4.38497E-18	RHSIM_Rhsim07G0207000
Stem vs Leaf	RHSIM_Rhsim13G0042600	-3.510138663	2.44209E-19	4.42765E-18	RHSIM_Rhsim13G0042600
Stem vs Leaf	RHSIM_Rhsim05G0088400	-1.761468036	2.46682E-19	4.46898E-18	RHSIM_Rhsim05G0088400
Stem vs Leaf	novel.2797	2.109794824	2.49138E-19	4.50993E-18	--
Stem vs Leaf	RHSIM_RhsimUnG0060100	1.605913399	2.61561E-19	4.7311E-18	RHSIM_RhsimUnG0060100
Stem vs Leaf	RHSIM_Rhsim05G0190000	-3.870944607	2.68959E-19	4.86109E-18	RHSIM_Rhsim05G0190000
Stem vs Leaf	RHSIM_Rhsim03G0207200	2.427496248	2.73531E-19	4.93985E-18	RHSIM_Rhsim03G0207200
Stem vs Leaf	RHSIM_Rhsim02G0035000	-10.83098732	2.87741E-19	5.1924E-18	RHSIM_Rhsim02G0035000
Stem vs Leaf	RHSIM_Rhsim13G0053200	-1.296594129	2.92984E-19	5.28288E-18	RHSIM_Rhsim13G0053200
Stem vs Leaf	RHSIM_Rhsim06G0022900	-2.215853843	2.95971E-19	5.33256E-18	RHSIM_Rhsim06G0022900
Stem vs Leaf	RHSIM_Rhsim08G0152900	3.438708833	3.05331E-19	5.49691E-18	RHSIM_Rhsim08G0152900

Comparison	Gene ID	log_2FC	P-value	FDR (padj)	Gene Name
Stem vs Leaf	RHSIM_Rhsim01G0054500	-5.918989425	3.19552E-19	5.74845E-18	RHSIM_Rhsim01G0054500
Stem vs Leaf	RHSIM_Rhsim08G0021000	2.909688188	3.22721E-19	5.80093E-18	RHSIM_Rhsim08G0021000
Stem vs Leaf	RHSIM_Rhsim06G0053200	-1.412955788	3.28055E-19	5.8922E-18	RHSIM_Rhsim06G0053200
Stem vs Leaf	RHSIM_Rhsim09G0132600	-1.517683667	3.28872E-19	5.90227E-18	RHSIM_Rhsim09G0132600
Stem vs Leaf	RHSIM_Rhsim07G0172700	-2.21491998	3.31359E-19	5.94228E-18	RHSIM_Rhsim07G0172700
Stem vs Leaf	RHSIM_Rhsim12G0131600	-3.792009802	3.31848E-19	5.94643E-18	RHSIM_Rhsim12G0131600
Stem vs Leaf	RHSIM_Rhsim09G0048000	2.087178638	3.43027E-19	6.14196E-18	RHSIM_Rhsim09G0048000
Stem vs Leaf	RHSIM_Rhsim09G0146100	2.246193933	3.43468E-19	6.1451E-18	RHSIM_Rhsim09G0146100
Stem vs Leaf	RHSIM_Rhsim10G0188000	-2.786447504	3.56127E-19	6.36663E-18	RHSIM_Rhsim10G0188000
Stem vs Leaf	RHSIM_Rhsim13G0123400	6.145566988	3.58949E-19	6.41212E-18	RHSIM_Rhsim13G0123400
Stem vs Leaf	RHSIM_Rhsim10G0014800	1.990585936	3.60714E-19	6.43865E-18	RHSIM_Rhsim10G0014800
Stem vs Leaf	RHSIM_Rhsim06G0109400	-2.772431949	3.61623E-19	6.44988E-18	RHSIM_Rhsim06G0109400
Stem vs Leaf	RHSIM_Rhsim07G0096500	-2.051263024	3.68129E-19	6.56084E-18	RHSIM_Rhsim07G0096500
Stem vs Leaf	novel.1120	1.757703371	3.71928E-19	6.62204E-18	--
Stem vs Leaf	RHSIM_RhsimUnG0153300	-1.853302667	3.72138E-19	6.62204E-18	RHSIM_RhsimUnG0153300
Stem vs Leaf	RHSIM_Rhsim06G0108600	-1.708271662	4.00686E-19	7.12454E-18	RHSIM_Rhsim06G0108600
Stem vs Leaf	RHSIM_RhsimUnG0122900	-1.410026657	4.03009E-19	7.16032E-18	RHSIM_RhsimUnG0122900
Stem vs Leaf	RHSIM_Rhsim12G0183100	-1.131094461	4.05179E-19	7.19332E-18	RHSIM_Rhsim12G0183100
Stem vs Leaf	RHSIM_Rhsim11G0114800	-4.505697646	4.12226E-19	7.3128E-18	RHSIM_Rhsim11G0114800
Stem vs Leaf	RHSIM_Rhsim03G0140300	1.380438489	4.27317E-19	7.57469E-18	RHSIM_Rhsim03G0140300
Stem vs Leaf	RHSIM_Rhsim07G0005600	-1.956207599	4.29647E-19	7.61014E-18	RHSIM_Rhsim07G0005600
Stem vs Leaf	RHSIM_Rhsim07G0031600	1.905560516	4.31001E-19	7.62825E-18	RHSIM_Rhsim07G0031600
Stem vs Leaf	RHSIM_Rhsim07G0165600	-1.913323468	4.44537E-19	7.86178E-18	RHSIM_Rhsim07G0165600
Stem vs Leaf	RHSIM_Rhsim07G0216100	-7.689376319	4.70087E-19	8.30727E-18	RHSIM_Rhsim07G0216100
Stem vs Leaf	RHSIM_Rhsim12G0154100	2.149749573	4.80553E-19	8.48573E-18	RHSIM_Rhsim12G0154100

Comparison	Gene ID	log_2FC	P-value	FDR (padj)	Gene Name
Stem vs Leaf	RHSIM_Rhsim03G0235800	-6.303974996	4.85673E-19	8.56956E-18	RHSIM_Rhsim03G0235800
Stem vs Leaf	RHSIM_Rhsim10G0056300	11.74566002	5.01695E-19	8.84549E-18	RHSIM_Rhsim10G0056300
Stem vs Leaf	RHSIM_Rhsim05G0054700	12.09565845	5.0382E-19	8.87618E-18	RHSIM_Rhsim05G0054700
Stem vs Leaf	novel.2029	4.001640819	5.17497E-19	9.11016E-18	--
Stem vs Leaf	RHSIM_Rhsim02G0236300	3.371717636	5.2904E-19	9.30251E-18	RHSIM_Rhsim02G0236300
Stem vs Leaf	RHSIM_RhsimPtG0005800	-2.982168688	5.29231E-19	9.30251E-18	RHSIM_RhsimPtG0005800
Stem vs Leaf	RHSIM_Rhsim04G0170700	-9.78407145	5.45091E-19	9.57399E-18	RHSIM_Rhsim04G0170700
Stem vs Leaf	RHSIM_Rhsim01G0191600	-1.446392968	5.65721E-19	9.92878E-18	RHSIM_Rhsim01G0191600
Stem vs Leaf	RHSIM_Rhsim01G0184700	3.781635538	5.807E-19	1.01839E-17	RHSIM_Rhsim01G0184700
Stem vs Leaf	novel.575	4.232370215	6.04189E-19	1.05878E-17	--
Stem vs Leaf	novel.1315	-10.78168556	6.16288E-19	1.07916E-17	--
Stem vs Leaf	RHSIM_Rhsim06G0049900	-3.429489135	6.3685E-19	1.11432E-17	RHSIM_Rhsim06G0049900
Stem vs Leaf	RHSIM_Rhsim12G0141700	5.4285387	6.69889E-19	1.17124E-17	RHSIM_Rhsim12G0141700
Stem vs Leaf	RHSIM_Rhsim03G0161300	-1.772321549	6.79249E-19	1.1867E-17	RHSIM_Rhsim03G0161300
Stem vs Leaf	RHSIM_Rhsim13G0088200	-8.381780379	6.96598E-19	1.21598E-17	RHSIM_Rhsim13G0088200
Stem vs Leaf	RHSIM_Rhsim03G0083300	1.362796774	6.97203E-19	1.21598E-17	RHSIM_Rhsim03G0083300
Stem vs Leaf	RHSIM_Rhsim06G0021400	-3.647583782	6.97587E-19	1.21598E-17	RHSIM_Rhsim06G0021400
Stem vs Leaf	novel.1114	-2.415783756	6.98335E-19	1.21636E-17	--
Stem vs Leaf	novel.4168	-10.766368	6.99915E-19	1.21819E-17	--
Stem vs Leaf	RHSIM_Rhsim01G0033300	1.502483807	7.05471E-19	1.22693E-17	RHSIM_Rhsim01G0033300
Stem vs Leaf	RHSIM_Rhsim11G0108000	7.44833079	7.08123E-19	1.23062E-17	RHSIM_Rhsim11G0108000
Stem vs Leaf	RHSIM_Rhsim05G0184000	-4.390166972	7.12746E-19	1.23772E-17	RHSIM_Rhsim05G0184000
Stem vs Leaf	RHSIM_RhsimUnG0115600	-10.87777503	7.1999E-19	1.24936E-17	RHSIM_RhsimUnG0115600
Stem vs Leaf	RHSIM_Rhsim03G0094400	-9.406653124	7.87195E-19	1.36494E-17	RHSIM_Rhsim03G0094400
Stem vs Leaf	RHSIM_Rhsim08G0075400	-3.449029924	7.96388E-19	1.37985E-17	RHSIM_Rhsim08G0075400

Comparison	Gene ID	log_2FC	P-value	FDR (padj)	Gene Name
Stem vs Leaf	RHSIM_Rhsim03G0265200	-1.075665467	8.09943E-19	1.40228E-17	RHSIM_Rhsim03G0265200
Stem vs Leaf	RHSIM_Rhsim12G0150400	10.85469822	8.13442E-19	1.40728E-17	RHSIM_Rhsim12G0150400
Stem vs Leaf	RHSIM_Rhsim03G0089100	2.992994066	8.25836E-19	1.42765E-17	RHSIM_Rhsim03G0089100
Stem vs Leaf	RHSIM_Rhsim05G0093200	-3.0617129	8.54734E-19	1.4765E-17	RHSIM_Rhsim05G0093200
Stem vs Leaf	RHSIM_Rhsim13G0131200	-2.309254322	8.58979E-19	1.48272E-17	RHSIM_Rhsim13G0131200
Stem vs Leaf	RHSIM_Rhsim01G0247500	-2.527745464	8.6405E-19	1.49036E-17	RHSIM_Rhsim01G0247500
Stem vs Leaf	RHSIM_Rhsim06G0066300	6.754668189	8.93442E-19	1.53875E-17	RHSIM_Rhsim06G0066300
Stem vs Leaf	RHSIM_Rhsim07G0008600	-1.984810778	9.01047E-19	1.55069E-17	RHSIM_Rhsim07G0008600
Stem vs Leaf	RHSIM_Rhsim06G0033800	-2.129899946	9.10522E-19	1.56583E-17	RHSIM_Rhsim06G0033800
Stem vs Leaf	RHSIM_Rhsim04G0240200	-6.490371523	9.22809E-19	1.58577E-17	RHSIM_Rhsim04G0240200
Stem vs Leaf	RHSIM_Rhsim04G0068500	2.881717027	9.37656E-19	1.61008E-17	RHSIM_Rhsim04G0068500
Stem vs Leaf	RHSIM_Rhsim13G0135000	5.571499113	9.39246E-19	1.61162E-17	RHSIM_Rhsim13G0135000
Stem vs Leaf	RHSIM_RhsimUnG0099700	7.036437189	9.81726E-19	1.68325E-17	RHSIM_RhsimUnG0099700
Stem vs Leaf	RHSIM_Rhsim02G0093900	-2.329261769	1.01623E-18	1.74112E-17	RHSIM_Rhsim02G0093900
Stem vs Leaf	RHSIM_Rhsim06G0207600	-1.993291958	1.01767E-18	1.74228E-17	RHSIM_Rhsim06G0207600
Stem vs Leaf	RHSIM_Rhsim04G0223100	-5.937993978	1.04154E-18	1.78183E-17	RHSIM_Rhsim04G0223100
Stem vs Leaf	RHSIM_Rhsim04G0077100	-10.76439621	1.05853E-18	1.80956E-17	RHSIM_Rhsim04G0077100
Stem vs Leaf	RHSIM_Rhsim09G0132300	4.983160414	1.06465E-18	1.81866E-17	RHSIM_Rhsim09G0132300
Stem vs Leaf	RHSIM_Rhsim02G0071700	-2.098416719	1.07411E-18	1.83347E-17	RHSIM_Rhsim02G0071700
Stem vs Leaf	novel.6181	-4.715822729	1.07547E-18	1.83443E-17	--
Stem vs Leaf	RHSIM_Rhsim13G0034400	4.258494845	1.08077E-18	1.8421E-17	RHSIM_Rhsim13G0034400
Stem vs Leaf	RHSIM_Rhsim06G0141400	8.336875608	1.09666E-18	1.8678E-17	RHSIM_Rhsim06G0141400
Stem vs Leaf	novel.5951	-7.535805108	1.10999E-18	1.88911E-17	--
Stem vs Leaf	RHSIM_Rhsim08G0237900	-2.751688564	1.14935E-18	1.95466E-17	RHSIM_Rhsim08G0237900
Stem vs Leaf	novel.5146	2.211948386	1.18664E-18	2.01658E-17	--

Comparison	Gene ID	log_2FC	P-value	FDR (padj)	Gene Name
Stem vs Leaf	RHSIM_Rhsim10G0163700	5.948120632	1.31513E-18	2.2333E-17	RHSIM_Rhsim10G0163700
Stem vs Leaf	RHSIM_Rhsim05G0234200	-5.258910576	1.32971E-18	2.25639E-17	RHSIM_Rhsim05G0234200
Stem vs Leaf	RHSIM_Rhsim12G0136700	-1.096154194	1.33764E-18	2.26708E-17	RHSIM_Rhsim12G0136700
Stem vs Leaf	RHSIM_Rhsim08G0057900	-11.16554985	1.33798E-18	2.26708E-17	RHSIM_Rhsim08G0057900
Stem vs Leaf	RHSIM_Rhsim01G0144700	-1.974292916	1.44853E-18	2.4526E-17	RHSIM_Rhsim01G0144700
Stem vs Leaf	RHSIM_Rhsim06G0160900	3.238611065	1.47213E-18	2.49073E-17	RHSIM_Rhsim06G0160900
Stem vs Leaf	RHSIM_Rhsim12G0055100	5.718074111	1.47515E-18	2.49401E-17	RHSIM_Rhsim12G0055100
Stem vs Leaf	RHSIM_Rhsim12G0160400	-2.76504283	1.51028E-18	2.55153E-17	RHSIM_Rhsim12G0160400
Stem vs Leaf	RHSIM_Rhsim03G0038900	-1.37395264	1.52319E-18	2.57146E-17	RHSIM_Rhsim03G0038900
Stem vs Leaf	RHSIM_Rhsim03G0086400	-1.933461786	1.59747E-18	2.69489E-17	RHSIM_Rhsim03G0086400
Stem vs Leaf	RHSIM_RhsimUnG0118600	-2.95319482	1.60038E-18	2.69781E-17	RHSIM_RhsimUnG0118600
Stem vs Leaf	RHSIM_Rhsim04G0061200	-2.520976917	1.60255E-18	2.6995E-17	RHSIM_Rhsim04G0061200
Stem vs Leaf	RHSIM_Rhsim01G0259600	-2.013807384	1.65412E-18	2.78434E-17	RHSIM_Rhsim01G0259600
Stem vs Leaf	RHSIM_Rhsim04G0130500	-1.845535554	1.67865E-18	2.82357E-17	RHSIM_Rhsim04G0130500
Stem vs Leaf	RHSIM_Rhsim11G0043600	-3.770279753	1.72079E-18	2.89233E-17	RHSIM_Rhsim11G0043600
Stem vs Leaf	RHSIM_Rhsim05G0104300	-1.249718614	1.76032E-18	2.95662E-17	RHSIM_Rhsim05G0104300
Stem vs Leaf	RHSIM_Rhsim09G0195900	-3.667789386	1.7665E-18	2.96484E-17	RHSIM_Rhsim09G0195900
Stem vs Leaf	RHSIM_Rhsim06G0037000	11.48532619	1.86337E-18	3.12514E-17	RHSIM_Rhsim06G0037000
Stem vs Leaf	RHSIM_Rhsim05G0074400	4.582891912	1.86718E-18	3.12926E-17	RHSIM_Rhsim05G0074400
Stem vs Leaf	RHSIM_Rhsim05G0095400	-1.573134671	1.97082E-18	3.30055E-17	RHSIM_Rhsim05G0095400
Stem vs Leaf	novel.1539	-2.405561455	1.99017E-18	3.33054E-17	--
Stem vs Leaf	RHSIM_Rhsim06G0073600	-1.021212777	1.99475E-18	3.33578E-17	RHSIM_Rhsim06G0073600
Stem vs Leaf	RHSIM_Rhsim06G0036100	-2.538865881	2.00899E-18	3.35715E-17	RHSIM_Rhsim06G0036100
Stem vs Leaf	RHSIM_Rhsim13G0222700	2.971338168	2.04514E-18	3.41509E-17	RHSIM_Rhsim13G0222700
Stem vs Leaf	RHSIM_Rhsim06G0009900	1.46560162	2.06491E-18	3.44561E-17	RHSIM_Rhsim06G0009900

Comparison	Gene ID	log_2FC	P-value	FDR (padj)	Gene Name
Stem vs Leaf	RHSIM_Rhsim04G0201800	-2.633189448	2.10891E-18	3.51648E-17	RHSIM_Rhsim04G0201800
Stem vs Leaf	RHSIM_Rhsim08G0226700	-6.57271438	2.11415E-18	3.52266E-17	RHSIM_Rhsim08G0226700
Stem vs Leaf	RHSIM_Rhsim05G0039000	2.187592784	2.13317E-18	3.55179E-17	RHSIM_Rhsim05G0039000
Stem vs Leaf	RHSIM_Rhsim09G0061300	-1.556426151	2.15984E-18	3.59361E-17	RHSIM_Rhsim09G0061300
Stem vs Leaf	RHSIM_Rhsim01G0290800	-2.420424598	2.25048E-18	3.74171E-17	RHSIM_Rhsim01G0290800
Stem vs Leaf	RHSIM_Rhsim06G0237700	1.542735091	2.25872E-18	3.75269E-17	RHSIM_Rhsim06G0237700
Stem vs Leaf	RHSIM_Rhsim04G0168700	-1.98664456	2.2833E-18	3.7908E-17	RHSIM_Rhsim04G0168700
Stem vs Leaf	RHSIM_Rhsim13G0094300	2.603589564	2.28936E-18	3.79812E-17	RHSIM_Rhsim13G0094300
Stem vs Leaf	RHSIM_Rhsim04G0242100	-1.747808112	2.39409E-18	3.96662E-17	RHSIM_Rhsim04G0242100
Stem vs Leaf	RHSIM_Rhsim13G0141900	4.040280548	2.39436E-18	3.96662E-17	RHSIM_Rhsim13G0141900
Stem vs Leaf	RHSIM_Rhsim03G0067900	3.774930277	2.41854E-18	4.0038E-17	RHSIM_Rhsim03G0067900
Stem vs Leaf	novel.5707	-4.974030672	2.44735E-18	4.04858E-17	--
Stem vs Leaf	RHSIM_Rhsim01G0188500	-3.73356912	2.53136E-18	4.18455E-17	RHSIM_Rhsim01G0188500
Stem vs Leaf	RHSIM_Rhsim13G0119400	-3.207210177	2.56769E-18	4.24156E-17	RHSIM_Rhsim13G0119400
Stem vs Leaf	RHSIM_Rhsim06G0239000	-1.881486634	2.78774E-18	4.60177E-17	RHSIM_Rhsim06G0239000
Stem vs Leaf	RHSIM_RhsimUnG0242100	3.156530958	2.94892E-18	4.86086E-17	RHSIM_RhsimUnG0242100
Stem vs Leaf	RHSIM_Rhsim08G0003400	-1.44432446	2.99386E-18	4.93141E-17	RHSIM_Rhsim08G0003400
Stem vs Leaf	RHSIM_Rhsim11G0055300	3.146701574	3.0319E-18	4.99051E-17	RHSIM_Rhsim11G0055300
Stem vs Leaf	RHSIM_Rhsim08G0007800	-2.166158341	3.06759E-18	5.04565E-17	RHSIM_Rhsim08G0007800
Stem vs Leaf	RHSIM_Rhsim10G0173800	-2.661123872	3.12543E-18	5.13712E-17	RHSIM_Rhsim10G0173800
Stem vs Leaf	RHSIM_Rhsim13G0199500	3.681992683	3.17533E-18	5.21541E-17	RHSIM_Rhsim13G0199500
Stem vs Leaf	RHSIM_Rhsim08G0062300	-7.772141301	3.42223E-18	5.61694E-17	RHSIM_Rhsim08G0062300
Stem vs Leaf	RHSIM_Rhsim02G0234800	5.187013817	3.56299E-18	5.84382E-17	RHSIM_Rhsim02G0234800
Stem vs Leaf	RHSIM_Rhsim07G0145700	-3.353981015	3.57997E-18	5.86749E-17	RHSIM_Rhsim07G0145700
Stem vs Leaf	RHSIM_Rhsim01G0174800	3.096197499	3.70719E-18	6.07167E-17	RHSIM_Rhsim01G0174800

Comparison	Gene ID	log_2FC	P-value	FDR (padj)	Gene Name
Stem vs Leaf	RHSIM_Rhsim07G0123800	-2.041155277	3.82682E-18	6.26315E-17	RHSIM_Rhsim07G0123800
Stem vs Leaf	RHSIM_Rhsim04G0250200	-2.201681594	4.01702E-18	6.56979E-17	RHSIM_Rhsim04G0250200
Stem vs Leaf	RHSIM_Rhsim13G0027900	-4.064184761	4.16808E-18	6.812E-17	RHSIM_Rhsim13G0027900
Stem vs Leaf	RHSIM_Rhsim05G0116200	-1.116934715	4.22185E-18	6.89241E-17	RHSIM_Rhsim05G0116200
Stem vs Leaf	novel.2600	-11.26513938	4.22326E-18	6.89241E-17	--
Stem vs Leaf	novel.3963	11.56300869	4.2984E-18	7.01008E-17	--
Stem vs Leaf	RHSIM_Rhsim13G0085200	-1.62593558	4.32482E-18	7.04818E-17	RHSIM_Rhsim13G0085200
Stem vs Leaf	novel.5618	-10.55596402	4.40456E-18	7.17305E-17	--
Stem vs Leaf	RHSIM_Rhsim06G0209000	-2.762032834	4.47262E-18	7.27875E-17	RHSIM_Rhsim06G0209000
Stem vs Leaf	RHSIM_Rhsim10G0084900	-4.31604372	4.51423E-18	7.34128E-17	RHSIM_Rhsim10G0084900
Stem vs Leaf	RHSIM_Rhsim03G0144000	-4.740006387	4.53546E-18	7.3706E-17	RHSIM_Rhsim03G0144000
Stem vs Leaf	RHSIM_Rhsim09G0108100	-2.238696547	4.69939E-18	7.63162E-17	RHSIM_Rhsim09G0108100
Stem vs Leaf	RHSIM_Rhsim13G0064900	-2.790034931	4.72779E-18	7.67233E-17	RHSIM_Rhsim13G0064900
Stem vs Leaf	RHSIM_Rhsim09G0061400	-1.408547254	5.06289E-18	8.21035E-17	RHSIM_Rhsim09G0061400
Stem vs Leaf	RHSIM_Rhsim07G0116300	-2.314232351	5.3283E-18	8.63469E-17	RHSIM_Rhsim07G0116300
Stem vs Leaf	RHSIM_Rhsim08G0198800	-1.56597409	5.98196E-18	9.68717E-17	RHSIM_Rhsim08G0198800
Stem vs Leaf	RHSIM_Rhsim13G0110400	-2.29925183	5.99724E-18	9.70508E-17	RHSIM_Rhsim13G0110400
Stem vs Leaf	RHSIM_Rhsim13G0152000	2.40141318	6.37757E-18	1.03133E-16	RHSIM_Rhsim13G0152000
Stem vs Leaf	RHSIM_Rhsim06G0079300	1.578313026	6.44192E-18	1.04101E-16	RHSIM_Rhsim06G0079300
Stem vs Leaf	RHSIM_Rhsim02G0169200	-2.07719076	6.5486E-18	1.0575E-16	RHSIM_Rhsim02G0169200
Stem vs Leaf	RHSIM_Rhsim13G0175600	3.78256818	6.75286E-18	1.08973E-16	RHSIM_Rhsim13G0175600
Stem vs Leaf	RHSIM_Rhsim06G0063800	-2.598291755	6.82662E-18	1.10086E-16	RHSIM_Rhsim06G0063800
Stem vs Leaf	RHSIM_RhsimUnG0168400	-11.21182745	6.83446E-18	1.10135E-16	RHSIM_RhsimUnG0168400
Stem vs Leaf	RHSIM_Rhsim01G0202500	-1.604371844	7.15538E-18	1.15226E-16	RHSIM_Rhsim01G0202500
Stem vs Leaf	RHSIM_Rhsim03G0052700	5.151279014	7.19444E-18	1.15774E-16	RHSIM_Rhsim03G0052700

Comparison	Gene ID	log_2FC	P-value	FDR (padj)	Gene Name
Stem vs Leaf	RHSIM_Rhsim04G0221300	-2.556122635	7.28343E-18	1.17124E-16	RHSIM_Rhsim04G0221300
Stem vs Leaf	RHSIM_Rhsim10G0126800	-1.477581906	7.40355E-18	1.18973E-16	RHSIM_Rhsim10G0126800
Stem vs Leaf	RHSIM_Rhsim03G0192000	-1.159205396	7.4147E-18	1.19069E-16	RHSIM_Rhsim03G0192000
Stem vs Leaf	RHSIM_Rhsim09G0198300	5.19243738	7.4397E-18	1.19388E-16	RHSIM_Rhsim09G0198300
Stem vs Leaf	RHSIM_Rhsim12G0057400	-1.363358724	7.47201E-18	1.19823E-16	RHSIM_Rhsim12G0057400
Stem vs Leaf	RHSIM_Rhsim10G0133100	-2.243830137	7.65675E-18	1.227E-16	RHSIM_Rhsim10G0133100
Stem vs Leaf	RHSIM_Rhsim13G0003700	-3.932642309	7.75085E-18	1.24121E-16	RHSIM_Rhsim13G0003700
Stem vs Leaf	RHSIM_Rhsim10G0003100	1.197078129	8.02575E-18	1.28434E-16	RHSIM_Rhsim10G0003100
Stem vs Leaf	RHSIM_Rhsim04G0195100	-1.107491847	8.08256E-18	1.29254E-16	RHSIM_Rhsim04G0195100
Stem vs Leaf	RHSIM_Rhsim10G0072700	-5.290960205	8.39945E-18	1.34228E-16	RHSIM_Rhsim10G0072700
Stem vs Leaf	RHSIM_Rhsim10G0156400	2.279978349	8.51816E-18	1.35983E-16	RHSIM_Rhsim10G0156400
Stem vs Leaf	RHSIM_Rhsim09G0146900	-2.993137005	8.52104E-18	1.35983E-16	RHSIM_Rhsim09G0146900
Stem vs Leaf	RHSIM_Rhsim06G0149300	-1.34745001	9.45273E-18	1.50642E-16	RHSIM_Rhsim06G0149300
Stem vs Leaf	RHSIM_Rhsim13G0199400	1.209320919	9.50311E-18	1.5134E-16	RHSIM_Rhsim13G0199400
Stem vs Leaf	RHSIM_Rhsim04G0155800	-2.905154921	9.54287E-18	1.51869E-16	RHSIM_Rhsim04G0155800
Stem vs Leaf	RHSIM_Rhsim03G0197200	-2.410436069	9.58282E-18	1.52399E-16	RHSIM_Rhsim03G0197200
Stem vs Leaf	RHSIM_Rhsim06G0108900	-9.412447921	9.60424E-18	1.52635E-16	RHSIM_Rhsim06G0108900
Stem vs Leaf	RHSIM_Rhsim05G0198100	4.649835833	9.63797E-18	1.53065E-16	RHSIM_Rhsim05G0198100
Stem vs Leaf	RHSIM_Rhsim02G0099100	1.03081446	9.69953E-18	1.53937E-16	RHSIM_Rhsim02G0099100
Stem vs Leaf	RHSIM_Rhsim08G0019100	4.169564827	9.78319E-18	1.55158E-16	RHSIM_Rhsim08G0019100
Stem vs Leaf	RHSIM_Rhsim03G0115100	2.351134528	1.02528E-17	1.62493E-16	RHSIM_Rhsim03G0115100
Stem vs Leaf	RHSIM_Rhsim07G0014500	-7.457897293	1.02952E-17	1.63053E-16	RHSIM_Rhsim07G0014500
Stem vs Leaf	novel.3658	-2.271144004	1.09437E-17	1.73205E-16	--
Stem vs Leaf	RHSIM_RhsimUnG0005900	-3.233620253	1.12648E-17	1.78165E-16	RHSIM_RhsimUnG0005900
Stem vs Leaf	RHSIM_Rhsim04G0157100	3.179245814	1.13796E-17	1.79858E-16	RHSIM_Rhsim04G0157100

Comparison	Gene ID	log_2FC	P-value	FDR (padj)	Gene Name
Stem vs Leaf	RHSIM_Rhsim13G0070200	2.688592695	1.17146E-17	1.85024E-16	RHSIM_Rhsim13G0070200
Stem vs Leaf	RHSIM_Rhsim13G0220700	-2.475002658	1.19005E-17	1.87832E-16	RHSIM_Rhsim13G0220700
Stem vs Leaf	RHSIM_Rhsim03G0167100	-3.185857751	1.19158E-17	1.87944E-16	RHSIM_Rhsim03G0167100
Stem vs Leaf	RHSIM_Rhsim05G0223000	-9.015681291	1.22125E-17	1.92493E-16	RHSIM_Rhsim05G0223000
Stem vs Leaf	RHSIM_Rhsim01G0245900	-1.753553967	1.2339E-17	1.94354E-16	RHSIM_Rhsim01G0245900
Stem vs Leaf	RHSIM_Rhsim09G0098100	1.337710619	1.24467E-17	1.95916E-16	RHSIM_Rhsim09G0098100
Stem vs Leaf	RHSIM_Rhsim02G0034900	-10.56195373	1.24725E-17	1.96189E-16	RHSIM_Rhsim02G0034900
Stem vs Leaf	RHSIM_RhsimUnG0205800	-1.553227509	1.25149E-17	1.9672E-16	RHSIM_RhsimUnG0205800
Stem vs Leaf	RHSIM_Rhsim08G0246100	2.925469486	1.26298E-17	1.98392E-16	RHSIM_Rhsim08G0246100
Stem vs Leaf	RHSIM_Rhsim11G0136600	-2.21457978	1.3122E-17	2.05984E-16	RHSIM_Rhsim11G0136600
Stem vs Leaf	RHSIM_Rhsim13G0181700	-4.807537154	1.33801E-17	2.09892E-16	RHSIM_Rhsim13G0181700
Stem vs Leaf	RHSIM_Rhsim05G0170000	1.44567176	1.36563E-17	2.14079E-16	RHSIM_Rhsim05G0170000
Stem vs Leaf	RHSIM_Rhsim08G0171600	-14.12777285	1.36763E-17	2.14247E-16	RHSIM_Rhsim08G0171600
Stem vs Leaf	RHSIM_Rhsim01G0182500	4.663813407	1.49946E-17	2.34735E-16	RHSIM_Rhsim01G0182500
Stem vs Leaf	RHSIM_Rhsim06G0026300	-1.476722517	1.50075E-17	2.34735E-16	RHSIM_Rhsim06G0026300
Stem vs Leaf	RHSIM_Rhsim01G0167000	-3.091938466	1.50147E-17	2.34735E-16	RHSIM_Rhsim01G0167000
Stem vs Leaf	RHSIM_RhsimUnG0113800	1.658652975	1.50424E-17	2.35008E-16	RHSIM_RhsimUnG0113800
Stem vs Leaf	RHSIM_Rhsim08G0002000	4.253221154	1.5091E-17	2.35607E-16	RHSIM_Rhsim08G0002000
Stem vs Leaf	RHSIM_Rhsim11G0163600	-9.135670608	1.57306E-17	2.45428E-16	RHSIM_Rhsim11G0163600
Stem vs Leaf	RHSIM_Rhsim03G0146900	-2.11248886	1.63407E-17	2.54774E-16	RHSIM_Rhsim03G0146900
Stem vs Leaf	RHSIM_Rhsim06G0175100	5.942514454	1.69143E-17	2.63538E-16	RHSIM_Rhsim06G0175100
Stem vs Leaf	RHSIM_Rhsim03G0174500	6.472523506	1.73224E-17	2.69715E-16	RHSIM_Rhsim03G0174500
Stem vs Leaf	RHSIM_Rhsim05G0049500	4.00139608	1.75513E-17	2.73094E-16	RHSIM_Rhsim05G0049500
Stem vs Leaf	RHSIM_Rhsim02G0032200	-3.877679275	1.7574E-17	2.73263E-16	RHSIM_Rhsim02G0032200
Stem vs Leaf	RHSIM_Rhsim06G0211200	-3.420960688	1.7587E-17	2.7328E-16	RHSIM_Rhsim06G0211200

Comparison	Gene ID	log_2FC	P-value	FDR (padj)	Gene Name
Stem vs Leaf	RHSIM_Rhsim13G0137100	1.657662337	1.87109E-17	2.90549E-16	RHSIM_Rhsim13G0137100
Stem vs Leaf	RHSIM_Rhsim12G0052000	-1.140876166	2.0064E-17	3.1135E-16	RHSIM_Rhsim12G0052000
Stem vs Leaf	RHSIM_Rhsim01G0235200	-2.625010785	2.01013E-17	3.1172E-16	RHSIM_Rhsim01G0235200
Stem vs Leaf	RHSIM_Rhsim11G0069300	3.862276758	2.0599E-17	3.19222E-16	RHSIM_Rhsim11G0069300
Stem vs Leaf	novel.5274	1.744772428	2.17424E-17	3.36714E-16	--
Stem vs Leaf	RHSIM_Rhsim11G0111300	-2.157180576	2.20553E-17	3.41332E-16	RHSIM_Rhsim11G0111300
Stem vs Leaf	novel.5152	-3.669336943	2.2317E-17	3.4515E-16	--
Stem vs Leaf	RHSIM_Rhsim12G0056700	-5.340919548	2.24208E-17	3.46523E-16	RHSIM_Rhsim12G0056700
Stem vs Leaf	RHSIM_RhsimUnG0108100	4.697038724	2.43495E-17	3.76079E-16	RHSIM_RhsimUnG0108100
Stem vs Leaf	RHSIM_Rhsim12G0149300	-10.32534367	2.46505E-17	3.80473E-16	RHSIM_Rhsim12G0149300
Stem vs Leaf	RHSIM_Rhsim08G0009900	2.381103948	2.47566E-17	3.81855E-16	RHSIM_Rhsim08G0009900
Stem vs Leaf	RHSIM_Rhsim04G0243200	-10.02999465	2.55738E-17	3.94195E-16	RHSIM_Rhsim04G0243200
Stem vs Leaf	RHSIM_Rhsim06G0020700	-2.611618326	2.64512E-17	4.07448E-16	RHSIM_Rhsim06G0020700
Stem vs Leaf	RHSIM_Rhsim12G0136400	1.54863006	2.64877E-17	4.07737E-16	RHSIM_Rhsim12G0136400
Stem vs Leaf	RHSIM_RhsimUnG0083600	-5.021447981	2.72707E-17	4.19511E-16	RHSIM_RhsimUnG0083600
Stem vs Leaf	RHSIM_Rhsim12G0135100	7.741299229	2.84169E-17	4.36851E-16	RHSIM_Rhsim12G0135100
Stem vs Leaf	RHSIM_Rhsim07G0224800	5.560191279	2.8659E-17	4.40278E-16	RHSIM_Rhsim07G0224800
Stem vs Leaf	RHSIM_Rhsim08G0177200	1.721183076	2.86801E-17	4.4031E-16	RHSIM_Rhsim08G0177200
Stem vs Leaf	RHSIM_Rhsim05G0049900	4.687232388	2.96803E-17	4.55362E-16	RHSIM_Rhsim05G0049900
Stem vs Leaf	RHSIM_Rhsim03G0098400	-1.682984774	3.03076E-17	4.64677E-16	RHSIM_Rhsim03G0098400
Stem vs Leaf	RHSIM_Rhsim08G0102300	-5.920886128	3.25163E-17	4.98209E-16	RHSIM_Rhsim08G0102300
Stem vs Leaf	RHSIM_Rhsim06G0148500	-2.829853352	3.34996E-17	5.12934E-16	RHSIM_Rhsim06G0148500
Stem vs Leaf	RHSIM_Rhsim03G0168800	-11.58169846	3.52294E-17	5.39062E-16	RHSIM_Rhsim03G0168800
Stem vs Leaf	RHSIM_Rhsim08G0239200	-11.40731911	3.60482E-17	5.51224E-16	RHSIM_Rhsim08G0239200
Stem vs Leaf	RHSIM_Rhsim01G0194400	-1.97784773	3.67626E-17	5.61775E-16	RHSIM_Rhsim01G0194400

Comparison	Gene ID	log_2FC	P-value	FDR (padj)	Gene Name
Stem vs Leaf	RHSIM_Rhsim06G0192300	7.060407588	3.74581E-17	5.72024E-16	RHSIM_Rhsim06G0192300
Stem vs Leaf	RHSIM_RhsimUnG0085400	-3.077752128	3.77866E-17	5.76658E-16	RHSIM_RhsimUnG0085400
Stem vs Leaf	RHSIM_Rhsim10G0185700	-1.814481156	4.07609E-17	6.21638E-16	RHSIM_Rhsim10G0185700
Stem vs Leaf	RHSIM_RhsimUnG0030200	-5.947973664	4.0863E-17	6.22783E-16	RHSIM_RhsimUnG0030200
Stem vs Leaf	RHSIM_Rhsim10G0118300	5.328192143	4.17571E-17	6.35918E-16	RHSIM_Rhsim10G0118300
Stem vs Leaf	RHSIM_Rhsim06G0232900	-1.8119969	4.17801E-17	6.35918E-16	RHSIM_Rhsim06G0232900
Stem vs Leaf	RHSIM_Rhsim10G0029400	-1.998614378	4.57488E-17	6.95864E-16	RHSIM_Rhsim10G0029400
Stem vs Leaf	novel.5337	1.597448365	4.69863E-17	7.14216E-16	--
Stem vs Leaf	RHSIM_Rhsim01G0024600	-2.023484056	5.10736E-17	7.75834E-16	RHSIM_Rhsim01G0024600
Stem vs Leaf	RHSIM_Rhsim04G0196000	-2.083823646	5.19389E-17	7.88459E-16	RHSIM_Rhsim04G0196000
Stem vs Leaf	RHSIM_Rhsim11G0144900	2.300201356	5.30777E-17	8.05215E-16	RHSIM_Rhsim11G0144900
Stem vs Leaf	RHSIM_Rhsim06G0055300	-2.082752268	5.51279E-17	8.35768E-16	RHSIM_Rhsim06G0055300
Stem vs Leaf	RHSIM_Rhsim10G0151800	-3.191985164	5.60729E-17	8.49536E-16	RHSIM_Rhsim10G0151800
Stem vs Leaf	RHSIM_Rhsim03G0184700	-6.150831177	5.79405E-17	8.77254E-16	RHSIM_Rhsim03G0184700
Stem vs Leaf	RHSIM_Rhsim04G0195600	2.174775739	5.81309E-17	8.79559E-16	RHSIM_Rhsim04G0195600
Stem vs Leaf	RHSIM_Rhsim12G0037800	-1.592698106	5.93187E-17	8.96943E-16	RHSIM_Rhsim12G0037800
Stem vs Leaf	RHSIM_Rhsim02G0237600	-2.544359956	6.07326E-17	9.1772E-16	RHSIM_Rhsim02G0237600
Stem vs Leaf	RHSIM_Rhsim13G0056000	5.075379049	6.087E-17	9.19193E-16	RHSIM_Rhsim13G0056000
Stem vs Leaf	RHSIM_Rhsim01G0269000	1.780186593	6.1026E-17	9.20945E-16	RHSIM_Rhsim01G0269000
Stem vs Leaf	RHSIM_Rhsim09G0135500	-4.835682894	6.2524E-17	9.42934E-16	RHSIM_Rhsim09G0135500
Stem vs Leaf	RHSIM_Rhsim10G0002700	3.821031449	6.33054E-17	9.54094E-16	RHSIM_Rhsim10G0002700
Stem vs Leaf	RHSIM_Rhsim04G0130700	-1.759466867	6.74074E-17	1.01525E-15	RHSIM_Rhsim04G0130700
Stem vs Leaf	RHSIM_Rhsim13G0103500	-1.956834328	6.87384E-17	1.03462E-15	RHSIM_Rhsim13G0103500
Stem vs Leaf	RHSIM_Rhsim04G0016100	-1.912281305	6.92766E-17	1.04204E-15	RHSIM_Rhsim04G0016100
Stem vs Leaf	RHSIM_Rhsim02G0031900	-2.27118697	7.17327E-17	1.07828E-15	RHSIM_Rhsim02G0031900

Comparison	Gene ID	log_2FC	P-value	FDR (padj)	Gene Name
Stem vs Leaf	RHSIM_Rhsim12G0127500	-1.967098499	7.35289E-17	1.10456E-15	RHSIM_Rhsim12G0127500
Stem vs Leaf	RHSIM_Rhsim02G0167800	6.212320426	8.68617E-17	1.304E-15	RHSIM_Rhsim02G0167800
Stem vs Leaf	RHSIM_Rhsim01G0280000	-1.203738008	8.77726E-17	1.31682E-15	RHSIM_Rhsim01G0280000
Stem vs Leaf	RHSIM_Rhsim02G0241200	-1.492898681	8.88369E-17	1.33192E-15	RHSIM_Rhsim02G0241200
Stem vs Leaf	RHSIM_Rhsim03G0185800	-7.072986069	8.94295E-17	1.33967E-15	RHSIM_Rhsim03G0185800
Stem vs Leaf	RHSIM_Rhsim06G0204400	-5.73870613	8.94703E-17	1.33967E-15	RHSIM_Rhsim06G0204400
Stem vs Leaf	RHSIM_Rhsim08G0075800	-1.156141214	8.98211E-17	1.34405E-15	RHSIM_Rhsim08G0075800
Stem vs Leaf	RHSIM_Rhsim10G0193100	-3.460780017	9.11955E-17	1.36373E-15	RHSIM_Rhsim10G0193100
Stem vs Leaf	RHSIM_Rhsim03G0176200	-1.906933507	9.1344E-17	1.36507E-15	RHSIM_Rhsim03G0176200
Stem vs Leaf	novel.2302	-2.043554878	9.27721E-17	1.38551E-15	--
Stem vs Leaf	RHSIM_Rhsim04G0108400	-1.189566316	9.51555E-17	1.42018E-15	RHSIM_Rhsim04G0108400
Stem vs Leaf	RHSIM_Rhsim09G0160400	1.194636803	1.00198E-16	1.49447E-15	RHSIM_Rhsim09G0160400
Stem vs Leaf	RHSIM_Rhsim13G0142400	-3.068649887	1.0256E-16	1.52871E-15	RHSIM_Rhsim13G0142400
Stem vs Leaf	RHSIM_Rhsim07G0122100	1.028710578	1.05743E-16	1.57514E-15	RHSIM_Rhsim07G0122100
Stem vs Leaf	RHSIM_Rhsim07G0040500	1.669539747	1.06024E-16	1.57831E-15	RHSIM_Rhsim07G0040500
Stem vs Leaf	RHSIM_Rhsim03G0077400	4.448787278	1.06253E-16	1.58069E-15	RHSIM_Rhsim03G0077400
Stem vs Leaf	RHSIM_Rhsim02G0255300	5.986464547	1.07904E-16	1.60422E-15	RHSIM_Rhsim02G0255300
Stem vs Leaf	RHSIM_Rhsim08G0244400	3.051086707	1.09531E-16	1.62735E-15	RHSIM_Rhsim08G0244400
Stem vs Leaf	RHSIM_RhsimUnG0095500	-3.035909753	1.09668E-16	1.62834E-15	RHSIM_RhsimUnG0095500
Stem vs Leaf	novel.526	-10.12309055	1.13588E-16	1.68546E-15	--
Stem vs Leaf	RHSIM_Rhsim06G0004100	2.649189629	1.17116E-16	1.7367E-15	RHSIM_Rhsim06G0004100
Stem vs Leaf	novel.3413	6.964202446	1.18068E-16	1.74968E-15	--
Stem vs Leaf	RHSIM_Rhsim08G0129700	-1.707769032	1.18826E-16	1.75978E-15	RHSIM_Rhsim08G0129700
Stem vs Leaf	RHSIM_Rhsim12G0076700	-1.372701889	1.19791E-16	1.77294E-15	RHSIM_Rhsim12G0076700
Stem vs Leaf	novel.3142	-7.806837609	1.23539E-16	1.82723E-15	--

Comparison	Gene ID	log_2FC	P-value	FDR (padj)	Gene Name
Stem vs Leaf	RHSIM_Rhsim02G0138600	-5.816934329	1.24174E-16	1.83545E-15	RHSIM_Rhsim02G0138600
Stem vs Leaf	RHSIM_Rhsim06G0167500	-5.369131752	1.26103E-16	1.86276E-15	RHSIM_Rhsim06G0167500
Stem vs Leaf	novel.1579	-1.482552402	1.28036E-16	1.89011E-15	--
Stem vs Leaf	RHSIM_Rhsim10G0164700	-2.341797506	1.29537E-16	1.91105E-15	RHSIM_Rhsim10G0164700
Stem vs Leaf	RHSIM_Rhsim08G0230000	-7.197452754	1.32204E-16	1.94914E-15	RHSIM_Rhsim08G0230000
Stem vs Leaf	RHSIM_Rhsim11G0176700	5.551747526	1.33626E-16	1.96884E-15	RHSIM_Rhsim11G0176700
Stem vs Leaf	RHSIM_Rhsim07G0163900	1.781971427	1.33848E-16	1.97086E-15	RHSIM_Rhsim07G0163900
Stem vs Leaf	novel.5254	3.268982496	1.3571E-16	1.997E-15	--
Stem vs Leaf	RHSIM_Rhsim13G0228400	-1.066184755	1.41089E-16	2.07483E-15	RHSIM_Rhsim13G0228400
Stem vs Leaf	RHSIM_Rhsim11G0047300	1.054402899	1.44407E-16	2.12226E-15	RHSIM_Rhsim11G0047300
Stem vs Leaf	RHSIM_Rhsim10G0028700	-3.462358613	1.48216E-16	2.17686E-15	RHSIM_Rhsim10G0028700
Stem vs Leaf	RHSIM_Rhsim01G0027000	1.655446645	1.54862E-16	2.27302E-15	RHSIM_Rhsim01G0027000
Stem vs Leaf	novel.320	-2.801721841	1.64672E-16	2.41546E-15	--
Stem vs Leaf	RHSIM_Rhsim08G0022100	-6.122387751	1.6728E-16	2.45216E-15	RHSIM_Rhsim08G0022100
Stem vs Leaf	RHSIM_Rhsim07G0188200	-1.391851563	1.68379E-16	2.46671E-15	RHSIM_Rhsim07G0188200
Stem vs Leaf	RHSIM_Rhsim04G0100500	-1.368025038	1.7114E-16	2.50556E-15	RHSIM_Rhsim04G0100500
Stem vs Leaf	RHSIM_Rhsim11G0075000	1.597826708	1.71846E-16	2.5143E-15	RHSIM_Rhsim11G0075000
Stem vs Leaf	RHSIM_Rhsim13G0037500	1.390587831	1.72996E-16	2.52951E-15	RHSIM_Rhsim13G0037500
Stem vs Leaf	RHSIM_Rhsim02G0089600	-2.041395633	1.73735E-16	2.53871E-15	RHSIM_Rhsim02G0089600
Stem vs Leaf	RHSIM_Rhsim11G0193400	-2.182992944	1.76025E-16	2.57054E-15	RHSIM_Rhsim11G0193400
Stem vs Leaf	RHSIM_Rhsim08G0115500	4.92186825	1.7668E-16	2.57848E-15	RHSIM_Rhsim08G0115500
Stem vs Leaf	novel.2269	2.929353862	1.7712E-16	2.58326E-15	--
Stem vs Leaf	RHSIM_Rhsim06G0047000	-2.039013219	1.80388E-16	2.62926E-15	RHSIM_Rhsim06G0047000
Stem vs Leaf	novel.1387	-3.431034155	1.84518E-16	2.68606E-15	--
Stem vs Leaf	RHSIM_Rhsim01G0089700	-2.514873358	1.88867E-16	2.74763E-15	RHSIM_Rhsim01G0089700

Comparison	Gene ID	log2FC	P-value	FDR (padj)	Gene Name
Stem vs Leaf	RHSIM_Rhsim09G0068500	2.789880497	1.90127E-16	2.76422E-15	RHSIM_Rhsim09G0068500
Stem vs Leaf	RHSIM_Rhsim06G0072500	5.99043235	1.9206E-16	2.79056E-15	RHSIM_Rhsim06G0072500
Stem vs Leaf	novel.1534	-3.275292166	1.94705E-16	2.82721E-15	--
Stem vs Leaf	RHSIM_Rhsim03G0089000	1.537814434	2.045E-16	2.96757E-15	RHSIM_Rhsim03G0089000
Stem vs Leaf	RHSIM_Rhsim08G0240300	-1.551114398	2.08966E-16	3.03047E-15	RHSIM_Rhsim08G0240300
Stem vs Leaf	RHSIM_Rhsim05G0002500	1.99324906	2.09367E-16	3.03438E-15	RHSIM_Rhsim05G0002500
Stem vs Leaf	RHSIM_RhsimUnG0197300	-5.593985978	2.14367E-16	3.10489E-15	RHSIM_RhsimUnG0197300
Stem vs Leaf	RHSIM_Rhsim07G0129600	6.125859336	2.19776E-16	3.18122E-15	RHSIM_Rhsim07G0129600
Stem vs Leaf	RHSIM_Rhsim03G0244600	-2.605399583	2.1999E-16	3.18233E-15	RHSIM_Rhsim03G0244600
Stem vs Leaf	RHSIM_Rhsim08G0239300	-6.203267219	2.25269E-16	3.25665E-15	RHSIM_Rhsim08G0239300
Stem vs Leaf	RHSIM_Rhsim07G0068000	1.06632721	2.26557E-16	3.27322E-15	RHSIM_Rhsim07G0068000
Stem vs Leaf	RHSIM_Rhsim07G0071500	3.829268874	2.27243E-16	3.28107E-15	RHSIM_Rhsim07G0071500
Stem vs Leaf	RHSIM_Rhsim11G0179300	2.527030642	2.34166E-16	3.37891E-15	RHSIM_Rhsim11G0179300
Stem vs Leaf	RHSIM_Rhsim12G0187000	10.11665704	2.3714E-16	3.41968E-15	RHSIM_Rhsim12G0187000
Stem vs Leaf	RHSIM_Rhsim05G0169700	-2.751997272	2.40727E-16	3.46925E-15	RHSIM_Rhsim05G0169700
Stem vs Leaf	novel.5894	-7.286191521	2.55256E-16	3.67633E-15	--
Stem vs Leaf	RHSIM_Rhsim06G0071400	-10.05762197	2.64632E-16	3.80898E-15	RHSIM_Rhsim06G0071400
Stem vs Leaf	RHSIM_Rhsim06G0188300	-7.848305319	2.72596E-16	3.92116E-15	RHSIM_Rhsim06G0188300
Stem vs Leaf	RHSIM_Rhsim03G0078400	1.374369135	2.74447E-16	3.94532E-15	RHSIM_Rhsim03G0078400
Stem vs Leaf	RHSIM_Rhsim13G0229500	3.017017421	2.808E-16	4.03414E-15	RHSIM_Rhsim13G0229500
Stem vs Leaf	RHSIM_Rhsim06G0051200	7.494614005	2.94656E-16	4.23056E-15	RHSIM_Rhsim06G0051200
Stem vs Leaf	RHSIM_Rhsim09G0110000	-1.874025715	3.03252E-16	4.35126E-15	RHSIM_Rhsim09G0110000
Stem vs Leaf	RHSIM_Rhsim10G0202700	-10.77029538	3.05961E-16	4.38741E-15	RHSIM_Rhsim10G0202700
Stem vs Leaf	RHSIM_Rhsim10G0037300	-3.18434109	3.18198E-16	4.56005E-15	RHSIM_Rhsim10G0037300
Stem vs Leaf	RHSIM_Rhsim08G0252300	-1.426158969	3.20466E-16	4.5897E-15	RHSIM_Rhsim08G0252300

Comparison	Gene ID	log_2FC	P-value	FDR (padj)	Gene Name
Stem vs Leaf	RHSIM_Rhsim02G0025500	4.799513742	3.35849E-16	4.80702E-15	RHSIM_Rhsim02G0025500
Stem vs Leaf	RHSIM_RhsimUnG0120600	-3.093629222	3.494E-16	4.99787E-15	RHSIM_RhsimUnG0120600
Stem vs Leaf	RHSIM_Rhsim12G0186800	10.05017043	3.51501E-16	5.02481E-15	RHSIM_Rhsim12G0186800
Stem vs Leaf	RHSIM_RhsimUnG0123500	2.945025775	3.52665E-16	5.03832E-15	RHSIM_RhsimUnG0123500
Stem vs Leaf	RHSIM_Rhsim12G0072500	-1.931905248	3.67344E-16	5.24477E-15	RHSIM_Rhsim12G0072500
Stem vs Leaf	novel.570	2.874954075	3.83182E-16	5.46752E-15	--
Stem vs Leaf	RHSIM_Rhsim10G0019900	-2.027673327	3.85495E-16	5.49712E-15	RHSIM_Rhsim10G0019900
Stem vs Leaf	RHSIM_Rhsim03G0057700	3.130413883	4.17649E-16	5.95195E-15	RHSIM_Rhsim03G0057700
Stem vs Leaf	RHSIM_Rhsim01G0287600	1.350481167	4.22387E-16	6.01575E-15	RHSIM_Rhsim01G0287600
Stem vs Leaf	RHSIM_Rhsim02G0031200	1.335720013	4.23929E-16	6.03398E-15	RHSIM_Rhsim02G0031200
Stem vs Leaf	RHSIM_Rhsim11G0025800	1.399969288	4.29542E-16	6.1101E-15	RHSIM_Rhsim11G0025800
Stem vs Leaf	RHSIM_Rhsim12G0173800	1.144617823	4.47357E-16	6.3596E-15	RHSIM_Rhsim12G0173800
Stem vs Leaf	RHSIM_RhsimUnG0131800	-6.545537176	4.52317E-16	6.42613E-15	RHSIM_RhsimUnG0131800
Stem vs Leaf	RHSIM_Rhsim12G0209000	-1.33561449	4.56085E-16	6.47568E-15	RHSIM_Rhsim12G0209000
Stem vs Leaf	RHSIM_Rhsim13G0233200	1.808782759	4.65897E-16	6.61092E-15	RHSIM_Rhsim13G0233200
Stem vs Leaf	novel.5485	2.057702139	4.69671E-16	6.66037E-15	--
Stem vs Leaf	RHSIM_Rhsim05G0170400	-2.077350792	4.83427E-16	6.85123E-15	RHSIM_Rhsim05G0170400
Stem vs Leaf	RHSIM_Rhsim12G0201200	-2.42899123	4.91972E-16	6.96804E-15	RHSIM_Rhsim12G0201200
Stem vs Leaf	RHSIM_Rhsim03G0004300	7.291298152	4.95911E-16	7.01952E-15	RHSIM_Rhsim03G0004300
Stem vs Leaf	RHSIM_Rhsim07G0004400	-2.31678467	5.0432E-16	7.13416E-15	RHSIM_Rhsim07G0004400
Stem vs Leaf	RHSIM_Rhsim04G0126500	3.20722471	5.10067E-16	7.21104E-15	RHSIM_Rhsim04G0126500
Stem vs Leaf	RHSIM_Rhsim08G0227000	9.929525654	5.10478E-16	7.21242E-15	RHSIM_Rhsim08G0227000
Stem vs Leaf	RHSIM_Rhsim09G0199900	-2.519303789	5.22053E-16	7.37145E-15	RHSIM_Rhsim09G0199900
Stem vs Leaf	RHSIM_Rhsim09G0024100	4.302548543	5.29209E-16	7.46791E-15	RHSIM_Rhsim09G0024100
Stem vs Leaf	RHSIM_Rhsim08G0241800	1.254762903	5.46081E-16	7.70127E-15	RHSIM_Rhsim08G0241800

Comparison	Gene ID	log_2FC	P-value	FDR (padj)	Gene Name
Stem vs Leaf	RHSIM_Rhsim06G0166100	-2.842922302	5.54783E-16	7.81922E-15	RHSIM_Rhsim06G0166100
Stem vs Leaf	RHSIM_Rhsim04G0202300	-1.832775993	5.65004E-16	7.9584E-15	RHSIM_Rhsim04G0202300
Stem vs Leaf	RHSIM_Rhsim08G0100800	3.767469565	5.83551E-16	8.21462E-15	RHSIM_Rhsim08G0100800
Stem vs Leaf	RHSIM_Rhsim03G0068000	1.48738527	5.84709E-16	8.22591E-15	RHSIM_Rhsim03G0068000
Stem vs Leaf	novel.4762	-4.933074856	5.87912E-16	8.26592E-15	--
Stem vs Leaf	RHSIM_Rhsim02G0236500	-10.75734403	5.97807E-16	8.39992E-15	RHSIM_Rhsim02G0236500
Stem vs Leaf	RHSIM_RhsimUnG0133400	3.832970492	6.09313E-16	8.55637E-15	RHSIM_RhsimUnG0133400
Stem vs Leaf	RHSIM_Rhsim05G0162500	-1.880666659	6.18453E-16	8.67943E-15	RHSIM_Rhsim05G0162500
Stem vs Leaf	RHSIM_Rhsim12G0143300	6.002840923	6.19546E-16	8.68949E-15	RHSIM_Rhsim12G0143300
Stem vs Leaf	RHSIM_Rhsim07G0001600	3.463041404	6.20048E-16	8.69123E-15	RHSIM_Rhsim07G0001600
Stem vs Leaf	RHSIM_Rhsim01G0262700	-1.844632951	6.28976E-16	8.80576E-15	RHSIM_Rhsim01G0262700
Stem vs Leaf	RHSIM_Rhsim06G0064200	3.063342249	6.28983E-16	8.80576E-15	RHSIM_Rhsim06G0064200
Stem vs Leaf	RHSIM_Rhsim13G0093700	-2.210573984	6.56499E-16	9.17983E-15	RHSIM_Rhsim13G0093700
Stem vs Leaf	RHSIM_Rhsim04G0170900	-1.95274052	6.62253E-16	9.25467E-15	RHSIM_Rhsim04G0170900
Stem vs Leaf	RHSIM_Rhsim03G0193700	-2.361254441	6.81138E-16	9.51281E-15	RHSIM_Rhsim03G0193700
Stem vs Leaf	RHSIM_Rhsim09G0055600	-8.613872847	6.82152E-16	9.5212E-15	RHSIM_Rhsim09G0055600
Stem vs Leaf	novel.1237	1.21344872	6.83087E-16	9.52848E-15	--
Stem vs Leaf	RHSIM_Rhsim02G0106400	-1.032117707	6.87016E-16	9.57749E-15	RHSIM_Rhsim02G0106400
Stem vs Leaf	novel.4634	-5.468245519	7.0743E-16	9.85611E-15	--
Stem vs Leaf	RHSIM_Rhsim01G0285400	-3.523971954	7.10464E-16	9.89241E-15	RHSIM_Rhsim01G0285400
Stem vs Leaf	RHSIM_Rhsim10G0133800	-3.557074642	7.14043E-16	9.93624E-15	RHSIM_Rhsim10G0133800
Stem vs Leaf	RHSIM_Rhsim07G0139000	-1.557972426	7.3778E-16	1.02604E-14	RHSIM_Rhsim07G0139000
Stem vs Leaf	RHSIM_Rhsim06G0152500	2.840426126	7.38638E-16	1.02661E-14	RHSIM_Rhsim06G0152500
Stem vs Leaf	RHSIM_Rhsim12G0200100	-6.735435487	7.45562E-16	1.03561E-14	RHSIM_Rhsim12G0200100
Stem vs Leaf	RHSIM_Rhsim03G0155500	-2.462470113	7.46668E-16	1.03652E-14	RHSIM_Rhsim03G0155500

Comparison	Gene ID	log_2FC	P-value	FDR (padj)	Gene Name
Stem vs Leaf	RHSIM_RhsimUnG0251300	2.197993594	7.65137E-16	1.06152E-14	RHSIM_RhsimUnG0251300
Stem vs Leaf	RHSIM_Rhsim10G0068300	-4.519784354	7.70177E-16	1.06787E-14	RHSIM_Rhsim10G0068300
Stem vs Leaf	RHSIM_Rhsim13G0042900	1.128152164	7.74417E-16	1.0731E-14	RHSIM_Rhsim13G0042900
Stem vs Leaf	RHSIM_Rhsim06G0186600	3.260874432	7.86341E-16	1.08897E-14	RHSIM_Rhsim06G0186600
Stem vs Leaf	RHSIM_RhsimUnG0129000	7.366697522	7.87637E-16	1.09011E-14	RHSIM_RhsimUnG0129000
Stem vs Leaf	RHSIM_Rhsim13G0076300	-1.316173103	7.92715E-16	1.09648E-14	RHSIM_Rhsim13G0076300
Stem vs Leaf	novel.3057	2.678484786	8.14316E-16	1.12568E-14	--
Stem vs Leaf	RHSIM_Rhsim05G0008600	3.908746081	8.42803E-16	1.16436E-14	RHSIM_Rhsim05G0008600
Stem vs Leaf	RHSIM_Rhsim02G0164900	-5.55412472	8.48026E-16	1.17088E-14	RHSIM_Rhsim02G0164900
Stem vs Leaf	RHSIM_Rhsim07G0204200	4.380844824	8.5118E-16	1.17453E-14	RHSIM_Rhsim07G0204200
Stem vs Leaf	RHSIM_Rhsim13G0091100	-4.679690568	8.54435E-16	1.17792E-14	RHSIM_Rhsim13G0091100
Stem vs Leaf	RHSIM_Rhsim09G0064200	-3.724124164	8.54662E-16	1.17792E-14	RHSIM_Rhsim09G0064200
Stem vs Leaf	RHSIM_Rhsim03G0172100	3.870545175	8.67414E-16	1.19478E-14	RHSIM_Rhsim03G0172100
Stem vs Leaf	RHSIM_Rhsim08G0224800	1.829085288	8.96231E-16	1.23374E-14	RHSIM_Rhsim08G0224800
Stem vs Leaf	RHSIM_Rhsim08G0005200	1.335421089	9.14679E-16	1.25838E-14	RHSIM_Rhsim08G0005200
Stem vs Leaf	RHSIM_Rhsim03G0001300	-4.41767156	9.41442E-16	1.29443E-14	RHSIM_Rhsim03G0001300
Stem vs Leaf	RHSIM_RhsimUnG0105400	5.27140907	9.58964E-16	1.31773E-14	RHSIM_RhsimUnG0105400
Stem vs Leaf	RHSIM_Rhsim13G0174300	2.166876629	9.65527E-16	1.32596E-14	RHSIM_Rhsim13G0174300
Stem vs Leaf	RHSIM_Rhsim07G0064800	-6.184893166	9.92689E-16	1.36245E-14	RHSIM_Rhsim07G0064800
Stem vs Leaf	RHSIM_Rhsim05G0180400	-2.64827779	1.01204E-15	1.38818E-14	RHSIM_Rhsim05G0180400
Stem vs Leaf	RHSIM_Rhsim09G0011700	-4.628098456	1.08882E-15	1.49261E-14	RHSIM_Rhsim09G0011700
Stem vs Leaf	RHSIM_Rhsim05G0041000	-3.670801313	1.09477E-15	1.49987E-14	RHSIM_Rhsim05G0041000
Stem vs Leaf	RHSIM_RhsimPtG0003500	-2.114881742	1.09864E-15	1.50428E-14	RHSIM_RhsimPtG0003500
Stem vs Leaf	RHSIM_Rhsim04G0050100	-1.771978483	1.12114E-15	1.53417E-14	RHSIM_Rhsim04G0050100
Stem vs Leaf	RHSIM_Rhsim01G0192200	-3.594510446	1.15911E-15	1.58519E-14	RHSIM_Rhsim01G0192200

Comparison	Gene ID	log_2FC	P-value	FDR (padj)	Gene Name
Stem vs Leaf	novel.4450	-5.214031114	1.18786E-15	1.62354E-14	--
Stem vs Leaf	RHSIM_Rhsim04G0207100	3.619217949	1.19872E-15	1.63742E-14	RHSIM_Rhsim04G0207100
Stem vs Leaf	RHSIM_Rhsim06G0133700	-3.602159235	1.21355E-15	1.6567E-14	RHSIM_Rhsim06G0133700
Stem vs Leaf	RHSIM_Rhsim01G0241700	2.642199084	1.2337E-15	1.68321E-14	RHSIM_Rhsim01G0241700
Stem vs Leaf	RHSIM_Rhsim01G0051700	-3.032974406	1.28406E-15	1.75089E-14	RHSIM_Rhsim01G0051700
Stem vs Leaf	RHSIM_Rhsim13G0104000	-1.701426219	1.31289E-15	1.78914E-14	RHSIM_Rhsim13G0104000
Stem vs Leaf	RHSIM_Rhsim12G0137200	-1.765144764	1.31817E-15	1.79526E-14	RHSIM_Rhsim12G0137200
Stem vs Leaf	RHSIM_Rhsim04G0171200	-1.191699648	1.32238E-15	1.79993E-14	RHSIM_Rhsim04G0171200
Stem vs Leaf	novel.4140	-1.123347265	1.32533E-15	1.80288E-14	--
Stem vs Leaf	novel.1829	-9.991832335	1.37953E-15	1.87551E-14	--
Stem vs Leaf	RHSIM_RhsimUnG0189800	-3.509061116	1.38897E-15	1.88723E-14	RHSIM_RhsimUnG0189800
Stem vs Leaf	RHSIM_Rhsim11G0016600	2.033686152	1.39702E-15	1.89704E-14	RHSIM_Rhsim11G0016600
Stem vs Leaf	RHSIM_Rhsim12G0207300	-1.655008792	1.4094E-15	1.91274E-14	RHSIM_Rhsim12G0207300
Stem vs Leaf	RHSIM_Rhsim06G0026900	5.902447685	1.44715E-15	1.96281E-14	RHSIM_Rhsim06G0026900
Stem vs Leaf	RHSIM_Rhsim12G0020600	3.62755474	1.46206E-15	1.98187E-14	RHSIM_Rhsim12G0020600
Stem vs Leaf	RHSIM_Rhsim10G0141100	5.036543402	1.47706E-15	2.00101E-14	RHSIM_Rhsim10G0141100
Stem vs Leaf	RHSIM_Rhsim12G0005400	3.773379809	1.52571E-15	2.06571E-14	RHSIM_Rhsim12G0005400
Stem vs Leaf	RHSIM_Rhsim06G0223100	-2.103907262	1.563E-15	2.11372E-14	RHSIM_Rhsim06G0223100
Stem vs Leaf	RHSIM_Rhsim07G0251700	-3.060387202	1.5683E-15	2.11964E-14	RHSIM_Rhsim07G0251700
Stem vs Leaf	RHSIM_Rhsim02G0090600	-6.942781762	1.5709E-15	2.12191E-14	RHSIM_Rhsim02G0090600
Stem vs Leaf	RHSIM_Rhsim03G0219600	-3.588372355	1.57994E-15	2.13204E-14	RHSIM_Rhsim03G0219600
Stem vs Leaf	RHSIM_Rhsim05G0097700	-9.926724757	1.58025E-15	2.13204E-14	RHSIM_Rhsim05G0097700
Stem vs Leaf	novel.170	-4.486103039	1.62876E-15	2.1962E-14	--
Stem vs Leaf	novel.4440	1.322812878	1.63794E-15	2.2073E-14	--
Stem vs Leaf	RHSIM_Rhsim02G0209700	1.800156069	1.64999E-15	2.22223E-14	RHSIM_Rhsim02G0209700

Comparison	Gene ID	log_2FC	P-value	FDR (padj)	Gene Name
Stem vs Leaf	RHSIM_Rhsim01G0288000	-2.368996339	1.70561E-15	2.29445E-14	RHSIM_Rhsim01G0288000
Stem vs Leaf	RHSIM_RhsimUnG0190300	-9.986298568	1.71657E-15	2.30785E-14	RHSIM_RhsimUnG0190300
Stem vs Leaf	RHSIM_Rhsim07G0170200	-2.029593908	1.75812E-15	2.36233E-14	RHSIM_Rhsim07G0170200
Stem vs Leaf	RHSIM_Rhsim07G0143700	-1.17515334	1.81173E-15	2.43295E-14	RHSIM_Rhsim07G0143700
Stem vs Leaf	RHSIM_Rhsim06G0093200	-1.523640558	1.82153E-15	2.44469E-14	RHSIM_Rhsim06G0093200
Stem vs Leaf	RHSIM_Rhsim10G0021100	-1.394210441	1.85942E-15	2.4941E-14	RHSIM_Rhsim10G0021100
Stem vs Leaf	RHSIM_Rhsim08G0146500	3.365284544	1.89977E-15	2.54673E-14	RHSIM_Rhsim08G0146500
Stem vs Leaf	RHSIM_Rhsim11G0129100	1.219529941	1.91128E-15	2.56068E-14	RHSIM_Rhsim11G0129100
Stem vs Leaf	RHSIM_Rhsim12G0144700	-2.310954713	1.91429E-15	2.56321E-14	RHSIM_Rhsim12G0144700
Stem vs Leaf	RHSIM_Rhsim01G0019600	-4.09846267	1.92991E-15	2.58263E-14	RHSIM_Rhsim01G0019600
Stem vs Leaf	RHSIM_Rhsim05G0165200	1.48763209	1.95956E-15	2.62078E-14	RHSIM_Rhsim05G0165200
Stem vs Leaf	RHSIM_Rhsim09G0084400	-1.411803241	1.96311E-15	2.62401E-14	RHSIM_Rhsim09G0084400
Stem vs Leaf	RHSIM_Rhsim11G0039900	-10.57663379	1.9919E-15	2.66095E-14	RHSIM_Rhsim11G0039900
Stem vs Leaf	RHSIM_Rhsim09G0184200	-2.960114523	2.09769E-15	2.80065E-14	RHSIM_Rhsim09G0184200
Stem vs Leaf	RHSIM_Rhsim04G0032700	-1.935773566	2.09992E-15	2.802E-14	RHSIM_Rhsim04G0032700
Stem vs Leaf	RHSIM_Rhsim06G0030300	2.307010213	2.20558E-15	2.94129E-14	RHSIM_Rhsim06G0030300
Stem vs Leaf	RHSIM_Rhsim08G0198600	-3.040486649	2.24326E-15	2.9898E-14	RHSIM_Rhsim08G0198600
Stem vs Leaf	RHSIM_RhsimUnG0124000	5.250354201	2.27455E-15	3.02976E-14	RHSIM_RhsimUnG0124000
Stem vs Leaf	RHSIM_Rhsim02G0074200	-8.483802113	2.27689E-15	3.03112E-14	RHSIM_Rhsim02G0074200
Stem vs Leaf	RHSIM_Rhsim07G0082500	-1.298727826	2.40078E-15	3.1942E-14	RHSIM_Rhsim07G0082500
Stem vs Leaf	RHSIM_Rhsim04G0019500	-1.352137562	2.42475E-15	3.22282E-14	RHSIM_Rhsim04G0019500
Stem vs Leaf	RHSIM_Rhsim13G0112300	-9.383484535	2.42509E-15	3.22282E-14	RHSIM_Rhsim13G0112300
Stem vs Leaf	RHSIM_Rhsim12G0098100	-2.096179935	2.42735E-15	3.22397E-14	RHSIM_Rhsim12G0098100
Stem vs Leaf	RHSIM_Rhsim01G0195600	1.598812599	2.49272E-15	3.30889E-14	RHSIM_Rhsim01G0195600
Stem vs Leaf	novel.3103	3.918654778	2.49945E-15	3.31591E-14	--

Comparison	Gene ID	log_2FC	P-value	FDR (padj)	Gene Name
Stem vs Leaf	RHSIM_Rhsim04G0078100	-9.651448926	2.54517E-15	3.37437E-14	RHSIM_Rhsim04G0078100
Stem vs Leaf	RHSIM_Rhsim02G0218800	4.472818831	2.54645E-15	3.37437E-14	RHSIM_Rhsim02G0218800
Stem vs Leaf	RHSIM_Rhsim04G0172100	-3.399755206	2.55667E-15	3.38598E-14	RHSIM_Rhsim04G0172100
Stem vs Leaf	RHSIM_Rhsim03G0254100	-3.935627749	2.56232E-15	3.39151E-14	RHSIM_Rhsim03G0254100
Stem vs Leaf	RHSIM_Rhsim12G0066600	-1.53198775	2.57482E-15	3.4061E-14	RHSIM_Rhsim12G0066600
Stem vs Leaf	RHSIM_Rhsim12G0210300	-3.003489611	2.69075E-15	3.5574E-14	RHSIM_Rhsim12G0210300
Stem vs Leaf	RHSIM_Rhsim11G0088600	-1.745336355	2.72859E-15	3.60536E-14	RHSIM_Rhsim11G0088600
Stem vs Leaf	RHSIM_Rhsim01G0207000	-2.275190736	2.74867E-15	3.62982E-14	RHSIM_Rhsim01G0207000
Stem vs Leaf	RHSIM_Rhsim08G0180100	3.585253404	2.81714E-15	3.71811E-14	RHSIM_Rhsim08G0180100
Stem vs Leaf	RHSIM_Rhsim12G0214000	2.794849863	2.9487E-15	3.88952E-14	RHSIM_Rhsim12G0214000
Stem vs Leaf	RHSIM_Rhsim13G0003300	2.746799347	3.03682E-15	4.00345E-14	RHSIM_Rhsim13G0003300
Stem vs Leaf	RHSIM_Rhsim10G0046800	-4.160957613	3.09911E-15	4.08325E-14	RHSIM_Rhsim10G0046800
Stem vs Leaf	RHSIM_Rhsim09G0206700	-9.539711705	3.12018E-15	4.10674E-14	RHSIM_Rhsim09G0206700
Stem vs Leaf	novel.2691	8.057837094	3.12051E-15	4.10674E-14	--
Stem vs Leaf	RHSIM_Rhsim11G0008700	-3.856377992	3.13479E-15	4.12317E-14	RHSIM_Rhsim11G0008700
Stem vs Leaf	RHSIM_Rhsim10G0008700	3.781791809	3.14855E-15	4.13892E-14	RHSIM_Rhsim10G0008700
Stem vs Leaf	RHSIM_Rhsim01G0285200	-2.825962586	3.18097E-15	4.17915E-14	RHSIM_Rhsim01G0285200
Stem vs Leaf	RHSIM_Rhsim13G0026100	-2.726193293	3.1918E-15	4.19098E-14	RHSIM_Rhsim13G0026100
Stem vs Leaf	RHSIM_Rhsim08G0203700	2.298324436	3.2193E-15	4.22469E-14	RHSIM_Rhsim08G0203700
Stem vs Leaf	RHSIM_Rhsim08G0080800	-1.755565551	3.26055E-15	4.27638E-14	RHSIM_Rhsim08G0080800
Stem vs Leaf	RHSIM_Rhsim06G0074400	-3.402928488	3.28713E-15	4.3088E-14	RHSIM_Rhsim06G0074400
Stem vs Leaf	RHSIM_Rhsim11G0166900	-6.545450502	3.31424E-15	4.34186E-14	RHSIM_Rhsim11G0166900
Stem vs Leaf	RHSIM_Rhsim06G0171700	2.623816106	3.3309E-15	4.36121E-14	RHSIM_Rhsim06G0171700
Stem vs Leaf	RHSIM_RhsimUnG0180200	-2.543591898	3.37454E-15	4.41583E-14	RHSIM_RhsimUnG0180200
Stem vs Leaf	RHSIM_RhsimUnG0038200	4.409722231	3.39924E-15	4.44563E-14	RHSIM_RhsimUnG0038200

Comparison	Gene ID	log2FC	P-value	FDR (padj)	Gene Name
Stem vs Leaf	RHSIM_RhsimUnG0086600	-3.825493676	3.42687E-15	4.47923E-14	RHSIM_RhsimUnG0086600
Stem vs Leaf	RHSIM_Rhsim03G0205700	-4.157086174	3.46938E-15	4.53222E-14	RHSIM_Rhsim03G0205700
Stem vs Leaf	RHSIM_Rhsim07G0056100	1.636938531	3.53028E-15	4.60917E-14	RHSIM_Rhsim07G0056100
Stem vs Leaf	RHSIM_Rhsim04G0014200	-1.742877222	3.5569E-15	4.64129E-14	RHSIM_Rhsim04G0014200
Stem vs Leaf	RHSIM_Rhsim05G0208400	-7.851551851	3.61932E-15	4.72006E-14	RHSIM_Rhsim05G0208400
Stem vs Leaf	RHSIM_Rhsim10G0210900	-1.599661655	3.64592E-15	4.75207E-14	RHSIM_Rhsim10G0210900
Stem vs Leaf	RHSIM_Rhsim01G0251700	2.661219921	3.65622E-15	4.7628E-14	RHSIM_Rhsim01G0251700
Stem vs Leaf	RHSIM_Rhsim05G0206000	-2.403119362	3.66106E-15	4.76642E-14	RHSIM_Rhsim05G0206000
Stem vs Leaf	RHSIM_Rhsim04G0198400	-1.38643266	3.67341E-15	4.77979E-14	RHSIM_Rhsim04G0198400
Stem vs Leaf	RHSIM_Rhsim04G0020100	3.117230231	3.80751E-15	4.95149E-14	RHSIM_Rhsim04G0020100
Stem vs Leaf	RHSIM_Rhsim12G0148800	-9.749562721	3.88837E-15	5.05369E-14	RHSIM_Rhsim12G0148800
Stem vs Leaf	RHSIM_Rhsim04G0131900	-1.464277424	3.89049E-15	5.05369E-14	RHSIM_Rhsim04G0131900
Stem vs Leaf	RHSIM_Rhsim12G0128800	1.344603326	3.97828E-15	5.16481E-14	RHSIM_Rhsim12G0128800
Stem vs Leaf	RHSIM_Rhsim04G0065900	-1.606585465	4.02055E-15	5.21676E-14	RHSIM_Rhsim04G0065900
Stem vs Leaf	RHSIM_RhsimUnG0026400	-7.432392661	4.11841E-15	5.34024E-14	RHSIM_RhsimUnG0026400
Stem vs Leaf	RHSIM_RhsimUnG0121000	-5.368649858	4.12035E-15	5.34024E-14	RHSIM_RhsimUnG0121000
Stem vs Leaf	RHSIM_RhsimUnG0212900	3.364181406	4.20211E-15	5.44314E-14	RHSIM_RhsimUnG0212900
Stem vs Leaf	RHSIM_Rhsim01G0055300	2.765302007	4.22182E-15	5.46474E-14	RHSIM_Rhsim01G0055300
Stem vs Leaf	RHSIM_Rhsim02G0206400	-4.14166908	4.22353E-15	5.46474E-14	RHSIM_Rhsim02G0206400
Stem vs Leaf	RHSIM_Rhsim01G0051900	-9.927376497	4.23871E-15	5.48131E-14	RHSIM_Rhsim01G0051900
Stem vs Leaf	RHSIM_Rhsim05G0168900	1.126477125	4.25591E-15	5.50046E-14	RHSIM_Rhsim05G0168900
Stem vs Leaf	RHSIM_Rhsim10G0052500	-7.230122011	4.28513E-15	5.53512E-14	RHSIM_Rhsim10G0052500
Stem vs Leaf	RHSIM_Rhsim09G0213700	3.236440523	4.35394E-15	5.62085E-14	RHSIM_Rhsim09G0213700
Stem vs Leaf	RHSIM_Rhsim06G0169400	1.201239558	4.42076E-15	5.70392E-14	RHSIM_Rhsim06G0169400
Stem vs Leaf	RHSIM_Rhsim02G0164500	-5.54768414	4.46816E-15	5.76185E-14	RHSIM_Rhsim02G0164500

Comparison	Gene ID	log_2FC	P-value	FDR (padj)	Gene Name
Stem vs Leaf	RHSIM_Rhsim04G0132400	-1.457572357	4.51948E-15	5.82477E-14	RHSIM_Rhsim04G0132400
Stem vs Leaf	RHSIM_Rhsim06G0064800	-1.777156072	4.61128E-15	5.93976E-14	RHSIM_Rhsim06G0064800
Stem vs Leaf	RHSIM_Rhsim05G0203700	2.540399451	4.67467E-15	6.01806E-14	RHSIM_Rhsim05G0203700
Stem vs Leaf	RHSIM_Rhsim13G0163700	-1.381142361	4.78528E-15	6.15701E-14	RHSIM_Rhsim13G0163700
Stem vs Leaf	RHSIM_Rhsim01G0077000	10.08132229	4.78825E-15	6.15739E-14	RHSIM_Rhsim01G0077000
Stem vs Leaf	RHSIM_Rhsim05G0095100	-1.745187127	4.82444E-15	6.20047E-14	RHSIM_Rhsim05G0095100
Stem vs Leaf	RHSIM_Rhsim10G0030700	-2.347519055	4.92346E-15	6.3242E-14	RHSIM_Rhsim10G0030700
Stem vs Leaf	RHSIM_Rhsim08G0080300	3.81552037	4.95717E-15	6.36395E-14	RHSIM_Rhsim08G0080300
Stem vs Leaf	RHSIM_Rhsim09G0061500	-1.087591653	4.97649E-15	6.3852E-14	RHSIM_Rhsim09G0061500
Stem vs Leaf	RHSIM_Rhsim05G0087900	2.75842487	5.00333E-15	6.41607E-14	RHSIM_Rhsim05G0087900
Stem vs Leaf	RHSIM_Rhsim09G0000500	1.078391081	5.0229E-15	6.43759E-14	RHSIM_Rhsim09G0000500
Stem vs Leaf	RHSIM_Rhsim08G0126000	3.946158704	5.05969E-15	6.48113E-14	RHSIM_Rhsim08G0126000
Stem vs Leaf	RHSIM_Rhsim01G0003900	2.486702794	5.14322E-15	6.58447E-14	RHSIM_Rhsim01G0003900
Stem vs Leaf	RHSIM_Rhsim09G0142900	-1.444296093	5.30543E-15	6.78836E-14	RHSIM_Rhsim09G0142900
Stem vs Leaf	RHSIM_Rhsim05G0227300	2.423684708	5.32396E-15	6.80521E-14	RHSIM_Rhsim05G0227300
Stem vs Leaf	RHSIM_Rhsim01G0138300	13.42430841	5.32451E-15	6.80521E-14	RHSIM_Rhsim01G0138300
Stem vs Leaf	RHSIM_Rhsim06G0001300	3.697074859	5.3737E-15	6.86428E-14	RHSIM_Rhsim06G0001300
Stem vs Leaf	RHSIM_Rhsim02G0026200	3.622545066	5.51474E-15	7.04054E-14	RHSIM_Rhsim02G0026200
Stem vs Leaf	RHSIM_Rhsim09G0113200	-3.056343662	5.60473E-15	7.15146E-14	RHSIM_Rhsim09G0113200
Stem vs Leaf	RHSIM_RhsimUnG0008100	5.151881286	5.64051E-15	7.19167E-14	RHSIM_RhsimUnG0008100
Stem vs Leaf	RHSIM_Rhsim06G0141500	1.430858434	5.64249E-15	7.19167E-14	RHSIM_Rhsim06G0141500
Stem vs Leaf	RHSIM_Rhsim08G0118300	2.755593549	5.76502E-15	7.34378E-14	RHSIM_Rhsim08G0118300
Stem vs Leaf	RHSIM_Rhsim12G0182100	4.082919372	6.01412E-15	7.65687E-14	RHSIM_Rhsim12G0182100
Stem vs Leaf	RHSIM_Rhsim01G0206300	5.120731689	6.16413E-15	7.84352E-14	RHSIM_Rhsim01G0206300
Stem vs Leaf	RHSIM_Rhsim10G0028000	-2.96483902	6.17327E-15	7.85082E-14	RHSIM_Rhsim10G0028000

Comparison	Gene ID	log_2FC	P-value	FDR (padj)	Gene Name
Stem vs Leaf	novel.5542	6.259039618	6.21151E-15	7.89509E-14	--
Stem vs Leaf	novel.5071	1.870158463	6.42784E-15	8.16442E-14	--
Stem vs Leaf	novel.3874	3.33873473	6.43049E-15	8.16442E-14	--
Stem vs Leaf	RHSIM_Rhsim04G0115700	-10.88018217	6.50607E-15	8.25583E-14	RHSIM_Rhsim04G0115700
Stem vs Leaf	RHSIM_RhsimUnG0139500	2.296825511	6.57261E-15	8.33567E-14	RHSIM_RhsimUnG0139500
Stem vs Leaf	RHSIM_Rhsim07G0056900	-2.31558311	6.58541E-15	8.34731E-14	RHSIM_Rhsim07G0056900
Stem vs Leaf	RHSIM_Rhsim13G0142600	-1.893482868	6.62374E-15	8.39129E-14	RHSIM_Rhsim13G0142600
Stem vs Leaf	RHSIM_Rhsim13G0123100	-5.519107468	6.7042E-15	8.48855E-14	RHSIM_Rhsim13G0123100
Stem vs Leaf	RHSIM_Rhsim10G0191400	1.620435562	6.85635E-15	8.67643E-14	RHSIM_Rhsim10G0191400
Stem vs Leaf	RHSIM_Rhsim02G0142400	-1.511909059	6.887E-15	8.71043E-14	RHSIM_Rhsim02G0142400
Stem vs Leaf	RHSIM_Rhsim05G0204200	-8.653501883	6.89454E-15	8.71519E-14	RHSIM_Rhsim05G0204200
Stem vs Leaf	RHSIM_Rhsim06G0031000	2.522225466	6.94024E-15	8.76814E-14	RHSIM_Rhsim06G0031000
Stem vs Leaf	RHSIM_Rhsim11G0016400	1.833671748	6.98987E-15	8.826E-14	RHSIM_Rhsim11G0016400
Stem vs Leaf	RHSIM_Rhsim07G0006600	-1.879457606	7.09963E-15	8.95969E-14	RHSIM_Rhsim07G0006600
Stem vs Leaf	RHSIM_Rhsim01G0271500	-1.655593311	7.15493E-15	9.02454E-14	RHSIM_Rhsim01G0271500
Stem vs Leaf	RHSIM_Rhsim03G0159800	-1.769210058	7.23485E-15	9.12034E-14	RHSIM_Rhsim03G0159800
Stem vs Leaf	RHSIM_Rhsim02G0145800	-1.974730488	7.5742E-15	9.54291E-14	RHSIM_Rhsim02G0145800
Stem vs Leaf	RHSIM_Rhsim07G0052500	-2.190377352	7.62987E-15	9.6078E-14	RHSIM_Rhsim07G0052500
Stem vs Leaf	RHSIM_Rhsim12G0069900	-1.332785757	7.69928E-15	9.6899E-14	RHSIM_Rhsim12G0069900
Stem vs Leaf	RHSIM_Rhsim13G0077300	-1.33223521	7.86023E-15	9.8855E-14	RHSIM_Rhsim13G0077300
Stem vs Leaf	RHSIM_Rhsim08G0217100	2.8557079	7.86327E-15	9.8855E-14	RHSIM_Rhsim08G0217100
Stem vs Leaf	RHSIM_Rhsim03G0010500	-2.514522072	7.9417E-15	9.97865E-14	RHSIM_Rhsim03G0010500
Stem vs Leaf	RHSIM_Rhsim11G0191400	3.396759105	8.00746E-15	1.00558E-13	RHSIM_Rhsim11G0191400
Stem vs Leaf	RHSIM_Rhsim10G0004900	-1.719953998	8.13483E-15	1.02102E-13	RHSIM_Rhsim10G0004900
Stem vs Leaf	RHSIM_Rhsim08G0179600	-3.7580838	8.53666E-15	1.07087E-13	RHSIM_Rhsim08G0179600

Comparison	Gene ID	log_2FC	P-value	FDR (padj)	Gene Name
Stem vs Leaf	RHSIM_Rhsim01G0002300	1.1379348	8.6352E-15	1.08264E-13	RHSIM_Rhsim01G0002300
Stem vs Leaf	RHSIM_Rhsim03G0180900	3.16217196	8.76697E-15	1.09856E-13	RHSIM_Rhsim03G0180900
Stem vs Leaf	RHSIM_Rhsim04G0229700	-2.248795899	8.83361E-15	1.10631E-13	RHSIM_Rhsim04G0229700
Stem vs Leaf	novel.2741	-1.344894151	8.85955E-15	1.10896E-13	--
Stem vs Leaf	RHSIM_Rhsim05G0076900	-1.371355604	8.97157E-15	1.12237E-13	RHSIM_Rhsim05G0076900
Stem vs Leaf	novel.736	-11.24430567	9.47222E-15	1.18436E-13	--
Stem vs Leaf	RHSIM_Rhsim04G0155700	-2.053086283	9.64278E-15	1.20503E-13	RHSIM_Rhsim04G0155700
Stem vs Leaf	RHSIM_Rhsim09G0153000	-10.80691343	9.6744E-15	1.20833E-13	RHSIM_Rhsim09G0153000
Stem vs Leaf	RHSIM_Rhsim01G0245400	-1.369178981	9.70011E-15	1.21088E-13	RHSIM_Rhsim01G0245400
Stem vs Leaf	RHSIM_Rhsim01G0288700	-3.443558403	9.76907E-15	1.21883E-13	RHSIM_Rhsim01G0288700
Stem vs Leaf	RHSIM_Rhsim12G0184900	7.009035644	9.81717E-15	1.22417E-13	RHSIM_Rhsim12G0184900
Stem vs Leaf	RHSIM_Rhsim04G0083100	-4.122307809	1.02576E-14	1.2784E-13	RHSIM_Rhsim04G0083100
Stem vs Leaf	RHSIM_Rhsim04G0057400	2.749247622	1.06938E-14	1.33204E-13	RHSIM_Rhsim04G0057400
Stem vs Leaf	novel.578	3.454452595	1.08236E-14	1.34748E-13	--
Stem vs Leaf	RHSIM_Rhsim13G0229800	-4.499235399	1.12368E-14	1.39817E-13	RHSIM_Rhsim13G0229800
Stem vs Leaf	RHSIM_Rhsim11G0113700	-4.224866025	1.14146E-14	1.41952E-13	RHSIM_Rhsim11G0113700
Stem vs Leaf	novel.1501	3.341250073	1.14574E-14	1.42408E-13	--
Stem vs Leaf	RHSIM_Rhsim07G0188600	-10.75719201	1.16154E-14	1.44293E-13	RHSIM_Rhsim07G0188600
Stem vs Leaf	RHSIM_Rhsim01G0237200	-1.989108357	1.16831E-14	1.45057E-13	RHSIM_Rhsim01G0237200
Stem vs Leaf	RHSIM_Rhsim12G0119400	-10.48114076	1.18407E-14	1.46934E-13	RHSIM_Rhsim12G0119400
Stem vs Leaf	RHSIM_Rhsim08G0009200	-4.547068255	1.19582E-14	1.48313E-13	RHSIM_Rhsim08G0009200
Stem vs Leaf	RHSIM_Rhsim06G0034000	-1.27027481	1.21811E-14	1.50996E-13	RHSIM_Rhsim06G0034000
Stem vs Leaf	RHSIM_Rhsim01G0102400	5.440293009	1.24093E-14	1.53742E-13	RHSIM_Rhsim01G0102400
Stem vs Leaf	RHSIM_Rhsim02G0144300	1.38557364	1.28881E-14	1.59588E-13	RHSIM_Rhsim02G0144300
Stem vs Leaf	RHSIM_Rhsim07G0217700	2.837846636	1.32252E-14	1.63674E-13	RHSIM_Rhsim07G0217700

Comparison	Gene ID	log_2FC	P-value	FDR (padj)	Gene Name
Stem vs Leaf	RHSIM_Rhsim01G0270000	-1.451577533	1.33767E-14	1.65461E-13	RHSIM_Rhsim01G0270000
Stem vs Leaf	RHSIM_Rhsim02G0247000	-3.03681343	1.34435E-14	1.66197E-13	RHSIM_Rhsim02G0247000
Stem vs Leaf	RHSIM_Rhsim10G0041600	-2.828630056	1.34851E-14	1.66623E-13	RHSIM_Rhsim10G0041600
Stem vs Leaf	RHSIM_Rhsim07G0241500	-3.04712878	1.3793E-14	1.70336E-13	RHSIM_Rhsim07G0241500
Stem vs Leaf	RHSIM_RhsimUnG0179200	-7.084811316	1.48249E-14	1.82981E-13	RHSIM_RhsimUnG0179200
Stem vs Leaf	RHSIM_Rhsim04G0096800	-1.73483237	1.49621E-14	1.84575E-13	RHSIM_Rhsim04G0096800
Stem vs Leaf	RHSIM_Rhsim13G0039600	-1.569647816	1.52143E-14	1.87586E-13	RHSIM_Rhsim13G0039600
Stem vs Leaf	RHSIM_Rhsim11G0080300	-8.224266414	1.54145E-14	1.89953E-13	RHSIM_Rhsim11G0080300
Stem vs Leaf	RHSIM_RhsimUnG0146700	3.292106873	1.61573E-14	1.99E-13	RHSIM_RhsimUnG0146700
Stem vs Leaf	RHSIM_RhsimUnG0240300	-5.019700839	1.66383E-14	2.04815E-13	RHSIM_RhsimUnG0240300
Stem vs Leaf	RHSIM_Rhsim09G0040800	-1.100658702	1.67643E-14	2.06255E-13	RHSIM_Rhsim09G0040800
Stem vs Leaf	RHSIM_Rhsim01G0252300	1.386505349	1.71039E-14	2.10321E-13	RHSIM_Rhsim01G0252300
Stem vs Leaf	RHSIM_Rhsim07G0086300	3.155433178	1.71793E-14	2.11136E-13	RHSIM_Rhsim07G0086300
Stem vs Leaf	RHSIM_Rhsim11G0196300	-1.963619299	1.72863E-14	2.1224E-13	RHSIM_Rhsim11G0196300
Stem vs Leaf	RHSIM_Rhsim11G0071100	-10.04037586	1.72876E-14	2.1224E-13	RHSIM_Rhsim11G0071100
Stem vs Leaf	RHSIM_Rhsim02G0241400	2.697058052	1.76983E-14	2.17167E-13	RHSIM_Rhsim02G0241400
Stem vs Leaf	RHSIM_Rhsim11G0076200	-9.965474324	1.7934E-14	2.19825E-13	RHSIM_Rhsim11G0076200
Stem vs Leaf	RHSIM_Rhsim13G0147500	4.845580948	1.81947E-14	2.22902E-13	RHSIM_Rhsim13G0147500
Stem vs Leaf	RHSIM_Rhsim09G0024200	10.03044276	1.90962E-14	2.33822E-13	RHSIM_Rhsim09G0024200
Stem vs Leaf	RHSIM_Rhsim12G0127900	1.454228647	1.92673E-14	2.35792E-13	RHSIM_Rhsim12G0127900
Stem vs Leaf	RHSIM_Rhsim05G0018700	-1.464803716	1.9639E-14	2.40213E-13	RHSIM_Rhsim05G0018700
Stem vs Leaf	RHSIM_Rhsim05G0206900	-2.140736608	2.04367E-14	2.49837E-13	RHSIM_Rhsim05G0206900
Stem vs Leaf	novel.1308	-9.563930072	2.05475E-14	2.51058E-13	--
Stem vs Leaf	RHSIM_Rhsim08G0106000	-6.569347097	2.08925E-14	2.55138E-13	RHSIM_Rhsim08G0106000
Stem vs Leaf	RHSIM_Rhsim13G0054100	-1.936661721	2.1485E-14	2.62236E-13	RHSIM_Rhsim13G0054100

Comparison	Gene ID	log_2FC	P-value	FDR (padj)	Gene Name
Stem vs Leaf	RHSIM_Rhsim13G0206000	-1.115341377	2.23436E-14	2.72571E-13	RHSIM_Rhsim13G0206000
Stem vs Leaf	RHSIM_Rhsim09G0024300	8.249756357	2.2502E-14	2.74358E-13	RHSIM_Rhsim09G0024300
Stem vs Leaf	novel.2602	-10.59026958	2.27142E-14	2.76799E-13	--
Stem vs Leaf	RHSIM_Rhsim03G0075800	-1.428564493	2.32663E-14	2.83377E-13	RHSIM_Rhsim03G0075800
Stem vs Leaf	RHSIM_Rhsim08G0057600	-8.70006399	2.36295E-14	2.87648E-13	RHSIM_Rhsim08G0057600
Stem vs Leaf	RHSIM_Rhsim09G0207400	2.218248898	2.52677E-14	3.07428E-13	RHSIM_Rhsim09G0207400
Stem vs Leaf	RHSIM_Rhsim04G0190700	-2.043460842	2.52861E-14	3.0749E-13	RHSIM_Rhsim04G0190700
Stem vs Leaf	RHSIM_RhsimUnG0112800	-2.13418578	2.55604E-14	3.10661E-13	RHSIM_RhsimUnG0112800
Stem vs Leaf	novel.2058	-5.267368321	2.56302E-14	3.11345E-13	--
Stem vs Leaf	RHSIM_Rhsim10G0100700	-2.206802712	2.59831E-14	3.15466E-13	RHSIM_Rhsim10G0100700
Stem vs Leaf	RHSIM_Rhsim08G0099800	-7.66451642	2.62357E-14	3.18365E-13	RHSIM_Rhsim08G0099800
Stem vs Leaf	RHSIM_Rhsim06G0047300	-10.43776507	2.64585E-14	3.209E-13	RHSIM_Rhsim06G0047300
Stem vs Leaf	novel.677	-7.189905835	2.74077E-14	3.32238E-13	--
Stem vs Leaf	RHSIM_Rhsim01G0124700	-1.494972787	2.76085E-14	3.34496E-13	RHSIM_Rhsim01G0124700
Stem vs Leaf	RHSIM_Rhsim09G0114100	-2.679249461	2.81915E-14	3.41379E-13	RHSIM_Rhsim09G0114100
Stem vs Leaf	RHSIM_Rhsim05G0110900	-1.540169314	2.85319E-14	3.4532E-13	RHSIM_Rhsim05G0110900
Stem vs Leaf	RHSIM_Rhsim06G0184000	3.665828082	2.93749E-14	3.55336E-13	RHSIM_Rhsim06G0184000
Stem vs Leaf	RHSIM_Rhsim11G0093300	-4.849384514	2.99567E-14	3.62184E-13	RHSIM_Rhsim11G0093300
Stem vs Leaf	RHSIM_Rhsim03G0217900	-2.67440032	2.99783E-14	3.62255E-13	RHSIM_Rhsim03G0217900
Stem vs Leaf	RHSIM_Rhsim13G0053400	2.883318117	3.07878E-14	3.71842E-13	RHSIM_Rhsim13G0053400
Stem vs Leaf	RHSIM_Rhsim06G0002400	1.888094209	3.10885E-14	3.75277E-13	RHSIM_Rhsim06G0002400
Stem vs Leaf	RHSIM_Rhsim04G0139900	-1.768509531	3.11325E-14	3.75611E-13	RHSIM_Rhsim04G0139900
Stem vs Leaf	RHSIM_Rhsim12G0113400	9.685930733	3.21632E-14	3.87843E-13	RHSIM_Rhsim12G0113400
Stem vs Leaf	RHSIM_Rhsim04G0175100	-10.26502864	3.25147E-14	3.91877E-13	RHSIM_Rhsim04G0175100
Stem vs Leaf	RHSIM_Rhsim01G0247100	-1.498123488	3.29581E-14	3.97013E-13	RHSIM_Rhsim01G0247100

Comparison	Gene ID	log_2FC	P-value	FDR (padj)	Gene Name
Stem vs Leaf	RHSIM_Rhsim07G0103500	9.420894636	3.35084E-14	4.03431E-13	RHSIM_Rhsim07G0103500
Stem vs Leaf	RHSIM_Rhsim05G0049700	-4.143738403	3.471E-14	4.1768E-13	RHSIM_Rhsim05G0049700
Stem vs Leaf	RHSIM_Rhsim13G0124500	4.728909587	3.48905E-14	4.19633E-13	RHSIM_Rhsim13G0124500
Stem vs Leaf	RHSIM_Rhsim03G0226300	-1.670456917	3.57441E-14	4.29451E-13	RHSIM_Rhsim03G0226300
Stem vs Leaf	RHSIM_Rhsim11G0016500	-2.145460928	3.58425E-14	4.30409E-13	RHSIM_Rhsim11G0016500
Stem vs Leaf	RHSIM_Rhsim01G0269200	-1.328423887	3.60454E-14	4.32619E-13	RHSIM_Rhsim01G0269200
Stem vs Leaf	RHSIM_Rhsim13G0019700	10.21196038	3.63286E-14	4.35792E-13	RHSIM_Rhsim13G0019700
Stem vs Leaf	RHSIM_Rhsim04G0014400	-1.440694612	3.63915E-14	4.36319E-13	RHSIM_Rhsim04G0014400
Stem vs Leaf	RHSIM_Rhsim08G0066200	-1.126655989	3.69599E-14	4.42904E-13	RHSIM_Rhsim08G0066200
Stem vs Leaf	RHSIM_Rhsim03G0087300	-4.930687234	3.71556E-14	4.45018E-13	RHSIM_Rhsim03G0087300
Stem vs Leaf	novel.4482	-4.670925361	3.74746E-14	4.48605E-13	--
Stem vs Leaf	RHSIM_Rhsim12G0113500	8.355210803	3.78833E-14	4.53027E-13	RHSIM_Rhsim12G0113500
Stem vs Leaf	RHSIM_RhsimUnG0087600	-3.756283368	3.84358E-14	4.59395E-13	RHSIM_RhsimUnG0087600
Stem vs Leaf	RHSIM_Rhsim11G0066500	-4.122120943	3.86067E-14	4.61199E-13	RHSIM_Rhsim11G0066500
Stem vs Leaf	RHSIM_Rhsim06G0159700	7.578918508	3.88267E-14	4.63587E-13	RHSIM_Rhsim06G0159700
Stem vs Leaf	RHSIM_Rhsim01G0246000	-2.699115901	3.93987E-14	4.70173E-13	RHSIM_Rhsim01G0246000
Stem vs Leaf	RHSIM_Rhsim11G0105700	2.314562514	3.9522E-14	4.714E-13	RHSIM_Rhsim11G0105700
Stem vs Leaf	RHSIM_Rhsim03G0054400	-4.686637186	3.99286E-14	4.76003E-13	RHSIM_Rhsim03G0054400
Stem vs Leaf	RHSIM_Rhsim08G0134100	-1.007472579	3.99864E-14	4.76446E-13	RHSIM_Rhsim08G0134100
Stem vs Leaf	novel.218	-5.934628305	4.0217E-14	4.78719E-13	--
Stem vs Leaf	RHSIM_Rhsim12G0101300	2.571173713	4.02268E-14	4.78719E-13	RHSIM_Rhsim12G0101300
Stem vs Leaf	RHSIM_Rhsim03G0200000	-1.062500061	4.02395E-14	4.78719E-13	RHSIM_Rhsim03G0200000
Stem vs Leaf	RHSIM_Rhsim10G0214500	4.555968721	4.07878E-14	4.84992E-13	RHSIM_Rhsim10G0214500
Stem vs Leaf	RHSIM_Rhsim02G0169800	-3.441650401	4.17935E-14	4.96693E-13	RHSIM_Rhsim02G0169800
Stem vs Leaf	RHSIM_Rhsim04G0188600	4.51249759	4.22474E-14	5.0183E-13	RHSIM_Rhsim04G0188600

Comparison	Gene ID	log_2FC	P-value	FDR (padj)	Gene Name
Stem vs Leaf	RHSIM_Rhsim08G0106300	-7.194959183	4.22975E-14	5.02165E-13	RHSIM_Rhsim08G0106300
Stem vs Leaf	RHSIM_Rhsim07G0104100	-9.573561418	4.30426E-14	5.10749E-13	RHSIM_Rhsim07G0104100
Stem vs Leaf	novel.4179	2.344348462	4.32511E-14	5.12959E-13	--
Stem vs Leaf	RHSIM_Rhsim06G0165200	-4.43026344	4.42394E-14	5.2441E-13	RHSIM_Rhsim06G0165200
Stem vs Leaf	RHSIM_Rhsim10G0105600	-7.171297174	4.67983E-14	5.54458E-13	RHSIM_Rhsim10G0105600
Stem vs Leaf	RHSIM_Rhsim01G0064800	-9.385338407	4.90944E-14	5.81064E-13	RHSIM_Rhsim01G0064800
Stem vs Leaf	RHSIM_Rhsim09G0032900	-1.865963457	4.92297E-14	5.82366E-13	RHSIM_Rhsim09G0032900
Stem vs Leaf	RHSIM_Rhsim04G0122300	-8.228394157	4.93481E-14	5.83467E-13	RHSIM_Rhsim04G0122300
Stem vs Leaf	RHSIM_RhsimUnG0053100	-3.965738197	4.94673E-14	5.84294E-13	RHSIM_RhsimUnG0053100
Stem vs Leaf	RHSIM_Rhsim06G0040300	6.923943794	4.94687E-14	5.84294E-13	RHSIM_Rhsim06G0040300
Stem vs Leaf	novel.4890	-8.176425614	4.98482E-14	5.88474E-13	--
Stem vs Leaf	RHSIM_Rhsim01G0198000	-4.503375344	4.9936E-14	5.8921E-13	RHSIM_Rhsim01G0198000
Stem vs Leaf	RHSIM_Rhsim04G0049600	-1.27006822	5.12887E-14	6.0486E-13	RHSIM_Rhsim04G0049600
Stem vs Leaf	RHSIM_Rhsim10G0172100	2.598119058	5.14865E-14	6.06882E-13	RHSIM_Rhsim10G0172100
Stem vs Leaf	RHSIM_Rhsim03G0263900	-9.812189422	5.27891E-14	6.21918E-13	RHSIM_Rhsim03G0263900
Stem vs Leaf	novel.4869	-9.952209431	5.49005E-14	6.46462E-13	--
Stem vs Leaf	RHSIM_Rhsim04G0118500	-2.352237415	5.52115E-14	6.49793E-13	RHSIM_Rhsim04G0118500
Stem vs Leaf	RHSIM_Rhsim01G0285300	-2.536884	5.55346E-14	6.53261E-13	RHSIM_Rhsim01G0285300
Stem vs Leaf	RHSIM_Rhsim04G0242900	5.716896314	5.70384E-14	6.70608E-13	RHSIM_Rhsim04G0242900
Stem vs Leaf	RHSIM_Rhsim02G0171500	-1.53758946	5.80264E-14	6.81876E-13	RHSIM_Rhsim02G0171500
Stem vs Leaf	RHSIM_Rhsim11G0111500	-2.508032436	5.85483E-14	6.87659E-13	RHSIM_Rhsim11G0111500
Stem vs Leaf	RHSIM_Rhsim01G0110100	-1.692364722	5.93227E-14	6.96399E-13	RHSIM_Rhsim01G0110100
Stem vs Leaf	RHSIM_Rhsim13G0165700	-4.502460645	5.94994E-14	6.98119E-13	RHSIM_Rhsim13G0165700
Stem vs Leaf	RHSIM_Rhsim13G0087000	3.08727361	5.99374E-14	7.029E-13	RHSIM_Rhsim13G0087000
Stem vs Leaf	RHSIM_Rhsim11G0087700	1.750365922	6.00403E-14	7.03669E-13	RHSIM_Rhsim11G0087700

Comparison	Gene ID	log_2FC	P-value	FDR (padj)	Gene Name
Stem vs Leaf	RHSIM_Rhsim06G0242100	-2.540019504	6.00641E-14	7.03669E-13	RHSIM_Rhsim06G0242100
Stem vs Leaf	novel.1493	2.624312492	6.05458E-14	7.08952E-13	--
Stem vs Leaf	RHSIM_Rhsim09G0180100	-1.417703645	6.24332E-14	7.30681E-13	RHSIM_Rhsim09G0180100
Stem vs Leaf	RHSIM_Rhsim06G0120400	-5.376040885	6.54644E-14	7.65767E-13	RHSIM_Rhsim06G0120400
Stem vs Leaf	RHSIM_Rhsim13G0228600	-8.290088767	6.64832E-14	7.7729E-13	RHSIM_Rhsim13G0228600
Stem vs Leaf	RHSIM_Rhsim01G0293000	-1.089740999	6.6654E-14	7.78892E-13	RHSIM_Rhsim01G0293000
Stem vs Leaf	RHSIM_Rhsim08G0239700	-1.24681435	6.6738E-14	7.79478E-13	RHSIM_Rhsim08G0239700
Stem vs Leaf	RHSIM_Rhsim02G0254400	-2.756742116	6.68389E-14	7.80261E-13	RHSIM_Rhsim02G0254400
Stem vs Leaf	RHSIM_Rhsim03G0241300	-2.723823028	6.68796E-14	7.8034E-13	RHSIM_Rhsim03G0241300
Stem vs Leaf	RHSIM_Rhsim06G0196600	3.702720389	6.76216E-14	7.886E-13	RHSIM_Rhsim06G0196600
Stem vs Leaf	novel.4020	4.637453966	6.90843E-14	8.0525E-13	--
Stem vs Leaf	RHSIM_Rhsim06G0028500	-1.210437959	6.91767E-14	8.0592E-13	RHSIM_Rhsim06G0028500
Stem vs Leaf	RHSIM_Rhsim11G0149700	1.200371838	7.17303E-14	8.34825E-13	RHSIM_Rhsim11G0149700
Stem vs Leaf	RHSIM_Rhsim03G0000200	-1.068205091	7.28109E-14	8.46974E-13	RHSIM_Rhsim03G0000200
Stem vs Leaf	RHSIM_Rhsim08G0007000	-2.757515177	7.35144E-14	8.54726E-13	RHSIM_Rhsim08G0007000
Stem vs Leaf	RHSIM_Rhsim10G0174200	4.326021957	7.40445E-14	8.60455E-13	RHSIM_Rhsim10G0174200
Stem vs Leaf	novel.1954	2.487565654	7.4711E-14	8.67762E-13	--
Stem vs Leaf	RHSIM_Rhsim07G0016900	-3.287471613	7.50036E-14	8.70722E-13	RHSIM_Rhsim07G0016900
Stem vs Leaf	RHSIM_Rhsim02G0005200	3.719225789	7.55231E-14	8.76311E-13	RHSIM_Rhsim02G0005200
Stem vs Leaf	RHSIM_Rhsim03G0081700	1.362523128	7.55925E-14	8.76675E-13	RHSIM_Rhsim03G0081700
Stem vs Leaf	RHSIM_Rhsim11G0093500	-1.650373864	7.63184E-14	8.84648E-13	RHSIM_Rhsim11G0093500
Stem vs Leaf	RHSIM_Rhsim13G0226400	2.672153038	7.77508E-14	9.00799E-13	RHSIM_Rhsim13G0226400
Stem vs Leaf	RHSIM_Rhsim05G0075200	-1.81520752	7.82922E-14	9.06615E-13	RHSIM_Rhsim05G0075200
Stem vs Leaf	RHSIM_Rhsim04G0050500	5.323873279	7.843E-14	9.07756E-13	RHSIM_Rhsim04G0050500
Stem vs Leaf	RHSIM_Rhsim11G0134600	2.81953719	8.17318E-14	9.45496E-13	RHSIM_Rhsim11G0134600

Comparison	Gene ID	log_2FC	P-value	FDR (padj)	Gene Name
Stem vs Leaf	RHSIM_Rhsim01G0283900	2.761196796	8.48253E-14	9.8079E-13	RHSIM_Rhsim01G0283900
Stem vs Leaf	RHSIM_Rhsim08G0010000	6.035902762	8.51788E-14	9.84383E-13	RHSIM_Rhsim08G0010000
Stem vs Leaf	RHSIM_Rhsim01G0259800	1.375979425	8.60774E-14	9.94269E-13	RHSIM_Rhsim01G0259800
Stem vs Leaf	RHSIM_Rhsim06G0234600	1.0794266	8.70814E-14	1.00536E-12	RHSIM_Rhsim06G0234600
Stem vs Leaf	novel.3077	2.116890797	8.72662E-14	1.00699E-12	--
Stem vs Leaf	RHSIM_Rhsim05G0011500	2.611567043	8.75676E-14	1.00996E-12	RHSIM_Rhsim05G0011500
Stem vs Leaf	RHSIM_Rhsim04G0079300	-5.594471974	8.81605E-14	1.01629E-12	RHSIM_Rhsim04G0079300
Stem vs Leaf	RHSIM_Rhsim04G0014800	2.074415285	8.85047E-14	1.01964E-12	RHSIM_Rhsim04G0014800
Stem vs Leaf	RHSIM_Rhsim01G0050300	2.597647736	8.85392E-14	1.01964E-12	RHSIM_Rhsim01G0050300
Stem vs Leaf	RHSIM_Rhsim06G0007500	-1.483967374	8.89492E-14	1.02385E-12	RHSIM_Rhsim06G0007500
Stem vs Leaf	RHSIM_Rhsim13G0092400	1.328482539	9.01308E-14	1.03693E-12	RHSIM_Rhsim13G0092400
Stem vs Leaf	RHSIM_Rhsim02G0231600	3.914456444	9.05709E-14	1.04147E-12	RHSIM_Rhsim02G0231600
Stem vs Leaf	RHSIM_Rhsim03G0078300	-1.301424047	9.15531E-14	1.05224E-12	RHSIM_Rhsim03G0078300
Stem vs Leaf	novel.5271	2.076864619	9.21715E-14	1.05882E-12	--
Stem vs Leaf	RHSIM_Rhsim06G0226600	2.690158149	9.41198E-14	1.08067E-12	RHSIM_Rhsim06G0226600
Stem vs Leaf	RHSIM_Rhsim01G0280700	-1.406331157	9.50333E-14	1.09061E-12	RHSIM_Rhsim01G0280700
Stem vs Leaf	RHSIM_Rhsim06G0057500	4.386734617	9.51452E-14	1.09135E-12	RHSIM_Rhsim06G0057500
Stem vs Leaf	RHSIM_Rhsim13G0136900	1.077171912	9.89034E-14	1.1339E-12	RHSIM_Rhsim13G0136900
Stem vs Leaf	RHSIM_Rhsim06G0018300	-9.775793965	9.97923E-14	1.14352E-12	RHSIM_Rhsim06G0018300
Stem vs Leaf	RHSIM_Rhsim06G0169900	9.544046372	1.01873E-13	1.16678E-12	RHSIM_Rhsim06G0169900
Stem vs Leaf	RHSIM_Rhsim07G0124000	-1.853477185	1.02723E-13	1.17593E-12	RHSIM_Rhsim07G0124000
Stem vs Leaf	RHSIM_Rhsim09G0132200	9.718738549	1.03333E-13	1.18232E-12	RHSIM_Rhsim09G0132200
Stem vs Leaf	RHSIM_Rhsim07G0139900	3.846099608	1.05179E-13	1.20285E-12	RHSIM_Rhsim07G0139900
Stem vs Leaf	RHSIM_Rhsim01G0116400	-3.075427311	1.05285E-13	1.20347E-12	RHSIM_Rhsim01G0116400
Stem vs Leaf	RHSIM_Rhsim11G0185100	-4.109252955	1.05916E-13	1.21008E-12	RHSIM_Rhsim11G0185100

Comparison	Gene ID	log_2FC	P-value	FDR (padj)	Gene Name
Stem vs Leaf	RHSIM_Rhsim03G0070300	-3.815191154	1.07692E-13	1.22976E-12	RHSIM_Rhsim03G0070300
Stem vs Leaf	RHSIM_Rhsim02G0153500	-1.283547259	1.07919E-13	1.23174E-12	RHSIM_Rhsim02G0153500
Stem vs Leaf	RHSIM_Rhsim04G0060200	-4.950969007	1.08289E-13	1.23536E-12	RHSIM_Rhsim04G0060200
Stem vs Leaf	RHSIM_Rhsim10G0099400	2.438180025	1.11633E-13	1.27287E-12	RHSIM_Rhsim10G0099400
Stem vs Leaf	RHSIM_RhsimUnG0173800	-2.070832172	1.13819E-13	1.29715E-12	RHSIM_RhsimUnG0173800
Stem vs Leaf	RHSIM_Rhsim11G0080500	-6.42078592	1.14664E-13	1.30614E-12	RHSIM_Rhsim11G0080500
Stem vs Leaf	RHSIM_Rhsim13G0009900	-2.901548346	1.15896E-13	1.31952E-12	RHSIM_Rhsim13G0009900
Stem vs Leaf	RHSIM_Rhsim10G0212700	-1.347982974	1.16284E-13	1.32328E-12	RHSIM_Rhsim10G0212700
Stem vs Leaf	RHSIM_Rhsim13G0202500	-1.579377144	1.20759E-13	1.37331E-12	RHSIM_Rhsim13G0202500
Stem vs Leaf	RHSIM_Rhsim06G0167800	1.649673343	1.20799E-13	1.37331E-12	RHSIM_Rhsim06G0167800
Stem vs Leaf	RHSIM_Rhsim11G0178300	3.410246162	1.23565E-13	1.40406E-12	RHSIM_Rhsim11G0178300
Stem vs Leaf	RHSIM_Rhsim08G0167000	-2.907528434	1.2447E-13	1.41365E-12	RHSIM_Rhsim08G0167000
Stem vs Leaf	RHSIM_Rhsim03G0174400	-1.190069154	1.2608E-13	1.43122E-12	RHSIM_Rhsim03G0174400
Stem vs Leaf	RHSIM_Rhsim04G0235200	-1.857346137	1.27014E-13	1.44112E-12	RHSIM_Rhsim04G0235200
Stem vs Leaf	RHSIM_Rhsim12G0114000	-1.780062992	1.32073E-13	1.49778E-12	RHSIM_Rhsim12G0114000
Stem vs Leaf	RHSIM_Rhsim10G0187000	3.172158223	1.34107E-13	1.52009E-12	RHSIM_Rhsim10G0187000
Stem vs Leaf	RHSIM_Rhsim03G0163700	-3.528752419	1.35483E-13	1.53494E-12	RHSIM_Rhsim03G0163700
Stem vs Leaf	RHSIM_Rhsim03G0169200	-1.011894985	1.35676E-13	1.53637E-12	RHSIM_Rhsim03G0169200
Stem vs Leaf	RHSIM_Rhsim09G0170400	4.71716985	1.35869E-13	1.5378E-12	RHSIM_Rhsim09G0170400
Stem vs Leaf	RHSIM_Rhsim03G0191500	-1.581969554	1.3862E-13	1.56817E-12	RHSIM_Rhsim03G0191500
Stem vs Leaf	RHSIM_Rhsim11G0055900	-8.383178466	1.40171E-13	1.58486E-12	RHSIM_Rhsim11G0055900
Stem vs Leaf	RHSIM_Rhsim01G0257200	-1.393343773	1.40233E-13	1.58486E-12	RHSIM_Rhsim01G0257200
Stem vs Leaf	RHSIM_Rhsim12G0070500	-4.102781738	1.40767E-13	1.59011E-12	RHSIM_Rhsim12G0070500
Stem vs Leaf	RHSIM_Rhsim09G0154800	2.386582946	1.43004E-13	1.6146E-12	RHSIM_Rhsim09G0154800
Stem vs Leaf	RHSIM_Rhsim12G0017600	1.500571041	1.44067E-13	1.6258E-12	RHSIM_Rhsim12G0017600

Comparison	Gene ID	log_2FC	P-value	FDR (padj)	Gene Name
Stem vs Leaf	novel.5900	-9.95330059	1.46063E-13	1.64751E-12	--
Stem vs Leaf	RHSIM_Rhsim10G0026900	-2.387961934	1.47036E-13	1.65768E-12	RHSIM_Rhsim10G0026900
Stem vs Leaf	RHSIM_Rhsim01G0089900	-1.546227866	1.48E-13	1.66773E-12	RHSIM_Rhsim01G0089900
Stem vs Leaf	RHSIM_Rhsim06G0083200	-1.574373723	1.48686E-13	1.67464E-12	RHSIM_Rhsim06G0083200
Stem vs Leaf	RHSIM_Rhsim08G0012800	-7.185246592	1.49442E-13	1.68233E-12	RHSIM_Rhsim08G0012800
Stem vs Leaf	RHSIM_Rhsim02G0132300	-1.975974338	1.53186E-13	1.72365E-12	RHSIM_Rhsim02G0132300
Stem vs Leaf	RHSIM_Rhsim02G0085600	3.457541875	1.53303E-13	1.72411E-12	RHSIM_Rhsim02G0085600
Stem vs Leaf	RHSIM_Rhsim12G0065100	-1.062638781	1.54678E-13	1.73873E-12	RHSIM_Rhsim12G0065100
Stem vs Leaf	RHSIM_Rhsim03G0154300	-2.987684812	1.55658E-13	1.74889E-12	RHSIM_Rhsim03G0154300
Stem vs Leaf	RHSIM_Rhsim01G0189000	1.786421272	1.56421E-13	1.75661E-12	RHSIM_Rhsim01G0189000
Stem vs Leaf	novel.2306	1.936417933	1.62188E-13	1.82049E-12	--
Stem vs Leaf	RHSIM_Rhsim11G0040800	5.35995813	1.62279E-13	1.82062E-12	RHSIM_Rhsim11G0040800
Stem vs Leaf	RHSIM_Rhsim08G0028800	3.852029566	1.74677E-13	1.95876E-12	RHSIM_Rhsim08G0028800
Stem vs Leaf	RHSIM_Rhsim03G0128600	1.720622875	1.83222E-13	2.05359E-12	RHSIM_Rhsim03G0128600
Stem vs Leaf	RHSIM_Rhsim05G0060900	2.918416281	1.86027E-13	2.08401E-12	RHSIM_Rhsim05G0060900
Stem vs Leaf	RHSIM_Rhsim08G0075300	-3.634689009	1.89523E-13	2.12214E-12	RHSIM_Rhsim08G0075300
Stem vs Leaf	RHSIM_Rhsim07G0072400	2.111581621	1.90299E-13	2.12979E-12	RHSIM_Rhsim07G0072400
Stem vs Leaf	RHSIM_Rhsim10G0126200	-1.452597554	1.91254E-13	2.13945E-12	RHSIM_Rhsim10G0126200
Stem vs Leaf	RHSIM_Rhsim07G0108600	-2.228226969	1.95487E-13	2.18574E-12	RHSIM_Rhsim07G0108600
Stem vs Leaf	RHSIM_Rhsim04G0062100	-2.097307656	1.9779E-13	2.21042E-12	RHSIM_Rhsim04G0062100
Stem vs Leaf	novel.5534	-9.126088788	2.00994E-13	2.24513E-12	--
Stem vs Leaf	RHSIM_Rhsim11G0077500	7.640166194	2.03721E-13	2.27449E-12	RHSIM_Rhsim11G0077500
Stem vs Leaf	RHSIM_Rhsim01G0248700	1.804552429	2.07441E-13	2.3149E-12	RHSIM_Rhsim01G0248700
Stem vs Leaf	RHSIM_Rhsim03G0142900	1.059540111	2.10262E-13	2.34525E-12	RHSIM_Rhsim03G0142900
Stem vs Leaf	RHSIM_Rhsim02G0009100	-1.835300397	2.10863E-13	2.35081E-12	RHSIM_Rhsim02G0009100

Comparison	Gene ID	log_2FC	P-value	FDR (padj)	Gene Name
Stem vs Leaf	RHSIM_Rhsim11G0196000	-3.892355583	2.11684E-13	2.35882E-12	RHSIM_Rhsim11G0196000
Stem vs Leaf	RHSIM_Rhsim06G0237100	-2.855229527	2.13061E-13	2.37302E-12	RHSIM_Rhsim06G0237100
Stem vs Leaf	RHSIM_Rhsim07G0123700	-1.327609704	2.15567E-13	2.39977E-12	RHSIM_Rhsim07G0123700
Stem vs Leaf	RHSIM_Rhsim04G0205600	9.453280694	2.16716E-13	2.4114E-12	RHSIM_Rhsim04G0205600
Stem vs Leaf	RHSIM_Rhsim02G0086700	-9.209876437	2.17341E-13	2.41719E-12	RHSIM_Rhsim02G0086700
Stem vs Leaf	RHSIM_Rhsim04G0136700	4.983878127	2.19324E-13	2.43807E-12	RHSIM_Rhsim04G0136700
Stem vs Leaf	RHSIM_Rhsim02G0236600	-4.324948594	2.20072E-13	2.4452E-12	RHSIM_Rhsim02G0236600
Stem vs Leaf	RHSIM_Rhsim01G0206500	-1.420083807	2.23204E-13	2.4788E-12	RHSIM_Rhsim01G0206500
Stem vs Leaf	RHSIM_Rhsim05G0013100	-2.043894414	2.31556E-13	2.57031E-12	RHSIM_Rhsim05G0013100
Stem vs Leaf	RHSIM_Rhsim11G0080000	-9.111500209	2.32845E-13	2.58337E-12	RHSIM_Rhsim11G0080000
Stem vs Leaf	RHSIM_Rhsim13G0109500	-8.381748554	2.33762E-13	2.5923E-12	RHSIM_Rhsim13G0109500
Stem vs Leaf	RHSIM_Rhsim01G0229000	3.76505894	2.34622E-13	2.60059E-12	RHSIM_Rhsim01G0229000
Stem vs Leaf	novel.2966	5.379087556	2.44516E-13	2.70896E-12	--
Stem vs Leaf	RHSIM_Rhsim07G0217300	1.379954116	2.48746E-13	2.75449E-12	RHSIM_Rhsim07G0217300
Stem vs Leaf	novel.4898	-11.09163947	2.51572E-13	2.78445E-12	--
Stem vs Leaf	RHSIM_Rhsim04G0158900	3.103910733	2.53575E-13	2.80527E-12	RHSIM_Rhsim04G0158900
Stem vs Leaf	RHSIM_Rhsim10G0035200	-1.917427261	2.54313E-13	2.81208E-12	RHSIM_Rhsim10G0035200
Stem vs Leaf	RHSIM_Rhsim05G0229600	-1.018434638	2.55363E-13	2.82234E-12	RHSIM_Rhsim05G0229600
Stem vs Leaf	RHSIM_Rhsim12G0005100	-3.360564959	2.60852E-13	2.88162E-12	RHSIM_Rhsim12G0005100
Stem vs Leaf	RHSIM_Rhsim05G0017000	-3.725163063	2.68632E-13	2.96614E-12	RHSIM_Rhsim05G0017000
Stem vs Leaf	RHSIM_Rhsim07G0244600	-1.021971022	2.69164E-13	2.9706E-12	RHSIM_Rhsim07G0244600
Stem vs Leaf	RHSIM_Rhsim02G0079100	-6.273049334	2.73392E-13	3.01582E-12	RHSIM_Rhsim02G0079100
Stem vs Leaf	RHSIM_Rhsim01G0241000	5.485859158	2.7752E-13	3.05989E-12	RHSIM_Rhsim01G0241000
Stem vs Leaf	RHSIM_Rhsim08G0010600	-5.67614819	2.79774E-13	3.08327E-12	RHSIM_Rhsim08G0010600
Stem vs Leaf	RHSIM_Rhsim08G0034000	-2.707090531	2.94937E-13	3.24881E-12	RHSIM_Rhsim08G0034000

Comparison	Gene ID	log_2FC	P-value	FDR (padj)	Gene Name
Stem vs Leaf	RHSIM_Rhsim08G0102400	-2.006034958	2.97786E-13	3.27864E-12	RHSIM_Rhsim08G0102400
Stem vs Leaf	RHSIM_Rhsim09G0028800	2.08727053	3.00329E-13	3.30505E-12	RHSIM_Rhsim09G0028800
Stem vs Leaf	RHSIM_Rhsim03G0248000	-2.733065784	3.09347E-13	3.40267E-12	RHSIM_Rhsim03G0248000
Stem vs Leaf	RHSIM_Rhsim09G0197500	-2.331023376	3.11147E-13	3.42084E-12	RHSIM_Rhsim09G0197500
Stem vs Leaf	RHSIM_Rhsim01G0069300	-2.590254419	3.11545E-13	3.42358E-12	RHSIM_Rhsim01G0069300
Stem vs Leaf	RHSIM_Rhsim01G0023400	-1.435862944	3.18723E-13	3.50079E-12	RHSIM_Rhsim01G0023400
Stem vs Leaf	RHSIM_Rhsim03G0230400	1.705060269	3.19707E-13	3.50992E-12	RHSIM_Rhsim03G0230400
Stem vs Leaf	RHSIM_Rhsim07G0012000	-9.28531635	3.23241E-13	3.54703E-12	RHSIM_Rhsim07G0012000
Stem vs Leaf	RHSIM_Rhsim10G0047200	-3.13560735	3.27359E-13	3.59051E-12	RHSIM_Rhsim10G0047200
Stem vs Leaf	RHSIM_Rhsim05G0220000	-4.926168181	3.30446E-13	3.62092E-12	RHSIM_Rhsim05G0220000
Stem vs Leaf	RHSIM_Rhsim03G0216700	6.346808661	3.31281E-13	3.62835E-12	RHSIM_Rhsim03G0216700
Stem vs Leaf	RHSIM_Rhsim07G0257200	-1.920435738	3.33419E-13	3.65002E-12	RHSIM_Rhsim07G0257200
Stem vs Leaf	RHSIM_Rhsim02G0144600	2.108049289	3.34122E-13	3.65599E-12	RHSIM_Rhsim02G0144600
Stem vs Leaf	RHSIM_Rhsim03G0178700	2.08844133	3.41485E-13	3.73478E-12	RHSIM_Rhsim03G0178700
Stem vs Leaf	RHSIM_Rhsim08G0108400	-9.108583704	3.66267E-13	4.00391E-12	RHSIM_Rhsim08G0108400
Stem vs Leaf	RHSIM_Rhsim06G0084700	1.315692776	3.75974E-13	4.10808E-12	RHSIM_Rhsim06G0084700
Stem vs Leaf	RHSIM_Rhsim13G0090000	-2.516858992	3.82986E-13	4.18272E-12	RHSIM_Rhsim13G0090000
Stem vs Leaf	RHSIM_Rhsim03G0204000	-2.489214657	3.84358E-13	4.19571E-12	RHSIM_Rhsim03G0204000
Stem vs Leaf	RHSIM_Rhsim02G0212300	-1.264157238	3.91311E-13	4.26959E-12	RHSIM_Rhsim02G0212300
Stem vs Leaf	RHSIM_Rhsim05G0133700	-3.34755506	4.04891E-13	4.41566E-12	RHSIM_Rhsim05G0133700
Stem vs Leaf	RHSIM_Rhsim11G0087300	-1.532758856	4.07741E-13	4.44464E-12	RHSIM_Rhsim11G0087300
Stem vs Leaf	novel.760	-6.798469017	4.11263E-13	4.48092E-12	--
Stem vs Leaf	novel.3275	6.775168008	4.22042E-13	4.59618E-12	--
Stem vs Leaf	RHSIM_Rhsim11G0040600	-2.560143339	4.28196E-13	4.66101E-12	RHSIM_Rhsim11G0040600
Stem vs Leaf	RHSIM_Rhsim08G0010700	-2.258619256	4.30219E-13	4.68082E-12	RHSIM_Rhsim08G0010700

Comparison	Gene ID	log2FC	P-value	FDR (padj)	Gene Name
Stem vs Leaf	RHSIM_Rhsim06G0199700	-2.253705754	4.33324E-13	4.71237E-12	RHSIM_Rhsim06G0199700
Stem vs Leaf	RHSIM_Rhsim06G0091400	-1.637030294	4.36638E-13	4.74617E-12	RHSIM_Rhsim06G0091400
Stem vs Leaf	RHSIM_Rhsim06G0013000	2.966944551	4.37622E-13	4.75463E-12	RHSIM_Rhsim06G0013000
Stem vs Leaf	RHSIM_Rhsim08G0197800	4.354888686	4.43521E-13	4.81418E-12	RHSIM_Rhsim08G0197800
Stem vs Leaf	novel.3243	-9.337461439	4.43974E-13	4.81682E-12	--
Stem vs Leaf	RHSIM_Rhsim13G0171300	-1.77459188	4.50174E-13	4.88004E-12	RHSIM_Rhsim13G0171300
Stem vs Leaf	RHSIM_Rhsim08G0036900	-1.361025079	4.50225E-13	4.88004E-12	RHSIM_Rhsim08G0036900
Stem vs Leaf	RHSIM_Rhsim01G0044000	-2.448061159	4.56252E-13	4.94305E-12	RHSIM_Rhsim01G0044000
Stem vs Leaf	RHSIM_Rhsim08G0216200	-2.462900784	4.69438E-13	5.08352E-12	RHSIM_Rhsim08G0216200
Stem vs Leaf	novel.42	-1.202917745	4.73782E-13	5.12816E-12	--
Stem vs Leaf	novel.3173	-1.426441114	4.77932E-13	5.17064E-12	--
Stem vs Leaf	RHSIM_Rhsim09G0035900	2.529792783	4.91748E-13	5.31762E-12	RHSIM_Rhsim09G0035900
Stem vs Leaf	RHSIM_Rhsim05G0022300	-3.03941281	4.9415E-13	5.34108E-12	RHSIM_Rhsim05G0022300
Stem vs Leaf	novel.783	-1.73973924	4.96272E-13	5.36151E-12	--
Stem vs Leaf	RHSIM_Rhsim13G0190600	-6.841877274	4.96818E-13	5.36489E-12	RHSIM_Rhsim13G0190600
Stem vs Leaf	RHSIM_Rhsim03G0076800	7.097009205	5.05126E-13	5.45205E-12	RHSIM_Rhsim03G0076800
Stem vs Leaf	RHSIM_Rhsim02G0022300	-1.672399726	5.1016E-13	5.50381E-12	RHSIM_Rhsim02G0022300
Stem vs Leaf	RHSIM_RhsimUnG0127800	-8.04263736	5.11972E-13	5.52077E-12	RHSIM_RhsimUnG0127800
Stem vs Leaf	RHSIM_Rhsim13G0191500	-1.863599817	5.12951E-13	5.52874E-12	RHSIM_Rhsim13G0191500
Stem vs Leaf	novel.402	2.592079077	5.14338E-13	5.5411E-12	--
Stem vs Leaf	RHSIM_Rhsim05G0080500	4.511222431	5.21615E-13	5.61687E-12	RHSIM_Rhsim05G0080500
Stem vs Leaf	RHSIM_Rhsim13G0044300	-1.932953932	5.26615E-13	5.66806E-12	RHSIM_Rhsim13G0044300
Stem vs Leaf	novel.1843	5.940588447	5.35991E-13	5.76629E-12	--
Stem vs Leaf	RHSIM_Rhsim10G0151500	1.022551051	5.4024E-13	5.80928E-12	RHSIM_Rhsim10G0151500
Stem vs Leaf	RHSIM_Rhsim06G0027900	-1.919772005	5.50518E-13	5.91705E-12	RHSIM_Rhsim06G0027900

Comparison	Gene ID	log_2FC	P-value	FDR (padj)	Gene Name
Stem vs Leaf	RHSIM_Rhsim01G0060000	1.78681584	5.51032E-13	5.9198E-12	RHSIM_Rhsim01G0060000
Stem vs Leaf	RHSIM_Rhsim10G0118700	-1.309407366	5.55681E-13	5.96696E-12	RHSIM_Rhsim10G0118700
Stem vs Leaf	RHSIM_Rhsim08G0162600	-3.992154611	5.56395E-13	5.97185E-12	RHSIM_Rhsim08G0162600
Stem vs Leaf	RHSIM_Rhsim10G0210600	-2.558902125	5.65286E-13	6.06446E-12	RHSIM_Rhsim10G0210600
Stem vs Leaf	RHSIM_Rhsim07G0118800	-1.339067862	5.78779E-13	6.20632E-12	RHSIM_Rhsim07G0118800
Stem vs Leaf	RHSIM_Rhsim10G0186700	-2.316905831	5.83056E-13	6.24732E-12	RHSIM_Rhsim10G0186700
Stem vs Leaf	RHSIM_Rhsim13G0220900	1.349105716	5.83145E-13	6.24732E-12	RHSIM_Rhsim13G0220900
Stem vs Leaf	RHSIM_Rhsim03G0210900	4.719756327	5.90201E-13	6.31997E-12	RHSIM_Rhsim03G0210900
Stem vs Leaf	RHSIM_Rhsim06G0083300	2.888368481	5.98088E-13	6.40146E-12	RHSIM_Rhsim06G0083300
Stem vs Leaf	RHSIM_Rhsim07G0045100	-9.136663034	6.02515E-13	6.44585E-12	RHSIM_Rhsim07G0045100
Stem vs Leaf	RHSIM_Rhsim01G0009700	-2.018447254	6.04114E-13	6.45996E-12	RHSIM_Rhsim01G0009700
Stem vs Leaf	RHSIM_Rhsim02G0007600	-3.199406194	6.09915E-13	6.51896E-12	RHSIM_Rhsim02G0007600
Stem vs Leaf	RHSIM_Rhsim06G0141000	9.77085423	6.22635E-13	6.65183E-12	RHSIM_Rhsim06G0141000
Stem vs Leaf	novel.4685	-3.138307518	6.24647E-13	6.67024E-12	--
Stem vs Leaf	RHSIM_Rhsim03G0270300	-4.117461965	6.2537E-13	6.67486E-12	RHSIM_Rhsim03G0270300
Stem vs Leaf	novel.3441	-4.320666431	6.28577E-13	6.70598E-12	--
Stem vs Leaf	novel.2252	2.835515992	6.29884E-13	6.71682E-12	--
Stem vs Leaf	RHSIM_Rhsim03G0262700	-2.304932524	6.32138E-13	6.73773E-12	RHSIM_Rhsim03G0262700
Stem vs Leaf	novel.1217	6.863269422	6.35563E-13	6.77111E-12	--
Stem vs Leaf	RHSIM_Rhsim13G0059800	-4.090913511	6.37196E-13	6.78537E-12	RHSIM_Rhsim13G0059800
Stem vs Leaf	RHSIM_Rhsim04G0078500	-4.262506316	6.37689E-13	6.78749E-12	RHSIM_Rhsim04G0078500
Stem vs Leaf	RHSIM_Rhsim09G0185500	3.00246247	6.50103E-13	6.91642E-12	RHSIM_Rhsim09G0185500
Stem vs Leaf	RHSIM_Rhsim10G0005000	-2.719247481	6.83766E-13	7.27121E-12	RHSIM_Rhsim10G0005000
Stem vs Leaf	RHSIM_Rhsim07G0056200	4.904108994	6.94695E-13	7.38402E-12	RHSIM_Rhsim07G0056200
Stem vs Leaf	RHSIM_Rhsim02G0079000	-9.278265286	7.06939E-13	7.5085E-12	RHSIM_Rhsim02G0079000

Comparison	Gene ID	log_2FC	P-value	FDR (padj)	Gene Name
Stem vs Leaf	RHSIM_Rhsim07G0126300	1.466004449	7.07058E-13	7.5085E-12	RHSIM_Rhsim07G0126300
Stem vs Leaf	RHSIM_Rhsim06G0065800	3.751813548	7.17758E-13	7.61861E-12	RHSIM_Rhsim06G0065800
Stem vs Leaf	RHSIM_Rhsim11G0075300	1.761376898	7.25168E-13	7.69373E-12	RHSIM_Rhsim11G0075300
Stem vs Leaf	RHSIM_Rhsim06G0218100	6.212357469	7.38533E-13	7.83192E-12	RHSIM_Rhsim06G0218100
Stem vs Leaf	RHSIM_Rhsim01G0129200	-3.590994052	7.3988E-13	7.84259E-12	RHSIM_Rhsim01G0129200
Stem vs Leaf	RHSIM_Rhsim01G0292100	2.173559163	7.46871E-13	7.91305E-12	RHSIM_Rhsim01G0292100
Stem vs Leaf	RHSIM_Rhsim02G0107000	1.127346257	7.52858E-13	7.97282E-12	RHSIM_Rhsim02G0107000
Stem vs Leaf	RHSIM_Rhsim11G0101600	2.99663264	7.5988E-13	8.04349E-12	RHSIM_Rhsim11G0101600
Stem vs Leaf	RHSIM_Rhsim04G0216200	1.236348844	7.70444E-13	8.15157E-12	RHSIM_Rhsim04G0216200
Stem vs Leaf	RHSIM_Rhsim03G0035800	-1.989992857	7.76691E-13	8.21389E-12	RHSIM_Rhsim03G0035800
Stem vs Leaf	RHSIM_Rhsim13G0174000	2.220477225	7.77257E-13	8.21611E-12	RHSIM_Rhsim13G0174000
Stem vs Leaf	novel.2086	1.701705505	7.78465E-13	8.2251E-12	--
Stem vs Leaf	RHSIM_Rhsim06G0000500	-1.533721429	7.85539E-13	8.29604E-12	RHSIM_Rhsim06G0000500
Stem vs Leaf	RHSIM_Rhsim08G0098600	6.868167481	7.99262E-13	8.43711E-12	RHSIM_Rhsim08G0098600
Stem vs Leaf	RHSIM_Rhsim02G0062900	-5.300790089	8.27841E-13	8.73154E-12	RHSIM_Rhsim02G0062900
Stem vs Leaf	RHSIM_Rhsim12G0188400	4.338811836	8.27912E-13	8.73154E-12	RHSIM_Rhsim12G0188400
Stem vs Leaf	novel.2694	-2.811314342	8.39796E-13	8.85281E-12	--
Stem vs Leaf	RHSIM_Rhsim13G0091600	1.328170371	8.57797E-13	9.03844E-12	RHSIM_Rhsim13G0091600
Stem vs Leaf	RHSIM_Rhsim01G0187700	-3.536525286	8.60922E-13	9.06722E-12	RHSIM_Rhsim01G0187700
Stem vs Leaf	RHSIM_Rhsim07G0037800	-2.159167021	8.65958E-13	9.1161E-12	RHSIM_Rhsim07G0037800
Stem vs Leaf	RHSIM_Rhsim03G0195300	-4.286238577	8.69822E-13	9.15259E-12	RHSIM_Rhsim03G0195300
Stem vs Leaf	RHSIM_Rhsim06G0184100	-1.165463045	8.74316E-13	9.19568E-12	RHSIM_Rhsim06G0184100
Stem vs Leaf	novel.1319	2.083058026	8.7664E-13	9.21591E-12	--
Stem vs Leaf	RHSIM_Rhsim10G0183600	2.493581804	8.8067E-13	9.25406E-12	RHSIM_Rhsim10G0183600
Stem vs Leaf	RHSIM_Rhsim03G0195200	-5.312460496	8.93307E-13	9.38257E-12	RHSIM_Rhsim03G0195200

Comparison	Gene ID	log_2FC	P-value	FDR (padj)	Gene Name
Stem vs Leaf	RHSIM_Rhsim03G0003700	-9.058611077	8.96921E-13	9.41624E-12	RHSIM_Rhsim03G0003700
Stem vs Leaf	RHSIM_Rhsim08G0031900	3.020248517	9.04794E-13	9.49457E-12	RHSIM_Rhsim08G0031900
Stem vs Leaf	RHSIM_Rhsim08G0197200	1.258567074	9.12075E-13	9.56661E-12	RHSIM_Rhsim08G0197200
Stem vs Leaf	RHSIM_Rhsim13G0119600	-1.250399957	9.34264E-13	9.7949E-12	RHSIM_Rhsim13G0119600
Stem vs Leaf	RHSIM_Rhsim08G0191800	7.193939604	9.47833E-13	9.93263E-12	RHSIM_Rhsim08G0191800
Stem vs Leaf	RHSIM_Rhsim13G0078600	-3.43781621	9.5219E-13	9.97375E-12	RHSIM_Rhsim13G0078600
Stem vs Leaf	RHSIM_Rhsim10G0006600	-1.821022812	9.73609E-13	1.01935E-11	RHSIM_Rhsim10G0006600
Stem vs Leaf	RHSIM_Rhsim06G0008700	4.305009761	1.03615E-12	1.08434E-11	RHSIM_Rhsim06G0008700
Stem vs Leaf	RHSIM_Rhsim04G0174900	-2.079429335	1.03922E-12	1.08705E-11	RHSIM_Rhsim04G0174900
Stem vs Leaf	RHSIM_Rhsim12G0159600	-2.018096184	1.04649E-12	1.09416E-11	RHSIM_Rhsim12G0159600
Stem vs Leaf	novel.3414	8.943804343	1.0637E-12	1.1114E-11	--
Stem vs Leaf	RHSIM_Rhsim06G0206000	-2.643489256	1.06394E-12	1.1114E-11	RHSIM_Rhsim06G0206000
Stem vs Leaf	RHSIM_Rhsim13G0044800	1.566420582	1.0646E-12	1.11158E-11	RHSIM_Rhsim13G0044800
Stem vs Leaf	RHSIM_Rhsim05G0039500	-1.016095773	1.0724E-12	1.11922E-11	RHSIM_Rhsim05G0039500
Stem vs Leaf	RHSIM_Rhsim04G0050200	-4.081982374	1.0744E-12	1.1208E-11	RHSIM_Rhsim04G0050200
Stem vs Leaf	RHSIM_Rhsim06G0057700	-2.446111084	1.08251E-12	1.12875E-11	RHSIM_Rhsim06G0057700
Stem vs Leaf	RHSIM_RhsimUnG0043700	3.388950514	1.09438E-12	1.14061E-11	RHSIM_RhsimUnG0043700
Stem vs Leaf	RHSIM_Rhsim08G0024100	-3.200951269	1.1401E-12	1.18718E-11	RHSIM_Rhsim08G0024100
Stem vs Leaf	novel.6094	-2.376595991	1.16443E-12	1.21198E-11	--
Stem vs Leaf	novel.3082	4.742911026	1.1688E-12	1.21597E-11	--
Stem vs Leaf	RHSIM_Rhsim13G0028700	1.292273592	1.17462E-12	1.22148E-11	RHSIM_Rhsim13G0028700
Stem vs Leaf	RHSIM_Rhsim07G0099100	-9.002995771	1.18094E-12	1.2275E-11	RHSIM_Rhsim07G0099100
Stem vs Leaf	RHSIM_Rhsim06G0198600	-2.777742525	1.19628E-12	1.24288E-11	RHSIM_Rhsim06G0198600
Stem vs Leaf	novel.525	-7.805773171	1.21365E-12	1.26036E-11	--
Stem vs Leaf	RHSIM_Rhsim12G0029300	1.989316315	1.21674E-12	1.263E-11	RHSIM_Rhsim12G0029300

Comparison	Gene ID	log_2FC	P-value	FDR (padj)	Gene Name
Stem vs Leaf	RHSIM_Rhsim09G0143900	-3.826083272	1.29484E-12	1.34346E-11	RHSIM_Rhsim09G0143900
Stem vs Leaf	RHSIM_RhsimUnG0018100	-8.936449331	1.30583E-12	1.35425E-11	RHSIM_RhsimUnG0018100
Stem vs Leaf	RHSIM_Rhsim13G0115800	-2.182453124	1.31495E-12	1.3631E-11	RHSIM_Rhsim13G0115800
Stem vs Leaf	RHSIM_Rhsim05G0177600	6.771725622	1.31572E-12	1.36328E-11	RHSIM_Rhsim05G0177600
Stem vs Leaf	RHSIM_Rhsim01G0237500	-2.873479441	1.32284E-12	1.37004E-11	RHSIM_Rhsim01G0237500
Stem vs Leaf	RHSIM_Rhsim06G0102900	1.349616732	1.3445E-12	1.39185E-11	RHSIM_Rhsim06G0102900
Stem vs Leaf	RHSIM_Rhsim02G0220400	-3.690101235	1.35166E-12	1.39864E-11	RHSIM_Rhsim02G0220400
Stem vs Leaf	RHSIM_Rhsim04G0011600	-1.168070602	1.37613E-12	1.42332E-11	RHSIM_Rhsim04G0011600
Stem vs Leaf	RHSIM_Rhsim05G0214700	-3.724107534	1.40397E-12	1.45147E-11	RHSIM_Rhsim05G0214700
Stem vs Leaf	RHSIM_Rhsim01G0268800	4.276798024	1.40595E-12	1.45285E-11	RHSIM_Rhsim01G0268800
Stem vs Leaf	RHSIM_Rhsim10G0155200	3.561211761	1.40724E-12	1.45354E-11	RHSIM_Rhsim10G0155200
Stem vs Leaf	RHSIM_Rhsim11G0028200	3.583793056	1.42828E-12	1.47461E-11	RHSIM_Rhsim11G0028200
Stem vs Leaf	RHSIM_Rhsim13G0183800	-9.885059544	1.43637E-12	1.4823E-11	RHSIM_Rhsim13G0183800
Stem vs Leaf	RHSIM_Rhsim10G0191200	-3.370808565	1.44721E-12	1.49282E-11	RHSIM_Rhsim10G0191200
Stem vs Leaf	novel.2	1.180782534	1.49941E-12	1.54597E-11	--
Stem vs Leaf	RHSIM_Rhsim03G0029500	-9.065456265	1.52668E-12	1.57338E-11	RHSIM_Rhsim03G0029500
Stem vs Leaf	RHSIM_Rhsim01G0193200	-4.483815838	1.54256E-12	1.58904E-11	RHSIM_Rhsim01G0193200
Stem vs Leaf	RHSIM_Rhsim04G0015900	-1.039907727	1.55105E-12	1.59706E-11	RHSIM_Rhsim04G0015900
Stem vs Leaf	RHSIM_Rhsim13G0179300	2.023298038	1.55366E-12	1.59904E-11	RHSIM_Rhsim13G0179300
Stem vs Leaf	RHSIM_Rhsim12G0191200	-4.985721653	1.55516E-12	1.59987E-11	RHSIM_Rhsim12G0191200
Stem vs Leaf	RHSIM_RhsimUnG0190100	-2.931548573	1.58329E-12	1.62808E-11	RHSIM_RhsimUnG0190100
Stem vs Leaf	RHSIM_Rhsim03G0267700	1.889614601	1.5915E-12	1.63579E-11	RHSIM_Rhsim03G0267700
Stem vs Leaf	RHSIM_Rhsim13G0099300	9.122118977	1.61338E-12	1.65755E-11	RHSIM_Rhsim13G0099300
Stem vs Leaf	RHSIM_Rhsim01G0064700	-10.15823662	1.6221E-12	1.66576E-11	RHSIM_Rhsim01G0064700
Stem vs Leaf	RHSIM_Rhsim04G0121900	1.065787297	1.62696E-12	1.67001E-11	RHSIM_Rhsim04G0121900

Comparison	Gene ID	log_2FC	P-value	FDR (padj)	Gene Name
Stem vs Leaf	RHSIM_Rhsim07G0075100	-1.398895544	1.64469E-12	1.68685E-11	RHSIM_Rhsim07G0075100
Stem vs Leaf	RHSIM_Rhsim07G0125300	-1.212957975	1.64483E-12	1.68685E-11	RHSIM_Rhsim07G0125300
Stem vs Leaf	RHSIM_Rhsim06G0238600	-1.806736022	1.65758E-12	1.69917E-11	RHSIM_Rhsim06G0238600
Stem vs Leaf	RHSIM_Rhsim05G0111800	-1.234756021	1.66631E-12	1.70736E-11	RHSIM_Rhsim05G0111800
Stem vs Leaf	novel.4097	-3.22867237	1.74193E-12	1.78404E-11	--
Stem vs Leaf	RHSIM_Rhsim01G0187900	-1.916057134	1.74275E-12	1.78409E-11	RHSIM_Rhsim01G0187900
Stem vs Leaf	RHSIM_RhsimUnG0077000	-1.496782713	1.74761E-12	1.78827E-11	RHSIM_RhsimUnG0077000
Stem vs Leaf	RHSIM_Rhsim10G0102400	4.049763156	1.75206E-12	1.79203E-11	RHSIM_Rhsim10G0102400
Stem vs Leaf	RHSIM_Rhsim04G0077800	-8.692920862	1.75756E-12	1.79686E-11	RHSIM_Rhsim04G0077800
Stem vs Leaf	RHSIM_Rhsim04G0181400	-2.704990033	1.76572E-12	1.8044E-11	RHSIM_Rhsim04G0181400
Stem vs Leaf	RHSIM_Rhsim08G0248500	-2.397686884	1.83685E-12	1.87626E-11	RHSIM_Rhsim08G0248500
Stem vs Leaf	RHSIM_Rhsim04G0150300	2.558011803	1.83844E-12	1.87705E-11	RHSIM_Rhsim04G0150300
Stem vs Leaf	RHSIM_Rhsim13G0164100	2.025135047	1.87027E-12	1.9087E-11	RHSIM_Rhsim13G0164100
Stem vs Leaf	RHSIM_Rhsim07G0210000	2.653260189	1.87699E-12	1.91471E-11	RHSIM_Rhsim07G0210000
Stem vs Leaf	RHSIM_Rhsim12G0048700	-7.71530976	1.91613E-12	1.95378E-11	RHSIM_Rhsim12G0048700
Stem vs Leaf	RHSIM_Rhsim02G0028600	-2.370532694	1.92664E-12	1.96362E-11	RHSIM_Rhsim02G0028600
Stem vs Leaf	RHSIM_Rhsim09G0170200	2.723053146	1.94538E-12	1.98185E-11	RHSIM_Rhsim09G0170200
Stem vs Leaf	RHSIM_Rhsim10G0016800	1.8324196	1.96045E-12	1.99631E-11	RHSIM_Rhsim10G0016800
Stem vs Leaf	RHSIM_Rhsim06G0121500	1.629111861	1.97375E-12	2.00897E-11	RHSIM_Rhsim06G0121500
Stem vs Leaf	RHSIM_Rhsim08G0117900	5.47810884	1.99499E-12	2.0297E-11	RHSIM_Rhsim08G0117900
Stem vs Leaf	RHSIM_Rhsim13G0061600	2.425176703	2.01336E-12	2.04748E-11	RHSIM_Rhsim13G0061600
Stem vs Leaf	RHSIM_Rhsim01G0238100	2.627811433	2.03293E-12	2.06647E-11	RHSIM_Rhsim01G0238100
Stem vs Leaf	RHSIM_Rhsim05G0214800	-1.17418918	2.05724E-12	2.09026E-11	RHSIM_Rhsim05G0214800
Stem vs Leaf	RHSIM_Rhsim03G0140800	11.78084545	2.08274E-12	2.11523E-11	RHSIM_Rhsim03G0140800
Stem vs Leaf	RHSIM_Rhsim08G0040600	1.583842161	2.09778E-12	2.12957E-11	RHSIM_Rhsim08G0040600

Comparison	Gene ID	log$_2$FC	P-value	FDR (padj)	Gene Name
Stem vs Leaf	RHSIM_Rhsim08G0168800	3.606079406	2.11747E-12	2.14862E-11	RHSIM_Rhsim08G0168800
Stem vs Leaf	RHSIM_Rhsim09G0185300	-9.507292529	2.14514E-12	2.17573E-11	RHSIM_Rhsim09G0185300
Stem vs Leaf	RHSIM_Rhsim04G0241800	-3.025010986	2.17593E-12	2.20599E-11	RHSIM_Rhsim04G0241800
Stem vs Leaf	RHSIM_Rhsim07G0128900	-1.591615203	2.19702E-12	2.22639E-11	RHSIM_Rhsim07G0128900
Stem vs Leaf	RHSIM_Rhsim07G0095100	-2.151252782	2.24307E-12	2.27206E-11	RHSIM_Rhsim07G0095100
Stem vs Leaf	RHSIM_Rhsim03G0141800	-1.909231407	2.24913E-12	2.2772E-11	RHSIM_Rhsim03G0141800
Stem vs Leaf	RHSIM_Rhsim10G0207400	2.656878792	2.28384E-12	2.31132E-11	RHSIM_Rhsim10G0207400
Stem vs Leaf	RHSIM_Rhsim02G0035300	3.922483608	2.32077E-12	2.34767E-11	RHSIM_Rhsim02G0035300
Stem vs Leaf	RHSIM_Rhsim07G0026400	-5.946736442	2.35895E-12	2.38407E-11	RHSIM_Rhsim07G0026400
Stem vs Leaf	RHSIM_Rhsim07G0002400	1.817021947	2.35986E-12	2.38407E-11	RHSIM_Rhsim07G0002400
Stem vs Leaf	RHSIM_Rhsim01G0278000	-3.692818588	2.38969E-12	2.41315E-11	RHSIM_Rhsim01G0278000
Stem vs Leaf	RHSIM_Rhsim13G0165900	-3.332157439	2.39108E-12	2.41349E-11	RHSIM_Rhsim13G0165900
Stem vs Leaf	RHSIM_Rhsim10G0179900	-9.032695746	2.42063E-12	2.44225E-11	RHSIM_Rhsim10G0179900
Stem vs Leaf	RHSIM_Rhsim05G0203000	-3.208853859	2.47147E-12	2.49246E-11	RHSIM_Rhsim05G0203000
Stem vs Leaf	RHSIM_Rhsim12G0161700	-3.221568466	2.49271E-12	2.51277E-11	RHSIM_Rhsim12G0161700
Stem vs Leaf	RHSIM_Rhsim05G0232700	6.58612557	2.50213E-12	2.52117E-11	RHSIM_Rhsim05G0232700
Stem vs Leaf	RHSIM_Rhsim09G0109900	-2.344636618	2.50731E-12	2.52528E-11	RHSIM_Rhsim09G0109900
Stem vs Leaf	novel.1767	-4.36410524	2.5252E-12	2.54219E-11	--
Stem vs Leaf	RHSIM_Rhsim06G0149000	-1.000414916	2.61434E-12	2.63078E-11	RHSIM_Rhsim06G0149000
Stem vs Leaf	RHSIM_Rhsim02G0084500	3.1794283	2.61784E-12	2.63315E-11	RHSIM_Rhsim02G0084500
Stem vs Leaf	RHSIM_Rhsim04G0079700	-5.828196268	2.63219E-12	2.64643E-11	RHSIM_Rhsim04G0079700
Stem vs Leaf	RHSIM_Rhsim07G0080000	-2.654091905	2.652E-12	2.66518E-11	RHSIM_Rhsim07G0080000
Stem vs Leaf	RHSIM_Rhsim09G0021400	-6.386360019	2.65568E-12	2.66772E-11	RHSIM_Rhsim09G0021400
Stem vs Leaf	novel.3546	2.137530278	2.69286E-12	2.70389E-11	--
Stem vs Leaf	RHSIM_Rhsim01G0262300	-1.438111418	2.76858E-12	2.77871E-11	RHSIM_Rhsim01G0262300

Comparison	Gene ID	log_2FC	P-value	FDR (padj)	Gene Name
Stem vs Leaf	RHSIM_Rhsim10G0068700	-1.810210695	2.89574E-12	2.90506E-11	RHSIM_Rhsim10G0068700
Stem vs Leaf	RHSIM_Rhsim07G0225400	3.039640075	2.89832E-12	2.90639E-11	RHSIM_Rhsim07G0225400
Stem vs Leaf	RHSIM_Rhsim01G0215100	10.15952797	2.90104E-12	2.90785E-11	RHSIM_Rhsim01G0215100
Stem vs Leaf	RHSIM_Rhsim13G0096400	1.059483596	2.9054E-12	2.91096E-11	RHSIM_Rhsim13G0096400
Stem vs Leaf	RHSIM_Rhsim09G0075000	1.752109934	2.93259E-12	2.93692E-11	RHSIM_Rhsim09G0075000
Stem vs Leaf	RHSIM_Rhsim09G0051400	-1.04856972	2.93873E-12	2.94173E-11	RHSIM_Rhsim09G0051400
Stem vs Leaf	RHSIM_Rhsim12G0181800	-2.410542563	2.93994E-12	2.94173E-11	RHSIM_Rhsim12G0181800
Stem vs Leaf	RHSIM_Rhsim03G0172900	-2.123409122	3.00265E-12	3.00317E-11	RHSIM_Rhsim03G0172900
Stem vs Leaf	novel.5885	6.110563226	3.02793E-12	3.02714E-11	--
Stem vs Leaf	RHSIM_Rhsim07G0048600	-2.337139711	3.04728E-12	3.04516E-11	RHSIM_Rhsim07G0048600
Stem vs Leaf	RHSIM_Rhsim07G0067700	2.096794976	3.07358E-12	3.07012E-11	RHSIM_Rhsim07G0067700
Stem vs Leaf	RHSIM_Rhsim08G0091400	-3.216553798	3.08905E-12	3.08424E-11	RHSIM_Rhsim08G0091400
Stem vs Leaf	RHSIM_Rhsim09G0202700	2.474511131	3.11403E-12	3.10783E-11	RHSIM_Rhsim09G0202700
Stem vs Leaf	RHSIM_Rhsim04G0172200	-1.757884049	3.11962E-12	3.11206E-11	RHSIM_Rhsim04G0172200
Stem vs Leaf	RHSIM_Rhsim07G0089600	-3.90837944	3.13509E-12	3.12614E-11	RHSIM_Rhsim07G0089600
Stem vs Leaf	RHSIM_Rhsim09G0128900	-1.206454338	3.18861E-12	3.17813E-11	RHSIM_Rhsim09G0128900
Stem vs Leaf	RHSIM_RhsimUnG0185400	-2.099863454	3.21313E-12	3.20118E-11	RHSIM_RhsimUnG0185400
Stem vs Leaf	RHSIM_Rhsim06G0165800	-1.293361778	3.24374E-12	3.23028E-11	RHSIM_Rhsim06G0165800
Stem vs Leaf	RHSIM_Rhsim09G0027800	-1.801266378	3.26853E-12	3.25356E-11	RHSIM_Rhsim09G0027800
Stem vs Leaf	RHSIM_Rhsim08G0031200	-1.299024192	3.34287E-12	3.32613E-11	RHSIM_Rhsim08G0031200
Stem vs Leaf	RHSIM_Rhsim08G0171200	-1.076489357	3.35045E-12	3.33223E-11	RHSIM_Rhsim08G0171200
Stem vs Leaf	RHSIM_Rhsim08G0121700	2.217945314	3.3571E-12	3.3374E-11	RHSIM_Rhsim08G0121700
Stem vs Leaf	RHSIM_Rhsim06G0215000	-5.383534687	3.4072E-12	3.38575E-11	RHSIM_Rhsim06G0215000
Stem vs Leaf	RHSIM_Rhsim10G0118400	-1.903593157	3.41365E-12	3.3907E-11	RHSIM_Rhsim10G0118400
Stem vs Leaf	novel.5910	-4.334593505	3.49391E-12	3.46743E-11	--

Comparison	Gene ID	log_2FC	P-value	FDR (padj)	Gene Name
Stem vs Leaf	RHSIM_Rhsim09G0189800	-2.026158501	3.53983E-12	3.51149E-11	RHSIM_Rhsim09G0189800
Stem vs Leaf	RHSIM_Rhsim07G0153500	-1.361094643	3.55695E-12	3.5263E-11	RHSIM_Rhsim07G0153500
Stem vs Leaf	RHSIM_Rhsim01G0073700	-1.956397952	3.55783E-12	3.5263E-11	RHSIM_Rhsim01G0073700
Stem vs Leaf	RHSIM_Rhsim06G0236300	3.91261565	3.63586E-12	3.6021E-11	RHSIM_Rhsim06G0236300
Stem vs Leaf	RHSIM_Rhsim04G0052300	-3.454295507	3.66873E-12	3.63293E-11	RHSIM_Rhsim04G0052300
Stem vs Leaf	RHSIM_Rhsim09G0087600	-3.573753521	3.67013E-12	3.63293E-11	RHSIM_Rhsim09G0087600
Stem vs Leaf	RHSIM_RhsimUnG0056800	-9.352035807	3.70332E-12	3.6642E-11	RHSIM_RhsimUnG0056800
Stem vs Leaf	RHSIM_Rhsim07G0096000	-1.897176955	3.71121E-12	3.67044E-11	RHSIM_Rhsim07G0096000
Stem vs Leaf	RHSIM_Rhsim08G0192800	3.656034753	3.71469E-12	3.6723E-11	RHSIM_Rhsim08G0192800
Stem vs Leaf	RHSIM_Rhsim09G0069000	-1.631725142	3.76825E-12	3.72365E-11	RHSIM_Rhsim09G0069000
Stem vs Leaf	RHSIM_Rhsim07G0037500	4.16942859	3.90758E-12	3.859E-11	RHSIM_Rhsim07G0037500
Stem vs Leaf	RHSIM_Rhsim12G0187600	2.250244398	3.90857E-12	3.859E-11	RHSIM_Rhsim12G0187600
Stem vs Leaf	RHSIM_Rhsim08G0216500	1.128967711	3.9107E-12	3.85945E-11	RHSIM_Rhsim08G0216500
Stem vs Leaf	RHSIM_Rhsim01G0052400	-2.039727633	3.96621E-12	3.91255E-11	RHSIM_Rhsim01G0052400
Stem vs Leaf	RHSIM_Rhsim04G0023000	-2.191239709	3.97294E-12	3.91752E-11	RHSIM_Rhsim04G0023000
Stem vs Leaf	RHSIM_Rhsim01G0179000	-1.899213272	4.04568E-12	3.98754E-11	RHSIM_Rhsim01G0179000
Stem vs Leaf	RHSIM_Rhsim09G0003600	-1.495691533	4.10759E-12	4.04682E-11	RHSIM_Rhsim09G0003600
Stem vs Leaf	novel.1103	-3.355817968	4.20366E-12	4.1397E-11	--
Stem vs Leaf	RHSIM_Rhsim05G0208200	1.337896886	4.2128E-12	4.14694E-11	RHSIM_Rhsim05G0208200
Stem vs Leaf	RHSIM_Rhsim03G0211800	-1.145094803	4.23118E-12	4.16325E-11	RHSIM_Rhsim03G0211800
Stem vs Leaf	RHSIM_RhsimUnG0008000	5.642004899	4.29327E-12	4.22254E-11	RHSIM_RhsimUnG0008000
Stem vs Leaf	RHSIM_Rhsim08G0003800	-9.022055654	4.31354E-12	4.24067E-11	RHSIM_Rhsim08G0003800
Stem vs Leaf	RHSIM_Rhsim07G0014600	-2.392948042	4.32579E-12	4.25089E-11	RHSIM_Rhsim07G0014600
Stem vs Leaf	novel.4141	1.288057317	4.33214E-12	4.25532E-11	--
Stem vs Leaf	RHSIM_Rhsim03G0190200	-2.287192601	4.39793E-12	4.3181E-11	RHSIM_Rhsim03G0190200

Comparison	Gene ID	log_2FC	P-value	FDR (padj)	Gene Name
Stem vs Leaf	RHSIM_Rhsim08G0160800	4.263622241	4.48384E-12	4.40058E-11	RHSIM_Rhsim08G0160800
Stem vs Leaf	RHSIM_Rhsim02G0090500	-4.641828686	4.54523E-12	4.45894E-11	RHSIM_Rhsim02G0090500
Stem vs Leaf	RHSIM_Rhsim12G0005000	-2.965389487	4.55135E-12	4.46303E-11	RHSIM_Rhsim12G0005000
Stem vs Leaf	RHSIM_Rhsim03G0085600	-4.133991959	4.78684E-12	4.69196E-11	RHSIM_Rhsim03G0085600
Stem vs Leaf	RHSIM_Rhsim01G0235100	-3.211106703	4.81799E-12	4.71938E-11	RHSIM_Rhsim01G0235100
Stem vs Leaf	RHSIM_Rhsim06G0012300	-1.813492967	4.81892E-12	4.71938E-11	RHSIM_Rhsim06G0012300
Stem vs Leaf	RHSIM_Rhsim02G0053700	-2.351578858	4.87435E-12	4.77165E-11	RHSIM_Rhsim02G0053700
Stem vs Leaf	RHSIM_Rhsim07G0013000	-1.827409178	4.88628E-12	4.78129E-11	RHSIM_Rhsim07G0013000
Stem vs Leaf	RHSIM_Rhsim01G0003800	-3.010743334	4.91492E-12	4.80727E-11	RHSIM_Rhsim01G0003800
Stem vs Leaf	RHSIM_Rhsim11G0027000	-1.823623798	4.96873E-12	4.85785E-11	RHSIM_Rhsim11G0027000
Stem vs Leaf	RHSIM_Rhsim05G0032000	3.114559186	5.11623E-12	4.99781E-11	RHSIM_Rhsim05G0032000
Stem vs Leaf	RHSIM_Rhsim12G0113000	1.258271138	5.19509E-12	5.0727E-11	RHSIM_Rhsim12G0113000
Stem vs Leaf	RHSIM_Rhsim11G0071200	-1.199019385	5.29329E-12	5.1664E-11	RHSIM_Rhsim11G0071200
Stem vs Leaf	RHSIM_Rhsim08G0100700	-1.495264997	5.36394E-12	5.23092E-11	RHSIM_Rhsim08G0100700
Stem vs Leaf	RHSIM_Rhsim02G0254900	4.336324135	5.44369E-12	5.30645E-11	RHSIM_Rhsim02G0254900
Stem vs Leaf	RHSIM_Rhsim02G0143300	-1.49498989	5.44987E-12	5.31022E-11	RHSIM_Rhsim02G0143300
Stem vs Leaf	RHSIM_Rhsim11G0132800	2.586869845	5.4625E-12	5.32028E-11	RHSIM_Rhsim11G0132800
Stem vs Leaf	RHSIM_Rhsim09G0145900	1.196680915	5.51614E-12	5.37026E-11	RHSIM_Rhsim09G0145900
Stem vs Leaf	RHSIM_Rhsim09G0044600	-2.066318158	5.60933E-12	5.45407E-11	RHSIM_Rhsim09G0044600
Stem vs Leaf	RHSIM_Rhsim11G0031500	-2.7459171	5.67927E-12	5.51975E-11	RHSIM_Rhsim11G0031500
Stem vs Leaf	RHSIM_Rhsim08G0236500	2.255183608	5.71319E-12	5.55037E-11	RHSIM_Rhsim08G0236500
Stem vs Leaf	RHSIM_Rhsim07G0023400	9.142122316	5.71962E-12	5.55428E-11	RHSIM_Rhsim07G0023400
Stem vs Leaf	RHSIM_Rhsim03G0160200	-3.062966065	5.72726E-12	5.55935E-11	RHSIM_Rhsim03G0160200
Stem vs Leaf	RHSIM_Rhsim04G0162800	-1.263472227	5.76075E-12	5.58951E-11	RHSIM_Rhsim04G0162800
Stem vs Leaf	RHSIM_Rhsim12G0010500	-5.053995108	5.88629E-12	5.70891E-11	RHSIM_Rhsim12G0010500

Comparison	Gene ID	log_2FC	P-value	FDR (padj)	Gene Name
Stem vs Leaf	RHSIM_RhsimUnG0065500	5.122187764	5.89842E-12	5.71826E-11	RHSIM_RhsimUnG0065500
Stem vs Leaf	RHSIM_Rhsim09G0209100	-1.452880348	5.95347E-12	5.76921E-11	RHSIM_Rhsim09G0209100
Stem vs Leaf	RHSIM_Rhsim06G0164400	-2.43400113	5.96331E-12	5.77631E-11	RHSIM_Rhsim06G0164400
Stem vs Leaf	RHSIM_Rhsim13G0181500	-2.099375609	5.98135E-12	5.79136E-11	RHSIM_Rhsim13G0181500
Stem vs Leaf	RHSIM_Rhsim06G0054400	-1.136482594	6.01927E-12	5.82562E-11	RHSIM_Rhsim06G0054400
Stem vs Leaf	RHSIM_Rhsim10G0113200	-1.227435849	6.13663E-12	5.93671E-11	RHSIM_Rhsim10G0113200
Stem vs Leaf	RHSIM_Rhsim11G0122200	-1.988985342	6.16031E-12	5.95712E-11	RHSIM_Rhsim11G0122200
Stem vs Leaf	RHSIM_Rhsim12G0035300	2.180543226	6.16706E-12	5.96115E-11	RHSIM_Rhsim12G0035300
Stem vs Leaf	RHSIM_Rhsim10G0060300	1.406787168	6.1947E-12	5.98535E-11	RHSIM_Rhsim10G0060300
Stem vs Leaf	RHSIM_Rhsim07G0141500	-2.010197227	6.20123E-12	5.98915E-11	RHSIM_Rhsim07G0141500
Stem vs Leaf	RHSIM_Rhsim13G0211700	2.605741098	6.26257E-12	6.04586E-11	RHSIM_Rhsim13G0211700
Stem vs Leaf	RHSIM_Rhsim02G0086500	-6.719015354	6.35906E-12	6.13644E-11	RHSIM_Rhsim02G0086500
Stem vs Leaf	RHSIM_Rhsim09G0210100	2.717551136	6.37436E-12	6.14863E-11	RHSIM_Rhsim09G0210100
Stem vs Leaf	RHSIM_Rhsim10G0118200	1.544925331	6.40565E-12	6.17622E-11	RHSIM_Rhsim10G0118200
Stem vs Leaf	novel.1318	1.077953081	6.42139E-12	6.18486E-11	--
Stem vs Leaf	RHSIM_Rhsim04G0202800	2.507742721	6.42303E-12	6.18486E-11	RHSIM_Rhsim04G0202800
Stem vs Leaf	RHSIM_Rhsim08G0185600	3.742674328	6.42535E-12	6.18486E-11	RHSIM_Rhsim08G0185600
Stem vs Leaf	RHSIM_Rhsim07G0067100	5.851385629	6.46636E-12	6.22175E-11	RHSIM_Rhsim07G0067100
Stem vs Leaf	RHSIM_Rhsim06G0028900	3.650903734	6.51194E-12	6.26299E-11	RHSIM_Rhsim06G0028900
Stem vs Leaf	RHSIM_Rhsim11G0088200	-2.106705778	6.52092E-12	6.26744E-11	RHSIM_Rhsim11G0088200
Stem vs Leaf	RHSIM_Rhsim07G0186600	-9.13807148	6.52201E-12	6.26744E-11	RHSIM_Rhsim07G0186600
Stem vs Leaf	RHSIM_Rhsim06G0193000	4.531535815	6.55277E-12	6.29438E-11	RHSIM_Rhsim06G0193000
Stem vs Leaf	RHSIM_Rhsim02G0203700	-2.29007143	6.62758E-12	6.36358E-11	RHSIM_Rhsim02G0203700
Stem vs Leaf	RHSIM_Rhsim04G0120900	-1.340208896	6.77166E-12	6.49922E-11	RHSIM_Rhsim04G0120900
Stem vs Leaf	RHSIM_Rhsim10G0064000	2.64853067	6.82253E-12	6.54531E-11	RHSIM_Rhsim10G0064000

Comparison	Gene ID	log_2FC	P-value	FDR (padj)	Gene Name
Stem vs Leaf	RHSIM_Rhsim06G0166800	3.063483721	6.92118E-12	6.63719E-11	RHSIM_Rhsim06G0166800
Stem vs Leaf	RHSIM_Rhsim03G0246300	2.250166796	7.04972E-12	6.75764E-11	RHSIM_Rhsim03G0246300
Stem vs Leaf	RHSIM_RhsimUnG0249700	3.088335252	7.1082E-12	6.81087E-11	RHSIM_RhsimUnG0249700
Stem vs Leaf	RHSIM_Rhsim07G0123000	8.820234644	7.16827E-12	6.86557E-11	RHSIM_Rhsim07G0123000
Stem vs Leaf	RHSIM_Rhsim06G0142200	3.504419238	7.3203E-12	7.00827E-11	RHSIM_Rhsim06G0142200
Stem vs Leaf	RHSIM_Rhsim12G0194800	-1.704656589	7.34356E-12	7.02762E-11	RHSIM_Rhsim12G0194800
Stem vs Leaf	RHSIM_RhsimUnG0194200	-3.271087206	7.4238E-12	7.10146E-11	RHSIM_RhsimUnG0194200
Stem vs Leaf	RHSIM_Rhsim10G0139000	-3.315565619	7.51768E-12	7.18827E-11	RHSIM_Rhsim10G0139000
Stem vs Leaf	RHSIM_Rhsim03G0157000	-8.728711172	7.61428E-12	7.27762E-11	RHSIM_Rhsim03G0157000
Stem vs Leaf	RHSIM_Rhsim01G0220700	-2.043319044	7.71383E-12	7.36971E-11	RHSIM_Rhsim01G0220700
Stem vs Leaf	RHSIM_Rhsim13G0193900	-1.832458079	7.81076E-12	7.45923E-11	RHSIM_Rhsim13G0193900
Stem vs Leaf	novel.3793	7.410844737	7.95928E-12	7.59792E-11	--
Stem vs Leaf	RHSIM_Rhsim10G0109500	3.903211524	8.14969E-12	7.77645E-11	RHSIM_Rhsim10G0109500
Stem vs Leaf	novel.1500	1.334642806	8.2945E-12	7.91136E-11	--
Stem vs Leaf	RHSIM_Rhsim12G0004000	3.818922637	8.4096E-12	8.01782E-11	RHSIM_Rhsim12G0004000
Stem vs Leaf	RHSIM_Rhsim04G0132500	-1.575218783	8.57045E-12	8.16781E-11	RHSIM_Rhsim04G0132500
Stem vs Leaf	RHSIM_Rhsim07G0158200	-1.57812373	8.62064E-12	8.2113E-11	RHSIM_Rhsim07G0158200
Stem vs Leaf	RHSIM_Rhsim09G0195800	-1.884114314	8.62619E-12	8.2113E-11	RHSIM_Rhsim09G0195800
Stem vs Leaf	RHSIM_Rhsim04G0202000	-2.980259402	8.62678E-12	8.2113E-11	RHSIM_Rhsim04G0202000
Stem vs Leaf	RHSIM_Rhsim08G0201300	-2.330202313	8.63792E-12	8.21851E-11	RHSIM_Rhsim08G0201300
Stem vs Leaf	RHSIM_Rhsim03G0052000	-6.5055521	8.92858E-12	8.48804E-11	RHSIM_Rhsim03G0052000
Stem vs Leaf	RHSIM_Rhsim02G0049200	-1.797641339	8.93421E-12	8.48989E-11	RHSIM_Rhsim02G0049200
Stem vs Leaf	RHSIM_Rhsim13G0169300	-1.913794157	8.96839E-12	8.51886E-11	RHSIM_Rhsim13G0169300
Stem vs Leaf	RHSIM_Rhsim04G0046500	-1.202264981	9.01209E-12	8.55685E-11	RHSIM_Rhsim04G0046500
Stem vs Leaf	RHSIM_Rhsim03G0253400	1.057850326	9.04515E-12	8.58469E-11	RHSIM_Rhsim03G0253400

Comparison	Gene ID	log_2FC	P-value	FDR (padj)	Gene Name
Stem vs Leaf	RHSIM_RhsimUnG0210400	-8.619328283	9.13684E-12	8.66815E-11	RHSIM_RhsimUnG0210400
Stem vs Leaf	RHSIM_Rhsim10G0140100	-3.978604151	9.27067E-12	8.7915E-11	RHSIM_Rhsim10G0140100
Stem vs Leaf	RHSIM_Rhsim03G0018400	-1.364038346	9.76921E-12	9.25665E-11	RHSIM_Rhsim03G0018400
Stem vs Leaf	RHSIM_Rhsim01G0275900	-9.404390356	9.9285E-12	9.40371E-11	RHSIM_Rhsim01G0275900
Stem vs Leaf	RHSIM_Rhsim01G0249100	2.554792589	9.93533E-12	9.40436E-11	RHSIM_Rhsim01G0249100
Stem vs Leaf	RHSIM_RhsimUnG0202600	-3.05707132	9.93734E-12	9.40436E-11	RHSIM_RhsimUnG0202600
Stem vs Leaf	RHSIM_Rhsim10G0035500	4.833377207	1.00312E-11	9.48933E-11	RHSIM_Rhsim10G0035500
Stem vs Leaf	RHSIM_Rhsim08G0080600	-1.254047672	1.00604E-11	9.51302E-11	RHSIM_Rhsim08G0080600
Stem vs Leaf	RHSIM_Rhsim11G0088000	-3.0892949	1.00874E-11	9.53463E-11	RHSIM_Rhsim11G0088000
Stem vs Leaf	RHSIM_RhsimUnG0062800	-1.108105802	1.03264E-11	9.75257E-11	RHSIM_RhsimUnG0062800
Stem vs Leaf	novel.1789	-1.555784008	1.03385E-11	9.75991E-11	--
Stem vs Leaf	RHSIM_RhsimUnG0254400	-3.268509669	1.04876E-11	9.89663E-11	RHSIM_RhsimUnG0254400
Stem vs Leaf	RHSIM_Rhsim13G0179100	-7.209726564	1.05317E-11	9.93423E-11	RHSIM_Rhsim13G0179100
Stem vs Leaf	RHSIM_Rhsim08G0058300	-9.528281079	1.06416E-11	1.00338E-10	RHSIM_Rhsim08G0058300
Stem vs Leaf	RHSIM_Rhsim05G0086300	-3.036665734	1.06687E-11	1.00552E-10	RHSIM_Rhsim05G0086300
Stem vs Leaf	RHSIM_Rhsim01G0012400	-8.705502442	1.07614E-11	1.01384E-10	RHSIM_Rhsim01G0012400
Stem vs Leaf	RHSIM_Rhsim01G0109600	-2.358517179	1.07771E-11	1.01491E-10	RHSIM_Rhsim01G0109600
Stem vs Leaf	RHSIM_Rhsim11G0100200	1.050925292	1.08254E-11	1.01903E-10	RHSIM_Rhsim11G0100200
Stem vs Leaf	RHSIM_Rhsim03G0111900	-1.824159316	1.09129E-11	1.02686E-10	RHSIM_Rhsim03G0111900
Stem vs Leaf	RHSIM_Rhsim12G0148700	-1.396898904	1.12336E-11	1.0566E-10	RHSIM_Rhsim12G0148700
Stem vs Leaf	RHSIM_Rhsim01G0000700	-1.627003236	1.12658E-11	1.05919E-10	RHSIM_Rhsim01G0000700
Stem vs Leaf	RHSIM_Rhsim11G0052100	-1.077236822	1.12877E-11	1.06082E-10	RHSIM_Rhsim11G0052100
Stem vs Leaf	RHSIM_Rhsim09G0035700	3.305047599	1.1306E-11	1.06211E-10	RHSIM_Rhsim09G0035700
Stem vs Leaf	RHSIM_Rhsim06G0216100	-7.720166433	1.13979E-11	1.07031E-10	RHSIM_Rhsim06G0216100
Stem vs Leaf	RHSIM_Rhsim08G0015700	1.935556343	1.15222E-11	1.08154E-10	RHSIM_Rhsim08G0015700

Comparison	Gene ID	log_2FC	P-value	FDR (padj)	Gene Name
Stem vs Leaf	novel.1783	-9.020022774	1.15711E-11	1.08568E-10	--
Stem vs Leaf	RHSIM_Rhsim08G0060100	-6.021059059	1.16664E-11	1.09374E-10	RHSIM_Rhsim08G0060100
Stem vs Leaf	novel.6125	6.127791935	1.18516E-11	1.11065E-10	--
Stem vs Leaf	RHSIM_Rhsim12G0192900	-1.021212442	1.18973E-11	1.11447E-10	RHSIM_Rhsim12G0192900
Stem vs Leaf	RHSIM_Rhsim04G0028000	-2.917193585	1.19164E-11	1.11581E-10	RHSIM_Rhsim04G0028000
Stem vs Leaf	RHSIM_Rhsim01G0205500	-3.563788427	1.20553E-11	1.12836E-10	RHSIM_Rhsim01G0205500
Stem vs Leaf	RHSIM_Rhsim01G0188800	-4.180381225	1.24479E-11	1.16416E-10	RHSIM_Rhsim01G0188800
Stem vs Leaf	RHSIM_Rhsim02G0111100	-8.406668379	1.24905E-11	1.16767E-10	RHSIM_Rhsim02G0111100
Stem vs Leaf	RHSIM_Rhsim12G0105500	1.174643228	1.25326E-11	1.17114E-10	RHSIM_Rhsim12G0105500
Stem vs Leaf	RHSIM_Rhsim10G0050900	-3.181393143	1.25941E-11	1.1764E-10	RHSIM_Rhsim10G0050900
Stem vs Leaf	RHSIM_Rhsim12G0104400	-2.066346265	1.26633E-11	1.18239E-10	RHSIM_Rhsim12G0104400
Stem vs Leaf	RHSIM_Rhsim09G0184000	-1.455905048	1.2811E-11	1.19569E-10	RHSIM_Rhsim09G0184000
Stem vs Leaf	RHSIM_Rhsim03G0217200	-6.399529864	1.30227E-11	1.2147E-10	RHSIM_Rhsim03G0217200
Stem vs Leaf	RHSIM_Rhsim02G0210200	8.453266528	1.30251E-11	1.2147E-10	RHSIM_Rhsim02G0210200
Stem vs Leaf	RHSIM_Rhsim06G0064000	-1.147116216	1.30771E-11	1.21905E-10	RHSIM_Rhsim06G0064000
Stem vs Leaf	RHSIM_Rhsim04G0202600	2.524593085	1.3148E-11	1.22516E-10	RHSIM_Rhsim04G0202600
Stem vs Leaf	RHSIM_RhsimUnG0088600	8.918849525	1.31635E-11	1.22611E-10	RHSIM_RhsimUnG0088600
Stem vs Leaf	RHSIM_Rhsim07G0081100	-5.454535288	1.31963E-11	1.22867E-10	RHSIM_Rhsim07G0081100
Stem vs Leaf	RHSIM_Rhsim08G0123000	4.499131418	1.32504E-11	1.2332E-10	RHSIM_Rhsim08G0123000
Stem vs Leaf	RHSIM_Rhsim09G0121500	-8.191417107	1.33495E-11	1.24193E-10	RHSIM_Rhsim09G0121500
Stem vs Leaf	RHSIM_Rhsim01G0263800	8.762324043	1.34417E-11	1.25E-10	RHSIM_Rhsim01G0263800
Stem vs Leaf	RHSIM_Rhsim12G0036400	5.769680203	1.34857E-11	1.25359E-10	RHSIM_Rhsim12G0036400
Stem vs Leaf	RHSIM_Rhsim01G0010000	-2.344993702	1.35239E-11	1.25663E-10	RHSIM_Rhsim01G0010000
Stem vs Leaf	RHSIM_Rhsim08G0119600	-1.937297126	1.35538E-11	1.25891E-10	RHSIM_Rhsim08G0119600
Stem vs Leaf	RHSIM_Rhsim05G0177800	-4.886814686	1.40109E-11	1.30084E-10	RHSIM_Rhsim05G0177800

Comparison	Gene ID	log_2FC	P-value	FDR (padj)	Gene Name
Stem vs Leaf	RHSIM_Rhsim08G0227200	2.629781101	1.41407E-11	1.31236E-10	RHSIM_Rhsim08G0227200
Stem vs Leaf	RHSIM_Rhsim04G0060400	-3.738136675	1.45E-11	1.34517E-10	RHSIM_Rhsim04G0060400
Stem vs Leaf	RHSIM_Rhsim02G0041100	-4.735102422	1.46976E-11	1.36295E-10	RHSIM_Rhsim02G0041100
Stem vs Leaf	RHSIM_Rhsim13G0173500	-2.480941279	1.48739E-11	1.3784E-10	RHSIM_Rhsim13G0173500
Stem vs Leaf	RHSIM_Rhsim02G0212700	-2.316220568	1.48762E-11	1.3784E-10	RHSIM_Rhsim02G0212700
Stem vs Leaf	novel.1990	2.339447302	1.49016E-11	1.38019E-10	--
Stem vs Leaf	RHSIM_Rhsim11G0039500	-8.880490961	1.5151E-11	1.40273E-10	RHSIM_Rhsim11G0039500
Stem vs Leaf	RHSIM_Rhsim08G0102900	-4.83405943	1.53117E-11	1.41704E-10	RHSIM_Rhsim08G0102900
Stem vs Leaf	RHSIM_Rhsim13G0183900	-9.648045938	1.53414E-11	1.41922E-10	RHSIM_Rhsim13G0183900
Stem vs Leaf	RHSIM_Rhsim03G0236800	1.87980627	1.59292E-11	1.47301E-10	RHSIM_Rhsim03G0236800
Stem vs Leaf	novel.4629	-9.104812001	1.60251E-11	1.48128E-10	--
Stem vs Leaf	RHSIM_Rhsim04G0197500	-5.096166232	1.61538E-11	1.49258E-10	RHSIM_Rhsim04G0197500
Stem vs Leaf	RHSIM_Rhsim08G0193500	-3.159960367	1.62071E-11	1.4969E-10	RHSIM_Rhsim08G0193500
Stem vs Leaf	RHSIM_Rhsim04G0006900	-1.708636764	1.62295E-11	1.49837E-10	RHSIM_Rhsim04G0006900
Stem vs Leaf	RHSIM_Rhsim04G0109300	-3.103244711	1.62607E-11	1.50065E-10	RHSIM_Rhsim04G0109300
Stem vs Leaf	RHSIM_Rhsim04G0006000	2.498269135	1.63902E-11	1.51199E-10	RHSIM_Rhsim04G0006000
Stem vs Leaf	RHSIM_Rhsim09G0089200	1.36789482	1.66518E-11	1.53551E-10	RHSIM_Rhsim09G0089200
Stem vs Leaf	RHSIM_Rhsim09G0003200	-1.7695298	1.71868E-11	1.58421E-10	RHSIM_Rhsim09G0003200
Stem vs Leaf	RHSIM_Rhsim08G0109000	4.834812087	1.72982E-11	1.59384E-10	RHSIM_Rhsim08G0109000
Stem vs Leaf	RHSIM_RhsimUnG0146600	-5.036091454	1.75131E-11	1.613E-10	RHSIM_RhsimUnG0146600
Stem vs Leaf	RHSIM_Rhsim08G0108000	-5.74399958	1.76361E-11	1.62305E-10	RHSIM_Rhsim08G0108000
Stem vs Leaf	RHSIM_Rhsim12G0057300	-1.984218264	1.76364E-11	1.62305E-10	RHSIM_Rhsim12G0057300
Stem vs Leaf	RHSIM_Rhsim11G0021500	-1.936509843	1.77283E-11	1.63086E-10	RHSIM_Rhsim11G0021500
Stem vs Leaf	RHSIM_Rhsim13G0119900	2.110958595	1.7764E-11	1.63349E-10	RHSIM_Rhsim13G0119900
Stem vs Leaf	RHSIM_Rhsim05G0044400	3.817138231	1.79168E-11	1.64689E-10	RHSIM_Rhsim05G0044400

Comparison	Gene ID	log_2FC	P-value	FDR (padj)	Gene Name
Stem vs Leaf	RHSIM_Rhsim10G0072800	1.421437274	1.7965E-11	1.65066E-10	RHSIM_Rhsim10G0072800
Stem vs Leaf	RHSIM_Rhsim09G0047100	4.549949129	1.80737E-11	1.65999E-10	RHSIM_Rhsim09G0047100
Stem vs Leaf	RHSIM_Rhsim10G0195500	-1.591633281	1.81171E-11	1.66331E-10	RHSIM_Rhsim10G0195500
Stem vs Leaf	RHSIM_Rhsim02G0203100	3.103687884	1.81901E-11	1.66934E-10	RHSIM_Rhsim02G0203100
Stem vs Leaf	RHSIM_Rhsim06G0181200	-3.574894175	1.8562E-11	1.7028E-10	RHSIM_Rhsim06G0181200
Stem vs Leaf	RHSIM_Rhsim13G0232800	2.982491833	1.87609E-11	1.72036E-10	RHSIM_Rhsim13G0232800
Stem vs Leaf	RHSIM_Rhsim03G0254600	1.445207621	1.938E-11	1.77643E-10	RHSIM_Rhsim03G0254600
Stem vs Leaf	RHSIM_Rhsim04G0241600	-1.229682429	1.97602E-11	1.81056E-10	RHSIM_Rhsim04G0241600
Stem vs Leaf	RHSIM_Rhsim01G0185200	-1.709680786	1.98449E-11	1.8176E-10	RHSIM_Rhsim01G0185200
Stem vs Leaf	RHSIM_Rhsim05G0203100	-1.589832668	1.98751E-11	1.81964E-10	RHSIM_Rhsim05G0203100
Stem vs Leaf	RHSIM_Rhsim10G0098900	3.202987315	2.01536E-11	1.8444E-10	RHSIM_Rhsim10G0098900
Stem vs Leaf	RHSIM_Rhsim07G0110300	-1.480281282	2.02779E-11	1.85504E-10	RHSIM_Rhsim07G0110300
Stem vs Leaf	RHSIM_Rhsim02G0016400	7.345272988	2.03508E-11	1.86097E-10	RHSIM_Rhsim02G0016400
Stem vs Leaf	RHSIM_RhsimMtG0000800	-2.748840908	2.0443E-11	1.86865E-10	RHSIM_RhsimMtG0000800
Stem vs Leaf	novel.3141	-9.076427656	2.0467E-11	1.87011E-10	--
Stem vs Leaf	RHSIM_Rhsim07G0135800	-9.018223154	2.05213E-11	1.8737E-10	RHSIM_Rhsim07G0135800
Stem vs Leaf	novel.5601	-1.957388767	2.05226E-11	1.8737E-10	--
Stem vs Leaf	RHSIM_Rhsim11G0105800	2.633406026	2.07156E-11	1.89058E-10	RHSIM_Rhsim11G0105800
Stem vs Leaf	RHSIM_Rhsim07G0154000	1.906240851	2.07326E-11	1.89138E-10	RHSIM_Rhsim07G0154000
Stem vs Leaf	RHSIM_Rhsim13G0174100	2.00946119	2.10062E-11	1.91558E-10	RHSIM_Rhsim13G0174100
Stem vs Leaf	RHSIM_Rhsim09G0180700	3.959088616	2.11363E-11	1.92668E-10	RHSIM_Rhsim09G0180700
Stem vs Leaf	RHSIM_Rhsim05G0001300	1.703479615	2.13032E-11	1.94113E-10	RHSIM_Rhsim05G0001300
Stem vs Leaf	RHSIM_Rhsim03G0050100	-1.623827135	2.14469E-11	1.95344E-10	RHSIM_Rhsim03G0050100
Stem vs Leaf	RHSIM_Rhsim05G0184300	-4.91308434	2.15883E-11	1.96555E-10	RHSIM_Rhsim05G0184300
Stem vs Leaf	RHSIM_Rhsim09G0014700	8.963201458	2.17363E-11	1.97824E-10	RHSIM_Rhsim09G0014700

Comparison	Gene ID	log_2FC	P-value	FDR (padj)	Gene Name
Stem vs Leaf	RHSIM_Rhsim09G0100700	3.022480316	2.23074E-11	2.02942E-10	RHSIM_Rhsim09G0100700
Stem vs Leaf	novel.792	-2.365093758	2.24639E-11	2.04285E-10	--
Stem vs Leaf	RHSIM_Rhsim03G0162000	-4.280710505	2.26115E-11	2.05546E-10	RHSIM_Rhsim03G0162000
Stem vs Leaf	RHSIM_Rhsim09G0032200	-2.40907072	2.28644E-11	2.07763E-10	RHSIM_Rhsim09G0032200
Stem vs Leaf	RHSIM_Rhsim11G0098400	-1.943698986	2.33195E-11	2.11815E-10	RHSIM_Rhsim11G0098400
Stem vs Leaf	novel.523	-5.990894502	2.33366E-11	2.11887E-10	--
Stem vs Leaf	RHSIM_Rhsim13G0088100	-8.840574706	2.35146E-11	2.13335E-10	RHSIM_Rhsim13G0088100
Stem vs Leaf	RHSIM_Rhsim08G0168900	3.837460477	2.51357E-11	2.27952E-10	RHSIM_Rhsim08G0168900
Stem vs Leaf	RHSIM_Rhsim10G0181400	2.51670102	2.53161E-11	2.29498E-10	RHSIM_Rhsim10G0181400
Stem vs Leaf	RHSIM_Rhsim13G0175300	2.329674092	2.59953E-11	2.35563E-10	RHSIM_Rhsim13G0175300
Stem vs Leaf	RHSIM_Rhsim13G0186400	8.472930266	2.60465E-11	2.35864E-10	RHSIM_Rhsim13G0186400
Stem vs Leaf	RHSIM_Rhsim03G0005800	-1.10659422	2.6049E-11	2.35864E-10	RHSIM_Rhsim03G0005800
Stem vs Leaf	RHSIM_Rhsim12G0023300	-1.162904731	2.64393E-11	2.39151E-10	RHSIM_Rhsim12G0023300
Stem vs Leaf	RHSIM_Rhsim04G0080600	-6.081845039	2.64432E-11	2.39151E-10	RHSIM_Rhsim04G0080600
Stem vs Leaf	RHSIM_Rhsim13G0172800	6.122326949	2.64707E-11	2.39306E-10	RHSIM_Rhsim13G0172800
Stem vs Leaf	RHSIM_Rhsim10G0053300	-8.511623333	2.65848E-11	2.40244E-10	RHSIM_Rhsim10G0053300
Stem vs Leaf	RHSIM_RhsimUnG0212700	2.280551295	2.69213E-11	2.43189E-10	RHSIM_RhsimUnG0212700
Stem vs Leaf	novel.5556	-4.886325937	2.71543E-11	2.45197E-10	--
Stem vs Leaf	RHSIM_Rhsim11G0051400	4.260293637	2.71718E-11	2.45259E-10	RHSIM_Rhsim11G0051400
Stem vs Leaf	RHSIM_Rhsim10G0100200	1.076246477	2.73788E-11	2.47031E-10	RHSIM_Rhsim10G0100200
Stem vs Leaf	RHSIM_Rhsim06G0240900	3.007730961	2.74049E-11	2.4717E-10	RHSIM_Rhsim06G0240900
Stem vs Leaf	RHSIM_RhsimUnG0121800	-4.212562156	2.74483E-11	2.47464E-10	RHSIM_RhsimUnG0121800
Stem vs Leaf	RHSIM_Rhsim08G0207600	2.403785439	2.74829E-11	2.47679E-10	RHSIM_Rhsim08G0207600
Stem vs Leaf	RHSIM_Rhsim05G0098000	-2.642314694	2.82931E-11	2.54881E-10	RHSIM_Rhsim05G0098000
Stem vs Leaf	RHSIM_Rhsim12G0041000	-1.877535625	2.8349E-11	2.55285E-10	RHSIM_Rhsim12G0041000

Comparison	Gene ID	log_2FC	P-value	FDR (padj)	Gene Name
Stem vs Leaf	RHSIM_Rhsim03G0162100	-3.929332704	2.88753E-11	2.59923E-10	RHSIM_Rhsim03G0162100
Stem vs Leaf	novel.5498	-3.049454718	2.9151E-11	2.62302E-10	--
Stem vs Leaf	RHSIM_Rhsim03G0208700	-1.33225989	2.92553E-11	2.63138E-10	RHSIM_Rhsim03G0208700
Stem vs Leaf	novel.1794	-1.566418521	2.93754E-11	2.64115E-10	--
Stem vs Leaf	RHSIM_Rhsim06G0112100	-7.75491234	2.98447E-11	2.68125E-10	RHSIM_Rhsim06G0112100
Stem vs Leaf	RHSIM_Rhsim10G0139200	-5.851218694	3.05449E-11	2.74202E-10	RHSIM_Rhsim10G0139200
Stem vs Leaf	RHSIM_Rhsim09G0108500	-2.193826253	3.11897E-11	2.79881E-10	RHSIM_Rhsim09G0108500
Stem vs Leaf	RHSIM_RhsimUnG0253800	3.18835742	3.14537E-11	2.82141E-10	RHSIM_RhsimUnG0253800
Stem vs Leaf	RHSIM_Rhsim09G0135000	-2.246704298	3.17534E-11	2.84718E-10	RHSIM_Rhsim09G0135000
Stem vs Leaf	RHSIM_Rhsim09G0208500	2.192830005	3.19251E-11	2.86146E-10	RHSIM_Rhsim09G0208500
Stem vs Leaf	RHSIM_Rhsim08G0185900	3.621020873	3.26426E-11	2.92464E-10	RHSIM_Rhsim08G0185900
Stem vs Leaf	RHSIM_Rhsim13G0083400	4.28407508	3.26686E-11	2.92583E-10	RHSIM_Rhsim13G0083400
Stem vs Leaf	RHSIM_Rhsim09G0187900	3.537508392	3.28696E-11	2.94269E-10	RHSIM_Rhsim09G0187900
Stem vs Leaf	RHSIM_Rhsim07G0023500	-1.201610225	3.29654E-11	2.95011E-10	RHSIM_Rhsim07G0023500
Stem vs Leaf	RHSIM_Rhsim01G0170600	-1.085453974	3.334E-11	2.98248E-10	RHSIM_Rhsim01G0170600
Stem vs Leaf	RHSIM_Rhsim11G0021900	-1.548151309	3.36826E-11	3.01196E-10	RHSIM_Rhsim11G0021900
Stem vs Leaf	RHSIM_Rhsim06G0218800	-1.814036252	3.42654E-11	3.06289E-10	RHSIM_Rhsim06G0218800
Stem vs Leaf	RHSIM_Rhsim01G0192300	2.151152889	3.50079E-11	3.12804E-10	RHSIM_Rhsim01G0192300
Stem vs Leaf	RHSIM_Rhsim08G0193800	2.637665384	3.53843E-11	3.16045E-10	RHSIM_Rhsim08G0193800
Stem vs Leaf	RHSIM_Rhsim09G0208800	-1.536695483	3.54822E-11	3.16797E-10	RHSIM_Rhsim09G0208800
Stem vs Leaf	RHSIM_Rhsim08G0002400	-1.721938081	3.5551E-11	3.17288E-10	RHSIM_Rhsim08G0002400
Stem vs Leaf	novel.647	-2.470310772	3.62045E-11	3.22995E-10	--
Stem vs Leaf	RHSIM_Rhsim05G0018600	-5.153015827	3.7042E-11	3.30339E-10	RHSIM_Rhsim05G0018600
Stem vs Leaf	RHSIM_Rhsim03G0010700	-1.59088953	3.71965E-11	3.31589E-10	RHSIM_Rhsim03G0010700
Stem vs Leaf	RHSIM_Rhsim13G0077900	1.230073615	3.73162E-11	3.32443E-10	RHSIM_Rhsim13G0077900

Comparison	Gene ID	log_2FC	P-value	FDR (padj)	Gene Name
Stem vs Leaf	RHSIM_Rhsim13G0195500	3.523181849	3.73212E-11	3.32443E-10	RHSIM_Rhsim13G0195500
Stem vs Leaf	RHSIM_RhsimUnG0065400	-1.945439199	3.7652E-11	3.3526E-10	RHSIM_RhsimUnG0065400
Stem vs Leaf	RHSIM_Rhsim09G0012100	-2.456310714	3.79662E-11	3.37927E-10	RHSIM_Rhsim09G0012100
Stem vs Leaf	RHSIM_Rhsim12G0036500	3.018419928	3.80556E-11	3.38461E-10	RHSIM_Rhsim12G0036500
Stem vs Leaf	RHSIM_Rhsim04G0246900	1.271166638	3.81668E-11	3.3932E-10	RHSIM_Rhsim04G0246900
Stem vs Leaf	RHSIM_Rhsim01G0176000	-3.330354195	3.85079E-11	3.4222E-10	RHSIM_Rhsim01G0176000
Stem vs Leaf	RHSIM_Rhsim04G0122900	-1.91626839	3.89242E-11	3.45653E-10	RHSIM_Rhsim04G0122900
Stem vs Leaf	RHSIM_Rhsim07G0083300	1.227085881	3.90912E-11	3.47002E-10	RHSIM_Rhsim07G0083300
Stem vs Leaf	RHSIM_Rhsim06G0097000	-1.980482591	4.03507E-11	3.57906E-10	RHSIM_Rhsim06G0097000
Stem vs Leaf	RHSIM_Rhsim10G0069900	-8.59730375	4.09359E-11	3.62957E-10	RHSIM_Rhsim10G0069900
Stem vs Leaf	RHSIM_RhsimUnG0033300	-1.495712016	4.13512E-11	3.66499E-10	RHSIM_RhsimUnG0033300
Stem vs Leaf	RHSIM_Rhsim12G0164600	5.520043711	4.14632E-11	3.6735E-10	RHSIM_Rhsim12G0164600
Stem vs Leaf	RHSIM_Rhsim06G0008400	1.524861777	4.19231E-11	3.71282E-10	RHSIM_Rhsim06G0008400
Stem vs Leaf	RHSIM_Rhsim10G0137100	1.229330232	4.22105E-11	3.73684E-10	RHSIM_Rhsim10G0137100
Stem vs Leaf	RHSIM_Rhsim09G0200600	-3.287403561	4.32091E-11	3.8223E-10	RHSIM_Rhsim09G0200600
Stem vs Leaf	RHSIM_Rhsim09G0022700	3.497725325	4.36155E-11	3.85677E-10	RHSIM_Rhsim09G0022700
Stem vs Leaf	RHSIM_Rhsim07G0071400	4.482251725	4.37045E-11	3.86316E-10	RHSIM_Rhsim07G0071400
Stem vs Leaf	RHSIM_Rhsim04G0119900	1.106802686	4.43083E-11	3.91354E-10	RHSIM_Rhsim04G0119900
Stem vs Leaf	RHSIM_Rhsim06G0104100	2.084669316	4.44299E-11	3.92277E-10	RHSIM_Rhsim06G0104100
Stem vs Leaf	RHSIM_Rhsim08G0207100	4.532604879	4.55414E-11	4.01936E-10	RHSIM_Rhsim08G0207100
Stem vs Leaf	RHSIM_Rhsim06G0194400	-2.847672479	4.60086E-11	4.05904E-10	RHSIM_Rhsim06G0194400
Stem vs Leaf	novel.2929	-8.58985181	4.60278E-11	4.05918E-10	--
Stem vs Leaf	RHSIM_Rhsim07G0094100	3.953179394	4.73776E-11	4.17662E-10	RHSIM_Rhsim07G0094100
Stem vs Leaf	RHSIM_Rhsim12G0199600	1.31614616	4.78797E-11	4.21928E-10	RHSIM_Rhsim12G0199600
Stem vs Leaf	RHSIM_Rhsim06G0111300	-8.506025068	4.79531E-11	4.22413E-10	RHSIM_Rhsim06G0111300

Comparison	Gene ID	log_2FC	P-value	FDR (padj)	Gene Name
Stem vs Leaf	RHSIM_Rhsim10G0005100	7.843313731	4.82568E-11	4.24925E-10	RHSIM_Rhsim10G0005100
Stem vs Leaf	RHSIM_Rhsim02G0252500	1.061687061	4.86349E-11	4.27928E-10	RHSIM_Rhsim02G0252500
Stem vs Leaf	RHSIM_Rhsim09G0082600	4.214828559	4.95285E-11	4.35624E-10	RHSIM_Rhsim09G0082600
Stem vs Leaf	RHSIM_RhsimUnG0122800	-3.388410904	4.96572E-11	4.3659E-10	RHSIM_RhsimUnG0122800
Stem vs Leaf	RHSIM_Rhsim04G0156400	-1.196947458	5.04113E-11	4.42881E-10	RHSIM_Rhsim04G0156400
Stem vs Leaf	RHSIM_Rhsim04G0095200	-8.694676251	5.09658E-11	4.47582E-10	RHSIM_Rhsim04G0095200
Stem vs Leaf	RHSIM_Rhsim05G0169000	-1.457665534	5.13806E-11	4.51053E-10	RHSIM_Rhsim05G0169000
Stem vs Leaf	RHSIM_Rhsim02G0042500	-1.845748658	5.1505E-11	4.51973E-10	RHSIM_Rhsim02G0042500
Stem vs Leaf	RHSIM_Rhsim04G0023200	-3.78708844	5.18224E-11	4.54585E-10	RHSIM_Rhsim04G0023200
Stem vs Leaf	RHSIM_Rhsim13G0159200	-7.220092924	5.34212E-11	4.68432E-10	RHSIM_Rhsim13G0159200
Stem vs Leaf	RHSIM_Rhsim11G0170900	9.112028286	5.39718E-11	4.73079E-10	RHSIM_Rhsim11G0170900
Stem vs Leaf	RHSIM_Rhsim03G0180200	-8.293335381	5.40847E-11	4.73889E-10	RHSIM_Rhsim03G0180200
Stem vs Leaf	RHSIM_Rhsim04G0076100	-2.9231267	5.41586E-11	4.74356E-10	RHSIM_Rhsim04G0076100
Stem vs Leaf	RHSIM_Rhsim08G0194500	2.154355737	5.45235E-11	4.77371E-10	RHSIM_Rhsim08G0194500
Stem vs Leaf	RHSIM_Rhsim03G0146700	3.091998919	5.46916E-11	4.78661E-10	RHSIM_Rhsim03G0146700
Stem vs Leaf	RHSIM_RhsimUnG0151800	-5.095535846	5.47794E-11	4.79247E-10	RHSIM_RhsimUnG0151800
Stem vs Leaf	novel.4899	-6.271753557	5.49204E-11	4.80298E-10	--
Stem vs Leaf	novel.2983	-5.229544274	5.55758E-11	4.85846E-10	--
Stem vs Leaf	RHSIM_Rhsim03G0173200	1.205044412	5.58439E-11	4.88004E-10	RHSIM_Rhsim03G0173200
Stem vs Leaf	RHSIM_Rhsim06G0094300	4.700301626	5.58741E-11	4.88083E-10	RHSIM_Rhsim06G0094300
Stem vs Leaf	RHSIM_Rhsim06G0069000	5.684437798	5.59721E-11	4.88754E-10	RHSIM_Rhsim06G0069000
Stem vs Leaf	RHSIM_Rhsim09G0116300	2.239333485	5.60423E-11	4.89182E-10	RHSIM_Rhsim09G0116300
Stem vs Leaf	RHSIM_Rhsim10G0181600	1.779825812	5.63281E-11	4.9149E-10	RHSIM_Rhsim10G0181600
Stem vs Leaf	RHSIM_Rhsim07G0014200	-1.191011684	5.68314E-11	4.9533E-10	RHSIM_Rhsim07G0014200
Stem vs Leaf	RHSIM_Rhsim06G0195100	2.57079608	5.68327E-11	4.9533E-10	RHSIM_Rhsim06G0195100

Comparison	Gene ID	log_2FC	P-value	FDR (padj)	Gene Name
Stem vs Leaf	RHSIM_Rhsim11G0045800	-1.783696356	5.74095E-11	5.00168E-10	RHSIM_Rhsim11G0045800
Stem vs Leaf	RHSIM_Rhsim03G0160000	5.2675646	5.78749E-11	5.03842E-10	RHSIM_Rhsim03G0160000
Stem vs Leaf	novel.3561	9.460315419	5.80698E-11	5.0517E-10	--
Stem vs Leaf	RHSIM_Rhsim12G0199800	-2.468036007	5.80714E-11	5.0517E-10	RHSIM_Rhsim12G0199800
Stem vs Leaf	RHSIM_Rhsim02G0023100	-2.315263343	5.87684E-11	5.11041E-10	RHSIM_Rhsim02G0023100
Stem vs Leaf	RHSIM_Rhsim11G0163500	-7.596088178	5.88595E-11	5.1164E-10	RHSIM_Rhsim11G0163500
Stem vs Leaf	RHSIM_Rhsim01G0147400	-1.579953376	5.91199E-11	5.1371E-10	RHSIM_Rhsim01G0147400
Stem vs Leaf	RHSIM_Rhsim03G0209100	3.97327879	5.92129E-11	5.14325E-10	RHSIM_Rhsim03G0209100
Stem vs Leaf	RHSIM_Rhsim08G0146300	-1.04252123	6.03633E-11	5.24119E-10	RHSIM_Rhsim08G0146300
Stem vs Leaf	RHSIM_Rhsim03G0052900	1.47509809	6.04387E-11	5.24576E-10	RHSIM_Rhsim03G0052900
Stem vs Leaf	RHSIM_Rhsim08G0088900	1.659522697	6.09689E-11	5.28979E-10	RHSIM_Rhsim08G0088900
Stem vs Leaf	RHSIM_Rhsim06G0215300	-3.230402983	6.17197E-11	5.35291E-10	RHSIM_Rhsim06G0215300
Stem vs Leaf	RHSIM_Rhsim03G0232900	-3.54677583	6.19408E-11	5.37007E-10	RHSIM_Rhsim03G0232900
Stem vs Leaf	RHSIM_Rhsim11G0049500	-1.088387427	6.20968E-11	5.38157E-10	RHSIM_Rhsim11G0049500
Stem vs Leaf	RHSIM_Rhsim10G0025500	3.098290237	6.23141E-11	5.39837E-10	RHSIM_Rhsim10G0025500
Stem vs Leaf	RHSIM_Rhsim03G0049400	-2.03884013	6.29856E-11	5.45449E-10	RHSIM_Rhsim03G0049400
Stem vs Leaf	RHSIM_Rhsim11G0019900	1.207637616	6.34894E-11	5.49605E-10	RHSIM_Rhsim11G0019900
Stem vs Leaf	RHSIM_Rhsim02G0026400	-5.139039076	6.46489E-11	5.59433E-10	RHSIM_Rhsim02G0026400
Stem vs Leaf	novel.4760	-8.381183473	6.49775E-11	5.62065E-10	--
Stem vs Leaf	RHSIM_Rhsim09G0116000	-1.935315861	6.54974E-11	5.6635E-10	RHSIM_Rhsim09G0116000
Stem vs Leaf	RHSIM_Rhsim07G0247100	-1.824366756	6.56743E-11	5.67666E-10	RHSIM_Rhsim07G0247100
Stem vs Leaf	novel.4538	1.323020203	6.5804E-11	5.68575E-10	--
Stem vs Leaf	RHSIM_Rhsim10G0037000	3.800745156	6.71382E-11	5.79885E-10	RHSIM_Rhsim10G0037000
Stem vs Leaf	RHSIM_Rhsim11G0044200	2.399801316	6.73227E-11	5.81261E-10	RHSIM_Rhsim11G0044200
Stem vs Leaf	RHSIM_Rhsim03G0088500	1.162086603	6.78821E-11	5.85871E-10	RHSIM_Rhsim03G0088500

Comparison	Gene ID	log_2FC	P-value	FDR (padj)	Gene Name
Stem vs Leaf	RHSIM_Rhsim07G0170300	-2.125768549	6.82086E-11	5.88468E-10	RHSIM_Rhsim07G0170300
Stem vs Leaf	RHSIM_Rhsim03G0119600	2.433698134	6.85285E-11	5.91007E-10	RHSIM_Rhsim03G0119600
Stem vs Leaf	RHSIM_Rhsim02G0116300	-5.424802535	6.96201E-11	6.00197E-10	RHSIM_Rhsim02G0116300
Stem vs Leaf	novel.4902	1.687483897	6.97883E-11	6.01422E-10	--
Stem vs Leaf	novel.4830	9.759085023	6.98412E-11	6.01653E-10	--
Stem vs Leaf	RHSIM_Rhsim05G0076600	8.915759388	7.14088E-11	6.14927E-10	RHSIM_Rhsim05G0076600
Stem vs Leaf	RHSIM_Rhsim10G0187800	-1.970695794	7.1716E-11	6.17342E-10	RHSIM_Rhsim10G0187800
Stem vs Leaf	RHSIM_Rhsim04G0009300	5.835865086	7.21515E-11	6.20859E-10	RHSIM_Rhsim04G0009300
Stem vs Leaf	RHSIM_Rhsim01G0029200	5.842150357	7.51202E-11	6.46023E-10	RHSIM_Rhsim01G0029200
Stem vs Leaf	RHSIM_Rhsim08G0015500	4.217516722	7.5132E-11	6.46023E-10	RHSIM_Rhsim08G0015500
Stem vs Leaf	RHSIM_Rhsim10G0197300	-1.508016718	7.53661E-11	6.47794E-10	RHSIM_Rhsim10G0197300
Stem vs Leaf	RHSIM_Rhsim13G0190400	-8.471833517	7.6275E-11	6.55362E-10	RHSIM_Rhsim13G0190400
Stem vs Leaf	RHSIM_Rhsim03G0094900	-8.592726749	7.78133E-11	6.68256E-10	RHSIM_Rhsim03G0094900
Stem vs Leaf	RHSIM_Rhsim01G0267800	-1.283331851	7.78337E-11	6.68256E-10	RHSIM_Rhsim01G0267800
Stem vs Leaf	RHSIM_Rhsim04G0172500	3.866992299	7.85623E-11	6.74009E-10	RHSIM_Rhsim04G0172500
Stem vs Leaf	RHSIM_Rhsim07G0055000	3.449403792	8.00068E-11	6.86147E-10	RHSIM_Rhsim07G0055000
Stem vs Leaf	RHSIM_Rhsim02G0183100	-6.274497206	8.09112E-11	6.93387E-10	RHSIM_Rhsim02G0183100
Stem vs Leaf	RHSIM_Rhsim06G0227000	-1.940825984	8.11627E-11	6.95283E-10	RHSIM_Rhsim06G0227000
Stem vs Leaf	RHSIM_Rhsim12G0160500	-1.439851005	8.28514E-11	7.09487E-10	RHSIM_Rhsim12G0160500
Stem vs Leaf	RHSIM_Rhsim13G0075500	3.423030977	8.41604E-11	7.20428E-10	RHSIM_Rhsim13G0075500
Stem vs Leaf	RHSIM_Rhsim03G0218200	-4.902653532	8.52838E-11	7.29773E-10	RHSIM_Rhsim03G0218200
Stem vs Leaf	RHSIM_Rhsim08G0074900	-4.300398544	8.58469E-11	7.34319E-10	RHSIM_Rhsim08G0074900
Stem vs Leaf	RHSIM_Rhsim03G0170500	3.191299386	8.60946E-11	7.36165E-10	RHSIM_Rhsim03G0170500
Stem vs Leaf	RHSIM_Rhsim11G0109900	6.503610153	8.67781E-11	7.41734E-10	RHSIM_Rhsim11G0109900
Stem vs Leaf	RHSIM_Rhsim04G0220400	-1.175219411	8.71873E-11	7.44955E-10	RHSIM_Rhsim04G0220400

Comparison	Gene ID	log_2FC	P-value	FDR (padj)	Gene Name
Stem vs Leaf	RHSIM_Rhsim06G0050600	-2.02503675	8.84112E-11	7.55132E-10	RHSIM_Rhsim06G0050600
Stem vs Leaf	RHSIM_Rhsim12G0195800	-1.21229644	8.86807E-11	7.57154E-10	RHSIM_Rhsim12G0195800
Stem vs Leaf	RHSIM_Rhsim01G0230700	2.758581146	8.9707E-11	7.65633E-10	RHSIM_Rhsim01G0230700
Stem vs Leaf	RHSIM_Rhsim13G0132700	3.950335104	8.99445E-11	7.67197E-10	RHSIM_Rhsim13G0132700
Stem vs Leaf	RHSIM_Rhsim05G0231400	-3.28684362	8.99569E-11	7.67197E-10	RHSIM_Rhsim05G0231400
Stem vs Leaf	RHSIM_Rhsim10G0083700	-8.587909428	9.15908E-11	7.80843E-10	RHSIM_Rhsim10G0083700
Stem vs Leaf	RHSIM_Rhsim13G0013400	3.284817478	9.17331E-11	7.81767E-10	RHSIM_Rhsim13G0013400
Stem vs Leaf	RHSIM_Rhsim05G0058500	-9.352948554	9.21622E-11	7.85133E-10	RHSIM_Rhsim05G0058500
Stem vs Leaf	RHSIM_Rhsim10G0107900	-2.302042106	9.27445E-11	7.89802E-10	RHSIM_Rhsim10G0107900
Stem vs Leaf	RHSIM_Rhsim12G0202400	-9.491519842	9.36243E-11	7.97E-10	RHSIM_Rhsim12G0202400
Stem vs Leaf	RHSIM_Rhsim09G0133000	2.502169158	9.82804E-11	8.36327E-10	RHSIM_Rhsim09G0133000
Stem vs Leaf	RHSIM_Rhsim13G0154400	-1.44327577	1.00531E-10	8.55161E-10	RHSIM_Rhsim13G0154400
Stem vs Leaf	RHSIM_RhsimUnG0197600	-1.941291187	1.01526E-10	8.63306E-10	RHSIM_RhsimUnG0197600
Stem vs Leaf	RHSIM_Rhsim09G0037500	1.890707427	1.01836E-10	8.65629E-10	RHSIM_Rhsim09G0037500
Stem vs Leaf	RHSIM_RhsimUnG0166600	-2.469900349	1.02393E-10	8.70035E-10	RHSIM_RhsimUnG0166600
Stem vs Leaf	RHSIM_RhsimUnG0136600	-1.803046751	1.0351E-10	8.79202E-10	RHSIM_RhsimUnG0136600
Stem vs Leaf	RHSIM_Rhsim13G0166700	3.332864476	1.06319E-10	9.02733E-10	RHSIM_Rhsim13G0166700
Stem vs Leaf	RHSIM_Rhsim04G0155500	-2.043241405	1.06684E-10	9.05499E-10	RHSIM_Rhsim04G0155500
Stem vs Leaf	RHSIM_Rhsim04G0221700	-7.467662839	1.07242E-10	9.09896E-10	RHSIM_Rhsim04G0221700
Stem vs Leaf	RHSIM_Rhsim06G0197700	-1.459632508	1.08881E-10	9.23467E-10	RHSIM_Rhsim06G0197700
Stem vs Leaf	RHSIM_Rhsim07G0258700	-1.464233256	1.10764E-10	9.38742E-10	RHSIM_Rhsim07G0258700
Stem vs Leaf	RHSIM_Rhsim09G0022400	2.481922175	1.11767E-10	9.46895E-10	RHSIM_Rhsim09G0022400
Stem vs Leaf	RHSIM_Rhsim07G0098000	-8.379427905	1.11944E-10	9.48047E-10	RHSIM_Rhsim07G0098000
Stem vs Leaf	RHSIM_Rhsim13G0095200	-1.721382402	1.13229E-10	9.58578E-10	RHSIM_Rhsim13G0095200
Stem vs Leaf	RHSIM_Rhsim05G0159300	-1.916498372	1.14638E-10	9.70153E-10	RHSIM_Rhsim05G0159300

Comparison	Gene ID	log_2FC	P-value	FDR (padj)	Gene Name
Stem vs Leaf	RHSIM_Rhsim13G0001400	-2.621189987	1.14879E-10	9.71829E-10	RHSIM_Rhsim13G0001400
Stem vs Leaf	RHSIM_Rhsim01G0283300	1.316328153	1.15219E-10	9.74348E-10	RHSIM_Rhsim01G0283300
Stem vs Leaf	RHSIM_Rhsim10G0082900	1.696811395	1.16184E-10	9.82149E-10	RHSIM_Rhsim10G0082900
Stem vs Leaf	RHSIM_Rhsim03G0070600	-2.111908224	1.1723E-10	9.90632E-10	RHSIM_Rhsim03G0070600
Stem vs Leaf	RHSIM_Rhsim04G0074100	-1.339338358	1.17744E-10	9.94611E-10	RHSIM_Rhsim04G0074100
Stem vs Leaf	novel.5647	3.020156754	1.18159E-10	9.97751E-10	--
Stem vs Leaf	RHSIM_Rhsim13G0076700	4.33768096	1.20888E-10	1.02042E-09	RHSIM_Rhsim13G0076700
Stem vs Leaf	RHSIM_Rhsim04G0079800	-8.122630135	1.21607E-10	1.02612E-09	RHSIM_Rhsim04G0079800
Stem vs Leaf	RHSIM_Rhsim08G0060900	-8.86157723	1.25272E-10	1.05665E-09	RHSIM_Rhsim08G0060900
Stem vs Leaf	novel.1228	7.164623693	1.2662E-10	1.06763E-09	--
Stem vs Leaf	RHSIM_Rhsim06G0213100	-4.97606362	1.27392E-10	1.07375E-09	RHSIM_Rhsim06G0213100
Stem vs Leaf	RHSIM_Rhsim09G0112700	2.558799674	1.2765E-10	1.07552E-09	RHSIM_Rhsim09G0112700
Stem vs Leaf	RHSIM_Rhsim11G0078100	3.796034483	1.27815E-10	1.07652E-09	RHSIM_Rhsim11G0078100
Stem vs Leaf	novel.703	-8.513697674	1.28292E-10	1.07976E-09	--
Stem vs Leaf	RHSIM_RhsimUnG0197900	-2.165091315	1.29047E-10	1.08555E-09	RHSIM_RhsimUnG0197900
Stem vs Leaf	RHSIM_Rhsim10G0173100	-1.792849659	1.34923E-10	1.13432E-09	RHSIM_Rhsim10G0173100
Stem vs Leaf	RHSIM_Rhsim04G0005900	-2.327433912	1.35557E-10	1.13882E-09	RHSIM_Rhsim04G0005900
Stem vs Leaf	RHSIM_Rhsim03G0216500	8.666592023	1.36172E-10	1.14357E-09	RHSIM_Rhsim03G0216500
Stem vs Leaf	RHSIM_Rhsim03G0081300	3.897684594	1.37505E-10	1.15392E-09	RHSIM_Rhsim03G0081300
Stem vs Leaf	RHSIM_RhsimUnG0087700	-3.480394548	1.40096E-10	1.17523E-09	RHSIM_RhsimUnG0087700
Stem vs Leaf	novel.1136	-1.557892528	1.41961E-10	1.19002E-09	--
Stem vs Leaf	novel.672	-9.381869873	1.42401E-10	1.19326E-09	--
Stem vs Leaf	RHSIM_Rhsim02G0058100	-5.693620833	1.42546E-10	1.19405E-09	RHSIM_Rhsim02G0058100
Stem vs Leaf	novel.5621	-2.172848702	1.43862E-10	1.20463E-09	--
Stem vs Leaf	RHSIM_Rhsim11G0024800	-1.385359091	1.44448E-10	1.20866E-09	RHSIM_Rhsim11G0024800

Comparison	Gene ID	log_2FC	P-value	FDR (padj)	Gene Name
Stem vs Leaf	RHSIM_Rhsim03G0263400	4.302646799	1.44668E-10	1.21006E-09	RHSIM_Rhsim03G0263400
Stem vs Leaf	RHSIM_Rhsim01G0238700	-2.097518879	1.47877E-10	1.23645E-09	RHSIM_Rhsim01G0238700
Stem vs Leaf	RHSIM_Rhsim02G0032800	-2.777775687	1.49088E-10	1.24613E-09	RHSIM_Rhsim02G0032800
Stem vs Leaf	RHSIM_Rhsim09G0170700	1.766079339	1.53376E-10	1.28151E-09	RHSIM_Rhsim09G0170700
Stem vs Leaf	RHSIM_Rhsim08G0033300	1.267984879	1.54218E-10	1.28807E-09	RHSIM_Rhsim08G0033300
Stem vs Leaf	novel.4861	2.795084254	1.55545E-10	1.29869E-09	--
Stem vs Leaf	RHSIM_Rhsim13G0096000	3.926222921	1.59846E-10	1.33363E-09	RHSIM_Rhsim13G0096000
Stem vs Leaf	RHSIM_Rhsim07G0037400	3.856705135	1.60471E-10	1.33836E-09	RHSIM_Rhsim07G0037400
Stem vs Leaf	RHSIM_Rhsim08G0101400	3.407034276	1.64839E-10	1.3738E-09	RHSIM_Rhsim08G0101400
Stem vs Leaf	RHSIM_Rhsim08G0185700	3.395000016	1.65123E-10	1.37566E-09	RHSIM_Rhsim08G0185700
Stem vs Leaf	RHSIM_Rhsim09G0114700	2.738837502	1.67903E-10	1.39833E-09	RHSIM_Rhsim09G0114700
Stem vs Leaf	RHSIM_Rhsim06G0239900	3.782048961	1.69617E-10	1.41208E-09	RHSIM_Rhsim06G0239900
Stem vs Leaf	RHSIM_Rhsim04G0021600	-1.376675725	1.70259E-10	1.41646E-09	RHSIM_Rhsim04G0021600
Stem vs Leaf	RHSIM_Rhsim01G0039100	3.158822597	1.70266E-10	1.41646E-09	RHSIM_Rhsim01G0039100
Stem vs Leaf	RHSIM_Rhsim06G0208100	-1.540183015	1.71326E-10	1.42477E-09	RHSIM_Rhsim06G0208100
Stem vs Leaf	RHSIM_Rhsim06G0040500	-1.816975255	1.71968E-10	1.42959E-09	RHSIM_Rhsim06G0040500
Stem vs Leaf	RHSIM_Rhsim11G0159600	-10.71035873	1.73733E-10	1.44374E-09	RHSIM_Rhsim11G0159600
Stem vs Leaf	RHSIM_Rhsim03G0194300	-3.314709178	1.75264E-10	1.45594E-09	RHSIM_Rhsim03G0194300
Stem vs Leaf	RHSIM_Rhsim10G0190300	-2.298497097	1.76348E-10	1.46442E-09	RHSIM_Rhsim10G0190300
Stem vs Leaf	RHSIM_Rhsim10G0066900	4.455424818	1.76651E-10	1.4664E-09	RHSIM_Rhsim10G0066900
Stem vs Leaf	RHSIM_RhsimUnG0209700	2.59414938	1.78703E-10	1.4829E-09	RHSIM_RhsimUnG0209700
Stem vs Leaf	RHSIM_Rhsim03G0085000	1.498722343	1.79824E-10	1.49114E-09	RHSIM_Rhsim03G0085000
Stem vs Leaf	RHSIM_Rhsim04G0103800	6.862563985	1.83079E-10	1.51704E-09	RHSIM_Rhsim04G0103800
Stem vs Leaf	novel.4839	5.39306617	1.85546E-10	1.53692E-09	--
Stem vs Leaf	RHSIM_Rhsim09G0200300	2.561618915	1.85763E-10	1.53817E-09	RHSIM_Rhsim09G0200300

Comparison	Gene ID	log_2FC	P-value	FDR (padj)	Gene Name
Stem vs Leaf	RHSIM_RhsimMtG0002100	-2.719382974	1.87408E-10	1.55124E-09	RHSIM_RhsimMtG0002100
Stem vs Leaf	RHSIM_Rhsim08G0210900	1.292196216	1.89924E-10	1.57149E-09	RHSIM_Rhsim08G0210900
Stem vs Leaf	RHSIM_Rhsim10G0007300	3.990179876	1.91575E-10	1.58458E-09	RHSIM_Rhsim10G0007300
Stem vs Leaf	RHSIM_Rhsim05G0196500	4.664882293	1.93441E-10	1.59945E-09	RHSIM_Rhsim05G0196500
Stem vs Leaf	RHSIM_Rhsim06G0028000	-1.365477833	1.96197E-10	1.62107E-09	RHSIM_Rhsim06G0028000
Stem vs Leaf	RHSIM_Rhsim08G0066700	2.687535071	1.98996E-10	1.64361E-09	RHSIM_Rhsim08G0066700
Stem vs Leaf	novel.4175	-5.096265371	1.99168E-10	1.64444E-09	--
Stem vs Leaf	RHSIM_Rhsim09G0069900	-2.096391306	2.02746E-10	1.67338E-09	RHSIM_Rhsim09G0069900
Stem vs Leaf	RHSIM_Rhsim07G0174200	-3.01711861	2.04797E-10	1.6897E-09	RHSIM_Rhsim07G0174200
Stem vs Leaf	RHSIM_Rhsim01G0106800	5.80178889	2.07379E-10	1.7104E-09	RHSIM_Rhsim01G0106800
Stem vs Leaf	RHSIM_Rhsim12G0140400	2.859382357	2.08E-10	1.7149E-09	RHSIM_Rhsim12G0140400
Stem vs Leaf	RHSIM_Rhsim13G0009800	1.354771375	2.08293E-10	1.71671E-09	RHSIM_Rhsim13G0009800
Stem vs Leaf	RHSIM_Rhsim07G0244300	6.202019561	2.13587E-10	1.75971E-09	RHSIM_Rhsim07G0244300
Stem vs Leaf	RHSIM_Rhsim11G0096100	-3.800644184	2.1538E-10	1.77384E-09	RHSIM_Rhsim11G0096100
Stem vs Leaf	novel.3794	8.219257713	2.15705E-10	1.77588E-09	--
Stem vs Leaf	RHSIM_Rhsim08G0221900	1.244127006	2.17485E-10	1.7899E-09	RHSIM_Rhsim08G0221900
Stem vs Leaf	RHSIM_Rhsim12G0025900	-1.128889285	2.1894E-10	1.80123E-09	RHSIM_Rhsim12G0025900
Stem vs Leaf	novel.2265	-9.153273324	2.19053E-10	1.80152E-09	--
Stem vs Leaf	RHSIM_Rhsim13G0209200	-1.240296027	2.22755E-10	1.83131E-09	RHSIM_Rhsim13G0209200
Stem vs Leaf	RHSIM_Rhsim07G0026800	1.431499356	2.26305E-10	1.85983E-09	RHSIM_Rhsim07G0026800
Stem vs Leaf	RHSIM_Rhsim13G0114700	-2.85233021	2.29589E-10	1.88615E-09	RHSIM_Rhsim13G0114700
Stem vs Leaf	RHSIM_Rhsim13G0196300	-4.297529964	2.30962E-10	1.89675E-09	RHSIM_Rhsim13G0196300
Stem vs Leaf	RHSIM_Rhsim07G0079900	-1.704500395	2.3175E-10	1.90254E-09	RHSIM_Rhsim07G0079900
Stem vs Leaf	RHSIM_Rhsim06G0021700	-2.43149546	2.39961E-10	1.96925E-09	RHSIM_Rhsim06G0021700
Stem vs Leaf	novel.3092	-7.477465238	2.40644E-10	1.97416E-09	--

Comparison	Gene ID	log_2FC	P-value	FDR (padj)	Gene Name
Stem vs Leaf	RHSIM_Rhsim12G0105100	-1.356947249	2.41611E-10	1.98138E-09	RHSIM_Rhsim12G0105100
Stem vs Leaf	novel.1508	-9.018164995	2.42489E-10	1.98788E-09	--
Stem vs Leaf	novel.1652	5.228931988	2.43945E-10	1.9991E-09	--
Stem vs Leaf	RHSIM_Rhsim07G0008700	1.534389816	2.50759E-10	2.05421E-09	RHSIM_Rhsim07G0008700
Stem vs Leaf	RHSIM_Rhsim10G0067200	2.291853582	2.53753E-10	2.078E-09	RHSIM_Rhsim10G0067200
Stem vs Leaf	novel.4912	-8.463640083	2.57353E-10	2.10673E-09	--
Stem vs Leaf	RHSIM_Rhsim09G0185800	-3.542829866	2.59674E-10	2.12497E-09	RHSIM_Rhsim09G0185800
Stem vs Leaf	novel.660	-8.902506566	2.66049E-10	2.17637E-09	--
Stem vs Leaf	RHSIM_Rhsim07G0193500	-1.486034186	2.66745E-10	2.18129E-09	RHSIM_Rhsim07G0193500
Stem vs Leaf	novel.5075	-8.334055504	2.71709E-10	2.22109E-09	--
Stem vs Leaf	RHSIM_Rhsim08G0218300	1.240123863	2.74218E-10	2.24081E-09	RHSIM_Rhsim08G0218300
Stem vs Leaf	RHSIM_RhsimUnG0233800	5.115537413	2.75865E-10	2.25267E-09	RHSIM_RhsimUnG0233800
Stem vs Leaf	RHSIM_Rhsim03G0163800	-2.672355714	2.77428E-10	2.26383E-09	RHSIM_Rhsim03G0163800
Stem vs Leaf	RHSIM_Rhsim10G0066600	5.738489647	2.77785E-10	2.26594E-09	RHSIM_Rhsim10G0066600
Stem vs Leaf	RHSIM_Rhsim02G0238800	1.332841788	2.78139E-10	2.26802E-09	RHSIM_Rhsim02G0238800
Stem vs Leaf	RHSIM_Rhsim08G0057800	-8.957033943	2.80596E-10	2.28725E-09	RHSIM_Rhsim08G0057800
Stem vs Leaf	RHSIM_Rhsim13G0200000	1.331453299	2.93251E-10	2.38787E-09	RHSIM_Rhsim13G0200000
Stem vs Leaf	RHSIM_Rhsim02G0218300	4.000614241	3.0242E-10	2.46079E-09	RHSIM_Rhsim02G0218300
Stem vs Leaf	RHSIM_Rhsim06G0031200	2.978856373	3.02639E-10	2.46171E-09	RHSIM_Rhsim06G0031200
Stem vs Leaf	novel.2458	-5.513668575	3.04062E-10	2.47241E-09	--
Stem vs Leaf	RHSIM_Rhsim07G0049400	5.056344667	3.10888E-10	2.52702E-09	RHSIM_Rhsim07G0049400
Stem vs Leaf	RHSIM_Rhsim08G0245600	1.604121003	3.11325E-10	2.52968E-09	RHSIM_Rhsim08G0245600
Stem vs Leaf	RHSIM_Rhsim02G0135200	3.483739552	3.15361E-10	2.56157E-09	RHSIM_Rhsim02G0135200
Stem vs Leaf	RHSIM_Rhsim13G0028300	-3.657430255	3.16432E-10	2.56936E-09	RHSIM_Rhsim13G0028300
Stem vs Leaf	RHSIM_Rhsim10G0174300	7.012940281	3.16893E-10	2.5722E-09	RHSIM_Rhsim10G0174300

Comparison	Gene ID	log_2FC	P-value	FDR (padj)	Gene Name
Stem vs Leaf	RHSIM_Rhsim03G0091000	-1.284074466	3.18888E-10	2.58749E-09	RHSIM_Rhsim03G0091000
Stem vs Leaf	RHSIM_Rhsim08G0014300	4.032631113	3.21035E-10	2.60399E-09	RHSIM_Rhsim08G0014300
Stem vs Leaf	RHSIM_Rhsim13G0235500	1.399084763	3.21813E-10	2.60938E-09	RHSIM_Rhsim13G0235500
Stem vs Leaf	RHSIM_Rhsim03G0233400	6.688752387	3.2367E-10	2.62351E-09	RHSIM_Rhsim03G0233400
Stem vs Leaf	novel.2399	5.680427178	3.27728E-10	2.65547E-09	--
Stem vs Leaf	novel.618	7.676163347	3.36183E-10	2.72302E-09	--
Stem vs Leaf	RHSIM_Rhsim13G0125900	-1.0635601	3.37423E-10	2.73115E-09	RHSIM_Rhsim13G0125900
Stem vs Leaf	RHSIM_Rhsim01G0000500	-1.109671968	3.39913E-10	2.75034E-09	RHSIM_Rhsim01G0000500
Stem vs Leaf	RHSIM_Rhsim09G0065900	-1.400114504	3.44141E-10	2.78357E-09	RHSIM_Rhsim09G0065900
Stem vs Leaf	RHSIM_Rhsim03G0154400	3.955578925	3.45762E-10	2.79513E-09	RHSIM_Rhsim03G0154400
Stem vs Leaf	novel.2237	1.517885299	3.45813E-10	2.79513E-09	--
Stem vs Leaf	RHSIM_Rhsim01G0016100	-1.234630108	3.47786E-10	2.81009E-09	RHSIM_Rhsim01G0016100
Stem vs Leaf	RHSIM_Rhsim05G0226800	-3.057152443	3.49944E-10	2.82654E-09	RHSIM_Rhsim05G0226800
Stem vs Leaf	RHSIM_Rhsim09G0093000	-8.712468081	3.51193E-10	2.83563E-09	RHSIM_Rhsim09G0093000
Stem vs Leaf	RHSIM_Rhsim09G0114200	3.619518844	3.52495E-10	2.84515E-09	RHSIM_Rhsim09G0114200
Stem vs Leaf	RHSIM_RhsimUnG0120300	2.049150974	3.57368E-10	2.88347E-09	RHSIM_RhsimUnG0120300
Stem vs Leaf	RHSIM_Rhsim02G0104300	4.754055914	3.57572E-10	2.88411E-09	RHSIM_Rhsim02G0104300
Stem vs Leaf	RHSIM_Rhsim11G0117600	-8.263534041	3.60522E-10	2.90688E-09	RHSIM_Rhsim11G0117600
Stem vs Leaf	RHSIM_Rhsim07G0161300	1.836366136	3.61192E-10	2.91127E-09	RHSIM_Rhsim07G0161300
Stem vs Leaf	RHSIM_Rhsim03G0205500	-1.692012359	3.63321E-10	2.9274E-09	RHSIM_Rhsim03G0205500
Stem vs Leaf	RHSIM_Rhsim01G0275100	7.885849635	3.72257E-10	2.9973E-09	RHSIM_Rhsim01G0275100
Stem vs Leaf	RHSIM_Rhsim01G0280800	-2.716260721	3.7306E-10	3.00272E-09	RHSIM_Rhsim01G0280800
Stem vs Leaf	RHSIM_RhsimPtG0005000	-2.820155049	3.77937E-10	3.04091E-09	RHSIM_RhsimPtG0005000
Stem vs Leaf	RHSIM_Rhsim11G0133100	-8.147745987	3.78274E-10	3.04256E-09	RHSIM_Rhsim11G0133100
Stem vs Leaf	RHSIM_Rhsim05G0230100	-5.449079361	3.80641E-10	3.06054E-09	RHSIM_Rhsim05G0230100

Comparison	Gene ID	log_2FC	P-value	FDR (padj)	Gene Name
Stem vs Leaf	RHSIM_Rhsim09G0105500	4.481703956	3.86814E-10	3.108E-09	RHSIM_Rhsim09G0105500
Stem vs Leaf	RHSIM_Rhsim06G0119400	-8.304737744	3.87494E-10	3.11238E-09	RHSIM_Rhsim06G0119400
Stem vs Leaf	RHSIM_Rhsim12G0141500	-1.285960291	3.91743E-10	3.14541E-09	RHSIM_Rhsim12G0141500
Stem vs Leaf	RHSIM_Rhsim04G0126400	-1.344622357	3.95861E-10	3.17737E-09	RHSIM_Rhsim04G0126400
Stem vs Leaf	RHSIM_Rhsim11G0139300	-2.07571953	3.96393E-10	3.18053E-09	RHSIM_Rhsim11G0139300
Stem vs Leaf	novel.5153	-1.736552042	3.96658E-10	3.18155E-09	--
Stem vs Leaf	RHSIM_Rhsim02G0244900	1.446576661	3.97611E-10	3.18808E-09	RHSIM_Rhsim02G0244900
Stem vs Leaf	RHSIM_Rhsim10G0093100	-2.222521157	3.98812E-10	3.1966E-09	RHSIM_Rhsim10G0093100
Stem vs Leaf	RHSIM_RhsimUnG0156800	-2.592680086	4.05276E-10	3.24728E-09	RHSIM_RhsimUnG0156800
Stem vs Leaf	RHSIM_Rhsim06G0185600	5.935103843	4.06586E-10	3.25664E-09	RHSIM_Rhsim06G0185600
Stem vs Leaf	novel.2124	-2.338535962	4.13514E-10	3.31098E-09	--
Stem vs Leaf	RHSIM_Rhsim09G0205100	3.324788583	4.18872E-10	3.35156E-09	RHSIM_Rhsim09G0205100
Stem vs Leaf	RHSIM_Rhsim07G0039800	-3.982474399	4.19548E-10	3.35501E-09	RHSIM_Rhsim07G0039800
Stem vs Leaf	RHSIM_RhsimUnG0081900	-4.042822603	4.19594E-10	3.35501E-09	RHSIM_RhsimUnG0081900
Stem vs Leaf	RHSIM_Rhsim09G0125500	3.617060817	4.25092E-10	3.39779E-09	RHSIM_Rhsim09G0125500
Stem vs Leaf	novel.524	-8.374036747	4.27113E-10	3.41275E-09	--
Stem vs Leaf	RHSIM_Rhsim06G0035500	-1.394013019	4.29466E-10	3.43037E-09	RHSIM_Rhsim06G0035500
Stem vs Leaf	RHSIM_Rhsim08G0184400	1.211132237	4.31201E-10	3.44303E-09	RHSIM_Rhsim08G0184400
Stem vs Leaf	RHSIM_Rhsim03G0161600	1.020125315	4.37204E-10	3.48976E-09	RHSIM_Rhsim03G0161600
Stem vs Leaf	RHSIM_RhsimUnG0035300	8.728358193	4.37446E-10	3.49048E-09	RHSIM_RhsimUnG0035300
Stem vs Leaf	RHSIM_Rhsim08G0196100	-2.03355267	4.37706E-10	3.49135E-09	RHSIM_Rhsim08G0196100
Stem vs Leaf	RHSIM_Rhsim13G0216400	-1.512849781	4.42641E-10	3.52949E-09	RHSIM_Rhsim13G0216400
Stem vs Leaf	novel.5360	-2.823400396	4.494E-10	3.58214E-09	--
Stem vs Leaf	novel.1279	8.141022155	4.52896E-10	3.60876E-09	--
Stem vs Leaf	RHSIM_Rhsim03G0148500	2.712746575	4.56384E-10	3.63404E-09	RHSIM_Rhsim03G0148500

Comparison	Gene ID	log2FC	P-value	FDR (padj)	Gene Name
Stem vs Leaf	RHSIM_Rhsim01G0258400	1.247545473	4.57646E-10	3.64283E-09	RHSIM_Rhsim01G0258400
Stem vs Leaf	RHSIM_Rhsim12G0177000	-7.489506594	4.58047E-10	3.64476E-09	RHSIM_Rhsim12G0177000
Stem vs Leaf	novel.1259	1.058428284	4.58499E-10	3.6471E-09	--
Stem vs Leaf	RHSIM_Rhsim01G0071400	-1.022761304	4.59208E-10	3.65148E-09	RHSIM_Rhsim01G0071400
Stem vs Leaf	RHSIM_Rhsim07G0231700	8.622188682	4.60514E-10	3.6606E-09	RHSIM_Rhsim07G0231700
Stem vs Leaf	RHSIM_Rhsim09G0042000	8.805599914	4.63417E-10	3.6824E-09	RHSIM_Rhsim09G0042000
Stem vs Leaf	RHSIM_Rhsim09G0181600	-1.052682523	4.65698E-10	3.69925E-09	RHSIM_Rhsim09G0181600
Stem vs Leaf	RHSIM_Rhsim08G0181200	3.537329808	4.71331E-10	3.74271E-09	RHSIM_Rhsim08G0181200
Stem vs Leaf	RHSIM_RhsimUnG0018400	2.075002371	4.76945E-10	3.78598E-09	RHSIM_RhsimUnG0018400
Stem vs Leaf	RHSIM_Rhsim09G0163900	-2.829704615	4.86739E-10	3.86108E-09	RHSIM_Rhsim09G0163900
Stem vs Leaf	RHSIM_Rhsim04G0011700	4.482484301	4.8739E-10	3.86491E-09	RHSIM_Rhsim04G0011700
Stem vs Leaf	RHSIM_Rhsim04G0060500	-8.089731705	4.87954E-10	3.86805E-09	RHSIM_Rhsim04G0060500
Stem vs Leaf	RHSIM_Rhsim07G0016400	-1.522145573	4.90215E-10	3.88464E-09	RHSIM_Rhsim07G0016400
Stem vs Leaf	RHSIM_Rhsim06G0011900	1.304002209	4.92334E-10	3.89995E-09	RHSIM_Rhsim06G0011900
Stem vs Leaf	RHSIM_Rhsim01G0283700	-1.895756663	4.92486E-10	3.89995E-09	RHSIM_Rhsim01G0283700
Stem vs Leaf	RHSIM_Rhsim12G0005500	-3.929104669	4.93624E-10	3.90761E-09	RHSIM_Rhsim12G0005500
Stem vs Leaf	novel.4818	1.760120404	4.96299E-10	3.92744E-09	--
Stem vs Leaf	RHSIM_Rhsim05G0069500	-2.77332534	4.98151E-10	3.94075E-09	RHSIM_Rhsim05G0069500
Stem vs Leaf	RHSIM_Rhsim06G0139300	9.823207056	5.01107E-10	3.96277E-09	RHSIM_Rhsim06G0139300
Stem vs Leaf	RHSIM_Rhsim07G0091600	-1.435154139	5.06299E-10	4.00245E-09	RHSIM_Rhsim07G0091600
Stem vs Leaf	RHSIM_Rhsim05G0178600	1.241271399	5.12106E-10	4.04558E-09	RHSIM_Rhsim05G0178600
Stem vs Leaf	RHSIM_Rhsim08G0146900	1.894768672	5.12637E-10	4.04839E-09	RHSIM_Rhsim08G0146900
Stem vs Leaf	RHSIM_Rhsim03G0246900	-2.594467952	5.1375E-10	4.05579E-09	RHSIM_Rhsim03G0246900
Stem vs Leaf	RHSIM_Rhsim13G0062200	9.367370791	5.16869E-10	4.07902E-09	RHSIM_Rhsim13G0062200
Stem vs Leaf	RHSIM_Rhsim09G0192800	-1.884260763	5.18563E-10	4.09099E-09	RHSIM_Rhsim09G0192800

Comparison	Gene ID	log_2FC	P-value	FDR (padj)	Gene Name
Stem vs Leaf	RHSIM_Rhsim08G0210600	1.313428643	5.21726E-10	4.11453E-09	RHSIM_Rhsim08G0210600
Stem vs Leaf	RHSIM_Rhsim12G0124100	2.593635466	5.23539E-10	4.12741E-09	RHSIM_Rhsim12G0124100
Stem vs Leaf	novel.4487	-8.16348382	5.26421E-10	4.14872E-09	--
Stem vs Leaf	RHSIM_Rhsim11G0012400	-8.409733833	5.28774E-10	4.16584E-09	RHSIM_Rhsim11G0012400
Stem vs Leaf	novel.3056	5.210215038	5.31769E-10	4.188E-09	--
Stem vs Leaf	novel.424	1.987768788	5.3353E-10	4.20043E-09	--
Stem vs Leaf	RHSIM_Rhsim11G0048800	1.475257576	5.37872E-10	4.23317E-09	RHSIM_Rhsim11G0048800
Stem vs Leaf	novel.2151	2.787654921	5.39379E-10	4.24358E-09	--
Stem vs Leaf	RHSIM_Rhsim09G0110100	-1.914593944	5.4246E-10	4.26345E-09	RHSIM_Rhsim09G0110100
Stem vs Leaf	RHSIM_Rhsim08G0167700	2.159526147	5.42962E-10	4.26594E-09	RHSIM_Rhsim08G0167700
Stem vs Leaf	RHSIM_Rhsim13G0100000	9.17093143	5.43354E-10	4.26757E-09	RHSIM_Rhsim13G0100000
Stem vs Leaf	RHSIM_Rhsim09G0213800	-1.421684663	5.43956E-10	4.27084E-09	RHSIM_Rhsim09G0213800
Stem vs Leaf	RHSIM_Rhsim03G0136600	-1.330591022	5.53201E-10	4.34195E-09	RHSIM_Rhsim03G0136600
Stem vs Leaf	RHSIM_Rhsim03G0251900	2.298175301	5.56711E-10	4.36801E-09	RHSIM_Rhsim03G0251900
Stem vs Leaf	novel.3445	-1.945238218	5.57916E-10	4.37598E-09	--
Stem vs Leaf	RHSIM_Rhsim04G0173200	-8.375237576	5.6075E-10	4.39671E-09	RHSIM_Rhsim04G0173200
Stem vs Leaf	RHSIM_Rhsim04G0116300	2.844055385	5.64338E-10	4.42334E-09	RHSIM_Rhsim04G0116300
Stem vs Leaf	RHSIM_Rhsim06G0142000	1.705978413	5.71127E-10	4.47503E-09	RHSIM_Rhsim06G0142000
Stem vs Leaf	RHSIM_Rhsim03G0125000	1.454508934	5.8611E-10	4.59086E-09	RHSIM_Rhsim03G0125000
Stem vs Leaf	novel.5081	-9.430556603	5.90548E-10	4.62405E-09	--
Stem vs Leaf	novel.1353	-6.83137625	5.93783E-10	4.6478E-09	--
Stem vs Leaf	RHSIM_Rhsim13G0067800	2.402596173	6.00354E-10	4.69764E-09	RHSIM_Rhsim13G0067800
Stem vs Leaf	RHSIM_Rhsim11G0168700	-1.835478272	6.09697E-10	4.76913E-09	RHSIM_Rhsim11G0168700
Stem vs Leaf	RHSIM_Rhsim01G0152800	-1.152448491	6.11348E-10	4.78042E-09	RHSIM_Rhsim01G0152800
Stem vs Leaf	RHSIM_Rhsim07G0084600	-8.019916623	6.23095E-10	4.87062E-09	RHSIM_Rhsim07G0084600

Comparison	Gene ID	log_2FC	P-value	FDR (padj)	Gene Name
Stem vs Leaf	RHSIM_Rhsim01G0041100	-8.296292439	6.23539E-10	4.87244E-09	RHSIM_Rhsim01G0041100
Stem vs Leaf	RHSIM_Rhsim13G0184800	-3.646567097	6.24091E-10	4.87511E-09	RHSIM_Rhsim13G0184800
Stem vs Leaf	RHSIM_Rhsim13G0021000	-1.360099147	6.2534E-10	4.88321E-09	RHSIM_Rhsim13G0021000
Stem vs Leaf	novel.1856	-1.223476859	6.3485E-10	4.95579E-09	--
Stem vs Leaf	RHSIM_Rhsim12G0130100	-2.275721012	6.35882E-10	4.96216E-09	RHSIM_Rhsim12G0130100
Stem vs Leaf	RHSIM_Rhsim01G0234800	3.542387282	6.39576E-10	4.9893E-09	RHSIM_Rhsim01G0234800
Stem vs Leaf	RHSIM_Rhsim02G0108100	-3.696335668	6.408E-10	4.99716E-09	RHSIM_Rhsim02G0108100
Stem vs Leaf	RHSIM_Rhsim03G0172600	-2.277440077	6.45599E-10	5.03288E-09	RHSIM_Rhsim03G0172600
Stem vs Leaf	RHSIM_Rhsim12G0024900	1.684040465	6.52384E-10	5.08405E-09	RHSIM_Rhsim12G0024900
Stem vs Leaf	RHSIM_Rhsim08G0229400	2.459833923	6.6073E-10	5.14735E-09	RHSIM_Rhsim08G0229400
Stem vs Leaf	RHSIM_Rhsim08G0014900	-1.247155105	6.61516E-10	5.15173E-09	RHSIM_Rhsim08G0014900
Stem vs Leaf	RHSIM_Rhsim06G0054800	-1.905942296	6.6195E-10	5.15337E-09	RHSIM_Rhsim06G0054800
Stem vs Leaf	RHSIM_Rhsim06G0034200	3.194223924	6.68443E-10	5.20216E-09	RHSIM_Rhsim06G0034200
Stem vs Leaf	RHSIM_RhsimUnG0053500	-6.411840886	6.7295E-10	5.2337E-09	RHSIM_RhsimUnG0053500
Stem vs Leaf	novel.4584	1.337432059	6.75982E-10	5.2542E-09	--
Stem vs Leaf	novel.3845	-3.69999257	6.76041E-10	5.2542E-09	--
Stem vs Leaf	RHSIM_Rhsim12G0144400	-1.47200788	6.85379E-10	5.32498E-09	RHSIM_Rhsim12G0144400
Stem vs Leaf	RHSIM_Rhsim04G0044200	-3.266415097	6.87344E-10	5.33844E-09	RHSIM_Rhsim04G0044200
Stem vs Leaf	novel.5158	-1.391925227	6.91138E-10	5.3661E-09	--
Stem vs Leaf	novel.3705	1.144155511	6.96882E-10	5.40888E-09	--
Stem vs Leaf	RHSIM_Rhsim08G0009000	5.639353216	6.97952E-10	5.41536E-09	RHSIM_Rhsim08G0009000
Stem vs Leaf	RHSIM_Rhsim07G0215200	1.460602786	7.09254E-10	5.5012E-09	RHSIM_Rhsim07G0215200
Stem vs Leaf	novel.5543	2.088615497	7.1185E-10	5.51941E-09	--
Stem vs Leaf	RHSIM_Rhsim10G0026600	-6.06516092	7.12081E-10	5.51941E-09	RHSIM_Rhsim10G0026600
Stem vs Leaf	RHSIM_Rhsim11G0137700	-10.02625142	7.14705E-10	5.53788E-09	RHSIM_Rhsim11G0137700

Comparison	Gene ID	log_2FC	P-value	FDR (padj)	Gene Name
Stem vs Leaf	RHSIM_Rhsim03G0173100	2.88216552	7.26167E-10	5.62292E-09	RHSIM_Rhsim03G0173100
Stem vs Leaf	RHSIM_RhsimUnG0235800	2.676934714	7.30622E-10	5.65551E-09	RHSIM_RhsimUnG0235800
Stem vs Leaf	RHSIM_Rhsim10G0104100	-1.680321389	7.38891E-10	5.7176E-09	RHSIM_Rhsim10G0104100
Stem vs Leaf	novel.96	-8.324681499	7.39265E-10	5.71857E-09	--
Stem vs Leaf	RHSIM_Rhsim08G0075500	-1.705663512	7.40948E-10	5.72967E-09	RHSIM_Rhsim08G0075500
Stem vs Leaf	RHSIM_Rhsim10G0195200	1.124416151	7.58137E-10	5.86062E-09	RHSIM_Rhsim10G0195200
Stem vs Leaf	RHSIM_Rhsim01G0210200	-1.395520335	7.77684E-10	6.00971E-09	RHSIM_Rhsim01G0210200
Stem vs Leaf	RHSIM_Rhsim13G0093500	2.348417299	7.82721E-10	6.04661E-09	RHSIM_Rhsim13G0093500
Stem vs Leaf	RHSIM_RhsimUnG0105900	6.308510322	7.83012E-10	6.04683E-09	RHSIM_RhsimUnG0105900
Stem vs Leaf	RHSIM_Rhsim08G0161400	1.031567118	7.8839E-10	6.08428E-09	RHSIM_Rhsim08G0161400
Stem vs Leaf	RHSIM_Rhsim12G0017000	-1.6967389	7.96078E-10	6.14156E-09	RHSIM_Rhsim12G0017000
Stem vs Leaf	novel.5831	-5.119971432	7.97165E-10	6.14788E-09	--
Stem vs Leaf	RHSIM_Rhsim06G0155400	1.379598342	7.97615E-10	6.1493E-09	RHSIM_Rhsim06G0155400
Stem vs Leaf	RHSIM_RhsimUnG0057300	-8.688721619	8.03316E-10	6.19118E-09	RHSIM_RhsimUnG0057300
Stem vs Leaf	RHSIM_RhsimUnG0156400	-4.532592566	8.05426E-10	6.20536E-09	RHSIM_RhsimUnG0156400
Stem vs Leaf	novel.2907	-5.472929859	8.14066E-10	6.26983E-09	--
Stem vs Leaf	RHSIM_RhsimUnG0223500	-8.137523737	8.15876E-10	6.28167E-09	RHSIM_RhsimUnG0223500
Stem vs Leaf	RHSIM_Rhsim10G0171200	-2.335583681	8.19936E-10	6.31082E-09	RHSIM_Rhsim10G0171200
Stem vs Leaf	RHSIM_Rhsim13G0190700	-6.810238656	8.22119E-10	6.32551E-09	RHSIM_Rhsim13G0190700
Stem vs Leaf	RHSIM_Rhsim02G0088200	1.28007623	8.24724E-10	6.34178E-09	RHSIM_Rhsim02G0088200
Stem vs Leaf	RHSIM_Rhsim09G0072600	1.500655761	8.24783E-10	6.34178E-09	RHSIM_Rhsim09G0072600
Stem vs Leaf	RHSIM_Rhsim02G0234500	-2.12959641	8.27726E-10	6.36228E-09	RHSIM_Rhsim02G0234500
Stem vs Leaf	RHSIM_Rhsim05G0161500	2.132958657	8.35121E-10	6.41698E-09	RHSIM_Rhsim05G0161500
Stem vs Leaf	RHSIM_Rhsim13G0045400	1.149487106	8.36142E-10	6.42268E-09	RHSIM_Rhsim13G0045400
Stem vs Leaf	RHSIM_Rhsim08G0099300	2.79868172	8.48909E-10	6.51858E-09	RHSIM_Rhsim08G0099300

Comparison	Gene ID	log_2FC	P-value	FDR (padj)	Gene Name
Stem vs Leaf	RHSIM_Rhsim02G0109900	-1.285555803	8.5256E-10	6.54443E-09	RHSIM_Rhsim02G0109900
Stem vs Leaf	RHSIM_Rhsim04G0186900	-2.598010479	8.54597E-10	6.55789E-09	RHSIM_Rhsim04G0186900
Stem vs Leaf	RHSIM_Rhsim12G0143800	4.193153748	8.59593E-10	6.59403E-09	RHSIM_Rhsim12G0143800
Stem vs Leaf	RHSIM_Rhsim01G0061700	3.316978479	8.70448E-10	6.67508E-09	RHSIM_Rhsim01G0061700
Stem vs Leaf	novel.4832	-2.306782407	8.72612E-10	6.68945E-09	--
Stem vs Leaf	RHSIM_Rhsim07G0174700	-1.435756354	8.76742E-10	6.71887E-09	RHSIM_Rhsim07G0174700
Stem vs Leaf	RHSIM_Rhsim02G0006300	3.364335803	8.81938E-10	6.75644E-09	RHSIM_Rhsim02G0006300
Stem vs Leaf	RHSIM_Rhsim02G0200100	-1.459222351	8.84444E-10	6.77339E-09	RHSIM_Rhsim02G0200100
Stem vs Leaf	RHSIM_Rhsim12G0164800	-1.282584259	8.88563E-10	6.80268E-09	RHSIM_Rhsim12G0164800
Stem vs Leaf	RHSIM_Rhsim04G0247500	-1.128333032	8.99452E-10	6.88375E-09	RHSIM_Rhsim04G0247500
Stem vs Leaf	RHSIM_Rhsim02G0165300	-5.234135191	9.07164E-10	6.94047E-09	RHSIM_Rhsim02G0165300
Stem vs Leaf	RHSIM_Rhsim11G0106800	6.41429849	9.11598E-10	6.97207E-09	RHSIM_Rhsim11G0106800
Stem vs Leaf	RHSIM_Rhsim06G0157200	4.908430425	9.19874E-10	7.03304E-09	RHSIM_Rhsim06G0157200
Stem vs Leaf	RHSIM_Rhsim07G0121500	-3.35716971	9.24654E-10	7.0649E-09	RHSIM_Rhsim07G0121500
Stem vs Leaf	RHSIM_Rhsim03G0036200	-9.285214536	9.28807E-10	7.09428E-09	RHSIM_Rhsim03G0036200
Stem vs Leaf	RHSIM_Rhsim08G0246600	-1.035993777	9.4334E-10	7.20289E-09	RHSIM_Rhsim08G0246600
Stem vs Leaf	RHSIM_Rhsim06G0180800	-2.636389053	9.43929E-10	7.205E-09	RHSIM_Rhsim06G0180800
Stem vs Leaf	RHSIM_Rhsim12G0158600	-1.650519028	9.50286E-10	7.25112E-09	RHSIM_Rhsim12G0158600
Stem vs Leaf	RHSIM_Rhsim05G0060400	-3.916256619	9.56519E-10	7.29385E-09	RHSIM_Rhsim05G0060400
Stem vs Leaf	RHSIM_Rhsim03G0225300	-1.407903027	9.57484E-10	7.2988E-09	RHSIM_Rhsim03G0225300
Stem vs Leaf	RHSIM_Rhsim02G0068600	5.141505362	9.77287E-10	7.44483E-09	RHSIM_Rhsim02G0068600
Stem vs Leaf	RHSIM_Rhsim09G0187300	-2.554313363	9.81958E-10	7.47794E-09	RHSIM_Rhsim09G0187300
Stem vs Leaf	RHSIM_Rhsim13G0124400	5.723397362	9.95473E-10	7.57836E-09	RHSIM_Rhsim13G0124400
Stem vs Leaf	RHSIM_Rhsim06G0069600	5.02388582	9.98393E-10	7.59729E-09	RHSIM_Rhsim06G0069600
Stem vs Leaf	RHSIM_Rhsim12G0016400	-3.320326838	9.9862E-10	7.59729E-09	RHSIM_Rhsim12G0016400

Comparison	Gene ID	log_2FC	P-value	FDR (padj)	Gene Name
Stem vs Leaf	novel.309	-1.738004225	1.00304E-09	7.62839E-09	--
Stem vs Leaf	novel.50	-2.983447848	1.01494E-09	7.71635E-09	--
Stem vs Leaf	RHSIM_Rhsim10G0140400	-3.80585346	1.0181E-09	7.73782E-09	RHSIM_Rhsim10G0140400
Stem vs Leaf	RHSIM_Rhsim06G0179400	2.698581485	1.02198E-09	7.76472E-09	RHSIM_Rhsim06G0179400
Stem vs Leaf	RHSIM_Rhsim04G0118100	1.880985194	1.03928E-09	7.89022E-09	RHSIM_Rhsim04G0118100
Stem vs Leaf	RHSIM_Rhsim13G0103200	8.481974925	1.03952E-09	7.89022E-09	RHSIM_Rhsim13G0103200
Stem vs Leaf	RHSIM_Rhsim13G0038500	-1.350580067	1.04234E-09	7.90902E-09	RHSIM_Rhsim13G0038500
Stem vs Leaf	RHSIM_Rhsim12G0023700	6.585436665	1.04493E-09	7.92544E-09	RHSIM_Rhsim12G0023700
Stem vs Leaf	RHSIM_Rhsim03G0032700	-3.597324646	1.04519E-09	7.92544E-09	RHSIM_Rhsim03G0032700
Stem vs Leaf	RHSIM_Rhsim10G0022200	-1.423612472	1.05248E-09	7.97811E-09	RHSIM_Rhsim10G0022200
Stem vs Leaf	RHSIM_Rhsim10G0035800	5.121087282	1.06761E-09	8.0894E-09	RHSIM_Rhsim10G0035800
Stem vs Leaf	RHSIM_Rhsim13G0174400	2.028760977	1.06787E-09	8.0894E-09	RHSIM_Rhsim13G0174400
Stem vs Leaf	RHSIM_Rhsim08G0018400	1.216752165	1.07583E-09	8.14702E-09	RHSIM_Rhsim08G0018400
Stem vs Leaf	RHSIM_Rhsim07G0038100	-5.148733548	1.08202E-09	8.19125E-09	RHSIM_Rhsim07G0038100
Stem vs Leaf	RHSIM_Rhsim09G0107200	1.184255777	1.09852E-09	8.31069E-09	RHSIM_Rhsim09G0107200
Stem vs Leaf	RHSIM_Rhsim13G0114900	3.039214503	1.10906E-09	8.38768E-09	RHSIM_Rhsim13G0114900
Stem vs Leaf	RHSIM_Rhsim04G0200100	2.206032367	1.10978E-09	8.39038E-09	RHSIM_Rhsim04G0200100
Stem vs Leaf	RHSIM_Rhsim08G0197500	1.287683603	1.12267E-09	8.48505E-09	RHSIM_Rhsim08G0197500
Stem vs Leaf	RHSIM_Rhsim09G0122900	-2.415475947	1.12572E-09	8.5053E-09	RHSIM_Rhsim09G0122900
Stem vs Leaf	RHSIM_Rhsim13G0070900	-7.743049679	1.12752E-09	8.51608E-09	RHSIM_Rhsim13G0070900
Stem vs Leaf	RHSIM_Rhsim02G0041500	-2.557767259	1.13296E-09	8.55438E-09	RHSIM_Rhsim02G0041500
Stem vs Leaf	novel.4021	5.602956773	1.14236E-09	8.62252E-09	--
Stem vs Leaf	RHSIM_RhsimUnG0090500	-2.257774827	1.14278E-09	8.62285E-09	RHSIM_RhsimUnG0090500
Stem vs Leaf	novel.3058	5.053837974	1.14384E-09	8.62805E-09	--
Stem vs Leaf	novel.2982	-8.297892207	1.14845E-09	8.66001E-09	--

Comparison	Gene ID	log_2FC	P-value	FDR (padj)	Gene Name
Stem vs Leaf	RHSIM_Rhsim02G0235900	2.506343536	1.14936E-09	8.66401E-09	RHSIM_Rhsim02G0235900
Stem vs Leaf	RHSIM_Rhsim04G0046300	2.345553468	1.15412E-09	8.69707E-09	RHSIM_Rhsim04G0046300
Stem vs Leaf	RHSIM_Rhsim13G0166800	-1.50074323	1.15452E-09	8.69725E-09	RHSIM_Rhsim13G0166800
Stem vs Leaf	RHSIM_Rhsim08G0212900	2.474272843	1.18522E-09	8.92554E-09	RHSIM_Rhsim08G0212900
Stem vs Leaf	RHSIM_Rhsim08G0228100	-6.864747778	1.20395E-09	9.06071E-09	RHSIM_Rhsim08G0228100
Stem vs Leaf	RHSIM_Rhsim01G0182000	-4.253009772	1.21807E-09	9.16396E-09	RHSIM_Rhsim01G0182000
Stem vs Leaf	RHSIM_Rhsim03G0052100	-7.884096252	1.21889E-09	9.1671E-09	RHSIM_Rhsim03G0052100
Stem vs Leaf	RHSIM_Rhsim02G0143400	-1.551622889	1.26102E-09	9.48089E-09	RHSIM_Rhsim02G0143400
Stem vs Leaf	RHSIM_Rhsim03G0134500	-1.544977581	1.26432E-09	9.50258E-09	RHSIM_Rhsim03G0134500
Stem vs Leaf	RHSIM_Rhsim12G0214400	4.169537144	1.2662E-09	9.51364E-09	RHSIM_Rhsim12G0214400
Stem vs Leaf	novel.5685	3.484595635	1.27068E-09	9.54418E-09	--
Stem vs Leaf	RHSIM_Rhsim12G0090600	-1.172612722	1.28815E-09	9.67223E-09	RHSIM_Rhsim12G0090600
Stem vs Leaf	RHSIM_Rhsim08G0100100	-4.207901271	1.28933E-09	9.67792E-09	RHSIM_Rhsim08G0100100
Stem vs Leaf	RHSIM_Rhsim10G0131300	1.529904367	1.29035E-09	9.68247E-09	RHSIM_Rhsim10G0131300
Stem vs Leaf	RHSIM_Rhsim05G0072700	2.459990272	1.29985E-09	9.75055E-09	RHSIM_Rhsim05G0072700
Stem vs Leaf	RHSIM_Rhsim04G0032800	-2.661936617	1.31109E-09	9.83165E-09	RHSIM_Rhsim04G0032800
Stem vs Leaf	RHSIM_Rhsim13G0086900	-1.234183323	1.31528E-09	9.85988E-09	RHSIM_Rhsim13G0086900
Stem vs Leaf	RHSIM_Rhsim03G0070200	1.114759596	1.32047E-09	9.89555E-09	RHSIM_Rhsim03G0070200
Stem vs Leaf	RHSIM_Rhsim12G0040600	-1.398041139	1.32154E-09	9.90039E-09	RHSIM_Rhsim12G0040600
Stem vs Leaf	RHSIM_Rhsim11G0154100	1.28117445	1.32593E-09	9.93003E-09	RHSIM_Rhsim11G0154100
Stem vs Leaf	RHSIM_Rhsim12G0177700	1.348229016	1.32766E-09	9.93976E-09	RHSIM_Rhsim12G0177700
Stem vs Leaf	RHSIM_Rhsim10G0079800	1.310817046	1.33801E-09	1.0014E-08	RHSIM_Rhsim10G0079800
Stem vs Leaf	RHSIM_Rhsim10G0168700	5.902863908	1.34456E-09	1.00598E-08	RHSIM_Rhsim10G0168700
Stem vs Leaf	RHSIM_Rhsim09G0058700	-5.950033239	1.3513E-09	1.01069E-08	RHSIM_Rhsim09G0058700
Stem vs Leaf	RHSIM_Rhsim08G0045300	1.67220156	1.38172E-09	1.03311E-08	RHSIM_Rhsim08G0045300

Comparison	Gene ID	log_2FC	P-value	FDR (padj)	Gene Name
Stem vs Leaf	RHSIM_RhsimUnG0223700	2.551792824	1.38712E-09	1.03681E-08	RHSIM_RhsimUnG0223700
Stem vs Leaf	RHSIM_Rhsim13G0035300	-6.05848493	1.39275E-09	1.04068E-08	RHSIM_Rhsim13G0035300
Stem vs Leaf	RHSIM_Rhsim12G0164000	1.151018216	1.40061E-09	1.04621E-08	RHSIM_Rhsim12G0164000
Stem vs Leaf	RHSIM_Rhsim07G0121000	3.529674758	1.43248E-09	1.06933E-08	RHSIM_Rhsim07G0121000
Stem vs Leaf	RHSIM_Rhsim01G0186600	2.360049784	1.43833E-09	1.07334E-08	RHSIM_Rhsim01G0186600
Stem vs Leaf	RHSIM_Rhsim02G0182200	-1.957572476	1.45402E-09	1.08435E-08	RHSIM_Rhsim02G0182200
Stem vs Leaf	RHSIM_Rhsim10G0155800	4.945065574	1.45495E-09	1.0847E-08	RHSIM_Rhsim10G0155800
Stem vs Leaf	RHSIM_Rhsim03G0198800	-6.025067649	1.46727E-09	1.09353E-08	RHSIM_Rhsim03G0198800
Stem vs Leaf	RHSIM_Rhsim03G0142500	-2.189910554	1.47375E-09	1.098E-08	RHSIM_Rhsim03G0142500
Stem vs Leaf	RHSIM_Rhsim04G0138800	1.602870784	1.47843E-09	1.10113E-08	RHSIM_Rhsim04G0138800
Stem vs Leaf	novel.1978	-1.148383763	1.4802E-09	1.10209E-08	--
Stem vs Leaf	RHSIM_Rhsim12G0009800	-3.488295492	1.49655E-09	1.1139E-08	RHSIM_Rhsim12G0009800
Stem vs Leaf	RHSIM_Rhsim02G0189300	-4.398355158	1.5024E-09	1.11785E-08	RHSIM_Rhsim02G0189300
Stem vs Leaf	novel.3702	1.088497418	1.50282E-09	1.11785E-08	--
Stem vs Leaf	RHSIM_Rhsim08G0070700	4.079261541	1.53435E-09	1.14086E-08	RHSIM_Rhsim08G0070700
Stem vs Leaf	RHSIM_Rhsim01G0009900	2.893136705	1.53475E-09	1.14086E-08	RHSIM_Rhsim01G0009900
Stem vs Leaf	RHSIM_RhsimUnG0211400	-2.45815947	1.53681E-09	1.14203E-08	RHSIM_RhsimUnG0211400
Stem vs Leaf	RHSIM_Rhsim13G0018900	-1.530577857	1.56484E-09	1.1621E-08	RHSIM_Rhsim13G0018900
Stem vs Leaf	RHSIM_Rhsim03G0008400	2.391654909	1.5682E-09	1.16422E-08	RHSIM_Rhsim03G0008400
Stem vs Leaf	RHSIM_Rhsim04G0094100	-8.214675269	1.57843E-09	1.17106E-08	RHSIM_Rhsim04G0094100
Stem vs Leaf	RHSIM_Rhsim07G0215600	-1.071186793	1.58608E-09	1.17636E-08	RHSIM_Rhsim07G0215600
Stem vs Leaf	novel.3037	9.401899754	1.61335E-09	1.19621E-08	--
Stem vs Leaf	RHSIM_Rhsim13G0234200	-1.197692032	1.6233E-09	1.20291E-08	RHSIM_Rhsim13G0234200
Stem vs Leaf	RHSIM_RhsimUnG0131200	-2.842283953	1.62344E-09	1.20291E-08	RHSIM_RhsimUnG0131200
Stem vs Leaf	RHSIM_Rhsim13G0149800	1.377047828	1.64066E-09	1.21528E-08	RHSIM_Rhsim13G0149800

Comparison	Gene ID	log_2FC	P-value	FDR (padj)	Gene Name
Stem vs Leaf	novel.79	-8.049211392	1.65935E-09	1.22873E-08	--
Stem vs Leaf	RHSIM_RhsimUnG0255600	-3.5458677	1.66386E-09	1.23167E-08	RHSIM_RhsimUnG0255600
Stem vs Leaf	RHSIM_Rhsim04G0225200	-1.544372994	1.68319E-09	1.24558E-08	RHSIM_Rhsim04G0225200
Stem vs Leaf	RHSIM_Rhsim13G0187900	1.963160258	1.70982E-09	1.26488E-08	RHSIM_Rhsim13G0187900
Stem vs Leaf	RHSIM_Rhsim04G0108200	-1.454870444	1.71523E-09	1.26848E-08	RHSIM_Rhsim04G0108200
Stem vs Leaf	RHSIM_Rhsim11G0135800	-1.215820734	1.74354E-09	1.289E-08	RHSIM_Rhsim11G0135800
Stem vs Leaf	RHSIM_Rhsim05G0121700	-1.113116083	1.75123E-09	1.29386E-08	RHSIM_Rhsim05G0121700
Stem vs Leaf	RHSIM_Rhsim04G0010200	-8.361016542	1.76049E-09	1.30028E-08	RHSIM_Rhsim04G0010200
Stem vs Leaf	RHSIM_Rhsim08G0039500	1.122619292	1.76998E-09	1.30645E-08	RHSIM_Rhsim08G0039500
Stem vs Leaf	RHSIM_RhsimUnG0131300	-3.569329013	1.80143E-09	1.32882E-08	RHSIM_RhsimUnG0131300
Stem vs Leaf	RHSIM_Rhsim07G0144800	-1.475533481	1.8097E-09	1.33449E-08	RHSIM_Rhsim07G0144800
Stem vs Leaf	novel.579	-1.571753466	1.81197E-09	1.33574E-08	--
Stem vs Leaf	RHSIM_Rhsim12G0209400	-2.768171837	1.8159E-09	1.3382E-08	RHSIM_Rhsim12G0209400
Stem vs Leaf	RHSIM_Rhsim02G0213700	-3.776877386	1.82781E-09	1.34655E-08	RHSIM_Rhsim02G0213700
Stem vs Leaf	novel.2863	-3.581152203	1.83109E-09	1.34853E-08	--
Stem vs Leaf	RHSIM_Rhsim13G0096900	7.945693241	1.84143E-09	1.35571E-08	RHSIM_Rhsim13G0096900
Stem vs Leaf	RHSIM_Rhsim08G0063800	2.505382159	1.84614E-09	1.35875E-08	RHSIM_Rhsim08G0063800
Stem vs Leaf	RHSIM_Rhsim12G0040300	2.289561448	1.85172E-09	1.36242E-08	RHSIM_Rhsim12G0040300
Stem vs Leaf	RHSIM_Rhsim03G0098800	3.137339121	1.85389E-09	1.36358E-08	RHSIM_Rhsim03G0098800
Stem vs Leaf	RHSIM_Rhsim08G0096100	-2.469002864	1.90469E-09	1.40006E-08	RHSIM_Rhsim08G0096100
Stem vs Leaf	RHSIM_Rhsim04G0187000	-2.671576659	1.91211E-09	1.40506E-08	RHSIM_Rhsim04G0187000
Stem vs Leaf	RHSIM_Rhsim07G0029000	-1.000489111	1.93017E-09	1.41788E-08	RHSIM_Rhsim07G0029000
Stem vs Leaf	RHSIM_Rhsim05G0192200	-3.89637717	1.93491E-09	1.42091E-08	RHSIM_Rhsim05G0192200
Stem vs Leaf	RHSIM_Rhsim09G0206900	-2.113380219	1.93934E-09	1.42371E-08	RHSIM_Rhsim09G0206900
Stem vs Leaf	RHSIM_Rhsim01G0001300	1.401257962	1.95889E-09	1.4376E-08	RHSIM_Rhsim01G0001300

Comparison	Gene ID	log_2FC	P-value	FDR (padj)	Gene Name
Stem vs Leaf	RHSIM_Rhsim01G0096400	-2.195733867	1.9796E-09	1.45234E-08	RHSIM_Rhsim01G0096400
Stem vs Leaf	RHSIM_Rhsim04G0198200	-3.669077608	1.98194E-09	1.45359E-08	RHSIM_Rhsim04G0198200
Stem vs Leaf	RHSIM_Rhsim07G0165300	-2.314868864	2.00573E-09	1.47057E-08	RHSIM_Rhsim07G0165300
Stem vs Leaf	RHSIM_Rhsim08G0081400	-1.893128674	2.01704E-09	1.47792E-08	RHSIM_Rhsim08G0081400
Stem vs Leaf	RHSIM_Rhsim08G0217700	-8.340825246	2.02437E-09	1.48282E-08	RHSIM_Rhsim08G0217700
Stem vs Leaf	RHSIM_Rhsim06G0031700	-2.165183707	2.02585E-09	1.48344E-08	RHSIM_Rhsim06G0031700
Stem vs Leaf	RHSIM_RhsimMtG0002300	-4.315293997	2.02682E-09	1.48367E-08	RHSIM_RhsimMtG0002300
Stem vs Leaf	RHSIM_Rhsim12G0174100	2.121713183	2.02859E-09	1.4845E-08	RHSIM_Rhsim12G0174100
Stem vs Leaf	RHSIM_Rhsim07G0187500	-1.463651827	2.03249E-09	1.48688E-08	RHSIM_Rhsim07G0187500
Stem vs Leaf	RHSIM_Rhsim04G0076500	-6.812314837	2.0338E-09	1.48736E-08	RHSIM_Rhsim04G0076500
Stem vs Leaf	RHSIM_Rhsim06G0022800	1.483097492	2.03751E-09	1.48961E-08	RHSIM_Rhsim06G0022800
Stem vs Leaf	RHSIM_Rhsim11G0125800	3.606199046	2.05692E-09	1.50332E-08	RHSIM_Rhsim11G0125800
Stem vs Leaf	RHSIM_Rhsim12G0047800	3.16804584	2.15773E-09	1.5755E-08	RHSIM_Rhsim12G0047800
Stem vs Leaf	RHSIM_RhsimUnG0232300	-6.184112092	2.17522E-09	1.58777E-08	RHSIM_RhsimUnG0232300
Stem vs Leaf	RHSIM_Rhsim08G0044000	3.156165662	2.18221E-09	1.59236E-08	RHSIM_Rhsim08G0044000
Stem vs Leaf	RHSIM_Rhsim01G0279600	8.316802648	2.21914E-09	1.6188E-08	RHSIM_Rhsim01G0279600
Stem vs Leaf	RHSIM_Rhsim06G0055200	3.407062747	2.22832E-09	1.62498E-08	RHSIM_Rhsim06G0055200
Stem vs Leaf	RHSIM_Rhsim12G0156400	-5.662863537	2.23345E-09	1.62796E-08	RHSIM_Rhsim12G0156400
Stem vs Leaf	RHSIM_Rhsim03G0237900	-2.889743484	2.23382E-09	1.62796E-08	RHSIM_Rhsim03G0237900
Stem vs Leaf	novel.2755	8.278616721	2.23735E-09	1.62975E-08	--
Stem vs Leaf	RHSIM_Rhsim03G0073700	-8.405569772	2.23768E-09	1.62975E-08	RHSIM_Rhsim03G0073700
Stem vs Leaf	novel.1384	1.225215804	2.24074E-09	1.63146E-08	--
Stem vs Leaf	RHSIM_Rhsim12G0128900	8.944835912	2.24638E-09	1.63505E-08	RHSIM_Rhsim12G0128900
Stem vs Leaf	RHSIM_Rhsim07G0012600	-4.871093676	2.28706E-09	1.66413E-08	RHSIM_Rhsim07G0012600
Stem vs Leaf	RHSIM_Rhsim12G0166000	-4.331171485	2.29175E-09	1.66702E-08	RHSIM_Rhsim12G0166000

Comparison	Gene ID	log2FC	P-value	FDR (padj)	Gene Name
Stem vs Leaf	RHSIM_Rhsim13G0096300	3.0643956	2.29421E-09	1.66828E-08	RHSIM_Rhsim13G0096300
Stem vs Leaf	novel.3288	-1.504717351	2.29799E-09	1.6705E-08	--
Stem vs Leaf	RHSIM_Rhsim02G0145200	1.377157712	2.31534E-09	1.68258E-08	RHSIM_Rhsim02G0145200
Stem vs Leaf	RHSIM_Rhsim02G0231900	1.91106601	2.31745E-09	1.68359E-08	RHSIM_Rhsim02G0231900
Stem vs Leaf	RHSIM_Rhsim06G0220600	2.769689764	2.33287E-09	1.69426E-08	RHSIM_Rhsim06G0220600
Stem vs Leaf	novel.2642	8.434807864	2.35888E-09	1.7126E-08	--
Stem vs Leaf	RHSIM_Rhsim11G0095500	-1.03279566	2.37314E-09	1.72241E-08	RHSIM_Rhsim11G0095500
Stem vs Leaf	RHSIM_Rhsim12G0075200	-2.123706363	2.37437E-09	1.72277E-08	RHSIM_Rhsim12G0075200
Stem vs Leaf	RHSIM_Rhsim03G0074700	-2.667681596	2.37674E-09	1.72394E-08	RHSIM_Rhsim03G0074700
Stem vs Leaf	RHSIM_Rhsim08G0063900	-1.196575926	2.38742E-09	1.73114E-08	RHSIM_Rhsim08G0063900
Stem vs Leaf	novel.2862	3.194870883	2.39587E-09	1.73617E-08	--
Stem vs Leaf	RHSIM_Rhsim11G0157200	-4.031517933	2.40189E-09	1.73999E-08	RHSIM_Rhsim11G0157200
Stem vs Leaf	RHSIM_RhsimUnG0131600	-5.507248743	2.43342E-09	1.76228E-08	RHSIM_RhsimUnG0131600
Stem vs Leaf	RHSIM_Rhsim13G0229200	-8.376046185	2.43884E-09	1.76565E-08	RHSIM_Rhsim13G0229200
Stem vs Leaf	RHSIM_Rhsim09G0188900	2.297894012	2.47741E-09	1.79301E-08	RHSIM_Rhsim09G0188900
Stem vs Leaf	RHSIM_Rhsim03G0119000	5.515402493	2.50752E-09	1.81371E-08	RHSIM_Rhsim03G0119000
Stem vs Leaf	novel.1566	-1.620273813	2.50758E-09	1.81371E-08	--
Stem vs Leaf	novel.1089	1.786950277	2.51685E-09	1.81984E-08	--
Stem vs Leaf	RHSIM_Rhsim07G0060900	-2.449736641	2.53747E-09	1.83417E-08	RHSIM_Rhsim07G0060900
Stem vs Leaf	RHSIM_Rhsim01G0238200	2.082573331	2.54047E-09	1.83577E-08	RHSIM_Rhsim01G0238200
Stem vs Leaf	RHSIM_Rhsim01G0064400	-8.278512892	2.54516E-09	1.83858E-08	RHSIM_Rhsim01G0064400
Stem vs Leaf	RHSIM_Rhsim04G0013300	-5.032279252	2.57642E-09	1.85999E-08	RHSIM_Rhsim04G0013300
Stem vs Leaf	RHSIM_Rhsim03G0130900	-1.755549207	2.59509E-09	1.8723E-08	RHSIM_Rhsim03G0130900
Stem vs Leaf	RHSIM_Rhsim07G0102900	4.061280082	2.59811E-09	1.87389E-08	RHSIM_Rhsim07G0102900
Stem vs Leaf	novel.2639	2.070335763	2.61972E-09	1.88889E-08	--

Comparison	Gene ID	log_2FC	P-value	FDR (padj)	Gene Name
Stem vs Leaf	RHSIM_Rhsim03G0252700	-1.861754795	2.63108E-09	1.89649E-08	RHSIM_Rhsim03G0252700
Stem vs Leaf	RHSIM_RhsimUnG0079800	-2.614285332	2.63688E-09	1.90007E-08	RHSIM_RhsimUnG0079800
Stem vs Leaf	RHSIM_Rhsim03G0166500	-1.578749003	2.63811E-09	1.90037E-08	RHSIM_Rhsim03G0166500
Stem vs Leaf	RHSIM_Rhsim08G0026800	1.812476803	2.65017E-09	1.90845E-08	RHSIM_Rhsim08G0026800
Stem vs Leaf	RHSIM_Rhsim08G0104300	1.709421156	2.65492E-09	1.91127E-08	RHSIM_Rhsim08G0104300
Stem vs Leaf	RHSIM_Rhsim09G0166000	2.34846991	2.67712E-09	1.92666E-08	RHSIM_Rhsim09G0166000
Stem vs Leaf	RHSIM_Rhsim07G0056600	-3.882781199	2.71369E-09	1.95237E-08	RHSIM_Rhsim07G0056600
Stem vs Leaf	novel.4435	-9.411987676	2.71679E-09	1.95399E-08	--
Stem vs Leaf	RHSIM_Rhsim13G0134900	-1.855115957	2.73164E-09	1.96405E-08	RHSIM_Rhsim13G0134900
Stem vs Leaf	RHSIM_Rhsim08G0073400	3.8001826	2.79704E-09	2.01045E-08	RHSIM_Rhsim08G0073400
Stem vs Leaf	RHSIM_Rhsim03G0102600	-7.940536999	2.80084E-09	2.01255E-08	RHSIM_Rhsim03G0102600
Stem vs Leaf	RHSIM_Rhsim04G0216700	1.629355116	2.82225E-09	2.0273E-08	RHSIM_Rhsim04G0216700
Stem vs Leaf	RHSIM_Rhsim03G0150300	-8.700992172	2.84577E-09	2.04293E-08	RHSIM_Rhsim03G0150300
Stem vs Leaf	RHSIM_Rhsim04G0046400	-1.48717447	2.86079E-09	2.05307E-08	RHSIM_Rhsim04G0046400
Stem vs Leaf	RHSIM_Rhsim10G0039400	6.136233507	2.86625E-09	2.05635E-08	RHSIM_Rhsim10G0039400
Stem vs Leaf	RHSIM_Rhsim02G0071400	-4.054180496	2.88754E-09	2.06969E-08	RHSIM_Rhsim02G0071400
Stem vs Leaf	RHSIM_Rhsim12G0167600	1.70293883	2.92547E-09	2.09622E-08	RHSIM_Rhsim12G0167600
Stem vs Leaf	RHSIM_Rhsim08G0215400	-1.829647928	2.94131E-09	2.10692E-08	RHSIM_Rhsim08G0215400
Stem vs Leaf	RHSIM_RhsimUnG0007700	-8.865596132	2.95929E-09	2.11914E-08	RHSIM_RhsimUnG0007700
Stem vs Leaf	RHSIM_Rhsim04G0167800	-1.910212326	2.97278E-09	2.12813E-08	RHSIM_Rhsim04G0167800
Stem vs Leaf	RHSIM_Rhsim08G0044200	-1.29869631	2.98751E-09	2.13802E-08	RHSIM_Rhsim08G0044200
Stem vs Leaf	RHSIM_Rhsim01G0118100	1.260878883	3.01166E-09	2.15463E-08	RHSIM_Rhsim01G0118100
Stem vs Leaf	RHSIM_Rhsim05G0231700	-1.244842706	3.01769E-09	2.15827E-08	RHSIM_Rhsim05G0231700
Stem vs Leaf	RHSIM_Rhsim11G0052000	-1.804530708	3.04151E-09	2.17464E-08	RHSIM_Rhsim11G0052000
Stem vs Leaf	RHSIM_Rhsim12G0136300	-1.83480338	3.05055E-09	2.18043E-08	RHSIM_Rhsim12G0136300

Comparison	Gene ID	log_2FC	P-value	FDR (padj)	Gene Name
Stem vs Leaf	RHSIM_Rhsim02G0206500	-8.143996767	3.06106E-09	2.18726E-08	RHSIM_Rhsim02G0206500
Stem vs Leaf	RHSIM_RhsimUnG0015300	-4.613712999	3.07777E-09	2.19852E-08	RHSIM_RhsimUnG0015300
Stem vs Leaf	RHSIM_RhsimUnG0228600	-1.82180354	3.09027E-09	2.20676E-08	RHSIM_RhsimUnG0228600
Stem vs Leaf	RHSIM_Rhsim12G0163800	-4.688546204	3.11941E-09	2.22688E-08	RHSIM_Rhsim12G0163800
Stem vs Leaf	RHSIM_Rhsim06G0155900	-8.981178545	3.13725E-09	2.23892E-08	RHSIM_Rhsim06G0155900
Stem vs Leaf	RHSIM_Rhsim01G0100100	-4.174107072	3.14849E-09	2.24625E-08	RHSIM_Rhsim01G0100100
Stem vs Leaf	RHSIM_Rhsim11G0191000	4.198705337	3.20439E-09	2.28471E-08	RHSIM_Rhsim11G0191000
Stem vs Leaf	RHSIM_Rhsim06G0003200	-2.845089639	3.21397E-09	2.29084E-08	RHSIM_Rhsim06G0003200
Stem vs Leaf	novel.6098	-2.659425886	3.21732E-09	2.29252E-08	--
Stem vs Leaf	RHSIM_Rhsim08G0217400	1.854728673	3.27394E-09	2.3307E-08	RHSIM_Rhsim08G0217400
Stem vs Leaf	RHSIM_Rhsim01G0210100	2.268427965	3.28555E-09	2.33824E-08	RHSIM_Rhsim01G0210100
Stem vs Leaf	RHSIM_Rhsim01G0249200	2.440315072	3.29776E-09	2.3462E-08	RHSIM_Rhsim01G0249200
Stem vs Leaf	RHSIM_Rhsim13G0012900	5.545558935	3.31653E-09	2.35811E-08	RHSIM_Rhsim13G0012900
Stem vs Leaf	RHSIM_Rhsim01G0141500	-1.82617817	3.32493E-09	2.36334E-08	RHSIM_Rhsim01G0141500
Stem vs Leaf	RHSIM_Rhsim07G0124600	-2.406049473	3.3494E-09	2.38001E-08	RHSIM_Rhsim07G0124600
Stem vs Leaf	RHSIM_Rhsim03G0223000	3.997473185	3.3694E-09	2.39348E-08	RHSIM_Rhsim03G0223000
Stem vs Leaf	novel.5097	2.933624217	3.40358E-09	2.41702E-08	--
Stem vs Leaf	novel.4096	-8.494586859	3.40879E-09	2.41997E-08	--
Stem vs Leaf	RHSIM_Rhsim04G0234700	2.194574355	3.43663E-09	2.43898E-08	RHSIM_Rhsim04G0234700
Stem vs Leaf	RHSIM_Rhsim01G0218600	-3.591674465	3.45415E-09	2.45066E-08	RHSIM_Rhsim01G0218600
Stem vs Leaf	RHSIM_Rhsim10G0082700	1.66166636	3.47918E-09	2.46766E-08	RHSIM_Rhsim10G0082700
Stem vs Leaf	RHSIM_Rhsim05G0050000	-3.252613907	3.48558E-09	2.47144E-08	RHSIM_Rhsim05G0050000
Stem vs Leaf	RHSIM_Rhsim07G0257900	3.564394727	3.48799E-09	2.47239E-08	RHSIM_Rhsim07G0257900
Stem vs Leaf	RHSIM_Rhsim08G0203800	2.917650911	3.49665E-09	2.47776E-08	RHSIM_Rhsim08G0203800
Stem vs Leaf	RHSIM_Rhsim08G0173600	-6.009647198	3.50377E-09	2.48204E-08	RHSIM_Rhsim08G0173600

Comparison	Gene ID	log_2FC	P-value	FDR (padj)	Gene Name
Stem vs Leaf	RHSIM_Rhsim07G0041800	-4.213534969	3.52084E-09	2.49337E-08	RHSIM_Rhsim07G0041800
Stem vs Leaf	RHSIM_Rhsim02G0248600	2.16316959	3.54093E-09	2.50683E-08	RHSIM_Rhsim02G0248600
Stem vs Leaf	novel.1609	-7.740775443	3.54519E-09	2.50907E-08	--
Stem vs Leaf	RHSIM_Rhsim13G0195000	-3.406727345	3.58746E-09	2.53743E-08	RHSIM_Rhsim13G0195000
Stem vs Leaf	RHSIM_Rhsim07G0215900	1.432468413	3.5935E-09	2.54092E-08	RHSIM_Rhsim07G0215900
Stem vs Leaf	RHSIM_Rhsim03G0199300	-2.356696419	3.61149E-09	2.55286E-08	RHSIM_Rhsim03G0199300
Stem vs Leaf	novel.1287	3.097018483	3.63479E-09	2.56854E-08	--
Stem vs Leaf	RHSIM_Rhsim06G0175900	-2.014354095	3.6477E-09	2.57687E-08	RHSIM_Rhsim06G0175900
Stem vs Leaf	RHSIM_Rhsim11G0110200	4.79199314	3.65375E-09	2.58036E-08	RHSIM_Rhsim11G0110200
Stem vs Leaf	RHSIM_Rhsim02G0000600	11.44540095	3.69366E-09	2.60694E-08	RHSIM_Rhsim02G0000600
Stem vs Leaf	RHSIM_Rhsim10G0060600	2.408383505	3.70765E-09	2.61601E-08	RHSIM_Rhsim10G0060600
Stem vs Leaf	novel.4641	2.290496322	3.71995E-09	2.62389E-08	--
Stem vs Leaf	RHSIM_Rhsim08G0206500	4.162124959	3.73081E-09	2.63074E-08	RHSIM_Rhsim08G0206500
Stem vs Leaf	RHSIM_Rhsim01G0033000	-1.060156896	3.79707E-09	2.67665E-08	RHSIM_Rhsim01G0033000
Stem vs Leaf	RHSIM_Rhsim07G0239900	-5.188875542	3.83013E-09	2.69913E-08	RHSIM_Rhsim07G0239900
Stem vs Leaf	RHSIM_Rhsim03G0259900	-6.587340814	3.84961E-09	2.71203E-08	RHSIM_Rhsim03G0259900
Stem vs Leaf	RHSIM_Rhsim09G0191600	-2.937089675	3.86207E-09	2.71997E-08	RHSIM_Rhsim09G0191600
Stem vs Leaf	RHSIM_Rhsim06G0001000	2.412290614	3.92936E-09	2.76652E-08	RHSIM_Rhsim06G0001000
Stem vs Leaf	RHSIM_Rhsim04G0020400	2.478453973	4.01792E-09	2.82715E-08	RHSIM_Rhsim04G0020400
Stem vs Leaf	RHSIM_Rhsim07G0077500	3.027485505	4.0201E-09	2.82781E-08	RHSIM_Rhsim07G0077500
Stem vs Leaf	RHSIM_Rhsim07G0226100	8.286030587	4.0311E-09	2.83469E-08	RHSIM_Rhsim07G0226100
Stem vs Leaf	RHSIM_Rhsim13G0110600	-2.239509014	4.05461E-09	2.85035E-08	RHSIM_Rhsim13G0110600
Stem vs Leaf	RHSIM_Rhsim10G0188300	-2.079079173	4.07734E-09	2.86545E-08	RHSIM_Rhsim10G0188300
Stem vs Leaf	novel.5778	-2.843522811	4.08187E-09	2.86776E-08	--
Stem vs Leaf	RHSIM_Rhsim04G0021200	-3.452100215	4.11841E-09	2.89255E-08	RHSIM_Rhsim04G0021200

Comparison	Gene ID	log_2FC	P-value	FDR (padj)	Gene Name
Stem vs Leaf	RHSIM_Rhsim03G0099600	-1.1067689	4.12728E-09	2.8979E-08	RHSIM_Rhsim03G0099600
Stem vs Leaf	RHSIM_Rhsim04G0166000	3.466360694	4.14138E-09	2.90691E-08	RHSIM_Rhsim04G0166000
Stem vs Leaf	RHSIM_Rhsim13G0217500	1.891108068	4.19354E-09	2.94263E-08	RHSIM_Rhsim13G0217500
Stem vs Leaf	RHSIM_Rhsim09G0145700	2.04366912	4.23799E-09	2.97291E-08	RHSIM_Rhsim09G0145700
Stem vs Leaf	RHSIM_Rhsim12G0214600	-1.444697466	4.24101E-09	2.97413E-08	RHSIM_Rhsim12G0214600
Stem vs Leaf	RHSIM_Rhsim12G0188300	-7.895122	4.25427E-09	2.98252E-08	RHSIM_Rhsim12G0188300
Stem vs Leaf	RHSIM_Rhsim03G0046400	-1.247182189	4.25601E-09	2.98283E-08	RHSIM_Rhsim03G0046400
Stem vs Leaf	RHSIM_Rhsim02G0123600	1.035934707	4.2796E-09	2.99845E-08	RHSIM_Rhsim02G0123600
Stem vs Leaf	RHSIM_Rhsim13G0052300	1.723105421	4.28173E-09	2.99903E-08	RHSIM_Rhsim13G0052300
Stem vs Leaf	novel.4387	-4.31072432	4.32814E-09	3.03062E-08	--
Stem vs Leaf	RHSIM_Rhsim07G0036000	1.187418009	4.35272E-09	3.0469E-08	RHSIM_Rhsim07G0036000
Stem vs Leaf	RHSIM_Rhsim13G0040300	-2.940249173	4.42378E-09	3.09476E-08	RHSIM_Rhsim13G0040300
Stem vs Leaf	novel.3116	-5.057637802	4.43546E-09	3.102E-08	--
Stem vs Leaf	RHSIM_Rhsim02G0119200	-2.159876832	4.48365E-09	3.13474E-08	RHSIM_Rhsim02G0119200
Stem vs Leaf	RHSIM_Rhsim13G0213200	-1.462109169	4.51122E-09	3.15306E-08	RHSIM_Rhsim13G0213200
Stem vs Leaf	RHSIM_Rhsim06G0066700	4.636443387	4.52865E-09	3.16429E-08	RHSIM_Rhsim06G0066700
Stem vs Leaf	RHSIM_Rhsim01G0221100	3.057939282	4.53196E-09	3.16564E-08	RHSIM_Rhsim01G0221100
Stem vs Leaf	novel.4637	1.276999297	4.54595E-09	3.17445E-08	--
Stem vs Leaf	RHSIM_Rhsim11G0166600	2.935480844	4.55204E-09	3.17774E-08	RHSIM_Rhsim11G0166600
Stem vs Leaf	RHSIM_Rhsim10G0097900	-7.87028534	4.58097E-09	3.19697E-08	RHSIM_Rhsim10G0097900
Stem vs Leaf	RHSIM_Rhsim07G0108300	-8.38924461	4.62972E-09	3.23001E-08	RHSIM_Rhsim07G0108300
Stem vs Leaf	RHSIM_Rhsim09G0174100	-4.018732679	4.64946E-09	3.24182E-08	RHSIM_Rhsim09G0174100
Stem vs Leaf	RHSIM_Rhsim11G0101000	-3.550519964	4.65319E-09	3.24344E-08	RHSIM_Rhsim11G0101000
Stem vs Leaf	RHSIM_Rhsim03G0059500	-1.888750646	4.68366E-09	3.26369E-08	RHSIM_Rhsim03G0059500
Stem vs Leaf	RHSIM_Rhsim09G0135100	-5.999664789	4.72564E-09	3.29195E-08	RHSIM_Rhsim09G0135100

Comparison	Gene ID	log_2FC	P-value	FDR (padj)	Gene Name
Stem vs Leaf	RHSIM_Rhsim09G0172300	1.82085438	4.88542E-09	3.40222E-08	RHSIM_Rhsim09G0172300
Stem vs Leaf	RHSIM_Rhsim08G0173100	-1.097558587	4.90059E-09	3.41176E-08	RHSIM_Rhsim08G0173100
Stem vs Leaf	RHSIM_Rhsim10G0084800	-3.352404777	4.90551E-09	3.41334E-08	RHSIM_Rhsim10G0084800
Stem vs Leaf	RHSIM_Rhsim01G0101900	-2.678471753	4.90582E-09	3.41334E-08	RHSIM_Rhsim01G0101900
Stem vs Leaf	RHSIM_Rhsim08G0059300	-9.533104163	4.90793E-09	3.41378E-08	RHSIM_Rhsim08G0059300
Stem vs Leaf	RHSIM_Rhsim04G0123100	4.111407742	4.92826E-09	3.42688E-08	RHSIM_Rhsim04G0123100
Stem vs Leaf	RHSIM_Rhsim02G0193600	-1.04784281	4.93334E-09	3.42938E-08	RHSIM_Rhsim02G0193600
Stem vs Leaf	RHSIM_Rhsim07G0090500	1.890576716	5.01914E-09	3.48692E-08	RHSIM_Rhsim07G0090500
Stem vs Leaf	novel.4761	-3.652013346	5.05371E-09	3.50988E-08	--
Stem vs Leaf	RHSIM_Rhsim09G0024400	6.222555787	5.07426E-09	3.52309E-08	RHSIM_Rhsim09G0024400
Stem vs Leaf	RHSIM_Rhsim05G0094700	8.724245647	5.08979E-09	3.5328E-08	RHSIM_Rhsim05G0094700
Stem vs Leaf	RHSIM_Rhsim02G0178300	-2.042411199	5.18409E-09	3.59609E-08	RHSIM_Rhsim02G0178300
Stem vs Leaf	RHSIM_Rhsim03G0050300	-1.612086627	5.20508E-09	3.60957E-08	RHSIM_Rhsim03G0050300
Stem vs Leaf	RHSIM_Rhsim09G0209500	-2.171943094	5.2336E-09	3.62825E-08	RHSIM_Rhsim09G0209500
Stem vs Leaf	RHSIM_Rhsim04G0243400	-7.759843789	5.25113E-09	3.63931E-08	RHSIM_Rhsim04G0243400
Stem vs Leaf	RHSIM_Rhsim05G0233100	-1.50614026	5.28113E-09	3.659E-08	RHSIM_Rhsim05G0233100
Stem vs Leaf	RHSIM_Rhsim05G0064300	-1.690668158	5.31061E-09	3.67832E-08	RHSIM_Rhsim05G0064300
Stem vs Leaf	RHSIM_Rhsim07G0085700	2.161532394	5.34247E-09	3.69928E-08	RHSIM_Rhsim07G0085700
Stem vs Leaf	RHSIM_Rhsim12G0057500	-1.183251803	5.34899E-09	3.70268E-08	RHSIM_Rhsim12G0057500
Stem vs Leaf	RHSIM_Rhsim02G0143100	1.096926068	5.37839E-09	3.72191E-08	RHSIM_Rhsim02G0143100
Stem vs Leaf	RHSIM_Rhsim09G0199000	-1.65361066	5.43926E-09	3.7629E-08	RHSIM_Rhsim09G0199000
Stem vs Leaf	RHSIM_Rhsim01G0074500	-1.184270375	5.4701E-09	3.78294E-08	RHSIM_Rhsim01G0074500
Stem vs Leaf	novel.4900	-8.292012515	5.4715E-09	3.78294E-08	--
Stem vs Leaf	RHSIM_Rhsim04G0234400	3.413033518	5.47699E-09	3.78559E-08	RHSIM_Rhsim04G0234400
Stem vs Leaf	RHSIM_Rhsim02G0195700	-1.718194143	5.51646E-09	3.81173E-08	RHSIM_Rhsim02G0195700

Comparison	Gene ID	log_2FC	P-value	FDR (padj)	Gene Name
Stem vs Leaf	novel.353	-1.972084733	5.54684E-09	3.83158E-08	--
Stem vs Leaf	RHSIM_Rhsim13G0096800	-4.441715735	5.65109E-09	3.90125E-08	RHSIM_Rhsim13G0096800
Stem vs Leaf	RHSIM_Rhsim10G0009000	-1.898345928	5.69681E-09	3.93164E-08	RHSIM_Rhsim10G0009000
Stem vs Leaf	novel.5813	-6.158308829	5.78165E-09	3.9878E-08	--
Stem vs Leaf	RHSIM_Rhsim08G0030300	2.186502658	5.78929E-09	3.99187E-08	RHSIM_Rhsim08G0030300
Stem vs Leaf	novel.5469	-6.097311566	5.79327E-09	3.99342E-08	--
Stem vs Leaf	RHSIM_Rhsim02G0043300	1.059467213	5.80849E-09	4.00271E-08	RHSIM_Rhsim02G0043300
Stem vs Leaf	RHSIM_Rhsim07G0234800	-1.233281459	5.8408E-09	4.02258E-08	RHSIM_Rhsim07G0234800
Stem vs Leaf	RHSIM_Rhsim02G0091100	-2.098000332	5.8523E-09	4.02929E-08	RHSIM_Rhsim02G0091100
Stem vs Leaf	novel.5093	3.764470082	5.87035E-09	4.04051E-08	--
Stem vs Leaf	RHSIM_Rhsim01G0133700	8.234675029	5.87279E-09	4.04099E-08	RHSIM_Rhsim01G0133700
Stem vs Leaf	RHSIM_Rhsim06G0162400	2.560445316	5.8772E-09	4.04239E-08	RHSIM_Rhsim06G0162400
Stem vs Leaf	RHSIM_RhsimUnG0118100	6.795885984	5.87834E-09	4.04239E-08	RHSIM_RhsimUnG0118100
Stem vs Leaf	novel.5544	-6.672547202	5.95856E-09	4.09633E-08	--
Stem vs Leaf	RHSIM_Rhsim09G0164200	1.258705256	5.99921E-09	4.12305E-08	RHSIM_Rhsim09G0164200
Stem vs Leaf	RHSIM_Rhsim07G0241000	-2.7930701	6.06572E-09	4.16752E-08	RHSIM_Rhsim07G0241000
Stem vs Leaf	RHSIM_Rhsim07G0143500	-1.150663565	6.07987E-09	4.176E-08	RHSIM_Rhsim07G0143500
Stem vs Leaf	RHSIM_Rhsim02G0047900	6.759676403	6.10658E-09	4.19309E-08	RHSIM_Rhsim02G0047900
Stem vs Leaf	RHSIM_Rhsim08G0195900	1.183493986	6.12811E-09	4.20662E-08	RHSIM_Rhsim08G0195900
Stem vs Leaf	RHSIM_Rhsim02G0002100	1.690212641	6.22499E-09	4.27057E-08	RHSIM_Rhsim02G0002100
Stem vs Leaf	RHSIM_Rhsim07G0125500	-1.560048333	6.2382E-09	4.27836E-08	RHSIM_Rhsim07G0125500
Stem vs Leaf	novel.3562	8.916924421	6.31063E-09	4.32675E-08	--
Stem vs Leaf	RHSIM_Rhsim02G0176200	-2.969120926	6.32203E-09	4.33328E-08	RHSIM_Rhsim02G0176200
Stem vs Leaf	RHSIM_Rhsim10G0192800	1.020917861	6.35327E-09	4.3534E-08	RHSIM_Rhsim10G0192800
Stem vs Leaf	RHSIM_Rhsim05G0215900	-2.333904223	6.3634E-09	4.35904E-08	RHSIM_Rhsim05G0215900

Comparison	Gene ID	log_2FC	P-value	FDR (padj)	Gene Name
Stem vs Leaf	novel.3120	-1.002332047	6.38735E-09	4.37415E-08	--
Stem vs Leaf	RHSIM_Rhsim03G0169500	1.316544735	6.4084E-09	4.38726E-08	RHSIM_Rhsim03G0169500
Stem vs Leaf	RHSIM_Rhsim12G0018000	-1.332272136	6.48183E-09	4.43621E-08	RHSIM_Rhsim12G0018000
Stem vs Leaf	RHSIM_Rhsim11G0138200	-2.712655054	6.50351E-09	4.44973E-08	RHSIM_Rhsim11G0138200
Stem vs Leaf	RHSIM_Rhsim03G0157900	-7.652997367	6.54744E-09	4.47846E-08	RHSIM_Rhsim03G0157900
Stem vs Leaf	RHSIM_Rhsim02G0066700	2.233337096	6.6245E-09	4.52982E-08	RHSIM_Rhsim02G0066700
Stem vs Leaf	RHSIM_Rhsim11G0129000	-7.363495512	6.6695E-09	4.55924E-08	RHSIM_Rhsim11G0129000
Stem vs Leaf	RHSIM_Rhsim05G0078500	1.07416185	6.69886E-09	4.57795E-08	RHSIM_Rhsim05G0078500
Stem vs Leaf	RHSIM_Rhsim02G0097800	3.320883604	6.71772E-09	4.58948E-08	RHSIM_Rhsim02G0097800
Stem vs Leaf	RHSIM_Rhsim03G0194400	-2.616876324	6.73685E-09	4.60118E-08	RHSIM_Rhsim03G0194400
Stem vs Leaf	RHSIM_Rhsim01G0289000	9.486875059	6.83377E-09	4.666E-08	RHSIM_Rhsim01G0289000
Stem vs Leaf	RHSIM_Rhsim09G0176400	-1.765958398	6.84922E-09	4.67516E-08	RHSIM_Rhsim09G0176400
Stem vs Leaf	novel.5854	-1.214725424	6.90761E-09	4.71362E-08	--
Stem vs Leaf	RHSIM_Rhsim07G0065500	-1.387465562	6.99958E-09	4.77355E-08	RHSIM_Rhsim07G0065500
Stem vs Leaf	RHSIM_Rhsim12G0124500	-1.715576798	7.02101E-09	4.78675E-08	RHSIM_Rhsim12G0124500
Stem vs Leaf	RHSIM_Rhsim08G0234400	-1.51026679	7.04426E-09	4.80118E-08	RHSIM_Rhsim08G0234400
Stem vs Leaf	novel.746	1.340779728	7.08188E-09	4.8254E-08	--
Stem vs Leaf	RHSIM_Rhsim02G0132700	1.019021994	7.11111E-09	4.84259E-08	RHSIM_Rhsim02G0132700
Stem vs Leaf	RHSIM_Rhsim08G0177900	-2.601404541	7.11253E-09	4.84259E-08	RHSIM_Rhsim08G0177900
Stem vs Leaf	RHSIM_Rhsim01G0222000	-3.186457678	7.12103E-09	4.84634E-08	RHSIM_Rhsim01G0222000
Stem vs Leaf	RHSIM_Rhsim06G0188000	7.589547606	7.12379E-09	4.84678E-08	RHSIM_Rhsim06G0188000
Stem vs Leaf	RHSIM_Rhsim12G0152000	1.380932603	7.12841E-09	4.8485E-08	RHSIM_Rhsim12G0152000
Stem vs Leaf	RHSIM_Rhsim08G0015300	1.815529768	7.26835E-09	4.94076E-08	RHSIM_Rhsim08G0015300
Stem vs Leaf	RHSIM_Rhsim13G0223500	3.089541552	7.27105E-09	4.94114E-08	RHSIM_Rhsim13G0223500
Stem vs Leaf	novel.534	-4.301670344	7.2822E-09	4.94726E-08	--

Comparison	Gene ID	log_2FC	P-value	FDR (padj)	Gene Name
Stem vs Leaf	RHSIM_Rhsim02G0021000	-1.885147213	7.31914E-09	4.96942E-08	RHSIM_Rhsim02G0021000
Stem vs Leaf	RHSIM_Rhsim08G0113800	-3.146686075	7.33426E-09	4.97822E-08	RHSIM_Rhsim08G0113800
Stem vs Leaf	RHSIM_Rhsim11G0058400	3.883613013	7.36474E-09	4.99744E-08	RHSIM_Rhsim11G0058400
Stem vs Leaf	RHSIM_Rhsim06G0107800	1.48850276	7.43198E-09	5.04158E-08	RHSIM_Rhsim06G0107800
Stem vs Leaf	RHSIM_Rhsim01G0234700	-1.809146091	7.46292E-09	5.06108E-08	RHSIM_Rhsim01G0234700
Stem vs Leaf	RHSIM_Rhsim08G0248600	-1.482628574	7.52542E-09	5.10197E-08	RHSIM_Rhsim08G0248600
Stem vs Leaf	RHSIM_Rhsim10G0173400	3.261649634	7.54323E-09	5.11253E-08	RHSIM_Rhsim10G0173400
Stem vs Leaf	RHSIM_Rhsim01G0258800	-1.758169615	7.55127E-09	5.11648E-08	RHSIM_Rhsim01G0258800
Stem vs Leaf	RHSIM_Rhsim12G0138800	-2.71400107	7.63129E-09	5.16918E-08	RHSIM_Rhsim12G0138800
Stem vs Leaf	RHSIM_Rhsim12G0041700	-3.540103033	7.69309E-09	5.20951E-08	RHSIM_Rhsim12G0041700
Stem vs Leaf	RHSIM_Rhsim11G0167700	-3.228605102	7.69784E-09	5.21025E-08	RHSIM_Rhsim11G0167700
Stem vs Leaf	RHSIM_Rhsim08G0234000	-3.503498304	7.69871E-09	5.21025E-08	RHSIM_Rhsim08G0234000
Stem vs Leaf	novel.1467	-7.554475836	7.75202E-09	5.24479E-08	--
Stem vs Leaf	RHSIM_Rhsim05G0159600	-1.535507777	7.81886E-09	5.28846E-08	RHSIM_Rhsim05G0159600
Stem vs Leaf	RHSIM_Rhsim06G0190900	3.591342067	7.84106E-09	5.30192E-08	RHSIM_Rhsim06G0190900
Stem vs Leaf	RHSIM_Rhsim05G0182400	1.321455275	7.878E-09	5.32533E-08	RHSIM_Rhsim05G0182400
Stem vs Leaf	RHSIM_Rhsim12G0209900	1.891028711	7.88555E-09	5.32887E-08	RHSIM_Rhsim12G0209900
Stem vs Leaf	RHSIM_Rhsim12G0018700	3.321715231	8.00947E-09	5.41103E-08	RHSIM_Rhsim12G0018700
Stem vs Leaf	RHSIM_Rhsim03G0162500	-2.425924934	8.02195E-09	5.41787E-08	RHSIM_Rhsim03G0162500
Stem vs Leaf	RHSIM_Rhsim02G0112300	1.939036109	8.03402E-09	5.42444E-08	RHSIM_Rhsim02G0112300
Stem vs Leaf	RHSIM_RhsimUnG0115300	2.526212108	8.17748E-09	5.51968E-08	RHSIM_RhsimUnG0115300
Stem vs Leaf	RHSIM_Rhsim04G0096900	2.634533443	8.33221E-09	5.61918E-08	RHSIM_Rhsim04G0096900
Stem vs Leaf	RHSIM_Rhsim09G0184600	3.646963176	8.34851E-09	5.62852E-08	RHSIM_Rhsim09G0184600
Stem vs Leaf	novel.2921	-4.716854144	8.37782E-09	5.64663E-08	--
Stem vs Leaf	RHSIM_Rhsim03G0099700	-1.592328957	8.39846E-09	5.65889E-08	RHSIM_Rhsim03G0099700

Comparison	Gene ID	log_2FC	P-value	FDR (padj)	Gene Name
Stem vs Leaf	RHSIM_Rhsim10G0020900	1.574210999	8.42714E-09	5.67655E-08	RHSIM_Rhsim10G0020900
Stem vs Leaf	RHSIM_Rhsim02G0077100	-4.521375369	8.4435E-09	5.68591E-08	RHSIM_Rhsim02G0077100
Stem vs Leaf	RHSIM_Rhsim13G0149700	7.579480973	8.52802E-09	5.74115E-08	RHSIM_Rhsim13G0149700
Stem vs Leaf	RHSIM_Rhsim05G0056800	2.151294455	8.57682E-09	5.77232E-08	RHSIM_Rhsim05G0056800
Stem vs Leaf	RHSIM_Rhsim03G0260000	-5.298241734	8.58911E-09	5.7789E-08	RHSIM_Rhsim03G0260000
Stem vs Leaf	RHSIM_Rhsim02G0106600	2.358472709	8.74209E-09	5.88012E-08	RHSIM_Rhsim02G0106600
Stem vs Leaf	RHSIM_Rhsim12G0149200	-7.802083713	8.74584E-09	5.88092E-08	RHSIM_Rhsim12G0149200
Stem vs Leaf	RHSIM_Rhsim10G0052700	-5.644679464	8.75098E-09	5.88266E-08	RHSIM_Rhsim10G0052700
Stem vs Leaf	RHSIM_Rhsim08G0230400	-2.01038989	8.81503E-09	5.92399E-08	RHSIM_Rhsim08G0230400
Stem vs Leaf	novel.1873	-3.127866533	8.91627E-09	5.99028E-08	--
Stem vs Leaf	RHSIM_Rhsim03G0046200	1.514428538	8.9479E-09	6.00977E-08	RHSIM_Rhsim03G0046200
Stem vs Leaf	RHSIM_Rhsim06G0166200	-1.120434639	9.14497E-09	6.13856E-08	RHSIM_Rhsim06G0166200
Stem vs Leaf	RHSIM_Rhsim04G0166600	-1.020607403	9.17189E-09	6.15483E-08	RHSIM_Rhsim04G0166600
Stem vs Leaf	RHSIM_Rhsim10G0012500	-2.361148592	9.20617E-09	6.17604E-08	RHSIM_Rhsim10G0012500
Stem vs Leaf	novel.698	-7.832405192	9.23065E-09	6.19066E-08	--
Stem vs Leaf	RHSIM_Rhsim02G0253200	3.050381706	9.23661E-09	6.19285E-08	RHSIM_Rhsim02G0253200
Stem vs Leaf	RHSIM_Rhsim03G0097800	4.380134288	9.3504E-09	6.26733E-08	RHSIM_Rhsim03G0097800
Stem vs Leaf	RHSIM_Rhsim09G0181000	-3.840089274	9.3586E-09	6.271E-08	RHSIM_Rhsim09G0181000
Stem vs Leaf	RHSIM_Rhsim12G0190700	-1.558455207	9.48113E-09	6.35125E-08	RHSIM_Rhsim12G0190700
Stem vs Leaf	RHSIM_Rhsim04G0213900	1.911557478	9.54597E-09	6.39283E-08	RHSIM_Rhsim04G0213900
Stem vs Leaf	RHSIM_Rhsim13G0005000	-1.230327112	9.61816E-09	6.43931E-08	RHSIM_Rhsim13G0005000
Stem vs Leaf	RHSIM_RhsimUnG0109400	-1.440816595	9.66751E-09	6.47046E-08	RHSIM_RhsimUnG0109400
Stem vs Leaf	RHSIM_Rhsim06G0201700	-1.9060757	9.74712E-09	6.52185E-08	RHSIM_Rhsim06G0201700
Stem vs Leaf	RHSIM_Rhsim05G0190800	-1.162106732	9.84532E-09	6.58565E-08	RHSIM_Rhsim05G0190800
Stem vs Leaf	RHSIM_Rhsim06G0004000	-1.288117084	1.00731E-08	6.73411E-08	RHSIM_Rhsim06G0004000

Comparison	Gene ID	log_2FC	P-value	FDR (padj)	Gene Name
Stem vs Leaf	RHSIM_Rhsim10G0067900	-3.427190674	1.01928E-08	6.81218E-08	RHSIM_Rhsim10G0067900
Stem vs Leaf	RHSIM_Rhsim04G0144300	1.719338746	1.0205E-08	6.81829E-08	RHSIM_Rhsim04G0144300
Stem vs Leaf	RHSIM_Rhsim13G0044200	-4.778952349	1.02347E-08	6.8362E-08	RHSIM_Rhsim13G0044200
Stem vs Leaf	RHSIM_Rhsim12G0091100	2.844115	1.0246E-08	6.84176E-08	RHSIM_Rhsim12G0091100
Stem vs Leaf	RHSIM_Rhsim09G0123100	-3.663924153	1.02709E-08	6.8544E-08	RHSIM_Rhsim09G0123100
Stem vs Leaf	RHSIM_Rhsim02G0248700	-1.70925888	1.04713E-08	6.98615E-08	RHSIM_Rhsim02G0248700
Stem vs Leaf	RHSIM_Rhsim10G0036100	1.459681399	1.04755E-08	6.98691E-08	RHSIM_Rhsim10G0036100
Stem vs Leaf	RHSIM_Rhsim02G0233500	1.890455186	1.05498E-08	7.03236E-08	RHSIM_Rhsim02G0233500
Stem vs Leaf	RHSIM_Rhsim06G0169000	8.035389734	1.06756E-08	7.11421E-08	RHSIM_Rhsim06G0169000
Stem vs Leaf	novel.4193	-8.291757026	1.07398E-08	7.15493E-08	--
Stem vs Leaf	RHSIM_Rhsim03G0192700	-2.183128164	1.07621E-08	7.16769E-08	RHSIM_Rhsim03G0192700
Stem vs Leaf	RHSIM_RhsimUnG0079400	-7.554997348	1.09434E-08	7.28631E-08	RHSIM_RhsimUnG0079400
Stem vs Leaf	RHSIM_Rhsim01G0221900	1.632476529	1.09629E-08	7.29723E-08	RHSIM_Rhsim01G0221900
Stem vs Leaf	RHSIM_RhsimUnG0010900	7.920069944	1.11248E-08	7.40282E-08	RHSIM_RhsimUnG0010900
Stem vs Leaf	RHSIM_Rhsim09G0115600	-2.17101478	1.11964E-08	7.44619E-08	RHSIM_Rhsim09G0115600
Stem vs Leaf	RHSIM_Rhsim02G0247900	-3.907222959	1.12554E-08	7.48169E-08	RHSIM_Rhsim02G0247900
Stem vs Leaf	RHSIM_Rhsim07G0215400	-2.316850407	1.12563E-08	7.48169E-08	RHSIM_Rhsim07G0215400
Stem vs Leaf	novel.1657	-7.883806472	1.12706E-08	7.48904E-08	--
Stem vs Leaf	RHSIM_Rhsim08G0043900	1.579086646	1.12967E-08	7.50424E-08	RHSIM_Rhsim08G0043900
Stem vs Leaf	RHSIM_Rhsim12G0124800	-2.980170364	1.1419E-08	7.58107E-08	RHSIM_Rhsim12G0124800
Stem vs Leaf	RHSIM_Rhsim02G0069700	-1.592542406	1.15979E-08	7.69764E-08	RHSIM_Rhsim02G0069700
Stem vs Leaf	novel.3770	1.967370342	1.17176E-08	7.77488E-08	--
Stem vs Leaf	RHSIM_Rhsim09G0120100	-1.582667834	1.18129E-08	7.83359E-08	RHSIM_Rhsim09G0120100
Stem vs Leaf	RHSIM_Rhsim06G0067000	1.371765784	1.18373E-08	7.84749E-08	RHSIM_Rhsim06G0067000
Stem vs Leaf	RHSIM_Rhsim03G0215800	3.929421358	1.18428E-08	7.84885E-08	RHSIM_Rhsim03G0215800

Comparison	Gene ID	log_2FC	P-value	FDR (padj)	Gene Name
Stem vs Leaf	RHSIM_Rhsim02G0235400	-4.913700516	1.1856E-08	7.85538E-08	RHSIM_Rhsim02G0235400
Stem vs Leaf	RHSIM_Rhsim05G0010100	-7.845757728	1.18717E-08	7.86351E-08	RHSIM_Rhsim05G0010100
Stem vs Leaf	RHSIM_Rhsim03G0264100	-1.007022012	1.19739E-08	7.92666E-08	RHSIM_Rhsim03G0264100
Stem vs Leaf	RHSIM_RhsimUnG0200100	-7.863848587	1.20764E-08	7.98993E-08	RHSIM_RhsimUnG0200100
Stem vs Leaf	RHSIM_Rhsim11G0063500	-7.538582877	1.21737E-08	8.05195E-08	RHSIM_Rhsim11G0063500
Stem vs Leaf	RHSIM_Rhsim04G0185800	3.603311987	1.21835E-08	8.05612E-08	RHSIM_Rhsim04G0185800
Stem vs Leaf	RHSIM_Rhsim06G0222000	-8.195468357	1.23098E-08	8.13731E-08	RHSIM_Rhsim06G0222000
Stem vs Leaf	RHSIM_Rhsim03G0176100	1.232140389	1.232E-08	8.14175E-08	RHSIM_Rhsim03G0176100
Stem vs Leaf	novel.134	4.305958444	1.25983E-08	8.32325E-08	--
Stem vs Leaf	novel.2522	-2.455300802	1.26772E-08	8.37301E-08	--
Stem vs Leaf	RHSIM_RhsimUnG0158600	-2.114247467	1.27554E-08	8.42223E-08	RHSIM_RhsimUnG0158600
Stem vs Leaf	RHSIM_Rhsim08G0137600	4.056984326	1.28186E-08	8.46155E-08	RHSIM_Rhsim08G0137600
Stem vs Leaf	novel.1517	5.883839526	1.28691E-08	8.4924E-08	--
Stem vs Leaf	RHSIM_Rhsim06G0068800	-2.922506414	1.2905E-08	8.5137E-08	RHSIM_Rhsim06G0068800
Stem vs Leaf	novel.5715	-4.288120746	1.30453E-08	8.60376E-08	--
Stem vs Leaf	RHSIM_Rhsim08G0155700	-5.882364087	1.30789E-08	8.62349E-08	RHSIM_Rhsim08G0155700
Stem vs Leaf	RHSIM_Rhsim01G0165900	-5.226353439	1.3349E-08	8.79904E-08	RHSIM_Rhsim01G0165900
Stem vs Leaf	RHSIM_Rhsim05G0112700	6.015437605	1.34011E-08	8.83087E-08	RHSIM_Rhsim05G0112700
Stem vs Leaf	RHSIM_Rhsim08G0015100	1.595030645	1.34516E-08	8.86162E-08	RHSIM_Rhsim08G0015100
Stem vs Leaf	RHSIM_Rhsim03G0105900	-3.551557857	1.34769E-08	8.87573E-08	RHSIM_Rhsim03G0105900
Stem vs Leaf	RHSIM_Rhsim08G0061000	-12.92137936	1.34845E-08	8.87819E-08	RHSIM_Rhsim08G0061000
Stem vs Leaf	RHSIM_Rhsim03G0262000	-3.089265638	1.35031E-08	8.88787E-08	RHSIM_Rhsim03G0262000
Stem vs Leaf	RHSIM_Rhsim02G0000500	2.983468015	1.36301E-08	8.96892E-08	RHSIM_Rhsim02G0000500
Stem vs Leaf	RHSIM_Rhsim10G0096900	-5.490883265	1.37747E-08	9.06151E-08	RHSIM_Rhsim10G0096900
Stem vs Leaf	RHSIM_Rhsim07G0187000	1.60724932	1.3928E-08	9.15971E-08	RHSIM_Rhsim07G0187000

Comparison	Gene ID	log_2FC	P-value	FDR (padj)	Gene Name
Stem vs Leaf	RHSIM_Rhsim05G0037700	-4.791696206	1.40674E-08	9.24876E-08	RHSIM_Rhsim05G0037700
Stem vs Leaf	RHSIM_Rhsim13G0055900	5.060718563	1.42152E-08	9.3433E-08	RHSIM_Rhsim13G0055900
Stem vs Leaf	novel.3147	8.422498341	1.42367E-08	9.35473E-08	--
Stem vs Leaf	novel.4851	2.25702664	1.42994E-08	9.39323E-08	--
Stem vs Leaf	RHSIM_Rhsim13G0025700	-1.216926121	1.43831E-08	9.44555E-08	RHSIM_Rhsim13G0025700
Stem vs Leaf	RHSIM_Rhsim12G0074700	-1.676431114	1.43907E-08	9.44787E-08	RHSIM_Rhsim12G0074700
Stem vs Leaf	RHSIM_Rhsim05G0161600	2.262019093	1.45788E-08	9.56864E-08	RHSIM_Rhsim05G0161600
Stem vs Leaf	RHSIM_Rhsim11G0161100	3.074924401	1.46441E-08	9.60874E-08	RHSIM_Rhsim11G0161100
Stem vs Leaf	RHSIM_Rhsim06G0113000	-8.03001592	1.47594E-08	9.67887E-08	RHSIM_Rhsim06G0113000
Stem vs Leaf	RHSIM_Rhsim02G0019900	-1.21704789	1.47711E-08	9.68377E-08	RHSIM_Rhsim02G0019900
Stem vs Leaf	RHSIM_Rhsim02G0251700	-2.467930115	1.47767E-08	9.68473E-08	RHSIM_Rhsim02G0251700
Stem vs Leaf	RHSIM_Rhsim02G0135100	3.918735396	1.47944E-08	9.69357E-08	RHSIM_Rhsim02G0135100
Stem vs Leaf	novel.4068	3.243380671	1.49358E-08	9.78065E-08	--
Stem vs Leaf	RHSIM_Rhsim01G0205800	2.537373994	1.50187E-08	9.82774E-08	RHSIM_Rhsim01G0205800
Stem vs Leaf	RHSIM_Rhsim11G0026000	-1.371943428	1.50205E-08	9.82774E-08	RHSIM_Rhsim11G0026000
Stem vs Leaf	RHSIM_Rhsim03G0096300	-1.601983022	1.5221E-08	9.95329E-08	RHSIM_Rhsim03G0096300
Stem vs Leaf	RHSIM_RhsimPtG0003100	-1.923682241	1.52657E-08	9.97964E-08	RHSIM_RhsimPtG0003100
Stem vs Leaf	RHSIM_Rhsim08G0159800	-3.696786779	1.53789E-08	1.00508E-07	RHSIM_Rhsim08G0159800
Stem vs Leaf	RHSIM_Rhsim11G0121800	1.045413172	1.55883E-08	1.01848E-07	RHSIM_Rhsim11G0121800
Stem vs Leaf	novel.2518	-3.082149185	1.56264E-08	1.02039E-07	--
Stem vs Leaf	RHSIM_Rhsim01G0238000	-1.527478452	1.57449E-08	1.02783E-07	RHSIM_Rhsim01G0238000
Stem vs Leaf	RHSIM_Rhsim05G0086800	3.090786665	1.57634E-08	1.02875E-07	RHSIM_Rhsim05G0086800
Stem vs Leaf	RHSIM_Rhsim09G0043700	-1.633919334	1.59332E-08	1.03954E-07	RHSIM_Rhsim09G0043700
Stem vs Leaf	RHSIM_Rhsim02G0135700	5.643036507	1.59832E-08	1.0425E-07	RHSIM_Rhsim02G0135700
Stem vs Leaf	RHSIM_Rhsim07G0031300	-1.690025362	1.61098E-08	1.05046E-07	RHSIM_Rhsim07G0031300

Comparison	Gene ID	log_2FC	P-value	FDR (padj)	Gene Name
Stem vs Leaf	RHSIM_Rhsim04G0101200	-5.787478832	1.62745E-08	1.06091E-07	RHSIM_Rhsim04G0101200
Stem vs Leaf	RHSIM_Rhsim08G0190700	-1.446193991	1.62849E-08	1.06128E-07	RHSIM_Rhsim08G0190700
Stem vs Leaf	novel.5545	-4.838473495	1.6449E-08	1.07137E-07	--
Stem vs Leaf	RHSIM_Rhsim11G0187900	-11.97780776	1.65306E-08	1.07608E-07	RHSIM_Rhsim11G0187900
Stem vs Leaf	RHSIM_Rhsim05G0003700	4.52926107	1.65816E-08	1.07909E-07	RHSIM_Rhsim05G0003700
Stem vs Leaf	novel.1591	2.569650614	1.65979E-08	1.07985E-07	--
Stem vs Leaf	RHSIM_Rhsim03G0230500	3.088805653	1.66229E-08	1.08117E-07	RHSIM_Rhsim03G0230500
Stem vs Leaf	RHSIM_Rhsim03G0045900	-2.09852203	1.67179E-08	1.08704E-07	RHSIM_Rhsim03G0045900
Stem vs Leaf	novel.4849	-1.275796428	1.67296E-08	1.08749E-07	--
Stem vs Leaf	RHSIM_Rhsim13G0079600	-1.687092981	1.67379E-08	1.08773E-07	RHSIM_Rhsim13G0079600
Stem vs Leaf	novel.2514	1.866308978	1.68785E-08	1.09656E-07	--
Stem vs Leaf	RHSIM_Rhsim06G0047900	-2.068461648	1.70867E-08	1.10946E-07	RHSIM_Rhsim06G0047900
Stem vs Leaf	RHSIM_Rhsim08G0191900	3.156556206	1.72782E-08	1.12158E-07	RHSIM_Rhsim08G0191900
Stem vs Leaf	RHSIM_Rhsim02G0028300	3.379746892	1.73515E-08	1.12602E-07	RHSIM_Rhsim02G0028300
Stem vs Leaf	RHSIM_Rhsim10G0167700	2.083812805	1.76569E-08	1.14551E-07	RHSIM_Rhsim10G0167700
Stem vs Leaf	RHSIM_Rhsim03G0139800	1.255070059	1.76816E-08	1.14679E-07	RHSIM_Rhsim03G0139800
Stem vs Leaf	RHSIM_Rhsim07G0197600	-5.514023974	1.77128E-08	1.14849E-07	RHSIM_Rhsim07G0197600
Stem vs Leaf	RHSIM_Rhsim06G0185800	5.637637945	1.78079E-08	1.15433E-07	RHSIM_Rhsim06G0185800
Stem vs Leaf	RHSIM_Rhsim03G0065200	-3.68860553	1.78514E-08	1.15675E-07	RHSIM_Rhsim03G0065200
Stem vs Leaf	RHSIM_Rhsim12G0163700	7.785911849	1.78552E-08	1.15675E-07	RHSIM_Rhsim12G0163700
Stem vs Leaf	RHSIM_RhsimUnG0209400	-3.279518586	1.7999E-08	1.16574E-07	RHSIM_RhsimUnG0209400
Stem vs Leaf	RHSIM_Rhsim02G0063500	-3.905113875	1.8082E-08	1.17045E-07	RHSIM_Rhsim02G0063500
Stem vs Leaf	RHSIM_Rhsim08G0062900	1.046110776	1.81642E-08	1.17545E-07	RHSIM_Rhsim08G0062900
Stem vs Leaf	RHSIM_Rhsim09G0184100	-1.705449237	1.81856E-08	1.1765E-07	RHSIM_Rhsim09G0184100
Stem vs Leaf	RHSIM_Rhsim06G0103600	-4.928898549	1.8235E-08	1.17936E-07	RHSIM_Rhsim06G0103600

Comparison	Gene ID	log_2FC	P-value	FDR (padj)	Gene Name
Stem vs Leaf	RHSIM_Rhsim04G0117900	2.171114283	1.82523E-08	1.18015E-07	RHSIM_Rhsim04G0117900
Stem vs Leaf	RHSIM_RhsimUnG0075600	2.958836418	1.82942E-08	1.18253E-07	RHSIM_RhsimUnG0075600
[truncated: 486,596 more chars]
