# Supplementary material for: Integration of Transcriptomics and Metabolomics Reveals Organ-Specific Biosynthesis and Accumulation of Pharmacologically Active Flavonoids in Rhododendron yedoense var. poukhanense
Source: Plants (Basel). 2026 Aug 11;15(16):2441. doi: 10.3390/plants15162441 (PMC13516736; doi:10.3390/plants15162441)
Supplement: Supplementary file 1 [file plants-15-02441-s001.zip › Table S6.pdf]

Table S6. Primer amplification efficiencies for 12 validated genes in *R. yedoense* var. *poukhanense* qRT-PCR analysis.

Gene	Slope	Intercept	R_squared	Efficiency_%
CHS	-3.353	21.196	0.9979	98.7
CHI	-3.3077	23.6407	0.9978	100.6
F3H_FLS	-3.3201	24.3747	0.9984	100.1
DFR	-3.3716	22.7867	0.9985	98
ANS	-3.3616	23.6707	0.9981	98.4
MYB	-3.3001	20.7727	0.999	100.9
PAL	-3.3859	23.956	0.9976	97.4
4CL	-3.3082	24.66	0.9978	100.6
TPS	-3.332	25.9273	0.9982	99.6
CYP450	-3.3397	21.4607	0.9975	99.3

Gene	Slope	Intercept	R_squared	Efficiency_%
WRKY	-3.354	21.996	0.9969	98.7
bHLH	-3.3297	20.2047	0.9979	99.7
